# SNRPD1 conveys prognostic value on breast cancer survival and is required for anthracycline sensitivity

**DOI:** 10.1186/s12885-023-10860-z

**Published:** 2023-04-25

**Authors:** Xiaofeng Dai, Linhan Cai, Zhifa Zhang, Jitian Li

**Affiliations:** 1 The First Affiliated Hospital of Xi’an Jiaotong University, Xi’an 710061, China; 2 Wuxi School of Medicine, Jiangnan University, Wuxi, 214122 China; 3 Henan Luoyang Orthopedic Hospital (Henan Provincial Orthopedic Hospital), Henan University of Chinese Medicine, Zhengzhou, 450000 China

**Keywords:** *SNRPD1*, Clinical outcome, Prognostic value, Anthracycline resistance, Triple negative breast cancer

## Abstract

**Background:**

Cancers harboring spliceosome mutations are highly sensitive to additional perturbations on the spliceosome that leads to the development of onco-therapeutics targeting the spliceosome and opens novel opportunities for managing aggressive tumors lacking effective treatment options such as triple negative breast cancers. Being the core spliceosome associated proteins, *SNRPD1* and *SNRPE* have been both proposed as therapeutic targets for breast cancer management. Yet, their differences regarding their prognostic and therapeutic use as well as roles during carcinogenesis are largely unreported.

**Methods:**

We conducted *in silico* analysis at gene expression and genetic levels to differentiate the clinical relevance of *SNRPD1* and *SNRPE*, and explored their differential functionalities and molecular mechanistic associations with cancer in vitro.

**Results:**

We showed that high SNRPD1 gene expression was prognostic of poor breast cancer survival whereas SNRPE was not. The SNRPD1 expression quantitative trait loci, rs6733100, was found independently prognostic of breast cancer survival using TCGA data. Silencing either SNRPD1 or SNRPE independently suppressed the growth of breast cancer cells, but decreased migration was only observed in SNRPD1-silenced cells. Knocking down SNRPD1 but not SNRPE triggers doxorubicin resistance in triple negative breast cancer cells. Gene enrichment and network analyses revealed the dynamic regulatory role of SNRPD1 on cell cycle and genome stability, and the preventive role of SNRPE against cancer stemness that may neutralize its promotive role on cancer cell proliferation.

**Conclusion:**

Our results differentiated the functionalities of *SNRPD1* and *SNRPE* at both prognostic and therapeutic levels, and preliminarily explained the driving mechanism that requires additional explorations and validations.

**Supplementary Information:**

The online version contains supplementary material available at 10.1186/s12885-023-10860-z.

## Background

Breast cancers are highly heterogeneous, with the triple negative breast cancer (TNBC) subtype being the most difficult to treat. This particular cohort of malignant cells have higher invasiveness, recurrence rate and poorer patient survival as compared with the other subtypes, and are featured by low expression of many surface markers that render them insensitive to existing hormonal or targeted therapies such as Tamoxifen and Herceptin [1, 2]. This leaves chemotherapies the first-line approach against TNBCs that are known with strong adverse effects as a result of lack of tumor specificity. Intensive effort has been devoted to establish targeted therapies against TNBCs. Dominant strategies include tumor angiogenesis inhibitors such as Ruxolitinib [3] and poly ADP-ribose polymerase (PARP) inhibitors such as Olaparib [4]. Tumor angiogenesis inhibitors are typically accompanied with multifaceted toxicities such as liver damage, anaemia, neutropenia, and thrombocytopenia [5–7]. The efficacy of PAPR inhibitors is limited to TNBCs harboring BRCA1 mutations [8] and is adversely affected by many factors such as interactions between PARP1 and Fra-1 (an AP-1 member over-expressed in TNBCs) [4]. Thus, identifying novel targets towards improved TNBC management represents a global concern for researchers working on breast cancers.

Accurate splicing is essential to ensure normal cell functionality, alteration of which affects many cellular processes and is associated with a plethora of diseases including human cancers [9, 10]. Cancer transcriptomes are featured by abnormal RNA splicing as a result of, e.g., recurrent mutations to RNA splicing factors. Thus, cancers harboring spliceosome mutations are highly sensitive to additional perturbations on the spliceosome due to, at least partially, elevated pre-mRNA synthesis and consequent spliceosome burden [11]. This has led to the development of onco-therapeutics targeting the spliceosome [12–14] that opens novel opportunities for managing aggressive tumors lacking effective treatment options such as TNBCs [11], and a clinical trial on small-molecule modulators of the spliceosome (NCT02841540).

The spliceosome is a large dynamic macromolecular ribonucleoprotein (RNP) complex catalyzing the splicing of precursor mRNA into mRNA in eukaryotic cells, deregulation of which is associated with the generation of novel mRNA isoforms and protein variants. There are two types of spliceosomes in eukaryotic cells, i.e., U2-dependent (dominant) and U12-dependent spliceosomes. The U2 spliceosome processes approximately 95.5% of all U2-type introns, and the U12 spliceosome functions in the splicing of rare U12-type introns that occur at a frequency of around 0.35% in all human introns [11]. Both types of spliceosomes contain 5 uridine-rich (U-rich) small nuclear RNP (snRNP) units that differs in the types of snRNPs included [15]. While both spliceosomes contain the U5 snRNP, the U2-dependent spliceosome contains U1, U2, U4, U6 snRNPs and the U12-dependent spliceosome contains U11, U12, U4atac and U6atac snRNPs. Each snRNP is comprised of an RNA component, Sm proteins (i.e., SNRPB/B’, SNRPD1, SNRPD2, SNRPD3, SNRPE, SNRPF, SNRPG) that form a 7-member ring core structure encompassing RNA, and varieties of other associated proteins [11].

SNRPD1 and SNRPE, among other Sm proteins, were shown overexpressed in a subset of highly aggressive breast cancers, and were proposed as novel onco-therapeutic targets since the depletion of either one halted breast cancer growth without affecting the quasi-normal MCF10A cells [16]. However, we observed different effects of SNRPD1 and SNRPE on breast cancer cells that motivated us to investigate their differential roles and mechanisms associated with carcinogenesis.

The prognostic value of *SNRPD1* on breast cancer outcome was identified with statistical significance using multiple public data sets which, however, was not found for *SNRPE*. The rare allele of one quantitative trait loci (eQTL) of *SNRPD1*, rs6733100, was revealed to confer a protective effect on patient outcome using data retrieved from The Cancer Genome Atlas (TCGA). Though suppressed expression of either SNRPD1 or SNRPE led to reduced tumor cell proliferation, SNRPD1 promoted tumor cell migration and sensitized tumor cells to chemotherapy, SNRPE suppressed cancer stemness, and both proteins differ in their dynamic regulations on cell cycle progression. Our results suggest the prognostic value of SNRPD1 but not SNRPE on breast cancer survival, and warrant the use of SNPRD1 in the onco-therapeutic design given its unveiled roles in sensitizing TNBC cells to anthracycline-type of chemotherapies.

## Methods

### Computational analysis

The following datasets containing information on *SNRPD1* and *SNRPE* and being available at the time when this study was conducted were used in this study.

#### GSE24450 gene expression data set

The GSE24450 data set, retrieved from Gene Expression Omnibus (GEO) [17], is comprised of 183 primary breast tumor samples containing 39 breast cancer death or distant metastasis events (supplementary Table 1). The maximum follow-up time on disease specific death or distant metastasis was 60 months.

Gene expression profiling was conducted at SCIBLU Genomics Centre, Lund University, Sweden using Illumina HumanHT-12_V3 Expression Bead Chips that contain 24660 Entrez Gene entities following the manufacturer recommendations (http://www.illumina.com).

After quality control and quantile normalization of the microarray raw data, the gene expression matrix was obtained by averaging the probes mapped to the same Entrez Gene IDs. All analyses were processed using ‘BioConductor’ in R (https://www.r-project.org).

#### GSE1456 gene expression data set

The GSE1456 (GPL96) data set was retrieved from GEO, which is comprised of 159 samples including 40 breast cancer death or relapsed events [18] (supplementary Table 1). The maximum follow-up time on disease free survival was 102 months.

RNA was extracted following the RNeasy mini protocol (Qiagen, Hilden, Germany) [18]. All tumor specimens were assessed using the Affymetrix Human Genome U133A array at Bristol-Myers Squibb (Princeton, New Jersey) [18].

Data was normalized using the global mean method, natural-log-transformed and scaled by adjusting the average intensity of the signal to a target value of log 500 [18].

#### GSE4922 gene expression data set

The GSE4922 (GPL96) data set was retrieved from GEO, and comprised of 249 samples including 89 breast cancer specific death or relapsed events [19] (supplementary Table 1). The maximum follow-up time on disease free survival was 153 months and truncated at 10 years in the analysis.

RNA was extracted using the RNeasy mini protocol (Qiagen, Hilden, Germany). Tumor samples were profiled using the Affymetrix Human Genome U133A chips at the Genome Institute of Singapore [19].

Data was normalized using the global mean method, natural-log-transformed and scaled by adjusting the mean signal to a target value of log 500 [19].

#### TCGA gene expression data set

The primary solid breast tumor mRNA expression data of level 3 was retrieved from TCGA (http://cancergenome.nih.gov) on 21^st^ November 2015 (supplementary Table 1). The data included 514 samples, among which 512 had recorded information on overall survival including 53 death events. The mRNA data was lowess-normalized, and the ratio between the two channels was log2-transformed. The maximum follow-up time on overall survival was 226.5 months, which was truncated at 10 years in the analysis.

#### Kaplan Meier plotter data set

The prognostic values of the evaluated genes were confirmed using the online tool, Kaplan Meier Plotter [20], which included 4142 breast cancer samples. Gene expression data and clinical information were downloaded from GEO, EGA and TCGA, and integrated simultaneously. Only Affymetrix HG-U133A, HG-U133 Plus 2.0 and HG-U133A 2.0 were included.

#### TCGA genotype data set

The primary solid tumor genotyping data was retrieved from TCGA at http://tcga.cancer.gov/dataportal on 15^th^ January, 2015 (supplementary Table 1). The data set consisted of 906600 SNPs genotyped in 504 samples with an average age at diagnosis of 59.6 years. The follow up data for these cases included 53 overall death events. The TCGA genotype data on 906600 SNPs was produced using the Affymetrix Genome Wide Human SNP array 6.0. The genotypes and confidence scores for each sample and each SNP were generated from the raw data using the birdseed algorithm by fitting two-dimensional Gaussians to the SNP data and coding genotypes with confidence score > 0.1 as the missing data.

#### TCGA copy number variation data set

Breast cancer CNV data, which was comprised of putative copy-number calls determined using GISTIC 2.0 (-2, -1, 0, 1, 2 represent homozygous deletion, hemizygous deletion, neutral, gain and over-amplification, respectively), was downloaded from TCGA via cBio [21] on 15^th^ January, 2016 (supplementary Table 1). The data set includes 889 samples from the TCGA provisional study, among which 502 overlap with the genotype and gene expression data.

#### Mass spectrometry proteomic data set

Mass spectrometry data was retrieved from the supplementary file of Johansson H.J. et al. [22] that includes 45 samples and 9995 proteins. The sample cohort covers 5 subtypes, i.e., luminal A, luminal B, HER2 positive, triple negative/basal-like, and normal-like, each containing 9 samples. This proteomic data was produced using HiRIEF nanoLC-MS/MS.

#### Gene expression association analysis

Cox regression analysis was conducted on patient survival for disease specific death and distant metastasis or relapse at the mRNA level for *SNRPD1* and *SNRPE* using GSE24450, GSE1456 and GSE4922 data sets. The data was binarized by the median into high and low expression. Meta-analysis was conducted using the fixed-effects model for homogenous data (*SNRPD1*) and the random-effects model whenever heterogeneity exists (*SNRPE*) by the R package ‘meta’.

Kaplan Meier Plotter (which includes information from TCGA) was employed to check such associations using the log rank test, where the 5-year and 10-year relapse free survival were conducted with median being used as the sample splitting point. The prognostic values of *SNRPD1* expression on the clinical outcome among breast cancer patients with and without chemotherapy treatment were examined.

#### Histopathological association analysis

Histopathological association of *SNRPD1* gene expression was analysed using GSE24450 data. Samples were binarized into high and low expression by the median, and the associations with histopathological markers including ER, PR, TP53, HER2, tumor (T), node (N), Ki67 expression, grade, and different subtype classifications, were analysed separately by Chi-square test in R. The kruskal test was conducted to examine potential differences between the expression of genes in tumors categorized by different histopathological markers.

#### eQTL analysis

The primary solid tumor genotype and level 3 TCGA gene expression data were used for eQTL analysis. TCGA CNV data was retrieved from cBio cancer genomics portal (http://www.cbioportal.org/public-portal/) [21] and was used as the covariate. In total, 502 samples that have available information on genotyping, gene expression and CNV were used in the analysis. The gene expression and genotype data were fitted into a linear model in the eQTL analysis, with and without CNV being adjusted as the covariate. SNPs with *p* < 0.005 were selected for further analysis on its independent association with patient clinical outcome.

Tagging SNPs for these eQTL loci were retrieved using SNAP (Proxy Search) [23], where Caucasion samples (CEU) from 1000 Genomes Pilot 1 were used, the distance limit was set to 500 and ${\mathrm{r}}^{2}$ was set above 0.8. These SNPs together with their tagging SNPs were mapped to the TCGA genotype data for the survival analysis.

#### Genetic association analysis

Genetic association study with 10-year overall death as the endpoint was conducted on the eQTL SNPs using TCGA data. For each SNP, the data was fit into an additive, dominant and recessive model using a Cox regression model using the ‘R’ software. SNPs showing statistically significant associations with patient survival and having consistent clinical effects with the expression of corresponding genes were reported.

The gene encompassing or closest to the eQTL SNP (within 500 kb) was retrieved using GRAIL [24], where CEU samples (HapMap release 21) or Human Genome Assembly 17 were used with the ‘Functional Data source’ being set to PubMed April 2011.

#### Correlation analysis

Genes and proteins highly associated with *SNRPD1* or *SNRPE* regarding expression were analysed using the gene expression data retrieved from TCGA and the mass spectrometry data retrieved from [22]. Genes having the Pearson correlation scores above 0.5 or below -0.5 and *p*<0.001 were considered significantly correlated with *SNRPD1* or *SNRPE*. A gene or protein was considered significantly associated with *SNRPD1* or *SNRPE* if the absolute value of the correlation score was > 0.5 and the *p* $\le$ 0.001. A gene or protein was considered differentially correlated with *SNRPD1* or *SNRPE* if the absolute value of the difference between its correlation scores with *SNRPD1* or *SNRPE* was $\ge$ 0.3.

### Experimental studies

#### Cell culture

The luminal cell line MCF7 and the TNBC cell lines MDAMB231, HCC1937 were included in this study. All cells were purchased from ATCC, mycoplasma tested. MCF7 and MDAMB231 were cultivated using DMEM containing 10% fetal bovine serum (FBS) (Gibco), and HCC1937 was cultured using RPMI-1640 supplemented with 10% FBS.

Cells were stored in liquid nitrogen in frozen solution that contains 90% FBS and 10% DMSO. Immediately prior to transfection, cells were thawed and washed using the corresponding culture medium and the cell number was counted using a hemocytometer (Thermo).

#### Gene silencing

Two siRNAs (GenePharma) were designed for *SNRPD1* (s13229 and s13230) and *SNRPE* (s13237 and s13239)*,* respectively, and pooled together before usage. GenePharma Silencer Select Negative Control was used as the negative control. The siRNA products were ordered from Sigma-Aldrich (supplementary Table 2).

#### CRISPR sgRNA construction

Three enhancer sgRNAs (supplementary Table 3) were designed for SNRPD1 or SNRPE using http://crispr.mit.edu/, and synthesized from GENEWIZ. The sgRNAs were constructed into the sgRNA CRISPR lentivector (#CS30453, ABcam), separately, using BbsI (NEB). One dCas9 synergistic activation mediator lentivector (K015) was purchased from ABcam.

#### Quantitative real-time PCR

After siRNA transfection, cells were collected and total RNA was extracted using TRIzol reagent (TianGen) 2 days post-transfection. The cDNA was synthesized using PrimeScriptRT reverse transcriptase (Takara). The quantitative real-time PCR (q-PCR) system contains 5μL 2×SYBR Premix Ex Taq, 0.4μL, 10μM forward and reverse primers (supplementary Table 4), 0.2μL ROX Reference Dye, 2μL cDNA and 2μL ddH_2_O. The q-PCR experiments were conducted using ABI Stepone plus Real-Time PCR System (ABI). Detailed procedure for Q-PCR includes 5min initial denaturation at 95ºC, 5s denaturation at 95ºC for 45 cycles, 30s annealing at 57ºC, and 15s extension at 72ºC. The absorbance values were recorded at the extension stage, and the relative expression levels were computed using the 2^-△△Ct^ approach.

Student’s t-test was computed using R, where the *p* values were assessed as the two-tailed probability at 95% confidence from the standard Gaussian distribution.

#### Western blot

Cultured cells were washed twice using prechilled PBS and lysed in RIPA lysis buffer supplemented with protease inhibitors for 5min, centrifuged at 12,000 g for 10min followed by supernatant collection. The protein concentration was examined using the BCA Protein Assay Kit (Tiangen). Proteins (50μg) per lane were resolved by SDS-PAGE and transferred to PVDF membrane. After blocking with 5% non-fat dried milk powder in TBS plus Tween-20 buffer, the membrane was incubated using the appropriate primary antibodies at 4ºC overnight followed by secondary antibodies for 2h at the room temperature. Antibody binding was visualized by developing the blot using enhanced chemiluminescence reagent. The bands were visualized using OmegaLumG (UVP) followed by analysis using the Image J software. The primary and secondary antibodies used were listed in supplementary Table 5.

#### Doxorubicin resistance assay

Doxorubicin (#D1515, Sigma-Aldrich) at 8 concentrations (i.e., 0.83nM, 10nM, 25nM, 100nM, 250nM, 1000nM, 2500nM, 10000nM) were used in this study. ‘SiRNA+Doxorubicin’ and ‘negative control+Doxorubicin’ were designed for each gene in each 96-well plate, where ‘Doxorubicin’ has 8 concentrations and each sample has 6 replicates. Two siRNAs were designed for each gene and pooled together before usage.

Doxorubicin was added to cells 24h after siRNA transfection, and 10 μL/well CKK-8 was added to cells 96h after siRNA transfection. Luminescence was detected using EZ Read 800 microplate Reader after cells were incubated at 37ºC for 2h.

The dose curves in response to doxorubicin treatment were drawn for each cell line and half-maximum inhibitory concentration (IC50) values were obtained by fitting data to a 4-parameter log-logistic model (LL.4) using the ‘drc’ package in R. Statistical significance on IC50 shift was assessed by student’s t-test using R.

#### Proliferation assay

8000 cells/well were added to 100μL culture medium and seeded in a 96-well plate (Nalgene # 167008). Cells were incubated overnight until they achieved 30%-50% confluence prior to transfection. 50μL Optimem medium (Gibco) containing *SNRPD1*, *SNRPE* or control siRNAs was added to 750nL siRNA-mate (GenePharma) per well and mixed for 15-20min prior to transfection. The mixed content was transferred to 96-well plate, with the final siRNA concentration being 20nM. After transfection, cells were incubated at 37ºC in the presence of 5%CO_2_ (HERA Cell 150i, Thermo Scientific). For cell proliferation measurement, 10μL per well of CKK-8 (Dojindo) was added 48h post-transfection, and luminescence was detected using EZ Read 800 microplate Reader (Biochrom) after cell incubation at 37ºC for 2h.

Student’s t-test was conducted using R to assess the statistical significance, and the *p* values were computed as the two-tailed probability at 95% confidence from a standard normal distribution.

#### Wound healing assay

After siRNA (knock down) or sgRNA (enhance) transfection following the manufacture’s protocol, cells were incubated for 48h until the form of confluent monolayers. Medium was refreshed by serum-free medium before wounding. Wounds were made using a pipette tip, and photographs were taken immediately (time 0) and 12h, 24h and 36h after the wounds were made. Altered distance as measured between the two edges of wounded area were computed at each time point. Results were presented as the migration area.

The student’s t-test was used to assess the statistical significance in R, where the *p* values were computed as the two-tailed probability at 95% confidence from the standard Gaussian distribution.

#### Flow cytometry

Cell flow cytometry was performed 48h after siRNA transfection. Cells were collected using EDTA-free trypsin, washed twice using 0.5 ml PBS, suspended in 70% pre-cold ethanol, and stored in 4ºC overnight. Ethanol was removed and cells were re-suspended in PBS the next day. Cells were supplemented with 0.05 mg/ml Propidium Iodide (PI) and kept in darkness on ice for 30 min prior to cell cycle detection using BD Accuri™ C6 flow cytometer. The analysis was performed using flowJo v10 (Tree Star, USA).

In the ALDH analysis, cells were incubated with the antibodies in recommended concentrations at 37 °C for 0.5 h, followed by PBS washing for two times. Cells were sorted and analyzed by BD Accuri™ C6 flow cytometer. The ALDEFLUOR Kit (StemCell Technologies) was used to test ALDH activity following the manufacturer’s protocol. The diethylaminobenzaldehyde (DEAB), a specific ALDH inhibitor, was used as negative control. Flow cytometry data processing were performed using flowJo v10 (Tree Star, USA).

## Results

To investigate the differential roles of *SNRPD1* and *SNRPE* on breast cancer prognosis and therapeutics as well as the potential driving mechanisms, we conducted a series of *in silico* analyses and in vitro experiments that are summarized in supplementary Figure 1.

### SNRPD1 over-expression is prognostic of poor breast cancer survival

**Fig. 1:**
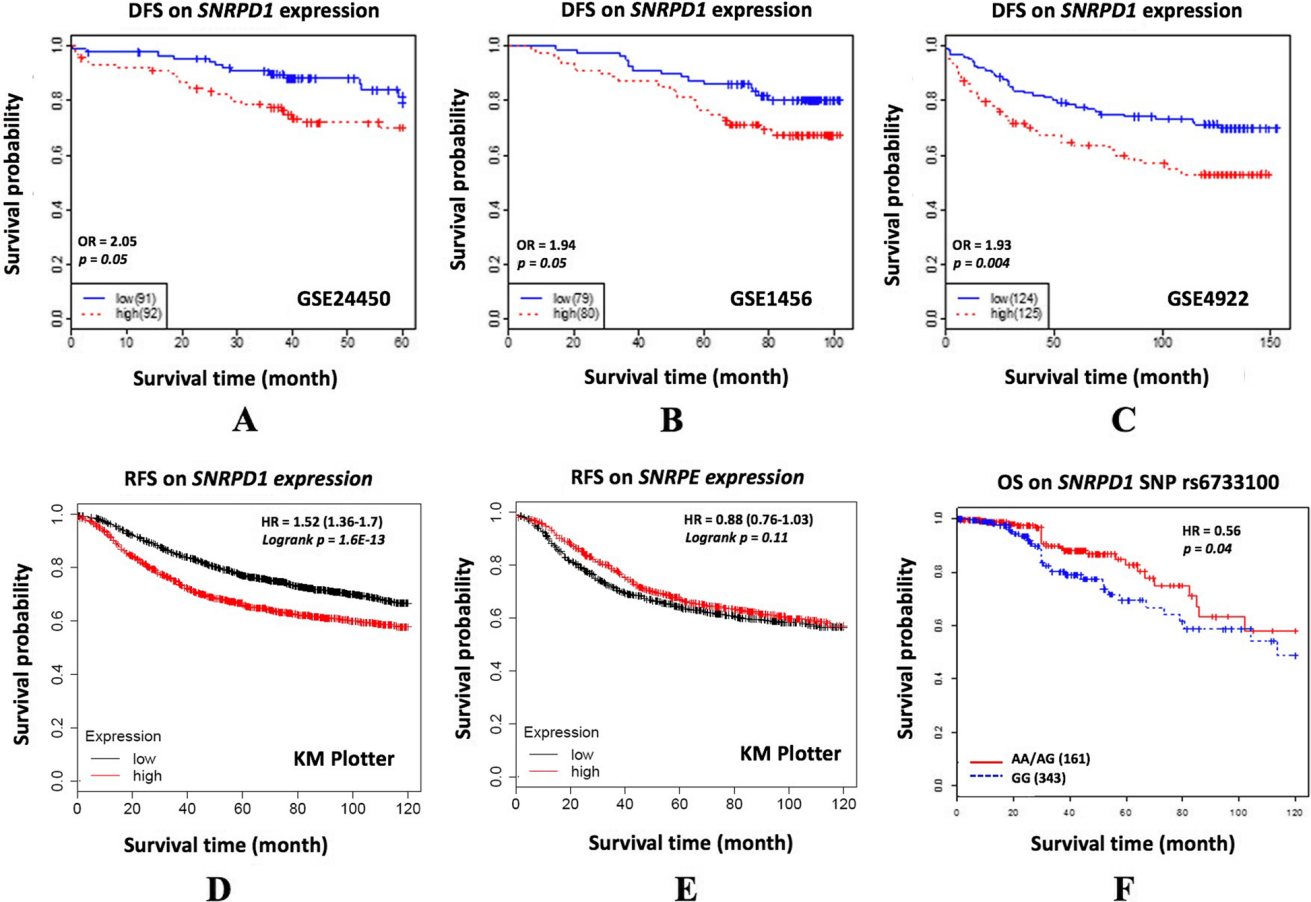
Kaplan-meier plots on the association between *SNRPD1* and patient survival. Survival analysis on (**A**) *SNRPD1* gene expression using GSE24450 data, (**B**) *SNRPD1* gene expression using GSE1456 data, (**C**) *SNRPD1* gene expression using GSE4922 data, (**D**) *SNRPD1* gene expression using Kaplan Meier Plotter (202690_s_at), (**E**) *SNRPE* gene expression using Kaplan Meier Plotter (231112_at), (**F**) *SNRPD1* eQTL SNP rs6733100 using TCGA data. DFS, RFS and OS each is short for disease free survival, relapse free survival, and overall survival, respectively

High *SNRPD1* gene expression was significantly associated with increased hazard on breast cancer specific and distant metastasis (GSE24450) or relapse free survival (GSE1456, GSE4922) in three independent patient series (*p*<0.0001, HR=1.89 from meta-analysis, Fig. 1). However, no significant association was found between *SNRPE* expression and patient survival (*p*>0.05, HR=0.93, Table 1).

**Table 1:** Summarized statistics of the association between *SNRPD1* and *SNRPE* gene expression and patient clinical outcome. The ‘p_cox’ and ‘HR’ are the log-likelihood *p* value and hazard ratio, respectively, from the Cox regression model. The number of patients in each data set is shown in ‘sample’ with the number of events given in the brackets. ‘Meta’ represents the meta-analysis

Data set	*SNRPD1*		*SNRPE*		SNRPESample
	**p_cox**	**HR**	**p_cox**	**HR**	
GSE24450	0.05	1.89	0.04	0.51	183 (39)
GSE1456	0.05	1.86	0.91	1.04	159 (40)
GSE4922	0.004	1.85	0.14	1.37	249 (89)
Meta	6.85E-05	1.89	0.82	0.93	

**Fig. 2:**
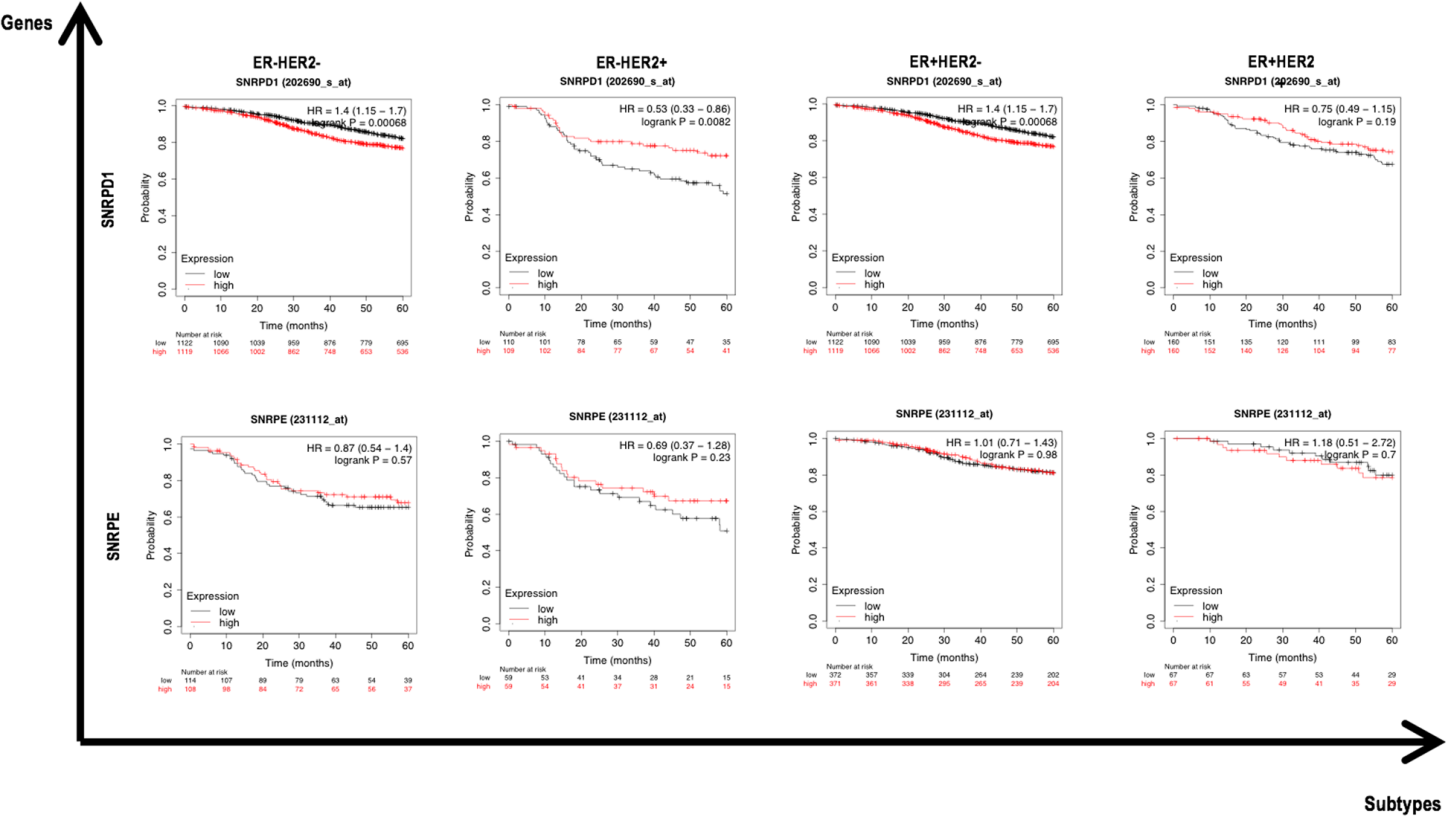
Relapse free survival of *SNRPD1 and SNRPE* gene expression among breast cancer patients as stratified by the status of ER and HER2. The plots were drawn using Kaplan-Meier Plotter, and 202690_s_at and 231112_at were used as the probe for *SNRPD1* and *SNRPE*, respectively

Analyses on 10-year breast cancer relapse free survival (RFS) using median for data binarization on 3951 breast tumors from Kaplan-Meier plotter (encompassing data from GEO, EGA and TCGA) [20] showed significant association between *SNRPD1* over-expression and increased hazard (probe 202690_s_at); whereas no consistent prognostic value was observed for *SNRPE* (probe 231112_at). Importantly, while *SNRPD1* over-expression is prognostic of poor RFS for ER-HER2- (corresponding to TNBC) and ER+HER2- (mostly referring to luminal A) tumors as well as suggests favorable RFS outcome for ER-HER2+ tumors (i.e., HER2+ breast cancers), high level of *SNRPE* conveys no prognostic value for none of these breast cancer subtypes (Fig. 2).

No significant association was found between *SNRPD1* expression and the histopathological parameters using GSE24450 data (supplementary Table 6).

### Expression quantitative trait loci of SNRPD1

Given the positive association between *SNRPD1* over-expression and poor breast cancer survival, we investigated the expression quantitative trait loci (eQTL) of *SNRPD1* using data retrieved from TCGA. 19638 SNPs (2.16% of the total SNPs tested) were found significantly associated with *SNRPD1* expression (*p* ≤ 0.005). These include 5394 and 5185 SNPs from the analysis without and with copy number variation (CNV) adjustment, respectively, and 9059 SNPs significant from both (supplementary Table 7).

The association of these eQTLs with patient clinical outcome was examined using TCGA data, with one SNP (rs6733100) found having an independent prognostic value on patient 10-year overall survival. This SNP was significantly associated with *SNRPD1* expression with (*p<*0.01) or without (*p<*0.01) CNV adjustment. The minor allele of rs6733100, A, with a frequency of 0.1753, showed significant protective effect on patient 10-year overall survival in the dominant model (Fig. 1F, *p* <0.05, HR = 0.56). It is a trans-eQTL (genomic location is 2:36293207) and about 62 kb upstream of the closest transcriptional start site of *CRIM1*.

### *SNRPD1 and SNRPE promote the proliferation of breast cancer *cells

**Fig. 3:**
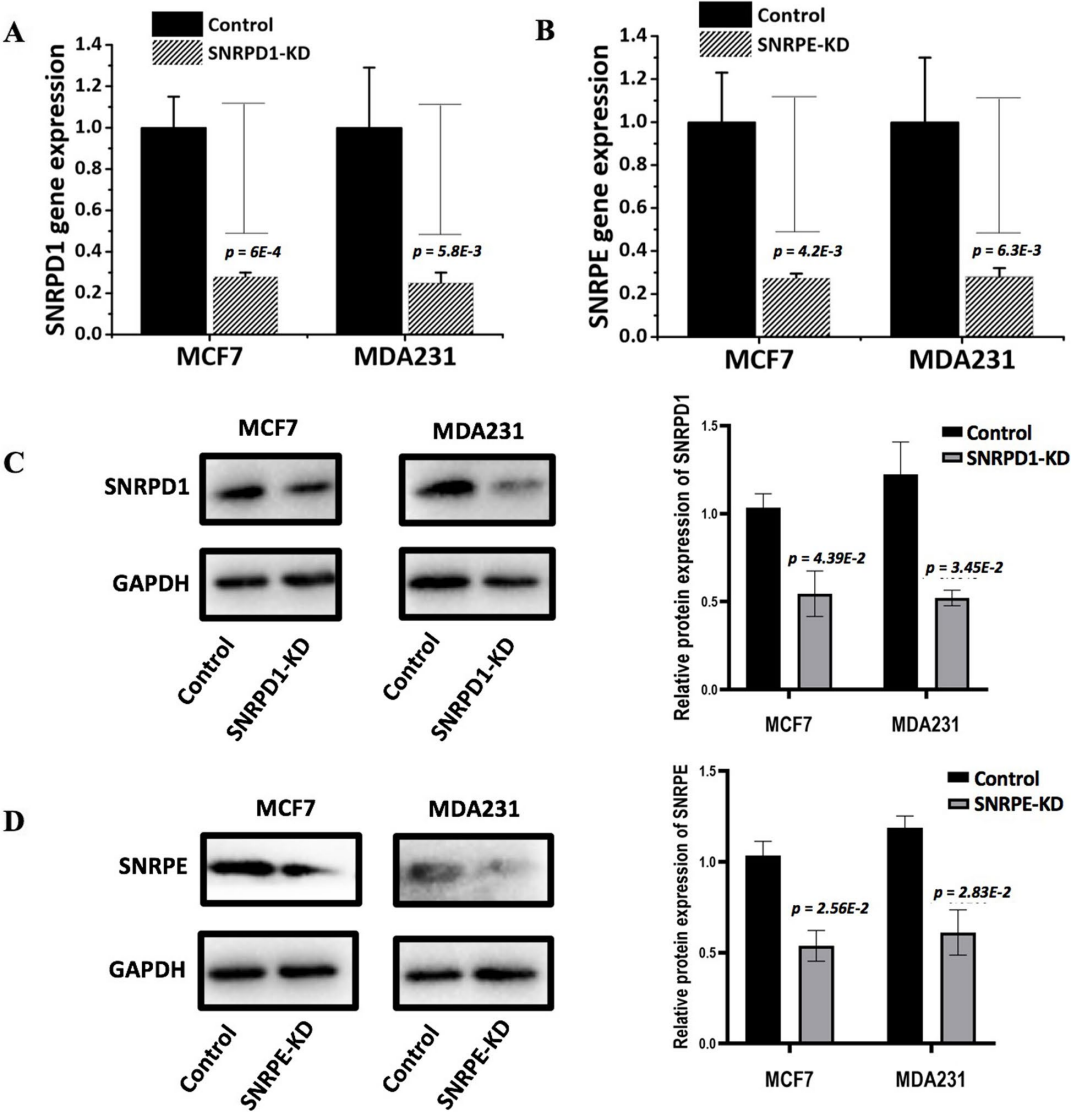
Boxplots showing the knockdown efficiencies of *SNRPD1* or *SNRPE* and their effects on cell viability in MCF7 and MDAMB231. The knockdown efficiencies of (**A**) *SNRPD1* and (**B**) *SNRPE*, as measured by qPCR, the knockdown efficiencies of (**C**) *SNRPD1* and* (****D****) SNRPE*, as measured by western blot*.* Student’s t-test *p* values were computed for MDAMB231 and MCF7 as compared with each control. The control of each cell line was used as the normalizing factor and shown as 1 in the plots. ‘-KD’ is short for ‘knockdown’, and ‘-C’ represents ‘control’

**Fig. 4:**
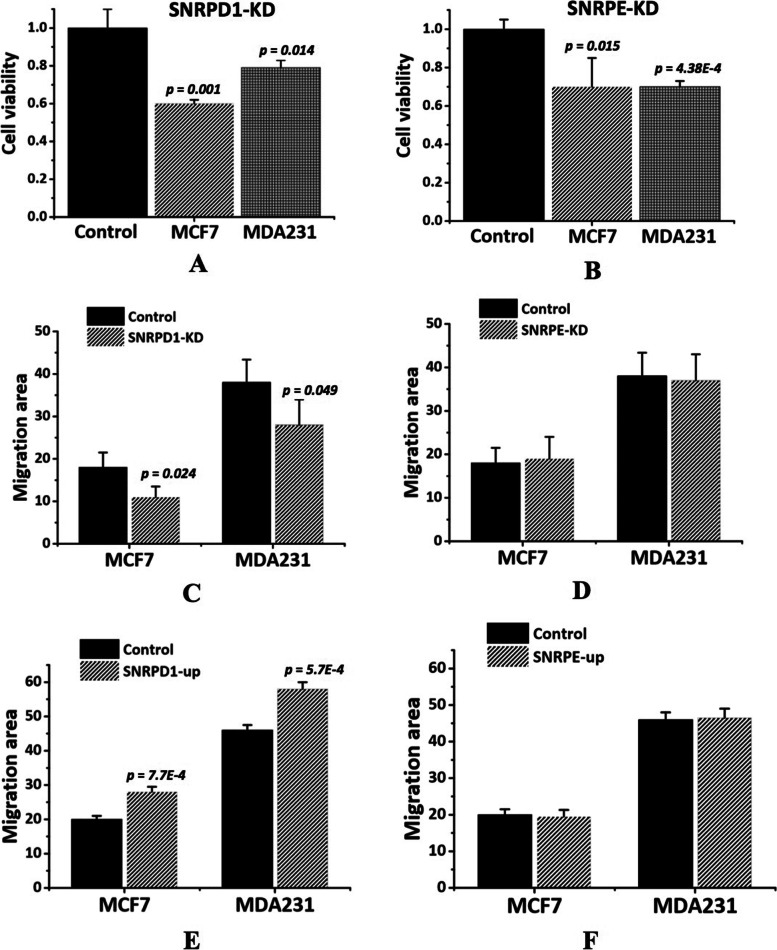
Effect on cell viability and migration by modulating *SNRPD1* or *SNRPE* in MCF7 and MDAMB231 cells. The effect on cell viability after knocking down (**A**) *SNRPD1* and (**B**) *SNRPE* at 48 hours after transfection. The effect on cell migration after knocking down (**C**) *SNRPD1*, (**D**) *SNRPE* for 12 hours (after wounding was made), and over-expressing (**E**) *SNRPD1*, (**F**) *SNRPE* for 12 hours (after wounding was made). Student’s t-test *p* values were computed for MDAMB231 and MCF7 as compared with each control. ‘-KD’ is short for ‘knockdown’, ‘-OE’ symbolizes ‘over-expression’, and ‘-C’ represents ‘control’. ‘GenePharma Silencer Select Negative Control’ was used as the negative control for gene silencing

Given the differential associations of *SNRPD1* and *SNRPE* on breast cancer survival, we explored their functionalities on cancer cell growth using in vitro assays. Both *SNRPD1* and *SNRPE* have been effectively knocked down as shown at the mRNA level (reduced to 1/4 of the control in both MDAMB231 and MCF7 cells for both genes, Fig 3A and B) and at the protein level (reduced to 68% in MDAMB231 and to 65% in MCF7 for *SNRPD1*; to 30% in MDAMB231 and to 47% in MCF7 for *SNRPE*, Fig. 3C and D). Significantly decreased cell viability was observed for tumor cells when either *SNRPD1* (reduced approximately 20% for MDAMB231 and 40% for MCF7) or *SNRPE* (reduced around 30% for MDAMB231and MCF7) was knocked down regardless of the tumor subtype (Fig. 4A and B).

### SNRPD1 promotes the migrative ability of breast cancer cells

We next examined the effects of *SNRPD1* and *SNRPE* on the migrative abilities of breast cancer cells. Significant recession or enhancement on cell migration was observed at 12 h (after scratches were made) in tumor cells with low or high *SNRPD1* expression (reduced 40% in MCF7 and almost 30% in MDAMB231 for siRNA-mediated *SNRPD1* silencing, enhanced 30% in MCF7 and 20% in MDAMB231 for sgRNA-mediated *SNRPD1* enhancing, Fig. 4C and E, supplementary Figure 2) but not in cells with altered *SNRPE* expression (Fig. 4D and F). *SNRPD1* and *SNRPE* were both effectively over-expressed in MDAMB231 and MCF7 cells as examined at both gene and protein expression levels. That is, *p*<0.0001 (MDAMB231) and *p*<0.005 (MCF7) for *SNRPD1*, and *p*<0.001 (MDAMB231) and *p*<0.0001 (MCF7) for *SNRPE* at the gene expression level (supplementary Figures 3A, 3B); and those at the protein expression level were increased to 3.5 folds (MDAMB231) and 2.2 folds (MCF7) for *SNRPD1*, and 3.8 folds (MDAMB231) and 2.9 folds (MCF7) for *SNRPE* (supplementary Figures 3C, 3D).

### SNRPD1 sensitizes breast cancer cells to doxorubicin

**Fig. 5:**
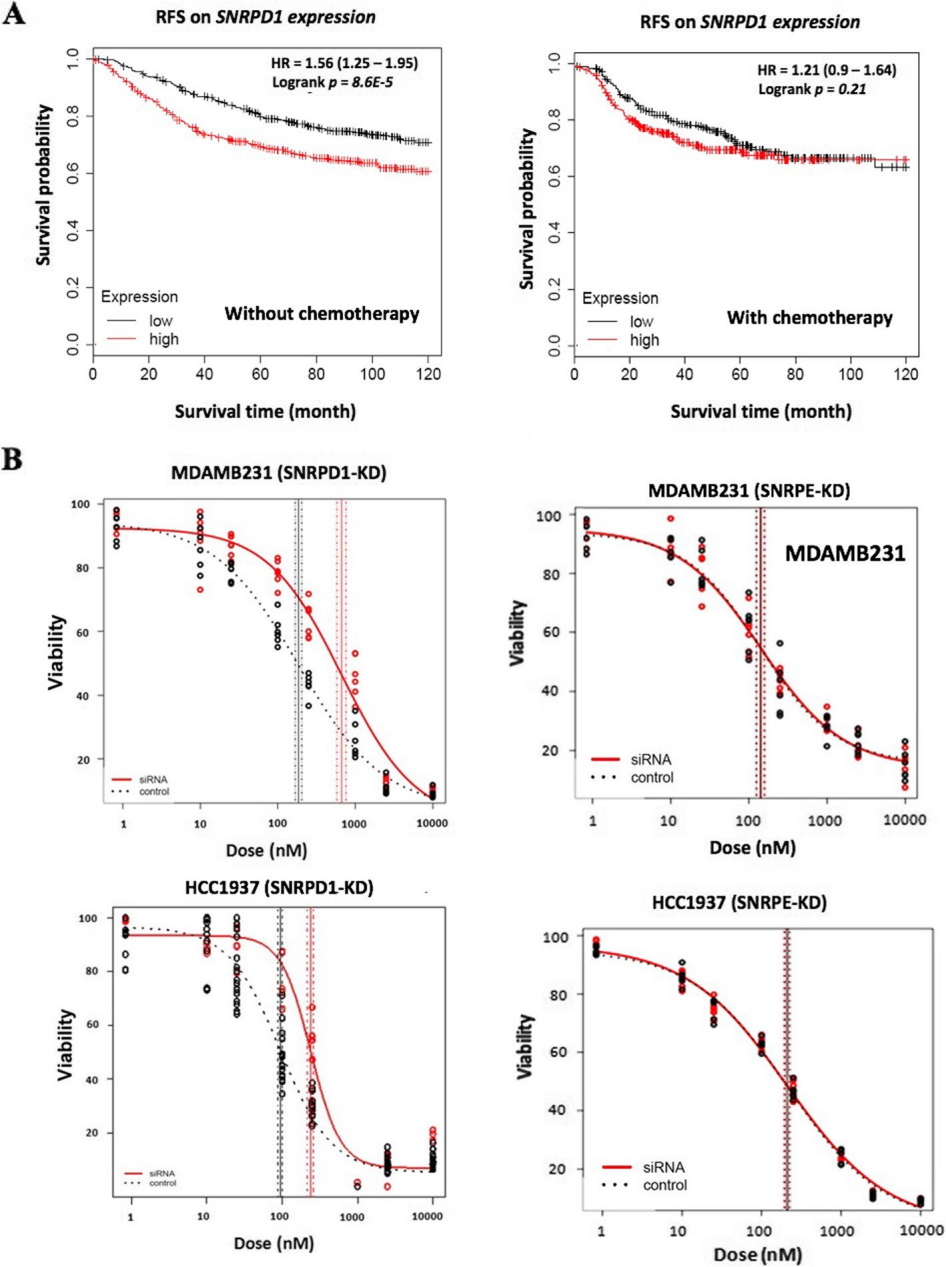
Response of *SNRPD1* and *SNRPE* to chemotherapy. Relapse free survival of *SNRPD1* gene expression among breast cancer patients (**A**) without and (**B**) with chemotherapy treatment from Kaplan Meier Plotter (202690_s_at). Dose-response curves of (**C**) *SNRPD1* and (**D**) *SNRPE* siRNA-transfected breast cancer cells in response to doxorubicin in MDAMB231 and HCC1937 cells. ‘GenePharma Silencer Select Negative Control’ was used as the negative control for gene silencing

Enlightened by the oncogenic roles unveiled for *SNRPD1* and *SNRPE*, we investigated their impacts on the efficacy of doxorubicin, an anthracycline type of chemotherapy canonically used for TNBC treatment. Patients with high *SNRPD1* expression have poorer RFS as compared with those harboring low *SNRPD1* expression (1010 patients, HR = 1.56, *p*<0.0001 for 10 years), and such a survival disadvantage vanished after receiving chemotherapies (602 patients, HR=1.21, *p*>0.05 for 10 years, Fig. 5A), suggestive of the role of *SNRPD1* in sensitizing breast cancer cells to chemotherapies.

We next examined the sensitivity of TNBCs in response to doxorubicin, a common form of adjuvant chemotherapy for breast cancer treatment especially among TNBCs, after silencing *SNRPD1* or *SNRPE* using MDAMB231 and HCC1937 as the cell models. The drug response curves were significantly right-ward shifted when *SNRPD1* was silenced (*p*<0.00001 in MDAMB231, *p*<E-9 in HCC1937) that was not observed when *SNRPE* was knocked down (Table 2, Fig. 5B), implicative of TNBC resistance developed in response to doxorubicin after silencing *SNRPD1*.

**Table 2:** IC50 of *SNRPD1* or *SNRPE* silenced cells in response to doxorubicin treatment in triple negative breast cancer cells. ‘Gene’ shows the gene targeted by siRNA. ‘IC50’ and ‘SE’ each lists the estimate and standard error of the IC50 values. The *p*-value shows the significance of the difference between siRNA transfected and control cells in each cell line

**Cell line**	**Gene**	**Type**	**IC50**	**SE**	**p**
MDAMB231	*SNRPD1*	siRNA	676.83	23.09	5.50E-6
	*SNRPD1*	control	177.82	21.98	
HCC1937	*SNRPD1*	siRNA	239.49	22.17	4.35E-10
	*SNRPD1*	control	93.81	7.29	
MDAMB231	*SNRPE*	siRNA	143.82	20.8	0.7861
	*SNRPE*	control	139.84	21.23	
HCC1937	*SNRPE*	siRNA	208.39	21.03	0.8199
	*SNRPE*	control	211.59	19.16	

### SNRPD1 and SNRPE differ in cell cycle genes they regulate

Provided with the different roles of SNRPD1 and SNRPE in cell migrative abilities and drug sensitivities that may drive their differential prognostic values on breast cancer survival, we examined their correlated genes to investigate differences on their molecular mechanisms.

**Fig. 6:**
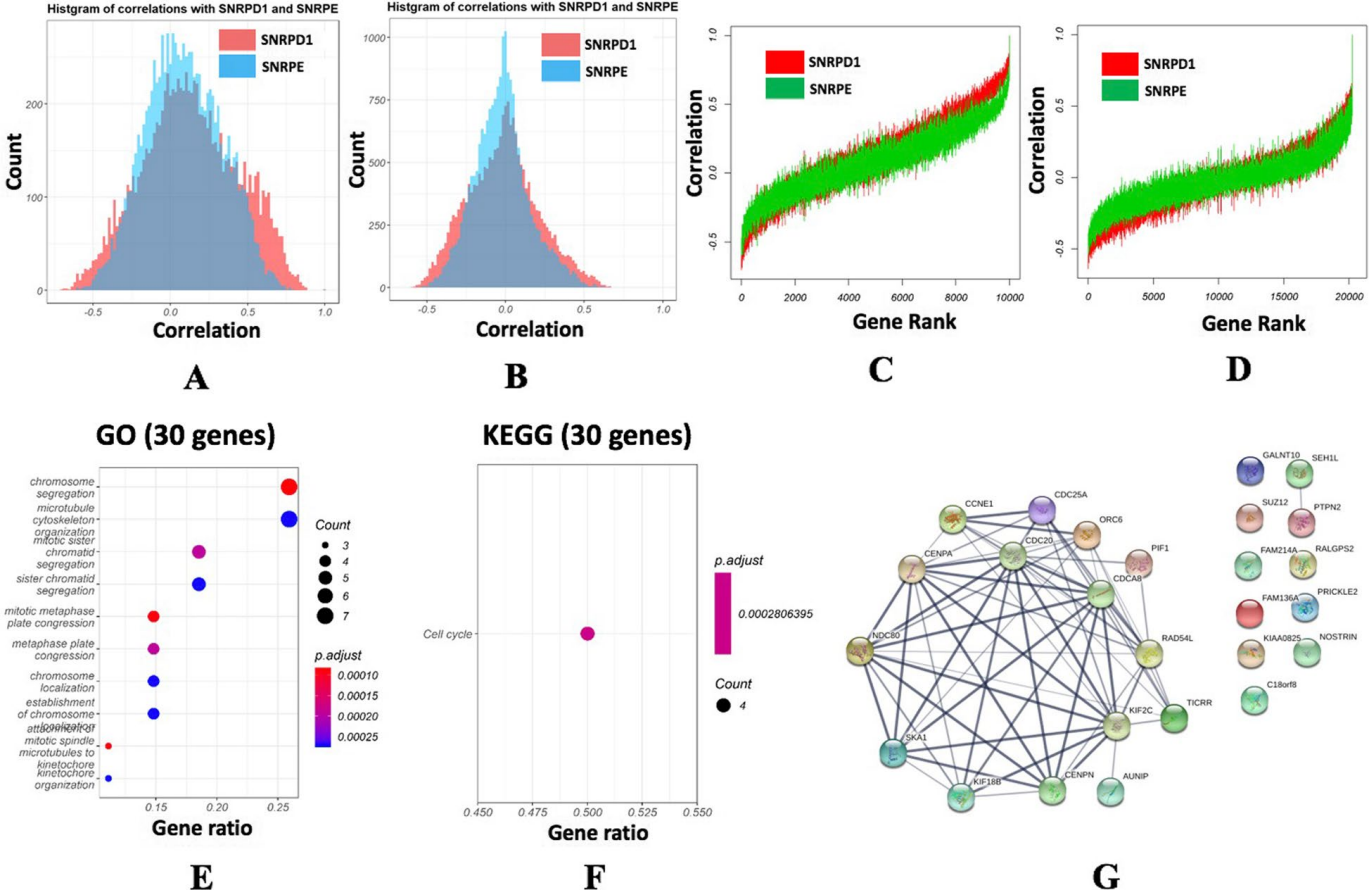
Exploration on genes having differential correlations with *SNPRD1* and *SNRPE*. **A** histograms of correlations of genes associated with *SNRPD1* and *SNRPE* using (**A**) TCGA gene expression data and (**B**) MS protein data. Sorted correlations of genes associated with *SNRPD1* and *SNRPE* using (**C**) TCGA gene expression data and (**D**) MS protein data. (**E**) Gene Ontology and (**F**) KEGG pathways enriched by the 30 differentially correlated genes/proteins with *SNRPD1* and *SNRPE*. **G** Protein-protein interaction network among the 30 differentially correlated genes/proteins with *SNRPD1* and *SNRPE* constructed using STRING

Genes and proteins highly associated with *SNRPD1* or *SNRPE* expression were analysed using TCGA gene expression data (supplementary Table 8) and mass spectrometry (MS) data retrieved from Johansson H.J. et al. [22] (supplementary Table 9). Correlations of these genes or proteins follow the Gaussian distribution, where the kurtosis and skewness for *SNRPD1* were 0.22 and 0.49, and for *SNRPE* were 0.4 and 0.36, respectively, using the TCGA gene expression data (Fig. 6A); and those for *SNRPD1* were 1.04 and 0.71, and for *SNRPE* were 1.16 and 0.93, respectively, using the MS protein data (Fig. 6B).

Genes highly correlated with *SNRPD1* had, in general, higher absolute correlation scores than those with *SNRPE* (Fig. 6C and D). There were 29 genes and 1 protein differentially associated with *SNRPD1* and *SNRPE* (supplementary Table 10), which were subjected to Gene Ontology (GO) and KEGG enrichment analyses. The results showed that these genes/proteins were significantly enriched in ‘chromatin segregation’, ‘microtubule cytoskeleton organization’ and ‘cell cycle’ (Fig. 6E and F ).

Among these 30 nodes, 63 pairs of protein-protein interactions (PPIs) were constructed using STRING (functional protein association networks) [25], 23 pairs out of which had the combined scores above 0.95 (Fig. 6G, supplementary Table 11). Among these 23 PPIs with high confidence, 10 pairs were experimentally validated (Table 3), with interactions between CENPN and CENPA being assigned with the highest experimental score (0.837), text mining score (0.851) and combined score (0.999). Interestingly, CENPN and CENPA were not highly correlated regarding their expression profiles (co-expression score being 0.68) and shared no homology, suggesting the involvement of additional players in their interactions. Thus, we selected for the follow-up q-PCR experimental validations.

**Table 3:** Protein-protein interactions among genes/proteins differentially associated with SNRPD1 and SNRPE. Predictions were conducted using STRING. Node pairs with ‘combined score’>0.95 were selected. The full results are available in supplementary Table 11

**Node1**	**Node2**	**Combined Score**	**Experimental Proof**	**Text Mining**	**Coexpression**	**Homology**
**CENPN**	**CENPA**	**0.999**	**0.837**	**0.851**	0.68	0
CDC20	NDC80	0.999	0	0.687	0.975	0
CDCA8	CDC20	0.999	0	0.599	0.988	0
CDC20	KIF2C	0.998	0.078	0.579	0.958	0
CDCA8	NDC80	0.998	0	0.567	0.96	0
SKA1	NDC80	0.997	0.354	0.701	0.876	0
KIF2C	NDC80	0.997	0.18	0.691	0.917	0
CDCA8	KIF2C	0.997	0.114	0.658	0.931	0
KIF18B	KIF2C	0.994	0.613	0.82	0.827	0.662
CENPA	NDC80	0.994	0.166	0.721	0.788	0
KIF2C	CENPA	0.993	0.08	0.67	0.818	0
CDCA8	CENPA	0.991	0	0.627	0.793	0
CENPN	NDC80	0.991	0	0.531	0.837	0
CDC25A	CCNE1	0.99	0.472	0.715	0.422	0
CDC20	CENPA	0.99	0.091	0.581	0.781	0
CENPN	CDC20	0.987	0	0.299	0.838	0
CENPN	CDCA8	0.986	0	0.3	0.822	0
CDC20	SKA1	0.985	0	0.293	0.811	0
CDCA8	SKA1	0.984	0	0.415	0.761	0
CENPN	KIF2C	0.983	0	0.41	0.748	0
KIF2C	SKA1	0.982	0	0.578	0.623	0
CENPA	SKA1	0.978	0	0.53	0.571	0
CENPN	SKA1	0.969	0	0.434	0.506	0

**Fig. 7:**
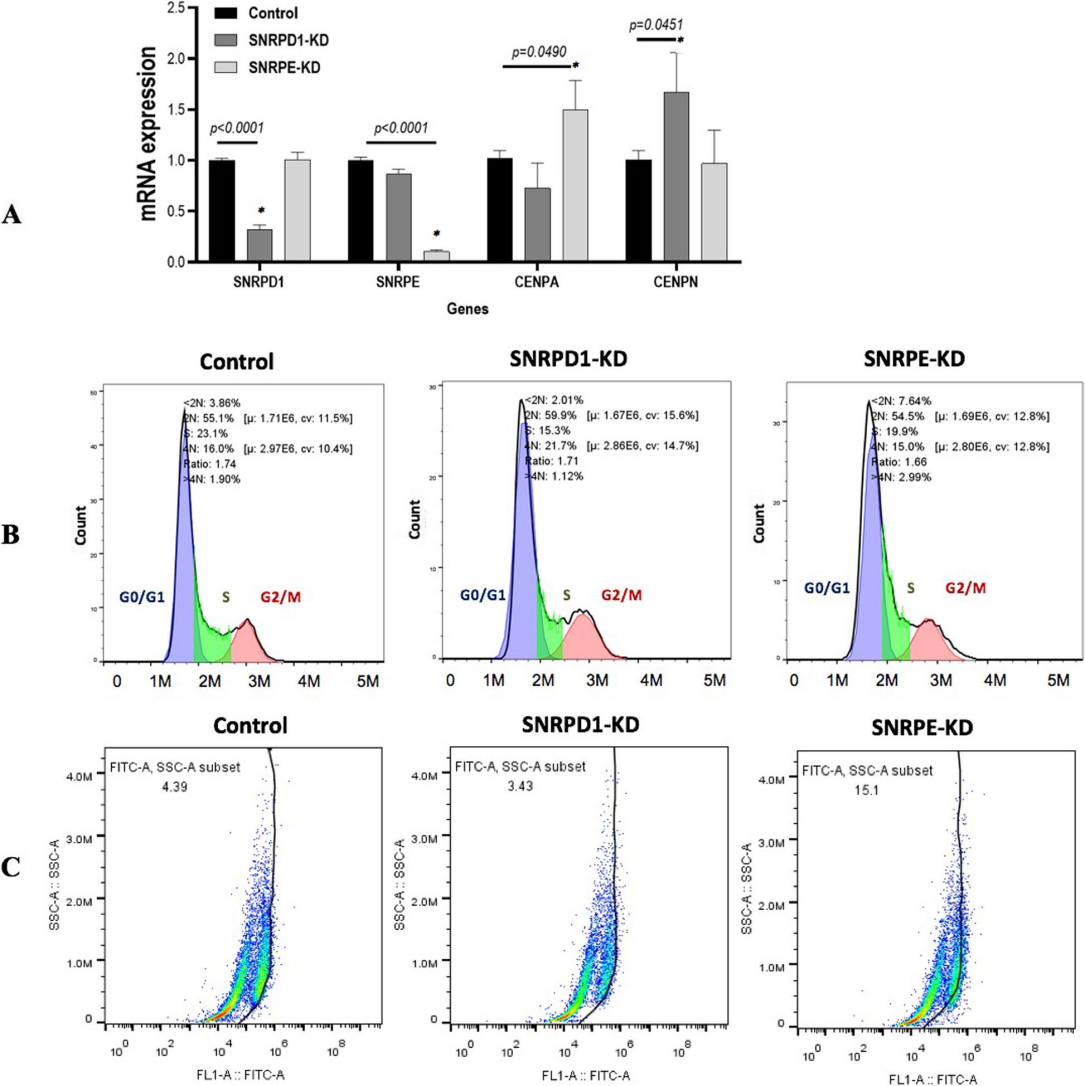
Experimental validation results. expression of (**A**) *CENPA* and (**B**) *CENPN*, (**C**) flow cytometry results showing (**C**) cell cycle alteration and (**D**) cancer stem cell percentage after knocking down *SNRPD1* or *SNRPE *in vitro in MDAMB231 cells. ‘GenePharma Silencer Select Negative Control’ was used as the negative control for gene silencing

We next explored whether *SNRPD1* or *SNRPE* affected *CENPN* or *CENPA*. The q-PCR results showed that *CENPA* and *CENPN* were differentially modulated on *SNRPD1* and *SNRPE* silencing (Fig. 7A and B). While knocking down *SNRPD1* significantly enhanced *CENPN* expression (*p*<0.05), silencing *SNRPE* increased *CENPA* expression with statistical significance (*p*<0.05). Silencing *SNRPD1* altered the cell cycle profile by reducing the S phase from 23.1% to 15.3% (Fig. 7B), and knocking down *SNRPE* considerably enhanced ALDH1 percentage from 4.39% to 15.1%, where MDAMB231 was used as the modelling cell line (Fig. 7C).

## Discussion

We differentiate SNRPD1 from SNRPE regarding its clinical implications and the potential underlying mechanism in this study, whereas both genes were proposed as therapeutic targets sharing similar carcinogenetic roles [16]. Specifically, *SNRPD1* over-expression is prognostic of poor breast cancer survival whereas *SNRPE* is not. Interestingly, high level of *SNRPD1* conveys poor prognostic value on ER-HER2- (mostly TNBC) and ER+HER2- (corresponding to luminal A) tumors, but implicates favorable clinical outcome on ER-HER2+ (HER2+ tumors) breast cancers. Though we could not exclude the possibility of having an inaccurate observation due to the small sample cohort (i.e., 219 ER-HER2+ cases in Fig. 2), the higher RFS, in general, for HER2+ patients (both ER-HER2+, ER+HER2+) together with the significantly poor RFS for HER2- patients harboring *SNRPD1* over-expression suggested the prominent role of HER2 in stratifying breast cancers towards the sensitivity of tumors to SNRPD1 status.

We, in addition, identified one trans-eQTL (rs6733100) of *SNRPD1* independently prognostic of breast cancer survival, with the rare allele conferring a protective effect. Given that genes can be regulated *in trans* by elements on other chromosomes [26], it is possible that somatic non-coding changes of distal regulatory elements can affect breast cancer progression by altering the levels and functions of *SNRPD1*. Thus, *CRIM1*, a gene located adjacent to the eQTL variant of *SNRPD1* and known with a regulatory role on epithelial-mesenchymal transition (EMT) and lung cancer metastasis [27], may be responsible for the observed role of *SNRPD1* on breast cancer cell migration.

Among the spliceosome-related proteins, silencing *SNRPD1* or *SNRPE* remarkably reduced the viabilities of breast, lung and melanoma cancer cells as a result of autophagy [16]. Consistent with these results, we did not observe any difference regarding their roles in cancer cell proliferation but identified a unique role of *SNRPD1* in promoting tumor cell migration whereas *SNRPE* could not. One might argue that the observed influence of *SNRPD1* on cell migration might be inflated by its role on cell proliferation. Yet, the data was collected at 12h that was within the time duration cells take to complete one life cycle (24h) and cells were cultured in serum-free medium to remove the influence of cell proliferation on migration. In addition, no significant correlation was found between *SNRPD1* expression and KI67 status (a proliferation marker) using GSE24450 (supplementary Table 6). These results collectively suggested that the prognostic value observed for *SNRPD1* was driven by its promotive roles on cancer cell migration instead of proliferation.

CENPA is a genomic marker for centromere activity that contributes to the assembly of active kinetochores, overexpression of which promotes genome instability [28]. Specific recognition of centromere-specific histone variant CENPA-containing chromatin by CENPN, a protein dynamically bound to kinetochores during S and G_2_ and absent from kinetochores during mitosis and G_1_ [29]_,_ is essential in the assembly of the kinetochore complex at centromeres prior to cell division [30]. The fact that silencing *SNRPD1* or *SNRPE* led to specific increase of *CENPN* and *CENPA*, respectively, implicated the driving functionality of *SNRPD1* on cell cycle that adversely afflicted cells’ genome stability. Flow cytometry assays further consolidated this hypothesis as the S phase of cells was substantially reduced on silencing *SNRPD1* (Fig. 7B). Interestingly, increased percentage of ALDH1, a cancer stem cell marker, was observed in cells deficient of *SNRPE* expression (Fig. 7C), suggestive of the suppressive role of *SNRPE* on cancer stemness that neutralizes its promotive role on cancer cell proliferation.

Doxorubicin resistance in response to *SNRPD1* silencing was observed in TNBC lines (Fig. 5). It is possible that the observed reduction on cells’ sensitivity to chemotherapies was inflated by cells’ slowed growth. However, as *SNRPE* that reduced cells’ viabilities in a comparable level to *SNRPD1* was used as the control gene in this study and silencing *SNRPE* did not cause curve shift, the observed altered dose response curve after silencing *SNRPD1* should reflect its true impact on cells’ drug response. Thus, *SNRPD1* over-expression may be a favourable sign on the treatment response of TNBC patients receiving doxorubicin-based chemotherapies. However, this does not imply that patients with high level of *SNRPD1* have, in general, better clinical outcome than those with low *SNRPD1* expression given the promotive role of *SNRPD1* on cancer cell proliferation and migration. As TNBCs are featured by fast growth and thus having been conventionally treated using chemotherapies including doxorubicin, the resistance gained for such tumors on silencing *SNRPD1* implicated a reduced chemo-sensitivity of tumors as a result of halted cell cycle progression. Previous reports have suggested therapies targeting spliceosome core machinery such as *SNRPD1* and *SNRPE*^16^. However, drugs targeting *SNRPD1* may not create desirable therapeutic outcome if coupled with anthracycline-like chemotherapies in cancer treatment.

This study is limited by the number of cell lines used for in vitro investigations and lack of *in vivo* validations. We used MDAMB231 and HCC1937 as the TNBC lines and MCF7 as the luminal breast cancer cells in investigating the effects of *SNRPD1* and *SNRPE* on cancer cell proliferation, apoptosis, migration and drug sensitivity; however, cells representative of other breast cancer subtypes such as HER2-positive tumors were not included. In addition, the key message that *SNRPD1* enhances TNBC sensitivity to doxorubicin should be validated *in vivo* by, e.g., treating *SNRPD1*-deficient TNBC-carrying mice with doxorubicin and comparing the growth of these tumors with those harboring the wildtype *SNRPD1*. However, these were not conducted due to the limited number of cell lines available and lack of the animal resource at the time of investigation that awaits to be performed towards generalized conclusions. Additionally, whether the sensitizing role identified for *SNRPD1* on TNBC cells in response to doxorubicin could be extended to other types of anthracycline-like chemotherapies needs to be investigated using other therapeutics.

## Conclusions

We showed, in this study, that *SNRPD1* over-expression was prognostic of poor breast cancer survival, promoted cancer cell proliferation and migration due to, possibly, reduced cell cycle S phase and enhanced genome stability. We also differentiated *SNRPD1* and *SNRPE* regarding their roles on breast cancer progression and onco-therapeutic implications. One eQTL of *SNRPD1*, rs6733100, was found independently prognostic of breast cancer patient survival. We warranted the combined use of drugs targeting *SNRPD1* and anthracycline type of chemotherapies in breast cancer management that may generate undesirable therapeutic outcome as targeting *SNRPD1* triggered the resistance of TNBC cells to doxorubicin. Our results differentiated *SNRPD1* from *SNRPE* at both prognostic and therapeutic levels and preliminarily explained the potential driving mechanisms, with the aim of advancing our therapeutic control of breast cancers via targeting these two genes.

## Supplementary Information

**Additional file 1: Supplementary Table 1.** Data sets description for computational prediction at the mRNA and genetic levels. In eQTL analysis, the number of samples overlapping among different types of data and used in the analysis is shown in 'Overlap'. In GEX survival (gene expression association analysis) and SNP survival (association analysis at the genetic level), the sample sizes are shown with the number of events listed in the brackets.

**Additional file 2: Supplementary Table 2.** Information on siRNAs purchased for knocking down *SNRPD1 *and *SNRPE *and main reagents used in the study.

**Additional file 3: Supplementary Table 3.** Information on the sgRNA primers used in the experiments.

**Additional file 4: Supplementary Table 4.** Information on the qPCR primers used in the experiments.

**Additional file 5: Supplementary Table 5. **Information on all antibodies used in the experiments.

**Additional file 6:** **Supplementary Table 6.** Histopathological association analysis on *SNRPD1* gene expression using GSE24450 data.

**Additional file 7: Supplementary Table 7.** The eQTL SNPs of SNRPD1 using TCGA data. 'P_TCGA' and 'P_TCGA_coCNV' each shows the p value obtained using TCGA data without and with copy number variation being adjusted.

**Additional file 8: Supplementary Table 8.** Genes highly correlated with *SNRPD1* or *SNRPE*.

**Additional file 9: Supplementary Table 9.** Genes differentially correlated with *SNRPD1* and *SNRPE*.

**Additional file 10:** **Supplementary Table 10.** Genes differentially correlated with SNRPD1 and SNRPE using both TCGA gene expression and MS protein expression data.

**Additional file 11:** **Supplementary Table 11.** Protein-protein interactions among genes differentially correlated with SNRPD1 and SNRPE. Protein-protein interactions were constructed using STRING.

**Additional file 12:** **Supplementary Figure 1.** Work flow and logic of this study. This study is comprised of investigations on the ‘phenomenon’ of SNRPD1 and SNRPE relevant to breast cancer prognosis and therapeutics, and ‘mechanism’ capable of explaining the observed phenomenon. In each set of investigations, both *in silico* dry lab analysis and in vitro wet lab experiments were conducted. ‘Green’ and ‘purple’ each represents dry lab analysis and wet lab assays conducted for SNRPD1 and SNRPE, respectively. ‘Black’ represents the analysis or assays. Statements in the brackets are conclusions drawn on SNRPD1 (‘green’) or SNRPE (‘purple’) from the corresponding series of analysis or experiments.

**Additional file 13:** **Supplementary Figure 2.** Cell migration after modulating *SNRPD1* at different time points in MDAMB231 and MCF7 cells. (A) Knocking down *SNRPD1*, (B) Over-expressing *SNRPD1*.

**Additional file 14:** **Supplementary Figure 3.** The efficiency of over-expressing *SNRPD1* or *SNRPE* in MDAMB231 and MCF7 cells. (A) Over-expressing *SNRPD1* or (B) *SNRPE* as tested at the mRNA level. (C) Over-expressing* SNRPD1* or (D) *SNRPE* as tested at the protein level.

## Data Availability

The datasets generated during and/or analysed during the current study are available from public repository, all detailed information is recorded in Material and methods.

## Abbreviations

TNBC: Triple negative breast cancer
PARP: Poly ADP-ribose polymerase
RNP: Ribonucleoprotein
snRNP: small nuclear RNP
eQTL: expression Quantitative Trait Loci
TCGA: The Cancer Genome Atlas
GEO: Gene Expression Omnibus
T: Tumor
N: Node
CEU: Caucasion samples
q-PCR: quantitative real-time PCR
PI: Propidium Iodide
DEAB: Diethylaminobenzaldehyde
RFS: Relapse free survival
MS: Mass spectrometry
GO: Gene Ontology
PPIs: Protein-protein interactions
