# Supplementary material for: SNRPD1 conveys prognostic value on breast cancer survival and is required for anthracycline sensitivity
Source: BMC Cancer. 2023 Apr 25;23:376. doi: 10.1186/s12885-023-10860-z (PMC10126993; doi:10.1186/s12885-023-10860-z)
Supplement: Supplementary file 1 — Additional file 1: Supplementary Table 1. Data sets description for computational prediction at the mRNA and genetic levels. In eQTL analysis, the number of samples overlapping among different types of data and used in the analysis is shown in 'Overlap'. In GEX survival (gene expression association analysis) and SNP survival (association analysis at the genetic level), the sample sizes are shown with the number of events listed in the brackets. [file 12885_2023_10860_MOESM1_ESM.docx]

**Supplementary Tables 1-5**

**Supplementary Table 1. Data sets description for computational prediction at the transcriptional and genetic levels. In eQTL analysis, the number of samples overlapping among different types of data and used in the analysis is shown in 'Overlap'.** In GEX survival (gene expression association analysis) and SNP survival (association analysis at the genetic level), the sample sizes are shown with the number of events listed in the brackets.

| **Analysis** | **GEX survival** | |  | **eQTL analysis** | |  | **SNP survival** |
| --- | --- | --- | --- | --- | --- | --- | --- |
| **Data source** | **GSE24450** | **GSE1456** | **GSE4922** | **TCGA** | **TCGA** | **TCGA** | **TCGA** |
| Data type | GEX | GEX | GEX | SNP | GEX | CNV | SNP |
| Number | 183 (39) | 159 (40) | 249 (89) | 504 | 514 | 889 | 504 (53) |
| Overlap |  |  |  | 502 | | |  |

**Supplementary Table 2. Information on siRNAs purchased for knocking down SNRPD1 and SNRPE and main reagents used in the study.**

| **Type** | **Product** | **Gene** | | **Catalog No.** | **Company** |
| --- | --- | --- | --- | --- | --- |
| siRNA | s13229 | SNRPD1 | | 93540 | GenePharma |
| siRNA | s13230 | SNRPD1 | | 94067 | GenePharma |
| siRNA | s13237 | SNRPE | | 94074 | GenePharma |
| siRNA | s13239 | SNRPE | | 94072 | GenePharma |
| Negative control | GenePharma Silencer Select Negative Control | |  | 93642 | GenePharma |
| Drug | Doxorubicin |  | | D1515 | Sigma-Aldrich |

**Supplementary Table 3. Information on the sgRNA primers used in the experiment.**

| **Gene** | **Forward primer** | **Reverse primer** |
| --- | --- | --- |
| SNRPD1-sgRNA-1 | CACCGAATGCGACAGTCCTATTAAT | AAACATTAATAGGACTGTCGCATTC |
| SNRPD1-sgRNA-2 | CACCGAGGCAACCTCCAGCCGCCAG | AAACCTGGCGGCTGGAGGTTGCCTC |
| SNRPD1-sgRNA-3 | CACCGTCGATACCCTCAGCCGTCGC | AAACGCGACGGCTGAGGGTATCGAC |
| SNRPE-sgRNA-1 | CACCGTGCAGCGAAGAAACGTGACT | AAACAGTCACGTTTCTTCGCTGCAC |
| SNRPE-sgRNA-2 | CACCGCAGTTTGGCCTCCCTCGGAG | AAACCTCCGAGGGAGGCCAAACTGC |
| SNRPE-sgRNA-3 | CACCGGTGGCGCTGTAGGACCGGC | AAACGCCGGTCCTACAGCGCCACC |

**Supplementary Table 4. Information on the qPCR primers used in the experiment.**

| **Gene** | **Forward primer** | **Reverse primer** | **Product length** | **Gene accession number** |
| --- | --- | --- | --- | --- |
| SNRPD1 | AGTCGGTCAGTGTTCGGTTG | TTCATGCTGACATCCACACCT | 168 | [NM_001291916.2](https://www.ncbi.nlm.nih.gov/entrez/viewer.fcgi?db=nucleotide&id=1676317790) |
| SNRPE | TAGATCGCGGATTCAGGTG | AGCATGATCCGACCCAGTT | 241 | [NM_001304464.2](https://www.ncbi.nlm.nih.gov/entrez/viewer.fcgi?db=nucleotide&id=1676319654) |
| CENPA | GCCTGGCAGCAGAAGCATT | AAAGTCCAGACAGCATCGCA | 255 | [NM_001042426.2](https://www.ncbi.nlm.nih.gov/entrez/viewer.fcgi?db=nucleotide&id=1890268226) |
| CENPN | TTCTAAGAAGAGCGGCGTGG | TCCCAAACTTTCTGGTGCTGA | 89 | [NM_001100624.3](https://www.ncbi.nlm.nih.gov/entrez/viewer.fcgi?db=nucleotide&id=1887789575) |
| GAPDH | GACAGTCAGCCGCATCTTCT | GCGCCCAATACGACCAAATC | 104 | [NM_001357943.2](https://www.ncbi.nlm.nih.gov/entrez/viewer.fcgi?db=nucleotide&id=1676440496) |

**Supplementary Table 5. Information of all antibodies used in the experiments.**

| **Antibody** | **Catolog number** | **Dilution ratio** |
| --- | --- | --- |
| SNRPD1 | sc-166650 (Santa Cruz) | 1:1000 |
| SNRPE | 20407-1-AP (Proteintech) | 1:1000 |
| GAPDH | AC001 (ABclonal) | 1:5000 |
| HRP-labeled goat anti-rabbit IgG(H+L) | A0208 (Beyotime) | 1:5000 |
