# Supplementary material for: SNRPD1 conveys prognostic value on breast cancer survival and is required for anthracycline sensitivity
Source: BMC Cancer. 2023 Apr 25;23:376. doi: 10.1186/s12885-023-10860-z (PMC10126993; doi:10.1186/s12885-023-10860-z)
Supplement: Supplementary file 2 — Additional file 2: Supplementary Table 2. Information on siRNAs purchased for knocking down SNRPD1 and SNRPE and main reagents used in the study. [file 12885_2023_10860_MOESM2_ESM.docx]

**Supplementary Table 2. Information on siRNAs purchased for knocking down SNRPD1 and SNRPE and main reagents used in the study.**

| **Type** | **Product** | **Gene** | | **Catalog No.** | **Company** |
| --- | --- | --- | --- | --- | --- |
| siRNA | s13229 | SNRPD1 | | 93540 | GenePharma |
| siRNA | s13230 | SNRPD1 | | 94067 | GenePharma |
| siRNA | s13237 | SNRPE | | 94074 | GenePharma |
| siRNA | s13239 | SNRPE | | 94072 | GenePharma |
| Negative control | GenePharma Silencer Select Negative Control | |  | 93642 | GenePharma |
| Drug | Doxorubicin |  | | D1515 | Sigma-Aldrich |
