# Supplementary material for: SNRPD1 conveys prognostic value on breast cancer survival and is required for anthracycline sensitivity
Source: BMC Cancer. 2023 Apr 25;23:376. doi: 10.1186/s12885-023-10860-z (PMC10126993; doi:10.1186/s12885-023-10860-z)
Supplement: Supplementary file 3 — Additional file 3: Supplementary Table 3. Information on the sgRNA primers used in the experiments. [file 12885_2023_10860_MOESM3_ESM.docx]

**Supplementary Table 3. Information on the sgRNA primers used in the experiment.**

| **Gene** | **Forward primer** | **Reverse primer** |
| --- | --- | --- |
| SNRPD1-sgRNA-1 | CACCGAATGCGACAGTCCTATTAAT | AAACATTAATAGGACTGTCGCATTC |
| SNRPD1-sgRNA-2 | CACCGAGGCAACCTCCAGCCGCCAG | AAACCTGGCGGCTGGAGGTTGCCTC |
| SNRPD1-sgRNA-3 | CACCGTCGATACCCTCAGCCGTCGC | AAACGCGACGGCTGAGGGTATCGAC |
| SNRPE-sgRNA-1 | CACCGTGCAGCGAAGAAACGTGACT | AAACAGTCACGTTTCTTCGCTGCAC |
| SNRPE-sgRNA-2 | CACCGCAGTTTGGCCTCCCTCGGAG | AAACCTCCGAGGGAGGCCAAACTGC |
| SNRPE-sgRNA-3 | CACCGGTGGCGCTGTAGGACCGGC | AAACGCCGGTCCTACAGCGCCACC |
