# Supplementary material for: SNRPD1 conveys prognostic value on breast cancer survival and is required for anthracycline sensitivity
Source: BMC Cancer. 2023 Apr 25;23:376. doi: 10.1186/s12885-023-10860-z (PMC10126993; doi:10.1186/s12885-023-10860-z)
Supplement: Supplementary file 4 — Additional file 4: Supplementary Table 4. Information on the qPCR primers used in the experiments. [file 12885_2023_10860_MOESM4_ESM.docx]

**Supplementary Table 4. Information on the qPCR primers used in the experiment.**

| **Gene** | **Forward primer** | **Reverse primer** | **Product length** | **Gene accession number** |
| --- | --- | --- | --- | --- |
| SNRPD1 | AGTCGGTCAGTGTTCGGTTG | TTCATGCTGACATCCACACCT | 168 | [NM_001291916.2](https://www.ncbi.nlm.nih.gov/entrez/viewer.fcgi?db=nucleotide&id=1676317790) |
| SNRPE | TAGATCGCGGATTCAGGTG | AGCATGATCCGACCCAGTT | 241 | [NM_001304464.2](https://www.ncbi.nlm.nih.gov/entrez/viewer.fcgi?db=nucleotide&id=1676319654) |
| CENPA | GCCTGGCAGCAGAAGCATT | AAAGTCCAGACAGCATCGCA | 255 | [NM_001042426.2](https://www.ncbi.nlm.nih.gov/entrez/viewer.fcgi?db=nucleotide&id=1890268226) |
| CENPN | TTCTAAGAAGAGCGGCGTGG | TCCCAAACTTTCTGGTGCTGA | 89 | [NM_001100624.3](https://www.ncbi.nlm.nih.gov/entrez/viewer.fcgi?db=nucleotide&id=1887789575) |
| GAPDH | GACAGTCAGCCGCATCTTCT | GCGCCCAATACGACCAAATC | 104 | [NM_001357943.2](https://www.ncbi.nlm.nih.gov/entrez/viewer.fcgi?db=nucleotide&id=1676440496) |
