# Supplementary material for: SNRPD1 conveys prognostic value on breast cancer survival and is required for anthracycline sensitivity
Source: BMC Cancer. 2023 Apr 25;23:376. doi: 10.1186/s12885-023-10860-z (PMC10126993; doi:10.1186/s12885-023-10860-z)
Supplement: Supplementary file 5 — Additional file 5: Supplementary Table 5. Information on all antibodies used in the experiments. [file 12885_2023_10860_MOESM5_ESM.docx]

**Supplementary Table 5. Information of all antibodies used in the experiments.**

| **Antibody** | **Catolog number** | **Dilution ratio** |
| --- | --- | --- |
| SNRPD1 | sc-166650 (Santa Cruz) | 1:1000 |
| SNRPE | 20407-1-AP (Proteintech) | 1:1000 |
| GAPDH | AC001 (ABclonal) | 1:5000 |
| HRP-labeled goat anti-rabbit IgG(H+L) | A0208 (Beyotime) | 1:5000 |
