# Supplementary material for: SNRPD1 conveys prognostic value on breast cancer survival and is required for anthracycline sensitivity
Source: BMC Cancer. 2023 Apr 25;23:376. doi: 10.1186/s12885-023-10860-z (PMC10126993; doi:10.1186/s12885-023-10860-z)
Supplement: Supplementary file 6 — Additional file 6: Supplementary Table 6. Histopathological association analysis on SNRPD1 gene expression using GSE24450 data. [file 12885_2023_10860_MOESM6_ESM.docx]

**Supplementary Table 6. Histopathological association analysis on *SNRPD1* gene expressionusing GSE24450 data.** p.kruskal = kruskal test of difference between categories, using SNRPD1expression as a continuous variable. p.chisq and p.emp have been calculated from median-split SNRPD1 data.

| **SNRPD1** | **all** | | **low** | | **high** | | **binary** | | **continuous** |
| --- | --- | --- | --- | --- | --- | --- | --- | --- | --- |
|  | **No.** | **(%)** | **No.** | **(%)** | **No.** | **(%)** | **p.chisq** | **p.emp** | **p.kruskal** |
| ER |  |  |  |  |  |  |  |  |  |
| **-** | 45 | 25.3 % | 24 | 27.0 % | 21 | 23.6 % | 0.73 | 0.73 | 0.87 |
| **+** | 133 | 74.7 % | 65 | 73.0 % | 68 | 76.4 % |  |  |  |
| **PR** |  |  |  |  |  |  |  |  |  |
| **-** | 67 | 37.6 % | 37 | 41.6 % | 30 | 33.7 % | 0.35 | 0.35 | 0.43 |
| **+** | 111 | 62.4 % | 52 | 58.4 % | 59 | 66.3 % |  |  |  |
| **TP53** |  |  |  |  |  |  |  |  |  |
| **-** | 113 | 66.9 % | 56 | 69.1 % | 57 | 64.8 % | 0.66 | 0.62 | 0.41 |
| **+** | 56 | 33.1 % | 25 | 30.9 % | 31 | 35.2 % |  |  |  |
| **HER2** |  |  |  |  |  |  |  |  |  |
| **-** | 96 | 82.8 % | 46 | 80.7 % | 50 | 84.7 % | 0.74 | 0.63 | 0.57 |
| **+** | 20 | 17.2 % | 11 | 19.3 % | 9 | 15.3 % |  |  |  |
| **T** |  |  |  |  |  |  |  |  |  |
| **1** | 78 | 43.8 % | 40 | 44.9 % | 38 | 42.7 % | 0.13 | 0.12 | 0.19 |
| **2** | 89 | 50.0 % | 47 | 52.8 % | 42 | 47.2 % |  |  |  |
| **3** | 7 | 3.9 % | 2 | 2.2 % | 5 | 5.6 % |  |  |  |
| **4** | 4 | 2.2 % | 0 | 0.0 % | 4 | 4.5 % |  |  |  |
| **N** |  |  |  |  |  |  |  |  |  |
| **-** | 82 | 46.1 % | 41 | 46.6 % | 41 | 45.6 % | 0.99 | 1.00 | 0.95 |
| **+** | 96 | 53.9 % | 47 | 53.4 % | 49 | 54.4 % |  |  |  |
| **KI67** |  |  |  |  |  |  |  |  |  |
| **1** | 13 | 7.4 % | 7 | 8.0 % | 6 | 6.8 % | 0.44 | 0.44 | 0.14 |
| **2** | 58 | 33.1 % | 33 | 37.9 % | 25 | 28.4 % |  |  |  |
| **3** | 51 | 29.1 % | 25 | 28.7 % | 26 | 29.5 % |  |  |  |
| **4** | 53 | 30.3 % | 22 | 25.3 % | 31 | 35.2 % |  |  |  |
| **Grade** |  |  |  |  |  |  |  |  |  |
| **1** | 25 |  | 15 | 17.4 % | 10 | 11.6 % | 0.36 | 0.37 | 0.20 |
| **2** | 77 |  | 40 | 46.5 % | 37 | 43.0 % |  |  |  |
| **3** | 70 |  | 31 | 36.0 % | 39 | 45.3 % |  |  |  |
| **Subtype** |  |  |  |  |  |  |  |  |  |
| **HER2+** | 8 | 6.9 % | 6 | 10.5 % | 2 | 3.4 % | 0.58 | 0.60 | 0.45 |
| **LumA** | 78 | 67.2 % | 36 | 63.2 % | 42 | 71.2 % |  |  |  |
| **LumB** | 12 | 10.3 % | 5 | 8.8 % | 7 | 11.9 % |  |  |  |
| **TNG0** | 4 | 3.4 % | 2 | 3.5 % | 2 | 3.4 % |  |  |  |
| **TNG1** | 10 | 8.6 % | 5 | 8.8 % | 5 | 8.5 % |  |  |  |
| **TNG2** | 4 | 3.4 % | 3 | 5.3 % | 1 | 1.7 % |  |  |  |
|  |  |  |  |  |  |  |  |  |  |
