# Supplementary material for: SNRPD1 conveys prognostic value on breast cancer survival and is required for anthracycline sensitivity
Source: BMC Cancer. 2023 Apr 25;23:376. doi: 10.1186/s12885-023-10860-z (PMC10126993; doi:10.1186/s12885-023-10860-z)
Supplement: Supplementary file 7 — Additional file 7: Supplementary Table 7. The eQTL SNPs of SNRPD1 using TCGA data. 'P_TCGA' and 'P_TCGA_coCNV' each shows the p value obtained using TCGA data without and with copy number variation being adjusted. [file 12885_2023_10860_MOESM7_ESM.docx]

| **SNP** | **Source** | **P_TCGA** | **P_TCGA_coCNV** |
| --- | --- | --- | --- |
| rs17667773 | TCGA&TCGA_coCNV | 4.61E-09 | 2.29E-08 |
| rs12557755 | TCGA&TCGA_coCNV | 2.50E-08 | 2.57E-08 |
| rs12688489 | TCGA&TCGA_coCNV | 4.19E-08 | 9.50E-06 |
| rs7879816 | TCGA&TCGA_coCNV | 3.98E-07 | 1.82E-03 |
| rs10847437 | TCGA&TCGA_coCNV | 4.32E-07 | 1.42E-03 |
| rs12479730 | TCGA&TCGA_coCNV | 5.15E-07 | 4.01E-03 |
| rs17735153 | TCGA&TCGA_coCNV | 5.43E-07 | 5.65E-07 |
| rs9962576 | TCGA&TCGA_coCNV | 6.02E-07 | 2.65E-06 |
| rs11059321 | TCGA&TCGA_coCNV | 6.04E-07 | 1.27E-03 |
| rs16989471 | TCGA&TCGA_coCNV | 6.07E-07 | 5.75E-06 |
| rs17667604 | TCGA&TCGA_coCNV | 6.34E-07 | 2.00E-06 |
| rs4687314 | TCGA&TCGA_coCNV | 7.49E-07 | 6.02E-05 |
| rs17127997 | TCGA&TCGA_coCNV | 8.88E-07 | 9.90E-06 |
| rs6776353 | TCGA&TCGA_coCNV | 1.02E-06 | 1.48E-04 |
| rs4317913 | TCGA&TCGA_coCNV | 1.13E-06 | 2.43E-04 |
| rs5982676 | TCGA&TCGA_coCNV | 1.26E-06 | 7.66E-08 |
| rs940902 | TCGA&TCGA_coCNV | 1.49E-06 | 4.46E-05 |
| rs2734224 | TCGA&TCGA_coCNV | 1.49E-06 | 1.59E-04 |
| rs9403784 | TCGA&TCGA_coCNV | 1.51E-06 | 1.16E-06 |
| rs17726596 | TCGA&TCGA_coCNV | 1.87E-06 | 2.99E-03 |
| rs17132406 | TCGA&TCGA_coCNV | 1.88E-06 | 3.16E-05 |
| rs4958116 | TCGA&TCGA_coCNV | 2.27E-06 | 3.52E-04 |
| rs4772659 | TCGA&TCGA_coCNV | 2.46E-06 | 9.35E-04 |
| rs2469760 | TCGA&TCGA_coCNV | 2.46E-06 | 1.21E-03 |
| rs2681360 | TCGA&TCGA_coCNV | 2.59E-06 | 6.73E-07 |
| rs10932625 | TCGA&TCGA_coCNV | 2.69E-06 | 2.16E-04 |
| rs9321072 | TCGA&TCGA_coCNV | 2.77E-06 | 7.84E-07 |
| rs17002059 | TCGA&TCGA_coCNV | 2.97E-06 | 1.34E-06 |
| rs10847435 | TCGA&TCGA_coCNV | 3.37E-06 | 6.56E-04 |
| rs4871998 | TCGA&TCGA_coCNV | 3.41E-06 | 2.32E-04 |
| rs7005502 | TCGA&TCGA_coCNV | 3.87E-06 | 1.82E-05 |
| rs1542485 | TCGA&TCGA_coCNV | 3.97E-06 | 4.79E-04 |
| rs983225 | TCGA&TCGA_coCNV | 3.97E-06 | 1.28E-05 |
| rs6740800 | TCGA&TCGA_coCNV | 4.16E-06 | 1.82E-06 |
| rs3763509 | TCGA&TCGA_coCNV | 4.68E-06 | 6.50E-05 |
| rs4141423 | TCGA&TCGA_coCNV | 4.92E-06 | 1.40E-05 |
| rs7878400 | TCGA&TCGA_coCNV | 5.02E-06 | 2.00E-04 |
| rs10120153 | TCGA&TCGA_coCNV | 5.28E-06 | 6.17E-04 |
| rs1464941 | TCGA&TCGA_coCNV | 5.42E-06 | 4.86E-04 |
| rs10120692 | TCGA&TCGA_coCNV | 5.52E-06 | 1.28E-05 |
| rs2367977 | TCGA&TCGA_coCNV | 5.94E-06 | 1.57E-04 |
| rs2130777 | TCGA&TCGA_coCNV | 5.94E-06 | 5.59E-04 |

**Supplementary Table 7. eQTL SNPs of SNRPD1 using TCGA data.** 'P_TCGA' and 'P_TCGA_coCNV' each shows the p value obtained using TCGA data without and with copy number variation being adjusted.

| rs12047817 | TCGA&TCGA_coCNV | 6.06E-06 | 3.26E-06 |
| --- | --- | --- | --- |
| rs9565647 | TCGA&TCGA_coCNV | 6.66E-06 | 3.62E-05 |
| rs16922439 | TCGA&TCGA_coCNV | 6.98E-06 | 1.59E-05 |
| rs10502197 | TCGA&TCGA_coCNV | 7.10E-06 | 4.73E-05 |
| rs915508 | TCGA&TCGA_coCNV | 8.46E-06 | 1.73E-03 |
| rs12403921 | TCGA&TCGA_coCNV | 8.95E-06 | 7.70E-04 |
| rs2469745 | TCGA&TCGA_coCNV | 8.97E-06 | 2.57E-03 |
| rs11001272 | TCGA&TCGA_coCNV | 9.02E-06 | 1.97E-03 |
| rs10516129 | TCGA&TCGA_coCNV | 9.05E-06 | 5.93E-04 |
| rs732954 | TCGA&TCGA_coCNV | 9.24E-06 | 4.90E-05 |
| rs9825263 | TCGA&TCGA_coCNV | 9.37E-06 | 2.25E-04 |
| rs11257625 | TCGA&TCGA_coCNV | 9.93E-06 | 9.97E-04 |
| rs1461237 | TCGA&TCGA_coCNV | 9.97E-06 | 1.44E-04 |
| rs2387716 | TCGA&TCGA_coCNV | 1.09E-05 | 7.32E-05 |
| rs7555650 | TCGA&TCGA_coCNV | 1.10E-05 | 1.89E-05 |
| rs12054909 | TCGA&TCGA_coCNV | 1.12E-05 | 2.70E-04 |
| rs226962 | TCGA&TCGA_coCNV | 1.12E-05 | 3.25E-04 |
| rs1522271 | TCGA&TCGA_coCNV | 1.24E-05 | 2.08E-04 |
| rs7827627 | TCGA&TCGA_coCNV | 1.27E-05 | 9.58E-06 |
| rs732401 | TCGA&TCGA_coCNV | 1.28E-05 | 1.02E-04 |
| rs3733101 | TCGA&TCGA_coCNV | 1.29E-05 | 9.49E-06 |
| rs7310068 | TCGA&TCGA_coCNV | 1.29E-05 | 1.15E-03 |
| rs11167641 | TCGA&TCGA_coCNV | 1.32E-05 | 3.23E-03 |
| rs10779492 | TCGA&TCGA_coCNV | 1.32E-05 | 1.79E-04 |
| rs7666210 | TCGA | 1.36E-05 | #N/A |
| rs12138644 | TCGA&TCGA_coCNV | 1.46E-05 | 4.23E-05 |
| rs203989 | TCGA&TCGA_coCNV | 1.53E-05 | 8.13E-05 |
| rs6578075 | TCGA&TCGA_coCNV | 5.70E-06 | 3.26E-04 |
| rs9535561 | TCGA&TCGA_coCNV | 1.56E-05 | 1.71E-03 |
| rs17012607 | TCGA&TCGA_coCNV | 1.58E-05 | 8.55E-05 |
| rs2126071 | TCGA&TCGA_coCNV | 1.61E-05 | 4.08E-04 |
| rs4424038 | TCGA&TCGA_coCNV | 1.68E-05 | 8.78E-05 |
| rs1698302 | TCGA&TCGA_coCNV | 1.71E-05 | 2.05E-03 |
| rs11193409 | TCGA&TCGA_coCNV | 1.71E-05 | 8.10E-04 |
| rs12480265 | TCGA&TCGA_coCNV | 1.74E-05 | 1.26E-04 |
| rs16870549 | TCGA&TCGA_coCNV | 1.75E-05 | 9.48E-06 |
| rs2268888 | TCGA&TCGA_coCNV | 1.78E-05 | 2.27E-06 |
| rs7137489 | TCGA&TCGA_coCNV | 1.82E-05 | 1.14E-03 |
| rs1382375 | TCGA&TCGA_coCNV | 1.89E-05 | 7.33E-04 |
| rs11113585 | TCGA&TCGA_coCNV | 1.90E-05 | 2.93E-06 |
| rs10060188 | TCGA | 1.92E-05 | #N/A |
| rs10959836 | TCGA&TCGA_coCNV | 2.00E-05 | 1.81E-03 |
| rs2383685 | TCGA&TCGA_coCNV | 2.00E-05 | 3.84E-03 |
| rs663906 | TCGA&TCGA_coCNV | 2.04E-05 | 1.07E-04 |
| rs6601006 | TCGA&TCGA_coCNV | 2.08E-05 | 3.12E-03 |
| rs16899048 | TCGA&TCGA_coCNV | 2.09E-05 | 1.87E-05 |

| rs7658172 | TCGA&TCGA_coCNV | 2.10E-05 | 9.73E-04 |
| --- | --- | --- | --- |
| rs10960002 | TCGA&TCGA_coCNV | 2.16E-05 | 2.25E-03 |
| rs4782548 | TCGA&TCGA_coCNV | 2.17E-05 | 2.17E-03 |
| rs1578477 | TCGA&TCGA_coCNV | 2.17E-05 | 4.00E-05 |
| rs11741924 | TCGA&TCGA_coCNV | 2.17E-05 | 1.32E-03 |
| rs10494487 | TCGA&TCGA_coCNV | 2.25E-05 | 1.59E-04 |
| rs7880554 | TCGA&TCGA_coCNV | 2.40E-05 | 1.18E-03 |
| rs5945467 | TCGA&TCGA_coCNV | 2.44E-05 | 1.09E-03 |
| rs950047 | TCGA | 2.45E-05 | #N/A |
| rs12339186 | TCGA&TCGA_coCNV | 2.54E-05 | 1.27E-04 |
| rs10959780 | TCGA&TCGA_coCNV | 2.55E-05 | 9.21E-03 |
| rs9838353 | TCGA&TCGA_coCNV | 2.58E-05 | 1.47E-03 |
| rs3924516 | TCGA&TCGA_coCNV | 2.58E-05 | 1.19E-04 |
| rs10493967 | TCGA&TCGA_coCNV | 2.59E-05 | 8.06E-06 |
| rs16885278 | TCGA&TCGA_coCNV | 2.59E-05 | 1.93E-06 |
| rs4318806 | TCGA&TCGA_coCNV | 2.63E-05 | 4.52E-04 |
| rs10814875 | TCGA&TCGA_coCNV | 2.66E-05 | 8.29E-06 |
| rs10919349 | TCGA&TCGA_coCNV | 2.76E-05 | 3.88E-04 |
| rs17679194 | TCGA&TCGA_coCNV | 2.81E-05 | 1.22E-03 |
| rs2484714 | TCGA&TCGA_coCNV | 2.87E-05 | 2.63E-04 |
| rs2836074 | TCGA&TCGA_coCNV | 2.91E-05 | 7.74E-05 |
| rs7329329 | TCGA&TCGA_coCNV | 2.92E-05 | 9.26E-05 |
| rs10224486 | TCGA&TCGA_coCNV | 2.93E-05 | 6.30E-05 |
| rs12004716 | TCGA&TCGA_coCNV | 2.96E-05 | 4.40E-06 |
| rs6793796 | TCGA&TCGA_coCNV | 3.08E-05 | 8.14E-04 |
| rs1039118 | TCGA&TCGA_coCNV | 3.08E-05 | 3.33E-04 |
| rs16925767 | TCGA&TCGA_coCNV | 3.09E-05 | 3.23E-04 |
| rs10477756 | TCGA&TCGA_coCNV | 3.11E-05 | 3.42E-03 |
| rs3134902 | TCGA&TCGA_coCNV | 3.13E-05 | 5.93E-04 |
| rs2292225 | TCGA&TCGA_coCNV | 3.21E-05 | 5.73E-05 |
| rs1356619 | TCGA&TCGA_coCNV | 3.22E-05 | 2.89E-05 |
| rs17094892 | TCGA&TCGA_coCNV | 3.24E-05 | 2.32E-03 |
| rs226946 | TCGA&TCGA_coCNV | 3.31E-05 | 3.15E-04 |
| rs6994029 | TCGA&TCGA_coCNV | 3.34E-05 | 1.76E-03 |
| rs418216 | TCGA&TCGA_coCNV | 3.34E-05 | 4.37E-04 |
| rs16926916 | TCGA&TCGA_coCNV | 3.39E-05 | 4.31E-06 |
| rs10847436 | TCGA | 3.42E-05 | #N/A |
| rs17161896 | TCGA | 3.42E-05 | #N/A |
| rs17350305 | TCGA&TCGA_coCNV | 3.47E-05 | 2.67E-04 |
| rs17460149 | TCGA&TCGA_coCNV | 3.48E-05 | 3.50E-04 |
| rs1514395 | TCGA&TCGA_coCNV | 3.59E-05 | 1.76E-04 |
| rs11794209 | TCGA&TCGA_coCNV | 3.61E-05 | 1.26E-04 |
| rs413912 | TCGA | 3.64E-05 | #N/A |
| rs4093501 | TCGA&TCGA_coCNV | 3.65E-05 | 7.37E-07 |
| rs2068824 | TCGA&TCGA_coCNV | 3.68E-05 | 1.17E-03 |
| rs17239028 | TCGA&TCGA_coCNV | 3.71E-05 | 5.59E-04 |

| rs181797 | TCGA&TCGA_coCNV | 3.73E-05 | 3.88E-03 |
| --- | --- | --- | --- |
| rs6787251 | TCGA&TCGA_coCNV | 3.82E-05 | 2.04E-04 |
| rs3794602 | TCGA&TCGA_coCNV | 3.82E-05 | 9.83E-03 |
| rs4680693 | TCGA&TCGA_coCNV | 3.83E-05 | 6.86E-05 |
| rs739143 | TCGA&TCGA_coCNV | 3.83E-05 | 3.67E-04 |
| rs2276347 | TCGA&TCGA_coCNV | 3.89E-05 | 5.54E-07 |
| rs1826834 | TCGA&TCGA_coCNV | 3.95E-05 | 8.33E-04 |
| rs2782444 | TCGA&TCGA_coCNV | 3.97E-05 | 2.40E-04 |
| rs17107347 | TCGA&TCGA_coCNV | 3.99E-05 | 1.42E-03 |
| rs9573115 | TCGA&TCGA_coCNV | 4.00E-05 | 1.15E-04 |
| rs9558750 | TCGA | 4.01E-05 | #N/A |
| rs16869562 | TCGA&TCGA_coCNV | 4.01E-05 | 3.43E-03 |
| rs11952867 | TCGA&TCGA_coCNV | 9.15E-06 | 1.11E-04 |
| rs1889318 | TCGA&TCGA_coCNV | 4.12E-05 | 5.90E-04 |
| rs12477370 | TCGA&TCGA_coCNV | 4.12E-05 | 2.78E-04 |
| rs17086776 | TCGA&TCGA_coCNV | 4.13E-05 | 2.28E-04 |
| rs3752134 | TCGA&TCGA_coCNV | 4.23E-05 | 5.51E-06 |
| rs4105175 | TCGA&TCGA_coCNV | 4.25E-05 | 5.03E-03 |
| rs12545909 | TCGA&TCGA_coCNV | 4.33E-05 | 3.19E-03 |
| rs9388573 | TCGA&TCGA_coCNV | 4.42E-05 | 4.76E-06 |
| rs4761003 | TCGA&TCGA_coCNV | 4.43E-05 | 6.70E-03 |
| rs9942422 | TCGA&TCGA_coCNV | 4.44E-05 | 5.79E-03 |
| rs615857 | TCGA&TCGA_coCNV | 4.45E-05 | 4.62E-04 |
| rs1257939 | TCGA&TCGA_coCNV | 4.51E-05 | 1.30E-05 |
| rs5746757 | TCGA&TCGA_coCNV | 4.62E-05 | 3.85E-03 |
| rs10169054 | TCGA&TCGA_coCNV | 4.63E-05 | 1.20E-04 |
| rs1828952 | TCGA&TCGA_coCNV | 4.63E-05 | 8.72E-05 |
| rs12118519 | TCGA&TCGA_coCNV | 4.70E-05 | 1.46E-04 |
| rs6128677 | TCGA&TCGA_coCNV | 4.84E-05 | 8.41E-04 |
| rs11167640 | TCGA&TCGA_coCNV | 4.86E-05 | 5.40E-05 |
| rs10510749 | TCGA&TCGA_coCNV | 4.89E-05 | 4.14E-04 |
| rs10488260 | TCGA&TCGA_coCNV | 4.90E-05 | 3.21E-04 |
| rs981545 | TCGA&TCGA_coCNV | 4.95E-05 | 6.27E-03 |
| rs1518863 | TCGA&TCGA_coCNV | 4.96E-05 | 1.25E-03 |
| rs6482819 | TCGA&TCGA_coCNV | 8.21E-06 | 3.65E-03 |
| rs6651478 | TCGA&TCGA_coCNV | 1.58E-05 | 2.48E-05 |
| rs16874658 | TCGA&TCGA_coCNV | 5.27E-05 | 1.45E-03 |
| rs2962617 | TCGA&TCGA_coCNV | 5.30E-05 | 2.56E-03 |
| rs10820589 | TCGA&TCGA_coCNV | 5.31E-05 | 2.04E-05 |
| rs7063594 | TCGA&TCGA_coCNV | 5.33E-05 | 2.20E-03 |
| rs16847635 | TCGA&TCGA_coCNV | 5.36E-05 | 1.78E-05 |
| rs10859374 | TCGA&TCGA_coCNV | 5.39E-05 | 1.70E-03 |
| rs8137767 | TCGA&TCGA_coCNV | 5.41E-05 | 5.95E-04 |
| rs11120300 | TCGA&TCGA_coCNV | 5.45E-05 | 3.18E-04 |
| rs5915408 | TCGA&TCGA_coCNV | 5.46E-05 | 6.39E-05 |
| rs17176454 | TCGA | 5.52E-05 | #N/A |

| rs6569485 | TCGA&TCGA_coCNV | 5.53E-05 | 1.75E-04 |
| --- | --- | --- | --- |
| rs214338 | TCGA&TCGA_coCNV | 5.58E-05 | 8.87E-04 |
| rs11229079 | TCGA&TCGA_coCNV | 5.71E-05 | 2.52E-03 |
| rs12007555 | TCGA&TCGA_coCNV | 5.73E-05 | 3.81E-04 |
| rs6627954 | TCGA&TCGA_coCNV | 5.77E-05 | 1.73E-04 |
| rs909657 | TCGA&TCGA_coCNV | 5.87E-05 | 3.62E-05 |
| rs9375505 | TCGA&TCGA_coCNV | 5.90E-05 | 2.79E-05 |
| rs227389 | TCGA&TCGA_coCNV | 5.96E-05 | 3.59E-03 |
| rs10912505 | TCGA&TCGA_coCNV | 6.06E-05 | 2.78E-03 |
| rs17330993 | TCGA&TCGA_coCNV | 6.11E-05 | 2.47E-04 |
| rs12596612 | TCGA&TCGA_coCNV | 6.13E-05 | 7.46E-04 |
| rs16995902 | TCGA&TCGA_coCNV | 6.22E-05 | 1.73E-03 |
| rs7901891 | TCGA&TCGA_coCNV | 6.23E-05 | 3.77E-04 |
| rs10163088 | TCGA&TCGA_coCNV | 6.27E-05 | 9.54E-05 |
| rs10919402 | TCGA&TCGA_coCNV | 6.37E-05 | 4.63E-05 |
| rs6527779 | TCGA&TCGA_coCNV | 6.40E-05 | 1.27E-03 |
| rs5935017 | TCGA | 6.41E-05 | #N/A |
| rs17223741 | TCGA&TCGA_coCNV | 6.47E-05 | 4.09E-05 |
| rs10959919 | TCGA&TCGA_coCNV | 6.56E-05 | 2.60E-03 |
| rs7711124 | TCGA&TCGA_coCNV | 6.62E-05 | 2.21E-03 |
| rs4867263 | TCGA&TCGA_coCNV | 6.62E-05 | 5.47E-04 |
| rs7712903 | TCGA | 6.64E-05 | #N/A |
| rs2062694 | TCGA&TCGA_coCNV | 6.72E-05 | 3.18E-03 |
| rs5928167 | TCGA&TCGA_coCNV | 6.75E-05 | 2.68E-04 |
| rs2287677 | TCGA&TCGA_coCNV | 5.25E-08 | 4.21E-03 |
| rs16835186 | TCGA&TCGA_coCNV | 6.82E-05 | 1.80E-04 |
| rs16935671 | TCGA&TCGA_coCNV | 6.82E-05 | 3.13E-04 |
| rs17046816 | TCGA&TCGA_coCNV | 6.86E-05 | 7.99E-04 |
| rs2468600 | TCGA&TCGA_coCNV | 6.88E-05 | 2.86E-03 |
| rs4256872 | TCGA&TCGA_coCNV | 6.91E-05 | 2.17E-04 |
| rs17048515 | TCGA&TCGA_coCNV | 6.93E-05 | 1.41E-04 |
| rs12420625 | TCGA&TCGA_coCNV | 6.95E-05 | 4.29E-05 |
| rs7332050 | TCGA | 7.00E-05 | #N/A |
| rs845558 | TCGA&TCGA_coCNV | 7.03E-05 | 6.68E-04 |
| rs4036623 | TCGA&TCGA_coCNV | 7.04E-05 | 9.58E-03 |
| rs12141935 | TCGA&TCGA_coCNV | 7.08E-05 | 3.13E-04 |
| rs7752612 | TCGA&TCGA_coCNV | 7.11E-05 | 8.48E-04 |
| rs896379 | TCGA&TCGA_coCNV | 7.12E-05 | 8.34E-03 |
| rs1536348 | TCGA | 7.24E-05 | #N/A |
| rs16835884 | TCGA&TCGA_coCNV | 7.28E-05 | 2.48E-04 |
| rs9370618 | TCGA&TCGA_coCNV | 7.34E-05 | 1.56E-03 |
| rs9610191 | TCGA&TCGA_coCNV | 7.34E-05 | 2.17E-03 |
| rs16900150 | TCGA&TCGA_coCNV | 7.35E-05 | 1.78E-04 |
| rs5975122 | TCGA&TCGA_coCNV | 7.39E-05 | 3.32E-04 |
| rs9569425 | TCGA&TCGA_coCNV | 7.50E-05 | 7.00E-05 |
| rs11576079 | TCGA&TCGA_coCNV | 7.52E-05 | 2.92E-03 |

| rs2461491 | TCGA&TCGA_coCNV | 7.53E-05 | 1.06E-03 |
| --- | --- | --- | --- |
| rs4569502 | TCGA&TCGA_coCNV | 7.55E-05 | 8.94E-04 |
| rs2809394 | TCGA&TCGA_coCNV | 7.62E-05 | 1.09E-03 |
| rs1017490 | TCGA&TCGA_coCNV | 7.63E-05 | 1.54E-04 |
| rs10172914 | TCGA&TCGA_coCNV | 7.65E-05 | 9.46E-04 |
| rs9401947 | TCGA&TCGA_coCNV | 7.74E-05 | 3.85E-05 |
| rs1522270 | TCGA&TCGA_coCNV | 7.76E-05 | 7.62E-04 |
| rs4694463 | TCGA&TCGA_coCNV | 7.82E-05 | 1.05E-04 |
| rs12848700 | TCGA&TCGA_coCNV | 7.93E-05 | 1.34E-04 |
| rs1883836 | TCGA&TCGA_coCNV | 7.95E-05 | 5.38E-05 |
| rs10814874 | TCGA&TCGA_coCNV | 7.96E-05 | 9.68E-05 |
| rs9372991 | TCGA&TCGA_coCNV | 7.99E-05 | 8.72E-05 |
| rs17010669 | TCGA | 8.00E-05 | #N/A |
| rs12239437 | TCGA&TCGA_coCNV | 8.07E-05 | 2.46E-05 |
| rs17270989 | TCGA&TCGA_coCNV | 8.08E-05 | 2.64E-03 |
| rs4566491 | TCGA&TCGA_coCNV | 8.10E-05 | 1.62E-04 |
| rs7832586 | TCGA&TCGA_coCNV | 1.81E-05 | 6.52E-03 |
| rs16936690 | TCGA&TCGA_coCNV | 8.25E-05 | 3.36E-05 |
| rs4578264 | TCGA&TCGA_coCNV | 8.35E-05 | 3.47E-04 |
| rs17066159 | TCGA&TCGA_coCNV | 8.35E-05 | 6.70E-03 |
| rs9809054 | TCGA&TCGA_coCNV | 8.36E-05 | 1.97E-04 |
| rs11768503 | TCGA&TCGA_coCNV | 8.40E-05 | 3.75E-03 |
| rs4846868 | TCGA&TCGA_coCNV | 8.48E-05 | 2.44E-04 |
| rs2067049 | TCGA&TCGA_coCNV | 8.49E-05 | 5.51E-04 |
| rs7298205 | TCGA&TCGA_coCNV | 8.50E-05 | 1.53E-03 |
| rs10753427 | TCGA&TCGA_coCNV | 8.51E-05 | 3.04E-04 |
| rs7307386 | TCGA&TCGA_coCNV | 8.52E-05 | 6.96E-04 |
| rs16962308 | TCGA&TCGA_coCNV | 2.31E-05 | 1.81E-05 |
| rs4687304 | TCGA&TCGA_coCNV | 8.56E-05 | 7.28E-04 |
| rs12202842 | TCGA | 8.56E-05 | #N/A |
| rs4872486 | TCGA | 8.59E-05 | #N/A |
| rs674313 | TCGA&TCGA_coCNV | 8.62E-05 | 3.39E-04 |
| rs10834893 | TCGA&TCGA_coCNV | 8.13E-05 | 5.17E-05 |
| rs10783884 | TCGA&TCGA_coCNV | 8.73E-05 | 4.71E-06 |
| rs4901995 | TCGA | 8.75E-05 | #N/A |
| rs2127065 | TCGA&TCGA_coCNV | 8.78E-05 | 8.63E-04 |
| rs12622476 | TCGA&TCGA_coCNV | 8.80E-05 | 3.04E-04 |
| rs4095657 | TCGA&TCGA_coCNV | 8.81E-05 | 2.60E-05 |
| rs11193402 | TCGA&TCGA_coCNV | 8.81E-05 | 2.78E-04 |
| rs1461062 | TCGA&TCGA_coCNV | 8.83E-05 | 3.31E-04 |
| rs41464149 | TCGA&TCGA_coCNV | 7.29E-06 | 1.27E-05 |
| rs1535836 | TCGA&TCGA_coCNV | 8.93E-05 | 1.26E-03 |
| rs12654214 | TCGA&TCGA_coCNV | 8.95E-05 | 8.06E-03 |
| rs2926862 | TCGA&TCGA_coCNV | 8.98E-05 | 5.38E-04 |
| rs17098289 | TCGA&TCGA_coCNV | 9.00E-05 | 1.92E-03 |
| rs1474581 | TCGA | 9.08E-05 | #N/A |

| rs17559587 | TCGA&TCGA_coCNV | 9.17E-05 | 3.27E-03 |
| --- | --- | --- | --- |
| rs10491950 | TCGA&TCGA_coCNV | 9.21E-05 | 5.08E-03 |
| rs7575757 | TCGA&TCGA_coCNV | 5.80E-05 | 1.11E-04 |
| rs17655028 | TCGA&TCGA_coCNV | 9.25E-05 | 1.40E-04 |
| rs6925601 | TCGA&TCGA_coCNV | 9.34E-05 | 8.65E-06 |
| rs867157 | TCGA&TCGA_coCNV | 9.36E-05 | 4.62E-03 |
| rs940901 | TCGA&TCGA_coCNV | 9.40E-05 | 4.58E-03 |
| rs9840881 | TCGA&TCGA_coCNV | 9.46E-05 | 6.27E-04 |
| rs12555006 | TCGA&TCGA_coCNV | 9.47E-05 | 4.20E-04 |
| rs10959855 | TCGA&TCGA_coCNV | 9.56E-05 | 1.39E-03 |
| rs7300729 | TCGA&TCGA_coCNV | 9.57E-05 | 1.49E-05 |
| rs7843993 | TCGA&TCGA_coCNV | 9.60E-05 | 5.27E-04 |
| rs10095762 | TCGA | 9.64E-05 | #N/A |
| rs7517588 | TCGA&TCGA_coCNV | 9.64E-05 | 1.27E-03 |
| rs17816294 | TCGA&TCGA_coCNV | 9.70E-05 | 1.84E-04 |
| rs5985683 | TCGA&TCGA_coCNV | 9.73E-05 | 8.16E-05 |
| rs1536897 | TCGA&TCGA_coCNV | 9.78E-05 | 6.62E-04 |
| rs1455321 | TCGA | 9.81E-05 | #N/A |
| rs3862499 | TCGA&TCGA_coCNV | 9.87E-05 | 1.35E-03 |
| rs11715989 | TCGA&TCGA_coCNV | 9.91E-05 | 1.15E-04 |
| rs12555370 | TCGA&TCGA_coCNV | 1.00E-04 | 4.65E-04 |
| rs17569335 | TCGA&TCGA_coCNV | 1.00E-04 | 4.08E-04 |
| rs781356 | TCGA&TCGA_coCNV | 1.00E-04 | 4.30E-05 |
| rs12601854 | TCGA&TCGA_coCNV | 1.00E-04 | 1.45E-04 |
| rs2210142 | TCGA&TCGA_coCNV | 1.01E-04 | 1.70E-04 |
| rs10018064 | TCGA&TCGA_coCNV | 1.01E-04 | 1.56E-04 |
| rs287030 | TCGA&TCGA_coCNV | 1.02E-04 | 8.28E-04 |
| rs6417786 | TCGA&TCGA_coCNV | 1.04E-04 | 1.88E-03 |
| rs2239305 | TCGA&TCGA_coCNV | 1.04E-04 | 7.92E-05 |
| rs1427203 | TCGA | 1.05E-04 | #N/A |
| rs193563 | TCGA&TCGA_coCNV | 1.05E-04 | 9.32E-05 |
| rs11095825 | TCGA&TCGA_coCNV | 1.05E-04 | 2.19E-03 |
| rs883428 | TCGA&TCGA_coCNV | 1.06E-04 | 6.57E-06 |
| rs17655151 | TCGA&TCGA_coCNV | 1.06E-04 | 7.50E-05 |
| rs7537355 | TCGA&TCGA_coCNV | 1.06E-04 | 9.02E-04 |
| rs12953436 | TCGA&TCGA_coCNV | 1.07E-04 | 2.11E-04 |
| rs4744451 | TCGA | 1.07E-04 | #N/A |
| rs1522264 | TCGA&TCGA_coCNV | 1.08E-04 | 6.49E-04 |
| rs4672500 | TCGA&TCGA_coCNV | 1.08E-04 | 7.37E-04 |
| rs6478342 | TCGA&TCGA_coCNV | 1.08E-04 | 3.35E-03 |
| rs16918020 | TCGA&TCGA_coCNV | 1.09E-04 | 8.79E-05 |
| rs2283656 | TCGA&TCGA_coCNV | 1.09E-04 | 7.55E-03 |
| rs3747302 | TCGA&TCGA_coCNV | 1.09E-04 | 1.84E-04 |
| rs10773423 | TCGA&TCGA_coCNV | 1.09E-04 | 2.61E-03 |
| rs3131260 | TCGA&TCGA_coCNV | 1.09E-04 | 8.14E-05 |
| rs11252334 | TCGA&TCGA_coCNV | 1.09E-04 | 3.08E-04 |

| rs16900159 | TCGA&TCGA_coCNV | 1.10E-04 | 3.79E-04 |
| --- | --- | --- | --- |
| rs2274588 | TCGA&TCGA_coCNV&SCANdb | 1.10E-04 | 4.15E-04 |
| rs4489621 | TCGA&TCGA_coCNV | 1.10E-04 | 9.03E-04 |
| rs4904889 | TCGA&TCGA_coCNV | 1.11E-04 | 7.33E-04 |
| rs3784598 | TCGA&TCGA_coCNV | 1.12E-04 | 7.54E-04 |
| rs10791774 | TCGA&TCGA_coCNV | 1.12E-04 | 8.06E-04 |
| rs7201755 | TCGA&TCGA_coCNV | 1.12E-04 | 3.13E-04 |
| rs11741039 | TCGA&TCGA_coCNV | 1.12E-04 | 1.24E-03 |
| rs10960023 | TCGA&TCGA_coCNV | 1.12E-04 | 1.08E-03 |
| rs12034091 | TCGA&TCGA_coCNV | 1.12E-04 | 2.38E-05 |
| rs2863380 | TCGA&TCGA_coCNV | 1.13E-04 | 7.55E-05 |
| rs16856373 | TCGA&TCGA_coCNV | 1.14E-04 | 7.57E-03 |
| rs1347990 | TCGA&TCGA_coCNV | 1.14E-04 | 1.99E-04 |
| rs9388559 | TCGA&TCGA_coCNV | 1.14E-04 | 1.13E-04 |
| rs11193388 | TCGA&TCGA_coCNV | 1.15E-04 | 2.15E-03 |
| rs17010618 | TCGA&TCGA_coCNV | 1.15E-04 | 1.82E-04 |
| rs10821978 | TCGA&TCGA_coCNV | 1.15E-04 | 1.78E-05 |
| rs4954000 | TCGA&TCGA_coCNV | 1.16E-04 | 8.71E-04 |
| rs2326841 | TCGA&TCGA_coCNV | 1.16E-04 | 3.42E-05 |
| rs2811593 | TCGA | 1.17E-04 | #N/A |
| rs9536340 | TCGA | 1.18E-04 | #N/A |
| rs12548309 | TCGA&TCGA_coCNV | 1.18E-04 | 1.77E-04 |
| rs11193416 | TCGA&TCGA_coCNV | 1.19E-04 | 9.24E-04 |
| rs17500814 | TCGA&TCGA_coCNV | 1.19E-04 | 5.73E-04 |
| rs6749308 | TCGA&TCGA_coCNV | 1.19E-04 | 2.64E-04 |
| rs11528238 | TCGA&TCGA_coCNV | 1.19E-04 | 4.40E-04 |
| rs1570427 | TCGA&TCGA_coCNV | 1.19E-04 | 7.58E-03 |
| rs7112278 | TCGA&TCGA_coCNV | 1.19E-04 | 3.84E-04 |
| rs4521229 | TCGA&TCGA_coCNV | 1.22E-04 | 1.62E-03 |
| rs2925534 | TCGA&TCGA_coCNV | 1.22E-04 | 8.86E-04 |
| rs2842229 | TCGA&TCGA_coCNV | 1.22E-04 | 1.75E-04 |
| rs16986518 | TCGA&TCGA_coCNV | 1.23E-04 | 3.13E-03 |
| rs4365459 | TCGA&TCGA_coCNV | 1.23E-04 | 5.98E-04 |
| rs17786841 | TCGA&TCGA_coCNV | 1.23E-04 | 1.98E-04 |
| rs9955940 | TCGA&TCGA_coCNV | 1.24E-04 | 7.97E-04 |
| rs10799635 | TCGA&TCGA_coCNV | 1.24E-04 | 9.60E-06 |
| rs376451 | TCGA&TCGA_coCNV | 1.24E-04 | 2.33E-04 |
| rs13177415 | TCGA&TCGA_coCNV | 1.24E-04 | 1.27E-03 |
| rs4829476 | TCGA | 1.25E-04 | #N/A |
| rs7949651 | TCGA&TCGA_coCNV | 1.25E-04 | 1.23E-04 |
| rs17244990 | TCGA&TCGA_coCNV | 1.25E-04 | 2.18E-04 |
| rs13327039 | TCGA&TCGA_coCNV | 1.26E-04 | 6.28E-04 |
| rs7098140 | TCGA&TCGA_coCNV | 1.28E-04 | 1.19E-03 |
| rs2005080 | TCGA&TCGA_coCNV | 1.29E-04 | 9.08E-04 |
| rs10483438 | TCGA&TCGA_coCNV | 1.30E-04 | 6.95E-05 |
| rs1572748 | TCGA&TCGA_coCNV | 1.32E-04 | 1.10E-03 |

| rs353211 | TCGA&TCGA_coCNV | 1.32E-04 | 4.23E-04 |
| --- | --- | --- | --- |
| rs2073458 | TCGA&TCGA_coCNV | 1.33E-04 | 6.25E-04 |
| rs4560872 | TCGA | 1.34E-04 | #N/A |
| rs1421029 | TCGA&TCGA_coCNV | 1.35E-04 | 1.05E-03 |
| rs884255 | TCGA&TCGA_coCNV | 1.37E-04 | 2.05E-04 |
| rs1482337 | TCGA | 1.38E-04 | #N/A |
| rs7089721 | TCGA&TCGA_coCNV | 1.40E-04 | 4.04E-04 |
| rs7080019 | TCGA&TCGA_coCNV | 1.41E-04 | 6.91E-04 |
| rs7908075 | TCGA&TCGA_coCNV | 1.42E-04 | 3.43E-03 |
| rs17165662 | TCGA&TCGA_coCNV | 1.45E-04 | 1.77E-03 |
| rs11149597 | TCGA&TCGA_coCNV | 1.45E-04 | 7.19E-03 |
| rs9430071 | TCGA&TCGA_coCNV | 1.45E-04 | 1.93E-05 |
| rs1330905 | TCGA&TCGA_coCNV | 1.45E-04 | 7.87E-03 |
| rs10784961 | TCGA&TCGA_coCNV | 1.45E-04 | 6.69E-04 |
| rs1012043 | TCGA | 1.46E-04 | #N/A |
| rs1360367 | TCGA&TCGA_coCNV | 1.46E-04 | 2.03E-03 |
| rs1464081 | TCGA&TCGA_coCNV | 1.47E-04 | 4.79E-04 |
| rs3115829 | TCGA&TCGA_coCNV | 1.48E-04 | 6.67E-04 |
| rs6599397 | TCGA&TCGA_coCNV | 1.48E-04 | 6.12E-03 |
| rs7202456 | TCGA&TCGA_coCNV | 1.48E-04 | 9.26E-06 |
| rs3756417 | TCGA&TCGA_coCNV | 1.48E-04 | 3.58E-03 |
| rs959057 | TCGA | 1.48E-04 | #N/A |
| rs11774982 | TCGA | 1.48E-04 | #N/A |
| rs10501600 | TCGA&TCGA_coCNV | 1.48E-04 | 5.67E-05 |
| rs11740914 | TCGA&TCGA_coCNV | 1.49E-04 | 2.35E-04 |
| rs10047875 | TCGA&TCGA_coCNV | 1.49E-04 | 1.50E-03 |
| rs17025935 | TCGA&TCGA_coCNV | 1.49E-04 | 3.07E-03 |
| rs158869 | TCGA&TCGA_coCNV | 1.50E-04 | 3.32E-03 |
| rs7731439 | TCGA&TCGA_coCNV | 1.50E-04 | 5.16E-03 |
| rs17719247 | TCGA&TCGA_coCNV | 1.51E-04 | 3.55E-04 |
| rs12196524 | TCGA | 1.51E-04 | #N/A |
| rs13100243 | TCGA&TCGA_coCNV | 1.52E-04 | 9.04E-04 |
| rs628117 | TCGA&TCGA_coCNV | 1.52E-04 | 2.07E-03 |
| rs1409477 | TCGA&TCGA_coCNV | 1.52E-04 | 3.19E-03 |
| rs17022421 | TCGA&TCGA_coCNV | 1.53E-04 | 7.23E-04 |
| rs17692727 | TCGA&TCGA_coCNV | 1.53E-04 | 5.79E-04 |
| rs7704961 | TCGA&TCGA_coCNV | 1.53E-04 | 4.18E-05 |
| rs7711859 | TCGA&TCGA_coCNV | 1.53E-04 | 5.07E-03 |
| rs3829488 | TCGA | 1.53E-04 | #N/A |
| rs11252333 | TCGA&TCGA_coCNV | 1.54E-04 | 1.53E-04 |
| rs16923166 | TCGA&TCGA_coCNV | 1.54E-04 | 1.78E-04 |
| rs4687306 | TCGA&TCGA_coCNV | 1.54E-04 | 2.24E-03 |
| rs11984280 | TCGA&TCGA_coCNV | 1.55E-04 | 8.10E-04 |
| rs4753937 | TCGA&TCGA_coCNV | 1.55E-04 | 6.79E-03 |
| rs12821878 | TCGA&TCGA_coCNV | 1.55E-04 | 1.07E-04 |
| rs16851177 | TCGA&TCGA_coCNV | 1.57E-04 | 7.04E-04 |

| rs1737352 | TCGA&TCGA_coCNV | 1.58E-04 | 3.73E-04 |
| --- | --- | --- | --- |
| rs12022869 | TCGA&TCGA_coCNV | 1.59E-04 | 1.09E-05 |
| rs6814371 | TCGA&TCGA_coCNV | 1.59E-04 | 5.76E-04 |
| rs1505863 | TCGA&TCGA_coCNV | 1.59E-04 | 2.34E-03 |
| rs1489651 | TCGA&TCGA_coCNV | 1.60E-04 | 5.52E-05 |
| rs403473 | TCGA | 1.60E-04 | #N/A |
| rs2796466 | TCGA&TCGA_coCNV | 1.62E-04 | 5.19E-03 |
| rs717026 | TCGA&TCGA_coCNV | 1.62E-04 | 2.95E-04 |
| rs11132847 | TCGA&TCGA_coCNV | 1.63E-04 | 5.84E-05 |
| rs226956 | TCGA&TCGA_coCNV | 1.64E-04 | 3.56E-03 |
| rs2469757 | TCGA&TCGA_coCNV | 1.64E-04 | 7.63E-03 |
| rs2885819 | TCGA&TCGA_coCNV | 1.65E-04 | 4.30E-05 |
| rs534775 | TCGA&TCGA_coCNV | 1.65E-04 | 2.26E-05 |
| rs16855866 | TCGA&TCGA_coCNV | 1.65E-04 | 1.58E-04 |
| rs16835587 | TCGA&TCGA_coCNV | 1.66E-04 | 7.94E-05 |
| rs7059518 | TCGA&TCGA_coCNV | 1.66E-04 | 2.57E-03 |
| rs17775017 | TCGA&TCGA_coCNV | 1.66E-04 | 1.51E-03 |
| rs7677031 | TCGA&TCGA_coCNV | 1.66E-04 | 4.06E-04 |
| rs12433054 | TCGA&TCGA_coCNV | 1.67E-04 | 1.11E-03 |
| rs2784920 | TCGA&TCGA_coCNV | 1.67E-04 | 1.46E-03 |
| rs2570738 | TCGA&TCGA_coCNV | 1.67E-04 | 8.41E-04 |
| rs1424183 | TCGA&TCGA_coCNV | 1.68E-04 | 8.55E-05 |
| rs17502774 | TCGA&TCGA_coCNV | 1.68E-04 | 2.13E-03 |
| rs1635561 | TCGA | 1.72E-04 | #N/A |
| rs1007681 | TCGA&TCGA_coCNV | 1.73E-04 | 2.10E-03 |
| rs7709884 | TCGA&TCGA_coCNV&SCANdb | 4.78E-05 | 1.43E-04 |
| rs7638757 | TCGA&TCGA_coCNV | 1.75E-04 | 7.55E-04 |
| rs16901407 | TCGA&TCGA_coCNV | 1.76E-04 | 3.53E-03 |
| rs1106414 | TCGA&TCGA_coCNV | 1.76E-04 | 5.02E-04 |
| rs468738 | TCGA&TCGA_coCNV | 1.76E-04 | 2.55E-05 |
| rs10158776 | TCGA&TCGA_coCNV | 1.76E-04 | 5.40E-05 |
| rs17339362 | TCGA&TCGA_coCNV | 1.77E-04 | 8.98E-05 |
| rs7967221 | TCGA&TCGA_coCNV | 1.80E-04 | 4.96E-03 |
| rs1344999 | TCGA&TCGA_coCNV | 1.80E-04 | 6.38E-04 |
| rs10043478 | TCGA&TCGA_coCNV | 1.80E-04 | 1.02E-03 |
| rs11145655 | TCGA&TCGA_coCNV | 1.81E-04 | 3.84E-03 |
| rs9532806 | TCGA&TCGA_coCNV | 1.81E-04 | 2.79E-03 |
| rs4514489 | TCGA&TCGA_coCNV | 1.82E-04 | 9.89E-04 |
| rs2512483 | TCGA | 1.82E-04 | #N/A |
| rs3849159 | TCGA&TCGA_coCNV | 1.82E-04 | 5.40E-05 |
| rs11164835 | TCGA | 1.82E-04 | #N/A |
| rs1556542 | TCGA&TCGA_coCNV | 3.87E-05 | 1.96E-04 |
| rs6483581 | TCGA&TCGA_coCNV | 1.86E-04 | 2.38E-03 |
| rs9969160 | TCGA&TCGA_coCNV | 1.86E-04 | 2.73E-04 |
| rs10260934 | TCGA&TCGA_coCNV | 1.86E-04 | 1.12E-03 |
| rs979676 | TCGA&TCGA_coCNV | 1.87E-04 | 1.16E-03 |

| rs7306617 | TCGA&TCGA_coCNV | 1.87E-04 | 1.32E-05 |
| --- | --- | --- | --- |
| rs9460164 | TCGA&TCGA_coCNV | 1.88E-04 | 3.43E-04 |
| rs16881465 | TCGA | 3.62E-06 | #N/A |
| rs1338966 | TCGA&TCGA_coCNV | 1.89E-04 | 2.99E-03 |
| rs17154115 | TCGA&TCGA_coCNV | 1.89E-04 | 1.08E-03 |
| rs10068082 | TCGA | 1.89E-04 | #N/A |
| rs9290711 | TCGA&TCGA_coCNV | 1.89E-04 | 1.93E-03 |
| rs17647701 | TCGA&TCGA_coCNV | 1.90E-04 | 8.96E-04 |
| rs10733410 | TCGA&TCGA_coCNV | 1.90E-04 | 6.38E-04 |
| rs12643432 | TCGA&TCGA_coCNV | 1.91E-04 | 5.75E-04 |
| rs3780567 | TCGA | 1.91E-04 | #N/A |
| rs6639134 | TCGA&TCGA_coCNV | 1.91E-04 | 9.96E-05 |
| rs16996779 | TCGA&TCGA_coCNV | 1.91E-04 | 5.18E-04 |
| rs7085245 | TCGA&TCGA_coCNV | 1.92E-04 | 1.64E-03 |
| rs9353972 | TCGA&TCGA_coCNV | 1.92E-04 | 2.05E-04 |
| rs347759 | TCGA&TCGA_coCNV | 1.92E-04 | 6.40E-04 |
| rs16890168 | TCGA&TCGA_coCNV | 1.93E-04 | 4.55E-05 |
| rs6856198 | TCGA&TCGA_coCNV | 1.93E-04 | 1.05E-03 |
| rs12660221 | TCGA&TCGA_coCNV | 1.94E-04 | 3.99E-05 |
| rs7073422 | TCGA&TCGA_coCNV | 1.94E-04 | 1.03E-03 |
| rs13323642 | TCGA&TCGA_coCNV | 1.95E-04 | 3.36E-03 |
| rs11533806 | TCGA&TCGA_coCNV | 1.95E-04 | 1.55E-03 |
| rs4459767 | TCGA&TCGA_coCNV | 1.96E-04 | 1.57E-04 |
| rs1431207 | TCGA&TCGA_coCNV | 1.96E-04 | 5.60E-04 |
| rs4336225 | TCGA&TCGA_coCNV | 1.96E-04 | 5.22E-04 |
| rs1215616 | TCGA&TCGA_coCNV | 1.97E-04 | 1.56E-03 |
| rs4610776 | TCGA | 1.97E-04 | #N/A |
| rs10229171 | TCGA&TCGA_coCNV | 1.98E-04 | 1.99E-04 |
| rs11195324 | TCGA&TCGA_coCNV | 1.98E-04 | 1.14E-03 |
| rs17661302 | TCGA&TCGA_coCNV | 1.98E-04 | 9.57E-05 |
| rs38880 | TCGA&TCGA_coCNV | 2.01E-04 | 9.88E-04 |
| rs2412716 | TCGA&TCGA_coCNV | 2.02E-04 | 1.44E-03 |
| rs12514205 | TCGA&TCGA_coCNV | 2.03E-04 | 4.41E-05 |
| rs12630527 | TCGA&TCGA_coCNV | 2.03E-04 | 1.22E-03 |
| rs1148994 | TCGA&TCGA_coCNV | 2.05E-04 | 8.46E-04 |
| rs4791876 | TCGA&TCGA_coCNV | 2.05E-04 | 2.55E-03 |
| rs6779680 | TCGA&TCGA_coCNV | 2.08E-04 | 2.93E-03 |
| rs1874537 | TCGA&TCGA_coCNV | 4.21E-05 | 3.40E-03 |
| rs859522 | TCGA&TCGA_coCNV | 2.10E-04 | 8.20E-05 |
| rs11905535 | TCGA&TCGA_coCNV | 2.10E-04 | 6.95E-04 |
| rs6502938 | TCGA&TCGA_coCNV | 2.11E-04 | 1.33E-03 |
| rs3770464 | TCGA&TCGA_coCNV | 2.13E-04 | 4.67E-04 |
| rs6506726 | TCGA&TCGA_coCNV | 2.13E-04 | 2.93E-03 |
| rs4379029 | TCGA&TCGA_coCNV | 2.13E-04 | 1.48E-04 |
| rs11023369 | TCGA&TCGA_coCNV | 2.14E-04 | 6.83E-04 |
| rs2943656 | TCGA&TCGA_coCNV | 2.14E-04 | 1.03E-04 |

| rs12395928 | TCGA | 2.14E-04 | #N/A |
| --- | --- | --- | --- |
| rs16839276 | TCGA&TCGA_coCNV | 2.14E-04 | 6.27E-03 |
| rs41330845 | TCGA&TCGA_coCNV | 2.15E-04 | 1.92E-03 |
| rs288033 | TCGA&TCGA_coCNV | 2.15E-04 | 8.77E-03 |
| rs4971827 | TCGA&TCGA_coCNV | 2.15E-04 | 8.34E-03 |
| rs11140297 | TCGA&TCGA_coCNV | 2.16E-04 | 4.39E-03 |
| rs5920897 | TCGA&TCGA_coCNV | 2.17E-04 | 8.93E-07 |
| rs3793480 | TCGA&TCGA_coCNV | 2.17E-04 | 8.73E-07 |
| rs11217699 | TCGA&TCGA_coCNV | 2.18E-04 | 3.84E-03 |
| rs11708271 | TCGA&TCGA_coCNV | 2.18E-04 | 2.27E-04 |
| rs10049886 | TCGA&TCGA_coCNV | 2.18E-04 | 9.73E-04 |
| rs11996159 | TCGA&TCGA_coCNV | 2.18E-04 | 8.71E-07 |
| rs17092344 | TCGA | 2.19E-04 | #N/A |
| rs17039220 | TCGA&TCGA_coCNV | 2.19E-04 | 9.13E-07 |
| rs17071867 | TCGA&TCGA_coCNV | 2.20E-04 | 1.21E-04 |
| rs17000759 | TCGA&TCGA_coCNV | 2.20E-04 | 9.21E-07 |
| rs4834190 | TCGA&TCGA_coCNV | 2.20E-04 | 4.80E-03 |
| rs10925413 | TCGA&TCGA_coCNV | 2.20E-04 | 1.95E-03 |
| rs11182568 | TCGA&TCGA_coCNV | 2.21E-04 | 4.01E-03 |
| rs16973832 | TCGA&TCGA_coCNV | 2.21E-04 | 8.92E-07 |
| rs5933947 | TCGA&TCGA_coCNV | 2.22E-04 | 7.42E-05 |
| rs2052480 | TCGA&TCGA_coCNV | 2.22E-04 | 3.55E-03 |
| rs16993711 | TCGA&TCGA_coCNV | 2.22E-04 | 6.24E-04 |
| rs2088285 | TCGA&TCGA_coCNV | 2.23E-04 | 3.46E-03 |
| rs17161065 | TCGA&TCGA_coCNV | 2.24E-04 | 3.15E-03 |
| rs4802826 | TCGA&TCGA_coCNV | 2.25E-04 | 1.52E-03 |
| rs11663191 | TCGA&TCGA_coCNV | 2.25E-04 | 2.01E-03 |
| rs41453849 | TCGA&TCGA_coCNV | 2.25E-04 | 8.89E-07 |
| rs7044326 | TCGA&TCGA_coCNV | 2.25E-04 | 1.10E-03 |
| rs10508670 | TCGA&TCGA_coCNV | 2.26E-04 | 9.66E-04 |
| rs12688523 | TCGA&TCGA_coCNV | 2.26E-04 | 5.21E-04 |
| rs9671429 | TCGA&TCGA_coCNV | 2.26E-04 | 4.75E-03 |
| rs8179620 | TCGA&TCGA_coCNV | 2.27E-04 | 6.22E-04 |
| rs12154943 | TCGA&TCGA_coCNV | 2.28E-04 | 5.11E-05 |
| rs10043237 | TCGA | 2.28E-04 | #N/A |
| rs710824 | TCGA&TCGA_coCNV | 2.29E-04 | 4.95E-03 |
| rs7529288 | TCGA&TCGA_coCNV | 2.30E-04 | 1.26E-03 |
| rs1985147 | TCGA&TCGA_coCNV | 2.30E-04 | 2.65E-03 |
| rs2587813 | TCGA&TCGA_coCNV | 2.30E-04 | 7.19E-04 |
| rs10518414 | TCGA&TCGA_coCNV | 2.31E-04 | 4.81E-04 |
| rs12507264 | TCGA&TCGA_coCNV | 2.33E-04 | 3.46E-03 |
| rs2303534 | TCGA&TCGA_coCNV | 2.33E-04 | 1.80E-04 |
| rs2640779 | TCGA&TCGA_coCNV | 2.33E-04 | 5.15E-03 |
| rs2224204 | TCGA&TCGA_coCNV | 2.33E-04 | 1.16E-03 |
| rs728668 | TCGA&TCGA_coCNV | 2.35E-04 | 8.38E-04 |
| rs9881064 | TCGA | 2.35E-04 | #N/A |

| rs1461060 | TCGA&TCGA_coCNV | 2.35E-04 | 1.09E-03 |
| --- | --- | --- | --- |
| rs2449348 | TCGA&TCGA_coCNV | 2.35E-04 | 6.65E-03 |
| rs2227331 | TCGA&TCGA_coCNV | 2.37E-04 | 4.05E-04 |
| rs11098708 | TCGA&TCGA_coCNV | 2.37E-04 | 4.57E-03 |
| rs4921893 | TCGA | 2.37E-04 | #N/A |
| rs1557622 | TCGA&TCGA_coCNV | 2.37E-04 | 9.41E-04 |
| rs10063716 | TCGA | 4.28E-05 | #N/A |
| rs1493389 | TCGA&TCGA_coCNV | 2.37E-04 | 1.84E-03 |
| rs3212617 | TCGA | 2.40E-04 | #N/A |
| rs11227619 | TCGA&TCGA_coCNV | 2.40E-04 | 2.43E-04 |
| rs2299841 | TCGA&TCGA_coCNV | 2.40E-04 | 1.19E-03 |
| rs12285109 | TCGA&TCGA_coCNV | 2.41E-04 | 4.54E-04 |
| rs2220064 | TCGA&TCGA_coCNV | 2.41E-04 | 2.14E-04 |
| rs7640013 | TCGA&TCGA_coCNV | 2.42E-04 | 2.49E-03 |
| rs16830513 | TCGA | 2.43E-04 | #N/A |
| rs4285039 | TCGA&TCGA_coCNV | 2.43E-04 | 1.36E-03 |
| rs1544767 | TCGA&TCGA_coCNV | 2.46E-04 | 6.79E-05 |
| rs17089822 | TCGA&TCGA_coCNV | 2.46E-04 | 1.39E-03 |
| rs11115262 | TCGA&TCGA_coCNV | 2.46E-04 | 1.74E-04 |
| rs2053736 | TCGA&TCGA_coCNV | 2.47E-04 | 5.23E-04 |
| rs1484802 | TCGA&TCGA_coCNV | 2.48E-04 | 9.20E-03 |
| rs6529399 | TCGA&TCGA_coCNV | 2.48E-04 | 8.14E-05 |
| rs17215981 | TCGA&TCGA_coCNV | 2.49E-04 | 7.21E-03 |
| rs10904184 | TCGA&TCGA_coCNV | 2.50E-04 | 2.04E-04 |
| rs10089292 | TCGA&TCGA_coCNV | 2.50E-04 | 5.12E-04 |
| rs315891 | TCGA&TCGA_coCNV | 2.52E-04 | 3.53E-06 |
| rs17754062 | TCGA | 2.53E-04 | #N/A |
| rs17091392 | TCGA&TCGA_coCNV | 2.53E-04 | 6.49E-03 |
| rs7327469 | TCGA&TCGA_coCNV | 2.54E-04 | 2.24E-05 |
| rs9966010 | TCGA | 2.54E-04 | #N/A |
| rs10479287 | TCGA&TCGA_coCNV | 7.03E-08 | 3.35E-03 |
| rs17604548 | TCGA&TCGA_coCNV | 2.54E-04 | 5.20E-03 |
| rs684458 | TCGA&TCGA_coCNV | 2.54E-04 | 3.39E-03 |
| rs6901726 | TCGA&TCGA_coCNV | 2.54E-04 | 9.14E-05 |
| rs17135168 | TCGA&TCGA_coCNV | 2.54E-04 | 2.42E-03 |
| rs9637390 | TCGA&TCGA_coCNV | 2.55E-04 | 5.86E-04 |
| rs10456788 | TCGA | 2.55E-04 | #N/A |
| rs10919410 | TCGA&TCGA_coCNV | 2.59E-04 | 9.50E-04 |
| rs7959718 | TCGA&TCGA_coCNV | 2.59E-04 | 1.35E-03 |
| rs7208330 | TCGA | 2.59E-04 | #N/A |
| rs6835296 | TCGA&TCGA_coCNV | 2.59E-04 | 1.52E-03 |
| rs10232716 | TCGA&TCGA_coCNV | 2.59E-04 | 2.14E-04 |
| rs3801438 | TCGA&TCGA_coCNV | 2.59E-04 | 1.10E-03 |
| rs5907231 | TCGA&TCGA_coCNV | 2.61E-04 | 4.46E-06 |
| rs4293213 | TCGA&TCGA_coCNV | 2.61E-04 | 1.33E-03 |
| rs1515108 | TCGA&TCGA_coCNV | 2.61E-04 | 2.72E-04 |

| rs6639121 | TCGA&TCGA_coCNV | 2.61E-04 | 4.67E-05 |
| --- | --- | --- | --- |
| rs10812391 | TCGA&TCGA_coCNV | 2.61E-04 | 8.72E-03 |
| rs10959767 | TCGA&TCGA_coCNV | 2.63E-04 | 4.79E-03 |
| rs1021571 | TCGA&TCGA_coCNV | 2.64E-04 | 8.08E-03 |
| rs725925 | TCGA&TCGA_coCNV | 2.64E-04 | 1.87E-03 |
| rs16969478 | TCGA&TCGA_coCNV | 2.65E-04 | 8.82E-03 |
| rs994769 | TCGA&TCGA_coCNV | 2.65E-04 | 9.97E-03 |
| rs2196552 | TCGA&TCGA_coCNV | 2.65E-04 | 1.17E-03 |
| rs7277117 | TCGA&TCGA_coCNV | 2.68E-04 | 3.85E-04 |
| rs4243365 | TCGA&TCGA_coCNV | 2.68E-04 | 1.47E-04 |
| rs2703842 | TCGA&TCGA_coCNV | 2.68E-04 | 2.44E-04 |
| rs11718240 | TCGA&TCGA_coCNV | 2.69E-04 | 1.79E-03 |
| rs1479291 | TCGA&TCGA_coCNV | 2.69E-04 | 1.83E-03 |
| rs6671808 | TCGA&TCGA_coCNV | 2.70E-04 | 2.45E-03 |
| rs906695 | TCGA&TCGA_coCNV | 2.70E-04 | 5.47E-03 |
| rs9799 | TCGA&TCGA_coCNV | 2.70E-04 | 7.79E-03 |
| rs9460753 | TCGA&TCGA_coCNV | 2.71E-04 | 3.79E-04 |
| rs12566259 | TCGA&TCGA_coCNV | 2.71E-04 | 1.18E-03 |
| rs17666880 | TCGA&TCGA_coCNV | 2.71E-04 | 3.64E-04 |
| rs11167894 | TCGA&TCGA_coCNV | 2.71E-04 | 2.54E-04 |
| rs6596833 | TCGA&TCGA_coCNV | 2.73E-04 | 3.45E-04 |
| rs12203173 | TCGA&TCGA_coCNV | 2.73E-04 | 3.68E-03 |
| rs12525847 | TCGA&TCGA_coCNV | 2.73E-04 | 5.34E-04 |
| rs927839 | TCGA&TCGA_coCNV | 2.74E-04 | 3.00E-03 |
| rs9940754 | TCGA&TCGA_coCNV | 2.75E-04 | 6.13E-03 |
| rs16856914 | TCGA&TCGA_coCNV | 2.76E-04 | 2.77E-04 |
| rs2843143 | TCGA&TCGA_coCNV | 2.76E-04 | 4.73E-03 |
| rs1339655 | TCGA | 2.76E-04 | #N/A |
| rs17783121 | TCGA | 2.77E-04 | #N/A |
| rs4958358 | TCGA&TCGA_coCNV | 2.78E-04 | 2.55E-03 |
| rs17066184 | TCGA | 2.80E-04 | #N/A |
| rs7827093 | TCGA&TCGA_coCNV | 2.80E-04 | 6.72E-03 |
| rs10937783 | TCGA&TCGA_coCNV | 2.80E-04 | 6.30E-04 |
| rs2722824 | TCGA&TCGA_coCNV | 2.81E-04 | 1.15E-04 |
| rs1330875 | TCGA&TCGA_coCNV | 2.81E-04 | 5.62E-05 |
| rs2623069 | TCGA&TCGA_coCNV | 2.81E-04 | 1.32E-03 |
| rs872694 | TCGA | 2.81E-04 | #N/A |
| rs1202397 | TCGA | 2.81E-04 | #N/A |
| rs917427 | TCGA&TCGA_coCNV | 2.82E-04 | 7.08E-03 |
| rs877339 | TCGA&TCGA_coCNV | 2.82E-04 | 9.61E-03 |
| rs11770453 | TCGA&TCGA_coCNV | 2.84E-04 | 1.93E-03 |
| rs10874753 | TCGA&TCGA_coCNV | 2.84E-04 | 9.34E-03 |
| rs7646617 | TCGA&TCGA_coCNV | 2.85E-04 | 5.57E-04 |
| rs7661544 | TCGA&TCGA_coCNV | 2.85E-04 | 3.57E-04 |
| rs3777997 | TCGA&TCGA_coCNV | 2.85E-04 | 6.10E-04 |
| rs11775425 | TCGA&TCGA_coCNV | 2.86E-04 | 7.94E-05 |

| rs193567 | TCGA&TCGA_coCNV | 2.86E-04 | 1.39E-03 |
| --- | --- | --- | --- |
| rs9864987 | TCGA&TCGA_coCNV | 2.88E-04 | 5.50E-04 |
| rs12008697 | TCGA&TCGA_coCNV | 2.89E-04 | 3.68E-03 |
| rs2403428 | TCGA&TCGA_coCNV | 2.90E-04 | 2.55E-03 |
| rs11142072 | TCGA&TCGA_coCNV | 2.90E-04 | 1.10E-03 |
| rs16978509 | TCGA&TCGA_coCNV | 2.92E-04 | 2.25E-03 |
| rs922234 | TCGA&TCGA_coCNV | 2.93E-04 | 7.88E-03 |
| rs1705244 | TCGA&TCGA_coCNV | 2.94E-04 | 1.83E-04 |
| rs2711730 | TCGA | 2.94E-04 | #N/A |
| rs17098386 | TCGA&TCGA_coCNV | 2.95E-04 | 2.90E-04 |
| rs8101572 | TCGA&TCGA_coCNV | 2.95E-04 | 1.74E-03 |
| rs2949942 | TCGA | 1.09E-05 | #N/A |
| rs12049789 | TCGA&TCGA_coCNV | 2.96E-04 | 7.41E-03 |
| rs12266759 | TCGA&TCGA_coCNV | 2.96E-04 | 8.73E-04 |
| rs1456564 | TCGA&TCGA_coCNV | 2.97E-04 | 5.74E-03 |
| rs17274134 | TCGA&TCGA_coCNV | 2.97E-04 | 8.79E-04 |
| rs933945 | TCGA&TCGA_coCNV | 3.70E-05 | 1.35E-04 |
| rs6842047 | TCGA&TCGA_coCNV | 2.98E-04 | 4.39E-03 |
| rs7285304 | TCGA&TCGA_coCNV | 2.99E-04 | 5.27E-03 |
| rs10018657 | TCGA&TCGA_coCNV | 3.00E-04 | 7.10E-05 |
| rs10967319 | TCGA&TCGA_coCNV | 1.28E-04 | 1.10E-03 |
| rs11683049 | TCGA&TCGA_coCNV | 3.01E-04 | 2.96E-03 |
| rs4328228 | TCGA&TCGA_coCNV | 3.02E-04 | 2.70E-03 |
| rs1456557 | TCGA | 3.02E-04 | #N/A |
| rs11155977 | TCGA&TCGA_coCNV | 3.02E-04 | 3.42E-03 |
| rs382108 | TCGA&TCGA_coCNV | 3.03E-04 | 6.65E-03 |
| rs17083159 | TCGA&TCGA_coCNV | 3.04E-04 | 3.33E-04 |
| rs17085176 | TCGA&TCGA_coCNV | 3.04E-04 | 4.83E-04 |
| rs3098377 | TCGA&TCGA_coCNV | 3.04E-04 | 7.56E-04 |
| rs3923107 | TCGA&TCGA_coCNV | 3.06E-04 | 4.35E-03 |
| rs908963 | TCGA&TCGA_coCNV | 3.08E-04 | 1.38E-03 |
| rs194594 | TCGA&TCGA_coCNV | 1.42E-04 | 5.13E-05 |
| rs4674022 | TCGA&TCGA_coCNV | 1.51E-05 | 2.69E-05 |
| rs10829383 | TCGA | 3.12E-04 | #N/A |
| rs893219 | TCGA&TCGA_coCNV | 3.12E-04 | 1.49E-03 |
| rs12809491 | TCGA | 3.14E-04 | #N/A |
| rs1981320 | TCGA&TCGA_coCNV | 3.15E-04 | 8.46E-03 |
| rs2821186 | TCGA&TCGA_coCNV | 3.16E-04 | 2.41E-03 |
| rs1498364 | TCGA&TCGA_coCNV | 3.16E-04 | 7.52E-03 |
| rs166031 | TCGA&TCGA_coCNV | 3.17E-04 | 6.55E-03 |
| rs12307364 | TCGA&TCGA_coCNV | 3.17E-04 | 1.78E-03 |
| rs7650354 | TCGA&TCGA_coCNV | 3.17E-04 | 3.69E-04 |
| rs10800620 | TCGA&TCGA_coCNV | 3.18E-04 | 3.25E-03 |
| rs11186481 | TCGA&TCGA_coCNV | 3.18E-04 | 4.03E-05 |
| rs4243302 | TCGA&TCGA_coCNV | 3.19E-04 | 4.35E-03 |
| rs2327891 | TCGA&TCGA_coCNV | 3.19E-04 | 5.77E-04 |

| rs12686293 | TCGA | 3.21E-04 | #N/A |
| --- | --- | --- | --- |
| rs6752820 | TCGA | 3.21E-04 | #N/A |
| rs10990984 | TCGA&TCGA_coCNV | 3.22E-04 | 4.62E-04 |
| rs10919392 | TCGA&TCGA_coCNV | 3.22E-04 | 2.91E-04 |
| rs6724844 | TCGA | 3.22E-04 | #N/A |
| rs392621 | TCGA | 3.23E-04 | #N/A |
| rs12407228 | TCGA&TCGA_coCNV | 3.23E-04 | 1.32E-04 |
| rs7142257 | TCGA&TCGA_coCNV | 3.24E-04 | 5.21E-03 |
| rs16859399 | TCGA&TCGA_coCNV | 3.24E-04 | 8.77E-04 |
| rs6042678 | TCGA&TCGA_coCNV | 3.25E-04 | 3.14E-03 |
| rs1410377 | TCGA&TCGA_coCNV | 3.27E-04 | 1.48E-03 |
| rs757404 | TCGA&TCGA_coCNV | 3.27E-04 | 5.42E-03 |
| rs10962689 | TCGA | 3.28E-04 | #N/A |
| rs12521548 | TCGA&TCGA_coCNV | 3.30E-04 | 4.92E-04 |
| rs12444846 | TCGA&TCGA_coCNV | 3.30E-04 | 5.66E-03 |
| rs17756667 | TCGA&TCGA_coCNV | 3.32E-04 | 7.12E-05 |
| rs1887013 | TCGA&TCGA_coCNV | 3.32E-04 | 4.71E-04 |
| rs962879 | TCGA&TCGA_coCNV | 3.32E-04 | 3.41E-04 |
| rs1076595 | TCGA&TCGA_coCNV | 3.33E-04 | 5.22E-03 |
| rs12630162 | TCGA&TCGA_coCNV | 3.33E-04 | 3.82E-03 |
| rs12833762 | TCGA&TCGA_coCNV | 2.48E-04 | 2.17E-03 |
| rs11055973 | TCGA&TCGA_coCNV | 3.35E-04 | 1.52E-03 |
| rs6443545 | TCGA&TCGA_coCNV | 3.36E-04 | 2.59E-04 |
| rs7023389 | TCGA&TCGA_coCNV | 3.36E-04 | 4.05E-05 |
| rs10068523 | TCGA&TCGA_coCNV | 7.65E-05 | 8.99E-03 |
| rs13065535 | TCGA&TCGA_coCNV | 3.38E-04 | 1.71E-04 |
| rs2240188 | TCGA | 3.39E-04 | #N/A |
| rs515917 | TCGA&TCGA_coCNV | 3.39E-04 | 1.25E-03 |
| rs12089663 | TCGA&TCGA_coCNV | 3.39E-04 | 4.18E-03 |
| rs16918030 | TCGA&TCGA_coCNV | 3.39E-04 | 1.60E-04 |
| rs935374 | TCGA&TCGA_coCNV | 3.40E-04 | 1.01E-04 |
| rs12237673 | TCGA&TCGA_coCNV | 3.41E-04 | 2.48E-03 |
| rs16982422 | TCGA&TCGA_coCNV | 3.41E-04 | 2.97E-05 |
| rs11077162 | TCGA&TCGA_coCNV | 3.42E-04 | 3.26E-03 |
| rs12497918 | TCGA&TCGA_coCNV | 3.44E-04 | 2.78E-03 |
| rs16831030 | TCGA&TCGA_coCNV | 3.44E-04 | 5.84E-04 |
| rs10228205 | TCGA&TCGA_coCNV | 3.45E-04 | 1.86E-03 |
| rs1917804 | TCGA&TCGA_coCNV | 3.45E-04 | 5.93E-04 |
| rs10489487 | TCGA&TCGA_coCNV | 1.30E-04 | 8.62E-04 |
| rs1931963 | TCGA&TCGA_coCNV | 3.47E-04 | 2.08E-03 |
| rs11038071 | TCGA&TCGA_coCNV | 3.47E-04 | 3.81E-04 |
| rs6879775 | TCGA | 3.47E-04 | #N/A |
| rs11788585 | TCGA | 3.47E-04 | #N/A |
| rs6055611 | TCGA | 3.49E-04 | #N/A |
| rs17044751 | TCGA&TCGA_coCNV | 3.50E-04 | 7.16E-04 |
| rs6776913 | TCGA&TCGA_coCNV | 3.51E-04 | 3.03E-05 |

| rs277493 | TCGA&TCGA_coCNV | 3.52E-04 | 2.26E-03 |
| --- | --- | --- | --- |
| rs2280521 | TCGA | 3.52E-04 | #N/A |
| rs9572798 | TCGA&TCGA_coCNV | 3.52E-04 | 9.91E-04 |
| rs12175343 | TCGA&TCGA_coCNV | 3.54E-04 | 2.83E-04 |
| rs1443841 | TCGA&TCGA_coCNV | 3.55E-04 | 9.39E-05 |
| rs10820587 | TCGA&TCGA_coCNV | 3.56E-04 | 1.90E-05 |
| rs10751958 | TCGA&TCGA_coCNV | 3.57E-04 | 4.36E-04 |
| rs9600969 | TCGA&TCGA_coCNV | 3.57E-04 | 3.28E-04 |
| rs10960243 | TCGA&TCGA_coCNV | 3.58E-04 | 7.45E-04 |
| rs16902818 | TCGA&TCGA_coCNV | 1.54E-04 | 3.41E-03 |
| rs4302389 | TCGA&TCGA_coCNV | 3.59E-04 | 9.87E-05 |
| rs1943506 | TCGA&TCGA_coCNV | 3.60E-04 | 1.43E-05 |
| rs2183762 | TCGA&TCGA_coCNV | 3.60E-04 | 2.60E-04 |
| rs10508305 | TCGA&TCGA_coCNV | 3.61E-04 | 3.82E-04 |
| rs3800043 | TCGA&TCGA_coCNV | 3.61E-04 | 1.59E-03 |
| rs4658411 | TCGA&TCGA_coCNV | 3.62E-04 | 9.39E-04 |
| rs1134975 | TCGA&TCGA_coCNV | 3.62E-04 | 1.37E-03 |
| rs17214952 | TCGA | 3.64E-04 | #N/A |
| rs10124782 | TCGA&TCGA_coCNV | 3.64E-04 | 1.29E-06 |
| rs1494464 | TCGA&TCGA_coCNV | 5.30E-05 | 4.94E-04 |
| rs9669517 | TCGA&TCGA_coCNV | 3.66E-04 | 6.65E-05 |
| rs12544427 | TCGA | 3.66E-04 | #N/A |
| rs6984908 | TCGA | 3.67E-04 | #N/A |
| rs6867978 | TCGA | 3.68E-04 | #N/A |
| rs2202370 | TCGA&TCGA_coCNV | 3.69E-04 | 1.92E-04 |
| rs1535989 | TCGA&TCGA_coCNV | 3.69E-04 | 4.48E-04 |
| rs1994803 | TCGA | 3.69E-04 | #N/A |
| rs16871000 | TCGA&TCGA_coCNV | 3.69E-04 | 1.86E-03 |
| rs5975496 | TCGA&TCGA_coCNV | 3.70E-04 | 4.30E-03 |
| rs3809682 | TCGA&TCGA_coCNV | 3.71E-04 | 5.49E-04 |
| rs16999857 | TCGA&TCGA_coCNV | 3.71E-04 | 4.49E-04 |
| rs9644986 | TCGA | 3.72E-04 | #N/A |
| rs1837235 | TCGA&TCGA_coCNV | 3.73E-04 | 1.91E-03 |
| rs2285549 | TCGA&TCGA_coCNV | 3.75E-04 | 3.16E-03 |
| rs4888570 | TCGA | 3.75E-04 | #N/A |
| rs10269684 | TCGA&TCGA_coCNV | 3.75E-04 | 3.29E-05 |
| rs4967959 | TCGA&TCGA_coCNV | 3.75E-04 | 4.42E-03 |
| rs16885908 | TCGA&TCGA_coCNV | 3.76E-04 | 5.97E-04 |
| rs2292984 | TCGA&TCGA_coCNV | 3.76E-04 | 3.42E-03 |
| rs404134 | TCGA&TCGA_coCNV | 3.77E-04 | 6.65E-04 |
| rs4287338 | TCGA&TCGA_coCNV | 3.78E-04 | 6.87E-04 |
| rs10131830 | TCGA&TCGA_coCNV | 3.78E-04 | 4.73E-03 |
| rs12745968 | TCGA | 3.80E-04 | #N/A |
| rs7797205 | TCGA&TCGA_coCNV | 3.81E-04 | 1.07E-03 |
| rs2554452 | TCGA&TCGA_coCNV | 3.82E-04 | 1.27E-03 |
| rs9854741 | TCGA&TCGA_coCNV | 3.83E-04 | 1.16E-04 |

| rs10193646 | TCGA | 3.83E-04 | #N/A |
| --- | --- | --- | --- |
| rs11732581 | TCGA&TCGA_coCNV | 3.84E-04 | 3.07E-06 |
| rs12469875 | TCGA&TCGA_coCNV | 3.84E-04 | 1.76E-03 |
| rs1999523 | TCGA&TCGA_coCNV | 3.85E-04 | 9.16E-04 |
| rs4373009 | TCGA&TCGA_coCNV | 3.85E-04 | 4.72E-03 |
| rs7882770 | TCGA | 3.87E-04 | #N/A |
| rs13200680 | TCGA | 3.87E-04 | #N/A |
| rs12021779 | TCGA&TCGA_coCNV | 3.89E-04 | 3.78E-06 |
| rs4465122 | TCGA&TCGA_coCNV | 3.90E-04 | 1.74E-03 |
| rs2637253 | TCGA&TCGA_coCNV | 3.90E-04 | 3.46E-03 |
| rs7408371 | TCGA&TCGA_coCNV | 3.91E-04 | 2.39E-03 |
| rs2009949 | TCGA&TCGA_coCNV | 3.91E-04 | 3.49E-03 |
| rs2095593 | TCGA | 3.92E-04 | #N/A |
| rs41390547 | TCGA&TCGA_coCNV | 3.92E-04 | 6.00E-04 |
| rs17775981 | TCGA&TCGA_coCNV | 3.94E-04 | 8.82E-03 |
| rs6639120 | TCGA&TCGA_coCNV | 3.94E-04 | 2.67E-05 |
| rs1721643 | TCGA | 3.94E-04 | #N/A |
| rs17137030 | TCGA&TCGA_coCNV | 3.95E-04 | 9.20E-03 |
| rs13229244 | TCGA&TCGA_coCNV | 3.96E-04 | 1.27E-03 |
| rs2852571 | TCGA&TCGA_coCNV | 3.96E-04 | 3.40E-03 |
| rs11692663 | TCGA | 3.96E-04 | #N/A |
| rs17120442 | TCGA&TCGA_coCNV | 3.97E-04 | 3.31E-04 |
| rs440862 | TCGA&TCGA_coCNV | 3.98E-04 | 2.41E-05 |
| rs12043127 | TCGA&TCGA_coCNV | 3.99E-04 | 4.79E-04 |
| rs10051144 | TCGA&TCGA_coCNV | 1.34E-04 | 7.57E-03 |
| rs10492058 | TCGA&TCGA_coCNV | 4.00E-04 | 8.98E-04 |
| rs16837372 | TCGA&TCGA_coCNV | 4.00E-04 | 2.40E-03 |
| rs3134905 | TCGA&TCGA_coCNV | 4.01E-04 | 1.77E-04 |
| rs4791947 | TCGA | 4.01E-04 | #N/A |
| rs632876 | TCGA&TCGA_coCNV | 4.02E-04 | 5.58E-03 |
| rs1652546 | TCGA&TCGA_coCNV | 1.66E-09 | 6.47E-03 |
| rs7723610 | TCGA&TCGA_coCNV | 4.03E-04 | 7.12E-04 |
| rs12666634 | TCGA&TCGA_coCNV | 4.03E-04 | 1.02E-03 |
| rs7947750 | TCGA&TCGA_coCNV | 4.03E-04 | 4.19E-03 |
| rs6443355 | TCGA&TCGA_coCNV | 4.04E-04 | 9.19E-04 |
| rs7714121 | TCGA&TCGA_coCNV | 4.05E-04 | 2.15E-03 |
| rs7775785 | TCGA&TCGA_coCNV | 4.05E-04 | 2.94E-04 |
| rs7036247 | TCGA | 4.06E-04 | #N/A |
| rs13160661 | TCGA | 1.85E-04 | #N/A |
| rs1996484 | TCGA&TCGA_coCNV | 4.08E-04 | 5.85E-04 |
| rs9555504 | TCGA | 4.08E-04 | #N/A |
| rs11108215 | TCGA | 4.09E-04 | #N/A |
| rs1969262 | TCGA&TCGA_coCNV | 4.09E-04 | 3.01E-03 |
| rs12392507 | TCGA&TCGA_coCNV | 4.09E-04 | 1.08E-04 |
| rs9806973 | TCGA&TCGA_coCNV | 4.09E-04 | 9.29E-03 |
| rs9944190 | TCGA&TCGA_coCNV | 3.25E-05 | 2.42E-03 |

| rs1736502 | TCGA&TCGA_coCNV | 4.10E-04 | 5.86E-03 |
| --- | --- | --- | --- |
| rs12026870 | TCGA&TCGA_coCNV | 4.10E-04 | 2.52E-03 |
| rs10874746 | TCGA | 4.10E-04 | #N/A |
| rs16910059 | TCGA&TCGA_coCNV | 4.12E-04 | 3.92E-04 |
| rs877474 | TCGA&TCGA_coCNV | 4.12E-04 | 5.08E-05 |
| rs11563068 | TCGA&TCGA_coCNV | 4.13E-04 | 1.19E-03 |
| rs11155978 | TCGA&TCGA_coCNV | 4.14E-04 | 7.48E-03 |
| rs1498090 | TCGA | 4.15E-04 | #N/A |
| rs17164441 | TCGA&TCGA_coCNV | 6.56E-05 | 3.20E-03 |
| rs402731 | TCGA | 4.17E-04 | #N/A |
| rs17068642 | TCGA&TCGA_coCNV | 4.17E-04 | 2.49E-05 |
| rs1467375 | TCGA&TCGA_coCNV | 1.04E-04 | 4.38E-04 |
| rs4782791 | TCGA&TCGA_coCNV | 4.18E-04 | 3.88E-04 |
| rs17141754 | TCGA&TCGA_coCNV | 4.19E-04 | 4.75E-03 |
| rs2327113 | TCGA&TCGA_coCNV | 4.19E-04 | 2.72E-03 |
| rs1824906 | TCGA&TCGA_coCNV | 4.20E-04 | 9.45E-03 |
| rs41482346 | TCGA&TCGA_coCNV | 4.21E-04 | 3.42E-04 |
| rs2775107 | TCGA&TCGA_coCNV | 4.22E-04 | 1.45E-03 |
| rs2185288 | TCGA | 4.22E-04 | #N/A |
| rs9636057 | TCGA&TCGA_coCNV | 4.22E-04 | 4.04E-03 |
| rs11612950 | TCGA | 4.23E-04 | #N/A |
| rs11646393 | TCGA&TCGA_coCNV | 4.23E-04 | 5.58E-05 |
| rs9415798 | TCGA | 4.23E-04 | #N/A |
| rs4774683 | TCGA&TCGA_coCNV | 4.25E-04 | 7.34E-03 |
| rs10512871 | TCGA&TCGA_coCNV | 4.25E-04 | 1.30E-03 |
| rs4366301 | TCGA&TCGA_coCNV | 4.25E-04 | 8.68E-04 |
| rs17749214 | TCGA&TCGA_coCNV | 4.26E-04 | 1.06E-03 |
| rs9863132 | TCGA&TCGA_coCNV | 4.26E-04 | 4.19E-03 |
| rs17007452 | TCGA&TCGA_coCNV | 4.26E-04 | 2.17E-04 |
| rs6074121 | TCGA&TCGA_coCNV | 4.27E-04 | 1.14E-03 |
| rs11219984 | TCGA&TCGA_coCNV | 4.28E-04 | 8.64E-04 |
| rs3788448 | TCGA | 4.28E-04 | #N/A |
| rs17148666 | TCGA&TCGA_coCNV | 4.28E-04 | 3.78E-03 |
| rs10401509 | TCGA&TCGA_coCNV | 4.28E-04 | 2.81E-04 |
| rs17153509 | TCGA&TCGA_coCNV | 4.29E-04 | 4.38E-05 |
| rs12066638 | TCGA | 4.29E-04 | #N/A |
| rs17282391 | TCGA&TCGA_coCNV | 4.29E-04 | 3.08E-04 |
| rs835363 | TCGA&TCGA_coCNV | 4.30E-04 | 7.13E-03 |
| rs1030687 | TCGA&TCGA_coCNV | 4.30E-04 | 7.53E-03 |
| rs6974138 | TCGA&TCGA_coCNV | 4.31E-04 | 2.84E-03 |
| rs524673 | TCGA | 4.32E-04 | #N/A |
| rs12366990 | TCGA&TCGA_coCNV | 4.32E-04 | 9.66E-03 |
| rs7844699 | TCGA&TCGA_coCNV | 4.32E-04 | 2.10E-03 |
| rs4837030 | TCGA&TCGA_coCNV | 4.32E-04 | 3.01E-04 |
| rs6878185 | TCGA&TCGA_coCNV | 4.08E-05 | 3.15E-04 |
| rs6743040 | TCGA&TCGA_coCNV | 4.34E-04 | 6.44E-04 |

| rs7937777 | TCGA&TCGA_coCNV | 4.35E-04 | 6.99E-04 |
| --- | --- | --- | --- |
| rs4600212 | TCGA&TCGA_coCNV | 7.83E-05 | 5.58E-04 |
| rs6571386 | TCGA&TCGA_coCNV | 4.35E-04 | 3.98E-04 |
| rs6959590 | TCGA&TCGA_coCNV | 4.36E-04 | 5.04E-04 |
| rs4141998 | TCGA&TCGA_coCNV | 4.36E-04 | 8.70E-04 |
| rs17015323 | TCGA&TCGA_coCNV | 4.37E-04 | 6.60E-04 |
| rs10894339 | TCGA | 4.38E-04 | #N/A |
| rs9520715 | TCGA&TCGA_coCNV | 4.38E-04 | 1.08E-04 |
| rs926029 | TCGA&TCGA_coCNV | 3.96E-04 | 7.05E-05 |
| rs853050 | TCGA&TCGA_coCNV | 4.40E-04 | 4.44E-06 |
| rs16890561 | TCGA&TCGA_coCNV | 4.40E-04 | 1.21E-03 |
| rs1328378 | TCGA&TCGA_coCNV | 4.40E-04 | 4.58E-03 |
| rs7974472 | TCGA | 4.41E-04 | #N/A |
| rs10409302 | TCGA&TCGA_coCNV | 4.41E-04 | 1.12E-03 |
| rs16963806 | TCGA&TCGA_coCNV | 4.41E-04 | 2.67E-03 |
| rs10983521 | TCGA&TCGA_coCNV | 2.60E-04 | 7.41E-04 |
| rs4817907 | TCGA&TCGA_coCNV | 4.42E-04 | 3.95E-04 |
| rs1333147 | TCGA&TCGA_coCNV | 4.42E-04 | 1.65E-04 |
| rs12470665 | TCGA&TCGA_coCNV | 4.42E-04 | 8.89E-03 |
| rs9289410 | TCGA | 4.43E-04 | #N/A |
| rs2113955 | TCGA&TCGA_coCNV | 4.44E-04 | 5.37E-03 |
| rs12612547 | TCGA&TCGA_coCNV | 4.46E-04 | 3.71E-04 |
| rs11637212 | TCGA&TCGA_coCNV | 4.48E-04 | 7.29E-03 |
| rs1993791 | TCGA&TCGA_coCNV | 4.49E-04 | 4.91E-05 |
| rs245832 | TCGA&TCGA_coCNV | 4.50E-04 | 6.80E-03 |
| rs1343251 | TCGA&TCGA_coCNV | 4.51E-04 | 4.59E-03 |
| rs2564044 | TCGA&TCGA_coCNV | 4.52E-04 | 4.09E-03 |
| rs10280789 | TCGA&TCGA_coCNV | 4.53E-04 | 4.38E-03 |
| rs6549006 | TCGA&TCGA_coCNV | 4.53E-04 | 3.47E-04 |
| rs16861309 | TCGA&TCGA_coCNV | 4.54E-04 | 3.69E-04 |
| rs153016 | TCGA&TCGA_coCNV | 4.54E-04 | 4.68E-04 |
| rs10050738 | TCGA | 4.54E-04 | #N/A |
| rs7671122 | TCGA&TCGA_coCNV | 4.54E-04 | 5.63E-03 |
| rs3893296 | TCGA&TCGA_coCNV | 4.55E-04 | 1.04E-04 |
| rs2255723 | TCGA | 4.57E-04 | #N/A |
| rs17709973 | TCGA&TCGA_coCNV | 4.58E-04 | 3.06E-03 |
| rs10246702 | TCGA&TCGA_coCNV | 4.58E-04 | 3.96E-04 |
| rs1588004 | TCGA&TCGA_coCNV | 4.60E-04 | 1.69E-04 |
| rs17752117 | TCGA&TCGA_coCNV | 4.62E-04 | 5.67E-03 |
| rs7030785 | TCGA&TCGA_coCNV | 4.66E-04 | 8.76E-04 |
| rs10938003 | TCGA&TCGA_coCNV | 3.42E-04 | 3.89E-04 |
| rs17181453 | TCGA&TCGA_coCNV | 4.68E-04 | 4.20E-04 |
| rs10195932 | TCGA&TCGA_coCNV | 4.68E-04 | 3.69E-03 |
| rs2990634 | TCGA&TCGA_coCNV | 4.68E-04 | 2.10E-04 |
| rs4899651 | TCGA&TCGA_coCNV | 4.69E-04 | 1.49E-03 |
| rs9379178 | TCGA&TCGA_coCNV | 4.69E-04 | 3.18E-03 |

| rs6731869 | TCGA&TCGA_coCNV | 4.70E-04 | 1.59E-04 |
| --- | --- | --- | --- |
| rs9451340 | TCGA&TCGA_coCNV | 4.70E-04 | 1.45E-03 |
| rs574249 | TCGA&TCGA_coCNV | 4.72E-04 | 4.03E-04 |
| rs2289395 | TCGA&TCGA_coCNV | 4.72E-04 | 1.20E-04 |
| rs7587814 | TCGA | 4.73E-04 | #N/A |
| rs12052388 | TCGA&TCGA_coCNV | 4.73E-04 | 1.42E-03 |
| rs7642215 | TCGA&TCGA_coCNV | 4.73E-04 | 2.51E-03 |
| rs10786148 | TCGA | 4.75E-04 | #N/A |
| rs13380113 | TCGA&TCGA_coCNV | 4.76E-04 | 8.86E-03 |
| rs6439335 | TCGA | 4.76E-04 | #N/A |
| rs10503614 | TCGA | 4.76E-04 | #N/A |
| rs17048520 | TCGA&TCGA_coCNV | 4.77E-04 | 1.63E-03 |
| rs4645529 | TCGA | 4.77E-04 | #N/A |
| rs1522259 | TCGA&TCGA_coCNV | 4.79E-04 | 2.73E-03 |
| rs2011300 | TCGA&TCGA_coCNV | 4.79E-04 | 1.82E-04 |
| rs734742 | TCGA&TCGA_coCNV | 4.81E-04 | 2.18E-03 |
| rs1005639 | TCGA&TCGA_coCNV | 6.58E-05 | 4.22E-04 |
| rs7589890 | TCGA&TCGA_coCNV | 4.17E-04 | 3.72E-04 |
| rs10981870 | TCGA | 4.84E-04 | #N/A |
| rs12271807 | TCGA&TCGA_coCNV | 4.86E-04 | 8.01E-05 |
| rs16867495 | TCGA&TCGA_coCNV | 4.86E-04 | 7.42E-04 |
| rs10956109 | TCGA&TCGA_coCNV | 4.86E-04 | 1.74E-03 |
| rs12010971 | TCGA | 4.87E-04 | #N/A |
| rs7917744 | TCGA | 4.88E-04 | #N/A |
| rs4871759 | TCGA&TCGA_coCNV | 4.88E-04 | 8.86E-03 |
| rs970970 | TCGA&TCGA_coCNV | 4.88E-04 | 4.56E-03 |
| rs4519176 | TCGA&TCGA_coCNV | 4.89E-04 | 1.61E-04 |
| rs4608933 | TCGA&TCGA_coCNV | 4.89E-04 | 4.44E-03 |
| rs2364813 | TCGA&TCGA_coCNV | 4.90E-04 | 2.08E-03 |
| rs7142486 | TCGA&TCGA_coCNV | 4.89E-04 | 3.04E-03 |
| rs2881016 | TCGA&TCGA_coCNV | 4.91E-04 | 1.54E-04 |
| rs11787149 | TCGA | 4.92E-04 | #N/A |
| rs12545777 | TCGA&TCGA_coCNV | 4.93E-04 | 1.53E-04 |
| rs17172706 | TCGA&TCGA_coCNV | 4.93E-04 | 8.62E-04 |
| rs17836349 | TCGA&TCGA_coCNV | 4.94E-04 | 1.67E-05 |
| rs10809600 | TCGA&TCGA_coCNV | 4.94E-04 | 1.01E-03 |
| rs10131251 | TCGA&TCGA_coCNV | 4.94E-04 | 2.54E-03 |
| rs41343844 | TCGA&TCGA_coCNV | 4.95E-04 | 1.88E-03 |
| rs1266249 | TCGA&TCGA_coCNV | 4.95E-04 | 4.08E-04 |
| rs2478518 | TCGA&TCGA_coCNV | 4.95E-04 | 1.32E-03 |
| rs609031 | TCGA&TCGA_coCNV | 4.96E-04 | 4.22E-03 |
| rs16982439 | TCGA&TCGA_coCNV | 4.97E-04 | 1.34E-04 |
| rs859601 | TCGA&TCGA_coCNV | 4.97E-04 | 3.32E-03 |
| rs4588420 | TCGA | 4.97E-04 | #N/A |
| rs16856655 | TCGA | 4.99E-04 | #N/A |
| rs9405506 | TCGA&TCGA_coCNV | 4.99E-04 | 5.18E-04 |

| rs3895615 | TCGA&TCGA_coCNV | 5.01E-04 | 5.05E-03 |
| --- | --- | --- | --- |
| rs7543338 | TCGA&TCGA_coCNV | 5.01E-04 | 7.07E-04 |
| rs1579198 | TCGA&TCGA_coCNV | 5.02E-04 | 5.19E-04 |
| rs4749307 | TCGA&TCGA_coCNV | 5.03E-04 | 4.90E-04 |
| rs5976918 | TCGA&TCGA_coCNV | 2.01E-04 | 1.25E-03 |
| rs12289619 | TCGA&TCGA_coCNV | 2.49E-04 | 3.82E-04 |
| rs2839509 | TCGA&TCGA_coCNV | 5.06E-04 | 5.70E-03 |
| rs6640864 | TCGA&TCGA_coCNV | 5.06E-04 | 1.10E-04 |
| rs11720464 | TCGA | 5.06E-04 | #N/A |
| rs10967311 | TCGA | 5.07E-04 | #N/A |
| rs12279047 | TCGA&TCGA_coCNV | 5.08E-04 | 1.49E-03 |
| rs11579616 | TCGA&TCGA_coCNV | 5.09E-04 | 1.52E-04 |
| rs6505890 | TCGA&TCGA_coCNV | 5.09E-04 | 1.49E-03 |
| rs285586 | TCGA | 5.11E-04 | #N/A |
| rs6994132 | TCGA&TCGA_coCNV | 5.11E-04 | 1.58E-04 |
| rs12079851 | TCGA&TCGA_coCNV | 5.11E-04 | 5.71E-05 |
| rs2205637 | TCGA&TCGA_coCNV | 5.13E-04 | 3.04E-04 |
| rs1249426 | TCGA | 5.13E-04 | #N/A |
| rs12918211 | TCGA&TCGA_coCNV | 5.14E-04 | 1.23E-03 |
| rs1583358 | TCGA&TCGA_coCNV | 5.15E-04 | 2.60E-03 |
| rs17259513 | TCGA | 5.15E-04 | #N/A |
| rs17735284 | TCGA&TCGA_coCNV | 5.15E-04 | 3.20E-03 |
| rs10800526 | TCGA&TCGA_coCNV | 5.15E-04 | 6.51E-04 |
| rs10960001 | TCGA&TCGA_coCNV | 5.15E-04 | 7.94E-03 |
| rs11228915 | TCGA&TCGA_coCNV | 5.18E-04 | 2.71E-05 |
| rs2897728 | TCGA&TCGA_coCNV | 5.18E-04 | 9.54E-03 |
| rs16880221 | TCGA&TCGA_coCNV | 5.20E-04 | 9.76E-03 |
| rs4654669 | TCGA&TCGA_coCNV | 5.20E-04 | 1.16E-04 |
| rs17047803 | TCGA&TCGA_coCNV | 5.21E-04 | 7.57E-05 |
| rs1465377 | TCGA&TCGA_coCNV | 5.22E-04 | 3.35E-04 |
| rs3800036 | TCGA&TCGA_coCNV | 5.22E-04 | 2.43E-03 |
| rs11873891 | TCGA&TCGA_coCNV | 5.23E-04 | 1.36E-03 |
| rs11006896 | TCGA&TCGA_coCNV | 5.24E-04 | 2.07E-03 |
| rs17048702 | TCGA&TCGA_coCNV | 5.24E-04 | 2.36E-03 |
| rs3136788 | TCGA | 5.25E-04 | #N/A |
| rs6654951 | TCGA&TCGA_coCNV | 5.25E-04 | 1.66E-05 |
| rs440746 | TCGA | 5.26E-04 | #N/A |
| rs11215811 | TCGA&TCGA_coCNV | 5.26E-04 | 5.01E-04 |
| rs1233817 | TCGA&TCGA_coCNV | 5.26E-04 | 1.84E-04 |
| rs17714969 | TCGA&TCGA_coCNV | 6.96E-05 | 2.31E-03 |
| rs1328138 | TCGA&TCGA_coCNV | 5.27E-04 | 4.97E-04 |
| rs10959915 | TCGA | 5.28E-04 | #N/A |
| rs2146238 | TCGA | 5.30E-04 | #N/A |
| rs6918990 | TCGA&TCGA_coCNV&SCANdb | 5.30E-04 | 5.12E-04 |
| rs10984843 | TCGA&TCGA_coCNV | 5.30E-04 | 1.95E-03 |
| rs17410426 | TCGA&TCGA_coCNV | 5.31E-04 | 4.59E-03 |

| rs7228388 | TCGA&TCGA_coCNV | 5.10E-04 | 4.54E-03 |
| --- | --- | --- | --- |
| rs2744462 | TCGA | 5.32E-04 | #N/A |
| rs9686966 | TCGA&TCGA_coCNV | 5.32E-04 | 6.90E-04 |
| rs6136625 | TCGA&TCGA_coCNV | 5.32E-04 | 8.18E-04 |
| rs2240189 | TCGA&TCGA_coCNV | 5.33E-04 | 5.45E-03 |
| rs11187721 | TCGA&TCGA_coCNV | 5.33E-04 | 9.53E-03 |
| rs2008776 | TCGA&TCGA_coCNV | 3.97E-04 | 1.24E-03 |
| rs7083688 | TCGA&TCGA_coCNV | 4.91E-04 | 1.25E-03 |
| rs12079284 | TCGA&TCGA_coCNV | 5.35E-04 | 8.05E-03 |
| rs2984359 | TCGA&TCGA_coCNV | 5.36E-04 | 2.19E-03 |
| rs10490666 | TCGA&TCGA_coCNV | 5.36E-04 | 1.74E-03 |
| rs7902125 | TCGA&TCGA_coCNV | 5.36E-04 | 3.51E-03 |
| rs16969914 | TCGA&TCGA_coCNV | 5.37E-04 | 1.08E-03 |
| rs10966281 | TCGA&TCGA_coCNV | 5.37E-04 | 2.59E-04 |
| rs1352727 | TCGA&TCGA_coCNV | 5.38E-04 | 8.45E-04 |
| rs12556126 | TCGA | 5.38E-04 | #N/A |
| rs7816670 | TCGA | 5.39E-04 | #N/A |
| rs2896902 | TCGA&TCGA_coCNV | 5.41E-04 | 2.02E-03 |
| rs261579 | TCGA&TCGA_coCNV | 3.67E-04 | 2.42E-03 |
| rs1379858 | TCGA&TCGA_coCNV | 5.42E-04 | 7.62E-04 |
| rs10888170 | TCGA&TCGA_coCNV | 5.42E-04 | 4.76E-03 |
| rs17059514 | TCGA&TCGA_coCNV | 5.45E-04 | 8.81E-03 |
| rs12254584 | TCGA&TCGA_coCNV | 5.45E-04 | 8.10E-03 |
| rs6546618 | TCGA&TCGA_coCNV | 5.46E-04 | 6.77E-03 |
| rs1040421 | TCGA&TCGA_coCNV | 5.46E-04 | 5.34E-04 |
| rs9296528 | TCGA&TCGA_coCNV | 5.47E-04 | 7.76E-03 |
| rs3169601 | TCGA&TCGA_coCNV | 5.47E-04 | 9.15E-04 |
| rs1566860 | TCGA&TCGA_coCNV | 5.48E-04 | 3.18E-04 |
| rs16959631 | TCGA&TCGA_coCNV | 5.48E-04 | 1.02E-03 |
| rs9393310 | TCGA&TCGA_coCNV | 5.48E-04 | 1.37E-03 |
| rs12380100 | TCGA | 5.49E-04 | #N/A |
| rs4745763 | TCGA&TCGA_coCNV | 5.50E-04 | 4.80E-03 |
| rs17836920 | TCGA | 5.51E-04 | #N/A |
| rs12613255 | TCGA&TCGA_coCNV | 5.51E-04 | 1.12E-03 |
| rs17075432 | TCGA&TCGA_coCNV | 5.51E-04 | 8.05E-04 |
| rs10052871 | TCGA&TCGA_coCNV | 5.51E-04 | 3.15E-04 |
| rs6947354 | TCGA&TCGA_coCNV | 5.53E-04 | 2.08E-03 |
| rs11806573 | TCGA&TCGA_coCNV | 6.05E-05 | 4.05E-04 |
| rs10113577 | TCGA&TCGA_coCNV | 5.54E-04 | 7.04E-05 |
| rs11210819 | TCGA&TCGA_coCNV | 5.54E-04 | 3.99E-03 |
| rs4895312 | TCGA | 5.54E-04 | #N/A |
| rs12142378 | TCGA&TCGA_coCNV | 5.54E-04 | 1.31E-03 |
| rs10457749 | TCGA&TCGA_coCNV | 5.56E-04 | 1.09E-03 |
| rs1607456 | TCGA&TCGA_coCNV | 5.56E-04 | 3.46E-03 |
| rs10516419 | TCGA | 5.57E-04 | #N/A |
| rs9787476 | TCGA | 5.57E-04 | #N/A |

| rs843082 | TCGA&TCGA_coCNV | 5.57E-04 | 3.17E-05 |
| --- | --- | --- | --- |
| rs3113502 | TCGA&TCGA_coCNV | 5.58E-04 | 6.11E-03 |
| rs4717540 | TCGA&TCGA_coCNV | 5.58E-04 | 1.69E-03 |
| rs205036 | TCGA&TCGA_coCNV | 5.59E-04 | 4.57E-03 |
| rs6991023 | TCGA&TCGA_coCNV | 5.60E-04 | 3.39E-03 |
| rs9816355 | TCGA&TCGA_coCNV | 5.61E-04 | 1.29E-03 |
| rs7059897 | TCGA&TCGA_coCNV | 5.63E-04 | 7.22E-05 |
| rs10986153 | TCGA&TCGA_coCNV | 5.63E-04 | 1.32E-04 |
| rs12584499 | TCGA&TCGA_coCNV | 5.63E-04 | 1.71E-03 |
| rs11232183 | TCGA&TCGA_coCNV | 5.64E-04 | 4.60E-04 |
| rs1218421 | TCGA&TCGA_coCNV | 5.65E-04 | 8.14E-04 |
| rs10809558 | TCGA | 5.66E-04 | #N/A |
| rs4867264 | TCGA&TCGA_coCNV | 5.66E-04 | 7.38E-03 |
| rs378437 | TCGA&TCGA_coCNV | 5.66E-04 | 1.56E-03 |
| rs6078533 | TCGA | 5.66E-04 | #N/A |
| rs17054763 | TCGA&TCGA_coCNV | 5.67E-04 | 1.16E-03 |
| rs10410339 | TCGA&TCGA_coCNV | 5.67E-04 | 2.19E-03 |
| rs17329541 | TCGA&TCGA_coCNV | 5.67E-04 | 6.01E-04 |
| rs2172509 | TCGA&TCGA_coCNV | 5.67E-04 | 2.43E-03 |
| rs205349 | TCGA&TCGA_coCNV | 5.68E-04 | 5.47E-03 |
| rs12567583 | TCGA&TCGA_coCNV | 5.69E-04 | 1.78E-03 |
| rs2943634 | TCGA&TCGA_coCNV | 5.70E-04 | 2.44E-03 |
| rs7870942 | TCGA | 5.70E-04 | #N/A |
| rs17317101 | TCGA&TCGA_coCNV | 5.70E-04 | 1.37E-03 |
| rs13075270 | TCGA&TCGA_coCNV | 5.72E-04 | 1.02E-04 |
| rs7032595 | TCGA | 5.72E-04 | #N/A |
| rs13420923 | TCGA&TCGA_coCNV | 5.73E-04 | 2.58E-03 |
| rs8020010 | TCGA&TCGA_coCNV | 5.73E-04 | 7.51E-03 |
| rs6910007 | TCGA&TCGA_coCNV | 5.73E-04 | 2.86E-03 |
| rs13268974 | TCGA | 5.73E-04 | #N/A |
| rs17172514 | TCGA&TCGA_coCNV | 5.74E-04 | 3.66E-03 |
| rs2027020 | TCGA&TCGA_coCNV | 5.74E-04 | 9.56E-04 |
| rs9868121 | TCGA&TCGA_coCNV | 5.74E-04 | 4.19E-03 |
| rs10915476 | TCGA&TCGA_coCNV | 5.76E-04 | 5.91E-03 |
| rs11791066 | TCGA&TCGA_coCNV | 5.77E-04 | 1.08E-04 |
| rs17700926 | TCGA | 5.77E-04 | #N/A |
| rs657820 | TCGA&TCGA_coCNV | 5.79E-04 | 2.60E-03 |
| rs7833852 | TCGA&TCGA_coCNV | 5.79E-04 | 1.94E-03 |
| rs9555651 | TCGA&TCGA_coCNV | 5.79E-04 | 6.52E-03 |
| rs8064466 | TCGA | 5.79E-04 | #N/A |
| rs41401551 | TCGA&TCGA_coCNV | 5.79E-04 | 3.97E-03 |
| rs2811600 | TCGA | 5.79E-04 | #N/A |
| rs1515099 | TCGA&TCGA_coCNV | 2.04E-04 | 7.57E-04 |
| rs6963669 | TCGA&TCGA_coCNV | 5.80E-04 | 4.61E-03 |
| rs17706458 | TCGA&TCGA_coCNV | 9.22E-05 | 5.65E-05 |
| rs2138270 | TCGA | 5.81E-04 | #N/A |

| rs16830446 | TCGA&TCGA_coCNV | 5.81E-04 | 7.07E-03 |
| --- | --- | --- | --- |
| rs2836307 | TCGA&TCGA_coCNV | 5.81E-04 | 1.56E-04 |
| rs11639522 | TCGA | 5.82E-04 | #N/A |
| rs4581844 | TCGA&TCGA_coCNV | 5.83E-04 | 8.52E-03 |
| rs6566554 | TCGA | 5.85E-04 | #N/A |
| rs3177137 | TCGA&TCGA_coCNV | 5.86E-04 | 7.07E-04 |
| rs17158477 | TCGA | 5.88E-04 | #N/A |
| rs6593739 | TCGA | 5.89E-04 | #N/A |
| rs4519342 | TCGA | 5.89E-04 | #N/A |
| rs11018324 | TCGA&TCGA_coCNV | 5.89E-04 | 1.14E-03 |
| rs4561770 | TCGA&TCGA_coCNV | 5.89E-04 | 2.88E-03 |
| rs9943596 | TCGA&TCGA_coCNV | 5.90E-04 | 6.64E-04 |
| rs2015621 | TCGA&TCGA_coCNV | 5.36E-05 | 2.36E-03 |
| rs10037537 | TCGA&TCGA_coCNV | 5.90E-04 | 2.13E-03 |
| rs12050227 | TCGA&TCGA_coCNV | 5.91E-04 | 1.55E-04 |
| rs16835011 | TCGA&TCGA_coCNV | 5.92E-04 | 5.63E-05 |
| rs6548964 | TCGA&TCGA_coCNV | 5.93E-04 | 2.32E-04 |
| rs9793263 | TCGA&TCGA_coCNV | 5.94E-04 | 9.61E-05 |
| rs4958687 | TCGA&TCGA_coCNV | 5.95E-04 | 5.80E-03 |
| rs9393289 | TCGA | 5.97E-04 | #N/A |
| rs17007531 | TCGA&TCGA_coCNV | 5.97E-04 | 5.30E-05 |
| rs10510121 | TCGA&TCGA_coCNV | 5.99E-04 | 4.36E-03 |
| rs1523691 | TCGA&TCGA_coCNV | 1.52E-05 | 1.12E-05 |
| rs5949642 | TCGA&TCGA_coCNV | 6.00E-04 | 7.30E-03 |
| rs7619872 | TCGA&TCGA_coCNV | 6.00E-04 | 5.22E-03 |
| rs17033340 | TCGA&TCGA_coCNV | 6.01E-04 | 4.15E-05 |
| rs254653 | TCGA&TCGA_coCNV | 6.01E-04 | 6.05E-03 |
| rs12095632 | TCGA&TCGA_coCNV | 6.01E-04 | 5.13E-03 |
| rs6134506 | TCGA&TCGA_coCNV | 6.02E-04 | 6.15E-04 |
| rs2076500 | TCGA&TCGA_coCNV | 6.03E-04 | 1.43E-04 |
| rs1160648 | TCGA&TCGA_coCNV | 6.04E-04 | 4.99E-03 |
| rs2022245 | TCGA&TCGA_coCNV | 6.05E-04 | 5.52E-03 |
| rs10460901 | TCGA&TCGA_coCNV | 2.57E-05 | 9.72E-05 |
| rs1798076 | TCGA&TCGA_coCNV | 6.05E-04 | 3.01E-03 |
| rs9837086 | TCGA&TCGA_coCNV | 6.06E-04 | 2.53E-03 |
| rs17684771 | TCGA | 6.06E-04 | #N/A |
| rs1864198 | TCGA&TCGA_coCNV | 6.07E-04 | 5.70E-04 |
| rs427585 | TCGA | 6.08E-04 | #N/A |
| rs6930922 | TCGA&TCGA_coCNV | 6.09E-04 | 4.80E-03 |
| rs17799481 | TCGA | 6.09E-04 | #N/A |
| rs3775378 | TCGA&TCGA_coCNV | 6.10E-04 | 1.33E-03 |
| rs12865613 | TCGA&TCGA_coCNV | 6.10E-04 | 1.27E-03 |
| rs13024020 | TCGA | 6.11E-04 | #N/A |
| rs6078524 | TCGA | 6.11E-04 | #N/A |
| rs7702029 | TCGA&TCGA_coCNV | 6.11E-04 | 5.04E-04 |
| rs1933990 | TCGA&TCGA_coCNV | 6.11E-04 | 1.30E-05 |

| rs2487345 | TCGA&TCGA_coCNV | 6.12E-04 | 4.53E-03 |
| --- | --- | --- | --- |
| rs260007 | TCGA&TCGA_coCNV | 6.13E-04 | 9.02E-04 |
| rs12145296 | TCGA&TCGA_coCNV | 6.14E-04 | 1.86E-03 |
| rs13249337 | TCGA&TCGA_coCNV | 6.15E-04 | 7.37E-04 |
| rs4867974 | TCGA | 6.15E-04 | #N/A |
| rs6746045 | TCGA&TCGA_coCNV | 6.15E-04 | 6.94E-03 |
| rs2673141 | TCGA&TCGA_coCNV | 6.16E-04 | 4.08E-04 |
| rs1980234 | TCGA&TCGA_coCNV | 6.16E-04 | 1.44E-04 |
| rs2111286 | TCGA&TCGA_coCNV | 6.16E-04 | 2.31E-03 |
| rs17094881 | TCGA | 6.17E-04 | #N/A |
| rs7711280 | TCGA&TCGA_coCNV | 6.18E-04 | 8.04E-03 |
| rs4574375 | TCGA&TCGA_coCNV | 6.19E-04 | 1.19E-03 |
| rs303851 | TCGA | 6.20E-04 | #N/A |
| rs2016597 | TCGA&TCGA_coCNV | 6.20E-04 | 6.33E-04 |
| rs252929 | TCGA&TCGA_coCNV | 6.20E-04 | 3.69E-04 |
| rs6954657 | TCGA&TCGA_coCNV | 6.21E-04 | 2.55E-05 |
| rs11795259 | TCGA | 6.21E-04 | #N/A |
| rs12217530 | TCGA | 4.08E-04 | #N/A |
| rs12048671 | TCGA&TCGA_coCNV | 6.22E-04 | 7.52E-03 |
| rs11955894 | TCGA&TCGA_coCNV | 6.22E-04 | 5.76E-03 |
| rs10961679 | TCGA&TCGA_coCNV | 6.22E-04 | 1.64E-03 |
| rs582100 | TCGA | 6.23E-04 | #N/A |
| rs11019217 | TCGA&TCGA_coCNV | 6.23E-04 | 4.74E-03 |
| rs12258655 | TCGA&TCGA_coCNV | 5.85E-05 | 8.87E-04 |
| rs1389600 | TCGA&TCGA_coCNV | 6.23E-04 | 1.88E-05 |
| rs16835198 | TCGA&TCGA_coCNV | 6.24E-04 | 2.87E-04 |
| rs7698500 | TCGA&TCGA_coCNV | 6.24E-04 | 1.97E-03 |
| rs1907336 | TCGA&TCGA_coCNV | 6.25E-04 | 6.96E-03 |
| rs10944770 | TCGA | 6.25E-04 | #N/A |
| rs4330756 | TCGA&TCGA_coCNV | 6.27E-04 | 8.87E-03 |
| rs7737362 | TCGA&TCGA_coCNV | 5.03E-04 | 1.14E-03 |
| rs1825926 | TCGA&TCGA_coCNV | 4.31E-04 | 7.58E-04 |
| rs2711896 | TCGA&TCGA_coCNV | 6.28E-04 | 4.61E-03 |
| rs10141340 | TCGA&TCGA_coCNV | 6.28E-04 | 4.31E-03 |
| rs6743627 | TCGA&TCGA_coCNV | 4.93E-04 | 5.69E-03 |
| rs4744369 | TCGA | 6.28E-04 | #N/A |
| rs4638625 | TCGA | 6.29E-04 | #N/A |
| rs2826174 | TCGA | 6.29E-04 | #N/A |
| rs11658445 | TCGA | 6.30E-04 | #N/A |
| rs1544237 | TCGA&TCGA_coCNV | 6.30E-04 | 5.97E-04 |
| rs11770758 | TCGA&TCGA_coCNV | 6.30E-04 | 1.38E-03 |
| rs17568665 | TCGA | 6.32E-04 | #N/A |
| rs17617046 | TCGA&TCGA_coCNV | 6.33E-04 | 9.22E-04 |
| rs1602754 | TCGA | 6.33E-04 | #N/A |
| rs669812 | TCGA&TCGA_coCNV | 6.34E-04 | 3.69E-03 |
| rs10035181 | TCGA&TCGA_coCNV | 6.35E-04 | 7.14E-03 |

| rs7206890 | TCGA&TCGA_coCNV | 6.35E-04 | 2.06E-03 |
| --- | --- | --- | --- |
| rs2294185 | TCGA&TCGA_coCNV | 6.35E-04 | 3.90E-03 |
| rs1010619 | TCGA | 6.36E-04 | #N/A |
| rs13412059 | TCGA | 6.37E-04 | #N/A |
| rs10756771 | TCGA | 6.38E-04 | #N/A |
| rs9262216 | TCGA&TCGA_coCNV | 6.40E-04 | 4.32E-03 |
| rs2647410 | TCGA | 3.94E-04 | #N/A |
| rs10490558 | TCGA&TCGA_coCNV | 3.70E-04 | 3.01E-05 |
| rs160399 | TCGA&TCGA_coCNV | 6.41E-04 | 3.48E-03 |
| rs4865080 | TCGA&TCGA_coCNV | 6.41E-04 | 2.30E-04 |
| rs2064216 | TCGA&TCGA_coCNV | 6.42E-04 | 1.17E-03 |
| rs16844406 | TCGA&TCGA_coCNV | 6.43E-04 | 9.77E-04 |
| rs2994809 | TCGA | 6.43E-04 | #N/A |
| rs11726534 | TCGA | 6.43E-04 | #N/A |
| rs17033021 | TCGA&TCGA_coCNV | 6.43E-04 | 9.33E-03 |
| rs7884617 | TCGA&TCGA_coCNV | 6.44E-04 | 1.27E-04 |
| rs12768615 | TCGA&TCGA_coCNV | 6.44E-04 | 7.30E-04 |
| rs9464832 | TCGA | 6.44E-04 | #N/A |
| rs16979902 | TCGA&TCGA_coCNV | 6.45E-04 | 1.21E-03 |
| rs1344650 | TCGA | 6.46E-04 | #N/A |
| rs12144976 | TCGA&TCGA_coCNV | 6.46E-04 | 2.15E-03 |
| rs1414215 | TCGA&TCGA_coCNV | 6.46E-04 | 8.08E-03 |
| rs13330853 | TCGA | 6.47E-04 | #N/A |
| rs7213743 | TCGA | 6.47E-04 | #N/A |
| rs12001263 | TCGA&TCGA_coCNV | 6.47E-04 | 2.06E-03 |
| rs9384293 | TCGA&TCGA_coCNV | 6.48E-04 | 4.90E-03 |
| rs1079008 | TCGA&TCGA_coCNV | 6.51E-04 | 3.10E-03 |
| rs6883496 | TCGA&TCGA_coCNV | 6.52E-04 | 9.68E-04 |
| rs2511868 | TCGA&TCGA_coCNV | 6.52E-04 | 3.46E-03 |
| rs7334240 | TCGA&TCGA_coCNV | 6.54E-04 | 2.13E-03 |
| rs3886695 | TCGA | 6.54E-04 | #N/A |
| rs3751151 | TCGA&TCGA_coCNV | 6.55E-04 | 3.11E-04 |
| rs12372334 | TCGA | 6.57E-04 | #N/A |
| rs11698353 | TCGA&TCGA_coCNV | 6.57E-04 | 1.28E-03 |
| rs1554327 | TCGA | 6.57E-04 | #N/A |
| rs7684643 | TCGA | 6.58E-04 | #N/A |
| rs2656986 | TCGA | 6.59E-04 | #N/A |
| rs9464721 | TCGA | 6.59E-04 | #N/A |
| rs2461481 | TCGA&TCGA_coCNV | 6.61E-04 | 3.46E-03 |
| rs4707561 | TCGA | 6.61E-04 | #N/A |
| rs7855819 | TCGA | 6.61E-04 | #N/A |
| rs12100446 | TCGA&TCGA_coCNV | 6.44E-04 | 2.72E-03 |
| rs16829395 | TCGA&TCGA_coCNV | 6.65E-04 | 2.55E-03 |
| rs6604869 | TCGA&TCGA_coCNV | 6.65E-04 | 2.95E-04 |
| rs10881991 | TCGA&TCGA_coCNV | 6.65E-04 | 7.37E-04 |
| rs1323949 | TCGA&TCGA_coCNV | 6.67E-04 | 1.76E-05 |

| rs2120790 | TCGA&TCGA_coCNV | 6.67E-04 | 9.25E-04 |
| --- | --- | --- | --- |
| rs12420681 | TCGA&TCGA_coCNV | 6.67E-04 | 2.74E-03 |
| rs11883583 | TCGA | 6.68E-04 | #N/A |
| rs909798 | TCGA&TCGA_coCNV | 6.69E-04 | 5.03E-03 |
| rs2860914 | TCGA | 6.69E-04 | #N/A |
| rs9360548 | TCGA | 6.69E-04 | #N/A |
| rs6640865 | TCGA&TCGA_coCNV | 6.70E-04 | 3.12E-04 |
| rs6714303 | TCGA&TCGA_coCNV | 6.71E-04 | 1.47E-03 |
| rs4883189 | TCGA&TCGA_coCNV | 6.72E-04 | 3.89E-03 |
| rs2283781 | TCGA&TCGA_coCNV | 6.73E-04 | 3.09E-04 |
| rs16940197 | TCGA | 6.74E-04 | #N/A |
| rs17401856 | TCGA&TCGA_coCNV | 6.74E-04 | 3.88E-04 |
| rs285708 | TCGA | 6.75E-04 | #N/A |
| rs2270528 | TCGA&TCGA_coCNV | 6.76E-04 | 3.78E-03 |
| rs1257641 | TCGA&TCGA_coCNV | 4.66E-04 | 1.67E-03 |
| rs7552331 | TCGA&TCGA_coCNV | 6.78E-04 | 5.93E-03 |
| rs12457651 | TCGA&TCGA_coCNV | 6.78E-04 | 5.15E-04 |
| rs7017252 | TCGA | 6.78E-04 | #N/A |
| rs17048558 | TCGA&TCGA_coCNV | 6.79E-04 | 3.21E-03 |
| rs7789574 | TCGA | 6.79E-04 | #N/A |
| rs1129179 | TCGA&TCGA_coCNV | 6.80E-04 | 2.32E-03 |
| rs9544679 | TCGA&TCGA_coCNV | 6.80E-04 | 3.99E-06 |
| rs6907838 | TCGA&TCGA_coCNV | 6.81E-04 | 1.16E-03 |
| rs11180981 | TCGA | 6.81E-04 | #N/A |
| rs4481224 | TCGA&TCGA_coCNV | 6.82E-04 | 4.20E-03 |
| rs9862923 | TCGA&TCGA_coCNV | 6.82E-04 | 9.65E-04 |
| rs7317786 | TCGA&TCGA_coCNV | 6.83E-04 | 6.61E-03 |
| rs11012433 | TCGA&TCGA_coCNV | 6.84E-04 | 7.43E-03 |
| rs11215251 | TCGA&TCGA_coCNV | 6.87E-04 | 1.28E-03 |
| rs2363768 | TCGA | 6.87E-04 | #N/A |
| rs2587814 | TCGA&TCGA_coCNV | 6.87E-04 | 6.60E-04 |
| rs6880527 | TCGA&TCGA_coCNV | 6.87E-04 | 1.19E-04 |
| rs4741173 | TCGA | 6.88E-04 | #N/A |
| rs36010441 | TCGA&TCGA_coCNV | 6.88E-04 | 3.44E-04 |
| rs3851204 | TCGA&TCGA_coCNV | 6.88E-04 | 7.16E-03 |
| rs12039857 | TCGA&TCGA_coCNV | 6.88E-04 | 5.29E-05 |
| rs7012644 | TCGA&TCGA_coCNV | 6.88E-04 | 5.95E-03 |
| rs1014136 | TCGA | 6.89E-04 | #N/A |
| rs12650367 | TCGA&TCGA_coCNV | 6.89E-04 | 1.49E-03 |
| rs1192686 | TCGA&TCGA_coCNV | 6.90E-04 | 1.40E-03 |
| rs1186839 | TCGA | 6.91E-04 | #N/A |
| rs6566620 | TCGA&TCGA_coCNV | 2.04E-04 | 2.37E-06 |
| rs753453 | TCGA&TCGA_coCNV | 6.92E-04 | 2.22E-05 |
| rs16957962 | TCGA&TCGA_coCNV | 6.92E-04 | 1.08E-03 |
| rs2964003 | TCGA&TCGA_coCNV | 6.93E-04 | 1.34E-03 |
| rs4968430 | TCGA&TCGA_coCNV | 6.93E-04 | 2.14E-03 |

| rs11203984 | TCGA&TCGA_coCNV | 6.94E-04 | 2.85E-03 |
| --- | --- | --- | --- |
| rs11109228 | TCGA&TCGA_coCNV | 6.95E-04 | 2.64E-03 |
| rs1881496 | TCGA&TCGA_coCNV | 6.95E-04 | 3.12E-03 |
| rs2176774 | TCGA&TCGA_coCNV | 6.95E-04 | 2.98E-04 |
| rs12641124 | TCGA&TCGA_coCNV | 6.95E-04 | 2.25E-03 |
| rs10911124 | TCGA&TCGA_coCNV | 6.96E-04 | 2.94E-03 |
| rs9410428 | TCGA | 6.96E-04 | #N/A |
| rs16886284 | TCGA&TCGA_coCNV | 6.97E-04 | 3.96E-03 |
| rs1502821 | TCGA&TCGA_coCNV | 6.97E-04 | 1.69E-03 |
| rs3829773 | TCGA&TCGA_coCNV | 6.97E-04 | 3.97E-03 |
| rs11152375 | TCGA&TCGA_coCNV | 6.99E-04 | 8.81E-05 |
| rs7430 | TCGA | 6.99E-04 | #N/A |
| rs9262218 | TCGA&TCGA_coCNV | 7.00E-04 | 4.94E-03 |
| rs11778257 | TCGA&TCGA_coCNV | 7.00E-04 | 7.43E-03 |
| rs10894623 | TCGA&TCGA_coCNV | 1.95E-04 | 4.87E-03 |
| rs9588040 | TCGA&TCGA_coCNV | 7.01E-04 | 1.18E-03 |
| rs1763398 | TCGA | 7.02E-04 | #N/A |
| rs353972 | TCGA&TCGA_coCNV | 7.02E-04 | 5.79E-04 |
| rs16975014 | TCGA&TCGA_coCNV | 2.28E-04 | 3.79E-04 |
| rs9884478 | TCGA&TCGA_coCNV | 7.02E-04 | 1.47E-03 |
| rs1192680 | TCGA&TCGA_coCNV | 7.02E-04 | 1.46E-03 |
| rs4865116 | TCGA&TCGA_coCNV | 7.03E-04 | 2.62E-03 |
| rs10805076 | TCGA | 7.03E-04 | #N/A |
| rs6871181 | TCGA&TCGA_coCNV | 7.03E-04 | 1.16E-03 |
| rs4682329 | TCGA&TCGA_coCNV | 1.40E-04 | 1.07E-04 |
| rs1399626 | TCGA&TCGA_coCNV | 2.76E-04 | 9.55E-04 |
| rs17509537 | TCGA&TCGA_coCNV | 7.04E-04 | 9.77E-03 |
| rs3803982 | TCGA&TCGA_coCNV | 7.04E-04 | 4.50E-05 |
| rs4909033 | TCGA&TCGA_coCNV | 7.04E-04 | 2.17E-04 |
| rs11250574 | TCGA&TCGA_coCNV | 7.04E-04 | 5.24E-03 |
| rs6628107 | TCGA&TCGA_coCNV | 5.24E-04 | 1.21E-04 |
| rs1774554 | TCGA | 7.05E-04 | #N/A |
| rs17015268 | TCGA&TCGA_coCNV | 7.06E-04 | 9.46E-04 |
| rs10833350 | TCGA&TCGA_coCNV | 7.08E-04 | 2.00E-03 |
| rs1935385 | TCGA&TCGA_coCNV | 7.08E-04 | 7.12E-04 |
| rs11179065 | TCGA&TCGA_coCNV | 7.10E-04 | 1.05E-03 |
| rs10061845 | TCGA | 7.10E-04 | #N/A |
| rs1560772 | TCGA&TCGA_coCNV | 7.11E-04 | 3.96E-03 |
| rs6875010 | TCGA&TCGA_coCNV | 7.12E-04 | 4.81E-04 |
| rs10789970 | TCGA&TCGA_coCNV | 7.12E-04 | 9.37E-03 |
| rs17726865 | TCGA&TCGA_coCNV | 7.12E-04 | 6.42E-03 |
| rs900755 | TCGA&TCGA_coCNV | 7.12E-04 | 8.92E-03 |
| rs4850305 | TCGA&TCGA_coCNV | 2.89E-04 | 1.97E-03 |
| rs1861772 | TCGA&TCGA_coCNV | 7.14E-04 | 2.31E-03 |
| rs17135365 | TCGA&TCGA_coCNV | 7.14E-04 | 9.82E-04 |
| rs16839333 | TCGA | 7.14E-04 | #N/A |

| rs6069673 | TCGA&TCGA_coCNV | 7.15E-04 | 1.07E-04 |
| --- | --- | --- | --- |
| rs6692923 | TCGA&TCGA_coCNV | 7.16E-04 | 8.49E-04 |
| rs3206576 | TCGA | 7.18E-04 | #N/A |
| rs12579153 | TCGA&TCGA_coCNV | 7.18E-04 | 7.64E-05 |
| rs11724732 | TCGA&TCGA_coCNV | 7.18E-04 | 5.34E-03 |
| rs7917903 | TCGA&TCGA_coCNV | 7.18E-04 | 2.71E-03 |
| rs17098433 | TCGA&TCGA_coCNV | 7.19E-04 | 7.21E-04 |
| rs761557 | TCGA&TCGA_coCNV | 7.19E-04 | 4.18E-03 |
| rs5975125 | TCGA&TCGA_coCNV | 5.64E-04 | 1.48E-04 |
| rs4689122 | TCGA&TCGA_coCNV | 7.20E-04 | 3.71E-03 |
| rs12644678 | TCGA | 7.20E-04 | #N/A |
| rs2955160 | TCGA&TCGA_coCNV | 7.20E-04 | 3.78E-04 |
| rs1341643 | TCGA&TCGA_coCNV | 7.21E-04 | 1.22E-04 |
| rs12713551 | TCGA&TCGA_coCNV | 7.22E-04 | 7.18E-03 |
| rs1559017 | TCGA | 7.22E-04 | #N/A |
| rs17049765 | TCGA&TCGA_coCNV | 7.22E-04 | 9.44E-03 |
| rs6803445 | TCGA&TCGA_coCNV | 7.23E-04 | 2.86E-03 |
| rs11885046 | TCGA&TCGA_coCNV | 7.24E-04 | 1.55E-04 |
| rs11263287 | TCGA&TCGA_coCNV | 7.24E-04 | 6.14E-03 |
| rs9964535 | TCGA&TCGA_coCNV | 7.28E-04 | 7.68E-03 |
| rs10259090 | TCGA&TCGA_coCNV | 7.28E-04 | 7.21E-03 |
| rs11891876 | TCGA | 7.30E-04 | #N/A |
| rs2075005 | TCGA | 7.31E-04 | #N/A |
| rs2715483 | TCGA&TCGA_coCNV | 7.31E-04 | 3.07E-03 |
| rs6760279 | TCGA&TCGA_coCNV | 6.57E-04 | 3.97E-03 |
| rs2726887 | TCGA | 7.31E-04 | #N/A |
| rs1607136 | TCGA&TCGA_coCNV | 7.33E-04 | 1.19E-03 |
| rs10000268 | TCGA&TCGA_coCNV | 7.36E-04 | 8.78E-06 |
| rs917580 | TCGA&TCGA_coCNV | 7.36E-04 | 4.05E-04 |
| rs9657690 | TCGA&TCGA_coCNV | 7.36E-04 | 2.42E-04 |
| rs6588391 | TCGA | 7.36E-04 | #N/A |
| rs13421607 | TCGA&TCGA_coCNV | 7.37E-04 | 4.11E-05 |
| rs10505695 | TCGA&TCGA_coCNV | 7.37E-04 | 7.08E-03 |
| rs1186834 | TCGA | 7.38E-04 | #N/A |
| rs8090284 | TCGA&TCGA_coCNV | 7.39E-04 | 4.39E-03 |
| rs1995537 | TCGA&TCGA_coCNV | 7.39E-04 | 2.84E-03 |
| rs2829353 | TCGA | 7.39E-04 | #N/A |
| rs7714008 | TCGA&TCGA_coCNV | 7.39E-04 | 6.76E-03 |
| rs1270053 | TCGA&TCGA_coCNV | 7.40E-04 | 8.94E-04 |
| rs2597519 | TCGA&TCGA_coCNV | 5.51E-04 | 1.73E-04 |
| rs16900004 | TCGA | 7.41E-04 | #N/A |
| rs16927706 | TCGA&TCGA_coCNV | 7.41E-04 | 2.05E-04 |
| rs138531 | TCGA&TCGA_coCNV | 7.41E-04 | 5.31E-05 |
| rs2277615 | TCGA&TCGA_coCNV | 7.43E-04 | 3.14E-03 |
| rs10052344 | TCGA&TCGA_coCNV | 7.44E-04 | 7.30E-03 |
| rs7706147 | TCGA&TCGA_coCNV | 7.44E-04 | 3.52E-03 |

| rs12637657 | TCGA&TCGA_coCNV | 7.45E-04 | 1.11E-03 |
| --- | --- | --- | --- |
| rs2934857 | TCGA&TCGA_coCNV | 7.46E-04 | 2.88E-03 |
| rs17592094 | TCGA&TCGA_coCNV | 7.46E-04 | 5.64E-08 |
| rs16859905 | TCGA&TCGA_coCNV | 2.11E-04 | 8.69E-04 |
| rs9297197 | TCGA&TCGA_coCNV | 7.47E-04 | 1.35E-03 |
| rs247957 | TCGA&TCGA_coCNV | 7.48E-04 | 6.19E-04 |
| rs11800749 | TCGA&TCGA_coCNV | 7.48E-04 | 5.84E-03 |
| rs4884747 | TCGA | 7.48E-04 | #N/A |
| rs17038192 | TCGA&TCGA_coCNV | 7.49E-04 | 2.70E-03 |
| rs2074950 | TCGA&TCGA_coCNV | 7.50E-04 | 2.07E-03 |
| rs10882591 | TCGA&TCGA_coCNV | 7.50E-04 | 8.47E-03 |
| rs2577711 | TCGA&TCGA_coCNV | 7.50E-04 | 2.73E-03 |
| rs16897153 | TCGA&TCGA_coCNV | 7.51E-04 | 2.04E-03 |
| rs7820520 | TCGA | 7.51E-04 | #N/A |
| rs6915078 | TCGA&TCGA_coCNV | 7.51E-04 | 3.55E-04 |
| rs1075534 | TCGA | 7.51E-04 | #N/A |
| rs6520296 | TCGA&TCGA_coCNV | 7.51E-04 | 3.30E-04 |
| rs10121195 | TCGA&TCGA_coCNV | 7.52E-04 | 1.56E-04 |
| rs17125750 | TCGA&TCGA_coCNV | 1.04E-04 | 6.64E-05 |
| rs8120300 | TCGA&TCGA_coCNV | 7.52E-04 | 5.52E-03 |
| rs9388899 | TCGA&TCGA_coCNV | 7.53E-04 | 2.94E-04 |
| rs12894405 | TCGA&TCGA_coCNV | 7.53E-04 | 8.10E-04 |
| rs17066154 | TCGA | 7.54E-04 | #N/A |
| rs4838596 | TCGA | 7.55E-04 | #N/A |
| rs11720066 | TCGA&TCGA_coCNV | 7.55E-04 | 7.78E-05 |
| rs612300 | TCGA&TCGA_coCNV | 7.56E-04 | 5.11E-04 |
| rs7794500 | TCGA&TCGA_coCNV | 7.58E-04 | 1.99E-03 |
| rs17793574 | TCGA&TCGA_coCNV | 7.58E-04 | 4.08E-04 |
| rs458739 | TCGA | 2.31E-05 | #N/A |
| rs301665 | TCGA&TCGA_coCNV | 7.59E-04 | 6.53E-03 |
| rs1479353 | TCGA | 7.59E-04 | #N/A |
| rs2372571 | TCGA&TCGA_coCNV | 7.59E-04 | 3.07E-03 |
| rs16852801 | TCGA&TCGA_coCNV | 7.59E-04 | 2.35E-03 |
| rs17148521 | TCGA&TCGA_coCNV | 7.60E-04 | 5.25E-03 |
| rs10253805 | TCGA&TCGA_coCNV | 2.10E-04 | 5.04E-03 |
| rs4942703 | TCGA | 7.60E-04 | #N/A |
| rs2407887 | TCGA&TCGA_coCNV | 7.60E-04 | 5.34E-03 |
| rs17724508 | TCGA | 7.60E-04 | #N/A |
| rs2084732 | TCGA&TCGA_coCNV | 7.62E-04 | 1.71E-03 |
| rs1513895 | TCGA&TCGA_coCNV | 7.62E-04 | 1.09E-03 |
| rs825669 | TCGA | 7.63E-04 | #N/A |
| rs4867709 | TCGA | 7.63E-04 | #N/A |
| rs1024970 | TCGA&TCGA_coCNV | 7.64E-04 | 9.13E-03 |
| rs13263101 | TCGA | 7.66E-04 | #N/A |
| rs12967106 | TCGA&TCGA_coCNV | 7.68E-04 | 4.53E-04 |
| rs7253742 | TCGA&TCGA_coCNV | 7.68E-04 | 8.12E-03 |

| rs10045397 | TCGA | 7.70E-04 | #N/A |
| --- | --- | --- | --- |
| rs596631 | TCGA | 7.70E-04 | #N/A |
| rs7724949 | TCGA&TCGA_coCNV | 7.71E-04 | 7.31E-04 |
| rs17281180 | TCGA&TCGA_coCNV | 7.71E-04 | 6.63E-04 |
| rs33460 | TCGA&TCGA_coCNV | 7.71E-04 | 2.14E-03 |
| rs13048676 | TCGA&TCGA_coCNV | 7.72E-04 | 4.15E-03 |
| rs1587420 | TCGA&TCGA_coCNV | 7.72E-04 | 2.93E-03 |
| rs1834332 | TCGA | 7.73E-04 | #N/A |
| rs7212143 | TCGA&TCGA_coCNV | 7.74E-04 | 4.76E-04 |
| rs1333145 | TCGA&TCGA_coCNV | 7.74E-04 | 1.79E-04 |
| rs2473229 | TCGA&TCGA_coCNV | 7.75E-04 | 6.65E-03 |
| rs3113508 | TCGA&TCGA_coCNV | 7.75E-04 | 8.95E-03 |
| rs12416668 | TCGA&TCGA_coCNV | 7.76E-04 | 2.36E-04 |
| rs138541 | TCGA&TCGA_coCNV | 7.76E-04 | 1.32E-04 |
| rs1567461 | TCGA&TCGA_coCNV | 4.35E-04 | 3.81E-03 |
| rs10871321 | TCGA | 7.77E-04 | #N/A |
| rs7643394 | TCGA&TCGA_coCNV | 7.79E-04 | 4.71E-04 |
| rs16934493 | TCGA&TCGA_coCNV | 7.79E-04 | 9.57E-05 |
| rs10456773 | TCGA&TCGA_coCNV | 7.81E-04 | 7.09E-03 |
| rs4242415 | TCGA&TCGA_coCNV | 7.81E-04 | 2.39E-04 |
| rs4750687 | TCGA&TCGA_coCNV | 7.81E-04 | 1.65E-04 |
| rs10122903 | TCGA&TCGA_coCNV | 7.83E-04 | 4.40E-03 |
| rs10868680 | TCGA | 7.83E-04 | #N/A |
| rs17062349 | TCGA | 7.84E-04 | #N/A |
| rs153728 | TCGA | 7.84E-04 | #N/A |
| rs1370171 | TCGA&TCGA_coCNV | 7.85E-04 | 6.34E-03 |
| rs11196284 | TCGA&TCGA_coCNV | 4.76E-05 | 7.96E-06 |
| rs6867227 | TCGA&TCGA_coCNV | 7.86E-04 | 8.17E-03 |
| rs2193028 | TCGA | 7.86E-04 | #N/A |
| rs16929851 | TCGA&TCGA_coCNV | 7.86E-04 | 2.30E-03 |
| rs17636683 | TCGA&TCGA_coCNV | 7.87E-04 | 4.51E-04 |
| rs17836419 | TCGA&TCGA_coCNV | 7.88E-04 | 9.03E-05 |
| rs11723176 | TCGA | 7.88E-04 | #N/A |
| rs4955627 | TCGA&TCGA_coCNV | 7.90E-04 | 7.84E-03 |
| rs2039547 | TCGA&TCGA_coCNV | 7.90E-04 | 9.37E-03 |
| rs11725292 | TCGA | 7.91E-04 | #N/A |
| rs4701031 | TCGA | 7.92E-04 | #N/A |
| rs7953708 | TCGA | 7.93E-04 | #N/A |
| rs16962992 | TCGA | 7.94E-04 | #N/A |
| rs11103480 | TCGA&TCGA_coCNV | 7.95E-04 | 4.43E-04 |
| rs1926170 | TCGA&TCGA_coCNV | 7.95E-04 | 1.13E-03 |
| rs11023210 | TCGA&TCGA_coCNV | 7.96E-04 | 2.05E-05 |
| rs10959753 | TCGA | 7.96E-04 | #N/A |
| rs11955735 | TCGA&TCGA_coCNV | 7.96E-04 | 3.21E-03 |
| rs6640067 | TCGA&TCGA_coCNV | 7.96E-04 | 2.14E-04 |
| rs1608973 | TCGA&TCGA_coCNV | 7.97E-04 | 3.29E-03 |

| rs1186857 | TCGA | 7.97E-04 | #N/A |
| --- | --- | --- | --- |
| rs16894153 | TCGA | 7.98E-04 | #N/A |
| rs12465442 | TCGA&TCGA_coCNV | 7.98E-04 | 2.12E-03 |
| rs16966229 | TCGA&TCGA_coCNV | 7.99E-04 | 5.51E-03 |
| rs2671692 | TCGA | 7.99E-04 | #N/A |
| rs5979326 | TCGA&TCGA_coCNV | 8.00E-04 | 7.61E-04 |
| rs1815687 | TCGA | 8.01E-04 | #N/A |
| rs2973766 | TCGA&TCGA_coCNV | 8.02E-04 | 4.02E-04 |
| rs2972136 | TCGA&TCGA_coCNV | 4.23E-04 | 9.20E-04 |
| rs12898472 | TCGA&TCGA_coCNV | 6.07E-04 | 9.70E-03 |
| rs12596771 | TCGA&TCGA_coCNV | 8.06E-04 | 7.89E-05 |
| rs303854 | TCGA | 8.06E-04 | #N/A |
| rs11002433 | TCGA&TCGA_coCNV | 8.06E-04 | 1.58E-03 |
| rs910391 | TCGA&TCGA_coCNV | 9.25E-06 | 1.13E-04 |
| rs10008736 | TCGA&TCGA_coCNV | 8.07E-04 | 9.14E-04 |
| rs4548845 | TCGA&TCGA_coCNV | 8.09E-04 | 2.43E-03 |
| rs41476944 | TCGA&TCGA_coCNV | 8.10E-04 | 9.76E-03 |
| rs909481 | TCGA&TCGA_coCNV | 8.10E-04 | 8.66E-03 |
| rs331608 | TCGA&TCGA_coCNV | 4.85E-04 | 7.96E-04 |
| rs10417304 | TCGA&TCGA_coCNV | 8.12E-04 | 7.94E-03 |
| rs17160093 | TCGA | 6.58E-06 | #N/A |
| rs7198605 | TCGA&TCGA_coCNV | 8.13E-04 | 1.12E-04 |
| rs12522495 | TCGA&TCGA_coCNV | 8.13E-04 | 8.48E-03 |
| rs10880081 | TCGA&TCGA_coCNV | 8.13E-04 | 3.13E-03 |
| rs9904656 | TCGA&TCGA_coCNV | 2.16E-04 | 4.37E-05 |
| rs2223198 | TCGA&TCGA_coCNV | 8.14E-04 | 3.77E-03 |
| rs6529391 | TCGA&TCGA_coCNV | 8.14E-04 | 9.72E-04 |
| rs12467166 | TCGA&TCGA_coCNV | 8.15E-04 | 3.08E-03 |
| rs12535115 | TCGA&TCGA_coCNV | 8.16E-04 | 1.32E-03 |
| rs16878852 | TCGA | 8.17E-04 | #N/A |
| rs6722824 | TCGA&TCGA_coCNV | 8.17E-04 | 2.63E-03 |
| rs9557247 | TCGA&TCGA_coCNV | 8.18E-04 | 3.74E-03 |
| rs133937 | TCGA&TCGA_coCNV | 3.09E-04 | 2.93E-03 |
| rs160400 | TCGA&TCGA_coCNV | 8.18E-04 | 5.36E-03 |
| rs9515353 | TCGA | 8.18E-04 | #N/A |
| rs1894466 | TCGA&TCGA_coCNV | 8.18E-04 | 1.56E-04 |
| rs11115091 | TCGA&TCGA_coCNV | 8.19E-04 | 3.36E-04 |
| rs2052573 | TCGA&TCGA_coCNV | 8.20E-04 | 2.31E-03 |
| rs16836429 | TCGA&TCGA_coCNV | 8.21E-04 | 2.84E-04 |
| rs17137044 | TCGA&TCGA_coCNV | 8.21E-04 | 5.25E-04 |
| rs284512 | TCGA | 8.22E-04 | #N/A |
| rs833283 | TCGA&TCGA_coCNV | 8.24E-04 | 4.74E-03 |
| rs11845128 | TCGA&TCGA_coCNV | 8.24E-04 | 9.18E-03 |
| rs6974217 | TCGA&TCGA_coCNV | 8.28E-04 | 2.87E-03 |
| rs11763878 | TCGA&TCGA_coCNV | 1.71E-05 | 1.30E-03 |
| rs12472029 | TCGA&TCGA_coCNV | 8.28E-04 | 8.24E-05 |

| rs17655367 | TCGA&TCGA_coCNV | 8.30E-04 | 6.69E-03 |
| --- | --- | --- | --- |
| rs2295705 | TCGA&TCGA_coCNV | 8.31E-04 | 6.80E-04 |
| rs16905011 | TCGA | 8.31E-04 | #N/A |
| rs7139689 | TCGA | 8.31E-04 | #N/A |
| rs16826839 | TCGA&TCGA_coCNV | 8.31E-04 | 8.85E-04 |
| rs12687202 | TCGA&TCGA_coCNV | 8.31E-04 | 1.18E-03 |
| rs1495239 | TCGA&TCGA_coCNV | 8.33E-04 | 4.07E-03 |
| rs12432334 | TCGA&TCGA_coCNV | 4.96E-05 | 7.16E-04 |
| rs2505609 | TCGA | 8.35E-04 | #N/A |
| rs2665544 | TCGA&TCGA_coCNV | 8.35E-04 | 9.11E-03 |
| rs16925189 | TCGA&TCGA_coCNV | 8.36E-04 | 7.64E-05 |
| rs2121121 | TCGA&TCGA_coCNV | 8.38E-04 | 1.34E-03 |
| rs7251046 | TCGA&TCGA_coCNV | 8.38E-04 | 3.33E-04 |
| rs12350378 | TCGA&TCGA_coCNV | 8.38E-04 | 1.20E-03 |
| rs4239380 | TCGA&TCGA_coCNV | 8.38E-04 | 1.26E-03 |
| rs3927458 | TCGA&TCGA_coCNV | 8.39E-04 | 1.03E-03 |
| rs3777908 | TCGA | 8.39E-04 | #N/A |
| rs1828187 | TCGA&TCGA_coCNV | 8.40E-04 | 2.83E-03 |
| rs17734832 | TCGA&TCGA_coCNV | 8.40E-04 | 9.57E-05 |
| rs12807233 | TCGA&TCGA_coCNV | 8.40E-04 | 3.01E-03 |
| rs1111114 | TCGA | 8.40E-04 | #N/A |
| rs333327 | TCGA&TCGA_coCNV | 8.40E-04 | 1.88E-03 |
| rs2578187 | TCGA&TCGA_coCNV | 8.42E-04 | 3.32E-03 |
| rs946131 | TCGA&TCGA_coCNV | 8.44E-04 | 8.28E-03 |
| rs13221837 | TCGA&TCGA_coCNV | 8.44E-04 | 1.98E-03 |
| rs2264117 | TCGA&TCGA_coCNV | 8.44E-04 | 8.67E-03 |
| rs3931460 | TCGA&TCGA_coCNV | 8.44E-04 | 6.09E-03 |
| rs2154490 | TCGA&TCGA_coCNV | 8.45E-04 | 8.95E-03 |
| rs16921061 | TCGA&TCGA_coCNV | 8.46E-04 | 6.05E-03 |
| rs7315231 | TCGA&TCGA_coCNV | 8.49E-04 | 2.29E-03 |
| rs8055572 | TCGA&TCGA_coCNV | 8.49E-04 | 1.67E-03 |
| rs2238926 | TCGA | 8.50E-04 | #N/A |
| rs34151263 | TCGA&TCGA_coCNV | 8.51E-04 | 1.68E-04 |
| rs10236274 | TCGA&TCGA_coCNV | 8.51E-04 | 4.07E-04 |
| rs400690 | TCGA&TCGA_coCNV | 8.51E-04 | 1.51E-03 |
| rs1837447 | TCGA&TCGA_coCNV | 8.52E-04 | 3.40E-03 |
| rs12530946 | TCGA&TCGA_coCNV | 8.53E-04 | 3.40E-03 |
| rs6780434 | TCGA&TCGA_coCNV | 8.53E-04 | 8.68E-03 |
| rs2647404 | TCGA | 8.55E-04 | #N/A |
| rs1596398 | TCGA&TCGA_coCNV | 8.57E-04 | 5.26E-03 |
| rs1336465 | TCGA&TCGA_coCNV | 8.58E-04 | 2.44E-03 |
| rs925190 | TCGA&TCGA_coCNV | 8.59E-04 | 4.77E-05 |
| rs17023075 | TCGA&TCGA_coCNV | 8.59E-04 | 2.31E-04 |
| rs17561228 | TCGA | 8.62E-04 | #N/A |
| rs7705083 | TCGA | 8.63E-04 | #N/A |
| rs7097768 | TCGA&TCGA_coCNV | 8.64E-04 | 3.24E-04 |

| rs12540094 | TCGA&TCGA_coCNV | 8.66E-04 | 7.37E-03 |
| --- | --- | --- | --- |
| rs16975861 | TCGA&TCGA_coCNV | 8.67E-04 | 5.31E-03 |
| rs16960489 | TCGA&TCGA_coCNV | 8.69E-04 | 4.43E-03 |
| rs7990457 | TCGA&TCGA_coCNV | 8.70E-04 | 6.22E-05 |
| rs6885285 | TCGA&TCGA_coCNV | 6.95E-05 | 3.60E-04 |
| rs17400240 | TCGA&TCGA_coCNV | 8.71E-04 | 8.32E-03 |
| rs10041141 | TCGA&TCGA_coCNV | 8.71E-04 | 9.08E-03 |
| rs9531855 | TCGA&TCGA_coCNV | 8.72E-04 | 8.99E-04 |
| rs10875170 | TCGA&TCGA_coCNV | 8.72E-04 | 4.82E-03 |
| rs14151 | TCGA&TCGA_coCNV | 8.73E-04 | 3.13E-03 |
| rs16844885 | TCGA | 8.75E-04 | #N/A |
| rs1496961 | TCGA&TCGA_coCNV | 6.84E-04 | 7.64E-03 |
| rs12522239 | TCGA&TCGA_coCNV | 8.76E-04 | 1.80E-03 |
| rs9899116 | TCGA&TCGA_coCNV | 8.77E-04 | 8.01E-04 |
| rs9874501 | TCGA&TCGA_coCNV | 8.78E-04 | 2.12E-03 |
| rs10506421 | TCGA&TCGA_coCNV | 8.79E-04 | 6.58E-03 |
| rs10277756 | TCGA&TCGA_coCNV | 8.79E-04 | 2.38E-03 |
| rs8035872 | TCGA | 8.80E-04 | #N/A |
| rs969997 | TCGA&TCGA_coCNV | 2.51E-04 | 1.25E-04 |
| rs2955163 | TCGA&TCGA_coCNV | 8.82E-04 | 4.02E-04 |
| rs843078 | TCGA&TCGA_coCNV | 8.82E-04 | 1.29E-04 |
| rs7943556 | TCGA&TCGA_coCNV | 8.83E-04 | 1.89E-03 |
| rs1287173 | TCGA&TCGA_coCNV | 8.84E-04 | 5.83E-03 |
| rs10475816 | TCGA&TCGA_coCNV | 8.84E-04 | 1.31E-03 |
| rs1373250 | TCGA&TCGA_coCNV | 8.85E-04 | 6.20E-05 |
| rs11164339 | TCGA&TCGA_coCNV | 8.86E-04 | 2.85E-03 |
| rs10189295 | TCGA&TCGA_coCNV | 8.86E-04 | 7.47E-03 |
| rs1967424 | TCGA&TCGA_coCNV | 8.87E-04 | 2.47E-03 |
| rs4533166 | TCGA&TCGA_coCNV | 8.87E-04 | 4.58E-03 |
| rs3807541 | TCGA | 8.91E-04 | #N/A |
| rs4703858 | TCGA&TCGA_coCNV | 5.00E-04 | 1.96E-03 |
| rs2911428 | TCGA&TCGA_coCNV | 8.92E-04 | 1.31E-03 |
| rs13097357 | TCGA&TCGA_coCNV | 8.94E-04 | 3.70E-03 |
| rs11255671 | TCGA&TCGA_coCNV | 8.94E-04 | 6.96E-04 |
| rs3744695 | TCGA&TCGA_coCNV | 8.95E-04 | 7.00E-05 |
| rs11681155 | TCGA&TCGA_coCNV | 8.96E-04 | 1.52E-03 |
| rs870361 | TCGA | 8.97E-04 | #N/A |
| rs16942760 | TCGA&TCGA_coCNV | 8.98E-04 | 5.04E-03 |
| rs6078518 | TCGA | 8.98E-04 | #N/A |
| rs7719344 | TCGA | 8.98E-04 | #N/A |
| rs16943161 | TCGA | 8.99E-04 | #N/A |
| rs12452924 | TCGA&TCGA_coCNV | 8.99E-04 | 1.61E-04 |
| rs1515100 | TCGA&TCGA_coCNV | 3.76E-04 | 4.59E-04 |
| rs2065660 | TCGA&TCGA_coCNV | 8.99E-04 | 1.49E-03 |
| rs9410430 | TCGA&TCGA_coCNV | 9.00E-04 | 8.67E-03 |
| rs446551 | TCGA&TCGA_coCNV | 9.02E-04 | 8.77E-03 |

| rs10818700 | TCGA | 9.02E-04 | #N/A |
| --- | --- | --- | --- |
| rs10788010 | TCGA&TCGA_coCNV | 1.84E-04 | 5.54E-04 |
| rs520257 | TCGA&TCGA_coCNV | 9.02E-04 | 8.59E-04 |
| rs9466090 | TCGA&TCGA_coCNV | 9.02E-04 | 6.18E-04 |
| rs1542755 | TCGA&TCGA_coCNV | 9.04E-04 | 1.46E-03 |
| rs7566976 | TCGA | 9.04E-04 | #N/A |
| rs1403755 | TCGA | 9.04E-04 | #N/A |
| rs823530 | TCGA | 9.04E-04 | #N/A |
| rs6991512 | TCGA | 9.05E-04 | #N/A |
| rs739903 | TCGA | 9.05E-04 | #N/A |
| rs12309292 | TCGA | 9.05E-04 | #N/A |
| rs1359490 | TCGA | 9.05E-04 | #N/A |
| rs805754 | TCGA&TCGA_coCNV | 9.06E-04 | 1.69E-03 |
| rs17865274 | TCGA&TCGA_coCNV | 9.06E-04 | 3.01E-03 |
| rs9648516 | TCGA&TCGA_coCNV | 9.07E-04 | 1.49E-03 |
| rs7100550 | TCGA&TCGA_coCNV | 9.08E-04 | 1.68E-03 |
| rs10760670 | TCGA&TCGA_coCNV | 9.08E-04 | 2.48E-03 |
| rs16946278 | TCGA&TCGA_coCNV | 9.09E-04 | 3.82E-03 |
| rs832075 | TCGA&TCGA_coCNV | 9.10E-04 | 5.42E-04 |
| rs3747343 | TCGA&TCGA_coCNV | 9.10E-04 | 1.16E-04 |
| rs11825213 | TCGA | 9.11E-04 | #N/A |
| rs12552958 | TCGA&TCGA_coCNV | 9.11E-04 | 7.52E-05 |
| rs13325429 | TCGA | 9.12E-04 | #N/A |
| rs12610504 | TCGA&TCGA_coCNV | 9.12E-04 | 1.73E-03 |
| rs16880652 | TCGA&TCGA_coCNV | 9.13E-04 | 8.48E-03 |
| rs2256413 | TCGA&TCGA_coCNV | 9.13E-04 | 2.03E-03 |
| rs1084086 | TCGA&TCGA_coCNV | 6.77E-04 | 1.48E-03 |
| rs9965896 | TCGA | 9.14E-04 | #N/A |
| rs8050047 | TCGA&TCGA_coCNV | 2.58E-05 | 9.84E-05 |
| rs10935236 | TCGA&TCGA_coCNV | 5.73E-04 | 1.07E-03 |
| rs16901461 | TCGA | 9.17E-04 | #N/A |
| rs7779653 | TCGA&TCGA_coCNV | 9.18E-04 | 3.67E-04 |
| rs17070707 | TCGA&TCGA_coCNV | 9.18E-04 | 8.55E-05 |
| rs6755696 | TCGA&TCGA_coCNV | 9.19E-04 | 5.35E-06 |
| rs1024445 | TCGA&TCGA_coCNV | 9.19E-04 | 8.79E-03 |
| rs1013316 | TCGA | 9.21E-04 | #N/A |
| rs6694158 | TCGA&TCGA_coCNV | 9.24E-04 | 1.88E-03 |
| rs17462666 | TCGA&TCGA_coCNV | 9.27E-04 | 1.56E-03 |
| rs11045726 | TCGA&TCGA_coCNV | 9.28E-04 | 2.80E-04 |
| rs4743365 | TCGA&TCGA_coCNV | 9.28E-04 | 3.38E-03 |
| rs9398947 | TCGA&TCGA_coCNV | 9.29E-04 | 5.31E-04 |
| rs10997205 | TCGA&TCGA_coCNV | 9.31E-04 | 1.04E-03 |
| rs10174809 | TCGA&TCGA_coCNV | 9.31E-04 | 2.32E-03 |
| rs1192658 | TCGA&TCGA_coCNV | 9.31E-04 | 5.26E-04 |
| rs9926545 | TCGA&TCGA_coCNV | 9.33E-04 | 1.73E-03 |
| rs11719573 | TCGA | 9.35E-04 | #N/A |

| rs16935634 | TCGA&TCGA_coCNV | 9.35E-04 | 2.20E-03 |
| --- | --- | --- | --- |
| rs3738202 | TCGA&TCGA_coCNV | 9.36E-04 | 1.03E-04 |
| rs142464 | TCGA&TCGA_coCNV | 5.79E-04 | 3.58E-04 |
| rs1445846 | TCGA | 9.38E-04 | #N/A |
| rs13096142 | TCGA | 9.38E-04 | #N/A |
| rs737963 | TCGA&TCGA_coCNV | 2.52E-04 | 1.50E-04 |
| rs6800566 | TCGA | 9.38E-04 | #N/A |
| rs6627728 | TCGA&TCGA_coCNV | 9.39E-04 | 4.68E-04 |
| rs6495653 | TCGA | 9.39E-04 | #N/A |
| rs12000213 | TCGA | 9.40E-04 | #N/A |
| rs17728638 | TCGA | 9.40E-04 | #N/A |
| rs17491398 | TCGA&TCGA_coCNV | 9.41E-04 | 1.23E-03 |
| rs4823393 | TCGA | 9.41E-04 | #N/A |
| rs11756162 | TCGA&TCGA_coCNV | 6.44E-04 | 4.35E-03 |
| rs2051534 | TCGA&TCGA_coCNV | 9.43E-04 | 4.37E-03 |
| rs4750833 | TCGA&TCGA_coCNV | 9.44E-04 | 2.85E-03 |
| rs1033470 | TCGA | 9.44E-04 | #N/A |
| rs10488142 | TCGA&TCGA_coCNV | 1.72E-05 | 5.87E-04 |
| rs17108559 | TCGA&TCGA_coCNV | 9.45E-04 | 1.79E-04 |
| rs12694069 | TCGA | 9.45E-04 | #N/A |
| rs8101014 | TCGA&TCGA_coCNV | 9.47E-04 | 1.09E-03 |
| rs705958 | TCGA | 9.11E-04 | #N/A |
| rs1192655 | TCGA&TCGA_coCNV | 9.48E-04 | 6.65E-04 |
| rs16896641 | TCGA&TCGA_coCNV | 9.49E-04 | 1.48E-05 |
| rs1504753 | TCGA&TCGA_coCNV | 9.49E-04 | 9.72E-04 |
| rs12236297 | TCGA | 9.50E-04 | #N/A |
| rs2615279 | TCGA&TCGA_coCNV | 9.50E-04 | 2.71E-04 |
| rs16973228 | TCGA&TCGA_coCNV | 9.51E-04 | 1.58E-04 |
| rs2654588 | TCGA&TCGA_coCNV | 9.52E-04 | 8.27E-04 |
| rs664600 | TCGA | 9.52E-04 | #N/A |
| rs217566 | TCGA&TCGA_coCNV | 4.77E-04 | 2.29E-05 |
| rs2187254 | TCGA&TCGA_coCNV | 9.54E-04 | 1.22E-03 |
| rs11764301 | TCGA | 9.54E-04 | #N/A |
| rs13263161 | TCGA&TCGA_coCNV | 9.54E-04 | 5.19E-04 |
| rs2141791 | TCGA&TCGA_coCNV | 9.05E-04 | 4.61E-04 |
| rs7191187 | TCGA&TCGA_coCNV | 9.55E-04 | 1.54E-03 |
| rs11072304 | TCGA&TCGA_coCNV | 9.56E-04 | 2.22E-03 |
| rs4901691 | TCGA | 9.57E-04 | #N/A |
| rs8036106 | TCGA&TCGA_coCNV | 9.57E-04 | 4.80E-03 |
| rs7337873 | TCGA&TCGA_coCNV | 9.58E-04 | 1.12E-03 |
| rs12840363 | TCGA&TCGA_coCNV | 9.58E-04 | 8.98E-03 |
| rs6640815 | TCGA&TCGA_coCNV | 9.58E-04 | 1.96E-03 |
| rs576117 | TCGA&TCGA_coCNV | 9.60E-04 | 4.93E-03 |
| rs2193104 | TCGA | 2.24E-04 | #N/A |
| rs980876 | TCGA&TCGA_coCNV | 9.62E-04 | 3.61E-03 |
| rs9699067 | TCGA&TCGA_coCNV | 1.15E-04 | 5.63E-04 |

| rs4378950 | TCGA&TCGA_coCNV | 9.63E-04 | 2.45E-03 |
| --- | --- | --- | --- |
| rs16876180 | TCGA | 1.88E-04 | #N/A |
| rs829182 | TCGA&TCGA_coCNV | 9.64E-04 | 2.31E-03 |
| rs10890406 | TCGA&TCGA_coCNV | 9.64E-04 | 9.62E-04 |
| rs11161691 | TCGA&TCGA_coCNV | 9.65E-04 | 6.04E-04 |
| rs381682 | TCGA | 9.65E-04 | #N/A |
| rs4705504 | TCGA | 9.66E-04 | #N/A |
| rs9347547 | TCGA | 9.67E-04 | #N/A |
| rs1856800 | TCGA | 9.68E-04 | #N/A |
| rs9459326 | TCGA | 9.68E-04 | #N/A |
| rs17147479 | TCGA&TCGA_coCNV | 9.70E-04 | 4.48E-03 |
| rs17742766 | TCGA&TCGA_coCNV | 6.99E-04 | 2.05E-03 |
| rs6708567 | TCGA&TCGA_coCNV | 9.71E-04 | 1.33E-04 |
| rs10483088 | TCGA | 9.72E-04 | #N/A |
| rs12639596 | TCGA | 9.73E-04 | #N/A |
| rs534231 | TCGA | 9.73E-04 | #N/A |
| rs471882 | TCGA | 9.74E-04 | #N/A |
| rs3006161 | TCGA&TCGA_coCNV | 9.75E-04 | 5.34E-03 |
| rs6582179 | TCGA | 9.75E-04 | #N/A |
| rs2234039 | TCGA&TCGA_coCNV | 9.75E-04 | 1.42E-03 |
| rs2871957 | TCGA&TCGA_coCNV | 1.67E-04 | 2.65E-03 |
| rs16822574 | TCGA | 9.75E-04 | #N/A |
| rs10030258 | TCGA&TCGA_coCNV | 4.20E-04 | 7.89E-04 |
| rs12640825 | TCGA&TCGA_coCNV | 9.78E-04 | 1.93E-03 |
| rs2299300 | TCGA&TCGA_coCNV | 9.78E-04 | 2.25E-03 |
| rs12143139 | TCGA&TCGA_coCNV | 9.79E-04 | 6.83E-04 |
| rs2735469 | TCGA&TCGA_coCNV | 9.79E-04 | 1.82E-03 |
| rs607948 | TCGA&TCGA_coCNV | 9.79E-04 | 1.34E-04 |
| rs4287157 | TCGA | 9.79E-04 | #N/A |
| rs699811 | TCGA | 9.80E-04 | #N/A |
| rs11682655 | TCGA | 9.80E-04 | #N/A |
| rs2878800 | TCGA&TCGA_coCNV | 9.80E-04 | 9.65E-04 |
| rs334355 | TCGA&TCGA_coCNV | 9.81E-04 | 3.25E-03 |
| rs9415442 | TCGA&TCGA_coCNV | 9.82E-04 | 4.55E-03 |
| rs4903692 | TCGA&TCGA_coCNV | 9.83E-04 | 3.17E-03 |
| rs3099124 | TCGA | 9.83E-04 | #N/A |
| rs4664513 | TCGA | 9.83E-04 | #N/A |
| rs577362 | TCGA&TCGA_coCNV | 9.86E-04 | 5.47E-03 |
| rs17589451 | TCGA&TCGA_coCNV | 9.86E-04 | 3.29E-04 |
| rs301661 | TCGA&TCGA_coCNV | 9.86E-04 | 6.94E-03 |
| rs10232889 | TCGA&TCGA_coCNV | 9.87E-04 | 6.88E-03 |
| rs12741964 | TCGA&TCGA_coCNV | 2.44E-04 | 7.04E-04 |
| rs12861872 | TCGA&TCGA_coCNV | 9.88E-04 | 4.33E-03 |
| rs3908476 | TCGA&TCGA_coCNV | 9.88E-04 | 3.51E-03 |
| rs17067729 | TCGA&TCGA_coCNV | 9.88E-04 | 1.16E-03 |
| rs10834866 | TCGA&TCGA_coCNV | 9.89E-04 | 2.10E-03 |

| rs3763016 | TCGA&TCGA_coCNV | 9.89E-04 | 4.49E-03 |
| --- | --- | --- | --- |
| rs6623093 | TCGA&TCGA_coCNV | 9.90E-04 | 8.13E-03 |
| rs4838594 | TCGA&TCGA_coCNV | 9.91E-04 | 5.24E-03 |
| rs10496555 | TCGA&TCGA_coCNV | 9.91E-04 | 5.86E-03 |
| rs4904578 | TCGA | 9.92E-04 | #N/A |
| rs4890619 | TCGA&TCGA_coCNV | 9.93E-04 | 4.78E-03 |
| rs7255274 | TCGA&TCGA_coCNV | 9.94E-04 | 2.67E-03 |
| rs11997992 | TCGA | 9.94E-04 | #N/A |
| rs17027961 | TCGA | 9.95E-04 | #N/A |
| rs9494154 | TCGA | 9.96E-04 | #N/A |
| rs819787 | TCGA&TCGA_coCNV | 9.96E-04 | 4.93E-04 |
| rs17572590 | TCGA | 9.97E-04 | #N/A |
| rs17770324 | TCGA | 9.97E-04 | #N/A |
| rs2497993 | TCGA | 9.98E-04 | #N/A |
| rs16969305 | TCGA&TCGA_coCNV | 9.98E-04 | 2.90E-04 |
| rs7101051 | TCGA&TCGA_coCNV | 9.98E-04 | 1.98E-03 |
| rs1393561 | TCGA | 9.98E-04 | #N/A |
| rs2840706 | TCGA&TCGA_coCNV | 9.98E-04 | 1.34E-03 |
| rs11132122 | TCGA | 9.99E-04 | #N/A |
| rs1948559 | TCGA&TCGA_coCNV | 1.00E-03 | 3.23E-03 |
| rs2378965 | TCGA&TCGA_coCNV | 1.00E-03 | 1.19E-03 |
| rs8088963 | TCGA&TCGA_coCNV | 1.00E-03 | 1.13E-04 |
| rs12715983 | TCGA | 1.00E-03 | #N/A |
| rs12568906 | TCGA&TCGA_coCNV | 1.81E-04 | 8.82E-05 |
| rs2713541 | TCGA&TCGA_coCNV | 2.49E-04 | 7.36E-04 |
| rs9870592 | TCGA&TCGA_coCNV | 1.00E-03 | 4.88E-03 |
| rs16949513 | TCGA | 1.00E-03 | #N/A |
| rs17808062 | TCGA&TCGA_coCNV | 1.00E-03 | 2.93E-03 |
| rs4315271 | TCGA&TCGA_coCNV | 1.01E-03 | 2.09E-03 |
| rs6055570 | TCGA&TCGA_coCNV | 8.48E-04 | 4.51E-03 |
| rs1149000 | TCGA&TCGA_coCNV | 1.01E-03 | 1.74E-03 |
| rs10936485 | TCGA&TCGA_coCNV | 1.01E-03 | 2.40E-04 |
| rs9327909 | TCGA&TCGA_coCNV | 1.01E-03 | 3.96E-03 |
| rs10267232 | TCGA&TCGA_coCNV | 1.01E-03 | 8.95E-03 |
| rs17025127 | TCGA&TCGA_coCNV | 1.01E-03 | 1.45E-03 |
| rs7575738 | TCGA | 1.01E-03 | #N/A |
| rs10843454 | TCGA&TCGA_coCNV | 1.01E-03 | 1.69E-04 |
| rs4497524 | TCGA&TCGA_coCNV | 1.01E-03 | 4.57E-04 |
| rs17036802 | TCGA&TCGA_coCNV | 1.01E-03 | 1.36E-04 |
| rs2196066 | TCGA&TCGA_coCNV | 1.01E-03 | 9.22E-04 |
| rs11195245 | TCGA&TCGA_coCNV | 1.02E-03 | 2.74E-03 |
| rs2206230 | TCGA&TCGA_coCNV | 1.02E-03 | 1.06E-03 |
| rs16880017 | TCGA&TCGA_coCNV | 1.02E-03 | 1.03E-03 |
| rs38887 | TCGA&TCGA_coCNV | 1.02E-03 | 2.74E-03 |
| rs11970446 | TCGA&TCGA_coCNV | 1.02E-03 | 6.44E-03 |
| rs7053061 | TCGA&TCGA_coCNV | 1.02E-03 | 3.68E-03 |

| rs1713526 | TCGA | 6.47E-04 | #N/A |
| --- | --- | --- | --- |
| rs10981894 | TCGA&TCGA_coCNV | 1.02E-03 | 6.50E-03 |
| rs17055732 | TCGA | 1.02E-03 | #N/A |
| rs7877088 | TCGA&TCGA_coCNV | 1.02E-03 | 5.78E-03 |
| rs6998966 | TCGA&TCGA_coCNV | 1.02E-03 | 1.31E-03 |
| rs4951307 | TCGA&TCGA_coCNV | 2.51E-05 | 7.26E-04 |
| rs3766270 | TCGA&TCGA_coCNV | 1.02E-03 | 5.86E-03 |
| rs17044634 | TCGA&TCGA_coCNV | 1.77E-05 | 5.07E-04 |
| rs1578230 | TCGA | 1.02E-03 | #N/A |
| rs1886510 | TCGA&TCGA_coCNV | 1.02E-03 | 2.21E-03 |
| rs2277238 | TCGA | 1.02E-03 | #N/A |
| rs2136162 | TCGA&TCGA_coCNV | 1.02E-03 | 4.16E-03 |
| rs6991018 | TCGA&TCGA_coCNV | 1.02E-03 | 1.82E-04 |
| rs1822105 | TCGA | 1.02E-03 | #N/A |
| rs6774332 | TCGA&TCGA_coCNV | 1.03E-03 | 9.72E-06 |
| rs3014210 | TCGA&TCGA_coCNV | 1.03E-03 | 1.90E-03 |
| rs12457958 | TCGA&TCGA_coCNV | 1.03E-03 | 1.59E-03 |
| rs4276256 | TCGA&TCGA_coCNV | 1.03E-03 | 9.19E-04 |
| rs2235212 | TCGA | 1.03E-03 | #N/A |
| rs4678585 | TCGA&TCGA_coCNV | 9.68E-04 | 1.88E-05 |
| rs7110348 | TCGA&TCGA_coCNV | 1.03E-03 | 4.86E-03 |
| rs7081421 | TCGA | 9.46E-04 | #N/A |
| rs10918646 | TCGA | 1.04E-03 | #N/A |
| rs7519600 | TCGA&TCGA_coCNV | 1.04E-03 | 2.28E-03 |
| rs285742 | TCGA&TCGA_coCNV | 1.04E-03 | 2.47E-03 |
| rs10509318 | TCGA&TCGA_coCNV | 1.04E-03 | 8.06E-03 |
| rs1549602 | TCGA | 1.04E-03 | #N/A |
| rs12369406 | TCGA | 1.04E-03 | #N/A |
| rs41518446 | TCGA&TCGA_coCNV | 1.04E-03 | 2.92E-03 |
| rs13095617 | TCGA&TCGA_coCNV | 1.04E-03 | 2.04E-03 |
| rs12743512 | TCGA&TCGA_coCNV | 1.04E-03 | 1.45E-03 |
| rs2045562 | TCGA | 1.04E-03 | #N/A |
| rs2242080 | TCGA&TCGA_coCNV | 1.04E-03 | 3.22E-03 |
| rs11838472 | TCGA | 1.04E-03 | #N/A |
| rs6450868 | TCGA&TCGA_coCNV | 1.04E-03 | 4.03E-03 |
| rs13679 | TCGA&TCGA_coCNV | 1.04E-03 | 7.47E-03 |
| rs6977301 | TCGA | 1.04E-03 | #N/A |
| rs9920106 | TCGA | 1.04E-03 | #N/A |
| rs3104800 | TCGA | 1.04E-03 | #N/A |
| rs9573559 | TCGA&TCGA_coCNV | 1.05E-03 | 8.29E-04 |
| rs2829291 | TCGA&TCGA_coCNV | 1.05E-03 | 7.01E-03 |
| rs9933134 | TCGA&TCGA_coCNV | 1.05E-03 | 2.23E-03 |
| rs6731916 | TCGA | 1.05E-03 | #N/A |
| rs10211285 | TCGA | 1.05E-03 | #N/A |
| rs13402267 | TCGA&TCGA_coCNV | 1.05E-03 | 5.05E-04 |
| rs10251563 | TCGA&TCGA_coCNV | 6.87E-04 | 1.17E-04 |

| rs11974300 | TCGA&TCGA_coCNV | 1.05E-03 | 7.06E-03 |
| --- | --- | --- | --- |
| rs1253685 | TCGA | 9.30E-04 | #N/A |
| rs9327807 | TCGA&TCGA_coCNV | 1.05E-03 | 5.15E-04 |
| rs17089038 | TCGA&TCGA_coCNV | 1.05E-03 | 4.21E-03 |
| rs16970779 | TCGA&TCGA_coCNV | 7.96E-05 | 2.32E-03 |
| rs12120879 | TCGA&TCGA_coCNV | 1.05E-03 | 4.34E-04 |
| rs6629920 | TCGA&TCGA_coCNV | 1.05E-03 | 3.60E-03 |
| rs12135132 | TCGA&TCGA_coCNV | 1.05E-03 | 1.41E-03 |
| rs744731 | TCGA&TCGA_coCNV | 1.05E-03 | 4.73E-04 |
| rs1808256 | TCGA | 1.05E-03 | #N/A |
| rs2356057 | TCGA&TCGA_coCNV | 6.70E-04 | 3.21E-03 |
| rs286967 | TCGA&TCGA_coCNV | 1.06E-03 | 1.07E-03 |
| rs9405980 | TCGA&TCGA_coCNV | 1.06E-03 | 1.95E-03 |
| rs7296702 | TCGA&TCGA_coCNV | 1.06E-03 | 5.21E-04 |
| rs7558514 | TCGA&TCGA_coCNV | 1.06E-03 | 3.94E-03 |
| rs3910052 | TCGA | 1.06E-03 | #N/A |
| rs4535224 | TCGA&TCGA_coCNV | 1.06E-03 | 3.87E-03 |
| rs7909706 | TCGA&TCGA_coCNV | 6.31E-05 | 5.12E-04 |
| rs714007 | TCGA&TCGA_coCNV | 1.06E-03 | 1.66E-04 |
| rs9688761 | TCGA&TCGA_coCNV | 1.39E-04 | 9.46E-05 |
| rs7734308 | TCGA&TCGA_coCNV | 1.06E-03 | 6.33E-03 |
| rs13171173 | TCGA | 1.06E-03 | #N/A |
| rs17366956 | TCGA&TCGA_coCNV | 1.06E-03 | 4.75E-03 |
| rs11064652 | TCGA&TCGA_coCNV | 7.78E-04 | 1.02E-04 |
| rs1336460 | TCGA | 1.07E-03 | #N/A |
| rs7909742 | TCGA&TCGA_coCNV | 1.07E-03 | 4.98E-03 |
| rs4674021 | TCGA&TCGA_coCNV | 1.07E-03 | 1.92E-03 |
| rs12622144 | TCGA&TCGA_coCNV | 1.07E-03 | 1.24E-04 |
| rs10118338 | TCGA&TCGA_coCNV | 1.07E-03 | 3.81E-03 |
| rs9580644 | TCGA&TCGA_coCNV | 6.54E-05 | 9.85E-04 |
| rs12729918 | TCGA&TCGA_coCNV | 1.07E-03 | 5.76E-04 |
| rs12999477 | TCGA&TCGA_coCNV | 1.07E-03 | 1.42E-03 |
| rs36856 | TCGA&TCGA_coCNV | 7.48E-04 | 2.18E-03 |
| rs625185 | TCGA | 1.07E-03 | #N/A |
| rs41420254 | TCGA&TCGA_coCNV | 1.07E-03 | 1.45E-03 |
| rs12531361 | TCGA&TCGA_coCNV | 1.07E-03 | 1.33E-03 |
| rs12035516 | TCGA&TCGA_coCNV | 1.07E-03 | 7.20E-04 |
| rs4128341 | TCGA&TCGA_coCNV | 1.07E-03 | 2.60E-03 |
| rs1950252 | TCGA&TCGA_coCNV | 1.07E-03 | 7.72E-03 |
| rs11807012 | TCGA&TCGA_coCNV | 1.07E-03 | 7.00E-04 |
| rs6455336 | TCGA&TCGA_coCNV | 1.82E-05 | 5.91E-04 |
| rs10964495 | TCGA&TCGA_coCNV | 1.07E-03 | 4.20E-04 |
| rs17124895 | TCGA | 1.07E-03 | #N/A |
| rs6866903 | TCGA&TCGA_coCNV | 1.07E-03 | 1.97E-03 |
| rs9397973 | TCGA&TCGA_coCNV | 1.08E-03 | 6.02E-03 |
| rs6653896 | TCGA&TCGA_coCNV | 1.08E-03 | 5.96E-03 |

| rs5915918 | TCGA&TCGA_coCNV | 9.11E-04 | 7.42E-03 |
| --- | --- | --- | --- |
| rs612749 | TCGA&TCGA_coCNV | 1.08E-03 | 6.81E-03 |
| rs7059823 | TCGA | 1.08E-03 | #N/A |
| rs7325394 | TCGA&TCGA_coCNV | 1.08E-03 | 1.45E-03 |
| rs1542824 | TCGA&TCGA_coCNV | 5.20E-04 | 2.92E-03 |
| rs17795457 | TCGA&TCGA_coCNV | 1.08E-03 | 6.19E-04 |
| rs6864279 | TCGA | 1.08E-03 | #N/A |
| rs10103434 | TCGA | 1.07E-05 | #N/A |
| rs13402632 | TCGA&TCGA_coCNV | 1.08E-03 | 4.55E-04 |
| rs9636830 | TCGA&TCGA_coCNV | 1.08E-03 | 5.14E-03 |
| rs10045068 | TCGA&TCGA_coCNV | 1.08E-03 | 2.93E-03 |
| rs10023961 | TCGA | 1.08E-03 | #N/A |
| rs1375310 | TCGA&TCGA_coCNV | 1.08E-03 | 5.72E-03 |
| rs2251209 | TCGA&TCGA_coCNV | 1.08E-03 | 1.11E-03 |
| rs10774674 | TCGA&TCGA_coCNV | 1.08E-03 | 2.52E-03 |
| rs1148990 | TCGA&TCGA_coCNV | 1.08E-03 | 3.33E-03 |
| rs6588775 | TCGA&TCGA_coCNV | 1.08E-03 | 5.75E-05 |
| rs2758988 | TCGA | 1.09E-03 | #N/A |
| rs12652190 | TCGA&TCGA_coCNV | 1.09E-03 | 4.12E-03 |
| rs6758995 | TCGA | 1.09E-03 | #N/A |
| rs11266520 | TCGA&TCGA_coCNV | 1.09E-03 | 4.86E-03 |
| rs12698265 | TCGA&TCGA_coCNV | 1.09E-03 | 2.23E-03 |
| rs4615335 | TCGA&TCGA_coCNV | 3.48E-08 | 6.41E-03 |
| rs9933249 | TCGA | 1.09E-03 | #N/A |
| rs2775133 | TCGA&TCGA_coCNV | 1.09E-03 | 3.82E-04 |
| rs4669114 | TCGA&TCGA_coCNV | 1.09E-03 | 3.07E-03 |
| rs919165 | TCGA&TCGA_coCNV | 1.09E-03 | 1.65E-03 |
| rs17448220 | TCGA&TCGA_coCNV | 1.09E-03 | 2.08E-03 |
| rs3127072 | TCGA&TCGA_coCNV | 1.09E-03 | 4.73E-04 |
| rs2001557 | TCGA&TCGA_coCNV | 1.09E-03 | 5.88E-03 |
| rs9410427 | TCGA | 1.09E-03 | #N/A |
| rs17124681 | TCGA | 1.04E-03 | #N/A |
| rs2871771 | TCGA | 1.10E-03 | #N/A |
| rs7535558 | TCGA | 1.10E-03 | #N/A |
| rs2964131 | TCGA&TCGA_coCNV | 1.10E-03 | 4.67E-04 |
| rs706350 | TCGA&TCGA_coCNV | 1.10E-03 | 4.03E-03 |
| rs10259246 | TCGA | 1.10E-03 | #N/A |
| rs909003 | TCGA&TCGA_coCNV | 1.10E-03 | 6.19E-03 |
| rs17148547 | TCGA&TCGA_coCNV | 1.10E-03 | 8.36E-03 |
| rs17585550 | TCGA&TCGA_coCNV | 1.10E-03 | 9.63E-03 |
| rs3123808 | TCGA&TCGA_coCNV | 1.10E-03 | 3.67E-03 |
| rs1524771 | TCGA | 1.10E-03 | #N/A |
| rs10834894 | TCGA&TCGA_coCNV | 1.81E-04 | 4.40E-05 |
| rs5752851 | TCGA&TCGA_coCNV | 1.10E-03 | 2.01E-05 |
| rs7777425 | TCGA&TCGA_coCNV | 1.10E-03 | 1.73E-04 |
| rs324627 | TCGA&TCGA_coCNV | 1.10E-03 | 3.94E-03 |

| rs11769405 | TCGA&TCGA_coCNV | 2.85E-04 | 1.50E-05 |
| --- | --- | --- | --- |
| rs11100920 | TCGA&TCGA_coCNV | 1.10E-03 | 8.62E-03 |
| rs10915872 | TCGA | 1.10E-03 | #N/A |
| rs10037781 | TCGA | 1.10E-03 | #N/A |
| rs16866028 | TCGA&TCGA_coCNV | 1.11E-03 | 4.53E-03 |
| rs10799772 | TCGA&TCGA_coCNV | 1.11E-03 | 1.35E-03 |
| rs4902613 | TCGA&TCGA_coCNV | 1.11E-03 | 7.09E-03 |
| rs1513315 | TCGA | 1.11E-03 | #N/A |
| rs16933639 | TCGA&TCGA_coCNV | 1.11E-03 | 8.65E-03 |
| rs4413279 | TCGA&TCGA_coCNV | 1.11E-03 | 3.58E-03 |
| rs7909618 | TCGA | 1.11E-03 | #N/A |
| rs41437249 | TCGA&TCGA_coCNV | 1.11E-03 | 9.79E-03 |
| rs2357468 | TCGA | 1.11E-03 | #N/A |
| rs11078606 | TCGA&TCGA_coCNV | 1.11E-03 | 5.58E-04 |
| rs1862581 | TCGA&TCGA_coCNV | 1.11E-03 | 1.86E-03 |
| rs10125295 | TCGA&TCGA_coCNV | 1.11E-03 | 7.84E-03 |
| rs331609 | TCGA&TCGA_coCNV | 1.71E-04 | 3.95E-04 |
| rs2641977 | TCGA&TCGA_coCNV | 4.38E-04 | 5.66E-03 |
| rs6785855 | TCGA&TCGA_coCNV | 1.11E-03 | 9.54E-05 |
| rs13030483 | TCGA&TCGA_coCNV | 3.52E-04 | 1.30E-05 |
| rs5761552 | TCGA&TCGA_coCNV | 6.84E-04 | 1.98E-04 |
| rs7964728 | TCGA&TCGA_coCNV | 1.11E-03 | 1.75E-03 |
| rs11193591 | TCGA&TCGA_coCNV | 1.11E-03 | 9.25E-03 |
| rs17015781 | TCGA&TCGA_coCNV | 1.11E-03 | 1.24E-03 |
| rs328096 | TCGA&TCGA_coCNV | 1.12E-03 | 8.74E-05 |
| rs17154526 | TCGA&TCGA_coCNV | 1.12E-03 | 2.73E-03 |
| rs1116084 | TCGA | 1.12E-03 | #N/A |
| rs11726294 | TCGA&TCGA_coCNV | 1.12E-03 | 4.07E-04 |
| rs17053979 | TCGA | 1.12E-03 | #N/A |
| rs7727892 | TCGA&TCGA_coCNV | 1.12E-03 | 4.63E-03 |
| rs1340117 | TCGA&TCGA_coCNV | 1.12E-03 | 3.47E-03 |
| rs17171045 | TCGA&TCGA_coCNV | 1.12E-03 | 3.86E-03 |
| rs6743846 | TCGA | 1.12E-03 | #N/A |
| rs8121979 | TCGA | 1.12E-03 | #N/A |
| rs6639984 | TCGA&TCGA_coCNV | 1.12E-03 | 5.73E-03 |
| rs11031636 | TCGA | 1.13E-03 | #N/A |
| rs201813 | TCGA&TCGA_coCNV | 1.13E-03 | 3.45E-04 |
| rs1330896 | TCGA&TCGA_coCNV | 1.13E-03 | 1.60E-04 |
| rs10516108 | TCGA&TCGA_coCNV | 1.13E-03 | 6.18E-03 |
| rs4361779 | TCGA | 1.13E-03 | #N/A |
| rs16841463 | TCGA&TCGA_coCNV | 1.13E-03 | 7.31E-03 |
| rs480677 | TCGA&TCGA_coCNV | 3.73E-04 | 5.29E-04 |
| rs7591219 | TCGA&TCGA_coCNV | 2.89E-04 | 8.89E-05 |
| rs357265 | TCGA&TCGA_coCNV | 6.56E-04 | 2.79E-03 |
| rs10767813 | TCGA&TCGA_coCNV | 1.13E-03 | 9.19E-03 |
| rs354391 | TCGA | 1.13E-03 | #N/A |

| rs11168091 | TCGA&TCGA_coCNV | 1.13E-03 | 2.24E-03 |
| --- | --- | --- | --- |
| rs7595535 | TCGA&TCGA_coCNV | 1.13E-03 | 6.85E-03 |
| rs9651257 | TCGA | 1.14E-03 | #N/A |
| rs5992636 | TCGA&TCGA_coCNV | 1.14E-03 | 3.67E-03 |
| rs4480073 | TCGA&TCGA_coCNV | 1.14E-03 | 1.85E-04 |
| rs9419200 | TCGA&TCGA_coCNV | 4.23E-04 | 7.50E-04 |
| rs41389049 | TCGA&TCGA_coCNV | 1.14E-03 | 1.13E-03 |
| rs10959698 | TCGA | 1.14E-03 | #N/A |
| rs8019257 | TCGA&TCGA_coCNV | 1.14E-03 | 2.15E-06 |
| rs13429985 | TCGA | 1.14E-03 | #N/A |
| rs7754521 | TCGA | 1.14E-03 | #N/A |
| rs1447007 | TCGA&TCGA_coCNV | 1.14E-03 | 5.89E-04 |
| rs875115 | TCGA | 1.14E-03 | #N/A |
| rs17000509 | TCGA&TCGA_coCNV | 1.14E-03 | 7.28E-03 |
| rs3794550 | TCGA&TCGA_coCNV | 1.14E-03 | 6.87E-03 |
| rs9994192 | TCGA&TCGA_coCNV | 1.14E-03 | 8.14E-04 |
| rs9544725 | TCGA&TCGA_coCNV | 1.15E-03 | 3.51E-03 |
| rs4867981 | TCGA | 1.15E-03 | #N/A |
| rs2062313 | TCGA&TCGA_coCNV | 1.15E-03 | 2.51E-04 |
| rs254855 | TCGA&TCGA_coCNV | 1.15E-03 | 7.18E-05 |
| rs7312321 | TCGA&TCGA_coCNV | 1.15E-03 | 1.87E-03 |
| rs6639916 | TCGA&TCGA_coCNV | 4.30E-04 | 2.05E-04 |
| rs5929597 | TCGA | 1.15E-03 | #N/A |
| rs9850019 | TCGA&TCGA_coCNV | 1.15E-03 | 3.06E-03 |
| rs11185775 | TCGA&TCGA_coCNV | 1.15E-03 | 1.06E-04 |
| rs8050947 | TCGA&TCGA_coCNV | 1.15E-03 | 4.91E-03 |
| rs6982016 | TCGA&TCGA_coCNV | 3.48E-04 | 1.63E-03 |
| rs9401956 | TCGA&TCGA_coCNV | 1.16E-03 | 1.80E-04 |
| rs17761446 | TCGA&TCGA_coCNV | 1.16E-03 | 4.17E-03 |
| rs4491085 | TCGA&TCGA_coCNV | 1.16E-03 | 8.39E-04 |
| rs134809 | TCGA | 1.16E-03 | #N/A |
| rs7095441 | TCGA&TCGA_coCNV | 1.16E-03 | 2.82E-03 |
| rs10022418 | TCGA&TCGA_coCNV | 1.16E-03 | 1.20E-04 |
| rs16882320 | TCGA | 1.16E-03 | #N/A |
| rs10220570 | TCGA&TCGA_coCNV | 1.16E-03 | 9.72E-03 |
| rs2124223 | TCGA | 1.16E-03 | #N/A |
| rs7777388 | TCGA&TCGA_coCNV | 4.60E-04 | 8.05E-03 |
| rs1535618 | TCGA&TCGA_coCNV | 1.16E-03 | 7.44E-05 |
| rs2972144 | TCGA&TCGA_coCNV | 1.16E-03 | 7.00E-03 |
| rs16909019 | TCGA&TCGA_coCNV | 1.16E-03 | 3.74E-05 |
| rs11688970 | TCGA&TCGA_coCNV | 1.17E-03 | 3.97E-03 |
| rs10487231 | TCGA&TCGA_coCNV | 1.17E-03 | 1.48E-03 |
| rs979775 | TCGA&TCGA_coCNV | 1.17E-03 | 9.89E-04 |
| rs9882669 | TCGA&TCGA_coCNV | 1.17E-03 | 9.05E-04 |
| rs17779649 | TCGA&TCGA_coCNV | 2.62E-04 | 9.14E-03 |
| rs17076122 | TCGA&TCGA_coCNV | 1.17E-03 | 2.68E-04 |

| rs6771662 | TCGA&TCGA_coCNV | 1.17E-03 | 2.56E-03 |
| --- | --- | --- | --- |
| rs16841170 | TCGA | 1.17E-03 | #N/A |
| rs6553421 | TCGA&TCGA_coCNV | 1.17E-03 | 3.38E-04 |
| rs7231504 | TCGA&TCGA_coCNV | 1.17E-03 | 5.65E-03 |
| rs10832839 | TCGA | 1.17E-03 | #N/A |
| rs17136323 | TCGA&TCGA_coCNV | 1.17E-03 | 4.11E-03 |
| rs9709422 | TCGA&TCGA_coCNV | 1.17E-03 | 4.77E-05 |
| rs2993263 | TCGA&TCGA_coCNV | 1.17E-03 | 3.95E-03 |
| rs12373196 | TCGA&TCGA_coCNV | 1.17E-03 | 2.06E-03 |
| rs2293504 | TCGA&TCGA_coCNV | 1.17E-03 | 1.04E-03 |
| rs3759171 | TCGA&TCGA_coCNV | 1.18E-03 | 1.31E-04 |
| rs9307374 | TCGA&TCGA_coCNV | 1.18E-03 | 7.58E-03 |
| rs10022357 | TCGA&TCGA_coCNV | 1.18E-03 | 3.78E-04 |
| rs7891571 | TCGA&TCGA_coCNV | 1.18E-03 | 5.45E-03 |
| rs4837414 | TCGA | 1.18E-03 | #N/A |
| rs7061031 | TCGA&TCGA_coCNV | 1.18E-03 | 8.43E-03 |
| rs2380251 | TCGA&TCGA_coCNV | 5.28E-05 | 2.45E-04 |
| rs1890665 | TCGA&TCGA_coCNV | 1.18E-03 | 1.52E-03 |
| rs2218383 | TCGA&TCGA_coCNV | 1.18E-03 | 1.16E-04 |
| rs7018101 | TCGA&TCGA_coCNV | 1.18E-03 | 4.69E-03 |
| rs9653359 | TCGA&TCGA_coCNV | 1.18E-03 | 3.30E-03 |
| rs10248331 | TCGA&TCGA_coCNV | 1.18E-03 | 1.33E-03 |
| rs1511891 | TCGA | 1.18E-03 | #N/A |
| rs287553 | TCGA&TCGA_coCNV | 1.19E-03 | 1.58E-03 |
| rs9494706 | TCGA&TCGA_coCNV | 2.56E-04 | 1.46E-04 |
| rs377675 | TCGA&TCGA_coCNV | 1.19E-03 | 1.44E-03 |
| rs226945 | TCGA&TCGA_coCNV | 5.93E-04 | 6.10E-03 |
| rs6578285 | TCGA&TCGA_coCNV | 1.19E-03 | 1.07E-03 |
| rs1371473 | TCGA | 1.19E-03 | #N/A |
| rs10142979 | TCGA&TCGA_coCNV | 1.19E-03 | 1.69E-05 |
| rs1515104 | TCGA&TCGA_coCNV | 4.04E-04 | 1.32E-03 |
| rs2079574 | TCGA&TCGA_coCNV | 1.19E-03 | 9.80E-04 |
| rs10760673 | TCGA&TCGA_coCNV | 1.19E-03 | 1.67E-03 |
| rs4629431 | TCGA&TCGA_coCNV | 1.19E-03 | 9.44E-03 |
| rs710766 | TCGA | 1.19E-03 | #N/A |
| rs6691444 | TCGA&TCGA_coCNV | 1.19E-03 | 3.51E-04 |
| rs5749450 | TCGA&TCGA_coCNV | 1.19E-03 | 4.62E-03 |
| rs232331 | TCGA | 1.19E-03 | #N/A |
| rs16941156 | TCGA | 1.19E-03 | #N/A |
| rs11749163 | TCGA&TCGA_coCNV | 1.19E-03 | 7.13E-03 |
| rs10970695 | TCGA&TCGA_coCNV | 1.19E-03 | 8.18E-04 |
| rs7107881 | TCGA&TCGA_coCNV | 1.19E-03 | 1.04E-03 |
| rs16857299 | TCGA&TCGA_coCNV | 1.19E-03 | 9.71E-03 |
| rs7824253 | TCGA&TCGA_coCNV | 1.19E-03 | 2.39E-03 |
| rs7785891 | TCGA&TCGA_coCNV | 1.19E-03 | 1.10E-03 |
| rs7533882 | TCGA&TCGA_coCNV | 1.20E-03 | 2.28E-05 |

| rs17098037 | TCGA&TCGA_coCNV | 1.20E-03 | 3.38E-03 |
| --- | --- | --- | --- |
| rs17006180 | TCGA&TCGA_coCNV | 1.20E-03 | 7.37E-04 |
| rs12594730 | TCGA | 1.20E-03 | #N/A |
| rs1406340 | TCGA&TCGA_coCNV | 4.62E-04 | 5.56E-05 |
| rs10175677 | TCGA | 1.20E-03 | #N/A |
| rs6563235 | TCGA&TCGA_coCNV | 1.20E-03 | 2.61E-03 |
| rs8124792 | TCGA&TCGA_coCNV | 1.20E-03 | 7.31E-03 |
| rs2819666 | TCGA&TCGA_coCNV | 1.20E-03 | 1.97E-03 |
| rs10010369 | TCGA&TCGA_coCNV | 8.35E-04 | 2.11E-04 |
| rs6976924 | TCGA&TCGA_coCNV | 1.20E-03 | 8.95E-03 |
| rs7357476 | TCGA | 1.20E-03 | #N/A |
| rs2059776 | TCGA | 1.20E-03 | #N/A |
| rs41517646 | TCGA&TCGA_coCNV | 1.20E-03 | 5.60E-03 |
| rs12150739 | TCGA&TCGA_coCNV | 3.04E-04 | 2.41E-03 |
| rs2686172 | TCGA&TCGA_coCNV | 1.20E-03 | 1.80E-03 |
| rs10849622 | TCGA&TCGA_coCNV | 1.20E-03 | 6.72E-04 |
| rs12492326 | TCGA | 1.21E-03 | #N/A |
| rs12481262 | TCGA&TCGA_coCNV | 1.21E-03 | 5.97E-03 |
| rs6977882 | TCGA&TCGA_coCNV | 1.21E-03 | 7.15E-04 |
| rs12404337 | TCGA&TCGA_coCNV | 8.94E-05 | 4.85E-04 |
| rs3791318 | TCGA&TCGA_coCNV | 1.21E-03 | 9.29E-03 |
| rs16994496 | TCGA&TCGA_coCNV | 1.21E-03 | 6.65E-03 |
| rs7218098 | TCGA | 1.21E-03 | #N/A |
| rs10735241 | TCGA&TCGA_coCNV | 1.21E-03 | 8.15E-04 |
| rs1917366 | TCGA | 1.21E-03 | #N/A |
| rs891163 | TCGA&TCGA_coCNV | 1.21E-03 | 1.17E-03 |
| rs11655081 | TCGA&TCGA_coCNV | 1.21E-03 | 3.26E-03 |
| rs12471988 | TCGA&TCGA_coCNV | 1.21E-03 | 3.12E-03 |
| rs10511393 | TCGA&TCGA_coCNV | 4.46E-04 | 1.83E-03 |
| rs11752885 | TCGA | 1.22E-03 | #N/A |
| rs902011 | TCGA | 1.22E-03 | #N/A |
| rs759555 | TCGA | 1.22E-03 | #N/A |
| rs7397056 | TCGA&TCGA_coCNV | 1.22E-03 | 5.88E-03 |
| rs447813 | TCGA | 1.22E-03 | #N/A |
| rs1322227 | TCGA&TCGA_coCNV | 1.22E-03 | 2.93E-03 |
| rs7919666 | TCGA&TCGA_coCNV | 1.22E-03 | 1.45E-03 |
| rs12150846 | TCGA&TCGA_coCNV | 7.33E-05 | 1.48E-03 |
| rs17003332 | TCGA&TCGA_coCNV | 1.22E-03 | 7.55E-03 |
| rs17118598 | TCGA | 1.22E-03 | #N/A |
| rs3755633 | TCGA&TCGA_coCNV | 1.22E-03 | 4.20E-05 |
| rs6112263 | TCGA&TCGA_coCNV | 1.22E-03 | 1.85E-03 |
| rs4073748 | TCGA&TCGA_coCNV | 1.22E-03 | 8.08E-03 |
| rs7976730 | TCGA&TCGA_coCNV | 1.22E-03 | 1.22E-03 |
| rs10764038 | TCGA&TCGA_coCNV | 1.22E-03 | 7.41E-03 |
| rs2280320 | TCGA&TCGA_coCNV | 4.02E-04 | 7.70E-04 |
| rs10434401 | TCGA&TCGA_coCNV | 1.23E-03 | 8.25E-04 |

| rs1933323 | TCGA&TCGA_coCNV | 1.23E-03 | 3.83E-03 |
| --- | --- | --- | --- |
| rs4241994 | TCGA&TCGA_coCNV | 1.23E-03 | 2.12E-04 |
| rs2275276 | TCGA&TCGA_coCNV | 1.23E-03 | 1.06E-03 |
| rs10891537 | TCGA&TCGA_coCNV | 1.23E-03 | 7.17E-04 |
| rs6432553 | TCGA&TCGA_coCNV | 1.23E-03 | 4.94E-03 |
| rs5979741 | TCGA&TCGA_coCNV | 1.23E-03 | 8.49E-05 |
| rs11172828 | TCGA&TCGA_coCNV | 1.20E-03 | 2.00E-03 |
| rs10880034 | TCGA&TCGA_coCNV | 1.23E-03 | 1.76E-03 |
| rs2019203 | TCGA | 1.23E-03 | #N/A |
| rs7031666 | TCGA | 1.23E-03 | #N/A |
| rs16997662 | TCGA&TCGA_coCNV | 1.23E-03 | 6.02E-04 |
| rs17008485 | TCGA&TCGA_coCNV | 1.23E-03 | 1.32E-03 |
| rs7624916 | TCGA&TCGA_coCNV | 2.49E-04 | 2.35E-04 |
| rs1321703 | TCGA&TCGA_coCNV | 1.24E-03 | 9.81E-03 |
| rs2978536 | TCGA&TCGA_coCNV | 1.24E-03 | 3.83E-03 |
| rs12412103 | TCGA&TCGA_coCNV | 1.24E-03 | 4.50E-04 |
| rs4242157 | TCGA | 1.24E-03 | #N/A |
| rs4819363 | TCGA&TCGA_coCNV | 1.24E-03 | 1.39E-03 |
| rs4942235 | TCGA&TCGA_coCNV | 1.24E-03 | 8.34E-03 |
| rs4462702 | TCGA&TCGA_coCNV | 1.24E-03 | 5.30E-03 |
| rs4241447 | TCGA&TCGA_coCNV | 1.24E-03 | 4.88E-03 |
| rs7552892 | TCGA | 1.24E-03 | #N/A |
| rs12574167 | TCGA&TCGA_coCNV | 5.02E-04 | 2.17E-04 |
| rs3783557 | TCGA&TCGA_coCNV | 1.24E-03 | 2.19E-03 |
| rs6477647 | TCGA&TCGA_coCNV | 1.24E-03 | 5.09E-05 |
| rs13314266 | TCGA | 7.48E-04 | #N/A |
| rs5767523 | TCGA&TCGA_coCNV | 1.24E-03 | 1.15E-03 |
| rs12378383 | TCGA&TCGA_coCNV | 1.25E-03 | 4.67E-03 |
| rs6847357 | TCGA | 1.25E-03 | #N/A |
| rs9520714 | TCGA&TCGA_coCNV | 1.25E-03 | 5.21E-03 |
| rs13178162 | TCGA | 1.25E-03 | #N/A |
| rs9408748 | TCGA&TCGA_coCNV | 1.25E-03 | 3.50E-03 |
| rs4865885 | TCGA&TCGA_coCNV | 1.25E-03 | 6.92E-04 |
| rs10250672 | TCGA&TCGA_coCNV | 1.25E-03 | 8.61E-04 |
| rs10188771 | TCGA | 1.25E-03 | #N/A |
| rs17807611 | TCGA | 1.25E-03 | #N/A |
| rs10952532 | TCGA | 1.25E-03 | #N/A |
| rs4957633 | TCGA&TCGA_coCNV | 2.32E-05 | 2.30E-03 |
| rs9575968 | TCGA&TCGA_coCNV | 1.25E-03 | 8.11E-04 |
| rs12408490 | TCGA&TCGA_coCNV | 1.25E-03 | 1.92E-03 |
| rs1558225 | TCGA&TCGA_coCNV | 1.25E-03 | 5.76E-04 |
| rs10917746 | TCGA | 1.25E-03 | #N/A |
| rs13172099 | TCGA&TCGA_coCNV | 1.25E-03 | 4.11E-03 |
| rs1930111 | TCGA&TCGA_coCNV | 1.25E-03 | 5.02E-03 |
| rs17168234 | TCGA&TCGA_coCNV | 6.39E-04 | 6.36E-03 |
| rs2269251 | TCGA&TCGA_coCNV | 1.26E-03 | 1.62E-04 |

| rs2931355 | TCGA&TCGA_coCNV | 1.26E-03 | 1.59E-03 |
| --- | --- | --- | --- |
| rs210729 | TCGA&TCGA_coCNV | 1.26E-03 | 1.04E-03 |
| rs11038484 | TCGA | 1.26E-03 | #N/A |
| rs2143510 | TCGA&TCGA_coCNV | 1.26E-03 | 9.96E-03 |
| rs7158663 | TCGA&TCGA_coCNV | 1.26E-03 | 1.89E-03 |
| rs2397988 | TCGA&TCGA_coCNV | 9.93E-04 | 3.15E-03 |
| rs10880686 | TCGA | 1.26E-03 | #N/A |
| rs1572280 | TCGA&TCGA_coCNV | 1.27E-03 | 5.16E-03 |
| rs7585698 | TCGA | 1.27E-03 | #N/A |
| rs12705873 | TCGA | 1.27E-03 | #N/A |
| rs11927826 | TCGA | 1.27E-03 | #N/A |
| rs12356389 | TCGA&TCGA_coCNV | 1.27E-03 | 1.77E-03 |
| rs3865359 | TCGA | 1.27E-03 | #N/A |
| rs9547442 | TCGA&TCGA_coCNV | 1.27E-03 | 5.02E-04 |
| rs9881405 | TCGA&TCGA_coCNV | 1.27E-03 | 2.97E-03 |
| rs10501085 | TCGA&TCGA_coCNV | 1.27E-03 | 1.32E-03 |
| rs16964821 | TCGA&TCGA_coCNV | 1.27E-03 | 6.96E-04 |
| rs9542785 | TCGA&TCGA_coCNV | 1.27E-03 | 3.04E-04 |
| rs2429632 | TCGA&TCGA_coCNV | 1.27E-03 | 1.19E-03 |
| rs2947872 | TCGA&TCGA_coCNV | 1.27E-03 | 7.46E-03 |
| rs420537 | TCGA | 1.27E-03 | #N/A |
| rs10083784 | TCGA&TCGA_coCNV | 1.27E-03 | 3.26E-04 |
| rs823163 | TCGA&TCGA_coCNV | 2.93E-04 | 8.21E-04 |
| rs1449247 | TCGA | 1.27E-03 | #N/A |
| rs17087787 | TCGA&TCGA_coCNV | 1.27E-03 | 2.17E-03 |
| rs1148995 | TCGA&TCGA_coCNV | 1.27E-03 | 1.93E-03 |
| rs5977215 | TCGA | 1.27E-03 | #N/A |
| rs13079982 | TCGA | 1.28E-03 | #N/A |
| rs4123443 | TCGA | 6.73E-04 | #N/A |
| rs3115401 | TCGA | 1.28E-03 | #N/A |
| rs2640853 | TCGA&TCGA_coCNV | 6.42E-05 | 2.08E-04 |
| rs280709 | TCGA&TCGA_coCNV | 1.28E-03 | 1.22E-03 |
| rs483591 | TCGA&TCGA_coCNV | 1.28E-03 | 2.76E-03 |
| rs990174 | TCGA&TCGA_coCNV | 1.28E-03 | 7.67E-03 |
| rs1376544 | TCGA&TCGA_coCNV | 7.37E-04 | 4.10E-04 |
| rs10959622 | TCGA | 1.28E-03 | #N/A |
| rs16986504 | TCGA&TCGA_coCNV | 1.28E-03 | 1.80E-03 |
| rs16852352 | TCGA&TCGA_coCNV | 1.28E-03 | 3.80E-03 |
| rs7628097 | TCGA&TCGA_coCNV | 1.28E-03 | 2.20E-03 |
| rs1019680 | TCGA | 1.28E-03 | #N/A |
| rs12050655 | TCGA&TCGA_coCNV | 1.28E-03 | 1.15E-03 |
| rs12579421 | TCGA&TCGA_coCNV | 1.29E-03 | 4.42E-04 |
| rs17839791 | TCGA | 8.68E-04 | #N/A |
| rs12612759 | TCGA&TCGA_coCNV | 1.29E-03 | 9.20E-03 |
| rs4453901 | TCGA&TCGA_coCNV | 8.93E-04 | 5.20E-04 |
| rs12961224 | TCGA&TCGA_coCNV | 2.95E-04 | 1.35E-04 |

| rs13084780 | TCGA&TCGA_coCNV | 1.29E-03 | 2.78E-04 |
| --- | --- | --- | --- |
| rs1461241 | TCGA | 1.29E-03 | #N/A |
| rs4687319 | TCGA | 1.29E-03 | #N/A |
| rs771889 | TCGA&TCGA_coCNV | 1.29E-03 | 9.97E-03 |
| rs7880545 | TCGA&TCGA_coCNV | 1.29E-03 | 8.57E-04 |
| rs2087688 | TCGA&TCGA_coCNV | 1.29E-03 | 2.44E-03 |
| rs7657617 | TCGA&TCGA_coCNV | 6.52E-04 | 2.41E-03 |
| rs2968667 | TCGA&TCGA_coCNV | 1.29E-03 | 4.11E-03 |
| rs16855865 | TCGA&TCGA_coCNV | 1.29E-03 | 4.90E-03 |
| rs6437473 | TCGA&TCGA_coCNV | 1.29E-03 | 6.76E-04 |
| rs17014189 | TCGA&TCGA_coCNV | 1.29E-03 | 2.12E-03 |
| rs11855800 | TCGA&TCGA_coCNV | 1.29E-03 | 1.17E-04 |
| rs9677250 | TCGA&TCGA_coCNV | 1.29E-03 | 2.75E-03 |
| rs11713133 | TCGA&TCGA_coCNV | 1.29E-03 | 1.74E-04 |
| rs1357434 | TCGA&TCGA_coCNV | 1.29E-03 | 1.87E-05 |
| rs7631626 | TCGA&TCGA_coCNV | 1.29E-03 | 7.32E-03 |
| rs10829271 | TCGA | 1.30E-03 | #N/A |
| rs1150258 | TCGA&TCGA_coCNV | 1.30E-03 | 1.32E-03 |
| rs12618033 | TCGA | 1.30E-03 | #N/A |
| rs1484465 | TCGA&TCGA_coCNV | 1.30E-03 | 6.44E-04 |
| rs1909819 | TCGA&TCGA_coCNV | 1.30E-03 | 9.00E-03 |
| rs9525326 | TCGA&TCGA_coCNV | 1.30E-03 | 1.81E-03 |
| rs12128136 | TCGA&TCGA_coCNV | 1.30E-03 | 4.22E-03 |
| rs2897417 | TCGA&TCGA_coCNV | 1.30E-03 | 1.92E-03 |
| rs11991944 | TCGA&TCGA_coCNV | 1.30E-03 | 1.59E-03 |
| rs2268336 | TCGA | 1.30E-03 | #N/A |
| rs4900247 | TCGA | 1.31E-03 | #N/A |
| rs2065997 | TCGA&TCGA_coCNV | 1.31E-03 | 2.30E-03 |
| rs11631949 | TCGA&TCGA_coCNV | 1.31E-03 | 1.32E-03 |
| rs10919360 | TCGA&TCGA_coCNV | 1.31E-03 | 2.77E-03 |
| rs8032912 | TCGA&TCGA_coCNV | 1.31E-03 | 1.08E-03 |
| rs17502075 | TCGA&TCGA_coCNV | 1.31E-03 | 6.67E-03 |
| rs17054113 | TCGA&TCGA_coCNV | 2.22E-05 | 7.82E-05 |
| rs16970334 | TCGA&TCGA_coCNV | 1.31E-03 | 1.01E-03 |
| rs1348453 | TCGA&TCGA_coCNV | 1.31E-03 | 8.63E-05 |
| rs12556557 | TCGA&TCGA_coCNV | 1.31E-03 | 8.82E-04 |
| rs10888744 | TCGA&TCGA_coCNV | 1.31E-03 | 8.77E-04 |
| rs3753271 | TCGA | 1.31E-03 | #N/A |
| rs13006912 | TCGA | 1.32E-03 | #N/A |
| rs7752681 | TCGA | 1.32E-03 | #N/A |
| rs2033195 | TCGA&TCGA_coCNV | 1.32E-03 | 1.49E-03 |
| rs4286642 | TCGA&TCGA_coCNV | 2.53E-04 | 5.81E-03 |
| rs17026937 | TCGA&TCGA_coCNV | 1.32E-03 | 5.41E-03 |
| rs7778880 | TCGA&TCGA_coCNV | 1.32E-03 | 6.47E-03 |
| rs2388828 | TCGA&TCGA_coCNV | 7.67E-04 | 7.58E-03 |
| rs1404412 | TCGA | 1.32E-03 | #N/A |

| rs2595506 | TCGA | 1.32E-03 | #N/A |
| --- | --- | --- | --- |
| rs4351852 | TCGA | 1.32E-03 | #N/A |
| rs11695198 | TCGA&TCGA_coCNV | 1.32E-03 | 7.52E-03 |
| rs17450407 | TCGA&TCGA_coCNV | 1.32E-03 | 1.67E-03 |
| rs11145266 | TCGA | 1.32E-03 | #N/A |
| rs4381272 | TCGA&TCGA_coCNV | 1.32E-03 | 5.95E-04 |
| rs13317787 | TCGA&TCGA_coCNV | 1.32E-03 | 5.55E-04 |
| rs12458185 | TCGA&TCGA_coCNV | 1.33E-03 | 7.97E-04 |
| rs6916928 | TCGA | 1.33E-03 | #N/A |
| rs17085051 | TCGA&TCGA_coCNV | 1.33E-03 | 4.19E-03 |
| rs9881633 | TCGA&TCGA_coCNV | 1.33E-03 | 1.17E-03 |
| rs17814881 | TCGA&TCGA_coCNV | 1.33E-03 | 6.07E-03 |
| rs6748415 | TCGA&TCGA_coCNV | 1.33E-03 | 2.35E-05 |
| rs9332647 | TCGA&TCGA_coCNV | 1.33E-03 | 2.04E-03 |
| rs9543274 | TCGA | 1.33E-03 | #N/A |
| rs470212 | TCGA | 1.33E-03 | #N/A |
| rs691474 | TCGA&TCGA_coCNV | 1.33E-03 | 2.55E-03 |
| rs36894 | TCGA&TCGA_coCNV | 3.22E-05 | 7.94E-04 |
| rs10914625 | TCGA&TCGA_coCNV | 8.02E-04 | 2.72E-04 |
| rs12167294 | TCGA&TCGA_coCNV | 1.33E-03 | 1.03E-03 |
| rs1944478 | TCGA&TCGA_coCNV | 9.99E-05 | 1.04E-03 |
| rs6360 | TCGA | 1.33E-03 | #N/A |
| rs12028899 | TCGA&TCGA_coCNV | 1.33E-03 | 1.82E-03 |
| rs3770154 | TCGA | 1.33E-03 | #N/A |
| rs2167941 | TCGA | 1.34E-03 | #N/A |
| rs9360862 | TCGA&TCGA_coCNV | 1.34E-03 | 8.54E-04 |
| rs4690410 | TCGA&TCGA_coCNV | 1.34E-03 | 5.94E-03 |
| rs2961680 | TCGA&TCGA_coCNV | 1.34E-03 | 1.29E-03 |
| rs452044 | TCGA&TCGA_coCNV | 1.34E-03 | 3.75E-04 |
| rs6776608 | TCGA&TCGA_coCNV | 9.15E-04 | 4.64E-03 |
| rs1217061 | TCGA | 1.34E-03 | #N/A |
| rs989447 | TCGA&TCGA_coCNV | 7.82E-05 | 2.49E-04 |
| rs2391018 | TCGA&TCGA_coCNV | 1.34E-03 | 4.44E-03 |
| rs4636851 | TCGA&TCGA_coCNV | 1.34E-03 | 1.57E-03 |
| rs6563234 | TCGA&TCGA_coCNV | 1.34E-03 | 3.23E-04 |
| rs6640879 | TCGA&TCGA_coCNV | 1.34E-03 | 1.04E-04 |
| rs35963889 | TCGA&TCGA_coCNV | 1.34E-03 | 1.62E-03 |
| rs11001052 | TCGA&TCGA_coCNV | 1.35E-03 | 2.36E-03 |
| rs9362672 | TCGA | 1.35E-03 | #N/A |
| rs11726260 | TCGA&TCGA_coCNV | 1.35E-03 | 6.63E-03 |
| rs12288992 | TCGA&TCGA_coCNV | 1.35E-03 | 5.28E-04 |
| rs1999951 | TCGA&TCGA_coCNV | 1.35E-03 | 1.66E-03 |
| rs1951217 | TCGA&TCGA_coCNV | 1.35E-03 | 6.91E-03 |
| rs2128100 | TCGA | 1.35E-03 | #N/A |
| rs13078739 | TCGA&TCGA_coCNV | 1.35E-03 | 8.88E-03 |
| rs7133728 | TCGA | 1.35E-03 | #N/A |

| rs1762428 | TCGA&TCGA_coCNV | 1.35E-03 | 1.32E-04 |
| --- | --- | --- | --- |
| rs4890620 | TCGA&TCGA_coCNV | 1.35E-03 | 5.46E-03 |
| rs4279776 | TCGA&TCGA_coCNV | 1.35E-03 | 9.31E-04 |
| rs11866138 | TCGA&TCGA_coCNV | 1.35E-03 | 8.14E-04 |
| rs10498114 | TCGA&TCGA_coCNV | 1.35E-03 | 2.68E-03 |
| rs731154 | TCGA&TCGA_coCNV | 1.35E-03 | 9.12E-03 |
| rs10511616 | TCGA&TCGA_coCNV | 1.35E-03 | 6.33E-03 |
| rs1349131 | TCGA&TCGA_coCNV | 1.35E-03 | 8.79E-03 |
| rs17123832 | TCGA | 1.35E-03 | #N/A |
| rs6976037 | TCGA&TCGA_coCNV | 1.35E-03 | 2.45E-03 |
| rs2729420 | TCGA | 1.36E-03 | #N/A |
| rs9843961 | TCGA&TCGA_coCNV | 1.36E-03 | 9.19E-03 |
| rs2972284 | TCGA | 1.36E-03 | #N/A |
| rs1470261 | TCGA | 1.36E-03 | #N/A |
| rs2654596 | TCGA&TCGA_coCNV | 1.36E-03 | 7.93E-04 |
| rs1397250 | TCGA&TCGA_coCNV | 1.36E-03 | 4.46E-03 |
| rs12558336 | TCGA | 1.36E-03 | #N/A |
| rs193569 | TCGA&TCGA_coCNV | 1.36E-03 | 2.61E-03 |
| rs10831325 | TCGA&TCGA_coCNV | 1.36E-03 | 9.14E-03 |
| rs7886058 | TCGA&TCGA_coCNV | 1.36E-03 | 9.98E-04 |
| rs797013 | TCGA | 1.36E-03 | #N/A |
| rs2526775 | TCGA&TCGA_coCNV | 1.36E-03 | 3.44E-04 |
| rs12688731 | TCGA&TCGA_coCNV | 2.83E-05 | 1.19E-06 |
| rs2596668 | TCGA&TCGA_coCNV | 1.36E-03 | 2.06E-04 |
| rs4514513 | TCGA&TCGA_coCNV | 1.36E-03 | 6.11E-03 |
| rs2418999 | TCGA | 1.37E-03 | #N/A |
| rs11724690 | TCGA&TCGA_coCNV | 1.37E-03 | 2.73E-03 |
| rs1395255 | TCGA&TCGA_coCNV | 1.37E-03 | 2.73E-03 |
| rs2079615 | TCGA | 1.37E-03 | #N/A |
| rs4104099 | TCGA&TCGA_coCNV | 1.37E-03 | 8.02E-05 |
| rs1406967 | TCGA&TCGA_coCNV | 1.37E-03 | 3.24E-04 |
| rs6733994 | TCGA&TCGA_coCNV | 1.37E-03 | 7.99E-04 |
| rs5908325 | TCGA&TCGA_coCNV | 1.37E-03 | 6.37E-04 |
| rs6674898 | TCGA&TCGA_coCNV | 1.37E-03 | 2.55E-03 |
| rs1943400 | TCGA&TCGA_coCNV | 1.37E-03 | 9.12E-03 |
| rs5975124 | TCGA&TCGA_coCNV | 3.03E-04 | 2.69E-04 |
| rs2474516 | TCGA&TCGA_coCNV | 1.37E-03 | 1.61E-04 |
| rs2097307 | TCGA&TCGA_coCNV | 5.48E-05 | 5.00E-04 |
| rs2586440 | TCGA&TCGA_coCNV | 2.00E-04 | 7.13E-04 |
| rs13415904 | TCGA&TCGA_coCNV | 1.38E-03 | 2.35E-03 |
| rs11723966 | TCGA&TCGA_coCNV | 1.38E-03 | 3.69E-03 |
| rs1202296 | TCGA&TCGA_coCNV | 1.38E-03 | 8.52E-04 |
| rs10496447 | TCGA&TCGA_coCNV | 1.38E-03 | 2.05E-03 |
| rs1332182 | TCGA&TCGA_coCNV | 1.38E-03 | 1.54E-03 |
| rs4738922 | TCGA&TCGA_coCNV | 1.38E-03 | 3.89E-04 |
| rs1028777 | TCGA&TCGA_coCNV | 1.38E-03 | 8.81E-03 |

| rs2456830 | TCGA | 1.38E-03 | #N/A |
| --- | --- | --- | --- |
| rs3112609 | TCGA | 1.38E-03 | #N/A |
| rs17521702 | TCGA&TCGA_coCNV | 1.38E-03 | 6.94E-03 |
| rs13415097 | TCGA | 1.38E-03 | #N/A |
| rs12739200 | TCGA&TCGA_coCNV | 1.38E-03 | 4.67E-04 |
| rs4306056 | TCGA&TCGA_coCNV | 1.38E-03 | 2.78E-03 |
| rs7690592 | TCGA | 1.38E-03 | #N/A |
| rs11865085 | TCGA | 1.38E-03 | #N/A |
| rs1871859 | TCGA&TCGA_coCNV | 1.31E-03 | 8.07E-03 |
| rs11985836 | TCGA | 1.38E-03 | #N/A |
| rs17792619 | TCGA&TCGA_coCNV | 1.39E-03 | 9.54E-03 |
| rs17040831 | TCGA&TCGA_coCNV | 1.39E-03 | 6.52E-03 |
| rs4909741 | TCGA&TCGA_coCNV | 3.07E-05 | 2.13E-03 |
| rs3737891 | TCGA&TCGA_coCNV | 1.39E-03 | 3.33E-03 |
| rs7359996 | TCGA | 1.39E-03 | #N/A |
| rs3788785 | TCGA&TCGA_coCNV | 1.39E-03 | 3.51E-03 |
| rs6890675 | TCGA&TCGA_coCNV | 7.99E-04 | 3.10E-03 |
| rs10125792 | TCGA | 1.39E-03 | #N/A |
| rs9381398 | TCGA&TCGA_coCNV | 1.39E-03 | 5.35E-04 |
| rs17108926 | TCGA&TCGA_coCNV | 1.39E-03 | 5.33E-03 |
| rs7062338 | TCGA | 1.39E-03 | #N/A |
| rs2580759 | TCGA&TCGA_coCNV | 1.39E-03 | 4.41E-03 |
| rs16876093 | TCGA&TCGA_coCNV | 1.39E-03 | 5.31E-03 |
| rs252930 | TCGA&TCGA_coCNV | 1.39E-03 | 2.86E-04 |
| rs10495321 | TCGA&TCGA_coCNV | 1.84E-04 | 3.56E-04 |
| rs2082477 | TCGA&TCGA_coCNV | 1.39E-03 | 8.93E-03 |
| rs11975826 | TCGA | 1.40E-03 | #N/A |
| rs4300184 | TCGA | 1.40E-03 | #N/A |
| rs1330695 | TCGA&TCGA_coCNV | 1.40E-03 | 6.30E-04 |
| rs17667613 | TCGA&TCGA_coCNV | 1.40E-03 | 2.06E-04 |
| rs902254 | TCGA&TCGA_coCNV | 1.40E-03 | 7.24E-03 |
| rs9445549 | TCGA&TCGA_coCNV | 1.40E-03 | 1.20E-03 |
| rs9629043 | TCGA&TCGA_coCNV | 1.40E-03 | 2.74E-04 |
| rs7888253 | TCGA&TCGA_coCNV | 5.03E-04 | 4.94E-05 |
| rs570880 | TCGA&TCGA_coCNV | 1.40E-03 | 4.33E-03 |
| rs1127291 | TCGA&TCGA_coCNV | 8.26E-04 | 2.37E-04 |
| rs984441 | TCGA&TCGA_coCNV | 1.40E-03 | 5.15E-03 |
| rs2840697 | TCGA&TCGA_coCNV | 4.76E-04 | 3.04E-05 |
| rs12689800 | TCGA&TCGA_coCNV | 1.40E-03 | 4.53E-03 |
| rs2526776 | TCGA&TCGA_coCNV | 1.40E-03 | 1.65E-04 |
| rs12647543 | TCGA | 1.40E-03 | #N/A |
| rs10176699 | TCGA&TCGA_coCNV | 1.40E-03 | 4.63E-03 |
| rs2701789 | TCGA | 1.40E-03 | #N/A |
| rs17033491 | TCGA | 1.41E-03 | #N/A |
| rs10782964 | TCGA | 1.41E-03 | #N/A |
| rs28358582 | TCGA | 1.41E-03 | #N/A |

| rs1826864 | TCGA | 1.41E-03 | #N/A |
| --- | --- | --- | --- |
| rs9323934 | TCGA&TCGA_coCNV | 1.41E-03 | 5.40E-03 |
| rs7567647 | TCGA&TCGA_coCNV | 1.41E-03 | 5.52E-03 |
| rs4633248 | TCGA | 1.41E-03 | #N/A |
| rs1470675 | TCGA&TCGA_coCNV | 1.41E-03 | 2.77E-03 |
| rs1036552 | TCGA | 1.41E-03 | #N/A |
| rs16903104 | TCGA&TCGA_coCNV | 1.41E-03 | 1.03E-03 |
| rs7918731 | TCGA&TCGA_coCNV | 1.41E-03 | 2.52E-03 |
| rs7702336 | TCGA&TCGA_coCNV | 1.41E-03 | 8.44E-03 |
| rs3772775 | TCGA&TCGA_coCNV | 1.41E-03 | 4.15E-03 |
| rs13374478 | TCGA&TCGA_coCNV | 1.41E-03 | 1.23E-03 |
| rs2676629 | TCGA&TCGA_coCNV | 1.41E-03 | 1.35E-04 |
| rs4757150 | TCGA&TCGA_coCNV | 1.41E-03 | 1.56E-03 |
| rs17117362 | TCGA&TCGA_coCNV | 1.41E-03 | 2.90E-03 |
| rs11844095 | TCGA&TCGA_coCNV | 1.03E-03 | 1.69E-04 |
| rs1144257 | TCGA&TCGA_coCNV | 1.42E-03 | 3.46E-03 |
| rs9573572 | TCGA&TCGA_coCNV | 1.42E-03 | 4.26E-03 |
| rs3797446 | TCGA | 1.42E-03 | #N/A |
| rs3118124 | TCGA | 1.42E-03 | #N/A |
| rs9421478 | TCGA&TCGA_coCNV | 1.42E-03 | 8.27E-03 |
| rs6741506 | TCGA&TCGA_coCNV | 1.42E-03 | 2.19E-03 |
| rs5929602 | TCGA&TCGA_coCNV | 3.35E-04 | 7.28E-03 |
| rs28357987 | TCGA | 1.43E-03 | #N/A |
| rs10062917 | TCGA | 1.43E-03 | #N/A |
| rs4300741 | TCGA&TCGA_coCNV | 1.43E-03 | 1.49E-03 |
| rs290490 | TCGA | 1.43E-03 | #N/A |
| rs10732874 | TCGA&TCGA_coCNV | 1.43E-03 | 4.64E-03 |
| rs968243 | TCGA&TCGA_coCNV | 1.43E-03 | 1.14E-03 |
| rs11634458 | TCGA&TCGA_coCNV | 7.44E-04 | 1.11E-03 |
| rs1368023 | TCGA&TCGA_coCNV | 2.48E-04 | 8.93E-05 |
| rs7762018 | TCGA&SCANdb | 1.43E-03 | #N/A |
| rs13203110 | TCGA&TCGA_coCNV | 1.43E-03 | 4.20E-03 |
| rs1953334 | TCGA&TCGA_coCNV | 1.43E-03 | 6.59E-03 |
| rs6097809 | TCGA | 1.43E-03 | #N/A |
| rs12008519 | TCGA&TCGA_coCNV | 1.43E-03 | 3.88E-03 |
| rs1936663 | TCGA&TCGA_coCNV | 1.43E-03 | 1.28E-03 |
| rs1925612 | TCGA | 1.44E-03 | #N/A |
| rs5924676 | TCGA | 1.44E-03 | #N/A |
| rs4611156 | TCGA | 1.44E-03 | #N/A |
| rs1466137 | TCGA&TCGA_coCNV | 1.44E-03 | 5.37E-03 |
| rs12620430 | TCGA&TCGA_coCNV | 1.22E-03 | 1.60E-03 |
| rs8020084 | TCGA&TCGA_coCNV | 1.44E-03 | 6.24E-03 |
| rs16831005 | TCGA | 1.44E-03 | #N/A |
| rs2884887 | TCGA | 1.44E-03 | #N/A |
| rs17268667 | TCGA&TCGA_coCNV | 1.44E-03 | 8.79E-03 |
| rs17083580 | TCGA&TCGA_coCNV | 4.63E-04 | 1.44E-03 |

| rs17258178 | TCGA&TCGA_coCNV | 1.44E-03 | 3.44E-03 |
| --- | --- | --- | --- |
| rs10210743 | TCGA&TCGA_coCNV | 1.44E-03 | 2.89E-03 |
| rs160405 | TCGA | 1.44E-03 | #N/A |
| rs16878301 | TCGA&TCGA_coCNV | 1.45E-03 | 9.95E-03 |
| rs3867242 | TCGA&TCGA_coCNV | 1.45E-03 | 2.21E-03 |
| rs16964591 | TCGA&TCGA_coCNV | 8.21E-04 | 1.40E-04 |
| rs935614 | TCGA&TCGA_coCNV | 2.90E-04 | 1.50E-04 |
| rs668959 | TCGA&TCGA_coCNV | 1.45E-03 | 4.40E-04 |
| rs926817 | TCGA&TCGA_coCNV | 1.06E-03 | 6.26E-03 |
| rs7984459 | TCGA&TCGA_coCNV | 1.03E-03 | 6.42E-04 |
| rs6675898 | TCGA&TCGA_coCNV | 1.46E-03 | 8.41E-03 |
| rs4694116 | TCGA&TCGA_coCNV | 1.46E-03 | 1.46E-03 |
| rs12517065 | TCGA&TCGA_coCNV | 1.46E-03 | 3.87E-03 |
| rs9831534 | TCGA&TCGA_coCNV | 1.46E-03 | 7.93E-03 |
| rs17124881 | TCGA&TCGA_coCNV | 1.46E-03 | 1.30E-03 |
| rs2715464 | TCGA&TCGA_coCNV | 1.46E-03 | 9.14E-03 |
| rs5763001 | TCGA&TCGA_coCNV | 1.46E-03 | 7.25E-04 |
| rs12704071 | TCGA&TCGA_coCNV | 1.46E-03 | 2.31E-03 |
| rs4867979 | TCGA | 1.46E-03 | #N/A |
| rs7076612 | TCGA&TCGA_coCNV | 1.46E-03 | 3.24E-03 |
| rs7527646 | TCGA&TCGA_coCNV | 2.96E-05 | 2.52E-05 |
| rs10737393 | TCGA&TCGA_coCNV | 1.46E-03 | 9.47E-03 |
| rs11122319 | TCGA&TCGA_coCNV | 1.46E-03 | 2.85E-03 |
| rs469999 | TCGA&TCGA_coCNV | 1.47E-03 | 1.87E-03 |
| rs11985451 | TCGA&TCGA_coCNV | 1.47E-03 | 8.96E-03 |
| rs4646491 | TCGA&TCGA_coCNV | 1.47E-03 | 1.70E-03 |
| rs10838341 | TCGA | 1.47E-03 | #N/A |
| rs9457053 | TCGA | 1.47E-03 | #N/A |
| rs16881955 | TCGA&TCGA_coCNV | 1.47E-03 | 9.72E-03 |
| rs828005 | TCGA | 1.47E-03 | #N/A |
| rs6120401 | TCGA&TCGA_coCNV | 1.47E-03 | 5.60E-03 |
| rs6027343 | TCGA | 1.47E-03 | #N/A |
| rs6527253 | TCGA | 1.47E-03 | #N/A |
| rs1029898 | TCGA&TCGA_coCNV | 1.47E-03 | 1.75E-03 |
| rs6683472 | TCGA | 1.48E-03 | #N/A |
| rs12994120 | TCGA | 1.48E-03 | #N/A |
| rs5998500 | TCGA&TCGA_coCNV | 1.48E-03 | 4.78E-03 |
| rs1449881 | TCGA&TCGA_coCNV | 1.48E-03 | 8.61E-04 |
| rs1954578 | TCGA | 1.48E-03 | #N/A |
| rs130051 | TCGA&TCGA_coCNV | 1.48E-03 | 7.80E-03 |
| rs5747145 | TCGA&TCGA_coCNV | 1.48E-03 | 7.84E-04 |
| rs4478536 | TCGA | 1.48E-03 | #N/A |
| rs728528 | TCGA | 1.48E-03 | #N/A |
| rs4916290 | TCGA&TCGA_coCNV | 1.48E-03 | 1.48E-03 |
| rs2723579 | TCGA&TCGA_coCNV | 1.48E-03 | 2.66E-03 |
| rs2930073 | TCGA&TCGA_coCNV | 1.48E-03 | 8.98E-04 |

| rs10516561 | TCGA | 1.48E-03 | #N/A |
| --- | --- | --- | --- |
| rs17113118 | TCGA&TCGA_coCNV | 1.49E-03 | 3.56E-03 |
| rs4503250 | TCGA | 1.49E-03 | #N/A |
| rs17155051 | TCGA&TCGA_coCNV | 1.49E-03 | 4.94E-03 |
| rs7261570 | TCGA&TCGA_coCNV | 1.49E-03 | 6.30E-04 |
| rs6715576 | TCGA&TCGA_coCNV | 1.49E-03 | 8.84E-03 |
| rs9634473 | TCGA | 1.49E-03 | #N/A |
| rs10959847 | TCGA | 1.49E-03 | #N/A |
| rs2275750 | TCGA&TCGA_coCNV | 1.49E-03 | 5.98E-04 |
| rs4772144 | TCGA&TCGA_coCNV | 1.49E-03 | 7.86E-04 |
| rs17203677 | TCGA&TCGA_coCNV | 1.50E-03 | 9.50E-04 |
| rs6725546 | TCGA | 1.50E-03 | #N/A |
| rs1553477 | TCGA | 1.50E-03 | #N/A |
| rs12525646 | TCGA | 1.50E-03 | #N/A |
| rs4667547 | TCGA&TCGA_coCNV | 1.50E-03 | 8.16E-03 |
| rs17639812 | TCGA&TCGA_coCNV | 1.50E-03 | 4.95E-03 |
| rs16878553 | TCGA&TCGA_coCNV | 1.50E-03 | 4.13E-03 |
| rs2059398 | TCGA | 1.50E-03 | #N/A |
| rs3953455 | TCGA&TCGA_coCNV | 1.50E-03 | 2.28E-03 |
| rs8036772 | TCGA | 1.50E-03 | #N/A |
| rs1134115 | TCGA&TCGA_coCNV | 1.50E-03 | 4.67E-03 |
| rs4300285 | TCGA | 1.50E-03 | #N/A |
| rs7315441 | TCGA&TCGA_coCNV | 1.50E-03 | 2.54E-03 |
| rs2556506 | TCGA&TCGA_coCNV | 1.50E-03 | 5.84E-03 |
| rs6568921 | TCGA&TCGA_coCNV | 1.71E-04 | 6.18E-06 |
| rs2738286 | TCGA&TCGA_coCNV | 2.17E-04 | 2.23E-04 |
| rs4861158 | TCGA | 1.51E-03 | #N/A |
| rs6848028 | TCGA | 1.51E-03 | #N/A |
| rs1377497 | TCGA | 1.51E-03 | #N/A |
| rs10966472 | TCGA | 1.51E-03 | #N/A |
| rs521135 | TCGA&TCGA_coCNV | 1.30E-03 | 1.37E-04 |
| rs7324507 | TCGA&TCGA_coCNV | 1.43E-03 | 1.35E-03 |
| rs10738546 | TCGA&TCGA_coCNV | 1.51E-03 | 4.60E-03 |
| rs7820505 | TCGA | 1.51E-03 | #N/A |
| rs2328066 | TCGA | 1.51E-03 | #N/A |
| rs9584779 | TCGA&TCGA_coCNV | 1.51E-03 | 1.06E-03 |
| rs17599814 | TCGA | 1.51E-03 | #N/A |
| rs2451018 | TCGA&TCGA_coCNV | 1.52E-03 | 2.01E-03 |
| rs17659543 | TCGA | 1.52E-03 | #N/A |
| rs2122222 | TCGA | 1.52E-03 | #N/A |
| rs4082843 | TCGA&TCGA_coCNV | 1.52E-03 | 3.57E-03 |
| rs6543402 | TCGA&TCGA_coCNV | 1.52E-03 | 3.21E-04 |
| rs6596855 | TCGA&TCGA_coCNV | 1.52E-03 | 1.77E-03 |
| rs1202437 | TCGA | 1.52E-03 | #N/A |
| rs11962317 | TCGA&TCGA_coCNV | 1.52E-03 | 1.05E-03 |
| rs7930674 | TCGA&TCGA_coCNV | 1.52E-03 | 8.07E-05 |

| rs17100500 | TCGA&TCGA_coCNV | 1.52E-03 | 6.09E-05 |
| --- | --- | --- | --- |
| rs1876086 | TCGA | 1.53E-03 | #N/A |
| rs4141870 | TCGA&TCGA_coCNV | 1.53E-03 | 6.19E-03 |
| rs7032605 | TCGA | 1.53E-03 | #N/A |
| rs10508855 | TCGA&TCGA_coCNV | 1.53E-03 | 1.83E-03 |
| rs3767661 | TCGA&TCGA_coCNV | 1.53E-03 | 1.87E-03 |
| rs12186102 | TCGA&TCGA_coCNV | 1.53E-03 | 1.57E-03 |
| rs12184990 | TCGA | 1.53E-03 | #N/A |
| rs1401492 | TCGA&TCGA_coCNV | 1.53E-03 | 7.47E-04 |
| rs12364823 | TCGA&TCGA_coCNV | 1.53E-03 | 1.94E-03 |
| rs12233940 | TCGA&TCGA_coCNV | 1.53E-03 | 9.92E-03 |
| rs826380 | TCGA&TCGA_coCNV | 1.53E-03 | 6.41E-04 |
| rs9960257 | TCGA&TCGA_coCNV | 1.53E-03 | 1.29E-03 |
| rs10903712 | TCGA&SCANdb | 1.53E-03 | #N/A |
| rs3806963 | TCGA&TCGA_coCNV | 1.53E-03 | 2.39E-03 |
| rs10018821 | TCGA&TCGA_coCNV | 1.54E-03 | 2.53E-04 |
| rs17100835 | TCGA&TCGA_coCNV | 1.54E-03 | 2.83E-03 |
| rs2683545 | TCGA&TCGA_coCNV | 1.54E-03 | 7.98E-03 |
| rs1883941 | TCGA&TCGA_coCNV | 1.54E-03 | 9.91E-04 |
| rs10900946 | TCGA | 1.54E-03 | #N/A |
| rs11015707 | TCGA&TCGA_coCNV | 1.54E-03 | 5.90E-03 |
| rs791190 | TCGA&TCGA_coCNV | 1.54E-03 | 2.77E-03 |
| rs12917403 | TCGA | 1.54E-03 | #N/A |
| rs7221773 | TCGA | 1.24E-03 | #N/A |
| rs10926147 | TCGA&TCGA_coCNV | 1.54E-03 | 3.68E-05 |
| rs6425717 | TCGA&TCGA_coCNV | 1.54E-03 | 1.72E-03 |
| rs1550750 | TCGA&TCGA_coCNV | 4.81E-04 | 3.67E-03 |
| rs1898552 | TCGA&TCGA_coCNV | 1.54E-03 | 1.45E-04 |
| rs9303008 | TCGA&TCGA_coCNV | 1.54E-03 | 6.48E-04 |
| rs2768595 | TCGA | 1.54E-03 | #N/A |
| rs2874739 | TCGA&TCGA_coCNV | 4.38E-04 | 4.92E-05 |
| rs13221600 | TCGA | 1.55E-03 | #N/A |
| rs1122389 | TCGA&TCGA_coCNV | 1.55E-03 | 7.41E-05 |
| rs9457048 | TCGA | 1.55E-03 | #N/A |
| rs17311996 | TCGA | 1.55E-03 | #N/A |
| rs430610 | TCGA&TCGA_coCNV | 1.55E-03 | 6.24E-03 |
| rs1547763 | TCGA | 1.55E-03 | #N/A |
| rs16987103 | TCGA&TCGA_coCNV | 1.55E-03 | 1.89E-04 |
| rs6530155 | TCGA&TCGA_coCNV | 1.55E-03 | 5.93E-04 |
| rs10865331 | TCGA&TCGA_coCNV | 1.55E-03 | 7.84E-03 |
| rs41422848 | TCGA&TCGA_coCNV | 1.55E-03 | 9.84E-05 |
| rs1961563 | TCGA&TCGA_coCNV | 1.55E-03 | 2.19E-04 |
| rs9649574 | TCGA&TCGA_coCNV | 1.55E-03 | 9.67E-03 |
| rs16824341 | TCGA&TCGA_coCNV | 1.56E-03 | 3.43E-03 |
| rs8000797 | TCGA | 1.56E-03 | #N/A |
| rs12229073 | TCGA&TCGA_coCNV | 1.56E-03 | 3.36E-04 |

| rs6421418 | TCGA&TCGA_coCNV | 4.67E-04 | 5.41E-03 |
| --- | --- | --- | --- |
| rs6940448 | TCGA&TCGA_coCNV | 7.70E-04 | 3.39E-03 |
| rs2618022 | TCGA | 1.56E-03 | #N/A |
| rs4825066 | TCGA | 1.56E-03 | #N/A |
| rs10915342 | TCGA&TCGA_coCNV | 1.56E-03 | 3.91E-04 |
| rs9956521 | TCGA&TCGA_coCNV | 1.56E-03 | 3.85E-03 |
| rs17815215 | TCGA&TCGA_coCNV | 1.56E-03 | 5.37E-04 |
| rs7336486 | TCGA&TCGA_coCNV | 1.56E-03 | 1.69E-03 |
| rs12416305 | TCGA&TCGA_coCNV | 1.56E-03 | 8.37E-03 |
| rs1441890 | TCGA | 1.56E-03 | #N/A |
| rs7800951 | TCGA | 1.56E-03 | #N/A |
| rs7118973 | TCGA&TCGA_coCNV | 1.57E-03 | 4.79E-03 |
| rs11694068 | TCGA&TCGA_coCNV | 1.57E-03 | 6.19E-04 |
| rs10991378 | TCGA | 1.57E-03 | #N/A |
| rs7166322 | TCGA&TCGA_coCNV | 2.03E-05 | 2.45E-04 |
| rs559322 | TCGA&TCGA_coCNV | 2.70E-04 | 1.04E-03 |
| rs7901838 | TCGA&TCGA_coCNV | 1.57E-03 | 2.82E-03 |
| rs2935621 | TCGA | 1.57E-03 | #N/A |
| rs3759273 | TCGA | 1.57E-03 | #N/A |
| rs1145090 | TCGA&TCGA_coCNV | 1.57E-03 | 9.01E-03 |
| rs16929048 | TCGA&TCGA_coCNV | 1.58E-03 | 9.94E-04 |
| rs17484794 | TCGA&TCGA_coCNV | 1.58E-03 | 1.46E-03 |
| rs1461757 | TCGA&TCGA_coCNV | 1.58E-03 | 2.77E-03 |
| rs12060692 | TCGA&TCGA_coCNV | 1.58E-03 | 4.81E-03 |
| rs13023812 | TCGA&TCGA_coCNV | 1.58E-03 | 2.69E-03 |
| rs11870340 | TCGA&TCGA_coCNV | 1.58E-03 | 1.04E-03 |
| rs6640866 | TCGA&TCGA_coCNV | 1.58E-03 | 6.39E-04 |
| rs4446473 | TCGA&TCGA_coCNV | 1.58E-03 | 5.37E-04 |
| rs4701026 | TCGA | 1.58E-03 | #N/A |
| rs815611 | TCGA&TCGA_coCNV | 1.58E-03 | 3.81E-03 |
| rs7030591 | TCGA&TCGA_coCNV | 1.58E-03 | 1.04E-04 |
| rs16925018 | TCGA&TCGA_coCNV | 1.58E-03 | 6.78E-03 |
| rs1821990 | TCGA | 1.58E-03 | #N/A |
| rs11067031 | TCGA | 1.58E-03 | #N/A |
| rs2738402 | TCGA&TCGA_coCNV | 1.58E-03 | 6.40E-03 |
| rs10506457 | TCGA&TCGA_coCNV | 1.58E-03 | 4.21E-03 |
| rs11753937 | TCGA&TCGA_coCNV | 1.23E-03 | 1.57E-03 |
| rs1877879 | TCGA | 1.59E-03 | #N/A |
| rs1375272 | TCGA&TCGA_coCNV | 1.59E-03 | 6.47E-03 |
| rs12041192 | TCGA | 1.59E-03 | #N/A |
| rs12233783 | TCGA&TCGA_coCNV | 1.59E-03 | 9.05E-03 |
| rs6558383 | TCGA&TCGA_coCNV | 1.33E-03 | 9.28E-03 |
| rs17057168 | TCGA | 1.59E-03 | #N/A |
| rs5982920 | TCGA | 1.13E-03 | #N/A |
| rs17613220 | TCGA | 1.59E-03 | #N/A |
| rs13287096 | TCGA&TCGA_coCNV | 1.60E-03 | 2.93E-04 |

| rs3845847 | TCGA&TCGA_coCNV | 1.60E-03 | 8.24E-03 |
| --- | --- | --- | --- |
| rs36849 | TCGA&TCGA_coCNV | 7.92E-04 | 3.33E-03 |
| rs9540308 | TCGA&TCGA_coCNV | 1.60E-03 | 5.10E-03 |
| rs6618905 | TCGA | 1.60E-03 | #N/A |
| rs4462692 | TCGA | 1.60E-03 | #N/A |
| rs2124493 | TCGA&TCGA_coCNV | 1.60E-03 | 4.55E-03 |
| rs17254754 | TCGA | 1.60E-03 | #N/A |
| rs9470353 | TCGA | 1.60E-03 | #N/A |
| rs12425333 | TCGA | 1.60E-03 | #N/A |
| rs9496177 | TCGA&TCGA_coCNV | 1.60E-03 | 3.68E-03 |
| rs3127572 | TCGA&TCGA_coCNV | 1.60E-03 | 3.92E-03 |
| rs10199534 | TCGA | 1.60E-03 | #N/A |
| rs12754490 | TCGA | 1.60E-03 | #N/A |
| rs4762975 | TCGA | 1.60E-03 | #N/A |
| rs7077187 | TCGA&TCGA_coCNV | 1.60E-03 | 9.40E-03 |
| rs11001281 | TCGA&TCGA_coCNV | 1.60E-03 | 2.92E-03 |
| rs12467062 | TCGA&TCGA_coCNV | 1.60E-03 | 7.45E-03 |
| rs12606007 | TCGA&TCGA_coCNV | 1.60E-03 | 6.71E-03 |
| rs11241098 | TCGA&TCGA_coCNV | 4.67E-04 | 3.38E-04 |
| rs9348263 | TCGA&TCGA_coCNV | 1.61E-03 | 2.13E-04 |
| rs17312115 | TCGA&TCGA_coCNV | 1.61E-03 | 5.86E-03 |
| rs16906031 | TCGA&TCGA_coCNV | 1.61E-03 | 1.11E-03 |
| rs7656326 | TCGA&TCGA_coCNV | 1.61E-03 | 1.86E-03 |
| rs7529482 | TCGA&TCGA_coCNV | 1.35E-03 | 4.03E-03 |
| rs2169480 | TCGA&TCGA_coCNV | 1.61E-03 | 3.10E-03 |
| rs17047949 | TCGA | 1.61E-03 | #N/A |
| rs11750647 | TCGA&TCGA_coCNV | 1.61E-03 | 2.02E-03 |
| rs6502752 | TCGA | 1.61E-03 | #N/A |
| rs901782 | TCGA&TCGA_coCNV | 1.62E-03 | 1.87E-03 |
| rs6552929 | TCGA&TCGA_coCNV | 1.62E-03 | 3.71E-03 |
| rs11718475 | TCGA&TCGA_coCNV | 1.62E-03 | 5.45E-03 |
| rs2024528 | TCGA&TCGA_coCNV | 1.81E-04 | 3.35E-03 |
| rs2511317 | TCGA&TCGA_coCNV | 1.62E-03 | 1.55E-03 |
| rs1994526 | TCGA | 1.62E-03 | #N/A |
| rs3759272 | TCGA&TCGA_coCNV | 1.62E-03 | 9.49E-03 |
| rs17688984 | TCGA&TCGA_coCNV | 1.62E-03 | 4.60E-03 |
| rs9945182 | TCGA&TCGA_coCNV | 1.62E-03 | 2.51E-03 |
| rs3822679 | TCGA&TCGA_coCNV | 1.62E-03 | 9.35E-03 |
| rs17748352 | TCGA&TCGA_coCNV | 1.62E-03 | 8.41E-03 |
| rs9668315 | TCGA&TCGA_coCNV | 6.62E-04 | 2.01E-04 |
| rs12477247 | TCGA&TCGA_coCNV | 1.62E-03 | 6.29E-03 |
| rs12224049 | TCGA&TCGA_coCNV | 1.62E-03 | 5.83E-03 |
| rs7114068 | TCGA | 1.62E-03 | #N/A |
| rs9845424 | TCGA&TCGA_coCNV | 1.62E-03 | 4.14E-03 |
| rs391794 | TCGA | 1.62E-03 | #N/A |
| rs4790907 | TCGA | 1.62E-03 | #N/A |

| rs11842316 | TCGA&TCGA_coCNV | 1.62E-03 | 6.90E-04 |
| --- | --- | --- | --- |
| rs5962044 | TCGA | 1.62E-03 | #N/A |
| rs35850283 | TCGA | 1.63E-03 | #N/A |
| rs11818374 | TCGA&TCGA_coCNV | 8.35E-05 | 1.30E-03 |
| rs17231856 | TCGA | 1.63E-03 | #N/A |
| rs4849413 | TCGA&TCGA_coCNV | 1.63E-03 | 2.39E-03 |
| rs9378813 | TCGA&TCGA_coCNV | 5.78E-06 | 8.21E-04 |
| rs12579770 | TCGA | 1.63E-03 | #N/A |
| rs583368 | TCGA | 1.63E-03 | #N/A |
| rs1391344 | TCGA&TCGA_coCNV | 7.18E-04 | 2.13E-03 |
| rs7673643 | TCGA | 1.63E-03 | #N/A |
| rs1904756 | TCGA&TCGA_coCNV | 1.63E-03 | 7.81E-04 |
| rs11675731 | TCGA&TCGA_coCNV | 1.63E-03 | 1.35E-03 |
| rs7427616 | TCGA | 1.63E-03 | #N/A |
| rs1559063 | TCGA&TCGA_coCNV | 1.63E-03 | 2.44E-03 |
| rs7721185 | TCGA | 1.63E-03 | #N/A |
| rs7762368 | TCGA | 1.64E-03 | #N/A |
| rs16973105 | TCGA | 1.64E-03 | #N/A |
| rs483970 | TCGA&TCGA_coCNV | 1.64E-03 | 2.39E-04 |
| rs13192449 | TCGA&TCGA_coCNV | 1.64E-03 | 9.06E-05 |
| rs7301592 | TCGA&TCGA_coCNV | 1.64E-03 | 1.90E-03 |
| rs10016511 | TCGA&TCGA_coCNV | 1.64E-03 | 2.03E-03 |
| rs9570012 | TCGA | 1.64E-03 | #N/A |
| rs17167396 | TCGA&TCGA_coCNV | 1.64E-03 | 1.05E-03 |
| rs10820562 | TCGA&TCGA_coCNV | 1.64E-03 | 2.57E-04 |
| rs10483437 | TCGA&TCGA_coCNV | 1.64E-03 | 9.78E-04 |
| rs1844711 | TCGA&TCGA_coCNV | 1.64E-03 | 6.61E-03 |
| rs5934007 | TCGA | 1.64E-03 | #N/A |
| rs12913266 | TCGA&TCGA_coCNV | 1.65E-03 | 1.45E-03 |
| rs8028504 | TCGA&TCGA_coCNV | 1.65E-03 | 3.30E-03 |
| rs11152377 | TCGA&TCGA_coCNV | 1.65E-03 | 7.82E-03 |
| rs1035347 | TCGA | 1.65E-03 | #N/A |
| rs2008651 | TCGA&TCGA_coCNV | 1.47E-03 | 6.23E-04 |
| rs4685330 | TCGA | 1.65E-03 | #N/A |
| rs17484815 | TCGA&TCGA_coCNV | 1.65E-03 | 3.95E-04 |
| rs12960912 | TCGA&TCGA_coCNV | 1.65E-03 | 5.50E-04 |
| rs2876375 | TCGA | 1.65E-03 | #N/A |
| rs7335067 | TCGA | 1.65E-03 | #N/A |
| rs17380908 | TCGA | 1.65E-03 | #N/A |
| rs7084956 | TCGA&TCGA_coCNV | 1.65E-03 | 2.71E-03 |
| rs12236453 | TCGA | 1.65E-03 | #N/A |
| rs210881 | TCGA&TCGA_coCNV | 1.65E-03 | 1.18E-03 |
| rs2046370 | TCGA | 1.65E-03 | #N/A |
| rs732857 | TCGA&Genevar | 7.51E-04 | #N/A |
| rs842825 | TCGA | 1.65E-03 | #N/A |
| rs6733100 | TCGA&TCGA_coCNV | 1.65E-03 | 5.40E-03 |

| rs11640796 | TCGA | 1.65E-03 | #N/A |
| --- | --- | --- | --- |
| rs2479130 | TCGA | 1.65E-03 | #N/A |
| rs17130554 | TCGA&TCGA_coCNV | 1.66E-03 | 1.27E-03 |
| rs6540549 | TCGA | 9.85E-04 | #N/A |
| rs9356192 | TCGA | 1.66E-03 | #N/A |
| rs17075862 | TCGA&TCGA_coCNV | 6.00E-07 | 2.61E-04 |
| rs1543181 | TCGA | 1.66E-03 | #N/A |
| rs10180663 | TCGA&TCGA_coCNV | 1.66E-03 | 1.36E-03 |
| rs12007527 | TCGA&TCGA_coCNV | 1.66E-03 | 1.20E-03 |
| rs17071610 | TCGA&TCGA_coCNV | 1.66E-03 | 6.14E-03 |
| rs13036242 | TCGA | 1.66E-03 | #N/A |
| rs16866166 | TCGA&TCGA_coCNV | 1.66E-03 | 9.51E-03 |
| rs16910750 | TCGA&TCGA_coCNV | 1.66E-03 | 2.50E-03 |
| rs17029613 | TCGA&TCGA_coCNV | 1.66E-03 | 1.11E-03 |
| rs284552 | TCGA&TCGA_coCNV | 7.68E-04 | 4.00E-03 |
| rs12105874 | TCGA&TCGA_coCNV | 1.66E-03 | 1.87E-03 |
| rs2015294 | TCGA | 1.66E-03 | #N/A |
| rs11217400 | TCGA&TCGA_coCNV | 1.66E-03 | 5.92E-03 |
| rs17064560 | TCGA&TCGA_coCNV | 1.66E-03 | 2.50E-04 |
| rs2043442 | TCGA&TCGA_coCNV | 1.66E-03 | 3.09E-03 |
| rs17284138 | TCGA | 1.67E-03 | #N/A |
| rs5968907 | TCGA&TCGA_coCNV | 1.67E-03 | 1.78E-04 |
| rs3949912 | TCGA&TCGA_coCNV | 1.67E-03 | 6.60E-04 |
| rs7076755 | TCGA&TCGA_coCNV | 1.67E-03 | 3.17E-04 |
| rs7196352 | TCGA | 1.67E-03 | #N/A |
| rs1465876 | TCGA | 1.67E-03 | #N/A |
| rs617791 | TCGA | 1.67E-03 | #N/A |
| rs2275086 | TCGA&TCGA_coCNV | 1.67E-03 | 1.11E-03 |
| rs17635998 | TCGA&TCGA_coCNV | 1.08E-03 | 3.45E-03 |
| rs11677416 | TCGA&TCGA_coCNV | 1.67E-03 | 1.57E-03 |
| rs10003163 | TCGA | 1.67E-03 | #N/A |
| rs4881282 | TCGA&TCGA_coCNV | 1.67E-03 | 3.54E-03 |
| rs9423269 | TCGA&TCGA_coCNV | 1.67E-03 | 7.19E-04 |
| rs411148 | TCGA&TCGA_coCNV | 1.67E-03 | 1.75E-04 |
| rs8062954 | TCGA&TCGA_coCNV | 1.67E-03 | 1.28E-04 |
| rs6508462 | TCGA | 1.68E-03 | #N/A |
| rs5983137 | TCGA&TCGA_coCNV | 1.68E-03 | 1.89E-03 |
| rs2016731 | TCGA | 1.68E-03 | #N/A |
| rs6628003 | TCGA&TCGA_coCNV | 1.68E-03 | 2.75E-03 |
| rs2345523 | TCGA&TCGA_coCNV | 1.68E-03 | 1.73E-04 |
| rs3855895 | TCGA&TCGA_coCNV | 1.05E-04 | 8.19E-04 |
| rs9577491 | TCGA&TCGA_coCNV | 1.68E-03 | 7.64E-03 |
| rs4881280 | TCGA | 1.68E-03 | #N/A |
| rs2853079 | TCGA&TCGA_coCNV | 7.94E-04 | 2.09E-03 |
| rs2418412 | TCGA&TCGA_coCNV | 1.68E-03 | 5.42E-03 |
| rs2138157 | TCGA | 1.68E-03 | #N/A |

| rs4307359 | TCGA&TCGA_coCNV | 1.68E-03 | 8.97E-04 |
| --- | --- | --- | --- |
| rs10015804 | TCGA | 1.68E-03 | #N/A |
| rs1922876 | TCGA&TCGA_coCNV | 1.68E-03 | 1.62E-03 |
| rs7236950 | TCGA | 1.68E-03 | #N/A |
| rs332735 | TCGA&TCGA_coCNV | 1.68E-03 | 1.44E-04 |
| rs9800948 | TCGA | 1.23E-03 | #N/A |
| rs9962745 | TCGA&TCGA_coCNV | 1.68E-03 | 1.98E-03 |
| rs161901 | TCGA&TCGA_coCNV | 1.69E-03 | 6.26E-04 |
| rs1890835 | TCGA&TCGA_coCNV | 1.69E-03 | 7.09E-05 |
| rs3784985 | TCGA | 1.69E-03 | #N/A |
| rs6738130 | TCGA&TCGA_coCNV | 1.69E-03 | 1.07E-03 |
| rs2511866 | TCGA&TCGA_coCNV | 1.69E-03 | 3.64E-03 |
| rs17143731 | TCGA&TCGA_coCNV | 1.69E-03 | 3.58E-04 |
| rs2734219 | TCGA | 1.69E-03 | #N/A |
| rs2691170 | TCGA&TCGA_coCNV | 1.69E-03 | 7.53E-03 |
| rs1277733 | TCGA | 1.69E-03 | #N/A |
| rs6640869 | TCGA&TCGA_coCNV | 1.69E-03 | 1.57E-03 |
| rs7232909 | TCGA&TCGA_coCNV | 1.21E-03 | 1.37E-04 |
| rs16843885 | TCGA&TCGA_coCNV | 1.69E-03 | 3.57E-03 |
| rs7897456 | TCGA&TCGA_coCNV | 1.01E-04 | 2.53E-04 |
| rs444119 | TCGA | 1.69E-03 | #N/A |
| rs3820817 | TCGA | 1.69E-03 | #N/A |
| rs11198445 | TCGA | 1.69E-03 | #N/A |
| rs17046269 | TCGA | 1.69E-03 | #N/A |
| rs16892265 | TCGA&TCGA_coCNV | 1.69E-03 | 1.41E-03 |
| rs337691 | TCGA | 1.69E-03 | #N/A |
| rs1893857 | TCGA | 1.69E-03 | #N/A |
| rs9358568 | TCGA&TCGA_coCNV | 1.70E-03 | 1.12E-03 |
| rs4084880 | TCGA&TCGA_coCNV | 1.70E-03 | 4.72E-03 |
| rs585064 | TCGA&TCGA_coCNV | 1.70E-03 | 5.41E-03 |
| rs12446802 | TCGA&TCGA_coCNV | 1.70E-03 | 7.52E-03 |
| rs6670503 | TCGA | 1.70E-03 | #N/A |
| rs1539798 | TCGA&TCGA_coCNV | 1.70E-03 | 8.76E-04 |
| rs10490715 | TCGA&TCGA_coCNV | 1.70E-03 | 1.50E-03 |
| rs955697 | TCGA&TCGA_coCNV | 1.33E-03 | 1.73E-05 |
| rs4765680 | TCGA | 1.70E-03 | #N/A |
| rs11158957 | TCGA | 1.71E-03 | #N/A |
| rs2388894 | TCGA&TCGA_coCNV | 2.70E-04 | 4.08E-03 |
| rs1008020 | TCGA&TCGA_coCNV | 8.25E-04 | 9.76E-04 |
| rs10176378 | TCGA&TCGA_coCNV | 1.71E-03 | 8.69E-03 |
| rs4647534 | TCGA | 1.71E-03 | #N/A |
| rs253312 | TCGA&TCGA_coCNV | 1.44E-03 | 1.11E-03 |
| rs11131779 | TCGA&TCGA_coCNV | 1.71E-03 | 1.26E-03 |
| rs412139 | TCGA | 1.71E-03 | #N/A |
| rs9387946 | TCGA&TCGA_coCNV | 7.79E-05 | 3.90E-07 |
| rs1334972 | TCGA&TCGA_coCNV | 1.15E-03 | 8.07E-04 |

| rs5958468 | TCGA | 1.71E-03 | #N/A |
| --- | --- | --- | --- |
| rs1867347 | TCGA&TCGA_coCNV | 1.71E-03 | 2.40E-05 |
| rs12978632 | TCGA | 1.71E-03 | #N/A |
| rs1453177 | TCGA&TCGA_coCNV | 1.71E-03 | 4.69E-03 |
| rs834884 | TCGA&TCGA_coCNV | 1.71E-03 | 8.72E-03 |
| rs799814 | TCGA&TCGA_coCNV | 1.72E-03 | 1.91E-03 |
| rs3096277 | TCGA | 1.72E-03 | #N/A |
| rs1939357 | TCGA&TCGA_coCNV | 5.96E-04 | 1.33E-03 |
| rs7452286 | TCGA | 1.72E-03 | #N/A |
| rs2770257 | TCGA&TCGA_coCNV | 1.46E-03 | 4.78E-03 |
| rs8096380 | TCGA&TCGA_coCNV | 1.72E-03 | 4.33E-05 |
| rs7211778 | TCGA&TCGA_coCNV | 1.72E-03 | 2.24E-03 |
| rs1962294 | TCGA&TCGA_coCNV | 1.07E-03 | 1.12E-03 |
| rs7901744 | TCGA&TCGA_coCNV | 1.72E-03 | 3.10E-03 |
| rs10735648 | TCGA&TCGA_coCNV | 1.72E-03 | 3.65E-03 |
| rs10142446 | TCGA&TCGA_coCNV | 1.73E-03 | 3.31E-03 |
| rs10415354 | TCGA | 1.73E-03 | #N/A |
| rs11721753 | TCGA | 1.73E-03 | #N/A |
| rs750070 | TCGA&TCGA_coCNV | 1.73E-03 | 2.19E-04 |
| rs12960329 | TCGA&TCGA_coCNV | 1.73E-03 | 3.90E-03 |
| rs12543862 | TCGA&TCGA_coCNV | 1.73E-03 | 9.16E-03 |
| rs598014 | TCGA | 1.73E-03 | #N/A |
| rs12123478 | TCGA&TCGA_coCNV | 1.94E-04 | 1.68E-03 |
| rs6081984 | TCGA | 1.73E-03 | #N/A |
| rs6489686 | TCGA&TCGA_coCNV | 5.77E-04 | 6.92E-05 |
| rs1374869 | TCGA&TCGA_coCNV | 1.73E-03 | 7.55E-03 |
| rs10488726 | TCGA&TCGA_coCNV | 1.73E-03 | 1.35E-03 |
| rs10147025 | TCGA&TCGA_coCNV | 1.74E-03 | 7.15E-03 |
| rs4300034 | TCGA&TCGA_coCNV | 1.34E-03 | 1.09E-03 |
| rs263169 | TCGA&TCGA_coCNV | 1.74E-03 | 3.89E-03 |
| rs2729729 | TCGA&TCGA_coCNV | 1.74E-03 | 1.88E-04 |
| rs237646 | TCGA&TCGA_coCNV | 1.62E-04 | 7.63E-04 |
| rs832734 | TCGA&TCGA_coCNV | 1.74E-03 | 1.23E-03 |
| rs11937820 | TCGA | 1.74E-03 | #N/A |
| rs1410347 | TCGA | 1.74E-03 | #N/A |
| rs1748952 | TCGA&TCGA_coCNV | 1.74E-03 | 3.10E-03 |
| rs9354209 | TCGA | 1.74E-03 | #N/A |
| rs2273232 | TCGA | 1.75E-03 | #N/A |
| rs17147682 | TCGA&TCGA_coCNV | 1.75E-03 | 3.87E-03 |
| rs11033730 | TCGA&TCGA_coCNV | 1.75E-03 | 3.12E-03 |
| rs12956424 | TCGA | 7.11E-04 | #N/A |
| rs16905601 | TCGA&TCGA_coCNV | 1.75E-03 | 4.73E-03 |
| rs17374031 | TCGA | 1.75E-03 | #N/A |
| rs408705 | TCGA&TCGA_coCNV | 1.75E-03 | 6.85E-03 |
| rs4672779 | TCGA&TCGA_coCNV | 1.75E-03 | 5.96E-03 |
| rs13404407 | TCGA&TCGA_coCNV | 1.75E-03 | 1.07E-03 |

| rs6933481 | TCGA&TCGA_coCNV | 1.75E-03 | 5.36E-03 |
| --- | --- | --- | --- |
| rs1525547 | TCGA&TCGA_coCNV | 1.75E-03 | 9.61E-05 |
| rs4332781 | TCGA&TCGA_coCNV | 1.75E-03 | 5.78E-03 |
| rs10991253 | TCGA | 1.75E-03 | #N/A |
| rs10509319 | TCGA | 1.75E-03 | #N/A |
| rs1070500 | TCGA&TCGA_coCNV | 1.75E-03 | 4.29E-03 |
| rs9312277 | TCGA&TCGA_coCNV | 1.76E-03 | 5.32E-03 |
| rs11151807 | TCGA&TCGA_coCNV | 1.76E-03 | 1.64E-03 |
| rs12435767 | TCGA | 1.76E-03 | #N/A |
| rs867445 | TCGA&TCGA_coCNV | 1.76E-03 | 1.04E-03 |
| rs9453597 | TCGA&TCGA_coCNV | 1.76E-03 | 2.32E-03 |
| rs6079903 | TCGA&TCGA_coCNV | 1.76E-03 | 6.26E-03 |
| rs17804602 | TCGA&TCGA_coCNV | 1.22E-03 | 8.21E-03 |
| rs8066971 | TCGA&TCGA_coCNV | 1.76E-03 | 4.48E-03 |
| rs16888037 | TCGA&TCGA_coCNV | 1.76E-03 | 9.27E-04 |
| rs11798448 | TCGA&TCGA_coCNV | 1.76E-03 | 1.56E-04 |
| rs1952925 | TCGA | 1.76E-03 | #N/A |
| rs16963175 | TCGA&TCGA_coCNV | 1.76E-03 | 2.38E-03 |
| rs1598572 | TCGA | 1.76E-03 | #N/A |
| rs17701938 | TCGA | 1.77E-03 | #N/A |
| rs209650 | TCGA&TCGA_coCNV | 1.77E-03 | 1.76E-03 |
| rs2274608 | TCGA&TCGA_coCNV | 1.77E-03 | 3.18E-03 |
| rs4553282 | TCGA&TCGA_coCNV | 1.77E-03 | 3.17E-03 |
| rs16990704 | TCGA&TCGA_coCNV | 1.77E-03 | 1.13E-03 |
| rs4741498 | TCGA&TCGA_coCNV | 1.77E-03 | 1.71E-03 |
| rs1787074 | TCGA | 1.77E-03 | #N/A |
| rs7107545 | TCGA | 1.77E-03 | #N/A |
| rs7375343 | TCGA | 1.77E-03 | #N/A |
| rs4371733 | TCGA&TCGA_coCNV | 1.77E-03 | 6.39E-03 |
| rs8178872 | TCGA&TCGA_coCNV | 1.77E-03 | 2.71E-03 |
| rs4722614 | TCGA | 1.77E-03 | #N/A |
| rs11585646 | TCGA&TCGA_coCNV | 1.77E-03 | 2.26E-04 |
| rs2673140 | TCGA&TCGA_coCNV | 1.83E-04 | 7.11E-04 |
| rs1231018 | TCGA&TCGA_coCNV | 1.77E-03 | 1.42E-03 |
| rs5987755 | TCGA&TCGA_coCNV | 1.77E-03 | 2.44E-03 |
| rs1027123 | TCGA | 1.78E-03 | #N/A |
| rs6661231 | TCGA&TCGA_coCNV | 1.78E-03 | 9.19E-03 |
| rs11195258 | TCGA&TCGA_coCNV | 3.15E-05 | 3.15E-03 |
| rs17400210 | TCGA&TCGA_coCNV | 1.78E-03 | 1.66E-03 |
| rs41414644 | TCGA&TCGA_coCNV | 1.78E-03 | 3.01E-04 |
| rs779104 | TCGA | 1.78E-03 | #N/A |
| rs11790275 | TCGA&TCGA_coCNV | 1.78E-03 | 1.15E-03 |
| rs1557922 | TCGA&TCGA_coCNV | 1.78E-03 | 4.02E-04 |
| rs12770633 | TCGA&TCGA_coCNV | 1.78E-03 | 1.27E-03 |
| rs12471567 | TCGA&TCGA_coCNV | 1.78E-03 | 9.46E-03 |
| rs17092496 | TCGA&TCGA_coCNV | 1.78E-03 | 4.19E-03 |

| rs12983573 | TCGA&TCGA_coCNV | 1.78E-03 | 3.80E-03 |
| --- | --- | --- | --- |
| rs10507176 | TCGA&TCGA_coCNV | 1.79E-03 | 2.15E-04 |
| rs4557538 | TCGA | 1.79E-03 | #N/A |
| rs1040715 | TCGA&TCGA_coCNV | 1.79E-03 | 1.28E-03 |
| rs6883079 | TCGA | 1.19E-08 | #N/A |
| rs10827212 | TCGA&TCGA_coCNV | 1.79E-03 | 5.28E-03 |
| rs4674023 | TCGA&TCGA_coCNV | 1.75E-04 | 1.23E-04 |
| rs10262212 | TCGA | 1.79E-03 | #N/A |
| rs1361643 | TCGA | 1.79E-03 | #N/A |
| rs12054958 | TCGA&TCGA_coCNV | 1.79E-03 | 9.91E-04 |
| rs17532973 | TCGA&TCGA_coCNV | 1.79E-03 | 3.41E-03 |
| rs10159293 | TCGA&TCGA_coCNV | 1.79E-03 | 7.76E-03 |
| rs2215229 | TCGA | 1.79E-03 | #N/A |
| rs10516715 | TCGA&TCGA_coCNV | 1.30E-04 | 2.17E-04 |
| rs17585543 | TCGA&TCGA_coCNV | 1.13E-03 | 1.60E-03 |
| rs1024971 | TCGA&TCGA_coCNV | 1.80E-03 | 3.08E-03 |
| rs12957397 | TCGA&TCGA_coCNV | 1.80E-03 | 1.34E-03 |
| rs6894650 | TCGA&TCGA_coCNV | 1.80E-03 | 3.58E-03 |
| rs7061172 | TCGA | 1.80E-03 | #N/A |
| rs2401868 | TCGA | 1.80E-03 | #N/A |
| rs41429646 | TCGA | 1.80E-03 | #N/A |
| rs2935630 | TCGA&TCGA_coCNV | 2.78E-04 | 2.99E-03 |
| rs6055590 | TCGA | 1.80E-03 | #N/A |
| rs7071067 | TCGA&TCGA_coCNV | 1.80E-03 | 7.92E-03 |
| rs13391272 | TCGA&TCGA_coCNV | 1.80E-03 | 6.83E-04 |
| rs6713393 | TCGA | 1.80E-03 | #N/A |
| rs4459045 | TCGA&TCGA_coCNV | 1.81E-03 | 7.75E-04 |
| rs12008479 | TCGA&TCGA_coCNV | 1.81E-03 | 7.38E-03 |
| rs2276490 | TCGA&TCGA_coCNV | 6.46E-05 | 1.50E-03 |
| rs8099219 | TCGA&TCGA_coCNV | 1.81E-03 | 2.84E-04 |
| rs12881922 | TCGA&TCGA_coCNV | 1.81E-03 | 1.95E-03 |
| rs9947278 | TCGA | 1.81E-03 | #N/A |
| rs17042682 | TCGA&TCGA_coCNV | 1.81E-03 | 9.53E-03 |
| rs7901026 | TCGA&TCGA_coCNV | 1.81E-03 | 7.25E-03 |
| rs4766673 | TCGA&TCGA_coCNV | 1.81E-03 | 4.13E-03 |
| rs16982813 | TCGA&TCGA_coCNV | 1.81E-03 | 7.93E-04 |
| rs28357969 | TCGA | 1.81E-03 | #N/A |
| rs12308682 | TCGA | 1.81E-03 | #N/A |
| rs8067638 | TCGA&TCGA_coCNV | 1.81E-03 | 4.83E-03 |
| rs1243064 | TCGA&TCGA_coCNV | 1.81E-03 | 6.04E-04 |
| rs10757621 | TCGA&TCGA_coCNV | 1.81E-03 | 1.22E-03 |
| rs17059546 | TCGA&TCGA_coCNV | 1.81E-03 | 6.37E-03 |
| rs1564617 | TCGA | 1.81E-03 | #N/A |
| rs979058 | TCGA&TCGA_coCNV | 1.81E-03 | 1.97E-03 |
| rs12148407 | TCGA&TCGA_coCNV | 4.61E-04 | 1.35E-03 |
| rs2057778 | TCGA&TCGA_coCNV | 1.82E-03 | 3.51E-03 |

| rs2665792 | TCGA&TCGA_coCNV | 1.30E-03 | 3.74E-05 |
| --- | --- | --- | --- |
| rs1037892 | TCGA&TCGA_coCNV | 1.82E-03 | 5.98E-03 |
| rs9356193 | TCGA&TCGA_coCNV | 1.82E-03 | 5.62E-03 |
| rs1979022 | TCGA | 1.82E-03 | #N/A |
| rs7043100 | TCGA&TCGA_coCNV | 8.04E-04 | 4.66E-03 |
| rs984078 | TCGA&TCGA_coCNV | 1.82E-03 | 3.20E-04 |
| rs267567 | TCGA&TCGA_coCNV | 1.82E-03 | 8.50E-03 |
| rs11223184 | TCGA | 1.82E-03 | #N/A |
| rs12033947 | TCGA&TCGA_coCNV | 1.82E-03 | 7.97E-04 |
| rs10006777 | TCGA&TCGA_coCNV | 1.82E-03 | 3.32E-03 |
| rs7235033 | TCGA&TCGA_coCNV | 1.27E-03 | 7.65E-04 |
| rs11734396 | TCGA | 1.82E-03 | #N/A |
| rs10485167 | TCGA&TCGA_coCNV | 1.82E-03 | 6.50E-03 |
| rs17013008 | TCGA&TCGA_coCNV | 1.82E-03 | 2.68E-03 |
| rs910159 | TCGA&TCGA_coCNV | 1.82E-03 | 9.44E-03 |
| rs525292 | TCGA&TCGA_coCNV | 9.43E-04 | 4.80E-03 |
| rs10148202 | TCGA | 1.83E-03 | #N/A |
| rs12010107 | TCGA&TCGA_coCNV | 1.83E-03 | 1.95E-03 |
| rs3206554 | TCGA&TCGA_coCNV | 1.83E-03 | 4.25E-04 |
| rs11778831 | TCGA | 1.83E-03 | #N/A |
| rs4892983 | TCGA&TCGA_coCNV | 1.83E-03 | 2.53E-03 |
| rs4772729 | TCGA | 1.83E-03 | #N/A |
| rs7910407 | TCGA&TCGA_coCNV | 1.83E-03 | 2.88E-03 |
| rs2643277 | TCGA | 1.84E-03 | #N/A |
| rs7033765 | TCGA | 3.56E-04 | #N/A |
| rs1298961 | TCGA | 1.84E-03 | #N/A |
| rs12226117 | TCGA&TCGA_coCNV | 1.84E-03 | 8.89E-03 |
| rs1724305 | TCGA | 1.84E-03 | #N/A |
| rs487669 | TCGA&TCGA_coCNV | 1.84E-03 | 9.07E-03 |
| rs3924970 | TCGA&TCGA_coCNV | 1.84E-03 | 2.22E-03 |
| rs1875619 | TCGA&TCGA_coCNV | 1.84E-03 | 2.48E-03 |
| rs8095071 | TCGA&TCGA_coCNV | 1.84E-03 | 2.45E-03 |
| rs11146136 | TCGA&TCGA_coCNV | 1.85E-03 | 2.10E-03 |
| rs5766205 | TCGA&TCGA_coCNV | 1.85E-03 | 2.76E-03 |
| rs2825017 | TCGA | 1.85E-03 | #N/A |
| rs7586298 | TCGA&TCGA_coCNV | 1.85E-03 | 1.12E-04 |
| rs11573492 | TCGA&TCGA_coCNV | 1.85E-03 | 7.79E-04 |
| rs11934233 | TCGA | 1.85E-03 | #N/A |
| rs7747913 | TCGA | 1.85E-03 | #N/A |
| rs863284 | TCGA&TCGA_coCNV | 1.85E-03 | 2.41E-04 |
| rs11122576 | TCGA&TCGA_coCNV | 1.85E-03 | 2.49E-03 |
| rs12645461 | TCGA | 1.85E-03 | #N/A |
| rs10122864 | TCGA&TCGA_coCNV | 1.85E-03 | 1.39E-03 |
| rs12547939 | TCGA&TCGA_coCNV | 1.85E-03 | 1.81E-03 |
| rs6758955 | TCGA&TCGA_coCNV&SCANdb | 1.85E-03 | 2.96E-03 |
| rs17672913 | TCGA | 1.86E-03 | #N/A |

| rs10795123 | TCGA&TCGA_coCNV | 1.86E-03 | 1.16E-03 |
| --- | --- | --- | --- |
| rs2719135 | TCGA&TCGA_coCNV | 1.86E-03 | 2.92E-04 |
| rs10160394 | TCGA | 1.86E-03 | #N/A |
| rs4647669 | TCGA | 1.86E-03 | #N/A |
| rs11136314 | TCGA&TCGA_coCNV | 1.86E-03 | 3.62E-03 |
| rs11140025 | TCGA&TCGA_coCNV | 1.67E-03 | 6.39E-04 |
| rs16919588 | TCGA&TCGA_coCNV | 1.86E-03 | 6.40E-03 |
| rs2706859 | TCGA&TCGA_coCNV | 1.86E-03 | 2.10E-04 |
| rs213384 | TCGA&TCGA_coCNV | 3.20E-04 | 2.79E-05 |
| rs10993251 | TCGA&TCGA_coCNV | 1.86E-03 | 9.75E-03 |
| rs13426260 | TCGA&TCGA_coCNV | 1.86E-03 | 1.85E-03 |
| rs6739409 | TCGA | 1.86E-03 | #N/A |
| rs8109558 | TCGA&TCGA_coCNV | 1.86E-03 | 4.17E-03 |
| rs9599174 | TCGA | 1.86E-03 | #N/A |
| rs10242554 | TCGA&TCGA_coCNV | 1.86E-03 | 2.49E-03 |
| rs3130871 | TCGA&TCGA_coCNV | 1.87E-03 | 2.49E-03 |
| rs12667502 | TCGA | 1.87E-03 | #N/A |
| rs4791868 | TCGA&TCGA_coCNV | 1.87E-03 | 3.35E-03 |
| rs4448987 | TCGA | 1.87E-03 | #N/A |
| rs3735015 | TCGA&TCGA_coCNV | 1.87E-03 | 8.53E-04 |
| rs17687371 | TCGA | 1.87E-03 | #N/A |
| rs2136721 | TCGA | 1.87E-03 | #N/A |
| rs3857431 | TCGA | 1.87E-03 | #N/A |
| rs4457318 | TCGA | 1.87E-03 | #N/A |
| rs9547427 | TCGA&TCGA_coCNV | 1.87E-03 | 1.19E-03 |
| rs1590944 | TCGA&TCGA_coCNV | 1.87E-03 | 9.05E-03 |
| rs7311281 | TCGA | 1.87E-03 | #N/A |
| rs2711642 | TCGA | 1.87E-03 | #N/A |
| rs2238927 | TCGA | 1.87E-03 | #N/A |
| rs41525145 | TCGA&TCGA_coCNV | 1.87E-03 | 2.05E-03 |
| rs2943640 | TCGA | 1.87E-03 | #N/A |
| rs16937588 | TCGA&TCGA_coCNV | 1.50E-03 | 1.75E-03 |
| rs9852791 | TCGA&TCGA_coCNV | 1.87E-03 | 2.56E-03 |
| rs9825636 | TCGA&TCGA_coCNV | 1.87E-03 | 9.42E-05 |
| rs4128686 | TCGA | 1.87E-03 | #N/A |
| rs701325 | TCGA&TCGA_coCNV | 1.88E-03 | 3.02E-04 |
| rs913192 | TCGA | 1.88E-03 | #N/A |
| rs217866 | TCGA | 1.88E-03 | #N/A |
| rs11642728 | TCGA | 1.88E-03 | #N/A |
| rs11176507 | TCGA&TCGA_coCNV | 1.88E-03 | 2.51E-03 |
| rs12946675 | TCGA | 1.88E-03 | #N/A |
| rs41340545 | TCGA&TCGA_coCNV | 1.01E-03 | 2.40E-04 |
| rs10484798 | TCGA&TCGA_coCNV | 7.55E-04 | 6.68E-04 |
| rs16949001 | TCGA | 1.88E-03 | #N/A |
| rs2180052 | TCGA&TCGA_coCNV | 1.88E-03 | 3.68E-03 |
| rs13181334 | TCGA&TCGA_coCNV | 1.88E-03 | 3.40E-03 |

| rs16857272 | TCGA&TCGA_coCNV | 1.33E-03 | 3.12E-03 |
| --- | --- | --- | --- |
| rs4506249 | TCGA&TCGA_coCNV | 1.88E-03 | 3.15E-04 |
| rs2833735 | TCGA | 1.88E-03 | #N/A |
| rs2461317 | TCGA&TCGA_coCNV | 1.88E-03 | 2.52E-03 |
| rs2724225 | TCGA | 1.88E-03 | #N/A |
| rs4285174 | TCGA | 1.88E-03 | #N/A |
| rs7627060 | TCGA&TCGA_coCNV | 9.44E-04 | 4.67E-03 |
| rs11917735 | TCGA&TCGA_coCNV | 1.88E-03 | 2.57E-03 |
| rs4791934 | TCGA&TCGA_coCNV | 1.88E-03 | 9.69E-03 |
| rs10742722 | TCGA&TCGA_coCNV | 1.89E-03 | 6.20E-03 |
| rs3857597 | TCGA&TCGA_coCNV | 1.89E-03 | 9.95E-03 |
| rs781792 | TCGA&TCGA_coCNV | 1.89E-03 | 2.50E-03 |
| rs2044868 | TCGA&TCGA_coCNV | 6.38E-04 | 1.12E-04 |
| rs4988172 | TCGA&TCGA_coCNV | 1.89E-03 | 3.48E-03 |
| rs12962229 | TCGA | 1.89E-03 | #N/A |
| rs5981573 | TCGA&TCGA_coCNV | 1.89E-03 | 2.57E-03 |
| rs12084086 | TCGA&TCGA_coCNV | 1.89E-03 | 2.55E-03 |
| rs1421611 | TCGA&TCGA_coCNV | 1.89E-03 | 2.56E-03 |
| rs12663465 | TCGA&TCGA_coCNV | 1.89E-03 | 3.23E-03 |
| rs7919849 | TCGA&TCGA_coCNV | 1.79E-03 | 3.79E-04 |
| rs17100981 | TCGA | 1.71E-03 | #N/A |
| rs9634474 | TCGA | 1.90E-03 | #N/A |
| rs6660593 | TCGA | 1.90E-03 | #N/A |
| rs17149200 | TCGA | 1.90E-03 | #N/A |
| rs874616 | TCGA | 1.90E-03 | #N/A |
| rs13376677 | TCGA&TCGA_coCNV | 1.90E-03 | 1.18E-03 |
| rs12528837 | TCGA&TCGA_coCNV | 1.90E-03 | 8.54E-04 |
| rs17157380 | TCGA | 1.90E-03 | #N/A |
| rs1935519 | TCGA | 1.90E-03 | #N/A |
| rs17712675 | TCGA&TCGA_coCNV | 1.90E-03 | 3.82E-03 |
| rs17059502 | TCGA&TCGA_coCNV | 1.90E-03 | 2.54E-03 |
| rs16949877 | TCGA&TCGA_coCNV | 6.37E-04 | 2.13E-03 |
| rs9710693 | TCGA&TCGA_coCNV | 1.90E-03 | 9.64E-03 |
| rs16840598 | TCGA&TCGA_coCNV | 7.71E-04 | 5.91E-04 |
| rs10791126 | TCGA | 1.90E-03 | #N/A |
| rs12584425 | TCGA&TCGA_coCNV | 1.90E-03 | 5.61E-03 |
| rs1422831 | TCGA | 1.90E-03 | #N/A |
| rs2609194 | TCGA&TCGA_coCNV | 1.90E-03 | 2.61E-03 |
| rs17649193 | TCGA&TCGA_coCNV | 1.90E-03 | 4.11E-04 |
| rs4458410 | TCGA | 1.90E-03 | #N/A |
| rs41431846 | TCGA&TCGA_coCNV | 1.90E-03 | 3.18E-03 |
| rs1120015 | TCGA&TCGA_coCNV | 1.90E-03 | 2.13E-03 |
| rs10220835 | TCGA | 1.90E-03 | #N/A |
| rs8884 | TCGA&TCGA_coCNV | 1.90E-03 | 2.65E-03 |
| rs7395884 | TCGA | 1.91E-03 | #N/A |
| rs7051238 | TCGA&TCGA_coCNV | 1.91E-03 | 2.54E-03 |

| rs4828602 | TCGA&TCGA_coCNV | 1.08E-03 | 1.02E-04 |
| --- | --- | --- | --- |
| rs7186094 | TCGA&TCGA_coCNV | 1.91E-03 | 1.47E-03 |
| rs16940493 | TCGA&TCGA_coCNV | 1.91E-03 | 2.54E-03 |
| rs11082138 | TCGA&TCGA_coCNV | 1.91E-03 | 6.19E-03 |
| rs5982981 | TCGA&TCGA_coCNV | 1.91E-03 | 2.57E-03 |
| rs17700949 | TCGA&TCGA_coCNV | 1.91E-03 | 7.46E-03 |
| rs4411058 | TCGA&TCGA_coCNV | 1.34E-03 | 5.02E-03 |
| rs17108999 | TCGA&TCGA_coCNV | 1.91E-03 | 6.66E-03 |
| rs6994354 | TCGA&TCGA_coCNV | 1.91E-03 | 8.80E-03 |
| rs6911818 | TCGA&TCGA_coCNV | 1.91E-03 | 9.52E-04 |
| rs6881618 | TCGA&TCGA_coCNV | 1.91E-03 | 5.82E-03 |
| rs1901401 | TCGA | 1.91E-03 | #N/A |
| rs3857476 | TCGA&TCGA_coCNV | 5.35E-04 | 9.32E-04 |
| rs2943653 | TCGA&TCGA_coCNV | 1.79E-03 | 2.14E-03 |
| rs9422897 | TCGA&TCGA_coCNV | 1.91E-03 | 2.38E-04 |
| rs9486096 | TCGA | 1.91E-03 | #N/A |
| rs1438216 | TCGA | 1.91E-03 | #N/A |
| rs6442682 | TCGA&TCGA_coCNV | 1.92E-03 | 2.57E-03 |
| rs17671456 | TCGA | 1.92E-03 | #N/A |
| rs4872049 | TCGA | 1.92E-03 | #N/A |
| rs7931475 | TCGA | 1.92E-03 | #N/A |
| rs8035369 | TCGA | 1.92E-03 | #N/A |
| rs16864374 | TCGA&TCGA_coCNV | 1.92E-03 | 7.54E-03 |
| rs10264298 | TCGA&TCGA_coCNV | 1.92E-03 | 2.54E-03 |
| rs1370509 | TCGA&TCGA_coCNV | 1.92E-03 | 5.33E-03 |
| rs2493013 | TCGA&TCGA_coCNV | 1.92E-03 | 4.39E-03 |
| rs1932387 | TCGA&TCGA_coCNV | 1.92E-03 | 6.11E-03 |
| rs309543 | TCGA&TCGA_coCNV | 1.92E-03 | 6.44E-03 |
| rs10124985 | TCGA&TCGA_coCNV | 1.92E-03 | 1.65E-04 |
| rs7203001 | TCGA&TCGA_coCNV | 6.24E-04 | 4.41E-03 |
| rs17160264 | TCGA | 1.92E-03 | #N/A |
| rs1369704 | TCGA&TCGA_coCNV | 1.92E-03 | 5.52E-03 |
| rs4754159 | TCGA | 1.92E-03 | #N/A |
| rs10819759 | TCGA&TCGA_coCNV | 1.93E-03 | 8.38E-04 |
| rs3103089 | TCGA&TCGA_coCNV | 1.93E-03 | 9.32E-03 |
| rs11660136 | TCGA&TCGA_coCNV | 1.93E-03 | 9.69E-03 |
| rs1366608 | TCGA&TCGA_coCNV | 1.93E-03 | 3.36E-03 |
| rs3796809 | TCGA&TCGA_coCNV | 1.93E-03 | 1.42E-03 |
| rs6796318 | TCGA&TCGA_coCNV | 3.01E-04 | 3.18E-03 |
| rs28357968 | TCGA | 1.93E-03 | #N/A |
| rs6790156 | TCGA&TCGA_coCNV | 1.93E-03 | 8.98E-04 |
| rs2642032 | TCGA&TCGA_coCNV | 1.93E-03 | 3.94E-03 |
| rs2936207 | TCGA&TCGA_coCNV | 1.93E-03 | 5.68E-04 |
| rs16896406 | TCGA&TCGA_coCNV | 2.94E-04 | 1.28E-03 |
| rs7256278 | TCGA&TCGA_coCNV | 1.94E-03 | 3.40E-03 |
| rs11598540 | TCGA | 1.94E-03 | #N/A |

| rs10488762 | TCGA&TCGA_coCNV | 1.48E-03 | 1.34E-03 |
| --- | --- | --- | --- |
| rs1031895 | TCGA&TCGA_coCNV | 1.94E-03 | 7.29E-03 |
| rs10163575 | TCGA | 1.94E-03 | #N/A |
| rs12791760 | TCGA&TCGA_coCNV | 1.94E-03 | 2.50E-03 |
| rs516510 | TCGA&TCGA_coCNV | 1.94E-03 | 4.44E-03 |
| rs33971948 | TCGA | 1.94E-03 | #N/A |
| rs8008285 | TCGA | 1.94E-03 | #N/A |
| rs13168261 | TCGA&TCGA_coCNV | 1.94E-03 | 3.43E-03 |
| rs4144873 | TCGA&TCGA_coCNV | 1.94E-03 | 9.18E-04 |
| rs4841214 | TCGA&TCGA_coCNV | 1.94E-03 | 2.59E-03 |
| rs12198961 | TCGA | 1.94E-03 | #N/A |
| rs7004762 | TCGA | 1.94E-03 | #N/A |
| rs9308169 | TCGA | 1.94E-03 | #N/A |
| rs6583138 | TCGA&TCGA_coCNV | 1.59E-03 | 2.24E-03 |
| rs12255119 | TCGA&TCGA_coCNV | 9.50E-04 | 2.57E-03 |
| rs4844696 | TCGA&TCGA_coCNV | 1.94E-03 | 2.59E-03 |
| rs4370339 | TCGA | 1.94E-03 | #N/A |
| rs17318072 | TCGA | 1.94E-03 | #N/A |
| rs9946028 | TCGA&TCGA_coCNV | 1.94E-03 | 4.02E-03 |
| rs2596679 | TCGA&TCGA_coCNV | 1.95E-03 | 1.24E-03 |
| rs7020775 | TCGA&TCGA_coCNV | 1.95E-03 | 4.51E-04 |
| rs17678540 | TCGA | 1.95E-03 | #N/A |
| rs7706687 | TCGA&TCGA_coCNV | 1.95E-03 | 2.44E-04 |
| rs11208958 | TCGA | 1.95E-03 | #N/A |
| rs11225907 | TCGA&TCGA_coCNV | 1.95E-03 | 7.54E-03 |
| rs10075320 | TCGA&TCGA_coCNV | 1.95E-03 | 6.98E-03 |
| rs11897520 | TCGA | 1.95E-03 | #N/A |
| rs7928384 | TCGA&TCGA_coCNV | 1.95E-03 | 2.66E-03 |
| rs927840 | TCGA&TCGA_coCNV | 6.92E-05 | 1.13E-04 |
| rs9811805 | TCGA | 1.95E-03 | #N/A |
| rs10496813 | TCGA | 1.95E-03 | #N/A |
| rs10994530 | TCGA | 1.95E-03 | #N/A |
| rs3736124 | TCGA&TCGA_coCNV | 1.95E-03 | 3.49E-03 |
| rs9670069 | TCGA&TCGA_coCNV | 1.95E-03 | 5.29E-03 |
| rs1332963 | TCGA&TCGA_coCNV | 1.95E-03 | 6.42E-03 |
| rs7735726 | TCGA&TCGA_coCNV | 1.95E-03 | 6.93E-04 |
| rs1918231 | TCGA | 1.95E-03 | #N/A |
| rs9410435 | TCGA&TCGA_coCNV | 1.95E-03 | 2.90E-03 |
| rs10502776 | TCGA | 1.95E-03 | #N/A |
| rs6630085 | TCGA&TCGA_coCNV | 1.95E-03 | 9.83E-04 |
| rs7958063 | TCGA&TCGA_coCNV | 1.95E-03 | 5.76E-04 |
| rs17048711 | TCGA&TCGA_coCNV | 1.96E-03 | 2.99E-03 |
| rs11992973 | TCGA | 1.96E-03 | #N/A |
| rs12665517 | TCGA&TCGA_coCNV | 3.89E-04 | 1.63E-03 |
| rs302295 | TCGA&TCGA_coCNV | 1.96E-03 | 3.55E-03 |
| rs17030612 | TCGA&TCGA_coCNV | 1.96E-03 | 2.64E-04 |

| rs10895932 | TCGA | 1.96E-03 | #N/A |
| --- | --- | --- | --- |
| rs7999982 | TCGA&TCGA_coCNV | 4.50E-04 | 3.41E-04 |
| rs17139387 | TCGA&TCGA_coCNV | 1.96E-03 | 4.54E-03 |
| rs3771545 | TCGA&TCGA_coCNV | 4.23E-04 | 1.33E-03 |
| rs4962390 | TCGA&TCGA_coCNV | 1.96E-03 | 2.20E-03 |
| rs11929337 | TCGA&TCGA_coCNV | 1.96E-03 | 2.60E-03 |
| rs301653 | TCGA&SCANdb | 1.96E-03 | #N/A |
| rs2972147 | TCGA&TCGA_coCNV | 1.59E-03 | 7.54E-03 |
| rs1041296 | TCGA&TCGA_coCNV | 1.83E-03 | 8.13E-04 |
| rs1787595 | TCGA&TCGA_coCNV | 1.96E-03 | 3.17E-03 |
| rs6140786 | TCGA | 1.96E-03 | #N/A |
| rs41436851 | TCGA&TCGA_coCNV | 1.97E-03 | 3.36E-03 |
| rs883853 | TCGA&TCGA_coCNV | 1.97E-03 | 3.50E-03 |
| rs6650455 | TCGA | 1.97E-03 | #N/A |
| rs6443712 | TCGA&TCGA_coCNV | 1.97E-03 | 8.88E-03 |
| rs1363099 | TCGA&TCGA_coCNV | 1.97E-03 | 3.88E-03 |
| rs9935049 | TCGA&TCGA_coCNV | 1.97E-03 | 3.03E-03 |
| rs641021 | TCGA&TCGA_coCNV | 1.97E-03 | 3.17E-03 |
| rs6660910 | TCGA&TCGA_coCNV | 1.97E-03 | 2.59E-03 |
| rs9570619 | TCGA&TCGA_coCNV | 1.97E-03 | 3.52E-03 |
| rs4593315 | TCGA&TCGA_coCNV | 1.97E-03 | 4.38E-03 |
| rs2684836 | TCGA&TCGA_coCNV | 1.97E-03 | 2.44E-03 |
| rs10033473 | TCGA&TCGA_coCNV | 1.97E-03 | 2.08E-03 |
| rs1208907 | TCGA&TCGA_coCNV | 1.97E-03 | 3.60E-03 |
| rs10238991 | TCGA&TCGA_coCNV | 1.97E-03 | 1.94E-04 |
| rs1940071 | TCGA&TCGA_coCNV | 1.98E-03 | 8.05E-03 |
| rs41442545 | TCGA&TCGA_coCNV | 1.98E-03 | 5.87E-04 |
| rs3803839 | TCGA&TCGA_coCNV | 1.98E-03 | 9.59E-05 |
| rs9990746 | TCGA&TCGA_coCNV | 1.98E-03 | 2.62E-03 |
| rs9895675 | TCGA | 1.98E-03 | #N/A |
| rs10903752 | TCGA&TCGA_coCNV | 1.98E-03 | 5.37E-03 |
| rs4895303 | TCGA&TCGA_coCNV | 1.98E-03 | 3.10E-03 |
| rs12238155 | TCGA | 1.98E-03 | #N/A |
| rs1406367 | TCGA&TCGA_coCNV | 2.40E-04 | 9.20E-04 |
| rs2699985 | TCGA&TCGA_coCNV | 1.98E-03 | 2.43E-03 |
| rs7608475 | TCGA | 1.98E-03 | #N/A |
| rs7915341 | TCGA&TCGA_coCNV | 1.98E-03 | 8.30E-04 |
| rs4749306 | TCGA&TCGA_coCNV | 1.98E-03 | 2.24E-03 |
| rs4488249 | TCGA | 1.98E-03 | #N/A |
| rs6486141 | TCGA&TCGA_coCNV | 1.99E-03 | 2.65E-04 |
| rs333323 | TCGA&TCGA_coCNV | 1.99E-03 | 4.04E-03 |
| rs4639211 | TCGA | 1.99E-03 | #N/A |
| rs10993127 | TCGA | 1.99E-03 | #N/A |
| rs2560030 | TCGA | 1.89E-03 | #N/A |
| rs10471184 | TCGA&TCGA_coCNV | 9.97E-05 | 1.48E-04 |
| rs4129230 | TCGA | 1.99E-03 | #N/A |

| rs243291 | TCGA&TCGA_coCNV | 1.99E-03 | 1.77E-03 |
| --- | --- | --- | --- |
| rs1947303 | TCGA | 1.99E-03 | #N/A |
| rs3852402 | TCGA | 1.99E-03 | #N/A |
| rs1919986 | TCGA | 1.99E-03 | #N/A |
| rs7923066 | TCGA&TCGA_coCNV | 1.99E-03 | 1.81E-03 |
| rs9373384 | TCGA&TCGA_coCNV | 1.99E-03 | 6.45E-04 |
| rs7566576 | TCGA&TCGA_coCNV | 1.99E-03 | 8.62E-04 |
| rs2735958 | TCGA&TCGA_coCNV | 5.50E-04 | 2.55E-04 |
| rs10747472 | TCGA | 2.00E-03 | #N/A |
| rs5935224 | TCGA&TCGA_coCNV | 2.00E-03 | 2.91E-04 |
| rs41396744 | TCGA&TCGA_coCNV | 2.00E-03 | 1.89E-03 |
| rs6480548 | TCGA | 2.00E-03 | #N/A |
| rs4129790 | TCGA&TCGA_coCNV | 2.00E-03 | 1.05E-04 |
| rs2835028 | TCGA | 2.00E-03 | #N/A |
| rs12107059 | TCGA&TCGA_coCNV | 2.00E-03 | 4.80E-03 |
| rs284507 | TCGA | 2.00E-03 | #N/A |
| rs2588625 | TCGA&TCGA_coCNV | 2.00E-03 | 2.13E-03 |
| rs6711718 | TCGA&TCGA_coCNV | 2.00E-03 | 4.02E-03 |
| rs10431976 | TCGA | 2.00E-03 | #N/A |
| rs2887312 | TCGA&TCGA_coCNV | 2.00E-03 | 1.27E-03 |
| rs10496236 | TCGA | 2.01E-03 | #N/A |
| rs5767517 | TCGA&TCGA_coCNV | 2.01E-03 | 3.42E-04 |
| rs11078324 | TCGA&TCGA_coCNV | 2.01E-03 | 3.95E-03 |
| rs10782945 | TCGA | 2.01E-03 | #N/A |
| rs16982272 | TCGA&TCGA_coCNV | 2.01E-03 | 6.36E-04 |
| rs7200654 | TCGA&TCGA_coCNV | 2.01E-03 | 7.22E-03 |
| rs11928764 | TCGA&TCGA_coCNV | 2.01E-03 | 4.43E-03 |
| rs7815993 | TCGA | 2.01E-03 | #N/A |
| rs11893938 | TCGA | 2.01E-03 | #N/A |
| rs1462953 | TCGA&TCGA_coCNV | 2.02E-03 | 2.42E-03 |
| rs1368545 | TCGA | 2.02E-03 | #N/A |
| rs967773 | TCGA | 2.02E-03 | #N/A |
| rs7561493 | TCGA | 2.02E-03 | #N/A |
| rs594709 | TCGA | 2.02E-03 | #N/A |
| rs4294422 | TCGA | 2.02E-03 | #N/A |
| rs12251587 | TCGA | 1.92E-03 | #N/A |
| rs1430498 | TCGA&TCGA_coCNV | 2.02E-03 | 7.86E-03 |
| rs11945044 | TCGA&TCGA_coCNV | 2.02E-03 | 1.63E-03 |
| rs1077429 | TCGA | 2.02E-03 | #N/A |
| rs6676516 | TCGA&TCGA_coCNV | 2.02E-03 | 5.48E-03 |
| rs12053568 | TCGA | 2.02E-03 | #N/A |
| rs7659639 | TCGA&TCGA_coCNV | 8.89E-04 | 9.11E-04 |
| rs12517092 | TCGA&TCGA_coCNV | 2.02E-03 | 2.78E-03 |
| rs9922362 | TCGA&TCGA_coCNV | 7.93E-04 | 1.34E-04 |
| rs582318 | TCGA&TCGA_coCNV | 2.02E-03 | 9.27E-03 |
| rs6817370 | TCGA&TCGA_coCNV | 2.02E-03 | 4.95E-03 |

| rs1027126 | TCGA | 2.02E-03 | #N/A |
| --- | --- | --- | --- |
| rs1873149 | TCGA | 2.03E-03 | #N/A |
| rs10863593 | TCGA&TCGA_coCNV | 2.03E-03 | 3.64E-03 |
| rs876031 | TCGA&TCGA_coCNV | 2.03E-03 | 2.64E-03 |
| rs7894028 | TCGA | 2.03E-03 | #N/A |
| rs1496389 | TCGA | 2.03E-03 | #N/A |
| rs10848668 | TCGA&TCGA_coCNV | 2.03E-03 | 3.83E-04 |
| rs12091071 | TCGA | 2.03E-03 | #N/A |
| rs6647753 | TCGA&TCGA_coCNV | 2.02E-03 | 5.67E-03 |
| rs10772132 | TCGA&TCGA_coCNV | 2.03E-03 | 8.53E-04 |
| rs6530458 | TCGA&TCGA_coCNV | 2.03E-03 | 2.38E-03 |
| rs6881298 | TCGA&TCGA_coCNV | 2.03E-03 | 1.47E-04 |
| rs7724316 | TCGA&TCGA_coCNV | 2.03E-03 | 6.77E-03 |
| rs2569916 | TCGA&TCGA_coCNV | 2.03E-03 | 1.63E-04 |
| rs1496475 | TCGA | 2.03E-03 | #N/A |
| rs4971594 | TCGA | 2.04E-03 | #N/A |
| rs1116853 | TCGA&TCGA_coCNV | 2.04E-03 | 1.06E-03 |
| rs7066215 | TCGA&TCGA_coCNV | 2.04E-03 | 9.56E-04 |
| rs7923550 | TCGA&TCGA_coCNV | 2.04E-03 | 4.64E-03 |
| rs2857918 | TCGA | 2.04E-03 | #N/A |
| rs1406100 | TCGA&TCGA_coCNV | 2.04E-03 | 3.81E-03 |
| rs6043124 | TCGA&TCGA_coCNV | 2.04E-03 | 7.34E-03 |
| rs768056 | TCGA&TCGA_coCNV | 5.93E-04 | 1.37E-03 |
| rs1236783 | TCGA&TCGA_coCNV | 2.04E-03 | 8.45E-04 |
| rs17817179 | TCGA&TCGA_coCNV | 2.04E-03 | 7.59E-03 |
| rs17671389 | TCGA&TCGA_coCNV | 2.04E-03 | 6.55E-03 |
| rs7700615 | TCGA&TCGA_coCNV | 1.56E-03 | 7.99E-03 |
| rs17650849 | TCGA | 2.04E-03 | #N/A |
| rs4684713 | TCGA&TCGA_coCNV | 2.04E-03 | 2.98E-03 |
| rs10745620 | TCGA&TCGA_coCNV | 2.04E-03 | 5.39E-03 |
| rs1267996 | TCGA&TCGA_coCNV | 1.68E-03 | 6.15E-03 |
| rs6525504 | TCGA&TCGA_coCNV | 2.04E-03 | 4.53E-03 |
| rs16990197 | TCGA&TCGA_coCNV | 2.04E-03 | 4.05E-03 |
| rs2826967 | TCGA | 2.05E-03 | #N/A |
| rs275379 | TCGA&TCGA_coCNV | 2.05E-03 | 9.59E-03 |
| rs7898040 | TCGA&TCGA_coCNV | 2.05E-03 | 3.58E-03 |
| rs11215264 | TCGA | 2.05E-03 | #N/A |
| rs4143832 | TCGA&TCGA_coCNV | 1.12E-04 | 2.52E-03 |
| rs7519796 | TCGA&TCGA_coCNV | 2.05E-03 | 5.67E-03 |
| rs10919335 | TCGA&TCGA_coCNV | 2.05E-03 | 5.89E-03 |
| rs6844679 | TCGA&TCGA_coCNV | 2.05E-03 | 1.27E-03 |
| rs1027827 | TCGA | 2.05E-03 | #N/A |
| rs6470338 | TCGA&TCGA_coCNV | 2.05E-03 | 1.05E-03 |
| rs452191 | TCGA&TCGA_coCNV | 2.05E-03 | 2.62E-03 |
| rs10959841 | TCGA&TCGA_coCNV | 2.05E-03 | 3.85E-03 |
| rs1888736 | TCGA&TCGA_coCNV | 5.50E-04 | 5.53E-03 |

| rs7230859 | TCGA&TCGA_coCNV | 2.05E-03 | 2.06E-03 |
| --- | --- | --- | --- |
| rs16849078 | TCGA&TCGA_coCNV | 2.05E-03 | 3.55E-03 |
| rs737007 | TCGA&TCGA_coCNV | 2.05E-03 | 6.05E-03 |
| rs1656463 | TCGA | 2.05E-03 | #N/A |
| rs41534051 | TCGA&TCGA_coCNV | 2.06E-03 | 2.07E-03 |
| rs4954894 | TCGA | 2.06E-03 | #N/A |
| rs4857093 | TCGA&TCGA_coCNV | 2.24E-04 | 8.55E-04 |
| rs2423429 | TCGA | 2.06E-03 | #N/A |
| rs4792343 | TCGA&TCGA_coCNV | 2.06E-03 | 5.50E-03 |
| rs2930371 | TCGA&TCGA_coCNV | 2.06E-03 | 9.03E-03 |
| rs12544880 | TCGA&TCGA_coCNV | 2.06E-03 | 4.76E-03 |
| rs6946256 | TCGA | 2.06E-03 | #N/A |
| rs7557799 | TCGA | 2.01E-03 | #N/A |
| rs3219337 | TCGA&TCGA_coCNV | 2.06E-03 | 1.22E-03 |
| rs5024432 | TCGA&TCGA_coCNV | 2.06E-03 | 2.09E-03 |
| rs4664332 | TCGA&TCGA_coCNV | 2.06E-03 | 2.50E-03 |
| rs7603839 | TCGA | 2.06E-03 | #N/A |
| rs502055 | TCGA | 2.06E-03 | #N/A |
| rs13175785 | TCGA&TCGA_coCNV | 2.06E-03 | 3.30E-03 |
| rs1439818 | TCGA&TCGA_coCNV | 2.06E-03 | 4.70E-03 |
| rs7542629 | TCGA&TCGA_coCNV | 8.96E-05 | 3.64E-04 |
| rs13020676 | TCGA&TCGA_coCNV | 2.07E-03 | 3.14E-03 |
| rs7229537 | TCGA | 2.07E-03 | #N/A |
| rs929871 | TCGA&TCGA_coCNV | 2.07E-03 | 5.37E-03 |
| rs11748225 | TCGA&TCGA_coCNV | 2.07E-03 | 1.81E-03 |
| rs6537336 | TCGA&TCGA_coCNV | 2.07E-03 | 1.58E-03 |
| rs2270128 | TCGA&TCGA_coCNV | 2.07E-03 | 1.08E-03 |
| rs7547026 | TCGA | 2.07E-03 | #N/A |
| rs16936782 | TCGA&TCGA_coCNV | 2.07E-03 | 2.96E-04 |
| rs2278499 | TCGA&TCGA_coCNV | 2.07E-03 | 5.92E-05 |
| rs1491099 | TCGA&TCGA_coCNV | 2.07E-03 | 2.88E-03 |
| rs4699445 | TCGA | 2.07E-03 | #N/A |
| rs6981472 | TCGA&TCGA_coCNV | 1.90E-03 | 6.53E-03 |
| rs11827128 | TCGA&TCGA_coCNV | 2.07E-03 | 5.91E-03 |
| rs13258195 | TCGA | 2.07E-03 | #N/A |
| rs16871987 | TCGA | 2.07E-03 | #N/A |
| rs6573320 | TCGA&TCGA_coCNV | 2.08E-03 | 5.97E-03 |
| rs10894338 | TCGA | 2.08E-03 | #N/A |
| rs6021381 | TCGA&TCGA_coCNV | 2.08E-03 | 1.08E-03 |
| rs13110156 | TCGA | 2.08E-03 | #N/A |
| rs17586462 | TCGA | 2.08E-03 | #N/A |
| rs1971033 | TCGA&TCGA_coCNV | 1.84E-04 | 1.36E-03 |
| rs12148044 | TCGA&TCGA_coCNV | 2.08E-03 | 1.33E-03 |
| rs11097173 | TCGA&TCGA_coCNV | 2.08E-03 | 1.63E-03 |
| rs6548978 | TCGA&TCGA_coCNV | 2.08E-03 | 1.16E-03 |
| rs17626094 | TCGA | 2.08E-03 | #N/A |

| rs13318886 | TCGA&TCGA_coCNV | 2.08E-03 | 1.74E-03 |
| --- | --- | --- | --- |
| rs10824395 | TCGA | 2.08E-03 | #N/A |
| rs10401263 | TCGA&TCGA_coCNV | 5.51E-04 | 8.59E-04 |
| rs4427376 | TCGA | 2.08E-03 | #N/A |
| rs10495648 | TCGA&TCGA_coCNV | 2.08E-03 | 8.18E-03 |
| rs6505771 | TCGA | 2.08E-03 | #N/A |
| rs561514 | TCGA&TCGA_coCNV | 2.09E-03 | 8.36E-03 |
| rs1317685 | TCGA&TCGA_coCNV | 2.09E-03 | 5.34E-04 |
| rs17108463 | TCGA&TCGA_coCNV | 4.49E-05 | 1.75E-04 |
| rs7172932 | TCGA | 2.09E-03 | #N/A |
| rs11620632 | TCGA | 2.09E-03 | #N/A |
| rs16875617 | TCGA&TCGA_coCNV | 2.09E-03 | 6.79E-04 |
| rs7998006 | TCGA | 2.09E-03 | #N/A |
| rs10098473 | TCGA&TCGA_coCNV | 2.09E-03 | 1.11E-03 |
| rs4828290 | TCGA&TCGA_coCNV | 2.09E-03 | 5.86E-03 |
| rs10404936 | TCGA&TCGA_coCNV | 1.06E-03 | 6.55E-04 |
| rs10416663 | TCGA&TCGA_coCNV | 2.09E-03 | 3.42E-03 |
| rs6079764 | TCGA&TCGA_coCNV | 2.09E-03 | 6.26E-03 |
| rs6728344 | TCGA&TCGA_coCNV | 4.23E-05 | 1.24E-04 |
| rs7702627 | TCGA | 2.17E-04 | #N/A |
| rs1567623 | TCGA&TCGA_coCNV | 2.09E-03 | 4.38E-03 |
| rs12503004 | TCGA&TCGA_coCNV | 2.09E-03 | 1.77E-03 |
| rs4673221 | TCGA | 2.09E-03 | #N/A |
| rs7900125 | TCGA | 1.48E-03 | #N/A |
| rs17252752 | TCGA&TCGA_coCNV | 2.09E-03 | 4.70E-04 |
| rs2359437 | TCGA&TCGA_coCNV | 2.09E-03 | 2.16E-03 |
| rs1802618 | TCGA | 2.10E-03 | #N/A |
| rs7922685 | TCGA | 2.10E-03 | #N/A |
| rs4651377 | TCGA&TCGA_coCNV | 2.10E-03 | 3.01E-04 |
| rs13067058 | TCGA&TCGA_coCNV | 2.10E-03 | 7.91E-04 |
| rs356960 | TCGA | 2.10E-03 | #N/A |
| rs4649415 | TCGA&TCGA_coCNV | 2.10E-03 | 3.41E-03 |
| rs11103433 | TCGA&TCGA_coCNV | 4.34E-04 | 5.00E-04 |
| rs7082636 | TCGA | 2.10E-03 | #N/A |
| rs9476531 | TCGA&TCGA_coCNV | 2.11E-03 | 5.99E-03 |
| rs11683506 | TCGA | 2.11E-03 | #N/A |
| rs2806747 | TCGA | 2.11E-03 | #N/A |
| rs4287917 | TCGA&TCGA_coCNV | 2.11E-03 | 8.69E-03 |
| rs11913228 | TCGA | 2.11E-03 | #N/A |
| rs7149207 | TCGA | 1.71E-03 | #N/A |
| rs4746409 | TCGA | 2.11E-03 | #N/A |
| rs2665529 | TCGA&TCGA_coCNV | 2.11E-03 | 4.57E-03 |
| rs10186221 | TCGA&TCGA_coCNV | 2.11E-03 | 2.34E-03 |
| rs13209127 | TCGA&TCGA_coCNV | 2.11E-03 | 4.73E-04 |
| rs1779579 | TCGA&TCGA_coCNV | 4.86E-04 | 1.25E-03 |
| rs13396312 | TCGA | 2.12E-03 | #N/A |

| rs8038194 | TCGA&TCGA_coCNV | 2.12E-03 | 3.69E-03 |
| --- | --- | --- | --- |
| rs530516 | TCGA | 2.12E-03 | #N/A |
| rs627948 | TCGA&TCGA_coCNV | 2.12E-03 | 1.61E-03 |
| rs7923999 | TCGA | 2.12E-03 | #N/A |
| rs3912944 | TCGA | 2.12E-03 | #N/A |
| rs1438220 | TCGA&TCGA_coCNV | 2.12E-03 | 3.45E-03 |
| rs11846935 | TCGA&TCGA_coCNV | 2.12E-03 | 1.96E-03 |
| rs11009691 | TCGA&TCGA_coCNV | 2.12E-03 | 3.20E-04 |
| rs9398855 | TCGA&TCGA_coCNV | 2.12E-03 | 1.53E-05 |
| rs871799 | TCGA&TCGA_coCNV | 2.12E-03 | 3.76E-04 |
| rs7573808 | TCGA&TCGA_coCNV | 1.21E-04 | 1.86E-04 |
| rs11564446 | TCGA&TCGA_coCNV | 2.12E-03 | 3.45E-03 |
| rs17734263 | TCGA&TCGA_coCNV | 1.78E-03 | 9.39E-04 |
| rs12356076 | TCGA | 1.57E-03 | #N/A |
| rs2618811 | TCGA | 2.12E-03 | #N/A |
| rs16977758 | TCGA&TCGA_coCNV | 2.12E-03 | 1.24E-03 |
| rs12600564 | TCGA&TCGA_coCNV | 2.12E-03 | 3.28E-03 |
| rs2323394 | TCGA&TCGA_coCNV | 2.13E-03 | 1.37E-03 |
| rs10228883 | TCGA&TCGA_coCNV | 2.13E-03 | 1.44E-03 |
| rs361137 | TCGA&TCGA_coCNV | 2.13E-03 | 3.75E-03 |
| rs9508016 | TCGA&TCGA_coCNV | 2.13E-03 | 9.36E-03 |
| rs11062860 | TCGA&TCGA_coCNV | 2.13E-03 | 3.79E-03 |
| rs7020398 | TCGA&TCGA_coCNV | 2.13E-03 | 1.68E-04 |
| rs7645692 | TCGA&TCGA_coCNV | 2.13E-03 | 2.85E-03 |
| rs2419846 | TCGA&TCGA_coCNV | 2.13E-03 | 7.61E-03 |
| rs7679984 | TCGA | 2.13E-03 | #N/A |
| rs12669679 | TCGA&TCGA_coCNV | 2.13E-03 | 8.02E-03 |
| rs1810475 | TCGA&TCGA_coCNV | 2.13E-03 | 9.01E-03 |
| rs5759002 | TCGA | 2.13E-03 | #N/A |
| rs13166462 | TCGA&TCGA_coCNV | 2.13E-03 | 1.95E-03 |
| rs571610 | TCGA&TCGA_coCNV | 2.14E-03 | 9.68E-04 |
| rs6120365 | TCGA&TCGA_coCNV | 2.14E-03 | 1.84E-04 |
| rs916846 | TCGA&TCGA_coCNV | 6.30E-04 | 3.33E-04 |
| rs7355592 | TCGA&TCGA_coCNV | 2.14E-03 | 8.59E-03 |
| rs1493062 | TCGA&TCGA_coCNV | 4.59E-04 | 5.40E-04 |
| rs2029962 | TCGA | 2.14E-03 | #N/A |
| rs6639914 | TCGA&TCGA_coCNV | 6.96E-06 | 1.33E-04 |
| rs8093901 | TCGA | 2.14E-03 | #N/A |
| rs1891930 | TCGA&TCGA_coCNV | 2.14E-03 | 1.61E-03 |
| rs7645826 | TCGA | 2.15E-03 | #N/A |
| rs10208770 | TCGA&TCGA_coCNV | 2.15E-03 | 7.98E-04 |
| rs1666555 | TCGA | 2.15E-03 | #N/A |
| rs17160062 | TCGA&TCGA_coCNV | 7.42E-06 | 4.69E-05 |
| rs10487440 | TCGA&TCGA_coCNV | 2.15E-03 | 3.15E-04 |
| rs16853948 | TCGA&TCGA_coCNV | 2.15E-03 | 8.59E-03 |
| rs11869429 | TCGA&TCGA_coCNV | 2.15E-03 | 2.71E-03 |

| rs512365 | TCGA&TCGA_coCNV | 2.15E-03 | 1.37E-03 |
| --- | --- | --- | --- |
| rs6716648 | TCGA&TCGA_coCNV | 2.15E-03 | 4.63E-03 |
| rs7979969 | TCGA | 2.15E-03 | #N/A |
| rs9591258 | TCGA&TCGA_coCNV | 7.58E-04 | 6.21E-03 |
| rs2520341 | TCGA | 2.16E-03 | #N/A |
| rs1010618 | TCGA | 2.16E-03 | #N/A |
| rs35183060 | TCGA&TCGA_coCNV | 2.16E-03 | 4.16E-03 |
| rs6501265 | TCGA&TCGA_coCNV | 1.79E-03 | 1.56E-03 |
| rs6982320 | TCGA&TCGA_coCNV | 2.16E-03 | 1.23E-03 |
| rs9680296 | TCGA | 2.16E-03 | #N/A |
| rs1665643 | TCGA&TCGA_coCNV | 1.47E-03 | 1.17E-03 |
| rs1820184 | TCGA | 2.16E-03 | #N/A |
| rs1792367 | TCGA&TCGA_coCNV | 2.16E-03 | 2.57E-03 |
| rs2206979 | TCGA&TCGA_coCNV | 6.90E-04 | 9.68E-04 |
| rs16882622 | TCGA | 2.17E-03 | #N/A |
| rs13069521 | TCGA | 2.17E-03 | #N/A |
| rs4972834 | TCGA | 2.17E-03 | #N/A |
| rs4028824 | TCGA&TCGA_coCNV | 2.17E-03 | 1.36E-03 |
| rs1898536 | TCGA&TCGA_coCNV | 2.17E-03 | 3.20E-03 |
| rs488433 | TCGA&TCGA_coCNV | 2.17E-03 | 2.09E-03 |
| rs176382 | TCGA | 2.17E-03 | #N/A |
| rs16823211 | TCGA&TCGA_coCNV | 2.17E-03 | 6.50E-04 |
| rs17014088 | TCGA&TCGA_coCNV | 2.17E-03 | 2.49E-03 |
| rs11662857 | TCGA&TCGA_coCNV | 2.17E-03 | 8.35E-03 |
| rs2456050 | TCGA | 2.17E-03 | #N/A |
| rs1500819 | TCGA | 2.17E-03 | #N/A |
| rs2364814 | TCGA&TCGA_coCNV | 2.17E-03 | 3.33E-03 |
| rs6573313 | TCGA&TCGA_coCNV | 2.17E-03 | 6.70E-03 |
| rs8090615 | TCGA&TCGA_coCNV | 2.17E-03 | 1.62E-03 |
| rs1589379 | TCGA | 2.18E-03 | #N/A |
| rs6564542 | TCGA | 2.18E-03 | #N/A |
| rs6640875 | TCGA&TCGA_coCNV | 2.18E-03 | 2.47E-03 |
| rs17017356 | TCGA&TCGA_coCNV | 5.90E-05 | 7.82E-04 |
| rs690101 | TCGA | 2.18E-03 | #N/A |
| rs2000751 | TCGA&TCGA_coCNV | 1.15E-03 | 2.96E-04 |
| rs463235 | TCGA&TCGA_coCNV | 2.18E-03 | 6.37E-03 |
| rs2426280 | TCGA | 2.18E-03 | #N/A |
| rs12477017 | TCGA&TCGA_coCNV | 2.18E-03 | 3.17E-04 |
| rs17829167 | TCGA | 2.18E-03 | #N/A |
| rs10235086 | TCGA | 2.18E-03 | #N/A |
| rs11634215 | TCGA | 2.18E-03 | #N/A |
| rs6636651 | TCGA&TCGA_coCNV | 2.18E-03 | 7.13E-03 |
| rs2774247 | TCGA | 2.18E-03 | #N/A |
| rs7588725 | TCGA&TCGA_coCNV | 6.97E-04 | 3.21E-04 |
| rs17734148 | TCGA&TCGA_coCNV | 2.19E-03 | 7.76E-04 |
| rs12582634 | TCGA | 2.19E-03 | #N/A |

| rs7784122 | TCGA&TCGA_coCNV | 2.19E-03 | 5.94E-03 |
| --- | --- | --- | --- |
| rs1159226 | TCGA | 2.19E-03 | #N/A |
| rs17169055 | TCGA&TCGA_coCNV | 2.19E-03 | 3.93E-03 |
| rs6964012 | TCGA&TCGA_coCNV | 2.19E-03 | 1.10E-03 |
| rs2139490 | TCGA&TCGA_coCNV | 2.19E-03 | 2.77E-03 |
| rs1484799 | TCGA | 2.19E-03 | #N/A |
| rs4672505 | TCGA&TCGA_coCNV | 2.20E-03 | 6.48E-03 |
| rs16978838 | TCGA | 6.89E-04 | #N/A |
| rs1024766 | TCGA | 2.20E-03 | #N/A |
| rs1553530 | TCGA | 2.20E-03 | #N/A |
| rs690526 | TCGA&TCGA_coCNV | 2.20E-03 | 6.82E-03 |
| rs797347 | TCGA | 2.20E-03 | #N/A |
| rs16860809 | TCGA&TCGA_coCNV | 2.20E-03 | 8.55E-04 |
| rs4727027 | TCGA | 2.20E-03 | #N/A |
| rs2832648 | TCGA&TCGA_coCNV | 2.20E-03 | 4.77E-03 |
| rs3810367 | TCGA | 2.20E-03 | #N/A |
| rs267529 | TCGA&TCGA_coCNV | 2.20E-03 | 2.57E-03 |
| rs10483964 | TCGA | 2.20E-03 | #N/A |
| rs846547 | TCGA&TCGA_coCNV | 2.20E-03 | 3.52E-03 |
| rs6630097 | TCGA&TCGA_coCNV | 8.13E-05 | 1.77E-04 |
| rs13299931 | TCGA&TCGA_coCNV | 2.20E-03 | 7.94E-03 |
| rs6965352 | TCGA&TCGA_coCNV | 2.21E-03 | 4.57E-03 |
| rs713720 | TCGA&TCGA_coCNV | 2.21E-03 | 3.33E-03 |
| rs6842683 | TCGA&TCGA_coCNV | 5.81E-04 | 6.46E-03 |
| rs17071227 | TCGA&TCGA_coCNV | 1.27E-03 | 8.93E-04 |
| rs3905396 | TCGA&TCGA_coCNV | 2.21E-03 | 9.67E-04 |
| rs1264563 | TCGA | 2.21E-03 | #N/A |
| rs9466534 | TCGA | 2.21E-03 | #N/A |
| rs11589054 | TCGA | 2.21E-03 | #N/A |
| rs4674010 | TCGA | 2.21E-03 | #N/A |
| rs10913878 | TCGA&TCGA_coCNV | 2.21E-03 | 1.74E-03 |
| rs3096192 | TCGA&TCGA_coCNV | 2.09E-03 | 2.24E-03 |
| rs10511334 | TCGA&TCGA_coCNV | 1.73E-03 | 8.28E-04 |
| rs4765679 | TCGA | 2.22E-03 | #N/A |
| rs12244831 | TCGA&TCGA_coCNV | 2.20E-03 | 4.89E-03 |
| rs16926240 | TCGA&TCGA_coCNV | 2.22E-03 | 7.28E-04 |
| rs7848718 | TCGA&TCGA_coCNV | 2.22E-03 | 5.07E-03 |
| rs7884420 | TCGA | 3.44E-04 | #N/A |
| rs7684137 | TCGA&TCGA_coCNV | 2.22E-03 | 4.55E-03 |
| rs1996992 | TCGA&TCGA_coCNV | 2.22E-03 | 9.80E-03 |
| rs1598546 | TCGA&TCGA_coCNV | 2.22E-03 | 6.36E-03 |
| rs1482785 | TCGA&TCGA_coCNV | 2.89E-04 | 2.00E-04 |
| rs16972312 | TCGA | 2.23E-03 | #N/A |
| rs8183670 | TCGA&TCGA_coCNV | 2.23E-03 | 9.65E-04 |
| rs12598082 | TCGA | 2.23E-03 | #N/A |
| rs890747 | TCGA&TCGA_coCNV | 2.23E-03 | 2.74E-03 |

| rs12679450 | TCGA&TCGA_coCNV | 4.25E-05 | 5.23E-04 |
| --- | --- | --- | --- |
| rs6923632 | TCGA | 2.23E-03 | #N/A |
| rs713991 | TCGA&TCGA_coCNV | 2.23E-03 | 2.42E-03 |
| rs7888275 | TCGA | 2.23E-03 | #N/A |
| rs7097461 | TCGA&TCGA_coCNV | 2.23E-03 | 6.61E-03 |
| rs4767773 | TCGA&TCGA_coCNV | 2.23E-03 | 1.07E-04 |
| rs4796877 | TCGA&TCGA_coCNV | 1.64E-03 | 4.83E-04 |
| rs10774679 | TCGA&TCGA_coCNV | 2.23E-03 | 4.23E-03 |
| rs757683 | TCGA&TCGA_coCNV | 2.23E-03 | 2.86E-03 |
| rs4867260 | TCGA | 2.23E-03 | #N/A |
| rs10033980 | TCGA&TCGA_coCNV | 1.94E-04 | 1.51E-03 |
| rs31517 | TCGA&TCGA_coCNV | 2.24E-03 | 2.49E-03 |
| rs2184903 | TCGA | 2.24E-03 | #N/A |
| rs12336314 | TCGA&TCGA_coCNV | 2.24E-03 | 4.69E-03 |
| rs2447974 | TCGA&TCGA_coCNV | 2.24E-03 | 4.47E-03 |
| rs13314784 | TCGA | 2.24E-03 | #N/A |
| rs5979856 | TCGA | 2.24E-03 | #N/A |
| rs11906514 | TCGA&TCGA_coCNV | 2.24E-03 | 3.41E-03 |
| rs2194387 | TCGA&TCGA_coCNV | 4.69E-05 | 1.93E-04 |
| rs8087290 | TCGA&TCGA_coCNV | 6.29E-04 | 1.54E-03 |
| rs16835267 | TCGA | 2.24E-03 | #N/A |
| rs1455432 | TCGA&TCGA_coCNV | 2.24E-03 | 8.45E-03 |
| rs6466273 | TCGA | 2.25E-03 | #N/A |
| rs3741691 | TCGA&TCGA_coCNV | 2.25E-03 | 7.25E-04 |
| rs6447561 | TCGA&TCGA_coCNV | 2.25E-03 | 3.29E-03 |
| rs7003398 | TCGA&TCGA_coCNV | 3.08E-05 | 9.37E-05 |
| rs2279290 | TCGA&TCGA_coCNV | 2.25E-03 | 2.11E-03 |
| rs10241657 | TCGA&TCGA_coCNV | 2.25E-03 | 1.02E-03 |
| rs10209905 | TCGA&TCGA_coCNV | 2.25E-03 | 1.52E-03 |
| rs11884886 | TCGA&TCGA_coCNV | 2.25E-03 | 7.74E-03 |
| rs11153964 | TCGA&TCGA_coCNV | 1.79E-03 | 1.15E-03 |
| rs1953057 | TCGA | 2.25E-03 | #N/A |
| rs6544015 | TCGA | 2.25E-03 | #N/A |
| rs10847617 | TCGA&TCGA_coCNV | 2.25E-03 | 7.33E-03 |
| rs16999879 | TCGA&TCGA_coCNV | 2.25E-03 | 7.02E-03 |
| rs7149250 | TCGA | 2.26E-03 | #N/A |
| rs1013303 | TCGA&TCGA_coCNV | 2.26E-03 | 3.72E-03 |
| rs2812585 | TCGA&TCGA_coCNV | 2.26E-03 | 5.56E-04 |
| rs4959432 | TCGA&TCGA_coCNV | 1.77E-03 | 2.06E-03 |
| rs17108960 | TCGA&TCGA_coCNV | 2.26E-03 | 2.69E-03 |
| rs9504413 | TCGA | 2.26E-03 | #N/A |
| rs17143060 | TCGA&TCGA_coCNV | 2.26E-03 | 3.80E-03 |
| rs10510980 | TCGA&TCGA_coCNV | 2.26E-03 | 5.41E-03 |
| rs10142319 | TCGA&TCGA_coCNV | 2.26E-03 | 7.07E-03 |
| rs11082096 | TCGA&TCGA_coCNV | 2.26E-03 | 2.21E-03 |
| rs2872960 | TCGA | 2.26E-03 | #N/A |

| rs6441961 | TCGA | 2.26E-03 | #N/A |
| --- | --- | --- | --- |
| rs1981251 | TCGA | 2.27E-03 | #N/A |
| rs205991 | TCGA&TCGA_coCNV | 2.78E-04 | 9.33E-03 |
| rs11080134 | TCGA | 2.22E-03 | #N/A |
| rs12326981 | TCGA&TCGA_coCNV | 2.27E-03 | 6.22E-03 |
| rs1655018 | TCGA&TCGA_coCNV | 2.27E-03 | 7.97E-03 |
| rs34518007 | TCGA&TCGA_coCNV | 2.27E-03 | 5.15E-03 |
| rs1417584 | TCGA&TCGA_coCNV | 2.27E-03 | 8.28E-03 |
| rs11130400 | TCGA&TCGA_coCNV | 2.27E-03 | 3.93E-03 |
| rs10437049 | TCGA&TCGA_coCNV | 2.27E-03 | 5.37E-03 |
| rs12380409 | TCGA&TCGA_coCNV | 2.27E-03 | 3.39E-03 |
| rs8179278 | TCGA&TCGA_coCNV | 2.27E-03 | 3.98E-04 |
| rs2287655 | TCGA | 3.07E-04 | #N/A |
| rs1559648 | TCGA&TCGA_coCNV | 2.28E-03 | 1.96E-03 |
| rs3912262 | TCGA | 2.28E-03 | #N/A |
| rs4806796 | TCGA&TCGA_coCNV | 2.12E-04 | 2.43E-04 |
| rs1497213 | TCGA&TCGA_coCNV | 2.01E-03 | 5.16E-04 |
| rs12941198 | TCGA | 2.28E-03 | #N/A |
| rs10471161 | TCGA&TCGA_coCNV | 2.29E-03 | 1.12E-03 |
| rs10420505 | TCGA&TCGA_coCNV | 2.29E-03 | 4.84E-03 |
| rs4655864 | TCGA&TCGA_coCNV | 1.96E-03 | 1.04E-03 |
| rs16848510 | TCGA&TCGA_coCNV | 2.29E-03 | 3.24E-03 |
| rs16986006 | TCGA | 2.29E-03 | #N/A |
| rs5978122 | TCGA&TCGA_coCNV | 2.29E-03 | 4.32E-04 |
| rs9643322 | TCGA&TCGA_coCNV | 2.29E-03 | 1.53E-03 |
| rs6061479 | TCGA&TCGA_coCNV | 2.29E-03 | 3.52E-03 |
| rs1477595 | TCGA&TCGA_coCNV | 1.36E-03 | 5.05E-03 |
| rs7834117 | TCGA&TCGA_coCNV | 2.69E-04 | 1.52E-03 |
| rs2761970 | TCGA&TCGA_coCNV | 2.29E-03 | 3.83E-03 |
| rs1560233 | TCGA&TCGA_coCNV | 2.29E-03 | 3.39E-04 |
| rs13181739 | TCGA&TCGA_coCNV | 2.29E-03 | 9.88E-04 |
| rs922953 | TCGA | 2.29E-03 | #N/A |
| rs6933379 | TCGA | 2.29E-03 | #N/A |
| rs11067367 | TCGA&TCGA_coCNV | 2.30E-03 | 5.18E-04 |
| rs3757001 | TCGA&TCGA_coCNV | 2.30E-03 | 1.74E-04 |
| rs12264636 | TCGA&TCGA_coCNV | 2.30E-03 | 8.15E-03 |
| rs4542359 | TCGA&TCGA_coCNV | 7.65E-04 | 1.34E-03 |
| rs6753305 | TCGA | 2.30E-03 | #N/A |
| rs17122093 | TCGA&TCGA_coCNV | 2.30E-03 | 2.18E-03 |
| rs17145022 | TCGA&TCGA_coCNV | 4.91E-05 | 3.65E-04 |
| rs12625776 | TCGA&TCGA_coCNV | 2.30E-03 | 4.33E-03 |
| rs1165213 | TCGA | 2.30E-03 | #N/A |
| rs1047677 | TCGA | 2.30E-03 | #N/A |
| rs361492 | TCGA | 2.30E-03 | #N/A |
| rs11965813 | TCGA&TCGA_coCNV | 2.30E-03 | 7.79E-03 |
| rs1474347 | TCGA&TCGA_coCNV | 2.30E-03 | 6.76E-03 |

| rs12035180 | TCGA&TCGA_coCNV | 2.30E-03 | 9.85E-03 |
| --- | --- | --- | --- |
| rs10266032 | TCGA&TCGA_coCNV | 3.05E-04 | 4.84E-03 |
| rs1464670 | TCGA | 2.30E-03 | #N/A |
| rs3752339 | TCGA&TCGA_coCNV | 1.14E-03 | 4.71E-04 |
| rs10226770 | TCGA | 2.31E-03 | #N/A |
| rs10874869 | TCGA&TCGA_coCNV | 2.31E-03 | 2.86E-03 |
| rs1747060 | TCGA&TCGA_coCNV | 2.31E-03 | 3.97E-03 |
| rs3138031 | TCGA&TCGA_coCNV | 2.31E-03 | 1.52E-03 |
| rs16907201 | TCGA&TCGA_coCNV | 2.31E-03 | 3.18E-03 |
| rs7098685 | TCGA&TCGA_coCNV | 2.31E-03 | 6.74E-03 |
| rs12467760 | TCGA | 2.31E-03 | #N/A |
| rs11864056 | TCGA&TCGA_coCNV | 2.31E-03 | 7.00E-03 |
| rs10962541 | TCGA&TCGA_coCNV | 2.31E-03 | 7.81E-03 |
| rs17242358 | TCGA | 2.31E-03 | #N/A |
| rs2247957 | TCGA&TCGA_coCNV | 2.31E-03 | 3.43E-03 |
| rs2394102 | TCGA | 2.31E-03 | #N/A |
| rs2309322 | TCGA | 2.31E-03 | #N/A |
| rs7690662 | TCGA&TCGA_coCNV | 2.31E-03 | 1.81E-03 |
| rs17543506 | TCGA&TCGA_coCNV | 2.31E-03 | 6.46E-03 |
| rs10208392 | TCGA | 2.31E-03 | #N/A |
| rs2683686 | TCGA | 2.32E-03 | #N/A |
| rs9371915 | TCGA&TCGA_coCNV | 1.59E-03 | 2.94E-05 |
| rs2526098 | TCGA&TCGA_coCNV | 2.32E-03 | 5.27E-04 |
| rs10502454 | TCGA&TCGA_coCNV | 2.32E-03 | 9.32E-04 |
| rs545682 | TCGA&TCGA_coCNV | 1.33E-04 | 1.77E-03 |
| rs973331 | TCGA | 2.32E-03 | #N/A |
| rs9635995 | TCGA&TCGA_coCNV | 2.32E-03 | 1.23E-03 |
| rs16993942 | TCGA&TCGA_coCNV | 2.32E-03 | 2.05E-03 |
| rs7092839 | TCGA | 2.32E-03 | #N/A |
| rs41451053 | TCGA&TCGA_coCNV | 2.32E-03 | 2.19E-03 |
| rs17125659 | TCGA | 2.32E-03 | #N/A |
| rs6079275 | TCGA&TCGA_coCNV | 2.32E-03 | 5.75E-03 |
| rs2824176 | TCGA | 2.32E-03 | #N/A |
| rs4741165 | TCGA | 2.32E-03 | #N/A |
| rs6594664 | TCGA&TCGA_coCNV | 2.22E-03 | 4.54E-03 |
| rs1931897 | TCGA&TCGA_coCNV | 6.93E-04 | 1.18E-03 |
| rs10445012 | TCGA | 2.33E-03 | #N/A |
| rs13437419 | TCGA&TCGA_coCNV | 2.33E-03 | 1.28E-03 |
| rs16882008 | TCGA | 2.33E-03 | #N/A |
| rs3814399 | TCGA&TCGA_coCNV | 2.33E-03 | 4.65E-03 |
| rs291083 | TCGA&TCGA_coCNV | 2.33E-03 | 2.79E-03 |
| rs7233147 | TCGA&TCGA_coCNV | 1.87E-03 | 5.77E-04 |
| rs7611506 | TCGA&TCGA_coCNV | 2.33E-03 | 8.13E-04 |
| rs8101425 | TCGA&TCGA_coCNV | 2.33E-03 | 6.19E-03 |
| rs12618777 | TCGA&TCGA_coCNV | 2.33E-03 | 3.45E-03 |
| rs12566285 | TCGA | 2.33E-03 | #N/A |

| rs288180 | TCGA&TCGA_coCNV | 2.33E-03 | 1.12E-03 |
| --- | --- | --- | --- |
| rs269247 | TCGA&TCGA_coCNV | 2.33E-03 | 3.40E-03 |
| rs4744821 | TCGA&TCGA_coCNV | 2.34E-03 | 3.25E-03 |
| rs12704084 | TCGA&TCGA_coCNV | 2.34E-03 | 4.19E-03 |
| rs4920292 | TCGA&TCGA_coCNV | 2.34E-03 | 4.28E-03 |
| rs2873392 | TCGA | 2.34E-03 | #N/A |
| rs13318609 | TCGA&TCGA_coCNV | 2.34E-03 | 1.40E-03 |
| rs6627119 | TCGA&TCGA_coCNV | 1.07E-03 | 9.44E-05 |
| rs7167741 | TCGA&TCGA_coCNV | 2.34E-03 | 9.49E-03 |
| rs6589202 | TCGA&TCGA_coCNV | 2.34E-03 | 4.00E-03 |
| rs419728 | TCGA&TCGA_coCNV | 2.34E-03 | 1.89E-03 |
| rs16911499 | TCGA&TCGA_coCNV | 2.34E-03 | 2.08E-03 |
| rs4246952 | TCGA | 2.34E-03 | #N/A |
| rs10061730 | TCGA&TCGA_coCNV | 2.34E-03 | 7.53E-03 |
| rs1488758 | TCGA&TCGA_coCNV | 2.34E-03 | 4.07E-03 |
| rs1981694 | TCGA&TCGA_coCNV&SCANdb | 5.09E-05 | 1.19E-04 |
| rs2159594 | TCGA&TCGA_coCNV | 2.35E-03 | 4.31E-03 |
| rs10000713 | TCGA&TCGA_coCNV | 2.35E-03 | 5.65E-03 |
| rs3825541 | TCGA&TCGA_coCNV | 1.25E-03 | 2.26E-03 |
| rs2806703 | TCGA&TCGA_coCNV | 2.35E-03 | 1.08E-03 |
| rs11620762 | TCGA&TCGA_coCNV | 5.37E-04 | 6.98E-03 |
| rs11692675 | TCGA&TCGA_coCNV | 2.35E-03 | 2.16E-03 |
| rs8004872 | TCGA | 2.35E-03 | #N/A |
| rs9514112 | TCGA&TCGA_coCNV | 6.80E-04 | 6.65E-04 |
| rs16899892 | TCGA&TCGA_coCNV | 2.35E-03 | 6.78E-04 |
| rs7419909 | TCGA&TCGA_coCNV | 2.35E-03 | 2.28E-03 |
| rs5933938 | TCGA&TCGA_coCNV | 2.35E-03 | 4.01E-04 |
| rs1079926 | TCGA | 2.35E-03 | #N/A |
| rs1967103 | TCGA | 2.35E-03 | #N/A |
| rs9287990 | TCGA&TCGA_coCNV | 2.35E-03 | 1.23E-03 |
| rs12692783 | TCGA&TCGA_coCNV | 2.35E-03 | 9.38E-04 |
| rs5976399 | TCGA | 2.35E-03 | #N/A |
| rs17706358 | TCGA&TCGA_coCNV | 2.35E-03 | 7.79E-03 |
| rs11910797 | TCGA | 2.35E-03 | #N/A |
| rs16990920 | TCGA&TCGA_coCNV | 2.35E-03 | 3.68E-03 |
| rs11842534 | TCGA&TCGA_coCNV | 2.35E-03 | 4.70E-03 |
| rs10492787 | TCGA&TCGA_coCNV | 2.35E-03 | 2.35E-03 |
| rs7094085 | TCGA&TCGA_coCNV | 2.36E-03 | 1.85E-03 |
| rs10124468 | TCGA | 2.36E-03 | #N/A |
| rs2232613 | TCGA | 2.36E-03 | #N/A |
| rs2339689 | TCGA | 2.36E-03 | #N/A |
| rs654387 | TCGA | 1.79E-03 | #N/A |
| rs423626 | TCGA | 2.36E-03 | #N/A |
| rs6558867 | TCGA | 2.36E-03 | #N/A |
| rs12588875 | TCGA | 2.36E-03 | #N/A |
| rs7056793 | TCGA&TCGA_coCNV | 2.36E-03 | 3.93E-03 |

| rs2505611 | TCGA | 2.36E-03 | #N/A |
| --- | --- | --- | --- |
| rs6477360 | TCGA&TCGA_coCNV | 2.37E-03 | 6.55E-03 |
| rs4650680 | TCGA&TCGA_coCNV | 2.37E-03 | 2.20E-03 |
| rs1959066 | TCGA&TCGA_coCNV | 2.37E-03 | 9.64E-05 |
| rs1531261 | TCGA&TCGA_coCNV | 2.37E-03 | 3.54E-03 |
| rs7814135 | TCGA | 3.55E-06 | #N/A |
| rs4863488 | TCGA&TCGA_coCNV | 2.37E-03 | 2.72E-03 |
| rs4018641 | TCGA | 2.37E-03 | #N/A |
| rs6808673 | TCGA&TCGA_coCNV | 2.37E-03 | 7.48E-03 |
| rs1111451 | TCGA | 2.37E-03 | #N/A |
| rs16991385 | TCGA&TCGA_coCNV | 2.37E-03 | 4.19E-04 |
| rs16957052 | TCGA&TCGA_coCNV | 1.50E-04 | 4.36E-03 |
| rs893856 | TCGA&TCGA_coCNV | 2.37E-03 | 3.73E-03 |
| rs16867348 | TCGA&TCGA_coCNV | 2.37E-03 | 1.21E-03 |
| rs7791432 | TCGA&TCGA_coCNV | 2.37E-03 | 8.31E-04 |
| rs12556508 | TCGA&TCGA_coCNV | 2.38E-03 | 9.94E-03 |
| rs17004969 | TCGA&TCGA_coCNV | 1.72E-04 | 6.08E-05 |
| rs17116700 | TCGA&TCGA_coCNV | 2.38E-03 | 2.44E-04 |
| rs6768637 | TCGA&TCGA_coCNV | 2.38E-03 | 5.22E-03 |
| rs10060837 | TCGA&TCGA_coCNV | 2.38E-03 | 3.66E-03 |
| rs4628134 | TCGA&TCGA_coCNV | 4.61E-05 | 7.34E-05 |
| rs7042818 | TCGA | 2.38E-03 | #N/A |
| rs2875567 | TCGA&TCGA_coCNV | 2.38E-03 | 8.10E-04 |
| rs905900 | TCGA&TCGA_coCNV | 2.38E-03 | 5.75E-03 |
| rs11636033 | TCGA&TCGA_coCNV | 2.38E-03 | 5.13E-03 |
| rs17151696 | TCGA&TCGA_coCNV | 2.38E-03 | 1.68E-03 |
| rs16850490 | TCGA&TCGA_coCNV | 1.42E-04 | 1.79E-04 |
| rs2383682 | TCGA | 2.39E-03 | #N/A |
| rs10959751 | TCGA | 2.39E-03 | #N/A |
| rs1756985 | TCGA | 2.39E-03 | #N/A |
| rs40143 | TCGA | 2.39E-03 | #N/A |
| rs4327453 | TCGA | 2.39E-03 | #N/A |
| rs691772 | TCGA | 2.39E-03 | #N/A |
| rs7939459 | TCGA&TCGA_coCNV | 1.87E-03 | 7.56E-04 |
| rs5767026 | TCGA&TCGA_coCNV | 2.39E-03 | 5.85E-03 |
| rs17504296 | TCGA&TCGA_coCNV | 1.34E-03 | 6.40E-03 |
| rs7758499 | TCGA&TCGA_coCNV | 2.39E-03 | 1.94E-03 |
| rs8123337 | TCGA | 2.39E-03 | #N/A |
| rs269866 | TCGA&TCGA_coCNV | 2.39E-03 | 1.60E-03 |
| rs1903216 | TCGA&TCGA_coCNV | 2.40E-03 | 7.19E-03 |
| rs6718489 | TCGA&TCGA_coCNV | 8.74E-06 | 1.68E-03 |
| rs17124704 | TCGA | 2.40E-03 | #N/A |
| rs7676378 | TCGA&TCGA_coCNV | 2.40E-03 | 2.66E-03 |
| rs1700069 | TCGA&TCGA_coCNV | 2.40E-03 | 6.23E-03 |
| rs4880657 | TCGA | 2.40E-03 | #N/A |
| rs8027980 | TCGA | 2.40E-03 | #N/A |

| rs7048463 | TCGA&TCGA_coCNV | 2.40E-03 | 1.20E-03 |
| --- | --- | --- | --- |
| rs7550014 | TCGA&TCGA_coCNV | 2.40E-03 | 6.40E-03 |
| rs4700160 | TCGA&TCGA_coCNV | 2.40E-03 | 9.75E-03 |
| rs1505861 | TCGA | 2.40E-03 | #N/A |
| rs244503 | TCGA&TCGA_coCNV | 2.40E-03 | 4.49E-03 |
| rs2733005 | TCGA&TCGA_coCNV | 2.40E-03 | 7.62E-04 |
| rs4237195 | TCGA&TCGA_coCNV | 2.41E-03 | 1.46E-03 |
| rs10876255 | TCGA&TCGA_coCNV | 2.41E-03 | 6.41E-04 |
| rs1148993 | TCGA&TCGA_coCNV | 2.41E-03 | 1.68E-03 |
| rs6893551 | TCGA&TCGA_coCNV | 2.41E-03 | 1.85E-03 |
| rs12293478 | TCGA | 2.41E-03 | #N/A |
| rs11563240 | TCGA&TCGA_coCNV | 2.41E-03 | 1.98E-03 |
| rs7794632 | TCGA&TCGA_coCNV | 2.41E-03 | 3.10E-03 |
| rs4500591 | TCGA&TCGA_coCNV | 1.51E-03 | 4.63E-03 |
| rs17115116 | TCGA | 2.42E-03 | #N/A |
| rs17018021 | TCGA&TCGA_coCNV | 2.42E-03 | 7.02E-04 |
| rs2224912 | TCGA&TCGA_coCNV | 2.42E-03 | 2.87E-04 |
| rs9848635 | TCGA | 2.42E-03 | #N/A |
| rs17782618 | TCGA | 2.42E-03 | #N/A |
| rs17214868 | TCGA&TCGA_coCNV | 2.42E-03 | 8.33E-03 |
| rs8070572 | TCGA&TCGA_coCNV | 2.42E-03 | 3.56E-03 |
| rs6961094 | TCGA | 2.42E-03 | #N/A |
| rs2952016 | TCGA&TCGA_coCNV | 2.98E-12 | 9.77E-03 |
| rs716532 | TCGA&TCGA_coCNV | 2.42E-03 | 7.98E-04 |
| rs10149796 | TCGA | 2.42E-03 | #N/A |
| rs1951354 | TCGA&TCGA_coCNV | 2.42E-03 | 8.40E-04 |
| rs4955659 | TCGA&TCGA_coCNV | 1.32E-03 | 1.29E-03 |
| rs6477872 | TCGA&TCGA_coCNV | 2.42E-03 | 4.95E-03 |
| rs11016169 | TCGA | 2.43E-03 | #N/A |
| rs4635362 | TCGA&TCGA_coCNV | 2.43E-03 | 3.25E-03 |
| rs17008486 | TCGA&TCGA_coCNV | 2.43E-03 | 1.50E-03 |
| rs10106512 | TCGA&TCGA_coCNV | 2.43E-03 | 7.55E-03 |
| rs11858344 | TCGA&TCGA_coCNV | 1.47E-04 | 1.80E-04 |
| rs16862250 | TCGA | 2.43E-03 | #N/A |
| rs11646101 | TCGA | 2.43E-03 | #N/A |
| rs2598575 | TCGA | 2.43E-03 | #N/A |
| rs10044396 | TCGA&TCGA_coCNV | 2.43E-03 | 3.10E-03 |
| rs4954854 | TCGA&TCGA_coCNV | 5.79E-04 | 3.23E-03 |
| rs10882362 | TCGA | 2.43E-03 | #N/A |
| rs10506525 | TCGA&TCGA_coCNV | 2.43E-03 | 2.16E-03 |
| rs16957197 | TCGA&TCGA_coCNV | 2.43E-03 | 4.28E-03 |
| rs492107 | TCGA | 2.44E-03 | #N/A |
| rs2863390 | TCGA&TCGA_coCNV | 2.44E-03 | 5.64E-04 |
| rs10959870 | TCGA&TCGA_coCNV | 2.44E-03 | 8.63E-03 |
| rs2048108 | TCGA | 2.44E-03 | #N/A |
| rs9298792 | TCGA | 2.44E-03 | #N/A |

| rs10741446 | TCGA&TCGA_coCNV | 2.44E-03 | 9.23E-04 |
| --- | --- | --- | --- |
| rs771901 | TCGA | 2.44E-03 | #N/A |
| rs41404844 | TCGA&TCGA_coCNV | 2.44E-03 | 4.67E-04 |
| rs9573227 | TCGA&TCGA_coCNV | 2.44E-03 | 7.00E-04 |
| rs10509838 | TCGA&TCGA_coCNV | 2.44E-03 | 4.12E-03 |
| rs8124688 | TCGA&TCGA_coCNV | 2.44E-03 | 2.87E-03 |
| rs17115760 | TCGA&TCGA_coCNV | 2.44E-03 | 8.90E-03 |
| rs7283057 | TCGA&TCGA_coCNV | 2.44E-03 | 8.13E-03 |
| rs13095281 | TCGA&TCGA_coCNV | 2.44E-03 | 1.15E-03 |
| rs16953389 | TCGA | 2.44E-03 | #N/A |
| rs130002 | TCGA&TCGA_coCNV | 2.44E-03 | 4.35E-03 |
| rs12701651 | TCGA | 2.45E-03 | #N/A |
| rs202063 | TCGA | 2.45E-03 | #N/A |
| rs17169117 | TCGA&TCGA_coCNV | 2.45E-03 | 2.99E-03 |
| rs5953690 | TCGA | 2.45E-03 | #N/A |
| rs7564727 | TCGA&TCGA_coCNV | 2.45E-03 | 1.53E-03 |
| rs41349448 | TCGA | 2.45E-03 | #N/A |
| rs10809359 | TCGA | 2.45E-03 | #N/A |
| rs16973101 | TCGA&TCGA_coCNV | 2.45E-03 | 9.71E-03 |
| rs1926156 | TCGA&TCGA_coCNV | 2.45E-03 | 1.40E-03 |
| rs11630398 | TCGA | 2.46E-03 | #N/A |
| rs4784596 | TCGA&TCGA_coCNV | 2.46E-03 | 4.90E-04 |
| rs17395223 | TCGA | 2.46E-03 | #N/A |
| rs131686 | TCGA&TCGA_coCNV | 2.46E-03 | 4.93E-03 |
| rs10823864 | TCGA&TCGA_coCNV | 6.75E-04 | 1.05E-03 |
| rs7098330 | TCGA&TCGA_coCNV | 2.17E-04 | 4.64E-04 |
| rs6864342 | TCGA | 4.95E-04 | #N/A |
| rs1501674 | TCGA&TCGA_coCNV | 2.46E-03 | 9.85E-03 |
| rs2645072 | TCGA&TCGA_coCNV | 8.86E-04 | 1.68E-03 |
| rs5970762 | TCGA&TCGA_coCNV | 2.46E-03 | 9.91E-04 |
| rs9887668 | TCGA&TCGA_coCNV | 2.46E-03 | 3.82E-03 |
| rs11118573 | TCGA&TCGA_coCNV | 2.46E-03 | 7.24E-04 |
| rs4829989 | TCGA&TCGA_coCNV | 2.29E-03 | 3.05E-04 |
| rs4964246 | TCGA | 2.46E-03 | #N/A |
| rs16840680 | TCGA | 2.46E-03 | #N/A |
| rs7537542 | TCGA&TCGA_coCNV | 2.46E-03 | 9.52E-04 |
| rs241359 | TCGA&TCGA_coCNV | 2.46E-03 | 1.44E-03 |
| rs9460022 | TCGA&TCGA_coCNV | 2.46E-03 | 1.51E-03 |
| rs7812436 | TCGA | 2.46E-03 | #N/A |
| rs7842355 | TCGA | 2.47E-03 | #N/A |
| rs2400439 | TCGA | 2.47E-03 | #N/A |
| rs2232259 | TCGA&TCGA_coCNV | 2.47E-03 | 1.97E-03 |
| rs11584933 | TCGA | 2.47E-03 | #N/A |
| rs965018 | TCGA | 2.47E-03 | #N/A |
| rs3905501 | TCGA&TCGA_coCNV | 2.47E-03 | 4.31E-03 |
| rs352388 | TCGA&TCGA_coCNV | 1.62E-03 | 1.11E-03 |

| rs9953469 | TCGA | 2.47E-03 | #N/A |
| --- | --- | --- | --- |
| rs11112828 | TCGA&TCGA_coCNV | 2.47E-03 | 2.45E-03 |
| rs7209400 | TCGA | 2.47E-03 | #N/A |
| rs17401675 | TCGA&TCGA_coCNV | 2.47E-03 | 4.97E-04 |
| rs732380 | TCGA&TCGA_coCNV | 1.14E-03 | 9.29E-03 |
| rs4889773 | TCGA | 2.48E-03 | #N/A |
| rs17200818 | TCGA&TCGA_coCNV | 2.48E-03 | 2.57E-03 |
| rs7290477 | TCGA&TCGA_coCNV | 2.48E-03 | 1.35E-03 |
| rs2795407 | TCGA&TCGA_coCNV | 6.99E-05 | 3.60E-03 |
| rs4731209 | TCGA | 2.48E-03 | #N/A |
| rs6043560 | TCGA&TCGA_coCNV | 1.54E-05 | 7.26E-04 |
| rs17093557 | TCGA&TCGA_coCNV | 2.48E-03 | 5.34E-04 |
| rs4727028 | TCGA | 2.48E-03 | #N/A |
| rs12549679 | TCGA&TCGA_coCNV | 2.48E-03 | 2.12E-03 |
| rs11239035 | TCGA&TCGA_coCNV | 7.23E-04 | 2.19E-04 |
| rs6640826 | TCGA&TCGA_coCNV | 2.48E-03 | 4.17E-03 |
| rs8104210 | TCGA | 2.48E-03 | #N/A |
| rs12490903 | TCGA&TCGA_coCNV | 2.26E-03 | 2.09E-03 |
| rs7068140 | TCGA | 2.49E-03 | #N/A |
| rs1551299 | TCGA&TCGA_coCNV | 2.49E-03 | 2.64E-03 |
| rs1633141 | TCGA&TCGA_coCNV | 2.49E-03 | 5.83E-04 |
| rs10490004 | TCGA&TCGA_coCNV | 2.49E-03 | 2.98E-03 |
| rs11070682 | TCGA&TCGA_coCNV | 2.49E-03 | 9.03E-03 |
| rs2650406 | TCGA&TCGA_coCNV | 2.49E-03 | 6.41E-03 |
| rs9950196 | TCGA | 2.49E-03 | #N/A |
| rs542077 | TCGA | 2.49E-03 | #N/A |
| rs6503296 | TCGA | 2.49E-03 | #N/A |
| rs2630351 | TCGA | 2.49E-03 | #N/A |
| rs17652715 | TCGA | 2.49E-03 | #N/A |
| rs12954479 | TCGA&TCGA_coCNV | 1.02E-03 | 8.66E-05 |
| rs1515116 | TCGA | 1.42E-03 | #N/A |
| rs7525706 | TCGA | 2.50E-03 | #N/A |
| rs11225905 | TCGA&TCGA_coCNV | 2.50E-03 | 3.95E-03 |
| rs2248539 | TCGA&TCGA_coCNV | 2.50E-03 | 1.78E-04 |
| rs41531449 | TCGA | 2.48E-03 | #N/A |
| rs7900745 | TCGA&TCGA_coCNV | 1.26E-03 | 4.12E-04 |
| rs12353705 | TCGA&TCGA_coCNV | 9.78E-06 | 6.00E-04 |
| rs17260254 | TCGA | 2.50E-03 | #N/A |
| rs10492561 | TCGA | 2.50E-03 | #N/A |
| rs4944357 | TCGA&TCGA_coCNV | 2.50E-03 | 2.37E-03 |
| rs7913355 | TCGA&TCGA_coCNV | 2.50E-03 | 3.45E-03 |
| rs7771441 | TCGA | 2.50E-03 | #N/A |
| rs16976258 | TCGA | 2.50E-03 | #N/A |
| rs11646484 | TCGA | 2.51E-03 | #N/A |
| rs4847214 | TCGA&TCGA_coCNV | 2.51E-03 | 3.41E-03 |
| rs11063473 | TCGA&TCGA_coCNV | 2.51E-03 | 5.84E-03 |

| rs12059696 | TCGA&TCGA_coCNV | 2.51E-03 | 2.56E-03 |
| --- | --- | --- | --- |
| rs11778096 | TCGA&TCGA_coCNV | 2.51E-03 | 4.12E-03 |
| rs12268038 | TCGA | 2.51E-03 | #N/A |
| rs16976162 | TCGA&TCGA_coCNV | 2.51E-03 | 1.48E-04 |
| rs4236383 | TCGA&TCGA_coCNV | 2.51E-03 | 2.23E-03 |
| rs2298261 | TCGA | 2.51E-03 | #N/A |
| rs161803 | TCGA&TCGA_coCNV | 2.51E-03 | 1.87E-03 |
| rs12776241 | TCGA&TCGA_coCNV | 2.51E-03 | 3.47E-03 |
| rs9902437 | TCGA&TCGA_coCNV | 1.75E-03 | 4.31E-03 |
| rs2526787 | TCGA&TCGA_coCNV | 2.51E-03 | 2.32E-04 |
| rs10933727 | TCGA&TCGA_coCNV | 2.51E-03 | 7.59E-03 |
| rs2272790 | TCGA&TCGA_coCNV | 2.51E-03 | 3.16E-03 |
| rs3793646 | TCGA&TCGA_coCNV | 2.51E-03 | 2.06E-03 |
| rs7673523 | TCGA&TCGA_coCNV | 2.51E-03 | 2.18E-03 |
| rs1567050 | TCGA&TCGA_coCNV | 2.51E-03 | 1.08E-03 |
| rs10184505 | TCGA&TCGA_coCNV | 6.01E-06 | 1.15E-03 |
| rs17086235 | TCGA&TCGA_coCNV | 2.42E-04 | 1.32E-03 |
| rs10490560 | TCGA&TCGA_coCNV | 2.52E-03 | 1.54E-03 |
| rs2292763 | TCGA&TCGA_coCNV | 2.52E-03 | 1.13E-04 |
| rs10519667 | TCGA&TCGA_coCNV | 2.52E-03 | 1.34E-03 |
| rs4765457 | TCGA | 2.52E-03 | #N/A |
| rs7706780 | TCGA&TCGA_coCNV | 2.52E-03 | 3.96E-03 |
| rs17048557 | TCGA&TCGA_coCNV | 2.52E-03 | 2.10E-04 |
| rs12274310 | TCGA&TCGA_coCNV | 2.52E-03 | 7.38E-05 |
| rs1953067 | TCGA&TCGA_coCNV | 2.52E-03 | 9.79E-03 |
| rs9879195 | TCGA | 2.52E-03 | #N/A |
| rs875968 | TCGA | 1.16E-03 | #N/A |
| rs1705237 | TCGA&TCGA_coCNV | 2.52E-03 | 1.49E-03 |
| rs17109410 | TCGA&TCGA_coCNV | 2.52E-03 | 6.80E-04 |
| rs12489817 | TCGA&TCGA_coCNV | 2.52E-03 | 1.82E-03 |
| rs11765797 | TCGA | 2.52E-03 | #N/A |
| rs11872161 | TCGA&TCGA_coCNV | 2.53E-03 | 6.67E-03 |
| rs11255657 | TCGA | 2.53E-03 | #N/A |
| rs5742469 | TCGA&TCGA_coCNV | 2.53E-03 | 1.02E-04 |
| rs12688940 | TCGA&TCGA_coCNV | 1.80E-03 | 2.59E-03 |
| rs687033 | TCGA | 2.22E-03 | #N/A |
| rs4123914 | TCGA&TCGA_coCNV | 6.62E-04 | 1.97E-03 |
| rs6732658 | TCGA&TCGA_coCNV | 1.71E-03 | 4.39E-03 |
| rs10738245 | TCGA&TCGA_coCNV | 2.46E-03 | 9.94E-04 |
| rs177802 | TCGA&TCGA_coCNV | 2.53E-03 | 6.74E-04 |
| rs7258943 | TCGA&TCGA_coCNV | 2.53E-03 | 9.53E-04 |
| rs7821384 | TCGA&TCGA_coCNV | 3.72E-05 | 1.19E-03 |
| rs2962615 | TCGA | 2.21E-03 | #N/A |
| rs7966691 | TCGA&TCGA_coCNV | 2.53E-03 | 1.18E-03 |
| rs17641275 | TCGA | 2.25E-03 | #N/A |
| rs10270076 | TCGA&TCGA_coCNV | 2.53E-03 | 9.02E-04 |

| rs2262459 | TCGA&TCGA_coCNV | 4.76E-04 | 4.76E-04 |
| --- | --- | --- | --- |
| rs17127243 | TCGA | 1.14E-04 | #N/A |
| rs12586774 | TCGA&TCGA_coCNV | 2.53E-03 | 8.95E-05 |
| rs11014779 | TCGA&TCGA_coCNV | 2.54E-03 | 8.13E-04 |
| rs2279519 | TCGA&TCGA_coCNV | 2.54E-03 | 2.68E-03 |
| rs16878895 | TCGA | 6.08E-04 | #N/A |
| rs9963424 | TCGA&TCGA_coCNV | 2.54E-03 | 8.29E-03 |
| rs8134365 | TCGA&TCGA_coCNV | 1.10E-03 | 3.50E-03 |
| rs11197998 | TCGA | 2.54E-03 | #N/A |
| rs6069267 | TCGA | 2.54E-03 | #N/A |
| rs12701296 | TCGA | 2.54E-03 | #N/A |
| rs41461548 | TCGA&TCGA_coCNV | 2.55E-03 | 9.20E-03 |
| rs6886152 | TCGA | 2.55E-03 | #N/A |
| rs2741331 | TCGA&TCGA_coCNV | 2.55E-03 | 8.96E-03 |
| rs3740571 | TCGA | 2.55E-03 | #N/A |
| rs17676418 | TCGA&TCGA_coCNV | 2.55E-03 | 3.42E-04 |
| rs9577814 | TCGA | 2.55E-03 | #N/A |
| rs10280133 | TCGA | 2.55E-03 | #N/A |
| rs956527 | TCGA&TCGA_coCNV | 2.55E-03 | 3.42E-03 |
| rs10917033 | TCGA | 2.55E-03 | #N/A |
| rs2972180 | TCGA&TCGA_coCNV | 2.55E-03 | 4.69E-03 |
| rs11592979 | TCGA | 2.55E-03 | #N/A |
| rs1712684 | TCGA&TCGA_coCNV&SCANdb | 2.55E-03 | 1.73E-03 |
| rs17160305 | TCGA&TCGA_coCNV | 1.69E-03 | 6.42E-03 |
| rs4448295 | TCGA | 9.55E-10 | #N/A |
| rs5953667 | TCGA&TCGA_coCNV | 2.56E-03 | 3.11E-03 |
| rs9997878 | TCGA&TCGA_coCNV | 2.56E-03 | 8.33E-03 |
| rs9532874 | TCGA | 2.56E-03 | #N/A |
| rs4857072 | TCGA&TCGA_coCNV | 8.89E-04 | 7.87E-03 |
| rs12606922 | TCGA&TCGA_coCNV | 2.56E-03 | 1.54E-03 |
| rs11809114 | TCGA&TCGA_coCNV | 2.56E-03 | 2.17E-03 |
| rs16928572 | TCGA&TCGA_coCNV | 2.56E-03 | 4.64E-03 |
| rs2404602 | TCGA&TCGA_coCNV | 2.56E-03 | 2.71E-03 |
| rs7105655 | TCGA | 6.86E-04 | #N/A |
| rs6615763 | TCGA | 2.03E-03 | #N/A |
| rs12433751 | TCGA | 2.57E-03 | #N/A |
| rs10490074 | TCGA&TCGA_coCNV | 2.38E-03 | 2.74E-03 |
| rs17006064 | TCGA | 2.57E-03 | #N/A |
| rs6855984 | TCGA | 2.57E-03 | #N/A |
| rs12434181 | TCGA | 2.57E-03 | #N/A |
| rs11802587 | TCGA&TCGA_coCNV | 2.57E-03 | 4.03E-03 |
| rs1403629 | TCGA&TCGA_coCNV | 2.57E-03 | 2.68E-03 |
| rs6470642 | TCGA | 2.57E-03 | #N/A |
| rs17553846 | TCGA&TCGA_coCNV | 9.79E-04 | 3.91E-03 |
| rs10143711 | TCGA | 2.57E-03 | #N/A |
| rs297851 | TCGA&TCGA_coCNV | 2.57E-03 | 8.77E-03 |

| rs4655330 | TCGA&TCGA_coCNV | 2.57E-03 | 6.82E-03 |
| --- | --- | --- | --- |
| rs11249231 | TCGA&TCGA_coCNV | 2.94E-04 | 1.88E-04 |
| rs4743671 | TCGA&TCGA_coCNV | 2.57E-03 | 3.12E-04 |
| rs2495288 | TCGA | 2.58E-03 | #N/A |
| rs4116812 | TCGA&TCGA_coCNV | 2.58E-03 | 1.17E-03 |
| rs4388012 | TCGA | 2.58E-03 | #N/A |
| rs1746477 | TCGA&TCGA_coCNV | 2.58E-03 | 5.73E-03 |
| rs7932486 | TCGA&TCGA_coCNV | 2.58E-03 | 6.20E-03 |
| rs1553257 | TCGA | 2.58E-03 | #N/A |
| rs9535530 | TCGA&TCGA_coCNV | 1.38E-03 | 5.52E-05 |
| rs2277012 | TCGA&TCGA_coCNV | 7.66E-04 | 3.97E-03 |
| rs965408 | TCGA | 2.54E-03 | #N/A |
| rs2819657 | TCGA&TCGA_coCNV | 2.58E-03 | 2.86E-04 |
| rs10247762 | TCGA | 2.59E-03 | #N/A |
| rs6855992 | TCGA&TCGA_coCNV | 2.59E-03 | 1.90E-04 |
| rs16982495 | TCGA&TCGA_coCNV | 2.59E-03 | 1.53E-03 |
| rs11755212 | TCGA | 2.59E-03 | #N/A |
| rs12002677 | TCGA | 2.59E-03 | #N/A |
| rs17152950 | TCGA&TCGA_coCNV | 2.59E-03 | 1.54E-04 |
| rs10832276 | TCGA&TCGA_coCNV | 2.59E-03 | 5.27E-04 |
| rs4897235 | TCGA&TCGA_coCNV | 2.59E-03 | 1.33E-04 |
| rs815614 | TCGA&TCGA_coCNV | 2.60E-03 | 8.56E-03 |
| rs11021109 | TCGA | 2.60E-03 | #N/A |
| rs17287344 | TCGA&TCGA_coCNV | 3.58E-05 | 1.58E-05 |
| rs6719645 | TCGA&TCGA_coCNV | 2.60E-03 | 2.68E-03 |
| rs12303118 | TCGA&TCGA_coCNV | 2.60E-03 | 1.74E-03 |
| rs17166237 | TCGA | 2.60E-03 | #N/A |
| rs12783844 | TCGA&TCGA_coCNV | 2.60E-03 | 2.23E-03 |
| rs7183000 | TCGA&TCGA_coCNV | 2.60E-03 | 2.30E-03 |
| rs11208029 | TCGA&TCGA_coCNV | 2.60E-03 | 2.13E-03 |
| rs7553012 | TCGA | 2.60E-03 | #N/A |
| rs16968634 | TCGA&TCGA_coCNV | 9.28E-04 | 5.53E-04 |
| rs9290281 | TCGA&TCGA_coCNV | 2.60E-03 | 8.28E-04 |
| rs1667765 | TCGA&TCGA_coCNV | 2.60E-03 | 2.79E-03 |
| rs179409 | TCGA&TCGA_coCNV | 2.60E-03 | 8.31E-03 |
| rs983444 | TCGA&TCGA_coCNV | 2.61E-03 | 3.68E-03 |
| rs11038193 | TCGA&TCGA_coCNV | 2.61E-03 | 5.71E-03 |
| rs267541 | TCGA | 2.61E-03 | #N/A |
| rs7071050 | TCGA&TCGA_coCNV | 2.61E-03 | 7.93E-03 |
| rs13133452 | TCGA&TCGA_coCNV | 2.61E-03 | 1.92E-03 |
| rs17163792 | TCGA&TCGA_coCNV | 2.61E-03 | 1.62E-03 |
| rs11781914 | TCGA&TCGA_coCNV | 2.61E-03 | 4.99E-03 |
| rs5990014 | TCGA&TCGA_coCNV | 2.61E-03 | 9.62E-03 |
| rs1980977 | TCGA | 2.61E-03 | #N/A |
| rs12607414 | TCGA&TCGA_coCNV | 2.61E-03 | 6.48E-04 |
| rs4660357 | TCGA&TCGA_coCNV | 2.61E-03 | 3.59E-03 |

| rs3990898 | TCGA&TCGA_coCNV | 2.61E-03 | 7.83E-03 |
| --- | --- | --- | --- |
| rs1940676 | TCGA | 2.61E-03 | #N/A |
| rs17823706 | TCGA&TCGA_coCNV | 5.47E-04 | 2.72E-03 |
| rs7691351 | TCGA | 2.61E-03 | #N/A |
| rs7885958 | TCGA&TCGA_coCNV | 2.61E-03 | 9.83E-04 |
| rs7650541 | TCGA | 2.61E-03 | #N/A |
| rs4951457 | TCGA | 2.62E-03 | #N/A |
| rs10156133 | TCGA&TCGA_coCNV | 2.06E-03 | 1.73E-03 |
| rs750874 | TCGA&TCGA_coCNV | 2.62E-03 | 8.05E-03 |
| rs7584375 | TCGA&TCGA_coCNV | 2.62E-03 | 3.80E-03 |
| rs2159894 | TCGA&TCGA_coCNV | 5.15E-04 | 2.40E-03 |
| rs11983087 | TCGA&TCGA_coCNV | 2.62E-03 | 1.88E-04 |
| rs919222 | TCGA&TCGA_coCNV | 2.62E-03 | 5.29E-03 |
| rs1822770 | TCGA | 2.62E-03 | #N/A |
| rs16979266 | TCGA&TCGA_coCNV | 2.62E-03 | 7.29E-04 |
| rs11790784 | TCGA&TCGA_coCNV | 2.63E-03 | 1.48E-03 |
| rs12058117 | TCGA&TCGA_coCNV | 2.63E-03 | 1.17E-03 |
| rs12105087 | TCGA&TCGA_coCNV | 2.63E-03 | 6.29E-03 |
| rs1406689 | TCGA&TCGA_coCNV | 2.63E-03 | 4.28E-03 |
| rs6743093 | TCGA&TCGA_coCNV | 2.63E-03 | 9.21E-04 |
| rs9401955 | TCGA&TCGA_coCNV | 1.20E-03 | 6.90E-04 |
| rs7870426 | TCGA&TCGA_coCNV | 2.63E-03 | 1.45E-03 |
| rs3762177 | TCGA | 2.63E-03 | #N/A |
| rs1547832 | TCGA&TCGA_coCNV | 1.34E-03 | 5.41E-03 |
| rs7633314 | TCGA&TCGA_coCNV | 2.50E-03 | 6.86E-03 |
| rs1882368 | TCGA&TCGA_coCNV | 1.77E-03 | 8.36E-04 |
| rs5004162 | TCGA | 2.63E-03 | #N/A |
| rs1230398 | TCGA&TCGA_coCNV | 2.63E-03 | 7.73E-03 |
| rs10819635 | TCGA&TCGA_coCNV | 2.63E-03 | 4.93E-03 |
| rs942753 | TCGA | 2.63E-03 | #N/A |
| rs288016 | TCGA&TCGA_coCNV | 2.64E-03 | 4.64E-03 |
| rs10178684 | TCGA&TCGA_coCNV | 2.64E-03 | 2.56E-03 |
| rs12588364 | TCGA&TCGA_coCNV | 2.64E-03 | 8.01E-03 |
| rs2073457 | TCGA | 1.42E-03 | #N/A |
| rs10988783 | TCGA&TCGA_coCNV | 2.64E-03 | 9.13E-03 |
| rs179903 | TCGA | 2.64E-03 | #N/A |
| rs10237251 | TCGA | 2.64E-03 | #N/A |
| rs17668615 | TCGA&TCGA_coCNV | 2.64E-03 | 3.04E-04 |
| rs10134475 | TCGA&TCGA_coCNV | 2.64E-03 | 1.66E-04 |
| rs7525103 | TCGA&TCGA_coCNV | 2.65E-03 | 1.02E-03 |
| rs3737293 | TCGA&TCGA_coCNV | 2.65E-03 | 6.84E-03 |
| rs12238719 | TCGA&TCGA_coCNV | 2.65E-03 | 6.71E-03 |
| rs4890325 | TCGA&TCGA_coCNV | 1.68E-03 | 3.53E-03 |
| rs17054536 | TCGA&TCGA_coCNV | 2.65E-03 | 8.35E-03 |
| rs6007710 | TCGA | 2.65E-03 | #N/A |
| rs9531565 | TCGA | 2.65E-03 | #N/A |

| rs7756164 | TCGA | 2.65E-03 | #N/A |
| --- | --- | --- | --- |
| rs1866171 | TCGA&TCGA_coCNV | 2.65E-03 | 1.19E-03 |
| rs16865924 | TCGA&TCGA_coCNV | 2.45E-04 | 1.56E-03 |
| rs8099738 | TCGA | 2.65E-03 | #N/A |
| rs4463124 | TCGA&TCGA_coCNV | 2.65E-03 | 7.81E-04 |
| rs10003019 | TCGA | 2.65E-03 | #N/A |
| rs12660323 | TCGA&TCGA_coCNV | 2.65E-03 | 3.77E-03 |
| rs9623226 | TCGA&TCGA_coCNV | 2.65E-03 | 5.19E-03 |
| rs13013842 | TCGA | 2.65E-03 | #N/A |
| rs4336667 | TCGA | 2.65E-03 | #N/A |
| rs16860148 | TCGA&TCGA_coCNV | 2.66E-03 | 1.11E-03 |
| rs2372573 | TCGA&TCGA_coCNV | 2.66E-03 | 6.33E-03 |
| rs4762758 | TCGA | 2.66E-03 | #N/A |
| rs8108510 | TCGA | 2.66E-03 | #N/A |
| rs2859252 | TCGA | 2.66E-03 | #N/A |
| rs415395 | TCGA&TCGA_coCNV | 2.66E-03 | 7.15E-04 |
| rs10462966 | TCGA&TCGA_coCNV | 2.66E-03 | 5.54E-04 |
| rs1834615 | TCGA | 2.66E-03 | #N/A |
| rs6677953 | TCGA | 2.66E-03 | #N/A |
| rs1595322 | TCGA | 2.66E-03 | #N/A |
| rs10404881 | TCGA&TCGA_coCNV | 2.66E-03 | 1.39E-03 |
| rs17209962 | TCGA | 2.66E-03 | #N/A |
| rs7825417 | TCGA&TCGA_coCNV | 2.67E-03 | 3.64E-04 |
| rs7858416 | TCGA | 2.67E-03 | #N/A |
| rs16967818 | TCGA&TCGA_coCNV | 2.67E-03 | 2.14E-03 |
| rs540491 | TCGA&TCGA_coCNV | 2.67E-03 | 3.10E-03 |
| rs4871222 | TCGA&TCGA_coCNV | 2.67E-03 | 1.32E-03 |
| rs510588 | TCGA&TCGA_coCNV | 2.67E-03 | 1.75E-03 |
| rs6505812 | TCGA&TCGA_coCNV | 2.67E-03 | 4.29E-03 |
| rs10895454 | TCGA&TCGA_coCNV | 2.67E-03 | 4.23E-03 |
| rs12805390 | TCGA&TCGA_coCNV | 1.48E-03 | 9.27E-03 |
| rs17011820 | TCGA&TCGA_coCNV | 2.67E-03 | 3.07E-03 |
| rs8105549 | TCGA&TCGA_coCNV | 2.67E-03 | 7.62E-04 |
| rs1472253 | TCGA | 2.67E-03 | #N/A |
| rs6540802 | TCGA&TCGA_coCNV | 2.67E-03 | 2.27E-03 |
| rs11243789 | TCGA&TCGA_coCNV | 2.67E-03 | 1.01E-03 |
| rs12025482 | TCGA&TCGA_coCNV | 2.68E-03 | 1.31E-05 |
| rs1689001 | TCGA&TCGA_coCNV | 2.68E-03 | 5.88E-03 |
| rs1468811 | TCGA | 2.68E-03 | #N/A |
| rs1539907 | TCGA | 2.68E-03 | #N/A |
| rs11854132 | TCGA | 2.68E-03 | #N/A |
| rs9667144 | TCGA&TCGA_coCNV | 2.68E-03 | 2.87E-04 |
| rs1915117 | TCGA | 2.68E-03 | #N/A |
| rs2417927 | TCGA&TCGA_coCNV | 2.68E-03 | 4.56E-03 |
| rs789181 | TCGA | 2.68E-03 | #N/A |
| rs11643324 | TCGA | 2.68E-03 | #N/A |

| rs3812699 | TCGA&TCGA_coCNV | 2.68E-03 | 8.17E-03 |
| --- | --- | --- | --- |
| rs17111216 | TCGA | 2.68E-03 | #N/A |
| rs11879622 | TCGA&TCGA_coCNV | 8.67E-04 | 2.04E-03 |
| rs7935680 | TCGA&TCGA_coCNV | 1.78E-03 | 1.80E-03 |
| rs17138978 | TCGA&TCGA_coCNV | 2.69E-03 | 1.25E-03 |
| rs12472569 | TCGA&TCGA_coCNV | 2.69E-03 | 2.85E-03 |
| rs16859369 | TCGA | 2.69E-03 | #N/A |
| rs9865499 | TCGA | 2.69E-03 | #N/A |
| rs4900246 | TCGA | 2.69E-03 | #N/A |
| rs6521472 | TCGA&TCGA_coCNV | 2.69E-03 | 4.60E-03 |
| rs1519900 | TCGA&TCGA_coCNV | 2.69E-03 | 3.52E-04 |
| rs10942768 | TCGA | 2.70E-03 | #N/A |
| rs129974 | TCGA | 1.74E-03 | #N/A |
| rs10080206 | TCGA&TCGA_coCNV | 2.70E-03 | 4.19E-05 |
| rs1787520 | TCGA | 2.70E-03 | #N/A |
| rs9318163 | TCGA&TCGA_coCNV | 2.70E-03 | 1.87E-03 |
| rs8014970 | TCGA | 2.70E-03 | #N/A |
| rs7831345 | TCGA | 5.39E-09 | #N/A |
| rs2636810 | TCGA | 2.70E-03 | #N/A |
| rs12688829 | TCGA&TCGA_coCNV | 2.70E-03 | 7.20E-03 |
| rs1446660 | TCGA&TCGA_coCNV | 2.70E-03 | 6.91E-03 |
| rs5935215 | TCGA&TCGA_coCNV | 2.71E-03 | 1.04E-04 |
| rs7805713 | TCGA&TCGA_coCNV | 2.71E-03 | 3.08E-03 |
| rs16831046 | TCGA&TCGA_coCNV | 2.71E-03 | 8.21E-03 |
| rs1930096 | TCGA&TCGA_coCNV | 2.71E-03 | 2.97E-03 |
| rs12622882 | TCGA | 2.71E-03 | #N/A |
| rs4958359 | TCGA | 2.71E-03 | #N/A |
| rs6106042 | TCGA&TCGA_coCNV | 2.72E-03 | 8.93E-04 |
| rs1468571 | TCGA&TCGA_coCNV | 2.42E-04 | 8.16E-03 |
| rs2043272 | TCGA | 2.72E-03 | #N/A |
| rs11175794 | TCGA&TCGA_coCNV | 5.82E-04 | 6.93E-05 |
| rs16858353 | TCGA&TCGA_coCNV | 2.72E-03 | 5.43E-03 |
| rs7720261 | TCGA | 2.72E-03 | #N/A |
| rs10070138 | TCGA&TCGA_coCNV | 2.72E-03 | 1.66E-03 |
| rs17147976 | TCGA | 2.72E-03 | #N/A |
| rs1958697 | TCGA&TCGA_coCNV | 1.84E-03 | 3.39E-04 |
| rs2240187 | TCGA | 2.72E-03 | #N/A |
| rs267539 | TCGA&TCGA_coCNV | 2.72E-03 | 9.28E-03 |
| rs10161605 | TCGA | 2.72E-03 | #N/A |
| rs1341731 | TCGA | 2.72E-03 | #N/A |
| rs970127 | TCGA | 2.72E-03 | #N/A |
| rs159954 | TCGA | 2.72E-03 | #N/A |
| rs2673142 | TCGA&TCGA_coCNV | 5.87E-04 | 1.43E-03 |
| rs847800 | TCGA&TCGA_coCNV | 2.72E-03 | 1.55E-03 |
| rs9968624 | TCGA&TCGA_coCNV | 2.72E-03 | 2.29E-03 |
| rs10427892 | TCGA&TCGA_coCNV | 2.72E-03 | 4.24E-03 |

| rs10108738 | TCGA | 2.73E-03 | #N/A |
| --- | --- | --- | --- |
| rs12837981 | TCGA&TCGA_coCNV | 2.73E-03 | 5.11E-03 |
| rs11988913 | TCGA&TCGA_coCNV | 2.73E-03 | 1.74E-03 |
| rs4864045 | TCGA | 2.73E-03 | #N/A |
| rs9598627 | TCGA&TCGA_coCNV | 2.73E-03 | 7.60E-03 |
| rs1943229 | TCGA | 2.73E-03 | #N/A |
| rs104017 | TCGA&TCGA_coCNV | 1.22E-03 | 1.24E-03 |
| rs17026927 | TCGA&TCGA_coCNV | 2.73E-03 | 8.84E-03 |
| rs1337081 | TCGA&TCGA_coCNV | 2.73E-03 | 2.80E-03 |
| rs4639040 | TCGA&TCGA_coCNV | 2.73E-03 | 3.92E-03 |
| rs13118 | TCGA | 2.73E-03 | #N/A |
| rs17696544 | TCGA&TCGA_coCNV | 2.73E-03 | 6.66E-03 |
| rs17561451 | TCGA&TCGA_coCNV | 2.73E-03 | 7.05E-03 |
| rs2338802 | TCGA&TCGA_coCNV | 2.73E-03 | 8.81E-05 |
| rs11138417 | TCGA&TCGA_coCNV | 2.73E-03 | 7.18E-03 |
| rs6929041 | TCGA | 2.74E-03 | #N/A |
| rs5933948 | TCGA&TCGA_coCNV | 2.74E-03 | 3.56E-04 |
| rs680997 | TCGA&TCGA_coCNV | 4.31E-04 | 2.80E-04 |
| rs10056584 | TCGA&TCGA_coCNV | 2.74E-03 | 4.10E-03 |
| rs16871928 | TCGA | 2.74E-03 | #N/A |
| rs10520399 | TCGA | 2.74E-03 | #N/A |
| rs10824397 | TCGA | 2.74E-03 | #N/A |
| rs9472318 | TCGA&TCGA_coCNV | 2.74E-03 | 2.59E-03 |
| rs11868925 | TCGA | 2.74E-03 | #N/A |
| rs11937260 | TCGA | 2.74E-03 | #N/A |
| rs4829565 | TCGA | 2.74E-03 | #N/A |
| rs10494356 | TCGA&TCGA_coCNV | 2.74E-03 | 7.83E-03 |
| rs10999965 | TCGA&TCGA_coCNV | 2.73E-03 | 2.02E-03 |
| rs6428663 | TCGA&TCGA_coCNV | 2.74E-03 | 2.57E-03 |
| rs5997068 | TCGA | 2.74E-03 | #N/A |
| rs456639 | TCGA&TCGA_coCNV | 1.08E-03 | 5.51E-03 |
| rs6537565 | TCGA&TCGA_coCNV | 9.31E-04 | 3.82E-03 |
| rs12826619 | TCGA&TCGA_coCNV | 2.75E-03 | 5.72E-03 |
| rs2222630 | TCGA&TCGA_coCNV | 3.37E-04 | 1.06E-03 |
| rs1330007 | TCGA&TCGA_coCNV | 2.75E-03 | 7.98E-03 |
| rs8096009 | TCGA | 2.75E-03 | #N/A |
| rs4836962 | TCGA&TCGA_coCNV | 2.75E-03 | 3.29E-03 |
| rs9540207 | TCGA | 2.75E-03 | #N/A |
| rs9510774 | TCGA&TCGA_coCNV | 2.75E-03 | 4.14E-03 |
| rs13266670 | TCGA | 2.75E-03 | #N/A |
| rs2647236 | TCGA&TCGA_coCNV | 6.85E-04 | 8.65E-04 |
| rs10956453 | TCGA | 2.75E-03 | #N/A |
| rs2000639 | TCGA&TCGA_coCNV | 2.75E-03 | 1.89E-03 |
| rs290491 | TCGA | 2.75E-03 | #N/A |
| rs13147065 | TCGA&TCGA_coCNV | 2.75E-03 | 8.84E-03 |
| rs9543956 | TCGA | 2.75E-03 | #N/A |

| rs8092488 | TCGA&TCGA_coCNV | 2.75E-03 | 4.13E-03 |
| --- | --- | --- | --- |
| rs2361290 | TCGA | 2.75E-03 | #N/A |
| rs10759502 | TCGA | 2.76E-03 | #N/A |
| rs16876575 | TCGA | 2.76E-03 | #N/A |
| rs11006228 | TCGA | 2.76E-03 | #N/A |
| rs17094342 | TCGA&TCGA_coCNV | 2.76E-03 | 3.52E-03 |
| rs2456997 | TCGA&TCGA_coCNV | 2.76E-03 | 9.66E-03 |
| rs16928837 | TCGA | 2.76E-03 | #N/A |
| rs9462481 | TCGA&TCGA_coCNV | 2.76E-03 | 5.54E-03 |
| rs13152506 | TCGA&TCGA_coCNV | 2.76E-03 | 2.66E-03 |
| rs4915499 | TCGA&TCGA_coCNV | 2.76E-03 | 2.04E-03 |
| rs7133790 | TCGA | 2.77E-03 | #N/A |
| rs10172519 | TCGA&TCGA_coCNV | 2.77E-03 | 1.02E-03 |
| rs11176371 | TCGA&TCGA_coCNV | 2.77E-03 | 1.19E-03 |
| rs1536517 | TCGA&TCGA_coCNV | 2.77E-03 | 5.00E-03 |
| rs6536844 | TCGA&TCGA_coCNV | 1.81E-04 | 1.31E-03 |
| rs16985377 | TCGA | 2.77E-03 | #N/A |
| rs2467021 | TCGA | 2.77E-03 | #N/A |
| rs9564853 | TCGA&TCGA_coCNV | 2.77E-03 | 5.16E-04 |
| rs7858043 | TCGA&TCGA_coCNV | 2.77E-03 | 2.82E-03 |
| rs1735722 | TCGA | 2.77E-03 | #N/A |
| rs1602912 | TCGA&TCGA_coCNV | 2.95E-04 | 3.27E-03 |
| rs12206543 | TCGA&TCGA_coCNV | 2.77E-03 | 4.65E-03 |
| rs2964448 | TCGA | 2.78E-03 | #N/A |
| rs4750496 | TCGA&TCGA_coCNV | 2.78E-03 | 2.33E-03 |
| rs10842519 | TCGA&TCGA_coCNV | 1.55E-04 | 4.52E-04 |
| rs2484505 | TCGA&TCGA_coCNV | 2.78E-03 | 2.42E-03 |
| rs191241 | TCGA&TCGA_coCNV | 2.78E-03 | 8.81E-03 |
| rs17035243 | TCGA&TCGA_coCNV | 2.78E-03 | 8.17E-03 |
| rs17312192 | TCGA&TCGA_coCNV | 2.78E-03 | 3.38E-03 |
| rs11634467 | TCGA&TCGA_coCNV | 2.78E-03 | 1.16E-03 |
| rs2633358 | TCGA&TCGA_coCNV | 3.92E-04 | 1.77E-04 |
| rs7706423 | TCGA&TCGA_coCNV | 2.78E-03 | 2.48E-04 |
| rs4784227 | TCGA&TCGA_coCNV | 2.78E-03 | 8.84E-03 |
| rs9406426 | TCGA&TCGA_coCNV | 4.00E-04 | 3.34E-04 |
| rs893523 | TCGA&TCGA_coCNV | 2.78E-03 | 3.38E-03 |
| rs6937034 | TCGA&TCGA_coCNV | 2.79E-03 | 5.60E-03 |
| rs9940865 | TCGA | 1.74E-03 | #N/A |
| rs12612749 | TCGA&TCGA_coCNV | 2.79E-03 | 2.06E-03 |
| rs17813975 | TCGA&TCGA_coCNV | 2.79E-03 | 8.63E-03 |
| rs980394 | TCGA&TCGA_coCNV | 1.18E-03 | 9.83E-04 |
| rs3890144 | TCGA | 2.79E-03 | #N/A |
| rs1078589 | TCGA&TCGA_coCNV | 2.79E-03 | 6.28E-03 |
| rs10064546 | TCGA&TCGA_coCNV | 2.79E-03 | 8.41E-04 |
| rs6839872 | TCGA&TCGA_coCNV | 2.79E-03 | 3.26E-03 |
| rs7230555 | TCGA | 2.79E-03 | #N/A |

| rs10486156 | TCGA&TCGA_coCNV | 2.80E-03 | 6.19E-03 |
| --- | --- | --- | --- |
| rs1886405 | TCGA | 2.80E-03 | #N/A |
| rs11906230 | TCGA&TCGA_coCNV | 2.77E-03 | 7.15E-04 |
| rs1947455 | TCGA&TCGA_coCNV | 2.80E-03 | 2.72E-03 |
| rs11536949 | TCGA&TCGA_coCNV | 2.80E-03 | 5.60E-03 |
| rs17183295 | TCGA | 2.80E-03 | #N/A |
| rs9821459 | TCGA&TCGA_coCNV | 2.80E-03 | 4.81E-04 |
| rs13302855 | TCGA&TCGA_coCNV | 2.80E-03 | 3.95E-03 |
| rs12906896 | TCGA&TCGA_coCNV | 2.80E-03 | 4.65E-04 |
| rs7598498 | TCGA | 2.80E-03 | #N/A |
| rs595227 | TCGA | 2.80E-03 | #N/A |
| rs41336845 | TCGA&TCGA_coCNV | 2.80E-03 | 9.60E-03 |
| rs12419641 | TCGA | 2.80E-03 | #N/A |
| rs12507792 | TCGA&TCGA_coCNV | 2.11E-03 | 1.21E-04 |
| rs17559267 | TCGA&TCGA_coCNV | 2.80E-03 | 4.93E-03 |
| rs1856859 | TCGA&TCGA_coCNV | 2.26E-03 | 5.87E-03 |
| rs12211331 | TCGA&TCGA_coCNV | 2.52E-03 | 1.63E-03 |
| rs646512 | TCGA&TCGA_coCNV | 4.57E-04 | 6.64E-04 |
| rs12619223 | TCGA | 2.81E-03 | #N/A |
| rs9572009 | TCGA | 2.81E-03 | #N/A |
| rs17199574 | TCGA&TCGA_coCNV | 2.81E-03 | 4.43E-03 |
| rs4734840 | TCGA | 2.81E-03 | #N/A |
| rs17105543 | TCGA&TCGA_coCNV | 2.81E-03 | 2.32E-03 |
| rs6788008 | TCGA&TCGA_coCNV | 2.81E-03 | 3.57E-03 |
| rs1672741 | TCGA&TCGA_coCNV | 1.13E-04 | 2.38E-04 |
| rs13320647 | TCGA&TCGA_coCNV | 2.81E-03 | 8.73E-03 |
| rs535375 | TCGA&TCGA_coCNV | 1.62E-03 | 2.86E-03 |
| rs2843175 | TCGA&TCGA_coCNV | 2.81E-03 | 3.35E-03 |
| rs9398031 | TCGA&TCGA_coCNV | 2.81E-03 | 3.00E-03 |
| rs1404716 | TCGA | 2.81E-03 | #N/A |
| rs12149144 | TCGA | 2.81E-03 | #N/A |
| rs4844509 | TCGA&TCGA_coCNV | 2.81E-03 | 3.64E-04 |
| rs1874532 | TCGA&TCGA_coCNV | 2.81E-03 | 3.71E-03 |
| rs8054772 | TCGA&TCGA_coCNV | 2.81E-03 | 2.79E-03 |
| rs7810962 | TCGA&TCGA_coCNV | 2.81E-03 | 9.11E-04 |
| rs5007461 | TCGA&TCGA_coCNV | 2.82E-03 | 7.44E-03 |
| rs16949125 | TCGA&TCGA_coCNV | 2.82E-03 | 3.43E-03 |
| rs630765 | TCGA | 2.82E-03 | #N/A |
| rs7613456 | TCGA | 2.82E-03 | #N/A |
| rs259925 | TCGA | 2.82E-03 | #N/A |
| rs42398 | TCGA&TCGA_coCNV | 3.11E-06 | 3.08E-05 |
| rs2697299 | TCGA&TCGA_coCNV | 2.82E-03 | 7.55E-03 |
| rs535743 | TCGA&TCGA_coCNV | 2.82E-03 | 2.62E-03 |
| rs11033219 | TCGA&TCGA_coCNV | 2.82E-03 | 2.88E-03 |
| rs11844302 | TCGA | 2.82E-03 | #N/A |
| rs1513334 | TCGA | 2.82E-03 | #N/A |

| rs3852216 | TCGA&TCGA_coCNV | 2.82E-03 | 1.74E-04 |
| --- | --- | --- | --- |
| rs910559 | TCGA&TCGA_coCNV | 2.82E-03 | 3.30E-03 |
| rs10867023 | TCGA&TCGA_coCNV | 2.82E-03 | 3.58E-03 |
| rs9788041 | TCGA&TCGA_coCNV | 2.82E-03 | 2.68E-03 |
| rs17692633 | TCGA | 2.83E-03 | #N/A |
| rs138744 | TCGA&TCGA_coCNV | 2.83E-03 | 3.47E-03 |
| rs10007099 | TCGA&TCGA_coCNV | 2.83E-03 | 1.94E-03 |
| rs41464950 | TCGA&TCGA_coCNV | 3.35E-04 | 2.53E-03 |
| rs6000627 | TCGA | 2.83E-03 | #N/A |
| rs6600090 | TCGA&TCGA_coCNV | 2.83E-03 | 5.74E-04 |
| rs4242337 | TCGA&TCGA_coCNV | 2.83E-03 | 8.11E-03 |
| rs732096 | TCGA | 2.83E-03 | #N/A |
| rs8036295 | TCGA | 2.83E-03 | #N/A |
| rs13335336 | TCGA&TCGA_coCNV | 1.43E-03 | 1.49E-03 |
| rs2216543 | TCGA | 2.83E-03 | #N/A |
| rs12898759 | TCGA | 2.83E-03 | #N/A |
| rs16976281 | TCGA&TCGA_coCNV | 2.83E-03 | 3.18E-03 |
| rs16936992 | TCGA&TCGA_coCNV | 2.83E-03 | 1.10E-03 |
| rs17128533 | TCGA&TCGA_coCNV | 2.83E-03 | 1.65E-04 |
| rs4834262 | TCGA&TCGA_coCNV | 2.79E-04 | 5.89E-04 |
| rs1329742 | TCGA&TCGA_coCNV | 2.84E-03 | 5.90E-03 |
| rs2405496 | TCGA&TCGA_coCNV | 2.84E-03 | 2.18E-03 |
| rs4430122 | TCGA&TCGA_coCNV | 2.84E-03 | 1.18E-03 |
| rs17506154 | TCGA&TCGA_coCNV | 2.84E-03 | 9.23E-03 |
| rs17111827 | TCGA&TCGA_coCNV | 2.84E-03 | 5.97E-03 |
| rs11193394 | TCGA&TCGA_coCNV | 1.28E-03 | 4.40E-04 |
| rs12042637 | TCGA&TCGA_coCNV | 9.63E-05 | 1.01E-03 |
| rs1939361 | TCGA | 2.84E-03 | #N/A |
| rs12545507 | TCGA&TCGA_coCNV | 2.84E-03 | 3.41E-04 |
| rs1358685 | TCGA&TCGA_coCNV | 2.84E-03 | 4.57E-03 |
| rs16950604 | TCGA&TCGA_coCNV | 2.84E-03 | 3.14E-03 |
| rs13405263 | TCGA&TCGA_coCNV | 2.84E-03 | 9.02E-03 |
| rs214003 | TCGA&TCGA_coCNV | 7.44E-04 | 8.06E-04 |
| rs2440527 | TCGA&TCGA_coCNV | 2.84E-03 | 5.61E-03 |
| rs9935327 | TCGA&TCGA_coCNV | 2.84E-03 | 9.72E-04 |
| rs2285934 | TCGA&TCGA_coCNV | 2.84E-03 | 8.81E-03 |
| rs16971146 | TCGA | 2.84E-03 | #N/A |
| rs1475373 | TCGA&TCGA_coCNV | 2.84E-03 | 9.17E-03 |
| rs2417924 | TCGA | 2.84E-03 | #N/A |
| rs16893838 | TCGA&TCGA_coCNV | 2.46E-03 | 4.53E-04 |
| rs288172 | TCGA&TCGA_coCNV | 2.84E-03 | 6.23E-04 |
| rs17687280 | TCGA | 2.84E-03 | #N/A |
| rs17104044 | TCGA&TCGA_coCNV | 1.07E-04 | 4.39E-04 |
| rs287097 | TCGA | 2.85E-03 | #N/A |
| rs7536563 | TCGA | 2.85E-03 | #N/A |
| rs10927348 | TCGA&TCGA_coCNV | 2.85E-03 | 9.78E-03 |

| rs4891963 | TCGA&TCGA_coCNV | 2.85E-03 | 5.73E-03 |
| --- | --- | --- | --- |
| rs2823206 | TCGA&TCGA_coCNV | 2.85E-03 | 7.45E-03 |
| rs12047798 | TCGA&TCGA_coCNV | 1.08E-03 | 4.50E-03 |
| rs41322152 | TCGA&TCGA_coCNV | 1.99E-03 | 2.18E-03 |
| rs3790794 | TCGA&TCGA_coCNV | 2.56E-03 | 2.80E-03 |
| rs2913362 | TCGA&TCGA_coCNV | 2.85E-03 | 2.88E-03 |
| rs9923068 | TCGA&TCGA_coCNV | 2.85E-03 | 9.28E-04 |
| rs4277682 | TCGA | 2.85E-03 | #N/A |
| rs7656684 | TCGA | 2.85E-03 | #N/A |
| rs7551623 | TCGA&TCGA_coCNV | 2.86E-03 | 2.21E-03 |
| rs9520712 | TCGA&TCGA_coCNV | 2.86E-03 | 4.92E-03 |
| rs6565677 | TCGA&TCGA_coCNV | 2.86E-03 | 2.25E-03 |
| rs1394076 | TCGA&TCGA_coCNV | 2.86E-03 | 6.02E-03 |
| rs6609788 | TCGA&TCGA_coCNV | 2.86E-03 | 3.17E-03 |
| rs390961 | TCGA&TCGA_coCNV | 2.86E-03 | 1.00E-02 |
| rs9848122 | TCGA&TCGA_coCNV | 2.86E-03 | 5.09E-03 |
| rs2057115 | TCGA | 2.86E-03 | #N/A |
| rs5991683 | TCGA&TCGA_coCNV | 2.86E-03 | 9.94E-03 |
| rs17153170 | TCGA | 2.86E-03 | #N/A |
| rs1867579 | TCGA&TCGA_coCNV | 2.20E-03 | 7.59E-03 |
| rs6516405 | TCGA&TCGA_coCNV | 2.86E-03 | 2.19E-03 |
| rs205866 | TCGA | 2.86E-03 | #N/A |
| rs2501856 | TCGA&TCGA_coCNV | 2.70E-03 | 7.46E-03 |
| rs6667576 | TCGA&TCGA_coCNV | 2.87E-03 | 6.67E-03 |
| rs1371901 | TCGA | 2.87E-03 | #N/A |
| rs1438261 | TCGA&TCGA_coCNV | 2.97E-04 | 3.54E-03 |
| rs295128 | TCGA&TCGA_coCNV | 2.87E-03 | 4.39E-03 |
| rs7484876 | TCGA&TCGA_coCNV | 2.87E-03 | 6.68E-04 |
| rs17835169 | TCGA | 2.87E-03 | #N/A |
| rs1772414 | TCGA&TCGA_coCNV | 2.87E-03 | 1.07E-04 |
| rs12756720 | TCGA | 2.87E-03 | #N/A |
| rs9559702 | TCGA | 2.87E-03 | #N/A |
| rs12501994 | TCGA | 2.87E-03 | #N/A |
| rs4391609 | TCGA | 2.87E-03 | #N/A |
| rs1449857 | TCGA&TCGA_coCNV | 2.87E-03 | 7.51E-03 |
| rs12906801 | TCGA&TCGA_coCNV | 2.87E-03 | 5.23E-03 |
| rs9549540 | TCGA | 2.87E-03 | #N/A |
| rs1500609 | TCGA | 2.87E-03 | #N/A |
| rs2029747 | TCGA&TCGA_coCNV | 2.87E-03 | 1.92E-03 |
| rs239047 | TCGA | 2.88E-03 | #N/A |
| rs1872145 | TCGA&TCGA_coCNV | 2.88E-03 | 4.17E-03 |
| rs2254143 | TCGA | 2.88E-03 | #N/A |
| rs2073809 | TCGA | 4.60E-04 | #N/A |
| rs17214309 | TCGA&TCGA_coCNV | 4.74E-04 | 3.56E-04 |
| rs7432211 | TCGA | 2.88E-03 | #N/A |
| rs11686719 | TCGA&TCGA_coCNV | 2.88E-03 | 2.24E-05 |

| rs7089960 | TCGA&TCGA_coCNV | 2.88E-03 | 2.91E-03 |
| --- | --- | --- | --- |
| rs4543885 | TCGA&TCGA_coCNV | 2.88E-03 | 2.41E-03 |
| rs12562375 | TCGA | 2.88E-03 | #N/A |
| rs7211468 | TCGA&TCGA_coCNV | 2.88E-03 | 1.24E-03 |
| rs2925216 | TCGA&TCGA_coCNV | 1.87E-04 | 1.67E-04 |
| rs7079833 | TCGA | 2.88E-03 | #N/A |
| rs826375 | TCGA&TCGA_coCNV | 2.89E-03 | 6.42E-03 |
| rs17736249 | TCGA | 2.89E-03 | #N/A |
| rs1338121 | TCGA&TCGA_coCNV | 2.89E-03 | 2.64E-03 |
| rs12458739 | TCGA | 2.89E-03 | #N/A |
| rs8041472 | TCGA&TCGA_coCNV | 2.89E-03 | 9.97E-03 |
| rs7712770 | TCGA | 2.89E-03 | #N/A |
| rs10971709 | TCGA&TCGA_coCNV | 2.89E-03 | 9.88E-03 |
| rs17781266 | TCGA&TCGA_coCNV | 2.05E-03 | 2.57E-03 |
| rs5974238 | TCGA&TCGA_coCNV | 2.89E-03 | 2.36E-03 |
| rs3942487 | TCGA&TCGA_coCNV | 1.99E-03 | 5.72E-03 |
| rs288029 | TCGA&TCGA_coCNV | 2.89E-03 | 6.18E-03 |
| rs6934903 | TCGA | 2.89E-03 | #N/A |
| rs6862488 | TCGA | 2.89E-03 | #N/A |
| rs1556355 | TCGA&TCGA_coCNV | 2.89E-03 | 5.51E-03 |
| rs9676752 | TCGA&TCGA_coCNV | 2.90E-03 | 6.35E-03 |
| rs4905794 | TCGA&TCGA_coCNV | 1.22E-04 | 5.41E-03 |
| rs2281896 | TCGA&TCGA_coCNV | 2.82E-05 | 1.20E-04 |
| rs193570 | TCGA&TCGA_coCNV | 2.72E-03 | 2.21E-04 |
| rs12499138 | TCGA&TCGA_coCNV | 2.90E-03 | 8.22E-03 |
| rs17076336 | TCGA&TCGA_coCNV | 5.11E-04 | 1.91E-06 |
| rs5953789 | TCGA&TCGA_coCNV | 2.90E-03 | 1.05E-03 |
| rs503596 | TCGA | 2.91E-03 | #N/A |
| rs10079469 | TCGA | 2.91E-03 | #N/A |
| rs2181457 | TCGA | 2.91E-03 | #N/A |
| rs9636891 | TCGA&TCGA_coCNV | 2.91E-03 | 9.14E-04 |
| rs2271422 | TCGA&TCGA_coCNV | 2.91E-03 | 3.49E-04 |
| rs12552067 | TCGA&TCGA_coCNV | 2.91E-03 | 1.86E-03 |
| rs2475856 | TCGA&TCGA_coCNV | 2.91E-03 | 5.26E-04 |
| rs7740792 | TCGA | 2.91E-03 | #N/A |
| rs7252561 | TCGA | 2.91E-03 | #N/A |
| rs7213585 | TCGA&TCGA_coCNV | 2.33E-03 | 2.52E-03 |
| rs1492639 | TCGA | 2.91E-03 | #N/A |
| rs1056690 | TCGA | 6.31E-04 | #N/A |
| rs17279456 | TCGA&TCGA_coCNV | 2.91E-03 | 6.94E-03 |
| rs2232754 | TCGA | 2.91E-03 | #N/A |
| rs3808798 | TCGA&TCGA_coCNV | 2.92E-03 | 4.55E-03 |
| rs4825483 | TCGA | 2.92E-03 | #N/A |
| rs8043433 | TCGA | 2.92E-03 | #N/A |
| rs7885840 | TCGA&TCGA_coCNV | 2.92E-03 | 9.16E-03 |
| rs868584 | TCGA&TCGA_coCNV | 2.92E-03 | 3.94E-03 |

| rs825552 | TCGA&TCGA_coCNV | 2.92E-03 | 5.18E-03 |
| --- | --- | --- | --- |
| rs9844930 | TCGA&TCGA_coCNV | 2.92E-03 | 1.86E-03 |
| rs658347 | TCGA | 2.92E-03 | #N/A |
| rs1359652 | TCGA&TCGA_coCNV | 3.11E-04 | 3.71E-04 |
| rs766772 | TCGA&TCGA_coCNV | 2.92E-03 | 8.85E-03 |
| rs4791278 | TCGA&TCGA_coCNV | 2.92E-03 | 1.59E-03 |
| rs10509087 | TCGA&TCGA_coCNV | 1.68E-03 | 9.13E-04 |
| rs3756773 | TCGA&TCGA_coCNV | 2.92E-03 | 2.93E-03 |
| rs7123483 | TCGA&TCGA_coCNV | 2.92E-03 | 9.07E-04 |
| rs41516545 | TCGA&TCGA_coCNV | 1.03E-03 | 2.11E-03 |
| rs7661069 | TCGA | 2.92E-03 | #N/A |
| rs12781317 | TCGA&TCGA_coCNV | 2.92E-03 | 7.14E-04 |
| rs7813624 | TCGA&TCGA_coCNV | 9.65E-05 | 9.09E-03 |
| rs11598894 | TCGA&TCGA_coCNV | 4.61E-04 | 2.89E-04 |
| rs12328809 | TCGA&TCGA_coCNV | 2.93E-03 | 3.09E-03 |
| rs17641864 | TCGA&TCGA_coCNV | 1.96E-04 | 4.91E-04 |
| rs17255466 | TCGA | 2.93E-03 | #N/A |
| rs16844933 | TCGA&TCGA_coCNV | 2.93E-03 | 5.68E-05 |
| rs7837309 | TCGA&TCGA_coCNV | 2.93E-03 | 2.67E-03 |
| rs2042020 | TCGA | 2.93E-03 | #N/A |
| rs10504052 | TCGA&TCGA_coCNV | 2.93E-03 | 4.35E-03 |
| rs10949552 | TCGA&TCGA_coCNV | 2.93E-03 | 9.21E-03 |
| rs10125920 | TCGA | 2.93E-03 | #N/A |
| rs12758496 | TCGA | 2.93E-03 | #N/A |
| rs10046958 | TCGA | 2.93E-03 | #N/A |
| rs12409375 | TCGA&TCGA_coCNV | 2.93E-03 | 7.87E-03 |
| rs12701311 | TCGA | 2.93E-03 | #N/A |
| rs1708791 | TCGA | 2.93E-03 | #N/A |
| rs41395244 | TCGA | 2.94E-03 | #N/A |
| rs4723076 | TCGA&TCGA_coCNV | 2.94E-03 | 2.81E-03 |
| rs667537 | TCGA&TCGA_coCNV | 2.94E-03 | 5.80E-03 |
| rs4233262 | TCGA&TCGA_coCNV | 2.10E-03 | 4.83E-03 |
| rs6809864 | TCGA&TCGA_coCNV | 2.94E-03 | 3.00E-04 |
| rs2738348 | TCGA&TCGA_coCNV | 2.94E-03 | 5.89E-03 |
| rs3934005 | TCGA | 2.94E-03 | #N/A |
| rs4852403 | TCGA&TCGA_coCNV | 2.94E-03 | 8.24E-03 |
| rs2858638 | TCGA | 2.94E-03 | #N/A |
| rs2457071 | TCGA&TCGA_coCNV | 2.74E-03 | 3.93E-04 |
| rs11136029 | TCGA | 2.94E-03 | #N/A |
| rs527731 | TCGA | 2.94E-03 | #N/A |
| rs7894663 | TCGA | 2.94E-03 | #N/A |
| rs7590441 | TCGA&TCGA_coCNV | 1.25E-03 | 2.58E-04 |
| rs10900947 | TCGA&TCGA_coCNV | 2.95E-03 | 6.27E-03 |
| rs1004636 | TCGA&TCGA_coCNV | 2.04E-03 | 2.74E-03 |
| rs2730238 | TCGA | 2.95E-03 | #N/A |
| rs1088716 | TCGA&TCGA_coCNV | 1.61E-03 | 3.88E-03 |

| rs6598964 | TCGA | 2.95E-03 | #N/A |
| --- | --- | --- | --- |
| rs6647631 | TCGA&TCGA_coCNV | 2.36E-03 | 5.85E-03 |
| rs622019 | TCGA | 2.95E-03 | #N/A |
| rs2423457 | TCGA&TCGA_coCNV | 2.95E-03 | 7.60E-03 |
| rs6817297 | TCGA&TCGA_coCNV | 2.95E-03 | 1.03E-03 |
| rs1885170 | TCGA&TCGA_coCNV | 2.95E-03 | 8.08E-03 |
| rs10469051 | TCGA | 2.96E-03 | #N/A |
| rs6660335 | TCGA&TCGA_coCNV | 2.96E-03 | 1.82E-03 |
| rs490995 | TCGA&TCGA_coCNV | 2.96E-03 | 8.60E-04 |
| rs883848 | TCGA&TCGA_coCNV | 1.78E-03 | 7.42E-03 |
| rs6463813 | TCGA&TCGA_coCNV | 2.96E-03 | 8.78E-04 |
| rs11743246 | TCGA&TCGA_coCNV | 2.96E-03 | 3.89E-03 |
| rs9607261 | TCGA&TCGA_coCNV | 2.30E-03 | 4.72E-03 |
| rs2428207 | TCGA&TCGA_coCNV | 2.96E-03 | 4.92E-03 |
| rs11033170 | TCGA&TCGA_coCNV | 2.96E-03 | 9.18E-03 |
| rs4553849 | TCGA&TCGA_coCNV | 2.96E-03 | 3.66E-03 |
| rs6949567 | TCGA&TCGA_coCNV | 6.89E-04 | 3.79E-04 |
| rs11012444 | TCGA | 2.97E-03 | #N/A |
| rs10172811 | TCGA&TCGA_coCNV | 2.97E-03 | 3.14E-03 |
| rs726041 | TCGA&TCGA_coCNV | 2.97E-03 | 3.51E-03 |
| rs12195797 | TCGA&TCGA_coCNV | 2.97E-03 | 1.24E-03 |
| rs11773477 | TCGA | 2.97E-03 | #N/A |
| rs7610464 | TCGA&TCGA_coCNV | 2.97E-03 | 3.94E-03 |
| rs1888644 | TCGA | 2.97E-03 | #N/A |
| rs2871888 | TCGA&TCGA_coCNV | 2.45E-03 | 3.20E-03 |
| rs1556573 | TCGA&TCGA_coCNV | 2.54E-03 | 9.58E-03 |
| rs5905454 | TCGA | 2.97E-03 | #N/A |
| rs1421673 | TCGA | 2.97E-03 | #N/A |
| rs7967522 | TCGA | 2.97E-03 | #N/A |
| rs2191054 | TCGA | 2.97E-03 | #N/A |
| rs10120669 | TCGA | 2.97E-03 | #N/A |
| rs130016 | TCGA&TCGA_coCNV | 7.07E-04 | 2.73E-03 |
| rs11231144 | TCGA&TCGA_coCNV | 2.97E-03 | 2.25E-03 |
| rs12352914 | TCGA&TCGA_coCNV | 2.97E-03 | 1.93E-03 |
| rs9856983 | TCGA | 1.95E-04 | #N/A |
| rs9850727 | TCGA&TCGA_coCNV | 2.98E-03 | 1.80E-03 |
| rs6989297 | TCGA | 2.98E-03 | #N/A |
| rs10804383 | TCGA | 2.98E-03 | #N/A |
| rs12236817 | TCGA | 2.98E-03 | #N/A |
| rs2153487 | TCGA&TCGA_coCNV | 2.98E-03 | 1.73E-03 |
| rs1560899 | TCGA&TCGA_coCNV | 2.98E-03 | 5.59E-03 |
| rs1533593 | TCGA | 2.98E-03 | #N/A |
| rs6639131 | TCGA&TCGA_coCNV | 2.98E-03 | 1.30E-03 |
| rs1218426 | TCGA&TCGA_coCNV | 1.75E-04 | 3.54E-03 |
| rs11661651 | TCGA&TCGA_coCNV | 2.98E-03 | 3.40E-03 |
| rs7487651 | TCGA&TCGA_coCNV | 2.98E-03 | 6.48E-03 |

| rs138204 | TCGA | 2.98E-03 | #N/A |
| --- | --- | --- | --- |
| rs35359582 | TCGA&TCGA_coCNV | 2.98E-03 | 3.73E-03 |
| rs12295717 | TCGA | 2.98E-03 | #N/A |
| rs242835 | TCGA&TCGA_coCNV | 2.98E-03 | 9.95E-05 |
| rs2190921 | TCGA | 2.98E-03 | #N/A |
| rs7548847 | TCGA&TCGA_coCNV | 2.21E-04 | 3.11E-03 |
| rs6507721 | TCGA&TCGA_coCNV | 2.99E-03 | 2.55E-03 |
| rs1168550 | TCGA | 2.99E-03 | #N/A |
| rs7140514 | TCGA&TCGA_coCNV | 2.14E-03 | 8.11E-04 |
| rs4867978 | TCGA | 2.99E-03 | #N/A |
| rs4597582 | TCGA&TCGA_coCNV | 2.99E-03 | 4.25E-04 |
| rs6639135 | TCGA&TCGA_coCNV | 2.99E-03 | 2.06E-03 |
| rs6608959 | TCGA&TCGA_coCNV | 9.29E-05 | 9.38E-03 |
| rs972431 | TCGA&TCGA_coCNV | 2.99E-03 | 2.91E-04 |
| rs6936255 | TCGA&TCGA_coCNV | 2.99E-03 | 3.05E-03 |
| rs4652990 | TCGA&TCGA_coCNV | 2.99E-03 | 1.16E-03 |
| rs4919054 | TCGA&TCGA_coCNV | 2.99E-03 | 9.63E-04 |
| rs7819890 | TCGA&TCGA_coCNV | 3.00E-03 | 8.51E-03 |
| rs16957702 | TCGA | 3.00E-03 | #N/A |
| rs10821462 | TCGA&TCGA_coCNV | 3.00E-03 | 5.14E-03 |
| rs1391010 | TCGA | 3.00E-03 | #N/A |
| rs17154746 | TCGA&TCGA_coCNV | 3.00E-03 | 2.48E-03 |
| rs1517298 | TCGA | 3.00E-03 | #N/A |
| rs12642406 | TCGA | 3.00E-03 | #N/A |
| rs2235125 | TCGA&TCGA_coCNV | 3.00E-03 | 5.74E-03 |
| rs6489868 | TCGA&TCGA_coCNV | 3.00E-03 | 7.24E-03 |
| rs7208206 | TCGA | 3.00E-03 | #N/A |
| rs4606498 | TCGA&TCGA_coCNV | 5.20E-04 | 4.96E-04 |
| rs9819432 | TCGA&TCGA_coCNV | 3.00E-03 | 2.45E-03 |
| rs16968004 | TCGA | 3.00E-03 | #N/A |
| rs7857377 | TCGA | 3.00E-03 | #N/A |
| rs17478799 | TCGA&TCGA_coCNV | 3.00E-03 | 9.04E-03 |
| rs6633212 | TCGA&TCGA_coCNV | 3.00E-03 | 2.72E-03 |
| rs9934151 | TCGA | 3.00E-03 | #N/A |
| rs7780294 | TCGA | 3.01E-03 | #N/A |
| rs10810005 | TCGA | 6.25E-04 | #N/A |
| rs2468154 | TCGA | 3.01E-03 | #N/A |
| rs4239313 | TCGA&TCGA_coCNV | 3.01E-03 | 1.96E-03 |
| rs6845772 | TCGA | 3.01E-03 | #N/A |
| rs16963418 | TCGA&TCGA_coCNV | 3.76E-04 | 8.09E-04 |
| rs12703533 | TCGA&TCGA_coCNV | 3.01E-03 | 3.39E-03 |
| rs848210 | TCGA&TCGA_coCNV | 2.50E-03 | 2.30E-03 |
| rs10417223 | TCGA&TCGA_coCNV | 3.01E-03 | 4.33E-03 |
| rs1324652 | TCGA&TCGA_coCNV | 1.78E-03 | 6.93E-04 |
| rs1405337 | TCGA&TCGA_coCNV | 3.01E-03 | 2.61E-03 |
| rs41528247 | TCGA | 3.01E-03 | #N/A |

| rs9924445 | TCGA&TCGA_coCNV | 7.39E-05 | 1.67E-03 |
| --- | --- | --- | --- |
| rs12714442 | TCGA&TCGA_coCNV | 3.01E-03 | 3.39E-03 |
| rs12035082 | TCGA&TCGA_coCNV | 3.02E-03 | 1.21E-03 |
| rs10263975 | TCGA&TCGA_coCNV | 3.02E-03 | 8.82E-03 |
| rs6049831 | TCGA&TCGA_coCNV | 3.02E-03 | 7.40E-03 |
| rs16906942 | TCGA&TCGA_coCNV | 3.02E-03 | 8.41E-03 |
| rs9957243 | TCGA | 3.02E-03 | #N/A |
| rs291753 | TCGA | 3.02E-03 | #N/A |
| rs12265864 | TCGA | 3.02E-03 | #N/A |
| rs11218002 | TCGA&TCGA_coCNV | 3.02E-03 | 8.93E-03 |
| rs5976318 | TCGA&TCGA_coCNV | 3.02E-03 | 4.98E-03 |
| rs9376702 | TCGA | 3.02E-03 | #N/A |
| rs890699 | TCGA | 3.02E-03 | #N/A |
| rs1658007 | TCGA | 3.03E-03 | #N/A |
| rs12676398 | TCGA&TCGA_coCNV | 3.03E-03 | 1.75E-03 |
| rs7692283 | TCGA&TCGA_coCNV | 3.03E-03 | 3.44E-03 |
| rs703457 | TCGA&TCGA_coCNV | 1.66E-03 | 1.39E-03 |
| rs1557924 | TCGA&TCGA_coCNV | 3.03E-03 | 1.10E-03 |
| rs7602833 | TCGA&TCGA_coCNV | 3.03E-03 | 8.89E-03 |
| rs17105847 | TCGA&TCGA_coCNV | 3.03E-03 | 1.41E-03 |
| rs2512106 | TCGA&TCGA_coCNV | 1.12E-04 | 5.56E-04 |
| rs6092111 | TCGA | 3.03E-03 | #N/A |
| rs9920811 | TCGA&TCGA_coCNV | 9.79E-04 | 4.37E-03 |
| rs2057116 | TCGA&TCGA_coCNV | 3.03E-03 | 2.72E-04 |
| rs12140615 | TCGA | 3.03E-03 | #N/A |
| rs2579785 | TCGA&TCGA_coCNV | 2.39E-03 | 7.86E-03 |
| rs10832289 | TCGA&TCGA_coCNV | 3.04E-03 | 1.80E-04 |
| rs4933443 | TCGA&TCGA_coCNV | 3.04E-03 | 3.24E-03 |
| rs1225897 | TCGA&TCGA_coCNV | 3.04E-03 | 9.68E-04 |
| rs8016950 | TCGA&TCGA_coCNV | 3.04E-03 | 4.09E-04 |
| rs16930126 | TCGA&TCGA_coCNV | 3.04E-03 | 2.86E-04 |
| rs717620 | TCGA | 3.04E-03 | #N/A |
| rs17802224 | TCGA&TCGA_coCNV | 3.04E-03 | 4.05E-03 |
| rs338449 | TCGA&TCGA_coCNV | 3.04E-03 | 2.03E-03 |
| rs2478251 | TCGA | 3.04E-03 | #N/A |
| rs11874322 | TCGA | 3.04E-03 | #N/A |
| rs868381 | TCGA&TCGA_coCNV | 3.04E-03 | 3.35E-03 |
| rs2887208 | TCGA&TCGA_coCNV | 3.04E-03 | 6.86E-03 |
| rs10939036 | TCGA | 3.04E-03 | #N/A |
| rs4813802 | TCGA | 3.05E-03 | #N/A |
| rs6755193 | TCGA&TCGA_coCNV | 2.56E-03 | 2.10E-03 |
| rs1005886 | TCGA | 3.05E-03 | #N/A |
| rs5958668 | TCGA | 3.05E-03 | #N/A |
| rs16902709 | TCGA&TCGA_coCNV | 3.05E-03 | 5.41E-03 |
| rs1405124 | TCGA&TCGA_coCNV | 3.05E-03 | 8.99E-03 |
| rs2910164 | TCGA | 2.60E-03 | #N/A |

| rs11067038 | TCGA&TCGA_coCNV | 3.05E-03 | 3.24E-03 |
| --- | --- | --- | --- |
| rs8076417 | TCGA | 3.05E-03 | #N/A |
| rs446540 | TCGA | 3.05E-03 | #N/A |
| rs6850505 | TCGA&TCGA_coCNV | 2.00E-03 | 7.99E-04 |
| rs10959719 | TCGA | 3.05E-03 | #N/A |
| rs643091 | TCGA&TCGA_coCNV | 3.05E-03 | 1.33E-04 |
| rs992586 | TCGA | 3.05E-03 | #N/A |
| rs6490312 | TCGA&TCGA_coCNV | 2.77E-03 | 4.20E-04 |
| rs10827345 | TCGA&TCGA_coCNV | 3.05E-03 | 1.53E-03 |
| rs7049130 | TCGA&TCGA_coCNV | 3.05E-03 | 3.68E-03 |
| rs4820389 | TCGA&TCGA_coCNV | 5.29E-04 | 5.10E-03 |
| rs6536195 | TCGA | 3.06E-03 | #N/A |
| rs7161298 | TCGA&TCGA_coCNV | 3.06E-03 | 4.34E-03 |
| rs2028016 | TCGA&TCGA_coCNV | 3.06E-03 | 1.04E-03 |
| rs13426909 | TCGA&TCGA_coCNV | 3.06E-03 | 8.65E-03 |
| rs11234045 | TCGA | 3.06E-03 | #N/A |
| rs10848089 | TCGA&TCGA_coCNV | 3.06E-03 | 4.04E-03 |
| rs12249562 | TCGA&TCGA_coCNV | 1.10E-03 | 4.08E-03 |
| rs10821744 | TCGA | 3.06E-03 | #N/A |
| rs17578843 | TCGA | 3.06E-03 | #N/A |
| rs1202394 | TCGA | 3.06E-03 | #N/A |
| rs10879749 | TCGA&TCGA_coCNV | 2.60E-03 | 7.72E-03 |
| rs9600565 | TCGA&TCGA_coCNV | 3.06E-03 | 4.19E-04 |
| rs11218927 | TCGA | 3.06E-03 | #N/A |
| rs1701607 | TCGA&TCGA_coCNV | 3.06E-03 | 2.59E-03 |
| rs10864192 | TCGA | 3.06E-03 | #N/A |
| rs3857801 | TCGA&TCGA_coCNV | 3.07E-03 | 6.79E-03 |
| rs10959970 | TCGA | 3.07E-03 | #N/A |
| rs6776830 | TCGA | 3.07E-03 | #N/A |
| rs4868209 | TCGA | 3.07E-03 | #N/A |
| rs7546879 | TCGA&TCGA_coCNV | 3.07E-03 | 1.35E-03 |
| rs17768396 | TCGA&TCGA_coCNV | 3.07E-03 | 6.85E-03 |
| rs950155 | TCGA&TCGA_coCNV | 3.07E-03 | 5.50E-04 |
| rs12089286 | TCGA | 3.07E-03 | #N/A |
| rs910542 | TCGA | 3.07E-03 | #N/A |
| rs912081 | TCGA | 3.07E-03 | #N/A |
| rs1934113 | TCGA | 3.07E-03 | #N/A |
| rs933064 | TCGA&TCGA_coCNV | 3.07E-03 | 1.46E-03 |
| rs11562896 | TCGA&TCGA_coCNV | 3.07E-03 | 6.71E-04 |
| rs13084982 | TCGA&TCGA_coCNV | 1.80E-03 | 3.92E-03 |
| rs1557106 | TCGA&TCGA_coCNV | 3.07E-03 | 9.77E-03 |
| rs11229083 | TCGA&TCGA_coCNV | 3.07E-03 | 2.24E-03 |
| rs764276 | TCGA&TCGA_coCNV | 1.99E-03 | 2.70E-03 |
| rs7080125 | TCGA&TCGA_coCNV | 1.39E-03 | 8.12E-04 |
| rs11014665 | TCGA&TCGA_coCNV | 3.08E-03 | 2.29E-03 |
| rs581634 | TCGA&TCGA_coCNV | 3.08E-03 | 4.16E-04 |

| rs17724833 | TCGA | 3.08E-03 | #N/A |
| --- | --- | --- | --- |
| rs3851570 | TCGA&TCGA_coCNV | 3.08E-03 | 4.42E-03 |
| rs929196 | TCGA&TCGA_coCNV | 3.08E-03 | 5.12E-03 |
| rs12488591 | TCGA&TCGA_coCNV | 3.08E-03 | 6.59E-03 |
| rs8038299 | TCGA | 3.08E-03 | #N/A |
| rs4828878 | TCGA&TCGA_coCNV | 3.09E-03 | 4.30E-03 |
| rs2949941 | TCGA | 3.09E-03 | #N/A |
| rs2370386 | TCGA&TCGA_coCNV | 1.82E-03 | 3.85E-03 |
| rs12826979 | TCGA&TCGA_coCNV | 3.09E-03 | 7.50E-03 |
| rs12000864 | TCGA | 3.09E-03 | #N/A |
| rs12311156 | TCGA&TCGA_coCNV | 3.09E-03 | 4.63E-03 |
| rs180112 | TCGA&TCGA_coCNV | 3.09E-03 | 2.24E-03 |
| rs2856339 | TCGA | 3.09E-03 | #N/A |
| rs2918009 | TCGA | 3.09E-03 | #N/A |
| rs4785396 | TCGA&TCGA_coCNV | 1.68E-03 | 3.22E-04 |
| rs7875404 | TCGA&TCGA_coCNV | 3.09E-03 | 8.82E-03 |
| rs16874621 | TCGA&TCGA_coCNV | 3.09E-03 | 5.81E-03 |
| rs4281434 | TCGA&TCGA_coCNV | 3.09E-03 | 7.20E-03 |
| rs17673497 | TCGA&TCGA_coCNV | 3.10E-03 | 9.27E-03 |
| rs10989064 | TCGA&TCGA_coCNV | 3.10E-03 | 8.81E-04 |
| rs6491543 | TCGA&TCGA_coCNV | 2.93E-03 | 1.54E-03 |
| rs6544643 | TCGA&TCGA_coCNV | 3.10E-03 | 2.39E-03 |
| rs8140508 | TCGA | 3.10E-03 | #N/A |
| rs17072146 | TCGA&TCGA_coCNV | 3.10E-03 | 4.25E-03 |
| rs227412 | TCGA&TCGA_coCNV | 3.10E-03 | 6.50E-03 |
| rs721259 | TCGA&TCGA_coCNV | 1.75E-04 | 2.99E-03 |
| rs2754087 | TCGA | 3.10E-03 | #N/A |
| rs9821530 | TCGA&TCGA_coCNV | 3.10E-03 | 2.41E-03 |
| rs11184535 | TCGA&TCGA_coCNV | 3.10E-03 | 7.83E-03 |
| rs11193393 | TCGA&TCGA_coCNV | 3.10E-03 | 4.40E-03 |
| rs1929911 | TCGA | 3.10E-03 | #N/A |
| rs4684106 | TCGA | 3.11E-03 | #N/A |
| rs1175165 | TCGA | 3.11E-03 | #N/A |
| rs1402787 | TCGA | 3.11E-03 | #N/A |
| rs4958701 | TCGA | 3.11E-03 | #N/A |
| rs11663532 | TCGA | 3.11E-03 | #N/A |
| rs12733767 | TCGA&TCGA_coCNV | 3.11E-03 | 1.50E-03 |
| rs211915 | TCGA | 3.11E-03 | #N/A |
| rs353969 | TCGA&TCGA_coCNV | 3.11E-03 | 6.39E-03 |
| rs11850785 | TCGA&TCGA_coCNV | 1.43E-03 | 3.63E-03 |
| rs6475966 | TCGA&TCGA_coCNV | 8.84E-05 | 5.87E-03 |
| rs7932914 | TCGA | 3.11E-03 | #N/A |
| rs11983635 | TCGA&TCGA_coCNV | 3.11E-03 | 7.42E-05 |
| rs1977251 | TCGA&TCGA_coCNV | 3.11E-03 | 6.94E-03 |
| rs9408721 | TCGA&TCGA_coCNV | 3.12E-03 | 9.36E-03 |
| rs17749772 | TCGA&TCGA_coCNV | 2.49E-03 | 7.85E-03 |

| rs6496845 | TCGA | 3.12E-03 | #N/A |
| --- | --- | --- | --- |
| rs10038691 | TCGA&TCGA_coCNV | 3.12E-03 | 9.17E-03 |
| rs1367469 | TCGA | 3.12E-03 | #N/A |
| rs1146908 | TCGA&TCGA_coCNV | 3.12E-03 | 1.91E-04 |
| rs619130 | TCGA&TCGA_coCNV | 3.12E-03 | 1.86E-03 |
| rs12686673 | TCGA&TCGA_coCNV | 3.12E-03 | 3.39E-03 |
| rs2457047 | TCGA&TCGA_coCNV | 3.69E-04 | 2.30E-05 |
| rs10489391 | TCGA | 3.12E-03 | #N/A |
| rs1881279 | TCGA | 3.12E-03 | #N/A |
| rs10502453 | TCGA&TCGA_coCNV | 3.12E-03 | 4.16E-03 |
| rs4648478 | TCGA&TCGA_coCNV | 1.57E-03 | 3.19E-03 |
| rs1999527 | TCGA&TCGA_coCNV | 3.12E-03 | 7.74E-03 |
| rs1468885 | TCGA&TCGA_coCNV | 3.12E-03 | 3.72E-03 |
| rs34675716 | TCGA | 3.12E-03 | #N/A |
| rs1947305 | TCGA | 3.13E-03 | #N/A |
| rs2223134 | TCGA | 3.13E-03 | #N/A |
| rs12260815 | TCGA&TCGA_coCNV | 1.15E-05 | 1.56E-04 |
| rs11697413 | TCGA&TCGA_coCNV | 3.13E-03 | 2.43E-03 |
| rs10181842 | TCGA&TCGA_coCNV | 1.93E-03 | 4.57E-03 |
| rs2947237 | TCGA&TCGA_coCNV | 3.13E-03 | 6.40E-03 |
| rs12987374 | TCGA | 2.87E-03 | #N/A |
| rs9293878 | TCGA&TCGA_coCNV | 3.13E-03 | 8.80E-05 |
| rs13085005 | TCGA&TCGA_coCNV | 3.14E-03 | 6.40E-03 |
| rs897589 | TCGA | 3.14E-03 | #N/A |
| rs4831914 | TCGA&TCGA_coCNV | 3.14E-03 | 4.29E-03 |
| rs10777867 | TCGA&TCGA_coCNV | 3.14E-03 | 8.39E-04 |
| rs2634953 | TCGA | 3.14E-03 | #N/A |
| rs2160777 | TCGA&TCGA_coCNV | 3.14E-03 | 4.92E-03 |
| rs12683969 | TCGA&TCGA_coCNV | 3.14E-03 | 1.51E-03 |
| rs2275085 | TCGA&TCGA_coCNV | 3.14E-03 | 2.99E-03 |
| rs17012208 | TCGA&TCGA_coCNV | 1.63E-03 | 6.36E-03 |
| rs10029057 | TCGA&TCGA_coCNV | 2.86E-03 | 7.75E-04 |
| rs728637 | TCGA&TCGA_coCNV | 3.14E-03 | 3.52E-03 |
| rs4838598 | TCGA | 3.14E-03 | #N/A |
| rs1615480 | TCGA&TCGA_coCNV | 4.85E-04 | 9.72E-04 |
| rs497122 | TCGA | 3.15E-03 | #N/A |
| rs362351 | TCGA&TCGA_coCNV | 3.15E-03 | 4.67E-03 |
| rs7168655 | TCGA&TCGA_coCNV | 3.15E-03 | 7.14E-03 |
| rs12640173 | TCGA&TCGA_coCNV | 3.15E-03 | 5.09E-03 |
| rs968773 | TCGA | 3.15E-03 | #N/A |
| rs1166693 | TCGA&TCGA_coCNV | 1.62E-03 | 5.38E-03 |
| rs10757069 | TCGA&TCGA_coCNV | 3.15E-03 | 2.65E-03 |
| rs5963635 | TCGA | 3.15E-03 | #N/A |
| rs509859 | TCGA&TCGA_coCNV | 3.15E-03 | 5.15E-03 |
| rs4099169 | TCGA&TCGA_coCNV | 3.15E-03 | 9.25E-03 |
| rs6127905 | TCGA&TCGA_coCNV | 3.15E-03 | 1.06E-03 |

| rs7608438 | TCGA | 3.15E-03 | #N/A |
| --- | --- | --- | --- |
| rs6595364 | TCGA&TCGA_coCNV | 3.15E-03 | 3.79E-04 |
| rs10860347 | TCGA | 3.15E-03 | #N/A |
| rs12872407 | TCGA&TCGA_coCNV | 3.15E-03 | 1.23E-03 |
| rs10521754 | TCGA | 3.16E-03 | #N/A |
| rs6032826 | TCGA | 3.16E-03 | #N/A |
| rs1389970 | TCGA&TCGA_coCNV | 3.16E-03 | 1.85E-03 |
| rs2619829 | TCGA&TCGA_coCNV | 3.16E-03 | 2.04E-04 |
| rs16873624 | TCGA&TCGA_coCNV | 3.16E-03 | 2.26E-04 |
| rs3781386 | TCGA | 3.16E-03 | #N/A |
| rs17125797 | TCGA | 3.16E-03 | #N/A |
| rs10924024 | TCGA&TCGA_coCNV | 3.16E-03 | 1.54E-03 |
| rs4339935 | TCGA&TCGA_coCNV | 3.16E-03 | 5.62E-03 |
| rs363605 | TCGA&TCGA_coCNV | 1.95E-03 | 3.15E-03 |
| rs1504028 | TCGA&TCGA_coCNV | 3.16E-03 | 8.35E-04 |
| rs10504980 | TCGA&TCGA_coCNV | 3.16E-03 | 4.43E-03 |
| rs11772093 | TCGA | 3.16E-03 | #N/A |
| rs17608701 | TCGA&TCGA_coCNV | 3.16E-03 | 7.69E-03 |
| rs3847535 | TCGA | 3.16E-03 | #N/A |
| rs2892545 | TCGA&TCGA_coCNV | 3.16E-03 | 8.12E-04 |
| rs11751348 | TCGA&TCGA_coCNV | 3.16E-03 | 1.44E-03 |
| rs1511601 | TCGA&TCGA_coCNV | 3.16E-03 | 8.08E-04 |
| rs5934285 | TCGA&TCGA_coCNV | 1.54E-03 | 4.38E-03 |
| rs6639912 | TCGA&TCGA_coCNV | 3.17E-03 | 1.43E-03 |
| rs4145540 | TCGA&TCGA_coCNV | 3.17E-03 | 1.62E-03 |
| rs17044596 | TCGA&TCGA_coCNV | 3.17E-03 | 8.48E-04 |
| rs10794885 | TCGA&TCGA_coCNV | 3.17E-03 | 7.58E-03 |
| rs16913696 | TCGA&TCGA_coCNV | 3.17E-03 | 4.65E-03 |
| rs2043062 | TCGA | 3.17E-03 | #N/A |
| rs6753025 | TCGA&TCGA_coCNV | 3.17E-03 | 3.60E-03 |
| rs10485012 | TCGA | 1.41E-03 | #N/A |
| rs11242562 | TCGA | 3.17E-03 | #N/A |
| rs816589 | TCGA&TCGA_coCNV | 3.17E-03 | 7.74E-04 |
| rs13394306 | TCGA | 3.17E-03 | #N/A |
| rs16857313 | TCGA | 3.17E-03 | #N/A |
| rs781355 | TCGA&TCGA_coCNV | 3.17E-03 | 4.38E-03 |
| rs2836305 | TCGA&TCGA_coCNV | 3.17E-03 | 2.33E-03 |
| rs12523434 | TCGA | 3.18E-03 | #N/A |
| rs4962514 | TCGA&TCGA_coCNV | 1.05E-03 | 1.93E-03 |
| rs4872499 | TCGA | 3.18E-03 | #N/A |
| rs1816028 | TCGA&TCGA_coCNV | 3.03E-03 | 6.42E-03 |
| rs12689508 | TCGA&TCGA_coCNV | 2.13E-03 | 9.01E-03 |
| rs780874 | TCGA&TCGA_coCNV | 3.18E-03 | 8.39E-03 |
| rs9864494 | TCGA&TCGA_coCNV | 3.18E-03 | 6.94E-03 |
| rs136692 | TCGA&TCGA_coCNV | 3.18E-03 | 2.99E-03 |
| rs12468540 | TCGA&TCGA_coCNV | 3.18E-03 | 3.76E-03 |

| rs1711084 | TCGA&TCGA_coCNV | 3.18E-03 | 1.83E-03 |
| --- | --- | --- | --- |
| rs7968232 | TCGA&TCGA_coCNV | 3.18E-03 | 7.06E-03 |
| rs9547922 | TCGA | 3.18E-03 | #N/A |
| rs12009974 | TCGA | 3.18E-03 | #N/A |
| rs773229 | TCGA&TCGA_coCNV | 2.03E-03 | 6.54E-03 |
| rs4904003 | TCGA&TCGA_coCNV | 3.18E-03 | 9.21E-03 |
| rs5935230 | TCGA&TCGA_coCNV | 3.18E-03 | 2.50E-04 |
| rs2029055 | TCGA&TCGA_coCNV | 3.18E-03 | 5.11E-04 |
| rs10068400 | TCGA&TCGA_coCNV | 3.56E-04 | 2.54E-03 |
| rs12324363 | TCGA | 3.18E-03 | #N/A |
| rs6074409 | TCGA | 3.18E-03 | #N/A |
| rs1218345 | TCGA&TCGA_coCNV | 1.75E-03 | 1.21E-03 |
| rs7075670 | TCGA&TCGA_coCNV | 3.19E-03 | 2.68E-03 |
| rs2960355 | TCGA | 3.19E-03 | #N/A |
| rs38913 | TCGA&TCGA_coCNV | 3.19E-03 | 9.21E-03 |
| rs17157663 | TCGA | 3.19E-03 | #N/A |
| rs4950974 | TCGA&TCGA_coCNV | 1.88E-03 | 8.82E-04 |
| rs12647885 | TCGA&TCGA_coCNV | 3.19E-03 | 2.12E-03 |
| rs2903213 | TCGA | 2.11E-03 | #N/A |
| rs10194585 | TCGA | 3.19E-03 | #N/A |
| rs10171741 | TCGA | 3.19E-03 | #N/A |
| rs17082946 | TCGA&TCGA_coCNV | 3.20E-03 | 2.57E-03 |
| rs8090560 | TCGA&TCGA_coCNV | 3.20E-03 | 5.84E-03 |
| rs6515052 | TCGA&TCGA_coCNV | 3.20E-03 | 6.09E-04 |
| rs7988202 | TCGA | 3.20E-03 | #N/A |
| rs8084533 | TCGA&TCGA_coCNV | 1.77E-03 | 7.26E-03 |
| rs868032 | TCGA | 3.20E-03 | #N/A |
| rs7519593 | TCGA | 3.20E-03 | #N/A |
| rs4675439 | TCGA&TCGA_coCNV | 3.20E-03 | 8.56E-04 |
| rs743936 | TCGA&TCGA_coCNV | 3.20E-03 | 4.61E-03 |
| rs13027191 | TCGA&TCGA_coCNV | 3.20E-03 | 5.00E-03 |
| rs7250734 | TCGA&TCGA_coCNV | 1.98E-03 | 6.56E-03 |
| rs9672753 | TCGA&TCGA_coCNV | 3.20E-03 | 4.20E-03 |
| rs7732828 | TCGA&TCGA_coCNV | 1.01E-05 | 1.13E-04 |
| rs347776 | TCGA&TCGA_coCNV | 3.20E-03 | 2.29E-03 |
| rs3823529 | TCGA | 3.20E-03 | #N/A |
| rs7625229 | TCGA | 3.20E-03 | #N/A |
| rs2013092 | TCGA&TCGA_coCNV | 3.21E-03 | 5.72E-03 |
| rs10483400 | TCGA | 3.21E-03 | #N/A |
| rs1526795 | TCGA&TCGA_coCNV | 3.21E-03 | 2.40E-04 |
| rs11955149 | TCGA&TCGA_coCNV | 3.21E-03 | 7.84E-03 |
| rs16835685 | TCGA&TCGA_coCNV | 1.61E-03 | 1.86E-03 |
| rs2673148 | TCGA&TCGA_coCNV | 3.21E-03 | 6.21E-03 |
| rs9948446 | TCGA&TCGA_coCNV | 1.11E-03 | 3.07E-04 |
| rs2295134 | TCGA | 2.05E-03 | #N/A |
| rs6984769 | TCGA | 3.22E-03 | #N/A |

| rs804937 | TCGA&TCGA_coCNV | 3.22E-03 | 4.47E-03 |
| --- | --- | --- | --- |
| rs12131617 | TCGA&TCGA_coCNV | 7.66E-04 | 4.00E-03 |
| rs16841641 | TCGA&TCGA_coCNV | 3.22E-03 | 3.26E-03 |
| rs9505555 | TCGA | 3.22E-03 | #N/A |
| rs2887396 | TCGA&TCGA_coCNV | 3.22E-03 | 1.00E-03 |
| rs7167134 | TCGA | 3.22E-03 | #N/A |
| rs928799 | TCGA | 3.22E-03 | #N/A |
| rs4495324 | TCGA | 3.22E-03 | #N/A |
| rs8103177 | TCGA&TCGA_coCNV | 3.23E-03 | 4.18E-03 |
| rs498309 | TCGA | 3.23E-03 | #N/A |
| rs1882999 | TCGA | 3.23E-03 | #N/A |
| rs12100382 | TCGA&TCGA_coCNV | 2.41E-04 | 8.82E-04 |
| rs43004 | TCGA&TCGA_coCNV | 3.23E-03 | 2.14E-03 |
| rs8093149 | TCGA | 9.06E-04 | #N/A |
| rs16943178 | TCGA | 3.23E-03 | #N/A |
| rs9956540 | TCGA | 3.23E-03 | #N/A |
| rs2028525 | TCGA | 3.23E-03 | #N/A |
| rs797205 | TCGA&TCGA_coCNV | 1.43E-03 | 7.22E-03 |
| rs1799888 | TCGA | 3.23E-03 | #N/A |
| rs17547853 | TCGA&TCGA_coCNV | 3.23E-03 | 6.76E-03 |
| rs41487750 | TCGA | 3.23E-03 | #N/A |
| rs17094791 | TCGA&TCGA_coCNV | 1.52E-03 | 2.24E-04 |
| rs9485985 | TCGA&TCGA_coCNV | 2.82E-03 | 6.32E-03 |
| rs10507264 | TCGA&TCGA_coCNV | 3.23E-03 | 8.52E-04 |
| rs17109806 | TCGA | 3.23E-03 | #N/A |
| rs1650337 | TCGA | 3.24E-03 | #N/A |
| rs9849985 | TCGA | 4.23E-04 | #N/A |
| rs2765385 | TCGA&TCGA_coCNV | 3.24E-03 | 2.54E-03 |
| rs10476 | TCGA | 3.24E-03 | #N/A |
| rs11187728 | TCGA&TCGA_coCNV | 3.24E-03 | 4.08E-03 |
| rs6885620 | TCGA&TCGA_coCNV | 8.45E-04 | 1.58E-03 |
| rs41437345 | TCGA&TCGA_coCNV | 3.24E-03 | 1.08E-03 |
| rs12583257 | TCGA&TCGA_coCNV | 2.63E-03 | 2.45E-03 |
| rs9293625 | TCGA&TCGA_coCNV | 4.18E-04 | 4.01E-04 |
| rs5922313 | TCGA&TCGA_coCNV | 3.24E-03 | 5.22E-03 |
| rs12247405 | TCGA | 2.93E-03 | #N/A |
| rs17660913 | TCGA&TCGA_coCNV | 3.24E-03 | 7.91E-03 |
| rs17659742 | TCGA | 3.24E-03 | #N/A |
| rs1348439 | TCGA | 3.04E-03 | #N/A |
| rs16925619 | TCGA&TCGA_coCNV | 3.24E-03 | 1.62E-04 |
| rs6519839 | TCGA | 3.24E-03 | #N/A |
| rs470094 | TCGA | 3.24E-03 | #N/A |
| rs6630093 | TCGA&TCGA_coCNV | 4.94E-05 | 2.88E-04 |
| rs2760550 | TCGA | 3.24E-03 | #N/A |
| rs3751055 | TCGA | 3.25E-03 | #N/A |
| rs6761536 | TCGA | 3.25E-03 | #N/A |

| rs12359848 | TCGA&TCGA_coCNV | 3.25E-03 | 5.49E-03 |
| --- | --- | --- | --- |
| rs7669620 | TCGA&TCGA_coCNV | 3.25E-03 | 7.67E-03 |
| rs7655574 | TCGA | 3.25E-03 | #N/A |
| rs9666878 | TCGA | 3.25E-03 | #N/A |
| rs2382911 | TCGA&TCGA_coCNV | 1.15E-03 | 1.49E-03 |
| rs6767184 | TCGA&TCGA_coCNV | 1.34E-03 | 3.03E-03 |
| rs10022025 | TCGA&TCGA_coCNV | 3.26E-03 | 1.47E-03 |
| rs16828779 | TCGA | 3.26E-03 | #N/A |
| rs6435690 | TCGA | 3.26E-03 | #N/A |
| rs10484533 | TCGA | 3.26E-03 | #N/A |
| rs4871396 | TCGA&TCGA_coCNV | 3.26E-03 | 1.33E-03 |
| rs12421903 | TCGA&TCGA_coCNV | 3.26E-03 | 5.17E-03 |
| rs6801007 | TCGA&TCGA_coCNV | 3.26E-03 | 5.94E-03 |
| rs3927373 | TCGA&TCGA_coCNV | 2.74E-03 | 9.73E-03 |
| rs17734724 | TCGA | 3.26E-03 | #N/A |
| rs1135102 | TCGA&TCGA_coCNV | 3.27E-03 | 3.54E-03 |
| rs16843133 | TCGA&TCGA_coCNV | 3.27E-03 | 6.61E-03 |
| rs1368877 | TCGA&TCGA_coCNV | 1.80E-03 | 2.87E-03 |
| rs11059376 | TCGA&TCGA_coCNV | 3.27E-03 | 5.54E-03 |
| rs7670299 | TCGA&TCGA_coCNV | 3.27E-03 | 7.86E-03 |
| rs9890973 | TCGA&TCGA_coCNV | 3.10E-03 | 1.61E-03 |
| rs1572045 | TCGA&TCGA_coCNV | 3.27E-03 | 1.16E-03 |
| rs9852802 | TCGA&TCGA_coCNV | 3.09E-04 | 8.87E-03 |
| rs8012512 | TCGA&TCGA_coCNV | 3.28E-03 | 9.27E-03 |
| rs4814878 | TCGA | 3.28E-03 | #N/A |
| rs6693831 | TCGA&TCGA_coCNV | 7.03E-04 | 8.87E-04 |
| rs11070765 | TCGA&TCGA_coCNV | 3.28E-03 | 4.05E-03 |
| rs13334238 | TCGA&TCGA_coCNV | 3.28E-03 | 3.75E-03 |
| rs16949529 | TCGA&TCGA_coCNV | 3.28E-03 | 4.93E-03 |
| rs9897666 | TCGA&TCGA_coCNV | 3.28E-03 | 3.79E-03 |
| rs6783393 | TCGA&TCGA_coCNV | 3.28E-03 | 6.19E-03 |
| rs10957863 | TCGA&TCGA_coCNV | 9.99E-06 | 1.51E-05 |
| rs2485172 | TCGA | 3.28E-03 | #N/A |
| rs12028052 | TCGA&TCGA_coCNV | 3.28E-03 | 6.06E-03 |
| rs4831869 | TCGA | 6.83E-04 | #N/A |
| rs11722943 | TCGA | 3.29E-03 | #N/A |
| rs2733941 | TCGA&TCGA_coCNV | 3.29E-03 | 1.88E-03 |
| rs17161636 | TCGA&TCGA_coCNV | 3.29E-03 | 3.77E-03 |
| rs2150759 | TCGA&TCGA_coCNV | 3.29E-03 | 3.93E-04 |
| rs1636334 | TCGA&TCGA_coCNV | 3.29E-03 | 4.91E-03 |
| rs5970765 | TCGA&TCGA_coCNV | 3.29E-03 | 2.42E-03 |
| rs12548036 | TCGA&TCGA_coCNV | 7.62E-04 | 2.08E-03 |
| rs9957463 | TCGA&TCGA_coCNV | 3.96E-04 | 3.39E-05 |
| rs10190786 | TCGA&TCGA_coCNV | 3.29E-03 | 2.65E-03 |
| rs6527294 | TCGA&TCGA_coCNV | 3.29E-03 | 7.42E-03 |
| rs10944384 | TCGA | 3.29E-03 | #N/A |

| rs9954463 | TCGA&TCGA_coCNV | 3.29E-03 | 1.22E-04 |
| --- | --- | --- | --- |
| rs690214 | TCGA | 3.29E-03 | #N/A |
| rs4899339 | TCGA&TCGA_coCNV | 3.29E-03 | 5.65E-04 |
| rs7127843 | TCGA | 3.29E-03 | #N/A |
| rs6813350 | TCGA&TCGA_coCNV | 3.29E-03 | 8.74E-03 |
| rs10510628 | TCGA | 3.29E-03 | #N/A |
| rs2002028 | TCGA&TCGA_coCNV | 3.29E-03 | 4.56E-03 |
| rs3799307 | TCGA | 3.30E-03 | #N/A |
| rs9912202 | TCGA&TCGA_coCNV | 3.30E-03 | 2.66E-03 |
| rs11664751 | TCGA&TCGA_coCNV | 1.03E-03 | 2.86E-03 |
| rs12277747 | TCGA&TCGA_coCNV | 3.30E-03 | 2.35E-03 |
| rs2977591 | TCGA&TCGA_coCNV | 3.30E-03 | 3.79E-03 |
| rs793276 | TCGA&TCGA_coCNV | 2.41E-03 | 3.54E-03 |
| rs2453146 | TCGA&TCGA_coCNV | 3.30E-03 | 8.21E-03 |
| rs11063155 | TCGA&TCGA_coCNV | 3.30E-03 | 4.01E-03 |
| rs9556465 | TCGA | 3.30E-03 | #N/A |
| rs16933906 | TCGA&TCGA_coCNV | 3.30E-03 | 5.01E-04 |
| rs10487689 | TCGA | 3.30E-03 | #N/A |
| rs12238237 | TCGA | 1.46E-03 | #N/A |
| rs4983089 | TCGA | 3.30E-03 | #N/A |
| rs420901 | TCGA | 3.30E-03 | #N/A |
| rs8065829 | TCGA&TCGA_coCNV | 1.33E-03 | 5.68E-04 |
| rs10214572 | TCGA&TCGA_coCNV | 3.05E-03 | 9.16E-03 |
| rs16826777 | TCGA&TCGA_coCNV | 3.30E-03 | 7.64E-04 |
| rs187773 | TCGA | 3.31E-03 | #N/A |
| rs12823670 | TCGA&TCGA_coCNV | 3.31E-03 | 3.02E-03 |
| rs7145733 | TCGA | 3.31E-03 | #N/A |
| rs9544708 | TCGA&TCGA_coCNV | 3.31E-03 | 7.42E-03 |
| rs6757457 | TCGA&TCGA_coCNV | 3.31E-03 | 2.27E-03 |
| rs6508329 | TCGA | 3.31E-03 | #N/A |
| rs357399 | TCGA | 3.31E-03 | #N/A |
| rs2050376 | TCGA&TCGA_coCNV | 1.31E-03 | 4.23E-03 |
| rs17552044 | TCGA | 3.31E-03 | #N/A |
| rs8117244 | TCGA&TCGA_coCNV | 3.32E-03 | 8.55E-03 |
| rs16824406 | TCGA&TCGA_coCNV | 3.32E-03 | 2.94E-03 |
| rs2094799 | TCGA | 3.32E-03 | #N/A |
| rs7280689 | TCGA | 3.32E-03 | #N/A |
| rs1562855 | TCGA&TCGA_coCNV | 3.32E-03 | 3.45E-03 |
| rs2280656 | TCGA | 3.32E-03 | #N/A |
| rs10835129 | TCGA&TCGA_coCNV | 7.17E-04 | 4.27E-04 |
| rs269605 | TCGA&TCGA_coCNV | 3.32E-03 | 4.74E-03 |
| rs10513445 | TCGA&TCGA_coCNV | 3.32E-03 | 3.48E-03 |
| rs1541689 | TCGA | 3.32E-03 | #N/A |
| rs4689836 | TCGA&TCGA_coCNV | 1.92E-03 | 1.05E-03 |
| rs4726654 | TCGA&TCGA_coCNV | 3.32E-03 | 3.06E-03 |
| rs959243 | TCGA&TCGA_coCNV | 3.32E-03 | 2.55E-03 |

| rs4909743 | TCGA&TCGA_coCNV | 4.40E-04 | 6.65E-03 |
| --- | --- | --- | --- |
| rs180107 | TCGA | 3.32E-03 | #N/A |
| rs1466424 | TCGA | 3.32E-03 | #N/A |
| rs1625353 | TCGA | 3.33E-03 | #N/A |
| rs12664807 | TCGA&TCGA_coCNV | 3.33E-03 | 4.61E-03 |
| rs7313418 | TCGA | 3.33E-03 | #N/A |
| rs10465590 | TCGA | 3.33E-03 | #N/A |
| rs9650551 | TCGA&TCGA_coCNV | 2.25E-03 | 4.23E-03 |
| rs10469582 | TCGA | 3.33E-03 | #N/A |
| rs4664511 | TCGA | 3.33E-03 | #N/A |
| rs4740266 | TCGA | 3.33E-03 | #N/A |
| rs1949761 | TCGA | 3.33E-03 | #N/A |
| rs12074230 | TCGA&TCGA_coCNV | 2.32E-04 | 9.12E-04 |
| rs7286387 | TCGA | 3.33E-03 | #N/A |
| rs16905855 | TCGA&TCGA_coCNV | 3.33E-03 | 4.16E-03 |
| rs993248 | TCGA | 3.33E-03 | #N/A |
| rs12554101 | TCGA | 3.33E-03 | #N/A |
| rs11857014 | TCGA | 3.33E-03 | #N/A |
| rs2659051 | TCGA | 3.33E-03 | #N/A |
| rs601731 | TCGA | 3.33E-03 | #N/A |
| rs228956 | TCGA&TCGA_coCNV | 3.34E-03 | 3.16E-03 |
| rs4953858 | TCGA&TCGA_coCNV | 3.34E-03 | 6.71E-03 |
| rs623170 | TCGA&TCGA_coCNV | 3.34E-03 | 2.17E-04 |
| rs1537076 | TCGA | 3.34E-03 | #N/A |
| rs12589836 | TCGA&TCGA_coCNV | 4.65E-04 | 7.36E-04 |
| rs10832840 | TCGA&TCGA_coCNV | 3.34E-03 | 6.96E-03 |
| rs12595707 | TCGA | 3.34E-03 | #N/A |
| rs11158958 | TCGA | 3.34E-03 | #N/A |
| rs16878545 | TCGA&TCGA_coCNV | 3.12E-03 | 4.38E-04 |
| rs9878161 | TCGA | 3.34E-03 | #N/A |
| rs4926925 | TCGA&TCGA_coCNV | 3.34E-03 | 8.82E-04 |
| rs17038082 | TCGA | 3.34E-03 | #N/A |
| rs17082106 | TCGA&TCGA_coCNV | 3.34E-03 | 9.01E-03 |
| rs976156 | TCGA&TCGA_coCNV | 3.34E-03 | 3.65E-03 |
| rs558550 | TCGA&TCGA_coCNV | 1.29E-03 | 1.80E-03 |
| rs12439725 | TCGA | 3.34E-03 | #N/A |
| rs17618119 | TCGA&TCGA_coCNV | 3.34E-03 | 3.65E-03 |
| rs2340494 | TCGA | 3.34E-03 | #N/A |
| rs7537749 | TCGA&TCGA_coCNV | 3.35E-03 | 8.91E-03 |
| rs12081714 | TCGA&TCGA_coCNV | 3.35E-03 | 6.63E-03 |
| rs6004794 | TCGA | 3.35E-03 | #N/A |
| rs2759395 | TCGA&TCGA_coCNV | 3.35E-03 | 2.12E-03 |
| rs17726502 | TCGA | 3.35E-03 | #N/A |
| rs205986 | TCGA&TCGA_coCNV | 1.38E-03 | 4.15E-03 |
| rs17805310 | TCGA | 3.35E-03 | #N/A |
| rs2657763 | TCGA&TCGA_coCNV | 3.35E-03 | 2.29E-03 |

| rs38953 | TCGA&TCGA_coCNV | 2.39E-03 | 1.75E-03 |
| --- | --- | --- | --- |
| rs1861985 | TCGA&TCGA_coCNV | 3.35E-03 | 1.42E-03 |
| rs6965258 | TCGA&TCGA_coCNV | 3.35E-03 | 5.06E-03 |
| rs9529683 | TCGA | 3.35E-03 | #N/A |
| rs10909882 | TCGA&TCGA_coCNV | 3.35E-03 | 9.46E-04 |
| rs16942812 | TCGA&TCGA_coCNV | 1.59E-03 | 1.89E-03 |
| rs6580179 | TCGA&TCGA_coCNV | 2.77E-03 | 3.59E-03 |
| rs2271688 | TCGA&TCGA_coCNV | 3.35E-03 | 8.21E-03 |
| rs7684481 | TCGA&TCGA_coCNV | 3.36E-03 | 5.62E-03 |
| rs41515846 | TCGA | 3.36E-03 | #N/A |
| rs11827599 | TCGA&TCGA_coCNV | 2.27E-03 | 4.80E-03 |
| rs4437360 | TCGA&TCGA_coCNV | 3.36E-03 | 3.74E-03 |
| rs2000894 | TCGA&TCGA_coCNV | 3.36E-03 | 6.76E-04 |
| rs1952447 | TCGA | 3.36E-03 | #N/A |
| rs12056492 | TCGA&TCGA_coCNV | 6.07E-04 | 5.05E-04 |
| rs4371707 | TCGA | 2.54E-03 | #N/A |
| rs17204260 | TCGA | 3.36E-03 | #N/A |
| rs2444985 | TCGA | 3.36E-03 | #N/A |
| rs8005235 | TCGA | 3.36E-03 | #N/A |
| rs2458250 | TCGA&TCGA_coCNV | 2.34E-03 | 3.50E-03 |
| rs1377297 | TCGA&TCGA_coCNV | 3.37E-03 | 9.33E-03 |
| rs16896573 | TCGA&TCGA_coCNV | 3.37E-03 | 7.44E-03 |
| rs4361593 | TCGA&TCGA_coCNV | 3.37E-03 | 7.61E-03 |
| rs16958140 | TCGA&TCGA_coCNV | 3.37E-03 | 3.61E-04 |
| rs11815249 | TCGA&TCGA_coCNV | 3.37E-03 | 4.56E-04 |
| rs3734105 | TCGA&TCGA_coCNV | 3.37E-03 | 8.02E-03 |
| rs930794 | TCGA&TCGA_coCNV | 5.49E-04 | 2.50E-03 |
| rs12684404 | TCGA&TCGA_coCNV | 3.37E-03 | 3.06E-03 |
| rs7970750 | TCGA | 3.37E-03 | #N/A |
| rs6530475 | TCGA&TCGA_coCNV | 3.37E-03 | 3.16E-03 |
| rs6520091 | TCGA&TCGA_coCNV | 3.37E-03 | 3.35E-03 |
| rs675333 | TCGA&TCGA_coCNV | 6.19E-04 | 1.79E-03 |
| rs1592085 | TCGA | 3.38E-03 | #N/A |
| rs13429694 | TCGA&TCGA_coCNV | 3.13E-03 | 7.74E-03 |
| rs659682 | TCGA&TCGA_coCNV | 3.38E-03 | 8.63E-04 |
| rs820773 | TCGA&TCGA_coCNV | 3.38E-03 | 3.62E-03 |
| rs2072587 | TCGA&TCGA_coCNV | 2.04E-03 | 2.02E-04 |
| rs7597504 | TCGA&TCGA_coCNV | 3.38E-03 | 7.20E-03 |
| rs681023 | TCGA&TCGA_coCNV | 2.28E-03 | 2.52E-03 |
| rs6642610 | TCGA | 3.39E-03 | #N/A |
| rs407765 | TCGA&TCGA_coCNV | 3.39E-03 | 3.03E-03 |
| rs2391610 | TCGA&TCGA_coCNV | 3.39E-03 | 3.19E-03 |
| rs10857579 | TCGA&TCGA_coCNV | 7.31E-04 | 1.27E-03 |
| rs7333447 | TCGA&TCGA_coCNV | 3.39E-03 | 7.12E-05 |
| rs10929400 | TCGA&TCGA_coCNV | 1.10E-03 | 4.12E-03 |
| rs10864907 | TCGA | 3.39E-03 | #N/A |

| rs7854570 | TCGA | 3.39E-03 | #N/A |
| --- | --- | --- | --- |
| rs4789473 | TCGA&TCGA_coCNV | 3.39E-03 | 1.81E-03 |
| rs1312913 | TCGA | 3.39E-03 | #N/A |
| rs12054720 | TCGA | 3.39E-03 | #N/A |
| rs11607556 | TCGA&TCGA_coCNV | 1.91E-03 | 3.20E-03 |
| rs305347 | TCGA | 3.39E-03 | #N/A |
| rs2784568 | TCGA&TCGA_coCNV | 3.39E-03 | 1.82E-03 |
| rs10083806 | TCGA | 8.64E-04 | #N/A |
| rs12687833 | TCGA&TCGA_coCNV | 3.40E-03 | 3.10E-03 |
| rs17015006 | TCGA&TCGA_coCNV | 3.40E-03 | 1.97E-03 |
| rs2168252 | TCGA | 3.40E-03 | #N/A |
| rs9378381 | TCGA&TCGA_coCNV | 6.51E-06 | 8.14E-04 |
| rs4239061 | TCGA&TCGA_coCNV | 5.29E-04 | 1.98E-04 |
| rs7240208 | TCGA | 3.40E-03 | #N/A |
| rs5978480 | TCGA&TCGA_coCNV | 2.45E-03 | 9.32E-04 |
| rs288144 | TCGA&TCGA_coCNV | 3.40E-03 | 1.16E-03 |
| rs16895710 | TCGA&TCGA_coCNV | 3.40E-03 | 5.57E-03 |
| rs1592800 | TCGA&TCGA_coCNV | 1.30E-03 | 3.77E-03 |
| rs16849127 | TCGA&TCGA_coCNV | 3.40E-03 | 1.62E-03 |
| rs255166 | TCGA&TCGA_coCNV | 3.40E-03 | 5.86E-03 |
| rs1465635 | TCGA | 3.40E-03 | #N/A |
| rs819552 | TCGA&TCGA_coCNV | 3.40E-03 | 9.10E-03 |
| rs12818091 | TCGA&TCGA_coCNV | 3.41E-03 | 9.58E-03 |
| rs16916144 | TCGA | 3.41E-03 | #N/A |
| rs10928980 | TCGA&TCGA_coCNV | 3.41E-03 | 1.84E-03 |
| rs12086243 | TCGA&TCGA_coCNV | 3.41E-03 | 4.53E-03 |
| rs10892095 | TCGA | 3.41E-03 | #N/A |
| rs11012067 | TCGA | 3.41E-03 | #N/A |
| rs13084057 | TCGA&TCGA_coCNV | 3.41E-03 | 3.33E-03 |
| rs16960563 | TCGA&TCGA_coCNV | 3.41E-03 | 6.79E-03 |
| rs1249457 | TCGA | 3.41E-03 | #N/A |
| rs4738252 | TCGA | 3.41E-03 | #N/A |
| rs10510296 | TCGA&TCGA_coCNV | 1.86E-04 | 2.10E-03 |
| rs1545913 | TCGA | 3.42E-03 | #N/A |
| rs11677059 | TCGA&TCGA_coCNV | 2.30E-03 | 9.16E-03 |
| rs1609494 | TCGA | 3.42E-03 | #N/A |
| rs7232362 | TCGA | 3.42E-03 | #N/A |
| rs11058836 | TCGA | 3.42E-03 | #N/A |
| rs12911006 | TCGA&TCGA_coCNV | 3.42E-03 | 5.64E-03 |
| rs11098808 | TCGA | 3.42E-03 | #N/A |
| rs28519394 | TCGA&TCGA_coCNV | 3.42E-03 | 3.47E-03 |
| rs16917833 | TCGA&TCGA_coCNV | 2.70E-03 | 3.64E-04 |
| rs1422933 | TCGA&TCGA_coCNV | 3.42E-03 | 5.29E-03 |
| rs13397916 | TCGA&TCGA_coCNV | 2.33E-03 | 4.17E-04 |
| rs7751466 | TCGA&TCGA_coCNV | 3.43E-03 | 5.79E-03 |
| rs10174587 | TCGA&TCGA_coCNV | 6.86E-04 | 7.75E-03 |

| rs8065346 | TCGA&TCGA_coCNV | 3.43E-03 | 6.14E-03 |
| --- | --- | --- | --- |
| rs4810001 | TCGA | 3.43E-03 | #N/A |
| rs288173 | TCGA&TCGA_coCNV | 3.43E-03 | 2.15E-03 |
| rs16980240 | TCGA | 3.43E-03 | #N/A |
| rs2302511 | TCGA&TCGA_coCNV | 3.43E-03 | 8.66E-03 |
| rs2851752 | TCGA | 3.43E-03 | #N/A |
| rs6135815 | TCGA&TCGA_coCNV | 3.43E-03 | 7.78E-03 |
| rs10998479 | TCGA&TCGA_coCNV | 3.43E-03 | 2.62E-03 |
| rs11157800 | TCGA | 3.43E-03 | #N/A |
| rs12123324 | TCGA | 3.43E-03 | #N/A |
| rs7077462 | TCGA&TCGA_coCNV | 3.43E-03 | 9.45E-03 |
| rs4519741 | TCGA&TCGA_coCNV | 2.69E-03 | 5.10E-03 |
| rs2754085 | TCGA | 3.44E-03 | #N/A |
| rs10897789 | TCGA&TCGA_coCNV | 4.51E-04 | 7.49E-03 |
| rs2988048 | TCGA&TCGA_coCNV | 3.44E-03 | 3.23E-03 |
| rs3027111 | TCGA&TCGA_coCNV | 3.44E-03 | 9.36E-05 |
| rs2238925 | TCGA | 3.44E-03 | #N/A |
| rs7180354 | TCGA&TCGA_coCNV | 3.44E-03 | 3.94E-03 |
| rs10463031 | TCGA&TCGA_coCNV | 3.44E-03 | 6.46E-03 |
| rs16866111 | TCGA | 3.44E-03 | #N/A |
| rs12669427 | TCGA | 3.44E-03 | #N/A |
| rs4684958 | TCGA&TCGA_coCNV | 3.44E-03 | 1.06E-03 |
| rs4769421 | TCGA | 3.44E-03 | #N/A |
| rs2139417 | TCGA | 3.44E-03 | #N/A |
| rs10128526 | TCGA&TCGA_coCNV | 3.44E-03 | 7.22E-04 |
| rs16950093 | TCGA&TCGA_coCNV | 3.44E-03 | 9.71E-03 |
| rs8015152 | TCGA&TCGA_coCNV | 3.44E-03 | 7.71E-03 |
| rs2033790 | TCGA | 3.44E-03 | #N/A |
| rs5768453 | TCGA&TCGA_coCNV | 3.44E-03 | 1.89E-04 |
| rs4589200 | TCGA&TCGA_coCNV | 3.44E-03 | 3.75E-03 |
| rs2537752 | TCGA | 3.44E-03 | #N/A |
| rs9630611 | TCGA&TCGA_coCNV | 3.44E-03 | 4.19E-03 |
| rs10040819 | TCGA | 3.44E-03 | #N/A |
| rs6634816 | TCGA | 3.44E-03 | #N/A |
| rs1355342 | TCGA&TCGA_coCNV | 3.45E-03 | 5.26E-03 |
| rs996580 | TCGA&TCGA_coCNV | 3.45E-03 | 3.77E-03 |
| rs8017323 | TCGA | 3.45E-03 | #N/A |
| rs7770404 | TCGA&TCGA_coCNV | 3.80E-04 | 2.93E-03 |
| rs41462748 | TCGA | 3.45E-03 | #N/A |
| rs4397239 | TCGA | 3.45E-03 | #N/A |
| rs6560688 | TCGA&TCGA_coCNV | 3.45E-03 | 2.45E-04 |
| rs10824259 | TCGA&TCGA_coCNV | 3.45E-03 | 9.32E-03 |
| rs11170211 | TCGA | 3.45E-03 | #N/A |
| rs10830174 | TCGA&TCGA_coCNV | 3.45E-03 | 1.19E-03 |
| rs12328609 | TCGA&TCGA_coCNV | 1.13E-03 | 7.52E-04 |
| rs2913372 | TCGA&TCGA_coCNV | 3.46E-03 | 7.66E-03 |

| rs17050383 | TCGA | 3.46E-03 | #N/A |
| --- | --- | --- | --- |
| rs10009731 | TCGA | 3.46E-03 | #N/A |
| rs4958571 | TCGA&TCGA_coCNV | 3.46E-03 | 6.39E-03 |
| rs16874906 | TCGA&TCGA_coCNV | 3.46E-03 | 8.72E-03 |
| rs4985452 | TCGA | 8.83E-04 | #N/A |
| rs6545831 | TCGA&TCGA_coCNV | 3.46E-03 | 2.98E-03 |
| rs11187870 | TCGA&TCGA_coCNV | 1.52E-03 | 1.35E-03 |
| rs10853029 | TCGA&TCGA_coCNV | 3.46E-03 | 1.02E-03 |
| rs6742329 | TCGA&TCGA_coCNV | 1.61E-03 | 7.83E-03 |
| rs2526768 | TCGA&TCGA_coCNV | 3.23E-03 | 5.92E-04 |
| rs9654806 | TCGA | 3.47E-03 | #N/A |
| rs9559708 | TCGA | 3.47E-03 | #N/A |
| rs1628880 | TCGA | 3.47E-03 | #N/A |
| rs978956 | TCGA | 3.47E-03 | #N/A |
| rs6941408 | TCGA&TCGA_coCNV | 3.47E-03 | 6.70E-03 |
| rs688862 | TCGA | 3.47E-03 | #N/A |
| rs6674470 | TCGA&TCGA_coCNV | 3.47E-03 | 4.17E-03 |
| rs2175371 | TCGA&TCGA_coCNV | 4.40E-04 | 1.14E-04 |
| rs7937228 | TCGA&TCGA_coCNV | 3.47E-03 | 5.83E-03 |
| rs9613266 | TCGA | 3.47E-03 | #N/A |
| rs10515875 | TCGA&TCGA_coCNV | 3.47E-03 | 4.40E-03 |
| rs12909308 | TCGA | 3.47E-03 | #N/A |
| rs12940183 | TCGA | 3.47E-03 | #N/A |
| rs9567814 | TCGA | 3.48E-03 | #N/A |
| rs10094283 | TCGA&TCGA_coCNV | 3.48E-03 | 4.22E-04 |
| rs16840418 | TCGA&TCGA_coCNV | 4.73E-04 | 2.07E-03 |
| rs9844587 | TCGA&TCGA_coCNV | 1.97E-03 | 1.28E-03 |
| rs6039267 | TCGA | 2.82E-03 | #N/A |
| rs948217 | TCGA | 3.48E-03 | #N/A |
| rs1030394 | TCGA | 3.48E-03 | #N/A |
| rs2426204 | TCGA | 3.48E-03 | #N/A |
| rs2212866 | TCGA&TCGA_coCNV | 3.48E-03 | 1.79E-03 |
| rs7194919 | TCGA | 3.48E-03 | #N/A |
| rs6081526 | TCGA | 3.48E-03 | #N/A |
| rs10118987 | TCGA | 3.48E-03 | #N/A |
| rs7862101 | TCGA | 3.49E-03 | #N/A |
| rs7574895 | TCGA | 3.49E-03 | #N/A |
| rs13194192 | TCGA&TCGA_coCNV | 1.50E-05 | 1.54E-03 |
| rs425437 | TCGA&TCGA_coCNV | 3.49E-03 | 7.09E-03 |
| rs9355724 | TCGA&TCGA_coCNV | 3.49E-03 | 9.00E-04 |
| rs8129414 | TCGA | 3.49E-03 | #N/A |
| rs1445327 | TCGA&TCGA_coCNV | 3.49E-03 | 5.51E-03 |
| rs6822395 | TCGA | 3.49E-03 | #N/A |
| rs11987162 | TCGA&TCGA_coCNV | 3.49E-03 | 9.95E-03 |
| rs10514133 | TCGA | 3.49E-03 | #N/A |
| rs5754888 | TCGA&TCGA_coCNV | 3.49E-03 | 2.87E-03 |

| rs217222 | TCGA&TCGA_coCNV | 3.49E-03 | 2.83E-03 |
| --- | --- | --- | --- |
| rs6519574 | TCGA | 3.49E-03 | #N/A |
| rs17300302 | TCGA&TCGA_coCNV | 8.45E-04 | 4.79E-04 |
| rs16969168 | TCGA | 3.49E-03 | #N/A |
| rs10075893 | TCGA&TCGA_coCNV | 2.22E-03 | 6.49E-03 |
| rs6034240 | TCGA&TCGA_coCNV | 3.49E-03 | 2.95E-03 |
| rs7036855 | TCGA&TCGA_coCNV | 9.10E-04 | 7.62E-03 |
| rs2025890 | TCGA&TCGA_coCNV | 3.49E-03 | 9.41E-04 |
| rs5967760 | TCGA | 3.49E-03 | #N/A |
| rs7535600 | TCGA | 3.49E-03 | #N/A |
| rs4469659 | TCGA&TCGA_coCNV | 3.48E-03 | 1.86E-03 |
| rs7104313 | TCGA | 3.49E-03 | #N/A |
| rs7774223 | TCGA | 3.49E-03 | #N/A |
| rs9350243 | TCGA&TCGA_coCNV | 3.50E-03 | 3.25E-03 |
| rs10748248 | TCGA&TCGA_coCNV | 3.50E-03 | 6.30E-05 |
| rs7933499 | TCGA | 3.50E-03 | #N/A |
| rs2271489 | TCGA&TCGA_coCNV | 3.50E-03 | 7.01E-03 |
| rs9498061 | TCGA | 3.50E-03 | #N/A |
| rs6425196 | TCGA | 3.50E-03 | #N/A |
| rs7721816 | TCGA&TCGA_coCNV | 3.50E-03 | 6.75E-03 |
| rs252928 | TCGA&TCGA_coCNV | 3.50E-03 | 2.40E-04 |
| rs4881271 | TCGA | 3.50E-03 | #N/A |
| rs10873366 | TCGA&TCGA_coCNV | 3.51E-03 | 2.73E-03 |
| rs2659079 | TCGA&TCGA_coCNV | 2.08E-03 | 1.00E-03 |
| rs10059973 | TCGA | 3.51E-03 | #N/A |
| rs1373660 | TCGA | 3.51E-03 | #N/A |
| rs11199523 | TCGA&TCGA_coCNV | 1.80E-04 | 5.50E-03 |
| rs5923508 | TCGA&TCGA_coCNV | 3.51E-03 | 3.76E-04 |
| rs17000531 | TCGA&TCGA_coCNV | 3.51E-03 | 7.90E-03 |
| rs12944725 | TCGA | 3.51E-03 | #N/A |
| rs17660928 | TCGA&TCGA_coCNV | 3.51E-03 | 1.69E-03 |
| rs11065904 | TCGA | 3.51E-03 | #N/A |
| rs13242757 | TCGA&TCGA_coCNV | 3.51E-03 | 3.56E-03 |
| rs12297964 | TCGA | 3.51E-03 | #N/A |
| rs6129989 | TCGA&TCGA_coCNV | 3.51E-03 | 6.96E-03 |
| rs10447107 | TCGA&TCGA_coCNV | 4.78E-04 | 2.44E-03 |
| rs685959 | TCGA&TCGA_coCNV | 3.52E-03 | 1.51E-03 |
| rs17761197 | TCGA&TCGA_coCNV | 3.52E-03 | 9.16E-03 |
| rs12607268 | TCGA&TCGA_coCNV | 3.52E-03 | 8.17E-04 |
| rs6647951 | TCGA&TCGA_coCNV | 3.52E-03 | 6.41E-03 |
| rs4401102 | TCGA | 3.52E-03 | #N/A |
| rs1976005 | TCGA&TCGA_coCNV | 3.52E-03 | 1.06E-03 |
| rs1491474 | TCGA&TCGA_coCNV | 3.52E-03 | 4.02E-03 |
| rs7695680 | TCGA&TCGA_coCNV | 3.52E-03 | 6.34E-03 |
| rs9878954 | TCGA&TCGA_coCNV | 3.52E-03 | 6.46E-03 |
| rs4350455 | TCGA | 3.52E-03 | #N/A |

| rs7514979 | TCGA&TCGA_coCNV | 3.52E-03 | 8.79E-03 |
| --- | --- | --- | --- |
| rs9990094 | TCGA&TCGA_coCNV | 3.52E-03 | 6.31E-04 |
| rs1035572 | TCGA&TCGA_coCNV | 5.49E-04 | 7.99E-03 |
| rs13440974 | TCGA&TCGA_coCNV | 1.45E-05 | 1.55E-04 |
| rs11973006 | TCGA&TCGA_coCNV | 3.53E-03 | 6.99E-03 |
| rs1537346 | TCGA&TCGA_coCNV | 3.53E-03 | 8.21E-03 |
| rs10077610 | TCGA | 2.97E-04 | #N/A |
| rs4845488 | TCGA&TCGA_coCNV | 3.53E-03 | 9.10E-03 |
| rs7537410 | TCGA | 3.53E-03 | #N/A |
| rs12132899 | TCGA | 3.53E-03 | #N/A |
| rs10102276 | TCGA | 3.53E-03 | #N/A |
| rs17038697 | TCGA | 3.53E-03 | #N/A |
| rs1031501 | TCGA&TCGA_coCNV | 3.53E-03 | 8.76E-03 |
| rs17089980 | TCGA&TCGA_coCNV | 3.53E-03 | 6.12E-03 |
| rs5936212 | TCGA&TCGA_coCNV | 3.53E-03 | 7.81E-03 |
| rs3788449 | TCGA&TCGA_coCNV | 3.53E-03 | 7.24E-03 |
| rs276512 | TCGA&TCGA_coCNV | 3.36E-03 | 5.23E-03 |
| rs3134906 | TCGA | 3.53E-03 | #N/A |
| rs10962810 | TCGA | 3.53E-03 | #N/A |
| rs9601651 | TCGA | 3.54E-03 | #N/A |
| rs7591615 | TCGA | 3.29E-03 | #N/A |
| rs6860413 | TCGA&TCGA_coCNV | 3.54E-03 | 9.28E-03 |
| rs17029598 | TCGA&TCGA_coCNV | 3.54E-03 | 1.83E-04 |
| rs999503 | TCGA&TCGA_coCNV | 3.54E-03 | 9.78E-03 |
| rs2392540 | TCGA&TCGA_coCNV | 3.54E-03 | 3.51E-03 |
| rs12584904 | TCGA | 3.54E-03 | #N/A |
| rs690205 | TCGA&TCGA_coCNV | 3.54E-03 | 2.29E-03 |
| rs699127 | TCGA | 1.09E-03 | #N/A |
| rs10237218 | TCGA | 3.54E-03 | #N/A |
| rs9916623 | TCGA&TCGA_coCNV | 3.54E-03 | 7.58E-03 |
| rs1922697 | TCGA | 3.55E-03 | #N/A |
| rs1350208 | TCGA | 3.55E-03 | #N/A |
| rs11579340 | TCGA&TCGA_coCNV | 3.55E-03 | 9.42E-04 |
| rs12669177 | TCGA | 3.55E-03 | #N/A |
| rs7695632 | TCGA | 3.55E-03 | #N/A |
| rs4883947 | TCGA | 3.55E-03 | #N/A |
| rs7242031 | TCGA | 3.51E-03 | #N/A |
| rs612441 | TCGA&TCGA_coCNV&SCANdb | 3.55E-03 | 2.82E-03 |
| rs763218 | TCGA | 3.55E-03 | #N/A |
| rs11992634 | TCGA&TCGA_coCNV | 3.55E-03 | 8.95E-03 |
| rs2288593 | TCGA&TCGA_coCNV | 3.55E-03 | 5.72E-03 |
| rs1572067 | TCGA&TCGA_coCNV | 3.55E-03 | 9.09E-03 |
| rs13223326 | TCGA | 3.56E-03 | #N/A |
| rs4624285 | TCGA&TCGA_coCNV | 3.56E-03 | 4.54E-03 |
| rs954516 | TCGA&TCGA_coCNV | 2.00E-03 | 7.58E-04 |
| rs13202955 | TCGA | 3.56E-03 | #N/A |

| rs10959724 | TCGA&TCGA_coCNV | 3.56E-03 | 4.86E-03 |
| --- | --- | --- | --- |
| rs4954148 | TCGA | 3.56E-03 | #N/A |
| rs7999501 | TCGA&TCGA_coCNV | 1.01E-03 | 2.33E-03 |
| rs1531868 | TCGA | 3.56E-03 | #N/A |
| rs10509707 | TCGA | 3.56E-03 | #N/A |
| rs7144656 | TCGA | 3.56E-03 | #N/A |
| rs6702984 | TCGA&TCGA_coCNV | 3.56E-03 | 6.25E-04 |
| rs16959333 | TCGA&TCGA_coCNV | 2.37E-03 | 5.49E-03 |
| rs11775998 | TCGA | 3.56E-03 | #N/A |
| rs10038162 | TCGA&TCGA_coCNV | 3.56E-03 | 5.73E-03 |
| rs6431302 | TCGA | 3.56E-03 | #N/A |
| rs1952052 | TCGA | 3.56E-03 | #N/A |
| rs12631771 | TCGA | 3.56E-03 | #N/A |
| rs9576301 | TCGA&TCGA_coCNV | 1.21E-03 | 2.99E-03 |
| rs9580641 | TCGA | 1.81E-03 | #N/A |
| rs2551405 | TCGA&TCGA_coCNV | 3.57E-03 | 2.72E-03 |
| rs9990200 | TCGA | 3.57E-03 | #N/A |
| rs1086215 | TCGA | 3.57E-03 | #N/A |
| rs1529287 | TCGA&TCGA_coCNV | 3.57E-03 | 4.30E-03 |
| rs10086985 | TCGA | 3.57E-03 | #N/A |
| rs1579199 | TCGA&TCGA_coCNV | 3.57E-03 | 5.21E-03 |
| rs2437633 | TCGA&TCGA_coCNV | 7.98E-04 | 1.90E-03 |
| rs4531679 | TCGA&TCGA_coCNV | 3.57E-03 | 2.67E-03 |
| rs1013511 | TCGA&TCGA_coCNV | 3.57E-03 | 1.30E-03 |
| rs4522646 | TCGA | 3.57E-03 | #N/A |
| rs4331974 | TCGA | 3.57E-03 | #N/A |
| rs4711248 | TCGA&TCGA_coCNV | 1.70E-03 | 2.11E-04 |
| rs13004955 | TCGA&TCGA_coCNV | 3.58E-03 | 2.78E-03 |
| rs6781453 | TCGA | 3.58E-03 | #N/A |
| rs10186531 | TCGA&TCGA_coCNV | 3.58E-03 | 4.36E-03 |
| rs9924080 | TCGA | 3.58E-03 | #N/A |
| rs10803620 | TCGA&TCGA_coCNV | 3.58E-03 | 2.60E-03 |
| rs13269454 | TCGA | 8.56E-04 | #N/A |
| rs7975983 | TCGA | 3.58E-03 | #N/A |
| rs1433209 | TCGA&TCGA_coCNV | 3.58E-03 | 9.25E-04 |
| rs6083015 | TCGA&TCGA_coCNV | 3.58E-03 | 2.03E-03 |
| rs3793479 | TCGA | 3.58E-03 | #N/A |
| rs16999029 | TCGA&TCGA_coCNV | 3.58E-03 | 8.91E-03 |
| rs12994012 | TCGA&TCGA_coCNV | 3.58E-03 | 3.72E-03 |
| rs2293007 | TCGA&TCGA_coCNV | 3.58E-03 | 2.49E-04 |
| rs971251 | TCGA | 3.58E-03 | #N/A |
| rs4765458 | TCGA | 3.58E-03 | #N/A |
| rs17171730 | TCGA | 3.58E-03 | #N/A |
| rs11040901 | TCGA&TCGA_coCNV | 1.03E-04 | 3.64E-03 |
| rs11799544 | TCGA&TCGA_coCNV | 3.59E-03 | 7.92E-03 |
| rs9872865 | TCGA&TCGA_coCNV | 3.59E-03 | 2.96E-03 |

| rs997572 | TCGA&TCGA_coCNV | 3.59E-03 | 4.89E-03 |
| --- | --- | --- | --- |
| rs152711 | TCGA&TCGA_coCNV | 3.59E-03 | 2.35E-03 |
| rs1566813 | TCGA&TCGA_coCNV | 3.20E-04 | 9.10E-04 |
| rs1951215 | TCGA&TCGA_coCNV | 3.59E-03 | 3.99E-03 |
| rs11024394 | TCGA | 6.13E-04 | #N/A |
| rs3783076 | TCGA&TCGA_coCNV | 6.75E-04 | 1.39E-03 |
| rs10021925 | TCGA | 3.60E-03 | #N/A |
| rs1105892 | TCGA&TCGA_coCNV | 3.60E-03 | 1.54E-03 |
| rs9837367 | TCGA | 3.60E-03 | #N/A |
| rs7024058 | TCGA&TCGA_coCNV | 3.60E-03 | 7.95E-03 |
| rs7461638 | TCGA | 3.60E-03 | #N/A |
| rs7159 | TCGA | 3.60E-03 | #N/A |
| rs10519678 | TCGA | 3.60E-03 | #N/A |
| rs17085463 | TCGA&TCGA_coCNV | 3.60E-03 | 7.68E-03 |
| rs12578814 | TCGA&TCGA_coCNV | 3.60E-03 | 8.48E-03 |
| rs1720843 | TCGA | 3.60E-03 | #N/A |
| rs2083937 | TCGA | 3.60E-03 | #N/A |
| rs12197567 | TCGA&TCGA_coCNV | 3.60E-03 | 2.01E-03 |
| rs2301093 | TCGA | 3.60E-03 | #N/A |
| rs908487 | TCGA | 3.60E-03 | #N/A |
| rs7621590 | TCGA&TCGA_coCNV | 3.61E-03 | 1.70E-03 |
| rs9563616 | TCGA | 2.49E-04 | #N/A |
| rs11049633 | TCGA&TCGA_coCNV | 3.61E-03 | 1.23E-03 |
| rs2978550 | TCGA&TCGA_coCNV | 1.85E-03 | 7.79E-04 |
| rs2278204 | TCGA&TCGA_coCNV | 3.61E-03 | 2.77E-03 |
| rs610695 | TCGA&TCGA_coCNV | 3.61E-03 | 8.68E-04 |
| rs9834018 | TCGA&TCGA_coCNV | 3.61E-03 | 8.64E-03 |
| rs6940907 | TCGA | 3.61E-03 | #N/A |
| rs275853 | TCGA | 3.61E-03 | #N/A |
| rs7920137 | TCGA&TCGA_coCNV | 3.61E-03 | 2.58E-03 |
| rs9559755 | TCGA&TCGA_coCNV | 3.61E-03 | 8.34E-03 |
| rs6638767 | TCGA&TCGA_coCNV | 1.93E-03 | 5.31E-04 |
| rs8082057 | TCGA&TCGA_coCNV | 3.62E-03 | 3.98E-03 |
| rs2286485 | TCGA | 3.09E-03 | #N/A |
| rs4765451 | TCGA&TCGA_coCNV | 3.62E-03 | 8.26E-03 |
| rs2293356 | TCGA | 3.62E-03 | #N/A |
| rs6975435 | TCGA&TCGA_coCNV | 3.62E-03 | 3.40E-04 |
| rs1711390 | TCGA&TCGA_coCNV | 3.62E-03 | 4.07E-03 |
| rs12584910 | TCGA | 3.62E-03 | #N/A |
| rs8073681 | TCGA | 3.62E-03 | #N/A |
| rs9648104 | TCGA | 3.62E-03 | #N/A |
| rs6641073 | TCGA&TCGA_coCNV | 3.62E-03 | 4.12E-05 |
| rs11162323 | TCGA&TCGA_coCNV | 3.62E-03 | 1.08E-03 |
| rs13396027 | TCGA&TCGA_coCNV | 3.62E-03 | 8.77E-04 |
| rs1723722 | TCGA&TCGA_coCNV | 3.62E-03 | 6.05E-04 |
| rs4605656 | TCGA&TCGA_coCNV | 3.63E-03 | 2.06E-03 |

| rs2515479 | TCGA&TCGA_coCNV | 3.63E-03 | 2.34E-03 |
| --- | --- | --- | --- |
| rs7000722 | TCGA | 3.63E-03 | #N/A |
| rs1523681 | TCGA | 3.63E-03 | #N/A |
| rs4532319 | TCGA | 3.63E-03 | #N/A |
| rs1575125 | TCGA&TCGA_coCNV | 3.63E-03 | 7.88E-03 |
| rs348044 | TCGA | 3.63E-03 | #N/A |
| rs13400838 | TCGA&TCGA_coCNV | 3.63E-03 | 4.04E-03 |
| rs2190920 | TCGA | 3.63E-03 | #N/A |
| rs7814470 | TCGA&TCGA_coCNV | 1.84E-03 | 1.90E-03 |
| rs4472239 | TCGA&TCGA_coCNV | 3.18E-03 | 3.37E-03 |
| rs12061691 | TCGA&TCGA_coCNV | 3.63E-03 | 5.68E-03 |
| rs4307765 | TCGA&TCGA_coCNV | 1.08E-03 | 3.26E-04 |
| rs12062 | TCGA&TCGA_coCNV | 3.63E-03 | 4.19E-03 |
| rs11608740 | TCGA&TCGA_coCNV | 3.63E-03 | 1.27E-03 |
| rs6808139 | TCGA&TCGA_coCNV | 4.39E-04 | 1.15E-04 |
| rs12853128 | TCGA | 3.64E-03 | #N/A |
| rs282471 | TCGA&TCGA_coCNV | 3.64E-03 | 1.16E-03 |
| rs12325247 | TCGA&TCGA_coCNV | 2.39E-03 | 3.47E-03 |
| rs1893854 | TCGA&TCGA_coCNV | 3.64E-03 | 7.35E-03 |
| rs6745718 | TCGA | 2.98E-03 | #N/A |
| rs6640065 | TCGA&TCGA_coCNV | 3.06E-03 | 1.16E-03 |
| rs11552145 | TCGA&TCGA_coCNV | 3.64E-03 | 2.83E-03 |
| rs16910858 | TCGA | 3.64E-03 | #N/A |
| rs1942410 | TCGA | 3.64E-03 | #N/A |
| rs2252534 | TCGA&TCGA_coCNV | 3.64E-03 | 3.44E-03 |
| rs4075601 | TCGA&TCGA_coCNV | 3.64E-03 | 5.89E-05 |
| rs41490153 | TCGA | 3.64E-03 | #N/A |
| rs4647473 | TCGA&TCGA_coCNV | 2.05E-04 | 1.37E-03 |
| rs1933007 | TCGA&TCGA_coCNV | 3.65E-03 | 6.48E-03 |
| rs7787158 | TCGA&TCGA_coCNV | 3.65E-03 | 4.72E-04 |
| rs10136094 | TCGA&TCGA_coCNV | 3.65E-03 | 5.94E-03 |
| rs6838853 | TCGA | 3.65E-03 | #N/A |
| rs6055578 | TCGA&TCGA_coCNV | 3.08E-03 | 4.36E-03 |
| rs16969387 | TCGA | 3.65E-03 | #N/A |
| rs3798357 | TCGA&TCGA_coCNV | 3.65E-03 | 3.20E-03 |
| rs6662194 | TCGA | 4.03E-04 | #N/A |
| rs2144630 | TCGA&TCGA_coCNV | 3.65E-03 | 3.47E-03 |
| rs452542 | TCGA&TCGA_coCNV | 6.81E-04 | 4.48E-04 |
| rs9295431 | TCGA | 3.65E-03 | #N/A |
| rs277542 | TCGA | 3.65E-03 | #N/A |
| rs5916444 | TCGA | 3.65E-03 | #N/A |
| rs7093985 | TCGA | 3.50E-03 | #N/A |
| rs7229587 | TCGA | 3.65E-03 | #N/A |
| rs2843129 | TCGA | 3.65E-03 | #N/A |
| rs11591738 | TCGA&TCGA_coCNV | 3.66E-03 | 2.33E-03 |
| rs17071528 | TCGA&TCGA_coCNV | 1.27E-03 | 4.83E-04 |

| rs16881991 | TCGA | 3.66E-03 | #N/A |
| --- | --- | --- | --- |
| rs969667 | TCGA&TCGA_coCNV | 3.66E-03 | 2.01E-04 |
| rs4976801 | TCGA | 3.66E-03 | #N/A |
| rs4376516 | TCGA | 4.86E-04 | #N/A |
| rs4819958 | TCGA | 3.66E-03 | #N/A |
| rs1932746 | TCGA | 3.66E-03 | #N/A |
| rs9466087 | TCGA&TCGA_coCNV | 3.66E-03 | 1.02E-03 |
| rs6627734 | TCGA&TCGA_coCNV | 3.66E-03 | 2.70E-04 |
| rs9512230 | TCGA&TCGA_coCNV | 3.66E-03 | 2.55E-03 |
| rs1007854 | TCGA | 3.66E-03 | #N/A |
| rs7868612 | TCGA | 3.43E-03 | #N/A |
| rs2056314 | TCGA | 3.66E-03 | #N/A |
| rs2789371 | TCGA | 3.66E-03 | #N/A |
| rs10814873 | TCGA&TCGA_coCNV | 3.66E-03 | 3.15E-03 |
| rs4881404 | TCGA&TCGA_coCNV | 1.99E-03 | 9.89E-03 |
| rs1485741 | TCGA | 3.66E-03 | #N/A |
| rs12041799 | TCGA&TCGA_coCNV | 3.66E-03 | 5.04E-04 |
| rs11646827 | TCGA&TCGA_coCNV | 8.61E-05 | 9.14E-04 |
| rs1884540 | TCGA&TCGA_coCNV | 3.67E-03 | 4.59E-03 |
| rs11845021 | TCGA | 3.67E-03 | #N/A |
| rs7172774 | TCGA | 3.67E-03 | #N/A |
| rs12211654 | TCGA&TCGA_coCNV | 3.67E-03 | 3.60E-03 |
| rs13338287 | TCGA&TCGA_coCNV | 3.67E-03 | 4.50E-03 |
| rs11132115 | TCGA | 3.67E-03 | #N/A |
| rs6051457 | TCGA&TCGA_coCNV | 3.67E-03 | 2.28E-03 |
| rs10508045 | TCGA | 2.41E-03 | #N/A |
| rs4242756 | TCGA | 3.67E-03 | #N/A |
| rs13188158 | TCGA | 3.67E-03 | #N/A |
| rs10751957 | TCGA | 3.67E-03 | #N/A |
| rs2578376 | TCGA | 3.67E-03 | #N/A |
| rs2810142 | TCGA&TCGA_coCNV | 3.67E-03 | 5.04E-03 |
| rs160935 | TCGA | 3.68E-03 | #N/A |
| rs9945336 | TCGA&TCGA_coCNV | 3.68E-03 | 4.30E-03 |
| rs9574841 | TCGA&TCGA_coCNV | 3.68E-03 | 9.88E-03 |
| rs9861610 | TCGA&TCGA_coCNV | 3.68E-03 | 4.01E-03 |
| rs11620653 | TCGA&TCGA_coCNV | 2.47E-03 | 4.63E-03 |
| rs1451886 | TCGA&TCGA_coCNV | 3.68E-03 | 7.63E-03 |
| rs6812702 | TCGA | 3.68E-03 | #N/A |
| rs16842891 | TCGA&TCGA_coCNV | 3.68E-03 | 2.11E-03 |
| rs888821 | TCGA&TCGA_coCNV | 3.69E-03 | 3.81E-03 |
| rs2922333 | TCGA | 3.69E-03 | #N/A |
| rs17632741 | TCGA&TCGA_coCNV | 3.69E-03 | 4.26E-03 |
| rs4748977 | TCGA | 3.70E-03 | #N/A |
| rs1453109 | TCGA | 2.46E-03 | #N/A |
| rs13393540 | TCGA | 3.70E-03 | #N/A |
| rs16948315 | TCGA | 3.70E-03 | #N/A |

| rs553945 | TCGA | 3.70E-03 | #N/A |
| --- | --- | --- | --- |
| rs4787386 | TCGA&TCGA_coCNV | 3.70E-03 | 8.02E-03 |
| rs2762933 | TCGA | 3.70E-03 | #N/A |
| rs2231580 | TCGA | 3.70E-03 | #N/A |
| rs5748261 | TCGA | 2.46E-03 | #N/A |
| rs17473484 | TCGA&TCGA_coCNV | 3.70E-03 | 5.75E-03 |
| rs1926374 | TCGA&TCGA_coCNV | 3.70E-03 | 6.90E-03 |
| rs7870154 | TCGA | 3.70E-03 | #N/A |
| rs33378 | TCGA&TCGA_coCNV | 3.70E-03 | 8.46E-03 |
| rs3758930 | TCGA&TCGA_coCNV | 3.70E-03 | 3.56E-03 |
| rs7733844 | TCGA&TCGA_coCNV | 1.74E-04 | 4.06E-05 |
| rs1962150 | TCGA&TCGA_coCNV | 2.63E-03 | 4.05E-03 |
| rs1470130 | TCGA | 3.70E-03 | #N/A |
| rs2250538 | TCGA&TCGA_coCNV | 3.70E-03 | 4.28E-03 |
| rs4764074 | TCGA | 3.70E-03 | #N/A |
| rs7304294 | TCGA&TCGA_coCNV | 3.70E-03 | 2.53E-03 |
| rs11822525 | TCGA | 3.71E-03 | #N/A |
| rs7543472 | TCGA | 3.71E-03 | #N/A |
| rs10129057 | TCGA | 3.71E-03 | #N/A |
| rs4236773 | TCGA&TCGA_coCNV | 3.71E-03 | 5.86E-04 |
| rs2567675 | TCGA | 3.71E-03 | #N/A |
| rs10520647 | TCGA | 3.71E-03 | #N/A |
| rs11045743 | TCGA&TCGA_coCNV | 3.71E-03 | 5.05E-04 |
| rs13058500 | TCGA | 3.71E-03 | #N/A |
| rs6841433 | TCGA | 3.71E-03 | #N/A |
| rs6848890 | TCGA&TCGA_coCNV | 3.71E-03 | 8.47E-04 |
| rs10749429 | TCGA&TCGA_coCNV | 3.71E-03 | 7.52E-04 |
| rs7649455 | TCGA&TCGA_coCNV | 4.36E-04 | 1.25E-03 |
| rs1937713 | TCGA | 3.71E-03 | #N/A |
| rs1337059 | TCGA&TCGA_coCNV | 1.79E-03 | 9.77E-04 |
| rs1980770 | TCGA | 3.71E-03 | #N/A |
| rs6540481 | TCGA | 3.72E-03 | #N/A |
| rs7839182 | TCGA | 3.72E-03 | #N/A |
| rs17091846 | TCGA&TCGA_coCNV | 3.72E-03 | 2.13E-03 |
| rs979059 | TCGA&TCGA_coCNV | 1.66E-03 | 8.60E-04 |
| rs7966314 | TCGA | 3.72E-03 | #N/A |
| rs4984681 | TCGA&TCGA_coCNV | 1.03E-04 | 6.51E-04 |
| rs1387735 | TCGA&TCGA_coCNV | 5.23E-04 | 9.65E-03 |
| rs9579845 | TCGA&TCGA_coCNV | 3.72E-03 | 8.66E-03 |
| rs1489880 | TCGA&TCGA_coCNV | 3.72E-03 | 1.95E-03 |
| rs11657271 | TCGA | 3.72E-03 | #N/A |
| rs13379979 | TCGA&TCGA_coCNV | 3.72E-03 | 1.08E-03 |
| rs11736120 | TCGA&TCGA_coCNV | 3.72E-03 | 9.40E-03 |
| rs11992377 | TCGA | 3.73E-03 | #N/A |
| rs10119795 | TCGA | 3.73E-03 | #N/A |
| rs9301891 | TCGA&TCGA_coCNV | 1.23E-03 | 1.04E-03 |

| rs9909281 | TCGA&TCGA_coCNV | 1.07E-04 | 1.65E-03 |
| --- | --- | --- | --- |
| rs4830793 | TCGA&TCGA_coCNV | 3.73E-03 | 1.49E-03 |
| rs12121091 | TCGA&TCGA_coCNV | 8.92E-04 | 3.40E-03 |
| rs2178125 | TCGA | 3.73E-03 | #N/A |
| rs7085725 | TCGA&TCGA_coCNV | 1.37E-04 | 5.06E-04 |
| rs7074234 | TCGA | 3.73E-03 | #N/A |
| rs1470262 | TCGA | 3.73E-03 | #N/A |
| rs16983765 | TCGA&TCGA_coCNV | 3.21E-03 | 1.76E-04 |
| rs1850639 | TCGA&TCGA_coCNV | 3.73E-03 | 4.85E-04 |
| rs11755246 | TCGA | 3.73E-03 | #N/A |
| rs11151910 | TCGA&TCGA_coCNV | 3.73E-03 | 5.61E-03 |
| rs11194127 | TCGA | 3.73E-03 | #N/A |
| rs3892259 | TCGA | 3.73E-03 | #N/A |
| rs4355226 | TCGA | 3.73E-03 | #N/A |
| rs8029637 | TCGA | 3.74E-03 | #N/A |
| rs585978 | TCGA | 3.74E-03 | #N/A |
| rs12355916 | TCGA | 8.35E-04 | #N/A |
| rs17770006 | TCGA | 1.02E-03 | #N/A |
| rs11637642 | TCGA&TCGA_coCNV | 3.74E-03 | 4.56E-03 |
| rs17654757 | TCGA&TCGA_coCNV | 3.74E-03 | 1.09E-03 |
| rs180424 | TCGA&TCGA_coCNV | 3.74E-03 | 4.46E-03 |
| rs41323250 | TCGA&TCGA_coCNV | 1.66E-03 | 1.60E-03 |
| rs10224699 | TCGA&TCGA_coCNV | 3.74E-03 | 3.70E-03 |
| rs11973685 | TCGA&TCGA_coCNV | 3.74E-03 | 1.73E-03 |
| rs9446516 | TCGA&TCGA_coCNV | 3.74E-03 | 4.33E-03 |
| rs9363328 | TCGA | 3.74E-03 | #N/A |
| rs10735079 | TCGA&TCGA_coCNV | 3.75E-03 | 7.11E-03 |
| rs6898403 | TCGA | 1.05E-03 | #N/A |
| rs4827790 | TCGA | 3.75E-03 | #N/A |
| rs10441083 | TCGA&TCGA_coCNV | 3.75E-03 | 4.58E-03 |
| rs1405655 | TCGA&TCGA_coCNV | 3.93E-05 | 1.44E-03 |
| rs4607330 | TCGA | 1.62E-04 | #N/A |
| rs6830133 | TCGA&TCGA_coCNV | 3.75E-03 | 1.03E-03 |
| rs41330746 | TCGA | 3.75E-03 | #N/A |
| rs929531 | TCGA&TCGA_coCNV | 4.66E-04 | 4.52E-04 |
| rs16901360 | TCGA&TCGA_coCNV | 3.00E-03 | 3.05E-03 |
| rs9994358 | TCGA&TCGA_coCNV | 9.78E-04 | 5.96E-04 |
| rs17094439 | TCGA&TCGA_coCNV | 2.31E-03 | 2.06E-03 |
| rs1874536 | TCGA&TCGA_coCNV | 3.75E-03 | 1.16E-03 |
| rs11630261 | TCGA&TCGA_coCNV | 3.75E-03 | 3.84E-03 |
| rs8063813 | TCGA | 3.75E-03 | #N/A |
| rs2253775 | TCGA&TCGA_coCNV | 3.76E-03 | 7.70E-04 |
| rs6767394 | TCGA | 4.72E-04 | #N/A |
| rs4472109 | TCGA&TCGA_coCNV | 6.15E-04 | 4.92E-03 |
| rs9459212 | TCGA&TCGA_coCNV | 3.12E-03 | 1.19E-04 |
| rs10189317 | TCGA | 3.76E-03 | #N/A |

| rs2938082 | TCGA | 3.76E-03 | #N/A |
| --- | --- | --- | --- |
| rs9375539 | TCGA&TCGA_coCNV | 3.76E-03 | 5.38E-04 |
| rs2721762 | TCGA&TCGA_coCNV | 1.33E-03 | 4.87E-04 |
| rs3009503 | TCGA | 3.76E-03 | #N/A |
| rs1868165 | TCGA&TCGA_coCNV | 2.82E-03 | 4.32E-03 |
| rs603589 | TCGA | 3.76E-03 | #N/A |
| rs7602762 | TCGA | 3.76E-03 | #N/A |
| rs16922356 | TCGA | 3.76E-03 | #N/A |
| rs2059155 | TCGA | 3.76E-03 | #N/A |
| rs984761 | TCGA&TCGA_coCNV | 3.76E-03 | 2.16E-03 |
| rs906157 | TCGA&TCGA_coCNV | 3.77E-03 | 2.52E-03 |
| rs848519 | TCGA&TCGA_coCNV | 3.77E-03 | 3.06E-03 |
| rs13335487 | TCGA | 3.77E-03 | #N/A |
| rs16956576 | TCGA | 3.77E-03 | #N/A |
| rs10506353 | TCGA&TCGA_coCNV | 3.77E-03 | 1.16E-03 |
| rs10959965 | TCGA&TCGA_coCNV | 3.77E-03 | 9.44E-03 |
| rs11226019 | TCGA&TCGA_coCNV | 3.77E-03 | 1.48E-04 |
| rs2304983 | TCGA&TCGA_coCNV | 3.77E-03 | 6.61E-03 |
| rs11097059 | TCGA&TCGA_coCNV | 1.35E-03 | 4.99E-03 |
| rs17193833 | TCGA&TCGA_coCNV | 1.96E-04 | 1.84E-03 |
| rs12105006 | TCGA | 3.77E-03 | #N/A |
| rs8006577 | TCGA&TCGA_coCNV | 6.71E-05 | 3.23E-03 |
| rs6919620 | TCGA&TCGA_coCNV | 3.78E-03 | 9.80E-05 |
| rs2218054 | TCGA&TCGA_coCNV | 3.78E-03 | 7.57E-03 |
| rs1011850 | TCGA&TCGA_coCNV | 3.78E-03 | 9.71E-03 |
| rs7512301 | TCGA | 3.78E-03 | #N/A |
| rs10086626 | TCGA | 3.78E-03 | #N/A |
| rs41397045 | TCGA&TCGA_coCNV | 3.78E-03 | 5.38E-04 |
| rs2664132 | TCGA | 3.78E-03 | #N/A |
| rs1990286 | TCGA | 3.78E-03 | #N/A |
| rs10483734 | TCGA&TCGA_coCNV | 3.78E-03 | 6.82E-03 |
| rs10132049 | TCGA&TCGA_coCNV | 1.65E-03 | 4.23E-03 |
| rs9586965 | TCGA&TCGA_coCNV | 3.78E-03 | 5.75E-03 |
| rs17066530 | TCGA&TCGA_coCNV | 3.78E-03 | 4.42E-03 |
| rs3906744 | TCGA | 3.78E-03 | #N/A |
| rs16922833 | TCGA&TCGA_coCNV | 4.18E-04 | 1.32E-04 |
| rs7674947 | TCGA&TCGA_coCNV | 3.78E-03 | 4.66E-03 |
| rs964837 | TCGA&TCGA_coCNV | 8.24E-04 | 5.70E-03 |
| rs2290731 | TCGA&TCGA_coCNV | 4.42E-04 | 1.19E-03 |
| rs10817388 | TCGA | 3.79E-03 | #N/A |
| rs10892951 | TCGA | 3.79E-03 | #N/A |
| rs10502488 | TCGA | 3.79E-03 | #N/A |
| rs13066544 | TCGA&TCGA_coCNV | 3.79E-03 | 1.66E-03 |
| rs4829634 | TCGA | 3.79E-03 | #N/A |
| rs11775176 | TCGA | 3.79E-03 | #N/A |
| rs12450199 | TCGA | 5.84E-04 | #N/A |

| rs12958031 | TCGA | 5.17E-04 | #N/A |
| --- | --- | --- | --- |
| rs10807241 | TCGA&TCGA_coCNV | 3.79E-03 | 3.10E-03 |
| rs2417920 | TCGA | 3.79E-03 | #N/A |
| rs807094 | TCGA | 3.80E-03 | #N/A |
| rs7168796 | TCGA&TCGA_coCNV | 2.11E-04 | 6.03E-03 |
| rs1324041 | TCGA&TCGA_coCNV | 3.80E-03 | 8.80E-03 |
| rs3791423 | TCGA&TCGA_coCNV | 3.80E-03 | 6.89E-03 |
| rs10423465 | TCGA&TCGA_coCNV | 6.02E-04 | 1.38E-03 |
| rs6586 | TCGA&TCGA_coCNV | 3.80E-03 | 2.03E-03 |
| rs1485718 | TCGA | 3.80E-03 | #N/A |
| rs997171 | TCGA&TCGA_coCNV | 3.80E-03 | 1.24E-03 |
| rs17719769 | TCGA&TCGA_coCNV | 1.23E-03 | 1.41E-03 |
| rs7853940 | TCGA&TCGA_coCNV | 3.80E-03 | 3.26E-05 |
| rs10866333 | TCGA&TCGA_coCNV | 3.80E-03 | 3.49E-03 |
| rs7731574 | TCGA | 3.80E-03 | #N/A |
| rs686190 | TCGA | 3.80E-03 | #N/A |
| rs6789225 | TCGA | 3.81E-03 | #N/A |
| rs133945 | TCGA&TCGA_coCNV | 1.38E-04 | 4.32E-04 |
| rs17101521 | TCGA | 3.81E-03 | #N/A |
| rs6531413 | TCGA&TCGA_coCNV | 2.27E-04 | 4.66E-03 |
| rs389870 | TCGA&TCGA_coCNV | 3.81E-03 | 2.77E-03 |
| rs346172 | TCGA&TCGA_coCNV | 3.81E-03 | 9.97E-03 |
| rs11735367 | TCGA | 3.81E-03 | #N/A |
| rs2188622 | TCGA | 3.81E-03 | #N/A |
| rs11236661 | TCGA&TCGA_coCNV | 3.81E-03 | 2.81E-03 |
| rs17084873 | TCGA&TCGA_coCNV | 3.81E-03 | 6.12E-03 |
| rs6528687 | TCGA | 3.81E-03 | #N/A |
| rs17560428 | TCGA | 1.66E-03 | #N/A |
| rs3128341 | TCGA | 3.81E-03 | #N/A |
| rs8123503 | TCGA&TCGA_coCNV | 3.82E-03 | 3.05E-03 |
| rs11915033 | TCGA&TCGA_coCNV | 3.82E-03 | 1.83E-03 |
| rs11939291 | TCGA&TCGA_coCNV | 3.82E-03 | 8.68E-03 |
| rs9920411 | TCGA&TCGA_coCNV | 2.30E-03 | 1.35E-03 |
| rs5908146 | TCGA&TCGA_coCNV | 3.82E-03 | 2.10E-03 |
| rs11119445 | TCGA&TCGA_coCNV | 3.82E-03 | 9.35E-03 |
| rs10826385 | TCGA&TCGA_coCNV | 3.82E-03 | 5.97E-03 |
| rs11177921 | TCGA&TCGA_coCNV | 3.82E-03 | 6.29E-03 |
| rs4772121 | TCGA | 3.82E-03 | #N/A |
| rs17073310 | TCGA | 3.82E-03 | #N/A |
| rs7313721 | TCGA | 3.82E-03 | #N/A |
| rs6544586 | TCGA | 3.83E-03 | #N/A |
| rs752724 | TCGA&TCGA_coCNV | 3.83E-03 | 3.55E-03 |
| rs9549825 | TCGA | 3.83E-03 | #N/A |
| rs1009528 | TCGA | 3.83E-03 | #N/A |
| rs433778 | TCGA&TCGA_coCNV | 3.83E-03 | 6.33E-03 |
| rs2068368 | TCGA | 3.83E-03 | #N/A |

| rs1397253 | TCGA | 3.83E-03 | #N/A |
| --- | --- | --- | --- |
| rs6104699 | TCGA&TCGA_coCNV | 3.83E-03 | 2.56E-03 |
| rs670146 | TCGA | 3.83E-03 | #N/A |
| rs17067132 | TCGA&TCGA_coCNV | 3.83E-03 | 1.42E-03 |
| rs12590902 | TCGA&TCGA_coCNV | 7.34E-04 | 4.41E-04 |
| rs7152252 | TCGA | 3.83E-03 | #N/A |
| rs1701643 | TCGA&TCGA_coCNV | 3.84E-03 | 1.31E-03 |
| rs10488722 | TCGA&TCGA_coCNV | 5.73E-04 | 1.43E-03 |
| rs9377800 | TCGA&TCGA_coCNV | 3.84E-03 | 6.52E-03 |
| rs11776549 | TCGA&TCGA_coCNV | 2.11E-05 | 2.02E-12 |
| rs17361887 | TCGA&TCGA_coCNV | 3.84E-03 | 2.21E-03 |
| rs1414924 | TCGA&TCGA_coCNV | 3.84E-03 | 7.24E-03 |
| rs959244 | TCGA&TCGA_coCNV | 3.84E-03 | 5.47E-04 |
| rs16937736 | TCGA | 3.84E-03 | #N/A |
| rs354063 | TCGA&TCGA_coCNV | 3.84E-03 | 1.83E-03 |
| rs3735733 | TCGA | 3.84E-03 | #N/A |
| rs508371 | TCGA&TCGA_coCNV | 6.22E-04 | 6.18E-06 |
| rs11911346 | TCGA | 3.84E-03 | #N/A |
| rs10800619 | TCGA | 3.84E-03 | #N/A |
| rs7399173 | TCGA&TCGA_coCNV | 3.84E-03 | 4.39E-03 |
| rs2484918 | TCGA | 3.84E-03 | #N/A |
| rs2491006 | TCGA&TCGA_coCNV | 3.85E-03 | 2.04E-03 |
| rs768361 | TCGA | 1.95E-03 | #N/A |
| rs6878849 | TCGA | 3.85E-03 | #N/A |
| rs10829381 | TCGA | 3.85E-03 | #N/A |
| rs9878957 | TCGA | 3.85E-03 | #N/A |
| rs8008377 | TCGA | 3.85E-03 | #N/A |
| rs1917365 | TCGA | 3.85E-03 | #N/A |
| rs6762516 | TCGA&TCGA_coCNV | 3.85E-03 | 2.45E-03 |
| rs12920413 | TCGA&TCGA_coCNV | 7.36E-04 | 6.13E-04 |
| rs7134391 | TCGA&TCGA_coCNV | 3.85E-03 | 7.95E-03 |
| rs5743176 | TCGA&TCGA_coCNV | 1.80E-03 | 3.18E-04 |
| rs1126098 | TCGA&TCGA_coCNV | 2.27E-03 | 2.08E-03 |
| rs13270169 | TCGA | 3.85E-03 | #N/A |
| rs4921209 | TCGA&TCGA_coCNV | 3.85E-03 | 6.94E-03 |
| rs4556232 | TCGA&TCGA_coCNV | 3.86E-03 | 2.94E-03 |
| rs211917 | TCGA | 3.86E-03 | #N/A |
| rs1950431 | TCGA | 3.86E-03 | #N/A |
| rs1915220 | TCGA&TCGA_coCNV | 3.86E-03 | 1.34E-03 |
| rs7002869 | TCGA | 3.86E-03 | #N/A |
| rs1654794 | TCGA&TCGA_coCNV | 3.86E-03 | 5.94E-03 |
| rs568434 | TCGA | 3.86E-03 | #N/A |
| rs9969972 | TCGA&TCGA_coCNV | 1.93E-03 | 4.24E-04 |
| rs6538945 | TCGA | 3.86E-03 | #N/A |
| rs11052729 | TCGA | 2.92E-03 | #N/A |
| rs2033069 | TCGA | 3.86E-03 | #N/A |

| rs6596465 | TCGA | 3.87E-03 | #N/A |
| --- | --- | --- | --- |
| rs10742725 | TCGA | 3.87E-03 | #N/A |
| rs7316021 | TCGA | 3.87E-03 | #N/A |
| rs1456242 | TCGA | 3.87E-03 | #N/A |
| rs199431 | TCGA&TCGA_coCNV | 3.32E-03 | 2.54E-03 |
| rs472067 | TCGA | 3.87E-03 | #N/A |
| rs17424192 | TCGA&TCGA_coCNV | 1.47E-03 | 3.86E-04 |
| rs1898960 | TCGA&TCGA_coCNV | 3.87E-03 | 7.19E-04 |
| rs259994 | TCGA&TCGA_coCNV | 3.87E-03 | 3.29E-04 |
| rs1054743 | TCGA&TCGA_coCNV | 3.87E-03 | 8.09E-03 |
| rs7111701 | TCGA&SCANdb | 2.99E-03 | #N/A |
| rs4811706 | TCGA&TCGA_coCNV | 3.88E-03 | 7.31E-03 |
| rs2565661 | TCGA&TCGA_coCNV | 2.51E-04 | 3.12E-04 |
| rs4973518 | TCGA | 3.88E-03 | #N/A |
| rs3784599 | TCGA&TCGA_coCNV | 3.88E-03 | 1.87E-03 |
| rs2238087 | TCGA | 3.88E-03 | #N/A |
| rs8012955 | TCGA | 3.88E-03 | #N/A |
| rs7014409 | TCGA&TCGA_coCNV | 3.88E-03 | 7.25E-03 |
| rs2243632 | TCGA&TCGA_coCNV | 3.88E-03 | 4.27E-03 |
| rs7902635 | TCGA&TCGA_coCNV | 3.88E-03 | 5.22E-03 |
| rs4354102 | TCGA | 3.88E-03 | #N/A |
| rs581769 | TCGA&TCGA_coCNV | 3.89E-03 | 9.39E-03 |
| rs9313814 | TCGA&TCGA_coCNV | 3.84E-03 | 8.55E-03 |
| rs17561221 | TCGA | 3.89E-03 | #N/A |
| rs9926131 | TCGA&TCGA_coCNV | 3.89E-03 | 1.42E-03 |
| rs12216096 | TCGA&TCGA_coCNV | 3.89E-03 | 6.52E-03 |
| rs9487408 | TCGA | 3.89E-03 | #N/A |
| rs17594523 | TCGA | 2.68E-03 | #N/A |
| rs9394299 | TCGA | 3.89E-03 | #N/A |
| rs11080764 | TCGA&TCGA_coCNV | 3.89E-03 | 4.27E-03 |
| rs11016224 | TCGA&TCGA_coCNV | 1.12E-03 | 5.96E-04 |
| rs1466835 | TCGA | 2.28E-03 | #N/A |
| rs12761112 | TCGA | 3.89E-03 | #N/A |
| rs1463151 | TCGA | 3.89E-03 | #N/A |
| rs4478368 | TCGA&TCGA_coCNV | 3.89E-03 | 4.43E-03 |
| rs17075073 | TCGA&TCGA_coCNV | 3.56E-03 | 1.37E-04 |
| rs882083 | TCGA | 3.89E-03 | #N/A |
| rs17126807 | TCGA&TCGA_coCNV | 3.89E-03 | 2.70E-03 |
| rs7673317 | TCGA&TCGA_coCNV | 3.89E-03 | 2.82E-03 |
| rs6809775 | TCGA&TCGA_coCNV | 3.89E-03 | 4.51E-03 |
| rs12061474 | TCGA | 3.90E-03 | #N/A |
| rs643491 | TCGA | 3.90E-03 | #N/A |
| rs1202468 | TCGA | 3.90E-03 | #N/A |
| rs17705985 | TCGA | 3.90E-03 | #N/A |
| rs6702469 | TCGA&TCGA_coCNV | 3.90E-03 | 4.16E-04 |
| rs6464182 | TCGA | 3.90E-03 | #N/A |

| rs12820860 | TCGA | 1.88E-03 | #N/A |
| --- | --- | --- | --- |
| rs11168359 | TCGA&TCGA_coCNV | 1.73E-03 | 3.62E-04 |
| rs8099530 | TCGA&TCGA_coCNV | 3.90E-03 | 7.20E-03 |
| rs8068882 | TCGA | 3.90E-03 | #N/A |
| rs7718814 | TCGA | 3.04E-03 | #N/A |
| rs1547924 | TCGA&TCGA_coCNV | 3.58E-03 | 3.44E-03 |
| rs10908901 | TCGA&TCGA_coCNV | 3.90E-03 | 2.45E-03 |
| rs6472718 | TCGA&TCGA_coCNV | 3.90E-03 | 6.15E-03 |
| rs12261188 | TCGA&TCGA_coCNV | 3.90E-03 | 7.39E-04 |
| rs720889 | TCGA | 1.90E-03 | #N/A |
| rs10516463 | TCGA&TCGA_coCNV | 9.24E-04 | 2.22E-04 |
| rs17075051 | TCGA&TCGA_coCNV | 3.91E-03 | 3.50E-03 |
| rs3027634 | TCGA&TCGA_coCNV | 3.91E-03 | 7.96E-03 |
| rs4689990 | TCGA&TCGA_coCNV | 2.72E-03 | 2.48E-03 |
| rs5910125 | TCGA | 3.91E-03 | #N/A |
| rs11895045 | TCGA&TCGA_coCNV | 3.91E-03 | 1.24E-03 |
| rs6597699 | TCGA | 3.91E-03 | #N/A |
| rs760819 | TCGA&TCGA_coCNV | 1.69E-04 | 5.04E-03 |
| rs7527613 | TCGA&TCGA_coCNV | 3.91E-03 | 3.79E-03 |
| rs11130603 | TCGA&TCGA_coCNV | 3.56E-04 | 3.09E-04 |
| rs2274609 | TCGA&TCGA_coCNV | 3.91E-03 | 3.95E-03 |
| rs11415 | TCGA | 3.91E-03 | #N/A |
| rs13223359 | TCGA&TCGA_coCNV | 3.92E-03 | 7.42E-03 |
| rs12558682 | TCGA | 3.92E-03 | #N/A |
| rs11043075 | TCGA&TCGA_coCNV | 3.92E-03 | 4.95E-03 |
| rs4888974 | TCGA&TCGA_coCNV | 3.92E-03 | 7.76E-04 |
| rs12132872 | TCGA&TCGA_coCNV | 3.92E-03 | 2.13E-03 |
| rs12607048 | TCGA | 3.92E-03 | #N/A |
| rs6737832 | TCGA&TCGA_coCNV | 3.92E-03 | 6.09E-03 |
| rs235700 | TCGA&TCGA_coCNV | 7.20E-04 | 1.15E-03 |
| rs4308508 | TCGA&TCGA_coCNV | 3.92E-03 | 3.91E-03 |
| rs7464083 | TCGA | 3.92E-03 | #N/A |
| rs213093 | TCGA&TCGA_coCNV | 3.92E-03 | 2.24E-03 |
| rs1358713 | TCGA | 3.92E-03 | #N/A |
| rs17081078 | TCGA&TCGA_coCNV | 3.92E-03 | 1.71E-03 |
| rs825541 | TCGA&TCGA_coCNV | 3.92E-03 | 7.51E-03 |
| rs17689718 | TCGA&TCGA_coCNV | 3.92E-03 | 5.02E-03 |
| rs7330666 | TCGA&TCGA_coCNV | 3.93E-03 | 9.24E-03 |
| rs2267654 | TCGA | 3.93E-03 | #N/A |
| rs16832534 | TCGA&TCGA_coCNV | 3.93E-03 | 7.67E-03 |
| rs6698613 | TCGA | 3.93E-03 | #N/A |
| rs5908868 | TCGA&TCGA_coCNV | 3.93E-03 | 6.48E-03 |
| rs4398624 | TCGA | 3.93E-03 | #N/A |
| rs3792580 | TCGA&TCGA_coCNV | 3.93E-03 | 5.26E-05 |
| rs4697607 | TCGA | 3.93E-03 | #N/A |
| rs4578396 | TCGA&TCGA_coCNV | 5.32E-04 | 6.43E-03 |

| rs644992 | TCGA&TCGA_coCNV | 3.93E-03 | 7.85E-03 |
| --- | --- | --- | --- |
| rs2208989 | TCGA | 3.93E-03 | #N/A |
| rs11064933 | TCGA&TCGA_coCNV | 3.93E-03 | 6.63E-04 |
| rs1035778 | TCGA&TCGA_coCNV | 3.93E-03 | 1.24E-03 |
| rs12688712 | TCGA&TCGA_coCNV | 3.93E-03 | 8.86E-03 |
| rs2340456 | TCGA | 8.26E-04 | #N/A |
| rs6091949 | TCGA&TCGA_coCNV | 3.93E-03 | 4.51E-03 |
| rs4342964 | TCGA&TCGA_coCNV | 3.94E-03 | 3.27E-03 |
| rs2417269 | TCGA&TCGA_coCNV | 3.16E-03 | 1.15E-03 |
| rs1965 | TCGA | 3.94E-03 | #N/A |
| rs9557024 | TCGA&TCGA_coCNV | 3.94E-03 | 2.75E-03 |
| rs6542456 | TCGA&TCGA_coCNV | 3.94E-03 | 6.75E-03 |
| rs6863010 | TCGA&TCGA_coCNV | 3.94E-03 | 8.87E-03 |
| rs10781409 | TCGA&TCGA_coCNV | 3.94E-03 | 7.02E-03 |
| rs7230580 | TCGA | 3.94E-03 | #N/A |
| rs4541164 | TCGA&TCGA_coCNV | 1.01E-03 | 6.98E-03 |
| rs10495889 | TCGA&TCGA_coCNV | 3.94E-03 | 7.49E-04 |
| rs9949409 | TCGA&TCGA_coCNV | 3.94E-03 | 4.08E-03 |
| rs9554219 | TCGA&TCGA_coCNV | 3.95E-03 | 8.92E-03 |
| rs7961554 | TCGA&TCGA_coCNV | 3.95E-03 | 3.26E-04 |
| rs4684823 | TCGA | 3.95E-03 | #N/A |
| rs10954417 | TCGA | 3.95E-03 | #N/A |
| rs11884677 | TCGA&TCGA_coCNV | 3.95E-03 | 9.56E-04 |
| rs722165 | TCGA | 3.95E-03 | #N/A |
| rs2040416 | TCGA&TCGA_coCNV | 3.95E-03 | 3.82E-03 |
| rs7331435 | TCGA&TCGA_coCNV | 2.31E-03 | 1.54E-04 |
| rs289159 | TCGA&TCGA_coCNV | 3.95E-03 | 2.70E-03 |
| rs10813058 | TCGA&TCGA_coCNV | 3.95E-03 | 7.59E-03 |
| rs17083393 | TCGA&TCGA_coCNV | 3.95E-03 | 2.29E-03 |
| rs16856020 | TCGA&TCGA_coCNV | 3.95E-03 | 9.53E-03 |
| rs2421987 | TCGA&TCGA_coCNV | 2.15E-03 | 3.04E-03 |
| rs7143971 | TCGA&TCGA_coCNV | 3.95E-03 | 2.02E-03 |
| rs7301757 | TCGA&TCGA_coCNV | 3.95E-03 | 6.44E-03 |
| rs4830448 | TCGA&TCGA_coCNV | 3.96E-03 | 9.80E-04 |
| rs16968187 | TCGA | 1.28E-03 | #N/A |
| rs12738318 | TCGA&TCGA_coCNV | 3.96E-03 | 6.95E-03 |
| rs13431346 | TCGA&TCGA_coCNV | 3.96E-03 | 7.14E-03 |
| rs9994970 | TCGA&TCGA_coCNV | 3.96E-03 | 2.39E-04 |
| rs16893807 | TCGA&TCGA_coCNV | 2.18E-03 | 5.33E-03 |
| rs12454943 | TCGA&TCGA_coCNV | 3.96E-03 | 7.16E-04 |
| rs12658100 | TCGA | 3.96E-03 | #N/A |
| rs7299713 | TCGA&TCGA_coCNV | 3.96E-03 | 6.16E-04 |
| rs7101197 | TCGA&TCGA_coCNV | 3.97E-03 | 3.87E-03 |
| rs11026993 | TCGA&TCGA_coCNV | 3.92E-03 | 2.18E-03 |
| rs7354999 | TCGA&TCGA_coCNV | 3.97E-03 | 7.72E-03 |
| rs2494506 | TCGA | 3.97E-03 | #N/A |

| rs1703927 | TCGA | 3.97E-03 | #N/A |
| --- | --- | --- | --- |
| rs3848127 | TCGA | 3.97E-03 | #N/A |
| rs7707955 | TCGA&TCGA_coCNV | 3.97E-03 | 3.97E-03 |
| rs7566546 | TCGA&TCGA_coCNV | 3.68E-03 | 4.48E-03 |
| rs7991143 | TCGA | 3.97E-03 | #N/A |
| rs1347012 | TCGA&TCGA_coCNV | 3.97E-03 | 4.51E-03 |
| rs10882364 | TCGA | 3.97E-03 | #N/A |
| rs7906964 | TCGA&TCGA_coCNV | 3.97E-03 | 3.55E-03 |
| rs11144105 | TCGA&TCGA_coCNV | 1.27E-03 | 4.51E-03 |
| rs6899257 | TCGA&TCGA_coCNV | 3.97E-03 | 1.33E-03 |
| rs11650810 | TCGA | 3.97E-03 | #N/A |
| rs11782622 | TCGA&TCGA_coCNV | 3.97E-03 | 8.19E-03 |
| rs756452 | TCGA | 3.97E-03 | #N/A |
| rs4867560 | TCGA&TCGA_coCNV | 3.97E-03 | 6.56E-03 |
| rs618897 | TCGA&TCGA_coCNV | 9.55E-04 | 2.65E-03 |
| rs9581400 | TCGA&TCGA_coCNV | 1.01E-04 | 2.15E-03 |
| rs17092057 | TCGA&TCGA_coCNV | 3.98E-03 | 1.19E-03 |
| rs4680746 | TCGA | 3.98E-03 | #N/A |
| rs9303104 | TCGA | 3.98E-03 | #N/A |
| rs4688966 | TCGA | 3.98E-03 | #N/A |
| rs7155290 | TCGA | 3.98E-03 | #N/A |
| rs2155340 | TCGA | 3.98E-03 | #N/A |
| rs2924286 | TCGA | 3.98E-03 | #N/A |
| rs10989157 | TCGA&TCGA_coCNV | 1.76E-03 | 3.49E-03 |
| rs4420626 | TCGA | 1.08E-03 | #N/A |
| rs6867189 | TCGA&TCGA_coCNV | 3.98E-03 | 6.36E-03 |
| rs10124893 | TCGA&TCGA_coCNV | 3.99E-03 | 1.27E-03 |
| rs6769640 | TCGA&TCGA_coCNV | 3.99E-03 | 1.80E-03 |
| rs7137125 | TCGA&TCGA_coCNV | 2.61E-03 | 6.63E-03 |
| rs574570 | TCGA&TCGA_coCNV | 1.17E-03 | 6.31E-04 |
| rs217635 | TCGA | 3.99E-03 | #N/A |
| rs2027765 | TCGA | 3.99E-03 | #N/A |
| rs1561945 | TCGA | 3.99E-03 | #N/A |
| rs17644426 | TCGA&TCGA_coCNV | 2.65E-04 | 7.22E-03 |
| rs11223996 | TCGA&TCGA_coCNV | 4.00E-03 | 1.59E-03 |
| rs9446521 | TCGA&TCGA_coCNV | 4.00E-03 | 5.45E-03 |
| rs2474365 | TCGA&TCGA_coCNV | 4.00E-03 | 6.17E-03 |
| rs10889201 | TCGA | 4.00E-03 | #N/A |
| rs10808181 | TCGA&TCGA_coCNV | 4.00E-03 | 9.23E-03 |
| rs7454874 | TCGA&TCGA_coCNV | 4.00E-03 | 3.72E-03 |
| rs17131910 | TCGA&TCGA_coCNV | 1.44E-03 | 6.21E-03 |
| rs16902577 | TCGA&TCGA_coCNV | 1.21E-03 | 3.69E-04 |
| rs41458047 | TCGA&TCGA_coCNV | 4.00E-03 | 8.67E-04 |
| rs1531497 | TCGA&TCGA_coCNV | 4.00E-03 | 5.47E-03 |
| rs16860805 | TCGA&TCGA_coCNV | 4.00E-03 | 2.25E-03 |
| rs1620675 | TCGA&TCGA_coCNV | 6.22E-04 | 5.18E-03 |

| rs4801411 | TCGA&TCGA_coCNV | 6.84E-04 | 3.40E-03 |
| --- | --- | --- | --- |
| rs6703187 | TCGA | 1.64E-03 | #N/A |
| rs5990898 | TCGA&TCGA_coCNV | 4.01E-03 | 2.21E-03 |
| rs34319350 | TCGA&TCGA_coCNV | 4.01E-03 | 5.66E-03 |
| rs4868208 | TCGA | 4.01E-03 | #N/A |
| rs10513625 | TCGA | 4.01E-03 | #N/A |
| rs2643425 | TCGA&TCGA_coCNV | 1.31E-03 | 4.35E-05 |
| rs2962612 | TCGA&TCGA_coCNV | 2.66E-04 | 6.20E-03 |
| rs10787039 | TCGA | 4.01E-03 | #N/A |
| rs2699313 | TCGA&TCGA_coCNV | 4.01E-03 | 4.69E-03 |
| rs1339517 | TCGA | 4.01E-03 | #N/A |
| rs1997378 | TCGA&TCGA_coCNV | 4.01E-03 | 8.76E-04 |
| rs16856809 | TCGA&TCGA_coCNV | 1.76E-03 | 9.24E-03 |
| rs17646980 | TCGA&TCGA_coCNV | 4.02E-03 | 8.27E-03 |
| rs519175 | TCGA | 4.02E-03 | #N/A |
| rs3739795 | TCGA&TCGA_coCNV | 4.02E-03 | 1.17E-03 |
| rs16902928 | TCGA&TCGA_coCNV | 4.02E-03 | 3.23E-03 |
| rs2339706 | TCGA | 4.02E-03 | #N/A |
| rs11945509 | TCGA&TCGA_coCNV | 4.02E-03 | 8.30E-03 |
| rs639444 | TCGA | 4.02E-03 | #N/A |
| rs10187986 | TCGA&TCGA_coCNV | 3.06E-03 | 3.99E-03 |
| rs3850090 | TCGA | 4.02E-03 | #N/A |
| rs10927372 | TCGA&TCGA_coCNV | 4.02E-03 | 3.17E-03 |
| rs476241 | TCGA&TCGA_coCNV | 4.02E-03 | 3.58E-03 |
| rs8004548 | TCGA&TCGA_coCNV | 4.02E-03 | 1.20E-03 |
| rs7140732 | TCGA&TCGA_coCNV | 4.03E-03 | 9.28E-03 |
| rs13173916 | TCGA&TCGA_coCNV | 4.03E-03 | 8.76E-03 |
| rs570858 | TCGA&TCGA_coCNV | 4.03E-03 | 1.19E-04 |
| rs568089 | TCGA | 4.03E-03 | #N/A |
| rs10884533 | TCGA | 4.03E-03 | #N/A |
| rs4726540 | TCGA | 4.03E-03 | #N/A |
| rs4490352 | TCGA&TCGA_coCNV | 4.03E-03 | 4.03E-03 |
| rs12277199 | TCGA&TCGA_coCNV | 4.03E-03 | 9.61E-03 |
| rs10851223 | TCGA | 3.20E-03 | #N/A |
| rs7625148 | TCGA | 4.03E-03 | #N/A |
| rs606618 | TCGA | 4.04E-03 | #N/A |
| rs13406905 | TCGA&TCGA_coCNV | 4.04E-03 | 1.14E-03 |
| rs17131052 | TCGA | 4.04E-03 | #N/A |
| rs17145153 | TCGA&TCGA_coCNV | 1.08E-03 | 1.77E-03 |
| rs7648955 | TCGA&TCGA_coCNV | 2.67E-04 | 5.52E-04 |
| rs8066596 | TCGA | 4.04E-03 | #N/A |
| rs4885507 | TCGA&TCGA_coCNV | 4.04E-03 | 1.16E-03 |
| rs11614423 | TCGA | 4.04E-03 | #N/A |
| rs3792252 | TCGA&TCGA_coCNV | 9.34E-04 | 3.62E-03 |
| rs11594360 | TCGA | 4.04E-03 | #N/A |
| rs16933630 | TCGA&TCGA_coCNV | 2.53E-03 | 7.37E-03 |

| rs8074072 | TCGA&TCGA_coCNV | 3.41E-04 | 7.85E-04 |
| --- | --- | --- | --- |
| rs17458287 | TCGA | 4.04E-03 | #N/A |
| rs10494027 | TCGA | 4.04E-03 | #N/A |
| rs10196949 | TCGA&TCGA_coCNV | 4.05E-03 | 1.90E-03 |
| rs13160778 | TCGA | 4.05E-03 | #N/A |
| rs7893337 | TCGA | 4.05E-03 | #N/A |
| rs4654839 | TCGA&TCGA_coCNV | 4.05E-03 | 5.72E-03 |
| rs9986143 | TCGA&TCGA_coCNV | 4.05E-03 | 9.21E-04 |
| rs10484930 | TCGA&TCGA_coCNV | 4.05E-03 | 1.65E-03 |
| rs4646786 | TCGA | 4.05E-03 | #N/A |
| rs1225709 | TCGA&TCGA_coCNV | 1.82E-03 | 1.65E-03 |
| rs16831992 | TCGA&TCGA_coCNV | 4.05E-03 | 8.03E-03 |
| rs12686439 | TCGA&TCGA_coCNV | 4.05E-03 | 7.16E-03 |
| rs16870033 | TCGA | 4.05E-03 | #N/A |
| rs2992629 | TCGA | 4.05E-03 | #N/A |
| rs829237 | TCGA | 4.05E-03 | #N/A |
| rs759341 | TCGA&TCGA_coCNV | 3.55E-03 | 8.09E-03 |
| rs1524377 | TCGA&TCGA_coCNV | 4.05E-03 | 8.24E-04 |
| rs9400393 | TCGA | 4.05E-03 | #N/A |
| rs11242729 | TCGA&TCGA_coCNV | 4.05E-03 | 1.97E-03 |
| rs11151792 | TCGA | 4.05E-03 | #N/A |
| rs16859371 | TCGA&TCGA_coCNV | 4.05E-03 | 3.57E-03 |
| rs10860211 | TCGA&TCGA_coCNV | 4.06E-03 | 4.19E-03 |
| rs2123526 | TCGA | 4.06E-03 | #N/A |
| rs4322405 | TCGA&TCGA_coCNV | 4.06E-03 | 2.84E-03 |
| rs2944520 | TCGA | 4.06E-03 | #N/A |
| rs2943657 | TCGA&TCGA_coCNV | 1.29E-03 | 1.13E-03 |
| rs12216685 | TCGA&TCGA_coCNV | 4.06E-03 | 2.61E-03 |
| rs7762412 | TCGA | 4.06E-03 | #N/A |
| rs2618988 | TCGA&TCGA_coCNV | 4.06E-03 | 8.10E-04 |
| rs11069725 | TCGA | 4.06E-03 | #N/A |
| rs11150443 | TCGA | 4.06E-03 | #N/A |
| rs16926123 | TCGA&TCGA_coCNV | 3.77E-04 | 3.90E-03 |
| rs13389321 | TCGA | 4.06E-03 | #N/A |
| rs10485317 | TCGA&TCGA_coCNV | 3.91E-03 | 1.60E-03 |
| rs152377 | TCGA&TCGA_coCNV | 4.06E-03 | 6.61E-03 |
| rs16989109 | TCGA&TCGA_coCNV | 4.07E-03 | 8.91E-04 |
| rs7222805 | TCGA | 4.07E-03 | #N/A |
| rs7157903 | TCGA&TCGA_coCNV | 4.07E-03 | 1.57E-04 |
| rs912669 | TCGA&TCGA_coCNV | 4.07E-03 | 3.42E-03 |
| rs7273200 | TCGA&TCGA_coCNV | 2.20E-03 | 1.17E-03 |
| rs1928155 | TCGA | 4.07E-03 | #N/A |
| rs2733499 | TCGA&TCGA_coCNV | 4.07E-03 | 1.37E-03 |
| rs6142005 | TCGA&TCGA_coCNV | 4.07E-03 | 3.53E-03 |
| rs4320963 | TCGA | 4.07E-03 | #N/A |
| rs864708 | TCGA | 4.07E-03 | #N/A |

| rs11045758 | TCGA&TCGA_coCNV | 2.70E-03 | 3.68E-04 |
| --- | --- | --- | --- |
| rs487276 | TCGA | 4.08E-03 | #N/A |
| rs979278 | TCGA | 1.40E-03 | #N/A |
| rs12566963 | TCGA | 4.08E-03 | #N/A |
| rs9276964 | TCGA | 4.08E-03 | #N/A |
| rs11824255 | TCGA&TCGA_coCNV | 4.98E-04 | 4.11E-04 |
| rs2189090 | TCGA&TCGA_coCNV | 4.08E-03 | 1.31E-03 |
| rs10934643 | TCGA | 4.08E-03 | #N/A |
| rs4919557 | TCGA&TCGA_coCNV | 4.08E-03 | 7.24E-03 |
| rs2886671 | TCGA | 4.08E-03 | #N/A |
| rs3766779 | TCGA | 4.08E-03 | #N/A |
| rs4678103 | TCGA | 4.08E-03 | #N/A |
| rs17564624 | TCGA&TCGA_coCNV | 4.08E-03 | 6.92E-03 |
| rs7748298 | TCGA | 4.08E-03 | #N/A |
| rs12419137 | TCGA | 3.29E-03 | #N/A |
| rs7898970 | TCGA | 4.09E-03 | #N/A |
| rs10504839 | TCGA | 4.09E-03 | #N/A |
| rs11657249 | TCGA | 4.09E-03 | #N/A |
| rs4303371 | TCGA | 4.09E-03 | #N/A |
| rs458029 | TCGA | 1.84E-03 | #N/A |
| rs10490763 | TCGA&TCGA_coCNV | 4.09E-03 | 2.08E-03 |
| rs6577622 | TCGA&TCGA_coCNV | 4.09E-03 | 7.29E-03 |
| rs522732 | TCGA | 4.09E-03 | #N/A |
| rs1923964 | TCGA&TCGA_coCNV | 4.09E-03 | 5.48E-04 |
| rs739122 | TCGA | 4.09E-03 | #N/A |
| rs1074155 | TCGA | 4.09E-03 | #N/A |
| rs9407902 | TCGA | 4.09E-03 | #N/A |
| rs6594298 | TCGA&TCGA_coCNV | 4.09E-03 | 2.19E-03 |
| rs13118846 | TCGA | 4.09E-03 | #N/A |
| rs1670339 | TCGA&TCGA_coCNV | 4.09E-03 | 6.29E-03 |
| rs1523209 | TCGA | 4.09E-03 | #N/A |
| rs3844280 | TCGA | 4.09E-03 | #N/A |
| rs16949390 | TCGA | 4.09E-03 | #N/A |
| rs9826728 | TCGA | 4.09E-03 | #N/A |
| rs9366956 | TCGA&TCGA_coCNV | 4.10E-03 | 5.45E-03 |
| rs11218782 | TCGA&TCGA_coCNV | 4.10E-03 | 1.54E-03 |
| rs8041437 | TCGA | 4.10E-03 | #N/A |
| rs13203542 | TCGA | 4.10E-03 | #N/A |
| rs2911266 | TCGA&TCGA_coCNV | 9.18E-06 | 6.30E-04 |
| rs6692738 | TCGA&TCGA_coCNV | 3.53E-03 | 3.48E-03 |
| rs3138107 | TCGA&TCGA_coCNV | 7.62E-05 | 2.56E-03 |
| rs2699323 | TCGA&TCGA_coCNV | 4.10E-03 | 1.10E-03 |
| rs11753875 | TCGA&TCGA_coCNV | 1.09E-03 | 1.79E-03 |
| rs2141418 | TCGA | 4.10E-03 | #N/A |
| rs3845691 | TCGA&TCGA_coCNV | 5.17E-04 | 5.06E-03 |
| rs6753727 | TCGA&TCGA_coCNV | 4.10E-03 | 2.14E-03 |

| rs1353868 | TCGA | 4.10E-03 | #N/A |
| --- | --- | --- | --- |
| rs12436408 | TCGA | 4.11E-03 | #N/A |
| rs11132429 | TCGA&TCGA_coCNV | 2.06E-03 | 3.24E-03 |
| rs16864337 | TCGA | 4.11E-03 | #N/A |
| rs4920374 | TCGA&TCGA_coCNV | 7.72E-04 | 1.33E-03 |
| rs12579067 | TCGA&TCGA_coCNV | 4.11E-03 | 5.05E-03 |
| rs2913363 | TCGA&TCGA_coCNV | 4.11E-03 | 3.02E-03 |
| rs11575722 | TCGA&TCGA_coCNV | 4.11E-03 | 2.92E-03 |
| rs1393573 | TCGA&TCGA_coCNV | 4.11E-03 | 4.63E-03 |
| rs1441891 | TCGA | 4.11E-03 | #N/A |
| rs1112837 | TCGA | 4.11E-03 | #N/A |
| rs1569819 | TCGA&TCGA_coCNV | 4.12E-03 | 9.87E-03 |
| rs3093054 | TCGA | 4.12E-03 | #N/A |
| rs17726577 | TCGA | 4.12E-03 | #N/A |
| rs5953014 | TCGA&TCGA_coCNV | 4.12E-03 | 9.93E-04 |
| rs9894103 | TCGA&TCGA_coCNV | 4.12E-03 | 9.30E-04 |
| rs2731502 | TCGA&TCGA_coCNV | 5.36E-04 | 8.61E-05 |
| rs7244811 | TCGA&TCGA_coCNV | 4.12E-03 | 3.45E-03 |
| rs1319365 | TCGA | 2.69E-03 | #N/A |
| rs2938081 | TCGA&TCGA_coCNV | 4.12E-03 | 1.15E-03 |
| rs573894 | TCGA | 4.12E-03 | #N/A |
| rs2707879 | TCGA | 4.12E-03 | #N/A |
| rs11776828 | TCGA | 4.13E-03 | #N/A |
| rs17510191 | TCGA | 4.13E-03 | #N/A |
| rs4871463 | TCGA&TCGA_coCNV | 4.13E-03 | 1.93E-03 |
| rs1566158 | TCGA&TCGA_coCNV | 4.13E-03 | 9.41E-03 |
| rs10104849 | TCGA&TCGA_coCNV | 4.13E-03 | 9.85E-04 |
| rs7837914 | TCGA&TCGA_coCNV | 4.13E-03 | 2.06E-03 |
| rs1822470 | TCGA&TCGA_coCNV | 4.03E-04 | 4.03E-03 |
| rs1792361 | TCGA&TCGA_coCNV | 4.13E-03 | 3.31E-03 |
| rs921891 | TCGA&TCGA_coCNV | 3.46E-03 | 3.46E-03 |
| rs10280280 | TCGA&TCGA_coCNV | 4.13E-03 | 2.67E-03 |
| rs1396416 | TCGA&TCGA_coCNV | 4.13E-03 | 1.85E-03 |
| rs12650956 | TCGA&TCGA_coCNV | 4.13E-03 | 1.58E-03 |
| rs12624885 | TCGA&TCGA_coCNV | 4.13E-03 | 3.66E-03 |
| rs6858738 | TCGA | 4.13E-03 | #N/A |
| rs10788668 | TCGA&TCGA_coCNV | 4.13E-03 | 7.75E-03 |
| rs36845 | TCGA&TCGA_coCNV | 2.85E-03 | 9.41E-03 |
| rs944805 | TCGA | 4.14E-03 | #N/A |
| rs1408617 | TCGA | 4.14E-03 | #N/A |
| rs9324051 | TCGA | 4.14E-03 | #N/A |
| rs2949824 | TCGA&TCGA_coCNV | 4.14E-03 | 1.33E-03 |
| rs7178362 | TCGA&TCGA_coCNV | 3.80E-03 | 5.97E-04 |
| rs1148421 | TCGA&TCGA_coCNV | 7.81E-04 | 6.04E-04 |
| rs5931407 | TCGA&TCGA_coCNV | 4.14E-03 | 9.67E-04 |
| rs3788309 | TCGA&TCGA_coCNV | 4.14E-03 | 7.29E-04 |

| rs3774767 | TCGA&TCGA_coCNV | 4.14E-03 | 2.17E-03 |
| --- | --- | --- | --- |
| rs11780296 | TCGA&TCGA_coCNV | 4.14E-03 | 2.47E-03 |
| rs12492530 | TCGA&TCGA_coCNV | 4.14E-03 | 4.09E-03 |
| rs1769795 | TCGA&TCGA_coCNV | 4.14E-03 | 5.13E-03 |
| rs12786766 | TCGA&TCGA_coCNV | 4.14E-03 | 3.56E-03 |
| rs12820630 | TCGA&TCGA_coCNV | 4.14E-03 | 6.71E-03 |
| rs1175114 | TCGA | 4.14E-03 | #N/A |
| rs7919203 | TCGA&TCGA_coCNV | 1.86E-03 | 5.14E-03 |
| rs9522789 | TCGA | 4.15E-03 | #N/A |
| rs4850780 | TCGA | 4.15E-03 | #N/A |
| rs6448034 | TCGA | 4.15E-03 | #N/A |
| rs3733935 | TCGA | 4.15E-03 | #N/A |
| rs7984646 | TCGA | 4.15E-03 | #N/A |
| rs16891075 | TCGA&TCGA_coCNV | 4.15E-03 | 2.52E-03 |
| rs8109787 | TCGA | 4.15E-03 | #N/A |
| rs1797397 | TCGA | 4.15E-03 | #N/A |
| rs13200447 | TCGA&TCGA_coCNV | 4.15E-03 | 2.48E-03 |
| rs10260319 | TCGA&TCGA_coCNV | 4.15E-03 | 9.12E-03 |
| rs4655973 | TCGA&TCGA_coCNV | 4.15E-03 | 1.74E-04 |
| rs209384 | TCGA | 4.15E-03 | #N/A |
| rs7945912 | TCGA | 4.15E-03 | #N/A |
| rs7489681 | TCGA | 4.15E-03 | #N/A |
| rs4774271 | TCGA | 4.15E-03 | #N/A |
| rs10872876 | TCGA&TCGA_coCNV | 4.15E-03 | 2.78E-03 |
| rs4936770 | TCGA | 4.15E-03 | #N/A |
| rs9606655 | TCGA&TCGA_coCNV | 2.80E-06 | 3.20E-05 |
| rs5758010 | TCGA | 4.16E-03 | #N/A |
| rs6855490 | TCGA | 4.16E-03 | #N/A |
| rs9572197 | TCGA | 4.16E-03 | #N/A |
| rs1233811 | TCGA&TCGA_coCNV | 4.16E-03 | 8.67E-04 |
| rs9384514 | TCGA&TCGA_coCNV | 4.16E-03 | 9.37E-03 |
| rs2157903 | TCGA&TCGA_coCNV | 4.16E-03 | 1.81E-03 |
| rs41508450 | TCGA&TCGA_coCNV | 2.02E-03 | 9.52E-03 |
| rs6741439 | TCGA&TCGA_coCNV | 4.16E-03 | 7.86E-03 |
| rs10810618 | TCGA | 4.16E-03 | #N/A |
| rs311596 | TCGA&TCGA_coCNV | 4.16E-03 | 7.54E-03 |
| rs10121251 | TCGA&TCGA_coCNV | 4.16E-03 | 3.32E-04 |
| rs13417747 | TCGA&TCGA_coCNV | 1.80E-03 | 2.31E-03 |
| rs4710538 | TCGA&TCGA_coCNV | 4.17E-03 | 9.28E-03 |
| rs283358 | TCGA | 4.17E-03 | #N/A |
| rs659832 | TCGA | 4.17E-03 | #N/A |
| rs1343190 | TCGA&TCGA_coCNV | 8.91E-04 | 8.48E-04 |
| rs2936504 | TCGA | 4.17E-03 | #N/A |
| rs6725609 | TCGA | 4.17E-03 | #N/A |
| rs10161146 | TCGA | 4.17E-03 | #N/A |
| rs13411746 | TCGA | 4.17E-03 | #N/A |

| rs4528269 | TCGA&TCGA_coCNV | 4.17E-03 | 1.97E-03 |
| --- | --- | --- | --- |
| rs7248363 | TCGA&TCGA_coCNV | 4.17E-03 | 5.25E-03 |
| rs1544355 | TCGA&TCGA_coCNV | 4.17E-03 | 7.51E-03 |
| rs7741827 | TCGA&TCGA_coCNV | 1.59E-03 | 1.66E-03 |
| rs11193592 | TCGA&TCGA_coCNV | 2.25E-03 | 6.37E-03 |
| rs7653275 | TCGA&TCGA_coCNV | 4.18E-03 | 9.75E-03 |
| rs11259939 | TCGA&TCGA_coCNV | 4.18E-03 | 9.27E-04 |
| rs7514506 | TCGA&TCGA_coCNV | 4.18E-03 | 4.46E-03 |
| rs2157479 | TCGA&TCGA_coCNV | 4.18E-03 | 7.64E-03 |
| rs678004 | TCGA | 4.18E-03 | #N/A |
| rs1510978 | TCGA&TCGA_coCNV | 4.18E-03 | 1.93E-03 |
| rs11258934 | TCGA&TCGA_coCNV | 4.18E-03 | 8.54E-03 |
| rs9575095 | TCGA | 4.18E-03 | #N/A |
| rs2732529 | TCGA | 4.18E-03 | #N/A |
| rs7041951 | TCGA&TCGA_coCNV | 4.18E-03 | 4.82E-04 |
| rs4850056 | TCGA&TCGA_coCNV | 4.18E-03 | 1.73E-03 |
| rs17032095 | TCGA&TCGA_coCNV | 4.18E-03 | 1.07E-03 |
| rs4828603 | TCGA&TCGA_coCNV | 2.82E-03 | 4.06E-04 |
| rs35406 | TCGA&TCGA_coCNV | 4.18E-03 | 1.15E-03 |
| rs1333984 | TCGA&TCGA_coCNV | 2.73E-03 | 3.34E-03 |
| rs4432654 | TCGA&TCGA_coCNV | 4.19E-03 | 9.74E-03 |
| rs2125635 | TCGA | 4.19E-03 | #N/A |
| rs1477137 | TCGA&TCGA_coCNV | 4.19E-03 | 5.13E-03 |
| rs3797244 | TCGA&TCGA_coCNV | 4.19E-03 | 6.13E-03 |
| rs5934648 | TCGA | 4.19E-03 | #N/A |
| rs1272845 | TCGA | 4.19E-03 | #N/A |
| rs9387705 | TCGA | 4.19E-03 | #N/A |
| rs16909493 | TCGA&TCGA_coCNV | 4.19E-03 | 6.30E-04 |
| rs7130880 | TCGA&TCGA_coCNV | 4.19E-03 | 9.23E-03 |
| rs10957157 | TCGA&TCGA_coCNV | 3.00E-03 | 6.23E-03 |
| rs12203399 | TCGA&TCGA_coCNV | 3.66E-03 | 2.51E-03 |
| rs9961416 | TCGA | 1.77E-03 | #N/A |
| rs4847562 | TCGA | 4.20E-03 | #N/A |
| rs8072096 | TCGA&TCGA_coCNV | 4.20E-03 | 3.11E-03 |
| rs2304945 | TCGA | 4.20E-03 | #N/A |
| rs3755632 | TCGA&TCGA_coCNV | 4.20E-03 | 1.52E-03 |
| rs4887981 | TCGA | 4.20E-03 | #N/A |
| rs12558709 | TCGA&TCGA_coCNV | 3.41E-03 | 1.09E-03 |
| rs7081480 | TCGA | 4.20E-03 | #N/A |
| rs7949489 | TCGA | 4.20E-03 | #N/A |
| rs5954528 | TCGA&TCGA_coCNV | 4.20E-03 | 3.74E-03 |
| rs3103093 | TCGA&TCGA_coCNV | 4.20E-03 | 6.79E-03 |
| rs7967551 | TCGA&TCGA_coCNV | 4.20E-03 | 8.95E-03 |
| rs4865115 | TCGA | 4.21E-03 | #N/A |
| rs3814597 | TCGA&TCGA_coCNV | 4.21E-03 | 2.71E-04 |
| rs3943787 | TCGA&TCGA_coCNV | 4.21E-03 | 9.33E-04 |

| rs825672 | TCGA | 4.21E-03 | #N/A |
| --- | --- | --- | --- |
| rs6889835 | TCGA | 4.21E-03 | #N/A |
| rs151619 | TCGA&TCGA_coCNV | 4.21E-03 | 3.11E-03 |
| rs10834086 | TCGA | 4.21E-03 | #N/A |
| rs11892684 | TCGA | 2.25E-03 | #N/A |
| rs373182 | TCGA | 4.21E-03 | #N/A |
| rs2046471 | TCGA&TCGA_coCNV | 4.21E-03 | 6.91E-03 |
| rs7319342 | TCGA&TCGA_coCNV | 4.21E-03 | 1.98E-05 |
| rs6576752 | TCGA | 4.21E-03 | #N/A |
| rs2301179 | TCGA | 4.21E-03 | #N/A |
| rs17795505 | TCGA&TCGA_coCNV | 2.87E-03 | 6.90E-03 |
| rs1396418 | TCGA&TCGA_coCNV | 4.21E-03 | 1.28E-03 |
| rs4698436 | TCGA&TCGA_coCNV | 4.22E-03 | 6.78E-03 |
| rs514235 | TCGA | 4.22E-03 | #N/A |
| rs1036502 | TCGA | 4.22E-03 | #N/A |
| rs6590764 | TCGA | 4.22E-03 | #N/A |
| rs7121368 | TCGA&TCGA_coCNV | 4.22E-03 | 7.24E-03 |
| rs10948005 | TCGA&TCGA_coCNV | 4.22E-03 | 7.17E-03 |
| rs7200686 | TCGA&TCGA_coCNV | 4.22E-03 | 9.94E-04 |
| rs7753645 | TCGA&TCGA_coCNV | 4.22E-03 | 5.91E-04 |
| rs1558628 | TCGA | 4.23E-03 | #N/A |
| rs12526408 | TCGA | 4.23E-03 | #N/A |
| rs41348246 | TCGA | 4.23E-03 | #N/A |
| rs1923486 | TCGA&TCGA_coCNV | 4.23E-03 | 4.96E-03 |
| rs16958320 | TCGA | 4.23E-03 | #N/A |
| rs1407298 | TCGA | 4.23E-03 | #N/A |
| rs5907230 | TCGA&TCGA_coCNV | 4.23E-03 | 2.40E-03 |
| rs9686689 | TCGA&TCGA_coCNV | 4.23E-03 | 3.90E-03 |
| rs2225409 | TCGA | 4.23E-03 | #N/A |
| rs7424403 | TCGA&TCGA_coCNV | 1.20E-03 | 2.15E-03 |
| rs974251 | TCGA | 4.23E-03 | #N/A |
| rs8043993 | TCGA&TCGA_coCNV | 4.23E-03 | 9.87E-05 |
| rs2287833 | TCGA&TCGA_coCNV | 4.23E-03 | 8.80E-03 |
| rs2537781 | TCGA | 4.24E-03 | #N/A |
| rs9605337 | TCGA | 4.24E-03 | #N/A |
| rs16854671 | TCGA&TCGA_coCNV | 4.24E-03 | 2.85E-03 |
| rs637678 | TCGA | 4.24E-03 | #N/A |
| rs11221510 | TCGA&TCGA_coCNV | 4.24E-03 | 7.74E-03 |
| rs4760261 | TCGA | 4.24E-03 | #N/A |
| rs11631475 | TCGA | 4.24E-03 | #N/A |
| rs745806 | TCGA&TCGA_coCNV | 2.59E-03 | 6.01E-05 |
| rs2469202 | TCGA&TCGA_coCNV | 2.50E-03 | 3.77E-03 |
| rs10494620 | TCGA | 4.24E-03 | #N/A |
| rs6965873 | TCGA | 4.25E-03 | #N/A |
| rs5972475 | TCGA&TCGA_coCNV | 4.25E-03 | 3.46E-03 |
| rs11764174 | TCGA | 4.25E-03 | #N/A |

| rs2022251 | TCGA | 8.72E-04 | #N/A |
| --- | --- | --- | --- |
| rs2758960 | TCGA&TCGA_coCNV | 4.25E-03 | 4.68E-03 |
| rs9875758 | TCGA | 4.25E-03 | #N/A |
| rs9921035 | TCGA | 4.25E-03 | #N/A |
| rs2063584 | TCGA&TCGA_coCNV | 4.25E-03 | 2.32E-03 |
| rs3101338 | TCGA | 4.25E-03 | #N/A |
| rs2626703 | TCGA&TCGA_coCNV | 4.25E-03 | 2.81E-03 |
| rs10411879 | TCGA&TCGA_coCNV | 9.90E-04 | 1.09E-03 |
| rs12143543 | TCGA | 4.25E-03 | #N/A |
| rs7144633 | TCGA | 4.25E-03 | #N/A |
| rs747974 | TCGA&TCGA_coCNV | 4.25E-03 | 2.52E-03 |
| rs4372032 | TCGA&TCGA_coCNV | 4.25E-03 | 2.21E-03 |
| rs7870186 | TCGA | 4.25E-03 | #N/A |
| rs9634953 | TCGA | 4.26E-03 | #N/A |
| rs10034706 | TCGA&TCGA_coCNV | 6.16E-04 | 1.55E-03 |
| rs12044753 | TCGA | 4.26E-03 | #N/A |
| rs10017492 | TCGA | 4.26E-03 | #N/A |
| rs2811796 | TCGA | 4.26E-03 | #N/A |
| rs7498859 | TCGA | 4.26E-03 | #N/A |
| rs10505355 | TCGA | 4.26E-03 | #N/A |
| rs7147083 | TCGA | 4.26E-03 | #N/A |
| rs1244061 | TCGA | 4.26E-03 | #N/A |
| rs10089519 | TCGA | 4.26E-03 | #N/A |
| rs8037598 | TCGA | 4.26E-03 | #N/A |
| rs17278143 | TCGA&TCGA_coCNV | 4.26E-03 | 2.01E-03 |
| rs7242528 | TCGA&TCGA_coCNV | 4.26E-03 | 9.65E-03 |
| rs6421653 | TCGA&TCGA_coCNV | 3.79E-03 | 3.08E-03 |
| rs10799771 | TCGA&TCGA_coCNV | 4.26E-03 | 5.51E-04 |
| rs7071206 | TCGA&TCGA_coCNV | 3.75E-05 | 5.27E-05 |
| rs332105 | TCGA&TCGA_coCNV | 4.27E-03 | 2.60E-03 |
| rs3019218 | TCGA | 4.27E-03 | #N/A |
| rs12684019 | TCGA&TCGA_coCNV | 4.27E-03 | 1.47E-03 |
| rs808178 | TCGA | 4.27E-03 | #N/A |
| rs236827 | TCGA | 4.27E-03 | #N/A |
| rs2187705 | TCGA | 4.27E-03 | #N/A |
| rs17099282 | TCGA&TCGA_coCNV | 4.27E-03 | 5.93E-03 |
| rs7215691 | TCGA&TCGA_coCNV | 1.19E-04 | 1.82E-05 |
| rs10450317 | TCGA&TCGA_coCNV | 4.27E-03 | 4.03E-03 |
| rs9510773 | TCGA | 4.27E-03 | #N/A |
| rs5741800 | TCGA&TCGA_coCNV | 4.27E-03 | 5.84E-03 |
| rs11199345 | TCGA&TCGA_coCNV | 3.41E-03 | 2.80E-03 |
| rs16875899 | TCGA&TCGA_coCNV | 4.28E-03 | 1.56E-03 |
| rs7837991 | TCGA&TCGA_coCNV | 4.28E-03 | 2.63E-03 |
| rs9622678 | TCGA | 4.28E-03 | #N/A |
| rs2267179 | TCGA | 4.28E-03 | #N/A |
| rs3027475 | TCGA&TCGA_coCNV | 4.28E-03 | 5.63E-03 |

| rs16913237 | TCGA&TCGA_coCNV | 4.28E-03 | 4.77E-03 |
| --- | --- | --- | --- |
| rs11172486 | TCGA&TCGA_coCNV | 4.28E-03 | 4.51E-04 |
| rs9950823 | TCGA&TCGA_coCNV | 4.28E-03 | 3.24E-04 |
| rs4746998 | TCGA | 4.28E-03 | #N/A |
| rs1893673 | TCGA | 4.28E-03 | #N/A |
| rs228380 | TCGA | 4.28E-03 | #N/A |
| rs11206455 | TCGA | 4.28E-03 | #N/A |
| rs2064148 | TCGA | 4.28E-03 | #N/A |
| rs11128242 | TCGA | 4.28E-03 | #N/A |
| rs13087350 | TCGA&TCGA_coCNV | 4.29E-03 | 3.79E-03 |
| rs3790461 | TCGA | 4.29E-03 | #N/A |
| rs6833789 | TCGA&TCGA_coCNV | 1.50E-04 | 6.54E-03 |
| rs6922656 | TCGA&TCGA_coCNV | 1.42E-03 | 1.35E-03 |
| rs16869379 | TCGA&TCGA_coCNV | 4.29E-03 | 4.63E-03 |
| rs17648806 | TCGA&TCGA_coCNV | 4.29E-03 | 2.86E-03 |
| rs1870839 | TCGA&TCGA_coCNV | 4.29E-03 | 4.71E-03 |
| rs6627141 | TCGA&TCGA_coCNV | 4.30E-03 | 4.35E-03 |
| rs7213033 | TCGA | 4.30E-03 | #N/A |
| rs986508 | TCGA | 4.30E-03 | #N/A |
| rs13211282 | TCGA | 4.30E-03 | #N/A |
| rs16859071 | TCGA&TCGA_coCNV | 4.30E-03 | 2.12E-03 |
| rs7563869 | TCGA | 4.30E-03 | #N/A |
| rs2080161 | TCGA | 4.30E-03 | #N/A |
| rs1532107 | TCGA&TCGA_coCNV | 4.30E-03 | 1.60E-03 |
| rs2053797 | TCGA | 4.30E-03 | #N/A |
| rs16198 | TCGA | 1.81E-03 | #N/A |
| rs17677397 | TCGA&TCGA_coCNV | 4.30E-03 | 2.41E-03 |
| rs9682904 | TCGA | 1.32E-03 | #N/A |
| rs10080064 | TCGA | 4.30E-03 | #N/A |
| rs9366533 | TCGA&TCGA_coCNV | 4.30E-03 | 4.31E-03 |
| rs11936467 | TCGA | 4.30E-03 | #N/A |
| rs17060061 | TCGA&TCGA_coCNV | 2.43E-04 | 1.85E-03 |
| rs10981874 | TCGA | 4.31E-03 | #N/A |
| rs7943820 | TCGA | 4.31E-03 | #N/A |
| rs17040083 | TCGA | 4.31E-03 | #N/A |
| rs1965297 | TCGA | 2.44E-03 | #N/A |
| rs7144954 | TCGA&TCGA_coCNV | 4.31E-03 | 5.35E-03 |
| rs2107749 | TCGA&TCGA_coCNV | 4.31E-03 | 8.19E-03 |
| rs4828877 | TCGA&TCGA_coCNV | 4.31E-03 | 3.69E-03 |
| rs10508621 | TCGA | 4.31E-03 | #N/A |
| rs6959224 | TCGA&TCGA_coCNV | 4.31E-03 | 7.70E-03 |
| rs8115229 | TCGA | 4.31E-03 | #N/A |
| rs9323206 | TCGA&TCGA_coCNV | 1.85E-03 | 4.14E-03 |
| rs9864951 | TCGA&TCGA_coCNV | 4.32E-03 | 8.17E-03 |
| rs5984925 | TCGA | 4.32E-03 | #N/A |
| rs7901384 | TCGA | 4.32E-03 | #N/A |

| rs41480946 | TCGA&TCGA_coCNV | 3.62E-03 | 8.75E-04 |
| --- | --- | --- | --- |
| rs12645446 | TCGA&TCGA_coCNV | 4.32E-03 | 2.06E-03 |
| rs17277102 | TCGA&TCGA_coCNV | 2.49E-03 | 7.55E-03 |
| rs7448647 | TCGA&TCGA_coCNV | 4.32E-03 | 5.23E-03 |
| rs6583919 | TCGA | 4.32E-03 | #N/A |
| rs17169056 | TCGA | 4.32E-03 | #N/A |
| rs6878786 | TCGA&TCGA_coCNV | 4.33E-03 | 4.64E-03 |
| rs1391118 | TCGA | 4.33E-03 | #N/A |
| rs2738319 | TCGA&TCGA_coCNV | 4.33E-03 | 3.69E-03 |
| rs310456 | TCGA&TCGA_coCNV | 4.33E-03 | 5.07E-03 |
| rs2353320 | TCGA&TCGA_coCNV | 6.92E-04 | 5.77E-04 |
| rs1175154 | TCGA | 4.33E-03 | #N/A |
| rs1844461 | TCGA&TCGA_coCNV | 4.33E-03 | 9.74E-03 |
| rs8031463 | TCGA&TCGA_coCNV | 4.33E-03 | 5.21E-03 |
| rs3803781 | TCGA&TCGA_coCNV | 4.33E-03 | 9.62E-04 |
| rs2968403 | TCGA&TCGA_coCNV | 4.34E-03 | 3.80E-04 |
| rs12161672 | TCGA | 4.34E-03 | #N/A |
| rs1837476 | TCGA | 4.34E-03 | #N/A |
| rs12594990 | TCGA&TCGA_coCNV | 1.61E-03 | 7.41E-04 |
| rs17532798 | TCGA&TCGA_coCNV | 4.34E-03 | 2.02E-03 |
| rs12521501 | TCGA&TCGA_coCNV | 4.34E-03 | 1.65E-03 |
| rs2483837 | TCGA&TCGA_coCNV | 4.34E-03 | 8.80E-03 |
| rs13288811 | TCGA | 2.07E-03 | #N/A |
| rs16831044 | TCGA | 4.34E-03 | #N/A |
| rs1232582 | TCGA | 4.34E-03 | #N/A |
| rs16897452 | TCGA&TCGA_coCNV | 2.32E-03 | 5.42E-03 |
| rs16889894 | TCGA | 4.34E-03 | #N/A |
| rs16940401 | TCGA&TCGA_coCNV | 4.34E-03 | 3.08E-04 |
| rs7986486 | TCGA | 4.34E-03 | #N/A |
| rs6931694 | TCGA&TCGA_coCNV | 1.09E-03 | 4.36E-04 |
| rs1873465 | TCGA&TCGA_coCNV | 4.34E-03 | 2.69E-03 |
| rs9652283 | TCGA | 4.11E-03 | #N/A |
| rs6494364 | TCGA&TCGA_coCNV | 1.95E-03 | 4.87E-03 |
| rs4763989 | TCGA&TCGA_coCNV | 4.35E-03 | 7.61E-04 |
| rs12452943 | TCGA | 4.35E-03 | #N/A |
| rs28216 | TCGA | 2.81E-03 | #N/A |
| rs6999814 | TCGA | 4.35E-03 | #N/A |
| rs17026853 | TCGA&TCGA_coCNV | 4.35E-03 | 3.10E-03 |
| rs9567224 | TCGA | 3.80E-03 | #N/A |
| rs9918275 | TCGA&TCGA_coCNV | 8.86E-04 | 7.99E-04 |
| rs1246174 | TCGA | 4.35E-03 | #N/A |
| rs7623072 | TCGA | 4.35E-03 | #N/A |
| rs10990134 | TCGA&TCGA_coCNV | 2.54E-03 | 4.07E-03 |
| rs10828055 | TCGA | 5.14E-04 | #N/A |
| rs4760012 | TCGA&TCGA_coCNV | 4.35E-03 | 6.46E-04 |
| rs9882843 | TCGA&TCGA_coCNV | 4.36E-03 | 3.97E-03 |

| rs8029805 | TCGA&TCGA_coCNV | 2.25E-03 | 6.34E-03 |
| --- | --- | --- | --- |
| rs4084881 | TCGA&TCGA_coCNV | 4.57E-04 | 1.40E-04 |
| rs9858493 | TCGA&TCGA_coCNV | 1.28E-03 | 9.98E-03 |
| rs17190904 | TCGA | 1.23E-03 | #N/A |
| rs276821 | TCGA&TCGA_coCNV | 1.00E-03 | 1.60E-03 |
| rs6924819 | TCGA | 4.36E-03 | #N/A |
| rs5953916 | TCGA | 4.36E-03 | #N/A |
| rs7165549 | TCGA | 4.36E-03 | #N/A |
| rs11968404 | TCGA&TCGA_coCNV | 3.06E-03 | 1.16E-04 |
| rs16975868 | TCGA&TCGA_coCNV | 2.95E-03 | 4.80E-03 |
| rs7759008 | TCGA&TCGA_coCNV | 4.37E-03 | 6.61E-03 |
| rs8034994 | TCGA&TCGA_coCNV | 4.37E-03 | 2.04E-03 |
| rs17068081 | TCGA | 4.37E-03 | #N/A |
| rs10484884 | TCGA&TCGA_coCNV | 4.37E-03 | 7.62E-03 |
| rs6942866 | TCGA&TCGA_coCNV | 4.37E-03 | 1.56E-03 |
| rs9530208 | TCGA | 1.87E-03 | #N/A |
| rs195323 | TCGA | 4.37E-03 | #N/A |
| rs1513324 | TCGA&TCGA_coCNV | 4.37E-03 | 8.41E-03 |
| rs17040945 | TCGA | 4.37E-03 | #N/A |
| rs7253295 | TCGA&TCGA_coCNV | 4.37E-03 | 1.85E-03 |
| rs6070584 | TCGA | 4.37E-03 | #N/A |
| rs2684348 | TCGA | 4.37E-03 | #N/A |
| rs12206548 | TCGA&TCGA_coCNV | 1.44E-03 | 5.13E-04 |
| rs2967337 | TCGA&TCGA_coCNV | 1.63E-03 | 7.96E-03 |
| rs6004955 | TCGA&TCGA_coCNV | 4.38E-03 | 8.99E-03 |
| rs7918988 | TCGA | 4.38E-03 | #N/A |
| rs6909901 | TCGA&TCGA_coCNV | 4.38E-03 | 5.57E-03 |
| rs2206474 | TCGA | 4.38E-03 | #N/A |
| rs944281 | TCGA | 4.38E-03 | #N/A |
| rs12051806 | TCGA&TCGA_coCNV | 4.38E-03 | 4.27E-03 |
| rs2269972 | TCGA&TCGA_coCNV | 4.38E-03 | 2.84E-03 |
| rs2136843 | TCGA | 4.38E-03 | #N/A |
| rs10511425 | TCGA&TCGA_coCNV | 4.39E-03 | 6.05E-03 |
| rs7309076 | TCGA&TCGA_coCNV | 4.39E-03 | 4.20E-03 |
| rs9815886 | TCGA&TCGA_coCNV | 4.39E-03 | 2.81E-03 |
| rs6741384 | TCGA | 4.39E-03 | #N/A |
| rs6041265 | TCGA&TCGA_coCNV | 4.39E-03 | 2.05E-03 |
| rs4881702 | TCGA | 4.39E-03 | #N/A |
| rs2426256 | TCGA | 4.39E-03 | #N/A |
| rs1158037 | TCGA | 4.39E-03 | #N/A |
| rs11949864 | TCGA | 4.39E-03 | #N/A |
| rs1423415 | TCGA&TCGA_coCNV | 3.57E-03 | 6.51E-03 |
| rs2899617 | TCGA | 4.39E-03 | #N/A |
| rs11906842 | TCGA | 4.39E-03 | #N/A |
| rs9966287 | TCGA | 4.39E-03 | #N/A |
| rs4746263 | TCGA&TCGA_coCNV | 4.39E-03 | 5.34E-03 |

| rs9576472 | TCGA&TCGA_coCNV | 4.06E-03 | 5.66E-03 |
| --- | --- | --- | --- |
| rs1270639 | TCGA | 4.39E-03 | #N/A |
| rs1535593 | TCGA&TCGA_coCNV | 5.34E-04 | 9.99E-03 |
| rs12559996 | TCGA | 4.40E-03 | #N/A |
| rs1007342 | TCGA&TCGA_coCNV | 4.40E-03 | 2.33E-03 |
| rs13418880 | TCGA | 4.40E-03 | #N/A |
| rs10894815 | TCGA&TCGA_coCNV | 4.40E-03 | 9.33E-03 |
| rs1688378 | TCGA&TCGA_coCNV | 4.40E-03 | 3.58E-03 |
| rs41407148 | TCGA&TCGA_coCNV | 4.40E-03 | 4.16E-03 |
| rs1423710 | TCGA | 4.40E-03 | #N/A |
| rs5933954 | TCGA&TCGA_coCNV | 4.40E-03 | 5.80E-04 |
| rs4639865 | TCGA&TCGA_coCNV | 2.45E-03 | 1.34E-03 |
| rs491222 | TCGA&TCGA_coCNV | 4.40E-03 | 8.52E-03 |
| rs2141795 | TCGA&TCGA_coCNV | 4.40E-03 | 4.80E-04 |
| rs4627638 | TCGA | 4.40E-03 | #N/A |
| rs613280 | TCGA | 2.08E-03 | #N/A |
| rs209491 | TCGA&TCGA_coCNV | 4.41E-03 | 9.96E-03 |
| rs6541229 | TCGA&TCGA_coCNV | 4.41E-03 | 7.42E-03 |
| rs13223890 | TCGA&TCGA_coCNV | 4.41E-03 | 5.34E-03 |
| rs12436420 | TCGA | 4.41E-03 | #N/A |
| rs3768513 | TCGA&TCGA_coCNV | 4.41E-03 | 1.05E-03 |
| rs6820355 | TCGA | 4.41E-03 | #N/A |
| rs963375 | TCGA&TCGA_coCNV | 4.41E-03 | 4.16E-03 |
| rs6635025 | TCGA | 4.42E-03 | #N/A |
| rs10225755 | TCGA | 4.42E-03 | #N/A |
| rs10748367 | TCGA&TCGA_coCNV | 4.42E-03 | 6.03E-03 |
| rs11242884 | TCGA | 4.42E-03 | #N/A |
| rs13000706 | TCGA | 2.20E-03 | #N/A |
| rs6453412 | TCGA&TCGA_coCNV | 4.42E-03 | 3.75E-03 |
| rs17077691 | TCGA | 4.42E-03 | #N/A |
| rs8133006 | TCGA&TCGA_coCNV | 4.42E-03 | 1.47E-03 |
| rs17712289 | TCGA&TCGA_coCNV | 4.43E-03 | 7.95E-04 |
| rs998532 | TCGA&TCGA_coCNV | 2.67E-03 | 6.66E-04 |
| rs41491845 | TCGA&TCGA_coCNV | 3.60E-03 | 1.50E-04 |
| rs11777950 | TCGA&TCGA_coCNV | 4.43E-03 | 5.10E-03 |
| rs4722469 | TCGA&TCGA_coCNV | 4.43E-03 | 1.42E-03 |
| rs6730025 | TCGA&TCGA_coCNV | 4.43E-03 | 2.40E-03 |
| rs4626067 | TCGA&TCGA_coCNV | 4.43E-03 | 5.42E-03 |
| rs1499099 | TCGA&TCGA_coCNV | 8.96E-04 | 3.52E-03 |
| rs36020 | TCGA&TCGA_coCNV | 2.77E-04 | 7.28E-03 |
| rs7805672 | TCGA&TCGA_coCNV | 4.09E-03 | 3.83E-03 |
| rs17031 | TCGA | 4.44E-03 | #N/A |
| rs9532889 | TCGA | 4.44E-03 | #N/A |
| rs16895458 | TCGA&TCGA_coCNV | 8.37E-04 | 7.82E-04 |
| rs8079205 | TCGA | 4.44E-03 | #N/A |
| rs167602 | TCGA&TCGA_coCNV | 4.44E-03 | 4.43E-03 |

| rs11100667 | TCGA&TCGA_coCNV | 4.44E-03 | 5.12E-04 |
| --- | --- | --- | --- |
| rs11216357 | TCGA | 4.44E-03 | #N/A |
| rs7817569 | TCGA | 4.44E-03 | #N/A |
| rs10224717 | TCGA | 3.93E-03 | #N/A |
| rs2236550 | TCGA&TCGA_coCNV | 4.68E-04 | 5.64E-03 |
| rs286695 | TCGA&TCGA_coCNV | 1.24E-03 | 3.78E-03 |
| rs11754156 | TCGA&TCGA_coCNV | 1.06E-03 | 1.49E-03 |
| rs7221370 | TCGA&TCGA_coCNV | 4.45E-03 | 1.37E-03 |
| rs489650 | TCGA&TCGA_coCNV | 4.45E-03 | 2.68E-03 |
| rs3748093 | TCGA&TCGA_coCNV | 4.45E-03 | 6.88E-04 |
| rs12680290 | TCGA | 4.45E-03 | #N/A |
| rs4685235 | TCGA&TCGA_coCNV | 4.45E-03 | 5.51E-03 |
| rs6552890 | TCGA | 4.45E-03 | #N/A |
| rs12139940 | TCGA | 4.45E-03 | #N/A |
| rs11123314 | TCGA | 4.45E-03 | #N/A |
| rs10195563 | TCGA&TCGA_coCNV | 5.31E-04 | 1.41E-04 |
| rs6654754 | TCGA&TCGA_coCNV | 4.45E-03 | 1.77E-03 |
| rs10956062 | TCGA&TCGA_coCNV | 4.45E-03 | 6.81E-03 |
| rs1237262 | TCGA&TCGA_coCNV | 3.53E-03 | 2.42E-03 |
| rs7892407 | TCGA&TCGA_coCNV | 1.83E-03 | 6.52E-03 |
| rs1364288 | TCGA&TCGA_coCNV | 4.46E-03 | 3.19E-03 |
| rs9927612 | TCGA | 4.46E-03 | #N/A |
| rs1202392 | TCGA&TCGA_coCNV | 4.46E-03 | 2.61E-03 |
| rs2784921 | TCGA | 4.46E-03 | #N/A |
| rs10966191 | TCGA&TCGA_coCNV | 4.46E-03 | 5.07E-03 |
| rs9907049 | TCGA&TCGA_coCNV | 2.70E-03 | 8.73E-05 |
| rs17690697 | TCGA | 4.46E-03 | #N/A |
| rs17778162 | TCGA | 4.46E-03 | #N/A |
| rs17706512 | TCGA&TCGA_coCNV | 4.46E-03 | 3.52E-03 |
| rs1427293 | TCGA | 4.46E-03 | #N/A |
| rs262232 | TCGA&TCGA_coCNV | 3.32E-03 | 3.75E-03 |
| rs1329520 | TCGA&TCGA_coCNV | 1.73E-03 | 8.31E-03 |
| rs9392129 | TCGA&TCGA_coCNV | 1.09E-03 | 3.70E-04 |
| rs10488981 | TCGA&TCGA_coCNV | 1.69E-03 | 5.36E-03 |
| rs4831940 | TCGA&TCGA_coCNV | 4.46E-03 | 2.32E-03 |
| rs6580032 | TCGA | 3.80E-03 | #N/A |
| rs6853171 | TCGA&TCGA_coCNV | 4.47E-03 | 1.72E-03 |
| rs12690276 | TCGA | 4.47E-03 | #N/A |
| rs16880539 | TCGA&TCGA_coCNV | 2.90E-03 | 2.54E-03 |
| rs927142 | TCGA&TCGA_coCNV | 4.47E-03 | 6.29E-04 |
| rs9601381 | TCGA | 4.48E-03 | #N/A |
| rs7163729 | TCGA | 4.48E-03 | #N/A |
| rs7645220 | TCGA | 3.88E-03 | #N/A |
| rs12805212 | TCGA | 4.48E-03 | #N/A |
| rs10451925 | TCGA | 4.48E-03 | #N/A |
| rs4563896 | TCGA&TCGA_coCNV | 4.48E-03 | 5.90E-03 |

| rs2004850 | TCGA&TCGA_coCNV | 4.32E-03 | 3.49E-03 |
| --- | --- | --- | --- |
| rs1433465 | TCGA | 4.48E-03 | #N/A |
| rs12796470 | TCGA&TCGA_coCNV | 4.48E-03 | 3.97E-03 |
| rs6438276 | TCGA | 4.48E-03 | #N/A |
| rs1798096 | TCGA&TCGA_coCNV | 7.22E-06 | 1.53E-03 |
| rs11663406 | TCGA&TCGA_coCNV | 4.48E-03 | 8.57E-04 |
| rs11056990 | TCGA&TCGA_coCNV | 4.48E-03 | 2.12E-03 |
| rs5925599 | TCGA&TCGA_coCNV | 4.48E-03 | 2.81E-03 |
| rs41372147 | TCGA&TCGA_coCNV | 1.38E-03 | 5.19E-03 |
| rs12989809 | TCGA | 4.48E-03 | #N/A |
| rs16866737 | TCGA | 4.48E-03 | #N/A |
| rs13441235 | TCGA&TCGA_coCNV | 4.48E-03 | 2.21E-03 |
| rs4750910 | TCGA&TCGA_coCNV | 4.48E-03 | 7.97E-03 |
| rs11702521 | TCGA | 4.49E-03 | #N/A |
| rs7154230 | TCGA | 4.49E-03 | #N/A |
| rs4661471 | TCGA&TCGA_coCNV | 1.86E-03 | 4.02E-04 |
| rs35791994 | TCGA&TCGA_coCNV | 3.86E-04 | 2.65E-03 |
| rs382752 | TCGA&TCGA_coCNV | 4.49E-03 | 8.90E-03 |
| rs755041 | TCGA | 4.49E-03 | #N/A |
| rs11793523 | TCGA&TCGA_coCNV | 4.49E-03 | 1.27E-03 |
| rs10891753 | TCGA&TCGA_coCNV | 4.49E-03 | 9.62E-04 |
| rs9960267 | TCGA | 4.49E-03 | #N/A |
| rs7422754 | TCGA&TCGA_coCNV | 4.49E-03 | 3.65E-03 |
| rs2172609 | TCGA&TCGA_coCNV | 4.49E-03 | 8.50E-03 |
| rs9536586 | TCGA | 4.49E-03 | #N/A |
| rs356131 | TCGA | 4.49E-03 | #N/A |
| rs1423859 | TCGA | 6.88E-04 | #N/A |
| rs17262115 | TCGA&TCGA_coCNV | 4.49E-03 | 3.87E-03 |
| rs296857 | TCGA | 4.49E-03 | #N/A |
| rs3088126 | TCGA | 4.49E-03 | #N/A |
| rs16953199 | TCGA&TCGA_coCNV | 4.49E-03 | 1.77E-03 |
| rs17824941 | TCGA | 4.50E-03 | #N/A |
| rs12642225 | TCGA&TCGA_coCNV | 1.82E-04 | 7.13E-04 |
| rs242920 | TCGA&TCGA_coCNV | 4.50E-03 | 5.28E-03 |
| rs2876767 | TCGA&TCGA_coCNV | 4.50E-03 | 1.49E-03 |
| rs2763342 | TCGA | 4.50E-03 | #N/A |
| rs9401207 | TCGA | 4.50E-03 | #N/A |
| rs10258451 | TCGA | 3.78E-03 | #N/A |
| rs12591682 | TCGA&TCGA_coCNV | 4.50E-03 | 5.26E-03 |
| rs17756192 | TCGA&TCGA_coCNV | 4.50E-03 | 9.26E-04 |
| rs13375716 | TCGA&TCGA_coCNV | 4.50E-03 | 6.63E-03 |
| rs11780404 | TCGA | 4.50E-03 | #N/A |
| rs8110662 | TCGA&TCGA_coCNV | 4.50E-03 | 4.99E-04 |
| rs7912821 | TCGA&TCGA_coCNV | 4.50E-03 | 7.08E-03 |
| rs414219 | TCGA | 4.50E-03 | #N/A |
| rs599641 | TCGA | 4.50E-03 | #N/A |

| rs17468651 | TCGA | 4.50E-03 | #N/A |
| --- | --- | --- | --- |
| rs9799272 | TCGA&TCGA_coCNV | 4.50E-03 | 2.97E-03 |
| rs10170780 | TCGA&TCGA_coCNV | 1.70E-03 | 5.88E-03 |
| rs738383 | TCGA&TCGA_coCNV | 2.75E-03 | 2.27E-03 |
| rs3131282 | TCGA&TCGA_coCNV | 6.77E-05 | 1.70E-03 |
| rs2281252 | TCGA&TCGA_coCNV | 4.51E-03 | 9.60E-03 |
| rs5021934 | TCGA | 4.51E-03 | #N/A |
| rs2221061 | TCGA&TCGA_coCNV | 3.32E-03 | 5.30E-04 |
| rs8080915 | TCGA&TCGA_coCNV | 4.51E-03 | 4.32E-03 |
| rs9573220 | TCGA&TCGA_coCNV | 4.51E-03 | 2.58E-03 |
| rs652845 | TCGA&TCGA_coCNV | 1.97E-03 | 5.93E-03 |
| rs9883958 | TCGA&TCGA_coCNV | 4.51E-03 | 3.75E-03 |
| rs569578 | TCGA&TCGA_coCNV | 4.51E-03 | 5.67E-03 |
| rs1412243 | TCGA | 4.52E-03 | #N/A |
| rs4315770 | TCGA&TCGA_coCNV | 4.52E-03 | 9.24E-03 |
| rs706497 | TCGA | 4.52E-03 | #N/A |
| rs7703941 | TCGA | 4.52E-03 | #N/A |
| rs9954486 | TCGA&TCGA_coCNV | 2.78E-03 | 1.07E-03 |
| rs7773435 | TCGA&TCGA_coCNV | 4.52E-03 | 7.22E-03 |
| rs1400891 | TCGA | 4.52E-03 | #N/A |
| rs2103437 | TCGA | 4.52E-03 | #N/A |
| rs11749270 | TCGA | 2.72E-03 | #N/A |
| rs10112410 | TCGA&TCGA_coCNV | 4.52E-03 | 1.29E-03 |
| rs2442901 | TCGA&TCGA_coCNV | 4.52E-03 | 9.91E-03 |
| rs1257656 | TCGA&TCGA_coCNV | 4.52E-03 | 4.25E-03 |
| rs3967768 | TCGA | 4.52E-03 | #N/A |
| rs9359845 | TCGA&TCGA_coCNV | 4.52E-03 | 4.61E-03 |
| rs12434512 | TCGA | 4.52E-03 | #N/A |
| rs12209573 | TCGA&TCGA_coCNV | 4.53E-03 | 8.95E-03 |
| rs4987053 | TCGA&TCGA_coCNV | 4.53E-03 | 7.57E-03 |
| rs2785092 | TCGA&TCGA_coCNV | 2.03E-03 | 2.53E-03 |
| rs10519868 | TCGA | 4.53E-03 | #N/A |
| rs2180108 | TCGA&TCGA_coCNV | 4.53E-03 | 1.23E-03 |
| rs17366143 | TCGA&TCGA_coCNV | 2.89E-04 | 2.98E-04 |
| rs10501823 | TCGA | 4.53E-03 | #N/A |
| rs10511364 | TCGA&TCGA_coCNV | 4.53E-03 | 4.62E-03 |
| rs848515 | TCGA&TCGA_coCNV | 4.54E-03 | 8.70E-03 |
| rs2268897 | TCGA&TCGA_coCNV | 3.37E-03 | 7.18E-03 |
| rs6481930 | TCGA&TCGA_coCNV | 4.54E-03 | 5.18E-03 |
| rs6045666 | TCGA&TCGA_coCNV | 3.41E-03 | 1.61E-03 |
| rs1857129 | TCGA&TCGA_coCNV | 4.54E-03 | 2.35E-03 |
| rs10836541 | TCGA | 4.54E-03 | #N/A |
| rs826651 | TCGA | 4.54E-03 | #N/A |
| rs2663257 | TCGA&TCGA_coCNV | 4.15E-04 | 1.05E-03 |
| rs7498801 | TCGA | 4.55E-03 | #N/A |
| rs4802376 | TCGA&TCGA_coCNV | 1.46E-03 | 5.18E-03 |

| rs651327 | TCGA | 4.55E-03 | #N/A |
| --- | --- | --- | --- |
| rs406195 | TCGA | 4.55E-03 | #N/A |
| rs1480152 | TCGA&TCGA_coCNV | 4.55E-03 | 4.14E-03 |
| rs9825300 | TCGA | 4.55E-03 | #N/A |
| rs2257505 | TCGA | 4.55E-03 | #N/A |
| rs9655078 | TCGA&TCGA_coCNV | 3.62E-03 | 1.49E-03 |
| rs17042598 | TCGA&TCGA_coCNV | 4.55E-03 | 1.21E-03 |
| rs212584 | TCGA | 4.55E-03 | #N/A |
| rs4923411 | TCGA&TCGA_coCNV | 5.52E-04 | 3.70E-03 |
| rs998383 | TCGA | 4.55E-03 | #N/A |
| rs678941 | TCGA&TCGA_coCNV | 4.56E-03 | 1.45E-03 |
| rs969348 | TCGA | 4.56E-03 | #N/A |
| rs11952997 | TCGA&TCGA_coCNV | 5.74E-05 | 3.44E-03 |
| rs9285017 | TCGA&TCGA_coCNV | 4.56E-03 | 3.19E-03 |
| rs7299358 | TCGA&TCGA_coCNV | 4.56E-03 | 4.32E-03 |
| rs7394996 | TCGA | 4.56E-03 | #N/A |
| rs11769011 | TCGA&TCGA_coCNV | 4.56E-03 | 8.00E-03 |
| rs4145226 | TCGA&TCGA_coCNV | 4.56E-03 | 6.93E-04 |
| rs4843664 | TCGA&TCGA_coCNV | 4.56E-03 | 9.72E-04 |
| rs6630098 | TCGA&TCGA_coCNV | 6.45E-04 | 5.55E-04 |
| rs17621880 | TCGA | 3.97E-03 | #N/A |
| rs10805642 | TCGA&TCGA_coCNV | 4.57E-03 | 4.32E-04 |
| rs10965980 | TCGA&TCGA_coCNV | 4.57E-03 | 3.84E-03 |
| rs5977104 | TCGA&TCGA_coCNV | 9.06E-05 | 3.14E-03 |
| rs17810130 | TCGA&TCGA_coCNV | 4.57E-03 | 9.38E-04 |
| rs4890613 | TCGA | 4.57E-03 | #N/A |
| rs4844438 | TCGA&TCGA_coCNV | 4.57E-03 | 1.15E-03 |
| rs4894273 | TCGA | 4.57E-03 | #N/A |
| rs1373247 | TCGA&TCGA_coCNV | 4.57E-03 | 9.17E-04 |
| rs7852336 | TCGA&TCGA_coCNV | 4.57E-03 | 3.06E-04 |
| rs1559018 | TCGA | 4.57E-03 | #N/A |
| rs7414922 | TCGA&TCGA_coCNV | 4.57E-03 | 3.22E-03 |
| rs8016721 | TCGA&TCGA_coCNV | 2.63E-05 | 1.72E-05 |
| rs6579993 | TCGA | 4.57E-03 | #N/A |
| rs7701418 | TCGA | 4.58E-03 | #N/A |
| rs7900369 | TCGA | 4.58E-03 | #N/A |
| rs7255412 | TCGA&TCGA_coCNV | 3.23E-03 | 3.46E-04 |
| rs7579045 | TCGA&TCGA_coCNV | 8.40E-04 | 1.84E-04 |
| rs11158064 | TCGA | 5.96E-04 | #N/A |
| rs16990155 | TCGA&TCGA_coCNV | 4.58E-03 | 2.47E-03 |
| rs11098809 | TCGA | 4.58E-03 | #N/A |
| rs7789021 | TCGA | 4.58E-03 | #N/A |
| rs26346 | TCGA | 4.58E-03 | #N/A |
| rs10818805 | TCGA&TCGA_coCNV | 4.42E-04 | 3.95E-03 |
| rs16898558 | TCGA&TCGA_coCNV | 4.58E-03 | 4.92E-03 |
| rs769084 | TCGA&TCGA_coCNV | 5.24E-06 | 1.69E-04 |

| rs10122373 | TCGA | 4.59E-03 | #N/A |
| --- | --- | --- | --- |
| rs2274602 | TCGA | 4.59E-03 | #N/A |
| rs7163310 | TCGA&TCGA_coCNV | 4.59E-03 | 2.96E-03 |
| rs11680550 | TCGA | 2.04E-03 | #N/A |
| rs3781759 | TCGA&TCGA_coCNV | 4.59E-03 | 1.63E-03 |
| rs12467862 | TCGA | 4.59E-03 | #N/A |
| rs17034362 | TCGA | 4.59E-03 | #N/A |
| rs4690738 | TCGA | 1.93E-03 | #N/A |
| rs917124 | TCGA&TCGA_coCNV | 4.59E-03 | 5.11E-03 |
| rs17171848 | TCGA&TCGA_coCNV | 4.59E-03 | 1.45E-03 |
| rs585974 | TCGA | 4.59E-03 | #N/A |
| rs1431983 | TCGA | 4.59E-03 | #N/A |
| rs41479746 | TCGA&TCGA_coCNV | 3.95E-03 | 8.23E-04 |
| rs17824874 | TCGA&TCGA_coCNV | 4.59E-03 | 5.31E-03 |
| rs10857580 | TCGA&TCGA_coCNV | 1.79E-03 | 7.40E-03 |
| rs7601963 | TCGA | 4.59E-03 | #N/A |
| rs11207949 | TCGA&TCGA_coCNV | 1.80E-03 | 3.25E-04 |
| rs1998112 | TCGA&TCGA_coCNV | 4.59E-03 | 7.52E-03 |
| rs9955714 | TCGA | 4.59E-03 | #N/A |
| rs11739351 | TCGA | 4.59E-03 | #N/A |
| rs6102600 | TCGA&TCGA_coCNV | 1.10E-06 | 1.51E-04 |
| rs11038537 | TCGA&TCGA_coCNV | 4.60E-03 | 2.46E-03 |
| rs11936427 | TCGA&TCGA_coCNV | 4.60E-03 | 5.52E-03 |
| rs13231611 | TCGA&TCGA_coCNV | 4.60E-03 | 6.07E-03 |
| rs4954184 | TCGA | 4.60E-03 | #N/A |
| rs1440369 | TCGA | 4.60E-03 | #N/A |
| rs12476027 | TCGA&TCGA_coCNV | 4.60E-03 | 3.41E-03 |
| rs9548525 | TCGA&TCGA_coCNV | 2.58E-03 | 5.58E-03 |
| rs3101362 | TCGA&TCGA_coCNV | 4.61E-03 | 7.61E-03 |
| rs12259450 | TCGA&TCGA_coCNV | 4.61E-03 | 2.13E-03 |
| rs17241553 | TCGA&TCGA_coCNV | 4.61E-03 | 7.39E-03 |
| rs1869875 | TCGA | 4.61E-03 | #N/A |
| rs7818431 | TCGA&TCGA_coCNV | 4.61E-03 | 2.50E-03 |
| rs4142702 | TCGA | 4.61E-03 | #N/A |
| rs8111651 | TCGA | 4.61E-03 | #N/A |
| rs2369974 | TCGA&TCGA_coCNV | 4.61E-03 | 4.30E-03 |
| rs28526823 | TCGA&TCGA_coCNV | 4.61E-03 | 4.48E-03 |
| rs12552752 | TCGA | 3.86E-03 | #N/A |
| rs1276295 | TCGA&TCGA_coCNV | 4.62E-03 | 7.65E-03 |
| rs2861106 | TCGA | 2.59E-04 | #N/A |
| rs11594689 | TCGA&TCGA_coCNV | 7.01E-04 | 1.74E-03 |
| rs2588637 | TCGA | 4.62E-03 | #N/A |
| rs7681829 | TCGA&TCGA_coCNV | 4.62E-03 | 1.45E-03 |
| rs6840589 | TCGA | 3.82E-04 | #N/A |
| rs17264887 | TCGA | 4.62E-03 | #N/A |
| rs2504211 | TCGA&TCGA_coCNV | 4.62E-03 | 1.90E-03 |

| rs12966846 | TCGA | 4.62E-03 | #N/A |
| --- | --- | --- | --- |
| rs17000005 | TCGA | 1.29E-03 | #N/A |
| rs5994685 | TCGA | 4.62E-03 | #N/A |
| rs10276544 | TCGA | 3.06E-03 | #N/A |
| rs9397725 | TCGA&TCGA_coCNV | 4.62E-03 | 1.34E-04 |
| rs2345160 | TCGA | 4.62E-03 | #N/A |
| rs7998264 | TCGA&TCGA_coCNV | 4.62E-03 | 5.87E-05 |
| rs6554837 | TCGA | 4.63E-03 | #N/A |
| rs2428022 | TCGA | 4.63E-03 | #N/A |
| rs7848139 | TCGA | 4.63E-03 | #N/A |
| rs2021846 | TCGA | 4.63E-03 | #N/A |
| rs5929606 | TCGA | 4.63E-03 | #N/A |
| rs17805030 | TCGA&TCGA_coCNV | 1.94E-05 | 2.02E-04 |
| rs7763358 | TCGA | 4.63E-03 | #N/A |
| rs1947304 | TCGA | 4.56E-03 | #N/A |
| rs9593873 | TCGA&TCGA_coCNV | 4.63E-03 | 3.85E-03 |
| rs2833507 | TCGA&SCANdb | 4.63E-03 | #N/A |
| rs1930537 | TCGA | 4.63E-03 | #N/A |
| rs11082646 | TCGA | 4.63E-03 | #N/A |
| rs133946 | TCGA&TCGA_coCNV | 1.91E-04 | 1.92E-03 |
| rs5982640 | TCGA | 4.64E-03 | #N/A |
| rs7606803 | TCGA | 4.64E-03 | #N/A |
| rs6773675 | TCGA&TCGA_coCNV | 4.64E-03 | 1.68E-03 |
| rs1471859 | TCGA | 4.64E-03 | #N/A |
| rs7392389 | TCGA&TCGA_coCNV | 1.08E-03 | 6.09E-03 |
| rs935564 | TCGA&TCGA_coCNV | 7.45E-07 | 5.72E-03 |
| rs6579959 | TCGA | 4.65E-03 | #N/A |
| rs17271095 | TCGA | 4.65E-03 | #N/A |
| rs10079090 | TCGA | 4.65E-03 | #N/A |
| rs16918536 | TCGA | 4.65E-03 | #N/A |
| rs10270250 | TCGA&TCGA_coCNV | 4.43E-03 | 1.54E-03 |
| rs155523 | TCGA&TCGA_coCNV | 4.65E-03 | 6.23E-03 |
| rs16969025 | TCGA | 4.65E-03 | #N/A |
| rs1958055 | TCGA&TCGA_coCNV | 4.65E-03 | 2.71E-05 |
| rs12506596 | TCGA&TCGA_coCNV | 4.65E-03 | 2.35E-03 |
| rs16996176 | TCGA&TCGA_coCNV | 4.65E-03 | 4.13E-03 |
| rs7890904 | TCGA | 5.21E-04 | #N/A |
| rs232758 | TCGA | 4.65E-03 | #N/A |
| rs610601 | TCGA&TCGA_coCNV | 4.66E-03 | 3.41E-03 |
| rs7729142 | TCGA | 4.66E-03 | #N/A |
| rs10759563 | TCGA&TCGA_coCNV | 4.66E-03 | 2.06E-03 |
| rs7306469 | TCGA | 4.66E-03 | #N/A |
| rs10253249 | TCGA&TCGA_coCNV | 4.66E-03 | 9.69E-03 |
| rs5927688 | TCGA&TCGA_coCNV | 4.66E-03 | 8.80E-03 |
| rs13228990 | TCGA | 4.66E-03 | #N/A |
| rs17085440 | TCGA | 4.66E-03 | #N/A |

| rs6971943 | TCGA | 4.66E-03 | #N/A |
| --- | --- | --- | --- |
| rs10892188 | TCGA | 4.66E-03 | #N/A |
| rs1725261 | TCGA&TCGA_coCNV | 4.66E-03 | 3.96E-03 |
| rs12417208 | TCGA | 4.66E-03 | #N/A |
| rs4683553 | TCGA | 4.66E-03 | #N/A |
| rs256257 | TCGA | 4.66E-03 | #N/A |
| rs9517863 | TCGA&TCGA_coCNV | 3.24E-03 | 6.54E-03 |
| rs6492041 | TCGA | 4.67E-03 | #N/A |
| rs6533932 | TCGA | 4.67E-03 | #N/A |
| rs2619118 | TCGA | 3.72E-03 | #N/A |
| rs11865259 | TCGA&TCGA_coCNV | 4.67E-03 | 6.90E-03 |
| rs11885418 | TCGA&TCGA_coCNV | 6.56E-04 | 1.95E-04 |
| rs2393460 | TCGA | 4.67E-03 | #N/A |
| rs3784353 | TCGA | 4.67E-03 | #N/A |
| rs5937001 | TCGA&TCGA_coCNV | 4.67E-03 | 4.72E-03 |
| rs4145541 | TCGA&TCGA_coCNV | 3.91E-03 | 1.76E-04 |
| rs755800 | TCGA&TCGA_coCNV | 3.45E-03 | 4.46E-05 |
| rs7083680 | TCGA&TCGA_coCNV | 4.67E-03 | 2.12E-03 |
| rs2840705 | TCGA&TCGA_coCNV | 4.67E-03 | 1.30E-03 |
| rs1355551 | TCGA&TCGA_coCNV | 4.67E-03 | 6.67E-03 |
| rs2360667 | TCGA&TCGA_coCNV | 4.67E-03 | 4.94E-03 |
| rs4930820 | TCGA | 4.68E-03 | #N/A |
| rs17153063 | TCGA | 4.68E-03 | #N/A |
| rs2073968 | TCGA | 4.68E-03 | #N/A |
| rs12303876 | TCGA | 4.68E-03 | #N/A |
| rs1544694 | TCGA&TCGA_coCNV | 4.69E-03 | 3.31E-03 |
| rs2501849 | TCGA | 4.54E-03 | #N/A |
| rs11831827 | TCGA&TCGA_coCNV | 3.23E-03 | 1.88E-04 |
| rs3181227 | TCGA | 4.69E-03 | #N/A |
| rs12031789 | TCGA&TCGA_coCNV | 4.69E-03 | 1.00E-02 |
| rs7677172 | TCGA&TCGA_coCNV | 4.69E-03 | 8.50E-03 |
| rs2855981 | TCGA | 4.69E-03 | #N/A |
| rs2270293 | TCGA&TCGA_coCNV | 3.84E-03 | 7.01E-03 |
| rs1356656 | TCGA&TCGA_coCNV | 4.69E-03 | 2.68E-03 |
| rs4909161 | TCGA | 3.30E-03 | #N/A |
| rs2241445 | TCGA&TCGA_coCNV | 1.33E-03 | 3.74E-03 |
| rs9877107 | TCGA&TCGA_coCNV | 4.70E-03 | 4.45E-03 |
| rs11747262 | TCGA&TCGA_coCNV | 4.70E-03 | 8.83E-03 |
| rs2339512 | TCGA&TCGA_coCNV | 2.37E-03 | 1.11E-03 |
| rs6961522 | TCGA&TCGA_coCNV | 2.66E-03 | 9.59E-03 |
| rs7297453 | TCGA&TCGA_coCNV | 4.70E-03 | 4.12E-04 |
| rs7998812 | TCGA | 4.70E-03 | #N/A |
| rs12607847 | TCGA | 4.70E-03 | #N/A |
| rs2836259 | TCGA&TCGA_coCNV | 4.70E-03 | 5.54E-04 |
| rs13002288 | TCGA | 4.70E-03 | #N/A |
| rs6133736 | TCGA&TCGA_coCNV | 6.92E-04 | 9.19E-03 |

| rs1261225 | TCGA | 4.70E-03 | #N/A |
| --- | --- | --- | --- |
| rs698094 | TCGA | 4.70E-03 | #N/A |
| rs4566567 | TCGA&TCGA_coCNV | 4.70E-03 | 3.32E-03 |
| rs10163956 | TCGA | 4.70E-03 | #N/A |
| rs4804227 | TCGA | 4.70E-03 | #N/A |
| rs190133 | TCGA | 4.70E-03 | #N/A |
| rs12957137 | TCGA&TCGA_coCNV | 4.70E-03 | 3.92E-04 |
| rs17605233 | TCGA&TCGA_coCNV | 2.04E-03 | 2.09E-03 |
| rs7493621 | TCGA&TCGA_coCNV | 1.57E-03 | 9.25E-03 |
| rs17182853 | TCGA&TCGA_coCNV | 4.71E-03 | 4.22E-04 |
| rs10149693 | TCGA&TCGA_coCNV | 4.71E-03 | 8.10E-03 |
| rs6707204 | TCGA | 4.71E-03 | #N/A |
| rs12256983 | TCGA&TCGA_coCNV | 1.72E-03 | 1.32E-03 |
| rs7543069 | TCGA&TCGA_coCNV | 4.71E-03 | 1.70E-03 |
| rs2417470 | TCGA&TCGA_coCNV | 4.71E-03 | 4.63E-04 |
| rs12630843 | TCGA | 4.71E-03 | #N/A |
| rs7952778 | TCGA&TCGA_coCNV | 4.71E-03 | 4.58E-04 |
| rs4833395 | TCGA&TCGA_coCNV | 4.71E-03 | 4.77E-03 |
| rs3087213 | TCGA&TCGA_coCNV | 3.30E-03 | 2.90E-03 |
| rs972033 | TCGA&TCGA_coCNV | 1.76E-03 | 9.59E-03 |
| rs10515788 | TCGA&TCGA_coCNV | 4.71E-03 | 5.72E-03 |
| rs868097 | TCGA | 4.71E-03 | #N/A |
| rs2523188 | TCGA&TCGA_coCNV | 4.71E-03 | 2.84E-03 |
| rs12824952 | TCGA | 4.71E-03 | #N/A |
| rs13066557 | TCGA&TCGA_coCNV | 2.98E-03 | 2.25E-03 |
| rs1549028 | TCGA | 4.71E-03 | #N/A |
| rs6573038 | TCGA&TCGA_coCNV | 4.71E-03 | 3.17E-03 |
| rs1810084 | TCGA&TCGA_coCNV | 4.72E-03 | 1.19E-03 |
| rs10515168 | TCGA | 4.72E-03 | #N/A |
| rs2398490 | TCGA&TCGA_coCNV | 4.72E-03 | 6.52E-04 |
| rs10977037 | TCGA | 4.72E-03 | #N/A |
| rs7708391 | TCGA | 4.72E-03 | #N/A |
| rs9356632 | TCGA&TCGA_coCNV | 4.72E-03 | 2.96E-03 |
| rs193908 | TCGA | 4.38E-03 | #N/A |
| rs3901133 | TCGA | 4.72E-03 | #N/A |
| rs7302842 | TCGA&TCGA_coCNV | 4.72E-03 | 8.00E-03 |
| rs17729477 | TCGA&TCGA_coCNV | 3.93E-04 | 4.77E-04 |
| rs10832253 | TCGA&TCGA_coCNV | 4.72E-03 | 2.25E-03 |
| rs1354583 | TCGA&TCGA_coCNV | 4.72E-03 | 7.06E-04 |
| rs494801 | TCGA&TCGA_coCNV | 4.50E-04 | 4.66E-03 |
| rs3931570 | TCGA&TCGA_coCNV | 4.73E-03 | 8.32E-03 |
| rs41432549 | TCGA&TCGA_coCNV | 4.73E-03 | 5.40E-03 |
| rs16940295 | TCGA&TCGA_coCNV | 3.42E-03 | 8.89E-03 |
| rs6974087 | TCGA | 4.73E-03 | #N/A |
| rs7138022 | TCGA&TCGA_coCNV | 4.73E-03 | 9.48E-03 |
| rs1304757 | TCGA&TCGA_coCNV | 4.73E-03 | 1.78E-03 |

| rs760061 | TCGA&TCGA_coCNV | 4.74E-03 | 5.57E-03 |
| --- | --- | --- | --- |
| rs3766331 | TCGA | 4.74E-03 | #N/A |
| rs17026873 | TCGA&TCGA_coCNV | 9.95E-04 | 3.00E-03 |
| rs10744237 | TCGA&TCGA_coCNV | 4.74E-03 | 8.77E-03 |
| rs13085742 | TCGA&TCGA_coCNV | 4.74E-03 | 2.04E-03 |
| rs8010969 | TCGA&TCGA_coCNV | 4.74E-03 | 5.48E-03 |
| rs11250788 | TCGA&TCGA_coCNV | 4.74E-03 | 1.77E-03 |
| rs17702978 | TCGA&TCGA_coCNV | 4.74E-03 | 7.37E-04 |
| rs9533520 | TCGA | 4.74E-03 | #N/A |
| rs11907843 | TCGA | 4.74E-03 | #N/A |
| rs1157946 | TCGA&TCGA_coCNV | 2.42E-03 | 2.61E-03 |
| rs9619326 | TCGA | 4.75E-03 | #N/A |
| rs12618634 | TCGA | 4.75E-03 | #N/A |
| rs820774 | TCGA&TCGA_coCNV | 4.75E-03 | 4.11E-03 |
| rs16933101 | TCGA&TCGA_coCNV | 4.75E-03 | 3.28E-03 |
| rs10024036 | TCGA&TCGA_coCNV | 3.86E-03 | 3.00E-03 |
| rs1275592 | TCGA&TCGA_coCNV | 4.75E-03 | 6.79E-04 |
| rs2500536 | TCGA&TCGA_coCNV | 4.75E-03 | 1.38E-03 |
| rs4987774 | TCGA&TCGA_coCNV | 4.75E-03 | 4.56E-03 |
| rs4431713 | TCGA&TCGA_coCNV | 4.76E-03 | 6.07E-03 |
| rs11224686 | TCGA | 4.76E-03 | #N/A |
| rs8131767 | TCGA&TCGA_coCNV | 4.76E-03 | 2.06E-03 |
| rs16827545 | TCGA&TCGA_coCNV | 4.76E-03 | 6.36E-03 |
| rs17113771 | TCGA | 4.76E-03 | #N/A |
| rs2807056 | TCGA&TCGA_coCNV | 4.76E-03 | 9.60E-03 |
| rs9949765 | TCGA&TCGA_coCNV | 2.97E-03 | 6.70E-04 |
| rs2986261 | TCGA | 4.76E-03 | #N/A |
| rs1944685 | TCGA | 4.76E-03 | #N/A |
| rs1870738 | TCGA&TCGA_coCNV | 7.79E-04 | 4.30E-03 |
| rs10942234 | TCGA&TCGA_coCNV | 4.76E-03 | 1.23E-03 |
| rs2131880 | TCGA | 4.77E-03 | #N/A |
| rs10965403 | TCGA | 4.77E-03 | #N/A |
| rs12584525 | TCGA | 4.77E-03 | #N/A |
| rs10897633 | TCGA&TCGA_coCNV | 4.77E-03 | 9.86E-03 |
| rs1335898 | TCGA | 4.77E-03 | #N/A |
| rs17012378 | TCGA | 4.77E-03 | #N/A |
| rs7876150 | TCGA | 4.77E-03 | #N/A |
| rs4585955 | TCGA | 4.77E-03 | #N/A |
| rs2961609 | TCGA&TCGA_coCNV | 1.34E-04 | 3.89E-03 |
| rs12617382 | TCGA | 4.77E-03 | #N/A |
| rs3770879 | TCGA&TCGA_coCNV | 4.77E-03 | 2.55E-03 |
| rs10901590 | TCGA | 4.77E-03 | #N/A |
| rs10959776 | TCGA&TCGA_coCNV | 4.77E-03 | 7.55E-03 |
| rs4142090 | TCGA | 4.77E-03 | #N/A |
| rs9636797 | TCGA&TCGA_coCNV | 4.77E-03 | 1.35E-03 |
| rs8010024 | TCGA | 4.77E-03 | #N/A |

| rs1218384 | TCGA | 4.78E-03 | #N/A |
| --- | --- | --- | --- |
| rs9311041 | TCGA | 4.78E-03 | #N/A |
| rs7596636 | TCGA&TCGA_coCNV | 4.78E-03 | 8.90E-03 |
| rs7088654 | TCGA | 4.78E-03 | #N/A |
| rs10477429 | TCGA | 4.78E-03 | #N/A |
| rs10239554 | TCGA&TCGA_coCNV | 6.21E-04 | 8.59E-05 |
| rs17157967 | TCGA&TCGA_coCNV | 4.78E-03 | 7.82E-03 |
| rs4550138 | TCGA&TCGA_coCNV | 4.79E-04 | 1.69E-03 |
| rs4435247 | TCGA&TCGA_coCNV | 4.78E-03 | 8.66E-03 |
| rs277488 | TCGA&TCGA_coCNV | 4.78E-03 | 9.58E-03 |
| rs8072935 | TCGA&TCGA_coCNV | 2.84E-03 | 3.81E-03 |
| rs10050347 | TCGA&TCGA_coCNV | 5.97E-04 | 3.07E-04 |
| rs58129 | TCGA | 4.78E-03 | #N/A |
| rs17767375 | TCGA&TCGA_coCNV | 4.78E-03 | 8.44E-04 |
| rs3762404 | TCGA&TCGA_coCNV | 4.78E-03 | 5.86E-03 |
| rs11956960 | TCGA&TCGA_coCNV | 4.78E-03 | 4.89E-03 |
| rs7168072 | TCGA | 4.78E-03 | #N/A |
| rs10993849 | TCGA&TCGA_coCNV | 4.79E-03 | 7.05E-04 |
| rs2471064 | TCGA&TCGA_coCNV | 4.79E-03 | 2.48E-03 |
| rs667520 | TCGA | 4.79E-03 | #N/A |
| rs4792709 | TCGA&TCGA_coCNV | 4.79E-03 | 2.93E-03 |
| rs9873870 | TCGA&TCGA_coCNV | 5.12E-04 | 1.60E-04 |
| rs17325998 | TCGA | 4.79E-03 | #N/A |
| rs1461197 | TCGA&TCGA_coCNV | 4.80E-03 | 2.75E-04 |
| rs6510216 | TCGA | 4.80E-03 | #N/A |
| rs10960164 | TCGA&TCGA_coCNV | 4.80E-03 | 5.21E-03 |
| rs4692185 | TCGA | 4.80E-03 | #N/A |
| rs3790838 | TCGA&TCGA_coCNV | 4.80E-03 | 1.69E-03 |
| rs5759198 | TCGA | 4.80E-03 | #N/A |
| rs1573254 | TCGA | 4.80E-03 | #N/A |
| rs4703882 | TCGA&TCGA_coCNV | 4.80E-03 | 2.94E-03 |
| rs9889643 | TCGA&TCGA_coCNV | 1.42E-03 | 1.01E-03 |
| rs259971 | TCGA | 4.80E-03 | #N/A |
| rs41421244 | TCGA | 4.80E-03 | #N/A |
| rs11157804 | TCGA&TCGA_coCNV | 1.89E-03 | 2.93E-03 |
| rs2091218 | TCGA | 4.80E-03 | #N/A |
| rs11883900 | TCGA | 4.81E-03 | #N/A |
| rs11599399 | TCGA | 4.81E-03 | #N/A |
| rs1205233 | TCGA | 4.81E-03 | #N/A |
| rs2965886 | TCGA | 4.81E-03 | #N/A |
| rs11071746 | TCGA&TCGA_coCNV | 1.83E-03 | 2.71E-03 |
| rs7749539 | TCGA | 4.81E-03 | #N/A |
| rs1892259 | TCGA | 4.81E-03 | #N/A |
| rs453394 | TCGA&TCGA_coCNV | 4.81E-03 | 3.69E-03 |
| rs9604375 | TCGA&TCGA_coCNV | 4.81E-03 | 5.33E-03 |
| rs4147339 | TCGA&TCGA_coCNV | 4.81E-03 | 7.08E-04 |

| rs2955821 | TCGA&TCGA_coCNV | 4.81E-03 | 1.04E-03 |
| --- | --- | --- | --- |
| rs16884419 | TCGA&TCGA_coCNV | 7.61E-04 | 3.46E-03 |
| rs8036797 | TCGA&TCGA_coCNV | 4.81E-03 | 8.98E-03 |
| rs12582717 | TCGA&TCGA_coCNV | 4.42E-03 | 9.38E-03 |
| rs12154585 | TCGA&TCGA_coCNV | 4.81E-03 | 1.03E-04 |
| rs12656386 | TCGA | 4.82E-03 | #N/A |
| rs11691477 | TCGA&TCGA_coCNV | 1.38E-06 | 1.43E-04 |
| rs4302659 | TCGA&TCGA_coCNV | 4.82E-03 | 6.96E-03 |
| rs11893561 | TCGA | 4.82E-03 | #N/A |
| rs7192929 | TCGA&TCGA_coCNV | 4.82E-03 | 3.26E-03 |
| rs17130616 | TCGA&TCGA_coCNV | 2.51E-03 | 1.64E-03 |
| rs1511599 | TCGA&TCGA_coCNV | 4.82E-03 | 3.94E-03 |
| rs17102657 | TCGA&TCGA_coCNV | 4.82E-03 | 4.87E-03 |
| rs614018 | TCGA&TCGA_coCNV | 4.82E-03 | 3.40E-03 |
| rs1152952 | TCGA | 4.82E-03 | #N/A |
| rs34114 | TCGA&TCGA_coCNV | 4.82E-03 | 1.46E-03 |
| rs1119541 | TCGA | 4.82E-03 | #N/A |
| rs12443114 | TCGA | 4.82E-03 | #N/A |
| rs7729 | TCGA&TCGA_coCNV | 4.14E-03 | 2.53E-03 |
| rs11195323 | TCGA | 4.83E-03 | #N/A |
| rs1491959 | TCGA | 4.83E-03 | #N/A |
| rs6500224 | TCGA&TCGA_coCNV | 4.83E-03 | 1.01E-03 |
| rs997135 | TCGA&TCGA_coCNV | 4.83E-03 | 3.75E-04 |
| rs11860517 | TCGA&TCGA_coCNV | 4.83E-03 | 5.06E-03 |
| rs41461747 | TCGA&TCGA_coCNV | 4.83E-03 | 1.86E-03 |
| rs9927211 | TCGA | 4.83E-03 | #N/A |
| rs1495032 | TCGA&TCGA_coCNV | 4.83E-03 | 8.86E-03 |
| rs7780715 | TCGA&TCGA_coCNV | 4.83E-03 | 7.03E-03 |
| rs1265214 | TCGA | 4.83E-03 | #N/A |
| rs12681567 | TCGA | 4.83E-03 | #N/A |
| rs1877216 | TCGA | 8.99E-04 | #N/A |
| rs725574 | TCGA | 4.83E-03 | #N/A |
| rs4128317 | TCGA&TCGA_coCNV | 4.83E-03 | 2.83E-03 |
| rs1405123 | TCGA | 4.83E-03 | #N/A |
| rs11248496 | TCGA | 4.83E-03 | #N/A |
| rs13430107 | TCGA&TCGA_coCNV | 4.83E-03 | 4.11E-03 |
| rs11187866 | TCGA&TCGA_coCNV | 4.83E-03 | 2.48E-03 |
| rs291944 | TCGA&TCGA_coCNV | 4.83E-03 | 6.54E-03 |
| rs12449687 | TCGA&TCGA_coCNV | 2.64E-03 | 2.72E-04 |
| rs2274871 | TCGA&TCGA_coCNV | 4.84E-03 | 1.70E-03 |
| rs10872009 | TCGA&TCGA_coCNV | 4.84E-03 | 3.84E-03 |
| rs6734844 | TCGA&TCGA_coCNV | 4.84E-03 | 7.63E-03 |
| rs17123288 | TCGA | 4.06E-03 | #N/A |
| rs4772442 | TCGA&TCGA_coCNV | 4.84E-03 | 3.39E-03 |
| rs218018 | TCGA | 4.84E-03 | #N/A |
| rs7936807 | TCGA | 4.84E-03 | #N/A |

| rs6688532 | TCGA&TCGA_coCNV | 4.84E-03 | 2.51E-03 |
| --- | --- | --- | --- |
| rs12938663 | TCGA&TCGA_coCNV | 4.29E-03 | 1.16E-03 |
| rs2309319 | TCGA | 4.84E-03 | #N/A |
| rs9443394 | TCGA&TCGA_coCNV | 1.73E-03 | 1.11E-03 |
| rs4981658 | TCGA&TCGA_coCNV | 4.84E-03 | 8.30E-03 |
| rs4938056 | TCGA&TCGA_coCNV | 4.84E-03 | 9.43E-03 |
| rs11126067 | TCGA&TCGA_coCNV | 4.84E-03 | 2.39E-03 |
| rs13286950 | TCGA | 4.84E-03 | #N/A |
| rs1123992 | TCGA&TCGA_coCNV | 4.85E-03 | 9.24E-03 |
| rs1441138 | TCGA | 4.85E-03 | #N/A |
| rs2647400 | TCGA | 4.85E-03 | #N/A |
| rs41422951 | TCGA&TCGA_coCNV | 1.73E-03 | 9.39E-03 |
| rs2158587 | TCGA&TCGA_coCNV | 2.67E-03 | 4.74E-05 |
| rs7713313 | TCGA&TCGA_coCNV | 4.85E-03 | 8.56E-04 |
| rs10169639 | TCGA&TCGA_coCNV | 4.85E-03 | 9.16E-03 |
| rs682600 | TCGA | 4.85E-03 | #N/A |
| rs16980378 | TCGA | 4.85E-03 | #N/A |
| rs353936 | TCGA | 4.85E-03 | #N/A |
| rs4868215 | TCGA | 4.85E-03 | #N/A |
| rs17359526 | TCGA | 4.85E-03 | #N/A |
| rs2860258 | TCGA&TCGA_coCNV | 4.86E-03 | 7.40E-03 |
| rs6016295 | TCGA&TCGA_coCNV | 4.86E-03 | 1.69E-03 |
| rs1062073 | TCGA&TCGA_coCNV | 4.86E-03 | 7.36E-03 |
| rs7226130 | TCGA&TCGA_coCNV | 4.86E-03 | 6.89E-03 |
| rs382480 | TCGA | 4.86E-03 | #N/A |
| rs3134389 | TCGA&TCGA_coCNV | 4.86E-03 | 6.29E-03 |
| rs7602631 | TCGA&TCGA_coCNV | 4.86E-03 | 9.50E-03 |
| rs10244186 | TCGA | 4.86E-03 | #N/A |
| rs1458326 | TCGA | 4.86E-03 | #N/A |
| rs12437288 | TCGA&TCGA_coCNV | 1.45E-03 | 4.85E-03 |
| rs380322 | TCGA | 4.86E-03 | #N/A |
| rs11195166 | TCGA | 4.87E-03 | #N/A |
| rs4795348 | TCGA&TCGA_coCNV | 7.84E-04 | 4.43E-04 |
| rs431315 | TCGA | 4.87E-03 | #N/A |
| rs4625578 | TCGA&TCGA_coCNV | 4.87E-03 | 1.48E-03 |
| rs10497216 | TCGA | 4.87E-03 | #N/A |
| rs11701500 | TCGA | 4.87E-03 | #N/A |
| rs6431588 | TCGA | 4.87E-03 | #N/A |
| rs1554766 | TCGA | 7.05E-04 | #N/A |
| rs13269006 | TCGA&TCGA_coCNV | 1.20E-03 | 3.75E-03 |
| rs6566645 | TCGA&TCGA_coCNV | 4.87E-03 | 5.46E-03 |
| rs17067566 | TCGA&TCGA_coCNV | 2.20E-03 | 8.99E-03 |
| rs9561428 | TCGA | 4.87E-03 | #N/A |
| rs664954 | TCGA | 4.88E-03 | #N/A |
| rs6822997 | TCGA | 4.88E-03 | #N/A |
| rs2468118 | TCGA&TCGA_coCNV | 2.28E-03 | 9.46E-04 |

| rs7971089 | TCGA&TCGA_coCNV | 4.88E-03 | 7.61E-04 |
| --- | --- | --- | --- |
| rs11193517 | TCGA | 4.88E-03 | #N/A |
| rs7101546 | TCGA | 4.88E-03 | #N/A |
| rs41433250 | TCGA | 4.88E-03 | #N/A |
| rs2014418 | TCGA&TCGA_coCNV | 4.88E-03 | 2.38E-03 |
| rs13180151 | TCGA&TCGA_coCNV | 4.88E-03 | 1.96E-03 |
| rs3910054 | TCGA | 4.88E-03 | #N/A |
| rs7780365 | TCGA&TCGA_coCNV | 4.88E-03 | 3.28E-03 |
| rs12010547 | TCGA | 4.88E-03 | #N/A |
| rs2223848 | TCGA | 4.88E-03 | #N/A |
| rs11974093 | TCGA&TCGA_coCNV | 4.88E-03 | 3.58E-03 |
| rs3802032 | TCGA&TCGA_coCNV | 4.88E-03 | 1.95E-03 |
| rs10516406 | TCGA&TCGA_coCNV | 4.88E-03 | 3.44E-04 |
| rs12322094 | TCGA | 4.89E-03 | #N/A |
| rs12593682 | TCGA&TCGA_coCNV | 2.22E-03 | 8.45E-03 |
| rs12041754 | TCGA&TCGA_coCNV | 4.40E-03 | 3.95E-03 |
| rs2973588 | TCGA&TCGA_coCNV | 4.89E-03 | 1.99E-03 |
| rs9746347 | TCGA | 4.77E-03 | #N/A |
| rs636969 | TCGA&TCGA_coCNV | 4.89E-03 | 1.22E-03 |
| rs17603318 | TCGA&TCGA_coCNV | 4.89E-03 | 3.74E-03 |
| rs2791356 | TCGA&TCGA_coCNV | 4.89E-03 | 1.04E-03 |
| rs8233 | TCGA | 4.89E-03 | #N/A |
| rs17720336 | TCGA&TCGA_coCNV | 3.34E-03 | 3.56E-03 |
| rs17357759 | TCGA&TCGA_coCNV | 4.89E-03 | 1.76E-03 |
| rs12026288 | TCGA | 4.90E-03 | #N/A |
| rs11073502 | TCGA&TCGA_coCNV | 4.90E-03 | 4.50E-03 |
| rs1705392 | TCGA&TCGA_coCNV | 4.02E-04 | 8.86E-04 |
| rs922235 | TCGA | 4.90E-03 | #N/A |
| rs2619602 | TCGA | 4.90E-03 | #N/A |
| rs1003532 | TCGA | 4.90E-03 | #N/A |
| rs7023945 | TCGA | 4.90E-03 | #N/A |
| rs7521572 | TCGA&TCGA_coCNV | 4.90E-03 | 9.21E-04 |
| rs2169682 | TCGA&TCGA_coCNV | 4.90E-03 | 8.52E-03 |
| rs10127810 | TCGA&TCGA_coCNV | 4.90E-03 | 8.13E-03 |
| rs1414459 | TCGA | 3.85E-03 | #N/A |
| rs11771231 | TCGA&TCGA_coCNV | 4.90E-03 | 7.11E-03 |
| rs17792710 | TCGA&TCGA_coCNV | 7.41E-04 | 4.83E-03 |
| rs9306203 | TCGA&TCGA_coCNV | 8.94E-04 | 2.02E-03 |
| rs17827087 | TCGA&TCGA_coCNV | 4.90E-03 | 1.64E-03 |
| rs13148469 | TCGA | 4.91E-03 | #N/A |
| rs7979666 | TCGA&TCGA_coCNV | 4.91E-03 | 8.05E-03 |
| rs13263602 | TCGA&TCGA_coCNV | 4.91E-03 | 5.40E-03 |
| rs2745820 | TCGA&TCGA_coCNV | 4.91E-03 | 8.94E-03 |
| rs1275068 | TCGA&TCGA_coCNV | 4.91E-03 | 7.87E-03 |
| rs11122578 | TCGA&TCGA_coCNV | 4.91E-03 | 2.83E-03 |
| rs968916 | TCGA&TCGA_coCNV | 4.91E-03 | 1.46E-03 |

| rs2369861 | TCGA&TCGA_coCNV | 4.91E-03 | 6.70E-03 |
| --- | --- | --- | --- |
| rs4928097 | TCGA&TCGA_coCNV | 4.91E-03 | 3.05E-03 |
| rs8014139 | TCGA | 4.91E-03 | #N/A |
| rs10120094 | TCGA&TCGA_coCNV | 4.92E-03 | 4.39E-03 |
| rs17606249 | TCGA | 4.51E-03 | #N/A |
| rs2033533 | TCGA&TCGA_coCNV | 4.92E-03 | 8.22E-03 |
| rs12400933 | TCGA | 4.92E-03 | #N/A |
| rs12668460 | TCGA&TCGA_coCNV | 3.69E-04 | 1.79E-03 |
| rs7720671 | TCGA&TCGA_coCNV | 1.06E-04 | 2.45E-03 |
| rs7579206 | TCGA&TCGA_coCNV | 4.92E-03 | 7.78E-03 |
| rs1813496 | TCGA&SCANdb | 4.92E-03 | #N/A |
| rs4908744 | TCGA&TCGA_coCNV | 4.93E-03 | 6.31E-04 |
| rs16944230 | TCGA&TCGA_coCNV | 1.33E-03 | 1.68E-03 |
| rs7736500 | TCGA | 4.93E-03 | #N/A |
| rs1891536 | TCGA | 4.93E-03 | #N/A |
| rs16944691 | TCGA&TCGA_coCNV | 4.93E-03 | 6.99E-03 |
| rs13101986 | TCGA | 4.93E-03 | #N/A |
| rs1793576 | TCGA | 4.93E-03 | #N/A |
| rs7548516 | TCGA&TCGA_coCNV | 4.93E-03 | 8.73E-03 |
| rs4847017 | TCGA | 4.93E-03 | #N/A |
| rs12550768 | TCGA&TCGA_coCNV | 4.93E-03 | 4.17E-03 |
| rs4608898 | TCGA | 4.93E-03 | #N/A |
| rs17073814 | TCGA | 4.93E-03 | #N/A |
| rs10150234 | TCGA&TCGA_coCNV | 4.94E-03 | 5.47E-03 |
| rs12273073 | TCGA&TCGA_coCNV | 9.37E-04 | 9.07E-04 |
| rs2410666 | TCGA&TCGA_coCNV | 4.31E-03 | 8.75E-03 |
| rs10930892 | TCGA | 4.94E-03 | #N/A |
| rs7299571 | TCGA&TCGA_coCNV | 4.94E-03 | 9.28E-04 |
| rs2108763 | TCGA | 4.94E-03 | #N/A |
| rs593910 | TCGA | 4.94E-03 | #N/A |
| rs2298697 | TCGA | 4.94E-03 | #N/A |
| rs2987294 | TCGA&TCGA_coCNV | 4.94E-03 | 5.82E-03 |
| rs10157423 | TCGA | 4.94E-03 | #N/A |
| rs150987 | TCGA&TCGA_coCNV | 4.94E-03 | 1.59E-03 |
| rs12032641 | TCGA | 4.95E-03 | #N/A |
| rs7311343 | TCGA&TCGA_coCNV | 5.12E-04 | 3.51E-05 |
| rs4668312 | TCGA | 4.95E-03 | #N/A |
| rs7350332 | TCGA | 4.95E-03 | #N/A |
| rs5749770 | TCGA | 4.95E-03 | #N/A |
| rs17081908 | TCGA&TCGA_coCNV | 4.95E-03 | 2.97E-04 |
| rs440968 | TCGA&TCGA_coCNV | 4.95E-03 | 2.46E-03 |
| rs537266 | TCGA | 4.95E-03 | #N/A |
| rs942083 | TCGA&TCGA_coCNV | 4.95E-03 | 1.69E-03 |
| rs27043 | TCGA&TCGA_coCNV | 3.13E-06 | 9.67E-05 |
| rs11676617 | TCGA | 4.95E-03 | #N/A |
| rs899486 | TCGA | 4.95E-03 | #N/A |

| rs1567620 | TCGA | 4.96E-03 | #N/A |
| --- | --- | --- | --- |
| rs1606490 | TCGA | 4.96E-03 | #N/A |
| rs16968029 | TCGA&TCGA_coCNV | 4.96E-03 | 2.53E-03 |
| rs17819978 | TCGA | 4.96E-03 | #N/A |
| rs11077163 | TCGA | 4.96E-03 | #N/A |
| rs911664 | TCGA | 6.25E-04 | #N/A |
| rs2202978 | TCGA&TCGA_coCNV | 4.96E-03 | 4.64E-03 |
| rs4575288 | TCGA&TCGA_coCNV | 4.96E-03 | 5.26E-03 |
| rs17608159 | TCGA&TCGA_coCNV | 2.86E-03 | 6.00E-03 |
| rs7036143 | TCGA&TCGA_coCNV | 4.96E-03 | 1.45E-03 |
| rs11012463 | TCGA | 4.96E-03 | #N/A |
| rs2649712 | TCGA | 2.44E-03 | #N/A |
| rs10031664 | TCGA | 1.17E-03 | #N/A |
| rs6640804 | TCGA&TCGA_coCNV | 4.97E-03 | 3.51E-03 |
| rs297223 | TCGA&TCGA_coCNV | 4.97E-03 | 1.15E-03 |
| rs7993181 | TCGA&TCGA_coCNV | 4.97E-03 | 1.97E-04 |
| rs5770417 | TCGA | 4.97E-03 | #N/A |
| rs843072 | TCGA&TCGA_coCNV | 4.97E-03 | 1.78E-03 |
| rs9809665 | TCGA&TCGA_coCNV | 4.97E-03 | 7.69E-04 |
| rs13430741 | TCGA&TCGA_coCNV | 4.97E-03 | 6.55E-03 |
| rs17100963 | TCGA&TCGA_coCNV | 1.50E-03 | 6.42E-03 |
| rs17063226 | TCGA&TCGA_coCNV | 4.97E-03 | 8.56E-03 |
| rs998335 | TCGA&TCGA_coCNV | 4.97E-03 | 3.34E-03 |
| rs2529229 | TCGA | 4.98E-03 | #N/A |
| rs735447 | TCGA&TCGA_coCNV | 4.98E-03 | 4.37E-03 |
| rs6594004 | TCGA&TCGA_coCNV | 4.98E-03 | 2.97E-03 |
| rs490734 | TCGA | 4.98E-03 | #N/A |
| rs12943936 | TCGA&TCGA_coCNV | 4.98E-03 | 8.17E-03 |
| rs12143658 | TCGA | 4.98E-03 | #N/A |
| rs1353020 | TCGA&TCGA_coCNV | 4.98E-03 | 7.59E-03 |
| rs10800621 | TCGA | 4.98E-03 | #N/A |
| rs10065084 | TCGA&TCGA_coCNV | 9.27E-04 | 4.07E-03 |
| rs12537541 | TCGA&TCGA_coCNV | 4.98E-03 | 3.68E-03 |
| rs2798329 | TCGA&TCGA_coCNV | 4.98E-03 | 1.45E-03 |
| rs11683773 | TCGA | 4.98E-03 | #N/A |
| rs2185026 | TCGA | 4.98E-03 | #N/A |
| rs7656541 | TCGA&TCGA_coCNV | 4.99E-03 | 4.43E-03 |
| rs6864913 | TCGA | 7.33E-04 | #N/A |
| rs4631754 | TCGA | 4.99E-03 | #N/A |
| rs9426471 | TCGA | 4.99E-03 | #N/A |
| rs4476894 | TCGA | 4.99E-03 | #N/A |
| rs10493646 | TCGA&TCGA_coCNV | 4.99E-03 | 8.49E-03 |
| rs1373259 | TCGA | 4.99E-03 | #N/A |
| rs9319028 | TCGA | 4.99E-03 | #N/A |
| rs2748723 | TCGA&TCGA_coCNV | 4.01E-05 | 2.19E-05 |
| rs2167201 | TCGA&SCANdb | 4.99E-03 | #N/A |

| rs4766675 | TCGA | 4.99E-03 | #N/A |
| --- | --- | --- | --- |
| rs6939381 | TCGA | 4.99E-03 | #N/A |
| rs6019909 | TCGA&TCGA_coCNV | 4.99E-03 | 3.34E-04 |
| rs12269358 | TCGA&TCGA_coCNV | 4.99E-03 | 6.48E-03 |
| rs739391 | TCGA | 4.99E-03 | #N/A |
| rs11781480 | TCGA | 5.00E-03 | #N/A |
| rs1859336 | TCGA | 5.00E-03 | #N/A |
| rs9840094 | TCGA&TCGA_coCNV | 2.55E-04 | 7.82E-03 |
| rs17140778 | TCGA&TCGA_coCNV | 5.00E-03 | 4.56E-03 |
| rs6896713 | TCGA&TCGA_coCNV | 4.26E-03 | 4.72E-03 |
| rs10809491 | TCGA | 5.00E-03 | #N/A |
| rs1202422 | TCGA | 5.00E-03 | #N/A |
| rs6465477 | TCGA&TCGA_coCNV | 5.00E-03 | 2.62E-03 |
| rs4898690 | TCGA | 5.00E-03 | #N/A |
| rs7769173 | TCGA | 5.00E-03 | #N/A |
| rs1415428 | TCGA | 5.01E-03 | #N/A |
| rs2279758 | TCGA | 5.01E-03 | #N/A |
| rs6913263 | TCGA | 2.85E-03 | #N/A |
| rs1899979 | TCGA | 5.01E-03 | #N/A |
| rs16951812 | TCGA | 1.28E-03 | #N/A |
| rs2096858 | TCGA&TCGA_coCNV | 2.52E-03 | 3.67E-03 |
| rs2599684 | TCGA&TCGA_coCNV | 5.01E-03 | 7.06E-04 |
| rs7847592 | TCGA | 5.01E-03 | #N/A |
| rs10934991 | TCGA | 5.02E-03 | #N/A |
| rs869902 | TCGA&TCGA_coCNV | 5.02E-03 | 2.98E-03 |
| rs473688 | TCGA | 5.02E-03 | #N/A |
| rs2576709 | TCGA | 5.02E-03 | #N/A |
| rs12676460 | TCGA | 5.02E-03 | #N/A |
| rs11791477 | TCGA&TCGA_coCNV | 5.02E-03 | 3.94E-03 |
| rs17003353 | TCGA&TCGA_coCNV | 5.02E-03 | 2.93E-03 |
| rs11777237 | TCGA | 5.02E-03 | #N/A |
| rs12113120 | TCGA | 5.02E-03 | #N/A |
| rs187303 | TCGA&TCGA_coCNV | 5.02E-03 | 8.05E-04 |
| rs17776 | TCGA | 5.02E-03 | #N/A |
| rs10988422 | TCGA | 5.02E-03 | #N/A |
| rs17561867 | TCGA | 5.03E-03 | #N/A |
| rs12465045 | TCGA | 5.03E-03 | #N/A |
| rs11217604 | TCGA&TCGA_coCNV | 5.96E-04 | 5.21E-03 |
| rs9557021 | TCGA | 9.85E-04 | #N/A |
| rs5944705 | TCGA | 5.03E-03 | #N/A |
| rs3904174 | TCGA&TCGA_coCNV | 5.03E-03 | 1.72E-03 |
| rs4794281 | TCGA | 5.03E-03 | #N/A |
| rs616593 | TCGA | 2.74E-03 | #N/A |
| rs6621803 | TCGA&TCGA_coCNV | 1.67E-03 | 6.82E-03 |
| rs2658459 | TCGA&TCGA_coCNV | 5.03E-03 | 2.40E-03 |
| rs702607 | TCGA | 5.03E-03 | #N/A |

| rs6991079 | TCGA&TCGA_coCNV | 5.03E-03 | 4.22E-03 |
| --- | --- | --- | --- |
| rs2207016 | TCGA&TCGA_coCNV | 5.03E-03 | 4.02E-03 |
| rs9381493 | TCGA | 5.03E-03 | #N/A |
| rs1024811 | TCGA | 1.49E-03 | #N/A |
| rs2286830 | TCGA | 3.85E-03 | #N/A |
| rs883954 | TCGA | 3.12E-03 | #N/A |
| rs1981098 | TCGA | 5.04E-03 | #N/A |
| rs2706338 | TCGA | 2.58E-03 | #N/A |
| rs4680819 | TCGA | 5.04E-03 | #N/A |
| rs17584193 | TCGA&TCGA_coCNV | 4.11E-03 | 3.98E-03 |
| rs4670899 | TCGA | 5.04E-03 | #N/A |
| rs11685418 | TCGA&TCGA_coCNV | 5.04E-03 | 8.26E-04 |
| rs11144780 | TCGA | 5.04E-03 | #N/A |
| rs9422915 | TCGA&TCGA_coCNV | 5.04E-03 | 2.46E-04 |
| rs1316353 | TCGA | 5.04E-03 | #N/A |
| rs2530293 | TCGA | 5.04E-03 | #N/A |
| rs9406229 | TCGA&TCGA_coCNV | 5.04E-03 | 9.15E-04 |
| rs655229 | TCGA&TCGA_coCNV | 5.04E-03 | 4.18E-03 |
| rs1500258 | TCGA | 5.05E-03 | #N/A |
| rs1481964 | TCGA | 5.05E-03 | #N/A |
| rs10884068 | TCGA&TCGA_coCNV | 5.05E-03 | 1.99E-03 |
| rs11743722 | TCGA&TCGA_coCNV | 1.57E-05 | 2.48E-04 |
| rs2063480 | TCGA&TCGA_coCNV | 5.05E-03 | 2.45E-03 |
| rs6775392 | TCGA&TCGA_coCNV | 5.05E-03 | 2.94E-03 |
| rs10832986 | TCGA&TCGA_coCNV | 2.49E-03 | 2.40E-04 |
| rs4282001 | TCGA&TCGA_coCNV | 5.05E-03 | 3.55E-03 |
| rs7593 | TCGA&TCGA_coCNV | 3.05E-03 | 6.25E-03 |
| rs12223607 | TCGA | 5.06E-03 | #N/A |
| rs3092957 | TCGA | 5.06E-03 | #N/A |
| rs17096437 | TCGA&TCGA_coCNV | 5.06E-03 | 5.87E-04 |
| rs17054692 | TCGA | 5.06E-03 | #N/A |
| rs6754148 | TCGA&TCGA_coCNV&SCANdb | 5.06E-03 | 4.75E-03 |
| rs33388 | TCGA&TCGA_coCNV | 3.82E-03 | 4.64E-03 |
| rs9551683 | TCGA | 4.47E-03 | #N/A |
| rs2809369 | TCGA&TCGA_coCNV | 1.57E-03 | 9.13E-05 |
| rs1438047 | TCGA | 5.07E-03 | #N/A |
| rs13112518 | TCGA&TCGA_coCNV | 5.07E-03 | 4.21E-03 |
| rs13396426 | TCGA | 5.07E-03 | #N/A |
| rs7548781 | TCGA | 5.07E-03 | #N/A |
| rs11128331 | TCGA | 5.07E-03 | #N/A |
| rs4695963 | TCGA | 5.07E-03 | #N/A |
| rs1523692 | TCGA&TCGA_coCNV | 8.39E-04 | 8.08E-04 |
| rs1862871 | TCGA | 5.07E-03 | #N/A |
| rs12712854 | TCGA | 5.07E-03 | #N/A |
| rs7990838 | TCGA | 2.68E-03 | #N/A |
| rs17753166 | TCGA | 5.08E-03 | #N/A |

| rs2619685 | TCGA | 3.22E-04 | #N/A |
| --- | --- | --- | --- |
| rs9919217 | TCGA&TCGA_coCNV | 5.08E-03 | 8.56E-04 |
| rs1544278 | TCGA | 5.08E-03 | #N/A |
| rs12425190 | TCGA | 5.08E-03 | #N/A |
| rs1521652 | TCGA&TCGA_coCNV | 4.61E-03 | 1.88E-03 |
| rs246537 | TCGA&TCGA_coCNV | 4.09E-03 | 6.40E-04 |
| rs7563368 | TCGA | 5.08E-03 | #N/A |
| rs10508517 | TCGA&TCGA_coCNV | 5.08E-03 | 3.71E-03 |
| rs2118312 | TCGA&TCGA_coCNV | 5.08E-03 | 6.51E-04 |
| rs12643969 | TCGA | 2.41E-03 | #N/A |
| rs1013548 | TCGA&TCGA_coCNV | 5.09E-03 | 9.26E-04 |
| rs6990716 | TCGA | 5.09E-03 | #N/A |
| rs10876402 | TCGA&TCGA_coCNV | 5.09E-03 | 4.44E-03 |
| rs753647 | TCGA&TCGA_coCNV | 5.09E-03 | 3.31E-04 |
| rs10081878 | TCGA | 5.09E-03 | #N/A |
| rs2348942 | TCGA | 5.10E-03 | #N/A |
| rs7888254 | TCGA | 2.72E-03 | #N/A |
| rs7039618 | TCGA | 5.10E-03 | #N/A |
| rs11807829 | TCGA&TCGA_coCNV | 2.64E-03 | 2.30E-03 |
| rs11944591 | TCGA | 5.10E-03 | #N/A |
| rs562731 | TCGA&TCGA_coCNV | 2.83E-04 | 1.94E-05 |
| rs7281481 | TCGA | 5.10E-03 | #N/A |
| rs11602279 | TCGA&TCGA_coCNV | 1.55E-03 | 2.70E-03 |
| rs13030799 | TCGA | 5.11E-03 | #N/A |
| rs17290473 | TCGA&TCGA_coCNV | 5.11E-03 | 1.22E-03 |
| rs809317 | TCGA&TCGA_coCNV | 5.11E-03 | 8.87E-04 |
| rs560354 | TCGA | 5.11E-03 | #N/A |
| rs560096 | TCGA&TCGA_coCNV | 5.11E-03 | 2.39E-03 |
| rs11599308 | TCGA | 5.11E-03 | #N/A |
| rs459465 | TCGA&TCGA_coCNV | 5.11E-03 | 1.37E-03 |
| rs11629888 | TCGA | 5.11E-03 | #N/A |
| rs16868358 | TCGA&TCGA_coCNV | 5.11E-03 | 3.88E-03 |
| rs2250471 | TCGA&TCGA_coCNV | 5.11E-03 | 2.37E-03 |
| rs1984633 | TCGA&TCGA_coCNV | 5.11E-03 | 1.11E-03 |
| rs670712 | TCGA&TCGA_coCNV | 5.16E-05 | 2.24E-03 |
| rs5912124 | TCGA&TCGA_coCNV | 3.87E-03 | 9.95E-03 |
| rs41531346 | TCGA | 5.11E-03 | #N/A |
| rs9309919 | TCGA&TCGA_coCNV | 5.11E-03 | 3.26E-03 |
| rs17010022 | TCGA&TCGA_coCNV | 2.82E-04 | 1.68E-03 |
| rs9476934 | TCGA | 5.11E-03 | #N/A |
| rs12619731 | TCGA&TCGA_coCNV | 5.12E-03 | 1.81E-03 |
| rs6074876 | TCGA | 5.12E-03 | #N/A |
| rs17094879 | TCGA | 5.12E-03 | #N/A |
| rs2515580 | TCGA&TCGA_coCNV | 1.48E-03 | 4.82E-03 |
| rs1010293 | TCGA | 5.12E-03 | #N/A |
| rs2316100 | TCGA&TCGA_coCNV | 4.09E-03 | 1.51E-03 |

| rs670311 | TCGA | 5.13E-03 | #N/A |
| --- | --- | --- | --- |
| rs1917606 | TCGA | 5.13E-03 | #N/A |
| rs1941498 | TCGA&TCGA_coCNV | 5.13E-03 | 3.39E-03 |
| rs10515093 | TCGA | 5.13E-03 | #N/A |
| rs283940 | TCGA&TCGA_coCNV | 5.13E-03 | 1.76E-03 |
| rs12566454 | TCGA&TCGA_coCNV | 5.13E-03 | 2.26E-03 |
| rs41335945 | TCGA | 5.13E-03 | #N/A |
| rs10896468 | TCGA&TCGA_coCNV | 5.13E-03 | 1.17E-03 |
| rs7911075 | TCGA&TCGA_coCNV | 7.81E-04 | 6.65E-03 |
| rs6994620 | TCGA | 5.13E-03 | #N/A |
| rs11906910 | TCGA | 5.13E-03 | #N/A |
| rs2011013 | TCGA | 2.01E-03 | #N/A |
| rs41421144 | TCGA | 5.13E-03 | #N/A |
| rs940848 | TCGA | 4.40E-03 | #N/A |
| rs9975460 | TCGA | 5.13E-03 | #N/A |
| rs855534 | TCGA | 5.13E-03 | #N/A |
| rs13155212 | TCGA&TCGA_coCNV | 1.49E-03 | 7.85E-03 |
| rs4499421 | TCGA | 5.13E-03 | #N/A |
| rs1320561 | TCGA&TCGA_coCNV | 5.14E-03 | 3.43E-03 |
| rs9292274 | TCGA&TCGA_coCNV | 1.21E-03 | 5.22E-03 |
| rs876365 | TCGA&TCGA_coCNV | 5.14E-03 | 1.72E-03 |
| rs1479681 | TCGA | 5.14E-03 | #N/A |
| rs7163164 | TCGA&TCGA_coCNV | 5.14E-03 | 6.92E-04 |
| rs5932692 | TCGA&TCGA_coCNV | 9.98E-05 | 1.18E-03 |
| rs1393423 | TCGA | 5.14E-03 | #N/A |
| rs9973993 | TCGA&TCGA_coCNV | 5.14E-03 | 1.09E-03 |
| rs6079969 | TCGA&TCGA_coCNV | 5.00E-03 | 6.45E-03 |
| rs12596804 | TCGA | 5.14E-03 | #N/A |
| rs9918369 | TCGA | 5.14E-03 | #N/A |
| rs8051395 | TCGA | 5.14E-03 | #N/A |
| rs283943 | TCGA&TCGA_coCNV | 5.14E-03 | 1.59E-03 |
| rs11670672 | TCGA | 1.06E-03 | #N/A |
| rs7537687 | TCGA | 5.15E-03 | #N/A |
| rs2084229 | TCGA&TCGA_coCNV | 5.15E-03 | 1.23E-03 |
| rs11023094 | TCGA | 5.15E-03 | #N/A |
| rs2107098 | TCGA | 5.15E-03 | #N/A |
| rs10848219 | TCGA&TCGA_coCNV | 5.15E-03 | 2.57E-03 |
| rs17725634 | TCGA&TCGA_coCNV | 5.15E-03 | 1.31E-03 |
| rs1375178 | TCGA&TCGA_coCNV | 5.15E-03 | 4.08E-03 |
| rs8200 | TCGA | 4.98E-03 | #N/A |
| rs10850098 | TCGA | 5.15E-03 | #N/A |
| rs9874192 | TCGA&TCGA_coCNV | 5.15E-03 | 1.60E-03 |
| rs7736512 | TCGA | 5.15E-03 | #N/A |
| rs17080801 | TCGA&TCGA_coCNV | 5.15E-03 | 3.04E-04 |
| rs1003199 | TCGA | 5.16E-03 | #N/A |
| rs2195962 | TCGA | 5.16E-03 | #N/A |

| rs907311 | TCGA&TCGA_coCNV | 3.20E-03 | 2.62E-03 |
| --- | --- | --- | --- |
| rs8006004 | TCGA&TCGA_coCNV | 1.76E-03 | 7.10E-03 |
| rs1395470 | TCGA | 5.16E-03 | #N/A |
| rs7791345 | TCGA | 5.16E-03 | #N/A |
| rs7899985 | TCGA | 5.17E-03 | #N/A |
| rs10501814 | TCGA | 5.17E-03 | #N/A |
| rs17029516 | TCGA&TCGA_coCNV | 5.17E-03 | 6.85E-05 |
| rs1422794 | TCGA | 4.65E-03 | #N/A |
| rs11996527 | TCGA&TCGA_coCNV | 5.17E-03 | 6.99E-04 |
| rs39653 | TCGA | 5.17E-03 | #N/A |
| rs17517037 | TCGA&TCGA_coCNV | 5.01E-04 | 1.09E-03 |
| rs9863261 | TCGA&TCGA_coCNV | 5.17E-03 | 1.39E-03 |
| rs1744594 | TCGA&TCGA_coCNV | 3.34E-03 | 8.42E-03 |
| rs11226920 | TCGA&TCGA_coCNV | 5.18E-03 | 2.12E-03 |
| rs1370328 | TCGA&TCGA_coCNV | 3.34E-03 | 2.12E-03 |
| rs10892543 | TCGA&TCGA_coCNV | 4.09E-03 | 5.35E-03 |
| rs12062565 | TCGA&TCGA_coCNV | 5.18E-03 | 1.92E-03 |
| rs667483 | TCGA&TCGA_coCNV | 8.71E-04 | 1.38E-03 |
| rs231693 | TCGA&TCGA_coCNV | 5.18E-03 | 3.24E-03 |
| rs5939519 | TCGA&TCGA_coCNV | 5.18E-03 | 4.47E-03 |
| rs16827377 | TCGA&TCGA_coCNV | 1.42E-03 | 4.72E-04 |
| rs170568 | TCGA&TCGA_coCNV | 5.18E-03 | 1.85E-03 |
| rs41343849 | TCGA | 5.19E-03 | #N/A |
| rs1746098 | TCGA&TCGA_coCNV | 3.53E-03 | 3.27E-04 |
| rs11514908 | TCGA | 5.19E-03 | #N/A |
| rs1556089 | TCGA | 5.19E-03 | #N/A |
| rs1363094 | TCGA | 5.19E-03 | #N/A |
| rs7297872 | TCGA&TCGA_coCNV | 4.72E-03 | 3.51E-04 |
| rs12326994 | TCGA | 5.19E-03 | #N/A |
| rs625913 | TCGA | 5.09E-03 | #N/A |
| rs16944525 | TCGA | 5.19E-03 | #N/A |
| rs6496052 | TCGA | 5.19E-03 | #N/A |
| rs12141362 | TCGA&TCGA_coCNV | 5.19E-03 | 4.84E-03 |
| rs7216834 | TCGA&TCGA_coCNV | 5.19E-03 | 2.69E-04 |
| rs2233213 | TCGA&TCGA_coCNV | 5.19E-03 | 1.21E-03 |
| rs1079338 | TCGA&TCGA_coCNV | 4.49E-04 | 1.35E-03 |
| rs10213934 | TCGA | 5.19E-03 | #N/A |
| rs17837135 | TCGA | 5.19E-03 | #N/A |
| rs2585483 | TCGA&TCGA_coCNV | 5.20E-03 | 2.92E-04 |
| rs10407737 | TCGA&TCGA_coCNV | 2.97E-03 | 2.54E-03 |
| rs1470225 | TCGA | 5.20E-03 | #N/A |
| rs6458532 | TCGA&TCGA_coCNV | 3.54E-03 | 1.54E-03 |
| rs4237370 | TCGA&TCGA_coCNV | 5.20E-03 | 4.37E-03 |
| rs17656058 | TCGA&TCGA_coCNV | 7.28E-04 | 7.59E-03 |
| rs1218427 | TCGA&TCGA_coCNV | 5.20E-03 | 1.79E-03 |
| rs9792144 | TCGA&TCGA_coCNV | 5.20E-03 | 9.80E-04 |

| rs7873389 | TCGA | 2.09E-03 | #N/A |
| --- | --- | --- | --- |
| rs12601154 | TCGA&TCGA_coCNV | 5.20E-03 | 4.55E-03 |
| rs1202308 | TCGA | 5.20E-03 | #N/A |
| rs7695327 | TCGA&TCGA_coCNV | 5.20E-03 | 2.39E-03 |
| rs4488702 | TCGA&TCGA_coCNV | 2.80E-03 | 4.36E-03 |
| rs235930 | TCGA&TCGA_coCNV | 5.21E-03 | 2.87E-03 |
| rs6717961 | TCGA&TCGA_coCNV | 5.21E-03 | 1.60E-03 |
| rs16891157 | TCGA | 5.21E-03 | #N/A |
| rs11125637 | TCGA | 5.21E-03 | #N/A |
| rs1478594 | TCGA | 5.21E-03 | #N/A |
| rs6536514 | TCGA | 5.21E-03 | #N/A |
| rs10252256 | TCGA&TCGA_coCNV | 5.11E-04 | 2.25E-03 |
| rs3754674 | TCGA | 5.21E-03 | #N/A |
| rs995538 | TCGA | 5.21E-03 | #N/A |
| rs17465265 | TCGA | 5.21E-03 | #N/A |
| rs5963419 | TCGA | 5.22E-03 | #N/A |
| rs2614280 | TCGA&TCGA_coCNV | 5.22E-03 | 2.06E-03 |
| rs13431051 | TCGA&TCGA_coCNV | 2.16E-04 | 1.20E-03 |
| rs3743200 | TCGA&TCGA_coCNV | 5.22E-03 | 3.49E-03 |
| rs13267596 | TCGA | 5.22E-03 | #N/A |
| rs4739793 | TCGA&TCGA_coCNV | 2.44E-03 | 1.27E-03 |
| rs11737310 | TCGA | 2.26E-03 | #N/A |
| rs283935 | TCGA&TCGA_coCNV | 5.22E-03 | 2.79E-03 |
| rs6005500 | TCGA | 5.22E-03 | #N/A |
| rs6479732 | TCGA | 5.22E-03 | #N/A |
| rs10130835 | TCGA&TCGA_coCNV | 8.77E-04 | 3.21E-04 |
| rs1129301 | TCGA | 5.23E-03 | #N/A |
| rs2131967 | TCGA&TCGA_coCNV | 5.23E-03 | 3.51E-04 |
| rs2271239 | TCGA&TCGA_coCNV | 5.23E-03 | 2.15E-03 |
| rs10282325 | TCGA | 5.23E-03 | #N/A |
| rs610754 | TCGA&TCGA_coCNV | 5.23E-03 | 1.69E-03 |
| rs16980045 | TCGA | 5.23E-03 | #N/A |
| rs5921524 | TCGA&TCGA_coCNV | 4.66E-04 | 1.31E-03 |
| rs7707528 | TCGA | 5.23E-03 | #N/A |
| rs259920 | TCGA | 4.43E-03 | #N/A |
| rs3114477 | TCGA | 5.23E-03 | #N/A |
| rs10744281 | TCGA | 5.23E-03 | #N/A |
| rs161455 | TCGA | 5.24E-03 | #N/A |
| rs2957614 | TCGA | 5.24E-03 | #N/A |
| rs1214210 | TCGA | 5.24E-03 | #N/A |
| rs6563991 | TCGA | 5.24E-03 | #N/A |
| rs7827176 | TCGA&TCGA_coCNV | 5.24E-03 | 4.41E-04 |
| rs1127047 | TCGA&TCGA_coCNV | 5.24E-03 | 7.03E-04 |
| rs11180811 | TCGA&TCGA_coCNV | 6.21E-04 | 9.75E-04 |
| rs9327829 | TCGA | 5.24E-03 | #N/A |
| rs1731645 | TCGA | 5.24E-03 | #N/A |

| rs1212304 | TCGA | 5.24E-03 | #N/A |
| --- | --- | --- | --- |
| rs7430822 | TCGA | 5.24E-03 | #N/A |
| rs2291681 | TCGA | 5.24E-03 | #N/A |
| rs11884423 | TCGA&TCGA_coCNV | 5.25E-03 | 4.41E-03 |
| rs7702415 | TCGA&TCGA_coCNV | 5.25E-03 | 1.74E-03 |
| rs1563703 | TCGA | 5.25E-03 | #N/A |
| rs4744106 | TCGA | 5.25E-03 | #N/A |
| rs12232062 | TCGA&TCGA_coCNV | 5.25E-03 | 4.94E-04 |
| rs17068265 | TCGA | 5.25E-03 | #N/A |
| rs12196140 | TCGA&TCGA_coCNV | 5.25E-03 | 4.45E-04 |
| rs9634574 | TCGA | 5.26E-03 | #N/A |
| rs929853 | TCGA | 2.05E-03 | #N/A |
| rs6746495 | TCGA | 4.17E-03 | #N/A |
| rs17432784 | TCGA | 5.26E-03 | #N/A |
| rs17026836 | TCGA&TCGA_coCNV | 3.46E-05 | 9.38E-05 |
| rs2782516 | TCGA | 5.26E-03 | #N/A |
| rs6419561 | TCGA | 5.26E-03 | #N/A |
| rs673709 | TCGA | 5.26E-03 | #N/A |
| rs6134494 | TCGA | 5.26E-03 | #N/A |
| rs970552 | TCGA | 5.26E-03 | #N/A |
| rs9961444 | TCGA&TCGA_coCNV | 6.96E-04 | 6.64E-04 |
| rs7625422 | TCGA | 5.26E-03 | #N/A |
| rs4664544 | TCGA | 5.26E-03 | #N/A |
| rs7791651 | TCGA | 5.27E-03 | #N/A |
| rs9663811 | TCGA | 5.27E-03 | #N/A |
| rs4499607 | TCGA&TCGA_coCNV | 2.77E-03 | 1.84E-03 |
| rs2935620 | TCGA | 5.27E-03 | #N/A |
| rs5915365 | TCGA | 5.27E-03 | #N/A |
| rs17738017 | TCGA | 5.28E-03 | #N/A |
| rs4724508 | TCGA&TCGA_coCNV | 5.28E-03 | 2.65E-03 |
| rs17226450 | TCGA&TCGA_coCNV | 5.28E-03 | 1.41E-03 |
| rs10519381 | TCGA | 5.28E-03 | #N/A |
| rs8014949 | TCGA&TCGA_coCNV | 5.28E-03 | 2.43E-03 |
| rs17632797 | TCGA&TCGA_coCNV | 2.16E-03 | 2.58E-03 |
| rs37318 | TCGA&TCGA_coCNV | 5.28E-03 | 6.26E-04 |
| rs16878919 | TCGA | 2.80E-04 | #N/A |
| rs12893426 | TCGA | 5.28E-03 | #N/A |
| rs10842321 | TCGA&TCGA_coCNV | 4.61E-03 | 7.97E-04 |
| rs8073971 | TCGA&TCGA_coCNV | 5.28E-03 | 2.27E-03 |
| rs900158 | TCGA&TCGA_coCNV | 5.28E-03 | 1.28E-03 |
| rs17069009 | TCGA | 5.28E-03 | #N/A |
| rs10495513 | TCGA | 5.28E-03 | #N/A |
| rs401147 | TCGA | 3.08E-03 | #N/A |
| rs13075932 | TCGA&TCGA_coCNV | 4.37E-03 | 3.09E-03 |
| rs6003648 | TCGA&TCGA_coCNV | 5.29E-03 | 3.83E-03 |
| rs2826496 | TCGA | 5.29E-03 | #N/A |

| rs3946526 | TCGA | 4.52E-03 | #N/A |
| --- | --- | --- | --- |
| rs6714774 | TCGA | 5.29E-03 | #N/A |
| rs2620752 | TCGA&TCGA_coCNV | 5.29E-03 | 5.32E-04 |
| rs7814601 | TCGA&TCGA_coCNV | 3.53E-03 | 7.61E-03 |
| rs11079382 | TCGA&TCGA_coCNV | 5.30E-03 | 3.03E-03 |
| rs17427767 | TCGA | 5.30E-03 | #N/A |
| rs17020151 | TCGA&TCGA_coCNV | 1.18E-03 | 4.26E-04 |
| rs2986213 | TCGA&TCGA_coCNV | 1.34E-03 | 6.45E-03 |
| rs3817326 | TCGA&TCGA_coCNV | 4.88E-03 | 9.43E-03 |
| rs11647185 | TCGA | 5.30E-03 | #N/A |
| rs12776269 | TCGA&TCGA_coCNV | 1.17E-03 | 9.89E-03 |
| rs17287916 | TCGA | 5.30E-03 | #N/A |
| rs12132957 | TCGA&TCGA_coCNV | 5.30E-03 | 3.93E-03 |
| rs2186305 | TCGA | 2.46E-03 | #N/A |
| rs6728532 | TCGA | 1.29E-03 | #N/A |
| rs1539761 | TCGA&TCGA_coCNV | 3.34E-03 | 3.51E-04 |
| rs16865932 | TCGA | 4.27E-03 | #N/A |
| rs17084334 | TCGA | 5.31E-03 | #N/A |
| rs1520500 | TCGA&TCGA_coCNV | 5.31E-03 | 3.01E-03 |
| rs16838982 | TCGA | 5.31E-03 | #N/A |
| rs535690 | TCGA | 5.31E-03 | #N/A |
| rs37321 | TCGA&TCGA_coCNV | 5.31E-03 | 1.27E-03 |
| rs1110146 | TCGA | 5.31E-03 | #N/A |
| rs2428015 | TCGA | 5.31E-03 | #N/A |
| rs7153608 | TCGA&TCGA_coCNV | 5.32E-03 | 1.84E-03 |
| rs11175792 | TCGA&TCGA_coCNV | 6.09E-05 | 1.09E-04 |
| rs4946748 | TCGA | 5.32E-03 | #N/A |
| rs6493543 | TCGA&TCGA_coCNV | 2.58E-04 | 2.01E-03 |
| rs2228046 | TCGA | 5.32E-03 | #N/A |
| rs5749447 | TCGA | 5.32E-03 | #N/A |
| rs412681 | TCGA | 5.32E-03 | #N/A |
| rs7757198 | TCGA&TCGA_coCNV | 5.32E-03 | 2.58E-03 |
| rs1981626 | TCGA | 5.32E-03 | #N/A |
| rs218012 | TCGA | 5.32E-03 | #N/A |
| rs12003718 | TCGA&TCGA_coCNV | 1.53E-03 | 4.79E-04 |
| rs16915421 | TCGA&TCGA_coCNV | 5.32E-03 | 3.24E-03 |
| rs17072519 | TCGA&TCGA_coCNV | 5.32E-03 | 4.59E-04 |
| rs1422505 | TCGA&TCGA_coCNV | 5.33E-03 | 4.04E-03 |
| rs341137 | TCGA&TCGA_coCNV | 5.33E-03 | 2.14E-03 |
| rs502771 | TCGA | 5.33E-03 | #N/A |
| rs400857 | TCGA | 5.33E-03 | #N/A |
| rs4477859 | TCGA&TCGA_coCNV | 5.33E-03 | 2.38E-03 |
| rs1892721 | TCGA&TCGA_coCNV | 5.33E-03 | 2.58E-03 |
| rs10491490 | TCGA | 5.33E-03 | #N/A |
| rs9318198 | TCGA&TCGA_coCNV | 1.84E-03 | 5.38E-03 |
| rs4617684 | TCGA&TCGA_coCNV | 9.42E-04 | 9.06E-04 |

| rs2172849 | TCGA&TCGA_coCNV | 4.83E-04 | 1.36E-03 |
| --- | --- | --- | --- |
| rs16986759 | TCGA | 5.34E-03 | #N/A |
| rs456835 | TCGA | 5.34E-03 | #N/A |
| rs6083014 | TCGA&TCGA_coCNV | 5.34E-03 | 2.55E-03 |
| rs17050925 | TCGA&TCGA_coCNV | 5.34E-03 | 1.51E-03 |
| rs17821226 | TCGA | 4.21E-03 | #N/A |
| rs4766526 | TCGA | 5.35E-03 | #N/A |
| rs13298697 | TCGA&TCGA_coCNV | 5.35E-03 | 4.75E-03 |
| rs2726885 | TCGA&TCGA_coCNV | 3.13E-03 | 7.03E-03 |
| rs7219074 | TCGA&TCGA_coCNV | 1.29E-03 | 1.12E-03 |
| rs11678113 | TCGA&TCGA_coCNV | 2.98E-03 | 6.98E-03 |
| rs822518 | TCGA | 5.35E-03 | #N/A |
| rs12561781 | TCGA&TCGA_coCNV | 1.72E-03 | 7.22E-04 |
| rs2162683 | TCGA&TCGA_coCNV | 1.90E-03 | 4.16E-03 |
| rs2286069 | TCGA&TCGA_coCNV | 2.46E-03 | 1.31E-03 |
| rs17096966 | TCGA | 5.36E-03 | #N/A |
| rs11890049 | TCGA | 5.36E-03 | #N/A |
| rs16980592 | TCGA | 4.51E-04 | #N/A |
| rs9646423 | TCGA&TCGA_coCNV | 7.65E-04 | 4.97E-03 |
| rs4708157 | TCGA | 5.36E-03 | #N/A |
| rs9382980 | TCGA&TCGA_coCNV | 5.36E-03 | 3.07E-03 |
| rs7958091 | TCGA&TCGA_coCNV | 5.37E-03 | 1.71E-03 |
| rs10241470 | TCGA&TCGA_coCNV | 5.37E-03 | 3.21E-03 |
| rs11732162 | TCGA | 5.37E-03 | #N/A |
| rs4389183 | TCGA&TCGA_coCNV | 5.37E-03 | 9.87E-04 |
| rs5984630 | TCGA&TCGA_coCNV | 5.37E-03 | 6.96E-04 |
| rs346146 | TCGA&TCGA_coCNV | 1.90E-03 | 1.98E-03 |
| rs7461467 | TCGA | 5.37E-03 | #N/A |
| rs2869822 | TCGA | 5.37E-03 | #N/A |
| rs725670 | TCGA | 5.37E-03 | #N/A |
| rs2383474 | TCGA&TCGA_coCNV | 4.43E-04 | 2.18E-03 |
| rs3804621 | TCGA&TCGA_coCNV | 5.37E-03 | 4.82E-04 |
| rs13163662 | TCGA | 5.38E-03 | #N/A |
| rs10918486 | TCGA | 5.38E-03 | #N/A |
| rs2828777 | TCGA&TCGA_coCNV | 5.38E-03 | 4.12E-03 |
| rs9980699 | TCGA&TCGA_coCNV | 5.38E-03 | 1.70E-03 |
| rs7819140 | TCGA&TCGA_coCNV | 2.31E-03 | 3.78E-03 |
| rs869806 | TCGA | 5.38E-03 | #N/A |
| rs16843440 | TCGA | 5.15E-03 | #N/A |
| rs11873674 | TCGA | 4.75E-03 | #N/A |
| rs7722152 | TCGA | 5.38E-03 | #N/A |
| rs11854614 | TCGA&TCGA_coCNV | 5.38E-03 | 4.43E-03 |
| rs1618598 | TCGA&TCGA_coCNV | 5.39E-03 | 1.87E-03 |
| rs11164940 | TCGA&TCGA_coCNV | 5.39E-03 | 3.46E-03 |
| rs16943128 | TCGA&TCGA_coCNV | 2.76E-03 | 2.21E-03 |
| rs10823565 | TCGA | 5.39E-03 | #N/A |

| rs7003129 | TCGA | 1.89E-03 | #N/A |
| --- | --- | --- | --- |
| rs13102150 | TCGA | 5.39E-03 | #N/A |
| rs17005956 | TCGA | 1.43E-03 | #N/A |
| rs4859011 | TCGA&TCGA_coCNV | 5.39E-03 | 1.58E-03 |
| rs4789502 | TCGA&TCGA_coCNV | 5.39E-03 | 4.02E-03 |
| rs680789 | TCGA&TCGA_coCNV | 5.39E-03 | 6.51E-04 |
| rs10825687 | TCGA&TCGA_coCNV | 5.40E-03 | 2.12E-03 |
| rs10038913 | TCGA | 5.40E-03 | #N/A |
| rs17097516 | TCGA&TCGA_coCNV | 5.40E-03 | 1.22E-03 |
| rs6453360 | TCGA | 5.40E-03 | #N/A |
| rs10508785 | TCGA | 5.40E-03 | #N/A |
| rs2239352 | TCGA&TCGA_coCNV | 5.40E-03 | 1.65E-03 |
| rs6767544 | TCGA&TCGA_coCNV | 5.40E-03 | 2.80E-03 |
| rs6860106 | TCGA&TCGA_coCNV | 3.02E-04 | 2.95E-03 |
| rs11663430 | TCGA | 5.40E-03 | #N/A |
| rs12287079 | TCGA | 5.40E-03 | #N/A |
| rs4547126 | TCGA | 3.75E-03 | #N/A |
| rs6747618 | TCGA | 5.41E-03 | #N/A |
| rs7886331 | TCGA&TCGA_coCNV | 1.96E-03 | 4.90E-03 |
| rs7140387 | TCGA&TCGA_coCNV | 5.41E-03 | 4.88E-03 |
| rs6436407 | TCGA&TCGA_coCNV | 5.41E-03 | 1.71E-03 |
| rs17012514 | TCGA | 3.86E-03 | #N/A |
| rs430746 | TCGA | 4.92E-03 | #N/A |
| rs11159249 | TCGA | 4.77E-03 | #N/A |
| rs1015966 | TCGA | 5.41E-03 | #N/A |
| rs11099745 | TCGA | 5.41E-03 | #N/A |
| rs7195562 | TCGA&TCGA_coCNV | 5.41E-03 | 4.88E-03 |
| rs17252426 | TCGA | 5.41E-03 | #N/A |
| rs2122775 | TCGA&TCGA_coCNV | 7.28E-04 | 5.23E-03 |
| rs4344425 | TCGA | 5.41E-03 | #N/A |
| rs17016013 | TCGA | 2.51E-04 | #N/A |
| rs1529619 | TCGA | 5.42E-03 | #N/A |
| rs2848993 | TCGA | 1.36E-03 | #N/A |
| rs6490627 | TCGA | 5.42E-03 | #N/A |
| rs2293276 | TCGA&TCGA_coCNV | 1.92E-03 | 2.06E-03 |
| rs4612055 | TCGA | 5.42E-03 | #N/A |
| rs2112176 | TCGA&TCGA_coCNV | 5.42E-03 | 2.22E-03 |
| rs515246 | TCGA | 5.42E-03 | #N/A |
| rs2953029 | TCGA | 5.42E-03 | #N/A |
| rs1030326 | TCGA | 5.42E-03 | #N/A |
| rs4760953 | TCGA | 5.42E-03 | #N/A |
| rs750515 | TCGA&TCGA_coCNV | 2.50E-04 | 5.36E-05 |
| rs2025050 | TCGA | 5.42E-03 | #N/A |
| rs4768065 | TCGA | 5.42E-03 | #N/A |
| rs17016967 | TCGA | 5.43E-03 | #N/A |
| rs1428129 | TCGA&TCGA_coCNV | 5.43E-03 | 2.65E-03 |

| rs1047470 | TCGA | 5.43E-03 | #N/A |
| --- | --- | --- | --- |
| rs9494404 | TCGA | 5.43E-03 | #N/A |
| rs7051102 | TCGA | 5.43E-03 | #N/A |
| rs4297869 | TCGA | 5.43E-03 | #N/A |
| rs12229654 | TCGA | 5.43E-03 | #N/A |
| rs13181830 | TCGA&TCGA_coCNV | 1.63E-03 | 3.29E-03 |
| rs11573125 | TCGA&TCGA_coCNV | 4.38E-03 | 5.27E-03 |
| rs7426534 | TCGA&TCGA_coCNV | 2.47E-03 | 2.00E-03 |
| rs11245515 | TCGA&TCGA_coCNV | 5.44E-03 | 4.65E-03 |
| rs1360526 | TCGA&TCGA_coCNV | 4.13E-04 | 3.53E-03 |
| rs3766558 | TCGA&TCGA_coCNV | 5.44E-03 | 5.78E-04 |
| rs2928127 | TCGA | 5.44E-03 | #N/A |
| rs9671895 | TCGA | 5.44E-03 | #N/A |
| rs11893187 | TCGA&TCGA_coCNV | 5.44E-03 | 2.39E-04 |
| rs11694724 | TCGA&TCGA_coCNV | 1.17E-03 | 5.84E-03 |
| rs12889345 | TCGA | 5.44E-03 | #N/A |
| rs1999716 | TCGA | 5.44E-03 | #N/A |
| rs4849148 | TCGA&TCGA_coCNV | 2.26E-04 | 1.85E-03 |
| rs968026 | TCGA&TCGA_coCNV | 5.00E-04 | 8.14E-04 |
| rs10966611 | TCGA&TCGA_coCNV | 6.49E-04 | 4.91E-03 |
| rs2371475 | TCGA | 5.45E-03 | #N/A |
| rs11564216 | TCGA | 5.45E-03 | #N/A |
| rs1485264 | TCGA&TCGA_coCNV | 1.93E-03 | 4.50E-04 |
| rs6878430 | TCGA&TCGA_coCNV | 5.45E-03 | 9.97E-04 |
| rs12948120 | TCGA&TCGA_coCNV | 5.45E-03 | 8.86E-04 |
| rs2186598 | TCGA&TCGA_coCNV | 5.45E-03 | 3.16E-03 |
| rs9815711 | TCGA&TCGA_coCNV | 5.45E-03 | 4.43E-04 |
| rs473755 | TCGA&TCGA_coCNV | 5.45E-03 | 4.23E-03 |
| rs10887142 | TCGA&TCGA_coCNV | 3.95E-03 | 8.67E-03 |
| rs2829347 | TCGA&TCGA_coCNV | 5.45E-03 | 4.59E-03 |
| rs13288000 | TCGA | 4.03E-03 | #N/A |
| rs2289895 | TCGA | 5.45E-03 | #N/A |
| rs9369073 | TCGA&TCGA_coCNV | 5.45E-03 | 4.37E-03 |
| rs1510937 | TCGA | 4.30E-03 | #N/A |
| rs13192411 | TCGA&TCGA_coCNV | 9.33E-04 | 1.50E-03 |
| rs10999172 | TCGA | 5.45E-03 | #N/A |
| rs2811332 | TCGA&TCGA_coCNV | 2.57E-03 | 1.88E-03 |
| rs1382278 | TCGA | 5.46E-03 | #N/A |
| rs7682109 | TCGA | 5.46E-03 | #N/A |
| rs12465802 | TCGA&TCGA_coCNV | 3.25E-03 | 2.95E-04 |
| rs32477 | TCGA | 5.46E-03 | #N/A |
| rs4806798 | TCGA | 4.13E-03 | #N/A |
| rs560708 | TCGA&TCGA_coCNV | 5.46E-03 | 1.87E-03 |
| rs2237136 | TCGA | 5.46E-03 | #N/A |
| rs1992570 | TCGA&TCGA_coCNV | 1.79E-03 | 4.38E-03 |
| rs16993238 | TCGA | 5.46E-03 | #N/A |

| rs17102869 | TCGA | 5.46E-03 | #N/A |
| --- | --- | --- | --- |
| rs2050361 | TCGA | 2.90E-03 | #N/A |
| rs9531826 | TCGA&TCGA_coCNV | 8.34E-04 | 4.96E-03 |
| rs12150976 | TCGA | 5.46E-03 | #N/A |
| rs4713692 | TCGA&TCGA_coCNV | 3.44E-03 | 5.98E-03 |
| rs731091 | TCGA | 5.47E-03 | #N/A |
| rs1754070 | TCGA | 5.47E-03 | #N/A |
| rs1545133 | TCGA&TCGA_coCNV | 1.25E-04 | 6.54E-03 |
| rs3741165 | TCGA&TCGA_coCNV | 5.22E-05 | 3.45E-04 |
| rs1020334 | TCGA&TCGA_coCNV | 3.17E-03 | 2.92E-03 |
| rs7019796 | TCGA&TCGA_coCNV | 5.47E-03 | 3.83E-03 |
| rs13380978 | TCGA&TCGA_coCNV | 5.47E-03 | 4.54E-03 |
| rs10213025 | TCGA | 5.47E-03 | #N/A |
| rs10818293 | TCGA | 2.70E-03 | #N/A |
| rs28655007 | TCGA | 5.47E-03 | #N/A |
| rs561025 | TCGA&TCGA_coCNV | 3.96E-03 | 6.06E-03 |
| rs11651871 | TCGA&TCGA_coCNV | 5.47E-03 | 4.01E-03 |
| rs1385133 | TCGA&TCGA_coCNV | 5.47E-03 | 1.19E-03 |
| rs16943028 | TCGA | 5.47E-03 | #N/A |
| rs41383349 | TCGA&TCGA_coCNV | 4.30E-03 | 1.13E-03 |
| rs9579520 | TCGA | 5.48E-03 | #N/A |
| rs1413796 | TCGA&TCGA_coCNV | 1.85E-03 | 8.59E-03 |
| rs573857 | TCGA&TCGA_coCNV | 5.48E-03 | 2.22E-03 |
| rs1515367 | TCGA&TCGA_coCNV | 5.48E-03 | 4.79E-03 |
| rs945044 | TCGA&TCGA_coCNV | 5.48E-03 | 4.63E-03 |
| rs725455 | TCGA&TCGA_coCNV | 5.48E-03 | 4.77E-03 |
| rs12067303 | TCGA&TCGA_coCNV | 5.48E-03 | 4.73E-03 |
| rs5906360 | TCGA&TCGA_coCNV | 5.48E-03 | 2.78E-03 |
| rs7042293 | TCGA | 5.41E-03 | #N/A |
| rs2478517 | TCGA | 5.49E-03 | #N/A |
| rs912323 | TCGA&TCGA_coCNV | 5.49E-03 | 7.38E-04 |
| rs11972240 | TCGA | 5.49E-03 | #N/A |
| rs16827056 | TCGA&TCGA_coCNV | 3.45E-04 | 1.20E-03 |
| rs1871185 | TCGA&TCGA_coCNV | 5.49E-03 | 2.69E-03 |
| rs41402944 | TCGA&TCGA_coCNV | 3.43E-05 | 1.84E-03 |
| rs1061338 | TCGA | 5.49E-03 | #N/A |
| rs4671681 | TCGA | 5.49E-03 | #N/A |
| rs2963758 | TCGA&TCGA_coCNV | 5.49E-03 | 1.34E-03 |
| rs6626224 | TCGA | 5.49E-03 | #N/A |
| rs6421297 | TCGA&TCGA_coCNV | 5.49E-03 | 4.76E-04 |
| rs16886626 | TCGA&TCGA_coCNV | 2.46E-04 | 1.22E-04 |
| rs12594801 | TCGA | 5.50E-03 | #N/A |
| rs1372697 | TCGA&TCGA_coCNV | 2.16E-03 | 4.31E-03 |
| rs10943632 | TCGA&TCGA_coCNV | 1.83E-03 | 8.78E-06 |
| rs4693716 | TCGA&TCGA_coCNV | 5.50E-03 | 4.92E-03 |
| rs7422521 | TCGA&TCGA_coCNV | 3.49E-03 | 5.10E-04 |

| rs17244499 | TCGA | 5.50E-03 | #N/A |
| --- | --- | --- | --- |
| rs640934 | TCGA | 5.50E-03 | #N/A |
| rs2259727 | TCGA | 5.50E-03 | #N/A |
| rs11939510 | TCGA&TCGA_coCNV | 2.45E-03 | 4.71E-03 |
| rs12928238 | TCGA&TCGA_coCNV | 1.88E-03 | 6.57E-03 |
| rs16848183 | TCGA&TCGA_coCNV | 5.50E-03 | 2.24E-03 |
| rs11067371 | TCGA&TCGA_coCNV | 5.50E-03 | 2.60E-03 |
| rs12247568 | TCGA&TCGA_coCNV | 2.29E-03 | 1.72E-04 |
| rs17189116 | TCGA | 5.51E-03 | #N/A |
| rs11659253 | TCGA | 5.51E-03 | #N/A |
| rs5905410 | TCGA | 4.09E-03 | #N/A |
| rs2844463 | TCGA | 5.75E-04 | #N/A |
| rs1536661 | TCGA | 3.78E-03 | #N/A |
| rs10026773 | TCGA&TCGA_coCNV | 9.89E-04 | 9.16E-03 |
| rs2166060 | TCGA&TCGA_coCNV | 5.51E-03 | 2.69E-03 |
| rs41340145 | TCGA&TCGA_coCNV | 4.66E-03 | 2.26E-03 |
| rs9536789 | TCGA | 5.51E-03 | #N/A |
| rs12438080 | TCGA&TCGA_coCNV | 5.51E-03 | 3.77E-03 |
| rs1701644 | TCGA&TCGA_coCNV | 5.51E-03 | 1.10E-03 |
| rs6428094 | TCGA | 5.51E-03 | #N/A |
| rs3856510 | TCGA&TCGA_coCNV | 5.51E-03 | 1.55E-03 |
| rs12548772 | TCGA&TCGA_coCNV | 5.51E-03 | 3.68E-03 |
| rs6525572 | TCGA | 2.33E-03 | #N/A |
| rs3820387 | TCGA | 5.52E-03 | #N/A |
| rs4633449 | TCGA | 5.52E-03 | #N/A |
| rs13119187 | TCGA | 5.52E-03 | #N/A |
| rs1439095 | TCGA&TCGA_coCNV | 5.52E-03 | 5.00E-03 |
| rs2038252 | TCGA | 5.52E-03 | #N/A |
| rs6662186 | TCGA&TCGA_coCNV | 4.71E-03 | 4.86E-03 |
| rs7083479 | TCGA | 5.52E-03 | #N/A |
| rs11737981 | TCGA&TCGA_coCNV | 5.52E-03 | 1.32E-03 |
| rs10851449 | TCGA&TCGA_coCNV | 5.52E-03 | 3.14E-04 |
| rs2072019 | TCGA | 5.52E-03 | #N/A |
| rs2504327 | TCGA | 5.53E-03 | #N/A |
| rs1510336 | TCGA&TCGA_coCNV | 2.13E-03 | 7.45E-04 |
| rs11618138 | TCGA | 4.59E-03 | #N/A |
| rs4875134 | TCGA&TCGA_coCNV | 5.53E-03 | 6.46E-04 |
| rs11713583 | TCGA&TCGA_coCNV | 3.57E-03 | 5.93E-03 |
| rs9998162 | TCGA | 5.53E-03 | #N/A |
| rs41395644 | TCGA&TCGA_coCNV | 5.53E-03 | 3.32E-04 |
| rs12062276 | TCGA&TCGA_coCNV | 5.53E-03 | 3.64E-03 |
| rs7597220 | TCGA&TCGA_coCNV | 5.53E-03 | 1.13E-03 |
| rs9475342 | TCGA&TCGA_coCNV | 4.52E-03 | 1.85E-03 |
| rs12494548 | TCGA&TCGA_coCNV | 5.53E-03 | 3.86E-04 |
| rs4407429 | TCGA&TCGA_coCNV | 2.96E-03 | 1.96E-03 |
| rs11807765 | TCGA&TCGA_coCNV | 5.54E-03 | 6.58E-04 |

| rs17110253 | TCGA | 5.54E-03 | #N/A |
| --- | --- | --- | --- |
| rs2570558 | TCGA | 5.54E-03 | #N/A |
| rs17105879 | TCGA&TCGA_coCNV | 2.46E-03 | 5.82E-03 |
| rs3923330 | TCGA | 5.54E-03 | #N/A |
| rs2933190 | TCGA | 2.38E-03 | #N/A |
| rs900234 | TCGA | 5.54E-03 | #N/A |
| rs6023496 | TCGA | 5.54E-03 | #N/A |
| rs12632101 | TCGA&TCGA_coCNV | 5.54E-03 | 1.06E-03 |
| rs2158177 | TCGA | 5.54E-03 | #N/A |
| rs7038686 | TCGA | 5.54E-03 | #N/A |
| rs2900500 | TCGA&TCGA_coCNV | 5.55E-03 | 1.60E-03 |
| rs10877193 | TCGA&TCGA_coCNV | 2.04E-03 | 8.68E-04 |
| rs2330523 | TCGA&TCGA_coCNV | 1.02E-03 | 7.28E-04 |
| rs7802899 | TCGA&TCGA_coCNV | 4.80E-03 | 9.33E-06 |
| rs1451511 | TCGA | 9.64E-04 | #N/A |
| rs2056344 | TCGA&TCGA_coCNV | 5.55E-03 | 1.28E-03 |
| rs17071722 | TCGA&TCGA_coCNV | 5.55E-03 | 1.52E-03 |
| rs10777886 | TCGA&TCGA_coCNV | 5.56E-03 | 8.19E-04 |
| rs6568381 | TCGA | 5.56E-03 | #N/A |
| rs1405718 | TCGA | 5.56E-03 | #N/A |
| rs6874016 | TCGA&TCGA_coCNV | 7.12E-04 | 4.59E-03 |
| rs4475948 | TCGA | 5.56E-03 | #N/A |
| rs2389651 | TCGA | 5.56E-03 | #N/A |
| rs17349248 | TCGA&TCGA_coCNV | 2.05E-03 | 3.62E-03 |
| rs705768 | TCGA | 5.56E-03 | #N/A |
| rs11070911 | TCGA&TCGA_coCNV | 5.56E-03 | 3.90E-03 |
| rs2784912 | TCGA | 5.56E-03 | #N/A |
| rs12699152 | TCGA&TCGA_coCNV | 5.56E-03 | 3.83E-03 |
| rs3121494 | TCGA | 5.56E-03 | #N/A |
| rs16851773 | TCGA | 5.56E-03 | #N/A |
| rs315808 | TCGA&TCGA_coCNV | 5.57E-03 | 4.89E-03 |
| rs2733496 | TCGA&TCGA_coCNV | 5.57E-03 | 1.94E-03 |
| rs2835200 | TCGA | 5.57E-03 | #N/A |
| rs2819656 | TCGA&TCGA_coCNV | 5.57E-03 | 1.40E-03 |
| rs16867717 | TCGA | 5.57E-03 | #N/A |
| rs17502658 | TCGA&TCGA_coCNV | 2.94E-03 | 8.44E-03 |
| rs1361058 | TCGA | 5.57E-03 | #N/A |
| rs10083500 | TCGA&TCGA_coCNV | 5.57E-03 | 4.27E-03 |
| rs16941327 | TCGA&TCGA_coCNV | 5.57E-03 | 6.01E-04 |
| rs11099681 | TCGA | 5.58E-03 | #N/A |
| rs5980419 | TCGA | 5.58E-03 | #N/A |
| rs9637846 | TCGA&TCGA_coCNV | 5.58E-03 | 3.14E-03 |
| rs11194093 | TCGA | 1.97E-03 | #N/A |
| rs1466267 | TCGA | 5.58E-03 | #N/A |
| rs7278205 | TCGA | 2.61E-03 | #N/A |
| rs9343266 | TCGA | 5.58E-03 | #N/A |

| rs6637685 | TCGA | 5.58E-03 | #N/A |
| --- | --- | --- | --- |
| rs2034442 | TCGA | 5.58E-03 | #N/A |
| rs869804 | TCGA | 3.07E-03 | #N/A |
| rs2579762 | TCGA | 5.58E-03 | #N/A |
| rs9510775 | TCGA | 5.58E-03 | #N/A |
| rs7882344 | TCGA | 1.30E-03 | #N/A |
| rs7398686 | TCGA | 5.59E-03 | #N/A |
| rs17073897 | TCGA | 5.59E-03 | #N/A |
| rs17023809 | TCGA | 5.59E-03 | #N/A |
| rs2517014 | TCGA&TCGA_coCNV | 4.25E-08 | 4.47E-04 |
| rs13232226 | TCGA&TCGA_coCNV | 5.59E-03 | 2.03E-03 |
| rs4603445 | TCGA | 5.59E-03 | #N/A |
| rs1747717 | TCGA | 4.50E-03 | #N/A |
| rs6960293 | TCGA | 5.59E-03 | #N/A |
| rs10239108 | TCGA&TCGA_coCNV | 5.59E-03 | 3.33E-03 |
| rs11250154 | TCGA | 5.60E-03 | #N/A |
| rs11636597 | TCGA | 5.60E-03 | #N/A |
| rs5974406 | TCGA&TCGA_coCNV | 1.03E-03 | 2.17E-03 |
| rs12078021 | TCGA | 5.60E-03 | #N/A |
| rs7149354 | TCGA | 4.62E-03 | #N/A |
| rs12649149 | TCGA | 5.60E-03 | #N/A |
| rs10860831 | TCGA | 5.60E-03 | #N/A |
| rs28358579 | TCGA | 4.10E-03 | #N/A |
| rs1211751 | TCGA | 3.05E-03 | #N/A |
| rs13317384 | TCGA | 1.98E-03 | #N/A |
| rs2535668 | TCGA&TCGA_coCNV | 5.61E-03 | 3.31E-03 |
| rs9957517 | TCGA&TCGA_coCNV | 5.61E-03 | 4.31E-03 |
| rs7743259 | TCGA&TCGA_coCNV | 2.30E-03 | 5.75E-03 |
| rs16919133 | TCGA | 5.61E-03 | #N/A |
| rs13179216 | TCGA&TCGA_coCNV | 2.94E-03 | 8.03E-03 |
| rs16875595 | TCGA&TCGA_coCNV | 2.46E-03 | 2.19E-03 |
| rs1331319 | TCGA | 5.61E-03 | #N/A |
| rs2285444 | TCGA&TCGA_coCNV | 5.61E-03 | 4.59E-03 |
| rs2136844 | TCGA | 5.61E-03 | #N/A |
| rs1289174 | TCGA | 5.62E-03 | #N/A |
| rs311830 | TCGA | 5.62E-03 | #N/A |
| rs527914 | TCGA | 5.62E-03 | #N/A |
| rs1476291 | TCGA&TCGA_coCNV | 5.62E-03 | 2.67E-03 |
| rs9862024 | TCGA | 5.62E-03 | #N/A |
| rs6952190 | TCGA | 5.62E-03 | #N/A |
| rs4827095 | TCGA&TCGA_coCNV | 5.62E-03 | 1.22E-03 |
| rs16882366 | TCGA&TCGA_coCNV | 1.27E-04 | 4.52E-03 |
| rs4701032 | TCGA&TCGA_coCNV | 1.41E-04 | 3.99E-05 |
| rs6893901 | TCGA&TCGA_coCNV | 5.62E-03 | 3.51E-03 |
| rs1770206 | TCGA | 5.62E-03 | #N/A |
| rs10882356 | TCGA | 5.62E-03 | #N/A |

| rs1384031 | TCGA | 5.62E-03 | #N/A |
| --- | --- | --- | --- |
| rs10897634 | TCGA&TCGA_coCNV | 5.63E-03 | 3.32E-03 |
| rs12607008 | TCGA&TCGA_coCNV | 5.63E-03 | 3.54E-03 |
| rs6738592 | TCGA | 1.65E-03 | #N/A |
| rs4891316 | TCGA | 5.63E-03 | #N/A |
| rs17023069 | TCGA&TCGA_coCNV | 5.63E-03 | 1.21E-03 |
| rs2186343 | TCGA | 5.63E-03 | #N/A |
| rs13104683 | TCGA&TCGA_coCNV | 3.33E-03 | 5.73E-03 |
| rs931186 | TCGA | 1.38E-03 | #N/A |
| rs6870573 | TCGA | 5.63E-03 | #N/A |
| rs7066188 | TCGA | 5.63E-03 | #N/A |
| rs334940 | TCGA | 5.63E-03 | #N/A |
| rs7722980 | TCGA | 5.63E-03 | #N/A |
| rs301989 | TCGA | 5.64E-03 | #N/A |
| rs10029212 | TCGA&TCGA_coCNV | 2.23E-03 | 1.97E-03 |
| rs915832 | TCGA | 5.64E-03 | #N/A |
| rs7091811 | TCGA&TCGA_coCNV | 5.64E-03 | 1.80E-03 |
| rs9384483 | TCGA&TCGA_coCNV | 5.64E-03 | 2.36E-03 |
| rs2341422 | TCGA&TCGA_coCNV | 5.65E-03 | 3.75E-03 |
| rs9367924 | TCGA&TCGA_coCNV | 5.65E-03 | 1.62E-03 |
| rs7314888 | TCGA | 5.65E-03 | #N/A |
| rs11135694 | TCGA&Genevar | 2.38E-03 | #N/A |
| rs2564045 | TCGA | 5.65E-03 | #N/A |
| rs3859904 | TCGA | 5.65E-03 | #N/A |
| rs12713602 | TCGA | 5.65E-03 | #N/A |
| rs17054110 | TCGA&TCGA_coCNV | 1.39E-04 | 1.09E-03 |
| rs5960221 | TCGA | 5.65E-03 | #N/A |
| rs7334402 | TCGA | 5.65E-03 | #N/A |
| rs732601 | TCGA | 5.65E-03 | #N/A |
| rs17092753 | TCGA&TCGA_coCNV | 1.53E-03 | 6.61E-03 |
| rs17077968 | TCGA&TCGA_coCNV | 5.66E-03 | 8.02E-04 |
| rs1320211 | TCGA | 5.66E-03 | #N/A |
| rs747555 | TCGA&TCGA_coCNV | 1.57E-03 | 1.77E-03 |
| rs11016179 | TCGA | 5.66E-03 | #N/A |
| rs2399741 | TCGA | 5.66E-03 | #N/A |
| rs11114666 | TCGA&TCGA_coCNV | 5.66E-03 | 4.78E-03 |
| rs747911 | TCGA&TCGA_coCNV | 5.15E-03 | 5.33E-05 |
| rs6546637 | TCGA | 5.67E-03 | #N/A |
| rs554379 | TCGA&TCGA_coCNV | 8.27E-04 | 3.19E-03 |
| rs6803690 | TCGA&TCGA_coCNV | 1.79E-03 | 7.28E-04 |
| rs707 | TCGA | 5.67E-03 | #N/A |
| rs12510909 | TCGA | 5.67E-03 | #N/A |
| rs11935448 | TCGA | 5.67E-03 | #N/A |
| rs4399316 | TCGA | 5.67E-03 | #N/A |
| rs7954789 | TCGA | 5.67E-03 | #N/A |
| rs10006938 | TCGA&TCGA_coCNV | 1.18E-03 | 1.79E-03 |

| rs4984062 | TCGA | 5.67E-03 | #N/A |
| --- | --- | --- | --- |
| rs17041398 | TCGA&TCGA_coCNV | 5.67E-03 | 3.31E-03 |
| rs6884210 | TCGA&TCGA_coCNV | 1.44E-04 | 2.72E-03 |
| rs6094887 | TCGA | 5.68E-03 | #N/A |
| rs7339052 | TCGA | 5.68E-03 | #N/A |
| rs6811785 | TCGA&TCGA_coCNV | 1.05E-03 | 1.20E-03 |
| rs16832186 | TCGA | 4.71E-03 | #N/A |
| rs17067183 | TCGA&TCGA_coCNV | 5.68E-03 | 3.35E-03 |
| rs16929850 | TCGA&TCGA_coCNV | 5.68E-03 | 1.63E-03 |
| rs533308 | TCGA&TCGA_coCNV | 5.68E-03 | 2.26E-03 |
| rs2477758 | TCGA | 5.68E-03 | #N/A |
| rs1876331 | TCGA&TCGA_coCNV | 5.69E-03 | 3.77E-03 |
| rs1729340 | TCGA | 5.69E-03 | #N/A |
| rs1795871 | TCGA&TCGA_coCNV | 5.69E-03 | 4.00E-03 |
| rs10933137 | TCGA | 5.69E-03 | #N/A |
| rs1450365 | TCGA&TCGA_coCNV | 5.69E-03 | 3.12E-04 |
| rs10908303 | TCGA&TCGA_coCNV | 7.13E-05 | 1.74E-04 |
| rs3817821 | TCGA | 3.84E-03 | #N/A |
| rs16837097 | TCGA | 5.69E-03 | #N/A |
| rs16826120 | TCGA&TCGA_coCNV | 5.69E-03 | 1.47E-03 |
| rs12590407 | TCGA | 5.69E-03 | #N/A |
| rs535467 | TCGA&TCGA_coCNV | 6.92E-04 | 4.94E-04 |
| rs2707910 | TCGA | 4.80E-03 | #N/A |
| rs5925479 | TCGA | 5.69E-03 | #N/A |
| rs10166174 | TCGA&TCGA_coCNV | 5.70E-03 | 1.51E-03 |
| rs9946476 | TCGA&TCGA_coCNV | 5.70E-03 | 4.82E-03 |
| rs11217608 | TCGA&TCGA_coCNV | 4.31E-03 | 1.36E-03 |
| rs4148216 | TCGA&TCGA_coCNV | 8.12E-04 | 1.90E-03 |
| rs2304773 | TCGA&TCGA_coCNV | 5.70E-03 | 2.77E-03 |
| rs2164808 | TCGA&TCGA_coCNV | 3.25E-03 | 6.27E-03 |
| rs846589 | TCGA | 5.70E-03 | #N/A |
| rs12114398 | TCGA | 5.70E-03 | #N/A |
| rs4121751 | TCGA | 5.70E-03 | #N/A |
| rs988973 | TCGA&TCGA_coCNV | 5.66E-04 | 5.14E-03 |
| rs1440447 | TCGA&TCGA_coCNV | 5.70E-03 | 3.71E-03 |
| rs10493255 | TCGA&TCGA_coCNV | 5.70E-03 | 3.05E-03 |
| rs1027710 | TCGA | 3.72E-03 | #N/A |
| rs17011980 | TCGA | 5.70E-03 | #N/A |
| rs248426 | TCGA&TCGA_coCNV | 2.39E-03 | 5.34E-03 |
| rs12531680 | TCGA&TCGA_coCNV | 5.71E-03 | 3.54E-03 |
| rs7451287 | TCGA&TCGA_coCNV | 5.71E-03 | 3.82E-03 |
| rs7878853 | TCGA&TCGA_coCNV | 1.35E-05 | 4.08E-05 |
| rs13200541 | TCGA | 5.71E-03 | #N/A |
| rs1000256 | TCGA&TCGA_coCNV | 5.71E-03 | 2.49E-03 |
| rs6463213 | TCGA | 5.71E-03 | #N/A |
| rs696018 | TCGA | 1.93E-03 | #N/A |

| rs3864859 | TCGA | 5.71E-03 | #N/A |
| --- | --- | --- | --- |
| rs4933622 | TCGA&TCGA_coCNV | 1.68E-03 | 4.50E-04 |
| rs1987361 | TCGA | 5.71E-03 | #N/A |
| rs4908986 | TCGA | 5.71E-03 | #N/A |
| rs2050802 | TCGA | 5.72E-03 | #N/A |
| rs182075 | TCGA | 5.72E-03 | #N/A |
| rs1016735 | TCGA&TCGA_coCNV | 5.76E-04 | 1.61E-03 |
| rs359443 | TCGA | 5.72E-03 | #N/A |
| rs11863922 | TCGA | 2.50E-03 | #N/A |
| rs2882450 | TCGA&TCGA_coCNV | 5.72E-03 | 9.55E-04 |
| rs11485336 | TCGA | 5.72E-03 | #N/A |
| rs1327561 | TCGA&TCGA_coCNV | 5.72E-03 | 1.16E-03 |
| rs1345851 | TCGA | 5.72E-03 | #N/A |
| rs10161093 | TCGA&TCGA_coCNV | 5.72E-03 | 8.75E-04 |
| rs7673644 | TCGA | 5.72E-03 | #N/A |
| rs869297 | TCGA&TCGA_coCNV | 5.73E-03 | 5.92E-04 |
| rs11225203 | TCGA&TCGA_coCNV | 2.87E-04 | 8.57E-03 |
| rs1418043 | TCGA | 5.73E-03 | #N/A |
| rs17331452 | TCGA&TCGA_coCNV | 4.08E-03 | 3.05E-04 |
| rs4289997 | TCGA | 5.73E-03 | #N/A |
| rs625996 | TCGA&TCGA_coCNV | 3.58E-03 | 6.06E-03 |
| rs817691 | TCGA | 5.73E-03 | #N/A |
| rs10503257 | TCGA | 5.74E-03 | #N/A |
| rs10042752 | TCGA&TCGA_coCNV | 2.76E-04 | 1.62E-03 |
| rs17820733 | TCGA | 5.74E-03 | #N/A |
| rs4850266 | TCGA&TCGA_coCNV | 1.78E-03 | 9.41E-04 |
| rs4088114 | TCGA&TCGA_coCNV | 2.44E-04 | 6.99E-04 |
| rs6948900 | TCGA | 5.74E-03 | #N/A |
| rs218983 | TCGA&TCGA_coCNV | 5.74E-03 | 2.17E-03 |
| rs16882207 | TCGA&TCGA_coCNV | 4.56E-05 | 8.04E-03 |
| rs7571836 | TCGA&TCGA_coCNV | 5.74E-03 | 1.83E-04 |
| rs16943189 | TCGA&TCGA_coCNV | 1.42E-03 | 4.35E-03 |
| rs12058603 | TCGA | 5.74E-03 | #N/A |
| rs174719 | TCGA | 5.75E-03 | #N/A |
| rs16842128 | TCGA | 5.75E-03 | #N/A |
| rs925140 | TCGA | 2.44E-03 | #N/A |
| rs7906354 | TCGA&TCGA_coCNV | 1.93E-03 | 2.48E-03 |
| rs17827129 | TCGA | 5.75E-03 | #N/A |
| rs9504361 | TCGA&TCGA_coCNV | 5.67E-04 | 1.31E-04 |
| rs5991970 | TCGA&TCGA_coCNV | 5.75E-03 | 2.48E-03 |
| rs6775338 | TCGA&TCGA_coCNV | 5.75E-03 | 6.27E-04 |
| rs7553566 | TCGA | 5.76E-03 | #N/A |
| rs517060 | TCGA | 5.76E-03 | #N/A |
| rs3960630 | TCGA&TCGA_coCNV | 5.76E-03 | 4.54E-03 |
| rs2014213 | TCGA&TCGA_coCNV | 4.88E-04 | 1.57E-03 |
| rs13225884 | TCGA | 5.76E-03 | #N/A |

| rs11084466 | TCGA&TCGA_coCNV | 5.76E-03 | 1.18E-03 |
| --- | --- | --- | --- |
| rs6001679 | TCGA | 5.76E-03 | #N/A |
| rs12467524 | TCGA&TCGA_coCNV | 4.42E-03 | 4.93E-04 |
| rs610388 | TCGA&TCGA_coCNV | 9.49E-04 | 5.18E-03 |
| rs10514108 | TCGA&TCGA_coCNV | 5.76E-03 | 1.01E-03 |
| rs12969974 | TCGA | 2.62E-03 | #N/A |
| rs6550019 | TCGA | 5.76E-03 | #N/A |
| rs11618305 | TCGA | 5.76E-03 | #N/A |
| rs2899614 | TCGA&TCGA_coCNV | 5.76E-03 | 1.77E-03 |
| rs12943480 | TCGA&TCGA_coCNV | 1.99E-03 | 2.12E-04 |
| rs6123647 | TCGA | 4.93E-03 | #N/A |
| rs4768745 | TCGA&TCGA_coCNV | 2.03E-03 | 4.48E-03 |
| rs10520599 | TCGA&TCGA_coCNV | 4.90E-03 | 9.47E-04 |
| rs616413 | TCGA&TCGA_coCNV | 1.97E-03 | 4.91E-03 |
| rs12598065 | TCGA | 5.77E-03 | #N/A |
| rs389089 | TCGA&TCGA_coCNV | 2.84E-03 | 4.47E-03 |
| rs726486 | TCGA | 5.77E-03 | #N/A |
| rs4868699 | TCGA&TCGA_coCNV | 5.77E-03 | 3.62E-03 |
| rs8075583 | TCGA | 5.77E-03 | #N/A |
| rs423648 | TCGA | 5.77E-03 | #N/A |
| rs12403822 | TCGA | 5.77E-03 | #N/A |
| rs7842946 | TCGA&TCGA_coCNV | 5.78E-03 | 3.41E-03 |
| rs3803976 | TCGA | 5.78E-03 | #N/A |
| rs13060869 | TCGA&TCGA_coCNV | 3.02E-05 | 5.48E-05 |
| rs954478 | TCGA | 5.78E-03 | #N/A |
| rs4135081 | TCGA&TCGA_coCNV | 5.63E-03 | 2.79E-03 |
| rs12440026 | TCGA&TCGA_coCNV | 1.87E-04 | 6.80E-03 |
| rs7741995 | TCGA&TCGA_coCNV | 5.78E-03 | 4.63E-03 |
| rs334354 | TCGA | 5.78E-03 | #N/A |
| rs12719517 | TCGA | 5.79E-03 | #N/A |
| rs16959057 | TCGA&TCGA_coCNV | 4.10E-03 | 2.70E-03 |
| rs4858353 | TCGA | 5.79E-03 | #N/A |
| rs7211216 | TCGA | 5.79E-03 | #N/A |
| rs12225526 | TCGA&TCGA_coCNV | 5.79E-03 | 2.01E-03 |
| rs7217605 | TCGA&TCGA_coCNV | 5.79E-03 | 8.76E-04 |
| rs17760488 | TCGA | 5.79E-03 | #N/A |
| rs10144183 | TCGA | 5.79E-03 | #N/A |
| rs17575480 | TCGA | 5.79E-03 | #N/A |
| rs17661149 | TCGA&TCGA_coCNV | 1.78E-03 | 1.54E-03 |
| rs9566022 | TCGA | 5.79E-03 | #N/A |
| rs2069230 | TCGA | 5.79E-03 | #N/A |
| rs3770008 | TCGA | 5.79E-03 | #N/A |
| rs2106406 | TCGA | 5.80E-03 | #N/A |
[truncated: 550,010 more chars]
