# Supplementary material for: SNRPD1 conveys prognostic value on breast cancer survival and is required for anthracycline sensitivity
Source: BMC Cancer. 2023 Apr 25;23:376. doi: 10.1186/s12885-023-10860-z (PMC10126993; doi:10.1186/s12885-023-10860-z)
Supplement: Supplementary file 9 — Additional file 9: Supplementary Table 9. Genes differentially correlated with SNRPD1 and SNRPE. [file 12885_2023_10860_MOESM9_ESM.docx]

**Supplementary Table 9. Genes differentially correlated with *SNRPD1* and *SNRPE*.**

| **Genes** | ***SNRPD1*** | | ***SNRPE*** | | **Abs(sub)** |
| --- | --- | --- | --- | --- | --- |
|  | **Correlation** | ***p*** | **Correlation** | ***p*** | **abs(sub)** |
| SUPT20H | 0.5567229 | 7.14E-05 | 0.07784838 | 0.611243 | 0.48 |
| SIRT7 | 0.5863169 | 2.31E-05 | 0.12787298 | 0.402534 | 0.46 |
| GTF2H3 | 0.5701245 | 4.34E-05 | 0.11265856 | 0.461231 | 0.46 |
| DSN1 | 0.5341219 | 0.000158 | 0.09026838 | 0.555389 | 0.45 |
| DUS1L | 0.5255299 | 0.00021 | 0.09356028 | 0.540999 | 0.44 |
| CNOT10 | 0.6370394 | 2.54E-06 | 0.21747331 | 0.151275 | 0.42 |
| C10orf12 | 0.6468585 | 1.58E-06 | 0.22811254 | 0.131781 | 0.42 |
| ZCCHC2 | 0.5460702 | 0.000104 | 0.12891543 | 0.398678 | 0.42 |
| CNOT2 | 0.5081827 | 0.000365 | 0.09433687 | 0.53763 | 0.42 |
| AAR2 | 0.5038338 | 0.000418 | 0.09775696 | 0.522919 | 0.41 |
| TUBG1 | 0.5138226 | 0.000306 | 0.10987107 | 0.472463 | 0.41 |
| LIN37 | 0.69122 | 1.47E-07 | 0.2911494 | 0.052332 | 0.41 |
| CEP97 | 0.5678843 | 4.73E-05 | 0.17263622 | 0.256782 | 0.4 |
| BDP1 | 0.5706557 | 4.26E-05 | 0.1754942 | 0.24887 | 0.4 |
| BUB1 | 0.6026655 | 1.18E-05 | 0.21388061 | 0.158314 | 0.39 |
| EED | 0.6861019 | 1.97E-07 | 0.29735785 | 0.047286 | 0.39 |
| PRKRIP1 | 0.5177077 | 0.00027 | 0.12933905 | 0.397116 | 0.39 |
| FZR1 | 0.5169078 | 0.000277 | 0.12886077 | 0.398879 | 0.39 |
| MED10 | 0.704071 | 6.81E-08 | 0.31631143 | 0.034272 | 0.39 |
| MED29 | 0.517377 | 0.000273 | 0.133123 | 0.383331 | 0.39 |
| CEP76 | 0.5347227 | 0.000154 | 0.15229991 | 0.317915 | 0.39 |
| BTAF1 | 0.5184733 | 0.000264 | 0.13762038 | 0.367319 | 0.39 |
| VEGFB | -0.5347 | 0.000154 | -0.1567376 | 0.30385 | 0.38 |
| MED15 | 0.7000348 | 8.71E-08 | 0.32340827 | 0.030231 | 0.38 |
| CHD1 | 0.6235453 | 4.75E-06 | 0.25046718 | 0.09702 | 0.38 |
| MIS12 | 0.5132491 | 0.000312 | 0.14030636 | 0.357951 | 0.38 |
| POLR2D | 0.7352301 | 8.84E-09 | 0.36232123 | 0.01445 | 0.38 |
| TCOF1 | 0.6207187 | 5.39E-06 | 0.24804552 | 0.100403 | 0.38 |
| RPL37 | 0.5742067 | 3.72E-05 | 0.20290734 | 0.181289 | 0.38 |
| CNTN1 | -0.573581 | 3.81E-05 | -0.2027796 | 0.18157 | 0.38 |
| MRPL54 | 0.5621304 | 5.86E-05 | 0.19310069 | 0.203761 | 0.37 |
| OXA1L | 0.5591738 | 6.53E-05 | 0.19421304 | 0.201118 | 0.37 |
| RPS23 | 0.5405555 | 0.000126 | 0.17668613 | 0.245619 | 0.37 |
| RPS29 | 0.5253368 | 0.000211 | 0.16243689 | 0.28638 | 0.37 |
| LIN54 | 0.7385038 | 7.01E-09 | 0.3765703 | 0.010783 | 0.37 |
| LEO1 | 0.6555612 | 1.02E-06 | 0.29600401 | 0.048351 | 0.36 |
| SF3B5 | 0.5306055 | 0.000177 | 0.17296235 | 0.255871 | 0.36 |
| BOP1 | 0.6306152 | 3.43E-06 | 0.27307603 | 0.069529 | 0.36 |
| MED7 | 0.6037003 | 1.13E-05 | 0.24702786 | 0.101851 | 0.36 |
| LARP1 | 0.5463139 | 0.000104 | 0.19113357 | 0.208495 | 0.36 |
| FOXK2 | 0.5170608 | 0.000276 | 0.16230178 | 0.286786 | 0.36 |
| CTDSPL2 | 0.5776493 | 3.26E-05 | 0.2242674 | 0.138597 | 0.36 |
| KIF2A | 0.6075966 | 9.59E-06 | 0.25719645 | 0.088082 | 0.36 |
| COL16A1 | -0.596063 | 1.56E-05 | -0.2457797 | 0.103649 | 0.36 |
| MRPL51 | 0.5170097 | 0.000277 | 0.16768224 | 0.270892 | 0.35 |
| HELLS | 0.6751643 | 3.63E-07 | 0.32775956 | 0.027955 | 0.35 |
| POLE | 0.7027822 | 7.37E-08 | 0.35721737 | 0.015999 | 0.35 |
| SREK1IP1 | 0.7331814 | 1.02E-08 | 0.38764167 | 0.008514 | 0.35 |
| RBM27 | 0.7629103 | 1.12E-09 | 0.41758461 | 0.004316 | 0.35 |
| SDAD1 | 0.6362093 | 2.64E-06 | 0.29302359 | 0.050765 | 0.35 |
| BRD8 | 0.7003692 | 8.53E-08 | 0.3572722 | 0.015982 | 0.35 |
| HNRNPAB | 0.5692164 | 0.000045 | 0.22614687 | 0.135234 | 0.35 |
| TBCD | 0.5032587 | 0.000426 | 0.1610395 | 0.290601 | 0.35 |
| C18orf25 | 0.5945318 | 1.66E-05 | 0.25249354 | 0.094258 | 0.35 |
| G3BP1 | 0.6701986 | 4.76E-07 | 0.3282788 | 0.027693 | 0.35 |
| ZC3HAV1 | 0.5606138 | 6.19E-05 | 0.21914673 | 0.148076 | 0.35 |
| EIF3G | 0.6216734 | 5.17E-06 | 0.28177286 | 0.060766 | 0.34 |
| NFATC2IP | 0.5104123 | 0.000341 | 0.17300698 | 0.255746 | 0.34 |
| COL1A2 | -0.55989 | 6.36E-05 | -0.2225289 | 0.141763 | 0.34 |
| ZCCHC8 | 0.7204296 | 2.41E-08 | 0.38344965 | 0.00932 | 0.34 |
| RPL23 | 0.5236451 | 0.000223 | 0.18675426 | 0.219307 | 0.34 |
| RQCD1 | 0.6161097 | 6.62E-06 | 0.27976994 | 0.062701 | 0.34 |
| HTATSF1 | 0.71121 | 4.37E-08 | 0.37521518 | 0.011094 | 0.34 |
| JUNB | 0.6198124 | 5.62E-06 | 0.28455356 | 0.058159 | 0.34 |
| CHAF1B | 0.7068212 | 5.75E-08 | 0.37244706 | 0.011752 | 0.34 |
| CDC45 | 0.5393788 | 0.000132 | 0.20510222 | 0.176513 | 0.34 |
| COL14A1 | -0.548735 | 9.51E-05 | -0.215401 | 0.155306 | 0.34 |
| PWP1 | 0.6562379 | 9.87E-07 | 0.32384507 | 0.029996 | 0.34 |
| RNF113A | 0.6174129 | 6.25E-06 | 0.28538866 | 0.057393 | 0.34 |
| SMARCA5 | 0.7405678 | 6.05E-09 | 0.40877572 | 0.005305 | 0.34 |
| AZI1 | 0.5754367 | 3.55E-05 | 0.24439516 | 0.105671 | 0.34 |
| PPM1G | 0.5987918 | 1.39E-05 | 0.26797568 | 0.075115 | 0.34 |
| LLPH | 0.7230772 | 2.02E-08 | 0.39244959 | 0.007665 | 0.34 |
| MRPL19 | 0.5151184 | 0.000294 | 0.18501317 | 0.223711 | 0.34 |
| IGHMBP2 | 0.6386725 | 2.35E-06 | 0.308894 | 0.03896 | 0.33 |
| PID1 | -0.633375 | 3.02E-06 | -0.3040973 | 0.042263 | 0.33 |
| NSL1 | 0.5529867 | 8.17E-05 | 0.22408476 | 0.138927 | 0.33 |
| SUZ12 | 0.8065312 | 2.27E-11 | 0.47808736 | 0.000895 | 0.33 |
| CPSF3 | 0.662975 | 6.97E-07 | 0.3349937 | 0.024487 | 0.33 |
| USP1 | 0.671892 | 4.34E-07 | 0.34450595 | 0.020481 | 0.33 |
| RPL31 | 0.5128445 | 0.000316 | 0.1866061 | 0.219679 | 0.33 |
| GON4L | 0.6033706 | 1.15E-05 | 0.27722091 | 0.065235 | 0.33 |
| CDK9 | 0.5042248 | 0.000413 | 0.17878192 | 0.239974 | 0.33 |
| PHAX | 0.6427319 | 1.93E-06 | 0.3175144 | 0.033557 | 0.33 |
| WDHD1 | 0.7250283 | 1.78E-08 | 0.39984824 | 0.006502 | 0.33 |
| SAAL1 | 0.582502 | 2.69E-05 | 0.25834911 | 0.086618 | 0.33 |
| EIF2B1 | 0.5498063 | 9.15E-05 | 0.2261539 | 0.135221 | 0.33 |
| DDX39A | 0.568721 | 4.58E-05 | 0.24592225 | 0.103442 | 0.33 |
| ALKBH2 | 0.7197572 | 2.52E-08 | 0.39720678 | 0.006898 | 0.33 |
| ATXN2L | 0.6115099 | 8.1E-06 | 0.28995918 | 0.053347 | 0.33 |
| POLD1 | 0.7003626 | 8.54E-08 | 0.37887635 | 0.010272 | 0.33 |
| GCFC2 | 0.5745059 | 3.68E-05 | 0.25327137 | 0.093214 | 0.33 |
| DNAJC7 | 0.5592204 | 6.52E-05 | 0.23967139 | 0.1128 | 0.32 |
| BCL7A | 0.5439172 | 0.000113 | 0.22474051 | 0.137745 | 0.32 |
| ZNF263 | 0.5694977 | 4.45E-05 | 0.25312971 | 0.093404 | 0.32 |
| TSEN2 | 0.5488503 | 9.47E-05 | 0.23254047 | 0.124247 | 0.32 |
| MED4 | 0.7454651 | 4.24E-09 | 0.430294 | 0.003174 | 0.32 |
| DNMT1 | 0.716682 | 3.08E-08 | 0.40180269 | 0.006221 | 0.32 |
| OLFML3 | -0.686418 | 1.94E-07 | -0.3717514 | 0.011922 | 0.32 |
| MED21 | 0.7305355 | 1.22E-08 | 0.41589285 | 0.004492 | 0.32 |
| C11orf30 | 0.5610653 | 6.09E-05 | 0.24751451 | 0.101156 | 0.32 |
| SBNO1 | 0.7682001 | 7.29E-10 | 0.45506982 | 0.001685 | 0.32 |
| ATAD2 | 0.6296326 | 3.59E-06 | 0.31702363 | 0.033847 | 0.32 |
| PPP1R10 | 0.6455943 | 1.68E-06 | 0.33301635 | 0.025397 | 0.32 |
| TTF2 | 0.5295756 | 0.000183 | 0.21746612 | 0.151289 | 0.32 |
| PCNA | 0.6017959 | 1.23E-05 | 0.28970481 | 0.053566 | 0.32 |
| SKA3 | 0.6391638 | 2.29E-06 | 0.32715479 | 0.028263 | 0.32 |
| PAF1 | 0.7684221 | 7.16E-10 | 0.45685444 | 0.001607 | 0.32 |
| XRCC1 | 0.5232944 | 0.000226 | 0.21233455 | 0.161415 | 0.32 |
| BUB1B | 0.5763417 | 3.42E-05 | 0.26682071 | 0.076428 | 0.31 |
| MED1 | 0.5520513 | 8.45E-05 | 0.24258561 | 0.10836 | 0.31 |
| SPDL1 | 0.5188335 | 0.000261 | 0.20954934 | 0.167114 | 0.31 |
| GTF2E1 | 0.5611877 | 6.07E-05 | 0.2523719 | 0.094422 | 0.31 |
| CGGBP1 | 0.5186274 | 0.000263 | 0.20988604 | 0.166417 | 0.31 |
| RNF34 | 0.6549448 | 1.05E-06 | 0.34683433 | 0.019589 | 0.31 |
| PBK | 0.5995373 | 1.35E-05 | 0.29216088 | 0.051481 | 0.31 |
| WDR82 | 0.6766005 | 3.36E-07 | 0.36933246 | 0.012531 | 0.31 |
| VRK3 | 0.5243409 | 0.000218 | 0.21759164 | 0.151047 | 0.31 |
| OAS3 | 0.5375306 | 0.00014 | 0.23079717 | 0.127173 | 0.31 |
| NOSIP | 0.6104128 | 8.5E-06 | 0.30417019 | 0.042211 | 0.31 |
| SSH2 | 0.5115208 | 0.000329 | 0.20538705 | 0.1759 | 0.31 |
| PSMC4 | 0.5602897 | 6.27E-05 | 0.25425063 | 0.091913 | 0.31 |
| EZH2 | 0.6897163 | 1.6E-07 | 0.38401832 | 0.009207 | 0.31 |
| THOC3 | 0.6172511 | 6.3E-06 | 0.31190635 | 0.036997 | 0.31 |
| MLH1 | 0.6621523 | 7.28E-07 | 0.35706224 | 0.016049 | 0.31 |
| FAT4 | -0.601724 | 1.23E-05 | -0.296635 | 0.047852 | 0.31 |
| PCDHGC3 | -0.60695 | 9.86E-06 | -0.3018853 | 0.043861 | 0.31 |
| BAZ1A | 0.6653644 | 6.15E-07 | 0.36185627 | 0.014586 | 0.31 |
| LRWD1 | 0.7706449 | 5.96E-10 | 0.46814082 | 0.001183 | 0.31 |
| YEATS4 | 0.7308371 | 1.2E-08 | 0.42997289 | 0.0032 | 0.31 |
| HLTF | 0.5863254 | 2.31E-05 | 0.28561377 | 0.057188 | 0.31 |
| NSUN5 | 0.5339856 | 0.000158 | 0.23410765 | 0.121659 | 0.3 |
| CCNH | 0.7042962 | 6.72E-08 | 0.40446014 | 0.005857 | 0.3 |
| CEP250 | 0.5528348 | 8.21E-05 | 0.25319355 | 0.093318 | 0.3 |
| CTR9 | 0.828909 | 2.05E-12 | 0.53011365 | 0.00018 | 0.3 |
| CLK4 | 0.6725798 | 4.18E-07 | 0.37420083 | 0.011331 | 0.3 |
| HIVEP1 | 0.530323 | 0.000179 | 0.23227395 | 0.124691 | 0.3 |
| EXOSC9 | 0.6909471 | 1.49E-07 | 0.39312599 | 0.007552 | 0.3 |
| MTO1 | 0.5544721 | 7.75E-05 | 0.25667167 | 0.088756 | 0.3 |
| CMTR1 | 0.5748172 | 3.63E-05 | 0.27704044 | 0.065418 | 0.3 |
| ILKAP | 0.5688963 | 4.55E-05 | 0.27124072 | 0.0715 | 0.3 |
| BPTF | 0.7370685 | 7.76E-09 | 0.43969263 | 0.00251 | 0.3 |
| CLP1 | 0.625597 | 4.33E-06 | 0.32908795 | 0.027289 | 0.3 |
| GTF2E2 | 0.6271645 | 4.03E-06 | 0.33071963 | 0.02649 | 0.3 |
| DTL | 0.524193 | 0.000219 | 0.22787035 | 0.132203 | 0.3 |
| ZMAT2 | 0.7030833 | 7.24E-08 | 0.40705219 | 0.005519 | 0.3 |
| HMGB2 | 0.676184 | 3.44E-07 | 0.38037836 | 0.00995 | 0.3 |
| ALYREF | 0.6743071 | 3.81E-07 | 0.37856922 | 0.010339 | 0.3 |
| SYMPK | 0.7234979 | 1.97E-08 | 0.42786306 | 0.00337 | 0.3 |
| UFD1L | 0.5368764 | 0.000143 | 0.24224838 | 0.108867 | 0.3 |
| C12orf45 | 0.5339863 | 0.000158 | 0.23947874 | 0.113099 | 0.3 |
| MRPS33 | 0.6581411 | 8.96E-07 | 0.36365958 | 0.014066 | 0.3 |
| CCBL2 | -0.501444 | 0.00045 | -0.2070273 | 0.172399 | 0.3 |
| GTF2H1 | 0.6253969 | 4.37E-06 | 0.33136022 | 0.026181 | 0.3 |
| NUDT21 | 0.7573023 | 1.74E-09 | 0.46342544 | 0.001346 | 0.3 |
| ANAPC7 | 0.5698598 | 4.39E-05 | 0.27622985 | 0.066242 | 0.3 |
| POLD2 | 0.6572112 | 9.39E-07 | 0.36403046 | 0.013961 | 0.3 |
| MIER2 | 0.5202927 | 0.000249 | 0.22712976 | 0.133499 | 0.3 |
| UHRF1BP1 | 0.5180065 | 0.000268 | 0.22581301 | 0.135827 | 0.3 |
| CHAF1A | 0.7286362 | 1.39E-08 | 0.43646458 | 0.002723 | 0.3 |
| KLF13 | 0.6208396 | 5.36E-06 | 0.32868693 | 0.027489 | 0.3 |
| LIN52 | 0.5437798 | 0.000113 | 0.25174303 | 0.095274 | 0.3 |
| POLD3 | 0.738125 | 7.2E-09 | 0.44681821 | 0.002091 | 0.3 |
| GRWD1 | 0.6155139 | 6.8E-06 | 0.32486576 | 0.029452 | 0.3 |
| EXOSC3 | 0.6194405 | 5.71E-06 | 0.32951088 | 0.02708 | 0.29 |
| PRIM1 | 0.7259734 | 1.67E-08 | 0.4366058 | 0.002713 | 0.29 |
| SSX2IP | 0.533416 | 0.000161 | 0.24405074 | 0.106179 | 0.29 |
| MRPL16 | 0.5400234 | 0.000129 | 0.25098774 | 0.096305 | 0.29 |
| ZNHIT6 | 0.587128 | 2.24E-05 | 0.29844394 | 0.046445 | 0.29 |
| WDR47 | 0.6039036 | 1.12E-05 | 0.31555375 | 0.034728 | 0.29 |
| TEFM | 0.5073015 | 0.000376 | 0.21930347 | 0.147779 | 0.29 |
| GTF2B | 0.647316 | 1.54E-06 | 0.35958578 | 0.015264 | 0.29 |
| POC1A | 0.6302439 | 3.49E-06 | 0.34269644 | 0.021197 | 0.29 |
| SCAPER | 0.6766601 | 3.35E-07 | 0.38963543 | 0.008153 | 0.29 |
| RPL7A | 0.5655318 | 5.16E-05 | 0.2790283 | 0.06343 | 0.29 |
| COL2A1 | -0.565702 | 5.13E-05 | -0.2792299 | 0.063232 | 0.29 |
| GEMIN5 | 0.6834648 | 2.29E-07 | 0.39738888 | 0.00687 | 0.29 |
| ZNF668 | 0.5567771 | 7.13E-05 | 0.27142362 | 0.071301 | 0.29 |
| NUSAP1 | 0.6289452 | 3.71E-06 | 0.34361016 | 0.020833 | 0.29 |
| PABPC1 | 0.6339918 | 2.93E-06 | 0.34869119 | 0.018901 | 0.29 |
| DDX5 | 0.6979124 | 9.89E-08 | 0.41313615 | 0.004793 | 0.29 |
| DHX37 | 0.6848883 | 2.11E-07 | 0.40030716 | 0.006435 | 0.29 |
| PLXDC2 | -0.550569 | 8.91E-05 | -0.2662817 | 0.077047 | 0.29 |
| CENPI | 0.7167437 | 3.07E-08 | 0.43253763 | 0.003003 | 0.29 |
| RTF1 | 0.5967972 | 1.51E-05 | 0.31259489 | 0.036559 | 0.29 |
| MORF4L1 | 0.524324 | 0.000218 | 0.24036452 | 0.111732 | 0.29 |
| MRPL28 | 0.5145455 | 0.000299 | 0.23087189 | 0.127047 | 0.29 |
| BRD2 | 0.8436074 | 3.47E-13 | 0.55999362 | 6.34E-05 | 0.29 |
| TOPBP1 | 0.6585116 | 8.79E-07 | 0.37498921 | 0.011146 | 0.29 |
| PA2G4 | 0.5641646 | 5.43E-05 | 0.28156446 | 0.060965 | 0.29 |
| ZRSR2 | 0.6469942 | 1.57E-06 | 0.36462675 | 0.013793 | 0.29 |
| IL17B | -0.518558 | 0.000263 | -0.2365715 | 0.117673 | 0.29 |
| MED16 | 0.8288231 | 2.07E-12 | 0.54686469 | 0.000102 | 0.29 |
| SLC4A1AP | 0.5612043 | 6.06E-05 | 0.27928669 | 0.063176 | 0.29 |
| RPL4 | 0.5242394 | 0.000219 | 0.24246954 | 0.108535 | 0.29 |
| RFC4 | 0.7274452 | 1.51E-08 | 0.44573816 | 0.002151 | 0.29 |
| ZWILCH | 0.6641491 | 6.56E-07 | 0.38292457 | 0.009425 | 0.29 |
| LUC7L3 | 0.5765371 | 0.000034 | 0.29533372 | 0.048886 | 0.29 |
| RALY | 0.6308476 | 3.4E-06 | 0.34970729 | 0.018534 | 0.29 |
| HUS1 | 0.5624808 | 5.78E-05 | 0.28170954 | 0.060827 | 0.29 |
| ATAD2B | 0.6546858 | 1.07E-06 | 0.37413999 | 0.011345 | 0.29 |
| RBMS3 | -0.534181 | 0.000157 | -0.254403 | 0.091712 | 0.28 |
| MAK16 | 0.6228219 | 4.91E-06 | 0.34330026 | 0.020956 | 0.28 |
| MTF2 | 0.7210034 | 2.32E-08 | 0.44158038 | 0.002392 | 0.28 |
| HSPA12B | -0.594466 | 1.66E-05 | -0.3152715 | 0.0349 | 0.28 |
| MRPL11 | 0.5495975 | 9.22E-05 | 0.27047409 | 0.072336 | 0.28 |
| KNTC1 | 0.6941683 | 1.24E-07 | 0.41513452 | 0.004573 | 0.28 |
| MAMDC2 | -0.552871 | 0.000082 | -0.274076 | 0.068473 | 0.28 |
| CEP152 | 0.5333952 | 0.000161 | 0.25497503 | 0.090959 | 0.28 |
| KIAA1524 | 0.5066925 | 0.000383 | 0.22834053 | 0.131385 | 0.28 |
| MRPS31 | 0.544419 | 0.000111 | 0.26617191 | 0.077174 | 0.28 |
| ALKBH5 | 0.5096312 | 0.000349 | 0.23163009 | 0.125769 | 0.28 |
| RPS5 | 0.5329073 | 0.000164 | 0.25523407 | 0.09062 | 0.28 |
| MRPL14 | 0.5011701 | 0.000454 | 0.22374998 | 0.139534 | 0.28 |
| HIRIP3 | 0.6568496 | 9.57E-07 | 0.37946535 | 0.010145 | 0.28 |
| AGGF1 | 0.5517794 | 8.53E-05 | 0.27448546 | 0.068044 | 0.28 |
| FLRT2 | -0.575562 | 3.53E-05 | -0.2984961 | 0.046405 | 0.28 |
| POLR2L | 0.7769929 | 3.49E-10 | 0.50019939 | 0.000467 | 0.28 |
| KIF20B | 0.6196078 | 5.67E-06 | 0.3428427 | 0.021138 | 0.28 |
| TRAFD1 | 0.529471 | 0.000184 | 0.25284015 | 0.093792 | 0.28 |
| ANKLE2 | 0.5861135 | 2.33E-05 | 0.30955011 | 0.038526 | 0.28 |
| RPP38 | 0.7150022 | 3.43E-08 | 0.43910287 | 0.002548 | 0.28 |
| TAF4 | 0.5807457 | 2.89E-05 | 0.3049146 | 0.041685 | 0.28 |
| PLAC9 | -0.550513 | 8.92E-05 | -0.2747353 | 0.067784 | 0.28 |
| ERH | 0.707128 | 5.64E-08 | 0.43147189 | 0.003083 | 0.28 |
| TTI1 | 0.5409036 | 0.000125 | 0.26605786 | 0.077305 | 0.28 |
| POLA1 | 0.6962519 | 1.09E-07 | 0.42149039 | 0.003932 | 0.28 |
| ZWINT | 0.5605553 | 6.21E-05 | 0.28580402 | 0.057016 | 0.28 |
| MCM2 | 0.6256792 | 4.31E-06 | 0.35125967 | 0.017984 | 0.28 |
| CEP95 | 0.5422985 | 0.000119 | 0.26792674 | 0.075171 | 0.28 |
| MED24 | 0.6158057 | 6.71E-06 | 0.34184392 | 0.021542 | 0.28 |
| RFC5 | 0.7612758 | 1.27E-09 | 0.4873656 | 0.000685 | 0.28 |
| MSL3 | 0.5658918 | 5.09E-05 | 0.29255287 | 0.051155 | 0.28 |
| RSL24D1 | 0.7312362 | 1.17E-08 | 0.45845773 | 0.001539 | 0.28 |
| ZCRB1 | 0.5432393 | 0.000115 | 0.27079078 | 0.07199 | 0.28 |
| PNN | 0.8413481 | 4.61E-13 | 0.56913447 | 4.51E-05 | 0.28 |
| CEP350 | 0.5772046 | 3.31E-05 | 0.30532241 | 0.041399 | 0.28 |
| MRPL43 | 0.5212252 | 0.000241 | 0.24943562 | 0.09845 | 0.28 |
| RFC3 | 0.706825 | 5.75E-08 | 0.43552878 | 0.002787 | 0.28 |
| CCDC137 | 0.6709655 | 4.56E-07 | 0.39978639 | 0.006511 | 0.28 |
| NAA10 | 0.5262882 | 0.000205 | 0.25513998 | 0.090743 | 0.28 |
| CCP110 | 0.5856716 | 2.37E-05 | 0.3146578 | 0.035275 | 0.28 |
| GPATCH11 | 0.699465 | 9.01E-08 | 0.42931278 | 0.003252 | 0.28 |
| PCDH18 | -0.535797 | 0.000149 | -0.2663149 | 0.077009 | 0.27 |
| MRPL41 | 0.5050585 | 0.000403 | 0.23559496 | 0.119241 | 0.27 |
| YBX2 | 0.6002103 | 1.31E-05 | 0.33098368 | 0.026362 | 0.27 |
| PRPF38B | 0.6819622 | 2.49E-07 | 0.41347701 | 0.004755 | 0.27 |
| PSIP1 | 0.5133669 | 0.000311 | 0.24521914 | 0.104464 | 0.27 |
| TMPO | 0.6940409 | 1.24E-07 | 0.42597556 | 0.003528 | 0.27 |
| CPSF4 | 0.6116831 | 8.04E-06 | 0.34365223 | 0.020816 | 0.27 |
| ZFC3H1 | 0.7007703 | 8.33E-08 | 0.43307846 | 0.002963 | 0.27 |
| FEN1 | 0.6895432 | 1.62E-07 | 0.42228692 | 0.003858 | 0.27 |
| WDR76 | 0.5373996 | 0.000141 | 0.27022611 | 0.072608 | 0.27 |
| RTTN | 0.6118634 | 7.98E-06 | 0.34489745 | 0.020328 | 0.27 |
| TAF1 | 0.6777573 | 3.15E-07 | 0.41080475 | 0.005061 | 0.27 |
| ARID2 | 0.5275698 | 0.000196 | 0.26087914 | 0.08347 | 0.27 |
| PRPF4B | 0.7974929 | 5.49E-11 | 0.5308321 | 0.000176 | 0.27 |
| YY1AP1 | 0.5238827 | 0.000221 | 0.25724739 | 0.088017 | 0.27 |
| TNPO2 | 0.5511221 | 8.73E-05 | 0.28452376 | 0.058186 | 0.27 |
| MRPL9 | 0.5707176 | 4.25E-05 | 0.30422253 | 0.042174 | 0.27 |
| NCAPH2 | 0.6958525 | 1.12E-07 | 0.42996246 | 0.0032 | 0.27 |
| TRMT6 | 0.5097897 | 0.000348 | 0.24409902 | 0.106108 | 0.27 |
| RPS19 | 0.5054268 | 0.000398 | 0.24014567 | 0.112068 | 0.27 |
| CENPU | 0.6650042 | 6.27E-07 | 0.40035608 | 0.006428 | 0.27 |
| SNRPC | 0.6652116 | 6.2E-07 | 0.4010118 | 0.006334 | 0.27 |
| CD2BP2 | 0.5932082 | 1.75E-05 | 0.32902747 | 0.027319 | 0.27 |
| TCERG1 | 0.819469 | 5.88E-12 | 0.55531442 | 7.51E-05 | 0.27 |
| DR1 | 0.5658748 | 0.000051 | 0.30194789 | 0.043815 | 0.27 |
| HNRNPLL | 0.7133881 | 3.8E-08 | 0.44983005 | 0.001934 | 0.27 |
| PHF8 | 0.617861 | 6.13E-06 | 0.35440627 | 0.016911 | 0.27 |
| HIC1 | -0.559306 | 0.000065 | -0.2959443 | 0.048399 | 0.27 |
| SHCBP1 | 0.5208908 | 0.000244 | 0.2579362 | 0.08714 | 0.27 |
| KNSTRN | 0.6517367 | 1.24E-06 | 0.38885802 | 0.008292 | 0.27 |
| SMG7 | 0.6198104 | 5.62E-06 | 0.35706983 | 0.016046 | 0.27 |
| PARN | 0.6332803 | 3.03E-06 | 0.37132228 | 0.012028 | 0.27 |
| ESCO2 | 0.6616341 | 7.48E-07 | 0.40026424 | 0.006441 | 0.27 |
| ACTR8 | 0.6294421 | 3.62E-06 | 0.36827282 | 0.012807 | 0.27 |
| RC3H2 | 0.5235642 | 0.000224 | 0.26325795 | 0.080592 | 0.27 |
| EIF2B4 | 0.5554323 | 7.48E-05 | 0.29521556 | 0.04898 | 0.27 |
| PTBP3 | 0.575031 | 0.000036 | 0.31482656 | 0.035171 | 0.27 |
| EP300 | 0.5632196 | 5.63E-05 | 0.30310155 | 0.042977 | 0.27 |
| BAZ1B | 0.6996232 | 8.93E-08 | 0.43997052 | 0.002492 | 0.26 |
| SNRPA1 | 0.6937579 | 1.27E-07 | 0.43410658 | 0.002888 | 0.26 |
| LIMS2 | -0.505028 | 0.000403 | -0.24575 | 0.103692 | 0.26 |
| MCM3 | 0.6334677 | 0.000003 | 0.3744433 | 0.011274 | 0.26 |
| GPATCH8 | 0.5587262 | 6.64E-05 | 0.29975484 | 0.045447 | 0.26 |
| RAE1 | 0.6852611 | 2.07E-07 | 0.42632889 | 0.003498 | 0.26 |
| FMR1 | 0.7458543 | 4.12E-09 | 0.48744684 | 0.000683 | 0.26 |
| DPYSL2 | -0.574786 | 3.64E-05 | -0.3164899 | 0.034165 | 0.26 |
| GTF2H4 | 0.6100821 | 8.62E-06 | 0.3519805 | 0.017733 | 0.26 |
| SCAF1 | 0.6846415 | 2.14E-07 | 0.42663305 | 0.003472 | 0.26 |
| CPSF7 | 0.721444 | 2.26E-08 | 0.46384864 | 0.001331 | 0.26 |
| NSD1 | 0.5654979 | 5.17E-05 | 0.30826851 | 0.039379 | 0.26 |
| RECK | -0.567986 | 4.71E-05 | -0.3109748 | 0.037595 | 0.26 |
| NOP14 | 0.6930827 | 1.32E-07 | 0.43614312 | 0.002745 | 0.26 |
| OLFML1 | -0.595915 | 1.57E-05 | -0.3391771 | 0.022651 | 0.26 |
| ZNF451 | 0.5950368 | 1.63E-05 | 0.33849563 | 0.022942 | 0.26 |
| MRPS30 | 0.5210073 | 0.000243 | 0.26460758 | 0.078994 | 0.26 |
| PSMD4 | 0.5231691 | 0.000227 | 0.26682842 | 0.076419 | 0.26 |
| MYLK | -0.661152 | 7.67E-07 | -0.4048306 | 0.005808 | 0.26 |
| ASF1B | 0.5477826 | 9.83E-05 | 0.2915767 | 0.051971 | 0.26 |
| MCM4 | 0.5976883 | 1.46E-05 | 0.34171911 | 0.021593 | 0.26 |
| NFRKB | 0.6962051 | 1.1E-07 | 0.44043645 | 0.002463 | 0.26 |
| MCMBP | 0.6475679 | 1.52E-06 | 0.39202869 | 0.007737 | 0.26 |
| ORC4 | 0.6350894 | 2.78E-06 | 0.3796024 | 0.010116 | 0.26 |
| MCM6 | 0.6045169 | 1.09E-05 | 0.34918646 | 0.018721 | 0.26 |
| RAD51AP1 | 0.5628152 | 5.71E-05 | 0.30778001 | 0.039708 | 0.26 |
| RNF168 | 0.64065 | 2.14E-06 | 0.38567869 | 0.008884 | 0.26 |
| EMILIN1 | -0.57162 | 0.000041 | -0.3170011 | 0.033861 | 0.26 |
| DDX46 | 0.821447 | 4.74E-12 | 0.56716488 | 4.86E-05 | 0.26 |
| COL5A1 | -0.515334 | 0.000292 | -0.2614446 | 0.082779 | 0.26 |
| KRR1 | 0.6815035 | 2.56E-07 | 0.42808073 | 0.003352 | 0.26 |
| RPS13 | 0.5223436 | 0.000233 | 0.26926161 | 0.073674 | 0.26 |
| KRI1 | 0.6605393 | 7.91E-07 | 0.40764021 | 0.005445 | 0.26 |
| CCNB1 | 0.5583697 | 6.73E-05 | 0.30558323 | 0.041216 | 0.26 |
| EIF3B | 0.7171106 | 2.99E-08 | 0.46441408 | 0.00131 | 0.26 |
| PRIM2 | 0.6959639 | 1.11E-07 | 0.4435037 | 0.002278 | 0.26 |
| PRRC2B | 0.5073232 | 0.000375 | 0.25489921 | 0.091059 | 0.26 |
| SLIT2 | -0.544089 | 0.000112 | -0.2917788 | 0.051801 | 0.26 |
| KIF4A | 0.6437255 | 1.84E-06 | 0.39145719 | 0.007834 | 0.26 |
| TNK2 | 0.5522803 | 8.38E-05 | 0.30016386 | 0.045139 | 0.26 |
| NOC4L | 0.6338008 | 2.96E-06 | 0.38200522 | 0.009612 | 0.26 |
| DCN | -0.623003 | 4.87E-06 | -0.3714112 | 0.012006 | 0.26 |
| EXOSC7 | 0.6168495 | 6.41E-06 | 0.36531178 | 0.013603 | 0.26 |
| C1orf43 | 0.5616269 | 5.97E-05 | 0.31018086 | 0.038111 | 0.26 |
| CCNL1 | 0.789163 | 1.19E-10 | 0.53801118 | 0.000138 | 0.26 |
| MND1 | 0.5581456 | 6.78E-05 | 0.30712192 | 0.040155 | 0.26 |
| CNOT1 | 0.687868 | 1.78E-07 | 0.43688869 | 0.002694 | 0.26 |
| KIF11 | 0.6301123 | 3.51E-06 | 0.37929251 | 0.010182 | 0.26 |
| GCN1L1 | 0.5926389 | 1.79E-05 | 0.34195354 | 0.021497 | 0.26 |
| KDM5C | 0.6363747 | 2.62E-06 | 0.38591535 | 0.008838 | 0.26 |
| MFF | 0.5619473 | 0.000059 | 0.3115189 | 0.037244 | 0.26 |
| SCAF4 | 0.5635459 | 5.56E-05 | 0.31337651 | 0.036068 | 0.26 |
| SNRNP48 | 0.7299437 | 1.27E-08 | 0.48000823 | 0.000848 | 0.25 |
| TOPORS | 0.6222471 | 5.04E-06 | 0.37260979 | 0.011712 | 0.25 |
| KIF20A | 0.603052 | 1.16E-05 | 0.35345837 | 0.017228 | 0.25 |
| YBX1 | 0.5465104 | 0.000103 | 0.2972304 | 0.047385 | 0.25 |
| DDX41 | 0.7230633 | 2.03E-08 | 0.47410783 | 0.001002 | 0.25 |
| TBK1 | 0.6285881 | 3.77E-06 | 0.3796696 | 0.010101 | 0.25 |
| GINS3 | 0.5552375 | 7.53E-05 | 0.3063554 | 0.040681 | 0.25 |
| RPL6 | 0.5378185 | 0.000139 | 0.28910753 | 0.054083 | 0.25 |
| NPAT | 0.579794 | 2.99E-05 | 0.33115679 | 0.026279 | 0.25 |
| ZNF318 | 0.5666578 | 4.95E-05 | 0.31810854 | 0.033209 | 0.25 |
| CENPH | 0.6656394 | 6.06E-07 | 0.4171244 | 0.004363 | 0.25 |
| ANXA2 | -0.53156 | 0.000172 | -0.2831215 | 0.05949 | 0.25 |
| LIN9 | 0.7942567 | 7.45E-11 | 0.54595087 | 0.000105 | 0.25 |
| C11orf84 | 0.5031145 | 0.000427 | 0.25541147 | 0.090388 | 0.25 |
| HCFC2 | 0.5884719 | 2.12E-05 | 0.3409392 | 0.021913 | 0.25 |
| DIS3 | 0.5457102 | 0.000106 | 0.29835721 | 0.046512 | 0.25 |
| CAAP1 | 0.6567973 | 9.59E-07 | 0.40951673 | 0.005214 | 0.25 |
| GMNN | 0.5920606 | 1.84E-05 | 0.34506673 | 0.020263 | 0.25 |
| LMOD1 | -0.536719 | 0.000144 | -0.2897321 | 0.053542 | 0.25 |
| ERCC2 | 0.6758327 | 3.5E-07 | 0.42889011 | 0.003286 | 0.25 |
| ECT2 | 0.5736508 | 0.000038 | 0.3267512 | 0.028469 | 0.25 |
| RRP7A | 0.6393913 | 2.27E-06 | 0.39307474 | 0.007561 | 0.25 |
| SF3A2 | 0.8425407 | 3.97E-13 | 0.59631084 | 1.54E-05 | 0.25 |
| SAP30BP | 0.5851097 | 2.43E-05 | 0.33891489 | 0.022763 | 0.25 |
| SNRPF | 0.8231491 | 3.93E-12 | 0.57736543 | 3.29E-05 | 0.25 |
| KAT8 | 0.6378214 | 2.45E-06 | 0.3920467 | 0.007734 | 0.25 |
| MED6 | 0.7165678 | 3.1E-08 | 0.47107222 | 0.001091 | 0.25 |
| MCM5 | 0.5850547 | 2.43E-05 | 0.33970608 | 0.022427 | 0.25 |
| DLGAP5 | 0.5412358 | 0.000124 | 0.29592313 | 0.048415 | 0.25 |
| RFC1 | 0.7360012 | 8.37E-09 | 0.49095954 | 0.000616 | 0.25 |
| C1orf112 | 0.5065283 | 0.000385 | 0.26185608 | 0.082278 | 0.25 |
| PCGF5 | 0.6546505 | 1.07E-06 | 0.41076487 | 0.005066 | 0.25 |
| HNRNPL | 0.837273 | 7.62E-13 | 0.59375484 | 1.71E-05 | 0.25 |
| L3MBTL2 | 0.620013 | 5.57E-06 | 0.37654573 | 0.010789 | 0.25 |
| RRP8 | 0.6267346 | 4.11E-06 | 0.38348219 | 0.009313 | 0.25 |
| SSU72 | 0.6104668 | 8.48E-06 | 0.36732251 | 0.013058 | 0.25 |
| CENPF | 0.5000959 | 0.000469 | 0.25705954 | 0.088258 | 0.25 |
| MRPL39 | 0.5584437 | 6.71E-05 | 0.31545925 | 0.034786 | 0.25 |
| BRI3BP | 0.5703249 | 4.31E-05 | 0.32739782 | 0.028139 | 0.25 |
| CDK2AP1 | 0.6305169 | 3.45E-06 | 0.38764938 | 0.008513 | 0.25 |
| SPC24 | 0.6299828 | 3.54E-06 | 0.38732331 | 0.008573 | 0.25 |
| FBLN7 | -0.526704 | 0.000202 | -0.2843487 | 0.058348 | 0.25 |
| ZNHIT1 | 0.6889177 | 1.68E-07 | 0.44660726 | 0.002103 | 0.25 |
| MED25 | 0.5361514 | 0.000147 | 0.29399506 | 0.049967 | 0.25 |
| TAF9 | 0.5039985 | 0.000416 | 0.26218226 | 0.081884 | 0.25 |
| EFEMP2 | -0.502228 | 0.000439 | -0.2607486 | 0.08363 | 0.25 |
| DDX23 | 0.6857488 | 2.01E-07 | 0.44429718 | 0.002232 | 0.25 |
| CDYL | 0.584548 | 2.48E-05 | 0.34342244 | 0.020907 | 0.25 |
| TSEN34 | 0.5827515 | 2.67E-05 | 0.34162809 | 0.02163 | 0.25 |
| ZC3H18 | 0.6060736 | 1.02E-05 | 0.36501783 | 0.013685 | 0.25 |
| PSMD2 | 0.5220762 | 0.000235 | 0.28105986 | 0.061449 | 0.25 |
| ORC1 | 0.6915821 | 1.44E-07 | 0.45058264 | 0.001896 | 0.25 |
| INO80D | 0.5575332 | 6.93E-05 | 0.31658449 | 0.034108 | 0.25 |
| UBE2S | 0.589833 | 2.01E-05 | 0.34966113 | 0.01855 | 0.25 |
| MCRS1 | 0.7141498 | 3.62E-08 | 0.47427923 | 0.000997 | 0.24 |
| EIF3I | 0.6390921 | 2.3E-06 | 0.39962491 | 0.006535 | 0.24 |
| BLM | 0.6647578 | 6.35E-07 | 0.42540397 | 0.003578 | 0.24 |
| DDX28 | 0.5195642 | 0.000255 | 0.28025399 | 0.062229 | 0.24 |
| SMIM13 | 0.566822 | 4.92E-05 | 0.32758801 | 0.028042 | 0.24 |
| MRPS18C | 0.5216463 | 0.000238 | 0.28241255 | 0.060158 | 0.24 |
| USP39 | 0.7093113 | 4.92E-08 | 0.47030114 | 0.001114 | 0.24 |
| MRPL48 | 0.5392208 | 0.000132 | 0.30043289 | 0.044937 | 0.24 |
| TOP1 | 0.5273552 | 0.000197 | 0.28884287 | 0.054313 | 0.24 |
| SRSF9 | 0.6765351 | 3.37E-07 | 0.43813119 | 0.002611 | 0.24 |
| SURF2 | 0.5541406 | 7.84E-05 | 0.31584188 | 0.034554 | 0.24 |
| POLA2 | 0.6332975 | 3.03E-06 | 0.39504383 | 0.007238 | 0.24 |
| RRP12 | 0.6310885 | 3.36E-06 | 0.39306735 | 0.007562 | 0.24 |
| ANP32B | 0.5397917 | 0.00013 | 0.30206516 | 0.04373 | 0.24 |
| BUD31 | 0.725263 | 1.75E-08 | 0.4876718 | 0.000679 | 0.24 |
| SPC25 | 0.5395552 | 0.000131 | 0.30221652 | 0.043619 | 0.24 |
| INHBB | -0.525882 | 0.000207 | -0.2886046 | 0.054521 | 0.24 |
| RPL18 | 0.6195006 | 5.7E-06 | 0.38280664 | 0.009449 | 0.24 |
| MED27 | 0.7078845 | 5.38E-08 | 0.47133463 | 0.001083 | 0.24 |
| KPNA2 | 0.5901616 | 1.98E-05 | 0.35390804 | 0.017077 | 0.24 |
| KIF2C | 0.6339012 | 2.94E-06 | 0.39783141 | 0.006803 | 0.24 |
| RBM14 | 0.7339576 | 9.66E-09 | 0.49800518 | 0.000499 | 0.24 |
| HERPUD2 | 0.5114943 | 0.000329 | 0.2755717 | 0.066918 | 0.24 |
| NAF1 | 0.5416718 | 0.000122 | 0.30610763 | 0.040852 | 0.24 |
| MED8 | 0.8007647 | 4.01E-11 | 0.56534377 | 0.000052 | 0.24 |
| KDM2A | 0.6381425 | 2.41E-06 | 0.40274664 | 0.00609 | 0.24 |
| MED26 | 0.6421758 | 1.98E-06 | 0.40684082 | 0.005546 | 0.24 |
| ZC3H13 | 0.729097 | 1.35E-08 | 0.49478455 | 0.00055 | 0.24 |
| BAP1 | 0.7016944 | 7.87E-08 | 0.46816362 | 0.001182 | 0.24 |
| FTSJ3 | 0.6555864 | 1.02E-06 | 0.42208738 | 0.003876 | 0.24 |
| CDC7 | 0.6946168 | 1.2E-07 | 0.46124305 | 0.001428 | 0.24 |
| SLX4 | 0.5141175 | 0.000303 | 0.28074847 | 0.06175 | 0.24 |
| OGT | 0.6180841 | 6.07E-06 | 0.38480434 | 0.009053 | 0.24 |
| SPON1 | -0.628057 | 3.86E-06 | -0.3948006 | 0.007278 | 0.24 |
| RPP40 | 0.7241042 | 1.89E-08 | 0.4909741 | 0.000616 | 0.24 |
| RPL14 | 0.5981943 | 1.43E-05 | 0.36531234 | 0.013603 | 0.24 |
| SRFBP1 | 0.6481675 | 1.48E-06 | 0.41530675 | 0.004555 | 0.24 |
| YTHDF1 | 0.5878544 | 2.18E-05 | 0.35513311 | 0.016671 | 0.24 |
| IGFBP6 | -0.702919 | 7.31E-08 | -0.4702419 | 0.001116 | 0.24 |
| ORC5 | 0.7590429 | 1.52E-09 | 0.52639455 | 0.000204 | 0.24 |
| MRPS35 | 0.5308395 | 0.000176 | 0.29822723 | 0.046612 | 0.24 |
| GEMIN8 | 0.6524672 | 1.19E-06 | 0.41998319 | 0.004077 | 0.24 |
| RP9 | 0.6508442 | 1.3E-06 | 0.41953234 | 0.004121 | 0.24 |
| THAP11 | 0.5381226 | 0.000137 | 0.30699958 | 0.040239 | 0.24 |
| MAU2 | 0.6541887 | 1.1E-06 | 0.42316235 | 0.003777 | 0.24 |
| CPSF6 | 0.814266 | 1.02E-11 | 0.58338502 | 0.000026 | 0.24 |
| ZNF654 | 0.5343475 | 0.000156 | 0.30350523 | 0.042686 | 0.24 |
| C19orf53 | 0.5457231 | 0.000106 | 0.3148853 | 0.035135 | 0.24 |
| ALAS1 | 0.5166924 | 0.000279 | 0.28588095 | 0.056946 | 0.24 |
| SBNO2 | 0.5096736 | 0.000349 | 0.27901775 | 0.063441 | 0.24 |
| SYNPO2 | -0.597704 | 1.46E-05 | -0.3673123 | 0.013061 | 0.24 |
| GNL2 | 0.6981544 | 9.75E-08 | 0.46818864 | 0.001181 | 0.23 |
| GPS2 | 0.5529513 | 8.18E-05 | 0.3231553 | 0.030368 | 0.23 |
| PRC1 | 0.5924677 | 1.81E-05 | 0.36302315 | 0.014248 | 0.23 |
| CHERP | 0.8391066 | 6.09E-13 | 0.60988513 | 8.69E-06 | 0.23 |
| HGF | -0.554255 | 7.81E-05 | -0.3250909 | 0.029333 | 0.23 |
| EIF2AK2 | 0.5258447 | 0.000208 | 0.29684299 | 0.047689 | 0.23 |
| ABL2 | 0.537561 | 0.00014 | 0.30940653 | 0.03862 | 0.23 |
| TNRC6A | 0.6673562 | 5.54E-07 | 0.43929574 | 0.002535 | 0.23 |
| VRK1 | 0.6813922 | 2.57E-07 | 0.45346125 | 0.001758 | 0.23 |
| WDR33 | 0.740339 | 6.15E-09 | 0.512436 | 0.00032 | 0.23 |
| DHX8 | 0.7126176 | 3.99E-08 | 0.48516922 | 0.00073 | 0.23 |
| GSN | -0.575805 | 0.000035 | -0.3485502 | 0.018953 | 0.23 |
| CD248 | -0.527512 | 0.000196 | -0.3003677 | 0.044986 | 0.23 |
| MCM7 | 0.5871479 | 2.24E-05 | 0.36010042 | 0.015108 | 0.23 |
| RFC2 | 0.7352832 | 8.8E-09 | 0.50830721 | 0.000364 | 0.23 |
| CLASP1 | 0.5008629 | 0.000458 | 0.27395645 | 0.068598 | 0.23 |
| SNRNP200 | 0.8582037 | 4.92E-14 | 0.6314905 | 3.3E-06 | 0.23 |
| NCAPD3 | 0.6592085 | 8.48E-07 | 0.43268221 | 0.002992 | 0.23 |
| PRPF38A | 0.7902382 | 1.08E-10 | 0.56391436 | 5.48E-05 | 0.23 |
| DLC1 | -0.601685 | 1.23E-05 | -0.3758171 | 0.010955 | 0.23 |
| EIF2S2 | 0.7016249 | 7.91E-08 | 0.47582065 | 0.000955 | 0.23 |
| C4orf27 | 0.5849617 | 2.44E-05 | 0.35930783 | 0.015349 | 0.23 |
| PRRC2C | 0.5087178 | 0.000359 | 0.28309253 | 0.059517 | 0.23 |
| DDX27 | 0.6033495 | 1.15E-05 | 0.37779458 | 0.010509 | 0.23 |
| MSL2 | 0.5025948 | 0.000434 | 0.27708544 | 0.065372 | 0.23 |
| CDK13 | 0.5919857 | 1.84E-05 | 0.36695816 | 0.013155 | 0.23 |
| NSMCE4A | 0.6551167 | 1.04E-06 | 0.43015354 | 0.003185 | 0.23 |
| RBMX | 0.7364345 | 8.12E-09 | 0.51154831 | 0.000329 | 0.23 |
| ATR | 0.5580488 | 0.000068 | 0.33316467 | 0.025328 | 0.23 |
| SUGP2 | 0.5530473 | 8.15E-05 | 0.3282088 | 0.027728 | 0.23 |
| CD3EAP | 0.6082369 | 9.33E-06 | 0.38345526 | 0.009319 | 0.23 |
| IQGAP3 | 0.5547596 | 7.67E-05 | 0.33002697 | 0.026827 | 0.23 |
| SPARCL1 | -0.546543 | 0.000103 | -0.3218152 | 0.031102 | 0.23 |
| DDX20 | 0.6502731 | 1.33E-06 | 0.42564272 | 0.003557 | 0.23 |
| ATAD1 | 0.5470577 | 0.000101 | 0.32246053 | 0.030747 | 0.23 |
| SCYL2 | 0.5283528 | 0.000191 | 0.30376366 | 0.042501 | 0.23 |
| CHD4 | 0.8160826 | 8.45E-12 | 0.59156082 | 1.87E-05 | 0.23 |
| GTPBP4 | 0.6418525 | 2.02E-06 | 0.4173813 | 0.004337 | 0.23 |
| NUP43 | 0.5552948 | 7.52E-05 | 0.33086036 | 0.026422 | 0.23 |
| CXXC1 | 0.6500341 | 1.35E-06 | 0.42572899 | 0.00355 | 0.23 |
| EIF1AX | 0.5445811 | 0.00011 | 0.32056388 | 0.0318 | 0.23 |
| MRPL15 | 0.5222729 | 0.000233 | 0.29828883 | 0.046565 | 0.23 |
| NDNL2 | 0.5441369 | 0.000112 | 0.32032162 | 0.031937 | 0.23 |
| RRS1 | 0.6267435 | 4.1E-06 | 0.40366012 | 0.005965 | 0.23 |
| HAUS8 | 0.6550895 | 1.05E-06 | 0.43207564 | 0.003038 | 0.23 |
| TBC1D15 | 0.5637869 | 5.51E-05 | 0.34083938 | 0.021954 | 0.23 |
| HCFC1 | 0.7106183 | 4.53E-08 | 0.48816919 | 0.000669 | 0.23 |
| GZF1 | 0.6788127 | 2.97E-07 | 0.45636636 | 0.001628 | 0.23 |
| CHD8 | 0.7468983 | 3.82E-09 | 0.52463663 | 0.000216 | 0.23 |
| GUF1 | 0.5504474 | 8.95E-05 | 0.32826529 | 0.0277 | 0.23 |
| U2SURP | 0.8318943 | 1.45E-12 | 0.60979578 | 8.73E-06 | 0.23 |
| DDX54 | 0.69279 | 1.34E-07 | 0.47100876 | 0.001093 | 0.23 |
| HDAC2 | 0.6321669 | 3.19E-06 | 0.41068866 | 0.005075 | 0.23 |
| SKIV2L2 | 0.7960171 | 6.31E-11 | 0.57454611 | 3.67E-05 | 0.23 |
| FAM204A | 0.5146301 | 0.000298 | 0.29321177 | 0.050609 | 0.23 |
| UTP14A | 0.6591962 | 8.48E-07 | 0.43810632 | 0.002613 | 0.23 |
| PRPF6 | 0.765721 | 8.92E-10 | 0.5459137 | 0.000105 | 0.22 |
| ZC3H14 | 0.6558027 | 1.01E-06 | 0.43634918 | 0.002731 | 0.22 |
| RIF1 | 0.6115445 | 8.09E-06 | 0.39214056 | 0.007718 | 0.22 |
| DDX52 | 0.6681365 | 5.31E-07 | 0.448737 | 0.00199 | 0.22 |
| DIDO1 | 0.5060672 | 0.00039 | 0.28667035 | 0.056235 | 0.22 |
| ZNF48 | 0.5985351 | 1.41E-05 | 0.37919816 | 0.010202 | 0.22 |
| MBTD1 | 0.544791 | 0.000109 | 0.32574273 | 0.028992 | 0.22 |
| TNPO1 | 0.5213876 | 0.00024 | 0.30240425 | 0.043482 | 0.22 |
| XRN1 | 0.6499391 | 1.36E-06 | 0.43119698 | 0.003104 | 0.22 |
| CCNK | 0.5463121 | 0.000104 | 0.32761812 | 0.028027 | 0.22 |
| LYRM2 | 0.5022778 | 0.000439 | 0.28362303 | 0.059021 | 0.22 |
| GEMIN2 | 0.5207186 | 0.000245 | 0.30218407 | 0.043643 | 0.22 |
| FAM208B | 0.572751 | 3.93E-05 | 0.35429089 | 0.01695 | 0.22 |
| MRPS5 | 0.5543303 | 7.78E-05 | 0.33633349 | 0.023886 | 0.22 |
| POLR2E | 0.8469028 | 2.27E-13 | 0.62902698 | 3.7E-06 | 0.22 |
| CPSF2 | 0.7403406 | 6.15E-09 | 0.52252134 | 0.000231 | 0.22 |
| ZBTB11 | 0.672157 | 4.28E-07 | 0.45433918 | 0.001718 | 0.22 |
| NXT1 | 0.5575159 | 6.94E-05 | 0.3397406 | 0.022413 | 0.22 |
| EIF3D | 0.7251217 | 1.77E-08 | 0.50743117 | 0.000374 | 0.22 |
| ZNF207 | 0.7297768 | 1.29E-08 | 0.51229209 | 0.000321 | 0.22 |
| SMG5 | 0.5327177 | 0.000165 | 0.31533797 | 0.034859 | 0.22 |
| NAA25 | 0.5265696 | 0.000203 | 0.30921755 | 0.038745 | 0.22 |
| DDX55 | 0.6990328 | 9.25E-08 | 0.48178532 | 0.000805 | 0.22 |
| ADNP | 0.7043867 | 6.68E-08 | 0.4871585 | 0.000689 | 0.22 |
| MRGBP | 0.5259377 | 0.000207 | 0.30893774 | 0.038931 | 0.22 |
| UBTF | 0.6032688 | 1.15E-05 | 0.38629924 | 0.008766 | 0.22 |
| RBM23 | 0.5201743 | 0.00025 | 0.30324657 | 0.042872 | 0.22 |
| NSA2 | 0.5197016 | 0.000254 | 0.30303151 | 0.043027 | 0.22 |
| NELFE | 0.630248 | 3.49E-06 | 0.41361816 | 0.004739 | 0.22 |
| POP4 | 0.7798325 | 2.74E-10 | 0.56322041 | 5.63E-05 | 0.22 |
| ZGPAT | 0.5469666 | 0.000101 | 0.33041428 | 0.026638 | 0.22 |
| DHX16 | 0.8185013 | 6.53E-12 | 0.60200925 | 1.22E-05 | 0.22 |
| SLC25A25 | 0.5612003 | 6.06E-05 | 0.34471331 | 0.0204 | 0.22 |
| CHTF8 | 0.5161619 | 0.000284 | 0.29990659 | 0.045332 | 0.22 |
| ERI1 | 0.5110665 | 0.000334 | 0.29485362 | 0.049271 | 0.22 |
| C12orf43 | 0.6596983 | 8.27E-07 | 0.44354081 | 0.002276 | 0.22 |
| LMNB1 | 0.6266007 | 4.13E-06 | 0.41080474 | 0.005061 | 0.22 |
| FNBP4 | 0.7056255 | 6.19E-08 | 0.48998625 | 0.000634 | 0.22 |
| IGF2 | -0.509327 | 0.000353 | -0.2937534 | 0.050165 | 0.22 |
| HAT1 | 0.5423816 | 0.000119 | 0.32688071 | 0.028403 | 0.22 |
| RPL28 | 0.5210023 | 0.000243 | 0.3056065 | 0.0412 | 0.22 |
| PES1 | 0.6387074 | 2.34E-06 | 0.42334389 | 0.003761 | 0.22 |
| FAM111B | 0.5871504 | 2.24E-05 | 0.37187238 | 0.011892 | 0.22 |
| HELZ2 | 0.5927541 | 1.78E-05 | 0.37762302 | 0.010547 | 0.22 |
| PCOLCE | -0.564834 | 0.000053 | -0.3497212 | 0.018529 | 0.22 |
| PSMC1 | 0.5542074 | 7.82E-05 | 0.3393298 | 0.022586 | 0.22 |
| PCF11 | 0.7801005 | 2.67E-10 | 0.5653182 | 0.000052 | 0.22 |
| ORC2 | 0.8036651 | 3.01E-11 | 0.58895101 | 2.08E-05 | 0.22 |
| EIF2S3 | 0.6899317 | 1.58E-07 | 0.47536663 | 0.000967 | 0.22 |
| PRRC2A | 0.5457144 | 0.000106 | 0.33124056 | 0.026238 | 0.22 |
| KDM2B | 0.6932402 | 1.3E-07 | 0.47891334 | 0.000874 | 0.22 |
| TAOK2 | 0.5802322 | 2.94E-05 | 0.36600973 | 0.013412 | 0.22 |
| IGJ | -0.566718 | 4.94E-05 | -0.3528172 | 0.017446 | 0.22 |
| TDRD7 | 0.6301451 | 3.51E-06 | 0.41662917 | 0.004415 | 0.22 |
| THOC7 | 0.7710657 | 5.76E-10 | 0.5576038 | 6.92E-05 | 0.22 |
| IK | 0.76771 | 7.59E-10 | 0.55430228 | 7.79E-05 | 0.22 |
| METTL18 | 0.5585707 | 6.68E-05 | 0.3455362 | 0.020082 | 0.22 |
| ORC3 | 0.7404637 | 6.1E-09 | 0.52773997 | 0.000195 | 0.22 |
| EIF4A3 | 0.7893145 | 1.18E-10 | 0.57679354 | 3.37E-05 | 0.22 |
| MRPS24 | 0.5966387 | 1.52E-05 | 0.38441514 | 0.009129 | 0.22 |
| RBM34 | 0.6612904 | 7.61E-07 | 0.44914692 | 0.001969 | 0.22 |
| NOL7 | 0.7033527 | 7.12E-08 | 0.49133989 | 0.000609 | 0.22 |
| PRKRIR | 0.6538866 | 1.11E-06 | 0.44190563 | 0.002373 | 0.22 |
| ZNF593 | 0.5023241 | 0.000438 | 0.29034504 | 0.053016 | 0.22 |
| SREK1 | 0.5675606 | 4.79E-05 | 0.35567273 | 0.016495 | 0.22 |
| GLTSCR1 | 0.5275922 | 0.000196 | 0.31579992 | 0.034579 | 0.22 |
| RC3H1 | 0.5470389 | 0.000101 | 0.33541413 | 0.024297 | 0.22 |
| MED23 | 0.7591445 | 1.5E-09 | 0.54752931 | 9.92E-05 | 0.22 |
| TBC1D31 | 0.5097092 | 0.000348 | 0.29818874 | 0.046642 | 0.22 |
| KDM6A | 0.5996073 | 1.35E-05 | 0.38825476 | 0.008402 | 0.22 |
| MKI67 | 0.6387572 | 2.34E-06 | 0.4275492 | 0.003396 | 0.22 |
| LUM | -0.614493 | 7.11E-06 | -0.4033634 | 0.006005 | 0.22 |
| ATRIP | 0.6180352 | 6.08E-06 | 0.40699825 | 0.005526 | 0.22 |
| EIF2S1 | 0.7227666 | 2.07E-08 | 0.51173545 | 0.000327 | 0.22 |
| RBBP5 | 0.7056484 | 6.18E-08 | 0.49551307 | 0.000538 | 0.22 |
| SMC4 | 0.6768026 | 3.32E-07 | 0.46678334 | 0.001228 | 0.22 |
| DBR1 | 0.5051579 | 0.000401 | 0.29619708 | 0.048198 | 0.21 |
| S100A10 | -0.545729 | 0.000106 | -0.3369646 | 0.023607 | 0.21 |
| RPF1 | 0.6022112 | 1.21E-05 | 0.39347955 | 0.007493 | 0.21 |
| HERC2 | 0.5224249 | 0.000232 | 0.3140452 | 0.035652 | 0.21 |
| MRPL4 | 0.5297415 | 0.000182 | 0.3216009 | 0.031221 | 0.21 |
| CPXM1 | -0.541227 | 0.000124 | -0.333317 | 0.025257 | 0.21 |
| TDP1 | 0.5108752 | 0.000336 | 0.30297944 | 0.043065 | 0.21 |
| CKS2 | 0.5928501 | 1.78E-05 | 0.38505701 | 0.009004 | 0.21 |
| PIAS4 | 0.5377308 | 0.000139 | 0.33006393 | 0.026809 | 0.21 |
| ADH5 | -0.552275 | 8.38E-05 | -0.3447463 | 0.020387 | 0.21 |
| RBM39 | 0.7859909 | 1.59E-10 | 0.57847655 | 3.15E-05 | 0.21 |
| EIF4ENIF1 | 0.5332651 | 0.000162 | 0.3259016 | 0.028909 | 0.21 |
| CFD | -0.505346 | 0.000399 | -0.2980115 | 0.046779 | 0.21 |
| REEP4 | 0.509 | 0.000356 | 0.30173633 | 0.043971 | 0.21 |
| SF3A3 | 0.8282742 | 2.2E-12 | 0.62105818 | 5.31E-06 | 0.21 |
| VASN | -0.547492 | 9.93E-05 | -0.340379 | 0.022145 | 0.21 |
| DGCR14 | 0.6321144 | 3.2E-06 | 0.42512122 | 0.003602 | 0.21 |
| RPL34 | 0.5303149 | 0.000179 | 0.32335217 | 0.030262 | 0.21 |
| HAUS4 | 0.7084006 | 5.21E-08 | 0.50150203 | 0.000449 | 0.21 |
| SASS6 | 0.5895557 | 2.03E-05 | 0.38283928 | 0.009442 | 0.21 |
| ANK2 | -0.523404 | 0.000225 | -0.3171201 | 0.03379 | 0.21 |
| PPIE | 0.5499646 | 0.000091 | 0.34382471 | 0.020748 | 0.21 |
| NSRP1 | 0.6452074 | 1.71E-06 | 0.43908644 | 0.002549 | 0.21 |
| RXRB | 0.6188423 | 5.87E-06 | 0.41301584 | 0.004807 | 0.21 |
| GPATCH1 | 0.5712274 | 4.17E-05 | 0.36563534 | 0.013514 | 0.21 |
| UCKL1 | 0.6472933 | 1.55E-06 | 0.4417134 | 0.002384 | 0.21 |
| ZC3H10 | 0.5744186 | 3.69E-05 | 0.36920491 | 0.012564 | 0.21 |
| PSMD1 | 0.588688 | 0.000021 | 0.38350386 | 0.009309 | 0.21 |
| RSRC2 | 0.6844424 | 2.17E-07 | 0.47929349 | 0.000865 | 0.21 |
| FANCD2 | 0.5931735 | 1.75E-05 | 0.38807376 | 0.008435 | 0.21 |
| RMI1 | 0.6961575 | 1.1E-07 | 0.49120756 | 0.000612 | 0.21 |
| MATR3 | 0.705927 | 6.08E-08 | 0.50098334 | 0.000456 | 0.21 |
| MEAF6 | 0.6162698 | 6.58E-06 | 0.41135232 | 0.004997 | 0.21 |
| PLA2R1 | -0.505043 | 0.000403 | -0.3004448 | 0.044928 | 0.21 |
| DCAF13 | 0.5647729 | 5.31E-05 | 0.36025023 | 0.015063 | 0.21 |
| CSPP1 | 0.6200242 | 5.56E-06 | 0.41560582 | 0.004523 | 0.21 |
| BTD | -0.593655 | 1.72E-05 | -0.3892404 | 0.008224 | 0.21 |
| NSMCE2 | 0.5748729 | 3.62E-05 | 0.37084875 | 0.012146 | 0.21 |
| NR2C1 | 0.5011074 | 0.000454 | 0.29708719 | 0.047497 | 0.21 |
| C1orf131 | 0.5700737 | 4.35E-05 | 0.36610052 | 0.013387 | 0.21 |
| CKAP5 | 0.6095765 | 8.81E-06 | 0.40578274 | 0.005683 | 0.21 |
| NCAPG | 0.626073 | 4.23E-06 | 0.42241308 | 0.003846 | 0.21 |
| RPL7L1 | 0.5079763 | 0.000368 | 0.3043355 | 0.042094 | 0.21 |
| POLR3E | 0.6012649 | 1.25E-05 | 0.39765206 | 0.00683 | 0.21 |
| C19orf43 | 0.5933241 | 1.74E-05 | 0.38987846 | 0.00811 | 0.21 |
| YLPM1 | 0.5614849 | 0.00006 | 0.35820607 | 0.015689 | 0.21 |
| EFTUD2 | 0.8713741 | 6.94E-15 | 0.66811386 | 5.32E-07 | 0.21 |
| ING5 | 0.5989431 | 1.38E-05 | 0.39589529 | 0.007103 | 0.21 |
| RBM45 | 0.7179041 | 2.84E-08 | 0.51490516 | 0.000296 | 0.21 |
| CIT | 0.5881845 | 2.15E-05 | 0.38538662 | 0.00894 | 0.21 |
| RPL18A | 0.5320286 | 0.000169 | 0.32933772 | 0.027166 | 0.21 |
| SNRNP70 | 0.8494164 | 1.63E-13 | 0.64706618 | 1.56E-06 | 0.21 |
| CEP63 | 0.5066441 | 0.000383 | 0.30493864 | 0.041668 | 0.21 |
| PBRM1 | 0.5705723 | 4.27E-05 | 0.36889425 | 0.012645 | 0.21 |
| SF3B2 | 0.8079324 | 1.97E-11 | 0.60660481 | 0.00001 | 0.21 |
| GLTSCR2 | 0.7027278 | 7.39E-08 | 0.50147516 | 0.000449 | 0.21 |
| HNRNPA2B1 | 0.6639382 | 6.63E-07 | 0.46275752 | 0.001371 | 0.21 |
| RAD9A | 0.5524418 | 8.33E-05 | 0.35128081 | 0.017976 | 0.21 |
| EIF2B3 | 0.5211834 | 0.000242 | 0.32002912 | 0.032103 | 0.21 |
| NOP9 | 0.6631328 | 6.92E-07 | 0.46202745 | 0.001398 | 0.21 |
| KAT7 | 0.688662 | 1.7E-07 | 0.4878952 | 0.000675 | 0.21 |
| PINX1 | 0.6600387 | 8.12E-07 | 0.45947803 | 0.001498 | 0.21 |
| GPATCH4 | 0.7076956 | 5.44E-08 | 0.50733208 | 0.000375 | 0.21 |
| DDX42 | 0.6762447 | 3.43E-07 | 0.47630125 | 0.000942 | 0.2 |
| RNMT | 0.6279951 | 3.88E-06 | 0.42846366 | 0.00332 | 0.2 |
| NEMF | 0.552232 | 8.39E-05 | 0.35277624 | 0.01746 | 0.2 |
| LARP4B | 0.6027561 | 1.18E-05 | 0.40334851 | 0.006007 | 0.2 |
| GAR1 | 0.625937 | 4.26E-06 | 0.42659052 | 0.003476 | 0.2 |
| SKA1 | 0.6387588 | 2.34E-06 | 0.43981578 | 0.002502 | 0.2 |
| CSTF2 | 0.7308577 | 1.2E-08 | 0.53201939 | 0.000169 | 0.2 |
| TAF5 | 0.6688803 | 5.1E-07 | 0.47032119 | 0.001114 | 0.2 |
| EXOSC1 | 0.6948269 | 1.19E-07 | 0.49629587 | 0.000526 | 0.2 |
| SNRNP40 | 0.7472364 | 3.72E-09 | 0.54873866 | 0.000095 | 0.2 |
| RBM7 | 0.569423 | 4.46E-05 | 0.37146216 | 0.011994 | 0.2 |
| RBM25 | 0.8054553 | 2.52E-11 | 0.60764316 | 9.57E-06 | 0.2 |
| RAD18 | 0.6876596 | 1.8E-07 | 0.49046655 | 0.000625 | 0.2 |
| NCAPH | 0.6028533 | 1.17E-05 | 0.40590788 | 0.005666 | 0.2 |
| NCAPD2 | 0.6155328 | 6.79E-06 | 0.41871879 | 0.004201 | 0.2 |
| SMC2 | 0.6660592 | 5.93E-07 | 0.46932961 | 0.001145 | 0.2 |
| MED14 | 0.7582942 | 1.61E-09 | 0.56172102 | 5.95E-05 | 0.2 |
| DHX34 | 0.5640241 | 5.46E-05 | 0.36748615 | 0.013014 | 0.2 |
| CLASRP | 0.7327762 | 1.05E-08 | 0.53645851 | 0.000146 | 0.2 |
| F13A1 | -0.591301 | 1.89E-05 | -0.3952895 | 0.007199 | 0.2 |
| CCDC8 | -0.579935 | 2.98E-05 | -0.3840661 | 0.009197 | 0.2 |
| FIP1L1 | 0.7157576 | 3.27E-08 | 0.52013402 | 0.00025 | 0.2 |
| TNKS | 0.5768913 | 3.35E-05 | 0.38140961 | 0.009735 | 0.2 |
| AQR | 0.7457977 | 4.14E-09 | 0.55034944 | 8.98E-05 | 0.2 |
| TOP3A | 0.685715 | 2.02E-07 | 0.49029669 | 0.000629 | 0.2 |
| CAPRIN1 | 0.6321851 | 3.19E-06 | 0.43708761 | 0.002681 | 0.2 |
| POLR1E | 0.6699152 | 4.83E-07 | 0.47486544 | 0.000981 | 0.2 |
| SAMD1 | 0.5252152 | 0.000212 | 0.33020119 | 0.026742 | 0.2 |
| TAF11 | 0.6178083 | 6.14E-06 | 0.42305995 | 0.003786 | 0.2 |
| CNOT11 | 0.7416434 | 5.6E-09 | 0.54712809 | 0.000101 | 0.2 |
| EIF3A | 0.7804603 | 2.59E-10 | 0.58598599 | 2.35E-05 | 0.2 |
| MMP2 | -0.537619 | 0.00014 | -0.343248 | 0.020976 | 0.2 |
| PRPF8 | 0.8283549 | 2.18E-12 | 0.63404861 | 2.92E-06 | 0.2 |
| DCAF4 | 0.6108794 | 8.33E-06 | 0.41674276 | 0.004403 | 0.2 |
| ADAR | 0.7621385 | 1.19E-09 | 0.56814129 | 4.68E-05 | 0.2 |
| IGKV2-30 | -0.536286 | 0.000146 | -0.3425194 | 0.021268 | 0.2 |
| POLR2K | 0.6830267 | 2.35E-07 | 0.48954884 | 0.000643 | 0.2 |
| ANKRD11 | 0.519125 | 0.000258 | 0.32567634 | 0.029026 | 0.2 |
| PSMC2 | 0.5257029 | 0.000209 | 0.33236426 | 0.025703 | 0.2 |
| RAB3IL1 | -0.536757 | 0.000144 | -0.3436907 | 0.020801 | 0.2 |
| NUPL1 | 0.701252 | 8.09E-08 | 0.50831946 | 0.000364 | 0.2 |
| HAUS2 | 0.652599 | 1.19E-06 | 0.45967487 | 0.00149 | 0.2 |
| KIF22 | 0.6098823 | 8.69E-06 | 0.41704677 | 0.004371 | 0.2 |
| C17orf85 | 0.7573166 | 1.73E-09 | 0.56455679 | 5.35E-05 | 0.2 |
| CNOT7 | 0.6220503 | 5.08E-06 | 0.42981477 | 0.003212 | 0.2 |
| PPHLN1 | 0.5594543 | 6.46E-05 | 0.36727227 | 0.013071 | 0.2 |
| SAP30 | 0.5206335 | 0.000246 | 0.32879431 | 0.027435 | 0.2 |
| ERCC3 | 0.7455722 | 4.21E-09 | 0.55388107 | 7.91E-05 | 0.2 |
| WDFY2 | -0.50981 | 0.000347 | -0.3181824 | 0.033166 | 0.2 |
| POLR3D | 0.6750062 | 3.67E-07 | 0.48341883 | 0.000768 | 0.2 |
| SON | 0.5199689 | 0.000251 | 0.32840391 | 0.02763 | 0.2 |
| BCOR | 0.6390384 | 2.31E-06 | 0.44786973 | 0.002035 | 0.2 |
| RBM17 | 0.7663821 | 8.45E-10 | 0.57533923 | 3.56E-05 | 0.2 |
| HAUS6 | 0.63107 | 3.36E-06 | 0.44055448 | 0.002456 | 0.2 |
| PSMD3 | 0.5801432 | 2.95E-05 | 0.38972437 | 0.008137 | 0.2 |
| LRRC8B | 0.5663474 | 5.01E-05 | 0.37608778 | 0.010893 | 0.2 |
| SNRNP27 | 0.8068701 | 2.19E-11 | 0.61667952 | 6.46E-06 | 0.2 |
| PDS5A | 0.636616 | 2.59E-06 | 0.44649093 | 0.002109 | 0.2 |
| SNRPD3 | 0.8792357 | 1.94E-15 | 0.68919108 | 1.65E-07 | 0.2 |
| SSRP1 | 0.7139293 | 3.67E-08 | 0.52426829 | 0.000219 | 0.19 |
| SF1 | 0.7573432 | 1.73E-09 | 0.56769582 | 4.76E-05 | 0.19 |
| PAXIP1 | 0.560269 | 6.27E-05 | 0.37064794 | 0.012197 | 0.19 |
| RPAP2 | 0.7320013 | 1.11E-08 | 0.54238599 | 0.000119 | 0.19 |
| SMNDC1 | 0.6351866 | 2.77E-06 | 0.4456255 | 0.002157 | 0.19 |
| RPL24 | 0.5002206 | 0.000467 | 0.3110877 | 0.037522 | 0.19 |
| POLR2A | 0.8142215 | 1.03E-11 | 0.62548897 | 4.35E-06 | 0.19 |
| HNRNPA1 | 0.7081234 | 5.3E-08 | 0.51949203 | 0.000255 | 0.19 |
| AATF | 0.6370623 | 2.54E-06 | 0.44844483 | 0.002005 | 0.19 |
| GTF3C1 | 0.6533324 | 1.14E-06 | 0.46486293 | 0.001294 | 0.19 |
| SERPINA4 | -0.67079 | 4.61E-07 | -0.4824309 | 0.000791 | 0.19 |
| MRPS14 | 0.6891991 | 1.65E-07 | 0.50114313 | 0.000454 | 0.19 |
| THRAP3 | 0.7252536 | 1.75E-08 | 0.53727202 | 0.000142 | 0.19 |
| MED19 | 0.5740911 | 3.73E-05 | 0.3863399 | 0.008758 | 0.19 |
| WDR83 | 0.5360202 | 0.000148 | 0.34837666 | 0.019016 | 0.19 |
| NCAPG2 | 0.6247677 | 4.49E-06 | 0.43722523 | 0.002671 | 0.19 |
| SENP2 | 0.5706267 | 4.26E-05 | 0.38324835 | 0.00936 | 0.19 |
| WDR43 | 0.6370623 | 2.54E-06 | 0.45003868 | 0.001923 | 0.19 |
| CAPZA2 | -0.517448 | 0.000273 | -0.3304323 | 0.026629 | 0.19 |
| IL17RA | 0.5570877 | 7.05E-05 | 0.37009408 | 0.012337 | 0.19 |
| ANKMY2 | -0.501207 | 0.000453 | -0.3143245 | 0.03548 | 0.19 |
| BRD9 | 0.6197009 | 5.65E-06 | 0.43294185 | 0.002973 | 0.19 |
| UBAP2L | 0.6460855 | 1.64E-06 | 0.45933216 | 0.001504 | 0.19 |
| U2AF1 | 0.6998256 | 8.82E-08 | 0.51317029 | 0.000312 | 0.19 |
| CENPL | 0.6718202 | 4.36E-07 | 0.4859104 | 0.000715 | 0.19 |
| DHX9 | 0.7897918 | 1.13E-10 | 0.60424794 | 1.11E-05 | 0.19 |
| MDC1 | 0.5491996 | 9.35E-05 | 0.36378568 | 0.01403 | 0.19 |
| PCBP1 | 0.5262363 | 0.000205 | 0.34082454 | 0.02196 | 0.19 |
| KIAA0907 | 0.611361 | 8.15E-06 | 0.4262026 | 0.003509 | 0.19 |
| SRCAP | 0.7065045 | 5.86E-08 | 0.52152921 | 0.000239 | 0.19 |
| CDC73 | 0.7450976 | 4.36E-09 | 0.56037556 | 6.25E-05 | 0.19 |
| GKAP1 | 0.5508249 | 8.83E-05 | 0.36612711 | 0.01338 | 0.19 |
| HPS3 | 0.5009069 | 0.000457 | 0.31638246 | 0.034229 | 0.19 |
| QSER1 | 0.5982389 | 1.42E-05 | 0.41372266 | 0.004728 | 0.19 |
| NPM1 | 0.5552894 | 7.52E-05 | 0.37088389 | 0.012138 | 0.19 |
| ZMYM3 | 0.5817302 | 2.78E-05 | 0.39734742 | 0.006877 | 0.19 |
| SMC6 | 0.6937992 | 1.26E-07 | 0.50964672 | 0.000349 | 0.19 |
| AFF4 | 0.6204297 | 5.46E-06 | 0.43633904 | 0.002731 | 0.19 |
| INTS8 | 0.5841973 | 2.52E-05 | 0.40023433 | 0.006446 | 0.19 |
| DPH1 | 0.5694081 | 4.46E-05 | 0.38549036 | 0.00892 | 0.19 |
| KPNA1 | 0.56578 | 5.12E-05 | 0.38204407 | 0.009604 | 0.19 |
| SCML2 | 0.5089473 | 0.000357 | 0.32538486 | 0.029179 | 0.19 |
| ZC3H11A | 0.5432221 | 0.000115 | 0.36015944 | 0.01509 | 0.19 |
| RSL1D1 | 0.6462357 | 1.63E-06 | 0.4632372 | 0.001353 | 0.19 |
| NUP62 | 0.7503622 | 2.95E-09 | 0.56740301 | 4.81E-05 | 0.19 |
| SNRPD2 | 0.8639143 | 2.16E-14 | 0.68098569 | 2.63E-07 | 0.19 |
| LIMD1 | 0.5496957 | 9.19E-05 | 0.36678145 | 0.013203 | 0.19 |
| CCDC9 | 0.5517593 | 8.54E-05 | 0.36889287 | 0.012645 | 0.19 |
| FXR2 | 0.6274583 | 3.97E-06 | 0.44459845 | 0.002215 | 0.19 |
| PGLYRP2 | -0.629628 | 3.59E-06 | -0.447046 | 0.002079 | 0.19 |
| WTAP | 0.7469936 | 3.79E-09 | 0.56454214 | 5.36E-05 | 0.19 |
| NUF2 | 0.608095 | 9.39E-06 | 0.42580691 | 0.003543 | 0.19 |
| SRSF3 | 0.7985846 | 4.94E-11 | 0.61633885 | 6.56E-06 | 0.19 |
| WHSC1 | 0.647412 | 1.54E-06 | 0.46542284 | 0.001275 | 0.19 |
| ZNF281 | 0.5494841 | 9.26E-05 | 0.36759762 | 0.012985 | 0.19 |
| LRP1 | -0.566493 | 4.98E-05 | -0.3847335 | 0.009066 | 0.19 |
| FUBP1 | 0.6925737 | 1.36E-07 | 0.51086795 | 0.000336 | 0.19 |
| MASTL | 0.5972055 | 1.49E-05 | 0.41563302 | 0.00452 | 0.19 |
| PNO1 | 0.5970995 | 1.49E-05 | 0.41554653 | 0.004529 | 0.19 |
| RBM42 | 0.7286863 | 1.39E-08 | 0.54740614 | 9.96E-05 | 0.19 |
| INO80 | 0.7858859 | 1.6E-10 | 0.60461358 | 1.09E-05 | 0.19 |
| SLC39A14 | 0.5565046 | 0.000072 | 0.37529982 | 0.011074 | 0.19 |
| MSRB3 | -0.624656 | 4.51E-06 | -0.4434592 | 0.00228 | 0.19 |
| RPL7 | 0.5245694 | 0.000216 | 0.34343416 | 0.020902 | 0.19 |
| LTV1 | 0.5216615 | 0.000238 | 0.34059371 | 0.022056 | 0.19 |
| PTBP1 | 0.6096263 | 8.79E-06 | 0.42870189 | 0.003301 | 0.19 |
| POTEF | -0.507865 | 0.000369 | -0.327406 | 0.028135 | 0.19 |
| VCL | -0.550504 | 8.93E-05 | -0.3701045 | 0.012334 | 0.19 |
| CEP192 | 0.6477581 | 1.51E-06 | 0.46745607 | 0.001205 | 0.19 |
| UBR5 | 0.7573119 | 1.73E-09 | 0.57723206 | 3.31E-05 | 0.19 |
| PTCD3 | 0.5034727 | 0.000423 | 0.32340575 | 0.030233 | 0.19 |
| RNMTL1 | 0.5530012 | 8.17E-05 | 0.37320817 | 0.011567 | 0.18 |
| CHL1 | -0.604756 | 1.08E-05 | -0.4250724 | 0.003607 | 0.18 |
| CIRH1A | 0.6280054 | 3.87E-06 | 0.44898515 | 0.001977 | 0.18 |
| PODN | -0.579211 | 3.06E-05 | -0.4003252 | 0.006432 | 0.18 |
| ZNF740 | 0.5242588 | 0.000219 | 0.34575357 | 0.019999 | 0.18 |
| MPHOSPH6 | 0.5713651 | 4.14E-05 | 0.39294034 | 0.007583 | 0.18 |
| SMCHD1 | 0.6200112 | 5.57E-06 | 0.44172426 | 0.002384 | 0.18 |
| PHF5A | 0.6817006 | 2.53E-07 | 0.50353528 | 0.000422 | 0.18 |
| FAM32A | 0.5213367 | 0.000241 | 0.34343432 | 0.020902 | 0.18 |
| RPS2 | 0.5031336 | 0.000427 | 0.32538757 | 0.029177 | 0.18 |
| RPL27 | 0.5043427 | 0.000412 | 0.32660842 | 0.028543 | 0.18 |
| EHD3 | -0.521376 | 0.00024 | -0.343833 | 0.020745 | 0.18 |
| ARL6IP4 | 0.6916159 | 1.43E-07 | 0.51453733 | 0.000299 | 0.18 |
| EXOSC8 | 0.563484 | 5.57E-05 | 0.38668593 | 0.008693 | 0.18 |
| SRSF1 | 0.8073075 | 2.1E-11 | 0.63080562 | 3.4E-06 | 0.18 |
| NAT10 | 0.6756283 | 3.54E-07 | 0.49919476 | 0.000482 | 0.18 |
| ORC6 | 0.5507806 | 8.84E-05 | 0.37446555 | 0.011269 | 0.18 |
| SERPINA3 | -0.520519 | 0.000247 | -0.3443408 | 0.020545 | 0.18 |
| RNPS1 | 0.8332152 | 1.24E-12 | 0.65706379 | 9.46E-07 | 0.18 |
| TPX2 | 0.6124817 | 7.77E-06 | 0.43656785 | 0.002716 | 0.18 |
| GTF3C2 | 0.669248 | 5E-07 | 0.49338984 | 0.000574 | 0.18 |
| SUPT16H | 0.713905 | 3.68E-08 | 0.53805086 | 0.000138 | 0.18 |
| CDCA5 | 0.6165003 | 6.51E-06 | 0.44065815 | 0.002449 | 0.18 |
| EXOSC6 | 0.5305978 | 0.000177 | 0.3547603 | 0.016794 | 0.18 |
| HMGXB4 | 0.6543574 | 1.09E-06 | 0.47852845 | 0.000884 | 0.18 |
| INTS3 | 0.5059505 | 0.000392 | 0.33014102 | 0.026771 | 0.18 |
| CENPB | 0.5462914 | 0.000104 | 0.37091424 | 0.01213 | 0.18 |
| CNOT4 | 0.6414576 | 2.05E-06 | 0.46612185 | 0.00125 | 0.18 |
| VWA9 | 0.67764 | 3.17E-07 | 0.50233792 | 0.000438 | 0.18 |
| CCDC130 | 0.5913187 | 1.89E-05 | 0.41606513 | 0.004474 | 0.18 |
| CDK1 | 0.5640624 | 5.45E-05 | 0.38935779 | 0.008203 | 0.18 |
| TDP2 | 0.5108411 | 0.000336 | 0.33656863 | 0.023782 | 0.18 |
| PQBP1 | 0.6852928 | 2.07E-07 | 0.51111404 | 0.000333 | 0.18 |
| SPAG5 | 0.5071712 | 0.000377 | 0.33299559 | 0.025407 | 0.18 |
| MED22 | 0.7758452 | 3.85E-10 | 0.60178217 | 1.23E-05 | 0.18 |
| HAUS3 | 0.6934004 | 1.29E-07 | 0.51940449 | 0.000256 | 0.18 |
| YME1L1 | 0.5560574 | 7.31E-05 | 0.38257002 | 0.009497 | 0.18 |
| MLLT10 | 0.587956 | 2.17E-05 | 0.41453284 | 0.004639 | 0.18 |
| PLK1 | 0.5346896 | 0.000155 | 0.36140566 | 0.014718 | 0.18 |
| EIF4G2 | 0.5505895 | 0.000089 | 0.37763095 | 0.010545 | 0.18 |
| KIN | 0.5745202 | 3.67E-05 | 0.40196004 | 0.006199 | 0.18 |
| NDC80 | 0.5950953 | 1.62E-05 | 0.42256413 | 0.003832 | 0.18 |
| NOC3L | 0.5958365 | 1.57E-05 | 0.42367897 | 0.00373 | 0.18 |
| NKTR | 0.7025454 | 7.48E-08 | 0.53073326 | 0.000176 | 0.18 |
| POLR1A | 0.6392699 | 2.28E-06 | 0.46766218 | 0.001199 | 0.18 |
| YY1 | 0.6720973 | 4.29E-07 | 0.50071107 | 0.00046 | 0.18 |
| SF3B1 | 0.8327793 | 1.3E-12 | 0.66143161 | 7.56E-07 | 0.18 |
| RBMX2 | 0.7631481 | 1.1E-09 | 0.59196057 | 1.84E-05 | 0.18 |
| ZC3H8 | 0.6547006 | 1.07E-06 | 0.48358264 | 0.000765 | 0.18 |
| RPL30 | 0.5258381 | 0.000208 | 0.35502414 | 0.016707 | 0.18 |
| MED12 | 0.6982738 | 9.68E-08 | 0.52758544 | 0.000196 | 0.18 |
| TSR1 | 0.563447 | 5.58E-05 | 0.39291864 | 0.007587 | 0.18 |
| BMS1 | 0.6664427 | 5.81E-07 | 0.49602636 | 0.00053 | 0.18 |
| ELAVL1 | 0.715329 | 3.36E-08 | 0.54498946 | 0.000108 | 0.18 |
| SNRNP35 | 0.7933763 | 8.09E-11 | 0.62379082 | 4.7E-06 | 0.17 |
| CXorf56 | 0.6661582 | 5.9E-07 | 0.49675172 | 0.000519 | 0.17 |
| SMC5 | 0.6581775 | 8.94E-07 | 0.48901615 | 0.000653 | 0.17 |
| TUBGCP2 | 0.5069956 | 0.000379 | 0.33830108 | 0.023026 | 0.17 |
| MORC2 | 0.5506381 | 8.88E-05 | 0.38196203 | 0.009621 | 0.17 |
| RAN | 0.6783046 | 3.06E-07 | 0.50965661 | 0.000349 | 0.17 |
| SAP18 | 0.8624159 | 2.69E-14 | 0.69460549 | 1.2E-07 | 0.17 |
| RSRC1 | 0.741528 | 5.65E-09 | 0.57379509 | 3.78E-05 | 0.17 |
| ADNP2 | 0.6197061 | 5.64E-06 | 0.45198827 | 0.001828 | 0.17 |
| C9orf78 | 0.5987409 | 1.39E-05 | 0.43133664 | 0.003094 | 0.17 |
| SART1 | 0.8250524 | 3.17E-12 | 0.65779794 | 9.11E-07 | 0.17 |
| ANG | -0.545794 | 0.000105 | -0.3787165 | 0.010307 | 0.17 |
| TOMM70A | 0.5626089 | 5.76E-05 | 0.3956609 | 0.00714 | 0.17 |
| AHCTF1 | 0.6904367 | 1.54E-07 | 0.52367694 | 0.000223 | 0.17 |
| RPF2 | 0.6163603 | 6.55E-06 | 0.44983586 | 0.001934 | 0.17 |
| UTP20 | 0.6289211 | 3.71E-06 | 0.46278837 | 0.00137 | 0.17 |
| MDN1 | 0.5790536 | 3.08E-05 | 0.41304518 | 0.004803 | 0.17 |
| KDM1A | 0.6267199 | 4.11E-06 | 0.46094943 | 0.001439 | 0.17 |
| HNRNPM | 0.8409705 | 4.83E-13 | 0.6752415 | 3.62E-07 | 0.17 |
| TTK | 0.5795993 | 3.02E-05 | 0.41388598 | 0.00471 | 0.17 |
| RIOK3 | 0.6244075 | 4.57E-06 | 0.45880367 | 0.001525 | 0.17 |
| CCDC101 | 0.6126368 | 7.71E-06 | 0.44737253 | 0.002062 | 0.17 |
| KANSL3 | 0.6080733 | 9.4E-06 | 0.44282609 | 0.002318 | 0.17 |
| SERPINC1 | -0.620538 | 5.44E-06 | -0.4556509 | 0.001659 | 0.17 |
| AKAP12 | -0.617389 | 6.26E-06 | -0.4526489 | 0.001796 | 0.17 |
| MCM3AP | 0.6317195 | 3.26E-06 | 0.467004 | 0.00122 | 0.17 |
| SRRM2 | 0.5718218 | 4.07E-05 | 0.4071253 | 0.00551 | 0.17 |
| TFPT | 0.6070937 | 9.8E-06 | 0.44242306 | 0.002342 | 0.17 |
| CCNA2 | 0.5387971 | 0.000134 | 0.37425561 | 0.011318 | 0.17 |
| TWISTNB | 0.5935302 | 1.73E-05 | 0.42900365 | 0.003277 | 0.17 |
| SLC1A4 | 0.5114527 | 0.00033 | 0.3469914 | 0.01953 | 0.17 |
| SPEN | 0.6754575 | 3.58E-07 | 0.51116742 | 0.000333 | 0.17 |
| UTP11L | 0.6247518 | 4.5E-06 | 0.46068437 | 0.00145 | 0.17 |
| EIF3F | 0.6873886 | 1.83E-07 | 0.52336776 | 0.000225 | 0.17 |
| ARGLU1 | 0.690812 | 1.5E-07 | 0.52681208 | 0.000201 | 0.17 |
| CSTF3 | 0.8229081 | 4.03E-12 | 0.65928048 | 8.45E-07 | 0.17 |
| NCBP2 | 0.7171066 | 3E-08 | 0.55395297 | 7.89E-05 | 0.17 |
| ERCC6L | 0.6069435 | 9.86E-06 | 0.44382356 | 0.002259 | 0.17 |
| NOP16 | 0.6331289 | 3.05E-06 | 0.47009653 | 0.001121 | 0.17 |
| CACNA2D1 | -0.509157 | 0.000354 | -0.3463219 | 0.019782 | 0.17 |
| ZCCHC7 | 0.5836218 | 2.58E-05 | 0.42095826 | 0.003983 | 0.17 |
| USP34 | 0.6110044 | 8.28E-06 | 0.44847387 | 0.002003 | 0.17 |
| PRKDC | 0.7394226 | 6.57E-09 | 0.57692652 | 3.35E-05 | 0.17 |
| A1BG | -0.586988 | 2.25E-05 | -0.4248446 | 0.003627 | 0.17 |
| PPM1F | -0.623851 | 4.68E-06 | -0.4617728 | 0.001408 | 0.17 |
| RBM26 | 0.6090089 | 9.03E-06 | 0.44716327 | 0.002073 | 0.17 |
| RPL13A | 0.5710159 | 0.000042 | 0.40917281 | 0.005256 | 0.17 |
| NOL6 | 0.6252567 | 4.39E-06 | 0.46341831 | 0.001346 | 0.17 |
| SS18 | 0.6002712 | 1.31E-05 | 0.43848824 | 0.002588 | 0.17 |
| MYBBP1A | 0.6047492 | 1.08E-05 | 0.44308742 | 0.002302 | 0.17 |
| IGHV3-9 | -0.531722 | 0.000171 | -0.3703776 | 0.012265 | 0.17 |
| RPS11 | 0.541313 | 0.000123 | 0.38013965 | 0.010001 | 0.17 |
| ETV3 | 0.5583386 | 6.73E-05 | 0.39719745 | 0.0069 | 0.17 |
| FCF1 | 0.5287794 | 0.000188 | 0.36775012 | 0.012944 | 0.17 |
| RPRD1A | 0.6618777 | 7.38E-07 | 0.50092082 | 0.000457 | 0.17 |
| GTF3C4 | 0.6611513 | 7.67E-07 | 0.50046848 | 0.000463 | 0.17 |
| NUP54 | 0.8144454 | 1.01E-11 | 0.65382747 | 1.12E-06 | 0.17 |
| PRCC | 0.6296841 | 3.58E-06 | 0.46908697 | 0.001152 | 0.17 |
| CEP55 | 0.5915711 | 1.87E-05 | 0.43108655 | 0.003113 | 0.17 |
| ZNF24 | 0.5588058 | 6.62E-05 | 0.39860963 | 0.006685 | 0.17 |
| DDX10 | 0.6339591 | 2.94E-06 | 0.47392505 | 0.001007 | 0.17 |
| ORM1 | -0.613438 | 7.45E-06 | -0.453759 | 0.001744 | 0.16 |
| SETD2 | 0.5696046 | 4.43E-05 | 0.40993536 | 0.005164 | 0.16 |
| DAZAP1 | 0.742115 | 5.41E-09 | 0.58261328 | 2.68E-05 | 0.16 |
| NONO | 0.8694669 | 9.34E-15 | 0.70999039 | 4.72E-08 | 0.16 |
| AKAP8L | 0.7274041 | 1.51E-08 | 0.56798502 | 4.71E-05 | 0.16 |
| EBNA1BP2 | 0.6445398 | 1.77E-06 | 0.48588019 | 0.000715 | 0.16 |
| TIAL1 | 0.5920469 | 1.84E-05 | 0.43435724 | 0.00287 | 0.16 |
| RNF10 | 0.6177187 | 6.17E-06 | 0.46006067 | 0.001474 | 0.16 |
| TRIM33 | 0.5368846 | 0.000143 | 0.37956467 | 0.010124 | 0.16 |
| GGNBP2 | 0.5722513 | 4.01E-05 | 0.4149364 | 0.004595 | 0.16 |
| ERICH1 | 0.6231986 | 4.82E-06 | 0.46591824 | 0.001257 | 0.16 |
| CPSF1 | 0.8685484 | 1.08E-14 | 0.7114719 | 4.3E-08 | 0.16 |
| JAM3 | -0.59673 | 1.52E-05 | -0.4397651 | 0.002505 | 0.16 |
| CCDC14 | 0.5825343 | 2.69E-05 | 0.42569908 | 0.003552 | 0.16 |
| RCL1 | 0.6376306 | 2.47E-06 | 0.48092207 | 0.000826 | 0.16 |
| TRMT61A | 0.5371102 | 0.000142 | 0.38061232 | 0.009901 | 0.16 |
| ZMIZ2 | 0.5741087 | 3.73E-05 | 0.41767077 | 0.004307 | 0.16 |
| MGA | 0.7046017 | 6.59E-08 | 0.54847613 | 9.59E-05 | 0.16 |
| NOP2 | 0.6347617 | 2.83E-06 | 0.47870232 | 0.00088 | 0.16 |
| MBD1 | 0.6203498 | 5.48E-06 | 0.46441029 | 0.00131 | 0.16 |
| ZDHHC5 | 0.5646117 | 5.34E-05 | 0.40899215 | 0.005278 | 0.16 |
| WDR12 | 0.5301551 | 0.00018 | 0.37455659 | 0.011247 | 0.16 |
| CENPO | 0.5614735 | 0.00006 | 0.40590624 | 0.005667 | 0.16 |
| ZC3H4 | 0.6286055 | 3.77E-06 | 0.47312156 | 0.00103 | 0.16 |
| ANAPC1 | 0.5462134 | 0.000104 | 0.39078821 | 0.00795 | 0.16 |
| RRP15 | 0.6075281 | 9.62E-06 | 0.45215217 | 0.00182 | 0.16 |
| CNRIP1 | -0.632705 | 3.11E-06 | -0.4774061 | 0.000913 | 0.16 |
| ZFR | 0.6546135 | 1.07E-06 | 0.49980627 | 0.000473 | 0.16 |
| SERBP1 | 0.5494066 | 9.28E-05 | 0.39467717 | 0.007298 | 0.16 |
| ILF3 | 0.7516095 | 2.68E-09 | 0.5972048 | 1.49E-05 | 0.16 |
| VCPIP1 | 0.5743825 | 3.69E-05 | 0.42005113 | 0.00407 | 0.16 |
| AZGP1 | -0.517718 | 0.00027 | -0.3634204 | 0.014134 | 0.16 |
| TNPO3 | 0.6017279 | 1.23E-05 | 0.44768535 | 0.002045 | 0.16 |
| NIPBL | 0.6804172 | 2.72E-07 | 0.52649891 | 0.000203 | 0.16 |
| UPF3B | 0.5646422 | 5.34E-05 | 0.41087765 | 0.005052 | 0.16 |
| PUM2 | 0.7274521 | 1.51E-08 | 0.57376678 | 3.78E-05 | 0.16 |
| WDR3 | 0.6359377 | 2.67E-06 | 0.48226835 | 0.000794 | 0.16 |
| MBD3 | 0.5922362 | 1.82E-05 | 0.43865787 | 0.002577 | 0.16 |
| CWC22 | 0.6827393 | 2.39E-07 | 0.52921884 | 0.000186 | 0.16 |
| CDK7 | 0.7520042 | 2.61E-09 | 0.59848803 | 1.41E-05 | 0.16 |
| THOC2 | 0.7776154 | 3.31E-10 | 0.62438941 | 4.57E-06 | 0.16 |
| RPL22 | 0.560255 | 6.28E-05 | 0.40714063 | 0.005508 | 0.16 |
| CUL1 | 0.616345 | 6.55E-06 | 0.46325844 | 0.001352 | 0.16 |
| ZNF512B | 0.6185683 | 5.94E-06 | 0.46583371 | 0.00126 | 0.16 |
| CHEK1 | 0.5227424 | 0.00023 | 0.37036329 | 0.012269 | 0.16 |
| INCENP | 0.5611801 | 6.07E-05 | 0.40902176 | 0.005274 | 0.16 |
| EIF3H | 0.7485793 | 3.37E-09 | 0.59662424 | 1.52E-05 | 0.16 |
| PRPF3 | 0.7598222 | 1.43E-09 | 0.60803993 | 9.41E-06 | 0.16 |
| SETX | 0.5921175 | 1.83E-05 | 0.4403956 | 0.002466 | 0.16 |
| FBRS | 0.598537 | 1.41E-05 | 0.44689484 | 0.002087 | 0.16 |
| BIRC5 | 0.5850802 | 2.43E-05 | 0.43356191 | 0.002928 | 0.16 |
| NARS | 0.5864334 | 0.000023 | 0.43491625 | 0.002831 | 0.16 |
| KDM5A | 0.576798 | 3.36E-05 | 0.42528596 | 0.003588 | 0.16 |
| PHF6 | 0.6332809 | 3.03E-06 | 0.48231087 | 0.000793 | 0.16 |
| ACIN1 | 0.6771966 | 3.25E-07 | 0.52637107 | 0.000204 | 0.16 |
| SNW1 | 0.7802592 | 2.64E-10 | 0.6295358 | 3.61E-06 | 0.16 |
| KIAA1429 | 0.8076176 | 2.03E-11 | 0.65696645 | 9.51E-07 | 0.16 |
| SMEK1 | 0.5968422 | 1.51E-05 | 0.44626105 | 0.002122 | 0.16 |
| PDCD11 | 0.6543854 | 1.08E-06 | 0.50400426 | 0.000416 | 0.16 |
| RBM33 | 0.6136605 | 7.38E-06 | 0.46357598 | 0.00134 | 0.16 |
| MTA2 | 0.7131768 | 3.86E-08 | 0.56339442 | 5.59E-05 | 0.15 |
| CTNNBL1 | 0.7571122 | 1.76E-09 | 0.60746132 | 9.65E-06 | 0.15 |
| SUPT5H | 0.6763542 | 3.4E-07 | 0.52670632 | 0.000202 | 0.15 |
| SMAD4 | 0.5316185 | 0.000171 | 0.38209368 | 0.009594 | 0.15 |
| CKAP2 | 0.5019869 | 0.000442 | 0.35248618 | 0.017559 | 0.15 |
| PARG | 0.5197519 | 0.000253 | 0.37028145 | 0.012289 | 0.15 |
| CHTOP | 0.6773875 | 3.22E-07 | 0.52841246 | 0.000191 | 0.15 |
| CLPX | 0.5181718 | 0.000266 | 0.3695276 | 0.012481 | 0.15 |
| PPIG | 0.7436489 | 4.84E-09 | 0.5950669 | 1.62E-05 | 0.15 |
| KHSRP | 0.6576862 | 9.17E-07 | 0.50935549 | 0.000352 | 0.15 |
| TTF1 | 0.6602731 | 8.02E-07 | 0.51214528 | 0.000323 | 0.15 |
| SCARF2 | -0.568515 | 4.62E-05 | -0.4204243 | 0.004034 | 0.15 |
| TFAP2A | 0.605022 | 1.07E-05 | 0.45694887 | 0.001603 | 0.15 |
| GEMIN4 | 0.5777074 | 3.25E-05 | 0.42991452 | 0.003204 | 0.15 |
| MPND | 0.5006833 | 0.00046 | 0.35313807 | 0.017337 | 0.15 |
| EIF3E | 0.6606506 | 7.87E-07 | 0.51326891 | 0.000311 | 0.15 |
| CCDC59 | 0.642005 | 0.000002 | 0.49478531 | 0.00055 | 0.15 |
| L3HYPDH | -0.530738 | 0.000176 | -0.38383 | 0.009244 | 0.15 |
| KIAA1033 | 0.5569353 | 7.09E-05 | 0.41083072 | 0.005058 | 0.15 |
| BRD4 | 0.6075931 | 9.59E-06 | 0.4615498 | 0.001416 | 0.15 |
| KDM3A | 0.5200828 | 0.00025 | 0.37415943 | 0.011341 | 0.15 |
| SERPINA7 | -0.60121 | 1.26E-05 | -0.45552 | 0.001665 | 0.15 |
| CDC40 | 0.6593562 | 8.41E-07 | 0.51375612 | 0.000307 | 0.15 |
| PUS1 | 0.6627196 | 7.07E-07 | 0.51737362 | 0.000273 | 0.15 |
| STAG2 | 0.6791578 | 2.92E-07 | 0.53388966 | 0.000159 | 0.15 |
| DIMT1 | 0.5797082 | 0.00003 | 0.43460025 | 0.002853 | 0.15 |
| NFKBIL1 | 0.71223 | 4.09E-08 | 0.56729676 | 4.83E-05 | 0.15 |
| ANKRD28 | 0.5210537 | 0.000243 | 0.37636424 | 0.01083 | 0.15 |
| SETD1B | 0.5273245 | 0.000198 | 0.38264753 | 0.009481 | 0.15 |
| FARSA | 0.5276633 | 0.000195 | 0.38320573 | 0.009368 | 0.15 |
| CWC15 | 0.6347018 | 2.84E-06 | 0.49031257 | 0.000628 | 0.15 |
| TBL3 | 0.6622349 | 7.25E-07 | 0.51801553 | 0.000268 | 0.15 |
| EIF3L | 0.7295275 | 1.31E-08 | 0.58546187 | 2.39E-05 | 0.15 |
| DDX56 | 0.5796679 | 3.01E-05 | 0.43580352 | 0.002768 | 0.15 |
| GNL3 | 0.60376 | 1.13E-05 | 0.46024516 | 0.001467 | 0.15 |
| VPS72 | 0.6331312 | 3.05E-06 | 0.48990383 | 0.000636 | 0.15 |
| ZNF280C | 0.6302029 | 3.5E-06 | 0.48733532 | 0.000686 | 0.15 |
| U2AF2 | 0.7458971 | 4.11E-09 | 0.6032765 | 1.15E-05 | 0.15 |
| NAA30 | 0.5958214 | 1.57E-05 | 0.45325056 | 0.001768 | 0.15 |
| RPL37A | 0.5748354 | 3.63E-05 | 0.43229882 | 0.003021 | 0.15 |
| POLR1B | 0.6758584 | 3.5E-07 | 0.53361784 | 0.00016 | 0.15 |
| NUFIP1 | 0.5194316 | 0.000256 | 0.37731788 | 0.010615 | 0.15 |
| EXOSC5 | 0.5267842 | 0.000201 | 0.38473769 | 0.009066 | 0.15 |
| ZNF146 | 0.5850274 | 2.44E-05 | 0.44302518 | 0.002306 | 0.15 |
| UBE2T | 0.512215 | 0.000322 | 0.37022608 | 0.012303 | 0.15 |
| SRSF7 | 0.8305294 | 1.7E-12 | 0.68897103 | 1.67E-07 | 0.15 |
| TOMM40 | 0.5109069 | 0.000336 | 0.36940426 | 0.012513 | 0.15 |
| FAM76B | 0.6231793 | 4.83E-06 | 0.48183025 | 0.000804 | 0.15 |
| LRIF1 | 0.5446593 | 0.00011 | 0.4033593 | 0.006006 | 0.15 |
| PRKD1 | -0.524545 | 0.000217 | -0.3833126 | 0.009347 | 0.15 |
| DNTTIP2 | 0.6468584 | 1.58E-06 | 0.5056522 | 0.000395 | 0.15 |
| SUGP1 | 0.7064062 | 5.9E-08 | 0.56524077 | 5.22E-05 | 0.15 |
| SECISBP2 | 0.6045804 | 1.09E-05 | 0.46345833 | 0.001345 | 0.15 |
| JAGN1 | 0.512631 | 0.000318 | 0.65371755 | 1.12E-06 | 0.15 |
| WBP11 | 0.5488871 | 9.45E-05 | 0.40799747 | 0.005401 | 0.15 |
| FANCI | 0.5616272 | 5.97E-05 | 0.42105958 | 0.003973 | 0.15 |
| IGHA2 | -0.550985 | 8.78E-05 | -0.4105866 | 0.005087 | 0.15 |
| CDC20 | 0.558133 | 6.78E-05 | 0.41780764 | 0.004293 | 0.15 |
| SNRPG | 0.6088915 | 9.07E-06 | 0.46857647 | 0.001169 | 0.15 |
| TAF6 | 0.6084009 | 9.27E-06 | 0.4682755 | 0.001178 | 0.15 |
| C3orf17 | 0.6221362 | 5.06E-06 | 0.48206495 | 0.000799 | 0.15 |
| SVIP | 0.5422929 | 0.000119 | 0.40225811 | 0.006158 | 0.15 |
| FGF1 | -0.603546 | 1.14E-05 | -0.4635962 | 0.00134 | 0.14 |
| CMSS1 | 0.6148679 | 6.99E-06 | 0.47501884 | 0.000976 | 0.14 |
| MPHOSPH10 | 0.6565014 | 9.74E-07 | 0.51684023 | 0.000278 | 0.14 |
| PLXDC1 | -0.501421 | 0.00045 | -0.362044 | 0.014531 | 0.14 |
| COA1 | 0.5148887 | 0.000296 | 0.37555061 | 0.011016 | 0.14 |
| NOC2L | 0.6670775 | 5.62E-07 | 0.52789807 | 0.000194 | 0.14 |
| DHX33 | 0.6217326 | 5.15E-06 | 0.48277577 | 0.000783 | 0.14 |
| NIFK | 0.6204573 | 5.46E-06 | 0.48152619 | 0.000811 | 0.14 |
| DAXX | 0.663578 | 6.76E-07 | 0.52466313 | 0.000216 | 0.14 |
| HAUS1 | 0.6731167 | 4.06E-07 | 0.53422106 | 0.000157 | 0.14 |
| IGHV3-72 | -0.546153 | 0.000104 | -0.4073067 | 0.005487 | 0.14 |
| CSTF1 | 0.6370127 | 2.54E-06 | 0.49824883 | 0.000496 | 0.14 |
| CEBPZ | 0.6700061 | 4.81E-07 | 0.53170948 | 0.000171 | 0.14 |
| NAA50 | 0.5549538 | 7.61E-05 | 0.41672143 | 0.004405 | 0.14 |
| MNAT1 | 0.6524115 | 1.2E-06 | 0.5142226 | 0.000302 | 0.14 |
| NAA15 | 0.522281 | 0.000233 | 0.38416309 | 0.009178 | 0.14 |
| 5-Mar | 0.5468222 | 0.000102 | 0.40879466 | 0.005302 | 0.14 |
| MSH6 | 0.6262176 | 4.2E-06 | 0.48874814 | 0.000658 | 0.14 |
| KPNA4 | 0.5757219 | 3.51E-05 | 0.43844762 | 0.00259 | 0.14 |
| PAK1IP1 | 0.602766 | 1.18E-05 | 0.46570572 | 0.001265 | 0.14 |
| GTF2H5 | 0.6052173 | 1.06E-05 | 0.46842046 | 0.001174 | 0.14 |
| ZNF407 | 0.6607558 | 7.83E-07 | 0.52398918 | 0.000221 | 0.14 |
| HNRNPK | 0.7249606 | 1.79E-08 | 0.58834929 | 2.13E-05 | 0.14 |
| ABCB10 | 0.5243946 | 0.000218 | 0.38784555 | 0.008477 | 0.14 |
| ZKSCAN8 | 0.5944642 | 1.66E-05 | 0.45810547 | 0.001554 | 0.14 |
| ACTL6A | 0.7219812 | 2.18E-08 | 0.58563228 | 2.38E-05 | 0.14 |
| GPKOW | 0.7185422 | 2.73E-08 | 0.58234593 | 2.71E-05 | 0.14 |
| SLC7A1 | 0.6178715 | 6.13E-06 | 0.48168036 | 0.000808 | 0.14 |
| RPS7 | 0.560128 | 6.31E-05 | 0.42442984 | 0.003663 | 0.14 |
| PTGDS | -0.582205 | 2.72E-05 | -0.4465972 | 0.002103 | 0.14 |
| HEATR1 | 0.6050291 | 1.07E-05 | 0.46947717 | 0.00114 | 0.14 |
| ABCF2 | 0.5545982 | 7.71E-05 | 0.41927512 | 0.004146 | 0.14 |
| ZNF771 | 0.5643748 | 5.39E-05 | 0.42921762 | 0.00326 | 0.14 |
| ZCCHC17 | 0.5072177 | 0.000377 | 0.37210037 | 0.011836 | 0.14 |
| INTS1 | 0.5115609 | 0.000329 | 0.37645553 | 0.010809 | 0.14 |
| ZBED1 | 0.5216012 | 0.000238 | 0.38657671 | 0.008713 | 0.14 |
| IMP3 | 0.5139886 | 0.000304 | 0.37914086 | 0.010215 | 0.14 |
| SF3A1 | 0.8262742 | 2.77E-12 | 0.69159356 | 1.44E-07 | 0.14 |
| CDCA2 | 0.6172116 | 6.31E-06 | 0.48286605 | 0.000781 | 0.14 |
| POLR2C | 0.7585572 | 1.57E-09 | 0.62433143 | 4.58E-06 | 0.14 |
| ZNF326 | 0.6650393 | 6.26E-07 | 0.53116149 | 0.000174 | 0.14 |
| ZC3HC1 | 0.6676176 | 5.46E-07 | 0.53381008 | 0.000159 | 0.14 |
| SENP1 | 0.7000604 | 8.69E-08 | 0.56635649 | 5.01E-05 | 0.14 |
| RPL13 | 0.5325279 | 0.000166 | 0.39900863 | 0.006626 | 0.14 |
| SERPINA1 | -0.690328 | 1.55E-07 | -0.5569036 | 7.09E-05 | 0.14 |
| PDCD7 | 0.7192909 | 2.6E-08 | 0.58593997 | 2.35E-05 | 0.14 |
| DDX18 | 0.6098876 | 8.69E-06 | 0.47654747 | 0.000935 | 0.14 |
| MRVI1 | -0.542295 | 0.000119 | -0.4089693 | 0.005281 | 0.14 |
| NNT | 0.5187819 | 0.000261 | 0.38556986 | 0.008905 | 0.14 |
| PHF14 | 0.5676641 | 4.77E-05 | 0.4345098 | 0.002859 | 0.14 |
| ATF1 | 0.5623863 | 0.000058 | 0.42923701 | 0.003258 | 0.14 |
| NCL | 0.6793328 | 2.89E-07 | 0.54622527 | 0.000104 | 0.14 |
| APEX2 | 0.5691852 | 0.000045 | 0.43618873 | 0.002742 | 0.14 |
| C11orf57 | 0.6353414 | 2.75E-06 | 0.50264423 | 0.000434 | 0.14 |
| SENP3 | 0.6076576 | 9.57E-06 | 0.4749941 | 0.000977 | 0.14 |
| KIFC1 | 0.6066138 | 0.00001 | 0.47414433 | 0.001001 | 0.14 |
| ABCF1 | 0.5744786 | 3.68E-05 | 0.44234697 | 0.002346 | 0.14 |
| AMBP | -0.513874 | 0.000306 | -0.3818115 | 0.009652 | 0.14 |
| DFNA5 | -0.50508 | 0.000402 | -0.3732383 | 0.01156 | 0.14 |
| RBM28 | 0.6201933 | 5.52E-06 | 0.48840415 | 0.000665 | 0.14 |
| HNRNPH1 | 0.6432274 | 1.89E-06 | 0.51161627 | 0.000328 | 0.14 |
| CDCA8 | 0.5749729 | 3.61E-05 | 0.44347713 | 0.002279 | 0.14 |
| SART3 | 0.6373484 | 2.5E-06 | 0.50612999 | 0.000389 | 0.14 |
| CDH11 | -0.510563 | 0.000339 | -0.3797472 | 0.010085 | 0.14 |
| MED11 | 0.7016164 | 7.91E-08 | 0.57111817 | 4.18E-05 | 0.14 |
| FARSB | 0.5299885 | 0.000181 | 0.39970929 | 0.006522 | 0.14 |
| FAM210A | 0.5532809 | 8.08E-05 | 0.42301333 | 0.003791 | 0.14 |
| REXO1 | 0.6972256 | 1.03E-07 | 0.5673462 | 4.82E-05 | 0.13 |
| ESCO1 | 0.5985357 | 1.41E-05 | 0.46910238 | 0.001152 | 0.13 |
| ALDH1A1 | -0.569274 | 4.49E-05 | -0.4399515 | 0.002494 | 0.13 |
| NOLC1 | 0.5869165 | 2.26E-05 | 0.4576679 | 0.001572 | 0.13 |
| CENPQ | 0.5527168 | 8.25E-05 | 0.42357031 | 0.00374 | 0.13 |
| RPP30 | 0.6080764 | 9.4E-06 | 0.47923072 | 0.000867 | 0.13 |
| WDR5 | 0.6801479 | 2.76E-07 | 0.55135349 | 8.66E-05 | 0.13 |
| PIK3C3 | 0.5647346 | 5.32E-05 | 0.43613403 | 0.002746 | 0.13 |
| TRNAU1AP | 0.5635548 | 5.56E-05 | 0.69210999 | 1.39E-07 | 0.13 |
| PLIN3 | -0.511209 | 0.000332 | -0.3827713 | 0.009456 | 0.13 |
| 7-Mar | 0.6426611 | 1.94E-06 | 0.51428488 | 0.000302 | 0.13 |
| TADA3 | 0.6679935 | 5.35E-07 | 0.53982809 | 0.00013 | 0.13 |
| DEK | 0.5349751 | 0.000153 | 0.40682037 | 0.005549 | 0.13 |
| HNRNPU | 0.7945504 | 7.25E-11 | 0.666449 | 5.81E-07 | 0.13 |
| MSH2 | 0.6448928 | 1.74E-06 | 0.51692018 | 0.000277 | 0.13 |
| CDC5L | 0.6958993 | 1.12E-07 | 0.56798854 | 4.71E-05 | 0.13 |
| ZRANB2 | 0.6242596 | 4.6E-06 | 0.49639037 | 0.000524 | 0.13 |
| TTR | -0.615858 | 6.7E-06 | -0.4884419 | 0.000664 | 0.13 |
| DDX59 | 0.625129 | 4.42E-06 | 0.49779711 | 0.000503 | 0.13 |
| ATN1 | 0.607422 | 9.66E-06 | 0.48014438 | 0.000844 | 0.13 |
| MEN1 | 0.5953765 | 0.000016 | 0.46810921 | 0.001184 | 0.13 |
| RPE | -0.510561 | 0.000339 | -0.3833094 | 0.009348 | 0.13 |
| RBM19 | 0.659408 | 8.39E-07 | 0.53236374 | 0.000167 | 0.13 |
| CACTIN | 0.6114158 | 8.13E-06 | 0.48486168 | 0.000737 | 0.13 |
| CHAMP1 | 0.5937882 | 1.71E-05 | 0.46746862 | 0.001205 | 0.13 |
| BRIX1 | 0.6455699 | 1.68E-06 | 0.5192584 | 0.000257 | 0.13 |
| ZNF384 | 0.5354469 | 0.000151 | 0.40928745 | 0.005242 | 0.13 |
| SS18L2 | 0.5894319 | 2.04E-05 | 0.46338883 | 0.001347 | 0.13 |
| SETD5 | 0.5010905 | 0.000455 | 0.626972 | 4.06E-06 | 0.13 |
| MRTO4 | 0.6430362 | 1.9E-06 | 0.51716972 | 0.000275 | 0.13 |
| WDR89 | 0.5378228 | 0.000139 | 0.41209052 | 0.004912 | 0.13 |
| SURF6 | 0.6107275 | 8.38E-06 | 0.48509513 | 0.000732 | 0.13 |
| RPS18 | 0.5051 | 0.000402 | 0.3795309 | 0.010131 | 0.13 |
| TAF3 | 0.5148775 | 0.000296 | 0.38943751 | 0.008188 | 0.13 |
| SAFB2 | 0.634499 | 2.86E-06 | 0.50912637 | 0.000355 | 0.13 |
| SLU7 | 0.6964201 | 1.08E-07 | 0.5711016 | 4.19E-05 | 0.13 |
| BRPF1 | 0.7123264 | 4.07E-08 | 0.58716627 | 2.24E-05 | 0.13 |
| SFRP4 | -0.594959 | 1.63E-05 | -0.469974 | 0.001124 | 0.13 |
| NOL11 | 0.6177537 | 6.16E-06 | 0.49300258 | 0.00058 | 0.13 |
| POLR2B | 0.8028763 | 3.26E-11 | 0.67813847 | 3.09E-07 | 0.13 |
| ESF1 | 0.5543016 | 7.79E-05 | 0.42958561 | 0.00323 | 0.13 |
| WRNIP1 | 0.6823048 | 2.45E-07 | 0.5576465 | 0.000069 | 0.13 |
| DDX47 | 0.5061189 | 0.00039 | 0.38171122 | 0.009672 | 0.13 |
| PAPD4 | 0.5292295 | 0.000186 | 0.40487795 | 0.005801 | 0.13 |
| YTHDF2 | 0.6974054 | 1.02E-07 | 0.57313197 | 3.87E-05 | 0.13 |
| SF3B4 | 0.6888065 | 1.69E-07 | 0.56461281 | 5.34E-05 | 0.13 |
| SLC25A24 | 0.5502016 | 9.02E-05 | 0.42615082 | 0.003513 | 0.13 |
| TFIP11 | 0.7595336 | 1.46E-09 | 0.63572792 | 2.7E-06 | 0.13 |
| WDR46 | 0.6345923 | 2.85E-06 | 0.51104342 | 0.000334 | 0.13 |
| DNAJC9 | 0.521407 | 0.00024 | 0.39813788 | 0.006756 | 0.13 |
| ORM2 | -0.609151 | 8.97E-06 | -0.4859638 | 0.000714 | 0.13 |
| MBD2 | 0.6506154 | 1.31E-06 | 0.5276624 | 0.000195 | 0.13 |
| NGDN | 0.6622328 | 7.25E-07 | 0.53945239 | 0.000131 | 0.13 |
| RNF138 | 0.5556992 | 7.41E-05 | 0.43347184 | 0.002934 | 0.13 |
| NUP188 | 0.7295966 | 1.3E-08 | 0.60746904 | 9.64E-06 | 0.13 |
| NOL10 | 0.6089341 | 9.06E-06 | 0.48700696 | 0.000692 | 0.13 |
| UTP6 | 0.605419 | 1.05E-05 | 0.48374963 | 0.000761 | 0.13 |
| NUP88 | 0.7090665 | 5E-08 | 0.58745018 | 2.21E-05 | 0.13 |
| CEP78 | 0.6146371 | 7.07E-06 | 0.4930438 | 0.000579 | 0.13 |
| USP36 | 0.654058 | 1.1E-06 | 0.53254143 | 0.000166 | 0.13 |
| PAPOLG | 0.6002071 | 1.31E-05 | 0.47880824 | 0.000877 | 0.13 |
| EXOSC2 | 0.6426304 | 1.94E-06 | 0.5213719 | 0.00024 | 0.13 |
| ARID4B | 0.7200574 | 2.47E-08 | 0.59882499 | 1.39E-05 | 0.13 |
| C1orf35 | 0.5348481 | 0.000154 | 0.41394258 | 0.004703 | 0.13 |
| RAB35 | 0.5971726 | 1.49E-05 | 0.47629605 | 0.000942 | 0.13 |
| CABIN1 | 0.671245 | 4.5E-07 | 0.55049406 | 8.93E-05 | 0.13 |
| UTP3 | 0.6948967 | 1.18E-07 | 0.57445425 | 3.68E-05 | 0.13 |
| SNRPB2 | 0.8344951 | 1.06E-12 | 0.71428525 | 3.59E-08 | 0.13 |
| IGHV3OR16-12 | -0.522907 | 0.000229 | -0.4027314 | 0.006092 | 0.13 |
| IGHV5-51 | -0.545408 | 0.000107 | -0.425276 | 0.003589 | 0.13 |
| NCBP1 | 0.6386648 | 2.35E-06 | 0.51878297 | 0.000261 | 0.12 |
| RPS8 | 0.5550453 | 7.59E-05 | 0.43539662 | 0.002797 | 0.12 |
| IGHA1 | -0.536916 | 0.000143 | -0.4173312 | 0.004342 | 0.12 |
| PHF3 | 0.6513401 | 1.26E-06 | 0.53198692 | 0.000169 | 0.12 |
| RBP4 | -0.554396 | 7.77E-05 | -0.4352442 | 0.002807 | 0.12 |
| ACTR6 | 0.6882293 | 1.75E-07 | 0.56933577 | 4.48E-05 | 0.12 |
| HIRA | 0.6852249 | 2.07E-07 | 0.56666304 | 4.95E-05 | 0.12 |
| SENP6 | 0.6402797 | 2.17E-06 | 0.521756 | 0.000237 | 0.12 |
| POP5 | 0.6172697 | 6.29E-06 | 0.49879418 | 0.000488 | 0.12 |
| SYF2 | 0.7025499 | 7.48E-08 | 0.58415349 | 2.52E-05 | 0.12 |
| SMIM12 | 0.5572209 | 7.01E-05 | 0.43900413 | 0.002554 | 0.12 |
| PATL1 | 0.6059743 | 1.03E-05 | 0.48777356 | 0.000677 | 0.12 |
| NUP85 | 0.7089798 | 5.03E-08 | 0.59080606 | 1.93E-05 | 0.12 |
| EHMT2 | 0.5717011 | 4.09E-05 | 0.4536433 | 0.00175 | 0.12 |
| BUD13 | 0.6428501 | 1.92E-06 | 0.52522175 | 0.000212 | 0.12 |
| LRG1 | -0.586687 | 2.28E-05 | -0.4691153 | 0.001151 | 0.12 |
| WRN | 0.5994238 | 1.36E-05 | 0.4826159 | 0.000786 | 0.12 |
| INPP1 | -0.513723 | 0.000307 | -0.3971046 | 0.006914 | 0.12 |
| SMC1A | 0.7732954 | 4.78E-10 | 0.65669274 | 9.64E-07 | 0.12 |
| THOC1 | 0.734901 | 9.04E-09 | 0.61838246 | 5.99E-06 | 0.12 |
| USP3 | 0.5543175 | 7.79E-05 | 0.43789334 | 0.002627 | 0.12 |
| SAFB | 0.7521343 | 2.58E-09 | 0.63580721 | 2.69E-06 | 0.12 |
| RRP1B | 0.6058977 | 1.03E-05 | 0.48968507 | 0.00064 | 0.12 |
| SRRT | 0.7603303 | 1.37E-09 | 0.6442123 | 1.8E-06 | 0.12 |
| TTC14 | 0.5196101 | 0.000254 | 0.4038764 | 0.005935 | 0.12 |
| SNIP1 | 0.5761725 | 3.45E-05 | 0.4605094 | 0.001457 | 0.12 |
| RBBP6 | 0.6623983 | 7.19E-07 | 0.54716362 | 0.0001 | 0.12 |
| ZMAT5 | 0.6737732 | 3.92E-07 | 0.5586423 | 6.66E-05 | 0.12 |
| XPO1 | 0.5847124 | 2.47E-05 | 0.4696021 | 0.001136 | 0.12 |
| BBX | 0.5566635 | 7.16E-05 | 0.44180805 | 0.002379 | 0.12 |
| HNRNPC | 0.8528474 | 1.03E-13 | 0.73821719 | 7.16E-09 | 0.12 |
| RBM15 | 0.7629104 | 1.12E-09 | 0.6482917 | 1.47E-06 | 0.12 |
| ATRN | -0.500858 | 0.000458 | -0.3867989 | 0.008671 | 0.12 |
| SNRPA | 0.7974794 | 5.49E-11 | 0.68343324 | 2.3E-07 | 0.12 |
| GATAD2A | 0.7292145 | 1.34E-08 | 0.61519616 | 6.89E-06 | 0.12 |
| USP9X | 0.5013586 | 0.000451 | 0.38741171 | 0.008557 | 0.12 |
| HDAC3 | 0.5300678 | 0.00018 | 0.41651745 | 0.004427 | 0.12 |
| UTP18 | 0.6049947 | 1.07E-05 | 0.49162867 | 0.000604 | 0.12 |
| ILF2 | 0.7107373 | 4.5E-08 | 0.59764735 | 1.46E-05 | 0.12 |
| GATAD2B | 0.6168963 | 6.4E-06 | 0.50439414 | 0.000411 | 0.12 |
| SMARCA4 | 0.6320538 | 3.21E-06 | 0.51962972 | 0.000254 | 0.12 |
| C1RL | -0.548419 | 9.61E-05 | -0.4361704 | 0.002743 | 0.12 |
| DPH2 | 0.5377312 | 0.000139 | 0.42549169 | 0.00357 | 0.12 |
| PRPF4 | 0.7816817 | 2.33E-10 | 0.66944302 | 4.95E-07 | 0.12 |
| FXR1 | 0.6392769 | 2.28E-06 | 0.52712377 | 0.000199 | 0.12 |
| SFPQ | 0.8278108 | 2.32E-12 | 0.71572292 | 3.28E-08 | 0.12 |
| BCLAF1 | 0.6809633 | 2.64E-07 | 0.56918286 | 0.000045 | 0.12 |
| SETD1A | 0.5342466 | 0.000157 | 0.42293347 | 0.003798 | 0.12 |
| ASPM | 0.5889698 | 2.08E-05 | 0.47765778 | 0.000906 | 0.12 |
| ISG20L2 | 0.6237565 | 4.7E-06 | 0.51249079 | 0.000319 | 0.12 |
| CORO2B | -0.502496 | 0.000436 | -0.3916114 | 0.007808 | 0.12 |
| CEP85 | 0.6938297 | 1.26E-07 | 0.58312514 | 2.63E-05 | 0.12 |
| WDR74 | 0.6220946 | 5.07E-06 | 0.51171969 | 0.000327 | 0.12 |
| NDC1 | 0.6806503 | 2.68E-07 | 0.57053689 | 4.28E-05 | 0.12 |
| PLTP | -0.575302 | 3.57E-05 | -0.4653623 | 0.001277 | 0.11 |
| MCM10 | 0.5714556 | 4.13E-05 | 0.46197557 | 0.0014 | 0.11 |
| DIEXF | 0.5171445 | 0.000275 | 0.40803443 | 0.005396 | 0.11 |
| RPL5 | 0.5948702 | 1.64E-05 | 0.48578502 | 0.000717 | 0.11 |
| RPL21 | 0.5230537 | 0.000227 | 0.41411201 | 0.004685 | 0.11 |
| PPIL4 | 0.5919831 | 1.84E-05 | 0.48313633 | 0.000775 | 0.11 |
| RSBN1 | 0.6018836 | 1.22E-05 | 0.49327503 | 0.000575 | 0.11 |
| NFYA | 0.5677186 | 4.76E-05 | 0.45937941 | 0.001502 | 0.11 |
| KIAA0947 | 0.5468342 | 0.000102 | 0.4386758 | 0.002575 | 0.11 |
| HPX | -0.585306 | 2.41E-05 | -0.4772755 | 0.000916 | 0.11 |
| ZMYM1 | 0.5928281 | 1.78E-05 | 0.48509178 | 0.000732 | 0.11 |
| TGFBR2 | -0.521868 | 0.000236 | -0.4144457 | 0.004648 | 0.11 |
| FAM120C | 0.5426999 | 0.000117 | 0.43543489 | 0.002794 | 0.11 |
| CWF19L2 | 0.603427 | 1.15E-05 | 0.49666074 | 0.00052 | 0.11 |
| DHX57 | 0.5362166 | 0.000147 | 0.42949161 | 0.003238 | 0.11 |
| WDR36 | 0.6115989 | 8.07E-06 | 0.50509724 | 0.000402 | 0.11 |
| RPRD2 | 0.6633458 | 6.84E-07 | 0.55685501 | 7.11E-05 | 0.11 |
| RPS24 | 0.5436317 | 0.000114 | 0.4372605 | 0.002669 | 0.11 |
| RARS2 | 0.5110803 | 0.000334 | 0.40480992 | 0.00581 | 0.11 |
| FRA10AC1 | 0.6298529 | 3.56E-06 | 0.52363913 | 0.000223 | 0.11 |
| TPR | 0.7658966 | 8.79E-10 | 0.6599016 | 8.18E-07 | 0.11 |
| UPF1 | 0.5334435 | 0.000161 | 0.42745051 | 0.003404 | 0.11 |
| PARP2 | 0.6079742 | 9.44E-06 | 0.50230928 | 0.000438 | 0.11 |
| UTP23 | 0.5935053 | 1.73E-05 | 0.48831348 | 0.000666 | 0.11 |
| AGT | -0.59859 | 0.000014 | -0.4934374 | 0.000573 | 0.11 |
| EXOSC4 | 0.6478906 | 1.5E-06 | 0.54311298 | 0.000116 | 0.11 |
| RAVER1 | 0.5243468 | 0.000218 | 0.41963299 | 0.004111 | 0.11 |
| EXD2 | 0.5579385 | 6.83E-05 | 0.45327131 | 0.001767 | 0.11 |
| NUP153 | 0.5874299 | 2.21E-05 | 0.48315614 | 0.000774 | 0.11 |
| ZBTB43 | 0.5955446 | 1.59E-05 | 0.49135062 | 0.000609 | 0.11 |
| FBXO11 | 0.6185805 | 5.93E-06 | 0.51439167 | 0.000301 | 0.11 |
| ANKRD26 | 0.536797 | 0.000144 | 0.43266663 | 0.002994 | 0.11 |
| ING4 | 0.5980791 | 1.43E-05 | 0.49396377 | 0.000564 | 0.11 |
| SRSF5 | 0.6341153 | 2.91E-06 | 0.53003778 | 0.000181 | 0.11 |
| SRSF4 | 0.6553699 | 1.03E-06 | 0.55139242 | 8.65E-05 | 0.11 |
| MEPCE | 0.5752814 | 3.57E-05 | 0.47143727 | 0.00108 | 0.11 |
| NUP214 | 0.7744186 | 4.35E-10 | 0.67065429 | 4.64E-07 | 0.11 |
| AKAP17A | 0.6624042 | 7.18E-07 | 0.55865346 | 6.66E-05 | 0.11 |
| DDX49 | 0.5450592 | 0.000108 | 0.44141739 | 0.002402 | 0.11 |
| KCTD12 | -0.667132 | 5.6E-07 | -0.5634949 | 5.57E-05 | 0.11 |
| INTS10 | 0.5685307 | 4.61E-05 | 0.46565794 | 0.001266 | 0.11 |
| TRMT1 | 0.521162 | 0.000242 | 0.41905011 | 0.004168 | 0.11 |
| ZBTB1 | 0.6075606 | 9.61E-06 | 0.50586655 | 0.000393 | 0.11 |
| REXO4 | 0.5754368 | 3.55E-05 | 0.47384066 | 0.001009 | 0.11 |
| RPL11 | 0.5460178 | 0.000105 | 0.44450339 | 0.00222 | 0.11 |
| HECA | 0.5110369 | 0.000334 | 0.40982935 | 0.005177 | 0.11 |
| SLIT3 | -0.533907 | 0.000159 | -0.4331356 | 0.002959 | 0.11 |
| PRPF40A | 0.7580809 | 1.63E-09 | 0.65740554 | 9.3E-07 | 0.11 |
| SFSWAP | 0.6519842 | 1.22E-06 | 0.55148857 | 8.62E-05 | 0.11 |
| C2orf44 | 0.5165505 | 0.000281 | 0.41665324 | 0.004412 | 0.1 |
| SUV39H1 | 0.6142819 | 7.18E-06 | 0.51471066 | 0.000298 | 0.1 |
| KIAA1468 | 0.5412794 | 0.000123 | 0.44184464 | 0.002376 | 0.1 |
| TRRAP | 0.7364076 | 8.13E-09 | 0.63719045 | 2.52E-06 | 0.1 |
| PPP1R35 | 0.6415796 | 2.04E-06 | 0.54241352 | 0.000119 | 0.1 |
| AHSG | -0.599064 | 1.38E-05 | -0.5002216 | 0.000467 | 0.1 |
| BCAS2 | 0.629065 | 3.69E-06 | 0.53037084 | 0.000179 | 0.1 |
| THOC5 | 0.7654397 | 9.12E-10 | 0.66676968 | 5.71E-07 | 0.1 |
| POLR3K | 0.5330515 | 0.000163 | 0.43456308 | 0.002856 | 0.1 |
| RPS27A | 0.5205315 | 0.000247 | 0.42218294 | 0.003867 | 0.1 |
| TPK1 | -0.529705 | 0.000183 | -0.4315091 | 0.003081 | 0.1 |
| ZNF691 | 0.5028272 | 0.000431 | 0.40477985 | 0.005814 | 0.1 |
| RRP36 | 0.5445978 | 0.00011 | 0.44685948 | 0.002089 | 0.1 |
| FKTN | 0.5103615 | 0.000341 | 0.41279655 | 0.004831 | 0.1 |
| RBM22 | 0.8025885 | 3.35E-11 | 0.70504257 | 6.42E-08 | 0.1 |
| NAA35 | 0.5215761 | 0.000239 | 0.42428867 | 0.003676 | 0.1 |
| WDR70 | 0.531984 | 0.000169 | 0.43488221 | 0.002833 | 0.1 |
| NDUFS8 | 0.505014 | 0.000403 | 0.40795331 | 0.005406 | 0.1 |
| KAT2A | 0.533423 | 0.000161 | 0.63044736 | 3.46E-06 | 0.1 |
| GTF3C3 | 0.6180668 | 6.07E-06 | 0.5212061 | 0.000242 | 0.1 |
| SRRM1 | 0.7404196 | 6.12E-09 | 0.64384546 | 1.83E-06 | 0.1 |
| ZMYND8 | 0.6345502 | 2.86E-06 | 0.53857623 | 0.000135 | 0.1 |
| RPL3 | 0.5668388 | 4.92E-05 | 0.47098793 | 0.001093 | 0.1 |
| SERPIND1 | -0.505031 | 0.000403 | -0.4092764 | 0.005243 | 0.1 |
| POGZ | 0.6223826 | 0.000005 | 0.5266792 | 0.000202 | 0.1 |
| ZFYVE21 | -0.514044 | 0.000304 | -0.4186135 | 0.004212 | 0.1 |
| PUF60 | 0.6463725 | 1.62E-06 | 0.55115753 | 8.72E-05 | 0.1 |
| RBBP4 | 0.5512534 | 8.69E-05 | 0.45655308 | 0.00162 | 0.1 |
| RAD21 | 0.7595051 | 1.46E-09 | 0.66486548 | 6.31E-07 | 0.1 |
| ATRX | 0.5260333 | 0.000206 | 0.43166559 | 0.003069 | 0.1 |
| NOM1 | 0.5622468 | 5.83E-05 | 0.46817636 | 0.001182 | 0.1 |
| DPY30 | 0.5833084 | 2.61E-05 | 0.48940974 | 0.000645 | 0.1 |
| BRMS1L | 0.5292304 | 0.000186 | 0.43550528 | 0.002789 | 0.1 |
| TMEM209 | 0.6911658 | 1.47E-07 | 0.59748748 | 1.47E-05 | 0.1 |
| XRCC6 | 0.6262572 | 4.2E-06 | 0.53274505 | 0.000165 | 0.1 |
| NELFA | 0.5613759 | 6.02E-05 | 0.46808774 | 0.001185 | 0.1 |
| NUP50 | 0.5750226 | 0.000036 | 0.4817653 | 0.000806 | 0.1 |
| TOMM5 | 0.5749813 | 3.61E-05 | 0.48173592 | 0.000807 | 0.1 |
| SVEP1 | -0.62192 | 5.11E-06 | -0.5287071 | 0.000189 | 0.1 |
| KDM4A | 0.6214562 | 5.22E-06 | 0.52826487 | 0.000192 | 0.1 |
| ZNF638 | 0.7155627 | 3.31E-08 | 0.62250632 | 4.98E-06 | 0.1 |
| POLR3A | 0.6369735 | 2.55E-06 | 0.54392645 | 0.000113 | 0.1 |
| LAS1L | 0.563136 | 5.64E-05 | 0.47013675 | 0.001119 | 0.1 |
| PLRG1 | 0.6194048 | 5.72E-06 | 0.52657646 | 0.000203 | 0.1 |
| TRMT1L | 0.5535763 | 0.00008 | 0.46084793 | 0.001443 | 0.1 |
| HNMT | -0.524825 | 0.000215 | -0.4321708 | 0.003031 | 0.1 |
| SUDS3 | 0.7144553 | 3.55E-08 | 0.62213403 | 5.06E-06 | 0.1 |
| FARS2 | 0.5505183 | 8.92E-05 | 0.4582393 | 0.001548 | 0.1 |
| CDK11A | 0.6197886 | 5.62E-06 | 0.52765385 | 0.000196 | 0.1 |
| SMC3 | 0.6994922 | 9E-08 | 0.60769808 | 9.55E-06 | 0.1 |
| NOP10 | 0.5216682 | 0.000238 | 0.42990809 | 0.003205 | 0.1 |
| UTP15 | 0.6282565 | 3.83E-06 | 0.53680214 | 0.000144 | 0.1 |
| PSEN1 | 0.574983 | 3.61E-05 | 0.48373264 | 0.000761 | 0.1 |
| THOC6 | 0.7181987 | 2.79E-08 | 0.62726772 | 4.01E-06 | 0.1 |
| RIOK1 | 0.5974763 | 1.47E-05 | 0.50657316 | 0.000384 | 0.1 |
| PHC2 | 0.5098966 | 0.000346 | 0.41910958 | 0.004162 | 0.1 |
| ANKRD54 | 0.6607483 | 7.83E-07 | 0.57019319 | 4.33E-05 | 0.1 |
| GTPBP1 | 0.5921456 | 1.83E-05 | 0.50174966 | 0.000446 | 0.1 |
| POT1 | 0.5567228 | 7.14E-05 | 0.46645383 | 0.001239 | 0.1 |
| API5 | 0.7183431 | 2.76E-08 | 0.62807495 | 3.86E-06 | 0.1 |
| HP | -0.528433 | 0.000191 | -0.4384677 | 0.002589 | 0.09 |
| ROR1 | -0.579165 | 3.07E-05 | -0.4896652 | 0.00064 | 0.09 |
| WDR75 | 0.6395614 | 2.25E-06 | 0.55009722 | 9.06E-05 | 0.09 |
| MTA3 | 0.5367505 | 0.000144 | 0.44784601 | 0.002036 | 0.09 |
| MNT | 0.5471558 | 0.0001 | 0.45878286 | 0.001526 | 0.09 |
| DIS3L | 0.5842404 | 2.51E-05 | 0.49642756 | 0.000524 | 0.09 |
| ZFX | 0.5113773 | 0.000331 | 0.4237493 | 0.003724 | 0.09 |
| NUCKS1 | 0.5269882 | 0.0002 | 0.4394213 | 0.002527 | 0.09 |
| DDX21 | 0.604986 | 1.07E-05 | 0.51783881 | 0.000269 | 0.09 |
| OXNAD1 | 0.5029356 | 0.00043 | 0.41589442 | 0.004492 | 0.09 |
| TAF5L | 0.63858 | 2.36E-06 | 0.55181708 | 8.52E-05 | 0.09 |
| DDX31 | 0.5892927 | 2.05E-05 | 0.50253228 | 0.000435 | 0.09 |
| DKC1 | 0.6635605 | 6.76E-07 | 0.57696419 | 3.34E-05 | 0.09 |
| TAF12 | 0.5106394 | 0.000338 | 0.42423573 | 0.00368 | 0.09 |
| HMGB3 | 0.5903938 | 1.96E-05 | 0.5041518 | 0.000414 | 0.09 |
| NHP2 | 0.5105334 | 0.00034 | 0.42449226 | 0.003658 | 0.09 |
| ZW10 | 0.509389 | 0.000352 | 0.42343425 | 0.003752 | 0.09 |
| CHD2 | 0.639238 | 2.29E-06 | 0.55356369 | 0.00008 | 0.09 |
| DCUN1D5 | 0.6047668 | 1.08E-05 | 0.51953731 | 0.000255 | 0.09 |
| EDRF1 | 0.6276449 | 3.94E-06 | 0.54244545 | 0.000118 | 0.09 |
| TRIM24 | 0.5759809 | 3.47E-05 | 0.49080856 | 0.000619 | 0.09 |
| WIZ | 0.6380232 | 2.42E-06 | 0.5541463 | 7.84E-05 | 0.09 |
| POGK | 0.5821756 | 2.73E-05 | 0.49865555 | 0.00049 | 0.09 |
| RNF20 | 0.6642473 | 6.52E-07 | 0.58079459 | 2.88E-05 | 0.09 |
| CIR1 | 0.5927107 | 1.79E-05 | 0.5094077 | 0.000352 | 0.09 |
| MAD2L1BP | 0.514771 | 0.000297 | 0.43186718 | 0.003053 | 0.09 |
| NELFB | 0.5428067 | 0.000117 | 0.46080074 | 0.001445 | 0.09 |
| FOXJ3 | 0.5974092 | 1.47E-05 | 0.51568884 | 0.000288 | 0.09 |
| EIF3C | 0.5742179 | 3.72E-05 | 0.49257234 | 0.000588 | 0.09 |
| CCDC12 | 0.5374753 | 0.000141 | 0.45604174 | 0.001642 | 0.09 |
| XAB2 | 0.7382688 | 7.13E-09 | 0.65707761 | 9.46E-07 | 0.09 |
| NUP35 | 0.6058947 | 1.03E-05 | 0.52470875 | 0.000215 | 0.09 |
| HNRNPA0 | 0.5917707 | 1.86E-05 | 0.51077649 | 0.000337 | 0.09 |
| SMARCB1 | 0.6949192 | 1.18E-07 | 0.77555901 | 3.95E-10 | 0.09 |
| PRPF19 | 0.753119 | 2.39E-09 | 0.67331857 | 4.02E-07 | 0.08 |
| ZNF292 | 0.5072215 | 0.000377 | 0.4278117 | 0.003374 | 0.08 |
| MKRN1 | 0.6215273 | 5.2E-06 | 0.54238456 | 0.000119 | 0.08 |
| CCDC86 | 0.5298703 | 0.000182 | 0.4508647 | 0.001882 | 0.08 |
| AFM | -0.596737 | 1.52E-05 | -0.5178945 | 0.000269 | 0.08 |
| AAAS | 0.6340888 | 2.92E-06 | 0.71274832 | 3.96E-08 | 0.08 |
| PNISR | 0.7067632 | 5.77E-08 | 0.62815998 | 3.85E-06 | 0.08 |
| PPWD1 | 0.6057387 | 1.04E-05 | 0.52727071 | 0.000198 | 0.08 |
| DGCR8 | 0.5805359 | 2.91E-05 | 0.50310233 | 0.000428 | 0.08 |
| SPTY2D1 | 0.6641216 | 6.57E-07 | 0.58700994 | 2.25E-05 | 0.08 |
| SMG1 | 0.5712367 | 4.17E-05 | 0.49420984 | 0.00056 | 0.08 |
| XPO6 | 0.5574596 | 6.95E-05 | 0.48047352 | 0.000836 | 0.08 |
| CSRP2BP | 0.6036309 | 1.14E-05 | 0.52665687 | 0.000202 | 0.08 |
| NOL12 | 0.5275679 | 0.000196 | 0.45129395 | 0.001861 | 0.08 |
| CFI | -0.549219 | 9.34E-05 | -0.4730638 | 0.001032 | 0.08 |
| RRP1 | 0.5650378 | 5.26E-05 | 0.48892862 | 0.000654 | 0.08 |
| DHX40 | 0.5592424 | 6.51E-05 | 0.48356098 | 0.000765 | 0.08 |
| WAPAL | 0.5357675 | 0.000149 | 0.46042485 | 0.00146 | 0.08 |
| EXOSC10 | 0.6406848 | 2.13E-06 | 0.56539001 | 5.19E-05 | 0.08 |
| TF | -0.591156 | 0.000019 | -0.5158758 | 0.000287 | 0.08 |
| BTBD10 | 0.5093666 | 0.000352 | 0.43483296 | 0.002836 | 0.08 |
| ALB | -0.570771 | 4.24E-05 | -0.4964287 | 0.000524 | 0.08 |
| ATG13 | 0.6026194 | 1.19E-05 | 0.52839355 | 0.000191 | 0.08 |
| TADA2B | 0.5584078 | 6.72E-05 | 0.63228131 | 3.18E-06 | 0.08 |
| PPP1R8 | 0.5968977 | 1.51E-05 | 0.67042874 | 4.7E-07 | 0.08 |
| CDK11B | 0.6949953 | 1.18E-07 | 0.76828767 | 7.24E-10 | 0.08 |
| HNRNPH3 | 0.6268401 | 4.09E-06 | 0.55402262 | 7.87E-05 | 0.08 |
| NUP160 | 0.7077098 | 5.44E-08 | 0.63563624 | 2.71E-06 | 0.08 |
| LDB1 | 0.7048657 | 6.49E-08 | 0.63310465 | 3.06E-06 | 0.08 |
| HNRNPF | 0.7128343 | 3.94E-08 | 0.6411545 | 2.08E-06 | 0.08 |
| PSMD8 | 0.5561521 | 7.29E-05 | 0.48477061 | 0.000739 | 0.08 |
| NUP210 | 0.7256401 | 1.71E-08 | 0.65434789 | 1.09E-06 | 0.08 |
| YEATS2 | 0.629868 | 3.55E-06 | 0.55861712 | 6.66E-05 | 0.08 |
| RBM10 | 0.7183622 | 2.76E-08 | 0.64721709 | 1.55E-06 | 0.08 |
| BPHL | 0.5220591 | 0.000235 | 0.45138032 | 0.001857 | 0.08 |
| DHX15 | 0.6807406 | 2.67E-07 | 0.61009499 | 8.61E-06 | 0.08 |
| TRA2B | 0.7562274 | 1.89E-09 | 0.6860797 | 1.97E-07 | 0.08 |
| MTIF2 | 0.5699896 | 4.37E-05 | 0.50013395 | 0.000468 | 0.07 |
| LYAR | 0.5508955 | 0.000088 | 0.48141362 | 0.000814 | 0.07 |
| RNPC3 | 0.7698101 | 6.39E-10 | 0.70054184 | 8.44E-08 | 0.07 |
| ZNF507 | 0.5163843 | 0.000282 | 0.44749667 | 0.002055 | 0.07 |
| KHDRBS1 | 0.7543998 | 2.17E-09 | 0.68598133 | 1.99E-07 | 0.07 |
| PAXBP1 | 0.5635724 | 5.55E-05 | 0.49525338 | 0.000542 | 0.07 |
| PRPF18 | 0.538726 | 0.000135 | 0.47053042 | 0.001107 | 0.07 |
| KNG1 | -0.553807 | 7.93E-05 | -0.486158 | 0.00071 | 0.07 |
| DDX50 | 0.6175944 | 6.2E-06 | 0.54995051 | 0.000091 | 0.07 |
| SRSF2 | 0.6395078 | 2.26E-06 | 0.57209567 | 4.03E-05 | 0.07 |
| ZNF800 | 0.5050353 | 0.000403 | 0.43789875 | 0.002626 | 0.07 |
| CELF1 | 0.7072988 | 5.58E-08 | 0.64022523 | 2.18E-06 | 0.07 |
| CP | -0.530426 | 0.000178 | -0.4635226 | 0.001342 | 0.07 |
| ORAI1 | 0.5450713 | 0.000108 | 0.47824179 | 0.000891 | 0.07 |
| SERPING1 | -0.571265 | 4.16E-05 | -0.5046072 | 0.000408 | 0.07 |
| RRP9 | 0.559119 | 6.54E-05 | 0.49356325 | 0.000571 | 0.07 |
| FSTL1 | -0.529553 | 0.000184 | -0.4648095 | 0.001296 | 0.07 |
| INTS12 | 0.5727399 | 3.93E-05 | 0.5082595 | 0.000365 | 0.07 |
| CCAR1 | 0.729222 | 1.34E-08 | 0.66474205 | 6.36E-07 | 0.07 |
| ZNF397 | 0.5547516 | 7.67E-05 | 0.49042073 | 0.000626 | 0.07 |
| FUS | 0.5642379 | 5.42E-05 | 0.50034114 | 0.000465 | 0.07 |
| LARS | 0.5121859 | 0.000322 | 0.4483727 | 0.002009 | 0.07 |
| POLR3F | 0.5826788 | 2.67E-05 | 0.64636061 | 1.62E-06 | 0.07 |
| ARL14EP | 0.5720055 | 4.05E-05 | 0.50925268 | 0.000353 | 0.07 |
| TXLNG | 0.541844 | 0.000121 | 0.47942815 | 0.000862 | 0.07 |
| EPB41L2 | -0.510772 | 0.000337 | -0.4490781 | 0.001972 | 0.07 |
| DAB2 | -0.587632 | 0.000022 | -0.5263378 | 0.000204 | 0.07 |
| RNF40 | 0.6318843 | 3.24E-06 | 0.57062049 | 4.26E-05 | 0.07 |
| ABT1 | 0.596858 | 1.51E-05 | 0.53568437 | 0.000149 | 0.07 |
| HNRNPA3 | 0.6705494 | 4.67E-07 | 0.60948144 | 8.84E-06 | 0.07 |
| AFG3L2 | 0.5376672 | 0.00014 | 0.47730112 | 0.000915 | 0.07 |
| GLE1 | 0.786637 | 1.5E-10 | 0.72632184 | 1.63E-08 | 0.07 |
| SCLT1 | 0.598973 | 1.38E-05 | 0.53887057 | 0.000134 | 0.07 |
| PWP2 | 0.6032389 | 1.15E-05 | 0.5431678 | 0.000116 | 0.07 |
| UBN2 | 0.6134669 | 7.44E-06 | 0.55362905 | 7.98E-05 | 0.06 |
| NXF1 | 0.8072079 | 2.12E-11 | 0.74756416 | 3.63E-09 | 0.06 |
| ZMYM4 | 0.6797622 | 2.82E-07 | 0.62025986 | 5.51E-06 | 0.06 |
| CHD1L | 0.6083975 | 9.27E-06 | 0.54896963 | 9.43E-05 | 0.06 |
| PSMD6 | 0.5206576 | 0.000246 | 0.46149142 | 0.001418 | 0.06 |
| NOL8 | 0.6514367 | 1.26E-06 | 0.59230163 | 1.82E-05 | 0.06 |
| FAF1 | 0.635788 | 2.69E-06 | 0.57667169 | 3.38E-05 | 0.06 |
| BUB3 | 0.6730139 | 4.09E-07 | 0.73208486 | 1.1E-08 | 0.06 |
| NUP98 | 0.7733021 | 4.78E-10 | 0.71427 | 3.6E-08 | 0.06 |
| TCF20 | 0.5892709 | 2.06E-05 | 0.53031049 | 0.000179 | 0.06 |
| CCDC94 | 0.6453842 | 1.7E-06 | 0.58661788 | 2.29E-05 | 0.06 |
| SIN3A | 0.6709443 | 4.57E-07 | 0.61218778 | 7.87E-06 | 0.06 |
| CLMP | -0.508458 | 0.000362 | -0.4498066 | 0.001935 | 0.06 |
| NACA | 0.5109335 | 0.000335 | 0.45229732 | 0.001813 | 0.06 |
| SMARCE1 | 0.599191 | 1.37E-05 | 0.54066141 | 0.000126 | 0.06 |
| FUBP3 | 0.6430623 | 1.9E-06 | 0.58466965 | 2.47E-05 | 0.06 |
| DDX17 | 0.6928696 | 1.33E-07 | 0.63467084 | 2.84E-06 | 0.06 |
| ASUN | 0.5744433 | 3.68E-05 | 0.51635197 | 0.000282 | 0.06 |
| RBM12B | 0.6446328 | 1.76E-06 | 0.58670186 | 2.28E-05 | 0.06 |
| DMAP1 | 0.708672 | 5.12E-08 | 0.65107515 | 1.28E-06 | 0.06 |
| POLR3C | 0.7458457 | 4.12E-09 | 0.68826912 | 1.74E-07 | 0.06 |
| DDX51 | 0.7186358 | 2.71E-08 | 0.66127611 | 7.62E-07 | 0.06 |
| MED17 | 0.7894353 | 1.16E-10 | 0.73224061 | 1.09E-08 | 0.06 |
| TAF6L | 0.6499948 | 1.35E-06 | 0.59343453 | 1.74E-05 | 0.06 |
| NDUFS7 | 0.506588 | 0.000384 | 0.45026089 | 0.001912 | 0.06 |
| C1D | 0.5124985 | 0.000319 | 0.4570328 | 0.001599 | 0.06 |
| SF3B3 | 0.8196262 | 5.78E-12 | 0.76436599 | 9.94E-10 | 0.06 |
| MPHOSPH8 | 0.5843122 | 2.51E-05 | 0.52915726 | 0.000186 | 0.06 |
| NKAP | 0.5406052 | 0.000126 | 0.48581938 | 0.000717 | 0.06 |
| FBXL19 | 0.6663517 | 5.84E-07 | 0.61237336 | 7.8E-06 | 0.06 |
| NUP37 | 0.7360956 | 8.31E-09 | 0.68224535 | 2.45E-07 | 0.06 |
| KAT6A | 0.5690572 | 4.52E-05 | 0.51532811 | 0.000292 | 0.06 |
| POLR2H | 0.656413 | 9.78E-07 | 0.60274675 | 1.18E-05 | 0.06 |
| SMARCD2 | 0.7215211 | 2.24E-08 | 0.66819204 | 5.3E-07 | 0.06 |
| DPF2 | 0.5535376 | 8.01E-05 | 0.50037247 | 0.000465 | 0.06 |
| GC | -0.604628 | 1.09E-05 | -0.5516697 | 8.56E-05 | 0.06 |
| RBM6 | 0.6239315 | 4.67E-06 | 0.5712786 | 4.16E-05 | 0.06 |
| HAUS5 | 0.6140824 | 7.24E-06 | 0.56171662 | 5.95E-05 | 0.06 |
| CCNL2 | 0.535794 | 0.000149 | 0.48345257 | 0.000768 | 0.06 |
| EWSR1 | 0.5717349 | 4.09E-05 | 0.5195815 | 0.000255 | 0.06 |
| SMEK2 | 0.550944 | 8.79E-05 | 0.49898396 | 0.000485 | 0.06 |
| FETUB | -0.545127 | 0.000108 | -0.4931929 | 0.000577 | 0.06 |
| PRPF31 | 0.7460732 | 4.06E-09 | 0.69431129 | 1.23E-07 | 0.06 |
| CHRAC1 | 0.5455155 | 0.000106 | 0.49397139 | 0.000564 | 0.06 |
| TOP2B | 0.6192055 | 5.77E-06 | 0.56804388 | 0.000047 | 0.06 |
| METTL8 | 0.5163703 | 0.000282 | 0.46554783 | 0.00127 | 0.06 |
| RAD54L2 | 0.500692 | 0.00046 | 0.4507417 | 0.001888 | 0.05 |
| TFPI | -0.540473 | 0.000127 | -0.4913724 | 0.000609 | 0.05 |
| CPSF3L | 0.5317931 | 0.00017 | 0.48279256 | 0.000782 | 0.05 |
| RFX1 | 0.5056602 | 0.000395 | 0.45680989 | 0.001609 | 0.05 |
| RPL15 | 0.5138138 | 0.000306 | 0.46500962 | 0.001289 | 0.05 |
| NUP205 | 0.7197938 | 2.51E-08 | 0.67105844 | 4.54E-07 | 0.05 |
| TRA2A | 0.5629123 | 5.69E-05 | 0.51468061 | 0.000298 | 0.05 |
| RPA1 | 0.5693499 | 4.47E-05 | 0.52119929 | 0.000242 | 0.05 |
| NUP107 | 0.7251794 | 1.76E-08 | 0.77154205 | 5.53E-10 | 0.05 |
| RPL35A | 0.5725648 | 3.96E-05 | 0.52688768 | 0.000201 | 0.05 |
| TARDBP | 0.7865138 | 1.52E-10 | 0.74110403 | 5.82E-09 | 0.05 |
| HMG20A | 0.573583 | 3.81E-05 | 0.52818041 | 0.000192 | 0.05 |
| URB1 | 0.6499982 | 1.35E-06 | 0.60460395 | 1.09E-05 | 0.05 |
| NARG2 | 0.5363602 | 0.000146 | 0.49193834 | 0.000599 | 0.05 |
| MFAP1 | 0.7472742 | 3.71E-09 | 0.70392103 | 6.87E-08 | 0.05 |
| SMARCAD1 | 0.5251887 | 0.000212 | 0.48505456 | 0.000733 | 0.05 |
| ARHGEF11 | 0.5551205 | 7.57E-05 | 0.5153099 | 0.000292 | 0.04 |
| RANBP2 | 0.686458 | 1.93E-07 | 0.64736967 | 1.54E-06 | 0.04 |
| SUV39H2 | 0.5070338 | 0.000379 | 0.46809117 | 0.001184 | 0.04 |
| IGLL1 | -0.542235 | 0.000119 | -0.5043869 | 0.000411 | 0.04 |
| TOMM20 | 0.5778932 | 3.23E-05 | 0.54082238 | 0.000125 | 0.04 |
| DROSHA | 0.5437748 | 0.000113 | 0.50741799 | 0.000374 | 0.04 |
| SRSF11 | 0.6369854 | 2.55E-06 | 0.67319782 | 4.05E-07 | 0.04 |
| HNRNPUL1 | 0.5597069 | 0.000064 | 0.52363139 | 0.000223 | 0.04 |
| FAM111A | 0.5130433 | 0.000314 | 0.47716282 | 0.000919 | 0.04 |
| TAF2 | 0.5349981 | 0.000153 | 0.499656 | 0.000475 | 0.04 |
| PARP1 | 0.5750455 | 0.000036 | 0.54035759 | 0.000127 | 0.04 |
| SRBD1 | 0.5940233 | 1.69E-05 | 0.5596458 | 6.42E-05 | 0.04 |
| ZBTB33 | 0.5335907 | 0.00016 | 0.49926203 | 0.000481 | 0.04 |
| WDR18 | 0.5571098 | 7.04E-05 | 0.5236257 | 0.000223 | 0.04 |
| RCC2 | 0.5580476 | 0.000068 | 0.52457218 | 0.000216 | 0.04 |
| SLC25A44 | 0.6038527 | 1.13E-05 | 0.63726382 | 2.51E-06 | 0.04 |
| EPCAM | 0.5196535 | 0.000254 | 0.48650935 | 0.000702 | 0.04 |
| NUP133 | 0.7415785 | 5.63E-09 | 0.70874148 | 5.1E-08 | 0.04 |
| NUP93 | 0.6857697 | 2.01E-07 | 0.65320264 | 1.15E-06 | 0.04 |
| SAP130 | 0.5820793 | 2.74E-05 | 0.54964817 | 0.000092 | 0.04 |
| RPS6 | 0.5295024 | 0.000184 | 0.49771359 | 0.000504 | 0.04 |
| PUM1 | 0.5663859 | 0.00005 | 0.53496321 | 0.000153 | 0.04 |
| NUPL2 | 0.5414905 | 0.000122 | 0.5107675 | 0.000337 | 0.04 |
| DDX39B | 0.5406875 | 0.000126 | 0.51042818 | 0.000341 | 0.04 |
| TLK1 | 0.5077415 | 0.00037 | 0.47780719 | 0.000902 | 0.03 |
| POLR1C | 0.5769043 | 3.35E-05 | 0.54877336 | 9.49E-05 | 0.03 |
| MTR | -0.505664 | 0.000395 | -0.4779241 | 0.000899 | 0.03 |
| NUP155 | 0.6297929 | 3.57E-06 | 0.60244307 | 1.19E-05 | 0.03 |
| GTF3C5 | 0.5306102 | 0.000177 | 0.5032842 | 0.000425 | 0.03 |
| TIA1 | 0.5561454 | 7.29E-05 | 0.5289231 | 0.000187 | 0.03 |
| XRCC5 | 0.5759445 | 3.48E-05 | 0.54883799 | 9.47E-05 | 0.03 |
| RUVBL2 | 0.5467332 | 0.000102 | 0.52005531 | 0.000251 | 0.03 |
| ZNF646 | 0.629438 | 3.63E-06 | 0.60310381 | 1.16E-05 | 0.03 |
| POLR3B | 0.6283454 | 3.81E-06 | 0.60361088 | 1.14E-05 | 0.03 |
| ATXN7L3 | 0.639747 | 2.23E-06 | 0.66417819 | 6.55E-07 | 0.03 |
| OFD1 | 0.5689884 | 4.54E-05 | 0.59323333 | 1.75E-05 | 0.03 |
| TOR2A | 0.5504896 | 8.93E-05 | 0.52756285 | 0.000196 | 0.03 |
| PSMD14 | 0.5541627 | 7.83E-05 | 0.53177133 | 0.00017 | 0.03 |
| RPS16 | 0.513039 | 0.000314 | 0.49084599 | 0.000618 | 0.03 |
| IQCB1 | 0.5175166 | 0.000272 | 0.49603397 | 0.00053 | 0.03 |
| IGHV2-5 | -0.518562 | 0.000263 | -0.4971428 | 0.000513 | 0.03 |
| SERPINA6 | -0.521189 | 0.000242 | -0.4998716 | 0.000472 | 0.03 |
| LRRC47 | 0.5027268 | 0.000433 | 0.52312171 | 0.000227 | 0.03 |
| NSMCE1 | 0.5968529 | 1.51E-05 | 0.5767165 | 3.38E-05 | 0.03 |
| OGG1 | 0.5102632 | 0.000342 | 0.49059705 | 0.000623 | 0.02 |
| IGHV1-69 | -0.504666 | 0.000408 | -0.4850832 | 0.000732 | 0.02 |
| EP400 | 0.7421647 | 5.39E-09 | 0.72307891 | 2.02E-08 | 0.02 |
| KIAA0753 | 0.5095552 | 0.00035 | 0.49127006 | 0.000611 | 0.02 |
| INTS4 | 0.5481044 | 9.72E-05 | 0.52998944 | 0.000181 | 0.02 |
| PTPRM | -0.576581 | 3.39E-05 | -0.5946483 | 1.65E-05 | 0.02 |
| SMPD4 | 0.5232537 | 0.000226 | 0.50558752 | 0.000396 | 0.02 |
| SRSF6 | 0.5621614 | 5.85E-05 | 0.54492186 | 0.000109 | 0.02 |
| SUPT7L | 0.6036256 | 1.14E-05 | 0.61961122 | 5.67E-06 | 0.02 |
| NPHP3 | -0.500624 | 0.000461 | -0.5165763 | 0.00028 | 0.02 |
| CRNKL1 | 0.6525311 | 1.19E-06 | 0.63734504 | 2.5E-06 | 0.02 |
| TOMM22 | 0.540558 | 0.000126 | 0.55503739 | 7.59E-05 | 0.02 |
| SEH1L | 0.5857929 | 2.36E-05 | 0.57133957 | 4.15E-05 | 0.02 |
| ARID1A | 0.6814645 | 2.56E-07 | 0.66762969 | 5.46E-07 | 0.02 |
| MAD1L1 | 0.7241199 | 1.89E-08 | 0.7112694 | 4.35E-08 | 0.02 |
| PCGF6 | 0.6010441 | 1.27E-05 | 0.58824107 | 2.14E-05 | 0.02 |
| HNRNPR | 0.7164576 | 3.12E-08 | 0.70399397 | 6.84E-08 | 0.02 |
| EIF3K | 0.568356 | 4.64E-05 | 0.55614136 | 7.29E-05 | 0.02 |
| NOL9 | 0.5766377 | 3.39E-05 | 0.58846166 | 2.12E-05 | 0.02 |
| TOE1 | 0.5386181 | 0.000135 | 0.52837391 | 0.000191 | 0.02 |
| NOP58 | 0.5238211 | 0.000222 | 0.53378743 | 0.000159 | 0.01 |
| ZBTB9 | 0.5513167 | 8.67E-05 | 0.56097815 | 6.11E-05 | 0.01 |
| TP63 | -0.565922 | 5.09E-05 | -0.5748387 | 3.63E-05 | 0.01 |
| SMU1 | 0.7931538 | 8.26E-11 | 0.78536139 | 1.68E-10 | 0.01 |
| RLIM | 0.5020119 | 0.000442 | 0.49469018 | 0.000552 | 0.01 |
| LBH | -0.548392 | 9.62E-05 | -0.554797 | 7.66E-05 | 0.01 |
| NOP56 | 0.538715 | 0.000135 | 0.54485985 | 0.000109 | 0.01 |
| PHF21A | 0.5636709 | 5.53E-05 | 0.56951935 | 4.45E-05 | 0.01 |
| KLHL20 | 0.5143233 | 0.000301 | 0.52002654 | 0.000251 | 0.01 |
| URB2 | 0.6113758 | 8.15E-06 | 0.6059152 | 1.03E-05 | 0.01 |
| KDSR | 0.5027074 | 0.000433 | 0.49741658 | 0.000508 | 0.01 |
| XRN2 | 0.5274667 | 0.000197 | 0.53199469 | 0.000169 | 0.01 |
| USP48 | 0.5088895 | 0.000357 | 0.50622804 | 0.000388 | 0.01 |
| TFAP4 | 0.5180828 | 0.000267 | 0.52063289 | 0.000246 | 0.01 |
| NCOA5 | 0.5436473 | 0.000114 | 0.54502321 | 0.000108 | 0.01 |
| ING3 | 0.5396094 | 0.000131 | 0.54079969 | 0.000125 | 0.01 |
| USP20 | 0.5971261 | 1.49E-05 | 0.59614168 | 1.55E-05 | 0.01 |
| TCHP | 0.6312981 | 3.33E-06 | 0.63100064 | 3.37E-06 | 0.01 |
| PPP3R1 | -0.081137 | 0.59622 | -0.5283986 | 0.000191 | 0.45 |
| MPPE1 | 0.2808972 | 0.061606 | 0.57175137 | 4.08E-05 | 0.3 |
| BRD2 | 0.8436074 | 3.47E-13 | 0.55999362 | 6.34E-05 | 0.29 |
| OTUD3 | 0.2846955 | 0.058028 | 0.56689186 | 4.91E-05 | 0.29 |
| MED16 | 0.8288231 | 2.07E-12 | 0.54686469 | 0.000102 | 0.29 |
| ZCCHC4 | 0.2372394 | 0.11661 | 0.51620313 | 0.000284 | 0.28 |
| POLR2L | 0.7769929 | 3.49E-10 | 0.50019939 | 0.000467 | 0.28 |
| ALKBH3 | 0.2885903 | 0.054534 | 0.56340174 | 5.59E-05 | 0.28 |
| SPIDR | 0.2726965 | 0.069933 | 0.54674727 | 0.000102 | 0.28 |
| PNN | 0.8413481 | 4.61E-13 | 0.56913447 | 4.51E-05 | 0.28 |
| PRPF4B | 0.7974929 | 5.49E-11 | 0.5308321 | 0.000176 | 0.27 |
| TCERG1 | 0.819469 | 5.88E-12 | 0.55531442 | 7.51E-05 | 0.27 |
| DNAJC11 | 0.2725929 | 0.070043 | 0.52929892 | 0.000185 | 0.26 |
| DDX46 | 0.821447 | 4.74E-12 | 0.56716488 | 4.86E-05 | 0.26 |
| CCNL1 | 0.789163 | 1.19E-10 | 0.53801118 | 0.000138 | 0.26 |
| LIN9 | 0.7942567 | 7.45E-11 | 0.54595087 | 0.000105 | 0.25 |
| SF3A2 | 0.8425407 | 3.97E-13 | 0.59631084 | 1.54E-05 | 0.25 |
| SNRPF | 0.8231491 | 3.93E-12 | 0.57736543 | 3.29E-05 | 0.25 |
| HNRNPL | 0.837273 | 7.62E-13 | 0.59375484 | 1.71E-05 | 0.25 |
| HLA-DPA1 | -0.355338 | 0.016604 | -0.5933788 | 1.74E-05 | 0.24 |
| MED8 | 0.8007647 | 4.01E-11 | 0.56534377 | 0.000052 | 0.24 |
| ORC5 | 0.7590429 | 1.52E-09 | 0.52639455 | 0.000204 | 0.24 |
| CPSF6 | 0.814266 | 1.02E-11 | 0.58338502 | 0.000026 | 0.24 |
| CHERP | 0.8391066 | 6.09E-13 | 0.60988513 | 8.69E-06 | 0.23 |
| WDR33 | 0.740339 | 6.15E-09 | 0.512436 | 0.00032 | 0.23 |
| RFC2 | 0.7352832 | 8.8E-09 | 0.50830721 | 0.000364 | 0.23 |
| SNRNP200 | 0.8582037 | 4.92E-14 | 0.6314905 | 3.3E-06 | 0.23 |
| PRPF38A | 0.7902382 | 1.08E-10 | 0.56391436 | 5.48E-05 | 0.23 |
| RBMX | 0.7364345 | 8.12E-09 | 0.51154831 | 0.000329 | 0.23 |
| CHD4 | 0.8160826 | 8.45E-12 | 0.59156082 | 1.87E-05 | 0.23 |
| CHD8 | 0.7468983 | 3.82E-09 | 0.52463663 | 0.000216 | 0.23 |
| U2SURP | 0.8318943 | 1.45E-12 | 0.60979578 | 8.73E-06 | 0.23 |
| SKIV2L2 | 0.7960171 | 6.31E-11 | 0.57454611 | 3.67E-05 | 0.23 |
| PRPF6 | 0.765721 | 8.92E-10 | 0.5459137 | 0.000105 | 0.22 |
| POLR2E | 0.8469028 | 2.27E-13 | 0.62902698 | 3.7E-06 | 0.22 |
| CPSF2 | 0.7403406 | 6.15E-09 | 0.52252134 | 0.000231 | 0.22 |
| EIF3D | 0.7251217 | 1.77E-08 | 0.50743117 | 0.000374 | 0.22 |
| ZNF207 | 0.7297768 | 1.29E-08 | 0.51229209 | 0.000321 | 0.22 |
| POP4 | 0.7798325 | 2.74E-10 | 0.56322041 | 5.63E-05 | 0.22 |
| DHX16 | 0.8185013 | 6.53E-12 | 0.60200925 | 1.22E-05 | 0.22 |
| PCF11 | 0.7801005 | 2.67E-10 | 0.5653182 | 0.000052 | 0.22 |
| ORC2 | 0.8036651 | 3.01E-11 | 0.58895101 | 2.08E-05 | 0.22 |
| VPS26A | 0.3157845 | 0.034589 | 0.53043327 | 0.000178 | 0.22 |
| THOC7 | 0.7710657 | 5.76E-10 | 0.5576038 | 6.92E-05 | 0.22 |
| IK | 0.76771 | 7.59E-10 | 0.55430228 | 7.79E-05 | 0.22 |
| ORC3 | 0.7404637 | 6.1E-09 | 0.52773997 | 0.000195 | 0.22 |
| EIF4A3 | 0.7893145 | 1.18E-10 | 0.57679354 | 3.37E-05 | 0.22 |
| MED23 | 0.7591445 | 1.5E-09 | 0.54752931 | 9.92E-05 | 0.22 |
| EIF2S1 | 0.7227666 | 2.07E-08 | 0.51173545 | 0.000327 | 0.22 |
| RBM39 | 0.7859909 | 1.59E-10 | 0.57847655 | 3.15E-05 | 0.21 |
| SF3A3 | 0.8282742 | 2.2E-12 | 0.62105818 | 5.31E-06 | 0.21 |
| HAUS4 | 0.7084006 | 5.21E-08 | 0.50150203 | 0.000449 | 0.21 |
| MATR3 | 0.705927 | 6.08E-08 | 0.50098334 | 0.000456 | 0.21 |
| EFTUD2 | 0.8713741 | 6.94E-15 | 0.66811386 | 5.32E-07 | 0.21 |
| RBM45 | 0.7179041 | 2.84E-08 | 0.51490516 | 0.000296 | 0.21 |
| SNRNP70 | 0.8494164 | 1.63E-13 | 0.64706618 | 1.56E-06 | 0.21 |
| SF3B2 | 0.8079324 | 1.97E-11 | 0.60660481 | 0.00001 | 0.21 |
| GLTSCR2 | 0.7027278 | 7.39E-08 | 0.50147516 | 0.000449 | 0.21 |
| GPATCH4 | 0.7076956 | 5.44E-08 | 0.50733208 | 0.000375 | 0.21 |
| CSTF2 | 0.7308577 | 1.2E-08 | 0.53201939 | 0.000169 | 0.2 |
| SNRNP40 | 0.7472364 | 3.72E-09 | 0.54873866 | 0.000095 | 0.2 |
| RBM25 | 0.8054553 | 2.52E-11 | 0.60764316 | 9.57E-06 | 0.2 |
| HBP1 | 0.3220527 | 0.030971 | 0.51867161 | 0.000262 | 0.2 |
| MED14 | 0.7582942 | 1.61E-09 | 0.56172102 | 5.95E-05 | 0.2 |
| TOMM6 | 0.323906 | 0.029963 | 0.52039791 | 0.000248 | 0.2 |
| CLASRP | 0.7327762 | 1.05E-08 | 0.53645851 | 0.000146 | 0.2 |
| FIP1L1 | 0.7157576 | 3.27E-08 | 0.52013402 | 0.00025 | 0.2 |
| AQR | 0.7457977 | 4.14E-09 | 0.55034944 | 8.98E-05 | 0.2 |
| CNOT11 | 0.7416434 | 5.6E-09 | 0.54712809 | 0.000101 | 0.2 |
| EIF3A | 0.7804603 | 2.59E-10 | 0.58598599 | 2.35E-05 | 0.2 |
| PRPF8 | 0.8283549 | 2.18E-12 | 0.63404861 | 2.92E-06 | 0.2 |
| ADAR | 0.7621385 | 1.19E-09 | 0.56814129 | 4.68E-05 | 0.2 |
| NUPL1 | 0.701252 | 8.09E-08 | 0.50831946 | 0.000364 | 0.2 |
| C17orf85 | 0.7573166 | 1.73E-09 | 0.56455679 | 5.35E-05 | 0.2 |
| ERCC3 | 0.7455722 | 4.21E-09 | 0.55388107 | 7.91E-05 | 0.2 |
| ZBTB48 | 0.4950368 | 0.000546 | 0.6864861 | 1.93E-07 | 0.2 |
| RBM17 | 0.7663821 | 8.45E-10 | 0.57533923 | 3.56E-05 | 0.2 |
| SNRNP27 | 0.8068701 | 2.19E-11 | 0.61667952 | 6.46E-06 | 0.2 |
| SNRPD3 | 0.8792357 | 1.94E-15 | 0.68919108 | 1.65E-07 | 0.2 |
| SSRP1 | 0.7139293 | 3.67E-08 | 0.52426829 | 0.000219 | 0.19 |
| SF1 | 0.7573432 | 1.73E-09 | 0.56769582 | 4.76E-05 | 0.19 |
| RPAP2 | 0.7320013 | 1.11E-08 | 0.54238599 | 0.000119 | 0.19 |
| POLR2A | 0.8142215 | 1.03E-11 | 0.62548897 | 4.35E-06 | 0.19 |
| HNRNPA1 | 0.7081234 | 5.3E-08 | 0.51949203 | 0.000255 | 0.19 |
| MRPS14 | 0.6891991 | 1.65E-07 | 0.50114313 | 0.000454 | 0.19 |
| THRAP3 | 0.7252536 | 1.75E-08 | 0.53727202 | 0.000142 | 0.19 |
| U2AF1 | 0.6998256 | 8.82E-08 | 0.51317029 | 0.000312 | 0.19 |
| KLHL12 | 0.3519792 | 0.017733 | 0.53765615 | 0.00014 | 0.19 |
| DHX9 | 0.7897918 | 1.13E-10 | 0.60424794 | 1.11E-05 | 0.19 |
| SRCAP | 0.7065045 | 5.86E-08 | 0.52152921 | 0.000239 | 0.19 |
| CDC73 | 0.7450976 | 4.36E-09 | 0.56037556 | 6.25E-05 | 0.19 |
| SMC6 | 0.6937992 | 1.26E-07 | 0.50964672 | 0.000349 | 0.19 |
| NUP62 | 0.7503622 | 2.95E-09 | 0.56740301 | 4.81E-05 | 0.19 |
| SNRPD2 | 0.8639143 | 2.16E-14 | 0.68098569 | 2.63E-07 | 0.19 |
| DCTD | -0.338221 | 0.02306 | -0.5209756 | 0.000243 | 0.19 |
| ZFAT | 0.4932014 | 0.000577 | 0.67583111 | 3.5E-07 | 0.19 |
| WTAP | 0.7469936 | 3.79E-09 | 0.56454214 | 5.36E-05 | 0.19 |
| SRSF3 | 0.7985846 | 4.94E-11 | 0.61633885 | 6.56E-06 | 0.19 |
| FUBP1 | 0.6925737 | 1.36E-07 | 0.51086795 | 0.000336 | 0.19 |
| RBM42 | 0.7286863 | 1.39E-08 | 0.54740614 | 9.96E-05 | 0.19 |
| INO80 | 0.7858859 | 1.6E-10 | 0.60461358 | 1.09E-05 | 0.19 |
| UBR5 | 0.7573119 | 1.73E-09 | 0.57723206 | 3.31E-05 | 0.19 |
| ABHD4 | -0.388974 | 0.008271 | -0.5675644 | 4.79E-05 | 0.18 |
| PHF5A | 0.6817006 | 2.53E-07 | 0.50353528 | 0.000422 | 0.18 |
| ARL6IP4 | 0.6916159 | 1.43E-07 | 0.51453733 | 0.000299 | 0.18 |
| SRSF1 | 0.8073075 | 2.1E-11 | 0.63080562 | 3.4E-06 | 0.18 |
| RNPS1 | 0.8332152 | 1.24E-12 | 0.65706379 | 9.46E-07 | 0.18 |
| SUPT16H | 0.713905 | 3.68E-08 | 0.53805086 | 0.000138 | 0.18 |
| VWA9 | 0.67764 | 3.17E-07 | 0.50233792 | 0.000438 | 0.18 |
| PQBP1 | 0.6852928 | 2.07E-07 | 0.51111404 | 0.000333 | 0.18 |
| MED22 | 0.7758452 | 3.85E-10 | 0.60178217 | 1.23E-05 | 0.18 |
| HAUS3 | 0.6934004 | 1.29E-07 | 0.51940449 | 0.000256 | 0.18 |
| NKTR | 0.7025454 | 7.48E-08 | 0.53073326 | 0.000176 | 0.18 |
| YY1 | 0.6720973 | 4.29E-07 | 0.50071107 | 0.00046 | 0.18 |
| SF3B1 | 0.8327793 | 1.3E-12 | 0.66143161 | 7.56E-07 | 0.18 |
| RBMX2 | 0.7631481 | 1.1E-09 | 0.59196057 | 1.84E-05 | 0.18 |
| MED12 | 0.6982738 | 9.68E-08 | 0.52758544 | 0.000196 | 0.18 |
| ELAVL1 | 0.715329 | 3.36E-08 | 0.54498946 | 0.000108 | 0.18 |
| SNRNP35 | 0.7933763 | 8.09E-11 | 0.62379082 | 4.7E-06 | 0.17 |
| RAN | 0.6783046 | 3.06E-07 | 0.50965661 | 0.000349 | 0.17 |
| SAP18 | 0.8624159 | 2.69E-14 | 0.69460549 | 1.2E-07 | 0.17 |
| RSRC1 | 0.741528 | 5.65E-09 | 0.57379509 | 3.78E-05 | 0.17 |
| SART1 | 0.8250524 | 3.17E-12 | 0.65779794 | 9.11E-07 | 0.17 |
| AHCTF1 | 0.6904367 | 1.54E-07 | 0.52367694 | 0.000223 | 0.17 |
| HNRNPM | 0.8409705 | 4.83E-13 | 0.6752415 | 3.62E-07 | 0.17 |
| SPEN | 0.6754575 | 3.58E-07 | 0.51116742 | 0.000333 | 0.17 |
| EIF3F | 0.6873886 | 1.83E-07 | 0.52336776 | 0.000225 | 0.17 |
| ARGLU1 | 0.690812 | 1.5E-07 | 0.52681208 | 0.000201 | 0.17 |
| CSTF3 | 0.8229081 | 4.03E-12 | 0.65928048 | 8.45E-07 | 0.17 |
| NCBP2 | 0.7171066 | 3E-08 | 0.55395297 | 7.89E-05 | 0.17 |
| PRKDC | 0.7394226 | 6.57E-09 | 0.57692652 | 3.35E-05 | 0.17 |
| RPRD1A | 0.6618777 | 7.38E-07 | 0.50092082 | 0.000457 | 0.17 |
| GTF3C4 | 0.6611513 | 7.67E-07 | 0.50046848 | 0.000463 | 0.17 |
| NUP54 | 0.8144454 | 1.01E-11 | 0.65382747 | 1.12E-06 | 0.17 |
| DAZAP1 | 0.742115 | 5.41E-09 | 0.58261328 | 2.68E-05 | 0.16 |
| NONO | 0.8694669 | 9.34E-15 | 0.70999039 | 4.72E-08 | 0.16 |
| AKAP8L | 0.7274041 | 1.51E-08 | 0.56798502 | 4.71E-05 | 0.16 |
| CPSF1 | 0.8685484 | 1.08E-14 | 0.7114719 | 4.3E-08 | 0.16 |
| TAF9B | 0.3494303 | 0.018633 | 0.50586683 | 0.000393 | 0.16 |
| MGA | 0.7046017 | 6.59E-08 | 0.54847613 | 9.59E-05 | 0.16 |
| ILF3 | 0.7516095 | 2.68E-09 | 0.5972048 | 1.49E-05 | 0.16 |
| NIPBL | 0.6804172 | 2.72E-07 | 0.52649891 | 0.000203 | 0.16 |
| PUM2 | 0.7274521 | 1.51E-08 | 0.57376678 | 3.78E-05 | 0.16 |
| CWC22 | 0.6827393 | 2.39E-07 | 0.52921884 | 0.000186 | 0.16 |
| CDK7 | 0.7520042 | 2.61E-09 | 0.59848803 | 1.41E-05 | 0.16 |
| THOC2 | 0.7776154 | 3.31E-10 | 0.62438941 | 4.57E-06 | 0.16 |
| EIF3H | 0.7485793 | 3.37E-09 | 0.59662424 | 1.52E-05 | 0.16 |
| PRPF3 | 0.7598222 | 1.43E-09 | 0.60803993 | 9.41E-06 | 0.16 |
| ACIN1 | 0.6771966 | 3.25E-07 | 0.52637107 | 0.000204 | 0.16 |
| SNW1 | 0.7802592 | 2.64E-10 | 0.6295358 | 3.61E-06 | 0.16 |
| KIAA1429 | 0.8076176 | 2.03E-11 | 0.65696645 | 9.51E-07 | 0.16 |
| PDCD11 | 0.6543854 | 1.08E-06 | 0.50400426 | 0.000416 | 0.16 |
| MTA2 | 0.7131768 | 3.86E-08 | 0.56339442 | 5.59E-05 | 0.15 |
| CTNNBL1 | 0.7571122 | 1.76E-09 | 0.60746132 | 9.65E-06 | 0.15 |
| SUPT5H | 0.6763542 | 3.4E-07 | 0.52670632 | 0.000202 | 0.15 |
| CHTOP | 0.6773875 | 3.22E-07 | 0.52841246 | 0.000191 | 0.15 |
| PPIG | 0.7436489 | 4.84E-09 | 0.5950669 | 1.62E-05 | 0.15 |
| KHSRP | 0.6576862 | 9.17E-07 | 0.50935549 | 0.000352 | 0.15 |
| TTF1 | 0.6602731 | 8.02E-07 | 0.51214528 | 0.000323 | 0.15 |
| EIF3E | 0.6606506 | 7.87E-07 | 0.51326891 | 0.000311 | 0.15 |
| CDC40 | 0.6593562 | 8.41E-07 | 0.51375612 | 0.000307 | 0.15 |
| PUS1 | 0.6627196 | 7.07E-07 | 0.51737362 | 0.000273 | 0.15 |
| STAG2 | 0.6791578 | 2.92E-07 | 0.53388966 | 0.000159 | 0.15 |
| NFKBIL1 | 0.71223 | 4.09E-08 | 0.56729676 | 4.83E-05 | 0.15 |
| TBL3 | 0.6622349 | 7.25E-07 | 0.51801553 | 0.000268 | 0.15 |
| EIF3L | 0.7295275 | 1.31E-08 | 0.58546187 | 2.39E-05 | 0.15 |
| IGKV3D-20 | -0.451628 | 0.001845 | -0.5947462 | 1.65E-05 | 0.15 |
| U2AF2 | 0.7458971 | 4.11E-09 | 0.6032765 | 1.15E-05 | 0.15 |
| POLR1B | 0.6758584 | 3.5E-07 | 0.53361784 | 0.00016 | 0.15 |
| SRSF7 | 0.8305294 | 1.7E-12 | 0.68897103 | 1.67E-07 | 0.15 |
| DNTTIP2 | 0.6468584 | 1.58E-06 | 0.5056522 | 0.000395 | 0.15 |
| SUGP1 | 0.7064062 | 5.9E-08 | 0.56524077 | 5.22E-05 | 0.15 |
| JAGN1 | 0.512631 | 0.000318 | 0.65371755 | 1.12E-06 | 0.15 |
| BRMS1 | 0.3844654 | 0.009119 | 0.52553386 | 0.00021 | 0.15 |
| ZSWIM7 | 0.4580917 | 0.001554 | 0.59909007 | 1.37E-05 | 0.15 |
| MPHOSPH10 | 0.6565014 | 9.74E-07 | 0.51684023 | 0.000278 | 0.14 |
| NOC2L | 0.6670775 | 5.62E-07 | 0.52789807 | 0.000194 | 0.14 |
| DAXX | 0.663578 | 6.76E-07 | 0.52466313 | 0.000216 | 0.14 |
| HAUS1 | 0.6731167 | 4.06E-07 | 0.53422106 | 0.000157 | 0.14 |
| CEBPZ | 0.6700061 | 4.81E-07 | 0.53170948 | 0.000171 | 0.14 |
| MNAT1 | 0.6524115 | 1.2E-06 | 0.5142226 | 0.000302 | 0.14 |
| ZNF407 | 0.6607558 | 7.83E-07 | 0.52398918 | 0.000221 | 0.14 |
| HNRNPK | 0.7249606 | 1.79E-08 | 0.58834929 | 2.13E-05 | 0.14 |
| ACTL6A | 0.7219812 | 2.18E-08 | 0.58563228 | 2.38E-05 | 0.14 |
| GPKOW | 0.7185422 | 2.73E-08 | 0.58234593 | 2.71E-05 | 0.14 |
| SF3A1 | 0.8262742 | 2.77E-12 | 0.69159356 | 1.44E-07 | 0.14 |
| MTCH2 | 0.3803424 | 0.009958 | 0.51478112 | 0.000297 | 0.14 |
| POLR2C | 0.7585572 | 1.57E-09 | 0.62433143 | 4.58E-06 | 0.14 |
| ZNF326 | 0.6650393 | 6.26E-07 | 0.53116149 | 0.000174 | 0.14 |
| ZC3HC1 | 0.6676176 | 5.46E-07 | 0.53381008 | 0.000159 | 0.14 |
| SENP1 | 0.7000604 | 8.69E-08 | 0.56635649 | 5.01E-05 | 0.14 |
| SERPINA1 | -0.690328 | 1.55E-07 | -0.5569036 | 7.09E-05 | 0.14 |
| PDCD7 | 0.7192909 | 2.6E-08 | 0.58593997 | 2.35E-05 | 0.14 |
| NCL | 0.6793328 | 2.89E-07 | 0.54622527 | 0.000104 | 0.14 |
| C11orf57 | 0.6353414 | 2.75E-06 | 0.50264423 | 0.000434 | 0.14 |
| HNRNPH1 | 0.6432274 | 1.89E-06 | 0.51161627 | 0.000328 | 0.14 |
| SART3 | 0.6373484 | 2.5E-06 | 0.50612999 | 0.000389 | 0.14 |
| MED11 | 0.7016164 | 7.91E-08 | 0.57111817 | 4.18E-05 | 0.14 |
| REXO1 | 0.6972256 | 1.03E-07 | 0.5673462 | 4.82E-05 | 0.13 |
| WDR5 | 0.6801479 | 2.76E-07 | 0.55135349 | 8.66E-05 | 0.13 |
| TRNAU1AP | 0.5635548 | 5.56E-05 | 0.69210999 | 1.39E-07 | 0.13 |
| 7-Mar | 0.6426611 | 1.94E-06 | 0.51428488 | 0.000302 | 0.13 |
| TADA3 | 0.6679935 | 5.35E-07 | 0.53982809 | 0.00013 | 0.13 |
| HNRNPU | 0.7945504 | 7.25E-11 | 0.666449 | 5.81E-07 | 0.13 |
| MSH2 | 0.6448928 | 1.74E-06 | 0.51692018 | 0.000277 | 0.13 |
| CDC5L | 0.6958993 | 1.12E-07 | 0.56798854 | 4.71E-05 | 0.13 |
| C9orf114 | 0.4099682 | 0.00516 | 0.53717645 | 0.000142 | 0.13 |
| RBM19 | 0.659408 | 8.39E-07 | 0.53236374 | 0.000167 | 0.13 |
| BRIX1 | 0.6455699 | 1.68E-06 | 0.5192584 | 0.000257 | 0.13 |
| SETD5 | 0.5010905 | 0.000455 | 0.626972 | 4.06E-06 | 0.13 |
| MRTO4 | 0.6430362 | 1.9E-06 | 0.51716972 | 0.000275 | 0.13 |
| SAFB2 | 0.634499 | 2.86E-06 | 0.50912637 | 0.000355 | 0.13 |
| SLU7 | 0.6964201 | 1.08E-07 | 0.5711016 | 4.19E-05 | 0.13 |
| BRPF1 | 0.7123264 | 4.07E-08 | 0.58716627 | 2.24E-05 | 0.13 |
| POLR2B | 0.8028763 | 3.26E-11 | 0.67813847 | 3.09E-07 | 0.13 |
| WRNIP1 | 0.6823048 | 2.45E-07 | 0.5576465 | 0.000069 | 0.13 |
| YTHDF2 | 0.6974054 | 1.02E-07 | 0.57313197 | 3.87E-05 | 0.13 |
| SF3B4 | 0.6888065 | 1.69E-07 | 0.56461281 | 5.34E-05 | 0.13 |
| TFIP11 | 0.7595336 | 1.46E-09 | 0.63572792 | 2.7E-06 | 0.13 |
| WDR46 | 0.6345923 | 2.85E-06 | 0.51104342 | 0.000334 | 0.13 |
| MBD2 | 0.6506154 | 1.31E-06 | 0.5276624 | 0.000195 | 0.13 |
| NGDN | 0.6622328 | 7.25E-07 | 0.53945239 | 0.000131 | 0.13 |
| NUP188 | 0.7295966 | 1.3E-08 | 0.60746904 | 9.64E-06 | 0.13 |
| NUP88 | 0.7090665 | 5E-08 | 0.58745018 | 2.21E-05 | 0.13 |
| USP36 | 0.654058 | 1.1E-06 | 0.53254143 | 0.000166 | 0.13 |
| EXOSC2 | 0.6426304 | 1.94E-06 | 0.5213719 | 0.00024 | 0.13 |
| ARID4B | 0.7200574 | 2.47E-08 | 0.59882499 | 1.39E-05 | 0.13 |
| ZNF639 | 0.4160597 | 0.004475 | 0.53722533 | 0.000142 | 0.13 |
| CABIN1 | 0.671245 | 4.5E-07 | 0.55049406 | 8.93E-05 | 0.13 |
| UTP3 | 0.6948967 | 1.18E-07 | 0.57445425 | 3.68E-05 | 0.13 |
| SNRPB2 | 0.8344951 | 1.06E-12 | 0.71428525 | 3.59E-08 | 0.13 |
| NCBP1 | 0.6386648 | 2.35E-06 | 0.51878297 | 0.000261 | 0.12 |
| PHF3 | 0.6513401 | 1.26E-06 | 0.53198692 | 0.000169 | 0.12 |
| ACTR6 | 0.6882293 | 1.75E-07 | 0.56933577 | 4.48E-05 | 0.12 |
| HIRA | 0.6852249 | 2.07E-07 | 0.56666304 | 4.95E-05 | 0.12 |
| SENP6 | 0.6402797 | 2.17E-06 | 0.521756 | 0.000237 | 0.12 |
| SYF2 | 0.7025499 | 7.48E-08 | 0.58415349 | 2.52E-05 | 0.12 |
| SNX3 | -0.439922 | 0.002495 | -0.558198 | 6.77E-05 | 0.12 |
| NUP85 | 0.7089798 | 5.03E-08 | 0.59080606 | 1.93E-05 | 0.12 |
| BUD13 | 0.6428501 | 1.92E-06 | 0.52522175 | 0.000212 | 0.12 |
| SMC1A | 0.7732954 | 4.78E-10 | 0.65669274 | 9.64E-07 | 0.12 |
| THOC1 | 0.734901 | 9.04E-09 | 0.61838246 | 5.99E-06 | 0.12 |
| SAFB | 0.7521343 | 2.58E-09 | 0.63580721 | 2.69E-06 | 0.12 |
| SRRT | 0.7603303 | 1.37E-09 | 0.6442123 | 1.8E-06 | 0.12 |
| RBBP6 | 0.6623983 | 7.19E-07 | 0.54716362 | 0.0001 | 0.12 |
| ZMAT5 | 0.6737732 | 3.92E-07 | 0.5586423 | 6.66E-05 | 0.12 |
| HNRNPC | 0.8528474 | 1.03E-13 | 0.73821719 | 7.16E-09 | 0.12 |
| RBM15 | 0.7629104 | 1.12E-09 | 0.6482917 | 1.47E-06 | 0.12 |
| RBM5 | 0.4462924 | 0.00212 | 0.56088501 | 6.13E-05 | 0.12 |
| SNRPA | 0.7974794 | 5.49E-11 | 0.68343324 | 2.3E-07 | 0.12 |
| GATAD2A | 0.7292145 | 1.34E-08 | 0.61519616 | 6.89E-06 | 0.12 |
| ERCC6 | 0.4392726 | 0.002537 | 0.55318611 | 8.11E-05 | 0.12 |
| SNUPN | 0.4546132 | 0.001705 | 0.56780421 | 4.74E-05 | 0.12 |
| ILF2 | 0.7107373 | 4.5E-08 | 0.59764735 | 1.46E-05 | 0.12 |
| GATAD2B | 0.6168963 | 6.4E-06 | 0.50439414 | 0.000411 | 0.12 |
| SMARCA4 | 0.6320538 | 3.21E-06 | 0.51962972 | 0.000254 | 0.12 |
| PRPF4 | 0.7816817 | 2.33E-10 | 0.66944302 | 4.95E-07 | 0.12 |
| FXR1 | 0.6392769 | 2.28E-06 | 0.52712377 | 0.000199 | 0.12 |
| SFPQ | 0.8278108 | 2.32E-12 | 0.71572292 | 3.28E-08 | 0.12 |
| BCLAF1 | 0.6809633 | 2.64E-07 | 0.56918286 | 0.000045 | 0.12 |
| ISG20L2 | 0.6237565 | 4.7E-06 | 0.51249079 | 0.000319 | 0.12 |
| CEP85 | 0.6938297 | 1.26E-07 | 0.58312514 | 2.63E-05 | 0.12 |
| WDR74 | 0.6220946 | 5.07E-06 | 0.51171969 | 0.000327 | 0.12 |
| NDC1 | 0.6806503 | 2.68E-07 | 0.57053689 | 4.28E-05 | 0.12 |
| WDR36 | 0.6115989 | 8.07E-06 | 0.50509724 | 0.000402 | 0.11 |
| RPRD2 | 0.6633458 | 6.84E-07 | 0.55685501 | 7.11E-05 | 0.11 |
| FRA10AC1 | 0.6298529 | 3.56E-06 | 0.52363913 | 0.000223 | 0.11 |
| TPR | 0.7658966 | 8.79E-10 | 0.6599016 | 8.18E-07 | 0.11 |
| PARP2 | 0.6079742 | 9.44E-06 | 0.50230928 | 0.000438 | 0.11 |
| EXOSC4 | 0.6478906 | 1.5E-06 | 0.54311298 | 0.000116 | 0.11 |
| FBXO11 | 0.6185805 | 5.93E-06 | 0.51439167 | 0.000301 | 0.11 |
| SRSF5 | 0.6341153 | 2.91E-06 | 0.53003778 | 0.000181 | 0.11 |
| SRSF4 | 0.6553699 | 1.03E-06 | 0.55139242 | 8.65E-05 | 0.11 |
| NUP214 | 0.7744186 | 4.35E-10 | 0.67065429 | 4.64E-07 | 0.11 |
| AKAP17A | 0.6624042 | 7.18E-07 | 0.55865346 | 6.66E-05 | 0.11 |
| KCTD12 | -0.667132 | 5.6E-07 | -0.5634949 | 5.57E-05 | 0.11 |
| ZBTB1 | 0.6075606 | 9.61E-06 | 0.50586655 | 0.000393 | 0.11 |
| PRPF40A | 0.7580809 | 1.63E-09 | 0.65740554 | 9.3E-07 | 0.11 |
| SFSWAP | 0.6519842 | 1.22E-06 | 0.55148857 | 8.62E-05 | 0.11 |
| ZMYND11 | 0.4310943 | 0.003112 | 0.53148785 | 0.000172 | 0.11 |
| MINPP1 | -0.494918 | 0.000548 | -0.5945013 | 1.66E-05 | 0.1 |
| SUV39H1 | 0.6142819 | 7.18E-06 | 0.51471066 | 0.000298 | 0.1 |
| TRRAP | 0.7364076 | 8.13E-09 | 0.63719045 | 2.52E-06 | 0.1 |
| PPP1R35 | 0.6415796 | 2.04E-06 | 0.54241352 | 0.000119 | 0.1 |
| AHSG | -0.599064 | 1.38E-05 | -0.5002216 | 0.000467 | 0.1 |
| BCAS2 | 0.629065 | 3.69E-06 | 0.53037084 | 0.000179 | 0.1 |
| THOC5 | 0.7654397 | 9.12E-10 | 0.66676968 | 5.71E-07 | 0.1 |
| AASDH | 0.4223697 | 0.00385 | 0.52040923 | 0.000248 | 0.1 |
| RBM22 | 0.8025885 | 3.35E-11 | 0.70504257 | 6.42E-08 | 0.1 |
| KAT2A | 0.533423 | 0.000161 | 0.63044736 | 3.46E-06 | 0.1 |
| GTF3C3 | 0.6180668 | 6.07E-06 | 0.5212061 | 0.000242 | 0.1 |
| SRRM1 | 0.7404196 | 6.12E-09 | 0.64384546 | 1.83E-06 | 0.1 |
| ZMYND8 | 0.6345502 | 2.86E-06 | 0.53857623 | 0.000135 | 0.1 |
| POGZ | 0.6223826 | 0.000005 | 0.5266792 | 0.000202 | 0.1 |
| PUF60 | 0.6463725 | 1.62E-06 | 0.55115753 | 8.72E-05 | 0.1 |
| RAD21 | 0.7595051 | 1.46E-09 | 0.66486548 | 6.31E-07 | 0.1 |
| TMEM209 | 0.6911658 | 1.47E-07 | 0.59748748 | 1.47E-05 | 0.1 |
| XRCC6 | 0.6262572 | 4.2E-06 | 0.53274505 | 0.000165 | 0.1 |
| SVEP1 | -0.62192 | 5.11E-06 | -0.5287071 | 0.000189 | 0.1 |
| KDM4A | 0.6214562 | 5.22E-06 | 0.52826487 | 0.000192 | 0.1 |
| ZNF638 | 0.7155627 | 3.31E-08 | 0.62250632 | 4.98E-06 | 0.1 |
| POLR3A | 0.6369735 | 2.55E-06 | 0.54392645 | 0.000113 | 0.1 |
| PLRG1 | 0.6194048 | 5.72E-06 | 0.52657646 | 0.000203 | 0.1 |
| SUDS3 | 0.7144553 | 3.55E-08 | 0.62213403 | 5.06E-06 | 0.1 |
| CDK11A | 0.6197886 | 5.62E-06 | 0.52765385 | 0.000196 | 0.1 |
| SMC3 | 0.6994922 | 9E-08 | 0.60769808 | 9.55E-06 | 0.1 |
| UTP15 | 0.6282565 | 3.83E-06 | 0.53680214 | 0.000144 | 0.1 |
| THOC6 | 0.7181987 | 2.79E-08 | 0.62726772 | 4.01E-06 | 0.1 |
| RIOK1 | 0.5974763 | 1.47E-05 | 0.50657316 | 0.000384 | 0.1 |
| ANKRD54 | 0.6607483 | 7.83E-07 | 0.57019319 | 4.33E-05 | 0.1 |
| GTPBP1 | 0.5921456 | 1.83E-05 | 0.50174966 | 0.000446 | 0.1 |
| API5 | 0.7183431 | 2.76E-08 | 0.62807495 | 3.86E-06 | 0.1 |
| SMIM8 | 0.4161759 | 0.004462 | 0.50598388 | 0.000391 | 0.09 |
| WDR75 | 0.6395614 | 2.25E-06 | 0.55009722 | 9.06E-05 | 0.09 |
| LGALS3 | -0.423148 | 0.003778 | -0.5105298 | 0.00034 | 0.09 |
| DDX21 | 0.604986 | 1.07E-05 | 0.51783881 | 0.000269 | 0.09 |
| TAF5L | 0.63858 | 2.36E-06 | 0.55181708 | 8.52E-05 | 0.09 |
| DDX31 | 0.5892927 | 2.05E-05 | 0.50253228 | 0.000435 | 0.09 |
| DKC1 | 0.6635605 | 6.76E-07 | 0.57696419 | 3.34E-05 | 0.09 |
| HMGB3 | 0.5903938 | 1.96E-05 | 0.5041518 | 0.000414 | 0.09 |
| CHD2 | 0.639238 | 2.29E-06 | 0.55356369 | 0.00008 | 0.09 |
| DCUN1D5 | 0.6047668 | 1.08E-05 | 0.51953731 | 0.000255 | 0.09 |
| EDRF1 | 0.6276449 | 3.94E-06 | 0.54244545 | 0.000118 | 0.09 |
| KPNA6 | 0.4946105 | 0.000553 | 0.57958896 | 3.02E-05 | 0.09 |
| PEX11B | 0.4186022 | 0.004213 | 0.50255153 | 0.000435 | 0.09 |
| WIZ | 0.6380232 | 2.42E-06 | 0.5541463 | 7.84E-05 | 0.09 |
| RNF20 | 0.6642473 | 6.52E-07 | 0.58079459 | 2.88E-05 | 0.09 |
| CIR1 | 0.5927107 | 1.79E-05 | 0.5094077 | 0.000352 | 0.09 |
| FOXJ3 | 0.5974092 | 1.47E-05 | 0.51568884 | 0.000288 | 0.09 |
| XAB2 | 0.7382688 | 7.13E-09 | 0.65707761 | 9.46E-07 | 0.09 |
| NUP35 | 0.6058947 | 1.03E-05 | 0.52470875 | 0.000215 | 0.09 |
| HNRNPA0 | 0.5917707 | 1.86E-05 | 0.51077649 | 0.000337 | 0.09 |
| SMARCB1 | 0.6949192 | 1.18E-07 | 0.77555901 | 3.95E-10 | 0.09 |
| PRPF19 | 0.753119 | 2.39E-09 | 0.67331857 | 4.02E-07 | 0.08 |
| MKRN1 | 0.6215273 | 5.2E-06 | 0.54238456 | 0.000119 | 0.08 |
| AFM | -0.596737 | 1.52E-05 | -0.5178945 | 0.000269 | 0.08 |
| AAAS | 0.6340888 | 2.92E-06 | 0.71274832 | 3.96E-08 | 0.08 |
| PNISR | 0.7067632 | 5.77E-08 | 0.62815998 | 3.85E-06 | 0.08 |
| PPWD1 | 0.6057387 | 1.04E-05 | 0.52727071 | 0.000198 | 0.08 |
| DGCR8 | 0.5805359 | 2.91E-05 | 0.50310233 | 0.000428 | 0.08 |
| SPTY2D1 | 0.6641216 | 6.57E-07 | 0.58700994 | 2.25E-05 | 0.08 |
| CSRP2BP | 0.6036309 | 1.14E-05 | 0.52665687 | 0.000202 | 0.08 |
| EXOSC10 | 0.6406848 | 2.13E-06 | 0.56539001 | 5.19E-05 | 0.08 |
| TF | -0.591156 | 0.000019 | -0.5158758 | 0.000287 | 0.08 |
| RREB1 | 0.4483194 | 0.002012 | 0.52334327 | 0.000225 | 0.08 |
| ATG13 | 0.6026194 | 1.19E-05 | 0.52839355 | 0.000191 | 0.08 |
| TADA2B | 0.5584078 | 6.72E-05 | 0.63228131 | 3.18E-06 | 0.08 |
| PPP1R8 | 0.5968977 | 1.51E-05 | 0.67042874 | 4.7E-07 | 0.08 |
| CDK11B | 0.6949953 | 1.18E-07 | 0.76828767 | 7.24E-10 | 0.08 |
| HNRNPH3 | 0.6268401 | 4.09E-06 | 0.55402262 | 7.87E-05 | 0.08 |
| NUP160 | 0.7077098 | 5.44E-08 | 0.63563624 | 2.71E-06 | 0.08 |
| LDB1 | 0.7048657 | 6.49E-08 | 0.63310465 | 3.06E-06 | 0.08 |
| HNRNPF | 0.7128343 | 3.94E-08 | 0.6411545 | 2.08E-06 | 0.08 |
| NUP210 | 0.7256401 | 1.71E-08 | 0.65434789 | 1.09E-06 | 0.08 |
| YEATS2 | 0.629868 | 3.55E-06 | 0.55861712 | 6.66E-05 | 0.08 |
| RBM10 | 0.7183622 | 2.76E-08 | 0.64721709 | 1.55E-06 | 0.08 |
| DHX15 | 0.6807406 | 2.67E-07 | 0.61009499 | 8.61E-06 | 0.08 |
| TRA2B | 0.7562274 | 1.89E-09 | 0.6860797 | 1.97E-07 | 0.08 |
| MTIF2 | 0.5699896 | 4.37E-05 | 0.50013395 | 0.000468 | 0.07 |
| RNPC3 | 0.7698101 | 6.39E-10 | 0.70054184 | 8.44E-08 | 0.07 |
| KHDRBS1 | 0.7543998 | 2.17E-09 | 0.68598133 | 1.99E-07 | 0.07 |
| DDX50 | 0.6175944 | 6.2E-06 | 0.54995051 | 0.000091 | 0.07 |
| SRSF2 | 0.6395078 | 2.26E-06 | 0.57209567 | 4.03E-05 | 0.07 |
| CELF1 | 0.7072988 | 5.58E-08 | 0.64022523 | 2.18E-06 | 0.07 |
| SERPING1 | -0.571265 | 4.16E-05 | -0.5046072 | 0.000408 | 0.07 |
| GTF3C6 | 0.4454291 | 0.002168 | 0.51161223 | 0.000328 | 0.07 |
| INTS12 | 0.5727399 | 3.93E-05 | 0.5082595 | 0.000365 | 0.07 |
| CCAR1 | 0.729222 | 1.34E-08 | 0.66474205 | 6.36E-07 | 0.07 |
| FUS | 0.5642379 | 5.42E-05 | 0.50034114 | 0.000465 | 0.07 |
| POLR3F | 0.5826788 | 2.67E-05 | 0.64636061 | 1.62E-06 | 0.07 |
| ARL14EP | 0.5720055 | 4.05E-05 | 0.50925268 | 0.000353 | 0.07 |
| MYSM1 | 0.4456812 | 0.002154 | 0.50830539 | 0.000364 | 0.07 |
| DAB2 | -0.587632 | 0.000022 | -0.5263378 | 0.000204 | 0.07 |
| RNF40 | 0.6318843 | 3.24E-06 | 0.57062049 | 4.26E-05 | 0.07 |
| ABT1 | 0.596858 | 1.51E-05 | 0.53568437 | 0.000149 | 0.07 |
| GNAI3 | 0.4762927 | 0.000942 | 0.53742361 | 0.000141 | 0.07 |
| HNRNPA3 | 0.6705494 | 4.67E-07 | 0.60948144 | 8.84E-06 | 0.07 |
| GLE1 | 0.786637 | 1.5E-10 | 0.72632184 | 1.63E-08 | 0.07 |
| SCLT1 | 0.598973 | 1.38E-05 | 0.53887057 | 0.000134 | 0.07 |
| PWP2 | 0.6032389 | 1.15E-05 | 0.5431678 | 0.000116 | 0.07 |
| UBN2 | 0.6134669 | 7.44E-06 | 0.55362905 | 7.98E-05 | 0.06 |
| NXF1 | 0.8072079 | 2.12E-11 | 0.74756416 | 3.63E-09 | 0.06 |
| PTPRF | 0.4581644 | 0.001551 | 0.51779843 | 0.00027 | 0.06 |
| ZMYM4 | 0.6797622 | 2.82E-07 | 0.62025986 | 5.51E-06 | 0.06 |
| CHD1L | 0.6083975 | 9.27E-06 | 0.54896963 | 9.43E-05 | 0.06 |
| NOL8 | 0.6514367 | 1.26E-06 | 0.59230163 | 1.82E-05 | 0.06 |
| FAF1 | 0.635788 | 2.69E-06 | 0.57667169 | 3.38E-05 | 0.06 |
| BUB3 | 0.6730139 | 4.09E-07 | 0.73208486 | 1.1E-08 | 0.06 |
| NUP98 | 0.7733021 | 4.78E-10 | 0.71427 | 3.6E-08 | 0.06 |
| TCF20 | 0.5892709 | 2.06E-05 | 0.53031049 | 0.000179 | 0.06 |
| CCDC94 | 0.6453842 | 1.7E-06 | 0.58661788 | 2.29E-05 | 0.06 |
| SIN3A | 0.6709443 | 4.57E-07 | 0.61218778 | 7.87E-06 | 0.06 |
| SMARCE1 | 0.599191 | 1.37E-05 | 0.54066141 | 0.000126 | 0.06 |
| FUBP3 | 0.6430623 | 1.9E-06 | 0.58466965 | 2.47E-05 | 0.06 |
| DDX17 | 0.6928696 | 1.33E-07 | 0.63467084 | 2.84E-06 | 0.06 |
| ASUN | 0.5744433 | 3.68E-05 | 0.51635197 | 0.000282 | 0.06 |
| RBM12B | 0.6446328 | 1.76E-06 | 0.58670186 | 2.28E-05 | 0.06 |
| DMAP1 | 0.708672 | 5.12E-08 | 0.65107515 | 1.28E-06 | 0.06 |
| POLR3C | 0.7458457 | 4.12E-09 | 0.68826912 | 1.74E-07 | 0.06 |
| DDX51 | 0.7186358 | 2.71E-08 | 0.66127611 | 7.62E-07 | 0.06 |
| MED17 | 0.7894353 | 1.16E-10 | 0.73224061 | 1.09E-08 | 0.06 |
| TAF6L | 0.6499948 | 1.35E-06 | 0.59343453 | 1.74E-05 | 0.06 |
| SF3B3 | 0.8196262 | 5.78E-12 | 0.76436599 | 9.94E-10 | 0.06 |
| MPHOSPH8 | 0.5843122 | 2.51E-05 | 0.52915726 | 0.000186 | 0.06 |
| SESN1 | -0.449029 | 0.001975 | -0.5041275 | 0.000414 | 0.06 |
| FBXL19 | 0.6663517 | 5.84E-07 | 0.61237336 | 7.8E-06 | 0.06 |
| NUP37 | 0.7360956 | 8.31E-09 | 0.68224535 | 2.45E-07 | 0.06 |
| KAT6A | 0.5690572 | 4.52E-05 | 0.51532811 | 0.000292 | 0.06 |
| POLR2H | 0.656413 | 9.78E-07 | 0.60274675 | 1.18E-05 | 0.06 |
| SMARCD2 | 0.7215211 | 2.24E-08 | 0.66819204 | 5.3E-07 | 0.06 |
| DPF2 | 0.5535376 | 8.01E-05 | 0.50037247 | 0.000465 | 0.06 |
| GC | -0.604628 | 1.09E-05 | -0.5516697 | 8.56E-05 | 0.06 |
| RBM6 | 0.6239315 | 4.67E-06 | 0.5712786 | 4.16E-05 | 0.06 |
| HAUS5 | 0.6140824 | 7.24E-06 | 0.56171662 | 5.95E-05 | 0.06 |
| EWSR1 | 0.5717349 | 4.09E-05 | 0.5195815 | 0.000255 | 0.06 |
| PRPF31 | 0.7460732 | 4.06E-09 | 0.69431129 | 1.23E-07 | 0.06 |
| TOP2B | 0.6192055 | 5.77E-06 | 0.56804388 | 0.000047 | 0.06 |
| WDR48 | 0.4782138 | 0.000892 | 0.52764259 | 0.000196 | 0.05 |
| NUP205 | 0.7197938 | 2.51E-08 | 0.67105844 | 4.54E-07 | 0.05 |
| TRA2A | 0.5629123 | 5.69E-05 | 0.51468061 | 0.000298 | 0.05 |
| RPA1 | 0.5693499 | 4.47E-05 | 0.52119929 | 0.000242 | 0.05 |
| FBL | 0.4582599 | 0.001547 | 0.50481465 | 0.000406 | 0.05 |
| NUP107 | 0.7251794 | 1.76E-08 | 0.77154205 | 5.53E-10 | 0.05 |
| RPL35A | 0.5725648 | 3.96E-05 | 0.52688768 | 0.000201 | 0.05 |
| TARDBP | 0.7865138 | 1.52E-10 | 0.74110403 | 5.82E-09 | 0.05 |
| HMG20A | 0.573583 | 3.81E-05 | 0.52818041 | 0.000192 | 0.05 |
| URB1 | 0.6499982 | 1.35E-06 | 0.60460395 | 1.09E-05 | 0.05 |
| MFAP1 | 0.7472742 | 3.71E-09 | 0.70392103 | 6.87E-08 | 0.05 |
| MRPS28 | 0.4726263 | 0.001044 | 0.51471134 | 0.000298 | 0.05 |
| HNRNPD | 0.4900012 | 0.000634 | 0.52993767 | 0.000181 | 0.04 |
| ARHGEF11 | 0.5551205 | 7.57E-05 | 0.5153099 | 0.000292 | 0.04 |
| RANBP2 | 0.686458 | 1.93E-07 | 0.64736967 | 1.54E-06 | 0.04 |
| MAP1A | -0.486559 | 0.000701 | -0.5251162 | 0.000213 | 0.04 |
| IGLL1 | -0.542235 | 0.000119 | -0.5043869 | 0.000411 | 0.04 |
| ZCCHC3 | 0.4687531 | 0.001163 | 0.50593109 | 0.000392 | 0.04 |
| TOMM20 | 0.5778932 | 3.23E-05 | 0.54082238 | 0.000125 | 0.04 |
| DROSHA | 0.5437748 | 0.000113 | 0.50741799 | 0.000374 | 0.04 |
| SRSF11 | 0.6369854 | 2.55E-06 | 0.67319782 | 4.05E-07 | 0.04 |
| HNRNPUL1 | 0.5597069 | 0.000064 | 0.52363139 | 0.000223 | 0.04 |
| PARP1 | 0.5750455 | 0.000036 | 0.54035759 | 0.000127 | 0.04 |
| SRBD1 | 0.5940233 | 1.69E-05 | 0.5596458 | 6.42E-05 | 0.04 |
| WDR18 | 0.5571098 | 7.04E-05 | 0.5236257 | 0.000223 | 0.04 |
| RCC2 | 0.5580476 | 0.000068 | 0.52457218 | 0.000216 | 0.04 |
| SLC25A44 | 0.6038527 | 1.13E-05 | 0.63726382 | 2.51E-06 | 0.04 |
| NUP133 | 0.7415785 | 5.63E-09 | 0.70874148 | 5.1E-08 | 0.04 |
| NUP93 | 0.6857697 | 2.01E-07 | 0.65320264 | 1.15E-06 | 0.04 |
| SAP130 | 0.5820793 | 2.74E-05 | 0.54964817 | 0.000092 | 0.04 |
| PUM1 | 0.5663859 | 0.00005 | 0.53496321 | 0.000153 | 0.04 |
| TRIM8 | 0.4904392 | 0.000626 | 0.5217331 | 0.000237 | 0.04 |
| NUPL2 | 0.5414905 | 0.000122 | 0.5107675 | 0.000337 | 0.04 |
| DDX39B | 0.5406875 | 0.000126 | 0.51042818 | 0.000341 | 0.04 |
| KIAA0368 | 0.4981823 | 0.000497 | 0.52703148 | 0.0002 | 0.03 |
| POLR1C | 0.5769043 | 3.35E-05 | 0.54877336 | 9.49E-05 | 0.03 |
| METTL9 | 0.4811398 | 0.00082 | 0.50873777 | 0.000359 | 0.03 |
| NUP155 | 0.6297929 | 3.57E-06 | 0.60244307 | 1.19E-05 | 0.03 |
| GTF3C5 | 0.5306102 | 0.000177 | 0.5032842 | 0.000425 | 0.03 |
| TIA1 | 0.5561454 | 7.29E-05 | 0.5289231 | 0.000187 | 0.03 |
| XRCC5 | 0.5759445 | 3.48E-05 | 0.54883799 | 9.47E-05 | 0.03 |
| RUVBL2 | 0.5467332 | 0.000102 | 0.52005531 | 0.000251 | 0.03 |
| ZNF646 | 0.629438 | 3.63E-06 | 0.60310381 | 1.16E-05 | 0.03 |
| POLR3B | 0.6283454 | 3.81E-06 | 0.60361088 | 1.14E-05 | 0.03 |
| ATXN7L3 | 0.639747 | 2.23E-06 | 0.66417819 | 6.55E-07 | 0.03 |
| OFD1 | 0.5689884 | 4.54E-05 | 0.59323333 | 1.75E-05 | 0.03 |
| NFYC | 0.4833678 | 0.00077 | 0.5071765 | 0.000377 | 0.03 |
| KAT5 | 0.4820093 | 0.0008 | 0.50557067 | 0.000396 | 0.03 |
| TOR2A | 0.5504896 | 8.93E-05 | 0.52756285 | 0.000196 | 0.03 |
| PSMD14 | 0.5541627 | 7.83E-05 | 0.53177133 | 0.00017 | 0.03 |
| LRRC47 | 0.5027268 | 0.000433 | 0.52312171 | 0.000227 | 0.03 |
| NSMCE1 | 0.5968529 | 1.51E-05 | 0.5767165 | 3.38E-05 | 0.03 |
| EP400 | 0.7421647 | 5.39E-09 | 0.72307891 | 2.02E-08 | 0.02 |
| INTS4 | 0.5481044 | 9.72E-05 | 0.52998944 | 0.000181 | 0.02 |
| PTPRM | -0.576581 | 3.39E-05 | -0.5946483 | 1.65E-05 | 0.02 |
| SMPD4 | 0.5232537 | 0.000226 | 0.50558752 | 0.000396 | 0.02 |
| SRSF6 | 0.5621614 | 5.85E-05 | 0.54492186 | 0.000109 | 0.02 |
| TARS2 | 0.4858686 | 0.000716 | 0.50191965 | 0.000443 | 0.02 |
| SUPT7L | 0.6036256 | 1.14E-05 | 0.61961122 | 5.67E-06 | 0.02 |
| NPHP3 | -0.500624 | 0.000461 | -0.5165763 | 0.00028 | 0.02 |
| CRNKL1 | 0.6525311 | 1.19E-06 | 0.63734504 | 2.5E-06 | 0.02 |
| TOMM22 | 0.540558 | 0.000126 | 0.55503739 | 7.59E-05 | 0.02 |
| SEH1L | 0.5857929 | 2.36E-05 | 0.57133957 | 4.15E-05 | 0.02 |
| ARID1A | 0.6814645 | 2.56E-07 | 0.66762969 | 5.46E-07 | 0.02 |
| MAD1L1 | 0.7241199 | 1.89E-08 | 0.7112694 | 4.35E-08 | 0.02 |
| PCGF6 | 0.6010441 | 1.27E-05 | 0.58824107 | 2.14E-05 | 0.02 |
| FBXL4 | 0.492778 | 0.000584 | 0.50548522 | 0.000397 | 0.02 |
| HNRNPR | 0.7164576 | 3.12E-08 | 0.70399397 | 6.84E-08 | 0.02 |
| EIF3K | 0.568356 | 4.64E-05 | 0.55614136 | 7.29E-05 | 0.02 |
| NOL9 | 0.5766377 | 3.39E-05 | 0.58846166 | 2.12E-05 | 0.02 |
| PCGF1 | 0.4985445 | 0.000491 | 0.50989634 | 0.000346 | 0.02 |
| TOE1 | 0.5386181 | 0.000135 | 0.52837391 | 0.000191 | 0.02 |
| NOP58 | 0.5238211 | 0.000222 | 0.53378743 | 0.000159 | 0.01 |
| ZBTB9 | 0.5513167 | 8.67E-05 | 0.56097815 | 6.11E-05 | 0.01 |
| AGO4 | 0.4994922 | 0.000477 | 0.50857721 | 0.000361 | 0.01 |
| TP63 | -0.565922 | 5.09E-05 | -0.5748387 | 3.63E-05 | 0.01 |
| TIMM22 | 0.4951253 | 0.000545 | 0.50321128 | 0.000426 | 0.01 |
| SMU1 | 0.7931538 | 8.26E-11 | 0.78536139 | 1.68E-10 | 0.01 |
| LBH | -0.548392 | 9.62E-05 | -0.554797 | 7.66E-05 | 0.01 |
| NOP56 | 0.538715 | 0.000135 | 0.54485985 | 0.000109 | 0.01 |
| PHF21A | 0.5636709 | 5.53E-05 | 0.56951935 | 4.45E-05 | 0.01 |
| KLHL20 | 0.5143233 | 0.000301 | 0.52002654 | 0.000251 | 0.01 |
| URB2 | 0.6113758 | 8.15E-06 | 0.6059152 | 1.03E-05 | 0.01 |
| XRN2 | 0.5274667 | 0.000197 | 0.53199469 | 0.000169 | 0.01 |
| USP48 | 0.5088895 | 0.000357 | 0.50622804 | 0.000388 | 0.01 |
| TFAP4 | 0.5180828 | 0.000267 | 0.52063289 | 0.000246 | 0.01 |
| NCOA5 | 0.5436473 | 0.000114 | 0.54502321 | 0.000108 | 0.01 |
| ING3 | 0.5396094 | 0.000131 | 0.54079969 | 0.000125 | 0.01 |
| USP20 | 0.5971261 | 1.49E-05 | 0.59614168 | 1.55E-05 | 0.01 |
| TCHP | 0.6312981 | 3.33E-06 | 0.63100064 | 3.37E-06 | 0.01 |
