# Supplementary material for: SNRPD1 conveys prognostic value on breast cancer survival and is required for anthracycline sensitivity
Source: BMC Cancer. 2023 Apr 25;23:376. doi: 10.1186/s12885-023-10860-z (PMC10126993; doi:10.1186/s12885-023-10860-z)
Supplement: Supplementary file 10 — Additional file 10: Supplementary Table 10. Genes differentially correlated with SNRPD1 and SNRPE using both TCGA gene expression and MS protein expression data. [file 12885_2023_10860_MOESM10_ESM.docx]

**Supplementary Table 10. Genes differentially correlated with SNRPD1 and SNRPE using both TCGA gene expression and MS protein expression data.**

|  | ***SNRPD1*** |  | ***SNRPE*** |  |  |  |
| --- | --- | --- | --- | --- | --- | --- |
|  | **Correlation** | ***p*** | **Correlation** | ***p*** | **abs(sub)** |  |
| SUZ12 | 0.806531 | 2.27E-11 | 0.4780874 | 0.000895 | 0.33 | protein |
| SKA1 | 0.640138 | 4.63E-127 | 0.2996091 | 4.17E-24 | 0.35 | gene |
| NDC80 | 0.628276 | 4.34E-121 | 0.3185312 | 3.39E-27 | 0.31 | gene |
| FAM136A | 0.622391 | 3.19E-118 | 0.3131845 | 2.67E-26 | 0.31 | gene |
| CDC20 | 0.615365 | 7.06E-115 | 0.3246075 | 3.09E-28 | 0.3 | gene |
| **CENPA** | **0.613253** | **6.88E-114** | **0.3005791** | **2.93E-24** | **0.32** | gene |
| LOC115567 | 0.601697 | 1.31E-108 | 0.2187939 | 2.59E-13 | 0.39 | gene |
| LOC116044 | 0.589817 | 2.12E-103 | 0.2955372 | 1.80E-23 | 0.3 | gene |
| SEH1L | 0.586253 | 7.02E-102 | 0.2589203 | 3.35E-18 | 0.33 | gene |
| PTPN2 | 0.586055 | 8.51E-102 | 0.2022278 | 1.50E-11 | 0.39 | gene |
| CDC25A | 0.579705 | 3.90E-99 | 0.2746209 | 2.31E-20 | 0.31 | gene |
| ORC6 | 0.578132 | 1.74E-98 | 0.2551968 | 1.04E-17 | 0.33 | gene |
| AUNIP | 0.56938 | 6.23E-95 | 0.2489202 | 6.72E-17 | 0.33 | gene |
| KIF2C | 0.561173 | 1.08E-91 | 0.2697455 | 1.12E-19 | 0.3 | gene |
| CCNE1 | 0.556533 | 6.63E-90 | 0.2441909 | 2.65E-16 | 0.32 | gene |
| RAD54L | 0.556419 | 7.33E-90 | 0.241273 | 6.10E-16 | 0.32 | gene |
| CDCA8 | 0.554885 | 2.82E-89 | 0.2527197 | 2.18E-17 | 0.31 | gene |
| PIF1 | 0.552891 | 1.61E-88 | 0.2554611 | 9.59E-18 | 0.3 | gene |
| **CENPN** | **0.542139** | **1.58E-84** | **0.2520665** | **2.65E-17** | **0.3** | gene |
| MPP.2 | 0.541596 | 2.50E-84 | 0.2447489 | 2.26E-16 | 0.3 | gene |
| LOC115722 | 0.53698 | 1.16E-82 | 0.1799803 | 2.07E-09 | 0.36 | gene |
| KIF18B | 0.512706 | 2.62E-74 | 0.2225383 | 9.89E-14 | 0.3 | gene |
| C18ORF8 | 0.503719 | 2.21E-71 | 0.1130453 | 0.00018 | 0.4 | gene |
| TICRR | 0.500981 | 1.65E-70 | 0.1975684 | 4.42E-11 | 0.31 | gene |
| RALGPS2 | -0.500465 | 2.41E-70 | -0.155349 | 2.45E-07 | 0.35 | gene |
| NOSTRIN | -0.505033 | 8.34E-72 | -0.155699 | 2.30E-07 | 0.35 | gene |
| FAM214A | -0.524376 | 3.06E-78 | -0.218284 | 2.95E-13 | 0.31 | gene |
| PRICKLE2 | -0.539509 | 1.43E-83 | -0.240221 | 8.22E-16 | 0.3 | gene |
| GALNT10 | -0.562859 | 2.37E-92 | -0.252343 | 2.44E-17 | 0.32 | gene |
| KIAA0825 | -0.564546 | 5.16E-93 | -0.246432 | 1.39E-16 | 0.32 | gene |
