# Supplementary material for: SNRPD1 conveys prognostic value on breast cancer survival and is required for anthracycline sensitivity
Source: BMC Cancer. 2023 Apr 25;23:376. doi: 10.1186/s12885-023-10860-z (PMC10126993; doi:10.1186/s12885-023-10860-z)
Supplement: Supplementary file 11 — Additional file 11: Supplementary Table 11. Protein-protein interactions among genes differentially correlated with SNRPD1 and SNRPE. Protein-protein interactions were constructed using STRING. [file 12885_2023_10860_MOESM11_ESM.docx]

**Supplementary Table 11. Protein-protein interactions among genes differentially correlated with SNRPD1 and SNRPE. Protein-protein interactions were constructed using STRING.**

| **Node1** | **Node2** | **Node1_string_internal_id** | **Node2_string_internal_id** | **Node1_external_id** | **Node2_external_id** | **Neighborhood_on_chromosome** | **Gene_fusion** | **Phylogenetic_cooccurrence** | **Homology** | | **Coexpression** | **Experimentally_determined_interaction** | **Database_annotated** | **Automated_textmining** | **Combined_score** |
| --- | --- | --- | --- | --- | --- | --- | --- | --- | --- | --- | --- | --- | --- | --- | --- |
| AUNIP | KIF2C | 4450090 | 4443901 | 9606.ENSP00000443647 | 9606.ENSP00000361298 | 0 | 0 | 0 | 0 | 0.562 | | 0 | 0 | 0 | 0.562 |
| CCNE1 | ORC6 | 4435118 | 4433062 | 9606.ENSP00000262643 | 9606.ENSP00000219097 | 0 | 0 | 0 | 0 | 0.297 | | 0 | 0.9 | 0.307 | 0.947 |
| CDC20 | NDC80 | 4443940 | 4434910 | 9606.ENSP00000361540 | 9606.ENSP00000261597 | 0 | 0 | 0 | 0 | 0.975 | | 0 | 0.9 | 0.687 | 0.999 |
| CDC20 | KIF2C | 4443940 | 4443901 | 9606.ENSP00000361540 | 9606.ENSP00000361298 | 0 | 0 | 0 | 0 | 0.958 | | 0.078 | 0.9 | 0.579 | 0.998 |
| CDC20 | CENPA | 4443940 | 4440745 | 9606.ENSP00000361540 | 9606.ENSP00000336868 | 0 | 0 | 0 | 0 | 0.781 | | 0.091 | 0.9 | 0.581 | 0.99 |
| CDC20 | SKA1 | 4443940 | 4436620 | 9606.ENSP00000361540 | 9606.ENSP00000285116 | 0 | 0 | 0 | 0 | 0.811 | | 0 | 0.9 | 0.293 | 0.985 |
| CDC20 | CDC25A | 4443940 | 4437985 | 9606.ENSP00000361540 | 9606.ENSP00000303706 | 0 | 0 | 0 | 0 | 0.575 | | 0.43 | 0 | 0.593 | 0.893 |
| CDC20 | CCNE1 | 4443940 | 4435118 | 9606.ENSP00000361540 | 9606.ENSP00000262643 | 0 | 0 | 0 | 0 | 0.388 | | 0.185 | 0 | 0.503 | 0.73 |
| CDC20 | RAD54L | 4443940 | 4443854 | 9606.ENSP00000361540 | 9606.ENSP00000361043 | 0 | 0 | 0 | 0 | 0.594 | | 0.085 | 0 | 0.294 | 0.715 |
| CDC20 | PIF1 | 4443940 | 4439990 | 9606.ENSP00000361540 | 9606.ENSP00000328174 | 0 | 0 | 0 | 0 | 0.522 | | 0.11 | 0 | 0.223 | 0.641 |
| CDC20 | ORC6 | 4443940 | 4433062 | 9606.ENSP00000361540 | 9606.ENSP00000219097 | 0 | 0 | 0 | 0 | 0.315 | | 0 | 0 | 0.374 | 0.553 |
| CDC20 | TICRR | 4443940 | 4435810 | 9606.ENSP00000361540 | 9606.ENSP00000268138 | 0 | 0 | 0 | 0 | 0.433 | | 0 | 0 | 0.128 | 0.485 |
| CDC25A | CCNE1 | 4437985 | 4435118 | 9606.ENSP00000303706 | 9606.ENSP00000262643 | 0 | 0 | 0 | 0 | 0.422 | | 0.472 | 0.9 | 0.715 | 0.99 |
| CDC25A | TICRR | 4437985 | 4435810 | 9606.ENSP00000303706 | 9606.ENSP00000268138 | 0 | 0 | 0 | 0 | 0.542 | | 0 | 0 | 0.256 | 0.645 |
| CDCA8 | CDC20 | 4444097 | 4443940 | 9606.ENSP00000362146 | 9606.ENSP00000361540 | 0 | 0 | 0 | 0 | 0.988 | | 0 | 0.9 | 0.599 | 0.999 |
| CDCA8 | NDC80 | 4444097 | 4434910 | 9606.ENSP00000362146 | 9606.ENSP00000261597 | 0 | 0 | 0 | 0 | 0.96 | | 0 | 0.9 | 0.567 | 0.998 |
| CDCA8 | KIF2C | 4444097 | 4443901 | 9606.ENSP00000362146 | 9606.ENSP00000361298 | 0 | 0 | 0 | 0 | 0.931 | | 0.114 | 0.9 | 0.658 | 0.997 |
| CDCA8 | CENPA | 4444097 | 4440745 | 9606.ENSP00000362146 | 9606.ENSP00000336868 | 0 | 0 | 0 | 0 | 0.793 | | 0 | 0.9 | 0.627 | 0.991 |
| CDCA8 | SKA1 | 4444097 | 4436620 | 9606.ENSP00000362146 | 9606.ENSP00000285116 | 0 | 0 | 0 | 0 | 0.761 | | 0 | 0.9 | 0.415 | 0.984 |
| CDCA8 | RAD54L | 4444097 | 4443854 | 9606.ENSP00000362146 | 9606.ENSP00000361043 | 0 | 0 | 0 | 0 | 0.809 | | 0 | 0 | 0.25 | 0.851 |
| CDCA8 | CDC25A | 4444097 | 4437985 | 9606.ENSP00000362146 | 9606.ENSP00000303706 | 0 | 0 | 0 | 0 | 0.579 | | 0 | 0 | 0.162 | 0.632 |
| CDCA8 | TICRR | 4444097 | 4435810 | 9606.ENSP00000362146 | 9606.ENSP00000268138 | 0 | 0 | 0 | 0 | 0.583 | | 0 | 0 | 0.093 | 0.606 |
| CDCA8 | CCNE1 | 4444097 | 4435118 | 9606.ENSP00000362146 | 9606.ENSP00000262643 | 0 | 0 | 0 | 0 | 0.306 | | 0 | 0 | 0.314 | 0.503 |
| CDCA8 | ORC6 | 4444097 | 4433062 | 9606.ENSP00000362146 | 9606.ENSP00000219097 | 0 | 0 | 0 | 0 | 0.248 | | 0 | 0 | 0.284 | 0.439 |
| CENPA | NDC80 | 4440745 | 4434910 | 9606.ENSP00000336868 | 9606.ENSP00000261597 | 0 | 0 | 0 | 0 | 0.788 | | 0.166 | 0.9 | 0.721 | 0.994 |
| CENPA | SKA1 | 4440745 | 4436620 | 9606.ENSP00000336868 | 9606.ENSP00000285116 | 0 | 0 | 0 | 0 | 0.571 | | 0 | 0.9 | 0.53 | 0.978 |
| CENPA | CDC25A | 4440745 | 4437985 | 9606.ENSP00000336868 | 9606.ENSP00000303706 | 0 | 0 | 0 | 0 | 0.531 | | 0 | 0 | 0.268 | 0.642 |
| CENPA | ORC6 | 4440745 | 4433062 | 9606.ENSP00000336868 | 9606.ENSP00000219097 | 0 | 0 | 0 | 0 | 0.232 | | 0 | 0 | 0.403 | 0.522 |
| CENPA | CCNE1 | 4440745 | 4435118 | 9606.ENSP00000336868 | 9606.ENSP00000262643 | 0 | 0 | 0 | 0 | 0.23 | | 0.126 | 0 | 0.293 | 0.483 |
| CENPN | CENPA | 4446136 | 4440745 | 9606.ENSP00000377007 | 9606.ENSP00000336868 | 0 | 0 | 0 | 0 | 0.68 | | 0.837 | 0.9 | 0.851 | 0.999 |
| CENPN | NDC80 | 4446136 | 4434910 | 9606.ENSP00000377007 | 9606.ENSP00000261597 | 0 | 0 | 0 | 0 | 0.837 | | 0 | 0.9 | 0.531 | 0.991 |
| CENPN | CDC20 | 4446136 | 4443940 | 9606.ENSP00000377007 | 9606.ENSP00000361540 | 0 | 0 | 0 | 0 | 0.838 | | 0 | 0.9 | 0.299 | 0.987 |
| CENPN | CDCA8 | 4446136 | 4444097 | 9606.ENSP00000377007 | 9606.ENSP00000362146 | 0 | 0 | 0 | 0 | 0.822 | | 0 | 0.9 | 0.3 | 0.986 |
| CENPN | KIF2C | 4446136 | 4443901 | 9606.ENSP00000377007 | 9606.ENSP00000361298 | 0 | 0 | 0 | 0 | 0.748 | | 0 | 0.9 | 0.41 | 0.983 |
| CENPN | SKA1 | 4446136 | 4436620 | 9606.ENSP00000377007 | 9606.ENSP00000285116 | 0 | 0 | 0 | 0 | 0.506 | | 0 | 0.9 | 0.434 | 0.969 |
| CENPN | RAD54L | 4446136 | 4443854 | 9606.ENSP00000377007 | 9606.ENSP00000361043 | 0 | 0 | 0 | 0 | 0.378 | | 0 | 0 | 0.356 | 0.582 |
| KIF18B | KIF2C | 4450792 | 4443901 | 9606.ENSP00000465992 | 9606.ENSP00000361298 | 0 | 0 | 0 | 0.662 | 0.827 | | 0.613 | 0.9 | 0.82 | 0.994 |
| KIF18B | CDC20 | 4450792 | 4443940 | 9606.ENSP00000465992 | 9606.ENSP00000361540 | 0 | 0 | 0 | 0 | 0.895 | | 0.185 | 0 | 0.099 | 0.916 |
| KIF18B | CDCA8 | 4450792 | 4444097 | 9606.ENSP00000465992 | 9606.ENSP00000362146 | 0 | 0 | 0 | 0 | 0.869 | | 0 | 0 | 0.15 | 0.884 |
| KIF18B | NDC80 | 4450792 | 4434910 | 9606.ENSP00000465992 | 9606.ENSP00000261597 | 0 | 0 | 0 | 0 | 0.527 | | 0.269 | 0 | 0.243 | 0.715 |
| KIF18B | CENPA | 4450792 | 4440745 | 9606.ENSP00000465992 | 9606.ENSP00000336868 | 0 | 0 | 0 | 0 | 0.545 | | 0.08 | 0 | 0.167 | 0.621 |
| KIF18B | CENPN | 4450792 | 4446136 | 9606.ENSP00000465992 | 9606.ENSP00000377007 | 0 | 0 | 0 | 0 | 0.554 | | 0 | 0 | 0.065 | 0.565 |
| KIF18B | SKA1 | 4450792 | 4436620 | 9606.ENSP00000465992 | 9606.ENSP00000285116 | 0 | 0 | 0 | 0 | 0.334 | | 0 | 0 | 0.33 | 0.535 |
| KIF2C | NDC80 | 4443901 | 4434910 | 9606.ENSP00000361298 | 9606.ENSP00000261597 | 0 | 0 | 0 | 0 | 0.917 | | 0.18 | 0.9 | 0.691 | 0.997 |
| KIF2C | CENPA | 4443901 | 4440745 | 9606.ENSP00000361298 | 9606.ENSP00000336868 | 0 | 0 | 0 | 0 | 0.818 | | 0.08 | 0.9 | 0.67 | 0.993 |
| KIF2C | SKA1 | 4443901 | 4436620 | 9606.ENSP00000361298 | 9606.ENSP00000285116 | 0 | 0 | 0 | 0 | 0.623 | | 0 | 0.9 | 0.578 | 0.982 |
| KIF2C | RAD54L | 4443901 | 4443854 | 9606.ENSP00000361298 | 9606.ENSP00000361043 | 0 | 0 | 0 | 0 | 0.803 | | 0.056 | 0 | 0.197 | 0.837 |
| KIF2C | CDC25A | 4443901 | 4437985 | 9606.ENSP00000361298 | 9606.ENSP00000303706 | 0 | 0 | 0 | 0 | 0.592 | | 0.087 | 0 | 0.174 | 0.666 |
| KIF2C | TICRR | 4443901 | 4435810 | 9606.ENSP00000361298 | 9606.ENSP00000268138 | 0 | 0 | 0 | 0 | 0.601 | | 0 | 0 | 0.082 | 0.619 |
| KIF2C | CCNE1 | 4443901 | 4435118 | 9606.ENSP00000361298 | 9606.ENSP00000262643 | 0 | 0 | 0 | 0 | 0.322 | | 0.15 | 0 | 0.194 | 0.495 |
| NDC80 | ORC6 | 4434910 | 4433062 | 9606.ENSP00000261597 | 9606.ENSP00000219097 | 0 | 0 | 0 | 0 | 0.604 | | 0 | 0 | 0.268 | 0.698 |
| RAD54L | CDC25A | 4443854 | 4437985 | 9606.ENSP00000361043 | 9606.ENSP00000303706 | 0 | 0 | 0 | 0 | 0.561 | | 0 | 0 | 0.39 | 0.721 |
| RAD54L | CENPA | 4443854 | 4440745 | 9606.ENSP00000361043 | 9606.ENSP00000336868 | 0 | 0 | 0 | 0 | 0.548 | | 0.176 | 0 | 0.175 | 0.666 |
| RAD54L | PIF1 | 4443854 | 4439990 | 9606.ENSP00000361043 | 9606.ENSP00000328174 | 0.052 | 0 | 0 | 0 | 0.135 | | 0.26 | 0 | 0.427 | 0.606 |
| RAD54L | TICRR | 4443854 | 4435810 | 9606.ENSP00000361043 | 9606.ENSP00000268138 | 0 | 0 | 0 | 0 | 0.571 | | 0 | 0 | 0 | 0.571 |
| RAD54L | NDC80 | 4443854 | 4434910 | 9606.ENSP00000361043 | 9606.ENSP00000261597 | 0.046 | 0 | 0 | 0 | 0.415 | | 0.056 | 0 | 0.056 | 0.436 |
| RAD54L | CCNE1 | 4443854 | 4435118 | 9606.ENSP00000361043 | 9606.ENSP00000262643 | 0 | 0 | 0 | 0 | 0.244 | | 0.157 | 0 | 0.145 | 0.408 |
| RAD54L | ORC6 | 4443854 | 4433062 | 9606.ENSP00000361043 | 9606.ENSP00000219097 | 0 | 0 | 0 | 0 | 0.244 | | 0 | 0 | 0.241 | 0.401 |
| SEH1L | PTPN2 | 4446956 | 4438733 | 9606.ENSP00000382779 | 9606.ENSP00000311857 | 0 | 0 | 0 | 0 | 0.088 | | 0 | 0 | 0.487 | 0.512 |
| SKA1 | NDC80 | 4436620 | 4434910 | 9606.ENSP00000285116 | 9606.ENSP00000261597 | 0 | 0 | 0 | 0 | 0.876 | | 0.354 | 0.9 | 0.701 | 0.997 |
| SKA1 | ORC6 | 4436620 | 4433062 | 9606.ENSP00000285116 | 9606.ENSP00000219097 | 0 | 0 | 0 | 0 | 0.343 | | 0 | 0 | 0.291 | 0.514 |
| TICRR | ORC6 | 4435810 | 4433062 | 9606.ENSP00000268138 | 9606.ENSP00000219097 | 0 | 0 | 0 | 0 | 0.124 | | 0 | 0 | 0.478 | 0.523 |
| TICRR | NDC80 | 4435810 | 4434910 | 9606.ENSP00000268138 | 9606.ENSP00000261597 | 0 | 0 | 0 | 0 | 0.428 | | 0 | 0 | 0 | 0.428 |
