# Supplementary material for: SNRPD1 conveys prognostic value on breast cancer survival and is required for anthracycline sensitivity
Source: BMC Cancer. 2023 Apr 25;23:376. doi: 10.1186/s12885-023-10860-z (PMC10126993; doi:10.1186/s12885-023-10860-z)
Supplement: Supplementary file 12 — Additional file 12: Supplementary Figure 1. Work flow and logic of this study. This study is comprised of investigations on the ‘phenomenon’ of SNRPD1 and SNRPE relevant to breast cancer prognosis and therapeutics, and ‘mechanism’ capable of explaining the observed phenomenon. In each set of investigations, both in silico dry lab analysis and in vitro wet lab experiments were conducted. ‘Green’ and ‘purple’ each represents dry lab analysis and wet lab assays conducted for SNRPD1 and SNRPE, respectively. ‘Black’ represents the analysis or assays. Statements in the brackets are conclusions drawn on SNRPD1 (‘green’) or SNRPE (‘purple’) from the corresponding series of analysis or experiments. [file 12885_2023_10860_MOESM12_ESM.docx]

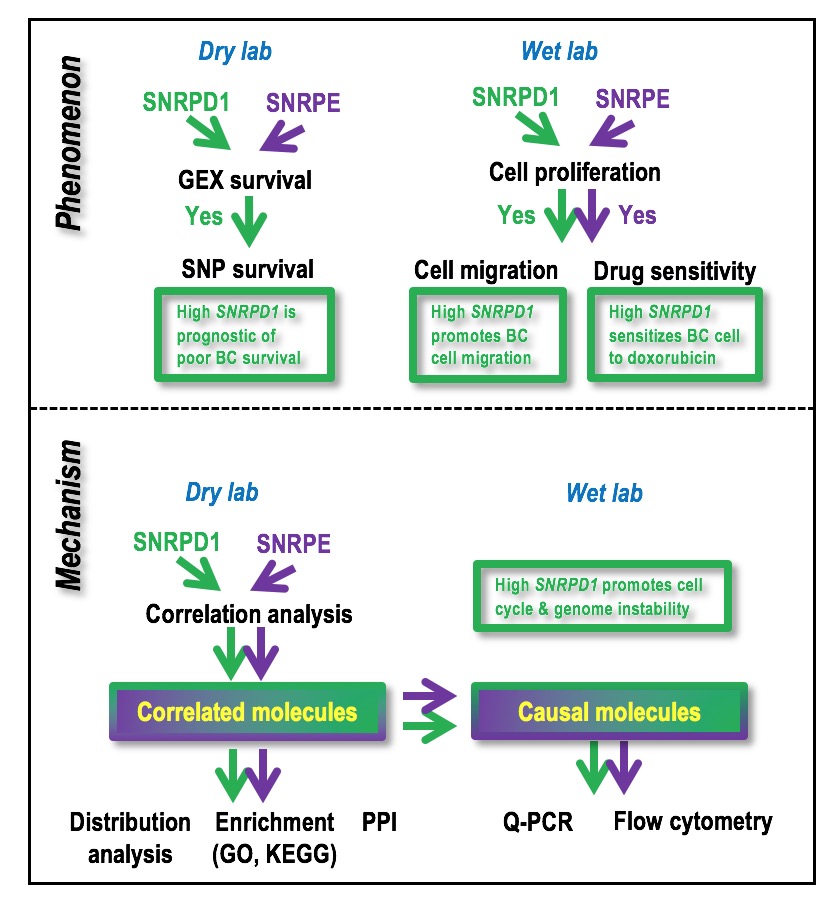
**Supplementary figures**

## Supplementary Figure 1. Work flow and logic of this study. This study is comprised of investigations on the ‘phenomenon’ of SNRPD1 and SNRPE relevant to breast cancer prognosis and therapeutics, and ‘mechanism’ capable of explaining the observed phenomenon. In each set of investigations, both *in silico* dry lab analysis and *in vitro* wet lab experiments were conducted. ‘Green’ and ‘purple’ each represents dry lab analysis and wet lab assays conducted for SNRPD1 and SNRPE, respectively. ‘Black’ represents the analysis or assays. Statements in the brackets are conclusions drawn on SNRPD1 (‘green’) or SNRPE (‘purple’) from the corresponding series of analysis or experiments.


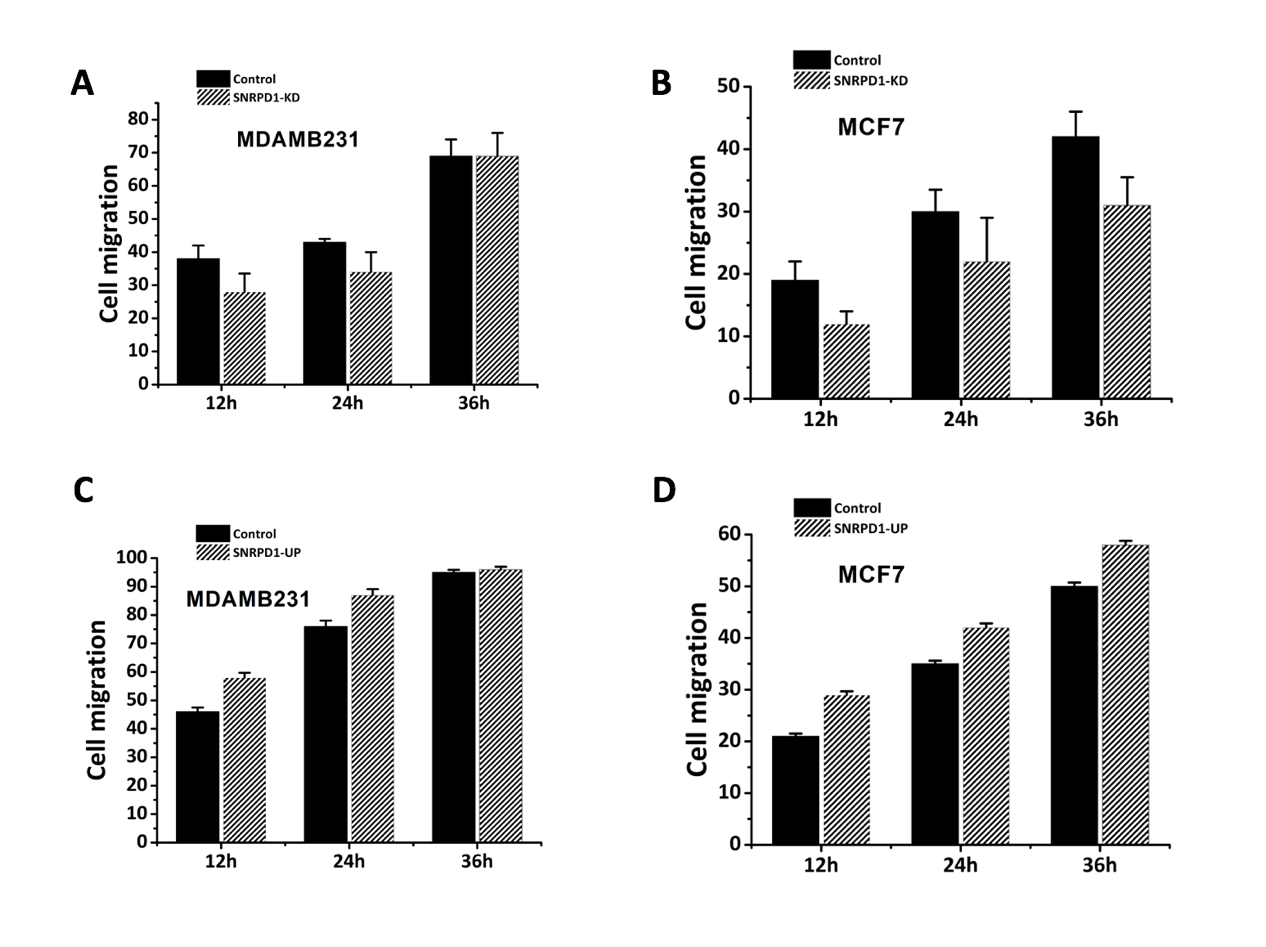


**Supplementary Figure 2. Cell migration after modulating *SNRPD1* at different time points in MDAMB231 and MCF7 cells. (A)** Knocking down *SNRPD1*, **(B)** Over-expressing *SNRPD1*.


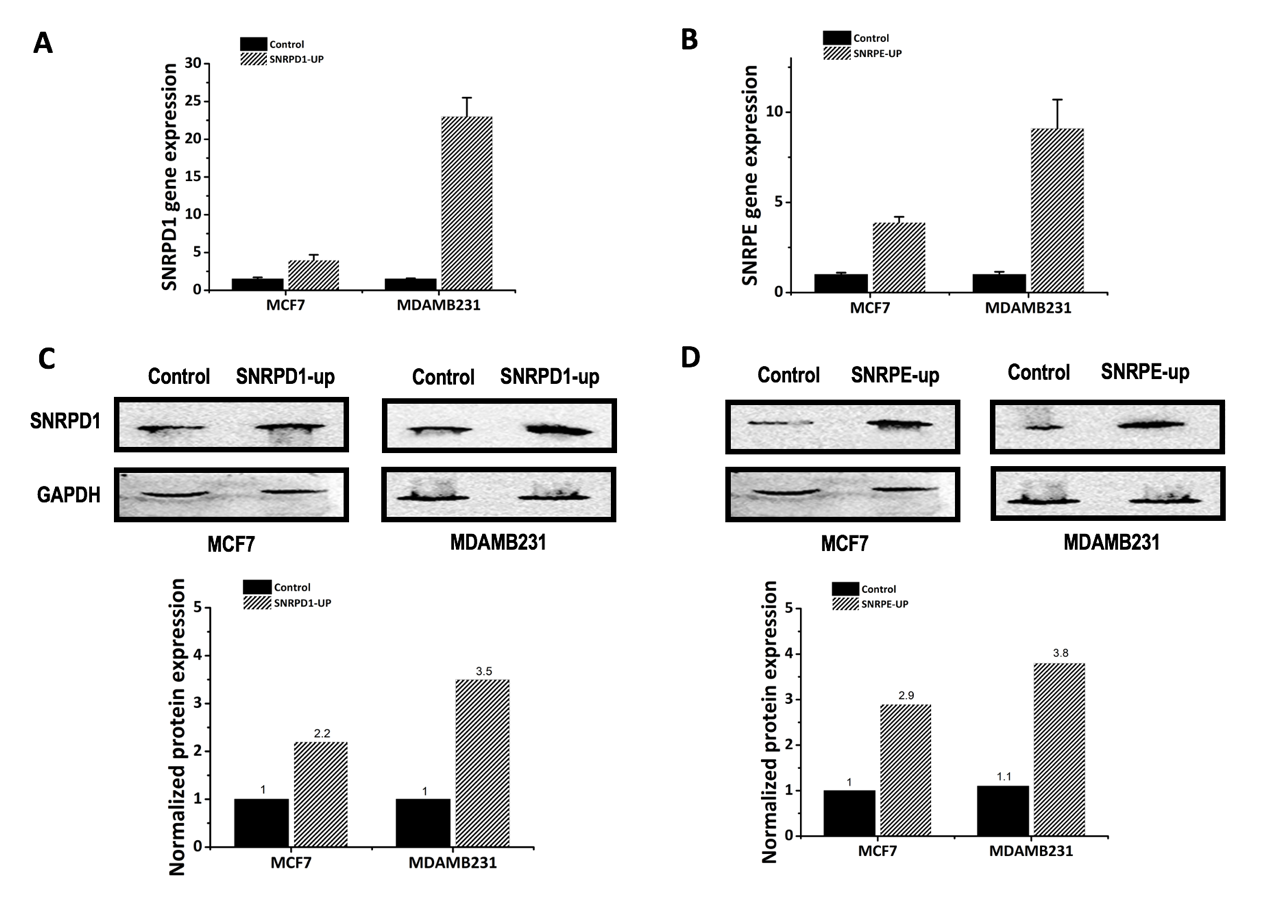


**Supplementary Figure 3. The efficiency of over-expressing SNRPD1 or SNRPE in MDAMB231 and MCF7 cells. (A)** Over-expressing SNRPD1 or **(B)** SNRPE as tested at the transcriptional level. **(C)** Over-expressing SNRPD1 or **(D)** SNRPE as tested at the translational level.
