# Supplementary material for: SNRPD1 conveys prognostic value on breast cancer survival and is required for anthracycline sensitivity
Source: BMC Cancer. 2023 Apr 25;23:376. doi: 10.1186/s12885-023-10860-z (PMC10126993; doi:10.1186/s12885-023-10860-z)
Supplement: Supplementary file 13 — Additional file 13: Supplementary Figure 2. Cell migration after modulating SNRPD1 at different time points in MDAMB231 and MCF7 cells. (A) Knocking down SNRPD1, (B) Over-expressing SNRPD1. [file 12885_2023_10860_MOESM13_ESM.docx]

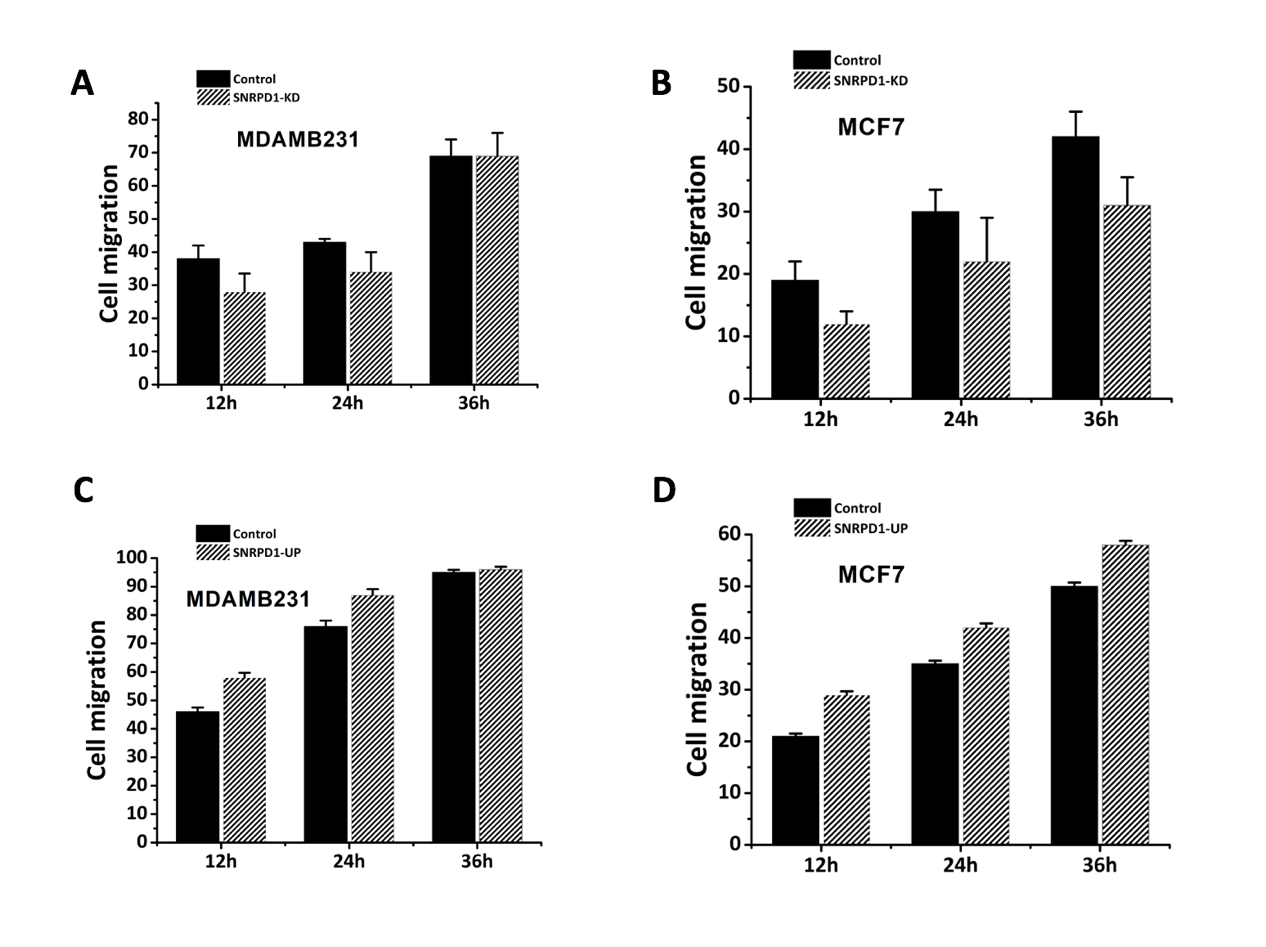


**Supplementary Figure 2. Cell migration after modulating *SNRPD1* at different time points in MDAMB231 and MCF7 cells. (A)** Knocking down *SNRPD1*, **(B)** Over-expressing *SNRPD1*.
