# Supplementary material for: SNRPD1 conveys prognostic value on breast cancer survival and is required for anthracycline sensitivity
Source: BMC Cancer. 2023 Apr 25;23:376. doi: 10.1186/s12885-023-10860-z (PMC10126993; doi:10.1186/s12885-023-10860-z)
Supplement: Supplementary file 14 — Additional file 14: Supplementary Figure 3. The efficiency of over-expressing SNRPD1 or SNRPE in MDAMB231 and MCF7 cells. (A) Over-expressing SNRPD1 or (B) SNRPE as tested at the mRNA level. (C) Over-expressing SNRPD1 or (D) SNRPE as tested at the protein level. [file 12885_2023_10860_MOESM14_ESM.docx]

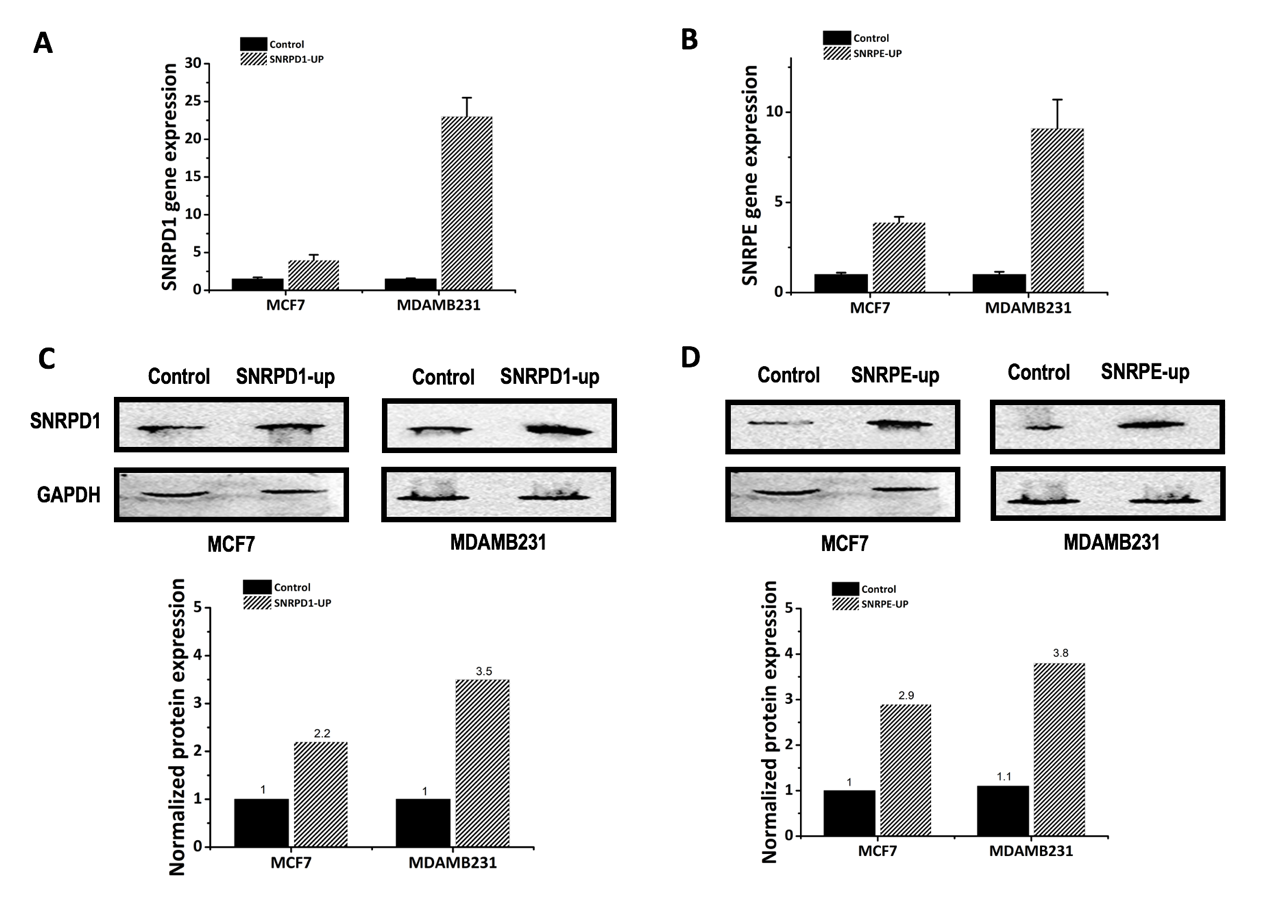


**Supplementary Figure 3. The efficiency of over-expressing SNRPD1 or SNRPE in MDAMB231 and MCF7 cells. (A)** Over-expressing SNRPD1 or **(B)** SNRPE as tested at the transcriptional level. **(C)** Over-expressing SNRPD1 or **(D)** SNRPE as tested at the translational level.
