# Supplementary material for: SNRPD1 conveys prognostic value on breast cancer survival and is required for anthracycline sensitivity
Source: BMC Cancer. 2023 Apr 25;23:376. doi: 10.1186/s12885-023-10860-z (PMC10126993; doi:10.1186/s12885-023-10860-z)
Supplement: Supplementary file 8 — Additional file 8: Supplementary Table 8. Genes highly correlated with SNRPD1 or SNRPE. [file 12885_2023_10860_MOESM8_ESM.docx]

**Supplementary Table 8. Genes highly correlated with *SNRPD1* or *SNRPE*.**

| **Genes** | ***SNRPD1*** |  | ***SNRPE*** |  | **Abs(sub)** |
| --- | --- | --- | --- | --- | --- |
|  | **Correlation** | ***p*** | **Correlation** | ***p*** | **abs(sub)** |
| LOC100130426 | -0.07520139 | 0.01288701 | -0.025747813 | 0.3951006 | 0.05 |
| UBE2Q2P3 | -0.096013602 | 0.00148308 | -0.157134272 | 1.78E-07 | 0.07 |
| UBE2Q2P3.1 | -0.096013602 | 0.00148308 | -0.157134272 | 1.78E-07 | 0.07 |
| LOC149767 | 0.337625298 | 1.51E-30 | 0.206974505 | 4.85E-12 | 0.14 |
| TIMM23 | 0.539189071 | 1.86E-83 | 0.459451292 | 3.52E-58 | 0.08 |
| LOC155060 | -0.051524274 | 0.08864245 | -0.019246554 | 0.525016 | 0.04 |
| RNU12.2P | -0.225011327 | 5.18E-14 | -0.165652817 | 3.62E-08 | 0.06 |
| SSX9 | 0.049194152 | 0.10405415 | 0.00167246 | 0.9559559 | 0.05 |
| LOC317712 | 0.034000823 | 0.26138465 | -0.013028023 | 0.6670218 | 0.05 |
| CXORF67 | 0.147102552 | 1.04E-06 | 0.01229624 | 0.6846907 | 0.14 |
| EFCAB8 | 0.024403103 | 0.42025618 | 0.000440418 | 0.9883961 | 0.03 |
| SRP14P1 | -0.052718919 | 0.08148505 | 0.075008986 | 0.01312 | 0.13 |
| LOC391343 | -0.141624385 | 2.59E-06 | -0.091350689 | 0.0025031 | 0.06 |
| TRIM75P | -0.019592231 | 0.51759902 | -0.069570429 | 0.0214373 | 0.05 |
| SPATA31B1P | 0.014671154 | 0.62802573 | -0.011000764 | 0.7163911 | 0.03 |
| LOC286106 | -0.00527859 | 0.86161821 | -0.034516137 | 0.2542211 | 0.03 |
| SDR16C6P | -0.001678873 | 0.95578713 | -0.005098817 | 0.866286 | 0.01 |
| LOC553137 | -0.023726689 | 0.43325715 | -0.065906048 | 0.029349 | 0.05 |
| KIAA1618 | -0.255408915 | 9.74E-18 | -0.33004325 | 3.46E-29 | 0.08 |
| LOC645851 | -0.170727923 | 1.35E-08 | -0.184285074 | 8.34E-10 | 0.02 |
| RGPD7 | -0.273825395 | 2.99E-20 | -0.307452911 | 2.33E-25 | 0.04 |
| HSPB1P1 | 0.075442643 | 0.01260006 | 0.209874036 | 2.40E-12 | 0.14 |
| PPBPL1 | -0.001052934 | 0.97226253 | 0.001207705 | 0.9681874 | 0.01 |
| LOC594835 | -0.037447681 | 0.21606547 | -0.040023806 | 0.1860933 | 0.01 |
| ANKRD20A20P | -0.07094738 | 0.01898466 | -0.077730132 | 0.0101478 | 0.01 |
| TMPRSS11E2 | -0.042435355 | 0.16092647 | -0.032050544 | 0.2897509 | 0.02 |
| LOC100132347 | 0.131429663 | 1.31E-05 | 0.158355749 | 1.42E-07 | 0.03 |
| EFCAB12 | -0.328638296 | 6.11E-29 | -0.053087132 | 0.0793759 | 0.28 |
| LOC147680 | -0.065621922 | 0.03005589 | 0.03070562 | 0.3104767 | 0.1 |
| A1CF | 0.067028523 | 0.02669447 | 0.026691031 | 0.3780107 | 0.05 |
| RBFOX1 | -0.086978423 | 0.00400561 | -0.043267318 | 0.152868 | 0.05 |
| GGACT | 0.007410971 | 0.80666257 | 0.05378845 | 0.0754814 | 0.05 |
| A2ML1 | 0.372568294 | 2.55E-37 | 0.117615223 | 9.72E-05 | 0.26 |
| A2M | -0.161455172 | 8.01E-08 | -0.202594512 | 1.38E-11 | 0.05 |
| P1 | -0.058149359 | 0.05462131 | -0.109204439 | 0.000298 | 0.06 |
| A4GNT | -0.208140439 | 3.66E-12 | -0.249195523 | 6.20E-17 | 0.05 |
| NPSR1.AS1 | -0.046176543 | 0.12708734 | -0.089119994 | 0.0031895 | 0.05 |
| AAAS | 0.061309525 | 0.04271215 | 0.161736874 | 7.60E-08 | 0.11 |
| LOC391856 | 0.200073904 | 2.48E-11 | 0.040603981 | 0.1797883 | 0.16 |
| AACS | -0.065912105 | 0.02933413 | -0.116439265 | 0.0001142 | 0.06 |
| AADACL2 | 0.066394421 | 0.02816742 | -0.040928648 | 0.1763299 | 0.11 |
| LOC649285 | -0.040957508 | 0.17602492 | -0.045880134 | 0.1295484 | 0.01 |
| AADACL4 | 0.028865916 | 0.34037237 | -0.045494428 | 0.132806 | 0.08 |
| AADAC | -0.009883508 | 0.74413058 | -0.05512106 | 0.0685108 | 0.05 |
| AADAT | 0.280345744 | 3.46E-21 | 0.061562047 | 0.0418633 | 0.22 |
| AAGAB | -0.170359536 | 1.45E-08 | -0.097275449 | 0.0012822 | 0.08 |
| DKFZp686K16132 | -0.343641887 | 1.18E-31 | -0.314392135 | 1.68E-26 | 0.03 |
| AAMP | 0.154988884 | 2.61E-07 | 0.118782431 | 8.27E-05 | 0.04 |
| AANAT | 0.129992343 | 1.63E-05 | -0.031971074 | 0.2909491 | 0.17 |
| LOC221410 | 0.145573333 | 1.34E-06 | 0.065110446 | 0.0313652 | 0.09 |
| AARSD1 | 0.221876358 | 1.17E-13 | 0.234922946 | 3.60E-15 | 0.02 |
| AARS | 0.294471272 | 2.64E-23 | 0.031236064 | 0.3021885 | 0.27 |
| AASDHPPT | -0.049465052 | 0.10215995 | -0.030285843 | 0.317141 | 0.02 |
| AASDH | -0.267200262 | 2.53E-19 | -0.231269282 | 9.76E-15 | 0.04 |
| AASS | -0.374397017 | 1.07E-37 | -0.271963491 | 5.48E-20 | 0.11 |
| AATF | 0.284095524 | 9.74E-22 | 0.24905135 | 6.47E-17 | 0.04 |
| LOC100292717 | -0.058590854 | 0.05280733 | -0.168671064 | 2.02E-08 | 0.12 |
| NPD009 | -0.465202521 | 8.68E-60 | -0.214482582 | 7.70E-13 | 0.26 |
| PRO2543 | -0.333083408 | 9.95E-30 | -0.2551581 | 1.05E-17 | 0.08 |
| ABCA11P | -0.162131848 | 7.06E-08 | -0.026367232 | 0.3838256 | 0.14 |
| ICR5 | -0.2049395 | 7.90E-12 | 0.027410876 | 0.365279 | 0.24 |
| ABCA13 | 0.242413748 | 4.41E-16 | 0.012929582 | 0.6693882 | 0.23 |
| ABCA17P | 0.157809864 | 1.57E-07 | 0.1266697 | 2.67E-05 | 0.04 |
| TGD | -0.342801851 | 1.69E-31 | -0.338070797 | 1.25E-30 | 0.01 |
| ABCA2 | -0.321762315 | 9.55E-28 | -0.276141128 | 1.40E-20 | 0.05 |
| ABCA3 | -0.230889107 | 1.08E-14 | -0.139552348 | 3.64E-06 | 0.1 |
| STGD1 | -0.09217648 | 0.00228526 | -0.011123489 | 0.713366 | 0.09 |
| HSA275973 | -0.210068403 | 2.29E-12 | -0.29942889 | 4.46E-24 | 0.09 |
| ABCA6 | -0.416693457 | 3.79E-47 | -0.301507629 | 2.09E-24 | 0.12 |
| ABCA7 | -0.191928738 | 1.58E-10 | -0.214945382 | 6.86E-13 | 0.03 |
| ABCA8 | -0.405911715 | 1.32E-44 | -0.274710778 | 2.24E-20 | 0.14 |
| ABCA9 | -0.411180731 | 7.75E-46 | -0.273578908 | 3.24E-20 | 0.14 |
| ABCB10 | -0.065956932 | 0.02922397 | 0.148116035 | 8.73E-07 | 0.22 |
| PFIC2 | -0.235929241 | 2.73E-15 | -0.18480319 | 7.47E-10 | 0.06 |
| ABCB1 | -0.356769953 | 3.75E-34 | -0.295051343 | 2.14E-23 | 0.07 |
| ABCB4 | -0.318811946 | 3.04E-27 | -0.226378902 | 3.61E-14 | 0.1 |
| ABCB5 | -0.26083629 | 1.86E-18 | -0.158124318 | 1.48E-07 | 0.11 |
| ABCB6 | 0.242572731 | 4.22E-16 | 0.089567122 | 0.0030396 | 0.16 |
| ABCB7 | -0.159228958 | 1.21E-07 | -0.098666586 | 0.00109 | 0.07 |
| ABCB8 | -0.047298654 | 0.11809848 | 0.067897783 | 0.0247835 | 0.12 |
| ABCB9 | 0.106685787 | 0.00041065 | 0.062941177 | 0.0374749 | 0.05 |
| ABCC10 | 0.109848346 | 0.00027422 | -0.047978025 | 0.1129032 | 0.16 |
| EWWD | -0.386097885 | 3.55E-40 | -0.122183954 | 5.12E-05 | 0.27 |
| ABCC12 | -0.157944102 | 1.53E-07 | 0.04356649 | 0.1500464 | 0.21 |
| ABCC13 | -0.109915106 | 0.00027186 | 0.031801856 | 0.2935115 | 0.15 |
| ABCC1 | 0.130823319 | 1.43E-05 | -0.043339133 | 0.1521871 | 0.18 |
| ABCC2 | -0.030192114 | 0.31864166 | -0.036349934 | 0.2298402 | 0.01 |
| ABCC3 | -0.265185654 | 4.78E-19 | -0.137238032 | 5.28E-06 | 0.13 |
| ABCC4 | -0.030505959 | 0.31363487 | -0.145196096 | 1.43E-06 | 0.12 |
| ABCC5 | -0.10500009 | 0.00050705 | -0.055656113 | 0.0658649 | 0.05 |
| ABCC6P1 | -0.189235696 | 2.86E-10 | -0.014933856 | 0.6218825 | 0.18 |
| ABCC6P2 | -0.077402732 | 0.01047053 | 0.142293752 | 2.32E-06 | 0.22 |
| PXE | -0.261690177 | 1.42E-18 | -0.036935133 | 0.2224213 | 0.23 |
| ABCC8 | -0.357692342 | 2.48E-34 | -0.095585056 | 0.0015576 | 0.27 |
| ABCC9 | -0.397736031 | 9.65E-43 | -0.331010944 | 2.33E-29 | 0.07 |
| ABCD1 | 0.176743508 | 4.03E-09 | -0.023661383 | 0.4345246 | 0.21 |
| ABCD2 | -0.159700663 | 1.11E-07 | -0.237017962 | 2.02E-15 | 0.08 |
| ABCD3 | -0.295276825 | 1.98E-23 | -0.207026799 | 4.79E-12 | 0.09 |
| ABCD4 | -0.225032158 | 5.16E-14 | -0.107709369 | 0.0003608 | 0.12 |
| ABCE1 | 0.221237319 | 1.39E-13 | 0.068866623 | 0.0227942 | 0.16 |
| ABCF1 | 0.359861643 | 9.29E-35 | 0.170137942 | 1.52E-08 | 0.19 |
| ABCF2 | 0.313236444 | 2.62E-26 | 0.189191765 | 2.89E-10 | 0.13 |
| ABCF3 | 0.19212904 | 1.51E-10 | 0.124265047 | 3.80E-05 | 0.07 |
| ABCG1 | -0.348670162 | 1.35E-32 | -0.19554752 | 7.00E-11 | 0.16 |
| ABCG2 | -0.42351908 | 8.36E-49 | -0.290451769 | 1.09E-22 | 0.14 |
| ABCG4 | 0.015296731 | 0.61343997 | -0.038954941 | 0.1981338 | 0.06 |
| ABCG5 | 0.199076361 | 3.12E-11 | 0.234054884 | 4.57E-15 | 0.04 |
| LOC115232 | 0.140302588 | 3.22E-06 | 0.146888306 | 1.08E-06 | 0.01 |
| ABHD10 | 0.003555636 | 0.90652949 | 0.080689518 | 0.0076091 | 0.08 |
| ABHD11 | 0.189435019 | 2.74E-10 | 0.240335788 | 7.96E-16 | 0.06 |
| ABHD12B | -0.15673941 | 1.91E-07 | -0.065681318 | 0.0299069 | 0.1 |
| C20orf22 | 0.165647094 | 3.62E-08 | 0.178728074 | 2.68E-09 | 0.02 |
| ABHD13 | -0.164792617 | 4.27E-08 | -0.138380067 | 4.39E-06 | 0.03 |
| ABHD14A | -0.038631602 | 0.20188588 | 0.09727555 | 0.0012822 | 0.14 |
| ABHD14B | -0.112513526 | 0.00019351 | 0.027963403 | 0.3556903 | 0.15 |
| ABHD15 | -0.344981251 | 6.66E-32 | -0.227581156 | 2.63E-14 | 0.12 |
| ABHD1 | -0.157676668 | 1.61E-07 | 0.028493048 | 0.346649 | 0.19 |
| LOC654057 | -0.366001127 | 5.56E-36 | -0.339510406 | 6.84E-31 | 0.03 |
| ABHD3 | -0.007170564 | 0.81281616 | -0.08067681 | 0.0076187 | 0.08 |
| ABHD4 | -0.244662307 | 2.32E-16 | -0.123324927 | 4.35E-05 | 0.13 |
| ABHD5 | 0.013569955 | 0.654054 | 0.031693511 | 0.29516 | 0.02 |
| ABHD6 | -0.208471442 | 3.38E-12 | -0.239669166 | 9.60E-16 | 0.04 |
| ABHD8 | 0.029027025 | 0.33768301 | -0.034259737 | 0.2577682 | 0.07 |
| ABI1 | 0.079831623 | 0.00827905 | -0.009073446 | 0.7644548 | 0.09 |
| ABI2 | -0.138470261 | 4.33E-06 | -0.198068405 | 3.94E-11 | 0.06 |
| FLJ21551 | -0.3496103 | 8.94E-33 | -0.292254821 | 5.77E-23 | 0.06 |
| ABI3 | -0.063306813 | 0.03637929 | -0.104454102 | 0.0005425 | 0.05 |
| LOC116063 | -0.350400861 | 6.33E-33 | -0.312369359 | 3.64E-26 | 0.04 |
| ABL2 | -0.157141486 | 1.77E-07 | -0.027748956 | 0.3593929 | 0.13 |
| ABLIM1 | -0.146835917 | 1.09E-06 | -0.100853248 | 0.0008409 | 0.05 |
| ABLIM2 | -0.120888325 | 6.16E-05 | -0.141712677 | 2.56E-06 | 0.03 |
| ABLIM3 | -0.464125323 | 1.74E-59 | -0.305722572 | 4.43E-25 | 0.16 |
| ABO | -0.217537784 | 3.57E-13 | -0.19037888 | 2.23E-10 | 0.03 |
| AOC1 | 0.122598545 | 4.83E-05 | 0.017718244 | 0.5584465 | 0.11 |
| ABRA | 0.023315673 | 0.44126926 | -0.006051127 | 0.8416163 | 0.03 |
| ABR | -0.366074646 | 5.37E-36 | -0.36220413 | 3.19E-35 | 0.01 |
| ABT1 | 0.466943192 | 2.79E-60 | 0.398663262 | 5.96E-43 | 0.07 |
| ABTB1 | -0.084511245 | 0.00517724 | -0.057822542 | 0.0559973 | 0.03 |
| ABTB2 | 0.277081939 | 1.03E-20 | 0.008449354 | 0.7802251 | 0.27 |
| ACAA1 | -0.060549826 | 0.04535398 | 0.007962338 | 0.7925951 | 0.07 |
| LOC147548 | 0.256737156 | 6.52E-18 | 0.062862963 | 0.0377129 | 0.2 |
| ACACA | -0.20846004 | 3.39E-12 | -0.143692826 | 1.84E-06 | 0.07 |
| ACACB | -0.409697471 | 1.73E-45 | -0.328315629 | 6.96E-29 | 0.09 |
| ACAD10 | -0.099929386 | 0.0009389 | 0.030804257 | 0.3089243 | 0.14 |
| ACAD11 | -0.175594586 | 5.09E-09 | -0.115481359 | 0.0001301 | 0.07 |
| ACAD8 | -0.211992837 | 1.43E-12 | -0.054005104 | 0.0743103 | 0.16 |
| ACAD9 | 0.295157417 | 2.07E-23 | 0.178243538 | 2.96E-09 | 0.12 |
| ACADL | -0.064876208 | 0.03198096 | -0.081957969 | 0.0067073 | 0.02 |
| ACADM | -0.177997879 | 3.12E-09 | -0.192958945 | 1.25E-10 | 0.02 |
| LOC654185 | -0.40661881 | 9.03E-45 | -0.232398224 | 7.18E-15 | 0.18 |
| ACADS | -0.142901693 | 2.10E-06 | -0.05726846 | 0.0583959 | 0.09 |
| ACADVL | -0.013652105 | 0.65209717 | 0.005510483 | 0.8556043 | 0.02 |
| ACAN | 0.091774849 | 0.00238893 | -0.045970916 | 0.1287908 | 0.14 |
| ACAP1 | 0.044960273 | 0.13742155 | -0.06590002 | 0.0293639 | 0.12 |
| ACAP2 | -0.305227157 | 5.33E-25 | -0.333353653 | 8.90E-30 | 0.03 |
| ACAP3 | -0.006915072 | 0.81936853 | -0.031852628 | 0.2927411 | 0.03 |
| ACAT1 | -0.009936983 | 0.74279497 | 0.04446496 | 0.1418109 | 0.06 |
| ACAT2 | 0.324022706 | 3.90E-28 | 0.210433339 | 2.10E-12 | 0.12 |
| ACBD3 | -0.252884789 | 2.08E-17 | 0.039554861 | 0.1913076 | 0.3 |
| ACBD4 | -0.222812311 | 9.21E-14 | -0.151505644 | 4.85E-07 | 0.08 |
| ACBD5 | -0.153664936 | 3.31E-07 | -0.16362881 | 5.32E-08 | 0.01 |
| ACBD6 | 0.352469745 | 2.54E-33 | 0.56974049 | 4.47E-95 | 0.22 |
| ACBD7 | 0.308943492 | 1.33E-25 | 0.058125845 | 0.0547194 | 0.26 |
| LOC654199 | -0.05178759 | 0.08702288 | -0.053181503 | 0.0788426 | 0.01 |
| ASIC1 | 0.125961597 | 2.96E-05 | 0.083761136 | 0.0055903 | 0.05 |
| ASIC3 | 0.008361504 | 0.78245249 | 0.049290799 | 0.1033752 | 0.05 |
| ASIC4 | 0.102260649 | 0.0007097 | 0.07233215 | 0.0167688 | 0.03 |
| ASIC5 | -0.035837131 | 0.23648438 | -0.020650918 | 0.4952249 | 0.02 |
| ACCSL | 0.04825122 | 0.11086544 | 0.001771055 | 0.9533622 | 0.05 |
| ACCS | -0.055352725 | 0.06735465 | -0.128592045 | 2.01E-05 | 0.08 |
| ACD | 0.295700647 | 1.70E-23 | 0.120901818 | 6.14E-05 | 0.18 |
| DKFZP434A014 | 0.170556272 | 1.40E-08 | 0.03126185 | 0.3017894 | 0.14 |
| ASAH3 | 0.016690966 | 0.58148322 | -0.119210606 | 7.79E-05 | 0.14 |
| ACER2 | -0.547755025 | 1.36E-86 | -0.393108204 | 1.04E-41 | 0.16 |
| ACER3 | -0.060182683 | 0.04667922 | -0.104381479 | 0.0005474 | 0.05 |
| LOC654142 | -0.231125409 | 1.01E-14 | -0.242557856 | 4.23E-16 | 0.02 |
| ACEE | 0.038247663 | 0.20640802 | -0.168169908 | 2.23E-08 | 0.21 |
| ACIN1 | -0.130471542 | 1.51E-05 | -0.028071602 | 0.3538313 | 0.11 |
| ACLY | -0.152802382 | 3.86E-07 | -0.10256189 | 0.0006842 | 0.06 |
| ACMSD | -0.166658855 | 2.98E-08 | -0.026656843 | 0.3786221 | 0.15 |
| SDHAF3 | 0.171945961 | 1.06E-08 | 0.137983215 | 4.68E-06 | 0.04 |
| IREB1 | -0.316979685 | 6.20E-27 | -0.274031598 | 2.80E-20 | 0.05 |
| LOC115645 | 0.02863432 | 0.34426229 | -0.078792518 | 0.0091608 | 0.11 |
| ACOT11 | 0.162373836 | 6.74E-08 | -0.036966383 | 0.22203 | 0.2 |
| ACOT12 | -0.025106957 | 0.40697385 | -0.044802041 | 0.1388123 | 0.02 |
| ACOT13 | 0.352490258 | 2.52E-33 | 0.339353821 | 7.31E-31 | 0.02 |
| LOC145480 | -0.133412152 | 9.63E-06 | -0.005522323 | 0.8552975 | 0.13 |
| ACOT2 | -0.236558855 | 2.29E-15 | -0.133432972 | 9.59E-06 | 0.11 |
| ACOT4 | -0.275625746 | 1.66E-20 | -0.00517311 | 0.8643564 | 0.28 |
| ACOT6 | -0.08818518 | 0.0035251 | 0.051073105 | 0.0914738 | 0.14 |
| ACOT7 | 0.441565125 | 2.26E-53 | 0.129798087 | 1.67E-05 | 0.32 |
| ACOT8 | 0.261054399 | 1.74E-18 | 0.29648638 | 1.29E-23 | 0.04 |
| ACOT9 | 0.167047359 | 2.77E-08 | 0.062803088 | 0.0378959 | 0.11 |
| ACOX1 | -0.071802945 | 0.01758733 | -0.157719799 | 1.60E-07 | 0.09 |
| ACOX2 | -0.333363127 | 8.87E-30 | -0.055298593 | 0.0676234 | 0.28 |
| ACOX3 | -0.229905968 | 1.41E-14 | -0.079035502 | 0.0089474 | 0.16 |
| ACOXL | 0.041631639 | 0.16901171 | -0.037274478 | 0.2181985 | 0.08 |
| ACP1 | 0.402860477 | 6.63E-44 | 0.346639544 | 3.26E-32 | 0.06 |
| LOC96117 | -0.023433023 | 0.43897311 | -0.037887245 | 0.2107196 | 0.02 |
| ACP5 | -0.084439601 | 0.00521547 | -0.048536235 | 0.1087705 | 0.04 |
| LOC116107 | -0.04293306 | 0.15606803 | 0.194826165 | 8.25E-11 | 0.24 |
| PXYLP1 | -0.093355877 | 0.00200416 | -0.069013225 | 0.0225056 | 0.03 |
| ACPP | -0.19795369 | 4.05E-11 | -0.120296926 | 6.69E-05 | 0.08 |
| ACPT | -0.047675554 | 0.1151936 | 0.013234039 | 0.6620801 | 0.07 |
| ACRBP | -0.099138862 | 0.00103102 | -0.033442753 | 0.2692984 | 0.07 |
| NAAR1 | 0.020328406 | 0.50198571 | 0.014994194 | 0.6204752 | 0.01 |
| ACRV1 | 0.3866339 | 2.72E-40 | 0.135174641 | 7.31E-06 | 0.26 |
| ACR | 0.102868493 | 0.00065913 | -0.070630473 | 0.0195263 | 0.18 |
| ACSBG1 | 0.03302467 | 0.2753336 | -0.081010839 | 0.0073709 | 0.12 |
| ACSBG2 | -0.173743843 | 7.40E-09 | -0.079014552 | 0.0089656 | 0.1 |
| ACSF2 | -0.168604022 | 2.05E-08 | -0.004662654 | 0.8776299 | 0.17 |
| ACSF3 | 0.020577316 | 0.49676353 | -0.00483712 | 0.8730892 | 0.03 |
| FACL1 | -0.120402468 | 6.59E-05 | -0.190848533 | 2.01E-10 | 0.08 |
| PRO2194 | -0.170407784 | 1.44E-08 | -0.118396023 | 8.72E-05 | 0.06 |
| MRX63 | -0.046469829 | 0.12468814 | -0.211834058 | 1.49E-12 | 0.17 |
| ACSL5 | -0.04155264 | 0.16982256 | -0.079184736 | 0.0088186 | 0.04 |
| ACSL6 | 0.095356353 | 0.00159883 | -0.080582679 | 0.0076898 | 0.18 |
| ACSM1 | -0.212284314 | 1.33E-12 | -0.007916665 | 0.7937579 | 0.21 |
| ACSM2A | -0.030062724 | 0.32072097 | -0.00086978 | 0.9770859 | 0.03 |
| ACSM2B | 0.009462418 | 0.75467441 | 0.025122609 | 0.4066814 | 0.02 |
| ACSM3 | -0.045214071 | 0.13521335 | -0.087431242 | 0.0038187 | 0.05 |
| ACSM4 | 0.017747232 | 0.55780292 | 0.011346314 | 0.7078849 | 0.01 |
| ACSM5 | -0.423097886 | 1.06E-48 | -0.270995113 | 7.50E-20 | 0.16 |
| LOC89850 | -0.153026178 | 3.71E-07 | -0.082941617 | 0.0060754 | 0.08 |
| ACSS2 | -0.031310731 | 0.30103374 | -0.082509948 | 0.0063458 | 0.06 |
| ACSS3 | -0.268993746 | 1.43E-19 | -0.165275741 | 3.89E-08 | 0.11 |
| LOC115745 | 0.09619934 | 0.00145179 | 0.091876563 | 0.0023623 | 0.01 |
| ACTA2 | -0.09930159 | 0.0010114 | -0.057840116 | 0.0559226 | 0.05 |
| ACTBL2 | 0.042488474 | 0.16040256 | -0.059699064 | 0.0484744 | 0.11 |
| LOC115571 | 0.24974868 | 5.27E-17 | 0.249265705 | 6.07E-17 | 0.01 |
| ACTC1 | 0.016807153 | 0.57885559 | -0.017136877 | 0.5714292 | 0.04 |
| DFNA26 | 0.308161826 | 1.79E-25 | 0.251896513 | 2.79E-17 | 0.06 |
| LOC115664 | 0.09814765 | 0.00115832 | -0.018399553 | 0.5434165 | 0.12 |
| ACTL6A | 0.414272825 | 1.44E-46 | 0.259507117 | 2.80E-18 | 0.16 |
| ACTL6B | 0.013670181 | 0.65166694 | -0.083549995 | 0.0057118 | 0.1 |
| ACTL7A | -0.058073957 | 0.05493625 | -0.03429069 | 0.2573382 | 0.03 |
| ACTL7B | -0.075321295 | 0.01274368 | -0.07145507 | 0.0181442 | 0.01 |
| ACTL8 | 0.390565335 | 3.78E-41 | 0.246608671 | 1.32E-16 | 0.15 |
| ACTL9 | -0.022267375 | 0.46208343 | -0.032171815 | 0.2879289 | 0.01 |
| ACTN1 | -0.05693393 | 0.05988496 | -0.176522768 | 4.22E-09 | 0.12 |
| ACTN2 | 0.13886825 | 4.06E-06 | 0.155817933 | 2.25E-07 | 0.02 |
| ACTN3 | 0.000104336 | 0.99725092 | -0.030780426 | 0.3092989 | 0.04 |
| FSGS1 | 0.089568587 | 0.00303908 | -0.131680011 | 1.26E-05 | 0.23 |
| ACTR10 | -0.114572976 | 0.00014704 | 0.049818899 | 0.0997271 | 0.17 |
| ACTR1A | 0.138320697 | 4.44E-06 | 0.069666312 | 0.021258 | 0.07 |
| LOC130640 | -0.009345193 | 0.75761788 | -0.008694556 | 0.7740179 | 0.01 |
| ACTR2 | -0.012194018 | 0.68717289 | -0.113469068 | 0.0001705 | 0.11 |
| ACTR3B | 0.41830484 | 1.55E-47 | 0.26134743 | 1.58E-18 | 0.16 |
| LOC155033 | 0.194843196 | 8.21E-11 | 0.062963746 | 0.0374065 | 0.14 |
| ACTR3 | 0.169449174 | 1.74E-08 | 0.061057494 | 0.0435738 | 0.11 |
| ACTR5 | 0.253393469 | 1.79E-17 | 0.263746676 | 7.51E-19 | 0.02 |
| ACTR6 | 0.173606729 | 7.61E-09 | 0.176488604 | 4.25E-09 | 0.01 |
| ACTR8 | -0.285522275 | 5.98E-22 | -0.292797273 | 4.77E-23 | 0.01 |
| ACTRT1 | 0.02543551 | 0.40086036 | 0.051185194 | 0.0907636 | 0.03 |
| ACTRT2 | -0.009080939 | 0.76426601 | -0.003157314 | 0.9169604 | 0.01 |
| ACVR1B | -0.351827905 | 3.38E-33 | -0.177065388 | 3.77E-09 | 0.18 |
| ACVR1C | -0.30546 | 4.89E-25 | -0.291731873 | 6.94E-23 | 0.02 |
| FOP | -0.404416041 | 2.91E-44 | -0.312966338 | 2.90E-26 | 0.1 |
| ACVR2A | -0.134189524 | 8.53E-06 | -0.144765801 | 1.54E-06 | 0.02 |
| ACVR2B | -0.1254569 | 3.19E-05 | -0.094722548 | 0.0017183 | 0.04 |
| ACVRL1 | -0.18625914 | 5.46E-10 | -0.164976219 | 4.12E-08 | 0.03 |
| ACY1 | 0.010295162 | 0.73386912 | 0.115900541 | 0.0001229 | 0.11 |
| ACY3 | 0.063754813 | 0.03507412 | 0.026041222 | 0.3897351 | 0.04 |
| ACYP1 | 0.379537483 | 8.96E-39 | 0.218195129 | 3.02E-13 | 0.17 |
| LOC115706 | 0.239913146 | 8.96E-16 | 0.094095772 | 0.0018444 | 0.15 |
| ADAD1 | 0.111435078 | 0.00022303 | 0.037691508 | 0.2130882 | 0.08 |
| ADAD2 | -0.040622228 | 0.17959261 | -0.024060199 | 0.4268181 | 0.02 |
| ADAL | -0.299167713 | 4.90E-24 | -0.229914252 | 1.41E-14 | 0.07 |
| ADAM10 | -0.418672048 | 1.27E-47 | -0.40858085 | 3.16E-45 | 0.02 |
| ADAM11 | 0.208512749 | 3.35E-12 | 0.071771407 | 0.0176372 | 0.14 |
| CAR10 | -0.308174228 | 1.78E-25 | -0.190286963 | 2.27E-10 | 0.12 |
| ADAM15 | 0.127896042 | 2.23E-05 | 0.287512145 | 3.02E-22 | 0.16 |
| LOC129645 | -0.085775919 | 0.00454277 | -0.256340422 | 7.35E-18 | 0.18 |
| ADAM18 | 0.056369699 | 0.06246758 | -0.014574038 | 0.6303033 | 0.08 |
| ADAM19 | -0.012444117 | 0.68110589 | -0.157653951 | 1.61E-07 | 0.15 |
| ADAM20 | -0.171550277 | 1.15E-08 | -0.119443489 | 7.54E-05 | 0.06 |
| ADAM21P1 | -0.103592749 | 0.00060325 | -0.019688522 | 0.5155426 | 0.09 |
| ADAM21 | -0.07153652 | 0.01801248 | 0.073706697 | 0.0147969 | 0.15 |
| MDC2 | -0.339681721 | 6.36E-31 | -0.27383737 | 2.98E-20 | 0.07 |
| ADAM23 | -0.261443983 | 1.54E-18 | -0.26029263 | 2.20E-18 | 0.01 |
| EMDCII | -0.271803108 | 5.78E-20 | -0.281934383 | 2.03E-21 | 0.02 |
| ADAM29 | -0.095922343 | 0.00149867 | -0.098399914 | 0.0011246 | 0.01 |
| ADAM2 | -0.004273686 | 0.88776772 | -0.038947535 | 0.1982192 | 0.04 |
| ADAM30 | 0.006006565 | 0.84276738 | -0.000348786 | 0.9908103 | 0.01 |
| ADAM32 | 0.144020236 | 1.75E-06 | 0.024899781 | 0.4108572 | 0.12 |
| DJ964F7.1 | -0.340434133 | 4.63E-31 | -0.184797931 | 7.48E-10 | 0.16 |
| ADAM3A | 0.036716009 | 0.22517896 | 0.029630646 | 0.3277285 | 0.01 |
| ADAM5 | -0.007998637 | 0.79167129 | -0.063336121 | 0.0362927 | 0.06 |
| ADAM6 | 0.037028113 | 0.2212585 | -0.009829647 | 0.7454766 | 0.05 |
| ADAM7 | 0.011378504 | 0.70709435 | -0.009497388 | 0.753797 | 0.03 |
| ADAM8 | 0.040154632 | 0.18465751 | 0.015349144 | 0.6122247 | 0.03 |
| ADAM9 | -0.136655352 | 5.79E-06 | -0.217739386 | 3.39E-13 | 0.09 |
| ADAMDEC1 | 0.144795949 | 1.53E-06 | -0.058993726 | 0.0511962 | 0.21 |
| WMS | -0.228000804 | 2.35E-14 | -0.149740504 | 6.60E-07 | 0.08 |
| ADAMTS12 | -0.370652497 | 6.31E-37 | -0.313656366 | 2.23E-26 | 0.06 |
| DKFZp434C2322 | -0.137094186 | 5.40E-06 | -0.028986466 | 0.3383588 | 0.11 |
| ADAMTS14 | -0.131805924 | 1.23E-05 | -0.059711567 | 0.0484273 | 0.08 |
| ADAMTS15 | -0.473642019 | 3.33E-62 | -0.245801074 | 1.67E-16 | 0.23 |
| ADAMTS16 | -0.254155402 | 1.42E-17 | -0.094961844 | 0.0016722 | 0.16 |
| ADAMTS17 | -0.101485988 | 0.00077936 | -0.114206025 | 0.0001545 | 0.02 |
| ADAMTS18 | -0.193777952 | 1.04E-10 | -0.160809627 | 9.03E-08 | 0.04 |
| ADAMTS19 | -0.104223591 | 0.0005582 | -0.03577895 | 0.2372467 | 0.07 |
| ADAMTS1 | -0.064796798 | 0.03219204 | -0.107264819 | 0.0003817 | 0.05 |
| ADAMTS20 | -0.051429084 | 0.08923386 | -0.063527408 | 0.0357315 | 0.02 |
| ADAMTS2 | -0.327241416 | 1.07E-28 | -0.289128653 | 1.72E-22 | 0.04 |
| KIAA0366 | 0.053102808 | 0.07928711 | -0.077571004 | 0.0103036 | 0.14 |
| ADAMTS4 | 0.028089871 | 0.35351805 | 0.036258735 | 0.231012 | 0.01 |
| ADAMTS5 | -0.228660571 | 1.97E-14 | -0.19232861 | 1.44E-10 | 0.04 |
| LOC345667 | -0.302074938 | 1.70E-24 | -0.178723881 | 2.68E-09 | 0.13 |
| DKFZP434H204 | -0.053345141 | 0.07792461 | -0.105928298 | 0.0004516 | 0.06 |
| ADAMTS8 | -0.131383913 | 1.32E-05 | -0.101036479 | 0.0008226 | 0.04 |
| ADAMTS9 | -0.093899286 | 0.00188563 | -0.210245124 | 2.20E-12 | 0.12 |
| ADAMTSL1 | -0.368106043 | 2.09E-36 | -0.247014507 | 1.17E-16 | 0.13 |
| ADAMTSL2 | -0.222954232 | 8.88E-14 | -0.098312554 | 0.0011362 | 0.13 |
| ADAMTSL3 | -0.229452325 | 1.59E-14 | -0.287810945 | 2.72E-22 | 0.06 |
| LOC105371437 | -0.076847648 | 0.01103851 | -0.081848243 | 0.0067813 | 0.01 |
| ADAMTSL5 | -0.343181394 | 1.44E-31 | -0.114348687 | 0.0001515 | 0.23 |
| ADAP1 | -0.107782787 | 0.00035741 | -0.14716455 | 1.03E-06 | 0.04 |
| ADAP2 | -0.171975308 | 1.05E-08 | -0.180245151 | 1.96E-09 | 0.01 |
| ADARB1 | -0.048702257 | 0.10756474 | -0.203273893 | 1.17E-11 | 0.16 |
| LOC100130729 | -0.096240084 | 0.00144501 | -0.098910442 | 0.0010592 | 0.01 |
| IFI4 | -0.091402962 | 0.00248873 | 0.029624207 | 0.3278337 | 0.13 |
| ADAT1 | -0.327551657 | 9.48E-29 | -0.378001838 | 1.89E-38 | 0.06 |
| ADAT2 | 0.341690396 | 2.72E-31 | 0.160989104 | 8.74E-08 | 0.19 |
| ADAT3 | 0.095289097 | 0.00161113 | 0.118116282 | 9.07E-05 | 0.03 |
| ADA | 0.301601225 | 2.02E-24 | 0.188068318 | 3.69E-10 | 0.12 |
| ADCK1 | 0.079277673 | 0.00873918 | 0.123530361 | 4.22E-05 | 0.05 |
| ADCK2 | 0.00627628 | 0.83580534 | 0.066156591 | 0.0287376 | 0.06 |
| ADCK4 | 0.011102374 | 0.71388616 | 0.080830485 | 0.0075038 | 0.07 |
| ADCK5 | 0.277130095 | 1.01E-20 | 0.185364771 | 6.62E-10 | 0.1 |
| ADCY10 | -0.08041471 | 0.00781822 | 0.074985009 | 0.0131493 | 0.16 |
| DFNB44 | -0.322587218 | 6.89E-28 | -0.179266601 | 2.40E-09 | 0.15 |
| ADCY2 | -0.063191056 | 0.03672317 | -0.009023612 | 0.7657106 | 0.06 |
| AC3 | 0.091923208 | 0.00235014 | -0.242917369 | 3.82E-16 | 0.34 |
| ADCY4 | -0.300305727 | 3.24E-24 | -0.20928653 | 2.77E-12 | 0.1 |
| ADCY5 | -0.391923904 | 1.90E-41 | -0.236319545 | 2.45E-15 | 0.16 |
| ADCY6 | -0.415986837 | 5.60E-47 | -0.248645873 | 7.28E-17 | 0.17 |
| ADCY7 | -0.023893108 | 0.43003714 | -0.196455022 | 5.70E-11 | 0.18 |
| ADCY8 | 0.040448601 | 0.18146112 | -0.044094158 | 0.1451667 | 0.09 |
| ADCY9 | -0.440291385 | 4.85E-53 | -0.28244128 | 1.71E-21 | 0.16 |
| ADCYAP1R1 | -0.367204499 | 3.18E-36 | -0.33693503 | 2.02E-30 | 0.04 |
| ADCYAP1 | -0.04982602 | 0.09967867 | -0.156011102 | 2.18E-07 | 0.11 |
| AZIN2 | -0.24681245 | 1.24E-16 | -0.180555753 | 1.83E-09 | 0.07 |
| ADD1 | -0.475108251 | 1.25E-62 | -0.376461153 | 3.97E-38 | 0.1 |
| LOC130935 | 0.092623103 | 0.00217482 | -0.141037785 | 2.86E-06 | 0.24 |
| ADD3 | -0.167112644 | 2.73E-08 | -0.253477888 | 1.74E-17 | 0.09 |
| ADH1A | -0.312755895 | 3.14E-26 | -0.228566816 | 2.02E-14 | 0.09 |
| ADH1B | -0.273034589 | 3.87E-20 | -0.178945497 | 2.56E-09 | 0.1 |
| ADH1C | -0.190655891 | 2.09E-10 | -0.108955969 | 0.0003077 | 0.09 |
| ADH4 | -0.09739798 | 0.00126407 | -0.117818372 | 9.45E-05 | 0.03 |
| FDH | 0.122673159 | 4.78E-05 | -0.026617827 | 0.3793206 | 0.15 |
| ADH6 | 0.014229924 | 0.63840175 | 0.06541231 | 0.0305867 | 0.06 |
| ADH7 | 0.054233034 | 0.0730943 | -0.073116332 | 0.0156174 | 0.13 |
| ADHFE1 | -0.238843611 | 1.21E-15 | -0.157363032 | 1.70E-07 | 0.09 |
| ADI1 | 0.276011287 | 1.46E-20 | 0.272990597 | 3.93E-20 | 0.01 |
| ADIG | 0.02652348 | 0.38101286 | 0.003252658 | 0.9144622 | 0.03 |
| APM.1 | -0.282568007 | 1.64E-21 | -0.183824931 | 9.20E-10 | 0.1 |
| ADIPOR1 | -0.074453219 | 0.01381394 | 0.335742312 | 3.31E-30 | 0.42 |
| ADIPOR2 | -0.008682707 | 0.77431753 | -0.014512071 | 0.6317584 | 0.01 |
| ADK | 0.204014675 | 9.84E-12 | 0.193451028 | 1.12E-10 | 0.02 |
| ADM2 | 0.17438457 | 6.51E-09 | 0.077213589 | 0.0106611 | 0.1 |
| ADM | 0.177349097 | 3.56E-09 | 0.01795515 | 0.5531974 | 0.16 |
| ADNP2 | -0.073229231 | 0.01545745 | -0.180435772 | 1.88E-09 | 0.11 |
| LOC256440 | -0.20020026 | 2.41E-11 | -0.22111256 | 1.43E-13 | 0.03 |
| ADORA1 | 0.164372626 | 4.62E-08 | 0.106930169 | 0.0003982 | 0.06 |
| ADORA2A | -0.015180887 | 0.61612968 | 0.029788982 | 0.3251492 | 0.05 |
| ADORA2B | 0.169290481 | 1.79E-08 | 0.049335674 | 0.1030611 | 0.12 |
| ADORA3 | -0.209512375 | 2.63E-12 | -0.059487375 | 0.0492782 | 0.16 |
| ADO | 0.144969394 | 1.49E-06 | 0.09451891 | 0.0017583 | 0.06 |
| ADPGK | 0.051663715 | 0.0877818 | -0.065661794 | 0.0299558 | 0.12 |
| ADPRHL1 | -0.104010663 | 0.00057305 | 0.073930585 | 0.0144959 | 0.18 |
| ADPRHL2 | 0.360794462 | 6.08E-35 | 0.175207693 | 5.51E-09 | 0.19 |
| ADPRH | 0.022636453 | 0.45469359 | -0.165635329 | 3.63E-08 | 0.19 |
| ADRA1A | -0.219447488 | 2.19E-13 | -0.267318016 | 2.43E-19 | 0.05 |
| ADRA1B | -0.006860547 | 0.82076855 | -0.058234193 | 0.0542688 | 0.06 |
| ADRA1D | -0.062831781 | 0.03780814 | -0.11619067 | 0.0001181 | 0.06 |
| LOC92480 | -0.323348737 | 5.10E-28 | -0.140715334 | 3.01E-06 | 0.19 |
| ADRA2B | 0.171499727 | 1.16E-08 | 0.018896648 | 0.5325787 | 0.16 |
| ADRA2C | -0.080075177 | 0.00808369 | -0.005132345 | 0.8654151 | 0.08 |
| ADRB1 | -0.092387358 | 0.00223249 | -0.019732001 | 0.5146155 | 0.08 |
| ADRB2 | -0.342577891 | 1.86E-31 | -0.225597417 | 4.44E-14 | 0.12 |
| LOC157462 | -0.045262069 | 0.13479884 | -0.07071884 | 0.0193739 | 0.03 |
| ADRBK1 | 0.005240359 | 0.86261046 | -0.146298351 | 1.19E-06 | 0.16 |
| ADRBK2 | -0.303655516 | 9.52E-25 | -0.240799819 | 6.98E-16 | 0.07 |
| ADRM1 | 0.433180539 | 3.24E-51 | 0.245922631 | 1.61E-16 | 0.19 |
| ADSL | 0.433278057 | 3.06E-51 | 0.273974319 | 2.85E-20 | 0.16 |
| ADSSL1 | -0.198797645 | 3.33E-11 | -0.071143799 | 0.0186556 | 0.13 |
| ADSS | -0.053368603 | 0.07779371 | 0.275488291 | 1.74E-20 | 0.33 |
| AEBP1 | -0.22624472 | 3.74E-14 | -0.099796514 | 0.0009538 | 0.13 |
| AEBP2 | 0.00305639 | 0.9196056 | -0.084296639 | 0.0052925 | 0.09 |
| AEN | 0.225750778 | 4.27E-14 | 0.032857441 | 0.2777732 | 0.2 |
| AES | 0.117506657 | 9.87E-05 | 0.151559233 | 4.80E-07 | 0.04 |
| AFAP1L1 | -0.260361907 | 2.15E-18 | -0.294879595 | 2.28E-23 | 0.04 |
| AFAP1L2 | -0.225137552 | 5.01E-14 | -0.20597449 | 6.17E-12 | 0.02 |
| AFAP1 | -0.275017004 | 2.03E-20 | -0.298124143 | 7.14E-24 | 0.03 |
| AKR7A2P1 | -0.026705543 | 0.37775138 | -0.117294096 | 0.0001016 | 0.1 |
| PBM1 | -0.544824897 | 1.64E-85 | -0.396224562 | 2.11E-42 | 0.15 |
| AFF2 | -0.133742148 | 9.14E-06 | -0.192681559 | 1.33E-10 | 0.06 |
| AFF3 | -0.401543845 | 1.33E-43 | -0.275141764 | 1.94E-20 | 0.13 |
| AFF4 | -0.521370538 | 3.26E-77 | -0.376284485 | 4.32E-38 | 0.15 |
| FLJ45200 | 0.232949798 | 6.18E-15 | 0.033215885 | 0.272562 | 0.2 |
| SCA28 | 0.460403612 | 1.91E-58 | 0.053866849 | 0.0750559 | 0.41 |
| AFMID | -0.068059176 | 0.02444205 | 0.064946071 | 0.0317962 | 0.14 |
| AFM | 0.015754957 | 0.60285181 | -0.012474911 | 0.6803603 | 0.03 |
| AFP | -0.183576486 | 9.70E-10 | -0.076561314 | 0.011342 | 0.11 |
| AFTPH | -0.274714396 | 2.24E-20 | -0.207589104 | 4.18E-12 | 0.07 |
| C11ORF96 | -0.041671703 | 0.1686016 | -0.004682442 | 0.8771147 | 0.04 |
| AGAP11 | -0.351987861 | 3.15E-33 | -0.251709097 | 2.95E-17 | 0.11 |
| AGAP1 | -0.228827624 | 1.88E-14 | -0.210185956 | 2.23E-12 | 0.02 |
| KIAA0167 | -0.00789617 | 0.79427984 | -0.159420801 | 1.17E-07 | 0.16 |
| LOC100134082 | 0.177416304 | 3.51E-09 | 0.026701324 | 0.3778268 | 0.16 |
| AGAP4 | 0.0680372 | 0.02448831 | 0.062337598 | 0.0393449 | 0.01 |
| LOC118704 | -0.300630637 | 2.88E-24 | -0.201696374 | 1.70E-11 | 0.1 |
| AGAP6 | 0.113082587 | 0.00017945 | 0.053676963 | 0.0760899 | 0.06 |
| AGAP7P | 0.123048458 | 4.52E-05 | 0.063529382 | 0.0357258 | 0.06 |
| AGAP8 | -0.030932546 | 0.30691281 | 0.019347079 | 0.5228535 | 0.06 |
| AGA | -0.091917593 | 0.0023516 | -0.010913659 | 0.7185409 | 0.09 |
| LOC728206 | 0.069746807 | 0.02110851 | 0.004587489 | 0.8795875 | 0.07 |
| AGBL2 | -0.29471774 | 2.42E-23 | -0.183148292 | 1.06E-09 | 0.12 |
| FLJ12983 | -0.018782566 | 0.53505624 | 0.041158841 | 0.1739081 | 0.06 |
| AGBL4 | -0.13268816 | 1.08E-05 | 0.003107431 | 0.9182677 | 0.14 |
| AGBL5 | 0.257916004 | 4.55E-18 | 0.023256205 | 0.4424355 | 0.24 |
| AGER | 0.159362104 | 1.18E-07 | 0.093495937 | 0.001973 | 0.07 |
| AGFG1 | -0.023582406 | 0.43606011 | -0.13171774 | 1.25E-05 | 0.11 |
| RAB.R | -0.276991077 | 1.06E-20 | -0.152197253 | 4.29E-07 | 0.13 |
| HSU84971 | -0.320906102 | 1.34E-27 | -0.117136504 | 0.0001038 | 0.21 |
| AGK | 0.13287682 | 1.05E-05 | 0.099473215 | 0.0009911 | 0.04 |
| AGL | -0.15606069 | 2.16E-07 | -0.097299788 | 0.0012786 | 0.06 |
| LOC100507192 | 0.201961081 | 1.60E-11 | 0.077140916 | 0.0107351 | 0.13 |
| MGC5423 | 0.15415621 | 3.03E-07 | 0.077562306 | 0.0103121 | 0.08 |
| AGPAT2 | 0.059850196 | 0.0479073 | 0.096998357 | 0.001324 | 0.04 |
| AGPAT3 | -0.123473823 | 4.26E-05 | -0.036088032 | 0.2332168 | 0.09 |
| AGPAT4 | 0.078642161 | 0.00929505 | -0.00954479 | 0.7526082 | 0.09 |
| AGPAT5 | 0.063276344 | 0.03646953 | 0.053664626 | 0.0761575 | 0.01 |
| AGPAT6 | 0.045237789 | 0.1350084 | -0.034628028 | 0.2526839 | 0.08 |
| AGPAT9 | 0.004170307 | 0.89046529 | -0.167359244 | 2.61E-08 | 0.18 |
| HYKK | -0.045172518 | 0.135573 | -0.055755519 | 0.0653827 | 0.02 |
| AGPS | -0.180225482 | 1.96E-09 | -0.164236397 | 4.74E-08 | 0.02 |
| AGR2 | -0.395674236 | 2.80E-42 | -0.090744229 | 0.0026749 | 0.31 |
| AGR3 | -0.393516018 | 8.45E-42 | -0.058008732 | 0.0552099 | 0.34 |
| LOC348522 | -0.119717951 | 7.26E-05 | -0.138272203 | 4.47E-06 | 0.02 |
| AGRP | 0.129032643 | 1.88E-05 | 0.079227965 | 0.0087816 | 0.05 |
| AGTPBP1 | 0.121517601 | 5.63E-05 | -0.169352768 | 1.77E-08 | 0.3 |
| AGTR1B | -0.343514767 | 1.25E-31 | -0.269456796 | 1.23E-19 | 0.08 |
| AGTR2 | -0.17902935 | 2.52E-09 | -0.128614883 | 2.00E-05 | 0.06 |
| AGTRAP | 0.181573438 | 1.48E-09 | 0.154590962 | 2.81E-07 | 0.03 |
| AGT | -0.038635119 | 0.20184479 | 0.136759316 | 5.69E-06 | 0.18 |
| ETNPPL | -0.063547862 | 0.03567198 | 0.024481821 | 0.4187582 | 0.09 |
| PHYKPL | -0.271283368 | 6.83E-20 | -0.19084223 | 2.01E-10 | 0.09 |
| AGXT2 | -0.119150221 | 7.86E-05 | -0.062170651 | 0.0398759 | 0.06 |
| AGXT | -0.084551969 | 0.00515562 | -0.028743411 | 0.3424265 | 0.06 |
| AHCTF1 | 0.1013375 | 0.00079342 | 0.200671825 | 2.16E-11 | 0.1 |
| LOC115541 | -0.335186162 | 4.17E-30 | -0.279933318 | 3.97E-21 | 0.06 |
| AHCYL2 | -0.352794166 | 2.20E-33 | -0.351670507 | 3.62E-33 | 0.01 |
| AHCY | 0.416913516 | 3.36E-47 | 0.300126051 | 3.46E-24 | 0.12 |
| AHDC1 | -0.132876642 | 1.05E-05 | -0.237138472 | 1.95E-15 | 0.11 |
| AHI1 | -0.104955295 | 0.00050988 | -0.073940165 | 0.0144831 | 0.04 |
| AHNAK2 | -0.150905296 | 5.39E-07 | -0.17924941 | 2.41E-09 | 0.03 |
| AHNAKRS | -0.637237547 | 1.41E-125 | -0.500389832 | 2.54E-70 | 0.14 |
| LOC256948 | 0.001702549 | 0.95516429 | -0.087060025 | 0.0039713 | 0.09 |
| AHR | -0.309140091 | 1.24E-25 | -0.273337488 | 3.51E-20 | 0.04 |
| AHSA1 | 0.50507958 | 8.06E-72 | 0.353318391 | 1.75E-33 | 0.16 |
| AHSA2 | -0.144115325 | 1.72E-06 | -0.126102511 | 2.90E-05 | 0.02 |
| AHSG | 0.159685075 | 1.11E-07 | 0.058128053 | 0.0547101 | 0.11 |
| AHSP | -0.010296227 | 0.73384264 | 0.010396317 | 0.7313548 | 0.03 |
| AICDA | 0.044771051 | 0.13908589 | -0.069824568 | 0.0209649 | 0.12 |
| LOC92615 | 0.204823279 | 8.12E-12 | 0.480173926 | 4.04E-64 | 0.28 |
| AIF1L | 0.280052972 | 3.81E-21 | 0.099210025 | 0.0010224 | 0.19 |
| LOC116399 | 0.014272571 | 0.63739572 | -0.051675631 | 0.0877086 | 0.07 |
| NAMSD | 0.281678556 | 2.21E-21 | 0.208803102 | 3.12E-12 | 0.08 |
| MGC13000 | 0.17891067 | 2.58E-09 | 0.011037045 | 0.7154964 | 0.17 |
| AIFM3 | 0.010561958 | 0.72724372 | 0.122531284 | 4.87E-05 | 0.12 |
| LOC51638 | 0.022194742 | 0.46354558 | 0.108355919 | 0.0003322 | 0.09 |
| FLJ38020 | 0.275419427 | 1.78E-20 | 0.214965622 | 6.83E-13 | 0.07 |
| ST4 | -0.070180986 | 0.0203177 | -0.037560587 | 0.2146831 | 0.04 |
| AIM2 | 0.139799624 | 3.49E-06 | -0.054022935 | 0.0742146 | 0.2 |
| AIMP1 | 0.197454905 | 4.54E-11 | 0.18265733 | 1.18E-09 | 0.02 |
| AIMP2 | 0.451365104 | 5.69E-56 | 0.343784774 | 1.11E-31 | 0.11 |
| AIPL1 | 0.188417889 | 3.42E-10 | 0.041787771 | 0.1674177 | 0.15 |
| AIP | 0.235994497 | 2.68E-15 | 0.160540158 | 9.50E-08 | 0.08 |
| AIRE | -0.004848602 | 0.8727905 | -0.071701353 | 0.0177484 | 0.07 |
| AJAP1 | -0.173164732 | 8.31E-09 | -0.19600071 | 6.32E-11 | 0.03 |
| AK1 | 0.087227814 | 0.0039017 | 0.071339422 | 0.0183328 | 0.02 |
| AK2 | 0.465206497 | 8.65E-60 | 0.280929649 | 2.84E-21 | 0.19 |
| AK4 | 0.157523097 | 1.65E-07 | -0.002443216 | 0.935695 | 0.16 |
| AK3 | -0.128014354 | 2.19E-05 | -0.14138609 | 2.70E-06 | 0.02 |
| AK5 | -0.339770001 | 6.13E-31 | -0.191256998 | 1.83E-10 | 0.15 |
| AK7 | -0.261510105 | 1.51E-18 | -0.217266505 | 3.82E-13 | 0.05 |
| AKAP10 | -0.439072994 | 1.00E-52 | -0.45209816 | 3.61E-56 | 0.02 |
| AKAP11 | -0.474432313 | 1.96E-62 | -0.398641371 | 6.03E-43 | 0.08 |
| GRAVIN | -0.475395754 | 1.03E-62 | -0.364193683 | 1.28E-35 | 0.12 |
| LBC | -0.435981519 | 6.27E-52 | -0.524764613 | 2.25E-78 | 0.09 |
| AKAP14 | 0.025693974 | 0.39608997 | 0.064897903 | 0.0319235 | 0.04 |
| PRKA1 | 0.108805735 | 0.00031364 | 0.073836898 | 0.0146212 | 0.04 |
| PRKA2 | -0.312348108 | 3.67E-26 | -0.452527335 | 2.76E-56 | 0.15 |
| AKAP3 | 0.099455515 | 0.00099316 | -0.06963955 | 0.0213079 | 0.17 |
| AKAP4 | -0.024107484 | 0.42590973 | -0.044850145 | 0.1383883 | 0.03 |
| AKAP5 | -0.333141689 | 9.72E-30 | -0.153922299 | 3.16E-07 | 0.18 |
| AKAP6 | -0.213564292 | 9.68E-13 | -0.273901605 | 2.92E-20 | 0.07 |
| LOC51743 | 0.052280552 | 0.084055 | -0.014738675 | 0.6264443 | 0.07 |
| AKAP8L | 0.186362713 | 5.34E-10 | 0.103244991 | 0.0006295 | 0.09 |
| AKAP8 | 0.043491207 | 0.1507527 | 0.005896803 | 0.8456042 | 0.04 |
| AKAP450 | -0.511411905 | 6.99E-74 | -0.375524996 | 6.22E-38 | 0.14 |
| AK9 | -0.205356123 | 7.15E-12 | -0.10552788 | 0.0004748 | 0.1 |
| AKIRIN1 | 0.060914647 | 0.04406864 | -0.102495431 | 0.0006898 | 0.17 |
| AKIRIN2 | 0.339091732 | 8.16E-31 | 0.213272829 | 1.04E-12 | 0.13 |
| AKNAD1 | -0.028756683 | 0.34220355 | 0.032949551 | 0.2764277 | 0.07 |
| LOC158267 | -0.148536735 | 8.12E-07 | -0.247392057 | 1.05E-16 | 0.1 |
| AKR1A1 | 0.085839448 | 0.00451284 | 0.140833645 | 2.95E-06 | 0.06 |
| AKR1B11 | -0.021088844 | 0.48612298 | -0.003091608 | 0.9186824 | 0.02 |
| AKR1B15 | 0.105678492 | 0.00046597 | -0.021360206 | 0.4805286 | 0.13 |
| AKR1B1 | 0.122971638 | 4.58E-05 | -0.020837554 | 0.4913347 | 0.15 |
| AKR1C1 | -0.018555586 | 0.54000283 | -0.074106138 | 0.0142636 | 0.06 |
| TDD | -0.060246318 | 0.04644722 | -0.088200876 | 0.0035192 | 0.03 |
| HSD17B5 | -0.131807446 | 1.23E-05 | -0.116403098 | 0.0001148 | 0.02 |
| AKR1C4 | 0.049027413 | 0.10523381 | -0.022496368 | 0.4574906 | 0.08 |
| AKR1C8P | -0.030361638 | 0.31593078 | -0.025535745 | 0.3990063 | 0.01 |
| AKR1D1 | -0.060692756 | 0.0448467 | 0.051531862 | 0.0885954 | 0.12 |
| AKR1E2 | 0.228124008 | 2.27E-14 | 0.086223755 | 0.0043355 | 0.15 |
| LOC115578 | -0.03442964 | 0.2554139 | -0.040420556 | 0.1817643 | 0.01 |
| AKR7A3 | -0.233786458 | 4.92E-15 | -0.088982886 | 0.0032369 | 0.15 |
| AKR7L | -0.135908041 | 6.51E-06 | -0.065247256 | 0.0310103 | 0.08 |
| AKT1S1 | 0.199013293 | 3.17E-11 | 0.216110233 | 5.12E-13 | 0.02 |
| AKT1 | -0.072997379 | 0.01578743 | -0.02392249 | 0.4294701 | 0.05 |
| AKT2 | -0.034779182 | 0.25061751 | -0.11562043 | 0.0001276 | 0.09 |
| AKT3 | -0.157975797 | 1.52E-07 | -0.188381232 | 3.45E-10 | 0.04 |
| AKTIP | 0.06367667 | 0.03529885 | -0.031630983 | 0.2961142 | 0.1 |
| ALAD | -0.465195006 | 8.72E-60 | -0.255881937 | 8.44E-18 | 0.21 |
| ALAS1 | 0.072513198 | 0.01649656 | -0.058561137 | 0.0529278 | 0.14 |
| LOC115365 | -0.095255037 | 0.00161739 | -0.067431876 | 0.0257925 | 0.03 |
| ALB | -0.100723909 | 0.00085403 | -0.090836407 | 0.0026481 | 0.01 |
| ALCAM | -0.376450752 | 3.99E-38 | -0.191783805 | 1.63E-10 | 0.19 |
| ALDH16A1 | 0.007121381 | 0.81407652 | 0.036652593 | 0.2259816 | 0.03 |
| LOC118736 | 0.079382914 | 0.00865004 | 0.172220679 | 1.00E-08 | 0.1 |
| ALDH1A1 | -0.253467552 | 1.75E-17 | -0.174752016 | 6.04E-09 | 0.08 |
| ALDH1A2 | -0.286226898 | 4.70E-22 | -0.201930373 | 1.61E-11 | 0.09 |
| LOC145757 | -0.074425855 | 0.01384893 | -0.129340353 | 1.79E-05 | 0.06 |
| ALDH1B1 | 0.08924435 | 0.00314717 | 0.004688403 | 0.8769595 | 0.09 |
| ALDH1L1 | -0.167582559 | 2.50E-08 | -0.137412112 | 5.13E-06 | 0.04 |
| ALDH1L2 | -0.123437446 | 4.28E-05 | -0.176754735 | 4.02E-09 | 0.06 |
| ALDH2 | -0.113053343 | 0.00018015 | -0.061288946 | 0.042782 | 0.06 |
| ALDH3A1 | 0.089230656 | 0.00315181 | -0.069982744 | 0.0206755 | 0.16 |
| ALDH3A2 | -0.156507478 | 1.99E-07 | -0.111597837 | 0.0002183 | 0.05 |
| LOC115175 | -0.05106931 | 0.09149787 | -0.085320968 | 0.0047624 | 0.04 |
| ALDH3B2 | -0.207609258 | 4.16E-12 | -0.073189768 | 0.0155132 | 0.14 |
| ALDH4A1 | -0.134631544 | 7.96E-06 | -0.068038304 | 0.024486 | 0.07 |
| ALDH5A1 | -0.004787005 | 0.8743931 | -0.071681064 | 0.0177807 | 0.07 |
| LOC100506517 | -0.516688335 | 1.24E-75 | -0.305304042 | 5.18E-25 | 0.22 |
| EPD | 0.111789846 | 0.00021288 | 0.120814144 | 6.22E-05 | 0.01 |
| ALDH8A1 | 0.00272801 | 0.9282185 | 0.008879757 | 0.7693391 | 0.01 |
| ALDH9A1 | -0.077739948 | 0.0101383 | 0.184504661 | 7.96E-10 | 0.27 |
| LOC115339 | 0.085589222 | 0.00463177 | 0.127054893 | 2.52E-05 | 0.05 |
| LOC138369 | -0.031914141 | 0.29180953 | -0.06621876 | 0.0285876 | 0.04 |
| ALDOC | 0.15794126 | 1.53E-07 | 0.003879362 | 0.8980638 | 0.16 |
| ALG10B | -0.325218181 | 2.42E-28 | -0.216499094 | 4.64E-13 | 0.11 |
| ALG10 | -0.16194534 | 7.31E-08 | -0.095930436 | 0.0014973 | 0.07 |
| ALG11 | -0.043128386 | 0.15419199 | -0.06001858 | 0.047282 | 0.02 |
| ALG12 | -0.037797077 | 0.2118083 | 0.006963445 | 0.818127 | 0.05 |
| LOC728848 | -0.248655021 | 7.27E-17 | -0.117738367 | 9.56E-05 | 0.14 |
| LOC116072 | 0.071060679 | 0.01879421 | 0.256681198 | 6.63E-18 | 0.19 |
| ALG1L2 | 0.081769311 | 0.00683506 | 0.084747262 | 0.0050531 | 0.01 |
| ALG1L | 0.137963739 | 4.70E-06 | 0.170098022 | 1.53E-08 | 0.04 |
| ALG1 | -0.117178333 | 0.0001032 | 0.007200874 | 0.8120397 | 0.13 |
| ALG2 | -0.125324757 | 3.25E-05 | -0.042132997 | 0.1639332 | 0.09 |
| LOC131416 | 0.546692045 | 3.36E-86 | 0.39612852 | 2.21E-42 | 0.16 |
| ALG5 | 0.052056637 | 0.08539278 | 0.164570874 | 4.45E-08 | 0.12 |
| LOC94752 | 0.265598575 | 4.20E-19 | 0.248019173 | 8.75E-17 | 0.02 |
| ALG8 | 0.060041873 | 0.04719605 | 0.174115636 | 6.87E-09 | 0.12 |
| ALG9 | 0.02139992 | 0.47971283 | 0.056491904 | 0.0619005 | 0.04 |
| ALKBH1 | -0.210632986 | 2.00E-12 | -0.012940865 | 0.6691168 | 0.2 |
| ALKBH2 | 0.480983691 | 2.32E-64 | 0.418819045 | 1.17E-47 | 0.07 |
| LOC143968 | -0.012159403 | 0.68801418 | -0.034161412 | 0.2591375 | 0.03 |
| ALKBH4 | 0.257247816 | 5.58E-18 | 0.269426639 | 1.24E-19 | 0.02 |
| ALKBH5 | -0.031704466 | 0.29499305 | -0.108354469 | 0.0003323 | 0.08 |
| ALKBH6 | 0.276048468 | 1.44E-20 | 0.234787383 | 3.74E-15 | 0.05 |
| ALKBH7 | 0.034729491 | 0.25129551 | 0.129031227 | 1.88E-05 | 0.1 |
| ALKBH8 | -0.286780155 | 3.88E-22 | -0.235895665 | 2.75E-15 | 0.06 |
| ALK | 0.062916203 | 0.03755077 | -0.04492223 | 0.1377549 | 0.11 |
| ALLC | 0.001285439 | 0.966141 | -0.017451974 | 0.5643749 | 0.02 |
| ALMS1P | -0.21616222 | 5.05E-13 | -0.191234918 | 1.84E-10 | 0.03 |
| KIAA0328 | -0.175683075 | 5.00E-09 | -0.246239077 | 1.47E-16 | 0.08 |
| ALOX12B | 0.196226402 | 6.00E-11 | -0.062451665 | 0.0389856 | 0.26 |
| ALOX12P2 | 0.121625158 | 5.55E-05 | -0.015557242 | 0.6074103 | 0.14 |
| ALOX12 | 0.060988699 | 0.04381153 | -0.026822696 | 0.3756618 | 0.09 |
| ALOX15B | -0.148768461 | 7.80E-07 | 0.00360019 | 0.9053637 | 0.16 |
| ALOX15 | -0.066820569 | 0.02717003 | 0.001109758 | 0.9707663 | 0.07 |
| ALOX5AP | -0.125268804 | 3.28E-05 | -0.146050787 | 1.24E-06 | 0.03 |
| ALOX5 | -0.200849284 | 2.07E-11 | -0.240779174 | 7.02E-16 | 0.04 |
| ALOXE3 | -0.016868836 | 0.57746286 | -0.094095971 | 0.0018443 | 0.08 |
| ALPI | 0.045713228 | 0.13095038 | 0.056000112 | 0.0642086 | 0.02 |
| KIAA1527 | -0.195039488 | 7.86E-11 | -0.148579355 | 8.06E-07 | 0.05 |
| ALPK2 | -0.034950821 | 0.24828542 | -0.081588329 | 0.0069596 | 0.05 |
| ALPK3 | -0.34212389 | 2.26E-31 | -0.328699634 | 5.96E-29 | 0.02 |
| ALPL | 0.065796919 | 0.02961883 | -0.041377046 | 0.1716353 | 0.11 |
| LOC115984 | 0.199195219 | 3.04E-11 | 0.074767138 | 0.0134181 | 0.13 |
| LOC129564 | 0.206304128 | 5.70E-12 | 0.046746538 | 0.122457 | 0.16 |
| ALS2CL | -0.078236817 | 0.00966585 | -0.010024891 | 0.7406011 | 0.07 |
| ALS2CR11 | -0.224064753 | 6.64E-14 | -0.15997676 | 1.05E-07 | 0.07 |
| ALS2CR12 | -0.124344618 | 3.75E-05 | -0.056063243 | 0.0639084 | 0.07 |
| TMEM237 | 0.087358982 | 0.00384803 | 0.009337595 | 0.7578088 | 0.08 |
| LOC644870 | -0.477630306 | 2.28E-63 | -0.414181576 | 1.51E-46 | 0.07 |
| ALS2CR6 | -0.223173725 | 8.38E-14 | -0.219452474 | 2.19E-13 | 0.01 |
| ALX1 | 0.188368743 | 3.46E-10 | 0.08479252 | 0.0050296 | 0.11 |
| FND | 0.045568362 | 0.13217672 | -0.140214183 | 3.27E-06 | 0.19 |
| PFM | -0.244373525 | 2.52E-16 | -0.29351117 | 3.70E-23 | 0.05 |
| SLC35G5 | 0.02532943 | 0.40282817 | 0.079495629 | 0.0085555 | 0.06 |
| LOC146741 | -0.186317075 | 5.40E-10 | -0.16994888 | 1.57E-08 | 0.02 |
| SLC35G3 | 0.023732224 | 0.43314984 | 0.053681989 | 0.0760624 | 0.03 |
| AMACR | -0.269186995 | 1.34E-19 | -0.115820449 | 0.0001242 | 0.16 |
| LOC51269 | 0.114664924 | 0.00014523 | 0.059621849 | 0.0487663 | 0.06 |
| AMBP | -0.117436415 | 9.96E-05 | 0.028941884 | 0.3391025 | 0.15 |
| AMBRA1 | -0.26539778 | 4.47E-19 | -0.317136881 | 5.83E-27 | 0.06 |
| LOC221268 | 0.349690157 | 8.64E-33 | 0.134054037 | 8.71E-06 | 0.22 |
| AMDHD1 | -0.056786117 | 0.06055283 | -0.10045608 | 0.0008818 | 0.05 |
| AMDHD2 | -0.052034381 | 0.08552669 | -0.018349025 | 0.5445242 | 0.04 |
| AMELX | 0.047014752 | 0.12032424 | 0.004924287 | 0.8708221 | 0.05 |
| AMELY | 0.009477262 | 0.75430192 | 0.020525621 | 0.4978457 | 0.02 |
| AMFR | -0.206322877 | 5.67E-12 | -0.216002847 | 5.26E-13 | 0.01 |
| AMHR2 | -0.078276255 | 0.0096292 | -0.032679404 | 0.2803867 | 0.05 |
| AMH | -0.038389506 | 0.20472888 | 0.029568109 | 0.3287509 | 0.07 |
| AMICA1 | -0.039153052 | 0.19586021 | -0.138442993 | 4.35E-06 | 0.1 |
| AMIGO1 | -0.348781454 | 1.28E-32 | -0.378527891 | 1.46E-38 | 0.03 |
| AMIGO2 | -0.222228313 | 1.07E-13 | -0.082524947 | 0.0063362 | 0.14 |
| AMIGO3 | 0.02978557 | 0.32520459 | 0.071491111 | 0.0180858 | 0.05 |
| AMMECR1L | -0.141846604 | 2.50E-06 | -0.216135656 | 5.09E-13 | 0.08 |
| LOC286505 | 0.022688849 | 0.45364991 | 0.044542869 | 0.1411135 | 0.03 |
| AMN1 | -0.119555371 | 7.42E-05 | -0.049995699 | 0.098529 | 0.07 |
| AMN | 0.272942232 | 3.99E-20 | 0.08171239 | 0.006874 | 0.2 |
| JEAP | -0.144191571 | 1.70E-06 | -0.204150236 | 9.53E-12 | 0.06 |
| KIAA0989 | -0.29981667 | 3.87E-24 | -0.278879436 | 5.65E-21 | 0.03 |
| KIAA1071 | -0.148561782 | 8.08E-07 | -0.04535131 | 0.1340308 | 0.11 |
| AMPD1 | -0.147753213 | 9.29E-07 | -0.143817638 | 1.81E-06 | 0.01 |
| AMPD2 | 0.154370566 | 2.92E-07 | 0.005688449 | 0.8509946 | 0.15 |
| AMPD3 | -0.152284862 | 4.23E-07 | -0.320963024 | 1.31E-27 | 0.17 |
| AMPH | -0.33601268 | 2.96E-30 | -0.162768294 | 6.26E-08 | 0.18 |
| AMTN | 0.212803377 | 1.17E-12 | 0.052496663 | 0.08278 | 0.17 |
| AMT | -0.110690973 | 0.00024581 | -0.062110565 | 0.0400685 | 0.05 |
| AMY1A | 0.341665118 | 2.75E-31 | 0.125548921 | 3.15E-05 | 0.22 |
| AMY2A | 0.088062431 | 0.00357146 | 0.044408653 | 0.1423166 | 0.05 |
| AMY2B | -0.090993244 | 0.00260309 | -0.0822985 | 0.0064822 | 0.01 |
| AMZ1 | -0.113881452 | 0.00016132 | -0.093863626 | 0.0018932 | 0.03 |
| AMZ2P1 | 0.051638657 | 0.08793596 | 0.104228276 | 0.0005579 | 0.06 |
| AMZ2 | 0.179973901 | 2.07E-09 | 0.15488098 | 2.67E-07 | 0.03 |
| ANAPC10 | 0.23174677 | 8.58E-15 | 0.124010829 | 3.94E-05 | 0.11 |
| ANAPC11 | 0.420137112 | 5.60E-48 | 0.384457656 | 8.02E-40 | 0.04 |
| ANAPC13 | -0.046734578 | 0.1225528 | 0.156229012 | 2.09E-07 | 0.21 |
| ANAPC16 | 0.101748194 | 0.0007551 | 0.165631612 | 3.64E-08 | 0.07 |
| LOC100134261 | -0.126202793 | 2.86E-05 | -0.238423632 | 1.36E-15 | 0.12 |
| ANAPC2 | -0.122453883 | 4.93E-05 | -0.05116031 | 0.0909209 | 0.08 |
| ANAPC4 | -0.046103103 | 0.12769369 | 0.021963892 | 0.4682097 | 0.07 |
| PDL.108 | 0.217599455 | 3.51E-13 | 0.124429232 | 3.71E-05 | 0.1 |
| ANAPC7 | 0.389468981 | 6.57E-41 | 0.401839611 | 1.13E-43 | 0.02 |
| ANGEL1 | -0.31181327 | 4.50E-26 | -0.258773364 | 3.50E-18 | 0.06 |
| ANGEL2 | -0.25753714 | 5.11E-18 | 0.177855746 | 3.21E-09 | 0.44 |
| ANGPT1 | -0.065954489 | 0.02922997 | -0.202578887 | 1.38E-11 | 0.14 |
| ANGPT2 | -0.132880076 | 1.05E-05 | -0.154745646 | 2.73E-07 | 0.03 |
| ANGPT4 | -0.400344259 | 2.48E-43 | -0.322770985 | 6.41E-28 | 0.08 |
| ANGPTL1 | -0.40370058 | 4.26E-44 | -0.257845785 | 4.65E-18 | 0.15 |
| ANGPTL2 | -0.265376264 | 4.50E-19 | -0.188201243 | 3.59E-10 | 0.08 |
| FHBL2 | 0.018277821 | 0.54608714 | -0.015546121 | 0.6076672 | 0.04 |
| PGAR | 0.064108548 | 0.03407197 | -0.012676032 | 0.6754983 | 0.08 |
| ANGPTL5 | -0.16442165 | 4.58E-08 | -0.107747744 | 0.000359 | 0.06 |
| ANGPTL6 | -0.011290112 | 0.70926597 | -0.097103271 | 0.001308 | 0.09 |
| LOC149217 | -0.198855151 | 3.29E-11 | -0.185118374 | 6.98E-10 | 0.02 |
| ANG | -0.178085497 | 3.06E-09 | 0.032909312 | 0.2770149 | 0.22 |
| LOC157847 | 0.012275231 | 0.68520055 | -0.167262016 | 2.66E-08 | 0.18 |
| LQT4 | -0.311253287 | 5.57E-26 | -0.248933394 | 6.70E-17 | 0.07 |
| ANK3 | -0.221009502 | 1.47E-13 | -0.098108699 | 0.0011636 | 0.13 |
| ANKAR | -0.306725602 | 3.05E-25 | -0.23755688 | 1.73E-15 | 0.07 |
| ANKDD1A | 0.056787145 | 0.06054816 | -0.054608103 | 0.0711287 | 0.12 |
| ANKFN1 | -0.26078631 | 1.89E-18 | -0.051729465 | 0.0873783 | 0.21 |
| ANKFY1 | -0.410062634 | 1.42E-45 | -0.402875599 | 6.58E-44 | 0.01 |
| ANKHD1.EIF4EBP3 | -0.486475825 | 5.22E-66 | -0.290064948 | 1.24E-22 | 0.2 |
| ANKHD1 | -0.294114812 | 2.99E-23 | -0.205988126 | 6.15E-12 | 0.09 |
| ANKH | -0.070992866 | 0.018908 | -0.193972883 | 9.99E-11 | 0.13 |
| ANKIB1 | -0.211720897 | 1.53E-12 | -0.268248696 | 1.81E-19 | 0.06 |
| ANKK1 | -0.116148899 | 0.0001188 | -0.104465535 | 0.0005418 | 0.02 |
| ANKLE1 | 0.37607935 | 4.77E-38 | 0.077908801 | 0.0099755 | 0.3 |
| ANKLE2 | 0.084901505 | 0.0049734 | -0.136692254 | 5.75E-06 | 0.23 |
| ANKMY1 | -0.276041661 | 1.45E-20 | -0.033576898 | 0.2673813 | 0.25 |
| LOC116364 | -0.473884216 | 2.83E-62 | -0.239339871 | 1.05E-15 | 0.24 |
| ANKRA2 | -0.482366037 | 8.99E-65 | -0.198375317 | 3.67E-11 | 0.29 |
| ANKRD10 | -0.124819987 | 3.50E-05 | -0.005481273 | 0.8563614 | 0.12 |
| LOC92821 | 0.049317937 | 0.10318518 | -0.243617137 | 3.13E-16 | 0.3 |
| FLJ20053 | -0.292215366 | 5.85E-23 | -0.33655135 | 2.37E-30 | 0.05 |
| ANKRD13A | -0.127148242 | 2.49E-05 | -0.275590141 | 1.68E-20 | 0.15 |
| ANKRD13B | 0.32550618 | 2.16E-28 | 0.182573308 | 1.20E-09 | 0.15 |
| ANKRD13C | -0.147709358 | 9.36E-07 | -0.21540062 | 6.12E-13 | 0.07 |
| ANKRD13D | 0.137998171 | 4.67E-06 | 0.100305596 | 0.0008978 | 0.04 |
| ANKRD16 | 0.227340078 | 2.80E-14 | 0.231127828 | 1.01E-14 | 0.01 |
| FLJ22206 | -0.23362011 | 5.15E-15 | -0.313922547 | 2.01E-26 | 0.09 |
| ANKRD19P | 0.246248165 | 1.47E-16 | 0.246008889 | 1.57E-16 | 0.01 |
| ANKRD1 | 0.144678915 | 1.56E-06 | -0.015282786 | 0.6137635 | 0.16 |
| ANKRD20A3 | -0.068774099 | 0.02297798 | -0.123161417 | 4.45E-05 | 0.06 |
| LOC392331 | -0.127613456 | 2.32E-05 | -0.118860304 | 8.18E-05 | 0.01 |
| ANKRD20B | -0.053888491 | 0.07493881 | -0.092976764 | 0.0020909 | 0.04 |
| ANKRD22 | 0.08438751 | 0.00524342 | 0.074241949 | 0.0140861 | 0.02 |
| ANKRD23 | 0.065749967 | 0.02973555 | -0.038188438 | 0.2071121 | 0.11 |
| ANKRD24 | -0.278318707 | 6.81E-21 | -0.138697621 | 4.18E-06 | 0.14 |
| ANKRD26P1 | 0.07748699 | 0.01038662 | -0.014777782 | 0.6255291 | 0.1 |
| LOC101929149 | -0.179092834 | 2.49E-09 | -0.218805378 | 2.59E-13 | 0.04 |
| ANKRD27 | 0.228701443 | 1.95E-14 | 0.00100592 | 0.9735006 | 0.23 |
| ANKRD28 | -0.158947701 | 1.27E-07 | -0.193628715 | 1.08E-10 | 0.04 |
| ANKRD29 | -0.295141327 | 2.08E-23 | -0.322797348 | 6.34E-28 | 0.03 |
| ANKRD2 | -0.026820539 | 0.37570023 | -0.013910218 | 0.6459645 | 0.02 |
| ANKRD30A | -0.462979328 | 3.66E-59 | -0.199384205 | 2.91E-11 | 0.27 |
| ANKRD30B | -0.278634886 | 6.13E-21 | -0.081779117 | 0.0068284 | 0.2 |
| ANKRD31 | -0.243329753 | 3.40E-16 | -0.212918059 | 1.14E-12 | 0.04 |
| BRCTD1 | -0.111040804 | 0.00023484 | -0.003078668 | 0.9190216 | 0.11 |
| C12orf7 | -0.057626043 | 0.05683839 | -0.075522021 | 0.0125069 | 0.02 |
| ANKRD34A | -0.056201675 | 0.06325421 | 0.054180644 | 0.0733724 | 0.12 |
| ANKRD34B | 0.119673764 | 7.30E-05 | -0.006554451 | 0.8286384 | 0.13 |
| ANKRD34C | -0.237193092 | 1.92E-15 | -0.074720625 | 0.0134761 | 0.17 |
| LOC101060345 | 0.025120271 | 0.40672503 | -0.046605608 | 0.1235894 | 0.08 |
| ANKRD36BP1 | -0.173409312 | 7.92E-09 | -0.252746223 | 2.17E-17 | 0.08 |
| ANKRD36B | -0.189494687 | 2.70E-10 | -0.25175584 | 2.91E-17 | 0.07 |
| ANKRD36 | -0.029056284 | 0.33719606 | -0.146107274 | 1.23E-06 | 0.12 |
| ANKRD37 | 0.062689027 | 0.03824673 | 0.031988614 | 0.2906844 | 0.04 |
| ANKRD39 | 0.398230275 | 7.47E-43 | 0.29426934 | 2.83E-23 | 0.11 |
| ANKRD40 | -0.185805538 | 6.02E-10 | -0.144645581 | 1.57E-06 | 0.05 |
| LOC729814 | -0.371041114 | 5.25E-37 | -0.157224616 | 1.75E-07 | 0.22 |
| SOWAHA | -0.333684478 | 7.77E-30 | -0.074375381 | 0.0139137 | 0.26 |
| ANKRD44 | -0.360590055 | 6.67E-35 | -0.427194629 | 1.03E-49 | 0.07 |
| ANKRD45 | 0.209836768 | 2.43E-12 | 0.033142238 | 0.2736272 | 0.18 |
| ANKRD46 | 0.118532525 | 8.56E-05 | -0.015862314 | 0.6003831 | 0.14 |
| ANKRD49 | 0.006332578 | 0.83435373 | 0.006434092 | 0.8317377 | 0.01 |
| ANKRD50 | -0.377998344 | 1.89E-38 | -0.240848496 | 6.88E-16 | 0.14 |
| LOC100131489 | -0.301167336 | 2.37E-24 | -0.332997212 | 1.03E-29 | 0.04 |
| ANKRD53 | -0.006358439 | 0.83368712 | -0.062317927 | 0.0394072 | 0.06 |
| ANKRD54 | 0.336738447 | 2.19E-30 | 0.215856541 | 5.46E-13 | 0.13 |
| ANKRD55 | -0.066169565 | 0.02870628 | -0.116487765 | 0.0001134 | 0.06 |
| SOWAHB | -0.354479335 | 1.04E-33 | -0.248477598 | 7.65E-17 | 0.11 |
| SOWAHC | -0.164696171 | 4.35E-08 | -0.22857024 | 2.02E-14 | 0.07 |
| LOC648756 | 0.081625431 | 0.00693394 | -0.006296996 | 0.8352711 | 0.09 |
| ANKEF1 | -0.264887312 | 5.25E-19 | -0.136183092 | 6.24E-06 | 0.13 |
| ANKRD6 | -0.180565874 | 1.83E-09 | -0.186476479 | 5.21E-10 | 0.01 |
| ANKRD7 | 0.24512349 | 2.03E-16 | 0.057177169 | 0.0587992 | 0.19 |
| ANKRD9 | 0.026280313 | 0.38539578 | 0.03200098 | 0.2904978 | 0.01 |
| ANKS1A | -0.054097171 | 0.07381716 | -0.142042128 | 2.42E-06 | 0.09 |
| ANKS1B | -0.321292133 | 1.15E-27 | -0.149338511 | 7.07E-07 | 0.18 |
| ANKS3 | 0.069987486 | 0.02066689 | 0.114068143 | 0.0001573 | 0.05 |
| ANKS4B | 0.030458748 | 0.31438471 | 0.063927795 | 0.034581 | 0.04 |
| ANKS6 | 0.224259798 | 6.31E-14 | 0.01777626 | 0.5571588 | 0.21 |
| ANKZF1 | 0.130591344 | 1.48E-05 | 0.108066667 | 0.0003447 | 0.03 |
| ANLN | 0.363427636 | 1.82E-35 | 0.139921537 | 3.43E-06 | 0.23 |
| ANO10 | -0.090076639 | 0.0028765 | 0.04649725 | 0.1244656 | 0.14 |
| ANO1 | -0.270218495 | 9.63E-20 | -0.132954918 | 1.03E-05 | 0.14 |
| C12ORF3 | -0.153536045 | 3.39E-07 | -0.177115254 | 3.74E-09 | 0.03 |
| ANO3 | -0.069204983 | 0.02213283 | -0.082105724 | 0.0066088 | 0.02 |
| ANO4 | -0.001081583 | 0.97150816 | -0.086459971 | 0.0042297 | 0.09 |
| LGMD2L | -0.144171365 | 1.70E-06 | -0.185093004 | 7.02E-10 | 0.05 |
| ANO6 | -0.416550637 | 4.10E-47 | -0.312840202 | 3.05E-26 | 0.11 |
| NGEP | 0.12513995 | 3.34E-05 | 0.025998688 | 0.3905102 | 0.1 |
| ANO8 | -0.169613465 | 1.68E-08 | -0.112709597 | 0.0001886 | 0.06 |
| ANO9 | 0.017634167 | 0.56031516 | 0.019663458 | 0.5160775 | 0.01 |
| ANP32A | 0.212726245 | 1.19E-12 | 0.216093028 | 5.14E-13 | 0.01 |
| ANP32B | 0.550463837 | 1.32E-87 | 0.35041879 | 6.28E-33 | 0.21 |
| ANP32C | 0.086830622 | 0.00406838 | 0.049998004 | 0.0985135 | 0.04 |
| ANP32D | 0.026567902 | 0.3802155 | 0.009731531 | 0.7479306 | 0.02 |
| ANP32E | 0.389859018 | 5.40E-41 | 0.242651686 | 4.12E-16 | 0.15 |
| ANPEP | -0.061676576 | 0.04148303 | -0.119844747 | 7.13E-05 | 0.06 |
| LOC220695 | -0.266649822 | 3.01E-19 | -0.202939665 | 1.27E-11 | 0.07 |
| ANTXR2 | -0.309699431 | 1.00E-25 | -0.273082283 | 3.81E-20 | 0.04 |
| ANTXRL | 0.288289507 | 2.30E-22 | 0.134246719 | 8.45E-06 | 0.16 |
| LOC143160 | -0.301730816 | 1.93E-24 | -0.208981434 | 2.99E-12 | 0.1 |
| ANX14 | -0.017873061 | 0.5550135 | -0.031307067 | 0.3010903 | 0.02 |
| ANXA11 | -0.11459449 | 0.00014661 | -0.067382296 | 0.0259019 | 0.05 |
| ANXA13 | -0.108099086 | 0.0003433 | 0.039505358 | 0.1918643 | 0.15 |
| ANXA1 | 0.050234237 | 0.0969307 | -0.084396444 | 0.0052386 | 0.14 |
| LOC90722 | 0.006242309 | 0.83668152 | 0.022076997 | 0.4659213 | 0.02 |
| ANXA2P2 | -0.034786146 | 0.25052259 | -0.020702124 | 0.494156 | 0.02 |
| LOC221025 | 0.085297479 | 0.00477402 | 0.051097438 | 0.0913192 | 0.04 |
| ANXA2 | 0.077362732 | 0.01051057 | 0.088374244 | 0.0034548 | 0.02 |
| ANXA3 | -0.011026389 | 0.7157591 | 0.030443207 | 0.3146318 | 0.05 |
| ANXA4 | 0.057050186 | 0.05936396 | 0.015795602 | 0.6019166 | 0.05 |
| ANXA5 | 0.017134651 | 0.5714792 | -0.030081951 | 0.3204114 | 0.05 |
| ANXA6 | -0.285691645 | 5.64E-22 | -0.252417837 | 2.39E-17 | 0.04 |
| ANXA7 | -0.112383784 | 0.00019686 | -0.092816898 | 0.0021284 | 0.02 |
| LOC142972 | 0.162809796 | 6.21E-08 | 0.003811442 | 0.8998391 | 0.16 |
| ANXA8L2 | 0.099474942 | 0.00099088 | -0.059330988 | 0.0498792 | 0.16 |
| ANXA8 | 0.151785564 | 4.62E-07 | 0.001917328 | 0.9495153 | 0.15 |
| ANXA9 | -0.353105989 | 1.92E-33 | -0.032839322 | 0.2780385 | 0.33 |
| AOAH | -0.13720051 | 5.31E-06 | -0.217610394 | 3.50E-13 | 0.09 |
| AOC2 | -0.012693754 | 0.67507057 | -0.170017795 | 1.55E-08 | 0.16 |
| AOC3 | -0.39409027 | 6.30E-42 | -0.266502558 | 3.15E-19 | 0.13 |
| AOX1 | -0.292690685 | 4.95E-23 | -0.174662643 | 6.15E-09 | 0.12 |
| AOX2P | -0.277361783 | 9.35E-21 | -0.093498208 | 0.0019725 | 0.19 |
| AP1AR | -0.274471887 | 2.42E-20 | -0.148255174 | 8.52E-07 | 0.13 |
| AP1B1 | 0.012983561 | 0.66809019 | -0.111689115 | 0.0002157 | 0.13 |
| AP1G1 | -0.194352647 | 9.18E-11 | -0.249470732 | 5.72E-17 | 0.06 |
| AP1G2 | -0.163076817 | 5.91E-08 | 0.027743123 | 0.3594939 | 0.2 |
| AP1M1 | 0.176776838 | 4.00E-09 | 0.084529301 | 0.0051676 | 0.1 |
| AP1M2 | -0.013844172 | 0.64753151 | 0.145811438 | 1.29E-06 | 0.16 |
| EKV3 | 0.32304142 | 5.76E-28 | 0.361868261 | 3.72E-35 | 0.04 |
| AP1S2 | -0.087987063 | 0.00360021 | -0.124031229 | 3.93E-05 | 0.04 |
| AP1S3 | -0.047249439 | 0.11848199 | -0.09570489 | 0.0015364 | 0.05 |
| AP2A1 | -0.073091123 | 0.01565327 | -0.106063752 | 0.000444 | 0.04 |
| AP2A2 | -0.168385321 | 2.14E-08 | -0.098631692 | 0.0010944 | 0.07 |
| AP2B1 | -0.277919492 | 7.77E-21 | -0.133611866 | 9.33E-06 | 0.15 |
| AP2M1 | 0.345030648 | 6.52E-32 | 0.169672744 | 1.66E-08 | 0.18 |
| AP2S1 | 0.374381694 | 1.08E-37 | 0.353032743 | 1.98E-33 | 0.03 |
| AP3B1 | -0.359217525 | 1.24E-34 | -0.16238528 | 6.73E-08 | 0.2 |
| AP3B2 | 0.0227692 | 0.45205201 | -0.119266357 | 7.73E-05 | 0.15 |
| AP3D1 | -0.140773133 | 2.98E-06 | -0.192040671 | 1.54E-10 | 0.06 |
| AP3M1 | -0.200042485 | 2.50E-11 | -0.142047721 | 2.42E-06 | 0.06 |
| AP3M2 | 0.045407917 | 0.13354529 | -0.052762946 | 0.0812305 | 0.1 |
| AP3S1 | 0.069640396 | 0.02130636 | 0.197246566 | 4.76E-11 | 0.13 |
| AP3S2 | -0.124262526 | 3.80E-05 | -0.100265511 | 0.0009021 | 0.03 |
| AP4B1 | 0.207316213 | 4.47E-12 | 0.062876286 | 0.0376723 | 0.15 |
| AP4E1 | -0.30693882 | 2.82E-25 | -0.347923618 | 1.87E-32 | 0.05 |
| AP4M1 | 0.197141842 | 4.87E-11 | 0.269323348 | 1.28E-19 | 0.08 |
| AP4S1 | -0.128347407 | 2.08E-05 | 0.024510517 | 0.4182129 | 0.16 |
| APAF1 | -0.272742575 | 4.26E-20 | -0.247166883 | 1.12E-16 | 0.03 |
| APBA1 | -0.016670497 | 0.58194671 | -0.142124099 | 2.39E-06 | 0.13 |
| APBA2 | 0.299579692 | 4.22E-24 | 0.076413301 | 0.0115018 | 0.23 |
| APBA3 | 0.054583255 | 0.07125758 | -0.029079301 | 0.3368133 | 0.09 |
| APBB1IP | -0.1607425 | 9.15E-08 | -0.194102005 | 9.71E-11 | 0.04 |
| APBB1 | -0.2483991 | 7.83E-17 | -0.187732012 | 3.97E-10 | 0.07 |
| APBB2 | -0.457471336 | 1.24E-57 | -0.292348587 | 5.58E-23 | 0.17 |
| APBB3 | -0.188088425 | 3.68E-10 | -0.051478621 | 0.0889257 | 0.14 |
| APC2 | -0.037238546 | 0.21864289 | -0.119266634 | 7.73E-05 | 0.09 |
| APCDD1L | 0.039918498 | 0.18725502 | -0.0668746 | 0.0270458 | 0.11 |
| HTS | -0.029219754 | 0.33448377 | -0.089264046 | 0.0031405 | 0.07 |
| APCS | 0.020764731 | 0.49285067 | 0.032333874 | 0.2855062 | 0.02 |
| APC | -0.443684589 | 6.30E-54 | -0.36997897 | 8.67E-37 | 0.08 |
| OPH | -0.149872183 | 6.45E-07 | -0.014808053 | 0.6248212 | 0.14 |
| APEX1 | 0.328008087 | 7.89E-29 | 0.348814911 | 1.27E-32 | 0.03 |
| APEX2 | 0.338794492 | 9.25E-31 | 0.271020843 | 7.44E-20 | 0.07 |
| APH1A | 0.217365098 | 3.73E-13 | 0.400048541 | 2.90E-43 | 0.19 |
| APH1B | -0.497693207 | 1.81E-69 | -0.291214425 | 8.32E-23 | 0.21 |
| LOC63324 | 0.02799653 | 0.35512049 | -0.034998901 | 0.2476349 | 0.07 |
| APIP | 0.15636761 | 2.04E-07 | 0.103121721 | 0.0006391 | 0.06 |
| APITD1 | 0.275842581 | 1.54E-20 | 0.226438876 | 3.56E-14 | 0.05 |
| APLF | 0.127887366 | 2.23E-05 | 0.013872111 | 0.6468685 | 0.12 |
| APLNR | -0.231142396 | 1.01E-14 | -0.156289374 | 2.07E-07 | 0.08 |
| APLN | 0.084841584 | 0.00500422 | -0.012406798 | 0.6820099 | 0.1 |
| APLP1 | 0.138753799 | 4.14E-06 | -0.063756152 | 0.0350703 | 0.21 |
| APLP2 | -0.146095255 | 1.23E-06 | -0.201548574 | 1.76E-11 | 0.06 |
| APOA1BP | 0.315639905 | 1.04E-26 | 0.493657974 | 3.28E-68 | 0.18 |
| APOA1 | 0.043352772 | 0.15205801 | 0.084599145 | 0.0051307 | 0.05 |
| APOA2 | 0.109512101 | 0.0002864 | 0.065440086 | 0.0305159 | 0.05 |
| APOA4 | -0.023174107 | 0.44404837 | -0.040089096 | 0.1853757 | 0.02 |
| APOA5 | -0.056165421 | 0.063425 | 0.011849695 | 0.6955586 | 0.07 |
| LOC220567 | -0.090051132 | 0.00288447 | -0.052385733 | 0.0834325 | 0.04 |
| APOBEC1 | 0.1081258 | 0.00034214 | 0.140950329 | 2.90E-06 | 0.04 |
| APOBEC2 | -0.059162229 | 0.05053461 | -0.105321542 | 0.0004872 | 0.05 |
| APOBEC3A | 0.270683348 | 8.29E-20 | 0.081548597 | 0.0069873 | 0.19 |
| APOBEC3B | 0.281704134 | 2.19E-21 | 0.06635423 | 0.0282631 | 0.22 |
| APOBEC3C | -0.166393998 | 3.14E-08 | -0.287644945 | 2.88E-22 | 0.13 |
| APOBEC3D | -0.150784584 | 5.50E-07 | -0.227924764 | 2.40E-14 | 0.08 |
| APOBEC3F | -0.030929148 | 0.30696597 | -0.144915699 | 1.50E-06 | 0.12 |
| APOBEC3G | -0.055073725 | 0.068749 | -0.144301279 | 1.67E-06 | 0.09 |
| APOBEC3H | 0.04134999 | 0.17191591 | -0.004745876 | 0.8754635 | 0.05 |
| APOBEC4 | -0.081705222 | 0.00687895 | 0.078886432 | 0.0090778 | 0.17 |
| APOB | -0.227216805 | 2.89E-14 | -0.233392947 | 5.48E-15 | 0.01 |
| APOC1P1 | 0.032953659 | 0.27636776 | 0.026673317 | 0.3783274 | 0.01 |
| APOC1 | 0.181209942 | 1.60E-09 | 0.174106738 | 6.88E-09 | 0.01 |
| APOC2 | 0.064022672 | 0.03431298 | 0.068006499 | 0.0245531 | 0.01 |
| APOC3 | 0.019831989 | 0.51248664 | 0.037853796 | 0.211123 | 0.02 |
| APOC4 | 0.046624078 | 0.12344054 | 0.099692305 | 0.0009657 | 0.06 |
| APOD | -0.19193699 | 1.58E-10 | -0.012164497 | 0.6878904 | 0.18 |
| AD2 | 0.082057276 | 0.00664094 | 0.065355948 | 0.0307308 | 0.02 |
| APOF | -0.295773305 | 1.66E-23 | -0.043586839 | 0.149856 | 0.26 |
| APOH | -0.004327113 | 0.88637412 | 0.010360983 | 0.7322327 | 0.02 |
| FSGS4 | -0.042441029 | 0.16087044 | -0.076917265 | 0.0109658 | 0.04 |
| APOL2 | -0.017767488 | 0.5573534 | -0.063667056 | 0.0353266 | 0.05 |
| LOC129128 | -0.126019025 | 2.94E-05 | -0.134896683 | 7.64E-06 | 0.01 |
| APOL4 | -0.07733928 | 0.01053411 | -0.076031554 | 0.0119232 | 0.01 |
| APOL5 | -0.19426635 | 9.36E-11 | -0.083582752 | 0.0056928 | 0.12 |
| APOL6 | -0.100733818 | 0.00085302 | -0.169309667 | 1.78E-08 | 0.07 |
| APOLD1 | -0.255881644 | 8.45E-18 | -0.227612824 | 2.60E-14 | 0.03 |
| LOC257553 | -0.028680456 | 0.34348513 | 0.136305782 | 6.12E-06 | 0.17 |
| APOOL | -0.33900741 | 8.45E-31 | -0.346207981 | 3.92E-32 | 0.01 |
| APOO | 0.401798923 | 1.16E-43 | 0.342031082 | 2.35E-31 | 0.06 |
| APPBP2 | -0.259424381 | 2.87E-18 | -0.16465836 | 4.38E-08 | 0.1 |
| APPL1 | -0.306953173 | 2.81E-25 | -0.236877273 | 2.10E-15 | 0.08 |
| APPL2 | -0.481953701 | 1.19E-64 | -0.278412674 | 6.60E-21 | 0.21 |
| APP | -0.061708958 | 0.04137604 | -0.13497604 | 7.54E-06 | 0.08 |
| LOC115988 | 0.412527459 | 3.73E-46 | 0.291827479 | 6.71E-23 | 0.13 |
| AOA | 0.324199391 | 3.63E-28 | 0.391027008 | 2.99E-41 | 0.07 |
| AQP10 | -0.198105543 | 3.91E-11 | -0.093834828 | 0.0018993 | 0.11 |
| AQP11 | -0.162628277 | 6.43E-08 | -0.087031845 | 0.0039831 | 0.08 |
| AQP12 | 0.057510845 | 0.05733635 | 0.028078315 | 0.3537162 | 0.03 |
| LOC130985 | 0.083756071 | 0.0055932 | 0.012256552 | 0.685654 | 0.08 |
| CO | -0.307537954 | 2.25E-25 | -0.245330018 | 1.91E-16 | 0.07 |
| AQP2 | 0.072550039 | 0.01644164 | 0.050741104 | 0.0936032 | 0.03 |
| LOC116088 | -0.081848135 | 0.00678142 | -0.000845484 | 0.9777258 | 0.09 |
| HMIWC2 | 0.070092075 | 0.0204775 | -0.048701351 | 0.1075713 | 0.12 |
| PPKB | 0.193947108 | 1.01E-10 | 0.138334924 | 4.43E-06 | 0.06 |
| AQP6 | 0.134563211 | 8.05E-06 | 0.048435308 | 0.1095088 | 0.09 |
| AQP7P1 | -0.220926385 | 1.50E-13 | -0.177000556 | 3.82E-09 | 0.05 |
| AQP7P3 | -0.094517266 | 0.00175867 | -0.071660177 | 0.017814 | 0.03 |
| AQP7L | -0.277716473 | 8.31E-21 | -0.173116264 | 8.40E-09 | 0.11 |
| AQP8 | 0.04947599 | 0.10208404 | -0.032128208 | 0.2885832 | 0.09 |
| AQP9 | 0.240512254 | 7.57E-16 | 0.072616322 | 0.0163432 | 0.17 |
| LVRN | -0.208872238 | 3.07E-12 | -0.15528113 | 2.48E-07 | 0.06 |
| AQR | -0.337959751 | 1.31E-30 | -0.273221447 | 3.64E-20 | 0.07 |
| ARAF | -0.111058322 | 0.0002343 | 0.033454566 | 0.2691292 | 0.15 |
| ARAP1 | -0.216997044 | 4.09E-13 | -0.214091786 | 8.49E-13 | 0.01 |
| ARAP2 | -0.122898808 | 4.62E-05 | -0.211914068 | 1.46E-12 | 0.09 |
| ARAP3 | -0.165042459 | 4.07E-08 | -0.190615083 | 2.11E-10 | 0.03 |
| ARCN1 | -0.179339742 | 2.36E-09 | -0.133565922 | 9.40E-06 | 0.05 |
| ARC | -0.001376588 | 0.96374169 | -0.032575967 | 0.2819127 | 0.04 |
| LOC115165 | -0.133012804 | 1.02E-05 | 0.00334755 | 0.9119768 | 0.14 |
| ARF1 | 0.302404785 | 1.51E-24 | 0.495608546 | 8.12E-69 | 0.2 |
| LOC115999 | -0.228524039 | 2.04E-14 | -0.090448747 | 0.0027625 | 0.14 |
| ARF4 | -0.07791715 | 0.0099675 | 0.038220472 | 0.2067311 | 0.12 |
| ARF5 | 0.390634579 | 3.65E-41 | 0.383683968 | 1.18E-39 | 0.01 |
| LOC63379 | -0.025909644 | 0.39213574 | 0.011694638 | 0.6993471 | 0.04 |
| ARFGAP1 | 0.177481033 | 3.47E-09 | 0.125527574 | 3.16E-05 | 0.06 |
| ARFGAP2 | 0.051678716 | 0.08768961 | 0.030336005 | 0.3163397 | 0.03 |
| ARFGAP3 | -0.107796474 | 0.00035679 | -0.082361349 | 0.0064413 | 0.03 |
| DKFZP434L057 | -0.17034997 | 1.45E-08 | -0.177023967 | 3.81E-09 | 0.01 |
| ARFGEF2 | -0.348552364 | 1.42E-32 | -0.360069185 | 8.45E-35 | 0.02 |
| LOC102724121 | -0.34317256 | 1.45E-31 | -0.174519792 | 6.33E-09 | 0.17 |
| ARFIP2 | -0.207638833 | 4.14E-12 | 0.000926419 | 0.9755942 | 0.21 |
| LOC115076 | 0.147555576 | 9.61E-07 | 0.059651989 | 0.0486522 | 0.09 |
| ARG1 | 0.010630517 | 0.72554445 | -0.04699258 | 0.1204994 | 0.06 |
| ARG2 | 0.120330643 | 6.66E-05 | 0.049573816 | 0.1014072 | 0.08 |
| ARGFXP2 | -0.174179695 | 6.78E-09 | -0.127063223 | 2.52E-05 | 0.05 |
| ARGFX | 0.00826054 | 0.78501452 | -0.007141031 | 0.8135729 | 0.02 |
| ARGLU1 | -0.126291033 | 2.82E-05 | -0.155428125 | 2.42E-07 | 0.03 |
| ARHGAP10 | 0.073392395 | 0.01522887 | -0.085316783 | 0.0047645 | 0.16 |
| ARHGAP11A | 0.362422719 | 2.89E-35 | 0.110100415 | 0.0002654 | 0.26 |
| ARHGAP11B | 0.118906517 | 8.13E-05 | -0.113755188 | 0.0001641 | 0.24 |
| FLJ10971 | -0.380237147 | 6.37E-39 | -0.277656815 | 8.48E-21 | 0.11 |
| ARHGAP15 | -0.060003932 | 0.04733613 | -0.104726472 | 0.0005246 | 0.05 |
| LOC100129892 | 0.0345065 | 0.25435381 | -0.160128894 | 1.02E-07 | 0.2 |
| ARHGAP18 | -0.268270379 | 1.80E-19 | -0.218340976 | 2.91E-13 | 0.05 |
| ARHGAP19 | -0.131512525 | 1.29E-05 | -0.124940204 | 3.44E-05 | 0.01 |
| ARHGAP1 | -0.411692802 | 5.87E-46 | -0.233007482 | 6.08E-15 | 0.18 |
| ARHGAP20 | -0.448655638 | 3.04E-55 | -0.368403381 | 1.81E-36 | 0.09 |
| ARHGAP21 | 0.018110718 | 0.54976371 | -0.257836197 | 4.66E-18 | 0.28 |
| LOC101930357 | 0.20521083 | 7.40E-12 | 0.02913632 | 0.3358663 | 0.18 |
| ARHGAP23 | -0.23015686 | 1.32E-14 | -0.243101752 | 3.63E-16 | 0.02 |
| ARHGAP24 | -0.279661295 | 4.35E-21 | -0.249928657 | 5.00E-17 | 0.03 |
| ARHGAP25 | -0.088803034 | 0.00329991 | -0.204309843 | 9.17E-12 | 0.12 |
| ARHGAP26 | -0.165844016 | 3.49E-08 | -0.14413953 | 1.71E-06 | 0.03 |
| SH3D20 | -0.010075876 | 0.73932957 | -0.092124943 | 0.0022983 | 0.09 |
| LOC388462 | -0.137780384 | 4.84E-06 | -0.142133936 | 2.39E-06 | 0.01 |
| ARHGAP29 | -0.354015035 | 1.28E-33 | -0.193075181 | 1.22E-10 | 0.17 |
| ARHGAP30 | -0.067546783 | 0.02554041 | -0.151090433 | 5.21E-07 | 0.09 |
| ARHGAP31 | -0.238564923 | 1.31E-15 | -0.328738445 | 5.87E-29 | 0.1 |
| MGC1892 | -0.424002777 | 6.36E-49 | -0.215204369 | 6.43E-13 | 0.21 |
| DKFZp434A1010 | 0.099647683 | 0.00097081 | 0.03686298 | 0.2233267 | 0.07 |
| ARHGAP36 | -0.099733391 | 0.000961 | -0.123550318 | 4.21E-05 | 0.03 |
| ARHGAP39 | 0.17475886 | 6.03E-09 | 0.072487635 | 0.0165348 | 0.11 |
| LOC100129602 | -0.250751195 | 3.92E-17 | -0.294449872 | 2.66E-23 | 0.05 |
| ARHGAP4 | 0.050714013 | 0.09377873 | -0.059930652 | 0.0476077 | 0.12 |
| GFI2 | -0.286924481 | 3.69E-22 | -0.277920959 | 7.77E-21 | 0.01 |
| ARHGAP6 | -0.420855179 | 3.74E-48 | -0.277194138 | 9.88E-21 | 0.15 |
| ARHGAP8 | -0.003311515 | 0.91292049 | 0.149838835 | 6.48E-07 | 0.16 |
| ARHGAP9 | -0.036604126 | 0.22659636 | -0.14577659 | 1.30E-06 | 0.11 |
| ARHGDIA | 0.244840422 | 2.20E-16 | 0.135536786 | 6.91E-06 | 0.11 |
| ARHGDIB | -0.16378334 | 5.17E-08 | 0.05026204 | 0.0967458 | 0.22 |
| ARHGDIG | 0.208289367 | 3.53E-12 | 0.152889083 | 3.80E-07 | 0.06 |
| ARHGEF10L | 0.012400596 | 0.68216016 | -0.183280956 | 1.03E-09 | 0.2 |
| ARHGEF10 | -0.250627609 | 4.07E-17 | -0.182054833 | 1.34E-09 | 0.07 |
| ARHGEF11 | -0.161284861 | 8.27E-08 | -0.055014506 | 0.069048 | 0.11 |
| PRO2792 | -0.455156994 | 5.33E-57 | -0.368810623 | 1.50E-36 | 0.09 |
| ARHGEF15 | -0.371994149 | 3.35E-37 | -0.257745236 | 4.79E-18 | 0.12 |
| ARHGEF16 | -0.108916109 | 0.00030923 | -0.030899776 | 0.3074258 | 0.08 |
| ARHGEF17 | -0.398703665 | 5.84E-43 | -0.361463905 | 4.48E-35 | 0.04 |
| ARHGEF18 | -0.178683023 | 2.71E-09 | -0.193687659 | 1.07E-10 | 0.02 |
| ARHGEF19 | 0.283323497 | 1.27E-21 | 0.227348192 | 2.79E-14 | 0.06 |
| ARHGEF1 | 0.04878003 | 0.10700354 | -0.012209197 | 0.6868041 | 0.07 |
| ARHGEF2 | 0.063894122 | 0.03467649 | 0.155811457 | 2.26E-07 | 0.1 |
| ARHGEF33 | 0.059810978 | 0.04805393 | -0.016275195 | 0.5909314 | 0.08 |
| ARHGEF35 | -0.18867837 | 3.23E-10 | -0.132017323 | 1.19E-05 | 0.06 |
| ARHGEF37 | -0.246755498 | 1.26E-16 | -0.175590984 | 5.10E-09 | 0.08 |
| ARHGEF38 | -0.457156481 | 1.51E-57 | -0.127660944 | 2.31E-05 | 0.33 |
| ARHGEF3 | -0.387080925 | 2.18E-40 | -0.195748263 | 6.69E-11 | 0.2 |
| ARHGEF4 | 0.188340849 | 3.48E-10 | 0.033571922 | 0.2674523 | 0.16 |
| ARHGEF5 | -0.106802456 | 0.00040466 | -0.071249716 | 0.0184802 | 0.04 |
| ARHGEF6 | -0.235203523 | 3.33E-15 | -0.335296618 | 3.99E-30 | 0.11 |
| ARHGEF7 | -0.156410716 | 2.02E-07 | -0.189776614 | 2.54E-10 | 0.04 |
| ARHGEF9 | 0.103113039 | 0.00063975 | -0.089284589 | 0.0031336 | 0.2 |
| ARID1A | -0.362029416 | 3.46E-35 | -0.375527595 | 6.21E-38 | 0.02 |
| RP11.419L10.1 | -0.286986317 | 3.62E-22 | -0.276062335 | 1.44E-20 | 0.02 |
| ARID2 | -0.394007956 | 6.57E-42 | -0.24567745 | 1.73E-16 | 0.15 |
| ARID3A | -0.141829446 | 2.51E-06 | -0.081398041 | 0.0070928 | 0.07 |
| ARID3B | -0.164278469 | 4.71E-08 | -0.250798868 | 3.86E-17 | 0.09 |
| ARID3C | 0.203978608 | 9.92E-12 | 0.000126027 | 0.9966794 | 0.21 |
| ARID4A | -0.522939029 | 9.52E-78 | -0.402246924 | 9.16E-44 | 0.13 |
| ARID4B | -0.283946323 | 1.02E-21 | -0.029465476 | 0.3304333 | 0.26 |
| ARID5A | -0.019713702 | 0.51500559 | 0.001743635 | 0.9540835 | 0.03 |
| ARID5B | -0.282048098 | 1.95E-21 | -0.196270558 | 5.94E-11 | 0.09 |
| ARI | -0.252227224 | 2.53E-17 | -0.30906337 | 1.27E-25 | 0.06 |
| ARIH2 | -0.027638806 | 0.36130409 | -0.107203624 | 0.0003847 | 0.08 |
| ARL10A | -0.244618157 | 2.35E-16 | -0.326630919 | 1.37E-28 | 0.09 |
| ARL11 | -0.029603048 | 0.32817943 | -0.004900704 | 0.8714354 | 0.03 |
| ARL13A | 0.127840394 | 2.24E-05 | 0.025741859 | 0.3952099 | 0.11 |
| ARL13B | -0.256083888 | 7.94E-18 | -0.263645799 | 7.75E-19 | 0.01 |
| ARL14 | 0.144345856 | 1.65E-06 | 0.037032045 | 0.2212094 | 0.11 |
| ARL15 | -0.479400154 | 6.85E-64 | -0.329995159 | 3.52E-29 | 0.15 |
| ARL16 | 0.402489338 | 8.06E-44 | 0.268173272 | 1.85E-19 | 0.14 |
| ARL17A | -0.182001814 | 1.35E-09 | -0.147365796 | 9.92E-07 | 0.04 |
| ARL17B | -0.103734948 | 0.00059281 | -0.075964403 | 0.0119987 | 0.03 |
| ARL1 | -0.241742101 | 5.34E-16 | -0.050460952 | 0.0954309 | 0.2 |
| ARL2BP | -0.029173474 | 0.33525022 | -0.069272466 | 0.0220029 | 0.05 |
| ARL2 | 0.277491678 | 8.96E-21 | 0.181576364 | 1.48E-09 | 0.1 |
| ARL3 | -0.1078001 | 0.00035663 | 0.009950343 | 0.7424614 | 0.12 |
| ARL4A | -0.07350556 | 0.01507209 | 0.030874934 | 0.307815 | 0.11 |
| ARL4C | 0.074544708 | 0.01369752 | -0.045360379 | 0.1339529 | 0.12 |
| ARL4D | -0.066731124 | 0.02737681 | 0.044142467 | 0.1447261 | 0.12 |
| ARL5A | -0.106940385 | 0.00039767 | -0.109186875 | 0.0002987 | 0.01 |
| ARL5B | -0.016537155 | 0.58497026 | -0.168987558 | 1.90E-08 | 0.16 |
| LOC100131933 | 0.02559634 | 0.39788793 | -0.044377621 | 0.1425959 | 0.07 |
| ARL6IP | 0.250563653 | 4.14E-17 | 0.152287426 | 4.23E-07 | 0.1 |
| ARL6IP4 | 0.220338808 | 1.75E-13 | 0.197848927 | 4.14E-11 | 0.03 |
| ARL6IP5 | -0.29552087 | 1.81E-23 | -0.048387284 | 0.1098614 | 0.25 |
| ARL6IP6 | 0.076768795 | 0.01112137 | -0.084785046 | 0.0050335 | 0.17 |
| LOC100506362 | -0.050488478 | 0.09525004 | -0.019734852 | 0.5145547 | 0.04 |
| ARL8A | 0.194671836 | 8.54E-11 | 0.501161581 | 1.45E-70 | 0.31 |
| ARL8B | -0.095630627 | 0.00154955 | -0.080962851 | 0.0074061 | 0.02 |
| ARL9 | 0.437349124 | 2.79E-52 | 0.209715299 | 2.50E-12 | 0.23 |
| ARMC10 | 0.247065853 | 1.16E-16 | 0.308822783 | 1.39E-25 | 0.07 |
| ARMC1 | 0.137125926 | 5.37E-06 | 0.007506416 | 0.8042228 | 0.13 |
| ARMC2 | -0.083025189 | 0.00602421 | 0.053021825 | 0.0797467 | 0.14 |
| ARMC3 | -0.106937774 | 0.0003978 | 0.140078136 | 3.34E-06 | 0.25 |
| ARMC4 | -0.031996522 | 0.29056507 | -0.069768072 | 0.0210692 | 0.04 |
| ARMC5 | 0.017954213 | 0.55321811 | 0.042356217 | 0.1617094 | 0.03 |
| ARMC6 | 0.391768988 | 2.06E-41 | 0.238916782 | 1.19E-15 | 0.16 |
| ARMC7 | 0.088491878 | 0.00341163 | 0.044501606 | 0.1414825 | 0.05 |
| HSPC056 | -0.05509823 | 0.06862559 | -0.148231272 | 8.56E-07 | 0.1 |
| ARMC9 | -0.309579237 | 1.05E-25 | -0.149198058 | 7.24E-07 | 0.17 |
| ARMCX1 | -0.205732638 | 6.54E-12 | -0.050961623 | 0.0921844 | 0.16 |
| ARMCX2 | -0.209784465 | 2.46E-12 | -0.050137154 | 0.0975787 | 0.16 |
| ARMCX3 | -0.367859452 | 2.34E-36 | -0.159205055 | 1.22E-07 | 0.21 |
| ARMCX5 | -0.297383829 | 9.32E-24 | -0.181182959 | 1.61E-09 | 0.12 |
| LOC115858 | -0.097981266 | 0.00118106 | 0.006699637 | 0.8249034 | 0.11 |
| ARMS2 | 0.002222757 | 0.94148657 | 0.006175761 | 0.8383985 | 0.01 |
| ARNT2 | -0.232935621 | 6.21E-15 | -0.197008595 | 5.02E-11 | 0.04 |
| ARNTL2 | 0.153193902 | 3.60E-07 | -0.087835555 | 0.0036586 | 0.25 |
| ARNTL | -0.290390018 | 1.11E-22 | -0.35285039 | 2.15E-33 | 0.07 |
| ARNT | -0.28339647 | 1.23E-21 | -0.078709406 | 0.0092348 | 0.21 |
| ARPC1A | 0.312105318 | 4.03E-26 | 0.295974428 | 1.54E-23 | 0.02 |
| ARPC1B | 0.144797574 | 1.53E-06 | 0.092095209 | 0.0023059 | 0.06 |
| LOC220721 | 0.180762562 | 1.76E-09 | 0.062815833 | 0.0378569 | 0.12 |
| ARPC3 | 0.304372079 | 7.31E-25 | 0.362949948 | 2.27E-35 | 0.06 |
| ARPC4 | 0.389989638 | 5.06E-41 | 0.297941533 | 7.62E-24 | 0.1 |
| ARPC5L | 0.4659011 | 5.51E-60 | 0.338615524 | 9.97E-31 | 0.13 |
| ARPC5 | 0.367642595 | 2.59E-36 | 0.471034855 | 1.89E-61 | 0.11 |
| ACTRT3 | 0.124048761 | 3.92E-05 | 0.049600251 | 0.1012249 | 0.08 |
| ARPP19 | -0.239341854 | 1.05E-15 | -0.176908444 | 3.90E-09 | 0.07 |
| FLJ32997 | -0.074746697 | 0.01344355 | -0.075350185 | 0.0127094 | 0.01 |
| ARR3 | -0.081953155 | 0.00671055 | -0.069076497 | 0.022382 | 0.02 |
| ARRB1 | -0.287565649 | 2.96E-22 | -0.243468727 | 3.26E-16 | 0.05 |
| ARRB2 | 0.256929142 | 6.15E-18 | 0.056909862 | 0.0599933 | 0.21 |
| ARRDC1 | -0.018133177 | 0.54926885 | 0.073008889 | 0.0157709 | 0.1 |
| ARRDC2 | 0.034617566 | 0.25282733 | -0.013409794 | 0.6578758 | 0.05 |
| ARRDC3 | -0.213558151 | 9.70E-13 | -0.233854798 | 4.83E-15 | 0.03 |
| ARRDC4 | -0.291249123 | 8.22E-23 | -0.271796789 | 5.79E-20 | 0.02 |
| ARRDC5 | -0.006079769 | 0.84087656 | -0.067401296 | 0.0258599 | 0.07 |
| LOC100506900 | -0.0983656 | 0.00112914 | -0.104898975 | 0.0005135 | 0.01 |
| ARSB | -0.106735251 | 0.0004081 | -0.211146875 | 1.76E-12 | 0.11 |
| ARSD | -0.354517608 | 1.03E-33 | -0.113433202 | 0.0001713 | 0.25 |
| ARSE | 0.034248879 | 0.25791914 | 0.034471447 | 0.2548369 | 0.01 |
| ARSF | 0.105238774 | 0.00049223 | -0.003346525 | 0.9120036 | 0.11 |
| ARSG | -0.414536569 | 1.24E-46 | -0.176410664 | 4.31E-09 | 0.24 |
| ARSH | -0.161378766 | 8.12E-08 | -0.020291609 | 0.5027602 | 0.15 |
| LOC134458 | -0.007194062 | 0.81221417 | 0.016715183 | 0.5809351 | 0.03 |
| LOC102723905 | -0.168275052 | 2.18E-08 | -0.186184922 | 5.55E-10 | 0.02 |
| ARSK | -0.156035924 | 2.17E-07 | 0.010612889 | 0.7259812 | 0.17 |
| ART1 | -0.186157913 | 5.58E-10 | -0.107082424 | 0.0003906 | 0.08 |
| ART3 | 0.381544565 | 3.36E-39 | 0.131276611 | 1.34E-05 | 0.26 |
| DO | -0.116593668 | 0.00011181 | -0.191985774 | 1.56E-10 | 0.08 |
| ART5 | -0.064186642 | 0.03385405 | -0.044087836 | 0.1452245 | 0.03 |
| ARTN | 0.174564157 | 6.28E-09 | 0.104146815 | 0.0005635 | 0.08 |
| ARV1 | 0.035648704 | 0.2389595 | 0.397623014 | 1.02E-42 | 0.37 |
| ARVCF | -0.070967636 | 0.01895049 | -0.043121245 | 0.1542603 | 0.03 |
| ARX | -0.099092587 | 0.00103666 | -0.048850402 | 0.1064977 | 0.06 |
| AR | -0.566232738 | 1.11E-93 | -0.27758242 | 8.69E-21 | 0.29 |
| LOC115695 | 0.110776782 | 0.00024307 | 0.100245924 | 0.0009042 | 0.02 |
| ASAH1 | -0.445051633 | 2.75E-54 | -0.172436753 | 9.62E-09 | 0.28 |
| ASAH2C | -0.117719339 | 9.58E-05 | -0.136631144 | 5.81E-06 | 0.02 |
| ASAH2 | -0.200242395 | 2.38E-11 | -0.141128322 | 2.81E-06 | 0.06 |
| CLMP | -0.251315061 | 3.32E-17 | -0.270153422 | 9.84E-20 | 0.02 |
| ASAP1.IT1 | 0.01056924 | 0.72706317 | 0.030393314 | 0.3154259 | 0.02 |
| ASAP1 | -0.070637279 | 0.01951455 | -0.20919686 | 2.83E-12 | 0.14 |
| ASAP2 | 0.04087577 | 0.17688978 | -0.164715586 | 4.33E-08 | 0.21 |
| DDEFL1 | -0.212812511 | 1.17E-12 | -0.241714693 | 5.38E-16 | 0.03 |
| ASB10 | 0.13177709 | 1.24E-05 | 0.020909263 | 0.4898445 | 0.12 |
| ASB11 | -0.043659346 | 0.14917879 | -0.038629955 | 0.2019051 | 0.01 |
| ASB12 | 0.155827332 | 2.25E-07 | 0.058526572 | 0.0530683 | 0.1 |
| ASB13 | -0.077869225 | 0.01001344 | 0.00068618 | 0.9819219 | 0.08 |
| ASB14 | -0.296214993 | 1.42E-23 | -0.179431322 | 2.32E-09 | 0.12 |
| ASB15 | -0.118796191 | 8.25E-05 | -0.086053618 | 0.0044132 | 0.04 |
| LOC146903 | -0.298900791 | 5.39E-24 | -0.181918497 | 1.38E-09 | 0.12 |
| ASB17 | -0.014434153 | 0.63359013 | 0.014514993 | 0.6316898 | 0.03 |
| ASB18 | 0.045375398 | 0.133824 | -0.048787902 | 0.1069469 | 0.1 |
| ASB1 | -0.267207681 | 2.52E-19 | -0.257621701 | 4.98E-18 | 0.01 |
| ASB2 | -0.011412626 | 0.70625667 | -0.102887413 | 0.0006576 | 0.1 |
| ASB3 | -0.024388259 | 0.42053901 | -0.091010685 | 0.0025981 | 0.07 |
| ASB4 | -0.056687124 | 0.06100355 | -0.084316207 | 0.0052819 | 0.03 |
| ASB5 | 0.008138701 | 0.78810937 | -0.062507478 | 0.0388108 | 0.08 |
| FLJ20548 | 0.112179722 | 0.00020223 | 0.042286402 | 0.1624025 | 0.07 |
| FLJ22551 | -0.125039619 | 3.39E-05 | -0.241877034 | 5.14E-16 | 0.12 |
| MGC5540 | -0.349623365 | 8.89E-33 | -0.166418451 | 3.13E-08 | 0.19 |
| MGC4954 | 0.063117238 | 0.0369439 | 0.07798413 | 0.0099036 | 0.02 |
| ASCC1 | 0.160273545 | 9.98E-08 | 0.008570267 | 0.7771625 | 0.16 |
| ASCC2 | 0.228038029 | 2.33E-14 | 0.163125808 | 5.85E-08 | 0.07 |
| ASCC3 | -0.167490578 | 2.54E-08 | -0.135241632 | 7.23E-06 | 0.04 |
| ASCL1 | -0.03334792 | 0.27065934 | -0.09117684 | 0.0025513 | 0.06 |
| ASCL2 | 0.099100641 | 0.00103568 | 0.136185018 | 6.23E-06 | 0.04 |
| ASCL3 | -0.119370459 | 7.62E-05 | -0.125987689 | 2.95E-05 | 0.01 |
| ASCL4 | -0.130196297 | 1.58E-05 | -0.003820235 | 0.8996092 | 0.13 |
| ASF1A | 0.238609528 | 1.29E-15 | 0.209824354 | 2.43E-12 | 0.03 |
| ASF1B | 0.494175695 | 2.27E-68 | 0.289368991 | 1.58E-22 | 0.21 |
| FMR1.AS1 | -0.079879128 | 0.00824062 | -0.154741164 | 2.73E-07 | 0.08 |
| ASGR1 | 0.222029337 | 1.13E-13 | 0.067417692 | 0.0258238 | 0.16 |
| ASGR2 | 0.017492927 | 0.5634611 | -0.085597516 | 0.0046278 | 0.11 |
| ASH1L | -0.433239963 | 3.13E-51 | -0.238490566 | 1.34E-15 | 0.2 |
| ASH2L | 0.050747551 | 0.0935615 | -0.048250231 | 0.1108728 | 0.1 |
| ASIP | 0.071205808 | 0.01855271 | 0.036819845 | 0.2238692 | 0.04 |
| LOC154808 | -0.138213986 | 4.51E-06 | -0.016473216 | 0.5864227 | 0.13 |
| ASMTL | -0.132429863 | 1.12E-05 | -0.077646574 | 0.0102293 | 0.06 |
| ASMTY | 0.016344312 | 0.58935585 | -0.053487258 | 0.0771345 | 0.07 |
| ASNA1 | 0.116855918 | 0.00010787 | 0.177547978 | 3.42E-09 | 0.07 |
| ASNSD1 | 0.133621736 | 9.32E-06 | 0.082733726 | 0.0062043 | 0.06 |
| ASNS | 0.414517246 | 1.26E-46 | 0.199182064 | 3.05E-11 | 0.22 |
| ASPA | -0.369902529 | 8.98E-37 | -0.25614666 | 7.79E-18 | 0.12 |
| ASPDH | -0.148821854 | 7.73E-07 | -0.165045087 | 4.07E-08 | 0.02 |
| C14orf76 | 0.030315507 | 0.31666696 | -0.024986771 | 0.409224 | 0.06 |
| ASPHD1 | 0.171651012 | 1.12E-08 | -0.002342445 | 0.9383419 | 0.18 |
| ASPHD2 | -0.10904179 | 0.00030428 | -0.126467644 | 2.75E-05 | 0.02 |
| LOC56921 | -0.174881878 | 5.89E-09 | -0.106441047 | 0.0004235 | 0.07 |
| FLJ10517 | 0.355208077 | 7.54E-34 | 0.241446494 | 5.81E-16 | 0.12 |
| ASPN | -0.334387095 | 5.81E-30 | -0.112050636 | 0.0002057 | 0.23 |
| ASPRV1 | -0.066842737 | 0.02711899 | -0.122385845 | 4.98E-05 | 0.06 |
| LOC100131505 | 0.2490652 | 6.44E-17 | 0.247613012 | 9.85E-17 | 0.01 |
| ASRGL1 | 0.026688167 | 0.37806191 | 0.044627506 | 0.1403588 | 0.02 |
| ASS1 | 0.195507484 | 7.07E-11 | 0.129468663 | 1.76E-05 | 0.07 |
| ASTE1 | -0.195325644 | 7.37E-11 | -0.015629135 | 0.605751 | 0.18 |
| ASTL | -0.03517639 | 0.2452437 | 0.003337623 | 0.9122367 | 0.04 |
| ASTN1 | -0.153985906 | 3.13E-07 | -0.156314477 | 2.06E-07 | 0.01 |
| LOC100291924 | -0.337850487 | 1.37E-30 | -0.181677183 | 1.45E-09 | 0.16 |
| ASXL1 | -0.041183335 | 0.17365182 | -0.1521228 | 4.35E-07 | 0.12 |
| ASXL2 | -0.423711858 | 7.49E-49 | -0.482621472 | 7.54E-65 | 0.06 |
| ASXL3 | -0.179764535 | 2.16E-09 | -0.193132463 | 1.21E-10 | 0.02 |
| ANKL1 | 0.052918147 | 0.0803383 | 0.014957812 | 0.6213236 | 0.04 |
| ATAD1 | -0.125298226 | 3.27E-05 | -0.107964234 | 0.0003493 | 0.02 |
| ATAD2B | -0.101655563 | 0.00076359 | -0.199470949 | 2.85E-11 | 0.1 |
| MGC5254 | 0.352693615 | 2.30E-33 | 0.127842786 | 2.24E-05 | 0.23 |
| ATAD3A | 0.386290793 | 3.23E-40 | 0.140871631 | 2.94E-06 | 0.25 |
| ATAD3B | 0.31954149 | 2.29E-27 | 0.143251732 | 1.98E-06 | 0.18 |
| ATAD3C | -0.026607872 | 0.37949892 | 0.019166258 | 0.5267466 | 0.05 |
| ATAD5 | 0.191270936 | 1.83E-10 | 0.026363855 | 0.3838865 | 0.17 |
| ATCAY | 0.109604483 | 0.000283 | -0.014751716 | 0.6261391 | 0.13 |
| ATE1 | -0.414832672 | 1.06E-46 | -0.41898353 | 1.06E-47 | 0.01 |
| ATF1 | -0.287591121 | 2.93E-22 | -0.166421847 | 3.12E-08 | 0.13 |
| ATF2 | -0.397792078 | 9.37E-43 | -0.405823689 | 1.38E-44 | 0.01 |
| ATF3 | -5.35E-05 | 0.99859036 | -0.011523209 | 0.7035443 | 0.02 |
| ATF4 | 0.389993728 | 5.05E-41 | 0.231053657 | 1.03E-14 | 0.16 |
| ATF5 | 0.375632802 | 5.91E-38 | 0.16603431 | 3.37E-08 | 0.21 |
| LOC87886 | 0.027103322 | 0.37068558 | 0.078063904 | 0.009828 | 0.06 |
| ATF6 | -0.270376621 | 9.15E-20 | -0.073460716 | 0.015134 | 0.2 |
| ATF7IP2 | -0.104641256 | 0.00053012 | -0.042228831 | 0.1629757 | 0.07 |
| ATF7IP | -0.16004392 | 1.04E-07 | -0.244603433 | 2.36E-16 | 0.09 |
| ATF7 | -0.452872423 | 2.23E-56 | -0.313647939 | 2.24E-26 | 0.14 |
| ATG10 | 0.090297994 | 0.00280818 | 0.199201512 | 3.03E-11 | 0.11 |
| ATG12 | -0.198072066 | 3.94E-11 | 0.007450615 | 0.8056489 | 0.21 |
| FLJ22677 | -0.381024288 | 4.34E-39 | -0.139822346 | 3.48E-06 | 0.25 |
| ATG16L2 | -0.13587466 | 6.55E-06 | -0.089845816 | 0.0029494 | 0.05 |
| ATG2A | -0.221438064 | 1.32E-13 | -0.239916567 | 8.95E-16 | 0.02 |
| ATG2B | -0.555169025 | 2.20E-89 | -0.427919678 | 6.82E-50 | 0.13 |
| ATG3 | 0.33358366 | 8.10E-30 | 0.241872595 | 5.15E-16 | 0.1 |
| ATG4A | -0.112340837 | 0.00019797 | -0.027372951 | 0.365943 | 0.09 |
| DKFZp586D1822 | 0.188723999 | 3.20E-10 | 0.093941913 | 0.0018766 | 0.1 |
| ATG4C | -0.029149318 | 0.33565073 | -0.151060521 | 5.24E-07 | 0.13 |
| ATG4D | 0.227946248 | 2.38E-14 | 0.149116328 | 7.35E-07 | 0.08 |
| ATG5 | 0.153770101 | 3.25E-07 | 0.154227302 | 3.00E-07 | 0.01 |
| ATG7 | 0.034483079 | 0.25467652 | 0.130498085 | 1.51E-05 | 0.1 |
| ATG9A | 0.20553668 | 6.85E-12 | -0.038247604 | 0.2064087 | 0.25 |
| ATG9B | 0.266947899 | 2.74E-19 | 0.102771721 | 0.000667 | 0.17 |
| ATHL1 | -0.054338948 | 0.0725348 | 0.021352018 | 0.4806969 | 0.08 |
| ATIC | 0.201252033 | 1.88E-11 | 0.300314051 | 3.23E-24 | 0.1 |
| ATL1 | -0.089469161 | 0.00307186 | -0.172162688 | 1.02E-08 | 0.09 |
| ATL2 | 0.261553996 | 1.49E-18 | 0.063648718 | 0.0353795 | 0.2 |
| ATL3 | -0.13545559 | 7.00E-06 | -0.182722553 | 1.16E-09 | 0.05 |
| ATMIN | -0.021523632 | 0.47717643 | -0.138924064 | 4.03E-06 | 0.12 |
| ATM | -0.408453927 | 3.38E-45 | -0.383978518 | 1.02E-39 | 0.03 |
| ATN1 | 0.056504842 | 0.06184076 | -0.079072779 | 0.0089151 | 0.14 |
| ATOH1 | 0.112886523 | 0.00018418 | 0.029500025 | 0.3298663 | 0.09 |
| ATOH7 | 0.401221701 | 1.57E-43 | 0.108875831 | 0.0003108 | 0.3 |
| ATOH8 | -0.210759135 | 1.94E-12 | -0.18646843 | 5.22E-10 | 0.03 |
| ATOX1 | 0.16425725 | 4.73E-08 | 0.311394735 | 5.28E-26 | 0.15 |
| LOC105376701 | -0.173315846 | 8.07E-09 | -0.090700786 | 0.0026876 | 0.09 |
| FLJ21477 | 0.19459518 | 8.69E-11 | 0.002269572 | 0.9402564 | 0.2 |
| ATP10D | -0.345244869 | 5.94E-32 | -0.357229058 | 3.05E-34 | 0.02 |
| FLJ21858 | 0.002440679 | 0.93576168 | -0.201126412 | 1.94E-11 | 0.21 |
| ATP11B | 0.00384585 | 0.89893967 | -0.180014301 | 2.05E-09 | 0.19 |
| ATP11C | 0.074270887 | 0.01404855 | -0.065814738 | 0.0295746 | 0.15 |
| ATP12A | 0.119255092 | 7.74E-05 | -0.055404951 | 0.0670962 | 0.18 |
| ATP13A1 | 0.145362959 | 1.39E-06 | 0.097427176 | 0.0012598 | 0.05 |
| ATP13A2 | -0.116489891 | 0.00011341 | -0.195335639 | 7.35E-11 | 0.08 |
| ATP13A3 | -0.039593583 | 0.19087306 | -0.116670623 | 0.0001106 | 0.08 |
| ATP13A4 | -0.187239394 | 4.42E-10 | -0.110892681 | 0.0002394 | 0.08 |
| ATP13A5 | 0.036349488 | 0.22984593 | -0.00064749 | 0.982941 | 0.04 |
| LOC148767 | -0.016916006 | 0.57639889 | -0.069309798 | 0.0219314 | 0.06 |
| LOC116200 | -0.423228426 | 9.85E-49 | -0.285449649 | 6.13E-22 | 0.14 |
| MGC13276 | -0.025920768 | 0.39193245 | -0.083848179 | 0.0055409 | 0.06 |
| MGC25056 | -0.380657081 | 5.19E-39 | -0.142429716 | 2.27E-06 | 0.24 |
| LOC127872 | -0.166159629 | 3.29E-08 | 0.047088496 | 0.119743 | 0.22 |
| ATP1B2 | -0.278778935 | 5.84E-21 | -0.16964371 | 1.67E-08 | 0.11 |
| ATP1B3 | 0.386279445 | 3.25E-40 | 0.135702424 | 6.73E-06 | 0.26 |
| ATP1B4 | -0.071414716 | 0.01820983 | -0.071637596 | 0.0178501 | 0.01 |
| ATP2A1 | 0.314209156 | 1.80E-26 | 0.102573712 | 0.0006832 | 0.22 |
| DAR | -0.116814888 | 0.00010848 | -0.171252306 | 1.22E-08 | 0.06 |
| ATP2A3 | -0.230037994 | 1.36E-14 | -0.217792435 | 3.35E-13 | 0.02 |
| ATP2B1 | -0.260025776 | 2.38E-18 | -0.25626348 | 7.52E-18 | 0.01 |
| ATP2B2 | 0.053892741 | 0.07491583 | -0.0427945 | 0.1574093 | 0.1 |
| SCAX1 | 0.214472063 | 7.72E-13 | 0.033970263 | 0.2618138 | 0.19 |
| MXRA1.withdrawn | -0.217501907 | 3.60E-13 | -0.088530605 | 0.0033975 | 0.13 |
| BCPM | 0.053741725 | 0.07573596 | -0.050440525 | 0.0955652 | 0.11 |
| ATP2C2 | -0.238048728 | 1.51E-15 | -0.164447936 | 4.56E-08 | 0.08 |
| ATP4A | 0.087433248 | 0.00381794 | -0.015279434 | 0.6138413 | 0.11 |
| ATP4B | 0.122440451 | 4.94E-05 | 0.038525803 | 0.2031248 | 0.09 |
| ATP5AL2 | 0.368406519 | 1.81E-36 | 0.124642419 | 3.59E-05 | 0.25 |
| ATP5B | 0.200884524 | 2.05E-11 | 0.131876634 | 1.22E-05 | 0.07 |
| ATP5CL1 | 0.569306995 | 6.67E-95 | 0.436584872 | 4.39E-52 | 0.14 |
| ATP5D | 0.208025666 | 3.77E-12 | 0.2254368 | 4.63E-14 | 0.02 |
| LOC145000 | 0.360130894 | 8.22E-35 | 0.38915942 | 7.68E-41 | 0.03 |
| ATP5E | 0.423199665 | 1.00E-48 | 0.438557715 | 1.36E-52 | 0.02 |
| ATP5F1 | 0.312550321 | 3.40E-26 | 0.216470241 | 4.68E-13 | 0.1 |
| ATP5G1 | 0.387014339 | 2.25E-40 | 0.408717753 | 2.93E-45 | 0.03 |
| ATP5G2 | 0.137810554 | 4.81E-06 | 0.383673553 | 1.18E-39 | 0.25 |
| ATP5G3 | 0.472349486 | 7.89E-62 | 0.382345436 | 2.27E-39 | 0.1 |
| ATP5H | 0.401728967 | 1.20E-43 | 0.394807157 | 4.36E-42 | 0.01 |
| ATP5I | 0.35263079 | 2.37E-33 | 0.407455113 | 5.77E-45 | 0.06 |
| ATP5J2 | 0.535543585 | 3.78E-82 | 0.49018107 | 3.88E-67 | 0.05 |
| ATP5J | 0.402234336 | 9.22E-44 | 0.433823762 | 2.23E-51 | 0.04 |
| ATP5L2 | 0.122659639 | 4.78E-05 | 0.107977087 | 0.0003487 | 0.02 |
| ATP5L | 0.426778688 | 1.31E-49 | 0.393344528 | 9.22E-42 | 0.04 |
| ATP5O | 0.412166807 | 4.54E-46 | 0.460952206 | 1.35E-58 | 0.05 |
| ATP5SL | -0.020317594 | 0.5022132 | 0.030469815 | 0.3142088 | 0.06 |
| ATP5S | -0.144905788 | 1.50E-06 | 0.020906051 | 0.4899111 | 0.17 |
| ATP6AP1L | -0.266836231 | 2.84E-19 | -0.031303573 | 0.3011443 | 0.24 |
| ATP6AP1 | -0.072466385 | 0.01656659 | -0.056628106 | 0.0612736 | 0.02 |
| MRXE | -0.134410842 | 8.24E-06 | -0.060002606 | 0.047341 | 0.08 |
| ATP6V0A1 | -0.21803373 | 3.15E-13 | -0.185082205 | 7.04E-10 | 0.04 |
| WSS | 0.053190749 | 0.07879045 | -0.106378702 | 0.0004268 | 0.16 |
| RTA1C | 0.120897441 | 6.15E-05 | 0.065754584 | 0.0297241 | 0.06 |
| ATP6V0B | 0.314332999 | 1.72E-26 | 0.208712452 | 3.19E-12 | 0.11 |
| ATP6V0C | 0.138121618 | 4.58E-06 | 0.132880173 | 1.05E-05 | 0.01 |
| LOC100132855 | -0.026503083 | 0.38137931 | -0.097334814 | 0.0012734 | 0.08 |
| FLJ38708 | -0.073482602 | 0.01510378 | -0.083242519 | 0.005893 | 0.01 |
| ATP6V0E1 | 0.080999385 | 0.00737931 | 0.186377718 | 5.33E-10 | 0.11 |
| ATP6V0E2 | 0.023416722 | 0.43929166 | 0.102285494 | 0.0007076 | 0.08 |
| ATP6V1A | -0.20889164 | 3.05E-12 | -0.233078864 | 5.97E-15 | 0.03 |
| LOC130937 | 0.058163233 | 0.05456352 | -0.019811256 | 0.5129277 | 0.08 |
| ATP6V1B2 | -0.139188889 | 3.86E-06 | -0.145151995 | 1.44E-06 | 0.01 |
| ATP6V1C1 | 0.0376949 | 0.21304695 | -0.050811104 | 0.093151 | 0.09 |
| ATP6V1C2 | 0.406974013 | 7.47E-45 | 0.193134022 | 1.21E-10 | 0.22 |
| ATP6V1D | -0.043756865 | 0.1482717 | 0.148098217 | 8.75E-07 | 0.2 |
| ATP6V1E1 | 0.093118258 | 0.00205811 | 0.113738907 | 0.0001644 | 0.03 |
| ATP6V1E2 | 0.295363083 | 1.92E-23 | 0.115857802 | 0.0001236 | 0.18 |
| LOC154989 | 0.410512682 | 1.11E-45 | 0.354274854 | 1.14E-33 | 0.06 |
| ATP6V1G1 | -0.075439076 | 0.01260426 | -0.004106347 | 0.8921349 | 0.08 |
| ATP6V1G2 | -0.131273295 | 1.34E-05 | -0.045508705 | 0.1326843 | 0.09 |
| ATP6V1G3 | 0.077904117 | 0.00997997 | 0.065923303 | 0.0293066 | 0.02 |
| ATP6V1H | 0.079692511 | 0.00839252 | 0.028805411 | 0.3413859 | 0.06 |
| ATP7A | -0.50533671 | 6.66E-72 | -0.378334482 | 1.61E-38 | 0.13 |
| ATP7B | -0.439312592 | 8.70E-53 | -0.161516568 | 7.92E-08 | 0.28 |
| ATP8A1 | -0.184572629 | 7.85E-10 | -0.242109629 | 4.81E-16 | 0.06 |
| ATP8A2 | -0.17745315 | 3.49E-09 | -0.038420908 | 0.2043585 | 0.14 |
| ATP8B1 | -0.413064894 | 2.78E-46 | -0.237207745 | 1.91E-15 | 0.18 |
| ATP8B2 | -0.452529717 | 2.76E-56 | -0.30679612 | 2.97E-25 | 0.15 |
| ATP8B3 | -0.198995395 | 3.18E-11 | -0.113912116 | 0.0001607 | 0.09 |
| ATP8B4 | -0.256237863 | 7.58E-18 | -0.31912418 | 2.69E-27 | 0.07 |
| ATP8B5P | 0.18151571 | 1.50E-09 | 0.3378306 | 1.39E-30 | 0.16 |
| LOC654090 | -0.200602758 | 2.19E-11 | -0.233458339 | 5.38E-15 | 0.04 |
| ATP9B | -0.274795444 | 2.18E-20 | -0.361064223 | 5.38E-35 | 0.09 |
| ATPAF1 | -0.023198856 | 0.44356179 | 0.074774665 | 0.0134087 | 0.1 |
| ATPAF2 | -0.042409489 | 0.16118205 | 0.036869104 | 0.2232497 | 0.08 |
| DPH6 | 0.010078678 | 0.73925972 | -0.058974907 | 0.0512705 | 0.07 |
| ATPIF1 | 0.2069523 | 4.88E-12 | 0.167080793 | 2.75E-08 | 0.04 |
| ATRIP | -0.062252384 | 0.03961518 | 0.110746082 | 0.000244 | 0.18 |
| ATRNL1 | -0.109521677 | 0.00028604 | -0.102905361 | 0.0006562 | 0.01 |
| KIAA0548 | -0.342148719 | 2.24E-31 | -0.315574753 | 1.07E-26 | 0.03 |
| JMS | -0.443527235 | 6.92E-54 | -0.364696729 | 1.02E-35 | 0.08 |
| ATR | -0.16026536 | 9.99E-08 | -0.17376514 | 7.37E-09 | 0.02 |
| SCA10 | 6.56E-05 | 0.9982703 | 0.023134295 | 0.4448317 | 0.03 |
| LOC100131476 | -0.291138675 | 8.54E-23 | -0.437726083 | 2.23E-52 | 0.15 |
| ATXN1 | -0.49148404 | 1.54E-67 | -0.302768221 | 1.32E-24 | 0.19 |
| ATXN2L | 0.19191353 | 1.58E-10 | -0.012334821 | 0.6837547 | 0.21 |
| TNRC13 | -0.235554587 | 3.02E-15 | -0.2204297 | 1.71E-13 | 0.02 |
| ATXN3L | 0.002870106 | 0.92449043 | -0.014039614 | 0.6428992 | 0.02 |
| ATXN3 | -0.132696988 | 1.08E-05 | -0.090822992 | 0.002652 | 0.05 |
| LOC168835 | -0.235052948 | 3.47E-15 | -0.20472465 | 8.31E-12 | 0.04 |
| ATXN7L2 | 0.369847638 | 9.22E-37 | 0.205816244 | 6.41E-12 | 0.17 |
| ATXN7L3B | -0.050258281 | 0.09677075 | -0.139634197 | 3.59E-06 | 0.09 |
| ATXN7L3 | 0.027464695 | 0.36433802 | -0.0527556 | 0.0812729 | 0.09 |
| ATXN7 | -0.328480063 | 6.52E-29 | -0.311891969 | 4.37E-26 | 0.02 |
| ATXN8OS | -0.039000119 | 0.19761364 | -0.005895895 | 0.8456277 | 0.04 |
| AUH | -0.263301137 | 8.63E-19 | -0.154096574 | 3.07E-07 | 0.11 |
| AUP1 | 0.407345143 | 6.12E-45 | 0.3191563 | 2.66E-27 | 0.09 |
| AURKAIP1 | 0.284349047 | 8.93E-22 | 0.191739245 | 1.65E-10 | 0.1 |
| AURKAPS1 | 0.440162332 | 5.24E-53 | 0.318528846 | 3.39E-27 | 0.13 |
| AURKA | 0.52565441 | 1.11E-78 | 0.288832305 | 1.91E-22 | 0.24 |
| AURKB | 0.659625627 | 1.82E-137 | 0.378841167 | 1.26E-38 | 0.29 |
| AURKC | 0.064789462 | 0.0322116 | 0.019733923 | 0.5145745 | 0.05 |
| AUTS2 | -0.142203352 | 2.36E-06 | -0.09463603 | 0.0017352 | 0.05 |
| AVEN | 0.345987615 | 4.32E-32 | 0.255331823 | 9.97E-18 | 0.1 |
| FLJ12386 | -0.130111747 | 1.60E-05 | -0.230833525 | 1.10E-14 | 0.11 |
| AVL9 | -0.301806171 | 1.88E-24 | -0.296028227 | 1.51E-23 | 0.01 |
| AVPI1 | 0.105530463 | 0.00047466 | -0.020308444 | 0.5024058 | 0.13 |
| AVPR1A | -0.385083888 | 5.88E-40 | -0.391457051 | 2.41E-41 | 0.01 |
| AVPR1B | 0.091251153 | 0.00253056 | -0.024462774 | 0.4191204 | 0.12 |
| AVPR2 | -0.261768008 | 1.39E-18 | -0.160343867 | 9.85E-08 | 0.11 |
| AVP | 0.065777635 | 0.02966672 | 0.010117057 | 0.7383031 | 0.06 |
| AWAT1 | -0.08267983 | 0.00623809 | -0.005585629 | 0.8536572 | 0.08 |
| AWAT2 | 0.172458509 | 9.57E-09 | 0.050762471 | 0.093465 | 0.13 |
| AXIN1 | 0.248243649 | 8.20E-17 | 0.132427441 | 1.12E-05 | 0.12 |
| AXIN2 | -0.406149483 | 1.16E-44 | -0.299869362 | 3.80E-24 | 0.11 |
| AXL | -0.348215099 | 1.64E-32 | -0.273951487 | 2.87E-20 | 0.08 |
| LOC90053 | -0.254634174 | 1.23E-17 | -0.024408361 | 0.420156 | 0.24 |
| CEP131 | 0.298230957 | 6.87E-24 | 0.120013873 | 6.96E-05 | 0.18 |
| AZ2 | -0.255199492 | 1.04E-17 | -0.177674307 | 3.33E-09 | 0.08 |
| AZIN1 | 0.122523121 | 4.88E-05 | 0.040144817 | 0.1847649 | 0.09 |
| AZU1 | -0.13510834 | 7.39E-06 | -0.016813632 | 0.5787092 | 0.12 |
| B2M | 0.022165496 | 0.46413503 | -0.037938821 | 0.2100986 | 0.07 |
| P | 0.022030151 | 0.46686837 | -0.078251835 | 0.0096519 | 0.11 |
| B3GALNT2 | 0.153161514 | 3.62E-07 | 0.257209433 | 5.65E-18 | 0.11 |
| B3GALT1 | -0.169912045 | 1.58E-08 | -0.184361022 | 8.21E-10 | 0.02 |
| B3GALT2 | -0.172625706 | 9.26E-09 | -0.168444018 | 2.11E-08 | 0.01 |
| B3GALT4 | -0.078839663 | 0.00911901 | -0.048188564 | 0.1113302 | 0.04 |
| B3GALT5 | -0.179267741 | 2.40E-09 | -0.058697406 | 0.0523771 | 0.13 |
| B3GALT6 | 0.205983929 | 6.15E-12 | 0.160974629 | 8.76E-08 | 0.05 |
| B3GALTL | -0.090416738 | 0.00277214 | -0.069877711 | 0.0208673 | 0.03 |
| CD57.withdrawn | 0.104539419 | 0.00053685 | -0.040888867 | 0.176751 | 0.15 |
| B3GAT2 | 0.00838689 | 0.78180868 | -0.149513992 | 6.86E-07 | 0.16 |
| B3GAT3 | 0.286519959 | 4.25E-22 | 0.179208372 | 2.43E-09 | 0.11 |
| B4GAT1 | -0.110296193 | 0.00025876 | -0.039714533 | 0.1895203 | 0.08 |
| HSA6077 | -0.143633572 | 1.86E-06 | -0.221437791 | 1.32E-13 | 0.08 |
| B3GNT3 | 0.094205789 | 0.00182165 | 0.010489178 | 0.7290491 | 0.09 |
| B3GNT4 | 0.398884097 | 5.32E-43 | 0.21578345 | 5.56E-13 | 0.19 |
| B3GNT5 | 0.254955566 | 1.12E-17 | -0.009446315 | 0.7550785 | 0.27 |
| LOC119818 | -0.062387334 | 0.03918788 | -0.055130096 | 0.0684654 | 0.01 |
| B3GNT7 | 0.258057233 | 4.36E-18 | 0.049827692 | 0.0996673 | 0.21 |
| B3GNT8 | -0.204221606 | 9.37E-12 | -0.10050381 | 0.0008768 | 0.11 |
| B3GNT9 | -0.212919082 | 1.14E-12 | -0.20299691 | 1.25E-11 | 0.01 |
| B3GNTL1 | 0.326716086 | 1.33E-28 | 0.252786583 | 2.14E-17 | 0.08 |
| B4GALNT1 | 0.217042029 | 4.05E-13 | -0.002189264 | 0.9423667 | 0.22 |
| B4GALNT2 | -0.008503578 | 0.77885125 | 0.101372272 | 0.0007901 | 0.11 |
| B4GALNT3 | -0.151522766 | 4.83E-07 | -0.194970622 | 7.98E-11 | 0.05 |
| B4GALNT4 | 0.105362001 | 0.00048473 | 0.112776251 | 0.0001869 | 0.01 |
| B4GALT1 | -0.141028166 | 2.86E-06 | -0.092724289 | 0.0021505 | 0.05 |
| B4GALT2 | 0.414371677 | 1.36E-46 | 0.221867073 | 1.18E-13 | 0.2 |
| B4GALT3 | 0.170572724 | 1.39E-08 | 0.446438639 | 1.18E-54 | 0.28 |
| B4GALT4 | 0.079353627 | 0.00867476 | -0.029782866 | 0.3252485 | 0.11 |
| B4GALT5 | 0.091462003 | 0.00247264 | -0.035935802 | 0.2351955 | 0.13 |
| B4GALT6 | 0.061560923 | 0.04186704 | -0.236357837 | 2.42E-15 | 0.3 |
| LOC202179 | 0.023049652 | 0.44649978 | 0.085372422 | 0.0047371 | 0.07 |
| B9D1 | 0.110432267 | 0.00025422 | 0.082130183 | 0.0065926 | 0.03 |
| B9D2 | -0.001882825 | 0.95042261 | 0.078232449 | 0.0096699 | 0.09 |
| BAALC | 0.037381372 | 0.21688029 | -0.107297146 | 0.0003801 | 0.15 |
| BAAT | 0.231952095 | 8.11E-15 | 0.060343249 | 0.0460957 | 0.18 |
| BACE1 | -0.236176039 | 2.55E-15 | -0.330544112 | 2.82E-29 | 0.1 |
| BACE2 | 0.104585788 | 0.00053377 | 0.05379474 | 0.0754472 | 0.06 |
| BACH1 | -0.240267875 | 8.11E-16 | -0.292291633 | 5.70E-23 | 0.06 |
| BACH2 | -0.178834429 | 2.62E-09 | -0.210252567 | 2.19E-12 | 0.04 |
| BAD | 0.101676827 | 0.00076164 | 0.115854295 | 0.0001237 | 0.02 |
| BAG1 | -0.036567281 | 0.22706452 | 0.017133053 | 0.5715151 | 0.06 |
| LOC135059 | 0.217121693 | 3.97E-13 | 0.034563487 | 0.2535698 | 0.19 |
| BAG3 | -0.093846841 | 0.00189678 | -0.080360962 | 0.0078597 | 0.02 |
| SODD | -0.228098211 | 2.29E-14 | -0.325178694 | 2.46E-28 | 0.1 |
| KIAA0873 | -0.055074468 | 0.06874525 | -0.086837742 | 0.0040653 | 0.04 |
| BAGE2 | -0.310428986 | 7.61E-26 | -0.311228947 | 5.62E-26 | 0.01 |
| BAGE | 0.086369648 | 0.0042699 | -0.001620103 | 0.9573333 | 0.09 |
| FLJ23058 | -0.020683118 | 0.49455256 | -0.199787629 | 2.65E-11 | 0.18 |
| BAHD1 | -0.242413728 | 4.41E-16 | -0.225596444 | 4.44E-14 | 0.02 |
| ADGRB1 | -0.066891626 | 0.02700672 | -0.047478219 | 0.1167074 | 0.02 |
| ADGRB2 | -0.352604401 | 2.40E-33 | -0.215939453 | 5.35E-13 | 0.14 |
| ADGRB3 | -0.309873339 | 9.38E-26 | -0.286239788 | 4.67E-22 | 0.03 |
| LOC100128461 | 0.197875332 | 4.12E-11 | 0.215745912 | 5.61E-13 | 0.02 |
| BAIAP2L2 | 0.36109808 | 5.29E-35 | 0.150895994 | 5.39E-07 | 0.22 |
| BAIAP2 | 0.033012056 | 0.27551712 | -0.009566603 | 0.7520613 | 0.05 |
| BAIAP3 | -0.276056146 | 1.44E-20 | -0.02839102 | 0.3483792 | 0.25 |
| BAK1 | 0.336993276 | 1.97E-30 | 0.095507778 | 0.0015714 | 0.25 |
| BAMBI | 0.078537482 | 0.00938957 | 0.143751735 | 1.83E-06 | 0.07 |
| BANF1 | 0.53843551 | 3.48E-83 | 0.459388419 | 3.66E-58 | 0.08 |
| BANF2 | 0.115047805 | 0.00013793 | 0.063965377 | 0.0344746 | 0.06 |
| BANK1 | -0.116736798 | 0.00010964 | -0.204589247 | 8.58E-12 | 0.09 |
| BANP | 0.149808304 | 6.52E-07 | 0.041898617 | 0.1662929 | 0.11 |
| BAP1 | -0.075955625 | 0.01200863 | -0.112507063 | 0.0001937 | 0.04 |
| BARD1 | -0.191434013 | 1.76E-10 | -0.275013131 | 2.03E-20 | 0.09 |
| BARHL1 | 0.023284071 | 0.44188878 | -0.00718235 | 0.8125142 | 0.04 |
| BARHL2 | 0.214373284 | 7.92E-13 | 0.079857074 | 0.0082584 | 0.14 |
| BARX1 | 0.197496223 | 4.49E-11 | 0.055737498 | 0.0654699 | 0.15 |
| BARX2 | 0.121613384 | 5.55E-05 | 0.073821119 | 0.0146424 | 0.05 |
| BPIFA4P | -0.0134959 | 0.65582 | 0.079922421 | 0.0082057 | 0.1 |
| BASP1 | -0.075515278 | 0.01251478 | -0.137478376 | 5.08E-06 | 0.07 |
| DDX39B | 0.167320734 | 2.63E-08 | 0.184489681 | 7.99E-10 | 0.02 |
| LQFBS.1 | -0.435361936 | 9.03E-52 | -0.450736716 | 8.40E-56 | 0.02 |
| PRRC2C | -0.116884649 | 0.00010745 | -0.00855819 | 0.7774682 | 0.11 |
| LOC154578 | 0.039501733 | 0.19190509 | -0.068835189 | 0.0228565 | 0.11 |
| BAG6 | 0.222160811 | 1.09E-13 | 0.122207884 | 5.10E-05 | 0.1 |
| LOC91959 | 0.203546151 | 1.10E-11 | 0.175697348 | 4.99E-09 | 0.03 |
| ABHD16A | 0.032873286 | 0.27754145 | 0.076881407 | 0.0110032 | 0.05 |
| BATF2 | 0.146392567 | 1.17E-06 | 0.018944391 | 0.5315436 | 0.13 |
| BATF3 | 0.200336921 | 2.33E-11 | 0.044096598 | 0.1451444 | 0.16 |
| BATF | -0.068658172 | 0.02321009 | 0.028783729 | 0.3417496 | 0.1 |
| BAX | 0.363096079 | 2.12E-35 | 0.303836835 | 8.90E-25 | 0.06 |
| BAZ1A | 0.057725708 | 0.05641048 | 0.029498648 | 0.3298889 | 0.03 |
| BAZ1B | 0.006488622 | 0.83033322 | -0.144167047 | 1.70E-06 | 0.16 |
| BAZ2A | -0.508248859 | 7.58E-73 | -0.418141492 | 1.70E-47 | 0.1 |
| BAZ2B | -0.495806875 | 7.04E-69 | -0.389241298 | 7.37E-41 | 0.11 |
| BBC3 | -0.050219084 | 0.09703161 | 0.054989355 | 0.0691753 | 0.11 |
| BBOX1 | 0.134002409 | 8.78E-06 | -0.003720403 | 0.9022193 | 0.14 |
| BBS10 | -0.300482414 | 3.04E-24 | -0.246324118 | 1.43E-16 | 0.06 |
| BBS12 | -0.275585374 | 1.68E-20 | -0.304317313 | 7.46E-25 | 0.03 |
| FLJ23590 | -0.474345039 | 2.08E-62 | -0.279794645 | 4.16E-21 | 0.2 |
| BBS2 | -0.301288459 | 2.27E-24 | -0.231932514 | 8.15E-15 | 0.07 |
| BBS4 | -0.48107626 | 2.18E-64 | -0.261126086 | 1.70E-18 | 0.22 |
| BBS5 | -0.338368807 | 1.11E-30 | -0.131916528 | 1.21E-05 | 0.21 |
| BBS7 | 0.014744927 | 0.62629795 | -0.123796185 | 4.06E-05 | 0.14 |
| BBS9 | -0.305768333 | 4.36E-25 | -0.213790776 | 9.15E-13 | 0.1 |
| BBX | -0.314585799 | 1.56E-26 | -0.284311165 | 9.05E-22 | 0.04 |
| LU | -0.376752129 | 3.45E-38 | -0.162210626 | 6.95E-08 | 0.22 |
| MGC13038 | 0.012574511 | 0.67795086 | -0.069989101 | 0.020664 | 0.09 |
| BCAP29 | -0.21989707 | 1.96E-13 | 0.021758158 | 0.4723883 | 0.25 |
| BCAP31 | 0.349775274 | 8.32E-33 | 0.230075078 | 1.35E-14 | 0.12 |
| LOC100131601 | -0.042001117 | 0.16525781 | -0.105068313 | 0.0005028 | 0.07 |
| BCAR3 | -0.281405008 | 2.42E-21 | -0.250641581 | 4.05E-17 | 0.04 |
| BCAR4 | 0.156124331 | 2.13E-07 | 0.162329316 | 6.80E-08 | 0.01 |
| BCAS1 | -0.417814611 | 2.04E-47 | -0.147576588 | 9.57E-07 | 0.28 |
| BCAS2 | 0.266000095 | 3.70E-19 | 0.199852583 | 2.61E-11 | 0.07 |
| BCAS3 | -0.244323154 | 2.55E-16 | -0.156536953 | 1.98E-07 | 0.09 |
| BCAS4 | -0.020489985 | 0.49859244 | 0.093008394 | 0.0020835 | 0.12 |
| BCAT1 | 0.039539817 | 0.19147668 | -0.060535443 | 0.0454053 | 0.11 |
| BCAT2 | -0.029045382 | 0.33737743 | 0.094297651 | 0.0018029 | 0.13 |
| BCCIP | 0.296357166 | 1.35E-23 | 0.362509551 | 2.78E-35 | 0.07 |
| BCDIN3D | -0.211242437 | 1.72E-12 | 0.03845346 | 0.203975 | 0.25 |
| BCHE | -0.1495839 | 6.78E-07 | -0.073337733 | 0.0153051 | 0.08 |
| OVD1A | -0.096889693 | 0.00134076 | -0.081428033 | 0.0070717 | 0.02 |
| LOC101928714 | -0.301288213 | 2.27E-24 | -0.198253067 | 3.78E-11 | 0.11 |
| BCKDK | 0.062104038 | 0.04008944 | 0.153666565 | 3.31E-07 | 0.1 |
| BCL10 | -0.11534606 | 0.00013248 | -0.09977004 | 0.0009568 | 0.02 |
| FLJ10173 | 0.305183153 | 5.42E-25 | 0.077209775 | 0.010665 | 0.23 |
| BCL11B | 0.049846207 | 0.09954133 | -0.094499792 | 0.0017621 | 0.15 |
| BCL2A1 | 0.310767239 | 6.69E-26 | 0.08006487 | 0.0080919 | 0.24 |
| BCL2L10 | 0.202673057 | 1.35E-11 | 0.054758717 | 0.0703517 | 0.15 |
| LOC150819 | -0.300663747 | 2.85E-24 | -0.184976123 | 7.20E-10 | 0.12 |
| LOC92647 | 0.375565384 | 6.10E-38 | 0.292992967 | 4.45E-23 | 0.09 |
| ZD77D08 | -0.100280639 | 0.00090046 | -0.115553647 | 0.0001288 | 0.02 |
| LOC101928224 | 0.157017102 | 1.81E-07 | 0.069822465 | 0.0209688 | 0.09 |
| LOC148280 | -0.106291562 | 0.00043153 | -0.019790456 | 0.5133704 | 0.09 |
| BCL2L1 | -0.236370764 | 2.41E-15 | -0.083865751 | 0.005531 | 0.16 |
| BCL2L2 | -0.335446912 | 3.75E-30 | -0.356695248 | 3.88E-34 | 0.03 |
| BCL2 | -0.2873277 | 3.21E-22 | -0.265304647 | 4.60E-19 | 0.03 |
| LOC105372417 | -0.036857169 | 0.2233997 | 0.042189725 | 0.1633659 | 0.08 |
| ZNF62 | -0.318226587 | 3.82E-27 | -0.21992867 | 1.94E-13 | 0.1 |
| BCL6 | -0.205418423 | 7.05E-12 | -0.244648886 | 2.33E-16 | 0.04 |
| BCL7A | 0.169498286 | 1.72E-08 | 0.032323315 | 0.2856636 | 0.14 |
| BCL7B | 0.24105757 | 6.49E-16 | 0.23176699 | 8.53E-15 | 0.01 |
| BCL7C | 0.331813594 | 1.68E-29 | 0.355505783 | 6.60E-34 | 0.03 |
| LOC727870 | -0.219010765 | 2.45E-13 | -0.119733388 | 7.24E-05 | 0.1 |
| BCL9L | -0.258233406 | 4.13E-18 | -0.25170967 | 2.95E-17 | 0.01 |
| BCL9 | -0.247598726 | 9.89E-17 | 0.053062144 | 0.0795176 | 0.31 |
| BCLAF1 | -0.204360516 | 9.06E-12 | -0.143507001 | 1.90E-06 | 0.07 |
| BCDO | 0.083234691 | 0.00589771 | -0.066497884 | 0.0279224 | 0.15 |
| BCO2 | -0.088721483 | 0.00332887 | -0.092547175 | 0.0021932 | 0.01 |
| BCORL1 | -0.280196541 | 3.64E-21 | -0.336479499 | 2.44E-30 | 0.06 |
| BCORP1 | 0.028974516 | 0.33855803 | 0.024392228 | 0.4204634 | 0.01 |
| MAA2 | -0.349727811 | 8.49E-33 | -0.189767411 | 2.55E-10 | 0.16 |
| BCR | 0.070399851 | 0.01992894 | -0.059481396 | 0.0493011 | 0.13 |
| BJS | 0.387637601 | 1.65E-40 | 0.33063415 | 2.72E-29 | 0.06 |
| BCYRN1 | 0.026894145 | 0.37439095 | 0.0139479 | 0.6450712 | 0.02 |
| BDH1 | 0.06859587 | 0.02333567 | 0.11206954 | 0.0002052 | 0.05 |
| BDH2 | -0.007491766 | 0.80459714 | 0.121476429 | 5.66E-05 | 0.13 |
| BDKRB1 | -0.001668714 | 0.95605441 | 0.019106239 | 0.5280421 | 0.03 |
| BDKRB2 | -0.241135216 | 6.35E-16 | -0.127940024 | 2.21E-05 | 0.12 |
| BDNF.AS | -0.237053465 | 2.00E-15 | -0.141725451 | 2.55E-06 | 0.1 |
| OCD1 | -0.073035884 | 0.01573221 | -0.117788835 | 9.49E-05 | 0.05 |
| TAF3B1 | -0.41187449 | 5.32E-46 | -0.340177209 | 5.16E-31 | 0.08 |
| SCA31 | -0.200945831 | 2.02E-11 | -0.131040229 | 1.39E-05 | 0.07 |
| BECN1 | -0.33349193 | 8.41E-30 | -0.187300629 | 4.36E-10 | 0.15 |
| BEGAIN | -0.087074423 | 0.00396532 | -0.079744527 | 0.0083499 | 0.01 |
| BEND2 | 0.040633142 | 0.17947563 | -0.006825736 | 0.8216627 | 0.05 |
| BEND3 | 0.310710596 | 6.84E-26 | 0.174092882 | 6.90E-09 | 0.14 |
| BEND4 | -0.014059762 | 0.64242239 | -0.140201743 | 3.27E-06 | 0.13 |
| BEND5 | -0.134773697 | 7.79E-06 | -0.062928929 | 0.0375121 | 0.08 |
| LOC154180 | 0.02511648 | 0.40679587 | -0.087888799 | 0.003638 | 0.12 |
| BEND7 | 0.0068384 | 0.82133735 | -0.165960654 | 3.41E-08 | 0.18 |
| BEST1 | -0.226423032 | 3.57E-14 | -0.202326142 | 1.47E-11 | 0.03 |
| BEST2 | 0.190062061 | 2.39E-10 | 0.038916409 | 0.1985782 | 0.16 |
| BEST3 | -0.121715055 | 5.47E-05 | 0.012761139 | 0.673445 | 0.14 |
| BEST4 | 0.102723336 | 0.00067089 | 0.038135492 | 0.207743 | 0.07 |
| BET1L | -0.265347543 | 4.54E-19 | -0.096749568 | 0.0013626 | 0.17 |
| BET1 | 0.017672142 | 0.55947076 | 0.167627807 | 2.48E-08 | 0.15 |
| TRAPPC3L | -0.118038855 | 9.17E-05 | -0.085306691 | 0.0047695 | 0.04 |
| BEX1 | 0.019683392 | 0.51565209 | -0.063568484 | 0.035612 | 0.09 |
| BEX2 | 0.10751261 | 0.00036989 | 0.031727373 | 0.2946441 | 0.08 |
| BEX4 | -0.144411079 | 1.63E-06 | -0.010927893 | 0.7181895 | 0.14 |
| BEX5 | -0.065757623 | 0.02971649 | 0.043473201 | 0.150922 | 0.11 |
| LINC00293 | 0.022742479 | 0.45258304 | 0.00845413 | 0.7801041 | 0.02 |
| BFAR | 0.116563501 | 0.00011227 | 0.119763835 | 7.21E-05 | 0.01 |
| BFSP1 | 0.224504601 | 5.92E-14 | 0.276241428 | 1.35E-20 | 0.06 |
| BFSP2 | 0.030753905 | 0.3097161 | 0.05447546 | 0.0718188 | 0.03 |
| BGLAP | 0.223177865 | 8.38E-14 | 0.298880588 | 5.43E-24 | 0.08 |
| BGN | -0.100720746 | 0.00085436 | -0.0633303 | 0.0363098 | 0.04 |
| BHLHA15 | -0.070576815 | 0.01961936 | -0.216518724 | 4.62E-13 | 0.15 |
| BHLHB9 | -0.173454085 | 7.85E-09 | -0.144275392 | 1.67E-06 | 0.03 |
| BHLHE22 | -0.173438379 | 7.87E-09 | -0.167011109 | 2.79E-08 | 0.01 |
| BHLHE23 | 0.000275314 | 0.99274603 | -0.011014712 | 0.7160471 | 0.02 |
| BHLHE40 | -0.415003166 | 9.62E-47 | -0.274661609 | 2.28E-20 | 0.15 |
| BHLHE41 | -0.163457179 | 5.50E-08 | -0.149896144 | 6.42E-07 | 0.02 |
| FLJ20001 | -0.222335295 | 1.04E-13 | -0.151718097 | 4.67E-07 | 0.08 |
| LOC116122 | -0.153588967 | 3.36E-07 | -0.127923351 | 2.22E-05 | 0.03 |
| BICC1 | -0.375502777 | 6.29E-38 | -0.379001826 | 1.16E-38 | 0.01 |
| BICD1 | -0.087468543 | 0.00380371 | -0.26262392 | 1.07E-18 | 0.18 |
| BICD2 | -0.078240535 | 0.00966239 | -0.2106755 | 1.98E-12 | 0.14 |
| BID | 0.413866797 | 1.79E-46 | 0.303650801 | 9.54E-25 | 0.12 |
| BIK | -0.064524698 | 0.03292439 | 0.157548757 | 1.65E-07 | 0.23 |
| BIN1 | 0.056823984 | 0.06038115 | -0.1203286 | 6.66E-05 | 0.18 |
| LOC51722 | 0.022036941 | 0.46673104 | -0.138612206 | 4.23E-06 | 0.17 |
| BIN3 | 0.068794554 | 0.02293724 | 0.151351091 | 4.98E-07 | 0.09 |
| BIRC2 | -0.07740118 | 0.01047208 | -0.191733264 | 1.65E-10 | 0.12 |
| BIRC3 | -0.04002749 | 0.18605276 | -0.137358298 | 5.18E-06 | 0.1 |
| BIRC5 | 0.636055126 | 5.63E-125 | 0.370431646 | 7.00E-37 | 0.27 |
| BIRC6 | -0.353815943 | 1.40E-33 | -0.418993804 | 1.06E-47 | 0.07 |
| LIVIN | 0.0504588 | 0.09544502 | 0.083472519 | 0.005757 | 0.04 |
| BIRC8 | 0.056326937 | 0.062667 | 0.044909175 | 0.1378695 | 0.02 |
| BIVM | 0.046240885 | 0.12655795 | -0.048233669 | 0.1109955 | 0.1 |
| BLCAP | 0.0164859 | 0.58613441 | 0.054519038 | 0.0715915 | 0.04 |
| LOC283156 | 0.041694852 | 0.16836498 | 0.032825521 | 0.2782406 | 0.01 |
| BLK | -0.044352418 | 0.1428231 | -0.126992743 | 2.55E-05 | 0.09 |
| BLMH | 0.173087302 | 8.44E-09 | 0.090295333 | 0.002809 | 0.09 |
| BLM | 0.463258729 | 3.06E-59 | 0.206366575 | 5.62E-12 | 0.26 |
| BLNK | -0.211089815 | 1.78E-12 | -0.10310406 | 0.0006405 | 0.11 |
| BLOC1S1 | 0.210816739 | 1.91E-12 | 0.338090825 | 1.24E-30 | 0.13 |
| BLOC1S2 | 0.137169321 | 5.33E-06 | 0.197714216 | 4.27E-11 | 0.07 |
| BLOC1S3 | 0.179255193 | 2.40E-09 | 0.164790505 | 4.27E-08 | 0.02 |
| BLVRA | -0.10649952 | 0.00042039 | 0.066245994 | 0.0285221 | 0.18 |
| FLR | -0.072523115 | 0.01648176 | 0.03181013 | 0.2933859 | 0.11 |
| BLZF1 | -0.224846832 | 5.41E-14 | 0.105511404 | 0.0004758 | 0.34 |
| BMF | -0.044014915 | 0.14589169 | -0.142284233 | 2.33E-06 | 0.1 |
| LOC115789 | -0.284886741 | 7.43E-22 | -0.011595651 | 0.7017696 | 0.28 |
| BMP10 | 0.019359837 | 0.52257938 | -0.075566372 | 0.0124551 | 0.1 |
| BMP15 | 0.021360329 | 0.48052609 | 0.02262271 | 0.4549675 | 0.01 |
| PCOLC | -0.018403548 | 0.54332895 | -0.048622571 | 0.1081422 | 0.04 |
| BMP2K | -0.404579683 | 2.67E-44 | -0.442615758 | 1.20E-53 | 0.04 |
| BMP2 | 0.04780461 | 0.11421193 | -0.14953003 | 6.84E-07 | 0.2 |
| BMP3 | -0.112574993 | 0.00019194 | -0.11935946 | 7.63E-05 | 0.01 |
| BMP4 | -0.354435564 | 1.06E-33 | -0.081179678 | 0.0072485 | 0.28 |
| BMP5 | 0.037204673 | 0.21906241 | -0.036742242 | 0.2248475 | 0.08 |
| VGR1 | -0.010778286 | 0.7218865 | -0.072205358 | 0.0169618 | 0.07 |
| BMP7 | 0.109299703 | 0.00029435 | 0.018225634 | 0.547234 | 0.1 |
| LOC643948 | -0.137248991 | 5.27E-06 | -0.138671579 | 4.19E-06 | 0.01 |
| BMP8B | -0.056400417 | 0.06232464 | -0.075262148 | 0.0128142 | 0.02 |
| BMPER | -0.183858983 | 9.14E-10 | -0.194067984 | 9.78E-11 | 0.02 |
| JIP | -0.067102205 | 0.0265277 | -0.147765225 | 9.27E-07 | 0.09 |
| BMPR1B | -0.252202057 | 2.55E-17 | -0.15529738 | 2.47E-07 | 0.1 |
| PPH1 | -0.46088242 | 1.41E-58 | -0.394661428 | 4.70E-42 | 0.07 |
| LOC654000 | -0.420420044 | 4.78E-48 | -0.29992967 | 3.72E-24 | 0.13 |
| BMS1P1 | -0.304147671 | 7.94E-25 | -0.218671373 | 2.68E-13 | 0.09 |
| LOC93065 | 0.342579881 | 1.86E-31 | 0.10917485 | 0.0002991 | 0.24 |
| BMX | -0.31991771 | 1.97E-27 | -0.207403257 | 4.38E-12 | 0.12 |
| BNC1 | 0.002349125 | 0.93816643 | -0.160184831 | 1.01E-07 | 0.17 |
| BNC2 | -0.406672692 | 8.77E-45 | -0.253968574 | 1.50E-17 | 0.16 |
| BNIP1 | 0.243294814 | 3.43E-16 | 0.319792401 | 2.07E-27 | 0.08 |
| BNIP2 | -0.014499355 | 0.6320572 | -0.15461844 | 2.79E-07 | 0.15 |
| BNIP3L | -0.394905929 | 4.15E-42 | -0.180818043 | 1.74E-09 | 0.22 |
| BNIP3 | 0.155953041 | 2.20E-07 | 0.075518209 | 0.0125114 | 0.09 |
| BNIPL | -0.207562203 | 4.21E-12 | 0.020980704 | 0.4883621 | 0.23 |
| BOC | -0.035303789 | 0.24353734 | -0.126586349 | 2.70E-05 | 0.1 |
| BOD1L1 | -0.455857378 | 3.43E-57 | -0.467377503 | 2.10E-60 | 0.02 |
| BOD1 | 0.136691501 | 5.75E-06 | 0.072258877 | 0.0168801 | 0.07 |
| BOK | -0.056337122 | 0.06261946 | -0.02349362 | 0.4377901 | 0.04 |
| BOLA1 | 0.330155458 | 3.30E-29 | 0.447814541 | 5.09E-55 | 0.12 |
| LOC197202 | 0.478860624 | 9.89E-64 | 0.448219225 | 3.97E-55 | 0.04 |
| BOLA3 | 0.556505827 | 6.79E-90 | 0.423250805 | 9.73E-49 | 0.14 |
| BOLL | 0.135233439 | 7.24E-06 | 0.063274952 | 0.0364737 | 0.08 |
| LOC727967 | 0.434858629 | 1.21E-51 | 0.20181041 | 1.65E-11 | 0.24 |
| BPESC1 | 0.064890901 | 0.03194203 | 0.005685128 | 0.8510805 | 0.06 |
| BPGM | 0.068922658 | 0.02268349 | 0.105521366 | 0.0004752 | 0.04 |
| MCNAA | 0.140726693 | 3.01E-06 | 0.278099173 | 7.32E-21 | 0.14 |
| BPIFB2 | -0.288053666 | 2.50E-22 | -0.009543966 | 0.7526289 | 0.28 |
| BPIFC | 0.084525344 | 0.00516974 | -0.042132584 | 0.1639373 | 0.13 |
| BPIFB6 | -0.078325957 | 0.00958319 | 0.08234882 | 0.0064495 | 0.17 |
| BPI | 0.238018143 | 1.53E-15 | 0.024461247 | 0.4191494 | 0.22 |
| BPNT1 | 0.065810036 | 0.02958629 | 0.392558095 | 1.38E-41 | 0.33 |
| LOC348241 | -0.241666409 | 5.46E-16 | -0.270991103 | 7.51E-20 | 0.03 |
| VCY2 | 0.046354027 | 0.1256312 | 0.099642827 | 0.0009714 | 0.06 |
| BRAF | -0.115186907 | 0.00013536 | -0.21921833 | 2.33E-13 | 0.11 |
| BRAP | -0.001719085 | 0.95472928 | -0.02140322 | 0.4796451 | 0.02 |
| BRCA1 | 0.022410992 | 0.45919994 | 0.012021392 | 0.6913723 | 0.02 |
| BRCA2 | 0.028414461 | 0.34798119 | -0.131011625 | 1.39E-05 | 0.16 |
| BRCC3 | -0.006149567 | 0.83907457 | -0.01359969 | 0.6533454 | 0.01 |
| DKFZP434B094 | -0.071840674 | 0.01752783 | -0.19679128 | 5.28E-11 | 0.13 |
| KIAA9001 | -0.127982709 | 2.20E-05 | -0.155530195 | 2.37E-07 | 0.03 |
| LOC115719 | -0.266170239 | 3.50E-19 | -0.210642499 | 1.99E-12 | 0.06 |
| LOC90616 | -0.052621502 | 0.08205059 | -0.224920776 | 5.31E-14 | 0.18 |
| BRD7P3 | 0.099874663 | 0.00094502 | 0.021332772 | 0.4810926 | 0.08 |
| BRD7 | 0.281856938 | 2.08E-21 | 0.082288719 | 0.0064885 | 0.2 |
| BRD8 | -0.203636851 | 1.08E-11 | -0.114974331 | 0.0001393 | 0.09 |
| BRD9 | 0.187994249 | 3.75E-10 | 0.092622196 | 0.002175 | 0.1 |
| BRDT | -0.046105546 | 0.12767349 | 0.000640377 | 0.9831284 | 0.05 |
| BREA2 | 0.153480804 | 3.42E-07 | 0.099667474 | 0.0009685 | 0.06 |
| BRE | 0.081571256 | 0.00697151 | 0.106815412 | 0.000404 | 0.03 |
| LOC90137 | 0.045561672 | 0.13223356 | 0.018816879 | 0.5343104 | 0.03 |
| BRF2 | 0.147658236 | 9.44E-07 | 0.013880494 | 0.6466696 | 0.14 |
| BRI3BP | 0.162440675 | 6.66E-08 | -0.026188991 | 0.3870497 | 0.19 |
| BRI3 | 0.401603947 | 1.28E-43 | 0.294145624 | 2.96E-23 | 0.11 |
| BRIP1 | 0.133055132 | 1.02E-05 | -0.060951096 | 0.0439419 | 0.2 |
| BRIX1 | 0.57998053 | 3.00E-99 | 0.453471 | 1.53E-56 | 0.13 |
| BRMS1L | 0.118509773 | 8.59E-05 | 0.00764599 | 0.8006584 | 0.12 |
| BRMS1 | 0.355742198 | 5.94E-34 | 0.290696321 | 9.97E-23 | 0.07 |
| MPC1 | 0.177192049 | 3.68E-09 | 0.242506115 | 4.30E-16 | 0.07 |
| MPC2 | 0.163496378 | 5.46E-08 | 0.362757103 | 2.48E-35 | 0.2 |
| BRPF1 | 0.093735731 | 0.00192061 | -0.195924618 | 6.43E-11 | 0.29 |
| BRPF3 | -0.36106371 | 5.38E-35 | -0.275859579 | 1.54E-20 | 0.09 |
| BRS3 | 0.084504732 | 0.0051807 | 0.039347054 | 0.1936523 | 0.05 |
| BRSK1 | 0.070736583 | 0.01934347 | 0.054498785 | 0.0716971 | 0.02 |
| BRSK2 | 0.126256647 | 2.84E-05 | 0.011640541 | 0.7006706 | 0.12 |
| WDR9 | -0.300951227 | 2.56E-24 | -0.197902921 | 4.09E-11 | 0.11 |
| BRWD3 | -0.256815554 | 6.36E-18 | -0.264265247 | 6.38E-19 | 0.01 |
| BSCL2 | 0.085293034 | 0.00477622 | 0.126026956 | 2.94E-05 | 0.05 |
| BSDC1 | -0.203096498 | 1.22E-11 | -0.226125649 | 3.86E-14 | 0.03 |
| OK | 0.247085755 | 1.15E-16 | 0.209353684 | 2.73E-12 | 0.04 |
| DFNB73 | 0.199706893 | 2.70E-11 | 0.081944271 | 0.0067165 | 0.12 |
| BSN | -0.21244851 | 1.28E-12 | -0.261613656 | 1.46E-18 | 0.05 |
| BSPH1 | 0.023669712 | 0.4343628 | -0.006985424 | 0.817563 | 0.04 |
| BSPRY | -0.127221948 | 2.46E-05 | 0.057975706 | 0.0553489 | 0.19 |
| BST1 | -0.067407492 | 0.02584627 | -0.04283812 | 0.1569861 | 0.03 |
| LOC100129681 | 0.027751354 | 0.35935137 | 0.016692295 | 0.5814531 | 0.02 |
| BSX | 0.012336925 | 0.68370371 | -0.038503521 | 0.2033864 | 0.06 |
| BTAF1 | -0.348703254 | 1.33E-32 | -0.416703417 | 3.77E-47 | 0.07 |
| BTBD10 | -0.072287432 | 0.01683667 | -0.093620885 | 0.0019455 | 0.03 |
| BTBD11 | -0.202691329 | 1.34E-11 | -0.136891556 | 5.57E-06 | 0.07 |
| KIAA1987 | -0.209442049 | 2.67E-12 | -0.170576228 | 1.39E-08 | 0.04 |
| BTBD16 | 0.121274306 | 5.83E-05 | 0.07569991 | 0.0123003 | 0.05 |
| BTBD17 | 0.072370631 | 0.01671062 | 0.009673137 | 0.7493923 | 0.07 |
| LOC648278 | -0.089924341 | 0.00292438 | -0.140237245 | 3.26E-06 | 0.06 |
| BTBD19 | -0.391786068 | 2.04E-41 | -0.349458246 | 9.56E-33 | 0.05 |
| FLJ20724 | -0.163125599 | 5.85E-08 | -0.234870806 | 3.65E-15 | 0.08 |
| BTBD2 | 0.026736478 | 0.37719894 | 0.003016371 | 0.9206547 | 0.03 |
| BTBD3 | 0.16188068 | 7.40E-08 | 0.004934477 | 0.8705571 | 0.16 |
| BTBD6 | 0.179726978 | 2.18E-09 | 0.144111031 | 1.72E-06 | 0.04 |
| BTBD7 | -0.449157807 | 2.23E-55 | -0.339967785 | 5.64E-31 | 0.11 |
| BTBD8 | -0.378268816 | 1.66E-38 | -0.36198176 | 3.54E-35 | 0.02 |
| BTBD9 | -0.410940747 | 8.83E-46 | -0.251073947 | 3.56E-17 | 0.16 |
| BTC | -0.103543854 | 0.00060688 | -0.22767307 | 2.56E-14 | 0.13 |
| BTD | -0.399724805 | 3.43E-43 | -0.109016758 | 0.0003053 | 0.3 |
| BTF3P11 | -0.026104164 | 0.38858988 | 0.094234245 | 0.0018158 | 0.13 |
| BTF3L4 | 0.389359045 | 6.95E-41 | 0.261288762 | 1.61E-18 | 0.13 |
| NACB | -0.098064331 | 0.00116966 | 0.132346337 | 1.13E-05 | 0.24 |
| BTG1 | -0.020039631 | 0.50808043 | 0.059459667 | 0.0493843 | 0.08 |
| BTG2 | -0.394468415 | 5.19E-42 | -0.07092617 | 0.0190205 | 0.33 |
| BTG3 | 0.32719557 | 1.09E-28 | 0.139759849 | 3.52E-06 | 0.19 |
| LOC101928620 | -0.014283389 | 0.63714064 | -0.033721814 | 0.2653209 | 0.02 |
| BTK | -0.121319285 | 5.79E-05 | -0.217745769 | 3.39E-13 | 0.1 |
| BTLA | -0.003548895 | 0.9067059 | -0.129840122 | 1.66E-05 | 0.13 |
| BTN1A1 | 0.061483127 | 0.04212704 | -0.073264121 | 0.0154083 | 0.14 |
| BTN2A1 | 0.01726576 | 0.56853874 | -0.138326218 | 4.43E-06 | 0.16 |
| BTN2A2 | 0.065402028 | 0.03061293 | -0.118680479 | 8.39E-05 | 0.19 |
| BTN2A3P | -0.21947324 | 2.18E-13 | -0.247583774 | 9.94E-17 | 0.03 |
| BTN3A1 | -0.117744513 | 9.55E-05 | -0.155848884 | 2.24E-07 | 0.04 |
| BTN3A2 | 0.034706693 | 0.25160701 | -0.032222003 | 0.2871772 | 0.07 |
| BTN3A3 | -0.099470264 | 0.00099143 | -0.112029212 | 0.0002063 | 0.02 |
| BTNL2 | 0.014194209 | 0.63924476 | -0.100211538 | 0.0009079 | 0.12 |
| BTNL3 | -0.046720093 | 0.12266889 | 0.016236242 | 0.5918202 | 0.07 |
| BTNL8 | 0.015935803 | 0.59869577 | 0.008057379 | 0.7901769 | 0.01 |
| BTNL9 | -0.361762024 | 3.91E-35 | -0.237529851 | 1.75E-15 | 0.13 |
| MGC4643 | -0.357631653 | 2.54E-34 | -0.197416054 | 4.58E-11 | 0.17 |
| BUB1B | 0.42406036 | 6.15E-49 | 0.205379241 | 7.11E-12 | 0.22 |
| LOC55563_2 | 0.443476777 | 7.14E-54 | 0.194438361 | 9.00E-11 | 0.25 |
| BUB3 | 0.098256282 | 0.00114369 | 0.118474646 | 8.63E-05 | 0.03 |
| BUD13 | 0.289262118 | 1.64E-22 | 0.140376581 | 3.18E-06 | 0.15 |
| BUD31 | 0.499018791 | 6.90E-70 | 0.443544098 | 6.85E-54 | 0.06 |
| BVES | -0.078738699 | 0.00920863 | -0.254037852 | 1.47E-17 | 0.18 |
| BYSL | 0.574060354 | 8.09E-97 | 0.371727522 | 3.80E-37 | 0.21 |
| BZRAP1 | -0.083767388 | 0.00558675 | -0.115575804 | 0.0001284 | 0.04 |
| BZW1 | -0.150940822 | 5.35E-07 | -0.053357688 | 0.0778546 | 0.1 |
| BZW2 | 0.177682416 | 3.33E-09 | 0.313365715 | 2.49E-26 | 0.14 |
| C10ORF105 | -0.168140977 | 2.24E-08 | -0.203514538 | 1.11E-11 | 0.04 |
| C10ORF107 | -0.185532689 | 6.39E-10 | -0.042705084 | 0.1582795 | 0.15 |
| PRR26 | -0.047180058 | 0.1190243 | -0.166199897 | 3.26E-08 | 0.12 |
| C10ORF10 | 0.001046721 | 0.97242614 | -0.075137511 | 0.0129639 | 0.08 |
| IDI2.AS1 | 0.158242194 | 1.45E-07 | 0.038204582 | 0.20692 | 0.13 |
| C10ORF111 | 0.330722803 | 2.62E-29 | 0.112691427 | 0.000189 | 0.22 |
| C10ORF113 | 0.084138015 | 0.0053792 | 0.079385047 | 0.0086482 | 0.01 |
| C10orf114 | 0.222808998 | 9.22E-14 | 0.044871539 | 0.1382001 | 0.18 |
| ADIRF | -0.225665689 | 4.36E-14 | -0.037396421 | 0.2166952 | 0.19 |
| FLJ35301 | -0.455613481 | 4.00E-57 | -0.440706462 | 3.78E-53 | 0.02 |
| MCMBP | 0.183496965 | 9.87E-10 | 0.060549703 | 0.0453544 | 0.13 |
| C10ORF11 | 0.053615916 | 0.07642478 | 0.153615424 | 3.34E-07 | 0.1 |
| C10ORF120 | -0.031524232 | 0.29774806 | -0.037097583 | 0.2203925 | 0.01 |
| TEX36 | 0.052053369 | 0.08541243 | 0.114883835 | 0.000141 | 0.07 |
| FUOM | 0.275740035 | 1.60E-20 | 0.129222001 | 1.83E-05 | 0.15 |
| C10ORF128 | -0.333871253 | 7.19E-30 | -0.356084825 | 5.10E-34 | 0.03 |
| ACSM6 | -0.121296883 | 5.81E-05 | -0.053956609 | 0.0745712 | 0.07 |
| C10ORF12 | -0.269299398 | 1.29E-19 | -0.332724292 | 1.15E-29 | 0.07 |
| C10ORF131 | 0.05647989 | 0.0619561 | 0.033885471 | 0.2630071 | 0.03 |
| FLJ21617 | -0.067687026 | 0.02523563 | -0.116641123 | 0.0001111 | 0.05 |
| SKIDA1 | 0.030815812 | 0.30874274 | -0.002851126 | 0.9249883 | 0.04 |
| FAM208B | 0.015362959 | 0.6119046 | -0.117958072 | 9.27E-05 | 0.14 |
| C10ORF25 | 0.116553816 | 0.00011242 | -0.028392562 | 0.348353 | 0.15 |
| WBP1L | -0.483723027 | 3.53E-65 | -0.268992443 | 1.43E-19 | 0.22 |
| TBATA | 0.010624336 | 0.72569759 | 0.01015094 | 0.7374589 | 0.01 |
| LOC142820 | -0.137033139 | 5.45E-06 | -0.122006018 | 5.25E-05 | 0.02 |
| C10ORF2 | 0.428926673 | 3.82E-50 | 0.217596645 | 3.52E-13 | 0.22 |
| C10ORF32 | -0.393227337 | 9.79E-42 | -0.124882329 | 3.47E-05 | 0.27 |
| LOC115039 | 0.369535156 | 1.07E-36 | 0.327881811 | 8.30E-29 | 0.05 |
| LINC01553 | -0.000746726 | 0.98032701 | 0.019644275 | 0.516487 | 0.03 |
| C10orf41 | 0.090999464 | 0.00260132 | 0.064285285 | 0.0335805 | 0.03 |
| CACUL1 | -0.105641315 | 0.00046813 | -0.029668829 | 0.3271053 | 0.08 |
| PROSER2 | 0.076910572 | 0.01097278 | 0.088411226 | 0.0034411 | 0.02 |
| FRA10AC1 | 0.094086407 | 0.00184633 | 0.198271978 | 3.76E-11 | 0.11 |
| C10orf50 | 0.014662889 | 0.62821943 | -0.04124703 | 0.1729868 | 0.06 |
| C10ORF53 | 0.149469318 | 6.91E-07 | 0.082210666 | 0.0065396 | 0.07 |
| C10ORF54 | -0.114195633 | 0.00015468 | -0.209472591 | 2.65E-12 | 0.1 |
| C10ORF55 | -0.090900844 | 0.00262954 | -0.1284318 | 2.06E-05 | 0.04 |
| TMEM254 | -0.120965372 | 6.09E-05 | 0.092827373 | 0.002126 | 0.22 |
| FAM213A | 0.146152051 | 1.22E-06 | 0.12877495 | 1.95E-05 | 0.02 |
| C10ORF62 | 0.043805288 | 0.14782284 | 0.000962344 | 0.9746481 | 0.05 |
| C10ORF67 | 0.086986806 | 0.00400208 | 0.111468007 | 0.0002221 | 0.03 |
| CCDC7 | -0.174113237 | 6.87E-09 | -0.027718794 | 0.3599156 | 0.15 |
| C10ORF71 | 0.016521339 | 0.58532936 | -0.040838393 | 0.1772863 | 0.06 |
| VSTM4 | -0.3794309 | 9.44E-39 | -0.250217733 | 4.59E-17 | 0.13 |
| OLMALINC | 0.027672656 | 0.36071609 | 0.08746067 | 0.0038069 | 0.06 |
| C10ORF76 | -0.257690371 | 4.88E-18 | -0.207277992 | 4.51E-12 | 0.06 |
| SFR1 | 0.13225811 | 1.15E-05 | 0.195408876 | 7.23E-11 | 0.07 |
| FLJ36006 | -0.376159968 | 4.59E-38 | -0.122804477 | 4.69E-05 | 0.26 |
| PLEKHS1 | -0.090710682 | 0.00268473 | 0.051058519 | 0.0915665 | 0.15 |
| C10ORF82 | -0.138539952 | 4.28E-06 | -0.06332165 | 0.0363354 | 0.08 |
| LOC220479 | -0.047628279 | 0.11555485 | 0.062871172 | 0.0376879 | 0.12 |
| C10ORF88 | -0.041032456 | 0.17523466 | -0.027070652 | 0.3712628 | 0.02 |
| C10ORF90 | 0.115161583 | 0.00013582 | -0.030300271 | 0.3169104 | 0.15 |
| C10ORF91 | 0.209532243 | 2.61E-12 | 0.038859082 | 0.1992408 | 0.18 |
| C10orf93 | -0.239564778 | 9.89E-16 | -0.07150877 | 0.0180573 | 0.17 |
| C10ORF95 | 0.118330503 | 8.80E-05 | 0.017778298 | 0.5571136 | 0.11 |
| CCDC172 | -0.01158324 | 0.70207351 | -0.009836745 | 0.7452992 | 0.01 |
| C10ORF99 | 0.177956701 | 3.14E-09 | -0.001970614 | 0.9481142 | 0.18 |
| TMEM258 | 0.477993969 | 1.78E-63 | 0.514742863 | 5.53E-75 | 0.04 |
| C11ORF16 | 0.102527914 | 0.00068704 | 0.082628412 | 0.0062705 | 0.02 |
| AKIP1 | 0.020382667 | 0.50084483 | 0.028054466 | 0.3541253 | 0.01 |
| C11orf1 | 0.071041238 | 0.01882677 | 0.176940629 | 3.87E-09 | 0.11 |
| TEX40 | 0.156728218 | 1.91E-07 | 0.095597598 | 0.0015554 | 0.07 |
| C11ORF21 | -0.11639807 | 0.00011484 | -0.216211012 | 4.99E-13 | 0.1 |
| C11ORF24 | 0.153922951 | 3.16E-07 | 0.122288271 | 5.05E-05 | 0.04 |
| VPS51 | 0.056806289 | 0.06046132 | 0.047901809 | 0.1134769 | 0.01 |
| C11ORF30 | -0.245097792 | 2.04E-16 | -0.275506391 | 1.73E-20 | 0.04 |
| C17orf10 | 0.315259825 | 1.20E-26 | 0.391462102 | 2.40E-41 | 0.08 |
| PLET1 | 0.060017845 | 0.04728472 | -0.068113911 | 0.0243272 | 0.13 |
| LMNTD2 | -0.207732177 | 4.04E-12 | -0.055376534 | 0.0672367 | 0.16 |
| MRGPRG.AS1 | 0.196846073 | 5.21E-11 | 0.103076701 | 0.0006426 | 0.1 |
| C11ORF40 | -0.009472372 | 0.75442464 | 0.007622135 | 0.8012673 | 0.02 |
| KIAA1549L | 0.081999489 | 0.00667949 | -0.058972292 | 0.0512809 | 0.15 |
| C11ORF42 | -0.235004599 | 3.52E-15 | -0.307819069 | 2.03E-25 | 0.08 |
| C11ORF45 | 0.043406916 | 0.15154646 | -0.02718848 | 0.3691836 | 0.08 |
| ARL14EP | -0.065040636 | 0.03154765 | -0.005473602 | 0.8565602 | 0.06 |
| LBHD1 | 0.511166133 | 8.42E-74 | 0.454815179 | 6.60E-57 | 0.06 |
| C11ORF49 | 0.104122298 | 0.00056522 | 0.14419842 | 1.69E-06 | 0.05 |
| ANAPC15 | 0.356270936 | 4.69E-34 | 0.313827169 | 2.09E-26 | 0.05 |
| C11ORF52 | -0.171383974 | 1.19E-08 | -0.012714216 | 0.6745768 | 0.16 |
| C11ORF53 | -0.052059404 | 0.08537614 | -0.042179193 | 0.1634711 | 0.01 |
| LOC115897 | -0.199579875 | 2.78E-11 | -0.081781381 | 0.0068268 | 0.12 |
| C11ORF57 | 0.042065785 | 0.16460728 | 0.031236882 | 0.3021758 | 0.02 |
| C11ORF58 | -0.116153868 | 0.00011872 | -0.053872869 | 0.0750233 | 0.07 |
| LAMTOR1 | 0.07878513 | 0.00916732 | 0.135730281 | 6.70E-06 | 0.06 |
| MSANTD2 | -0.15742364 | 1.68E-07 | -0.052575356 | 0.0823196 | 0.11 |
| C11ORF63 | -0.311779872 | 4.56E-26 | -0.228845155 | 1.87E-14 | 0.09 |
| LINC00301 | -0.036892451 | 0.22295656 | -0.026998688 | 0.3725362 | 0.01 |
| C11ORF65 | -0.186450641 | 5.24E-10 | -0.090643877 | 0.0027044 | 0.1 |
| PPP1R32 | -0.085615891 | 0.00461896 | -0.000263581 | 0.9930552 | 0.09 |
| FLJ21035 | 0.070169877 | 0.02033761 | 0.164172551 | 4.80E-08 | 0.1 |
| C11ORF68 | 0.098168478 | 0.0011555 | 0.092333309 | 0.0022459 | 0.01 |
| C11ORF70 | -0.05425462 | 0.07297998 | 0.025438958 | 0.4007965 | 0.08 |
| C11ORF71 | 0.082918947 | 0.00608929 | 0.010466483 | 0.7296124 | 0.08 |
| HSPC179 | 0.39373137 | 7.57E-42 | 0.328228228 | 7.22E-29 | 0.07 |
| C11ORF74 | 0.262896911 | 9.79E-19 | 0.308375323 | 1.65E-25 | 0.05 |
| SMCO4 | 0.272657503 | 4.38E-20 | 0.132809853 | 1.06E-05 | 0.14 |
| C11ORF80 | 0.13847224 | 4.33E-06 | 0.154145054 | 3.04E-07 | 0.02 |
| FLJ13936 | 0.409226559 | 2.23E-45 | 0.194460468 | 8.96E-11 | 0.22 |
| UQCC3 | 0.337841945 | 1.38E-30 | 0.313168217 | 2.69E-26 | 0.03 |
| C11ORF84 | 0.401386505 | 1.44E-43 | 0.224315783 | 6.22E-14 | 0.18 |
| C11ORF85 | 0.062877736 | 0.03766786 | 0.024824707 | 0.4122699 | 0.04 |
| LOC91681 | 0.395035742 | 3.88E-42 | 0.151829182 | 4.58E-07 | 0.25 |
| LOC143891 | -0.108117408 | 0.0003425 | -0.118415333 | 8.70E-05 | 0.02 |
| C11ORF88 | -0.071695625 | 0.01775752 | -0.010763831 | 0.722244 | 0.07 |
| VSTM5 | 0.28395267 | 1.02E-21 | 0.00383004 | 0.8993529 | 0.29 |
| COLCA1 | 0.060223781 | 0.04652927 | -0.04578789 | 0.1303218 | 0.11 |
| COLCA2 | 0.155383374 | 2.44E-07 | -0.028144993 | 0.3525739 | 0.19 |
| C11ORF94 | 0.041200426 | 0.1734732 | -0.052138208 | 0.0849035 | 0.1 |
| C11ORF95 | -0.006551481 | 0.82871489 | -0.028844923 | 0.3407238 | 0.03 |
| MGC10781 | 0.179910568 | 2.10E-09 | 0.135980467 | 6.44E-06 | 0.05 |
| C12ORF10 | 0.050291739 | 0.09654853 | 0.172674388 | 9.17E-09 | 0.13 |
| FLJ10630 | 0.486090195 | 6.83E-66 | 0.257097955 | 5.84E-18 | 0.23 |
| CCER1 | 0.038112418 | 0.20801834 | -0.001182386 | 0.968854 | 0.04 |
| TMEM263 | -0.241948784 | 5.04E-16 | -0.033535984 | 0.267965 | 0.21 |
| FAM216A | 0.387130292 | 2.12E-40 | 0.107000051 | 0.0003947 | 0.29 |
| METTL25 | -0.281211048 | 2.59E-21 | -0.044448691 | 0.1419569 | 0.24 |
| HNF1A.AS1 | 0.027141714 | 0.37000799 | 0.036823972 | 0.2238172 | 0.01 |
| C12ORF29 | 0.019016244 | 0.52998763 | 0.078702397 | 0.009241 | 0.06 |
| RHNO1 | 0.462607848 | 4.65E-59 | 0.309630309 | 1.03E-25 | 0.16 |
| FAM222A | -0.026974248 | 0.37296931 | -0.062286536 | 0.0395067 | 0.04 |
| FLJ20696 | -0.231608453 | 8.90E-15 | -0.300547348 | 2.97E-24 | 0.07 |
| LINC01559 | 0.13703725 | 5.45E-06 | 0.045659902 | 0.1314008 | 0.1 |
| SPX | -0.031748438 | 0.29432351 | -0.091336032 | 0.0025071 | 0.06 |
| C12ORF40 | 0.020606091 | 0.49616168 | 0.000666701 | 0.982435 | 0.02 |
| FLJ12670 | 0.077774156 | 0.01010512 | 0.09790697 | 0.0011913 | 0.03 |
| C12ORF42 | -0.00695591 | 0.81832035 | -0.057346672 | 0.0580523 | 0.06 |
| LOC115455 | -0.061976408 | 0.04050132 | -0.020995737 | 0.4880505 | 0.05 |
| ATG101 | 0.274823699 | 2.16E-20 | 0.296261398 | 1.39E-23 | 0.03 |
| C12ORF45 | 0.485174102 | 1.29E-65 | 0.505233053 | 7.19E-72 | 0.03 |
| MAPKAPK5.AS1 | 0.414111083 | 1.57E-46 | 0.368103832 | 2.09E-36 | 0.05 |
| PARPBP | 0.432096262 | 6.10E-51 | 0.24416272 | 2.68E-16 | 0.19 |
| C12ORF49 | -0.068515767 | 0.02349799 | -0.109229719 | 0.000297 | 0.05 |
| C12ORF4 | 0.173247105 | 8.18E-09 | 0.073451632 | 0.0151466 | 0.1 |
| C12ORF50 | 0.067324011 | 0.02603109 | 0.030465529 | 0.3142769 | 0.04 |
| HECTD4 | -0.502757836 | 4.48E-71 | -0.444014767 | 5.16E-54 | 0.06 |
| RITA1 | 0.218563678 | 2.75E-13 | 0.211741471 | 1.52E-12 | 0.01 |
| PIANP | -0.094952658 | 0.00167398 | -0.086305895 | 0.0042985 | 0.01 |
| C12ORF54 | 0.076294192 | 0.01163187 | 0.019374215 | 0.5222705 | 0.06 |
| C12ORF56 | 0.17912094 | 2.47E-09 | 0.042741989 | 0.1579199 | 0.14 |
| C12ORF57 | 0.15325753 | 3.56E-07 | 0.207481141 | 4.30E-12 | 0.06 |
| TMEM52B | -0.039359658 | 0.19350952 | -0.058268102 | 0.0541284 | 0.02 |
| TIGAR | 0.063276082 | 0.03647031 | -0.01077284 | 0.7220212 | 0.08 |
| C12ORF60 | 0.144052668 | 1.74E-06 | 0.098799023 | 0.0010731 | 0.05 |
| LINC01465 | 0.035232898 | 0.24448581 | 0.002753026 | 0.927562 | 0.04 |
| COX14 | 0.198948833 | 3.22E-11 | 0.332437311 | 1.30E-29 | 0.14 |
| C12orf63 | -0.220888893 | 1.52E-13 | -0.118235578 | 8.92E-05 | 0.11 |
| C12ORF65 | 0.310821375 | 6.56E-26 | 0.115442739 | 0.0001308 | 0.2 |
| C12ORF66 | -0.212348344 | 1.31E-12 | -0.142641618 | 2.19E-06 | 0.07 |
| CCDC184 | -0.097745721 | 0.00121396 | -0.12831651 | 2.09E-05 | 0.04 |
| LOC387843 | -0.1242597 | 3.80E-05 | -0.080107532 | 0.008058 | 0.05 |
| SMCO2 | 0.193475179 | 1.12E-10 | 0.083801231 | 0.0055675 | 0.11 |
| C12ORF71 | -0.060664286 | 0.04494736 | -0.129061749 | 1.87E-05 | 0.07 |
| METTL20 | -0.387265653 | 1.99E-40 | -0.233662874 | 5.09E-15 | 0.16 |
| LOC732143 | 0.439304247 | 8.74E-53 | 0.368466768 | 1.76E-36 | 0.08 |
| C12ORF74 | -0.124486583 | 3.68E-05 | -0.053405811 | 0.0775865 | 0.08 |
| C12ORF75 | 0.103522398 | 0.00060848 | 0.056223892 | 0.0631497 | 0.05 |
| C12ORF76 | -0.017536577 | 0.56248795 | 0.011393164 | 0.7067344 | 0.03 |
| C12ORF77 | 0.000985736 | 0.97403208 | -0.074656493 | 0.0135565 | 0.08 |
| C13orf15 | 0.027064994 | 0.37136282 | 0.029234234 | 0.3342442 | 0.01 |
| TEX29 | 0.158760859 | 1.32E-07 | 0.119519855 | 7.46E-05 | 0.04 |
| KIAA0226L | 0.023205775 | 0.44342582 | -0.123145584 | 4.46E-05 | 0.15 |
| SPRYD7 | -0.021118031 | 0.48551957 | 0.008175023 | 0.7871864 | 0.03 |
| PROSER1 | 0.12589563 | 2.99E-05 | -0.00140082 | 0.9631039 | 0.13 |
| TEX26 | -0.068120483 | 0.02431342 | -0.098129769 | 0.0011607 | 0.04 |
| TEX30 | 0.522273174 | 1.61E-77 | 0.342945703 | 1.59E-31 | 0.18 |
| SPACA7 | -0.038782379 | 0.20012986 | -0.004228605 | 0.8889439 | 0.04 |
| LINC00346 | -0.001074879 | 0.97168469 | -0.134929411 | 7.60E-06 | 0.14 |
| FAM216B | -0.11296332 | 0.00018231 | -0.061861155 | 0.0408763 | 0.06 |
| LACC1 | -0.124387217 | 3.73E-05 | -0.156641696 | 1.94E-07 | 0.04 |
| MEDAG | -0.193068755 | 1.22E-10 | -0.159001997 | 1.26E-07 | 0.04 |
| BORA | 0.38510446 | 5.82E-40 | 0.16919924 | 1.82E-08 | 0.22 |
| ATP11AUN | 0.128910052 | 1.91E-05 | 0.01789236 | 0.5545863 | 0.12 |
| LOC145053 | 0.012972954 | 0.66834517 | -0.110055873 | 0.000267 | 0.13 |
| MZT1 | 0.271311595 | 6.77E-20 | 0.186961644 | 4.69E-10 | 0.09 |
| CCDC169 | 0.062280355 | 0.03952629 | 0.127568774 | 2.34E-05 | 0.07 |
| METTL21C | 0.071140792 | 0.01866056 | 0.030415893 | 0.3150664 | 0.05 |
| TMEM260 | -0.235117067 | 3.41E-15 | -0.012883702 | 0.6704922 | 0.23 |
| NRDE2 | -0.354585265 | 9.95E-34 | -0.261020634 | 1.75E-18 | 0.1 |
| DNAAF2 | 0.141381839 | 2.70E-06 | 0.125809634 | 3.03E-05 | 0.02 |
| C14ORF105 | -0.009890199 | 0.74396343 | -0.014069203 | 0.642199 | 0.01 |
| MIS18BP1 | -0.089915876 | 0.00292707 | -0.181308094 | 1.57E-09 | 0.1 |
| TMEM251 | 0.018820481 | 0.53423219 | 0.129129337 | 1.85E-05 | 0.12 |
| VRTN | 0.314768019 | 1.46E-26 | 0.111296257 | 0.0002271 | 0.21 |
| GPATCH2L | -0.375173848 | 7.36E-38 | -0.40989191 | 1.56E-45 | 0.04 |
| C14ORF119 | 0.155591616 | 2.35E-07 | 0.325300756 | 2.34E-28 | 0.17 |
| DTD2 | -0.012724971 | 0.67431731 | 0.053649739 | 0.0762391 | 0.07 |
| ARHGAP5.AS1 | 0.126050677 | 2.93E-05 | -0.038391827 | 0.2047015 | 0.17 |
| GSKIP | -0.218730776 | 2.64E-13 | -0.036374727 | 0.2295224 | 0.19 |
| LOC100132684 | -0.379038899 | 1.14E-38 | -0.209652987 | 2.54E-12 | 0.17 |
| PCNXL4 | -0.386171876 | 3.42E-40 | -0.258596779 | 3.70E-18 | 0.13 |
| VCPKMT | 0.049674044 | 0.10071743 | 0.130295487 | 1.55E-05 | 0.09 |
| LINC00341 | -0.252435114 | 2.38E-17 | -0.262229572 | 1.21E-18 | 0.01 |
| C14ORF142 | 0.135219267 | 7.26E-06 | 0.208165331 | 3.64E-12 | 0.08 |
| EFCAB11 | -0.043042288 | 0.15501682 | 0.19960222 | 2.77E-11 | 0.25 |
| C14orf61 | 0.114788317 | 0.00014284 | -0.057756409 | 0.0562792 | 0.18 |
| SPTSSA | 0.091094321 | 0.00257444 | 0.201085686 | 1.96E-11 | 0.11 |
| NOXRED1 | -0.321698609 | 9.79E-28 | -0.156518496 | 1.98E-07 | 0.17 |
| L3HYPDH | -0.096328515 | 0.00143039 | -0.064435209 | 0.0331683 | 0.04 |
| APOPT1 | 0.425633206 | 2.52E-49 | 0.376011745 | 4.93E-38 | 0.05 |
| SLIRP | 0.406921405 | 7.68E-45 | 0.489823249 | 4.99E-67 | 0.09 |
| C14ORF159 | -0.263164986 | 9.00E-19 | -0.185284224 | 6.74E-10 | 0.08 |
| CCDC177 | -0.012275951 | 0.68518308 | -0.00700468 | 0.817069 | 0.01 |
| LINC00596 | 0.004817448 | 0.87360099 | -0.024201973 | 0.4240979 | 0.03 |
| LRRC74A | 0.153742232 | 3.27E-07 | 0.071142075 | 0.0186584 | 0.09 |
| C14ORF166 | 0.296051547 | 1.50E-23 | 0.418360769 | 1.51E-47 | 0.13 |
| DHRS4.AS1 | -0.239841732 | 9.14E-16 | -0.103191857 | 0.0006336 | 0.14 |
| C14ORF169 | 0.132652191 | 1.08E-05 | 0.156609682 | 1.95E-07 | 0.03 |
| FAM15A | -0.429417334 | 2.88E-50 | -0.214909185 | 6.92E-13 | 0.22 |
| TMEM253 | 0.061117727 | 0.04336655 | 0.097222622 | 0.0012901 | 0.04 |
| C14ORF177 | -0.017678739 | 0.55932413 | -0.023327466 | 0.4410382 | 0.01 |
| C14ORF178 | -0.097479414 | 0.00125217 | -0.1316265 | 1.27E-05 | 0.04 |
| IFT43 | 0.078868454 | 0.0090936 | 0.282903985 | 1.46E-21 | 0.21 |
| C14ORF180 | -0.249891383 | 5.05E-17 | -0.160434754 | 9.68E-08 | 0.09 |
| C14orf181 | 0.140377272 | 3.18E-06 | 0.120517006 | 6.49E-05 | 0.02 |
| LINC01588 | 0.121557209 | 5.60E-05 | -0.001211844 | 0.9680785 | 0.13 |
| LOC100506499 | -0.083196401 | 0.00592065 | -0.022961262 | 0.4482455 | 0.07 |
| C14orf184 | -0.185236211 | 6.81E-10 | 0.008142632 | 0.7880095 | 0.2 |
| IGBP1P1 | -0.123208913 | 4.42E-05 | -0.003905382 | 0.8973839 | 0.12 |
| C14ORF1 | 0.232062966 | 7.87E-15 | 0.251490279 | 3.15E-17 | 0.02 |
| NOP9 | -0.296373919 | 1.34E-23 | -0.339868409 | 5.88E-31 | 0.05 |
| LINC01551 | 0.159225158 | 1.21E-07 | 0.036307452 | 0.2303855 | 0.13 |
| C14ORF28 | -0.252768595 | 2.15E-17 | -0.090225823 | 0.0028303 | 0.17 |
| C14ORF2 | 0.448093275 | 4.29E-55 | 0.516974269 | 9.95E-76 | 0.07 |
| C14orf33 | -0.146983375 | 1.06E-06 | -0.011134657 | 0.7130909 | 0.14 |
| LOC647582 | 0.127781504 | 2.26E-05 | 0.03006203 | 0.3207321 | 0.1 |
| C14ORF37 | -0.350072818 | 7.30E-33 | -0.259692429 | 2.64E-18 | 0.1 |
| C14ORF39 | -0.074009545 | 0.01439096 | 0.018111572 | 0.5497449 | 0.1 |
| LOC101928293 | -0.511492462 | 6.58E-74 | -0.473292035 | 4.21E-62 | 0.04 |
| CCDC176 | -0.498451905 | 1.04E-69 | -0.191010238 | 1.94E-10 | 0.31 |
| LINC00521 | 0.00970377 | 0.74862538 | -0.084524284 | 0.0051703 | 0.1 |
| SYNE3 | -0.340947831 | 3.73E-31 | -0.429826871 | 2.27E-50 | 0.09 |
| IRF2BPL | -0.266345815 | 3.31E-19 | -0.371127449 | 5.04E-37 | 0.11 |
| PPP1R36 | -0.187813507 | 3.90E-10 | -0.073736661 | 0.0147563 | 0.12 |
| LINC00238 | -0.066317179 | 0.02835154 | -0.006116107 | 0.8399383 | 0.07 |
| LINC01550 | -0.049775511 | 0.10002295 | -0.12837299 | 2.07E-05 | 0.08 |
| SLC25A47 | 0.172559486 | 9.38E-09 | 0.114574608 | 0.000147 | 0.06 |
| LINC00523 | 0.082715185 | 0.00621588 | 0.063174057 | 0.0367739 | 0.02 |
| LINC00239 | 0.174013032 | 7.01E-09 | 0.133622727 | 9.32E-06 | 0.05 |
| EXOC3L4 | 0.04263261 | 0.15898745 | -0.057327148 | 0.0581379 | 0.1 |
| C14ORF79 | -0.10938226 | 0.00029123 | 0.12597137 | 2.96E-05 | 0.24 |
| C14ORF80 | 0.275827208 | 1.55E-20 | 0.237140815 | 1.95E-15 | 0.04 |
| FAM181A.AS1 | 0.02918255 | 0.33509982 | 0.015224323 | 0.6151206 | 0.02 |
| C14ORF93 | -0.037541605 | 0.21491506 | 0.135014195 | 7.50E-06 | 0.18 |
| FAM219B | -0.200134411 | 2.44E-11 | -0.231779793 | 8.50E-15 | 0.04 |
| HMGN2P46 | 0.037747631 | 0.21240707 | 0.061543922 | 0.0419237 | 0.03 |
| KNSTRN | 0.525713825 | 1.06E-78 | 0.266377991 | 3.28E-19 | 0.26 |
| EMC7 | 0.061628947 | 0.04164081 | 0.10357886 | 0.0006043 | 0.05 |
| C15ORF26 | -0.052287323 | 0.08401482 | -0.004529375 | 0.8811015 | 0.05 |
| C15ORF27 | -0.032250455 | 0.28675156 | -0.044622836 | 0.1404003 | 0.02 |
| ANP32A.IT1 | -0.294821734 | 2.33E-23 | -0.237251155 | 1.89E-15 | 0.06 |
| KATNBL1 | 0.070788388 | 0.01925475 | 0.041563766 | 0.1697082 | 0.03 |
| NPAP1 | 0.072573346 | 0.01640698 | 0.013491992 | 0.6559132 | 0.06 |
| C15ORF32 | 0.005462746 | 0.85684163 | -0.016529581 | 0.5851422 | 0.03 |
| FAM227B | -0.365460159 | 7.14E-36 | -0.243346731 | 3.38E-16 | 0.13 |
| HEXA.AS1 | -0.39269075 | 1.29E-41 | -0.332280017 | 1.38E-29 | 0.07 |
| ST20.AS1 | -0.065573338 | 0.03017821 | -0.204802869 | 8.16E-12 | 0.14 |
| ARPIN | -0.250802882 | 3.86E-17 | -0.169863962 | 1.60E-08 | 0.09 |
| C15ORF39 | 0.120036608 | 6.94E-05 | -0.051377869 | 0.0895534 | 0.18 |
| C15ORF40 | 0.00264959 | 0.9302766 | 0.050549287 | 0.0948515 | 0.05 |
| C15ORF41 | 0.25187788 | 2.81E-17 | 0.06543034 | 0.0305407 | 0.19 |
| TICRR | 0.500981466 | 1.65E-70 | 0.197568389 | 4.42E-11 | 0.31 |
| C15ORF43 | -0.031221218 | 0.30241845 | -0.032976138 | 0.2760401 | 0.01 |
| VWA9 | 0.07249249 | 0.01652751 | 0.021898851 | 0.4695285 | 0.06 |
| C15ORF48 | -0.071744341 | 0.01768008 | -0.061009704 | 0.0437388 | 0.02 |
| LINC00593 | -0.294329508 | 2.77E-23 | -0.125347446 | 3.24E-05 | 0.17 |
| DNM1P46 | -0.304235651 | 7.69E-25 | -0.268107713 | 1.89E-19 | 0.04 |
| C15ORF52 | 0.015990336 | 0.59744509 | -0.131794766 | 1.24E-05 | 0.15 |
| C15ORF53 | 0.045646892 | 0.13151083 | -0.070113245 | 0.0204394 | 0.12 |
| C15ORF54 | 0.06177552 | 0.04115687 | -0.025998353 | 0.3905163 | 0.09 |
| NUTM1 | 0.16339442 | 5.56E-08 | -0.030324692 | 0.3165203 | 0.2 |
| C15ORF56 | 0.046353214 | 0.12563784 | -0.034334205 | 0.2567345 | 0.09 |
| C15ORF57 | -0.225301805 | 4.80E-14 | -0.10251627 | 0.000688 | 0.13 |
| GDPGP1 | -0.234795026 | 3.73E-15 | -0.144025699 | 1.74E-06 | 0.1 |
| C15ORF59 | -0.208924138 | 3.03E-12 | -0.108464511 | 0.0003277 | 0.11 |
| LINC00597 | -0.061933051 | 0.04064205 | -0.06625306 | 0.0285052 | 0.01 |
| REC114 | 0.021101313 | 0.48586514 | 0.025631981 | 0.397231 | 0.01 |
| C15ORF61 | 0.370303407 | 7.44E-37 | 0.250376058 | 4.38E-17 | 0.12 |
| C15ORF62 | -0.209664956 | 2.53E-12 | -0.144441056 | 1.63E-06 | 0.07 |
| HSPC136 | 0.265383493 | 4.49E-19 | 0.318714592 | 3.16E-27 | 0.06 |
| PRR35 | 0.141543085 | 2.63E-06 | 0.039054485 | 0.196989 | 0.11 |
| C16ORF13 | 0.299454394 | 4.41E-24 | 0.388119389 | 1.29E-40 | 0.09 |
| GAS8.AS1 | -0.236873098 | 2.10E-15 | -0.138224809 | 4.51E-06 | 0.1 |
| TSR3 | 0.245174267 | 2.00E-16 | 0.255570865 | 9.28E-18 | 0.02 |
| C16ORF45 | -0.309502934 | 1.08E-25 | -0.097392801 | 0.0012648 | 0.22 |
| C16ORF46 | -0.260662548 | 1.96E-18 | -0.186320746 | 5.39E-10 | 0.08 |
| ENKD1 | 0.118162089 | 9.01E-05 | 0.120106504 | 6.87E-05 | 0.01 |
| LOC100506442 | -0.292664037 | 5.00E-23 | -0.198274956 | 3.76E-11 | 0.1 |
| PAGR1 | 0.197366285 | 4.63E-11 | 0.271989176 | 5.44E-20 | 0.08 |
| LOC101929877 | -0.085369393 | 0.0047386 | -0.167206219 | 2.69E-08 | 0.09 |
| SPATA33 | 0.371687708 | 3.87E-37 | 0.240322301 | 7.99E-16 | 0.14 |
| LOC221190 | 0.317037576 | 6.06E-27 | 0.106906332 | 0.0003994 | 0.22 |
| C16ORF58 | -0.122662955 | 4.78E-05 | 0.044425124 | 0.1421686 | 0.17 |
| C16ORF59 | 0.506189044 | 3.53E-72 | 0.366567188 | 4.27E-36 | 0.14 |
| CDIP1 | 0.044555653 | 0.14099928 | -0.072546665 | 0.0164467 | 0.12 |
| CMC2 | 0.617259515 | 9.03E-116 | 0.386470907 | 2.95E-40 | 0.24 |
| C16ORF62 | -0.149703679 | 6.64E-07 | -0.109161778 | 0.0002996 | 0.05 |
| FOPNL | 0.103998252 | 0.00057392 | 0.162435733 | 6.67E-08 | 0.06 |
| METTL22 | 0.101578269 | 0.00077074 | 0.123810483 | 4.05E-05 | 0.03 |
| C16orf6 | -0.059649908 | 0.04866009 | -0.115904664 | 0.0001228 | 0.06 |
| C16ORF71 | -0.306046565 | 3.93E-25 | -0.075494226 | 0.0125394 | 0.24 |
| C16ORF72 | -0.210698506 | 1.96E-12 | -0.014724188 | 0.6267835 | 0.2 |
| MEIOB | 0.160468915 | 9.62E-08 | 0.069421318 | 0.0217188 | 0.1 |
| C16ORF74 | -0.020469114 | 0.49903007 | 0.109503528 | 0.0002867 | 0.13 |
| RMI2 | 0.524487685 | 2.81E-78 | 0.422300714 | 1.66E-48 | 0.11 |
| C16ORF78 | -0.019794688 | 0.51328029 | -0.002102355 | 0.9446509 | 0.02 |
| BRICD5 | 0.164112168 | 4.86E-08 | 0.110591655 | 0.000249 | 0.06 |
| VPS9D1 | 0.083842581 | 0.00554407 | -0.040473773 | 0.1811893 | 0.13 |
| CFAP20 | 0.46672038 | 3.23E-60 | 0.183531783 | 9.80E-10 | 0.29 |
| LINC00304 | -0.022254258 | 0.46234731 | -0.125557333 | 3.14E-05 | 0.11 |
| C16ORF82 | -0.006778988 | 0.82286374 | -0.021366362 | 0.4804021 | 0.02 |
| C16ORF86 | -0.095204213 | 0.00162678 | -0.110790985 | 0.0002426 | 0.02 |
| C16ORF87 | 0.353220678 | 1.82E-33 | 0.061412798 | 0.0423633 | 0.3 |
| KNOP1 | 0.219315402 | 2.27E-13 | 0.269786317 | 1.11E-19 | 0.06 |
| C16ORF89 | -0.204515836 | 8.73E-12 | -0.114182412 | 0.000155 | 0.1 |
| C16ORF90 | 0.19179591 | 1.63E-10 | 0.014328027 | 0.6360885 | 0.18 |
| C16ORF91 | 0.354326988 | 1.12E-33 | 0.298036306 | 7.37E-24 | 0.06 |
| C16ORF92 | 0.067956339 | 0.02465916 | 0.041282497 | 0.1726174 | 0.03 |
| C16ORF93 | -0.005901459 | 0.84548381 | 0.050305022 | 0.0964604 | 0.06 |
| C17ORF100 | 0.143507726 | 1.90E-06 | 0.001921712 | 0.9494 | 0.15 |
| OGFOD3 | 0.064674255 | 0.03252012 | -0.05304692 | 0.0796041 | 0.12 |
| C17ORF102 | 0.055432975 | 0.06695792 | 0.037720567 | 0.2127353 | 0.02 |
| NATD1 | -0.450434805 | 1.01E-55 | -0.377508948 | 2.39E-38 | 0.08 |
| C17ORF104 | 0.097783715 | 0.00120859 | -0.079236758 | 0.008774 | 0.18 |
| C17ORF105 | -0.064159825 | 0.03392875 | -0.070325898 | 0.0200596 | 0.01 |
| TEN1 | 0.323762958 | 4.32E-28 | 0.219144664 | 2.37E-13 | 0.11 |
| C17ORF107 | -0.252909413 | 2.06E-17 | -0.138151753 | 4.56E-06 | 0.12 |
| LYRM9 | -0.237936677 | 1.56E-15 | -0.069788247 | 0.0210319 | 0.17 |
| HID1 | -0.243423179 | 3.31E-16 | -0.004128061 | 0.891568 | 0.24 |
| MIEN1 | 0.143108099 | 2.03E-06 | 0.236048638 | 2.64E-15 | 0.1 |
| GID4 | -0.037418347 | 0.21642566 | -0.214935459 | 6.88E-13 | 0.18 |
| TEFM | 0.213840571 | 9.04E-13 | 0.247039556 | 1.16E-16 | 0.04 |
| LINC00324 | -0.284515889 | 8.44E-22 | -0.143311677 | 1.96E-06 | 0.15 |
| LOC101927036 | -0.120203488 | 6.78E-05 | -0.087510691 | 0.0037868 | 0.04 |
| C17ORF47 | -0.161107069 | 8.55E-08 | -0.086928119 | 0.0040269 | 0.08 |
| ADPRM | 0.035338257 | 0.24307712 | 0.095183996 | 0.0016305 | 0.06 |
| C17ORF49 | 0.349800312 | 8.23E-33 | 0.332911483 | 1.07E-29 | 0.02 |
| C17ORF50 | -0.017041598 | 0.57357053 | -0.010505554 | 0.7286427 | 0.01 |
| C17ORF51 | -0.020620296 | 0.49586473 | -0.22719563 | 2.91E-14 | 0.21 |
| C17ORF53 | 0.528659277 | 1.01E-79 | 0.305040272 | 5.71E-25 | 0.23 |
| LINC00469 | 0.01527944 | 0.61384111 | -0.031873314 | 0.2924276 | 0.05 |
| LINC00482 | 0.081993871 | 0.00668325 | 0.021608441 | 0.4754419 | 0.07 |
| ENTHD2 | 0.17029192 | 1.47E-08 | 0.072516219 | 0.0164921 | 0.1 |
| EFCAB13 | -0.291908286 | 6.52E-23 | -0.240267771 | 8.11E-16 | 0.06 |
| C17ORF58 | -0.055125898 | 0.06848647 | 0.063602846 | 0.0355123 | 0.12 |
| PRO2472 | -0.040812687 | 0.17755942 | -0.004115337 | 0.8919002 | 0.04 |
| MILR1 | -0.06471313 | 0.03241573 | -0.063171081 | 0.0367828 | 0.01 |
| TMEM256 | 0.283831613 | 1.07E-21 | 0.339744092 | 6.20E-31 | 0.06 |
| C17ORF62 | 0.095320565 | 0.00160536 | 0.069248489 | 0.022049 | 0.03 |
| FAM222B | 0.053721746 | 0.07584501 | 0.072836673 | 0.0160198 | 0.02 |
| C17ORF64 | 0.101668673 | 0.00076239 | 0.028172464 | 0.3521039 | 0.08 |
| ASB16.AS1 | -0.045314439 | 0.13434768 | 0.028202444 | 0.3515915 | 0.08 |
| HEATR9 | 0.029684049 | 0.32685707 | -0.043193008 | 0.1535751 | 0.08 |
| LOC100131091 | 0.232733284 | 6.56E-15 | 0.120132166 | 6.85E-05 | 0.12 |
| CTC1 | -0.41547022 | 7.45E-47 | -0.273355875 | 3.49E-20 | 0.15 |
| LOC147080 | 0.039339699 | 0.1937357 | 0.048293365 | 0.1105537 | 0.01 |
| C17ORF70 | 0.142835318 | 2.13E-06 | 0.033177626 | 0.273115 | 0.11 |
| SMG8 | -0.026338742 | 0.38433984 | -0.017630655 | 0.5603933 | 0.01 |
| PRR29 | -0.183880494 | 9.10E-10 | 0.002240138 | 0.9410298 | 0.19 |
| LINC00483 | 0.017063495 | 0.57307809 | 0.093416365 | 0.0019906 | 0.08 |
| C17ORF74 | 0.188237963 | 3.56E-10 | 0.15775516 | 1.59E-07 | 0.04 |
| C17ORF75 | 0.099197171 | 0.00102395 | 0.117897646 | 9.35E-05 | 0.02 |
| LRRC75A | -0.114413659 | 0.00015022 | -0.094451976 | 0.0017717 | 0.02 |
| C17ORF77 | -0.025043836 | 0.40815469 | -0.038294047 | 0.2058578 | 0.02 |
| C17ORF78 | -0.049648377 | 0.10089371 | 0.031834034 | 0.2930231 | 0.09 |
| COPRS | 0.289078933 | 1.75E-22 | 0.291512287 | 7.49E-23 | 0.01 |
| C17ORF80 | 0.085378668 | 0.00473404 | -0.064446077 | 0.0331386 | 0.15 |
| ELP5 | 0.25145468 | 3.18E-17 | 0.197917013 | 4.08E-11 | 0.06 |
| C17ORF82 | 0.002306588 | 0.93928393 | -0.066080861 | 0.0289213 | 0.07 |
| C17ORF85 | -0.194043152 | 9.84E-11 | -0.237595444 | 1.72E-15 | 0.05 |
| SNHG20 | 0.282123215 | 1.90E-21 | 0.115938351 | 0.0001223 | 0.17 |
| SCIMP | -0.145078533 | 1.46E-06 | -0.218257611 | 2.97E-13 | 0.08 |
| FAM215A | -0.104909683 | 0.00051277 | -0.13212204 | 1.17E-05 | 0.03 |
| C17ORF89 | 0.493971273 | 2.63E-68 | 0.379696849 | 8.29E-39 | 0.12 |
| OXLD1 | 0.302080727 | 1.70E-24 | 0.218411206 | 2.86E-13 | 0.09 |
| MIR22HG | -0.20225295 | 1.49E-11 | -0.12879305 | 1.95E-05 | 0.08 |
| LOC100132903 | 0.202072943 | 1.55E-11 | 0.232562864 | 6.87E-15 | 0.04 |
| METTL23 | 0.349773961 | 8.32E-33 | 0.313651614 | 2.23E-26 | 0.04 |
| C17ORF96 | 0.306288876 | 3.59E-25 | 0.124235813 | 3.81E-05 | 0.19 |
| C17ORF97 | -0.111701029 | 0.00021538 | 0.0467422 | 0.1224917 | 0.16 |
| C17ORF98 | 0.060443557 | 0.04573427 | 0.00548283 | 0.856321 | 0.06 |
| C17ORF99 | 0.06927671 | 0.0219948 | -0.062506712 | 0.0388132 | 0.14 |
| LOC115722 | 0.53698027 | 1.16E-82 | 0.179980251 | 2.07E-09 | 0.36 |
| AQP4.AS1 | -0.059244632 | 0.05021369 | 0.029785127 | 0.3252118 | 0.09 |
| LINC00526 | 0.1476616 | 9.43E-07 | -0.134860376 | 7.68E-06 | 0.29 |
| FAM210A | 0.385672322 | 4.39E-40 | 0.120718171 | 6.31E-05 | 0.27 |
| LDLRAD4 | -0.238229872 | 1.44E-15 | -0.171252254 | 1.22E-08 | 0.07 |
| LINC00305 | 0.002058269 | 0.94580974 | 0.084202667 | 0.0053437 | 0.09 |
| C18ORF21 | 0.654715517 | 9.03E-135 | 0.449922137 | 1.39E-55 | 0.21 |
| RBFA | 0.315348186 | 1.16E-26 | 0.159245802 | 1.21E-07 | 0.16 |
| C18ORF25 | 0.077739247 | 0.01013898 | -0.161872951 | 7.41E-08 | 0.24 |
| DYNAP | -0.001405184 | 0.96298903 | 0.051861451 | 0.0865729 | 0.06 |
| LINC00470 | -0.00857954 | 0.77692774 | 0.007205731 | 0.8119153 | 0.02 |
| C18ORF32 | 0.201231777 | 1.89E-11 | 0.182844517 | 1.13E-09 | 0.02 |
| LOC342710 | -0.190557149 | 2.14E-10 | -0.133945267 | 8.86E-06 | 0.06 |
| TMEM241 | 0.179017645 | 2.52E-09 | 0.090652821 | 0.0027017 | 0.09 |
| C18ORF54 | 0.274751504 | 2.21E-20 | 0.005194826 | 0.8637925 | 0.27 |
| TIMM21 | 0.508424451 | 6.65E-73 | 0.287215721 | 3.34E-22 | 0.23 |
| TYMSOS | 0.557123453 | 3.94E-90 | 0.275125826 | 1.95E-20 | 0.29 |
| SMIM21 | -0.074349275 | 0.01394726 | -0.046359257 | 0.1255885 | 0.03 |
| C18ORF8 | 0.503718974 | 2.21E-71 | 0.113045294 | 0.0001803 | 0.4 |
| MYDGF | 0.229002333 | 1.80E-14 | 0.199715076 | 2.69E-11 | 0.03 |
| C19ORF12 | 0.157692499 | 1.60E-07 | -0.005626463 | 0.8525996 | 0.17 |
| C19ORF18 | 0.004323747 | 0.88646191 | 0.079703751 | 0.0083833 | 0.08 |
| TPGS1 | -0.04697284 | 0.12065559 | -0.017536834 | 0.5624822 | 0.03 |
| MISP | -0.232299702 | 7.38E-15 | -0.013882412 | 0.6466241 | 0.22 |
| R3HDM4 | 0.187875143 | 3.85E-10 | 0.239105122 | 1.12E-15 | 0.06 |
| CIRBP.AS1 | 0.110090948 | 0.00026574 | 0.082899301 | 0.0061014 | 0.03 |
| C19ORF24 | 0.262316122 | 1.17E-18 | 0.30321239 | 1.12E-24 | 0.05 |
| C19ORF25 | 0.004842397 | 0.87295191 | 0.085220334 | 0.0048123 | 0.09 |
| C19ORF26 | -0.044648193 | 0.14017478 | -0.107034337 | 0.000393 | 0.07 |
| PP3501 | 0.334487127 | 5.58E-30 | 0.128167442 | 2.14E-05 | 0.21 |
| CACTIN | 0.082867897 | 0.00612079 | 0.026705486 | 0.3777524 | 0.06 |
| URI1 | 0.236414497 | 2.38E-15 | 0.109247882 | 0.0002963 | 0.13 |
| MIR7.3HG | 0.017442858 | 0.56457838 | -0.07196065 | 0.0173398 | 0.09 |
| LOC116344 | -0.087728773 | 0.00370031 | 0.002098173 | 0.9447608 | 0.09 |
| CSNK1G2.AS1 | -0.090261275 | 0.00281941 | -0.079216943 | 0.008791 | 0.02 |
| C19ORF35 | -0.175332346 | 5.37E-09 | -0.188286364 | 3.52E-10 | 0.02 |
| IZUMO4 | -0.19219288 | 1.49E-10 | -0.031782362 | 0.2938076 | 0.17 |
| C19ORF38 | 0.091383504 | 0.00249406 | -0.037361615 | 0.2171235 | 0.13 |
| SWSAP1 | 0.009962626 | 0.7421548 | 0.174086199 | 6.91E-09 | 0.17 |
| C19ORF40 | 0.204423352 | 8.93E-12 | 0.10258618 | 0.0006822 | 0.11 |
| IZUMO2 | 0.128769351 | 1.95E-05 | 0.05784047 | 0.0559211 | 0.08 |
| SMIM7 | 0.109510535 | 0.00028646 | 0.294203872 | 2.90E-23 | 0.19 |
| C19ORF43 | 0.451529593 | 5.14E-56 | 0.400551521 | 2.23E-43 | 0.06 |
| C19ORF44 | -0.039743552 | 0.18919677 | 0.075562863 | 0.0124592 | 0.12 |
| C19ORF45 | 0.01039662 | 0.73134723 | 0.184034991 | 8.80E-10 | 0.18 |
| SYNE4 | 0.003641371 | 0.9042864 | 0.149852354 | 6.47E-07 | 0.15 |
| C19ORF47 | 0.265703299 | 4.06E-19 | 0.134707825 | 7.87E-06 | 0.14 |
| C19ORF48 | 0.490837872 | 2.44E-67 | 0.399323207 | 4.23E-43 | 0.1 |
| KXD1 | 0.356723655 | 3.83E-34 | 0.267746493 | 2.12E-19 | 0.09 |
| CILD2 | -0.260064969 | 2.35E-18 | 0.009483202 | 0.7541529 | 0.27 |
| C19ORF52 | 0.337161767 | 1.83E-30 | 0.275872125 | 1.53E-20 | 0.07 |
| C19ORF53 | 0.419103247 | 9.96E-48 | 0.406058483 | 1.22E-44 | 0.02 |
| C19ORF54 | -0.103297785 | 0.00062546 | 0.00208927 | 0.9449948 | 0.11 |
| PROSER3 | -0.256666854 | 6.66E-18 | -0.266575382 | 3.08E-19 | 0.01 |
| WDR83OS | 0.263132788 | 9.10E-19 | 0.363584238 | 1.70E-35 | 0.11 |
| C19ORF57 | 0.288967208 | 1.82E-22 | 0.107674673 | 0.0003624 | 0.19 |
| MCEMP1 | 0.207740196 | 4.04E-12 | 0.091358391 | 0.0025009 | 0.12 |
| C19ORF60 | 0.235277037 | 3.26E-15 | 0.166456411 | 3.10E-08 | 0.07 |
| SMG9 | 0.177467891 | 3.47E-09 | 0.110465107 | 0.0002531 | 0.07 |
| BABAM1 | 0.355845808 | 5.67E-34 | 0.296867201 | 1.12E-23 | 0.06 |
| EMC10 | 0.082298008 | 0.00648247 | 0.098472567 | 0.0011151 | 0.02 |
| C19ORF66 | 0.07631261 | 0.01161168 | 0.030309282 | 0.3167664 | 0.05 |
| ERICH4 | -0.060529443 | 0.04542672 | -0.0566191 | 0.0613149 | 0.01 |
| TMEM259 | -0.02167341 | 0.47411544 | -0.032120273 | 0.2887024 | 0.02 |
| C19ORF70 | 0.251633635 | 3.02E-17 | 0.318218412 | 3.83E-27 | 0.07 |
| C19ORF71 | -0.159516662 | 1.15E-07 | -0.147518784 | 9.67E-07 | 0.02 |
| C19ORF73 | 0.272198581 | 5.08E-20 | 0.25502003 | 1.10E-17 | 0.02 |
| SIGLEC23P | 0.013285466 | 0.66084878 | -0.033831959 | 0.2637621 | 0.05 |
| ADM5 | 0.146924796 | 1.07E-06 | 0.093875693 | 0.0018906 | 0.06 |
| SMIM24 | 0.062210283 | 0.03974929 | 0.012533741 | 0.6789367 | 0.05 |
| C1D | 0.063424368 | 0.03603287 | 0.141093355 | 2.83E-06 | 0.08 |
| C1GALT1C1 | 0.021134612 | 0.48517696 | 0.09219172 | 0.0022814 | 0.08 |
| C1GALT1 | 0.067038067 | 0.02667282 | -0.093320712 | 0.0020121 | 0.17 |
| LOC51089 | 0.086862762 | 0.00405465 | -0.036479076 | 0.2281881 | 0.13 |
| HABP1 | 0.444987762 | 2.86E-54 | 0.360350755 | 7.44E-35 | 0.09 |
| C1QB | 0.038499206 | 0.20343706 | -0.071206787 | 0.0185511 | 0.11 |
| LOC90369 | 0.027702098 | 0.36020515 | -0.102409688 | 0.000697 | 0.14 |
| C1QL1 | 0.07407856 | 0.01429983 | -0.014566412 | 0.6304823 | 0.09 |
| C1QL2 | 0.261298813 | 1.61E-18 | 0.069989271 | 0.0206636 | 0.2 |
| C1QL3 | -0.12860122 | 2.00E-05 | -0.131802453 | 1.23E-05 | 0.01 |
| C1QL4 | 0.399511241 | 3.84E-43 | 0.126154404 | 2.88E-05 | 0.28 |
| ZSIG37 | -0.011981355 | 0.69234765 | -0.072883556 | 0.0159517 | 0.07 |
| C1QTNF2 | -0.093956503 | 0.00187353 | 0.006056024 | 0.8414898 | 0.11 |
| FLJ37576 | -0.293959022 | 3.16E-23 | -0.066534408 | 0.0278364 | 0.23 |
| C1QTNF4 | -0.125187429 | 3.32E-05 | -0.070736441 | 0.0193437 | 0.06 |
| C1QTNF6 | -0.289325411 | 1.61E-22 | -0.149690536 | 6.65E-07 | 0.14 |
| C1QTNF7 | -0.447995268 | 4.55E-55 | -0.241709847 | 5.39E-16 | 0.21 |
| C1QTNF8 | 0.005301755 | 0.86101708 | 0.036828834 | 0.223756 | 0.04 |
| C1QTNF9B | -0.082891297 | 0.00610633 | -0.005046221 | 0.8676525 | 0.08 |
| C1QTNF9 | -0.242803004 | 3.95E-16 | -0.178437803 | 2.85E-09 | 0.07 |
| C1RL | -0.102727807 | 0.00067053 | -0.108157317 | 0.0003408 | 0.01 |
| C1R | -0.022103102 | 0.46539399 | -0.094570454 | 0.0017481 | 0.08 |
| C1S | -0.062585375 | 0.03856789 | -0.111817992 | 0.0002121 | 0.05 |
| C1ORF100 | 0.060606351 | 0.04515279 | 0.008637316 | 0.7754656 | 0.06 |
| C1ORF101 | -0.234463736 | 4.08E-15 | -0.009256977 | 0.7598353 | 0.23 |
| LOC105378902 | -0.070250739 | 0.02019309 | -0.103218144 | 0.0006316 | 0.04 |
| RUSC1.AS1 | -0.011001065 | 0.7163837 | 0.156012972 | 2.17E-07 | 0.17 |
| C1ORF105 | 0.239091038 | 1.13E-15 | 0.192192745 | 1.49E-10 | 0.05 |
| C1ORF106 | 0.328547369 | 6.34E-29 | 0.243354427 | 3.37E-16 | 0.09 |
| DIEXF | 0.034249062 | 0.25791659 | 0.241763136 | 5.31E-16 | 0.21 |
| C1ORF109 | 0.060117902 | 0.0469164 | -0.130763143 | 1.45E-05 | 0.2 |
| C1ORF110 | 0.022708041 | 0.45326795 | -0.020390487 | 0.5006805 | 0.05 |
| C1ORF111 | -0.335215804 | 4.12E-30 | -0.224575143 | 5.81E-14 | 0.12 |
| C1ORF112 | 0.344486409 | 8.24E-32 | 0.395855624 | 2.55E-42 | 0.06 |
| SH3D21 | -0.317142869 | 5.82E-27 | -0.170706545 | 1.36E-08 | 0.15 |
| CCDC181 | -0.039382228 | 0.19325399 | 0.042168841 | 0.1635746 | 0.09 |
| C1ORF115 | -0.219851401 | 1.98E-13 | -0.013144874 | 0.6642171 | 0.21 |
| C1ORF116 | 0.065118841 | 0.03134333 | 0.140258129 | 3.24E-06 | 0.08 |
| C1ORF122 | 0.23224868 | 7.48E-15 | 0.160573067 | 9.44E-08 | 0.08 |
| LOC115355 | 0.249717481 | 5.32E-17 | 0.204523839 | 8.72E-12 | 0.05 |
| SPRTN | 0.032339197 | 0.28542685 | 0.173703238 | 7.46E-09 | 0.15 |
| AXDND1 | -0.010786142 | 0.7216922 | 0.085947114 | 0.0044625 | 0.1 |
| TMEM51.AS1 | -0.100626841 | 0.00086401 | -0.18658546 | 5.09E-10 | 0.09 |
| C1ORF127 | -0.081267972 | 0.00718519 | 0.061881683 | 0.0408093 | 0.15 |
| LOC96276 | 0.356896366 | 3.54E-34 | 0.211696634 | 1.54E-12 | 0.15 |
| MROH9 | 0.017176819 | 0.57053268 | 0.095897151 | 0.001503 | 0.08 |
| NCMAP | 0.109076719 | 0.00030291 | -0.043022813 | 0.1552038 | 0.16 |
| C1ORF131 | 0.322096946 | 8.37E-28 | 0.514363437 | 7.40E-75 | 0.2 |
| SERTAD4.AS1 | 0.142817485 | 2.13E-06 | 0.127349977 | 2.41E-05 | 0.02 |
| AUNIP | 0.569380339 | 6.23E-95 | 0.248920167 | 6.72E-17 | 0.33 |
| C1ORF141 | 0.04913045 | 0.10450359 | 0.047593739 | 0.1158194 | 0.01 |
| SZRD1 | 0.137388641 | 5.15E-06 | -0.089072968 | 0.0032057 | 0.23 |
| LOC148289 | 0.056687537 | 0.06100167 | 0.012999466 | 0.6677079 | 0.05 |
| SHCBP1L | 0.080201046 | 0.00798435 | 0.055567938 | 0.066295 | 0.03 |
| GCSAML | -0.429391204 | 2.92E-50 | -0.30450962 | 6.95E-25 | 0.13 |
| MINOS1 | 0.28295938 | 1.43E-21 | 0.296305447 | 1.37E-23 | 0.02 |
| PFN1P2 | -0.317719558 | 4.65E-27 | -0.199531374 | 2.81E-11 | 0.12 |
| METTL18 | 0.101782333 | 0.000752 | 0.424209599 | 5.65E-49 | 0.33 |
| LINC00303 | 0.120319417 | 6.67E-05 | 0.134100157 | 8.65E-06 | 0.02 |
| C1ORF158 | 0.020015093 | 0.50860009 | 0.096898512 | 0.0013394 | 0.08 |
| C1ORF159 | 0.239082131 | 1.13E-15 | 0.157705731 | 1.60E-07 | 0.09 |
| MAB21L3 | -0.13940117 | 3.73E-06 | -0.114108196 | 0.0001565 | 0.03 |
| C1ORF162 | 0.053135175 | 0.07910402 | -0.018976142 | 0.5308557 | 0.08 |
| LOC148971 | 0.269865478 | 1.08E-19 | 0.052523839 | 0.0826207 | 0.22 |
| C1ORF168 | -0.387421587 | 1.84E-40 | -0.170042158 | 1.54E-08 | 0.22 |
| PERM1 | 0.143894573 | 1.78E-06 | 0.10963954 | 0.0002817 | 0.04 |
| KDF1 | 0.19793365 | 4.06E-11 | 0.17656567 | 4.18E-09 | 0.03 |
| ERICH3 | -0.279179005 | 5.11E-21 | -0.172558446 | 9.39E-09 | 0.11 |
| C1ORF174 | 0.327380925 | 1.02E-28 | 0.146821837 | 1.09E-06 | 0.19 |
| MROH7 | -0.089005822 | 0.00322891 | -0.199602828 | 2.77E-11 | 0.12 |
| C1ORF177 | 0.046512492 | 0.12434209 | -0.014630754 | 0.6289728 | 0.07 |
| FLJ35487 | -0.097315181 | 0.00127628 | -0.002397371 | 0.9368991 | 0.1 |
| TSACC | 0.316227723 | 8.29E-27 | 0.427806416 | 7.27E-50 | 0.12 |
| FAM212B | -0.275797184 | 1.57E-20 | -0.334843394 | 4.81E-30 | 0.06 |
| C1ORF185 | 0.010319561 | 0.7332624 | -0.045296277 | 0.134504 | 0.06 |
| C1ORF186 | 0.036994048 | 0.22168401 | -0.059205668 | 0.0503652 | 0.1 |
| DRAXIN | 0.117475006 | 9.91E-05 | -0.045208128 | 0.1352647 | 0.17 |
| C1ORF189 | 0.001376574 | 0.96374206 | 0.133147255 | 1.00E-05 | 0.14 |
| LOC148922 | -0.269643675 | 1.16E-19 | -0.194199615 | 9.50E-11 | 0.08 |
| CFAP126 | -0.26022102 | 2.24E-18 | 0.019893222 | 0.5111852 | 0.29 |
| C1ORF194 | 0.069983487 | 0.02067416 | 0.123964518 | 3.97E-05 | 0.06 |
| MGC10710 | 0.235593321 | 2.99E-15 | 0.284390435 | 8.80E-22 | 0.05 |
| LOC648861 | 0.09563845 | 0.00154816 | -0.040923712 | 0.1763821 | 0.14 |
| STPG1 | -0.02879766 | 0.34151587 | -0.084075394 | 0.0054138 | 0.06 |
| ATP1A1.AS1 | -0.081727237 | 0.00686384 | -0.044480157 | 0.1416747 | 0.04 |
| C1ORF204 | -0.015673981 | 0.60471693 | 0.059894972 | 0.0477403 | 0.08 |
| C1ORF210 | -0.120287592 | 6.70E-05 | -0.022237516 | 0.4626842 | 0.1 |
| SMIM12 | 0.308520844 | 1.56E-25 | 0.08253774 | 0.006328 | 0.23 |
| ZNF436.AS1 | -0.017821885 | 0.55614715 | -0.074767106 | 0.0134181 | 0.06 |
| C1ORF216 | -0.086305971 | 0.00429844 | -0.150781474 | 5.50E-07 | 0.07 |
| C1ORF21 | -0.295796411 | 1.64E-23 | 0.016960373 | 0.575399 | 0.32 |
| C1ORF220 | -0.307916765 | 1.96E-25 | -0.106650615 | 0.0004125 | 0.21 |
| TEX38 | 0.080325511 | 0.0078872 | 0.023279594 | 0.4419766 | 0.06 |
| LOC100133912 | -0.213316668 | 1.03E-12 | -0.036575122 | 0.2269648 | 0.18 |
| SPATA45 | -0.035268452 | 0.2440098 | 0.084509773 | 0.005178 | 0.12 |
| C1ORF228 | -0.014932905 | 0.62190473 | 0.02961378 | 0.328004 | 0.05 |
| C1ORF229 | 0.007541707 | 0.80332116 | 0.076948511 | 0.0109333 | 0.07 |
| RIIAD1 | -0.026630159 | 0.37909971 | 0.285171 | 6.74E-22 | 0.32 |
| TRMT1L | -0.189530787 | 2.68E-10 | 0.106377147 | 0.0004269 | 0.3 |
| SWT1 | -0.239214292 | 1.09E-15 | 0.090710002 | 0.0026849 | 0.33 |
| C1ORF27 | -0.236162204 | 2.56E-15 | 0.197472099 | 4.52E-11 | 0.44 |
| C1orf31 | 0.489476529 | 6.37E-67 | 0.672081531 | 1.56E-144 | 0.19 |
| C1ORF35 | 0.331491533 | 1.91E-29 | 0.47506167 | 1.29E-62 | 0.15 |
| THEMIS2 | 0.103160115 | 0.00063608 | -0.104275391 | 0.0005546 | 0.21 |
| LOC91192 | 0.220429569 | 1.71E-13 | 0.435220935 | 9.81E-52 | 0.22 |
| TEX35 | 0.021823116 | 0.47106673 | 0.11245364 | 0.000195 | 0.1 |
| C1ORF50 | 0.270146867 | 9.86E-20 | 0.290823745 | 9.54E-23 | 0.03 |
| CIART | 0.236735949 | 2.18E-15 | 0.210329795 | 2.15E-12 | 0.03 |
| C1ORF52 | 0.327490769 | 9.72E-29 | 0.129219057 | 1.83E-05 | 0.2 |
| LOC375044 | 0.141888924 | 2.48E-06 | 0.276943539 | 1.07E-20 | 0.14 |
| C1ORF54 | 0.115395607 | 0.00013159 | 0.082736299 | 0.0062027 | 0.04 |
| SDE2 | -0.236404645 | 2.39E-15 | -0.038750737 | 0.2004974 | 0.2 |
| C1ORF56 | 0.035592473 | 0.23970165 | 0.297235168 | 9.83E-24 | 0.27 |
| NTPCR | 0.232655827 | 6.70E-15 | 0.47096652 | 1.98E-61 | 0.24 |
| BROX | -0.342361547 | 2.04E-31 | -0.199070358 | 3.13E-11 | 0.15 |
| HENMT1 | 0.284882962 | 7.44E-22 | 0.128408894 | 2.06E-05 | 0.16 |
| LOC100505765 | 0.290902563 | 9.28E-23 | 0.126062048 | 2.92E-05 | 0.17 |
| LOC101928189 | 0.041184248 | 0.17364228 | 0.058815732 | 0.0519029 | 0.02 |
| C1ORF64 | -0.410100905 | 1.39E-45 | -0.112266019 | 0.0001999 | 0.3 |
| CCDC185 | 0.12051971 | 6.48E-05 | 0.055688209 | 0.0657089 | 0.07 |
| RRNAD1 | -0.036093365 | 0.23314771 | 0.248152781 | 8.42E-17 | 0.29 |
| C1orf68 | 0.040600008 | 0.17983093 | -0.004767721 | 0.8748949 | 0.05 |
| IBA57 | 0.115009311 | 0.00013865 | 0.147466795 | 9.75E-07 | 0.04 |
| TMEM240 | -0.16152226 | 7.91E-08 | -0.104430935 | 0.0005441 | 0.06 |
| LOC105419998 | 0.220475253 | 1.69E-13 | 0.321855787 | 9.20E-28 | 0.11 |
| CHTOP | 0.25701518 | 5.99E-18 | 0.445697347 | 1.86E-54 | 0.19 |
| TCEANC2 | -0.09649001 | 0.00140405 | -0.193750311 | 1.05E-10 | 0.1 |
| C1orf84 | 0.024808115 | 0.41258244 | -0.11743128 | 9.97E-05 | 0.15 |
| GLMP | 0.21803609 | 3.15E-13 | 0.338036918 | 1.27E-30 | 0.13 |
| LOC100293374 | 0.151406253 | 4.93E-07 | 0.136756307 | 5.70E-06 | 0.02 |
| C1ORF87 | 0.01969202 | 0.51546801 | 0.002278112 | 0.9400321 | 0.02 |
| PIFO | -0.126767691 | 2.63E-05 | 0.039707256 | 0.1896015 | 0.17 |
| RSG1 | -0.317598851 | 4.88E-27 | -0.24716097 | 1.12E-16 | 0.08 |
| RP4.622L5 | 0.202973538 | 1.26E-11 | 0.133951242 | 8.85E-06 | 0.07 |
| LRRC71 | -0.037299373 | 0.21789098 | 0.094864733 | 0.0016908 | 0.14 |
| FAM213B | -0.097809474 | 0.00120497 | -0.081345058 | 0.0071303 | 0.02 |
| C1ORF94 | 0.243525625 | 3.21E-16 | 0.126529074 | 2.73E-05 | 0.12 |
| LOC91266 | -0.262913186 | 9.74E-19 | -0.192667927 | 1.34E-10 | 0.08 |
| CCSAP | 0.224024581 | 6.71E-14 | 0.212631702 | 1.22E-12 | 0.02 |
| LINC00467 | 0.018803403 | 0.53460328 | 0.383938968 | 1.04E-39 | 0.37 |
| C1ORF9 | -0.214573393 | 7.53E-13 | 0.107008252 | 0.0003943 | 0.33 |
| LAMP5 | -0.354293624 | 1.13E-33 | -0.113168782 | 0.0001774 | 0.25 |
| FAM209A | 0.125839498 | 3.02E-05 | 0.039574308 | 0.1910893 | 0.09 |
| FAM209B | 0.143095316 | 2.04E-06 | 0.053999521 | 0.0743403 | 0.09 |
| DKFZP434A1114 | 0.017866225 | 0.55516486 | 0.00467896 | 0.8772054 | 0.02 |
| OSER1 | 0.126503357 | 2.74E-05 | 0.043897197 | 0.1469738 | 0.09 |
| NOL4L | -0.088836494 | 0.0032881 | -0.124809263 | 3.51E-05 | 0.04 |
| BPIFB1 | -0.303145521 | 1.15E-24 | -0.059470366 | 0.0493433 | 0.25 |
| LOC149670 | -0.229043753 | 1.78E-14 | -0.237823826 | 1.61E-15 | 0.01 |
| TLDC2 | -0.153243345 | 3.57E-07 | -0.254019823 | 1.48E-17 | 0.11 |
| GID8 | 0.251174534 | 3.46E-17 | 0.118952865 | 8.08E-05 | 0.14 |
| OCSTAMP | 0.017285579 | 0.56809489 | -0.031219503 | 0.302445 | 0.05 |
| DZANK1 | -0.222193576 | 1.08E-13 | -0.066285284 | 0.0284279 | 0.16 |
| FLJ36113 | -0.205489001 | 6.93E-12 | -0.295319125 | 1.95E-23 | 0.09 |
| ACTL10 | 0.070188195 | 0.02030479 | 0.158006084 | 1.51E-07 | 0.09 |
| ABHD16B | 0.09659924 | 0.00138648 | -0.013721404 | 0.6504483 | 0.12 |
| C20ORF141 | 0.093395068 | 0.00199539 | 0.057644511 | 0.0567589 | 0.04 |
| C20ORF144 | -0.068400212 | 0.02373388 | -0.083058498 | 0.0060039 | 0.02 |
| RBBP8NL | 0.171656779 | 1.12E-08 | 0.230527926 | 1.19E-14 | 0.06 |
| CNBD2 | 0.051742415 | 0.08729903 | 0.0302529 | 0.3176679 | 0.03 |
| CCM2L | -0.186761368 | 4.90E-10 | -0.147588266 | 9.55E-07 | 0.04 |
| SPATA25 | -0.028818396 | 0.34116822 | -0.11587041 | 0.0001234 | 0.09 |
| LOC149920 | -0.019077002 | 0.52867374 | -0.019872473 | 0.511626 | 0.01 |
| C20ORF173 | -0.026250015 | 0.38594402 | -0.105232355 | 0.0004926 | 0.08 |
| FAM217B | -0.229065972 | 1.77E-14 | -0.25988881 | 2.49E-18 | 0.04 |
| BPIFB3 | 0.089581829 | 0.00303474 | 0.036349073 | 0.2298513 | 0.06 |
| BPIFB4 | 0.095296952 | 0.00160969 | 0.078493967 | 0.0094291 | 0.02 |
| NCOR1P1 | 0.086860252 | 0.00405572 | 0.028261138 | 0.3505897 | 0.06 |
| C20ORF194 | -0.506272074 | 3.32E-72 | -0.395899354 | 2.49E-42 | 0.12 |
| C20ORF195 | -0.156922903 | 1.84E-07 | -0.119013442 | 8.01E-05 | 0.04 |
| C20ORF196 | -0.008313775 | 0.78366334 | 0.011646137 | 0.7005337 | 0.02 |
| C20ORF197 | -0.154835079 | 2.69E-07 | -0.19602381 | 6.29E-11 | 0.05 |
| ZFAS1 | 0.325606088 | 2.07E-28 | 0.306705512 | 3.08E-25 | 0.02 |
| C20ORF166.AS1 | -0.063304953 | 0.03638479 | -0.091121319 | 0.0025668 | 0.03 |
| LKAAEAR1 | 0.07003302 | 0.02058425 | 0.076312944 | 0.0116113 | 0.01 |
| C20ORF202 | -0.012400736 | 0.68215678 | -0.093823977 | 0.0019017 | 0.09 |
| C20ORF203 | -0.052481404 | 0.08286948 | -0.148760341 | 7.81E-07 | 0.1 |
| MRGBP | 0.503772079 | 2.12E-71 | 0.341330875 | 3.17E-31 | 0.17 |
| C20ORF24 | 0.51066796 | 1.23E-73 | 0.404245757 | 3.19E-44 | 0.11 |
| CFAP61 | -0.329743568 | 3.90E-29 | -0.126842028 | 2.60E-05 | 0.21 |
| C20ORF27 | 0.435297197 | 9.38E-52 | 0.33521479 | 4.12E-30 | 0.11 |
| AP5S1 | -0.0464981 | 0.12445875 | -0.024224497 | 0.4236666 | 0.03 |
| TMEM230 | -0.045791066 | 0.1302951 | 0.062175704 | 0.0398597 | 0.11 |
| APMAP | -0.178641821 | 2.73E-09 | 0.007081524 | 0.8150982 | 0.19 |
| RTFDC1 | 0.202048083 | 1.56E-11 | 0.137607446 | 4.97E-06 | 0.07 |
| TMEM74B | 0.16674724 | 2.93E-08 | 0.044691743 | 0.139788 | 0.13 |
| LOC51609 | 0.086450837 | 0.00423376 | 0.088984819 | 0.0032362 | 0.01 |
| SLC52A3 | -0.139514262 | 3.66E-06 | 0.020702946 | 0.4941388 | 0.17 |
| LINC00261 | 0.055540147 | 0.0664311 | 0.028387687 | 0.3484358 | 0.03 |
| BPIFA2 | 0.036386976 | 0.22936546 | 0.114820118 | 0.0001422 | 0.08 |
| BPIFA3 | 0.031897513 | 0.29206115 | 0.099513913 | 0.0009863 | 0.07 |
| MGME1 | 0.304524011 | 6.91E-25 | 0.243891674 | 2.89E-16 | 0.07 |
| SCP2D1 | -0.040573571 | 0.18011479 | -0.032270998 | 0.2864445 | 0.01 |
| NDUFAF5 | 0.476792309 | 4.02E-63 | 0.364202873 | 1.28E-35 | 0.12 |
| LOC400848 | -0.028047281 | 0.35424865 | 0.094239078 | 0.0018148 | 0.13 |
| C20orf94 | -0.375423456 | 6.53E-38 | -0.351065388 | 4.72E-33 | 0.03 |
| FLJ40075 | -0.11373113 | 0.0001646 | 0.029378132 | 0.3318694 | 0.15 |
| URB1.AS1 | 0.260054921 | 2.36E-18 | 0.104028809 | 0.0005718 | 0.16 |
| ZNF295.AS1 | 0.090867763 | 0.00263906 | 0.080916494 | 0.0074402 | 0.01 |
| LOC732307 | 0.205030488 | 7.73E-12 | 0.111359255 | 0.0002253 | 0.1 |
| LINC00319 | -0.083955518 | 0.00548051 | -0.08566608 | 0.0045949 | 0.01 |
| UMODL1.AS1 | 0.093372266 | 0.00200049 | 0.05386321 | 0.0750756 | 0.04 |
| LINC00479 | 0.067682606 | 0.02524519 | 0.039587726 | 0.1909387 | 0.03 |
| LINC00323 | 0.022693912 | 0.45354912 | 0.057048513 | 0.0593714 | 0.04 |
| LINC00320 | 0.014375716 | 0.63496533 | -0.002705559 | 0.9288077 | 0.02 |
| C21orf15 | -0.177074678 | 3.77E-09 | -0.008666716 | 0.7747219 | 0.17 |
| TSPEAR | -0.076123965 | 0.01181997 | -0.082128984 | 0.0065934 | 0.01 |
| C21ORF2 | -0.01588195 | 0.59993202 | 0.024321375 | 0.4218148 | 0.05 |
| C21ORF33 | 0.293155563 | 4.20E-23 | 0.266994507 | 2.70E-19 | 0.03 |
| MIR99AHG | -0.260807792 | 1.87E-18 | -0.169113052 | 1.85E-08 | 0.1 |
| MIS18A | 0.498778515 | 8.22E-70 | 0.390050713 | 4.90E-41 | 0.11 |
| C21ORF62.AS1 | -0.135894391 | 6.53E-06 | -0.059853595 | 0.0478946 | 0.08 |
| LINC01548 | 0.06350845 | 0.03578682 | 0.00769606 | 0.7993807 | 0.06 |
| LOC140417 | 0.047099219 | 0.11965863 | 0.079589701 | 0.0084773 | 0.04 |
| YBEY | 0.114757 | 0.00014344 | 0.143773279 | 1.82E-06 | 0.03 |
| C21ORF58 | 0.300849428 | 2.66E-24 | 0.158977139 | 1.27E-07 | 0.15 |
| FLJ20467 | 0.19217804 | 1.49E-10 | 0.243321816 | 3.40E-16 | 0.06 |
| MGC10954 | -0.169025108 | 1.89E-08 | -0.217399407 | 3.70E-13 | 0.05 |
| EVA1C | -0.054837762 | 0.06994667 | -0.095863522 | 0.0015088 | 0.05 |
| LINC01547 | 0.060740061 | 0.04467986 | 0.049519978 | 0.1017793 | 0.02 |
| FAM207A | 0.36196347 | 3.57E-35 | 0.231972484 | 8.07E-15 | 0.13 |
| LINC00515 | 0.083535522 | 0.00572024 | 0.021999089 | 0.4674969 | 0.07 |
| MAP3K7CL | -0.183517461 | 9.82E-10 | -0.124168503 | 3.85E-05 | 0.06 |
| LOC100421426 | -0.161091411 | 8.57E-08 | -0.004349072 | 0.8858014 | 0.16 |
| LINC00310 | -0.001940436 | 0.9489077 | 0.064760931 | 0.0322878 | 0.07 |
| LINC00313 | 0.001578526 | 0.95842729 | -0.023281899 | 0.4419314 | 0.03 |
| LOC100131337 | -0.087372605 | 0.00384249 | -0.00514947 | 0.8649703 | 0.09 |
| TSPEAR.AS2 | -0.062625588 | 0.03844302 | -0.072518366 | 0.0164888 | 0.01 |
| C21ORF91 | 0.103554961 | 0.00060606 | -0.121682605 | 5.50E-05 | 0.23 |
| LINC00314 | -0.017179536 | 0.57047173 | 0.035344059 | 0.2429997 | 0.06 |
| RUNX1.IT1 | 0.04383949 | 0.14750644 | -0.09988301 | 0.0009441 | 0.15 |
| ANKRD30BP2 | 0.050868574 | 0.09278098 | 0.079357502 | 0.0086715 | 0.03 |
| GUCD1 | -0.122219596 | 5.10E-05 | -0.097066525 | 0.0013136 | 0.03 |
| C22ORF15 | -0.00011971 | 0.99684586 | 0.001265524 | 0.9666653 | 0.01 |
| C22ORF23 | 0.067570318 | 0.02548905 | -0.185105659 | 7.00E-10 | 0.26 |
| C22ORF24 | -0.102507894 | 0.00068871 | -0.125921704 | 2.98E-05 | 0.03 |
| TANGO2 | -0.156667628 | 1.93E-07 | -0.101089716 | 0.0008174 | 0.06 |
| PRR34 | -0.201225228 | 1.90E-11 | -0.208802623 | 3.12E-12 | 0.01 |
| MORC2.AS1 | 0.295935974 | 1.56E-23 | 0.262371628 | 1.15E-18 | 0.04 |
| RTCB | 0.078692506 | 0.00924989 | 0.031045062 | 0.3051558 | 0.05 |
| C22ORF29 | -0.108496363 | 0.00032632 | -0.064224293 | 0.0337494 | 0.05 |
| FLJ23059 | -0.206550624 | 5.37E-12 | -0.259736462 | 2.61E-18 | 0.06 |
| C22ORF31 | -0.029883765 | 0.32361141 | 0.010571304 | 0.727012 | 0.05 |
| SMDT1 | 0.051832577 | 0.08674858 | 0.184707291 | 7.62E-10 | 0.14 |
| TEX33 | 0.028778269 | 0.34184118 | 0.008057463 | 0.7901748 | 0.03 |
| C22ORF34 | -0.096968176 | 0.00132865 | -0.177078136 | 3.76E-09 | 0.09 |
| LRRC75B | 0.033706286 | 0.26554113 | 0.15793745 | 1.53E-07 | 0.13 |
| C22ORF39 | -0.019335413 | 0.52310424 | -0.000308992 | 0.9918587 | 0.02 |
| CDPF1 | 0.207845934 | 3.93E-12 | 0.232957127 | 6.17E-15 | 0.03 |
| SYCE3 | -0.070701857 | 0.01940315 | 0.172182876 | 1.01E-08 | 0.25 |
| C22ORF42 | -0.016370412 | 0.5887614 | 0.003515247 | 0.9075865 | 0.02 |
| LOC102724759 | -0.038787462 | 0.20007085 | 0.005425673 | 0.8578028 | 0.05 |
| ADORA2A.AS1 | -0.165242553 | 3.92E-08 | -0.170252268 | 1.48E-08 | 0.01 |
| LOC100130558 | -0.057573052 | 0.057067 | -0.13890044 | 4.04E-06 | 0.09 |
| C22orf9 | -0.021599205 | 0.4756306 | -0.032942996 | 0.2765233 | 0.02 |
| C2CD2L | -0.178465738 | 2.83E-09 | 0.068966109 | 0.022598 | 0.25 |
| C21ORF25 | -0.134296544 | 8.39E-06 | -0.051796917 | 0.0869659 | 0.09 |
| C2CD3 | -0.185304922 | 6.71E-10 | -0.199182219 | 3.05E-11 | 0.02 |
| C2CD4A | -0.106223559 | 0.00043523 | -0.027179923 | 0.3693344 | 0.08 |
| C2CD4B | -0.123796157 | 4.06E-05 | -0.049388045 | 0.1026956 | 0.08 |
| C2CD4C | 0.00355437 | 0.90656263 | -0.031042174 | 0.3052008 | 0.04 |
| C2CD4D | 0.287048089 | 3.54E-22 | 0.296337756 | 1.36E-23 | 0.01 |
| CYP4F30P | -0.189170532 | 2.90E-10 | -0.016701139 | 0.5812529 | 0.18 |
| C2ORF15 | -0.138121268 | 4.58E-06 | -0.038451638 | 0.2039965 | 0.1 |
| C2ORF16 | -0.281542699 | 2.31E-21 | -0.180107555 | 2.01E-09 | 0.11 |
| SLC35F6 | 0.00495929 | 0.86991202 | 0.004551111 | 0.8805352 | 0.01 |
| LOC130618 | 0.092689628 | 0.0021588 | 0.107992793 | 0.000348 | 0.02 |
| C2ORF27A | -0.155038041 | 2.59E-07 | 0.024889679 | 0.4110471 | 0.18 |
| LOC220698 | -0.024294499 | 0.42232803 | -0.070361422 | 0.0199967 | 0.05 |
| ATRAID | 0.258563325 | 3.73E-18 | 0.243314777 | 3.41E-16 | 0.02 |
| CNOT11 | 0.231186665 | 9.98E-15 | 0.281987826 | 1.99E-21 | 0.06 |
| CAMKMT | 0.311506532 | 5.06E-26 | 0.275227416 | 1.89E-20 | 0.04 |
| DRC1 | 0.000557525 | 0.98531098 | 0.026205164 | 0.3867565 | 0.03 |
| GCFC2 | 0.193496995 | 1.11E-10 | 0.127281507 | 2.44E-05 | 0.07 |
| C2ORF40 | -0.186964209 | 4.69E-10 | -0.149816787 | 6.51E-07 | 0.04 |
| C2ORF42 | -0.108885802 | 0.00031043 | -0.075790682 | 0.0121961 | 0.04 |
| LDAH | -0.086689365 | 0.0041292 | -0.078555183 | 0.0093735 | 0.01 |
| C2ORF44 | 0.156888263 | 1.86E-07 | 0.02577685 | 0.3945676 | 0.14 |
| C2ORF47 | 0.28551759 | 5.99E-22 | 0.255403393 | 9.76E-18 | 0.04 |
| C2ORF48 | 0.400165594 | 2.73E-43 | 0.227679216 | 2.56E-14 | 0.18 |
| C2ORF49 | 0.089681361 | 0.00300229 | 0.019093164 | 0.5283245 | 0.08 |
| C2ORF50 | -0.114073318 | 0.00015724 | -0.078788446 | 0.0091644 | 0.04 |
| TEX37 | 0.250232756 | 4.57E-17 | 0.099315081 | 0.0010098 | 0.16 |
| LINC00471 | 0.016962669 | 0.57534727 | -0.010962076 | 0.7173457 | 0.03 |
| PRR30 | 0.058679641 | 0.05244867 | 0.03000096 | 0.3217166 | 0.03 |
| C2ORF54 | 0.025294325 | 0.40348065 | 0.111223015 | 0.0002293 | 0.09 |
| KIAA1211L | -0.381708856 | 3.10E-39 | -0.144897794 | 1.51E-06 | 0.24 |
| NDUFAF7 | 0.323105455 | 5.61E-28 | 0.089881847 | 0.0029379 | 0.24 |
| C2ORF57 | -0.068616635 | 0.02329375 | -0.061126782 | 0.0433355 | 0.01 |
| CYP1B1.AS1 | -0.078828479 | 0.0091289 | -0.019708473 | 0.5151171 | 0.06 |
| TYW5 | -0.219386849 | 2.23E-13 | -0.166942584 | 2.83E-08 | 0.06 |
| C2ORF61 | 0.072850559 | 0.01599958 | -0.030742817 | 0.3098907 | 0.11 |
| CATIP | -0.079070372 | 0.00891715 | 0.009937564 | 0.7427805 | 0.09 |
| CLHC1 | -0.137101875 | 5.39E-06 | -0.04726805 | 0.1183368 | 0.09 |
| COA5 | 0.201132264 | 1.94E-11 | 0.286000053 | 5.08E-22 | 0.09 |
| M1AP | -0.180545645 | 1.84E-09 | -0.105384332 | 0.0004834 | 0.08 |
| C2ORF66 | -0.078833085 | 0.00912483 | -0.132065793 | 1.18E-05 | 0.06 |
| KANSL1L | 0.133117006 | 1.01E-05 | 0.05089696 | 0.0925987 | 0.09 |
| C2ORF68 | 0.265122995 | 4.87E-19 | 0.139306788 | 3.79E-06 | 0.13 |
| C2ORF69 | -0.106282176 | 0.00043204 | -0.165585574 | 3.67E-08 | 0.06 |
| C2ORF70 | 0.327845595 | 8.42E-29 | 0.160290268 | 9.95E-08 | 0.17 |
| C2ORF71 | 0.174153913 | 6.82E-09 | -0.020274249 | 0.5031258 | 0.2 |
| C2ORF72 | -0.045378127 | 0.1338006 | 0.017349648 | 0.5666611 | 0.07 |
| C2ORF73 | -0.221425492 | 1.32E-13 | -0.079935678 | 0.0081951 | 0.15 |
| C2ORF74 | 0.114954189 | 0.00013968 | -0.007651853 | 0.8005088 | 0.13 |
| C2ORF76 | 0.169171543 | 1.83E-08 | 0.264927507 | 5.18E-19 | 0.1 |
| CCDC173 | -0.232554445 | 6.88E-15 | -0.034233492 | 0.2581332 | 0.2 |
| C2ORF78 | 0.013909217 | 0.64598829 | 0.024719308 | 0.4142579 | 0.02 |
| PTRHD1 | 0.386420112 | 3.03E-40 | 0.380151315 | 6.65E-39 | 0.01 |
| PRADC1 | 0.280853282 | 2.92E-21 | 0.276795801 | 1.13E-20 | 0.01 |
| C2ORF80 | 0.118143231 | 9.04E-05 | 0.086416793 | 0.0042489 | 0.04 |
| C2ORF81 | -0.107456404 | 0.00037254 | 0.018363125 | 0.544215 | 0.13 |
| C2ORF82 | 0.129723898 | 1.69E-05 | 0.061388886 | 0.0424438 | 0.07 |
| C2ORF83 | 0.025161473 | 0.40595563 | -0.015368086 | 0.6117858 | 0.05 |
| FAM228A | -0.150476866 | 5.80E-07 | -0.168849309 | 1.95E-08 | 0.02 |
| RTP5 | -0.087619668 | 0.00374335 | -0.124835652 | 3.49E-05 | 0.04 |
| WDPCP | -0.185220178 | 6.83E-10 | -0.233608188 | 5.16E-15 | 0.05 |
| C2ORF88 | -0.004813401 | 0.87370627 | -0.13381043 | 9.05E-06 | 0.13 |
| TRABD2A | 0.027107854 | 0.37060556 | -0.072211473 | 0.0169525 | 0.1 |
| C2 | 0.040395907 | 0.18203104 | -0.022116656 | 0.4651203 | 0.07 |
| C3AR1 | -0.185842535 | 5.98E-10 | -0.210316772 | 2.16E-12 | 0.03 |
| C3P1 | 0.004518376 | 0.88138805 | 0.006678199 | 0.8254547 | 0.01 |
| BRK1 | 0.234240004 | 4.34E-15 | 0.298429018 | 6.39E-24 | 0.07 |
| C3ORF14 | -0.02288383 | 0.44977793 | 0.1476079 | 9.52E-07 | 0.18 |
| MAATS1 | -0.201122141 | 1.94E-11 | -0.109143675 | 0.0003003 | 0.1 |
| ANKUB1 | 0.022228392 | 0.46286787 | -0.009453612 | 0.7548954 | 0.04 |
| C3ORF17 | 0.125269491 | 3.28E-05 | 0.036099081 | 0.2330737 | 0.09 |
| C3ORF18 | -0.364720975 | 1.00E-35 | -0.15158425 | 4.78E-07 | 0.22 |
| CCDC174 | 0.003784537 | 0.90054243 | 0.057306935 | 0.0582267 | 0.06 |
| TIMMDC1 | 0.405440585 | 1.69E-44 | 0.324728307 | 2.94E-28 | 0.09 |
| C3ORF20 | -0.019228324 | 0.52540866 | -0.121387113 | 5.74E-05 | 0.11 |
| XXYLT1 | 0.266403648 | 3.25E-19 | 0.073973846 | 0.0144383 | 0.2 |
| C3ORF22 | 0.109635892 | 0.00028186 | 0.05186966 | 0.086523 | 0.06 |
| TCAIM | 0.096526985 | 0.00139808 | 0.155043484 | 2.59E-07 | 0.06 |
| FANCD2OS | 0.215242746 | 6.37E-13 | 0.236442803 | 2.36E-15 | 0.03 |
| CMSS1 | 0.57623262 | 1.05E-97 | 0.383329149 | 1.40E-39 | 0.2 |
| LINC01565 | -0.050011625 | 0.09842165 | -0.029620168 | 0.3278997 | 0.03 |
| C3ORF30 | 0.124653687 | 3.59E-05 | 0.002659015 | 0.9300292 | 0.13 |
| DKFZp434E0519 | 0.232877189 | 6.31E-15 | 0.235634491 | 2.96E-15 | 0.01 |
| SSUH2 | -0.086621386 | 0.00415876 | -0.023805167 | 0.431737 | 0.07 |
| C3ORF33 | 0.099354115 | 0.00100514 | 0.117197978 | 0.0001029 | 0.02 |
| CEP19 | -0.128011261 | 2.19E-05 | -0.144883819 | 1.51E-06 | 0.02 |
| C3ORF35 | -0.032641929 | 0.28093887 | 0.074136649 | 0.0142235 | 0.11 |
| C3ORF36 | -0.212835192 | 1.16E-12 | -0.14408033 | 1.73E-06 | 0.07 |
| HMCES | 0.427959801 | 6.66E-50 | 0.33480956 | 4.88E-30 | 0.1 |
| C3ORF38 | -0.095225122 | 0.00162291 | -0.015480053 | 0.6091941 | 0.08 |
| LOC102723588 | 0.054470231 | 0.07184615 | 0.004147387 | 0.8910635 | 0.06 |
| LINC00852 | 0.041872105 | 0.16656139 | 0.001938905 | 0.9489479 | 0.04 |
| SMCO1 | 0.134848951 | 7.69E-06 | 0.054386138 | 0.0722866 | 0.09 |
| LSMEM2 | 0.074850483 | 0.01331468 | 0.118111133 | 9.08E-05 | 0.05 |
| H1FX.AS1 | 0.113528766 | 0.0001691 | 0.118323453 | 8.81E-05 | 0.01 |
| PP2D1 | -0.17179628 | 1.09E-08 | -0.136346068 | 6.08E-06 | 0.04 |
| C3ORF49 | -0.123758478 | 4.08E-05 | -0.126941348 | 2.57E-05 | 0.01 |
| EGFEM1P | -0.016380494 | 0.58853185 | -0.095690198 | 0.001539 | 0.08 |
| ERC2.IT1 | 0.032004302 | 0.29044772 | 0.003732589 | 0.9019007 | 0.03 |
| C3ORF52 | -0.264458653 | 6.01E-19 | -0.145186882 | 1.44E-06 | 0.12 |
| FAM212A | -0.066065832 | 0.02895784 | -0.048646679 | 0.1079672 | 0.02 |
| LOC647046 | 0.070525489 | 0.01970871 | -0.010407785 | 0.7310699 | 0.09 |
| SPTSSB | -0.156976717 | 1.83E-07 | -0.04374627 | 0.14837 | 0.12 |
| C3ORF58 | 0.170236272 | 1.49E-08 | -0.067912671 | 0.0247518 | 0.24 |
| MB21D2 | -0.11487313 | 0.00014122 | -0.157381099 | 1.70E-07 | 0.05 |
| C3ORF62 | -0.104478373 | 0.00054091 | -0.056133558 | 0.0635754 | 0.05 |
| FAM208A | -0.395705883 | 2.75E-42 | -0.26506537 | 4.96E-19 | 0.14 |
| EOGT | -0.145115461 | 1.45E-06 | -0.243474459 | 3.26E-16 | 0.1 |
| IGF2BP2.AS1 | 0.138040846 | 4.64E-06 | 0.068684343 | 0.0231575 | 0.07 |
| LINC00488 | 0.17861083 | 2.75E-09 | 0.097943785 | 0.0011862 | 0.09 |
| C3ORF67 | 0.149412042 | 6.98E-07 | 0.049964776 | 0.0987377 | 0.1 |
| C3ORF70 | -0.099032983 | 0.00104397 | -0.172783868 | 8.97E-09 | 0.08 |
| ARIH2OS | 0.021778444 | 0.47197532 | 0.027978531 | 0.35543 | 0.01 |
| FOXL2NB | 0.041123228 | 0.17428111 | -0.086530951 | 0.0041984 | 0.13 |
| LINC00696 | -0.081411814 | 0.00708312 | -0.119056826 | 7.96E-05 | 0.04 |
| ELP6 | 0.172641431 | 9.23E-09 | 0.21880697 | 2.59E-13 | 0.05 |
| TOPAZ1 | 0.049296685 | 0.10333396 | -0.030321746 | 0.3165673 | 0.08 |
| C3ORF79 | 0.051534211 | 0.08858089 | 0.007936981 | 0.7932406 | 0.05 |
| C3 | -0.149784267 | 6.55E-07 | -0.159557997 | 1.14E-07 | 0.01 |
| C4A | -0.470914669 | 2.05E-61 | -0.129859297 | 1.66E-05 | 0.35 |
| C4BPA | 0.007358792 | 0.80799716 | -0.015422412 | 0.6105277 | 0.03 |
| C4BPB | -0.078309079 | 0.00959879 | 0.058298808 | 0.0540015 | 0.14 |
| NOP14.AS1 | -0.198342003 | 3.70E-11 | -0.102811582 | 0.0006637 | 0.1 |
| LINC00575 | -0.003387836 | 0.91092182 | 0.022561681 | 0.4561853 | 0.03 |
| WDFY3.AS2 | -0.523469844 | 6.27E-78 | -0.465384749 | 7.71E-60 | 0.06 |
| NOA1 | 0.176898421 | 3.91E-09 | 0.108757413 | 0.0003156 | 0.07 |
| C4ORF17 | 0.092877839 | 0.00211404 | 0.02810525 | 0.3532545 | 0.07 |
| C4ORF19 | 0.043963841 | 0.14636041 | -0.018617691 | 0.5386471 | 0.07 |
| LOC91431 | 0.207185458 | 4.61E-12 | 0.006995695 | 0.8172995 | 0.21 |
| C4ORF22 | -0.05006515 | 0.0980615 | 0.035051989 | 0.246918 | 0.09 |
| FLJ12891 | -0.173786172 | 7.34E-09 | -0.106311561 | 0.0004304 | 0.07 |
| C4ORF26 | 0.117757447 | 9.53E-05 | 0.006259749 | 0.8362317 | 0.12 |
| C4ORF27 | 0.327102564 | 1.14E-28 | 0.339288664 | 7.51E-31 | 0.02 |
| C4ORF29 | -0.366665255 | 4.08E-36 | -0.317903521 | 4.33E-27 | 0.05 |
| NDNF | -0.418818873 | 1.17E-47 | -0.21131171 | 1.69E-12 | 0.21 |
| C4ORF32 | -0.442267133 | 1.48E-53 | -0.180060872 | 2.03E-09 | 0.27 |
| C4ORF33 | -0.168040978 | 2.28E-08 | -0.044061292 | 0.1454671 | 0.13 |
| SMIM14 | -0.494897547 | 1.35E-68 | -0.233107464 | 5.92E-15 | 0.27 |
| CABS1 | 0.010584299 | 0.72668985 | 0.005744819 | 0.8495355 | 0.01 |
| C4ORF36 | -0.242724384 | 4.04E-16 | -0.16611379 | 3.31E-08 | 0.08 |
| STPG2 | -0.229881698 | 1.42E-14 | -0.19438228 | 9.11E-11 | 0.04 |
| WWC2.AS2 | -0.257871352 | 4.61E-18 | -0.215573754 | 5.86E-13 | 0.05 |
| FAM218A | 0.099092308 | 0.00103669 | -0.043074249 | 0.1547102 | 0.15 |
| C4ORF3 | -0.124847222 | 3.49E-05 | -0.091575997 | 0.0024418 | 0.04 |
| PRR27 | 0.248526427 | 7.54E-17 | 0.101335291 | 0.0007936 | 0.15 |
| TRAPPC11 | -0.336914911 | 2.03E-30 | -0.307950415 | 1.93E-25 | 0.03 |
| CTBP1.AS2 | -0.225573883 | 4.47E-14 | -0.135811848 | 6.61E-06 | 0.09 |
| TMA16 | 0.20181546 | 1.65E-11 | 0.158462867 | 1.39E-07 | 0.05 |
| MSANTD1 | 0.038318701 | 0.20556583 | 0.014354275 | 0.6354702 | 0.03 |
| C4ORF45 | 0.107871173 | 0.00035342 | -0.023793528 | 0.4319622 | 0.14 |
| C4ORF46 | 0.316240964 | 8.25E-27 | 0.118173333 | 9.00E-05 | 0.2 |
| C4ORF47 | 0.066366263 | 0.02823442 | 0.12434808 | 3.75E-05 | 0.06 |
| C4ORF48 | 0.272893779 | 4.05E-20 | 0.192080373 | 1.53E-10 | 0.09 |
| MGARP | -0.191527597 | 1.73E-10 | -0.159978074 | 1.05E-07 | 0.04 |
| LOC105374380 | -0.085950598 | 0.00446089 | -0.082239713 | 0.0065205 | 0.01 |
| LOC648996 | 0.222746642 | 9.37E-14 | 0.070823156 | 0.0191954 | 0.16 |
| SMIM20 | 0.147954856 | 8.97E-07 | 0.138774549 | 4.12E-06 | 0.01 |
| LINC01587 | 0.152640619 | 3.97E-07 | 0.176016091 | 4.68E-09 | 0.03 |
| FDCSP | 0.203790711 | 1.04E-11 | 0.000922443 | 0.9756989 | 0.21 |
| C5AR1 | -0.051346241 | 0.08975115 | -0.122811346 | 4.68E-05 | 0.08 |
| PRO1873 | 0.007337307 | 0.80854687 | 0.089236354 | 0.0031499 | 0.09 |
| C5ORF15 | -0.366026038 | 5.49E-36 | -0.157134504 | 1.78E-07 | 0.21 |
| DCANP1 | -0.014399566 | 0.6344039 | -0.149791581 | 6.54E-07 | 0.14 |
| C5ORF22 | -0.000734342 | 0.98065321 | -0.016641327 | 0.5826075 | 0.02 |
| C5orf23 | 0.057842881 | 0.05591082 | -0.005264974 | 0.8619716 | 0.07 |
| C5ORF24 | -0.349984345 | 7.59E-33 | -0.252979263 | 2.02E-17 | 0.1 |
| SIMC1 | 0.076576291 | 0.01132599 | -0.049770903 | 0.1000544 | 0.13 |
| LINC01554 | 0.166647393 | 2.99E-08 | 0.076952141 | 0.0109295 | 0.09 |
| C5ORF28 | -0.027039168 | 0.37181959 | 0.041506191 | 0.1703007 | 0.07 |
| C5ORF30 | -0.242954121 | 3.78E-16 | -0.026366058 | 0.3838468 | 0.22 |
| LOC116424 | -0.057100415 | 0.05914003 | 0.122948972 | 4.59E-05 | 0.19 |
| NADK2 | -0.13486345 | 7.68E-06 | 0.015855229 | 0.6005459 | 0.16 |
| C5ORF34 | 0.407081275 | 7.05E-45 | 0.244236976 | 2.62E-16 | 0.17 |
| SETD9 | -0.183382817 | 1.01E-09 | -0.056570848 | 0.0615365 | 0.13 |
| KIAA0825 | -0.564545916 | 5.16E-93 | -0.246432453 | 1.39E-16 | 0.32 |
| C5ORF38 | -0.07343409 | 0.01517094 | -0.006445869 | 0.8314343 | 0.07 |
| LOC257249 | 0.17291522 | 8.74E-09 | 0.077284219 | 0.0105896 | 0.1 |
| LOC202365 | 0.065141879 | 0.03128336 | -0.00969143 | 0.7489343 | 0.08 |
| CREBRF | -0.589143724 | 4.11E-103 | -0.512597552 | 2.84E-74 | 0.08 |
| LOC100132000 | -0.399609874 | 3.64E-43 | -0.337959653 | 1.31E-30 | 0.07 |
| SMIM15 | -0.135593714 | 6.84E-06 | 0.14223686 | 2.35E-06 | 0.28 |
| TRAPPC13 | -0.308594147 | 1.52E-25 | -0.073194984 | 0.0155058 | 0.24 |
| C5ORF45 | -0.073097527 | 0.01564414 | 0.045182931 | 0.1354828 | 0.12 |
| C5ORF46 | 0.27836392 | 6.70E-21 | 0.172798008 | 8.95E-09 | 0.11 |
| C5ORF47 | 0.01358277 | 0.65374858 | -0.024374779 | 0.4207959 | 0.04 |
| TEX43 | 0.088688601 | 0.00334061 | 0.030687337 | 0.310765 | 0.06 |
| C5ORF49 | -0.147623172 | 9.50E-07 | 0.048363351 | 0.1100375 | 0.2 |
| FAXDC2 | -0.317284581 | 5.51E-27 | -0.12698691 | 2.55E-05 | 0.2 |
| C5ORF51 | -0.181736469 | 1.43E-09 | -0.098947948 | 0.0010545 | 0.09 |
| C5ORF52 | 0.045651771 | 0.13146954 | 0.02938658 | 0.3317303 | 0.02 |
| IGIP | -0.482147028 | 1.05E-64 | -0.25093258 | 3.71E-17 | 0.24 |
| ZBED8 | -0.056780457 | 0.06057853 | 0.055172578 | 0.0682523 | 0.12 |
| EXOC3.AS1 | 0.154527123 | 2.84E-07 | 0.020183377 | 0.5050418 | 0.14 |
| C5ORF56 | -0.105926429 | 0.00045174 | -0.071513778 | 0.0180492 | 0.04 |
| C5ORF58 | -0.070050455 | 0.02055269 | -0.170480024 | 1.42E-08 | 0.11 |
| C5ORF60 | 0.081871922 | 0.00676531 | 0.088748815 | 0.0033191 | 0.01 |
| SMIM3 | -0.271694401 | 5.98E-20 | -0.234913583 | 3.61E-15 | 0.04 |
| C5 | -0.294018683 | 3.10E-23 | -0.191707522 | 1.66E-10 | 0.11 |
| LOC645793 | -0.140746753 | 3.00E-06 | -0.003484173 | 0.9083998 | 0.14 |
| ADTRP | 0.10883028 | 0.00031265 | 0.074558409 | 0.0136802 | 0.04 |
| MGC4614 | 0.229119235 | 1.74E-14 | -0.004428228 | 0.8837376 | 0.24 |
| DNPH1 | 0.41949107 | 8.03E-48 | 0.33822535 | 1.17E-30 | 0.09 |
| C6ORF10 | -0.01356567 | 0.65415611 | 0.012596531 | 0.6774186 | 0.03 |
| C6orf114 | 0.180582452 | 1.82E-09 | 0.02578484 | 0.394421 | 0.16 |
| ABRACL | 0.434593475 | 1.42E-51 | 0.416752174 | 3.67E-47 | 0.02 |
| C6ORF118 | 0.076957468 | 0.01092401 | 0.093455655 | 0.0019819 | 0.02 |
| C6ORF120 | -0.350796274 | 5.32E-33 | -0.071884389 | 0.0174591 | 0.28 |
| LOC154640 | -0.245232838 | 1.97E-16 | -0.158205506 | 1.46E-07 | 0.09 |
| LINC01558 | 0.060674239 | 0.04491215 | -0.058128174 | 0.0547096 | 0.12 |
| LOC654251 | 0.141960965 | 2.46E-06 | 0.04154383 | 0.1699132 | 0.11 |
| UQCC2 | 0.474252085 | 2.22E-62 | 0.454974451 | 5.98E-57 | 0.02 |
| CLPSL2 | -0.016159595 | 0.59357082 | 0.221063233 | 1.45E-13 | 0.24 |
| CLPSL1 | 0.119503765 | 7.48E-05 | 0.249871592 | 5.08E-17 | 0.14 |
| CCDC167 | 0.579123326 | 6.79E-99 | 0.480846574 | 2.55E-64 | 0.1 |
| OARD1 | 0.089853826 | 0.0029468 | 0.129971516 | 1.63E-05 | 0.05 |
| C6ORF132 | -0.081962479 | 0.00670429 | 0.009278156 | 0.7593027 | 0.1 |
| ATAT1 | 0.229857824 | 1.43E-14 | 0.222794793 | 9.25E-14 | 0.01 |
| C6ORF136 | 0.411013797 | 8.49E-46 | 0.304327604 | 7.43E-25 | 0.11 |
| PTCHD4 | -0.229568662 | 1.54E-14 | -0.157661169 | 1.61E-07 | 0.08 |
| C6ORF141 | 0.075742425 | 0.01225138 | -0.052808685 | 0.0809667 | 0.13 |
| MLIP | 0.103464858 | 0.00061279 | -0.041725287 | 0.1680543 | 0.15 |
| PXDC1 | 0.064444714 | 0.03314235 | 0.019991801 | 0.5090936 | 0.05 |
| FAM217A | -0.043998674 | 0.14604061 | -0.00788857 | 0.7944734 | 0.04 |
| C6orf147 | 0.194668092 | 8.55E-11 | 0.096616302 | 0.0013838 | 0.1 |
| MB21D1 | 0.116806668 | 0.0001086 | -0.070643035 | 0.0195046 | 0.19 |
| RRP36 | 0.454924026 | 6.17E-57 | 0.324398956 | 3.36E-28 | 0.14 |
| LRRC73 | -0.069487594 | 0.02159329 | 0.046193044 | 0.1269514 | 0.12 |
| LINC00472 | -0.283572739 | 1.16E-21 | -0.257309603 | 5.48E-18 | 0.03 |
| C6ORF15 | 0.224153327 | 6.49E-14 | 0.035156979 | 0.2455044 | 0.19 |
| SMIM8 | 0.263513871 | 8.07E-19 | 0.193600339 | 1.09E-10 | 0.07 |
| C6ORF163 | 0.037569033 | 0.21457994 | 0.038418824 | 0.2043831 | 0.01 |
| LINC01590 | 0.016442734 | 0.58711566 | 0.057093666 | 0.0591701 | 0.05 |
| C6ORF165 | -0.17174348 | 1.10E-08 | 0.05107577 | 0.0914568 | 0.23 |
| MMS22L | 0.170137874 | 1.52E-08 | 0.01059642 | 0.7263894 | 0.16 |
| FAXC | 0.155770757 | 2.27E-07 | 0.099722318 | 0.0009623 | 0.06 |
| TBC1D32 | -0.271775286 | 5.83E-20 | -0.175832463 | 4.85E-09 | 0.1 |
| SOGA3 | -0.135678335 | 6.75E-06 | -0.269921373 | 1.06E-19 | 0.14 |
| LINC00473 | 0.036864168 | 0.22331175 | -0.046367021 | 0.1255251 | 0.09 |
| CEP57L1 | 0.209209602 | 2.83E-12 | 0.172335982 | 9.81E-09 | 0.04 |
| METTL24 | -0.279387767 | 4.77E-21 | -0.287134085 | 3.44E-22 | 0.01 |
| TMEM244 | -0.178701442 | 2.70E-09 | -0.090594054 | 0.0027191 | 0.09 |
| SLC18B1 | 0.049806135 | 0.09981409 | 0.014871071 | 0.6233484 | 0.04 |
| LOC221759 | 0.206571297 | 5.35E-12 | -0.007288418 | 0.809798 | 0.22 |
| C6orf1 | 0.019213414 | 0.52572991 | 0.223521769 | 7.66E-14 | 0.21 |
| C6ORF201 | -0.46165259 | 8.60E-59 | -0.263643994 | 7.75E-19 | 0.2 |
| C6ORF203 | 0.329387013 | 4.51E-29 | 0.354815121 | 8.98E-34 | 0.03 |
| CEP85L | -0.221048557 | 1.45E-13 | -0.389897466 | 5.30E-41 | 0.17 |
| LINC00574 | -0.2929489 | 4.52E-23 | -0.189990556 | 2.42E-10 | 0.11 |
| ARMT1 | -0.235371313 | 3.18E-15 | -0.073795557 | 0.0146768 | 0.17 |
| LINC00271 | -0.313333982 | 2.52E-26 | -0.150016047 | 6.29E-07 | 0.17 |
| LINC00518 | 0.33444479 | 5.67E-30 | 0.071404671 | 0.0182262 | 0.27 |
| KHDC3L | 0.111633825 | 0.00021729 | -0.011281363 | 0.709481 | 0.13 |
| C6ORF222 | -0.023297043 | 0.44163442 | -0.006983397 | 0.817615 | 0.02 |
| C6ORF223 | 0.15433871 | 2.94E-07 | 0.030879535 | 0.3077429 | 0.13 |
| FAM229B | -0.020573875 | 0.49683551 | 0.164442981 | 4.56E-08 | 0.19 |
| C6ORF226 | 0.168208115 | 2.21E-08 | 0.086870564 | 0.0040513 | 0.09 |
| LINC00336 | -0.005194962 | 0.86378902 | 0.023852774 | 0.4308163 | 0.03 |
| C6ORF25 | -0.26264138 | 1.06E-18 | -0.16993444 | 1.58E-08 | 0.1 |
| C6orf26 | 0.302192193 | 1.63E-24 | 0.235276608 | 3.26E-15 | 0.07 |
| VWA7 | -0.076683558 | 0.01121156 | -0.198015259 | 3.99E-11 | 0.13 |
| TMEM242 | 0.039741326 | 0.18922157 | 0.059174281 | 0.0504876 | 0.02 |
| C6orf41 | 0.31095562 | 6.23E-26 | 0.231378677 | 9.48E-15 | 0.08 |
| LOC135662 | 0.117420599 | 9.98E-05 | 0.09445096 | 0.0017719 | 0.03 |
| C6ORF48 | 0.248977034 | 6.61E-17 | 0.219304003 | 2.28E-13 | 0.03 |
| LOC221713 | 0.219781447 | 2.01E-13 | 0.203639161 | 1.08E-11 | 0.02 |
| SDHAF4 | 0.186090828 | 5.67E-10 | 0.121691804 | 5.49E-05 | 0.07 |
| LOC389429 | 0.08857279 | 0.00338225 | 0.017153704 | 0.5710514 | 0.08 |
| AGPAT4.IT1 | 0.003394946 | 0.91073564 | -0.050628427 | 0.0943349 | 0.06 |
| C6ORF62 | 0.19957021 | 2.79E-11 | 0.268666191 | 1.58E-19 | 0.07 |
| SAYSD1 | 0.322897902 | 6.09E-28 | 0.282818717 | 1.50E-21 | 0.05 |
| ERMARD | -0.097124347 | 0.00130484 | 0.152922869 | 3.78E-07 | 0.26 |
| GINM1 | -0.259211773 | 3.06E-18 | -0.177973183 | 3.13E-09 | 0.09 |
| ARMC12 | 0.206746324 | 5.13E-12 | 0.119549828 | 7.43E-05 | 0.09 |
| C6ORF89 | -0.242034988 | 4.91E-16 | -0.220153943 | 1.83E-13 | 0.03 |
| C6orf94 | 0.037001181 | 0.22159487 | 0.072163252 | 0.0170263 | 0.04 |
| CCDC170 | -0.441995474 | 1.74E-53 | -0.19212553 | 1.51E-10 | 0.25 |
| C6 | -0.275216601 | 1.90E-20 | -0.167728016 | 2.43E-08 | 0.11 |
| SUGCT | 0.122228466 | 5.09E-05 | 0.111380475 | 0.0002246 | 0.02 |
| MPLKIP | 0.416399334 | 4.46E-47 | 0.334139939 | 6.44E-30 | 0.09 |
| C7ORF13 | 0.261271998 | 1.62E-18 | 0.155473555 | 2.40E-07 | 0.11 |
| PPP1R17 | 0.105267562 | 0.00049046 | -0.002030012 | 0.9465526 | 0.11 |
| TMEM243 | -0.026050674 | 0.38956299 | 0.051517734 | 0.088683 | 0.08 |
| C7ORF25 | 0.142171806 | 2.37E-06 | 0.184197402 | 8.50E-10 | 0.05 |
| C7ORF26 | 0.16191424 | 7.35E-08 | 0.178366668 | 2.89E-09 | 0.02 |
| BRAT1 | 0.122217088 | 5.10E-05 | 0.121488722 | 5.65E-05 | 0.01 |
| LOC115565 | 0.279648429 | 4.37E-21 | 0.244619622 | 2.35E-16 | 0.04 |
| DKFZP586I1023 | 0.128819644 | 1.94E-05 | 0.117176406 | 0.0001032 | 0.02 |
| ZBED6CL | 0.12699615 | 2.54E-05 | -0.018412634 | 0.5431299 | 0.15 |
| MALSU1 | 0.38139545 | 3.62E-39 | 0.359777302 | 9.65E-35 | 0.03 |
| C7ORF31 | -0.008706325 | 0.77372031 | -0.135956869 | 6.46E-06 | 0.13 |
| C7ORF33 | 0.13399926 | 8.79E-06 | 0.054566594 | 0.0713441 | 0.08 |
| C7ORF34 | -0.105044583 | 0.00050426 | -0.079964756 | 0.0081717 | 0.03 |
| YAE1D1 | 0.299019218 | 5.17E-24 | 0.383177677 | 1.51E-39 | 0.09 |
| SNHG15 | 0.452202387 | 3.38E-56 | 0.32790691 | 8.22E-29 | 0.13 |
| MTURN | -0.212182664 | 1.36E-12 | -0.084247264 | 0.0053194 | 0.13 |
| TMEM248 | -0.098842355 | 0.00106767 | -0.001685996 | 0.9555997 | 0.1 |
| C7ORF43 | -0.019816397 | 0.51281832 | 0.072817096 | 0.0160483 | 0.1 |
| COA1 | 0.131451368 | 1.30E-05 | 0.143684927 | 1.85E-06 | 0.02 |
| SSMEM1 | 0.107768009 | 0.00035808 | 0.025954715 | 0.3913124 | 0.09 |
| FAM221A | -0.139044107 | 3.95E-06 | -0.039442066 | 0.1925777 | 0.1 |
| LOC136466 | 0.250552992 | 4.16E-17 | 0.29376385 | 3.39E-23 | 0.05 |
| LOC654018 | 0.126880642 | 2.59E-05 | 0.04561292 | 0.1317986 | 0.09 |
| LINC00244 | 0.041765006 | 0.1676494 | 0.112015197 | 0.0002067 | 0.08 |
| C7ORF50 | 0.168020689 | 2.29E-08 | 0.193992079 | 9.95E-11 | 0.03 |
| NYAP1 | 0.033071006 | 0.27466021 | -0.121147715 | 5.93E-05 | 0.16 |
| NAT16 | 0.195723797 | 6.73E-11 | 0.066392828 | 0.0281712 | 0.13 |
| LSMEM1 | 0.200349053 | 2.33E-11 | 0.16192705 | 7.33E-08 | 0.04 |
| SND1.IT1 | 0.129466521 | 1.76E-05 | 0.104859357 | 0.000516 | 0.03 |
| C7ORF55 | 0.394619596 | 4.81E-42 | 0.247158897 | 1.12E-16 | 0.15 |
| C7ORF57 | 0.00053551 | 0.98589095 | 0.026017279 | 0.3901713 | 0.03 |
| CPED1 | -0.460585153 | 1.70E-58 | -0.416753169 | 3.67E-47 | 0.05 |
| LOC155131 | 0.226579378 | 3.43E-14 | 0.263121239 | 9.13E-19 | 0.04 |
| C7ORF60 | -0.028390521 | 0.34838767 | -0.153752931 | 3.26E-07 | 0.13 |
| C7ORF61 | 0.388837201 | 9.03E-41 | 0.129625368 | 1.72E-05 | 0.26 |
| CFAP69 | -0.43531311 | 9.29E-52 | -0.160775099 | 9.09E-08 | 0.28 |
| RBM48 | -0.020424582 | 0.49996448 | -0.002023628 | 0.9467204 | 0.02 |
| C7ORF65 | 0.040951057 | 0.17609306 | 0.006400578 | 0.8326012 | 0.04 |
| LOC392777 | 0.049509432 | 0.10185226 | 0.020367414 | 0.5011654 | 0.03 |
| LOC116148 | 0.245460845 | 1.84E-16 | 0.225347914 | 4.74E-14 | 0.03 |
| C7ORF69 | -0.166772764 | 2.92E-08 | -0.110238008 | 0.0002607 | 0.06 |
| FAM220A | 0.061134049 | 0.04331053 | 0.142929601 | 2.09E-06 | 0.09 |
| C7ORF71 | -0.060093339 | 0.04700659 | -0.132889793 | 1.04E-05 | 0.08 |
| C7ORF72 | 0.147423893 | 9.83E-07 | 0.085103488 | 0.0048708 | 0.07 |
| C7 | -0.326981706 | 1.19E-28 | -0.236811204 | 2.13E-15 | 0.1 |
| C8A | 0.040607367 | 0.17975197 | 0.063613864 | 0.0354804 | 0.03 |
| C8B | -0.056832179 | 0.06034405 | 0.00075073 | 0.9802216 | 0.06 |
| C8G | 0.258287071 | 4.06E-18 | 0.20093137 | 2.03E-11 | 0.06 |
| TMEM249 | 0.360959535 | 5.64E-35 | 0.244571094 | 2.38E-16 | 0.12 |
| FAM167A.AS1 | 0.051782381 | 0.08705468 | 0.020940498 | 0.4891961 | 0.04 |
| C8ORF22 | 0.19892834 | 3.23E-11 | 0.095889973 | 0.0015042 | 0.11 |
| HGH1 | 0.457160358 | 1.51E-57 | 0.329236647 | 4.80E-29 | 0.13 |
| C8ORF31 | -0.102044706 | 0.00072851 | -0.147212208 | 1.02E-06 | 0.05 |
| C8ORF33 | 0.313380267 | 2.48E-26 | 0.144577606 | 1.59E-06 | 0.17 |
| C8ORF34 | -0.115546391 | 0.00012893 | 0.020784248 | 0.4924441 | 0.14 |
| C8ORF37 | -0.147708685 | 9.36E-07 | -0.193673223 | 1.07E-10 | 0.05 |
| LOC101927002 | 0.271204487 | 7.01E-20 | 0.239752485 | 9.38E-16 | 0.04 |
| RBM12B.AS1 | -0.032971708 | 0.27610466 | -0.171397666 | 1.18E-08 | 0.14 |
| SMIM19 | 0.180325716 | 1.92E-09 | 0.18822133 | 3.57E-10 | 0.01 |
| TTI2 | 0.049426145 | 0.10243029 | 0.120384132 | 6.61E-05 | 0.08 |
| TDRP | -0.014986476 | 0.62065517 | 0.007715182 | 0.7988929 | 0.03 |
| C8ORF44 | 0.174190626 | 6.77E-09 | 0.102077283 | 0.0007256 | 0.08 |
| MCMDC2 | 0.12048176 | 6.52E-05 | 0.046059307 | 0.1280564 | 0.08 |
| C8ORF46 | 0.262566704 | 1.09E-18 | 0.047472345 | 0.1167527 | 0.22 |
| ERICH5 | 0.301836621 | 1.86E-24 | 0.076642345 | 0.0112554 | 0.23 |
| C8ORF48 | -0.108027694 | 0.00034644 | -0.026943157 | 0.3735207 | 0.09 |
| C8ORF4 | -0.254727086 | 1.20E-17 | -0.124059188 | 3.91E-05 | 0.14 |
| LOC116393 | 0.308929929 | 1.34E-25 | 0.144622468 | 1.58E-06 | 0.17 |
| LOC115231 | 0.25558494 | 9.24E-18 | 0.157277722 | 1.73E-07 | 0.1 |
| BAALC.AS2 | 0.219679645 | 2.07E-13 | 0.061946127 | 0.0405996 | 0.16 |
| C8ORF58 | -0.035064763 | 0.24674568 | 0.069436304 | 0.0216904 | 0.11 |
| C8ORF59 | 0.597078801 | 1.47E-106 | 0.528146069 | 1.52E-79 | 0.07 |
| LINC00588 | 0.058601183 | 0.0527655 | 0.041978011 | 0.1654907 | 0.02 |
| LOC286073 | 0.121323213 | 5.79E-05 | 0.12314816 | 4.46E-05 | 0.01 |
| C8ORF74 | 0.074294404 | 0.0140181 | 0.102304918 | 0.0007059 | 0.03 |
| LINC00589 | -0.243556806 | 3.18E-16 | -0.18260757 | 1.19E-09 | 0.07 |
| C8ORF76 | 0.501586132 | 1.06E-70 | 0.349541364 | 9.22E-33 | 0.16 |
| ZNF252P.AS1 | 0.143643293 | 1.86E-06 | 0.05747526 | 0.0574909 | 0.09 |
| C8orf79 | -0.352551066 | 2.45E-33 | -0.212267443 | 1.34E-12 | 0.15 |
| NUGGC | -0.080332958 | 0.00788142 | -0.150343071 | 5.94E-07 | 0.08 |
| DKFZP779L1068 | -0.189444711 | 2.73E-10 | -0.030625867 | 0.3117357 | 0.16 |
| SBSPON | -0.132045668 | 1.19E-05 | -0.070795508 | 0.0192426 | 0.07 |
| AARD | 0.116757872 | 0.00010933 | 0.045825088 | 0.1300095 | 0.08 |
| C8ORF86 | -0.074495945 | 0.01375946 | -0.08777507 | 0.0036822 | 0.02 |
| ARHGEF39 | 0.534764165 | 7.16E-82 | 0.353542214 | 1.58E-33 | 0.19 |
| C9orf102 | -0.399898669 | 3.13E-43 | -0.47014487 | 3.40E-61 | 0.08 |
| C9orf103 | -0.122179577 | 5.12E-05 | 0.034280319 | 0.2574822 | 0.16 |
| LOC286212 | -0.273010088 | 3.90E-20 | -0.144371426 | 1.65E-06 | 0.13 |
| LOC100134429 | -0.040518111 | 0.18071136 | 0.033598101 | 0.2670792 | 0.08 |
| LOC100133525 | -0.289023613 | 1.79E-22 | -0.237703121 | 1.67E-15 | 0.06 |
| DKFZP566D143 | 0.021575321 | 0.47611884 | 0.047238429 | 0.1185679 | 0.03 |
| C9ORF116 | -0.224959408 | 5.25E-14 | 0.063173847 | 0.0367745 | 0.29 |
| C9ORF117 | -0.215834145 | 5.49E-13 | -0.029904556 | 0.3232747 | 0.19 |
| SWI5 | 0.219694046 | 2.06E-13 | 0.214695951 | 7.30E-13 | 0.01 |
| EQTN | 0.148132835 | 8.70E-07 | -0.011198562 | 0.7115177 | 0.16 |
| FAM201A | 0.043758195 | 0.14825936 | -0.053917746 | 0.0747807 | 0.1 |
| TMEM261 | 0.352599842 | 2.40E-33 | 0.318507795 | 3.42E-27 | 0.04 |
| TMEM246 | -0.238022634 | 1.52E-15 | -0.199991599 | 2.53E-11 | 0.04 |
| FAM221B | -0.266777978 | 2.89E-19 | -0.002140505 | 0.9436482 | 0.27 |
| C9ORF129 | -0.464203223 | 1.66E-59 | -0.45176531 | 4.44E-56 | 0.02 |
| C9orf130 | 0.028426549 | 0.34777606 | -0.057396958 | 0.0578322 | 0.09 |
| C9ORF131 | -0.128948833 | 1.90E-05 | -0.20732072 | 4.46E-12 | 0.08 |
| C9ORF135 | 0.038539894 | 0.20295943 | 0.001519252 | 0.959987 | 0.04 |
| C9ORF139 | -0.151909588 | 4.52E-07 | -0.264824182 | 5.35E-19 | 0.12 |
| SAPCD2 | 0.469843556 | 4.15E-61 | 0.22118332 | 1.40E-13 | 0.25 |
| C9ORF142 | 0.285717536 | 5.59E-22 | 0.279139059 | 5.18E-21 | 0.01 |
| FAM205A | -0.233199317 | 5.77E-15 | -0.211629759 | 1.56E-12 | 0.03 |
| DKFZp434J193 | -0.022174531 | 0.46395289 | -0.058290612 | 0.0540354 | 0.04 |
| LURAP1L | -0.241900339 | 5.11E-16 | 0.001391856 | 0.9633398 | 0.25 |
| C9ORF152 | -0.426094451 | 1.94E-49 | -0.123848986 | 4.03E-05 | 0.31 |
| C9ORF153 | 0.164306994 | 4.68E-08 | 0.053088459 | 0.0793684 | 0.12 |
| C9ORF156 | -0.21817768 | 3.03E-13 | -0.037583775 | 0.2144 | 0.19 |
| C9ORF163 | -0.046886679 | 0.121339 | -0.001667145 | 0.9560957 | 0.05 |
| TOR4A | -0.095839677 | 0.00151293 | -0.182916338 | 1.12E-09 | 0.09 |
| CYSRT1 | -0.115531771 | 0.00012919 | 0.018838167 | 0.533848 | 0.14 |
| C9ORF16 | 0.267429833 | 2.35E-19 | 0.251566216 | 3.08E-17 | 0.02 |
| C9ORF170 | 0.310878705 | 6.42E-26 | 0.080906181 | 0.0074478 | 0.23 |
| C9ORF171 | -0.159971412 | 1.06E-07 | 0.001933139 | 0.9490995 | 0.17 |
| C9ORF172 | -0.349685624 | 8.65E-33 | -0.22926237 | 1.68E-14 | 0.13 |
| C9ORF173 | -0.073991224 | 0.01441524 | -0.044012527 | 0.1459136 | 0.03 |
| LOC169858 | -0.019132013 | 0.52748558 | -0.102131825 | 0.0007209 | 0.09 |
| RPP25L | 0.268855772 | 1.49E-19 | 0.320610763 | 1.50E-27 | 0.06 |
| C9ORF24 | 0.169512268 | 1.71E-08 | 0.080559596 | 0.0077073 | 0.09 |
| FAM219A | 0.192633038 | 1.35E-10 | -0.084026535 | 0.0054409 | 0.28 |
| LINC00474 | 0.00801465 | 0.79126384 | -0.005862107 | 0.8465013 | 0.02 |
| MSANTD3 | 0.383766909 | 1.13E-39 | 0.193724225 | 1.06E-10 | 0.2 |
| LOC116227 | 0.152813225 | 3.85E-07 | 0.11820864 | 8.95E-05 | 0.04 |
| C9orf3 | -0.024577857 | 0.41693489 | -0.05192368 | 0.0861952 | 0.03 |
| C9ORF40 | 0.467622311 | 1.79E-60 | 0.190743817 | 2.05E-10 | 0.28 |
| C9ORF41 | -0.374861351 | 8.55E-38 | -0.347895806 | 1.89E-32 | 0.03 |
| C9ORF43 | 0.054872476 | 0.06976941 | 0.051641802 | 0.0879166 | 0.01 |
| LINC00475 | -0.270496348 | 8.81E-20 | -0.161003151 | 8.71E-08 | 0.11 |
| MIR600HG | -0.121550466 | 5.60E-05 | -0.154457574 | 2.88E-07 | 0.04 |
| PLGRKT | -0.002256418 | 0.94060208 | 0.1673192 | 2.63E-08 | 0.17 |
| C9orf108 | -0.291202933 | 8.35E-23 | -0.230335434 | 1.26E-14 | 0.07 |
| FRRS1L | -0.047451514 | 0.1169135 | -0.066850923 | 0.0271002 | 0.02 |
| C9ORF50 | 0.036969209 | 0.22199465 | 0.05454083 | 0.071478 | 0.02 |
| CDKN2A.AS1 | 0.322493721 | 7.15E-28 | 0.08470067 | 0.0050774 | 0.24 |
| C9ORF57 | 0.003117757 | 0.91799704 | -0.011907142 | 0.6941569 | 0.02 |
| TMEM245 | -0.446148202 | 1.41E-54 | -0.38833169 | 1.16E-40 | 0.06 |
| C9ORF64 | -0.425630791 | 2.52E-49 | -0.279281214 | 4.94E-21 | 0.15 |
| C9ORF66 | -0.144008894 | 1.75E-06 | -0.037621276 | 0.2139427 | 0.11 |
| LOC105375966 | -0.331056891 | 2.29E-29 | -0.067040523 | 0.0266672 | 0.27 |
| C9ORF69 | 0.181938298 | 1.37E-09 | 0.127758923 | 2.27E-05 | 0.06 |
| FAM206A | -0.045516973 | 0.13261387 | 0.063387699 | 0.0361406 | 0.11 |
| GLIS3.AS1 | 0.237814019 | 1.61E-15 | 0.101490632 | 0.0007789 | 0.14 |
| TMEM252 | 0.026465923 | 0.38204751 | 0.034705689 | 0.2516207 | 0.01 |
| ALSFTD | 0.04413144 | 0.14482659 | -0.124071486 | 3.90E-05 | 0.17 |
| HSPC220 | 0.151024723 | 5.27E-07 | 0.081779093 | 0.0068284 | 0.07 |
| SPATA31E1 | 0.036728937 | 0.2250156 | -0.01151084 | 0.7038476 | 0.05 |
| CACFD1 | -0.214187568 | 8.29E-13 | 0.017683316 | 0.5592224 | 0.24 |
| INIP | -0.27497119 | 2.06E-20 | -0.204231118 | 9.35E-12 | 0.08 |
| CAAP1 | -0.046938242 | 0.12092965 | 0.072748591 | 0.0161484 | 0.12 |
| C9ORF84 | -0.114309715 | 0.00015233 | -0.028130446 | 0.3528229 | 0.09 |
| C9ORF85 | 0.107435524 | 0.00037352 | 0.062177427 | 0.0398542 | 0.05 |
| RABL6 | 0.137442167 | 5.11E-06 | 0.071926984 | 0.0173924 | 0.07 |
| C9ORF89 | 0.073384173 | 0.01524032 | 0.138989059 | 3.98E-06 | 0.07 |
| C9ORF91 | -0.31327408 | 2.58E-26 | -0.118973466 | 8.05E-05 | 0.2 |
| CCDC171 | -0.113781935 | 0.00016349 | -0.174792625 | 5.99E-09 | 0.07 |
| NMRK1 | -0.039939904 | 0.18701845 | 0.000963246 | 0.9746244 | 0.05 |
| STKLD1 | -0.092724832 | 0.00215036 | -0.068373217 | 0.0237893 | 0.03 |
| AK8 | -0.331892287 | 1.62E-29 | -0.043540943 | 0.1502858 | 0.29 |
| C9ORF9 | -0.130113431 | 1.60E-05 | 0.051042601 | 0.0916678 | 0.19 |
| C9 | -0.046017481 | 0.12840346 | -0.043758814 | 0.1482536 | 0.01 |
| LOC56059 | 0.08591616 | 0.00447693 | 0.061252387 | 0.0429062 | 0.03 |
| CA11 | -0.003039841 | 0.92003943 | -0.088880281 | 0.0032727 | 0.09 |
| FLJ20151 | -0.440019039 | 5.70E-53 | -0.149178455 | 7.27E-07 | 0.3 |
| CAXIV | 0.125192751 | 3.32E-05 | -0.023117755 | 0.4451574 | 0.15 |
| CA14 | -0.084494379 | 0.00518621 | 0.055911201 | 0.0646334 | 0.15 |
| CA1 | 0.049856071 | 0.09947428 | -0.035794911 | 0.2370374 | 0.09 |
| CA2 | -0.113882034 | 0.00016131 | -0.063329704 | 0.0363116 | 0.06 |
| CA3 | -0.028841232 | 0.34078561 | -0.086320869 | 0.0042917 | 0.06 |
| CA4 | -0.217710031 | 3.42E-13 | -0.175224511 | 5.49E-09 | 0.05 |
| CA5A | 0.05961345 | 0.04879818 | 0.048532773 | 0.1087958 | 0.02 |
| PRO2325 | 0.314957149 | 1.35E-26 | 0.173620099 | 7.59E-09 | 0.15 |
| CA5B | -0.329519312 | 4.28E-29 | -0.255597281 | 9.20E-18 | 0.08 |
| CA6 | 0.233745246 | 4.97E-15 | 0.072551985 | 0.0164387 | 0.17 |
| CA7 | 0.054200437 | 0.0732672 | 0.033094294 | 0.2743222 | 0.03 |
| CA8 | -0.142173248 | 2.37E-06 | -0.046382498 | 0.1253988 | 0.1 |
| CA9 | 0.366471964 | 4.47E-36 | 0.231737862 | 8.60E-15 | 0.14 |
| CAB39L | -0.333314635 | 9.05E-30 | -0.177015171 | 3.81E-09 | 0.16 |
| CAB39 | -0.356404447 | 4.42E-34 | -0.294623788 | 2.50E-23 | 0.07 |
| ADCK3 | 0.245853361 | 1.64E-16 | 0.319832431 | 2.04E-27 | 0.08 |
| CAIN | -0.245914643 | 1.61E-16 | -0.233034159 | 6.04E-15 | 0.02 |
| CABLES1 | 0.054671278 | 0.07080194 | -0.039250716 | 0.1947464 | 0.1 |
| CABLES2 | 0.317482219 | 5.10E-27 | 0.128580172 | 2.01E-05 | 0.19 |
| CABP1 | -0.055183656 | 0.0681968 | -0.067989447 | 0.0245891 | 0.02 |
| CABP2 | 0.070125541 | 0.02041722 | 0.027325196 | 0.3667802 | 0.05 |
| CABP4 | -0.328281193 | 7.06E-29 | -0.108396102 | 0.0003305 | 0.22 |
| CABP5 | 0.043194551 | 0.15356039 | 0.063035517 | 0.0371896 | 0.02 |
| CABP7 | 0.216285405 | 4.90E-13 | 0.083529711 | 0.0057236 | 0.14 |
| LOC115227 | 0.172756323 | 9.02E-09 | 0.017189867 | 0.57024 | 0.16 |
| CACHD1 | -0.186865469 | 4.79E-10 | -0.247297298 | 1.08E-16 | 0.07 |
| CACNA1A | 0.140048934 | 3.36E-06 | 0.072414563 | 0.0166444 | 0.07 |
| CACNA1B | 0.175253518 | 5.46E-09 | 0.028562534 | 0.3454738 | 0.15 |
| CACNA1C | -0.430610714 | 1.44E-50 | -0.333461036 | 8.52E-30 | 0.1 |
| CACNA1D | -0.462816209 | 4.07E-59 | -0.256875373 | 6.25E-18 | 0.21 |
| CACNA1E | -0.072274593 | 0.01685619 | -0.068580627 | 0.0233665 | 0.01 |
| CACNA1F | -0.283366249 | 1.25E-21 | -0.098501575 | 0.0011113 | 0.19 |
| CACNA1G | -0.357972212 | 2.18E-34 | -0.229539821 | 1.56E-14 | 0.13 |
| CACNA1H | -0.264495982 | 5.94E-19 | -0.124853351 | 3.49E-05 | 0.14 |
| CACNA1I | -0.070680805 | 0.0194394 | -0.087100026 | 0.0039546 | 0.02 |
| CACNA1S | -0.001065729 | 0.97192561 | -0.012104398 | 0.6893518 | 0.02 |
| CACNA2D1 | -0.459613017 | 3.17E-58 | -0.447766883 | 5.24E-55 | 0.02 |
| CACNA2D2 | -0.425809482 | 2.28E-49 | -0.267595062 | 2.23E-19 | 0.16 |
| CACNA2D3 | -0.206841578 | 5.01E-12 | -0.119157895 | 7.85E-05 | 0.09 |
| CACNA2D4 | -0.250396588 | 4.35E-17 | -0.198952621 | 3.21E-11 | 0.06 |
| CACNB1 | 0.172402307 | 9.68E-09 | -0.00172939 | 0.9544582 | 0.18 |
| CACNB2 | -0.199902302 | 2.58E-11 | -0.227742962 | 2.52E-14 | 0.03 |
| CACNB3 | 0.075628702 | 0.01238264 | 0.194004205 | 9.92E-11 | 0.12 |
| CACNB4 | -0.313036292 | 2.83E-26 | -0.238076148 | 1.50E-15 | 0.08 |
| CACNG1 | -0.152635261 | 3.97E-07 | -0.069932131 | 0.0207677 | 0.09 |
| CACNG2 | 0.051339304 | 0.08979457 | -0.056066098 | 0.0638949 | 0.11 |
| CACNG3 | 0.073421034 | 0.01518906 | 0.023897826 | 0.4299461 | 0.05 |
| CACNG4 | -0.198056486 | 3.95E-11 | -0.065550672 | 0.0302354 | 0.14 |
| CACNG5 | 0.121668528 | 5.51E-05 | 0.017880309 | 0.554853 | 0.11 |
| CACNG6 | 0.024236513 | 0.42343669 | -0.03815887 | 0.2074642 | 0.07 |
| CACNG7 | 0.006660665 | 0.82590564 | -0.039059805 | 0.196928 | 0.05 |
| CACNG8 | -0.071155733 | 0.01863573 | -0.078056916 | 0.0098346 | 0.01 |
| CACYBP | 0.53983318 | 1.09E-83 | 0.617022058 | 1.17E-115 | 0.08 |
| TSLC1 | 0.001355451 | 0.96429804 | -0.041069601 | 0.174844 | 0.05 |
| CADM2 | -0.198849221 | 3.29E-11 | -0.196973902 | 5.06E-11 | 0.01 |
| CADM3 | -0.205561323 | 6.81E-12 | -0.212257392 | 1.34E-12 | 0.01 |
| CADM4 | -0.00428447 | 0.8874864 | 0.042478582 | 0.1605 | 0.05 |
| CADPS2 | -0.389814963 | 5.52E-41 | -0.175583159 | 5.11E-09 | 0.22 |
| CADPS | -0.189769924 | 2.54E-10 | -0.162468107 | 6.62E-08 | 0.03 |
| CAD | 0.162365486 | 6.75E-08 | -0.037263342 | 0.2183362 | 0.2 |
| CAGE1 | 0.262403423 | 1.14E-18 | 0.109570823 | 0.0002842 | 0.16 |
| CALB1 | 0.068046142 | 0.02446948 | -0.049409837 | 0.1025438 | 0.12 |
| CALB2 | 0.183950399 | 8.96E-10 | 0.043559571 | 0.1501112 | 0.15 |
| LOC87044 | 0.17950535 | 2.28E-09 | 0.069927167 | 0.0207768 | 0.11 |
| CALCB | 0.336281398 | 2.65E-30 | 0.087539535 | 0.0037752 | 0.25 |
| CALCOCO1 | -0.518857051 | 2.32E-76 | -0.320900859 | 1.34E-27 | 0.2 |
| CALCOCO2 | -0.22477542 | 5.51E-14 | -0.109465298 | 0.0002881 | 0.12 |
| CALCRL | -0.42324401 | 9.76E-49 | -0.306250659 | 3.64E-25 | 0.12 |
| CTR | -0.289402931 | 1.57E-22 | -0.219870674 | 1.97E-13 | 0.07 |
| LOC55873 | -0.127191027 | 2.47E-05 | -0.153813975 | 3.23E-07 | 0.03 |
| LOC399807 | -0.030561215 | 0.31275874 | -0.082330458 | 0.0064614 | 0.06 |
| CALHM2 | -0.213147827 | 1.07E-12 | -0.152865675 | 3.82E-07 | 0.07 |
| CALHM3 | 0.149592425 | 6.77E-07 | 0.004400713 | 0.8844549 | 0.15 |
| CALM1 | 0.047123192 | 0.11947024 | -0.001670168 | 0.9560162 | 0.05 |
| CALM2 | 0.257157829 | 5.73E-18 | 0.361176339 | 5.11E-35 | 0.11 |
| CALM3 | 0.131674006 | 1.26E-05 | 0.031216924 | 0.302485 | 0.11 |
| CALML3 | -0.11303571 | 0.00018057 | -0.068598488 | 0.0233304 | 0.05 |
| CALML4 | 0.155006318 | 2.61E-07 | 0.088698838 | 0.0033369 | 0.07 |
| CALML5 | 0.109415141 | 0.00029 | 0.149418304 | 6.97E-07 | 0.05 |
| CALML6 | 0.040318391 | 0.18287182 | 0.109731126 | 0.0002784 | 0.07 |
| CALN1 | -0.290972191 | 9.05E-23 | -0.241717291 | 5.38E-16 | 0.05 |
| CALR3 | 0.013037586 | 0.66679207 | 0.037082164 | 0.2205845 | 0.03 |
| CALR | 0.397403847 | 1.15E-42 | 0.207754446 | 4.02E-12 | 0.19 |
| CALU | 0.025471803 | 0.40018844 | -0.106159719 | 0.0004387 | 0.14 |
| CALY | 0.007555835 | 0.80296029 | 0.013572243 | 0.6539994 | 0.01 |
| LOC105376417 | 0.003996 | 0.89501646 | -0.134191696 | 8.53E-06 | 0.14 |
| CAMK1G | 0.196196436 | 6.04E-11 | 0.002687204 | 0.9292894 | 0.2 |
| CAMK1 | -0.049186425 | 0.10410858 | -0.073499133 | 0.015081 | 0.03 |
| KIAA0968 | -0.120453562 | 6.54E-05 | -0.08309326 | 0.0059828 | 0.04 |
| CAMK2B | -0.00891964 | 0.76833258 | -0.099741282 | 0.0009601 | 0.1 |
| CAMK2D | -0.053689031 | 0.07602385 | -0.064279897 | 0.0335954 | 0.02 |
| CAMK2G | -0.023272991 | 0.44210611 | -0.141118995 | 2.82E-06 | 0.12 |
| CAMK2N1 | -0.24734037 | 1.07E-16 | -0.134334887 | 8.34E-06 | 0.12 |
| CAMK2N2 | 0.206097429 | 5.99E-12 | 0.102587935 | 0.000682 | 0.11 |
| CAMK4 | -0.142231736 | 2.35E-06 | -0.232450908 | 7.08E-15 | 0.1 |
| CAMKK1 | -0.117821501 | 9.45E-05 | 0.047909599 | 0.1134182 | 0.17 |
| LOC121657 | -0.130032681 | 1.62E-05 | -0.130575331 | 1.49E-05 | 0.01 |
| CAMKV | 0.409558078 | 1.86E-45 | 0.17489763 | 5.87E-09 | 0.24 |
| CAMLG | -0.126202154 | 2.86E-05 | 0.102405505 | 0.0006973 | 0.23 |
| CAMP | 0.156605408 | 1.95E-07 | 0.210028678 | 2.31E-12 | 0.06 |
| CAMSAP2 | -0.096177144 | 0.0014555 | 0.08479573 | 0.0050279 | 0.19 |
| CAMSAP1 | -0.108211953 | 0.0003384 | -0.238284719 | 1.42E-15 | 0.14 |
| LOC100505935 | 0.298380134 | 6.51E-24 | 0.20297086 | 1.26E-11 | 0.1 |
| CAMTA2 | -0.336061623 | 2.90E-30 | -0.301039093 | 2.48E-24 | 0.04 |
| KIAA0829 | -0.108350315 | 0.00033247 | -0.108495585 | 0.0003264 | 0.01 |
| CAND2 | -0.012918516 | 0.6696544 | -0.073974125 | 0.0144379 | 0.07 |
| LOC102724315 | -0.168911111 | 1.93E-08 | -0.06379043 | 0.0349721 | 0.11 |
| CANX | -0.122723205 | 4.74E-05 | -0.153416145 | 3.46E-07 | 0.04 |
| CAP1 | -0.098307912 | 0.0011368 | -0.073602504 | 0.0149389 | 0.03 |
| CAP2 | -0.172842337 | 8.87E-09 | -0.02957409 | 0.328653 | 0.15 |
| CAPG | 0.231253041 | 9.80E-15 | 0.137622766 | 4.96E-06 | 0.1 |
| CAPN10 | 0.07265947 | 0.01627944 | 0.032925985 | 0.2767715 | 0.04 |
| CAPN11 | -0.253884488 | 1.54E-17 | -0.115041048 | 0.0001381 | 0.14 |
| CAPN12 | 0.272218638 | 5.05E-20 | 0.170662205 | 1.37E-08 | 0.11 |
| CAPN13 | -0.356865667 | 3.59E-34 | -0.024982059 | 0.4093124 | 0.34 |
| CAPN14 | 0.059547039 | 0.04905056 | -0.011201923 | 0.711435 | 0.08 |
| CAPN1 | -0.015189676 | 0.61592545 | -0.091842588 | 0.0023711 | 0.08 |
| CAPN2 | -0.158231684 | 1.45E-07 | 0.024484293 | 0.4187112 | 0.19 |
| MGC10767 | -0.091384517 | 0.00249378 | -0.1265778 | 2.71E-05 | 0.04 |
| VRNI | -0.121028041 | 6.04E-05 | -0.180797338 | 1.74E-09 | 0.06 |
| CANPX | 0.059422346 | 0.0495274 | -0.051585998 | 0.0882606 | 0.12 |
| CAPN7 | -0.303593204 | 9.74E-25 | -0.23144557 | 9.31E-15 | 0.08 |
| LOC644151 | -0.456293762 | 2.60E-57 | -0.123399005 | 4.30E-05 | 0.34 |
| GC36 | -0.330756959 | 2.58E-29 | 0.058829749 | 0.0518469 | 0.39 |
| CAPNS1 | 0.177432449 | 3.50E-09 | 0.198680397 | 3.42E-11 | 0.03 |
| LOC115646 | 0.142380449 | 2.29E-06 | -0.023001221 | 0.4474558 | 0.17 |
| CAPRIN1 | -0.105079596 | 0.00050207 | -0.098996698 | 0.0010484 | 0.01 |
| CAPRIN2 | -0.068146454 | 0.0242591 | -0.171499872 | 1.16E-08 | 0.11 |
| CAPS2 | -0.30668752 | 3.10E-25 | -0.238456448 | 1.35E-15 | 0.07 |
| CAPSL | -0.108497242 | 0.00032628 | 0.049634657 | 0.100988 | 0.16 |
| CAPS | 0.230943664 | 1.07E-14 | 0.193216263 | 1.18E-10 | 0.04 |
| CAPZA1 | 0.065196884 | 0.03114057 | 0.030322669 | 0.3165526 | 0.04 |
| CAPZA2 | -0.239939558 | 8.90E-16 | -0.140736481 | 3.00E-06 | 0.1 |
| CAPZA3 | 0.034652962 | 0.2523422 | 0.022300678 | 0.4614139 | 0.02 |
| CAPZB | 0.123296473 | 4.37E-05 | 0.021202675 | 0.483772 | 0.11 |
| CARD10 | -0.329963082 | 3.57E-29 | -0.184117089 | 8.65E-10 | 0.15 |
| LOC102723892 | -0.159454053 | 1.16E-07 | -0.219798867 | 2.01E-13 | 0.07 |
| PSORS2 | -0.105278655 | 0.00048979 | 0.01378237 | 0.6489992 | 0.12 |
| LOC100421557 | 0.140658178 | 3.04E-06 | 0.076861524 | 0.011024 | 0.07 |
| CARD17 | 0.136550954 | 5.88E-06 | -0.008865998 | 0.7696864 | 0.15 |
| CARD18 | 0.071637097 | 0.01785094 | -0.019919619 | 0.5106247 | 0.1 |
| CARD6 | -0.136595889 | 5.84E-06 | -0.216661693 | 4.46E-13 | 0.09 |
| LOC102723980 | -0.292199078 | 5.89E-23 | -0.274302193 | 2.56E-20 | 0.02 |
| CARD9 | 0.173419883 | 7.90E-09 | 0.019873988 | 0.5115938 | 0.16 |
| CARHSP1 | 0.378698164 | 1.35E-38 | 0.313069771 | 2.79E-26 | 0.07 |
| LOC51254 | -0.1125127 | 0.00019353 | -0.04418287 | 0.1443584 | 0.07 |
| CARM1 | 0.331197848 | 2.16E-29 | 0.189349573 | 2.79E-10 | 0.15 |
| CARNS1 | -0.110538848 | 0.00025072 | -0.144574352 | 1.59E-06 | 0.04 |
| CARS2 | 0.305971041 | 4.04E-25 | 0.213891933 | 8.92E-13 | 0.1 |
| CARS | 0.248436686 | 7.75E-17 | 0.137015698 | 5.47E-06 | 0.12 |
| CARTPT | -0.079068062 | 0.00891915 | -0.086507951 | 0.0042085 | 0.01 |
| CASC1 | -0.362381366 | 2.95E-35 | -0.086827771 | 0.0040696 | 0.28 |
| CASC2 | -0.205483008 | 6.94E-12 | -0.047767454 | 0.1144939 | 0.16 |
| CASC3 | -0.079064369 | 0.00892235 | -0.015719256 | 0.6036738 | 0.07 |
| CASC4 | -0.480673854 | 2.87E-64 | -0.362291353 | 3.07E-35 | 0.12 |
| CASC5 | 0.03413837 | 0.25945908 | -0.123018201 | 4.54E-05 | 0.16 |
| LOC85885 | -0.398780353 | 5.61E-43 | -0.149813142 | 6.51E-07 | 0.25 |
| CASKIN1 | -0.06963972 | 0.02130762 | -0.116587243 | 0.0001119 | 0.05 |
| CASKIN2 | -0.083600228 | 0.0056827 | -0.213304358 | 1.03E-12 | 0.13 |
| FGS4 | 0.042500023 | 0.16028883 | -0.097087367 | 0.0013104 | 0.14 |
| CASP10 | -0.177419772 | 3.51E-09 | -0.206739762 | 5.13E-12 | 0.03 |
| CASP12 | -0.292867974 | 4.65E-23 | -0.171735221 | 1.11E-08 | 0.13 |
| CASP14 | 0.103389006 | 0.00061851 | 0.026489607 | 0.3816216 | 0.08 |
| CASP1 | -0.0090768 | 0.76437029 | -0.050242742 | 0.0968741 | 0.05 |
| CASP2 | 0.216372051 | 4.79E-13 | 0.086969145 | 0.0040095 | 0.13 |
| CASP3 | 0.137110268 | 5.38E-06 | 0.082170208 | 0.0065662 | 0.06 |
| CASP4 | -0.045282721 | 0.13462078 | 0.013620247 | 0.6528558 | 0.06 |
| CASP5 | 0.169430587 | 1.74E-08 | -0.041933282 | 0.1659423 | 0.22 |
| CASP6 | -0.009324125 | 0.75814725 | 0.209479724 | 2.65E-12 | 0.22 |
| CASP7 | -0.19027292 | 2.28E-10 | -0.141752348 | 2.54E-06 | 0.05 |
| FLJ11208 | -0.086393886 | 0.00425908 | -0.163635464 | 5.32E-08 | 0.08 |
| LOC116239 | -0.224537268 | 5.87E-14 | -0.103593163 | 0.0006032 | 0.13 |
| CASP9 | -0.195182945 | 7.61E-11 | -0.147494041 | 9.71E-07 | 0.05 |
| CASQ1 | -0.269079248 | 1.39E-19 | -0.095261608 | 0.0016162 | 0.18 |
| CASQ2 | -0.369361916 | 1.16E-36 | -0.22700423 | 3.06E-14 | 0.15 |
| FIH | 0.050664283 | 0.09410158 | 0.012485143 | 0.6801126 | 0.04 |
| CASS4 | -0.302365241 | 1.53E-24 | -0.393068038 | 1.06E-41 | 0.1 |
| LOC389311 | -0.328592897 | 6.23E-29 | -0.156377139 | 2.04E-07 | 0.18 |
| SRG | -0.455906336 | 3.33E-57 | -0.325164963 | 2.47E-28 | 0.14 |
| CATSPER1 | 0.084229597 | 0.00532899 | 0.025630105 | 0.3972656 | 0.06 |
| CATSPER2P1 | -0.119403771 | 7.58E-05 | -0.101041468 | 0.0008221 | 0.02 |
| CATSPER2 | -0.13124049 | 1.34E-05 | -0.043536909 | 0.1503236 | 0.09 |
| CATSPER3 | -0.082639049 | 0.00626379 | 0.10138008 | 0.0007894 | 0.19 |
| CATSPER4 | 0.010864025 | 0.71976696 | 0.012863167 | 0.6709866 | 0.01 |
| CATSPERB | -0.222645421 | 9.62E-14 | 0.035358667 | 0.2428049 | 0.26 |
| CATSPERG | -0.322458268 | 7.25E-28 | -0.145902328 | 1.27E-06 | 0.18 |
| CAT | -0.256602001 | 6.79E-18 | -0.141250168 | 2.76E-06 | 0.12 |
| CAV1 | -0.286758527 | 3.91E-22 | -0.239692122 | 9.54E-16 | 0.05 |
| CAV2 | -0.132198047 | 1.16E-05 | -0.170322903 | 1.46E-08 | 0.04 |
| CAV3 | -0.07185226 | 0.0175096 | -0.050005212 | 0.0984649 | 0.03 |
| DKFZp564C246 | -0.021947253 | 0.46854692 | 0.061610662 | 0.0417015 | 0.09 |
| CBFA2T2 | -0.222139826 | 1.10E-13 | -0.218476173 | 2.81E-13 | 0.01 |
| CBFA2T3 | -0.227388397 | 2.76E-14 | -0.202930049 | 1.27E-11 | 0.03 |
| CBFB | 0.252001663 | 2.70E-17 | 0.053575361 | 0.0766479 | 0.2 |
| CBLB | -0.246989929 | 1.18E-16 | -0.271719287 | 5.93E-20 | 0.03 |
| CBLC | -0.004375322 | 0.88511693 | 0.039332422 | 0.1938182 | 0.05 |
| CBLL1 | -0.098349439 | 0.00113128 | -0.066644856 | 0.0275775 | 0.04 |
| CBLN1 | -0.148551911 | 8.10E-07 | -0.124245893 | 3.81E-05 | 0.03 |
| LOC125688 | -0.046498306 | 0.12445707 | -0.054588682 | 0.0712294 | 0.01 |
| LOC651052 | -0.156827847 | 1.88E-07 | -0.178736506 | 2.68E-09 | 0.03 |
| CBLN4 | -0.45871743 | 5.61E-58 | -0.224517311 | 5.90E-14 | 0.24 |
| CBL | -0.235785455 | 2.84E-15 | -0.328726302 | 5.90E-29 | 0.1 |
| CBR1 | 0.228664486 | 1.97E-14 | 0.06603319 | 0.0290374 | 0.17 |
| CBR3 | 0.203318307 | 1.16E-11 | 0.243607773 | 3.14E-16 | 0.05 |
| CBR4 | -0.271572637 | 6.22E-20 | -0.144449663 | 1.62E-06 | 0.13 |
| CBS | 0.324170212 | 3.68E-28 | 0.110203072 | 0.0002619 | 0.22 |
| LOC57397 | 0.136448763 | 5.98E-06 | 0.180598805 | 1.82E-09 | 0.05 |
| LOC100653334 | 0.135820077 | 6.60E-06 | 0.237089548 | 1.98E-15 | 0.11 |
| CBWD3 | 0.068120671 | 0.02431302 | 0.105466111 | 0.0004785 | 0.04 |
| LOC375742 | -0.007707076 | 0.79909973 | -0.031129222 | 0.303846 | 0.03 |
| LOC100506607 | 0.18696901 | 4.69E-10 | 0.17908717 | 2.49E-09 | 0.01 |
| CBX1 | 0.239649122 | 9.65E-16 | 0.19583785 | 6.56E-11 | 0.05 |
| CDCA6 | 0.437478542 | 2.59E-52 | 0.228575704 | 2.01E-14 | 0.21 |
| HECH | 0.400438793 | 2.36E-43 | 0.428024833 | 6.42E-50 | 0.03 |
| CBX4 | -0.086834899 | 0.00406655 | 0.01279471 | 0.6726357 | 0.1 |
| CBX5 | -0.091740392 | 0.00239802 | -0.080089317 | 0.0080725 | 0.02 |
| CBX6 | -0.424212615 | 5.64E-49 | -0.526774447 | 4.56E-79 | 0.11 |
| CBX7 | -0.475417424 | 1.01E-62 | -0.412946476 | 2.97E-46 | 0.07 |
| LOC63441 | 0.216043276 | 5.21E-13 | 0.196564357 | 5.56E-11 | 0.02 |
| CBY1 | 0.230240338 | 1.29E-14 | 0.246103523 | 1.53E-16 | 0.02 |
| MRT3 | 0.060792134 | 0.04449681 | 0.012448796 | 0.6809926 | 0.05 |
| CC2D1B | 0.207799064 | 3.98E-12 | 0.008507439 | 0.7787535 | 0.2 |
| CC2D2A | -0.174015563 | 7.01E-09 | -0.147517744 | 9.67E-07 | 0.03 |
| CC2D2B | -0.219238267 | 2.32E-13 | -0.122563896 | 4.85E-05 | 0.1 |
| CCAR1 | 0.020912124 | 0.48978504 | -0.023696181 | 0.433849 | 0.05 |
| CCBE1 | -0.154583184 | 2.81E-07 | -0.09712041 | 0.0013054 | 0.06 |
| CCBL1 | -0.068917184 | 0.02269428 | 0.004038916 | 0.8938956 | 0.08 |
| CCBL2 | -0.091117322 | 0.00256796 | -0.044558161 | 0.1409769 | 0.05 |
| ACKR2 | -0.192809996 | 1.30E-10 | -0.098235526 | 0.0011465 | 0.1 |
| CCDC101 | 0.193939324 | 1.01E-10 | 0.256329098 | 7.38E-18 | 0.07 |
| CCDC102A | 0.197565689 | 4.42E-11 | 0.052527976 | 0.0825965 | 0.15 |
| CCDC102B | -0.051714431 | 0.08747044 | -0.114955399 | 0.0001397 | 0.07 |
| CCDC103 | -0.22847442 | 2.07E-14 | -0.076878177 | 0.0110066 | 0.16 |
| CFAP36 | 0.077692411 | 0.01018456 | 0.136669957 | 5.77E-06 | 0.06 |
| CCDC105 | 0.0752005 | 0.01288808 | 0.006140578 | 0.8393066 | 0.07 |
| CCDC106 | -0.070792809 | 0.01924719 | 0.002462832 | 0.9351799 | 0.08 |
| CCDC107 | 0.267382447 | 2.39E-19 | 0.184651498 | 7.72E-10 | 0.09 |
| MGC35338 | -0.025433844 | 0.40089121 | 0.05474018 | 0.0704469 | 0.09 |
| MCU | 0.098474259 | 0.00111485 | 0.010687822 | 0.7241251 | 0.09 |
| CCDC109B | 0.153191223 | 3.60E-07 | 0.108934453 | 0.0003085 | 0.05 |
| CCDC110 | -0.196251108 | 5.97E-11 | -0.068393622 | 0.0237474 | 0.13 |
| PRIMPOL | -0.314176404 | 1.83E-26 | -0.212471145 | 1.27E-12 | 0.11 |
| CCDC112 | 0.094867643 | 0.00169022 | 0.110699208 | 0.0002455 | 0.02 |
| FLJ23555 | -0.187802918 | 3.91E-10 | -0.045667159 | 0.1313394 | 0.15 |
| CCDC114 | -0.063569022 | 0.03561046 | 0.044692522 | 0.1397811 | 0.11 |
| CCDC115 | -0.097365763 | 0.00126881 | -0.026027992 | 0.3899761 | 0.08 |
| CCDC116 | -0.110408406 | 0.00025501 | -0.113584244 | 0.0001679 | 0.01 |
| CCDC117 | -0.021891915 | 0.46966928 | -0.062403008 | 0.0391385 | 0.05 |
| LOC147549 | -0.058042733 | 0.0550671 | 0.030155637 | 0.319227 | 0.09 |
| CCDC120 | -0.074645999 | 0.01356964 | -0.041340436 | 0.1720151 | 0.04 |
| CCDC121 | -0.194229335 | 9.43E-11 | -0.213977398 | 8.74E-13 | 0.02 |
| CCDC122 | -0.100390764 | 0.00088871 | -0.078040178 | 0.0098504 | 0.03 |
| CEP89 | 0.262835246 | 9.98E-19 | 0.130491416 | 1.51E-05 | 0.14 |
| CCDC124 | 0.337131328 | 1.86E-30 | 0.239931163 | 8.92E-16 | 0.1 |
| CCDC125 | -0.408682808 | 2.99E-45 | -0.054090303 | 0.0738539 | 0.36 |
| CCDC126 | -0.374651511 | 9.45E-38 | -0.207146038 | 4.66E-12 | 0.17 |
| CCDC127 | 0.066110693 | 0.02884882 | 0.190366232 | 2.23E-10 | 0.13 |
| CCDC129 | 0.10948108 | 0.00028755 | -0.052947922 | 0.080168 | 0.17 |
| CCDC12 | 0.249191158 | 6.21E-17 | 0.266386186 | 3.27E-19 | 0.02 |
| CCDC130 | 0.087782864 | 0.00367914 | 0.090447906 | 0.0027627 | 0.01 |
| FLJ23581 | -0.353824378 | 1.40E-33 | -0.173024113 | 8.55E-09 | 0.19 |
| CCDC134 | 0.110094255 | 0.00026563 | 0.021926981 | 0.4689579 | 0.09 |
| DRC7 | 0.11080756 | 0.0002421 | 0.136328153 | 6.10E-06 | 0.03 |
| CCDC136 | -0.09845485 | 0.00111739 | -0.227640869 | 2.59E-14 | 0.13 |
| CCDC137 | 0.409636437 | 1.79E-45 | 0.225653757 | 4.38E-14 | 0.19 |
| CCDC138 | 0.289380774 | 1.58E-22 | 0.138557597 | 4.27E-06 | 0.16 |
| CCDC13 | -0.092301962 | 0.00225372 | -0.050626211 | 0.0943493 | 0.05 |
| CCDC140 | 0.025330645 | 0.4028056 | -0.043523662 | 0.1504479 | 0.07 |
| CCDC141 | -0.157116957 | 1.78E-07 | -0.22922616 | 1.69E-14 | 0.08 |
| CCDC142 | -0.014396572 | 0.63447438 | -0.009696737 | 0.7488014 | 0.01 |
| CCDC144A | -0.289242196 | 1.66E-22 | -0.283168989 | 1.33E-21 | 0.01 |
| CCDC144B | -0.262439919 | 1.13E-18 | -0.29527514 | 1.98E-23 | 0.04 |
| LOC256207 | -0.000499392 | 0.98684245 | -0.149950944 | 6.36E-07 | 0.15 |
| CCDC144NL | -0.002510589 | 0.9339258 | -0.100827083 | 0.0008436 | 0.1 |
| CCDC146 | -0.295326038 | 1.95E-23 | -0.186148869 | 5.60E-10 | 0.11 |
| CFAP58 | 0.007491832 | 0.80459547 | 0.059063726 | 0.0509205 | 0.06 |
| CCDC148 | -0.344855607 | 7.03E-32 | -0.211699216 | 1.54E-12 | 0.14 |
| CCDC149 | -0.308453642 | 1.60E-25 | -0.160269259 | 9.98E-08 | 0.15 |
| CCDC14 | 0.151153363 | 5.16E-07 | 0.067237245 | 0.0262244 | 0.09 |
| CCDC150 | 0.466328687 | 4.17E-60 | 0.240293004 | 8.05E-16 | 0.23 |
| CCDC151 | -0.045994568 | 0.12859391 | 0.083277484 | 0.0058722 | 0.13 |
| CCDC152 | -0.23720706 | 1.91E-15 | -0.079444022 | 0.0085987 | 0.16 |
| CCDC153 | -0.108406599 | 0.00033009 | 0.105293669 | 0.0004889 | 0.22 |
| CCDC154 | -0.008053241 | 0.79028216 | 0.04816507 | 0.1115049 | 0.06 |
| CCDC155 | 0.165326621 | 3.85E-08 | 0.110833445 | 0.0002413 | 0.06 |
| CCDC157 | -0.186425686 | 5.27E-10 | -0.045309195 | 0.1343928 | 0.15 |
| CCDC158 | -0.371675472 | 3.89E-37 | -0.287255407 | 3.30E-22 | 0.09 |
| CCDC159 | -0.227906603 | 2.41E-14 | 0.009653525 | 0.7498834 | 0.24 |
| CCDC15 | -0.180394601 | 1.90E-09 | -0.076013631 | 0.0119433 | 0.11 |
| CCDC160 | -0.348670457 | 1.35E-32 | -0.02583587 | 0.3934857 | 0.33 |
| CCDC163P | -0.122965357 | 4.58E-05 | -0.267182537 | 2.54E-19 | 0.15 |
| CCDC17 | 0.071195736 | 0.01856938 | 0.081861142 | 0.0067726 | 0.02 |
| CCDC18 | 0.377777817 | 2.10E-38 | 0.069800421 | 0.0210094 | 0.31 |
| CFAP45 | -0.041698687 | 0.1683258 | 0.246153671 | 1.51E-16 | 0.29 |
| CEP85 | 0.097204928 | 0.00129271 | -0.161355389 | 8.16E-08 | 0.26 |
| CCDC22 | 0.071615862 | 0.01788494 | 0.10128457 | 0.0007985 | 0.03 |
| CCDC23 | 0.380646969 | 5.22E-39 | 0.273485538 | 3.34E-20 | 0.11 |
| CCDC24 | -0.150995177 | 5.30E-07 | 0.036937436 | 0.2223925 | 0.19 |
| CCDC25 | -0.18753859 | 4.14E-10 | -0.069817121 | 0.0209786 | 0.12 |
| CCDC27 | 0.032493386 | 0.28313499 | -0.054517288 | 0.0716006 | 0.09 |
| CCDC28A | -0.078551535 | 0.00937683 | 0.09684091 | 0.0013483 | 0.18 |
| CCDC28B | 0.321042692 | 1.27E-27 | 0.16340859 | 5.55E-08 | 0.16 |
| CCDC30 | -0.351941775 | 3.21E-33 | -0.141691915 | 2.57E-06 | 0.22 |
| LOC342097 | 0.009890129 | 0.74396518 | 0.091500414 | 0.0024622 | 0.09 |
| CCDC34 | 0.384414634 | 8.19E-40 | 0.227564441 | 2.64E-14 | 0.16 |
| CCDC36 | -0.370960155 | 5.46E-37 | -0.253461634 | 1.75E-17 | 0.12 |
| CCDC37 | 0.114740129 | 0.00014377 | 0.067618602 | 0.0253839 | 0.05 |
| CCDC38 | 0.086345191 | 0.00428084 | 0.022962642 | 0.4482182 | 0.07 |
| CCDC39 | -0.422688304 | 1.34E-48 | -0.258177121 | 4.20E-18 | 0.17 |
| LOC644822 | -0.121463765 | 5.67E-05 | -0.135995864 | 6.42E-06 | 0.02 |
| CCDC40 | -0.313498288 | 2.37E-26 | -0.138165245 | 4.55E-06 | 0.18 |
| CEP83 | 0.080481571 | 0.00776687 | 0.055300625 | 0.0676133 | 0.03 |
| CCDC42B | 0.137456672 | 5.09E-06 | 0.156403964 | 2.03E-07 | 0.02 |
| CCDC42 | 0.013303534 | 0.6604164 | -0.075125722 | 0.0129782 | 0.09 |
| CCDC43 | 0.116305485 | 0.00011629 | 0.121567937 | 5.59E-05 | 0.01 |
| CEP95 | 0.205437277 | 7.01E-12 | 0.117573134 | 9.78E-05 | 0.09 |
| CEP112 | -0.257123723 | 5.79E-18 | -0.244801623 | 2.23E-16 | 0.02 |
| CCDC47 | -0.022094193 | 0.46557392 | -0.059187558 | 0.0504358 | 0.04 |
| C3orf73 | -0.323023776 | 5.80E-28 | 0.007611735 | 0.8015328 | 0.34 |
| DFNA44 | -0.064194397 | 0.03383247 | -0.194777512 | 8.34E-11 | 0.14 |
| CCDC51 | 0.164908164 | 4.17E-08 | 0.168421667 | 2.12E-08 | 0.01 |
| SPICE1 | -0.27416169 | 2.68E-20 | -0.146587301 | 1.13E-06 | 0.13 |
| CCDC53 | 0.098056968 | 0.00117066 | 0.096386654 | 0.0014209 | 0.01 |
| CCDC54 | -0.115973298 | 0.00012167 | -0.044386862 | 0.1425127 | 0.08 |
| NSRP1 | -0.121362326 | 5.76E-05 | -0.068246328 | 0.0240512 | 0.06 |
| COA3 | 0.130917501 | 1.41E-05 | 0.286032483 | 5.02E-22 | 0.16 |
| FLJ23754 | -0.079747179 | 0.00834776 | -0.021469326 | 0.4782889 | 0.06 |
| CCDC58 | 0.603667148 | 1.71E-109 | 0.619755094 | 5.88E-117 | 0.02 |
| CCDC59 | 0.554780555 | 3.09E-89 | 0.411098094 | 8.11E-46 | 0.15 |
| CCDC60 | -0.089490708 | 0.00306473 | -0.004650637 | 0.8779429 | 0.09 |
| LOC732172 | 0.043524697 | 0.1504382 | 0.119086205 | 7.93E-05 | 0.08 |
| CCDC62 | -0.127747422 | 2.28E-05 | -0.229374239 | 1.63E-14 | 0.11 |
| CCDC63 | 0.115893496 | 0.000123 | 0.079515925 | 0.0085386 | 0.04 |
| CCDC64B | -0.066370231 | 0.02822497 | 0.060849315 | 0.0442965 | 0.13 |
| CCDC64 | -0.089350719 | 0.00311133 | -0.105616927 | 0.0004696 | 0.02 |
| CCDC65 | -0.250284151 | 4.50E-17 | -0.011901659 | 0.6942906 | 0.24 |
| CCDC66 | -0.176034625 | 4.66E-09 | -0.155985104 | 2.19E-07 | 0.03 |
| CCDC67 | 0.193655508 | 1.07E-10 | 0.061208088 | 0.0430572 | 0.14 |
| CCDC68 | -0.235320466 | 3.23E-15 | -0.216125055 | 5.10E-13 | 0.02 |
| CCDC69 | -0.25445506 | 1.30E-17 | -0.330076605 | 3.41E-29 | 0.08 |
| CCDC6 | -0.065304471 | 0.03086289 | 0.018398445 | 0.5434408 | 0.09 |
| CCDC70 | -0.039874184 | 0.18774548 | 0.054630772 | 0.0710113 | 0.1 |
| CCDC71 | -0.143611564 | 1.87E-06 | 0.000635357 | 0.9832606 | 0.15 |
| TMA7 | 0.346282924 | 3.80E-32 | 0.398320706 | 7.12E-43 | 0.06 |
| CCDC73 | 0.109867201 | 0.00027355 | 0.134558735 | 8.05E-06 | 0.03 |
| CCDC74A | -0.192659938 | 1.34E-10 | 0.011725386 | 0.6985952 | 0.21 |
| CCDC74B | -0.184874422 | 7.36E-10 | -0.012386143 | 0.6825104 | 0.18 |
| GPATCH11 | -0.26819482 | 1.84E-19 | -0.364996631 | 8.84E-36 | 0.1 |
| FLJ11219 | -0.092535256 | 0.00219615 | -0.101054862 | 0.0008208 | 0.01 |
| CCDC77 | 0.504815837 | 9.80E-72 | 0.257351523 | 5.41E-18 | 0.25 |
| CCDC78 | 0.009763026 | 0.74714259 | 0.153088785 | 3.67E-07 | 0.15 |
| CCDC79 | 0.018095839 | 0.55009166 | 0.021656777 | 0.4744548 | 0.01 |
| CCDC7.1 | -0.174113237 | 6.87E-09 | -0.027718794 | 0.3599156 | 0.15 |
| CCDC80 | -0.323051545 | 5.73E-28 | -0.218663932 | 2.68E-13 | 0.11 |
| CCDC81 | 0.134475352 | 8.16E-06 | 0.092568049 | 0.0021882 | 0.05 |
| HSPC048 | 0.077909153 | 0.00997515 | -0.125577361 | 3.14E-05 | 0.21 |
| CCDC83 | -0.129909458 | 1.65E-05 | -0.001344828 | 0.9645777 | 0.13 |
| CCDC84 | 0.162698511 | 6.34E-08 | 0.11151713 | 0.0002206 | 0.06 |
| CCDC85A | -0.265968536 | 3.73E-19 | -0.259019133 | 3.25E-18 | 0.01 |
| CCDC85B | 0.206392676 | 5.58E-12 | 0.126909977 | 2.58E-05 | 0.08 |
| CCDC85C | 0.065236779 | 0.03103735 | 0.045971476 | 0.1287861 | 0.02 |
| CCDC86 | 0.455883958 | 3.37E-57 | 0.256219017 | 7.63E-18 | 0.2 |
| CCDC87 | -0.212808937 | 1.17E-12 | -0.05712528 | 0.0590294 | 0.16 |
| CCDC88A | 0.011460644 | 0.70507844 | -0.25813581 | 4.26E-18 | 0.27 |
| DKFZp434G0920 | 0.038289367 | 0.2059133 | -0.05252433 | 0.0826179 | 0.1 |
| CCDC88C | -0.218159436 | 3.05E-13 | -0.184761178 | 7.54E-10 | 0.04 |
| CCDC89 | -0.141795705 | 2.52E-06 | -0.183967476 | 8.93E-10 | 0.05 |
| CCDC8 | -0.155007054 | 2.61E-07 | -0.116001358 | 0.0001212 | 0.04 |
| LOC100506556 | 0.479164764 | 8.04E-64 | 0.321377499 | 1.11E-27 | 0.16 |
| CCDC90B | 0.182996394 | 1.10E-09 | 0.144443902 | 1.63E-06 | 0.04 |
| CCDC91 | -0.069631115 | 0.02132369 | -0.067786562 | 0.0250212 | 0.01 |
| CCDC92 | 0.008099689 | 0.78910103 | 0.031618465 | 0.2963055 | 0.03 |
| MGC13033 | -0.267635737 | 2.20E-19 | -0.355417287 | 6.87E-34 | 0.09 |
| CCDC94 | 0.167979631 | 2.31E-08 | 0.224280859 | 6.28E-14 | 0.06 |
| CCDC96 | -0.25505546 | 1.08E-17 | -0.010537242 | 0.7278567 | 0.25 |
| CCDC97 | -0.190142145 | 2.34E-10 | -0.183770647 | 9.31E-10 | 0.01 |
| SPDL1 | 0.440851214 | 3.47E-53 | 0.201160784 | 1.93E-11 | 0.24 |
| CCDC9 | -0.019845714 | 0.51219478 | -0.026375996 | 0.3836675 | 0.01 |
| CCHCR1 | 0.376423387 | 4.04E-38 | 0.26525457 | 4.68E-19 | 0.12 |
| LOC92435 | -0.064381393 | 0.03331578 | 0.002835141 | 0.9254076 | 0.07 |
| CCKAR | -0.368443944 | 1.78E-36 | -0.215102276 | 6.60E-13 | 0.16 |
| CCKBR | 0.358600722 | 1.64E-34 | 0.092621371 | 0.0021752 | 0.27 |
| CCK | 0.170279131 | 1.47E-08 | 0.036168241 | 0.232179 | 0.14 |
| CCL11 | 0.079593596 | 0.00847404 | 0.070798702 | 0.0192371 | 0.01 |
| CCL13 | 0.21732593 | 3.77E-13 | -0.023703105 | 0.4337146 | 0.25 |
| CCL15.CCL14 | 0.017339798 | 0.56688145 | 0.045505901 | 0.1327082 | 0.03 |
| CCL14 | -0.288569323 | 2.09E-22 | -0.169767067 | 1.63E-08 | 0.12 |
| CCL15 | 0.028564907 | 0.34543367 | 0.094131539 | 0.001837 | 0.07 |
| CCL16 | -0.289424921 | 1.55E-22 | -0.173211133 | 8.24E-09 | 0.12 |
| CCL17 | 0.085870572 | 0.00449824 | 0.008569997 | 0.7771693 | 0.08 |
| CCL18 | 0.263181486 | 8.96E-19 | 0.030129064 | 0.3196538 | 0.24 |
| CCL19 | -0.022506145 | 0.45729505 | -0.076622734 | 0.0112763 | 0.06 |
| CCL1 | 0.121944963 | 5.30E-05 | -0.00824192 | 0.7854873 | 0.14 |
| CCL20 | 0.321385757 | 1.11E-27 | 0.163414752 | 5.54E-08 | 0.16 |
| CCL21 | -0.108671665 | 0.00031908 | -0.13018488 | 1.58E-05 | 0.03 |
| CCL22 | -0.067554485 | 0.02552359 | -0.060458706 | 0.0456799 | 0.01 |
| CCL23 | -0.015078203 | 0.61851816 | -0.098601621 | 0.0010983 | 0.09 |
| CCL24 | 0.020447908 | 0.49947491 | -0.105580762 | 0.0004717 | 0.13 |
| CCL25 | 0.23424085 | 4.34E-15 | 0.026807271 | 0.3759365 | 0.21 |
| LOC155403 | 0.18245667 | 1.23E-09 | 0.098650448 | 0.001092 | 0.09 |
| CCL27 | 0.09108541 | 0.00257696 | 0.066125558 | 0.0288128 | 0.03 |
| CCL28 | -0.159188034 | 1.22E-07 | -0.079844505 | 0.0082686 | 0.08 |
| CCL2 | 0.123176119 | 4.44E-05 | -0.036027031 | 0.2340083 | 0.16 |
| CCL3L1 | 0.018887523 | 0.53277665 | 0.012555229 | 0.6784171 | 0.01 |
| CCL3L3 | -0.173211969 | 8.24E-09 | -0.12993672 | 1.64E-05 | 0.05 |
| CCL3 | 0.130011784 | 1.62E-05 | 0.014115147 | 0.6411126 | 0.12 |
| CCL4L1 | 0.086153057 | 0.00436767 | 0.000638191 | 0.983186 | 0.09 |
| LAG1 | 0.12008715 | 6.89E-05 | -0.014905122 | 0.6225532 | 0.14 |
| TCP228 | 0.135279076 | 7.19E-06 | 0.010554806 | 0.7274211 | 0.13 |
| CCL7 | 0.334895781 | 4.71E-30 | 0.134771035 | 7.79E-06 | 0.21 |
| LOC96488 | 0.221586778 | 1.27E-13 | 0.040693892 | 0.1788256 | 0.19 |
| CCM2 | 0.208840955 | 3.09E-12 | 0.13025203 | 1.56E-05 | 0.08 |
| CCNA1 | 0.168822368 | 1.96E-08 | -0.001987764 | 0.9476633 | 0.18 |
| CCNA2 | 0.518609722 | 2.81E-76 | 0.264113903 | 6.69E-19 | 0.26 |
| CCNB1IP1 | 0.454482326 | 8.14E-57 | 0.307344314 | 2.42E-25 | 0.15 |
| CCNB1 | 0.575593123 | 1.92E-97 | 0.40237523 | 8.56E-44 | 0.18 |
| CCNB2 | 0.57709828 | 4.64E-98 | 0.300943299 | 2.57E-24 | 0.28 |
| CCNB3 | -0.028292879 | 0.35004866 | -0.078906785 | 0.0090599 | 0.06 |
| CCNC | 0.158514206 | 1.38E-07 | 0.167809622 | 2.39E-08 | 0.01 |
| LOC88572 | -0.242318124 | 4.53E-16 | -0.174909692 | 5.85E-09 | 0.07 |
| CCND2 | -0.191199144 | 1.86E-10 | -0.193410318 | 1.13E-10 | 0.01 |
| CCND3 | 0.169140717 | 1.84E-08 | 0.017773169 | 0.5572274 | 0.16 |
| CCNDBP1 | -0.497429207 | 2.19E-69 | -0.332921213 | 1.06E-29 | 0.17 |
| CCNE1 | 0.55653289 | 6.63E-90 | 0.244190921 | 2.65E-16 | 0.32 |
| CCNE2 | 0.336097881 | 2.86E-30 | 0.222065404 | 1.12E-13 | 0.12 |
| CCNF | 0.38782404 | 1.50E-40 | 0.173094365 | 8.43E-09 | 0.22 |
| CCNG1 | -0.270423826 | 9.02E-20 | -0.093614962 | 0.0019468 | 0.18 |
| CCNG2 | -0.421025128 | 3.40E-48 | -0.194596448 | 8.69E-11 | 0.23 |
| CCNH | -0.217552627 | 3.56E-13 | -0.028569342 | 0.3453587 | 0.19 |
| LOC649438 | -0.079128361 | 0.00886704 | -0.017042714 | 0.5735454 | 0.07 |
| CCNI | -0.353270662 | 1.78E-33 | -0.324163394 | 3.69E-28 | 0.03 |
| CCNJL | 0.057295656 | 0.05827624 | 0.059567162 | 0.048974 | 0.01 |
| CCNJ | 0.258522895 | 3.78E-18 | 0.052322289 | 0.0838075 | 0.21 |
| CCNK | 0.078437268 | 0.00948086 | 0.064559976 | 0.0328286 | 0.02 |
| CCNL1 | 0.015048142 | 0.61921815 | -0.063555923 | 0.0356485 | 0.08 |
| LOC643556 | -0.07085793 | 0.0191362 | -0.071321233 | 0.0183626 | 0.01 |
| LOC133400 | -0.175136299 | 5.59E-09 | 0.061299546 | 0.042746 | 0.24 |
| HIVE1 | -0.433947504 | 2.07E-51 | -0.444053725 | 5.04E-54 | 0.02 |
| CCNT2 | -0.357419641 | 2.80E-34 | -0.304665998 | 6.56E-25 | 0.06 |
| CCNYL1 | -0.060071249 | 0.04708783 | -0.114294786 | 0.0001526 | 0.06 |
| CCNY | 0.242654508 | 4.12E-16 | 0.094051074 | 0.0018537 | 0.15 |
| KIAA1254 | -0.534182217 | 1.15E-81 | -0.412790945 | 3.23E-46 | 0.13 |
| CCR10 | -0.07170935 | 0.01773567 | -0.092838929 | 0.0021232 | 0.03 |
| CCR1 | 0.047698045 | 0.11502204 | -0.116240196 | 0.0001173 | 0.17 |
| LOC90262 | -0.108220283 | 0.00033804 | -0.198968457 | 3.20E-11 | 0.1 |
| CCR3 | -0.015640135 | 0.60549728 | -0.028555245 | 0.3455969 | 0.02 |
| CCR4 | -0.225782772 | 4.23E-14 | -0.291424147 | 7.73E-23 | 0.07 |
| LOC727797 | -0.046072672 | 0.1279456 | -0.153860555 | 3.20E-07 | 0.11 |
| CCR6 | -0.204729189 | 8.30E-12 | -0.150525309 | 5.75E-07 | 0.06 |
| CCR7 | -0.02421023 | 0.42393976 | -0.109530887 | 0.0002857 | 0.09 |
| CCR8 | -0.002282889 | 0.93990655 | -0.160718027 | 9.19E-08 | 0.16 |
| GPR28 | -0.051496627 | 0.08881389 | -0.160268767 | 9.99E-08 | 0.11 |
| ACKR4 | -0.099427445 | 0.00099646 | -0.066717642 | 0.0274081 | 0.04 |
| LOC727811 | -0.134340264 | 8.33E-06 | -0.126897749 | 2.58E-05 | 0.01 |
| CCRN4L | -0.050996313 | 0.09196281 | -0.242224679 | 4.66E-16 | 0.2 |
| CCS | 0.128475411 | 2.04E-05 | 0.162332474 | 6.80E-08 | 0.04 |
| CCT2 | 0.291975537 | 6.37E-23 | 0.23922128 | 1.09E-15 | 0.06 |
| LOC115637 | 0.537368801 | 8.41E-83 | 0.592575276 | 1.37E-104 | 0.06 |
| CCT4 | 0.456532541 | 2.24E-57 | 0.283964726 | 1.02E-21 | 0.18 |
| CCT5 | 0.551139388 | 7.37E-88 | 0.308297181 | 1.70E-25 | 0.25 |
| CCT6A | 0.452693089 | 2.49E-56 | 0.288064676 | 2.49E-22 | 0.17 |
| CCT6B | -0.12548754 | 3.18E-05 | -0.046433074 | 0.1249869 | 0.08 |
| LOC641740 | 0.181952582 | 1.37E-09 | 0.13381539 | 9.04E-06 | 0.05 |
| CCT7 | 0.532921231 | 3.23E-81 | 0.277852432 | 7.95E-21 | 0.26 |
| CCT8L2 | -0.031916714 | 0.29177062 | 0.006658219 | 0.8259685 | 0.04 |
| C21orf112 | 0.433245628 | 3.12E-51 | 0.346321964 | 3.74E-32 | 0.09 |
| CD101 | 0.061987848 | 0.04046426 | -0.032353708 | 0.2852106 | 0.1 |
| LOC135227 | -0.062540316 | 0.03870822 | -0.117232559 | 0.0001024 | 0.06 |
| CD14 | 0.078109878 | 0.00978465 | -0.072217881 | 0.0169427 | 0.16 |
| CD151 | -0.026588204 | 0.37985143 | 0.095826657 | 0.0015152 | 0.13 |
| LOC101060629 | -0.086588831 | 0.00417298 | -0.041701657 | 0.1682955 | 0.05 |
| CD163B | 0.058923523 | 0.05147394 | -0.020515875 | 0.4980499 | 0.08 |
| CD163 | -0.033626837 | 0.26667004 | -0.20727835 | 4.51E-12 | 0.18 |
| CD164L2 | 0.142849154 | 2.12E-06 | 0.03904681 | 0.1970771 | 0.11 |
| CD164 | -0.316511864 | 7.43E-27 | -0.0270442 | 0.3717306 | 0.29 |
| CD177 | -0.064993622 | 0.03167103 | -0.067543529 | 0.0255475 | 0.01 |
| CD180 | -0.006504765 | 0.82991756 | -0.144155816 | 1.71E-06 | 0.14 |
| CD19 | 0.063779751 | 0.03500265 | -0.04787255 | 0.1136978 | 0.12 |
| CD1A | 0.102283957 | 0.0007077 | -0.031084579 | 0.3045403 | 0.14 |
| CD1B | 0.006061051 | 0.84135995 | -0.1277957 | 2.26E-05 | 0.14 |
| CD1C | -0.145341711 | 1.40E-06 | -0.206059518 | 6.04E-12 | 0.07 |
| CD1D | -0.119370407 | 7.62E-05 | -0.148124925 | 8.71E-07 | 0.03 |
| HSCDIEL | -0.106572607 | 0.00041655 | -0.162895386 | 6.11E-08 | 0.06 |
| CD200R1L | -0.042394626 | 0.16132904 | -0.118062121 | 9.14E-05 | 0.08 |
| CD200R1 | -0.190279873 | 2.27E-10 | -0.27333644 | 3.51E-20 | 0.09 |
| LOC131511 | -0.230348157 | 1.25E-14 | -0.216924538 | 4.17E-13 | 0.02 |
| CD207 | 0.022090464 | 0.46564923 | -0.05557218 | 0.0662743 | 0.08 |
| CD209 | -0.084773496 | 0.00503945 | -0.242399262 | 4.43E-16 | 0.16 |
| CD226 | -0.123081157 | 4.50E-05 | -0.235413662 | 3.14E-15 | 0.12 |
| FLJ22814 | -0.28383916 | 1.06E-21 | -0.204956487 | 7.87E-12 | 0.08 |
| CD244 | 0.000128745 | 0.99660778 | -0.105108863 | 0.0005002 | 0.11 |
| CD247 | 0.052574253 | 0.08232603 | -0.060245304 | 0.0464509 | 0.12 |
| CD248 | -0.160620139 | 9.36E-08 | -0.113687056 | 0.0001656 | 0.05 |
| CD24 | 0.106907749 | 0.00039932 | 0.120413972 | 6.58E-05 | 0.02 |
| CD274 | -0.129155966 | 1.84E-05 | -0.25190101 | 2.79E-17 | 0.13 |
| CD276 | 0.029101004 | 0.33645266 | -0.009489945 | 0.7539837 | 0.04 |
| CD27 | 0.047485183 | 0.11665375 | -0.051806039 | 0.0869103 | 0.1 |
| CD28 | -0.105754371 | 0.00046157 | -0.22735593 | 2.79E-14 | 0.13 |
| LOC221394 | -0.204182115 | 9.46E-12 | -0.094980957 | 0.0016686 | 0.11 |
| CD2BP2 | 0.076947745 | 0.01093411 | 0.126094788 | 2.91E-05 | 0.05 |
| CD2 | 0.044310617 | 0.14320045 | -0.046793162 | 0.1220842 | 0.1 |
| CD300A | 0.009556262 | 0.75232057 | -0.105751571 | 0.0004617 | 0.12 |
| LOC105371892 | -0.122732095 | 4.73E-05 | -0.163081775 | 5.90E-08 | 0.05 |
| CD300E | 0.004271403 | 0.88782729 | -0.175709051 | 4.98E-09 | 0.18 |
| CD300LB | -0.163222817 | 5.75E-08 | -0.167933598 | 2.33E-08 | 0.01 |
| CD300LD | -0.028988714 | 0.33832129 | -0.02589695 | 0.3923678 | 0.01 |
| CD300LF | 0.002899871 | 0.92370974 | -0.053162857 | 0.0789477 | 0.06 |
| CD300LG | -0.274587786 | 2.33E-20 | -0.213582777 | 9.64E-13 | 0.07 |
| CD302 | -0.38809007 | 1.31E-40 | -0.273313825 | 3.54E-20 | 0.12 |
| CD320 | 0.348551948 | 1.42E-32 | 0.193374238 | 1.14E-10 | 0.16 |
| LOC116196 | -0.150551448 | 5.73E-07 | -0.205927551 | 6.24E-12 | 0.06 |
| LOC199828 | -0.331585955 | 1.84E-29 | -0.223101609 | 8.54E-14 | 0.11 |
| CD36 | -0.273124267 | 3.76E-20 | -0.180172582 | 1.99E-09 | 0.1 |
| CD37 | -0.057998284 | 0.05525383 | -0.148044247 | 8.84E-07 | 0.1 |
| CD38 | 0.115728518 | 0.00012579 | -0.052759384 | 0.0812511 | 0.17 |
| CD3D | 0.088814813 | 0.00329575 | -0.003401197 | 0.910572 | 0.1 |
| CD3EAP | 0.176281152 | 4.43E-09 | 0.014942326 | 0.6216849 | 0.17 |
| CD3E | 0.024531306 | 0.41781812 | -0.063463708 | 0.0359176 | 0.09 |
| CD3G | -0.094029763 | 0.00185815 | -0.182042524 | 1.34E-09 | 0.09 |
| CD40LG | -0.072074699 | 0.01716274 | -0.141669397 | 2.58E-06 | 0.07 |
| CD40 | 0.045865016 | 0.1296749 | -0.109110291 | 0.0003016 | 0.16 |
| CD44 | -0.133404267 | 9.64E-06 | -0.187502617 | 4.18E-10 | 0.06 |
| MIC10 | -0.480637293 | 2.94E-64 | 0.000993075 | 0.9738388 | 0.49 |
| LOC105374030 | 0.142052993 | 2.42E-06 | -0.005294292 | 0.8612107 | 0.15 |
| CD48 | 0.010373519 | 0.7319212 | -0.083280438 | 0.0058704 | 0.1 |
| CD4 | -0.093891412 | 0.0018873 | -0.210495721 | 2.06E-12 | 0.12 |
| CD52 | 0.064565848 | 0.03281273 | -0.022256814 | 0.4622959 | 0.09 |
| CD53 | -0.041526975 | 0.17008662 | -0.164085018 | 4.88E-08 | 0.13 |
| CD55 | 0.000183088 | 0.99517596 | 0.098569051 | 0.0011025 | 0.1 |
| CD58 | 0.135210839 | 7.27E-06 | 0.129305378 | 1.80E-05 | 0.01 |
| MIC11 | -0.019812862 | 0.51289352 | -0.091326866 | 0.0025096 | 0.08 |
| LOC115919 | 0.089196604 | 0.00316338 | -0.024383619 | 0.4206274 | 0.12 |
| CD5 | -0.012866718 | 0.67090109 | -0.110022465 | 0.0002681 | 0.1 |
| CD63 | 0.007002697 | 0.81711984 | 0.183352967 | 1.02E-09 | 0.18 |
| CD68 | -0.043863012 | 0.14728913 | -0.122062761 | 5.21E-05 | 0.08 |
| CD69 | -0.111258969 | 0.00022823 | -0.148869735 | 7.67E-07 | 0.04 |
| CD6 | 0.039034844 | 0.1972145 | -0.042288108 | 0.1623855 | 0.09 |
| CD70 | 0.202661645 | 1.35E-11 | 0.040213863 | 0.1840102 | 0.17 |
| CD72 | 0.141122583 | 2.82E-06 | -0.005839235 | 0.8470928 | 0.15 |
| CD74 | -0.01984823 | 0.5121413 | -0.055768827 | 0.0653184 | 0.04 |
| CD79A | 0.05730016 | 0.05825644 | -0.04930344 | 0.1032867 | 0.11 |
| CD79B | 0.092446526 | 0.00221789 | -0.042416201 | 0.1611157 | 0.14 |
| CD7 | 0.169392585 | 1.75E-08 | 0.02796648 | 0.3556374 | 0.15 |
| CD80 | 0.041773016 | 0.16756785 | -0.093423272 | 0.0019891 | 0.14 |
| CD81 | 0.115719739 | 0.00012594 | 0.089095893 | 0.0031978 | 0.03 |
| CD82 | -0.009938461 | 0.74275809 | -0.093932114 | 0.0018787 | 0.09 |
| CD83 | 0.14031437 | 3.21E-06 | -0.02368038 | 0.4341557 | 0.17 |
| CD84 | -0.275417304 | 1.78E-20 | -0.35004008 | 7.41E-33 | 0.08 |
| CD86 | 0.00873194 | 0.77307277 | -0.114705328 | 0.0001444 | 0.13 |
| CD8A | 0.014383593 | 0.63477988 | -0.085443231 | 0.0047025 | 0.1 |
| LOC100509003 | 0.095326499 | 0.00160428 | -0.006683525 | 0.8253177 | 0.11 |
| MXRA4.withdrawn | -0.435519303 | 8.23E-52 | -0.37040797 | 7.08E-37 | 0.07 |
| CD96 | -0.024769143 | 0.41331721 | -0.119673813 | 7.30E-05 | 0.1 |
| ADGRE5 | 0.098493989 | 0.00111228 | -0.119248731 | 7.75E-05 | 0.22 |
| CD99L2 | -0.294680474 | 2.45E-23 | -0.255069612 | 1.08E-17 | 0.04 |
| MIC2Y | 0.056668811 | 0.06108724 | 0.123518523 | 4.23E-05 | 0.07 |
| CD9 | 0.021479133 | 0.47808791 | 0.050873229 | 0.0927511 | 0.03 |
| CDADC1 | -0.141634399 | 2.59E-06 | -0.04844183 | 0.1094609 | 0.1 |
| CDAN1 | -0.150721153 | 5.56E-07 | -0.098032797 | 0.001174 | 0.06 |
| CDA | -0.017679081 | 0.55931653 | 0.010362531 | 0.7321942 | 0.03 |
| CDC123 | 0.576675968 | 6.91E-98 | 0.350751717 | 5.42E-33 | 0.23 |
| CDC14A | -0.19441714 | 9.04E-11 | -0.31664646 | 7.05E-27 | 0.13 |
| CDC14B | -0.265914026 | 3.80E-19 | -0.275615127 | 1.66E-20 | 0.01 |
| CDC14C | -0.30532662 | 5.14E-25 | -0.321115236 | 1.23E-27 | 0.02 |
| CDC16 | 0.098804115 | 0.00107249 | 0.137466243 | 5.09E-06 | 0.04 |
| CDC20B | -0.129231179 | 1.82E-05 | 0.069830314 | 0.0209544 | 0.2 |
| CDC20 | 0.615364829 | 7.06E-115 | 0.324607489 | 3.09E-28 | 0.3 |
| CDC23 | -0.04659905 | 0.12364231 | -0.019162405 | 0.5268297 | 0.03 |
| CDC25A | 0.579704803 | 3.90E-99 | 0.274620922 | 2.31E-20 | 0.31 |
| CDC25B | 0.435582438 | 7.93E-52 | 0.123433274 | 4.28E-05 | 0.32 |
| CDC25C | 0.486706255 | 4.44E-66 | 0.312872575 | 3.01E-26 | 0.18 |
| CDC26 | 0.119941731 | 7.03E-05 | 0.113963774 | 0.0001596 | 0.01 |
| CDC27 | -0.094212544 | 0.00182026 | -0.140884956 | 2.93E-06 | 0.05 |
| CDC34 | 0.366680445 | 4.05E-36 | 0.286510766 | 4.26E-22 | 0.09 |
| CDC37L1 | -0.057322596 | 0.05815788 | 0.079799748 | 0.0083049 | 0.14 |
| CDC37 | 0.259249817 | 3.03E-18 | 0.179685444 | 2.20E-09 | 0.08 |
| CDC40 | -0.131731077 | 1.25E-05 | -0.091076982 | 0.0025793 | 0.05 |
| FLJ23347 | -0.127141222 | 2.49E-05 | -0.068429921 | 0.023673 | 0.06 |
| CDC42BPB | -0.468132958 | 1.28E-60 | -0.3566663 | 3.93E-34 | 0.12 |
| KAPPA.200 | -0.029748771 | 0.32580295 | -0.097613859 | 0.0012327 | 0.07 |
| LOC129136 | 0.204342419 | 9.10E-12 | 0.053873473 | 0.0750201 | 0.16 |
| CDC42EP2 | 0.137348759 | 5.18E-06 | 0.220345572 | 1.74E-13 | 0.09 |
| CDC42EP3 | -0.11063747 | 0.00024753 | -0.25780129 | 4.71E-18 | 0.15 |
| LOC91740 | 0.11818531 | 8.98E-05 | -0.025106043 | 0.4069909 | 0.15 |
| CDC42EP5 | 0.017548745 | 0.56221681 | 0.008065148 | 0.7899793 | 0.01 |
| CDC42SE1 | 0.044557139 | 0.14098601 | 0.157003061 | 1.82E-07 | 0.12 |
| CDC42SE2 | -0.139842888 | 3.47E-06 | -0.207118374 | 4.69E-12 | 0.07 |
| CDC42 | -0.110638264 | 0.0002475 | -0.077472548 | 0.010401 | 0.04 |
| CDC45 | 0.539190661 | 1.86E-83 | 0.309857289 | 9.44E-26 | 0.23 |
| CDC5L | 0.148942365 | 7.57E-07 | 0.016254423 | 0.5914053 | 0.14 |
| CDC6 | 0.40764599 | 5.21E-45 | 0.242064602 | 4.87E-16 | 0.17 |
| CDC73 | -0.159046941 | 1.25E-07 | 0.170123388 | 1.52E-08 | 0.33 |
| CDC7 | 0.465717326 | 6.21E-60 | 0.179988376 | 2.06E-09 | 0.29 |
| CDCA2 | 0.456744772 | 1.96E-57 | 0.225742253 | 4.28E-14 | 0.24 |
| CDCA3 | 0.655318665 | 4.24E-135 | 0.382920785 | 1.71E-39 | 0.28 |
| CDCA4 | 0.512013323 | 4.43E-74 | 0.283302785 | 1.27E-21 | 0.23 |
| CDCA5 | 0.568338806 | 1.62E-94 | 0.302323689 | 1.55E-24 | 0.27 |
| CDCA7L | 0.256027037 | 8.08E-18 | 0.173409992 | 7.92E-09 | 0.09 |
| CDCA7 | 0.425863569 | 2.21E-49 | 0.213371535 | 1.02E-12 | 0.22 |
| CDCA8 | 0.554884993 | 2.82E-89 | 0.252719688 | 2.18E-17 | 0.31 |
| CDCP1 | -0.0521168 | 0.08503166 | -0.020291069 | 0.5027715 | 0.04 |
| CDCP2 | 0.043119271 | 0.15427915 | -0.071793699 | 0.0176019 | 0.12 |
| CDH10 | -0.066338337 | 0.028301 | -0.001309923 | 0.9654965 | 0.07 |
| CDH11 | -0.360903236 | 5.78E-35 | -0.255502 | 9.47E-18 | 0.11 |
| CDH12 | -0.004796124 | 0.87415582 | 0.10311904 | 0.0006393 | 0.11 |
| CDH13 | -0.267269364 | 2.47E-19 | -0.211241423 | 1.72E-12 | 0.06 |
| CDH15 | 0.127245056 | 2.45E-05 | 0.085140504 | 0.0048522 | 0.05 |
| CDH16 | 0.12902399 | 1.88E-05 | -0.028914393 | 0.3395617 | 0.16 |
| CDH17 | -0.203192262 | 1.19E-11 | -0.166751722 | 2.93E-08 | 0.04 |
| CDH24 | -0.057612274 | 0.05689772 | -0.113371214 | 0.0001727 | 0.06 |
| CDH19 | 0.195411122 | 7.22E-11 | -0.038269648 | 0.2061471 | 0.24 |
| CDH1 | 0.034542054 | 0.25386447 | -0.095709669 | 0.0015356 | 0.14 |
| CDH20 | -0.133175431 | 9.98E-06 | -0.158536447 | 1.37E-07 | 0.03 |
| CDH22 | -0.138900443 | 4.04E-06 | -0.135032411 | 7.48E-06 | 0.01 |
| DFNB12 | -0.259668301 | 2.66E-18 | -0.23484946 | 3.67E-15 | 0.03 |
| CDH24.1 | -0.057612274 | 0.05689772 | -0.113371214 | 0.0001727 | 0.06 |
| CDH26 | 0.074094033 | 0.01427947 | 0.011420208 | 0.7060706 | 0.07 |
| CDH2 | 0.066547504 | 0.02780557 | -0.052503118 | 0.0827421 | 0.12 |
| CDH3 | 0.219486447 | 2.17E-13 | 0.055300468 | 0.067614 | 0.17 |
| LOC391260 | 0.11299468 | 0.00018156 | -0.046662009 | 0.1231352 | 0.16 |
| CDH5 | -0.384115495 | 9.50E-40 | -0.312191587 | 3.90E-26 | 0.08 |
| CDH6 | -0.401841643 | 1.13E-43 | -0.272250185 | 5.00E-20 | 0.13 |
| CDH7 | -0.067628983 | 0.02536139 | -0.11378026 | 0.0001635 | 0.05 |
| CDH8 | -0.198317506 | 3.72E-11 | -0.163202771 | 5.77E-08 | 0.04 |
| LOC51180 | 0.046444337 | 0.12489526 | 0.006028838 | 0.842192 | 0.05 |
| CDHR1 | 0.005270274 | 0.86183403 | -0.076931991 | 0.0109505 | 0.09 |
| CDHR2 | 0.047305062 | 0.11804861 | -0.004519444 | 0.8813602 | 0.06 |
| CDHR3 | -0.123539424 | 4.22E-05 | -0.123909334 | 4.00E-05 | 0.01 |
| CDHR4 | -0.095865812 | 0.00150841 | 0.05332992 | 0.0780096 | 0.15 |
| CDHR5 | 0.045708925 | 0.13098667 | -0.033536107 | 0.2679633 | 0.08 |
| CDIPT | 0.115897409 | 0.00012294 | 0.092114416 | 0.002301 | 0.03 |
| CDK10 | 0.148321804 | 8.43E-07 | 0.122584334 | 4.84E-05 | 0.03 |
| CDC2L2 | -0.018912607 | 0.53223255 | -0.115536213 | 0.0001291 | 0.1 |
| LOC100133692 | 0.152226991 | 4.27E-07 | 0.025743843 | 0.3951735 | 0.13 |
| CDK12 | -0.125639945 | 3.11E-05 | -0.117337078 | 0.000101 | 0.01 |
| CDK13 | -0.27822214 | 7.03E-21 | -0.301566302 | 2.05E-24 | 0.03 |
| CDK14 | -0.258200536 | 4.17E-18 | -0.143323007 | 1.96E-06 | 0.12 |
| CDK15 | -0.426788538 | 1.30E-49 | -0.332561926 | 1.23E-29 | 0.1 |
| CDK16 | 0.404981768 | 2.16E-44 | 0.239573992 | 9.86E-16 | 0.17 |
| CDK17 | -0.432247299 | 5.59E-51 | -0.317372684 | 5.32E-27 | 0.12 |
| CDK18 | 0.174976941 | 5.77E-09 | 0.120944691 | 6.11E-05 | 0.06 |
| CDK19 | 0.041121476 | 0.17429948 | 0.026734967 | 0.3772259 | 0.02 |
| CDK1 | 0.520437463 | 6.77E-77 | 0.337419842 | 1.65E-30 | 0.19 |
| CDK20 | -0.145766375 | 1.30E-06 | -0.001841583 | 0.9515072 | 0.15 |
| CDK2AP1 | 0.488578873 | 1.20E-66 | 0.292644309 | 5.03E-23 | 0.2 |
| CDK2AP2 | 0.194921086 | 8.07E-11 | 0.23548162 | 3.09E-15 | 0.05 |
| CDK2 | 0.308209058 | 1.75E-25 | 0.140494826 | 3.12E-06 | 0.17 |
| CDK3 | 0.214260431 | 8.14E-13 | 0.121038026 | 6.03E-05 | 0.1 |
| MGC14458 | 0.561896268 | 5.62E-92 | 0.376422135 | 4.04E-38 | 0.19 |
| CDK5R1 | 0.153427145 | 3.45E-07 | -0.04743209 | 0.1170636 | 0.21 |
| CDK5R2 | 0.134785398 | 7.77E-06 | -0.023075788 | 0.4459843 | 0.16 |
| HSPC167 | 0.312058025 | 4.10E-26 | 0.293047124 | 4.37E-23 | 0.02 |
| CDK5RAP2 | -0.15685173 | 1.87E-07 | -0.150126171 | 6.17E-07 | 0.01 |
| CDK5RAP3 | 0.032756898 | 0.27924707 | 0.114190489 | 0.0001548 | 0.09 |
| CDK5 | 0.274172786 | 2.67E-20 | 0.285425406 | 6.18E-22 | 0.02 |
| CDK6 | 0.019083653 | 0.52853002 | -0.099116148 | 0.0010338 | 0.12 |
| LOC116024 | 0.075804093 | 0.01218072 | 0.297588836 | 8.65E-24 | 0.23 |
| CDK8 | 0.074959443 | 0.01318057 | -0.063505171 | 0.0357964 | 0.14 |
| CDK9 | -0.005685626 | 0.85106764 | 0.06423984 | 0.0337063 | 0.07 |
| CDKAL1 | 0.31203708 | 4.13E-26 | 0.165659374 | 3.62E-08 | 0.15 |
| CDKL1 | -0.125561621 | 3.14E-05 | -0.174264747 | 6.67E-09 | 0.05 |
| CDKL2 | -0.146209221 | 1.21E-06 | -0.179380213 | 2.34E-09 | 0.04 |
| CDKL3 | -0.124746622 | 3.54E-05 | 0.067212924 | 0.0262788 | 0.2 |
| CDKL4 | 0.149685157 | 6.66E-07 | 0.059578791 | 0.0489298 | 0.1 |
| CDKL5 | -0.383159563 | 1.52E-39 | -0.400552957 | 2.23E-43 | 0.02 |
| CDKN1A | -0.175448967 | 5.25E-09 | -0.164474418 | 4.53E-08 | 0.02 |
| CDKN1B | -0.071357194 | 0.01830368 | -0.007788558 | 0.7970218 | 0.07 |
| IMAGE | -0.027705719 | 0.36014234 | -0.058993834 | 0.0511958 | 0.04 |
| CDKN2AIPNL | 0.057083042 | 0.0592174 | 0.143883478 | 1.79E-06 | 0.09 |
| CDKN2AIP | -0.338832645 | 9.10E-31 | -0.316180297 | 8.45E-27 | 0.03 |
| CDKN2A | 0.362373804 | 2.96E-35 | 0.146222603 | 1.21E-06 | 0.22 |
| CDKN2B.AS1 | 0.222949197 | 8.89E-14 | 0.007533881 | 0.8035211 | 0.22 |
| CDKN2B | -0.11033722 | 0.00025738 | -0.114480509 | 0.0001489 | 0.01 |
| LOC654235 | 0.31331358 | 2.54E-26 | 0.08169371 | 0.0068869 | 0.24 |
| CDKN2D | 0.416836504 | 3.50E-47 | 0.138338865 | 4.42E-06 | 0.28 |
| CDKN3 | 0.543467505 | 5.18E-85 | 0.373343723 | 1.76E-37 | 0.18 |
| ARMETL1 | -0.171360932 | 1.19E-08 | -0.159807765 | 1.09E-07 | 0.02 |
| LOC105379131 | -0.250871303 | 3.78E-17 | -0.148715115 | 7.87E-07 | 0.11 |
| CDON | -0.283632724 | 1.14E-21 | -0.215000698 | 6.77E-13 | 0.07 |
| CDR1 | -0.065829257 | 0.02953866 | -0.09931672 | 0.0010096 | 0.04 |
| CDR2L | 0.120780416 | 6.25E-05 | 0.064364203 | 0.033363 | 0.06 |
| CDR2 | -0.080493248 | 0.00775794 | -0.059082927 | 0.0508451 | 0.03 |
| CDRT15P1 | -0.032693391 | 0.28018074 | -0.069110534 | 0.0223157 | 0.04 |
| CDRT15 | 0.042158745 | 0.16367552 | 0.026707527 | 0.3777159 | 0.02 |
| CDRT1 | 0.001223368 | 0.96777506 | -0.06115835 | 0.0432272 | 0.07 |
| CDRT4 | -0.10562894 | 0.00046886 | -0.024399496 | 0.4203249 | 0.09 |
| CDS1 | -0.2446435 | 2.33E-16 | -0.107410178 | 0.0003747 | 0.14 |
| LOC149832 | -0.177022588 | 3.81E-09 | -0.091477183 | 0.0024685 | 0.09 |
| HTSS | -0.089420688 | 0.00308796 | 0.007700286 | 0.7992729 | 0.1 |
| LOC115990 | 0.567121602 | 4.95E-94 | 0.315641563 | 1.04E-26 | 0.26 |
| CDV3 | 0.00140366 | 0.96302913 | -0.066212984 | 0.0286015 | 0.07 |
| CDX1 | 0.039183187 | 0.19551605 | -0.031571977 | 0.2970166 | 0.08 |
| CDX2 | 0.251098608 | 3.54E-17 | 0.051624786 | 0.0880214 | 0.2 |
| CDX4 | 0.045189679 | 0.13542438 | 0.032629276 | 0.2811255 | 0.02 |
| LOC140148 | -0.036647272 | 0.22604902 | -0.012398784 | 0.6822041 | 0.03 |
| CDY1 | NA | NA | NA | NA | #VALUE! |
| LOC140007 | 0.059893488 | 0.04774588 | 0.035005794 | 0.2475417 | 0.03 |
| CDYL2 | -0.491639484 | 1.38E-67 | -0.384466446 | 7.98E-40 | 0.11 |
| DKFZP586C1622 | 0.039640425 | 0.19034831 | -0.068361186 | 0.023814 | 0.11 |
| CEACAM16 | -0.033045335 | 0.27503315 | -0.033426416 | 0.2695325 | 0.01 |
| CEACAM18 | -0.006042194 | 0.841847 | -0.03533923 | 0.2430641 | 0.03 |
| CEACAM19 | 0.334495449 | 5.56E-30 | 0.123106826 | 4.49E-05 | 0.22 |
| CEACAM1 | -0.122764102 | 4.71E-05 | -0.003687523 | 0.9030792 | 0.12 |
| CEACAM20 | -0.087061989 | 0.00397052 | -0.133014961 | 1.02E-05 | 0.05 |
| AC006129.2 | -0.071827303 | 0.0175489 | -0.135058779 | 7.45E-06 | 0.07 |
| CEACAM22P | 0.040476769 | 0.18115702 | -0.05195418 | 0.0860106 | 0.1 |
| CEACAM3 | 0.019535373 | 0.51881525 | -0.016568571 | 0.5842572 | 0.04 |
| CEACAM4 | 0.012030285 | 0.69115574 | -0.107564364 | 0.0003675 | 0.12 |
| CEACAM5 | -0.315255229 | 1.21E-26 | -0.080081192 | 0.0080789 | 0.24 |
| CEACAM6 | -0.323692043 | 4.45E-28 | -0.066526326 | 0.0278554 | 0.26 |
| CEACAM7 | -0.244012921 | 2.79E-16 | -0.024161791 | 0.4248678 | 0.22 |
| CEACAM8 | 0.0671781 | 0.02635686 | 0.009377484 | 0.7568067 | 0.06 |
| CEBPA | -0.019035758 | 0.52956545 | -0.007251876 | 0.8107335 | 0.02 |
| CEBPB | 0.419622733 | 7.46E-48 | 0.246110388 | 1.53E-16 | 0.18 |
| CEBPD | 0.03741354 | 0.21648473 | 0.02923701 | 0.3341983 | 0.01 |
| CEBPE | 0.001936846 | 0.94900208 | -0.028309117 | 0.3497721 | 0.04 |
| CEBPG | 0.460191449 | 2.19E-58 | 0.132752888 | 1.07E-05 | 0.33 |
| CEBPZ | 0.231404867 | 9.41E-15 | 0.095683934 | 0.0015401 | 0.14 |
| CECR1 | -0.150675743 | 5.61E-07 | -0.186020022 | 5.75E-10 | 0.04 |
| CECR2 | -0.088633104 | 0.0033605 | -0.098673509 | 0.0010891 | 0.02 |
| CECR4 | 0.141888459 | 2.48E-06 | 0.069456296 | 0.0216525 | 0.08 |
| CECR5 | 0.332211875 | 1.42E-29 | 0.240606072 | 7.37E-16 | 0.1 |
| CECR6 | -0.018134399 | 0.54924194 | -0.146087378 | 1.23E-06 | 0.13 |
| CECR7 | 0.15177281 | 4.63E-07 | -0.00804674 | 0.7904475 | 0.16 |
| CELA1 | -0.001439132 | 0.96209554 | -0.006978254 | 0.817747 | 0.01 |
| CELA2A | -0.042114822 | 0.16411528 | -0.052390285 | 0.0834056 | 0.02 |
| LOC63037 | -0.08100698 | 0.00737376 | -0.050361113 | 0.0960891 | 0.04 |
| CELA3A | -0.072256276 | 0.01688408 | -0.066657874 | 0.0275472 | 0.01 |
| CELA3B | -0.02466269 | 0.41532821 | -0.042370203 | 0.1615708 | 0.02 |
| CELF1 | -0.213203256 | 1.06E-12 | -0.290638383 | 1.02E-22 | 0.08 |
| CELF2 | -0.198113284 | 3.90E-11 | -0.265330181 | 4.57E-19 | 0.07 |
| CELF3 | 0.042031226 | 0.16495469 | 0.089946174 | 0.0029175 | 0.05 |
| CELF4 | 0.284149757 | 9.56E-22 | -0.006700583 | 0.8248791 | 0.3 |
| CELF5 | -0.052150055 | 0.08483258 | -0.003866388 | 0.8984029 | 0.05 |
| CELF6 | -0.173774696 | 7.36E-09 | -0.192956298 | 1.26E-10 | 0.02 |
| FLJ25862 | -0.018950462 | 0.53141201 | -0.008417386 | 0.7810355 | 0.02 |
| CELSR1 | -0.476644911 | 4.44E-63 | -0.228269581 | 2.19E-14 | 0.25 |
| CELSR2 | -0.239280265 | 1.07E-15 | -0.256812126 | 6.37E-18 | 0.02 |
| CELSR3 | 0.045417083 | 0.13346682 | -0.003102143 | 0.9184063 | 0.05 |
| CEL | 0.052333015 | 0.08374402 | 0.036494165 | 0.2279956 | 0.02 |
| CEMP1 | 0.081212381 | 0.00722499 | 0.094029295 | 0.0018582 | 0.02 |
| CEND1 | 0.043042169 | 0.15501796 | -0.103396816 | 0.0006179 | 0.15 |
| CENPA | 0.613253362 | 6.88E-114 | 0.300579125 | 2.93E-24 | 0.32 |
| CENPBD1 | 0.249644963 | 5.43E-17 | 0.052246242 | 0.0842589 | 0.2 |
| CENPB | 0.182500351 | 1.22E-09 | 0.140323486 | 3.21E-06 | 0.05 |
| CENPC | -0.320736729 | 1.43E-27 | -0.339046575 | 8.32E-31 | 0.02 |
| CENPE | 0.387681765 | 1.61E-40 | 0.143319449 | 1.96E-06 | 0.25 |
| PRO1779 | 0.383085493 | 1.58E-39 | 0.282735278 | 1.55E-21 | 0.11 |
| CENPH | 0.482407871 | 8.74E-65 | 0.397062536 | 1.37E-42 | 0.09 |
| CENPI | 0.262363468 | 1.16E-18 | 0.064645641 | 0.0325971 | 0.2 |
| CENPJ | 0.249169171 | 6.25E-17 | 0.071855841 | 0.017504 | 0.18 |
| CENPK | 0.311196828 | 5.69E-26 | 0.197893396 | 4.10E-11 | 0.12 |
| CENPL | 0.355153315 | 7.73E-34 | 0.369056324 | 1.34E-36 | 0.02 |
| CENPM | 0.535855936 | 2.93E-82 | 0.336250358 | 2.68E-30 | 0.2 |
| CENPN | 0.542138711 | 1.58E-84 | 0.252066464 | 2.65E-17 | 0.3 |
| CENPO | 0.399411011 | 4.04E-43 | 0.083866685 | 0.0055304 | 0.32 |
| CENPP | 0.037795787 | 0.2118239 | 0.131203632 | 1.35E-05 | 0.1 |
| CENPQ | 0.267387624 | 2.38E-19 | 0.186204525 | 5.53E-10 | 0.09 |
| CENPT | 0.219436191 | 2.20E-13 | 0.096691134 | 0.0013719 | 0.13 |
| CENPV | 0.295500903 | 1.83E-23 | 0.067271109 | 0.0261488 | 0.23 |
| CENPW | 0.623792905 | 6.72E-119 | 0.418580645 | 1.33E-47 | 0.21 |
| CNTRL | -0.173996716 | 7.04E-09 | -0.243718462 | 3.04E-16 | 0.07 |
| CEP120 | -0.453789946 | 1.26E-56 | -0.347665352 | 2.09E-32 | 0.11 |
| FLJ13621 | -0.126142468 | 2.89E-05 | -0.198780229 | 3.34E-11 | 0.08 |
| MCPH4 | 0.173814207 | 7.30E-09 | -0.024162349 | 0.4248571 | 0.2 |
| CEP164 | 0.007468596 | 0.80518931 | -0.045562612 | 0.1322256 | 0.06 |
| LOC86693 | -0.085068596 | 0.00488837 | -0.100434369 | 0.0008841 | 0.02 |
| CEP170 | -0.067308123 | 0.02606639 | -0.079088703 | 0.0089013 | 0.02 |
| CEP192 | 0.021413592 | 0.47943215 | -0.230750953 | 1.12E-14 | 0.26 |
| CEP250 | 0.158586381 | 1.36E-07 | -0.020862357 | 0.490819 | 0.18 |
| CEP290 | -0.298458456 | 6.33E-24 | -0.224620243 | 5.74E-14 | 0.08 |
| CEP350 | -0.285780046 | 5.47E-22 | -0.099325343 | 0.0010086 | 0.19 |
| CEP55 | 0.512415636 | 3.27E-74 | 0.2611256 | 1.70E-18 | 0.26 |
| CEP57 | 0.114075256 | 0.00015719 | -0.043290178 | 0.152651 | 0.16 |
| CEP63 | -0.140351719 | 3.19E-06 | -0.073616896 | 0.0149192 | 0.07 |
| CEP68 | -0.325311608 | 2.33E-28 | -0.318179811 | 3.89E-27 | 0.01 |
| CEP70 | 0.133121241 | 1.01E-05 | 0.093788864 | 0.0019092 | 0.04 |
| CEP72 | 0.18388631 | 9.08E-10 | -0.005519096 | 0.8553811 | 0.19 |
| CEP76 | 0.263115695 | 9.14E-19 | -0.054202047 | 0.0732587 | 0.32 |
| CEP78 | 0.2185964 | 2.73E-13 | 0.065178595 | 0.031188 | 0.16 |
| CEP97 | -0.273954347 | 2.87E-20 | -0.270357671 | 9.21E-20 | 0.01 |
| CEPT1 | -0.126524267 | 2.73E-05 | -0.083364272 | 0.0058207 | 0.05 |
| CER1 | 0.031651101 | 0.29580699 | -0.051149818 | 0.0909873 | 0.09 |
| CERCAM | -0.215286047 | 6.30E-13 | -0.100053382 | 0.0009252 | 0.12 |
| CERKL | -0.121128577 | 5.95E-05 | -0.051038527 | 0.0916937 | 0.08 |
| CERK | -0.00305215 | 0.91971675 | -0.10249018 | 0.0006902 | 0.1 |
| CES2 | -0.200685193 | 2.15E-11 | -0.100190051 | 0.0009102 | 0.11 |
| CES2.1 | -0.200685193 | 2.15E-11 | -0.100190051 | 0.0009102 | 0.11 |
| FLJ21736 | -0.009204396 | 0.76115788 | 0.066713853 | 0.0274169 | 0.08 |
| CES1P1 | -0.189110967 | 2.94E-10 | -0.126347171 | 2.80E-05 | 0.07 |
| CES5A | 0.033617732 | 0.26679963 | -0.047216346 | 0.1187404 | 0.09 |
| CES4A | -0.152617757 | 3.99E-07 | -0.121743382 | 5.45E-05 | 0.04 |
| CETN1 | 0.050590475 | 0.09458237 | -0.0215138 | 0.4773777 | 0.08 |
| CETN2 | 0.149533189 | 6.84E-07 | 0.290778367 | 9.69E-23 | 0.15 |
| CETN3 | 0.076914989 | 0.01096817 | 0.16871267 | 2.00E-08 | 0.1 |
| CETN4P | 0.094561552 | 0.00174988 | 0.006800226 | 0.822318 | 0.09 |
| CETP | 0.008582837 | 0.77684427 | -0.056579754 | 0.0614956 | 0.07 |
| CFB | -0.248138126 | 8.45E-17 | 0.008005962 | 0.7914849 | 0.26 |
| CFC1B | -0.040871805 | 0.17693181 | 0.01255005 | 0.6785423 | 0.06 |
| CFDP1 | 0.42361366 | 7.92E-49 | 0.278672265 | 6.05E-21 | 0.15 |
| CFD | -0.231820022 | 8.41E-15 | -0.113823907 | 0.0001626 | 0.12 |
| CFHR1 | -0.013396191 | 0.65820085 | -0.069721106 | 0.0211561 | 0.06 |
| FHR2 | -0.02539325 | 0.4016436 | -0.011138913 | 0.7129861 | 0.02 |
| CFHR3 | -0.075493244 | 0.0125406 | -0.090820578 | 0.0026527 | 0.02 |
| CFHR4 | 0.030062356 | 0.3207269 | -0.007338745 | 0.8085101 | 0.04 |
| CFHR5 | -0.009897542 | 0.74377999 | 0.057055316 | 0.0593411 | 0.07 |
| LOC115149 | -0.275791847 | 1.57E-20 | -0.210268171 | 2.18E-12 | 0.07 |
| CFI | -0.209221864 | 2.82E-12 | -0.170582081 | 1.39E-08 | 0.04 |
| CFL1 | 0.413630517 | 2.04E-46 | 0.315602453 | 1.06E-26 | 0.1 |
| CFL2 | -0.02113639 | 0.48514024 | -0.169920023 | 1.58E-08 | 0.15 |
| CFLAR | -0.117063544 | 0.00010484 | -0.233094561 | 5.94E-15 | 0.12 |
| CFL1P1 | -0.008885443 | 0.76919557 | -0.089187977 | 0.0031663 | 0.09 |
| CFP | 0.000859899 | 0.97734614 | -0.081543266 | 0.006991 | 0.09 |
| CFTR | -0.025932261 | 0.39172246 | -0.024610773 | 0.4163111 | 0.01 |
| N4BP2L2.IT2 | -0.167542578 | 2.52E-08 | -0.144562069 | 1.59E-06 | 0.03 |
| CGA | -0.018690478 | 0.53706037 | -0.086309232 | 0.004297 | 0.07 |
| CGB1 | 0.030291947 | 0.31704339 | 0.016104679 | 0.5948266 | 0.02 |
| LOC95509 | 0.10674311 | 0.00040769 | 0.063539048 | 0.0356976 | 0.05 |
| LOC113087 | 0.03576341 | 0.2374506 | -0.003160208 | 0.9168845 | 0.04 |
| LOC105376902 | -0.041535817 | 0.16999561 | -0.110007177 | 0.0002686 | 0.07 |
| LOC89982 | 0.035341499 | 0.24303387 | 0.005642393 | 0.852187 | 0.03 |
| CGB | 0.031840205 | 0.29292947 | -0.011197073 | 0.7115543 | 0.05 |
| CGGBP1 | -0.314181041 | 1.82E-26 | -0.212089637 | 1.40E-12 | 0.11 |
| CGNL1 | -0.384056061 | 9.78E-40 | -0.299832297 | 3.85E-24 | 0.09 |
| CGN | -0.145694005 | 1.32E-06 | -0.131774313 | 1.24E-05 | 0.02 |
| CGREF1 | 0.191006971 | 1.94E-10 | 0.210035355 | 2.31E-12 | 0.02 |
| CGRRF1 | -0.244521904 | 2.41E-16 | -0.061015947 | 0.0437172 | 0.19 |
| CH25H | -0.201284258 | 1.87E-11 | -0.177597054 | 3.38E-09 | 0.03 |
| CHAC1 | 0.318841944 | 3.00E-27 | 0.125953516 | 2.97E-05 | 0.2 |
| LOC129850 | 0.443220208 | 8.34E-54 | 0.240233827 | 8.19E-16 | 0.21 |
| CHADL | -0.038745378 | 0.20055975 | 0.040822959 | 0.1774502 | 0.08 |
| CHAD | -0.350723102 | 5.49E-33 | -0.149287558 | 7.13E-07 | 0.21 |
| CHAF1A | 0.440345556 | 4.69E-53 | 0.216543439 | 4.59E-13 | 0.23 |
| CHAF1B | 0.4624201 | 5.25E-59 | 0.243659658 | 3.09E-16 | 0.22 |
| CHAT | 0.152274248 | 4.24E-07 | 0.042173097 | 0.163532 | 0.12 |
| CHCHD10 | 0.272681301 | 4.34E-20 | 0.27921013 | 5.06E-21 | 0.01 |
| CHCHD1 | 0.364885022 | 9.31E-36 | 0.460995409 | 1.31E-58 | 0.1 |
| CHCHD2 | 0.392909883 | 1.15E-41 | 0.344436814 | 8.41E-32 | 0.05 |
| CHCHD3 | 0.463091004 | 3.41E-59 | 0.214234929 | 8.19E-13 | 0.25 |
| CHCHD4 | 0.429160064 | 3.34E-50 | 0.344565437 | 7.96E-32 | 0.09 |
| CHCHD5 | -0.021531588 | 0.47701356 | 0.167016923 | 2.79E-08 | 0.19 |
| CHCHD6 | 0.328392742 | 6.75E-29 | 0.254166313 | 1.42E-17 | 0.08 |
| CHCHD7 | 0.259632641 | 2.69E-18 | 0.153396343 | 3.47E-07 | 0.11 |
| COA4 | 0.367124417 | 3.30E-36 | 0.347878266 | 1.90E-32 | 0.02 |
| LOC86848 | 0.240777481 | 7.02E-16 | 0.242278157 | 4.59E-16 | 0.01 |
| CHD1 | -0.322159758 | 8.16E-28 | -0.242273003 | 4.59E-16 | 0.08 |
| LOC283680 | -0.46404748 | 1.84E-59 | -0.528241764 | 1.41E-79 | 0.07 |
| ZFH | -0.294340521 | 2.76E-23 | -0.255205219 | 1.04E-17 | 0.04 |
| CHD4 | -0.042983459 | 0.15558232 | -0.12423026 | 3.82E-05 | 0.09 |
| DKFZP434N231 | 0.09413634 | 0.00183597 | -0.047748049 | 0.1146414 | 0.15 |
| KIAA1335 | -0.453857037 | 1.20E-56 | -0.392640449 | 1.32E-41 | 0.07 |
| LOC286180 | 0.133196324 | 9.95E-06 | -0.06738829 | 0.0258887 | 0.21 |
| DUPLIN | -0.312247721 | 3.82E-26 | -0.272100479 | 5.25E-20 | 0.05 |
| LOC146249 | -0.431565188 | 8.31E-51 | -0.450843555 | 7.86E-56 | 0.02 |
| CHDH | -0.130459911 | 1.51E-05 | -0.098615215 | 0.0010966 | 0.04 |
| CHEK1 | 0.518512477 | 3.03E-76 | 0.296683001 | 1.20E-23 | 0.23 |
| CHEK2 | 0.557281407 | 3.43E-90 | 0.381861696 | 2.88E-39 | 0.18 |
| CHERP | 0.211999058 | 1.43E-12 | 0.085866968 | 0.0044999 | 0.13 |
| CHFR | 0.074763186 | 0.01342301 | -0.016269384 | 0.5910639 | 0.1 |
| CHGA | 0.003941619 | 0.89643707 | -0.131998601 | 1.20E-05 | 0.14 |
| CHGB | -0.049775073 | 0.10002594 | -0.146599785 | 1.13E-06 | 0.1 |
| GP39 | 0.201990238 | 1.59E-11 | 0.060003831 | 0.0473365 | 0.15 |
| YKL39 | 0.090889156 | 0.0026329 | 0.085617142 | 0.0046184 | 0.01 |
| CHIA | 0.204213123 | 9.39E-12 | 0.107421594 | 0.0003742 | 0.1 |
| CHIC1 | -0.425734556 | 2.38E-49 | -0.194723038 | 8.44E-11 | 0.24 |
| CHIC2 | 0.389576718 | 6.23E-41 | 0.124549965 | 3.64E-05 | 0.27 |
| CHID1 | 0.059864039 | 0.04785563 | 0.070333 | 0.020047 | 0.02 |
| CHIT1 | -0.02034085 | 0.50172395 | -0.07702271 | 0.0108565 | 0.06 |
| CHKA | 0.26260691 | 1.07E-18 | 0.186952813 | 4.70E-10 | 0.08 |
| CHKB.CPT1B | -0.130119513 | 1.59E-05 | -0.132374949 | 1.13E-05 | 0.01 |
| CHKB | 0.041161628 | 0.17387888 | 0.039753916 | 0.1890813 | 0.01 |
| CHL1 | -0.329826728 | 3.77E-29 | -0.237027912 | 2.01E-15 | 0.1 |
| CHML | 0.098871588 | 0.00106401 | 0.181012274 | 1.67E-09 | 0.09 |
| PRSM1 | 0.347242633 | 2.51E-32 | 0.183015406 | 1.09E-09 | 0.17 |
| LOC115918 | 0.27852599 | 6.35E-21 | 0.007512503 | 0.8040672 | 0.28 |
| CHMP2A | 0.041672529 | 0.16859315 | 0.173377926 | 7.97E-09 | 0.14 |
| DMT1 | -0.163414904 | 5.54E-08 | -0.099405403 | 0.0009991 | 0.07 |
| C14orf123 | 0.067635015 | 0.0253483 | 0.186349788 | 5.36E-10 | 0.12 |
| CTPP3 | 0.17339108 | 7.95E-09 | 0.19263196 | 1.35E-10 | 0.02 |
| CHMP4C | 0.262402142 | 1.14E-18 | 0.124994527 | 3.41E-05 | 0.14 |
| CHMP5 | -0.039442675 | 0.19257083 | 0.025320143 | 0.4030007 | 0.07 |
| CHMP6 | 0.177167481 | 3.70E-09 | 0.079386286 | 0.0086472 | 0.1 |
| CHMP7 | -0.166977893 | 2.81E-08 | -0.029029499 | 0.3376418 | 0.14 |
| CHM | -0.482544856 | 7.95E-65 | -0.419874667 | 6.48E-48 | 0.07 |
| DURS2 | 0.107754321 | 0.00035871 | -0.056417943 | 0.0622432 | 0.17 |
| LOC644086 | -0.365057834 | 8.60E-36 | -0.167273143 | 2.65E-08 | 0.2 |
| LOC116155 | 0.36516744 | 8.17E-36 | 0.084106873 | 0.0053964 | 0.29 |
| CHORDC1 | 0.338263956 | 1.16E-30 | 0.173044965 | 8.52E-09 | 0.17 |
| LOC115586 | 0.054425841 | 0.0720784 | 0.040856757 | 0.1770914 | 0.02 |
| CHPF2 | -0.023489876 | 0.43786314 | -0.032045026 | 0.289834 | 0.01 |
| CHPF | -0.021511559 | 0.47742363 | -0.061847474 | 0.040921 | 0.05 |
| CHPT1 | -0.025383773 | 0.40181938 | -0.038896624 | 0.1988067 | 0.02 |
| CHP1 | -0.191803326 | 1.62E-10 | -0.157904955 | 1.54E-07 | 0.04 |
| CHRAC1 | 0.450129721 | 1.22E-55 | 0.236247332 | 2.50E-15 | 0.22 |
| CHRDL1 | -0.162702934 | 6.34E-08 | -0.166934859 | 2.83E-08 | 0.01 |
| CHRDL2 | 0.213981271 | 8.73E-13 | -0.01286573 | 0.6709249 | 0.23 |
| LOC96177 | -0.375476341 | 6.37E-38 | -0.075166949 | 0.0129284 | 0.31 |
| LOC145926 | -0.042343603 | 0.16183445 | -0.155024927 | 2.60E-07 | 0.12 |
| LOC116076 | 0.163347368 | 5.61E-08 | 0.138807509 | 4.10E-06 | 0.03 |
| CHRM2 | 0.008540735 | 0.77791018 | -0.0094908 | 0.7539623 | 0.02 |
| LOC654136 | 0.129343705 | 1.79E-05 | -0.061575851 | 0.0418173 | 0.2 |
| CHRM4 | -0.238758046 | 1.24E-15 | -0.288514662 | 2.13E-22 | 0.05 |
| CHRM5 | -0.185964144 | 5.82E-10 | -0.236467232 | 2.35E-15 | 0.06 |
| CHRNA10 | 0.176617586 | 4.14E-09 | 0.176437461 | 4.29E-09 | 0.01 |
| CHRNA1 | -0.050368471 | 0.09604042 | 0.010430476 | 0.7305063 | 0.07 |
| CHRNA2 | -0.14532857 | 1.40E-06 | -0.139831352 | 3.48E-06 | 0.01 |
| CHRNA3 | 0.006793156 | 0.82249968 | -0.10267881 | 0.0006745 | 0.11 |
| CHRNA4 | 0.006960984 | 0.81819013 | -0.043882344 | 0.1471107 | 0.06 |
| CHRNA5 | 0.409184561 | 2.28E-45 | 0.13899538 | 3.98E-06 | 0.28 |
| CHRNA6 | -0.392512766 | 1.41E-41 | -0.231770652 | 8.52E-15 | 0.17 |
| CHRNA7 | -0.021400612 | 0.47969861 | -0.173713071 | 7.45E-09 | 0.16 |
| CHRNA9 | 0.022555674 | 0.45630527 | -0.039824112 | 0.1883008 | 0.07 |
| CHRNB1 | -0.013815407 | 0.64821444 | -0.077434082 | 0.0104392 | 0.07 |
| CHRNB2 | -0.064422224 | 0.03320386 | -0.058969844 | 0.0512905 | 0.01 |
| CHRNB3 | -0.012295673 | 0.68470445 | -0.043693231 | 0.1488631 | 0.04 |
| CHRNB4 | 0.10688495 | 0.00040047 | -0.027780367 | 0.3588491 | 0.14 |
| CHRND | 0.086109909 | 0.00438739 | 0.0011801 | 0.9689142 | 0.09 |
| CHRNE | -0.040692783 | 0.17883741 | -0.042825702 | 0.1571065 | 0.01 |
| CHRNG | 0.110289972 | 0.00025897 | -0.000766858 | 0.9797967 | 0.12 |
| CHST10 | -0.023640683 | 0.43492672 | 0.007739179 | 0.7982809 | 0.04 |
| CHST11 | 0.020456111 | 0.49930282 | -0.14370924 | 1.84E-06 | 0.17 |
| CHST12 | 0.070404206 | 0.01992127 | 0.022009379 | 0.4672887 | 0.05 |
| CHST13 | -0.043835234 | 0.14754578 | -0.017942574 | 0.5534754 | 0.03 |
| N4h | -0.060565639 | 0.04529762 | -0.070206982 | 0.0202712 | 0.01 |
| CHST15 | -0.49820127 | 1.25E-69 | -0.339146842 | 7.97E-31 | 0.16 |
| CHST1 | -0.15595765 | 2.20E-07 | -0.126285348 | 2.83E-05 | 0.03 |
| CHST2 | 0.176878956 | 3.92E-09 | -0.076653434 | 0.0112436 | 0.26 |
| CHST3 | 0.05584484 | 0.06495191 | -0.125182388 | 3.32E-05 | 0.19 |
| CHST4 | 0.22396961 | 6.81E-14 | 0.033484378 | 0.2687025 | 0.2 |
| LOC116365 | 0.263213161 | 8.87E-19 | 0.055165973 | 0.0682854 | 0.21 |
| CHST6 | 0.091686334 | 0.00241235 | -0.004325272 | 0.8864221 | 0.1 |
| CHST7 | -0.106613581 | 0.0004144 | -0.084096165 | 0.0054023 | 0.03 |
| CHST8 | -0.198021308 | 3.98E-11 | -0.050158758 | 0.0974342 | 0.15 |
| CHST9 | 0.053600275 | 0.07651077 | -0.065975971 | 0.0291773 | 0.12 |
| CHSY1 | -0.192233117 | 1.48E-10 | -0.230104642 | 1.34E-14 | 0.04 |
| CHSY3 | -0.29551216 | 1.82E-23 | -0.199174871 | 3.05E-11 | 0.1 |
| CHTF18 | 0.309419805 | 1.11E-25 | 0.231883121 | 8.26E-15 | 0.08 |
| CHTF8 | -0.082407917 | 0.00641126 | -0.120149522 | 6.83E-05 | 0.04 |
| CHUK | -0.149165006 | 7.29E-07 | -0.122744975 | 4.73E-05 | 0.03 |
| CHURC1 | -0.365430344 | 7.24E-36 | -0.144272303 | 1.67E-06 | 0.23 |
| CIAO1 | 0.186512073 | 5.17E-10 | 0.110854289 | 0.0002406 | 0.08 |
| CIAPIN1 | 0.407425414 | 5.87E-45 | 0.182798667 | 1.14E-09 | 0.23 |
| CIB1 | -0.034476909 | 0.25476158 | 0.163323059 | 5.64E-08 | 0.2 |
| DFNB48 | 0.281574482 | 2.29E-21 | 0.119397125 | 7.59E-05 | 0.17 |
| CIB3 | -0.095179767 | 0.00163131 | 0.146147728 | 1.22E-06 | 0.25 |
| CIB4 | 0.204311489 | 9.17E-12 | 0.119957149 | 7.02E-05 | 0.09 |
| CIC | -0.338478132 | 1.06E-30 | -0.358181434 | 1.99E-34 | 0.02 |
| CIDEA | -0.225616227 | 4.42E-14 | -0.144232191 | 1.68E-06 | 0.09 |
| CIDEB | 0.025313598 | 0.40312235 | -0.064557086 | 0.0328365 | 0.09 |
| CIDECP | 0.345505915 | 5.31E-32 | 0.289271007 | 1.64E-22 | 0.06 |
| CIDEC | -0.203114651 | 1.22E-11 | -0.125207018 | 3.31E-05 | 0.08 |
| CIITA | -0.105177383 | 0.000496 | -0.18462287 | 7.76E-10 | 0.08 |
| CILP2 | -0.251627292 | 3.02E-17 | -0.111841214 | 0.0002114 | 0.14 |
| CILP | -0.36172694 | 3.97E-35 | -0.160865179 | 8.94E-08 | 0.21 |
| MGC849 | 0.191407268 | 1.77E-10 | 0.29884758 | 5.50E-24 | 0.11 |
| CIR1 | 0.065942843 | 0.02925856 | 0.115064817 | 0.0001376 | 0.05 |
| CIRBP | -0.297768666 | 8.11E-24 | -0.04795507 | 0.1130758 | 0.25 |
| CIRH1A | 0.423347544 | 9.21E-49 | 0.144219783 | 1.69E-06 | 0.28 |
| CISD1 | 0.402224586 | 9.27E-44 | 0.209724592 | 2.49E-12 | 0.2 |
| WFS2 | 0.328576142 | 6.27E-29 | 0.200064806 | 2.48E-11 | 0.13 |
| CISD3 | 0.065915558 | 0.02932563 | 0.193793655 | 1.04E-10 | 0.13 |
| G18 | -0.296231891 | 1.41E-23 | 0.003890431 | 0.8977746 | 0.31 |
| CITED1 | -0.175384038 | 5.32E-09 | -0.080709466 | 0.0075941 | 0.1 |
| LOC154106 | -0.316174535 | 8.47E-27 | -0.069770284 | 0.0210651 | 0.25 |
| CITED4 | 0.114480557 | 0.00014888 | 0.185738465 | 6.11E-10 | 0.08 |
| CIT | 0.32400796 | 3.92E-28 | 0.113494475 | 0.0001699 | 0.22 |
| CIZ1 | 0.013978734 | 0.64434067 | -0.129810929 | 1.67E-05 | 0.15 |
| CKAP2L | 0.317728827 | 4.64E-27 | 0.088260989 | 0.0034967 | 0.23 |
| FLJ10749 | 0.263168941 | 8.99E-19 | 0.076339797 | 0.0115819 | 0.19 |
| LOC732190 | 0.1553065 | 2.47E-07 | 0.18543436 | 6.53E-10 | 0.04 |
| CKAP5 | 0.175436624 | 5.26E-09 | -0.042527443 | 0.160019 | 0.22 |
| CKB | 0.114061784 | 0.00015748 | 0.061259423 | 0.0428823 | 0.06 |
| HSPC224 | 0.376909043 | 3.20E-38 | 0.26656226 | 3.09E-19 | 0.12 |
| CKMT1A | 0.115669617 | 0.0001268 | 0.072097284 | 0.0171279 | 0.05 |
| CKMT1B | 0.124955085 | 3.43E-05 | 0.062047287 | 0.0402721 | 0.07 |
| CKMT2 | -0.160296919 | 9.93E-08 | -0.086137275 | 0.0043749 | 0.08 |
| CKM | 0.243104949 | 3.62E-16 | 0.081007793 | 0.0073732 | 0.17 |
| LOC88475 | 0.618571708 | 2.15E-116 | 0.561843136 | 5.90E-92 | 0.06 |
| CKS2 | 0.677988217 | 5.22E-148 | 0.45519649 | 5.20E-57 | 0.23 |
| CLASP1 | -0.159168806 | 1.22E-07 | -0.343250853 | 1.40E-31 | 0.19 |
| CLASP2 | -0.408609939 | 3.11E-45 | -0.295163875 | 2.06E-23 | 0.12 |
| CLCA1 | 0.003713487 | 0.90240021 | -0.027057281 | 0.3714992 | 0.04 |
| CLCA2 | -0.147016625 | 1.05E-06 | -0.038217954 | 0.206761 | 0.11 |
| CLCA3P | 0.059391171 | 0.04964722 | -0.016725642 | 0.5806984 | 0.08 |
| CLCA4 | -0.045629964 | 0.13165415 | -0.102263579 | 0.0007094 | 0.06 |
| CLCC1 | -0.034205537 | 0.25852235 | -0.092517659 | 0.0022004 | 0.06 |
| CLCF1 | -0.092447719 | 0.00221759 | -0.079273882 | 0.0087424 | 0.02 |
| CLCN1 | -0.139902724 | 3.44E-06 | 0.035038315 | 0.2471025 | 0.18 |
| EGI3 | 0.193740278 | 1.05E-10 | 0.099577717 | 0.0009789 | 0.1 |
| LOC133073 | -0.38190968 | 2.81E-39 | -0.362916581 | 2.30E-35 | 0.02 |
| CLCN4 | 0.122582189 | 4.84E-05 | -0.095262023 | 0.0016161 | 0.22 |
| LOC115066 | 0.205071643 | 7.65E-12 | 0.027986527 | 0.3552925 | 0.18 |
| CLCN6 | -0.45086101 | 7.78E-56 | -0.465608785 | 6.66E-60 | 0.02 |
| LOC116428 | -0.070053072 | 0.02054795 | -0.070951872 | 0.0189771 | 0.01 |
| CLCNKA | -0.122444922 | 4.93E-05 | 0.018509285 | 0.5410147 | 0.15 |
| CLCNKB | -0.057211378 | 0.05864782 | -0.018491664 | 0.5414 | 0.04 |
| CLC | 0.027737866 | 0.35958503 | -0.069872358 | 0.0208771 | 0.1 |
| CLDN10 | 0.055910963 | 0.06463452 | -0.00996828 | 0.7420137 | 0.07 |
| CLDN11 | -0.263630626 | 7.79E-19 | -0.17493489 | 5.82E-09 | 0.09 |
| LOC115383 | -0.31072019 | 6.81E-26 | -0.067111315 | 0.0265071 | 0.25 |
| CLDN14 | 0.139242063 | 3.82E-06 | 0.040063546 | 0.1856563 | 0.1 |
| CLDN15 | -0.008788414 | 0.77164567 | -0.009854954 | 0.7448441 | 0.01 |
| CLDN16 | 0.099232728 | 0.00101966 | -0.131029166 | 1.39E-05 | 0.24 |
| CLDN17 | -0.020857833 | 0.49091304 | -0.082650224 | 0.0062567 | 0.07 |
| SFTA5 | -0.099929506 | 0.00093888 | -0.14085707 | 2.94E-06 | 0.05 |
| CLDN19 | -0.282446978 | 1.70E-21 | -0.212293485 | 1.33E-12 | 0.08 |
| ILVASC | 0.025892399 | 0.39245105 | -0.066396171 | 0.0281633 | 0.1 |
| CLDN20 | -0.252790497 | 2.14E-17 | -0.207678039 | 4.10E-12 | 0.05 |
| CLDN22 | 0.173208857 | 8.24E-09 | 0.231992723 | 8.02E-15 | 0.06 |
| CLDN23 | 0.173426543 | 7.89E-09 | 0.038842344 | 0.1994346 | 0.14 |
| LOC120385 | -0.150008285 | 6.30E-07 | 0.047738333 | 0.1147152 | 0.2 |
| CLDN2 | -0.018781571 | 0.53507788 | 0.023417186 | 0.4392826 | 0.05 |
| CLDN3 | 0.137651971 | 4.94E-06 | 0.152602054 | 4.00E-07 | 0.02 |
| CLDN4 | 0.162705133 | 6.34E-08 | 0.14909719 | 7.37E-07 | 0.02 |
| CLDN5 | -0.216983902 | 4.11E-13 | -0.108213408 | 0.0003383 | 0.11 |
| CLDN6 | 0.291303852 | 8.06E-23 | 0.120191726 | 6.79E-05 | 0.18 |
| CLDN7 | 0.065893677 | 0.02937952 | 0.162953047 | 6.05E-08 | 0.1 |
| CLDN8 | -0.063054066 | 0.0371337 | -0.064417436 | 0.033217 | 0.01 |
| LOC100134406 | 0.233091687 | 5.95E-15 | 0.090506112 | 0.0027453 | 0.15 |
| HSPC174 | 0.103490089 | 0.0006109 | -0.001321062 | 0.9652033 | 0.11 |
| CLDND2 | 0.026243134 | 0.3860686 | -0.024035686 | 0.4272895 | 0.06 |
| CLEC10A | -0.108429467 | 0.00032912 | -0.199562562 | 2.79E-11 | 0.1 |
| CLEC11A | -0.136545838 | 5.89E-06 | -0.027749242 | 0.359388 | 0.11 |
| CLEC12A | -0.034340377 | 0.25664891 | -0.112167813 | 0.0002025 | 0.08 |
| CLEC12B | -0.074193032 | 0.0141498 | -0.112137671 | 0.0002034 | 0.04 |
| CLEC14A | -0.306610508 | 3.19E-25 | -0.169315101 | 1.78E-08 | 0.14 |
| CLEC16A | -0.219652532 | 2.08E-13 | -0.148331847 | 8.41E-07 | 0.08 |
| CLEC17A | -0.181400246 | 1.54E-09 | -0.234986673 | 3.54E-15 | 0.06 |
| CLEC18A | 0.093606206 | 0.00194874 | -0.02116961 | 0.4844542 | 0.12 |
| CLEC18B | 0.075127524 | 0.01297601 | -0.069298189 | 0.0219536 | 0.15 |
| CLEC18C | 0.057917809 | 0.05559325 | -0.025932584 | 0.3917166 | 0.09 |
| CLEC1A | -0.199169004 | 3.06E-11 | -0.203053765 | 1.23E-11 | 0.01 |
| CLEC1B | -0.051517441 | 0.08868479 | -0.021500244 | 0.4776554 | 0.04 |
| CLEC2A | -0.037336624 | 0.21743142 | -0.025363172 | 0.4022016 | 0.02 |
| CLEC2B | -0.058477935 | 0.05326643 | -0.017334592 | 0.5669979 | 0.05 |
| CLEC2D | 0.151369915 | 4.97E-07 | 0.089741898 | 0.0029827 | 0.07 |
| CLEC2L | 0.164519031 | 4.50E-08 | 0.021967478 | 0.4681371 | 0.15 |
| CLEC3A | -0.204671945 | 8.42E-12 | -0.003555692 | 0.906528 | 0.21 |
| CLEC3B | -0.209323947 | 2.75E-12 | -0.08270442 | 0.0062226 | 0.13 |
| CLEC4A | -0.023212201 | 0.44329955 | -0.139883034 | 3.45E-06 | 0.12 |
| CLEC4C | -0.021110003 | 0.4856855 | -0.129641503 | 1.71E-05 | 0.11 |
| CLEC.6 | 0.20926978 | 2.78E-12 | 0.019505505 | 0.5194547 | 0.19 |
| CLEC4E | 0.164477736 | 4.53E-08 | -0.032305384 | 0.2859311 | 0.2 |
| CLEC4F | 0.035159088 | 0.24547608 | -0.082617673 | 0.0062773 | 0.12 |
| LOC199676 | -0.012242999 | 0.68598308 | -0.086473945 | 0.0042235 | 0.08 |
| CLEC4G | -0.063374108 | 0.03618064 | -0.153465358 | 3.43E-07 | 0.1 |
| CLEC4M | -0.023258865 | 0.44238326 | -0.151041805 | 5.26E-07 | 0.13 |
| LOC154745 | -0.02000981 | 0.50871202 | -0.102405884 | 0.0006973 | 0.09 |
| CLEC6A | 0.112632308 | 0.00019049 | -0.056452941 | 0.0620809 | 0.17 |
| CLEC7A | 0.099532647 | 0.00098413 | -0.111249762 | 0.0002285 | 0.22 |
| CLEC9A | -0.240916832 | 6.75E-16 | -0.182541163 | 1.21E-09 | 0.06 |
| CLECL1 | -0.094038727 | 0.00185627 | -0.152515549 | 4.06E-07 | 0.06 |
| CLGN | -0.313040225 | 2.82E-26 | -0.058427752 | 0.0534715 | 0.26 |
| CLIC1 | 0.335094556 | 4.34E-30 | 0.317490615 | 5.08E-27 | 0.02 |
| CLIC2 | -0.157618017 | 1.63E-07 | -0.213337435 | 1.02E-12 | 0.06 |
| CLIC3 | 0.085403728 | 0.00472177 | 0.019395259 | 0.5218187 | 0.07 |
| CLIC4 | -0.037942978 | 0.21004863 | -0.176043992 | 4.65E-09 | 0.14 |
| CLIC5 | -0.340569093 | 4.37E-31 | -0.282049926 | 1.95E-21 | 0.06 |
| CLIC6 | -0.243473242 | 3.26E-16 | -0.105850185 | 0.0004561 | 0.14 |
| LOC100131045 | -0.241073908 | 6.46E-16 | -0.237278803 | 1.87E-15 | 0.01 |
| CLIP1 | -0.321106773 | 1.24E-27 | -0.378987172 | 1.17E-38 | 0.06 |
| LOC115441 | 0.02954286 | 0.32916428 | -0.183761333 | 9.33E-10 | 0.22 |
| LOC115700 | -0.175077301 | 5.66E-09 | -0.2510636 | 3.57E-17 | 0.08 |
| CLIP4 | 0.001718775 | 0.95473743 | -0.16385636 | 5.10E-08 | 0.17 |
| CLK1 | -0.276388726 | 1.29E-20 | -0.192914523 | 1.27E-10 | 0.09 |
| LOC646524 | 0.100105474 | 0.00091944 | 0.226538391 | 3.46E-14 | 0.13 |
| CLK2 | 0.173549204 | 7.70E-09 | 0.356678389 | 3.91E-34 | 0.19 |
| LOC102723772 | 0.07432294 | 0.01398122 | -0.030859774 | 0.3080527 | 0.11 |
| CLK4 | -0.320583869 | 1.52E-27 | -0.180312938 | 1.93E-09 | 0.15 |
| CLLU1OS | 0.189339584 | 2.80E-10 | 0.066820337 | 0.0271706 | 0.13 |
| CLLU1 | 0.138724296 | 4.16E-06 | 0.060931856 | 0.0440088 | 0.08 |
| KIAA0500 | -0.336000944 | 2.97E-30 | -0.207149845 | 4.65E-12 | 0.13 |
| CLN3 | -0.012421111 | 0.68166313 | 0.234083689 | 4.53E-15 | 0.25 |
| CLN5 | -0.333625657 | 7.96E-30 | -0.163221198 | 5.75E-08 | 0.18 |
| CLN6 | 0.402102957 | 9.88E-44 | 0.255183642 | 1.04E-17 | 0.15 |
| LOC101927714 | -0.262819324 | 1.00E-18 | -0.149070013 | 7.41E-07 | 0.12 |
| CLNK | -0.085324338 | 0.00476076 | -0.186882546 | 4.78E-10 | 0.11 |
| CLNS1A | 0.232281959 | 7.42E-15 | 0.197402932 | 4.59E-11 | 0.04 |
| CLOCK | -0.322014285 | 8.65E-28 | -0.364525691 | 1.10E-35 | 0.05 |
| CLP1 | 0.217197744 | 3.89E-13 | 0.212081891 | 1.40E-12 | 0.01 |
| CLPB | 0.179420563 | 2.32E-09 | 0.009225284 | 0.7606324 | 0.18 |
| CLPP | 0.352338575 | 2.70E-33 | 0.303373499 | 1.06E-24 | 0.05 |
| CLPS | 0.106004448 | 0.00044735 | 0.129516967 | 1.75E-05 | 0.03 |
| CLPTM1L | 0.265146148 | 4.84E-19 | 0.098817517 | 0.0010708 | 0.17 |
| CLPTM1 | 0.027786226 | 0.35874768 | -0.063072021 | 0.0370797 | 0.1 |
| CLPX | -0.129537268 | 1.74E-05 | -0.251297134 | 3.33E-17 | 0.13 |
| CLRN1.AS1 | -0.039985117 | 0.18651949 | 0.01719285 | 0.5701731 | 0.06 |
| CLRN1 | -0.117657358 | 9.66E-05 | -0.046535418 | 0.1241564 | 0.08 |
| LOC340003 | -0.033973701 | 0.26176551 | -0.044325658 | 0.1430646 | 0.02 |
| CLRN3 | -0.142610605 | 2.21E-06 | 0.025854004 | 0.3931536 | 0.17 |
| CLSPN | 0.186654096 | 5.02E-10 | -0.065988204 | 0.0291473 | 0.26 |
| CLSTN1 | -0.155359392 | 2.45E-07 | -0.231845709 | 8.35E-15 | 0.08 |
| CLSTN2 | -0.407344852 | 6.12E-45 | -0.20769311 | 4.08E-12 | 0.2 |
| CLSTN3 | -0.018589035 | 0.53927245 | -0.049939832 | 0.0989064 | 0.04 |
| LOC63271 | 0.489968571 | 4.51E-67 | 0.395703382 | 2.75E-42 | 0.1 |
| CLTB | 0.199892376 | 2.59E-11 | 0.120058181 | 6.92E-05 | 0.08 |
| CLTCL1 | 0.046541253 | 0.12410923 | -0.012641374 | 0.6763352 | 0.06 |
| CLTCL2 | -0.155789896 | 2.26E-07 | -0.082623087 | 0.0062739 | 0.08 |
| CLUAP1 | 0.017052365 | 0.57332835 | 0.022220547 | 0.4630258 | 0.01 |
| CLUL1 | -0.033101842 | 0.2742127 | 0.003977873 | 0.89549 | 0.04 |
| CLU | -0.192001651 | 1.55E-10 | -0.114821779 | 0.0001422 | 0.08 |
| CLVS1 | 0.225157086 | 4.99E-14 | 0.22162123 | 1.25E-13 | 0.01 |
| CLVS2 | -0.063632891 | 0.0354253 | -0.05826984 | 0.0541212 | 0.01 |
| CLB | 0.023404499 | 0.43953059 | 0.079323588 | 0.0087002 | 0.06 |
| CMA1 | -0.273481063 | 3.35E-20 | -0.225672904 | 4.35E-14 | 0.05 |
| CMAHP | -0.215068808 | 6.65E-13 | -0.036190205 | 0.2318954 | 0.18 |
| CMAS | 0.426146913 | 1.88E-49 | 0.257383642 | 5.35E-18 | 0.17 |
| CMBL | -0.315806886 | 9.76E-27 | -0.049991525 | 0.0985572 | 0.27 |
| CMC1 | 0.352394718 | 2.63E-33 | 0.389948097 | 5.16E-41 | 0.04 |
| CMIP | -0.124781506 | 3.52E-05 | -0.295451802 | 1.86E-23 | 0.18 |
| CMKLR1 | -0.195711555 | 6.75E-11 | -0.249176559 | 6.23E-17 | 0.06 |
| CMPK1 | 0.106537839 | 0.00041837 | -0.018062641 | 0.5508237 | 0.13 |
| CMPK2 | 0.073542061 | 0.01502182 | -0.070412598 | 0.0199065 | 0.15 |
| CMTM1 | -0.18083061 | 1.73E-09 | -0.306429925 | 3.41E-25 | 0.13 |
| CMTM2 | 0.106708475 | 0.00040948 | 0.04263078 | 0.1590054 | 0.07 |
| CMTM3 | -0.099319447 | 0.00100927 | -0.030086447 | 0.3203391 | 0.07 |
| CMTM4 | -0.116754285 | 0.00010938 | -0.149342135 | 7.07E-07 | 0.04 |
| CMTM5 | 0.106412428 | 0.00042502 | -0.007353239 | 0.8081392 | 0.12 |
| PRO2219 | -0.200777107 | 2.11E-11 | -0.213737187 | 9.27E-13 | 0.02 |
| CMTM7 | 0.270107419 | 9.98E-20 | 0.052341558 | 0.0836935 | 0.22 |
| CMTM8 | -0.001727608 | 0.95450508 | 0.090312101 | 0.0028039 | 0.1 |
| C5orf10 | -0.465990098 | 5.20E-60 | -0.265478283 | 4.36E-19 | 0.21 |
| GTSE1.AS1 | 0.141517441 | 2.64E-06 | -0.003664383 | 0.9036845 | 0.15 |
| CNBD1 | 0.037298128 | 0.21790635 | -0.021308331 | 0.4815954 | 0.06 |
| DM2 | 0.277630319 | 8.55E-21 | 0.159964769 | 1.06E-07 | 0.12 |
| CNDP1 | 0.180887004 | 1.71E-09 | 0.130284216 | 1.55E-05 | 0.06 |
| CNDP2 | 0.101059254 | 0.00082039 | 0.009007558 | 0.7661152 | 0.1 |
| CNFN | 0.149532217 | 6.84E-07 | 0.180825084 | 1.73E-09 | 0.04 |
| CNGA1 | 0.15365433 | 3.32E-07 | 0.071686907 | 0.0177714 | 0.09 |
| CNGA2 | 0.024193154 | 0.4242668 | 0.088707879 | 0.0033337 | 0.07 |
| ACHM2 | -0.229534342 | 1.56E-14 | -0.095591648 | 0.0015565 | 0.14 |
| CNGA4 | -0.138051024 | 4.63E-06 | -0.067612124 | 0.025398 | 0.08 |
| CNGB1 | 0.216165474 | 5.05E-13 | -0.016157509 | 0.5936185 | 0.24 |
| ACHM1 | -0.012436905 | 0.68128055 | -0.049231929 | 0.1037883 | 0.04 |
| CNIH2 | 0.427258748 | 9.96E-50 | 0.294745029 | 2.39E-23 | 0.14 |
| CNIH3 | -0.232546917 | 6.90E-15 | -0.080735382 | 0.0075747 | 0.16 |
| CNIH4 | 0.34229602 | 2.10E-31 | 0.57197068 | 5.68E-96 | 0.23 |
| CNIH1 | -0.082652088 | 0.00625556 | 0.09440413 | 0.0017813 | 0.18 |
| CNKSR1 | -0.009672985 | 0.74939609 | 0.10153125 | 0.0007751 | 0.12 |
| CNKSR2 | -0.002190615 | 0.94233121 | -0.194427067 | 9.02E-11 | 0.2 |
| CNKSR3 | 0.122361178 | 4.99E-05 | -0.05202806 | 0.0855647 | 0.18 |
| CNN1 | -0.192494419 | 1.39E-10 | -0.114972343 | 0.0001393 | 0.08 |
| CNN2 | -0.030982409 | 0.30613335 | 0.019275673 | 0.5243891 | 0.06 |
| CNN3 | -0.093916845 | 0.00188191 | -0.124051732 | 3.92E-05 | 0.04 |
| CNNM1 | 0.126847093 | 2.60E-05 | -0.063974775 | 0.034448 | 0.2 |
| CNNM2 | -0.14010324 | 3.33E-06 | -0.224336217 | 6.19E-14 | 0.09 |
| FLJ20018 | -0.159554788 | 1.14E-07 | -0.127598948 | 2.33E-05 | 0.04 |
| LOC619531 | -0.248849979 | 6.86E-17 | -0.15844703 | 1.40E-07 | 0.1 |
| CNOT10 | 0.250926684 | 3.72E-17 | 0.22376503 | 7.19E-14 | 0.03 |
| LOC51579 | -0.047994753 | 0.11277759 | -0.226853868 | 3.19E-14 | 0.18 |
| HSPC131 | -0.024854669 | 0.41170572 | 0.049686325 | 0.1006332 | 0.08 |
| KIAA0691 | 0.177785313 | 3.26E-09 | 0.090510776 | 0.0027439 | 0.09 |
| CLONE243 | -0.138409337 | 4.37E-06 | -0.079980438 | 0.0081592 | 0.06 |
| FLJ25459 | -0.352247889 | 2.81E-33 | -0.340100926 | 5.33E-31 | 0.02 |
| DKFZP564G1964 | -0.131326824 | 1.33E-05 | -0.033560611 | 0.2676136 | 0.1 |
| CNOT7 | 0.007311487 | 0.80920759 | 0.05608936 | 0.0637846 | 0.05 |
| CNOT8 | -0.212239267 | 1.34E-12 | -0.086791559 | 0.0040851 | 0.13 |
| BLOC1S4 | 0.135763118 | 6.66E-06 | 0.19617141 | 6.08E-11 | 0.07 |
| CNPY1 | 0.122583499 | 4.84E-05 | 0.063038689 | 0.03718 | 0.06 |
| CNPY2 | 0.34481567 | 7.15E-32 | 0.399913846 | 3.11E-43 | 0.06 |
| CNPY3 | 0.292458516 | 5.37E-23 | 0.239287558 | 1.07E-15 | 0.06 |
| CNPY4 | 0.037583133 | 0.21440782 | 0.054660121 | 0.0708596 | 0.02 |
| CNP | 0.18718926 | 4.47E-10 | 0.062167178 | 0.039887 | 0.13 |
| LOC154309 | -0.171537555 | 1.15E-08 | -0.242738938 | 4.02E-16 | 0.08 |
| CNR2 | -0.106362934 | 0.00042768 | -0.215024823 | 6.73E-13 | 0.11 |
| LOC116144 | -0.307921152 | 1.95E-25 | -0.189195128 | 2.89E-10 | 0.12 |
| LOC100134211 | -0.334428082 | 5.71E-30 | -0.049922105 | 0.0990263 | 0.29 |
| CNTD1 | -0.224072892 | 6.63E-14 | 0.001856119 | 0.951125 | 0.23 |
| CNTD2 | -0.077661143 | 0.01021509 | -0.023181394 | 0.4439051 | 0.06 |
| CNTFR | 0.043482508 | 0.15083446 | -0.107416189 | 0.0003744 | 0.16 |
| CNTF | -0.107957748 | 0.00034954 | -0.165619094 | 3.64E-08 | 0.06 |
| C9orf101 | 0.04380512 | 0.1478244 | -0.127831451 | 2.25E-05 | 0.18 |
| CNTN1 | -0.379155729 | 1.08E-38 | -0.286835605 | 3.81E-22 | 0.1 |
| CNTN2 | -0.051325855 | 0.08987881 | 0.069701148 | 0.0211932 | 0.13 |
| CNTN3 | -0.236970481 | 2.04E-15 | -0.13141796 | 1.31E-05 | 0.11 |
| CNTN4 | -0.251904367 | 2.78E-17 | -0.219422512 | 2.21E-13 | 0.04 |
| NB.2 | 0.051792358 | 0.08699377 | -0.039417685 | 0.192853 | 0.1 |
| CNTN6 | -0.102908557 | 0.00065592 | -0.094212202 | 0.0018203 | 0.01 |
| CNTNAP1 | -0.281180474 | 2.61E-21 | -0.264877681 | 5.27E-19 | 0.02 |
| CNTNAP2 | -0.086630339 | 0.00415485 | -0.008934373 | 0.7679609 | 0.08 |
| CNTNAP3 | 0.0189936 | 0.53047772 | -0.134215294 | 8.50E-06 | 0.16 |
| CNTNAP4 | 0.087433125 | 0.00381799 | -0.015825689 | 0.6012248 | 0.11 |
| CNTNAP5 | 0.002370578 | 0.9376029 | 0.020302114 | 0.502539 | 0.02 |
| CNTROB | 0.089711223 | 0.00299261 | 0.05996892 | 0.0474657 | 0.03 |
| COASY | -0.038251125 | 0.20636692 | 0.01692762 | 0.5761371 | 0.06 |
| COBLL1 | -0.347422039 | 2.32E-32 | -0.193761186 | 1.05E-10 | 0.16 |
| COBL | -0.163176339 | 5.80E-08 | -0.187904684 | 3.83E-10 | 0.03 |
| NELFB | 0.156787882 | 1.89E-07 | 0.077390782 | 0.0104825 | 0.08 |
| DFNA31 | 0.287179272 | 3.38E-22 | 0.057014514 | 0.0595234 | 0.24 |
| DKFZp762L1710 | -0.060473917 | 0.04562536 | -0.098456854 | 0.0011171 | 0.04 |
| LOC126726 | -0.251419103 | 3.22E-17 | 0.205145838 | 7.52E-12 | 0.46 |
| COG3 | -0.378001196 | 1.89E-38 | -0.23358198 | 5.20E-15 | 0.15 |
| COG4 | 0.210162011 | 2.24E-12 | 0.09880275 | 0.0010727 | 0.12 |
| COG5 | -0.323636543 | 4.55E-28 | -0.203034836 | 1.24E-11 | 0.13 |
| COG6 | -0.394704058 | 4.60E-42 | -0.267265437 | 2.48E-19 | 0.13 |
| LOC197425 | -0.24436754 | 2.52E-16 | -0.025884053 | 0.3926037 | 0.22 |
| COG8 | -0.051791542 | 0.08699875 | -0.100523785 | 0.0008747 | 0.05 |
| LOC96825 | 0.069045033 | 0.02244336 | 0.175047105 | 5.69E-09 | 0.11 |
| LOC93042 | -0.256979915 | 6.05E-18 | -0.071655685 | 0.0178212 | 0.19 |
| COL11A1 | -0.095195168 | 0.00162846 | -0.013277217 | 0.6610462 | 0.09 |
| DFNA13 | 0.269300483 | 1.29E-19 | 0.065345818 | 0.0307567 | 0.21 |
| COL12A1L | -0.328101162 | 7.60E-29 | -0.203948177 | 9.99E-12 | 0.13 |
| COL13A1 | 0.045522234 | 0.13256906 | 0.015006227 | 0.6201947 | 0.04 |
| COL14A1 | -0.4592002 | 4.13E-58 | -0.272556155 | 4.52E-20 | 0.19 |
| COL15A1 | -0.320101673 | 1.84E-27 | -0.304551263 | 6.84E-25 | 0.02 |
| COL16A1 | -0.213128162 | 1.08E-12 | -0.142779957 | 2.14E-06 | 0.08 |
| EBR2A | -0.224418263 | 6.06E-14 | -0.150415792 | 5.87E-07 | 0.08 |
| COL18A1_2 | -0.140802354 | 2.97E-06 | -0.125228155 | 3.30E-05 | 0.02 |
| COL19A1 | 0.043748681 | 0.14834766 | -0.157305503 | 1.72E-07 | 0.21 |
| OI4 | -0.282100402 | 1.92E-21 | -0.125763796 | 3.05E-05 | 0.16 |
| COL1A2 | -0.308984734 | 1.31E-25 | -0.147730604 | 9.32E-07 | 0.17 |
| COL20A1 | -0.003796986 | 0.90021698 | 0.02870493 | 0.3430733 | 0.04 |
| COL21A1 | -0.182956347 | 1.11E-09 | -0.159373563 | 1.18E-07 | 0.03 |
| LOC100293055 | 0.230930042 | 1.07E-14 | 0.049933213 | 0.0989511 | 0.19 |
| COL23A1 | 0.104876411 | 0.00051489 | 0.001607321 | 0.9576696 | 0.11 |
| COL24A1 | -0.291866166 | 6.62E-23 | -0.175506107 | 5.19E-09 | 0.12 |
| COL25A1 | -0.145026159 | 1.47E-06 | -0.186266574 | 5.46E-10 | 0.05 |
| MGC11337 | -0.049955378 | 0.09880124 | -0.139064351 | 3.94E-06 | 0.09 |
| LOC392630 | -0.149871712 | 6.45E-07 | -0.175031347 | 5.71E-09 | 0.03 |
| COL6A5 | 0.109150777 | 0.00030004 | -0.046974511 | 0.1206424 | 0.16 |
| AOM | 0.165946221 | 3.42E-08 | -0.001268377 | 0.9665902 | 0.17 |
| COL3A1 | -0.337452211 | 1.62E-30 | -0.186474448 | 5.22E-10 | 0.16 |
| COL4A1 | -0.095122081 | 0.00164206 | -0.196829433 | 5.23E-11 | 0.11 |
| COL4A2 | -0.021948568 | 0.46852028 | -0.171833549 | 1.08E-08 | 0.15 |
| COL4A3BP | -0.595619216 | 6.45E-106 | -0.334654411 | 5.20E-30 | 0.27 |
| COL4A3 | 0.048327523 | 0.11030151 | -0.10848607 | 0.0003267 | 0.16 |
| COL4A4 | 0.041715776 | 0.16815131 | -0.136920803 | 5.55E-06 | 0.18 |
| COL4A5 | -0.312181888 | 3.91E-26 | -0.082091268 | 0.0066184 | 0.24 |
| COL4A6 | -0.283076138 | 1.38E-21 | -0.131996859 | 1.20E-05 | 0.16 |
| COL5A1 | -0.285775497 | 5.48E-22 | -0.16309885 | 5.88E-08 | 0.13 |
| COL5A2 | -0.302221522 | 1.61E-24 | -0.161328649 | 8.20E-08 | 0.15 |
| COL5A3 | -0.193912307 | 1.01E-10 | -0.142671643 | 2.18E-06 | 0.06 |
| COL6A1 | -0.185599965 | 6.30E-10 | -0.135753296 | 6.67E-06 | 0.05 |
| COL6A2 | -0.160397862 | 9.75E-08 | -0.130687093 | 1.46E-05 | 0.03 |
| COL6A3 | -0.359047402 | 1.34E-34 | -0.245604212 | 1.77E-16 | 0.12 |
| LOC100131335 | -0.055699178 | 0.06565563 | 0.004997757 | 0.8689121 | 0.07 |
| COL6A6 | -0.237058922 | 1.99E-15 | -0.228383439 | 2.12E-14 | 0.01 |
| COL7A1 | -0.102766447 | 0.00066738 | -0.093754478 | 0.0019166 | 0.01 |
| COL8A1 | -0.364286643 | 1.23E-35 | -0.27346651 | 3.36E-20 | 0.1 |
| COL8A2 | -0.289020694 | 1.79E-22 | -0.137665393 | 4.93E-06 | 0.16 |
| COL9A1 | 0.243659926 | 3.09E-16 | 0.079187547 | 0.0088162 | 0.17 |
| EDM2 | -0.010778356 | 0.72188476 | 0.015278697 | 0.6138584 | 0.03 |
| COL9A3 | 0.264575155 | 5.79E-19 | 0.117533449 | 9.83E-05 | 0.15 |
| COLEC10 | -0.023271873 | 0.44212803 | -0.00392663 | 0.8968287 | 0.02 |
| COLEC11 | 0.159477222 | 1.16E-07 | 0.029919434 | 0.323034 | 0.13 |
| LOC125450 | -0.226782662 | 3.25E-14 | -0.210259184 | 2.19E-12 | 0.02 |
| COLQ | -0.045786327 | 0.13033492 | 0.011008051 | 0.7162114 | 0.06 |
| COMMD10 | -0.201727347 | 1.69E-11 | 0.068771885 | 0.0229824 | 0.28 |
| COMMD1 | 0.341093779 | 3.50E-31 | 0.339177431 | 7.87E-31 | 0.01 |
| COMMD2 | 0.218542613 | 2.77E-13 | 0.036469214 | 0.2283139 | 0.19 |
| COMMD3 | 0.089561333 | 0.00304146 | 0.336734627 | 2.19E-30 | 0.25 |
| COMMD4 | 0.297452661 | 9.09E-24 | 0.310420615 | 7.63E-26 | 0.02 |
| LOC157601 | 0.358340168 | 1.85E-34 | 0.292235487 | 5.81E-23 | 0.07 |
| COMMD6 | 0.230080031 | 1.35E-14 | 0.372437292 | 2.71E-37 | 0.15 |
| C20orf92 | 0.369955211 | 8.76E-37 | 0.372047823 | 3.26E-37 | 0.01 |
| COMMD8 | 0.091871113 | 0.0023637 | 0.121330825 | 5.78E-05 | 0.03 |
| COMMD9 | 0.263639723 | 7.76E-19 | 0.224578477 | 5.81E-14 | 0.04 |
| EDM1 | -0.20818569 | 3.62E-12 | -0.04920033 | 0.1040106 | 0.16 |
| COMTD1 | 0.250583719 | 4.12E-17 | 0.197114733 | 4.90E-11 | 0.06 |
| COMT | 0.191463396 | 1.75E-10 | 0.137629019 | 4.96E-06 | 0.06 |
| LOC127376 | -0.074990252 | 0.01314286 | 0.092597839 | 0.0021809 | 0.17 |
| DKFZp761K102 | -0.294930131 | 2.24E-23 | -0.15068241 | 5.60E-07 | 0.15 |
| COPB2 | -0.123030321 | 4.54E-05 | -0.025333888 | 0.4027454 | 0.1 |
| FLJ13241 | 0.279729058 | 4.25E-21 | 0.254379798 | 1.33E-17 | 0.03 |
| FLJ11781 | 0.042736169 | 0.15797656 | 0.098578103 | 0.0011014 | 0.06 |
| COPG1 | 0.040145053 | 0.18476236 | 0.086422431 | 0.0042464 | 0.05 |
| COPS2 | -0.074220442 | 0.01411408 | -0.050289638 | 0.0965625 | 0.03 |
| COPS3 | 0.3220876 | 8.40E-28 | 0.210426638 | 2.10E-12 | 0.12 |
| COPS4 | -0.169607739 | 1.68E-08 | -0.128996142 | 1.89E-05 | 0.05 |
| COPS5 | 0.388722858 | 9.57E-41 | 0.31161596 | 4.85E-26 | 0.08 |
| COPS6 | 0.367007456 | 3.48E-36 | 0.370426419 | 7.02E-37 | 0.01 |
| COPS7A | 0.101067977 | 0.00081954 | 0.167915984 | 2.34E-08 | 0.07 |
| COPS7B | 0.340344213 | 4.81E-31 | 0.196753244 | 5.32E-11 | 0.15 |
| COPS8 | -0.076299232 | 0.01162634 | 0.023958542 | 0.4287749 | 0.11 |
| COPZ1 | 0.01520583 | 0.61555012 | 0.270176282 | 9.76E-20 | 0.26 |
| COPZ2 | -0.20278882 | 1.31E-11 | -0.057272502 | 0.0583781 | 0.15 |
| COQ10A | 0.017250607 | 0.56887823 | -0.003634889 | 0.904456 | 0.03 |
| COQ10B | -0.170276343 | 1.48E-08 | -0.09887378 | 0.0010637 | 0.08 |
| COQ2 | 0.407618596 | 5.29E-45 | 0.123410862 | 4.29E-05 | 0.29 |
| LOC96592 | 0.434288591 | 1.70E-51 | 0.35793587 | 2.22E-34 | 0.08 |
| COQ4 | -0.078809406 | 0.00914579 | 0.003442651 | 0.9094867 | 0.09 |
| COQ5 | 0.050352073 | 0.09614882 | 0.178892715 | 2.59E-09 | 0.13 |
| LOC115747 | -0.204580299 | 8.60E-12 | -0.009453971 | 0.7548864 | 0.2 |
| LOC51672 | -0.154448915 | 2.88E-07 | 0.003863085 | 0.8984892 | 0.16 |
| COQ9 | 0.396866123 | 1.51E-42 | 0.209796674 | 2.45E-12 | 0.19 |
| CORIN | -0.236932073 | 2.06E-15 | -0.093007058 | 0.0020838 | 0.15 |
| CORO1A | 0.076561622 | 0.01134172 | -0.054786653 | 0.0702083 | 0.14 |
| CORO1B | -0.033601051 | 0.26703714 | 0.056092368 | 0.0637703 | 0.09 |
| CORO1C | 0.156089662 | 2.14E-07 | -0.015528082 | 0.6080839 | 0.18 |
| CORO2A | -0.309757228 | 9.80E-26 | -0.071961354 | 0.0173387 | 0.24 |
| CORO2B | -0.362649502 | 2.60E-35 | -0.249774013 | 5.23E-17 | 0.12 |
| CORO6 | -0.127356069 | 2.41E-05 | -0.046246728 | 0.12651 | 0.09 |
| CORO7 | 0.016071093 | 0.59559515 | -0.032936332 | 0.2766205 | 0.05 |
| CORT | 0.214758109 | 7.19E-13 | 0.137855747 | 4.78E-06 | 0.08 |
| LOC90755 | 0.274184122 | 2.66E-20 | 0.030590519 | 0.3122948 | 0.25 |
| COX10 | 0.192965959 | 1.25E-10 | 0.118813296 | 8.23E-05 | 0.08 |
| COX11 | 0.075747523 | 0.01224553 | 0.123971385 | 3.96E-05 | 0.05 |
| COX15 | -0.098979903 | 0.00105052 | -0.020936517 | 0.4892787 | 0.08 |
| COX16 | 0.182711487 | 1.17E-09 | 0.389093206 | 7.94E-41 | 0.21 |
| COX17 | 0.296812728 | 1.14E-23 | 0.344589532 | 7.88E-32 | 0.05 |
| COX18 | -0.038490587 | 0.20353834 | -0.034049474 | 0.2607024 | 0.01 |
| COX19 | 0.150953855 | 5.34E-07 | 0.112848913 | 0.0001851 | 0.04 |
| COX4I1 | 0.425637694 | 2.51E-49 | 0.337189933 | 1.81E-30 | 0.09 |
| COX4I2 | -0.090443819 | 0.00276398 | 0.054603159 | 0.0711543 | 0.15 |
| EMC8 | 0.51577762 | 2.50E-75 | 0.226992273 | 3.07E-14 | 0.29 |
| COX5A | 0.538619837 | 2.99E-83 | 0.402342062 | 8.71E-44 | 0.14 |
| COX5B | 0.429289776 | 3.10E-50 | 0.391604208 | 2.24E-41 | 0.04 |
| LOC112536 | 0.367872717 | 2.33E-36 | 0.405525709 | 1.62E-44 | 0.04 |
| COX6A2 | 0.127815384 | 2.25E-05 | 0.075557354 | 0.0124656 | 0.06 |
| COX6B1 | 0.460557284 | 1.73E-58 | 0.469756852 | 4.40E-61 | 0.01 |
| COX6B2 | -0.075007045 | 0.01312235 | -0.04274973 | 0.1578445 | 0.04 |
| COX6C | 0.115434843 | 0.0001309 | 0.115611038 | 0.0001278 | 0.01 |
| COX7A1 | -0.167831536 | 2.38E-08 | 0.006412004 | 0.8323068 | 0.18 |
| EB1 | 0.411344255 | 7.10E-46 | 0.359699521 | 1.00E-34 | 0.06 |
| COX7A2 | 0.420390764 | 4.86E-48 | 0.474709413 | 1.63E-62 | 0.06 |
| COX7B2 | 0.11386087 | 0.00016177 | 0.039909124 | 0.1873587 | 0.08 |
| COX7B | 0.48503439 | 1.42E-65 | 0.448686224 | 2.98E-55 | 0.04 |
| COX7C | 0.207423127 | 4.36E-12 | 0.459910298 | 2.62E-58 | 0.26 |
| COX8A | 0.276539612 | 1.23E-20 | 0.2912055 | 8.34E-23 | 0.02 |
| COX8C | 0.137680936 | 4.92E-06 | 0.039831346 | 0.1882205 | 0.1 |
| CCP110 | -0.214242284 | 8.18E-13 | -0.188332221 | 3.49E-10 | 0.03 |
| CPA1 | -0.213154227 | 1.07E-12 | -0.155031182 | 2.59E-07 | 0.06 |
| LOC136262 | 0.242176799 | 4.72E-16 | 0.087258406 | 0.0038891 | 0.16 |
| CPA3 | -0.433073975 | 3.45E-51 | -0.258165945 | 4.22E-18 | 0.18 |
| CPA4 | 0.203177994 | 1.20E-11 | 0.072597516 | 0.0163711 | 0.14 |
| CPA5 | 0.148225075 | 8.57E-07 | 0.137932516 | 4.72E-06 | 0.02 |
| CPA6 | -0.1743198 | 6.59E-09 | -0.043782102 | 0.1480376 | 0.14 |
| CPAMD8 | 0.012989432 | 0.66794907 | -0.025702049 | 0.3959415 | 0.04 |
| CPB1 | -0.210168912 | 2.24E-12 | -0.152449414 | 4.11E-07 | 0.06 |
| LOC51745 | -0.03678294 | 0.22433407 | -0.058230579 | 0.0542838 | 0.03 |
| CPD | -0.351767495 | 3.47E-33 | -0.232955837 | 6.17E-15 | 0.12 |
| CPEB1 | -0.331206652 | 2.15E-29 | -0.284833744 | 7.57E-22 | 0.05 |
| LOC254023 | -0.526578698 | 5.33E-79 | -0.364292721 | 1.22E-35 | 0.17 |
| CPEB3 | -0.557022487 | 4.31E-90 | -0.371794828 | 3.68E-37 | 0.19 |
| CPEB4 | -0.522699737 | 1.15E-77 | -0.412504919 | 3.78E-46 | 0.12 |
| CPE | -0.349967922 | 7.65E-33 | -0.233190884 | 5.79E-15 | 0.12 |
| CPLX1 | -0.27725148 | 9.70E-21 | -0.154051611 | 3.09E-07 | 0.13 |
| DKFZp547D155 | 0.015243016 | 0.61468651 | -0.092090112 | 0.0023072 | 0.11 |
| CPLX3 | 0.216938946 | 4.15E-13 | 0.138896408 | 4.04E-06 | 0.08 |
| CPLX4 | 0.096792436 | 0.00135591 | 0.036390341 | 0.2293224 | 0.07 |
| CPM | -0.042192189 | 0.16334128 | -0.170854973 | 1.32E-08 | 0.13 |
| CPN1 | 0.075904171 | 0.01206682 | 0.066643245 | 0.0275813 | 0.01 |
| CPN2 | -0.216093589 | 5.14E-13 | -0.110356876 | 0.0002567 | 0.11 |
| CPNE1 | 0.249480345 | 5.70E-17 | 0.204722926 | 8.32E-12 | 0.05 |
| CPNE2 | 0.190239688 | 2.29E-10 | -0.027693346 | 0.360357 | 0.22 |
| PRO1071 | -0.108274849 | 0.00033569 | -0.080114642 | 0.0080524 | 0.03 |
| CPNE4 | -0.003431739 | 0.90977235 | 0.03168149 | 0.2953433 | 0.04 |
| LOC100131043 | -0.033984886 | 0.26160839 | -0.119254181 | 7.74E-05 | 0.09 |
| CPNE6 | 0.061328177 | 0.04264896 | -0.021157622 | 0.4847017 | 0.09 |
| CPNE7 | 0.143467194 | 1.91E-06 | 0.24368716 | 3.07E-16 | 0.11 |
| CPNE8 | -0.077960205 | 0.00992639 | -0.170952156 | 1.29E-08 | 0.1 |
| CPNE9 | 0.046991951 | 0.12050441 | 0.057879658 | 0.0557548 | 0.02 |
| LOC131130 | 0.08899078 | 0.00323413 | 0.032518883 | 0.2827572 | 0.06 |
| CPO | -0.118166509 | 9.01E-05 | 0.033752004 | 0.264893 | 0.16 |
| CPPED1 | -0.143041959 | 2.05E-06 | -0.136972216 | 5.50E-06 | 0.01 |
| LOC130203 | -0.083724331 | 0.00561132 | -0.123270394 | 4.38E-05 | 0.04 |
| CPSF1 | 0.253401577 | 1.78E-17 | 0.096129645 | 0.0014635 | 0.16 |
| CPSF2 | -0.086392153 | 0.00425986 | -0.096882233 | 0.0013419 | 0.02 |
| CPSF3L | 0.130756204 | 1.45E-05 | 0.068789748 | 0.0229468 | 0.07 |
| CPSF3 | 0.546236627 | 4.96E-86 | 0.376357279 | 4.17E-38 | 0.17 |
| CPSF4L | 0.212388898 | 1.30E-12 | 0.136499203 | 5.93E-06 | 0.08 |
| CPSF4 | 0.392539598 | 1.39E-41 | 0.324181994 | 3.66E-28 | 0.07 |
| CPSF6 | 0.125116797 | 3.35E-05 | 0.095740503 | 0.0015302 | 0.03 |
| CPSF7 | 0.016334093 | 0.58958867 | -0.099612098 | 0.0009749 | 0.12 |
| CPT1A | -0.224685372 | 5.65E-14 | -0.141260991 | 2.75E-06 | 0.09 |
| LOC129264 | 0.021707466 | 0.47342094 | 0.072846981 | 0.0160048 | 0.06 |
| CPT1P | -0.006069163 | 0.84115047 | -0.007271687 | 0.8102263 | 0.01 |
| CPT2 | -0.096731818 | 0.00136544 | -0.079082303 | 0.0089068 | 0.02 |
| HVLP | -0.0734534 | 0.01514417 | -0.131747013 | 1.24E-05 | 0.06 |
| CPXCR1 | 0.029444019 | 0.33078571 | -0.012541704 | 0.6787441 | 0.05 |
| CPXM1 | -0.142366798 | 2.30E-06 | -0.078973412 | 0.0090015 | 0.07 |
| CPXM2 | -0.169520433 | 1.71E-08 | -0.189442573 | 2.73E-10 | 0.02 |
| CPZ | -0.247682842 | 9.65E-17 | -0.110540206 | 0.0002507 | 0.14 |
| CP | 0.051370335 | 0.08960045 | 0.025693452 | 0.3960996 | 0.03 |
| LOC204855 | 0.163846949 | 5.11E-08 | 0.00685248 | 0.8209757 | 0.16 |
| CR1 | -0.045042476 | 0.13670331 | -0.182024164 | 1.35E-09 | 0.14 |
| CR2 | 0.068652495 | 0.02322151 | -0.053652731 | 0.0762227 | 0.13 |
| CRABP1 | 0.316358462 | 7.88E-27 | 0.211330159 | 1.68E-12 | 0.11 |
| CRABP2 | 0.012519075 | 0.67929152 | 0.176001662 | 4.69E-09 | 0.17 |
| CRADD | -0.133504514 | 9.49E-06 | -0.097757731 | 0.0012123 | 0.04 |
| CRAMP1L | -0.258995888 | 3.27E-18 | -0.278580511 | 6.24E-21 | 0.02 |
| CRAT | -0.270344518 | 9.25E-20 | -0.014576643 | 0.6302422 | 0.26 |
| RP12 | -0.157204479 | 1.75E-07 | -0.01940974 | 0.5215079 | 0.14 |
| CRB2 | -0.156303489 | 2.06E-07 | -0.15264718 | 3.97E-07 | 0.01 |
| CRB3 | -0.014453382 | 0.63313788 | 0.127594411 | 2.33E-05 | 0.15 |
| MRT2A | -0.216789982 | 4.31E-13 | -0.116485618 | 0.0001135 | 0.11 |
| CRCP | 0.056118964 | 0.06364442 | 0.130534848 | 1.50E-05 | 0.08 |
| CRCT1 | 0.105864801 | 0.00045524 | 0.009159114 | 0.7622974 | 0.1 |
| CREB1 | -0.321970756 | 8.80E-28 | -0.273124593 | 3.76E-20 | 0.05 |
| CREB3L1 | -0.344624528 | 7.76E-32 | -0.056436888 | 0.0621553 | 0.29 |
| LOC155008 | -0.086013601 | 0.00443169 | -0.216611373 | 4.51E-13 | 0.14 |
| CREB3L3 | 0.088742819 | 0.00332127 | -0.022133407 | 0.4647823 | 0.12 |
| CREB3L4 | -0.232021504 | 7.96E-15 | 0.232799841 | 6.44E-15 | 0.47 |
| CREB3 | -0.045674567 | 0.13127678 | 0.087589603 | 0.0037553 | 0.14 |
| CREB5 | -0.068221259 | 0.02410323 | -0.142842841 | 2.12E-06 | 0.08 |
| CREBBP | -0.399779736 | 3.33E-43 | -0.348818203 | 1.26E-32 | 0.06 |
| CREBL2 | -0.438293698 | 1.59E-52 | -0.356816319 | 3.67E-34 | 0.09 |
| CREBZF | -0.149636722 | 6.72E-07 | -0.137423889 | 5.12E-06 | 0.02 |
| CREG1 | 0.035293522 | 0.24367455 | 0.190334742 | 2.25E-10 | 0.16 |
| CREG2 | -0.083042864 | 0.00601345 | -0.113453261 | 0.0001708 | 0.04 |
| CRELD1 | -0.135849575 | 6.57E-06 | -0.073215494 | 0.0154768 | 0.07 |
| CRELD2 | 0.231892025 | 8.24E-15 | 0.19002503 | 2.41E-10 | 0.05 |
| CREM | 0.223220357 | 8.28E-14 | 0.080509655 | 0.0077454 | 0.15 |
| CRHBP | -0.316651996 | 7.04E-27 | -0.226697574 | 3.32E-14 | 0.09 |
| LOC100131650 | 0.164328372 | 4.66E-08 | 0.031711978 | 0.2948786 | 0.14 |
| LOC100506602 | -0.208132564 | 3.67E-12 | -0.207754503 | 4.02E-12 | 0.01 |
| CRH | -0.012916575 | 0.66970111 | -0.066575126 | 0.0277407 | 0.06 |
| CRIM1 | -0.450995823 | 7.16E-56 | -0.399882532 | 3.16E-43 | 0.06 |
| CRIP1 | -0.185097366 | 7.01E-10 | 0.043190975 | 0.1535945 | 0.23 |
| CRIP2 | -0.089284169 | 0.00313371 | 0.108373476 | 0.0003315 | 0.2 |
| CRIP3 | 0.183184304 | 1.05E-09 | 0.128698174 | 1.97E-05 | 0.06 |
| CRIPAK | -0.277343704 | 9.41E-21 | -0.12995956 | 1.63E-05 | 0.15 |
| HSPC139 | 0.337517123 | 1.58E-30 | 0.292679886 | 4.97E-23 | 0.05 |
| CRISP1 | -0.015623778 | 0.60587455 | 0.078470589 | 0.0094504 | 0.1 |
| GAPDL5 | 0.115932283 | 0.00012236 | 0.087853798 | 0.0036515 | 0.03 |
| CRISP3 | -0.046603955 | 0.12360275 | 0.033670763 | 0.2660455 | 0.09 |
| CRISPLD1 | -0.006219446 | 0.83727133 | -0.116681621 | 0.0001105 | 0.12 |
| CRISPLD2 | -0.276842911 | 1.11E-20 | -0.175066718 | 5.67E-09 | 0.11 |
| CRKL | -0.090766778 | 0.00266834 | -0.118564185 | 8.52E-05 | 0.03 |
| CRK | -0.25828083 | 4.07E-18 | -0.278945483 | 5.52E-21 | 0.03 |
| CRLF1 | 0.092987169 | 0.00208844 | 0.043805828 | 0.1478178 | 0.05 |
| CRLF2 | -0.083561732 | 0.00570501 | -0.167929941 | 2.33E-08 | 0.09 |
| LOC125097 | 0.040575308 | 0.18009613 | -0.111519631 | 0.0002206 | 0.16 |
| CRLS1 | 0.278602907 | 6.19E-21 | 0.307681741 | 2.14E-25 | 0.03 |
| CRMP1 | -0.147133639 | 1.03E-06 | -0.203212697 | 1.19E-11 | 0.06 |
| CRNKL1 | -0.171002404 | 1.28E-08 | 0.052761766 | 0.0812373 | 0.23 |
| PDRC1 | 0.104798246 | 0.00051991 | 0.037448541 | 0.2160549 | 0.07 |
| LOC101060339 | 0.000101121 | 0.99733565 | -0.03530015 | 0.243586 | 0.04 |
| LOC729541 | -0.266595703 | 3.06E-19 | -0.273287238 | 3.57E-20 | 0.01 |
| CROCC | -0.051511477 | 0.08872177 | -0.084035602 | 0.0054358 | 0.04 |
| COT | -0.39779795 | 9.34E-43 | -0.127645712 | 2.31E-05 | 0.28 |
| CRP | -0.025861077 | 0.39302411 | -0.042469663 | 0.1605879 | 0.02 |
| CRTAC1 | -0.106161987 | 0.0004386 | -0.107655358 | 0.0003632 | 0.01 |
| CRTAM | -0.065729332 | 0.02978698 | -0.134362687 | 8.30E-06 | 0.07 |
| OI7 | -0.303250496 | 1.10E-24 | -0.100175629 | 0.0009118 | 0.21 |
| MECT1 | -0.168516537 | 2.08E-08 | -0.096912899 | 0.0013372 | 0.08 |
| CRTC2 | 0.26131458 | 1.60E-18 | 0.338653106 | 9.81E-31 | 0.08 |
| CRTC3 | -0.306345489 | 3.52E-25 | -0.330947862 | 2.39E-29 | 0.03 |
| CRX | 0.068704923 | 0.02311624 | 0.046165184 | 0.127181 | 0.03 |
| CRY1 | -0.171792591 | 1.09E-08 | -0.213638813 | 9.50E-13 | 0.05 |
| CRY2 | -0.536066269 | 2.46E-82 | -0.349359209 | 9.98E-33 | 0.19 |
| CRYAA | 0.142967117 | 2.08E-06 | 6.99E-05 | 0.9981595 | 0.15 |
| CRYAB | 0.158827607 | 1.30E-07 | 0.010148981 | 0.7375077 | 0.15 |
| CRYBA1 | -0.005250751 | 0.86234074 | 0.04078088 | 0.1778978 | 0.05 |
| CRYBA2 | 0.176263108 | 4.45E-09 | 0.055889624 | 0.0647368 | 0.13 |
| CRYBA4 | 0.025375695 | 0.40196923 | 0.011024094 | 0.7158157 | 0.02 |
| CRYBB1 | -0.08742843 | 0.00381988 | -0.042940265 | 0.1559985 | 0.05 |
| CRYBB2 | 0.264682917 | 5.60E-19 | 0.122046118 | 5.22E-05 | 0.15 |
| CRYBB3 | 0.171950044 | 1.06E-08 | 0.092100045 | 0.0023047 | 0.08 |
| CRYBG3 | -0.289747098 | 1.39E-22 | -0.281289225 | 2.52E-21 | 0.01 |
| CRYGA | 0.044121979 | 0.14491285 | -0.003037498 | 0.9201008 | 0.05 |
| CRYGB | 0.044167261 | 0.14450037 | 0.064775799 | 0.0322481 | 0.03 |
| CRYGC | 0.044080898 | 0.14528783 | 0.011754454 | 0.6978847 | 0.04 |
| PCC | -0.026157272 | 0.38762518 | 0.017985061 | 0.5525364 | 0.05 |
| LOC202871 | -0.084790357 | 0.00503071 | -0.058817827 | 0.0518945 | 0.03 |
| CRYGS | 0.133229441 | 9.90E-06 | 0.141257356 | 2.76E-06 | 0.01 |
| CRYL1 | -0.267152396 | 2.57E-19 | -0.104436232 | 0.0005437 | 0.17 |
| CRYM | -0.117147975 | 0.00010364 | 0.058499838 | 0.0531771 | 0.18 |
| LOC220679 | -0.113373464 | 0.00017264 | 0.091195992 | 0.0025459 | 0.21 |
| CRYZ | -0.088108194 | 0.00355411 | -0.047597128 | 0.1157934 | 0.05 |
| CSAD | -0.293651998 | 3.52E-23 | -0.079397381 | 0.0086379 | 0.22 |
| LOC101060231 | 0.218671263 | 2.68E-13 | 0.059941403 | 0.0475677 | 0.16 |
| CSAG2 | 0.250327049 | 4.44E-17 | 0.090283652 | 0.0028126 | 0.17 |
| CSAG3 | 0.298745196 | 5.71E-24 | 0.11037851 | 0.000256 | 0.19 |
| YBX3P1 | 0.197621046 | 4.37E-11 | -0.024171202 | 0.4246874 | 0.23 |
| YBX3 | 0.291190499 | 8.39E-23 | 0.094347255 | 0.0017928 | 0.2 |
| CSDC2 | -0.254725276 | 1.20E-17 | -0.098368037 | 0.0011288 | 0.16 |
| CSDE1 | -0.027805353 | 0.35841682 | -0.138942544 | 4.01E-06 | 0.12 |
| CSE1L | 0.369137697 | 1.29E-36 | 0.236211631 | 2.52E-15 | 0.14 |
| CSF1R | -0.219261947 | 2.30E-13 | -0.30590642 | 4.14E-25 | 0.09 |
| CSF1 | -0.281492562 | 2.35E-21 | -0.395071286 | 3.81E-42 | 0.12 |
| CSF2RA | -0.084628225 | 0.00511536 | -0.153573634 | 3.37E-07 | 0.07 |
| IL3RB | -0.132157374 | 1.17E-05 | -0.263249579 | 8.77E-19 | 0.14 |
| CSF2 | 0.198061056 | 3.95E-11 | -0.00507465 | 0.8669138 | 0.21 |
| CSF3R | -0.200258489 | 2.38E-11 | -0.090994519 | 0.0026027 | 0.11 |
| C17orf33 | -0.013350242 | 0.65929917 | -0.054989706 | 0.0691735 | 0.05 |
| CSGALNACT1 | -0.201717199 | 1.69E-11 | -0.18156512 | 1.48E-09 | 0.03 |
| CSGALNACT2 | -0.208445708 | 3.40E-12 | -0.268188364 | 1.85E-19 | 0.06 |
| LOC92338 | 0.051981026 | 0.08584838 | 0.030599374 | 0.3121546 | 0.03 |
| CSH2 | 0.131094737 | 1.37E-05 | 0.026131663 | 0.3880902 | 0.11 |
| LOC92339 | -0.004655368 | 0.87781967 | -0.007689427 | 0.79955 | 0.01 |
| CSK | 0.287497876 | 3.03E-22 | 0.035430022 | 0.2418548 | 0.26 |
| CSMD1 | -0.168597319 | 2.05E-08 | -0.088467528 | 0.0034205 | 0.09 |
| CSMD2 | -0.086805032 | 0.00407933 | -0.12126572 | 5.84E-05 | 0.04 |
| CSMD3 | 0.055790103 | 0.06521562 | -0.028980594 | 0.3384567 | 0.09 |
| CSN1S1 | -0.06513253 | 0.03130768 | -0.062359755 | 0.0392749 | 0.01 |
| CSN1S2AP | -0.017965966 | 0.55295833 | -0.027359478 | 0.3661791 | 0.01 |
| CSN2 | 0.043294525 | 0.15260978 | 0.004806115 | 0.8738958 | 0.04 |
| CSN3 | 0.157087929 | 1.79E-07 | 0.051269399 | 0.0902331 | 0.11 |
| CSNK1A1L | -0.279642943 | 4.38E-21 | -0.205297419 | 7.25E-12 | 0.08 |
| LOC100130789 | -0.098385703 | 0.00112649 | -0.086022917 | 0.0044274 | 0.02 |
| HLCDGP1 | -0.386288579 | 3.23E-40 | -0.152071823 | 4.39E-07 | 0.24 |
| CSNK1D | -0.041095785 | 0.17456899 | -0.031183057 | 0.3030101 | 0.01 |
| CSNK1E | 0.074306771 | 0.0140021 | -0.009718469 | 0.7482575 | 0.09 |
| CSNK1G1 | -0.378292361 | 1.64E-38 | -0.322626212 | 6.79E-28 | 0.06 |
| CSNK1G2 | 0.092952966 | 0.00209642 | -0.027669843 | 0.3607649 | 0.13 |
| CSNK1G3 | -0.343147075 | 1.46E-31 | -0.224772767 | 5.52E-14 | 0.12 |
| CSNK2A3 | -0.227334565 | 2.80E-14 | -0.282424412 | 1.72E-21 | 0.06 |
| CSNK2A1 | 0.054702256 | 0.07064215 | 0.009401687 | 0.7561989 | 0.05 |
| CSNK2A2 | 0.286677292 | 4.02E-22 | -0.005329559 | 0.8602957 | 0.3 |
| CSNK2B | 0.54458722 | 2.01E-85 | 0.449780583 | 1.52E-55 | 0.1 |
| LOC140144 | 0.054699488 | 0.07065641 | 0.026035956 | 0.389831 | 0.03 |
| CSPG4 | 0.033722748 | 0.26530762 | -0.146723111 | 1.11E-06 | 0.19 |
| CSPG5 | 0.154964385 | 2.63E-07 | 0.083296874 | 0.0058606 | 0.08 |
| CSPP1 | -0.045660902 | 0.13139231 | -0.060894352 | 0.0441393 | 0.02 |
| CSRNP1 | -0.349218813 | 1.06E-32 | -0.241820155 | 5.22E-16 | 0.11 |
| CSRNP2 | -0.246496264 | 1.36E-16 | -0.070279202 | 0.0201424 | 0.18 |
| CSRNP3 | -0.328542167 | 6.35E-29 | -0.29809105 | 7.22E-24 | 0.04 |
| CSRP1 | -0.02459547 | 0.41660103 | 0.082793681 | 0.0061668 | 0.11 |
| CSRP2BP | 0.015687622 | 0.60440257 | 0.053670641 | 0.0761245 | 0.04 |
| LMO5 | 0.295316175 | 1.95E-23 | 0.119871923 | 7.10E-05 | 0.18 |
| CSRP3 | -0.001232899 | 0.96752414 | -0.018263151 | 0.5464094 | 0.02 |
| LOC164378 | 0.067774382 | 0.02504738 | -0.036343445 | 0.2299234 | 0.11 |
| CST1 | -0.090128557 | 0.00286034 | 0.021595754 | 0.4757011 | 0.12 |
| CST2 | -0.296300109 | 1.37E-23 | -0.088489268 | 0.0034126 | 0.21 |
| CST3 | -0.194327969 | 9.23E-11 | -0.09962959 | 0.0009729 | 0.1 |
| CST4 | -0.214354913 | 7.95E-13 | 0.045620929 | 0.1317307 | 0.26 |
| CST5 | -0.197829703 | 4.16E-11 | -0.056974268 | 0.0597038 | 0.15 |
| CST6 | 0.059459674 | 0.04938425 | 0.111645794 | 0.0002169 | 0.06 |
| CST7 | 0.054708833 | 0.07060826 | -0.03166888 | 0.2955357 | 0.09 |
| CST8 | 0.040649642 | 0.1792989 | 0.030494813 | 0.3138118 | 0.02 |
| CST9L | -0.084114654 | 0.00539207 | -0.084750877 | 0.0050512 | 0.01 |
| CST9 | -0.190356948 | 2.24E-10 | -0.198994484 | 3.18E-11 | 0.01 |
| AREI | 0.164517932 | 4.50E-08 | 0.072381029 | 0.0166949 | 0.1 |
| CSTB | 0.447626853 | 5.71E-55 | 0.292254078 | 5.77E-23 | 0.16 |
| CSTF1 | 0.16923256 | 1.81E-08 | 0.18994006 | 2.45E-10 | 0.03 |
| CSTF2T | -0.212987598 | 1.12E-12 | -0.147371461 | 9.91E-07 | 0.07 |
| CSTF2 | 0.158573928 | 1.36E-07 | 0.085433713 | 0.0047071 | 0.08 |
| CSTF3 | 0.393595545 | 8.11E-42 | 0.278493008 | 6.42E-21 | 0.12 |
| LOC149915 | 0.069990784 | 0.02066089 | 0.013759914 | 0.6495328 | 0.06 |
| CST13P | -0.010731045 | 0.72305523 | 0.011404223 | 0.7064629 | 0.03 |
| CS | 0.053050873 | 0.07958162 | 0.052127355 | 0.0849684 | 0.01 |
| CT45A1 | 0.127463529 | 2.37E-05 | 0.019865981 | 0.511764 | 0.11 |
| LOC101060210 | 0.092578788 | 0.00218556 | 0.016647712 | 0.5824628 | 0.08 |
| CT45A3 | 0.084112266 | 0.00539339 | 0.012720345 | 0.6744289 | 0.08 |
| CT45A4 | 0.074469435 | 0.01379324 | 0.017003335 | 0.5744315 | 0.06 |
| CT45A5 | 0.116099792 | 0.0001196 | 0.023376794 | 0.4400725 | 0.1 |
| LOC101060208 | 0.071337557 | 0.01833582 | 0.004659405 | 0.8777145 | 0.07 |
| CT47A10 | NA | NA | NA | NA | #VALUE! |
| CT47A11 | 0.06147703 | 0.04214747 | -0.014561907 | 0.6305881 | 0.08 |
| CT47A1 | 0.024361084 | 0.42105707 | 0.000945863 | 0.9750822 | 0.03 |
| CT47A2 | -0.007163788 | 0.81298976 | 0.023807271 | 0.4316963 | 0.04 |
| CT47A6 | 0.001770198 | 0.95338477 | -0.036022641 | 0.2340653 | 0.04 |
| CT47A7 | 0.001770198 | 0.95338477 | -0.036022641 | 0.2340653 | 0.04 |
| CT47A9 | NA | NA | NA | NA | #VALUE! |
| CT47B1 | 0.022057573 | 0.46631386 | -0.023504701 | 0.437574 | 0.05 |
| CT62 | -0.465578565 | 6.79E-60 | -0.183481655 | 9.90E-10 | 0.29 |
| LOC116358 | 0.221683321 | 1.23E-13 | 0.094233897 | 0.0018159 | 0.13 |
| CTAG2 | 0.111650469 | 0.00021682 | 0.021429721 | 0.4791012 | 0.1 |
| LOC147575 | -0.292071655 | 6.16E-23 | -0.204890267 | 7.99E-12 | 0.09 |
| CTAGE4 | -0.002285156 | 0.93984699 | 0.086222351 | 0.0043362 | 0.09 |
| FLJ22404 | -0.263936136 | 7.08E-19 | -0.15570172 | 2.30E-07 | 0.11 |
| CTAGE6 | -0.157456039 | 1.67E-07 | -0.105182716 | 0.0004957 | 0.06 |
| CTAGE9 | -0.051886503 | 0.0864207 | 0.050416023 | 0.0957266 | 0.11 |
| CTBP1 | 0.1338105 | 9.05E-06 | 0.104191285 | 0.0005604 | 0.03 |
| LOC87435 | -0.056881687 | 0.06012031 | 0.045784969 | 0.1303463 | 0.11 |
| CTB | -0.328560173 | 6.31E-29 | -0.296537783 | 1.26E-23 | 0.04 |
| LOC164279 | 0.295256323 | 1.99E-23 | 0.094890872 | 0.0016858 | 0.21 |
| CTCF | -0.117013977 | 0.00010556 | -0.173472364 | 7.82E-09 | 0.06 |
| CTDP1 | 0.209868514 | 2.41E-12 | -0.013232721 | 0.6621116 | 0.23 |
| CTDSP1 | -0.248797571 | 6.97E-17 | -0.103343974 | 0.0006219 | 0.15 |
| LOC51589 | -0.431139595 | 1.06E-50 | -0.428570844 | 4.69E-50 | 0.01 |
| HSPC058 | -0.266702657 | 2.96E-19 | -0.282618532 | 1.61E-21 | 0.02 |
| CTDSPL | -0.291391751 | 7.82E-23 | -0.125076404 | 3.37E-05 | 0.17 |
| CTF1 | -0.105356644 | 0.00048505 | 0.035063007 | 0.2467694 | 0.15 |
| CTGF | -0.198905437 | 3.25E-11 | -0.157317116 | 1.72E-07 | 0.05 |
| CTHRC1 | -0.014737432 | 0.6264734 | 0.03415874 | 0.2591747 | 0.05 |
| CTH | -0.184379775 | 8.18E-10 | -0.064219081 | 0.0337639 | 0.13 |
| IDDM12 | 0.169073534 | 1.87E-08 | -0.012524864 | 0.6791515 | 0.19 |
| CTNNA1 | -0.256813108 | 6.37E-18 | -0.056752014 | 0.0607078 | 0.21 |
| CTNNA2 | -0.0154793 | 0.60921156 | -0.060403486 | 0.0458784 | 0.05 |
| CTNNA3 | -0.037990841 | 0.20947367 | -0.098312796 | 0.0011362 | 0.07 |
| CTNNAL1 | 0.025625565 | 0.39734922 | 0.002288672 | 0.9397546 | 0.03 |
| CTNNB1 | -0.243627008 | 3.12E-16 | -0.20525074 | 7.33E-12 | 0.04 |
| CTNNBIP1 | 0.180067644 | 2.03E-09 | 0.036537964 | 0.2274375 | 0.15 |
| CTNNBL1 | 0.31418884 | 1.82E-26 | 0.256952962 | 6.10E-18 | 0.06 |
| CTNND1 | -0.35611009 | 5.04E-34 | -0.287214872 | 3.34E-22 | 0.07 |
| CTNND2 | -0.123159697 | 4.45E-05 | -0.057841086 | 0.0559184 | 0.07 |
| CTNS | -0.095152762 | 0.00163633 | -0.162500035 | 6.59E-08 | 0.07 |
| LOC95807 | 0.003977865 | 0.89549017 | 0.140317979 | 3.21E-06 | 0.14 |
| CTPS1 | 0.526174062 | 7.36E-79 | 0.239166173 | 1.11E-15 | 0.29 |
| CTR9 | -0.324986085 | 2.66E-28 | -0.247708813 | 9.58E-17 | 0.08 |
| CTRB1 | -0.013273715 | 0.66113005 | 0.010869259 | 0.7196376 | 0.03 |
| CTRB2 | 0.045742608 | 0.13070273 | 0.059189764 | 0.0504272 | 0.02 |
| CTRC | 0.07682822 | 0.01105887 | 0.040326101 | 0.1827881 | 0.04 |
| CTRL | 0.333770515 | 7.50E-30 | 0.112482836 | 0.0001943 | 0.23 |
| PPGB | 0.110979515 | 0.00023673 | 0.076933641 | 0.0109488 | 0.04 |
| CTSB | -0.007272897 | 0.81019535 | -0.097308222 | 0.0012773 | 0.1 |
| JPD | 0.213070986 | 1.09E-12 | 0.017776183 | 0.5571605 | 0.2 |
| LOC196214 | -0.091819142 | 0.00237729 | -0.000522309 | 0.9862387 | 0.1 |
| CTSE | 0.053610041 | 0.07645707 | 0.023702279 | 0.4337306 | 0.03 |
| CTSF | -0.214497129 | 7.67E-13 | -0.101923358 | 0.0007393 | 0.12 |
| CTSG | -0.295541218 | 1.80E-23 | -0.21112974 | 1.77E-12 | 0.09 |
| CTSH | -0.014458936 | 0.63300727 | 0.012961228 | 0.6686271 | 0.03 |
| CTSK | -0.250641466 | 4.05E-17 | -0.109805716 | 0.0002757 | 0.15 |
| CTSL | 0.143809964 | 1.81E-06 | -0.010950438 | 0.717633 | 0.16 |
| CTSV | 0.427372412 | 9.33E-50 | 0.200417654 | 2.29E-11 | 0.23 |
| CTSL3P | 0.070031371 | 0.02058724 | 0.021538537 | 0.4768713 | 0.05 |
| CTSO | -0.470999229 | 1.93E-61 | -0.361123781 | 5.23E-35 | 0.11 |
| HSCATHEP | -0.016808518 | 0.57882474 | -0.088026304 | 0.0035852 | 0.08 |
| LYPN | 0.046671704 | 0.12305731 | -0.049435569 | 0.1023648 | 0.1 |
| CTSZ | 0.150688409 | 5.59E-07 | 0.032873241 | 0.2775421 | 0.12 |
| CTTNBP2NL | -0.128584846 | 2.01E-05 | -0.271909449 | 5.58E-20 | 0.15 |
| CTTNBP2 | -0.404109292 | 3.43E-44 | -0.332811783 | 1.11E-29 | 0.08 |
| CTTN | 0.006573201 | 0.82815587 | 0.031394404 | 0.2997432 | 0.03 |
| CTU1 | 0.396531602 | 1.80E-42 | 0.275354587 | 1.81E-20 | 0.13 |
| CTU2 | 0.37428786 | 1.12E-37 | 0.1574618 | 1.67E-07 | 0.22 |
| CTXN1 | 0.241076976 | 6.45E-16 | 0.271672314 | 6.03E-20 | 0.04 |
| DKFZp781M2440 | 0.284349593 | 8.93E-22 | 0.221304925 | 1.36E-13 | 0.07 |
| CTXN3 | -0.053334139 | 0.07798605 | -0.037483163 | 0.2156304 | 0.02 |
| LOC221070 | -0.145609803 | 1.34E-06 | -0.199517734 | 2.82E-11 | 0.06 |
| CUEDC1 | -0.313849661 | 2.07E-26 | -0.186642027 | 5.03E-10 | 0.13 |
| CUEDC2 | 0.272311022 | 4.90E-20 | 0.25694671 | 6.11E-18 | 0.02 |
| CUL1 | 0.250657023 | 4.03E-17 | 0.044735158 | 0.1394033 | 0.21 |
| LOC143070 | 0.126990648 | 2.55E-05 | -0.031510669 | 0.2979561 | 0.16 |
| CUL3 | -0.218267183 | 2.97E-13 | -0.096473013 | 0.0014068 | 0.13 |
| CUL4A | -0.052482197 | 0.08286482 | -0.029490196 | 0.3300276 | 0.03 |
| MRXSC | -0.148465686 | 8.22E-07 | -0.13473513 | 7.83E-06 | 0.02 |
| CUL5 | -0.227443145 | 2.73E-14 | -0.221629109 | 1.25E-13 | 0.01 |
| CUL7 | -0.170723654 | 1.35E-08 | -0.181949793 | 1.37E-09 | 0.02 |
| CUL9 | -0.269256891 | 1.31E-19 | -0.15001418 | 6.29E-07 | 0.12 |
| ACHAP | 0.399528865 | 3.80E-43 | 0.348993916 | 1.17E-32 | 0.06 |
| CUTC | 0.294586023 | 2.53E-23 | 0.192726395 | 1.32E-10 | 0.11 |
| LOC100506550 | -0.140438766 | 3.15E-06 | -0.231375499 | 9.48E-15 | 0.1 |
| CUX2 | -0.094065075 | 0.00185077 | -0.165401127 | 3.80E-08 | 0.08 |
| CUZD1 | 0.205023195 | 7.74E-12 | 0.051466273 | 0.0890024 | 0.16 |
| CWC15 | 0.413722472 | 1.94E-46 | 0.349099341 | 1.12E-32 | 0.07 |
| CWC22 | 0.053834658 | 0.0752304 | -0.031616841 | 0.2963303 | 0.09 |
| CWC25 | -0.031571311 | 0.29702678 | -0.057201253 | 0.0586926 | 0.03 |
| CWC27 | 0.119716882 | 7.26E-05 | 0.176600273 | 4.15E-09 | 0.06 |
| CWF19L1 | 0.242086028 | 4.84E-16 | 0.239477275 | 1.01E-15 | 0.01 |
| CWF19L2 | -0.181386539 | 1.54E-09 | -0.156608082 | 1.95E-07 | 0.03 |
| CWH43 | 0.133277781 | 9.83E-06 | 0.066729899 | 0.0273797 | 0.07 |
| CX3CL1 | 0.071462547 | 0.01813211 | -0.068469671 | 0.0235918 | 0.14 |
| CX3CR1 | -0.496118923 | 5.62E-69 | -0.362349451 | 2.99E-35 | 0.14 |
| CXADRP2 | -0.096126016 | 0.00146407 | -0.007428106 | 0.8062244 | 0.09 |
| CXADRP3 | -0.253604919 | 1.68E-17 | -0.045400434 | 0.1336094 | 0.21 |
| CXADR | -0.061641882 | 0.04159791 | -0.160674384 | 9.26E-08 | 0.1 |
| CXCL10 | 0.212702049 | 1.20E-12 | 0.056659066 | 0.0611318 | 0.16 |
| CXCL11 | 0.175313413 | 5.39E-09 | 0.050867165 | 0.09279 | 0.13 |
| CXCL12 | -0.357353401 | 2.88E-34 | -0.243304474 | 3.42E-16 | 0.12 |
| LOC115545 | 0.141453218 | 2.67E-06 | 0.040828954 | 0.1773866 | 0.11 |
| CXCL14 | -0.207469946 | 4.31E-12 | -0.060085165 | 0.0470366 | 0.15 |
| CXCL16 | 0.115662326 | 0.00012692 | 0.002624814 | 0.9309269 | 0.12 |
| CXCL17 | -0.028252561 | 0.35073596 | 0.15315651 | 3.62E-07 | 0.19 |
| FSP | 0.230868584 | 1.09E-14 | 0.008826323 | 0.7706881 | 0.23 |
| CXCL2 | 0.043737782 | 0.14844887 | -0.032252866 | 0.2867155 | 0.08 |
| CXCL3 | 0.20711091 | 4.70E-12 | 0.020946685 | 0.4890677 | 0.19 |
| CXCL5 | 0.293848872 | 3.29E-23 | 0.045251839 | 0.1348871 | 0.25 |
| CXCL6 | 0.147472398 | 9.74E-07 | -0.044411055 | 0.142295 | 0.2 |
| CXCL9 | 0.038386667 | 0.20476239 | -0.066617568 | 0.0276413 | 0.11 |
| CXCR1 | -0.156250429 | 2.08E-07 | -0.076182639 | 0.0117548 | 0.09 |
| CXCR2P1 | 0.144873595 | 1.51E-06 | -0.024524003 | 0.4179568 | 0.17 |
| CXCR2 | -0.183732951 | 9.39E-10 | -0.23443236 | 4.12E-15 | 0.06 |
| CXCR3 | 0.023855929 | 0.43075532 | -0.057729811 | 0.0563929 | 0.09 |
| CXCR4 | 0.108657805 | 0.00031964 | 0.067200132 | 0.0263074 | 0.05 |
| LOC115271 | 0.009388672 | 0.75652572 | -0.092539465 | 0.0021951 | 0.11 |
| CXCR6 | 0.032454828 | 0.28370694 | -0.091274622 | 0.0025241 | 0.13 |
| ACKR3 | -0.20276134 | 1.32E-11 | -0.125088717 | 3.37E-05 | 0.08 |
| CXXC1 | 0.320120175 | 1.82E-27 | 0.140799504 | 2.97E-06 | 0.18 |
| CXXC4 | -0.268350048 | 1.75E-19 | -0.157551306 | 1.65E-07 | 0.12 |
| CXXC5 | -0.409544516 | 1.88E-45 | -0.22835069 | 2.14E-14 | 0.19 |
| TMEM257 | 0.001210221 | 0.96812118 | -0.002006057 | 0.9471823 | 0.01 |
| CXORF21 | -0.174071947 | 6.93E-09 | -0.263169026 | 8.99E-19 | 0.09 |
| CXorf22 | -0.016163869 | 0.59347313 | -0.009925497 | 0.7430818 | 0.01 |
| CXORF23 | -0.413278057 | 2.48E-46 | -0.24013233 | 8.43E-16 | 0.18 |
| PBDC1 | 0.316576015 | 7.25E-27 | 0.281810217 | 2.11E-21 | 0.04 |
| HYPM | -0.002923236 | 0.92309693 | 0.015645415 | 0.6053755 | 0.02 |
| CXorf30 | 0.110905861 | 0.00023901 | 0.122043418 | 5.22E-05 | 0.02 |
| LOC286399 | -0.348386878 | 1.53E-32 | -0.242862621 | 3.88E-16 | 0.11 |
| CXORF38 | -0.28004295 | 3.83E-21 | -0.329017439 | 5.24E-29 | 0.05 |
| FLJ16423 | -0.098096333 | 0.00116529 | 0.000837566 | 0.9779344 | 0.1 |
| LOC642980 | -0.041661668 | 0.16870426 | -0.057538711 | 0.0572156 | 0.02 |
| PIH1D3 | 0.003653472 | 0.90396984 | 0.09168611 | 0.0024124 | 0.09 |
| NKAPP1 | -0.104369563 | 0.00054824 | -0.117765411 | 9.52E-05 | 0.02 |
| CT55 | 0.146171428 | 1.22E-06 | 0.080153905 | 0.0080214 | 0.07 |
| CXORF49B | 0.373138569 | 1.94E-37 | 0.119677377 | 7.30E-05 | 0.26 |
| CXorf50B | 0.006349337 | 0.83392173 | -0.023659008 | 0.4345707 | 0.04 |
| CXORF51A | 0.100713299 | 0.00085512 | 0.021043869 | 0.4870535 | 0.08 |
| CXORF56 | -0.094354532 | 0.00179132 | -0.241249628 | 6.14E-16 | 0.15 |
| CXORF57 | -0.033399311 | 0.26992125 | -0.190365958 | 2.23E-10 | 0.16 |
| CXORF58 | -0.02256802 | 0.45605873 | -0.026570535 | 0.3801683 | 0.01 |
| LOC158730 | 0.092668526 | 0.00216387 | 0.087570265 | 0.003763 | 0.01 |
| CT83 | 0.413151541 | 2.65E-46 | 0.156177629 | 2.11E-07 | 0.26 |
| PRR32 | 0.11853606 | 8.56E-05 | 0.12373836 | 4.10E-05 | 0.01 |
| CXORF65 | 0.07177874 | 0.01762558 | 0.010970694 | 0.717133 | 0.07 |
| CXORF66 | 0.001363701 | 0.96408089 | -0.003247983 | 0.9145847 | 0.01 |
| CYB561D1 | -0.231681521 | 8.73E-15 | -0.259426709 | 2.87E-18 | 0.03 |
| LOC101928965 | -0.190206338 | 2.31E-10 | 0.07511294 | 0.0129936 | 0.27 |
| CYB561 | 0.010108564 | 0.73851477 | 0.059012372 | 0.0511226 | 0.05 |
| CYB5A | -0.159177424 | 1.22E-07 | 0.100087464 | 0.0009214 | 0.26 |
| CYB5B | 0.262640939 | 1.06E-18 | 0.106009436 | 0.0004471 | 0.16 |
| CYB5D1 | -0.342627713 | 1.82E-31 | -0.142924114 | 2.09E-06 | 0.2 |
| CYB5D2 | -0.274874424 | 2.12E-20 | -0.095113655 | 0.0016436 | 0.18 |
| CYB5R1 | -0.31165809 | 4.78E-26 | 0.113781189 | 0.0001635 | 0.43 |
| CYB5R2 | 0.17158699 | 1.14E-08 | 0.200261716 | 2.37E-11 | 0.03 |
| CYB5R3 | -0.050145452 | 0.09752316 | -0.151576348 | 4.79E-07 | 0.11 |
| CYB5R4 | -0.094770026 | 0.00170904 | -0.060791296 | 0.0444998 | 0.04 |
| CYB5RL | -0.368447691 | 1.78E-36 | -0.367091565 | 3.35E-36 | 0.01 |
| CYBASC3 | 0.052887843 | 0.08051188 | -0.001823173 | 0.9519914 | 0.06 |
| LOC115214 | 0.218748832 | 2.62E-13 | 0.070420944 | 0.0198918 | 0.15 |
| CYBB | -0.146270022 | 1.20E-06 | -0.263928791 | 7.09E-19 | 0.12 |
| CYBRD1 | -0.530818999 | 1.77E-80 | -0.318971218 | 2.86E-27 | 0.22 |
| CYC1 | 0.467482239 | 1.96E-60 | 0.312631012 | 3.30E-26 | 0.16 |
| CYCSP52 | -0.068179262 | 0.02419063 | -0.123714636 | 4.11E-05 | 0.06 |
| CYCS | 0.444812522 | 3.18E-54 | 0.360153649 | 8.13E-35 | 0.09 |
| CYFIP1 | -0.285147754 | 6.80E-22 | -0.256514337 | 6.97E-18 | 0.03 |
| PRO1331 | -0.150626271 | 5.65E-07 | -0.18707526 | 4.58E-10 | 0.04 |
| HGb | -0.195984937 | 6.34E-11 | -0.151449905 | 4.90E-07 | 0.05 |
| LOC157542 | 0.28560487 | 5.81E-22 | 0.190606325 | 2.12E-10 | 0.1 |
| CYLC1 | 0.029303591 | 0.33309823 | 0.041104864 | 0.1744737 | 0.02 |
| CYLC2 | 0.042869712 | 0.15668018 | 0.044328397 | 0.1430398 | 0.01 |
| HSPC057 | -0.204812863 | 8.14E-12 | -0.336210495 | 2.73E-30 | 0.14 |
| LOC649462 | -0.00603832 | 0.84194707 | 0.004725282 | 0.8759995 | 0.02 |
| CYP11A1 | -0.075432026 | 0.01261257 | -0.055237205 | 0.0679291 | 0.03 |
| CYP11B1 | 0.035563797 | 0.24008075 | -0.023849652 | 0.4308766 | 0.06 |
| CYP11B2 | 0.066362264 | 0.02824395 | 0.064506977 | 0.0329726 | 0.01 |
| CYP17A1 | -0.057841157 | 0.05591815 | -0.090827844 | 0.0026506 | 0.04 |
| CYP19A1 | -0.077555203 | 0.01031913 | -0.067750936 | 0.0250978 | 0.01 |
| CYP1A1 | -0.046934977 | 0.12095553 | -0.074653239 | 0.0135605 | 0.03 |
| CYP1A2 | -0.059218153 | 0.05031663 | -0.065010448 | 0.0316268 | 0.01 |
| CYP1B1 | -0.056063986 | 0.0639049 | -0.044851922 | 0.1383727 | 0.02 |
| CYP20A1 | -0.302114615 | 1.68E-24 | -0.136030279 | 6.39E-06 | 0.17 |
| CYP21A2 | -0.336909921 | 2.04E-30 | 0.014494142 | 0.6321797 | 0.36 |
| CYP24A1 | 0.076386646 | 0.01153082 | -0.073879872 | 0.0145636 | 0.16 |
| CYP26A1 | -0.163718414 | 5.23E-08 | -0.013971258 | 0.6445178 | 0.15 |
| CYP26B1 | -0.035419042 | 0.24200087 | -0.226814428 | 3.22E-14 | 0.2 |
| CYP26C1 | -0.178105436 | 3.05E-09 | -0.146741697 | 1.10E-06 | 0.04 |
| CYP27A1 | 0.102948207 | 0.00065276 | 0.068852472 | 0.0228222 | 0.04 |
| PDDR | 0.173366387 | 7.99E-09 | 0.061967908 | 0.0405289 | 0.12 |
| CYP27C1 | 0.226709292 | 3.31E-14 | 0.000842766 | 0.9777974 | 0.23 |
| LOC90330 | 0.019739339 | 0.5144591 | 0.018782932 | 0.5350483 | 0.01 |
| LOC82212 | -0.179400733 | 2.33E-09 | -0.067363707 | 0.0259431 | 0.12 |
| CYP2A7 | -0.169107682 | 1.86E-08 | -0.076362189 | 0.0115575 | 0.1 |
| CYP2B | -0.171804253 | 1.09E-08 | -0.01677826 | 0.5795085 | 0.16 |
| CYP2B7P | -0.324363039 | 3.40E-28 | -0.043628522 | 0.1494664 | 0.29 |
| CYP2C18 | 0.268559062 | 1.64E-19 | 0.059165957 | 0.0505201 | 0.21 |
| CYP2C19 | 0.068881577 | 0.02276459 | 0.027410426 | 0.3652869 | 0.05 |
| CYP2C8 | -0.066643572 | 0.02758053 | 0.099652183 | 0.0009703 | 0.17 |
| CYP2C9 | 0.030840785 | 0.30835065 | 0.028912029 | 0.3396012 | 0.01 |
| CYP2D6 | 0.170760755 | 1.34E-08 | 0.105118443 | 0.0004996 | 0.07 |
| CYP2D7P2 | 0.15766555 | 1.61E-07 | 0.054674771 | 0.0707839 | 0.11 |
| CYP2E1 | -0.127711704 | 2.29E-05 | -0.079252587 | 0.0087605 | 0.05 |
| LOC147756 | 0.221447582 | 1.31E-13 | 0.130857622 | 1.43E-05 | 0.1 |
| CYP2J2 | 0.010137791 | 0.73778649 | 0.04556301 | 0.1322222 | 0.04 |
| CYP2R1 | -0.056957307 | 0.05977989 | 0.066664306 | 0.0275322 | 0.13 |
| CYP2S1 | 0.04798009 | 0.11288769 | -0.159989538 | 1.05E-07 | 0.21 |
| CYP2U1 | -0.30781955 | 2.03E-25 | -0.326789462 | 1.29E-28 | 0.02 |
| CYP2W1 | 0.189350204 | 2.79E-10 | 0.054060384 | 0.0740139 | 0.14 |
| CYP39A1 | 0.118394062 | 8.73E-05 | -0.041030875 | 0.1752513 | 0.16 |
| CYP3A43 | -0.02083001 | 0.49149168 | -0.081055641 | 0.0073383 | 0.07 |
| CYP3A3 | -0.112822426 | 0.00018575 | -0.170590989 | 1.39E-08 | 0.06 |
| CYP3A5 | -0.19113725 | 1.88E-10 | -0.085502228 | 0.0046738 | 0.11 |
| CYP3A7 | -0.08700848 | 0.00399296 | -0.06684671 | 0.0271099 | 0.03 |
| CYP46A1 | -0.244144912 | 2.69E-16 | -0.225768309 | 4.25E-14 | 0.02 |
| CYP4A11 | -0.255320701 | 1.00E-17 | 0.003377561 | 0.9111909 | 0.26 |
| CYP4A22 | -0.248667853 | 7.24E-17 | -0.015759047 | 0.6027577 | 0.24 |
| CYP4B1 | -0.320076668 | 1.85E-27 | -0.122012518 | 5.25E-05 | 0.2 |
| CYP4F11 | -0.220646183 | 1.61E-13 | -0.085411076 | 0.0047182 | 0.14 |
| CYP4F12 | -0.153045548 | 3.70E-07 | -0.099108983 | 0.0010347 | 0.06 |
| CYP4F22 | -0.265357317 | 4.53E-19 | -0.124175779 | 3.85E-05 | 0.15 |
| CYP4F2 | -0.088628899 | 0.00336202 | -0.084155592 | 0.0053695 | 0.01 |
| CYP4F3 | -0.015711429 | 0.60385406 | -0.137239583 | 5.27E-06 | 0.13 |
| CYP4F8 | -0.231292758 | 9.70E-15 | -0.104996968 | 0.0005072 | 0.13 |
| CYP4V2 | -0.362517616 | 2.77E-35 | -0.28371912 | 1.11E-21 | 0.08 |
| CYP4X1 | -0.348647761 | 1.36E-32 | -0.028239899 | 0.350952 | 0.33 |
| CYP4Z1 | -0.330711306 | 2.63E-29 | -0.004793353 | 0.8742279 | 0.33 |
| CYP4Z2P | -0.428489402 | 4.91E-50 | -0.063813483 | 0.0349062 | 0.37 |
| LOC154652 | -0.087164605 | 0.0039278 | -0.02854801 | 0.3457192 | 0.06 |
| CYP7A1 | -0.158100617 | 1.49E-07 | -0.165243554 | 3.92E-08 | 0.01 |
| CYP7B1 | -0.061385292 | 0.04245594 | -0.236967003 | 2.04E-15 | 0.18 |
| CYP8B1 | -0.150567211 | 5.71E-07 | -0.123484158 | 4.25E-05 | 0.03 |
| CYR61 | -0.150121478 | 6.17E-07 | -0.1315067 | 1.29E-05 | 0.02 |
| CYS1 | -0.231079796 | 1.03E-14 | -0.072513337 | 0.0164964 | 0.16 |
| CYSLTR1 | -0.430591474 | 1.46E-50 | -0.228205206 | 2.22E-14 | 0.21 |
| CYSLTR2 | -0.296757497 | 1.17E-23 | -0.334318066 | 5.98E-30 | 0.04 |
| CYTH1 | 0.054210621 | 0.07321315 | -0.096013114 | 0.0014832 | 0.16 |
| CYTH2 | -0.131105305 | 1.37E-05 | 0.001093863 | 0.9711848 | 0.14 |
| CYTH3 | -0.224370493 | 6.13E-14 | -0.253257837 | 1.86E-17 | 0.03 |
| CYTH4 | -0.115930011 | 0.00012239 | -0.210951487 | 1.85E-12 | 0.1 |
| CYTIP | -0.067769904 | 0.025057 | -0.17073424 | 1.35E-08 | 0.11 |
| CYTL1 | -0.268444648 | 1.70E-19 | -0.175710681 | 4.98E-09 | 0.1 |
| SPECC1L | -0.288155637 | 2.41E-22 | -0.345968701 | 4.35E-32 | 0.06 |
| SPECC1 | -0.038305944 | 0.20571689 | -0.139743896 | 3.53E-06 | 0.11 |
| CYYR1 | -0.073199475 | 0.01549946 | -0.124274729 | 3.79E-05 | 0.06 |
| CYorf15B | 0.037573522 | 0.21452514 | 0.022542696 | 0.4565645 | 0.02 |
| CYorf15B.1 | 0.037573522 | 0.21452514 | 0.022542696 | 0.4565645 | 0.02 |
| D2HGDH | -0.081391164 | 0.0070977 | 0.064649543 | 0.0325866 | 0.15 |
| LOC105374360 | -0.10062879 | 0.00086381 | -0.12138859 | 5.73E-05 | 0.03 |
| DAAM1 | -0.376805504 | 3.36E-38 | -0.319025877 | 2.80E-27 | 0.06 |
| DAAM2 | -0.320708558 | 1.45E-27 | -0.367591587 | 2.65E-36 | 0.05 |
| DAB1 | -0.178461494 | 2.83E-09 | -0.202284549 | 1.48E-11 | 0.03 |
| AF9Q34 | -0.141203958 | 2.78E-06 | -0.230887021 | 1.08E-14 | 0.09 |
| DAB2 | -0.363693911 | 1.61E-35 | -0.316466814 | 7.56E-27 | 0.05 |
| DACH1 | -0.448336162 | 3.69E-55 | -0.213615361 | 9.56E-13 | 0.24 |
| DACH2 | -0.028523369 | 0.34613583 | -0.097389354 | 0.0012653 | 0.07 |
| DACT1 | -0.285663712 | 5.70E-22 | -0.157696706 | 1.60E-07 | 0.13 |
| DACT2 | 0.027908812 | 0.35663058 | -0.082395796 | 0.0064191 | 0.12 |
| DACT3 | -0.283352242 | 1.25E-21 | -0.140268002 | 3.24E-06 | 0.15 |
| DAD1P1 | 0.127692281 | 2.29E-05 | 0.057289655 | 0.0583026 | 0.08 |
| LOC116180 | 0.184545628 | 7.89E-10 | 0.348240298 | 1.63E-32 | 0.17 |
| DAG1 | -0.233384165 | 5.49E-15 | -0.166969703 | 2.81E-08 | 0.07 |
| DAGLA | -0.144247848 | 1.68E-06 | -0.251661076 | 2.99E-17 | 0.11 |
| LOC155335 | -0.05398382 | 0.07442471 | 0.033708417 | 0.2655109 | 0.09 |
| TKFC | -0.110363539 | 0.0002565 | 0.042149366 | 0.1637693 | 0.16 |
| DALRD3 | -0.277732676 | 8.27E-21 | 0.004529236 | 0.8811051 | 0.29 |
| DAND5 | 0.201661296 | 1.71E-11 | 0.156957654 | 1.83E-07 | 0.05 |
| DAOA | 0.013061539 | 0.66621684 | 0.017142107 | 0.5713118 | 0.01 |
| DAO | 0.007940954 | 0.79313946 | -0.032201513 | 0.2874839 | 0.05 |
| DAP3 | 0.351485629 | 3.93E-33 | 0.560443647 | 2.06E-91 | 0.21 |
| DAPK1 | 0.083184046 | 0.00592807 | -0.101840168 | 0.0007468 | 0.19 |
| DAPK2 | 0.064235477 | 0.03371838 | -0.022116004 | 0.4651335 | 0.09 |
| DAPK3 | 0.063007819 | 0.03727317 | 0.091375236 | 0.0024963 | 0.03 |
| DAPL1 | 0.008457797 | 0.78001119 | 0.002311009 | 0.9391678 | 0.01 |
| DAPP1 | -0.009789868 | 0.74647121 | -0.035561192 | 0.2401152 | 0.03 |
| DAP | 0.000787062 | 0.97926457 | -0.014071268 | 0.6421502 | 0.02 |
| ACKR1 | -0.21727332 | 3.82E-13 | -0.150492266 | 5.79E-07 | 0.07 |
| DARS2 | 0.150851014 | 5.44E-07 | 0.298279288 | 6.75E-24 | 0.15 |
| DARS | 0.206194665 | 5.85E-12 | 0.098035228 | 0.0011736 | 0.11 |
| DAXX | 0.302951372 | 1.23E-24 | 0.19885093 | 3.29E-11 | 0.11 |
| DAZ1 | 0.072020669 | 0.01724643 | 0.03141799 | 0.2993801 | 0.05 |
| DAZ2 | 0.05929871 | 0.05000402 | 0.042549102 | 0.1598062 | 0.02 |
| DAZ3 | NA | NA | NA | NA | #VALUE! |
| DAZ4 | NA | NA | NA | NA | #VALUE! |
| DAZAP1 | 0.485085837 | 1.37E-65 | 0.3555103 | 6.59E-34 | 0.13 |
| DAZAP2 | -0.34741245 | 2.33E-32 | -0.081458303 | 0.0070504 | 0.27 |
| DAZL | 0.077041 | 0.01083763 | -0.062619779 | 0.038461 | 0.14 |
| DBCCR1 | -0.02553357 | 0.39904646 | -0.117613124 | 9.72E-05 | 0.1 |
| DBF4B | 0.273350858 | 3.49E-20 | 0.061451921 | 0.0422317 | 0.22 |
| DBF4 | 0.492272767 | 8.81E-68 | 0.300061499 | 3.54E-24 | 0.2 |
| DBH | -0.140558547 | 3.09E-06 | -0.169203361 | 1.82E-08 | 0.03 |
| DBI | 0.280163266 | 3.68E-21 | 0.326710513 | 1.33E-28 | 0.05 |
| DBN1 | 0.240902502 | 6.78E-16 | 0.223075411 | 8.60E-14 | 0.02 |
| DBNDD1 | 0.304488661 | 7.00E-25 | 0.209701094 | 2.51E-12 | 0.1 |
| DBNDD2 | -0.066194531 | 0.02864601 | 0.000649194 | 0.9828961 | 0.07 |
| DBNL | 0.045128434 | 0.13595536 | 0.02809701 | 0.3533957 | 0.02 |
| DBP | -0.107822587 | 0.00035561 | -0.044471425 | 0.141753 | 0.07 |
| DBR1 | 0.040774317 | 0.17796764 | 0.014797066 | 0.6250781 | 0.03 |
| DBT | -0.196916009 | 5.13E-11 | -0.23410335 | 4.51E-15 | 0.04 |
| DBX1 | 0.110382196 | 0.00025588 | 0.015668326 | 0.6048473 | 0.1 |
| DBX2 | -0.304973557 | 5.85E-25 | -0.167018668 | 2.78E-08 | 0.14 |
| DCAF10 | -0.227589007 | 2.62E-14 | -0.116568439 | 0.0001122 | 0.12 |
| DCAF11 | -0.207494867 | 4.28E-12 | -0.163688072 | 5.26E-08 | 0.05 |
| DCAF12L1 | -0.012758196 | 0.67351595 | -0.026919259 | 0.3739449 | 0.02 |
| DCAF12L2 | -0.151941486 | 4.49E-07 | -0.139092743 | 3.92E-06 | 0.02 |
| DCAF12 | 0.053569726 | 0.07667895 | -0.100873482 | 0.0008389 | 0.16 |
| DCAF13 | 0.534869577 | 6.57E-82 | 0.31900207 | 2.82E-27 | 0.22 |
| DCAF15 | 0.294654999 | 2.47E-23 | 0.138154461 | 4.56E-06 | 0.16 |
| DCAF16 | -0.223315096 | 8.08E-14 | -0.265409724 | 4.45E-19 | 0.05 |
| DCAF17 | -0.252851344 | 2.10E-17 | -0.254419556 | 1.31E-17 | 0.01 |
| WDR21B | 0.052706942 | 0.08155441 | 0.023957844 | 0.4287883 | 0.03 |
| WDR21C | 0.079353665 | 0.00867473 | 0.041444169 | 0.1709407 | 0.04 |
| DCAF4 | 0.226412598 | 3.58E-14 | 0.167319636 | 2.63E-08 | 0.06 |
| DCAF5 | -0.475647058 | 8.69E-63 | -0.315044191 | 1.31E-26 | 0.17 |
| DCAF6 | -0.036965625 | 0.2220395 | 0.22114705 | 1.42E-13 | 0.26 |
| DCAF7 | -0.02114578 | 0.48494629 | -0.148070933 | 8.80E-07 | 0.13 |
| DCAF8L1 | 0.087297456 | 0.00387312 | 0.027823295 | 0.3581066 | 0.06 |
| FLJ32742 | 0.062220335 | 0.03971724 | 0.030332628 | 0.3163936 | 0.04 |
| DCAF8 | -0.264101772 | 6.72E-19 | 0.095338219 | 0.0016021 | 0.36 |
| DCAKD | -0.089946447 | 0.00291739 | -0.120021209 | 6.96E-05 | 0.04 |
| DCBLD1 | 0.110634162 | 0.00024763 | -0.010128194 | 0.7380256 | 0.13 |
| DCBLD2 | 0.007990183 | 0.79188643 | -0.072207687 | 0.0169583 | 0.09 |
| DCC | -0.020729891 | 0.49357682 | -0.115985048 | 0.0001215 | 0.1 |
| DCDC5 | -0.343242038 | 1.40E-31 | -0.204925337 | 7.92E-12 | 0.14 |
| DCDC2B | -0.072805526 | 0.01606515 | -0.047394856 | 0.1173516 | 0.03 |
| DFNB66 | -0.227468302 | 2.71E-14 | -0.12299519 | 4.56E-05 | 0.11 |
| DCD | -0.013995286 | 0.64394861 | 0.053771956 | 0.0755712 | 0.07 |
| LOC144129 | -0.349479046 | 9.47E-33 | -0.286935893 | 3.68E-22 | 0.07 |
| CDH27 | 0.051750773 | 0.08724788 | -0.110785485 | 0.0002428 | 0.17 |
| ECI1 | 0.135151936 | 7.34E-06 | 0.218255295 | 2.98E-13 | 0.09 |
| DCK | 0.008583951 | 0.77681607 | -0.133703037 | 9.20E-06 | 0.15 |
| DCLK1 | -0.369988583 | 8.63E-37 | -0.265980736 | 3.72E-19 | 0.11 |
| DCLK2 | 0.05237663 | 0.08348621 | -0.01035713 | 0.7323285 | 0.07 |
| DCLK3 | -0.068487667 | 0.02355517 | -0.090827446 | 0.0026507 | 0.03 |
| DCLRE1A | 0.030629038 | 0.31168556 | -0.01590438 | 0.599417 | 0.05 |
| DCLRE1B | 0.272493899 | 4.62E-20 | 0.004139045 | 0.8912813 | 0.27 |
| SCIDA | 0.353218875 | 1.83E-33 | 0.169463512 | 1.73E-08 | 0.19 |
| DCN | -0.31991246 | 1.98E-27 | -0.165152612 | 3.98E-08 | 0.16 |
| DCP1A | -0.361210344 | 5.03E-35 | -0.344702801 | 7.50E-32 | 0.02 |
| DCP1B | -0.018968825 | 0.5310142 | 0.059735881 | 0.0483357 | 0.08 |
| LOC105379125 | -0.214339553 | 7.98E-13 | -0.101433194 | 0.0007843 | 0.12 |
| DCPS | 0.398828327 | 5.47E-43 | 0.244693977 | 2.30E-16 | 0.16 |
| DCST1 | -0.109490229 | 0.00028721 | 0.025953665 | 0.3913316 | 0.14 |
| DCST2 | -0.006343689 | 0.83406733 | 0.181763652 | 1.42E-09 | 0.19 |
| DCTD | -0.158231528 | 1.45E-07 | -0.149348293 | 7.06E-07 | 0.01 |
| DCTN1 | -0.083944291 | 0.0054868 | -0.155984335 | 2.19E-07 | 0.08 |
| DCTN2 | 0.166291738 | 3.20E-08 | 0.126640827 | 2.68E-05 | 0.04 |
| DCTN3 | 0.187125572 | 4.53E-10 | 0.238516046 | 1.33E-15 | 0.06 |
| DCTN4 | -0.339407338 | 7.14E-31 | -0.167588494 | 2.49E-08 | 0.18 |
| LOC100506587 | 0.077212159 | 0.01066253 | 0.0718204 | 0.0175598 | 0.01 |
| DCTN6 | -0.145989167 | 1.25E-06 | -0.02500906 | 0.4088061 | 0.13 |
| DCTPP1 | 0.241289679 | 6.07E-16 | 0.304313986 | 7.47E-25 | 0.07 |
| DCT | -0.107151931 | 0.00038718 | -0.1694771 | 1.73E-08 | 0.07 |
| RP42 | -0.044635375 | 0.14028876 | -0.126615586 | 2.69E-05 | 0.09 |
| FLJ20092 | 0.082179549 | 0.00656002 | 0.157306031 | 1.72E-07 | 0.08 |
| DCUN1D3 | -0.317528901 | 5.01E-27 | -0.237363895 | 1.83E-15 | 0.09 |
| DCUN1D4 | 0.021151747 | 0.48482305 | 0.019235587 | 0.5252522 | 0.01 |
| DCUN1D5 | 0.373115247 | 1.97E-37 | 0.31389933 | 2.03E-26 | 0.06 |
| DCXR | 0.122857666 | 4.65E-05 | 0.213799138 | 9.13E-13 | 0.1 |
| DCX | 0.093067863 | 0.00206972 | -0.084250089 | 0.0053178 | 0.18 |
| DDA1 | 0.351549805 | 3.82E-33 | 0.162810754 | 6.21E-08 | 0.19 |
| DDAH1 | -0.125311672 | 3.26E-05 | -0.084664771 | 0.0050962 | 0.05 |
| DDAH2 | 0.084809998 | 0.00502054 | 0.164237517 | 4.74E-08 | 0.08 |
| DDB1 | -0.035030803 | 0.24720389 | -0.097082185 | 0.0013112 | 0.07 |
| LOC113427 | -0.167841507 | 2.37E-08 | -0.142010767 | 2.44E-06 | 0.03 |
| DDC | 0.265337429 | 4.56E-19 | 0.069766148 | 0.0210727 | 0.2 |
| DDHD1 | -0.076046275 | 0.01190671 | -0.227926259 | 2.40E-14 | 0.16 |
| DDHD2 | -0.043666571 | 0.14911144 | -0.218043788 | 3.14E-13 | 0.18 |
| DDI1 | -0.110503879 | 0.00025187 | -0.029680337 | 0.3269176 | 0.09 |
| DDI2 | -0.429499939 | 2.75E-50 | -0.472588269 | 6.73E-62 | 0.05 |
| LOC92982 | 0.302807539 | 1.30E-24 | 0.21900901 | 2.46E-13 | 0.09 |
| DDIT4L | 0.023731862 | 0.43315686 | -0.046896179 | 0.1212635 | 0.08 |
| DDIT4 | 0.196506172 | 5.63E-11 | 0.078062152 | 0.0098296 | 0.12 |
| LOC115996 | 0.483105263 | 5.40E-65 | 0.238650386 | 1.28E-15 | 0.25 |
| DDOST | 0.310974494 | 6.19E-26 | 0.167832639 | 2.38E-08 | 0.15 |
| DDO | -0.362820572 | 2.41E-35 | -0.192509117 | 1.39E-10 | 0.18 |
| NEP | -0.129746395 | 1.69E-05 | -0.064745683 | 0.0323285 | 0.07 |
| DDR2 | -0.330577534 | 2.78E-29 | -0.382310509 | 2.31E-39 | 0.06 |
| DDRGK1 | 0.050562599 | 0.09476448 | 0.116527808 | 0.0001128 | 0.07 |
| DDTL | 0.141740672 | 2.55E-06 | 0.104156255 | 0.0005629 | 0.04 |
| DDT | 0.310108658 | 8.59E-26 | 0.285135617 | 6.83E-22 | 0.03 |
| DDX10 | 0.321805227 | 9.39E-28 | 0.20585301 | 6.35E-12 | 0.12 |
| DDX11L2 | 0.037151511 | 0.219722 | 0.044638001 | 0.1402654 | 0.01 |
| LOC87030 | 0.379751782 | 8.07E-39 | 0.130501753 | 1.50E-05 | 0.25 |
| DDX12P | 0.392175983 | 1.67E-41 | 0.173640915 | 7.56E-09 | 0.22 |
| DDX17 | -0.333555493 | 8.19E-30 | -0.172037179 | 1.04E-08 | 0.17 |
| DDX18 | 0.125425026 | 3.21E-05 | 0.021026419 | 0.4874149 | 0.11 |
| LOC116087 | 0.182038778 | 1.34E-09 | -0.048013706 | 0.1126354 | 0.24 |
| DDX19B | 0.131787386 | 1.24E-05 | -0.042763672 | 0.1577089 | 0.18 |
| DDX1 | 0.248644587 | 7.29E-17 | 0.157054465 | 1.80E-07 | 0.1 |
| DKFZp434H052 | 0.084680401 | 0.00508798 | -0.007734122 | 0.7984099 | 0.1 |
| DDX21 | -0.05916703 | 0.05051586 | -0.233586857 | 5.19E-15 | 0.18 |
| DDX23 | 0.01886365 | 0.53329472 | -0.007287276 | 0.8098273 | 0.03 |
| DDX24 | -0.172200697 | 1.01E-08 | -0.18070155 | 1.78E-09 | 0.01 |
| DDX25 | -0.007025998 | 0.81652215 | -0.071696681 | 0.0177558 | 0.07 |
| LOC654032 | 0.092093612 | 0.00230631 | -0.042226868 | 0.1629952 | 0.14 |
| DDX27 | 0.356051252 | 5.17E-34 | 0.171194924 | 1.23E-08 | 0.19 |
| DDX28 | 0.447876118 | 4.90E-55 | 0.258681574 | 3.60E-18 | 0.19 |
| DDX31 | -0.026508699 | 0.3812784 | 0.006463072 | 0.8309912 | 0.04 |
| DDX39A | 0.581087766 | 1.04E-99 | 0.386148922 | 3.46E-40 | 0.2 |
| LOC728964 | -0.345736323 | 4.81E-32 | -0.338915961 | 8.79E-31 | 0.01 |
| DDX3Y | 0.068284372 | 0.02397241 | 0.036505128 | 0.2278558 | 0.04 |
| LOC220782 | 0.209381808 | 2.71E-12 | 0.207283883 | 4.50E-12 | 0.01 |
| DDX42 | 0.008688936 | 0.77416001 | -0.069611727 | 0.0213599 | 0.08 |
| DDX43 | -0.042169013 | 0.16357284 | -0.097834115 | 0.0012015 | 0.06 |
| LOC51139 | -0.195691617 | 6.78E-11 | -0.168879568 | 1.94E-08 | 0.03 |
| DDX47 | 0.312227238 | 3.85E-26 | 0.241422873 | 5.85E-16 | 0.08 |
| FLJ10432 | 0.466573674 | 3.55E-60 | 0.330277856 | 3.14E-29 | 0.14 |
| DDX4 | 0.097043931 | 0.00131705 | 0.012477971 | 0.6802862 | 0.09 |
| DDX50 | 0.114318225 | 0.00015216 | 0.044728635 | 0.1394611 | 0.07 |
| DDX51 | 0.215524525 | 5.93E-13 | 0.142152055 | 2.38E-06 | 0.08 |
| FLJ10790 | 0.087610486 | 0.00374699 | 0.052141805 | 0.0848819 | 0.04 |
| LOC340567 | -0.011469641 | 0.70485777 | -0.026489967 | 0.3816151 | 0.02 |
| DDX54 | 0.203782774 | 1.04E-11 | 0.121163878 | 5.92E-05 | 0.09 |
| DDX55 | 0.289577634 | 1.47E-22 | 0.174876872 | 5.89E-09 | 0.12 |
| DDX56 | 0.35790948 | 2.25E-34 | 0.343715583 | 1.15E-31 | 0.02 |
| DDX58 | -0.039138302 | 0.19602883 | -0.041258378 | 0.1728685 | 0.01 |
| DDX59 | -0.018862007 | 0.53333038 | 0.295594958 | 1.77E-23 | 0.32 |
| DDX5 | -0.057861664 | 0.05583107 | -0.104887138 | 0.0005142 | 0.05 |
| DDX60L | -0.135626133 | 6.81E-06 | -0.190126451 | 2.35E-10 | 0.06 |
| FLJ10787 | -0.173288869 | 8.11E-09 | -0.209247248 | 2.80E-12 | 0.04 |
| DDX6 | -0.165346849 | 3.84E-08 | -0.105310388 | 0.0004879 | 0.07 |
| DEAF1 | -0.002072299 | 0.94544093 | 0.023789766 | 0.4320351 | 0.03 |
| 1-Dec | 0.017220438 | 0.5695544 | -0.058713416 | 0.0523128 | 0.08 |
| DECR1 | 0.288441252 | 2.19E-22 | 0.294104465 | 3.00E-23 | 0.01 |
| PDCR | 0.086510277 | 0.00420748 | 0.214310652 | 8.04E-13 | 0.13 |
| LOC125937 | 0.126518217 | 2.73E-05 | 0.097770613 | 0.0012104 | 0.03 |
| DEDD | 0.28037134 | 3.43E-21 | 0.454493215 | 8.08E-57 | 0.18 |
| DEF6 | 0.093967462 | 0.00187122 | 0.002532832 | 0.9333418 | 0.1 |
| DEF8 | 0.144347762 | 1.65E-06 | -0.05091933 | 0.0924552 | 0.2 |
| DEFA1B | 0.064704091 | 0.03243998 | 0.029485239 | 0.3301089 | 0.04 |
| DEFA4 | -0.032100081 | 0.28900574 | -0.063024928 | 0.0372215 | 0.04 |
| DEFA5 | 0.008088653 | 0.78938162 | 0.019157486 | 0.5269359 | 0.02 |
| DEFA6 | -0.006066876 | 0.84120951 | 0.01872967 | 0.536207 | 0.03 |
| DEFB103A | 0.067241664 | 0.02621452 | 0.016378952 | 0.588567 | 0.06 |
| DEFB104A | 0.008381195 | 0.78195309 | 0.043697071 | 0.1488274 | 0.04 |
| DEFB105A | -0.007479655 | 0.80490667 | 0.023859764 | 0.4306812 | 0.04 |
| DEFB106A | -0.007479655 | 0.80490667 | 0.023859764 | 0.4306812 | 0.04 |
| DEFB107A | 0.021293875 | 0.48189283 | 0.020870849 | 0.4906425 | 0.01 |
| DEFB108B | -0.038714962 | 0.20091365 | -0.03202237 | 0.2901753 | 0.01 |
| DEFB109P1B | -0.092936375 | 0.0021003 | -0.06308202 | 0.0370496 | 0.03 |
| DEFB109P1 | 0.001589019 | 0.95815119 | -0.019355671 | 0.5226689 | 0.03 |
| DEFB111 | -0.011971836 | 0.6925796 | -0.002587502 | 0.9319064 | 0.01 |
| DEFB112 | NA | NA | NA | NA | #VALUE! |
| DEFB113 | -0.02025629 | 0.50350412 | -0.006573087 | 0.8281588 | 0.02 |
| DEFB114 | NA | NA | NA | NA | #VALUE! |
| DEFB115 | 0.072899947 | 0.01592794 | 0.052450952 | 0.0830483 | 0.03 |
| DEFB116 | NA | NA | NA | NA | #VALUE! |
| DEFB118 | -0.050974494 | 0.09210215 | -0.070864461 | 0.0191251 | 0.02 |
| DEFB120 | -0.030720329 | 0.31024488 | -0.026356632 | 0.3840169 | 0.01 |
| DEFB121 | -0.046428709 | 0.12502238 | -0.025733744 | 0.395359 | 0.03 |
| DEFB122 | NA | NA | NA | NA | #VALUE! |
| DEFB123 | -0.040331865 | 0.18272547 | -0.006949902 | 0.8184745 | 0.04 |
| DEFB124 | 0.045887714 | 0.129485 | -0.016794252 | 0.5791471 | 0.07 |
| DEFB125 | 0.051286062 | 0.09012842 | 0.010220241 | 0.7357332 | 0.05 |
| DEFB126 | 0.077597026 | 0.01027794 | 0.022547478 | 0.456469 | 0.06 |
| DEFB127 | -0.007331535 | 0.80869455 | -0.010506495 | 0.7286194 | 0.01 |
| LOC642293 | -0.000728621 | 0.98080392 | 0.01771462 | 0.558527 | 0.02 |
| LOC149788 | 0.026419288 | 0.38288709 | -0.011164311 | 0.7123607 | 0.04 |
| DEFB130 | -0.007150581 | 0.81332818 | 0.019865437 | 0.5117755 | 0.03 |
| LOC650444 | 0.059685681 | 0.04852491 | 0.060891667 | 0.0441487 | 0.01 |
| DEFB132 | -0.365817006 | 6.05E-36 | -0.195951561 | 6.39E-11 | 0.17 |
| DEFB134 | 0.021678216 | 0.47401739 | -0.003393943 | 0.9107619 | 0.03 |
| DEFB135 | 0.015077388 | 0.61853713 | -0.009091262 | 0.764006 | 0.03 |
| DEFB136 | 0.001236957 | 0.96741731 | 0.017926936 | 0.5538213 | 0.02 |
| DEFB1 | 0.157364696 | 1.70E-07 | 0.067681546 | 0.0252475 | 0.09 |
| LOC115252 | 0.078593617 | 0.00933878 | 0.005622686 | 0.8526974 | 0.08 |
| DEGS1 | 0.04597334 | 0.12877057 | 0.311260749 | 5.55E-26 | 0.27 |
| DEGS2 | -0.349605197 | 8.96E-33 | -0.028258046 | 0.3506424 | 0.33 |
| DEK | 0.346237278 | 3.87E-32 | 0.16234449 | 6.78E-08 | 0.19 |
| EXO5 | 0.304834128 | 6.16E-25 | 0.155470239 | 2.40E-07 | 0.15 |
| FLJ21129 | -0.019870867 | 0.51166013 | -0.087261712 | 0.0038878 | 0.07 |
| DENND1B | -0.342623832 | 1.83E-31 | -0.206938292 | 4.90E-12 | 0.14 |
| DENND1C | -0.136205284 | 6.21E-06 | -0.252861952 | 2.09E-17 | 0.12 |
| DENND2A | -0.079415179 | 0.00862288 | -0.124505181 | 3.67E-05 | 0.05 |
| DENND2C | -0.252543975 | 2.30E-17 | -0.357720579 | 2.44E-34 | 0.11 |
| DENND2D | -0.233938344 | 4.72E-15 | -0.118800325 | 8.25E-05 | 0.12 |
| DENND3 | -0.136647821 | 5.79E-06 | -0.267924191 | 2.01E-19 | 0.14 |
| DENND4A | -0.420953038 | 3.54E-48 | -0.473408024 | 3.90E-62 | 0.06 |
| DENND4B | 0.033447962 | 0.26922379 | 0.002809899 | 0.9260698 | 0.04 |
| LOC100420355 | -0.463997203 | 1.90E-59 | -0.329659601 | 4.04E-29 | 0.14 |
| DENND5A | -0.363390428 | 1.85E-35 | -0.375617746 | 5.95E-38 | 0.02 |
| DENND5B | -0.390969193 | 3.08E-41 | -0.380962266 | 4.47E-39 | 0.02 |
| LOC196411 | 0.244704022 | 2.29E-16 | 0.221299297 | 1.36E-13 | 0.03 |
| LOC94594 | 0.444106799 | 4.88E-54 | 0.238443504 | 1.35E-15 | 0.21 |
| DEPDC1 | 0.440966428 | 3.24E-53 | 0.185668065 | 6.21E-10 | 0.26 |
| DEPDC4 | 0.004282285 | 0.8875434 | 0.040303027 | 0.1830388 | 0.04 |
| DEPDC5 | -0.21423151 | 8.20E-13 | -0.246051934 | 1.55E-16 | 0.04 |
| DEPTOR | -0.2347675 | 3.76E-15 | -0.060612795 | 0.0451299 | 0.18 |
| DEPDC7 | 0.144120933 | 1.72E-06 | 0.057897718 | 0.0556783 | 0.09 |
| DERA | 0.312783839 | 3.11E-26 | 0.190125693 | 2.35E-10 | 0.13 |
| PRO2577 | 0.256109092 | 7.88E-18 | 0.127206291 | 2.47E-05 | 0.13 |
| DERL2 | 0.207102592 | 4.71E-12 | 0.279993621 | 3.89E-21 | 0.08 |
| C22orf14 | 0.068545662 | 0.0234373 | 0.013891852 | 0.6464001 | 0.06 |
| DES | -0.047041004 | 0.12011706 | -0.076888059 | 0.0109963 | 0.03 |
| DET1 | -0.180670428 | 1.79E-09 | -0.116144524 | 0.0001189 | 0.07 |
| DEXI | 0.209103707 | 2.90E-12 | 0.218483666 | 2.81E-13 | 0.01 |
| DFFA | 0.16688314 | 2.86E-08 | -0.099898306 | 0.0009424 | 0.27 |
| DFFB | 0.222549169 | 9.86E-14 | 0.052358459 | 0.0835935 | 0.18 |
| DFNA5 | -0.08356319 | 0.00570416 | -0.118368777 | 8.76E-05 | 0.04 |
| WI | -0.1330257 | 1.02E-05 | -0.074127807 | 0.0142351 | 0.06 |
| DFNB59 | 0.023488447 | 0.43789102 | 0.049680322 | 0.1006743 | 0.03 |
| LOC100131064 | 0.232765202 | 6.50E-15 | 0.213533447 | 9.76E-13 | 0.02 |
| DGAT2L6 | -0.126025536 | 2.94E-05 | -0.048184519 | 0.1113603 | 0.08 |
| DGAT2 | 0.178484971 | 2.82E-09 | 0.122712874 | 4.75E-05 | 0.06 |
| DGCR10 | 0.223630747 | 7.44E-14 | 0.113462274 | 0.0001706 | 0.12 |
| DGCR11 | -0.007052129 | 0.81585197 | 0.06222459 | 0.0397037 | 0.07 |
| DGCR14 | 0.313074635 | 2.78E-26 | 0.212255493 | 1.34E-12 | 0.11 |
| KIAA0163 | -0.122897978 | 4.62E-05 | -0.110548358 | 0.0002504 | 0.02 |
| LOC100506454 | 0.248721974 | 7.12E-17 | 0.046788786 | 0.1221191 | 0.21 |
| DGCR6L | 0.146744752 | 1.10E-06 | 0.146207531 | 1.21E-06 | 0.01 |
| DGCR6 | 0.127338119 | 2.42E-05 | 0.158968069 | 1.27E-07 | 0.04 |
| FLJ22127 | 0.098506644 | 0.00111063 | -0.009962935 | 0.7421471 | 0.11 |
| DGCR9 | 0.201163779 | 1.92E-11 | 0.039519878 | 0.1917009 | 0.17 |
| DGKA | -0.168834949 | 1.96E-08 | -0.152395434 | 4.15E-07 | 0.02 |
| DGKB | -0.133667255 | 9.25E-06 | -0.131115473 | 1.37E-05 | 0.01 |
| DGKD | -0.41026598 | 1.27E-45 | -0.180661703 | 1.79E-09 | 0.23 |
| DGKE | -0.085106017 | 0.00486951 | -0.169225177 | 1.81E-08 | 0.09 |
| DGKG | 0.145896795 | 1.27E-06 | -0.090595899 | 0.0027186 | 0.24 |
| LOC283508 | -0.176283151 | 4.43E-09 | -0.130837922 | 1.43E-05 | 0.05 |
| LOC100128727 | -0.323838215 | 4.20E-28 | -0.244956865 | 2.13E-16 | 0.08 |
| DGKK | -0.081892093 | 0.00675167 | -0.020116925 | 0.5064453 | 0.07 |
| DGKQ | -0.195527401 | 7.04E-11 | -0.123012668 | 4.55E-05 | 0.08 |
| DGKZ | 0.252107335 | 2.62E-17 | 0.060817203 | 0.0444089 | 0.2 |
| LOC129820 | 0.572318622 | 4.11E-96 | 0.428434575 | 5.07E-50 | 0.15 |
| DCE | -0.292234883 | 5.81E-23 | -0.122380779 | 4.98E-05 | 0.17 |
| SLOS | 0.318210768 | 3.84E-27 | 0.15268845 | 3.94E-07 | 0.17 |
| DHDDS | -0.243265963 | 3.46E-16 | -0.316580203 | 7.24E-27 | 0.08 |
| DHDH | 0.279975108 | 3.92E-21 | 0.19746415 | 4.53E-11 | 0.09 |
| HOGA1 | -0.224837785 | 5.43E-14 | -0.18452475 | 7.93E-10 | 0.05 |
| DHFRL1 | -0.324643431 | 3.04E-28 | -0.244700979 | 2.29E-16 | 0.08 |
| DHFR | 0.135889492 | 6.53E-06 | 0.02217327 | 0.4639783 | 0.12 |
| GDXYM | -0.255838085 | 8.56E-18 | -0.153229611 | 3.58E-07 | 0.11 |
| DHODH | 0.346842122 | 2.98E-32 | 0.255850106 | 8.53E-18 | 0.1 |
| DHPS | 0.118539718 | 8.55E-05 | 0.152721922 | 3.91E-07 | 0.04 |
| DHRS11 | 0.268474048 | 1.68E-19 | 0.193789911 | 1.04E-10 | 0.08 |
| DHRS12 | -0.141750958 | 2.54E-06 | 0.053584842 | 0.0765957 | 0.2 |
| DHRS13 | 0.188808285 | 3.14E-10 | 0.197676149 | 4.31E-11 | 0.01 |
| DHRS1 | -0.037986255 | 0.20952872 | -0.052053873 | 0.0854094 | 0.02 |
| DHRS2 | -0.099521541 | 0.00098543 | -0.053337447 | 0.0779676 | 0.05 |
| DHRS3 | 0.111346544 | 0.00022563 | -0.013713244 | 0.6506424 | 0.13 |
| DHRS4L1 | -0.060103872 | 0.0469679 | 0.050485359 | 0.0952705 | 0.12 |
| DHRS4L2 | 0.037887796 | 0.21071292 | 0.122394599 | 4.97E-05 | 0.09 |
| DHRS4 | 0.000710699 | 0.98127599 | 0.079981637 | 0.0081582 | 0.08 |
| LOC51636 | -0.008795294 | 0.77147188 | 0.109134345 | 0.0003007 | 0.12 |
| DHRS7C | -0.040182991 | 0.18434735 | -0.023023554 | 0.4470148 | 0.02 |
| DHRS7 | -0.195775436 | 6.65E-11 | -0.057586305 | 0.0570098 | 0.14 |
| DHRS9 | -0.176378513 | 4.34E-09 | -0.183140202 | 1.06E-09 | 0.01 |
| DHRSX | 0.000652774 | 0.98280182 | 0.158678901 | 1.34E-07 | 0.16 |
| DHTKD1 | 0.231293537 | 9.70E-15 | 0.170505998 | 1.41E-08 | 0.07 |
| DHX15 | -0.122908023 | 4.62E-05 | -0.125389647 | 3.22E-05 | 0.01 |
| DHX16 | 0.123200361 | 4.43E-05 | -0.003705505 | 0.9026089 | 0.13 |
| DHX29 | -0.385204723 | 5.54E-40 | -0.12923862 | 1.82E-05 | 0.26 |
| DHX30 | 0.002561266 | 0.9325952 | -0.029322059 | 0.3327935 | 0.04 |
| DHX32 | -0.101687005 | 0.0007607 | 0.003274531 | 0.9138892 | 0.11 |
| DHX33 | -0.202167233 | 1.52E-11 | -0.310047958 | 8.78E-26 | 0.11 |
| DHX34 | 0.271681606 | 6.01E-20 | 0.243432405 | 3.30E-16 | 0.03 |
| DHX35 | -0.047883427 | 0.11361564 | -0.023516141 | 0.4373509 | 0.03 |
| DHX36 | 0.014168072 | 0.63986199 | -0.099692397 | 0.0009657 | 0.12 |
| MGC4322 | 0.200789757 | 2.10E-11 | 0.08254637 | 0.0063226 | 0.12 |
| DHX38 | 0.148908514 | 7.62E-07 | -0.103589484 | 0.0006035 | 0.26 |
| DHX40P | -0.019408072 | 0.52154366 | -0.033025347 | 0.2753238 | 0.02 |
| DHX40 | -0.167228971 | 2.67E-08 | -0.089210871 | 0.0031585 | 0.08 |
| FLJ32861 | 0.147202979 | 1.02E-06 | 0.052604484 | 0.0821497 | 0.1 |
| DHX58 | -0.142730323 | 2.16E-06 | -0.080448552 | 0.0077922 | 0.07 |
| DHX8 | -0.203955005 | 9.98E-12 | -0.213527956 | 9.77E-13 | 0.01 |
| DHX9 | 0.020795907 | 0.4922014 | 0.074636478 | 0.0135816 | 0.06 |
| DIABLO | 0.386260642 | 3.28E-40 | 0.366898851 | 3.66E-36 | 0.02 |
| DIAPH1 | -0.07121056 | 0.01854485 | -0.180078033 | 2.03E-09 | 0.11 |
| DIA2 | 0.092720647 | 0.00215136 | -0.066398106 | 0.0281587 | 0.16 |
| LOC101926912 | 0.380328354 | 6.10E-39 | 0.142779514 | 2.14E-06 | 0.24 |
| MNG1 | -0.424664045 | 4.37E-49 | -0.300663421 | 2.85E-24 | 0.13 |
| DIDO1 | -0.255395305 | 9.78E-18 | -0.265364027 | 4.52E-19 | 0.01 |
| DIMT1 | 0.068468831 | 0.02359356 | 0.049575236 | 0.1013974 | 0.02 |
| DIO1 | -0.096789467 | 0.00135638 | -0.007250456 | 0.8107699 | 0.09 |
| DIO2 | -0.204656899 | 8.45E-12 | -0.12117761 | 5.91E-05 | 0.09 |
| DIO3OS | -0.0430685 | 0.15476535 | -0.049011898 | 0.1053441 | 0.01 |
| DIO3 | -0.078775864 | 0.00917555 | -0.132248355 | 1.15E-05 | 0.06 |
| DIP2A | -0.229187812 | 1.71E-14 | -0.228122529 | 2.27E-14 | 0.01 |
| DIP2B | -0.301826037 | 1.86E-24 | -0.229150142 | 1.73E-14 | 0.08 |
| DIP2C | -0.324963274 | 2.68E-28 | -0.351163896 | 4.52E-33 | 0.03 |
| DIRAS1 | 0.216124218 | 5.10E-13 | 0.057876088 | 0.0557699 | 0.16 |
| DIRAS2 | 0.007035112 | 0.81628839 | -0.127942412 | 2.21E-05 | 0.14 |
| DIRAS3 | -0.203323238 | 1.16E-11 | -0.038057194 | 0.2086785 | 0.17 |
| DIRC1 | -0.05645004 | 0.06209431 | 0.010104007 | 0.7386283 | 0.07 |
| DIRC2 | -0.120453648 | 6.54E-05 | -0.038476196 | 0.2037075 | 0.09 |
| DIRC3 | -0.021476048 | 0.47815114 | 0.000842339 | 0.9778086 | 0.03 |
| FAM6A | -0.010659375 | 0.72482958 | 0.022975544 | 0.4479631 | 0.04 |
| DIS3L | -0.253344352 | 1.81E-17 | -0.268836654 | 1.50E-19 | 0.02 |
| DIS3 | -0.135627997 | 6.81E-06 | -0.15255236 | 4.03E-07 | 0.02 |
| DISC1 | -0.161732644 | 7.60E-08 | -0.213828353 | 9.07E-13 | 0.06 |
| DISC2 | 0.009521186 | 0.7532001 | -0.018379051 | 0.5438658 | 0.03 |
| DISP1 | -0.300106498 | 3.48E-24 | -0.013338844 | 0.6595717 | 0.29 |
| DISP2 | -0.094955617 | 0.00167342 | -0.1377085 | 4.89E-06 | 0.05 |
| DIXDC1 | -0.526157294 | 7.46E-79 | -0.326987373 | 1.19E-28 | 0.2 |
| DKC1 | 0.579152319 | 6.60E-99 | 0.290556404 | 1.05E-22 | 0.29 |
| DKFZP434H168 | -0.018532249 | 0.54051272 | -0.112430616 | 0.0001956 | 0.1 |
| DKFZP434K028 | 0.072458412 | 0.01657854 | 0.072888453 | 0.0159446 | 0.01 |
| DKFZP434L187 | 0.139501443 | 3.67E-06 | -0.011705946 | 0.6990706 | 0.16 |
| DKFZP586I1420 | -0.265786533 | 3.95E-19 | -0.106644556 | 0.0004128 | 0.16 |
| LOC100130358 | 0.025185626 | 0.40550499 | 0.17650138 | 4.24E-09 | 0.16 |
| LOC100128529 | 0.037953244 | 0.20992521 | -0.043290248 | 0.1526504 | 0.09 |
| DKFZP434L192 | -0.029738436 | 0.32597113 | -0.03547777 | 0.2412206 | 0.01 |
| DKFZp566F0947 | 0.014495291 | 0.63215269 | 0.02485518 | 0.4116961 | 0.02 |
| PHF2P1 | 0.127686935 | 2.30E-05 | 0.019109346 | 0.527975 | 0.11 |
| NCR3LG1 | -0.227472168 | 2.70E-14 | -0.323886133 | 4.12E-28 | 0.1 |
| AP5B1 | 0.000272339 | 0.99282443 | -0.238943236 | 1.18E-15 | 0.24 |
| DKFZP779M0652 | -0.016817493 | 0.57862201 | -0.031958737 | 0.2911354 | 0.02 |
| DKK1 | 0.188962275 | 3.04E-10 | 0.141417219 | 2.68E-06 | 0.05 |
| DKK2 | -0.366611041 | 4.19E-36 | -0.25032071 | 4.45E-17 | 0.12 |
| DKK3 | -0.314302384 | 1.74E-26 | -0.192744773 | 1.32E-10 | 0.13 |
| DKK4 | -0.07011253 | 0.02044064 | 0.019958593 | 0.5097977 | 0.1 |
| DKKL1 | 0.207229389 | 4.56E-12 | 0.223284796 | 8.15E-14 | 0.02 |
| DLAT | 0.093302508 | 0.00201617 | 0.015846694 | 0.600742 | 0.08 |
| DLC1 | -0.3461986 | 3.94E-32 | -0.320390312 | 1.64E-27 | 0.03 |
| DLD | 0.058633729 | 0.05263388 | 0.051291177 | 0.0900963 | 0.01 |
| DLEC1 | -0.163971651 | 4.99E-08 | -0.131066683 | 1.38E-05 | 0.04 |
| DLEU1 | 0.296367965 | 1.34E-23 | 0.2653687 | 4.51E-19 | 0.04 |
| DLEU2L | -0.198686582 | 3.42E-11 | -0.188282188 | 3.52E-10 | 0.02 |
| DLEU2 | 0.144289435 | 1.67E-06 | 0.121034167 | 6.03E-05 | 0.03 |
| DLEU7 | -0.116405717 | 0.00011472 | -0.162210047 | 6.95E-08 | 0.05 |
| DLG1 | -0.079309413 | 0.00871221 | -0.080841092 | 0.0074959 | 0.01 |
| DLG2 | -0.293530104 | 3.68E-23 | -0.214426041 | 7.81E-13 | 0.08 |
| DLG3 | -0.112344882 | 0.00019787 | 0.05413313 | 0.0736253 | 0.17 |
| DLG4 | -0.31631385 | 8.02E-27 | -0.258855335 | 3.42E-18 | 0.06 |
| DLG5 | -0.334906466 | 4.69E-30 | -0.24990035 | 5.04E-17 | 0.09 |
| DLGAP1 | 0.05008546 | 0.09792512 | -0.136054655 | 6.36E-06 | 0.19 |
| DLGAP2 | -0.272667823 | 4.36E-20 | -0.162970692 | 6.03E-08 | 0.11 |
| DLGAP3 | 0.19204996 | 1.54E-10 | -0.103770715 | 0.0005902 | 0.3 |
| DLGAP4 | 0.088791606 | 0.00330396 | 0.004718605 | 0.8761733 | 0.09 |
| DLGAP5 | 0.45610488 | 2.93E-57 | 0.235722195 | 2.89E-15 | 0.23 |
| DLK1 | -0.003794003 | 0.90029496 | -0.020809856 | 0.491911 | 0.02 |
| DLK2 | 0.013402161 | 0.6580582 | -0.051668303 | 0.0877536 | 0.07 |
| DLL1 | -0.313129463 | 2.73E-26 | -0.274072901 | 2.76E-20 | 0.04 |
| DLL3 | 0.36863282 | 1.63E-36 | 0.174917423 | 5.84E-09 | 0.2 |
| DLL4 | -0.278303046 | 6.84E-21 | -0.186612544 | 5.06E-10 | 0.1 |
| DLST | -0.133401633 | 9.64E-06 | -0.135513277 | 6.93E-06 | 0.01 |
| DLX1 | -0.013820157 | 0.64810165 | 0.035940575 | 0.2351333 | 0.05 |
| DLX2 | -0.193704195 | 1.06E-10 | -0.08736621 | 0.0038451 | 0.11 |
| DLX3 | 0.081882687 | 0.00675803 | 0.044510053 | 0.1414069 | 0.04 |
| DLX4 | 0.166176937 | 3.27E-08 | 0.0932612 | 0.0020255 | 0.08 |
| DLX5 | 0.209655078 | 2.54E-12 | 0.05766325 | 0.0566783 | 0.16 |
| DLX6.AS1 | 0.254181818 | 1.41E-17 | -0.018777745 | 0.5351611 | 0.28 |
| DLX6 | 0.296318797 | 1.36E-23 | 0.033061068 | 0.2748045 | 0.27 |
| DMAP1 | 0.162264748 | 6.88E-08 | 0.120446373 | 6.55E-05 | 0.05 |
| LOC100130706 | -0.050339253 | 0.09623364 | 0.011021054 | 0.7158907 | 0.07 |
| DMBX1 | 0.088268694 | 0.00349386 | 0.123055251 | 4.52E-05 | 0.04 |
| DMC1 | 0.092699267 | 0.00215648 | -0.012336722 | 0.6837086 | 0.11 |
| DMD | -0.064033823 | 0.03428161 | -0.201579412 | 1.75E-11 | 0.14 |
| DMGDH | -0.256380787 | 7.26E-18 | -0.236806553 | 2.14E-15 | 0.02 |
| DMKN | -0.020308203 | 0.50241083 | 0.002704456 | 0.9288366 | 0.03 |
| DMP1 | -0.038165395 | 0.20738649 | -0.095597388 | 0.0015554 | 0.06 |
| DMPK | -0.115354898 | 0.00013232 | -0.005450896 | 0.8571488 | 0.11 |
| DMRT1 | 0.366868894 | 3.71E-36 | 0.175555698 | 5.13E-09 | 0.2 |
| DMRT2 | -0.152367875 | 4.17E-07 | -0.100408324 | 0.0008869 | 0.06 |
| DMRT3 | 0.108991631 | 0.00030625 | 0.013036341 | 0.666822 | 0.1 |
| DMO | 0.109554943 | 0.00028482 | 0.003398926 | 0.9106314 | 0.11 |
| DMRTA2 | 0.304019847 | 8.32E-25 | 0.063352881 | 0.0362432 | 0.25 |
| DMRTB1 | 0.093262255 | 0.00202526 | 0.041744621 | 0.1678571 | 0.06 |
| DMRTC1B | -0.221451939 | 1.31E-13 | -0.166532706 | 3.06E-08 | 0.06 |
| DMRTC1 | -0.133925999 | 8.89E-06 | -0.125259333 | 3.28E-05 | 0.01 |
| DMRTC2 | -0.056603412 | 0.06138687 | 0.102664955 | 0.0006757 | 0.16 |
| DMTF1 | -0.177811418 | 3.24E-09 | -0.148361691 | 8.37E-07 | 0.03 |
| DMWD | -0.093644587 | 0.00194036 | -0.080932656 | 0.0074283 | 0.02 |
| DMXL1 | -0.532396664 | 4.94E-81 | -0.436559265 | 4.46E-52 | 0.1 |
| DMXL2 | -0.352813284 | 2.19E-33 | -0.34039103 | 4.72E-31 | 0.02 |
| DNA2 | 0.33987901 | 5.86E-31 | 0.215452677 | 6.04E-13 | 0.13 |
| DNAH10 | -0.213873311 | 8.97E-13 | -0.209893552 | 2.39E-12 | 0.01 |
| LOC221835 | 0.153567544 | 3.37E-07 | 0.013358576 | 0.6590999 | 0.15 |
| DNAH12 | -0.092839304 | 0.00212314 | -0.009292952 | 0.7589308 | 0.09 |
| C1orf67 | 0.152155708 | 4.33E-07 | 0.367953422 | 2.24E-36 | 0.22 |
| DNAH17 | 0.230149445 | 1.32E-14 | 0.057642863 | 0.056766 | 0.18 |
| DNAH1 | -0.320825548 | 1.38E-27 | -0.292872179 | 4.64E-23 | 0.03 |
| DNAH2 | -0.087262043 | 0.00388762 | 0.03546384 | 0.2414055 | 0.13 |
| DNAH3 | 0.080195428 | 0.00798876 | 0.042443302 | 0.160848 | 0.04 |
| KTGNR | -0.42917395 | 3.31E-50 | -0.150438689 | 5.84E-07 | 0.28 |
| DNAH6 | -0.259540419 | 2.77E-18 | -0.120840745 | 6.20E-05 | 0.14 |
| DNAH7 | -0.485354361 | 1.14E-65 | -0.240089899 | 8.53E-16 | 0.25 |
| LOC221461 | 0.002423624 | 0.93620959 | 0.027572873 | 0.3624511 | 0.03 |
| DNAH9 | -0.277485702 | 8.97E-21 | -0.167956752 | 2.32E-08 | 0.11 |
| ICS1 | -0.131098286 | 1.37E-05 | -0.056346673 | 0.0625749 | 0.08 |
| DNAI2 | -0.005835567 | 0.84718771 | -0.03878861 | 0.2000575 | 0.04 |
| DNAJA1 | 0.306901793 | 2.86E-25 | 0.153780271 | 3.24E-07 | 0.16 |
| DNJ3 | 0.188697957 | 3.22E-10 | 0.012626791 | 0.6766875 | 0.18 |
| DNAJA3 | 0.180266132 | 1.95E-09 | 0.104899837 | 0.0005134 | 0.08 |
| DNAJA4 | -0.109574189 | 0.00028411 | -0.079759441 | 0.0083378 | 0.03 |
| DNAJB11 | 0.543892613 | 3.62E-85 | 0.32884991 | 5.61E-29 | 0.22 |
| DNAJB12 | -0.017479025 | 0.56377122 | 0.009003324 | 0.766222 | 0.03 |
| DNAJB13 | 0.099137105 | 0.00103123 | 0.132720246 | 1.07E-05 | 0.04 |
| DNAJB14 | -0.44936154 | 1.97E-55 | -0.389181216 | 7.60E-41 | 0.07 |
| DNAJB1 | 0.125468248 | 3.19E-05 | 0.105161069 | 0.000497 | 0.03 |
| LOC130621 | 0.091696703 | 0.00240959 | 0.08078814 | 0.0075353 | 0.02 |
| DNAJB3 | 0.060336973 | 0.04611838 | 0.028821917 | 0.3411092 | 0.04 |
| DNAJB4 | -0.239386143 | 1.04E-15 | -0.25660843 | 6.78E-18 | 0.02 |
| DNAJB5 | -0.120163227 | 6.82E-05 | -0.171935586 | 1.06E-08 | 0.06 |
| LOC155110 | 0.207549008 | 4.23E-12 | 0.110627732 | 0.0002478 | 0.1 |
| DNAJB7 | -0.022094397 | 0.4655698 | -0.106097592 | 0.0004422 | 0.09 |
| DNAJB8 | 0.002879083 | 0.92425498 | -0.01918544 | 0.5263329 | 0.03 |
| DKFZP564F1862 | -0.275377722 | 1.80E-20 | -0.243295396 | 3.43E-16 | 0.04 |
| DNAJC10 | -0.173629983 | 7.57E-09 | -0.182504381 | 1.22E-09 | 0.01 |
| DNAJC11 | 0.222850148 | 9.12E-14 | 0.019168405 | 0.5267003 | 0.21 |
| DNAJC12 | -0.294722395 | 2.41E-23 | -0.076783199 | 0.0111062 | 0.22 |
| DNAJC13 | -0.348851504 | 1.25E-32 | -0.253939449 | 1.52E-17 | 0.1 |
| DNAJC14 | -0.229683776 | 1.50E-14 | -0.114807549 | 0.0001425 | 0.12 |
| DNAJC15 | 0.108225086 | 0.00033783 | 0.14568116 | 1.32E-06 | 0.04 |
| DNAJC16 | -0.420116578 | 5.66E-48 | -0.313168604 | 2.69E-26 | 0.11 |
| DNAJC17 | -0.013061874 | 0.6662088 | 0.133739547 | 9.15E-06 | 0.15 |
| DNAJC18 | -0.272521425 | 4.58E-20 | -0.307136831 | 2.62E-25 | 0.04 |
| DNAJC19 | 0.14263468 | 2.20E-06 | 0.236928589 | 2.07E-15 | 0.1 |
| DNAJC1 | -0.092398708 | 0.00222968 | 0.119287504 | 7.71E-05 | 0.22 |
| DNAJC21 | 0.006032119 | 0.84210724 | -0.0198639 | 0.5118082 | 0.03 |
| DNAJC22 | -0.266507878 | 3.15E-19 | -0.095805006 | 0.0015189 | 0.18 |
| DNAJC24 | -0.167213911 | 2.68E-08 | -0.156380167 | 2.04E-07 | 0.02 |
| LOC138778 | -0.049728433 | 0.1003447 | 0.014439263 | 0.6334699 | 0.07 |
| DNAJC25 | -0.101340227 | 0.00079316 | 0.006001296 | 0.8429035 | 0.11 |
| DNAJC27 | -0.412313095 | 4.19E-46 | -0.37998661 | 7.20E-39 | 0.04 |
| DNAJC28 | -0.196810046 | 5.26E-11 | -0.057181845 | 0.0587785 | 0.14 |
| DNAJC2 | 0.45358063 | 1.43E-56 | 0.342646841 | 1.81E-31 | 0.12 |
| DNAJC30 | 0.117440304 | 9.96E-05 | 0.152974577 | 3.74E-07 | 0.04 |
| LOC144871 | -0.236124955 | 2.58E-15 | -0.228734159 | 1.93E-14 | 0.01 |
| DNAJC4 | 0.091692476 | 0.00241071 | 0.145316002 | 1.40E-06 | 0.06 |
| DNAJC5B | 0.071309689 | 0.01838151 | 0.021576805 | 0.4760885 | 0.05 |
| DNAJC5G | 0.141433304 | 2.68E-06 | 0.067934447 | 0.0247056 | 0.08 |
| DNAJC5 | -0.037116274 | 0.22015997 | -0.176451877 | 4.28E-09 | 0.14 |
| DNAJC6 | 0.025479676 | 0.40004276 | -0.231620535 | 8.87E-15 | 0.26 |
| DNAJC7 | 0.284322864 | 9.01E-22 | 0.247527693 | 1.01E-16 | 0.04 |
| DNAJC8 | 0.316334073 | 7.96E-27 | 0.202979277 | 1.26E-11 | 0.12 |
| LOC196753 | 0.554907812 | 2.77E-89 | 0.340907991 | 3.79E-31 | 0.22 |
| DNAL1 | -0.453042692 | 2.00E-56 | -0.227276304 | 2.85E-14 | 0.23 |
| DNAL4 | 0.089795543 | 0.00296545 | 0.13457007 | 8.04E-06 | 0.05 |
| DNALI1 | -0.348644579 | 1.36E-32 | -0.114129461 | 0.0001561 | 0.24 |
| DNASE1L1 | -0.195428488 | 7.20E-11 | -0.253066607 | 1.97E-17 | 0.06 |
| DNASE1L2 | -0.006973595 | 0.81786653 | 0.049822727 | 0.0997011 | 0.06 |
| DNASE1L3 | -0.206680405 | 5.21E-12 | -0.224662066 | 5.68E-14 | 0.02 |
| DNASE1 | 0.090447889 | 0.00276275 | 0.099447647 | 0.0009941 | 0.01 |
| DNASE2B | -0.185731906 | 6.12E-10 | -0.080110511 | 0.0080557 | 0.11 |
| DNASE2 | 0.135605984 | 6.83E-06 | 0.054058719 | 0.0740228 | 0.09 |
| DND1 | -0.151327015 | 5.00E-07 | -0.044952093 | 0.1374932 | 0.11 |
| DNER | 0.24195036 | 5.03E-16 | 0.016315666 | 0.5900086 | 0.23 |
| LOC196337 | -0.127622147 | 2.32E-05 | -0.179629585 | 2.22E-09 | 0.06 |
| DNLZ | 0.3044402 | 7.13E-25 | 0.27661278 | 1.20E-20 | 0.03 |
| DNM1L | 0.242254325 | 4.62E-16 | 0.041718252 | 0.168126 | 0.21 |
| DNM1DN8.2 | 0.087165235 | 0.00392754 | -0.075735526 | 0.0122593 | 0.17 |
| DNM1 | -0.125102667 | 3.36E-05 | -0.179786734 | 2.15E-09 | 0.06 |
| DNM2 | 0.033773853 | 0.26458362 | -0.201218497 | 1.90E-11 | 0.24 |
| DNM3 | -0.150935531 | 5.36E-07 | -0.100068696 | 0.0009235 | 0.06 |
| DNMBP | -0.421676846 | 2.36E-48 | -0.287378193 | 3.16E-22 | 0.14 |
| DNMT1 | 0.213515828 | 9.80E-13 | -0.045986512 | 0.1286609 | 0.26 |
| DNMT3A | 0.327727466 | 8.83E-29 | 0.173629648 | 7.57E-09 | 0.16 |
| DNMT3B | 0.417077219 | 3.07E-47 | 0.227278882 | 2.85E-14 | 0.19 |
| DNMT3L | 0.179689894 | 2.20E-09 | 0.054908579 | 0.0695854 | 0.13 |
| DNPEP | 0.093509357 | 0.00197001 | 0.137148174 | 5.35E-06 | 0.05 |
| DNTTIP1 | 0.387814218 | 1.51E-40 | 0.240772155 | 7.03E-16 | 0.15 |
| DNTTIP2 | 0.307197495 | 2.56E-25 | 0.157142688 | 1.77E-07 | 0.16 |
| DNTT | 0.043560319 | 0.15010423 | -0.03080259 | 0.3089505 | 0.08 |
| DOC2A | -0.042741038 | 0.15792916 | 0.049698757 | 0.1005479 | 0.1 |
| DOC2BL | -0.341428682 | 3.04E-31 | -0.290785649 | 9.67E-23 | 0.06 |
| DOCK10 | -0.361259454 | 4.92E-35 | -0.348569252 | 1.41E-32 | 0.02 |
| DOCK11 | -0.25628784 | 7.47E-18 | -0.333760444 | 7.53E-30 | 0.08 |
| DOCK1 | -0.482322179 | 9.27E-65 | -0.437154896 | 3.13E-52 | 0.05 |
| DOCK2 | -0.199392796 | 2.90E-11 | -0.284094068 | 9.74E-22 | 0.09 |
| DOCK3 | 0.20549467 | 6.92E-12 | -0.122580295 | 4.84E-05 | 0.33 |
| FLJ34238 | -0.363661424 | 1.64E-35 | -0.337769417 | 1.42E-30 | 0.03 |
| LOC286061 | -0.329061883 | 5.15E-29 | -0.324798711 | 2.86E-28 | 0.01 |
| DOCK6 | -0.238223526 | 1.44E-15 | -0.219807755 | 2.00E-13 | 0.02 |
| DOCK7 | -0.039534001 | 0.19154206 | -0.246866435 | 1.22E-16 | 0.21 |
| DOCK8 | -0.303467535 | 1.02E-24 | -0.293989571 | 3.13E-23 | 0.01 |
| DOCK9 | -0.438003481 | 1.89E-52 | -0.400512375 | 2.27E-43 | 0.04 |
| DOHH | 0.236908633 | 2.08E-15 | 0.154976995 | 2.62E-07 | 0.09 |
| DOK1 | -0.231498692 | 9.17E-15 | -0.074050116 | 0.0143373 | 0.16 |
| DOK2 | 0.066657255 | 0.02754861 | -0.09409932 | 0.0018436 | 0.17 |
| DOK3 | 0.047001935 | 0.1204255 | -0.083597411 | 0.0056843 | 0.14 |
| DOK4 | -0.008670719 | 0.77462071 | -0.101838896 | 0.0007469 | 0.1 |
| DOK5 | 0.047219233 | 0.11871785 | -0.068073422 | 0.0244121 | 0.12 |
| DOK6 | -0.363903916 | 1.46E-35 | -0.274755826 | 2.21E-20 | 0.09 |
| DOK7 | -0.170590659 | 1.39E-08 | -0.075774883 | 0.0122141 | 0.1 |
| DOLK | -0.042710129 | 0.1582303 | 0.064817736 | 0.0321363 | 0.11 |
| LOC89888 | 0.016052377 | 0.59602365 | 0.079455127 | 0.0085894 | 0.07 |
| DXO | 0.249549814 | 5.59E-17 | 0.200511125 | 2.24E-11 | 0.05 |
| C21ORF60 | 0.435697392 | 7.41E-52 | 0.253660431 | 1.65E-17 | 0.19 |
| DOPEY1 | -0.327989946 | 7.94E-29 | -0.285512302 | 6.00E-22 | 0.05 |
| DOPEY2 | -0.367036989 | 3.43E-36 | -0.127810941 | 2.25E-05 | 0.24 |
| DOT1L | 0.061698003 | 0.04141221 | -0.071359531 | 0.0182999 | 0.14 |
| DPAGT1 | 0.097714763 | 0.00121834 | 0.181367317 | 1.55E-09 | 0.09 |
| DPCD | 0.187307554 | 4.36E-10 | 0.332419983 | 1.31E-29 | 0.15 |
| DPCR1 | -0.093677612 | 0.00193319 | -0.058322101 | 0.0539055 | 0.04 |
| DPEP1 | -0.185033426 | 7.11E-10 | -0.086057931 | 0.0044113 | 0.1 |
| DPEP2 | -0.102409674 | 0.00069698 | -0.122360965 | 4.99E-05 | 0.02 |
| DPEP3 | -0.134180774 | 8.54E-06 | -0.085406578 | 0.0047204 | 0.05 |
| DPF1 | 0.28267462 | 1.58E-21 | 0.061575066 | 0.0418199 | 0.23 |
| DPF2 | 0.152189793 | 4.30E-07 | 0.091989387 | 0.002333 | 0.07 |
| DPF3 | 0.01448612 | 0.63236825 | -0.075705797 | 0.0122935 | 0.1 |
| DPH1 | -0.095294934 | 0.00161006 | -0.042873883 | 0.1566398 | 0.06 |
| DPH2 | 0.372981441 | 2.10E-37 | 0.129938678 | 1.64E-05 | 0.25 |
| DPH3P1 | 0.01421382 | 0.63878179 | -0.178574267 | 2.77E-09 | 0.2 |
| DPH3 | 0.056279532 | 0.0628887 | 0.034699438 | 0.2517062 | 0.03 |
| DPH5 | 0.299860927 | 3.81E-24 | 0.248128774 | 8.48E-17 | 0.06 |
| DPM1 | 0.327493925 | 9.71E-29 | 0.234536636 | 4.00E-15 | 0.1 |
| DPM2 | 0.282049681 | 1.95E-21 | 0.357851237 | 2.31E-34 | 0.08 |
| DPM3 | 0.301115357 | 2.41E-24 | 0.366025159 | 5.49E-36 | 0.07 |
| DPP10 | 0.110416808 | 0.00025473 | 0.048958986 | 0.105721 | 0.07 |
| DPP3 | 0.14183022 | 2.51E-06 | 0.141486693 | 2.65E-06 | 0.01 |
| DPP4 | -0.21246358 | 1.27E-12 | -0.159171635 | 1.22E-07 | 0.06 |
| DPP6 | -0.061961062 | 0.04055108 | -0.099804646 | 0.0009529 | 0.04 |
| DPP7 | 0.004240164 | 0.8886423 | 0.074750625 | 0.0134387 | 0.08 |
| DPP8 | -0.467041529 | 2.62E-60 | -0.462260399 | 5.82E-59 | 0.01 |
| DPP9 | -0.092186584 | 0.0022827 | -0.088455583 | 0.0034249 | 0.01 |
| DPPA2 | 0.144341052 | 1.65E-06 | 0.083216357 | 0.0059087 | 0.07 |
| DPPA3 | 0.056961366 | 0.05976166 | -0.008864363 | 0.7697277 | 0.07 |
| DPPA4 | -0.031402337 | 0.29962103 | -0.126169232 | 2.87E-05 | 0.1 |
| DPPA5 | 0.108487383 | 0.00032669 | 0.060283811 | 0.046311 | 0.05 |
| DPRXP4 | -0.081181177 | 0.00724742 | -0.124060321 | 3.91E-05 | 0.05 |
| DPRX | -0.06175115 | 0.041237 | -0.06313668 | 0.0368857 | 0.01 |
| DPT | -0.234658128 | 3.87E-15 | -0.129077027 | 1.87E-05 | 0.11 |
| DPY19L1 | -0.13668789 | 5.76E-06 | -0.177946831 | 3.15E-09 | 0.05 |
| LOC100420070 | -0.004300188 | 0.88707639 | -0.039672673 | 0.1899877 | 0.04 |
| DPY19L2P2 | 0.045717615 | 0.13091337 | -0.147183684 | 1.02E-06 | 0.2 |
| DPY19L2P4 | -0.146255352 | 1.20E-06 | -0.086008006 | 0.0044343 | 0.07 |
| DPY19L2 | -0.058174043 | 0.05451854 | -0.173354587 | 8.00E-09 | 0.12 |
| DPY19L3 | -0.452206634 | 3.37E-56 | -0.348512312 | 1.44E-32 | 0.11 |
| DPY19L4 | -0.107290093 | 0.00038047 | -0.122962777 | 4.58E-05 | 0.02 |
| LOC130447 | 0.533985264 | 1.35E-81 | 0.515270221 | 3.69E-75 | 0.02 |
| DPYD | -0.344644688 | 7.69E-32 | -0.309620692 | 1.03E-25 | 0.04 |
| DPYSL2 | -0.052361935 | 0.083573 | -0.180119202 | 2.01E-09 | 0.13 |
| DPYSL3 | -0.138340791 | 4.42E-06 | -0.096523461 | 0.0013986 | 0.05 |
| DPYSL4 | -0.004938923 | 0.87044152 | -0.099211738 | 0.0010222 | 0.1 |
| DPYSL5 | 0.306919338 | 2.84E-25 | 0.023249263 | 0.4425717 | 0.29 |
| DPYS | 0.067849955 | 0.0248855 | -0.049774053 | 0.1000329 | 0.12 |
| DQX1 | -0.101413602 | 0.00078619 | 0.082503481 | 0.0063499 | 0.19 |
| DR1 | -0.02633029 | 0.38449247 | -0.141880124 | 2.49E-06 | 0.12 |
| DRAM1 | 0.065996762 | 0.02912639 | -0.123518697 | 4.23E-05 | 0.19 |
| DRAM2 | -0.098557813 | 0.00110398 | 0.053777064 | 0.0755434 | 0.16 |
| LOC119810 | 0.322681899 | 6.64E-28 | 0.23147126 | 9.24E-15 | 0.1 |
| DRD1 | -0.227672758 | 2.56E-14 | -0.193374358 | 1.14E-10 | 0.04 |
| DRD2 | 0.056815858 | 0.06041796 | -0.140880921 | 2.93E-06 | 0.2 |
| DRD3 | -0.01165646 | 0.70028104 | -0.071738283 | 0.0176897 | 0.07 |
| DRD4 | -0.001473378 | 0.96119425 | 0.056656606 | 0.0611431 | 0.06 |
| DRD5 | 0.068466989 | 0.02359732 | -0.048789234 | 0.1069373 | 0.12 |
| DRG1 | 0.421122773 | 3.22E-48 | 0.31646422 | 7.57E-27 | 0.11 |
| DRG2 | 0.162169488 | 7.01E-08 | 0.141027789 | 2.86E-06 | 0.03 |
| DRGX | 0.058457842 | 0.05334847 | -0.038883292 | 0.1989608 | 0.1 |
| DRP2 | -0.02033879 | 0.50176727 | -0.050520985 | 0.0950368 | 0.04 |
| DSC1 | -0.140157961 | 3.30E-06 | -0.18979101 | 2.53E-10 | 0.05 |
| DSC2 | 0.26577844 | 3.96E-19 | 0.048675854 | 0.1077558 | 0.22 |
| DSC3 | 0.095319081 | 0.00160563 | -0.07723296 | 0.0106414 | 0.18 |
| DSCAML1 | -0.162910479 | 6.10E-08 | -0.146942114 | 1.07E-06 | 0.02 |
| DSCAM | -0.069016442 | 0.02249926 | -0.059807011 | 0.0480688 | 0.01 |
| DSCC1 | 0.579386815 | 5.28E-99 | 0.313172086 | 2.68E-26 | 0.27 |
| DSCR10 | 0.054734315 | 0.0704771 | -0.006062846 | 0.8413136 | 0.07 |
| DSCR3 | -0.140583461 | 3.08E-06 | -0.20339305 | 1.14E-11 | 0.07 |
| DSCR4 | 0.079698857 | 0.00838731 | 0.025583118 | 0.3981318 | 0.06 |
| RIPPLY3 | 0.15908501 | 1.24E-07 | 0.105128981 | 0.000499 | 0.06 |
| DSCR8 | 0.050494995 | 0.09520727 | 0.043106253 | 0.1544037 | 0.01 |
| DSCR9 | 0.237644267 | 1.69E-15 | 0.105734269 | 0.0004627 | 0.14 |
| DSEL | -0.270662135 | 8.35E-20 | -0.276383134 | 1.29E-20 | 0.01 |
| DSE | -0.212129414 | 1.38E-12 | -0.245516201 | 1.81E-16 | 0.04 |
| DSG1 | 0.111657175 | 0.00021663 | -0.059583443 | 0.0489121 | 0.18 |
| DSG2 | 0.038642571 | 0.20175776 | -0.095150247 | 0.0016368 | 0.14 |
| DSG3 | 0.054079454 | 0.07391186 | -0.090502992 | 0.0027462 | 0.15 |
| DSG4 | -0.116540988 | 0.00011262 | -0.107868586 | 0.0003535 | 0.01 |
| DSN1 | 0.39232654 | 1.55E-41 | 0.307051401 | 2.70E-25 | 0.09 |
| DSPP | 0.021972063 | 0.46804422 | -0.019946124 | 0.5100622 | 0.05 |
| LOC154189 | -0.038262364 | 0.20623353 | -0.013732847 | 0.6501762 | 0.03 |
| DSTN | -0.191068941 | 1.91E-10 | 0.043538342 | 0.1503102 | 0.24 |
| DSTYK | -0.110750879 | 0.0002439 | 0.129089849 | 1.86E-05 | 0.24 |
| DST | -0.396936783 | 1.46E-42 | -0.368355111 | 1.86E-36 | 0.03 |
| DTD1 | 0.350609065 | 5.77E-33 | 0.199501657 | 2.83E-11 | 0.16 |
| DTHD1 | -0.15992268 | 1.06E-07 | -0.148413341 | 8.29E-07 | 0.02 |
| DTL | 0.335477604 | 3.70E-30 | 0.340479458 | 4.54E-31 | 0.01 |
| DTNA | 0.03105926 | 0.30493458 | -0.035604409 | 0.239544 | 0.07 |
| DTNBP1 | 0.271490163 | 6.39E-20 | 0.311275139 | 5.52E-26 | 0.04 |
| DTNB | 0.276939483 | 1.08E-20 | 0.209483951 | 2.64E-12 | 0.07 |
| DTWD1 | -0.150598729 | 5.68E-07 | -0.036889514 | 0.2229934 | 0.12 |
| DTWD2 | -0.469531642 | 5.10E-61 | -0.215331965 | 6.23E-13 | 0.26 |
| DTX1 | -0.112141503 | 0.00020325 | -0.114509186 | 0.0001483 | 0.01 |
| DTX2 | 0.106788628 | 0.00040536 | 0.114766502 | 0.0001433 | 0.01 |
| DTX3L | -0.141207295 | 2.78E-06 | -0.091654431 | 0.0024208 | 0.05 |
| DTX3 | -0.142489065 | 2.25E-06 | 0.043768269 | 0.1481659 | 0.19 |
| DTX4 | 0.035200982 | 0.24491367 | -0.019314208 | 0.5235601 | 0.06 |
| LOC643109 | 0.491370116 | 1.67E-67 | 0.387307951 | 1.94E-40 | 0.11 |
| CTDNEP1 | 0.275987897 | 1.47E-20 | 0.180450332 | 1.87E-09 | 0.1 |
| DUOX1 | -0.001914662 | 0.94958542 | -0.020057166 | 0.5077093 | 0.02 |
| DUOX2 | 0.024260956 | 0.42296914 | -0.053568296 | 0.0766868 | 0.08 |
| DUOXA1 | 0.067629457 | 0.02536036 | 0.008696629 | 0.7739655 | 0.06 |
| DUOXA2 | 0.128028519 | 2.18E-05 | 0.001667236 | 0.9560933 | 0.13 |
| LOC105378361 | -0.039897663 | 0.1874855 | -0.034700727 | 0.2516886 | 0.01 |
| DUS1L | 0.420000296 | 6.04E-48 | 0.275388516 | 1.79E-20 | 0.15 |
| DUS2 | 0.405270212 | 1.85E-44 | 0.196944721 | 5.10E-11 | 0.21 |
| LOC92365 | 0.156927158 | 1.84E-07 | 0.096796915 | 0.0013552 | 0.07 |
| DUS4L | 0.147186471 | 1.02E-06 | 0.201136902 | 1.94E-11 | 0.06 |
| DUSP10 | -0.198923577 | 3.24E-11 | 0.038039381 | 0.2088917 | 0.24 |
| DUSP11 | 0.19830857 | 3.73E-11 | 0.10925022 | 0.0002962 | 0.09 |
| DUSP12 | 0.318365479 | 3.62E-27 | 0.436776549 | 3.92E-52 | 0.12 |
| DUSP13 | -0.03696939 | 0.22199239 | 0.028080316 | 0.3536819 | 0.07 |
| LOC116242 | 0.195307752 | 7.40E-11 | 0.089960574 | 0.0029129 | 0.11 |
| C20orf57 | -0.072130613 | 0.01707651 | 0.001698164 | 0.9552796 | 0.08 |
| DUSP16 | -0.314144454 | 1.85E-26 | -0.159298797 | 1.19E-07 | 0.16 |
| LOC91443 | -0.366495308 | 4.42E-36 | -0.310724544 | 6.80E-26 | 0.06 |
| DUSP19 | -0.199484439 | 2.84E-11 | -0.191936892 | 1.58E-10 | 0.01 |
| DUSP1 | -0.274879438 | 2.12E-20 | -0.196377911 | 5.80E-11 | 0.08 |
| DUSP21 | -0.03750423 | 0.21537231 | -0.017011248 | 0.5742534 | 0.03 |
| LOC100134291 | 0.053288812 | 0.07823961 | 0.013044667 | 0.666622 | 0.05 |
| DUSP23 | 0.250287607 | 4.49E-17 | 0.427522127 | 8.56E-50 | 0.18 |
| DUSP26 | -0.0691846 | 0.0221722 | -0.142910629 | 2.10E-06 | 0.08 |
| DUSP27 | -0.243646406 | 3.10E-16 | -0.191949472 | 1.57E-10 | 0.06 |
| DUSP28 | -0.165033789 | 4.08E-08 | 0.006540201 | 0.8290053 | 0.18 |
| DUSP2 | 0.292971948 | 4.48E-23 | 0.150980646 | 5.32E-07 | 0.15 |
| DUSP3 | -0.329076403 | 5.12E-29 | -0.263588206 | 7.89E-19 | 0.07 |
| DUSP4 | -0.434739785 | 1.30E-51 | -0.233931276 | 4.73E-15 | 0.21 |
| FLJ31401 | 0.112688264 | 0.00018909 | 0.011109275 | 0.7137161 | 0.11 |
| DUSP5 | -0.20015382 | 2.43E-11 | -0.148163278 | 8.66E-07 | 0.06 |
| DUSP6 | -0.281401632 | 2.42E-21 | -0.123374304 | 4.32E-05 | 0.16 |
| DUSP7 | 0.121666567 | 5.51E-05 | -0.136408867 | 6.02E-06 | 0.26 |
| DUSP8 | -0.082350775 | 0.00644819 | 0.015678442 | 0.6046141 | 0.1 |
| DUSP9 | 0.389382875 | 6.87E-41 | 0.235051418 | 3.47E-15 | 0.16 |
| DUT | 0.349690281 | 8.63E-33 | 0.319904925 | 1.98E-27 | 0.03 |
| DUX4L1 | NA | NA | NA | NA | #VALUE! |
| DUXA | -0.005619025 | 0.8527922 | -0.012795356 | 0.6726201 | 0.01 |
| DVL1 | 0.155276154 | 2.48E-07 | 0.003603087 | 0.9052879 | 0.16 |
| DVL2 | 0.101988359 | 0.0007335 | -0.007618696 | 0.8013551 | 0.11 |
| DVL3 | 0.164337963 | 4.65E-08 | -0.039677312 | 0.1899358 | 0.21 |
| DIVA | 0.024132739 | 0.42542503 | 0.01649623 | 0.5858997 | 0.01 |
| DYDC1 | 0.052817458 | 0.08091621 | 0.087822254 | 0.0036638 | 0.04 |
| DYDC2 | 0.116908741 | 0.00010709 | 0.174779569 | 6.01E-09 | 0.06 |
| DYM | 0.226805709 | 3.23E-14 | 0.0187648 | 0.5354426 | 0.21 |
| CDC23.1 | -0.04659905 | 0.12364231 | -0.019162405 | 0.5268297 | 0.03 |
| DYNC1I1 | 0.052294981 | 0.08396939 | -0.08711259 | 0.0039494 | 0.14 |
| DYNC1I2 | -0.357627183 | 2.55E-34 | -0.251676995 | 2.98E-17 | 0.11 |
| DYNC1LI1 | 0.016609947 | 0.58331878 | -0.006248409 | 0.8365242 | 0.03 |
| DYNC1LI2 | -0.103994815 | 0.00057417 | -0.226292182 | 3.70E-14 | 0.13 |
| DYNC2H1 | -0.460770564 | 1.51E-58 | -0.344561071 | 7.98E-32 | 0.12 |
| DYNC2LI1 | -0.16908239 | 1.86E-08 | 0.063666565 | 0.035328 | 0.24 |
| DYNLL1 | 0.49105602 | 2.09E-67 | 0.454256255 | 9.38E-57 | 0.04 |
| DYNLL2 | -0.138105599 | 4.59E-06 | -0.160154334 | 1.02E-07 | 0.03 |
| DNLC2A | 0.341870967 | 2.52E-31 | 0.361923973 | 3.63E-35 | 0.03 |
| DNLC2B | -0.305615941 | 4.61E-25 | -0.004769737 | 0.8748424 | 0.31 |
| DYNLT1 | 0.345943949 | 4.40E-32 | 0.445334899 | 2.31E-54 | 0.1 |
| DYNLT3 | -0.206502754 | 5.44E-12 | -0.114343452 | 0.0001516 | 0.1 |
| DYRK1A | -0.103611885 | 0.00060184 | -0.162876212 | 6.14E-08 | 0.06 |
| DYRK1B | -0.10998271 | 0.00026949 | -0.028518466 | 0.3462188 | 0.09 |
| DYRK2 | -0.023886502 | 0.43016469 | -0.090140769 | 0.0028566 | 0.07 |
| DYRK3 | -0.007984163 | 0.79203961 | 0.082277262 | 0.006496 | 0.1 |
| LOC116342 | 0.44718955 | 7.46E-55 | 0.324647442 | 3.04E-28 | 0.13 |
| PPP1R27 | 0.25609666 | 7.91E-18 | 0.117605903 | 9.73E-05 | 0.14 |
| DYSF | -0.011535506 | 0.70324296 | -0.152177853 | 4.31E-07 | 0.15 |
| DYTN | -0.035040772 | 0.24706933 | -0.050182023 | 0.0972788 | 0.02 |
| DYX1C1 | -0.127856044 | 2.24E-05 | 0.079903766 | 0.0082207 | 0.21 |
| DZIP1L | -0.113869412 | 0.00016158 | -0.174702277 | 6.10E-09 | 0.07 |
| DZIP1 | 0.089549465 | 0.00304536 | -0.07047348 | 0.0197996 | 0.17 |
| DZIP3 | -0.256757652 | 6.48E-18 | -0.203529632 | 1.10E-11 | 0.06 |
| E2F1 | 0.549414674 | 3.27E-87 | 0.288103201 | 2.46E-22 | 0.27 |
| E2F2 | 0.476295883 | 5.61E-63 | 0.177626007 | 3.36E-09 | 0.3 |
| E2F3 | 0.370723086 | 6.11E-37 | 0.159228235 | 1.21E-07 | 0.22 |
| E2F4 | 0.311405141 | 5.26E-26 | 0.074124514 | 0.0142394 | 0.24 |
| E2F5 | 0.314616817 | 1.54E-26 | 0.149542975 | 6.83E-07 | 0.17 |
| E2F6 | 0.320154508 | 1.80E-27 | 0.165869336 | 3.47E-08 | 0.16 |
| E2F7 | 0.302503729 | 1.45E-24 | 0.070305145 | 0.0200964 | 0.24 |
| E2F8 | 0.308902814 | 1.35E-25 | 0.093979004 | 0.0018688 | 0.22 |
| E4F1 | 0.054887994 | 0.06969029 | 0.088130556 | 0.0035457 | 0.04 |
| EAF1 | -0.223430722 | 7.84E-14 | -0.202649314 | 1.36E-11 | 0.03 |
| EAF2 | 0.131937715 | 1.21E-05 | 0.079634751 | 0.00844 | 0.06 |
| EAPP | -0.135758119 | 6.67E-06 | 0.050852842 | 0.0928821 | 0.19 |
| EARS2 | -0.06133717 | 0.04261851 | 0.030340369 | 0.3162701 | 0.1 |
| EBAG9 | 0.237632489 | 1.70E-15 | 0.137619098 | 4.96E-06 | 0.11 |
| EBF1 | -0.3523343 | 2.70E-33 | -0.252820884 | 2.12E-17 | 0.1 |
| EBF2 | -0.498546435 | 9.73E-70 | -0.398067666 | 8.12E-43 | 0.11 |
| EBF3 | -0.322999384 | 5.85E-28 | -0.248072722 | 8.62E-17 | 0.08 |
| EBF4 | -0.170404445 | 1.44E-08 | -0.110428435 | 0.0002544 | 0.06 |
| EBI3 | -0.07809401 | 0.00979959 | -0.121735414 | 5.46E-05 | 0.05 |
| EBNA1BP2 | 0.498005398 | 1.44E-69 | 0.311384691 | 5.30E-26 | 0.19 |
| EBPL | 0.356173547 | 4.90E-34 | 0.42118424 | 3.11E-48 | 0.07 |
| CDPX2 | 0.535477064 | 3.99E-82 | 0.395031596 | 3.89E-42 | 0.15 |
| ECD | -0.126076471 | 2.91E-05 | -0.02975436 | 0.325712 | 0.1 |
| ECE1 | -0.350264074 | 6.72E-33 | -0.278277659 | 6.90E-21 | 0.08 |
| ECE2 | 0.598320118 | 4.17E-107 | 0.39549789 | 3.06E-42 | 0.21 |
| ECEL1 | 0.225722075 | 4.30E-14 | 0.033074702 | 0.2746065 | 0.2 |
| ECH1 | 0.095985562 | 0.00148785 | 0.150241322 | 6.05E-07 | 0.06 |
| ECHDC1 | 0.086448843 | 0.00423465 | -0.0569262 | 0.0599197 | 0.15 |
| ECHDC2 | -0.12266894 | 4.78E-05 | -0.041246978 | 0.1729874 | 0.09 |
| ECHDC3 | 0.125123882 | 3.35E-05 | 0.070629532 | 0.019528 | 0.06 |
| ECHS1 | 0.230319841 | 1.26E-14 | 0.297853408 | 7.87E-24 | 0.07 |
| ECM1 | -0.279283771 | 4.93E-21 | -0.098069016 | 0.001169 | 0.19 |
| ECM2 | -0.432891764 | 3.84E-51 | -0.258218804 | 4.15E-18 | 0.18 |
| ECSCR | -0.186228657 | 5.50E-10 | -0.072014422 | 0.0172561 | 0.12 |
| ECSIT | 0.157511185 | 1.66E-07 | 0.152136503 | 4.34E-07 | 0.01 |
| C6orf91 | -0.206284795 | 5.73E-12 | 0.018613757 | 0.5387329 | 0.23 |
| ECT2 | 0.191558378 | 1.71E-10 | 0.102290667 | 0.0007071 | 0.09 |
| EDA2R | -0.453546638 | 1.46E-56 | -0.333560244 | 8.18E-30 | 0.12 |
| EDARADD | -0.14911462 | 7.35E-07 | -0.002690564 | 0.9292012 | 0.15 |
| EDAR | 0.062522454 | 0.03876397 | -0.115246524 | 0.0001343 | 0.18 |
| LOC220890 | -0.178558981 | 2.78E-09 | -0.293459836 | 3.77E-23 | 0.12 |
| EDC3 | 0.03091702 | 0.30715578 | -0.01700984 | 0.5742851 | 0.05 |
| EDC4 | 0.11573713 | 0.00012564 | -0.025161151 | 0.4059616 | 0.15 |
| EDDM3A | 0.030923753 | 0.3070504 | 0.009897323 | 0.7437855 | 0.03 |
| EDDM3B | -0.035328124 | 0.24321236 | -0.038903862 | 0.1987231 | 0.01 |
| EDEM1 | -0.416703166 | 3.77E-47 | -0.344000382 | 1.01E-31 | 0.08 |
| LOC149963 | 0.208143758 | 3.66E-12 | 0.192408757 | 1.42E-10 | 0.02 |
| LOC87240 | -0.430301973 | 1.73E-50 | -0.053672495 | 0.0761144 | 0.38 |
| EDF1 | 0.228176954 | 2.24E-14 | 0.25535314 | 9.91E-18 | 0.03 |
| EDIL3 | -0.430974828 | 1.17E-50 | -0.362555702 | 2.72E-35 | 0.07 |
| EDN1 | 0.020991222 | 0.48814411 | -0.017890347 | 0.5546308 | 0.04 |
| EDN2 | 0.084747385 | 0.00505302 | 0.083921366 | 0.0054997 | 0.01 |
| EDN3 | -0.050427627 | 0.09565016 | -0.054210281 | 0.073215 | 0.01 |
| EDNRA | -0.312867641 | 3.01E-26 | -0.169214761 | 1.82E-08 | 0.15 |
| EDNRB | -0.387158279 | 2.09E-40 | -0.305753573 | 4.38E-25 | 0.09 |
| EEA1 | -0.432922741 | 3.77E-51 | -0.345510614 | 5.30E-32 | 0.09 |
| EED | 0.324146083 | 3.71E-28 | 0.224213893 | 6.39E-14 | 0.1 |
| EEF1A1P9 | 0.05738738 | 0.05787406 | 0.134113813 | 8.63E-06 | 0.08 |
| EEF1A1L14 | 0.052796089 | 0.0810393 | 0.143195276 | 2.00E-06 | 0.1 |
| STN | -0.22729788 | 2.83E-14 | -0.122178177 | 5.13E-05 | 0.11 |
| EEF1B2 | 0.289053778 | 1.77E-22 | 0.282293154 | 1.79E-21 | 0.01 |
| LOC100422652 | -0.30032298 | 3.22E-24 | -0.209529472 | 2.61E-12 | 0.1 |
| LOC100130834 | 0.272590126 | 4.47E-20 | 0.228425239 | 2.10E-14 | 0.05 |
| EEF1E1 | 0.473511555 | 3.64E-62 | 0.375201959 | 7.26E-38 | 0.1 |
| EEF1G | 0.312444018 | 3.54E-26 | 0.249113422 | 6.35E-17 | 0.07 |
| LOC101060570 | -0.333779035 | 7.47E-30 | -0.226678673 | 3.34E-14 | 0.11 |
| EEF2 | -0.164165706 | 4.81E-08 | -0.113696524 | 0.0001654 | 0.06 |
| LOC105374097 | 0.171996847 | 1.05E-08 | 0.158099832 | 1.49E-07 | 0.02 |
| EEPD1 | -0.149882074 | 6.44E-07 | -0.115604216 | 0.0001279 | 0.04 |
| LOC100134776 | 0.16794228 | 2.33E-08 | 0.140191683 | 3.28E-06 | 0.03 |
| EFCAB1 | -0.103872551 | 0.00058287 | -0.062436147 | 0.0390343 | 0.05 |
| EFCAB2 | -0.122805984 | 4.69E-05 | 0.070935517 | 0.0190047 | 0.2 |
| EFCAB3 | 0.018458204 | 0.54213208 | 0.049129727 | 0.1045087 | 0.04 |
| CRACR2B | -0.100377767 | 0.00089009 | 0.136830941 | 5.63E-06 | 0.24 |
| FLJ33046 | -0.353062796 | 1.96E-33 | -0.264393147 | 6.13E-19 | 0.09 |
| EFCAB5 | 0.050946238 | 0.09228284 | 0.003849808 | 0.8988362 | 0.05 |
| EFCAB6 | -0.495039054 | 1.22E-68 | -0.26948316 | 1.22E-19 | 0.23 |
| EFCAB7 | -0.071217779 | 0.01853291 | -0.08752727 | 0.0037802 | 0.02 |
| DHRD | -0.170736288 | 1.35E-08 | -0.213408836 | 1.01E-12 | 0.05 |
| EFEMP2 | -0.20690136 | 4.94E-12 | -0.12377732 | 4.07E-05 | 0.09 |
| LOC121883 | -0.135008344 | 7.50E-06 | 0.067004036 | 0.0267501 | 0.21 |
| MICU3 | -0.273044649 | 3.86E-20 | -0.256128792 | 7.84E-18 | 0.02 |
| EFHB | -0.198822801 | 3.31E-11 | -0.065801666 | 0.029607 | 0.14 |
| EJM1 | -0.32174214 | 9.63E-28 | -0.110764866 | 0.0002435 | 0.22 |
| FLJ22601 | -0.149581817 | 6.78E-07 | -0.00689416 | 0.8199054 | 0.15 |
| FLJ13612 | -0.121966774 | 5.28E-05 | -0.009968109 | 0.7420179 | 0.12 |
| MGC4342 | 0.161013161 | 8.70E-08 | 0.053062578 | 0.0795152 | 0.11 |
| EFNA1 | 0.089807804 | 0.00296151 | 0.320998667 | 1.29E-27 | 0.24 |
| LOC100506781 | 0.106462091 | 0.00042238 | 0.029825288 | 0.3245596 | 0.08 |
| EFNA3 | 0.168818357 | 1.96E-08 | 0.286216156 | 4.71E-22 | 0.12 |
| EFNA4 | 0.231853794 | 8.33E-15 | 0.404366102 | 2.99E-44 | 0.18 |
| EFNA5 | 0.125512319 | 3.17E-05 | 0.114473388 | 0.000149 | 0.02 |
| EFNB1 | 0.10009169 | 0.00092095 | -0.056930433 | 0.0599007 | 0.16 |
| EFNB2 | -0.204980634 | 7.82E-12 | -0.247350396 | 1.06E-16 | 0.05 |
| LOC146750 | -0.051716694 | 0.08745657 | -0.13815544 | 4.56E-06 | 0.09 |
| EFR3A | -0.234417718 | 4.14E-15 | -0.247602575 | 9.88E-17 | 0.02 |
| EFR3B | -0.150763987 | 5.52E-07 | -0.158146417 | 1.48E-07 | 0.01 |
| EFS | 0.135651267 | 6.78E-06 | 0.106422925 | 0.0004245 | 0.03 |
| EFTUD1 | -0.126938766 | 2.57E-05 | -0.100495205 | 0.0008777 | 0.03 |
| EFTUD2 | 0.275570598 | 1.69E-20 | 0.129734951 | 1.69E-05 | 0.15 |
| EGFL6 | 0.105135835 | 0.00049857 | -0.019878887 | 0.5114897 | 0.13 |
| EGFL7 | -0.084587998 | 0.00513656 | 0.006761229 | 0.8233201 | 0.1 |
| EGFL8 | 0.101459183 | 0.00078188 | 0.147958686 | 8.97E-07 | 0.05 |
| EGFLAM | -0.283848321 | 1.06E-21 | -0.184911728 | 7.30E-10 | 0.1 |
| EGFR | -0.039979803 | 0.18657808 | -0.228277276 | 2.18E-14 | 0.19 |
| EGF | -0.24504587 | 2.07E-16 | -0.012878857 | 0.6706089 | 0.24 |
| SM.20 | 0.051226917 | 0.09050046 | 0.096404865 | 0.0014179 | 0.05 |
| LOC115774 | -0.074461692 | 0.01380312 | 0.001225713 | 0.9677133 | 0.08 |
| FLJ21620 | -0.111141856 | 0.00023176 | 0.020771505 | 0.4927096 | 0.14 |
| EGOT | -0.273096519 | 3.80E-20 | -0.119035326 | 7.98E-05 | 0.16 |
| EGR1 | -0.256453236 | 7.10E-18 | -0.166604457 | 3.02E-08 | 0.09 |
| EGR2 | -0.182352675 | 1.26E-09 | -0.207024603 | 4.79E-12 | 0.03 |
| EGR3 | -0.33290567 | 1.07E-29 | -0.195780851 | 6.64E-11 | 0.14 |
| EGR4 | 0.140887672 | 2.93E-06 | 0.053559446 | 0.0767356 | 0.09 |
| EHBP1L1 | -0.018713765 | 0.53655321 | -0.172989167 | 8.61E-09 | 0.16 |
| EHBP1 | 0.078361593 | 0.00955033 | -0.172779589 | 8.98E-09 | 0.26 |
| EHD1 | 0.101082853 | 0.00081807 | 0.026026137 | 0.3900099 | 0.08 |
| LOC115390 | -0.352071063 | 3.03E-33 | -0.275220677 | 1.89E-20 | 0.08 |
| EHD3 | -0.334821632 | 4.85E-30 | -0.371718948 | 3.81E-37 | 0.04 |
| EHD4 | -0.258734098 | 3.54E-18 | -0.259525972 | 2.78E-18 | 0.01 |
| EHF | -0.18874152 | 3.19E-10 | -0.057800224 | 0.0560923 | 0.14 |
| EHHADH | -0.188991914 | 3.02E-10 | -0.118415921 | 8.70E-05 | 0.08 |
| EHMT1 | -0.325052338 | 2.59E-28 | -0.24577408 | 1.68E-16 | 0.08 |
| NG36 | 0.324767467 | 2.90E-28 | 0.295411923 | 1.89E-23 | 0.03 |
| EI24 | 0.253232958 | 1.87E-17 | 0.261214527 | 1.65E-18 | 0.01 |
| EID1 | -0.321402392 | 1.10E-27 | -0.172359989 | 9.76E-09 | 0.15 |
| EID2B | -0.028651268 | 0.34397668 | -0.056157249 | 0.0634636 | 0.03 |
| EID2 | 0.286994857 | 3.61E-22 | 0.216317057 | 4.86E-13 | 0.08 |
| EID3 | -0.078233101 | 0.00966931 | -0.150140916 | 6.15E-07 | 0.08 |
| EIF1AD | 0.303696938 | 9.38E-25 | 0.174567102 | 6.27E-09 | 0.13 |
| EIF4C_2 | 0.036408072 | 0.2290954 | 0.028098246 | 0.3533745 | 0.01 |
| EIF1AY | 0.049633074 | 0.10099893 | 0.036043962 | 0.2337884 | 0.02 |
| EIF1B | -0.036234173 | 0.23132837 | -0.004500612 | 0.881851 | 0.04 |
| EIF1 | 0.242009079 | 4.95E-16 | 0.206335658 | 5.66E-12 | 0.04 |
| EIF2AK1 | 0.155034192 | 2.59E-07 | 0.189456925 | 2.73E-10 | 0.04 |
| EIF2AK2 | 0.080092456 | 0.00806999 | -0.059202324 | 0.0503782 | 0.14 |
| EIF2AK3 | -0.367729458 | 2.49E-36 | -0.292184553 | 5.92E-23 | 0.08 |
| EIF2AK4 | -0.251486934 | 3.15E-17 | -0.28604587 | 5.00E-22 | 0.04 |
| EIF2A | 0.073774178 | 0.01470561 | 0.172625417 | 9.26E-09 | 0.1 |
| EIF2B1 | 0.335524874 | 3.63E-30 | 0.326690213 | 1.34E-28 | 0.01 |
| EIF2B2 | 0.03777002 | 0.2121358 | 0.092816564 | 0.0021285 | 0.06 |
| EIF2B3 | 0.43016716 | 1.87E-50 | 0.296125476 | 1.46E-23 | 0.14 |
| EIF2B4 | 0.240521881 | 7.55E-16 | 0.202026738 | 1.57E-11 | 0.04 |
| EIF2B5 | 0.164475138 | 4.53E-08 | 0.203017739 | 1.25E-11 | 0.04 |
| GERP95 | -0.097852954 | 0.00119888 | -0.321574998 | 1.03E-27 | 0.23 |
| AGO2 | 0.304806484 | 6.23E-25 | 0.028286991 | 0.350149 | 0.28 |
| AGO3 | -0.067347662 | 0.02597861 | -0.276605405 | 1.20E-20 | 0.21 |
| AGO4 | -0.339145887 | 7.98E-31 | -0.481785036 | 1.34E-64 | 0.15 |
| EIF2S1 | 0.135834709 | 6.59E-06 | 0.10551373 | 0.0004756 | 0.04 |
| EIF2S2 | 0.482245525 | 9.77E-65 | 0.360401267 | 7.27E-35 | 0.13 |
| EIF2S3 | 0.172497622 | 9.50E-09 | 0.200521798 | 2.23E-11 | 0.03 |
| EIF3A | -0.203511462 | 1.11E-11 | -0.263963433 | 7.01E-19 | 0.07 |
| EIF3B | 0.459821557 | 2.78E-58 | 0.300065282 | 3.54E-24 | 0.16 |
| EIF3CL | 0.207515217 | 4.26E-12 | 0.196408309 | 5.76E-11 | 0.02 |
| LOC197365 | 0.064616718 | 0.03267514 | -0.158533766 | 1.37E-07 | 0.23 |
| EIF3D | 0.228660287 | 1.97E-14 | 0.179045539 | 2.51E-09 | 0.05 |
| EIF3E | 0.369549809 | 1.06E-36 | 0.265681073 | 4.09E-19 | 0.11 |
| EIF3F | 0.223628998 | 7.45E-14 | 0.221907979 | 1.16E-13 | 0.01 |
| EIF3G | 0.188917271 | 3.07E-10 | 0.167586638 | 2.49E-08 | 0.03 |
| EIF3H | 0.273494676 | 3.33E-20 | 0.274002363 | 2.82E-20 | 0.01 |
| EIF3IP1 | 0.036751775 | 0.22472719 | 0.093259196 | 0.002026 | 0.06 |
| EIF3I | 0.378472157 | 1.50E-38 | 0.257979848 | 4.46E-18 | 0.13 |
| EIF3J | 0.159092958 | 1.24E-07 | 0.103051915 | 0.0006445 | 0.06 |
| PRO1474 | 0.363159136 | 2.06E-35 | 0.308029101 | 1.88E-25 | 0.06 |
| EIF3L | 0.007311114 | 0.80921714 | 0.023735206 | 0.433092 | 0.02 |
| LOC63319 | 0.401871827 | 1.12E-43 | 0.37761067 | 2.28E-38 | 0.03 |
| EIF4A1 | 0.418891362 | 1.12E-47 | 0.27558464 | 1.68E-20 | 0.15 |
| BM.010 | -0.030118106 | 0.3198299 | -0.135145367 | 7.34E-06 | 0.11 |
| EIF4A3 | 0.376565312 | 3.77E-38 | 0.177422527 | 3.51E-09 | 0.2 |
| PRO1843 | -0.260656591 | 1.96E-18 | -0.092077525 | 0.0023104 | 0.17 |
| EIF4E1B | -0.07280921 | 0.01605978 | 0.066617523 | 0.0276414 | 0.14 |
| EIF4E2 | 0.226027564 | 3.97E-14 | 0.243717514 | 3.04E-16 | 0.02 |
| EIF4E3 | -0.434884674 | 1.19E-51 | -0.274975034 | 2.05E-20 | 0.16 |
| EIF4EBP1 | 0.463161165 | 3.25E-59 | 0.25738846 | 5.35E-18 | 0.21 |
| EIF4EBP2 | -0.098158168 | 0.0011569 | -0.055999785 | 0.0642102 | 0.05 |
| EIF4EBP3 | 0.043380344 | 0.15179734 | 0.220282544 | 1.77E-13 | 0.18 |
| EIF4ENIF1 | -0.080589871 | 0.00768434 | -0.153238521 | 3.57E-07 | 0.08 |
| EIF4E | 0.083683386 | 0.00563478 | 0.027909705 | 0.3566152 | 0.06 |
| EIF4G1 | 0.109842779 | 0.00027442 | -0.095399068 | 0.0015911 | 0.21 |
| EIF4G2 | -0.169528821 | 1.71E-08 | -0.151021546 | 5.28E-07 | 0.02 |
| EIF4G3 | -0.401851934 | 1.13E-43 | -0.370903563 | 5.61E-37 | 0.04 |
| LOC155284 | -0.013401119 | 0.65808308 | -0.132226555 | 1.16E-05 | 0.12 |
| EIF5A2 | -0.057829322 | 0.05596846 | -0.087007662 | 0.0039933 | 0.03 |
| EIF5AP1 | 0.574043602 | 8.21E-97 | 0.392395299 | 1.50E-41 | 0.19 |
| EIF5A | 0.577492039 | 3.20E-98 | 0.411554383 | 6.33E-46 | 0.17 |
| LOC51590 | 0.44413405 | 4.80E-54 | 0.334471434 | 5.61E-30 | 0.11 |
| EIF5 | -0.231659277 | 8.78E-15 | -0.14521332 | 1.43E-06 | 0.09 |
| EIF6 | 0.473608152 | 3.41E-62 | 0.385348749 | 5.16E-40 | 0.09 |
| ELAC1 | 0.163541179 | 5.41E-08 | 0.006291986 | 0.8354003 | 0.16 |
| FLJ10530 | 0.186712215 | 4.95E-10 | 0.094286578 | 0.0018051 | 0.1 |
| SERP1 | -0.136227393 | 6.19E-06 | -0.013072835 | 0.6659456 | 0.13 |
| ELAVL1 | 0.519850511 | 1.07E-76 | 0.326176637 | 1.65E-28 | 0.2 |
| ELAVL2 | 0.095724083 | 0.00153308 | 0.026026647 | 0.3900006 | 0.07 |
| ELAVL3 | 0.107197631 | 0.00038495 | -0.024324923 | 0.421747 | 0.14 |
| ELAVL4 | -0.030061756 | 0.32073657 | -0.15215015 | 4.33E-07 | 0.13 |
| ELF1 | -0.487407429 | 2.72E-66 | -0.368041573 | 2.15E-36 | 0.12 |
| ELF2 | -0.308103542 | 1.82E-25 | -0.301792132 | 1.89E-24 | 0.01 |
| ELF3 | 0.032698362 | 0.28010759 | 0.149201002 | 7.24E-07 | 0.12 |
| ELF4 | 0.028230218 | 0.35111721 | -0.223211173 | 8.30E-14 | 0.26 |
| ELF5 | 0.22756848 | 2.64E-14 | 0.136500323 | 5.93E-06 | 0.1 |
| LOC346355 | -0.229934358 | 1.40E-14 | -0.171087436 | 1.26E-08 | 0.06 |
| RP1.63G5.5 | -0.1184038 | 8.72E-05 | -0.126560331 | 2.71E-05 | 0.01 |
| ELK1 | 0.204067422 | 9.72E-12 | -0.010687811 | 0.7241254 | 0.22 |
| ELK3 | -0.54222467 | 1.47E-84 | -0.512254566 | 3.69E-74 | 0.03 |
| ELK4 | -0.156328913 | 2.05E-07 | -0.075417534 | 0.0126297 | 0.09 |
| ELL2 | -0.326629228 | 1.37E-28 | -0.094468595 | 0.0017684 | 0.24 |
| ELL3 | 0.220524891 | 1.66E-13 | 0.190955854 | 1.96E-10 | 0.03 |
| ELL | -0.079397016 | 0.00863816 | -0.145300354 | 1.41E-06 | 0.07 |
| ELMO1 | -0.137266165 | 5.25E-06 | -0.183859775 | 9.14E-10 | 0.05 |
| ELMO2 | -0.218704297 | 2.65E-13 | -0.224877373 | 5.37E-14 | 0.01 |
| ELMO3 | -0.029894349 | 0.32344001 | 0.086221144 | 0.0043367 | 0.12 |
| LOC643923 | -0.174815677 | 5.96E-09 | -0.192821481 | 1.29E-10 | 0.02 |
| ELMOD2 | -0.34067552 | 4.18E-31 | -0.127061513 | 2.52E-05 | 0.22 |
| ELMOD3 | -0.231027093 | 1.04E-14 | -0.139303464 | 3.79E-06 | 0.1 |
| ELN | -0.214064972 | 8.55E-13 | -0.132761626 | 1.06E-05 | 0.09 |
| ELOF1 | 0.274779217 | 2.19E-20 | 0.272998394 | 3.92E-20 | 0.01 |
| ELOVL1 | 0.316858126 | 6.50E-27 | 0.11466218 | 0.0001453 | 0.21 |
| ELOVL2 | -0.203871791 | 1.02E-11 | -0.141827617 | 2.51E-06 | 0.07 |
| MGC21435 | 0.039299359 | 0.19419344 | -0.066904111 | 0.0269781 | 0.11 |
| STGD2 | 0.191329567 | 1.80E-10 | -0.031953641 | 0.2912124 | 0.23 |
| ELOVL5 | -0.403966045 | 3.70E-44 | -0.179591015 | 2.24E-09 | 0.23 |
| ELOVL6 | 0.221968438 | 1.15E-13 | 0.109653981 | 0.0002812 | 0.12 |
| ELOVL7 | 0.104733426 | 0.0005241 | -0.137772851 | 4.84E-06 | 0.25 |
| DBIL5P | 0.182985142 | 1.10E-09 | 0.123432855 | 4.28E-05 | 0.06 |
| ELP2 | -0.116099888 | 0.0001196 | -0.066984167 | 0.0267953 | 0.05 |
| ELP3 | -0.181353098 | 1.55E-09 | -0.04390698 | 0.1468836 | 0.14 |
| ELP4 | 0.077840459 | 0.0100411 | 0.071699454 | 0.0177514 | 0.01 |
| ELSPBP1 | -0.052505314 | 0.08272926 | -0.001269164 | 0.9665694 | 0.06 |
| ADGRL4 | -0.364623438 | 1.05E-35 | -0.234070096 | 4.55E-15 | 0.14 |
| EMB | -0.259339554 | 2.94E-18 | -0.194878668 | 8.15E-11 | 0.07 |
| EMCN | -0.341472007 | 2.98E-31 | -0.192601286 | 1.36E-10 | 0.15 |
| EMD | 0.369004366 | 1.37E-36 | 0.22855819 | 2.02E-14 | 0.15 |
| EME1 | 0.4467994 | 9.47E-55 | 0.316979241 | 6.20E-27 | 0.13 |
| LOC390668 | -0.223690811 | 7.33E-14 | -0.197682604 | 4.30E-11 | 0.03 |
| EMG1 | 0.559072653 | 7.00E-91 | 0.44807236 | 4.34E-55 | 0.12 |
| EMID1 | 0.069377022 | 0.02180301 | 0.059714432 | 0.0484165 | 0.01 |
| COL26A1 | 0.20386877 | 1.02E-11 | 0.0531841 | 0.0788279 | 0.16 |
| DKFZP586M121 | -0.172358851 | 9.77E-09 | -0.137761113 | 4.85E-06 | 0.04 |
| EMILIN2 | 0.03456179 | 0.25359311 | -0.132109547 | 1.18E-05 | 0.17 |
| EMILIN3 | 0.187874937 | 3.85E-10 | 0.061239357 | 0.0429506 | 0.13 |
| EML1 | -0.267317073 | 2.44E-19 | -0.28445462 | 8.61E-22 | 0.02 |
| EML2 | -0.262927373 | 9.70E-19 | -0.176531583 | 4.21E-09 | 0.09 |
| EML3 | -0.119974223 | 7.00E-05 | -0.193172509 | 1.20E-10 | 0.08 |
| EML4 | 0.16650306 | 3.08E-08 | -0.030019727 | 0.3214139 | 0.2 |
| EML5 | -0.038380175 | 0.20483903 | -0.156776487 | 1.89E-07 | 0.12 |
| EML6 | 0.012637957 | 0.67641774 | -0.078559545 | 0.0093696 | 0.1 |
| EMP1 | -0.200957245 | 2.02E-11 | -0.198121784 | 3.89E-11 | 0.01 |
| EMP2 | -0.047039571 | 0.12012836 | 0.048463002 | 0.1093058 | 0.1 |
| EMP3 | 0.080229789 | 0.00796182 | 0.029208716 | 0.3346665 | 0.06 |
| ADGRE1 | -0.063390209 | 0.03613324 | -0.10887905 | 0.0003107 | 0.05 |
| ADGRE2 | 0.002714854 | 0.92856374 | -0.217892547 | 3.26E-13 | 0.23 |
| ADGRE3 | -0.123586303 | 4.19E-05 | -0.163800081 | 5.15E-08 | 0.05 |
| EMR4 | -0.293518598 | 3.70E-23 | -0.310716419 | 6.82E-26 | 0.02 |
| EMX1 | -0.157996679 | 1.52E-07 | -0.063029068 | 0.037209 | 0.1 |
| LOC645902 | -0.300455516 | 3.07E-24 | -0.20432214 | 9.15E-12 | 0.1 |
| LOC115684 | -0.164240323 | 4.74E-08 | -0.090509155 | 0.0027444 | 0.08 |
| EN1 | 0.353292746 | 1.77E-33 | 0.127866109 | 2.24E-05 | 0.23 |
| AUT | 0.138001119 | 4.67E-06 | 0.059380482 | 0.0496884 | 0.08 |
| ENAH | -0.062298578 | 0.03946847 | 0.198492239 | 3.57E-11 | 0.27 |
| ENAM | -0.120416605 | 6.58E-05 | -0.141388993 | 2.70E-06 | 0.03 |
| ENC1 | -0.155169986 | 2.53E-07 | -0.044876009 | 0.1381608 | 0.12 |
| ENDOD1 | -0.085226395 | 0.00480927 | -0.151677954 | 4.70E-07 | 0.07 |
| ENDOG | 0.245162865 | 2.01E-16 | 0.218705006 | 2.65E-13 | 0.03 |
| ENDOU | -0.155671518 | 2.31E-07 | -0.082448638 | 0.0063851 | 0.08 |
| ENGASE | 0.001346588 | 0.96453134 | -0.020393621 | 0.5006147 | 0.03 |
| ENG | -0.16873949 | 1.99E-08 | -0.160170161 | 1.02E-07 | 0.01 |
| ENHO | 0.264477314 | 5.97E-19 | -0.091693693 | 0.0024104 | 0.36 |
| ENKUR | 0.082825305 | 0.00614718 | 0.049687415 | 0.1006257 | 0.04 |
| ENO1 | 0.436743426 | 4.00E-52 | 0.208416583 | 3.43E-12 | 0.23 |
| ENO2 | 0.016418612 | 0.58766435 | -0.123053136 | 4.52E-05 | 0.14 |
| ENO3 | 0.150468473 | 5.81E-07 | 0.11578093 | 0.0001249 | 0.04 |
| ENOPH1 | 0.254004814 | 1.49E-17 | 0.122133416 | 5.16E-05 | 0.14 |
| LOC100505523 | 0.040402555 | 0.18195906 | 0.064656385 | 0.0325682 | 0.03 |
| ENOX1 | -0.18797789 | 3.77E-10 | -0.032528072 | 0.2826211 | 0.16 |
| ENOX2 | 0.049655096 | 0.10084754 | -0.06449446 | 0.0330066 | 0.12 |
| ENPEP | -0.189213659 | 2.87E-10 | -0.187076051 | 4.58E-10 | 0.01 |
| ENPP1 | -0.40640606 | 1.01E-44 | -0.108731074 | 0.0003167 | 0.3 |
| ENPP2 | -0.235303276 | 3.24E-15 | -0.296469433 | 1.29E-23 | 0.07 |
| ENPP3 | -0.145299085 | 1.41E-06 | -0.011055713 | 0.7150361 | 0.14 |
| ENPP4 | -0.342410869 | 2.00E-31 | -0.19140874 | 1.77E-10 | 0.16 |
| ENPP5 | -0.361198213 | 5.06E-35 | -0.153092461 | 3.67E-07 | 0.21 |
| ENPP6 | 0.015310193 | 0.61312774 | -0.099857309 | 0.000947 | 0.12 |
| ENPP7 | -0.118821597 | 8.22E-05 | -0.067659713 | 0.0252947 | 0.06 |
| ENSA | 0.166812209 | 2.90E-08 | 0.448801001 | 2.78E-55 | 0.29 |
| ENTHD1 | 0.034705284 | 0.25162627 | -0.056306037 | 0.0627647 | 0.1 |
| ENTPD1 | -0.219627922 | 2.10E-13 | -0.204491283 | 8.79E-12 | 0.02 |
| ENTPD2 | 0.070031666 | 0.02058671 | 0.099052489 | 0.0010416 | 0.03 |
| ENTPD3 | -0.306696755 | 3.09E-25 | -0.028844098 | 0.3407376 | 0.28 |
| ENTPD4 | -0.38234957 | 2.27E-39 | -0.227706156 | 2.54E-14 | 0.16 |
| ENTPD5 | -0.501743835 | 9.44E-71 | -0.346957169 | 2.84E-32 | 0.16 |
| ENTPD6 | 0.106276542 | 0.00043234 | 0.1430183 | 2.06E-06 | 0.04 |
| ENTPD7 | -0.279889926 | 4.03E-21 | -0.2063775 | 5.60E-12 | 0.08 |
| ENTPD8 | -0.208200951 | 3.61E-12 | -0.090394021 | 0.002779 | 0.12 |
| ENY2 | 0.6291522 | 1.60E-121 | 0.476909651 | 3.71E-63 | 0.16 |
| EOMES | -0.110346971 | 0.00025706 | -0.166752488 | 2.93E-08 | 0.06 |
| EP300 | -0.399167218 | 4.59E-43 | -0.422235451 | 1.72E-48 | 0.03 |
| LOC732378 | -0.112574675 | 0.00019195 | -0.14029594 | 3.22E-06 | 0.03 |
| TNRC12 | -0.326299637 | 1.57E-28 | -0.347091843 | 2.68E-32 | 0.03 |
| EPAS1 | -0.388303977 | 1.18E-40 | -0.294914193 | 2.25E-23 | 0.1 |
| KIAA0338 | -0.170752625 | 1.34E-08 | -0.131289803 | 1.33E-05 | 0.04 |
| EPB41L2 | -0.188043235 | 3.71E-10 | -0.330829852 | 2.51E-29 | 0.15 |
| EPB41L3 | -0.261251755 | 1.63E-18 | -0.297149377 | 1.01E-23 | 0.04 |
| EPB41L4A | -0.451596265 | 4.93E-56 | -0.176201121 | 4.50E-09 | 0.28 |
| LOC87974 | -0.033313803 | 0.27115009 | -0.034937869 | 0.2484609 | 0.01 |
| FLJ12957 | -0.47083584 | 2.16E-61 | -0.309159595 | 1.23E-25 | 0.17 |
| EPB41 | -0.069694586 | 0.0212054 | -0.333082255 | 9.96E-30 | 0.27 |
| EPB42 | -0.163213484 | 5.76E-08 | -0.142950494 | 2.09E-06 | 0.03 |
| DMTN | -0.063919678 | 0.03460396 | -0.051634414 | 0.0879621 | 0.02 |
| EPC1 | -0.356088227 | 5.09E-34 | -0.405775203 | 1.42E-44 | 0.05 |
| EPC2 | -0.301575577 | 2.04E-24 | -0.35150134 | 3.90E-33 | 0.05 |
| EPCAM | 0.345790355 | 4.70E-32 | 0.282168415 | 1.87E-21 | 0.07 |
| EPDR1 | -0.137484789 | 5.07E-06 | -0.137185184 | 5.32E-06 | 0.01 |
| EPGN | -0.030250293 | 0.3177096 | -0.01104604 | 0.7152746 | 0.02 |
| FLJ16103 | -0.191125756 | 1.89E-10 | -0.12422155 | 3.82E-05 | 0.07 |
| EPHA1 | -0.042289935 | 0.16236733 | 0.066998338 | 0.0267631 | 0.11 |
| EPHA2 | 0.015109811 | 0.61778252 | -0.08817221 | 0.00353 | 0.11 |
| EPHA3 | -0.347788592 | 1.98E-32 | -0.213149496 | 1.07E-12 | 0.14 |
| EPHA4 | -0.159510361 | 1.15E-07 | -0.225282745 | 4.83E-14 | 0.07 |
| TYRO4 | -0.241166357 | 6.29E-16 | -0.169733295 | 1.64E-08 | 0.08 |
| EPHA6 | 0.03896942 | 0.197967 | -0.045039515 | 0.1367291 | 0.09 |
| EPHA7 | 0.122716582 | 4.75E-05 | 0.033809248 | 0.264083 | 0.09 |
| EPHA8 | 0.001294628 | 0.96589909 | -0.043204395 | 0.1534666 | 0.05 |
| EPHB1 | 0.094327627 | 0.00179677 | -0.074158457 | 0.014195 | 0.17 |
| ERK | 0.065764457 | 0.02969949 | -0.122899431 | 4.62E-05 | 0.19 |
| EPHB3 | 0.285337223 | 6.37E-22 | 0.180300612 | 1.93E-09 | 0.11 |
| EPHB4 | 0.02601394 | 0.39023212 | 0.063203539 | 0.036686 | 0.04 |
| EPHB6 | 0.147506814 | 9.69E-07 | -0.049003239 | 0.1054057 | 0.2 |
| EPHX1 | -0.091163027 | 0.00255513 | 0.12542838 | 3.20E-05 | 0.22 |
| EPHX2 | -0.291380712 | 7.85E-23 | -0.065514006 | 0.0303282 | 0.23 |
| EPHX3 | 0.240658118 | 7.26E-16 | 0.081288087 | 0.0071708 | 0.16 |
| EPHX4 | 0.198879338 | 3.27E-11 | 0.067611429 | 0.0253995 | 0.14 |
| EPM2AIP1 | -0.235581953 | 3.00E-15 | -0.266563508 | 3.09E-19 | 0.04 |
| EPM2A | -0.03686296 | 0.22332692 | -0.007729678 | 0.7985232 | 0.03 |
| EPN1 | 0.160666876 | 9.27E-08 | 0.086581017 | 0.0041764 | 0.08 |
| EPN2 | -0.186218455 | 5.51E-10 | -0.118781576 | 8.27E-05 | 0.07 |
| EPN3 | -0.06958317 | 0.02141343 | 0.112823677 | 0.0001857 | 0.19 |
| EPOR | -0.223325428 | 8.06E-14 | -0.062527994 | 0.0387467 | 0.17 |
| EPO | 0.09113519 | 0.00256294 | 0.188821343 | 3.13E-10 | 0.1 |
| LOC642584 | -0.138829104 | 4.09E-06 | -0.133524987 | 9.46E-06 | 0.01 |
| EPR1 | 0.448851932 | 2.69E-55 | 0.221165853 | 1.41E-13 | 0.23 |
| EPRS | 0.110059102 | 0.00026684 | 0.261926069 | 1.32E-18 | 0.16 |
| LOC105372294 | -0.031920964 | 0.29170632 | -0.073505863 | 0.0150717 | 0.05 |
| EPS15 | -0.244393833 | 2.50E-16 | -0.297799878 | 8.02E-24 | 0.06 |
| EPS8L1 | -0.180436513 | 1.88E-09 | 0.00674533 | 0.8237288 | 0.19 |
| EPS8L2 | 0.014587654 | 0.62998379 | 0.001061235 | 0.972044 | 0.02 |
| EPS8L3 | -0.026275507 | 0.38548272 | -0.041365211 | 0.171758 | 0.02 |
| EPS8 | -0.265918297 | 3.79E-19 | -0.206631714 | 5.27E-12 | 0.06 |
| EPSTI1 | 0.045574945 | 0.1321208 | -0.074420258 | 0.0138561 | 0.12 |
| EPT1 | 0.080077655 | 0.00808172 | -0.086629298 | 0.0041553 | 0.17 |
| EPX | -0.072444971 | 0.0165987 | -0.157859513 | 1.56E-07 | 0.09 |
| EPYC | -0.081324094 | 0.00714521 | -0.022520312 | 0.4570118 | 0.06 |
| ERAL1 | 0.26983371 | 1.09E-19 | 0.284182417 | 9.45E-22 | 0.02 |
| ERAP1 | -0.331418352 | 1.97E-29 | -0.257670238 | 4.91E-18 | 0.08 |
| LOC87126 | -0.074816682 | 0.01335653 | -0.005311177 | 0.8607726 | 0.07 |
| ERAS | 0.244263852 | 2.60E-16 | 0.117842627 | 9.42E-05 | 0.13 |
| ERBB2IP | -0.432688109 | 4.32E-51 | -0.254728929 | 1.20E-17 | 0.18 |
| ERBB2 | -0.164972506 | 4.12E-08 | 0.039462763 | 0.1923442 | 0.21 |
| ERBB3 | -0.311236463 | 5.60E-26 | -0.018303403 | 0.5455254 | 0.3 |
| ERBB4 | -0.459830332 | 2.76E-58 | -0.152293486 | 4.22E-07 | 0.31 |
| ERC1 | -0.15866696 | 1.34E-07 | -0.227147588 | 2.95E-14 | 0.07 |
| ERC2 | 0.048024037 | 0.11255795 | -0.135456027 | 6.99E-06 | 0.19 |
| ERCC1 | 0.075024757 | 0.01310074 | 0.136836468 | 5.62E-06 | 0.07 |
| TTD | -0.029998739 | 0.32175248 | 0.006321339 | 0.8346435 | 0.04 |
| ERCC3 | 0.062406543 | 0.03912738 | -0.065659669 | 0.0299612 | 0.13 |
| XPF | -0.2878994 | 2.64E-22 | -0.283250732 | 1.30E-21 | 0.01 |
| ERCC5 | -0.15727797 | 1.73E-07 | -0.118451037 | 8.66E-05 | 0.04 |
| ERCC6L | 0.396286856 | 2.04E-42 | 0.225236388 | 4.89E-14 | 0.18 |
| ERCC6 | -0.214845897 | 7.03E-13 | -0.249563906 | 5.56E-17 | 0.04 |
| ERCC8 | -0.115049007 | 0.00013791 | -0.030317742 | 0.3166313 | 0.09 |
| EREG | 0.025024255 | 0.40852142 | 0.018644762 | 0.5380567 | 0.01 |
| ERF | 0.064261633 | 0.03364591 | -0.01302419 | 0.6671139 | 0.08 |
| ERGIC1 | -0.357614458 | 2.56E-34 | -0.062191136 | 0.0398104 | 0.3 |
| ERGIC2 | 0.229852601 | 1.43E-14 | 0.200437055 | 2.28E-11 | 0.03 |
| ERGIC3 | 0.236206484 | 2.52E-15 | 0.261742699 | 1.40E-18 | 0.03 |
| ERG | -0.414668179 | 1.16E-46 | -0.3083061 | 1.69E-25 | 0.11 |
| ERH | 0.430115666 | 1.92E-50 | 0.466315073 | 4.20E-60 | 0.04 |
| ERI1 | 0.036011408 | 0.23421131 | 0.031941277 | 0.2913992 | 0.01 |
| ERI2 | -0.178072742 | 3.07E-09 | 0.005187793 | 0.8639752 | 0.19 |
| ERI3 | 0.495963959 | 6.29E-69 | 0.34323152 | 1.41E-31 | 0.16 |
| LOC286163 | -0.037663684 | 0.21342641 | -0.031395402 | 0.2997278 | 0.01 |
| ERLEC1 | -0.259840937 | 2.52E-18 | -0.191063502 | 1.91E-10 | 0.07 |
| ERLIN1 | -0.147613866 | 9.51E-07 | -0.161710952 | 7.63E-08 | 0.02 |
| ERLIN2 | -0.207051462 | 4.76E-12 | -0.195301254 | 7.41E-11 | 0.02 |
| RD | -0.354725468 | 9.35E-34 | -0.24311601 | 3.61E-16 | 0.12 |
| ERMN | -0.060217515 | 0.04655211 | -0.042293466 | 0.1623322 | 0.02 |
| ERMP1 | -0.236212435 | 2.52E-15 | -0.117632955 | 9.69E-05 | 0.12 |
| ERN1 | -0.47294091 | 5.32E-62 | -0.469346875 | 5.76E-61 | 0.01 |
| ERN2 | 0.110895407 | 0.00023934 | 0.042477813 | 0.1605076 | 0.07 |
| ERO1LB | -0.255763269 | 8.75E-18 | -0.088499094 | 0.003409 | 0.17 |
| ERO1L | -0.065861738 | 0.02945833 | -0.100092556 | 0.0009209 | 0.04 |
| ERP27 | -0.046099115 | 0.12772669 | 0.060023199 | 0.0472649 | 0.11 |
| ERP29 | 0.161476223 | 7.98E-08 | 0.153309581 | 3.53E-07 | 0.01 |
| ERP44 | 0.268520825 | 1.66E-19 | 0.144389564 | 1.64E-06 | 0.13 |
| ERRFI1 | 0.032756625 | 0.27925108 | -0.174747429 | 6.05E-09 | 0.21 |
| ERVFRD.1 | -0.262437529 | 1.13E-18 | -0.198784298 | 3.34E-11 | 0.07 |
| ESAM | -0.269702189 | 1.14E-19 | -0.143212816 | 2.00E-06 | 0.13 |
| ESCO1 | 0.210088789 | 2.28E-12 | -0.138595484 | 4.24E-06 | 0.35 |
| ESCO2 | 0.243138491 | 3.59E-16 | 0.15442557 | 2.89E-07 | 0.09 |
| ESD | 0.02445607 | 0.41924787 | 0.126359077 | 2.80E-05 | 0.11 |
| ESF1 | 0.034961161 | 0.24814543 | -0.030803681 | 0.3089333 | 0.07 |
| ESM1 | 0.080297581 | 0.0079089 | 0.089756909 | 0.0029779 | 0.01 |
| ESPL1 | 0.399227192 | 4.45E-43 | 0.199910652 | 2.58E-11 | 0.2 |
| ESPNL | 0.027368748 | 0.36601668 | -0.053759242 | 0.0756405 | 0.09 |
| ESPNP | -0.020730049 | 0.49357352 | -0.044087112 | 0.1452311 | 0.03 |
| DFNB36 | -0.132227208 | 1.16E-05 | 0.093510528 | 0.0019698 | 0.23 |
| ESR1 | -0.416600151 | 3.99E-47 | -0.142920775 | 2.10E-06 | 0.28 |
| ESR2 | 0.006888508 | 0.82005053 | -0.133739457 | 9.15E-06 | 0.15 |
| ESRP1 | 0.259366989 | 2.92E-18 | 0.222900458 | 9.00E-14 | 0.04 |
| FLJ22248 | 0.049559305 | 0.10150737 | 0.093334713 | 0.0020089 | 0.05 |
| ESRRA | 0.250547991 | 4.16E-17 | 0.150961323 | 5.33E-07 | 0.1 |
| DFNB35 | -0.136386516 | 6.04E-06 | -0.066683728 | 0.0274869 | 0.07 |
| ESRRG | -0.303477474 | 1.02E-24 | -0.073924555 | 0.0145039 | 0.23 |
| ESX1 | 0.239994405 | 8.76E-16 | 0.084202284 | 0.0053439 | 0.16 |
| ESYT1 | -0.254925045 | 1.13E-17 | -0.218425651 | 2.85E-13 | 0.04 |
| ESYT2 | -0.128672516 | 1.98E-05 | -0.297297118 | 9.61E-24 | 0.17 |
| ESYT3 | 0.24619543 | 1.49E-16 | 0.066743529 | 0.0273481 | 0.18 |
| ETAA1 | -0.163811937 | 5.14E-08 | -0.126657284 | 2.68E-05 | 0.04 |
| SUP45L1 | -0.059596584 | 0.04886217 | -0.066440031 | 0.0280592 | 0.01 |
| ETFA | 0.075521891 | 0.01250704 | 0.075622005 | 0.0123904 | 0.01 |
| ETFB | 0.098688328 | 0.00108719 | 0.077683617 | 0.0101931 | 0.03 |
| ETFDH | -0.283730251 | 1.10E-21 | -0.111074694 | 0.0002338 | 0.18 |
| LOC115699 | -0.023543581 | 0.43681613 | 0.055157218 | 0.0683293 | 0.08 |
| ETNK1 | -0.194342943 | 9.20E-11 | -0.095885238 | 0.0015051 | 0.1 |
| ETNK2 | -0.096652353 | 0.00137802 | 0.155306083 | 2.47E-07 | 0.26 |
| ETS1 | -0.171801171 | 1.09E-08 | -0.299957671 | 3.68E-24 | 0.13 |
| ETS2 | -0.073905379 | 0.01452947 | -0.173519898 | 7.74E-09 | 0.1 |
| ETV1 | -0.27082634 | 7.92E-20 | -0.190422517 | 2.20E-10 | 0.09 |
| ETV2 | 0.12514103 | 3.34E-05 | 0.181641198 | 1.46E-09 | 0.06 |
| ETV3L | 0.063936818 | 0.03455539 | -0.038975361 | 0.1978986 | 0.11 |
| ETV3 | -0.350095307 | 7.23E-33 | -0.360681446 | 6.40E-35 | 0.02 |
| ETV4 | 0.243444366 | 3.29E-16 | 0.188729878 | 3.20E-10 | 0.06 |
| ETV5 | 0.014716884 | 0.62695448 | -0.131632838 | 1.27E-05 | 0.15 |
| ETV6 | 0.193400734 | 1.14E-10 | 0.049261034 | 0.1035839 | 0.15 |
| TEL.2 | 0.24822731 | 8.23E-17 | 0.092867062 | 0.0021166 | 0.16 |
| EVC2 | -0.180609003 | 1.81E-09 | -0.150793207 | 5.49E-07 | 0.03 |
| EVCL | -0.448538646 | 3.26E-55 | -0.317323745 | 5.42E-27 | 0.14 |
| EVI2A | -0.169345132 | 1.77E-08 | -0.199435009 | 2.88E-11 | 0.04 |
| EVI2B | -0.160928023 | 8.84E-08 | -0.237973207 | 1.54E-15 | 0.08 |
| EVI5L | -0.100250987 | 0.00090365 | -0.099624281 | 0.0009735 | 0.01 |
| EVI5 | -0.397233152 | 1.25E-42 | -0.41073923 | 9.85E-46 | 0.02 |
| EVL | -0.308733998 | 1.44E-25 | -0.114477852 | 0.0001489 | 0.2 |
| EVPLL | -0.068389595 | 0.02375565 | 0.009223202 | 0.7606848 | 0.08 |
| EVPL | 0.068238086 | 0.02406829 | 0.03022276 | 0.3181505 | 0.04 |
| EVX1 | -0.093217848 | 0.00203534 | -0.009370936 | 0.7569712 | 0.09 |
| LOC129908 | -0.018937771 | 0.53168703 | 0.0064024 | 0.8325542 | 0.03 |
| EWSR1 | 0.266786969 | 2.88E-19 | 0.143795589 | 1.81E-06 | 0.13 |
| EXD1 | 0.215175593 | 6.48E-13 | 0.103127326 | 0.0006386 | 0.12 |
| EXD2 | -0.280115095 | 3.74E-21 | -0.199985764 | 2.53E-11 | 0.09 |
| EXD3 | 0.014262902 | 0.63762374 | 0.050925725 | 0.0924142 | 0.04 |
| EXO1 | 0.483487643 | 4.15E-65 | 0.376344593 | 4.20E-38 | 0.11 |
| EXOC1 | -0.162116959 | 7.08E-08 | -0.108943163 | 0.0003082 | 0.06 |
| EXOC2 | -0.140712287 | 3.01E-06 | -0.079919767 | 0.0082079 | 0.07 |
| EXOC3L2 | -0.156696138 | 1.92E-07 | -0.104233638 | 0.0005575 | 0.06 |
| EXOC3L1 | -0.079343308 | 0.00868349 | -0.0535413 | 0.0768357 | 0.03 |
| EXOC3 | -0.054635946 | 0.07098454 | -0.037601232 | 0.214187 | 0.02 |
| EXOC4 | -0.096996231 | 0.00132434 | 0.005843682 | 0.8469778 | 0.11 |
| PRO1912 | -0.195180985 | 7.61E-11 | -0.1732657 | 8.15E-09 | 0.03 |
| EXOC6B | -0.40788188 | 4.59E-45 | -0.447270372 | 7.10E-55 | 0.04 |
| EXOC6 | -0.342375619 | 2.03E-31 | -0.06891796 | 0.0226927 | 0.28 |
| EXOC7 | -0.040042722 | 0.18588519 | -0.179251659 | 2.41E-09 | 0.14 |
| EXOC8 | -0.246537798 | 1.35E-16 | 0.019175341 | 0.5265507 | 0.27 |
| EXOG | -0.019532853 | 0.51886919 | -0.111122388 | 0.0002323 | 0.1 |
| EXOSC10 | 0.062343435 | 0.03932645 | 0.026938107 | 0.3736103 | 0.04 |
| EXOSC1 | 0.356367558 | 4.49E-34 | 0.423638782 | 7.81E-49 | 0.07 |
| EXOSC2 | 0.442207876 | 1.53E-53 | 0.321084117 | 1.25E-27 | 0.13 |
| EXOSC3 | 0.313000062 | 2.86E-26 | 0.235769172 | 2.85E-15 | 0.08 |
| EXOSC4 | 0.475087835 | 1.27E-62 | 0.333360154 | 8.88E-30 | 0.15 |
| EXOSC5 | 0.437163132 | 3.12E-52 | 0.376536128 | 3.83E-38 | 0.07 |
| EXOSC6 | 0.352475573 | 2.54E-33 | 0.141820413 | 2.51E-06 | 0.22 |
| LOC115558 | 0.396313449 | 2.01E-42 | 0.374669322 | 9.37E-38 | 0.03 |
| EXOSC8 | 0.431272744 | 9.84E-51 | 0.411758297 | 5.67E-46 | 0.02 |
| EXOSC9 | 0.487830843 | 2.02E-66 | 0.314002802 | 1.95E-26 | 0.18 |
| EXPH5 | -0.105824225 | 0.00045755 | -0.086541861 | 0.0041936 | 0.02 |
| EXT1 | 0.173285896 | 8.12E-09 | 0.029929825 | 0.3228659 | 0.15 |
| EXT2 | 0.065900635 | 0.02936238 | -0.087120903 | 0.0039459 | 0.16 |
| EXTL1 | 0.014321381 | 0.63624512 | -0.119049059 | 7.97E-05 | 0.14 |
| EXTL2 | -0.078904394 | 0.00906197 | -0.164699137 | 4.34E-08 | 0.09 |
| EXTL3 | -0.088794875 | 0.0033028 | -0.157274798 | 1.73E-07 | 0.07 |
| EYA1 | -0.059531803 | 0.04910862 | -0.059531831 | 0.0491085 | 0.01 |
| EYA2 | 0.100128984 | 0.00091687 | 0.051089237 | 0.0913713 | 0.05 |
| EYA3 | -0.269959225 | 1.05E-19 | -0.397750511 | 9.58E-43 | 0.13 |
| DFNA10 | -0.217126759 | 3.96E-13 | -0.079835437 | 0.008276 | 0.14 |
| EYS | -0.295233983 | 2.01E-23 | -0.138024726 | 4.65E-06 | 0.16 |
| EZH1 | -0.413286552 | 2.46E-46 | -0.292883646 | 4.62E-23 | 0.13 |
| EZH2 | 0.513949534 | 1.01E-74 | 0.32410857 | 3.77E-28 | 0.19 |
| EZR | -0.085609559 | 0.004622 | 0.007626892 | 0.8011459 | 0.1 |
| F10 | -0.255114934 | 1.06E-17 | -0.142608621 | 2.21E-06 | 0.12 |
| F11R | 0.08858668 | 0.00337723 | 0.263437665 | 8.27E-19 | 0.18 |
| F11 | -0.005633265 | 0.85242341 | -0.071705522 | 0.0177418 | 0.07 |
| F12 | 0.184894037 | 7.33E-10 | 0.278413705 | 6.59E-21 | 0.1 |
| F13A1 | -0.36033823 | 7.48E-35 | -0.274504108 | 2.40E-20 | 0.09 |
| F13B | 0.098472501 | 0.00111508 | 0.095606595 | 0.0015538 | 0.01 |
| F2RL1 | -0.16868878 | 2.01E-08 | -0.075274269 | 0.0127997 | 0.1 |
| F2RL2 | -0.356551405 | 4.13E-34 | -0.161653985 | 7.72E-08 | 0.2 |
| F2RL3 | -0.081340247 | 0.00713374 | -0.013951777 | 0.6449794 | 0.07 |
| F2R | -0.438878059 | 1.13E-52 | -0.295793805 | 1.65E-23 | 0.15 |
| F2 | 0.006349351 | 0.83392136 | -0.015232162 | 0.6149385 | 0.03 |
| F3 | -0.287273372 | 3.28E-22 | -0.233211311 | 5.76E-15 | 0.06 |
| F5 | 0.035062958 | 0.24677001 | -0.124845363 | 3.49E-05 | 0.16 |
| F7 | -0.324507063 | 3.21E-28 | 0.017942952 | 0.5534671 | 0.35 |
| F8A1 | 0.199661229 | 2.73E-11 | 0.066967794 | 0.0268326 | 0.14 |
| F8 | -0.330394293 | 2.99E-29 | -0.341481743 | 2.97E-31 | 0.02 |
| F9 | -0.022089973 | 0.46565915 | 0.000810938 | 0.9786357 | 0.03 |
| FA2H | -0.096032445 | 0.00147987 | 0.043518828 | 0.1504933 | 0.14 |
| FAAH2 | -0.226934493 | 3.12E-14 | -0.060596435 | 0.045188 | 0.17 |
| FAAH | -0.216604387 | 4.52E-13 | -0.040234672 | 0.1837831 | 0.18 |
| FABP12 | 0.078258098 | 0.00964606 | 0.038620095 | 0.2020204 | 0.04 |
| FABP1 | 0.052400261 | 0.08334678 | 0.026086846 | 0.3889048 | 0.03 |
| FABP2 | -0.049913965 | 0.09908147 | -0.042673982 | 0.158583 | 0.01 |
| FABP3 | 0.044540391 | 0.14113562 | 0.112982271 | 0.0001819 | 0.07 |
| FABP4 | -0.194237771 | 9.42E-11 | -0.113311072 | 0.0001741 | 0.09 |
| FABP5P3 | 0.209389619 | 2.70E-12 | 0.100508779 | 0.0008763 | 0.11 |
| FABP5 | 0.316572025 | 7.26E-27 | 0.071191835 | 0.0185758 | 0.25 |
| FABP6 | 0.181956818 | 1.37E-09 | 0.155953068 | 2.20E-07 | 0.03 |
| FABP7 | 0.261985695 | 1.30E-18 | 0.034428199 | 0.2554338 | 0.23 |
| FABP9 | 0.001153314 | 0.96961945 | 0.002414015 | 0.936462 | 0.01 |
| FADD | 0.142961282 | 2.08E-06 | 0.146050133 | 1.24E-06 | 0.01 |
| FADS1 | 0.17076042 | 1.34E-08 | -0.010600262 | 0.7262942 | 0.19 |
| FADS2 | 0.2117318 | 1.52E-12 | 0.110490289 | 0.0002523 | 0.11 |
| FADS3 | 0.187407826 | 4.26E-10 | 0.058639446 | 0.0526108 | 0.13 |
| FADS6 | 0.279756261 | 4.21E-21 | 0.127291223 | 2.44E-05 | 0.16 |
| FAF1 | 0.430820209 | 1.28E-50 | 0.177624096 | 3.37E-09 | 0.26 |
| FAF2 | -0.251021962 | 3.62E-17 | -0.131579271 | 1.28E-05 | 0.12 |
| FAHD1 | 0.094350638 | 0.00179211 | 0.19616817 | 6.08E-11 | 0.11 |
| FAHD2A | 0.368859812 | 1.47E-36 | 0.155001792 | 2.61E-07 | 0.22 |
| FAHD2B | 0.018779889 | 0.53511444 | 0.127896847 | 2.23E-05 | 0.11 |
| FAH | -0.189736503 | 2.56E-10 | 0.034307509 | 0.2571047 | 0.23 |
| FAIM2 | -0.221709846 | 1.23E-13 | 0.021605435 | 0.4755033 | 0.25 |
| FCMR | -0.114582966 | 0.00014684 | -0.011691067 | 0.6994344 | 0.11 |
| FAIM | 0.339803169 | 6.05E-31 | 0.171051908 | 1.27E-08 | 0.17 |
| UBALD1 | 0.125327782 | 3.25E-05 | 0.148202947 | 8.60E-07 | 0.03 |
| LOC197455 | 0.44597071 | 1.57E-54 | 0.215129508 | 6.55E-13 | 0.24 |
| FAM101A | 0.216964414 | 4.13E-13 | 0.124286539 | 3.78E-05 | 0.1 |
| FAM101B | -0.071594694 | 0.01791889 | -0.158029302 | 1.51E-07 | 0.09 |
| FAM102A | -0.326341644 | 1.54E-28 | -0.218487536 | 2.80E-13 | 0.11 |
| FAM102B | -0.161143269 | 8.49E-08 | -0.193137647 | 1.21E-10 | 0.04 |
| LOC91280 | 0.214519237 | 7.63E-13 | 0.250920007 | 3.73E-17 | 0.04 |
| FAM104A | 0.173940141 | 7.12E-09 | 0.095506008 | 0.0015718 | 0.08 |
| FAM104B | 0.401408423 | 1.42E-43 | 0.392771691 | 1.23E-41 | 0.01 |
| FAM105A | -0.255566165 | 9.29E-18 | -0.261302617 | 1.61E-18 | 0.01 |
| OTULIN | 0.191182694 | 1.86E-10 | -0.027810788 | 0.3583229 | 0.22 |
| FAM106A | -0.167924442 | 2.34E-08 | -0.167016693 | 2.79E-08 | 0.01 |
| LOC220575 | -0.271671062 | 6.03E-20 | -0.262574594 | 1.08E-18 | 0.01 |
| FAM107A | -0.062058824 | 0.04023495 | -0.184363872 | 8.20E-10 | 0.13 |
| FAM107B | -0.198390617 | 3.66E-11 | -0.177752676 | 3.28E-09 | 0.03 |
| ABHD17A | 0.05991855 | 0.04765263 | 0.049174832 | 0.1041903 | 0.02 |
| ABHD17B | -0.02834804 | 0.3491097 | -0.0863026 | 0.0043 | 0.06 |
| ABHD17C | 0.12053884 | 6.47E-05 | 0.038324422 | 0.2054981 | 0.09 |
| FAM109A | 0.110591445 | 0.00024901 | 0.11109138 | 0.0002333 | 0.01 |
| FAM109B | -0.062872338 | 0.03768431 | 0.037625558 | 0.2138905 | 0.11 |
| LOC88248 | -0.052175731 | 0.08467913 | -0.087736578 | 0.0036972 | 0.04 |
| LOC115466 | 0.088526983 | 0.00339885 | 0.199283317 | 2.98E-11 | 0.12 |
| FAM110B | -0.193212678 | 1.19E-10 | -0.059664328 | 0.0486056 | 0.14 |
| LOC642145 | -0.392540849 | 1.39E-41 | -0.251760432 | 2.91E-17 | 0.15 |
| FAM111A | -0.192028289 | 1.54E-10 | -0.071003238 | 0.0188906 | 0.13 |
| FAM111B | 0.305715804 | 4.44E-25 | 0.170859592 | 1.32E-08 | 0.14 |
| PCED1A | -0.047430377 | 0.11707679 | 0.045413455 | 0.1334979 | 0.1 |
| PCED1B | -0.02378898 | 0.43205028 | 0.072465267 | 0.0165683 | 0.1 |
| FAM114A1 | -0.508763731 | 5.15E-73 | -0.453188572 | 1.83E-56 | 0.06 |
| X133K02 | -0.478075095 | 1.69E-63 | -0.200716876 | 2.14E-11 | 0.28 |
| TCAF1 | -0.431103429 | 1.09E-50 | -0.388437008 | 1.10E-40 | 0.05 |
| TCAF2 | -0.023412847 | 0.4393674 | -0.066198974 | 0.0286353 | 0.05 |
| DENND6A | -0.19810043 | 3.91E-11 | -0.144170234 | 1.70E-06 | 0.06 |
| DENND6B | -0.1215446 | 5.61E-05 | -0.004449426 | 0.883185 | 0.12 |
| FAM117A | -0.180317304 | 1.93E-09 | -0.189062144 | 2.97E-10 | 0.01 |
| ALS2CR13 | 0.037632086 | 0.21381101 | 0.052516293 | 0.0826649 | 0.02 |
| C22orf8 | 0.015343203 | 0.61236243 | -0.048979991 | 0.1055713 | 0.07 |
| FAM118B | 0.069069451 | 0.02239571 | 0.034590159 | 0.2532034 | 0.04 |
| METTL21A | 0.310816108 | 6.57E-26 | 0.187784859 | 3.93E-10 | 0.13 |
| METTL21B | -0.102280827 | 0.00070796 | -0.112193432 | 0.0002019 | 0.01 |
| FAM120AOS | -0.274587795 | 2.33E-20 | -0.071334013 | 0.0183416 | 0.21 |
| FAM120A | -0.349483642 | 9.45E-33 | -0.189603218 | 2.64E-10 | 0.16 |
| FAM120B | -0.448114615 | 4.23E-55 | -0.264044805 | 6.84E-19 | 0.19 |
| LOC158576 | -0.113870213 | 0.00016157 | -0.118060633 | 9.14E-05 | 0.01 |
| FAM122A | -0.352544901 | 2.46E-33 | -0.321832664 | 9.29E-28 | 0.04 |
| FAM122B | -0.035229704 | 0.24452861 | -0.082292984 | 0.0064857 | 0.05 |
| FAM122C | -0.080940267 | 0.00742267 | -0.117404821 | 0.0001 | 0.04 |
| AMER2 | 0.12639332 | 2.78E-05 | -0.018063492 | 0.550805 | 0.15 |
| AMER1 | 0.247125427 | 1.14E-16 | -0.032910496 | 0.2769977 | 0.29 |
| AMER3 | 0.005352302 | 0.85970569 | -0.098114217 | 0.0011629 | 0.11 |
| FAM124A | 0.048995447 | 0.10546118 | -0.064625995 | 0.0326501 | 0.12 |
| FAM124B | -0.344135797 | 9.57E-32 | -0.222793362 | 9.26E-14 | 0.13 |
| LOC100507535 | 0.144757575 | 1.54E-06 | 0.240812205 | 6.95E-16 | 0.1 |
| MVB12B | -0.205482641 | 6.94E-12 | -0.270623297 | 8.45E-20 | 0.07 |
| FAM126A | -0.102062672 | 0.00072693 | -0.230845238 | 1.09E-14 | 0.13 |
| FAM126B | -0.280999628 | 2.78E-21 | -0.312424221 | 3.57E-26 | 0.04 |
| FAM127A | 0.268200801 | 1.84E-19 | 0.175059642 | 5.68E-09 | 0.1 |
| MGC8471 | 0.436504341 | 4.60E-52 | 0.239184349 | 1.10E-15 | 0.2 |
| FAM127C | 0.266376245 | 3.28E-19 | 0.133662645 | 9.26E-06 | 0.14 |
| MZT2A | 0.11215864 | 0.00020279 | 0.183264913 | 1.04E-09 | 0.08 |
| LOC115241 | 0.133108824 | 1.01E-05 | 0.182350067 | 1.26E-09 | 0.05 |
| FAM129A | -0.357414224 | 2.81E-34 | -0.278427896 | 6.56E-21 | 0.08 |
| FAM129B | -0.023880588 | 0.43027892 | -0.126022796 | 2.94E-05 | 0.11 |
| FAM129C | -0.042923302 | 0.15616221 | -0.151312901 | 5.02E-07 | 0.11 |
| FAM131A | 0.082875468 | 0.00611611 | -0.01557365 | 0.6070314 | 0.1 |
| FAM131B | -0.324882175 | 2.77E-28 | -0.15889375 | 1.29E-07 | 0.17 |
| FAM131C | 0.39686792 | 1.51E-42 | 0.164892121 | 4.19E-08 | 0.24 |
| FAM132A | 0.112598222 | 0.00019135 | 0.113295949 | 0.0001744 | 0.01 |
| FAM133A | -0.121121333 | 5.96E-05 | -0.066503832 | 0.0279084 | 0.06 |
| FAM133B | 0.100113804 | 0.00091853 | 0.074873209 | 0.0132866 | 0.03 |
| FAM134A | -0.140770181 | 2.98E-06 | -0.070071288 | 0.020515 | 0.08 |
| FAM134B | -0.302558209 | 1.42E-24 | -0.130309683 | 1.55E-05 | 0.18 |
| FAM134C | -0.13729932 | 5.22E-06 | -0.03190906 | 0.2918864 | 0.11 |
| FAM135A | 0.026890526 | 0.37445526 | -0.26610115 | 3.58E-19 | 0.3 |
| FAM135B | -0.201143428 | 1.93E-11 | -0.094158427 | 0.0018314 | 0.11 |
| FAM136A | 0.622391438 | 3.19E-118 | 0.313184495 | 2.67E-26 | 0.31 |
| LOC643233 | 0.077821375 | 0.01005949 | 0.078137552 | 0.0097586 | 0.01 |
| FAM138B | -0.016542503 | 0.58484885 | 0.022640974 | 0.4546035 | 0.04 |
| FAM138D | 0.013435133 | 0.65727057 | 0.028727286 | 0.3426974 | 0.02 |
| FAM138E | 0.053867168 | 0.0750542 | 0.03749328 | 0.2155064 | 0.02 |
| LOC100132098 | 0.00339716 | 0.91067768 | -0.00285946 | 0.9247697 | 0.01 |
| FAM13A.AS1 | -0.244419949 | 2.48E-16 | -0.228776703 | 1.91E-14 | 0.02 |
| FAM13A | -0.193809087 | 1.04E-10 | -0.253461214 | 1.75E-17 | 0.06 |
| FAM13B | -0.49981422 | 3.87E-70 | -0.347130721 | 2.63E-32 | 0.16 |
| FAM13C | -0.348283411 | 1.60E-32 | -0.334012016 | 6.79E-30 | 0.02 |
| FAM149A | -0.198045598 | 3.96E-11 | -0.231255774 | 9.80E-15 | 0.04 |
| FAM149B1 | -0.241415954 | 5.86E-16 | -0.060318432 | 0.0461855 | 0.19 |
| FAM150A | 0.232467167 | 7.05E-15 | 0.079650081 | 0.0084274 | 0.16 |
| FAM150B | 0.160605704 | 9.38E-08 | -0.011123512 | 0.7133654 | 0.18 |
| FAM151A | -0.007064687 | 0.81552994 | -0.107266532 | 0.0003816 | 0.11 |
| FAM151B | -0.4011175 | 1.66E-43 | -0.084263345 | 0.0053106 | 0.32 |
| FAM153A | -0.052144335 | 0.0848668 | -0.11126938 | 0.0002279 | 0.06 |
| FAM153B | -0.148166337 | 8.65E-07 | -0.210669827 | 1.98E-12 | 0.07 |
| FAM153C | -0.123093996 | 4.50E-05 | -0.105718521 | 0.0004636 | 0.02 |
| SAXO1 | -0.197594472 | 4.39E-11 | -0.10849272 | 0.0003265 | 0.09 |
| SAXO2 | -0.288901417 | 1.86E-22 | -0.121661913 | 5.52E-05 | 0.17 |
| FAM155A | -0.174853595 | 5.92E-09 | -0.029467471 | 0.3304005 | 0.15 |
| FAM155B | -0.072441223 | 0.01660433 | -0.024607538 | 0.4163724 | 0.05 |
| FAM156A | 0.121572873 | 5.59E-05 | 0.050071698 | 0.0980175 | 0.08 |
| FAM157A | -0.277096998 | 1.02E-20 | -0.265595403 | 4.20E-19 | 0.02 |
| FAM157B | -0.242304366 | 4.55E-16 | -0.249715273 | 5.32E-17 | 0.01 |
| EMC9 | 0.378232895 | 1.69E-38 | 0.348479136 | 1.47E-32 | 0.03 |
| FAM159A | -0.034235262 | 0.25810854 | -0.035381469 | 0.242501 | 0.01 |
| FAM160A1 | -0.120221757 | 6.76E-05 | -0.235335006 | 3.21E-15 | 0.12 |
| FAM160A2 | -0.260208183 | 2.25E-18 | -0.146354799 | 1.18E-06 | 0.12 |
| FAM160B1 | -0.362585853 | 2.68E-35 | -0.222980956 | 8.82E-14 | 0.14 |
| FAM160B2 | -0.233548883 | 5.25E-15 | -0.053969469 | 0.0745019 | 0.18 |
| FAM161A | 0.130496337 | 1.51E-05 | -0.092032098 | 0.002322 | 0.23 |
| FAM161B | -0.608219105 | 1.46E-111 | -0.416707548 | 3.76E-47 | 0.2 |
| FAM162A | 0.158496028 | 1.38E-07 | 0.31372192 | 2.17E-26 | 0.16 |
| LOC154273 | -0.216494026 | 4.65E-13 | -0.112717033 | 0.0001884 | 0.11 |
| FAM163A | 0.132378907 | 1.13E-05 | -0.007073549 | 0.8153027 | 0.14 |
| LOC643750 | 0.119362846 | 7.63E-05 | 0.004744849 | 0.8754902 | 0.12 |
| ZC2HC1A | -0.067309253 | 0.02606388 | -0.110078374 | 0.0002662 | 0.05 |
| ZC2HC1C | -0.003426491 | 0.90990974 | 0.048602244 | 0.1082899 | 0.06 |
| SMIM11 | 0.246239367 | 1.47E-16 | 0.274434398 | 2.45E-20 | 0.03 |
| FAM166A | 0.002913503 | 0.92335221 | 0.025772922 | 0.3946397 | 0.03 |
| FAM166B | -0.005872373 | 0.84623587 | -0.004441957 | 0.8833797 | 0.01 |
| FAM167A | -0.16670169 | 2.96E-08 | -0.160786227 | 9.07E-08 | 0.01 |
| LOC115245 | 0.111865956 | 0.00021076 | 0.072772555 | 0.0161133 | 0.04 |
| FAM168A | -0.398454852 | 6.65E-43 | -0.449538783 | 1.76E-55 | 0.06 |
| FAM168B | -0.110389902 | 0.00025563 | -0.22474409 | 5.56E-14 | 0.12 |
| FAM169A | -0.198842356 | 3.30E-11 | -0.232195602 | 7.59E-15 | 0.04 |
| FAM169B | 0.159860545 | 1.08E-07 | 0.063985817 | 0.0344169 | 0.1 |
| FAM170A | -0.119792524 | 7.18E-05 | 0.022475796 | 0.4579021 | 0.15 |
| C10orf73 | -0.016418147 | 0.58767492 | -0.043788168 | 0.1479814 | 0.03 |
| FAM171A1 | 0.241698759 | 5.41E-16 | -0.008346151 | 0.7828419 | 0.26 |
| FAM171A2 | 0.277861499 | 7.92E-21 | 0.144681464 | 1.56E-06 | 0.14 |
| FAM171B | -0.174944343 | 5.81E-09 | -0.198990959 | 3.19E-11 | 0.03 |
| FAM172A | -0.414880548 | 1.03E-46 | -0.120677845 | 6.34E-05 | 0.3 |
| FAM173A | 0.090187876 | 0.00284198 | 0.212004481 | 1.42E-12 | 0.13 |
| FAM173B | -0.070408951 | 0.01991291 | 0.095884121 | 0.0015053 | 0.17 |
| FAM174A | -0.292579987 | 5.15E-23 | -0.055098603 | 0.0686237 | 0.24 |
| FAM174B | -0.301665706 | 1.97E-24 | -0.053265237 | 0.0783717 | 0.25 |
| FAM175A | -0.366683716 | 4.05E-36 | -0.210568392 | 2.03E-12 | 0.16 |
| LOC142931 | -0.075003764 | 0.01312635 | -0.109733051 | 0.0002783 | 0.04 |
| EVA1A | 0.075939686 | 0.01202663 | -0.000712462 | 0.9812295 | 0.08 |
| EVA1B | -0.186921499 | 4.74E-10 | -0.015635373 | 0.6056071 | 0.18 |
| FAM177A1 | -0.067565576 | 0.02549939 | -0.0442877 | 0.1434077 | 0.03 |
| FAM177B | -0.25161756 | 3.03E-17 | -0.067768413 | 0.0250602 | 0.19 |
| FLJ25012 | -0.334970721 | 4.56E-30 | -0.233471823 | 5.36E-15 | 0.11 |
| FAM178B | 0.202572423 | 1.38E-11 | 0.121746189 | 5.45E-05 | 0.09 |
| FAM179A | 0.114207094 | 0.00015444 | -0.003320408 | 0.9126876 | 0.12 |
| FAM179B | -0.549663881 | 2.64E-87 | -0.367768069 | 2.44E-36 | 0.19 |
| LOC392794 | -0.167727714 | 2.43E-08 | -0.1694125 | 1.75E-08 | 0.01 |
| FAM180B | -0.125766449 | 3.05E-05 | -0.113320934 | 0.0001739 | 0.02 |
| FAM181A | 0.080117579 | 0.0080501 | 0.046131132 | 0.127462 | 0.04 |
| FLJ37735 | 0.05974151 | 0.04831458 | 0.011623595 | 0.7010854 | 0.05 |
| C20orf91 | -0.122580764 | 4.84E-05 | -0.139646192 | 3.58E-06 | 0.02 |
| FAM182B | -0.080490977 | 0.00775967 | -0.066023727 | 0.0290605 | 0.02 |
| FAM183A | 0.010150082 | 0.73748028 | 0.084379117 | 0.0052479 | 0.08 |
| LOC392661 | -0.012051033 | 0.69065057 | -0.015553815 | 0.6074895 | 0.01 |
| FAM184A | 0.1178756 | 9.38E-05 | -0.054182703 | 0.0733614 | 0.18 |
| FAM184B | -0.231657182 | 8.79E-15 | -0.067257012 | 0.0261802 | 0.17 |
| FAM185A | -0.048095167 | 0.11202586 | 0.126317484 | 2.81E-05 | 0.18 |
| FAM186A | -0.025127376 | 0.4065923 | 0.125465525 | 3.19E-05 | 0.16 |
| FAM186B | -0.054435362 | 0.07202854 | -0.115999119 | 0.0001212 | 0.07 |
| FAM187B | -0.01055901 | 0.72731683 | -0.027995967 | 0.3551302 | 0.02 |
| FAM188A | -0.253479137 | 1.74E-17 | -0.120462819 | 6.54E-05 | 0.14 |
| LOC392651 | 0.069412311 | 0.02173589 | 0.078635592 | 0.009301 | 0.01 |
| FAM189A1 | -0.158844992 | 1.30E-07 | -0.092318763 | 0.0022495 | 0.07 |
| FAM189A2 | -0.103786467 | 0.00058907 | -0.032156664 | 0.2881561 | 0.08 |
| LOC115490 | 0.327281633 | 1.06E-28 | 0.387877012 | 1.46E-40 | 0.07 |
| TVP23A | -0.057205877 | 0.05867214 | -0.075663794 | 0.012342 | 0.02 |
| TVP23C | -0.03244329 | 0.28387825 | 0.035256578 | 0.2441687 | 0.07 |
| TVP23B | -0.170644164 | 1.37E-08 | -0.154351616 | 2.93E-07 | 0.02 |
| CCSER1 | -0.16086938 | 8.93E-08 | -0.214731711 | 7.24E-13 | 0.06 |
| LOC118783 | -0.160352004 | 9.83E-08 | -0.218591105 | 2.73E-13 | 0.06 |
| FAM192A | 0.2954914 | 1.83E-23 | 0.14226702 | 2.33E-06 | 0.16 |
| FAM193A | -0.340751944 | 4.05E-31 | -0.312389223 | 3.62E-26 | 0.03 |
| LOC654030 | 0.006032977 | 0.84208507 | 0.06002031 | 0.0472756 | 0.06 |
| ERICH6 | -0.04097067 | 0.17588595 | -0.003995439 | 0.8950311 | 0.04 |
| ERICH6B | 0.002986749 | 0.92143142 | -0.027974828 | 0.3554937 | 0.04 |
| FAM195A | 0.208597115 | 3.28E-12 | 0.282550024 | 1.65E-21 | 0.08 |
| FAM195B | 0.369131939 | 1.29E-36 | 0.306278885 | 3.61E-25 | 0.07 |
| FAM196A | -0.186896978 | 4.76E-10 | -0.181609913 | 1.47E-09 | 0.01 |
| FAM196B | -0.185143316 | 6.95E-10 | -0.199625115 | 2.75E-11 | 0.02 |
| FAM197Y2 | NA | NA | NA | NA | #VALUE! |
| FAM198A | -0.076720828 | 0.01117205 | -0.040361869 | 0.1823999 | 0.04 |
| FAM198B | -0.516382414 | 1.57E-75 | -0.284680917 | 7.97E-22 | 0.24 |
| FAM199X | -0.285343061 | 6.36E-22 | -0.158773258 | 1.32E-07 | 0.13 |
| FAM19A1 | -0.076266548 | 0.01166223 | -0.100698854 | 0.0008566 | 0.03 |
| FAM19A2 | -0.04728836 | 0.11817861 | 0.016621732 | 0.5830516 | 0.07 |
| FAM19A3 | 0.324524353 | 3.19E-28 | 0.106872707 | 0.0004011 | 0.22 |
| FAM19A4 | 0.265697971 | 4.07E-19 | 0.08280173 | 0.0061618 | 0.19 |
| FAM19A5 | 0.001319709 | 0.96523888 | -0.044475446 | 0.1417169 | 0.05 |
| FAM200A | -0.094452135 | 0.00177167 | 0.065352198 | 0.0307404 | 0.16 |
| FAM200B | 0.149650458 | 6.70E-07 | 0.071493501 | 0.018082 | 0.08 |
| FAM20A | 0.055923975 | 0.06457221 | -0.084642183 | 0.005108 | 0.15 |
| FAM20B | -0.054908154 | 0.06958761 | 0.185661103 | 6.21E-10 | 0.25 |
| LOC100132244 | 0.032279265 | 0.28632104 | -0.109447602 | 0.0002888 | 0.15 |
| LOC115460 | -0.221171531 | 1.41E-13 | -0.345390441 | 5.58E-32 | 0.13 |
| FAM21B | 0.037451555 | 0.21601793 | -0.003009969 | 0.9208226 | 0.05 |
| FAM21C | -0.142651557 | 2.19E-06 | -0.18348471 | 9.89E-10 | 0.05 |
| NUTM2A | -0.09770881 | 0.00121919 | 0.007711771 | 0.79898 | 0.11 |
| FAM22D | -0.308602182 | 1.51E-25 | -0.21261651 | 1.22E-12 | 0.1 |
| FAM22F | 0.003399063 | 0.91062786 | -0.05269891 | 0.0816009 | 0.06 |
| FAM22G | -0.015875429 | 0.60008178 | -0.043624064 | 0.149508 | 0.03 |
| TMEM236 | -0.0975496 | 0.00124199 | -0.214181756 | 8.30E-13 | 0.12 |
| FAM24A | 0.001436381 | 0.96216793 | -0.06106334 | 0.0435536 | 0.07 |
| FAM24B | 0.315227671 | 1.22E-26 | 0.174250466 | 6.69E-09 | 0.15 |
| FAM25A | 0.084484416 | 0.00519152 | 0.040443887 | 0.1815121 | 0.05 |
| FAM25B | 0.153880002 | 3.19E-07 | 0.066143182 | 0.0287701 | 0.09 |
| FAM26D | -0.037252103 | 0.21847515 | -0.012390288 | 0.68241 | 0.03 |
| FAM26E | -0.353803371 | 1.41E-33 | -0.236301279 | 2.46E-15 | 0.12 |
| FAM26F | 0.189740475 | 2.56E-10 | 0.050161486 | 0.097416 | 0.14 |
| FAM27A | 0.289848785 | 1.34E-22 | 0.152527396 | 4.05E-07 | 0.14 |
| FAM27B | 0.150074765 | 6.22E-07 | 0.034130887 | 0.2595636 | 0.12 |
| LOC100132429 | 0.335156896 | 4.22E-30 | 0.16831513 | 2.17E-08 | 0.17 |
| FAM27L | 0.089188639 | 0.00316609 | 0.049134024 | 0.1044783 | 0.05 |
| FAM32A | 0.362331788 | 3.01E-35 | 0.268231607 | 1.82E-19 | 0.1 |
| FAM35A | -0.160120191 | 1.03E-07 | -0.06952273 | 0.021527 | 0.1 |
| LOC100421578 | -0.148556802 | 8.09E-07 | -0.030804563 | 0.3089195 | 0.12 |
| FAM35BP | -0.083814177 | 0.00556016 | 0.030127692 | 0.3196758 | 0.12 |
| COX20 | -0.008335415 | 0.78311428 | 0.279401546 | 4.74E-21 | 0.29 |
| PIEZO1 | -0.040769026 | 0.17802399 | -0.122805362 | 4.69E-05 | 0.09 |
| LOC100421587 | -0.376037295 | 4.87E-38 | -0.321679827 | 9.87E-28 | 0.06 |
| FAM3A | 0.290149151 | 1.21E-22 | 0.178916214 | 2.58E-09 | 0.12 |
| FAM3B | -0.043977863 | 0.14623161 | -0.013159408 | 0.6638686 | 0.04 |
| FAM3C | 0.037221482 | 0.21885417 | -0.015648095 | 0.6053137 | 0.06 |
| FAM3D | 0.19968194 | 2.72E-11 | 0.070233026 | 0.0202247 | 0.13 |
| STRIP1 | -0.209509574 | 2.63E-12 | -0.312734535 | 3.17E-26 | 0.11 |
| STRIP2 | -0.093275278 | 0.00202232 | -0.089892069 | 0.0029346 | 0.01 |
| FAM41AY1 | -0.013343076 | 0.65947053 | -0.011838132 | 0.6958408 | 0.01 |
| FAM41C | 0.195176157 | 7.62E-11 | 0.207074067 | 4.74E-12 | 0.02 |
| FAM43A | -0.179966068 | 2.07E-09 | -0.232671713 | 6.67E-15 | 0.06 |
| FAM43B | -0.166265461 | 3.22E-08 | -0.103116446 | 0.0006395 | 0.07 |
| FAM45A | -0.074400835 | 0.01388099 | -0.00313248 | 0.9176112 | 0.08 |
| LOC115146 | -0.324440639 | 3.30E-28 | -0.333185007 | 9.54E-30 | 0.01 |
| FAM46A | -0.09424577 | 0.00181345 | -0.159488191 | 1.15E-07 | 0.07 |
| FAM46B | -0.116673724 | 0.00011059 | 0.011566506 | 0.7024834 | 0.13 |
| FAM46C | -0.354147465 | 1.21E-33 | -0.278418916 | 6.58E-21 | 0.08 |
| FAM46D | -0.017368848 | 0.56623181 | 0.006855239 | 0.8209049 | 0.03 |
| FAM47A | -0.124035551 | 3.92E-05 | -0.054061598 | 0.0740074 | 0.07 |
| FAM47B | -0.000296637 | 0.99218424 | 0.035873697 | 0.2360062 | 0.04 |
| FAM47C | 0.22221967 | 1.07E-13 | 0.078723458 | 0.0092222 | 0.15 |
| FAM47E | -0.403825708 | 3.98E-44 | -0.163263272 | 5.70E-08 | 0.25 |
| C13orf19 | -0.088194455 | 0.00352162 | -0.035080866 | 0.2465286 | 0.06 |
| SUPT20HL1 | -0.301634973 | 2.00E-24 | -0.220668322 | 1.60E-13 | 0.09 |
| FAM48B2 | -0.283142179 | 1.35E-21 | -0.255402849 | 9.76E-18 | 0.03 |
| FAM49A | 0.011912916 | 0.69401605 | -0.174658318 | 6.16E-09 | 0.19 |
| FAM49B | 0.308819536 | 1.40E-25 | 0.179899199 | 2.10E-09 | 0.13 |
| FAM50A | 0.302294054 | 1.57E-24 | 0.215244331 | 6.37E-13 | 0.09 |
| FAM50B | -0.005327646 | 0.8603453 | -0.006402016 | 0.8325641 | 0.01 |
| FAM53A | 0.103196746 | 0.00063324 | 0.085709628 | 0.0045742 | 0.02 |
| FAM53B | -0.037094179 | 0.22043492 | -0.184738083 | 7.57E-10 | 0.15 |
| FAM53C | 0.083651281 | 0.00565324 | -0.014960415 | 0.6212629 | 0.1 |
| MTFR2 | 0.57551936 | 2.06E-97 | 0.397829179 | 9.19E-43 | 0.18 |
| MTFR1L | -0.05359254 | 0.07655333 | -0.118434897 | 8.68E-05 | 0.07 |
| NXPE1 | -0.100666192 | 0.00085995 | -0.101899614 | 0.0007414 | 0.01 |
| LOC101928962 | -0.11467576 | 0.00014502 | -0.145998667 | 1.25E-06 | 0.04 |
| NXPE3 | -0.276496033 | 1.25E-20 | -0.245244395 | 1.96E-16 | 0.04 |
| NXPE4 | -0.188689727 | 3.22E-10 | -0.139348216 | 3.76E-06 | 0.05 |
| FAM57A | 0.333983523 | 6.87E-30 | 0.057717801 | 0.0564443 | 0.28 |
| FAM57B | -0.072573002 | 0.01640749 | -0.073660339 | 0.01486 | 0.01 |
| FAM58A | 0.4870175 | 3.58E-66 | 0.291026249 | 8.88E-23 | 0.2 |
| FAM58BP | 0.16157629 | 7.83E-08 | 0.030439991 | 0.314683 | 0.14 |
| GAREM | -0.122006169 | 5.25E-05 | -0.121744255 | 5.45E-05 | 0.01 |
| BRINP2 | -0.003034476 | 0.92018009 | 0.051877589 | 0.0864748 | 0.06 |
| BRINP3 | -0.031415212 | 0.29942283 | 0.121051693 | 6.02E-05 | 0.16 |
| FAM60A | 0.317572942 | 4.92E-27 | 0.124586619 | 3.62E-05 | 0.2 |
| FAM63A | -0.359689401 | 1.00E-34 | -0.078859328 | 0.0091016 | 0.29 |
| FAM63B | -0.55116619 | 7.20E-88 | -0.498391687 | 1.09E-69 | 0.06 |
| LOC116044 | 0.589817271 | 2.12E-103 | 0.295537214 | 1.80E-23 | 0.3 |
| FAM65A | -0.297760765 | 8.14E-24 | -0.339619617 | 6.53E-31 | 0.05 |
| Diff48 | -0.183429209 | 1.00E-09 | -0.208920247 | 3.03E-12 | 0.03 |
| FAM65C | -0.165284011 | 3.89E-08 | -0.15446729 | 2.87E-07 | 0.02 |
| FAM66A | -0.219865212 | 1.97E-13 | -0.198151527 | 3.87E-11 | 0.03 |
| FAM66C | -0.138612092 | 4.23E-06 | -0.139872909 | 3.45E-06 | 0.01 |
| LOC100133184 | 0.129440866 | 1.77E-05 | 0.156283797 | 2.07E-07 | 0.03 |
| FAM66E | -0.131594044 | 1.27E-05 | -0.195880667 | 6.49E-11 | 0.07 |
| FAM69A | -0.349505095 | 9.36E-33 | -0.322397848 | 7.43E-28 | 0.03 |
| FAM69B | 0.179539736 | 2.27E-09 | 0.17492975 | 5.83E-09 | 0.01 |
| FAM69C | 0.003296912 | 0.91330298 | 0.027004403 | 0.372435 | 0.03 |
| TMEM255A | -0.326852236 | 1.26E-28 | -0.232625246 | 6.75E-15 | 0.1 |
| LOC100291462 | -0.117144516 | 0.00010368 | -0.115580455 | 0.0001283 | 0.01 |
| FAM71A | 0.052438755 | 0.08312008 | -0.010072384 | 0.7394166 | 0.07 |
| FAM71B | 0.010676264 | 0.72441134 | -0.040313719 | 0.1829226 | 0.06 |
| FAM71C | -0.084350827 | 0.00526319 | -0.043062287 | 0.1548249 | 0.05 |
| FAM71D | 0.290564123 | 1.04E-22 | 0.437501225 | 2.55E-52 | 0.15 |
| FAM71E1 | 0.253457214 | 1.75E-17 | 0.148584476 | 8.05E-07 | 0.11 |
| MGC35045 | 0.019527845 | 0.51897638 | -0.016312656 | 0.5900772 | 0.04 |
| FAM71F1 | 0.012330002 | 0.6838716 | -0.015281916 | 0.6137837 | 0.03 |
| FAM71F2 | -0.158373713 | 1.42E-07 | -0.152353738 | 4.18E-07 | 0.01 |
| FAM72A | 0.44888601 | 2.63E-55 | 0.349354058 | 1.00E-32 | 0.1 |
| FAM72B | 0.583201834 | 1.36E-100 | 0.375108056 | 7.60E-38 | 0.21 |
| GCUD2 | 0.483895701 | 3.13E-65 | 0.325696896 | 2.00E-28 | 0.16 |
| FAM73A | -0.489255837 | 7.44E-67 | -0.447981588 | 4.59E-55 | 0.05 |
| FAM73B | -0.077363208 | 0.0105101 | -0.100184409 | 0.0009108 | 0.03 |
| FAM74A5 | 0.033315134 | 0.27113094 | -0.036329206 | 0.2301062 | 0.07 |
| FAM74A3 | -0.045515304 | 0.13262809 | -0.083005178 | 0.0060364 | 0.04 |
| FAM74A4 | -0.000773722 | 0.97961594 | -0.022737799 | 0.4526761 | 0.03 |
| SPATA31A2 | 0.132450015 | 1.12E-05 | 0.042472344 | 0.1605615 | 0.09 |
| SPATA31A3 | 0.077125198 | 0.01075118 | 0.032101967 | 0.2889774 | 0.05 |
| SPATA31A5 | 0.027590227 | 0.362149 | 8.14E-05 | 0.9978559 | 0.03 |
| SPATA31A6 | 0.044136118 | 0.14478396 | 0.017930257 | 0.5537478 | 0.03 |
| LOC286235 | -0.018107014 | 0.54984534 | -0.033198822 | 0.2728085 | 0.02 |
| FAM76A | -0.076836614 | 0.01105007 | -0.24212482 | 4.79E-16 | 0.17 |
| FAM76B | -0.01054994 | 0.72754175 | -0.072563796 | 0.0164212 | 0.07 |
| FAM78A | -0.092643844 | 0.00216981 | -0.169809426 | 1.62E-08 | 0.08 |
| FAM78B | 0.178276348 | 2.94E-09 | 0.132757532 | 1.07E-05 | 0.05 |
| FAM7A2 | -0.160219213 | 1.01E-07 | -0.168004156 | 2.30E-08 | 0.01 |
| ULK4P3 | -0.147245964 | 1.01E-06 | -0.143640491 | 1.86E-06 | 0.01 |
| FAM81A | 0.145622406 | 1.33E-06 | 0.077035547 | 0.0108433 | 0.07 |
| FAM81B | -0.205678733 | 6.62E-12 | 0.05679277 | 0.0605226 | 0.27 |
| RMDN2 | -0.353581386 | 1.56E-33 | -0.232647446 | 6.71E-15 | 0.13 |
| RMDN3 | -0.2626895 | 1.04E-18 | -0.101851172 | 0.0007458 | 0.17 |
| RMDN1 | 0.116590437 | 0.00011186 | 0.030074872 | 0.3205254 | 0.09 |
| FAM83A | 0.114459371 | 0.0001493 | 0.040459785 | 0.1813403 | 0.08 |
| FAM83B | 0.042284163 | 0.16242472 | -0.017767524 | 0.5573526 | 0.07 |
| FAM83C | 0.200108733 | 2.46E-11 | 0.03544902 | 0.2416023 | 0.17 |
| FAM83D | 0.443166165 | 8.61E-54 | 0.225339504 | 4.75E-14 | 0.22 |
| FAM83E | -0.195440662 | 7.18E-11 | -0.090021947 | 0.0028936 | 0.11 |
| FAM83F | 0.129197412 | 1.83E-05 | -0.029150968 | 0.3356234 | 0.16 |
| FAM83G | -0.14614811 | 1.22E-06 | -0.262427702 | 1.13E-18 | 0.12 |
| AI3 | 0.213581182 | 9.64E-13 | 0.136737228 | 5.71E-06 | 0.08 |
| LOC654112 | 0.1082641 | 0.00033615 | -0.053586121 | 0.0765887 | 0.17 |
| FAM84B | -0.145712983 | 1.31E-06 | -0.055252574 | 0.0678525 | 0.1 |
| EEF2KMT | 0.310442993 | 7.57E-26 | 0.378883628 | 1.23E-38 | 0.07 |
| FAM86B1 | 0.01869929 | 0.53686843 | 0.066201385 | 0.0286295 | 0.05 |
| LOC100509790 | -0.103584946 | 0.00060383 | -0.06793855 | 0.0246969 | 0.04 |
| LOC90960 | 0.147989546 | 8.92E-07 | 0.1737692 | 7.37E-09 | 0.03 |
| FAM86DP | 0.183447034 | 9.97E-10 | 0.113497764 | 0.0001698 | 0.07 |
| FAM89A | 0.100246285 | 0.00090415 | -0.029109227 | 0.3363161 | 0.13 |
| FAM89B | 0.329382791 | 4.52E-29 | 0.274049811 | 2.78E-20 | 0.06 |
| LOC221659 | -0.305744901 | 4.40E-25 | -0.140342784 | 3.20E-06 | 0.17 |
| LOC220509 | 0.174927228 | 5.83E-09 | 0.048130956 | 0.1117589 | 0.13 |
| LOC389613 | 0.078448366 | 0.00947072 | 0.054521854 | 0.0715768 | 0.03 |
| FAM91A1 | 0.062987689 | 0.03733401 | -0.073919473 | 0.0145107 | 0.14 |
| FAM92A1 | 0.257272561 | 5.54E-18 | 0.097084183 | 0.0013109 | 0.17 |
| FAM92A1P2 | 0.044531417 | 0.14121583 | 0.027808588 | 0.3583609 | 0.02 |
| FAM92B | 0.077148229 | 0.01072764 | 0.046520578 | 0.1242766 | 0.04 |
| LOC100132791 | -0.097617964 | 0.00123215 | -0.100993988 | 0.0008268 | 0.01 |
| FAM96A | 0.291078298 | 8.72E-23 | 0.210842237 | 1.90E-12 | 0.09 |
| FAM96B | 0.515521387 | 3.04E-75 | 0.375507648 | 6.27E-38 | 0.15 |
| FAM98A | 0.276294472 | 1.33E-20 | 0.196337157 | 5.85E-11 | 0.08 |
| FAM98B | -0.118410947 | 8.71E-05 | -0.223945729 | 6.85E-14 | 0.11 |
| FAM98C | 0.00363568 | 0.90443526 | 0.101132354 | 0.0008132 | 0.1 |
| FAM99A | -0.033326632 | 0.27096549 | -0.002108102 | 0.9444998 | 0.04 |
| FAM99B | 0.016515517 | 0.58546159 | -0.010730961 | 0.7230573 | 0.03 |
| LOC286475 | 0.116374535 | 0.0001152 | 0.094544726 | 0.0017532 | 0.03 |
| FAM9B | -0.018129536 | 0.54934906 | 0.000290252 | 0.9923525 | 0.02 |
| FAM9C | 0.016237221 | 0.59179782 | -0.014382289 | 0.6348106 | 0.04 |
| FANCH | 0.449561662 | 1.74E-55 | 0.13819007 | 4.53E-06 | 0.32 |
| FLJ34064 | 0.379713803 | 8.22E-39 | 0.163684335 | 5.27E-08 | 0.22 |
| FANCC | 0.282371803 | 1.75E-21 | 0.134460847 | 8.18E-06 | 0.15 |
| FANCD2 | 0.316451709 | 7.61E-27 | 0.163948603 | 5.01E-08 | 0.16 |
| FANCE | 0.378298501 | 1.63E-38 | 0.153767842 | 3.25E-07 | 0.23 |
| FANCF | 0.02250645 | 0.45728895 | 0.101138649 | 0.0008126 | 0.08 |
| FANCG | 0.418729532 | 1.23E-47 | 0.263633908 | 7.78E-19 | 0.16 |
| FANCI | 0.40758674 | 5.38E-45 | 0.210039722 | 2.31E-12 | 0.2 |
| FANCL | 0.449234464 | 2.13E-55 | 0.305116346 | 5.55E-25 | 0.15 |
| FANCM | -0.183200452 | 1.05E-09 | -0.167695085 | 2.44E-08 | 0.02 |
| FANK1 | -0.283468169 | 1.21E-21 | -0.04298544 | 0.1555633 | 0.25 |
| FAP | -0.249951267 | 4.96E-17 | -0.101919027 | 0.0007397 | 0.15 |
| FAR1 | -0.171994679 | 1.05E-08 | -0.194506306 | 8.86E-11 | 0.03 |
| FAR2 | 0.008981487 | 0.76677258 | -0.016084175 | 0.5952957 | 0.03 |
| FARP1 | -0.045011946 | 0.13696973 | -0.008495714 | 0.7790505 | 0.04 |
| FARP2 | -0.493898288 | 2.77E-68 | -0.363668507 | 1.63E-35 | 0.14 |
| FARS2 | 0.068478596 | 0.02357365 | 0.198678484 | 3.42E-11 | 0.14 |
| FARSA | 0.369617897 | 1.03E-36 | 0.211871365 | 1.47E-12 | 0.16 |
| FARSB | 0.518553154 | 2.93E-76 | 0.400352409 | 2.47E-43 | 0.12 |
| FASLG | 0.003802219 | 0.90008018 | -0.080294375 | 0.0079114 | 0.09 |
| FASN | -0.189375026 | 2.77E-10 | -0.089247827 | 0.003146 | 0.11 |
| FASTKD1 | 0.183162633 | 1.06E-09 | 0.093632791 | 0.0019429 | 0.09 |
| FASTKD2 | 0.066661908 | 0.02753776 | 0.060132376 | 0.0468633 | 0.01 |
| FASTKD3 | 0.100824645 | 0.0008438 | 0.166671918 | 2.98E-08 | 0.07 |
| FASTKD5 | -0.006958966 | 0.81824191 | 0.015105966 | 0.617872 | 0.03 |
| FASTK | 0.171103506 | 1.25E-08 | 0.234079749 | 4.54E-15 | 0.07 |
| FAS | -0.078943835 | 0.00902737 | -0.15038169 | 5.90E-07 | 0.08 |
| FAT1 | -0.057095648 | 0.05916125 | -0.211438798 | 1.64E-12 | 0.16 |
| FAT2 | -0.025840688 | 0.39339742 | -0.146597452 | 1.13E-06 | 0.13 |
| FAT3 | -0.386103996 | 3.54E-40 | -0.235752666 | 2.86E-15 | 0.16 |
| FAT4 | -0.467533898 | 1.90E-60 | -0.393920606 | 6.87E-42 | 0.08 |
| FATE1 | 0.180290548 | 1.94E-09 | 0.118225805 | 8.93E-05 | 0.07 |
| FAU | 0.35190304 | 3.27E-33 | 0.332395786 | 1.32E-29 | 0.02 |
| FBF1 | 0.02368427 | 0.43408015 | -0.060930421 | 0.0440138 | 0.09 |
| FBLIM1 | 0.101081909 | 0.00081817 | 0.078199053 | 0.0097011 | 0.03 |
| FBLL1 | 0.016641177 | 0.5826109 | -0.066835849 | 0.0271348 | 0.09 |
| FBLN1 | -0.192233669 | 1.48E-10 | -0.098176798 | 0.0011544 | 0.1 |
| FBLN2 | -0.161457274 | 8.01E-08 | -0.127776171 | 2.27E-05 | 0.04 |
| FBLN5 | -0.107907667 | 0.00035178 | -0.098327112 | 0.0011342 | 0.01 |
| FBLN7 | -0.228657489 | 1.97E-14 | -0.173243572 | 8.18E-09 | 0.06 |
| FBL | 0.518109414 | 4.14E-76 | 0.417484875 | 2.45E-47 | 0.11 |
| FBN1 | -0.396029894 | 2.33E-42 | -0.250156593 | 4.67E-17 | 0.15 |
| FBN2 | 0.068752773 | 0.02302053 | -0.02686898 | 0.3748383 | 0.1 |
| FBN3 | 0.314178171 | 1.83E-26 | 0.046710529 | 0.1227456 | 0.27 |
| FBP1 | -0.333559109 | 8.18E-30 | -0.121377542 | 5.74E-05 | 0.22 |
| FBP2 | -0.007343354 | 0.80839214 | -0.066661228 | 0.0275393 | 0.06 |
| LOC642031 | 0.238920112 | 1.18E-15 | 0.194183457 | 9.53E-11 | 0.05 |
| FBRS | 0.043372423 | 0.1518722 | 0.013647754 | 0.6522008 | 0.03 |
| FBXL12 | 0.076793505 | 0.01109535 | 0.053248409 | 0.0784662 | 0.03 |
| FBXL13 | -0.054624618 | 0.07104317 | -0.101900397 | 0.0007413 | 0.05 |
| FBXL14 | 0.126063278 | 2.92E-05 | 0.038981876 | 0.1978236 | 0.09 |
| MGC11279 | 0.038816397 | 0.19973521 | 0.027327211 | 0.3667449 | 0.02 |
| FBXL16 | -0.251909716 | 2.78E-17 | -0.142882307 | 2.11E-06 | 0.11 |
| FBXL17 | -0.476896094 | 3.74E-63 | -0.249179514 | 6.23E-17 | 0.23 |
| FBXL18 | -0.03575302 | 0.23758699 | -0.036002788 | 0.2343234 | 0.01 |
| FBXL19 | 0.240857796 | 6.86E-16 | 0.202344774 | 1.46E-11 | 0.04 |
| LOC90110 | -0.045550654 | 0.13232723 | 0.01913083 | 0.5275111 | 0.07 |
| FBXL21 | -0.008186477 | 0.78689539 | -0.029147986 | 0.3356728 | 0.03 |
| LOC390593 | -0.150245372 | 6.04E-07 | -0.170486497 | 1.42E-08 | 0.03 |
| FBXL2 | -0.176921634 | 3.89E-09 | -0.068148259 | 0.0242553 | 0.11 |
| FBXL3 | -0.457908435 | 9.38E-58 | -0.259273422 | 3.00E-18 | 0.2 |
| FBXL4 | -0.097995833 | 0.00117905 | -0.091265125 | 0.0025267 | 0.01 |
| FBXL5 | -0.470127402 | 3.44E-61 | -0.313249598 | 2.60E-26 | 0.16 |
| FBXL6 | 0.416077646 | 5.33E-47 | 0.338831995 | 9.10E-31 | 0.08 |
| FBXL7 | -0.362800188 | 2.43E-35 | -0.248122941 | 8.49E-17 | 0.12 |
| LOC115023 | -0.116593291 | 0.00011181 | -0.048418903 | 0.1096291 | 0.07 |
| FBXO10 | 0.039075312 | 0.1967501 | -0.095662102 | 0.001544 | 0.14 |
| VIT1 | -0.056981847 | 0.05966976 | -0.179373851 | 2.34E-09 | 0.13 |
| FBXO15 | -0.156560501 | 1.97E-07 | -0.034556425 | 0.2536669 | 0.13 |
| FBXO16 | -0.119569824 | 7.41E-05 | 0.103087409 | 0.0006418 | 0.23 |
| FLJ11798 | 0.175074211 | 5.66E-09 | -0.015512192 | 0.6084511 | 0.2 |
| FBXO18 | 0.165254061 | 3.91E-08 | 0.010500904 | 0.7287581 | 0.16 |
| FBXO21 | -0.208965977 | 3.00E-12 | -0.179440664 | 2.31E-09 | 0.03 |
| FBXO22.AS1 | 0.427458938 | 8.88E-50 | 0.380258307 | 6.31E-39 | 0.05 |
| FLJ13986 | 0.131177116 | 1.36E-05 | 0.066019021 | 0.029072 | 0.07 |
| FBXO24 | 0.109524144 | 0.00028595 | 0.142673946 | 2.18E-06 | 0.04 |
| FBXO25 | -0.102009028 | 0.00073167 | 0.035045486 | 0.2470057 | 0.14 |
| FBXO27 | 0.107880719 | 0.00035299 | 0.006593877 | 0.8276238 | 0.11 |
| FBXO28 | -0.140710621 | 3.01E-06 | 0.231912483 | 8.20E-15 | 0.38 |
| FBXO2 | -0.016535114 | 0.58501658 | -0.148035697 | 8.85E-07 | 0.14 |
| FBXO30 | -0.089287619 | 0.00313255 | -0.126576251 | 2.71E-05 | 0.04 |
| DKFZP434B027 | -0.042175077 | 0.16351223 | -0.173067913 | 8.48E-09 | 0.14 |
| FBXO32 | 0.119342707 | 7.65E-05 | 0.042142923 | 0.1638338 | 0.08 |
| FBXO33 | 0.044580564 | 0.14077697 | -0.189198231 | 2.88E-10 | 0.24 |
| DKFZp547C162 | -0.250512518 | 4.21E-17 | -0.182913777 | 1.12E-09 | 0.07 |
| FBXO36 | -0.441699221 | 2.08E-53 | -0.281574752 | 2.29E-21 | 0.17 |
| FLJ13962 | -0.503871511 | 1.97E-71 | -0.324053038 | 3.85E-28 | 0.18 |
| FBXO39 | -0.050266122 | 0.09671863 | -0.067776352 | 0.0250431 | 0.02 |
| FBXO3 | -0.358723212 | 1.56E-34 | -0.256911631 | 6.18E-18 | 0.11 |
| LOC116109 | -0.107659757 | 0.00036304 | -0.131850302 | 1.22E-05 | 0.03 |
| FBXO41 | 0.021099756 | 0.48589735 | -0.108280275 | 0.0003355 | 0.13 |
| FBXO42 | -0.085085773 | 0.0048797 | -0.170712994 | 1.35E-08 | 0.09 |
| FBXO43 | 0.307408706 | 2.37E-25 | 0.15923011 | 1.21E-07 | 0.15 |
| FBXO44 | 0.077141753 | 0.01073425 | -0.040459093 | 0.1813478 | 0.12 |
| FBXO45 | 0.306113167 | 3.84E-25 | 0.155902162 | 2.22E-07 | 0.16 |
| FBXO46 | 0.147348101 | 9.95E-07 | 0.169691937 | 1.65E-08 | 0.03 |
| FBXO47 | 0.047086039 | 0.1197623 | 0.037719505 | 0.2127482 | 0.01 |
| FBXO48 | -0.128718909 | 1.97E-05 | -0.236555028 | 2.29E-15 | 0.11 |
| FBXO4 | -0.219538015 | 2.14E-13 | 0.066178329 | 0.0286851 | 0.29 |
| FBXO5 | 0.401223101 | 1.57E-43 | 0.174619994 | 6.21E-09 | 0.23 |
| FBG2 | 0.127759492 | 2.27E-05 | 0.032763553 | 0.2791494 | 0.1 |
| FBXO7 | 0.057531601 | 0.05724636 | -0.048523605 | 0.1088627 | 0.11 |
| FBXO8 | -0.355272839 | 7.33E-34 | -0.209929791 | 2.37E-12 | 0.15 |
| FBXO9 | -0.142749085 | 2.16E-06 | -0.038450646 | 0.2040082 | 0.11 |
| FBXW10 | 0.188462755 | 3.39E-10 | 0.019113725 | 0.5278804 | 0.17 |
| FBXW11 | -0.357513205 | 2.68E-34 | -0.340513512 | 4.48E-31 | 0.02 |
| FBXO35 | -0.042848422 | 0.15688631 | -0.010180109 | 0.7367324 | 0.04 |
| FBXW2 | -0.340518144 | 4.47E-31 | -0.224623436 | 5.74E-14 | 0.12 |
| FBXW4 | -0.082392084 | 0.00642147 | 0.037922421 | 0.2102959 | 0.13 |
| FBXW5 | 0.036758739 | 0.2246393 | -0.011637585 | 0.700743 | 0.05 |
| FBXW7 | 0.021583305 | 0.4759556 | -0.109630497 | 0.0002821 | 0.14 |
| MGC33534 | -0.314702683 | 1.49E-26 | -0.227964275 | 2.37E-14 | 0.09 |
| FBXW9 | 0.155762892 | 2.28E-07 | 0.160383819 | 9.78E-08 | 0.01 |
| FCA.MR | 0.041953629 | 0.16573675 | -0.065616888 | 0.0300685 | 0.11 |
| FCAR | 0.020973317 | 0.48851529 | -0.188784654 | 3.16E-10 | 0.21 |
| FCER1A | -0.29114461 | 8.52E-23 | -0.228600252 | 2.00E-14 | 0.07 |
| FCER1G | 0.119299171 | 7.69E-05 | -0.001584265 | 0.9582763 | 0.13 |
| FCER2 | -0.024113381 | 0.42579652 | -0.105563571 | 0.0004727 | 0.09 |
| FCF1 | -0.115178053 | 0.00013552 | -0.065703225 | 0.0298522 | 0.05 |
| LOC100133944 | -0.202827064 | 1.30E-11 | -0.225500919 | 4.56E-14 | 0.03 |
| FCRI | 0.077133956 | 0.01074222 | -0.020625784 | 0.49575 | 0.1 |
| FCGR1B | 0.095375224 | 0.00159539 | 0.001869553 | 0.9507716 | 0.1 |
| LOC644410 | 0.066666671 | 0.02752666 | -0.024750779 | 0.4136637 | 0.1 |
| FCGR2A | -0.085135289 | 0.0048548 | -0.133356753 | 9.71E-06 | 0.05 |
| FCGR2B | -0.015856399 | 0.60051896 | -0.107311106 | 0.0003795 | 0.1 |
| FCGR2C | -0.180784869 | 1.75E-09 | -0.238128651 | 1.48E-15 | 0.06 |
| FCGR3A | -0.036859445 | 0.2233711 | -0.071334786 | 0.0183404 | 0.04 |
| FCGR3B | 0.0094381 | 0.75528472 | -0.056657494 | 0.061139 | 0.07 |
| FCGRT | -0.144382692 | 1.64E-06 | -0.164836868 | 4.23E-08 | 0.03 |
| FCHO1 | 0.108962487 | 0.00030739 | 0.014659356 | 0.6283022 | 0.1 |
| FCHO2 | -0.555087212 | 2.37E-89 | -0.317132755 | 5.84E-27 | 0.24 |
| FCHSD1 | -0.120881626 | 6.16E-05 | -0.127551351 | 2.34E-05 | 0.01 |
| FCHSD2 | -0.129430305 | 1.77E-05 | -0.258020686 | 4.41E-18 | 0.13 |
| FCN1 | 0.069560685 | 0.02145562 | -0.076863674 | 0.0110217 | 0.15 |
| FCN2 | -0.237193382 | 1.92E-15 | -0.058724625 | 0.0522677 | 0.18 |
| FCN3 | 0.201399964 | 1.82E-11 | 0.132833862 | 1.05E-05 | 0.07 |
| FCRL1 | -0.073766391 | 0.01471612 | -0.16515947 | 3.98E-08 | 0.1 |
| FCRL2 | -6.66E-05 | 0.99824544 | -0.105654469 | 0.0004674 | 0.11 |
| FCRL3 | -0.007847323 | 0.79552419 | -0.124896156 | 3.46E-05 | 0.12 |
| FCRL4 | 0.006967454 | 0.81802409 | -0.065663275 | 0.0299521 | 0.08 |
| FCRL5 | 0.038807248 | 0.1998413 | -0.08628385 | 0.0043084 | 0.13 |
| FCRL6 | -0.103146823 | 0.00063711 | -0.121140291 | 5.94E-05 | 0.02 |
| FCRLA | 0.006389288 | 0.83289208 | -0.112757323 | 0.0001874 | 0.12 |
| FCRLB | -0.027995548 | 0.35513738 | -0.086354704 | 0.0042766 | 0.06 |
| FDFT1 | -0.07051326 | 0.01973005 | 0.017650972 | 0.5599414 | 0.09 |
| FDPSP2 | -0.145074287 | 1.46E-06 | -0.042229963 | 0.1629644 | 0.11 |
| FDPS | 0.401238542 | 1.56E-43 | 0.423825132 | 7.03E-49 | 0.03 |
| FDX1L | 0.328065947 | 7.70E-29 | 0.280950161 | 2.82E-21 | 0.05 |
| FDX1 | 0.195385128 | 7.27E-11 | 0.118743513 | 8.31E-05 | 0.08 |
| FDXACB1 | -0.277245374 | 9.72E-21 | -0.006295719 | 0.8353041 | 0.28 |
| FDXR | 0.089633502 | 0.00301785 | 0.045928787 | 0.1291419 | 0.05 |
| FECH | 0.029807068 | 0.32485536 | -0.088193663 | 0.0035219 | 0.12 |
| FEM1A | 0.055752377 | 0.06539789 | -0.036330532 | 0.2300892 | 0.1 |
| FEM1B | -0.347509576 | 2.23E-32 | -0.383270933 | 1.44E-39 | 0.04 |
| FEM1C | -0.438936677 | 1.09E-52 | -0.145555175 | 1.35E-06 | 0.3 |
| FEN1 | 0.569938272 | 3.73E-95 | 0.329003201 | 5.27E-29 | 0.25 |
| FLJ13459 | 0.116012311 | 0.00012103 | 0.037599676 | 0.214206 | 0.08 |
| LOC100508640 | -0.248703602 | 7.16E-17 | -0.086992577 | 0.0039996 | 0.17 |
| FER1L6 | -0.227473704 | 2.70E-14 | -0.256927921 | 6.15E-18 | 0.03 |
| FERD3L | -0.09612215 | 0.00146472 | -0.076096722 | 0.0118503 | 0.03 |
| FERMT1 | 0.232333123 | 7.31E-15 | -0.000386543 | 0.9898155 | 0.24 |
| FERMT2 | -0.246504742 | 1.36E-16 | -0.214259112 | 8.14E-13 | 0.04 |
| FERMT3 | 0.0091017 | 0.76374307 | -0.122584635 | 4.84E-05 | 0.14 |
| FER | -0.330446513 | 2.93E-29 | -0.305184429 | 5.41E-25 | 0.03 |
| FES | -0.01472405 | 0.6267867 | -0.106011016 | 0.000447 | 0.1 |
| FETUB | 0.301987258 | 1.76E-24 | 0.103461148 | 0.0006131 | 0.2 |
| FEV | 0.084540653 | 0.00516162 | 0.020361475 | 0.5012902 | 0.07 |
| FEZ1 | -0.220334966 | 1.75E-13 | -0.140102164 | 3.33E-06 | 0.09 |
| LOC115841 | -0.15768711 | 1.61E-07 | -0.088015463 | 0.0035894 | 0.07 |
| LOC392779 | 0.122630907 | 4.80E-05 | 0.031632091 | 0.2960973 | 0.1 |
| FEZF2 | 0.013653295 | 0.65206886 | -0.024905141 | 0.4107565 | 0.04 |
| FFAR1 | -0.122058369 | 5.21E-05 | -0.094704585 | 0.0017218 | 0.03 |
| FFAR2 | -0.076485415 | 0.01142374 | 0.063309391 | 0.0363717 | 0.14 |
| FFAR3 | 0.003512057 | 0.90766994 | -0.050107564 | 0.0977769 | 0.06 |
| FGA | -0.023301142 | 0.44155406 | -0.005577409 | 0.8538702 | 0.02 |
| FGB | -0.063437669 | 0.03599385 | 0.051499756 | 0.0887945 | 0.12 |
| FGD1 | 0.323267241 | 5.26E-28 | -0.01636184 | 0.5889566 | 0.34 |
| FGD2 | -0.106784624 | 0.00040557 | -0.234958564 | 3.56E-15 | 0.13 |
| FGD3 | -0.28976273 | 1.38E-22 | -0.141043004 | 2.85E-06 | 0.15 |
| FGD4 | -0.257637561 | 4.96E-18 | -0.311978952 | 4.23E-26 | 0.06 |
| FGD5 | -0.356617879 | 4.01E-34 | -0.210290875 | 2.17E-12 | 0.15 |
| FGD6 | -0.316025733 | 8.97E-27 | -0.223855101 | 7.02E-14 | 0.1 |
| FGF10 | -0.379563823 | 8.85E-39 | -0.270931306 | 7.66E-20 | 0.11 |
| FGF11 | 0.166831123 | 2.89E-08 | 0.035032622 | 0.2471793 | 0.14 |
| LOC100505888 | -0.066470989 | 0.02798593 | -0.123293652 | 4.37E-05 | 0.06 |
| FGF13 | -0.104819034 | 0.00051857 | -0.011664906 | 0.7000744 | 0.1 |
| FGF14 | -0.334198257 | 6.28E-30 | -0.147379901 | 9.90E-07 | 0.19 |
| MF4 | -0.188192264 | 3.59E-10 | -0.138547764 | 4.28E-06 | 0.05 |
| FGF17 | 0.077664719 | 0.01021159 | -0.001752565 | 0.9538486 | 0.08 |
| FGF18 | -0.293020894 | 4.41E-23 | -0.173056486 | 8.50E-09 | 0.12 |
| FGF19 | -0.015231905 | 0.6149445 | -0.048457166 | 0.1093485 | 0.04 |
| FGF1 | -0.274432691 | 2.45E-20 | -0.184195291 | 8.51E-10 | 0.1 |
| FGF20 | -0.298429764 | 6.39E-24 | -0.157042925 | 1.80E-07 | 0.15 |
| FGF21 | 0.275981293 | 1.48E-20 | 0.036555687 | 0.227212 | 0.24 |
| FGF22 | -0.039491714 | 0.19201791 | -0.076514714 | 0.0113921 | 0.04 |
| FGF23 | 0.130512037 | 1.50E-05 | 0.035757162 | 0.2375326 | 0.1 |
| FGF2 | -0.204602689 | 8.56E-12 | -0.263531124 | 8.03E-19 | 0.06 |
| FGF3 | -0.044373165 | 0.14263608 | -0.014791449 | 0.6252095 | 0.03 |
| FGF4 | 0.020709134 | 0.49400971 | -0.065883623 | 0.0294043 | 0.09 |
| FGF5 | 0.050136463 | 0.09758331 | 0.008165045 | 0.7874399 | 0.05 |
| FGF6 | -0.014511598 | 0.63176955 | -0.02389172 | 0.4300639 | 0.01 |
| FGF7 | -0.250426641 | 4.31E-17 | -0.230439372 | 1.22E-14 | 0.02 |
| FGF8 | 0.222638158 | 9.64E-14 | 0.131474588 | 1.30E-05 | 0.1 |
| FGF9 | 0.106767036 | 0.00040647 | -0.020403428 | 0.5004087 | 0.13 |
| FGFBP1 | 0.257280203 | 5.52E-18 | -0.001763757 | 0.9535542 | 0.26 |
| FGFBP2 | 0.081574754 | 0.00696907 | -0.064511278 | 0.0329609 | 0.15 |
| FGFBP3 | 0.187094889 | 4.56E-10 | 0.001879986 | 0.9504973 | 0.19 |
| FGFR1OP2 | -0.049290769 | 0.10337541 | -0.233138717 | 5.87E-15 | 0.19 |
| FGFR1OP | 0.05571091 | 0.06559872 | 0.15746326 | 1.67E-07 | 0.11 |
| KAL2 | -0.11536605 | 0.00013212 | -0.209075563 | 2.92E-12 | 0.1 |
| FGFR2 | -0.098008393 | 0.00117733 | -0.047684177 | 0.1151278 | 0.06 |
| FGFR3 | -0.123295896 | 4.37E-05 | -0.115765833 | 0.0001252 | 0.01 |
| FGFR4 | 0.101215665 | 0.00080513 | 0.114875056 | 0.0001412 | 0.02 |
| FLJ20523 | -0.060018182 | 0.04728347 | -0.022646757 | 0.4544882 | 0.04 |
| FGGY | -0.181758428 | 1.42E-09 | -0.143741653 | 1.83E-06 | 0.04 |
| FGG | -0.033518503 | 0.26821469 | 0.018206148 | 0.5476626 | 0.06 |
| FGL1 | -0.013823637 | 0.64801904 | 0.068666141 | 0.0231941 | 0.09 |
| FGL2 | -0.260354779 | 2.15E-18 | -0.287544307 | 2.98E-22 | 0.03 |
| FGR | -0.054565641 | 0.07134904 | -0.17521905 | 5.50E-09 | 0.13 |
| FHAD1 | -0.26727187 | 2.47E-19 | -0.185317439 | 6.69E-10 | 0.09 |
| FHDC1 | -0.041999736 | 0.16527173 | 0.006116867 | 0.8399187 | 0.05 |
| FRA3B | -0.042412294 | 0.16115431 | 0.044455293 | 0.1418977 | 0.09 |
| FHL1 | -0.286469691 | 4.32E-22 | -0.242984875 | 3.75E-16 | 0.05 |
| FHL2 | -0.115435312 | 0.00013089 | -0.01574314 | 0.6031238 | 0.1 |
| LOC116426 | 0.220425241 | 1.71E-13 | 0.130422476 | 1.52E-05 | 0.1 |
| FHL5 | -0.430909919 | 1.21E-50 | -0.281257778 | 2.55E-21 | 0.15 |
| FHOD1 | -0.099372336 | 0.00100298 | -0.021880471 | 0.4699016 | 0.08 |
| FLJ22717 | -0.062870979 | 0.03768845 | -0.122335421 | 5.01E-05 | 0.06 |
| FH | 0.37365696 | 1.52E-37 | 0.549792158 | 2.36E-87 | 0.18 |
| FIBCD1 | -0.070998322 | 0.01889882 | -0.037959005 | 0.209856 | 0.04 |
| FIBIN | -0.212128745 | 1.38E-12 | -0.099579981 | 0.0009786 | 0.12 |
| FIBP | 0.271055278 | 7.36E-20 | 0.27485104 | 2.14E-20 | 0.01 |
| FICD | -0.47563075 | 8.79E-63 | -0.446852721 | 9.17E-55 | 0.03 |
| FIG4 | -0.162337784 | 6.79E-08 | -0.030397407 | 0.3153607 | 0.14 |
| FIGF | -0.12709326 | 2.51E-05 | -0.140595891 | 3.07E-06 | 0.02 |
| FIGLA | 0.169418987 | 1.75E-08 | 0.029126642 | 0.336027 | 0.15 |
| FIGNL1 | 0.24504362 | 2.08E-16 | 0.136920734 | 5.55E-06 | 0.11 |
| FIGNL2 | -0.209827331 | 2.43E-12 | -0.193866178 | 1.02E-10 | 0.02 |
| FIGN | 0.010677624 | 0.72437766 | -0.120886255 | 6.16E-05 | 0.14 |
| LOC102724841 | -0.336641535 | 2.28E-30 | -0.268396316 | 1.73E-19 | 0.07 |
| FILIP1 | -0.335546465 | 3.59E-30 | -0.310404358 | 7.68E-26 | 0.03 |
| FIP1L1 | 0.21357713 | 9.65E-13 | 0.011170295 | 0.7122134 | 0.21 |
| FIS1 | 0.083680459 | 0.00563646 | 0.176733355 | 4.04E-09 | 0.1 |
| FITM1 | -0.045743494 | 0.13069527 | -0.091182345 | 0.0025497 | 0.05 |
| FITM2 | -0.37787205 | 2.01E-38 | -0.294411779 | 2.69E-23 | 0.09 |
| FIZ1 | 0.127259039 | 2.45E-05 | 0.170980808 | 1.28E-08 | 0.05 |
| FJX1 | 0.250098265 | 4.75E-17 | 0.111992102 | 0.0002073 | 0.14 |
| FKBP10 | 0.013771893 | 0.64924813 | 0.037658757 | 0.2134863 | 0.03 |
| FKBP11 | 0.113363001 | 0.00017288 | 0.147442353 | 9.79E-07 | 0.04 |
| FKBP14 | -0.263734737 | 7.54E-19 | -0.240087332 | 8.53E-16 | 0.03 |
| FKBP15 | -0.293505623 | 3.71E-23 | -0.246660284 | 1.30E-16 | 0.05 |
| LOC100131140 | -0.013384248 | 0.65848624 | -0.049327188 | 0.1031205 | 0.04 |
| FKBP1A | 0.312330112 | 3.70E-26 | 0.25033929 | 4.43E-17 | 0.07 |
| FKBP1B | -0.097315273 | 0.00127627 | -0.127732481 | 2.28E-05 | 0.04 |
| FKBP2 | 0.161038059 | 8.66E-08 | 0.137430958 | 5.12E-06 | 0.03 |
| FKBP3 | 0.201610248 | 1.73E-11 | 0.307139779 | 2.62E-25 | 0.11 |
| FKBP4 | 0.145341788 | 1.40E-06 | 0.180435713 | 1.88E-09 | 0.04 |
| FKBP5 | 0.02005643 | 0.50772482 | -0.005285799 | 0.8614311 | 0.03 |
| MGC26475 | 0.078587535 | 0.00934427 | -0.018018539 | 0.551797 | 0.1 |
| FKBP7 | -0.112804914 | 0.00018619 | -0.019855132 | 0.5119946 | 0.1 |
| FKBP8 | 0.169420301 | 1.75E-08 | 0.148480343 | 8.20E-07 | 0.03 |
| FKBP9P1 | -0.19071177 | 2.07E-10 | -0.267803732 | 2.09E-19 | 0.08 |
| LOC155254 | -0.169893026 | 1.59E-08 | -0.200588773 | 2.20E-11 | 0.04 |
| FKBPL | 0.478380868 | 1.37E-63 | 0.423868442 | 6.86E-49 | 0.06 |
| FKRP | 0.060431159 | 0.04577882 | -0.019606077 | 0.5173031 | 0.09 |
| FKSG29 | -0.201349146 | 1.84E-11 | -0.200101717 | 2.46E-11 | 0.01 |
| ACTR3BP2 | -0.012608758 | 0.67712313 | -0.013141047 | 0.6643089 | 0.01 |
| FKSG83 | -0.028778497 | 0.34183736 | -0.010300103 | 0.7337462 | 0.02 |
| FKTN | -0.304695663 | 6.49E-25 | -0.191500489 | 1.74E-10 | 0.12 |
| FLAD1 | 0.456798939 | 1.89E-57 | 0.555239756 | 2.07E-89 | 0.1 |
| FLCN | -0.300050968 | 3.56E-24 | -0.279205358 | 5.06E-21 | 0.03 |
| FLG2 | -0.062284058 | 0.03951453 | -0.109728497 | 0.0002785 | 0.05 |
| FLG | -0.311697794 | 4.70E-26 | -0.32392418 | 4.05E-28 | 0.02 |
| FLI1 | -0.270080897 | 1.01E-19 | -0.309287659 | 1.17E-25 | 0.04 |
| FLII | -0.210155323 | 2.24E-12 | -0.164679494 | 4.36E-08 | 0.05 |
| FLJ10038 | -0.126496856 | 2.74E-05 | -0.147325955 | 9.99E-07 | 0.03 |
| EBLN2 | -0.394174819 | 6.03E-42 | -0.301359366 | 2.21E-24 | 0.1 |
| ARHGEF40 | -0.351457595 | 3.98E-33 | -0.257737101 | 4.81E-18 | 0.1 |
| FAM86B3P | -0.126708365 | 2.66E-05 | -0.052353434 | 0.0836232 | 0.08 |
| EPB41L4A.AS2 | -0.314194517 | 1.81E-26 | -0.116789975 | 0.0001088 | 0.2 |
| FLJ12825 | -0.029194779 | 0.33489725 | 0.189402307 | 2.76E-10 | 0.22 |
| KLF3.AS1 | -0.312073571 | 4.08E-26 | -0.175663218 | 5.02E-09 | 0.14 |
| LOC144382 | 0.183765896 | 9.32E-10 | 0.007591094 | 0.8020598 | 0.18 |
| BIN3.IT1 | -0.200319748 | 2.34E-11 | -0.140240567 | 3.25E-06 | 0.07 |
| FLJ16779 | -0.212788341 | 1.17E-12 | -0.057704048 | 0.0565032 | 0.16 |
| LOC645330 | -0.009134493 | 0.76291728 | -0.054482015 | 0.0717846 | 0.05 |
| FLJ23867 | 0.185451877 | 6.50E-10 | 0.210041901 | 2.31E-12 | 0.03 |
| LINC00905 | -0.025225275 | 0.4047659 | -0.023066096 | 0.4461754 | 0.01 |
| LINC01205 | 0.101773691 | 0.00075278 | 0.01949381 | 0.5197052 | 0.09 |
| FLJ25758 | 0.052556693 | 0.08242859 | 0.05592447 | 0.0645698 | 0.01 |
| FLJ26850 | 0.045045842 | 0.13667397 | -0.029840713 | 0.3243093 | 0.08 |
| FLJ30679 | 0.259094245 | 3.17E-18 | 0.018251107 | 0.5466741 | 0.25 |
| SATB2.AS1 | 0.032162485 | 0.28806884 | -0.070723733 | 0.0193655 | 0.11 |
| FLJ33360 | -0.14845099 | 8.24E-07 | -0.142051179 | 2.42E-06 | 0.01 |
| LOC644869 | -0.171516079 | 1.15E-08 | 0.057317755 | 0.0581791 | 0.23 |
| FLJ34503 | -0.24615749 | 1.50E-16 | -0.141827754 | 2.51E-06 | 0.11 |
| LOC286341 | 0.219971817 | 1.92E-13 | 0.117647856 | 9.68E-05 | 0.11 |
| ENDOV | 0.037790265 | 0.21189072 | 0.018337233 | 0.5447829 | 0.02 |
| LINC00957 | -0.218810484 | 2.58E-13 | -0.198560554 | 3.52E-11 | 0.03 |
| DLGAP1.AS1 | 0.016374518 | 0.58866792 | 0.051499542 | 0.0887958 | 0.04 |
| FLJ36000 | 0.161890729 | 7.38E-08 | 0.04839073 | 0.1098361 | 0.12 |
| CCDC71L | 0.219729841 | 2.04E-13 | 0.101537374 | 0.0007746 | 0.12 |
| FLJ36777 | -0.262291878 | 1.18E-18 | -0.079406526 | 0.0086302 | 0.19 |
| FLJ37201 | -0.039480712 | 0.19214186 | -0.112540676 | 0.0001928 | 0.08 |
| LOC100132760 | -0.102353278 | 0.00070177 | -0.042692455 | 0.1584027 | 0.06 |
| FLJ37453 | 0.105670276 | 0.00046644 | -0.067765609 | 0.0250662 | 0.18 |
| LOC100130434 | -0.15615235 | 2.12E-07 | -0.245260866 | 1.95E-16 | 0.09 |
| FLJ39582 | 0.013851753 | 0.64735157 | 0.001140895 | 0.9699464 | 0.02 |
| LOC100130417 | 0.010882925 | 0.71930004 | 0.003926365 | 0.8968356 | 0.01 |
| TAPT1.AS1 | -0.178001773 | 3.11E-09 | 0.013835162 | 0.6477454 | 0.2 |
| LINC00875 | -0.147687125 | 9.39E-07 | -0.117055371 | 0.000105 | 0.04 |
| EHMT1.IT1 | 0.020948642 | 0.48902708 | 0.019631464 | 0.5167606 | 0.01 |
| ANKRD36BP2 | 0.064446602 | 0.03313719 | -0.082991289 | 0.0060449 | 0.15 |
| SLC25A3P1 | -0.049062849 | 0.10498222 | -0.038982216 | 0.1978197 | 0.02 |
| KRT18P55 | 0.006983935 | 0.81760121 | 0.095149595 | 0.0016369 | 0.09 |
| WEE2.AS1 | -0.155407917 | 2.43E-07 | -0.019205283 | 0.5259052 | 0.14 |
| FLJ41941 | 0.21738016 | 3.72E-13 | 0.095762974 | 0.0015263 | 0.13 |
| SPATA41 | -0.1318073 | 1.23E-05 | -0.080372292 | 0.007851 | 0.06 |
| FLJ42393 | 0.04939019 | 0.10268064 | 0.058951821 | 0.0513618 | 0.01 |
| MGC4278 | -0.140604558 | 3.07E-06 | -0.085840699 | 0.0045122 | 0.06 |
| NR2F1.AS1 | -0.205276521 | 7.29E-12 | -0.063079574 | 0.037057 | 0.15 |
| MGC35434 | -0.159117138 | 1.24E-07 | -0.068541754 | 0.0234452 | 0.1 |
| LOC648271 | -0.055604169 | 0.06611801 | -0.129809495 | 1.67E-05 | 0.08 |
| LOC641798 | -0.101522816 | 0.00077591 | -0.100818121 | 0.0008445 | 0.01 |
| SPATA31D4 | 0.022676544 | 0.45389488 | -0.014345517 | 0.6356765 | 0.04 |
| MROH5 | 0.066830068 | 0.02714815 | 0.003327836 | 0.912493 | 0.07 |
| LOC100292385 | 0.010694925 | 0.72394928 | -0.063174149 | 0.0367736 | 0.08 |
| LINC00453 | 0.143324028 | 1.96E-06 | -0.020057396 | 0.5077044 | 0.17 |
| SPATA31D3 | 0.034002474 | 0.26136147 | 0.018559288 | 0.539922 | 0.02 |
| C5ORF63 | -0.263825485 | 7.32E-19 | -0.012182782 | 0.6874459 | 0.26 |
| FLJ44635 | -0.072790868 | 0.01608655 | 0.08376055 | 0.0055906 | 0.16 |
| FLJ45079 | -0.029998292 | 0.32175969 | -0.144276438 | 1.67E-06 | 0.12 |
| DICER1.AS1 | 0.116234879 | 0.00011742 | 0.14666021 | 1.12E-06 | 0.04 |
| LINC01000 | -0.298026855 | 7.39E-24 | -0.284434871 | 8.67E-22 | 0.02 |
| LINC01002 | 0.014901376 | 0.62264066 | 0.051935522 | 0.0861235 | 0.04 |
| GATA3.AS1 | -0.258439916 | 3.88E-18 | -0.041216892 | 0.1733012 | 0.22 |
| KRT8P41 | -0.312003398 | 4.19E-26 | -0.134382791 | 8.28E-06 | 0.18 |
| SPATA31D1 | 0.007632553 | 0.80100139 | -0.008151329 | 0.7877885 | 0.02 |
| LOC100420937 | -0.004381145 | 0.8849651 | -0.031066931 | 0.3048151 | 0.03 |
| BAIAP2.AS1 | -0.182011063 | 1.35E-09 | -0.091668695 | 0.002417 | 0.1 |
| LOC100131857 | -0.034842401 | 0.24975678 | -0.191267847 | 1.83E-10 | 0.16 |
| FLNB | -0.312969433 | 2.90E-26 | -0.283976278 | 1.01E-21 | 0.03 |
| FLNC | -0.320309993 | 1.69E-27 | -0.256243662 | 7.57E-18 | 0.07 |
| FLOT1 | 0.019801906 | 0.51312665 | 0.139441723 | 3.70E-06 | 0.12 |
| FLOT2 | 0.141328286 | 2.72E-06 | 0.061855771 | 0.0408939 | 0.08 |
| FLRT1 | 0.059399699 | 0.04961442 | -0.007273958 | 0.8101682 | 0.07 |
| KIAA0405 | -0.379866568 | 7.63E-39 | -0.269020254 | 1.41E-19 | 0.12 |
| FLRT3 | -0.264415601 | 6.09E-19 | -0.086572195 | 0.0041803 | 0.18 |
| FLT1 | -0.298444586 | 6.36E-24 | -0.27468239 | 2.26E-20 | 0.03 |
| FLT3LG | -0.045575372 | 0.13211717 | -0.076025298 | 0.0119302 | 0.04 |
| FLT3 | -0.193461568 | 1.12E-10 | -0.178361648 | 2.89E-09 | 0.02 |
| FLT4 | -0.31250959 | 3.45E-26 | -0.22829964 | 2.17E-14 | 0.09 |
| FLVCR1 | 0.011331838 | 0.70824053 | 0.226571186 | 3.43E-14 | 0.22 |
| FLVCR2 | 0.108351915 | 0.0003324 | -0.055836008 | 0.0649944 | 0.17 |
| FLYWCH1 | -0.171671756 | 1.12E-08 | -0.095319078 | 0.0016056 | 0.08 |
| FLYWCH2 | 0.054011073 | 0.07427826 | 0.191112132 | 1.89E-10 | 0.14 |
| DKFZP686C2281 | -0.477217167 | 3.01E-63 | -0.430087676 | 1.96E-50 | 0.05 |
| FMN2 | -0.119369599 | 7.62E-05 | -0.104832997 | 0.0005177 | 0.02 |
| FMNL1 | -0.051122784 | 0.09115848 | -0.166821418 | 2.89E-08 | 0.12 |
| FMNL2 | 0.066907808 | 0.02696965 | -0.135090809 | 7.41E-06 | 0.21 |
| FMNL3 | -0.362049525 | 3.43E-35 | -0.375347119 | 6.78E-38 | 0.02 |
| FMO1 | -0.05852112 | 0.05309046 | -0.073121896 | 0.0156095 | 0.02 |
| FMO2 | -0.107147607 | 0.00038739 | -0.149954533 | 6.36E-07 | 0.05 |
| FMO3 | -0.055625589 | 0.06601353 | -0.066559482 | 0.0277774 | 0.02 |
| FMO4 | -0.179052477 | 2.51E-09 | -0.012737617 | 0.6740122 | 0.17 |
| FMO5 | -0.466715958 | 3.24E-60 | -0.067857956 | 0.0248684 | 0.4 |
| FMO6P | 0.276496332 | 1.24E-20 | 0.105052873 | 0.0005037 | 0.18 |
| FMO9P | -0.113161744 | 0.00017757 | 0.067669875 | 0.0252727 | 0.19 |
| FMOD | -0.378480589 | 1.50E-38 | -0.114330139 | 0.0001519 | 0.27 |
| FMR1NB | 0.045752533 | 0.13061917 | -0.019449783 | 0.5206489 | 0.07 |
| FMR1 | 0.114459417 | 0.0001493 | -0.058241401 | 0.0542389 | 0.18 |
| FN1 | -0.207631601 | 4.14E-12 | -0.143294264 | 1.97E-06 | 0.07 |
| FN3KRP | 0.273611583 | 3.21E-20 | 0.139737412 | 3.53E-06 | 0.14 |
| FN3K | 0.097589198 | 0.00123628 | 0.091386845 | 0.0024931 | 0.01 |
| C1orf39 | -0.163209946 | 5.76E-08 | -0.129046293 | 1.87E-05 | 0.04 |
| FNBP1 | -0.108709283 | 0.00031754 | -0.184032862 | 8.80E-10 | 0.08 |
| FNBP4 | -0.054973023 | 0.06925805 | -0.109515704 | 0.0002863 | 0.06 |
| FNDC1 | -0.134780101 | 7.78E-06 | -0.146994681 | 1.06E-06 | 0.02 |
| FNDC3A | -0.479476523 | 6.50E-64 | -0.365771787 | 6.18E-36 | 0.12 |
| FNDC3B | -0.110441422 | 0.00025392 | -0.203432651 | 1.13E-11 | 0.1 |
| FNDC4 | 0.056830203 | 0.06035299 | 0.010307732 | 0.7335565 | 0.05 |
| FNDC5 | -0.14514894 | 1.44E-06 | -0.139924244 | 3.42E-06 | 0.01 |
| FNDC7 | 0.203106333 | 1.22E-11 | 0.025482099 | 0.3999979 | 0.18 |
| FNDC8 | 0.042936825 | 0.15603171 | -0.080803457 | 0.0075239 | 0.13 |
| FNIP1 | -0.513492823 | 1.44E-74 | -0.335542689 | 3.60E-30 | 0.18 |
| FNIP2 | -0.420177005 | 5.47E-48 | -0.425042481 | 3.52E-49 | 0.01 |
| FNTA | 0.251245794 | 3.39E-17 | 0.129280754 | 1.81E-05 | 0.13 |
| FNTB | -0.232652815 | 6.70E-15 | -0.017403298 | 0.5654619 | 0.22 |
| FOLH1B | 0.053556217 | 0.07675342 | 0.027231406 | 0.368428 | 0.03 |
| LOC93212 | 0.205108833 | 7.59E-12 | 0.080181125 | 0.008 | 0.13 |
| FOLR1 | 0.116193134 | 0.00011809 | 0.041019823 | 0.1753677 | 0.08 |
| FOLR2 | -0.143131636 | 2.02E-06 | -0.194250857 | 9.39E-11 | 0.06 |
| FOLR3 | 0.167285138 | 2.64E-08 | 0.071886846 | 0.0174553 | 0.1 |
| IZUMO1R | 0.085763405 | 0.00454868 | 0.021989045 | 0.4677003 | 0.07 |
| FOSB | -0.252551956 | 2.30E-17 | -0.179100537 | 2.48E-09 | 0.08 |
| FOSL1 | 0.277907191 | 7.80E-21 | 0.052989112 | 0.079933 | 0.23 |
| FLJ23306 | -0.311726788 | 4.65E-26 | -0.361161334 | 5.14E-35 | 0.05 |
| FOS | -0.258432178 | 3.89E-18 | -0.189386053 | 2.77E-10 | 0.07 |
| FOXA1 | -0.439893539 | 6.15E-53 | -0.101572255 | 0.0007713 | 0.34 |
| FOXA2 | 0.160783927 | 9.08E-08 | 0.058895218 | 0.0515863 | 0.11 |
| FOXA3 | -0.064658557 | 0.03256235 | -0.00273614 | 0.9280051 | 0.07 |
| FOXB1 | 0.028677012 | 0.34354311 | -0.054946445 | 0.0693929 | 0.09 |
| FOXB2 | 0.014729998 | 0.62664744 | -0.017597097 | 0.56114 | 0.04 |
| IRID1 | 0.310343457 | 7.86E-26 | 0.047537758 | 0.1162491 | 0.27 |
| LD | -0.057903548 | 0.05565357 | -0.189946319 | 2.45E-10 | 0.14 |
| FOXD1 | 0.186281279 | 5.44E-10 | 0.050588184 | 0.0945973 | 0.14 |
| FOXD2 | -0.118735613 | 8.32E-05 | -0.053162973 | 0.078947 | 0.07 |
| FOXD3 | 0.143458759 | 1.92E-06 | 0.085282936 | 0.0047812 | 0.06 |
| FOXD4L1 | 0.032065748 | 0.28952208 | 0.130774803 | 1.44E-05 | 0.1 |
| FOXD4L2 | -0.203644327 | 1.07E-11 | -0.082330686 | 0.0064612 | 0.13 |
| FOXD4L3 | 0.066886124 | 0.02701933 | 0.036593961 | 0.2267255 | 0.04 |
| FOXD4L5 | -0.057295413 | 0.05827731 | -0.008598761 | 0.7764412 | 0.05 |
| FOXD4L6 | 0.06070962 | 0.04478716 | 0.070531165 | 0.0196988 | 0.01 |
| FOXD4 | 0.036884385 | 0.22305782 | 0.115355833 | 0.0001323 | 0.08 |
| TITF2 | 0.050703879 | 0.09384445 | -0.069436354 | 0.0216903 | 0.13 |
| FOXE3 | 0.232693779 | 6.63E-15 | 0.10645557 | 0.0004227 | 0.13 |
| FOXF1 | -0.143715617 | 1.84E-06 | -0.174332635 | 6.58E-09 | 0.04 |
| FOXF2 | -0.095450268 | 0.00158179 | -0.078670725 | 0.0092694 | 0.02 |
| FKHL4 | 0.236604556 | 2.26E-15 | 0.036123311 | 0.2327599 | 0.21 |
| FOXH1 | 0.21779044 | 3.35E-13 | 0.19004132 | 2.40E-10 | 0.03 |
| FOXI1 | -0.011906715 | 0.69416728 | -0.024342498 | 0.4214116 | 0.02 |
| FOXI2 | -0.245630238 | 1.75E-16 | -0.208143511 | 3.66E-12 | 0.04 |
| LOC730270 | 0.203007429 | 1.25E-11 | 0.005761554 | 0.8491024 | 0.2 |
| FOXJ1 | -0.037980443 | 0.20959848 | 0.082562995 | 0.006312 | 0.13 |
| FOXJ2 | -0.120676377 | 6.34E-05 | -0.290898674 | 9.29E-23 | 0.18 |
| FOXJ3 | -0.177793725 | 3.25E-09 | -0.22706333 | 3.01E-14 | 0.05 |
| FLJ14977 | -0.068847937 | 0.0228312 | -0.209727447 | 2.49E-12 | 0.15 |
| DKFZp434D1428 | 0.421144988 | 3.18E-48 | 0.123524522 | 4.23E-05 | 0.3 |
| LOC124308 | 0.187371786 | 4.30E-10 | -0.000329653 | 0.9913144 | 0.19 |
| FOXL2 | 0.091763666 | 0.00239187 | -0.020997521 | 0.4880136 | 0.12 |
| MPP.2 | 0.541596182 | 2.50E-84 | 0.244748861 | 2.26E-16 | 0.3 |
| RONU | -0.229579721 | 1.54E-14 | -0.112281304 | 0.0001995 | 0.12 |
| FOXN2 | 0.04046626 | 0.18127043 | -0.156046041 | 2.16E-07 | 0.2 |
| LOC654111 | -0.245651234 | 1.74E-16 | -0.331885312 | 1.63E-29 | 0.09 |
| FOXN4 | 0.083558084 | 0.00570712 | 0.146515573 | 1.15E-06 | 0.07 |
| FOXO1 | -0.361963257 | 3.57E-35 | -0.316349036 | 7.91E-27 | 0.05 |
| FOXO3B | -0.140457677 | 3.14E-06 | -0.08318682 | 0.0059264 | 0.06 |
| FOXO3 | -0.181998278 | 1.35E-09 | -0.083216051 | 0.0059089 | 0.1 |
| FOXO4 | -0.20963949 | 2.55E-12 | -0.094605527 | 0.0017412 | 0.12 |
| LOC87246 | -0.433757553 | 2.31E-51 | -0.141037586 | 2.86E-06 | 0.3 |
| TNRC10 | -0.27657471 | 1.21E-20 | -0.265480701 | 4.35E-19 | 0.02 |
| JM2 | 0.098484568 | 0.00111351 | -0.083170183 | 0.0059364 | 0.19 |
| FOXP4 | 0.2471992 | 1.11E-16 | 0.114096361 | 0.0001568 | 0.14 |
| FOXQ1 | 0.136455264 | 5.97E-06 | 0.056963689 | 0.0597512 | 0.08 |
| FOXR1 | 0.035495248 | 0.24098868 | -0.020666659 | 0.4948962 | 0.06 |
| FOXR2 | 0.037094363 | 0.22043263 | 0.023208813 | 0.4433661 | 0.02 |
| FOXRED1 | 0.248952342 | 6.66E-17 | 0.210267583 | 2.18E-12 | 0.04 |
| FOXRED2 | 0.164921113 | 4.16E-08 | 0.009819408 | 0.7457326 | 0.16 |
| LOC115276 | -0.112420866 | 0.00019589 | 0.004360931 | 0.8854922 | 0.12 |
| FPGS | 0.068264512 | 0.02401351 | 0.069069998 | 0.0223946 | 0.01 |
| FPGT | -0.338961973 | 8.62E-31 | -0.148912476 | 7.61E-07 | 0.2 |
| FPR1 | -0.140257635 | 3.24E-06 | -0.19811838 | 3.90E-11 | 0.06 |
| LOC116243 | 0.17082062 | 1.33E-08 | -0.074578096 | 0.0136553 | 0.25 |
| FPR3 | 0.013023906 | 0.66712067 | -0.129491092 | 1.75E-05 | 0.15 |
| FRAS1 | -0.121659754 | 5.52E-05 | -0.245644922 | 1.75E-16 | 0.13 |
| FRAT1 | -0.170656229 | 1.37E-08 | -0.098492598 | 0.0011125 | 0.08 |
| LOC115429 | 0.438511412 | 1.40E-52 | 0.309121149 | 1.25E-25 | 0.13 |
| FREM1 | -0.299540285 | 4.28E-24 | -0.248329174 | 7.99E-17 | 0.06 |
| FREM2 | 0.129297475 | 1.80E-05 | -0.047333774 | 0.1178254 | 0.18 |
| C20orf80 | -0.261606437 | 1.46E-18 | -0.14954217 | 6.83E-07 | 0.12 |
| FRG1 | 0.204242342 | 9.32E-12 | 0.179197492 | 2.43E-09 | 0.03 |
| FRG2B | -0.035728024 | 0.23791536 | -0.066026287 | 0.0290542 | 0.04 |
| FRG2C | -0.023223937 | 0.443069 | -0.047801248 | 0.1142374 | 0.03 |
| FRG2 | 0.03167847 | 0.29538937 | -0.039588993 | 0.1909245 | 0.08 |
| FRK | -0.237767261 | 1.64E-15 | -0.117165394 | 0.0001034 | 0.13 |
| FRMD1 | -0.194905596 | 8.10E-11 | -0.106811097 | 0.0004042 | 0.09 |
| FRMD3 | -0.112772269 | 0.00018699 | -0.335995548 | 2.98E-30 | 0.23 |
| FRMD4A | 0.081329618 | 0.00714129 | -0.078815474 | 0.0091404 | 0.17 |
| FRMD4B | -0.439806703 | 6.48E-53 | -0.271307351 | 6.78E-20 | 0.17 |
| FRMD5 | 0.010709385 | 0.72359132 | -0.185067 | 7.06E-10 | 0.2 |
| FRMD6 | -0.456694522 | 2.02E-57 | -0.308957315 | 1.32E-25 | 0.15 |
| FRMD7 | -0.201705992 | 1.69E-11 | -0.146313376 | 1.19E-06 | 0.06 |
| FRMD8 | 0.151435195 | 4.91E-07 | 0.014593251 | 0.6298525 | 0.14 |
| FRMPD1 | -0.024058209 | 0.42685638 | -0.11192076 | 0.0002092 | 0.09 |
| LOC100420616 | -0.047316578 | 0.11795905 | -0.048018193 | 0.1126018 | 0.01 |
| DKFZp566K0524 | -0.299521472 | 4.31E-24 | -0.162331704 | 6.80E-08 | 0.14 |
| FRMPD4 | -0.070794996 | 0.01924346 | -0.143816121 | 1.81E-06 | 0.08 |
| FRRS1 | -0.306461163 | 3.37E-25 | -0.33655382 | 2.36E-30 | 0.04 |
| FRS2 | -0.363907389 | 1.46E-35 | -0.221205887 | 1.40E-13 | 0.15 |
| FRS3 | 0.147264718 | 1.01E-06 | 0.075483645 | 0.0125519 | 0.08 |
| FRYL | -0.386889395 | 2.40E-40 | -0.364387564 | 1.17E-35 | 0.03 |
| C13orf14 | -0.541317683 | 3.15E-84 | -0.362271787 | 3.10E-35 | 0.18 |
| FRZB | -0.24392099 | 2.87E-16 | -0.115839057 | 0.0001239 | 0.13 |
| FSCB | -0.007754181 | 0.79789832 | -0.008944336 | 0.7677095 | 0.01 |
| FSCN1 | 0.245208106 | 1.98E-16 | 0.037082993 | 0.2205742 | 0.21 |
| FSCN2 | -0.231660127 | 8.78E-15 | -0.194263424 | 9.36E-11 | 0.04 |
| FSCN3 | 0.111586579 | 0.00021864 | 0.006388006 | 0.8329251 | 0.11 |
| FSD1L | -0.099536664 | 0.00098367 | -0.167562924 | 2.51E-08 | 0.07 |
| FSD1 | 0.404873738 | 2.29E-44 | 0.103762372 | 0.0005908 | 0.31 |
| SPRYD1 | -0.011854824 | 0.69543337 | -0.092102434 | 0.0023041 | 0.09 |
| FSHB | 0.043607818 | 0.14965979 | 0.026384085 | 0.3835216 | 0.02 |
| FSHR | -0.071412594 | 0.01821328 | -0.037992723 | 0.2094511 | 0.04 |
| FSIP1 | -0.362321839 | 3.03E-35 | -0.108342487 | 0.0003328 | 0.26 |
| FSTL1 | -0.266323068 | 3.34E-19 | -0.197671838 | 4.32E-11 | 0.07 |
| FSTL3 | -0.173326815 | 8.05E-09 | -0.034781159 | 0.2505906 | 0.14 |
| FSTL4 | -0.208666826 | 3.22E-12 | -0.034502939 | 0.2544029 | 0.18 |
| FSTL5 | 0.106144218 | 0.00043958 | 3.72E-05 | 0.9990209 | 0.11 |
| FST | -0.183801119 | 9.25E-10 | -0.069089553 | 0.0223566 | 0.12 |
| FTCD | 0.244512404 | 2.42E-16 | 0.107404778 | 0.000375 | 0.14 |
| FTH1 | 0.147439177 | 9.80E-07 | 0.262695106 | 1.04E-18 | 0.12 |
| FTHL17 | 0.141873961 | 2.49E-06 | 0.058172718 | 0.054524 | 0.09 |
| FTH1P3 | 0.080632139 | 0.00765234 | 0.189622929 | 2.63E-10 | 0.11 |
| FTLP10 | 0.012185678 | 0.68737554 | -0.027037795 | 0.3718439 | 0.04 |
| PRO2760 | 0.19709991 | 4.92E-11 | 0.138232762 | 4.50E-06 | 0.06 |
| FTMT | 0.301587231 | 2.03E-24 | 0.169785104 | 1.62E-08 | 0.14 |
| FTO | -0.277554351 | 8.77E-21 | -0.31175395 | 4.60E-26 | 0.04 |
| FTSJ1 | 0.324086035 | 3.80E-28 | 0.215015738 | 6.74E-13 | 0.11 |
| FTSJ2 | 0.340466552 | 4.57E-31 | 0.230815275 | 1.10E-14 | 0.11 |
| FLJ20062 | 0.168584473 | 2.06E-08 | 0.128463836 | 2.05E-05 | 0.05 |
| CMTR2 | -0.191280895 | 1.82E-10 | -0.198511995 | 3.56E-11 | 0.01 |
| CMTR1 | -0.017820379 | 0.55618054 | -0.153293366 | 3.54E-07 | 0.14 |
| FUBP1 | -0.071546638 | 0.01799617 | -0.166976523 | 2.81E-08 | 0.1 |
| LOC158301 | -0.339541259 | 6.75E-31 | -0.307595731 | 2.21E-25 | 0.04 |
| FUCA1 | -0.390485163 | 3.94E-41 | -0.186206779 | 5.53E-10 | 0.21 |
| FUCA2 | 0.257642354 | 4.95E-18 | 0.235106276 | 3.42E-15 | 0.03 |
| FUK | 0.031500511 | 0.29811194 | 0.015040371 | 0.6193992 | 0.02 |
| FUNDC1 | 0.065942909 | 0.0292584 | 0.205286794 | 7.27E-12 | 0.14 |
| FUNDC2P2 | 0.014837285 | 0.62413779 | -0.033107916 | 0.2741246 | 0.05 |
| FUNDC2 | 0.182631652 | 1.19E-09 | 0.206721334 | 5.16E-12 | 0.03 |
| FURIN | 0.041248615 | 0.1729703 | -0.098201162 | 0.0011511 | 0.14 |
| FUS | 0.278877853 | 5.65E-21 | 0.295215574 | 2.02E-23 | 0.02 |
| FUT10 | -0.186329108 | 5.38E-10 | -0.192108181 | 1.52E-10 | 0.01 |
| FUT11 | -0.427520719 | 8.57E-50 | -0.398892758 | 5.29E-43 | 0.03 |
| FUT1 | 0.05962041 | 0.04877179 | -0.110599711 | 0.0002487 | 0.18 |
| FUT2 | -0.121778055 | 5.43E-05 | -0.056655349 | 0.0611488 | 0.07 |
| FUT3 | 0.195552485 | 7.00E-11 | 0.176173739 | 4.53E-09 | 0.02 |
| FUT4 | 0.069799498 | 0.02101113 | -0.068859899 | 0.0228075 | 0.14 |
| LOC116018 | 0.317989804 | 4.19E-27 | 0.092621258 | 0.0021753 | 0.23 |
| FUT6 | 0.000239629 | 0.99368624 | 0.071002644 | 0.0188916 | 0.08 |
| FUT7 | 0.105232455 | 0.00049261 | -0.049862469 | 0.0994308 | 0.16 |
| FUT8 | -0.393934562 | 6.82E-42 | -0.118490892 | 8.61E-05 | 0.28 |
| FUT9 | 0.059666371 | 0.04859784 | 0.123545125 | 4.21E-05 | 0.07 |
| FUZ | -0.051688467 | 0.08762973 | -0.024043371 | 0.4271417 | 0.03 |
| TIMM10B | -0.230022269 | 1.37E-14 | -0.147211421 | 1.02E-06 | 0.09 |
| FXN | 0.405544754 | 1.60E-44 | 0.227718065 | 2.53E-14 | 0.18 |
| FXR1 | 0.215444304 | 6.05E-13 | 0.121838174 | 5.38E-05 | 0.1 |
| FXR2 | 0.072014562 | 0.01725592 | -0.01722217 | 0.5695156 | 0.09 |
| FXYD1 | -0.208109973 | 3.69E-12 | -0.126075671 | 2.91E-05 | 0.09 |
| HOMG2 | 0.154199665 | 3.01E-07 | 0.010848311 | 0.7201553 | 0.15 |
| FXYD3 | -0.041266723 | 0.17278161 | 0.087961491 | 0.00361 | 0.13 |
| FXYD4 | 0.122012913 | 5.25E-05 | 0.072746484 | 0.0161515 | 0.05 |
| HSPC113 | 0.160373231 | 9.79E-08 | 0.101516656 | 0.0007765 | 0.06 |
| FXYD6 | 0.138316161 | 4.44E-06 | 0.011979323 | 0.6923972 | 0.13 |
| FXYD7 | 0.057678843 | 0.05661136 | -0.057835281 | 0.0559431 | 0.12 |
| FYB | -0.145650227 | 1.33E-06 | -0.236102343 | 2.60E-15 | 0.1 |
| FYCO1 | -0.639171401 | 1.45E-126 | -0.391063194 | 2.94E-41 | 0.25 |
| FYN | 0.007938951 | 0.79319047 | -0.12438856 | 3.73E-05 | 0.14 |
| LOC115217 | 0.021335372 | 0.48103912 | -0.128896951 | 1.92E-05 | 0.16 |
| FZD10 | -0.18822317 | 3.57E-10 | -0.113470585 | 0.0001704 | 0.08 |
| FZD1 | -0.16565639 | 3.62E-08 | -0.08674895 | 0.0041034 | 0.08 |
| FZD2 | 0.015259466 | 0.61430465 | 0.102184323 | 0.0007163 | 0.09 |
| FZD3 | -0.009094311 | 0.76392919 | 0.016468674 | 0.5865259 | 0.03 |
| LOC220055 | -0.446154118 | 1.40E-54 | -0.261695208 | 1.42E-18 | 0.19 |
| FZD5 | -0.017169613 | 0.57069439 | -0.151517986 | 4.84E-07 | 0.14 |
| FZD6 | 0.148731563 | 7.85E-07 | -0.145510628 | 1.36E-06 | 0.3 |
| FZD7 | 0.072886546 | 0.01594735 | -0.068626911 | 0.023273 | 0.15 |
| FZD8 | -0.066286752 | 0.02842435 | -0.068822945 | 0.0228808 | 0.01 |
| FZD9 | 0.404395275 | 2.95E-44 | 0.124233574 | 3.81E-05 | 0.29 |
| FZR1 | 0.033159212 | 0.27338146 | -0.022060616 | 0.4662524 | 0.06 |
| G0S2 | -0.032853161 | 0.27783587 | -0.04970835 | 0.1004822 | 0.02 |
| G2E3 | -0.048823908 | 0.10668794 | -0.072501146 | 0.0165146 | 0.03 |
| G3BP1 | 0.032073494 | 0.28940554 | 0.012676751 | 0.675481 | 0.02 |
| LOC115293 | -0.20591339 | 6.26E-12 | -0.203105449 | 1.22E-11 | 0.01 |
| G6PC2 | -0.287634278 | 2.89E-22 | -0.191476982 | 1.75E-10 | 0.1 |
| G6PC3 | -0.020168675 | 0.50535211 | 0.098223284 | 0.0011481 | 0.12 |
| G6PC | -0.00818798 | 0.78685723 | -0.01191284 | 0.6940179 | 0.01 |
| G6PD | 0.186891316 | 4.77E-10 | 0.090252441 | 0.0028221 | 0.1 |
| GAA | -0.009728581 | 0.74800441 | -0.056409032 | 0.0622846 | 0.05 |
| GAB1 | -0.41524763 | 8.41E-47 | -0.359652763 | 1.02E-34 | 0.06 |
| GAB2 | -0.150066808 | 6.23E-07 | -0.163583352 | 5.37E-08 | 0.02 |
| GAB3 | -0.217313463 | 3.78E-13 | -0.262534969 | 1.10E-18 | 0.05 |
| GAB4 | 0.057560769 | 0.0571201 | -0.00756069 | 0.8028363 | 0.07 |
| GABARAPL1 | -0.11490649 | 0.00014058 | -0.176642925 | 4.11E-09 | 0.07 |
| LOC90769 | 0.048328419 | 0.1102949 | -0.007123025 | 0.8140344 | 0.06 |
| GABARAPL3 | -0.028859293 | 0.34048322 | -0.152413848 | 4.13E-07 | 0.13 |
| LOC201246 | 0.154690595 | 2.76E-07 | 0.263178203 | 8.97E-19 | 0.11 |
| LOC221563 | 0.006227431 | 0.83706533 | 0.013777839 | 0.6491068 | 0.01 |
| GABBR2 | 0.359270752 | 1.21E-34 | 0.033574397 | 0.267417 | 0.33 |
| GABPA | -0.244482007 | 2.44E-16 | -0.201212103 | 1.90E-11 | 0.05 |
| GABPB1 | 0.155719169 | 2.29E-07 | 0.094461286 | 0.0017698 | 0.07 |
| GABPB2 | -0.095856493 | 0.00151002 | 0.18212772 | 1.32E-09 | 0.28 |
| GABRA1 | 0.035252895 | 0.24421801 | -0.087739416 | 0.0036961 | 0.13 |
| LOC115187 | -0.227642596 | 2.58E-14 | -0.178403947 | 2.87E-09 | 0.05 |
| GABRA3 | 0.213260567 | 1.04E-12 | 0.072617565 | 0.0163414 | 0.15 |
| GABRA4 | -0.096019054 | 0.00148215 | -0.088079325 | 0.003565 | 0.01 |
| GABRA5 | 0.316150921 | 8.54E-27 | 0.123107827 | 4.49E-05 | 0.2 |
| GABRA6 | 0.021080291 | 0.48629988 | 0.006370671 | 0.8333719 | 0.02 |
| GABRB1 | 0.010592195 | 0.72649411 | -0.022410251 | 0.4592148 | 0.04 |
| GABRB2 | -0.213948618 | 8.80E-13 | -0.111014749 | 0.0002356 | 0.11 |
| GABRB3 | -0.143023544 | 2.06E-06 | -0.150760867 | 5.52E-07 | 0.01 |
| GABRD | -0.076273837 | 0.01165422 | 0.051190742 | 0.0907286 | 0.13 |
| GABRE | 0.103878965 | 0.00058241 | 0.008187204 | 0.7868769 | 0.1 |
| GABRG1 | -0.175662396 | 5.02E-09 | -0.13551022 | 6.94E-06 | 0.05 |
| GABRG2 | -0.023454814 | 0.43854748 | -0.087257115 | 0.0038896 | 0.07 |
| GABRG3 | 0.161158056 | 8.46E-08 | -0.033692147 | 0.2657418 | 0.2 |
| GABRP | 0.214867999 | 6.99E-13 | -0.00484323 | 0.8729303 | 0.22 |
| GABRQ | 0.185257361 | 6.78E-10 | 0.077460255 | 0.0104132 | 0.11 |
| GABRR1 | -0.011655059 | 0.70031534 | -0.082744905 | 0.0061973 | 0.08 |
| GABRR2 | -0.116416109 | 0.00011455 | -0.18734521 | 4.32E-10 | 0.08 |
| GABRR3 | -0.044679299 | 0.13989847 | 0.044098801 | 0.1451243 | 0.09 |
| SCP | -0.149581773 | 6.78E-07 | -0.013076972 | 0.6658463 | 0.14 |
| GAD2 | 0.034641669 | 0.25249691 | 0.045028207 | 0.1368278 | 0.02 |
| GADD45A | -0.028322752 | 0.34953996 | -0.054548066 | 0.0714404 | 0.03 |
| DKFZP566B133 | -0.038704605 | 0.20103426 | -0.028238497 | 0.3509759 | 0.02 |
| PRG6 | 0.372423726 | 2.73E-37 | 0.296725231 | 1.18E-23 | 0.08 |
| GADD45G | -0.111217139 | 0.00022949 | 0.043627152 | 0.1494792 | 0.16 |
| GADL1 | -0.022711267 | 0.45320377 | -0.060172979 | 0.0467147 | 0.04 |
| GAGE10 | 0.103127459 | 0.00063862 | 0.073136615 | 0.0155885 | 0.03 |
| GAGE12D | 0.09825221 | 0.00114424 | 0.05770446 | 0.0565015 | 0.05 |
| GAGE12F | 0.033847294 | 0.26354559 | 0.016710821 | 0.5810338 | 0.02 |
| GAGE11 | 0.109282975 | 0.00029498 | 0.041597586 | 0.1693609 | 0.07 |
| GAGE13 | 0.081061415 | 0.00733407 | 0.040022062 | 0.1861125 | 0.05 |
| GAGE1 | 0.076573887 | 0.01132857 | 0.051956822 | 0.0859946 | 0.03 |
| LOC645093 | 0.053819073 | 0.07531499 | 0.05037567 | 0.0959929 | 0.01 |
| GAGE2B | 0.069188632 | 0.0221644 | 0.041484927 | 0.1705199 | 0.03 |
| GAGE2C | 0.075483331 | 0.01255223 | 0.027684436 | 0.3605116 | 0.05 |
| GAGE2D | 0.083936488 | 0.00549117 | 0.037769955 | 0.2121366 | 0.05 |
| LOC645021 | 0.064461198 | 0.03309733 | 0.008212313 | 0.7862391 | 0.06 |
| GAGE4 | 0.120119477 | 6.86E-05 | 0.071706705 | 0.0177399 | 0.05 |
| GAGE8 | 0.06935832 | 0.02183866 | 0.044640715 | 0.1402413 | 0.03 |
| GAK | -0.146655935 | 1.12E-06 | -0.111290395 | 0.0002273 | 0.04 |
| GAL3ST1 | 0.11488759 | 0.00014094 | 0.026644936 | 0.3788352 | 0.09 |
| GAL3ST2 | -0.099590156 | 0.00097745 | 0.033537313 | 0.2679461 | 0.14 |
| GAL3ST3 | 0.013264156 | 0.66135889 | -0.032774044 | 0.2789954 | 0.05 |
| GAL3ST4 | -0.075642824 | 0.01236627 | -0.041897091 | 0.1663083 | 0.04 |
| GALC | -0.451029779 | 7.01E-56 | -0.259167336 | 3.10E-18 | 0.2 |
| GALE | 0.143400876 | 1.93E-06 | 0.199789065 | 2.65E-11 | 0.06 |
| LOC124499 | 0.330457843 | 2.92E-29 | 0.183513621 | 9.83E-10 | 0.15 |
| GALK2 | 0.032011623 | 0.29033733 | 0.004307939 | 0.8868742 | 0.03 |
| GALM | 0.014621838 | 0.62918188 | 0.006555453 | 0.8286127 | 0.01 |
| LOC105371408 | 0.231707186 | 8.67E-15 | -0.032595137 | 0.2816294 | 0.27 |
| GALNT10 | -0.562858735 | 2.37E-92 | -0.252342757 | 2.44E-17 | 0.32 |
| GALNT11 | -0.132606532 | 1.09E-05 | -0.09335745 | 0.0020038 | 0.04 |
| GALNT12 | 0.05706688 | 0.05928946 | -0.034728044 | 0.2513153 | 0.1 |
| GALNT13 | 0.151239677 | 5.08E-07 | 0.025634546 | 0.3971838 | 0.13 |
| GALNT14 | 0.197450749 | 4.54E-11 | 0.132946871 | 1.03E-05 | 0.07 |
| GALNT1 | 0.02567053 | 0.39652124 | -0.017942421 | 0.5534788 | 0.05 |
| GALNT2 | -0.048092743 | 0.11204396 | 0.095424556 | 0.0015864 | 0.15 |
| LOC102724230 | 0.033395113 | 0.2699815 | -0.028848399 | 0.3406656 | 0.07 |
| GALNT4 | -0.37153667 | 4.16E-37 | -0.220597336 | 1.63E-13 | 0.16 |
| GALNT5 | -0.47595855 | 7.05E-63 | -0.266602858 | 3.05E-19 | 0.21 |
| GALNT6 | -0.31340655 | 2.45E-26 | -0.091436726 | 0.0024795 | 0.23 |
| GALNT7 | -0.462394848 | 5.33E-59 | -0.159223509 | 1.21E-07 | 0.31 |
| GALNT8 | -0.082942692 | 0.00607469 | -0.073507733 | 0.0150691 | 0.01 |
| LOC729185 | -0.057222191 | 0.05860003 | -0.132860991 | 1.05E-05 | 0.08 |
| GALNT16 | -0.172601058 | 9.31E-09 | -0.098724111 | 0.0010826 | 0.08 |
| GALNT15 | -0.383621749 | 1.21E-39 | -0.242282936 | 4.58E-16 | 0.15 |
| GALNT18 | 0.143054103 | 2.05E-06 | 0.033597676 | 0.2670852 | 0.11 |
| GALNTL5 | 0.09897828 | 0.00105072 | 0.019051297 | 0.5292294 | 0.08 |
| GALNTL6 | -0.3029783 | 1.22E-24 | -0.200089095 | 2.47E-11 | 0.11 |
| GALP | 0.067919303 | 0.02473775 | 0.04406327 | 0.145449 | 0.03 |
| GALR1 | -0.171212699 | 1.23E-08 | -0.145610572 | 1.34E-06 | 0.03 |
| GALR2 | 0.254180675 | 1.41E-17 | 0.112445455 | 0.0001953 | 0.15 |
| GALR3 | 0.11544066 | 0.00013079 | 0.045994711 | 0.1285927 | 0.07 |
| GALT | 0.161563434 | 7.85E-08 | 0.062106524 | 0.0400815 | 0.1 |
| GAL | 0.441588913 | 2.23E-53 | 0.197573725 | 4.41E-11 | 0.25 |
| GAMT | -0.201783957 | 1.66E-11 | 0.043971827 | 0.146287 | 0.25 |
| GANAB | 0.053339207 | 0.07795774 | -0.083673884 | 0.0056402 | 0.14 |
| GANC | -0.25518246 | 1.04E-17 | -0.336383401 | 2.54E-30 | 0.09 |
| GAN | -0.034905555 | 0.24889899 | -0.207184664 | 4.61E-12 | 0.18 |
| GAP43 | -0.093977833 | 0.00186904 | -0.093577062 | 0.0019551 | 0.01 |
| GAPDHS | -0.009926397 | 0.74305933 | -0.115914774 | 0.0001226 | 0.11 |
| GAPDH | 0.563393991 | 1.46E-92 | 0.389373297 | 6.90E-41 | 0.18 |
| GAPT | -0.351984328 | 3.15E-33 | -0.215299079 | 6.28E-13 | 0.14 |
| LOC158123 | -0.316061598 | 8.84E-27 | -0.29295503 | 4.51E-23 | 0.03 |
| GAR1 | 0.575987391 | 1.32E-97 | 0.431558807 | 8.34E-51 | 0.15 |
| GARNL3 | -0.288363134 | 2.25E-22 | -0.146758053 | 1.10E-06 | 0.15 |
| GARS | 0.390895897 | 3.20E-41 | 0.200201287 | 2.41E-11 | 0.2 |
| GART | 0.267612296 | 2.22E-19 | 0.08546966 | 0.0046896 | 0.19 |
| GAS1 | -0.077676288 | 0.01020029 | -0.084528946 | 0.0051678 | 0.01 |
| GAS2L1 | 0.015829071 | 0.60114701 | 0.081977214 | 0.0066944 | 0.07 |
| GAS2L2 | -0.133404757 | 9.64E-06 | -0.050170033 | 0.0973588 | 0.09 |
| GAS2L3 | -0.035020374 | 0.24734473 | -0.224021361 | 6.72E-14 | 0.19 |
| GAS2 | -0.061852345 | 0.04090512 | -0.12431734 | 3.77E-05 | 0.07 |
| GAS5 | 0.266483901 | 3.17E-19 | 0.454026751 | 1.08E-56 | 0.19 |
| GAS6 | -0.260456558 | 2.09E-18 | -0.233307332 | 5.61E-15 | 0.03 |
| GAS7 | -0.436567535 | 4.43E-52 | -0.311204959 | 5.67E-26 | 0.13 |
| GAS8 | 0.008044514 | 0.79050413 | -0.003099834 | 0.9184668 | 0.02 |
| GAST | 0.183873657 | 9.11E-10 | 0.063189216 | 0.0367287 | 0.13 |
| GATA1 | -0.229164722 | 1.72E-14 | -0.129687501 | 1.70E-05 | 0.1 |
| GATA2 | -0.278818595 | 5.76E-21 | -0.12920751 | 1.83E-05 | 0.15 |
| MGC5445 | -0.360366665 | 7.38E-35 | -0.078088485 | 0.0098048 | 0.29 |
| GATA4 | -0.037319838 | 0.21763843 | -0.047637578 | 0.1154837 | 0.02 |
| GATA5 | 0.058810351 | 0.05192436 | 0.031506934 | 0.2980134 | 0.03 |
| GATA6 | 0.140387265 | 3.18E-06 | 0.061720468 | 0.0413381 | 0.08 |
| FLJ22489 | -0.324609781 | 3.09E-28 | -0.147409057 | 9.85E-07 | 0.18 |
| GATAD2A | 0.322331376 | 7.63E-28 | 0.115655338 | 0.000127 | 0.21 |
| GATAD2B | -0.046941969 | 0.1209001 | 0.061871895 | 0.0408413 | 0.11 |
| GATC | -0.169280697 | 1.79E-08 | -0.304164448 | 7.89E-25 | 0.14 |
| GATM | -0.315415563 | 1.13E-26 | -0.167899699 | 2.35E-08 | 0.15 |
| GATSL1 | -0.280300616 | 3.51E-21 | -0.383715988 | 1.16E-39 | 0.11 |
| GATSL2 | -0.10800273 | 0.00034754 | -0.153698994 | 3.29E-07 | 0.05 |
| GATSL3 | -0.171054283 | 1.27E-08 | -0.071884196 | 0.0174594 | 0.1 |
| GATS | 0.01043725 | 0.73033813 | -0.190009712 | 2.41E-10 | 0.21 |
| GBA2 | -0.110665713 | 0.00024662 | -0.124400398 | 3.72E-05 | 0.02 |
| GBA3 | 0.007782802 | 0.79716859 | -0.064126074 | 0.034023 | 0.08 |
| LOC440693 | 0.314991394 | 1.34E-26 | 0.416756684 | 3.66E-47 | 0.11 |
| GBAS | 0.092324767 | 0.00224804 | 0.152828946 | 3.84E-07 | 0.07 |
| LOC115185 | 0.019060692 | 0.52902629 | 0.1806642 | 1.79E-09 | 0.17 |
| GBE1 | -0.175412416 | 5.29E-09 | -0.233573671 | 5.21E-15 | 0.06 |
| GBF1 | -0.484747531 | 1.74E-65 | -0.387569964 | 1.70E-40 | 0.1 |
| GBGT1 | 0.027080771 | 0.37108396 | -0.009404067 | 0.7561391 | 0.04 |
| GBP1 | 0.170107433 | 1.53E-08 | -0.013413141 | 0.6577959 | 0.19 |
| GBP2 | -0.071053577 | 0.0188061 | -0.061730911 | 0.0413036 | 0.01 |
| GBP3 | -0.169536343 | 1.71E-08 | -0.119718665 | 7.26E-05 | 0.05 |
| GBP4 | -0.047692105 | 0.11506733 | -0.139452477 | 3.70E-06 | 0.1 |
| GBP5 | 0.09936423 | 0.00100394 | -0.072116934 | 0.0170976 | 0.18 |
| GBP6 | 0.026412085 | 0.38301686 | -0.101560803 | 0.0007724 | 0.13 |
| GBP7 | -0.014107883 | 0.64128432 | -0.011962012 | 0.692819 | 0.01 |
| GBX1 | 0.062456612 | 0.03897004 | 0.010842688 | 0.7202942 | 0.06 |
| GBX2 | 0.110811839 | 0.00024196 | 0.054655792 | 0.0708819 | 0.06 |
| GCAT | 0.21168647 | 1.54E-12 | 0.213585642 | 9.63E-13 | 0.01 |
| GCA | 0.030703174 | 0.31051527 | -0.005359136 | 0.8595284 | 0.04 |
| GCC1 | -0.240338456 | 7.95E-16 | -0.181627665 | 1.46E-09 | 0.06 |
| GCC2 | -0.53649615 | 1.73E-82 | -0.463223737 | 3.13E-59 | 0.08 |
| GCDH | 0.150023221 | 6.28E-07 | 0.085146211 | 0.0048493 | 0.07 |
| GCSAM | -0.237420479 | 1.80E-15 | -0.252253815 | 2.51E-17 | 0.02 |
| PAXBP1 | 0.059206816 | 0.05036075 | -0.013422221 | 0.657579 | 0.08 |
| GCGR | 0.095900356 | 0.00150245 | -0.002580627 | 0.9320869 | 0.1 |
| GCG | 0.067223752 | 0.02625456 | 0.032607771 | 0.2814429 | 0.04 |
| GCH1 | 0.055481533 | 0.0667188 | -0.016775129 | 0.5795793 | 0.08 |
| GCHFR | 0.014663584 | 0.62820316 | 0.124752719 | 3.54E-05 | 0.12 |
| GCKR | -0.012240969 | 0.68603238 | -0.021744008 | 0.4726764 | 0.01 |
| GCK | -0.055268327 | 0.06777397 | -0.126014242 | 2.94E-05 | 0.08 |
| GCLC | -0.105419192 | 0.00048129 | -0.12384174 | 4.04E-05 | 0.02 |
| GCLM | 0.01350453 | 0.65561409 | -0.1998763 | 2.60E-11 | 0.22 |
| GCM1 | -0.223704592 | 7.30E-14 | -0.055492057 | 0.0666671 | 0.17 |
| GCM2 | -0.010390311 | 0.73150397 | -0.020909392 | 0.4898418 | 0.02 |
| GCN1L1 | -0.100920999 | 0.00083411 | -0.172651657 | 9.21E-09 | 0.08 |
| GCNT1 | -0.044660314 | 0.14006706 | -0.01550829 | 0.6085413 | 0.03 |
| II | 0.067651948 | 0.02531157 | -0.0056729 | 0.8513971 | 0.08 |
| GCNT3 | 0.096138173 | 0.00146203 | 0.126069597 | 2.92E-05 | 0.03 |
| GCNT4 | -0.414071005 | 1.60E-46 | -0.375456977 | 6.43E-38 | 0.04 |
| GCNT7 | -0.170593838 | 1.39E-08 | -0.143333929 | 1.96E-06 | 0.03 |
| GCOM1 | -0.125762213 | 3.05E-05 | -0.194152368 | 9.60E-11 | 0.07 |
| GCSH | 0.238421934 | 1.36E-15 | 0.035430454 | 0.2418491 | 0.21 |
| GC | -0.021629859 | 0.47500436 | -0.02060931 | 0.4960944 | 0.01 |
| GDAP1L1 | -0.014146329 | 0.64037565 | -0.061663362 | 0.0415268 | 0.05 |
| GDAP1 | -0.089595787 | 0.00303017 | -0.079320125 | 0.0087031 | 0.02 |
| GDAP2 | -0.066167125 | 0.02871217 | -0.153809224 | 3.23E-07 | 0.09 |
| GDA | 0.136101329 | 6.32E-06 | -0.009631245 | 0.7504415 | 0.15 |
| GDE1 | -0.224626776 | 5.73E-14 | -0.068169917 | 0.0242101 | 0.16 |
| PCAT4 | -0.016428394 | 0.58744181 | 0.004621848 | 0.8786926 | 0.03 |
| GDF10 | -0.250968732 | 3.67E-17 | -0.178605254 | 2.75E-09 | 0.08 |
| GDF11 | -0.010877734 | 0.71942826 | -0.067109568 | 0.0265111 | 0.06 |
| GDF15 | -0.236807244 | 2.14E-15 | -0.052069341 | 0.0853164 | 0.19 |
| GDF1 | 0.18131936 | 1.56E-09 | 0.013474927 | 0.6563205 | 0.17 |
| GDF2 | 0.032406068 | 0.28443134 | 0.022576327 | 0.4558929 | 0.01 |
| GDF3 | -0.175664114 | 5.02E-09 | -0.016233076 | 0.5918924 | 0.16 |
| BDC | 0.214190852 | 8.28E-13 | 0.023226425 | 0.4430201 | 0.2 |
| KFM | -0.183193717 | 1.05E-09 | -0.107515204 | 0.0003698 | 0.08 |
| GDF7 | -0.14826003 | 8.51E-07 | -0.200280113 | 2.36E-11 | 0.06 |
| GDF9 | -0.215289368 | 6.29E-13 | -0.022259256 | 0.4622467 | 0.2 |
| LOC116360 | 0.094929155 | 0.00167846 | 0.067356205 | 0.0259597 | 0.03 |
| GDI2 | 0.306397644 | 3.45E-25 | 0.200215016 | 2.40E-11 | 0.11 |
| GDNF | -0.042466065 | 0.16062342 | -0.017325073 | 0.5672109 | 0.03 |
| GDPD1 | -0.217044304 | 4.05E-13 | -0.117720887 | 9.58E-05 | 0.1 |
| GDPD2 | 0.100799417 | 0.00084635 | 0.030493845 | 0.3138272 | 0.08 |
| GDPD3 | 0.036466055 | 0.22835428 | -0.015989135 | 0.5974726 | 0.06 |
| GDPD4 | -0.114670997 | 0.00014511 | -0.15621392 | 2.10E-07 | 0.05 |
| GDPD5 | 0.047269597 | 0.11832479 | -0.000191674 | 0.9949497 | 0.05 |
| ARHGEF25 | -0.041318365 | 0.17224432 | -0.047795736 | 0.1142792 | 0.01 |
| DKFZP434B131 | 0.192122676 | 1.51E-10 | 0.060856758 | 0.0442705 | 0.14 |
| GEMIN5 | -0.112388236 | 0.00019674 | -0.050296209 | 0.0965189 | 0.07 |
| GEMIN6 | 0.596419565 | 2.87E-106 | 0.491706019 | 1.32E-67 | 0.11 |
| GEMIN7 | 0.312074538 | 4.08E-26 | 0.350671544 | 5.62E-33 | 0.04 |
| LOC149635 | 0.2871158 | 3.46E-22 | 0.130063576 | 1.61E-05 | 0.16 |
| GEMIN8 | -0.058602661 | 0.05275952 | 0.102262067 | 0.0007096 | 0.17 |
| GEM | -0.290269333 | 1.16E-22 | -0.240049066 | 8.63E-16 | 0.06 |
| GEN1 | 0.330276479 | 3.14E-29 | 0.163640839 | 5.31E-08 | 0.17 |
| GET4 | 0.213675562 | 9.42E-13 | 0.152891696 | 3.80E-07 | 0.07 |
| GFAP | -0.164190547 | 4.79E-08 | -0.185993342 | 5.79E-10 | 0.03 |
| GFER | 0.135675856 | 6.76E-06 | 0.183437074 | 9.99E-10 | 0.05 |
| GFI1B | -0.056895283 | 0.06005898 | -0.044502851 | 0.1414714 | 0.02 |
| GFI1 | 0.007976887 | 0.7922248 | -0.111861079 | 0.0002109 | 0.12 |
| GFM1 | -0.157164119 | 1.77E-07 | -0.074445995 | 0.0138232 | 0.09 |
| GFM2 | -0.256694083 | 6.60E-18 | -0.106820528 | 0.0004037 | 0.15 |
| GFOD1 | 0.022510101 | 0.45721595 | -0.115142682 | 0.0001362 | 0.14 |
| GFOD2 | 0.063394085 | 0.03612184 | -0.071787741 | 0.0176113 | 0.14 |
| GFPT1 | -0.152898602 | 3.79E-07 | -0.148267179 | 8.50E-07 | 0.01 |
| GFPT2 | -0.047935113 | 0.11322595 | -0.083667895 | 0.0056437 | 0.04 |
| GFRA1 | -0.3019668 | 1.77E-24 | -0.113309158 | 0.0001741 | 0.19 |
| GFRA2 | -0.054486553 | 0.07176091 | -0.112443012 | 0.0001953 | 0.06 |
| GFRA3 | 0.421793516 | 2.21E-48 | 0.177697711 | 3.31E-09 | 0.25 |
| GFRA4 | 0.043508223 | 0.15059284 | -0.012397921 | 0.682225 | 0.06 |
| GFRAL | -0.02711173 | 0.37053712 | -0.028630646 | 0.3443242 | 0.01 |
| GGA1 | 0.094428441 | 0.00177642 | 0.048914053 | 0.1060419 | 0.05 |
| GGA2 | -0.104739864 | 0.00052369 | -0.275696958 | 1.62E-20 | 0.18 |
| GGA3 | -0.001423241 | 0.96251378 | -0.122073873 | 5.20E-05 | 0.13 |
| GCTG | 0.314399609 | 1.68E-26 | 0.44383773 | 5.74E-54 | 0.13 |
| LOC129302 | -0.075452964 | 0.01258791 | -0.053555541 | 0.0767571 | 0.03 |
| GGH | 0.455377917 | 4.64E-57 | 0.234971902 | 3.55E-15 | 0.23 |
| GGNBP1 | -0.029774028 | 0.32539219 | -0.058691877 | 0.0523994 | 0.03 |
| FLJ21230 | -0.119154945 | 7.85E-05 | -0.111222144 | 0.0002293 | 0.01 |
| GGN | -0.143829189 | 1.80E-06 | -0.116678674 | 0.0001105 | 0.03 |
| GGPS1 | 0.001673225 | 0.95593573 | 0.438063177 | 1.83E-52 | 0.44 |
| GGT1 | 0.037993504 | 0.20944172 | 0.098377909 | 0.0011275 | 0.07 |
| GGT3 | 0.112542775 | 0.00019276 | 0.133608214 | 9.34E-06 | 0.03 |
| GGT5 | -0.213927001 | 8.85E-13 | -0.183501191 | 9.86E-10 | 0.04 |
| GGT6 | -0.081390462 | 0.00709819 | 0.108696187 | 0.0003181 | 0.2 |
| GGT7 | -0.128260421 | 2.11E-05 | -0.137768764 | 4.85E-06 | 0.01 |
| GGT8P | -0.037847966 | 0.21119334 | -0.026841542 | 0.3753263 | 0.02 |
| a1.3GTP | -0.157825646 | 1.57E-07 | -0.187543272 | 4.14E-10 | 0.03 |
| GGTLA3 | 0.102440859 | 0.00069434 | 0.148398737 | 8.31E-07 | 0.05 |
| LOC150238 | 0.153840597 | 3.21E-07 | 0.202419708 | 1.43E-11 | 0.05 |
| GH1 | 0.081363415 | 0.00711732 | -0.016096817 | 0.5950064 | 0.1 |
| GH2 | 0.095467204 | 0.00157874 | 0.047782425 | 0.1143802 | 0.05 |
| GHDC | -0.301765723 | 1.90E-24 | -0.157822782 | 1.57E-07 | 0.15 |
| GHITM | 0.058433292 | 0.05344885 | 0.042146884 | 0.1637942 | 0.02 |
| GHRHR | 0.203724375 | 1.05E-11 | 0.104023365 | 0.0005722 | 0.1 |
| GHRH | -0.139704441 | 3.55E-06 | 0.04769447 | 0.1150493 | 0.19 |
| GHRLOS | 0.097496401 | 0.0012497 | 0.045402382 | 0.1335927 | 0.06 |
| GHRL | 0.051720308 | 0.08743442 | 0.041941242 | 0.1658619 | 0.01 |
| GHR | -0.267783988 | 2.10E-19 | -0.200479274 | 2.26E-11 | 0.07 |
| LOC116061 | -0.04768895 | 0.11509139 | -0.025098667 | 0.4071288 | 0.03 |
| GIF | -0.041775476 | 0.1675428 | -0.009292673 | 0.7589378 | 0.04 |
| GIGYF1 | -0.089144671 | 0.0031811 | -0.019033788 | 0.5296081 | 0.08 |
| GIGYF2 | -0.488088926 | 1.69E-66 | -0.387457146 | 1.80E-40 | 0.11 |
| GIMAP1 | -0.135910726 | 6.51E-06 | -0.170468552 | 1.42E-08 | 0.04 |
| LOC115261 | -0.208933109 | 3.02E-12 | -0.152275298 | 4.24E-07 | 0.06 |
| GIMAP4 | -0.135491577 | 6.96E-06 | -0.18347084 | 9.92E-10 | 0.05 |
| GIMAP5 | -0.084799743 | 0.00502584 | -0.163399956 | 5.56E-08 | 0.08 |
| GIMAP6 | -0.350721029 | 5.50E-33 | -0.33012753 | 3.34E-29 | 0.03 |
| GIMAP7 | -0.178742964 | 2.67E-09 | -0.217554255 | 3.56E-13 | 0.04 |
| GIMAP8 | -0.305345523 | 5.10E-25 | -0.317400093 | 5.27E-27 | 0.02 |
| GIN1 | -0.265924484 | 3.79E-19 | -0.031179481 | 0.3030655 | 0.24 |
| GINS1 | 0.512175546 | 3.92E-74 | 0.327560151 | 9.45E-29 | 0.19 |
| GINS2 | 0.492966057 | 5.38E-68 | 0.290370229 | 1.12E-22 | 0.21 |
| LOC115591 | 0.431824543 | 7.14E-51 | 0.204909583 | 7.95E-12 | 0.23 |
| GINS4 | 0.390595999 | 3.72E-41 | 0.107425752 | 0.000374 | 0.29 |
| GIPC1 | 0.279889384 | 4.03E-21 | 0.232435869 | 7.11E-15 | 0.05 |
| GIPC2 | -0.298380359 | 6.51E-24 | -0.220255307 | 1.78E-13 | 0.08 |
| DFNB15 | -0.107288943 | 0.00038053 | -0.132964325 | 1.03E-05 | 0.03 |
| GIPR | -0.45436974 | 8.73E-57 | -0.248757742 | 7.05E-17 | 0.21 |
| GIP | -0.030342061 | 0.31624307 | 0.038378149 | 0.204863 | 0.07 |
| GIT1 | 0.28616014 | 4.80E-22 | 0.16133813 | 8.19E-08 | 0.13 |
| GIT2 | -0.429064106 | 3.53E-50 | -0.372556732 | 2.56E-37 | 0.06 |
| LOC644122 | 0.240155066 | 8.37E-16 | 0.274376771 | 2.50E-20 | 0.04 |
| GJA10 | 0.063364834 | 0.03620796 | 0.009126066 | 0.7631295 | 0.06 |
| HSS | -0.216523627 | 4.61E-13 | -0.105448449 | 0.0004795 | 0.12 |
| GJA3 | 0.007816523 | 0.79630906 | -0.039615766 | 0.1906244 | 0.05 |
| GJA4 | -0.206913542 | 4.92E-12 | -0.100480987 | 0.0008792 | 0.11 |
| GJA5 | -0.168941123 | 1.92E-08 | -0.115646864 | 0.0001272 | 0.06 |
| GJA8 | -0.029720736 | 0.32625927 | -0.069433334 | 0.021696 | 0.04 |
| GJA9 | 0.176361214 | 4.36E-09 | 0.091848629 | 0.0023696 | 0.09 |
| GJB1 | -0.223901777 | 6.93E-14 | 0.010816708 | 0.7209364 | 0.24 |
| GJB2 | 0.024373573 | 0.42081893 | 0.052595684 | 0.082201 | 0.03 |
| EKV | 0.325364574 | 2.28E-28 | 0.003414236 | 0.9102306 | 0.33 |
| GJB4 | 0.141589172 | 2.61E-06 | -0.068190019 | 0.0241682 | 0.21 |
| GJB5 | 0.151695743 | 4.69E-07 | 0.001793445 | 0.9527733 | 0.15 |
| ED2 | 0.275799817 | 1.57E-20 | 0.044004016 | 0.1459916 | 0.24 |
| GJB7 | 0.166957761 | 2.82E-08 | 0.031084605 | 0.3045399 | 0.14 |
| GJC1 | -0.047242037 | 0.11853975 | -0.134191766 | 8.53E-06 | 0.09 |
| GJC2 | 0.030007501 | 0.3216111 | 0.01874244 | 0.535929 | 0.02 |
| GJC3 | -0.099336664 | 0.00100721 | -0.025814886 | 0.3938701 | 0.08 |
| GJD2 | 0.122353602 | 5.00E-05 | -0.026740874 | 0.3771205 | 0.15 |
| GJD3 | -0.153721485 | 3.28E-07 | -0.294971462 | 2.21E-23 | 0.15 |
| GJD4 | 0.106322181 | 0.00042987 | 0.003393145 | 0.9107828 | 0.11 |
| GK2 | -0.001493797 | 0.9606569 | -0.011178458 | 0.7120125 | 0.01 |
| GK3P | -0.219384575 | 2.23E-13 | -0.208131235 | 3.67E-12 | 0.02 |
| GK5 | -0.250676609 | 4.01E-17 | -0.178932682 | 2.57E-09 | 0.08 |
| GKAP1 | 0.059827055 | 0.04799377 | 0.112103173 | 0.0002043 | 0.06 |
| GKN1 | 0.060656031 | 0.04497658 | 0.047322634 | 0.117912 | 0.02 |
| GKN2 | -0.006132428 | 0.83951699 | 0.018467243 | 0.5419343 | 0.03 |
| GK | -0.074959507 | 0.01318049 | -0.002835525 | 0.9253976 | 0.08 |
| GLA | -0.045619716 | 0.13174097 | 0.050656136 | 0.0941546 | 0.1 |
| GLB1L2 | 0.092812691 | 0.00212944 | 0.107483127 | 0.0003713 | 0.02 |
| FLJ90231 | -0.069036314 | 0.02246039 | -0.102434532 | 0.0006949 | 0.04 |
| GLB1L | -0.196638455 | 5.47E-11 | -0.092749314 | 0.0021445 | 0.11 |
| ELNR1 | -0.090318979 | 0.00280178 | -0.01442518 | 0.6338012 | 0.08 |
| GLCCI1 | -0.341785708 | 2.61E-31 | -0.141209693 | 2.78E-06 | 0.21 |
| GLCE | -0.303705897 | 9.34E-25 | -0.254920613 | 1.13E-17 | 0.05 |
| GLDC | 0.286998604 | 3.60E-22 | 0.051088685 | 0.0913748 | 0.24 |
| GLDN | -0.280300203 | 3.51E-21 | -0.195703605 | 6.76E-11 | 0.09 |
| GLE1 | -0.032021275 | 0.29019183 | -0.01993649 | 0.5102666 | 0.02 |
| GLG1 | -0.115451826 | 0.0001306 | -0.249848427 | 5.12E-17 | 0.14 |
| GLI1 | -0.267298337 | 2.45E-19 | -0.189903588 | 2.47E-10 | 0.08 |
| GLI2 | -0.280815965 | 2.95E-21 | -0.239298691 | 1.07E-15 | 0.05 |
| GLI3 | -0.434105907 | 1.89E-51 | -0.199500156 | 2.83E-11 | 0.24 |
| LOC116391 | 0.269967063 | 1.04E-19 | 0.238776498 | 1.23E-15 | 0.04 |
| GLIPR1L1 | -0.142435103 | 2.27E-06 | -0.081950234 | 0.0067125 | 0.07 |
| GLIPR1L2 | -0.342385585 | 2.02E-31 | -0.161155576 | 8.47E-08 | 0.19 |
| GLIPR1 | -0.048637047 | 0.10803709 | -0.162261076 | 6.89E-08 | 0.12 |
| GLIPR2 | 0.244210948 | 2.64E-16 | 0.083407519 | 0.0057952 | 0.17 |
| GLIS1 | -0.207430908 | 4.35E-12 | -0.082545852 | 0.0063229 | 0.13 |
| GLIS2 | -0.273863439 | 2.96E-20 | -0.278078273 | 7.37E-21 | 0.01 |
| LOC645856 | -0.220129213 | 1.84E-13 | -0.147857742 | 9.12E-07 | 0.08 |
| GLMN | 0.318709512 | 3.16E-27 | 0.211272685 | 1.71E-12 | 0.11 |
| GLO1 | 0.26119444 | 1.66E-18 | 0.25954733 | 2.76E-18 | 0.01 |
| GLOD4 | 0.087109572 | 0.00395066 | 0.068883357 | 0.0227611 | 0.02 |
| GLOD5 | -0.057571036 | 0.05707572 | 0.041617626 | 0.1691553 | 0.1 |
| GLP1R | 0.013726814 | 0.65031969 | -0.037886562 | 0.2107278 | 0.06 |
| GLP2R | -0.127362845 | 2.41E-05 | -0.202890354 | 1.28E-11 | 0.08 |
| GLRA1 | -0.016311195 | 0.59011052 | -0.039733432 | 0.1893095 | 0.03 |
| GLRA2 | 0.07651748 | 0.01138916 | -0.040027008 | 0.1860581 | 0.12 |
| GLRA3 | -0.173000631 | 8.59E-09 | -0.161167484 | 8.45E-08 | 0.02 |
| GLRA4 | -0.105274288 | 0.00049005 | -0.006691752 | 0.8251062 | 0.1 |
| GLRB | -0.306520738 | 3.30E-25 | -0.188566766 | 3.31E-10 | 0.12 |
| GLRX2 | 0.415452221 | 7.52E-47 | 0.62559343 | 8.95E-120 | 0.22 |
| TXNL3 | 0.473204739 | 4.46E-62 | 0.324686748 | 2.99E-28 | 0.15 |
| GLRX5 | 0.353635955 | 1.52E-33 | 0.362262061 | 3.11E-35 | 0.01 |
| GLRX | -0.009904363 | 0.74360961 | 0.110565502 | 0.0002499 | 0.13 |
| GA | -0.195722536 | 6.73E-11 | -0.085977513 | 0.0044484 | 0.11 |
| GLS | 0.087061167 | 0.00397086 | -0.154994615 | 2.61E-07 | 0.25 |
| GLT1D1 | 0.046251293 | 0.12647247 | -0.104796882 | 0.00052 | 0.16 |
| COLGALT1 | 0.358057829 | 2.10E-34 | 0.096611963 | 0.0013845 | 0.27 |
| COLGALT2 | 0.017197431 | 0.57007029 | -0.074421502 | 0.0138545 | 0.1 |
| GLT6D1 | 0.06180502 | 0.04106004 | 0.038998315 | 0.1976344 | 0.03 |
| LOC91870 | -0.272304987 | 4.91E-20 | -0.066835124 | 0.0271365 | 0.21 |
| GLT8D2 | -0.281869513 | 2.07E-21 | -0.136880398 | 5.58E-06 | 0.15 |
| LOC440743 | 0.192335316 | 1.44E-10 | 0.048557069 | 0.1086186 | 0.15 |
| LOC374763 | 0.022537506 | 0.45666819 | -0.042887541 | 0.1565077 | 0.07 |
| GLTP | 0.18396785 | 8.93E-10 | 0.052029878 | 0.0855538 | 0.14 |
| GLTSCR1 | -0.05957828 | 0.0489317 | -0.015783199 | 0.6022019 | 0.05 |
| GLTSCR2 | -0.049038216 | 0.10515707 | 0.042744849 | 0.1578921 | 0.1 |
| GLUD1 | -0.262892097 | 9.80E-19 | -0.101225577 | 0.0008042 | 0.17 |
| GLUD2 | -0.422122276 | 1.84E-48 | -0.367410038 | 2.89E-36 | 0.06 |
| GLUL | -0.393242041 | 9.72E-42 | -0.035704342 | 0.2382268 | 0.36 |
| GLYATL1 | -0.171872888 | 1.08E-08 | 0.018995032 | 0.5304467 | 0.2 |
| GLYATL2 | 0.123994481 | 3.95E-05 | 0.084912807 | 0.0049676 | 0.04 |
| C6orf140 | 0.073353695 | 0.01528281 | 0.089372618 | 0.003104 | 0.02 |
| GLYAT | -0.22947915 | 1.58E-14 | -0.180901267 | 1.71E-09 | 0.05 |
| GLYCAM1 | -0.069214408 | 0.02211465 | 0.015992154 | 0.5974034 | 0.09 |
| GLYCTK | -0.096950362 | 0.00133139 | -0.05173939 | 0.0873175 | 0.05 |
| GLYR1 | -0.29545588 | 1.86E-23 | -0.213087991 | 1.09E-12 | 0.09 |
| GM2A | -0.104689201 | 0.00052698 | -0.080982294 | 0.0073918 | 0.03 |
| GMCL1P1 | -0.206421893 | 5.54E-12 | -0.170805984 | 1.33E-08 | 0.04 |
| GMCL1 | -0.275473846 | 1.74E-20 | -0.296054279 | 1.50E-23 | 0.03 |
| GMDS | 0.297017373 | 1.06E-23 | 0.207053589 | 4.76E-12 | 0.09 |
| GMEB1 | -0.081561111 | 0.00697856 | -0.195891264 | 6.48E-11 | 0.12 |
| GMEB2 | 0.166839578 | 2.88E-08 | 0.142086909 | 2.40E-06 | 0.03 |
| GMFB | -0.217617266 | 3.50E-13 | -0.082396473 | 0.0064186 | 0.14 |
| GMFG | 0.074051225 | 0.01433587 | -0.005984885 | 0.8433276 | 0.09 |
| GMIP | -0.032774025 | 0.27899564 | -0.128601632 | 2.00E-05 | 0.1 |
| GML | 0.037129137 | 0.22000003 | -0.00525671 | 0.8621861 | 0.05 |
| GMNN | 0.498266794 | 1.19E-69 | 0.370313427 | 7.41E-37 | 0.13 |
| GMPPA | 0.142299593 | 2.32E-06 | 0.249847112 | 5.12E-17 | 0.11 |
| GMPPB | -0.164528177 | 4.49E-08 | -0.094438953 | 0.0017743 | 0.08 |
| GMPR2 | -0.284929641 | 7.32E-22 | -0.044734115 | 0.1394126 | 0.25 |
| GMPR | 0.118027484 | 9.18E-05 | 0.061463348 | 0.0421934 | 0.06 |
| GMPS | 0.410897397 | 9.04E-46 | 0.244634395 | 2.34E-16 | 0.17 |
| GNA.11 | -0.435358638 | 9.04E-52 | -0.4877079 | 2.21E-66 | 0.06 |
| LOC654140 | -0.200449576 | 2.27E-11 | -0.300370212 | 3.17E-24 | 0.1 |
| GNA13 | -0.114629546 | 0.00014592 | -0.147500817 | 9.70E-07 | 0.04 |
| GNA14 | -0.400290638 | 2.55E-43 | -0.162975031 | 6.02E-08 | 0.24 |
| LOC115973 | 0.103425295 | 0.00061577 | -0.09474032 | 0.0017148 | 0.2 |
| GNAI1 | 0.025356208 | 0.40233088 | -0.127686483 | 2.30E-05 | 0.16 |
| GNAI2 | -0.134761671 | 7.80E-06 | -0.101849675 | 0.0007459 | 0.04 |
| GNAI3 | 0.143736412 | 1.83E-06 | 0.022545598 | 0.4565065 | 0.13 |
| GNAL | -0.226136638 | 3.85E-14 | -0.332903398 | 1.07E-29 | 0.11 |
| GNAO1 | -0.142365032 | 2.30E-06 | -0.301300673 | 2.26E-24 | 0.16 |
| GNAQ | -0.276619094 | 1.20E-20 | -0.244464095 | 2.45E-16 | 0.04 |
| GNAS.AS1 | 0.043539077 | 0.15030332 | 0.096312896 | 0.001433 | 0.06 |
| PHP1B | 0.372499748 | 2.63E-37 | 0.275034564 | 2.01E-20 | 0.1 |
| GNAT1 | 0.219802178 | 2.00E-13 | 0.107637773 | 0.0003641 | 0.12 |
| LOC92084 | 0.03506512 | 0.24674086 | -0.076066185 | 0.0118844 | 0.12 |
| LOC392946 | -0.032742878 | 0.27945301 | 0.020236383 | 0.5039237 | 0.06 |
| GNAZ | 0.161319578 | 8.21E-08 | 0.014967681 | 0.6210934 | 0.15 |
| GNB1L | 0.337474838 | 1.61E-30 | 0.15194786 | 4.49E-07 | 0.19 |
| GNB1 | -0.059002088 | 0.05116318 | -0.226616315 | 3.39E-14 | 0.17 |
| GNB2L1 | 0.175310778 | 5.40E-09 | 0.228178055 | 2.24E-14 | 0.06 |
| GNB2 | 0.163044015 | 5.94E-08 | 0.154790592 | 2.71E-07 | 0.01 |
| GNB3 | 0.057980945 | 0.05532682 | -0.016798389 | 0.5790536 | 0.08 |
| GNB4 | 0.084099007 | 0.00540071 | -0.054181858 | 0.0733659 | 0.14 |
| GNB5 | -0.267099308 | 2.61E-19 | -0.348012358 | 1.80E-32 | 0.09 |
| DMRV | -0.13507918 | 7.42E-06 | -0.153997494 | 3.12E-07 | 0.02 |
| GNG10 | -0.065170425 | 0.03120918 | -0.006458467 | 0.8311098 | 0.06 |
| GNG11 | -0.206372465 | 5.61E-12 | -0.061962593 | 0.0405461 | 0.15 |
| GNG12 | -0.39665255 | 1.69E-42 | -0.227989689 | 2.36E-14 | 0.17 |
| LOC115963 | 0.056261364 | 0.06297383 | 0.086096988 | 0.0043933 | 0.03 |
| GNG2 | -0.22413713 | 6.52E-14 | -0.247318579 | 1.07E-16 | 0.03 |
| GNG3 | -0.055973538 | 0.06433533 | -0.072767662 | 0.0161205 | 0.02 |
| GNG4 | 0.195500549 | 7.08E-11 | -0.031477589 | 0.2984638 | 0.23 |
| GNG5 | 0.38743198 | 1.83E-40 | 0.353250636 | 1.80E-33 | 0.04 |
| GNG7 | -0.124790443 | 3.52E-05 | -0.091692541 | 0.0024107 | 0.04 |
| GNG8 | 0.108639873 | 0.00032038 | 0.095125194 | 0.0016415 | 0.02 |
| GNGT1 | 0.117852591 | 9.41E-05 | 0.054363888 | 0.0724036 | 0.07 |
| GNGT2 | 0.064306594 | 0.03352164 | 0.00499771 | 0.8689133 | 0.06 |
| GNL1 | 0.204429801 | 8.92E-12 | 0.122280079 | 5.05E-05 | 0.09 |
| GNL2 | 0.487173905 | 3.21E-66 | 0.294675719 | 2.45E-23 | 0.2 |
| GNL3L | 0.012302836 | 0.68453064 | -0.140765591 | 2.99E-06 | 0.16 |
| GNL3 | 0.2373098 | 1.86E-15 | 0.166030812 | 3.37E-08 | 0.08 |
| D2S69E | 0.159584824 | 1.13E-07 | 0.03720964 | 0.2190009 | 0.13 |
| GNMT | -0.202849545 | 1.30E-11 | -0.01523128 | 0.614959 | 0.19 |
| GNPAT | 0.13478817 | 7.77E-06 | 0.488810646 | 1.02E-66 | 0.36 |
| GNPDA1 | 0.11604932 | 0.00012042 | 0.22579474 | 4.22E-14 | 0.11 |
| GNPDA2 | -0.274196851 | 2.65E-20 | -0.172836602 | 8.88E-09 | 0.11 |
| GNPNAT1 | 0.015782446 | 0.60221925 | 0.124178715 | 3.84E-05 | 0.11 |
| GNPTAB | -0.149620618 | 6.73E-07 | -0.238107889 | 1.49E-15 | 0.09 |
| GNPTG | -0.039367651 | 0.193419 | 0.102785055 | 0.0006659 | 0.15 |
| GNRH1 | 0.108012067 | 0.00034713 | 0.068806978 | 0.0229125 | 0.04 |
| GNRH2 | 0.027978305 | 0.3554339 | 0.047776592 | 0.1144245 | 0.02 |
| GNRHR2 | 0.116297156 | 0.00011643 | 0.193463276 | 1.12E-10 | 0.08 |
| GNRHR | -0.322480492 | 7.19E-28 | -0.262249793 | 1.20E-18 | 0.07 |
| GNS | -0.387192755 | 2.06E-40 | -0.338325715 | 1.13E-30 | 0.05 |
| GOLGA1 | -0.423179496 | 1.01E-48 | -0.273733425 | 3.08E-20 | 0.15 |
| LOC92497 | -0.203078273 | 1.23E-11 | -0.016480099 | 0.5862662 | 0.19 |
| GOLGA2P3Y | -0.011694828 | 0.69934244 | 0.046463749 | 0.1247375 | 0.06 |
| GOLGA2 | -0.372796565 | 2.29E-37 | -0.286361812 | 4.48E-22 | 0.09 |
| GOLGA3 | -0.199210665 | 3.03E-11 | -0.184363766 | 8.21E-10 | 0.02 |
| GOLGA4 | -0.312975543 | 2.89E-26 | -0.297787969 | 8.06E-24 | 0.02 |
| GOLGA5 | -0.272993156 | 3.93E-20 | -0.098227458 | 0.0011476 | 0.18 |
| GOLGA6A | -0.096085806 | 0.00147084 | -0.129003777 | 1.89E-05 | 0.04 |
| LOC220538 | -0.160741369 | 9.15E-08 | -0.198980758 | 3.19E-11 | 0.04 |
| GOLGA6C | 0.033481977 | 0.26873687 | -0.023502572 | 0.4376155 | 0.06 |
| GOLGA6D | -0.014105598 | 0.64133833 | -0.036218193 | 0.2315343 | 0.03 |
| LOC642325 | -0.053081637 | 0.07940706 | -0.120729929 | 6.29E-05 | 0.07 |
| GOLGA6L1 | 0.141301121 | 2.74E-06 | 0.056988929 | 0.059638 | 0.09 |
| FLJ35171 | -0.178329114 | 2.91E-09 | -0.266273162 | 3.39E-19 | 0.09 |
| FLJ36131 | 0.134679426 | 7.90E-06 | 0.042209716 | 0.1631663 | 0.1 |
| LOC728636 | 0.181125058 | 1.63E-09 | 0.124504241 | 3.67E-05 | 0.06 |
| GOLGA7B | 0.001733866 | 0.95434047 | -0.068189197 | 0.0241699 | 0.07 |
| GOLGA7 | 0.191934809 | 1.58E-10 | 0.036030614 | 0.2339617 | 0.16 |
| GOLGA8A | 0.008386709 | 0.78181326 | -0.089319424 | 0.0031218 | 0.1 |
| GOLGA8B | -0.076364089 | 0.01155541 | -0.058875339 | 0.0516653 | 0.02 |
| GOLGA8C | 0.005557895 | 0.85437573 | -0.021122801 | 0.485421 | 0.03 |
| GOLGA8D | 0.009945352 | 0.74258603 | -0.040767662 | 0.1780385 | 0.06 |
| GOLGA8EP | 0.01891191 | 0.53224768 | -0.011954582 | 0.6930001 | 0.04 |
| FLJ32679 | -0.078229472 | 0.00967269 | -0.042383449 | 0.1614397 | 0.04 |
| GOLGA8G | -0.043417382 | 0.15144773 | -0.091515585 | 0.0024581 | 0.05 |
| GOLGA8IP | -0.235408221 | 3.15E-15 | -0.218799702 | 2.59E-13 | 0.02 |
| GOLGB1 | -0.515345177 | 3.49E-75 | -0.358669945 | 1.59E-34 | 0.16 |
| GOLIM4 | -0.342989575 | 1.56E-31 | -0.363272409 | 1.96E-35 | 0.03 |
| C9orf155 | -0.228725532 | 1.94E-14 | 0.037403502 | 0.2166081 | 0.27 |
| GOLPH3L | -0.206504056 | 5.43E-12 | 0.132847583 | 1.05E-05 | 0.34 |
| GOLPH3 | -0.097062523 | 0.00131422 | -0.028622917 | 0.3444545 | 0.07 |
| GOLT1A | 0.057870559 | 0.05579334 | 0.356877937 | 3.57E-34 | 0.3 |
| GOLT1B | 0.172565103 | 9.37E-09 | 0.122805099 | 4.69E-05 | 0.05 |
| DKFZp761I241 | -0.296667024 | 1.20E-23 | -0.111089583 | 0.0002333 | 0.19 |
| GOPC | -0.115296016 | 0.00013338 | -0.127090816 | 2.51E-05 | 0.02 |
| GORAB | -0.051149542 | 0.09098903 | 0.166237302 | 3.24E-08 | 0.22 |
| GORASP1 | -0.324847729 | 2.81E-28 | -0.226395084 | 3.60E-14 | 0.1 |
| GORASP2 | 0.042215916 | 0.16310445 | 0.060102608 | 0.0469725 | 0.02 |
| GOSR1 | -0.146026074 | 1.25E-06 | -0.09763709 | 0.0012294 | 0.05 |
| GOSR2 | -0.175874455 | 4.81E-09 | -0.16102782 | 8.67E-08 | 0.02 |
| GOT1L1 | 0.020540856 | 0.49752665 | 0.030341299 | 0.3162552 | 0.01 |
| GOT1 | 0.306445221 | 3.39E-25 | 0.221730339 | 1.22E-13 | 0.09 |
| GOT2 | 0.078437092 | 0.00948103 | 0.0590582 | 0.0509422 | 0.02 |
| GP1BA | -0.104307479 | 0.00055246 | -0.235242787 | 3.30E-15 | 0.14 |
| GP2 | -0.362461434 | 2.84E-35 | -0.101164826 | 0.0008101 | 0.27 |
| GP5 | -0.386653743 | 2.69E-40 | -0.327591469 | 9.33E-29 | 0.06 |
| GP6 | -0.094664924 | 0.00172952 | -0.096889184 | 0.0013408 | 0.01 |
| GP9 | 0.00284182 | 0.92523244 | 0.004570439 | 0.8800316 | 0.01 |
| LOC105371599 | -0.08456051 | 0.0051511 | -0.139270481 | 3.81E-06 | 0.06 |
| GPAA1 | 0.26165201 | 1.44E-18 | 0.196965337 | 5.07E-11 | 0.07 |
| GPAM | -0.326298313 | 1.57E-28 | -0.190709954 | 2.07E-10 | 0.14 |
| GPAT2 | -0.067592151 | 0.02544147 | -0.103546487 | 0.0006067 | 0.04 |
| GPATCH1 | 0.170163962 | 1.51E-08 | 0.087090668 | 0.0039585 | 0.09 |
| GPATCH2 | -0.169371811 | 1.76E-08 | 0.111074994 | 0.0002338 | 0.29 |
| GPATCH3 | 0.167641594 | 2.47E-08 | 0.052715939 | 0.0815023 | 0.12 |
| GPATCH4 | 0.244713349 | 2.28E-16 | 0.291493067 | 7.54E-23 | 0.05 |
| GPATCH8 | -0.355345991 | 7.09E-34 | -0.414282964 | 1.43E-46 | 0.06 |
| GPBAR1 | -0.011992112 | 0.69208553 | -0.093960439 | 0.0018727 | 0.09 |
| GPBP1L1 | -0.092595414 | 0.00218152 | -0.147608521 | 9.52E-07 | 0.06 |
| GPBP1 | -0.153186458 | 3.61E-07 | 0.02880454 | 0.3414005 | 0.19 |
| GPC1 | -0.140933307 | 2.91E-06 | -0.123905006 | 4.00E-05 | 0.02 |
| GPC2 | 0.368919897 | 1.43E-36 | 0.202440565 | 1.43E-11 | 0.17 |
| GPC3 | -0.16043037 | 9.69E-08 | -0.076950619 | 0.0109311 | 0.09 |
| GPC4 | -0.27612608 | 1.41E-20 | -0.009772139 | 0.7469146 | 0.27 |
| GPC5 | 0.072457699 | 0.01657961 | -0.026721325 | 0.3774695 | 0.1 |
| GPC6 | -0.134345569 | 8.32E-06 | -0.123241329 | 4.40E-05 | 0.02 |
| GPCPD1 | -0.312968828 | 2.90E-26 | -0.150355468 | 5.93E-07 | 0.17 |
| GPD1L | -0.434748272 | 1.29E-51 | -0.175447037 | 5.25E-09 | 0.26 |
| GPD1 | -0.247864361 | 9.16E-17 | -0.164505164 | 4.51E-08 | 0.09 |
| GPD2 | -0.242006836 | 4.95E-16 | -0.17127438 | 1.21E-08 | 0.08 |
| CMKRL2 | -0.136728928 | 5.72E-06 | -0.184760625 | 7.54E-10 | 0.05 |
| LOC92535 | 0.133262834 | 9.85E-06 | 0.082866198 | 0.0061218 | 0.06 |
| GPHB5 | -0.017351842 | 0.56661207 | -0.025239146 | 0.4045075 | 0.01 |
| GPHN | -0.063367379 | 0.03620046 | -0.114022993 | 0.0001583 | 0.06 |
| GPIHBP1 | -0.328168548 | 7.39E-29 | -0.179141315 | 2.46E-09 | 0.15 |
| GPI | 0.372778166 | 2.31E-37 | 0.243431532 | 3.30E-16 | 0.13 |
| GPKOW | 0.111938104 | 0.00020877 | 0.102249616 | 0.0007107 | 0.01 |
| GPLD1 | -0.137237352 | 5.28E-06 | -0.208621278 | 3.26E-12 | 0.08 |
| GPM6A | -0.128384783 | 2.07E-05 | -0.089047173 | 0.0032146 | 0.04 |
| GPM6B | 0.137984716 | 4.68E-06 | -0.017787812 | 0.5569026 | 0.16 |
| GPN1 | 0.420267957 | 5.20E-48 | 0.208329411 | 3.50E-12 | 0.22 |
| GPN2 | 0.060178937 | 0.04669291 | -0.035325889 | 0.2432422 | 0.1 |
| GPN3 | 0.067443613 | 0.02576665 | 0.132642984 | 1.08E-05 | 0.07 |
| GPNMB | -0.088307091 | 0.00347959 | -0.128758323 | 1.96E-05 | 0.05 |
| GPR101 | 0.030219708 | 0.31819937 | -0.007328507 | 0.808772 | 0.04 |
| FLJ22591 | -0.415955298 | 5.70E-47 | -0.322673006 | 6.66E-28 | 0.1 |
| GPR108 | -0.08705773 | 0.0039723 | 0.036707659 | 0.2252845 | 0.13 |
| HCAR2 | -0.153741295 | 3.27E-07 | -0.1738234 | 7.29E-09 | 0.03 |
| LOC91492 | -0.230292306 | 1.27E-14 | -0.194665426 | 8.55E-11 | 0.04 |
| FLJ22684 | 0.022574719 | 0.45592501 | 0.03725437 | 0.2184471 | 0.02 |
| ADGRF2 | 0.07045856 | 0.01982576 | -0.047912917 | 0.1133932 | 0.12 |
| ADGRG4 | -0.125444973 | 3.20E-05 | -0.166984548 | 2.80E-08 | 0.05 |
| ADGRF3 | 0.019816023 | 0.51282627 | -0.14404989 | 1.74E-06 | 0.17 |
| ADGRG5 | -0.111333604 | 0.00022602 | -0.19762156 | 4.37E-11 | 0.09 |
| ADGRF4 | 0.034138144 | 0.25946223 | -0.045306946 | 0.1344122 | 0.08 |
| ADGRF5 | -0.412945609 | 2.97E-46 | -0.356783534 | 3.73E-34 | 0.06 |
| GPR119 | 0.030306145 | 0.3168165 | 0.010837508 | 0.7204223 | 0.02 |
| LOC118923 | -0.194535699 | 8.81E-11 | -0.166671481 | 2.98E-08 | 0.03 |
| LOC196747 | -0.010171986 | 0.7369347 | -0.023073907 | 0.4460214 | 0.02 |
| ADGRA2 | -0.396337028 | 1.99E-42 | -0.322756515 | 6.45E-28 | 0.08 |
| ADGRA3 | 0.220473486 | 1.69E-13 | 0.024535233 | 0.4177436 | 0.2 |
| VIGR | 0.174069252 | 6.93E-09 | 0.059015117 | 0.0511118 | 0.12 |
| ADGRG7 | 0.074616832 | 0.01360635 | 0.072764863 | 0.0161246 | 0.01 |
| GPR12 | 0.15243286 | 4.12E-07 | -0.028678825 | 0.3435126 | 0.19 |
| GPR132 | -0.084254867 | 0.00531522 | -0.088066212 | 0.00357 | 0.01 |
| ADGRD1 | -0.294605978 | 2.51E-23 | -0.267569728 | 2.25E-19 | 0.03 |
| GPR135 | -0.293211332 | 4.12E-23 | -0.265685057 | 4.08E-19 | 0.03 |
| GPR137B | -0.186807082 | 4.85E-10 | 0.084748115 | 0.0050526 | 0.28 |
| GPR137C | 0.035110161 | 0.24613406 | 0.11413205 | 0.000156 | 0.08 |
| GPR137 | -0.01455459 | 0.63075985 | 0.078826968 | 0.0091302 | 0.1 |
| GPR139 | -0.188631166 | 3.27E-10 | -0.221837001 | 1.19E-13 | 0.04 |
| GPR141 | -0.327966211 | 8.02E-29 | -0.216032979 | 5.22E-13 | 0.12 |
| GPR142 | -0.198229929 | 3.80E-11 | -0.252219001 | 2.54E-17 | 0.06 |
| GPR143 | 0.024682408 | 0.41495527 | 0.01791352 | 0.554118 | 0.01 |
| ADGRD2 | -0.171187901 | 1.23E-08 | -0.135517166 | 6.93E-06 | 0.04 |
| GPR146 | -0.236772266 | 2.16E-15 | -0.178873745 | 2.60E-09 | 0.06 |
| GPR148 | -0.025082171 | 0.40743729 | -0.07984088 | 0.0082715 | 0.06 |
| GPR149 | 0.144416108 | 1.63E-06 | 0.066966249 | 0.0268361 | 0.08 |
| GPR150 | -0.065642506 | 0.0300042 | -0.135655875 | 6.78E-06 | 0.08 |
| GPR151 | 0.046885739 | 0.12134647 | -0.066400511 | 0.0281529 | 0.12 |
| LOC120052 | -0.343009121 | 1.55E-31 | -0.234355947 | 4.21E-15 | 0.11 |
| GPR153 | -0.101541541 | 0.00077416 | -0.053140576 | 0.0790735 | 0.05 |
| GPR155 | -0.325652627 | 2.03E-28 | -0.251101457 | 3.53E-17 | 0.08 |
| GPR156 | 0.238328877 | 1.40E-15 | 0.034025601 | 0.261037 | 0.21 |
| LOC148918 | -0.335122551 | 4.29E-30 | -0.363061312 | 2.16E-35 | 0.03 |
| GPR158 | 0.18546611 | 6.48E-10 | -0.112661155 | 0.0001898 | 0.3 |
| GPR15 | -0.151020674 | 5.28E-07 | -0.223077129 | 8.60E-14 | 0.08 |
| GPR160 | -0.416097064 | 5.27E-47 | -0.050300758 | 0.0964887 | 0.37 |
| GPR161 | 0.200517491 | 2.24E-11 | 0.075960593 | 0.012003 | 0.13 |
| GPR162 | -0.172826756 | 8.90E-09 | -0.043626252 | 0.1494876 | 0.13 |
| GPR171 | 0.014210347 | 0.63886377 | -0.08997877 | 0.0029072 | 0.11 |
| SLC52A2 | 0.461978555 | 6.97E-59 | 0.269749828 | 1.12E-19 | 0.2 |
| SLC52A1 | 0.002340993 | 0.93838007 | 0.028658979 | 0.3438468 | 0.03 |
| GPR173 | -0.090184051 | 0.00284316 | -0.281315931 | 2.50E-21 | 0.2 |
| GPR174 | -0.151607219 | 4.76E-07 | -0.2515048 | 3.13E-17 | 0.1 |
| GPR176 | -0.288466861 | 2.17E-22 | -0.364081478 | 1.35E-35 | 0.08 |
| LOC100290717 | -0.027288556 | 0.36742341 | -0.15107047 | 5.23E-07 | 0.13 |
| LOC115824 | -0.320063182 | 1.86E-27 | -0.301118178 | 2.41E-24 | 0.02 |
| LOC144874 | 0.100099507 | 0.0009201 | -0.02921294 | 0.3345965 | 0.13 |
| GPR182 | -0.204611395 | 8.54E-12 | -0.174213751 | 6.74E-09 | 0.04 |
| GPR183 | -0.030249394 | 0.317724 | -0.152225494 | 4.27E-07 | 0.13 |
| GPR18 | -0.00470064 | 0.87664096 | -0.112323158 | 0.0001984 | 0.11 |
| GPR19 | 0.421056196 | 3.34E-48 | 0.290970073 | 9.06E-23 | 0.14 |
| LOC220720 | -0.22788452 | 2.42E-14 | -0.109942064 | 0.0002709 | 0.12 |
| GPR20 | -0.340532724 | 4.44E-31 | -0.262991686 | 9.50E-19 | 0.08 |
| GPR21 | -0.276791764 | 1.13E-20 | -0.219701813 | 2.06E-13 | 0.06 |
| GPR22 | -0.037770337 | 0.21213195 | -0.003043634 | 0.91994 | 0.04 |
| GPR25 | -0.009215845 | 0.76086984 | -0.075793039 | 0.0121934 | 0.07 |
| LOC119586 | 0.02450588 | 0.41830099 | -0.078625053 | 0.0093104 | 0.11 |
| GPR27 | -0.243129404 | 3.60E-16 | -0.247780986 | 9.38E-17 | 0.01 |
| GPR31 | -0.186030153 | 5.74E-10 | -0.2206821 | 1.60E-13 | 0.04 |
| GPR32 | 0.023972136 | 0.42851291 | -0.01766135 | 0.5597107 | 0.05 |
| GPR34 | -0.438859457 | 1.14E-52 | -0.337229181 | 1.78E-30 | 0.11 |
| GPR35 | 0.145238265 | 1.42E-06 | 0.075466595 | 0.0125719 | 0.07 |
| GPR37L1 | 0.162293533 | 6.85E-08 | 0.074227458 | 0.014105 | 0.09 |
| GPR37 | 0.101590589 | 0.0007696 | -0.039525899 | 0.1916332 | 0.15 |
| GPR39 | -0.252010793 | 2.70E-17 | -0.027418272 | 0.3651496 | 0.23 |
| GPR3 | 0.294171496 | 2.93E-23 | 0.055940838 | 0.0644915 | 0.24 |
| PTGDR2 | -0.113038448 | 0.0001805 | -0.081266865 | 0.007186 | 0.04 |
| GPR45 | -0.064323511 | 0.03347499 | -0.138236465 | 4.50E-06 | 0.08 |
| GPR4 | -0.158447127 | 1.40E-07 | -0.141463303 | 2.66E-06 | 0.02 |
| GPR50 | -0.028154792 | 0.35240619 | -0.023027475 | 0.4469374 | 0.01 |
| GPR52 | -0.005107702 | 0.86605519 | 0.003995762 | 0.8950227 | 0.01 |
| GPR55 | 0.071259645 | 0.01846381 | -0.137634248 | 4.95E-06 | 0.21 |
| ADGRG1 | 0.137473383 | 5.08E-06 | 0.002524198 | 0.9335685 | 0.14 |
| GPR61 | 0.12048189 | 6.52E-05 | -0.028539274 | 0.3458669 | 0.15 |
| GPR62 | 0.023716407 | 0.43345657 | -0.041814111 | 0.1671499 | 0.07 |
| GPR63 | 0.196860867 | 5.20E-11 | -0.008040731 | 0.7906003 | 0.21 |
| ADGRG2 | 0.071853235 | 0.01750807 | -0.118491866 | 8.61E-05 | 0.2 |
| GPR65 | -0.129051588 | 1.87E-05 | -0.163740919 | 5.21E-08 | 0.04 |
| GPR68 | -0.206770185 | 5.10E-12 | -0.18564049 | 6.24E-10 | 0.03 |
| GPR6 | -0.043751478 | 0.14832169 | -0.009710188 | 0.7484647 | 0.04 |
| GPR75 | -0.190721344 | 2.06E-10 | -0.349316999 | 1.02E-32 | 0.16 |
| C5AR2 | -0.484429962 | 2.16E-65 | -0.229320212 | 1.65E-14 | 0.26 |
| GPR78 | -0.001271801 | 0.96650002 | -0.11829905 | 8.84E-05 | 0.12 |
| FKSG80 | -0.438640853 | 1.30E-52 | -0.247605792 | 9.87E-17 | 0.2 |
| GPR82 | -0.220981419 | 1.48E-13 | -0.275115754 | 1.96E-20 | 0.06 |
| GPR83 | 0.117296895 | 0.00010154 | -0.002554337 | 0.9327771 | 0.12 |
| EX33 | 0.165130266 | 4.00E-08 | -0.028562247 | 0.3454786 | 0.2 |
| GPR85 | -0.098835707 | 0.00106851 | -0.125838603 | 3.02E-05 | 0.03 |
| GPR87 | 0.091890343 | 0.00235868 | -0.009678005 | 0.7492704 | 0.11 |
| GPR88 | -0.188944928 | 3.05E-10 | -0.189441056 | 2.73E-10 | 0.01 |
| LOC100508578 | 0.184613597 | 7.78E-10 | 0.316693519 | 6.93E-27 | 0.14 |
| LOC101060247 | -0.03078009 | 0.30930415 | -0.024816083 | 0.4124323 | 0.01 |
| GPR89C | 0.222034185 | 1.13E-13 | 0.307462613 | 2.32E-25 | 0.09 |
| ADGRG3 | 0.162624805 | 6.43E-08 | 0.002439825 | 0.9357841 | 0.17 |
| LOC116067 | -0.255714733 | 8.88E-18 | -0.11167804 | 0.000216 | 0.15 |
| GPRASP1 | -0.405425035 | 1.71E-44 | -0.349407781 | 9.77E-33 | 0.06 |
| GPRASP2 | -0.370423856 | 7.03E-37 | -0.271965005 | 5.48E-20 | 0.1 |
| GPRC5A | -0.248436828 | 7.74E-17 | -0.013769982 | 0.6492935 | 0.24 |
| GPRC5B | 0.089546431 | 0.00304636 | -0.041960808 | 0.1656643 | 0.14 |
| GPRC5C | -0.310173953 | 8.38E-26 | -0.06703705 | 0.0266751 | 0.25 |
| GPRC5D | 0.032009081 | 0.29037565 | 0.043076285 | 0.1546907 | 0.02 |
| LOC135196 | 0.065529675 | 0.03028851 | 0.079769381 | 0.0083296 | 0.02 |
| GPRIN1 | 0.26171616 | 1.41E-18 | 0.087346841 | 0.003853 | 0.18 |
| LOC142964 | 0.077117631 | 0.01075892 | 0.007842197 | 0.7956548 | 0.07 |
| GPRIN3 | -0.360233644 | 7.84E-35 | -0.461969389 | 7.01E-59 | 0.11 |
| GPS1 | 0.372451471 | 2.70E-37 | 0.223176964 | 8.38E-14 | 0.15 |
| GPS2 | 0.214247977 | 8.17E-13 | 0.193292035 | 1.16E-10 | 0.03 |
| GPSM1 | 0.121111147 | 5.96E-05 | 0.064711671 | 0.0324196 | 0.06 |
| DFNB82 | 0.379094506 | 1.11E-38 | 0.108741733 | 0.0003162 | 0.28 |
| GPSM3 | -0.018857903 | 0.53341947 | -0.08547959 | 0.0046848 | 0.07 |
| GPT2 | 0.428590748 | 4.64E-50 | 0.096478973 | 0.0014058 | 0.34 |
| GPT | 0.093430422 | 0.00198751 | 0.082590806 | 0.0062943 | 0.02 |
| GPX1 | 0.168060136 | 2.28E-08 | 0.141681526 | 2.57E-06 | 0.03 |
| GPX2 | -0.082056715 | 0.00664131 | -0.061450157 | 0.0422376 | 0.03 |
| GPX3 | -0.133736162 | 9.15E-06 | -0.13292754 | 1.04E-05 | 0.01 |
| GPX4 | 0.120912246 | 6.13E-05 | 0.15874522 | 1.32E-07 | 0.04 |
| GPX5 | 0.008003166 | 0.79155603 | -0.000279125 | 0.9926456 | 0.01 |
| GPX6 | 0.080886878 | 0.00746202 | 0.030869978 | 0.3078927 | 0.06 |
| GPX6.1 | 0.080886878 | 0.00746202 | 0.030869978 | 0.3078927 | 0.06 |
| GPX8 | -0.193810475 | 1.04E-10 | 0.016251353 | 0.5914753 | 0.22 |
| GRAMD1A | 0.071269783 | 0.01844711 | 0.031526613 | 0.2977116 | 0.04 |
| LINC01059 | -0.021416047 | 0.47938176 | -0.180232344 | 1.96E-09 | 0.16 |
| GRAMD1C | -0.289468057 | 1.53E-22 | -0.24195752 | 5.02E-16 | 0.05 |
| GRAMD2 | -0.024472693 | 0.41893174 | -0.001626419 | 0.9571672 | 0.03 |
| GRAMD3 | -0.074886531 | 0.01327018 | -0.044586414 | 0.1407248 | 0.04 |
| GRAMD4 | -0.107715238 | 0.00036049 | -0.101937352 | 0.000738 | 0.01 |
| GRAP2 | -0.100872928 | 0.00083893 | -0.140495011 | 3.12E-06 | 0.04 |
| LOC105376802 | -0.250713037 | 3.96E-17 | -0.165312722 | 3.86E-08 | 0.09 |
| GRAP | -0.112688922 | 0.00018907 | -0.097522922 | 0.0012458 | 0.02 |
| GRASP | -0.122896468 | 4.62E-05 | -0.089580792 | 0.0030351 | 0.04 |
| RSS | -0.021720172 | 0.47316198 | -0.071512055 | 0.018052 | 0.05 |
| GRB14 | 0.154090689 | 3.07E-07 | 0.101173352 | 0.0008092 | 0.06 |
| FLJ20886 | -0.036536856 | 0.22745164 | -0.118018687 | 9.19E-05 | 0.09 |
| GRB7 | 0.000181214 | 0.99522534 | 0.117414836 | 9.99E-05 | 0.12 |
| GREB1L | -0.286448959 | 4.35E-22 | -0.192549147 | 1.37E-10 | 0.1 |
| GREB1 | -0.325364833 | 2.28E-28 | -0.161390624 | 8.11E-08 | 0.17 |
| GREMLIN | -0.131686741 | 1.26E-05 | -0.094913111 | 0.0016815 | 0.04 |
| GREM2 | -0.303190622 | 1.13E-24 | -0.187732123 | 3.97E-10 | 0.12 |
| GRHL1 | 0.040987064 | 0.17571296 | 0.055257092 | 0.06783 | 0.02 |
| DFNA28 | -0.007191005 | 0.81229248 | 0.0097542 | 0.7473634 | 0.02 |
| GRHL3 | 0.070293738 | 0.0201166 | 0.018350135 | 0.5444999 | 0.06 |
| GLYD | 0.179616774 | 2.23E-09 | 0.163027441 | 5.96E-08 | 0.02 |
| GRIA1 | -0.226022619 | 3.97E-14 | -0.251130394 | 3.50E-17 | 0.03 |
| GRIA2 | -0.166354983 | 3.16E-08 | -0.151724549 | 4.67E-07 | 0.02 |
| GRIA3 | -0.158829794 | 1.30E-07 | -0.216356088 | 4.81E-13 | 0.06 |
| GRIA4 | -0.237379615 | 1.82E-15 | -0.146748325 | 1.10E-06 | 0.1 |
| KIAA1220 | -0.274165542 | 2.68E-20 | -0.199515958 | 2.82E-11 | 0.08 |
| LOC641916 | -0.212564112 | 1.24E-12 | -0.116959214 | 0.0001064 | 0.1 |
| GRID2 | 0.026452979 | 0.38228043 | -0.052507412 | 0.082717 | 0.08 |
| GRIK1 | -0.228857006 | 1.87E-14 | -0.221085969 | 1.44E-13 | 0.01 |
| MRT6 | -0.135985739 | 6.43E-06 | -0.095475176 | 0.0015773 | 0.05 |
| GRIK3 | -0.305806171 | 4.30E-25 | -0.18350431 | 9.85E-10 | 0.13 |
| EAA1 | -0.267618457 | 2.21E-19 | -0.17991813 | 2.09E-09 | 0.09 |
| GRIK5 | -0.196981782 | 5.05E-11 | -0.093501474 | 0.0019718 | 0.11 |
| GRIN1 | -0.250428153 | 4.31E-17 | -0.113852851 | 0.0001619 | 0.14 |
| GRIN2A | -0.187718972 | 3.98E-10 | -0.21154256 | 1.60E-12 | 0.03 |
| GRIN2B | 0.021190377 | 0.48402568 | -0.062217075 | 0.0397276 | 0.09 |
| GRIN2C | 0.048669307 | 0.10780321 | -0.071955548 | 0.0173478 | 0.13 |
| EB11 | 0.091831842 | 0.00237396 | 0.029978123 | 0.3220853 | 0.07 |
| GRIN3A | -0.137122381 | 5.37E-06 | -0.152306194 | 4.21E-07 | 0.02 |
| LOC100508403 | 0.088883347 | 0.00327162 | -0.014797906 | 0.6250584 | 0.11 |
| GRINA | 0.190270218 | 2.28E-10 | 0.16849608 | 2.09E-08 | 0.03 |
| POLR2M | -0.415941533 | 5.74E-47 | -0.283665223 | 1.13E-21 | 0.14 |
| GRIP1 | -0.111811585 | 0.00021227 | -0.026949674 | 0.3734051 | 0.09 |
| GRIP2 | -0.162116012 | 7.08E-08 | -0.245681875 | 1.73E-16 | 0.09 |
| GRIPAP1 | -0.031216746 | 0.30248774 | -0.072836562 | 0.0160199 | 0.05 |
| GRK1 | 0.09679175 | 0.00135602 | -0.061976144 | 0.0405022 | 0.16 |
| GRK4 | -0.080422379 | 0.00781231 | -0.036339318 | 0.2299764 | 0.05 |
| LOC100131848 | -0.137501693 | 5.06E-06 | -0.222067566 | 1.12E-13 | 0.09 |
| GRK6 | 0.357168829 | 3.13E-34 | 0.11972902 | 7.25E-05 | 0.24 |
| GRK7 | -0.025439251 | 0.40079106 | -0.109655813 | 0.0002811 | 0.09 |
| LOC654215 | -0.461810492 | 7.77E-59 | -0.304278133 | 7.57E-25 | 0.16 |
| GRM1 | -0.151714887 | 4.67E-07 | -0.060419073 | 0.0458223 | 0.1 |
| GRM2 | 0.043146307 | 0.15402072 | -0.036594748 | 0.2267155 | 0.08 |
| GRM3 | -0.1558584 | 2.24E-07 | -0.196967119 | 5.07E-11 | 0.05 |
| GRM4 | -0.171094181 | 1.26E-08 | -0.045373784 | 0.1338378 | 0.13 |
| GRM5 | -0.164423225 | 4.58E-08 | -0.103344262 | 0.0006219 | 0.07 |
| GRM6 | 0.211185176 | 1.74E-12 | 0.035667603 | 0.2387104 | 0.18 |
| GRM7 | -0.238367683 | 1.38E-15 | -0.171135902 | 1.25E-08 | 0.07 |
| GRM8 | -0.107173147 | 0.00038614 | -0.078211387 | 0.0096895 | 0.03 |
| GRN | 0.000399349 | 0.98947814 | -0.046398605 | 0.1252675 | 0.05 |
| GRPEL1 | 0.32943202 | 4.43E-29 | 0.183718879 | 9.41E-10 | 0.15 |
| GRPEL2 | 0.226496059 | 3.50E-14 | 0.136766006 | 5.69E-06 | 0.09 |
| GRPR | -0.319519967 | 2.31E-27 | -0.088505068 | 0.0034068 | 0.24 |
| GRP | -0.221636765 | 1.25E-13 | -0.026239698 | 0.3861308 | 0.2 |
| FAM110D | -0.140595422 | 3.07E-06 | -0.122113499 | 5.17E-05 | 0.02 |
| GRSF1 | 0.185989879 | 5.79E-10 | 0.085844645 | 0.0045104 | 0.11 |
| GRTP1 | -0.068127455 | 0.02429882 | 0.037083539 | 0.2205674 | 0.11 |
| GRWD1 | 0.206448473 | 5.51E-12 | 0.222078709 | 1.11E-13 | 0.02 |
| DFNB25 | 0.051080811 | 0.09142479 | 0.041934049 | 0.1659345 | 0.01 |
| GRXCR2 | -0.062944663 | 0.03746435 | -0.077583528 | 0.0102912 | 0.02 |
| GSC2 | 0.046615872 | 0.12350666 | 0.011007268 | 0.7162307 | 0.04 |
| GSC | -0.245452624 | 1.84E-16 | -0.100074042 | 0.0009229 | 0.15 |
| GSDMA | 0.007827897 | 0.79601918 | -0.001407205 | 0.9629358 | 0.01 |
| GSDMB | 0.186989363 | 4.67E-10 | 0.153856711 | 3.20E-07 | 0.04 |
| GSDMC | 0.219294742 | 2.28E-13 | 0.131422208 | 1.31E-05 | 0.09 |
| GSDMD | 0.079997163 | 0.00814581 | 0.080355483 | 0.007864 | 0.01 |
| GSG1L | 0.070520292 | 0.01971778 | -0.07781752 | 0.0100632 | 0.15 |
| GSG1 | 0.009006178 | 0.76615004 | -0.00127481 | 0.9664208 | 0.02 |
| GSG2 | 0.419916971 | 6.33E-48 | 0.134615356 | 7.98E-06 | 0.29 |
| GSK3A | 0.254779475 | 1.18E-17 | 0.159425335 | 1.17E-07 | 0.1 |
| GSK3B | -0.164701467 | 4.34E-08 | -0.177372569 | 3.54E-09 | 0.02 |
| GSN | -0.157668284 | 1.61E-07 | -0.217520099 | 3.59E-13 | 0.06 |
| LOC146148 | -0.112451418 | 0.0001951 | -0.039767683 | 0.1889281 | 0.08 |
| FLJ10441 | -0.124612257 | 3.61E-05 | -0.108712704 | 0.0003174 | 0.02 |
| GSR | -0.297514663 | 8.89E-24 | -0.173651268 | 7.54E-09 | 0.13 |
| GSS | 0.131095198 | 1.37E-05 | 0.093896149 | 0.0018863 | 0.04 |
| LOC221360 | 0.205834457 | 6.38E-12 | 0.023887604 | 0.4301434 | 0.19 |
| LOC154371 | 0.224616714 | 5.75E-14 | 0.097239184 | 0.0012876 | 0.13 |
| GSTA3 | -0.053679623 | 0.07607534 | -0.02496371 | 0.4096566 | 0.03 |
| LOC202556 | 0.199679373 | 2.72E-11 | 0.115388187 | 0.0001317 | 0.09 |
| GSTA5 | 0.140323385 | 3.21E-06 | 0.11607386 | 0.00012 | 0.03 |
| GSTCD | -0.393500757 | 8.52E-42 | -0.207383762 | 4.40E-12 | 0.19 |
| LOC51064 | 0.037637783 | 0.21374163 | 0.088339347 | 0.0034676 | 0.06 |
| LOC92085 | -0.053020661 | 0.07975335 | -0.000695358 | 0.9816801 | 0.06 |
| GSTM2P1 | -0.101917342 | 0.00073983 | -0.06985158 | 0.0209153 | 0.04 |
| LOC82152 | -0.161461054 | 8.00E-08 | -0.052350423 | 0.083641 | 0.11 |
| GSTM3 | -0.218634493 | 2.70E-13 | -0.072095174 | 0.0171311 | 0.15 |
| LOC82153 | -0.105723979 | 0.00046332 | -0.009893309 | 0.7438857 | 0.1 |
| LOC82154 | -0.207134522 | 4.67E-12 | -0.14097309 | 2.89E-06 | 0.07 |
| GSTO1 | 0.401886833 | 1.11E-43 | 0.337555769 | 1.55E-30 | 0.07 |
| LOC102724376 | -0.032791745 | 0.27873566 | 0.157550472 | 1.65E-07 | 0.2 |
| GSTP1 | 0.278194525 | 7.09E-21 | 0.182457567 | 1.23E-09 | 0.1 |
| GSTT1 | -0.067902807 | 0.02477283 | -0.072851951 | 0.0159976 | 0.01 |
| LOC91334 | -0.029036156 | 0.337531 | 0.002696398 | 0.9290481 | 0.04 |
| LOC100420767 | 0.027855308 | 0.35755364 | -0.009099547 | 0.7637973 | 0.04 |
| LOC654020 | 0.043570718 | 0.15000685 | -0.015240513 | 0.6147446 | 0.06 |
| GSTZ1 | -0.03422407 | 0.2582643 | 0.085510449 | 0.0046698 | 0.12 |
| GSX1 | 0.065149776 | 0.03126282 | 0.037115757 | 0.2201664 | 0.03 |
| GSX2 | 0.002190751 | 0.94232762 | -0.043427903 | 0.1513485 | 0.05 |
| PRO0159 | 0.165151052 | 3.99E-08 | -0.00430899 | 0.8868468 | 0.17 |
| GTF2A1L | 0.161462237 | 8.00E-08 | 0.112930424 | 0.0001831 | 0.05 |
| GTF2A1 | -0.407028546 | 7.25E-45 | -0.434898667 | 1.19E-51 | 0.03 |
| GTF2A2 | 0.386887097 | 2.40E-40 | 0.37771882 | 2.16E-38 | 0.01 |
| GTF2B | 0.219195262 | 2.34E-13 | 0.094629224 | 0.0017365 | 0.13 |
| GTF2E1 | 0.039796095 | 0.18861204 | 0.031246319 | 0.3020297 | 0.01 |
| GTF2E2 | 0.304578342 | 6.77E-25 | 0.252667545 | 2.22E-17 | 0.06 |
| GTF2F1 | -0.002198077 | 0.94213511 | 0.084552488 | 0.0051553 | 0.09 |
| GTF2F2 | 0.377172283 | 2.82E-38 | 0.293401023 | 3.85E-23 | 0.09 |
| GTF2H1 | -0.046617939 | 0.12349001 | -0.10404655 | 0.0005705 | 0.06 |
| GTF2H2B | -0.097991598 | 0.00117964 | 0.036887354 | 0.2230205 | 0.14 |
| GTF2H2C | -0.151864453 | 4.55E-07 | 0.053305641 | 0.0781454 | 0.21 |
| GTF2H2 | 0.004892093 | 0.87165929 | 0.055029822 | 0.0689706 | 0.06 |
| GTF2H3 | -0.22895065 | 1.82E-14 | -0.271924872 | 5.55E-20 | 0.05 |
| GTF2H4 | 0.419704129 | 7.13E-48 | 0.336346652 | 2.58E-30 | 0.09 |
| GTF2H5 | 0.287861697 | 2.67E-22 | 0.428805974 | 4.10E-50 | 0.15 |
| LOC641758 | 0.036676379 | 0.22568029 | 0.021711343 | 0.4733419 | 0.02 |
| GTF3 | 0.127179462 | 2.48E-05 | 0.142361664 | 2.30E-06 | 0.02 |
| GTF2IRD2B | -0.358184898 | 1.98E-34 | -0.174782883 | 6.00E-09 | 0.19 |
| GTF2IRD2P1 | -0.313788723 | 2.12E-26 | -0.113823895 | 0.0001626 | 0.2 |
| LOC100133260 | -0.317247613 | 5.59E-27 | -0.103481323 | 0.0006116 | 0.22 |
| LOC732437 | -0.262400063 | 1.14E-18 | -0.292048738 | 6.21E-23 | 0.03 |
| GTF3A | 0.437873998 | 2.05E-52 | 0.375203018 | 7.26E-38 | 0.07 |
| GTF3C1 | -0.198443091 | 3.61E-11 | -0.118478572 | 8.63E-05 | 0.08 |
| GTF3C2 | 0.218678628 | 2.67E-13 | 0.046046434 | 0.1281631 | 0.18 |
| GTF3C3 | -0.105235072 | 0.00049245 | -0.18827127 | 3.53E-10 | 0.09 |
| GTF3C4 | -0.271375861 | 6.63E-20 | -0.297790576 | 8.05E-24 | 0.03 |
| GTF3C5 | 0.358647518 | 1.61E-34 | 0.203908773 | 1.01E-11 | 0.16 |
| GTF3C6 | 0.460982027 | 1.32E-58 | 0.458560389 | 6.20E-58 | 0.01 |
| GTPBP10 | -0.043890371 | 0.14703669 | 0.114932759 | 0.0001401 | 0.16 |
| HSPC018 | -0.105308291 | 0.00048798 | -0.26413211 | 6.65E-19 | 0.16 |
| GTPBP2 | 0.19968281 | 2.71E-11 | 0.028746354 | 0.342377 | 0.18 |
| GTPBP3 | 0.28709285 | 3.49E-22 | 0.19470623 | 8.47E-11 | 0.1 |
| GTPBP4 | 0.508341375 | 7.07E-73 | 0.271001592 | 7.48E-20 | 0.24 |
| MTG2 | 0.140630762 | 3.05E-06 | 0.093640896 | 0.0019412 | 0.05 |
| LOC131448 | 0.254676406 | 1.21E-17 | 0.144521649 | 1.60E-06 | 0.12 |
| GTSE1 | 0.455035437 | 5.75E-57 | 0.188437468 | 3.41E-10 | 0.27 |
| C20orf65 | 0.107365053 | 0.00037688 | -0.007658538 | 0.8003382 | 0.12 |
| GTSF1 | 0.113488684 | 0.00017001 | -0.007460303 | 0.8054013 | 0.13 |
| GUCA1A | 0.22937377 | 1.63E-14 | 0.045295484 | 0.1345108 | 0.19 |
| GUCA1B | 0.021544686 | 0.4767455 | -0.050342702 | 0.0962108 | 0.08 |
| GUCA1C | 0.003487059 | 0.90832422 | -0.060816753 | 0.0444105 | 0.07 |
| GUCA2A | 0.097196724 | 0.00129394 | 0.042345294 | 0.1618177 | 0.06 |
| GUCA2B | 0.069158278 | 0.02222312 | 0.058304443 | 0.0539783 | 0.02 |
| GUCY1A2 | -0.294261363 | 2.84E-23 | -0.159794925 | 1.09E-07 | 0.14 |
| GUCY1A3 | -0.166433934 | 3.12E-08 | -0.151456478 | 4.89E-07 | 0.02 |
| GUCY1B2 | 0.062745084 | 0.03807399 | 0.150761422 | 5.52E-07 | 0.09 |
| GUCY1B3 | -0.153857337 | 3.20E-07 | -0.156167957 | 2.11E-07 | 0.01 |
| GUCY2C | -0.040549209 | 0.18037667 | -0.135669935 | 6.76E-06 | 0.1 |
| CORD5 | -0.061496094 | 0.04208361 | 0.0510234 | 0.0917901 | 0.12 |
| GUCY2E | 0.044962561 | 0.13740152 | 0.008890905 | 0.7690577 | 0.04 |
| GUCY2F | 0.111560081 | 0.0002194 | 0.015003926 | 0.6202484 | 0.1 |
| GUCY2GP | 0.05474156 | 0.07043984 | 0.010102676 | 0.7386615 | 0.05 |
| GUF1 | -0.072158762 | 0.01703324 | -0.011982029 | 0.6923312 | 0.07 |
| GUK1 | 0.295545005 | 1.80E-23 | 0.361417335 | 4.58E-35 | 0.07 |
| GULP1 | -0.221507523 | 1.29E-13 | -0.199967381 | 2.54E-11 | 0.03 |
| GUSBP4 | -0.402467361 | 8.16E-44 | -0.152203803 | 4.29E-07 | 0.26 |
| LOC135125 | 0.275709571 | 1.61E-20 | 0.190512405 | 2.16E-10 | 0.09 |
| GUSBP1 | -0.026732276 | 0.37727395 | 0.031717173 | 0.2947995 | 0.06 |
| MGC22265 | -0.290791507 | 9.65E-23 | -0.002932963 | 0.9228418 | 0.29 |
| GUSB | -0.019848897 | 0.5121271 | 0.055847551 | 0.0649389 | 0.08 |
| FLJ13373 | -0.21975398 | 2.03E-13 | -0.303486572 | 1.01E-24 | 0.09 |
| GXYLT1 | -0.115123548 | 0.00013652 | -0.084362962 | 0.0052566 | 0.04 |
| LOC732216 | -0.276225996 | 1.36E-20 | -0.177421703 | 3.51E-09 | 0.1 |
| GYG1 | 0.168704815 | 2.01E-08 | 0.081557474 | 0.0069811 | 0.09 |
| GYG2 | -0.129674283 | 1.71E-05 | -0.064937154 | 0.0318198 | 0.07 |
| GYLTL1B | 0.238041591 | 1.52E-15 | 0.161486867 | 7.96E-08 | 0.08 |
| GYPA | 0.015013429 | 0.6200269 | -0.011990015 | 0.6921366 | 0.03 |
| GYPB | -0.006768071 | 0.82314429 | 0.00162792 | 0.9571277 | 0.01 |
| GYPC | 0.015239994 | 0.61475668 | -0.070284085 | 0.0201338 | 0.09 |
| GYPE | -0.232648804 | 6.71E-15 | -0.199504305 | 2.83E-11 | 0.04 |
| GYS1 | 0.025241305 | 0.4044673 | -0.057162523 | 0.0588641 | 0.09 |
| GYS2 | -0.036894558 | 0.22293011 | -0.110680598 | 0.0002461 | 0.08 |
| GZF1 | -0.072658573 | 0.01628077 | -0.163648106 | 5.30E-08 | 0.1 |
| GZMA | 0.0360138 | 0.23418021 | -0.037837685 | 0.2113175 | 0.08 |
| GZMB | 0.235965762 | 2.70E-15 | 0.024850367 | 0.4117867 | 0.22 |
| GZMH | 0.027952241 | 0.35588244 | -0.018145495 | 0.5489975 | 0.05 |
| GZMK | -0.049728423 | 0.10034476 | -0.087504817 | 0.0037891 | 0.04 |
| GZMM | 0.02328537 | 0.44186331 | -0.023301806 | 0.4415411 | 0.05 |
| MGC4485 | -0.135277975 | 7.19E-06 | -0.064804777 | 0.0321708 | 0.08 |
| H1F0 | -0.121087577 | 5.98E-05 | -0.038903174 | 0.1987311 | 0.09 |
| H1FNT | -0.083830843 | 0.00555071 | -0.113949609 | 0.0001599 | 0.04 |
| H1FOO | 0.094414257 | 0.00177927 | 0.038021467 | 0.2091064 | 0.06 |
| H1FX | 0.188883527 | 3.09E-10 | 0.172470826 | 9.55E-09 | 0.02 |
| H2AFB1 | 0.247821181 | 9.27E-17 | 0.150501656 | 5.78E-07 | 0.1 |
| H2AFJ | 0.050968408 | 0.09214104 | 0.023866871 | 0.4305439 | 0.03 |
| H2AFV | 0.083971364 | 0.00547165 | 0.066369119 | 0.0282276 | 0.02 |
| LOC115272 | 0.561485876 | 8.12E-92 | 0.3495308 | 9.26E-33 | 0.22 |
| H2AFY2 | 0.186839051 | 4.82E-10 | 0.060718728 | 0.044755 | 0.13 |
| H2AFY | 0.231017796 | 1.04E-14 | 0.25836722 | 3.97E-18 | 0.03 |
| H2AFZ | 0.635446201 | 1.15E-124 | 0.456401008 | 2.43E-57 | 0.18 |
| H2BFM | 0.175477003 | 5.22E-09 | 0.101630991 | 0.0007659 | 0.08 |
| H2BFWT | 0.135949051 | 6.47E-06 | 0.07202563 | 0.0172387 | 0.07 |
| H2BFXP | 0.345983189 | 4.32E-32 | 0.144753622 | 1.54E-06 | 0.21 |
| H3F3A | 0.313241583 | 2.61E-26 | 0.621745157 | 6.54E-118 | 0.31 |
| H3F3B | 0.26404914 | 6.83E-19 | 0.130810984 | 1.44E-05 | 0.14 |
| H3F3C | 0.140809266 | 2.97E-06 | -0.033366931 | 0.2703861 | 0.18 |
| GDH | -0.42299362 | 1.12E-48 | -0.38151643 | 3.41E-39 | 0.05 |
| HAAO | 0.067605733 | 0.02541192 | -0.023108336 | 0.4453429 | 0.1 |
| HABP2 | -0.143978378 | 1.76E-06 | 0.066490821 | 0.0279391 | 0.22 |
| HABP4 | 0.047236583 | 0.11858234 | -0.102402863 | 0.0006976 | 0.15 |
| HACE1 | -0.168690007 | 2.01E-08 | -0.201521806 | 1.77E-11 | 0.04 |
| HACL1 | -0.09222243 | 0.00227366 | -0.062049016 | 0.0402666 | 0.04 |
| HADHA | 0.058968322 | 0.05129655 | 0.010726957 | 0.7231564 | 0.05 |
| HADHB | -0.094605834 | 0.00174113 | 0.033967118 | 0.261858 | 0.13 |
| HADH | 0.094956688 | 0.00167322 | 0.108830339 | 0.0003127 | 0.02 |
| HAGHL | 0.089879012 | 0.00293877 | 0.178998311 | 2.53E-09 | 0.09 |
| HAGH | 0.07148094 | 0.0181023 | 0.079576344 | 0.0084883 | 0.01 |
| HAL | 0.078679921 | 0.00926116 | -0.014984219 | 0.6207078 | 0.1 |
| HAMP | 0.112634669 | 0.00019043 | 0.079852736 | 0.0082619 | 0.04 |
| HAND1 | 0.092046742 | 0.00231829 | 0.038509588 | 0.2033151 | 0.06 |
| HAND2 | -0.059426412 | 0.04951179 | -0.042106799 | 0.1641957 | 0.02 |
| HAO1 | 0.284079637 | 9.79E-22 | 0.102324274 | 0.0007042 | 0.19 |
| HAO2 | -0.130028312 | 1.62E-05 | -0.159946794 | 1.06E-07 | 0.03 |
| HAP1 | -0.02087945 | 0.49046374 | -0.080787684 | 0.0075356 | 0.06 |
| HAPLN1 | 0.115672867 | 0.00012674 | 0.039142562 | 0.1959801 | 0.08 |
| HAPLN2 | -0.007655334 | 0.80041993 | -0.002575935 | 0.9322101 | 0.01 |
| EXLD1 | 0.273688114 | 3.13E-20 | 0.056903213 | 0.0600232 | 0.22 |
| HAPLN4 | 0.046784687 | 0.12215188 | -0.032124092 | 0.288645 | 0.08 |
| HAR1A | -0.166041837 | 3.36E-08 | -0.158690511 | 1.34E-07 | 0.01 |
| HAR1B | -0.084263303 | 0.00531062 | -0.119783833 | 7.19E-05 | 0.04 |
| HARBI1 | 0.103524544 | 0.00060832 | 0.063781269 | 0.0349983 | 0.04 |
| LOC134023 | -0.064217266 | 0.03376892 | 0.096666043 | 0.0013758 | 0.17 |
| HARS | 0.190362997 | 2.23E-10 | 0.171307294 | 1.20E-08 | 0.02 |
| HAS1 | -0.058765523 | 0.05210368 | -0.068939118 | 0.0226511 | 0.02 |
| HAS2.AS1 | -0.020652068 | 0.49520087 | -0.131314155 | 1.33E-05 | 0.12 |
| HAS2 | -0.152603528 | 4.00E-07 | -0.20914568 | 2.87E-12 | 0.06 |
| HAS3 | -0.042526573 | 0.16002759 | -0.201232958 | 1.89E-11 | 0.16 |
| HAT1 | -0.035031789 | 0.24719057 | 0.007034265 | 0.8163101 | 0.05 |
| HAUS1 | 0.445276223 | 2.40E-54 | 0.218114299 | 3.08E-13 | 0.23 |
| HAUS2 | -0.050349626 | 0.09616501 | -0.072997268 | 0.0157876 | 0.03 |
| HAUS3 | -0.166226311 | 3.24E-08 | -0.112893663 | 0.000184 | 0.06 |
| HAUS4 | 0.111112417 | 0.00023265 | 0.150363571 | 5.92E-07 | 0.04 |
| HAUS5 | 0.304666882 | 6.56E-25 | 0.167433295 | 2.57E-08 | 0.14 |
| HAUS6 | 0.191898122 | 1.59E-10 | 0.039170553 | 0.1956603 | 0.16 |
| HAUS7 | 0.434711603 | 1.32E-51 | 0.29106261 | 8.77E-23 | 0.15 |
| HAUS8 | 0.565545045 | 2.08E-93 | 0.369093254 | 1.31E-36 | 0.2 |
| HAVCR1 | -0.081038746 | 0.00735057 | -0.09101892 | 0.0025958 | 0.01 |
| HAVCR2 | -0.055733877 | 0.06548743 | -0.126524151 | 2.73E-05 | 0.08 |
| HAX1 | 0.403739432 | 4.17E-44 | 0.564416444 | 5.80E-93 | 0.17 |
| HBA1 | -0.089649428 | 0.00301266 | -0.102239385 | 0.0007115 | 0.02 |
| HBA2 | -0.119844575 | 7.13E-05 | -0.100885135 | 0.0008377 | 0.02 |
| HBBP1 | 0.014222517 | 0.63857654 | -0.031477253 | 0.298469 | 0.05 |
| HBB | -0.204240841 | 9.32E-12 | -0.136504956 | 5.93E-06 | 0.07 |
| HBD | -0.028687508 | 0.34336643 | -0.102029651 | 0.0007298 | 0.08 |
| HBE1 | 0.053903112 | 0.07485977 | 0.043301567 | 0.152543 | 0.02 |
| HBEGF | -0.016833634 | 0.57825749 | -0.121337229 | 5.78E-05 | 0.11 |
| HBGR | -0.07349891 | 0.01508126 | -0.074935417 | 0.01321 | 0.01 |
| HBG2 | -0.040565499 | 0.18020152 | -0.028468608 | 0.3470629 | 0.02 |
| SNORD115.24 | NA | NA | NA | NA | #VALUE! |
| SNORD115.27 | NA | NA | NA | NA | #VALUE! |
| SNORD115.28 | NA | NA | NA | NA | #VALUE! |
| SNORD115.45 | NA | NA | NA | NA | #VALUE! |
| SNORD115.47 | NA | NA | NA | NA | #VALUE! |
| HBM | -0.045277112 | 0.13466912 | -0.051656442 | 0.0878265 | 0.01 |
| HBP1 | -0.502791867 | 4.37E-71 | -0.262195002 | 1.22E-18 | 0.25 |
| HBQ1 | 0.299500342 | 4.34E-24 | 0.059615332 | 0.048791 | 0.24 |
| HBS1L | 0.103486776 | 0.00061115 | 0.072795879 | 0.0160792 | 0.04 |
| LAMTOR5 | 0.460088899 | 2.34E-58 | 0.411503982 | 6.51E-46 | 0.05 |
| HBZ | 0.084500582 | 0.00518291 | 0.05721254 | 0.0586427 | 0.03 |
| MLS | 0.279020294 | 5.39E-21 | 0.10257497 | 0.0006831 | 0.18 |
| HCFC1R1 | 0.071283717 | 0.01842418 | 0.170656121 | 1.37E-08 | 0.1 |
| HFC1 | -0.062770015 | 0.03799738 | -0.227703246 | 2.54E-14 | 0.17 |
| HCFC2 | -0.476562287 | 4.69E-63 | -0.404742921 | 2.45E-44 | 0.08 |
| HCG11 | 0.013643524 | 0.65230148 | -0.106751264 | 0.0004073 | 0.13 |
| HCG18 | 0.127244462 | 2.45E-05 | 0.074148481 | 0.014208 | 0.06 |
| HCG22 | -0.202647414 | 1.36E-11 | -0.143746392 | 1.83E-06 | 0.06 |
| HCG26 | 0.045078341 | 0.13639086 | -0.04505566 | 0.1365884 | 0.1 |
| LOC282955 | 0.038875404 | 0.19905201 | -0.00982466 | 0.7456013 | 0.05 |
| HCG2P7 | -0.263842404 | 7.29E-19 | -0.191470914 | 1.75E-10 | 0.08 |
| HCG4B | -0.035735626 | 0.23781546 | -0.010584803 | 0.7266773 | 0.03 |
| HCG4 | -0.140795911 | 2.97E-06 | -0.151690297 | 4.69E-07 | 0.02 |
| HCG9 | -0.06623881 | 0.0285394 | -0.084434153 | 0.0052184 | 0.02 |
| HCK | 0.028840499 | 0.3407979 | -0.150302151 | 5.98E-07 | 0.18 |
| HCLS1 | -0.056522015 | 0.06176149 | -0.168771662 | 1.98E-08 | 0.12 |
| HCN1 | -0.171325833 | 1.20E-08 | -0.163810613 | 5.14E-08 | 0.01 |
| HCN2 | -0.06197009 | 0.04052181 | -0.153570584 | 3.37E-07 | 0.1 |
| HCN3 | 0.056254777 | 0.06300472 | 0.108016544 | 0.0003469 | 0.06 |
| HCN4 | 0.102085811 | 0.0007249 | -0.007600386 | 0.8018226 | 0.11 |
| LOC154638 | 0.033075872 | 0.27458956 | -0.049461384 | 0.1021854 | 0.09 |
| HCRTR1 | -0.012291839 | 0.68479749 | -0.099604089 | 0.0009758 | 0.09 |
| HCRTR2 | 0.15115787 | 5.15E-07 | 0.067117488 | 0.0264932 | 0.09 |
| HCRT | 0.041132926 | 0.17417947 | 0.025865188 | 0.3929489 | 0.02 |
| HCST | 0.089573666 | 0.00303741 | -0.016756468 | 0.5800012 | 0.11 |
| HDAC10 | -0.071521321 | 0.018037 | -0.039803998 | 0.1885242 | 0.04 |
| HDAC11 | -0.086813281 | 0.0040758 | -0.024185684 | 0.4244099 | 0.07 |
| HDAC1 | 0.234800448 | 3.72E-15 | 0.062534781 | 0.0387255 | 0.18 |
| HDAC2 | 0.413414989 | 2.30E-46 | 0.291364296 | 7.89E-23 | 0.13 |
| HDAC3 | 0.049179808 | 0.10415522 | 0.088355775 | 0.0034616 | 0.04 |
| LOC727809 | -0.199762844 | 2.66E-11 | -0.228803971 | 1.90E-14 | 0.03 |
| KIAA0600 | -0.020932364 | 0.48936487 | -0.108580666 | 0.0003228 | 0.09 |
| HDAC6 | -0.060433279 | 0.0457712 | -0.218359286 | 2.90E-13 | 0.16 |
| DKFZP586J0917 | -0.24444061 | 2.47E-16 | -0.126518329 | 2.73E-05 | 0.12 |
| WTS | 0.270573995 | 8.59E-20 | 0.2468856 | 1.22E-16 | 0.03 |
| HDAC9 | -0.103520218 | 0.00060865 | -0.159142861 | 1.23E-07 | 0.06 |
| HDC | -0.320866424 | 1.36E-27 | -0.288063647 | 2.49E-22 | 0.04 |
| HDDC2 | 0.42726433 | 9.92E-50 | 0.434819926 | 1.24E-51 | 0.01 |
| HDDC3 | 0.154499969 | 2.85E-07 | 0.167775474 | 2.41E-08 | 0.02 |
| HDGFL1 | 0.071615855 | 0.01788495 | 0.069437416 | 0.0216882 | 0.01 |
| HDGFRP2 | 0.234279033 | 4.30E-15 | 0.159855917 | 1.08E-07 | 0.08 |
| HDGFRP3 | -0.12340428 | 4.30E-05 | -0.169493485 | 1.72E-08 | 0.05 |
| HDGF | 0.538964276 | 2.24E-83 | 0.478154043 | 1.60E-63 | 0.07 |
| LOC116065 | 0.003256463 | 0.91436254 | 0.145024473 | 1.48E-06 | 0.15 |
| HDHD2 | 0.021778866 | 0.47196673 | 0.002177386 | 0.9426789 | 0.02 |
| HDHD3 | 0.087239687 | 0.00389681 | 0.210742322 | 1.94E-12 | 0.13 |
| PRO2900 | -0.145248333 | 1.42E-06 | -0.07629872 | 0.0116269 | 0.07 |
| HDX | -0.232503325 | 6.98E-15 | -0.107551699 | 0.0003681 | 0.13 |
| MGC72083 | 0.030350191 | 0.31611335 | 0.123325716 | 4.35E-05 | 0.1 |
| DNAAF5 | 0.163588842 | 5.36E-08 | 0.175960884 | 4.73E-09 | 0.02 |
| HEATR3 | 0.033053296 | 0.27491745 | -0.090558311 | 0.0027297 | 0.13 |
| HEATR4 | -0.219882872 | 1.96E-13 | -0.162055094 | 7.16E-08 | 0.06 |
| HEATR5A | -0.40364962 | 4.37E-44 | -0.337291615 | 1.74E-30 | 0.07 |
| HEATR5B | -0.462709245 | 4.36E-59 | -0.38612855 | 3.50E-40 | 0.08 |
| HEATR6 | -0.137503519 | 5.06E-06 | -0.063401823 | 0.0360991 | 0.08 |
| MROH1 | 0.051016174 | 0.09183612 | -0.09806773 | 0.0011692 | 0.15 |
| MROH2B | -0.063089448 | 0.03702729 | -0.038708439 | 0.2009896 | 0.03 |
| HEBP1 | -0.004774156 | 0.87472747 | 0.051040625 | 0.0916803 | 0.06 |
| HEBP2 | 0.363797294 | 1.54E-35 | 0.385205019 | 5.54E-40 | 0.03 |
| HECA | 0.006669564 | 0.82567677 | -0.07196902 | 0.0173268 | 0.08 |
| HECTD1 | -0.345796408 | 4.69E-32 | -0.331681463 | 1.77E-29 | 0.02 |
| HECTD2 | -0.401164593 | 1.62E-43 | -0.224654754 | 5.69E-14 | 0.18 |
| HECTD3 | -0.028677311 | 0.34353807 | -0.037001035 | 0.2215967 | 0.01 |
| LOC102724911 | -0.092596344 | 0.0021813 | -0.10799905 | 0.0003477 | 0.02 |
| HECW2 | -0.278156119 | 7.18E-21 | -0.280836167 | 2.93E-21 | 0.01 |
| HEG1 | -0.445245652 | 2.44E-54 | -0.362951698 | 2.27E-35 | 0.09 |
| HELB | -0.216575734 | 4.55E-13 | -0.208947955 | 3.01E-12 | 0.01 |
| LOC220513 | 0.372869713 | 2.21E-37 | 0.203397719 | 1.14E-11 | 0.17 |
| HELQ | -0.291756632 | 6.88E-23 | -0.25073483 | 3.94E-17 | 0.05 |
| HELT | 0.141753318 | 2.54E-06 | 0.02068293 | 0.4945565 | 0.13 |
| HELZ | -0.230533236 | 1.19E-14 | -0.29314119 | 4.22E-23 | 0.07 |
| HEMGN | -0.127169543 | 2.48E-05 | -0.186425737 | 5.27E-10 | 0.06 |
| LOC132224 | -0.255456264 | 9.60E-18 | -0.061471867 | 0.0421648 | 0.2 |
| HEPACAM2 | -0.329881942 | 3.69E-29 | -0.173853029 | 7.24E-09 | 0.16 |
| HEPACAM | -0.220060059 | 1.88E-13 | -0.175417102 | 5.28E-09 | 0.05 |
| HEPHL1 | 0.169290766 | 1.79E-08 | 0.047396204 | 0.1173412 | 0.13 |
| HEPH | -0.071227471 | 0.01851689 | -0.102738696 | 0.0006696 | 0.04 |
| HEPN1 | -0.232945127 | 6.19E-15 | -0.211113385 | 1.77E-12 | 0.03 |
| HERC1 | -0.492914735 | 5.58E-68 | -0.54442711 | 2.30E-85 | 0.06 |
| MN7 | -0.162238274 | 6.92E-08 | -0.171995289 | 1.05E-08 | 0.01 |
| HERC2P4 | -0.099972975 | 0.00093405 | -0.034055863 | 0.2606129 | 0.07 |
| LOC101929832 | -0.412441031 | 3.91E-46 | -0.528490962 | 1.16E-79 | 0.12 |
| KIAA0032 | -0.507298992 | 1.54E-72 | -0.515088174 | 4.24E-75 | 0.01 |
| KIAA1593 | -0.170443092 | 1.43E-08 | -0.152011065 | 4.44E-07 | 0.02 |
| HERC5 | 0.162756294 | 6.28E-08 | -0.036978729 | 0.2218756 | 0.2 |
| LOC101929118 | 0.031938597 | 0.29143973 | -0.106736061 | 0.0004081 | 0.14 |
| HERPUD1 | -0.145357472 | 1.39E-06 | -0.206428063 | 5.53E-12 | 0.07 |
| HERPUD2 | -0.269873614 | 1.08E-19 | -0.239292413 | 1.07E-15 | 0.04 |
| HES1 | -0.032179582 | 0.2878125 | 0.089088419 | 0.0032004 | 0.13 |
| HES2 | -0.085095434 | 0.00487483 | -0.020480213 | 0.4987973 | 0.07 |
| HES3 | 0.114442776 | 0.00014963 | 0.038416283 | 0.204413 | 0.08 |
| HES4 | 0.140117031 | 3.32E-06 | 0.080388862 | 0.0078382 | 0.06 |
| HES5 | -0.043017664 | 0.15525333 | 0.069291914 | 0.0219656 | 0.12 |
| HES6 | 0.325642787 | 2.04E-28 | 0.215393942 | 6.13E-13 | 0.12 |
| HES7 | 0.073001103 | 0.01578208 | -0.017762575 | 0.5574624 | 0.1 |
| ESRG | -0.082833197 | 0.00614228 | 0.000740087 | 0.9805019 | 0.09 |
| HESX1 | 0.110050054 | 0.00026715 | 0.030551457 | 0.3129133 | 0.08 |
| HEXA | -0.079957754 | 0.00817736 | -0.114255081 | 0.0001535 | 0.04 |
| HEXB | -0.14778397 | 9.24E-07 | 0.012268279 | 0.6853693 | 0.17 |
| HEXDC | 0.066031752 | 0.0290409 | 0.007768772 | 0.7975263 | 0.06 |
| HEXIM1 | -0.265464033 | 4.38E-19 | -0.112303511 | 0.000199 | 0.16 |
| HEXIM2 | -0.047937345 | 0.11320914 | 0.024760129 | 0.4134873 | 0.08 |
| LOC220853 | -0.05961972 | 0.0487744 | -0.038304947 | 0.2057287 | 0.03 |
| GRL | 0.015997812 | 0.59727372 | 0.00745959 | 0.8054195 | 0.01 |
| LOC115688 | -0.31540439 | 1.14E-26 | -0.17848867 | 2.82E-09 | 0.14 |
| HFE2 | -0.018462572 | 0.54203648 | -0.000673206 | 0.9822636 | 0.02 |
| HFE | -0.427846021 | 7.11E-50 | -0.20487611 | 8.02E-12 | 0.23 |
| HFM1 | 0.032043247 | 0.28986082 | -0.052197139 | 0.0845514 | 0.09 |
| HGC6.3 | 0.057806563 | 0.0560653 | 0.052660822 | 0.0818219 | 0.01 |
| HGD | -0.219614065 | 2.10E-13 | -0.010040451 | 0.7402129 | 0.21 |
| HGFAC | 0.091813516 | 0.00237876 | 0.065344714 | 0.0307596 | 0.03 |
| DFNB39 | -0.469837901 | 4.17E-61 | -0.328693733 | 5.98E-29 | 0.15 |
| FLJ22242 | -0.135699298 | 6.73E-06 | -0.154010131 | 3.11E-07 | 0.02 |
| HGS | 0.3135585 | 2.31E-26 | 0.154315271 | 2.95E-07 | 0.16 |
| HHATL | -0.068944091 | 0.02264127 | -0.07336049 | 0.0152733 | 0.01 |
| HHAT | -0.43330237 | 3.02E-51 | -0.027401551 | 0.3654422 | 0.41 |
| HHEX | -0.263953976 | 7.04E-19 | -0.179862491 | 2.12E-09 | 0.09 |
| HHIPL1 | -0.030431175 | 0.31482319 | -0.047558398 | 0.1160905 | 0.02 |
| HHIPL2 | 0.039810148 | 0.18845587 | 0.163651937 | 5.30E-08 | 0.13 |
| HHIP | -0.117309791 | 0.00010136 | -0.100764653 | 0.0008499 | 0.02 |
| HHLA1 | 0.099049866 | 0.0010419 | 0.063364831 | 0.036208 | 0.04 |
| HHLA2 | 0.056137666 | 0.06355602 | 0.029104781 | 0.3363899 | 0.03 |
| HHLA3 | 0.266597991 | 3.06E-19 | 0.214878294 | 6.98E-13 | 0.06 |
| HIAT1 | -0.22606866 | 3.92E-14 | -0.324919773 | 2.73E-28 | 0.1 |
| HIATL1 | 0.02048761 | 0.49864224 | -0.117319063 | 0.0001012 | 0.14 |
| HIATL2 | 0.057699841 | 0.05652128 | -0.05250719 | 0.0827183 | 0.12 |
| HIBADH | -0.027123272 | 0.37033338 | 0.039849009 | 0.1880245 | 0.07 |
| HIBCH | -4.04E-05 | 0.99893624 | 0.086579643 | 0.004177 | 0.09 |
| HIC1 | -0.238916777 | 1.19E-15 | -0.150499556 | 5.78E-07 | 0.09 |
| HIC2 | 0.014094455 | 0.64160181 | -0.144190102 | 1.70E-06 | 0.16 |
| FLJ22027 | -0.346665236 | 3.22E-32 | -0.194785126 | 8.32E-11 | 0.16 |
| LOC115049 | -0.027137993 | 0.37007362 | -0.07844482 | 0.009474 | 0.06 |
| HIF3A | 0.014117854 | 0.64104859 | -0.09035038 | 0.0027922 | 0.11 |
| HIGD1A | -0.176977776 | 3.84E-09 | -0.016959227 | 0.5754248 | 0.17 |
| HIGD1B | -0.230629633 | 1.16E-14 | -0.004837219 | 0.8730866 | 0.23 |
| HIGD1C | -0.050820265 | 0.09309191 | -0.016326053 | 0.5897719 | 0.04 |
| HIGD2A | 0.266577389 | 3.08E-19 | 0.336077258 | 2.88E-30 | 0.07 |
| HIGD2B | 0.042204219 | 0.16322117 | 0.103653364 | 0.0005988 | 0.07 |
| HILS1 | 0.092945237 | 0.00209822 | -0.002857304 | 0.9248263 | 0.1 |
| HINFP | 0.207913227 | 3.87E-12 | 0.160875642 | 8.92E-08 | 0.05 |
| HINT1 | 0.264753533 | 5.47E-19 | 0.38080173 | 4.84E-39 | 0.12 |
| HINT2 | 0.182901383 | 1.12E-09 | 0.23511166 | 3.42E-15 | 0.06 |
| HINT3 | -0.081607469 | 0.00694638 | 0.006687909 | 0.825205 | 0.09 |
| HIP1R | 0.044514221 | 0.14136963 | 0.054206797 | 0.0732334 | 0.01 |
| HIP1 | -0.421446827 | 2.69E-48 | -0.340823942 | 3.93E-31 | 0.09 |
| HIPK1 | -0.386333897 | 3.16E-40 | -0.443918897 | 5.46E-54 | 0.06 |
| HIPK2 | -0.243971361 | 2.83E-16 | -0.237431246 | 1.80E-15 | 0.01 |
| HIPK3 | -0.438761421 | 1.21E-52 | -0.4808613 | 2.53E-64 | 0.05 |
| HIPK4 | -0.182431846 | 1.24E-09 | -0.129145797 | 1.85E-05 | 0.06 |
| HIRA | 0.109052332 | 0.00030387 | -0.072378697 | 0.0166984 | 0.19 |
| HIRIP3 | 0.110578887 | 0.00024942 | 0.072493643 | 0.0165258 | 0.04 |
| HIST1H1A | 0.272867895 | 4.09E-20 | 0.11876129 | 8.29E-05 | 0.16 |
| HIST1H1B | 0.373572481 | 1.58E-37 | 0.183458466 | 9.95E-10 | 0.2 |
| HIST1H1C | 0.157892192 | 1.55E-07 | 0.106606418 | 0.0004148 | 0.06 |
| HIST1H1D | 0.031613711 | 0.29637817 | -0.075505556 | 0.0125262 | 0.11 |
| HIST1H1E | 0.209790437 | 2.45E-12 | 0.115298821 | 0.0001333 | 0.1 |
| HIST1H1T | 0.006996674 | 0.81727436 | -0.095131927 | 0.0016402 | 0.11 |
| HIST1H2AA | -0.012082094 | 0.68989455 | -0.034771438 | 0.2507231 | 0.03 |
| HIST1H2AB | 0.296168678 | 1.44E-23 | 0.135079273 | 7.42E-06 | 0.17 |
| HIST1H2AC | -0.091400739 | 0.00248934 | 0.006226192 | 0.8370973 | 0.1 |
| HIST1H2AD | 0.066763241 | 0.02730241 | -0.060923394 | 0.0440382 | 0.13 |
| HIST1H2AE | 0.185970217 | 5.82E-10 | 0.074742757 | 0.0134485 | 0.12 |
| HIST1H2AG | 0.143297825 | 1.97E-06 | -0.106135179 | 0.0004401 | 0.25 |
| HIST1H2AH | 0.407099255 | 6.98E-45 | 0.118010481 | 9.20E-05 | 0.29 |
| HIST1H2AJ | 0.238205077 | 1.45E-15 | 0.091960489 | 0.0023405 | 0.15 |
| HIST1H2AK | -0.040943344 | 0.17617456 | -0.123792241 | 4.07E-05 | 0.09 |
| HIST1H2AL | 0.273219502 | 3.65E-20 | 0.10190166 | 0.0007412 | 0.18 |
| HIST1H2AM | 0.278282507 | 6.89E-21 | 0.143253873 | 1.98E-06 | 0.14 |
| HIST1H2BA | 0.056159106 | 0.06345479 | 0.036137638 | 0.2325746 | 0.03 |
| HIST1H2BB | 0.127601478 | 2.33E-05 | 0.066513882 | 0.0278847 | 0.07 |
| HIST1H2BC | -0.078006232 | 0.00988261 | -0.089089794 | 0.0031999 | 0.02 |
| HIST1H2BD | 0.016221373 | 0.59215959 | 0.047625443 | 0.1155766 | 0.04 |
| HIST1H2BE | 0.220831202 | 1.54E-13 | 0.080001743 | 0.0081422 | 0.15 |
| HIST1H2BF | 0.08125171 | 0.00719681 | -0.097069264 | 0.0013132 | 0.18 |
| HIST1H2BG | 0.070986644 | 0.01891847 | -0.022535459 | 0.4567091 | 0.1 |
| HIST1H2BH | 0.208103093 | 3.70E-12 | 0.094612212 | 0.0017399 | 0.12 |
| HIST1H2BI | 0.2014524 | 1.80E-11 | 0.085615335 | 0.0046192 | 0.12 |
| HIST1H2BJ | 0.26401944 | 6.89E-19 | 0.004156608 | 0.8908228 | 0.26 |
| HIST1H2BK | 0.279475851 | 4.63E-21 | 0.178033304 | 3.09E-09 | 0.11 |
| HIST1H2BL | 0.28374175 | 1.10E-21 | 0.043501652 | 0.1506546 | 0.25 |
| HIST1H2BM | 0.231024364 | 1.04E-14 | 0.086347858 | 0.0042796 | 0.15 |
| MGC9388 | 0.121693685 | 5.49E-05 | 0.043948369 | 0.1465026 | 0.08 |
| HIST1H2BO | 0.309117883 | 1.25E-25 | 0.083227171 | 0.0059022 | 0.23 |
| HIST1H3A | 0.242668226 | 4.10E-16 | 0.010236984 | 0.7353165 | 0.24 |
| HIST1H3B | 0.340232115 | 5.04E-31 | 0.150432063 | 5.85E-07 | 0.19 |
| HIST1H3C | 0.267416575 | 2.36E-19 | 0.093600803 | 0.0019499 | 0.18 |
| HIST1H3D | 0.133731402 | 9.16E-06 | 0.008843101 | 0.7702645 | 0.13 |
| HIST1H3E | -0.130881329 | 1.42E-05 | -0.165011321 | 4.09E-08 | 0.04 |
| HIST1H3F | 0.307399787 | 2.37E-25 | 0.115911719 | 0.0001227 | 0.2 |
| HIST1H3G | 0.219623819 | 2.10E-13 | 0.065327097 | 0.0308048 | 0.16 |
| HIST1H3H | 0.103146365 | 0.00063715 | -0.002007342 | 0.9471486 | 0.11 |
| HIST1H3I | 0.281593255 | 2.27E-21 | 0.142277177 | 2.33E-06 | 0.14 |
| HIST1H3J | 0.229454316 | 1.59E-14 | 0.042614099 | 0.1591687 | 0.19 |
| HIST1H4A | 0.293606985 | 3.58E-23 | 0.19445744 | 8.96E-11 | 0.1 |
| HIST1H4B | 0.299227689 | 4.79E-24 | 0.151060683 | 5.24E-07 | 0.15 |
| HIST1H4C | 0.330876977 | 2.46E-29 | 0.177821276 | 3.23E-09 | 0.16 |
| HIST1H4D | 0.171759866 | 1.10E-08 | 0.02706597 | 0.3713456 | 0.15 |
| HIST1H4E | 0.212876013 | 1.15E-12 | 0.092216462 | 0.0022752 | 0.13 |
| HIST1H4F | 0.204720701 | 8.32E-12 | 0.111020366 | 0.0002355 | 0.1 |
| HIST1H4G | 0.062575123 | 0.03859978 | 0.000692573 | 0.9817534 | 0.07 |
| HIST1H4H | 0.118166207 | 9.01E-05 | 0.075310001 | 0.0127571 | 0.05 |
| HIST1H4I | 0.18606111 | 5.70E-10 | -0.000563638 | 0.98515 | 0.19 |
| HIST1H4J | 0.086000673 | 0.00443767 | 0.122014333 | 5.25E-05 | 0.04 |
| HIST1H4K | 0.013415125 | 0.65774846 | -0.004685189 | 0.8770432 | 0.02 |
| HIST1H4L | 0.185240165 | 6.80E-10 | 0.100999075 | 0.0008263 | 0.09 |
| HIST2H2AA3 | 0.129326441 | 1.80E-05 | 0.108024495 | 0.0003466 | 0.03 |
| HIST2H2AB | 0.223976304 | 6.80E-14 | 0.135671004 | 6.76E-06 | 0.09 |
| H2A | 0.302216588 | 1.61E-24 | 0.292474946 | 5.34E-23 | 0.01 |
| HIST2H2BA | -0.235596411 | 2.99E-15 | -0.322536047 | 7.03E-28 | 0.09 |
| H2B | -0.09355303 | 0.00196039 | -0.09904305 | 0.0010427 | 0.01 |
| HIST2H2BF | -0.10105931 | 0.00082039 | -0.138610535 | 4.23E-06 | 0.04 |
| H3FM | 0.185893018 | 5.91E-10 | 0.092846095 | 0.0021215 | 0.1 |
| HIST2H3D | 0.096080207 | 0.00147179 | 0.007633911 | 0.8009667 | 0.09 |
| H4F2 | 0.106453408 | 0.00042284 | 0.102505769 | 0.0006889 | 0.01 |
| HIST3H2A | 0.118708291 | 8.35E-05 | 0.006795287 | 0.8224449 | 0.12 |
| HIST3H2BB | 0.005880458 | 0.84602679 | -0.044176161 | 0.1444194 | 0.06 |
| HIST3H3 | -0.116716316 | 0.00010995 | -0.024484113 | 0.4187146 | 0.1 |
| HIST4H4 | -0.23124405 | 9.83E-15 | -0.296108084 | 1.47E-23 | 0.07 |
| LOC221701 | -0.228547195 | 2.03E-14 | -0.247877483 | 9.12E-17 | 0.02 |
| HIVEP2 | -0.135282728 | 7.19E-06 | -0.267446287 | 2.34E-19 | 0.14 |
| LOC86368 | -0.34674364 | 3.11E-32 | -0.433516279 | 2.66E-51 | 0.09 |
| HJURP | 0.532887396 | 3.32E-81 | 0.290046722 | 1.25E-22 | 0.25 |
| HK1 | -0.220216905 | 1.80E-13 | -0.197132148 | 4.88E-11 | 0.03 |
| HK2 | -0.093504666 | 0.00197105 | -0.109091044 | 0.0003024 | 0.02 |
| HK3 | 0.180394643 | 1.90E-09 | -0.030916853 | 0.3071584 | 0.22 |
| HKDC1 | 0.058203219 | 0.05439727 | -0.043936291 | 0.1466137 | 0.11 |
| HKR1 | -0.202709445 | 1.34E-11 | -0.063284594 | 0.0364451 | 0.14 |
| LOC100133382 | 0.1695406 | 1.70E-08 | 0.05370399 | 0.075942 | 0.12 |
| AS | 0.143686935 | 1.84E-06 | 0.026793012 | 0.3761906 | 0.12 |
| HLA.C | 0.040194076 | 0.18422622 | -0.017259979 | 0.5686682 | 0.06 |
| HLA.DMA | -0.000285246 | 0.99248435 | -0.043267656 | 0.1528648 | 0.05 |
| HLA.DMB | -0.110849402 | 0.00024078 | -0.168329339 | 2.16E-08 | 0.06 |
| HLA.DOA | -0.202508279 | 1.40E-11 | -0.258831666 | 3.44E-18 | 0.06 |
| HLA.DOB | 0.197007073 | 5.02E-11 | 0.027979628 | 0.3554111 | 0.17 |
| LOC221511 | -0.136897583 | 5.57E-06 | -0.164388596 | 4.61E-08 | 0.03 |
| LOC221510 | -0.089652027 | 0.00301182 | -0.127182566 | 2.48E-05 | 0.04 |
| HLA.DPB2 | 0.029330377 | 0.33265632 | -0.044638048 | 0.140265 | 0.08 |
| GSE | -0.03675807 | 0.22464775 | -0.112576241 | 0.0001919 | 0.08 |
| HLA.DQA2 | -0.052561378 | 0.08240121 | -0.121569085 | 5.59E-05 | 0.07 |
| LOC221518 | 0.02465525 | 0.41546897 | -0.025247798 | 0.4043464 | 0.05 |
| LOC222674 | -0.074693087 | 0.01351055 | -0.095571179 | 0.0015601 | 0.03 |
| HLA.DRA | -0.080275944 | 0.00792576 | -0.135780407 | 6.65E-06 | 0.06 |
| HLA.DRB1 | -0.015289745 | 0.61360204 | -0.06898252 | 0.0225657 | 0.06 |
| HLA.DRB5 | -0.032471389 | 0.28346119 | -0.079959205 | 0.0081762 | 0.05 |
| HLA.DRB6 | -0.003209779 | 0.9155856 | -0.058164403 | 0.0545587 | 0.06 |
| HLA.E | -0.022578938 | 0.45584078 | -0.142911123 | 2.10E-06 | 0.13 |
| HLA.F | 0.189433868 | 2.74E-10 | 0.038392275 | 0.2046962 | 0.16 |
| HLA.G | 0.096307687 | 0.00143382 | -0.034251354 | 0.2578847 | 0.14 |
| HLA.H | 0.124972048 | 3.43E-05 | -0.019058494 | 0.5290738 | 0.15 |
| LOC154452 | 0.006826954 | 0.82163138 | 0.025024869 | 0.4085099 | 0.02 |
| FLJ45422 | 0.041514948 | 0.17021047 | -0.05928969 | 0.0500389 | 0.11 |
| HLCS | -0.156224003 | 2.09E-07 | -0.132518364 | 1.11E-05 | 0.03 |
| HLF | -0.185223495 | 6.83E-10 | -0.134732715 | 7.84E-06 | 0.06 |
| HLTF | -0.061466724 | 0.04218203 | -0.053029173 | 0.0797049 | 0.01 |
| HLX | -0.0106598 | 0.72481906 | -0.021283673 | 0.4821029 | 0.02 |
| HM13 | 0.267652016 | 2.19E-19 | 0.198272517 | 3.76E-11 | 0.07 |
| HMBOX1 | -0.393653222 | 7.88E-42 | -0.224803985 | 5.47E-14 | 0.17 |
| HMBS | 0.491286781 | 1.77E-67 | 0.367634341 | 2.60E-36 | 0.13 |
| ARMD1 | -0.415592401 | 6.96E-47 | -0.273928794 | 2.89E-20 | 0.15 |
| FLJ10739 | -0.18118783 | 1.61E-09 | -0.247000813 | 1.18E-16 | 0.07 |
| HMG20B | 0.135513093 | 6.93E-06 | 0.219839654 | 1.99E-13 | 0.09 |
| HMGA1 | 0.502752533 | 4.50E-71 | 0.347728096 | 2.03E-32 | 0.16 |
| STQTL9 | 0.063053747 | 0.03713466 | -0.026336723 | 0.3843763 | 0.09 |
| HMGB1 | 0.376055106 | 4.82E-38 | 0.345809633 | 4.66E-32 | 0.04 |
| HMGB2 | 0.519683211 | 1.22E-76 | 0.268637673 | 1.60E-19 | 0.26 |
| HMGB3P1 | 0.084683072 | 0.00508658 | 0.05851956 | 0.0530968 | 0.03 |
| HMGB3 | 0.392812685 | 1.21E-41 | 0.254309374 | 1.36E-17 | 0.14 |
| HMGB4 | 0.082297684 | 0.00648268 | 0.036752128 | 0.2247227 | 0.05 |
| HMGCLL1 | -0.045427495 | 0.13337771 | -0.099407492 | 0.0009988 | 0.06 |
| HMGCL | -0.179968582 | 2.07E-09 | -0.025331537 | 0.402789 | 0.16 |
| HMGCR | -0.196546366 | 5.58E-11 | -0.119574258 | 7.40E-05 | 0.08 |
| HMGCS1 | -0.020310707 | 0.50235814 | -0.037221219 | 0.2188574 | 0.02 |
| HMGCS2 | -0.352547291 | 2.46E-33 | -0.050381155 | 0.0959566 | 0.31 |
| HMGN1 | 0.346584432 | 3.33E-32 | 0.358541374 | 1.69E-34 | 0.02 |
| MGC5629 | 0.341459134 | 3.00E-31 | 0.295476353 | 1.84E-23 | 0.05 |
| HMGN3 | 0.353061653 | 1.96E-33 | 0.334740713 | 5.02E-30 | 0.02 |
| HMGN4 | 0.265363415 | 4.52E-19 | 0.082871278 | 0.0061187 | 0.19 |
| HMGN5 | 0.059642141 | 0.04868948 | 0.144499558 | 1.61E-06 | 0.09 |
| HMGXB3 | -0.319810242 | 2.06E-27 | -0.225432118 | 4.64E-14 | 0.1 |
| HMGXB4 | -0.190387337 | 2.22E-10 | -0.174563386 | 6.28E-09 | 0.02 |
| HMHA1 | -0.092787378 | 0.00213545 | -0.142518355 | 2.24E-06 | 0.05 |
| HMHB1 | 0.075548724 | 0.01247569 | 0.064333153 | 0.0334484 | 0.02 |
| HMMR | 0.387435095 | 1.82E-40 | 0.213883584 | 8.94E-13 | 0.18 |
| HMOX1 | 0.059477575 | 0.04931572 | -0.023407965 | 0.4394628 | 0.09 |
| HMOX2 | 0.278739094 | 5.92E-21 | 0.260096139 | 2.33E-18 | 0.02 |
| HMP19 | -0.101773742 | 0.00075278 | -0.09314209 | 0.0020526 | 0.01 |
| HMSD | 0.286700772 | 3.99E-22 | 0.052742452 | 0.0813489 | 0.24 |
| HMX1 | 0.000528548 | 0.98607435 | 0.037880038 | 0.2108064 | 0.04 |
| HMX2 | 0.098035588 | 0.00117359 | 0.078907007 | 0.0090597 | 0.02 |
| HMX3 | 0.01632134 | 0.5898793 | -0.032393085 | 0.2846244 | 0.05 |
| HN1L | 0.044928588 | 0.13769917 | 0.196095294 | 6.19E-11 | 0.16 |
| HN1 | 0.58817907 | 1.06E-102 | 0.4337052 | 2.39E-51 | 0.16 |
| IDDM20 | -0.020265014 | 0.5033203 | 0.007036842 | 0.816244 | 0.03 |
| HNF1B | 0.029763516 | 0.32556311 | 0.032371475 | 0.284946 | 0.01 |
| MODY1 | 0.050927636 | 0.09240196 | 0.008029651 | 0.7908822 | 0.05 |
| HNF4G | 0.011449968 | 0.70534033 | -0.038888204 | 0.198904 | 0.06 |
| HNMT | -0.413256686 | 2.51E-46 | -0.102622719 | 0.0006792 | 0.32 |
| HNRNPA0 | 0.066770483 | 0.02728566 | 0.125748375 | 3.06E-05 | 0.06 |
| HNRNPA1L2 | 0.295134537 | 2.08E-23 | 0.253533627 | 1.71E-17 | 0.05 |
| HNRNPA1 | 0.408645653 | 3.05E-45 | 0.462511916 | 4.95E-59 | 0.06 |
| HNRNPA2B1 | 0.411110272 | 8.06E-46 | 0.33361339 | 8.00E-30 | 0.08 |
| HNRNPA3P1 | 0.466085293 | 4.88E-60 | 0.323867812 | 4.15E-28 | 0.15 |
| HNRNPA3 | 0.399025211 | 4.94E-43 | 0.256144388 | 7.80E-18 | 0.15 |
| HNRNPAB | 0.515838647 | 2.39E-75 | 0.420071482 | 5.80E-48 | 0.1 |
| HNRNPCL1 | 0.042998593 | 0.15543669 | 0.013389432 | 0.6583624 | 0.03 |
| HNRNPC | 0.411457469 | 6.67E-46 | 0.460502843 | 1.80E-58 | 0.05 |
| AUF1 | 0.523167399 | 7.96E-78 | 0.304388418 | 7.27E-25 | 0.22 |
| HNRNPF | 0.112436637 | 0.00019549 | 0.193388694 | 1.14E-10 | 0.09 |
| HNRNPH1 | 0.089186197 | 0.00316692 | 0.027355279 | 0.3662527 | 0.07 |
| HNRNPH2 | -0.366283979 | 4.87E-36 | -0.153062895 | 3.69E-07 | 0.22 |
| DKFZP586F0222 | 0.286239113 | 4.68E-22 | 0.071494543 | 0.0180803 | 0.22 |
| HNRNPK | 0.03872058 | 0.20084825 | 0.077017778 | 0.0108616 | 0.04 |
| LOC91538 | 0.491074284 | 2.06E-67 | 0.39915956 | 4.61E-43 | 0.1 |
| HNRNPM | 0.232305843 | 7.37E-15 | 0.130104066 | 1.60E-05 | 0.11 |
| HNRNPR | 0.094813523 | 0.00170063 | -0.112727226 | 0.0001881 | 0.21 |
| HNRNPUL1 | 0.102145512 | 0.00071967 | -0.056229401 | 0.0631238 | 0.16 |
| HNRNPUL2 | 0.088461587 | 0.00342269 | -0.115421329 | 0.0001311 | 0.21 |
| HNRNPU | 0.20463974 | 8.48E-12 | 0.375593752 | 6.02E-38 | 0.18 |
| HNRNPDL | 0.021932248 | 0.46885111 | 0.031202492 | 0.3027087 | 0.01 |
| HNRNPLL | 0.052767656 | 0.0812033 | -0.12036421 | 6.63E-05 | 0.18 |
| HOMER1 | -0.007371186 | 0.8076801 | -0.126100443 | 2.90E-05 | 0.12 |
| LOC145697 | 0.047721694 | 0.11484187 | -0.05985346 | 0.0478951 | 0.11 |
| HOMER3 | 0.316878623 | 6.45E-27 | 0.266434475 | 3.22E-19 | 0.06 |
| HOMEZ | -0.205221282 | 7.38E-12 | 0.012343413 | 0.6835464 | 0.22 |
| HOOK1 | -0.138150865 | 4.56E-06 | -0.107350429 | 0.0003776 | 0.04 |
| HOOK2 | -0.115907076 | 0.00012278 | -0.029985102 | 0.3219726 | 0.09 |
| LOC286170 | -0.159235065 | 1.21E-07 | -0.234205659 | 4.38E-15 | 0.08 |
| HOPX | -0.114840359 | 0.00014184 | -0.035084504 | 0.2464796 | 0.08 |
| HORMAD1 | 0.372185967 | 3.06E-37 | 0.132181961 | 1.16E-05 | 0.25 |
| HORMAD2 | 0.098345283 | 0.00113184 | 0.007107966 | 0.8144204 | 0.1 |
| HOTAIR | 0.074733484 | 0.01346004 | 0.22419448 | 6.42E-14 | 0.15 |
| LOC221882 | -0.075900317 | 0.01207119 | -0.046546191 | 0.1240693 | 0.03 |
| HOXA11.AS | 0.038155543 | 0.20750388 | 0.061785063 | 0.0411255 | 0.03 |
| HOXA11 | -0.001537053 | 0.95951859 | 0.030091871 | 0.3202518 | 0.04 |
| HOXA13 | -0.133274526 | 9.83E-06 | -0.0542816 | 0.0728373 | 0.08 |
| HOXA1 | 0.02148744 | 0.47791768 | -0.109520449 | 0.0002861 | 0.14 |
| HOXA2 | -0.118498025 | 8.60E-05 | -0.093359307 | 0.0020034 | 0.03 |
| HOXA3 | -0.070432016 | 0.01987235 | -0.056175371 | 0.0633781 | 0.02 |
| HOXA4 | -0.079846956 | 0.00826663 | -0.062254999 | 0.0396069 | 0.02 |
| HOXA5 | -0.195588848 | 6.94E-11 | -0.13976857 | 3.51E-06 | 0.06 |
| HOXA6 | -0.279301381 | 4.90E-21 | -0.261673662 | 1.43E-18 | 0.02 |
| HOXA7 | -0.210883208 | 1.88E-12 | -0.170081311 | 1.53E-08 | 0.05 |
| HOXA9 | -0.151716159 | 4.67E-07 | -0.118302735 | 8.84E-05 | 0.04 |
| HOXB13 | 0.151813809 | 4.59E-07 | 0.17957192 | 2.25E-09 | 0.03 |
| HOXB1 | -0.2021569 | 1.52E-11 | -0.011390524 | 0.7067992 | 0.2 |
| HOXB2 | -0.297884755 | 7.78E-24 | -0.006463884 | 0.8309703 | 0.3 |
| HOXB3 | -0.306019989 | 3.97E-25 | -0.041886075 | 0.1664199 | 0.27 |
| HOXB4 | -0.229947604 | 1.39E-14 | -0.041580072 | 0.1695407 | 0.19 |
| HOXB5 | -0.251932658 | 2.76E-17 | -0.031255907 | 0.3018813 | 0.23 |
| HOXB6 | -0.229900565 | 1.41E-14 | 0.000872326 | 0.9770189 | 0.24 |
| HOXB7 | -0.046488349 | 0.12453783 | 0.11213497 | 0.0002034 | 0.16 |
| HOXB8 | -0.104251389 | 0.00055629 | 0.02262001 | 0.4550214 | 0.13 |
| HOXB9 | 0.052133315 | 0.08493275 | 0.110223707 | 0.0002612 | 0.06 |
| HOXC10 | 0.029340427 | 0.33249063 | 0.199538195 | 2.81E-11 | 0.18 |
| LOC121345 | 0.094928202 | 0.00167864 | 0.261360616 | 1.58E-18 | 0.17 |
| HOXC12 | 0.031496832 | 0.29816839 | 0.036876841 | 0.2231525 | 0.01 |
| HOXC13 | 0.129789836 | 1.68E-05 | 0.234023724 | 4.61E-15 | 0.11 |
| HOX3 | -0.068863016 | 0.02280132 | 0.135794666 | 6.63E-06 | 0.21 |
| HOXC5 | -0.109264039 | 0.0002957 | 0.060438887 | 0.045751 | 0.17 |
| HOXC6 | -0.174436108 | 6.44E-09 | -0.006175838 | 0.8383965 | 0.17 |
| HOXC8 | -0.010639972 | 0.7253102 | 0.07502911 | 0.0130954 | 0.09 |
| HOXC9 | 0.092851308 | 0.0021203 | 0.224317099 | 6.22E-14 | 0.14 |
| HOXD10 | -0.100092354 | 0.00092088 | -0.088870405 | 0.0032762 | 0.02 |
| HOXD11 | 0.094975338 | 0.00166968 | 0.061741436 | 0.041269 | 0.04 |
| LOC150793 | 0.110840687 | 0.00024106 | 0.022844721 | 0.4505531 | 0.09 |
| BDE | 0.207689109 | 4.09E-12 | 0.059580114 | 0.0489247 | 0.15 |
| HOXD1 | -0.224691632 | 5.64E-14 | -0.03416268 | 0.2591198 | 0.2 |
| HOXD3 | -0.229213123 | 1.70E-14 | -0.080718598 | 0.0075873 | 0.15 |
| HOM4 | -0.207240172 | 4.55E-12 | -0.086043851 | 0.0044177 | 0.13 |
| HOXD8 | -0.246209964 | 1.48E-16 | -0.065541624 | 0.0302583 | 0.19 |
| HOXD9 | -0.12799068 | 2.20E-05 | -0.03466136 | 0.2522272 | 0.1 |
| HP1BP3 | -0.212070212 | 1.40E-12 | -0.259398297 | 2.89E-18 | 0.05 |
| HPCAL1 | 0.299471816 | 4.39E-24 | 0.148014179 | 8.88E-07 | 0.16 |
| HPCAL4 | -0.051639235 | 0.0879324 | -0.139515652 | 3.66E-06 | 0.09 |
| HPCA | 0.072008249 | 0.01726572 | -0.05828022 | 0.0540783 | 0.14 |
| HPDL | 0.474735451 | 1.60E-62 | 0.269517417 | 1.21E-19 | 0.21 |
| HPD | 0.021177907 | 0.48428299 | -0.008480416 | 0.779438 | 0.03 |
| HPGDS | -0.351693566 | 3.58E-33 | -0.204169295 | 9.48E-12 | 0.15 |
| HPGD | -0.160146112 | 1.02E-07 | -0.185760738 | 6.08E-10 | 0.03 |
| HPN | -0.242828834 | 3.92E-16 | -0.006605627 | 0.8273215 | 0.24 |
| HPRT1 | 0.516494976 | 1.44E-75 | 0.34686507 | 2.95E-32 | 0.17 |
| HPR | 0.033915948 | 0.26257776 | -0.020047759 | 0.5079084 | 0.06 |
| HPS1 | 0.008205487 | 0.78641251 | -0.022854303 | 0.4503631 | 0.04 |
| HPS3 | -0.134619743 | 7.98E-06 | -0.133275934 | 9.83E-06 | 0.01 |
| HPS4 | -0.223615904 | 7.47E-14 | -0.228552171 | 2.03E-14 | 0.01 |
| HPS5 | -0.162249446 | 6.90E-08 | -0.241600665 | 5.56E-16 | 0.08 |
| LOC143296 | 0.031109743 | 0.30414881 | 0.048961505 | 0.105703 | 0.02 |
| UFS | -0.182220111 | 1.29E-09 | -0.173058362 | 8.49E-09 | 0.01 |
| HPSE | 0.104673066 | 0.00052804 | -0.035649677 | 0.2389467 | 0.15 |
| HPVC1 | 0.014890469 | 0.62289533 | -0.009914565 | 0.7433548 | 0.03 |
| HPX | -0.326032973 | 1.75E-28 | 0.071768749 | 0.0176414 | 0.4 |
| HPYR1 | -0.084700184 | 0.00507763 | -0.055761011 | 0.0653561 | 0.03 |
| HP | -0.03970469 | 0.1896301 | -0.025727088 | 0.3954813 | 0.02 |
| HRASLS2 | -0.223951925 | 6.84E-14 | 0.009169449 | 0.7620373 | 0.24 |
| HRASLS5 | -0.240298845 | 8.04E-16 | -0.168501845 | 2.09E-08 | 0.08 |
| A.C1 | 0.284599327 | 8.20E-22 | 0.155990891 | 2.18E-07 | 0.13 |
| HRAS | 0.312747513 | 3.15E-26 | 0.2163557 | 4.81E-13 | 0.1 |
| HRCT1 | 0.235729585 | 2.88E-15 | 0.040792505 | 0.177774 | 0.2 |
| HRC | -0.279694866 | 4.30E-21 | -0.13399068 | 8.80E-06 | 0.15 |
| HRG | 0.0565324 | 0.06171359 | -0.01478009 | 0.6254751 | 0.08 |
| HRH1 | -0.159872445 | 1.07E-07 | -0.129716177 | 1.69E-05 | 0.04 |
| HRH2 | -0.272348289 | 4.84E-20 | -0.354506302 | 1.03E-33 | 0.09 |
| HRH3 | 0.227101724 | 2.98E-14 | 0.038980951 | 0.1978342 | 0.19 |
| HRH4 | -0.106898836 | 0.00039976 | -0.187985814 | 3.76E-10 | 0.09 |
| HRK | 0.427196914 | 1.03E-49 | 0.199662084 | 2.73E-11 | 0.23 |
| LOC100130312 | 0.157181791 | 1.76E-07 | -0.013746484 | 0.649852 | 0.18 |
| HRNR | -0.24519962 | 1.98E-16 | -0.232888193 | 6.29E-15 | 0.02 |
| LOC137671 | 0.45320676 | 1.81E-56 | 0.376136172 | 4.64E-38 | 0.08 |
| AU | 0.244382484 | 2.51E-16 | 0.064620353 | 0.0326653 | 0.18 |
| HS1BP3 | 0.14893893 | 7.58E-07 | 0.064016819 | 0.0343295 | 0.09 |
| HS2ST1 | -0.020684273 | 0.49452844 | -0.123669054 | 4.14E-05 | 0.11 |
| HS3ST1 | 0.067027257 | 0.02669734 | 0.011259807 | 0.710011 | 0.06 |
| HS3ST2 | -0.039635831 | 0.19039973 | -0.02175648 | 0.4724224 | 0.02 |
| HS3ST3A1 | -0.048607314 | 0.108253 | -0.135165965 | 7.32E-06 | 0.09 |
| HS3ST3B1 | -0.260395241 | 2.13E-18 | -0.385539088 | 4.69E-40 | 0.13 |
| HS3ST4 | -0.019366012 | 0.52244673 | -0.083063411 | 0.006001 | 0.07 |
| NBLA04021 | 0.044148484 | 0.1446713 | 0.002429526 | 0.9360546 | 0.05 |
| HS3ST6 | 0.143291478 | 1.97E-06 | 0.041576873 | 0.1695735 | 0.11 |
| HS6ST1 | 0.077579938 | 0.01029475 | -0.077919603 | 0.0099652 | 0.16 |
| HS6ST2 | -0.012659051 | 0.67590832 | 0.050309969 | 0.0964276 | 0.07 |
| HS6ST3 | -0.196648579 | 5.45E-11 | -0.046663224 | 0.1231255 | 0.15 |
| HSBP1L1 | 0.297237613 | 9.82E-24 | 0.184626616 | 7.76E-10 | 0.12 |
| HSBP1 | 0.307870015 | 1.99E-25 | 0.24497059 | 2.12E-16 | 0.07 |
| HSCB | 0.082055283 | 0.00664226 | 0.145392227 | 1.39E-06 | 0.07 |
| HSD11B1L | 0.217289838 | 3.80E-13 | 0.213572549 | 9.66E-13 | 0.01 |
| HSD11B1 | -0.037405098 | 0.21658849 | -0.13585016 | 6.57E-06 | 0.1 |
| HSD11B2 | 0.145688007 | 1.32E-06 | 0.10861609 | 0.0003214 | 0.04 |
| MRXS10 | 0.486781087 | 4.22E-66 | 0.412225234 | 4.40E-46 | 0.08 |
| HSD17B11 | -0.266764121 | 2.90E-19 | -0.219860005 | 1.97E-13 | 0.05 |
| HSD17B12 | 0.060719397 | 0.04475267 | -0.01356651 | 0.6541361 | 0.08 |
| HSD17B13 | -0.196945775 | 5.10E-11 | -0.166877497 | 2.86E-08 | 0.04 |
| HSD17B14 | -0.041769991 | 0.16759864 | -0.067107088 | 0.0265167 | 0.03 |
| HSD17B1 | -0.033798319 | 0.2642375 | -0.02406122 | 0.4267985 | 0.01 |
| HSD17B2 | 0.18021053 | 1.97E-09 | 0.135775567 | 6.65E-06 | 0.05 |
| HSD17B3 | 0.017131561 | 0.57154859 | -0.064191514 | 0.0338405 | 0.09 |
| HSD17B4 | -0.346076583 | 4.15E-32 | -0.099937275 | 0.000938 | 0.25 |
| HSD17B6 | 0.007901834 | 0.79413559 | 0.049759306 | 0.1001336 | 0.05 |
| HSD17B7P2 | -0.193345291 | 1.15E-10 | -0.006603148 | 0.8273853 | 0.19 |
| HSD17B7 | -0.041868089 | 0.1666021 | 0.217284641 | 3.81E-13 | 0.26 |
| HSD17B8 | -0.194319197 | 9.25E-11 | 0.023681503 | 0.4341339 | 0.22 |
| HSD3B1 | -0.051330886 | 0.08984729 | -0.030073847 | 0.3205419 | 0.03 |
| HSD3B2 | -0.071086072 | 0.01875176 | -0.092359812 | 0.0022393 | 0.03 |
| HSD3B7 | -0.037798145 | 0.21179538 | 0.043536301 | 0.1503293 | 0.09 |
| HSDL1 | -0.026613621 | 0.37939593 | -0.014330977 | 0.636019 | 0.02 |
| HSDL2 | -0.218353545 | 2.90E-13 | 0.000577428 | 0.9847867 | 0.22 |
| LOC642255 | 0.411981853 | 5.02E-46 | 0.172269206 | 9.94E-09 | 0.24 |
| HSF2BP | 0.085403883 | 0.00472169 | 0.145421526 | 1.38E-06 | 0.07 |
| HSF2 | 0.182460965 | 1.23E-09 | 0.089158285 | 0.0031764 | 0.1 |
| CTM | -0.020014805 | 0.5086062 | 0.04445136 | 0.141933 | 0.07 |
| HSF5 | -0.081072304 | 0.00732615 | -0.194246479 | 9.40E-11 | 0.12 |
| HSFX1 | 0.026001351 | 0.39046159 | 0.051611393 | 0.0881039 | 0.03 |
| HSFX2 | -0.241262379 | 6.12E-16 | -0.193686072 | 1.07E-10 | 0.05 |
| LOC140022 | 0.024629397 | 0.41595833 | 0.006461918 | 0.8310209 | 0.02 |
| HSFY1P1 | -0.009587427 | 0.75153939 | 0.012258068 | 0.6856172 | 0.03 |
| HSH2D | -0.119982399 | 6.99E-05 | -0.045271378 | 0.1347186 | 0.08 |
| HSN2 | -0.062104619 | 0.04008758 | -0.190165272 | 2.33E-10 | 0.13 |
| HSPCAL4 | 0.211603666 | 1.57E-12 | 0.179194312 | 2.43E-09 | 0.04 |
| LOC154342 | 0.330293382 | 3.12E-29 | 0.191611862 | 1.69E-10 | 0.14 |
| HSP90AB2P | 0.060677091 | 0.04490206 | -0.033987601 | 0.2615703 | 0.1 |
| HSP90AB4P | 0.206121906 | 5.95E-12 | 0.206431086 | 5.53E-12 | 0.01 |
| HSP90B1 | 0.31410525 | 1.88E-26 | 0.169435669 | 1.74E-08 | 0.15 |
| TRAP2 | 0.084942176 | 0.00495258 | 0.017618365 | 0.5606667 | 0.07 |
| HSPA12A | -0.32572714 | 1.97E-28 | -0.22730689 | 2.83E-14 | 0.1 |
| HSPA12B | -0.32933295 | 4.61E-29 | -0.194510299 | 8.86E-11 | 0.14 |
| HSPA13 | -0.1354609 | 6.99E-06 | -0.204058381 | 9.74E-12 | 0.07 |
| HSPA14 | 0.39475476 | 4.48E-42 | 0.192301727 | 1.45E-10 | 0.21 |
| HSPA1A | 0.074980059 | 0.01315532 | 0.076310663 | 0.0116138 | 0.01 |
| HSPA1B | 0.232146166 | 7.69E-15 | 0.203368391 | 1.15E-11 | 0.03 |
| HSPA1L | -0.166508864 | 3.07E-08 | -0.001766226 | 0.9534893 | 0.17 |
| HSPA2 | -0.22006228 | 1.87E-13 | -0.09110839 | 0.0025705 | 0.13 |
| HSPA4L | 0.090768451 | 0.00266785 | -0.136522877 | 5.91E-06 | 0.23 |
| HSPA4 | -0.01605804 | 0.59589399 | 0.082343773 | 0.0064527 | 0.1 |
| HSPA5 | 0.148384319 | 8.34E-07 | 0.027697505 | 0.3602848 | 0.13 |
| HSPA6 | 0.073174492 | 0.0155348 | 0.047770792 | 0.1144685 | 0.03 |
| HSPA7 | -0.033899775 | 0.26280554 | -0.086995119 | 0.0039986 | 0.06 |
| HSPA10 | 0.020616571 | 0.49594258 | -0.021331394 | 0.4811209 | 0.05 |
| LOC91471 | 0.06148374 | 0.04212498 | 0.065103838 | 0.0313824 | 0.01 |
| HSPB11 | 0.371568058 | 4.10E-37 | 0.263704251 | 7.61E-19 | 0.11 |
| CMT2F | 0.042686953 | 0.15845638 | 0.170525272 | 1.40E-08 | 0.13 |
| HSPB2 | -0.053602838 | 0.07649668 | -0.054954981 | 0.0693496 | 0.01 |
| LOC115842 | 0.140241176 | 3.25E-06 | -0.005127567 | 0.8655392 | 0.15 |
| HSPB6 | -0.240092736 | 8.52E-16 | -0.133911556 | 8.91E-06 | 0.11 |
| HSPB7 | -0.294722197 | 2.41E-23 | -0.212275372 | 1.33E-12 | 0.09 |
| LOC643737 | -0.157923932 | 1.54E-07 | -0.149098596 | 7.37E-07 | 0.01 |
| HSPB9 | 0.114947135 | 0.00013981 | 0.090102322 | 0.0028685 | 0.03 |
| HSPBAP1 | 0.251484101 | 3.15E-17 | 0.199742776 | 2.68E-11 | 0.06 |
| HSPBP1 | 0.343889418 | 1.06E-31 | 0.306730472 | 3.05E-25 | 0.04 |
| LINC00652 | 0.191500896 | 1.74E-10 | 0.165531443 | 3.71E-08 | 0.03 |
| LINC00339 | 0.135050335 | 7.45E-06 | 0.04924996 | 0.1036617 | 0.09 |
| LGALSL | 0.229369749 | 1.63E-14 | 0.00812054 | 0.788571 | 0.23 |
| HSPD1 | 0.482107236 | 1.07E-64 | 0.298722056 | 5.75E-24 | 0.19 |
| HSPE1 | 0.560174049 | 2.63E-91 | 0.507526277 | 1.30E-72 | 0.06 |
| HSPG2 | -0.42328352 | 9.55E-49 | -0.369508164 | 1.08E-36 | 0.06 |
| KIAA0201 | 0.190143861 | 2.34E-10 | 0.108171019 | 0.0003402 | 0.09 |
| HTATIP2 | 0.302412787 | 1.50E-24 | 0.134588416 | 8.01E-06 | 0.17 |
| HTATSF1 | 0.307622853 | 2.18E-25 | 0.086940522 | 0.0040216 | 0.23 |
| HCCAT5 | -0.005887021 | 0.84585711 | 0.037517635 | 0.2152082 | 0.05 |
| HTN1 | 0.190868406 | 2.00E-10 | 0.055909889 | 0.0646397 | 0.14 |
| HTN3 | 0.135617583 | 6.82E-06 | 0.081062988 | 0.0073329 | 0.06 |
| HTR1A | -0.121575627 | 5.58E-05 | -0.115554422 | 0.0001288 | 0.01 |
| HTR1B | -0.159022178 | 1.26E-07 | -0.153003938 | 3.72E-07 | 0.01 |
| HTR1D | 0.028430678 | 0.34770601 | -0.101820063 | 0.0007486 | 0.14 |
| HTR1E | -0.055278355 | 0.06772403 | -0.070054254 | 0.0205458 | 0.02 |
| HTR1F | -0.344560039 | 7.98E-32 | -0.285397415 | 6.24E-22 | 0.06 |
| HTR2A | -0.186470251 | 5.22E-10 | -0.241264265 | 6.12E-16 | 0.06 |
| HTR2B | -0.379748349 | 8.09E-39 | -0.224448401 | 6.01E-14 | 0.16 |
| HTR2C | 0.119724813 | 7.25E-05 | 0.006988252 | 0.8174904 | 0.12 |
| HTR3A | 0.182108058 | 1.32E-09 | 0.055901759 | 0.0646786 | 0.13 |
| HTR3B | 0.154132556 | 3.05E-07 | 0.057514127 | 0.0573221 | 0.1 |
| HTR3C | 0.101371854 | 0.00079015 | 0.041553331 | 0.1698155 | 0.06 |
| HTR3D | 0.035574281 | 0.2399421 | 0.026903463 | 0.3742254 | 0.01 |
| HTR3E | 0.189550931 | 2.67E-10 | 0.038167736 | 0.2073586 | 0.16 |
| HTR4 | -0.147982501 | 8.93E-07 | -0.095568605 | 0.0015606 | 0.06 |
| HTR5A | 0.097681487 | 0.00122307 | 0.038820191 | 0.1996912 | 0.06 |
| HTR6 | 0.266589087 | 3.07E-19 | 0.031948864 | 0.2912846 | 0.24 |
| HTR7P1 | -0.233234276 | 5.72E-15 | -0.164517203 | 4.50E-08 | 0.07 |
| HTR7 | -0.397719623 | 9.73E-43 | -0.307825114 | 2.03E-25 | 0.09 |
| HTRA1 | -0.32438591 | 3.37E-28 | -0.133881041 | 8.95E-06 | 0.2 |
| HTRA2 | 0.407023601 | 7.27E-45 | 0.341115701 | 3.47E-31 | 0.07 |
| HTRA3 | -0.176364394 | 4.36E-09 | -0.037978724 | 0.2096191 | 0.14 |
| HTRA4 | 0.089258042 | 0.00314254 | -0.010831041 | 0.7205821 | 0.11 |
| HD | -0.292912041 | 4.58E-23 | -0.313704445 | 2.19E-26 | 0.03 |
| HULC | 0.147074624 | 1.04E-06 | 0.141568391 | 2.62E-06 | 0.01 |
| HUNK | -0.125067494 | 3.38E-05 | -0.075603269 | 0.0124122 | 0.05 |
| HUS1B | 0.093536017 | 0.00196413 | 0.062766512 | 0.0380081 | 0.04 |
| HUS1 | 0.050581015 | 0.09464414 | 0.102507863 | 0.0006887 | 0.06 |
| FLJ20030 | -0.138175951 | 4.54E-06 | -0.267839088 | 2.06E-19 | 0.13 |
| HVCN1 | -0.011088459 | 0.71422902 | -0.102539917 | 0.000686 | 0.1 |
| HYAL1 | -0.097845432 | 0.00119993 | -0.1235664 | 4.20E-05 | 0.03 |
| HYAL2 | -0.054746025 | 0.07041689 | 0.003884795 | 0.8979219 | 0.06 |
| HYAL3 | 0.244146642 | 2.69E-16 | 0.172546123 | 9.41E-09 | 0.08 |
| HYAL4 | 0.046812818 | 0.12192725 | -0.064122474 | 0.034033 | 0.12 |
| HYALP1 | -0.03497817 | 0.24791524 | -0.036709275 | 0.2252641 | 0.01 |
| HYDIN | -0.207807981 | 3.97E-12 | -0.127919588 | 2.22E-05 | 0.08 |
| HYI | 0.020868827 | 0.49068451 | 0.051963267 | 0.0859557 | 0.04 |
| HYLS1 | 0.371576124 | 4.08E-37 | 0.319035785 | 2.79E-27 | 0.06 |
| HYMAI | 0.156655667 | 1.94E-07 | 0.003207139 | 0.9156548 | 0.16 |
| HYOU1 | 0.103758908 | 0.00059107 | -0.064564175 | 0.0328173 | 0.17 |
| LOC129644 | 0.327987718 | 7.95E-29 | 0.314259962 | 1.77E-26 | 0.02 |
| IAPP | 0.095788579 | 0.00152181 | 0.102239831 | 0.0007115 | 0.01 |
| IARS2 | -0.072430151 | 0.01662096 | 0.241812734 | 5.24E-16 | 0.32 |
| IARS | 0.042988901 | 0.15552995 | -0.043594175 | 0.1497873 | 0.09 |
| IBSP | 0.098296173 | 0.00113836 | -0.003147069 | 0.9172288 | 0.11 |
| IBTK | -0.380054656 | 6.97E-39 | -0.243668413 | 3.08E-16 | 0.14 |
| ALS2CR14 | -0.067569676 | 0.02549045 | -0.202446898 | 1.42E-11 | 0.14 |
| ICA1 | -0.200163569 | 2.43E-11 | 0.120805458 | 6.23E-05 | 0.33 |
| ICAM1 | 0.074246974 | 0.01407958 | -0.009697845 | 0.7487737 | 0.09 |
| ICAM2 | 0.076042044 | 0.01191145 | -4.22E-05 | 0.9988892 | 0.08 |
| ICAM3 | 0.068310869 | 0.02391766 | 0.047419347 | 0.1171621 | 0.03 |
| LW | 0.09622827 | 0.00144697 | -0.017858925 | 0.5553265 | 0.12 |
| ICAM5 | 0.067046012 | 0.0266548 | -0.061760709 | 0.0412056 | 0.13 |
| ICK | -0.176075139 | 4.62E-09 | -0.184874561 | 7.36E-10 | 0.01 |
| ICMT | -0.011906983 | 0.69416076 | -0.145260504 | 1.42E-06 | 0.14 |
| ICOSLG | 0.078581828 | 0.00934942 | -0.035604994 | 0.2395363 | 0.12 |
| ICOS | 0.111289295 | 0.00022733 | -0.044784995 | 0.1389627 | 0.16 |
| ICT1 | 0.43834647 | 1.55E-52 | 0.369477764 | 1.10E-36 | 0.07 |
| ID1 | 0.003524675 | 0.90733971 | -0.027461201 | 0.3643991 | 0.04 |
| ID2B | 0.061828521 | 0.04098305 | 0.083119571 | 0.0059669 | 0.03 |
| ID2 | 0.104958582 | 0.00050967 | 0.054560145 | 0.0713776 | 0.06 |
| ID3 | -0.076246257 | 0.01168457 | -0.046445069 | 0.1248893 | 0.03 |
| ID4 | 0.039658593 | 0.19014506 | -0.078982574 | 0.0089935 | 0.12 |
| IDE | -0.297380499 | 9.33E-24 | -0.277191991 | 9.89E-21 | 0.03 |
| IDH1 | -0.131991679 | 1.20E-05 | -0.008007559 | 0.7914443 | 0.13 |
| IDH2 | 0.267542424 | 2.27E-19 | 0.218598795 | 2.73E-13 | 0.05 |
| IDH3A | 0.064309912 | 0.03351249 | -0.103593026 | 0.0006032 | 0.17 |
| IDH3B | 0.28589577 | 5.26E-22 | 0.232198991 | 7.58E-15 | 0.06 |
| IDH3G | 0.341255413 | 3.27E-31 | 0.216010037 | 5.25E-13 | 0.13 |
| IDI1 | 0.167925221 | 2.34E-08 | 0.096747301 | 0.001363 | 0.08 |
| IDI2 | 0.416747523 | 3.68E-47 | 0.306928545 | 2.83E-25 | 0.11 |
| IDO | 0.208867393 | 3.07E-12 | 0.008891036 | 0.7690544 | 0.2 |
| IDO2 | 0.081599729 | 0.00695174 | -0.044893251 | 0.1380093 | 0.13 |
| IDS | -0.385994913 | 3.74E-40 | -0.407797925 | 4.80E-45 | 0.03 |
| IDUA | -0.180140047 | 2.00E-09 | -0.001050806 | 0.9723186 | 0.18 |
| IER2 | 0.033884771 | 0.26301697 | 0.071533391 | 0.0180175 | 0.04 |
| PRO2309 | 0.426567534 | 1.48E-49 | 0.302743886 | 1.33E-24 | 0.13 |
| LOC116252 | -0.090435031 | 0.00276662 | 0.016809625 | 0.5787997 | 0.11 |
| IER5L | 0.098188072 | 0.00115286 | 0.132004552 | 1.20E-05 | 0.04 |
| IER5 | 0.114512105 | 0.00014825 | 0.15949342 | 1.15E-07 | 0.05 |
| IFFO1 | -0.194268203 | 9.35E-11 | -0.178503407 | 2.81E-09 | 0.02 |
| IFFO2 | 0.073778221 | 0.01470015 | -0.226214371 | 3.77E-14 | 0.3 |
| IFI16 | -0.03211152 | 0.28883385 | -0.133633295 | 9.30E-06 | 0.11 |
| IFI27L1 | 0.275567858 | 1.69E-20 | 0.165115853 | 4.01E-08 | 0.12 |
| IFI27L2 | 0.051765105 | 0.08716024 | 0.044640573 | 0.1402425 | 0.01 |
| IFI27 | 0.155333211 | 2.46E-07 | 0.119165335 | 7.84E-05 | 0.04 |
| IFI30 | 0.215940159 | 5.34E-13 | 0.097014582 | 0.0013215 | 0.12 |
| IFI35 | -0.023417357 | 0.43927924 | 0.001289151 | 0.9660433 | 0.03 |
| LOC100129349 | 0.077505577 | 0.01036819 | -0.023957038 | 0.4288039 | 0.11 |
| IFI44 | 0.176527442 | 4.21E-09 | 0.039162197 | 0.1957557 | 0.14 |
| IFI6 | 0.14100048 | 2.87E-06 | 0.058303211 | 0.0539834 | 0.09 |
| IFIH1 | 0.083291534 | 0.0058638 | -0.031498656 | 0.2981404 | 0.12 |
| LOC387699 | 0.09059062 | 0.00272013 | 0.000843473 | 0.9777788 | 0.09 |
| IFIT1 | 0.027967527 | 0.35561934 | -0.014771255 | 0.6256818 | 0.05 |
| IFIT2 | -0.030658113 | 0.31122624 | -0.071530068 | 0.0180229 | 0.05 |
| IFIT3 | 0.028323007 | 0.3495356 | -0.043923504 | 0.1467314 | 0.08 |
| IFIT5 | -0.062679697 | 0.03827555 | -0.111862473 | 0.0002109 | 0.05 |
| IFITM1 | 0.055456656 | 0.06684122 | 0.07734455 | 0.0105288 | 0.03 |
| IFITM2 | -0.219615126 | 2.10E-13 | 0.002877065 | 0.9243079 | 0.23 |
| IFITM3 | 0.12945646 | 1.76E-05 | 0.148426906 | 8.27E-07 | 0.02 |
| LOC221559 | 0.029551336 | 0.32902547 | 0.051842563 | 0.0866878 | 0.03 |
| IFITM5 | -0.085410182 | 0.00471861 | 0.03170562 | 0.2949755 | 0.12 |
| LMNTD1 | -0.048867391 | 0.1063759 | -0.011224193 | 0.710887 | 0.04 |
| IFNA10 | 0.177270203 | 3.62E-09 | 0.08660349 | 0.0041666 | 0.1 |
| IFNA13 | 0.0181991 | 0.54781762 | -0.021284887 | 0.4820778 | 0.04 |
| IFNA14 | -0.064495892 | 0.03300274 | -0.025898031 | 0.392348 | 0.04 |
| IFNA16 | -0.02588189 | 0.39264326 | -0.037062809 | 0.2208257 | 0.02 |
| IFNA17 | 0.040267055 | 0.18343023 | -0.001061547 | 0.9720358 | 0.05 |
| LOC89955 | 0.051458439 | 0.08905114 | -0.010392979 | 0.7314377 | 0.07 |
| IFNA21 | -0.094137263 | 0.00183578 | -0.06677027 | 0.0272861 | 0.03 |
| LOC116281 | -0.066470053 | 0.02798814 | -0.044862765 | 0.1382773 | 0.03 |
| INFA4 | 0.130495576 | 1.51E-05 | 0.055020096 | 0.0690197 | 0.08 |
| LOC115837 | 0.017158494 | 0.57094392 | 0.042181146 | 0.1634516 | 0.03 |
| LOC116277 | 8.40E-05 | 0.99778566 | -0.01878271 | 0.5350531 | 0.02 |
| IFNA7 | 0.014617129 | 0.62929231 | -0.021443908 | 0.4788101 | 0.04 |
| LOC89954 | 0.008532707 | 0.77811349 | -0.039088596 | 0.1965978 | 0.05 |
| IFNAR1 | -0.302302036 | 1.56E-24 | -0.208924018 | 3.03E-12 | 0.1 |
| IFNAR2 | 0.079286156 | 0.00873196 | -0.154183984 | 3.02E-07 | 0.24 |
| IFNB1 | 0.228236699 | 2.21E-14 | 0.119825978 | 7.15E-05 | 0.11 |
| IFNE | 0.005505957 | 0.85572158 | -0.047735444 | 0.1147372 | 0.06 |
| IFNGR1 | -0.07959609 | 0.00847198 | -0.066728591 | 0.0273827 | 0.02 |
| IFNGR2 | 0.039073647 | 0.19676919 | 0.111847257 | 0.0002113 | 0.08 |
| IFNG | 0.164040318 | 4.92E-08 | -0.000512864 | 0.9864875 | 0.17 |
| IFNK | 0.021688184 | 0.47381408 | -0.005710679 | 0.8504191 | 0.03 |
| IFNW1 | -0.098972661 | 0.00105142 | -0.04604117 | 0.1282068 | 0.06 |
| IFRD1 | 0.389691055 | 5.88E-41 | 0.156726855 | 1.91E-07 | 0.24 |
| IFRD2 | 0.360083538 | 8.40E-35 | 0.281689083 | 2.20E-21 | 0.08 |
| IFT122 | -0.109711607 | 0.00027911 | 0.060080418 | 0.0470541 | 0.17 |
| IFT140 | -0.282356344 | 1.76E-21 | -0.098682368 | 0.0010879 | 0.19 |
| IFT172 | -0.231865075 | 8.30E-15 | -0.073903952 | 0.0145314 | 0.16 |
| IFT20 | 0.152442696 | 4.11E-07 | 0.29896711 | 5.27E-24 | 0.15 |
| IFT27 | -0.058430749 | 0.05345926 | 0.061003917 | 0.0437588 | 0.12 |
| LOC115235 | -0.215429311 | 6.08E-13 | -0.060573625 | 0.0452692 | 0.16 |
| IFT52 | 0.050813601 | 0.09313487 | 0.078095696 | 0.009798 | 0.03 |
| IFT57 | 0.148783074 | 7.78E-07 | 0.04530171 | 0.1344572 | 0.11 |
| IFT74 | -0.067670845 | 0.02527064 | 0.058890257 | 0.051606 | 0.13 |
| IFT80 | -0.124637865 | 3.60E-05 | -0.152230538 | 4.27E-07 | 0.03 |
| MGC4027 | -0.093419664 | 0.0019899 | -0.077385633 | 0.0104876 | 0.02 |
| IFT88 | -0.290360989 | 1.12E-22 | -0.02650426 | 0.3813582 | 0.27 |
| IGBP1 | -0.238746618 | 1.24E-15 | -0.025066877 | 0.4077234 | 0.22 |
| IGDCC3 | -0.220321115 | 1.75E-13 | -0.152114032 | 4.36E-07 | 0.07 |
| IGDCC4 | -0.311414763 | 5.24E-26 | -0.285758059 | 5.52E-22 | 0.03 |
| LOC51049 | -0.346755691 | 3.10E-32 | -0.251780149 | 2.89E-17 | 0.1 |
| IGF1 | -0.430926159 | 1.20E-50 | -0.273200893 | 3.67E-20 | 0.16 |
| IGF2.AS | -0.226271535 | 3.72E-14 | -0.07560225 | 0.0124133 | 0.16 |
| IGF2BP1 | 0.105481452 | 0.00047757 | 0.007972238 | 0.7923431 | 0.1 |
| IGF2BP2 | 0.251023668 | 3.62E-17 | -0.000867142 | 0.9771554 | 0.26 |
| IGF2BP3 | 0.336305315 | 2.62E-30 | -0.015049399 | 0.6191889 | 0.36 |
| IGF2R | -0.268142677 | 1.87E-19 | -0.28249383 | 1.68E-21 | 0.02 |
| IGF2 | -0.318336915 | 3.66E-27 | -0.136467899 | 5.96E-06 | 0.19 |
| IGFALS | -0.275346426 | 1.82E-20 | -0.05357923 | 0.0766266 | 0.23 |
| IGFBP1 | -0.054017167 | 0.07424555 | -0.056911191 | 0.0599873 | 0.01 |
| IGFBP2 | -0.057528465 | 0.05725995 | 0.074007386 | 0.0143938 | 0.14 |
| IGFBP3 | 0.023156396 | 0.44439674 | -0.053996249 | 0.0743579 | 0.08 |
| IGFBP4 | -0.327139897 | 1.12E-28 | -0.170879983 | 1.31E-08 | 0.16 |
| IGFBP5 | -0.215101312 | 6.60E-13 | -0.049197514 | 0.1040305 | 0.17 |
| IGFBP6 | -0.186864627 | 4.79E-10 | -0.144407031 | 1.64E-06 | 0.05 |
| IGFBP7 | -0.201930692 | 1.61E-11 | -0.110309082 | 0.0002583 | 0.1 |
| IGFBPL1 | 0.066373083 | 0.02821818 | -0.028411545 | 0.3480307 | 0.1 |
| IGFL1 | 0.107080253 | 0.00039071 | -0.057820087 | 0.0560077 | 0.17 |
| IGFL2 | -0.005577286 | 0.85387335 | 0.007899297 | 0.7942002 | 0.02 |
| IGFL3 | -0.01211308 | 0.68914065 | 0.05039328 | 0.0958766 | 0.07 |
| IGFL4 | -0.130134642 | 1.59E-05 | -0.109849566 | 0.0002742 | 0.03 |
| IGFN1 | -0.242803769 | 3.95E-16 | -0.142088322 | 2.40E-06 | 0.11 |
| IGHMBP2 | -0.031263148 | 0.3017693 | -0.099525901 | 0.0009849 | 0.07 |
| JCHAIN | -0.084183372 | 0.00535428 | -0.076076415 | 0.011873 | 0.01 |
| IGLL1 | 0.050766742 | 0.09343738 | -0.025302207 | 0.4033341 | 0.08 |
| IGLL3P | 0.024608644 | 0.41635138 | 0.054234216 | 0.073088 | 0.03 |
| IGLON5 | 0.113530108 | 0.00016907 | -0.016522029 | 0.5853137 | 0.14 |
| IGSF10 | -0.237910176 | 1.57E-15 | -0.258048538 | 4.37E-18 | 0.03 |
| IGSF11 | 0.024837641 | 0.41202626 | -0.120994333 | 6.06E-05 | 0.15 |
| IGCD1 | -0.069663224 | 0.02126378 | -0.142162496 | 2.37E-06 | 0.08 |
| IGSF21 | -0.238603543 | 1.29E-15 | -0.081769874 | 0.0068347 | 0.16 |
| IGSF22 | -0.330584893 | 2.77E-29 | -0.096509821 | 0.0014008 | 0.24 |
| IGSF3 | -0.15828298 | 1.44E-07 | -0.035137827 | 0.2457619 | 0.13 |
| LOC102724786 | -0.029659533 | 0.32725693 | 0.15737272 | 1.70E-07 | 0.19 |
| IGSF6 | -0.12432966 | 3.76E-05 | -0.180865464 | 1.72E-09 | 0.06 |
| IGSF8 | 0.022500537 | 0.45740719 | 0.178416705 | 2.86E-09 | 0.16 |
| IGSF9B | -0.455462916 | 4.40E-57 | -0.253974611 | 1.50E-17 | 0.21 |
| IGSF9 | 0.124065518 | 3.91E-05 | 0.334972085 | 4.56E-30 | 0.22 |
| IHH | 0.087246192 | 0.00389414 | 0.029889824 | 0.3235133 | 0.06 |
| LOC387877 | -0.010870155 | 0.71961552 | -0.035850131 | 0.2363143 | 0.03 |
| DYS | -0.301047374 | 2.47E-24 | -0.185662254 | 6.21E-10 | 0.12 |
| IKBKB | -0.281398115 | 2.43E-21 | -0.113648608 | 0.0001664 | 0.17 |
| IKBKE | 0.15792139 | 1.54E-07 | 0.271594628 | 6.18E-20 | 0.12 |
| IP2 | 0.106283354 | 0.00043197 | 0.041058834 | 0.1749572 | 0.07 |
| IKZF1 | -0.120688287 | 6.33E-05 | -0.213931238 | 8.84E-13 | 0.1 |
| IKZF2 | -0.277207344 | 9.84E-21 | -0.14303987 | 2.05E-06 | 0.14 |
| IKZF3 | -0.169907003 | 1.59E-08 | -0.26553794 | 4.28E-19 | 0.1 |
| IKZF4 | -0.404899393 | 2.25E-44 | -0.434899135 | 1.18E-51 | 0.03 |
| IKZF5 | -0.360380059 | 7.34E-35 | -0.285268168 | 6.52E-22 | 0.08 |
| IK | -0.059235052 | 0.05025092 | 0.082259996 | 0.0065073 | 0.15 |
| IL10RA | -0.128931756 | 1.91E-05 | -0.236882822 | 2.09E-15 | 0.11 |
| IL10RB | 0.099047813 | 0.00104215 | -0.009205481 | 0.7611306 | 0.11 |
| IL10 | 0.00374362 | 0.90161224 | -0.078997574 | 0.0089804 | 0.09 |
| IL11RA | -0.112407732 | 0.00019623 | -0.048016445 | 0.1126149 | 0.07 |
| IL11 | 0.137870928 | 4.77E-06 | 0.089280344 | 0.003135 | 0.05 |
| IL12A | 0.164844536 | 4.23E-08 | 0.119173609 | 7.83E-05 | 0.05 |
| BHR1 | 0.032847397 | 0.27792023 | -0.056592675 | 0.0614362 | 0.09 |
| IL12RB1 | 0.005543693 | 0.8547437 | -0.141216517 | 2.77E-06 | 0.15 |
| IL12RB2 | 0.299171323 | 4.89E-24 | 0.054230633 | 0.073107 | 0.25 |
| IL13RA1 | -0.402343574 | 8.70E-44 | -0.163440436 | 5.52E-08 | 0.24 |
| IL13RA2 | -0.02029934 | 0.5025974 | -0.029758855 | 0.3256389 | 0.01 |
| IL13 | 0.050390437 | 0.09589535 | -0.066443604 | 0.0280507 | 0.12 |
| IL15RA | 0.144897677 | 1.51E-06 | -0.021235842 | 0.4830881 | 0.17 |
| IL15 | 0.047797989 | 0.11426213 | -0.028657599 | 0.34387 | 0.08 |
| IL16 | -0.266397531 | 3.26E-19 | -0.268608715 | 1.61E-19 | 0.01 |
| LOC63189 | 0.089375501 | 0.00310303 | 0.039932033 | 0.1871054 | 0.05 |
| IL17B | -0.074527824 | 0.01371894 | -0.066585373 | 0.0277167 | 0.01 |
| IL17C | 0.165607094 | 3.65E-08 | 0.053044044 | 0.0796204 | 0.12 |
| IL27 | -0.140652058 | 3.04E-06 | -0.173156305 | 8.33E-09 | 0.04 |
| IL17F | 0.143670261 | 1.85E-06 | 0.037706132 | 0.2129105 | 0.11 |
| LOC100506204 | -0.251204056 | 3.43E-17 | -0.321327376 | 1.13E-27 | 0.08 |
| IL17RB | 0.056781981 | 0.06057161 | 0.017397292 | 0.5655961 | 0.04 |
| IL17RC | 0.020175956 | 0.50519841 | 0.010058957 | 0.7397514 | 0.02 |
| IL17RD | -0.088042389 | 0.00357909 | -0.05461911 | 0.0710717 | 0.04 |
| IL17REL | -0.161304179 | 8.24E-08 | -0.156199385 | 2.10E-07 | 0.01 |
| IL17RE | 0.10712722 | 0.00038839 | 0.12762962 | 2.32E-05 | 0.03 |
| IL18BP | 0.103196334 | 0.00063327 | -0.048967921 | 0.1056573 | 0.16 |
| IL18R1 | -0.040961747 | 0.17598015 | -0.169565661 | 1.70E-08 | 0.13 |
| IL18RAP | 0.059775501 | 0.04818689 | -0.078685219 | 0.0092564 | 0.14 |
| IL18 | 0.080525429 | 0.00773335 | 0.08239055 | 0.0064225 | 0.01 |
| IL19 | 0.042929272 | 0.15610459 | 0.091522597 | 0.0024562 | 0.05 |
| IL1A | 0.125654007 | 3.10E-05 | -0.036214799 | 0.2315781 | 0.17 |
| IL1B | -0.002961124 | 0.92210335 | -0.116741504 | 0.0001096 | 0.12 |
| IL1F10 | 0.053853042 | 0.07513072 | -0.021527269 | 0.477102 | 0.08 |
| IL36RN | 0.289071499 | 1.76E-22 | 0.108518334 | 0.0003254 | 0.19 |
| IL36A | 0.043873786 | 0.14718969 | -0.059208505 | 0.0503542 | 0.11 |
| IL37 | 0.170238133 | 1.49E-08 | 0.076168386 | 0.0117706 | 0.1 |
| IL36B | 0.012314368 | 0.68425084 | -0.047580293 | 0.1159224 | 0.06 |
| IL36G | 0.260669472 | 1.95E-18 | 0.024417407 | 0.4199837 | 0.24 |
| IL1R1 | -0.257223089 | 5.62E-18 | -0.209601792 | 2.57E-12 | 0.05 |
| IL1R2 | 0.251345491 | 3.29E-17 | 0.029299277 | 0.3331694 | 0.23 |
| MRX34 | -0.18358185 | 9.69E-10 | -0.224897986 | 5.34E-14 | 0.05 |
| IL1RAPL2 | -0.238108688 | 1.49E-15 | -0.135599443 | 6.84E-06 | 0.11 |
| IL1RAP | -0.144460156 | 1.62E-06 | -0.257171848 | 5.71E-18 | 0.12 |
| IL1RL1 | -0.364846584 | 9.48E-36 | -0.315227995 | 1.22E-26 | 0.05 |
| LOC115605 | 0.103669928 | 0.00059757 | -0.048706229 | 0.107536 | 0.16 |
| IL1RN | 0.020635136 | 0.4955546 | -0.021562881 | 0.4763733 | 0.05 |
| IL20RA | -0.280473935 | 3.31E-21 | -0.061790885 | 0.0411064 | 0.22 |
| IL20RB | 0.321417743 | 1.09E-27 | 0.195334584 | 7.35E-11 | 0.13 |
| IL20 | -0.158325878 | 1.43E-07 | 0.035113073 | 0.2460949 | 0.2 |
| IL21R | -0.006236686 | 0.83682657 | -0.114388845 | 0.0001507 | 0.11 |
| IL21 | 0.021069188 | 0.48652957 | -0.069854674 | 0.0209096 | 0.1 |
| IL22RA1 | 0.129655939 | 1.71E-05 | -0.031201496 | 0.3027241 | 0.17 |
| IL22RA2 | 0.237640807 | 1.69E-15 | 0.042131157 | 0.1639516 | 0.2 |
| ILTIF | 0.057929229 | 0.05554497 | 0.006731138 | 0.8240936 | 0.06 |
| IL23A | 0.251658029 | 3.00E-17 | 0.045751285 | 0.1306297 | 0.21 |
| IL23R | -0.097960575 | 0.00118392 | -0.129110669 | 1.86E-05 | 0.04 |
| IL24 | -0.014004296 | 0.64373524 | 0.072369172 | 0.0167128 | 0.09 |
| IL25 | -0.072087737 | 0.0171426 | 0.034078893 | 0.2602905 | 0.11 |
| IL26 | 0.087854247 | 0.00365137 | -0.027306167 | 0.3671142 | 0.12 |
| IL27RA | 0.195679575 | 6.80E-11 | 0.018613834 | 0.5387313 | 0.18 |
| IL27.1 | -0.140652058 | 3.04E-06 | -0.173156305 | 8.33E-09 | 0.04 |
| IFNL2 | 0.176907363 | 3.90E-09 | 0.076447786 | 0.0114644 | 0.11 |
| IFNL3 | 0.126232581 | 2.85E-05 | 0.039689483 | 0.1897999 | 0.09 |
| IFNLR1 | -0.09245239 | 0.00221644 | -0.239028975 | 1.15E-15 | 0.15 |
| IFNL1 | 0.220150939 | 1.83E-13 | 0.117594574 | 9.75E-05 | 0.11 |
| IL2RA | 0.118647441 | 8.43E-05 | -0.102850017 | 0.0006606 | 0.23 |
| IL2RB | -0.058129606 | 0.05470367 | -0.214347795 | 7.97E-13 | 0.16 |
| IL2RG | 0.106790147 | 0.00040528 | -0.033438335 | 0.2693617 | 0.15 |
| IL2 | -0.037205371 | 0.21905377 | -0.075243399 | 0.0128366 | 0.04 |
| IL31RA | 0.055738061 | 0.06546717 | -0.058031533 | 0.0551141 | 0.12 |
| IL31 | 0.000449927 | 0.98814561 | -0.031909117 | 0.2918855 | 0.04 |
| IL32 | 0.274889661 | 2.11E-20 | 0.13719441 | 5.31E-06 | 0.14 |
| IL33 | -0.26204738 | 1.28E-18 | -0.209361432 | 2.72E-12 | 0.06 |
| IL34 | 0.122912712 | 4.61E-05 | -0.041925157 | 0.1660244 | 0.17 |
| IL3RA | -0.058937222 | 0.05141964 | -0.053140157 | 0.0790759 | 0.01 |
| IL3 | 0.118757753 | 8.30E-05 | 0.010488178 | 0.7290739 | 0.11 |
| IL4I1 | 0.233590339 | 5.19E-15 | 0.03156769 | 0.2970822 | 0.21 |
| IL4R | -0.170922746 | 1.30E-08 | -0.160715513 | 9.19E-08 | 0.02 |
| IL4 | 0.002316488 | 0.93902384 | -0.008213177 | 0.7862172 | 0.02 |
| IL5RA | -0.131007647 | 1.39E-05 | -0.115327551 | 0.0001328 | 0.02 |
| IL5 | 0.013946528 | 0.64510376 | -0.017425692 | 0.5649617 | 0.04 |
| IL6R | -0.275534081 | 1.71E-20 | -0.305159321 | 5.46E-25 | 0.03 |
| IL6ST | -0.565221025 | 2.80E-93 | -0.439450934 | 8.01E-53 | 0.13 |
| IL6 | 0.018486855 | 0.54150518 | -0.109566709 | 0.0002844 | 0.13 |
| IL7R | -0.197105652 | 4.91E-11 | -0.294201118 | 2.90E-23 | 0.1 |
| IL7 | -0.032812796 | 0.27842703 | -0.133059959 | 1.02E-05 | 0.11 |
| CXCL8 | 0.196286406 | 5.92E-11 | 0.038636844 | 0.2018246 | 0.16 |
| IL9R | -0.175146825 | 5.58E-09 | -0.105129394 | 0.000499 | 0.08 |
| IL9 | -0.042222208 | 0.1630417 | -0.040379987 | 0.1822035 | 0.01 |
| DFNB42 | -0.25410231 | 1.44E-17 | -0.036662762 | 0.2258527 | 0.22 |
| ILDR2 | -0.278630826 | 6.13E-21 | -0.260584219 | 2.01E-18 | 0.02 |
| ILF2 | 0.473459133 | 3.77E-62 | 0.442594779 | 1.22E-53 | 0.04 |
| FLJ20011 | 0.333076972 | 9.98E-30 | 0.134593941 | 8.01E-06 | 0.2 |
| ILKAP | 0.361349857 | 4.72E-35 | 0.276095183 | 1.42E-20 | 0.09 |
| ILK.2 | -0.103280492 | 0.00062678 | 0.011770941 | 0.6974818 | 0.12 |
| ILVBL | 0.162907524 | 6.10E-08 | 0.130530891 | 1.50E-05 | 0.04 |
| IMMP1L | 0.343910607 | 1.05E-31 | 0.411282961 | 7.34E-46 | 0.07 |
| IMMP2L.IT1 | 0.069956092 | 0.02072403 | 0.176662934 | 4.10E-09 | 0.11 |
| IMMT | 0.245189098 | 1.99E-16 | 0.125959457 | 2.96E-05 | 0.12 |
| IMP3 | 0.230930872 | 1.07E-14 | 0.238488771 | 1.34E-15 | 0.01 |
| IMP4 | 0.380213179 | 6.45E-39 | 0.35127222 | 4.31E-33 | 0.03 |
| SPPL2C | -0.217205069 | 3.88E-13 | -0.066945858 | 0.0268826 | 0.16 |
| IMPA1 | 0.091963455 | 0.00233972 | -0.047883469 | 0.1136153 | 0.14 |
| IMPA2 | 0.496365835 | 4.71E-69 | 0.210190313 | 2.22E-12 | 0.29 |
| IMPACT | -0.103782224 | 0.00058938 | -0.192432317 | 1.41E-10 | 0.09 |
| IMPAD1 | 0.000711646 | 0.98125105 | -0.042442922 | 0.1608518 | 0.05 |
| IMPDH1 | 0.217885766 | 3.27E-13 | 0.139624546 | 3.60E-06 | 0.08 |
| IMPDH2 | 0.069738575 | 0.02112375 | 0.20296227 | 1.26E-11 | 0.14 |
| IMPG1 | -0.371433267 | 4.37E-37 | -0.236614702 | 2.25E-15 | 0.14 |
| IMPG2 | -0.234497347 | 4.05E-15 | -0.25432364 | 1.35E-17 | 0.02 |
| INADL | -0.398886453 | 5.31E-43 | -0.216223428 | 4.98E-13 | 0.19 |
| INA | 0.331068754 | 2.27E-29 | 0.05286209 | 0.0806596 | 0.28 |
| INCA1 | -0.051804249 | 0.08692122 | -0.099983261 | 0.0009329 | 0.05 |
| INCENP | 0.239670197 | 9.60E-16 | -0.04511833 | 0.1360431 | 0.29 |
| INE1 | -0.287299913 | 3.25E-22 | -0.205958632 | 6.19E-12 | 0.09 |
| INE2 | 0.013777154 | 0.64912313 | 0.111795414 | 0.0002127 | 0.1 |
| INF2 | -0.034261398 | 0.25774508 | 0.008090543 | 0.7893336 | 0.05 |
| ING1 | 0.161861725 | 7.42E-08 | 0.060527384 | 0.0454341 | 0.11 |
| ING2 | 0.078338417 | 0.00957169 | 0.120732531 | 6.29E-05 | 0.05 |
| ING3 | -0.036311523 | 0.23033325 | -0.17625779 | 4.45E-09 | 0.14 |
| ING4 | 0.22402319 | 6.72E-14 | 0.122909361 | 4.62E-05 | 0.11 |
| LOC654207 | -0.0365755 | 0.22696004 | 0.001812746 | 0.9522656 | 0.04 |
| LOC158826 | 0.312376033 | 3.63E-26 | 0.131997811 | 1.20E-05 | 0.19 |
| INHA | 0.114096672 | 0.00015674 | -0.015163964 | 0.616523 | 0.13 |
| INHBA | -0.248877986 | 6.81E-17 | -0.22654302 | 3.46E-14 | 0.03 |
| INHBB | -0.24526163 | 1.95E-16 | -0.112215631 | 0.0002013 | 0.14 |
| INHBC | 0.051556394 | 0.0884436 | -0.065082465 | 0.0314382 | 0.12 |
| INHBE | 0.236196225 | 2.53E-15 | 0.038939741 | 0.198309 | 0.2 |
| INMT | -0.300269481 | 3.28E-24 | -0.189639339 | 2.62E-10 | 0.12 |
| INO80B | 0.144030937 | 1.74E-06 | 0.10101938 | 0.0008243 | 0.05 |
| INO80C | 0.55596573 | 1.09E-89 | 0.304567107 | 6.80E-25 | 0.26 |
| INO80D | -0.323387852 | 5.02E-28 | -0.281711195 | 2.18E-21 | 0.05 |
| INO80E | 0.254528694 | 1.27E-17 | 0.27984898 | 4.08E-21 | 0.03 |
| INO80 | -0.41424222 | 1.46E-46 | -0.383085598 | 1.58E-39 | 0.04 |
| INPP1 | -0.090487542 | 0.00275085 | -0.222740476 | 9.39E-14 | 0.14 |
| INPP4A | -0.196942013 | 5.10E-11 | -0.379793237 | 7.91E-39 | 0.19 |
| INPP4B | -0.502683063 | 4.73E-71 | -0.248879221 | 6.80E-17 | 0.26 |
| INPP5A | -0.13464684 | 7.94E-06 | -0.039911553 | 0.1873318 | 0.1 |
| INPP5B | -0.107512454 | 0.0003699 | -0.162910172 | 6.10E-08 | 0.06 |
| INPP5D | -0.200114275 | 2.46E-11 | -0.265993166 | 3.70E-19 | 0.07 |
| JBTS1 | 0.093787819 | 0.00190941 | -0.004517499 | 0.8814109 | 0.1 |
| INPP5F | -0.117047676 | 0.00010507 | -0.226628849 | 3.38E-14 | 0.11 |
| INPP5J | -0.369184696 | 1.26E-36 | -0.118634745 | 8.44E-05 | 0.26 |
| INPP5K | -0.062475546 | 0.03891069 | -0.057488333 | 0.0574341 | 0.01 |
| INPPL1 | -0.130875542 | 1.42E-05 | -0.17271639 | 9.09E-09 | 0.05 |
| INS.IGF2 | -0.199754572 | 2.67E-11 | -0.148269483 | 8.50E-07 | 0.06 |
| INSC | -0.033618809 | 0.26678429 | -0.065442298 | 0.0305103 | 0.04 |
| INSIG1 | 0.083326213 | 0.0058432 | 0.004846126 | 0.8728549 | 0.08 |
| LOC129941 | -0.210993764 | 1.83E-12 | -0.125663997 | 3.10E-05 | 0.09 |
| INSL3 | 0.163034592 | 5.95E-08 | -0.001608707 | 0.9576332 | 0.17 |
| INSL4 | 0.110918067 | 0.00023863 | 0.066074882 | 0.0289358 | 0.05 |
| INSL5 | 0.044903795 | 0.1379167 | 0.097239474 | 0.0012875 | 0.06 |
| INSL6 | 0.115736206 | 0.00012566 | 0.032657813 | 0.2807047 | 0.09 |
| IA1 | -0.011734411 | 0.6983746 | -0.006601764 | 0.8274209 | 0.01 |
| INSM2 | 0.126389419 | 2.78E-05 | -0.008653692 | 0.7750514 | 0.14 |
| INSRR | -0.216796277 | 4.31E-13 | -0.211613093 | 1.57E-12 | 0.01 |
| INSR | -0.236161072 | 2.56E-15 | -0.116668965 | 0.0001107 | 0.12 |
| INS | 0.03349595 | 0.26853703 | 0.04011675 | 0.1850724 | 0.01 |
| INTS10 | 0.122797562 | 4.69E-05 | 0.12594197 | 2.97E-05 | 0.01 |
| INTS12 | -0.105284019 | 0.00048946 | 0.035450385 | 0.2415842 | 0.15 |
| INTS1 | 0.003503952 | 0.90788206 | -0.053865498 | 0.0750632 | 0.06 |
| LOC86656 | -0.135697761 | 6.73E-06 | -0.172831932 | 8.89E-09 | 0.04 |
| INTS3 | -0.070811075 | 0.01921601 | 0.206176553 | 5.88E-12 | 0.28 |
| LOC107986705 | -0.10935951 | 0.00029209 | -0.089057449 | 0.0032111 | 0.03 |
| INTS4P2 | -0.173437841 | 7.87E-09 | -0.160585488 | 9.42E-08 | 0.02 |
| INTS4 | -0.057710874 | 0.056474 | -0.024153796 | 0.4250211 | 0.04 |
| INTS5 | 0.020635673 | 0.49554337 | 0.011016323 | 0.7160074 | 0.01 |
| DKFZP434B105 | -0.255985092 | 8.19E-18 | -0.161631132 | 7.75E-08 | 0.1 |
| INTS7 | 0.083826314 | 0.00555328 | 0.306614051 | 3.18E-25 | 0.23 |
| INTS8 | 0.338184236 | 1.19E-30 | 0.267537086 | 2.27E-19 | 0.08 |
| INTS9 | -0.074668244 | 0.0135417 | 0.095705607 | 0.0015363 | 0.18 |
| INTU | -0.344202628 | 9.30E-32 | -0.190936898 | 1.97E-10 | 0.16 |
| NPHP2 | -0.145845474 | 1.28E-06 | -0.276827589 | 1.12E-20 | 0.14 |
| IP6K1 | -0.325057611 | 2.58E-28 | -0.288107202 | 2.46E-22 | 0.04 |
| IP6K2 | 0.014170053 | 0.63981519 | 0.101671357 | 0.0007621 | 0.09 |
| IP6K3 | 0.029031486 | 0.33760872 | -0.079558611 | 0.008503 | 0.11 |
| IPCEF1 | -0.079028865 | 0.00895317 | -0.22238286 | 1.03E-13 | 0.15 |
| IPMK | -0.275637979 | 1.65E-20 | -0.365977511 | 5.62E-36 | 0.1 |
| IPO11 | -0.324039373 | 3.87E-28 | -0.162010214 | 7.22E-08 | 0.17 |
| IPO13 | 0.124802692 | 3.51E-05 | -0.04558812 | 0.1320089 | 0.18 |
| IPO4 | 0.099627745 | 0.00097311 | 0.169854262 | 1.60E-08 | 0.08 |
| IPO5 | 0.143127689 | 2.02E-06 | 0.01448016 | 0.6325083 | 0.13 |
| IPO7 | -0.070106733 | 0.02045108 | -0.130707594 | 1.46E-05 | 0.07 |
| IPO8 | -0.126861384 | 2.60E-05 | -0.175357957 | 5.34E-09 | 0.05 |
| IPO9 | 0.096455088 | 0.00140971 | 0.348427913 | 1.50E-32 | 0.26 |
| IPPK | -0.162042909 | 7.17E-08 | -0.183863672 | 9.13E-10 | 0.03 |
| IPP | -0.382879091 | 1.75E-39 | -0.386573483 | 2.80E-40 | 0.01 |
| IPW | -0.371778591 | 3.71E-37 | -0.22639913 | 3.59E-14 | 0.15 |
| IQCA1 | -0.269027782 | 1.41E-19 | -0.11691289 | 0.000107 | 0.16 |
| IQCB1 | 0.182718179 | 1.16E-09 | 0.055979396 | 0.0643074 | 0.13 |
| IQCC | 0.03684994 | 0.22349057 | 0.098470625 | 0.0011153 | 0.07 |
| IQCD | -0.148641049 | 7.98E-07 | 0.062897964 | 0.0376062 | 0.22 |
| DKFZp434P1219 | -0.220573624 | 1.64E-13 | -0.078611652 | 0.0093225 | 0.15 |
| IQCF1 | -0.088805304 | 0.00329911 | 0.057576047 | 0.0570541 | 0.15 |
| IQCF2 | 0.023571302 | 0.43627626 | 0.007138598 | 0.8136353 | 0.02 |
| IQCF3 | 0.003877656 | 0.89810844 | 0.04950751 | 0.1018656 | 0.05 |
| IQCF5 | 0.033391683 | 0.27003072 | 0.025957323 | 0.3912648 | 0.01 |
| IQCF6 | -0.111245562 | 0.00022864 | -0.022112526 | 0.4652037 | 0.09 |
| IQCG | 0.269346346 | 1.27E-19 | 0.131845318 | 1.23E-05 | 0.14 |
| IQCH | -0.394778839 | 4.43E-42 | -0.260337766 | 2.17E-18 | 0.14 |
| IQCJ | -0.158150687 | 1.47E-07 | -0.088900288 | 0.0032657 | 0.07 |
| IQCK | -0.154992862 | 2.61E-07 | 0.041773241 | 0.1675656 | 0.2 |
| IQGAP1 | -0.336531948 | 2.38E-30 | -0.333924701 | 7.04E-30 | 0.01 |
| IQGAP2 | -0.483896502 | 3.13E-65 | -0.212225421 | 1.35E-12 | 0.28 |
| IQGAP3 | 0.392619022 | 1.33E-41 | 0.274411679 | 2.47E-20 | 0.12 |
| IQSEC1 | -0.417781356 | 2.08E-47 | -0.413078452 | 2.76E-46 | 0.01 |
| LOC100134137 | -0.260379905 | 2.14E-18 | -0.234220081 | 4.37E-15 | 0.03 |
| IQSEC3 | -0.172072111 | 1.03E-08 | -0.261262202 | 1.63E-18 | 0.09 |
| IQUB | -0.390997817 | 3.04E-41 | -0.16958127 | 1.69E-08 | 0.23 |
| SIMPL | 0.086556032 | 0.00418736 | 0.113300195 | 0.0001743 | 0.03 |
| IRAK1 | 0.331583626 | 1.84E-29 | -0.017507023 | 0.5631468 | 0.35 |
| IRAK2 | -0.144226181 | 1.69E-06 | -0.216307693 | 4.87E-13 | 0.08 |
| IRAK3 | -0.287544504 | 2.98E-22 | -0.236627577 | 2.25E-15 | 0.06 |
| IRAK4 | -0.287817987 | 2.71E-22 | -0.241919162 | 5.08E-16 | 0.05 |
| LOC146012 | -0.261114857 | 1.70E-18 | -0.336585916 | 2.33E-30 | 0.08 |
| IRF1 | 0.027918032 | 0.35647166 | -0.028692184 | 0.3432878 | 0.06 |
| IRF2BP1 | 0.050924515 | 0.09242196 | 0.082263511 | 0.006505 | 0.04 |
| LOC199907 | 0.008250777 | 0.78526239 | 0.230710927 | 1.14E-14 | 0.23 |
| IRF2 | -0.262636341 | 1.06E-18 | -0.305997688 | 4.00E-25 | 0.05 |
| IRF3 | 0.199348975 | 2.93E-11 | 0.129296097 | 1.81E-05 | 0.08 |
| LOC154397 | -0.003606348 | 0.90520261 | -0.12158951 | 5.57E-05 | 0.12 |
| IRF5 | 0.03984938 | 0.18802041 | -0.117432771 | 9.97E-05 | 0.16 |
| PPS | -0.187613253 | 4.08E-10 | -0.030161315 | 0.3191358 | 0.16 |
| IRF7 | 0.006626191 | 0.8267924 | 0.042341789 | 0.1618524 | 0.04 |
| IRF8 | -0.172138199 | 1.02E-08 | -0.241070166 | 6.46E-16 | 0.07 |
| IRF9 | 0.012992516 | 0.66787495 | -0.000176843 | 0.9953405 | 0.02 |
| IRGC | 0.048642976 | 0.10799408 | 0.009934045 | 0.7428683 | 0.04 |
| IRGM | -0.035223674 | 0.24460941 | -0.121640098 | 5.53E-05 | 0.09 |
| IRGQ | 0.065128311 | 0.03131867 | -0.006672807 | 0.8255934 | 0.08 |
| IRS1 | -0.41399321 | 1.67E-46 | -0.24993208 | 4.99E-17 | 0.17 |
| LOC115972 | -0.155253385 | 2.49E-07 | -0.242523317 | 4.28E-16 | 0.09 |
| IRS4 | 0.058383831 | 0.05365157 | -0.089874583 | 0.0029402 | 0.15 |
| IRX1 | 0.055806932 | 0.06513445 | -0.024137356 | 0.4253364 | 0.08 |
| IRX2 | -0.109489325 | 0.00028724 | -0.022044157 | 0.4665851 | 0.09 |
| IRX3 | 0.024693971 | 0.41473669 | 0.102847109 | 0.0006609 | 0.08 |
| IRX4 | 0.002953341 | 0.92230744 | 0.003319411 | 0.9127137 | 0.01 |
| IRX5 | -0.082036163 | 0.006655 | 0.044281471 | 0.143464 | 0.13 |
| IRX6 | 0.156518044 | 1.99E-07 | 0.021644699 | 0.4747014 | 0.14 |
| LOC642170 | 0.039920486 | 0.18723305 | 0.054995278 | 0.0691453 | 0.02 |
| ISCA1 | -0.048330332 | 0.1102808 | 0.012392989 | 0.6823445 | 0.07 |
| ISCA2 | 0.078658114 | 0.00928072 | 0.24970291 | 5.34E-17 | 0.18 |
| ISCU | -0.149235066 | 7.20E-07 | -0.007027059 | 0.8164949 | 0.15 |
| G1P2 | 0.223608165 | 7.49E-14 | 0.119318139 | 7.67E-05 | 0.11 |
| ISG20L2 | 0.073106966 | 0.0156307 | 0.129660449 | 1.71E-05 | 0.06 |
| ISG20 | 0.232227029 | 7.53E-15 | 0.065312458 | 0.0308424 | 0.17 |
| ISL1 | 0.003511886 | 0.90767443 | 0.003401362 | 0.9105677 | 0.01 |
| ISL2 | 0.20842823 | 3.42E-12 | 0.043286548 | 0.1526855 | 0.17 |
| ISLR2 | -0.3434805 | 1.27E-31 | -0.231899938 | 8.23E-15 | 0.12 |
| ISLR | -0.094857569 | 0.00169215 | -0.027746271 | 0.3594394 | 0.07 |
| ISM1 | -0.228770926 | 1.91E-14 | -0.193632505 | 1.08E-10 | 0.04 |
| THSD3 | 0.083876175 | 0.00552509 | -0.007101676 | 0.8145816 | 0.1 |
| ISOC1 | -0.02774422 | 0.35947494 | -0.034311544 | 0.2570487 | 0.01 |
| ISOC2 | 0.268780383 | 1.53E-19 | 0.275313782 | 1.84E-20 | 0.01 |
| LOC100132254 | -0.198600927 | 3.49E-11 | -0.191633405 | 1.69E-10 | 0.01 |
| ISX | -0.031022291 | 0.30551085 | 0.034027964 | 0.2610039 | 0.07 |
| ISY1 | 0.361400753 | 4.61E-35 | 0.177405799 | 3.52E-09 | 0.19 |
| ISYNA1 | 0.124640333 | 3.59E-05 | 0.113436984 | 0.0001712 | 0.02 |
| ITCH | -0.253252332 | 1.86E-17 | -0.30300301 | 1.21E-24 | 0.05 |
| LOC146360 | -0.171119963 | 1.25E-08 | -0.244968363 | 2.12E-16 | 0.08 |
| ITFG2 | 0.121746435 | 5.45E-05 | 0.046962935 | 0.120734 | 0.08 |
| ITFG3 | 0.064414608 | 0.03322471 | 0.081667441 | 0.0069049 | 0.02 |
| ITGA10 | -0.197379964 | 4.61E-11 | -0.148207918 | 8.59E-07 | 0.05 |
| ITGA11 | -0.246308044 | 1.44E-16 | -0.110878399 | 0.0002399 | 0.14 |
| ITGA1 | -0.347363664 | 2.38E-32 | -0.337324337 | 1.71E-30 | 0.02 |
| ITGA2B | 0.270523863 | 8.73E-20 | 0.129364442 | 1.79E-05 | 0.15 |
| ITGA2 | -0.312409888 | 3.59E-26 | -0.216828698 | 4.27E-13 | 0.1 |
| MSK18 | -0.379995016 | 7.17E-39 | -0.274254812 | 2.60E-20 | 0.11 |
| ITGA4 | -0.274605706 | 2.32E-20 | -0.354610803 | 9.84E-34 | 0.09 |
| ITGA5 | -0.124565622 | 3.63E-05 | -0.12768152 | 2.30E-05 | 0.01 |
| ITGA6 | -0.098612034 | 0.00109697 | -0.184553102 | 7.88E-10 | 0.09 |
| ITGA7 | -0.212160943 | 1.37E-12 | -0.194498542 | 8.88E-11 | 0.02 |
| ITGA8 | -0.486148897 | 6.56E-66 | -0.413659413 | 2.01E-46 | 0.08 |
| ITGA4L | -0.224532885 | 5.88E-14 | -0.299029835 | 5.15E-24 | 0.08 |
| LOC197414 | -0.135523119 | 6.92E-06 | -0.106619479 | 0.0004141 | 0.03 |
| ITGAE | 0.294226163 | 2.88E-23 | 0.207866574 | 3.91E-12 | 0.09 |
| ITGAL | -0.136776772 | 5.68E-06 | -0.118469995 | 8.64E-05 | 0.02 |
| ITGAM | -0.180832237 | 1.73E-09 | -0.151790775 | 4.61E-07 | 0.03 |
| VTNR | -0.44703134 | 8.22E-55 | -0.333789448 | 7.44E-30 | 0.12 |
| ITGAX | -0.08987943 | 0.00293864 | -0.186784081 | 4.88E-10 | 0.1 |
| ITGB1BP1 | 0.361556475 | 4.29E-35 | 0.224175962 | 6.45E-14 | 0.14 |
| ITGB1BP2 | 0.254531656 | 1.27E-17 | 0.120973332 | 6.08E-05 | 0.14 |
| NMRK2 | -0.011915323 | 0.69395734 | -0.047892296 | 0.1135487 | 0.04 |
| LOC143058 | -0.145113472 | 1.45E-06 | -0.187951809 | 3.79E-10 | 0.05 |
| ITGB2 | -0.032712737 | 0.27989609 | -0.166321122 | 3.19E-08 | 0.14 |
| ITGB3BP | 0.363994566 | 1.40E-35 | 0.323357521 | 5.08E-28 | 0.05 |
| ITGB3 | -0.399806568 | 3.29E-43 | -0.391260456 | 2.66E-41 | 0.01 |
| ITGB4 | -0.09395402 | 0.00187406 | -0.159055652 | 1.25E-07 | 0.07 |
| ITGB5 | -0.421081226 | 3.30E-48 | -0.183442353 | 9.98E-10 | 0.24 |
| ITGB6 | -0.128384678 | 2.07E-05 | -0.013469847 | 0.6564417 | 0.12 |
| ITGB7 | 0.086586323 | 0.00417408 | -0.042493158 | 0.1603564 | 0.13 |
| ITGB8 | 0.00521053 | 0.86338482 | -0.078933602 | 0.0090363 | 0.09 |
| ITGBL1 | -0.359681933 | 1.01E-34 | -0.155317174 | 2.47E-07 | 0.21 |
| ITIH1 | 0.047201915 | 0.11885325 | -0.047714038 | 0.1149002 | 0.1 |
| ITIH2 | -0.164130954 | 4.84E-08 | -0.035799564 | 0.2369764 | 0.13 |
| ITIH3 | -0.277318633 | 9.49E-21 | -0.167735153 | 2.42E-08 | 0.11 |
| ITIHL1 | -0.174942331 | 5.81E-09 | -0.126650366 | 2.68E-05 | 0.05 |
| ITIH6 | -0.336395216 | 2.52E-30 | -0.199045022 | 3.15E-11 | 0.14 |
| ITIH5 | -0.294872404 | 2.29E-23 | -0.244389272 | 2.51E-16 | 0.06 |
| ITK | -0.069202611 | 0.02213741 | -0.166811335 | 2.90E-08 | 0.1 |
| ITLN1 | 0.17753086 | 3.43E-09 | 0.086602098 | 0.0041672 | 0.1 |
| ITLN2 | 0.155193023 | 2.52E-07 | 0.013188034 | 0.6631823 | 0.15 |
| ITM2A | -0.275715458 | 1.61E-20 | -0.232384553 | 7.21E-15 | 0.05 |
| BRI | -0.363247816 | 1.98E-35 | -0.203024045 | 1.24E-11 | 0.17 |
| ITM2C | 0.147118324 | 1.04E-06 | -0.044081407 | 0.1452832 | 0.2 |
| ITPA | 0.377323819 | 2.62E-38 | 0.369061673 | 1.33E-36 | 0.01 |
| LOC100130685 | -0.07925186 | 0.00876116 | 0.022617443 | 0.4550726 | 0.11 |
| ITPKA | 0.003473228 | 0.90868624 | -0.053400156 | 0.077618 | 0.06 |
| ITPKB | -0.005873024 | 0.84621902 | 0.069119057 | 0.0222992 | 0.08 |
| ITPKC | 0.005555227 | 0.85444484 | -0.020383979 | 0.5008173 | 0.03 |
| SCA29 | -0.536658986 | 1.51E-82 | -0.379961853 | 7.29E-39 | 0.16 |
| ITPR2 | -0.243963127 | 2.83E-16 | -0.270465434 | 8.90E-20 | 0.03 |
| LOC221498 | -0.047113695 | 0.11954484 | -0.230777085 | 1.12E-14 | 0.19 |
| ITPRIPL1 | -0.083161359 | 0.00594171 | -0.224204233 | 6.41E-14 | 0.15 |
| ITPRIPL2 | -0.252951843 | 2.04E-17 | -0.056452098 | 0.0620848 | 0.2 |
| ITPRIP | -0.065221574 | 0.03107665 | -0.149300315 | 7.12E-07 | 0.09 |
| ITSN1 | -0.30303639 | 1.20E-24 | -0.288511824 | 2.13E-22 | 0.02 |
| SH3D1B | -0.349526933 | 9.27E-33 | -0.410048692 | 1.43E-45 | 0.07 |
| IVD | -0.368734152 | 1.55E-36 | -0.189110508 | 2.94E-10 | 0.18 |
| IVL | 0.182510784 | 1.22E-09 | 0.078526116 | 0.0093999 | 0.11 |
| IVNS1ABP | 0.197556046 | 4.43E-11 | 0.221712541 | 1.23E-13 | 0.03 |
| IWS1 | 0.130073926 | 1.61E-05 | 0.028717161 | 0.3428676 | 0.11 |
| C6orf71 | -0.211807505 | 1.50E-12 | -0.003859837 | 0.8985741 | 0.21 |
| IZUMO1 | 0.019566616 | 0.51814674 | -0.087140286 | 0.0039379 | 0.11 |
| JAG1 | -0.301708317 | 1.94E-24 | -0.269040655 | 1.41E-19 | 0.04 |
| JAG2 | 0.012434481 | 0.68133925 | -0.044836443 | 0.138509 | 0.06 |
| JAGN1 | 0.461716223 | 8.25E-59 | 0.36286518 | 2.36E-35 | 0.1 |
| JAK1 | -0.376750609 | 3.45E-38 | -0.412131758 | 4.63E-46 | 0.04 |
| JAK2 | -0.296414089 | 1.32E-23 | -0.31479267 | 1.44E-26 | 0.02 |
| JAK3 | 0.061545312 | 0.0419191 | -0.085106254 | 0.0048694 | 0.15 |
| JAKMIP1 | 0.024451482 | 0.41933517 | -0.027451422 | 0.36457 | 0.06 |
| JAKMIP2 | -0.022372691 | 0.45996796 | -0.133184079 | 9.97E-06 | 0.12 |
| C10orf14 | -0.302732114 | 1.34E-24 | -0.198043046 | 3.96E-11 | 0.11 |
| JAM2 | -0.349286819 | 1.03E-32 | -0.234537986 | 4.00E-15 | 0.12 |
| JAM3 | -0.368260106 | 1.94E-36 | -0.222362187 | 1.04E-13 | 0.15 |
| JARID2 | 0.009386274 | 0.75658595 | 0.040970563 | 0.1758871 | 0.04 |
| JAZF1 | -0.22514805 | 5.00E-14 | -0.270324201 | 9.31E-20 | 0.05 |
| JDP2 | -0.153798891 | 3.23E-07 | -0.099101006 | 0.0010356 | 0.06 |
| KDM7A | -0.23955303 | 9.92E-16 | -0.190638994 | 2.10E-10 | 0.05 |
| JKAMP | -0.33325819 | 9.26E-30 | -0.113131681 | 0.0001783 | 0.23 |
| LOC439979 | -0.342986282 | 1.57E-31 | -0.352314366 | 2.72E-33 | 0.01 |
| JMJD4 | 0.438222072 | 1.66E-52 | 0.467084725 | 2.54E-60 | 0.03 |
| KDM8 | -0.185505188 | 6.43E-10 | -0.087393391 | 0.0038341 | 0.1 |
| JMJD6 | 0.425430036 | 2.83E-49 | 0.193819933 | 1.03E-10 | 0.24 |
| JMJD7.PLA2G4B | -0.196185374 | 6.06E-11 | -0.117713371 | 9.59E-05 | 0.08 |
| JMJD8 | -0.032693845 | 0.28017405 | 0.106682734 | 0.0004108 | 0.14 |
| JMY | -0.266793165 | 2.88E-19 | -0.198269066 | 3.76E-11 | 0.07 |
| LOC129143 | 0.17992397 | 2.09E-09 | -0.062199276 | 0.0397844 | 0.25 |
| JOSD2 | 0.081156299 | 0.00726534 | 0.147419052 | 9.83E-07 | 0.07 |
| JPH1 | 0.17997973 | 2.07E-09 | 0.102617141 | 0.0006796 | 0.08 |
| JPH2 | -0.047648845 | 0.11539759 | -0.148491538 | 8.18E-07 | 0.11 |
| TNRC22 | -0.059616403 | 0.04878698 | -0.099398214 | 0.0009999 | 0.04 |
| JPH4 | -0.19345017 | 1.12E-10 | -0.190972381 | 1.95E-10 | 0.01 |
| JRKL | 0.035314108 | 0.24339951 | -0.084231388 | 0.005328 | 0.12 |
| JRK | 0.034161968 | 0.2591297 | -0.093188459 | 0.002042 | 0.13 |
| JSRP1 | 0.097326364 | 0.00127463 | 0.030965668 | 0.3063949 | 0.07 |
| JTB | 0.293593985 | 3.60E-23 | 0.54399542 | 3.32E-85 | 0.26 |
| AJUBA | -0.112157454 | 0.00020282 | 0.013740546 | 0.6499932 | 0.13 |
| LOC115910 | -0.1493654 | 7.04E-07 | -0.086019916 | 0.0044288 | 0.07 |
| JUND | 0.050382849 | 0.09594545 | 0.130627348 | 1.48E-05 | 0.09 |
| JUN | -0.158438864 | 1.40E-07 | -0.076129834 | 0.0118134 | 0.09 |
| JUP | 0.003295566 | 0.91333825 | 0.057978298 | 0.055338 | 0.06 |
| KAAG1 | 0.020930034 | 0.48941324 | 0.068938262 | 0.0226527 | 0.05 |
| ANOS1 | -0.398219958 | 7.51E-43 | -0.277810823 | 8.06E-21 | 0.13 |
| CHDS5 | -0.018245113 | 0.54680581 | -0.123281561 | 4.38E-05 | 0.11 |
| LOC100505905 | -0.089630444 | 0.00301885 | -0.120780579 | 6.25E-05 | 0.04 |
| MXRA3.withdrawn | -0.452289371 | 3.20E-56 | -0.292194028 | 5.90E-23 | 0.17 |
| LOC100996774 | -0.171194155 | 1.23E-08 | -0.134404772 | 8.25E-06 | 0.04 |
| LOC388637 | -0.001146617 | 0.96979578 | -0.018691069 | 0.5370475 | 0.02 |
| DFNB89 | 0.527685124 | 2.20E-79 | 0.280125912 | 3.72E-21 | 0.25 |
| KAT2A | 0.249925282 | 5.00E-17 | 0.186434856 | 5.26E-10 | 0.07 |
| KAT2B | -0.398984976 | 5.05E-43 | -0.37887585 | 1.24E-38 | 0.03 |
| KAT5 | 0.028884649 | 0.34005896 | -0.005316673 | 0.86063 | 0.04 |
| KATNA1 | 0.357347701 | 2.89E-34 | 0.239948985 | 8.87E-16 | 0.12 |
| KATNAL1 | -0.227178873 | 2.92E-14 | -0.239458153 | 1.02E-15 | 0.02 |
| KATNAL2 | 0.190212943 | 2.31E-10 | 0.06422905 | 0.0337362 | 0.13 |
| KATNB1 | 0.195132547 | 7.69E-11 | 0.019422555 | 0.5212329 | 0.18 |
| KAZALD1 | -0.156486821 | 2.00E-07 | 0.003323542 | 0.9126055 | 0.16 |
| KAZN | -0.005357908 | 0.85956025 | -0.141418956 | 2.68E-06 | 0.14 |
| KLHL41 | -0.209612748 | 2.56E-12 | -0.031615583 | 0.2963496 | 0.18 |
| KBTBD11 | -0.109958921 | 0.00027033 | -0.153792147 | 3.24E-07 | 0.05 |
| KBTBD12 | 0.216000666 | 5.26E-13 | 0.045426697 | 0.1333845 | 0.18 |
| KBTBD13 | -0.09968482 | 0.00096655 | -0.082099363 | 0.006613 | 0.02 |
| KBTBD2 | -0.149126407 | 7.34E-07 | -0.160776033 | 9.09E-08 | 0.02 |
| KBTBD3 | -0.28066264 | 3.11E-21 | -0.006169856 | 0.8385509 | 0.28 |
| HSPC252 | -0.45349867 | 1.51E-56 | -0.340926757 | 3.76E-31 | 0.12 |
| KLHL40 | -0.040019144 | 0.18614463 | -0.040253509 | 0.1835778 | 0.01 |
| KBTBD6 | -0.018343918 | 0.54463623 | -0.059989961 | 0.0473878 | 0.05 |
| KBTBD7 | -0.396642112 | 1.70E-42 | -0.362770615 | 2.46E-35 | 0.04 |
| KBTBD8 | -0.074394406 | 0.01388923 | -0.210962621 | 1.84E-12 | 0.14 |
| KC6 | 0.110175597 | 0.00026284 | 0.021730195 | 0.4729578 | 0.09 |
| KCMF1 | 0.493290537 | 4.27E-68 | 0.250433032 | 4.31E-17 | 0.25 |
| KCNA10 | 0.025771117 | 0.39467282 | -0.05792441 | 0.0555653 | 0.09 |
| KCNA1 | -0.228194793 | 2.23E-14 | -0.200598001 | 2.19E-11 | 0.03 |
| KCNA2 | -0.004274503 | 0.88774641 | -0.131107771 | 1.37E-05 | 0.13 |
| KCNA3 | -0.202228632 | 1.50E-11 | -0.282552033 | 1.64E-21 | 0.09 |
| KCNA4 | 0.007214041 | 0.81170243 | -0.105026148 | 0.0005054 | 0.12 |
| KCNA5 | -0.198969978 | 3.20E-11 | -0.156820748 | 1.88E-07 | 0.05 |
| KCNA6 | -0.210943573 | 1.85E-12 | -0.178843068 | 2.62E-09 | 0.04 |
| KCNA7 | 0.090599546 | 0.00271748 | -0.08014928 | 0.0080251 | 0.18 |
| KCNAB1 | -0.12467769 | 3.58E-05 | -0.123303529 | 4.36E-05 | 0.01 |
| KCNAB2 | -0.125852312 | 3.01E-05 | -0.295086682 | 2.12E-23 | 0.17 |
| KCNAB3 | 0.003339032 | 0.91219983 | -0.057343271 | 0.0580672 | 0.07 |
| KCNB1 | -0.134275587 | 8.42E-06 | -0.303872421 | 8.79E-25 | 0.17 |
| KCNB2 | 0.172478552 | 9.54E-09 | 0.013775221 | 0.649169 | 0.16 |
| KCNC1 | -0.107744308 | 0.00035916 | -0.213168208 | 1.07E-12 | 0.11 |
| KCNC2 | -0.174977647 | 5.77E-09 | -0.123619588 | 4.17E-05 | 0.06 |
| KCNC3 | -0.077336506 | 0.0105369 | -0.087037437 | 0.0039808 | 0.01 |
| KCNC4 | 0.03601518 | 0.23416228 | -0.076813997 | 0.0110738 | 0.12 |
| KCND1 | -0.135154587 | 7.33E-06 | -0.188967655 | 3.03E-10 | 0.06 |
| KCND2 | -0.334630189 | 5.26E-30 | -0.285507016 | 6.01E-22 | 0.05 |
| KCND3 | -0.528388312 | 1.26E-79 | -0.386053296 | 3.63E-40 | 0.15 |
| KCNE5 | 0.240672726 | 7.23E-16 | 0.073277089 | 0.0153901 | 0.17 |
| KCNE1 | -0.206613152 | 5.29E-12 | -0.115982681 | 0.0001215 | 0.1 |
| KCNE2 | 0.310643232 | 7.02E-26 | 0.227144651 | 2.95E-14 | 0.09 |
| KCNE3 | -0.020517561 | 0.49801455 | -0.101006805 | 0.0008256 | 0.09 |
| KCNE4 | -0.347183235 | 2.57E-32 | -0.177759027 | 3.27E-09 | 0.17 |
| KCNF1 | 0.007358581 | 0.80800256 | 0.056084939 | 0.0638055 | 0.05 |
| KCNG1 | 0.285767861 | 5.50E-22 | 0.105625313 | 0.0004691 | 0.19 |
| KCNG2 | 0.007556159 | 0.80295201 | -0.069909176 | 0.0208097 | 0.08 |
| KCNG3 | 0.188899749 | 3.08E-10 | 0.00848044 | 0.7794374 | 0.19 |
| KCNG4 | 0.034240794 | 0.25803159 | -0.043474639 | 0.1509085 | 0.08 |
| KCNH1 | -0.305559928 | 4.71E-25 | -0.056787254 | 0.0605477 | 0.25 |
| KCNH2 | 0.130983345 | 1.40E-05 | -0.033596748 | 0.2670984 | 0.17 |
| KCNH3 | 0.134203124 | 8.51E-06 | 0.009004946 | 0.7661811 | 0.13 |
| KCNH4 | -0.055582033 | 0.06622613 | -0.172248287 | 9.98E-09 | 0.12 |
| KCNH5 | 0.141719474 | 2.55E-06 | 0.039313434 | 0.1940336 | 0.11 |
| KCNH6 | -0.042881382 | 0.15656726 | -0.049116386 | 0.104603 | 0.01 |
| KCNH7 | -0.013172574 | 0.66355291 | -0.070442744 | 0.0198535 | 0.06 |
| KCNH8 | 0.052213996 | 0.08445086 | -0.123456732 | 4.27E-05 | 0.18 |
| KCNIP1 | -0.106279054 | 0.0004322 | -0.202298408 | 1.47E-11 | 0.1 |
| KCNIP2 | -0.190857312 | 2.00E-10 | -0.134140098 | 8.60E-06 | 0.06 |
| LOC115312 | 0.061736755 | 0.04128439 | -0.046535012 | 0.1241597 | 0.11 |
| KCNIP4 | -0.205519735 | 6.88E-12 | -0.126217438 | 2.85E-05 | 0.08 |
| KCNJ10 | 0.14531569 | 1.40E-06 | 0.041946713 | 0.1658066 | 0.11 |
| KCNJ11 | -0.30860596 | 1.51E-25 | -0.022082542 | 0.4658093 | 0.29 |
| LOC115321 | -0.20173485 | 1.68E-11 | -0.174488804 | 6.37E-09 | 0.03 |
| KCNJ13 | -0.175372904 | 5.33E-09 | -0.008779494 | 0.771871 | 0.17 |
| KCNJ14 | 0.119345379 | 7.65E-05 | 0.018194502 | 0.5479188 | 0.11 |
| KCNJ15 | -0.04509243 | 0.13626826 | -0.1117036 | 0.0002153 | 0.07 |
| KCNJ16 | -0.079124395 | 0.00887046 | -0.066144567 | 0.0287667 | 0.02 |
| LOC115473 | 0.073384377 | 0.01524003 | -0.000488234 | 0.9871364 | 0.08 |
| KCNJ2 | -0.104167938 | 0.00056205 | -0.184554065 | 7.88E-10 | 0.09 |
| KCNJ3 | -0.226305878 | 3.68E-14 | -0.148159622 | 8.66E-07 | 0.08 |
| KCNJ4 | 0.224938514 | 5.28E-14 | 0.086900869 | 0.0040384 | 0.14 |
| KCNJ5 | -0.285403104 | 6.23E-22 | -0.256197667 | 7.68E-18 | 0.03 |
| KCNJ6 | 0.091535129 | 0.00245283 | -0.04623208 | 0.1266303 | 0.14 |
| KCNJ8 | -0.141980152 | 2.45E-06 | -0.100531158 | 0.0008739 | 0.05 |
| KCNJ9 | 0.102479618 | 0.00069108 | -0.000759238 | 0.9799975 | 0.11 |
| KCNK10 | -0.13095272 | 1.40E-05 | -0.165747751 | 3.56E-08 | 0.04 |
| KCNK12 | 0.336343652 | 2.58E-30 | 0.045405614 | 0.133565 | 0.3 |
| LOC115619 | -0.138775297 | 4.12E-06 | -0.123681619 | 4.13E-05 | 0.02 |
| KCNK14 | -0.298816587 | 5.56E-24 | -0.102213652 | 0.0007138 | 0.2 |
| KCNK16 | -0.02113108 | 0.48524994 | 0.024925966 | 0.4103652 | 0.05 |
| KCNK17 | 0.013728126 | 0.65028851 | 0.006076593 | 0.8409586 | 0.01 |
| KCNK18 | 0.071323077 | 0.01835955 | -0.011704203 | 0.6991132 | 0.09 |
| KCNK1 | 0.120332719 | 6.66E-05 | 0.186461723 | 5.23E-10 | 0.07 |
| KCNK2 | -0.048000025 | 0.11273802 | 0.004948216 | 0.8701999 | 0.06 |
| KCNK3 | -0.013496759 | 0.65579952 | -0.121964704 | 5.28E-05 | 0.11 |
| KCNK4 | -0.04869963 | 0.10758374 | -0.030809506 | 0.3088418 | 0.02 |
| KCNK5 | 0.314255913 | 1.77E-26 | 0.10573343 | 0.0004628 | 0.21 |
| KCNK6 | -0.292324994 | 5.63E-23 | -0.105749438 | 0.0004619 | 0.19 |
| KCNK7 | 0.253564526 | 1.70E-17 | 0.091118954 | 0.0025675 | 0.17 |
| KCNK9 | 0.197662528 | 4.32E-11 | -0.013049849 | 0.6664975 | 0.22 |
| KCNMA1 | -0.35531788 | 7.18E-34 | -0.240432921 | 7.74E-16 | 0.12 |
| KCNMB1 | -0.003485341 | 0.90836918 | -0.099417121 | 0.0009977 | 0.1 |
| KCNMB2 | -0.051386584 | 0.08949894 | -0.067340522 | 0.0259944 | 0.02 |
| KCNMB3 | 0.031101623 | 0.30427511 | 0.01561646 | 0.6060434 | 0.02 |
| KCNMB4 | -0.097393923 | 0.00126467 | -0.042738584 | 0.157953 | 0.06 |
| KCNN1 | 0.117303064 | 0.00010145 | 0.042401379 | 0.1612622 | 0.08 |
| KCNN2 | 0.031558059 | 0.29722969 | 0.038682577 | 0.201291 | 0.01 |
| KCNN3 | -0.10379854 | 0.0005882 | -0.194906983 | 8.10E-11 | 0.1 |
| KCNN4 | 0.302408967 | 1.50E-24 | 0.11501296 | 0.0001386 | 0.19 |
| KCNQ1DN | 0.090071405 | 0.00287813 | 0.003891917 | 0.8977357 | 0.09 |
| KCNQ1OT1 | -0.155454045 | 2.41E-07 | -0.056751502 | 0.0607101 | 0.1 |
| KCNQ1 | -0.192673197 | 1.34E-10 | -0.152115002 | 4.36E-07 | 0.05 |
| EBN1 | 0.116509889 | 0.0001131 | 0.017645897 | 0.5600543 | 0.1 |
| EBN2 | -0.131580481 | 1.28E-05 | -0.179042004 | 2.51E-09 | 0.05 |
| KCNQ4 | 0.225635018 | 4.40E-14 | 0.056744962 | 0.0607399 | 0.17 |
| KCNQ5 | 0.003003282 | 0.92099792 | -0.080752255 | 0.0075621 | 0.09 |
| KCNRG | 0.024933869 | 0.41021677 | 0.008796624 | 0.7714383 | 0.02 |
| KCNS1 | 0.185846991 | 5.97E-10 | 0.008325018 | 0.7833781 | 0.18 |
| KCNS2 | -0.344131688 | 9.59E-32 | -0.256998759 | 6.02E-18 | 0.09 |
| LOC129330 | -0.145398309 | 1.39E-06 | 0.019807939 | 0.5129983 | 0.17 |
| KCNT1 | 0.004348554 | 0.88581496 | -0.010544212 | 0.7276838 | 0.02 |
| KCNT2 | -0.185428004 | 6.53E-10 | -0.213262794 | 1.04E-12 | 0.03 |
| KCNU1 | -0.106919033 | 0.00039875 | -0.022278138 | 0.461867 | 0.09 |
| KCNV1 | 0.26623559 | 3.43E-19 | 0.06828703 | 0.0239669 | 0.2 |
| KCNV2 | 0.049734686 | 0.10030191 | -0.007365237 | 0.8078323 | 0.06 |
| KCP | 0.101891809 | 0.00074211 | 0.077199187 | 0.0106757 | 0.03 |
| KCTD10 | -0.196063915 | 6.23E-11 | -0.205950348 | 6.20E-12 | 0.01 |
| KCTD11 | -0.151211226 | 5.11E-07 | -0.167453537 | 2.56E-08 | 0.02 |
| KCTD12 | -0.361644532 | 4.13E-35 | -0.3200466 | 1.88E-27 | 0.05 |
| KCTD13 | 0.223903628 | 6.93E-14 | 0.224910629 | 5.32E-14 | 0.01 |
| KCTD14 | 0.08137332 | 0.00711031 | -0.042928827 | 0.1561089 | 0.13 |
| KCTD15 | -0.012175367 | 0.68762615 | -0.034932086 | 0.2485392 | 0.03 |
| KCTD16 | -0.298752478 | 5.69E-24 | -0.210319098 | 2.16E-12 | 0.09 |
| KCTD17 | 0.109822082 | 0.00027516 | -0.010481148 | 0.7292484 | 0.13 |
| KCTD18 | -0.368228184 | 1.97E-36 | -0.324435188 | 3.31E-28 | 0.05 |
| KCTD19 | -0.008026435 | 0.79096403 | -0.071067384 | 0.018783 | 0.07 |
| KCTD1 | 0.112577292 | 0.00019188 | 0.072099019 | 0.0171252 | 0.05 |
| KCTD20 | -0.264070803 | 6.78E-19 | -0.274232479 | 2.62E-20 | 0.02 |
| KCTD21 | -0.296764179 | 1.16E-23 | -0.121307043 | 5.80E-05 | 0.18 |
| KCTD2 | -0.11977911 | 7.20E-05 | -0.12066931 | 6.35E-05 | 0.01 |
| KCTD3 | -0.314352536 | 1.71E-26 | 0.042634389 | 0.15897 | 0.36 |
| KCTD4 | -0.036034206 | 0.23391509 | -0.077810844 | 0.0100697 | 0.05 |
| KCTD5 | 0.33508106 | 4.36E-30 | 0.159475063 | 1.16E-07 | 0.18 |
| KCTD6 | -0.222610218 | 9.71E-14 | -0.043904811 | 0.1469036 | 0.18 |
| KCTD7 | -0.299970945 | 3.66E-24 | -0.31972874 | 2.12E-27 | 0.02 |
| KCTD8 | 0.022845411 | 0.45053938 | -0.05756314 | 0.0571099 | 0.09 |
| KCTD9 | -0.070263046 | 0.02017117 | -0.146564523 | 1.14E-06 | 0.08 |
| KDELC1 | 0.056454911 | 0.06207174 | 7.16E-05 | 0.9981135 | 0.06 |
| KDELC2 | -0.179391789 | 2.34E-09 | -0.239381168 | 1.04E-15 | 0.06 |
| KDELR1 | 0.148956537 | 7.55E-07 | 0.19744619 | 4.54E-11 | 0.05 |
| KDELR2 | 0.096612721 | 0.00138433 | 0.199177622 | 3.05E-11 | 0.11 |
| KDELR3 | -0.019599207 | 0.51744988 | 0.109636373 | 0.0002818 | 0.13 |
| KDM1A | 0.453496201 | 1.51E-56 | 0.227186007 | 2.92E-14 | 0.23 |
| KDM1B | 0.025607678 | 0.3976789 | -0.014236321 | 0.6382508 | 0.04 |
| KDM2A | -0.126164712 | 2.88E-05 | -0.209917226 | 2.38E-12 | 0.09 |
| KDM2B | 0.314525261 | 1.60E-26 | 0.000191175 | 0.9949629 | 0.32 |
| KDM3A | -0.103177554 | 0.00063472 | -0.18962265 | 2.63E-10 | 0.09 |
| KDM3B | -0.428850434 | 3.99E-50 | -0.330546356 | 2.81E-29 | 0.1 |
| KDM4A | -0.094130705 | 0.00183713 | -0.261627499 | 1.45E-18 | 0.17 |
| KDM4B | -0.358752353 | 1.53E-34 | -0.190690763 | 2.08E-10 | 0.17 |
| KDM4C | -0.163717965 | 5.23E-08 | -0.181147266 | 1.62E-09 | 0.02 |
| KDM4E | -0.035036624 | 0.24712531 | -0.102737926 | 0.0006697 | 0.07 |
| KDM4D | 0.074680518 | 0.0135263 | -0.041203021 | 0.1734461 | 0.12 |
| KDM5A | -0.101917859 | 0.00073978 | -0.235070534 | 3.46E-15 | 0.14 |
| KDM5B | -0.135970217 | 6.45E-06 | 0.263742743 | 7.52E-19 | 0.4 |
| KDM5C | -0.111318724 | 0.00022646 | -0.218257299 | 2.97E-13 | 0.11 |
| HY | 0.050909831 | 0.09251609 | 0.034219187 | 0.2583323 | 0.02 |
| KDM6A | -0.471332784 | 1.55E-61 | -0.386434205 | 3.01E-40 | 0.09 |
| KDM6B | -0.278049583 | 7.44E-21 | -0.241593226 | 5.57E-16 | 0.04 |
| KDR | -0.363019273 | 2.20E-35 | -0.312700587 | 3.21E-26 | 0.06 |
| KDSR | 0.018010035 | 0.55198478 | -0.135374571 | 7.08E-06 | 0.16 |
| KEAP1 | 0.123340179 | 4.34E-05 | 0.078410778 | 0.0095051 | 0.05 |
| KEL | 0.054619371 | 0.07107034 | 0.003933506 | 0.896649 | 0.06 |
| KERA | -0.427532314 | 8.51E-50 | -0.208241138 | 3.57E-12 | 0.22 |
| KGFLP1 | -0.053247491 | 0.07847134 | -0.043368716 | 0.1519072 | 0.01 |
| KGFLP2 | -0.147264158 | 1.01E-06 | -0.093334262 | 0.002009 | 0.06 |
| KHDC1L | 0.236900665 | 2.08E-15 | 0.080175012 | 0.0080048 | 0.16 |
| KHDC1 | 0.212492506 | 1.26E-12 | 0.109431185 | 0.0002894 | 0.11 |
| KHDRBS1 | 0.371537412 | 4.16E-37 | 0.243661798 | 3.09E-16 | 0.13 |
| KHDRBS2 | -0.139707735 | 3.55E-06 | -0.126399449 | 2.78E-05 | 0.02 |
| KHDRBS3 | 0.210381979 | 2.12E-12 | 0.103054382 | 0.0006443 | 0.11 |
| KHK | 0.299386977 | 4.52E-24 | 0.085742976 | 0.0045584 | 0.22 |
| KHNYN | -0.300780655 | 2.73E-24 | -0.210126712 | 2.26E-12 | 0.1 |
| KHSRP | 0.270780046 | 8.04E-20 | 0.056343378 | 0.0625903 | 0.22 |
| KIAA0020 | 0.436458879 | 4.73E-52 | 0.287120573 | 3.45E-22 | 0.15 |
| KIAA0040 | -0.405851858 | 1.36E-44 | -0.02311537 | 0.4452044 | 0.39 |
| KIAA0087 | -0.328347897 | 6.87E-29 | -0.235017898 | 3.51E-15 | 0.1 |
| EMC1 | 0.049013537 | 0.10533247 | -0.187223996 | 4.44E-10 | 0.24 |
| KIAA0100 | -0.233903037 | 4.76E-15 | -0.214851387 | 7.02E-13 | 0.02 |
| KIAA0101 | 0.45146543 | 5.35E-56 | 0.358179703 | 1.99E-34 | 0.1 |
| DANCR | 0.38601434 | 3.70E-40 | 0.263001352 | 9.48E-19 | 0.13 |
| FAM30A | -0.000443754 | 0.98830826 | -0.092764175 | 0.002141 | 0.1 |
| KIAA0141 | -0.420050728 | 5.87E-48 | -0.215897553 | 5.40E-13 | 0.21 |
| SPIDR | 0.035387085 | 0.24242622 | 0.018169484 | 0.5484694 | 0.02 |
| IST1 | 0.048907783 | 0.1060867 | -0.068631457 | 0.0232639 | 0.12 |
| GSE1 | -0.24258747 | 4.20E-16 | -0.203483922 | 1.12E-11 | 0.04 |
| KIAA0195 | -0.013932259 | 0.64544198 | -0.082214794 | 0.0065369 | 0.07 |
| KIAA0196 | -0.115020518 | 0.00013844 | -0.150041693 | 6.26E-07 | 0.04 |
| KIAA0226 | -0.062205169 | 0.03976561 | -0.192357473 | 1.43E-10 | 0.14 |
| KIAA0232 | -0.510803162 | 1.11E-73 | -0.312742615 | 3.16E-26 | 0.2 |
| LOC105375062 | -0.372878201 | 2.20E-37 | -0.340470518 | 4.56E-31 | 0.04 |
| SUSD6 | -0.566862841 | 6.27E-94 | -0.39307694 | 1.06E-41 | 0.18 |
| KIAA0284 | -0.089938488 | 0.00291991 | -0.163275862 | 5.69E-08 | 0.08 |
| AREL1 | -0.116742249 | 0.00010956 | -0.194207996 | 9.48E-11 | 0.08 |
| KIAA0319L | -0.463555107 | 2.52E-59 | -0.260543356 | 2.03E-18 | 0.21 |
| DYX2 | 0.131271172 | 1.34E-05 | 0.020639703 | 0.4954592 | 0.12 |
| KIAA0355 | -0.42528594 | 3.07E-49 | -0.397757776 | 9.54E-43 | 0.03 |
| KIAA0368 | -0.14549614 | 1.36E-06 | -0.141874516 | 2.49E-06 | 0.01 |
| KIAA0391 | -0.05821311 | 0.05435621 | -0.000997927 | 0.973711 | 0.06 |
| TTI1 | 0.100906889 | 0.00083552 | 0.01810637 | 0.5498595 | 0.09 |
| KIAA0408 | -0.228836426 | 1.88E-14 | -0.172581751 | 9.34E-09 | 0.06 |
| AP5Z1 | 0.041165233 | 0.17384116 | 0.040946244 | 0.1761439 | 0.01 |
| CTIF | -0.020707364 | 0.49404662 | -0.139656295 | 3.58E-06 | 0.12 |
| A.362G6.1 | -0.46358519 | 2.47E-59 | -0.34438744 | 8.59E-32 | 0.12 |
| C1orf84.1 | 0.024808115 | 0.41258244 | -0.11743128 | 9.97E-05 | 0.15 |
| EFCAB14 | -0.569681536 | 4.72E-95 | -0.476198397 | 5.99E-63 | 0.1 |
| TP73.AS1 | -0.229757408 | 1.47E-14 | -0.175065882 | 5.67E-09 | 0.06 |
| KIAA0513 | -0.289880218 | 1.33E-22 | -0.291574824 | 7.33E-23 | 0.01 |
| C2CD5 | -0.224554391 | 5.84E-14 | -0.15145963 | 4.89E-07 | 0.08 |
| KIAA0556 | -0.412339968 | 4.13E-46 | -0.187245508 | 4.41E-10 | 0.23 |
| CEP104 | -0.247754982 | 9.45E-17 | -0.42119068 | 3.10E-48 | 0.18 |
| VWA8 | -0.392491843 | 1.42E-41 | -0.208488967 | 3.37E-12 | 0.19 |
| KIAA0586 | -0.265497965 | 4.33E-19 | -0.171863188 | 1.08E-08 | 0.1 |
| PPP1R26 | -0.235933626 | 2.72E-15 | -0.152318933 | 4.20E-07 | 0.09 |
| ATG13 | -0.072109649 | 0.01710879 | -0.205934423 | 6.23E-12 | 0.14 |
| CLUHP3 | -0.303918708 | 8.64E-25 | -0.27530064 | 1.85E-20 | 0.03 |
| CLUH | 0.225732386 | 4.29E-14 | 0.059863521 | 0.0478576 | 0.17 |
| TESPA1 | -0.116586535 | 0.00011192 | -0.202281676 | 1.48E-11 | 0.09 |
| KIAA0753 | -0.20008137 | 2.48E-11 | -0.113533751 | 0.000169 | 0.09 |
| LOC649183 | -0.266721384 | 2.94E-19 | -0.368325581 | 1.88E-36 | 0.11 |
| UFL1 | -0.319603093 | 2.23E-27 | -0.1705221 | 1.41E-08 | 0.15 |
| MTCL1 | -0.111081056 | 0.00023361 | -0.223694812 | 7.32E-14 | 0.12 |
| ATG14 | -0.401400554 | 1.43E-43 | -0.264902009 | 5.22E-19 | 0.14 |
| MAU2 | -0.241180767 | 6.26E-16 | -0.243973578 | 2.82E-16 | 0.01 |
| LOC654077 | 0.129008878 | 1.88E-05 | 0.018614616 | 0.5387142 | 0.12 |
| KIAA0895 | -0.129639601 | 1.71E-05 | -0.099011013 | 0.0010467 | 0.04 |
| KIAA0907 | 0.092792656 | 0.00213419 | 0.177104546 | 3.74E-09 | 0.09 |
| LOC196752 | -0.32867529 | 6.02E-29 | -0.296179362 | 1.43E-23 | 0.04 |
| KIAA0922 | 0.05861708 | 0.05270117 | -0.081859266 | 0.0067739 | 0.15 |
| ICE1 | -0.13167443 | 1.26E-05 | -0.247495705 | 1.02E-16 | 0.12 |
| CEP162 | -0.164948195 | 4.14E-08 | -0.073328532 | 0.015318 | 0.1 |
| TRAPPC8 | -0.167780093 | 2.40E-08 | -0.317443731 | 5.18E-27 | 0.15 |
| KIAA1024 | -0.106775312 | 0.00040604 | -0.166737782 | 2.94E-08 | 0.06 |
| LOC121052 | -0.356009181 | 5.27E-34 | -0.319735519 | 2.12E-27 | 0.04 |
| KIAA1045 | 0.15054587 | 5.73E-07 | -0.074596822 | 0.0136316 | 0.23 |
| KIAA1107 | -0.261573261 | 1.48E-18 | -0.259438194 | 2.86E-18 | 0.01 |
| KIAA1109 | -0.513367282 | 1.58E-74 | -0.508275588 | 7.43E-73 | 0.01 |
| LOC92278 | -0.268466154 | 1.69E-19 | -0.167020133 | 2.78E-08 | 0.11 |
| KIAA1147 | -0.339552326 | 6.72E-31 | -0.242115894 | 4.80E-16 | 0.1 |
| KIAA1161 | -0.086028781 | 0.00442468 | -0.097828234 | 0.0012023 | 0.02 |
| FLJ21022 | -0.227534077 | 2.66E-14 | -0.163523027 | 5.43E-08 | 0.07 |
| CEMIP | -0.08881326 | 0.0032963 | -0.12370085 | 4.12E-05 | 0.04 |
| KIAA1210 | -0.211822444 | 1.49E-12 | -0.058270638 | 0.0541179 | 0.16 |
| KIAA1211 | -0.171523953 | 1.15E-08 | -0.183397972 | 1.01E-09 | 0.02 |
| KIAA1217 | -0.363663306 | 1.64E-35 | -0.292665976 | 4.99E-23 | 0.08 |
| NWD2 | -0.131154214 | 1.36E-05 | -0.153341631 | 3.51E-07 | 0.03 |
| C6orf92 | -0.518873491 | 2.29E-76 | -0.314470162 | 1.63E-26 | 0.21 |
| LOC100132731 | -0.142991161 | 2.07E-06 | 0.098376787 | 0.0011277 | 0.25 |
| DKFZP727C091 | -0.398441407 | 6.69E-43 | -0.34640051 | 3.61E-32 | 0.06 |
| PALD1 | -0.238526458 | 1.32E-15 | -0.248132947 | 8.46E-17 | 0.01 |
| KIF1BP | -0.256151725 | 7.78E-18 | -0.169932882 | 1.58E-08 | 0.09 |
| KANSL3 | -0.228895266 | 1.85E-14 | -0.178385626 | 2.88E-09 | 0.06 |
| KIAA1324L | -0.346118546 | 4.08E-32 | -0.341341085 | 3.15E-31 | 0.01 |
| KIAA1324 | -0.374150416 | 1.20E-37 | -0.102079151 | 0.0007255 | 0.28 |
| LOC102724402 | -0.21941525 | 2.21E-13 | -0.21453712 | 7.60E-13 | 0.01 |
| FAM214A | -0.52437623 | 3.06E-78 | -0.218284373 | 2.95E-13 | 0.31 |
| CEP126 | -0.412886202 | 3.07E-46 | -0.209842437 | 2.42E-12 | 0.21 |
| MAP10 | -0.008682192 | 0.77433054 | -0.000504172 | 0.9867165 | 0.01 |
| KIAA1407 | -0.366342197 | 4.74E-36 | -0.138532026 | 4.29E-06 | 0.23 |
| UNC79 | -0.12857049 | 2.01E-05 | -0.234650868 | 3.88E-15 | 0.11 |
| KIAA1429 | -0.031603878 | 0.2965285 | -0.025366284 | 0.4021439 | 0.01 |
| CFAP97 | -0.338069659 | 1.25E-30 | -0.317658309 | 4.76E-27 | 0.03 |
| RIC1 | -0.35865347 | 1.61E-34 | -0.315187649 | 1.24E-26 | 0.05 |
| KIAA1462 | -0.428124141 | 6.06E-50 | -0.295878609 | 1.60E-23 | 0.14 |
| KIAA1467 | -0.271784448 | 5.81E-20 | -0.104639687 | 0.0005302 | 0.17 |
| KIAA1468 | -0.069824488 | 0.02096508 | -0.184947701 | 7.24E-10 | 0.12 |
| LOC100293817 | -0.036419661 | 0.22894713 | -0.110396214 | 0.0002554 | 0.08 |
| KIAA1522 | -0.288817764 | 1.92E-22 | -0.157580458 | 1.64E-07 | 0.14 |
| KIAA1524 | 0.332734709 | 1.15E-29 | 0.114581098 | 0.0001469 | 0.22 |
| LOC100499484.C9ORF174 | -0.060018659 | 0.04728171 | -0.082925225 | 0.0060854 | 0.03 |
| LOC100133766 | -0.359800197 | 9.55E-35 | -0.250603682 | 4.09E-17 | 0.11 |
| FAM214B | -0.102371928 | 0.00070018 | -0.054432402 | 0.072044 | 0.05 |
| CAMSAP3 | -0.007936923 | 0.79324209 | 0.02986789 | 0.3238686 | 0.04 |
| LOC286025 | -0.077992598 | 0.00989556 | -0.107192582 | 0.0003852 | 0.03 |
| KIAA1586 | 0.095047998 | 0.00165595 | 0.019444502 | 0.5207621 | 0.08 |
| FLJ11122 | -0.329963201 | 3.57E-29 | -0.124584794 | 3.62E-05 | 0.21 |
| TLDC1 | 0.236781612 | 2.15E-15 | 0.023034959 | 0.4467897 | 0.22 |
| KIAA1614 | -0.197102816 | 4.92E-11 | -0.24803237 | 8.72E-17 | 0.06 |
| LOC654033 | -0.284719807 | 7.87E-22 | -0.382235944 | 2.40E-39 | 0.1 |
| KIAA1644 | -0.251484014 | 3.15E-17 | -0.243703286 | 3.05E-16 | 0.01 |
| LOC729869 | -0.330848356 | 2.49E-29 | -0.310630718 | 7.05E-26 | 0.03 |
| KIAA1683 | -0.3259242 | 1.82E-28 | -0.215929552 | 5.36E-13 | 0.11 |
| GPALPP1 | -0.306551996 | 3.26E-25 | -0.223976985 | 6.80E-14 | 0.09 |
| CEP44 | -0.224505824 | 5.92E-14 | -0.177034724 | 3.80E-09 | 0.05 |
| KIAA1715 | -0.271185609 | 7.05E-20 | -0.27376732 | 3.05E-20 | 0.01 |
| LOC100506644 | -0.029717463 | 0.32631257 | -0.068920599 | 0.0226875 | 0.04 |
| CIPC | -0.40536447 | 1.76E-44 | -0.254071801 | 1.46E-17 | 0.16 |
| LOC199991 | -0.00686021 | 0.82077719 | 0.00218849 | 0.9423871 | 0.01 |
| KIAA1755 | 0.025853982 | 0.39315398 | -0.046863699 | 0.1215218 | 0.08 |
| FOCAD | -0.18955567 | 2.67E-10 | -0.107842448 | 0.0003547 | 0.09 |
| KIAA1804 | 0.256960713 | 6.09E-18 | 0.126576091 | 2.71E-05 | 0.14 |
| MSANTD4 | -0.087738859 | 0.00369635 | -0.134898093 | 7.63E-06 | 0.05 |
| KIAA1841 | 0.186101417 | 5.65E-10 | 0.010452643 | 0.7299559 | 0.18 |
| KIAA1875 | 0.065598938 | 0.03011371 | 0.036542035 | 0.2273857 | 0.03 |
| PSMG3.AS1 | -0.370999169 | 5.36E-37 | -0.235955816 | 2.71E-15 | 0.14 |
| MGC33953 | -0.251636084 | 3.02E-17 | -0.18898975 | 3.02E-10 | 0.07 |
| LOC100507678 | 0.113077568 | 0.00017957 | -0.09574772 | 0.0015289 | 0.21 |
| FLJ39294 | -0.289173068 | 1.70E-22 | -0.274715392 | 2.24E-20 | 0.02 |
| CCAR2 | -0.11368844 | 0.00016554 | -0.014806348 | 0.624861 | 0.1 |
| CCDC183 | -0.290938546 | 9.16E-23 | -0.153465447 | 3.43E-07 | 0.14 |
| KIAA2013 | 0.322545613 | 7.01E-28 | 0.084678732 | 0.0050889 | 0.24 |
| KIAA2018 | -0.382972759 | 1.67E-39 | -0.319074344 | 2.74E-27 | 0.07 |
| KIAA2022 | -0.187293212 | 4.37E-10 | -0.159924328 | 1.06E-07 | 0.03 |
| KIAA2026 | -0.304455506 | 7.09E-25 | -0.308963053 | 1.32E-25 | 0.01 |
| KIDINS220 | -0.337438428 | 1.63E-30 | -0.387488446 | 1.78E-40 | 0.06 |
| KIF11 | 0.426622433 | 1.43E-49 | 0.217183658 | 3.91E-13 | 0.21 |
| KIF12 | -0.269185665 | 1.34E-19 | -0.072724444 | 0.0161838 | 0.2 |
| KIF13A | -0.484775101 | 1.70E-65 | -0.459453 | 3.51E-58 | 0.03 |
| KIF13B | -0.547809211 | 1.29E-86 | -0.26356507 | 7.95E-19 | 0.29 |
| KIF14 | 0.375753301 | 5.58E-38 | 0.287667752 | 2.86E-22 | 0.09 |
| KIF15 | 0.413988244 | 1.68E-46 | 0.21826651 | 2.97E-13 | 0.2 |
| KIF16B | -0.486925493 | 3.81E-66 | -0.247073843 | 1.15E-16 | 0.24 |
| KIF17 | -0.226922533 | 3.13E-14 | -0.139212066 | 3.84E-06 | 0.09 |
| KIF18A | 0.448253661 | 3.89E-55 | 0.224750129 | 5.55E-14 | 0.23 |
| KIF18B | 0.512705625 | 2.62E-74 | 0.222538319 | 9.89E-14 | 0.3 |
| FLJ37300 | 0.069014742 | 0.02250259 | -0.021957709 | 0.468335 | 0.1 |
| KIF1A | 0.264481014 | 5.96E-19 | 0.054333479 | 0.0725636 | 0.22 |
| KIF1B | -0.054297434 | 0.07275368 | -0.264702779 | 5.56E-19 | 0.22 |
| KIF1C | 0.000452751 | 0.98807121 | -0.109357628 | 0.0002922 | 0.11 |
| KIF20A | 0.458021423 | 8.73E-58 | 0.253719296 | 1.62E-17 | 0.21 |
| KIF20B | 0.163517248 | 5.44E-08 | 0.009685 | 0.7490953 | 0.16 |
| KIF21A | -0.032840386 | 0.27802287 | -0.083796273 | 0.0055703 | 0.06 |
| KIF21B | -0.079424142 | 0.00861535 | -0.091420592 | 0.0024839 | 0.02 |
| KIF22 | 0.449995363 | 1.33E-55 | 0.369925474 | 8.89E-37 | 0.09 |
| KIF23 | 0.454170425 | 9.90E-57 | 0.182325861 | 1.26E-09 | 0.28 |
| KIF24 | 0.105370896 | 0.00048419 | 0.064327786 | 0.0334632 | 0.05 |
| KIF25 | 0.184602914 | 7.80E-10 | 0.093150479 | 0.0020507 | 0.1 |
| KIF26A | -0.254138007 | 1.43E-17 | -0.249068915 | 6.43E-17 | 0.01 |
| KIF26B | -0.259831156 | 2.53E-18 | -0.169206605 | 1.82E-08 | 0.1 |
| KIF27 | -0.378884319 | 1.23E-38 | -0.339082063 | 8.19E-31 | 0.04 |
| KIF2A | -0.171839693 | 1.08E-08 | -0.243550637 | 3.19E-16 | 0.08 |
| KIF2B | -0.025182498 | 0.40556333 | 0.000990672 | 0.9739021 | 0.03 |
| KIF2C | 0.561172964 | 1.08E-91 | 0.269745505 | 1.12E-19 | 0.3 |
| KIF3A | -0.396254133 | 2.07E-42 | -0.227938935 | 2.39E-14 | 0.17 |
| KIF3B | -0.305020937 | 5.75E-25 | -0.159609043 | 1.13E-07 | 0.15 |
| KIF3C | 0.140320226 | 3.21E-06 | -0.075160913 | 0.0129357 | 0.22 |
| LOC158832 | 0.4512715 | 6.03E-56 | 0.235829974 | 2.80E-15 | 0.22 |
| KIF4B | 0.320175134 | 1.78E-27 | 0.091081145 | 0.0025782 | 0.23 |
| KIF5A | -0.188570576 | 3.31E-10 | -0.243811943 | 2.96E-16 | 0.06 |
| LOC221012 | -0.172790984 | 8.96E-09 | -0.204622992 | 8.52E-12 | 0.04 |
| KIF5C | -0.333787293 | 7.45E-30 | -0.21221164 | 1.35E-12 | 0.13 |
| KIF6 | -0.044737752 | 0.13938037 | -0.025938631 | 0.3916061 | 0.02 |
| KIF7 | 0.024834375 | 0.41208777 | -0.08956889 | 0.003039 | 0.12 |
| KIF9 | -0.036661755 | 0.2258655 | 0.21059799 | 2.01E-12 | 0.25 |
| KIFAP3 | -0.051207954 | 0.09062 | 0.170306426 | 1.47E-08 | 0.23 |
| KIFC1 | 0.558310215 | 1.38E-90 | 0.296118059 | 1.47E-23 | 0.27 |
| KIFC2 | 0.240131759 | 8.43E-16 | 0.162628642 | 6.43E-08 | 0.08 |
| KIFC3 | 0.050426223 | 0.09565941 | -0.089346921 | 0.0031126 | 0.14 |
| KLLN | -0.16979499 | 1.62E-08 | -0.195985435 | 6.34E-11 | 0.03 |
| KIN | 0.385805088 | 4.11E-40 | 0.253440467 | 1.76E-17 | 0.14 |
| KIR2DL1 | 0.071323475 | 0.01835889 | -0.030650615 | 0.3113447 | 0.11 |
| KIR2DL3 | 0.091796052 | 0.00238335 | -0.025144155 | 0.4062789 | 0.12 |
| KIR2DL4 | 0.197219723 | 4.79E-11 | 0.00249738 | 0.9342726 | 0.2 |
| KIR2DS4 | 0.112847108 | 0.00018515 | -0.003714079 | 0.9023847 | 0.12 |
| LOC115656 | 0.039694073 | 0.18974862 | -0.065682656 | 0.0299036 | 0.11 |
| KIR3DL2 | 0.080402727 | 0.00782745 | -0.021551699 | 0.476602 | 0.11 |
| KIR3DL3 | 0.040024037 | 0.18609076 | -0.024798319 | 0.4127671 | 0.07 |
| KIR3DP1 | 0.017077239 | 0.57276909 | 0.00480757 | 0.873858 | 0.02 |
| KIR3DX1 | 0.064499499 | 0.03299292 | -0.068558352 | 0.0234116 | 0.14 |
| KIRREL2 | 0.133639112 | 9.29E-06 | -0.056684118 | 0.0610173 | 0.2 |
| KIRREL3 | 0.1951565 | 7.65E-11 | -0.050121353 | 0.0976845 | 0.25 |
| LOC348416 | -0.426558544 | 1.49E-49 | -0.449148606 | 2.24E-55 | 0.03 |
| GPR54 | -0.016878723 | 0.57723978 | 0.040750119 | 0.1782255 | 0.06 |
| KISS1 | 0.19751876 | 4.47E-11 | 0.313498334 | 2.37E-26 | 0.12 |
| KITLG | -0.35437839 | 1.09E-33 | -0.18277278 | 1.15E-09 | 0.18 |
| KIT | -0.039306875 | 0.19410809 | -0.079858281 | 0.0082575 | 0.05 |
| KLB | -0.176954857 | 3.86E-09 | -0.176693507 | 4.07E-09 | 0.01 |
| KLC1 | -0.035660956 | 0.23879801 | -0.178594416 | 2.76E-09 | 0.15 |
| KLC2 | 0.103590463 | 0.00060342 | -0.043567176 | 0.15004 | 0.15 |
| KLC3 | 0.181681799 | 1.45E-09 | 0.087713852 | 0.0037062 | 0.1 |
| LOC116139 | -0.031121788 | 0.30396153 | 0.009595855 | 0.7513282 | 0.05 |
| KLF10 | -0.209547227 | 2.60E-12 | -0.30811244 | 1.82E-25 | 0.1 |
| KLF11 | 0.056681522 | 0.06102915 | -0.067104215 | 0.0265232 | 0.13 |
| KLF12 | -0.254023632 | 1.48E-17 | -0.272871232 | 4.08E-20 | 0.02 |
| KLF13 | -0.279275348 | 4.95E-21 | -0.400175814 | 2.71E-43 | 0.13 |
| KLF14 | 0.09709619 | 0.0013091 | -0.024468783 | 0.4190061 | 0.13 |
| KLF15 | -0.101093518 | 0.00081703 | -0.122800878 | 4.69E-05 | 0.03 |
| KLF16 | 0.227490958 | 2.69E-14 | 0.163755864 | 5.20E-08 | 0.07 |
| KLF17 | -0.118268693 | 8.88E-05 | -0.055392341 | 0.0671586 | 0.07 |
| INLU | 0.286185217 | 4.76E-22 | 0.180346184 | 1.92E-09 | 0.11 |
| KLF2 | -0.236742577 | 2.18E-15 | -0.077715732 | 0.0101618 | 0.16 |
| KLF3 | -0.402754177 | 7.01E-44 | -0.290968863 | 9.06E-23 | 0.12 |
| KLF4 | -0.319499213 | 2.32E-27 | -0.201637282 | 1.72E-11 | 0.12 |
| KLF5 | 0.157518806 | 1.66E-07 | 0.003241662 | 0.9147503 | 0.16 |
| KLF6 | -0.203042044 | 1.24E-11 | -0.225037054 | 5.15E-14 | 0.03 |
| KLF7 | -0.343036649 | 1.53E-31 | -0.361024164 | 5.47E-35 | 0.02 |
| LOC105373216 | -0.241789129 | 5.27E-16 | -0.140229228 | 3.26E-06 | 0.11 |
| KLF9 | -0.429746727 | 2.38E-50 | -0.245554687 | 1.79E-16 | 0.19 |
| KLHDC10 | -0.339162088 | 7.92E-31 | -0.356778877 | 3.73E-34 | 0.02 |
| KLHDC1 | -0.484581586 | 1.95E-65 | -0.323404838 | 4.98E-28 | 0.17 |
| LOC122774 | -0.355287795 | 7.28E-34 | -0.130785828 | 1.44E-05 | 0.23 |
| KLHDC3 | 0.467608671 | 1.81E-60 | 0.308324185 | 1.68E-25 | 0.16 |
| LOC100130068 | 0.31384031 | 2.08E-26 | 0.199604338 | 2.76E-11 | 0.12 |
| KLHL42 | -0.143246297 | 1.99E-06 | -0.208624312 | 3.26E-12 | 0.07 |
| KLHDC7A | -0.367969311 | 2.22E-36 | -0.152258503 | 4.25E-07 | 0.22 |
| KLHDC7B | 0.207083735 | 4.73E-12 | 0.021194001 | 0.4839509 | 0.19 |
| KLHDC8A | 0.065288166 | 0.03090483 | 0.033398102 | 0.2699386 | 0.04 |
| KLHDC8B | -0.013931149 | 0.6454683 | -0.009400804 | 0.7562211 | 0.01 |
| KLHDC9 | -0.113381445 | 0.00017246 | 0.183911442 | 9.04E-10 | 0.3 |
| FLJ32662 | -0.042739068 | 0.15794834 | -0.026862472 | 0.374954 | 0.02 |
| KLHL11 | -0.388086812 | 1.32E-40 | -0.408256345 | 3.76E-45 | 0.03 |
| KLHL12 | -0.142918828 | 2.10E-06 | 0.292441989 | 5.40E-23 | 0.44 |
| KLHL13 | -0.02805853 | 0.35405558 | -0.072747528 | 0.0161499 | 0.05 |
| KLHL14 | -0.196916843 | 5.13E-11 | -0.178144113 | 3.02E-09 | 0.02 |
| KLHL15 | -0.06983749 | 0.02094116 | -0.174777681 | 6.01E-09 | 0.11 |
| KLHL17 | 0.154624934 | 2.79E-07 | 0.07093782 | 0.0190008 | 0.09 |
| FLJ13703 | -0.233256739 | 5.68E-15 | -0.244344429 | 2.54E-16 | 0.02 |
| KLHL1 | -0.037728272 | 0.21264182 | 0.046662935 | 0.1231278 | 0.09 |
| KLHL20 | -0.416182695 | 5.03E-47 | -0.180913761 | 1.70E-09 | 0.24 |
| KLHL21 | -0.046788101 | 0.1221246 | -0.261556642 | 1.49E-18 | 0.22 |
| KLHL22 | -0.015411871 | 0.61077175 | -0.047668963 | 0.1152439 | 0.04 |
| KLHL23 | -0.111014972 | 0.00023563 | -0.283010882 | 1.41E-21 | 0.18 |
| FLJ22673 | -0.115589041 | 0.00012819 | -0.146344763 | 1.18E-06 | 0.04 |
| KLHL25 | 0.127216406 | 2.46E-05 | -0.003711797 | 0.9024444 | 0.14 |
| KLHL26 | -0.221081721 | 1.44E-13 | -0.086505565 | 0.0042096 | 0.14 |
| KLHL28 | -0.482970524 | 5.93E-65 | -0.356168573 | 4.91E-34 | 0.13 |
| KLHL29 | -0.168881521 | 1.94E-08 | -0.235694799 | 2.91E-15 | 0.07 |
| KLHL2 | -0.28998932 | 1.28E-22 | -0.320930358 | 1.33E-27 | 0.04 |
| KLHL30 | -0.03918019 | 0.19555025 | -0.073620277 | 0.0149146 | 0.04 |
| LOC391932 | -0.073103311 | 0.0156359 | -0.073272177 | 0.015397 | 0.01 |
| KLHL32 | -0.017617226 | 0.56069205 | -0.053353902 | 0.0778757 | 0.04 |
| KLHL33 | -0.386363227 | 3.11E-40 | -0.307220956 | 2.54E-25 | 0.08 |
| KLHL34 | 0.142604223 | 2.21E-06 | -0.073812586 | 0.0146539 | 0.22 |
| LOC143568 | 0.015562283 | 0.60729391 | 0.067251861 | 0.0261917 | 0.06 |
| KLHL36 | 0.006727115 | 0.82419698 | -0.129339599 | 1.79E-05 | 0.14 |
| LOC649830 | -0.11153638 | 0.00022009 | -0.087619369 | 0.0037435 | 0.03 |
| KLHL3 | -0.453896866 | 1.17E-56 | -0.341401518 | 3.07E-31 | 0.12 |
| KLHL4 | -0.371833887 | 3.61E-37 | -0.266026434 | 3.67E-19 | 0.11 |
| KLHL5 | -0.242179954 | 4.72E-16 | -0.293662104 | 3.51E-23 | 0.06 |
| KLHL6 | -0.15479283 | 2.71E-07 | -0.244280386 | 2.59E-16 | 0.09 |
| KLHL7 | 0.123826665 | 4.04E-05 | 0.012606139 | 0.6771864 | 0.12 |
| KLHL8 | -0.230476438 | 1.21E-14 | -0.275259657 | 1.87E-20 | 0.05 |
| LOC158014 | -0.320924517 | 1.33E-27 | -0.169399923 | 1.75E-08 | 0.16 |
| KLK10 | 0.137238441 | 5.27E-06 | 0.024824293 | 0.4122776 | 0.12 |
| KLK11 | -0.033168216 | 0.27325115 | 0.012468972 | 0.6805041 | 0.05 |
| KLK12 | 0.012372545 | 0.68284003 | 0.059453723 | 0.049407 | 0.05 |
| KLK13 | 0.024160292 | 0.42489657 | 0.011062047 | 0.71488 | 0.02 |
| KLK14 | 0.089105723 | 0.00319444 | 0.027453161 | 0.3645396 | 0.07 |
| KLK15 | 0.090757488 | 0.00267105 | 0.0563947 | 0.0623512 | 0.04 |
| KLK1 | 0.06052376 | 0.04544702 | 0.025165195 | 0.4058862 | 0.04 |
| LOC115292 | -0.154455153 | 2.88E-07 | -0.06531727 | 0.03083 | 0.09 |
| LOC147857 | -0.164101775 | 4.87E-08 | -0.058209596 | 0.0543708 | 0.11 |
| KLK4 | -0.274223397 | 2.63E-20 | -0.037390283 | 0.2167707 | 0.24 |
| KLK5 | 0.114869391 | 0.00014129 | -0.009700686 | 0.7487026 | 0.13 |
| KLK6 | 0.210121593 | 2.26E-12 | 0.038943933 | 0.1982607 | 0.18 |
| KLK7 | 0.131173672 | 1.36E-05 | -0.023204056 | 0.4434596 | 0.16 |
| LOC116193 | 0.181279693 | 1.58E-09 | 0.031422293 | 0.2993139 | 0.15 |
| KLK9 | 0.244057878 | 2.76E-16 | 0.057544793 | 0.0571892 | 0.19 |
| KLKB1 | -0.012846887 | 0.67137864 | -0.092834714 | 0.0021242 | 0.08 |
| LOC100132624 | -0.28247429 | 1.69E-21 | -0.059643421 | 0.0486846 | 0.23 |
| KLRAP1 | -0.189434761 | 2.74E-10 | -0.109758282 | 0.0002774 | 0.08 |
| PPP1R21 | -0.043628736 | 0.14946438 | -0.105626389 | 0.000469 | 0.07 |
| KLRB1 | -0.057211558 | 0.05864702 | -0.091615935 | 0.0024311 | 0.04 |
| KLRC1 | 0.063782833 | 0.03499383 | -0.04228017 | 0.1624644 | 0.11 |
| KLRC2 | 0.056382389 | 0.0624085 | -0.071736527 | 0.0176925 | 0.13 |
| KLRC3 | 0.025802286 | 0.39410111 | -0.098283952 | 0.00114 | 0.13 |
| KLRC4 | -0.184225263 | 8.45E-10 | -0.221154647 | 1.42E-13 | 0.04 |
| KLRD1 | 0.002305059 | 0.93932409 | -0.079692421 | 0.0083926 | 0.09 |
| KLRF1 | -0.090718568 | 0.00268242 | -0.099626196 | 0.0009733 | 0.01 |
| KLRG1 | -0.070531672 | 0.01969793 | -0.087683309 | 0.0037182 | 0.02 |
| KLRG2 | 0.293611252 | 3.58E-23 | 0.17525357 | 5.46E-09 | 0.12 |
| KLRK1 | -0.03152 | 0.29781296 | -0.102386289 | 0.000699 | 0.08 |
| KL | -0.421017641 | 3.42E-48 | -0.322665822 | 6.68E-28 | 0.1 |
| KMO | -0.048133552 | 0.11173955 | 0.146829977 | 1.09E-06 | 0.2 |
| KNCN | -0.012733808 | 0.67410413 | 0.057319771 | 0.0581703 | 0.08 |
| RASGEF2 | -0.191982836 | 1.56E-10 | -0.012327642 | 0.6839289 | 0.18 |
| BDK | 0.01562738 | 0.60579145 | 0.022658292 | 0.4542584 | 0.01 |
| LOC65669 | 0.239216306 | 1.09E-15 | 0.091591143 | 0.0024378 | 0.15 |
| KPNA1 | -0.102152692 | 0.00071905 | -0.092356686 | 0.0022401 | 0.01 |
| LOC643995 | 0.513116122 | 1.92E-74 | 0.281849576 | 2.08E-21 | 0.24 |
| KPNA3 | -0.0103963 | 0.73135518 | -0.050624725 | 0.094359 | 0.05 |
| MGC12217 | 0.208202359 | 3.61E-12 | 0.098140143 | 0.0011593 | 0.12 |
| KPNA5 | -0.193205732 | 1.19E-10 | -0.248427402 | 7.77E-17 | 0.06 |
| KPNA6 | -0.138312825 | 4.44E-06 | -0.289584406 | 1.47E-22 | 0.16 |
| KPNA7 | -0.010341303 | 0.73272185 | 0.070813048 | 0.0192126 | 0.09 |
| KPNB1 | 0.088493018 | 0.00341121 | -0.01157999 | 0.7021531 | 0.11 |
| KPRP | 0.092271648 | 0.00226131 | 0.034417092 | 0.2555873 | 0.06 |
| KPTN | 0.205217828 | 7.39E-12 | 0.247912095 | 9.03E-17 | 0.05 |
| KRAS | -0.099025847 | 0.00104485 | -0.065966691 | 0.0292 | 0.04 |
| LOC392836 | 0.057649947 | 0.05673551 | -0.009877086 | 0.744291 | 0.07 |
| KRBA2 | -0.435632349 | 7.70E-52 | -0.427081436 | 1.10E-49 | 0.01 |
| KRCC1 | -0.106125968 | 0.00044059 | -0.10316066 | 0.000636 | 0.01 |
| KREMEN1 | -0.234851305 | 3.67E-15 | -0.248904372 | 6.75E-17 | 0.02 |
| KREMEN2 | 0.284058729 | 9.86E-22 | 0.192756685 | 1.31E-10 | 0.1 |
| KRI1 | 0.249292507 | 6.03E-17 | 0.20019208 | 2.41E-11 | 0.05 |
| KRIT1 | -0.189247601 | 2.85E-10 | -0.215457196 | 6.03E-13 | 0.03 |
| KRR1 | -0.050098399 | 0.09783831 | -0.11185349 | 0.0002111 | 0.07 |
| KRT10 | 0.143054091 | 2.05E-06 | 0.264053421 | 6.82E-19 | 0.13 |
| KRT12 | -0.05370004 | 0.07596363 | 0.021360188 | 0.480529 | 0.08 |
| KRT13 | 0.067314881 | 0.02605137 | -0.079619893 | 0.0084523 | 0.15 |
| NFJ | 0.018148923 | 0.54892206 | -0.060665183 | 0.0449442 | 0.08 |
| KRT15 | 0.037372631 | 0.21698787 | -0.061897472 | 0.0407578 | 0.1 |
| KRT16 | 0.268134357 | 1.88E-19 | 0.056189468 | 0.0633117 | 0.22 |
| PCHC1 | 0.123692488 | 4.12E-05 | -0.02527962 | 0.4037542 | 0.15 |
| KRT18 | -0.102836955 | 0.00066167 | 0.113885325 | 0.0001612 | 0.22 |
| KRT19 | -0.033269471 | 0.2717887 | 0.071481665 | 0.0181011 | 0.11 |
| KRT1 | -0.091141703 | 0.00256111 | -0.065046099 | 0.0315333 | 0.03 |
| KRT20 | 0.008967695 | 0.76712038 | 0.017517436 | 0.5629146 | 0.01 |
| KRT222 | -0.303854507 | 8.85E-25 | -0.141565844 | 2.62E-06 | 0.17 |
| KRT23 | 0.039884861 | 0.18762722 | -0.018661994 | 0.537681 | 0.06 |
| FLJ20261 | -0.031946804 | 0.29131569 | 0.037706354 | 0.2129078 | 0.07 |
| KRT25 | 0.016759233 | 0.57993865 | 0.032859239 | 0.2777469 | 0.02 |
| KRT26 | 0.062322075 | 0.03939402 | 0.030874747 | 0.307818 | 0.04 |
| KRT27 | -0.059137962 | 0.05062944 | -0.015724879 | 0.6035443 | 0.05 |
| KRT28 | 0.009826659 | 0.7455513 | 0.070949955 | 0.0189803 | 0.07 |
| KRT2 | 0.104652033 | 0.00052941 | 0.083035331 | 0.006018 | 0.03 |
| KRT31 | -0.014532133 | 0.63128719 | -0.063000911 | 0.037294 | 0.05 |
| KRT32 | -0.106500527 | 0.00042034 | -0.021599152 | 0.4756317 | 0.09 |
| KRT33A | -0.013025473 | 0.66708303 | -0.091141244 | 0.0025612 | 0.08 |
| KRT33B | 0.125416264 | 3.21E-05 | 0.027827634 | 0.3580317 | 0.1 |
| KRT34 | 0.182375436 | 1.25E-09 | 0.020147626 | 0.5057966 | 0.17 |
| KRT35 | -0.060220595 | 0.04654088 | -0.071891281 | 0.0174483 | 0.02 |
| KRT36 | 0.035045208 | 0.24700945 | 0.041361122 | 0.1718004 | 0.01 |
| KRT37 | -0.16400738 | 4.95E-08 | -0.018972113 | 0.530943 | 0.15 |
| KRT38 | 0.145868026 | 1.28E-06 | -0.024098573 | 0.4260808 | 0.17 |
| LOC728765 | 0.020119669 | 0.50638732 | 0.028277688 | 0.3503075 | 0.01 |
| KRT3 | 0.198349152 | 3.69E-11 | 0.045169428 | 0.1355998 | 0.16 |
| LOC728760 | 0.02201111 | 0.46725363 | 0.032783626 | 0.2788548 | 0.02 |
| KRT4 | 0.119669409 | 7.31E-05 | -0.019303072 | 0.5237996 | 0.14 |
| KRT5 | 0.090852467 | 0.00264348 | -0.056465954 | 0.0620206 | 0.15 |
| LOC93086 | 0.224138544 | 6.52E-14 | -0.006103177 | 0.8402721 | 0.24 |
| KRTL1 | 0.196245776 | 5.98E-11 | 0.015075807 | 0.6185739 | 0.19 |
| LOC95009 | 0.219956359 | 1.93E-13 | 0.001644914 | 0.9566806 | 0.22 |
| KRT71 | -0.013642966 | 0.65231474 | 0.018482477 | 0.541601 | 0.04 |
| KRT72 | 0.187544482 | 4.14E-10 | 0.065787756 | 0.0296416 | 0.13 |
| KRT73 | -0.023457041 | 0.438504 | -0.041008969 | 0.175482 | 0.02 |
| KRT74 | 0.128605914 | 2.00E-05 | -0.003963859 | 0.895856 | 0.14 |
| KRT75 | 0.206918223 | 4.92E-12 | 0.04214067 | 0.1638564 | 0.17 |
| KRT76 | 0.070566115 | 0.01963796 | -0.009075364 | 0.7644065 | 0.08 |
| LOC387860 | 0.09995013 | 0.00093659 | -0.02448765 | 0.4186474 | 0.13 |
| KRT78 | 0.275315794 | 1.84E-20 | 0.073209027 | 0.015486 | 0.21 |
| KRT6L | 0.205386688 | 7.10E-12 | 0.074202906 | 0.0141369 | 0.14 |
| KRT7 | 0.135352366 | 7.11E-06 | 0.146001619 | 1.25E-06 | 0.02 |
| KRT80 | 0.06950867 | 0.02155351 | 0.00861522 | 0.7760247 | 0.07 |
| KRT81 | 0.231866344 | 8.30E-15 | 0.091181082 | 0.0025501 | 0.15 |
| KRT82 | 0.146273219 | 1.19E-06 | 0.057419072 | 0.0577356 | 0.09 |
| LOC652010 | 0.321874783 | 9.14E-28 | 0.145090664 | 1.46E-06 | 0.18 |
| KRT84 | 0.157976229 | 1.52E-07 | 0.073615227 | 0.0149215 | 0.09 |
| KRT85 | 0.186525585 | 5.16E-10 | 0.055437698 | 0.0669346 | 0.14 |
| LOC650428 | 0.218661997 | 2.68E-13 | 0.111859717 | 0.0002109 | 0.11 |
| LOC115042 | -0.014727273 | 0.62671124 | 0.139428522 | 3.71E-06 | 0.16 |
| KRT9 | 0.260399297 | 2.12E-18 | 0.045598311 | 0.1319225 | 0.22 |
| LOC115775 | 0.102844057 | 0.0006611 | -0.008785536 | 0.7717184 | 0.12 |
| LOC644385 | -0.005028051 | 0.8681247 | -0.063315137 | 0.0363547 | 0.06 |
| LOC644392 | 0.063691708 | 0.03525551 | -0.0064329 | 0.8317684 | 0.08 |
| KRTAP10.10 | 0.040685365 | 0.1789167 | 0.033930063 | 0.2623791 | 0.01 |
| KRTAP10.11 | 0.004448772 | 0.88320203 | -0.014572048 | 0.63035 | 0.02 |
| KRTAP10.12 | -0.004813292 | 0.87370911 | -0.001978274 | 0.9479128 | 0.01 |
| KRTAP10.1 | -0.021715961 | 0.47324781 | 0.016943226 | 0.5757853 | 0.04 |
| KRTAP10.2 | -0.033852496 | 0.26347218 | -0.02194403 | 0.4686122 | 0.02 |
| KRTAP10.3 | -0.031411527 | 0.29947955 | -0.023323861 | 0.4411088 | 0.01 |
| KRTAP10.4 | 0.014825853 | 0.62440501 | 0.035792408 | 0.2370702 | 0.03 |
| KRTAP10.5 | -0.014753608 | 0.62609478 | -0.025013245 | 0.4087277 | 0.02 |
| KRTAP10.6 | 0.025260576 | 0.40410853 | 0.035047086 | 0.2469841 | 0.01 |
| KRTAP10.7 | 0.026249907 | 0.38594598 | 0.00065444 | 0.9827579 | 0.03 |
| KRTAP10.8 | 0.038401367 | 0.20458892 | -0.007220986 | 0.8115246 | 0.05 |
| KRTAP10.9 | 0.024940362 | 0.41009484 | -0.003388712 | 0.9108989 | 0.03 |
| KRTAP11.1 | 0.08244491 | 0.00638745 | 0.068886064 | 0.0227557 | 0.02 |
| KRTAP12.1 | 0.020755189 | 0.4930495 | 0.004633735 | 0.878383 | 0.02 |
| KRTAP12.2 | 0.06127539 | 0.042828 | 0.091932088 | 0.0023478 | 0.04 |
| KRTAP12.3 | 0.011216958 | 0.71106501 | -0.011748324 | 0.6980345 | 0.03 |
| KRTAP12.4 | 0.009990356 | 0.7414627 | 0.038464205 | 0.2038486 | 0.03 |
| KRTAP13.1 | 0.00246587 | 0.9351001 | -0.000158035 | 0.9958361 | 0.01 |
| KRTAP13.2 | 0.004082433 | 0.89275925 | -0.011393505 | 0.706726 | 0.02 |
| KRTAP13.3 | NA | NA | NA | NA | #VALUE! |
| KRTAP13.4 | -0.0638542 | 0.03479004 | -0.055048335 | 0.0688771 | 0.01 |
| KRTAP15.1 | 0.016535218 | 0.58501423 | -0.003331678 | 0.9123924 | 0.02 |
| KRTAP17.1 | 0.165116001 | 4.01E-08 | 0.10337522 | 0.0006196 | 0.07 |
| KRTAP19.1 | 0.090239952 | 0.00282595 | 0.034404874 | 0.2557561 | 0.06 |
| KRTAP19.2 | -0.007830864 | 0.79594358 | -0.005458118 | 0.8569616 | 0.01 |
| KRTAP19.3 | 0.068581539 | 0.02336464 | -0.028241765 | 0.3509201 | 0.1 |
| KRTAP19.4 | NA | NA | NA | NA | #VALUE! |
| KRTAP19.5 | 0.023709384 | 0.43359279 | 0.032544226 | 0.282382 | 0.01 |
| KRTAP19.6 | 0.07823549 | 0.00966708 | 0.0160572 | 0.5959132 | 0.07 |
| KRTAP19.7 | NA | NA | NA | NA | #VALUE! |
| KRTAP19.8 | 0.01067362 | 0.72447681 | -0.0037041 | 0.9026457 | 0.02 |
| LOC728943 | 0.052201446 | 0.08452567 | 0.017271358 | 0.5684134 | 0.04 |
| KRTAP2.2 | 0.050332266 | 0.0962799 | 0.104671375 | 0.0005281 | 0.06 |
| KAP2.1B | -0.009652384 | 0.74991198 | -0.03155955 | 0.2972069 | 0.03 |
| KRTAP20.1 | NA | NA | NA | NA | #VALUE! |
| KRTAP20.2 | 0.002141264 | 0.9436282 | 0.035803411 | 0.236926 | 0.04 |
| KRTAP20.3 | NA | NA | NA | NA | #VALUE! |
| KRTAP20.4 | 0.094262954 | 0.00180994 | 0.060512557 | 0.0454871 | 0.04 |
| KRTAP21.1 | 0.002923033 | 0.92310225 | 0.02943459 | 0.3309407 | 0.03 |
| KRTAP21.2 | -0.014935742 | 0.62183852 | 0.005527185 | 0.8551715 | 0.03 |
| KRTAP22.1 | NA | NA | NA | NA | #VALUE! |
| KRTAP23.1 | NA | NA | NA | NA | #VALUE! |
| KRTAP24.1 | -0.087849287 | 0.00365329 | -0.030438745 | 0.3147028 | 0.06 |
| KRTAP25.1 | -0.007830864 | 0.79594358 | -0.005458118 | 0.8569616 | 0.01 |
| KRTAP26.1 | 0.054101906 | 0.07379187 | 0.06002871 | 0.0472446 | 0.01 |
| LOC140256 | 0.013829627 | 0.64787681 | -0.008722212 | 0.7733187 | 0.03 |
| LOC644398 | -0.027446313 | 0.36465925 | 0.071714687 | 0.0177272 | 0.1 |
| LOC644406 | -0.064865147 | 0.03201029 | -0.072210302 | 0.0169543 | 0.01 |
| KRTAP3.3 | -0.151474082 | 4.88E-07 | -0.076205641 | 0.0117294 | 0.08 |
| KRTAP4.11 | 0.055883508 | 0.06476615 | 0.035776179 | 0.237283 | 0.03 |
| LOC644308 | 0.022764156 | 0.45215222 | 0.000362777 | 0.9904417 | 0.03 |
| KRTAP4.1 | 0.076004462 | 0.01195362 | 0.039710286 | 0.1895677 | 0.04 |
| KRTAP4.2 | 0.05781314 | 0.0560373 | 0.029375086 | 0.3319196 | 0.03 |
| LOC100508111 | -0.060586661 | 0.04522279 | -0.01968413 | 0.5156363 | 0.05 |
| KRTAP4.13 | 0.003397982 | 0.91065615 | -0.012045797 | 0.690778 | 0.02 |
| KRTAP4.5 | -0.028866765 | 0.34035816 | 0.010829175 | 0.7206282 | 0.04 |
| KRTAP4.7 | 0.044123816 | 0.1448961 | 0.026855533 | 0.3750774 | 0.02 |
| LOC100134267 | 0.002218436 | 0.94160012 | 0.024895562 | 0.4109365 | 0.03 |
| KRTAP4.9 | 0.022102109 | 0.46541406 | 0.021050778 | 0.4869105 | 0.01 |
| KRTAP5.10 | 0.145186983 | 1.44E-06 | 0.176772315 | 4.01E-09 | 0.04 |
| KRTAP5.11 | 0.067321358 | 0.02603698 | 0.048547361 | 0.1086894 | 0.02 |
| KRN1L | 0.029697395 | 0.32663952 | 0.107737624 | 0.0003595 | 0.08 |
| KRTAP5.2 | -0.035042665 | 0.24704377 | -0.033041874 | 0.2750835 | 0.01 |
| KRTAP5.3 | 0.113605276 | 0.00016739 | 0.050070947 | 0.0980226 | 0.07 |
| KRTAP5.4 | -0.035882614 | 0.23588965 | -0.039513356 | 0.1917743 | 0.01 |
| KRTAP5.5 | 0.082862686 | 0.00612401 | 0.060750329 | 0.0446437 | 0.03 |
| KRTAP5.6 | 0.114600249 | 0.0001465 | 0.040172082 | 0.1844666 | 0.08 |
| KRTAP5.7 | -0.118230252 | 8.93E-05 | -0.01360375 | 0.6532487 | 0.11 |
| KRTAP5.8 | 0.126456016 | 2.76E-05 | 0.169086098 | 1.86E-08 | 0.05 |
| KRTAP5.9 | 0.01403475 | 0.64301428 | 0.159945184 | 1.06E-07 | 0.15 |
| KRTAP6.1 | -0.007830864 | 0.79594358 | -0.005458118 | 0.8569616 | 0.01 |
| KRTAP6.2 | -0.017157224 | 0.57097243 | -0.006979522 | 0.8177144 | 0.02 |
| KRTAP6.3 | 0.034571158 | 0.25346439 | 0.022989102 | 0.4476952 | 0.02 |
| KRTAP7.1 | 0.087385196 | 0.00383738 | 0.000305828 | 0.9919421 | 0.09 |
| KRTAP8.1 | 0.054393584 | 0.07224755 | 0.001237104 | 0.9674134 | 0.06 |
| KRTAP9.2 | 0.001107341 | 0.97082992 | 0.007776919 | 0.7973186 | 0.01 |
| KRTAP9.4 | 0.064862052 | 0.0320185 | -0.012380788 | 0.6826402 | 0.08 |
| LOC100996762 | -0.013051931 | 0.66644755 | 0.004818779 | 0.8735664 | 0.02 |
| KRTAP9.5 | 0.069494637 | 0.02157999 | -0.010200398 | 0.7362272 | 0.08 |
| KRTCAP2 | 0.428987139 | 3.69E-50 | 0.577907614 | 2.16E-98 | 0.15 |
| KRTCAP3 | 0.238969002 | 1.17E-15 | 0.18545874 | 6.49E-10 | 0.06 |
| KRTDAP | 0.221396446 | 1.33E-13 | 0.056084327 | 0.0638084 | 0.17 |
| KSR1 | -0.07967267 | 0.00840881 | -0.261905262 | 1.33E-18 | 0.19 |
| LOC341537 | -0.262247349 | 1.20E-18 | -0.204602836 | 8.56E-12 | 0.06 |
| POGLUT1 | -0.015949655 | 0.59837797 | -0.100738621 | 0.0008525 | 0.09 |
| KTI12 | 0.412163287 | 4.55E-46 | 0.235838146 | 2.80E-15 | 0.18 |
| CG1 | -0.224675709 | 5.66E-14 | -0.12346201 | 4.26E-05 | 0.11 |
| KYNU | 0.064757985 | 0.03229564 | 0.02188777 | 0.4697534 | 0.05 |
| KY | -0.080213638 | 0.00797447 | -0.073736957 | 0.0147559 | 0.01 |
| MIC5 | 0.148384945 | 8.33E-07 | -0.059348607 | 0.0498112 | 0.21 |
| L1TD1 | -0.141406934 | 2.69E-06 | -0.070943776 | 0.0189907 | 0.08 |
| L2HGDH | -0.063607943 | 0.03549753 | -0.073405803 | 0.0152102 | 0.01 |
| L3MBTL2 | 0.081804799 | 0.00681086 | 0.057883988 | 0.0557364 | 0.03 |
| L3MBTL3 | -0.069898644 | 0.02082896 | -0.160836964 | 8.99E-08 | 0.1 |
| LOC105372015 | 0.187430326 | 4.24E-10 | -0.049015963 | 0.1053152 | 0.24 |
| L3MBTL1 | -0.225485091 | 4.58E-14 | -0.164544542 | 4.47E-08 | 0.07 |
| LACE.1 | 0.027607823 | 0.36184282 | 0.065777223 | 0.0296677 | 0.04 |
| LACRT | -0.131987877 | 1.20E-05 | -0.014997792 | 0.6203914 | 0.12 |
| LACTB2 | 0.261339684 | 1.59E-18 | 0.147134474 | 1.03E-06 | 0.12 |
| LACTB | -0.099821788 | 0.00095097 | -0.084469882 | 0.0051993 | 0.02 |
| LAD1 | 0.277461129 | 9.05E-21 | 0.317669573 | 4.74E-27 | 0.05 |
| LAG3 | 0.276153228 | 1.39E-20 | 0.097051509 | 0.0013159 | 0.18 |
| LAGE3 | 0.398614682 | 6.12E-43 | 0.438625993 | 1.31E-52 | 0.05 |
| LAIR1 | -0.066916601 | 0.02694952 | -0.16181767 | 7.48E-08 | 0.1 |
| LAIR2 | 0.156673474 | 1.93E-07 | -0.002557612 | 0.9326911 | 0.16 |
| LALBA | 0.147951369 | 8.98E-07 | 0.027496876 | 0.3637761 | 0.13 |
| LAMA1 | -0.055598389 | 0.06614623 | -0.082591437 | 0.0062939 | 0.03 |
| LAMA2 | -0.440993967 | 3.18E-53 | -0.284030061 | 9.95E-22 | 0.16 |
| LOC201382 | -0.23820172 | 1.45E-15 | -0.353580232 | 1.56E-33 | 0.12 |
| LAMA4 | -0.289782547 | 1.37E-22 | -0.23450348 | 4.04E-15 | 0.06 |
| LAMA5 | -0.270686313 | 8.28E-20 | -0.250935668 | 3.71E-17 | 0.02 |
| CLM | -0.215150846 | 6.52E-13 | -0.210646856 | 1.99E-12 | 0.01 |
| LAMB2P1 | 0.029614008 | 0.32800032 | 0.043664029 | 0.1491351 | 0.02 |
| LAMB2 | -0.439110905 | 9.81E-53 | -0.301632681 | 2.00E-24 | 0.14 |
| LAMB3 | -0.021303245 | 0.48169999 | -0.021121552 | 0.4854468 | 0.01 |
| LOC392775 | -0.055091265 | 0.06866065 | -0.03884561 | 0.1993968 | 0.02 |
| LAMC1 | -0.166644337 | 2.99E-08 | -0.20941116 | 2.69E-12 | 0.05 |
| LAMC2 | 0.008369952 | 0.78223822 | -0.052958294 | 0.0801088 | 0.07 |
| LAMC3 | -0.04348409 | 0.1508196 | -0.125109431 | 3.36E-05 | 0.09 |
| LAMP1 | 0.003739636 | 0.90171642 | -0.091633045 | 0.0024265 | 0.1 |
| LAMP2 | -0.034674683 | 0.25204482 | -0.07164629 | 0.0178362 | 0.04 |
| LAMP3 | 0.223371808 | 7.96E-14 | -0.022420289 | 0.4590136 | 0.25 |
| LANCL1 | -0.319912183 | 1.98E-27 | -0.246126591 | 1.52E-16 | 0.08 |
| LANCL2 | 0.24630095 | 1.44E-16 | 0.09892709 | 0.0010571 | 0.15 |
| LANCL3 | 0.169008288 | 1.89E-08 | -0.063853257 | 0.0347927 | 0.24 |
| LAP3 | -0.008646483 | 0.77523373 | -0.121077142 | 5.99E-05 | 0.12 |
| LAPTM4A | -0.025089109 | 0.40730753 | 0.0380784 | 0.2084248 | 0.07 |
| LAPTM4B | 0.419681003 | 7.22E-48 | 0.218252425 | 2.98E-13 | 0.21 |
| LAPTM5 | -0.006849209 | 0.82105975 | -0.097837395 | 0.0012011 | 0.1 |
| LOC105373005 | -0.234373898 | 4.19E-15 | -0.160636752 | 9.33E-08 | 0.08 |
| LARP1B | -0.149190182 | 7.25E-07 | -0.219171075 | 2.36E-13 | 0.07 |
| LARP1 | -0.095701055 | 0.00153712 | -0.083537107 | 0.0057193 | 0.02 |
| LARP4B | 0.245699372 | 1.72E-16 | 0.06772365 | 0.0251566 | 0.18 |
| LARP4 | -0.240691012 | 7.20E-16 | -0.147492056 | 9.71E-07 | 0.1 |
| LARP6 | 0.068004227 | 0.02455785 | -0.175338444 | 5.37E-09 | 0.25 |
| DKFZP564K112 | 0.022998961 | 0.44750045 | 0.004640218 | 0.8782142 | 0.02 |
| LARS2 | -0.118613147 | 8.47E-05 | -0.150299288 | 5.99E-07 | 0.04 |
| FLJ10595 | 0.032071978 | 0.28942835 | 0.076115066 | 0.0118299 | 0.05 |
| LAS1L | 0.402776205 | 6.93E-44 | 0.285679515 | 5.67E-22 | 0.12 |
| LASP1 | -0.306761969 | 3.01E-25 | -0.198676259 | 3.43E-11 | 0.11 |
| CERS1 | 0.221072461 | 1.45E-13 | 0.033277619 | 0.2716713 | 0.19 |
| CERS2 | -0.198505625 | 3.56E-11 | 0.192048758 | 1.54E-10 | 0.4 |
| CERS3 | 0.073479675 | 0.01510782 | -0.127724695 | 2.28E-05 | 0.21 |
| CERS4 | -0.166527648 | 3.06E-08 | 0.050988437 | 0.0920131 | 0.22 |
| CERS5 | -0.04998746 | 0.09858459 | 0.117080327 | 0.0001046 | 0.17 |
| CERS6 | -0.402139469 | 9.69E-44 | -0.23866541 | 1.27E-15 | 0.17 |
| HSPC046 | -0.103884057 | 0.00058205 | -0.147483779 | 9.73E-07 | 0.05 |
| LATS1 | -0.416473563 | 4.28E-47 | -0.381282909 | 3.82E-39 | 0.04 |
| LATS2 | -0.286170228 | 4.79E-22 | -0.310925827 | 6.30E-26 | 0.03 |
| LAT | 0.084816914 | 0.00501696 | -0.01109103 | 0.7141657 | 0.1 |
| LAX1 | -0.112556118 | 0.00019242 | -0.21585211 | 5.46E-13 | 0.11 |
| LAYN | -0.211116965 | 1.77E-12 | -0.166495523 | 3.08E-08 | 0.05 |
| LOC654198 | -0.074476788 | 0.01378386 | -0.145846246 | 1.28E-06 | 0.08 |
| LBP | 0.062952754 | 0.03743981 | 0.056353424 | 0.0625434 | 0.01 |
| LBR | 0.296392285 | 1.33E-23 | 0.144784563 | 1.54E-06 | 0.16 |
| LBX1 | 0.041548975 | 0.16986026 | 0.041222847 | 0.1732391 | 0.01 |
| LBX2 | 0.02284268 | 0.45059353 | 0.057928521 | 0.055548 | 0.04 |
| LBXCOR1 | -0.150880278 | 5.41E-07 | -0.199800046 | 2.64E-11 | 0.05 |
| C21orf13 | -0.24902271 | 6.52E-17 | 0.053990685 | 0.0743878 | 0.31 |
| LCA5 | -0.443159412 | 8.65E-54 | -0.301833842 | 1.86E-24 | 0.15 |
| LCAT | 0.021070159 | 0.48650948 | 0.00139668 | 0.9632129 | 0.02 |
| LCE1A | 0.035073906 | 0.24662241 | -0.001058032 | 0.9721283 | 0.04 |
| LCE1B | 0.048174034 | 0.11143821 | 0.024057688 | 0.4268664 | 0.03 |
| LCE1C | 0.033542663 | 0.26786968 | 0.003148283 | 0.917197 | 0.04 |
| LCE1D | 0.017538958 | 0.56243488 | 0.017944266 | 0.553438 | 0.01 |
| LCE1E | 0.036741254 | 0.22486002 | 0.023656057 | 0.434628 | 0.02 |
| LCE1F | 0.032564364 | 0.28208418 | -0.009302421 | 0.7586927 | 0.05 |
| LCE2A | 0.111086978 | 0.00023343 | 0.059844067 | 0.0479302 | 0.06 |
| LCE2B | 0.002149418 | 0.94341389 | -0.012012619 | 0.691586 | 0.02 |
| LCE2C | 0.021874816 | 0.47001637 | -0.006482757 | 0.8304843 | 0.03 |
| LCE2D | 0.013628375 | 0.65266217 | -0.013147332 | 0.6641581 | 0.03 |
| LCE3A | 0.135971217 | 6.45E-06 | 0.08308459 | 0.0059881 | 0.06 |
| LCE3B | NA | NA | NA | NA | #VALUE! |
| LCE3C | 0.022174155 | 0.46396048 | -0.020507156 | 0.4982326 | 0.05 |
| LCE3D | 0.076921313 | 0.01096159 | -0.002109922 | 0.944452 | 0.08 |
| LCE3E | 0.06288204 | 0.03765474 | -0.014949373 | 0.6215205 | 0.08 |
| LCE4A | -0.021104425 | 0.48580081 | -0.043259618 | 0.1529412 | 0.03 |
| LCE5A | 0.068760924 | 0.02300426 | 0.010278269 | 0.7342893 | 0.06 |
| LCE6A | -0.008197914 | 0.78660487 | -0.023315191 | 0.4412787 | 0.02 |
| LCK | 0.0814767 | 0.00703751 | -0.04243815 | 0.1608989 | 0.13 |
| LCLAT1 | 0.184149309 | 8.59E-10 | 0.116591819 | 0.0001118 | 0.07 |
| LOC51628 | 0.274782212 | 2.19E-20 | 0.25207558 | 2.65E-17 | 0.03 |
| LOC123734 | -0.28440951 | 8.75E-22 | -0.106983624 | 0.0003955 | 0.18 |
| LCN10 | -0.122983253 | 4.57E-05 | -0.124528518 | 3.65E-05 | 0.01 |
| LCN12 | -0.026408536 | 0.38308083 | 0.010193921 | 0.7363885 | 0.04 |
| LCN15 | 0.041712123 | 0.1681886 | 0.021838387 | 0.4707563 | 0.02 |
| LCN1 | 0.118090834 | 9.10E-05 | 0.088165438 | 0.0035325 | 0.03 |
| LCN2 | 0.166151539 | 3.29E-08 | 0.106408585 | 0.0004252 | 0.06 |
| LCN6 | -0.200350573 | 2.33E-11 | -0.08137461 | 0.0071094 | 0.12 |
| LCN8 | 0.008320989 | 0.78348031 | -0.073073353 | 0.0156786 | 0.09 |
| LCN9 | -0.001593546 | 0.95803209 | 0.016076618 | 0.5954687 | 0.02 |
| LCNL1 | -0.039257456 | 0.19466974 | -0.084763814 | 0.0050445 | 0.05 |
| LCORL | -0.138021144 | 4.65E-06 | -0.249800616 | 5.19E-17 | 0.12 |
| C10orf12 | -0.478990737 | 9.05E-64 | -0.435619786 | 7.75E-52 | 0.05 |
| LOC220083 | -0.006348122 | 0.83395304 | -0.162316354 | 6.82E-08 | 0.16 |
| LCP2 | -0.092621791 | 0.00217514 | -0.213416909 | 1.00E-12 | 0.13 |
| LCTL | 0.285214915 | 6.64E-22 | 0.055598187 | 0.0661472 | 0.23 |
| LCT | 0.304371816 | 7.31E-25 | 0.158051413 | 1.50E-07 | 0.15 |
| LDB1 | -0.191460181 | 1.75E-10 | -0.140366779 | 3.19E-06 | 0.06 |
| LDB2 | -0.403012646 | 6.12E-44 | -0.254505309 | 1.28E-17 | 0.15 |
| LDB3 | -0.297958863 | 7.58E-24 | -0.239988789 | 8.77E-16 | 0.06 |
| LDHAL6A | -0.259262096 | 3.01E-18 | -0.269902787 | 1.07E-19 | 0.02 |
| LDHAL6B | -0.070054074 | 0.02054614 | -0.161453972 | 8.01E-08 | 0.1 |
| LDHA | 0.252898715 | 2.07E-17 | 0.166194232 | 3.26E-08 | 0.09 |
| LDHB | 0.40293481 | 6.38E-44 | 0.096133529 | 0.0014628 | 0.31 |
| LDHC | 0.097402652 | 0.00126339 | 0.095337115 | 0.0016023 | 0.01 |
| LDHD | -0.300986908 | 2.53E-24 | -0.172281708 | 9.92E-09 | 0.13 |
| LDLRAD1 | -0.235043256 | 3.48E-15 | -0.031717846 | 0.2947892 | 0.21 |
| LDLRAD2 | 0.017390807 | 0.56574099 | 0.053869306 | 0.0750426 | 0.04 |
| LDLRAD3 | -0.158753891 | 1.32E-07 | -0.216752941 | 4.35E-13 | 0.06 |
| LDLRAP1 | -0.181658697 | 1.46E-09 | -0.116557477 | 0.0001124 | 0.07 |
| LDLR | -0.118601906 | 8.48E-05 | -0.141863263 | 2.49E-06 | 0.03 |
| LDOC1L | -0.281035332 | 2.74E-21 | -0.282571508 | 1.63E-21 | 0.01 |
| LOC116340 | 0.199708679 | 2.70E-11 | 0.095645385 | 0.0015469 | 0.11 |
| LEAP2 | -0.017948306 | 0.55334871 | 0.178872446 | 2.60E-09 | 0.2 |
| MYETS1 | 0.047364582 | 0.11758625 | 0.030220121 | 0.3181928 | 0.02 |
| LECT2 | -0.013932618 | 0.64543348 | 0.022440578 | 0.4586072 | 0.04 |
| LEF1 | -0.219302697 | 2.28E-13 | -0.116651351 | 0.0001109 | 0.11 |
| LEFTY1 | 0.079029891 | 0.00895228 | 0.11620314 | 0.0001179 | 0.04 |
| LEFTY2 | -0.051023151 | 0.09179165 | -0.050321858 | 0.0963488 | 0.01 |
| LOC152084 | -0.051326398 | 0.08987541 | -0.006533595 | 0.8291753 | 0.05 |
| LELP1 | 0.037318892 | 0.21765009 | 0.070551928 | 0.0196626 | 0.04 |
| LEMD1 | 0.33315605 | 9.66E-30 | 0.100858049 | 0.0008404 | 0.24 |
| LOC135687 | 0.29540593 | 1.89E-23 | 0.175990256 | 4.70E-09 | 0.12 |
| LEMD3 | -0.201587803 | 1.74E-11 | -0.223181758 | 8.37E-14 | 0.03 |
| LENEP | -0.083177839 | 0.0059318 | 0.071176945 | 0.0186005 | 0.16 |
| LOC126011 | 0.072646541 | 0.01629853 | 0.089509458 | 0.0030585 | 0.02 |
| LENG8 | -0.189448627 | 2.73E-10 | -0.19853187 | 3.54E-11 | 0.01 |
| LOC126445 | 0.042419605 | 0.16108205 | 0.163918852 | 5.04E-08 | 0.13 |
| LEO1 | -0.20370648 | 1.06E-11 | -0.055247961 | 0.0678755 | 0.15 |
| P3H1 | 0.066963538 | 0.0268423 | 0.029293702 | 0.3332615 | 0.04 |
| P3H2 | -0.057451789 | 0.05759303 | -0.164398546 | 4.60E-08 | 0.11 |
| P3H3 | -0.076054646 | 0.01189733 | -0.010471922 | 0.7294774 | 0.07 |
| LEPROTL1 | -0.0547853 | 0.07021526 | 0.024878295 | 0.4112612 | 0.08 |
| LEPROT | -0.493936732 | 2.69E-68 | -0.516403527 | 1.54E-75 | 0.03 |
| LEPR | -0.391983554 | 1.84E-41 | -0.319768382 | 2.09E-27 | 0.08 |
| LEP | -0.190919577 | 1.97E-10 | -0.167144364 | 2.72E-08 | 0.03 |
| LETM1 | 0.005015336 | 0.86845516 | -0.059133652 | 0.0506463 | 0.07 |
| LETM2 | -0.173191104 | 8.27E-09 | -0.069942421 | 0.020749 | 0.11 |
| LETMD1 | -0.13822264 | 4.51E-06 | -0.029538813 | 0.3292306 | 0.11 |
| LOC651477 | 0.020071988 | 0.5073956 | -0.028635237 | 0.3442468 | 0.05 |
| LOC392618 | -0.244162454 | 2.68E-16 | -0.053722587 | 0.0758404 | 0.2 |
| LGALS12 | -0.216442581 | 4.71E-13 | -0.125948512 | 2.97E-05 | 0.1 |
| LGALS13 | 0.019320654 | 0.52342152 | -0.017137489 | 0.5714155 | 0.04 |
| LGALS14 | -0.016271146 | 0.59102374 | 0.009744065 | 0.747617 | 0.03 |
| LGALS1 | 0.179773144 | 2.16E-09 | 0.127847083 | 2.24E-05 | 0.06 |
| LGALS2 | 0.054283928 | 0.07282501 | -0.057672373 | 0.0566391 | 0.12 |
| LGALS3BP | 0.027146769 | 0.36991883 | -0.014872926 | 0.6233051 | 0.05 |
| LGALS3 | -0.06771468 | 0.02517591 | 0.081690329 | 0.0068892 | 0.15 |
| LGALS4 | 0.005446261 | 0.857269 | 0.042960905 | 0.1557995 | 0.04 |
| LOC654184 | 0.157960768 | 1.53E-07 | 0.057388849 | 0.0578676 | 0.11 |
| LGALS7 | 0.121886053 | 5.34E-05 | 0.049089007 | 0.1047968 | 0.08 |
| LGALS8 | -0.341898432 | 2.49E-31 | 0.047334672 | 0.1178184 | 0.39 |
| LGALS9B | -0.230079436 | 1.35E-14 | -0.047803086 | 0.1142235 | 0.19 |
| LGALS9C | -0.058159636 | 0.0545785 | 0.097610459 | 0.0012332 | 0.16 |
| LOC116410 | 0.071579812 | 0.01794279 | 0.066710804 | 0.027424 | 0.01 |
| EPT | -0.07121064 | 0.01854471 | -0.082606155 | 0.0062846 | 0.02 |
| LGI2 | -0.135651345 | 6.78E-06 | -0.210148016 | 2.25E-12 | 0.08 |
| LGI3 | 0.012450044 | 0.68096235 | -0.091791335 | 0.0023846 | 0.11 |
| LGI4 | -0.182708731 | 1.17E-09 | -0.153097819 | 3.66E-07 | 0.03 |
| LGMN | 0.029139048 | 0.33582109 | -0.044511055 | 0.141398 | 0.08 |
| LGR4 | -0.084353511 | 0.00526174 | -0.243855373 | 2.92E-16 | 0.16 |
| LGR5 | 0.075524002 | 0.01250457 | -0.066561815 | 0.0277719 | 0.15 |
| LGR6 | 0.085051615 | 0.00489695 | 0.047291933 | 0.1181508 | 0.04 |
| LGSN | 0.05603942 | 0.06402157 | -0.033233388 | 0.2723092 | 0.09 |
| EIF2D | 0.09153626 | 0.00245253 | 0.379136485 | 1.09E-38 | 0.29 |
| LHB | 0.148261821 | 8.51E-07 | 0.130353196 | 1.54E-05 | 0.02 |
| LOC129287 | -0.164583295 | 4.44E-08 | -0.113805378 | 0.000163 | 0.06 |
| LOC286465 | 0.060672554 | 0.04491811 | -0.040550103 | 0.1803671 | 0.11 |
| LHFPL2 | -0.195889288 | 6.48E-11 | -0.249792506 | 5.20E-17 | 0.06 |
| LHFPL4 | 0.024242517 | 0.42332182 | -0.029825669 | 0.3245534 | 0.06 |
| LHFPL4.1 | 0.024242517 | 0.42332182 | -0.029825669 | 0.3245534 | 0.06 |
| DFNB67 | 0.126494769 | 2.74E-05 | 0.156290165 | 2.07E-07 | 0.03 |
| LHFP | -0.375009787 | 7.96E-38 | -0.264326595 | 6.26E-19 | 0.12 |
| LHPP | 0.138698498 | 4.18E-06 | 0.05263887 | 0.0819495 | 0.09 |
| LHX1 | -0.017461311 | 0.56416647 | 0.038437961 | 0.2041575 | 0.06 |
| LHX2 | 0.209023024 | 2.96E-12 | 0.133869827 | 8.96E-06 | 0.08 |
| LHX3 | 0.217610668 | 3.50E-13 | 0.076372898 | 0.0115458 | 0.15 |
| LHX4 | -0.131434474 | 1.31E-05 | -0.035559308 | 0.2401401 | 0.1 |
| LHX5 | 0.1254309 | 3.20E-05 | 0.015484563 | 0.6090899 | 0.11 |
| LHX6 | -0.069191332 | 0.02215919 | -0.076452638 | 0.0114592 | 0.01 |
| LOC148864 | 0.101154308 | 0.00081109 | 0.055819518 | 0.0650738 | 0.05 |
| LHX9 | 0.033051496 | 0.27494361 | 0.03880495 | 0.1998679 | 0.01 |
| LIAS | -0.051756992 | 0.08720984 | 0.023735667 | 0.4330831 | 0.08 |
| LOC105374734 | -0.404804964 | 2.37E-44 | -0.355525444 | 6.54E-34 | 0.05 |
| LIF | -0.047973019 | 0.11294082 | -0.084427168 | 0.0052221 | 0.04 |
| LIG1 | 0.285650664 | 5.72E-22 | 0.182445962 | 1.23E-09 | 0.11 |
| LIG3 | 0.004285043 | 0.88747147 | -0.00576863 | 0.8489194 | 0.02 |
| LIG4 | -0.250320863 | 4.45E-17 | -0.253282288 | 1.85E-17 | 0.01 |
| LILRA1 | -0.388634951 | 1.00E-40 | -0.383405217 | 1.35E-39 | 0.01 |
| LILRA2 | -0.347351932 | 2.39E-32 | -0.168464118 | 2.10E-08 | 0.18 |
| LILRA3 | 0.246486848 | 1.37E-16 | 0.05628291 | 0.0628729 | 0.2 |
| LILRA4 | -0.118716261 | 8.35E-05 | -0.07913265 | 0.0088633 | 0.04 |
| LILRA5 | 0.160814734 | 9.02E-08 | 0.002312482 | 0.9391291 | 0.16 |
| LILRA6 | 0.070863745 | 0.01912632 | -0.058444171 | 0.0534043 | 0.13 |
| LILRB1 | -0.072095045 | 0.01713132 | -0.197470307 | 4.52E-11 | 0.13 |
| LILRB2 | 0.06247894 | 0.03890006 | -0.113445985 | 0.000171 | 0.18 |
| LILRB3 | 0.085978876 | 0.00444777 | -0.0778551 | 0.010027 | 0.17 |
| LILRB4 | 0.0059379 | 0.8445418 | -0.091628347 | 0.0024278 | 0.1 |
| LILRB5 | -0.046783324 | 0.12216277 | -0.150859979 | 5.43E-07 | 0.11 |
| LILRP2 | 0.107594573 | 0.00036606 | -0.008104592 | 0.7889764 | 0.12 |
| LIM2 | 0.052188009 | 0.08460583 | -0.013210874 | 0.662635 | 0.07 |
| LIMA1 | -0.547934305 | 1.16E-86 | -0.316756458 | 6.76E-27 | 0.24 |
| LIMCH1 | -0.313756485 | 2.15E-26 | -0.147097812 | 1.04E-06 | 0.17 |
| LIMD1 | -0.334217254 | 6.23E-30 | -0.263420159 | 8.31E-19 | 0.08 |
| LIMD2 | 0.251837212 | 2.84E-17 | 0.080097429 | 0.008066 | 0.18 |
| LIME1 | 0.162807755 | 6.21E-08 | 0.033866117 | 0.26328 | 0.13 |
| LIMK1 | 0.103995099 | 0.00057415 | -0.013706764 | 0.6507965 | 0.12 |
| LIMK2 | -0.014717248 | 0.62694596 | -0.098688483 | 0.0010872 | 0.09 |
| LIMS1 | -0.28156912 | 2.29E-21 | -0.407170527 | 6.72E-45 | 0.13 |
| LIMS2 | -0.169997759 | 1.56E-08 | -0.104612756 | 0.000532 | 0.07 |
| LIMS3.LOC440895 | -0.176837408 | 3.95E-09 | -0.106231357 | 0.0004348 | 0.08 |
| LOC115448 | -0.178153373 | 3.02E-09 | -0.0888228 | 0.0032929 | 0.09 |
| LIN28A | 0.022004371 | 0.46739002 | 0.085948622 | 0.0044618 | 0.07 |
| LIN28B | 0.31034262 | 7.86E-26 | 0.110101887 | 0.0002654 | 0.21 |
| LIN37 | 0.388340821 | 1.16E-40 | 0.280193233 | 3.64E-21 | 0.11 |
| LIN52 | -0.301404999 | 2.17E-24 | -0.296270417 | 1.39E-23 | 0.01 |
| LIN54 | -0.072831136 | 0.01602784 | -0.223331407 | 8.05E-14 | 0.16 |
| LIN7A | -0.285380304 | 6.28E-22 | -0.248616638 | 7.35E-17 | 0.04 |
| LIN7B | 0.173873392 | 7.21E-09 | 0.121426212 | 5.70E-05 | 0.06 |
| LIN7C | -0.040262617 | 0.18347857 | -0.039512506 | 0.1917838 | 0.01 |
| LIN9 | 0.315902334 | 9.40E-27 | 0.331216643 | 2.14E-29 | 0.02 |
| LINGO1 | -0.030514569 | 0.31349823 | 0.081860155 | 0.0067733 | 0.12 |
| LINGO2 | -0.018481539 | 0.54162149 | -0.111723137 | 0.0002148 | 0.1 |
| LINGO3 | 0.063964617 | 0.03447674 | -0.030046738 | 0.3209785 | 0.1 |
| LINGO4 | -0.243208712 | 3.52E-16 | -0.047594766 | 0.1158115 | 0.2 |
| LINS | -0.119227341 | 7.77E-05 | -0.114571026 | 0.0001471 | 0.01 |
| LIPA | -0.338500761 | 1.05E-30 | -0.259250418 | 3.03E-18 | 0.08 |
| LIPC | -0.145765029 | 1.30E-06 | -0.149483477 | 6.90E-07 | 0.01 |
| LIPE | -0.353529429 | 1.59E-33 | -0.226456602 | 3.54E-14 | 0.13 |
| LIPF | 0.019622066 | 0.51696142 | 0.004787869 | 0.8743706 | 0.02 |
| EDL | 0.088232571 | 0.00350734 | -0.048557443 | 0.1086159 | 0.14 |
| HTL2 | -0.364403259 | 1.16E-35 | -0.187728934 | 3.97E-10 | 0.18 |
| LIPI | -0.100886405 | 0.00083758 | -0.059098648 | 0.0507834 | 0.05 |
| LIPJ | 0.037863287 | 0.21100844 | -0.01977628 | 0.5136722 | 0.06 |
| LIPL2 | 0.074461382 | 0.01380351 | -0.046678095 | 0.123006 | 0.13 |
| LIPM | 0.013174434 | 0.66350833 | -0.021326334 | 0.481225 | 0.04 |
| LIPL4 | 0.016331328 | 0.58965168 | -0.0884714 | 0.0034191 | 0.11 |
| LIPT1 | -0.038351914 | 0.20517292 | 0.149675533 | 6.67E-07 | 0.19 |
| LIPT2 | 0.162387667 | 6.73E-08 | 0.040677036 | 0.1790058 | 0.13 |
| LITAF | -0.004073148 | 0.89300169 | -0.014542123 | 0.6310526 | 0.02 |
| LIX1L | -0.022220139 | 0.46303403 | -0.034294708 | 0.2572824 | 0.02 |
| LIX1 | 0.128329531 | 2.09E-05 | 0.007539191 | 0.8033854 | 0.13 |
| LLGL1 | -0.125545967 | 3.15E-05 | -0.204859072 | 8.05E-12 | 0.08 |
| LLGL2 | 0.06867839 | 0.02316946 | 0.025956776 | 0.3912748 | 0.05 |
| LLPH | 0.244762998 | 2.25E-16 | 0.325207154 | 2.43E-28 | 0.09 |
| LMAN1L | 0.124885713 | 3.47E-05 | 0.086443532 | 0.004237 | 0.04 |
| LMAN1 | -0.027346362 | 0.36640904 | -0.125444185 | 3.20E-05 | 0.1 |
| MGC11139 | -0.07054235 | 0.01967932 | -0.014348672 | 0.6356022 | 0.06 |
| LMAN2 | 0.019881395 | 0.51143643 | 0.130533953 | 1.50E-05 | 0.12 |
| LMBR1L | -0.023927638 | 0.42937077 | 0.0522261 | 0.0843788 | 0.08 |
| LMBR1 | -0.165071393 | 4.05E-08 | -0.13783453 | 4.80E-06 | 0.03 |
| LOC135131 | -0.296704903 | 1.19E-23 | -0.199171375 | 3.06E-11 | 0.1 |
| LMBRD2 | -0.475951343 | 7.08E-63 | -0.374620237 | 9.60E-38 | 0.11 |
| LMCD1 | -0.171270204 | 1.21E-08 | -0.04551803 | 0.1326049 | 0.13 |
| LMF1 | -0.150462564 | 5.82E-07 | -0.026546204 | 0.3806048 | 0.13 |
| LMF2 | 0.065301789 | 0.03086978 | -0.04486015 | 0.1383003 | 0.12 |
| LMLN | -0.372285202 | 2.92E-37 | -0.166796841 | 2.91E-08 | 0.21 |
| CMT2B1 | 0.104993935 | 0.00050744 | 0.096056372 | 0.0014758 | 0.01 |
| LMNB1 | 0.46931119 | 5.90E-61 | 0.266408548 | 3.25E-19 | 0.21 |
| LMNB2 | 0.45797558 | 8.99E-58 | 0.18055523 | 1.83E-09 | 0.28 |
| LMO1 | 0.288543787 | 2.11E-22 | 0.123808399 | 4.06E-05 | 0.17 |
| LMO2 | -0.164207336 | 4.77E-08 | -0.128358067 | 2.08E-05 | 0.04 |
| LMO3 | 0.043104792 | 0.15441768 | -0.05598498 | 0.0642808 | 0.1 |
| LMO4 | 0.272748308 | 4.25E-20 | 0.110019082 | 0.0002682 | 0.17 |
| LMO7 | -0.086496817 | 0.00421342 | -0.154428354 | 2.89E-07 | 0.07 |
| LMOD1 | -0.34384349 | 1.08E-31 | -0.202319261 | 1.47E-11 | 0.15 |
| LOC154866 | -0.047470298 | 0.11676853 | 0.02046379 | 0.4991417 | 0.07 |
| LOC131744 | -0.00963771 | 0.75027954 | 0.083608444 | 0.0056779 | 0.1 |
| LMTK2 | -0.385343172 | 5.17E-40 | -0.453916438 | 1.16E-56 | 0.07 |
| LMTK3 | -0.156958263 | 1.83E-07 | -0.05164736 | 0.0878824 | 0.11 |
| LMX1A | -0.148031863 | 8.85E-07 | 0.000875209 | 0.9769429 | 0.15 |
| LMX1B | -0.394137226 | 6.15E-42 | -0.168196395 | 2.22E-08 | 0.23 |
| LNP1 | 0.160929396 | 8.83E-08 | 0.034977262 | 0.2479275 | 0.13 |
| FLJ39485 | -0.432426147 | 5.03E-51 | -0.36322352 | 2.00E-35 | 0.07 |
| LNX | -0.268835306 | 1.50E-19 | -0.13757988 | 5.00E-06 | 0.14 |
| LNX2 | -0.397014448 | 1.40E-42 | -0.224691981 | 5.64E-14 | 0.18 |
| ZBTB11.AS1 | 0.148868348 | 7.67E-07 | 0.056227107 | 0.0631346 | 0.1 |
| HAVCR1P1 | -0.038018527 | 0.20914162 | -0.022778937 | 0.4518586 | 0.02 |
| ANKRD26P3 | 0.070346112 | 0.02002378 | 0.040719019 | 0.1785572 | 0.03 |
| LOC100124692 | 0.031549087 | 0.29736712 | -0.101491299 | 0.0007789 | 0.14 |
| FAM86JP | 0.11310289 | 0.00017897 | 0.168167007 | 2.23E-08 | 0.06 |
| LOC100126784 | -0.248915071 | 6.73E-17 | -0.246027436 | 1.56E-16 | 0.01 |
| SLCO4A1.AS1 | 0.286574883 | 4.17E-22 | 0.095566464 | 0.001561 | 0.2 |
| LOC100421155 | 0.087468798 | 0.00380361 | 0.003282507 | 0.9136803 | 0.09 |
| LOC100128076 | 0.076663977 | 0.01123237 | 0.010590222 | 0.726543 | 0.07 |
| LOC100420299 | -0.228085367 | 2.30E-14 | -0.250172572 | 4.65E-17 | 0.03 |
| TMPO.AS1 | 0.41728703 | 2.73E-47 | 0.170471303 | 1.42E-08 | 0.25 |
| LOC100128239 | -0.197031953 | 5.00E-11 | -0.0390019 | 0.1975932 | 0.16 |
| LOC100128288 | -0.058121302 | 0.05473832 | -0.0954823 | 0.001576 | 0.04 |
| DLG5.AS1 | 0.140290998 | 3.23E-06 | 0.088681905 | 0.003343 | 0.06 |
| BET1P1 | -0.030298908 | 0.31693213 | -0.030593687 | 0.3122446 | 0.01 |
| LOC144678 | -0.051810019 | 0.08688603 | -0.090017887 | 0.0028949 | 0.04 |
| LOC100128573 | -0.070606237 | 0.0195683 | -0.037719845 | 0.2127441 | 0.04 |
| ACVR2B.AS1 | 0.076318542 | 0.01160518 | -0.069788578 | 0.0210313 | 0.15 |
| HPN.AS1 | -0.176309428 | 4.40E-09 | -0.049801431 | 0.0998462 | 0.13 |
| SRRM2.AS1 | -0.092055288 | 0.0023161 | 0.050471376 | 0.0953624 | 0.15 |
| GPR158.AS1 | 0.128066424 | 2.17E-05 | -0.051315923 | 0.0899411 | 0.18 |
| LOC100134539 | 0.123106662 | 4.49E-05 | 0.066898578 | 0.0269908 | 0.06 |
| LOC100128425 | -0.300209373 | 3.36E-24 | -0.272856168 | 4.10E-20 | 0.03 |
| MAPT.AS1 | -0.2570723 | 5.89E-18 | -0.076508002 | 0.0113994 | 0.19 |
| LOC100129034 | -0.52588557 | 9.26E-79 | -0.285118064 | 6.87E-22 | 0.25 |
| LOC100129055 | -0.060277613 | 0.04633348 | -0.003059278 | 0.9195299 | 0.06 |
| LOC100130355 | 0.054262785 | 0.07293679 | 0.097343828 | 0.001272 | 0.05 |
| GABPB1.AS1 | -0.127868405 | 2.24E-05 | -0.173468315 | 7.82E-09 | 0.05 |
| LOC100129534 | -0.261173625 | 1.67E-18 | -0.096749078 | 0.0013627 | 0.17 |
| LOC100129550 | -0.399470755 | 3.92E-43 | -0.294520473 | 2.59E-23 | 0.11 |
| LOC100129637 | 0.166462555 | 3.10E-08 | 0.037160451 | 0.219611 | 0.13 |
| ARRDC3.AS1 | -0.369811505 | 9.38E-37 | -0.272482649 | 4.63E-20 | 0.1 |
| LINC01126 | -0.120830716 | 6.21E-05 | -0.248772981 | 7.02E-17 | 0.13 |
| LOC199725 | 0.026027854 | 0.38997858 | 0.013443838 | 0.6570627 | 0.02 |
| URAHP | -0.053027882 | 0.07971228 | -0.056943064 | 0.0598439 | 0.01 |
| LOC100130093 | -0.228383441 | 2.12E-14 | -0.022069663 | 0.4660695 | 0.21 |
| MAPT.IT1 | -0.250246918 | 4.55E-17 | -0.161457489 | 8.01E-08 | 0.09 |
| LOC100130238 | 0.013244444 | 0.66183088 | 0.018713924 | 0.5365498 | 0.01 |
| LOC100130264 | 0.02212629 | 0.46492591 | 0.093246891 | 0.0020287 | 0.08 |
| CCDC166 | 0.252383205 | 2.41E-17 | 0.075728361 | 0.0122675 | 0.18 |
| LOC100130331 | 0.043678484 | 0.14900044 | -0.014016118 | 0.6434553 | 0.06 |
| LINC00552 | 0.171715696 | 1.11E-08 | 0.037479509 | 0.2156751 | 0.14 |
| LOC147299 | -0.153000531 | 3.73E-07 | -0.042470035 | 0.1605843 | 0.12 |
| NFYC.AS1 | 0.076698251 | 0.01119597 | -0.068722555 | 0.0230809 | 0.15 |
| LINC00910 | -0.17610561 | 4.59E-09 | -0.10907216 | 0.0003031 | 0.07 |
| LOC100130691 | -0.11800943 | 9.20E-05 | -0.133481447 | 9.52E-06 | 0.02 |
| AGAP2.AS1 | 0.103588309 | 0.00060358 | 0.013202026 | 0.662847 | 0.1 |
| LOC100130872 | -0.228703537 | 1.95E-14 | -0.106007169 | 0.0004472 | 0.13 |
| SNRPGP15 | 0.447765355 | 5.24E-55 | 0.493476152 | 3.74E-68 | 0.05 |
| SMIM6 | -0.047438558 | 0.11701357 | 0.026478058 | 0.3818292 | 0.08 |
| LOC100144415 | -0.270880037 | 7.78E-20 | -0.253068702 | 1.97E-17 | 0.02 |
| CCDC183.AS1 | -0.33564367 | 3.45E-30 | -0.288526749 | 2.12E-22 | 0.05 |
| LOC643293 | -0.203973216 | 9.94E-12 | -0.156663199 | 1.93E-07 | 0.05 |
| LOC100131496 | -0.026873538 | 0.37475722 | -0.101762801 | 0.0007538 | 0.08 |
| LINC00887 | -0.064523481 | 0.03292769 | 0.031168333 | 0.3032385 | 0.1 |
| MZF1.AS1 | -0.154096399 | 3.07E-07 | -0.084618517 | 0.0051205 | 0.07 |
| FAM83A.AS1 | 0.159547238 | 1.14E-07 | 0.090210516 | 0.002835 | 0.07 |
| LOC100132111 | -0.199971158 | 2.54E-11 | -0.170059893 | 1.54E-08 | 0.03 |
| LOC100132215 | 0.194392062 | 9.09E-11 | -0.020782335 | 0.492484 | 0.22 |
| NPIPB5 | -0.076827431 | 0.0110597 | -0.073770964 | 0.0147099 | 0.01 |
| LOC100132287 | -0.069569261 | 0.02143952 | -0.059510631 | 0.0491894 | 0.02 |
| TEKT4P2 | -0.092331703 | 0.00224631 | -0.032551148 | 0.2822796 | 0.06 |
| LINC01512 | 0.032965403 | 0.27619655 | 0.053239984 | 0.0785135 | 0.03 |
| PAXIP1.AS2 | -0.526454322 | 5.89E-79 | -0.412891392 | 3.06E-46 | 0.12 |
| LOC100420824 | 0.261923051 | 1.33E-18 | 0.157263327 | 1.73E-07 | 0.11 |
| LOC100132831 | 0.007124744 | 0.81399032 | 0.013746054 | 0.6498623 | 0.01 |
| LOC100134744 | -0.185709168 | 6.15E-10 | -0.075867251 | 0.0121087 | 0.11 |
| LOC100133050 | -0.133713376 | 9.19E-06 | -0.022573775 | 0.4559438 | 0.12 |
| LINC01001 | -0.012496407 | 0.67984 | 0.008951941 | 0.7675177 | 0.03 |
| RSU1P2 | 0.115576321 | 0.00012841 | 0.084539725 | 0.0051621 | 0.04 |
| LOC100133331 | -0.166017269 | 3.38E-08 | -0.187474586 | 4.20E-10 | 0.03 |
| LOC100133469 | 0.037710422 | 0.21285845 | -0.018044344 | 0.5512274 | 0.06 |
| MRPL23.AS1 | -0.068149 | 0.02425378 | -0.145372794 | 1.39E-06 | 0.08 |
| LINC01134 | 0.021948229 | 0.46852714 | 0.001735817 | 0.9542891 | 0.03 |
| LOC100133669 | 0.208109192 | 3.69E-12 | 0.068044792 | 0.0244723 | 0.15 |
| LOC100133893 | 0.030175021 | 0.31891585 | -0.017795364 | 0.5567351 | 0.05 |
| LOC100133920 | -0.034874966 | 0.2493142 | -0.020622537 | 0.4958179 | 0.02 |
| UXT.AS1 | -0.041914783 | 0.1661293 | -0.072387859 | 0.0166846 | 0.04 |
| LOC100133985 | 0.356056196 | 5.16E-34 | 0.207229927 | 4.56E-12 | 0.15 |
| MAP3K14.AS1 | -0.193128126 | 1.21E-10 | -0.277172198 | 9.96E-21 | 0.09 |
| JHDM1D.AS1 | 0.046993864 | 0.12048928 | 0.082018668 | 0.0066667 | 0.04 |
| LINC01119 | 0.071743129 | 0.01768201 | -0.013635084 | 0.6525024 | 0.09 |
| LOC100134368 | -0.082624546 | 0.00627295 | 0.053780989 | 0.075522 | 0.14 |
| LOC100131931 | 0.271963834 | 5.48E-20 | 0.172500551 | 9.49E-09 | 0.1 |
| LOC100134868 | -0.100274682 | 0.0009011 | -0.078855551 | 0.009105 | 0.03 |
| LOC101928825 | 0.263087769 | 9.22E-19 | 0.203282382 | 1.17E-11 | 0.06 |
| LINC00930 | -0.116712857 | 0.00011 | -0.016192165 | 0.5928266 | 0.11 |
| TEX36.AS1 | -0.009630086 | 0.75047052 | -0.014783247 | 0.6254013 | 0.01 |
| LOC100170939 | -0.428560398 | 4.72E-50 | -0.226993517 | 3.07E-14 | 0.21 |
| HECTD2.AS1 | -0.227606835 | 2.61E-14 | 0.006149912 | 0.8390657 | 0.24 |
| LINC00426 | 0.024115045 | 0.42576458 | -0.0626756 | 0.0382882 | 0.09 |
| DCTN1.AS1 | -0.066078862 | 0.02892614 | 0.031696336 | 0.295117 | 0.1 |
| RAMP2.AS1 | -0.261958238 | 1.31E-18 | -0.081579496 | 0.0069658 | 0.19 |
| LOC100133194 | 0.144929338 | 1.50E-06 | -0.032705853 | 0.2799974 | 0.18 |
| LOC100190940 | -0.051751999 | 0.08724038 | 0.004374015 | 0.885151 | 0.06 |
| LOC100190986 | -0.395157498 | 3.65E-42 | -0.337764397 | 1.42E-30 | 0.06 |
| ZFHX4.AS1 | -0.28615293 | 4.82E-22 | -0.125549015 | 3.15E-05 | 0.17 |
| PP12613 | 0.042679714 | 0.15852705 | -0.059214073 | 0.0503325 | 0.11 |
| LOC100192426 | 0.047175391 | 0.11906085 | 0.026355405 | 0.384039 | 0.03 |
| LINC00704 | 0.097749217 | 0.00121346 | 0.045410022 | 0.1335273 | 0.06 |
| KMT2E.AS1 | 0.269320223 | 1.29E-19 | 0.156535941 | 1.98E-07 | 0.12 |
| PCED1B.AS1 | -0.101633378 | 0.00076564 | -0.150179333 | 6.11E-07 | 0.05 |
| LOC100240726 | -0.011014338 | 0.71605631 | -0.138219152 | 4.51E-06 | 0.13 |
| LOC100240734 | -0.017763766 | 0.55743598 | 0.149691325 | 6.65E-07 | 0.17 |
| LOC100240735 | -0.153005779 | 3.72E-07 | -0.110396851 | 0.0002554 | 0.05 |
| LOC100268168 | -0.09054413 | 0.00273394 | -0.056203464 | 0.0632458 | 0.04 |
| LOC100270710 | 0.194764789 | 8.36E-11 | 0.096207011 | 0.0014505 | 0.1 |
| LOC100270746 | 0.233397826 | 5.47E-15 | 0.171671214 | 1.12E-08 | 0.07 |
| LOC100270804 | 0.007547586 | 0.80317099 | 0.009709562 | 0.7484804 | 0.01 |
| LINC00899 | 0.052143327 | 0.08487283 | -0.070125908 | 0.0204166 | 0.13 |
| LOC100271831 | 0.17648896 | 4.25E-09 | 0.152188469 | 4.30E-07 | 0.03 |
| LOC100271832 | 0.015676751 | 0.60465309 | 0.012391456 | 0.6823817 | 0.01 |
| SMG1P3 | -0.239871213 | 9.07E-16 | -0.314202356 | 1.81E-26 | 0.08 |
| LOC100421524 | 0.122432235 | 4.94E-05 | 0.039139942 | 0.1960101 | 0.09 |
| LOC100272216 | -0.39548312 | 3.08E-42 | -0.311118916 | 5.86E-26 | 0.09 |
| LOC100272217 | 0.045829425 | 0.1299731 | -0.08578206 | 0.0045399 | 0.14 |
| LINC00894 | -0.202709519 | 1.34E-11 | -0.164031253 | 4.93E-08 | 0.04 |
| LINC00875.1 | -0.147687125 | 9.39E-07 | -0.117055371 | 0.000105 | 0.04 |
| BCDIN3D.AS1 | -0.244961554 | 2.13E-16 | -0.120701555 | 6.32E-05 | 0.13 |
| TIPARP.AS1 | 0.085417403 | 0.00471508 | 0.062771727 | 0.0379921 | 0.03 |
| LOC100291195 | -0.037199221 | 0.21912999 | -0.032537219 | 0.2824857 | 0.01 |
| LOC100293913 | 0.004600767 | 0.87924163 | 0.066088965 | 0.0289016 | 0.07 |
| LOC100288778 | 0.182953834 | 1.11E-09 | 0.168530676 | 2.08E-08 | 0.02 |
| LOC286260 | 0.051584202 | 0.08827173 | -0.054323254 | 0.0726175 | 0.11 |
| RASAL2.AS1 | 0.208486709 | 3.37E-12 | 0.103351812 | 0.0006213 | 0.11 |
| LINC00882 | -0.125618488 | 3.12E-05 | -0.018243399 | 0.5468435 | 0.11 |
| BRE.AS1 | 0.061940093 | 0.04061917 | -0.023863539 | 0.4306082 | 0.09 |
| SLC25A5.AS1 | -0.013899752 | 0.64621276 | -0.054594524 | 0.0711991 | 0.05 |
| LOC113230 | -0.187719644 | 3.98E-10 | -0.008700612 | 0.7738648 | 0.18 |
| LOC115110 | -0.328626401 | 6.14E-29 | -0.313728475 | 2.17E-26 | 0.02 |
| LINC01257 | 0.016911257 | 0.57650597 | -0.000954307 | 0.9748598 | 0.02 |
| LINC00284 | 0.210159474 | 2.24E-12 | 0.108980045 | 0.0003067 | 0.11 |
| METTL21EP | -0.149715928 | 6.62E-07 | -0.148745899 | 7.83E-07 | 0.01 |
| LINC00661 | 0.011116371 | 0.71354133 | 0.056445266 | 0.0621164 | 0.05 |
| LINC00628 | -0.015640891 | 0.60547983 | 0.173480786 | 7.80E-09 | 0.19 |
| ZNF300P1 | -0.294308167 | 2.79E-23 | -0.151970964 | 4.47E-07 | 0.15 |
| LOC143188 | -0.00938075 | 0.75672469 | 0.030034406 | 0.3211772 | 0.04 |
| LOC143666 | 0.206635736 | 5.26E-12 | 0.085070816 | 0.0048872 | 0.13 |
| LOC144438 | -0.179289501 | 2.39E-09 | -0.108395989 | 0.0003305 | 0.08 |
| CEP83.AS1 | 0.208798315 | 3.12E-12 | 0.018300034 | 0.5455993 | 0.2 |
| A2M.AS1 | 0.057197323 | 0.05870997 | -0.053548954 | 0.0767935 | 0.12 |
| LINC00934 | 0.008288016 | 0.78431707 | 0.037380144 | 0.2168954 | 0.03 |
| LINC00410 | -0.001062761 | 0.97200379 | -0.021032398 | 0.4872911 | 0.02 |
| LOC145474 | 0.226605324 | 3.40E-14 | 0.178153079 | 3.02E-09 | 0.05 |
| LOC145783 | 0.215226293 | 6.39E-13 | 0.095120441 | 0.0016424 | 0.13 |
| LINC00924 | -0.277277114 | 9.62E-21 | -0.231033845 | 1.04E-14 | 0.05 |
| DRAIC | -0.385908229 | 3.90E-40 | -0.049837134 | 0.099603 | 0.34 |
| LOC145845 | -0.100758462 | 0.00085051 | -0.042596799 | 0.1593382 | 0.06 |
| SSTR5.AS1 | -0.021797442 | 0.4715888 | -0.033427731 | 0.2695137 | 0.02 |
| FRG2DP | 0.023635157 | 0.43503413 | 0.038412122 | 0.2044621 | 0.02 |
| LOC220595 | 0.230844628 | 1.09E-14 | 0.128926122 | 1.91E-05 | 0.11 |
| ILF3.AS1 | 0.151587998 | 4.78E-07 | -0.01100731 | 0.7162297 | 0.17 |
| TPM3P9 | 0.120750848 | 6.28E-05 | 0.049099971 | 0.1047192 | 0.08 |
| LINC00906 | -0.192885109 | 1.28E-10 | -0.105615299 | 0.0004697 | 0.09 |
| LINC00662 | -0.074086637 | 0.0142892 | -0.152085581 | 4.38E-07 | 0.08 |
| LOC148413 | 0.131906704 | 1.21E-05 | 0.08248788 | 0.0063599 | 0.05 |
| LOC148696 | -0.469625398 | 4.80E-61 | -0.237951139 | 1.55E-15 | 0.24 |
| LOC148709 | 0.156794747 | 1.89E-07 | 0.306753158 | 3.02E-25 | 0.15 |
| GCSAML.AS1 | -0.033435701 | 0.26939944 | -0.068611065 | 0.023305 | 0.04 |
| LINC01341 | -0.123084465 | 4.50E-05 | 0.065344004 | 0.0307614 | 0.19 |
| CHIAP2 | -0.075940869 | 0.01202529 | -0.070312087 | 0.020084 | 0.01 |
| LINC00654 | -0.163618207 | 5.33E-08 | -0.212899201 | 1.14E-12 | 0.05 |
| LINC00895 | -0.0020389 | 0.9463189 | -0.037947953 | 0.2099888 | 0.04 |
| LINC00896 | 0.097486083 | 0.0012512 | 0.099431085 | 0.000996 | 0.01 |
| PRR34.AS1 | 0.208551415 | 3.32E-12 | 0.104694635 | 0.0005266 | 0.11 |
| TISP43 | -0.025188458 | 0.40545217 | 0.01689049 | 0.5769743 | 0.05 |
| LINC01102 | 0.083065371 | 0.00599976 | 0.10012838 | 0.0009169 | 0.02 |
| LINC01105 | 0.037366368 | 0.21706498 | 0.072373526 | 0.0167063 | 0.04 |
| LOC150776 | -0.046316215 | 0.12594033 | 0.012285522 | 0.6849508 | 0.06 |
| WTH3DI | -0.176773339 | 4.01E-09 | -0.257853101 | 4.64E-18 | 0.09 |
| LINC01106 | 0.120084359 | 6.89E-05 | -0.039592182 | 0.1908888 | 0.16 |
| LOC151162 | -0.059985541 | 0.04740415 | -0.249425323 | 5.79E-17 | 0.19 |
| LOC151174 | 0.029068441 | 0.33699387 | -0.012326771 | 0.68395 | 0.05 |
| LINC00608 | 0.050489464 | 0.09524357 | 0.007962778 | 0.7925839 | 0.05 |
| LBX2.AS1 | -0.050252402 | 0.09680984 | -0.028366994 | 0.3487874 | 0.03 |
| LINC00635 | 0.0870948 | 0.00395681 | 0.044264933 | 0.1436137 | 0.05 |
| LINC00691 | 0.084346572 | 0.00526549 | -0.029473504 | 0.3303015 | 0.12 |
| NCBP2.AS2 | 0.551625357 | 4.84E-88 | 0.316481689 | 7.52E-27 | 0.24 |
| LOC152225 | -0.163734133 | 5.22E-08 | 0.023653474 | 0.4346782 | 0.19 |
| SLC25A48 | -0.144563116 | 1.59E-06 | -0.029469064 | 0.3303744 | 0.12 |
| LOC153684 | -0.349138451 | 1.10E-32 | -0.234248861 | 4.33E-15 | 0.12 |
| LOC153910 | 0.236842703 | 2.12E-15 | 0.150711969 | 5.57E-07 | 0.09 |
| LOC154449 | 0.046771583 | 0.12225663 | 0.003404997 | 0.9104725 | 0.05 |
| LOC154761 | -0.078884891 | 0.00907912 | -0.100880395 | 0.0008382 | 0.03 |
| LINC00689 | -0.016465015 | 0.58660907 | -0.005962283 | 0.8439116 | 0.02 |
| LINC00964 | 0.211242709 | 1.72E-12 | 0.117863404 | 9.39E-05 | 0.1 |
| LINC00599 | 2.11E-05 | 0.99944397 | -0.051739865 | 0.0873146 | 0.06 |
| LINC00961 | 0.135204631 | 7.28E-06 | -0.059688094 | 0.0485158 | 0.2 |
| USP27X.AS1 | -0.229560216 | 1.55E-14 | -0.269963483 | 1.05E-19 | 0.05 |
| LINC00889 | -0.085702976 | 0.00457736 | -0.026745436 | 0.377039 | 0.06 |
| LOC220573 | -0.135047457 | 7.46E-06 | -0.091037856 | 0.0025904 | 0.05 |
| SEPHS1P1 | -0.25095161 | 3.69E-17 | -0.099135912 | 0.0010314 | 0.16 |
| NBPF24 | -0.284542495 | 8.36E-22 | -0.129490748 | 1.75E-05 | 0.16 |
| LOC200726 | -0.003751268 | 0.90141225 | -0.071110373 | 0.0187112 | 0.07 |
| AADACP1 | 0.041426641 | 0.17112186 | -0.073645628 | 0.01488 | 0.12 |
| LOC100130230 | -0.166396457 | 3.14E-08 | -0.268108827 | 1.89E-19 | 0.11 |
| PAXIP1.AS1 | 0.117491466 | 9.89E-05 | 0.002505321 | 0.9340641 | 0.12 |
| TMEM254.AS1 | 0.018776817 | 0.53518125 | 0.010395367 | 0.7313784 | 0.01 |
| CTAGE10P | -0.286778489 | 3.88E-22 | -0.218562722 | 2.75E-13 | 0.07 |
| LOC86618 | -0.017300936 | 0.56775107 | -0.049411192 | 0.1025344 | 0.04 |
| LOC220729 | 0.056999575 | 0.05959031 | -0.064894727 | 0.0319319 | 0.13 |
| LOC100129789 | 0.155487844 | 2.39E-07 | 0.038693888 | 0.2011591 | 0.12 |
| LOC221122 | -0.08421708 | 0.00533583 | -0.008595891 | 0.7765139 | 0.08 |
| LOC654172 | -0.039035469 | 0.19720732 | -0.078186042 | 0.0097132 | 0.04 |
| SMIM13 | 0.270802194 | 7.98E-20 | 0.0735228 | 0.0150483 | 0.2 |
| TOB2P1 | -0.124551003 | 3.64E-05 | 0.023268519 | 0.4421938 | 0.15 |
| PSMD5.AS1 | -0.154584853 | 2.81E-07 | -0.095184389 | 0.0016305 | 0.06 |
| TTC41P | -0.234715241 | 3.81E-15 | -0.091743761 | 0.0023971 | 0.15 |
| LINC00710 | -0.027159182 | 0.36969995 | -0.008461058 | 0.7799285 | 0.02 |
| LINC00925 | -0.169606913 | 1.68E-08 | -0.073935463 | 0.0144894 | 0.1 |
| LINC00879 | 0.028754183 | 0.34224552 | 0.004670979 | 0.8774132 | 0.03 |
| LINC01018 | 0.071143456 | 0.01865613 | -0.086584696 | 0.0041748 | 0.16 |
| LOC101929324 | 0.19381157 | 1.04E-10 | 0.063835966 | 0.034842 | 0.13 |
| LINC01366 | -0.186318883 | 5.39E-10 | -0.195637693 | 6.86E-11 | 0.01 |
| PP7080 | 0.214081568 | 8.51E-13 | 0.062886693 | 0.0376406 | 0.16 |
| DKFZP434A062 | 0.148539785 | 8.12E-07 | 0.114952993 | 0.0001397 | 0.04 |
| PDCD4.AS1 | -0.032035943 | 0.28997083 | -0.004568166 | 0.8800908 | 0.03 |
| ZMIZ1.AS1 | 0.011913853 | 0.69399318 | 0.033940461 | 0.2622328 | 0.03 |
| LOC283070 | 0.114051458 | 0.0001577 | 0.001442209 | 0.9620146 | 0.12 |
| MIR4697HG | -0.371869906 | 3.55E-37 | -0.182536998 | 1.21E-09 | 0.19 |
| LINC00294 | 0.117489948 | 9.89E-05 | -0.104248272 | 0.0005565 | 0.23 |
| C1RL.AS1 | -0.132916201 | 1.04E-05 | -0.150224148 | 6.06E-07 | 0.02 |
| LOC283332 | 0.047837829 | 0.11396031 | 0.022706622 | 0.4532962 | 0.03 |
| TRHDE.AS1 | -0.268027372 | 1.94E-19 | -0.206156214 | 5.91E-12 | 0.07 |
| LINC00592 | 0.309062255 | 1.27E-25 | 0.150010384 | 6.29E-07 | 0.16 |
| LINC00926 | -0.14372011 | 1.83E-06 | -0.196193971 | 6.05E-11 | 0.06 |
| LOC283731 | -0.302822684 | 1.29E-24 | -0.18589081 | 5.92E-10 | 0.12 |
| LINC00928 | 0.051257405 | 0.09030853 | -0.060092029 | 0.0470114 | 0.12 |
| LOC283856 | -0.155293727 | 2.48E-07 | -0.289490832 | 1.52E-22 | 0.14 |
| LINC00922 | -0.28478776 | 7.69E-22 | -0.045776107 | 0.1304208 | 0.24 |
| LINC01566 | -0.000535658 | 0.98588703 | -0.019783833 | 0.5135114 | 0.02 |
| LOC283922 | -0.234145911 | 4.46E-15 | -0.375783635 | 5.50E-38 | 0.15 |
| TMEM235 | 0.049699781 | 0.10054091 | 0.005128111 | 0.865525 | 0.05 |
| LOC284009 | 0.082786598 | 0.00617125 | 0.019382108 | 0.522101 | 0.07 |
| LOC284023 | -0.05244606 | 0.08307711 | -0.015246615 | 0.614603 | 0.04 |
| YWHAEP7 | -0.195993211 | 6.33E-11 | -0.170547998 | 1.40E-08 | 0.03 |
| LOC100421421 | -0.279665386 | 4.34E-21 | -0.188712761 | 3.21E-10 | 0.1 |
| CYP4F35P | -0.192260882 | 1.47E-10 | -0.031118566 | 0.3040116 | 0.17 |
| LOC100132894 | -0.241169863 | 6.28E-16 | -0.145859961 | 1.28E-06 | 0.1 |
| LOC284379 | 0.11009995 | 0.00026543 | 0.022412555 | 0.4591686 | 0.09 |
| LOC439924 | -0.229031455 | 1.78E-14 | -0.018267642 | 0.5463107 | 0.22 |
| LOC649125 | -0.250355465 | 4.41E-17 | -0.359152251 | 1.28E-34 | 0.11 |
| LINC01226 | -0.082284545 | 0.00649124 | -0.045550069 | 0.1323322 | 0.04 |
| LOC284578 | 0.22268911 | 9.51E-14 | 0.007839331 | 0.7957278 | 0.22 |
| LOC284632 | 0.052801115 | 0.08101033 | -0.028212979 | 0.3514116 | 0.09 |
| LOC284661 | 0.036105895 | 0.2329854 | -0.01318932 | 0.6631515 | 0.05 |
| LINC01142 | 0.035033286 | 0.24717036 | -0.000703863 | 0.981456 | 0.04 |
| LINC00494 | -0.036986967 | 0.22177253 | -0.106037692 | 0.0004455 | 0.07 |
| LOC284788 | -0.039548054 | 0.19138411 | -0.055698432 | 0.0656593 | 0.02 |
| LOC284798 | -0.045045679 | 0.13667539 | -0.081141334 | 0.0072761 | 0.04 |
| LOC100130166 | 0.099298343 | 0.00101179 | 0.085749079 | 0.0045555 | 0.02 |
| TTC28.AS1 | -0.064599056 | 0.03272285 | -0.013900202 | 0.6462021 | 0.06 |
| STARD7.AS1 | -0.111664911 | 0.00021641 | -0.280579288 | 3.20E-21 | 0.17 |
| LINC00486 | 0.053151986 | 0.07900906 | 0.025686343 | 0.3962303 | 0.03 |
| LOC100133003 | 0.181925833 | 1.38E-09 | -0.016445911 | 0.5870434 | 0.2 |
| TUSC7 | 0.015220532 | 0.61520861 | 0.03155346 | 0.2973001 | 0.02 |
| LINC00636 | 0.104573821 | 0.00053457 | -0.019489379 | 0.5198002 | 0.13 |
| LOC131908 | 0.170803345 | 1.33E-08 | 0.173400708 | 7.93E-09 | 0.01 |
| LINC00606 | 0.035781825 | 0.23720898 | 0.042603816 | 0.1592694 | 0.01 |
| LINC00620 | 0.032597226 | 0.28159856 | -0.001541425 | 0.9594035 | 0.04 |
| LINC00698 | -0.024515596 | 0.41811643 | -0.029455579 | 0.3305958 | 0.01 |
| LOC644624 | -0.20058762 | 2.20E-11 | -0.072375927 | 0.0167026 | 0.13 |
| RPL34.AS1 | -0.135414819 | 7.04E-06 | -0.254335535 | 1.35E-17 | 0.12 |
| LINC01098 | 0.090101043 | 0.00286889 | 0.025048857 | 0.4080607 | 0.07 |
| LINC01096 | 0.154721917 | 2.74E-07 | 0.049420729 | 0.102468 | 0.11 |
| LOC285593 | -0.267420239 | 2.36E-19 | -0.229342379 | 1.64E-14 | 0.04 |
| LOC285627 | 0.023667311 | 0.43440942 | 0.044916823 | 0.1378024 | 0.03 |
| LOC285629 | 0.306628508 | 3.17E-25 | 0.09816982 | 0.0011553 | 0.21 |
| LOC285692 | -0.013113346 | 0.66497337 | 0.02099934 | 0.4879759 | 0.04 |
| LOC285696 | -0.205313048 | 7.23E-12 | -0.242580678 | 4.21E-16 | 0.04 |
| LOC285733 | -0.037378632 | 0.21691402 | -0.099139081 | 0.001031 | 0.07 |
| LINC00326 | 0.059091602 | 0.05081103 | 0.087900905 | 0.0036333 | 0.03 |
| LOC285740 | 0.084798977 | 0.00502624 | 0.066986676 | 0.0267896 | 0.02 |
| LOC285768 | 0.054053539 | 0.07405054 | -0.070606361 | 0.0195681 | 0.13 |
| LY86.AS1 | -0.058024167 | 0.05514503 | -0.099266567 | 0.0010156 | 0.05 |
| PACRG.AS1 | 0.139809012 | 3.49E-06 | 0.021263281 | 0.4825228 | 0.12 |
| HLA.F.AS1 | -0.147926464 | 9.02E-07 | -0.186792554 | 4.87E-10 | 0.04 |
| LOC285847 | 0.014677271 | 0.6278824 | -0.011856973 | 0.6953809 | 0.03 |
| INHBA.AS1 | -0.016832144 | 0.57829112 | -0.046599174 | 0.1236413 | 0.03 |
| SLC26A4.AS1 | 0.082050404 | 0.00664551 | -0.086236376 | 0.0043298 | 0.17 |
| LINC01591 | 0.052488146 | 0.08282992 | 0.000290291 | 0.9923514 | 0.06 |
| FAM183CP | -0.033637606 | 0.26651683 | -0.041723269 | 0.1680748 | 0.01 |
| LOC286238 | -0.078752261 | 0.00919655 | -0.000216784 | 0.9942882 | 0.08 |
| LOC286359 | -0.045237743 | 0.1350088 | -0.010224572 | 0.7356254 | 0.04 |
| LOC286367 | -0.068528403 | 0.02347232 | -0.05616953 | 0.0634056 | 0.02 |
| FIRRE | 0.310939612 | 6.27E-26 | 0.112577501 | 0.0001919 | 0.2 |
| CPS1.IT1 | 0.004800224 | 0.87404913 | 0.004672931 | 0.8773624 | 0.01 |
| LINC00705 | 0.025405022 | 0.40142533 | -0.004729052 | 0.8759013 | 0.04 |
| KRTAP5.AS1 | -0.154599898 | 2.80E-07 | -0.077933494 | 0.0099519 | 0.08 |
| LINC00936 | -0.148475464 | 8.21E-07 | -0.225082712 | 5.09E-14 | 0.08 |
| LINC01089 | -0.102020788 | 0.00073063 | -0.014996141 | 0.6204299 | 0.09 |
| LOC339047 | 0.083860055 | 0.00553419 | 0.047203814 | 0.1188384 | 0.04 |
| KRT17P5 | 0.023977523 | 0.42840914 | 0.020896105 | 0.4901177 | 0.01 |
| LINC00667 | -0.058338968 | 0.053836 | -0.251967369 | 2.73E-17 | 0.2 |
| LINC01140 | -0.09065028 | 0.00270249 | -0.145286992 | 1.41E-06 | 0.06 |
| LINC01139 | 0.119282836 | 7.71E-05 | -0.00288538 | 0.9240898 | 0.13 |
| LOC339568 | 0.012364455 | 0.68303615 | -0.038557215 | 0.2027563 | 0.06 |
| LOC100131404 | 0.515336972 | 3.51E-75 | 0.268952896 | 1.45E-19 | 0.25 |
| LINC00298 | -0.039538159 | 0.19149532 | -0.039816675 | 0.1883834 | 0.01 |
| LOC340017 | -0.108438312 | 0.00032875 | -0.035929378 | 0.2352793 | 0.08 |
| LOC340074 | -0.034913172 | 0.24879566 | -0.029543126 | 0.3291599 | 0.01 |
| LINC01020 | 0.214607028 | 7.47E-13 | 0.122813204 | 4.68E-05 | 0.1 |
| LOC340357 | 0.109110718 | 0.00030159 | 0.046745956 | 0.1224617 | 0.07 |
| GAS2L1P2 | 0.026937 | 0.37362995 | 0.022042396 | 0.4666207 | 0.01 |
| LOC341056 | 0.272697255 | 4.32E-20 | 0.139471839 | 3.69E-06 | 0.14 |
| C16ORF96 | -0.138900028 | 4.04E-06 | -0.26097557 | 1.78E-18 | 0.13 |
| LINC00883 | -0.355615972 | 6.28E-34 | -0.221244858 | 1.38E-13 | 0.14 |
| LOC344967 | 0.044863784 | 0.13826831 | 0.017799458 | 0.5566443 | 0.03 |
| LOC169986 | 0.020512368 | 0.49812334 | 0.128251603 | 2.11E-05 | 0.11 |
| LINC00442 | 0.124447804 | 3.70E-05 | 0.046291889 | 0.1261395 | 0.08 |
| ANKRD18DP | 0.0932783 | 0.00202163 | -0.038737683 | 0.2006492 | 0.14 |
| FAM86EP | -0.107557876 | 0.00036777 | 0.100541383 | 0.0008729 | 0.21 |
| LINC00265 | -0.287635702 | 2.89E-22 | -0.152968539 | 3.75E-07 | 0.14 |
| LINC00965 | -0.272293927 | 4.93E-20 | -0.180910143 | 1.70E-09 | 0.1 |
| NANOGNB | 0.193070536 | 1.22E-10 | 0.091980227 | 0.0023354 | 0.11 |
| LOC196478 | -0.101864191 | 0.0007446 | -0.066304517 | 0.0283818 | 0.04 |
| TPTE2P6 | 0.098010381 | 0.00117705 | 0.011743177 | 0.6981603 | 0.09 |
| FAM228B | 0.044018874 | 0.14585541 | 0.099119462 | 0.0010334 | 0.06 |
| LRRC37A6P | -0.158743911 | 1.32E-07 | -0.126213064 | 2.86E-05 | 0.04 |
| PTCHD3P1 | 0.042370585 | 0.16156704 | -0.071587539 | 0.0179304 | 0.12 |
| LOC101929847 | 0.300060907 | 3.54E-24 | 0.102856169 | 0.0006601 | 0.2 |
| LOC388242 | 0.075868742 | 0.01210703 | 0.022391782 | 0.459585 | 0.06 |
| LINC00671 | -0.175281983 | 5.43E-09 | -0.136531882 | 5.90E-06 | 0.04 |
| AATK.AS1 | -0.055606989 | 0.06610425 | -0.119107844 | 7.90E-05 | 0.07 |
| SMIM1 | -0.142910374 | 2.10E-06 | 0.059570699 | 0.0489605 | 0.21 |
| LOC729003 | -0.271357262 | 6.67E-20 | -0.199341146 | 2.94E-11 | 0.08 |
| LINC00493 | 0.349950398 | 7.71E-33 | 0.476513045 | 4.85E-63 | 0.13 |
| LOC648273 | 0.444572378 | 3.68E-54 | 0.323616805 | 4.58E-28 | 0.13 |
| TMEM247 | 0.056054829 | 0.06394837 | 0.016727242 | 0.5806622 | 0.04 |
| LOC649930 | 0.381356242 | 3.69E-39 | 0.289448393 | 1.54E-22 | 0.1 |
| LOC389033 | -0.236037132 | 2.65E-15 | -0.020582521 | 0.4966546 | 0.22 |
| LOC256159 | 0.069104705 | 0.02232707 | -0.013314887 | 0.6601448 | 0.09 |
| LOC351912 | -0.494956875 | 1.30E-68 | -0.429439875 | 2.84E-50 | 0.07 |
| LOC392622 | 0.193984368 | 9.97E-11 | 0.152061495 | 4.40E-07 | 0.05 |
| NUPR1L | -0.037539706 | 0.21493828 | 0.082810878 | 0.0061561 | 0.13 |
| LOC286031 | 0.167847902 | 2.37E-08 | 0.04032646 | 0.1827842 | 0.13 |
| LOC389705 | 0.142904209 | 2.10E-06 | 0.13897676 | 3.99E-06 | 0.01 |
| LOC286209 | 0.048187593 | 0.11133742 | -0.012733171 | 0.6741195 | 0.07 |
| LOC100293156 | -0.054710538 | 0.07059948 | -0.132148446 | 1.17E-05 | 0.08 |
| OACYLP | -0.012741926 | 0.67390832 | -0.023396152 | 0.4396938 | 0.02 |
| LOC391322 | -0.026775108 | 0.37650975 | -0.068532743 | 0.0234635 | 0.05 |
| LOC392196 | -0.152438488 | 4.12E-07 | -0.078771749 | 0.0091792 | 0.08 |
| LOC646434 | -0.031779655 | 0.29384878 | -0.039385381 | 0.1932183 | 0.01 |
| LOC399815 | 0.299947542 | 3.69E-24 | 0.118505891 | 8.59E-05 | 0.19 |
| LOC100507145 | -0.232004675 | 8.00E-15 | -0.150561055 | 5.72E-07 | 0.09 |
| LINC00938 | -0.214623079 | 7.44E-13 | -0.157816915 | 1.57E-07 | 0.06 |
| LOC400043 | -0.19492116 | 8.07E-11 | 0.001392499 | 0.9633229 | 0.2 |
| LINC00909 | 0.209105603 | 2.90E-12 | 0.039235128 | 0.1949239 | 0.17 |
| LGALS17A | 0.048712105 | 0.10749355 | 0.04289067 | 0.1564775 | 0.01 |
| LINC01144 | 0.151072381 | 5.23E-07 | -0.018956735 | 0.5312761 | 0.18 |
| LOC400760 | 0.140462247 | 3.14E-06 | -0.003371027 | 0.911362 | 0.15 |
| LOC400794 | 0.065289744 | 0.03090077 | 0.123829535 | 4.04E-05 | 0.06 |
| C1ORF140 | -0.078134387 | 0.00976161 | -0.064340489 | 0.0334282 | 0.02 |
| LOC100131579 | -0.022089158 | 0.46567562 | 0.01728988 | 0.5679986 | 0.04 |
| LOC387593 | 0.021288317 | 0.48200724 | -0.096263202 | 0.0014412 | 0.12 |
| MIRLET7BHG | -0.148048116 | 8.83E-07 | -0.095764114 | 0.0015261 | 0.06 |
| LOC400940 | 0.015700653 | 0.60410232 | 0.042355147 | 0.16172 | 0.03 |
| LOC401010 | 0.357258453 | 3.01E-34 | 0.132743627 | 1.07E-05 | 0.23 |
| LOC401052 | 0.083862365 | 0.00553289 | 0.146384926 | 1.17E-06 | 0.07 |
| MBNL1.AS1 | 0.019983396 | 0.50927179 | -0.043838641 | 0.1475143 | 0.07 |
| LOC401127 | 0.007862296 | 0.79514269 | -0.096732589 | 0.0013653 | 0.11 |
| LOC100134751 | 0.009879295 | 0.74423584 | -0.068662739 | 0.0232009 | 0.08 |
| LINC00998 | 0.310928892 | 6.30E-26 | 0.327692705 | 8.96E-29 | 0.02 |
| ATP6V0E2.AS1 | 0.054484888 | 0.07176961 | -0.006844581 | 0.8211786 | 0.07 |
| LOC100129963 | -0.228670616 | 1.96E-14 | -0.237315165 | 1.86E-15 | 0.01 |
| ZNF674.AS1 | 0.128869514 | 1.92E-05 | -0.059025111 | 0.0510724 | 0.19 |
| LOC402377 | -0.347495215 | 2.25E-32 | -0.320358754 | 1.66E-27 | 0.03 |
| LOC402644 | 0.074284217 | 0.01403128 | 0.050190181 | 0.0972243 | 0.03 |
| LOC286017 | 0.121626421 | 5.54E-05 | 0.029571102 | 0.3287019 | 0.1 |
| CNTFR.AS1 | -0.004569332 | 0.88006048 | -0.126691999 | 2.66E-05 | 0.13 |
| LOC440040 | 0.060308204 | 0.04622252 | 0.041665818 | 0.1686618 | 0.02 |
| LOC440173 | -0.046302319 | 0.12605408 | -0.130214362 | 1.57E-05 | 0.09 |
| SMG1P2 | -0.066033567 | 0.02903647 | -0.160283366 | 9.96E-08 | 0.1 |
| CDIPT.AS1 | 0.159388727 | 1.17E-07 | 0.176316549 | 4.40E-09 | 0.02 |
| LOC440461 | 0.05018339 | 0.09726965 | -0.041986123 | 0.1654089 | 0.1 |
| HNRNPCL2 | 0.077617216 | 0.01025811 | 0.026062349 | 0.3893505 | 0.06 |
| LOC644781 | 0.043056612 | 0.15487935 | -0.086502368 | 0.004211 | 0.13 |
| FAR2P1 | -0.110521412 | 0.00025129 | 0.00199993 | 0.9473434 | 0.12 |
| LINC01124 | -0.278610134 | 6.18E-21 | -0.078797445 | 0.0091564 | 0.2 |
| LOC100507618 | 0.143164187 | 2.01E-06 | 0.101348902 | 0.0007923 | 0.05 |
| SMIM4 | 0.184945156 | 7.25E-10 | 0.228291777 | 2.17E-14 | 0.05 |
| GUSBP5 | -0.141286735 | 2.74E-06 | -0.15584122 | 2.24E-07 | 0.02 |
| LOC648198 | 0.149713572 | 6.63E-07 | 0.015108544 | 0.617812 | 0.14 |
| LINC00602 | 0.068686275 | 0.02315364 | -0.013873468 | 0.6468363 | 0.09 |
| LOC100130672 | -0.224284499 | 6.27E-14 | -0.141774122 | 2.53E-06 | 0.09 |
| ZNRF2P1 | -0.386984639 | 2.28E-40 | -0.472008239 | 9.90E-62 | 0.09 |
| CTAGE15 | -0.109037398 | 0.00030445 | -0.022618403 | 0.4550534 | 0.09 |
| LOC441454 | -0.266132216 | 3.54E-19 | -0.232814599 | 6.41E-15 | 0.04 |
| LOC441455 | 0.067546075 | 0.02554196 | 0.031275775 | 0.301574 | 0.04 |
| LOC441601 | -0.005283447 | 0.86149217 | -0.084611011 | 0.0051244 | 0.08 |
| LOC100132349 | 0.099517718 | 0.00098587 | 0.082130081 | 0.0065926 | 0.02 |
| ANKRD65 | -0.091821651 | 0.00237663 | -0.050264385 | 0.0967302 | 0.05 |
| TUBBP6 | 0.156853264 | 1.87E-07 | 0.038673638 | 0.2013952 | 0.12 |
| PTGER4P2.CDK2AP2P2 | -0.162869782 | 6.14E-08 | -0.056518149 | 0.0617793 | 0.11 |
| UQCRBP1 | 0.504513284 | 1.23E-71 | 0.440059436 | 5.57E-53 | 0.07 |
| XRCC6P5 | 0.108075563 | 0.00034433 | 0.063428634 | 0.0360204 | 0.05 |
| LOC493754 | 0.074170404 | 0.01417935 | 0.136614252 | 5.83E-06 | 0.07 |
| LOC494141 | 0.030392175 | 0.31544406 | 0.006291396 | 0.8354155 | 0.03 |
| LOC101927260 | 0.224709146 | 5.61E-14 | 0.277381191 | 9.29E-21 | 0.06 |
| LOC541473 | -0.034084562 | 0.26021118 | -0.080899163 | 0.0074529 | 0.05 |
| UBA6.AS1 | -0.059081988 | 0.05084875 | -0.061685431 | 0.0414538 | 0.01 |
| LINC01420 | 0.081149504 | 0.00727024 | 0.15160304 | 4.77E-07 | 0.08 |
| MIR31HG | -0.061643422 | 0.04159281 | -0.080706511 | 0.0075963 | 0.02 |
| C19ORF80 | -0.147059358 | 1.05E-06 | -0.080463769 | 0.0077805 | 0.07 |
| PGM5.AS1 | -0.181071023 | 1.65E-09 | -0.133946957 | 8.86E-06 | 0.05 |
| SMG1P5 | -0.067547176 | 0.02553956 | -0.111252028 | 0.0002284 | 0.05 |
| LOC606724 | 0.180210185 | 1.97E-09 | 0.107469579 | 0.0003719 | 0.08 |
| LOC101929910 | -0.076725276 | 0.01116734 | -0.02887786 | 0.3401725 | 0.05 |
| SCART1 | -0.26503115 | 5.02E-19 | -0.191703496 | 1.66E-10 | 0.08 |
| SMG1P1 | -0.274531792 | 2.38E-20 | -0.302292538 | 1.57E-24 | 0.03 |
| LOC641367 | -0.140812027 | 2.96E-06 | -0.097402824 | 0.0012634 | 0.05 |
| LOC199824 | -0.100324202 | 0.0008958 | 0.066909071 | 0.0269668 | 0.17 |
| LOC649461 | 0.038799243 | 0.19993415 | -0.08005403 | 0.0081005 | 0.12 |
| LOC642814 | -0.119630289 | 7.35E-05 | -0.059129577 | 0.0506622 | 0.07 |
| LOC642846 | 0.398289345 | 7.24E-43 | 0.189059795 | 2.97E-10 | 0.21 |
| LOC642852 | -0.150134024 | 6.16E-07 | -0.10368805 | 0.0005962 | 0.05 |
| LOC642929 | 0.0389819 | 0.19782329 | 0.002197235 | 0.9421572 | 0.04 |
| SMIM5 | 0.112492548 | 0.00019405 | 0.015168821 | 0.6164101 | 0.1 |
| LOC116000 | 0.477093764 | 3.28E-63 | 0.477640801 | 2.26E-63 | 0.01 |
| BRDTP1 | -0.008474281 | 0.77959349 | 0.002084949 | 0.9451084 | 0.02 |
| CCDC168 | 0.181767084 | 1.42E-09 | -0.052090095 | 0.0851918 | 0.24 |
| SCGB1B2P | 0.030029518 | 0.32125603 | 0.047930859 | 0.113258 | 0.02 |
| LOC647903 | -0.172261499 | 9.96E-09 | -0.097396915 | 0.0012642 | 0.08 |
| LOC647710 | 0.019806688 | 0.51302491 | -0.00961314 | 0.7508951 | 0.03 |
| LOC643923.1 | -0.174815677 | 5.96E-09 | -0.192821481 | 1.29E-10 | 0.02 |
| LOC641717 | 0.064456915 | 0.03310902 | 0.047770868 | 0.1144679 | 0.02 |
| LOC644145 | 0.019732279 | 0.51460956 | 0.056566477 | 0.0615566 | 0.04 |
| LOC129048 | 0.140730082 | 3.00E-06 | 0.130403426 | 1.53E-05 | 0.02 |
| LOC644172 | -0.0878078 | 0.00366942 | -0.092147479 | 0.0022926 | 0.01 |
| SMIM10 | -0.043016029 | 0.15526904 | -0.088700943 | 0.0033362 | 0.05 |
| LOC644669 | 0.077171999 | 0.01070339 | 0.061208037 | 0.0430574 | 0.02 |
| LOC644936 | 0.143402271 | 1.93E-06 | 0.143205163 | 2.00E-06 | 0.01 |
| LOC645166 | 0.068726524 | 0.02307299 | 0.106283962 | 0.0004319 | 0.04 |
| LOC649350 | 0.287309984 | 3.23E-22 | 0.14886576 | 7.67E-07 | 0.14 |
| LOC100421556 | 0.035219292 | 0.24466815 | 0.127152002 | 2.49E-05 | 0.1 |
| LOC648120 | -0.307337686 | 2.43E-25 | -0.128393262 | 2.07E-05 | 0.18 |
| LOC648965 | 0.123205387 | 4.42E-05 | 0.159516731 | 1.15E-07 | 0.04 |
| LOC645752 | -0.176571305 | 4.18E-09 | -0.170832442 | 1.32E-08 | 0.01 |
| LOC646214 | -0.266829199 | 2.84E-19 | -0.154370207 | 2.92E-07 | 0.12 |
| LOC646471 | -0.371603823 | 4.03E-37 | -0.385739258 | 4.25E-40 | 0.02 |
| C3ORF84 | 0.058215791 | 0.05434509 | 0.040978023 | 0.1758083 | 0.02 |
| LYPD8 | 0.097455282 | 0.00125568 | 0.024112338 | 0.4258166 | 0.08 |
| LOC641956 | -0.228999506 | 1.80E-14 | -0.169491785 | 1.72E-08 | 0.06 |
| LOC646813 | -0.025919932 | 0.39194772 | -0.036113553 | 0.2328862 | 0.02 |
| FAM227A | -0.099198954 | 0.00102373 | -0.007334174 | 0.808627 | 0.1 |
| LINC00598 | -0.278054195 | 7.43E-21 | -0.259783602 | 2.57E-18 | 0.02 |
| LOC646999 | -0.123577605 | 4.19E-05 | -0.172625116 | 9.26E-09 | 0.05 |
| EMBP1 | -0.149040431 | 7.44E-07 | -0.122605107 | 4.82E-05 | 0.03 |
| CTAGE11P | -0.227538716 | 2.66E-14 | -0.095150694 | 0.0016367 | 0.14 |
| GMNC | 0.030785225 | 0.30922341 | -0.014535922 | 0.6311982 | 0.05 |
| LOC647859 | -0.110305149 | 0.00025846 | -0.052736142 | 0.0813854 | 0.06 |
| LINC00669 | 0.379054949 | 1.13E-38 | 0.157416547 | 1.69E-07 | 0.23 |
| LINC00657 | -0.348813349 | 1.27E-32 | -0.239704898 | 9.50E-16 | 0.11 |
| LL22NC03.63E9.3 | 0.24415575 | 2.68E-16 | 0.1011427 | 0.0008122 | 0.15 |
| ACTG1P4 | -0.22412911 | 6.53E-14 | -0.2236685 | 7.37E-14 | 0.01 |
| HNRNPCL3 | 0.215016335 | 6.74E-13 | 0.186956561 | 4.70E-10 | 0.03 |
| LOC650293 | 0.068864459 | 0.02279846 | 0.040797539 | 0.1777205 | 0.03 |
| LOC100131558 | -0.030961438 | 0.30646101 | 0.056039196 | 0.0640226 | 0.09 |
| LOC642300 | -0.336343042 | 2.58E-30 | -0.414029648 | 1.64E-46 | 0.08 |
| LRRC37A16P | -0.177189347 | 3.68E-09 | -0.143113401 | 2.03E-06 | 0.04 |
| LOC124216 | -0.264589933 | 5.76E-19 | -0.251071637 | 3.56E-17 | 0.02 |
| FAM86FP | 0.067944575 | 0.0246841 | 0.147888509 | 9.07E-07 | 0.08 |
| LOC653501 | -0.306519283 | 3.30E-25 | -0.219293289 | 2.28E-13 | 0.09 |
| DUX4L6 | 0.017849729 | 0.55553021 | 0.002545316 | 0.933014 | 0.02 |
| DUX4L5 | NA | NA | NA | NA | #VALUE! |
| SPCS2P4 | 0.130460038 | 1.51E-05 | 0.150425575 | 5.86E-07 | 0.02 |
| LOC654127 | -0.249264825 | 6.07E-17 | -0.299808654 | 3.88E-24 | 0.06 |
| LOC653786 | 0.047374797 | 0.11750704 | 0.09254725 | 0.0021932 | 0.05 |
| LOC654342 | 0.061966984 | 0.04053188 | 0.061803826 | 0.041064 | 0.01 |
| PAX8.AS1 | -0.105218308 | 0.00049348 | -0.09051324 | 0.0027432 | 0.02 |
| CD27.AS1 | 0.239610669 | 9.76E-16 | 0.199411526 | 2.89E-11 | 0.05 |
| LHFPL3.AS2 | -0.034578209 | 0.25336752 | 0.010905614 | 0.7187396 | 0.05 |
| ANP32AP1 | 0.052066579 | 0.08533302 | 0.015992551 | 0.5973943 | 0.04 |
| LOC101930033 | 0.183544981 | 9.77E-10 | 0.101812154 | 0.0007493 | 0.09 |
| LOC727896 | 0.258806141 | 3.47E-18 | 0.064683361 | 0.0324956 | 0.2 |
| LOC727924 | 0.083030789 | 0.0060208 | 0.00282246 | 0.9257403 | 0.09 |
| LOC728024 | -0.182938892 | 1.11E-09 | -0.258089593 | 4.32E-18 | 0.08 |
| NUTM2A.AS1 | 0.055169336 | 0.06826853 | 0.046760775 | 0.1223431 | 0.01 |
| MIR143HG | -0.30536557 | 5.06E-25 | -0.206959022 | 4.87E-12 | 0.1 |
| DKFZp686A05212 | -0.013462324 | 0.65662134 | 0.030066239 | 0.3206644 | 0.05 |
| LOC728323 | -0.369209541 | 1.24E-36 | -0.315419529 | 1.13E-26 | 0.06 |
| LOC100291139 | -0.099945064 | 0.00093715 | -0.124134201 | 3.87E-05 | 0.03 |
| DUX4L2 | 0.011256878 | 0.71008307 | 0.018059795 | 0.5508865 | 0.01 |
| LOC728554 | 0.262472536 | 1.12E-18 | 0.261585536 | 1.47E-18 | 0.01 |
| PCAT18 | -0.277554129 | 8.77E-21 | -0.049711685 | 0.1004594 | 0.23 |
| LOC728613 | -0.200473462 | 2.26E-11 | -0.087653006 | 0.0037301 | 0.12 |
| LOC100421635 | 0.073521151 | 0.0150506 | 0.011589385 | 0.701923 | 0.07 |
| LOC439963 | 0.304561713 | 6.82E-25 | 0.147900724 | 9.06E-07 | 0.16 |
| ZBED3.AS1 | -0.015367834 | 0.61179165 | -0.006770798 | 0.8230742 | 0.01 |
| LOC155036 | 0.161429312 | 8.05E-08 | 0.12341928 | 4.29E-05 | 0.04 |
| PIN4P1 | 0.328681261 | 6.01E-29 | 0.298320647 | 6.65E-24 | 0.04 |
| C1GALT1C1L | 0.11113317 | 0.00023202 | 0.011409835 | 0.7063252 | 0.1 |
| LOC101929794 | 0.041265128 | 0.17279822 | -0.007267595 | 0.8103311 | 0.05 |
| LOC728875 | 0.181849838 | 1.40E-09 | 0.127435706 | 2.38E-05 | 0.06 |
| LOC101060582 | -0.160136567 | 1.02E-07 | -0.209626816 | 2.55E-12 | 0.05 |
| RPEL1 | -0.175803999 | 4.88E-09 | -0.234787753 | 3.74E-15 | 0.06 |
| OIP5.AS1 | -0.060634399 | 0.04505324 | -0.069249533 | 0.022047 | 0.01 |
| RGPD4.AS1 | 0.004239002 | 0.88867263 | 0.021586101 | 0.4758985 | 0.02 |
| GTF2IRD1P1 | 0.080755597 | 0.00755957 | 0.14656383 | 1.14E-06 | 0.07 |
| KATNBL1P6 | -0.095686182 | 0.00153974 | -0.179402197 | 2.33E-09 | 0.09 |
| FAHD2CP | 0.158197046 | 1.46E-07 | 0.230082024 | 1.34E-14 | 0.08 |
| FAM86HP | 0.021915752 | 0.46918565 | 0.172454753 | 9.58E-09 | 0.16 |
| LOC732373 | 0.073805929 | 0.01466281 | -0.034829636 | 0.2499304 | 0.11 |
| LOC729603 | -0.101545539 | 0.00077379 | -0.127888786 | 2.23E-05 | 0.03 |
| LOC729609 | -0.113067651 | 0.00017981 | -0.037997336 | 0.2093957 | 0.08 |
| LOC645901 | -0.037096421 | 0.22040702 | -0.009739863 | 0.7477221 | 0.03 |
| LINC00847 | -0.062410642 | 0.03911448 | -0.068973422 | 0.0225836 | 0.01 |
| SEC14L1P1 | -0.286027415 | 5.03E-22 | -0.333798996 | 7.41E-30 | 0.05 |
| MEF2BNB.MEF2B | 0.062498647 | 0.03883837 | -0.138548631 | 4.28E-06 | 0.21 |
| MEF2BNB | 0.179541276 | 2.26E-09 | 0.265540381 | 4.27E-19 | 0.09 |
| LOC221362 | 0.101190087 | 0.00080761 | -0.086946496 | 0.0040191 | 0.19 |
| LOC730668 | -0.175539711 | 5.15E-09 | -0.149467104 | 6.92E-07 | 0.03 |
| LOC100144416 | 0.009393118 | 0.75641406 | -0.033426886 | 0.2695258 | 0.05 |
| LINC01300 | -0.005778784 | 0.84865661 | -0.056292489 | 0.062828 | 0.06 |
| LINC00202.2 | 0.18403542 | 8.80E-10 | 0.082319968 | 0.0064682 | 0.11 |
| DKFZp434O0320 | 0.01767382 | 0.55943347 | 0.00957917 | 0.7517463 | 0.01 |
| CEBPA.AS1 | 0.077383389 | 0.01048988 | 0.012637311 | 0.6764334 | 0.07 |
| LOC116216 | 0.230835082 | 1.10E-14 | 0.102385327 | 0.000699 | 0.13 |
| LOC81691 | 0.1906876 | 2.08E-10 | 0.147935501 | 9.00E-07 | 0.05 |
| AFAP1.AS1 | 0.245871883 | 1.63E-16 | 0.044959124 | 0.1374316 | 0.21 |
| LINC00839 | 0.276099589 | 1.42E-20 | 0.062350869 | 0.039303 | 0.22 |
| LINC01101 | 0.033115876 | 0.27400921 | 0.054504519 | 0.0716672 | 0.03 |
| JMJD1C.AS1 | 0.126284915 | 2.83E-05 | 0.087022982 | 0.0039869 | 0.04 |
| LOC90110.1 | -0.045550654 | 0.13232723 | 0.01913083 | 0.5275111 | 0.07 |
| LOC90246 | -0.203093553 | 1.22E-11 | -0.007656808 | 0.8003823 | 0.2 |
| AOC4P | -0.26230887 | 1.18E-18 | -0.252662598 | 2.22E-17 | 0.01 |
| LOC90784 | -0.056801188 | 0.06048445 | -0.029030723 | 0.3376214 | 0.03 |
| LOC90834 | -0.312216384 | 3.86E-26 | -0.331040989 | 2.30E-29 | 0.02 |
| RAPGEF4.AS1 | 0.06873307 | 0.0230599 | 0.00654007 | 0.8290086 | 0.07 |
| LOC100128388 | -0.138976387 | 3.99E-06 | -0.134237223 | 8.47E-06 | 0.01 |
| LOC91450 | 0.124489357 | 3.67E-05 | 0.028427292 | 0.3477635 | 0.1 |
| LINC00923 | 0.000483483 | 0.98726158 | -0.084452132 | 0.0052088 | 0.09 |
| LINC01278 | -0.068319949 | 0.02389893 | -0.020170941 | 0.5053043 | 0.05 |
| MAFG.AS1 | 0.256209744 | 7.65E-18 | 0.209620453 | 2.56E-12 | 0.05 |
| LINC00950 | -0.332857291 | 1.09E-29 | -0.170754732 | 1.34E-08 | 0.17 |
| LOC641864 | 0.159733482 | 1.10E-07 | -0.047873324 | 0.1136919 | 0.21 |
| LOC93623 | 0.094422601 | 0.00177759 | 0.150063068 | 6.24E-07 | 0.06 |
| BMS1P20 | 0.119706887 | 7.27E-05 | 3.65E-05 | 0.9990391 | 0.12 |
| LOH12CR1 | 0.146910883 | 1.07E-06 | 0.02666586 | 0.3784608 | 0.13 |
| LOH12CR2 | 0.047833882 | 0.11399019 | -0.019700146 | 0.5152947 | 0.07 |
| LOH3CR2A | -0.278535446 | 6.33E-21 | -0.158871736 | 1.29E-07 | 0.12 |
| LONP1 | 0.273709352 | 3.11E-20 | 0.149437998 | 6.95E-07 | 0.13 |
| LONP2 | -0.282242342 | 1.83E-21 | -0.238138403 | 1.47E-15 | 0.05 |
| LONRF1 | -0.100963598 | 0.00082986 | -0.044528386 | 0.1412429 | 0.06 |
| LONRF2 | -0.346940609 | 2.86E-32 | -0.197911993 | 4.08E-11 | 0.15 |
| LONRF3 | -0.130568118 | 1.49E-05 | -0.140980583 | 2.88E-06 | 0.02 |
| LOR | 0.099942409 | 0.00093745 | 0.042694395 | 0.1583838 | 0.06 |
| LOC100130816 | -0.320742209 | 1.43E-27 | -0.273941967 | 2.88E-20 | 0.05 |
| LOXL1 | -0.237264409 | 1.88E-15 | -0.074114738 | 0.0142523 | 0.17 |
| LOXL2 | -0.075040406 | 0.01308168 | -0.103559823 | 0.0006057 | 0.03 |
| LOXL3 | 0.015611073 | 0.60616767 | -0.050855773 | 0.0928633 | 0.07 |
| LOC115463 | 0.079863817 | 0.00825299 | -0.072862327 | 0.0159825 | 0.16 |
| LOX | -0.186908487 | 4.75E-10 | -0.158808101 | 1.31E-07 | 0.03 |
| LPAL2 | -0.056254811 | 0.06300456 | -0.113760993 | 0.0001639 | 0.06 |
| LPAR1 | -0.354286806 | 1.14E-33 | -0.226688512 | 3.33E-14 | 0.13 |
| LPAR2 | 0.337811259 | 1.40E-30 | 0.263937533 | 7.07E-19 | 0.08 |
| LPAR3 | 0.070259018 | 0.02017834 | 0.013394195 | 0.6582485 | 0.06 |
| LPAR4 | -0.325488663 | 2.17E-28 | -0.226469803 | 3.53E-14 | 0.1 |
| LPAR5 | -0.173433572 | 7.88E-09 | -0.244045618 | 2.77E-16 | 0.08 |
| LPAR6 | -0.331676618 | 1.77E-29 | -0.148458702 | 8.23E-07 | 0.19 |
| LOC654241 | 0.056363171 | 0.06249799 | 0.03674498 | 0.224813 | 0.02 |
| LOC100128216 | 0.111625719 | 0.00021752 | 0.10478618 | 0.0005207 | 0.01 |
| LPCAT2 | -0.21742322 | 3.68E-13 | -0.256714186 | 6.56E-18 | 0.04 |
| LPCAT3 | -0.074872791 | 0.01328713 | -0.058394041 | 0.0536097 | 0.02 |
| FLJ10257 | 0.116417695 | 0.00011453 | -0.014644289 | 0.6286554 | 0.14 |
| LPGAT1 | -0.217283705 | 3.81E-13 | 0.106064057 | 0.000444 | 0.33 |
| LOC101928805 | -0.051639298 | 0.08793201 | -0.033569701 | 0.2674839 | 0.02 |
| ADGRL2 | -0.11168711 | 0.00021578 | -0.223904836 | 6.93E-14 | 0.12 |
| ADGRL3 | -0.169650224 | 1.67E-08 | -0.164806409 | 4.26E-08 | 0.01 |
| LPIN1 | 0.186646644 | 5.03E-10 | -0.154556743 | 2.82E-07 | 0.35 |
| LPIN2 | -0.176514303 | 4.22E-09 | -0.268929773 | 1.46E-19 | 0.1 |
| LOC391248 | -0.080308344 | 0.00790053 | -0.059018207 | 0.0510996 | 0.03 |
| LPL | -0.143363896 | 1.95E-06 | -0.151749493 | 4.65E-07 | 0.01 |
| LPO | 0.314941626 | 1.36E-26 | 0.094667605 | 0.001729 | 0.23 |
| LPPR1 | -0.044210022 | 0.14411168 | 0.015882752 | 0.5999136 | 0.07 |
| LPPR2 | -0.248432786 | 7.75E-17 | -0.048238841 | 0.1109571 | 0.21 |
| LPPR3 | -0.174798971 | 5.98E-09 | -0.022082696 | 0.4658061 | 0.16 |
| LPPR4 | -0.248036755 | 8.71E-17 | -0.301304494 | 2.25E-24 | 0.06 |
| LPPR5 | -0.23337046 | 5.51E-15 | -0.092630961 | 0.0021729 | 0.15 |
| LPP | -0.330149509 | 3.31E-29 | -0.266067997 | 3.62E-19 | 0.07 |
| LPXN | -0.023686019 | 0.43404619 | -0.145849505 | 1.28E-06 | 0.13 |
| FLVCR1.AS1 | 0.093316943 | 0.00201291 | 0.158709575 | 1.33E-07 | 0.07 |
| LOC101927965 | 0.071647283 | 0.01783465 | 0.085897252 | 0.0044858 | 0.02 |
| LRBA | -0.437681258 | 2.29E-52 | -0.333641018 | 7.91E-30 | 0.11 |
| LRCH1 | -0.394318408 | 5.61E-42 | -0.388872623 | 8.87E-41 | 0.01 |
| LRCH2 | -0.268447232 | 1.70E-19 | -0.200465214 | 2.26E-11 | 0.07 |
| LOC101060069 | -0.311315112 | 5.44E-26 | -0.402425108 | 8.34E-44 | 0.1 |
| LRCH4 | -0.047813597 | 0.11414381 | -0.043752985 | 0.1483077 | 0.01 |
| PIDD1 | 0.072656856 | 0.0162833 | 0.100266665 | 0.000902 | 0.03 |
| LRFN1 | 0.283413699 | 1.23E-21 | 0.19307155 | 1.22E-10 | 0.1 |
| LRFN2 | -0.10755447 | 0.00036793 | 0.10238868 | 0.0006988 | 0.21 |
| LRFN3 | -0.03047042 | 0.31419921 | -0.032571674 | 0.2819761 | 0.01 |
| LRFN4 | 0.329057535 | 5.16E-29 | 0.109795387 | 0.0002761 | 0.22 |
| LRFN5 | -0.259104956 | 3.16E-18 | -0.212931709 | 1.13E-12 | 0.05 |
| LRG1 | -0.311182886 | 5.72E-26 | 0.040083967 | 0.185432 | 0.36 |
| LRGUK | -0.221094378 | 1.44E-13 | -0.022567066 | 0.4560778 | 0.2 |
| LRIG1 | -0.465781214 | 5.95E-60 | -0.197798681 | 4.19E-11 | 0.27 |
| LRIG2 | -0.292459898 | 5.37E-23 | -0.37803926 | 1.85E-38 | 0.09 |
| LRIG3 | -0.076035081 | 0.01191925 | -0.167281115 | 2.65E-08 | 0.1 |
| LRIT1 | 0.058158221 | 0.05458439 | -0.00744922 | 0.8056846 | 0.07 |
| LRIT2 | 0.096130861 | 0.00146325 | 0.012722728 | 0.6743714 | 0.09 |
| LRIT3 | -0.101719572 | 0.00075772 | -0.004872982 | 0.8721564 | 0.1 |
| LRMP | -0.177185807 | 3.68E-09 | -0.259139506 | 3.13E-18 | 0.09 |
| LRP10 | -0.511877756 | 4.91E-74 | -0.309212299 | 1.20E-25 | 0.21 |
| LRP11 | 0.124935776 | 3.44E-05 | 0.113933214 | 0.0001602 | 0.02 |
| FLJ12929 | 0.069936509 | 0.02075975 | -0.131947256 | 1.21E-05 | 0.21 |
| LRPDIT | -0.232206618 | 7.57E-15 | -0.123431734 | 4.28E-05 | 0.11 |
| LRP1 | -0.469865152 | 4.09E-61 | -0.367879871 | 2.32E-36 | 0.11 |
| LRP2BP | -0.347223943 | 2.53E-32 | -0.211241085 | 1.72E-12 | 0.14 |
| LRP2 | -0.312950694 | 2.92E-26 | -0.233392608 | 5.48E-15 | 0.08 |
| LRP3 | 0.128020283 | 2.19E-05 | 0.067127606 | 0.0264704 | 0.07 |
| LRP4 | -0.191489036 | 1.74E-10 | -0.207693228 | 4.08E-12 | 0.02 |
| LOC100130773 | -0.044135581 | 0.14478885 | 0.027459932 | 0.3644212 | 0.08 |
| HBM.1 | -0.045277112 | 0.13466912 | -0.051656442 | 0.0878265 | 0.01 |
| LRP6 | -0.033686286 | 0.26582499 | -0.159827848 | 1.08E-07 | 0.13 |
| LRP8 | 0.415724723 | 6.47E-47 | 0.059965961 | 0.0474767 | 0.36 |
| LOC100290567 | -0.060486867 | 0.04557897 | -0.052869896 | 0.0806148 | 0.01 |
| LRPPRC | 0.206718717 | 5.16E-12 | 0.085170796 | 0.004837 | 0.13 |
| LRRC10B | -0.015041055 | 0.61938324 | 0.085320899 | 0.0047625 | 0.11 |
| LRRC10 | 0.032075644 | 0.28937319 | 0.020182887 | 0.5050521 | 0.02 |
| LRRC14B | 0.014157602 | 0.64010932 | 0.029506829 | 0.3297548 | 0.02 |
| LRRC14 | 0.163795539 | 5.16E-08 | 0.076655828 | 0.011241 | 0.09 |
| LRRC15 | -0.287466164 | 3.06E-22 | -0.117123721 | 0.000104 | 0.18 |
| LOC340186 | -0.105648582 | 0.00046771 | -0.057392542 | 0.0578515 | 0.05 |
| LRRC16B | 0.049519527 | 0.10178237 | -0.039607524 | 0.1907168 | 0.09 |
| LRRC17 | -0.407350504 | 6.11E-45 | -0.143885864 | 1.78E-06 | 0.27 |
| LRRC18 | -0.204967839 | 7.84E-12 | -0.10046636 | 0.0008807 | 0.11 |
| LRRC19 | 0.064166983 | 0.03390879 | 0.066701694 | 0.0274451 | 0.01 |
| LOC732300 | -0.095965045 | 0.00149136 | -0.003471581 | 0.9087294 | 0.1 |
| LRRC20 | 0.044705561 | 0.13966552 | 0.099681142 | 0.000967 | 0.06 |
| LRRC23 | 0.077664258 | 0.01021204 | 0.112120107 | 0.0002038 | 0.04 |
| LRRC24 | -0.013533122 | 0.65493212 | -0.014310817 | 0.6364941 | 0.01 |
| LRRC25 | -0.060309257 | 0.04621871 | -0.178758765 | 2.66E-09 | 0.12 |
| LRRC26 | -0.279716998 | 4.27E-21 | 0.018500744 | 0.5412014 | 0.3 |
| LRRC27 | -0.126634347 | 2.68E-05 | 0.030789612 | 0.3091544 | 0.16 |
| LRRC28 | 0.079354663 | 0.00867389 | 0.001932137 | 0.9491259 | 0.08 |
| LRRC29 | -0.045177294 | 0.13553163 | -0.047035785 | 0.1201582 | 0.01 |
| FLJ11857 | -0.236112473 | 2.59E-15 | -0.219715852 | 2.05E-13 | 0.02 |
| LRRC30 | 0.011111823 | 0.71365335 | -0.008108348 | 0.7888809 | 0.02 |
| LRRC31 | -0.397274948 | 1.22E-42 | -0.016973056 | 0.5751133 | 0.39 |
| LRRC32 | -0.401492616 | 1.36E-43 | -0.225453973 | 4.61E-14 | 0.18 |
| NRROS | -0.09814855 | 0.0011582 | -0.184360432 | 8.21E-10 | 0.09 |
| LRRC34 | 0.020437533 | 0.49969264 | -0.078678257 | 0.0092627 | 0.1 |
| LRRC36 | -0.103397195 | 0.00061789 | 0.063251096 | 0.0365445 | 0.17 |
| LOC100294335 | -0.267765117 | 2.11E-19 | -0.156451069 | 2.01E-07 | 0.12 |
| LOC728627 | -0.247221748 | 1.10E-16 | -0.080506888 | 0.0077475 | 0.17 |
| LOC100421586 | -0.141521335 | 2.64E-06 | -0.202312842 | 1.47E-11 | 0.07 |
| LRRC37A | -0.268139014 | 1.87E-19 | -0.162230354 | 6.93E-08 | 0.11 |
| LOC645435 | -0.020713554 | 0.49391752 | -0.03557246 | 0.2399662 | 0.02 |
| LRRC37B | -0.005472301 | 0.85659394 | -0.058550218 | 0.0529722 | 0.06 |
| LRRC39 | -0.003936294 | 0.89657621 | 0.012695394 | 0.675031 | 0.02 |
| LRRC3B | -0.138175974 | 4.54E-06 | -0.096929431 | 0.0013346 | 0.05 |
| DKFZP434C128 | -0.077646994 | 0.01022893 | -0.163760773 | 5.19E-08 | 0.09 |
| LRRC40 | 0.013675046 | 0.65155116 | -0.043592322 | 0.1498047 | 0.06 |
| LRRC41 | 0.076173556 | 0.01176489 | -0.129907801 | 1.65E-05 | 0.21 |
| LRRC42 | 0.248237069 | 8.21E-17 | 0.095058628 | 0.0016539 | 0.16 |
| LRRC43 | -0.05018485 | 0.0972599 | 0.052440049 | 0.0831125 | 0.11 |
| LRRC45 | 0.309176489 | 1.22E-25 | 0.237602338 | 1.71E-15 | 0.08 |
| LRRC46 | -0.249874688 | 5.08E-17 | 0.046985033 | 0.1205591 | 0.3 |
| LRRC47 | 0.206899053 | 4.94E-12 | 0.084180048 | 0.0053561 | 0.13 |
| LRRC48 | -0.365465076 | 7.12E-36 | -0.086869122 | 0.0040519 | 0.28 |
| LRRC49 | -0.152662446 | 3.96E-07 | -0.168109254 | 2.25E-08 | 0.02 |
| LRRC4B | -0.228952489 | 1.82E-14 | -0.212375338 | 1.30E-12 | 0.02 |
| LRRC4C | -0.327092273 | 1.14E-28 | -0.181887161 | 1.39E-09 | 0.15 |
| LRRC4 | -0.161915207 | 7.35E-08 | -0.158478381 | 1.39E-07 | 0.01 |
| DNAAF1 | -0.314913967 | 1.38E-26 | -0.101743553 | 0.0007555 | 0.22 |
| LRRC52 | 0.072470818 | 0.01655994 | 0.188623819 | 3.27E-10 | 0.12 |
| LRRC55 | -0.113549412 | 0.00016864 | -0.151762148 | 4.64E-07 | 0.04 |
| LRRC56 | -0.273692266 | 3.13E-20 | -0.086227383 | 0.0043339 | 0.19 |
| LRRC57 | -0.151793128 | 4.61E-07 | -0.181003641 | 1.67E-09 | 0.03 |
| LRRC58 | -0.137967747 | 4.69E-06 | -0.164755839 | 4.30E-08 | 0.03 |
| LRRC59 | 0.214069944 | 8.54E-13 | 0.148005588 | 8.89E-07 | 0.07 |
| LRRC61 | 0.265401401 | 4.46E-19 | 0.118573719 | 8.51E-05 | 0.15 |
| LRRC66 | 0.092215583 | 0.00227539 | -0.018595926 | 0.539122 | 0.12 |
| PPP1R42 | 0.0308466 | 0.3082594 | 0.035531684 | 0.2405058 | 0.01 |
| LRRC69 | 0.158983194 | 1.27E-07 | 0.134299619 | 8.38E-06 | 0.03 |
| LRRC6 | -0.203645528 | 1.07E-11 | 0.007266396 | 0.8103618 | 0.22 |
| LRRC70 | -0.334180264 | 6.33E-30 | -0.193854662 | 1.03E-10 | 0.15 |
| LRRC7 | 0.109758766 | 0.00027742 | 0.013773522 | 0.6492094 | 0.1 |
| LRRC8A | -0.203091371 | 1.22E-11 | -0.216723475 | 4.39E-13 | 0.02 |
| LRRC8B | 0.033627595 | 0.26665925 | -0.091438315 | 0.0024791 | 0.13 |
| LRRC8C | -0.080403463 | 0.00782689 | -0.274230862 | 2.62E-20 | 0.2 |
| LRRC8D | 0.249647146 | 5.43E-17 | 0.074001814 | 0.0144012 | 0.18 |
| LRRC8E | -0.173751496 | 7.39E-09 | -0.041335044 | 0.1720711 | 0.14 |
| LRRCC1 | 0.14857686 | 8.06E-07 | 0.093966941 | 0.0018713 | 0.06 |
| LOC86706 | -0.472833955 | 5.72E-62 | -0.335420178 | 3.79E-30 | 0.14 |
| FLJ20248 | 0.10868934 | 0.00031836 | 0.044704841 | 0.1396719 | 0.07 |
| LRRIQ1 | -0.037485417 | 0.21560274 | 0.018747734 | 0.5358138 | 0.06 |
| LOC654248 | -0.007565808 | 0.80270557 | -0.090638431 | 0.002706 | 0.09 |
| LRRIQ4 | 0.068853351 | 0.02282047 | 0.114679723 | 0.0001449 | 0.05 |
| FLJ27465 | -0.193731863 | 1.06E-10 | -0.30501437 | 5.76E-25 | 0.12 |
| FLJ45829 | -0.385658214 | 4.42E-40 | -0.234125628 | 4.48E-15 | 0.16 |
| FLJ13211 | -0.149571504 | 6.79E-07 | -0.147181449 | 1.02E-06 | 0.01 |
| LRRN2 | -0.158051506 | 1.50E-07 | 0.013456092 | 0.6567701 | 0.18 |
| LRRN3 | -0.313231905 | 2.62E-26 | -0.236916028 | 2.07E-15 | 0.08 |
| LRRN4CL | -0.31907297 | 2.75E-27 | -0.18758938 | 4.10E-10 | 0.14 |
| C20orf75 | 0.148227174 | 8.56E-07 | -0.081977918 | 0.0066939 | 0.24 |
| LRRTM1 | -0.03596677 | 0.23479203 | -0.061080403 | 0.0434949 | 0.03 |
| LRRTM2 | -0.355547432 | 6.48E-34 | -0.287663931 | 2.86E-22 | 0.07 |
| LRRTM3 | -0.007539721 | 0.80337189 | -0.052926868 | 0.0802884 | 0.05 |
| LRRTM4 | 0.049960364 | 0.09876754 | -0.01170251 | 0.6991546 | 0.07 |
| LRSAM1 | -0.10666131 | 0.00041192 | -0.012670055 | 0.6756426 | 0.1 |
| LRTM1 | -0.079122516 | 0.00887208 | -0.024382098 | 0.4206564 | 0.06 |
| LOC644564 | -0.007373742 | 0.80761472 | 0.014576421 | 0.6302474 | 0.03 |
| DFNB63 | -0.294954431 | 2.22E-23 | -0.012711834 | 0.6746343 | 0.29 |
| LRWD1 | 0.252019993 | 2.69E-17 | 0.211508357 | 1.61E-12 | 0.05 |
| LSAMP | -0.14259688 | 2.21E-06 | -0.198163356 | 3.86E-11 | 0.06 |
| LSG1 | 0.242597358 | 4.19E-16 | 0.072628735 | 0.0163249 | 0.17 |
| LSM10 | 0.370694522 | 6.19E-37 | 0.272220634 | 5.04E-20 | 0.1 |
| LSM11 | -0.178644346 | 2.73E-09 | -0.140029367 | 3.37E-06 | 0.04 |
| LSM12 | 0.308442178 | 1.61E-25 | 0.270082522 | 1.01E-19 | 0.04 |
| LOC91161 | 0.20750319 | 4.27E-12 | 0.054075537 | 0.0739328 | 0.16 |
| C20orf40 | 0.202068371 | 1.56E-11 | 0.090099042 | 0.0028695 | 0.12 |
| LSM1 | 0.253545522 | 1.71E-17 | 0.136750816 | 5.70E-06 | 0.12 |
| LSM2 | 0.643306036 | 1.06E-128 | 0.533860495 | 1.50E-81 | 0.11 |
| LOC115781 | 0.453127524 | 1.90E-56 | 0.491757908 | 1.27E-67 | 0.04 |
| LSM4 | 0.558202096 | 1.52E-90 | 0.423792954 | 7.16E-49 | 0.14 |
| LSM5 | 0.427165581 | 1.05E-49 | 0.505306359 | 6.81E-72 | 0.08 |
| LSM6 | 0.546210417 | 5.07E-86 | 0.352534684 | 2.47E-33 | 0.2 |
| LSM7 | 0.45341826 | 1.58E-56 | 0.371538469 | 4.15E-37 | 0.09 |
| NAA38 | 0.264827857 | 5.35E-19 | 0.304367672 | 7.32E-25 | 0.04 |
| LSP1 | -0.044134139 | 0.14480199 | -0.063279836 | 0.0364592 | 0.02 |
| LSR | 0.290484707 | 1.07E-22 | 0.176942238 | 3.87E-09 | 0.12 |
| LSS | 0.044525402 | 0.14126962 | -0.07280579 | 0.0160648 | 0.12 |
| SLCO1B7 | -0.051735715 | 0.08734004 | -0.097308951 | 0.0012772 | 0.05 |
| LST1 | 0.06764837 | 0.02531933 | 0.004789957 | 0.8743163 | 0.07 |
| LTA4H | 0.045696549 | 0.13109112 | 0.051173556 | 0.0908372 | 0.01 |
| LTA | 0.148864429 | 7.67E-07 | -0.025704522 | 0.395896 | 0.18 |
| LTB4R2 | -0.209686363 | 2.52E-12 | -0.217792447 | 3.35E-13 | 0.01 |
| LTB4R | 0.024709177 | 0.41444931 | 0.006700439 | 0.8248828 | 0.02 |
| LTBP1 | 0.044278311 | 0.1434926 | -0.054834888 | 0.0699614 | 0.1 |
| C14orf141 | -0.405539977 | 1.60E-44 | -0.288557638 | 2.10E-22 | 0.12 |
| DKFZP586M2123 | -0.327212966 | 1.09E-28 | -0.296699051 | 1.19E-23 | 0.04 |
| LTBP4 | -0.340106924 | 5.32E-31 | -0.238189779 | 1.45E-15 | 0.11 |
| LTBR | 0.381418653 | 3.58E-39 | 0.152200579 | 4.29E-07 | 0.23 |
| LTB | 0.154681261 | 2.76E-07 | -0.009786987 | 0.7465433 | 0.17 |
| LTC4S | -0.234145294 | 4.46E-15 | -0.098691571 | 0.0010868 | 0.14 |
| LTF | -0.141519777 | 2.64E-06 | -0.006888091 | 0.8200612 | 0.14 |
| LTK | 0.118877255 | 8.16E-05 | 0.016595068 | 0.5836562 | 0.11 |
| LTV1 | 0.447022038 | 8.27E-55 | 0.298870237 | 5.45E-24 | 0.15 |
| LOC51625 | -0.055940627 | 0.06449254 | -0.103885092 | 0.000582 | 0.05 |
| LUC7L3 | 0.05309183 | 0.07934929 | 0.102478536 | 0.0006912 | 0.05 |
| LUC7L | 0.08422313 | 0.00533252 | 0.187805908 | 3.91E-10 | 0.11 |
| LUM | -0.256993758 | 6.03E-18 | -0.098951044 | 0.0010541 | 0.16 |
| LOC148894 | -0.333290508 | 9.14E-30 | -0.439556162 | 7.52E-53 | 0.11 |
| LUZP2 | -0.273606858 | 3.21E-20 | -0.120677129 | 6.34E-05 | 0.16 |
| LUZP4 | 0.118893822 | 8.14E-05 | -0.023895466 | 0.4299916 | 0.15 |
| LUZP6 | -0.059532217 | 0.04910704 | -0.128833204 | 1.94E-05 | 0.07 |
| LXN | -0.041727738 | 0.16802925 | -0.048647524 | 0.1079611 | 0.01 |
| LY6D | 0.270130501 | 9.91E-20 | 0.026727633 | 0.3773568 | 0.25 |
| LY6E | 0.291720027 | 6.97E-23 | 0.133693565 | 9.21E-06 | 0.16 |
| LY6G5B | 0.155922917 | 2.21E-07 | 0.068065579 | 0.0244286 | 0.09 |
| LY6G5C | -0.099884194 | 0.00094395 | 0.052529977 | 0.0825848 | 0.16 |
| LY6G6C | -0.106904148 | 0.0003995 | -0.06210315 | 0.0400923 | 0.05 |
| LY6G6D | 0.146105591 | 1.23E-06 | 0.023866276 | 0.4305554 | 0.13 |
| LY6G6E | 0.000694205 | 0.98171047 | -0.045562654 | 0.1322252 | 0.05 |
| LY6G6F | -0.036459148 | 0.22844247 | -0.05793299 | 0.0555291 | 0.03 |
| LY6H | -0.102083319 | 0.00072512 | -0.01377828 | 0.6490964 | 0.09 |
| LY6K | 0.21583671 | 5.49E-13 | 0.120265161 | 6.72E-05 | 0.1 |
| LY75 | -0.142974859 | 2.08E-06 | -0.212901225 | 1.14E-12 | 0.07 |
| LY86 | -0.130725625 | 1.45E-05 | -0.068190258 | 0.0241677 | 0.07 |
| LY96 | 0.037134553 | 0.2199327 | 0.02830162 | 0.3498998 | 0.01 |
| LY9 | -0.048155371 | 0.11157705 | -0.134945469 | 7.58E-06 | 0.09 |
| LYAR | 0.591715679 | 3.22E-104 | 0.341695264 | 2.71E-31 | 0.26 |
| LYG1 | 0.288542851 | 2.11E-22 | 0.151565681 | 4.80E-07 | 0.14 |
| LYG2 | 0.034068144 | 0.26044097 | 0.03487039 | 0.2493764 | 0.01 |
| LYL1 | -0.077142381 | 0.01073361 | -0.091256323 | 0.0025291 | 0.02 |
| LYNX1 | 0.054141088 | 0.07358286 | -0.078162239 | 0.0097355 | 0.14 |
| LYN | 0.081590566 | 0.0069581 | -0.139814973 | 3.49E-06 | 0.23 |
| LYPD1 | 0.28744968 | 3.08E-22 | 0.102195862 | 0.0007153 | 0.19 |
| LYPD2 | 0.106653591 | 0.00041232 | 0.014737609 | 0.6264693 | 0.1 |
| LYPD3 | -0.164943024 | 4.15E-08 | -0.174243699 | 6.69E-09 | 0.01 |
| LYPD4 | -0.060886724 | 0.04416592 | 0.129567824 | 1.73E-05 | 0.2 |
| LYPD5 | 0.157735044 | 1.59E-07 | -0.044403349 | 0.1423644 | 0.21 |
| LYPD6B | -0.207062811 | 4.75E-12 | -0.179543658 | 2.26E-09 | 0.03 |
| LYPD6 | -0.282402705 | 1.73E-21 | -0.221030899 | 1.46E-13 | 0.07 |
| LYPLA1 | -0.020671173 | 0.49480191 | 0.01675579 | 0.5800165 | 0.04 |
| LOC154607 | 0.173627868 | 7.58E-09 | 0.139501636 | 3.67E-06 | 0.04 |
| LYPLA2 | 0.266685449 | 2.98E-19 | 0.250283262 | 4.50E-17 | 0.02 |
| LYPLAL1 | 0.100050248 | 0.0009255 | 0.525150638 | 1.66E-78 | 0.43 |
| LYRM1 | 0.077082408 | 0.01079504 | 0.220276345 | 1.77E-13 | 0.15 |
| LYRM2 | 0.106580772 | 0.00041612 | 0.09721875 | 0.0012906 | 0.01 |
| LYRM4 | 0.392549837 | 1.38E-41 | 0.40435168 | 3.01E-44 | 0.02 |
| LYRM5 | -0.107335944 | 0.00037827 | 0.016761327 | 0.5798913 | 0.13 |
| LYRM7 | -0.301343709 | 2.22E-24 | -0.139751548 | 3.52E-06 | 0.17 |
| LYSMD1 | 0.130251813 | 1.56E-05 | 0.288372424 | 2.24E-22 | 0.16 |
| LYSMD2 | 0.185057209 | 7.07E-10 | 0.039140873 | 0.1959994 | 0.15 |
| LYSMD3 | -0.441153711 | 2.89E-53 | -0.27971125 | 4.28E-21 | 0.17 |
| LYSMD4 | 0.175586677 | 5.10E-09 | 0.136030631 | 6.39E-06 | 0.04 |
| LYST.IT1 | -0.45905632 | 4.52E-58 | -0.266819085 | 2.85E-19 | 0.2 |
| LYVE1 | -0.19841752 | 3.64E-11 | -0.264379579 | 6.16E-19 | 0.07 |
| LYZL1 | 0.22622998 | 3.76E-14 | 0.105083972 | 0.0005018 | 0.13 |
| LYZL2 | 0.151614924 | 4.76E-07 | 0.052837078 | 0.0808033 | 0.1 |
| LYZL4 | -0.037770511 | 0.21212984 | 0.012270952 | 0.6853044 | 0.06 |
| LYZL6 | 0.018284074 | 0.54594979 | 0.009939116 | 0.7427417 | 0.01 |
| LYZ | 0.034728297 | 0.25131182 | -0.092411438 | 0.0022265 | 0.13 |
| LZIC | -0.121465736 | 5.67E-05 | -0.132869503 | 1.05E-05 | 0.02 |
| LZTFL1 | -0.382061009 | 2.61E-39 | -0.163362062 | 5.60E-08 | 0.22 |
| LZTR1 | -0.07431911 | 0.01398616 | -0.022877292 | 0.4499074 | 0.06 |
| LZTS1 | -0.19360685 | 1.09E-10 | -0.1916911 | 1.66E-10 | 0.01 |
| LZTS2 | 0.133286436 | 9.81E-06 | 0.108359969 | 0.0003321 | 0.03 |
| M6PR | 0.080593657 | 0.00768147 | -0.013075428 | 0.6658834 | 0.1 |
| MAB21L1 | -0.436767034 | 3.94E-52 | -0.266122317 | 3.56E-19 | 0.18 |
| MAB21L2 | -0.162711383 | 6.33E-08 | -0.084462649 | 0.0052031 | 0.08 |
| MACC1 | -0.160489921 | 9.58E-08 | -0.035385374 | 0.242449 | 0.13 |
| MACF1 | -0.401948327 | 1.07E-43 | -0.515254314 | 3.74E-75 | 0.12 |
| MACROD1 | 0.216918146 | 4.18E-13 | 0.18057864 | 1.82E-09 | 0.04 |
| MACROD2 | -0.216002162 | 5.26E-13 | -0.142141741 | 2.38E-06 | 0.08 |
| MAD1L1 | 0.213127591 | 1.08E-12 | 0.094796902 | 0.0017038 | 0.12 |
| MAD2L1BP | 0.19993694 | 2.56E-11 | 0.268855324 | 1.49E-19 | 0.07 |
| MAD2L1 | 0.539645241 | 1.27E-83 | 0.276907633 | 1.09E-20 | 0.27 |
| MAD2L2 | 0.434413589 | 1.58E-51 | 0.23513053 | 3.40E-15 | 0.2 |
| MADCAM1 | 0.009471104 | 0.75445644 | -0.054741434 | 0.0704405 | 0.07 |
| MADD | -0.406817935 | 8.12E-45 | -0.331543868 | 1.87E-29 | 0.08 |
| LOC101928676 | 0.037844027 | 0.21124089 | -0.007227785 | 0.8113504 | 0.05 |
| MAEL | 0.022040576 | 0.46665753 | 0.026987198 | 0.3727398 | 0.01 |
| MAF1 | 0.39029651 | 4.33E-41 | 0.223952611 | 6.84E-14 | 0.17 |
| MAFA | 0.074808799 | 0.01336631 | -0.016627648 | 0.5829175 | 0.1 |
| MGC39981 | -0.233300816 | 5.62E-15 | -0.170330095 | 1.46E-08 | 0.07 |
| MAFF | 0.073122878 | 0.01560805 | -0.113590788 | 0.0001677 | 0.19 |
| MAFG | 0.033591858 | 0.26716811 | -0.220868975 | 1.52E-13 | 0.26 |
| FLJ32205 | -0.071919962 | 0.01740339 | -0.080493755 | 0.0077575 | 0.01 |
| MAF | -0.244774524 | 2.24E-16 | -0.231910892 | 8.20E-15 | 0.02 |
| MAGEA10 | 0.159413645 | 1.17E-07 | 0.053268592 | 0.0783529 | 0.11 |
| MAGEA11 | 0.167295111 | 2.64E-08 | 0.071665066 | 0.0178062 | 0.1 |
| MAGEA12 | 0.14253987 | 2.23E-06 | 0.032970544 | 0.2761216 | 0.11 |
| MAGEA1 | 0.050542007 | 0.09489917 | 0.015578813 | 0.6069122 | 0.04 |
| LOC115914 | 0.188305009 | 3.51E-10 | 0.050287731 | 0.0965751 | 0.14 |
| MAGEA3 | 0.220540809 | 1.66E-13 | 0.062206197 | 0.0397623 | 0.16 |
| MAGEA4 | 0.239356871 | 1.05E-15 | 0.103543608 | 0.0006069 | 0.14 |
| MAGEA5 | 0.179415317 | 2.32E-09 | 0.055777319 | 0.0652773 | 0.13 |
| LOC158510 | 0.221173226 | 1.41E-13 | 0.05847786 | 0.0532667 | 0.17 |
| LOC115203 | -0.10102916 | 0.00082336 | -0.069291514 | 0.0219664 | 0.04 |
| LOC115202 | 0.18692735 | 4.73E-10 | 0.075873117 | 0.0121021 | 0.12 |
| MAGEB10 | 0.050447536 | 0.0955191 | -0.023316405 | 0.4412549 | 0.08 |
| MAGEB16 | -0.016875779 | 0.57730619 | -0.002274365 | 0.9401305 | 0.02 |
| MAGEB18 | 0.020893101 | 0.49018012 | 0.044680927 | 0.139884 | 0.03 |
| MAGEL1 | 0.303479712 | 1.02E-24 | 0.100527066 | 0.0008744 | 0.21 |
| MAGEB2 | 0.061222991 | 0.04300635 | -0.035918631 | 0.2354195 | 0.1 |
| MAGEB3 | 0.060700533 | 0.04481924 | 0.005541057 | 0.854812 | 0.06 |
| MAGEB4 | 0.332152516 | 1.46E-29 | 0.133801025 | 9.06E-06 | 0.2 |
| MAGEB6 | 0.078327013 | 0.00958222 | 0.040796153 | 0.1777352 | 0.04 |
| MAGEC1 | -0.025882486 | 0.39263235 | 0.006471237 | 0.8307809 | 0.04 |
| MAGEC2 | 0.042201691 | 0.1632464 | 0.011630214 | 0.7009234 | 0.04 |
| MAGEC3 | 0.041968879 | 0.16558284 | -0.005308718 | 0.8608364 | 0.05 |
| MAGED1 | 0.000297608 | 0.99215865 | 0.082333994 | 0.0064591 | 0.09 |
| MAGED2 | -0.317364261 | 5.34E-27 | -0.128581883 | 2.01E-05 | 0.19 |
| MAGED4B | 0.09900391 | 0.00104755 | 0.023506132 | 0.4375461 | 0.08 |
| MAGED4 | 0.282077231 | 1.93E-21 | 0.216519139 | 4.62E-13 | 0.07 |
| MAGEE1 | -0.208069502 | 3.73E-12 | -0.269990252 | 1.04E-19 | 0.07 |
| MAGEE2 | -0.153889934 | 3.18E-07 | -0.160091355 | 1.03E-07 | 0.01 |
| LOC115083 | 0.316892004 | 6.41E-27 | 0.124142048 | 3.86E-05 | 0.2 |
| MAGEH1 | -0.084381323 | 0.00524675 | -0.033185094 | 0.273007 | 0.06 |
| MAGEL2 | -0.28487059 | 7.47E-22 | -0.123769854 | 4.08E-05 | 0.17 |
| DKFZP434B203 | -0.419870781 | 6.49E-48 | -0.399676417 | 3.52E-43 | 0.03 |
| MAGI2 | -0.235343455 | 3.20E-15 | -0.228195093 | 2.23E-14 | 0.01 |
| MAGI3 | -0.375086304 | 7.68E-38 | -0.239313958 | 1.06E-15 | 0.14 |
| MAGIX | 0.129097058 | 1.86E-05 | 0.152533364 | 4.05E-07 | 0.03 |
| MAGOHB | 0.567464514 | 3.62E-94 | 0.496702724 | 3.69E-69 | 0.08 |
| MAGOH | 0.630120504 | 5.32E-122 | 0.418207635 | 1.64E-47 | 0.22 |
| MAGT1 | -0.135953192 | 6.47E-06 | -0.141305153 | 2.73E-06 | 0.01 |
| MAG | -0.252279006 | 2.49E-17 | -0.092493814 | 0.0022063 | 0.16 |
| RBM13 | 0.15775143 | 1.59E-07 | 0.095449856 | 0.0015819 | 0.07 |
| MAK | -0.248912629 | 6.74E-17 | -0.095108686 | 0.0016446 | 0.16 |
| MAL2 | 0.092830564 | 0.0021252 | 0.174101302 | 6.89E-09 | 0.09 |
| PRO1073 | -0.166886698 | 2.86E-08 | -0.054247684 | 0.0730167 | 0.12 |
| MALL | 0.099530676 | 0.00098436 | -0.083036394 | 0.0060174 | 0.19 |
| MALT1 | 0.094798723 | 0.00170349 | -0.16826879 | 2.19E-08 | 0.27 |
| MAL | 0.101505109 | 0.00077757 | -0.012038397 | 0.6909582 | 0.12 |
| MAMDC2 | -0.371248152 | 4.77E-37 | -0.310977408 | 6.18E-26 | 0.07 |
| MAMDC4 | -0.16176691 | 7.56E-08 | -0.115371275 | 0.000132 | 0.05 |
| MAML1 | -0.339548847 | 6.73E-31 | -0.340630871 | 4.26E-31 | 0.01 |
| MAML2 | -0.137867953 | 4.77E-06 | -0.295027862 | 2.16E-23 | 0.16 |
| MAML3 | -0.506804234 | 2.23E-72 | -0.352153938 | 2.92E-33 | 0.16 |
| MAMLD1 | 0.094139313 | 0.00183535 | -0.12885125 | 1.93E-05 | 0.23 |
| MAMSTR | 0.268647573 | 1.59E-19 | 0.074940279 | 0.0132041 | 0.2 |
| MAN9 | -0.410814673 | 9.45E-46 | -0.337550279 | 1.56E-30 | 0.08 |
| MAN1A2 | -0.357161447 | 3.14E-34 | -0.350489645 | 6.08E-33 | 0.01 |
| DKFZP434I213 | 0.007558305 | 0.80289718 | 0.009384627 | 0.7566273 | 0.01 |
| MAN1C1 | -0.425147662 | 3.32E-49 | -0.332879361 | 1.08E-29 | 0.1 |
| LOC101927004 | -0.460361817 | 1.97E-58 | -0.384756097 | 6.92E-40 | 0.08 |
| PRO2198 | -0.253460058 | 1.75E-17 | -0.38383464 | 1.09E-39 | 0.14 |
| MAN2B1 | 0.082696357 | 0.0062277 | 0.003425901 | 0.9099252 | 0.08 |
| MAN2B2 | -0.532741838 | 3.73E-81 | -0.3046204 | 6.67E-25 | 0.23 |
| MAN2C1 | -0.142824565 | 2.13E-06 | -0.154795616 | 2.71E-07 | 0.02 |
| MANBAL | 0.090511888 | 0.00274356 | 0.150783289 | 5.50E-07 | 0.07 |
| MANBA | -0.216067516 | 5.18E-13 | -0.101311533 | 0.0007959 | 0.12 |
| MANEAL | 0.073346726 | 0.01529255 | 0.005687581 | 0.851017 | 0.07 |
| MANEA | -0.262677559 | 1.05E-18 | -0.223419829 | 7.86E-14 | 0.04 |
| MANF | 0.430646784 | 1.42E-50 | 0.331168198 | 2.18E-29 | 0.1 |
| MANSC1 | -0.124869149 | 3.48E-05 | 0.021443252 | 0.4788236 | 0.15 |
| MAOA | -0.332139496 | 1.47E-29 | -0.263514169 | 8.07E-19 | 0.07 |
| MAOB | -0.078024551 | 0.00986523 | 0.001831883 | 0.9517623 | 0.08 |
| MAP1L | -0.390353141 | 4.21E-41 | -0.310017912 | 8.88E-26 | 0.09 |
| MAP1B | -0.196582116 | 5.54E-11 | -0.217162129 | 3.93E-13 | 0.03 |
| METAP1D | -0.111330289 | 0.00022611 | -0.069383398 | 0.0217909 | 0.05 |
| MAP1LC3A | 0.112664722 | 0.00018968 | 0.077107331 | 0.0107695 | 0.04 |
| LOC652245 | 0.072141661 | 0.01705951 | -0.089995725 | 0.0029018 | 0.17 |
| MAP1LC3B | 0.007894322 | 0.79432691 | -0.120802591 | 6.23E-05 | 0.13 |
| MAP1LC3C | -0.259246389 | 3.03E-18 | -0.235249119 | 3.29E-15 | 0.03 |
| MAP1S | 0.067796981 | 0.02499888 | 0.0262807 | 0.3853888 | 0.05 |
| MAP2K1 | 0.022358049 | 0.46026175 | -0.225274494 | 4.84E-14 | 0.25 |
| LOC85511 | 0.265255156 | 4.68E-19 | 0.166693881 | 2.96E-08 | 0.1 |
| LOC92079 | 0.242949727 | 3.79E-16 | 0.106553079 | 0.0004176 | 0.14 |
| MAP2K4 | -0.252470135 | 2.35E-17 | -0.165541331 | 3.70E-08 | 0.09 |
| MAP2K5 | -0.242187606 | 4.71E-16 | -0.204137903 | 9.55E-12 | 0.04 |
| MAP2K6 | 0.112862698 | 0.00018476 | 0.121797091 | 5.41E-05 | 0.01 |
| LOC199677 | 0.088755097 | 0.0033169 | 0.013827993 | 0.6479156 | 0.08 |
| MAP2 | 0.078011036 | 0.00987805 | -0.141451134 | 2.67E-06 | 0.22 |
| MAP3K10 | 0.030976527 | 0.30622523 | -0.049578738 | 0.1013732 | 0.09 |
| MAP3K11 | 0.118784101 | 8.27E-05 | 0.049362402 | 0.1028744 | 0.07 |
| MAP3K12 | -0.42297652 | 1.14E-48 | -0.261940089 | 1.32E-18 | 0.17 |
| MAP3K13 | -0.310554344 | 7.25E-26 | -0.335429512 | 3.77E-30 | 0.03 |
| MAP3K14 | -0.082599125 | 0.00628904 | -0.145139376 | 1.45E-06 | 0.07 |
| MAP3K15 | -0.012333881 | 0.68377752 | -0.093932351 | 0.0018786 | 0.09 |
| MAP3K1 | -0.483214599 | 5.01E-65 | -0.232351992 | 7.28E-15 | 0.26 |
| LOC100506904 | -0.359062431 | 1.33E-34 | -0.408055905 | 4.18E-45 | 0.05 |
| MAP3K3 | -0.317186665 | 5.72E-27 | -0.386358105 | 3.12E-40 | 0.07 |
| MAP3K4 | -0.101646257 | 0.00076445 | -0.080468655 | 0.0077768 | 0.03 |
| MAP3K5 | -0.196162528 | 6.09E-11 | -0.246749915 | 1.27E-16 | 0.06 |
| MAP3K6 | -0.01291724 | 0.66968511 | -0.053981037 | 0.0744397 | 0.05 |
| MAP3K7 | -0.082196031 | 0.00654918 | -0.14021743 | 3.27E-06 | 0.06 |
| ESTF | -0.094432161 | 0.00177567 | -0.179152518 | 2.46E-09 | 0.09 |
| MAP3K9 | -0.251155471 | 3.48E-17 | -0.231557362 | 9.03E-15 | 0.02 |
| MAP4K1 | 0.049180754 | 0.10414855 | -0.098713616 | 0.001084 | 0.15 |
| MAP4K2 | 0.107627119 | 0.00036455 | 0.007186401 | 0.8124104 | 0.11 |
| MAP4K3 | -0.336230065 | 2.70E-30 | -0.245931064 | 1.61E-16 | 0.1 |
| FLJ20373 | 0.046209445 | 0.12681641 | -0.09694874 | 0.0013316 | 0.15 |
| KHS1 | -0.201338313 | 1.85E-11 | -0.246847302 | 1.23E-16 | 0.05 |
| MAP4 | -0.191251013 | 1.83E-10 | -0.259589936 | 2.73E-18 | 0.07 |
| MAP6D1 | 0.173096142 | 8.43E-09 | 0.01077962 | 0.7218535 | 0.17 |
| MAP6 | -0.137603297 | 4.98E-06 | -0.16546512 | 3.75E-08 | 0.03 |
| MAP7D1 | 0.04591372 | 0.12926769 | -0.129736987 | 1.69E-05 | 0.18 |
| FLJ14503 | 0.278312123 | 6.82E-21 | 0.084665827 | 0.0050956 | 0.2 |
| FLJ12401 | 0.035083143 | 0.24649792 | -0.156622546 | 1.95E-07 | 0.2 |
| MAP7 | -0.167945798 | 2.33E-08 | -0.017074672 | 0.5728268 | 0.16 |
| MAP9 | -0.213586702 | 9.63E-13 | -0.175977629 | 4.71E-09 | 0.04 |
| MAPK10 | -0.1227971 | 4.69E-05 | -0.027252992 | 0.3680483 | 0.1 |
| MAPK11 | -0.086702634 | 0.00412345 | -0.114708908 | 0.0001444 | 0.03 |
| LOC100509672 | 0.153996338 | 3.12E-07 | -0.03392426 | 0.2624608 | 0.19 |
| MAPK13 | 0.073709063 | 0.01479372 | 0.055139588 | 0.0684177 | 0.02 |
| LOC101929346 | -0.100721101 | 0.00085432 | -0.154615914 | 2.80E-07 | 0.06 |
| MAPK15 | 0.113516288 | 0.00016939 | 0.176645885 | 4.11E-09 | 0.07 |
| MAPK1IP1L | -0.028789202 | 0.34165775 | -0.023651863 | 0.4347095 | 0.01 |
| MAPK1 | -0.199631238 | 2.75E-11 | -0.268936213 | 1.45E-19 | 0.07 |
| MAPK3 | -0.108218659 | 0.00033811 | -0.097117271 | 0.0013059 | 0.02 |
| MAPK4 | 0.205378123 | 7.11E-12 | -0.065964896 | 0.0292044 | 0.28 |
| MAPK6 | -0.13531227 | 7.15E-06 | -0.147711379 | 9.35E-07 | 0.02 |
| MAPK7 | 0.051888641 | 0.08640771 | -0.031401041 | 0.299641 | 0.09 |
| MAPK8IP1 | -0.074409375 | 0.01387004 | -0.106702015 | 0.0004098 | 0.04 |
| JIP2 | -0.101091195 | 0.00081726 | -0.142183978 | 2.37E-06 | 0.05 |
| FLJ00027 | -0.107066042 | 0.00039141 | -0.146516059 | 1.15E-06 | 0.04 |
| MAPK8 | -0.178836847 | 2.62E-09 | -0.298709717 | 5.78E-24 | 0.12 |
| JNK2 | -0.240798712 | 6.98E-16 | -0.087223443 | 0.0039035 | 0.16 |
| LOC79182 | 0.170567507 | 1.39E-08 | 0.097254137 | 0.0012854 | 0.08 |
| MAPKAPK2 | -0.012942634 | 0.66907425 | 0.217675356 | 3.45E-13 | 0.24 |
| MAPKAPK3 | -0.03521905 | 0.24467139 | 0.006935834 | 0.8188356 | 0.05 |
| MAPKAPK5 | 0.249908333 | 5.03E-17 | 0.149268297 | 7.16E-07 | 0.11 |
| MAPKBP1 | -0.439026085 | 1.03E-52 | -0.42470017 | 4.28E-49 | 0.02 |
| LOC100132990 | -0.144346556 | 1.65E-06 | -0.080289713 | 0.007915 | 0.07 |
| MAPRE1 | 0.256728302 | 6.53E-18 | 0.133978951 | 8.81E-06 | 0.13 |
| LOC51683 | 0.011408167 | 0.70636611 | -0.211279594 | 1.70E-12 | 0.23 |
| MAPRE3 | -0.041352114 | 0.17189387 | -0.086228568 | 0.0043334 | 0.05 |
| LOC147077 | -0.375844613 | 5.34E-38 | -0.137987942 | 4.68E-06 | 0.24 |
| 10-Mar | -0.132866644 | 1.05E-05 | -0.016840333 | 0.5781062 | 0.12 |
| 11-Mar | -0.079567975 | 0.00849527 | -0.039316432 | 0.1939996 | 0.05 |
| 1-Mar | -0.209578752 | 2.58E-12 | -0.331762284 | 1.71E-29 | 0.13 |
| 2-Mar | -0.121150474 | 5.93E-05 | 0.010942869 | 0.7178198 | 0.14 |
| LOC153277 | 0.005664415 | 0.85161679 | -0.068543051 | 0.0234426 | 0.08 |
| 4-Mar | 0.050252646 | 0.09680822 | -0.032567738 | 0.2820343 | 0.09 |
| 5-Mar | -0.021794619 | 0.47164623 | 0.044288079 | 0.1434042 | 0.07 |
| 6-Mar | -0.301918864 | 1.80E-24 | -0.223136346 | 8.47E-14 | 0.08 |
| 7-Mar | -0.31542892 | 1.13E-26 | -0.207491649 | 4.28E-12 | 0.11 |
| 8-Mar | -0.484099845 | 2.72E-65 | -0.472495138 | 7.16E-62 | 0.02 |
| 9-Mar | 0.108555605 | 0.00032385 | 0.102665442 | 0.0006756 | 0.01 |
| MARCKSL1 | 0.385143679 | 5.71E-40 | 0.365886636 | 5.86E-36 | 0.02 |
| LOC154339 | 0.049045671 | 0.10510413 | -0.009103583 | 0.7636956 | 0.06 |
| MARCO | 0.307608801 | 2.20E-25 | 0.067372474 | 0.0259237 | 0.25 |
| MARK1 | 0.124559354 | 3.64E-05 | 0.071723188 | 0.0177137 | 0.06 |
| MARK2 | -0.026473266 | 0.38191541 | -0.096246154 | 0.001444 | 0.07 |
| MARK3 | 0.141379709 | 2.70E-06 | 0.075982572 | 0.0119782 | 0.07 |
| MARK4 | 0.060855706 | 0.0442742 | -0.109485487 | 0.0002874 | 0.18 |
| MARS2 | 0.113619399 | 0.00016707 | -0.032495406 | 0.283105 | 0.15 |
| MARS | 0.316179118 | 8.45E-27 | 0.133404672 | 9.64E-06 | 0.19 |
| MARVELD1 | -0.168582433 | 2.06E-08 | -0.288563352 | 2.10E-22 | 0.12 |
| DFNB49 | -0.294425043 | 2.68E-23 | -0.111964117 | 0.0002081 | 0.19 |
| MARVELD3 | 0.061662511 | 0.04152957 | 0.058043559 | 0.0550636 | 0.01 |
| LOC154544 | -0.327475426 | 9.78E-29 | -0.258418861 | 3.90E-18 | 0.07 |
| MAS1 | -0.029420451 | 0.3311731 | -0.049533974 | 0.1016824 | 0.03 |
| MASP1 | -0.193625597 | 1.08E-10 | -0.209836914 | 2.43E-12 | 0.02 |
| MASP2 | -0.416457759 | 4.32E-47 | -0.463877283 | 2.05E-59 | 0.05 |
| MAST1 | 0.313008808 | 2.86E-26 | 0.036246689 | 0.2311671 | 0.28 |
| MAST2 | 0.014939379 | 0.62175366 | -0.105520943 | 0.0004752 | 0.13 |
| MAST3 | -0.228654858 | 1.97E-14 | -0.194092578 | 9.73E-11 | 0.04 |
| MAST4 | -0.585739781 | 1.16E-101 | -0.476764323 | 4.09E-63 | 0.11 |
| MASTL | 0.35482073 | 8.96E-34 | 0.134784287 | 7.77E-06 | 0.23 |
| MAT1A | 0.173633027 | 7.57E-09 | 0.103793367 | 0.0005886 | 0.07 |
| MAT2A | -0.155089835 | 2.57E-07 | -0.06134437 | 0.0425942 | 0.1 |
| TGR | -0.225583254 | 4.46E-14 | -0.137390881 | 5.15E-06 | 0.09 |
| MATK | 0.117836306 | 9.43E-05 | -0.017866832 | 0.5551514 | 0.14 |
| LOC115278 | 0.161007253 | 8.71E-08 | -0.048068018 | 0.1122287 | 0.21 |
| MATN2 | -0.115997834 | 0.00012127 | -0.138030597 | 4.65E-06 | 0.03 |
| MATN3 | -0.397826738 | 9.20E-43 | -0.135197589 | 7.28E-06 | 0.27 |
| MATN4 | 0.319657261 | 2.18E-27 | 0.040809698 | 0.1775912 | 0.28 |
| LOC133338 | -0.280710571 | 3.06E-21 | -0.118674637 | 8.39E-05 | 0.17 |
| MAVS | -0.272483965 | 4.63E-20 | -0.204492063 | 8.78E-12 | 0.07 |
| MAX | -0.071116398 | 0.01870117 | 0.009122324 | 0.7632237 | 0.09 |
| MAZ | 0.369978319 | 8.67E-37 | 0.288112202 | 2.45E-22 | 0.09 |
| MBD1 | 0.127128401 | 2.50E-05 | -0.133280218 | 9.82E-06 | 0.27 |
| MBD2 | 0.3784935 | 1.49E-38 | 0.141132123 | 2.81E-06 | 0.24 |
| MBD3L | 0.10043142 | 0.00088441 | 0.003797081 | 0.9002145 | 0.1 |
| LOC147790 | 0.060235473 | 0.04648669 | 0.019996316 | 0.5089979 | 0.05 |
| LOC201531 | 0.072024673 | 0.01724022 | 0.029075027 | 0.3368844 | 0.05 |
| MBD3 | 0.200508702 | 2.24E-11 | 0.101639404 | 0.0007651 | 0.1 |
| MBD4 | 0.174267912 | 6.66E-09 | 0.126842393 | 2.60E-05 | 0.05 |
| MBD5 | -0.43131183 | 9.62E-51 | -0.388245071 | 1.22E-40 | 0.05 |
| MBD6 | -0.26052805 | 2.04E-18 | -0.05686997 | 0.0601732 | 0.21 |
| MBIP | 0.018100004 | 0.54999986 | -0.053105894 | 0.0792696 | 0.08 |
| MBL1P | -0.060940484 | 0.04397879 | -0.072272052 | 0.0168601 | 0.02 |
| MBL2 | -0.023420387 | 0.43922001 | -0.019195985 | 0.5261056 | 0.01 |
| LOC392757 | 0.306564691 | 3.24E-25 | 0.281705468 | 2.19E-21 | 0.03 |
| MBLAC2 | -0.270914278 | 7.70E-20 | -0.225278871 | 4.83E-14 | 0.05 |
| MBNL1 | -0.158513901 | 1.38E-07 | -0.160087502 | 1.03E-07 | 0.01 |
| PRO2032 | -0.208767053 | 3.15E-12 | -0.195862694 | 6.52E-11 | 0.02 |
| LOC100132018 | -0.207530596 | 4.24E-12 | -0.265989784 | 3.71E-19 | 0.06 |
| MBOAT1 | -0.07334642 | 0.01529297 | -0.061762219 | 0.0412006 | 0.02 |
| MBOAT2 | -0.03514745 | 0.24563249 | 0.048928758 | 0.1059368 | 0.09 |
| MBOAT4 | -0.119572445 | 7.41E-05 | -0.130714618 | 1.46E-05 | 0.02 |
| MBOAT7 | -0.025759787 | 0.39488077 | 0.023344029 | 0.4407138 | 0.05 |
| MBP | -0.079091608 | 0.00889877 | -0.344908229 | 6.87E-32 | 0.27 |
| MBTD1 | -0.048068661 | 0.11222391 | -0.025524685 | 0.3992106 | 0.03 |
| MBTPS1 | -0.244265278 | 2.60E-16 | -0.276408536 | 1.28E-20 | 0.04 |
| BRESEK | -0.295802664 | 1.64E-23 | -0.231442952 | 9.31E-15 | 0.07 |
| MB | -0.021927222 | 0.46895303 | 0.142331375 | 2.31E-06 | 0.17 |
| MC1R | 0.031171066 | 0.30319612 | 0.041581703 | 0.1695239 | 0.02 |
| MC2R | -0.031728241 | 0.29463091 | -0.038005234 | 0.209301 | 0.01 |
| MC3R | 0.089771031 | 0.00297332 | 0.000627162 | 0.9834765 | 0.09 |
| MC4R | 0.018360958 | 0.5442625 | -0.046639212 | 0.1233187 | 0.07 |
| MC5R | 0.015461479 | 0.60962372 | 0.06538942 | 0.0306451 | 0.05 |
| MCAM | -0.065393609 | 0.03063443 | -0.198198899 | 3.82E-11 | 0.14 |
| SLC25A51 | 0.115011162 | 0.00013861 | 0.011078157 | 0.7144829 | 0.11 |
| SLC25A52 | 0.082019371 | 0.0066662 | -0.036526016 | 0.2275897 | 0.12 |
| LOC100421021 | -0.000385231 | 0.98985009 | -0.024159131 | 0.4249188 | 0.03 |
| SLC25A53 | 0.170870589 | 1.31E-08 | -0.04106656 | 0.1748759 | 0.22 |
| MGC47838 | 0.302239312 | 1.60E-24 | 0.291815555 | 6.74E-23 | 0.02 |
| MCCC1 | 0.332152034 | 1.46E-29 | 0.115087446 | 0.0001372 | 0.22 |
| MCCC2 | -0.317229396 | 5.63E-27 | -0.092564342 | 0.0021891 | 0.23 |
| MCCD1 | -0.155454755 | 2.40E-07 | -0.042262955 | 0.1626357 | 0.12 |
| MCC | -0.417687714 | 2.19E-47 | -0.270967112 | 7.57E-20 | 0.15 |
| MCEE | -0.04081819 | 0.17750093 | 0.087189206 | 0.0039176 | 0.13 |
| MCF2L2 | 0.150793597 | 5.49E-07 | -0.079607204 | 0.0084628 | 0.24 |
| MCF2L | -0.287656026 | 2.87E-22 | -0.133708677 | 9.19E-06 | 0.16 |
| MCF2 | 0.11475578 | 0.00014347 | -0.034842692 | 0.2497528 | 0.15 |
| MCFD2 | -0.247623989 | 9.82E-17 | -0.325652618 | 2.03E-28 | 0.08 |
| MCHR1 | -0.033547639 | 0.26779866 | -0.125397493 | 3.22E-05 | 0.1 |
| MCHR2 | 0.088034793 | 0.00358198 | 0.027092336 | 0.3708796 | 0.07 |
| MCL1 | -0.120132438 | 6.85E-05 | 0.053005246 | 0.0798411 | 0.18 |
| LOC115720 | 0.500982574 | 1.65E-70 | 0.246105608 | 1.53E-16 | 0.26 |
| MCM2 | 0.485656608 | 9.23E-66 | 0.250145997 | 4.69E-17 | 0.24 |
| MCM3AP.AS1 | -0.051579599 | 0.08830016 | -0.122058527 | 5.21E-05 | 0.08 |
| MCM3AP | -0.205193978 | 7.43E-12 | -0.211746637 | 1.52E-12 | 0.01 |
| MCM3 | 0.516431295 | 1.51E-75 | 0.289456162 | 1.54E-22 | 0.23 |
| MCM4 | 0.327441321 | 9.91E-29 | 0.090220461 | 0.0028319 | 0.24 |
| MCM5 | 0.414996924 | 9.66E-47 | 0.147171779 | 1.03E-06 | 0.27 |
| MCM6 | 0.451753158 | 4.47E-56 | 0.220059197 | 1.88E-13 | 0.24 |
| MCM7 | 0.536926315 | 1.21E-82 | 0.290950773 | 9.12E-23 | 0.25 |
| MCM8 | 0.134905151 | 7.63E-06 | 0.025057698 | 0.4078952 | 0.11 |
| C6orf61 | -0.172217403 | 1.00E-08 | -0.101998114 | 0.0007326 | 0.08 |
| ML4 | 0.115133888 | 0.00013633 | 0.084003407 | 0.0054538 | 0.04 |
| MCOLN2 | 0.173993121 | 7.04E-09 | 0.016256887 | 0.591349 | 0.16 |
| MCOLN3 | 0.071466219 | 0.01812615 | -0.140071648 | 3.34E-06 | 0.22 |
| MCPH1 | -0.182010693 | 1.35E-09 | -0.113613643 | 0.0001672 | 0.07 |
| MCRS1 | 0.26101605 | 1.76E-18 | 0.290976923 | 9.04E-23 | 0.03 |
| MCTP1 | -0.407362738 | 6.07E-45 | -0.37645476 | 3.98E-38 | 0.04 |
| MCTP2 | -0.009044405 | 0.76518652 | -0.113724116 | 0.0001648 | 0.11 |
| MCTS1 | 0.292183425 | 5.92E-23 | 0.376047219 | 4.84E-38 | 0.09 |
| MDC1 | 0.115524362 | 0.00012932 | -0.067722248 | 0.0251596 | 0.19 |
| MDFIC | -0.151257263 | 5.06E-07 | -0.182041041 | 1.34E-09 | 0.04 |
| MDFI | 0.211026737 | 1.81E-12 | 0.135664396 | 6.77E-06 | 0.08 |
| MDGA1 | -0.309980338 | 9.01E-26 | -0.301158283 | 2.38E-24 | 0.01 |
| MDGA2 | 0.253920831 | 1.52E-17 | 0.068952966 | 0.0226238 | 0.19 |
| MDH1B | -0.120710506 | 6.31E-05 | 0.034024561 | 0.2610516 | 0.16 |
| MDH1 | 0.476716601 | 4.23E-63 | 0.285844733 | 5.35E-22 | 0.2 |
| MDH2 | 0.400562331 | 2.22E-43 | 0.334072266 | 6.62E-30 | 0.07 |
| MDK | 0.196076852 | 6.21E-11 | 0.12618139 | 2.87E-05 | 0.07 |
| MDM1 | -0.186765772 | 4.90E-10 | -0.125937489 | 2.97E-05 | 0.07 |
| MGC5370 | -0.160661499 | 9.28E-08 | -0.14537366 | 1.39E-06 | 0.02 |
| MDM4 | -0.179343435 | 2.36E-09 | 0.230298757 | 1.27E-14 | 0.41 |
| MDN1 | -0.222340159 | 1.04E-13 | -0.362737073 | 2.50E-35 | 0.15 |
| MDP1 | -0.008872836 | 0.76951378 | 0.1040017 | 0.0005737 | 0.12 |
| MDS2 | 0.040586357 | 0.17997747 | -0.04321371 | 0.1533779 | 0.09 |
| ME1 | 0.189014073 | 3.00E-10 | 0.03552042 | 0.240655 | 0.16 |
| ME2 | 0.333117495 | 9.81E-30 | 0.017501121 | 0.5632784 | 0.32 |
| ME3 | -0.132949845 | 1.03E-05 | -0.121202353 | 5.89E-05 | 0.02 |
| MEA1 | 0.453988277 | 1.11E-56 | 0.380221926 | 6.42E-39 | 0.08 |
| MEAF6 | -0.012888227 | 0.67038331 | 0.026936459 | 0.3736396 | 0.04 |
| MDS1 | -0.36361964 | 1.67E-35 | -0.259145808 | 3.12E-18 | 0.11 |
| RTT | -0.360551687 | 6.79E-35 | -0.411481899 | 6.58E-46 | 0.06 |
| MECR | 0.331350297 | 2.03E-29 | 0.220630262 | 1.62E-13 | 0.12 |
| MED10 | 0.280971831 | 2.80E-21 | 0.16803032 | 2.29E-08 | 0.12 |
| LOC201304 | 0.1218131 | 5.40E-05 | 0.192771873 | 1.31E-10 | 0.08 |
| MED12L | -0.201659028 | 1.71E-11 | -0.244255701 | 2.60E-16 | 0.05 |
| FGS1 | -0.209401891 | 2.70E-12 | -0.232283827 | 7.41E-15 | 0.03 |
| MED13L | -0.425572761 | 2.61E-49 | -0.41407749 | 1.60E-46 | 0.02 |
| MED13 | -0.247196189 | 1.11E-16 | -0.270765348 | 8.08E-20 | 0.03 |
| MED14 | -0.099285832 | 0.00101328 | -0.197083225 | 4.94E-11 | 0.1 |
| DKFZP586E2117 | 0.034522736 | 0.25413026 | 0.001868905 | 0.9507887 | 0.04 |
| MED16 | -0.128020893 | 2.19E-05 | -0.012490249 | 0.679989 | 0.12 |
| MED17 | 0.15523252 | 2.50E-07 | 0.029397051 | 0.331558 | 0.13 |
| LOC100292920 | -0.020357312 | 0.50137777 | -0.001725534 | 0.9545596 | 0.02 |
| MED19 | 0.232178607 | 7.63E-15 | 0.259371321 | 2.92E-18 | 0.03 |
| MED1 | -0.106442716 | 0.00042341 | -0.107239055 | 0.0003829 | 0.01 |
| LOC88724 | 0.146437327 | 1.16E-06 | 0.121445549 | 5.69E-05 | 0.03 |
| MED21 | 0.036894096 | 0.22293591 | 0.028810728 | 0.3412968 | 0.01 |
| LOC157888 | -0.086054468 | 0.00441285 | -0.120343119 | 6.65E-05 | 0.04 |
| MED23 | -0.359935758 | 8.98E-35 | -0.206206002 | 5.84E-12 | 0.16 |
| MED24 | 0.059069182 | 0.05089904 | 0.090633187 | 0.0027075 | 0.04 |
| CMT2B2 | 0.065150886 | 0.03125994 | 0.061259641 | 0.0428815 | 0.01 |
| MED26 | 0.314631665 | 1.53E-26 | 0.148101902 | 8.75E-07 | 0.17 |
| LOC100134189 | 0.436572923 | 4.42E-52 | 0.323658518 | 4.51E-28 | 0.12 |
| MED28 | 0.010297276 | 0.73381656 | 0.021467751 | 0.4783212 | 0.02 |
| MED29 | -0.127596665 | 2.33E-05 | -0.110272784 | 0.0002595 | 0.02 |
| MED30 | 0.554076172 | 5.73E-89 | 0.308390488 | 1.64E-25 | 0.25 |
| MED31 | 0.106456167 | 0.00042269 | 0.130083808 | 1.60E-05 | 0.03 |
| MED4 | 0.122037664 | 5.23E-05 | 0.143559268 | 1.88E-06 | 0.03 |
| MED6 | 0.275219212 | 1.90E-20 | 0.25923781 | 3.04E-18 | 0.02 |
| MED7 | -0.089737729 | 0.00298405 | 0.196377797 | 5.80E-11 | 0.29 |
| LOC115853 | 0.359701046 | 9.99E-35 | 0.208108144 | 3.69E-12 | 0.16 |
| MED9 | 0.132171719 | 1.17E-05 | 0.061746377 | 0.0412527 | 0.08 |
| MEF2A | -0.203604364 | 1.08E-11 | -0.376841013 | 3.31E-38 | 0.18 |
| MEF2B | 0.133941428 | 8.87E-06 | 0.038873378 | 0.1990754 | 0.1 |
| MEF2C | -0.306202141 | 3.71E-25 | -0.330120938 | 3.35E-29 | 0.03 |
| MEF2D | -0.450259717 | 1.13E-55 | -0.159309497 | 1.19E-07 | 0.3 |
| MEFV | -0.076174286 | 0.01176409 | -0.184508909 | 7.95E-10 | 0.11 |
| MEG3 | -0.22123644 | 1.39E-13 | -0.078649665 | 0.0092883 | 0.15 |
| LOC101929345 | 0.015751272 | 0.60293664 | -0.020260319 | 0.5034192 | 0.04 |
| MEGF10 | -0.065184581 | 0.03117246 | -0.101440569 | 0.0007836 | 0.04 |
| MEGF11 | 0.074801268 | 0.01337566 | -0.091776603 | 0.0023885 | 0.17 |
| MEGF6 | -0.272334742 | 4.86E-20 | -0.1395331 | 3.65E-06 | 0.14 |
| C19orf49 | -0.281155478 | 2.63E-21 | -0.33968232 | 6.36E-31 | 0.06 |
| MEGF9 | -0.40941522 | 2.01E-45 | -0.207689634 | 4.08E-12 | 0.21 |
| LOC654123 | 0.059371908 | 0.04972138 | -0.034930169 | 0.2485652 | 0.1 |
| LOC105376430 | 0.294264455 | 2.84E-23 | 0.150468246 | 5.81E-07 | 0.15 |
| MEIS1 | -0.167005858 | 2.79E-08 | -0.094652077 | 0.001732 | 0.08 |
| MEIS2 | -0.199092133 | 3.11E-11 | -0.191993399 | 1.56E-10 | 0.01 |
| MEIS3P1 | -0.45553401 | 4.20E-57 | -0.214344088 | 7.97E-13 | 0.25 |
| MEIS3 | -0.31361217 | 2.27E-26 | -0.178848019 | 2.61E-09 | 0.14 |
| MELK | 0.525573768 | 1.19E-78 | 0.270791042 | 8.01E-20 | 0.26 |
| C2orf4 | 0.518308755 | 3.54E-76 | 0.380825707 | 4.78E-39 | 0.14 |
| LOC196204 | 0.257956396 | 4.50E-18 | 0.249874689 | 5.08E-17 | 0.01 |
| MEOX1 | -0.153013492 | 3.72E-07 | -0.167282123 | 2.65E-08 | 0.02 |
| MEOX2 | -0.310561512 | 7.24E-26 | -0.210746946 | 1.94E-12 | 0.1 |
| MEP1A | 0.077788682 | 0.01009107 | -0.026150807 | 0.3877425 | 0.11 |
| MEP1B | 0.082283999 | 0.0064916 | -0.042144288 | 0.1638202 | 0.13 |
| MEPCE | 0.266899336 | 2.78E-19 | 0.308602451 | 1.51E-25 | 0.05 |
| MEPE | -0.069138081 | 0.02226226 | -0.059738698 | 0.0483251 | 0.01 |
| MERTK | -0.206247266 | 5.78E-12 | -0.290305116 | 1.14E-22 | 0.09 |
| MESDC1 | 0.116711415 | 0.00011002 | 0.166948263 | 2.82E-08 | 0.06 |
| MESDC2 | 0.040446307 | 0.18148591 | 0.097825595 | 0.0012027 | 0.06 |
| MESP1 | 0.207948506 | 3.84E-12 | 0.127461159 | 2.37E-05 | 0.09 |
| MESP2 | 0.165903831 | 3.45E-08 | 0.13971418 | 3.54E-06 | 0.03 |
| MESTIT1 | -0.249908135 | 5.03E-17 | -0.207830964 | 3.95E-12 | 0.05 |
| MEST | -0.038311968 | 0.20564554 | 0.053067558 | 0.0794869 | 0.1 |
| METAP1 | 0.25889854 | 3.37E-18 | 0.105885342 | 0.0004541 | 0.16 |
| METAP2 | 0.193869015 | 1.02E-10 | 0.160729454 | 9.17E-08 | 0.04 |
| METRNL | 0.062577305 | 0.03859299 | -0.037440891 | 0.2161488 | 0.11 |
| LOC100507933 | -0.120955578 | 6.10E-05 | 0.090048626 | 0.0028853 | 0.22 |
| METTL16 | -0.125108556 | 3.36E-05 | -0.168221531 | 2.21E-08 | 0.05 |
| METTL17 | 0.217981593 | 3.19E-13 | 0.221053955 | 1.45E-13 | 0.01 |
| METTL15 | -0.275267881 | 1.87E-20 | -0.075355486 | 0.0127031 | 0.2 |
| C10orf138 | 0.090749664 | 0.00267333 | -0.025931984 | 0.3917275 | 0.12 |
| NTMT1 | 0.299556215 | 4.25E-24 | 0.166422364 | 3.12E-08 | 0.14 |
| METTL11B | -0.16378264 | 5.17E-08 | -0.085093053 | 0.004876 | 0.08 |
| METTL12 | 0.189654249 | 2.61E-10 | 0.122019617 | 5.24E-05 | 0.07 |
| LOC88158 | 0.085593085 | 0.00462992 | 0.321620799 | 1.01E-27 | 0.24 |
| METTL14 | -0.374739211 | 9.06E-38 | -0.317572766 | 4.92E-27 | 0.06 |
| METTL1 | 0.447222705 | 7.31E-55 | 0.448245245 | 3.91E-55 | 0.01 |
| METTL2A | 0.158262681 | 1.44E-07 | 0.143164395 | 2.01E-06 | 0.02 |
| METTL2B | 0.01433577 | 0.6359061 | -0.014840409 | 0.6240648 | 0.03 |
| METTL3 | 0.057807489 | 0.05606136 | 0.079078651 | 0.00891 | 0.03 |
| METTL4 | 0.313207817 | 2.65E-26 | 0.014261177 | 0.6376644 | 0.3 |
| METTL5 | 0.495082321 | 1.18E-68 | 0.502163257 | 6.93E-71 | 0.01 |
| METTL6 | 0.080050615 | 0.0081032 | 0.13621436 | 6.21E-06 | 0.06 |
| METTL7A | -0.146759799 | 1.10E-06 | -0.174133333 | 6.85E-09 | 0.03 |
| METTL7B | 0.248526556 | 7.54E-17 | 0.090042479 | 0.0028872 | 0.16 |
| METTL8 | 0.077186191 | 0.01068894 | 0.020390654 | 0.500677 | 0.06 |
| METTL9 | 0.175865179 | 4.82E-09 | 0.165566924 | 3.68E-08 | 0.02 |
| MET | 0.009284457 | 0.75914432 | -0.149481251 | 6.90E-07 | 0.16 |
| MEX3A | 0.416287183 | 4.75E-47 | 0.374207097 | 1.17E-37 | 0.05 |
| MEX3B | -0.007209275 | 0.8118245 | -0.055250665 | 0.067862 | 0.05 |
| MEX3C | 0.094668118 | 0.00172889 | -0.128936705 | 1.91E-05 | 0.23 |
| MEX3D | 0.069226001 | 0.02209231 | 0.112443219 | 0.0001953 | 0.05 |
| MFAP1 | -0.210724323 | 1.95E-12 | -0.159076544 | 1.24E-07 | 0.06 |
| MFAP2 | 0.158764002 | 1.32E-07 | 0.139011283 | 3.97E-06 | 0.02 |
| LOC86609 | 0.023047465 | 0.44654292 | -0.01965701 | 0.5162151 | 0.05 |
| MFAP3 | -0.459541459 | 3.32E-58 | -0.261602953 | 1.46E-18 | 0.2 |
| MFAP4 | -0.332591595 | 1.22E-29 | -0.185949207 | 5.84E-10 | 0.15 |
| MFAP5 | -0.164317809 | 4.67E-08 | -0.05842724 | 0.0534736 | 0.11 |
| MFF | 0.297266193 | 9.72E-24 | 0.244570908 | 2.38E-16 | 0.06 |
| SPAG10 | 0.150337575 | 5.95E-07 | -0.018004562 | 0.5521056 | 0.17 |
| MFHAS1 | -0.17898092 | 2.54E-09 | -0.331369569 | 2.01E-29 | 0.16 |
| MGC4856 | 0.1992022 | 3.03E-11 | -0.074806289 | 0.0133694 | 0.28 |
| MFN1 | -0.054101819 | 0.07379234 | -0.13242396 | 1.12E-05 | 0.08 |
| MFN2 | -0.241722768 | 5.37E-16 | -0.404918072 | 2.23E-44 | 0.17 |
| MFNG | -0.192047116 | 1.54E-10 | -0.206615722 | 5.29E-12 | 0.02 |
| MFRP | -0.120600705 | 6.41E-05 | 0.019240531 | 0.5251457 | 0.14 |
| MFSD10 | 0.170216983 | 1.49E-08 | 0.199508603 | 2.83E-11 | 0.03 |
| MFSD11 | -0.04634288 | 0.12572227 | -0.028234029 | 0.3510522 | 0.02 |
| MFSD1 | -0.161978479 | 7.26E-08 | -0.173465152 | 7.83E-09 | 0.02 |
| MFSD2A | 0.088119875 | 0.0035497 | 0.150314461 | 5.97E-07 | 0.07 |
| MFSD2B | 0.492712993 | 6.44E-68 | 0.398810156 | 5.53E-43 | 0.1 |
| MFSD3 | 0.240426523 | 7.75E-16 | 0.176566438 | 4.18E-09 | 0.07 |
| MFSD4 | 0.145810015 | 1.29E-06 | 0.069073254 | 0.0223883 | 0.08 |
| MFSD5 | 0.049124715 | 0.10454413 | 0.203520216 | 1.11E-11 | 0.16 |
| MFSD6L | -0.042047472 | 0.1647913 | 0.03133824 | 0.300609 | 0.08 |
| MFSD6 | -0.227581641 | 2.63E-14 | -0.143094045 | 2.04E-06 | 0.09 |
| MFSD7 | -0.296212939 | 1.42E-23 | -0.090324069 | 0.0028002 | 0.21 |
| MFSD8 | -0.265481297 | 4.35E-19 | -0.25393937 | 1.52E-17 | 0.02 |
| MFSD9 | -0.171745724 | 1.10E-08 | -0.257974304 | 4.47E-18 | 0.09 |
| MGAM | -0.169752425 | 1.64E-08 | -0.059781169 | 0.0481656 | 0.11 |
| LOC102724833 | -0.025569877 | 0.3983761 | -0.074974553 | 0.0131621 | 0.05 |
| LOC145450 | -0.150272032 | 6.01E-07 | -0.111587004 | 0.0002186 | 0.04 |
| MGAT3 | 0.00282361 | 0.92571013 | -0.240245526 | 8.16E-16 | 0.25 |
| MGAT4A | -0.346728735 | 3.13E-32 | -0.251826959 | 2.85E-17 | 0.1 |
| MGAT4B | 0.191278709 | 1.82E-10 | 0.050113382 | 0.0977379 | 0.15 |
| MGAT4C | 0.023628024 | 0.43517277 | -0.057161773 | 0.0588674 | 0.09 |
| LOC101928538 | 0.011502882 | 0.70404264 | -0.112709017 | 0.0001886 | 0.13 |
| MGAT5 | -0.383862991 | 1.08E-39 | -0.480411636 | 3.43E-64 | 0.1 |
| MGA | -0.263722477 | 7.56E-19 | -0.285956866 | 5.15E-22 | 0.03 |
| MGC12916 | 0.074078158 | 0.01430036 | -0.153044577 | 3.70E-07 | 0.23 |
| FOXD2.AS1 | 0.212929778 | 1.13E-12 | 0.071015702 | 0.0188696 | 0.15 |
| FAM222A.AS1 | -0.095519671 | 0.00156931 | 0.012783542 | 0.6729049 | 0.11 |
| MGC15885 | -0.05477078 | 0.07028975 | -0.065418747 | 0.0305703 | 0.02 |
| MGC16025 | 0.121237151 | 5.86E-05 | 0.090661299 | 0.0026992 | 0.04 |
| MIR503HG | 0.012519533 | 0.67928043 | 0.036534942 | 0.227476 | 0.03 |
| MGC16142 | 0.149702498 | 6.64E-07 | -0.024338461 | 0.4214886 | 0.18 |
| MGC16275 | 0.230542351 | 1.19E-14 | 0.107582802 | 0.0003666 | 0.13 |
| MGC16384 | -0.404264904 | 3.16E-44 | -0.3546546 | 9.65E-34 | 0.05 |
| TUBA3FP | 0.002077802 | 0.94529629 | -0.124228102 | 3.82E-05 | 0.13 |
| LOC286286 | -0.063520664 | 0.0357512 | -0.227907247 | 2.41E-14 | 0.17 |
| LINC00638 | -0.081125866 | 0.00728732 | 0.015179019 | 0.6161731 | 0.1 |
| SNAI3.AS1 | -0.051366806 | 0.08962251 | -0.083668515 | 0.0056433 | 0.04 |
| C7ORF62 | -0.058313607 | 0.05394049 | 0.001762505 | 0.9535871 | 0.07 |
| MGC27382 | 0.122604067 | 4.82E-05 | -0.014616506 | 0.6293069 | 0.14 |
| CENPBD1P1 | -0.232099355 | 7.79E-15 | -0.191882306 | 1.60E-10 | 0.05 |
| MGC2889 | 0.332030929 | 1.53E-29 | 0.071128664 | 0.0186807 | 0.27 |
| MZB1 | 0.004108728 | 0.89207271 | -0.102895382 | 0.000657 | 0.11 |
| LOC439937 | 0.004519812 | 0.88135064 | 0.031404874 | 0.299582 | 0.03 |
| ZNF205.AS1 | -0.223288905 | 8.14E-14 | -0.017658767 | 0.5597681 | 0.21 |
| NIM1K | -0.041834934 | 0.16693841 | -0.153607637 | 3.35E-07 | 0.12 |
| LINC00626 | -0.013908761 | 0.64599911 | 0.038760612 | 0.2003827 | 0.06 |
| LOC101928703 | -0.207134334 | 4.67E-12 | -0.202058962 | 1.56E-11 | 0.01 |
| MGC57346 | 0.01079307 | 0.72152087 | -0.00891602 | 0.7684239 | 0.02 |
| C8ORF82 | 0.343899304 | 1.06E-31 | 0.23135078 | 9.55E-15 | 0.12 |
| MGC72080 | 0.470172788 | 3.34E-61 | 0.312875609 | 3.00E-26 | 0.16 |
| STEAP1B | 0.091241291 | 0.0025333 | 0.056590863 | 0.0614445 | 0.04 |
| KIAA0679 | -0.291932012 | 6.47E-23 | -0.137644352 | 4.94E-06 | 0.16 |
| MGLL | -0.310060388 | 8.74E-26 | -0.215086656 | 6.62E-13 | 0.1 |
| MGMT | 0.110481943 | 0.00025259 | 0.163549514 | 5.40E-08 | 0.06 |
| MGP | -0.064017733 | 0.03432689 | 0.030396894 | 0.3153689 | 0.1 |
| MGRN1 | -0.163442539 | 5.51E-08 | -0.126687718 | 2.66E-05 | 0.04 |
| MGST1 | 0.07860963 | 0.00932433 | 0.172114061 | 1.03E-08 | 0.1 |
| MGST2 | -0.075776774 | 0.01221198 | 0.073337866 | 0.0153049 | 0.15 |
| GST.III | 0.350152929 | 7.05E-33 | 0.50266542 | 4.79E-71 | 0.16 |
| MIA2 | -0.126442017 | 2.76E-05 | -0.088032112 | 0.003583 | 0.04 |
| MIA3 | -0.352459996 | 2.55E-33 | 0.044497812 | 0.1415165 | 0.4 |
| C22orf35 | -0.210694816 | 1.97E-12 | -0.211739523 | 1.52E-12 | 0.01 |
| MIA | 0.263008204 | 9.46E-19 | 0.067528128 | 0.0255812 | 0.2 |
| MIB1 | -0.097816328 | 0.00120401 | -0.232017088 | 7.97E-15 | 0.14 |
| MIB2 | 0.029375062 | 0.33191997 | -0.094487822 | 0.0017645 | 0.13 |
| MICAL1 | 0.00362297 | 0.90476775 | -0.067452146 | 0.0257479 | 0.08 |
| MICAL2 | -0.331381528 | 2.00E-29 | -0.307766761 | 2.07E-25 | 0.03 |
| LOC642566 | 0.067188973 | 0.02633247 | -0.057328159 | 0.0581335 | 0.13 |
| MICALCL | -0.500707977 | 2.01E-70 | -0.336398112 | 2.52E-30 | 0.17 |
| MICALL1 | 0.269322755 | 1.28E-19 | -0.052174799 | 0.0846847 | 0.33 |
| MICALL2 | -0.116210079 | 0.00011782 | -0.041656741 | 0.1687547 | 0.08 |
| MICA | -0.124050584 | 3.92E-05 | -0.00577784 | 0.848681 | 0.12 |
| MICB | -0.037269959 | 0.21825434 | -0.146813993 | 1.09E-06 | 0.11 |
| MID1IP1 | -0.054375315 | 0.0723435 | 0.096951202 | 0.0013313 | 0.16 |
| ZNFXY | 0.14041896 | 3.16E-06 | -0.065974803 | 0.0291802 | 0.21 |
| LOC286440 | -0.378375721 | 1.57E-38 | -0.234288175 | 4.29E-15 | 0.15 |
| MIDN | -0.265516164 | 4.31E-19 | -0.010472395 | 0.7294656 | 0.26 |
| MIER1 | -0.265337028 | 4.56E-19 | -0.280221299 | 3.61E-21 | 0.02 |
| MIER2 | 0.062164102 | 0.03989684 | 0.071466597 | 0.0181255 | 0.01 |
| MIER3 | -0.374102176 | 1.23E-37 | -0.257532058 | 5.12E-18 | 0.12 |
| MIF4GD | -0.017995568 | 0.5523043 | 0.010062876 | 0.7396537 | 0.03 |
| MIF | 0.272156403 | 5.15E-20 | 0.271747433 | 5.88E-20 | 0.01 |
| MIIP | 0.423655948 | 7.74E-49 | 0.246619153 | 1.32E-16 | 0.18 |
| MIMT1 | 0.210663164 | 1.98E-12 | 0.041552575 | 0.1698232 | 0.17 |
| MINA | 0.123827141 | 4.04E-05 | 0.084678664 | 0.0050889 | 0.04 |
| MINK1 | -0.283182091 | 1.33E-21 | -0.347486211 | 2.26E-32 | 0.07 |
| DKFZp564L2016 | -0.19031567 | 2.26E-10 | -0.074610995 | 0.0136137 | 0.12 |
| MIOS | -0.32754744 | 9.50E-29 | -0.172769771 | 9.00E-09 | 0.16 |
| MIOX | 0.249728867 | 5.30E-17 | 0.148489595 | 8.19E-07 | 0.11 |
| MIPEP | -0.115615156 | 0.00012774 | 0.107228899 | 0.0003834 | 0.23 |
| TMIP | -0.227596118 | 2.62E-14 | -0.02547849 | 0.4000647 | 0.21 |
| MIP | 0.036648818 | 0.22602941 | -0.077918431 | 0.0099663 | 0.12 |
| MIR155HG | 0.154374601 | 2.92E-07 | -0.003326402 | 0.9125306 | 0.16 |
| MIR17HG | 0.265174953 | 4.79E-19 | 0.172502968 | 9.49E-09 | 0.1 |
| LOC116046 | 0.01035335 | 0.73242242 | 0.000835096 | 0.9779994 | 0.01 |
| MITD1 | 0.274120714 | 2.72E-20 | 0.270966071 | 7.57E-20 | 0.01 |
| WS2A | -0.36714448 | 3.27E-36 | -0.22046723 | 1.69E-13 | 0.15 |
| MIXL1 | -0.002200453 | 0.94207266 | -0.12362477 | 4.16E-05 | 0.13 |
| NIFK | 0.490632648 | 2.82E-67 | 0.396140466 | 2.20E-42 | 0.1 |
| MKI67 | 0.3755823 | 6.05E-38 | 0.115706125 | 0.0001262 | 0.26 |
| MKKS | 0.231796175 | 8.46E-15 | 0.242144262 | 4.76E-16 | 0.02 |
| MKL1 | -0.069890535 | 0.02084381 | -0.207391597 | 4.39E-12 | 0.14 |
| NPD001 | -0.428508198 | 4.86E-50 | -0.323395563 | 5.00E-28 | 0.11 |
| MKLN1 | -0.334605965 | 5.31E-30 | -0.260362081 | 2.15E-18 | 0.08 |
| MKNK1 | -0.080678856 | 0.00761712 | -0.184576107 | 7.84E-10 | 0.11 |
| MKNK2 | -0.285953403 | 5.16E-22 | -0.152870423 | 3.81E-07 | 0.14 |
| MKRN1 | 0.014581918 | 0.6301184 | 0.079664273 | 0.0084157 | 0.07 |
| MKRN2 | 0.05038792 | 0.09591197 | -0.109683105 | 0.0002801 | 0.17 |
| MKRN3 | 0.263797109 | 7.39E-19 | 0.048102802 | 0.1119689 | 0.22 |
| MKS1 | 0.029063757 | 0.33707175 | 0.113377334 | 0.0001726 | 0.09 |
| MKX | -0.103518816 | 0.00060875 | -0.108795562 | 0.000314 | 0.01 |
| MLANA | -0.284663833 | 8.02E-22 | -0.205995155 | 6.14E-12 | 0.08 |
| LOC654039 | 0.04162716 | 0.16905761 | 0.004877339 | 0.872043 | 0.04 |
| LOC144732 | -0.259228162 | 3.05E-18 | -0.170417188 | 1.44E-08 | 0.09 |
| CENPU | 0.411503681 | 6.51E-46 | 0.249775727 | 5.23E-17 | 0.17 |
| MLF1 | 0.28098221 | 2.79E-21 | 0.253389899 | 1.79E-17 | 0.03 |
| MLF2 | 0.48843475 | 1.33E-66 | 0.356576209 | 4.09E-34 | 0.14 |
| MLH1 | -0.180427654 | 1.88E-09 | -0.119138257 | 7.87E-05 | 0.07 |
| MLH3 | -0.309243484 | 1.19E-25 | -0.121262153 | 5.84E-05 | 0.19 |
| MLKL | 0.171670772 | 1.12E-08 | 0.071348559 | 0.0183178 | 0.11 |
| TNRC21 | -0.448843421 | 2.70E-55 | -0.459368656 | 3.71E-58 | 0.02 |
| FLJ12625 | -0.438183995 | 1.70E-52 | -0.380837364 | 4.75E-39 | 0.06 |
| KMT2B | -0.028980496 | 0.3384583 | -0.162915283 | 6.09E-08 | 0.14 |
| FLJ10078 | -0.421006164 | 3.44E-48 | -0.355478519 | 6.68E-34 | 0.07 |
| MLLT10 | -0.017315099 | 0.56743407 | -0.182500154 | 1.22E-09 | 0.17 |
| LOC149430 | 0.273540784 | 3.28E-20 | 0.09494842 | 0.0016748 | 0.18 |
| MLLT1 | 0.037313026 | 0.21772247 | 0.013912462 | 0.6459114 | 0.03 |
| MLLT3 | -0.255613217 | 9.16E-18 | -0.144241891 | 1.68E-06 | 0.12 |
| LOC654167 | -0.140399534 | 3.17E-06 | -0.201496385 | 1.78E-11 | 0.07 |
| MLLT6 | -0.179420076 | 2.32E-09 | -0.160871015 | 8.93E-08 | 0.02 |
| FLJ11783 | -0.378765995 | 1.30E-38 | -0.372493586 | 2.64E-37 | 0.01 |
| MLNR | -0.052901146 | 0.08043564 | -0.053440947 | 0.0773912 | 0.01 |
| MLN | -0.007834885 | 0.7958411 | -0.004071328 | 0.8930492 | 0.01 |
| LOC129863 | -0.464300311 | 1.56E-59 | -0.113307789 | 0.0001742 | 0.36 |
| MLST8 | 0.28211349 | 1.91E-21 | 0.226892155 | 3.15E-14 | 0.06 |
| MLXIPL | -0.107777085 | 0.00035767 | -0.014108844 | 0.6412616 | 0.1 |
| MLXIP | -0.076314832 | 0.01160924 | -0.288372064 | 2.24E-22 | 0.22 |
| MLX | 0.177191051 | 3.68E-09 | 0.075715437 | 0.0122824 | 0.11 |
| MLYCD | -0.068320356 | 0.02389809 | -0.078330332 | 0.0095792 | 0.02 |
| MMAA | -0.233070349 | 5.98E-15 | -0.131510841 | 1.29E-05 | 0.11 |
| MGC20496 | 0.249678821 | 5.38E-17 | 0.261646721 | 1.44E-18 | 0.02 |
| MMACHC | 0.244866073 | 2.19E-16 | 0.164469584 | 4.54E-08 | 0.09 |
| MMADHC | 0.045964178 | 0.12884686 | 0.022182087 | 0.4638006 | 0.03 |
| MMD2 | 0.14803732 | 8.85E-07 | 0.041301139 | 0.1724234 | 0.11 |
| MMD | 0.003853473 | 0.89874044 | -0.018290483 | 0.545809 | 0.03 |
| MMEL1 | -0.26736664 | 2.40E-19 | -0.007403811 | 0.8068457 | 0.26 |
| MME | -0.166848221 | 2.88E-08 | -0.145277257 | 1.41E-06 | 0.03 |
| MMGT1 | -0.012548085 | 0.67858981 | -0.153561915 | 3.37E-07 | 0.15 |
| MMP10 | -0.112061894 | 0.00020539 | -0.050281038 | 0.0966196 | 0.07 |
| MMP11 | -0.082889398 | 0.00610751 | -0.001646539 | 0.9566378 | 0.09 |
| MMP12 | 0.282356005 | 1.76E-21 | 0.002542365 | 0.9330915 | 0.28 |
| MMP13 | -0.077354824 | 0.01051851 | -0.015714116 | 0.6037922 | 0.07 |
| MMP14 | -0.103506256 | 0.00060969 | -0.101017935 | 0.0008245 | 0.01 |
| MMP15 | 0.067418208 | 0.02582263 | 0.076815577 | 0.0110721 | 0.01 |
| MMP16 | -0.366351543 | 4.72E-36 | -0.249328995 | 5.96E-17 | 0.12 |
| MMP17 | -0.253957876 | 1.51E-17 | -0.07978704 | 0.0083153 | 0.18 |
| MMP18 | -0.071390712 | 0.01824894 | -0.027143062 | 0.3699842 | 0.05 |
| LOC115169 | 0.237059001 | 1.99E-15 | 0.09421979 | 0.0018188 | 0.15 |
| MMP20 | 0.203195854 | 1.19E-11 | 0.026019284 | 0.3901347 | 0.18 |
| MMP21 | -0.235676863 | 2.92E-15 | -0.197068171 | 4.96E-11 | 0.04 |
| LOC653409 | -0.067319027 | 0.02604216 | -0.017397056 | 0.5656014 | 0.05 |
| MMP23B | -0.231168529 | 1.00E-14 | -0.104907828 | 0.0005129 | 0.13 |
| MMP24 | 0.086260786 | 0.00431879 | 0.046640116 | 0.1233114 | 0.04 |
| MMP25 | 0.107538286 | 0.00036869 | -0.080376937 | 0.0078474 | 0.19 |
| LOC115735 | -0.056809862 | 0.06044513 | -0.090081669 | 0.0028749 | 0.04 |
| MMP27 | -0.281438348 | 2.39E-21 | -0.155707937 | 2.30E-07 | 0.13 |
| MMP28 | -0.259351702 | 2.93E-18 | -0.166508852 | 3.07E-08 | 0.1 |
| MMP2 | -0.275329056 | 1.83E-20 | -0.148728668 | 7.86E-07 | 0.13 |
| MMP3 | -0.089308707 | 0.00312544 | -0.053528189 | 0.0769081 | 0.04 |
| MMP7 | 0.22494688 | 5.27E-14 | 0.042788005 | 0.1574724 | 0.19 |
| MMP8 | 0.06117566 | 0.043168 | -0.024911047 | 0.4106455 | 0.09 |
| MMP9 | 0.147284343 | 1.01E-06 | -0.020535981 | 0.4976287 | 0.17 |
| MMRN1 | -0.412410253 | 3.97E-46 | -0.311069894 | 5.97E-26 | 0.11 |
| MMRN2 | -0.423701662 | 7.54E-49 | -0.273530385 | 3.29E-20 | 0.16 |
| MMS19 | -0.009421171 | 0.7557097 | -0.038621717 | 0.2020014 | 0.03 |
| MN1 | -0.351167136 | 4.52E-33 | -0.189488156 | 2.71E-10 | 0.17 |
| MNAT1 | 0.058327469 | 0.05388335 | 0.139959273 | 3.41E-06 | 0.09 |
| MND1 | 0.571451819 | 9.19E-96 | 0.314220652 | 1.80E-26 | 0.26 |
| MNDA | -0.102381583 | 0.00069936 | -0.184209692 | 8.48E-10 | 0.09 |
| MNS1 | -0.04863353 | 0.10806261 | -0.046566291 | 0.1239068 | 0.01 |
| MNT | -0.227289758 | 2.84E-14 | -0.213088713 | 1.09E-12 | 0.02 |
| SCRA1 | 0.117721686 | 9.58E-05 | 0.149252586 | 7.18E-07 | 0.04 |
| MOAP1 | -0.326003254 | 1.77E-28 | -0.199659983 | 2.73E-11 | 0.13 |
| MOB2 | 0.08361882 | 0.00567196 | 0.105074031 | 0.0005024 | 0.03 |
| MOB1B | -0.170404796 | 1.44E-08 | -0.23395002 | 4.70E-15 | 0.07 |
| MOB1A | -0.145914117 | 1.27E-06 | -0.24522396 | 1.97E-16 | 0.1 |
| MOB3A | -0.114865966 | 0.00014135 | -0.063892305 | 0.0346816 | 0.06 |
| MOB3B | 0.070448887 | 0.01984273 | -0.124803594 | 3.51E-05 | 0.2 |
| MOB3C | -0.236835816 | 2.12E-15 | -0.264785975 | 5.42E-19 | 0.03 |
| MOB4 | -0.003242753 | 0.91472169 | 0.019867211 | 0.5117378 | 0.03 |
| MOBP | 0.125463542 | 3.19E-05 | 0.018249074 | 0.5467188 | 0.11 |
| MOCOS | 0.225432803 | 4.64E-14 | 0.045677797 | 0.1312495 | 0.18 |
| MOCOD | -0.283365503 | 1.25E-21 | -0.176022842 | 4.67E-09 | 0.11 |
| MOCS2 | -0.058983305 | 0.05123733 | 0.0927414 | 0.0021464 | 0.16 |
| MOCS3 | 0.047959955 | 0.11303902 | 0.092729331 | 0.0021493 | 0.05 |
| MOGAT1 | 0.111096828 | 0.00023313 | 0.025726986 | 0.3954831 | 0.09 |
| LOC220021 | -0.191446198 | 1.76E-10 | -0.124273056 | 3.79E-05 | 0.07 |
| MOGAT3 | 0.063271565 | 0.03648371 | 0.027295908 | 0.3672943 | 0.04 |
| LOC130949 | 0.275327581 | 1.83E-20 | 0.120304276 | 6.68E-05 | 0.16 |
| MOG | 0.217634397 | 3.48E-13 | 0.045387955 | 0.1337163 | 0.18 |
| MON1A | 0.152985495 | 3.74E-07 | 0.129770332 | 1.68E-05 | 0.03 |
| MON1B | -0.165053992 | 4.06E-08 | -0.236113813 | 2.59E-15 | 0.08 |
| MON2 | -0.504059079 | 1.72E-71 | -0.344302158 | 8.91E-32 | 0.16 |
| MORC1 | 0.045213923 | 0.13521464 | -0.026868081 | 0.3748542 | 0.08 |
| MORC2 | 0.304243547 | 7.66E-25 | 0.194413825 | 9.05E-11 | 0.11 |
| MORC3 | -0.427232439 | 1.01E-49 | -0.343625188 | 1.19E-31 | 0.09 |
| LOC158602 | -0.028088122 | 0.35354803 | 0.046144204 | 0.1273541 | 0.08 |
| MORF4L1 | 0.046938841 | 0.1209249 | -0.018074145 | 0.55057 | 0.07 |
| MORF4L2 | 0.022867681 | 0.4500979 | 0.102550452 | 0.0006852 | 0.08 |
| MORF4 | -0.151203623 | 5.11E-07 | -0.202282933 | 1.48E-11 | 0.06 |
| MORN1 | -0.298020645 | 7.41E-24 | -0.162586023 | 6.48E-08 | 0.14 |
| LOC732175 | 0.346899914 | 2.91E-32 | 0.467034381 | 2.63E-60 | 0.13 |
| MORN3 | 0.108070028 | 0.00034458 | 0.083316237 | 0.0058491 | 0.03 |
| MORN4 | -0.117115502 | 0.0001041 | -0.024809467 | 0.412557 | 0.1 |
| MORN5 | 0.079105048 | 0.00888715 | 0.113417851 | 0.0001716 | 0.04 |
| 1-Mar | 0.094510431 | 0.00176003 | 0.312424023 | 3.57E-26 | 0.22 |
| 2-Mar | -0.31475853 | 1.46E-26 | 0.00560159 | 0.8532438 | 0.33 |
| MOSPD1 | 0.180526044 | 1.84E-09 | 0.104159058 | 0.0005627 | 0.08 |
| MOSPD2 | -0.367885764 | 2.31E-36 | -0.399747242 | 3.39E-43 | 0.04 |
| MOSPD3 | 0.199323871 | 2.95E-11 | 0.283554094 | 1.17E-21 | 0.09 |
| MOS | 0.012180629 | 0.68749825 | 0.031133188 | 0.3037843 | 0.02 |
| MOV10L1 | -0.038949502 | 0.19819651 | -0.022729407 | 0.452843 | 0.02 |
| MOV10 | 0.02772256 | 0.35985031 | 0.003146546 | 0.9172425 | 0.03 |
| MOXD1 | -0.020884438 | 0.4903601 | -0.105526941 | 0.0004749 | 0.09 |
| MPDU1 | 0.086349873 | 0.00427874 | 0.2179533 | 3.21E-13 | 0.14 |
| MPDZ | -0.340819192 | 3.93E-31 | -0.281757545 | 2.15E-21 | 0.06 |
| MPEG1 | -0.187845375 | 3.88E-10 | -0.287394349 | 3.14E-22 | 0.1 |
| MPG | 0.092758894 | 0.00214223 | 0.16481346 | 4.25E-08 | 0.08 |
| MPHOSPH10 | 0.433792742 | 2.27E-51 | 0.274811899 | 2.17E-20 | 0.16 |
| MPHOSPH6 | 0.265119875 | 4.88E-19 | 0.165467286 | 3.75E-08 | 0.1 |
| MPHOSPH8 | -0.435659197 | 7.58E-52 | -0.363315164 | 1.92E-35 | 0.08 |
| MPHOSPH9 | -0.141873272 | 2.49E-06 | -0.201402642 | 1.82E-11 | 0.06 |
| MPI | -0.077824747 | 0.01005624 | -0.018923568 | 0.5319949 | 0.06 |
| MPL | 0.019721912 | 0.51483055 | -0.100861854 | 0.00084 | 0.13 |
| MPND | 0.156864073 | 1.86E-07 | 0.196140743 | 6.12E-11 | 0.04 |
| MPO | 0.076003114 | 0.01195514 | -0.027956995 | 0.3558006 | 0.11 |
| MPP1 | -0.034843929 | 0.249736 | -0.18043692 | 1.88E-09 | 0.15 |
| MPP2 | -0.11309327 | 0.0001792 | -0.035453979 | 0.2415365 | 0.08 |
| MPP3 | -0.064819411 | 0.03213181 | -0.002115327 | 0.9443099 | 0.07 |
| DLG6 | -0.017183104 | 0.57039168 | -0.016366468 | 0.5888512 | 0.01 |
| MPP5 | -0.353267108 | 1.79E-33 | -0.188910883 | 3.07E-10 | 0.17 |
| MPP6 | 0.230500384 | 1.20E-14 | 0.057807792 | 0.0560601 | 0.18 |
| MPP7 | -0.409222952 | 2.23E-45 | -0.192773619 | 1.31E-10 | 0.22 |
| MPPE1 | -0.052209524 | 0.08447751 | -0.075904376 | 0.0120666 | 0.03 |
| MPPED1 | -0.102018546 | 0.00073082 | -0.140526132 | 3.11E-06 | 0.04 |
| MPPED2 | -0.16268437 | 6.36E-08 | -0.192260704 | 1.47E-10 | 0.03 |
| MPRIP | -0.09367036 | 0.00193476 | -0.220092089 | 1.86E-13 | 0.13 |
| MPST | 0.300614458 | 2.90E-24 | 0.218952605 | 2.49E-13 | 0.09 |
| MPV17L2 | 0.335248224 | 4.07E-30 | 0.242550878 | 4.24E-16 | 0.1 |
| MPV17L | -0.213013652 | 1.11E-12 | -0.045945865 | 0.1289995 | 0.17 |
| MPV17 | 0.198182894 | 3.84E-11 | 0.249246241 | 6.11E-17 | 0.06 |
| FLJ21047 | 0.149514277 | 6.86E-07 | 0.218478791 | 2.81E-13 | 0.07 |
| MPZL2 | 0.106908317 | 0.00039929 | 0.079436557 | 0.0086049 | 0.03 |
| MPZL3 | -0.253585098 | 1.69E-17 | -0.310823089 | 6.55E-26 | 0.06 |
| MPZ | 0.205572002 | 6.79E-12 | 0.051113631 | 0.0912165 | 0.16 |
| MR1 | -0.385806577 | 4.11E-40 | -0.082924597 | 0.0060858 | 0.31 |
| MRAP2 | 0.020702915 | 0.49413944 | -0.052875625 | 0.0805819 | 0.08 |
| MRAP | -0.223127519 | 8.49E-14 | -0.131845727 | 1.23E-05 | 0.1 |
| LOC654181 | 0.139476995 | 3.68E-06 | -0.081736524 | 0.0068575 | 0.23 |
| LOC101928757 | -0.09589505 | 0.00150337 | -0.239906042 | 8.98E-16 | 0.15 |
| MRC2 | -0.185379379 | 6.60E-10 | -0.149888528 | 6.43E-07 | 0.04 |
| MRE11A | -0.042250909 | 0.16275566 | -0.122192006 | 5.12E-05 | 0.08 |
| MREG | -0.292355046 | 5.57E-23 | -0.077437705 | 0.0104356 | 0.22 |
| MRFAP1L1 | -0.089850759 | 0.00294778 | 0.008372255 | 0.7821798 | 0.1 |
| MRFAP1 | -0.007574704 | 0.80247837 | 0.054162091 | 0.073471 | 0.07 |
| LOC159948 | -0.02645737 | 0.38220141 | -0.049729603 | 0.1003367 | 0.03 |
| MRGPRE | -0.127439228 | 2.38E-05 | -0.22402915 | 6.71E-14 | 0.1 |
| MRGPRF | -0.236368393 | 2.41E-15 | -0.14739799 | 9.87E-07 | 0.09 |
| MRGPRG | 0.036518722 | 0.22768258 | -0.012100093 | 0.6894566 | 0.05 |
| MRGPRX1 | -0.001784347 | 0.95301262 | 0.007331866 | 0.8086861 | 0.01 |
| MRGPRX2 | -0.248318738 | 8.02E-17 | -0.222672643 | 9.55E-14 | 0.03 |
| MRGPRX3 | -0.042227953 | 0.16298442 | -0.078773843 | 0.0091773 | 0.04 |
| MRGPRX4 | -0.1158523 | 0.00012369 | -0.092016588 | 0.002326 | 0.03 |
| MRI1 | -0.052050354 | 0.08543057 | -0.018160449 | 0.5486683 | 0.04 |
| MRM1 | 0.134833149 | 7.71E-06 | 0.185838527 | 5.98E-10 | 0.06 |
| C18orf3 | -0.145711234 | 1.31E-06 | -0.09214495 | 0.0022932 | 0.06 |
| MRP63 | 0.25255202 | 2.30E-17 | 0.288746655 | 1.97E-22 | 0.04 |
| MRPL10 | 0.033896768 | 0.26284789 | 0.099934697 | 0.0009383 | 0.07 |
| LOC101928090 | 0.548172322 | 9.49E-87 | 0.407911581 | 4.52E-45 | 0.15 |
| MRPL12 | 0.538114104 | 4.54E-83 | 0.324792102 | 2.87E-28 | 0.22 |
| MRPL13 | 0.481110864 | 2.13E-64 | 0.40667536 | 8.76E-45 | 0.08 |
| MRPL14 | 0.535444843 | 4.10E-82 | 0.444612321 | 3.59E-54 | 0.1 |
| MRPL15 | 0.552407797 | 2.45E-88 | 0.383346989 | 1.39E-39 | 0.17 |
| MRPL16 | 0.394799283 | 4.38E-42 | 0.336675934 | 2.25E-30 | 0.06 |
| MRPL17 | 0.442955608 | 9.78E-54 | 0.435997498 | 6.21E-52 | 0.01 |
| LOC96273 | 0.468287825 | 1.16E-60 | 0.436093798 | 5.86E-52 | 0.04 |
| MRPL19 | 0.157448162 | 1.68E-07 | 0.108151663 | 0.000341 | 0.05 |
| MRPL1 | 0.483197593 | 5.07E-65 | 0.452478192 | 2.85E-56 | 0.04 |
| LOC642393 | 0.347259464 | 2.49E-32 | 0.270722258 | 8.19E-20 | 0.08 |
| MRPL21 | 0.377169638 | 2.82E-38 | 0.329118941 | 5.03E-29 | 0.05 |
| MRPL22 | 0.343731394 | 1.14E-31 | 0.441135155 | 2.92E-53 | 0.1 |
| L23MRP | 0.347539657 | 2.20E-32 | 0.379608565 | 8.66E-39 | 0.04 |
| MRPL24 | 0.290401478 | 1.11E-22 | 0.545859117 | 6.83E-86 | 0.26 |
| MRPL27 | 0.252578025 | 2.28E-17 | 0.356169065 | 4.91E-34 | 0.11 |
| MRPL28 | 0.329872202 | 3.70E-29 | 0.296107198 | 1.47E-23 | 0.04 |
| MRPL2 | 0.506431881 | 2.95E-72 | 0.339776635 | 6.11E-31 | 0.17 |
| MRPL30 | 0.147300758 | 1.00E-06 | 0.126684294 | 2.66E-05 | 0.03 |
| MRPL32 | 0.322660489 | 6.70E-28 | 0.356781646 | 3.73E-34 | 0.04 |
| MRPL33 | 0.388259117 | 1.21E-40 | 0.291007109 | 8.94E-23 | 0.1 |
| MRPL34 | 0.2659026 | 3.81E-19 | 0.216244117 | 4.95E-13 | 0.05 |
| MRPL35 | 0.177069325 | 3.77E-09 | 0.207553918 | 4.22E-12 | 0.04 |
| MRPL36 | 0.484829883 | 1.64E-65 | 0.406738848 | 8.47E-45 | 0.08 |
| MRPL37 | 0.527025394 | 3.73E-79 | 0.302935128 | 1.24E-24 | 0.23 |
| LOC201293 | 0.458191003 | 7.84E-58 | 0.308504458 | 1.57E-25 | 0.15 |
| MRPL39 | 0.379760234 | 8.04E-39 | 0.280494706 | 3.29E-21 | 0.1 |
| MRPL3 | 0.408499437 | 3.30E-45 | 0.377554855 | 2.34E-38 | 0.04 |
| MRPL40 | 0.248327203 | 8.00E-17 | 0.286373758 | 4.46E-22 | 0.04 |
| LOC138179 | 0.15296906 | 3.75E-07 | 0.247785604 | 9.37E-17 | 0.1 |
| MRPL42P5 | -0.148451749 | 8.24E-07 | -0.018270723 | 0.546243 | 0.14 |
| MRPL42 | 0.250674881 | 4.01E-17 | 0.256565033 | 6.87E-18 | 0.01 |
| MRPL43 | 0.130538549 | 1.50E-05 | 0.252568207 | 2.28E-17 | 0.13 |
| MRPL44 | 0.191551817 | 1.72E-10 | 0.263952403 | 7.04E-19 | 0.08 |
| LOC201219 | 0.046441088 | 0.12492168 | 0.145958595 | 1.26E-06 | 0.1 |
| LOC123541 | 0.29218354 | 5.92E-23 | 0.212947191 | 1.13E-12 | 0.08 |
| MRPL47 | 0.599345856 | 1.47E-107 | 0.49421858 | 2.20E-68 | 0.11 |
| MRPL48 | 0.48403058 | 2.85E-65 | 0.388232639 | 1.22E-40 | 0.1 |
| MRPL49 | 0.005909354 | 0.84527971 | -0.043318864 | 0.152379 | 0.05 |
| MRPL4 | 0.405486503 | 1.65E-44 | 0.267426791 | 2.35E-19 | 0.14 |
| LOC96800 | 0.227438158 | 2.73E-14 | 0.233221272 | 5.74E-15 | 0.01 |
| MRPL51 | 0.599880309 | 8.48E-108 | 0.490071405 | 4.19E-67 | 0.11 |
| MRPL52 | 0.420791497 | 3.88E-48 | 0.409856362 | 1.59E-45 | 0.02 |
| MRPL53 | 0.22853451 | 2.04E-14 | 0.321067577 | 1.26E-27 | 0.1 |
| MRPL54 | 0.148354408 | 8.38E-07 | 0.23235984 | 7.26E-15 | 0.09 |
| MRPL55 | 0.313524028 | 2.35E-26 | 0.492550479 | 7.23E-68 | 0.18 |
| MRPL9 | 0.498416452 | 1.07E-69 | 0.583317129 | 1.21E-100 | 0.09 |
| MRPS10 | 0.294784097 | 2.36E-23 | 0.263840749 | 7.29E-19 | 0.04 |
| FLJ22512 | 0.379005517 | 1.16E-38 | 0.297304265 | 9.59E-24 | 0.09 |
| MRPS12 | 0.466327648 | 4.17E-60 | 0.373823035 | 1.40E-37 | 0.1 |
| MRPS14 | 0.067729017 | 0.02514499 | 0.484365285 | 2.26E-65 | 0.42 |
| MRPS15 | 0.535492979 | 3.94E-82 | 0.402142196 | 9.68E-44 | 0.14 |
| MRPS16 | 0.355046752 | 8.10E-34 | 0.374405345 | 1.06E-37 | 0.02 |
| MRPS17 | 0.565022846 | 3.35E-93 | 0.515905923 | 2.27E-75 | 0.05 |
| MRPS18A | 0.439343506 | 8.54E-53 | 0.329510047 | 4.29E-29 | 0.11 |
| MRPS18B | 0.306098905 | 3.86E-25 | 0.300775876 | 2.73E-24 | 0.01 |
| MRPS18C | 0.410954773 | 8.76E-46 | 0.365212368 | 8.01E-36 | 0.05 |
| MRPS21 | 0.368317347 | 1.89E-36 | 0.420595574 | 4.33E-48 | 0.06 |
| MRPS22 | 0.429422313 | 2.87E-50 | 0.323923681 | 4.06E-28 | 0.11 |
| MRPS23 | 0.346756488 | 3.10E-32 | 0.332122485 | 1.48E-29 | 0.02 |
| MRPS24 | 0.508945119 | 4.50E-73 | 0.461751669 | 8.07E-59 | 0.05 |
| MRPS25 | 0.429243331 | 3.18E-50 | 0.345117804 | 6.28E-32 | 0.09 |
| NY.BR.87 | 0.374243965 | 1.15E-37 | 0.358245231 | 1.93E-34 | 0.02 |
| MRPS27 | -0.146090871 | 1.23E-06 | 0.082276306 | 0.0064966 | 0.23 |
| MRPS28 | 0.350445749 | 6.20E-33 | 0.313209875 | 2.64E-26 | 0.04 |
| MRPS2 | 0.379915341 | 7.45E-39 | 0.309146698 | 1.23E-25 | 0.08 |
| LOC51331 | -0.147489352 | 9.72E-07 | -0.059487271 | 0.0492786 | 0.09 |
| LOC112759 | 0.124505502 | 3.67E-05 | 0.154067971 | 3.08E-07 | 0.03 |
| LOC65515 | 0.421646706 | 2.40E-48 | 0.424208242 | 5.66E-49 | 0.01 |
| MRPS34 | 0.267507622 | 2.29E-19 | 0.300263574 | 3.29E-24 | 0.04 |
| MRPS35 | 0.219723644 | 2.04E-13 | 0.29002037 | 1.26E-22 | 0.08 |
| MRPS36 | 0.020141631 | 0.50592325 | 0.247825893 | 9.26E-17 | 0.23 |
| MRPS5 | 0.579924592 | 3.16E-99 | 0.401960264 | 1.06E-43 | 0.18 |
| MRPS6 | 0.338741791 | 9.45E-31 | 0.348397583 | 1.52E-32 | 0.01 |
| MRPS7 | 0.373195694 | 1.89E-37 | 0.25781666 | 4.69E-18 | 0.12 |
| MRPS9 | 0.506372671 | 3.08E-72 | 0.425643559 | 2.50E-49 | 0.09 |
| MRRF | 0.141295104 | 2.74E-06 | 0.184858701 | 7.38E-10 | 0.05 |
| MRS2P2 | 0.159767285 | 1.10E-07 | 0.225530307 | 4.52E-14 | 0.07 |
| MRS2L | 0.27001738 | 1.03E-19 | 0.254106332 | 1.44E-17 | 0.02 |
| MRTO4 | 0.589850898 | 2.05E-103 | 0.332194684 | 1.43E-29 | 0.26 |
| IRAG | -0.343627969 | 1.19E-31 | -0.235899545 | 2.75E-15 | 0.11 |
| MS4A10 | 0.031702065 | 0.29502964 | -0.004373135 | 0.8851739 | 0.04 |
| MS4A12 | 0.002759007 | 0.92740511 | -0.011910093 | 0.6940849 | 0.02 |
| MS4A13 | 0.079787525 | 0.00831487 | 0.048602178 | 0.1082903 | 0.04 |
| MS4A16 | -0.290510956 | 1.06E-22 | -0.131865549 | 1.22E-05 | 0.16 |
| MS4A15 | -0.072383302 | 0.0166915 | 0.015410473 | 0.6108041 | 0.09 |
| MS4A1 | -0.024275796 | 0.42268544 | -0.112911747 | 0.0001836 | 0.09 |
| IGER | -0.41641917 | 4.41E-47 | -0.225076487 | 5.10E-14 | 0.2 |
| MS4A3 | -0.057130245 | 0.05900738 | -0.089691338 | 0.002999 | 0.04 |
| MS4A4 | -0.03985308 | 0.18797938 | -0.137191366 | 5.31E-06 | 0.1 |
| TETM4 | 0.039943293 | 0.18698102 | 0.009059359 | 0.7648097 | 0.04 |
| LOC64166 | -0.125535475 | 3.15E-05 | -0.174803479 | 5.98E-09 | 0.05 |
| LOC120712 | -0.06427755 | 0.03360187 | -0.071577061 | 0.0179472 | 0.01 |
| MS4A7 | -0.385667001 | 4.40E-40 | -0.221126364 | 1.43E-13 | 0.17 |
| MS4A8 | -0.21220044 | 1.36E-12 | -0.111158497 | 0.0002313 | 0.11 |
| MSC | -0.024455178 | 0.41926485 | -0.040150133 | 0.1847067 | 0.02 |
| MSGN1 | 0.066923041 | 0.02693479 | 0.057600339 | 0.0569492 | 0.01 |
| MSH2 | 0.319712602 | 2.14E-27 | 0.064680481 | 0.0325034 | 0.26 |
| LOC134472 | -0.330740055 | 2.60E-29 | -0.115975512 | 0.0001216 | 0.22 |
| MSH4 | -0.026946271 | 0.37346546 | -0.022960708 | 0.4482564 | 0.01 |
| MSH5 | 0.211458214 | 1.63E-12 | 0.223649081 | 7.41E-14 | 0.02 |
| MSH6 | 0.343107851 | 1.49E-31 | 0.114027274 | 0.0001582 | 0.23 |
| MSI1 | 0.363558492 | 1.72E-35 | 0.079091366 | 0.008899 | 0.29 |
| MSI2 | -0.288485195 | 2.15E-22 | -0.142790386 | 2.14E-06 | 0.15 |
| MSL1 | -0.004837568 | 0.87307754 | 0.065871713 | 0.0294337 | 0.08 |
| MSL2 | -0.149372352 | 7.03E-07 | -0.150620962 | 5.66E-07 | 0.01 |
| MSL3L2 | 0.184429047 | 8.09E-10 | 0.104338922 | 0.0005503 | 0.09 |
| DKFZP586J1822 | 0.286803737 | 3.85E-22 | 0.083066244 | 0.0059992 | 0.21 |
| MSLNL | 0.051745473 | 0.08728031 | 0.081995758 | 0.006682 | 0.04 |
| MSLN | 0.325721226 | 1.98E-28 | 0.114225526 | 0.0001541 | 0.22 |
| MSMB | 0.021534108 | 0.47696196 | 0.106481259 | 0.0004214 | 0.09 |
| MSMP | -0.31904069 | 2.78E-27 | -0.352686247 | 2.31E-33 | 0.04 |
| MSN | 0.074440931 | 0.01382964 | -0.157154319 | 1.77E-07 | 0.24 |
| MSR1 | -0.192767241 | 1.31E-10 | -0.164340638 | 4.65E-08 | 0.03 |
| MSRA | -0.013575404 | 0.65392411 | 0.02070355 | 0.4941262 | 0.04 |
| MSRB2 | 0.239241076 | 1.08E-15 | 0.343432033 | 1.29E-31 | 0.11 |
| DFNB74 | -0.366387656 | 4.64E-36 | -0.27070063 | 8.25E-20 | 0.1 |
| MSTP1 | -0.032120957 | 0.28869209 | -0.01278634 | 0.6728374 | 0.02 |
| MST1L | 0.036405804 | 0.22912441 | -0.003244415 | 0.9146782 | 0.04 |
| MST1R | -0.126935258 | 2.57E-05 | -0.03506388 | 0.2467576 | 0.1 |
| MST1 | -0.105125875 | 0.00049919 | -0.014235685 | 0.6382658 | 0.1 |
| STK26 | 0.156092988 | 2.14E-07 | 0.020230495 | 0.5040478 | 0.14 |
| MSTN | -0.180847831 | 1.72E-09 | -0.140032735 | 3.37E-06 | 0.05 |
| MSTO1 | 0.226963919 | 3.09E-14 | 0.422822353 | 1.24E-48 | 0.2 |
| MSTO2 | 0.03530209 | 0.24356005 | 0.152963474 | 3.75E-07 | 0.12 |
| MSX1 | -0.045249663 | 0.13490588 | 0.003048435 | 0.9198141 | 0.05 |
| LOC100418958 | -0.2182526 | 2.98E-13 | -0.093211036 | 0.0020369 | 0.13 |
| MSX2 | -0.33767934 | 1.48E-30 | -0.083624621 | 0.0056686 | 0.26 |
| MT1S | -0.068548923 | 0.02343069 | -0.056356104 | 0.0625309 | 0.02 |
| MT1Q | 0.107435008 | 0.00037355 | 0.045419241 | 0.1334483 | 0.07 |
| LOC390733 | -0.039953628 | 0.18686689 | 0.025825492 | 0.3936758 | 0.07 |
| MTD | 0.132219097 | 1.16E-05 | -0.047295307 | 0.1181245 | 0.18 |
| MT1F | 0.265294139 | 4.62E-19 | 0.053208215 | 0.0786921 | 0.22 |
| MGC12386 | 0.214655886 | 7.38E-13 | 0.055321288 | 0.0675106 | 0.16 |
| LOC727730 | 0.388697019 | 9.69E-41 | 0.153780824 | 3.24E-07 | 0.24 |
| MTE | 0.067220832 | 0.0262611 | 0.009300355 | 0.7587447 | 0.06 |
| MT1L | 0.134736991 | 7.83E-06 | 0.006962056 | 0.8181626 | 0.13 |
| MT1M | 0.064790192 | 0.03220965 | -0.063137657 | 0.0368827 | 0.13 |
| MT1X | 0.234535451 | 4.00E-15 | 0.069218856 | 0.0221061 | 0.17 |
| MT2A | 0.230287388 | 1.27E-14 | 0.096931013 | 0.0013344 | 0.14 |
| MT3 | 0.244193043 | 2.65E-16 | 0.06311277 | 0.0369573 | 0.19 |
| MT4 | -0.003288082 | 0.91353428 | -0.03354304 | 0.2678643 | 0.04 |
| MTA1 | 0.190233114 | 2.30E-10 | 0.188254659 | 3.55E-10 | 0.01 |
| MTA2 | 0.349652311 | 8.78E-33 | 0.072342911 | 0.0167525 | 0.28 |
| LOC100130921 | -0.02614844 | 0.38778552 | 0.118689557 | 8.38E-05 | 0.15 |
| LGMBF | 0.183810352 | 9.23E-10 | 0.119455179 | 7.53E-05 | 0.07 |
| MGC21051 | 0.352495871 | 2.51E-33 | 0.152637785 | 3.97E-07 | 0.2 |
| MTCH1 | 0.332314431 | 1.37E-29 | 0.138528145 | 4.29E-06 | 0.2 |
| MTCH2 | 0.41849947 | 1.39E-47 | 0.310589638 | 7.16E-26 | 0.11 |
| CMC4 | 0.391568947 | 2.28E-41 | 0.266662851 | 3.00E-19 | 0.13 |
| MTCP1 | 0.132854676 | 1.05E-05 | -0.019054317 | 0.5291641 | 0.16 |
| MTDH | 0.157048803 | 1.80E-07 | 0.041932915 | 0.165946 | 0.12 |
| LOC116032 | 0.574883655 | 3.74E-97 | 0.373794848 | 1.42E-37 | 0.21 |
| MTERF4 | -0.232404459 | 7.17E-15 | -0.057521793 | 0.0572889 | 0.18 |
| MTERF2 | -0.152006391 | 4.44E-07 | 0.043048799 | 0.1549543 | 0.2 |
| MTERF1 | 0.081161563 | 0.00726155 | 0.161516168 | 7.92E-08 | 0.09 |
| MTF1 | -0.26867342 | 1.58E-19 | -0.347121534 | 2.64E-32 | 0.08 |
| MTF2 | 0.160508783 | 9.55E-08 | 0.040956411 | 0.1760365 | 0.12 |
| MTFMT | 0.120128971 | 6.85E-05 | -0.102238718 | 0.0007116 | 0.23 |
| MTFR1 | 0.280350853 | 3.45E-21 | 0.153097838 | 3.66E-07 | 0.13 |
| MTG1 | 0.239040237 | 1.15E-15 | 0.261659875 | 1.44E-18 | 0.03 |
| FLJ21145 | 0.370089548 | 8.23E-37 | 0.085952347 | 0.0044601 | 0.29 |
| MTHFD1 | 0.277383447 | 9.28E-21 | 0.155032364 | 2.59E-07 | 0.13 |
| FLJ13105 | 0.116785406 | 0.00010892 | 0.043544406 | 0.1502534 | 0.08 |
| MTHFD2 | 0.365296043 | 7.70E-36 | 0.169525621 | 1.71E-08 | 0.2 |
| MTHFR | -0.437251343 | 2.96E-52 | -0.319962784 | 1.94E-27 | 0.12 |
| MTHFSD | 0.193523425 | 1.11E-10 | 0.14815027 | 8.68E-07 | 0.05 |
| MTHFS | 0.182908696 | 1.12E-09 | 0.1985825 | 3.50E-11 | 0.02 |
| MTIF2 | 0.385772639 | 4.18E-40 | 0.188950465 | 3.05E-10 | 0.2 |
| MTIF3 | 0.071580738 | 0.0179413 | 0.246241889 | 1.47E-16 | 0.18 |
| MTL5 | -0.061988394 | 0.04046249 | 0.109410264 | 0.0002902 | 0.18 |
| MTM1 | -0.354393069 | 1.08E-33 | -0.378167914 | 1.74E-38 | 0.03 |
| MTMR10 | -0.474690659 | 1.65E-62 | -0.409703795 | 1.72E-45 | 0.07 |
| MTMR11 | -0.069916861 | 0.02079564 | 0.060281863 | 0.0463181 | 0.14 |
| MTMR12 | -0.114968903 | 0.0001394 | -0.172472318 | 9.55E-09 | 0.06 |
| MTMR14 | 0.230220201 | 1.30E-14 | 0.214779324 | 7.15E-13 | 0.02 |
| FAN1 | -0.337532419 | 1.57E-30 | -0.266998014 | 2.69E-19 | 0.08 |
| LOC654001 | -0.120074025 | 6.90E-05 | -0.152572031 | 4.02E-07 | 0.04 |
| MTMR2 | 0.207146156 | 4.66E-12 | 0.011706963 | 0.6990457 | 0.2 |
| MTMR3 | -0.451163662 | 6.45E-56 | -0.432651394 | 4.41E-51 | 0.02 |
| MTMR4 | -0.010134587 | 0.73786632 | -0.055370276 | 0.0672677 | 0.05 |
| MTMR6 | -0.283269126 | 1.29E-21 | -0.275669716 | 1.63E-20 | 0.01 |
| MTMR7 | 0.024473428 | 0.41891776 | 0.009338792 | 0.7577787 | 0.02 |
| MTMR8 | 0.041680335 | 0.16851334 | -0.119579529 | 7.40E-05 | 0.17 |
| LOC642940 | -0.076615867 | 0.01128365 | -0.142045672 | 2.42E-06 | 0.07 |
| MTMR9 | -0.411536835 | 6.39E-46 | -0.272268687 | 4.97E-20 | 0.14 |
| MTNR1A | 0.126069472 | 2.92E-05 | -0.063456645 | 0.0359383 | 0.19 |
| MTNR1B | -0.023644382 | 0.43485485 | -0.030933198 | 0.3069026 | 0.01 |
| LOC115005 | -0.012969056 | 0.66843887 | -0.015904136 | 0.5994226 | 0.01 |
| FRAP2 | -0.220290069 | 1.77E-13 | -0.378713518 | 1.34E-38 | 0.16 |
| LOC129091 | 0.386865186 | 2.42E-40 | 0.287929204 | 2.61E-22 | 0.1 |
| MTPAP | 0.337781611 | 1.41E-30 | 0.172475147 | 9.54E-09 | 0.17 |
| MTRF1L | 0.006938166 | 0.81877573 | 0.020376893 | 0.5009662 | 0.02 |
| MTRF1 | -0.052629096 | 0.08200639 | -0.011394915 | 0.7066914 | 0.05 |
| MTRR | -0.094160944 | 0.00183088 | -0.084690431 | 0.0050827 | 0.01 |
| MTR | -0.292988794 | 4.46E-23 | -0.105269827 | 0.0004903 | 0.19 |
| MTSS1L | 0.215177375 | 6.47E-13 | -0.038971496 | 0.1979431 | 0.26 |
| MTSS1 | -0.047406649 | 0.11726032 | -0.049677281 | 0.1006952 | 0.01 |
| MTTP | 0.182644037 | 1.18E-09 | 0.012531678 | 0.6789866 | 0.18 |
| DKFZP586D1519 | -0.477744914 | 2.11E-63 | -0.189756319 | 2.55E-10 | 0.29 |
| MTUS2 | -0.106311072 | 0.00043047 | -0.060740356 | 0.0446788 | 0.05 |
| MTVR2 | 0.068964483 | 0.02260117 | 0.015140877 | 0.6170599 | 0.06 |
| LOC149532 | 0.344767317 | 7.30E-32 | 0.55624692 | 8.54E-90 | 0.22 |
| MTX2 | 0.270690046 | 8.27E-20 | 0.32347492 | 4.85E-28 | 0.06 |
| MTX3 | -0.408729669 | 2.91E-45 | -0.237712087 | 1.66E-15 | 0.18 |
| LOC100130716 | 0.080129002 | 0.00804107 | 0.069855499 | 0.0209081 | 0.02 |
| FLJ20063 | 0.097426022 | 0.00125996 | 0.063632089 | 0.0354276 | 0.04 |
| MUC15 | 0.136964534 | 5.51E-06 | -0.012348818 | 0.6834153 | 0.15 |
| LOC100293231 | 0.161841611 | 7.45E-08 | 0.027220755 | 0.3686154 | 0.14 |
| LOC392765 | 0.054557063 | 0.07139362 | 0.058300348 | 0.0539952 | 0.01 |
| LOC115186 | -0.34725071 | 2.50E-32 | 0.048312372 | 0.1104133 | 0.4 |
| MUC20 | -0.010371921 | 0.73196089 | 0.048758063 | 0.1071618 | 0.06 |
| MUC21 | 0.046300814 | 0.1260664 | 0.043051999 | 0.1549236 | 0.01 |
| LOC100294420 | -0.079272165 | 0.00874386 | -0.103981588 | 0.0005751 | 0.03 |
| HSA276359 | -0.006747079 | 0.82368382 | -0.079605607 | 0.0084641 | 0.08 |
| MUC5B | 0.05289537 | 0.08046873 | 0.082723543 | 0.0062106 | 0.03 |
| MUC6 | -0.130864155 | 1.42E-05 | -0.059193283 | 0.0504135 | 0.08 |
| MGC34772 | -0.095666996 | 0.00154312 | -0.062322596 | 0.0393924 | 0.04 |
| MUCL1 | -0.187289992 | 4.37E-10 | -0.051477477 | 0.0889328 | 0.14 |
| AP5M1 | -0.244083465 | 2.74E-16 | -0.11817263 | 9.00E-05 | 0.13 |
| MUL1 | -0.253490933 | 1.73E-17 | -0.229113101 | 1.74E-14 | 0.03 |
| MUM1L1 | -0.275068976 | 1.99E-20 | -0.099004976 | 0.0010474 | 0.18 |
| FLJ22283 | -0.189364491 | 2.78E-10 | -0.048015206 | 0.1126242 | 0.15 |
| MURC | 0.309928982 | 9.19E-26 | 0.074840556 | 0.013327 | 0.24 |
| MUS81 | 0.178698768 | 2.70E-09 | 0.076288467 | 0.0116382 | 0.11 |
| MUSK | -0.018401713 | 0.54336915 | -0.128630994 | 1.99E-05 | 0.12 |
| MUSTN1 | -0.224334887 | 6.19E-14 | -0.101942085 | 0.0007376 | 0.13 |
| BLOC1S5 | -0.073294024 | 0.01536632 | -0.025225364 | 0.4047642 | 0.05 |
| MUTYH | 0.383368961 | 1.37E-39 | 0.162219018 | 6.94E-08 | 0.23 |
| MUT | -0.214233802 | 8.20E-13 | -0.158334764 | 1.43E-07 | 0.06 |
| MVD | 0.307743431 | 2.09E-25 | 0.156056672 | 2.16E-07 | 0.16 |
| MVK | 0.254665662 | 1.22E-17 | 0.189836847 | 2.51E-10 | 0.07 |
| MVP | -0.237903452 | 1.57E-15 | -0.165470176 | 3.75E-08 | 0.08 |
| MX1 | 0.12388037 | 4.01E-05 | 0.046726931 | 0.1226141 | 0.08 |
| LOC100131190 | 0.086674029 | 0.00413585 | -0.048930739 | 0.1059226 | 0.14 |
| MXD1 | -0.023454323 | 0.43855706 | -0.177161232 | 3.70E-09 | 0.16 |
| MXD3 | 0.434763084 | 1.28E-51 | 0.344155064 | 9.49E-32 | 0.1 |
| MXD4 | -0.265979206 | 3.72E-19 | -0.166241112 | 3.23E-08 | 0.1 |
| MXI1 | 0.048564933 | 0.10856135 | 0.013107733 | 0.665108 | 0.04 |
| LOC91006 | -0.33478339 | 4.93E-30 | -0.245167137 | 2.00E-16 | 0.09 |
| FLJ46603 | -0.007565865 | 0.80270412 | -0.100258216 | 0.0009029 | 0.1 |
| MXRA8 | -0.207002757 | 4.82E-12 | -0.05764204 | 0.0567695 | 0.15 |
| MYADML2 | -0.02379303 | 0.43197186 | -0.053068113 | 0.0794838 | 0.03 |
| MYADML | 0.100730892 | 0.00085332 | 0.046917505 | 0.1210941 | 0.06 |
| MYADM | -0.260452558 | 2.09E-18 | -0.221454085 | 1.31E-13 | 0.04 |
| MYBBP1A | 0.248634277 | 7.31E-17 | 0.085150495 | 0.0048472 | 0.17 |
| LOC645895 | 0.297482639 | 8.99E-24 | 0.072947606 | 0.0158591 | 0.23 |
| MYBL2 | 0.557455008 | 2.94E-90 | 0.306299366 | 3.58E-25 | 0.26 |
| MYBPC1 | -0.021101011 | 0.48587138 | 0.027438777 | 0.364791 | 0.05 |
| MYBPC2 | 0.077502183 | 0.01037156 | -0.05183207 | 0.0867517 | 0.13 |
| MYBPC3 | 0.061047085 | 0.0436097 | -0.047052931 | 0.120023 | 0.11 |
| MYBPHL | 0.107831484 | 0.0003552 | 0.091130483 | 0.0025643 | 0.02 |
| MYBPH | 0.175843072 | 4.84E-09 | 0.165394918 | 3.80E-08 | 0.02 |
| MYB | -0.311859705 | 4.42E-26 | -0.039066 | 0.1968569 | 0.28 |
| MYCBP2 | -0.421994341 | 1.97E-48 | -0.468391975 | 1.08E-60 | 0.05 |
| MYCBPAP | -0.244148698 | 2.69E-16 | -0.052452093 | 0.0830416 | 0.2 |
| MYCBP | 0.01259512 | 0.67745271 | 0.207452638 | 4.32E-12 | 0.2 |
| MYCL | -0.029931043 | 0.32284617 | 0.061832716 | 0.0409693 | 0.1 |
| MYCNOS | 0.132067504 | 1.18E-05 | 0.148703248 | 7.89E-07 | 0.02 |
| MYCN | 0.08472531 | 0.00506452 | 0.044642272 | 0.1402274 | 0.05 |
| MYCT1 | -0.368934458 | 1.42E-36 | -0.23730228 | 1.86E-15 | 0.14 |
| MYC | 0.278458808 | 6.50E-21 | 0.050471253 | 0.0953632 | 0.23 |
| MYD88 | 0.011009013 | 0.71618766 | -0.065138255 | 0.0312928 | 0.08 |
| FLJ11213 | -0.098599671 | 0.00109857 | -0.015709317 | 0.6039027 | 0.09 |
| MYEOV2 | 0.180282603 | 1.94E-09 | 0.302410677 | 1.50E-24 | 0.13 |
| MYEOV | 0.068941539 | 0.02264629 | -0.020010684 | 0.5086935 | 0.09 |
| MYF5 | 0.016587484 | 0.58382818 | -0.004195228 | 0.8898149 | 0.03 |
| MYF6 | -0.000728585 | 0.98080485 | -0.105722745 | 0.0004634 | 0.11 |
| LOC112557 | -0.226873965 | 3.17E-14 | -0.220980211 | 1.48E-13 | 0.01 |
| LOC100506151 | -0.386703376 | 2.63E-40 | -0.282944849 | 1.44E-21 | 0.11 |
| MYH13 | -0.09054462 | 0.0027338 | -0.070185984 | 0.0203087 | 0.03 |
| MYH14 | -0.054443988 | 0.07198338 | -0.103322241 | 0.0006236 | 0.05 |
| MYH15 | 0.212528429 | 1.25E-12 | 0.0737237 | 0.0147739 | 0.14 |
| MYH5.withdrawn | 0.110903716 | 0.00023908 | -0.016746251 | 0.5802322 | 0.13 |
| MYH1 | -0.160225245 | 1.01E-07 | -0.119838753 | 7.14E-05 | 0.05 |
| IBM3 | -0.05462334 | 0.07104978 | -0.049828662 | 0.0996607 | 0.01 |
| MYH3 | -0.002828239 | 0.92558869 | -0.074921741 | 0.0132268 | 0.08 |
| MYH4 | -0.190929778 | 1.97E-10 | -0.151456963 | 4.89E-07 | 0.04 |
| MYH6 | 0.117517361 | 9.85E-05 | -0.029442901 | 0.3308041 | 0.15 |
| LOC92771 | -0.076367524 | 0.01155166 | -0.034146646 | 0.2593435 | 0.05 |
| MPD1 | 0.145096546 | 1.46E-06 | 0.031885452 | 0.2922437 | 0.12 |
| MYH8 | -0.091591562 | 0.00243765 | -0.072395561 | 0.016673 | 0.02 |
| DFNA17 | -0.186979252 | 4.68E-10 | -0.292726094 | 4.89E-23 | 0.11 |
| MYL10 | 0.000755681 | 0.98009114 | 0.00198078 | 0.9478469 | 0.01 |
| MYL12A | 0.344441868 | 8.39E-32 | 0.199899802 | 2.58E-11 | 0.15 |
| MYL12B | 0.423453408 | 8.67E-49 | 0.332829443 | 1.10E-29 | 0.1 |
| MYL1 | 0.034084754 | 0.26020849 | 0.006820141 | 0.8218064 | 0.03 |
| MYL2 | 0.022084116 | 0.46577747 | 0.01764138 | 0.5601547 | 0.01 |
| MYL3 | -0.234481764 | 4.06E-15 | -0.095114177 | 0.0016435 | 0.14 |
| MYL4 | 0.097040679 | 0.00131754 | 0.093576175 | 0.0019553 | 0.01 |
| MYL5 | -0.074873742 | 0.01328595 | 0.027405828 | 0.3653674 | 0.11 |
| MYL6B | 0.349827944 | 8.13E-33 | 0.255740278 | 8.81E-18 | 0.1 |
| MYL6 | 0.198968603 | 3.20E-11 | 0.272761644 | 4.23E-20 | 0.08 |
| MYL7 | -0.049588624 | 0.10130505 | -0.023616543 | 0.435396 | 0.03 |
| MYL9 | -0.037071023 | 0.22072333 | 0.028093277 | 0.3534597 | 0.07 |
| MYLIP | -0.394072942 | 6.36E-42 | -0.156740745 | 1.91E-07 | 0.24 |
| MYLK2 | 0.31299887 | 2.87E-26 | 0.200532712 | 2.23E-11 | 0.12 |
| MYLK3 | 0.111205254 | 0.00022984 | 0.000910545 | 0.9760123 | 0.12 |
| MYLK4 | 0.024986915 | 0.40922128 | -0.182633279 | 1.18E-09 | 0.21 |
| KRP | -0.244720372 | 2.28E-16 | -0.287338961 | 3.20E-22 | 0.05 |
| LOC105371169 | 0.213748088 | 9.25E-13 | 0.170939992 | 1.29E-08 | 0.05 |
| MYNN | -0.09133646 | 0.00250698 | -0.064752784 | 0.0323095 | 0.03 |
| KIAA0799 | 0.11398109 | 0.00015919 | -0.032961995 | 0.2762462 | 0.15 |
| MYO15 | -0.186245465 | 5.48E-10 | -0.05750264 | 0.057372 | 0.13 |
| LOC732111 | -0.238788599 | 1.23E-15 | -0.162704992 | 6.34E-08 | 0.08 |
| MYO16 | -0.091970675 | 0.00233786 | -0.106215247 | 0.0004357 | 0.02 |
| LOC399700 | -0.259564129 | 2.75E-18 | -0.366925498 | 3.62E-36 | 0.11 |
| MYO18B | -0.24610762 | 1.53E-16 | -0.094232619 | 0.0018161 | 0.16 |
| MYO19 | 0.213642634 | 9.50E-13 | 0.090526247 | 0.0027393 | 0.13 |
| DFNA48 | 0.004733792 | 0.87577797 | -0.078169628 | 0.0097286 | 0.09 |
| LOC92451 | -0.138351356 | 4.41E-06 | -0.162439662 | 6.66E-08 | 0.03 |
| MYO1C | -0.344833505 | 7.09E-32 | -0.1851017 | 7.01E-10 | 0.16 |
| KIAA0727 | -0.300110635 | 3.48E-24 | -0.209781472 | 2.46E-12 | 0.1 |
| MYO1E | -0.036973899 | 0.22193597 | -0.196234975 | 5.99E-11 | 0.16 |
| MYO1F | -0.110007547 | 0.00026863 | -0.184374953 | 8.19E-10 | 0.08 |
| LOC392665 | -0.013124633 | 0.66470257 | -0.202430436 | 1.43E-11 | 0.19 |
| LOC732239 | 0.160365202 | 9.81E-08 | 0.070880634 | 0.0190976 | 0.09 |
| DFNB30 | 0.044197192 | 0.14422821 | 0.004650435 | 0.8779481 | 0.04 |
| LOC130673 | -0.048811288 | 0.10677863 | -0.026501341 | 0.3814106 | 0.03 |
| LOC116110 | -0.331426842 | 1.96E-29 | -0.483730645 | 3.51E-65 | 0.16 |
| MYO5B | -0.046510416 | 0.12435892 | -0.165295822 | 3.88E-08 | 0.12 |
| MYO5C | -0.486428703 | 5.39E-66 | -0.268784854 | 1.53E-19 | 0.22 |
| DFNB37 | -0.355616827 | 6.28E-34 | -0.157504735 | 1.66E-07 | 0.2 |
| MYO7A | -0.018893469 | 0.53264766 | -0.186209585 | 5.52E-10 | 0.17 |
| MYO7B | -0.06478424 | 0.03222553 | -0.102243562 | 0.0007112 | 0.04 |
| FLJ11061 | -0.452272374 | 3.24E-56 | -0.477376455 | 2.71E-63 | 0.03 |
| CELIAC4 | -0.033079143 | 0.27454209 | -0.187124415 | 4.53E-10 | 0.16 |
| LOC124689 | -0.438910831 | 1.10E-52 | -0.301022619 | 2.50E-24 | 0.14 |
| MYOC | -0.2406386 | 7.30E-16 | -0.204651615 | 8.46E-12 | 0.04 |
| MYOD1 | 0.136169375 | 6.25E-06 | 0.066932299 | 0.0269136 | 0.07 |
| MYOF | -0.520672226 | 5.63E-77 | -0.492998812 | 5.26E-68 | 0.03 |
| MYOG | 0.00181114 | 0.95230789 | 0.052156218 | 0.0847957 | 0.06 |
| MYOM1 | -0.251056881 | 3.58E-17 | -0.279263468 | 4.97E-21 | 0.03 |
| MYOM2 | -0.119689037 | 7.29E-05 | -0.162577226 | 6.49E-08 | 0.05 |
| MYOM3 | -0.277274346 | 9.63E-21 | -0.210634758 | 2.00E-12 | 0.07 |
| MYOT | -0.087721606 | 0.00370312 | -0.153937796 | 3.15E-07 | 0.07 |
| MYOZ1 | 0.025963981 | 0.39114327 | -0.072345685 | 0.0167483 | 0.1 |
| C4orf5 | -0.115010085 | 0.00013863 | -0.143243947 | 1.99E-06 | 0.03 |
| MYOZ3 | -0.185872615 | 5.94E-10 | -0.083447165 | 0.0057719 | 0.11 |
| MYPN | 0.002843404 | 0.92519088 | -0.034251701 | 0.2578799 | 0.04 |
| MYPOP | 0.306681415 | 3.10E-25 | 0.209491366 | 2.64E-12 | 0.1 |
| MYRIP | -0.38260932 | 2.00E-39 | -0.216367344 | 4.80E-13 | 0.17 |
| MYSM1 | -0.451032635 | 6.99E-56 | -0.448296173 | 3.79E-55 | 0.01 |
| MOF | 0.121607045 | 5.56E-05 | 0.163663121 | 5.29E-08 | 0.05 |
| KAT7 | -0.11986137 | 7.11E-05 | -0.08496396 | 0.0049415 | 0.04 |
| KAT6A | -0.226235055 | 3.75E-14 | -0.282981989 | 1.42E-21 | 0.06 |
| KAT6B | -0.406004176 | 1.25E-44 | -0.31916178 | 2.65E-27 | 0.09 |
| LOC102724128 | 0.063334955 | 0.0362961 | -0.039710461 | 0.1895657 | 0.11 |
| MYT1 | -0.106164628 | 0.00043846 | -0.128214996 | 2.12E-05 | 0.03 |
| MZF1 | -0.242085769 | 4.84E-16 | -0.126092903 | 2.91E-05 | 0.12 |
| FLJ31821 | -0.233481562 | 5.35E-15 | -0.312667083 | 3.25E-26 | 0.08 |
| N4BP2L1 | -0.244876324 | 2.18E-16 | -0.202708046 | 1.34E-11 | 0.05 |
| LOC88523 | -0.210754523 | 1.94E-12 | -0.085608741 | 0.0046224 | 0.13 |
| N4BP2 | -0.392644281 | 1.32E-41 | -0.453027033 | 2.02E-56 | 0.07 |
| N4BP3 | -0.190176846 | 2.33E-10 | -0.070993562 | 0.0189068 | 0.12 |
| N6AMT1 | -0.20190304 | 1.62E-11 | -0.00772758 | 0.7985767 | 0.2 |
| N6AMT2 | 0.172349589 | 9.78E-09 | 0.325198491 | 2.44E-28 | 0.16 |
| NAA10 | 0.510979127 | 9.70E-74 | 0.357470055 | 2.74E-34 | 0.16 |
| NAA11 | 0.05520612 | 0.06808441 | -0.009759544 | 0.7472297 | 0.07 |
| NAA15 | 0.1583268 | 1.43E-07 | -0.003525679 | 0.9073134 | 0.17 |
| PRO2435 | -0.17662109 | 4.13E-09 | -0.16759216 | 2.49E-08 | 0.01 |
| NAA20 | 0.210076284 | 2.29E-12 | 0.290353782 | 1.12E-22 | 0.09 |
| NAA25 | 0.058529662 | 0.05305571 | -0.043256484 | 0.152971 | 0.11 |
| NAA30 | -0.280279921 | 3.54E-21 | -0.201202331 | 1.91E-11 | 0.08 |
| NAA35 | -0.162307854 | 6.83E-08 | -0.111369767 | 0.0002249 | 0.06 |
| LSM8 | 0.349172389 | 1.08E-32 | 0.384820751 | 6.70E-40 | 0.04 |
| NAA40 | 0.048965557 | 0.10567413 | -0.074738156 | 0.0134542 | 0.13 |
| NAA50 | 0.183271161 | 1.04E-09 | 0.062730878 | 0.0381177 | 0.13 |
| NAAA | -0.055390873 | 0.06716582 | -0.046924914 | 0.1210354 | 0.01 |
| NAALAD2 | -0.187512684 | 4.17E-10 | -0.116643171 | 0.0001111 | 0.08 |
| NAALADL1 | -0.18637706 | 5.33E-10 | -0.142341918 | 2.31E-06 | 0.05 |
| NAALADL2 | -0.328588754 | 6.24E-29 | -0.13859613 | 4.24E-06 | 0.19 |
| NAB1 | -0.073720914 | 0.01477765 | -0.143359196 | 1.95E-06 | 0.07 |
| NAB2 | 0.116350718 | 0.00011558 | 0.093564907 | 0.0019578 | 0.03 |
| NACA2 | 0.140184459 | 3.28E-06 | 0.247812117 | 9.30E-17 | 0.11 |
| NACAD | -0.162967539 | 6.03E-08 | -0.162838927 | 6.18E-08 | 0.01 |
| NACAP1 | 0.22548859 | 4.57E-14 | 0.389089055 | 7.96E-41 | 0.17 |
| NACA | 0.239762574 | 9.35E-16 | 0.360985723 | 5.57E-35 | 0.13 |
| NACC1 | 0.199405801 | 2.89E-11 | 0.14031043 | 3.22E-06 | 0.06 |
| NACC2 | -0.292020248 | 6.27E-23 | -0.381057249 | 4.27E-39 | 0.09 |
| NADK | -0.091526843 | 0.00245507 | -0.200029239 | 2.51E-11 | 0.11 |
| NADSYN1 | -0.022188829 | 0.46366472 | 0.01497461 | 0.6209319 | 0.04 |
| NAE1 | 0.429754154 | 2.37E-50 | 0.235178603 | 3.35E-15 | 0.2 |
| NAF1 | -0.092400126 | 0.00222933 | -0.149748659 | 6.59E-07 | 0.06 |
| NAGA | -0.118082981 | 9.11E-05 | -0.163110339 | 5.87E-08 | 0.05 |
| NAGK | 0.140114431 | 3.32E-06 | 0.120311488 | 6.68E-05 | 0.02 |
| NAGLU | -0.181757671 | 1.43E-09 | -0.087401297 | 0.0038309 | 0.1 |
| NAGPA | 0.137790137 | 4.83E-06 | 0.132873542 | 1.05E-05 | 0.01 |
| NAGS | -0.138214971 | 4.51E-06 | -0.174100987 | 6.89E-09 | 0.04 |
| NAIF1 | 0.119101388 | 7.91E-05 | 0.114237465 | 0.0001538 | 0.01 |
| NAIP | -0.394849328 | 4.27E-42 | -0.260727527 | 1.92E-18 | 0.14 |
| NALCN | -0.125906077 | 2.99E-05 | -0.06033689 | 0.0461187 | 0.07 |
| NAMPT | -0.004385792 | 0.88484392 | -0.050195154 | 0.0971911 | 0.05 |
| NANOG | 0.148111857 | 8.73E-07 | 0.08237507 | 0.0064325 | 0.07 |
| NANOS1 | 0.22720834 | 2.90E-14 | 0.194534739 | 8.81E-11 | 0.04 |
| NANOS2 | 0.14554572 | 1.35E-06 | 0.08991525 | 0.0029273 | 0.06 |
| NANOS3 | 0.214975829 | 6.81E-13 | 0.162751412 | 6.28E-08 | 0.06 |
| FLJ23755 | 0.264245636 | 6.42E-19 | 0.103858524 | 0.0005839 | 0.17 |
| NANS | 0.052749918 | 0.08130575 | 0.201645884 | 1.72E-11 | 0.15 |
| LOC64165 | 0.296417131 | 1.32E-23 | 0.244826059 | 2.21E-16 | 0.06 |
| NAP1L2 | -0.295306653 | 1.96E-23 | -0.088064313 | 0.0035707 | 0.21 |
| NAP1L3 | -0.338146681 | 1.21E-30 | -0.201915446 | 1.61E-11 | 0.14 |
| NAP1L4 | 0.128826799 | 1.94E-05 | 0.186389021 | 5.31E-10 | 0.06 |
| NAP1L5 | -0.174575282 | 6.26E-09 | -0.193140048 | 1.20E-10 | 0.02 |
| LOC286486 | -0.122118911 | 5.17E-05 | -0.029771462 | 0.3254339 | 0.1 |
| NAPA | -0.104270977 | 0.00055495 | 0.0125118 | 0.6794675 | 0.12 |
| NAPB | -0.060101961 | 0.04697492 | -0.129677698 | 1.70E-05 | 0.07 |
| NAPEPLD | -0.349751304 | 8.41E-33 | -0.170687549 | 1.36E-08 | 0.18 |
| NAPG | -0.004163601 | 0.89064031 | -0.117571683 | 9.78E-05 | 0.12 |
| NAPRT | 0.07193666 | 0.01737728 | 0.100883399 | 0.0008379 | 0.03 |
| NAPSA | 0.005803403 | 0.84801969 | -0.053501032 | 0.0770582 | 0.06 |
| NAPSB | -0.125971814 | 2.96E-05 | -0.102015365 | 0.0007311 | 0.03 |
| NARFL | 0.117068459 | 0.00010477 | 0.18600183 | 5.78E-10 | 0.07 |
| NARF | 0.485437775 | 1.08E-65 | 0.255032084 | 1.09E-17 | 0.24 |
| ICE2 | -0.330727775 | 2.61E-29 | -0.232754866 | 6.52E-15 | 0.1 |
| NARS2 | 0.137310813 | 5.21E-06 | 0.105743383 | 0.0004622 | 0.04 |
| NARS | 0.281924393 | 2.03E-21 | 0.042939989 | 0.1560012 | 0.24 |
| MGC20372 | 0.537110766 | 1.04E-82 | 0.26372483 | 7.56E-19 | 0.28 |
| NAT10 | 0.291718009 | 6.97E-23 | 0.156740034 | 1.91E-07 | 0.14 |
| NAT14 | 0.268798485 | 1.52E-19 | 0.260103673 | 2.33E-18 | 0.01 |
| NAA60 | -0.019510122 | 0.51935587 | 0.003405465 | 0.9104602 | 0.03 |
| NAT1 | -0.394784042 | 4.42E-42 | -0.007430902 | 0.8061529 | 0.39 |
| NAT2 | -0.166730097 | 2.94E-08 | 0.052050218 | 0.0854314 | 0.22 |
| NAT6 | -0.061917153 | 0.04069376 | -0.013402997 | 0.6580382 | 0.05 |
| NAT8B | 0.058475811 | 0.05327509 | 0.103827048 | 0.0005861 | 0.05 |
| NAT8L | 0.099834453 | 0.00094954 | -0.069676981 | 0.0212382 | 0.17 |
| NAT8 | -0.214655278 | 7.38E-13 | -0.143013291 | 2.06E-06 | 0.08 |
| NAT9 | 0.37054697 | 6.63E-37 | 0.220776303 | 1.56E-13 | 0.15 |
| NAV1 | -0.254263067 | 1.38E-17 | -0.194630874 | 8.62E-11 | 0.06 |
| FLJ11030_2 | -0.114914476 | 0.00014043 | -0.127124656 | 2.50E-05 | 0.02 |
| LOC121318 | -0.45954111 | 3.32E-58 | -0.353215736 | 1.83E-33 | 0.11 |
| NBAS | -0.273260149 | 3.60E-20 | -0.241952762 | 5.03E-16 | 0.04 |
| ALS2CR16 | -0.457306229 | 1.37E-57 | -0.409122928 | 2.36E-45 | 0.05 |
| NBEAL2 | -0.196102584 | 6.18E-11 | -0.132440711 | 1.12E-05 | 0.07 |
| FLJ10197 | -0.518694202 | 2.63E-76 | -0.3312902 | 2.08E-29 | 0.19 |
| LOC115581 | -0.179396544 | 2.33E-09 | -0.157130076 | 1.78E-07 | 0.03 |
| HAND2.AS1 | -0.238811328 | 1.22E-15 | -0.155196583 | 2.52E-07 | 0.09 |
| NBN | 0.019043647 | 0.52939484 | -0.043000126 | 0.1554219 | 0.07 |
| NBPF10 | -0.437394135 | 2.72E-52 | -0.376416378 | 4.05E-38 | 0.07 |
| NBPF14 | -0.339033056 | 8.36E-31 | -0.3331245 | 9.79E-30 | 0.01 |
| NBPF16 | -0.32913584 | 5.00E-29 | -0.14417552 | 1.70E-06 | 0.19 |
| NBPF16.1 | -0.32913584 | 5.00E-29 | -0.14417552 | 1.70E-06 | 0.19 |
| NBPF1 | -0.282169801 | 1.87E-21 | -0.297075296 | 1.04E-23 | 0.02 |
| NBPF22P | -0.157817719 | 1.57E-07 | -0.098583874 | 0.0011006 | 0.06 |
| NBPF3 | -0.349266961 | 1.04E-32 | -0.295268588 | 1.99E-23 | 0.06 |
| NBPF4 | -0.185166889 | 6.91E-10 | -0.140581675 | 3.08E-06 | 0.05 |
| NBPF6 | -0.210070128 | 2.29E-12 | -0.150448486 | 5.83E-07 | 0.06 |
| NBPF7 | -0.268674655 | 1.58E-19 | -0.0828155 | 0.0061533 | 0.19 |
| NBPF9 | -0.334967082 | 4.57E-30 | -0.339342554 | 7.34E-31 | 0.01 |
| NBR1 | -0.441050836 | 3.08E-53 | -0.329936537 | 3.61E-29 | 0.12 |
| NBR2 | -0.141881129 | 2.49E-06 | -0.052255233 | 0.0842054 | 0.09 |
| NCALD | 0.041348555 | 0.1719308 | -0.024228865 | 0.423583 | 0.07 |
| NCAM1 | -0.005513478 | 0.85552667 | -0.112604872 | 0.0001912 | 0.11 |
| NCAM2 | -0.335606227 | 3.51E-30 | -0.122328413 | 5.02E-05 | 0.22 |
| NCAN | 0.29405218 | 3.06E-23 | 0.074940943 | 0.0132033 | 0.22 |
| NCAPD2 | 0.439925531 | 6.03E-53 | 0.130256371 | 1.56E-05 | 0.31 |
| NCAPD3 | 0.108660986 | 0.00031951 | -0.022613925 | 0.4551427 | 0.14 |
| NCAPG2 | 0.291821313 | 6.72E-23 | 0.057038218 | 0.0594174 | 0.24 |
| NCAPG | 0.533000984 | 3.02E-81 | 0.302428584 | 1.49E-24 | 0.24 |
| NCAPH2 | 0.340315363 | 4.87E-31 | 0.189286441 | 2.83E-10 | 0.16 |
| BRRN1 | 0.496789196 | 3.47E-69 | 0.256002491 | 8.14E-18 | 0.25 |
| NCBP1 | 0.081149105 | 0.00727053 | -0.019205578 | 0.5258988 | 0.11 |
| NCBP2 | 0.329904508 | 3.66E-29 | 0.308842722 | 1.38E-25 | 0.03 |
| NCCRP1 | 0.172009011 | 1.05E-08 | 0.059132722 | 0.0506499 | 0.12 |
| NCDN | -0.16820427 | 2.21E-08 | -0.160931568 | 8.83E-08 | 0.01 |
| NCEH1 | -0.382217579 | 2.42E-39 | -0.25387366 | 1.55E-17 | 0.13 |
| NCF1B | 0.018542682 | 0.54028475 | -0.106853541 | 0.0004021 | 0.13 |
| LOC4687 | 0.023168259 | 0.44416337 | -0.078558371 | 0.0093706 | 0.11 |
| NCF1 | 0.054469449 | 0.07185024 | -0.091355877 | 0.0025016 | 0.15 |
| NCF2 | 0.045701518 | 0.13104917 | -0.082348807 | 0.0064495 | 0.13 |
| NCF4 | 0.04744097 | 0.11699494 | -0.080692838 | 0.0076066 | 0.13 |
| NCK1 | 0.078041959 | 0.00984874 | -0.095181086 | 0.0016311 | 0.18 |
| LOC115075 | 0.252265104 | 2.50E-17 | 0.00069487 | 0.981693 | 0.26 |
| NCKAP1L | -0.15289281 | 3.80E-07 | -0.251269262 | 3.36E-17 | 0.1 |
| KIAA0587 | -0.125803008 | 3.03E-05 | -0.208744232 | 3.16E-12 | 0.09 |
| NCKAP5L | -0.135598847 | 6.84E-06 | -0.201343462 | 1.84E-11 | 0.07 |
| FLJ34870 | -0.278284976 | 6.88E-21 | -0.222525516 | 9.92E-14 | 0.06 |
| NCKIPSD | 0.00156666 | 0.95873953 | 0.01802049 | 0.5517539 | 0.02 |
| NCLN | 0.298730311 | 5.74E-24 | 0.18038658 | 1.90E-09 | 0.12 |
| NCL | 0.487197599 | 3.15E-66 | 0.218842092 | 2.56E-13 | 0.27 |
| NCOA1 | -0.404227114 | 3.22E-44 | -0.432780498 | 4.09E-51 | 0.03 |
| NCOA2 | -0.39502773 | 3.90E-42 | -0.421880541 | 2.11E-48 | 0.03 |
| NCOA3 | -0.22176963 | 1.21E-13 | -0.251504734 | 3.14E-17 | 0.03 |
| NCOA4 | -0.258175226 | 4.21E-18 | -0.279527948 | 4.55E-21 | 0.03 |
| NCOA5 | 0.106188821 | 0.00043713 | 0.147402048 | 9.86E-07 | 0.05 |
| NCOA6 | -0.05617513 | 0.06337922 | -0.163297833 | 5.67E-08 | 0.11 |
| NCOA7 | 0.067068781 | 0.02660324 | -0.060506074 | 0.0455102 | 0.13 |
| NCOR1 | -0.339688937 | 6.34E-31 | -0.393162264 | 1.01E-41 | 0.06 |
| NCOR2 | -0.261072713 | 1.73E-18 | -0.368805102 | 1.50E-36 | 0.11 |
| NCR1 | -0.00708126 | 0.81510499 | -0.143159083 | 2.01E-06 | 0.14 |
| NCR2 | 0.049256823 | 0.10361348 | -0.048992592 | 0.1054815 | 0.1 |
| LOC154576 | 0.070220339 | 0.02024731 | -0.084065024 | 0.0054195 | 0.16 |
| LINC00028 | 0.143528264 | 1.89E-06 | 0.031368812 | 0.3001375 | 0.12 |
| LINC00029 | 0.04580189 | 0.13020417 | 0.000474507 | 0.9874981 | 0.05 |
| LINC00032 | 0.114646824 | 0.00014559 | 0.011253139 | 0.710175 | 0.11 |
| LINC00051 | -0.027708099 | 0.36010106 | -0.024965907 | 0.4096153 | 0.01 |
| LINC00052 | -0.018431695 | 0.54271242 | 0.088087416 | 0.003562 | 0.11 |
| BBIP1 | -0.02957389 | 0.32865629 | -0.012018464 | 0.6914436 | 0.02 |
| SPACA6P | 0.084118111 | 0.00539016 | 0.047180972 | 0.1190171 | 0.04 |
| LOC158856 | -0.122684452 | 4.77E-05 | -0.144027305 | 1.74E-06 | 0.03 |
| LOC649999 | 0.054449949 | 0.07195219 | -0.10626081 | 0.0004332 | 0.17 |
| LINC00092 | 0.293575085 | 3.62E-23 | 0.033443522 | 0.2692874 | 0.27 |
| DNMBP.AS1 | -0.382387115 | 2.23E-39 | -0.134089382 | 8.66E-06 | 0.25 |
| LOC100132749 | -0.119929352 | 7.05E-05 | 0.01017756 | 0.7367959 | 0.14 |
| FBXL19.AS1 | -0.194027976 | 9.87E-11 | -0.086490585 | 0.0042162 | 0.11 |
| KCNIP4.IT1 | -0.007391978 | 0.80714829 | -0.006298162 | 0.8352411 | 0.01 |
| LOC100133191 | 0.100868721 | 0.00083935 | 0.053541143 | 0.0768366 | 0.05 |
| LINC00685 | 0.035883241 | 0.23588146 | 0.163268218 | 5.70E-08 | 0.13 |
| C21orf9 | -0.288924432 | 1.85E-22 | -0.098198936 | 0.0011514 | 0.2 |
| LINC00111 | -0.048960174 | 0.10571251 | -0.117900491 | 9.34E-05 | 0.07 |
| LINC00112 | 0.034934561 | 0.24850571 | 0.008281551 | 0.7844812 | 0.03 |
| LINC00113 | 0.26008896 | 2.34E-18 | 0.128519872 | 2.03E-05 | 0.14 |
| LINC00114 | -0.022373766 | 0.4599464 | -0.023145355 | 0.444614 | 0.01 |
| LINC00115 | 0.155744302 | 2.28E-07 | -0.034750707 | 0.2510059 | 0.2 |
| LINC00116 | 0.246548997 | 1.34E-16 | 0.26463186 | 5.69E-19 | 0.02 |
| NPHP3.AS1 | 0.107342711 | 0.00037794 | 0.034288122 | 0.2573738 | 0.08 |
| AKIRIN2.AS1 | -0.12603865 | 2.93E-05 | -0.32555931 | 2.11E-28 | 0.2 |
| LINC00152 | 0.272844558 | 4.12E-20 | 0.307641681 | 2.17E-25 | 0.04 |
| CHODL.AS1 | 0.072321158 | 0.01678547 | -0.020263515 | 0.5033519 | 0.1 |
| LINC00158 | 0.173641814 | 7.56E-09 | 0.035575403 | 0.2399273 | 0.14 |
| NCRNA00159 | 0.048035064 | 0.11247533 | 0.026172311 | 0.3873523 | 0.03 |
| LINC00160 | -0.179766487 | 2.16E-09 | 0.085623602 | 0.0046153 | 0.27 |
| LINC00161 | 0.005901876 | 0.84547304 | 0.016457138 | 0.5867881 | 0.02 |
| NLC1.C | 0.185148852 | 6.94E-10 | -0.002477994 | 0.9347817 | 0.19 |
| ANKRD30BL | 0.144167493 | 1.70E-06 | 0.075307461 | 0.0127601 | 0.07 |
| LOC143840 | 0.042219376 | 0.16306994 | -0.004448318 | 0.8832139 | 0.05 |
| CRYM.AS1 | 0.033074595 | 0.27460811 | 0.004810332 | 0.8737861 | 0.03 |
| HCG8 | -0.176531015 | 4.21E-09 | -0.206528335 | 5.40E-12 | 0.03 |
| LOC100291250 | -0.142041782 | 2.42E-06 | -0.082707449 | 0.0062207 | 0.06 |
| LINC00174 | -0.173919695 | 7.15E-09 | -0.08110877 | 0.0072997 | 0.1 |
| COL18A1.AS1 | -0.119383663 | 7.60E-05 | -0.071956177 | 0.0173468 | 0.05 |
| LINC00176 | -0.040429906 | 0.18166317 | 0.105009281 | 0.0005065 | 0.15 |
| A1BG.AS1 | -0.184929963 | 7.27E-10 | -0.120699885 | 6.32E-05 | 0.07 |
| FTX | -0.438715325 | 1.24E-52 | -0.284265118 | 9.19E-22 | 0.16 |
| JPX | -0.068797371 | 0.02293163 | 0.053171416 | 0.0788994 | 0.13 |
| NCRNA00185 | 0.023740952 | 0.43298064 | 0.03191437 | 0.2918061 | 0.01 |
| LRRC75A.AS1 | 0.236638328 | 2.24E-15 | 0.259384848 | 2.90E-18 | 0.03 |
| LINC00189 | -0.030747508 | 0.3098168 | -0.111078443 | 0.0002337 | 0.09 |
| LINC00200 | -0.030272251 | 0.31735829 | -0.032552828 | 0.2822548 | 0.01 |
| HNRNPU.AS1 | 0.006867006 | 0.82060268 | 0.240375693 | 7.87E-16 | 0.24 |
| LOC119151 | 0.011968681 | 0.6926565 | 0.002165985 | 0.9429785 | 0.01 |
| ITPK1.AS1 | -0.057579191 | 0.05704048 | -0.037267237 | 0.218288 | 0.03 |
| FAM223B | 0.00820918 | 0.78631872 | 0.057057116 | 0.059333 | 0.05 |
| LINC00207 | 0.02387636 | 0.43036057 | -0.005217404 | 0.8632064 | 0.03 |
| EPB41L4A.AS1 | 0.026009377 | 0.39031529 | 0.141580656 | 2.61E-06 | 0.12 |
| FAM224B | 0.046165036 | 0.1271822 | 0.031777109 | 0.2938875 | 0.02 |
| LINC00235 | -0.103500918 | 0.00061009 | -0.179369677 | 2.35E-09 | 0.08 |
| NCS1 | 0.328282279 | 7.06E-29 | 0.11715135 | 0.0001036 | 0.22 |
| NCSTN | -0.129161022 | 1.84E-05 | 0.128444777 | 2.05E-05 | 0.26 |
| NDC80 | 0.628275586 | 4.34E-121 | 0.318531243 | 3.39E-27 | 0.31 |
| LOC115942 | -0.076253914 | 0.01167614 | -0.116908278 | 0.0001071 | 0.05 |
| NDEL1 | -0.107382041 | 0.00037607 | -0.213149997 | 1.07E-12 | 0.11 |
| NDFIP1 | -0.297490037 | 8.97E-24 | -0.087713477 | 0.0037063 | 0.21 |
| NDFIP2 | -0.162884588 | 6.13E-08 | -0.130441461 | 1.52E-05 | 0.04 |
| MAGEG1 | -0.069795662 | 0.0210182 | -0.080492579 | 0.0077584 | 0.02 |
| NDN | -0.245235052 | 1.96E-16 | -0.083514304 | 0.0057326 | 0.17 |
| NDOR1 | -0.041360615 | 0.17180567 | 0.042100634 | 0.1642575 | 0.09 |
| EVR2 | -0.095491657 | 0.00157434 | -0.070080828 | 0.0204978 | 0.03 |
| HMSNL | 0.247564602 | 9.99E-17 | -0.019275084 | 0.5244018 | 0.27 |
| NDRG2 | 0.040161212 | 0.18458551 | -0.012096174 | 0.6895519 | 0.06 |
| NDRG3 | 0.000807185 | 0.97873454 | 0.001865323 | 0.9508829 | 0.01 |
| NDRG4 | 0.147506056 | 9.69E-07 | -0.06377412 | 0.0350188 | 0.22 |
| NDST1 | -0.481148498 | 2.07E-64 | -0.337563435 | 1.55E-30 | 0.15 |
| NDST2 | -0.412537836 | 3.71E-46 | -0.396180173 | 2.16E-42 | 0.02 |
| NDST3 | 0.022016786 | 0.46713878 | -0.03593735 | 0.2351753 | 0.06 |
| NDST4 | -0.07555116 | 0.01247284 | -0.077581767 | 0.010293 | 0.01 |
| LOC101927147 | 0.037176253 | 0.21941484 | 0.194185263 | 9.53E-11 | 0.16 |
| NDUFA11 | 0.409056592 | 2.44E-45 | 0.371116343 | 5.07E-37 | 0.04 |
| NDUFA12 | 0.423432388 | 8.78E-49 | 0.446314292 | 1.27E-54 | 0.03 |
| LOC112763 | 0.287782633 | 2.75E-22 | 0.319328055 | 2.48E-27 | 0.04 |
| NDUFA1 | 0.441859081 | 1.89E-53 | 0.439409071 | 8.21E-53 | 0.01 |
| NDUFA2 | 0.134914841 | 7.61E-06 | 0.338572711 | 1.01E-30 | 0.21 |
| NDUFA3 | 0.250567869 | 4.14E-17 | 0.350874029 | 5.14E-33 | 0.11 |
| NDUFA4L2 | 0.125748496 | 3.06E-05 | 0.036847384 | 0.2235227 | 0.09 |
| NDUFA4 | 0.321591286 | 1.02E-27 | 0.394192412 | 5.98E-42 | 0.08 |
| FLJ12147 | -0.005872679 | 0.84622795 | 0.197726482 | 4.26E-11 | 0.21 |
| NDUFA6 | 0.35564429 | 6.21E-34 | 0.317700675 | 4.69E-27 | 0.04 |
| NDUFA7 | 0.312056529 | 4.10E-26 | 0.364204523 | 1.27E-35 | 0.06 |
| NDUFA8 | 0.395795321 | 2.63E-42 | 0.393841993 | 7.15E-42 | 0.01 |
| NDUFS2L | 0.559248042 | 5.99E-91 | 0.385936574 | 3.85E-40 | 0.18 |
| NDUFAB1 | 0.470945351 | 2.00E-61 | 0.461975849 | 6.98E-59 | 0.01 |
| NDUFAF1 | -0.07869291 | 0.00924953 | 0.063551841 | 0.0356604 | 0.15 |
| NDUFAF2 | 0.389776473 | 5.63E-41 | 0.401049314 | 1.72E-43 | 0.02 |
| MGC10527 | 0.120562073 | 6.45E-05 | 0.131619618 | 1.27E-05 | 0.02 |
| NDUFAF4 | 0.536609725 | 1.57E-82 | 0.41021486 | 1.31E-45 | 0.13 |
| NDUFB10 | 0.307744568 | 2.09E-25 | 0.359901681 | 9.12E-35 | 0.06 |
| NDUFB11 | 0.445364455 | 2.27E-54 | 0.47137989 | 1.50E-61 | 0.03 |
| NDUFB1 | 0.275063743 | 2.00E-20 | 0.412848183 | 3.13E-46 | 0.14 |
| NDUFB2 | 0.363432608 | 1.82E-35 | 0.42468783 | 4.31E-49 | 0.07 |
| NDUFB3 | 0.386060642 | 3.62E-40 | 0.436133323 | 5.73E-52 | 0.06 |
| LOC727762 | 0.263519225 | 8.06E-19 | 0.368032922 | 2.16E-36 | 0.11 |
| LOC96666 | 0.323445898 | 4.90E-28 | 0.370464665 | 6.90E-37 | 0.05 |
| NDUFB6 | 0.378031514 | 1.86E-38 | 0.441331317 | 2.60E-53 | 0.07 |
| NDUFB7 | 0.325437112 | 2.22E-28 | 0.292416216 | 5.45E-23 | 0.04 |
| NDUFB8 | 0.332358945 | 1.34E-29 | 0.351549362 | 3.82E-33 | 0.02 |
| NDUFB9 | 0.575947403 | 1.37E-97 | 0.410511617 | 1.11E-45 | 0.17 |
| NDUFC1 | 0.258124067 | 4.27E-18 | 0.31543247 | 1.13E-26 | 0.06 |
| NDUFC2 | 0.09254462 | 0.00219387 | 0.194749734 | 8.39E-11 | 0.11 |
| PRO1304 | -0.159683812 | 1.11E-07 | -0.162411877 | 6.70E-08 | 0.01 |
| NDUFS2 | 0.177793742 | 3.25E-09 | 0.467561878 | 1.86E-60 | 0.29 |
| NDUFS3 | 0.479315981 | 7.25E-64 | 0.419079638 | 1.01E-47 | 0.07 |
| NDUFS4 | 0.075940122 | 0.01202613 | 0.265456245 | 4.39E-19 | 0.19 |
| NDUFS5 | 0.567893761 | 2.44E-94 | 0.466528906 | 3.66E-60 | 0.11 |
| NDUFS6 | 0.366251699 | 4.95E-36 | 0.335174983 | 4.19E-30 | 0.04 |
| NDUFS7 | 0.130507373 | 1.50E-05 | 0.152145659 | 4.33E-07 | 0.03 |
| NDUFS8 | 0.300915223 | 2.60E-24 | 0.270466068 | 8.89E-20 | 0.04 |
| NDUFV1 | 0.2690215 | 1.41E-19 | 0.185498683 | 6.44E-10 | 0.09 |
| NDUFV2 | 0.399909306 | 3.12E-43 | 0.360254159 | 7.77E-35 | 0.04 |
| LOC100509698 | 0.307073155 | 2.68E-25 | 0.363554797 | 1.72E-35 | 0.06 |
| LOC100292444 | -0.298783979 | 5.63E-24 | -0.115340154 | 0.0001326 | 0.19 |
| LOC220996 | -0.284923598 | 7.34E-22 | -0.173193765 | 8.27E-09 | 0.12 |
| NEB | 0.000381758 | 0.98994159 | -0.101871983 | 0.0007439 | 0.11 |
| NECAB1 | -0.113862909 | 0.00016173 | -0.132292623 | 1.14E-05 | 0.02 |
| NECAB2 | 0.096000103 | 0.00148537 | 0.073731692 | 0.014763 | 0.03 |
| NECAB3 | -0.092795433 | 0.00213353 | 0.043075974 | 0.1546937 | 0.14 |
| NECAP1 | -0.11630682 | 0.00011627 | -0.123581075 | 4.19E-05 | 0.01 |
| NECAP2 | 0.064472737 | 0.03306585 | -0.059970127 | 0.0474612 | 0.13 |
| NEDD1 | -0.125342869 | 3.24E-05 | -0.143452652 | 1.92E-06 | 0.02 |
| NEDD4L | -0.168040157 | 2.28E-08 | -0.166208015 | 3.26E-08 | 0.01 |
| NEDD4 | -0.494052421 | 2.48E-68 | -0.326903105 | 1.23E-28 | 0.17 |
| NEDD8 | 0.314352299 | 1.71E-26 | 0.389308491 | 7.13E-41 | 0.08 |
| HEF1 | -0.303925965 | 8.62E-25 | -0.189035597 | 2.99E-10 | 0.12 |
| NEFH | -0.154746905 | 2.73E-07 | -0.099241555 | 0.0010186 | 0.06 |
| NEFL | -0.282059414 | 1.94E-21 | -0.180252668 | 1.95E-09 | 0.11 |
| NEFM | -0.240916824 | 6.75E-16 | -0.180517296 | 1.85E-09 | 0.07 |
| NEGR1 | -0.379752392 | 8.07E-39 | -0.248686674 | 7.20E-17 | 0.14 |
| NEIL1 | -0.250822432 | 3.84E-17 | -0.050883728 | 0.0926836 | 0.2 |
| NEIL2 | -0.031454236 | 0.29882262 | 0.027932811 | 0.356217 | 0.06 |
| NEIL3 | 0.453024127 | 2.03E-56 | 0.222594233 | 9.75E-14 | 0.24 |
| NEK10 | -0.460243772 | 2.12E-58 | -0.144568443 | 1.59E-06 | 0.32 |
| NEK11 | -0.37421186 | 1.17E-37 | -0.058390052 | 0.053626 | 0.32 |
| NEK1 | -0.407105892 | 6.96E-45 | -0.365721128 | 6.33E-36 | 0.05 |
| NEK2 | 0.439658831 | 7.07E-53 | 0.422778131 | 1.27E-48 | 0.02 |
| NEK3 | 0.002225441 | 0.94141605 | 0.039244053 | 0.1948223 | 0.04 |
| NEK4 | -0.354434623 | 1.06E-33 | -0.291518797 | 7.48E-23 | 0.07 |
| LOC100506859 | -0.489573008 | 5.95E-67 | -0.162070266 | 7.14E-08 | 0.33 |
| SID6.1512 | -0.186214418 | 5.52E-10 | -0.067050353 | 0.026645 | 0.12 |
| LOC148565 | -0.28330773 | 1.27E-21 | -0.067490299 | 0.0256641 | 0.22 |
| NEK8 | -0.18756456 | 4.12E-10 | -0.087956257 | 0.003612 | 0.1 |
| NEK9 | -0.542753291 | 9.45E-85 | -0.389967428 | 5.11E-41 | 0.16 |
| LOC254617 | 0.24586633 | 1.64E-16 | 0.008190223 | 0.7868002 | 0.24 |
| IDH3GL | -0.046661734 | 0.12313746 | -0.049026319 | 0.1052416 | 0.01 |
| NELL2 | -0.136737968 | 5.71E-06 | -0.074860324 | 0.0133025 | 0.07 |
| NENF | 0.249303283 | 6.01E-17 | 0.48666619 | 4.57E-66 | 0.24 |
| NEO1 | -0.326731247 | 1.32E-28 | -0.277659955 | 8.47E-21 | 0.05 |
| NES | 0.111071422 | 0.0002339 | 0.011955824 | 0.6929699 | 0.1 |
| NET1 | 0.103144691 | 0.00063728 | 0.040616129 | 0.179658 | 0.07 |
| NETO1 | -0.023826461 | 0.43132503 | -0.060952739 | 0.0439362 | 0.04 |
| LOC115228 | 0.301392747 | 2.18E-24 | 0.007890559 | 0.7944228 | 0.3 |
| NEU1 | 0.045111612 | 0.13610149 | 0.144406454 | 1.64E-06 | 0.1 |
| NEU2 | 0.07527169 | 0.0128028 | -0.012010219 | 0.6916444 | 0.09 |
| NEU3 | -0.362999567 | 2.22E-35 | -0.369513907 | 1.08E-36 | 0.01 |
| NEU4 | 0.066574835 | 0.02774138 | 0.140487504 | 3.13E-06 | 0.08 |
| NEURL1B | -0.096347343 | 0.0014273 | -0.193521633 | 1.11E-10 | 0.1 |
| NEURL2 | -0.002090834 | 0.94495372 | -0.013249615 | 0.661707 | 0.02 |
| NEURL3 | 0.134632956 | 7.96E-06 | 0.088313694 | 0.0034771 | 0.05 |
| NEURL4 | -0.083628502 | 0.00566637 | -0.093073976 | 0.0020683 | 0.01 |
| NEURL1 | -0.059031203 | 0.05104842 | -0.050953813 | 0.0922344 | 0.01 |
| NEUROD1 | 0.03795519 | 0.20990182 | 0.001535608 | 0.9595566 | 0.04 |
| NEUROD2 | 0.06986984 | 0.02088174 | 0.107556915 | 0.0003678 | 0.04 |
| NEUROD4 | 0.052691011 | 0.08164674 | -0.046055513 | 0.1280878 | 0.1 |
| NEUROD6 | -0.013970402 | 0.64453804 | -0.016672732 | 0.5818961 | 0.01 |
| NEUROG1 | 0.064930077 | 0.03183844 | 0.014460885 | 0.6329615 | 0.06 |
| NEUROG2 | -0.054047344 | 0.07408373 | -0.024696351 | 0.4146917 | 0.03 |
| NEUROG3 | 0.009539222 | 0.7527478 | -0.001494549 | 0.9606371 | 0.02 |
| NEXN | -0.177462199 | 3.48E-09 | -0.194048966 | 9.83E-11 | 0.02 |
| NF1P2 | 0.069716119 | 0.0211654 | 0.028420359 | 0.3478811 | 0.05 |
| NF1 | -0.360368388 | 7.38E-35 | -0.290356958 | 1.12E-22 | 0.08 |
| LOC654093 | 0.10950806 | 0.00028655 | -0.044475324 | 0.141718 | 0.16 |
| NFAM1 | -0.09542027 | 0.00158722 | -0.087996857 | 0.0035965 | 0.01 |
| NFASC | -0.038355467 | 0.20513093 | -0.051274726 | 0.0901996 | 0.02 |
| NFAT5 | -0.286515663 | 4.25E-22 | -0.396087274 | 2.26E-42 | 0.11 |
| NFATC1 | -0.020574516 | 0.49682211 | -0.144984997 | 1.48E-06 | 0.13 |
| NFATC2IP | -0.052808201 | 0.08096952 | -0.050682913 | 0.0939805 | 0.01 |
| NFATC2 | -0.479062807 | 8.62E-64 | -0.525477313 | 1.28E-78 | 0.05 |
| NFATC3 | 0.043086191 | 0.1545958 | -0.127314883 | 2.43E-05 | 0.18 |
| NFATC4 | -0.155107997 | 2.56E-07 | 0.014761237 | 0.6259162 | 0.17 |
| NFE2L1 | -0.250666065 | 4.02E-17 | -0.27902021 | 5.39E-21 | 0.03 |
| NFE2L2 | -0.213216296 | 1.06E-12 | -0.21044875 | 2.09E-12 | 0.01 |
| NFE2L3 | 0.275024252 | 2.02E-20 | 0.127628532 | 2.32E-05 | 0.15 |
| NFE2 | 0.036058341 | 0.23360181 | 0.061670369 | 0.0415036 | 0.03 |
| NFIA | -0.276341863 | 1.31E-20 | 0.025313974 | 0.4031154 | 0.31 |
| NFIB | 0.16991362 | 1.58E-08 | 0.055524851 | 0.0665061 | 0.12 |
| NFIC | -0.486988786 | 3.65E-66 | -0.431862411 | 6.99E-51 | 0.06 |
| NFIL3 | 0.190585492 | 2.13E-10 | 0.080838342 | 0.0074979 | 0.11 |
| NFIX | -0.062138885 | 0.03997761 | -0.080864269 | 0.0074787 | 0.02 |
| NFKB1 | -0.308956832 | 1.32E-25 | -0.304714366 | 6.44E-25 | 0.01 |
| NFKB2 | 0.210589266 | 2.02E-12 | 0.077579822 | 0.0102949 | 0.14 |
| NFKBIA | 0.098329919 | 0.00113387 | 0.0838418 | 0.0055445 | 0.02 |
| NFKBIB | 0.290309824 | 1.14E-22 | 0.207891096 | 3.89E-12 | 0.09 |
| NFKBID | 0.02057342 | 0.49684504 | -0.103952419 | 0.0005772 | 0.13 |
| NFKBIE | 0.339872526 | 5.87E-31 | 0.150197592 | 6.09E-07 | 0.19 |
| LOC221331 | 0.223103186 | 8.54E-14 | 0.16473917 | 4.31E-08 | 0.06 |
| TONSL | 0.453780259 | 1.26E-56 | 0.227666655 | 2.57E-14 | 0.23 |
| NFKBIZ | -0.224807644 | 5.47E-14 | -0.089971484 | 0.0029095 | 0.14 |
| NFRKB | -0.157749292 | 1.59E-07 | -0.143461547 | 1.92E-06 | 0.02 |
| NFS1 | 0.121062806 | 6.01E-05 | 0.169642088 | 1.67E-08 | 0.05 |
| NFU1 | 0.105481415 | 0.00047757 | 0.038263132 | 0.2062244 | 0.07 |
| NFX1 | -0.153785736 | 3.24E-07 | -0.138297499 | 4.45E-06 | 0.02 |
| NFXL1 | 0.033375031 | 0.27026979 | -0.11452059 | 0.0001481 | 0.15 |
| NFYA | 0.184477784 | 8.01E-10 | 0.111408357 | 0.0002238 | 0.08 |
| NFYB | 0.132035361 | 1.19E-05 | 0.100329158 | 0.0008953 | 0.04 |
| null | 0.215355504 | 6.19E-13 | 0.096580473 | 0.0013895 | 0.12 |
| NGB | 0.130902271 | 1.42E-05 | 0.043712568 | 0.1486832 | 0.09 |
| NGDN | 0.379164172 | 1.07E-38 | 0.331483668 | 1.92E-29 | 0.05 |
| NGEF | 0.084604589 | 0.00512781 | 0.166463945 | 3.10E-08 | 0.09 |
| NGFRAP1 | 0.126600002 | 2.70E-05 | 0.158033334 | 1.51E-07 | 0.04 |
| NGFR | -0.182874657 | 1.13E-09 | -0.165431108 | 3.78E-08 | 0.02 |
| NGF | 0.015780078 | 0.60227374 | -0.00541785 | 0.8580057 | 0.03 |
| NGLY1 | -0.065659826 | 0.02996076 | -0.043044666 | 0.154994 | 0.03 |
| LOC116217 | 0.078760726 | 0.00918901 | 0.0612733 | 0.0428351 | 0.02 |
| SLC9B1 | -0.123098463 | 4.49E-05 | -0.045289714 | 0.1345605 | 0.08 |
| SLC9B2 | 0.103013388 | 0.00064758 | -0.06337724 | 0.0361714 | 0.17 |
| NHEG1 | -0.081865562 | 0.00676961 | -0.047895767 | 0.1135225 | 0.04 |
| NHEJ1 | 0.275055924 | 2.00E-20 | 0.036509323 | 0.2278023 | 0.24 |
| NHLH1 | 0.276963336 | 1.07E-20 | 0.098703491 | 0.0010853 | 0.18 |
| LOC90888 | -0.129707917 | 1.70E-05 | -0.095770301 | 0.001525 | 0.04 |
| NHLRC1 | 0.142670583 | 2.18E-06 | 0.209637522 | 2.55E-12 | 0.07 |
| FLJ20147 | -0.34824386 | 1.62E-32 | -0.381945201 | 2.76E-39 | 0.04 |
| NHLRC3 | -0.459128269 | 4.32E-58 | -0.226826125 | 3.21E-14 | 0.24 |
| NHLRC4 | -0.245553334 | 1.79E-16 | -0.058457536 | 0.0533497 | 0.19 |
| SSFA1 | 0.390474678 | 3.96E-41 | 0.340439012 | 4.62E-31 | 0.06 |
| NHP2 | 0.422029725 | 1.94E-48 | 0.422243015 | 1.72E-48 | 0.01 |
| C6orf63 | 0.182001955 | 1.35E-09 | 0.058636987 | 0.0526207 | 0.13 |
| NHSL2 | -0.516691398 | 1.24E-75 | -0.394389032 | 5.41E-42 | 0.13 |
| NHS | -0.239021462 | 1.15E-15 | -0.279126968 | 5.20E-21 | 0.05 |
| NICN1 | -0.240358447 | 7.90E-16 | -0.167553877 | 2.51E-08 | 0.08 |
| NID1 | -0.331770546 | 1.71E-29 | -0.27590656 | 1.51E-20 | 0.06 |
| NID2 | -0.273148138 | 3.73E-20 | -0.203986068 | 9.90E-12 | 0.07 |
| ALS2CR1 | 0.245420664 | 1.86E-16 | 0.169398226 | 1.75E-08 | 0.08 |
| NINJ1 | -0.14711127 | 1.04E-06 | 0.04234566 | 0.161814 | 0.19 |
| LOC121626 | 0.078911726 | 0.00905553 | 0.014108471 | 0.6412704 | 0.07 |
| FLJ11792 | 0.082243031 | 0.00651836 | -0.047182058 | 0.1190086 | 0.13 |
| NIN | -0.320464091 | 1.59E-27 | -0.30537556 | 5.04E-25 | 0.02 |
| NIP7 | 0.46500328 | 9.87E-60 | 0.161108334 | 8.54E-08 | 0.31 |
| NIPA1 | -0.206001932 | 6.13E-12 | -0.210032688 | 2.31E-12 | 0.01 |
| NIPA2 | 0.105047955 | 0.00050404 | -0.007428377 | 0.8062175 | 0.12 |
| NIPAL1 | 0.023793836 | 0.43195627 | -0.151272996 | 5.05E-07 | 0.18 |
| NIPAL2 | -0.227982847 | 2.36E-14 | -0.210984049 | 1.83E-12 | 0.02 |
| NIPAL3 | -0.190941287 | 1.97E-10 | -0.309669154 | 1.01E-25 | 0.12 |
| NIPAL4 | 0.236733871 | 2.18E-15 | 0.042468706 | 0.1605974 | 0.2 |
| NIPBL | -0.348574553 | 1.41E-32 | -0.323819226 | 4.23E-28 | 0.03 |
| NIPSNAP1 | 0.208376413 | 3.46E-12 | 0.14922454 | 7.21E-07 | 0.06 |
| LOC93242 | 0.029280223 | 0.33348405 | -0.034221434 | 0.258301 | 0.07 |
| LOC286367.1 | -0.068528403 | 0.02347232 | -0.05616953 | 0.0634056 | 0.02 |
| NISCH | -0.42504208 | 3.53E-49 | -0.314226477 | 1.79E-26 | 0.12 |
| NIT1 | -0.009762704 | 0.74715064 | 0.286952338 | 3.66E-22 | 0.3 |
| NIT2 | 0.404527936 | 2.75E-44 | 0.442635974 | 1.19E-53 | 0.04 |
| NKAIN1 | -0.071464422 | 0.01812907 | -0.031942021 | 0.291388 | 0.04 |
| NKAIN2 | -0.225128728 | 5.03E-14 | -0.150901139 | 5.39E-07 | 0.08 |
| NKAIN3 | -0.009862325 | 0.74465987 | -0.004463826 | 0.8828097 | 0.01 |
| NKAIN4 | -0.102582164 | 0.00068252 | 0.023959705 | 0.4287525 | 0.13 |
| NKAPL | -0.246986599 | 1.18E-16 | -0.209824555 | 2.43E-12 | 0.04 |
| NKAP | 0.295687173 | 1.71E-23 | 0.214204884 | 8.25E-13 | 0.09 |
| NKD1 | -0.120059727 | 6.92E-05 | -0.03501393 | 0.2474318 | 0.09 |
| NKD2 | -0.079411413 | 0.00862605 | -0.051169381 | 0.0908636 | 0.03 |
| NKG7 | 0.107903619 | 0.00035196 | -0.007127687 | 0.8139149 | 0.12 |
| NKIRAS1 | -0.068434615 | 0.02366344 | -0.037751135 | 0.2123646 | 0.04 |
| NKIRAS2 | 0.310622122 | 7.07E-26 | 0.100014969 | 0.0009294 | 0.22 |
| NKPD1 | 0.173260131 | 8.16E-09 | 0.058512648 | 0.0531249 | 0.12 |
| NKRF | 0.375650338 | 5.86E-38 | 0.195803475 | 6.61E-11 | 0.18 |
| NKTR | -0.377598951 | 2.29E-38 | -0.285659543 | 5.70E-22 | 0.1 |
| NKX1.2 | 0.275452603 | 1.76E-20 | -0.035679612 | 0.2385523 | 0.32 |
| BCH | 0.123833245 | 4.04E-05 | 0.034198289 | 0.2586233 | 0.09 |
| NKX2.2 | -0.051967621 | 0.08592936 | 0.137530252 | 5.04E-06 | 0.19 |
| NKX2.3 | 0.217329598 | 3.76E-13 | 0.054686764 | 0.070722 | 0.17 |
| NKX2.4 | 0.082539025 | 0.00632723 | 0.017347915 | 0.5666999 | 0.07 |
| NKX2.5 | 0.33418629 | 6.31E-30 | 0.123315205 | 4.35E-05 | 0.22 |
| NKX2.6 | 0.067260744 | 0.02617192 | 0.005245146 | 0.8624862 | 0.07 |
| NKX2.8 | -0.021464961 | 0.4783784 | -0.13034694 | 1.54E-05 | 0.11 |
| NKX3.1 | -0.274980611 | 2.05E-20 | -0.128584789 | 2.01E-05 | 0.15 |
| NKX3.2 | 0.003795587 | 0.90025355 | 0.039522208 | 0.1916747 | 0.04 |
| NKX6.1 | -0.076562154 | 0.01134115 | -0.105409246 | 0.0004819 | 0.03 |
| LOC159201 | 0.063336001 | 0.03629301 | 0.014700187 | 0.6273455 | 0.05 |
| NKX6.3 | -0.019190064 | 0.52623323 | -0.0181595 | 0.5486891 | 0.01 |
| NLE1 | 0.220210448 | 1.80E-13 | 0.191301401 | 1.81E-10 | 0.03 |
| NLGN1 | -0.17430603 | 6.61E-09 | -0.154748188 | 2.73E-07 | 0.02 |
| NLGN2 | -0.102274434 | 0.00070851 | -0.170490073 | 1.41E-08 | 0.07 |
| NLGN3 | -0.220949444 | 1.49E-13 | -0.120260881 | 6.72E-05 | 0.11 |
| NLGN4X | -0.190459112 | 2.19E-10 | -0.070204548 | 0.0202755 | 0.13 |
| NLGN4Y | -0.080488075 | 0.00776189 | -0.032515374 | 0.2828092 | 0.05 |
| NLK | -0.117592783 | 9.75E-05 | -0.021717874 | 0.4732088 | 0.1 |
| NLN | -0.013499423 | 0.65573595 | -0.055788403 | 0.0652238 | 0.05 |
| NLRC3 | -0.130612754 | 1.48E-05 | -0.217999346 | 3.18E-13 | 0.09 |
| NLRC4 | -0.065364243 | 0.03070954 | -0.171676385 | 1.12E-08 | 0.11 |
| NLRC5 | 0.031509216 | 0.29797836 | -0.129166831 | 1.84E-05 | 0.17 |
| NLRP10 | 0.08794265 | 0.00361724 | -0.059201738 | 0.0503805 | 0.15 |
| NLRP11 | -0.036344349 | 0.22991185 | -0.055743824 | 0.0654393 | 0.02 |
| PYPAF7 | -0.292116969 | 6.06E-23 | -0.103166967 | 0.0006355 | 0.19 |
| NLRP13 | -0.213099444 | 1.09E-12 | -0.130368126 | 1.54E-05 | 0.09 |
| NLRP14 | -0.108869127 | 0.0003111 | -0.16677242 | 2.92E-08 | 0.06 |
| PP1044 | -0.228649444 | 1.98E-14 | -0.28756869 | 2.96E-22 | 0.06 |
| NLRP2 | -0.015982422 | 0.59762651 | -0.034863256 | 0.2494733 | 0.02 |
| FCU | -0.296599624 | 1.23E-23 | -0.330440826 | 2.94E-29 | 0.04 |
| NLRP4 | -0.182008405 | 1.35E-09 | -0.175505992 | 5.19E-09 | 0.01 |
| NLRP5 | -0.214230493 | 8.20E-13 | -0.098652838 | 0.0010917 | 0.12 |
| NLRP6 | -0.217104062 | 3.98E-13 | -0.153068485 | 3.68E-07 | 0.07 |
| HYDM | 0.037582641 | 0.21441383 | -0.076680425 | 0.0112149 | 0.12 |
| NLRP8 | -0.259278101 | 3.00E-18 | -0.112283825 | 0.0001995 | 0.15 |
| NLRP9 | 0.101254742 | 0.00080136 | 0.040106374 | 0.1851862 | 0.07 |
| NLRX1 | -0.092082284 | 0.0023092 | -0.079491035 | 0.0085593 | 0.02 |
| NMBR | -0.12639288 | 2.78E-05 | -0.126351729 | 2.80E-05 | 0.01 |
| NMB | 0.31387892 | 2.05E-26 | 0.117413331 | 9.99E-05 | 0.2 |
| NMD3 | -0.08070999 | 0.00759372 | -0.099978567 | 0.0009334 | 0.02 |
| NME1.NME2 | 0.231740385 | 8.59E-15 | 0.132082612 | 1.18E-05 | 0.1 |
| NB | 0.504954494 | 8.84E-72 | 0.471517101 | 1.37E-61 | 0.04 |
| NME2P1 | 0.413558239 | 2.12E-46 | 0.416955333 | 3.28E-47 | 0.01 |
| NME2 | 0.41581542 | 6.16E-47 | 0.430554378 | 1.49E-50 | 0.02 |
| NME3 | -0.014498366 | 0.63208044 | 0.132289245 | 1.14E-05 | 0.15 |
| NME4 | 0.188824059 | 3.13E-10 | 0.32911455 | 5.04E-29 | 0.15 |
| NME5 | -0.176042085 | 4.65E-09 | -0.020868013 | 0.4907014 | 0.16 |
| NME6 | -0.083564484 | 0.00570341 | 0.050993175 | 0.0919828 | 0.14 |
| NME7 | -0.043524058 | 0.15044419 | 0.291452895 | 7.65E-23 | 0.34 |
| NMI | 0.23174702 | 8.57E-15 | 0.135061612 | 7.44E-06 | 0.1 |
| NMNAT1 | -0.225559335 | 4.49E-14 | -0.228755341 | 1.92E-14 | 0.01 |
| NMNAT2 | -0.065286964 | 0.03090793 | -0.141742413 | 2.54E-06 | 0.08 |
| NMNAT3 | -0.078256662 | 0.00964739 | -0.053993697 | 0.0743716 | 0.03 |
| NMRAL1 | 0.263246407 | 8.78E-19 | 0.319438088 | 2.38E-27 | 0.06 |
| NMS | 0.023559035 | 0.43651511 | -0.001526097 | 0.9598069 | 0.03 |
| NMT1 | -0.10448885 | 0.00054021 | -0.202719816 | 1.34E-11 | 0.1 |
| NMT2 | 0.118784167 | 8.27E-05 | -0.087842433 | 0.003656 | 0.21 |
| NMUR1 | 0.026043493 | 0.38969374 | -0.114944681 | 0.0001399 | 0.15 |
| NMUR2 | -0.061674527 | 0.04148981 | -0.018775739 | 0.5352047 | 0.05 |
| NMU | 0.412710203 | 3.38E-46 | 0.232745911 | 6.53E-15 | 0.18 |
| NNAT | -0.27735751 | 9.36E-21 | -0.203328277 | 1.16E-11 | 0.08 |
| NNMT | -0.005878265 | 0.84608349 | 0.022424634 | 0.4589266 | 0.03 |
| NNT | -0.110839547 | 0.00024109 | -0.179747791 | 2.17E-09 | 0.07 |
| LOC90015 | 0.385196866 | 5.56E-40 | 0.151430084 | 4.91E-07 | 0.24 |
| NOBOX | 0.045917782 | 0.12923377 | -0.010357442 | 0.7323207 | 0.06 |
| NOC2L | 0.390712894 | 3.51E-41 | 0.170133521 | 1.52E-08 | 0.23 |
| NOC3L | 0.107339737 | 0.00037809 | -0.026728577 | 0.37734 | 0.14 |
| NOC4L | 0.330304657 | 3.11E-29 | 0.323362667 | 5.07E-28 | 0.01 |
| NOD1 | -0.067686003 | 0.02523784 | -0.034876381 | 0.249295 | 0.04 |
| IBD1 | 0.053618751 | 0.07640921 | -0.022391115 | 0.4595984 | 0.08 |
| NODAL | 0.066184028 | 0.02867135 | -0.02709969 | 0.3707497 | 0.1 |
| NOG | -0.070722769 | 0.01936719 | -0.078220358 | 0.0096812 | 0.01 |
| NOL10 | 0.502779991 | 4.41E-71 | 0.388422824 | 1.11E-40 | 0.12 |
| NOL11 | 0.280623471 | 3.15E-21 | 0.189242651 | 2.86E-10 | 0.1 |
| NOL12 | 0.324947611 | 2.70E-28 | 0.285411955 | 6.21E-22 | 0.04 |
| NOL3 | 0.058829435 | 0.05184818 | 0.100013364 | 0.0009296 | 0.05 |
| NOL4 | 0.290732027 | 9.85E-23 | 0.065161855 | 0.0312314 | 0.23 |
| NOL6 | 0.051158133 | 0.09093468 | 0.010231733 | 0.7354472 | 0.05 |
| LOC135792 | 0.503567806 | 2.47E-71 | 0.399262437 | 4.37E-43 | 0.11 |
| NOL8 | -0.045442089 | 0.13325289 | 0.011421073 | 0.7060493 | 0.06 |
| NOL9 | -0.102291876 | 0.00070702 | -0.25818021 | 4.20E-18 | 0.16 |
| NOLC1 | 0.330490194 | 2.88E-29 | 0.135006607 | 7.51E-06 | 0.2 |
| NOM1 | 0.085120461 | 0.00486224 | 0.024655876 | 0.4154571 | 0.07 |
| NOMO1 | 0.022313728 | 0.46115167 | -0.01862532 | 0.5384807 | 0.05 |
| NOMO2 | -0.082732905 | 0.00620478 | -0.190223411 | 2.30E-10 | 0.11 |
| NOMO3 | 0.08194661 | 0.00671494 | 0.040904244 | 0.1765881 | 0.05 |
| NRB54 | 0.325153055 | 2.48E-28 | 0.192522633 | 1.38E-10 | 0.14 |
| NOP10 | 0.428596919 | 4.62E-50 | 0.409875131 | 1.57E-45 | 0.02 |
| NOP14 | 0.11714617 | 0.00010366 | 0.015114844 | 0.6176654 | 0.11 |
| NOP16 | 0.592543155 | 1.41E-104 | 0.435387217 | 8.89E-52 | 0.16 |
| NOP2 | 0.578001737 | 1.97E-98 | 0.331527336 | 1.89E-29 | 0.25 |
| NOP56 | 0.540109157 | 8.65E-84 | 0.402746914 | 7.04E-44 | 0.14 |
| NOP58 | 0.620547387 | 2.46E-117 | 0.485757534 | 8.61E-66 | 0.14 |
| NOS1AP | -0.336033182 | 2.94E-30 | -0.18417681 | 8.54E-10 | 0.16 |
| NOS1 | -0.11656543 | 0.00011224 | -0.198682076 | 3.42E-11 | 0.09 |
| NOS2 | 0.022661017 | 0.45420411 | -0.085903415 | 0.0044829 | 0.11 |
| NOS3L | -0.164571296 | 4.45E-08 | -0.192541043 | 1.38E-10 | 0.03 |
| NOSIP | 0.231642968 | 8.82E-15 | 0.186468117 | 5.22E-10 | 0.05 |
| NOSTRIN | -0.505033195 | 8.34E-72 | -0.15569879 | 2.30E-07 | 0.35 |
| FLJ20005 | 0.044162854 | 0.14454048 | -0.133270506 | 9.84E-06 | 0.18 |
| NOTCH2NL | 0.026701485 | 0.37782389 | 0.174222544 | 6.72E-09 | 0.15 |
| NOTCH2 | -0.357772493 | 2.39E-34 | -0.314044232 | 1.92E-26 | 0.05 |
| CADASIL | -0.149000449 | 7.50E-07 | -0.083902093 | 0.0055105 | 0.07 |
| NOTCH4 | -0.406706279 | 8.62E-45 | -0.287583096 | 2.94E-22 | 0.12 |
| NOTO | 0.005986303 | 0.84329091 | -0.013588952 | 0.6536013 | 0.02 |
| NOTUM | 0.162898396 | 6.11E-08 | 0.042005789 | 0.1652107 | 0.13 |
| NOVA1 | -0.283606931 | 1.15E-21 | -0.221022621 | 1.46E-13 | 0.07 |
| NOVA2 | -0.101068199 | 0.00081951 | -0.059846356 | 0.0479216 | 0.05 |
| NOV | -0.161843648 | 7.45E-08 | -0.185677226 | 6.19E-10 | 0.03 |
| MOX1 | 0.146449559 | 1.16E-06 | 0.214171524 | 8.32E-13 | 0.07 |
| GP91.3 | -0.017294769 | 0.56788913 | -0.020694301 | 0.4943192 | 0.01 |
| NOX4 | -0.175680285 | 5.01E-09 | -0.02813745 | 0.352703 | 0.15 |
| NOX5 | -0.034971421 | 0.24800655 | -0.07026821 | 0.020162 | 0.04 |
| NOXA1 | -0.127176695 | 2.48E-05 | 0.04130475 | 0.1723858 | 0.17 |
| NOXO1 | 0.337802936 | 1.40E-30 | 0.155357872 | 2.45E-07 | 0.19 |
| NPAS1 | -0.214268089 | 8.13E-13 | -0.066868377 | 0.0270601 | 0.15 |
| NPAS2 | -0.153686982 | 3.30E-07 | -0.065751462 | 0.0297318 | 0.09 |
| NPAS3 | -0.069004377 | 0.02252289 | -0.194552419 | 8.77E-11 | 0.13 |
| NPAS4 | -0.02753534 | 0.3631051 | -0.14639465 | 1.17E-06 | 0.12 |
| E14 | -0.28532669 | 6.39E-22 | -0.307040465 | 2.72E-25 | 0.03 |
| NPBWR1 | 0.105663621 | 0.00046683 | -0.065170838 | 0.0312081 | 0.18 |
| NPBWR2 | 0.001248041 | 0.9671255 | -0.023698774 | 0.4337986 | 0.03 |
| NPB | 0.252956039 | 2.04E-17 | 0.065112474 | 0.0313599 | 0.19 |
| NPC1L1 | -0.075005183 | 0.01312462 | -0.051815029 | 0.0868555 | 0.03 |
| NPC1 | 0.142884366 | 2.11E-06 | -0.228877769 | 1.86E-14 | 0.38 |
| NPC2 | 0.137684117 | 4.91E-06 | 0.119288083 | 7.71E-05 | 0.02 |
| DKFZP586J0523 | -0.195984449 | 6.34E-11 | -0.008808308 | 0.7711432 | 0.19 |
| NPEPL1 | 0.104005317 | 0.00057343 | 0.027271461 | 0.3677237 | 0.08 |
| NPEPPS | -0.228794049 | 1.90E-14 | -0.150123927 | 6.17E-07 | 0.08 |
| NPFFR1 | 0.031979301 | 0.29082492 | -0.128125124 | 2.15E-05 | 0.17 |
| NPGPR | -0.192777644 | 1.31E-10 | -0.007698678 | 0.799314 | 0.19 |
| NPFF | 0.063017156 | 0.03724497 | 0.144619084 | 1.58E-06 | 0.09 |
| NPHP1 | -0.146105647 | 1.23E-06 | 0.028914043 | 0.3395676 | 0.18 |
| NPHP3 | -0.360806179 | 6.05E-35 | -0.285660595 | 5.70E-22 | 0.08 |
| NPHP4 | -0.143131507 | 2.02E-06 | -0.18605083 | 5.71E-10 | 0.05 |
| NPHS1 | 0.033995513 | 0.26145919 | -0.077276367 | 0.0105975 | 0.12 |
| NPHS2 | -0.027842726 | 0.35777093 | -0.003050878 | 0.9197501 | 0.03 |
| NPIPB3 | 0.194504065 | 8.87E-11 | 0.216135258 | 5.09E-13 | 0.03 |
| LOC220542 | -0.025993719 | 0.39060076 | -0.105938853 | 0.000451 | 0.08 |
| NPLOC4 | 0.240849292 | 6.88E-16 | -0.013392662 | 0.6582852 | 0.26 |
| NPL | 0.069168729 | 0.02220289 | -0.0210047 | 0.4878648 | 0.1 |
| NPM1 | 0.402703786 | 7.20E-44 | 0.346673631 | 3.21E-32 | 0.06 |
| LOC286056 | 0.253358362 | 1.80E-17 | 0.05877831 | 0.0520525 | 0.2 |
| LOC220476 | 0.502979357 | 3.80E-71 | 0.356182396 | 4.88E-34 | 0.15 |
| NPNT | -0.364267483 | 1.24E-35 | -0.100147954 | 0.0009148 | 0.27 |
| LOC90230 | -0.082125329 | 0.00659579 | -0.081215544 | 0.0072227 | 0.01 |
| NPPB | 0.079452009 | 0.00859196 | -0.019137822 | 0.5273602 | 0.1 |
| NPPC | 0.146796762 | 1.09E-06 | -0.013045542 | 0.666601 | 0.16 |
| NPR1 | -0.232260627 | 7.46E-15 | -0.22136707 | 1.34E-13 | 0.02 |
| NPR2 | -0.218554829 | 2.76E-13 | -0.223884825 | 6.96E-14 | 0.01 |
| C5orf23.1 | 0.057842881 | 0.05591082 | -0.005264974 | 0.8619716 | 0.07 |
| NPRL2 | 0.019746314 | 0.51431046 | 0.177074611 | 3.77E-09 | 0.16 |
| NPRL3 | 0.178615323 | 2.74E-09 | 0.166359655 | 3.16E-08 | 0.02 |
| NPSR1 | 0.041205707 | 0.17341803 | -0.035838834 | 0.2364621 | 0.08 |
| NPS | -0.009743048 | 0.74764242 | -0.003244727 | 0.91467 | 0.01 |
| NPTN | -0.314061994 | 1.91E-26 | -0.232112895 | 7.76E-15 | 0.09 |
| NPTX1 | -0.03940426 | 0.19300478 | -0.173770557 | 7.36E-09 | 0.14 |
| NPTX2 | -0.028485887 | 0.34677024 | -0.059579483 | 0.0489271 | 0.04 |
| NPTXR | -0.176257643 | 4.45E-09 | -0.258023613 | 4.40E-18 | 0.09 |
| NPVF | 0.050040831 | 0.098225 | 0.029209984 | 0.3346455 | 0.03 |
| NPW | 0.259747187 | 2.60E-18 | 0.142141719 | 2.38E-06 | 0.12 |
| NPY1R | -0.105295764 | 0.00048875 | -0.07779725 | 0.0100828 | 0.03 |
| NPY2R | -0.263964807 | 7.01E-19 | -0.223526657 | 7.65E-14 | 0.05 |
| NPY5R | -0.033694638 | 0.26570642 | -0.099200292 | 0.0010236 | 0.07 |
| LOC100420029 | -0.180315625 | 1.93E-09 | -0.183045134 | 1.09E-09 | 0.01 |
| NPY | 0.015310536 | 0.61311978 | -0.079063183 | 0.0089234 | 0.1 |
| NQO1 | -0.060897242 | 0.04412926 | 0.12641747 | 2.77E-05 | 0.19 |
| NQO2 | 0.227462337 | 2.71E-14 | 0.216838248 | 4.26E-13 | 0.02 |
| DSS | 0.10218662 | 0.0007161 | -0.021427995 | 0.4791366 | 0.13 |
| NR0B2 | 0.059623108 | 0.04876156 | -0.077039128 | 0.0108396 | 0.14 |
| LOC115736 | -0.089807618 | 0.00296157 | -0.052139071 | 0.0848983 | 0.04 |
| NR1D2 | -0.221236234 | 1.39E-13 | -0.268625262 | 1.61E-19 | 0.05 |
| NR1H2 | 0.030936361 | 0.30685313 | 0.066632771 | 0.0276058 | 0.04 |
| NR1H3 | 0.078758112 | 0.00919134 | 0.072191048 | 0.0169837 | 0.01 |
| NR1H4 | 0.089955745 | 0.00291445 | -0.010711077 | 0.7235494 | 0.11 |
| NR1I2 | 0.16570047 | 3.59E-08 | 0.03264258 | 0.2809293 | 0.14 |
| NR1I3 | 0.29965396 | 4.11E-24 | 0.368523889 | 1.72E-36 | 0.07 |
| NR2C1 | 0.029336261 | 0.3325593 | -0.163338059 | 5.62E-08 | 0.2 |
| NR2C2AP | 0.526434639 | 5.98E-79 | 0.434125284 | 1.87E-51 | 0.1 |
| NR2C2 | -0.346979462 | 2.81E-32 | -0.385767403 | 4.19E-40 | 0.04 |
| NR2E1 | 0.319593186 | 2.24E-27 | 0.063146573 | 0.036856 | 0.26 |
| NR2E3 | -0.252521653 | 2.32E-17 | -0.124509008 | 3.66E-05 | 0.13 |
| NR2F1 | -0.335689845 | 3.39E-30 | -0.202617618 | 1.37E-11 | 0.14 |
| NR2F2 | -0.298207053 | 6.93E-24 | -0.096711167 | 0.0013687 | 0.21 |
| NR2F6 | 0.27443176 | 2.45E-20 | 0.326191102 | 1.64E-28 | 0.06 |
| NR3C1 | -0.349209558 | 1.07E-32 | -0.398210922 | 7.54E-43 | 0.05 |
| NR3C2 | -0.26992131 | 1.06E-19 | -0.293427325 | 3.82E-23 | 0.03 |
| LOC115357 | -0.081934135 | 0.00672333 | -0.079647383 | 0.0084296 | 0.01 |
| NR4A2 | -0.327953667 | 8.06E-29 | -0.215603798 | 5.82E-13 | 0.12 |
| NR4A3 | -0.094881365 | 0.00168759 | -0.192127399 | 1.51E-10 | 0.1 |
| NR5A1 | 0.138293868 | 4.46E-06 | -0.028496743 | 0.3465864 | 0.17 |
| CPF | -0.325229228 | 2.41E-28 | -0.276901004 | 1.09E-20 | 0.05 |
| NR6A1 | -0.09097055 | 0.00260956 | -0.169532575 | 1.71E-08 | 0.08 |
| NRADDP | 0.160510724 | 9.55E-08 | 0.16437422 | 4.62E-08 | 0.01 |
| NRAP | -0.004745982 | 0.87546071 | 0.014777088 | 0.6255454 | 0.02 |
| NRARP | 0.207418557 | 4.36E-12 | 0.121755263 | 5.44E-05 | 0.09 |
| NRAS | 0.059727777 | 0.04836624 | -0.07336144 | 0.015272 | 0.14 |
| NRBF2 | 0.16849717 | 2.09E-08 | 0.033810123 | 0.2640706 | 0.14 |
| NRBP1 | 0.206649666 | 5.25E-12 | 0.096405141 | 0.0014178 | 0.12 |
| NRBP2 | 0.162600485 | 6.46E-08 | -0.04809065 | 0.1120596 | 0.22 |
| NRCAM | -0.084336843 | 0.00527074 | -0.192572492 | 1.37E-10 | 0.11 |
| NRD1 | 0.247460265 | 1.03E-16 | 0.056631281 | 0.061259 | 0.2 |
| NRF1 | 0.074968126 | 0.01316993 | 0.096650515 | 0.0013783 | 0.03 |
| NRG1.IT2 | -0.147545151 | 9.62E-07 | -0.111010002 | 0.0002358 | 0.04 |
| NRG2 | 0.068369949 | 0.023796 | -0.104278539 | 0.0005544 | 0.18 |
| NRG3 | 0.091222036 | 0.00253865 | 0.072048071 | 0.0172039 | 0.02 |
| NRG4 | 0.223356173 | 8.00E-14 | 0.165627314 | 3.64E-08 | 0.06 |
| NRGN | 0.174631021 | 6.19E-09 | 0.180111347 | 2.01E-09 | 0.01 |
| NRIP1 | -0.4052736 | 1.85E-44 | -0.297272172 | 9.70E-24 | 0.11 |
| NRIP2 | -0.357165166 | 3.14E-34 | -0.24999285 | 4.90E-17 | 0.11 |
| NRIP3 | -0.151912926 | 4.51E-07 | -0.167877794 | 2.36E-08 | 0.02 |
| NRK | -0.312402929 | 3.60E-26 | -0.100492356 | 0.000878 | 0.22 |
| NRL | -0.022478223 | 0.45785357 | 0.010490598 | 0.7290139 | 0.04 |
| NRM | 0.451484944 | 5.28E-56 | 0.267942132 | 2.00E-19 | 0.19 |
| NRN1L | -0.008841694 | 0.77030001 | -0.015508628 | 0.6085335 | 0.01 |
| NRN1 | -0.011766186 | 0.69759802 | -0.088943847 | 0.0032505 | 0.08 |
| NRP1 | -0.344338803 | 8.77E-32 | -0.290457898 | 1.08E-22 | 0.06 |
| PRO2714 | -0.178743179 | 2.67E-09 | -0.241819206 | 5.23E-16 | 0.07 |
| NRSN1 | -0.016754748 | 0.58004005 | -0.09569517 | 0.0015382 | 0.08 |
| NRSN2 | -0.023062912 | 0.44623822 | 0.091263572 | 0.0025271 | 0.12 |
| NRTN | 0.432304238 | 5.40E-51 | 0.201645106 | 1.72E-11 | 0.24 |
| NRXN1 | -0.079285181 | 0.00873279 | -0.150913466 | 5.38E-07 | 0.08 |
| NRXN2 | -0.210810327 | 1.91E-12 | -0.180372702 | 1.90E-09 | 0.04 |
| NRXN3 | -0.358008368 | 2.15E-34 | -0.263634228 | 7.78E-19 | 0.1 |
| NSA2 | -0.0254182 | 0.40118106 | 0.132154727 | 1.17E-05 | 0.16 |
| STO | -0.292083381 | 6.13E-23 | -0.329893126 | 3.67E-29 | 0.04 |
| NSDHL | 0.390126025 | 4.72E-41 | 0.270351816 | 9.23E-20 | 0.12 |
| p47 | 0.230000003 | 1.37E-14 | 0.185870463 | 5.94E-10 | 0.05 |
| NSF | -0.233405714 | 5.46E-15 | -0.128891437 | 1.92E-05 | 0.11 |
| NSL1 | -0.102679728 | 0.00067447 | 0.317624068 | 4.83E-27 | 0.43 |
| NSMAF | 0.218045485 | 3.14E-13 | 0.032536237 | 0.2825003 | 0.19 |
| NSMCE1 | 0.092426514 | 0.00222282 | 0.29575489 | 1.67E-23 | 0.21 |
| NSMCE2 | 0.439718594 | 6.83E-53 | 0.309886242 | 9.34E-26 | 0.13 |
| NSMCE4A | 0.129594271 | 1.73E-05 | 0.065299775 | 0.030875 | 0.07 |
| NSUN2 | 0.311505946 | 5.06E-26 | 0.099974769 | 0.0009338 | 0.22 |
| NSUN3 | -0.154206804 | 3.01E-07 | -0.10653503 | 0.0004185 | 0.05 |
| NSUN4 | 0.02192521 | 0.46899382 | -0.051923405 | 0.0861969 | 0.08 |
| LOC222178 | 0.222151147 | 1.09E-13 | 0.176749978 | 4.03E-09 | 0.05 |
| NSUN5P2 | 0.196266804 | 5.95E-11 | 0.129590487 | 1.73E-05 | 0.07 |
| NSUN5 | 0.375286754 | 6.97E-38 | 0.362347813 | 2.99E-35 | 0.02 |
| NSUN6 | 0.06660817 | 0.02766327 | 0.06896383 | 0.0226024 | 0.01 |
| NSUN7 | -0.063521226 | 0.03574956 | -0.001966675 | 0.9482178 | 0.07 |
| NT5C1A | -0.079616523 | 0.00845508 | -0.114444551 | 0.0001496 | 0.04 |
| NT5C1B | -0.067119605 | 0.02648845 | -0.090911601 | 0.0026264 | 0.03 |
| NT5C2 | 0.181675177 | 1.45E-09 | 0.000702543 | 0.9814908 | 0.19 |
| NT5C3B | 0.292308519 | 5.66E-23 | 0.208223982 | 3.59E-12 | 0.09 |
| NT5C3A | 0.216401321 | 4.76E-13 | 0.213513917 | 9.80E-13 | 0.01 |
| UMPH2 | 0.247490107 | 1.02E-16 | 0.139180664 | 3.86E-06 | 0.11 |
| NT5DC1 | -0.256672658 | 6.65E-18 | -0.085805173 | 0.004529 | 0.18 |
| NT5DC2 | 0.426952992 | 1.19E-49 | 0.218666121 | 2.68E-13 | 0.21 |
| NT5DC3 | 0.086117126 | 0.00438409 | -0.035093686 | 0.2463559 | 0.13 |
| NT5E | -0.148700074 | 7.89E-07 | -0.190569885 | 2.13E-10 | 0.05 |
| NT5M | 0.179994082 | 2.06E-09 | 0.116755647 | 0.0001094 | 0.07 |
| NTAN1 | 0.314236891 | 1.78E-26 | 0.309895961 | 9.30E-26 | 0.01 |
| NTF3 | -0.031117819 | 0.30402322 | -0.066874653 | 0.0270457 | 0.04 |
| NTF4 | -0.057381841 | 0.05789828 | -0.096721973 | 0.001367 | 0.04 |
| NTHL1 | 0.296401439 | 1.32E-23 | 0.324214797 | 3.61E-28 | 0.03 |
| NTM | -0.259889366 | 2.49E-18 | -0.095615996 | 0.0015521 | 0.17 |
| NTN1 | 0.002657065 | 0.9300804 | -0.069966818 | 0.0207045 | 0.08 |
| NTN3 | -0.03785658 | 0.21108938 | -0.123424452 | 4.29E-05 | 0.09 |
| PRO3091 | -0.494022223 | 2.53E-68 | -0.1991045 | 3.10E-11 | 0.3 |
| NTN5 | -0.097128334 | 0.00130424 | -0.236682831 | 2.21E-15 | 0.14 |
| NTNG1 | -0.134948425 | 7.57E-06 | 0.003324228 | 0.9125875 | 0.14 |
| NTNG1.1 | -0.134948425 | 7.57E-06 | 0.003324228 | 0.9125875 | 0.14 |
| TRK | -0.113660299 | 0.00016616 | -0.126835993 | 2.61E-05 | 0.02 |
| NTRK2 | -0.296462095 | 1.30E-23 | -0.341953816 | 2.43E-31 | 0.05 |
| NTRK3 | -0.069512942 | 0.02154546 | -0.134044781 | 8.72E-06 | 0.07 |
| NTSR1 | 0.184337138 | 8.25E-10 | 0.060259315 | 0.0464 | 0.13 |
| NTSR2 | 0.205823352 | 6.40E-12 | 0.055271106 | 0.0677601 | 0.16 |
| NTS | -0.072866227 | 0.01597682 | -0.170500819 | 1.41E-08 | 0.1 |
| LOC732183 | -0.424175899 | 5.76E-49 | -0.274047992 | 2.78E-20 | 0.16 |
| NUAK2 | -0.131626504 | 1.27E-05 | 0.056744206 | 0.0607433 | 0.19 |
| NUB1 | 0.026560239 | 0.38035296 | -0.080389814 | 0.0078374 | 0.11 |
| NUBP1 | 0.096857391 | 0.00134578 | 0.184354391 | 8.22E-10 | 0.09 |
| NUBP2 | 0.189451284 | 2.73E-10 | 0.208506392 | 3.35E-12 | 0.02 |
| NUBPL | -0.272136639 | 5.18E-20 | -0.092896598 | 0.0021096 | 0.18 |
| NUCB1 | -0.02431704 | 0.42189753 | -0.058606033 | 0.0527459 | 0.04 |
| NUCB2 | -0.170792908 | 1.33E-08 | -0.065237856 | 0.0310346 | 0.11 |
| LOC100130123 | 0.107513584 | 0.00036984 | 0.431489105 | 8.68E-51 | 0.33 |
| NUDCD1 | 0.395196049 | 3.58E-42 | 0.181469153 | 1.51E-09 | 0.22 |
| NUDCD2 | 0.302389596 | 1.52E-24 | 0.301173992 | 2.36E-24 | 0.01 |
| NUDCD3 | -0.210193199 | 2.22E-12 | -0.088138779 | 0.0035426 | 0.13 |
| NUDC | 0.364915839 | 9.18E-36 | 0.238059221 | 1.51E-15 | 0.13 |
| LOC139770 | -0.129574402 | 1.73E-05 | -0.082014283 | 0.0066696 | 0.05 |
| NUDT11 | 0.08064776 | 0.00764055 | -0.027651439 | 0.3610846 | 0.11 |
| NUDT12 | -0.376649399 | 3.62E-38 | -0.143681005 | 1.85E-06 | 0.24 |
| LOC51055 | -0.16888114 | 1.94E-08 | 0.004475583 | 0.8825032 | 0.18 |
| NUDT14 | 0.116183659 | 0.00011824 | 0.147261666 | 1.01E-06 | 0.04 |
| NUDT15 | 0.311428783 | 5.21E-26 | 0.165469319 | 3.75E-08 | 0.15 |
| NUDT16L1 | -0.021279141 | 0.48219616 | 0.122482924 | 4.91E-05 | 0.15 |
| NUDT16P1 | -0.263364198 | 8.46E-19 | -0.205086871 | 7.63E-12 | 0.06 |
| NUDT16 | -0.405939425 | 1.30E-44 | -0.209085654 | 2.91E-12 | 0.2 |
| LOC101060409 | -0.040545495 | 0.18041661 | 0.079894578 | 0.0082282 | 0.13 |
| NUDT18 | -0.175721415 | 4.96E-09 | 0.001123679 | 0.9703997 | 0.18 |
| NUDT19 | 0.289195582 | 1.68E-22 | 0.185971048 | 5.81E-10 | 0.11 |
| LOC115206 | 0.525039058 | 1.81E-78 | 0.415587451 | 6.98E-47 | 0.11 |
| NUDT21 | 0.106865709 | 0.00040144 | -0.102919844 | 0.000655 | 0.21 |
| NUDT22 | 0.025908729 | 0.39215246 | 0.0897372 | 0.0029842 | 0.07 |
| NUDT2 | 0.066411924 | 0.02812584 | 0.195759214 | 6.68E-11 | 0.13 |
| DIPP | 0.431427801 | 9.00E-51 | 0.343576558 | 1.22E-31 | 0.09 |
| NUDT4 | -0.297562189 | 8.74E-24 | -0.10277562 | 0.0006666 | 0.2 |
| NUDT5 | 0.590893018 | 7.29E-104 | 0.394537863 | 5.01E-42 | 0.2 |
| NUDT6 | -0.05388088 | 0.07497998 | 0.043386642 | 0.1517379 | 0.1 |
| NUDT7 | 0.011237375 | 0.71056275 | 0.004185756 | 0.8900621 | 0.01 |
| NUDT8 | 0.181208272 | 1.60E-09 | 0.152175085 | 4.31E-07 | 0.03 |
| NUDT9P1 | 0.015993003 | 0.59738396 | -0.143733723 | 1.83E-06 | 0.16 |
| NUDT9 | 0.029915572 | 0.32309645 | 0.078971357 | 0.0090033 | 0.05 |
| NUF2 | 0.52112702 | 3.95E-77 | 0.40203685 | 1.02E-43 | 0.12 |
| NUFIP1 | 0.216244174 | 4.95E-13 | 0.146151782 | 1.22E-06 | 0.08 |
| NUFIP2 | -0.169635128 | 1.67E-08 | -0.094868154 | 0.0016901 | 0.08 |
| NUMA1 | -0.42272903 | 1.31E-48 | -0.301407248 | 2.17E-24 | 0.13 |
| NUMBL | 0.174886408 | 5.88E-09 | 0.056605651 | 0.0613766 | 0.12 |
| NUMB | -0.385155995 | 5.67E-40 | -0.270645562 | 8.39E-20 | 0.12 |
| NUP107 | 0.285770178 | 5.49E-22 | 0.193745813 | 1.05E-10 | 0.1 |
| NUP133 | -0.099163927 | 0.00102797 | 0.234785899 | 3.74E-15 | 0.34 |
| NUP153 | -0.04553452 | 0.13246448 | -0.155125425 | 2.55E-07 | 0.11 |
| NUP155 | 0.004142194 | 0.89119908 | -0.069305683 | 0.0219393 | 0.08 |
| FLJ22583 | 0.068515213 | 0.02349912 | -0.106709647 | 0.0004094 | 0.18 |
| NUP188 | 0.049484245 | 0.10202679 | -0.117373593 | 0.0001005 | 0.17 |
| NUP205 | 0.169813186 | 1.62E-08 | -0.026861327 | 0.3749744 | 0.2 |
| NUP210L | -0.097275931 | 0.00128211 | -0.040253573 | 0.1835771 | 0.06 |
| FLJ22389 | 0.078518124 | 0.00940715 | -0.038034477 | 0.2089505 | 0.12 |
| NUP214 | -0.232671598 | 6.67E-15 | -0.193787021 | 1.04E-10 | 0.04 |
| NUP35 | 0.301756264 | 1.91E-24 | 0.266616544 | 3.04E-19 | 0.04 |
| NUP37 | 0.454741714 | 6.92E-57 | 0.352465549 | 2.55E-33 | 0.11 |
| NUP43 | 0.012957737 | 0.66871105 | 0.126376544 | 2.79E-05 | 0.12 |
| NUP50 | 0.048141437 | 0.1116808 | -0.050972231 | 0.0921166 | 0.1 |
| NUP54 | 0.147537977 | 9.64E-07 | 0.049218189 | 0.103885 | 0.1 |
| NUP62CL | -0.0495962 | 0.10125282 | 0.013507527 | 0.6555426 | 0.07 |
| DKFZp547L134 | 0.253415419 | 1.77E-17 | 0.053235635 | 0.0785379 | 0.21 |
| NUP85 | 0.466854261 | 2.96E-60 | 0.267472838 | 2.32E-19 | 0.2 |
| NUP88 | 0.263638267 | 7.77E-19 | 0.087222305 | 0.003904 | 0.18 |
| NUP93 | 0.459540867 | 3.32E-58 | 0.170437515 | 1.43E-08 | 0.29 |
| NUP98 | -0.170433496 | 1.43E-08 | -0.203144532 | 1.21E-11 | 0.04 |
| PRO2463 | -0.088619707 | 0.00336532 | -0.144519581 | 1.61E-06 | 0.06 |
| NUPL2 | 0.23985341 | 9.11E-16 | 0.201709123 | 1.69E-11 | 0.04 |
| NUPR1 | 0.135084654 | 7.41E-06 | 0.153193149 | 3.60E-07 | 0.02 |
| NUS1 | 0.223707631 | 7.29E-14 | 0.104374578 | 0.0005479 | 0.12 |
| BM037 | 0.412573261 | 3.64E-46 | 0.216097612 | 5.14E-13 | 0.2 |
| NUTF2 | 0.583057348 | 1.56E-100 | 0.404849927 | 2.31E-44 | 0.18 |
| NVL | 0.00188665 | 0.95032203 | 0.383163868 | 1.52E-39 | 0.39 |
| NWD1 | -0.010854257 | 0.72000832 | -0.032789252 | 0.2787722 | 0.03 |
| NXF1 | -0.021535505 | 0.47693337 | -0.05753842 | 0.0572168 | 0.04 |
| NXF2B | 0.049011828 | 0.10534462 | 0.041798375 | 0.1673098 | 0.01 |
| LOC100130328 | 0.12864008 | 1.99E-05 | 0.057887841 | 0.0557201 | 0.08 |
| NXF3 | -0.03882972 | 0.19958081 | -0.002621789 | 0.9310064 | 0.04 |
| NXF4 | 0.088732955 | 0.00332478 | -0.008408301 | 0.7812658 | 0.1 |
| NXF5 | -0.100966578 | 0.00082957 | -0.008722068 | 0.7733223 | 0.1 |
| NXNL1 | -0.156778655 | 1.89E-07 | -0.014413482 | 0.6340764 | 0.15 |
| NXNL2 | -0.355324532 | 7.16E-34 | -0.1123168 | 0.0001986 | 0.25 |
| NXN | 0.193886699 | 1.02E-10 | 0.065121406 | 0.0313366 | 0.13 |
| FLJ34336 | -0.234698758 | 3.83E-15 | -0.032816439 | 0.2783736 | 0.21 |
| NXPH2 | 0.046631711 | 0.12337906 | 0.043203339 | 0.1534767 | 0.01 |
| NXPH3 | -0.266360337 | 3.30E-19 | -0.216787235 | 4.32E-13 | 0.05 |
| NXPH4 | 0.243318535 | 3.41E-16 | 0.124073082 | 3.90E-05 | 0.12 |
| NXT1 | 0.547570927 | 1.59E-86 | 0.431772061 | 7.37E-51 | 0.12 |
| NXT2 | 0.13749901 | 5.06E-06 | 0.054863269 | 0.0698164 | 0.09 |
| NYNRIN | -0.059710566 | 0.04843106 | -0.154425074 | 2.89E-07 | 0.1 |
| NYX | 0.065525458 | 0.03029918 | 0.043323366 | 0.1523364 | 0.03 |
| OAF | -0.099011128 | 0.00104666 | -0.073186678 | 0.0155176 | 0.03 |
| OAS1 | 0.041277761 | 0.17266666 | -0.010037675 | 0.7402822 | 0.06 |
| OAS2 | -0.004969954 | 0.86963479 | -0.03385203 | 0.2634787 | 0.03 |
| OAS3 | -0.028162275 | 0.35227818 | -0.143544965 | 1.89E-06 | 0.12 |
| OASL | 0.182065865 | 1.34E-09 | 0.074778824 | 0.0134035 | 0.11 |
| OAT | 0.002351502 | 0.938104 | 0.009183332 | 0.7616879 | 0.01 |
| OAZ1 | 0.212801271 | 1.17E-12 | 0.232678244 | 6.66E-15 | 0.02 |
| OAZ2 | -0.017405487 | 0.56541297 | -0.10420015 | 0.0005598 | 0.09 |
| OAZ3 | 0.005360941 | 0.85948159 | 0.295056521 | 2.14E-23 | 0.29 |
| OBFC1 | -0.289090741 | 1.75E-22 | -0.143581542 | 1.88E-06 | 0.15 |
| NABP1 | 0.081300996 | 0.00716164 | 0.022292371 | 0.4615809 | 0.06 |
| NABP2 | 0.370813602 | 5.85E-37 | 0.397393972 | 1.15E-42 | 0.03 |
| OBP2A | 0.050358561 | 0.09610592 | 0.170380692 | 1.45E-08 | 0.13 |
| OBP2B | 0.09265585 | 0.00216692 | 0.202921291 | 1.27E-11 | 0.12 |
| OBSCN | -0.0532155 | 0.07865112 | -0.044352018 | 0.1428267 | 0.01 |
| OBSL1 | 0.148347313 | 8.39E-07 | 0.010696535 | 0.7239094 | 0.14 |
| OC90 | 0.183512274 | 9.84E-10 | 0.166828101 | 2.89E-08 | 0.02 |
| HCL3 | 0.295979311 | 1.54E-23 | 0.072688499 | 0.0162367 | 0.23 |
| OCEL1 | -0.141010773 | 2.87E-06 | 0.03354152 | 0.267886 | 0.18 |
| OCIAD1 | -0.045155257 | 0.13572261 | 0.058616024 | 0.0527054 | 0.11 |
| OCIAD2 | 0.408411373 | 3.46E-45 | 0.374234298 | 1.15E-37 | 0.04 |
| OCLM | -0.296330129 | 1.36E-23 | -0.115094737 | 0.0001371 | 0.19 |
| OCLN | -0.26303647 | 9.37E-19 | -0.153403304 | 3.47E-07 | 0.11 |
| OCM2 | 0.010722457 | 0.72326779 | 0.0381062 | 0.2080926 | 0.03 |
| LOC115566 | -0.002173649 | 0.94277707 | 0.04486569 | 0.1382515 | 0.05 |
| OCRL | -0.126846598 | 2.60E-05 | -0.116636874 | 0.0001112 | 0.02 |
| ODAM | 0.225763395 | 4.25E-14 | 0.039147778 | 0.1959205 | 0.19 |
| ODC1 | 0.333228319 | 9.38E-30 | 0.095565908 | 0.0015611 | 0.24 |
| ODF1 | 0.040519874 | 0.18069237 | -0.015769159 | 0.602525 | 0.06 |
| ODF2L | 0.078945218 | 0.00902616 | 0.044899075 | 0.1379581 | 0.04 |
| ODF2 | 0.018721776 | 0.5363788 | 0.081933222 | 0.0067239 | 0.07 |
| ODF3B | 0.023253694 | 0.44248474 | 0.057208932 | 0.0586586 | 0.04 |
| ODF3L1 | -0.06199591 | 0.04043815 | -0.063759545 | 0.0350605 | 0.01 |
| ODF3L2 | 0.022466188 | 0.45809442 | -0.01201603 | 0.6915029 | 0.04 |
| ODF3 | -0.056857175 | 0.060231 | -0.065142402 | 0.031282 | 0.01 |
| ODF4 | 0.049753069 | 0.10017622 | 0.096110708 | 0.0014666 | 0.05 |
| TENM1 | -0.1473914 | 9.88E-07 | -0.206266657 | 5.75E-12 | 0.06 |
| TENM2 | -0.221018041 | 1.47E-13 | -0.173332742 | 8.04E-09 | 0.05 |
| TENM3 | -0.322415383 | 7.38E-28 | -0.263149736 | 9.05E-19 | 0.06 |
| TENM4 | -0.33969483 | 6.33E-31 | -0.258088025 | 4.32E-18 | 0.09 |
| SGBS2 | 0.011881903 | 0.69477257 | 0.007057768 | 0.8157073 | 0.01 |
| OGDHL | 0.229381224 | 1.62E-14 | 0.001054823 | 0.9722128 | 0.23 |
| LOC135860 | -0.145150622 | 1.44E-06 | -0.246009541 | 1.57E-16 | 0.11 |
| OGFOD1 | 0.167604225 | 2.49E-08 | -0.025695674 | 0.3960587 | 0.2 |
| OGFOD2 | 0.069002856 | 0.02252587 | 0.12188087 | 5.35E-05 | 0.06 |
| OGFRL1 | 0.153383343 | 3.48E-07 | -0.022672839 | 0.4539687 | 0.18 |
| LOC51781 | 0.151637578 | 4.74E-07 | 0.082097465 | 0.0066142 | 0.07 |
| LOC115798 | 0.138260207 | 4.48E-06 | 0.038944908 | 0.1982495 | 0.1 |
| OGN | -0.451829616 | 4.26E-56 | -0.206145748 | 5.92E-12 | 0.25 |
| OGT | -0.189780369 | 2.54E-10 | -0.162955512 | 6.04E-08 | 0.03 |
| OIP5 | 0.539551873 | 1.38E-83 | 0.313627351 | 2.25E-26 | 0.23 |
| OIT3 | 0.094932458 | 0.00167783 | -0.024581445 | 0.4168669 | 0.12 |
| LOC89690 | 0.252536723 | 2.31E-17 | 0.234561096 | 3.98E-15 | 0.02 |
| OLAH | 0.292318967 | 5.64E-23 | 0.058612362 | 0.0527203 | 0.24 |
| NOE1 | -0.205767048 | 6.48E-12 | -0.189783995 | 2.54E-10 | 0.02 |
| OLFM2 | 0.159821866 | 1.08E-07 | 0.107909385 | 0.0003517 | 0.06 |
| OLFM3 | 0.10787952 | 0.00035304 | -0.018160484 | 0.5486675 | 0.13 |
| OLFM4 | 0.016043981 | 0.59621592 | 0.029871081 | 0.3238169 | 0.02 |
| OLFML1 | -0.346974129 | 2.82E-32 | -0.223157212 | 8.42E-14 | 0.13 |
| OLFML2A | -0.301091391 | 2.44E-24 | -0.235981465 | 2.69E-15 | 0.07 |
| OLFML2B | -0.271036584 | 7.40E-20 | -0.166364039 | 3.16E-08 | 0.11 |
| OLFML3 | -0.307929799 | 1.95E-25 | -0.160824542 | 9.01E-08 | 0.15 |
| OLIG1 | 0.198113778 | 3.90E-11 | 0.026269002 | 0.3856004 | 0.18 |
| OLIG2 | 0.247079942 | 1.15E-16 | 0.09067361 | 0.0026956 | 0.16 |
| OLIG3 | 0.039366248 | 0.19343488 | 0.06602552 | 0.0290561 | 0.03 |
| OLR1 | -0.126904296 | 2.58E-05 | -0.084041459 | 0.0054326 | 0.05 |
| OMA1 | -0.078952135 | 0.0090201 | 0.078984677 | 0.0089917 | 0.16 |
| OMD | -0.419025767 | 1.04E-47 | -0.184428315 | 8.09E-10 | 0.24 |
| OMG | -0.075135285 | 0.01296664 | -0.127190241 | 2.47E-05 | 0.06 |
| OMP | -0.022453442 | 0.45834959 | -0.083970218 | 0.0054723 | 0.07 |
| ONECUT1 | 0.024160025 | 0.4249017 | -0.028870162 | 0.3403013 | 0.06 |
| ONECUT2 | 0.212927866 | 1.13E-12 | 0.116365462 | 0.0001153 | 0.1 |
| ONECUT3 | 0.058282023 | 0.05407085 | 0.033322954 | 0.2710184 | 0.03 |
| LOC648473 | 0.096243449 | 0.00144445 | -0.024828253 | 0.4122031 | 0.13 |
| KIAA0567 | 0.001332684 | 0.96489732 | -0.091101453 | 0.0025724 | 0.1 |
| OPA3 | -0.099788235 | 0.00095477 | -0.178659748 | 2.72E-09 | 0.08 |
| OPALIN | 0.03230653 | 0.285914 | -0.008860879 | 0.7698156 | 0.05 |
| LOC100128095 | -0.224543502 | 5.86E-14 | -0.11801736 | 9.19E-05 | 0.11 |
| MRX60 | -0.274548931 | 2.36E-20 | -0.355098102 | 7.92E-34 | 0.09 |
| DKFZp434H244 | -0.02350814 | 0.43750692 | -0.004525636 | 0.8811989 | 0.02 |
| OPN1LW | 0.023369118 | 0.44022267 | 0.0121678 | 0.6878101 | 0.02 |
| OPN1MW | -0.019951804 | 0.50994173 | -0.02885627 | 0.3405338 | 0.01 |
| OPN1SW | 0.055153027 | 0.06835028 | -0.007212627 | 0.8117386 | 0.07 |
| OPN3 | 0.182240358 | 1.29E-09 | 0.226959264 | 3.10E-14 | 0.05 |
| OPN4 | -0.127228885 | 2.46E-05 | -0.067139141 | 0.0264444 | 0.07 |
| OPN5 | 0.103696877 | 0.00059559 | 0.029704408 | 0.3265253 | 0.08 |
| OPRD1 | 0.000922009 | 0.97571036 | -0.079542602 | 0.0085163 | 0.09 |
| OPRK1 | 0.339371053 | 7.25E-31 | 0.053225009 | 0.0785976 | 0.29 |
| OPRL1 | -0.158072391 | 1.50E-07 | -0.115063176 | 0.0001376 | 0.05 |
| OPRM1 | 0.016893176 | 0.57691372 | 0.011039636 | 0.7154325 | 0.01 |
| OPTC | -0.007127598 | 0.81391717 | 0.003345543 | 0.9120293 | 0.02 |
| GLC1E | 0.156868432 | 1.86E-07 | -0.087459927 | 0.0038072 | 0.25 |
| OR10A2 | -0.016185686 | 0.59297463 | 0.015764306 | 0.6026366 | 0.04 |
| OR10A3 | 0.115617451 | 0.0001277 | 0.015289106 | 0.6136169 | 0.11 |
| OR10A4 | -0.022890439 | 0.44964701 | -0.016706371 | 0.5811345 | 0.01 |
| OR10A1 | -0.015511721 | 0.60846201 | -0.037810712 | 0.2116434 | 0.03 |
| OR10A6 | -0.021282595 | 0.48212504 | -0.046321232 | 0.1258993 | 0.03 |
| OR10A7 | 0.003732921 | 0.90189199 | -0.012697244 | 0.6749863 | 0.02 |
| OR10AD1 | -0.08326933 | 0.00587702 | -0.151818821 | 4.59E-07 | 0.07 |
| OR10AG1 | 0.046589397 | 0.12372019 | 0.02275787 | 0.4522771 | 0.03 |
| OR10C2 | -0.014639457 | 0.62876871 | -0.009577484 | 0.7517886 | 0.01 |
| OR10G2 | -0.068164948 | 0.02422048 | -0.027207983 | 0.3688402 | 0.05 |
| OR10G3 | 0.006840869 | 0.82127395 | 0.00885521 | 0.7699587 | 0.01 |
| OR10G4 | -0.077526038 | 0.01034794 | -0.087820596 | 0.0036644 | 0.02 |
| OR10G7 | -0.036201275 | 0.23175253 | -0.056897706 | 0.0600481 | 0.03 |
| OR10G8 | -0.021494344 | 0.47777624 | -0.064675514 | 0.0325167 | 0.05 |
| OR10G9 | -0.026435781 | 0.38259003 | -0.052043744 | 0.0854703 | 0.03 |
| OR10H1 | -0.125285135 | 3.27E-05 | -0.039343767 | 0.1936896 | 0.09 |
| OR10H2 | -0.065503985 | 0.03035356 | -0.018495598 | 0.541314 | 0.05 |
| OR10H3 | 0.010010863 | 0.74095101 | 0.007847842 | 0.7955109 | 0.01 |
| OR10H4 | -0.008692453 | 0.77407108 | 0.008296555 | 0.7841004 | 0.02 |
| OR10H5 | -0.080264703 | 0.00793453 | -0.003022436 | 0.9204957 | 0.08 |
| OR10J1 | -0.006413353 | 0.83227201 | 0.015316405 | 0.6129837 | 0.03 |
| OR10J3 | 0.019006062 | 0.53020798 | 0.070575678 | 0.0196213 | 0.06 |
| OR10J5 | 0.010744615 | 0.72271945 | 0.01338441 | 0.6584824 | 0.01 |
| OR10K1 | 0.012704671 | 0.67480712 | 0.030039642 | 0.3210928 | 0.02 |
| OR10K2 | -0.019049409 | 0.52927024 | -0.023784846 | 0.4321303 | 0.01 |
| OR10P1 | 0.049821432 | 0.0997099 | 0.0093667 | 0.7570776 | 0.05 |
| OR10Q1 | 0.074963913 | 0.01317509 | 0.069905458 | 0.0208165 | 0.01 |
| OR10R2 | 0.047295853 | 0.11812028 | 0.038742834 | 0.2005893 | 0.01 |
| OR10S1 | 0.033794556 | 0.26429071 | -0.022407258 | 0.4592748 | 0.06 |
| OR10T2 | -0.018815613 | 0.53433795 | 0.015442996 | 0.6100513 | 0.04 |
| OR10V1 | -0.162064974 | 7.15E-08 | -0.097534542 | 0.0012442 | 0.07 |
| OR10W1 | 0.073861886 | 0.01458766 | 0.073373 | 0.0152559 | 0.01 |
| OR10X1 | 0.031361932 | 0.30024359 | 0.003561092 | 0.9063867 | 0.03 |
| OR10Z1 | 0.006582947 | 0.82790507 | -0.009523495 | 0.7531422 | 0.02 |
| OR11A1 | 0.052283931 | 0.08403495 | 0.043426735 | 0.1513595 | 0.01 |
| OR11G2 | 0.03903665 | 0.19719376 | 0.028534099 | 0.3459544 | 0.02 |
| OR11H12 | 0.099026251 | 0.0010448 | 0.036035658 | 0.2338962 | 0.07 |
| OR11H1 | -0.011116897 | 0.71352837 | -0.057253118 | 0.0584635 | 0.05 |
| OR11H4 | -0.006756051 | 0.82345322 | -0.016051157 | 0.5960516 | 0.01 |
| OR11H6 | 0.02098957 | 0.48817835 | -0.036356887 | 0.229751 | 0.06 |
| OR11L1 | -0.073689819 | 0.01481984 | 0.04036836 | 0.1823295 | 0.12 |
| OR12D2 | 0.010658103 | 0.7248611 | -0.013019786 | 0.6672197 | 0.03 |
| OR12D3 | 0.014313431 | 0.63643247 | 0.018803627 | 0.5345984 | 0.01 |
| LOC220973 | 0.137926508 | 4.73E-06 | -0.010212376 | 0.735929 | 0.15 |
| OR13C2 | -0.062896135 | 0.03761182 | -0.014621692 | 0.6291853 | 0.05 |
| OR13C3 | 0.003114546 | 0.91808121 | -0.028543199 | 0.3458005 | 0.04 |
| OR13C4 | -0.000123001 | 0.99675913 | 0.009440984 | 0.7552123 | 0.01 |
| OR13C5 | -0.039054165 | 0.19699268 | -0.041457767 | 0.1708002 | 0.01 |
| OR13C8 | -0.019119762 | 0.52775006 | 0.005821387 | 0.8475545 | 0.03 |
| OR13C9 | -0.067207275 | 0.02629145 | -0.075137678 | 0.0129637 | 0.01 |
| OR13D1 | -0.053262359 | 0.07838789 | -0.049457234 | 0.1022142 | 0.01 |
| OR13F1 | 0.007300298 | 0.80949396 | -0.018961663 | 0.5311693 | 0.03 |
| OR13G1 | -0.129651192 | 1.71E-05 | 0.021605299 | 0.4755061 | 0.16 |
| OR13H1 | 0.083030205 | 0.00602116 | 0.014309856 | 0.6365167 | 0.07 |
| OR13J1 | -0.079665049 | 0.00841508 | -0.01173957 | 0.6982485 | 0.07 |
| OR5AT1 | -0.061203892 | 0.04307152 | 0.010694473 | 0.7239605 | 0.08 |
| OR5BF1 | -0.038230675 | 0.2066098 | -0.015806028 | 0.6016768 | 0.03 |
| OR14I1 | -0.008151994 | 0.78777155 | 0.000303445 | 0.9920049 | 0.01 |
| OR14J1 | -0.000768241 | 0.9797603 | -0.009669562 | 0.7494818 | 0.01 |
| OR1A1 | 0.029717872 | 0.32630591 | 0.011430141 | 0.7058268 | 0.02 |
| OR1A2 | -0.008801543 | 0.77131401 | -0.002304093 | 0.9393495 | 0.01 |
| OR1B1 | -0.164437768 | 4.57E-08 | -0.11823177 | 8.93E-05 | 0.05 |
| OR1C1 | -0.060495025 | 0.04554976 | 0.063355559 | 0.0362353 | 0.13 |
| OR1D2 | -0.009704594 | 0.74860474 | -0.014926935 | 0.622044 | 0.01 |
| OR1D4 | 0.024506248 | 0.418294 | -0.002764888 | 0.9272508 | 0.03 |
| OR1E9P | -0.018225974 | 0.54722655 | -0.049668205 | 0.1007575 | 0.04 |
| OR1E7 | 0.012064186 | 0.6903304 | -0.01730584 | 0.5676413 | 0.03 |
| OR1F10 | 0.152600502 | 4.00E-07 | -0.005953589 | 0.8441363 | 0.16 |
| OR1F11 | 0.077397282 | 0.01047598 | 2.90E-05 | 0.9992351 | 0.08 |
| OR1G2 | 0.009884069 | 0.74411658 | -0.00406559 | 0.893199 | 0.02 |
| OR1I1 | 0.031454262 | 0.29882222 | 0.044123436 | 0.1448996 | 0.02 |
| OR1J1 | 0.019795318 | 0.51326689 | 0.04360115 | 0.1497221 | 0.03 |
| OR1J5 | -0.000732431 | 0.98070354 | -0.046438619 | 0.1249418 | 0.05 |
| OR1J4 | 0.034084296 | 0.26021491 | -0.020495503 | 0.4984768 | 0.06 |
| LOC138880 | -0.023566242 | 0.43637477 | -0.020402223 | 0.500434 | 0.01 |
| OR1L2 | -0.033510555 | 0.26832827 | -0.030795087 | 0.3090684 | 0.01 |
| OR1L3 | -0.048601185 | 0.10829755 | -0.087575329 | 0.003761 | 0.04 |
| OR1L5 | 0.041242039 | 0.17303887 | 0.009648968 | 0.7499975 | 0.04 |
| OR1L7 | -0.077368476 | 0.01050481 | -0.014768916 | 0.6257366 | 0.07 |
| OR1L8 | -0.217748366 | 3.38E-13 | -0.162538035 | 6.54E-08 | 0.06 |
| OR1M1 | 0.081355639 | 0.00712283 | -0.011762376 | 0.6976911 | 0.1 |
| OR1N3 | 0.020115001 | 0.50648599 | 0.004517899 | 0.8814005 | 0.02 |
| OR1N2 | -0.065387848 | 0.03064916 | -0.091483825 | 0.0024667 | 0.03 |
| OR1Q3 | -0.069635133 | 0.02131619 | -0.090474636 | 0.0027547 | 0.03 |
| OR1S1 | 0.0121454 | 0.68835464 | 0.005309194 | 0.8608241 | 0.01 |
| OR1S2 | 0.010128714 | 0.73801265 | 0.026859151 | 0.3750131 | 0.02 |
| OR2A16P | -0.013812878 | 0.64827452 | -0.027916722 | 0.3564942 | 0.02 |
| LOC393051 | -0.004739252 | 0.87563586 | -0.062111744 | 0.0400647 | 0.06 |
| LOC402713 | -0.293552282 | 3.65E-23 | -0.231310488 | 9.65E-15 | 0.07 |
| OR2A24P | -0.090100283 | 0.00286913 | -0.059657662 | 0.0486308 | 0.04 |
| OR2A17P | -0.018352914 | 0.54443893 | -0.066426353 | 0.0280916 | 0.05 |
| OR2A10 | -0.207611028 | 4.16E-12 | -0.148223558 | 8.57E-07 | 0.06 |
| OR2A11P | -0.042998538 | 0.15543722 | -0.106894136 | 0.0004 | 0.07 |
| OR2A21 | -0.196975925 | 5.06E-11 | -0.144350217 | 1.65E-06 | 0.06 |
| OR2A22P | -0.161296568 | 8.25E-08 | -0.094482988 | 0.0017655 | 0.07 |
| OR2AE1 | -0.175193548 | 5.53E-09 | -0.155300475 | 2.47E-07 | 0.02 |
| OR2AG3 | -0.054842832 | 0.06992076 | -0.011490209 | 0.7043534 | 0.05 |
| OR2AG2 | 0.002180121 | 0.94260698 | -0.04331349 | 0.15243 | 0.05 |
| OR2AK1P | -0.033857599 | 0.26340016 | -0.0151938 | 0.6158296 | 0.02 |
| OR2AT4 | -0.01015329 | 0.73740038 | 0.006244698 | 0.8366199 | 0.02 |
| OR2B11 | -0.076286743 | 0.01164005 | -0.135297339 | 7.17E-06 | 0.06 |
| OR2B9 | 0.127622666 | 2.32E-05 | 0.069281202 | 0.0219862 | 0.06 |
| OR2B3 | 0.096176137 | 0.00145567 | 0.055433526 | 0.0669552 | 0.05 |
| OR2B6 | 0.323893889 | 4.10E-28 | 0.173170934 | 8.30E-09 | 0.16 |
| OR2C2P | -0.354609012 | 9.85E-34 | -0.268983291 | 1.43E-19 | 0.09 |
| OR2C5P | 0.030027717 | 0.32128505 | 0.016069117 | 0.5956404 | 0.02 |
| OR2D2 | 0.018187094 | 0.54808177 | 0.016538675 | 0.5849357 | 0.01 |
| OR2D3 | -0.063111959 | 0.03695973 | -0.036466935 | 0.228343 | 0.03 |
| OR2F5 | 0.006924586 | 0.81912431 | -0.0216854 | 0.4738709 | 0.03 |
| LOC402710 | -0.02696045 | 0.37321396 | -0.012349145 | 0.6834073 | 0.02 |
| OR2G2 | -0.012452191 | 0.68091037 | -0.020540608 | 0.4975318 | 0.01 |
| OR2G3 | -0.04092585 | 0.17635951 | -0.035234343 | 0.2444665 | 0.01 |
| OR2G6 | -0.058176441 | 0.05450856 | -0.047782984 | 0.114376 | 0.02 |
| OR2H8 | 0.118539179 | 8.55E-05 | -0.000385571 | 0.9898411 | 0.12 |
| OR2H2 | 0.229928199 | 1.40E-14 | 0.081488107 | 0.0070295 | 0.15 |
| OR2J2 | 0.072299617 | 0.01681815 | -0.020857065 | 0.490929 | 0.1 |
| OR2J3 | 0.147450897 | 9.78E-07 | 0.015274649 | 0.6139523 | 0.14 |
| OR2AN1P | 0.011946381 | 0.69320006 | 0.008134923 | 0.7882054 | 0.01 |
| OR2L13 | -0.28508643 | 6.94E-22 | -0.203317894 | 1.16E-11 | 0.09 |
| OR2L7P | -0.211464556 | 1.63E-12 | -0.14298391 | 2.07E-06 | 0.07 |
| OR2L12 | -0.071242165 | 0.01849263 | -0.051926141 | 0.0861803 | 0.02 |
| OR2L3 | -0.169851486 | 1.60E-08 | -0.102967208 | 0.0006512 | 0.07 |
| OR2L8 | 0.029072917 | 0.33691944 | 0.030539696 | 0.3130997 | 0.01 |
| OR2M1P | -0.015573061 | 0.60704502 | -0.013081943 | 0.665727 | 0.01 |
| OR2M2 | -0.019001824 | 0.53029969 | -0.006550358 | 0.8287438 | 0.02 |
| OR2M6 | -0.036301353 | 0.23046391 | -0.04037884 | 0.1822159 | 0.01 |
| HSTPCR100 | -0.191928453 | 1.58E-10 | -0.13886068 | 4.07E-06 | 0.06 |
| OR2M5 | -0.001254928 | 0.9669442 | -0.024531438 | 0.4178156 | 0.03 |
| OR2M7 | -0.01317674 | 0.66345305 | -0.001319615 | 0.9652414 | 0.02 |
| OR2S2 | 0.153942504 | 3.15E-07 | -0.060638568 | 0.0450385 | 0.22 |
| OR2T10 | 0.029936729 | 0.32275421 | 0.051845066 | 0.0866725 | 0.03 |
| OR2T11 | 0.032013261 | 0.29031264 | 0.014110861 | 0.6412139 | 0.02 |
| OR2T12 | 0.025777395 | 0.39455764 | 0.050576859 | 0.0946713 | 0.03 |
| OR2T1 | 0.062513418 | 0.03879219 | 0.087368227 | 0.0038443 | 0.03 |
| OR2T27 | -0.030439799 | 0.314686 | -0.005509225 | 0.8556369 | 0.03 |
| OR2T29 | 0.034330245 | 0.25678936 | 0.017108303 | 0.572071 | 0.02 |
| OR2T2 | -0.006889018 | 0.82003743 | -0.001708445 | 0.9550092 | 0.01 |
| OR2T33 | -0.1349561 | 7.57E-06 | -0.003415551 | 0.9101962 | 0.14 |
| OR2T34 | -0.053252546 | 0.07844296 | -0.010916454 | 0.7184719 | 0.05 |
| OR2T35 | -0.005980185 | 0.84344899 | -0.021541952 | 0.4768014 | 0.02 |
| OR2T3 | -0.044434722 | 0.14208234 | -0.038885301 | 0.1989376 | 0.01 |
| OR2T4 | -0.140377832 | 3.18E-06 | -0.098468043 | 0.0011157 | 0.05 |
| OR2T5 | -0.033638113 | 0.26650962 | -0.036832575 | 0.223709 | 0.01 |
| OR2T6 | -0.002076153 | 0.94533963 | 0.000903997 | 0.9761847 | 0.01 |
| OR2T8 | -0.221089511 | 1.44E-13 | -0.020262713 | 0.5033688 | 0.21 |
| OR2V3 | -0.014260437 | 0.63768189 | -0.019312812 | 0.5235902 | 0.01 |
| OR2W1 | -0.015540871 | 0.60778845 | -0.006130367 | 0.8395702 | 0.01 |
| OR2W8P | -0.29333544 | 3.94E-23 | -0.05413577 | 0.0736112 | 0.24 |
| OR2W5 | 0.020687239 | 0.49446656 | 0.008710697 | 0.7736098 | 0.02 |
| OR2Y1 | 0.036085273 | 0.23325257 | 0.000465085 | 0.9877463 | 0.04 |
| OR2Z2 | -0.028146871 | 0.35254174 | -0.026584629 | 0.3799155 | 0.01 |
| OR3A1 | -0.120984615 | 6.07E-05 | -0.183410951 | 1.00E-09 | 0.07 |
| OR3A2 | -0.152605446 | 4.00E-07 | -0.157365582 | 1.70E-07 | 0.01 |
| OR3A8P | 0.056896914 | 0.06005163 | 0.017135635 | 0.5714571 | 0.04 |
| OR3A5P | 0.008895229 | 0.76894858 | -0.033296267 | 0.2714026 | 0.05 |
| OR4A15 | -0.041634842 | 0.1689789 | -0.001111823 | 0.9707119 | 0.05 |
| OR4A16 | -0.013977167 | 0.64437779 | -0.040365522 | 0.1823603 | 0.03 |
| OR4A47 | 0.073992951 | 0.01441295 | -0.007181476 | 0.8125366 | 0.09 |
| OR4A5 | -0.050743896 | 0.09358515 | -0.044439622 | 0.1420383 | 0.01 |
| OR4B1 | 0.052067974 | 0.08532464 | -0.006219295 | 0.8372752 | 0.06 |
| OR4C11 | -0.0340628 | 0.26051579 | -0.011284977 | 0.7093922 | 0.03 |
| OR4C12 | -0.062002683 | 0.04041624 | -0.023507738 | 0.4375147 | 0.04 |
| OR4C13 | 0.006127547 | 0.83964297 | 0.024841509 | 0.4119534 | 0.02 |
| OR11.134 | 0.031452551 | 0.29884852 | 0.053287228 | 0.0782485 | 0.03 |
| OR4C16 | -0.019126312 | 0.52760866 | -0.015934723 | 0.5987205 | 0.01 |
| OR4C3 | -0.068924412 | 0.02268003 | -0.058482657 | 0.0532472 | 0.02 |
| OR4C45 | -0.073450731 | 0.01514787 | -0.069771813 | 0.0210622 | 0.01 |
| OR4C46 | -0.019262934 | 0.52466332 | -0.084726828 | 0.0050637 | 0.07 |
| OR4C6 | 0.074788913 | 0.013391 | -0.034122815 | 0.2596763 | 0.11 |
| OR4D10 | -0.065451579 | 0.03048664 | -0.004713821 | 0.8762978 | 0.07 |
| OR4D11 | 0.035067364 | 0.2467106 | -0.022581579 | 0.4557881 | 0.06 |
| OR4D4P | 0.004479115 | 0.88241117 | 0.002396256 | 0.9369284 | 0.01 |
| OR4D2 | -0.020346455 | 0.50160607 | -0.015489731 | 0.6089704 | 0.01 |
| OR4D5 | -0.011309556 | 0.70878805 | -0.02545538 | 0.4004924 | 0.02 |
| OR4D6 | -0.001399861 | 0.96312914 | -0.010684334 | 0.7242115 | 0.01 |
| OR4D9 | -0.00431701 | 0.88663763 | 0.019527275 | 0.5189886 | 0.03 |
| OR4E2 | 0.013665741 | 0.65177261 | 0.028714018 | 0.3429205 | 0.02 |
| OR4F15 | -0.050682434 | 0.09398363 | -0.039935105 | 0.1870715 | 0.02 |
| OR4F17 | 0.044353228 | 0.14281579 | 0.031418632 | 0.2993702 | 0.02 |
| OR4F21 | 0.016331037 | 0.58965832 | 0.002688443 | 0.9292569 | 0.02 |
| OR4F29 | -0.092982877 | 0.00208944 | -0.036988655 | 0.2217514 | 0.06 |
| OR4F18 | 0.038896834 | 0.19880431 | -0.0012147 | 0.9680033 | 0.05 |
| OR4F5 | -0.01808459 | 0.55033968 | -0.002973928 | 0.9217676 | 0.02 |
| OR4F12 | -0.017560257 | 0.56196035 | -0.030639897 | 0.311514 | 0.02 |
| OR4K13 | 0.003652214 | 0.90400275 | 0.003347197 | 0.911986 | 0.01 |
| OR4K14 | -0.004706218 | 0.87649574 | -0.010983849 | 0.7168084 | 0.01 |
| OR4K15 | -0.104478669 | 0.00054089 | -0.038482214 | 0.2036368 | 0.07 |
| OR4K17 | 0.05072576 | 0.09370259 | 0.060589598 | 0.0452123 | 0.01 |
| OR4K1 | 0.026884748 | 0.37455795 | -0.009359676 | 0.757254 | 0.04 |
| OR4K2 | 0.0193349 | 0.52311526 | 0.03931254 | 0.1940438 | 0.02 |
| OR4K5 | 0.00191446 | 0.94959074 | 0.032825268 | 0.2782443 | 0.04 |
| OR4L2P | -0.011727897 | 0.69853382 | 0.009898233 | 0.7437627 | 0.03 |
| OR4M1 | 0.048038264 | 0.11245137 | 0.103628455 | 0.0006006 | 0.06 |
| OR4M2 | 0.008449408 | 0.78022378 | -0.033368803 | 0.2703592 | 0.05 |
| OR4N2 | 0.036368167 | 0.22960645 | 0.004260328 | 0.8881162 | 0.04 |
| OR4N3P | 0.059516824 | 0.04916575 | 0.013195912 | 0.6629935 | 0.05 |
| OR4N4 | 0.055708237 | 0.06561169 | -0.00306258 | 0.9194433 | 0.06 |
| OR4N5 | 0.026951345 | 0.37337544 | 0.021864812 | 0.4702195 | 0.01 |
| OR4P3P | 0.021439807 | 0.47889423 | -0.015041841 | 0.6193649 | 0.04 |
| OR4Q4 | 0.045468739 | 0.1330252 | 0.023618029 | 0.4353671 | 0.03 |
| OR4S1 | -0.007254287 | 0.81067181 | 0.002000307 | 0.9473335 | 0.01 |
| OR4S2 | 0.043558912 | 0.15011741 | 0.029505862 | 0.3297706 | 0.02 |
| OR4X1 | 0.004850761 | 0.87273434 | -0.016775138 | 0.5795791 | 0.03 |
| OR4X2 | -0.011098729 | 0.71397595 | -0.037300189 | 0.2178809 | 0.03 |
| OR51A2 | -0.019772527 | 0.51375209 | -0.039465407 | 0.1923144 | 0.02 |
| OR51A4 | 0.014310721 | 0.63649634 | -0.005728936 | 0.8499466 | 0.03 |
| OR51A7 | 0.055203143 | 0.06809929 | 0.038314783 | 0.2056122 | 0.02 |
| OR51B1P | 0.017385573 | 0.56585797 | -0.040974983 | 0.1758404 | 0.06 |
| OR51B4 | -0.043970128 | 0.14630266 | -0.027923295 | 0.356381 | 0.02 |
| OR51B5 | -0.024756991 | 0.41354648 | -0.064367293 | 0.0333545 | 0.04 |
| OR51B6 | -0.018378039 | 0.543888 | -0.034972968 | 0.2479856 | 0.02 |
| OR51D1 | -0.03931696 | 0.19399361 | -0.006485124 | 0.8304233 | 0.04 |
| OR51E1 | -0.024947741 | 0.4099563 | 0.004033972 | 0.8940247 | 0.03 |
| OR51E3P | -0.173622942 | 7.58E-09 | -0.070451082 | 0.0198389 | 0.11 |
| OR51F1 | -0.005626965 | 0.85258657 | 0.006122158 | 0.8397821 | 0.02 |
| OR51F2 | -0.027071196 | 0.37125318 | -0.014583062 | 0.6300916 | 0.02 |
| OR51G1 | 0.008197238 | 0.78662205 | -0.025392246 | 0.4016622 | 0.04 |
| OR51G2 | -0.018775985 | 0.53519935 | -0.033036793 | 0.2751573 | 0.02 |
| OR51I1 | 0.010497148 | 0.72885133 | 0.012497291 | 0.6798186 | 0.01 |
| OR51I2 | 0.029164586 | 0.33539755 | -0.041328123 | 0.1721429 | 0.08 |
| OR51L1 | -0.001163535 | 0.96935034 | 0.009544996 | 0.752603 | 0.02 |
| OR51M1 | 0.000353942 | 0.99067444 | -0.020807208 | 0.4919661 | 0.03 |
| OR51Q1 | -0.089184545 | 0.00316749 | -0.012568277 | 0.6781016 | 0.08 |
| OR51S1 | -0.051999651 | 0.08573598 | -0.027461044 | 0.3644018 | 0.03 |
| OR51T1 | 0.000704985 | 0.9814265 | 0.006774915 | 0.8229684 | 0.01 |
| OR51V1 | 0.003769549 | 0.90093428 | 0.007132871 | 0.813782 | 0.01 |
| OR52A1 | 0.018262116 | 0.54643216 | 0.022193993 | 0.4635607 | 0.01 |
| OR52A4 | 0.027690201 | 0.36041155 | -0.00021895 | 0.9942311 | 0.03 |
| OR52A5 | 0.002628012 | 0.93084301 | 0.002970837 | 0.9218486 | 0.01 |
| OR52B2 | -0.039823906 | 0.18830308 | -0.061195431 | 0.0431004 | 0.03 |
| OR52B4 | -0.024095617 | 0.42613761 | 0.000222422 | 0.9941396 | 0.03 |
| OR52B6 | -0.070253583 | 0.02018802 | -0.011449608 | 0.7053492 | 0.06 |
| OR52D1 | 0.002216662 | 0.94164673 | -0.030119257 | 0.3198114 | 0.04 |
| OR52E2 | 0.077834435 | 0.0100469 | 0.021482943 | 0.4780098 | 0.06 |
| OR52E4 | 0.105358877 | 0.00048492 | 0.019684918 | 0.5156195 | 0.09 |
| OR52E6 | 0.072025544 | 0.01723887 | 0.032188716 | 0.2876756 | 0.04 |
| OR52E8 | -0.026556452 | 0.38042092 | -0.037389826 | 0.2167763 | 0.02 |
| OR52H1 | -0.068278337 | 0.02398489 | -0.076230256 | 0.0117022 | 0.01 |
| OR52I1 | -0.011132764 | 0.71313753 | -0.058426792 | 0.0534755 | 0.05 |
| OR52I2 | -0.02764398 | 0.36121417 | -0.052818088 | 0.0809126 | 0.03 |
| OR52J3 | 0.013678245 | 0.65147503 | -0.020633253 | 0.4955939 | 0.04 |
| OR52K1 | -0.048353047 | 0.11011338 | -0.027699175 | 0.3602558 | 0.03 |
| OR52K2 | -0.047541247 | 0.11622224 | -0.037204455 | 0.2190651 | 0.02 |
| OR52L1 | 0.08031005 | 0.00789921 | -0.0169001 | 0.5767576 | 0.1 |
| OR52M1 | -0.013182268 | 0.66332053 | -0.014541503 | 0.6310671 | 0.01 |
| OR52N1 | -0.037496792 | 0.21546339 | -0.117032897 | 0.0001053 | 0.08 |
| OR52N2 | 0.121833385 | 5.38E-05 | 0.017833378 | 0.5558925 | 0.11 |
| OR52N4 | -0.10400977 | 0.00057311 | -0.155756797 | 2.28E-07 | 0.06 |
| OR52N5 | -0.019987791 | 0.50917862 | -0.089466669 | 0.0030727 | 0.07 |
| OR52R1 | -0.038115709 | 0.20797906 | -0.02605647 | 0.3894575 | 0.02 |
| OR52W1 | -0.118500734 | 8.60E-05 | -0.19548577 | 7.10E-11 | 0.08 |
| OR56A1 | 0.05145598 | 0.08906644 | -0.03284857 | 0.2779031 | 0.09 |
| OR56A6 | 0.121944313 | 5.30E-05 | 0.030506601 | 0.3136247 | 0.1 |
| OR56A4 | 0.01383276 | 0.64780242 | -0.017304975 | 0.5676606 | 0.04 |
| OR56A5 | 0.133973314 | 8.82E-06 | 0.018783296 | 0.5350404 | 0.12 |
| OR56B1 | -0.061099038 | 0.04343076 | -0.152972825 | 3.74E-07 | 0.1 |
| OR56B4 | -0.19805977 | 3.95E-11 | -0.164498789 | 4.51E-08 | 0.04 |
| OR5A1 | -0.016235421 | 0.59183892 | -0.028396084 | 0.3482932 | 0.02 |
| OR5A2 | 0.001393979 | 0.96328394 | -0.01334578 | 0.6594059 | 0.02 |
| OR5AC2 | 0.012673012 | 0.67557124 | 0.068696001 | 0.0231341 | 0.06 |
| OR5AK2 | -0.046341215 | 0.12573587 | -0.051896232 | 0.0863616 | 0.01 |
| OR5AN1 | -0.052127728 | 0.0849662 | -0.065082257 | 0.0314388 | 0.02 |
| OR5AP2 | 0.001536708 | 0.95952767 | 0.048104331 | 0.1119575 | 0.05 |
| OR5AR1 | -0.030077346 | 0.32048555 | -0.04305135 | 0.1549298 | 0.02 |
| OR5AS1 | -0.00120051 | 0.96837684 | 0.00199781 | 0.9473992 | 0.01 |
| OR5AU1 | 0.21832389 | 2.92E-13 | 0.124626268 | 3.60E-05 | 0.1 |
| OR5B12 | 0.017498393 | 0.56333919 | 0.005964606 | 0.8438516 | 0.02 |
| OR5B20P | -0.002848582 | 0.92505503 | 0.038122696 | 0.2078956 | 0.05 |
| OR5B21 | -0.051306939 | 0.0899974 | -0.108735007 | 0.0003165 | 0.06 |
| OR5B2 | -0.020456707 | 0.4992903 | 0.010185064 | 0.736609 | 0.04 |
| OR5B13 | 0.01697167 | 0.5751445 | -0.005226139 | 0.8629796 | 0.03 |
| OR5C2P | -0.011107998 | 0.7137476 | -0.032749309 | 0.2793585 | 0.03 |
| OR5D13 | 0.003304146 | 0.9131135 | 0.004872291 | 0.8721743 | 0.01 |
| OR5D14 | 0.001705785 | 0.95507915 | -0.000226719 | 0.9940264 | 0.01 |
| OR5D16 | -0.000512857 | 0.98648774 | -0.012308299 | 0.6843981 | 0.02 |
| OR5D18 | -0.026734059 | 0.37724211 | -0.006382642 | 0.8330633 | 0.03 |
| LOC100421987 | 0.020104795 | 0.50670173 | -0.057462929 | 0.0575445 | 0.08 |
| LOC283186 | -0.036385266 | 0.22938736 | -0.068520316 | 0.0234887 | 0.04 |
| OR5H14 | 0.001970479 | 0.94811777 | 0.008369436 | 0.7822513 | 0.01 |
| OR5H15 | -0.014947819 | 0.62155674 | -0.015120439 | 0.6175352 | 0.01 |
| OR5H1 | -0.008910178 | 0.76857134 | -0.019455416 | 0.5205281 | 0.02 |
| OR5H2 | 0.009368825 | 0.75702421 | 0.029503758 | 0.3298051 | 0.03 |
| OR5H6 | 0.082560092 | 0.00631382 | 0.069212657 | 0.022118 | 0.02 |
| OR5I1 | 0.028227944 | 0.35115603 | -0.013422072 | 0.6575825 | 0.05 |
| OR5J2 | 0.0317476 | 0.29433625 | 0.036270266 | 0.2308636 | 0.01 |
| OR5K1 | -0.076435718 | 0.01147751 | -0.060216762 | 0.0465549 | 0.02 |
| OR5K2 | -0.210333508 | 2.15E-12 | -0.154960511 | 2.63E-07 | 0.06 |
| OR5K3 | 0.013678601 | 0.65146656 | -0.010152749 | 0.7374139 | 0.03 |
| OR5K4 | -0.042017762 | 0.16509018 | -0.051689253 | 0.0876249 | 0.01 |
| OR5L1 | 0.023375884 | 0.44009027 | 0.033804411 | 0.2641514 | 0.02 |
| OR5L2 | -0.024994929 | 0.40907101 | -0.034918687 | 0.2487209 | 0.01 |
| OR5M10 | 0.006054751 | 0.84152267 | -0.009139466 | 0.7627921 | 0.02 |
| OR5M11 | 0.058057847 | 0.05500373 | 0.041865413 | 0.1666292 | 0.02 |
| OR5M1 | 0.029729701 | 0.32611331 | 0.026387277 | 0.383464 | 0.01 |
| OR5M3 | 0.001406244 | 0.96296112 | 0.033158756 | 0.2733881 | 0.04 |
| OR5M8 | -0.012014587 | 0.69153805 | 0.022160823 | 0.4642293 | 0.04 |
| OR5M9 | 0.101716771 | 0.00075797 | 0.08800791 | 0.0035922 | 0.02 |
| OR5P2 | -0.058866759 | 0.05169945 | -0.079804832 | 0.0083008 | 0.03 |
| OR5P3 | 0.037625608 | 0.21388991 | 0.004553232 | 0.8804799 | 0.04 |
| OR5R1 | -0.008072701 | 0.78978724 | 0.026822434 | 0.3756665 | 0.04 |
| OR5T1 | 0.030879522 | 0.30774312 | -0.013881114 | 0.6466549 | 0.05 |
| OR5T2 | 0.014103755 | 0.64138192 | 0.045785979 | 0.1303378 | 0.04 |
| OR5T3 | 0.030115508 | 0.31987166 | 0.060738605 | 0.044685 | 0.04 |
| LOC105379569 | 0.013049061 | 0.66651648 | -0.004293259 | 0.8872571 | 0.02 |
| OR5W3P | 0.03413722 | 0.25947513 | -0.03519946 | 0.2449341 | 0.07 |
| OR6A2 | -0.003079316 | 0.91900462 | -0.02128245 | 0.482128 | 0.02 |
| LOC393045 | -0.011978543 | 0.69241618 | -0.024291048 | 0.422394 | 0.02 |
| OR6B2P | -0.127405456 | 2.39E-05 | -0.073870243 | 0.0145765 | 0.06 |
| OR6B3 | -0.138407587 | 4.38E-06 | -0.029384691 | 0.3317614 | 0.11 |
| OR6C1 | -0.020477383 | 0.49885665 | -0.023404935 | 0.4395221 | 0.01 |
| OR6C2 | 0.005465889 | 0.85676016 | 0.040074447 | 0.1855366 | 0.04 |
| OR6C3 | -0.010660729 | 0.72479606 | -0.001150078 | 0.9697046 | 0.01 |
| OR6C4 | -0.045768892 | 0.1304815 | -0.017144069 | 0.5712677 | 0.03 |
| OR6C65 | -0.009646114 | 0.75006903 | 0.004421748 | 0.8839065 | 0.02 |
| OR6C68 | 0.006220363 | 0.83724768 | -0.030475726 | 0.3141149 | 0.04 |
| OR6C6 | -0.025943687 | 0.39151377 | -0.026773987 | 0.3765297 | 0.01 |
| OR6C70 | 0.012028781 | 0.69119236 | 0.006542371 | 0.8289494 | 0.01 |
| OR6C74 | 0.003980033 | 0.89543356 | 0.008361803 | 0.7824449 | 0.01 |
| OR6C75 | 0.031325662 | 0.30080318 | 0.018550753 | 0.5401084 | 0.02 |
| OR6C76 | 0.035646994 | 0.23898204 | -0.013485147 | 0.6560766 | 0.05 |
| OR6F1 | -0.192928694 | 1.26E-10 | 0.002234046 | 0.9411899 | 0.2 |
| OR6K2 | 0.094030325 | 0.00185803 | 0.055141918 | 0.068406 | 0.04 |
| OR6K3 | -0.007161165 | 0.81305699 | -0.028710876 | 0.3429733 | 0.03 |
| OR6K6 | 0.021541506 | 0.47681057 | 0.004970008 | 0.8696334 | 0.02 |
| OR6M1 | 0.000172863 | 0.99544538 | 0.0017162 | 0.9548052 | 0.01 |
| OR6N1 | 0.021764765 | 0.47225374 | 0.003148314 | 0.9171962 | 0.02 |
| OR6N2 | 0.017081254 | 0.57267883 | -0.001456866 | 0.9616288 | 0.02 |
| OR6P1 | 0.008677473 | 0.77444989 | 0.002384976 | 0.9372247 | 0.01 |
| OR6Q1 | 0.013125454 | 0.66468289 | 0.025635836 | 0.39716 | 0.02 |
| OR6S1 | -0.037503991 | 0.21537524 | -0.033159521 | 0.273377 | 0.01 |
| OR6T1 | -0.037972607 | 0.20969257 | -0.079564737 | 0.008498 | 0.05 |
| LOC402708 | -0.032698398 | 0.28010705 | -0.005773117 | 0.8488032 | 0.03 |
| OR6W1P | -0.092394737 | 0.00223066 | -0.009996003 | 0.7413218 | 0.09 |
| OR6X1 | -0.004817444 | 0.8736011 | -0.008647977 | 0.7751959 | 0.01 |
| OR6Y1 | 0.05201492 | 0.08564391 | 0.031995232 | 0.2905845 | 0.03 |
| OR7A10 | -0.012371085 | 0.68287541 | 0.008428256 | 0.7807599 | 0.03 |
| OR7A17 | 0.02984653 | 0.32421494 | 0.029379415 | 0.3318483 | 0.01 |
| HSTPCR86P | 0.177968906 | 3.13E-09 | 0.00816734 | 0.7873816 | 0.17 |
| OR7C1 | 0.073564005 | 0.01499167 | 0.001199605 | 0.9684007 | 0.08 |
| OR7C3 | 0.035030007 | 0.24721464 | 0.021235067 | 0.4831041 | 0.02 |
| OR7D2 | -0.038170393 | 0.20732695 | -0.070747665 | 0.0193245 | 0.04 |
| OR7D4 | 0.018214038 | 0.54748903 | 0.095345013 | 0.0016009 | 0.08 |
| OR7E156P | 0.166773546 | 2.92E-08 | 0.127767796 | 2.27E-05 | 0.04 |
| OR7E24 | 0.132419689 | 1.12E-05 | 0.017895484 | 0.5545171 | 0.12 |
| LOC26636 | 0.263287623 | 8.67E-19 | 0.138431262 | 4.36E-06 | 0.13 |
| OR7E5P | 0.129662768 | 1.71E-05 | 0.055048613 | 0.0688757 | 0.08 |
| OR7E91P | 0.197564697 | 4.42E-11 | 0.16678673 | 2.91E-08 | 0.04 |
| OR7G1 | -0.026371569 | 0.38374736 | -0.057709001 | 0.056482 | 0.04 |
| OR7G2 | 0.037914093 | 0.21039616 | -0.022375162 | 0.4599184 | 0.07 |
| OR7G3 | -0.003614025 | 0.90500177 | -0.024540381 | 0.4176458 | 0.03 |
| OR8A1 | 0.03851406 | 0.2032626 | 0.003652112 | 0.9040054 | 0.04 |
| OR8B12 | 0.051377856 | 0.08955345 | -0.012109142 | 0.6892364 | 0.07 |
| OR8B2 | 0.031666784 | 0.29556763 | -0.00381922 | 0.8996358 | 0.04 |
| OR8B3 | 0.026975489 | 0.37294732 | -0.009932459 | 0.7429079 | 0.04 |
| OR8B4 | -0.007494043 | 0.80453896 | 0.00849952 | 0.778954 | 0.02 |
| OR8B8 | 0.032054554 | 0.28969057 | 0.021792698 | 0.4716853 | 0.02 |
| OR8D3 | 0.065643668 | 0.03000128 | 0.038502093 | 0.2034031 | 0.03 |
| OR8D2 | 0.036212299 | 0.23161033 | 0.005164178 | 0.8645883 | 0.04 |
| OR8D4 | 0.022741137 | 0.45260972 | 0.001807873 | 0.9523938 | 0.03 |
| OR8G4 | 0.026851543 | 0.37514839 | 0.012117885 | 0.6890238 | 0.02 |
| OR8G6 | 0.059331932 | 0.04987557 | 0.020458534 | 0.499252 | 0.04 |
| OR8H1 | -0.000575982 | 0.98482476 | 0.026127669 | 0.3881627 | 0.03 |
| OR8H2 | 0.042291778 | 0.16234901 | 0.016055504 | 0.5959521 | 0.03 |
| OR8H3 | -0.012367426 | 0.68296412 | -0.028022155 | 0.3546801 | 0.02 |
| OR8I2 | -0.009722025 | 0.74816849 | 0.012056752 | 0.6905113 | 0.03 |
| OR8J1 | -0.022115462 | 0.46514446 | -0.011392209 | 0.7067578 | 0.02 |
| OR8J3 | -0.006214453 | 0.83740013 | -0.009293785 | 0.7589098 | 0.01 |
| OR8N1P | 0.000697537 | 0.9816227 | -0.013371118 | 0.6588001 | 0.02 |
| OR8K3 | 0.015953504 | 0.59828967 | 0.036597479 | 0.2266808 | 0.03 |
| OR8K5 | 0.01434712 | 0.63563874 | 0.021002711 | 0.487906 | 0.01 |
| OR8S1 | 0.010151449 | 0.73744624 | -0.042168678 | 0.1635762 | 0.06 |
| OR8U1 | -0.03846577 | 0.20383017 | 0.002230069 | 0.9412944 | 0.05 |
| LOC402707 | -0.05016713 | 0.09737824 | 0.012510031 | 0.6795103 | 0.07 |
| LOC402698 | 0.060430865 | 0.04577987 | -0.006163988 | 0.8387024 | 0.07 |
| OR9G4 | 0.016267277 | 0.59111199 | -0.021587666 | 0.4758665 | 0.04 |
| OR9G9 | -0.007232596 | 0.81122722 | -0.03826809 | 0.2061656 | 0.04 |
| OR9I1 | -0.00284901 | 0.92504383 | 0.024272602 | 0.4227465 | 0.03 |
| OR9K2 | 0.024660504 | 0.41536955 | 0.041151015 | 0.17399 | 0.02 |
| OR9Q1 | 0.038090455 | 0.20828071 | 0.062163126 | 0.0399 | 0.03 |
| OR9Q2 | -0.007708159 | 0.79907211 | 0.007984057 | 0.7920423 | 0.02 |
| ORAI1 | 0.317180359 | 5.74E-27 | 0.140610362 | 3.06E-06 | 0.18 |
| FLJ14733 | -0.176548455 | 4.19E-09 | -0.076700209 | 0.0111939 | 0.1 |
| ORAI3 | -0.241295538 | 6.06E-16 | 0.021406232 | 0.4795832 | 0.27 |
| ORAOV1 | 0.145060655 | 1.47E-06 | 0.096426378 | 0.0014144 | 0.05 |
| ORC1 | 0.490814116 | 2.48E-67 | 0.174792512 | 5.99E-09 | 0.32 |
| ORC2 | -0.10851405 | 0.00032558 | -0.092069923 | 0.0023124 | 0.02 |
| ORC3 | 0.18172954 | 1.43E-09 | 0.158439481 | 1.40E-07 | 0.03 |
| ORC4 | 0.028289445 | 0.35010716 | 0.083341683 | 0.005834 | 0.06 |
| ORC5 | 0.143981228 | 1.76E-06 | 0.266100465 | 3.58E-19 | 0.13 |
| ORC6 | 0.578132365 | 1.74E-98 | 0.255196836 | 1.04E-17 | 0.33 |
| ORM1 | 0.156124641 | 2.13E-07 | 0.201232673 | 1.89E-11 | 0.05 |
| ORM2 | 0.200417887 | 2.29E-11 | 0.202581375 | 1.38E-11 | 0.01 |
| LOC51240 | 0.246148356 | 1.51E-16 | 0.13052177 | 1.50E-05 | 0.12 |
| ORMDL2 | 0.148668771 | 7.94E-07 | 0.330851892 | 2.48E-29 | 0.19 |
| LOC51242 | -0.092533911 | 0.00219648 | 0.034497656 | 0.2544756 | 0.13 |
| OS9 | -0.121324683 | 5.79E-05 | -0.067205268 | 0.0262959 | 0.06 |
| OSBP2 | -0.072482365 | 0.01654265 | -0.120458588 | 6.54E-05 | 0.05 |
| FLJ20363 | -0.244836633 | 2.20E-16 | -0.239864851 | 9.09E-16 | 0.01 |
| FLJ13164 | -0.086010812 | 0.00443298 | -0.212756736 | 1.18E-12 | 0.13 |
| FLJ10217 | -0.010134214 | 0.7378756 | -0.201810423 | 1.65E-11 | 0.2 |
| FLJ44790 | -0.022801051 | 0.45141947 | -0.150143729 | 6.15E-07 | 0.13 |
| OSBPL3 | 0.025524269 | 0.39921828 | -0.160918873 | 8.85E-08 | 0.19 |
| KIAA1534 | -0.288320337 | 2.28E-22 | -0.144334263 | 1.66E-06 | 0.15 |
| ORP6 | -0.178495805 | 2.81E-09 | -0.094259716 | 0.0018106 | 0.09 |
| FLJ20260 | -0.210665007 | 1.98E-12 | -0.17163922 | 1.13E-08 | 0.04 |
| KIAA1451 | -0.308779918 | 1.42E-25 | -0.289508342 | 1.51E-22 | 0.02 |
| FLJ12492 | -0.012271783 | 0.68528426 | -0.125612395 | 3.12E-05 | 0.12 |
| OSBP | -0.282504864 | 1.67E-21 | -0.254414711 | 1.31E-17 | 0.03 |
| OSCAR | 0.092772139 | 0.00213907 | 0.020880136 | 0.4904495 | 0.08 |
| OSCP1 | -0.194885111 | 8.14E-11 | -0.005905645 | 0.8453756 | 0.19 |
| OSGEPL1 | -0.096971979 | 0.00132806 | 0.119049577 | 7.97E-05 | 0.22 |
| OSGEP | 0.224291142 | 6.26E-14 | 0.189738821 | 2.56E-10 | 0.04 |
| OSGIN1 | 0.018562547 | 0.53985079 | -0.065752611 | 0.029729 | 0.09 |
| OSGIN2 | 0.161622662 | 7.76E-08 | 0.059882332 | 0.0477874 | 0.11 |
| OSMR | -0.338270049 | 1.15E-30 | -0.307187252 | 2.57E-25 | 0.04 |
| OSM | 0.104562606 | 0.00053531 | -0.015614755 | 0.6060827 | 0.13 |
| ODD | 0.025050061 | 0.40803814 | -0.059094541 | 0.0507995 | 0.09 |
| OSR2 | -0.064761095 | 0.03228733 | -0.039782132 | 0.1887673 | 0.03 |
| OST4 | 0.340828749 | 3.92E-31 | 0.37673463 | 3.48E-38 | 0.04 |
| SLC51B | 0.041508441 | 0.1702775 | 0.067725392 | 0.0251528 | 0.03 |
| OSTCP1 | 0.017913288 | 0.55412318 | 0.021530442 | 0.477037 | 0.01 |
| OSTC | 0.300857616 | 2.65E-24 | 0.280211469 | 3.62E-21 | 0.03 |
| OSTF1 | 0.110391526 | 0.00025557 | 7.06E-06 | 0.999814 | 0.12 |
| OSTM1 | -0.25797148 | 4.48E-18 | -0.136128956 | 6.29E-06 | 0.13 |
| OSTN | 0.048800817 | 0.10685393 | -0.042160704 | 0.1636559 | 0.1 |
| SLC51A | -0.011604072 | 0.70156338 | 0.040616025 | 0.1796591 | 0.06 |
| OTC | -0.177886742 | 3.19E-09 | -0.086188004 | 0.0043518 | 0.1 |
| OTOA | -0.069702637 | 0.02119044 | -0.00391474 | 0.8971394 | 0.07 |
| OTOF | 0.097718762 | 0.00121778 | -0.049431095 | 0.1023959 | 0.15 |
| OTOL1 | 0.071778773 | 0.01762553 | 0.086165256 | 0.0043621 | 0.02 |
| OTOP1 | 0.150404459 | 5.88E-07 | -0.021139536 | 0.4850752 | 0.18 |
| OTOP2 | 0.101919614 | 0.00073962 | 0.014054593 | 0.6425447 | 0.09 |
| OTOP3 | 0.035315043 | 0.24338701 | 0.014468376 | 0.6327853 | 0.03 |
| OTOR | -0.151678674 | 4.70E-07 | 0.067653054 | 0.0253092 | 0.22 |
| OTOS | 0.194665769 | 8.55E-11 | 0.088792777 | 0.0033035 | 0.11 |
| OTP | 0.187817691 | 3.90E-10 | 0.105119276 | 0.0004996 | 0.09 |
| OTUB1 | 0.28482705 | 7.59E-22 | 0.267006429 | 2.69E-19 | 0.02 |
| OTUB2 | 0.004371474 | 0.88521727 | 0.114293791 | 0.0001527 | 0.11 |
| OTUD1 | -0.208454481 | 3.39E-12 | -0.139762828 | 3.52E-06 | 0.07 |
| OTUD3 | -0.095448852 | 0.00158205 | -0.202741322 | 1.33E-11 | 0.11 |
| OTUD4 | -0.19457496 | 8.73E-11 | -0.305197453 | 5.39E-25 | 0.12 |
| OTUD5 | 0.076136333 | 0.01180621 | 0.064312628 | 0.033505 | 0.02 |
| OTUD6A | -0.010671898 | 0.72451944 | -0.0213823 | 0.4800747 | 0.02 |
| OTUD6B | 0.219908388 | 1.95E-13 | 0.03429614 | 0.2572625 | 0.19 |
| OTUD7A | -0.266884391 | 2.79E-19 | -0.318355889 | 3.63E-27 | 0.06 |
| OTUD7B | -0.365951106 | 5.69E-36 | -0.039646476 | 0.1902806 | 0.33 |
| OTX1 | 0.272234691 | 5.02E-20 | 0.110791343 | 0.0002426 | 0.17 |
| OTX2 | 0.144358608 | 1.65E-06 | 0.052550642 | 0.082464 | 0.1 |
| OVCA2 | 0.292624766 | 5.07E-23 | 0.314910009 | 1.38E-26 | 0.03 |
| OVCH1 | -0.102871968 | 0.00065885 | -0.070751862 | 0.0193173 | 0.04 |
| OVCH2 | -0.174009724 | 7.02E-09 | -0.192295775 | 1.45E-10 | 0.02 |
| OVGP1 | -0.096518552 | 0.00139944 | 0.051450197 | 0.0891024 | 0.15 |
| OVOL1 | -0.022184701 | 0.46374791 | 0.005232351 | 0.8628184 | 0.03 |
| OVOL2 | 0.009027535 | 0.76561171 | 0.22646662 | 3.53E-14 | 0.22 |
| OXA1L | 0.110281411 | 0.00025926 | 0.220651358 | 1.61E-13 | 0.12 |
| OXCT1 | 0.08927308 | 0.00313745 | -0.088508412 | 0.0034056 | 0.18 |
| LOC115690 | 0.086717411 | 0.00411705 | 0.108997962 | 0.000306 | 0.03 |
| OXER1 | 0.156421412 | 2.02E-07 | 0.01994732 | 0.5100368 | 0.14 |
| OXGR1 | 0.251042807 | 3.60E-17 | 0.131256935 | 1.34E-05 | 0.12 |
| OXNAD1 | 0.150912185 | 5.38E-07 | 0.088859629 | 0.00328 | 0.07 |
| OXR1 | -0.127547506 | 2.34E-05 | -0.218557577 | 2.75E-13 | 0.1 |
| OXSM | 0.221916803 | 1.16E-13 | 0.35182556 | 3.38E-33 | 0.13 |
| OXSR1 | -0.02091974 | 0.4896269 | -0.16562478 | 3.64E-08 | 0.15 |
| OXTR | -0.131431193 | 1.31E-05 | -0.11150189 | 0.0002211 | 0.02 |
| OXT | 0.002253189 | 0.94068691 | 0.023004979 | 0.4473816 | 0.03 |
| P2RX1 | -0.09481477 | 0.00170039 | -0.175851311 | 4.83E-09 | 0.09 |
| DFNA41 | 0.102073391 | 0.00072599 | 0.070294683 | 0.0201149 | 0.04 |
| P2RX3 | -0.00478326 | 0.87449055 | -0.033657878 | 0.2662286 | 0.03 |
| P2RX4 | -0.209500197 | 2.63E-12 | -0.071390532 | 0.0182492 | 0.14 |
| P2RX5 | 0.178220873 | 2.98E-09 | -0.018068957 | 0.5506844 | 0.2 |
| P2RX6P | -0.036410962 | 0.22905842 | -0.002734724 | 0.9280423 | 0.04 |
| P2X6 | -0.108127074 | 0.00034208 | -0.120675247 | 6.34E-05 | 0.02 |
| P2RX7 | -0.346754237 | 3.10E-32 | -0.419340244 | 8.73E-48 | 0.08 |
| P2RY10 | -0.111975855 | 0.00020774 | -0.159720538 | 1.11E-07 | 0.05 |
| P2RY11 | 0.045815423 | 0.13009057 | 0.01043744 | 0.7303334 | 0.04 |
| P2RY12 | -0.427571262 | 8.32E-50 | -0.359843231 | 9.37E-35 | 0.07 |
| P2RY13 | -0.290472764 | 1.08E-22 | -0.282612561 | 1.61E-21 | 0.01 |
| P2RY14 | -0.159167404 | 1.22E-07 | -0.213945211 | 8.81E-13 | 0.06 |
| LOC115371 | -0.290846705 | 9.46E-23 | -0.338503203 | 1.05E-30 | 0.05 |
| P2RY2 | -0.224517505 | 5.90E-14 | -0.06430412 | 0.0335285 | 0.17 |
| P2RY4 | -0.292792031 | 4.78E-23 | -0.271656243 | 6.06E-20 | 0.03 |
| P2RY6 | 0.203274997 | 1.17E-11 | 0.005297972 | 0.8611152 | 0.2 |
| P2RY8 | -0.132147569 | 1.17E-05 | -0.17484859 | 5.93E-09 | 0.05 |
| P4HA1 | 0.102749926 | 0.00066873 | 0.11128197 | 0.0002275 | 0.01 |
| P4HA2 | -0.001645847 | 0.956656 | 0.006947448 | 0.8185375 | 0.01 |
| P4HA3 | -0.250138673 | 4.70E-17 | -0.076628833 | 0.0112698 | 0.18 |
| ERBA2L | 0.250440674 | 4.30E-17 | 0.099573429 | 0.0009794 | 0.16 |
| P4HTM | -0.315762066 | 9.93E-27 | -0.131350529 | 1.32E-05 | 0.19 |
| POTEM | 0.007079027 | 0.81516225 | -0.030872244 | 0.3078572 | 0.04 |
| LOC651973 | 0.390331564 | 4.26E-41 | 0.340213635 | 5.08E-31 | 0.06 |
| PA2G4 | 0.526848957 | 4.30E-79 | 0.419748807 | 6.95E-48 | 0.11 |
| PAAF1 | -0.071225812 | 0.01851963 | 0.075404568 | 0.012645 | 0.15 |
| PABPC1L2A | 0.009965678 | 0.7420786 | 0.009581971 | 0.7516761 | 0.01 |
| LOC647480 | -0.026814437 | 0.3758089 | -0.012508253 | 0.6795533 | 0.02 |
| PABPC1L | 0.009112875 | 0.76346164 | 0.047358375 | 0.1176344 | 0.04 |
| PABPC1P2 | 0.15644983 | 2.01E-07 | 0.117211106 | 0.0001027 | 0.04 |
| PABPC2 | 0.396910318 | 1.48E-42 | 0.23980479 | 9.24E-16 | 0.16 |
| LOC144785 | 0.26131196 | 1.60E-18 | 0.100633504 | 0.0008633 | 0.17 |
| PABPC4L | 0.027088395 | 0.37094924 | -0.123250893 | 4.40E-05 | 0.16 |
| PABPC4 | 0.347818386 | 1.95E-32 | 0.230389583 | 1.24E-14 | 0.12 |
| PABPC5 | -0.269994554 | 1.04E-19 | -0.21160781 | 1.57E-12 | 0.06 |
| PABPN1L | -0.011731853 | 0.69843713 | -0.01514997 | 0.6168484 | 0.01 |
| PABP2 | 0.419810892 | 6.71E-48 | 0.346555206 | 3.38E-32 | 0.08 |
| PACRGL | -0.111199005 | 0.00023003 | -0.09148695 | 0.0024659 | 0.02 |
| LPRS2 | -0.078819569 | 0.00913679 | -0.066037406 | 0.0290271 | 0.02 |
| PACS1 | -0.087302641 | 0.003871 | -0.073242614 | 0.0154386 | 0.02 |
| PACS2 | 0.065682406 | 0.02990421 | 0.023653142 | 0.4346846 | 0.05 |
| PACSIN1 | -0.014280559 | 0.63720736 | 0.086124404 | 0.0043808 | 0.11 |
| LOC129172 | -0.277043236 | 1.04E-20 | -0.270295133 | 9.40E-20 | 0.01 |
| PACSIN3 | 0.236024512 | 2.66E-15 | 0.212692977 | 1.20E-12 | 0.03 |
| PADI1 | 0.083090867 | 0.00598429 | 0.015659441 | 0.6050521 | 0.07 |
| PADI2 | 0.216178285 | 5.03E-13 | 0.130247889 | 1.56E-05 | 0.09 |
| PADI3 | 0.026125387 | 0.38820419 | 0.008054649 | 0.7902463 | 0.02 |
| PADI4 | 0.009050458 | 0.765034 | -0.057873802 | 0.0557796 | 0.07 |
| PADI6 | 0.001821519 | 0.95203493 | -0.000733979 | 0.9806628 | 0.01 |
| PAEP | 0.097947498 | 0.00118573 | 0.042066459 | 0.1646005 | 0.06 |
| PAF1 | 0.100650002 | 0.00086162 | 0.105937226 | 0.0004511 | 0.01 |
| PAFAH1B1 | -0.2525989 | 2.26E-17 | -0.344332435 | 8.80E-32 | 0.1 |
| PAFAH1B2 | -0.347450127 | 2.29E-32 | -0.39564602 | 2.84E-42 | 0.05 |
| PAFAH1B3 | 0.260330381 | 2.17E-18 | 0.364136174 | 1.32E-35 | 0.11 |
| PAFAH2 | -0.384327144 | 8.55E-40 | -0.189499891 | 2.70E-10 | 0.2 |
| FLJ37858 | -0.136068529 | 6.35E-06 | -0.259102983 | 3.17E-18 | 0.13 |
| PAGE1 | 0.08327387 | 0.00587432 | 0.066819588 | 0.0271723 | 0.02 |
| PAGE2B | 0.142467947 | 2.26E-06 | 0.053089874 | 0.0793604 | 0.09 |
| GAGEC2 | 0.099625131 | 0.00097341 | 0.018664546 | 0.5376254 | 0.09 |
| GAGED1 | 0.026782623 | 0.37637576 | 0.056253339 | 0.0630115 | 0.03 |
| JM27 | -0.002561027 | 0.93260148 | 0.025899859 | 0.3923146 | 0.03 |
| PAGE5 | 0.061819501 | 0.04101258 | 0.022920207 | 0.4490576 | 0.04 |
| PAH | -0.067509499 | 0.02562197 | -0.038655546 | 0.2016063 | 0.03 |
| LOC132947 | 0.417349227 | 2.64E-47 | 0.262553299 | 1.09E-18 | 0.16 |
| PAIP1 | 0.161380453 | 8.12E-08 | 0.14717519 | 1.03E-06 | 0.02 |
| PAIP2B | -0.114055059 | 0.00015762 | -0.174544867 | 6.30E-09 | 0.07 |
| PAIP2 | 0.023292291 | 0.44172759 | 0.114769017 | 0.0001432 | 0.1 |
| PAK1IP1 | 0.495297041 | 1.02E-68 | 0.291611996 | 7.23E-23 | 0.21 |
| PAK1 | -0.029349708 | 0.33233764 | -0.079308733 | 0.0087128 | 0.05 |
| PAK2 | -0.095606914 | 0.00155375 | -0.12344958 | 4.27E-05 | 0.03 |
| MRX30 | -0.044385956 | 0.14252088 | -0.22637217 | 3.62E-14 | 0.19 |
| LOC115291 | 0.055365044 | 0.06729362 | 0.085021298 | 0.0049123 | 0.03 |
| PAK6 | 0.029238327 | 0.33417651 | -0.031520911 | 0.297799 | 0.07 |
| PAK7 | -0.125606749 | 3.12E-05 | -0.139874428 | 3.45E-06 | 0.02 |
| FLJ40214 | 0.013216212 | 0.66250713 | -0.012908742 | 0.6698896 | 0.03 |
| PNCA1 | -0.312700891 | 3.21E-26 | -0.225731158 | 4.29E-14 | 0.09 |
| PALM2.AKAP2 | -0.126338864 | 2.80E-05 | -0.250120327 | 4.72E-17 | 0.13 |
| PALM2 | 0.179929729 | 2.09E-09 | 0.045161809 | 0.1356658 | 0.14 |
| PALM3 | 0.060994677 | 0.04379082 | 0.119026866 | 7.99E-05 | 0.06 |
| LOC116203 | -0.185347241 | 6.65E-10 | -0.165987221 | 3.40E-08 | 0.02 |
| PALM | -0.289011381 | 1.79E-22 | -0.149925204 | 6.39E-07 | 0.14 |
| PAMR1 | -0.271733444 | 5.91E-20 | -0.188681964 | 3.23E-10 | 0.09 |
| PAM | -0.076267334 | 0.01166137 | -0.106309034 | 0.0004306 | 0.04 |
| PAN2 | -0.261218787 | 1.65E-18 | -0.20552491 | 6.87E-12 | 0.06 |
| PAN3 | -0.227073835 | 3.01E-14 | -0.16520916 | 3.94E-08 | 0.07 |
| PanK | 0.211793539 | 1.50E-12 | -0.021224201 | 0.4833281 | 0.24 |
| NBIA1 | 0.037150453 | 0.21973515 | -0.016571053 | 0.5842009 | 0.06 |
| PANK3 | -0.322399553 | 7.42E-28 | -0.219989196 | 1.91E-13 | 0.11 |
| PANK4 | -0.055490471 | 0.06667486 | -0.179779744 | 2.16E-09 | 0.13 |
| PANX1 | 0.05059664 | 0.09454214 | 0.029793939 | 0.3250686 | 0.03 |
| LOC115765 | 0.212912624 | 1.14E-12 | 0.021753092 | 0.4724914 | 0.2 |
| PANX3 | 0.102165956 | 0.00071789 | 0.013566935 | 0.654126 | 0.09 |
| PAOX | -0.047279038 | 0.11825122 | -0.03731605 | 0.2176852 | 0.01 |
| PAPD4 | -0.435967483 | 6.32E-52 | -0.208915555 | 3.04E-12 | 0.23 |
| PAPD5 | -0.085593755 | 0.00462959 | -0.171390276 | 1.18E-08 | 0.09 |
| PAPD7 | 0.290932677 | 9.18E-23 | 0.030946363 | 0.3066967 | 0.26 |
| PAPLN | -0.282431812 | 1.71E-21 | -0.247646956 | 9.76E-17 | 0.04 |
| PAPL | 0.286899289 | 3.73E-22 | 0.068011377 | 0.0245428 | 0.22 |
| MGC5378 | -0.080289589 | 0.00791513 | -0.037284198 | 0.2180784 | 0.05 |
| PAPOLB | -0.137570789 | 5.00E-06 | -0.147592828 | 9.55E-07 | 0.02 |
| PAPOLG | -0.098684766 | 0.00108764 | -0.184395456 | 8.15E-10 | 0.09 |
| PAPPA2 | -0.319052886 | 2.77E-27 | -0.299043758 | 5.12E-24 | 0.03 |
| PAPPA | -0.266812668 | 2.86E-19 | -0.278749118 | 5.90E-21 | 0.02 |
| PAPSS1 | 0.242594103 | 4.19E-16 | -0.019835384 | 0.5124145 | 0.27 |
| PAPSS2 | -0.095136722 | 0.00163932 | -0.083807757 | 0.0055638 | 0.02 |
| PAQR3 | 0.032210319 | 0.28735205 | -0.104912588 | 0.0005126 | 0.14 |
| PAQR4 | 0.285917178 | 5.22E-22 | 0.271808425 | 5.77E-20 | 0.02 |
| PAQR5 | -0.000113133 | 0.99701915 | -0.074962581 | 0.0131767 | 0.08 |
| PAQR6 | 0.130447438 | 1.52E-05 | 0.188874548 | 3.10E-10 | 0.06 |
| LOC255358 | 0.004191805 | 0.8899042 | -0.12171909 | 5.47E-05 | 0.13 |
| C6orf33 | -0.003489853 | 0.90825109 | -0.086809407 | 0.0040775 | 0.09 |
| PAQR9 | 0.145121358 | 1.45E-06 | 0.004910835 | 0.8711719 | 0.15 |
| PWARSN | -0.333188541 | 9.53E-30 | -0.221663444 | 1.24E-13 | 0.12 |
| D15S227E | -0.214627045 | 7.43E-13 | -0.144313443 | 1.66E-06 | 0.08 |
| PWAR4 | -0.051991792 | 0.08578339 | -0.070471351 | 0.0198033 | 0.02 |
| PWAR5 | -0.483512368 | 4.08E-65 | -0.399477766 | 3.90E-43 | 0.09 |
| ALS2CR19 | -0.516469885 | 1.47E-75 | -0.467318301 | 2.18E-60 | 0.05 |
| PARD3 | 0.020178992 | 0.50513432 | -0.126589702 | 2.70E-05 | 0.15 |
| PARD6A | 0.090878051 | 0.0026361 | 0.060806107 | 0.0444478 | 0.04 |
| PARD6B | -0.213005931 | 1.11E-12 | -0.106524198 | 0.0004191 | 0.11 |
| PARD6G | 0.107268326 | 0.00038152 | 0.005864236 | 0.8464463 | 0.11 |
| LOC727726 | -0.066172244 | 0.02869981 | -0.142110185 | 2.40E-06 | 0.08 |
| PRKN | -0.329503565 | 4.30E-29 | -0.235121382 | 3.41E-15 | 0.1 |
| PARK7 | 0.227085342 | 3.00E-14 | 0.215907326 | 5.39E-13 | 0.02 |
| PARL | 0.446218662 | 1.35E-54 | 0.326389709 | 1.51E-28 | 0.12 |
| PARM1 | -0.190406689 | 2.21E-10 | -0.235076581 | 3.45E-15 | 0.05 |
| PARN | 0.081276281 | 0.00717926 | 0.174304175 | 6.61E-09 | 0.1 |
| PARP10 | 0.022042534 | 0.46661794 | 0.004370638 | 0.8852391 | 0.02 |
| C12ORF6 | -0.347965213 | 1.83E-32 | -0.323594104 | 4.62E-28 | 0.03 |
| PARP12 | 0.150321102 | 5.96E-07 | 0.050065568 | 0.0980587 | 0.11 |
| PARP14 | -0.103764631 | 0.00059066 | -0.142509649 | 2.24E-06 | 0.04 |
| PARP15 | -0.156604579 | 1.95E-07 | -0.246655041 | 1.30E-16 | 0.1 |
| PARP16 | -0.13903812 | 3.95E-06 | -0.180613626 | 1.81E-09 | 0.05 |
| PARP1 | 0.144458934 | 1.62E-06 | 0.358447335 | 1.76E-34 | 0.22 |
| PARP2 | 0.332954798 | 1.05E-29 | 0.262675462 | 1.05E-18 | 0.08 |
| DKFZP566G0224 | -0.36548426 | 7.06E-36 | -0.213141598 | 1.08E-12 | 0.16 |
| VPARP | -0.182644288 | 1.18E-09 | -0.216658831 | 4.46E-13 | 0.04 |
| LOC56966 | 0.147725268 | 9.33E-07 | -0.104661551 | 0.0005288 | 0.26 |
| PARP8 | -0.319225015 | 2.59E-27 | -0.276207219 | 1.37E-20 | 0.05 |
| PARP9 | -0.142843363 | 2.12E-06 | -0.109543568 | 0.0002852 | 0.04 |
| PARS2 | 0.286309538 | 4.56E-22 | 0.115622255 | 0.0001276 | 0.18 |
| PART1 | 0.143275379 | 1.98E-06 | 0.028507218 | 0.3464091 | 0.12 |
| MXRA2.withdrawn | -0.335628736 | 3.47E-30 | -0.246956934 | 1.19E-16 | 0.09 |
| PARVB | 0.278394951 | 6.64E-21 | 0.13975243 | 3.52E-06 | 0.14 |
| PARVG | -0.029686508 | 0.32681698 | -0.133229691 | 9.90E-06 | 0.11 |
| PASD1 | 0.125095361 | 3.36E-05 | 0.040315093 | 0.1829077 | 0.09 |
| PASK | 0.05343959 | 0.07739876 | -0.042797623 | 0.157379 | 0.1 |
| PATE1 | 0.006817592 | 0.82187187 | -0.016609547 | 0.5833279 | 0.03 |
| PATE2 | 0.154277617 | 2.97E-07 | 0.004830636 | 0.8732579 | 0.15 |
| PATE3 | 0.07488085 | 0.01327718 | 0.004226157 | 0.8890078 | 0.08 |
| PATE4 | 0.004246056 | 0.88848857 | -0.03859992 | 0.2022563 | 0.05 |
| PATL1 | 0.119671158 | 7.30E-05 | -0.064372593 | 0.0333399 | 0.19 |
| PATL2 | 0.057877518 | 0.05576383 | -0.054018732 | 0.0742371 | 0.12 |
| PATZ1 | -0.185471848 | 6.47E-10 | 0.0718633 | 0.0174922 | 0.26 |
| PAWR | -0.206155826 | 5.91E-12 | -0.219473477 | 2.18E-13 | 0.02 |
| PAX1 | -0.0034357 | 0.90966864 | 0.010329805 | 0.7330077 | 0.02 |
| PAX2 | -0.209632669 | 2.55E-12 | -0.054814539 | 0.0700655 | 0.16 |
| PAX3 | -0.061409568 | 0.04237413 | -0.083766731 | 0.0055871 | 0.03 |
| PAX4 | 0.0615534 | 0.04189212 | 0.001241408 | 0.9673001 | 0.07 |
| PAX5 | -0.01548119 | 0.60916784 | -0.145592997 | 1.34E-06 | 0.14 |
| PAX6 | 0.060762127 | 0.04460222 | -0.071269271 | 0.018448 | 0.14 |
| PAX7 | 0.038408121 | 0.20450925 | 0.078876765 | 0.0090863 | 0.05 |
| PAX8 | -0.089594363 | 0.00303063 | 0.001576448 | 0.958482 | 0.1 |
| PAX9 | -0.227740599 | 2.52E-14 | 0.013789287 | 0.6488349 | 0.25 |
| PAXIP1 | -0.096768008 | 0.00135974 | -0.108688383 | 0.0003184 | 0.02 |
| PBK | 0.457457027 | 1.25E-57 | 0.283320106 | 1.27E-21 | 0.18 |
| PBLD | -0.310406295 | 7.67E-26 | -0.124446678 | 3.70E-05 | 0.19 |
| PBOV1 | -0.230958013 | 1.06E-14 | -0.021187632 | 0.4840823 | 0.21 |
| FLJ11064 | -0.343037062 | 1.53E-31 | -0.304238125 | 7.68E-25 | 0.04 |
| LOC100131938 | -0.312293693 | 3.75E-26 | -0.031192574 | 0.3028624 | 0.29 |
| PBX2 | 0.119936323 | 7.04E-05 | 0.081924539 | 0.0067298 | 0.04 |
| PBX3 | -0.244078698 | 2.74E-16 | -0.249408208 | 5.82E-17 | 0.01 |
| PBX4 | 0.169607664 | 1.68E-08 | 0.042079846 | 0.1644661 | 0.13 |
| PBXIP1 | -0.183155579 | 1.06E-09 | 0.069763669 | 0.0210773 | 0.26 |
| PCA3 | 0.00944133 | 0.75520365 | 0.000220982 | 0.9941776 | 0.01 |
| PCBD1 | 0.205788234 | 6.45E-12 | 0.330985613 | 2.35E-29 | 0.13 |
| PCBD2 | -0.320375765 | 1.65E-27 | -0.070065454 | 0.0205256 | 0.26 |
| PCBP1 | 0.298718562 | 5.76E-24 | 0.178356184 | 2.89E-09 | 0.13 |
| PCBP2 | -0.023688861 | 0.43399102 | 0.030774933 | 0.3093853 | 0.06 |
| PCBP3.OT1 | 0.131184779 | 1.36E-05 | -0.01698617 | 0.574818 | 0.15 |
| MCG10 | 0.290752621 | 9.78E-23 | 0.183645891 | 9.56E-10 | 0.11 |
| PCCA | -0.270492718 | 8.82E-20 | -0.094274039 | 0.0018077 | 0.18 |
| PCCB | 0.133895127 | 8.93E-06 | 0.256510255 | 6.98E-18 | 0.13 |
| PCDH10 | -0.131140804 | 1.36E-05 | -0.139214634 | 3.84E-06 | 0.01 |
| LOC93452 | 0.054169707 | 0.07343051 | -0.102826276 | 0.0006625 | 0.16 |
| LOC349451 | 0.06866842 | 0.02318949 | -0.05467215 | 0.0707974 | 0.13 |
| PCDH12 | -0.317330927 | 5.41E-27 | -0.212923176 | 1.14E-12 | 0.11 |
| DFNB23 | 0.089792721 | 0.00296635 | 0.011690888 | 0.6994388 | 0.08 |
| LOC144997 | -0.24689111 | 1.22E-16 | -0.2378436 | 1.60E-15 | 0.01 |
| PCDH18 | -0.268179569 | 1.85E-19 | -0.271158503 | 7.11E-20 | 0.01 |
| EFMR | -0.317166275 | 5.77E-27 | -0.288053056 | 2.50E-22 | 0.03 |
| PCDH1 | -0.444028708 | 5.11E-54 | -0.255743496 | 8.81E-18 | 0.19 |
| FLJ22218 | -0.233508338 | 5.31E-15 | -0.097492782 | 0.0012502 | 0.14 |
| LOC115364 | -0.341492476 | 2.96E-31 | -0.177702358 | 3.31E-09 | 0.17 |
| PCDH8 | 0.249833478 | 5.14E-17 | 0.024342071 | 0.4214198 | 0.23 |
| LOC57123 | -0.119024332 | 8.00E-05 | -0.261162464 | 1.68E-18 | 0.15 |
| CNRS8 | -0.215135085 | 6.54E-13 | -0.105959279 | 0.0004499 | 0.11 |
| CNRS7 | -0.201335728 | 1.85E-11 | -0.16151545 | 7.92E-08 | 0.04 |
| PCDHA12 | -0.262590896 | 1.08E-18 | -0.188380724 | 3.45E-10 | 0.08 |
| CNRS5 | -0.17217658 | 1.01E-08 | -0.154905195 | 2.65E-07 | 0.02 |
| PCDHA1 | 0.006519132 | 0.82954765 | 0.07234696 | 0.0167464 | 0.07 |
| PCDHA2 | -0.178966108 | 2.55E-09 | -0.102165196 | 0.000718 | 0.08 |
| PCDHA3 | -0.273215007 | 3.65E-20 | -0.167494643 | 2.54E-08 | 0.11 |
| PCDHA4 | -0.251517686 | 3.12E-17 | -0.162627123 | 6.43E-08 | 0.09 |
| PCDHA5 | -0.164822694 | 4.24E-08 | -0.095974993 | 0.0014897 | 0.07 |
| PCDHA6 | -0.248753143 | 7.06E-17 | -0.150093308 | 6.20E-07 | 0.1 |
| PCDHA7 | -0.128388843 | 2.07E-05 | -0.082276265 | 0.0064966 | 0.05 |
| PCDHA8 | -0.128263919 | 2.11E-05 | -0.085990633 | 0.0044423 | 0.05 |
| PCDHA9 | -0.180999769 | 1.67E-09 | -0.132499791 | 1.11E-05 | 0.05 |
| LOC133740 | -0.191707363 | 1.66E-10 | -0.278136345 | 7.23E-21 | 0.09 |
| LOC116041 | -0.285442955 | 6.14E-22 | -0.378077731 | 1.82E-38 | 0.1 |
| PCDHB10 | -0.024337081 | 0.42151498 | -0.117009867 | 0.0001056 | 0.1 |
| PCDHB11 | -0.435947013 | 6.39E-52 | -0.298145054 | 7.08E-24 | 0.14 |
| PCDHB12 | -0.392628141 | 1.33E-41 | -0.243051641 | 3.68E-16 | 0.15 |
| LOC115663 | -0.31050903 | 7.38E-26 | -0.15874869 | 1.32E-07 | 0.16 |
| PCDHB14 | -0.201328858 | 1.85E-11 | -0.153313208 | 3.53E-07 | 0.05 |
| PCDHB15 | -0.341995796 | 2.39E-31 | -0.284429324 | 8.69E-22 | 0.06 |
| PCDHB16 | -0.207347577 | 4.44E-12 | -0.103020756 | 0.000647 | 0.11 |
| LOC112446 | -0.036124526 | 0.23274421 | -0.017814034 | 0.5563212 | 0.02 |
| LOC112442 | -0.372541496 | 2.58E-37 | -0.227283249 | 2.84E-14 | 0.15 |
| PCDHB19P | -0.258616995 | 3.67E-18 | -0.150835382 | 5.45E-07 | 0.11 |
| PCDHB1 | -0.128780136 | 1.95E-05 | -0.118573287 | 8.51E-05 | 0.02 |
| PCDHB2 | 0.000996228 | 0.9737558 | -0.013198691 | 0.6629269 | 0.02 |
| PCDHB3 | -0.164947871 | 4.14E-08 | -0.105613134 | 0.0004698 | 0.06 |
| PCDHB4 | -0.308949944 | 1.33E-25 | -0.193575194 | 1.09E-10 | 0.12 |
| PCDHB5 | -0.278335551 | 6.77E-21 | -0.176536217 | 4.21E-09 | 0.11 |
| PCDHB6 | -0.171411036 | 1.18E-08 | -0.121179477 | 5.91E-05 | 0.06 |
| LOC115667 | -0.238608571 | 1.29E-15 | -0.148060914 | 8.81E-07 | 0.1 |
| PCDHB8 | -0.233003185 | 6.09E-15 | -0.14121253 | 2.78E-06 | 0.1 |
| PCDHB9 | 0.127063618 | 2.52E-05 | -0.003028563 | 0.9203351 | 0.14 |
| PCDHGA10 | -0.319648287 | 2.19E-27 | -0.276668202 | 1.18E-20 | 0.05 |
| PCDHGA11 | -0.32767412 | 9.03E-29 | -0.353342627 | 1.73E-33 | 0.03 |
| CDH21 | -0.41205195 | 4.83E-46 | -0.398173624 | 7.69E-43 | 0.02 |
| PCDHGA1 | -0.15089734 | 5.39E-07 | -0.164124861 | 4.85E-08 | 0.02 |
| PCDHGA2 | -0.42961597 | 2.57E-50 | -0.332069343 | 1.51E-29 | 0.1 |
| PCDHGA3 | -0.399609267 | 3.64E-43 | -0.297643993 | 8.48E-24 | 0.11 |
| PCDHGA4 | -0.333855144 | 7.24E-30 | -0.264085157 | 6.75E-19 | 0.07 |
| PCDHGA5 | -0.412532381 | 3.72E-46 | -0.283740332 | 1.10E-21 | 0.13 |
| PCDHGA6 | -0.416908257 | 3.37E-47 | -0.336443918 | 2.47E-30 | 0.09 |
| PCDHGA7 | -0.300740856 | 2.77E-24 | -0.24248476 | 4.32E-16 | 0.06 |
| PCDHGA8 | -0.114186843 | 0.00015486 | -0.101641563 | 0.0007649 | 0.02 |
| PCDHGA9 | -0.450787031 | 8.14E-56 | -0.410026058 | 1.45E-45 | 0.05 |
| PCDHGB1 | -0.123489847 | 4.25E-05 | -0.149420065 | 6.97E-07 | 0.03 |
| PCDHGB2 | -0.316086871 | 8.76E-27 | -0.306967407 | 2.79E-25 | 0.01 |
| PCDHGB3 | -0.327423111 | 9.99E-29 | -0.300335743 | 3.21E-24 | 0.03 |
| PCDHGB4 | -0.269721083 | 1.13E-19 | -0.300141201 | 3.44E-24 | 0.04 |
| PCDHGB5 | -0.338930304 | 8.73E-31 | -0.28751424 | 3.01E-22 | 0.06 |
| PCDHGB6 | -0.409647877 | 1.78E-45 | -0.393113602 | 1.04E-41 | 0.02 |
| PCDHGB7 | -0.477106725 | 3.25E-63 | -0.452147433 | 3.50E-56 | 0.03 |
| PCDHGB8P | -0.173841126 | 7.26E-09 | -0.180156713 | 1.99E-09 | 0.01 |
| LOC94378 | -0.212566805 | 1.24E-12 | -0.189996395 | 2.42E-10 | 0.03 |
| PCDHGC4 | -0.199958558 | 2.55E-11 | -0.262026137 | 1.28E-18 | 0.07 |
| PCDHGC5 | -0.307976495 | 1.91E-25 | -0.284283671 | 9.13E-22 | 0.03 |
| CFAP221 | -0.114349456 | 0.00015152 | 0.031521087 | 0.2977963 | 0.15 |
| KIAA0824 | -0.337324943 | 1.71E-30 | -0.284785435 | 7.69E-22 | 0.06 |
| PCGEM1 | 0.049781126 | 0.09998463 | 0.072431985 | 0.0166182 | 0.03 |
| PCGF1 | 0.430485377 | 1.55E-50 | 0.351656906 | 3.64E-33 | 0.08 |
| PCGF2 | -0.149734204 | 6.60E-07 | 0.036991683 | 0.2217136 | 0.19 |
| RNF3A | -0.195751705 | 6.69E-11 | -0.114599726 | 0.0001465 | 0.09 |
| PCGF5 | -0.240389987 | 7.83E-16 | -0.299195035 | 4.85E-24 | 0.06 |
| PCGF6 | 0.325461362 | 2.20E-28 | 0.141888801 | 2.48E-06 | 0.19 |
| LOC105370376 | 0.183736605 | 9.38E-10 | 0.156958368 | 1.83E-07 | 0.03 |
| C20orf67 | -0.00024755 | 0.99347754 | -0.070302227 | 0.0201015 | 0.08 |
| PCK1 | -0.243626302 | 3.12E-16 | -0.152079635 | 4.38E-07 | 0.1 |
| PCK2 | 0.054392216 | 0.07225473 | 0.035365841 | 0.2427093 | 0.02 |
| ACZ | -0.329032197 | 5.21E-29 | -0.262108128 | 1.25E-18 | 0.07 |
| PCM1 | -0.368541698 | 1.70E-36 | -0.175609525 | 5.08E-09 | 0.2 |
| PCMT1 | 0.232047773 | 7.90E-15 | 0.19666307 | 5.44E-11 | 0.04 |
| PCMTD1 | -0.188936277 | 3.05E-10 | -0.094345588 | 0.0017931 | 0.1 |
| PCMTD2 | -0.219934196 | 1.94E-13 | -0.202317232 | 1.47E-11 | 0.02 |
| LOC100420696 | -0.012763064 | 0.67339857 | 0.000176914 | 0.9953386 | 0.02 |
| PCNA | 0.551301349 | 6.41E-88 | 0.349328525 | 1.01E-32 | 0.21 |
| PCNP | -0.002422301 | 0.93624434 | -0.014410927 | 0.6341365 | 0.02 |
| KEN | 0.000665052 | 0.98247842 | -0.155882656 | 2.23E-07 | 0.16 |
| PCNXL2 | -0.097132959 | 0.00130354 | -0.060459324 | 0.0456777 | 0.04 |
| PCNXL3 | -0.002016722 | 0.94690197 | -0.038271402 | 0.2061263 | 0.04 |
| PCNXL1 | -0.430300742 | 1.73E-50 | -0.472185833 | 8.80E-62 | 0.05 |
| PCOLCE2 | 0.078480043 | 0.0094418 | -0.05270913 | 0.0815417 | 0.14 |
| PCOLCE | -0.119908686 | 7.07E-05 | -0.039991912 | 0.1864446 | 0.08 |
| C1QTNF9B.AS1 | 0.041758047 | 0.16772028 | 0.186983657 | 4.67E-10 | 0.15 |
| PCP2 | -0.19498971 | 7.95E-11 | 0.008542956 | 0.777854 | 0.21 |
| PCP4L1 | 0.134667575 | 7.92E-06 | 0.076232233 | 0.0117 | 0.06 |
| PCP4 | 0.161835594 | 7.46E-08 | 0.103672763 | 0.0005974 | 0.06 |
| PCSK1N | 0.297123631 | 1.02E-23 | 0.05636305 | 0.0624985 | 0.25 |
| PCSK1 | -0.032139231 | 0.28841771 | -0.17670319 | 4.06E-09 | 0.15 |
| PCSK2 | 0.038552981 | 0.20280598 | -0.000572865 | 0.9849069 | 0.04 |
| PCSK4 | -0.316863021 | 6.49E-27 | -0.076313702 | 0.0116105 | 0.25 |
| LOC100290671 | -0.392772742 | 1.23E-41 | -0.323362994 | 5.07E-28 | 0.07 |
| PCSK6 | -0.286371634 | 4.47E-22 | -0.196628281 | 5.48E-11 | 0.09 |
| LOC95070 | -0.091677129 | 0.00241479 | -0.103134258 | 0.0006381 | 0.02 |
| HCHOLA3 | 0.039259933 | 0.19464156 | -0.02122375 | 0.4833374 | 0.07 |
| PCTP | 0.045714486 | 0.13093976 | 0.066678747 | 0.0274985 | 0.03 |
| PCYOX1L | -0.147301348 | 1.00E-06 | 0.000696692 | 0.981645 | 0.15 |
| LOC130931 | -0.369305173 | 1.19E-36 | -0.352882047 | 2.12E-33 | 0.02 |
| PCYT1A | -0.135062619 | 7.44E-06 | -0.283749874 | 1.10E-21 | 0.15 |
| PCYT1B | 0.032096817 | 0.28905481 | -0.018256582 | 0.5465538 | 0.06 |
| PCYT2 | 0.355357223 | 7.05E-34 | 0.211124152 | 1.77E-12 | 0.15 |
| PC | 0.00708472 | 0.81501628 | -0.106644773 | 0.0004128 | 0.12 |
| PDAP1 | 0.443738374 | 6.09E-54 | 0.312626156 | 3.30E-26 | 0.14 |
| PDCD10 | 0.197079323 | 4.94E-11 | 0.09777931 | 0.0012092 | 0.1 |
| PDCD11 | 0.205977658 | 6.16E-12 | 0.041200133 | 0.1734763 | 0.17 |
| PDCD1LG2 | 0.010112536 | 0.73841578 | -0.124338942 | 3.76E-05 | 0.14 |
| SLEB2 | 0.113519541 | 0.00016931 | -0.046868093 | 0.1214868 | 0.17 |
| PDCD2L | 0.513812387 | 1.13E-74 | 0.38501642 | 6.08E-40 | 0.13 |
| PDCD2 | 0.302835847 | 1.29E-24 | 0.331415971 | 1.97E-29 | 0.03 |
| PDCD4 | -0.168801178 | 1.97E-08 | 0.058583702 | 0.0528363 | 0.23 |
| PDCD5 | 0.667704417 | 5.19E-142 | 0.479528066 | 6.28E-64 | 0.19 |
| PDCD6IP | -0.353769133 | 1.43E-33 | -0.218518306 | 2.78E-13 | 0.14 |
| LOC100129491 | 0.312044578 | 4.12E-26 | 0.318714661 | 3.16E-27 | 0.01 |
| PDCD7 | 0.197982685 | 4.02E-11 | -0.020542733 | 0.4974873 | 0.22 |
| PDCL2 | 0.216830667 | 4.27E-13 | 0.089574882 | 0.003037 | 0.13 |
| PDCL3 | 0.494718407 | 1.54E-68 | 0.435693228 | 7.43E-52 | 0.06 |
| DKFZp564M1863 | -0.126707934 | 2.66E-05 | -0.049017839 | 0.1053019 | 0.08 |
| PDC | 0.043502107 | 0.15065028 | 0.061705566 | 0.0413872 | 0.02 |
| PDDC1 | 0.041826429 | 0.16702476 | -0.004766799 | 0.8749189 | 0.05 |
| PDE10A | -0.243315829 | 3.41E-16 | -0.23119674 | 9.95E-15 | 0.02 |
| PDE11A | -0.414728003 | 1.12E-46 | -0.214481195 | 7.70E-13 | 0.21 |
| LOC102724626 | -0.218772876 | 2.61E-13 | -0.134750491 | 7.81E-06 | 0.09 |
| PDE1A | -0.420110259 | 5.68E-48 | -0.236270495 | 2.48E-15 | 0.19 |
| PDE1B | -0.205794957 | 6.44E-12 | -0.144780453 | 1.54E-06 | 0.07 |
| PDE1C | -0.170589062 | 1.39E-08 | -0.279398234 | 4.75E-21 | 0.11 |
| LOC119865 | -0.398827829 | 5.47E-43 | -0.315678951 | 1.02E-26 | 0.09 |
| PDE3A | -0.420552205 | 4.44E-48 | -0.396480183 | 1.85E-42 | 0.03 |
| PDE3B | -0.047508597 | 0.11647338 | -0.172774441 | 8.99E-09 | 0.13 |
| PDE4A | -0.332515191 | 1.26E-29 | -0.13179987 | 1.23E-05 | 0.21 |
| PDE4B | -0.035114129 | 0.24608065 | 0.042844119 | 0.156928 | 0.08 |
| PDE4C | -0.084908622 | 0.00496976 | -0.054608592 | 0.0711262 | 0.04 |
| PDE4DIP | -0.152869923 | 3.81E-07 | -0.02798885 | 0.3552525 | 0.13 |
| LOC654081 | -0.151409738 | 4.93E-07 | -0.076216524 | 0.0117174 | 0.08 |
| PDE5A | -0.226628193 | 3.38E-14 | -0.22026284 | 1.78E-13 | 0.01 |
| PDE6A | 0.082285252 | 0.00649078 | 0.029602969 | 0.3281807 | 0.06 |
| PDE6B | -0.146419111 | 1.17E-06 | 0.012504584 | 0.6796421 | 0.16 |
| PDE6C | -0.094822127 | 0.00169897 | -0.108766639 | 0.0003152 | 0.02 |
| PDE6D | 0.225447552 | 4.62E-14 | 0.176137633 | 4.56E-09 | 0.05 |
| PDE6G | 0.12932942 | 1.80E-05 | -0.003861203 | 0.8985384 | 0.14 |
| PDE6H | 0.13088017 | 1.42E-05 | 0.057262753 | 0.0584211 | 0.08 |
| PDE7A | 0.391924565 | 1.90E-41 | 0.170028801 | 1.55E-08 | 0.23 |
| PDE7B | -0.354650054 | 9.67E-34 | -0.295742244 | 1.68E-23 | 0.06 |
| PDE8A | -0.110599027 | 0.00024877 | -0.270652101 | 8.38E-20 | 0.17 |
| ADSD | -0.235628212 | 2.96E-15 | -0.117378067 | 0.0001004 | 0.12 |
| LOC100132338 | 0.173177676 | 8.29E-09 | 0.094772516 | 0.0017086 | 0.08 |
| LOC86077 | 0.452931268 | 2.15E-56 | 0.365000672 | 8.83E-36 | 0.09 |
| PDGFA | -0.11408071 | 0.00015708 | -0.192056401 | 1.53E-10 | 0.08 |
| PDGFB | -0.187081711 | 4.57E-10 | -0.22792834 | 2.39E-14 | 0.05 |
| LOC51158 | -0.352288737 | 2.76E-33 | -0.240200677 | 8.26E-16 | 0.12 |
| PDGFD | -0.50114524 | 1.46E-70 | -0.258254365 | 4.10E-18 | 0.25 |
| PDGFRA | -0.213854037 | 9.01E-13 | -0.177867901 | 3.20E-09 | 0.04 |
| PDGFRB | -0.408663552 | 3.02E-45 | -0.279496567 | 4.60E-21 | 0.13 |
| PDGFRL | -0.263335367 | 8.54E-19 | -0.092238214 | 0.0022697 | 0.18 |
| PDHA1 | 0.504149722 | 1.60E-71 | 0.262446315 | 1.13E-18 | 0.25 |
| PDHA2 | 0.01983886 | 0.51234052 | -0.006696367 | 0.8249875 | 0.03 |
| PDHB | -0.06514195 | 0.03128317 | 0.098251786 | 0.0011443 | 0.17 |
| PDHX | 0.165153797 | 3.98E-08 | 0.041341062 | 0.1720086 | 0.13 |
| PDI | 0.310903982 | 6.36E-26 | 0.169526982 | 1.71E-08 | 0.15 |
| GRP58P | 0.13988929 | 3.44E-06 | 0.073946188 | 0.0144751 | 0.07 |
| PDIA3 | 0.221233939 | 1.39E-13 | 0.157725262 | 1.59E-07 | 0.07 |
| LOC220831 | 0.231616572 | 8.88E-15 | 0.133157676 | 1.00E-05 | 0.1 |
| PDIA5 | 0.130046882 | 1.61E-05 | 0.135217257 | 7.26E-06 | 0.01 |
| PDIA6 | 0.39197426 | 1.85E-41 | 0.156161309 | 2.12E-07 | 0.24 |
| PDIK1L | -0.207909087 | 3.87E-12 | -0.156677826 | 1.93E-07 | 0.06 |
| PDILT | 0.006416846 | 0.83218201 | 0.059313201 | 0.049948 | 0.06 |
| PDK1 | 0.187912061 | 3.82E-10 | 0.038458019 | 0.2039214 | 0.15 |
| LOC100506500 | -0.217578865 | 3.53E-13 | -0.129041548 | 1.88E-05 | 0.09 |
| PDK3 | 0.18510223 | 7.01E-10 | 0.033748989 | 0.2649357 | 0.16 |
| DLX6.1 | 0.296318797 | 1.36E-23 | 0.033061068 | 0.2748045 | 0.27 |
| CLIM1 | -0.103005102 | 0.00064824 | 0.107753459 | 0.0003587 | 0.22 |
| FLJ34715 | -0.053547191 | 0.0768032 | -0.036740583 | 0.2248685 | 0.02 |
| PDLIM3 | -0.170685441 | 1.36E-08 | -0.100758583 | 0.0008505 | 0.07 |
| PDLIM4 | -0.06344895 | 0.03596079 | -0.005743555 | 0.8495682 | 0.06 |
| PDLIM5 | -0.23087106 | 1.09E-14 | -0.258649309 | 3.64E-18 | 0.03 |
| PDLIM7 | 0.001186183 | 0.96875404 | 0.036478294 | 0.2281981 | 0.04 |
| LOC157663 | -0.090746384 | 0.00267429 | -0.013079625 | 0.6657826 | 0.08 |
| LOC100505926 | -0.284221131 | 9.33E-22 | -0.351992117 | 3.14E-33 | 0.07 |
| PDPK1 | -0.461397844 | 1.01E-58 | -0.429689586 | 2.46E-50 | 0.04 |
| PDPN | -0.163811446 | 5.14E-08 | -0.12140865 | 5.72E-05 | 0.05 |
| PDPR | -0.340978951 | 3.68E-31 | -0.419166868 | 9.62E-48 | 0.08 |
| C20orf126 | 0.26720284 | 2.53E-19 | 0.272224363 | 5.04E-20 | 0.01 |
| PDS5A | -0.124093907 | 3.89E-05 | -0.151516727 | 4.84E-07 | 0.03 |
| AS3 | -0.351312688 | 4.24E-33 | -0.240760101 | 7.06E-16 | 0.12 |
| PDSS1 | 0.594816484 | 1.45E-105 | 0.427141913 | 1.06E-49 | 0.17 |
| PDSS2 | -0.103703512 | 0.00059511 | -0.004639416 | 0.8782351 | 0.1 |
| IUF1 | 0.284277685 | 9.15E-22 | 0.050000072 | 0.0984995 | 0.24 |
| PDXDC1 | -0.208756247 | 3.16E-12 | -0.058686477 | 0.0524211 | 0.16 |
| PDXDC2P | -0.111904103 | 0.00020971 | -0.098414516 | 0.0011227 | 0.02 |
| LOC85572 | 0.25421675 | 1.40E-17 | 0.081634871 | 0.0069274 | 0.18 |
| DJ37E16.5 | 0.438447973 | 1.46E-52 | 0.162476986 | 6.61E-08 | 0.28 |
| SCA23 | 0.114041416 | 0.00015791 | 0.087478419 | 0.0037997 | 0.03 |
| PDZD11 | 0.220479461 | 1.68E-13 | 0.309735232 | 9.88E-26 | 0.09 |
| PDZD2 | -0.350430169 | 6.24E-33 | -0.228947656 | 1.82E-14 | 0.13 |
| PDZD3 | -0.034498589 | 0.25446278 | 0.066009842 | 0.0290944 | 0.11 |
| LU1 | -0.074927437 | 0.01321984 | -0.106043184 | 0.0004452 | 0.04 |
| DFNB57 | 0.050453851 | 0.09547756 | -0.042772994 | 0.1576183 | 0.1 |
| PDZD8 | -0.337638917 | 1.50E-30 | -0.37994744 | 7.34E-39 | 0.05 |
| PDZD9 | 0.015519258 | 0.60828782 | -0.031088303 | 0.3044824 | 0.05 |
| PDZK1IP1 | 0.026110437 | 0.38847586 | 0.126448989 | 2.76E-05 | 0.11 |
| PDZK1P1 | -0.166652139 | 2.99E-08 | -0.065822341 | 0.0295558 | 0.11 |
| LOC115142 | -0.282065494 | 1.94E-21 | -0.162155557 | 7.02E-08 | 0.12 |
| PDZRN3 | -0.236769783 | 2.16E-15 | -0.201202732 | 1.91E-11 | 0.04 |
| PDZRN4 | -0.299417771 | 4.47E-24 | -0.14364894 | 1.86E-06 | 0.16 |
| LOC116081 | -0.039657098 | 0.19016179 | 0.065642956 | 0.0300031 | 0.11 |
| PEAR1 | -0.518125613 | 4.08E-76 | -0.340819417 | 3.93E-31 | 0.18 |
| LOC100506570 | 0.235867144 | 2.77E-15 | 0.188364231 | 3.46E-10 | 0.05 |
| PEBP4 | -0.246208322 | 1.48E-16 | -0.054739065 | 0.0704527 | 0.2 |
| LOC100506961 | -0.173117201 | 8.39E-09 | -0.110974549 | 0.0002369 | 0.07 |
| ECI2 | -0.198564493 | 3.52E-11 | 0.0589058 | 0.0515443 | 0.26 |
| PECR | -0.072718057 | 0.01619319 | -0.016002725 | 0.5971611 | 0.06 |
| PEFLIN | 0.173553116 | 7.69E-09 | 0.066301328 | 0.0283894 | 0.11 |
| LOC115497 | 0.109437789 | 0.00028916 | 0.095948092 | 0.0014943 | 0.02 |
| PEG3.AS1 | -0.130226775 | 1.57E-05 | -0.241756873 | 5.32E-16 | 0.12 |
| PEG3 | -0.078021111 | 0.00986849 | -0.139785502 | 3.50E-06 | 0.07 |
| LOC57334 | 0.068086158 | 0.02438537 | -0.097481196 | 0.0012519 | 0.17 |
| LOC93480 | -0.298600766 | 6.01E-24 | -0.269658237 | 1.15E-19 | 0.03 |
| PELI3 | 0.032614775 | 0.28133946 | -0.029966822 | 0.3222678 | 0.07 |
| PRO1770 | -0.083611122 | 0.0056764 | -0.059912681 | 0.0476745 | 0.03 |
| LOC116052 | 0.350483892 | 6.10E-33 | 0.264680245 | 5.60E-19 | 0.09 |
| PEMT | 0.171989363 | 1.05E-08 | 0.10360513 | 0.0006023 | 0.07 |
| PENK | -0.174381094 | 6.51E-09 | -0.134482539 | 8.15E-06 | 0.04 |
| MGC10905 | 0.033633949 | 0.26656884 | -0.042651669 | 0.158801 | 0.08 |
| PER1 | -0.224347211 | 6.17E-14 | -0.226814496 | 3.22E-14 | 0.01 |
| PER2 | -0.484267203 | 2.42E-65 | -0.359150987 | 1.28E-34 | 0.13 |
| PER3 | -0.367973081 | 2.22E-36 | -0.367582462 | 2.66E-36 | 0.01 |
| PER4 | 0.096092328 | 0.00146974 | 0.002455363 | 0.935376 | 0.1 |
| PERP | 0.151203683 | 5.11E-07 | 0.164631778 | 4.40E-08 | 0.02 |
| PES1 | 0.420905931 | 3.64E-48 | 0.296593494 | 1.24E-23 | 0.13 |
| GATB | 0.125638927 | 3.11E-05 | 0.061985618 | 0.0404715 | 0.07 |
| PEX10 | 0.016722753 | 0.58076377 | -0.044644631 | 0.1402065 | 0.07 |
| PEX11A | -0.320766666 | 1.41E-27 | -0.052918384 | 0.0803369 | 0.27 |
| PEX11B | 0.001666118 | 0.95612269 | 0.331185135 | 2.17E-29 | 0.33 |
| PEX11G | -0.159277323 | 1.20E-07 | 0.008260404 | 0.785018 | 0.17 |
| PEX12 | -0.319841732 | 2.03E-27 | -0.189729961 | 2.57E-10 | 0.14 |
| PEX13 | 0.136706812 | 5.74E-06 | 0.100459618 | 0.0008814 | 0.04 |
| PEX14 | 0.132482122 | 1.11E-05 | 0.046633611 | 0.1233638 | 0.09 |
| PEX16 | 0.164073448 | 4.89E-08 | 0.218651767 | 2.69E-13 | 0.06 |
| PEX19 | -0.30822089 | 1.75E-25 | 0.049811522 | 0.0997774 | 0.36 |
| ZWS1 | -0.340481433 | 4.54E-31 | -0.219180647 | 2.35E-13 | 0.13 |
| PEX26 | -0.269739325 | 1.12E-19 | -0.332264173 | 1.39E-29 | 0.07 |
| PEX2 | 0.017571217 | 0.56171623 | 0.053146667 | 0.0790391 | 0.04 |
| PEX3 | 0.020029775 | 0.50828913 | 0.022481941 | 0.4577792 | 0.01 |
| PEX5L | -0.162495963 | 6.59E-08 | -0.189685187 | 2.59E-10 | 0.03 |
| PEX5 | 0.15495623 | 2.63E-07 | 0.038646534 | 0.2017115 | 0.12 |
| PEX6 | 0.077236765 | 0.01063757 | 0.051727354 | 0.0873913 | 0.03 |
| PEX7 | -0.170024326 | 1.55E-08 | 0.120306191 | 6.68E-05 | 0.3 |
| PF4V1 | 0.238302749 | 1.41E-15 | 0.087960029 | 0.0036106 | 0.16 |
| PF4 | 0.115826495 | 0.00012413 | 0.012996924 | 0.667769 | 0.11 |
| KIAA0361 | -0.129121558 | 1.85E-05 | -0.131757558 | 1.24E-05 | 0.01 |
| PFDN1 | 0.15713749 | 1.77E-07 | 0.267967736 | 1.98E-19 | 0.12 |
| PFDN2 | 0.560618931 | 1.77E-91 | 0.560226919 | 2.51E-91 | 0.01 |
| PFDN4 | 0.508281435 | 7.40E-73 | 0.397671773 | 9.97E-43 | 0.12 |
| PFDN5 | 0.133582253 | 9.37E-06 | 0.266173875 | 3.50E-19 | 0.14 |
| PFDN6 | 0.519958343 | 9.83E-77 | 0.453120457 | 1.91E-56 | 0.07 |
| PFKFB1 | -0.223993686 | 6.77E-14 | -0.075821429 | 0.0121609 | 0.15 |
| PFKFB2 | -0.021722058 | 0.47312355 | 0.099255051 | 0.001017 | 0.13 |
| PFKFB3 | -0.303233476 | 1.11E-24 | -0.167430721 | 2.57E-08 | 0.14 |
| PFKFB4 | 0.050679977 | 0.09399959 | -0.046536137 | 0.1241506 | 0.1 |
| PFKL | 0.198074734 | 3.93E-11 | 0.083462958 | 0.0057626 | 0.12 |
| PFKX | -0.058937359 | 0.0514191 | -0.11746388 | 9.92E-05 | 0.06 |
| PFKP | 0.277115537 | 1.01E-20 | 0.13057627 | 1.49E-05 | 0.15 |
| PFN1 | 0.516062527 | 2.01E-75 | 0.370920526 | 5.56E-37 | 0.15 |
| LOC85837 | 0.351885506 | 3.29E-33 | 0.01717854 | 0.5704941 | 0.34 |
| PFN3 | 0.060509889 | 0.04549659 | 0.042924259 | 0.156153 | 0.02 |
| PFN4 | 0.118247683 | 8.91E-05 | 0.088998355 | 0.0032315 | 0.03 |
| LOC101929842 | -0.108054372 | 0.00034527 | -0.129414551 | 1.77E-05 | 0.03 |
| PGA4 | 0.000505916 | 0.98667058 | -0.03130307 | 0.3011521 | 0.04 |
| LOC220000 | -0.008399038 | 0.78150062 | -0.044711442 | 0.1396134 | 0.04 |
| PGAM1 | 0.30924613 | 1.19E-25 | 0.153937909 | 3.15E-07 | 0.16 |
| PGAM2 | -0.067512596 | 0.02561519 | -0.01206504 | 0.6903096 | 0.06 |
| PGAM3 | -0.010851805 | 0.72006891 | -0.175604206 | 5.08E-09 | 0.17 |
| PGAM5 | 0.291407734 | 7.77E-23 | 0.053343676 | 0.0779328 | 0.24 |
| PGAP1 | -0.182847858 | 1.13E-09 | -0.229298993 | 1.66E-14 | 0.05 |
| PGAP2 | 0.083517036 | 0.005731 | 0.202334763 | 1.46E-11 | 0.12 |
| PGAP3 | -0.074943974 | 0.01319953 | 0.100484942 | 0.0008788 | 0.18 |
| PGBD1 | -0.009837549 | 0.74527909 | -0.07250599 | 0.0165073 | 0.07 |
| PGBD2 | -0.09240299 | 0.00222862 | 0.15699696 | 1.82E-07 | 0.25 |
| PGBD3 | -0.146814806 | 1.09E-06 | -0.14993383 | 6.38E-07 | 0.01 |
| PGBD4 | -0.26321088 | 8.88E-19 | -0.23061963 | 1.16E-14 | 0.04 |
| PGBD5 | 0.260088986 | 2.34E-18 | -0.026474416 | 0.3818947 | 0.29 |
| CPQ | -0.0295536 | 0.3289884 | -0.150452968 | 5.83E-07 | 0.13 |
| PGC | -0.036674448 | 0.22570474 | 0.0139942 | 0.6439743 | 0.06 |
| PGD | 0.252973245 | 2.02E-17 | 0.063149502 | 0.0368473 | 0.19 |
| PGF | -0.006792194 | 0.82252442 | -0.034199836 | 0.2586018 | 0.03 |
| LOC91374 | -0.356104533 | 5.05E-34 | -0.108129211 | 0.000342 | 0.25 |
| PGK1 | 0.225797143 | 4.21E-14 | 0.083012099 | 0.0060322 | 0.15 |
| PGK2 | -0.030617091 | 0.31187443 | 0.089238021 | 0.0031493 | 0.12 |
| PGLS | 0.211309911 | 1.69E-12 | 0.244264593 | 2.60E-16 | 0.04 |
| PGLYRP1 | 0.014129105 | 0.64078267 | -0.016094422 | 0.5950612 | 0.04 |
| PGLYRP2 | -0.249968577 | 4.94E-17 | -0.079677039 | 0.0084052 | 0.18 |
| PGLYRP3 | 0.193200762 | 1.19E-10 | 0.053227074 | 0.078586 | 0.14 |
| PGLYRP4 | 0.246895295 | 1.21E-16 | 0.136883889 | 5.58E-06 | 0.12 |
| PGM1 | 0.152216885 | 4.28E-07 | -0.067698058 | 0.0252118 | 0.22 |
| PGM2L1 | -0.143379904 | 1.94E-06 | -0.023117447 | 0.4451634 | 0.13 |
| PGM2 | -0.040382095 | 0.18218064 | -0.111902101 | 0.0002098 | 0.08 |
| PGM3 | -0.105377797 | 0.00048378 | -0.026482542 | 0.3817486 | 0.08 |
| PGM5P2 | -0.300160806 | 3.42E-24 | -0.332293879 | 1.38E-29 | 0.04 |
| PGM5 | -0.378564931 | 1.44E-38 | -0.271896316 | 5.60E-20 | 0.11 |
| PGPEP1L | 0.343842878 | 1.09E-31 | 0.114050178 | 0.0001577 | 0.23 |
| PGPEP1 | -0.468587283 | 9.50E-61 | -0.246603767 | 1.32E-16 | 0.23 |
| MGC4692 | 0.332950362 | 1.05E-29 | 0.273744684 | 3.07E-20 | 0.06 |
| PGRMC1 | 0.168135895 | 2.24E-08 | 0.210832478 | 1.90E-12 | 0.05 |
| PGRMC2 | -0.164971207 | 4.12E-08 | -0.056366593 | 0.062482 | 0.11 |
| PGR | -0.387489599 | 1.77E-40 | -0.167677241 | 2.45E-08 | 0.22 |
| DKFZp762M186 | 0.122033019 | 5.23E-05 | -0.096881156 | 0.0013421 | 0.22 |
| PHACTR1 | -0.178777198 | 2.65E-09 | -0.218858826 | 2.55E-13 | 0.05 |
| PHACTR2 | -0.178151052 | 3.02E-09 | -0.210425535 | 2.10E-12 | 0.04 |
| PHACTR3 | 0.095316636 | 0.00160608 | 0.01606859 | 0.5956524 | 0.08 |
| PHACTR4 | -0.115580932 | 0.00012833 | -0.262462154 | 1.12E-18 | 0.15 |
| PHAX | -0.265450338 | 4.40E-19 | -0.179652294 | 2.21E-09 | 0.09 |
| PHB2 | 0.441187536 | 2.83E-53 | 0.271166434 | 7.10E-20 | 0.18 |
| PHB | 0.342633971 | 1.82E-31 | 0.334334206 | 5.94E-30 | 0.01 |
| PHC1 | -0.044238238 | 0.14385564 | -0.120248376 | 6.74E-05 | 0.08 |
| PHC2 | 0.15245361 | 4.10E-07 | -0.013428432 | 0.6574306 | 0.17 |
| FLJ12967 | -0.455940426 | 3.25E-57 | -0.3702318 | 7.70E-37 | 0.09 |
| PHEX | -0.085873406 | 0.00449691 | 0.008525258 | 0.7783021 | 0.1 |
| PHF10 | 0.034400833 | 0.25581202 | -0.018394957 | 0.5435172 | 0.06 |
| PHF11 | -0.009723861 | 0.74812253 | 0.094284669 | 0.0018055 | 0.11 |
| PHF12 | -0.293417508 | 3.83E-23 | -0.163976823 | 4.98E-08 | 0.13 |
| PHF13 | 0.035415512 | 0.24204782 | -0.097778108 | 0.0012094 | 0.14 |
| PHF14 | 0.136866023 | 5.60E-06 | 0.212968214 | 1.12E-12 | 0.08 |
| JADE2 | -0.483216077 | 5.01E-65 | -0.346640282 | 3.25E-32 | 0.14 |
| JADE3 | -0.004865973 | 0.87233866 | -0.094020974 | 0.00186 | 0.09 |
| JADE1 | -0.175039366 | 5.70E-09 | -0.216012709 | 5.25E-13 | 0.05 |
| PHF19 | 0.335962563 | 3.02E-30 | 0.090712209 | 0.0026843 | 0.25 |
| PHF1 | -0.169595229 | 1.69E-08 | -0.085574667 | 0.0046388 | 0.09 |
| PHF20L1 | 0.093676208 | 0.00193349 | -0.082443057 | 0.0063886 | 0.18 |
| PHF20 | -0.342447892 | 1.97E-31 | -0.184770943 | 7.52E-10 | 0.16 |
| LOC143677 | -0.082943686 | 0.00607408 | -0.194638748 | 8.60E-11 | 0.12 |
| PHF21B | -0.119078742 | 7.94E-05 | -0.185285517 | 6.74E-10 | 0.07 |
| PHF23 | 0.055690774 | 0.06569642 | 0.159769733 | 1.10E-07 | 0.11 |
| KIAA0662 | -0.388701816 | 9.67E-41 | -0.372533919 | 2.59E-37 | 0.02 |
| KIAA0244 | -0.319461263 | 2.36E-27 | -0.312354358 | 3.66E-26 | 0.01 |
| PHF5A | 0.500711923 | 2.01E-70 | 0.292823667 | 4.72E-23 | 0.21 |
| LOC220901 | 0.021487898 | 0.47790831 | -0.034282623 | 0.2574502 | 0.06 |
| PHF7 | -0.302143283 | 1.66E-24 | -0.21953849 | 2.14E-13 | 0.09 |
| MRXSSD | -0.284383718 | 8.82E-22 | -0.208352993 | 3.48E-12 | 0.08 |
| LOC115871 | 0.364648396 | 1.04E-35 | 0.204564713 | 8.63E-12 | 0.17 |
| PHGR1 | -0.178568644 | 2.77E-09 | -0.127723003 | 2.28E-05 | 0.06 |
| PHIP | -0.205677033 | 6.62E-12 | -0.119435914 | 7.55E-05 | 0.09 |
| PHKA1 | -0.065990087 | 0.02914273 | -0.03774951 | 0.2123843 | 0.03 |
| PHKA2 | -0.152791756 | 3.87E-07 | -0.118551578 | 8.54E-05 | 0.04 |
| PHKB | -0.248598754 | 7.39E-17 | -0.209830497 | 2.43E-12 | 0.04 |
| PHKG1 | -0.050113977 | 0.09773388 | -0.150073034 | 6.23E-07 | 0.1 |
| PHKG2 | 0.163791155 | 5.16E-08 | 0.171972995 | 1.05E-08 | 0.01 |
| PHLDA1 | -0.01024955 | 0.73500382 | -0.178312156 | 2.92E-09 | 0.17 |
| PHLDA2 | 0.21787823 | 3.27E-13 | 0.182360966 | 1.26E-09 | 0.04 |
| PHLDA3 | -0.10519282 | 0.00049505 | 0.068872866 | 0.0227818 | 0.18 |
| PHLDB1 | -0.263847071 | 7.28E-19 | -0.194880114 | 8.15E-11 | 0.07 |
| PHLDB2 | -0.42301041 | 1.11E-48 | -0.306112437 | 3.84E-25 | 0.12 |
| LOC654204 | -0.144781044 | 1.54E-06 | -0.089837038 | 0.0029522 | 0.06 |
| PHLPP1 | 0.156514037 | 1.99E-07 | -0.125663443 | 3.10E-05 | 0.29 |
| PHLPP2 | -0.238618473 | 1.29E-15 | -0.354019411 | 1.28E-33 | 0.12 |
| PHOSPHO1 | -0.049223823 | 0.10384533 | -0.097998897 | 0.0011786 | 0.05 |
| PHOSPHO2 | -0.300834889 | 2.67E-24 | 0.014765727 | 0.6258112 | 0.32 |
| FEOM2 | 0.130672559 | 1.47E-05 | 0.050488133 | 0.0952523 | 0.09 |
| PHOX2B | 0.191531035 | 1.72E-10 | 0.103419185 | 0.0006162 | 0.09 |
| PHPT1 | 0.037713982 | 0.21281523 | 0.195020255 | 7.89E-11 | 0.16 |
| PHRF1 | -0.109332305 | 0.00029311 | -0.18053803 | 1.84E-09 | 0.08 |
| PHTF1 | 0.061720507 | 0.04133794 | -0.113377866 | 0.0001725 | 0.18 |
| PHTF2 | -0.043736095 | 0.14846454 | -0.056784549 | 0.0605599 | 0.02 |
| PHYHD1 | -0.327385376 | 1.01E-28 | -0.110667706 | 0.0002466 | 0.22 |
| PHYHIPL | 0.053206724 | 0.0787005 | -0.036803178 | 0.224079 | 0.1 |
| DYRK1AIP3 | -0.25236616 | 2.43E-17 | -0.173765764 | 7.37E-09 | 0.08 |
| PHYH | 0.198537256 | 3.54E-11 | 0.115622998 | 0.0001276 | 0.09 |
| PI15 | -0.080189284 | 0.00799359 | -0.157529008 | 1.65E-07 | 0.08 |
| PI16 | -0.18465989 | 7.70E-10 | -0.154312374 | 2.95E-07 | 0.04 |
| PI3 | 0.253514716 | 1.72E-17 | 0.036083801 | 0.2332716 | 0.22 |
| PI4K2A | -0.175632508 | 5.05E-09 | -0.294962208 | 2.21E-23 | 0.12 |
| PI4K2B | -0.02776608 | 0.35909635 | -0.017700865 | 0.5588325 | 0.02 |
| PI4KAP1 | -0.034037176 | 0.26087477 | -0.090587344 | 0.0027211 | 0.06 |
| LOC220686 | -0.027785289 | 0.35876389 | -0.084121111 | 0.0053885 | 0.06 |
| PI4KA | -0.377922893 | 1.96E-38 | -0.353648605 | 1.51E-33 | 0.03 |
| PI4KB | 0.059299335 | 0.0500016 | 0.186391865 | 5.31E-10 | 0.13 |
| DDXBP1 | -0.349615055 | 8.92E-33 | -0.398331515 | 7.08E-43 | 0.05 |
| PIAS2 | 0.102940211 | 0.00065339 | -0.112200101 | 0.0002017 | 0.22 |
| PIAS3 | -0.246453679 | 1.38E-16 | 0.055290262 | 0.0676648 | 0.31 |
| PIAS4 | -0.119092333 | 7.92E-05 | -0.093135405 | 0.0020542 | 0.03 |
| PIBF1 | -0.175561137 | 5.13E-09 | -0.096848308 | 0.0013472 | 0.08 |
| PICALM | -0.078988388 | 0.00898842 | -0.169673777 | 1.66E-08 | 0.1 |
| PICK1 | 0.133255029 | 9.86E-06 | 0.06684013 | 0.027125 | 0.07 |
| PID1 | -0.120290263 | 6.70E-05 | -0.145922317 | 1.27E-06 | 0.03 |
| PIF1 | 0.552891044 | 1.61E-88 | 0.25546107 | 9.59E-18 | 0.3 |
| PIGA | 0.039222118 | 0.19507209 | 0.028427723 | 0.3477561 | 0.02 |
| PIGB | -0.200235017 | 2.39E-11 | 0.038633067 | 0.2018688 | 0.24 |
| PIGC | 0.241558623 | 5.63E-16 | 0.49203376 | 1.04E-67 | 0.26 |
| PIGF | 0.166527 | 3.06E-08 | 0.276546427 | 1.22E-20 | 0.12 |
| PIGG | -0.371563037 | 4.11E-37 | -0.174576816 | 6.26E-09 | 0.2 |
| PIGH | -0.189180331 | 2.90E-10 | 0.042272079 | 0.1625449 | 0.24 |
| PIGK | -0.354079745 | 1.25E-33 | -0.271776793 | 5.83E-20 | 0.09 |
| LOC102724483 | -0.056001328 | 0.06420283 | -0.138267416 | 4.47E-06 | 0.09 |
| PIGM | -0.032748965 | 0.27936358 | 0.295716581 | 1.69E-23 | 0.33 |
| PIGN | -0.140818512 | 2.96E-06 | -0.090975085 | 0.0026083 | 0.05 |
| PIGO | -0.265244478 | 4.69E-19 | -0.164663512 | 4.37E-08 | 0.11 |
| DSCR5 | 0.150516884 | 5.76E-07 | 0.167264966 | 2.66E-08 | 0.02 |
| LOC146326 | -0.1419755 | 2.45E-06 | 0.034661774 | 0.2522215 | 0.18 |
| PIGR | -0.157854831 | 1.56E-07 | 0.020892909 | 0.4901841 | 0.18 |
| LOC90402 | -0.209190195 | 2.84E-12 | -0.123292659 | 4.37E-05 | 0.09 |
| PIGT | -0.181632615 | 1.46E-09 | -0.03807964 | 0.20841 | 0.15 |
| PIGU | 0.389137797 | 7.77E-41 | 0.400396141 | 2.42E-43 | 0.02 |
| LOC115569 | -0.271134154 | 7.17E-20 | -0.171163076 | 1.24E-08 | 0.1 |
| PIGW | 0.242139731 | 4.77E-16 | 0.072531717 | 0.0164689 | 0.17 |
| PIGX | 0.00988127 | 0.74418651 | 0.085754674 | 0.0045528 | 0.08 |
| PIGY | 0.315852588 | 9.59E-27 | 0.157349019 | 1.71E-07 | 0.16 |
| PIGZ | -0.010924198 | 0.71828072 | -0.105241841 | 0.000492 | 0.1 |
| PIH1D1 | 0.214186439 | 8.29E-13 | 0.235294266 | 3.25E-15 | 0.03 |
| PIH1D2 | -0.222487916 | 1.00E-13 | 0.058834533 | 0.0518278 | 0.29 |
| PIK3AP1 | -0.01775343 | 0.55766535 | -0.170043777 | 1.54E-08 | 0.16 |
| PIK3C2A | -0.41763378 | 2.25E-47 | -0.395106257 | 3.74E-42 | 0.03 |
| PIK3C2B | -0.346199797 | 3.94E-32 | -0.105080441 | 0.000502 | 0.25 |
| LOC121529 | -0.054959484 | 0.06932673 | -0.056444241 | 0.0621212 | 0.01 |
| PIK3C3 | -0.032077302 | 0.28934825 | -0.165142652 | 3.99E-08 | 0.14 |
| PIK3CA | -0.181727488 | 1.43E-09 | -0.292673738 | 4.98E-23 | 0.12 |
| PIK3CB | -0.185191974 | 6.87E-10 | -0.238413331 | 1.37E-15 | 0.06 |
| PIK3CD | -0.03543998 | 0.24172247 | -0.195986805 | 6.34E-11 | 0.17 |
| PIK3CG | -0.345626883 | 5.04E-32 | -0.395275515 | 3.43E-42 | 0.05 |
| PIK3IP1 | -0.432111314 | 6.05E-51 | -0.35476588 | 9.18E-34 | 0.08 |
| PIK3R1 | -0.365602764 | 6.68E-36 | -0.257400648 | 5.33E-18 | 0.11 |
| PIK3R2 | 0.051886782 | 0.086419 | 0.042417952 | 0.1610984 | 0.01 |
| PIK3R3 | -0.132420165 | 1.12E-05 | -0.071681673 | 0.0177797 | 0.07 |
| PIK3R4 | -0.321219403 | 1.18E-27 | -0.22056219 | 1.65E-13 | 0.11 |
| PIK3R5 | -0.173589424 | 7.64E-09 | -0.248262648 | 8.15E-17 | 0.08 |
| PIK3R6 | -0.090396855 | 0.00277814 | -0.154572861 | 2.82E-07 | 0.07 |
| CFD.1 | -0.231820022 | 8.41E-15 | -0.113823907 | 0.0001626 | 0.12 |
| PILRA | 0.079333139 | 0.0086921 | -0.058137894 | 0.0546691 | 0.14 |
| PILRB | -0.016100281 | 0.59492717 | 0.033693929 | 0.2657165 | 0.05 |
| PIM1 | 0.227303405 | 2.83E-14 | -0.067182527 | 0.0263469 | 0.3 |
| PIM2 | 0.151423096 | 4.92E-07 | -0.009983683 | 0.7416292 | 0.17 |
| PIM3 | 0.123847737 | 4.03E-05 | 0.021314711 | 0.4814641 | 0.11 |
| PIN1P1 | 0.051107656 | 0.09125439 | 0.054997329 | 0.0691349 | 0.01 |
| PIN1 | 0.140645913 | 3.05E-06 | 0.101702221 | 0.0007593 | 0.04 |
| PIN4 | 0.352473005 | 2.54E-33 | 0.388876379 | 8.86E-41 | 0.04 |
| PINK1 | -0.254077998 | 1.45E-17 | -0.315317545 | 1.18E-26 | 0.07 |
| PINX1 | 0.301673624 | 1.97E-24 | 0.206802722 | 5.06E-12 | 0.1 |
| LOC392943 | -0.2642633 | 6.39E-19 | -0.197716356 | 4.27E-11 | 0.07 |
| PIP4K2A | -0.157553338 | 1.64E-07 | -0.0767667 | 0.0111236 | 0.09 |
| PIP4K2B | -0.178051966 | 3.08E-09 | -0.1102392 | 0.0002607 | 0.07 |
| PIP4K2C | -0.144699378 | 1.56E-06 | -0.116566531 | 0.0001122 | 0.03 |
| PIP5K1A | -0.017973963 | 0.5527816 | 0.092504241 | 0.0022037 | 0.12 |
| PIP5K1B | -0.125446678 | 3.20E-05 | -0.088133182 | 0.0035447 | 0.04 |
| PIP5K1C | -0.203124681 | 1.21E-11 | -0.204048463 | 9.76E-12 | 0.01 |
| LOC100422366 | -0.090791344 | 0.00266119 | -0.083010231 | 0.0060333 | 0.01 |
| PIP5KL1 | -0.113547393 | 0.00016868 | -0.046415697 | 0.1251283 | 0.07 |
| PIPOX | 0.012959787 | 0.66866174 | -0.083810248 | 0.0055624 | 0.1 |
| PIPSL | 0.258401772 | 3.92E-18 | 0.353331474 | 1.74E-33 | 0.1 |
| PIP | -0.342508728 | 1.92E-31 | -0.028630967 | 0.3443188 | 0.32 |
| LOC649488 | 0.044692081 | 0.13978506 | -0.026557104 | 0.3804092 | 0.08 |
| PIR | 0.428623699 | 4.55E-50 | 0.245528838 | 1.80E-16 | 0.19 |
| DKFZP566G2246_2 | -0.133687906 | 9.22E-06 | -0.137329094 | 5.20E-06 | 0.01 |
| PISRT1 | -0.008944371 | 0.76770866 | 0.009746576 | 0.7475541 | 0.02 |
| PITPNA | -0.004924836 | 0.87080782 | -0.070265987 | 0.0201659 | 0.07 |
| PITPNB | 0.215606512 | 5.81E-13 | 0.065345341 | 0.030758 | 0.16 |
| PITPNC1 | 0.125097394 | 3.36E-05 | 0.025707698 | 0.3958376 | 0.1 |
| PITPNM1 | -0.116759625 | 0.0001093 | -0.180367179 | 1.91E-09 | 0.07 |
| PITPNM2 | 0.1612827 | 8.27E-08 | -0.095213122 | 0.0016251 | 0.26 |
| PITPNM3 | 0.029908281 | 0.32321445 | -0.168752139 | 1.99E-08 | 0.2 |
| KIAA1104 | 0.181777819 | 1.42E-09 | 0.068600386 | 0.0233265 | 0.12 |
| PITX1 | 0.154583958 | 2.81E-07 | 0.206106368 | 5.98E-12 | 0.06 |
| PITX2 | -0.06083774 | 0.04433702 | -0.047242118 | 0.1185391 | 0.02 |
| PITX3 | 0.027161968 | 0.36965083 | 0.032498386 | 0.2830609 | 0.01 |
| PIWIL1 | 0.010532536 | 0.72797338 | -0.01776322 | 0.5574481 | 0.03 |
| PIWIL2 | -0.276377522 | 1.29E-20 | -0.247852112 | 9.19E-17 | 0.03 |
| PIWIL3 | 0.046182549 | 0.12703786 | 0.038568805 | 0.2026205 | 0.01 |
| PIWIL4 | 0.104242492 | 0.0005569 | 0.002244255 | 0.9409217 | 0.11 |
| PJA1 | -0.148452105 | 8.24E-07 | -0.227357642 | 2.79E-14 | 0.08 |
| PJA2 | -0.58482195 | 2.83E-101 | -0.370836832 | 5.79E-37 | 0.22 |
| LOC136290 | -0.272517568 | 4.58E-20 | -0.100129774 | 0.0009168 | 0.18 |
| PKD1L2 | -0.152025494 | 4.43E-07 | -0.172951327 | 8.68E-09 | 0.03 |
| PKD1L3 | -0.171347217 | 1.19E-08 | -0.220029018 | 1.89E-13 | 0.05 |
| LOC116058 | -0.253607581 | 1.67E-17 | -0.320345281 | 1.67E-27 | 0.07 |
| PKDL_2 | 0.097859012 | 0.00119803 | 0.114864052 | 0.0001414 | 0.02 |
| PKD2L2 | -0.217145667 | 3.94E-13 | -0.165715841 | 3.58E-08 | 0.06 |
| PKD2 | -0.342652198 | 1.81E-31 | -0.424395692 | 5.09E-49 | 0.09 |
| PKDCC | -0.072709968 | 0.01620507 | -0.197035929 | 4.99E-11 | 0.13 |
| PKDREJ | -0.014228405 | 0.63843759 | -0.133162724 | 1.00E-05 | 0.12 |
| PKHD1L1 | -0.080505765 | 0.00774837 | -0.171877493 | 1.07E-08 | 0.1 |
| TIGM1 | -0.167059108 | 2.76E-08 | -0.058831122 | 0.0518414 | 0.11 |
| PKIA | -0.040978623 | 0.17580201 | -0.078871763 | 0.0090907 | 0.04 |
| PKIB | -0.240482938 | 7.63E-16 | -0.038956923 | 0.198111 | 0.21 |
| PKIG | 0.069609734 | 0.02136367 | 0.043070949 | 0.1547419 | 0.03 |
| PKLR | 0.06538363 | 0.03065994 | 0.146835514 | 1.09E-06 | 0.09 |
| PKM | 0.157381442 | 1.70E-07 | 0.02976726 | 0.3255022 | 0.13 |
| PKMYT1 | 0.49594451 | 6.38E-69 | 0.312271255 | 3.78E-26 | 0.19 |
| PKN1 | 0.149561491 | 6.80E-07 | 0.098008423 | 0.0011773 | 0.06 |
| PKN2 | -0.231686524 | 8.72E-15 | -0.284295915 | 9.09E-22 | 0.06 |
| PKN3 | -0.051768809 | 0.0871376 | -0.043302081 | 0.1525381 | 0.01 |
| PKNOX1 | -0.232774011 | 6.49E-15 | -0.044137842 | 0.1447682 | 0.19 |
| PKNOX2 | -0.11139843 | 0.0002241 | -0.104984239 | 0.000508 | 0.01 |
| PKP1 | 0.194076007 | 9.77E-11 | 0.030749773 | 0.3097811 | 0.17 |
| PKP2 | -0.11462333 | 0.00014605 | -0.098543301 | 0.0011059 | 0.02 |
| PKP3 | 0.228063859 | 2.31E-14 | 0.181812031 | 1.41E-09 | 0.05 |
| PKP4 | -0.231876251 | 8.28E-15 | -0.202003011 | 1.58E-11 | 0.03 |
| LOC641845 | 0.435860847 | 6.73E-52 | 0.345992367 | 4.31E-32 | 0.09 |
| PLA1A | -0.075524829 | 0.01250361 | -0.053578129 | 0.0766327 | 0.03 |
| PLA2G10 | -0.163978056 | 4.98E-08 | 0.002445854 | 0.9356258 | 0.17 |
| PLA2G12A | -0.26739971 | 2.37E-19 | -0.11691753 | 0.000107 | 0.16 |
| PLA2G12B | 0.061758562 | 0.04121261 | 0.023320438 | 0.4411759 | 0.04 |
| PLA2G15 | -0.000498238 | 0.98687287 | -0.001291745 | 0.965975 | 0.01 |
| PLA2G16 | -0.202369434 | 1.45E-11 | -0.033740074 | 0.265062 | 0.17 |
| PLA2G1B | 0.021274127 | 0.48229939 | 0.016341975 | 0.5894091 | 0.01 |
| PLA2G2A | -0.140258808 | 3.24E-06 | -0.021056834 | 0.4867852 | 0.12 |
| LOC105376824 | -0.130751503 | 1.45E-05 | -0.008558392 | 0.7774631 | 0.13 |
| SPLA2S | 0.052977337 | 0.08000013 | -0.072211451 | 0.0169525 | 0.13 |
| PLA2G2E | NA | NA | NA | NA | #VALUE! |
| PLA2G2F | 0.056841432 | 0.06030218 | 0.010845975 | 0.720213 | 0.05 |
| PLA2G3 | -0.082215054 | 0.00653669 | 0.007032599 | 0.8163528 | 0.09 |
| PLA2G4A | 0.038835238 | 0.19951688 | -0.117267041 | 0.000102 | 0.16 |
| LOC112491 | -0.104028579 | 0.00057179 | -0.105454429 | 0.0004792 | 0.01 |
| PLA2G4D | 0.047780375 | 0.11439577 | -0.018054658 | 0.5509998 | 0.07 |
| LOC388117 | 0.05369935 | 0.0759674 | -0.045973539 | 0.1287689 | 0.1 |
| PLA2G4F | -0.327447718 | 9.89E-29 | -0.139407653 | 3.72E-06 | 0.19 |
| PLA2G5 | -0.088585765 | 0.00337756 | -0.063661296 | 0.0353432 | 0.03 |
| PLA2G6 | -0.05278848 | 0.08108316 | -0.039533491 | 0.1915478 | 0.02 |
| PLA2G7 | 0.211781623 | 1.51E-12 | 0.038268302 | 0.2061631 | 0.18 |
| PLA2R1 | -0.515321713 | 3.55E-75 | -0.395861685 | 2.54E-42 | 0.12 |
| PLA2P | 0.158910381 | 1.28E-07 | 0.10386304 | 0.0005836 | 0.06 |
| OOSP2 | 0.003110976 | 0.91817477 | -0.000389927 | 0.9897264 | 0.01 |
| PLAC1 | 0.012208055 | 0.68683184 | 0.113313071 | 0.000174 | 0.11 |
| TINCR | 0.109903558 | 0.00027227 | 0.022060675 | 0.4662512 | 0.09 |
| PLAC4 | -0.046044379 | 0.12818016 | -0.012241618 | 0.6860166 | 0.04 |
| PLAC8L1 | 0.303721919 | 9.29E-25 | 0.239969589 | 8.82E-16 | 0.07 |
| PLAC8 | 0.064068854 | 0.03418319 | -0.069407035 | 0.0217459 | 0.14 |
| PLAC9 | -0.215948448 | 5.33E-13 | -0.081683396 | 0.0068939 | 0.14 |
| PLAG1 | -0.03418748 | 0.25877394 | -0.125211077 | 3.31E-05 | 0.1 |
| PLAGL1 | -0.01292642 | 0.66946426 | -0.136423978 | 6.00E-06 | 0.13 |
| PLAGL2 | 0.002072681 | 0.9454309 | -0.057265117 | 0.0584107 | 0.06 |
| PLAT | -0.339993448 | 5.58E-31 | -0.190813927 | 2.02E-10 | 0.15 |
| PLAUR | 0.222843246 | 9.14E-14 | 0.084252582 | 0.0053165 | 0.14 |
| LOC115418 | 0.014364686 | 0.63522505 | -0.005836252 | 0.84717 | 0.03 |
| PLB1 | -0.149054218 | 7.43E-07 | -0.174376568 | 6.52E-09 | 0.03 |
| PLBD1 | 0.091522516 | 0.00245624 | -0.038221961 | 0.2067134 | 0.13 |
| PLBD2 | -0.431407144 | 9.11E-51 | -0.422011487 | 1.96E-48 | 0.01 |
| PLCB1 | -0.211977921 | 1.43E-12 | -0.195242429 | 7.51E-11 | 0.02 |
| PLCB2 | -0.055344542 | 0.06739521 | -0.139846062 | 3.47E-06 | 0.09 |
| PLCB3 | 0.147904273 | 9.05E-07 | 0.089739066 | 0.0029836 | 0.06 |
| PLCB4 | 0.106880872 | 0.00040067 | 0.076463414 | 0.0114475 | 0.04 |
| PLCD1 | -0.167430919 | 2.57E-08 | -0.175041238 | 5.70E-09 | 0.01 |
| PLCD3 | -0.185266503 | 6.76E-10 | -0.194180723 | 9.54E-11 | 0.01 |
| PLCD4 | -0.178229178 | 2.97E-09 | -0.126253786 | 2.84E-05 | 0.06 |
| PLCE1 | -0.027857454 | 0.35751659 | -0.100965942 | 0.0008296 | 0.08 |
| PLCG1 | 0.036485181 | 0.22811019 | -0.116999174 | 0.0001058 | 0.16 |
| RP11.960L18.1 | 0.061845832 | 0.04092641 | -0.167560266 | 2.51E-08 | 0.23 |
| PLCH1 | 0.277885553 | 7.86E-21 | 0.14635461 | 1.18E-06 | 0.14 |
| PLCH2 | -0.20838339 | 3.45E-12 | -0.202733865 | 1.33E-11 | 0.01 |
| PLCL1 | -0.3280709 | 7.69E-29 | -0.118074785 | 9.12E-05 | 0.21 |
| PLCL2 | -0.187704032 | 4.00E-10 | -0.250419774 | 4.32E-17 | 0.07 |
| PLCXD1 | 0.268279719 | 1.79E-19 | 0.165315012 | 3.86E-08 | 0.11 |
| PLCXD2 | -0.164786758 | 4.27E-08 | -0.230872386 | 1.09E-14 | 0.07 |
| PLCXD3 | -0.328971916 | 5.34E-29 | -0.234222917 | 4.36E-15 | 0.1 |
| PLCZ1 | -0.069163359 | 0.02221328 | -0.056940875 | 0.0598537 | 0.02 |
| PLD1 | -0.04786135 | 0.11378242 | -0.116565804 | 0.0001122 | 0.07 |
| PLD2 | -0.106582161 | 0.00041604 | -0.055562273 | 0.0663228 | 0.06 |
| PLD3 | 0.040550667 | 0.18036099 | -0.054103662 | 0.0737825 | 0.1 |
| PLD4 | -0.359214624 | 1.25E-34 | -0.250400978 | 4.35E-17 | 0.11 |
| PLD5 | -0.085670841 | 0.00459267 | -0.125012591 | 3.41E-05 | 0.04 |
| PLD6 | 0.098201568 | 0.00115104 | 0.058814252 | 0.0519088 | 0.04 |
| BLOC1S6 | -0.372407745 | 2.75E-37 | -0.252194373 | 2.55E-17 | 0.13 |
| EBS1 | -0.187496098 | 4.18E-10 | -0.275479467 | 1.74E-20 | 0.09 |
| PLEK2 | 0.057710902 | 0.05647388 | 0.128214982 | 2.12E-05 | 0.08 |
| PLEKHA1 | -0.110920959 | 0.00023854 | -0.182253037 | 1.28E-09 | 0.08 |
| PLEKHA2 | -0.132909121 | 1.04E-05 | -0.192825171 | 1.29E-10 | 0.06 |
| FLJ20067 | -0.283277505 | 1.29E-21 | -0.196710812 | 5.38E-11 | 0.09 |
| PLEKHA4 | -0.038474024 | 0.20373308 | -0.040038344 | 0.1859333 | 0.01 |
| PLEKHA5 | -0.171776123 | 1.10E-08 | -0.173549425 | 7.70E-09 | 0.01 |
| PLEKHA6 | -0.27724925 | 9.71E-21 | 0.085032105 | 0.0049068 | 0.37 |
| PLEKHA7 | -0.362618687 | 2.64E-35 | -0.376892908 | 3.22E-38 | 0.02 |
| PLEKHA8 | 0.072629299 | 0.01632402 | 0.104529039 | 0.0005375 | 0.04 |
| PLEKHA8P1 | 0.214240484 | 8.18E-13 | 0.25090051 | 3.75E-17 | 0.04 |
| PLEKHB1 | 0.146118098 | 1.23E-06 | 0.072835243 | 0.0160219 | 0.08 |
| PLEKHB2 | -0.350790069 | 5.33E-33 | -0.281064603 | 2.72E-21 | 0.07 |
| PLEKHF1 | 0.218687068 | 2.67E-13 | 0.038964663 | 0.1980218 | 0.18 |
| PLEKHF2 | -0.230034281 | 1.36E-14 | -0.08329814 | 0.0058599 | 0.15 |
| PLEKHG1 | 0.001581905 | 0.95833839 | -0.190465085 | 2.18E-10 | 0.2 |
| PLEKHG2 | 0.014573484 | 0.63031633 | -0.164676003 | 4.36E-08 | 0.18 |
| PLEKHG3 | -0.218797125 | 2.59E-13 | -0.099803494 | 0.000953 | 0.12 |
| PLEKHG4B | 0.153518867 | 3.40E-07 | 0.069379122 | 0.021799 | 0.09 |
| PLEKHG4 | 0.033637637 | 0.26651638 | -0.04614035 | 0.1273859 | 0.08 |
| PLEKHG5 | -0.143630999 | 1.86E-06 | -0.239754019 | 9.37E-16 | 0.1 |
| PLEKHG6 | 0.127302244 | 2.43E-05 | 0.062558997 | 0.03865 | 0.07 |
| PLEKHG7 | -0.101403989 | 0.0007871 | -0.020398045 | 0.5005217 | 0.09 |
| PLEKHH1 | -0.136760872 | 5.69E-06 | -0.150567494 | 5.71E-07 | 0.02 |
| PLEKHH2 | -0.327685755 | 8.98E-29 | -0.170244329 | 1.48E-08 | 0.16 |
| PLEKHH3 | -0.134823044 | 7.73E-06 | -0.021695738 | 0.4736601 | 0.12 |
| PLEKHJ1 | 0.285801919 | 5.43E-22 | 0.228754913 | 1.92E-14 | 0.06 |
| PLEKHM1P | -0.188077917 | 3.68E-10 | -0.208303188 | 3.52E-12 | 0.03 |
| PLEKHM1 | -0.422912 | 1.18E-48 | -0.366790652 | 3.85E-36 | 0.06 |
| PLEKHM2 | -0.008318 | 0.78355615 | -0.233324105 | 5.58E-15 | 0.23 |
| LOC100131989 | -0.291373957 | 7.87E-23 | -0.437520232 | 2.52E-52 | 0.15 |
| PLEKHN1 | 0.006421556 | 0.83206066 | -0.061078694 | 0.0435008 | 0.07 |
| PLEKHO1 | 0.279189985 | 5.09E-21 | 0.171321812 | 1.20E-08 | 0.11 |
| PLEKHO2 | -0.160483385 | 9.60E-08 | -0.223850101 | 7.03E-14 | 0.07 |
| PLEK | -0.060748996 | 0.04464841 | -0.192757284 | 1.31E-10 | 0.14 |
| PLGLA | -0.062295347 | 0.03947872 | -0.033954381 | 0.262037 | 0.03 |
| PLGLB2 | -0.129887331 | 1.65E-05 | -0.015079153 | 0.618496 | 0.12 |
| PLG | -0.024967007 | 0.40959473 | -0.017709409 | 0.5586427 | 0.01 |
| PLIN1 | -0.313850875 | 2.07E-26 | -0.189340806 | 2.80E-10 | 0.13 |
| PLIN2 | 0.212400545 | 1.29E-12 | 0.109807014 | 0.0002757 | 0.11 |
| PLIN3 | -0.098806152 | 0.00107223 | -0.046530731 | 0.1241944 | 0.06 |
| PLIN4 | -0.246862244 | 1.23E-16 | -0.175808542 | 4.88E-09 | 0.08 |
| PLIN5 | -0.21642166 | 4.73E-13 | 0.014409392 | 0.6341727 | 0.24 |
| KIZ | -0.19401521 | 9.90E-11 | -0.068631277 | 0.0232642 | 0.13 |
| PLK1 | 0.556438098 | 7.21E-90 | 0.281205831 | 2.59E-21 | 0.28 |
| PLK2 | -0.309935254 | 9.17E-26 | -0.118801327 | 8.25E-05 | 0.2 |
| PLK3 | -0.136722402 | 5.73E-06 | -0.136125296 | 6.29E-06 | 0.01 |
| SAK | 0.401413195 | 1.42E-43 | 0.168251183 | 2.19E-08 | 0.24 |
| PLK5 | -0.079959393 | 0.00817604 | 0.030074228 | 0.3205358 | 0.12 |
| PLLP | -0.052033065 | 0.08553461 | 0.035868328 | 0.2360763 | 0.09 |
| PLN | -0.330493724 | 2.88E-29 | -0.171548068 | 1.15E-08 | 0.16 |
| LOC100130069 | 0.31399935 | 1.95E-26 | 0.063904079 | 0.0346482 | 0.26 |
| PLOD2 | 0.1368068 | 5.65E-06 | 0.004984774 | 0.8692495 | 0.14 |
| PLOD3 | 0.311489547 | 5.09E-26 | 0.141889957 | 2.48E-06 | 0.17 |
| PLP1 | -0.045119523 | 0.13603276 | -0.062203006 | 0.0397725 | 0.02 |
| PLP2 | 0.402920826 | 6.42E-44 | 0.265902161 | 3.81E-19 | 0.14 |
| PLRG1 | 0.169766391 | 1.63E-08 | 0.064077248 | 0.0341596 | 0.11 |
| PLS1 | -0.224228676 | 6.36E-14 | -0.13635548 | 6.07E-06 | 0.09 |
| PLS3 | 0.013324921 | 0.65990475 | -0.111843808 | 0.0002114 | 0.13 |
| PLSCR1 | 0.153207382 | 3.59E-07 | -0.026256943 | 0.3858186 | 0.18 |
| PLSCR2 | 0.219507571 | 2.16E-13 | 0.076006892 | 0.0119509 | 0.15 |
| PLSCR3 | 0.170495149 | 1.41E-08 | 0.098535292 | 0.0011069 | 0.08 |
| PLSCR4 | -0.497279708 | 2.43E-69 | -0.390052475 | 4.90E-41 | 0.11 |
| PLSCR5 | 0.247569482 | 9.98E-17 | 0.11669066 | 0.0001103 | 0.14 |
| PLTP | 0.108344656 | 0.00033271 | -0.015222538 | 0.615162 | 0.13 |
| BPIFA1 | 0.029970042 | 0.32221581 | 0.038092565 | 0.2082555 | 0.01 |
| PLVAP | -0.209190057 | 2.84E-12 | -0.138659185 | 4.20E-06 | 0.08 |
| PLXDC1 | -0.384335188 | 8.52E-40 | -0.250618193 | 4.08E-17 | 0.14 |
| PLXDC2 | -0.194758671 | 8.37E-11 | -0.209482432 | 2.64E-12 | 0.02 |
| DKFZp564A176 | 0.02074675 | 0.49322537 | -0.210441699 | 2.09E-12 | 0.24 |
| PLXNA2 | -0.214172106 | 8.32E-13 | -0.217653846 | 3.47E-13 | 0.01 |
| SEX | -0.174289059 | 6.63E-09 | -0.163041434 | 5.95E-08 | 0.02 |
| PLXNA4 | -0.308086321 | 1.84E-25 | -0.227484788 | 2.70E-14 | 0.09 |
| PLXNB1 | -0.196847575 | 5.21E-11 | -0.108620823 | 0.0003212 | 0.09 |
| PLXNB2 | -0.249074887 | 6.42E-17 | -0.195182089 | 7.61E-11 | 0.06 |
| PLXNB3 | 0.033844762 | 0.26358133 | 0.019264736 | 0.5246245 | 0.02 |
| PLXNC1 | -0.462437786 | 5.19E-59 | -0.438716997 | 1.24E-52 | 0.03 |
| PLXND1 | -0.181683533 | 1.45E-09 | -0.20844477 | 3.40E-12 | 0.03 |
| PM20D1 | -0.148424077 | 8.28E-07 | -0.137891918 | 4.75E-06 | 0.02 |
| PM20D2 | 0.178483115 | 2.82E-09 | -0.078192077 | 0.0097076 | 0.26 |
| APR | 0.002908871 | 0.92347368 | -0.059798645 | 0.0481001 | 0.07 |
| PMCHL1 | -0.042735516 | 0.15798293 | 0.005895682 | 0.8456332 | 0.05 |
| PMCHL2 | -0.050314646 | 0.09639661 | 0.027800286 | 0.3585045 | 0.08 |
| PMCH | 0.122197987 | 5.11E-05 | 0.002906007 | 0.9235488 | 0.12 |
| PMEPA1 | -0.156662078 | 1.93E-07 | -0.070894587 | 0.019074 | 0.09 |
| PMF1 | 0.285800655 | 5.44E-22 | 0.488557881 | 1.22E-66 | 0.21 |
| PMFBP1 | 0.157769438 | 1.58E-07 | -0.110705543 | 0.0002453 | 0.27 |
| PML | 0.126797061 | 2.62E-05 | -0.071915569 | 0.0174103 | 0.2 |
| PMM1 | 0.177129164 | 3.72E-09 | 0.057023694 | 0.0594824 | 0.13 |
| PMM2 | 0.252380483 | 2.42E-17 | 0.349812571 | 8.18E-33 | 0.1 |
| PMP22 | -0.2444007 | 2.50E-16 | -0.074432012 | 0.013841 | 0.17 |
| PMP2 | -0.040252228 | 0.18359174 | -0.071950229 | 0.0173561 | 0.04 |
| PMPCA | 0.267942873 | 2.00E-19 | 0.246395619 | 1.40E-16 | 0.03 |
| PMPCB | 0.064603719 | 0.03271025 | 0.157046596 | 1.80E-07 | 0.1 |
| PMS1 | 0.065142833 | 0.03128088 | 0.073465394 | 0.0151276 | 0.01 |
| PMS2CL | -0.152501106 | 4.07E-07 | -0.126625902 | 2.69E-05 | 0.03 |
| LOC100420315 | -0.19620244 | 6.04E-11 | -0.180538022 | 1.84E-09 | 0.02 |
| PMS2P1 | 0.199578339 | 2.78E-11 | 0.321475158 | 1.07E-27 | 0.13 |
| LOC100101441 | -0.121821732 | 5.39E-05 | -0.005044599 | 0.8676947 | 0.12 |
| PMS2P3 | 0.008088122 | 0.78939511 | -0.020750227 | 0.4931529 | 0.03 |
| PMS2P4 | -0.062390812 | 0.03917692 | -0.055565036 | 0.0663092 | 0.01 |
| MGC34222 | 0.069165172 | 0.02220977 | 0.129461688 | 1.76E-05 | 0.07 |
| LOC91271 | -0.027360112 | 0.36616802 | 0.091334556 | 0.0025075 | 0.12 |
| PMVK | 0.1290559 | 1.87E-05 | 0.324387512 | 3.37E-28 | 0.2 |
| PNCK | 0.0496487 | 0.10089149 | 0.086770994 | 0.004094 | 0.04 |
| BRP17 | 0.128588138 | 2.01E-05 | 0.229427045 | 1.60E-14 | 0.11 |
| PNKP | 0.082487635 | 0.00636005 | 0.10124317 | 0.0008025 | 0.02 |
| PNLDC1 | 0.096448973 | 0.0014107 | 0.057278915 | 0.0583499 | 0.04 |
| PNLIPRP1 | 0.024391383 | 0.42047947 | 0.016308192 | 0.590179 | 0.01 |
| PNLIPRP2 | -0.008069988 | 0.78985624 | 0.103991935 | 0.0005744 | 0.12 |
| PNLIPRP3 | 0.002129711 | 0.94393185 | -0.041170316 | 0.173788 | 0.05 |
| PNLIP | -0.029250629 | 0.33397308 | 0.025028258 | 0.4084464 | 0.06 |
| PNMA1 | -0.174702466 | 6.10E-09 | -0.189742355 | 2.56E-10 | 0.02 |
| MM2 | -0.294272142 | 2.83E-23 | -0.25896508 | 3.30E-18 | 0.04 |
| LOC115917 | 0.227503371 | 2.68E-14 | 0.054506467 | 0.071657 | 0.18 |
| PNMA5 | 0.103551345 | 0.00060633 | -0.061705233 | 0.0413883 | 0.17 |
| PNMA6C | 0.07797526 | 0.00991205 | 0.054179803 | 0.0733768 | 0.03 |
| PNMAL1 | 0.083876715 | 0.00552479 | -0.022945945 | 0.4485484 | 0.11 |
| PNMAL2 | -0.128394043 | 2.07E-05 | -0.122253553 | 5.07E-05 | 0.01 |
| PNMT | -0.049809366 | 0.09979208 | 0.05646064 | 0.0620452 | 0.11 |
| PNN | 0.057125522 | 0.05902836 | -0.061565061 | 0.0418532 | 0.12 |
| KHRBP1 | 0.428873217 | 3.94E-50 | 0.202671108 | 1.35E-11 | 0.23 |
| PNOC | 0.019442805 | 0.52079849 | -0.077245777 | 0.0106284 | 0.1 |
| PNPLA1 | 0.203725333 | 1.05E-11 | 0.066410266 | 0.0281298 | 0.14 |
| PNPLA2 | -0.265736495 | 4.02E-19 | -0.086523241 | 0.0042018 | 0.18 |
| PNPLA3 | 0.12750439 | 2.36E-05 | 0.023213385 | 0.4432763 | 0.11 |
| PNPLA4 | -0.280449401 | 3.34E-21 | -0.120504975 | 6.50E-05 | 0.16 |
| PNPLA5 | 0.180447589 | 1.88E-09 | 0.077635345 | 0.0102403 | 0.11 |
| PNPLA6 | 0.060423994 | 0.04580457 | -0.023995764 | 0.4280578 | 0.09 |
| FLJ31318 | -0.367044683 | 3.42E-36 | -0.215246722 | 6.36E-13 | 0.16 |
| LOC392776 | -0.29289583 | 4.60E-23 | -0.322076401 | 8.44E-28 | 0.03 |
| PNPO | -0.049871055 | 0.0993725 | 0.047985942 | 0.1128437 | 0.1 |
| DFNB70 | 0.394520637 | 5.06E-42 | 0.169560503 | 1.70E-08 | 0.23 |
| PNP | 0.418570766 | 1.34E-47 | 0.228267981 | 2.19E-14 | 0.2 |
| PNRC1 | 0.087596673 | 0.00375248 | 0.018344861 | 0.5446156 | 0.07 |
| LOC115275 | -0.142411065 | 2.28E-06 | -0.075819914 | 0.0121627 | 0.07 |
| POC1A | 0.482091129 | 1.09E-64 | 0.356121615 | 5.01E-34 | 0.13 |
| LOC91413 | -0.15535081 | 2.45E-07 | -0.082546267 | 0.0063226 | 0.08 |
| POC5 | -0.165718692 | 3.58E-08 | 0.02327013 | 0.4421622 | 0.19 |
| LOC101928772 | -0.184761482 | 7.54E-10 | -0.0669153 | 0.0269525 | 0.12 |
| PODN | -0.380458578 | 5.72E-39 | -0.175340067 | 5.36E-09 | 0.21 |
| PODXL2 | 0.256186011 | 7.70E-18 | 0.179695225 | 2.19E-09 | 0.08 |
| PODXL | -0.06823811 | 0.02406824 | -0.210665605 | 1.98E-12 | 0.15 |
| POF1B | -0.164321106 | 4.67E-08 | -0.150854925 | 5.43E-07 | 0.02 |
| POFUT1 | -0.20009855 | 2.47E-11 | -0.156110433 | 2.14E-07 | 0.05 |
| POFUT2 | -0.073764322 | 0.01471891 | -0.118051582 | 9.15E-05 | 0.05 |
| LOC200137 | 0.113158488 | 0.00017765 | 0.276119216 | 1.41E-20 | 0.17 |
| POGZ | -0.132623151 | 1.09E-05 | 0.109979889 | 0.0002696 | 0.25 |
| NSX | 0.062443368 | 0.03901161 | -0.050756867 | 0.0935012 | 0.12 |
| POLA2 | 0.447027541 | 8.24E-55 | 0.241283303 | 6.08E-16 | 0.21 |
| POLB | 0.142960134 | 2.08E-06 | 0.197173958 | 4.84E-11 | 0.06 |
| POLD1 | 0.387173854 | 2.08E-40 | 0.179993928 | 2.06E-09 | 0.21 |
| POLD2 | 0.45602063 | 3.09E-57 | 0.360234139 | 7.84E-35 | 0.1 |
| POLD3 | 0.137965617 | 4.70E-06 | 0.033685248 | 0.2658397 | 0.11 |
| POLD4 | -0.087337072 | 0.00385694 | 0.083526211 | 0.0057257 | 0.18 |
| POLDIP2 | 0.275330299 | 1.83E-20 | 0.289139114 | 1.72E-22 | 0.02 |
| POLDIP3 | 0.031310724 | 0.30103385 | -0.164579158 | 4.44E-08 | 0.2 |
| POLE2 | 0.391625163 | 2.21E-41 | 0.265023022 | 5.03E-19 | 0.13 |
| POLE3 | 0.267202811 | 2.53E-19 | 0.132626577 | 1.09E-05 | 0.14 |
| POLE4 | 0.307943186 | 1.94E-25 | 0.305780214 | 4.34E-25 | 0.01 |
| FLJ21434 | 0.237182055 | 1.93E-15 | 0.126242954 | 2.84E-05 | 0.12 |
| POLG2 | 0.168208794 | 2.21E-08 | 0.17658175 | 4.17E-09 | 0.01 |
| POLG | -0.00570372 | 0.85059925 | -0.254558585 | 1.26E-17 | 0.25 |
| POLH | -0.019005647 | 0.53021695 | -0.062100036 | 0.0401023 | 0.05 |
| POLI | -0.231849561 | 8.34E-15 | -0.178648628 | 2.72E-09 | 0.06 |
| POLK | -0.532197542 | 5.81E-81 | -0.319848194 | 2.03E-27 | 0.22 |
| POLL | -0.065119839 | 0.03134073 | 0.043281778 | 0.1527307 | 0.11 |
| LOC100132763 | -0.268811719 | 1.51E-19 | -0.112524604 | 0.0001932 | 0.16 |
| POLN | -0.365185089 | 8.11E-36 | -0.162952838 | 6.05E-08 | 0.21 |
| POLQ | 0.380535255 | 5.51E-39 | 0.156400538 | 2.03E-07 | 0.23 |
| POLR1A | 0.005548895 | 0.85460891 | -0.194320184 | 9.24E-11 | 0.2 |
| POLR1B | 0.10712631 | 0.00038844 | 0.01703669 | 0.5736809 | 0.1 |
| POLR1C | 0.567590664 | 3.22E-94 | 0.426402285 | 1.62E-49 | 0.15 |
| POLR1C.1 | 0.567590664 | 3.22E-94 | 0.426402285 | 1.62E-49 | 0.15 |
| POLR1E | 0.412521914 | 3.74E-46 | 0.244668277 | 2.31E-16 | 0.17 |
| POLR2A | -0.387696989 | 1.60E-40 | -0.340979422 | 3.68E-31 | 0.05 |
| POLR2B | 0.033482385 | 0.26873104 | -0.108427441 | 0.0003292 | 0.15 |
| POLR2C | 0.263852332 | 7.26E-19 | 0.065818858 | 0.0295644 | 0.2 |
| POLR2D | 0.41869039 | 1.25E-47 | 0.219357787 | 2.25E-13 | 0.2 |
| POLR2E | 0.130863304 | 1.42E-05 | 0.075849757 | 0.0121286 | 0.06 |
| POLR2F | 0.53720204 | 9.65E-83 | 0.339373468 | 7.25E-31 | 0.2 |
| POLR2G | 0.419575393 | 7.66E-48 | 0.352404974 | 2.62E-33 | 0.07 |
| POLR2H | 0.550603247 | 1.17E-87 | 0.527806114 | 2.00E-79 | 0.03 |
| POLR2I | 0.468432732 | 1.05E-60 | 0.402824033 | 6.76E-44 | 0.07 |
| POLR2J2 | 0.091260901 | 0.00252785 | 0.022735985 | 0.4527121 | 0.07 |
| POLR2J3 | 0.204934888 | 7.91E-12 | 0.13236736 | 1.13E-05 | 0.08 |
| POLR2J4 | 0.112055568 | 0.00020557 | 0.112556887 | 0.0001924 | 0.01 |
| POLR2J | 0.418795747 | 1.18E-47 | 0.462682913 | 4.43E-59 | 0.05 |
| POLR2K | 0.47211012 | 9.25E-62 | 0.332834858 | 1.10E-29 | 0.14 |
| POLR2L | 0.114680581 | 0.00014493 | 0.206135507 | 5.94E-12 | 0.1 |
| POLR3A | -0.128767256 | 1.95E-05 | -0.242690468 | 4.08E-16 | 0.12 |
| POLR3B | -0.007571834 | 0.80255166 | 0.001208824 | 0.968158 | 0.01 |
| POLR3C | 0.201844462 | 1.64E-11 | 0.340443733 | 4.61E-31 | 0.14 |
| POLR3D | 0.179703652 | 2.19E-09 | 0.089499002 | 0.003062 | 0.1 |
| POLR3E | -0.081585437 | 0.00696166 | -0.062478623 | 0.038901 | 0.02 |
| POLR3F | 0.258092336 | 4.31E-18 | 0.231941623 | 8.13E-15 | 0.03 |
| POLR3GL | -0.078813365 | 0.00914228 | 0.118861708 | 8.18E-05 | 0.2 |
| POLR3G | 0.422237226 | 1.72E-48 | 0.188491534 | 3.37E-10 | 0.24 |
| POLR3H | 0.109490529 | 0.0002872 | 0.07037997 | 0.019964 | 0.04 |
| POLR3K | 0.25627937 | 7.49E-18 | 0.288748047 | 1.97E-22 | 0.04 |
| POLRMT | 0.120944754 | 6.11E-05 | 0.11339952 | 0.000172 | 0.01 |
| LOC392733 | -0.108282774 | 0.00033535 | -0.154266136 | 2.98E-07 | 0.05 |
| POM121L10P | 0.056485025 | 0.06193235 | 0.016232099 | 0.5919147 | 0.05 |
| POM121L12 | -0.022558658 | 0.45624568 | -0.022812915 | 0.451184 | 0.01 |
| LOC157446 | -0.034196637 | 0.25864634 | -0.060822417 | 0.0443907 | 0.03 |
| POM121L2 | 0.152595686 | 4.00E-07 | -0.020769318 | 0.4927551 | 0.18 |
| POM121L4P | -0.03546307 | 0.24141572 | -0.096835555 | 0.0013492 | 0.07 |
| POM121L8P | -0.003172662 | 0.91655816 | -0.026252405 | 0.3859008 | 0.03 |
| UNQ2565 | -0.004223754 | 0.88907047 | -0.092856125 | 0.0021192 | 0.09 |
| POM121 | -0.059553162 | 0.04902725 | -0.131051873 | 1.38E-05 | 0.08 |
| POMC | 0.243213651 | 3.51E-16 | 0.083906598 | 0.005508 | 0.16 |
| MEB | -0.086956149 | 0.00401502 | -0.105295256 | 0.0004888 | 0.02 |
| POMP | 0.431919982 | 6.76E-51 | 0.467279963 | 2.24E-60 | 0.04 |
| POMT1 | -0.022112675 | 0.46520071 | 0.01436268 | 0.6352723 | 0.04 |
| POMT2 | -0.212506438 | 1.26E-12 | -0.106091676 | 0.0004425 | 0.11 |
| LOC154946 | -0.005161213 | 0.86466535 | 0.077638095 | 0.0102376 | 0.09 |
| PON1 | 0.018564065 | 0.53981763 | 0.069949726 | 0.0207356 | 0.06 |
| PON2 | -0.139617264 | 3.60E-06 | 0.062631581 | 0.0384244 | 0.21 |
| PON3 | -0.056191733 | 0.06330101 | 0.005680789 | 0.8511929 | 0.07 |
| KIAA0061 | 0.45977666 | 2.86E-58 | 0.182399003 | 1.25E-09 | 0.28 |
| POP4 | 0.402026958 | 1.03E-43 | 0.296833841 | 1.13E-23 | 0.11 |
| LOC115451 | 0.352439905 | 2.58E-33 | 0.363779361 | 1.55E-35 | 0.02 |
| LOC155123 | 0.517734964 | 5.53E-76 | 0.415521944 | 7.24E-47 | 0.11 |
| POPDC2 | -0.153868878 | 3.19E-07 | -0.155362246 | 2.45E-07 | 0.01 |
| POPDC3 | 0.25647977 | 7.05E-18 | -0.005398144 | 0.8585167 | 0.27 |
| DHOF | -0.034455168 | 0.25506147 | 0.008859787 | 0.7698432 | 0.05 |
| POR | 0.252150868 | 2.59E-17 | 0.09794847 | 0.0011856 | 0.16 |
| POSTN | -0.303808657 | 9.00E-25 | -0.149327733 | 7.08E-07 | 0.16 |
| POT1 | -0.091013695 | 0.00259727 | 0.010234999 | 0.7353659 | 0.11 |
| POTEA | 0.015740884 | 0.60317578 | 0.001250977 | 0.9670482 | 0.02 |
| LOC339010 | -0.119326716 | 7.67E-05 | -0.025804634 | 0.3940581 | 0.1 |
| POTEC | -0.108255704 | 0.00033651 | -0.024243694 | 0.4232993 | 0.09 |
| POTED | -0.199417673 | 2.89E-11 | -0.047932736 | 0.1132438 | 0.16 |
| POTEE | -0.084133021 | 0.00538195 | -0.106846778 | 0.0004024 | 0.03 |
| POTEF | -0.217941696 | 3.22E-13 | -0.160710147 | 9.20E-08 | 0.06 |
| POTEG | 0.019056965 | 0.52910686 | -0.042269424 | 0.1625713 | 0.07 |
| POTEH | -0.142078147 | 2.41E-06 | -0.111873442 | 0.0002106 | 0.04 |
| POU1F1 | -0.02059956 | 0.49629825 | -0.078509909 | 0.0094146 | 0.06 |
| POU2AF1 | 0.053674866 | 0.07610139 | -0.068231116 | 0.0240828 | 0.13 |
| POU2F1 | 0.130823808 | 1.43E-05 | 0.158050124 | 1.50E-07 | 0.03 |
| POU2F2 | -0.047178811 | 0.11903407 | -0.197108535 | 4.91E-11 | 0.15 |
| POU2F3 | 0.024372942 | 0.42083096 | 0.016041517 | 0.5962724 | 0.01 |
| POU3F1 | 0.00771609 | 0.79886979 | -0.042291564 | 0.1623511 | 0.06 |
| POU3F2 | 0.142416849 | 2.28E-06 | 0.039889057 | 0.1875808 | 0.11 |
| POU3F3 | -0.002118446 | 0.94422795 | -0.070083547 | 0.0204929 | 0.07 |
| POU3F4 | 0.098902727 | 0.00106011 | 0.009768989 | 0.7469934 | 0.09 |
| POU4F1 | 0.350153368 | 7.05E-33 | 0.080458023 | 0.0077849 | 0.27 |
| BRN3B | 0.088118036 | 0.00355039 | -0.02992037 | 0.3230188 | 0.12 |
| POU4F3 | -0.068932282 | 0.02266452 | -0.123555389 | 4.21E-05 | 0.06 |
| POU5F1B | 0.221664959 | 1.24E-13 | 0.075071682 | 0.0130437 | 0.15 |
| POU5F1 | 0.381093366 | 4.20E-39 | 0.081453274 | 0.0070539 | 0.3 |
| POU5F2 | 0.08814432 | 0.00354047 | 0.11009339 | 0.0002657 | 0.03 |
| POU6F1 | -0.193903227 | 1.02E-10 | -0.275681642 | 1.63E-20 | 0.09 |
| WTSL | 0.062505967 | 0.03881548 | 0.01407735 | 0.6420063 | 0.05 |
| PP14571 | -0.238577865 | 1.30E-15 | -0.086538745 | 0.004195 | 0.16 |
| PPA1 | 0.374652534 | 9.45E-38 | 0.355265959 | 7.35E-34 | 0.02 |
| LOC92033 | 0.050027282 | 0.09831619 | 0.249132929 | 6.31E-17 | 0.2 |
| PPAN.P2RY11 | -0.079181643 | 0.00882122 | -0.045966052 | 0.1288313 | 0.04 |
| MGC14226 | 0.38090515 | 4.60E-39 | 0.233786411 | 4.92E-15 | 0.15 |
| PPAP2A | -0.243519298 | 3.22E-16 | -0.142030031 | 2.43E-06 | 0.11 |
| PPAP2B | -0.359952784 | 8.91E-35 | -0.385359753 | 5.13E-40 | 0.03 |
| PPAP2C | 0.259865004 | 2.50E-18 | 0.261018699 | 1.75E-18 | 0.01 |
| PPAPDC1A | -0.070906149 | 0.01905438 | 0.063945899 | 0.0345297 | 0.14 |
| PPAPDC1B | -0.098047962 | 0.0011719 | -0.083555036 | 0.0057089 | 0.02 |
| PPAPDC2 | -0.228018198 | 2.34E-14 | -0.02913931 | 0.3358167 | 0.2 |
| FLJ14662 | -0.219741547 | 2.04E-13 | -0.140972851 | 2.89E-06 | 0.08 |
| LOC439932 | -0.039572856 | 0.19110559 | -0.240397125 | 7.82E-16 | 0.21 |
| PPARD | 0.155938617 | 2.20E-07 | 0.051197008 | 0.0906891 | 0.11 |
| PPARGC1A | -0.083943152 | 0.00548744 | -0.135175659 | 7.31E-06 | 0.06 |
| PPARGC1B | -0.072258932 | 0.01688003 | -0.213023031 | 1.11E-12 | 0.15 |
| PPARG2 | -0.164172864 | 4.80E-08 | -0.159724826 | 1.10E-07 | 0.01 |
| PPAT | 0.241322034 | 6.02E-16 | 0.060303668 | 0.046239 | 0.19 |
| PPBPP2 | 0.023736867 | 0.43305982 | -0.046871316 | 0.1214612 | 0.08 |
| LOC90374 | 0.14494402 | 1.50E-06 | 0.023822364 | 0.4314043 | 0.13 |
| LOC146003 | 0.253053026 | 1.98E-17 | 0.278228324 | 7.01E-21 | 0.03 |
| PPCS | 0.026843044 | 0.37529961 | 0.17504274 | 5.70E-09 | 0.15 |
| PPDPF | 0.065230141 | 0.0310545 | 0.119309183 | 7.68E-05 | 0.06 |
| PPEF1 | -0.073211693 | 0.01548219 | 0.066588733 | 0.0277088 | 0.14 |
| PPEF2 | -0.099831823 | 0.00094984 | -0.066061349 | 0.0289688 | 0.04 |
| PPFIA1 | -0.104346472 | 0.0005498 | -0.168597569 | 2.05E-08 | 0.07 |
| PPFIA2 | -0.359716451 | 9.92E-35 | -0.274975713 | 2.05E-20 | 0.09 |
| PPFIA3 | 0.163593808 | 5.36E-08 | 0.057556936 | 0.0571367 | 0.11 |
| LOC101059970 | 0.173087029 | 8.44E-09 | 0.129968959 | 1.63E-05 | 0.05 |
| PPFIBP1 | -0.346588039 | 3.33E-32 | -0.283495434 | 1.19E-21 | 0.07 |
| PPFIBP2 | -0.149880712 | 6.44E-07 | 0.056452733 | 0.0620818 | 0.21 |
| HSPC206 | 0.123672553 | 4.14E-05 | 0.162973789 | 6.02E-08 | 0.04 |
| PPIAL4A | 0.039492303 | 0.19201128 | 0.030000841 | 0.3217186 | 0.01 |
| PPIAL4C | 0.404377249 | 2.97E-44 | 0.304428312 | 7.16E-25 | 0.1 |
| PPIAL4D | 0.220955463 | 1.49E-13 | 0.234046572 | 4.58E-15 | 0.02 |
| PPIAL4E | 0.090835091 | 0.0026485 | 0.056184435 | 0.0633354 | 0.04 |
| PPIAL4G | 0.339020224 | 8.41E-31 | 0.26928666 | 1.30E-19 | 0.07 |
| PPIA | 0.544907756 | 1.53E-85 | 0.458799117 | 5.33E-58 | 0.09 |
| PPIB | 0.382563215 | 2.04E-39 | 0.244514625 | 2.42E-16 | 0.14 |
| PPIC | -0.082693051 | 0.00622978 | 0.076773287 | 0.0111166 | 0.16 |
| PPID | 0.245658192 | 1.74E-16 | 0.114861003 | 0.0001414 | 0.14 |
| PPIEL | -0.066902239 | 0.0269824 | -0.031520987 | 0.2977978 | 0.04 |
| PPIE | 0.30365984 | 9.50E-25 | 0.299332411 | 4.61E-24 | 0.01 |
| PPIF | 0.3001585 | 3.42E-24 | 0.152872031 | 3.81E-07 | 0.15 |
| PPIG | 0.005598425 | 0.85332578 | -0.018555655 | 0.5400013 | 0.03 |
| PPIH | 0.560013028 | 3.03E-91 | 0.474261801 | 2.20E-62 | 0.09 |
| PPIL1 | 0.496879469 | 3.25E-69 | 0.289926897 | 1.30E-22 | 0.21 |
| PPIL2 | -0.049442628 | 0.10231569 | 0.084076395 | 0.0054132 | 0.14 |
| PPIL3 | 0.009368912 | 0.75702201 | 0.065287605 | 0.0309063 | 0.06 |
| PPIL4 | -0.025399782 | 0.40152247 | -0.060556013 | 0.0453319 | 0.04 |
| LRR1 | 0.488721491 | 1.08E-66 | 0.364405556 | 1.16E-35 | 0.13 |
| PPIL6 | -0.106568917 | 0.00041674 | 0.03298168 | 0.2759594 | 0.14 |
| PPIP5K1 | -0.411398157 | 6.89E-46 | -0.285127386 | 6.85E-22 | 0.13 |
| PPIP5K2 | -0.38049912 | 5.61E-39 | -0.239325977 | 1.06E-15 | 0.15 |
| PPL | -0.18829655 | 3.51E-10 | -0.203067114 | 1.23E-11 | 0.02 |
| PPM1A | -0.378059451 | 1.84E-38 | -0.187211371 | 4.45E-10 | 0.2 |
| PPM1B | -0.152258406 | 4.25E-07 | -0.166572635 | 3.03E-08 | 0.02 |
| PPM1D | -0.054636572 | 0.0709813 | -0.124243805 | 3.81E-05 | 0.07 |
| PPM1E | 0.145121123 | 1.45E-06 | -0.062660724 | 0.0383342 | 0.21 |
| PPM1F | -0.077606589 | 0.01026855 | -0.161316451 | 8.22E-08 | 0.09 |
| PPM1G | 0.577891404 | 2.19E-98 | 0.308233405 | 1.74E-25 | 0.27 |
| ARHCL1 | -0.168939503 | 1.92E-08 | -0.038902759 | 0.1987359 | 0.14 |
| PPM1J | -0.093081082 | 0.00206667 | 0.04283428 | 0.1570233 | 0.14 |
| PPM1K | -0.031098449 | 0.30432449 | -0.174756593 | 6.04E-09 | 0.15 |
| PPM1L | -0.11360402 | 0.00016741 | -0.325258002 | 2.38E-28 | 0.22 |
| PPM1M | -0.271281683 | 6.84E-20 | -0.209158481 | 2.86E-12 | 0.07 |
| PPM1N | 0.103051279 | 0.00064459 | -0.027665551 | 0.3608395 | 0.14 |
| PPME1 | 0.153348626 | 3.50E-07 | 0.091336863 | 0.0025069 | 0.07 |
| VP | 0.060236699 | 0.04648222 | 0.342416063 | 2.00E-31 | 0.29 |
| PPP1CA | 0.303293056 | 1.09E-24 | 0.258361282 | 3.97E-18 | 0.05 |
| PPP1CB | 0.186591937 | 5.09E-10 | 0.035804929 | 0.2369061 | 0.16 |
| PPP1CC | 0.262505371 | 1.11E-18 | 0.171628836 | 1.13E-08 | 0.1 |
| PPP1R10 | 0.153498079 | 3.41E-07 | 0.161656882 | 7.71E-08 | 0.01 |
| HCGV | 0.098619069 | 0.00109607 | 0.189678622 | 2.60E-10 | 0.1 |
| PPP1R12A | -0.267972791 | 1.98E-19 | -0.324306265 | 3.48E-28 | 0.06 |
| PPP1R12B | -0.411138506 | 7.93E-46 | -0.224058179 | 6.66E-14 | 0.19 |
| PPP1R12C | 0.024713092 | 0.41437535 | -0.044170299 | 0.1444727 | 0.07 |
| LOC145215 | -0.208653602 | 3.23E-12 | -0.069519376 | 0.0215333 | 0.14 |
| PPP1R13L | -0.037424973 | 0.21634426 | 0.033191419 | 0.2729156 | 0.08 |
| PPP1R14A | 0.050558473 | 0.09479145 | 0.019141826 | 0.5272738 | 0.04 |
| PPP1R14B | 0.546410027 | 4.28E-86 | 0.453194722 | 1.82E-56 | 0.1 |
| PPP1R14C | 0.340526819 | 4.45E-31 | 0.091266499 | 0.0025263 | 0.25 |
| PPP1R14D | -0.094079746 | 0.00184771 | 0.0515747 | 0.0883304 | 0.15 |
| PPP1R15A | -0.031950212 | 0.2912642 | -0.093066015 | 0.0020701 | 0.07 |
| PPP1R15B | -0.212617439 | 1.22E-12 | 0.164785237 | 4.27E-08 | 0.38 |
| PPP1R16A | 0.21383832 | 9.04E-13 | 0.155418867 | 2.42E-07 | 0.06 |
| PPP1R16B | -0.232727213 | 6.57E-15 | -0.296581043 | 1.24E-23 | 0.07 |
| PPP1R1A | 0.006906837 | 0.81957996 | 0.080674243 | 0.0076206 | 0.08 |
| PPP1R1B | 0.047295641 | 0.11812192 | 0.175817853 | 4.87E-09 | 0.13 |
| PPP1R1C | -0.030532285 | 0.31321724 | -0.015539935 | 0.6078101 | 0.02 |
| LOC135673 | -0.096500442 | 0.00140236 | -0.210978039 | 1.83E-12 | 0.12 |
| PPP1R2P3 | -0.203943795 | 1.00E-11 | -0.20786204 | 3.92E-12 | 0.01 |
| PPP1R2P9 | 0.026602706 | 0.37959149 | -0.03072842 | 0.3101174 | 0.06 |
| PPP1R2 | -0.149959161 | 6.35E-07 | -0.068025013 | 0.024514 | 0.09 |
| PPP1R3A | 0.024496698 | 0.41847544 | -0.025891917 | 0.3924598 | 0.06 |
| LOC286044 | -0.348046176 | 1.77E-32 | -0.202489915 | 1.41E-11 | 0.15 |
| PPP1R3C | -0.360746526 | 6.21E-35 | -0.142136961 | 2.39E-06 | 0.22 |
| PPP1R3D | -0.115566015 | 0.00012859 | -0.079491072 | 0.0085593 | 0.04 |
| PPP1R3E | -0.268624269 | 1.61E-19 | -0.227389735 | 2.76E-14 | 0.05 |
| LOC158569 | 0.013140698 | 0.66431725 | -0.102958129 | 0.000652 | 0.12 |
| PPP1R3G | -0.051736074 | 0.08733784 | -0.075934871 | 0.0120321 | 0.03 |
| PPP1R7 | 0.19242439 | 1.41E-10 | 0.302278314 | 1.58E-24 | 0.11 |
| PRO2047 | 0.318608215 | 3.29E-27 | 0.082837411 | 0.0061397 | 0.24 |
| PPP1R9A | -0.16350308 | 5.45E-08 | -0.090472785 | 0.0027553 | 0.08 |
| PPP1R9B | -0.061934719 | 0.04063663 | -0.052357701 | 0.083598 | 0.01 |
| PPP2CA | -0.072347648 | 0.01674536 | 0.127647022 | 2.31E-05 | 0.2 |
| PPP2CB | -0.252223086 | 2.53E-17 | -0.148489246 | 8.19E-07 | 0.11 |
| PPP2R1A | 0.171596563 | 1.14E-08 | 0.17893901 | 2.57E-09 | 0.01 |
| LOC101928690 | -0.055182483 | 0.06820267 | -0.089774487 | 0.0029722 | 0.04 |
| PPP2R2A | -0.257146984 | 5.75E-18 | -0.084577543 | 0.0051421 | 0.18 |
| SCA12 | -0.060119875 | 0.04690916 | -0.146584278 | 1.13E-06 | 0.09 |
| PPP2R2C | -0.00577905 | 0.84864973 | -0.03880614 | 0.1998541 | 0.04 |
| PPP2R2D | 0.158087041 | 1.49E-07 | 0.175079231 | 5.66E-09 | 0.02 |
| PPP2R3A | -0.07912023 | 0.00887405 | -0.099665021 | 0.0009688 | 0.03 |
| LOC102724991 | 0.32232064 | 7.66E-28 | 0.159418522 | 1.17E-07 | 0.17 |
| PPP2R3C | 0.119086507 | 7.93E-05 | 0.023580301 | 0.4361011 | 0.1 |
| PPP2R4 | -0.082647267 | 0.0062586 | 0.044372799 | 0.1426394 | 0.13 |
| PPP2R5A | -0.238624956 | 1.29E-15 | 0.19083137 | 2.01E-10 | 0.43 |
| PPP2R5B | 0.005649744 | 0.85199668 | -0.0542691 | 0.0729034 | 0.06 |
| LOC100132532 | -0.056148206 | 0.06350624 | -0.030941711 | 0.3067694 | 0.03 |
| PPP2R5D | 0.328592562 | 6.23E-29 | 0.111084147 | 0.0002335 | 0.22 |
| LOC63385 | -0.228732902 | 1.93E-14 | -0.186883738 | 4.77E-10 | 0.05 |
| PPP3CA | -0.090747662 | 0.00267392 | -0.117405856 | 0.0001 | 0.03 |
| PPP3CB | -0.276076765 | 1.43E-20 | -0.112109198 | 0.0002041 | 0.17 |
| PPP3CC | -0.184384759 | 8.17E-10 | -0.194981906 | 7.96E-11 | 0.02 |
| PPP3R1 | 0.202677884 | 1.35E-11 | 0.018970087 | 0.5309869 | 0.19 |
| PPP3R2 | -0.012566768 | 0.67813804 | -0.061162644 | 0.0432125 | 0.05 |
| LOC116414 | 0.357121888 | 3.20E-34 | 0.406125217 | 1.17E-44 | 0.05 |
| PPP4R1L | -0.133933029 | 8.88E-06 | -0.219007523 | 2.46E-13 | 0.09 |
| PPP4R1 | 0.06896372 | 0.02260267 | -0.172773477 | 8.99E-09 | 0.25 |
| PPP4R2 | -0.31485865 | 1.41E-26 | -0.331427287 | 1.96E-29 | 0.02 |
| PPP4R4 | -0.108791168 | 0.00031423 | -0.214680867 | 7.33E-13 | 0.11 |
| PPP5C | 0.303690144 | 9.40E-25 | 0.087177364 | 0.0039225 | 0.22 |
| LOC115568 | -0.215663355 | 5.73E-13 | -0.117776751 | 9.50E-05 | 0.1 |
| DESI2 | 0.236286082 | 2.47E-15 | 0.255980649 | 8.20E-18 | 0.02 |
| DJ347H13.4 | 0.381021119 | 4.35E-39 | 0.262576087 | 1.08E-18 | 0.12 |
| LOC143298 | 0.225660441 | 4.37E-14 | 0.08249507 | 0.0063553 | 0.15 |
| PPT1 | -0.110440769 | 0.00025394 | -0.024511018 | 0.4182034 | 0.09 |
| LOC116323 | -0.055459489 | 0.06682727 | -0.075513476 | 0.0125169 | 0.03 |
| PPTC7 | -0.342430149 | 1.98E-31 | -0.44444743 | 3.97E-54 | 0.11 |
| LOC133986 | -0.178604063 | 2.75E-09 | -0.096015436 | 0.0014828 | 0.09 |
| PPY2P | 0.149098789 | 7.37E-07 | 0.092156064 | 0.0022904 | 0.06 |
| NPY4R | 0.150266097 | 6.02E-07 | -0.064407444 | 0.0332443 | 0.22 |
| PPY | 0.026326722 | 0.38455691 | 0.007182538 | 0.8125094 | 0.02 |
| MRX55 | 0.435977219 | 6.28E-52 | 0.301962929 | 1.77E-24 | 0.14 |
| PQLC1 | 0.309836451 | 9.51E-26 | 0.061315195 | 0.0426929 | 0.25 |
| PQLC2 | 0.141319888 | 2.73E-06 | 0.056831894 | 0.0603453 | 0.09 |
| PQLC3 | -0.15372452 | 3.28E-07 | -0.146263609 | 1.20E-06 | 0.01 |
| PRAC1 | 0.110171908 | 0.00026297 | 0.031351516 | 0.3004042 | 0.08 |
| PRAF2 | 0.171306495 | 1.20E-08 | 0.165664377 | 3.61E-08 | 0.01 |
| PRAM1 | -0.105191615 | 0.00049512 | -0.115390295 | 0.0001317 | 0.02 |
| PRAMEF10 | -0.001072611 | 0.9717444 | -0.013551221 | 0.6545006 | 0.02 |
| PRAMEF11 | 0.077476102 | 0.01039743 | 0.028242124 | 0.350914 | 0.05 |
| LOC649289 | 0.060467331 | 0.04564897 | -0.004869782 | 0.8722396 | 0.07 |
| LOC100507851 | 0.048899411 | 0.10614659 | 0.025375432 | 0.4019741 | 0.03 |
| PRAMEF14 | 0.003716966 | 0.90230923 | -0.015746989 | 0.6030352 | 0.02 |
| PRAMEF16 | 0.03830972 | 0.20567217 | 0.03847623 | 0.2037071 | 0.01 |
| PRAMEF17 | 0.048168149 | 0.11148198 | 0.036214179 | 0.2315861 | 0.02 |
| LOC391003 | 0.042915168 | 0.15624074 | 0.049045749 | 0.1051036 | 0.01 |
| LOC93189 | 0.052014418 | 0.08564693 | -0.002624268 | 0.9309413 | 0.06 |
| PRAMEF20 | 0.048229459 | 0.11102669 | 0.007810257 | 0.7964687 | 0.05 |
| PRAMEF22 | 0.033847361 | 0.26354465 | 0.038579662 | 0.2024934 | 0.01 |
| PRAMEF2 | 0.038227935 | 0.20664235 | -0.013682563 | 0.6513723 | 0.06 |
| PRAMEF3 | NA | NA | NA | NA | #VALUE! |
| PRAMEF4 | 0.027140496 | 0.37002947 | -0.021504716 | 0.4775638 | 0.05 |
| PRAMEF23 | 0.018248253 | 0.54673679 | 0.002495581 | 0.9343199 | 0.02 |
| PRAMEF6 | 0.040363477 | 0.18238245 | 0.002699366 | 0.9289702 | 0.04 |
| PRAMEF24 | 0.039973297 | 0.18664984 | -0.029662357 | 0.3272109 | 0.07 |
| PRAMEF9 | 0.018996846 | 0.53040744 | -0.002267499 | 0.9403109 | 0.03 |
| MAPE | 0.343331322 | 1.35E-31 | 0.189233688 | 2.86E-10 | 0.16 |
| PRAP1 | 0.146314492 | 1.19E-06 | 0.052125238 | 0.0849811 | 0.1 |
| PRB1 | 0.176348292 | 4.37E-09 | 0.048093397 | 0.1120391 | 0.13 |
| PRB2 | 0.194596458 | 8.69E-11 | 0.05235633 | 0.0836061 | 0.15 |
| LOC648979 | -0.000370073 | 0.99024944 | -0.018676106 | 0.5373735 | 0.02 |
| PRB4 | 0.132578057 | 1.09E-05 | 0.044949695 | 0.1375142 | 0.09 |
| PRC1 | 0.43890845 | 1.11E-52 | 0.183060305 | 1.08E-09 | 0.26 |
| PRCC | 0.352010421 | 3.12E-33 | 0.516650269 | 1.28E-75 | 0.17 |
| PRCD | -0.085807625 | 0.00452781 | -0.118199362 | 8.97E-05 | 0.04 |
| LOC120474 | -0.18190643 | 1.38E-09 | -0.130526853 | 1.50E-05 | 0.06 |
| PRDM10 | -0.362220085 | 3.17E-35 | -0.309667089 | 1.01E-25 | 0.06 |
| LOC100507338 | -0.219682399 | 2.07E-13 | -0.366100768 | 5.30E-36 | 0.15 |
| PRDM12 | 0.161773412 | 7.55E-08 | 0.112483848 | 0.0001943 | 0.05 |
| PRDM13 | 0.400288888 | 2.56E-43 | 0.129708715 | 1.70E-05 | 0.28 |
| PRDM14 | 0.009903016 | 0.74364326 | 0.016183999 | 0.5930131 | 0.01 |
| C21orf83 | 0.071986192 | 0.01730003 | 0.032773003 | 0.2790106 | 0.04 |
| LOC100290519 | -0.339276825 | 7.55E-31 | -0.217005716 | 4.09E-13 | 0.13 |
| PRDM1 | -0.173647369 | 7.55E-09 | -0.238631506 | 1.28E-15 | 0.07 |
| LOC654202 | -0.495843379 | 6.86E-69 | -0.449108185 | 2.30E-55 | 0.05 |
| PRDM4 | -0.197681543 | 4.31E-11 | -0.142353601 | 2.30E-06 | 0.06 |
| PRDM5 | -0.368584143 | 1.67E-36 | -0.334193092 | 6.30E-30 | 0.04 |
| PRDM6 | -0.253806282 | 1.58E-17 | -0.055366393 | 0.0672869 | 0.2 |
| LOC92805 | 0.137949221 | 4.71E-06 | 0.057387537 | 0.0578734 | 0.09 |
| PRDM8 | -0.213906705 | 8.89E-13 | -0.220580304 | 1.64E-13 | 0.01 |
| MSBP3 | 0.13157906 | 1.28E-05 | 0.029073131 | 0.3369159 | 0.11 |
| PRDX1 | 0.428876517 | 3.93E-50 | 0.303236392 | 1.11E-24 | 0.13 |
| PRDX2 | 0.251651348 | 3.00E-17 | 0.271341297 | 6.71E-20 | 0.02 |
| PRO1748 | -0.104890023 | 0.00051403 | 0.043690968 | 0.1488842 | 0.15 |
| PRDX4 | 0.496700115 | 3.70E-69 | 0.345372863 | 5.63E-32 | 0.16 |
| PRDX5 | 0.332775563 | 1.13E-29 | 0.29848049 | 6.28E-24 | 0.04 |
| PRDX6 | 0.443766462 | 5.99E-54 | 0.604377793 | 8.17E-110 | 0.17 |
| PRORSD1P | -0.092876002 | 0.00211448 | -0.118130962 | 9.05E-05 | 0.03 |
| LOC115725 | 0.373357566 | 1.75E-37 | 0.207789476 | 3.99E-12 | 0.17 |
| PRELID1 | 0.471872222 | 1.08E-61 | 0.390977767 | 3.07E-41 | 0.09 |
| PRELID2 | -0.083171982 | 0.00593532 | -0.023199648 | 0.4435462 | 0.06 |
| PRELP | -0.167069777 | 2.76E-08 | -0.124850637 | 3.49E-05 | 0.05 |
| PREPL | -0.35648299 | 4.26E-34 | -0.224295137 | 6.26E-14 | 0.14 |
| LOC51709 | 0.220048285 | 1.88E-13 | 0.073636822 | 0.014892 | 0.15 |
| LOC149730 | -0.363224156 | 2.00E-35 | -0.210283279 | 2.17E-12 | 0.16 |
| PREX2 | -0.534428585 | 9.43E-82 | -0.428446761 | 5.04E-50 | 0.11 |
| PRF1 | 0.061157614 | 0.04322976 | -0.087448461 | 0.0038118 | 0.15 |
| PRG1 | -0.020058702 | 0.50767673 | -0.021654187 | 0.4745077 | 0.01 |
| LOC87065 | 0.272857258 | 4.10E-20 | 0.104832551 | 0.0005177 | 0.17 |
| PRG3 | -0.022195641 | 0.46352747 | -0.022473306 | 0.457952 | 0.01 |
| PRG4 | -0.199825674 | 2.63E-11 | -0.134662988 | 7.92E-06 | 0.07 |
| PRH1 | 0.151752364 | 4.64E-07 | 0.080539403 | 0.0077227 | 0.08 |
| PRH2 | 0.178636923 | 2.73E-09 | 0.153080694 | 3.67E-07 | 0.03 |
| PRHOXNB | -0.099934031 | 0.00093838 | -0.042034475 | 0.164922 | 0.06 |
| LOC200213 | -0.120957874 | 6.10E-05 | -0.233484844 | 5.34E-15 | 0.12 |
| PRICKLE1 | -0.045409287 | 0.13353356 | -0.084244384 | 0.0053209 | 0.04 |
| PRICKLE2 | -0.539509107 | 1.43E-83 | -0.240221378 | 8.22E-16 | 0.3 |
| PRICKLE3 | -0.004952735 | 0.87008243 | -0.065152689 | 0.0312553 | 0.07 |
| DKFZp761H221 | -0.343201642 | 1.43E-31 | -0.215742868 | 5.62E-13 | 0.13 |
| PRIM1 | 0.210636827 | 1.99E-12 | 0.125968084 | 2.96E-05 | 0.09 |
| PRIM2 | 0.387957131 | 1.40E-40 | 0.215439558 | 6.06E-13 | 0.18 |
| PRIMA1 | 0.090914397 | 0.00262564 | 0.014442006 | 0.6334054 | 0.08 |
| PRINS | -0.064600579 | 0.03271874 | 0.00203738 | 0.9463589 | 0.07 |
| PRKAA1 | -0.268886522 | 1.48E-19 | -0.234040139 | 4.59E-15 | 0.04 |
| PRKAA2 | -0.237893273 | 1.58E-15 | -0.159177282 | 1.22E-07 | 0.08 |
| PRKAB1 | -0.271478097 | 6.42E-20 | -0.104443605 | 0.0005432 | 0.17 |
| PRKAB2 | -0.194773951 | 8.34E-11 | -0.027706438 | 0.3601299 | 0.17 |
| PRKACA | 0.053881855 | 0.0749747 | -0.003214875 | 0.9154521 | 0.06 |
| PRKACB | -0.198968322 | 3.20E-11 | -0.193362199 | 1.15E-10 | 0.01 |
| PRKACG | -0.050912423 | 0.09249947 | -0.028597068 | 0.3448906 | 0.03 |
| PRKAG1 | -0.079569008 | 0.00849442 | 0.090310857 | 0.0028043 | 0.17 |
| PRKAG2 | -0.024220741 | 0.42373854 | -0.141053836 | 2.85E-06 | 0.12 |
| PRKAG3 | 0.260168332 | 2.28E-18 | 0.12663617 | 2.68E-05 | 0.14 |
| LOC102724296 | -0.307139791 | 2.62E-25 | -0.317619375 | 4.84E-27 | 0.02 |
| LOC645590 | 0.139659992 | 3.57E-06 | -0.030978378 | 0.3061963 | 0.18 |
| PRKAR2A | -0.424595522 | 4.54E-49 | -0.427383767 | 9.27E-50 | 0.01 |
| PRKAR2B | -0.176501416 | 4.24E-09 | -0.045661024 | 0.1313913 | 0.14 |
| LOC146784 | -0.110034201 | 0.0002677 | -0.273819668 | 3.00E-20 | 0.17 |
| PRKCB | -0.11103393 | 0.00023505 | -0.219778838 | 2.02E-13 | 0.11 |
| PRKCDBP | 0.092846372 | 0.00212147 | 0.026630505 | 0.3790935 | 0.07 |
| PRKCD | -0.121829028 | 5.39E-05 | -0.013980629 | 0.6442958 | 0.11 |
| PRKCE | -0.303443099 | 1.03E-24 | -0.28649736 | 4.28E-22 | 0.02 |
| SCA14 | 0.088989968 | 0.00323441 | -0.021890914 | 0.4696896 | 0.12 |
| MGC5363 | -0.331875872 | 1.63E-29 | -0.269808711 | 1.10E-19 | 0.07 |
| PRKCI | 0.050351815 | 0.09615053 | -0.03491861 | 0.2487219 | 0.09 |
| PRKCQ | 0.075064937 | 0.01305185 | -0.081296493 | 0.0071648 | 0.16 |
| PRKCSH | 0.256643964 | 6.70E-18 | 0.178374342 | 2.88E-09 | 0.08 |
| PRKCZ | 0.072894954 | 0.01593517 | 0.011935342 | 0.6934692 | 0.07 |
| PRKD1 | -0.236169258 | 2.55E-15 | -0.233785577 | 4.92E-15 | 0.01 |
| HSPC187 | -0.047616415 | 0.11564565 | -0.00447544 | 0.882507 | 0.05 |
| EPK2 | 0.018457413 | 0.54214938 | -0.124681101 | 3.57E-05 | 0.15 |
| PRKDC | 0.092662786 | 0.00216525 | -0.086425843 | 0.0042449 | 0.18 |
| PRKG1 | -0.491244438 | 1.83E-67 | -0.418175898 | 1.67E-47 | 0.08 |
| PRKG2 | -0.218806661 | 2.59E-13 | -0.176472189 | 4.26E-09 | 0.05 |
| LOC90645 | 0.252257362 | 2.51E-17 | 0.217844235 | 3.30E-13 | 0.04 |
| PRKRIP1 | 0.136715275 | 5.73E-06 | 0.192872124 | 1.28E-10 | 0.06 |
| PRKRIR | 0.111574663 | 0.00021898 | -0.002679276 | 0.9294975 | 0.12 |
| PRKX | 0.300600637 | 2.91E-24 | 0.075099471 | 0.01301 | 0.23 |
| PRKY | 0.202093534 | 1.55E-11 | 0.08354405 | 0.0057153 | 0.12 |
| GR3 | -0.094709924 | 0.00172072 | -0.124463173 | 3.69E-05 | 0.03 |
| PRLH | -0.01022806 | 0.73553863 | 0.038685585 | 0.2012559 | 0.05 |
| PRLR | -0.377811638 | 2.07E-38 | -0.132293858 | 1.14E-05 | 0.25 |
| PRL | -0.003853846 | 0.8987307 | -0.011301119 | 0.7089954 | 0.01 |
| PRM1 | 0.090128778 | 0.00286027 | 0.056523741 | 0.0617535 | 0.04 |
| PRM2 | 0.098648261 | 0.00109232 | 0.080660472 | 0.007631 | 0.02 |
| PRM3 | 0.065224904 | 0.03106804 | -0.006742561 | 0.8238 | 0.08 |
| PRMT9 | -0.298961837 | 5.28E-24 | -0.323902383 | 4.09E-28 | 0.03 |
| PRMT1 | 0.494023754 | 2.53E-68 | 0.292224528 | 5.83E-23 | 0.21 |
| PRMT2 | 0.162553398 | 6.52E-08 | 0.078044694 | 0.0098462 | 0.09 |
| PRMT3 | 0.127112958 | 2.50E-05 | 0.062265819 | 0.0395725 | 0.07 |
| HRMT1L5 | 0.108438514 | 0.00032874 | 0.11234051 | 0.000198 | 0.01 |
| PRMT6 | 0.072817207 | 0.01604812 | 0.017034921 | 0.5737207 | 0.06 |
| PRMT7 | 0.181295264 | 1.57E-09 | 0.204672812 | 8.42E-12 | 0.03 |
| PRMT8 | -0.107227255 | 0.00038351 | -0.093651001 | 0.001939 | 0.02 |
| PRND | -0.310792857 | 6.63E-26 | -0.150064794 | 6.23E-07 | 0.17 |
| LOC115483 | 0.100848032 | 0.00084144 | -0.129830907 | 1.67E-05 | 0.24 |
| PRNT | -0.036268193 | 0.23089031 | -0.03937331 | 0.1933549 | 0.01 |
| PRO0611 | -0.205404261 | 7.07E-12 | -0.209187068 | 2.84E-12 | 0.01 |
| PRO0628 | -0.033260917 | 0.27191205 | -0.010036484 | 0.7403119 | 0.03 |
| FOXN3.AS2 | -0.015440071 | 0.61011903 | -0.06097796 | 0.0438487 | 0.05 |
| PROCA1 | -0.037436784 | 0.21619923 | 0.01608414 | 0.5952965 | 0.06 |
| PROCR | -0.130333543 | 1.54E-05 | -0.16699308 | 2.80E-08 | 0.04 |
| PROC | -0.085530342 | 0.00466017 | 0.039071804 | 0.1967903 | 0.13 |
| PRODH2 | 0.006419232 | 0.83212054 | -0.008616645 | 0.7759887 | 0.02 |
| SCZD4 | -0.12281616 | 4.68E-05 | 0.065571523 | 0.0301828 | 0.19 |
| PROK1 | -0.052290324 | 0.08399701 | -0.003761613 | 0.9011418 | 0.05 |
| PROK2 | 0.202372478 | 1.45E-11 | 0.049327733 | 0.1031167 | 0.16 |
| ZAQ | -0.077003008 | 0.01087685 | -0.074586928 | 0.0136441 | 0.01 |
| KAL3 | 0.166809246 | 2.90E-08 | -0.031189204 | 0.3029147 | 0.2 |
| PROL1 | -0.157318517 | 1.72E-07 | -0.071142918 | 0.018657 | 0.09 |
| STGD4 | 0.156706537 | 1.92E-07 | 0.119770894 | 7.20E-05 | 0.04 |
| LOC200480 | -0.24798408 | 8.84E-17 | -0.082988109 | 0.0060469 | 0.17 |
| PROP1 | -0.063028782 | 0.03720989 | -0.055037604 | 0.0689312 | 0.01 |
| PROS1 | -0.272101193 | 5.24E-20 | -0.268231779 | 1.82E-19 | 0.01 |
| PROSC | -0.034742393 | 0.25111935 | -0.111988505 | 0.0002074 | 0.08 |
| PROX1 | -0.215504484 | 5.96E-13 | -0.309483283 | 1.09E-25 | 0.1 |
| PROX2 | -0.185747569 | 6.10E-10 | -0.208154068 | 3.65E-12 | 0.03 |
| PROZ | -0.098299919 | 0.00113786 | 0.034580685 | 0.2533335 | 0.14 |
| PRPF18 | 0.270720548 | 8.19E-20 | 0.204007852 | 9.85E-12 | 0.07 |
| PRPF19 | 0.218773508 | 2.61E-13 | 0.228696373 | 1.95E-14 | 0.01 |
| PRPF31 | 0.284628922 | 8.12E-22 | 0.214325134 | 8.01E-13 | 0.08 |
| MGC3320 | 0.387722705 | 1.58E-40 | 0.149353749 | 7.05E-07 | 0.24 |
| PRPF38B | 0.050464467 | 0.09540776 | -0.128812142 | 1.94E-05 | 0.18 |
| FLJ11128 | -0.139863299 | 3.46E-06 | -0.031780785 | 0.2938316 | 0.11 |
| PRPF3 | 0.349674713 | 8.69E-33 | 0.316048488 | 8.89E-27 | 0.04 |
| HYPA | -0.00740396 | 0.80684185 | -0.110674773 | 0.0002463 | 0.11 |
| PRPF40B | -0.007010352 | 0.81692347 | 0.117415981 | 9.99E-05 | 0.13 |
| PRPF4B | -0.183418901 | 1.00E-09 | -0.147922603 | 9.02E-07 | 0.04 |
| PRPF4 | 0.108522333 | 0.00032524 | 0.108827826 | 0.0003128 | 0.01 |
| PRPF6 | 0.140097548 | 3.33E-06 | 0.124993546 | 3.41E-05 | 0.02 |
| PRPF8 | -0.125532587 | 3.16E-05 | -0.24480085 | 2.23E-16 | 0.12 |
| PRPH2 | -0.199796752 | 2.64E-11 | -0.142622543 | 2.20E-06 | 0.06 |
| NEF4 | 0.100295661 | 0.00089885 | -0.045263128 | 0.1347897 | 0.15 |
| LOC91802 | -0.045190101 | 0.13542073 | -0.053242584 | 0.0784989 | 0.01 |
| CMTX5 | -0.000243276 | 0.99359016 | 0.071497897 | 0.0180749 | 0.08 |
| PRPS2 | 0.223957904 | 6.83E-14 | 0.148253775 | 8.52E-07 | 0.08 |
| PRPSAP1 | 0.335814616 | 3.21E-30 | 0.108226911 | 0.0003378 | 0.23 |
| PRPSAP2 | 0.235962923 | 2.70E-15 | 0.174308176 | 6.61E-09 | 0.07 |
| PRR11 | 0.161833404 | 7.46E-08 | -0.051853399 | 0.0866219 | 0.22 |
| PRR12 | -0.317908963 | 4.32E-27 | -0.281449637 | 2.39E-21 | 0.04 |
| PRR13 | 0.023530683 | 0.43706746 | 0.259137496 | 3.13E-18 | 0.24 |
| PRR14 | 0.200857603 | 2.07E-11 | 0.23652045 | 2.31E-15 | 0.04 |
| PRR15L | -0.214250535 | 8.16E-13 | 0.088224073 | 0.0035105 | 0.31 |
| PRR15 | -0.401830372 | 1.14E-43 | -0.081400142 | 0.0070914 | 0.33 |
| PRR16 | -0.074072358 | 0.014308 | -0.066610461 | 0.0276579 | 0.01 |
| PRR18 | 0.243395765 | 3.33E-16 | 0.070098552 | 0.0204658 | 0.18 |
| PRR19 | 0.260854949 | 1.85E-18 | 0.311820092 | 4.49E-26 | 0.06 |
| PRR20A | NA | NA | NA | NA | #VALUE! |
| PRR20B | NA | NA | NA | NA | #VALUE! |
| PRR20C | -0.018037988 | 0.55136769 | -0.006803131 | 0.8222434 | 0.02 |
| PRR20D | NA | NA | NA | NA | #VALUE! |
| LOC391495 | -0.008085455 | 0.78946294 | -0.065891681 | 0.0293844 | 0.06 |
| PRR22 | 0.18665368 | 5.02E-10 | 0.20992126 | 2.38E-12 | 0.03 |
| LOC131355 | 0.054135507 | 0.07361261 | 0.035937759 | 0.23517 | 0.02 |
| PRR23B | 0.009519781 | 0.75323533 | 0.038033132 | 0.2089666 | 0.03 |
| PRR23C | 0.01534126 | 0.61240746 | 0.022468503 | 0.4580481 | 0.01 |
| INAFM1 | 0.084890747 | 0.00497892 | 0.161772881 | 7.55E-08 | 0.08 |
| LOC342454 | 0.012163811 | 0.68790704 | 0.030604398 | 0.3120752 | 0.02 |
| PRR3 | 0.398399205 | 6.84E-43 | 0.269331309 | 1.28E-19 | 0.13 |
| PRR4 | 0.206466432 | 5.48E-12 | 0.075994809 | 0.0119645 | 0.14 |
| PRR5.ARHGAP8 | -0.015047018 | 0.61924434 | 0.085235636 | 0.0048047 | 0.11 |
| PRR5L | -0.223949413 | 6.85E-14 | -0.17130208 | 1.20E-08 | 0.06 |
| LOC86335 | 0.146849713 | 1.08E-06 | 0.126005949 | 2.94E-05 | 0.03 |
| PRR7 | 0.350999944 | 4.86E-33 | 0.268084613 | 1.91E-19 | 0.09 |
| PRRC1 | -0.369141384 | 1.28E-36 | -0.145788189 | 1.30E-06 | 0.23 |
| PRRG1 | 0.010798669 | 0.72138241 | -0.096040883 | 0.0014784 | 0.11 |
| PRRG2 | -0.184505643 | 7.96E-10 | -0.028024717 | 0.3546361 | 0.16 |
| PRRG3 | -0.162476478 | 6.61E-08 | -0.227634206 | 2.59E-14 | 0.07 |
| LOC100506702 | -0.178417518 | 2.86E-09 | -0.162181251 | 6.99E-08 | 0.02 |
| PRRT1 | -0.087483581 | 0.00379767 | -0.06782004 | 0.0249495 | 0.02 |
| ICCA | -0.110245817 | 0.00026046 | -0.063099707 | 0.0369965 | 0.05 |
| PRRT3 | -0.250780989 | 3.88E-17 | -0.065423832 | 0.0305573 | 0.19 |
| LOC648962 | -0.055616182 | 0.0660594 | -0.076576549 | 0.0113257 | 0.03 |
| PRRX1 | -0.188143386 | 3.63E-10 | -0.109478013 | 0.0002877 | 0.08 |
| PRRX2 | 0.168049027 | 2.28E-08 | 0.112610901 | 0.000191 | 0.06 |
| PRSS12 | 0.093865008 | 0.00189291 | -0.117123175 | 0.000104 | 0.22 |
| PRSS16 | -0.010963733 | 0.71730481 | 0.005280997 | 0.8615557 | 0.02 |
| PRSS1 | 0.161475069 | 7.98E-08 | -0.019339871 | 0.5230084 | 0.19 |
| ESP.1 | -0.042144911 | 0.16381393 | 0.007771413 | 0.7974589 | 0.05 |
| PRSS22 | -0.0710808 | 0.01876057 | -0.017351684 | 0.5666156 | 0.06 |
| LOC102724764 | -0.343817091 | 1.10E-31 | -0.248554813 | 7.48E-17 | 0.1 |
| PRSS27 | 0.266459356 | 3.20E-19 | 0.223272243 | 8.17E-14 | 0.05 |
| PRSS30P | -0.038395429 | 0.20465897 | -0.038962384 | 0.198048 | 0.01 |
| PRSS33 | 0.360579404 | 6.70E-35 | 0.114727737 | 0.000144 | 0.25 |
| PRSS35 | -0.27097089 | 7.56E-20 | -0.193767541 | 1.05E-10 | 0.08 |
| PRSS36 | 0.08337171 | 0.00581627 | 0.092984066 | 0.0020892 | 0.01 |
| PRSS37 | -0.116325082 | 0.00011598 | -0.122767901 | 4.71E-05 | 0.01 |
| PRSS38 | 0.00685534 | 0.82090228 | 0.032701777 | 0.2800573 | 0.03 |
| LOC86247 | 0.309669403 | 1.01E-25 | 0.073883458 | 0.0145588 | 0.24 |
| PRSS41 | 0.12983197 | 1.67E-05 | 0.043416754 | 0.1514537 | 0.09 |
| PRSS42 | 0.052510449 | 0.08269916 | -0.001171023 | 0.9691532 | 0.06 |
| TESSP5 | 0.043222197 | 0.15329707 | 0.031458847 | 0.2987518 | 0.02 |
| PRSS48 | 0.057245191 | 0.0584985 | 0.046580971 | 0.1237882 | 0.02 |
| PRSS50 | 0.028071111 | 0.35383974 | -0.001228435 | 0.9676417 | 0.03 |
| PRSS53 | 0.305396594 | 5.00E-25 | 0.109301534 | 0.0002943 | 0.2 |
| LOC92917 | 0.133297878 | 9.80E-06 | 0.067665001 | 0.0252833 | 0.07 |
| PRSS55 | 0.130834025 | 1.43E-05 | 0.029114398 | 0.3362302 | 0.11 |
| PRSS8 | -0.043429755 | 0.15133107 | 0.180409189 | 1.89E-09 | 0.23 |
| PRSS57 | -0.104677482 | 0.00052775 | -0.06541595 | 0.0305774 | 0.04 |
| PRTFDC1 | 0.244594766 | 2.36E-16 | -0.016889845 | 0.5769889 | 0.27 |
| LOC145786 | -0.324249014 | 3.56E-28 | -0.28781153 | 2.72E-22 | 0.04 |
| PRTN3 | -0.089457888 | 0.0030756 | -0.010994349 | 0.7165494 | 0.08 |
| KIAA0367 | -0.220484438 | 1.68E-13 | -0.292474786 | 5.34E-23 | 0.08 |
| LOC91961 | 0.015133967 | 0.61722056 | 0.262307372 | 1.18E-18 | 0.25 |
| PRX | -0.352315728 | 2.72E-33 | -0.302723787 | 1.34E-24 | 0.05 |
| LOC115412 | -0.037897076 | 0.21060109 | -0.004523729 | 0.8812486 | 0.04 |
| PSAPL1 | 0.232381259 | 7.22E-15 | 0.0140243 | 0.6432616 | 0.22 |
| PSAP | -0.162885055 | 6.13E-08 | -0.186797816 | 4.86E-10 | 0.03 |
| PSAT1 | 0.410638846 | 1.04E-45 | 0.092415593 | 0.0022255 | 0.32 |
| LOC90297 | 0.095118433 | 0.00164274 | 0.136535719 | 5.90E-06 | 0.05 |
| PSD2 | -0.084444795 | 0.00521269 | -0.208536889 | 3.33E-12 | 0.13 |
| DKFZp761K1423 | -0.331835535 | 1.66E-29 | -0.102960388 | 0.0006518 | 0.23 |
| PSD4 | -0.421018775 | 3.42E-48 | -0.320593998 | 1.51E-27 | 0.11 |
| PSD | -0.148792253 | 7.77E-07 | -0.153052124 | 3.69E-07 | 0.01 |
| PSEN1 | -0.239562497 | 9.89E-16 | -0.120282325 | 6.70E-05 | 0.12 |
| PSEN2 | 0.021406375 | 0.4795803 | 0.311369301 | 5.33E-26 | 0.29 |
| PSENEN | 0.286475392 | 4.31E-22 | 0.394963212 | 4.03E-42 | 0.11 |
| PSG10 | -0.015416864 | 0.61065617 | -0.057603245 | 0.0569367 | 0.05 |
| PSG11 | -0.023339313 | 0.44080615 | -0.016600319 | 0.5835371 | 0.01 |
| LOC91730 | -0.063006968 | 0.03727574 | -0.095077129 | 0.0016505 | 0.04 |
| PSG2 | -0.024598412 | 0.41654527 | -0.00466196 | 0.877648 | 0.02 |
| PSG3 | -0.048161532 | 0.11153121 | -0.095514572 | 0.0015702 | 0.05 |
| LOC91051 | -0.114679819 | 0.00014494 | -0.164847273 | 4.22E-08 | 0.06 |
| PSG5 | -0.108912604 | 0.00030937 | -0.122589223 | 4.83E-05 | 0.02 |
| PSG6 | -0.00566367 | 0.85163609 | -0.030999238 | 0.3058706 | 0.03 |
| PSG7 | -0.022312575 | 0.46117485 | -0.027337378 | 0.3665666 | 0.01 |
| PSG8 | -0.06336514 | 0.03620705 | -0.111211901 | 0.0002296 | 0.05 |
| LOC91052 | -0.059827781 | 0.04799106 | -0.109451509 | 0.0002886 | 0.05 |
| MCTS2P | -0.001481623 | 0.96097726 | -0.100843584 | 0.0008419 | 0.1 |
| LOC93428 | 0.304978542 | 5.84E-25 | 0.022981965 | 0.4478362 | 0.29 |
| LOC92137 | -0.078721832 | 0.00922368 | -0.18979142 | 2.53E-10 | 0.12 |
| PSKH2 | -0.115002587 | 0.00013877 | -0.067887605 | 0.0248052 | 0.05 |
| PSMA1 | 0.394015324 | 6.55E-42 | 0.28741741 | 3.12E-22 | 0.11 |
| PSMA2 | 0.460112001 | 2.31E-58 | 0.463973057 | 1.93E-59 | 0.01 |
| PSMA3 | 0.438970242 | 1.07E-52 | 0.4191308 | 9.81E-48 | 0.02 |
| PSMA4 | 0.548627868 | 6.42E-87 | 0.3780749 | 1.82E-38 | 0.18 |
| PSMA5 | 0.487730735 | 2.17E-66 | 0.36534047 | 7.54E-36 | 0.13 |
| LOC196877 | 0.343555351 | 1.23E-31 | 0.316770319 | 6.72E-27 | 0.03 |
| PSMA7 | 0.526504547 | 5.66E-79 | 0.326154121 | 1.66E-28 | 0.21 |
| PSMA8 | 0.108507511 | 0.00032585 | -0.00839466 | 0.7816117 | 0.12 |
| PSMB10 | 0.200236561 | 2.39E-11 | 0.156291731 | 2.07E-07 | 0.05 |
| PSMB11 | 0.048121476 | 0.11182956 | 0.033122512 | 0.273913 | 0.02 |
| PSMB1 | 0.473468649 | 3.74E-62 | 0.4424858 | 1.30E-53 | 0.04 |
| PSMB2 | 0.524587131 | 2.59E-78 | 0.269298411 | 1.29E-19 | 0.26 |
| LOC220568 | 0.3819161 | 2.80E-39 | 0.367922893 | 2.27E-36 | 0.02 |
| LOC115890 | 0.554468108 | 4.07E-89 | 0.58104061 | 1.09E-99 | 0.03 |
| PSMB5 | 0.459541319 | 3.32E-58 | 0.396041665 | 2.31E-42 | 0.07 |
| LOC124972 | 0.436167039 | 5.62E-52 | 0.330450598 | 2.93E-29 | 0.11 |
| PSMB7 | 0.519509138 | 1.40E-76 | 0.417505444 | 2.42E-47 | 0.11 |
| PSMB8 | 0.190219523 | 2.30E-10 | 0.135682522 | 6.75E-06 | 0.06 |
| LOC92051 | 0.2165786 | 4.55E-13 | 0.096576942 | 0.0013901 | 0.13 |
| PSMC1 | 0.468008883 | 1.39E-60 | 0.436984721 | 3.46E-52 | 0.04 |
| PSMC2 | 0.38453298 | 7.72E-40 | 0.29036603 | 1.12E-22 | 0.1 |
| PSMC3IP | 0.488477112 | 1.29E-66 | 0.252169901 | 2.57E-17 | 0.24 |
| PSMC3 | 0.46774328 | 1.65E-60 | 0.30028079 | 3.27E-24 | 0.17 |
| PSMC4 | 0.40587391 | 1.34E-44 | 0.303967635 | 8.49E-25 | 0.11 |
| PSMC5 | 0.26968516 | 1.14E-19 | 0.221127868 | 1.43E-13 | 0.05 |
| LOC63380 | 0.106723169 | 0.00040872 | 0.191091303 | 1.90E-10 | 0.09 |
| PSMD10 | 0.244114265 | 2.71E-16 | 0.183993749 | 8.88E-10 | 0.07 |
| PSMD11 | 0.234801041 | 3.72E-15 | 0.086527277 | 0.0042 | 0.15 |
| PSMD12 | 0.3046101 | 6.69E-25 | 0.158567007 | 1.37E-07 | 0.15 |
| HSPC027 | 0.41030034 | 1.25E-45 | 0.360033556 | 8.59E-35 | 0.06 |
| PSMD14 | 0.505809748 | 4.68E-72 | 0.334119791 | 6.49E-30 | 0.18 |
| PSMD1 | 0.217504645 | 3.60E-13 | 0.070243013 | 0.0202069 | 0.15 |
| PSMD2 | 0.33537392 | 3.86E-30 | 0.110778872 | 0.000243 | 0.23 |
| PSMD3 | 0.171361228 | 1.19E-08 | 0.164994755 | 4.11E-08 | 0.01 |
| PSMD4 | 0.469007409 | 7.21E-61 | 0.575781447 | 1.61E-97 | 0.11 |
| PSMD5 | -0.199103003 | 3.10E-11 | -0.067660784 | 0.0252924 | 0.14 |
| PSMD6 | 0.153926049 | 3.16E-07 | 0.174314629 | 6.60E-09 | 0.03 |
| PSMD7 | 0.408710697 | 2.94E-45 | 0.180077505 | 2.03E-09 | 0.23 |
| PSMD8 | 0.314802639 | 1.44E-26 | 0.172536988 | 9.43E-09 | 0.15 |
| PSMD9 | 0.311884703 | 4.38E-26 | 0.221366255 | 1.34E-13 | 0.1 |
| PSME1 | 0.064506492 | 0.03297389 | 0.141249924 | 2.76E-06 | 0.08 |
| PSME2 | 0.339646921 | 6.46E-31 | 0.243916089 | 2.87E-16 | 0.1 |
| PSME3 | 0.091084067 | 0.00257734 | 0.012306964 | 0.6844305 | 0.08 |
| PSME4 | 0.244495181 | 2.43E-16 | 0.08213289 | 0.0065908 | 0.17 |
| PI31 | 0.121630577 | 5.54E-05 | 0.110834972 | 0.0002412 | 0.02 |
| PSMG1 | 0.596261493 | 3.37E-106 | 0.354705083 | 9.44E-34 | 0.25 |
| HCCA3 | 0.607777469 | 2.33E-111 | 0.432250631 | 5.58E-51 | 0.18 |
| PSMG3 | 0.495383149 | 9.55E-69 | 0.427326247 | 9.58E-50 | 0.07 |
| C6orf86 | 0.440654367 | 3.90E-53 | 0.3967544 | 1.60E-42 | 0.05 |
| C6orf16 | 0.109815561 | 0.00027539 | 0.158723757 | 1.33E-07 | 0.05 |
| C6orf17 | 0.296442314 | 1.31E-23 | 0.121028579 | 6.03E-05 | 0.18 |
| LOC100133342 | 0.16836285 | 2.15E-08 | 0.034172301 | 0.2589856 | 0.14 |
| PSPC1 | 0.219125038 | 2.38E-13 | 0.113000184 | 0.0001814 | 0.11 |
| PSPH | 0.283629281 | 1.14E-21 | 0.147454051 | 9.77E-07 | 0.14 |
| LOC147775 | 0.123622192 | 4.17E-05 | 0.181863939 | 1.39E-09 | 0.06 |
| PSRC1 | 0.567823919 | 2.60E-94 | 0.282468385 | 1.69E-21 | 0.29 |
| PSTK | 0.286064047 | 4.97E-22 | 0.174466734 | 6.40E-09 | 0.12 |
| PSTPIP1 | 0.068111212 | 0.02433283 | -0.058164077 | 0.05456 | 0.13 |
| PSTPIP2 | 0.143880169 | 1.79E-06 | 0.006492084 | 0.8302441 | 0.14 |
| LOC148456 | -0.282817633 | 1.50E-21 | -0.378812689 | 1.27E-38 | 0.1 |
| PTAR1 | -0.293554642 | 3.65E-23 | -0.331137326 | 2.21E-29 | 0.04 |
| LOC63477 | 0.305664152 | 4.53E-25 | 0.225973517 | 4.02E-14 | 0.08 |
| PTBP2 | 0.117898574 | 9.35E-05 | 0.01953503 | 0.5188226 | 0.1 |
| PTCD1 | 0.237287182 | 1.87E-15 | 0.162568493 | 6.50E-08 | 0.08 |
| PTCD2 | -0.323189155 | 5.43E-28 | -0.131623981 | 1.27E-05 | 0.2 |
| PTCD3 | 0.253529852 | 1.71E-17 | 0.105584241 | 0.0004715 | 0.15 |
| NBCCS | -0.00039947 | 0.98947495 | -0.149574124 | 6.79E-07 | 0.15 |
| PTCH2 | -0.182685334 | 1.17E-09 | -0.216280073 | 4.91E-13 | 0.04 |
| PTCHD1 | 0.182860835 | 1.13E-09 | 0.006947378 | 0.8185393 | 0.18 |
| PTCHD2 | -0.078177489 | 0.00972121 | -0.00575886 | 0.8491721 | 0.08 |
| PTCHD3 | -0.07921753 | 0.00879048 | -0.027203797 | 0.3689139 | 0.06 |
| LOC89959 | -0.023124107 | 0.44503229 | -0.070466127 | 0.0198125 | 0.05 |
| PTDSS1 | 0.47952892 | 6.27E-64 | 0.167461744 | 2.56E-08 | 0.32 |
| LOC100131598 | 0.16449314 | 4.52E-08 | 0.064126511 | 0.0340217 | 0.11 |
| PTENP1 | -0.221145891 | 1.42E-13 | -0.140077528 | 3.34E-06 | 0.09 |
| BZS | -0.293564199 | 3.64E-23 | -0.178693975 | 2.70E-09 | 0.12 |
| PTER | -0.290520507 | 1.06E-22 | -0.176799635 | 3.98E-09 | 0.12 |
| PTF1A | 0.06505586 | 0.03150779 | 0.00296019 | 0.9221278 | 0.07 |
| AS1 | -0.194898625 | 8.11E-11 | -0.189355986 | 2.79E-10 | 0.01 |
| PTGDS | -0.078868735 | 0.00909335 | -0.077159795 | 0.0107158 | 0.01 |
| PTGER1 | 0.073745973 | 0.01474371 | 0.044479743 | 0.1416784 | 0.03 |
| LOC63381 | -0.14069302 | 3.02E-06 | -0.223885044 | 6.96E-14 | 0.09 |
| PTGER3 | -0.381654 | 3.19E-39 | -0.234275381 | 4.30E-15 | 0.15 |
| PTGER4 | -0.259702125 | 2.63E-18 | -0.266762584 | 2.90E-19 | 0.01 |
| PTGES2 | 0.373587831 | 1.57E-37 | 0.212005949 | 1.42E-12 | 0.17 |
| PTGES3 | 0.290313893 | 1.14E-22 | 0.311255028 | 5.56E-26 | 0.03 |
| PTGES | 0.022433367 | 0.45875161 | -0.022030104 | 0.4668693 | 0.05 |
| PTGFRN | -0.229268882 | 1.67E-14 | -0.139892217 | 3.44E-06 | 0.09 |
| PTGFR | -0.076775266 | 0.01111455 | -0.177911642 | 3.17E-09 | 0.11 |
| PTGIR | -0.0178704 | 0.55507241 | -0.030598362 | 0.3121707 | 0.02 |
| PTGIS | -0.087244677 | 0.00389476 | -0.122096953 | 5.19E-05 | 0.04 |
| PTGR1 | -0.240284414 | 8.07E-16 | -0.12088166 | 6.16E-05 | 0.12 |
| PTGR2 | -0.202486725 | 1.41E-11 | -0.0289581 | 0.3388319 | 0.18 |
| PTGS1 | -0.255211807 | 1.03E-17 | -0.235566126 | 3.01E-15 | 0.02 |
| PTGS2 | 0.026268108 | 0.38561658 | -0.101188531 | 0.0008078 | 0.13 |
| PTH1R | -0.247077245 | 1.15E-16 | -0.110556682 | 0.0002501 | 0.14 |
| PTH2R | 0.004123573 | 0.89168517 | -0.047902783 | 0.1134696 | 0.06 |
| PTH2 | 0.094721466 | 0.00171847 | 0.088059531 | 0.0035726 | 0.01 |
| PTHLH | -0.164804397 | 4.26E-08 | -0.103349593 | 0.0006215 | 0.07 |
| PTH | 0.035810324 | 0.23683541 | -0.024061332 | 0.4267964 | 0.06 |
| PTK2B | -0.200300879 | 2.35E-11 | -0.213080515 | 1.09E-12 | 0.02 |
| PTK2 | 0.137076549 | 5.41E-06 | 0.054654219 | 0.0708901 | 0.09 |
| PTK6 | -0.278869271 | 5.67E-21 | -0.109021639 | 0.0003051 | 0.17 |
| PTK7 | 0.213152422 | 1.07E-12 | 0.111790025 | 0.0002129 | 0.11 |
| LOC91418 | 0.562708659 | 2.71E-92 | 0.414665571 | 1.16E-46 | 0.15 |
| PTMS | 0.29159662 | 7.27E-23 | 0.262323504 | 1.17E-18 | 0.03 |
| PTN | -0.173461573 | 7.83E-09 | -0.127233986 | 2.46E-05 | 0.05 |
| DKFZP586I111 | 0.193577992 | 1.09E-10 | 0.212115447 | 1.39E-12 | 0.02 |
| PTP4A1 | -0.024088356 | 0.42627707 | -0.106481989 | 0.0004213 | 0.09 |
| PTP4A2 | -0.265160448 | 4.82E-19 | -0.101697185 | 0.0007598 | 0.17 |
| PTP4A3 | 0.247707217 | 9.59E-17 | 0.163655667 | 5.30E-08 | 0.09 |
| PTPDC1 | -0.228798668 | 1.90E-14 | -0.240855218 | 6.87E-16 | 0.02 |
| HACD3 | -0.263349139 | 8.50E-19 | -0.145635544 | 1.33E-06 | 0.12 |
| HACD4 | -0.161271799 | 8.29E-08 | -0.062660322 | 0.0383354 | 0.1 |
| HACD1 | 0.222766203 | 9.32E-14 | 0.032201893 | 0.2874782 | 0.2 |
| HACD2 | -0.29108373 | 8.71E-23 | -0.350146237 | 7.07E-33 | 0.06 |
| PTPMT1 | 0.37066698 | 6.27E-37 | 0.237850926 | 1.60E-15 | 0.14 |
| PRO1847 | -0.123657718 | 4.14E-05 | -0.242160025 | 4.74E-16 | 0.12 |
| PTPN12 | -0.023086385 | 0.44577545 | -0.098268136 | 0.0011421 | 0.08 |
| PTPN13 | -0.295713965 | 1.69E-23 | -0.224771746 | 5.52E-14 | 0.08 |
| PTPN14 | -0.154631126 | 2.79E-07 | -0.143910277 | 1.78E-06 | 0.02 |
| PTPN18 | -0.056342466 | 0.06259452 | 0.034316243 | 0.2569835 | 0.1 |
| PTPN1 | -0.12013595 | 6.84E-05 | -0.234590858 | 3.94E-15 | 0.12 |
| PTPN20A | 0.003314684 | 0.91283751 | -0.02111204 | 0.4856434 | 0.03 |
| PTPN20 | 0.067666101 | 0.02528091 | -0.067895101 | 0.0247892 | 0.14 |
| PTPN21 | -0.35864347 | 1.61E-34 | -0.321705167 | 9.77E-28 | 0.04 |
| PTPN22 | -0.048973483 | 0.10561763 | -0.180621237 | 1.81E-09 | 0.14 |
| PTPN23 | -0.27802344 | 7.51E-21 | -0.227448866 | 2.72E-14 | 0.06 |
| PTPN2 | 0.586054752 | 8.51E-102 | 0.202227752 | 1.50E-11 | 0.39 |
| PTPN3 | -0.188068311 | 3.69E-10 | -0.05085664 | 0.0928577 | 0.14 |
| PTPN4 | -0.096925517 | 0.00133522 | -0.1960662 | 6.23E-11 | 0.1 |
| PTPN5 | -0.171573016 | 1.14E-08 | -0.101735988 | 0.0007562 | 0.07 |
| SHP.1L | 0.075006925 | 0.01312249 | -0.036201577 | 0.2317486 | 0.12 |
| PTPN7 | 0.033015377 | 0.27546879 | -0.10890455 | 0.0003097 | 0.15 |
| PTPN9 | -0.207723119 | 4.05E-12 | -0.312143382 | 3.97E-26 | 0.11 |
| PTPRA | -0.298412287 | 6.43E-24 | -0.22065463 | 1.61E-13 | 0.08 |
| PTPRB | -0.532447345 | 4.74E-81 | -0.375229453 | 7.17E-38 | 0.16 |
| PTPRCAP | 0.070699994 | 0.01940635 | -0.012677818 | 0.6754552 | 0.09 |
| PTPRC | -0.172069413 | 1.03E-08 | -0.281117176 | 2.67E-21 | 0.11 |
| PTPRD | -0.174304894 | 6.61E-09 | -0.121813674 | 5.40E-05 | 0.06 |
| LOC119466 | -0.393683304 | 7.76E-42 | -0.372551277 | 2.57E-37 | 0.03 |
| PTPRF | -0.155676297 | 2.31E-07 | -0.174881277 | 5.89E-09 | 0.02 |
| PTPRG | -0.457094517 | 1.57E-57 | -0.396381159 | 1.94E-42 | 0.07 |
| PTPRH | -0.107544888 | 0.00036838 | -0.092259006 | 0.0022645 | 0.02 |
| PTPRJ | -0.291276779 | 8.14E-23 | -0.290357027 | 1.12E-22 | 0.01 |
| PTPRK | -0.020973228 | 0.48851714 | -0.02974782 | 0.3258184 | 0.01 |
| PTPRM | -0.382753938 | 1.86E-39 | -0.345986693 | 4.32E-32 | 0.04 |
| PTPRN2 | -0.317223345 | 5.64E-27 | -0.261387123 | 1.57E-18 | 0.06 |
| PTPRN | -0.060523034 | 0.04544961 | -0.10135637 | 0.0007916 | 0.05 |
| PTPRO | -0.148627282 | 7.99E-07 | -0.226403688 | 3.59E-14 | 0.08 |
| DFNB84 | -0.106836753 | 0.00040291 | -0.141316857 | 2.73E-06 | 0.04 |
| PTPRR | -0.118952536 | 8.08E-05 | -0.094770609 | 0.0017089 | 0.03 |
| PTPRS | -0.078341183 | 0.00956914 | 0.008199967 | 0.7865527 | 0.09 |
| PTPRT | -0.424593863 | 4.55E-49 | -0.205234218 | 7.36E-12 | 0.22 |
| PTPRU | -0.0349932 | 0.24771196 | -0.167813157 | 2.39E-08 | 0.14 |
| PTPRVP | 0.039556195 | 0.19129265 | 0.118002079 | 9.21E-05 | 0.08 |
| PTPRZ1 | 0.011676937 | 0.69978005 | -0.121493924 | 5.65E-05 | 0.14 |
| PTRF | -0.163805519 | 5.15E-08 | -0.149065019 | 7.41E-07 | 0.02 |
| PTRH1 | 0.025659889 | 0.3967171 | 0.185127763 | 6.97E-10 | 0.16 |
| PTRH2 | 0.401576645 | 1.30E-43 | 0.424803375 | 4.04E-49 | 0.03 |
| PTS | 0.408851024 | 2.73E-45 | 0.327087079 | 1.14E-28 | 0.09 |
| PTTG1IP | 0.078046305 | 0.00984463 | 0.045116693 | 0.1360573 | 0.04 |
| PTTG1 | 0.653704884 | 3.19E-134 | 0.448134228 | 4.18E-55 | 0.21 |
| PTTG2 | 0.278813979 | 5.77E-21 | 0.139656246 | 3.58E-06 | 0.14 |
| PTTG3P | 0.522074183 | 1.88E-77 | 0.372832778 | 2.25E-37 | 0.15 |
| PTX3 | 0.180266673 | 1.95E-09 | -0.066758124 | 0.0273143 | 0.25 |
| PTX4 | -0.061319245 | 0.04267921 | -0.144711949 | 1.55E-06 | 0.09 |
| PUF60 | 0.465193303 | 8.73E-60 | 0.275864256 | 1.53E-20 | 0.19 |
| PUM1 | -0.295121738 | 2.09E-23 | -0.317678152 | 4.73E-27 | 0.03 |
| PUM2 | -0.131395593 | 1.31E-05 | -0.135489778 | 6.96E-06 | 0.01 |
| PURA | -0.322464769 | 7.24E-28 | -0.273149787 | 3.73E-20 | 0.05 |
| PURB | -0.060848282 | 0.04430015 | -0.067006564 | 0.0267443 | 0.01 |
| PURG | -0.236008466 | 2.67E-15 | -0.184585918 | 7.83E-10 | 0.06 |
| PUS10 | 0.017602678 | 0.5610158 | -0.038114742 | 0.2079906 | 0.06 |
| PUS1 | 0.491448379 | 1.58E-67 | 0.40644192 | 9.92E-45 | 0.09 |
| PUS3 | 0.177130686 | 3.72E-09 | 0.215399119 | 6.12E-13 | 0.04 |
| PUS7L | -0.219019796 | 2.45E-13 | -0.176687019 | 4.08E-09 | 0.05 |
| PUS7 | 0.342910266 | 1.62E-31 | 0.179018897 | 2.52E-09 | 0.17 |
| PUSL1 | 0.37775046 | 2.13E-38 | 0.213875905 | 8.96E-13 | 0.17 |
| PVALB | -0.090958579 | 0.00261298 | -0.00743837 | 0.805962 | 0.09 |
| PVRIG | -0.078408219 | 0.00950748 | -0.131340642 | 1.32E-05 | 0.06 |
| ED4 | 0.153501947 | 3.41E-07 | 0.034314388 | 0.2570093 | 0.12 |
| LOC125936 | -0.205999937 | 6.13E-12 | -0.053485607 | 0.0771436 | 0.16 |
| LOC100506575 | -0.334218706 | 6.23E-30 | -0.337058194 | 1.91E-30 | 0.01 |
| LOC115830 | 0.074744931 | 0.01344576 | 0.304607869 | 6.70E-25 | 0.23 |
| PVR | 0.358141102 | 2.02E-34 | 0.074197176 | 0.0141444 | 0.29 |
| PVT1 | 0.343076295 | 1.51E-31 | 0.270127221 | 9.92E-20 | 0.08 |
| PWP1 | 0.324486272 | 3.24E-28 | 0.281546551 | 2.31E-21 | 0.05 |
| PWP2 | 0.365520035 | 6.94E-36 | 0.199554811 | 2.80E-11 | 0.17 |
| PWRN1 | 0.010096405 | 0.73881782 | -0.004091361 | 0.8925261 | 0.02 |
| PWRN2 | -0.000508063 | 0.98661401 | -0.014334352 | 0.6359395 | 0.02 |
| PWWP2A | -0.325804225 | 1.91E-28 | -0.186310343 | 5.40E-10 | 0.14 |
| PWWP2B | 0.034941575 | 0.24841066 | 0.073137343 | 0.0155875 | 0.04 |
| PXDNL | -0.004433385 | 0.88360313 | -0.060024653 | 0.0472596 | 0.06 |
| KIAA0230 | -0.156110493 | 2.14E-07 | -0.168104313 | 2.26E-08 | 0.02 |
| PXK | -0.469223614 | 6.25E-61 | -0.455154633 | 5.34E-57 | 0.02 |
| PXMP2 | 0.196544449 | 5.58E-11 | 0.19059952 | 2.12E-10 | 0.01 |
| PXMP4 | -0.275062079 | 2.00E-20 | -0.136668265 | 5.78E-06 | 0.14 |
| LOC732318 | -0.142754773 | 2.15E-06 | -0.043595717 | 0.1497729 | 0.1 |
| PXT1 | 0.146048461 | 1.24E-06 | -0.057174545 | 0.0588108 | 0.21 |
| PYCARD | -0.0405159 | 0.18073517 | 0.16797454 | 2.31E-08 | 0.21 |
| PYCR1 | 0.314090804 | 1.89E-26 | 0.2962549 | 1.40E-23 | 0.02 |
| PYCR2 | 0.088396671 | 0.00344649 | 0.342037426 | 2.35E-31 | 0.26 |
| LOC116398 | 0.312701163 | 3.21E-26 | 0.265057631 | 4.98E-19 | 0.05 |
| PYDC1 | -0.059886593 | 0.04777155 | 0.155189372 | 2.52E-07 | 0.22 |
| PYDC2 | 0.052201912 | 0.08452289 | 0.055525794 | 0.0665015 | 0.01 |
| PYGB | 0.096576527 | 0.00139012 | -0.004616003 | 0.8788448 | 0.11 |
| PYGL | -0.163537683 | 5.42E-08 | -0.05337774 | 0.0777428 | 0.12 |
| PYGM | -0.172189386 | 1.01E-08 | -0.144733453 | 1.55E-06 | 0.03 |
| PYGO1 | -0.255507552 | 9.46E-18 | -0.402088294 | 9.96E-44 | 0.15 |
| PYGO2 | 0.093886733 | 0.0018883 | 0.349533927 | 9.25E-33 | 0.26 |
| PYHIN1 | -0.019971073 | 0.50953306 | -0.103756322 | 0.0005913 | 0.09 |
| PYROXD1 | 0.050688797 | 0.09394232 | 0.110430932 | 0.0002543 | 0.06 |
| PYROXD2 | -0.134519568 | 8.10E-06 | -0.082147622 | 0.0065811 | 0.06 |
| PYY2 | 0.187702633 | 4.00E-10 | 0.101458264 | 0.000782 | 0.09 |
| PYY | -0.066631678 | 0.02760831 | -0.009306032 | 0.758602 | 0.06 |
| PZP | 0.024970622 | 0.4095269 | -0.052201704 | 0.0845241 | 0.08 |
| LZTS3 | 0.135444894 | 7.01E-06 | 0.040194711 | 0.1842193 | 0.1 |
| QARS | 0.049821484 | 0.09970954 | 0.141818041 | 2.51E-06 | 0.1 |
| QDPR | -0.178161252 | 3.01E-09 | -0.003977787 | 0.8954922 | 0.18 |
| LOC154412 | -0.083956883 | 0.00547975 | -0.16896028 | 1.91E-08 | 0.09 |
| QPCTL | 0.230192268 | 1.31E-14 | 0.132933859 | 1.04E-05 | 0.1 |
| LOC130739 | 0.055972023 | 0.06434256 | -0.114183844 | 0.0001549 | 0.18 |
| LOC101929880 | 0.07770014 | 0.01017702 | 0.09318728 | 0.0020423 | 0.02 |
| QRFPR | 0.099022906 | 0.00104521 | -0.026295399 | 0.385123 | 0.13 |
| QRFP | 0.231864285 | 8.31E-15 | 0.133909152 | 8.91E-06 | 0.1 |
| QRICH1 | -0.355571478 | 6.41E-34 | -0.261588391 | 1.47E-18 | 0.1 |
| QRICH2 | 0.304433832 | 7.14E-25 | 0.034150753 | 0.2592862 | 0.28 |
| QRSL1 | 0.162243304 | 6.91E-08 | 0.12660916 | 2.69E-05 | 0.04 |
| QSER1 | 0.005923089 | 0.84492463 | -0.102718927 | 0.0006713 | 0.11 |
| QSOX1 | -0.130948826 | 1.41E-05 | 0.045198672 | 0.1353466 | 0.18 |
| QSOX2 | 0.231495283 | 9.18E-15 | 0.027062533 | 0.3714063 | 0.21 |
| QTRT1 | 0.207851965 | 3.93E-12 | 0.194988604 | 7.95E-11 | 0.02 |
| QTRTD1 | 0.037866237 | 0.21097286 | -0.01799628 | 0.5522886 | 0.06 |
| R3HCC1 | -0.00039574 | 0.98957322 | 0.087217817 | 0.0039058 | 0.09 |
| R3HDM1 | 0.223393249 | 7.92E-14 | 0.020178921 | 0.5051358 | 0.21 |
| LOC51220 | -0.257518842 | 5.14E-18 | -0.251439262 | 3.20E-17 | 0.01 |
| R3HDML | 0.016225925 | 0.59205567 | -0.038531911 | 0.2030531 | 0.06 |
| RAB10 | 0.039814865 | 0.18840348 | -0.040140185 | 0.1848157 | 0.08 |
| RAB11A | -0.16715024 | 2.71E-08 | -0.074659125 | 0.0135531 | 0.1 |
| RAB11B | 0.067697388 | 0.02521324 | 0.047260181 | 0.1183982 | 0.03 |
| LOC392278 | -0.224857615 | 5.40E-14 | -0.18965579 | 2.61E-10 | 0.04 |
| RAB11FIP2 | -0.373769129 | 1.44E-37 | -0.323744582 | 4.36E-28 | 0.06 |
| RAB11FIP3 | -0.209937791 | 2.37E-12 | -0.138545658 | 4.28E-06 | 0.08 |
| MGC11316 | -0.124907379 | 3.46E-05 | -0.090506986 | 0.002745 | 0.04 |
| DKFZP434H018 | -0.353086757 | 1.94E-33 | -0.328945797 | 5.40E-29 | 0.03 |
| RAB12 | 0.316491656 | 7.49E-27 | -0.014480411 | 0.6325024 | 0.34 |
| RAB13 | 0.142113303 | 2.39E-06 | 0.312204585 | 3.88E-26 | 0.18 |
| RAB14 | -0.337435243 | 1.64E-30 | -0.193025433 | 1.24E-10 | 0.15 |
| RAB15 | -0.042472009 | 0.16056481 | -0.02447866 | 0.4188183 | 0.02 |
| RAB17 | -0.298783342 | 5.63E-24 | -0.076529819 | 0.0113759 | 0.23 |
| RAB18 | -0.133467557 | 9.54E-06 | -0.080980227 | 0.0073933 | 0.06 |
| LOC392798 | -0.460234085 | 2.13E-58 | -0.262195941 | 1.22E-18 | 0.2 |
| RAB1A | 0.128313457 | 2.09E-05 | 0.099613476 | 0.0009748 | 0.03 |
| RAB1B | -0.015849238 | 0.6006835 | 0.103101031 | 0.0006407 | 0.12 |
| RAB20 | -0.042711029 | 0.15822152 | 0.091433408 | 0.0024804 | 0.14 |
| RAB21 | -0.245005634 | 2.10E-16 | -0.198525456 | 3.55E-11 | 0.05 |
| RAB22A | 0.035751276 | 0.23760989 | 0.008626661 | 0.7757352 | 0.03 |
| RAB23 | -0.00159003 | 0.95812461 | -0.142569029 | 2.22E-06 | 0.15 |
| RAB24 | 0.040806915 | 0.17762078 | 0.131140421 | 1.37E-05 | 0.1 |
| RAB25 | 0.117809079 | 9.46E-05 | 0.377600481 | 2.29E-38 | 0.26 |
| RAB26 | -0.149747565 | 6.59E-07 | 0.058161933 | 0.0545689 | 0.21 |
| RAB27A | -0.2488076 | 6.95E-17 | -0.262527787 | 1.10E-18 | 0.02 |
| RAB27B | -0.484566675 | 1.97E-65 | -0.270701272 | 8.24E-20 | 0.22 |
| RAB28 | -0.099213589 | 0.00102196 | -0.090711711 | 0.0026844 | 0.01 |
| LHX | 0.014041499 | 0.64285455 | -0.030167382 | 0.3190384 | 0.05 |
| RAB2B | -0.17694654 | 3.87E-09 | -0.177154982 | 3.71E-09 | 0.01 |
| LOC115828 | -0.397169011 | 1.29E-42 | -0.229265166 | 1.68E-14 | 0.17 |
| RAB31 | -0.256815922 | 6.36E-18 | -0.207928891 | 3.86E-12 | 0.05 |
| RAB32 | 0.138351717 | 4.41E-06 | 0.142477141 | 2.25E-06 | 0.01 |
| RAB33A | 0.052870797 | 0.08060965 | -0.042463731 | 0.1606464 | 0.1 |
| RAB33B | -0.424462535 | 4.90E-49 | -0.289264578 | 1.64E-22 | 0.14 |
| RAB34 | 0.12175299 | 5.45E-05 | 0.190181488 | 2.32E-10 | 0.07 |
| RAB35 | 0.066418903 | 0.02810928 | -0.088477047 | 0.003417 | 0.16 |
| RAB36 | -0.055886535 | 0.06475163 | -0.02560199 | 0.3977837 | 0.04 |
| RAB37 | -0.270200152 | 9.69E-20 | -0.261541867 | 1.49E-18 | 0.01 |
| LOC119637 | -0.036047141 | 0.23374716 | 0.018866838 | 0.5332255 | 0.06 |
| RAB39B | -0.029667473 | 0.3271274 | -0.247537576 | 1.01E-16 | 0.22 |
| RAB39A | -0.107019033 | 0.00039374 | -0.254720658 | 1.20E-17 | 0.15 |
| RAB3A | -0.038502679 | 0.20339626 | 0.016357604 | 0.5890531 | 0.06 |
| RAB3B | -0.081565871 | 0.00697525 | -0.165080801 | 4.04E-08 | 0.09 |
| RAB3C | -0.130071211 | 1.61E-05 | -0.180100614 | 2.02E-09 | 0.06 |
| GOV | -0.137572547 | 5.00E-06 | 0.076239943 | 0.0116915 | 0.22 |
| WARBM1.withdrawn | -0.349260732 | 1.04E-32 | -0.3253695 | 2.28E-28 | 0.03 |
| DKFZP434D245 | -0.271464134 | 6.45E-20 | 0.046245645 | 0.1265189 | 0.32 |
| RAB3IL1 | 0.005836515 | 0.84716317 | -0.012196392 | 0.6871152 | 0.02 |
| RAB3IP | -0.044965399 | 0.13737667 | 0.014551354 | 0.6308358 | 0.06 |
| RAB40AL | 0.023609985 | 0.43552354 | 0.004008313 | 0.8946949 | 0.02 |
| RAB40A | -0.103040475 | 0.00064545 | -0.046410495 | 0.1251706 | 0.06 |
| RAB40B | 0.280584847 | 3.19E-21 | 0.147152309 | 1.03E-06 | 0.14 |
| RAB40C | 0.010768289 | 0.72213378 | 0.131290649 | 1.33E-05 | 0.13 |
| RAB41 | -0.072292345 | 0.0168292 | -0.081789561 | 0.0068212 | 0.01 |
| RAB42 | 0.046596004 | 0.12366688 | -0.096053619 | 0.0014763 | 0.15 |
| RAB43 | -0.295709058 | 1.70E-23 | -0.285010648 | 7.12E-22 | 0.02 |
| RAB4A | 0.074962368 | 0.01317698 | 0.391342402 | 2.55E-41 | 0.32 |
| RAB4B | 0.006151275 | 0.83903048 | 0.038135054 | 0.2077482 | 0.04 |
| RAB5A | -0.091735772 | 0.00239924 | -0.076492858 | 0.0114157 | 0.02 |
| RAB5B | -0.331854003 | 1.65E-29 | -0.157537684 | 1.65E-07 | 0.18 |
| RAB5C | -0.336104534 | 2.85E-30 | -0.18853848 | 3.33E-10 | 0.15 |
| RAB6A | -0.029633446 | 0.32768278 | -0.079275645 | 0.0087409 | 0.05 |
| RAB6B | 0.153908073 | 3.17E-07 | 0.034425167 | 0.2554757 | 0.12 |
| RAB6C | -0.269985329 | 1.04E-19 | -0.143649978 | 1.86E-06 | 0.13 |
| PRO2706 | 0.103777344 | 0.00058973 | 0.068772801 | 0.0229806 | 0.04 |
| RAB29 | 0.065400979 | 0.03061561 | 0.200536177 | 2.23E-11 | 0.14 |
| RAB8A | 0.131904766 | 1.21E-05 | 0.089643675 | 0.0030145 | 0.05 |
| RAB8B | -0.35326173 | 1.79E-33 | -0.310334799 | 7.88E-26 | 0.05 |
| RAB9A | 0.124496697 | 3.67E-05 | 0.087635062 | 0.0037372 | 0.04 |
| RAB9BP1 | 0.036102906 | 0.23302411 | 0.041595008 | 0.1693873 | 0.01 |
| RAB9B | -0.028812255 | 0.34127116 | -0.125463591 | 3.19E-05 | 0.1 |
| RABAC1 | 0.080593342 | 0.00768171 | 0.131668512 | 1.26E-05 | 0.06 |
| RABEP1 | -0.470351437 | 2.97E-61 | -0.215282918 | 6.30E-13 | 0.26 |
| RABEP2 | 0.002313731 | 0.93909625 | 0.07985249 | 0.0082621 | 0.08 |
| RABEPK | 0.163414628 | 5.54E-08 | 0.219291083 | 2.28E-13 | 0.06 |
| HHL | -0.186227004 | 5.50E-10 | -0.112382425 | 0.0001969 | 0.08 |
| RABGAP1 | -0.217945238 | 3.22E-13 | -0.262633897 | 1.06E-18 | 0.05 |
| RABGEF1 | -0.009037384 | 0.76536347 | -0.040596111 | 0.1798728 | 0.04 |
| RABGGTA | 0.163119291 | 5.86E-08 | 0.158472402 | 1.39E-07 | 0.01 |
| RABGGTB | 0.366312252 | 4.81E-36 | 0.252319957 | 2.46E-17 | 0.12 |
| RABIF | 0.223008053 | 8.75E-14 | 0.566713275 | 7.19E-94 | 0.35 |
| RABL2A | -0.081262172 | 0.00718934 | 0.068754253 | 0.0230176 | 0.16 |
| RABL2B | -0.151017634 | 5.28E-07 | -0.032897641 | 0.2771854 | 0.12 |
| RABL3 | -0.403088437 | 5.88E-44 | -0.177321557 | 3.58E-09 | 0.23 |
| LOC222226 | -0.057239887 | 0.0585219 | 0.192806542 | 1.30E-10 | 0.26 |
| LOC155330 | 0.155467738 | 2.40E-07 | 0.219272294 | 2.30E-13 | 0.07 |
| LOC129132 | -0.041143173 | 0.17407211 | -0.128837735 | 1.93E-05 | 0.09 |
| RAC3 | 0.368500572 | 1.73E-36 | 0.253665226 | 1.65E-17 | 0.12 |
| RACGAP1P | 0.370381676 | 7.17E-37 | 0.300921475 | 2.59E-24 | 0.07 |
| LOC85722 | 0.364305768 | 1.22E-35 | 0.196557563 | 5.57E-11 | 0.17 |
| RAD17 | -0.279278307 | 4.94E-21 | -0.135306185 | 7.16E-06 | 0.15 |
| RAD18 | 0.095258381 | 0.00161678 | 0.079679121 | 0.0084035 | 0.02 |
| RAD1 | 0.124907988 | 3.46E-05 | 0.117934647 | 9.30E-05 | 0.01 |
| LOC649632 | -0.042433851 | 0.16094132 | 0.013043964 | 0.6666389 | 0.06 |
| RAD21 | 0.245754456 | 1.69E-16 | 0.077017365 | 0.010862 | 0.17 |
| LOC115906 | 0.422021732 | 1.94E-48 | 0.288465448 | 2.17E-22 | 0.14 |
| RAD23B | 0.011688826 | 0.69948925 | 0.044109796 | 0.145024 | 0.04 |
| RAD50 | -0.393447641 | 8.75E-42 | -0.240761391 | 7.05E-16 | 0.16 |
| RAD51AP1 | 0.502486121 | 5.47E-71 | 0.236828585 | 2.12E-15 | 0.27 |
| RAD51AP2 | 0.100426785 | 0.0008849 | 0.008572174 | 0.7771142 | 0.1 |
| RAD51C | 0.219840501 | 1.98E-13 | 0.189640797 | 2.62E-10 | 0.04 |
| RAD51B | 0.104605235 | 0.00053249 | 0.067694667 | 0.0252191 | 0.04 |
| RAD51D | 0.016414448 | 0.58775908 | -0.087883329 | 0.0036401 | 0.11 |
| RAD51 | 0.523361147 | 6.83E-78 | 0.282704435 | 1.56E-21 | 0.25 |
| LOC100130219 | -0.038667718 | 0.20146424 | -0.009364463 | 0.7571338 | 0.03 |
| FSBP | 0.442167283 | 1.57E-53 | 0.242138091 | 4.77E-16 | 0.21 |
| RAD54L2 | -0.345116862 | 6.28E-32 | -0.427318171 | 9.62E-50 | 0.09 |
| RAD54L | 0.556419318 | 7.33E-90 | 0.241272959 | 6.10E-16 | 0.32 |
| RAD9A | 0.19637668 | 5.80E-11 | 0.153545375 | 3.38E-07 | 0.05 |
| RAD9B | 0.139443896 | 3.70E-06 | -0.095284937 | 0.0016119 | 0.24 |
| RADIL | -0.10053605 | 0.00087343 | -0.118386764 | 8.74E-05 | 0.02 |
| RAE1 | 0.342209746 | 2.18E-31 | 0.262848469 | 9.94E-19 | 0.08 |
| RAET1E | -0.12815786 | 2.14E-05 | 0.039491718 | 0.1920179 | 0.17 |
| RAET1G | 0.145766743 | 1.30E-06 | 0.055103231 | 0.0686004 | 0.1 |
| RAET1K | 0.288788858 | 1.94E-22 | 0.168120028 | 2.25E-08 | 0.13 |
| RAET1L | 0.274526867 | 2.38E-20 | 0.034376209 | 0.2561527 | 0.25 |
| RAF1 | 0.09752408 | 0.00124568 | -0.051661461 | 0.0877957 | 0.15 |
| SLC50A1 | 0.141020708 | 2.86E-06 | 0.437751552 | 2.20E-52 | 0.3 |
| RAG1 | -0.291645586 | 7.15E-23 | -0.297511488 | 8.90E-24 | 0.01 |
| RAG2 | 0.016092995 | 0.59509389 | -0.045974198 | 0.1287634 | 0.07 |
| MOK | -0.064141807 | 0.03397901 | 0.071363798 | 0.0182929 | 0.14 |
| NORPEG | -0.044875599 | 0.13816441 | -0.14014351 | 3.31E-06 | 0.1 |
| DKFZp434A139 | -0.327024036 | 1.17E-28 | -0.384072003 | 9.70E-40 | 0.06 |
| RAI2 | -0.402484283 | 8.08E-44 | -0.263135441 | 9.09E-19 | 0.14 |
| LOC155232 | 0.123223761 | 4.41E-05 | 0.128125802 | 2.15E-05 | 0.01 |
| RALBP1 | 0.123534438 | 4.22E-05 | -0.224813514 | 5.46E-14 | 0.35 |
| RALB | -0.326869135 | 1.25E-28 | -0.11123767 | 0.0002289 | 0.22 |
| KIAA0884 | -0.388858383 | 8.94E-41 | -0.207990206 | 3.80E-12 | 0.19 |
| C20orf74 | -0.532213787 | 5.73E-81 | -0.410742178 | 9.83E-46 | 0.13 |
| RALGAPB | -0.309739198 | 9.87E-26 | -0.245587065 | 1.77E-16 | 0.07 |
| LOC158216 | -0.118432409 | 8.68E-05 | -0.186273033 | 5.45E-10 | 0.07 |
| RALGPS1 | -0.379507328 | 9.09E-39 | -0.251577038 | 3.07E-17 | 0.13 |
| RALGPS2 | -0.500465005 | 2.41E-70 | -0.155348799 | 2.45E-07 | 0.35 |
| RALYL | 0.072122638 | 0.01708878 | -0.033278326 | 0.2716611 | 0.11 |
| RALY | 0.455708141 | 3.77E-57 | 0.348704069 | 1.33E-32 | 0.11 |
| RAMP1 | -0.016564208 | 0.58435623 | -0.039000868 | 0.197605 | 0.03 |
| RAMP2 | -0.166103067 | 3.32E-08 | 0.032984275 | 0.2759216 | 0.2 |
| RAMP3 | -0.178444786 | 2.84E-09 | -0.107924221 | 0.000351 | 0.08 |
| RANBP10 | -0.214707552 | 7.28E-13 | -0.281234214 | 2.57E-21 | 0.07 |
| RANBP17 | 0.146623301 | 1.13E-06 | 0.081484516 | 0.007032 | 0.07 |
| RANBP1 | 0.636860699 | 2.20E-125 | 0.383730479 | 1.15E-39 | 0.26 |
| RANBP2 | -0.255337939 | 9.95E-18 | -0.319270993 | 2.54E-27 | 0.07 |
| RANBP3L | -0.397289286 | 1.22E-42 | -0.260396105 | 2.13E-18 | 0.14 |
| RANBP3 | 0.119100089 | 7.91E-05 | 0.068048161 | 0.0244652 | 0.06 |
| RANBP6 | -0.256920093 | 6.16E-18 | -0.139294865 | 3.79E-06 | 0.12 |
| RANBP9 | 0.024893147 | 0.41098193 | -0.010154544 | 0.7373691 | 0.04 |
| SD | 0.325850768 | 1.88E-28 | 0.166210769 | 3.25E-08 | 0.16 |
| HSPC236 | 0.15219617 | 4.29E-07 | 0.157918443 | 1.54E-07 | 0.01 |
| RAN | 0.601703446 | 1.30E-108 | 0.398602352 | 6.16E-43 | 0.21 |
| RAP1A | -0.046793713 | 0.12207977 | -0.132478087 | 1.11E-05 | 0.09 |
| RAP1B | -0.054237052 | 0.07307301 | 0.056184313 | 0.063336 | 0.12 |
| RAP1GAP2 | -0.079496405 | 0.00855483 | -0.091162341 | 0.0025553 | 0.02 |
| RAP1GAP | -0.099411336 | 0.00099836 | -0.068277635 | 0.0239863 | 0.04 |
| RAP1GDS1 | -0.032370727 | 0.28495717 | -0.097069183 | 0.0013132 | 0.07 |
| RAP2A | 0.17873665 | 2.68E-09 | -0.045480446 | 0.1329253 | 0.23 |
| RAP2B | 0.271158452 | 7.11E-20 | 0.08546017 | 0.0046942 | 0.19 |
| RAP2C | -0.171480274 | 1.16E-08 | -0.027936247 | 0.3561578 | 0.15 |
| RAPGEF1 | -0.251927233 | 2.77E-17 | -0.479386793 | 6.91E-64 | 0.23 |
| RAPGEF2 | -0.400591508 | 2.18E-43 | -0.463057862 | 3.48E-59 | 0.07 |
| RAPGEF3 | -0.449124133 | 2.27E-55 | -0.169998249 | 1.56E-08 | 0.28 |
| RAPGEF4 | -0.386506086 | 2.90E-40 | -0.162440983 | 6.66E-08 | 0.23 |
| LOC641947 | -0.28134966 | 2.47E-21 | -0.240944977 | 6.70E-16 | 0.05 |
| RAPGEF6 | -0.465942628 | 5.36E-60 | -0.458740463 | 5.53E-58 | 0.01 |
| RAPGEFL1 | 0.06532005 | 0.03082285 | 0.071841628 | 0.0175263 | 0.01 |
| ALS2CR18 | -0.363804878 | 1.53E-35 | -0.307801442 | 2.04E-25 | 0.06 |
| MGC3597 | 0.208060179 | 3.73E-12 | 0.083397218 | 0.0058012 | 0.13 |
| RARA | -0.281764429 | 2.15E-21 | -0.04866732 | 0.1078176 | 0.24 |
| LOC100130354 | -0.056294174 | 0.06282015 | -0.157277956 | 1.73E-07 | 0.11 |
| RARG | -0.009169243 | 0.76204249 | -0.070557531 | 0.0196529 | 0.07 |
| RARRES1 | 0.211555323 | 1.59E-12 | 0.047358381 | 0.1176344 | 0.17 |
| LOC115256 | -0.023358027 | 0.44043974 | 0.036443566 | 0.2286415 | 0.06 |
| RARRES3 | -0.203447264 | 1.13E-11 | -0.015645346 | 0.6053771 | 0.19 |
| PRO1992 | 0.05351427 | 0.07698502 | 0.054677609 | 0.0707693 | 0.01 |
| MGC8641 | 0.074584525 | 0.01364712 | 0.163080634 | 5.90E-08 | 0.09 |
| RASA1 | -0.454336965 | 8.92E-57 | -0.303596295 | 9.73E-25 | 0.16 |
| RASA2 | -0.312431494 | 3.56E-26 | -0.387906246 | 1.44E-40 | 0.08 |
| RASA3 | -0.200349585 | 2.33E-11 | -0.280766822 | 3.00E-21 | 0.09 |
| RASA4CP | -0.071542737 | 0.01800246 | -0.109179291 | 0.0002989 | 0.04 |
| RASA4 | -0.15140907 | 4.93E-07 | -0.197867028 | 4.13E-11 | 0.05 |
| RASAL1 | 0.226557023 | 3.45E-14 | 0.103350867 | 0.0006214 | 0.13 |
| RASAL2 | -0.146424277 | 1.16E-06 | -0.102183062 | 0.0007164 | 0.05 |
| RASAL3 | -0.073542712 | 0.01502093 | -0.133538829 | 9.44E-06 | 0.06 |
| RASD1 | -0.085270311 | 0.00478747 | -0.058370286 | 0.0537072 | 0.03 |
| RASD2 | 0.241685832 | 5.43E-16 | 0.139941366 | 3.42E-06 | 0.11 |
| RASEF | -0.517402474 | 7.15E-76 | -0.40080793 | 1.95E-43 | 0.12 |
| RASGEF1A | 0.069203975 | 0.02213478 | -0.174995509 | 5.75E-09 | 0.25 |
| RASGEF1B | 0.067325268 | 0.0260283 | -0.127237733 | 2.46E-05 | 0.2 |
| RASGEF1C | 0.358907347 | 1.43E-34 | 0.104756731 | 0.0005226 | 0.26 |
| LOC145899 | 0.12301397 | 4.55E-05 | -0.013492875 | 0.6558922 | 0.14 |
| LOC89993 | -0.408504226 | 3.29E-45 | -0.261734644 | 1.41E-18 | 0.15 |
| RASGRP1 | 0.008347617 | 0.78280475 | -0.14751359 | 9.68E-07 | 0.16 |
| RASGRP2 | -0.087520152 | 0.003783 | -0.148153064 | 8.67E-07 | 0.07 |
| RASGRP3 | -0.048159771 | 0.11154431 | -0.160439921 | 9.67E-08 | 0.12 |
| RASGRP4 | -0.366750445 | 3.92E-36 | -0.329518161 | 4.28E-29 | 0.04 |
| RASIP1 | -0.110741571 | 0.00024419 | -0.092834552 | 0.0021243 | 0.02 |
| RASL10A | 0.027139693 | 0.37004365 | 0.107121802 | 0.0003887 | 0.08 |
| MGC47540 | 0.246390549 | 1.41E-16 | 0.07298433 | 0.0158062 | 0.18 |
| LOC254109 | -0.173515476 | 7.75E-09 | 0.025006379 | 0.4088564 | 0.2 |
| MGC4499 | -0.209932126 | 2.37E-12 | -0.063082514 | 0.0370481 | 0.15 |
| RASL12 | -0.089849341 | 0.00294823 | -0.105187448 | 0.0004954 | 0.02 |
| RASSF10 | -0.082481627 | 0.00636389 | -0.049105902 | 0.1046772 | 0.04 |
| RASSF1 | 0.05697604 | 0.05969581 | 0.080578678 | 0.0076928 | 0.03 |
| RASSF2 | -0.148162539 | 8.66E-07 | -0.244798882 | 2.23E-16 | 0.1 |
| RASSF3 | -0.323491025 | 4.82E-28 | -0.39414967 | 6.11E-42 | 0.08 |
| RASSF4 | 0.20276658 | 1.32E-11 | 0.01001567 | 0.7408311 | 0.2 |
| RASSF5 | -0.151513923 | 4.84E-07 | -0.122853941 | 4.65E-05 | 0.03 |
| RASSF6 | -0.347764529 | 2.00E-32 | -0.270319948 | 9.32E-20 | 0.08 |
| RASSF7 | 0.111729025 | 0.00021459 | 0.176710036 | 4.06E-09 | 0.07 |
| RASSF8 | -0.31595329 | 9.22E-27 | -0.274262715 | 2.59E-20 | 0.05 |
| RASSF9 | -0.142274805 | 2.33E-06 | -0.219135399 | 2.38E-13 | 0.08 |
| RAVER1 | 0.29359966 | 3.59E-23 | 0.205137593 | 7.53E-12 | 0.09 |
| RAVER2 | 0.163382449 | 5.58E-08 | -0.090005814 | 0.0028987 | 0.26 |
| RAX2 | -0.010433419 | 0.73043326 | -0.048854505 | 0.1064683 | 0.04 |
| RAX | 0.274990821 | 2.04E-20 | 0.090911593 | 0.0026264 | 0.19 |
| RB1CC1 | 4.04E-05 | 0.99893493 | -0.100836766 | 0.0008426 | 0.11 |
| RB1 | -0.457538099 | 1.19E-57 | -0.280408725 | 3.39E-21 | 0.18 |
| RBAK | -0.231098746 | 1.02E-14 | -0.212476851 | 1.27E-12 | 0.02 |
| RBBP4 | -0.006371963 | 0.83333857 | -0.141989304 | 2.44E-06 | 0.14 |
| RBBP5 | -0.223844779 | 7.04E-14 | -0.001785793 | 0.9529746 | 0.23 |
| RBBP6 | -0.041634538 | 0.16898201 | -0.100310621 | 0.0008972 | 0.06 |
| RBBP7 | 0.188250993 | 3.55E-10 | 0.184435302 | 8.08E-10 | 0.01 |
| SCKL2 | 0.091328771 | 0.00250909 | -0.082780539 | 0.006175 | 0.18 |
| RBBP9 | -0.125493426 | 3.17E-05 | -0.037129303 | 0.219998 | 0.09 |
| RBCK1 | 0.013198398 | 0.66293394 | 0.100831097 | 0.0008431 | 0.09 |
| RBKS | -0.198146615 | 3.87E-11 | 0.018721097 | 0.5363936 | 0.22 |
| RBL1 | 0.040193406 | 0.18423355 | -0.128089509 | 2.16E-05 | 0.17 |
| LOC123947 | -0.382687043 | 1.92E-39 | -0.418805878 | 1.18E-47 | 0.04 |
| RBM10 | 0.260424128 | 2.11E-18 | 0.22547013 | 4.59E-14 | 0.04 |
| RBM11 | -0.164109573 | 4.86E-08 | -0.105329204 | 0.0004867 | 0.06 |
| RBM12B | -0.100533013 | 0.00087375 | -0.131440867 | 1.30E-05 | 0.04 |
| RBM12 | -0.216236031 | 4.96E-13 | -0.191814089 | 1.62E-10 | 0.03 |
| TMEM137 | 0.270053305 | 1.02E-19 | 0.195521947 | 7.05E-11 | 0.08 |
| RBM15B | -0.033258119 | 0.2719524 | -0.015114049 | 0.6176839 | 0.02 |
| RBM15 | 0.196919617 | 5.13E-11 | 0.05412313 | 0.0736786 | 0.15 |
| SCAF8 | -0.260183391 | 2.27E-18 | -0.180270023 | 1.95E-09 | 0.08 |
| RBM17 | 0.557625714 | 2.53E-90 | 0.270545649 | 8.67E-20 | 0.29 |
| RBM18 | -0.24905381 | 6.46E-17 | -0.206322444 | 5.68E-12 | 0.05 |
| RBM19 | -0.00864971 | 0.77515211 | -0.038940508 | 0.1983002 | 0.04 |
| RBM20 | -0.143085686 | 2.04E-06 | 0.00046121 | 0.9878484 | 0.15 |
| RBM22 | -0.065388517 | 0.03064745 | 0.098841544 | 0.0010678 | 0.17 |
| RBM23 | -0.061410821 | 0.04236991 | -0.080958006 | 0.0074096 | 0.02 |
| LOC154247 | -0.140733191 | 3.00E-06 | -0.133926596 | 8.89E-06 | 0.01 |
| RBM25 | -0.134014173 | 8.77E-06 | -0.094495707 | 0.001763 | 0.04 |
| C13orf10 | -0.189467589 | 2.72E-10 | -0.183118985 | 1.07E-09 | 0.01 |
| RBM27 | -0.273070111 | 3.83E-20 | -0.210594511 | 2.02E-12 | 0.07 |
| RBM28 | 0.370180763 | 7.88E-37 | 0.125501335 | 3.17E-05 | 0.25 |
| RBM33 | -0.136248658 | 6.17E-06 | -0.24302717 | 3.70E-16 | 0.11 |
| RBM34 | 0.4317954 | 7.27E-51 | 0.614365135 | 2.08E-114 | 0.19 |
| RBM38 | 0.435906301 | 6.55E-52 | 0.188544131 | 3.33E-10 | 0.25 |
| RBM39 | -0.128936983 | 1.91E-05 | 0.013282105 | 0.6609292 | 0.15 |
| RBM3 | 0.000154556 | 0.99592772 | 0.140800072 | 2.97E-06 | 0.15 |
| RBM41 | -0.20063922 | 2.17E-11 | -0.174764947 | 6.03E-09 | 0.03 |
| RBM42 | 0.284365129 | 8.88E-22 | 0.207603632 | 4.17E-12 | 0.08 |
| RBM43 | -0.556647649 | 6.00E-90 | -0.396844028 | 1.53E-42 | 0.16 |
| RBM44 | -0.253901025 | 1.53E-17 | -0.286608937 | 4.12E-22 | 0.04 |
| RBM45 | -0.031892757 | 0.29213314 | 0.103803121 | 0.0005879 | 0.14 |
| RBM46 | 0.132500704 | 1.11E-05 | 0.030131016 | 0.3196224 | 0.11 |
| RBM47 | -0.372013526 | 3.32E-37 | -0.087821134 | 0.0036642 | 0.29 |
| RBM4B | 0.112862118 | 0.00018478 | 0.169604436 | 1.68E-08 | 0.06 |
| RBM4 | 0.195599569 | 6.92E-11 | 0.2595626 | 2.75E-18 | 0.07 |
| RBM5 | -0.388170045 | 1.26E-40 | -0.190230357 | 2.30E-10 | 0.2 |
| LOC646559 | -0.065934798 | 0.02927832 | -0.046279911 | 0.1262377 | 0.02 |
| LOC51120 | -0.126607791 | 2.69E-05 | -0.129302371 | 1.80E-05 | 0.01 |
| LOC88607 | 0.34228848 | 2.11E-31 | 0.371934542 | 3.44E-37 | 0.03 |
| RBFOX2 | -0.283466056 | 1.21E-21 | -0.380447002 | 5.75E-39 | 0.1 |
| RBMS1 | 0.008021624 | 0.7910864 | -0.113010505 | 0.0001812 | 0.13 |
| RBMS2 | -0.271831395 | 5.72E-20 | -0.248049653 | 8.67E-17 | 0.03 |
| RBMS3 | -0.447392316 | 6.59E-55 | -0.334054618 | 6.67E-30 | 0.12 |
| RBMX2 | 0.44463319 | 3.54E-54 | 0.372008468 | 3.33E-37 | 0.08 |
| RBMXL1 | -0.100618277 | 0.00086489 | -0.233119207 | 5.90E-15 | 0.14 |
| RBMXL2 | 0.064570723 | 0.03279952 | 0.006851043 | 0.8210126 | 0.06 |
| RBMXL3 | -0.020910644 | 0.48981577 | -0.014811966 | 0.6247297 | 0.01 |
| RBMX | 0.283592052 | 1.16E-21 | 0.277795131 | 8.10E-21 | 0.01 |
| RBMY1A1 | 0.052561108 | 0.08240279 | 0.047305235 | 0.1180473 | 0.01 |
| RBMY1A3P | 0.028294824 | 0.35001551 | 0.042638561 | 0.1589292 | 0.02 |
| RBMY1B | NA | NA | NA | NA | #VALUE! |
| RBMY1E | 0.028294824 | 0.35001551 | 0.042638561 | 0.1589292 | 0.02 |
| RBMY1F | 0.028294824 | 0.35001551 | 0.042638561 | 0.1589292 | 0.02 |
| RBMY1J | 0.049306416 | 0.10326582 | 0.049738793 | 0.1002738 | 0.01 |
| RBMY2EP | NA | NA | NA | NA | #VALUE! |
| RBMY2FP | 0.047427844 | 0.11709637 | 0.049437208 | 0.1023534 | 0.01 |
| RBMY3P | 0.028294824 | 0.35001551 | 0.042638561 | 0.1589292 | 0.02 |
| RBP1 | 0.215626967 | 5.78E-13 | 0.063591898 | 0.035544 | 0.16 |
| RBP2 | 0.070933758 | 0.01900767 | 0.045671441 | 0.1313032 | 0.03 |
| RBP3 | 0.042884556 | 0.15653658 | -0.033702428 | 0.2655959 | 0.08 |
| RBP4 | -0.104869824 | 0.00051532 | -0.071695582 | 0.0177576 | 0.04 |
| RBP5 | -0.061486119 | 0.04211701 | 0.006385068 | 0.8330008 | 0.07 |
| RBP7 | 0.057182576 | 0.05877525 | -0.046171285 | 0.1271307 | 0.11 |
| RBPJL | 0.146793624 | 1.09E-06 | -0.013608432 | 0.6531372 | 0.17 |
| KBF2 | 0.135305713 | 7.16E-06 | 0.021842302 | 0.4706768 | 0.12 |
| RBPMS2 | 0.06411144 | 0.03406388 | 0.046447082 | 0.1248729 | 0.02 |
| RBPMS | -0.203314298 | 1.16E-11 | 0.028498411 | 0.3465582 | 0.24 |
| RBX1 | 0.43904934 | 1.02E-52 | 0.424273211 | 5.45E-49 | 0.02 |
| RC3H1 | -0.229546818 | 1.55E-14 | -0.037806723 | 0.2116916 | 0.2 |
| RC3H2 | -0.291035076 | 8.86E-23 | -0.44370834 | 6.21E-54 | 0.16 |
| RCAN1 | 0.085222476 | 0.00481122 | 0.002367041 | 0.9376958 | 0.09 |
| LOC221402 | -0.277436838 | 9.12E-21 | -0.200845056 | 2.07E-11 | 0.08 |
| RCAN3 | -0.173106803 | 8.41E-09 | -0.315389765 | 1.15E-26 | 0.15 |
| RCBTB1 | -0.366822333 | 3.79E-36 | -0.268378281 | 1.74E-19 | 0.1 |
| RCBTB2 | -0.305182633 | 5.42E-25 | -0.260534173 | 2.04E-18 | 0.05 |
| SNHG3.RCC1 | 0.429013651 | 3.63E-50 | 0.224540445 | 5.87E-14 | 0.21 |
| RCC2 | 0.487406361 | 2.73E-66 | 0.242242481 | 4.63E-16 | 0.25 |
| RCCD1 | 0.501045901 | 1.57E-70 | 0.260341364 | 2.16E-18 | 0.25 |
| RCE1 | 0.227028531 | 3.04E-14 | 0.154932439 | 2.64E-07 | 0.08 |
| PRO1996 | -0.231801736 | 8.45E-15 | -0.136123789 | 6.30E-06 | 0.1 |
| LOC94533 | 0.285015885 | 7.11E-22 | 0.211816046 | 1.49E-12 | 0.08 |
| RCN1 | 0.242720752 | 4.04E-16 | 0.154149314 | 3.04E-07 | 0.09 |
| RCN2 | 0.255862544 | 8.49E-18 | 0.072998832 | 0.0157853 | 0.19 |
| RCN3 | 0.095184241 | 0.00163048 | 0.068371072 | 0.0237937 | 0.03 |
| RCOR1 | -0.15410684 | 3.06E-07 | -0.181509127 | 1.50E-09 | 0.03 |
| RCOR2 | 0.425043428 | 3.52E-49 | 0.225715644 | 4.31E-14 | 0.2 |
| RCOR3 | -0.314892149 | 1.39E-26 | 0.09227349 | 0.0022608 | 0.41 |
| LOC105371602 | -0.078165061 | 0.00973284 | -0.180938065 | 1.69E-09 | 0.11 |
| RCVRN | -0.265190892 | 4.77E-19 | -0.131632648 | 1.27E-05 | 0.14 |
| C1orf36 | -0.319697618 | 2.15E-27 | -0.155058156 | 2.58E-07 | 0.17 |
| NELFE | 0.487074576 | 3.44E-66 | 0.388252937 | 1.21E-40 | 0.1 |
| RDH10 | 0.275574138 | 1.69E-20 | 0.129728073 | 1.69E-05 | 0.15 |
| RDH11 | 0.057815116 | 0.05602889 | 0.053417519 | 0.0775214 | 0.01 |
| RDH12 | 0.148147679 | 8.68E-07 | 0.063941094 | 0.0345433 | 0.09 |
| RDH13 | 0.073389723 | 0.01523259 | 0.120551406 | 6.46E-05 | 0.05 |
| RDH14 | 0.260001764 | 2.40E-18 | 0.220505987 | 1.67E-13 | 0.04 |
| RDH16 | 0.122970542 | 4.58E-05 | 0.117827105 | 9.44E-05 | 0.01 |
| LOC196409 | -0.11430152 | 0.0001525 | -0.075752724 | 0.0122396 | 0.04 |
| RDH8 | 0.072283722 | 0.01684231 | -0.052872736 | 0.0805985 | 0.13 |
| RDM1 | 0.35607969 | 5.11E-34 | 0.265153672 | 4.83E-19 | 0.1 |
| DFNB24 | -0.039215291 | 0.19514989 | -0.134011835 | 8.77E-06 | 0.1 |
| REC8L1 | 0.080943013 | 0.00742065 | -0.003494604 | 0.9081267 | 0.09 |
| RECK | -0.323059399 | 5.72E-28 | -0.234083776 | 4.53E-15 | 0.09 |
| RTS | 0.499852023 | 3.76E-70 | 0.275905776 | 1.51E-20 | 0.23 |
| RECQL5 | 0.100095734 | 0.00092051 | 0.052800642 | 0.0810131 | 0.05 |
| RECQL | -0.112610558 | 0.00019104 | -0.209697334 | 2.51E-12 | 0.1 |
| REEP1 | -0.378971127 | 1.18E-38 | -0.122348484 | 5.00E-05 | 0.26 |
| REEP2 | 0.158371321 | 1.42E-07 | 0.003659543 | 0.9038111 | 0.16 |
| REEP3 | -0.208600104 | 3.28E-12 | -0.130278733 | 1.56E-05 | 0.08 |
| FLJ22246 | 0.32575136 | 1.96E-28 | 0.237041042 | 2.00E-15 | 0.09 |
| REEP5 | -0.261182406 | 1.67E-18 | -0.029014994 | 0.3378834 | 0.24 |
| REEP6 | -0.376771459 | 3.42E-38 | -0.030080422 | 0.3204361 | 0.35 |
| REG1A | 0.062142951 | 0.03996458 | -0.003888178 | 0.8978335 | 0.07 |
| REG1B | -0.010026291 | 0.74056613 | -0.063889107 | 0.0346907 | 0.06 |
| REG1P | -0.018802574 | 0.53462131 | -0.015433517 | 0.6102707 | 0.01 |
| INGAP | -0.02529792 | 0.4034138 | -0.04331251 | 0.1524392 | 0.02 |
| REG3G | -0.022417306 | 0.4590734 | -0.026463109 | 0.3820981 | 0.01 |
| REG4 | -0.044672285 | 0.13996074 | 0.117058647 | 0.0001049 | 0.17 |
| RELA | 0.097958235 | 0.00118424 | 0.012186391 | 0.6873582 | 0.09 |
| RELB | 0.185714268 | 6.14E-10 | 0.031946505 | 0.2913202 | 0.16 |
| RELL1 | -0.141830481 | 2.51E-06 | -0.114306036 | 0.0001524 | 0.03 |
| RELL2 | 0.150095301 | 6.20E-07 | -0.054478237 | 0.0718043 | 0.21 |
| RELN | -0.191371445 | 1.79E-10 | -0.213308344 | 1.03E-12 | 0.03 |
| RELT | 0.263880267 | 7.20E-19 | -0.011229448 | 0.7107577 | 0.28 |
| REL | -0.405773861 | 1.42E-44 | -0.392477637 | 1.43E-41 | 0.02 |
| REM1 | 0.070695863 | 0.01941347 | -0.049809624 | 0.0997903 | 0.13 |
| REM2 | -0.029905575 | 0.32325825 | 0.089637935 | 0.0030164 | 0.12 |
| RENBP | 0.106296465 | 0.00043126 | 0.058720703 | 0.0522835 | 0.05 |
| REN | 0.033470324 | 0.26890361 | 0.134682559 | 7.90E-06 | 0.11 |
| REP15 | -0.03446564 | 0.25491699 | -0.057922384 | 0.0555739 | 0.03 |
| LOC155035 | 0.020063131 | 0.507583 | 0.108302553 | 0.0003345 | 0.09 |
| REPS1 | 0.196080202 | 6.21E-11 | 0.142162111 | 2.38E-06 | 0.06 |
| REPS2 | -0.272759032 | 4.24E-20 | -0.116873612 | 0.0001076 | 0.16 |
| RER1 | 0.100805373 | 0.00084575 | 0.092165751 | 0.002288 | 0.01 |
| KIAA0458 | -0.441912205 | 1.83E-53 | -0.387698159 | 1.60E-40 | 0.06 |
| RERGL | -0.16596492 | 3.41E-08 | -0.079941967 | 0.00819 | 0.09 |
| RERG | -0.303503843 | 1.01E-24 | -0.141935088 | 2.47E-06 | 0.17 |
| RESP18 | 0.00141392 | 0.9627591 | -0.006741068 | 0.8238383 | 0.01 |
| LOC132936 | -0.419605345 | 7.53E-48 | -0.44707615 | 8.00E-55 | 0.03 |
| RETNLB | 0.079515574 | 0.00853884 | 0.035424906 | 0.2419229 | 0.05 |
| RETN | 0.216153239 | 5.07E-13 | 0.104396839 | 0.0005464 | 0.12 |
| RETSAT | -0.423710273 | 7.50E-49 | -0.276168825 | 1.39E-20 | 0.15 |
| RET | -0.277644473 | 8.51E-21 | -0.132150522 | 1.17E-05 | 0.15 |
| REV1 | -0.200580811 | 2.20E-11 | -0.23592663 | 2.73E-15 | 0.04 |
| REV3L | -0.269454697 | 1.23E-19 | -0.349745961 | 8.43E-33 | 0.09 |
| REXO1L1P | 0.0507349 | 0.09364339 | 0.035100689 | 0.2462616 | 0.02 |
| REXO1 | 0.068664558 | 0.02319725 | -0.002761654 | 0.9273357 | 0.08 |
| REXO2 | 0.189277498 | 2.83E-10 | 0.170782882 | 1.34E-08 | 0.02 |
| REXO4 | 0.315719667 | 1.01E-26 | 0.166520261 | 3.07E-08 | 0.15 |
| MHCBFB | -0.182178695 | 1.30E-09 | -0.241774098 | 5.29E-16 | 0.06 |
| RFC2 | 0.569641014 | 4.90E-95 | 0.448421841 | 3.50E-55 | 0.13 |
| RFC3 | 0.378390224 | 1.56E-38 | 0.208291568 | 3.53E-12 | 0.18 |
| RFC4 | 0.599637472 | 1.09E-107 | 0.392658454 | 1.31E-41 | 0.21 |
| RFC5 | 0.320490308 | 1.58E-27 | 0.160568992 | 9.44E-08 | 0.16 |
| RFESD | 0.046491631 | 0.1245112 | 0.224461032 | 5.99E-14 | 0.18 |
| RFFL | -0.236931192 | 2.06E-15 | -0.209938439 | 2.37E-12 | 0.03 |
| RFK | -0.040425003 | 0.18171618 | -0.05762398 | 0.0568473 | 0.02 |
| RFNG | 0.209475077 | 2.65E-12 | 0.147957235 | 8.97E-07 | 0.07 |
| RFPL1S | -0.203132475 | 1.21E-11 | -0.175748839 | 4.94E-09 | 0.03 |
| LOC91366 | -0.141507122 | 2.65E-06 | -0.047091947 | 0.1197158 | 0.1 |
| RFPL2 | 0.093407156 | 0.00199269 | 0.049142456 | 0.1044188 | 0.05 |
| RFPL3S | 0.236447796 | 2.36E-15 | 0.126221976 | 2.85E-05 | 0.12 |
| RFPL3 | 0.093195582 | 0.00204041 | 0.050503016 | 0.0951546 | 0.05 |
| RFPL4 | -0.135273106 | 7.20E-06 | -0.116441728 | 0.0001142 | 0.02 |
| RFPL4B | -0.242104452 | 4.82E-16 | -0.14425619 | 1.68E-06 | 0.1 |
| RFT1 | 0.126674679 | 2.67E-05 | 0.169955433 | 1.57E-08 | 0.05 |
| RFTN1 | -0.148857779 | 7.68E-07 | -0.166436805 | 3.12E-08 | 0.02 |
| RFTN2 | -0.370672894 | 6.25E-37 | -0.267601086 | 2.22E-19 | 0.11 |
| RFWD2 | 0.293378906 | 3.88E-23 | 0.569638663 | 4.91E-95 | 0.28 |
| RFWD3 | 0.268907714 | 1.47E-19 | 0.034431552 | 0.2553875 | 0.24 |
| RFX1 | -0.211529998 | 1.60E-12 | -0.140172925 | 3.29E-06 | 0.08 |
| RFX2 | -0.137350247 | 5.18E-06 | -0.111556565 | 0.0002195 | 0.03 |
| RFX3 | -0.186703548 | 4.96E-10 | -0.18253132 | 1.21E-09 | 0.01 |
| NYD.SP10 | 0.263939364 | 7.07E-19 | 0.068622734 | 0.0232814 | 0.2 |
| RFX5 | 0.08847975 | 0.00341605 | 0.047524691 | 0.1163495 | 0.05 |
| RFX6 | -0.182216929 | 1.29E-09 | -0.078735808 | 0.0092112 | 0.11 |
| RFX7 | -0.388010431 | 1.37E-40 | -0.35989267 | 9.16E-35 | 0.03 |
| LOC728988 | -0.186079718 | 5.68E-10 | -0.110803162 | 0.0002422 | 0.08 |
| RFXANK | 0.373126772 | 1.96E-37 | 0.376011475 | 4.93E-38 | 0.01 |
| RFXAP | 0.07029455 | 0.02011516 | -0.023931235 | 0.4293014 | 0.1 |
| TRMT10C | 0.444218548 | 4.56E-54 | 0.471850819 | 1.10E-61 | 0.03 |
| TRMT10A | -0.15842248 | 1.40E-07 | 0.00675125 | 0.8235766 | 0.17 |
| TRMT10B | -0.103290082 | 0.00062605 | -0.075326918 | 0.012737 | 0.03 |
| RGAG1 | -0.189206364 | 2.88E-10 | -0.094131298 | 0.001837 | 0.1 |
| RGAG4 | -0.291536843 | 7.43E-23 | -0.194999315 | 7.93E-11 | 0.1 |
| RGL1 | -0.202505678 | 1.40E-11 | -0.231839739 | 8.36E-15 | 0.03 |
| HKE1.5 | -0.168925655 | 1.92E-08 | 0.042016873 | 0.1650991 | 0.22 |
| RGL3 | -0.22322019 | 8.28E-14 | 0.010696817 | 0.7239025 | 0.24 |
| RGL4 | -0.046113515 | 0.12760759 | -0.130110441 | 1.60E-05 | 0.09 |
| RGMA | 0.198926521 | 3.23E-11 | -0.010667854 | 0.7246196 | 0.21 |
| RGMB | -0.33583761 | 3.18E-30 | -0.127200065 | 2.47E-05 | 0.21 |
| ARHGEF28 | -0.224077506 | 6.62E-14 | -0.232363256 | 7.25E-15 | 0.01 |
| RGN | 0.021191675 | 0.48399889 | -0.064003165 | 0.0343679 | 0.09 |
| RGP1 | -0.382037336 | 2.64E-39 | -0.449114267 | 2.29E-55 | 0.07 |
| RGPD1 | -0.161502849 | 7.94E-08 | -0.226334037 | 3.66E-14 | 0.07 |
| LOC220706 | -0.385766235 | 4.19E-40 | -0.460799814 | 1.49E-58 | 0.08 |
| RGPD4 | -0.410018647 | 1.45E-45 | -0.463572662 | 2.49E-59 | 0.06 |
| RGPD5 | -0.311683452 | 4.73E-26 | -0.354323024 | 1.12E-33 | 0.05 |
| RGPD6 | -0.307786568 | 2.05E-25 | -0.369072636 | 1.33E-36 | 0.07 |
| LOC220692 | 0.024104659 | 0.42596398 | -0.079681622 | 0.0084015 | 0.11 |
| RGR | 0.3138399 | 2.08E-26 | 0.075283868 | 0.0127883 | 0.24 |
| RGS10 | 0.276435511 | 1.27E-20 | 0.276241878 | 1.35E-20 | 0.01 |
| RGS11 | -0.19309833 | 1.22E-10 | 0.00732962 | 0.8087436 | 0.21 |
| RGS12 | -0.209478268 | 2.65E-12 | -0.154705366 | 2.75E-07 | 0.06 |
| RGS13 | -0.239727584 | 9.44E-16 | -0.214077053 | 8.52E-13 | 0.03 |
| RGS14 | -0.05947591 | 0.04932209 | 0.060754336 | 0.0446296 | 0.13 |
| RGS16 | 0.005793961 | 0.84826394 | -0.023839992 | 0.4310634 | 0.03 |
| RGS17 | -0.146450085 | 1.16E-06 | -0.080475765 | 0.0077713 | 0.07 |
| RGS18 | -0.203787498 | 1.04E-11 | -0.252644267 | 2.23E-17 | 0.05 |
| RGS19 | 0.20806081 | 3.73E-12 | 0.103637615 | 0.0005999 | 0.11 |
| RGS1 | -0.041175095 | 0.17373799 | -0.08298999 | 0.0060457 | 0.05 |
| RGS20 | 0.300188524 | 3.38E-24 | 0.042507783 | 0.1602124 | 0.26 |
| RGS21 | 0.013358988 | 0.65909005 | -0.008149662 | 0.7878308 | 0.03 |
| RGS22 | -0.325100496 | 2.54E-28 | -0.154890092 | 2.66E-07 | 0.18 |
| RGS2 | 0.114107956 | 0.00015651 | 0.025944786 | 0.3914937 | 0.09 |
| FLJ20370 | -0.116184711 | 0.00011822 | -0.165781506 | 3.53E-08 | 0.05 |
| SCZD9 | -0.170273923 | 1.48E-08 | -0.131598243 | 1.27E-05 | 0.04 |
| LOC100505773 | -0.418958172 | 1.08E-47 | -0.247106724 | 1.14E-16 | 0.18 |
| RGS6 | 0.052814722 | 0.08093196 | -0.017375016 | 0.5660939 | 0.08 |
| RGS7BP | -0.246816186 | 1.24E-16 | -0.224924035 | 5.30E-14 | 0.03 |
| RGS7 | -0.175538365 | 5.15E-09 | -0.099995329 | 0.0009316 | 0.08 |
| RGS8 | -0.025465474 | 0.40030557 | -0.076144427 | 0.0117972 | 0.06 |
| RGS9BP | 0.266679126 | 2.98E-19 | 0.110201982 | 0.0002619 | 0.16 |
| RGS9 | -0.019082124 | 0.52856305 | -0.18481856 | 7.45E-10 | 0.17 |
| RGSL2 | 0.106238787 | 0.0004344 | 0.065097825 | 0.0313981 | 0.05 |
| RHAG | -0.022893099 | 0.44959432 | 0.032834525 | 0.2781087 | 0.06 |
| RHBDD1 | -0.434701189 | 1.33E-51 | -0.297349288 | 9.43E-24 | 0.14 |
| RHBDD2 | 0.003065339 | 0.91937099 | 0.028591739 | 0.3449806 | 0.03 |
| RHBDD3 | 0.187447731 | 4.23E-10 | 0.149547028 | 6.82E-07 | 0.04 |
| RHBDF1 | -0.153221881 | 3.58E-07 | 0.024395713 | 0.420397 | 0.18 |
| TOC | 0.312652567 | 3.27E-26 | 0.028997329 | 0.3381777 | 0.29 |
| RHBDL1 | -0.186555294 | 5.13E-10 | 0.014293019 | 0.6369136 | 0.21 |
| RHBDL2 | 0.237013568 | 2.02E-15 | 0.089407273 | 0.0030924 | 0.15 |
| RHBDL3 | -0.215250497 | 6.36E-13 | -0.05916242 | 0.0505339 | 0.16 |
| RHBG | 0.087961978 | 0.00360982 | 0.143860169 | 1.79E-06 | 0.06 |
| LOC51688 | 0.048336949 | 0.110232 | 0.049786646 | 0.099947 | 0.01 |
| C15orf6 | 0.325333575 | 2.31E-28 | 0.09505283 | 0.001655 | 0.24 |
| RhDVa.TT. | -0.051695629 | 0.08758577 | -0.050059867 | 0.098097 | 0.01 |
| RHEBL1 | 0.357082892 | 3.26E-34 | 0.144035685 | 1.74E-06 | 0.22 |
| RHEB | 0.521200304 | 3.73E-77 | 0.319111818 | 2.70E-27 | 0.21 |
| RHOA | -0.140706758 | 3.02E-06 | -0.022365656 | 0.4601091 | 0.12 |
| RHOBTB1 | -0.158201302 | 1.46E-07 | -0.10833363 | 0.0003332 | 0.05 |
| RHOBTB2 | -0.426660604 | 1.40E-49 | -0.278924489 | 5.56E-21 | 0.15 |
| RHOBTB3 | -0.168796446 | 1.97E-08 | -0.09990433 | 0.0009417 | 0.07 |
| RHOB | -0.359376207 | 1.16E-34 | -0.059421558 | 0.0495304 | 0.3 |
| RHOC | -0.049222951 | 0.10385146 | 0.119700727 | 7.27E-05 | 0.17 |
| RHOD | 0.09661858 | 0.0013834 | 0.101182286 | 0.0008084 | 0.01 |
| RHOF | 0.091297645 | 0.00251768 | -0.08433462 | 0.0052719 | 0.18 |
| RHOG | 0.232263496 | 7.45E-15 | 0.161325834 | 8.20E-08 | 0.08 |
| RHOH | -0.243966794 | 2.83E-16 | -0.110193258 | 0.0002622 | 0.14 |
| RASL7B | -0.384419213 | 8.17E-40 | -0.293915973 | 3.21E-23 | 0.1 |
| RASL7A | 0.024798267 | 0.41276805 | -0.08925798 | 0.0031426 | 0.12 |
| RHOT1 | -0.18126052 | 1.58E-09 | -0.14431893 | 1.66E-06 | 0.04 |
| RHOT2 | -0.00231814 | 0.93898043 | 0.072289209 | 0.016834 | 0.08 |
| RHOU | -0.12240529 | 4.96E-05 | -0.075323094 | 0.0127415 | 0.05 |
| LOC123758 | 0.107928848 | 0.00035083 | 0.171212659 | 1.23E-08 | 0.07 |
| RHOXF1 | -0.054116637 | 0.07371323 | -0.030708673 | 0.3104286 | 0.03 |
| LOC115216 | 0.021496212 | 0.47773797 | 0.034140057 | 0.2594355 | 0.02 |
| RHO | 0.007808149 | 0.79652246 | 0.022532998 | 0.4567583 | 0.02 |
| RHPN1 | 0.077744675 | 0.01013371 | 0.15747458 | 1.67E-07 | 0.08 |
| RHPN2 | 0.004514553 | 0.88148769 | -0.024303543 | 0.4221553 | 0.03 |
| RIBC1 | -0.156246356 | 2.08E-07 | 0.023587427 | 0.4359624 | 0.18 |
| RIBC2 | 0.24231727 | 4.54E-16 | 0.205066797 | 7.66E-12 | 0.04 |
| RIC3 | -0.095694139 | 0.00153834 | -0.20166917 | 1.71E-11 | 0.11 |
| RIC8A | -0.092586355 | 0.00218372 | -0.037380551 | 0.2168904 | 0.06 |
| RIC8B | -0.139557155 | 3.63E-06 | -0.174536095 | 6.31E-09 | 0.04 |
| FLJ13735 | -0.171110502 | 1.25E-08 | -0.130611922 | 1.48E-05 | 0.05 |
| RICTOR | -0.37531429 | 6.88E-38 | -0.343377278 | 1.32E-31 | 0.04 |
| DKFZP434D193 | -0.228015719 | 2.34E-14 | -0.350033648 | 7.43E-33 | 0.13 |
| RILPL1 | 0.088455628 | 0.00342486 | 0.031383188 | 0.299916 | 0.06 |
| RILPL2 | -0.152776741 | 3.88E-07 | -0.068589051 | 0.0233494 | 0.09 |
| RILP | 0.062865226 | 0.037706 | 0.023221158 | 0.4431236 | 0.04 |
| LOC102724399 | -0.14457056 | 1.59E-06 | -0.198909633 | 3.25E-11 | 0.06 |
| RIMBP3C | -0.150044037 | 6.26E-07 | -0.188284304 | 3.52E-10 | 0.04 |
| DKFZP434H0735 | -0.248626672 | 7.33E-17 | -0.285176753 | 6.73E-22 | 0.04 |
| RIMKLA | -0.164110348 | 4.86E-08 | -0.128808972 | 1.94E-05 | 0.04 |
| LOC100506112 | -0.113867464 | 0.00016163 | -0.13656596 | 5.87E-06 | 0.03 |
| CORD7 | -0.108659057 | 0.00031959 | 0.041027382 | 0.1752881 | 0.15 |
| LOC100506793 | 0.128881654 | 1.92E-05 | -0.144556917 | 1.60E-06 | 0.28 |
| RIMS3 | 0.004038019 | 0.89391903 | -0.127139288 | 2.49E-05 | 0.14 |
| LOC200225 | -0.139276965 | 3.80E-06 | -0.086636691 | 0.0041521 | 0.06 |
| RIN1 | 0.006303316 | 0.83510817 | -0.00972066 | 0.7482026 | 0.02 |
| RIN2 | -0.353539898 | 1.58E-33 | -0.136773197 | 5.68E-06 | 0.22 |
| FLJ11700 | 0.073975391 | 0.01443625 | -0.106292505 | 0.0004315 | 0.19 |
| RING1 | 0.109666771 | 0.00028074 | 0.147224691 | 1.02E-06 | 0.04 |
| RINL | 0.025254977 | 0.40421274 | -0.029387669 | 0.3317124 | 0.06 |
| RINT1 | 0.064241958 | 0.03370041 | 0.052073702 | 0.0852902 | 0.02 |
| RIOK1 | 0.462867602 | 3.93E-59 | 0.247564981 | 9.99E-17 | 0.22 |
| RIOK2 | -0.141138683 | 2.81E-06 | 0.096612392 | 0.0013844 | 0.24 |
| RIOK3 | 0.209396022 | 2.70E-12 | -0.120064807 | 6.91E-05 | 0.33 |
| RIPK1 | -0.279884409 | 4.04E-21 | -0.141586609 | 2.61E-06 | 0.14 |
| RIPK2 | 0.457468828 | 1.24E-57 | 0.242196484 | 4.69E-16 | 0.22 |
| RIPK3 | -0.30942827 | 1.11E-25 | -0.15357815 | 3.36E-07 | 0.16 |
| RIPK4 | 0.147886824 | 9.08E-07 | 0.025842227 | 0.3933692 | 0.13 |
| RIPPLY1 | 0.077794833 | 0.01008512 | 0.109107693 | 0.0003017 | 0.04 |
| RIPPLY2 | 0.19224007 | 1.47E-10 | 0.04246476 | 0.1606363 | 0.15 |
| RIT1 | -0.079996056 | 0.0081467 | 0.131766932 | 1.24E-05 | 0.22 |
| RIT2 | -0.007631852 | 0.80101928 | 0.053398236 | 0.0776286 | 0.07 |
| RLBP1 | 0.318908113 | 2.93E-27 | 0.104378129 | 0.0005477 | 0.22 |
| RLF | -0.079071449 | 0.00891622 | -0.231995567 | 8.02E-15 | 0.16 |
| LOC340532 | -0.132209856 | 1.16E-05 | -0.167979595 | 2.31E-08 | 0.04 |
| RLN1 | -0.058770682 | 0.05208301 | -0.006753289 | 0.8235242 | 0.06 |
| LOC101929382 | -0.084233407 | 0.00532691 | -0.042805868 | 0.1572989 | 0.05 |
| RLN3 | 0.068171209 | 0.02420742 | -0.02254043 | 0.4566098 | 0.1 |
| RLTPR | 0.174642346 | 6.18E-09 | -0.034652932 | 0.2523426 | 0.21 |
| RMI1 | 0.002549129 | 0.93291386 | 0.018517272 | 0.5408401 | 0.02 |
| RMND1 | 0.059374657 | 0.04971079 | 0.07851436 | 0.0094106 | 0.02 |
| RMND5A | 0.057342972 | 0.05806849 | -0.139821114 | 3.48E-06 | 0.2 |
| RMND5B | -0.171281789 | 1.21E-08 | 0.002835376 | 0.9254015 | 0.18 |
| CHH | 0.240535163 | 7.52E-16 | 0.163141922 | 5.84E-08 | 0.08 |
| RMST | -0.067536938 | 0.02556193 | -0.04610917 | 0.1276435 | 0.03 |
| RNASE10 | 0.137391411 | 5.15E-06 | 0.017473392 | 0.5638969 | 0.12 |
| RNASE11 | -0.017615846 | 0.56072275 | 0.000760944 | 0.9799525 | 0.02 |
| RNASE12 | -0.012957147 | 0.66872522 | -0.014638449 | 0.6287923 | 0.01 |
| RNASE13 | -0.004177502 | 0.8902775 | -0.041511351 | 0.1702475 | 0.04 |
| RNASE1 | 0.132513374 | 1.11E-05 | 0.036642682 | 0.2261072 | 0.1 |
| RNASE2 | 0.130754094 | 1.45E-05 | 0.042520506 | 0.1600873 | 0.09 |
| RNASE3 | 0.052770605 | 0.08118628 | -0.006591565 | 0.8276833 | 0.06 |
| LOC122654 | -0.415557767 | 7.10E-47 | -0.149154457 | 7.30E-07 | 0.27 |
| RNASE6 | -0.105930275 | 0.00045152 | -0.1500892 | 6.21E-07 | 0.05 |
| RNASE7 | -0.139278592 | 3.80E-06 | -0.151341066 | 4.99E-07 | 0.02 |
| RNASE8 | -0.075084477 | 0.01302813 | -0.039837033 | 0.1881574 | 0.04 |
| LOC122650 | -0.002774749 | 0.92699208 | -0.04331138 | 0.15245 | 0.05 |
| RNASEH1 | 0.346582722 | 3.34E-32 | 0.131939693 | 1.21E-05 | 0.22 |
| RNASEH2A | 0.593574157 | 5.04E-105 | 0.427168826 | 1.05E-49 | 0.17 |
| RNASEH2B | 0.310201714 | 8.29E-26 | 0.257879325 | 4.60E-18 | 0.06 |
| RNASEH2C | 0.250011254 | 4.88E-17 | 0.230595832 | 1.17E-14 | 0.02 |
| RNASEK | 0.220138685 | 1.84E-13 | 0.175918146 | 4.77E-09 | 0.05 |
| HPC1 | -0.470728919 | 2.31E-61 | -0.167401961 | 2.59E-08 | 0.31 |
| DROSHA | 0.063540568 | 0.03569322 | -0.019148487 | 0.52713 | 0.09 |
| LOC100131869 | 0.146937787 | 1.07E-06 | 0.217918942 | 3.24E-13 | 0.08 |
| RND1 | -0.251431536 | 3.20E-17 | 0.014202876 | 0.6390401 | 0.27 |
| RND2 | 0.063726463 | 0.03515551 | 0.066373567 | 0.028217 | 0.01 |
| RND3 | -0.06049315 | 0.04555647 | -0.169525357 | 1.71E-08 | 0.11 |
| RNF103 | -0.373328747 | 1.78E-37 | -0.176934324 | 3.88E-09 | 0.2 |
| RNF10 | -0.082594255 | 0.00629213 | 0.041037821 | 0.1751782 | 0.13 |
| RNF111 | -0.479118058 | 8.30E-64 | -0.447904117 | 4.82E-55 | 0.04 |
| RNF112 | 0.161870074 | 7.41E-08 | 0.030277542 | 0.3172737 | 0.14 |
| RNF113A | 0.316125745 | 8.63E-27 | 0.248478851 | 7.65E-17 | 0.07 |
| RNF113B | -0.066551002 | 0.02779734 | -0.018556329 | 0.5399866 | 0.05 |
| LOC116140 | 0.229926649 | 1.40E-14 | 0.12654329 | 2.72E-05 | 0.11 |
| RNF115 | -0.186329119 | 5.38E-10 | -0.000642606 | 0.9830697 | 0.19 |
| RNF11 | -0.312178923 | 3.92E-26 | -0.216156159 | 5.06E-13 | 0.1 |
| RNF121 | 0.016304722 | 0.59025808 | -0.017515204 | 0.5629643 | 0.04 |
| RNF122 | 0.189959952 | 2.44E-10 | 0.085048771 | 0.0048984 | 0.11 |
| RNF123 | -0.269878097 | 1.07E-19 | -0.177823746 | 3.23E-09 | 0.1 |
| RNF125 | -0.276801048 | 1.13E-20 | -0.218958192 | 2.49E-13 | 0.06 |
| RNF126P1 | 0.051677071 | 0.08769971 | 0.04798481 | 0.1128522 | 0.01 |
| RNF126 | 0.232206767 | 7.57E-15 | 0.1367891 | 5.67E-06 | 0.1 |
| RNF128 | -0.075678436 | 0.01232508 | -0.140590805 | 3.07E-06 | 0.07 |
| RNF130 | 0.133712134 | 9.19E-06 | 0.169025876 | 1.89E-08 | 0.04 |
| RNF133 | -0.206390708 | 5.58E-12 | -0.130746129 | 1.45E-05 | 0.08 |
| RNF135 | -0.087871935 | 0.00364452 | 0.063309127 | 0.0363724 | 0.16 |
| RNF138P1 | 0.066880125 | 0.02703309 | -0.14073193 | 3.00E-06 | 0.21 |
| RNF138 | 0.407175615 | 6.70E-45 | 0.109131887 | 0.0003008 | 0.3 |
| RNF139 | -0.005508952 | 0.85564395 | 0.067542479 | 0.0255498 | 0.08 |
| RNF13 | -0.120438888 | 6.56E-05 | -0.081571515 | 0.0069713 | 0.04 |
| RNF141 | -0.381646422 | 3.20E-39 | -0.210464656 | 2.08E-12 | 0.18 |
| RNF144A | -0.058064194 | 0.05497713 | -0.11011312 | 0.000265 | 0.06 |
| RNF144B | -0.224383435 | 6.11E-14 | -0.010870543 | 0.7196059 | 0.22 |
| RNF145 | 0.077976536 | 0.00991083 | -0.107123808 | 0.0003886 | 0.19 |
| RNF146 | -0.091792183 | 0.00238437 | -0.030386471 | 0.3155349 | 0.07 |
| RNF148 | -0.129263163 | 1.81E-05 | -0.002702094 | 0.9288986 | 0.13 |
| RNF149 | 0.177764972 | 3.27E-09 | 0.21141458 | 1.65E-12 | 0.04 |
| RNF14 | -0.265516427 | 4.31E-19 | -0.113953943 | 0.0001598 | 0.16 |
| RNF150 | -0.092000132 | 0.00233026 | -0.300117444 | 3.47E-24 | 0.21 |
| RNF151 | 0.147529968 | 9.65E-07 | 0.115693853 | 0.0001264 | 0.04 |
| RNF152 | -0.111073869 | 0.00023383 | -0.117076722 | 0.0001047 | 0.01 |
| RNF157 | -0.177473921 | 3.47E-09 | -0.213781034 | 9.17E-13 | 0.04 |
| LTN1 | -0.374538866 | 9.98E-38 | -0.302761605 | 1.32E-24 | 0.08 |
| RNF165 | 0.063504636 | 0.03579795 | -0.073040771 | 0.0157252 | 0.14 |
| RNF166 | 0.168061511 | 2.28E-08 | 0.011941347 | 0.6933228 | 0.16 |
| RNF167 | 0.145731941 | 1.31E-06 | 0.119308542 | 7.68E-05 | 0.03 |
| RNF168 | -0.21795365 | 3.21E-13 | -0.350574509 | 5.86E-33 | 0.14 |
| RNF169 | -0.282715825 | 1.56E-21 | -0.343461285 | 1.28E-31 | 0.07 |
| RNF170 | -0.179498204 | 2.29E-09 | -0.117637007 | 9.69E-05 | 0.07 |
| RNF175 | 0.091319219 | 0.00251173 | 0.001650029 | 0.956546 | 0.09 |
| RNF17 | 0.069346419 | 0.02186137 | 0.049825866 | 0.0996797 | 0.02 |
| RNF180 | -0.233986381 | 4.66E-15 | -0.154340477 | 2.94E-07 | 0.08 |
| RNF181 | 0.317587738 | 4.90E-27 | 0.393212086 | 9.87E-42 | 0.08 |
| RNF182 | 0.218077427 | 3.11E-13 | -0.041348082 | 0.1719357 | 0.26 |
| RNF183 | -0.069199697 | 0.02214304 | 0.132925273 | 1.04E-05 | 0.21 |
| RNF185 | -0.313318047 | 2.54E-26 | -0.203087925 | 1.22E-11 | 0.12 |
| RNF186 | -0.073517841 | 0.01505516 | -0.064162147 | 0.0339223 | 0.01 |
| RNF187 | 0.223668813 | 7.37E-14 | 0.431641788 | 7.95E-51 | 0.21 |
| DORFIN | -0.067964868 | 0.02464109 | -0.09095288 | 0.0026146 | 0.03 |
| RNF19B | 0.017311186 | 0.56752163 | -0.21329186 | 1.04E-12 | 0.24 |
| RNF207 | -0.01459769 | 0.62974832 | -0.043426796 | 0.151359 | 0.03 |
| RNF208 | 0.006537671 | 0.82907039 | 0.108645583 | 0.0003201 | 0.11 |
| RNF20 | -0.204233408 | 9.34E-12 | -0.132236095 | 1.15E-05 | 0.08 |
| LOC100132787 | -0.09377875 | 0.00191135 | -0.090227936 | 0.0028296 | 0.01 |
| RNF213 | -0.167947134 | 2.33E-08 | -0.241951651 | 5.03E-16 | 0.08 |
| RNF214 | -0.069714493 | 0.02116842 | -0.121759212 | 5.44E-05 | 0.06 |
| RNF215 | -0.096648224 | 0.00137867 | -0.058468858 | 0.0533035 | 0.04 |
| LOC641911 | 0.032438155 | 0.2839545 | 0.110430065 | 0.0002543 | 0.08 |
| RNF216 | -0.166527773 | 3.06E-08 | -0.274312238 | 2.55E-20 | 0.11 |
| RNF217 | -0.177428921 | 3.50E-09 | -0.175905614 | 4.78E-09 | 0.01 |
| LOC86572 | 0.035535436 | 0.2404561 | -0.013025528 | 0.6670817 | 0.05 |
| RNF220 | 0.291217243 | 8.31E-23 | 0.140045681 | 3.36E-06 | 0.16 |
| RNF222 | 0.238314711 | 1.40E-15 | 0.03712729 | 0.220023 | 0.21 |
| RNF24 | 0.035214547 | 0.24473175 | -0.042245418 | 0.1628104 | 0.08 |
| RNF25 | 0.06366019 | 0.03534641 | 0.160510778 | 9.55E-08 | 0.1 |
| RNF26 | 0.207462091 | 4.32E-12 | 0.088354265 | 0.0034621 | 0.12 |
| DING | -0.161345705 | 8.17E-08 | 0.044352937 | 0.1428184 | 0.21 |
| RNF31 | -0.082136579 | 0.00658835 | -0.057250286 | 0.058476 | 0.03 |
| RNF32 | 0.104179431 | 0.00056125 | 0.113163972 | 0.0001775 | 0.01 |
| RNF34 | 0.086795954 | 0.00408323 | 0.043065223 | 0.1547968 | 0.05 |
| RNF38 | -0.231094101 | 1.02E-14 | -0.23540751 | 3.15E-15 | 0.01 |
| RNF39 | -0.161766887 | 7.56E-08 | -0.151548248 | 4.81E-07 | 0.02 |
| RNF40 | -0.023166351 | 0.4442009 | 0.008027865 | 0.7909276 | 0.04 |
| RNF41 | -0.212179731 | 1.36E-12 | -0.239621833 | 9.73E-16 | 0.03 |
| RNF43 | -0.194014399 | 9.90E-11 | -0.128586722 | 2.01E-05 | 0.07 |
| RNF44 | -0.101247268 | 0.00080208 | -0.114584521 | 0.0001468 | 0.02 |
| RNF4 | -0.205505671 | 6.90E-12 | -0.230603058 | 1.17E-14 | 0.03 |
| LOC157864 | 0.074111867 | 0.01425604 | 0.123914816 | 3.99E-05 | 0.05 |
| RNF5 | 0.273937593 | 2.88E-20 | 0.321324607 | 1.13E-27 | 0.05 |
| LOC102724232 | -0.169881101 | 1.59E-08 | -0.19387212 | 1.02E-10 | 0.03 |
| RNF7 | 0.325412302 | 2.24E-28 | 0.289536406 | 1.49E-22 | 0.04 |
| RNF8 | 0.458407968 | 6.83E-58 | 0.210939614 | 1.85E-12 | 0.25 |
| RNFT1 | -0.14939695 | 7.00E-07 | -0.135575556 | 6.86E-06 | 0.02 |
| RNFT2 | 0.484646955 | 1.86E-65 | 0.309062316 | 1.27E-25 | 0.18 |
| RNGTT | 0.099788818 | 0.0009547 | -0.010345577 | 0.7326156 | 0.12 |
| RNH1 | -0.072147012 | 0.01705129 | -0.024848125 | 0.4118289 | 0.05 |
| RNLS | -0.155630491 | 2.33E-07 | -0.088169031 | 0.0035312 | 0.07 |
| RNMTL1 | 0.281565344 | 2.29E-21 | 0.265012804 | 5.05E-19 | 0.02 |
| RNMT | 0.03182923 | 0.29309596 | -0.17662082 | 4.13E-09 | 0.21 |
| RNPC3 | -0.181787598 | 1.42E-09 | -0.216961899 | 4.13E-13 | 0.04 |
| RNPEPL1 | -0.020345947 | 0.50161674 | -0.041057704 | 0.174969 | 0.03 |
| RNPEP | -0.002028462 | 0.94659333 | 0.384183192 | 9.18E-40 | 0.39 |
| RNPS1 | 0.489227373 | 7.60E-67 | 0.337767541 | 1.42E-30 | 0.16 |
| RNU11 | 0.069940699 | 0.0207521 | 0.016093723 | 0.5950772 | 0.06 |
| MOPD1 | 0.060168024 | 0.0467328 | 0.038587064 | 0.2024067 | 0.03 |
| RNU5E.1 | NA | NA | NA | NA | #VALUE! |
| RNU6ATAC | 0.053012429 | 0.07980019 | -0.032642175 | 0.2809352 | 0.09 |
| RNY4 | NA | NA | NA | NA | #VALUE! |
| RNY5 | NA | NA | NA | NA | #VALUE! |
| LAMTOR2 | 0.243075677 | 3.65E-16 | 0.486206866 | 6.30E-66 | 0.25 |
| ROBO1 | -0.102889095 | 0.00065748 | -0.218595316 | 2.73E-13 | 0.12 |
| ROBO2 | -0.189966075 | 2.44E-10 | -0.128041603 | 2.18E-05 | 0.07 |
| HGPPS | -0.054229551 | 0.07311276 | -0.083551097 | 0.0057112 | 0.03 |
| ROBO4 | -0.278186687 | 7.11E-21 | -0.218068866 | 3.12E-13 | 0.07 |
| ROCK1 | -0.192553134 | 1.37E-10 | -0.408845642 | 2.74E-45 | 0.22 |
| ROCK2 | -0.245220643 | 1.97E-16 | -0.40227553 | 9.02E-44 | 0.16 |
| PTBP3 | -0.060443442 | 0.04573469 | -0.166965118 | 2.81E-08 | 0.11 |
| ROGDI | -0.100724262 | 0.000854 | 0.052704518 | 0.0815685 | 0.16 |
| ROM1 | 0.098718543 | 0.00108333 | -0.047164209 | 0.1191485 | 0.15 |
| ROMO1 | 0.366876231 | 3.70E-36 | 0.370902191 | 5.61E-37 | 0.01 |
| ROPN1B | 0.293566248 | 3.63E-23 | 0.095926323 | 0.001498 | 0.2 |
| LOC101929379 | -0.210219589 | 2.21E-12 | 0.002812663 | 0.9259973 | 0.22 |
| ROPN1 | 0.342172896 | 2.21E-31 | 0.104053636 | 0.00057 | 0.24 |
| LOC148582 | -0.252278625 | 2.49E-17 | -0.351805264 | 3.41E-33 | 0.1 |
| ROR2 | -0.245735112 | 1.70E-16 | -0.109166376 | 0.0002994 | 0.14 |
| RORA | -0.444840201 | 3.13E-54 | -0.458601788 | 6.04E-58 | 0.02 |
| RORB | -0.268252499 | 1.81E-19 | -0.163794776 | 5.16E-08 | 0.11 |
| RORC | -0.384121159 | 9.47E-40 | -0.034823572 | 0.2500129 | 0.35 |
| ROS1 | 0.134675175 | 7.91E-06 | -0.032699888 | 0.2800851 | 0.17 |
| LINC00632 | -0.006333193 | 0.83433787 | -0.00096266 | 0.9746398 | 0.01 |
| RP1L1 | -0.010639097 | 0.72533187 | -0.189044794 | 2.98E-10 | 0.18 |
| RP1 | -0.182779264 | 1.15E-09 | -0.130227143 | 1.57E-05 | 0.06 |
| RP2 | -0.291685493 | 7.05E-23 | -0.295985559 | 1.54E-23 | 0.01 |
| RP9P | 0.420746277 | 3.98E-48 | 0.204338707 | 9.11E-12 | 0.22 |
| RP9 | 0.47178476 | 1.15E-61 | 0.282973127 | 1.43E-21 | 0.19 |
| MST075 | 0.135399729 | 7.06E-06 | -0.005396488 | 0.8585596 | 0.15 |
| RPA2 | 0.19893437 | 3.23E-11 | 0.082523495 | 0.0063371 | 0.12 |
| RPA3 | 0.270554692 | 8.64E-20 | 0.391985831 | 1.84E-41 | 0.13 |
| RPA4 | 0.204835279 | 8.10E-12 | 0.087760957 | 0.0036877 | 0.12 |
| RPAIN | 0.259901142 | 2.48E-18 | 0.201417898 | 1.81E-11 | 0.06 |
| RPAP1 | -0.089393471 | 0.00309703 | -0.086847577 | 0.0040611 | 0.01 |
| RPAP2 | 0.041649426 | 0.16882955 | -0.049311364 | 0.1032312 | 0.1 |
| RPAP3 | -0.007580394 | 0.80233307 | 0.06553989 | 0.0302627 | 0.08 |
| RPE65 | 0.076316113 | 0.01160784 | -0.025077837 | 0.4075184 | 0.11 |
| RPE | 0.115041605 | 0.00013804 | 0.047675477 | 0.1151942 | 0.07 |
| RPF1 | 0.138848986 | 4.08E-06 | 0.126180135 | 2.87E-05 | 0.02 |
| RPF2 | 0.503006662 | 3.73E-71 | 0.463005563 | 3.60E-59 | 0.05 |
| RPGRIP1L | -0.111965649 | 0.00020801 | -0.148616567 | 8.01E-07 | 0.04 |
| RPGRIP1 | -0.176355182 | 4.36E-09 | -0.063444494 | 0.0359738 | 0.12 |
| RP15 | -0.246587938 | 1.33E-16 | -0.214779809 | 7.15E-13 | 0.04 |
| RPH3AL | -0.326193875 | 1.64E-28 | -0.135370262 | 7.09E-06 | 0.2 |
| RPH3A | -0.058014179 | 0.055187 | -0.092851156 | 0.0021203 | 0.04 |
| RPIA | 0.59238218 | 1.66E-104 | 0.343764599 | 1.12E-31 | 0.25 |
| RPL10A | 0.193339896 | 1.15E-10 | 0.297213371 | 9.90E-24 | 0.11 |
| RPL10L | 0.097893238 | 0.00119326 | 0.05919042 | 0.0504246 | 0.04 |
| RPL10 | 0.204029992 | 9.80E-12 | 0.241728751 | 5.36E-16 | 0.04 |
| RPL11 | 0.263370872 | 8.44E-19 | 0.23916366 | 1.11E-15 | 0.03 |
| RPL12 | 0.277985251 | 7.60E-21 | 0.308239231 | 1.73E-25 | 0.04 |
| RPL13AP17 | -0.073236836 | 0.01544673 | -0.053352444 | 0.0778838 | 0.02 |
| RPL13AP20 | 0.209194746 | 2.84E-12 | 0.268372723 | 1.74E-19 | 0.06 |
| LOC647572 | 0.224944256 | 5.28E-14 | 0.258014889 | 4.42E-18 | 0.04 |
| LOC649250 | 0.106139167 | 0.00043986 | 0.140230007 | 3.26E-06 | 0.04 |
| RPL13A | 0.169077489 | 1.87E-08 | 0.201131222 | 1.94E-11 | 0.04 |
| RPL13L | -0.049477444 | 0.10207396 | -0.199555156 | 2.80E-11 | 0.16 |
| RPL13 | 0.345536359 | 5.24E-32 | 0.280674203 | 3.10E-21 | 0.07 |
| RPL14 | 0.305784227 | 4.33E-25 | 0.359498339 | 1.10E-34 | 0.06 |
| RPL15 | 0.159305066 | 1.19E-07 | 0.230353255 | 1.25E-14 | 0.08 |
| RPL17 | 0.354899457 | 8.65E-34 | 0.254490649 | 1.28E-17 | 0.11 |
| RPL18A | 0.362547433 | 2.73E-35 | 0.337702788 | 1.46E-30 | 0.03 |
| RPL18 | 0.286038247 | 5.01E-22 | 0.292509771 | 5.28E-23 | 0.01 |
| RPL19P12 | 0.15244795 | 4.11E-07 | 0.177692964 | 3.32E-09 | 0.03 |
| RPL19 | 0.244885205 | 2.17E-16 | 0.280752005 | 3.02E-21 | 0.04 |
| RPL21P44 | -0.118823106 | 8.22E-05 | -0.176099357 | 4.60E-09 | 0.06 |
| RPL21 | 0.195907449 | 6.46E-11 | 0.161549263 | 7.87E-08 | 0.04 |
| RPL22L1 | 0.444826638 | 3.15E-54 | 0.437524613 | 2.52E-52 | 0.01 |
| RPL22 | 0.234536164 | 4.00E-15 | 0.202257874 | 1.49E-11 | 0.04 |
| RPL23AP32 | 0.167522038 | 2.53E-08 | 0.147819421 | 9.18E-07 | 0.02 |
| LOC642096 | -0.232558889 | 6.88E-15 | -0.206575957 | 5.34E-12 | 0.03 |
| RPL23AP64 | -0.379259595 | 1.03E-38 | -0.324304291 | 3.49E-28 | 0.06 |
| RPL23AP7 | 0.144056388 | 1.73E-06 | 0.082730193 | 0.0062065 | 0.07 |
| RPL23AP82 | 0.19739771 | 4.60E-11 | 0.153572447 | 3.37E-07 | 0.05 |
| RPL23A | 0.307978869 | 1.91E-25 | 0.370453514 | 6.93E-37 | 0.07 |
| RPL23P8 | 0.129391356 | 1.78E-05 | 0.139078891 | 3.93E-06 | 0.01 |
| RPL23 | 0.211594905 | 1.58E-12 | 0.283082282 | 1.37E-21 | 0.08 |
| RPL24 | 0.400843065 | 1.91E-43 | 0.46552662 | 7.03E-60 | 0.07 |
| RPL26L1 | 0.421393949 | 2.77E-48 | 0.461859223 | 7.53E-59 | 0.05 |
| RPL26 | 0.243277346 | 3.45E-16 | 0.251794331 | 2.88E-17 | 0.01 |
| RPL27A | 0.419196419 | 9.46E-48 | 0.373318837 | 1.79E-37 | 0.05 |
| RPL27 | 0.340687192 | 4.16E-31 | 0.345317248 | 5.76E-32 | 0.01 |
| RPL28 | 0.286424342 | 4.39E-22 | 0.290741871 | 9.81E-23 | 0.01 |
| RPL29P2 | 0.137617857 | 4.97E-06 | 0.120323194 | 6.67E-05 | 0.02 |
| RPL29 | 0.231860742 | 8.31E-15 | 0.292081989 | 6.13E-23 | 0.07 |
| RPL30 | 0.556478603 | 6.96E-90 | 0.470450895 | 2.78E-61 | 0.09 |
| RPL31P11 | 0.145823819 | 1.29E-06 | 0.153467249 | 3.43E-07 | 0.01 |
| RPL31 | 0.293111874 | 4.27E-23 | 0.355430153 | 6.83E-34 | 0.07 |
| RPL32P3 | -0.170050089 | 1.54E-08 | -0.142811784 | 2.13E-06 | 0.03 |
| RPL32 | 0.363964006 | 1.42E-35 | 0.388052967 | 1.34E-40 | 0.03 |
| RPL34 | 0.225329264 | 4.77E-14 | 0.34005462 | 5.44E-31 | 0.12 |
| RPL35A | 0.452200839 | 3.39E-56 | 0.442140301 | 1.60E-53 | 0.02 |
| RPL35 | 0.380446435 | 5.75E-39 | 0.35457984 | 9.98E-34 | 0.03 |
| RPL36AL | 0.160376124 | 9.79E-08 | 0.259484968 | 2.82E-18 | 0.1 |
| RPL36A | 0.337184319 | 1.82E-30 | 0.231378512 | 9.48E-15 | 0.11 |
| RPL36 | 0.341419119 | 3.05E-31 | 0.330321 | 3.09E-29 | 0.02 |
| RPL37A | 0.36070312 | 6.34E-35 | 0.38875947 | 9.39E-41 | 0.03 |
| RPL37 | 0.37488602 | 8.45E-38 | 0.415745163 | 6.40E-47 | 0.05 |
| RPL38 | 0.520920919 | 4.64E-77 | 0.437332055 | 2.82E-52 | 0.09 |
| RPL39L | 0.365275266 | 7.78E-36 | 0.315752801 | 9.96E-27 | 0.05 |
| RPL39 | 0.432981987 | 3.64E-51 | 0.453542772 | 1.47E-56 | 0.03 |
| RPL3L | 0.051137844 | 0.09106308 | 0.008183645 | 0.7869674 | 0.05 |
| RPL3 | 0.05238181 | 0.08345563 | 0.099026115 | 0.0010448 | 0.05 |
| RPL41 | 0.114208533 | 0.00015441 | 0.294558216 | 2.56E-23 | 0.19 |
| RPL4 | 0.299148656 | 4.93E-24 | 0.244639453 | 2.33E-16 | 0.06 |
| LOC115268 | 0.315507097 | 1.10E-26 | 0.240717298 | 7.14E-16 | 0.08 |
| RPL6 | 0.299487428 | 4.36E-24 | 0.350640019 | 5.69E-33 | 0.06 |
| RPL7A | 0.213972231 | 8.75E-13 | 0.274736106 | 2.22E-20 | 0.07 |
| RPL7L1 | 0.074711046 | 0.01348808 | -0.043669438 | 0.1490847 | 0.12 |
| RPL7 | 0.321701363 | 9.78E-28 | 0.278800563 | 5.80E-21 | 0.05 |
| LOC115159 | 0.437512459 | 2.54E-52 | 0.329443252 | 4.41E-29 | 0.11 |
| RPL9 | 0.09687283 | 0.00134338 | 0.112204505 | 0.0002016 | 0.02 |
| RPLP0_3_1146 | -0.027137263 | 0.3700865 | 0.045963187 | 0.1288551 | 0.08 |
| RPLP0 | 0.348432595 | 1.50E-32 | 0.341687353 | 2.72E-31 | 0.01 |
| RPLP1 | 0.260383967 | 2.13E-18 | 0.269130915 | 1.37E-19 | 0.01 |
| RPLP2 | 0.39495797 | 4.04E-42 | 0.365090548 | 8.47E-36 | 0.03 |
| RPN1 | 0.418343017 | 1.52E-47 | 0.25069657 | 3.98E-17 | 0.17 |
| RPN2 | 0.15206778 | 4.39E-07 | 0.054354299 | 0.072454 | 0.1 |
| LOC115127 | -0.083465709 | 0.00576099 | -0.1010813 | 0.0008182 | 0.02 |
| RPP21 | 0.533857255 | 1.50E-81 | 0.48597205 | 7.41E-66 | 0.05 |
| RPP25 | 0.394183737 | 6.01E-42 | 0.189624465 | 2.63E-10 | 0.21 |
| RPP30 | 0.366754635 | 3.92E-36 | 0.390119795 | 4.74E-41 | 0.03 |
| RPP38 | 0.552863672 | 1.65E-88 | 0.420082374 | 5.77E-48 | 0.14 |
| RPP40 | 0.507128059 | 1.75E-72 | 0.425092426 | 3.43E-49 | 0.09 |
| RPPH1 | 0.24021616 | 8.23E-16 | 0.186896498 | 4.76E-10 | 0.06 |
| LOC105372067 | 0.175627919 | 5.06E-09 | 0.013012065 | 0.6674052 | 0.17 |
| DJ1057B20.2 | -0.163537282 | 5.42E-08 | -0.160322154 | 9.89E-08 | 0.01 |
| RPRD2 | -0.178805687 | 2.64E-09 | 0.001757608 | 0.9537159 | 0.19 |
| RPRML | 0.249007123 | 6.55E-17 | 0.139262716 | 3.81E-06 | 0.11 |
| LOC116440 | 0.200427725 | 2.28E-11 | 0.050806674 | 0.0931795 | 0.15 |
| RPS10P7 | -0.107658062 | 0.00036312 | 0.038326865 | 0.2054692 | 0.15 |
| RPS10 | 0.429604232 | 2.59E-50 | 0.410460205 | 1.15E-45 | 0.02 |
| RPS11 | 0.326567692 | 1.41E-28 | 0.342350347 | 2.05E-31 | 0.02 |
| RPS12 | 0.325419918 | 2.23E-28 | 0.350460598 | 6.16E-33 | 0.03 |
| RPS13 | 0.314444935 | 1.65E-26 | 0.362956874 | 2.26E-35 | 0.05 |
| RPS14 | 0.201168039 | 1.92E-11 | 0.337746086 | 1.44E-30 | 0.14 |
| RPS15AP10 | -0.33620087 | 2.74E-30 | -0.223924343 | 6.89E-14 | 0.12 |
| RPS15A | 0.365350252 | 7.51E-36 | 0.392512886 | 1.41E-41 | 0.03 |
| RPS15 | 0.187730717 | 3.97E-10 | 0.226775824 | 3.25E-14 | 0.04 |
| RPS16 | 0.416615279 | 3.96E-47 | 0.369993956 | 8.61E-37 | 0.05 |
| RPS17 | 0.375210745 | 7.23E-38 | 0.348014975 | 1.79E-32 | 0.03 |
| RPS18 | 0.373039623 | 2.04E-37 | 0.379231469 | 1.04E-38 | 0.01 |
| RPS19BP1 | 0.353037653 | 1.98E-33 | 0.224828489 | 5.44E-14 | 0.13 |
| DBA | 0.442166442 | 1.57E-53 | 0.371465916 | 4.30E-37 | 0.08 |
| RPS20 | 0.493949074 | 2.67E-68 | 0.443271822 | 8.08E-54 | 0.06 |
| RPS21 | 0.472643034 | 6.49E-62 | 0.446690567 | 1.01E-54 | 0.03 |
| RPS23 | 0.207507065 | 4.27E-12 | 0.31459553 | 1.56E-26 | 0.11 |
| RPS24 | 0.415690166 | 6.60E-47 | 0.400686223 | 2.08E-43 | 0.02 |
| RPS25 | 0.296287844 | 1.38E-23 | 0.27734905 | 9.39E-21 | 0.02 |
| RPS26P11 | 0.205793949 | 6.44E-12 | 0.231755926 | 8.55E-15 | 0.03 |
| RPS26 | 0.173503834 | 7.77E-09 | 0.209958884 | 2.35E-12 | 0.04 |
| RPS27A | 0.453245593 | 1.76E-56 | 0.393815493 | 7.25E-42 | 0.06 |
| RPS27L | -0.068849316 | 0.02282846 | 0.091580788 | 0.0024405 | 0.17 |
| RPS27 | 0.120465703 | 6.53E-05 | 0.290611838 | 1.03E-22 | 0.18 |
| RPS28 | 0.060550673 | 0.04535096 | 0.079746274 | 0.0083485 | 0.02 |
| RPS29 | 0.359718029 | 9.91E-35 | 0.378772922 | 1.30E-38 | 0.02 |
| RPS2P32 | 0.308299314 | 1.70E-25 | 0.136251938 | 6.17E-06 | 0.18 |
| LOC124057 | 0.416100487 | 5.26E-47 | 0.394381717 | 5.43E-42 | 0.03 |
| RPS3A | 0.184865159 | 7.37E-10 | 0.217791829 | 3.35E-13 | 0.04 |
| RPS3 | 0.285717056 | 5.59E-22 | 0.244508326 | 2.42E-16 | 0.05 |
| RPS4X | 0.17499665 | 5.75E-09 | 0.249219564 | 6.16E-17 | 0.08 |
| RPS4Y1 | 0.074688091 | 0.01351681 | 0.023611444 | 0.4354951 | 0.06 |
| RPS4Y2 | 0.004306781 | 0.88690442 | 0.011252776 | 0.710184 | 0.01 |
| RPS5 | 0.237460008 | 1.78E-15 | 0.265803036 | 3.93E-19 | 0.03 |
| RPS6KA1 | 0.055116458 | 0.06853391 | -0.104358705 | 0.000549 | 0.16 |
| RPS6KA2 | -0.41810226 | 1.74E-47 | -0.355260007 | 7.37E-34 | 0.07 |
| CLS | -0.140668326 | 3.03E-06 | -0.31193737 | 4.29E-26 | 0.18 |
| RPS6KA4 | 0.133572318 | 9.39E-06 | -0.006315457 | 0.8347951 | 0.14 |
| RPS6KA5 | -0.439080696 | 9.98E-53 | -0.324560211 | 3.15E-28 | 0.12 |
| RPS6KA6 | -0.24674031 | 1.27E-16 | -0.230621612 | 1.16E-14 | 0.02 |
| STK14A | -0.062919571 | 0.03754054 | -0.021054679 | 0.4868298 | 0.05 |
| RPS6KB2 | 0.308794459 | 1.41E-25 | 0.193698262 | 1.06E-10 | 0.12 |
| RPS6KC1 | -0.153592835 | 3.35E-07 | 0.15387319 | 3.19E-07 | 0.31 |
| RPS6KL1 | -0.167148652 | 2.72E-08 | -0.019177881 | 0.5264959 | 0.15 |
| LOC92956 | 0.300887451 | 2.62E-24 | 0.332383268 | 1.33E-29 | 0.04 |
| RPS7 | 0.528610161 | 1.05E-79 | 0.417836662 | 2.01E-47 | 0.12 |
| RPS8 | 0.345452402 | 5.44E-32 | 0.29227172 | 5.74E-23 | 0.06 |
| RPS9 | 0.223344519 | 8.02E-14 | 0.228332359 | 2.15E-14 | 0.01 |
| RPSAP52 | -0.091127605 | 0.00256507 | 0.040516909 | 0.1807243 | 0.14 |
| RPSAP58 | 0.414315866 | 1.40E-46 | 0.345176507 | 6.12E-32 | 0.07 |
| RPSAP9 | 0.278628178 | 6.14E-21 | 0.234680454 | 3.85E-15 | 0.05 |
| RPSA | 0.410877653 | 9.14E-46 | 0.355869116 | 5.61E-34 | 0.06 |
| RPTN | 0.058320897 | 0.05391043 | -0.023525588 | 0.4371668 | 0.09 |
| LOC654218 | -0.017434638 | 0.56476189 | -0.211115255 | 1.77E-12 | 0.2 |
| RPUSD1 | 0.357876994 | 2.28E-34 | 0.271419033 | 6.54E-20 | 0.09 |
| FLJ31409 | 0.234862935 | 3.66E-15 | 0.220194558 | 1.81E-13 | 0.02 |
| LOC132086 | 0.539472521 | 1.47E-83 | 0.372841948 | 2.24E-37 | 0.17 |
| RPUSD4 | 0.103544792 | 0.00060681 | 0.112603168 | 0.0001912 | 0.01 |
| RQCD1 | -0.018540235 | 0.5403382 | -0.215550627 | 5.89E-13 | 0.2 |
| RRAD | -0.081251136 | 0.00719723 | -0.063753949 | 0.0350766 | 0.02 |
| LOC115960 | 0.055020225 | 0.06901906 | 0.083609626 | 0.0056773 | 0.03 |
| RRAGB | -0.140538686 | 3.10E-06 | -0.065242535 | 0.0310225 | 0.08 |
| RRAGC | -0.235317239 | 3.23E-15 | -0.347380694 | 2.36E-32 | 0.12 |
| RRAGD | 0.155453073 | 2.41E-07 | 0.008233572 | 0.7856993 | 0.15 |
| RRAS2 | 0.216381696 | 4.78E-13 | 0.006236985 | 0.8368189 | 0.22 |
| RRAS | 0.011317103 | 0.70860258 | 0.101763992 | 0.0007537 | 0.1 |
| RRBP1 | 0.067662721 | 0.02528823 | -0.00953336 | 0.7528948 | 0.08 |
| RREB1 | -0.513649053 | 1.28E-74 | -0.401751722 | 1.19E-43 | 0.12 |
| RRH | -0.116165039 | 0.00011854 | 0.02402363 | 0.4275214 | 0.15 |
| LOC143509 | 0.183443137 | 9.98E-10 | 0.127609723 | 2.32E-05 | 0.06 |
| RRM2B | -0.195382801 | 7.27E-11 | -0.234564199 | 3.97E-15 | 0.04 |
| RRM2 | 0.384252979 | 8.87E-40 | 0.205940939 | 6.22E-12 | 0.18 |
| LOC94431 | 0.036485899 | 0.22810102 | -0.058102754 | 0.0548158 | 0.1 |
| RRN3P2 | 0.102404455 | 0.00069742 | -0.027402021 | 0.365434 | 0.13 |
| RRN3P3 | -0.068401114 | 0.02373203 | -0.061103502 | 0.0434154 | 0.01 |
| DKFZP566E104 | -0.038580894 | 0.20247896 | -0.021783431 | 0.4718738 | 0.02 |
| RRP12 | 0.314425052 | 1.66E-26 | 0.229773485 | 1.46E-14 | 0.09 |
| RRP15 | 0.18968033 | 2.59E-10 | 0.419567342 | 7.69E-48 | 0.23 |
| RRP1B | 0.304603507 | 6.71E-25 | 0.099938834 | 0.0009378 | 0.21 |
| RRP1 | 0.491340686 | 1.71E-67 | 0.321612195 | 1.01E-27 | 0.17 |
| RRP7A | 0.303614865 | 9.66E-25 | 0.193220225 | 1.18E-10 | 0.12 |
| RRP7BP | 0.060304363 | 0.04623644 | -0.015728769 | 0.6034547 | 0.08 |
| RRP8 | 0.071697399 | 0.01775469 | 0.196664895 | 5.43E-11 | 0.13 |
| RRP9 | 0.42803806 | 6.37E-50 | 0.312919659 | 2.95E-26 | 0.12 |
| LOC90810 | 0.496171724 | 5.41E-69 | 0.322700868 | 6.59E-28 | 0.18 |
| RS1 | 0.091539927 | 0.00245154 | 0.091950572 | 0.0023431 | 0.01 |
| RSAD1 | -0.00230982 | 0.939199 | 0.118963143 | 8.06E-05 | 0.13 |
| RSAD2 | 0.038089094 | 0.20829698 | -0.04128492 | 0.1725921 | 0.08 |
| RSBN1L | -0.3263392 | 1.54E-28 | -0.251829648 | 2.85E-17 | 0.08 |
| RSBN1 | -0.291239151 | 8.25E-23 | -0.332768735 | 1.13E-29 | 0.05 |
| RSC1A1 | -0.377358941 | 2.57E-38 | -0.407363264 | 6.06E-45 | 0.04 |
| RSF1 | -0.237192917 | 1.92E-15 | -0.28544311 | 6.14E-22 | 0.05 |
| RSL1D1 | 0.272895395 | 4.05E-20 | 0.278508724 | 6.39E-21 | 0.01 |
| RSL24D1 | 0.147532172 | 9.65E-07 | 0.256795935 | 6.40E-18 | 0.11 |
| RSPH10B2 | 0.084520675 | 0.00517222 | 0.012022956 | 0.6913342 | 0.08 |
| RSPH1 | -0.275863828 | 1.53E-20 | 0.016726222 | 0.5806853 | 0.3 |
| RSPH3 | -0.377879834 | 2.00E-38 | -0.24836743 | 7.90E-17 | 0.13 |
| RSPH4A | -0.145417315 | 1.38E-06 | -0.098833134 | 0.0010688 | 0.05 |
| RSPH6A | 0.142044524 | 2.42E-06 | 0.085085842 | 0.0048797 | 0.06 |
| RSPH9 | 0.006885629 | 0.82012446 | 0.013544175 | 0.6546686 | 0.01 |
| RSPO1 | -0.214859348 | 7.01E-13 | -0.154357471 | 2.93E-07 | 0.07 |
| RSPO2 | 0.16893425 | 1.92E-08 | 0.062489449 | 0.0388672 | 0.11 |
| RSPO3 | -0.090930018 | 0.00262116 | -0.126862349 | 2.60E-05 | 0.04 |
| RSPO4 | 0.24821478 | 8.26E-17 | 0.007304194 | 0.8093942 | 0.25 |
| RSPRY1 | -0.340691017 | 4.15E-31 | -0.381030358 | 4.33E-39 | 0.05 |
| RSRC1 | -0.033388257 | 0.27007989 | -0.135764032 | 6.66E-06 | 0.11 |
| RSRC2 | -0.026525568 | 0.38097535 | 0.032944953 | 0.2764947 | 0.06 |
| FLJ31034 | 0.264504229 | 5.92E-19 | 0.113186438 | 0.000177 | 0.16 |
| RTBDN | 0.045569562 | 0.13216652 | -0.049704917 | 0.1005057 | 0.1 |
| RTCA | 0.275174002 | 1.92E-20 | 0.123584658 | 4.19E-05 | 0.16 |
| RSPH14 | 0.238986143 | 1.16E-15 | 0.09082856 | 0.0026504 | 0.15 |
| DKFZP434C013 | -0.048305099 | 0.11046701 | -0.096750123 | 0.0013626 | 0.05 |
| RTF1 | -0.264923858 | 5.19E-19 | -0.181780609 | 1.42E-09 | 0.09 |
| RTKN2 | 0.320996095 | 1.29E-27 | 0.198995394 | 3.18E-11 | 0.13 |
| RTKN | 0.448708702 | 2.94E-55 | 0.25499878 | 1.10E-17 | 0.2 |
| LOC161464 | 0.101871087 | 0.00074398 | 0.098810046 | 0.0010717 | 0.01 |
| NSP | -0.305260378 | 5.26E-25 | -0.252551741 | 2.30E-17 | 0.06 |
| RTN2 | -0.249343402 | 5.94E-17 | -0.073587602 | 0.0149593 | 0.18 |
| RTN3 | 0.124582458 | 3.63E-05 | 0.087804839 | 0.0036706 | 0.04 |
| RTN4IP1 | 0.331190572 | 2.16E-29 | 0.281783443 | 2.13E-21 | 0.05 |
| RTN4RL1 | -0.317610456 | 4.85E-27 | -0.151362513 | 4.97E-07 | 0.17 |
| RTN4RL2 | -0.097471037 | 0.00125339 | -0.007190673 | 0.812301 | 0.1 |
| RTN4R | 0.210656407 | 1.98E-12 | 0.124167921 | 3.85E-05 | 0.09 |
| NSP.CL | -0.167322356 | 2.63E-08 | -0.096583143 | 0.0013891 | 0.08 |
| RTP1 | 0.066602428 | 0.02767672 | -0.04828439 | 0.11062 | 0.12 |
| RTP2 | 0.016751181 | 0.58012071 | -0.026436173 | 0.382583 | 0.05 |
| RTP3 | 0.174505871 | 6.35E-09 | 0.076867901 | 0.0110173 | 0.1 |
| LOC115063 | 0.176168494 | 4.53E-09 | 0.046093812 | 0.1277706 | 0.14 |
| FLJ39085 | 0.066575397 | 0.02774006 | -0.069421558 | 0.0217183 | 0.14 |
| RUFY1 | -0.065703081 | 0.02985251 | 0.124188859 | 3.84E-05 | 0.19 |
| RUFY2 | -0.410178284 | 1.33E-45 | -0.315581732 | 1.06E-26 | 0.1 |
| RUFY3 | -0.101572571 | 0.00077127 | -0.086108911 | 0.0043878 | 0.02 |
| RUFY4 | 0.124854603 | 3.48E-05 | -0.015159589 | 0.6166247 | 0.15 |
| RUNDC1 | -0.397541253 | 1.07E-42 | -0.17833528 | 2.91E-09 | 0.22 |
| RUNDC2A | -0.47783331 | 1.99E-63 | -0.454704831 | 7.08E-57 | 0.03 |
| RUNDC2C | -0.350653121 | 5.66E-33 | -0.285679812 | 5.67E-22 | 0.07 |
| RUNDC3A | 0.025899888 | 0.3923141 | -0.072829498 | 0.0160302 | 0.1 |
| RUNDC3B | -0.282565036 | 1.64E-21 | -0.156255953 | 2.08E-07 | 0.13 |
| RUNX1T1 | -0.542040672 | 1.72E-84 | -0.403497739 | 4.74E-44 | 0.14 |
| RA | -0.378891967 | 1.23E-38 | -0.21703964 | 4.05E-13 | 0.17 |
| RUNX2 | -0.254729697 | 1.20E-17 | -0.147080815 | 1.04E-06 | 0.11 |
| RUNX3 | 0.113813576 | 0.0001628 | -0.071460004 | 0.0181362 | 0.19 |
| RUSC1 | 0.113275477 | 0.00017491 | 0.323754064 | 4.34E-28 | 0.22 |
| RUSC2 | -0.140002118 | 3.38E-06 | -0.220521422 | 1.67E-13 | 0.09 |
| RUVBL1 | 0.520727986 | 5.39E-77 | 0.408597274 | 3.13E-45 | 0.12 |
| RUVBL2 | 0.409613654 | 1.81E-45 | 0.342343095 | 2.06E-31 | 0.07 |
| RWDD1 | 0.234064745 | 4.56E-15 | 0.341935005 | 2.45E-31 | 0.11 |
| RWDD2A | -0.119236599 | 7.76E-05 | 0.076903827 | 0.0109798 | 0.2 |
| RWDD2B | 0.136168722 | 6.25E-06 | 0.175354321 | 5.35E-09 | 0.04 |
| RWDD3 | 0.253675672 | 1.64E-17 | 0.186236629 | 5.49E-10 | 0.07 |
| RWDD4 | 0.05904012 | 0.05101331 | 0.024646286 | 0.4156386 | 0.04 |
| RXFP1 | 0.031094594 | 0.30438446 | -0.088319423 | 0.003475 | 0.12 |
| RXFP2 | -0.030985991 | 0.30607741 | -0.005617654 | 0.8528277 | 0.03 |
| RXFP3 | -0.21885435 | 2.55E-13 | -0.234100784 | 4.51E-15 | 0.02 |
| RXFP4 | 0.086301553 | 0.00430042 | 0.017883847 | 0.5547747 | 0.07 |
| LOC101928454 | -0.332659871 | 1.18E-29 | -0.21832328 | 2.92E-13 | 0.12 |
| RXRB | 0.053992111 | 0.07438013 | 0.043250841 | 0.1530246 | 0.02 |
| RXRG | -0.066589195 | 0.02770771 | 0.028116214 | 0.3530666 | 0.1 |
| RYBP | -0.319050402 | 2.77E-27 | -0.16981714 | 1.61E-08 | 0.15 |
| JTK5A.withdrawn | 0.036622634 | 0.22636146 | 0.008884214 | 0.7692266 | 0.03 |
| CCO | 0.120481082 | 6.52E-05 | -0.06465325 | 0.0325766 | 0.19 |
| ARVD2 | -0.120712082 | 6.31E-05 | -0.093335009 | 0.0020088 | 0.03 |
| RYR3 | 0.008981277 | 0.76677788 | -0.129296649 | 1.81E-05 | 0.14 |
| S100A10 | 0.290668954 | 1.01E-22 | 0.196320181 | 5.88E-11 | 0.1 |
| S100A11 | 0.35313382 | 1.90E-33 | 0.441597594 | 2.22E-53 | 0.09 |
| S100A12 | 0.128871958 | 1.92E-05 | -0.011468536 | 0.7048849 | 0.15 |
| S100A13 | 0.119581624 | 7.40E-05 | 0.297469194 | 9.03E-24 | 0.18 |
| S100A14 | 0.005563119 | 0.85424038 | 0.129570423 | 1.73E-05 | 0.13 |
| S100A16 | 0.043536344 | 0.15032894 | 0.166894623 | 2.85E-08 | 0.13 |
| S100A1 | 0.114847361 | 0.00014171 | 0.162897441 | 6.11E-08 | 0.05 |
| S100A2 | 0.260774724 | 1.89E-18 | 0.072232964 | 0.0169196 | 0.19 |
| S100A3 | 0.22782863 | 2.46E-14 | 0.102548558 | 0.0006853 | 0.13 |
| S100A4 | 0.186633627 | 5.04E-10 | 0.086970087 | 0.0040091 | 0.1 |
| S100A5 | 0.179094681 | 2.48E-09 | 0.062614482 | 0.0384775 | 0.12 |
| PRA | 0.241678801 | 5.44E-16 | 0.145042069 | 1.47E-06 | 0.1 |
| S100A7A | 0.040275196 | 0.18334159 | 0.10474169 | 0.0005236 | 0.07 |
| LOC648586 | NA | NA | NA | NA | #VALUE! |
| S100A7 | 0.117908013 | 9.33E-05 | 0.163469277 | 5.49E-08 | 0.05 |
| S100A8 | 0.252345468 | 2.44E-17 | 0.169075717 | 1.87E-08 | 0.09 |
| S100A9 | 0.19050717 | 2.16E-10 | 0.146265467 | 1.20E-06 | 0.05 |
| S100B | 0.143148795 | 2.02E-06 | -0.02605799 | 0.3894298 | 0.17 |
| S100G | -0.022853173 | 0.45038548 | -0.016806177 | 0.5788776 | 0.01 |
| S100PBP | -0.182006304 | 1.35E-09 | -0.125812962 | 3.03E-05 | 0.06 |
| S100P | 0.038295847 | 0.20583651 | 0.136620766 | 5.82E-06 | 0.1 |
| S100Z | -0.240339652 | 7.95E-16 | -0.147166662 | 1.03E-06 | 0.1 |
| S1PR1 | -0.373578498 | 1.58E-37 | -0.315617859 | 1.05E-26 | 0.06 |
| DFNB68 | 0.217648453 | 3.47E-13 | 0.078703341 | 0.0092402 | 0.14 |
| S1PR3 | -0.275902145 | 1.51E-20 | -0.226333984 | 3.66E-14 | 0.05 |
| S1PR4 | 0.03881685 | 0.19972996 | -0.062218522 | 0.039723 | 0.11 |
| S1PR5 | 0.164711082 | 4.33E-08 | 0.003144468 | 0.917297 | 0.17 |
| SAA1 | 0.022351982 | 0.46038351 | -0.036843132 | 0.2235762 | 0.06 |
| SAA2 | 0.015702458 | 0.60406074 | -0.046014689 | 0.1284267 | 0.07 |
| SAA3P | 0.14080281 | 2.97E-06 | 0.015405889 | 0.6109102 | 0.13 |
| SAA4 | 0.116821328 | 0.00010838 | 0.002684536 | 0.9293594 | 0.12 |
| SAAL1 | 0.409057587 | 2.44E-45 | 0.272987765 | 3.93E-20 | 0.14 |
| SAC3D1 | 0.484410221 | 2.19E-65 | 0.340472261 | 4.56E-31 | 0.15 |
| SACM1L | -0.325870528 | 1.86E-28 | -0.243418014 | 3.31E-16 | 0.09 |
| FLJ33280 | -0.077682166 | 0.01019455 | -0.190319699 | 2.25E-10 | 0.12 |
| HSPC140 | 0.33035647 | 3.04E-29 | 0.121262297 | 5.84E-05 | 0.21 |
| SAFB2 | 0.016313822 | 0.59005063 | -0.004856157 | 0.872594 | 0.03 |
| SAFB | 0.276726247 | 1.15E-20 | 0.176558988 | 4.19E-09 | 0.11 |
| SAGE1 | 0.046134135 | 0.12743721 | -0.035417292 | 0.2420241 | 0.09 |
| SAG | -0.089183066 | 0.00316799 | 0.088247659 | 0.0035017 | 0.18 |
| SALL1 | -0.268632878 | 1.60E-19 | -0.182037824 | 1.34E-09 | 0.09 |
| SALL2 | -0.329558265 | 4.21E-29 | -0.150200969 | 6.09E-07 | 0.18 |
| SALL3 | -0.065803259 | 0.0296031 | -0.08620269 | 0.0043451 | 0.03 |
| SALL4 | -0.123087314 | 4.50E-05 | -0.10763731 | 0.0003641 | 0.02 |
| SAMD10 | 0.260330423 | 2.17E-18 | 0.206719185 | 5.16E-12 | 0.06 |
| SAMD11 | -0.087811381 | 0.00366802 | 0.005524456 | 0.8552422 | 0.1 |
| SAMD12 | -0.183637569 | 9.58E-10 | -0.042827542 | 0.1570887 | 0.15 |
| SAMD13 | 0.018840206 | 0.53380372 | 0.004363897 | 0.8854148 | 0.02 |
| SAMD14 | -0.09307987 | 0.00206695 | -0.085297159 | 0.0047742 | 0.01 |
| SAMD1 | 0.270539138 | 8.69E-20 | 0.208368084 | 3.47E-12 | 0.07 |
| SAMD3 | -0.02334905 | 0.44061548 | -0.079888345 | 0.0082332 | 0.06 |
| DKFZP434H0350 | -0.219573469 | 2.13E-13 | -0.261377477 | 1.57E-18 | 0.05 |
| SAMD4B | -0.092540942 | 0.00219476 | -0.244594037 | 2.36E-16 | 0.16 |
| SAMD5 | -0.143903298 | 1.78E-06 | -0.157513489 | 1.66E-07 | 0.02 |
| SAMD7 | 0.067065485 | 0.0266107 | -0.010939749 | 0.7178968 | 0.08 |
| SAMD8 | -0.385380987 | 5.07E-40 | -0.424074029 | 6.11E-49 | 0.04 |
| SAMD9L | -0.156689322 | 1.92E-07 | -0.141808066 | 2.52E-06 | 0.02 |
| SAMD9 | -0.170066397 | 1.54E-08 | -0.198275071 | 3.76E-11 | 0.03 |
| SAMHD1 | -0.231034032 | 1.04E-14 | -0.329116383 | 5.04E-29 | 0.1 |
| SAMM50 | 0.133314944 | 9.77E-06 | 0.133042409 | 1.02E-05 | 0.01 |
| SAMSN1 | -0.101092397 | 0.00081714 | -0.2081273 | 3.67E-12 | 0.11 |
| SAP130 | -0.039889162 | 0.1875796 | -0.157010031 | 1.82E-07 | 0.12 |
| SAP18 | 0.178394686 | 2.87E-09 | 0.234035394 | 4.59E-15 | 0.06 |
| SAP30BP | 0.406164645 | 1.15E-44 | 0.227877675 | 2.43E-14 | 0.18 |
| SAP30L | -0.357284336 | 2.97E-34 | -0.243444803 | 3.29E-16 | 0.12 |
| SAP30 | 0.35041509 | 6.29E-33 | 0.245404951 | 1.87E-16 | 0.11 |
| PPP6R1 | 0.0691145 | 0.02230803 | -0.089769976 | 0.0029737 | 0.16 |
| PPP6R2 | -0.096062079 | 0.00147485 | -0.137378266 | 5.16E-06 | 0.05 |
| PPP6R3 | -0.125514113 | 3.16E-05 | -0.092077032 | 0.0023105 | 0.04 |
| LOC115038 | -0.014942047 | 0.62169141 | 0.057797513 | 0.0561038 | 0.08 |
| SAR1B | -0.193827362 | 1.03E-10 | 0.010107163 | 0.7385497 | 0.21 |
| SARDH | -0.181428265 | 1.53E-09 | -0.05339446 | 0.0776497 | 0.13 |
| SARM1 | -0.197081224 | 4.94E-11 | -0.271187398 | 7.05E-20 | 0.08 |
| SARNP | 0.436507202 | 4.59E-52 | 0.480693954 | 2.83E-64 | 0.05 |
| SARSM | 0.320286165 | 1.71E-27 | 0.199323058 | 2.95E-11 | 0.13 |
| SARS | 0.120379778 | 6.61E-05 | -0.04915916 | 0.1043008 | 0.17 |
| SART1 | 0.2704978 | 8.80E-20 | 0.116594173 | 0.0001118 | 0.16 |
| SART3 | -0.046145838 | 0.12734059 | -0.121212047 | 5.88E-05 | 0.08 |
| SASH1 | -0.267807614 | 2.08E-19 | -0.111948373 | 0.0002085 | 0.16 |
| SASH3 | -0.074257462 | 0.01406597 | -0.166452724 | 3.11E-08 | 0.1 |
| SASS6 | 0.210962473 | 1.84E-12 | 0.056692857 | 0.0609774 | 0.16 |
| KFSD | 0.084309241 | 0.00528568 | 0.068920982 | 0.0226868 | 0.02 |
| SAT2 | 0.147383737 | 9.89E-07 | 0.148464501 | 8.22E-07 | 0.01 |
| SATB1 | -0.131024601 | 1.39E-05 | -0.18332714 | 1.02E-09 | 0.06 |
| SATB2 | -0.237059016 | 1.99E-15 | -0.232835975 | 6.38E-15 | 0.01 |
| SATL1 | -0.133712309 | 9.19E-06 | -0.156798518 | 1.89E-07 | 0.03 |
| SAV1 | -0.109314659 | 0.00029378 | -0.14451139 | 1.61E-06 | 0.04 |
| SBDSP1 | 0.003622707 | 0.90477463 | 0.0291176 | 0.3361771 | 0.03 |
| SDS | -0.005862025 | 0.84650345 | -0.079795106 | 0.0083087 | 0.08 |
| SBF1P1 | -0.001422253 | 0.96253979 | -0.058069967 | 0.054953 | 0.06 |
| SBF1 | -0.070204538 | 0.02027555 | -0.256036121 | 8.06E-18 | 0.19 |
| CMT4B2 | -0.386652127 | 2.70E-40 | -0.359443262 | 1.12E-34 | 0.03 |
| LOC112868 | 0.202531165 | 1.40E-11 | 0.204424022 | 8.93E-12 | 0.01 |
| LOC649015 | 0.049174074 | 0.10419564 | -0.015943504 | 0.5985191 | 0.07 |
| SBNO1 | -0.295670143 | 1.72E-23 | -0.359471688 | 1.11E-34 | 0.07 |
| SBNO2 | -0.153065198 | 3.68E-07 | -0.184967673 | 7.21E-10 | 0.04 |
| SBSN | 0.330389652 | 3.00E-29 | 0.098253836 | 0.001144 | 0.24 |
| MSMO1 | 0.026414993 | 0.38296448 | 0.028776811 | 0.3418657 | 0.01 |
| SC5D | -0.292970477 | 4.48E-23 | -0.189861802 | 2.49E-10 | 0.11 |
| P3H4 | 0.050974925 | 0.0920994 | 0.080163718 | 0.0080137 | 0.03 |
| SCAF1 | 0.088188962 | 0.00352368 | 0.045380035 | 0.1337842 | 0.05 |
| SCAI | -0.164549395 | 4.47E-08 | -0.155958766 | 2.20E-07 | 0.01 |
| SCAMP1 | -0.324434547 | 3.31E-28 | -0.103005163 | 0.0006482 | 0.23 |
| SCAMP2 | -0.19862028 | 3.47E-11 | -0.170344106 | 1.46E-08 | 0.03 |
| LOC115489 | 0.214554102 | 7.57E-13 | 0.48128584 | 1.89E-64 | 0.27 |
| SCAMP4 | -0.204576431 | 8.61E-12 | -0.045241286 | 0.1349782 | 0.16 |
| SCAMP5 | 0.021700213 | 0.47356881 | -0.126908737 | 2.58E-05 | 0.15 |
| LOC115570 | 0.168333724 | 2.16E-08 | 0.163594086 | 5.36E-08 | 0.01 |
| SCAND2P | -0.201554449 | 1.76E-11 | -0.145396126 | 1.39E-06 | 0.06 |
| ZBED9 | -0.105529087 | 0.00047474 | -0.13850772 | 4.31E-06 | 0.04 |
| SCAPER | -0.366568481 | 4.27E-36 | -0.393172002 | 1.01E-41 | 0.03 |
| SCAP | -0.048836034 | 0.10660085 | -0.075116725 | 0.0129891 | 0.03 |
| SCARA3 | -0.127524191 | 2.35E-05 | -0.118728709 | 8.33E-05 | 0.01 |
| SCARA5 | -0.220691989 | 1.59E-13 | -0.262245192 | 1.20E-18 | 0.05 |
| SCARB1 | 0.271553771 | 6.26E-20 | 0.132541372 | 1.10E-05 | 0.14 |
| SCARB2 | -0.295283257 | 1.97E-23 | -0.202796109 | 1.31E-11 | 0.1 |
| SCARF1 | -0.267391864 | 2.38E-19 | -0.249323622 | 5.97E-17 | 0.02 |
| SCARF2 | -0.188671136 | 3.24E-10 | -0.10500201 | 0.0005069 | 0.09 |
| SCARNA10 | 0.195237958 | 7.51E-11 | 0.068689984 | 0.0231462 | 0.13 |
| SCARNA11 | -0.012087009 | 0.68977493 | -0.004983085 | 0.8692934 | 0.01 |
| SCARNA12 | 0.379598029 | 8.70E-39 | 0.150564563 | 5.72E-07 | 0.23 |
| SCARNA14 | 0.001833331 | 0.95172427 | -0.011182318 | 0.7119175 | 0.02 |
| SCARNA15 | 0.010691018 | 0.72404602 | 0.007256247 | 0.8106216 | 0.01 |
| SCARNA16 | 0.196272977 | 5.94E-11 | 0.090416048 | 0.0027723 | 0.11 |
| SCARNA17 | 0.123212452 | 4.42E-05 | 0.040545748 | 0.1804139 | 0.09 |
| SCARNA18 | 0.013821684 | 0.64806539 | -0.004337329 | 0.8861077 | 0.02 |
| SCARNA1 | 0.015332892 | 0.61260143 | -0.029910293 | 0.3231819 | 0.05 |
| SCARNA20 | 0.059959889 | 0.04749917 | 0.028316933 | 0.349639 | 0.04 |
| SCARNA21 | 0.075745634 | 0.01224769 | 0.06150061 | 0.0420685 | 0.02 |
| SCARNA22 | 0.038903799 | 0.19872385 | 0.04496263 | 0.1374009 | 0.01 |
| SCARNA23 | 0.05119623 | 0.09069397 | 0.023528012 | 0.4371195 | 0.03 |
| SCARNA27 | 0.037772534 | 0.21210536 | -0.021049803 | 0.4869307 | 0.06 |
| SCARNA2 | 0.127900904 | 2.22E-05 | 0.085278607 | 0.0047834 | 0.05 |
| SCARNA3 | 0.048567975 | 0.10853919 | 0.024204194 | 0.4240554 | 0.03 |
| SCARNA4 | 0.026104175 | 0.38858968 | 0.021144643 | 0.4849698 | 0.01 |
| SCARNA5 | -0.031582593 | 0.2968541 | 0.011361734 | 0.7075062 | 0.05 |
| SCARNA6 | -0.14057332 | 3.08E-06 | -0.083696824 | 0.0056271 | 0.06 |
| SCARNA7 | 0.147274736 | 1.01E-06 | 0.099327537 | 0.0010083 | 0.05 |
| SCARNA8 | 0.019853199 | 0.51203565 | 0.00272703 | 0.9282442 | 0.02 |
| SCARNA9L | 0.041554572 | 0.1698027 | -0.000611277 | 0.983895 | 0.05 |
| SCARNA9 | 0.05092403 | 0.09242507 | -0.018613553 | 0.5387374 | 0.07 |
| SCCPDH | -0.20423069 | 9.35E-12 | 0.107648881 | 0.0003635 | 0.32 |
| SCD5 | 0.001288559 | 0.96605886 | -0.128953001 | 1.90E-05 | 0.14 |
| SCDOS | -0.018412543 | 0.54313188 | -0.057038828 | 0.0594147 | 0.04 |
| SCEL | 0.169851442 | 1.60E-08 | -0.018224524 | 0.5472584 | 0.19 |
| SCFD1 | -0.063028489 | 0.03721077 | 0.060619323 | 0.0451067 | 0.13 |
| SCFD2 | 0.092166578 | 0.00228776 | 0.038495017 | 0.2034863 | 0.06 |
| SCG2 | -0.237641348 | 1.69E-15 | -0.216467965 | 4.68E-13 | 0.03 |
| SCG3 | 0.067091441 | 0.02655201 | -0.044156401 | 0.1445992 | 0.12 |
| SCG5 | -0.040708489 | 0.17866962 | -0.034515469 | 0.2542303 | 0.01 |
| SCGB1A1 | 0.097255144 | 0.0012852 | 0.048859572 | 0.106432 | 0.05 |
| LOC144152 | -0.006739243 | 0.82388524 | 0.105160764 | 0.000497 | 0.12 |
| LOC86801 | 0.026719347 | 0.37750481 | 0.028634924 | 0.3442521 | 0.01 |
| SCGB1D2 | -0.274172432 | 2.67E-20 | -0.028702418 | 0.3431156 | 0.25 |
| SCGB1D4 | 0.037365597 | 0.21707446 | 0.033238449 | 0.2722362 | 0.01 |
| SCGB2A1 | -0.203486592 | 1.11E-11 | -0.002529898 | 0.9334188 | 0.21 |
| SCGB2A2 | -0.302221175 | 1.61E-24 | -0.04232078 | 0.1620609 | 0.26 |
| SCGB3A1 | -0.044980873 | 0.13724129 | 0.064592261 | 0.0327412 | 0.11 |
| SCGB3A2 | 0.186388235 | 5.31E-10 | 0.004960206 | 0.8698882 | 0.19 |
| SCGB2B2 | -0.096378402 | 0.00142221 | 0.010605712 | 0.7261591 | 0.11 |
| SCGN | -0.066825158 | 0.02715946 | 0.025574915 | 0.3982831 | 0.1 |
| SCHIP1 | 0.240490329 | 7.62E-16 | 0.035780276 | 0.2372293 | 0.21 |
| SCIN | -0.128095113 | 2.16E-05 | -0.003336791 | 0.9122585 | 0.13 |
| SCLT1 | 0.256677425 | 6.64E-18 | -0.010795456 | 0.7214619 | 0.27 |
| SCLY | 0.020824277 | 0.49161095 | 0.027343729 | 0.3664552 | 0.01 |
| SCMH1 | 0.027091873 | 0.37088781 | -0.020856223 | 0.4909465 | 0.05 |
| SCML1 | -0.039793208 | 0.18864414 | -0.123631357 | 4.16E-05 | 0.09 |
| SCML2 | 0.303446424 | 1.03E-24 | 0.059667995 | 0.0485917 | 0.25 |
| SCML4 | -0.052844472 | 0.08076083 | -0.119671857 | 7.30E-05 | 0.07 |
| SCN10A | -0.02416533 | 0.42479999 | 0.000197979 | 0.9947836 | 0.03 |
| LOC105377643 | -0.180337866 | 1.92E-09 | -0.230771676 | 1.12E-14 | 0.06 |
| 3-Feb | 0.226653133 | 3.36E-14 | 0.077712682 | 0.0101648 | 0.15 |
| SCN1B | -0.32601701 | 1.76E-28 | -0.216032533 | 5.22E-13 | 0.11 |
| SCN2A | -0.094211126 | 0.00182055 | -0.101979119 | 0.0007343 | 0.01 |
| SCN2B | -0.41799532 | 1.84E-47 | -0.22662019 | 3.39E-14 | 0.2 |
| SCN3A | -0.308133408 | 1.80E-25 | -0.262808087 | 1.01E-18 | 0.05 |
| SCN3B | -0.327516828 | 9.62E-29 | -0.289007973 | 1.80E-22 | 0.04 |
| HYKPP | -0.329995251 | 3.52E-29 | -0.220364064 | 1.74E-13 | 0.11 |
| SCN4B | -0.417795949 | 2.06E-47 | -0.273873406 | 2.95E-20 | 0.15 |
| SCN5A | -0.046782358 | 0.12217049 | -0.148089171 | 8.77E-07 | 0.11 |
| SCN7A | -0.527769092 | 2.06E-79 | -0.348268229 | 1.61E-32 | 0.18 |
| SCN8A | -0.174915998 | 5.84E-09 | -0.187866649 | 3.86E-10 | 0.02 |
| ETHA | -0.209487092 | 2.64E-12 | -0.275903627 | 1.51E-20 | 0.07 |
| SCNM1 | 0.547046795 | 2.48E-86 | 0.579399464 | 5.22E-99 | 0.04 |
| SCNN1A | -0.4487472 | 2.87E-55 | -0.167132631 | 2.72E-08 | 0.29 |
| LOC197426 | 0.145498499 | 1.36E-06 | -0.026909758 | 0.3741136 | 0.18 |
| SCNN1D | -0.070916146 | 0.01903746 | -0.024337773 | 0.4215018 | 0.05 |
| SCNN1G | 0.139669104 | 3.57E-06 | -0.023311788 | 0.4413454 | 0.17 |
| SCO1 | 0.121679631 | 5.50E-05 | 0.114960565 | 0.0001396 | 0.01 |
| MYP6 | 0.266561492 | 3.09E-19 | 0.221450555 | 1.31E-13 | 0.05 |
| SCOC | -0.119090274 | 7.92E-05 | -0.029949376 | 0.3225497 | 0.09 |
| SCP2 | -0.28071907 | 3.05E-21 | -0.140135524 | 3.31E-06 | 0.15 |
| SCPEP1 | 0.068043949 | 0.02447409 | -0.002126697 | 0.9440111 | 0.08 |
| SCRG1 | 0.245796666 | 1.67E-16 | 0.046260841 | 0.1263941 | 0.2 |
| SCRIB | 0.224182868 | 6.44E-14 | 0.06023778 | 0.0464783 | 0.17 |
| SCRN1 | -0.146814865 | 1.09E-06 | -0.190865965 | 2.00E-10 | 0.05 |
| SCRN2 | -0.074849902 | 0.0133154 | 0.005175728 | 0.8642884 | 0.09 |
| SCRN3 | -0.354566623 | 1.00E-33 | -0.171681302 | 1.12E-08 | 0.19 |
| SCRT1 | 0.0766826 | 0.01121258 | -0.079315497 | 0.008707 | 0.16 |
| LOC391226 | 0.225103081 | 5.06E-14 | 0.085118925 | 0.004863 | 0.14 |
| SCTR | -0.154929645 | 2.64E-07 | -0.118484179 | 8.62E-05 | 0.04 |
| SCT | 0.03276183 | 0.27917465 | 0.129988821 | 1.63E-05 | 0.1 |
| SCUBE1 | -0.296908247 | 1.10E-23 | -0.133978213 | 8.81E-06 | 0.17 |
| SCUBE2 | -0.460668458 | 1.62E-58 | -0.2103536 | 2.14E-12 | 0.26 |
| SCUBE3 | 0.007994618 | 0.79177356 | 0.04868641 | 0.1076794 | 0.05 |
| SCX | 0.282361521 | 1.75E-21 | 0.212712332 | 1.20E-12 | 0.07 |
| GKLP | 0.163717746 | 5.23E-08 | 0.083349011 | 0.0058297 | 0.09 |
| SCYL2 | -0.23566181 | 2.94E-15 | -0.267365206 | 2.40E-19 | 0.04 |
| SCYL3 | -0.295904517 | 1.58E-23 | 0.119751849 | 7.22E-05 | 0.42 |
| SDAD1 | 0.057641415 | 0.05677222 | -0.020473922 | 0.4989292 | 0.08 |
| SDC1 | 0.07835873 | 0.00955296 | 0.138461254 | 4.34E-06 | 0.07 |
| LOC157526 | 0.070248419 | 0.02019722 | -0.054492515 | 0.0717298 | 0.13 |
| SDC3 | -0.092806714 | 0.00213085 | -0.1461718 | 1.22E-06 | 0.06 |
| SDC4P | 0.043602397 | 0.14971047 | 0.011332472 | 0.708225 | 0.04 |
| SDC4 | -0.153326948 | 3.52E-07 | -0.126846075 | 2.60E-05 | 0.03 |
| SDCBP2 | 0.088191625 | 0.00352268 | -0.108984511 | 0.0003065 | 0.2 |
| SDCBP | 0.052174115 | 0.08468878 | 0.046182105 | 0.1270415 | 0.01 |
| FLJ10051 | -0.229878931 | 1.42E-14 | -0.213186309 | 1.06E-12 | 0.02 |
| SDCCAG3 | 0.32069454 | 1.45E-27 | 0.252409402 | 2.40E-17 | 0.07 |
| LOC102724256 | -0.059008309 | 0.05113864 | 0.193139073 | 1.21E-10 | 0.26 |
| SDF2L1 | 0.269332286 | 1.28E-19 | 0.213900621 | 8.91E-13 | 0.06 |
| SDF2 | 0.070793268 | 0.01924641 | 0.212325434 | 1.32E-12 | 0.15 |
| LOC51767 | 0.002151975 | 0.94334669 | -0.085350037 | 0.0047481 | 0.09 |
| SDHAF1 | 0.32606551 | 1.72E-28 | 0.239391662 | 1.04E-15 | 0.09 |
| SDHAF2 | 0.328178912 | 7.36E-29 | 0.317854146 | 4.41E-27 | 0.02 |
| LOC642749 | 0.003405652 | 0.91045534 | -0.0830989 | 0.0059794 | 0.09 |
| SDHAL2 | 0.024841479 | 0.41195402 | -0.020225921 | 0.5041443 | 0.05 |
| SDHACL | -0.038519531 | 0.20319837 | 0.037935045 | 0.210144 | 0.08 |
| SDHA | 0.148091645 | 8.76E-07 | 0.067638705 | 0.0253403 | 0.09 |
| PCHC | 0.359695146 | 1.00E-34 | 0.153367468 | 3.49E-07 | 0.21 |
| PGL3 | 0.130150812 | 1.59E-05 | 0.452042232 | 3.74E-56 | 0.33 |
| LOC91899 | 0.04259381 | 0.15936746 | 0.059114831 | 0.05072 | 0.02 |
| SDK1 | -0.142053651 | 2.42E-06 | -0.14741856 | 9.83E-07 | 0.01 |
| LOC100134174 | 0.036378129 | 0.22947879 | -0.146986873 | 1.06E-06 | 0.19 |
| SDPR | -0.394445606 | 5.25E-42 | -0.299464336 | 4.40E-24 | 0.1 |
| SDR16C5 | -0.261620689 | 1.46E-18 | -0.08559368 | 0.0046296 | 0.18 |
| SDR39U1 | 0.133404468 | 9.64E-06 | 0.142067637 | 2.41E-06 | 0.01 |
| SDR42E1 | 0.040000756 | 0.18634713 | -0.021698426 | 0.4736053 | 0.07 |
| SDR9C7 | 0.116256247 | 0.00011708 | -0.010768537 | 0.7221276 | 0.13 |
| SDSL | -0.068758155 | 0.02300979 | 0.070281286 | 0.0201387 | 0.14 |
| SDS.1 | -0.005862025 | 0.84650345 | -0.079795106 | 0.0083087 | 0.08 |
| SEBOX | -0.020379418 | 0.50091312 | -0.009102579 | 0.7637209 | 0.02 |
| SEC11A | 0.283180407 | 1.33E-21 | 0.227417942 | 2.74E-14 | 0.06 |
| SEC11C | 0.280098863 | 3.76E-21 | 0.133552142 | 9.42E-06 | 0.15 |
| SEC13 | 0.37683689 | 3.31E-38 | 0.220340392 | 1.75E-13 | 0.16 |
| SEC14L1 | -0.118986888 | 8.04E-05 | -0.349601984 | 8.97E-33 | 0.24 |
| SEC14L2 | -0.319530158 | 2.30E-27 | -0.125863126 | 3.01E-05 | 0.2 |
| SEC14L3 | 0.05108922 | 0.09137139 | -0.029968756 | 0.3222366 | 0.09 |
| SEC14L4 | 0.260989228 | 1.77E-18 | 0.042448445 | 0.1607972 | 0.22 |
| SEC14L5 | -0.057981331 | 0.05532519 | -0.164105607 | 4.86E-08 | 0.11 |
| SEC16A | -0.465944353 | 5.35E-60 | -0.216911998 | 4.18E-13 | 0.25 |
| SEC16B | -0.276062168 | 1.44E-20 | -0.017215163 | 0.5696727 | 0.26 |
| SEC1P | -0.356997237 | 3.38E-34 | -0.27802664 | 7.50E-21 | 0.08 |
| SEC22A | 0.151151017 | 5.16E-07 | 0.22519303 | 4.94E-14 | 0.08 |
| SEC22B | -0.140476727 | 3.13E-06 | -0.026066127 | 0.3892817 | 0.12 |
| SEC22C | -0.255603191 | 9.19E-18 | -0.103217634 | 0.0006316 | 0.16 |
| SEC23A | -0.274124036 | 2.71E-20 | -0.219897499 | 1.96E-13 | 0.06 |
| CDAN2 | -0.177842276 | 3.22E-09 | -0.082275279 | 0.0064973 | 0.1 |
| SEC23IP | -0.378563092 | 1.44E-38 | -0.23383701 | 4.85E-15 | 0.15 |
| SEC24A | -0.397983995 | 8.48E-43 | -0.288950054 | 1.83E-22 | 0.11 |
| SEC24B | -0.362023068 | 3.47E-35 | -0.337762381 | 1.43E-30 | 0.03 |
| SEC24C | -0.18517958 | 6.89E-10 | -0.164931625 | 4.16E-08 | 0.03 |
| SEC24D | -0.322676584 | 6.65E-28 | -0.272001504 | 5.42E-20 | 0.06 |
| ABP125 | -0.236971756 | 2.04E-15 | -0.175986984 | 4.70E-09 | 0.07 |
| SEC31B | -0.151104069 | 5.20E-07 | -0.153698243 | 3.29E-07 | 0.01 |
| LOC51080 | 0.176248747 | 4.46E-09 | 0.094465327 | 0.001769 | 0.09 |
| LOC88207 | 0.226546097 | 3.46E-14 | 0.046466464 | 0.1247155 | 0.19 |
| SEC61B | 0.427237342 | 1.01E-49 | 0.417063553 | 3.09E-47 | 0.02 |
| SEC61G | 0.459182003 | 4.17E-58 | 0.456699748 | 2.02E-57 | 0.01 |
| FLJ32803 | -0.259629881 | 2.69E-18 | -0.11132464 | 0.0002263 | 0.15 |
| PRO2507 | 0.059446174 | 0.04943598 | 0.067521214 | 0.0255963 | 0.01 |
| SECISBP2L | -0.437176738 | 3.09E-52 | -0.429445227 | 2.83E-50 | 0.01 |
| LOC100131231 | -0.232738694 | 6.55E-15 | -0.069829281 | 0.0209563 | 0.17 |
| SECTM1 | 0.12534039 | 3.25E-05 | 0.045071546 | 0.13645 | 0.09 |
| SEH1L | 0.586253162 | 7.02E-102 | 0.258920275 | 3.35E-18 | 0.33 |
| SEL1L2 | -0.129931207 | 1.64E-05 | -0.089627203 | 0.0030199 | 0.05 |
| SEL1L3 | 0.070822016 | 0.01919735 | -0.125995865 | 2.95E-05 | 0.2 |
| SEL1L | -0.512376232 | 3.36E-74 | -0.330187016 | 3.26E-29 | 0.19 |
| LOC86701 | -0.23541209 | 3.14E-15 | 0.114528263 | 0.0001479 | 0.35 |
| SELE | -0.072040574 | 0.01721556 | -0.133337892 | 9.74E-06 | 0.07 |
| SELK | 0.276266055 | 1.34E-20 | 0.263212511 | 8.87E-19 | 0.02 |
| LNHR | -0.029387702 | 0.33171186 | -0.111179116 | 0.0002306 | 0.09 |
| SELM | -0.053522386 | 0.07694016 | 0.05460566 | 0.0711414 | 0.11 |
| SELO | 0.112978384 | 0.00018195 | 0.067181071 | 0.0263502 | 0.05 |
| SELPLG | -0.125454294 | 3.19E-05 | -0.12666278 | 2.67E-05 | 0.01 |
| SELP | -0.257683839 | 4.89E-18 | -0.207370265 | 4.41E-12 | 0.06 |
| VIMP | 0.227640825 | 2.59E-14 | 0.118038597 | 9.17E-05 | 0.11 |
| SELT | -0.170525666 | 1.40E-08 | -0.0399959 | 0.1864006 | 0.14 |
| SELV | 0.09487163 | 0.00168945 | 0.014913141 | 0.622366 | 0.08 |
| SEMA3A | -0.171896513 | 1.07E-08 | -0.22642043 | 3.57E-14 | 0.06 |
| SEMA3B | -0.297926911 | 7.66E-24 | -0.143135364 | 2.02E-06 | 0.16 |
| LOC222200 | -0.452087991 | 3.63E-56 | -0.212755352 | 1.18E-12 | 0.24 |
| SEMA3D | -0.241457613 | 5.79E-16 | -0.211001307 | 1.82E-12 | 0.04 |
| SEMA3E | -0.325490161 | 2.17E-28 | -0.059529474 | 0.0491175 | 0.27 |
| SEMA3F | -0.267998252 | 1.96E-19 | -0.037984968 | 0.2095442 | 0.24 |
| SEMA3G | -0.417070794 | 3.08E-47 | -0.311526342 | 5.02E-26 | 0.11 |
| SEMA4A | 0.004717585 | 0.87619986 | 0.106204255 | 0.0004363 | 0.11 |
| SEMA4B | -0.281572148 | 2.29E-21 | -0.098465477 | 0.001116 | 0.19 |
| SEMA4C | 0.037846079 | 0.21121612 | -0.056050096 | 0.0639708 | 0.1 |
| C9orf164 | 0.058043137 | 0.05506541 | -0.160359404 | 9.82E-08 | 0.22 |
| PRO2353 | -0.019991091 | 0.50910868 | -0.133934494 | 8.87E-06 | 0.12 |
| FLJ20590 | 0.008771184 | 0.77208099 | -0.006468141 | 0.8308607 | 0.02 |
| SEMA5A | -0.439717341 | 6.83E-53 | -0.316793814 | 6.66E-27 | 0.13 |
| SEMA5B | 0.03719562 | 0.21917463 | -0.055708347 | 0.0656111 | 0.1 |
| SEMA6A | -0.105688439 | 0.00046539 | -0.041914921 | 0.1661279 | 0.07 |
| SEMA6B | -0.19424339 | 9.40E-11 | -0.169846793 | 1.61E-08 | 0.03 |
| SEMA6C | 0.019402976 | 0.52165303 | 0.042370697 | 0.1615659 | 0.03 |
| SEMA6D | -0.429802131 | 2.31E-50 | -0.309475402 | 1.09E-25 | 0.13 |
| SEMA7A | 0.118087358 | 9.11E-05 | -0.079012394 | 0.0089675 | 0.2 |
| SEMG1 | 0.010007008 | 0.74104718 | 0.01498149 | 0.6207714 | 0.01 |
| SEMG2 | -0.020690204 | 0.49440467 | 0.017004138 | 0.5744135 | 0.04 |
| SENP1 | -0.06664333 | 0.0275811 | -0.174334088 | 6.57E-09 | 0.11 |
| SENP2 | -0.131277359 | 1.34E-05 | -0.213473769 | 9.90E-13 | 0.09 |
| SENP3 | 0.3201354 | 1.81E-27 | 0.090595213 | 0.0027188 | 0.23 |
| SENP5 | 0.225226653 | 4.90E-14 | 0.000601859 | 0.9841431 | 0.23 |
| SENP6 | -0.124796028 | 3.51E-05 | -0.135168716 | 7.32E-06 | 0.02 |
| SENP7 | -0.439697368 | 6.91E-53 | -0.343001414 | 1.56E-31 | 0.1 |
| SENP8 | -0.14214183 | 2.38E-06 | -0.099284095 | 0.0010135 | 0.05 |
| 15-Sep | 0.118018263 | 9.19E-05 | 0.058982507 | 0.0512405 | 0.06 |
| LOC88214 | 0.463562654 | 2.51E-59 | 0.243963448 | 2.83E-16 | 0.22 |
| SEPHS2 | 0.036174349 | 0.2321001 | 0.125641101 | 3.11E-05 | 0.09 |
| SEPN1 | -0.279939516 | 3.96E-21 | -0.302582925 | 1.41E-24 | 0.03 |
| SEPP1 | -0.398071828 | 8.11E-43 | -0.227972715 | 2.37E-14 | 0.18 |
| SEPSECS | -0.369920057 | 8.91E-37 | -0.225154988 | 4.99E-14 | 0.15 |
| 10-Sep | -0.323450363 | 4.90E-28 | -0.387025938 | 2.24E-40 | 0.07 |
| 11-Sep | -0.180990111 | 1.67E-09 | -0.209645535 | 2.54E-12 | 0.03 |
| 12-Sep | -0.076466146 | 0.01144456 | 0.114055983 | 0.0001576 | 0.2 |
| 14-Sep | 0.013163877 | 0.66376141 | -0.013280199 | 0.6609749 | 0.03 |
| 1-Sep | 0.054156184 | 0.07350247 | -0.042557234 | 0.1597263 | 0.1 |
| 2-Sep | -0.289821561 | 1.35E-22 | -0.173058329 | 8.49E-09 | 0.12 |
| 3-Sep | 0.341889131 | 2.50E-31 | 0.102504611 | 0.000689 | 0.24 |
| 4-Sep | -0.017862962 | 0.55523712 | -0.081175328 | 0.0072516 | 0.07 |
| 5-Sep | 0.094651642 | 0.00173212 | 0.073658106 | 0.014863 | 0.03 |
| 6-Sep | -0.06206406 | 0.04021808 | -0.110113379 | 0.000265 | 0.05 |
| SEPT7P9 | -0.062640276 | 0.0383975 | -0.111303205 | 0.0002269 | 0.05 |
| SEPT7P2 | -0.209335187 | 2.74E-12 | -0.085946712 | 0.0044627 | 0.13 |
| 7-Sep | -0.115447791 | 0.00013067 | -0.135979728 | 6.44E-06 | 0.03 |
| 8-Sep | -0.354143088 | 1.21E-33 | -0.155319875 | 2.46E-07 | 0.2 |
| 9-Sep | 0.149550779 | 6.82E-07 | -0.071765487 | 0.0176466 | 0.23 |
| SEPW1 | 0.176732224 | 4.04E-09 | 0.263200202 | 8.91E-19 | 0.09 |
| MSRB1 | 0.195132027 | 7.70E-11 | 0.224016128 | 6.73E-14 | 0.03 |
| SERAC1 | -0.022928756 | 0.44888844 | -0.082217099 | 0.0065353 | 0.06 |
| SERBP1 | 0.395135039 | 3.69E-42 | 0.16410233 | 4.87E-08 | 0.24 |
| SERF1A | 0.107931266 | 0.00035072 | 0.172667407 | 9.18E-09 | 0.07 |
| LOC88287 | 0.211115235 | 1.77E-12 | 0.340914485 | 3.78E-31 | 0.13 |
| SERGEF | 0.044628239 | 0.14035225 | 0.101043793 | 0.0008219 | 0.06 |
| SERHL2 | -0.252135843 | 2.60E-17 | -0.133835268 | 9.01E-06 | 0.12 |
| BK126B4.1 | -0.128204157 | 2.13E-05 | -0.077059174 | 0.0108189 | 0.06 |
| SERINC1 | -0.508569251 | 5.96E-73 | -0.344495721 | 8.20E-32 | 0.17 |
| SERINC2 | -0.062973134 | 0.03737806 | 0.089269398 | 0.0031387 | 0.16 |
| SERINC3 | -0.401237224 | 1.56E-43 | -0.351047769 | 4.76E-33 | 0.06 |
| SERINC4 | -0.185386677 | 6.59E-10 | -0.118412626 | 8.70E-05 | 0.07 |
| SERINC5 | -0.426535675 | 1.51E-49 | -0.399869052 | 3.18E-43 | 0.03 |
| SERP1.1 | -0.136227393 | 6.19E-06 | -0.013072835 | 0.6659456 | 0.13 |
| C13orf21 | -0.226627395 | 3.38E-14 | -0.09444912 | 0.0017723 | 0.14 |
| SERPINA10 | -0.100572884 | 0.0008696 | -0.154315852 | 2.95E-07 | 0.06 |
| SERPINA11 | -0.372615412 | 2.49E-37 | -0.068930489 | 0.0226681 | 0.31 |
| SERPINA12 | -0.096124556 | 0.00146431 | -0.056821179 | 0.0603939 | 0.04 |
| SERPINA13P | -0.021164068 | 0.48456865 | 0.009045259 | 0.765165 | 0.04 |
| SERPINA1 | -0.165980241 | 3.40E-08 | -0.129638605 | 1.71E-05 | 0.04 |
| SERPINA3 | -0.142291631 | 2.32E-06 | -0.004400048 | 0.8844722 | 0.14 |
| SERPINA4 | -0.085614388 | 0.00461969 | 0.129337677 | 1.79E-05 | 0.22 |
| SERPINA5 | -0.225864087 | 4.14E-14 | -0.04144514 | 0.1709306 | 0.19 |
| SERPINA6 | -0.173090633 | 8.44E-09 | -0.142914919 | 2.10E-06 | 0.04 |
| SERPINA7 | -0.008630004 | 0.77565064 | -0.013870944 | 0.6468961 | 0.01 |
| SERPINA9 | -0.132152828 | 1.17E-05 | -0.085967132 | 0.0044532 | 0.05 |
| SERPINB10 | 0.087055697 | 0.00397315 | -0.007779247 | 0.7972592 | 0.1 |
| SERPINB11 | 0.091350109 | 0.00250322 | -0.007462676 | 0.8053406 | 0.1 |
| LOC115015 | 0.147153523 | 1.03E-06 | -0.004707669 | 0.876458 | 0.16 |
| HSHUR7SEQ | 0.130731255 | 1.45E-05 | -0.025380781 | 0.4018749 | 0.16 |
| SERPINB1 | -0.279893858 | 4.02E-21 | -0.137660233 | 4.93E-06 | 0.15 |
| LOC125818 | 0.080814294 | 0.00751581 | -0.024245089 | 0.4232726 | 0.11 |
| LOC115016 | 0.239076876 | 1.13E-15 | 0.036800435 | 0.2241136 | 0.21 |
| SERPINB4 | 0.254522577 | 1.27E-17 | 0.061865144 | 0.0408633 | 0.2 |
| LOC221232 | 0.157452743 | 1.68E-07 | -0.018080895 | 0.5504212 | 0.18 |
| SERPINB6 | -0.101670537 | 0.00076221 | 0.041335019 | 0.1720713 | 0.15 |
| SERPINB7 | 0.179385999 | 2.34E-09 | 0.052496718 | 0.0827796 | 0.13 |
| SERPINB8 | -0.036866461 | 0.22328294 | -0.198502993 | 3.57E-11 | 0.17 |
| LOC116279 | -0.084653208 | 0.00510223 | -0.261345226 | 1.59E-18 | 0.18 |
| SERPINC1 | -0.120759358 | 6.27E-05 | 0.058164864 | 0.0545567 | 0.18 |
| SERPIND1 | -0.11849262 | 8.61E-05 | -0.061817342 | 0.0410197 | 0.06 |
| SERPINE1 | -0.232569149 | 6.86E-15 | -0.213739216 | 9.27E-13 | 0.02 |
| LOC130339 | 0.074970538 | 0.01316698 | -0.012328822 | 0.6839002 | 0.09 |
| SERPINE3 | -0.053854804 | 0.07512117 | -0.059580995 | 0.0489214 | 0.01 |
| SERPINF1 | -0.185819492 | 6.01E-10 | -0.098017312 | 0.0011761 | 0.09 |
| PLI | 0.022471774 | 0.45798262 | -0.069278183 | 0.021992 | 0.1 |
| SERPING1 | -0.094697427 | 0.00172316 | -0.074639666 | 0.0135776 | 0.03 |
| LOC119842 | 0.223948522 | 6.85E-14 | 0.11515666 | 0.0001359 | 0.11 |
| SERPINI1 | -0.206619552 | 5.29E-12 | -0.14845718 | 8.23E-07 | 0.06 |
| SERPINI2 | 0.006809558 | 0.82207828 | 0.082832083 | 0.006143 | 0.08 |
| SERTAD1 | -0.00220566 | 0.94193583 | 0.049677934 | 0.1006907 | 0.06 |
| SERTAD2 | -0.007432013 | 0.80612453 | -0.127870626 | 2.23E-05 | 0.13 |
| SERTAD3 | -0.113694037 | 0.00016542 | 0.061352522 | 0.0425666 | 0.18 |
| SERTAD4 | -0.097049231 | 0.00131624 | -0.078031803 | 0.0098584 | 0.02 |
| SESN1 | -0.346993074 | 2.79E-32 | -0.142339369 | 2.31E-06 | 0.21 |
| SESN2 | -0.126709293 | 2.65E-05 | -0.171707592 | 1.11E-08 | 0.05 |
| SESN3 | -0.251238267 | 3.39E-17 | -0.330511126 | 2.86E-29 | 0.08 |
| SESTD1 | -0.181104494 | 1.63E-09 | -0.248723123 | 7.12E-17 | 0.07 |
| SETBP1 | -0.402678044 | 7.30E-44 | -0.387041286 | 2.22E-40 | 0.02 |
| SETD1A | 0.065630632 | 0.03003401 | -0.022697588 | 0.453476 | 0.09 |
| SETD1B | -0.282707712 | 1.56E-21 | -0.295551528 | 1.79E-23 | 0.02 |
| FLJ22472 | -0.336245738 | 2.69E-30 | -0.345901573 | 4.48E-32 | 0.01 |
| SETD3 | -0.09982557 | 0.00095054 | -0.059817131 | 0.0480309 | 0.05 |
| SETD4 | 0.073333383 | 0.01531119 | 0.128475041 | 2.04E-05 | 0.06 |
| LOC115800 | -0.099944722 | 0.00093719 | -0.222951282 | 8.88E-14 | 0.13 |
| SETD6 | 0.32010844 | 1.83E-27 | 0.160064678 | 1.04E-07 | 0.17 |
| SETD7 | -0.490388837 | 3.35E-67 | -0.345295878 | 5.82E-32 | 0.15 |
| SETD8 | 0.315204603 | 1.23E-26 | 0.094601698 | 0.0017419 | 0.23 |
| SETDB1 | 0.150728903 | 5.55E-07 | 0.307529825 | 2.26E-25 | 0.16 |
| SETDB2 | -0.294551652 | 2.56E-23 | -0.292322221 | 5.64E-23 | 0.01 |
| SETMAR | 0.061718747 | 0.04134375 | 0.219986583 | 1.91E-13 | 0.16 |
| SCAR1 | -0.470715246 | 2.33E-61 | -0.455388183 | 4.61E-57 | 0.02 |
| SET | 0.367437574 | 2.85E-36 | 0.201767772 | 1.67E-11 | 0.17 |
| SEZ6L2 | -0.112789803 | 0.00018656 | -0.175601953 | 5.09E-09 | 0.07 |
| SEZ6L | -0.263338126 | 8.53E-19 | -0.229383511 | 1.62E-14 | 0.04 |
| SEZ6 | 0.003014402 | 0.92070636 | -0.088121679 | 0.003549 | 0.1 |
| SF1 | 0.040176321 | 0.18442026 | -0.032245228 | 0.2868297 | 0.08 |
| SF3A1 | -0.26918447 | 1.34E-19 | -0.326307471 | 1.56E-28 | 0.06 |
| LOC116218 | 0.305084743 | 5.62E-25 | 0.199529866 | 2.81E-11 | 0.11 |
| SF3A3 | 0.399497528 | 3.86E-43 | 0.16599289 | 3.39E-08 | 0.24 |
| SF3B6 | 0.597740098 | 7.53E-107 | 0.441545414 | 2.29E-53 | 0.16 |
| SF3B1 | -0.291533433 | 7.44E-23 | -0.274102581 | 2.73E-20 | 0.02 |
| SF3B2 | 0.227685231 | 2.56E-14 | 0.052019138 | 0.0856185 | 0.18 |
| SF3B3 | 0.310873617 | 6.43E-26 | 0.061037078 | 0.0436442 | 0.25 |
| SF3B4 | 0.344040181 | 9.97E-32 | 0.520247495 | 7.85E-77 | 0.18 |
| SF3B5 | 0.446972408 | 8.52E-55 | 0.423361437 | 9.14E-49 | 0.03 |
| SUGP1 | 0.180801214 | 1.74E-09 | 0.198106691 | 3.91E-11 | 0.02 |
| SFI1 | -0.186242828 | 5.48E-10 | -0.075415444 | 0.0126321 | 0.12 |
| SFMBT1 | -0.273567159 | 3.26E-20 | -0.293734355 | 3.42E-23 | 0.03 |
| SFMBT2 | -0.332404945 | 1.32E-29 | -0.379994055 | 7.17E-39 | 0.05 |
| SFN | 0.227719072 | 2.53E-14 | 0.112664177 | 0.0001897 | 0.12 |
| LOC100506168 | 0.46716005 | 2.42E-60 | 0.323962718 | 3.99E-28 | 0.15 |
| SFRP1 | 0.090325207 | 0.00279988 | -0.079669726 | 0.0084112 | 0.17 |
| SFRP2 | -0.257166525 | 5.72E-18 | -0.069333263 | 0.0218865 | 0.19 |
| LOC222113 | -0.314091044 | 1.89E-26 | -0.152198891 | 4.29E-07 | 0.17 |
| SFRP5 | 0.031823045 | 0.29318981 | -0.082113449 | 0.0066037 | 0.12 |
| SRSF11 | -0.120968489 | 6.09E-05 | -0.127145244 | 2.49E-05 | 0.01 |
| SREK1IP1 | -0.090403832 | 0.00277603 | 0.106041959 | 0.0004453 | 0.2 |
| SRRP86 | -0.409999542 | 1.47E-45 | -0.202276427 | 1.48E-11 | 0.21 |
| LOC642558 | 0.168439276 | 2.11E-08 | 0.0779986 | 0.0098899 | 0.1 |
| SRSF12 | 0.325323266 | 2.32E-28 | 0.061422008 | 0.0423323 | 0.27 |
| SUGP2 | -0.153456385 | 3.44E-07 | -0.187814656 | 3.90E-10 | 0.04 |
| SCAF4 | 0.162804176 | 6.22E-08 | -0.008075895 | 0.789706 | 0.18 |
| CLASRP | 0.072250902 | 0.01689227 | 0.032570424 | 0.2819946 | 0.04 |
| XE7Y | 0.015834514 | 0.6010219 | -0.02516035 | 0.4059766 | 0.05 |
| PNISR | -0.119508599 | 7.47E-05 | -0.1040279 | 0.0005718 | 0.02 |
| SRSF1 | 0.325049547 | 2.59E-28 | 0.288780991 | 1.94E-22 | 0.04 |
| SRSF8 | -0.012509171 | 0.67953114 | -0.015778354 | 0.6023134 | 0.01 |
| SCAF11 | -0.421210399 | 3.07E-48 | -0.334175787 | 6.34E-30 | 0.09 |
| SRSF2 | 0.435364837 | 9.01E-52 | 0.283034767 | 1.40E-21 | 0.16 |
| SRSF3 | 0.562532871 | 3.17E-92 | 0.498823814 | 7.95E-70 | 0.07 |
| SRSF4 | 0.212705175 | 1.20E-12 | -0.039316002 | 0.1940045 | 0.26 |
| SRSF5 | -0.272089417 | 5.26E-20 | -0.192241296 | 1.47E-10 | 0.08 |
| SRSF6 | -0.131200459 | 1.35E-05 | 0.057913439 | 0.0556117 | 0.19 |
| SRSF7 | 0.518934022 | 2.18E-76 | 0.435636379 | 7.68E-52 | 0.09 |
| SFSWAP | 0.05220059 | 0.08453078 | 0.056761387 | 0.0606652 | 0.01 |
| SRSF9 | 0.360991078 | 5.56E-35 | 0.347805704 | 1.96E-32 | 0.02 |
| SFT2D1 | 0.256695548 | 6.60E-18 | 0.376840274 | 3.31E-38 | 0.13 |
| SFT2D2 | 0.280737021 | 3.03E-21 | 0.249119735 | 6.34E-17 | 0.04 |
| SFT2D3 | 0.201802372 | 1.66E-11 | 0.181136006 | 1.62E-09 | 0.03 |
| SFTA1P | 0.139801615 | 3.49E-06 | 0.094501403 | 0.0017618 | 0.05 |
| SFTA2 | 0.17650079 | 4.24E-09 | 0.144839931 | 1.52E-06 | 0.04 |
| SFTA3 | -0.003140233 | 0.91740799 | -0.020302644 | 0.5025279 | 0.02 |
| SFTP1 | 0.063600639 | 0.0355187 | -0.042504071 | 0.160249 | 0.11 |
| LOC644676 | -0.01798366 | 0.55256735 | 0.067587448 | 0.0254517 | 0.09 |
| SFTPB | 0.120818451 | 6.22E-05 | 0.015268993 | 0.6140835 | 0.11 |
| SFTPC | 0.127183353 | 2.47E-05 | -0.019036788 | 0.5295432 | 0.15 |
| SFTPD | -0.036998819 | 0.22162438 | -0.091933476 | 0.0023475 | 0.06 |
| SFXN1 | -0.06026662 | 0.04637341 | -0.081788315 | 0.0068221 | 0.03 |
| SFXN2 | -0.169058772 | 1.87E-08 | -0.019500317 | 0.5195658 | 0.15 |
| SFXN3 | -0.266741683 | 2.92E-19 | -0.22356418 | 7.57E-14 | 0.05 |
| SFXN4 | 0.383561947 | 1.25E-39 | 0.444556003 | 3.71E-54 | 0.07 |
| LOC129811 | -0.195362381 | 7.30E-11 | -0.175418808 | 5.28E-09 | 0.02 |
| SGCA | -0.220405384 | 1.72E-13 | -0.140529342 | 3.10E-06 | 0.08 |
| SGCB | -0.024901687 | 0.41082141 | -0.156708651 | 1.92E-07 | 0.14 |
| SGCD | -0.399869159 | 3.18E-43 | -0.250196947 | 4.62E-17 | 0.15 |
| SGCE | 0.013580842 | 0.65379452 | -0.004412672 | 0.8841431 | 0.02 |
| MAM | -0.191175392 | 1.87E-10 | -0.144220966 | 1.69E-06 | 0.05 |
| SGCZ | 0.007056148 | 0.8157489 | -0.038445255 | 0.2040716 | 0.05 |
| ARHGEF26 | -0.060596485 | 0.04518785 | 0.049176349 | 0.1041796 | 0.11 |
| SGIP1 | -0.16613334 | 3.30E-08 | -0.096022898 | 0.0014815 | 0.08 |
| POMK | -0.180423414 | 1.88E-09 | -0.291884534 | 6.57E-23 | 0.12 |
| SGK1 | -0.129804731 | 1.67E-05 | -0.19949062 | 2.84E-11 | 0.07 |
| SGK223 | -0.291537293 | 7.43E-23 | -0.170541851 | 1.40E-08 | 0.13 |
| FLJ34483 | -0.390489362 | 3.93E-41 | -0.405777312 | 1.41E-44 | 0.02 |
| LOC51178 | -0.180543827 | 1.84E-09 | -0.137995584 | 4.67E-06 | 0.05 |
| SGK3 | -0.116380663 | 0.00011511 | -0.14014227 | 3.31E-06 | 0.03 |
| LOC645851.1 | -0.170727923 | 1.35E-08 | -0.184285074 | 8.34E-10 | 0.02 |
| SGMS1 | -0.244737032 | 2.27E-16 | -0.077428052 | 0.0104453 | 0.17 |
| SGMS2 | -0.185666152 | 6.21E-10 | -0.221572712 | 1.27E-13 | 0.04 |
| SGOL1 | 0.433185778 | 3.23E-51 | 0.219961014 | 1.92E-13 | 0.22 |
| SGOL2 | 0.316866628 | 6.48E-27 | 0.181897306 | 1.38E-09 | 0.14 |
| SGPL1 | -0.203473195 | 1.12E-11 | -0.114160525 | 0.0001554 | 0.09 |
| SGPP1 | -0.211243934 | 1.72E-12 | -0.241566172 | 5.62E-16 | 0.04 |
| SGPP2 | 0.043839041 | 0.14751059 | -0.004665013 | 0.8775685 | 0.05 |
| SGSH | -0.189490143 | 2.71E-10 | -0.116953838 | 0.0001064 | 0.08 |
| SGSM1 | -0.310743631 | 6.75E-26 | -0.252905419 | 2.07E-17 | 0.06 |
| SGSM2 | -0.207054251 | 4.76E-12 | -0.156952068 | 1.83E-07 | 0.06 |
| SGSM3 | 0.017755406 | 0.5576215 | 0.008182811 | 0.7869885 | 0.01 |
| SGTA | 0.299936855 | 3.71E-24 | 0.191976276 | 1.56E-10 | 0.11 |
| SGTB | -0.142908702 | 2.10E-06 | -0.2932241 | 4.10E-23 | 0.16 |
| SH2B1 | 0.020519191 | 0.49798041 | -0.047469412 | 0.1167754 | 0.07 |
| SH2B2 | 0.315431511 | 1.13E-26 | 0.136368675 | 6.06E-06 | 0.18 |
| SH2B3 | -0.330710202 | 2.63E-29 | -0.406814467 | 8.13E-45 | 0.08 |
| SH2D1A | -0.002011636 | 0.94703567 | -0.092548813 | 0.0021928 | 0.1 |
| SH2D1B | -0.032600446 | 0.28155101 | -0.105604118 | 0.0004703 | 0.08 |
| SH2D2A | 0.327866271 | 8.35E-29 | 0.171704668 | 1.11E-08 | 0.16 |
| SH2D3A | -0.03310615 | 0.27415024 | 0.00664949 | 0.8261931 | 0.04 |
| SH2D3C | -0.200815957 | 2.09E-11 | -0.199232962 | 3.01E-11 | 0.01 |
| SH2D4A | -0.199924289 | 2.57E-11 | 0.073614804 | 0.0149221 | 0.28 |
| SH2D4B | 0.180395917 | 1.90E-09 | 0.02341123 | 0.439399 | 0.16 |
| SH2D5 | 0.160329023 | 9.87E-08 | 0.005120438 | 0.8657243 | 0.16 |
| LOC101928082 | 0.091806823 | 0.00238052 | 0.099767571 | 0.0009571 | 0.01 |
| SH2D7 | 0.143367617 | 1.95E-06 | -0.042678531 | 0.1585386 | 0.19 |
| SH3BGRL2 | -0.229004189 | 1.80E-14 | -0.107118696 | 0.0003888 | 0.13 |
| SH3BGRL3 | 0.345796662 | 4.69E-32 | 0.223678642 | 7.35E-14 | 0.13 |
| LOC96022 | -0.321998712 | 8.70E-28 | -0.030140329 | 0.3194728 | 0.3 |
| SH3GBR | 0.049610181 | 0.1011565 | -0.006628748 | 0.8267266 | 0.06 |
| SH3BP1 | 0.343129807 | 1.47E-31 | 0.038090303 | 0.2082825 | 0.31 |
| SH3BP2 | 0.026723545 | 0.37742983 | -0.16125613 | 8.31E-08 | 0.19 |
| SH3BP4 | -0.431636939 | 7.97E-51 | -0.189984995 | 2.43E-10 | 0.25 |
| SH3BP5L | 0.091412369 | 0.00248616 | 0.32495339 | 2.69E-28 | 0.24 |
| SH3BP5 | -0.025638452 | 0.39711182 | -0.16244578 | 6.65E-08 | 0.14 |
| SH3D19 | -0.470197848 | 3.29E-61 | -0.360483953 | 7.00E-35 | 0.11 |
| SH3D20.1 | -0.010075876 | 0.73932957 | -0.092124943 | 0.0022983 | 0.09 |
| EEN | 0.221785777 | 1.20E-13 | 0.152465884 | 4.10E-07 | 0.07 |
| SH3GL2 | 0.128387268 | 2.07E-05 | 0.057484679 | 0.05745 | 0.08 |
| SH3GL3 | 0.423531111 | 8.30E-49 | 0.139325145 | 3.77E-06 | 0.29 |
| SH3GLB1 | -0.139393905 | 3.73E-06 | -0.252245677 | 2.52E-17 | 0.12 |
| SH3GLB2 | -0.078110296 | 0.00978425 | -0.025615378 | 0.3975369 | 0.06 |
| SH3KBP1 | 0.050081341 | 0.09795277 | -0.171239484 | 1.22E-08 | 0.23 |
| SH3PXD2A | -0.296949925 | 1.09E-23 | -0.285729675 | 5.57E-22 | 0.02 |
| LOC220776 | -0.130815253 | 1.43E-05 | -0.221505064 | 1.29E-13 | 0.1 |
| SH3RF1 | -0.396575228 | 1.76E-42 | -0.252424673 | 2.38E-17 | 0.15 |
| SH3RF2 | -0.181577055 | 1.48E-09 | -0.077012733 | 0.0108668 | 0.11 |
| SH3RF3 | -0.174483792 | 6.38E-09 | -0.169796212 | 1.62E-08 | 0.01 |
| SH3TC1 | -0.238860317 | 1.20E-15 | -0.249247107 | 6.11E-17 | 0.02 |
| SH3TC2 | -0.009857601 | 0.74477792 | -0.067622879 | 0.0253746 | 0.06 |
| SH3YL1 | 0.024466912 | 0.41904167 | 0.142252164 | 2.34E-06 | 0.12 |
| SHANK1 | -0.375891937 | 5.22E-38 | -0.27362978 | 3.19E-20 | 0.11 |
| LOC654128 | -0.276921314 | 1.08E-20 | -0.138414333 | 4.37E-06 | 0.14 |
| LOC284940 | -0.224263318 | 6.31E-14 | -0.296332446 | 1.36E-23 | 0.08 |
| SHARPIN | 0.323802101 | 4.26E-28 | 0.263856223 | 7.25E-19 | 0.06 |
| SHBG | 0.061538915 | 0.04194045 | 0.045297211 | 0.134496 | 0.02 |
| SHB | 0.074300125 | 0.0140107 | 0.077583645 | 0.0102911 | 0.01 |
| SHC1 | -0.158184035 | 1.47E-07 | 0.082576477 | 0.0063034 | 0.25 |
| LOC400665 | -0.285934822 | 5.19E-22 | -0.088934173 | 0.0032538 | 0.2 |
| SHC3 | 0.11780414 | 9.47E-05 | -0.029559051 | 0.3288992 | 0.15 |
| SHC4 | 0.177356267 | 3.56E-09 | -0.030492883 | 0.3138424 | 0.21 |
| SHCBP1 | 0.40107457 | 1.69E-43 | 0.157693762 | 1.60E-07 | 0.25 |
| SHD | 0.094451796 | 0.00177174 | -0.056043934 | 0.0640001 | 0.16 |
| SHE | -0.422461942 | 1.52E-48 | -0.287990735 | 2.56E-22 | 0.14 |
| SHFM1 | 0.598113634 | 5.15E-107 | 0.535387607 | 4.30E-82 | 0.07 |
| SHF | 0.071093082 | 0.01874006 | 0.005174569 | 0.8643185 | 0.07 |
| SHH | 0.031569003 | 0.2970621 | 0.066017538 | 0.0290756 | 0.04 |
| C13orf13 | -0.192046363 | 1.54E-10 | -0.021161459 | 0.4846225 | 0.18 |
| SHISA3 | -0.070775767 | 0.01927633 | -0.145427901 | 1.38E-06 | 0.08 |
| SHISA4 | -0.181331366 | 1.56E-09 | 0.048943336 | 0.1058327 | 0.24 |
| SHISA5 | -0.01352439 | 0.65514037 | 0.079141594 | 0.0088556 | 0.1 |
| SHISA6 | -0.407726908 | 4.99E-45 | -0.273962515 | 2.86E-20 | 0.14 |
| SHISA7 | -0.076108014 | 0.01183773 | -0.181170958 | 1.61E-09 | 0.11 |
| LOC732120 | -0.093483237 | 0.00197579 | -0.058053611 | 0.0550215 | 0.04 |
| SHKBP1 | 0.112759581 | 0.00018731 | 0.082517918 | 0.0063407 | 0.04 |
| SHMT1 | 0.045953063 | 0.12893947 | 0.051067401 | 0.09151 | 0.01 |
| SHMT2 | 0.513265219 | 1.71E-74 | 0.323311339 | 5.17E-28 | 0.19 |
| SHOC2 | -0.279839886 | 4.10E-21 | -0.123305302 | 4.36E-05 | 0.16 |
| SHOX2 | -0.111544289 | 0.00021986 | -0.128243577 | 2.11E-05 | 0.02 |
| SHOX | -0.10720216 | 0.00038473 | -0.078798346 | 0.0091556 | 0.03 |
| SHPK | -0.312531104 | 3.43E-26 | -0.253850464 | 1.56E-17 | 0.06 |
| SHPRH | -0.379089025 | 1.11E-38 | -0.390797311 | 3.36E-41 | 0.02 |
| SHQ1 | -0.274954726 | 2.07E-20 | -0.036068313 | 0.2334725 | 0.24 |
| SHROOM1 | -0.36024115 | 7.82E-35 | -0.02963136 | 0.3277168 | 0.34 |
| SHROOM2 | -0.20350443 | 1.11E-11 | -0.145767471 | 1.30E-06 | 0.06 |
| SHROOM3 | -0.36289232 | 2.33E-35 | -0.169308798 | 1.78E-08 | 0.2 |
| SHROOM4 | -0.423105498 | 1.06E-48 | -0.338959182 | 8.63E-31 | 0.09 |
| SIAE | -0.321341369 | 1.13E-27 | -0.137291991 | 5.23E-06 | 0.19 |
| SIAH1 | 0.096024182 | 0.00148128 | -0.066488709 | 0.0279441 | 0.17 |
| SIAH2 | -0.131080318 | 1.38E-05 | -0.01760364 | 0.5609944 | 0.12 |
| SIAH3 | -0.183083613 | 1.08E-09 | -0.216327241 | 4.85E-13 | 0.04 |
| SIDT1 | -0.482569409 | 7.82E-65 | -0.20785158 | 3.93E-12 | 0.28 |
| SIDT2 | -0.457219944 | 1.45E-57 | -0.363243946 | 1.98E-35 | 0.1 |
| SIGIRR | -0.110762431 | 0.00024353 | 0.009333463 | 0.7579126 | 0.13 |
| SIGLEC10 | -0.094719701 | 0.00171882 | -0.186137874 | 5.61E-10 | 0.1 |
| LOC147864 | -0.126783019 | 2.63E-05 | -0.224447553 | 6.01E-14 | 0.1 |
| SIGLEC12 | 0.100409399 | 0.00088674 | -0.10860532 | 0.0003218 | 0.21 |
| SIGLEC14 | -0.045367 | 0.13389605 | -0.081029535 | 0.0073573 | 0.04 |
| SIGLEC15 | -0.263130934 | 9.10E-19 | -0.0810083 | 0.0073728 | 0.19 |
| LOC100420984 | 0.016506881 | 0.58565774 | -0.093002542 | 0.0020849 | 0.11 |
| SIGLEC1 | 0.014196826 | 0.63918297 | -0.101197848 | 0.0008069 | 0.12 |
| SIGLEC5 | -0.172934389 | 8.71E-09 | -0.24881231 | 6.94E-17 | 0.08 |
| SIGLEC6 | -0.389424626 | 6.72E-41 | -0.347641075 | 2.11E-32 | 0.05 |
| LOC100421066 | 0.001669105 | 0.95604412 | -0.114228963 | 0.000154 | 0.12 |
| SIGLEC8 | -0.33781717 | 1.39E-30 | -0.162409927 | 6.70E-08 | 0.18 |
| LOC147644 | -0.073531316 | 0.0150366 | -0.175929248 | 4.76E-09 | 0.11 |
| SIGLEC17P | -0.38764338 | 1.64E-40 | -0.252799381 | 2.13E-17 | 0.14 |
| SIGMAR1 | 0.389345588 | 7.00E-41 | 0.190935935 | 1.97E-10 | 0.2 |
| SNF1LK | -0.023122315 | 0.44506758 | -0.159379261 | 1.18E-07 | 0.14 |
| SIK2 | -0.300038826 | 3.57E-24 | -0.348292314 | 1.59E-32 | 0.05 |
| FLJ12240 | -0.372890155 | 2.19E-37 | -0.365185803 | 8.10E-36 | 0.01 |
| SIKE1 | 0.090322727 | 0.00280063 | -0.080203717 | 0.0079823 | 0.18 |
| MSS | -0.042646307 | 0.15885347 | 0.151725679 | 4.67E-07 | 0.2 |
| D12S53E | -0.223266666 | 8.18E-14 | -0.146950118 | 1.07E-06 | 0.08 |
| SIM1 | -0.1093084 | 0.00029402 | -0.127030035 | 2.53E-05 | 0.02 |
| SIM2 | 0.205755122 | 6.50E-12 | 0.057519144 | 0.0573004 | 0.15 |
| SIN3A | -0.325698802 | 2.00E-28 | -0.359357425 | 1.17E-34 | 0.04 |
| SIN3B | -0.116975611 | 0.00010611 | -0.208392995 | 3.45E-12 | 0.1 |
| GEMIN2 | 0.375197358 | 7.28E-38 | 0.323958045 | 4.00E-28 | 0.06 |
| SIPA1L1 | -0.196999699 | 5.03E-11 | -0.262318534 | 1.17E-18 | 0.07 |
| SIPA1L2 | -0.138263792 | 4.48E-06 | -0.106608582 | 0.0004147 | 0.04 |
| SIPA1L3 | -0.324033827 | 3.88E-28 | -0.294729824 | 2.40E-23 | 0.03 |
| SIPA1 | 0.00743291 | 0.80610158 | -0.020613812 | 0.4960003 | 0.03 |
| PTPNS1 | 0.026593256 | 0.37976086 | -0.158262894 | 1.44E-07 | 0.19 |
| LOC93149 | -0.087552308 | 0.00377015 | -0.270182009 | 9.75E-20 | 0.19 |
| SIRPB2 | -0.217102048 | 3.99E-13 | -0.278938898 | 5.54E-21 | 0.07 |
| LOC149803 | 0.02221087 | 0.4632207 | -0.08214907 | 0.0065801 | 0.11 |
| LOC100131600 | 0.093204236 | 0.00203844 | -0.047240333 | 0.1185531 | 0.15 |
| SIRT1 | -0.304402995 | 7.23E-25 | -0.268448227 | 1.70E-19 | 0.04 |
| SIRT2 | 0.111897457 | 0.00020989 | 0.038962683 | 0.1980446 | 0.08 |
| SIRT3 | -0.087461619 | 0.0038065 | 0.107048174 | 0.0003923 | 0.2 |
| SIRT4 | 0.093025942 | 0.00207942 | 0.131792317 | 1.24E-05 | 0.04 |
| SIRT5 | 0.344332605 | 8.80E-32 | 0.223325082 | 8.06E-14 | 0.13 |
| SIRT6 | 0.230656313 | 1.15E-14 | 0.235240836 | 3.30E-15 | 0.01 |
| SIRT7 | 0.363940757 | 1.44E-35 | 0.243637466 | 3.11E-16 | 0.13 |
| SIT1 | 0.029475516 | 0.33026845 | -0.0733402 | 0.0153017 | 0.11 |
| LOC220525 | 0.286220875 | 4.71E-22 | 0.278360438 | 6.71E-21 | 0.01 |
| DFNA23 | -0.025053128 | 0.40798072 | 0.045076153 | 0.1364099 | 0.08 |
| SIX2 | 0.097054287 | 0.00131547 | 0.07369355 | 0.0148148 | 0.03 |
| HPE2 | 0.181618252 | 1.47E-09 | 0.084315877 | 0.0052821 | 0.1 |
| SIX4 | -0.074350066 | 0.01394624 | -0.008632016 | 0.7755997 | 0.07 |
| SIX5 | -0.034559718 | 0.2536216 | 0.092029452 | 0.0023227 | 0.13 |
| SIX6 | 0.080963026 | 0.00740595 | 0.001273144 | 0.9664647 | 0.08 |
| SI | 0.031292103 | 0.30132155 | 0.033856261 | 0.263419 | 0.01 |
| SKA1 | 0.640137824 | 4.63E-127 | 0.299609118 | 4.17E-24 | 0.35 |
| SKA2 | 0.269717121 | 1.13E-19 | 0.193052189 | 1.23E-10 | 0.08 |
| LOC116303 | 0.557053878 | 4.19E-90 | 0.365793284 | 6.12E-36 | 0.2 |
| SKAP1 | -0.177204226 | 3.67E-09 | 0.010457227 | 0.7298421 | 0.19 |
| SKAP2 | -0.253127336 | 1.93E-17 | -0.340988513 | 3.66E-31 | 0.09 |
| SKIL | -0.301551934 | 2.06E-24 | -0.270508699 | 8.77E-20 | 0.04 |
| SKINTL | -0.161460787 | 8.00E-08 | -0.083646086 | 0.0056562 | 0.08 |
| SKIV2L2 | -0.252951747 | 2.04E-17 | -0.045484967 | 0.1328867 | 0.21 |
| LOC220821 | 0.014854087 | 0.62374516 | 0.028669971 | 0.3436616 | 0.02 |
| SKI | -0.155298153 | 2.47E-07 | -0.306797855 | 2.97E-25 | 0.16 |
| SKP1 | -0.186824869 | 4.84E-10 | 0.031073318 | 0.3047156 | 0.22 |
| LOC86997 | 0.299719049 | 4.01E-24 | 0.03867233 | 0.2014104 | 0.27 |
| SLA2 | 0.031562459 | 0.29716231 | -0.0726338 | 0.0163174 | 0.11 |
| SLAIN1 | 0.099315395 | 0.00100975 | -0.116358042 | 0.0001155 | 0.22 |
| FLJ21611 | -0.255074949 | 1.08E-17 | -0.22963633 | 1.52E-14 | 0.03 |
| SLAMF1 | 0.003720425 | 0.90221877 | -0.123307496 | 4.36E-05 | 0.13 |
| SLAMF6 | -0.013540312 | 0.65476067 | -0.123085114 | 4.50E-05 | 0.11 |
| LOC126807 | 0.023869457 | 0.43049391 | -0.09091935 | 0.0026242 | 0.12 |
| SBBI42 | 0.098543516 | 0.00110583 | -0.088905525 | 0.0032639 | 0.19 |
| SLAMF9 | 0.059251135 | 0.05018844 | 0.151384455 | 4.95E-07 | 0.1 |
| LOC157759 | -0.069235412 | 0.02207418 | -0.181729187 | 1.43E-09 | 0.12 |
| LOC132996 | 0.438570019 | 1.35E-52 | 0.270980472 | 7.54E-20 | 0.17 |
| SLC10A1 | 0.004652706 | 0.877889 | -0.054762187 | 0.0703339 | 0.06 |
| SLC10A2 | 0.110033395 | 0.00026773 | 0.015161504 | 0.6165802 | 0.1 |
| SLC10A3 | 0.243261318 | 3.46E-16 | 0.135595078 | 6.84E-06 | 0.11 |
| SLC10A4 | 0.177391351 | 3.53E-09 | 0.082029171 | 0.0066597 | 0.1 |
| SLC10A5 | 0.219568828 | 2.13E-13 | 0.192101138 | 1.52E-10 | 0.03 |
| SLC10A6 | -0.274766705 | 2.20E-20 | -0.265972329 | 3.73E-19 | 0.01 |
| SLC10A7 | -0.274083286 | 2.75E-20 | -0.232077779 | 7.84E-15 | 0.05 |
| SLC11A1 | 0.160993039 | 8.73E-08 | 0.043581691 | 0.1499041 | 0.12 |
| SLC11A2 | -0.163282078 | 5.68E-08 | -0.104769711 | 0.0005218 | 0.06 |
| SLC12A1 | 0.047223083 | 0.11868777 | 0.090975788 | 0.0026081 | 0.05 |
| SLC12A2 | -0.287593267 | 2.93E-22 | -0.094837355 | 0.001696 | 0.2 |
| SLC12A3 | -0.017371221 | 0.56617877 | -0.10100412 | 0.0008258 | 0.09 |
| SLC12A4 | -0.308169826 | 1.78E-25 | -0.314666846 | 1.51E-26 | 0.01 |
| SLC12A5 | 0.056500577 | 0.06186047 | -0.10363165 | 0.0006004 | 0.17 |
| ACCPN | -0.40479262 | 2.39E-44 | -0.442232065 | 1.51E-53 | 0.04 |
| DKFZP434F076 | -0.065680097 | 0.02990999 | -0.154668361 | 2.77E-07 | 0.09 |
| FLJ23188 | 0.051547841 | 0.08849651 | 0.138719005 | 4.16E-06 | 0.09 |
| SLC12A9 | 0.059153135 | 0.05057013 | 0.091790145 | 0.0023849 | 0.04 |
| SLC13A1 | -0.081560573 | 0.00697893 | -0.048797379 | 0.1068787 | 0.04 |
| SLC13A2 | -0.175290798 | 5.42E-09 | 0.080966642 | 0.0074033 | 0.26 |
| SLC13A3 | 0.047495955 | 0.11657074 | -0.008537495 | 0.7779922 | 0.06 |
| SLC13A4 | -0.063463207 | 0.03591904 | 0.018695989 | 0.5369403 | 0.09 |
| SLC13A5 | 0.001618798 | 0.95736768 | -0.136780249 | 5.67E-06 | 0.14 |
| RACH1 | -0.167654211 | 2.46E-08 | -0.07947878 | 0.0085696 | 0.09 |
| SLC14A2 | -0.32077189 | 1.41E-27 | -0.215076783 | 6.64E-13 | 0.11 |
| SLC15A1 | 0.329969703 | 3.56E-29 | 0.115762457 | 0.0001252 | 0.22 |
| SLC15A2 | 0.123788273 | 4.07E-05 | 0.107386784 | 0.0003758 | 0.02 |
| SLC15A3 | 0.027445283 | 0.36467726 | -0.011627432 | 0.7009915 | 0.04 |
| SLC15A4 | 0.005026546 | 0.86816383 | -0.060731878 | 0.0447087 | 0.07 |
| SLC16A10 | 0.304331327 | 7.42E-25 | 0.064029026 | 0.0342951 | 0.25 |
| SLC16A11 | -0.058145871 | 0.05463584 | -0.082108268 | 0.0066071 | 0.03 |
| LOC196023 | -0.370770628 | 5.97E-37 | -0.232352674 | 7.27E-15 | 0.14 |
| SLC16A13 | 0.057571858 | 0.05707216 | -0.030007689 | 0.3216081 | 0.09 |
| SLC16A14 | -0.174918084 | 5.84E-09 | -0.091295851 | 0.0025182 | 0.09 |
| SLC16A1 | 0.240976451 | 6.64E-16 | 0.106769206 | 0.0004064 | 0.14 |
| MRX22 | -0.256181153 | 7.71E-18 | -0.156772253 | 1.90E-07 | 0.1 |
| SLC16A3 | 0.192070834 | 1.53E-10 | -0.001531334 | 0.9596691 | 0.2 |
| SLC16A4 | -0.308105472 | 1.82E-25 | -0.138891363 | 4.05E-06 | 0.17 |
| SLC16A5 | -0.268791093 | 1.52E-19 | -0.224193494 | 6.42E-14 | 0.05 |
| SLC16A6 | -0.433731904 | 2.35E-51 | -0.223782482 | 7.15E-14 | 0.21 |
| SLC16A7 | -0.281526289 | 2.32E-21 | -0.379352493 | 9.80E-39 | 0.1 |
| SLC16A8 | 0.048903414 | 0.10611795 | -0.018055915 | 0.5509721 | 0.07 |
| SLC16A9 | -0.183773963 | 9.30E-10 | -0.077980074 | 0.0099075 | 0.11 |
| SLC17A1 | 0.011403456 | 0.70648175 | 0.019179353 | 0.5264642 | 0.01 |
| SLC17A2 | -0.00341393 | 0.91023859 | -0.034070375 | 0.2604097 | 0.04 |
| SLC17A3 | 0.035017142 | 0.24738838 | 0.049706163 | 0.1004972 | 0.02 |
| SLC17A4 | 0.018860142 | 0.53337087 | 0.006084041 | 0.8407663 | 0.02 |
| SIASD | -0.232626869 | 6.75E-15 | -0.051472577 | 0.0889632 | 0.19 |
| SLC17A6 | 0.214388887 | 7.88E-13 | 0.066028957 | 0.0290477 | 0.15 |
| SLC17A7 | -0.199754189 | 2.67E-11 | -0.157851283 | 1.56E-07 | 0.05 |
| DFNA25 | 0.022472743 | 0.45796322 | -0.00732078 | 0.8089698 | 0.03 |
| SLC17A9 | 0.068114515 | 0.02432591 | -0.072628356 | 0.0163254 | 0.15 |
| SLC18A1 | -0.06196332 | 0.04054376 | -0.135252126 | 7.22E-06 | 0.08 |
| SLC18A2 | -0.437310352 | 2.86E-52 | -0.32054381 | 1.54E-27 | 0.12 |
| SLC18A3 | 0.212105686 | 1.39E-12 | 0.059033592 | 0.051039 | 0.16 |
| LOC101928717 | 0.39774586 | 9.60E-43 | 0.264351293 | 6.21E-19 | 0.14 |
| SLC19A2 | -0.245988475 | 1.58E-16 | 0.026907414 | 0.3741552 | 0.28 |
| SLC19A3 | -0.09637603 | 0.00142259 | -0.100584671 | 0.0008684 | 0.01 |
| SLC1A1 | -0.223672825 | 7.36E-14 | -0.003059203 | 0.9195319 | 0.23 |
| SLC1A2 | -0.267471466 | 2.32E-19 | -0.166458836 | 3.10E-08 | 0.11 |
| SLC1A3 | -0.101502405 | 0.00077782 | -0.173219728 | 8.22E-09 | 0.08 |
| SLC1A4 | -0.192462706 | 1.40E-10 | -0.1609613 | 8.78E-08 | 0.04 |
| LOC220610 | 0.161564988 | 7.85E-08 | 0.051040092 | 0.0916837 | 0.12 |
| SLC1A6 | 0.255984601 | 8.19E-18 | 0.081020624 | 0.0073638 | 0.18 |
| SLC1A7 | -0.362310104 | 3.04E-35 | -0.193552417 | 1.10E-10 | 0.17 |
| SLC20A1 | -0.108694625 | 0.00031814 | -0.0819794 | 0.0066929 | 0.03 |
| LOC100507493 | -0.099479731 | 0.00099032 | -0.09397616 | 0.0018694 | 0.01 |
| SLC22A10 | -0.131393246 | 1.31E-05 | -0.018629109 | 0.5383981 | 0.12 |
| SLC22A11 | -0.193488529 | 1.11E-10 | -0.122475724 | 4.91E-05 | 0.08 |
| SLC22A12 | -0.118457007 | 8.65E-05 | -0.109669826 | 0.0002806 | 0.01 |
| SLC22A13 | -0.100224832 | 0.00090647 | -0.072585521 | 0.0163889 | 0.03 |
| SLC22A14 | 0.104551997 | 0.00053601 | 0.044267526 | 0.1435902 | 0.07 |
| SLC22A15 | -0.245678562 | 1.73E-16 | -0.225880639 | 4.12E-14 | 0.02 |
| LOC115715 | 0.310081086 | 8.68E-26 | 0.090572101 | 0.0027256 | 0.22 |
| SLC22A17 | -0.222335372 | 1.04E-13 | -0.143443432 | 1.92E-06 | 0.08 |
| BWSCR1B | 0.019482748 | 0.51994226 | 0.083918002 | 0.0055015 | 0.07 |
| IMPT1 | -0.215761443 | 5.59E-13 | 0.02800558 | 0.3549649 | 0.25 |
| SLC22A1 | 0.181529348 | 1.50E-09 | 0.0252531 | 0.4042477 | 0.16 |
| SLC22A20 | 0.139689468 | 3.56E-06 | -0.00666223 | 0.8258654 | 0.15 |
| SLC22A23 | -0.337000792 | 1.96E-30 | -0.085753948 | 0.0045532 | 0.26 |
| SLC22A24 | -0.065101977 | 0.03138729 | -0.013676313 | 0.651521 | 0.06 |
| SLC22A25 | -0.079306796 | 0.00871443 | 0.032205075 | 0.2874306 | 0.12 |
| SLC22A2 | -0.003793418 | 0.90031025 | -0.00845375 | 0.7801137 | 0.01 |
| LOC253271 | -0.12224009 | 5.08E-05 | -0.154986109 | 2.62E-07 | 0.04 |
| DFNB60 | -0.343125119 | 1.48E-31 | -0.205685864 | 6.61E-12 | 0.14 |
| SLC22A5 | -0.38380736 | 1.11E-39 | -0.140115359 | 3.32E-06 | 0.25 |
| SLC22A6 | 0.053052115 | 0.07957457 | 0.038238177 | 0.2065207 | 0.02 |
| SLC22A7 | -0.063834687 | 0.03484566 | -4.57E-06 | 0.9998796 | 0.07 |
| LOC144094 | 0.040511046 | 0.18078746 | 0.021579383 | 0.4760358 | 0.02 |
| LOC221106 | 0.0731551 | 0.01556229 | 0.075487265 | 0.0125476 | 0.01 |
| MGC22361 | -0.207307673 | 4.48E-12 | -0.0522848 | 0.0840298 | 0.16 |
| LOC102724905 | -0.193041811 | 1.23E-10 | -0.27046862 | 8.89E-20 | 0.08 |
| SLC23A3 | 0.130183511 | 1.58E-05 | 0.054178459 | 0.073384 | 0.08 |
| SLC24A1 | -0.49359812 | 3.43E-68 | -0.333519873 | 8.31E-30 | 0.17 |
| SLC24A2 | -0.426462551 | 1.57E-49 | -0.201059094 | 1.97E-11 | 0.23 |
| SLC24A3 | -0.080807063 | 0.00752119 | 0.075243484 | 0.0128365 | 0.16 |
| SLC24A4 | -0.104424645 | 0.00054452 | -0.185982576 | 5.80E-10 | 0.09 |
| SLC24A5 | 0.206858169 | 4.99E-12 | 0.353448291 | 1.65E-33 | 0.15 |
| SLC8B1 | -0.142245298 | 2.34E-06 | -0.101833839 | 0.0007473 | 0.05 |
| SLC25A10 | 0.390353756 | 4.21E-41 | 0.282901663 | 1.46E-21 | 0.11 |
| SLC25A11 | 0.279158096 | 5.15E-21 | 0.183955293 | 8.95E-10 | 0.1 |
| SLC25A12 | -0.202347437 | 1.46E-11 | -0.130049106 | 1.61E-05 | 0.08 |
| SLC25A13 | 0.225432009 | 4.64E-14 | 0.105000626 | 0.000507 | 0.13 |
| SLC25A14 | 0.130479777 | 1.51E-05 | 0.206145415 | 5.92E-12 | 0.08 |
| HHH | 0.097853838 | 0.00119875 | -0.010275453 | 0.7343593 | 0.11 |
| LOC142833 | -0.260369292 | 2.14E-18 | 0.00147946 | 0.9610342 | 0.27 |
| SLC25A17 | 0.110073401 | 0.00026635 | 0.117502079 | 9.87E-05 | 0.01 |
| SLC25A18 | -0.086169721 | 0.00436008 | 0.123153066 | 4.46E-05 | 0.21 |
| SLC25A19 | 0.572238647 | 4.42E-96 | 0.287834364 | 2.70E-22 | 0.29 |
| LOC115212 | 0.214892107 | 6.95E-13 | 0.224951293 | 5.27E-14 | 0.02 |
| SLC25A20 | -0.219412737 | 2.21E-13 | -0.109084628 | 0.0003026 | 0.12 |
| SLC25A21 | -0.085155986 | 0.00484442 | 0.002620847 | 0.9310311 | 0.09 |
| SLC25A22 | 0.26274057 | 1.03E-18 | 0.136185774 | 6.23E-06 | 0.13 |
| SLC25A23 | -0.035835082 | 0.23651119 | 0.051657975 | 0.0878171 | 0.09 |
| SLC25A24 | -0.33342425 | 8.65E-30 | -0.250985724 | 3.66E-17 | 0.09 |
| SLC25A25 | -0.085345 | 0.00475059 | -0.165942738 | 3.43E-08 | 0.09 |
| SLC25A26 | -0.026624156 | 0.37920723 | 0.080680192 | 0.0076161 | 0.11 |
| SLC25A27 | 0.051573863 | 0.0883356 | -0.078671067 | 0.0092691 | 0.14 |
| SLC25A28 | 0.265132051 | 4.86E-19 | 0.201196488 | 1.91E-11 | 0.07 |
| SLC25A29 | -0.024564615 | 0.41718602 | 0.144983332 | 1.49E-06 | 0.17 |
| SLC25A2 | -0.024360848 | 0.42106158 | 0.058337223 | 0.0538432 | 0.09 |
| SLC25A30 | -0.275022663 | 2.02E-20 | -0.284942577 | 7.29E-22 | 0.01 |
| SLC25A31 | 0.021795065 | 0.47163716 | -0.016841788 | 0.5780734 | 0.04 |
| SLC25A32 | 0.333208755 | 9.45E-30 | 0.071701744 | 0.0177478 | 0.27 |
| SLC25A33 | 0.409819295 | 1.62E-45 | 0.262383608 | 1.15E-18 | 0.15 |
| SLC25A34 | -0.090488713 | 0.0027505 | -0.062985345 | 0.0373411 | 0.03 |
| SLC25A35 | -0.160580182 | 9.43E-08 | -0.047476787 | 0.1167185 | 0.12 |
| SLC25A36 | -0.144416078 | 1.63E-06 | -0.109131997 | 0.0003008 | 0.04 |
| PRO1584 | 0.098818695 | 0.00107065 | 0.048605291 | 0.1082677 | 0.06 |
| SLC25A38 | -0.016580153 | 0.58399447 | -0.029532383 | 0.3293359 | 0.02 |
| SLC25A39 | 0.432363 | 5.22E-51 | 0.312432514 | 3.56E-26 | 0.12 |
| SLC25A3 | 0.316310059 | 8.03E-27 | 0.298873865 | 5.45E-24 | 0.02 |
| SLC25A40 | -0.333614287 | 8.00E-30 | -0.348765926 | 1.29E-32 | 0.02 |
| SLC25A41 | 0.063303896 | 0.03638792 | 0.029586544 | 0.3284493 | 0.04 |
| SLC25A42 | -0.172461184 | 9.57E-09 | -0.196804936 | 5.26E-11 | 0.03 |
| SLC25A43 | 0.115698681 | 0.0001263 | 0.01543152 | 0.6103169 | 0.11 |
| SLC25A44 | -0.01733965 | 0.56688475 | 0.207304118 | 4.48E-12 | 0.23 |
| SLC25A45 | -0.127127582 | 2.50E-05 | -0.024602209 | 0.4164733 | 0.11 |
| SLC25A46 | -0.419866684 | 6.51E-48 | -0.336633368 | 2.29E-30 | 0.09 |
| LOC115046 | 0.006793207 | 0.82249838 | 0.008764534 | 0.772249 | 0.01 |
| SLC25A5 | 0.400230629 | 2.64E-43 | 0.276586637 | 1.21E-20 | 0.13 |
| ANT3Y | 0.187909708 | 3.82E-10 | 0.136101063 | 6.32E-06 | 0.06 |
| SLC26A10 | 0.129353527 | 1.79E-05 | -0.01314303 | 0.6642613 | 0.15 |
| SLC26A11 | -0.120257904 | 6.73E-05 | -0.104989685 | 0.0005077 | 0.02 |
| SLC26A1 | -0.380733654 | 5.00E-39 | -0.24795463 | 8.92E-17 | 0.14 |
| SLC26A2 | -0.094906233 | 0.00168283 | -0.111648178 | 0.0002169 | 0.02 |
| CLD | -0.30439449 | 7.25E-25 | -0.100887184 | 0.0008375 | 0.21 |
| SLC26A4 | -0.130139184 | 1.59E-05 | -0.124332165 | 3.76E-05 | 0.01 |
| SLC26A5 | -0.302691317 | 1.36E-24 | -0.092748092 | 0.0021448 | 0.21 |
| DKFZP586E1422 | 0.117207796 | 0.00010279 | 0.075578472 | 0.012441 | 0.05 |
| SLC26A7 | 0.109122192 | 0.00030115 | -0.010897899 | 0.7189302 | 0.13 |
| SLC26A8 | 0.025721475 | 0.39558441 | 0.141003744 | 2.87E-06 | 0.12 |
| SLC26A9 | 0.323444662 | 4.91E-28 | 0.089538138 | 0.0030491 | 0.24 |
| SLC27A1 | -0.27206152 | 5.31E-20 | -0.065017937 | 0.0316072 | 0.21 |
| SLC27A2 | -0.180518979 | 1.85E-09 | -0.11653212 | 0.0001128 | 0.07 |
| MGC4365 | -0.053352088 | 0.07788584 | 0.070532504 | 0.0196965 | 0.13 |
| IPS | 0.086339326 | 0.00428347 | -0.014335117 | 0.6359215 | 0.11 |
| SLC27A5 | 0.200135336 | 2.44E-11 | 0.233278686 | 5.65E-15 | 0.04 |
| SLC27A6 | 0.052249611 | 0.08423885 | -0.094959854 | 0.0016726 | 0.15 |
| SLC28A1 | 0.286229887 | 4.69E-22 | 0.069293427 | 0.0219627 | 0.22 |
| SLC28A2 | -0.015877364 | 0.60003734 | -0.045588549 | 0.1320053 | 0.03 |
| SLC28A3 | -0.157663713 | 1.61E-07 | -0.17552782 | 5.16E-09 | 0.02 |
| LOC220811 | -0.114577729 | 0.00014694 | -0.073936846 | 0.0144875 | 0.05 |
| SLC29A2 | 0.205990903 | 6.14E-12 | 0.150842537 | 5.45E-07 | 0.06 |
| HJCD | -0.137634024 | 4.95E-06 | -0.048770965 | 0.1070688 | 0.09 |
| SLC29A4 | 0.178338433 | 2.91E-09 | 0.127966608 | 2.20E-05 | 0.06 |
| ATS | -0.447075994 | 8.00E-55 | -0.204352314 | 9.08E-12 | 0.25 |
| SLC2A11 | -0.094194239 | 0.00182402 | -0.133831372 | 9.02E-06 | 0.04 |
| SLC2A12 | 0.152118632 | 4.35E-07 | 0.013447558 | 0.6569739 | 0.14 |
| SLC2A13 | -0.338525935 | 1.04E-30 | -0.227645656 | 2.58E-14 | 0.12 |
| SLC2A14 | 0.011352356 | 0.70773649 | -0.159048794 | 1.25E-07 | 0.18 |
| SLC2A1 | 0.182112567 | 1.32E-09 | -0.04967345 | 0.1007215 | 0.24 |
| SLC2A2 | -0.029534854 | 0.32929541 | -0.02474657 | 0.4137432 | 0.01 |
| SLC2A3 | -0.073646817 | 0.01487838 | -0.19136061 | 1.79E-10 | 0.12 |
| SLC2A4RG | 0.200182925 | 2.42E-11 | 0.212827883 | 1.16E-12 | 0.02 |
| SLC2A4 | -0.176344986 | 4.37E-09 | -0.143431304 | 1.93E-06 | 0.04 |
| SLC2A5 | 0.113846822 | 0.00016207 | -0.055493938 | 0.0666578 | 0.17 |
| SLC2A6 | 0.27210601 | 5.24E-20 | 0.027781878 | 0.3588229 | 0.25 |
| SLC2A7 | 0.014076818 | 0.64201892 | -0.002146879 | 0.9434806 | 0.02 |
| SLC2A8 | 0.012569828 | 0.67806406 | 0.065233576 | 0.0310456 | 0.06 |
| SLC2A9 | -0.131680883 | 1.26E-05 | -0.120140693 | 6.84E-05 | 0.02 |
| SLC30A10 | 0.26250588 | 1.11E-18 | 0.144624591 | 1.58E-06 | 0.12 |
| SLC30A1 | -0.403926577 | 3.78E-44 | -0.169210002 | 1.82E-08 | 0.24 |
| MGC11303 | -0.030197676 | 0.31855248 | -0.027475202 | 0.3641545 | 0.01 |
| LOC63075 | 0.179950284 | 2.08E-09 | 0.08406272 | 0.0054208 | 0.1 |
| SLC30A4 | -0.445560942 | 2.02E-54 | -0.466855624 | 2.95E-60 | 0.03 |
| MGC5499 | -0.367554496 | 2.70E-36 | -0.160025413 | 1.04E-07 | 0.21 |
| FLJ31101 | -0.221935544 | 1.16E-13 | -0.273228274 | 3.64E-20 | 0.06 |
| SLC30A7 | -0.261904558 | 1.33E-18 | -0.160473187 | 9.61E-08 | 0.11 |
| SLC30A8 | -0.228196555 | 2.23E-14 | -0.041250185 | 0.1729539 | 0.19 |
| SLC30A9 | -0.190132358 | 2.35E-10 | -0.150612789 | 5.67E-07 | 0.04 |
| SLC31A1 | -0.04952697 | 0.10173087 | -0.058724428 | 0.0522685 | 0.01 |
| SLC31A2 | -0.186292203 | 5.43E-10 | -0.121669728 | 5.51E-05 | 0.07 |
| SLC32A1 | 0.066088663 | 0.02890231 | -0.054132919 | 0.0736264 | 0.13 |
| SLC33A1 | -0.113090339 | 0.00017926 | -0.14557543 | 1.34E-06 | 0.04 |
| SLC34A1 | 0.107335327 | 0.0003783 | 0.098776523 | 0.001076 | 0.01 |
| LOC133217 | 0.094442989 | 0.0017735 | 0.018774198 | 0.5352382 | 0.08 |
| SLC34A3 | -0.080777135 | 0.00754348 | 0.000585372 | 0.9845774 | 0.09 |
| SLC35A1 | -0.121067132 | 6.00E-05 | 0.058479933 | 0.0532583 | 0.18 |
| SLC35A2 | 0.170092401 | 1.53E-08 | 0.176581593 | 4.17E-09 | 0.01 |
| SLC35A3 | -0.436071114 | 5.94E-52 | -0.263538876 | 8.01E-19 | 0.18 |
| SLC35A4 | -0.089915286 | 0.00292725 | 0.032552545 | 0.282259 | 0.13 |
| LOC131453 | -0.282738028 | 1.54E-21 | -0.229222401 | 1.69E-14 | 0.06 |
| SLC35B1 | 0.301156374 | 2.38E-24 | 0.278079604 | 7.37E-21 | 0.03 |
| LOC221413 | 0.219859756 | 1.97E-13 | 0.148022863 | 8.87E-07 | 0.08 |
| SLC35B3 | -0.151345735 | 4.99E-07 | 0.064793721 | 0.0322002 | 0.22 |
| SLC35B4 | -0.017194098 | 0.57014505 | -0.096927537 | 0.0013349 | 0.08 |
| SLC35C1 | 0.155576883 | 2.35E-07 | 0.042022167 | 0.1650458 | 0.12 |
| SLC35C2 | 0.195542807 | 7.01E-11 | 0.173065475 | 8.48E-09 | 0.03 |
| SLC35D1 | -0.407635549 | 5.24E-45 | -0.312294856 | 3.75E-26 | 0.1 |
| SLC35D2 | 0.019716436 | 0.5149473 | 0.106232696 | 0.0004347 | 0.09 |
| FRCL1 | -0.052317253 | 0.08383736 | -0.068365565 | 0.023805 | 0.02 |
| SLC35E1 | -0.207217953 | 4.58E-12 | -0.261031148 | 1.75E-18 | 0.06 |
| SLC35E2B | -0.303712674 | 9.32E-25 | -0.256888762 | 6.22E-18 | 0.05 |
| LOC199993 | -0.394989909 | 3.97E-42 | -0.422951691 | 1.15E-48 | 0.03 |
| SLC35E3 | -0.216526642 | 4.61E-13 | -0.201850668 | 1.64E-11 | 0.02 |
| LOC101929728 | 0.146620442 | 1.13E-06 | -0.02895413 | 0.3388981 | 0.18 |
| SLC35F1 | -0.049436176 | 0.10236054 | -0.060790652 | 0.044502 | 0.02 |
| FLJ13018 | 0.270047095 | 1.02E-19 | 0.081293586 | 0.0071669 | 0.19 |
| SLC35F3 | 0.069780308 | 0.02104655 | -0.05213959 | 0.0848952 | 0.13 |
| SLC35F4 | -0.055007859 | 0.06908161 | -0.077587139 | 0.0102877 | 0.03 |
| SLC35F5 | -0.417521951 | 2.40E-47 | -0.239855203 | 9.11E-16 | 0.18 |
| LOC91974 | -0.075100981 | 0.01300813 | -0.179965508 | 2.07E-09 | 0.11 |
| SLC36A2 | 0.127355833 | 2.41E-05 | -0.007469169 | 0.8051747 | 0.14 |
| SLC36A3 | -0.008700882 | 0.77385794 | 0.038779354 | 0.200165 | 0.05 |
| SLC36A4 | 0.241684625 | 5.43E-16 | -0.007105548 | 0.8144823 | 0.25 |
| SLC37A1 | -0.103802271 | 0.00058793 | 0.035063583 | 0.2467616 | 0.14 |
| SLC37A2 | -0.233305339 | 5.61E-15 | -0.217978916 | 3.19E-13 | 0.02 |
| SLC37A3 | -0.061101632 | 0.04342185 | -0.024564698 | 0.4171845 | 0.04 |
| G6PT2 | 0.328528292 | 6.39E-29 | 0.318680393 | 3.20E-27 | 0.01 |
| SLC38A10 | -0.027601498 | 0.36195286 | -0.060022102 | 0.047269 | 0.04 |
| SLC38A11 | -0.390311871 | 4.30E-41 | -0.203626834 | 1.08E-11 | 0.19 |
| SLC38A1 | -0.211976691 | 1.43E-12 | -0.140541669 | 3.10E-06 | 0.08 |
| PRO1068 | -0.224725919 | 5.59E-14 | -0.225777077 | 4.24E-14 | 0.01 |
| SLC38A3 | 0.230135208 | 1.33E-14 | 0.114785053 | 0.0001429 | 0.12 |
| SLC38A4 | -0.275119061 | 1.96E-20 | -0.114628541 | 0.0001459 | 0.17 |
| SLC38A5 | 0.054953136 | 0.06935895 | 0.044912052 | 0.1378442 | 0.02 |
| SLC38A6 | -0.126162733 | 2.88E-05 | 0.132357999 | 1.13E-05 | 0.26 |
| SLC38A7 | -0.195647607 | 6.85E-11 | -0.335904911 | 3.10E-30 | 0.15 |
| LOC648732 | 0.141097761 | 2.83E-06 | 0.068152588 | 0.0242463 | 0.08 |
| SLC38A9 | -0.152331509 | 4.19E-07 | 0.002418163 | 0.936353 | 0.16 |
| SLC39A10 | -0.217565627 | 3.54E-13 | -0.038962848 | 0.1980427 | 0.18 |
| SLC39A11 | -0.07524291 | 0.01283722 | 0.173292484 | 8.10E-09 | 0.25 |
| SLC39A12 | 0.22028719 | 1.77E-13 | 0.063049011 | 0.0371489 | 0.16 |
| SLC39A13 | -0.048613436 | 0.10820851 | -0.051738111 | 0.0873254 | 0.01 |
| SLC39A14 | -0.084958552 | 0.00494422 | -0.211474317 | 1.62E-12 | 0.13 |
| SLC39A1 | 0.109045227 | 0.00030414 | 0.247462545 | 1.03E-16 | 0.14 |
| SLC39A2 | 0.083468632 | 0.00575928 | 0.062124287 | 0.0400244 | 0.03 |
| SLC39A3 | 0.235608017 | 2.98E-15 | 0.212244469 | 1.34E-12 | 0.03 |
| AEZ | 0.373017525 | 2.06E-37 | 0.288922978 | 1.85E-22 | 0.09 |
| SLC39A5 | -0.047624682 | 0.11558238 | -0.0199524 | 0.5099291 | 0.03 |
| SLC39A6 | -0.294451724 | 2.65E-23 | -0.152075091 | 4.39E-07 | 0.15 |
| SLC39A7 | 0.109238886 | 0.00029666 | 0.099187855 | 0.0010251 | 0.02 |
| SLC39A8 | 0.014202934 | 0.63903876 | -0.084847509 | 0.0050012 | 0.1 |
| SLC39A9 | -0.343811566 | 1.10E-31 | -0.115003937 | 0.0001387 | 0.23 |
| SLC3A1 | 0.001863069 | 0.95094218 | 0.07076583 | 0.0192933 | 0.07 |
| SLC3A2 | 0.328921576 | 5.45E-29 | 0.156501127 | 1.99E-07 | 0.18 |
| SLC11A3 | -0.422500189 | 1.49E-48 | -0.141122033 | 2.82E-06 | 0.29 |
| SLC41A1 | 0.042712385 | 0.1582083 | 0.09798408 | 0.0011807 | 0.06 |
| SLC41A2 | -0.243353695 | 3.37E-16 | -0.234597849 | 3.94E-15 | 0.01 |
| SLC41A3 | 0.20044584 | 2.27E-11 | 0.088698296 | 0.0033371 | 0.12 |
| SLC43A1 | -0.176225511 | 4.48E-09 | -0.033768606 | 0.2646579 | 0.15 |
| SLC43A2 | 0.048313877 | 0.1104022 | -0.036541126 | 0.2273973 | 0.09 |
| LOC144038 | 0.279150009 | 5.16E-21 | 0.078018922 | 0.0098706 | 0.21 |
| SLC44A1 | -0.273295195 | 3.56E-20 | -0.099317578 | 0.0010095 | 0.18 |
| SLC44A2 | 0.10777396 | 0.00035781 | -0.031195414 | 0.3028184 | 0.14 |
| SLC44A3 | -0.151240126 | 5.08E-07 | -0.008200797 | 0.7865316 | 0.15 |
| LOC87892 | -0.44645919 | 1.17E-54 | -0.105960331 | 0.0004498 | 0.35 |
| SLC44A5 | -0.235802635 | 2.82E-15 | -0.029354552 | 0.3322578 | 0.21 |
| SLC45A1 | -0.234961574 | 3.56E-15 | -0.145757926 | 1.30E-06 | 0.09 |
| SLC45A2 | -0.095434796 | 0.00158459 | -0.055395418 | 0.0671434 | 0.05 |
| SLC45A3 | 0.083854384 | 0.00553739 | 0.116915286 | 0.000107 | 0.04 |
| SLC45A4 | 0.115439756 | 0.00013081 | 0.023916525 | 0.4295852 | 0.1 |
| SLC46A1 | -0.326196791 | 1.64E-28 | -0.122452979 | 4.93E-05 | 0.21 |
| SLC46A2 | -0.295758864 | 1.67E-23 | -0.16224452 | 6.91E-08 | 0.14 |
| SLC46A3 | -0.355452029 | 6.76E-34 | -0.265482405 | 4.35E-19 | 0.09 |
| SLC47A1 | -0.08891076 | 0.00326202 | -0.14624379 | 1.20E-06 | 0.06 |
| SLC47A2 | -0.120143667 | 6.84E-05 | -0.132136763 | 1.17E-05 | 0.02 |
| SLC48A1 | -0.169784923 | 1.63E-08 | -0.056551602 | 0.0616251 | 0.12 |
| SLC4A10 | -0.061001656 | 0.04376667 | -0.116305562 | 0.0001163 | 0.06 |
| CDPD1 | 0.129538383 | 1.74E-05 | 0.105910811 | 0.0004526 | 0.03 |
| SLC4A1AP | 0.260803841 | 1.87E-18 | 0.012087843 | 0.6897547 | 0.25 |
| DI | -0.057605131 | 0.05692852 | -0.138320052 | 4.44E-06 | 0.09 |
| SLC4A2 | 0.037298104 | 0.21790665 | 0.031454299 | 0.2988216 | 0.01 |
| SLC4A3 | 0.101687742 | 0.00076063 | -0.04132287 | 0.1721975 | 0.15 |
| SLC4A4 | -0.193548012 | 1.10E-10 | -0.205144983 | 7.52E-12 | 0.02 |
| SLC4A5 | 0.342316697 | 2.08E-31 | 0.298619872 | 5.97E-24 | 0.05 |
| SLC4A6 | -0.378597927 | 1.41E-38 | -0.205694372 | 6.60E-12 | 0.18 |
| SLC4A8 | -0.384812281 | 6.73E-40 | -0.152350931 | 4.18E-07 | 0.24 |
| SLC4A9 | 0.103250479 | 0.00062909 | 0.062173118 | 0.039868 | 0.05 |
| SLC5A10 | 0.066086734 | 0.028907 | 0.026473789 | 0.381906 | 0.04 |
| SLC5A11 | 0.081860559 | 0.006773 | 0.043584884 | 0.1498743 | 0.04 |
| SLC5A12 | 0.041676827 | 0.1685492 | -0.001526404 | 0.9597988 | 0.05 |
| SLC5A1 | -0.049339602 | 0.10303369 | 0.048023551 | 0.1125616 | 0.1 |
| SLC5A2 | -0.044101738 | 0.14509752 | 0.002604049 | 0.931472 | 0.05 |
| SLC5A3 | -0.137969655 | 4.69E-06 | -0.187976897 | 3.77E-10 | 0.06 |
| SAAT1 | -0.46083842 | 1.45E-58 | -0.307482155 | 2.30E-25 | 0.16 |
| SLC5A5 | 0.121879187 | 5.35E-05 | 0.040964193 | 0.1759543 | 0.09 |
| SLC5A6 | 0.366548592 | 4.31E-36 | 0.239661395 | 9.62E-16 | 0.13 |
| SLC5A7 | -0.160759238 | 9.12E-08 | -0.103820621 | 0.0005866 | 0.06 |
| SLC5A8 | -0.020115127 | 0.50648331 | -0.031944878 | 0.2913448 | 0.02 |
| SLC5A9 | -0.168616576 | 2.04E-08 | -0.138360961 | 4.41E-06 | 0.04 |
| SLC6A10P | 0.114754797 | 0.00014349 | 0.012302866 | 0.6845299 | 0.11 |
| SLC6A11 | 0.253627424 | 1.66E-17 | 0.079520237 | 0.008535 | 0.18 |
| SLC6A12 | -0.011464953 | 0.70497275 | -0.137400602 | 5.14E-06 | 0.13 |
| HSU76343 | 0.097299549 | 0.0012786 | -0.006637001 | 0.8265143 | 0.11 |
| OQTLX | 0.158058165 | 1.50E-07 | 0.046530469 | 0.1241965 | 0.12 |
| SLC6A15 | 0.283921615 | 1.03E-21 | 0.044275604 | 0.1435171 | 0.24 |
| LOC95514 | 0.183577247 | 9.70E-10 | 0.117939747 | 9.29E-05 | 0.07 |
| LOC101928619 | 0.167238672 | 2.67E-08 | -0.039620681 | 0.1905694 | 0.21 |
| SLC6A18 | 0.033463726 | 0.26899806 | -0.037425887 | 0.216333 | 0.08 |
| HND | 0.19570629 | 6.76E-11 | 0.048823665 | 0.1066897 | 0.15 |
| SLC6A1 | -0.26231813 | 1.17E-18 | -0.135993354 | 6.43E-06 | 0.13 |
| LOC56960 | -0.080115046 | 0.0080521 | -0.038584393 | 0.202438 | 0.05 |
| SLC6A2 | 0.194377965 | 9.12E-11 | -0.060054851 | 0.0471482 | 0.26 |
| SLC6A3 | -0.076815454 | 0.01107227 | -0.037948798 | 0.2099786 | 0.04 |
| SLC6A4 | -0.172833864 | 8.88E-09 | -0.01706492 | 0.573046 | 0.16 |
| SLC6A5 | 0.008195155 | 0.78667496 | -0.014963269 | 0.6211963 | 0.03 |
| SLC6A6 | -0.309529068 | 1.07E-25 | -0.347847305 | 1.93E-32 | 0.04 |
| SLC6A7 | 0.044102002 | 0.14509511 | 0.022694592 | 0.4535356 | 0.03 |
| SLC6A8 | 0.27049928 | 8.80E-20 | 0.13495358 | 7.57E-06 | 0.14 |
| SLC6A9 | 0.120026813 | 6.95E-05 | 0.005145546 | 0.8650722 | 0.12 |
| FLJ20839 | -0.057023339 | 0.05948394 | -0.047589071 | 0.1158551 | 0.01 |
| SLC7A11 | 0.086359295 | 0.00427453 | -0.071633857 | 0.0178561 | 0.16 |
| SLC7A13 | -0.23971537 | 9.48E-16 | 0.007425957 | 0.8062794 | 0.25 |
| FLJ37228 | -0.08315923 | 0.005943 | -0.118533393 | 8.56E-05 | 0.04 |
| SLC7A1 | 0.157069319 | 1.80E-07 | -0.045165285 | 0.1356357 | 0.21 |
| SLC7A2 | -0.348452686 | 1.48E-32 | -0.12194907 | 5.30E-05 | 0.23 |
| SLC7A3 | -0.208171696 | 3.64E-12 | -0.140355599 | 3.19E-06 | 0.07 |
| SLC7A4 | -0.241525251 | 5.68E-16 | -0.097485497 | 0.0012513 | 0.15 |
| SLC7A5P1 | 0.06836422 | 0.02380777 | -0.04962942 | 0.1010241 | 0.12 |
| SLC7A5P2 | 0.082454312 | 0.00638141 | 0.035353775 | 0.2428701 | 0.05 |
| SLC7A5 | 0.423735366 | 7.40E-49 | 0.154306645 | 2.95E-07 | 0.27 |
| SLC7A6OS | -0.006317385 | 0.83474541 | -0.18156604 | 1.48E-09 | 0.18 |
| SLC7A6 | -0.018857832 | 0.53342102 | -0.10170559 | 0.000759 | 0.09 |
| SLC7A7 | -0.01892058 | 0.53205968 | -0.118091941 | 9.10E-05 | 0.1 |
| SLC7A8 | -0.44687624 | 9.04E-55 | -0.167621309 | 2.48E-08 | 0.28 |
| SLC7A9 | 0.089916086 | 0.002927 | 0.057327537 | 0.0581362 | 0.04 |
| SLC8A1 | -0.346841188 | 2.98E-32 | -0.377657408 | 2.23E-38 | 0.04 |
| SLC8A2 | -0.050920497 | 0.09244771 | -0.099038279 | 0.0010433 | 0.05 |
| SLC8A3 | -0.286855759 | 3.78E-22 | -0.303293813 | 1.09E-24 | 0.02 |
| SLC9C1 | -0.132048589 | 1.19E-05 | -0.125414117 | 3.21E-05 | 0.01 |
| SLC9C2 | 0.180102927 | 2.02E-09 | 0.094337379 | 0.0017948 | 0.09 |
| SLC9A1 | -0.358329476 | 1.86E-34 | -0.209982912 | 2.34E-12 | 0.15 |
| SLC9A2 | -0.06110695 | 0.04340357 | -0.014351466 | 0.6355364 | 0.05 |
| SLC9A3R1 | -0.133250443 | 9.87E-06 | -0.002140354 | 0.9436521 | 0.14 |
| SLC9A3R2 | -0.113111843 | 0.00017875 | 0.062724228 | 0.0381382 | 0.18 |
| SLC9A3 | -0.06814165 | 0.02426914 | -0.113071983 | 0.0001797 | 0.05 |
| SLC9A4 | 0.047852594 | 0.11384861 | -0.054469074 | 0.0718522 | 0.11 |
| SLC9A5 | -0.007828619 | 0.79600078 | 0.033422089 | 0.2695945 | 0.05 |
| SLC9A6 | 0.08094355 | 0.00742025 | -0.198862556 | 3.28E-11 | 0.28 |
| SLC9A7 | -0.314280248 | 1.76E-26 | -0.285140252 | 6.82E-22 | 0.03 |
| SLC9A8 | -0.26429202 | 6.33E-19 | -0.215456138 | 6.04E-13 | 0.05 |
| SLC9A9 | -0.215368578 | 6.17E-13 | -0.268846365 | 1.50E-19 | 0.06 |
| OATP.A | 0.108106583 | 0.00034297 | -0.022707959 | 0.4532696 | 0.14 |
| SLCO1B1 | 0.057109299 | 0.0591005 | 0.002659996 | 0.9300035 | 0.06 |
| SLCO1B3 | 0.139789448 | 3.50E-06 | 0.007481594 | 0.8048571 | 0.14 |
| SLCO1C1 | -0.327891291 | 8.27E-29 | -0.229309342 | 1.66E-14 | 0.1 |
| SLCO2A1 | -0.346833474 | 2.99E-32 | -0.225784775 | 4.23E-14 | 0.13 |
| SLCO2B1 | -0.307037987 | 2.72E-25 | -0.341212135 | 3.33E-31 | 0.04 |
| SLCO3A1 | -0.117559144 | 9.79E-05 | -0.259608114 | 2.71E-18 | 0.15 |
| SLCO4A1 | 0.203593713 | 1.09E-11 | -0.015837632 | 0.6009502 | 0.22 |
| PRO2176 | 0.150052929 | 6.25E-07 | -0.001978823 | 0.9478984 | 0.16 |
| SLCO5A1 | 0.272863493 | 4.09E-20 | 0.041194868 | 0.1735313 | 0.24 |
| SLCO6A1 | 0.11732251 | 0.00010118 | 0.058927414 | 0.0514585 | 0.06 |
| LOC649464 | 0.153321981 | 3.52E-07 | 0.062505064 | 0.0388183 | 0.1 |
| SLFN11 | -0.116673055 | 0.0001106 | -0.1890952 | 2.95E-10 | 0.08 |
| SLFN12L | -0.132454439 | 1.12E-05 | -0.228266657 | 2.19E-14 | 0.1 |
| SLFN12 | -0.239926585 | 8.93E-16 | -0.198951867 | 3.21E-11 | 0.05 |
| SLFN13 | -9.90E-05 | 0.99739143 | -0.014537609 | 0.6311586 | 0.02 |
| SLFN14 | -0.122517648 | 4.88E-05 | -0.169460123 | 1.73E-08 | 0.05 |
| SLFN5 | -0.292677154 | 4.97E-23 | -0.289262966 | 1.64E-22 | 0.01 |
| SLFNL1 | 0.162862004 | 6.15E-08 | 0.068949974 | 0.0226297 | 0.1 |
| SLIT1 | 0.017547264 | 0.5622498 | -0.118540639 | 8.55E-05 | 0.14 |
| SLIT2 | -0.493519104 | 3.63E-68 | -0.439214972 | 9.22E-53 | 0.06 |
| SLIT3 | -0.452636535 | 2.58E-56 | -0.316312745 | 8.03E-27 | 0.14 |
| SLITRK1 | 0.063191997 | 0.03672036 | -0.02051552 | 0.4980573 | 0.09 |
| SLITRK2 | 0.012059045 | 0.69045553 | -0.07286171 | 0.0159834 | 0.09 |
| SLITRK3 | -0.2128794 | 1.15E-12 | -0.124025976 | 3.93E-05 | 0.09 |
| SLITRK4 | -0.090597563 | 0.00271807 | -0.108530186 | 0.0003249 | 0.02 |
| SLITRK5 | 0.101192811 | 0.00080734 | 0.051592074 | 0.0882231 | 0.05 |
| SLITRK6 | -0.200697061 | 2.14E-11 | -0.011219765 | 0.710996 | 0.19 |
| SLK | -0.383705105 | 1.16E-39 | -0.348617017 | 1.38E-32 | 0.04 |
| SLMAP | -0.379067962 | 1.13E-38 | -0.254121852 | 1.44E-17 | 0.13 |
| SLMO1 | 0.441730211 | 2.05E-53 | 0.132961635 | 1.03E-05 | 0.31 |
| SLMO2 | 0.232861819 | 6.33E-15 | 0.130491592 | 1.51E-05 | 0.11 |
| SLN | 0.077386122 | 0.01048714 | 0.004875327 | 0.8720954 | 0.08 |
| SLPI | 0.129199542 | 1.83E-05 | 0.065719198 | 0.0298123 | 0.07 |
| FLJ10005 | -0.25219553 | 2.55E-17 | -0.280832099 | 2.94E-21 | 0.03 |
| SLU7 | -0.273007588 | 3.91E-20 | -0.118469699 | 8.64E-05 | 0.16 |
| MDM | 0.305169783 | 5.44E-25 | 0.174211135 | 6.74E-09 | 0.14 |
| DKFZP586M0622 | -0.237399288 | 1.81E-15 | -0.151941879 | 4.49E-07 | 0.09 |
| LOC654050 | 0.196290088 | 5.92E-11 | -0.003129767 | 0.9176823 | 0.2 |
| HSPC193 | -0.28792587 | 2.61E-22 | -0.310098838 | 8.62E-26 | 0.03 |
| SMAD4 | -0.055934282 | 0.06452289 | -0.17973244 | 2.18E-09 | 0.13 |
| SMAD5.AS1 | 0.146835715 | 1.09E-06 | 0.093124871 | 0.0020566 | 0.06 |
| SMAD5 | -0.376281562 | 4.33E-38 | -0.200160253 | 2.43E-11 | 0.18 |
| SMAD6 | -0.095581148 | 0.00155834 | -0.100224372 | 0.0009065 | 0.01 |
| SMAD7 | -0.086232639 | 0.00433152 | -0.248075319 | 8.61E-17 | 0.17 |
| SMAD9 | -0.293140761 | 4.22E-23 | -0.34647735 | 3.49E-32 | 0.06 |
| SMAGP | 0.33038941 | 3.00E-29 | 0.144368119 | 1.65E-06 | 0.19 |
| LOC642967 | 0.244499735 | 2.43E-16 | 0.125022287 | 3.40E-05 | 0.12 |
| SMAP2 | -0.223078912 | 8.59E-14 | -0.225958923 | 4.04E-14 | 0.01 |
| SMARCA1 | -0.147807963 | 9.20E-07 | -0.114394004 | 0.0001506 | 0.04 |
| SMARCA2 | -0.396653704 | 1.69E-42 | -0.339770379 | 6.13E-31 | 0.06 |
| SMARCA4 | 0.284567018 | 8.29E-22 | 0.126743988 | 2.64E-05 | 0.16 |
| SMARCA5 | -0.150737519 | 5.55E-07 | -0.2712322 | 6.95E-20 | 0.13 |
| SMARCAD1 | -0.283386643 | 1.24E-21 | -0.255734658 | 8.83E-18 | 0.03 |
| SMARCAL1 | 0.055453611 | 0.06685621 | 0.06429852 | 0.0335439 | 0.01 |
| SMARCB1 | 0.366103335 | 5.30E-36 | 0.20196779 | 1.59E-11 | 0.17 |
| SMARCC1 | 0.052602071 | 0.08216377 | 0.046687445 | 0.1229308 | 0.01 |
| LOC121127 | -0.264996182 | 5.07E-19 | -0.149576516 | 6.79E-07 | 0.12 |
| SMARCD1 | 0.118657084 | 8.41E-05 | 0.088850884 | 0.003283 | 0.03 |
| SMARCD2 | 0.293178499 | 4.17E-23 | 0.210759584 | 1.94E-12 | 0.09 |
| SMARCD3 | -0.002201565 | 0.94204346 | 0.019118983 | 0.5277669 | 0.03 |
| SMARCE1 | 0.045037274 | 0.13674868 | 0.153907729 | 3.17E-07 | 0.11 |
| SMC1A | 0.017425453 | 0.564967 | -0.154820429 | 2.69E-07 | 0.18 |
| Smc1l2 | 0.325057314 | 2.58E-28 | 0.142112815 | 2.39E-06 | 0.19 |
| SMC2 | 0.092865956 | 0.00211684 | -0.067672975 | 0.025266 | 0.17 |
| SMC3 | 0.006826942 | 0.82163168 | -0.144310465 | 1.66E-06 | 0.16 |
| SMC4 | 0.267597757 | 2.23E-19 | 0.115943586 | 0.0001222 | 0.16 |
| SMC5 | -0.165515726 | 3.72E-08 | -0.202155722 | 1.53E-11 | 0.04 |
| SMC6 | 0.193555024 | 1.10E-10 | 0.021786451 | 0.4718124 | 0.18 |
| SMCHD1 | -0.001120612 | 0.97048048 | -0.25737562 | 5.37E-18 | 0.26 |
| SMCP | 0.073507491 | 0.01506942 | 0.066660894 | 0.0275401 | 0.01 |
| SMCR5 | -0.090353656 | 0.00279123 | -0.076434417 | 0.0114789 | 0.02 |
| MIEF1 | -0.013806222 | 0.64843259 | -0.14869145 | 7.91E-07 | 0.14 |
| SMCR7 | -0.216276767 | 4.91E-13 | -0.069908742 | 0.0208105 | 0.15 |
| LOC162633 | -0.465017756 | 9.78E-60 | -0.402963012 | 6.28E-44 | 0.07 |
| SMEK1 | -0.106978543 | 0.00039576 | -0.054393664 | 0.0722471 | 0.06 |
| SMEK2 | 0.004638314 | 0.87826376 | -0.100707124 | 0.0008558 | 0.11 |
| SMEK3P | 0.084073526 | 0.0054148 | 0.042273504 | 0.1625307 | 0.05 |
| SMG1 | -0.298822668 | 5.55E-24 | -0.351965505 | 3.18E-33 | 0.06 |
| SMG5 | 0.256534351 | 6.93E-18 | 0.177681874 | 3.33E-09 | 0.08 |
| FLJ14069 | -0.327166308 | 1.11E-28 | -0.346184746 | 3.96E-32 | 0.02 |
| SMG7 | 0.068124507 | 0.02430499 | 0.229816174 | 1.44E-14 | 0.17 |
| LOC100133836 | 0.007719325 | 0.79878728 | 0.034326626 | 0.2568395 | 0.03 |
| LOC112600 | 0.227559667 | 2.64E-14 | 0.265718245 | 4.04E-19 | 0.04 |
| SMNDC1 | 0.290440399 | 1.09E-22 | 0.153821472 | 3.22E-07 | 0.14 |
| SMOC1 | 0.2111762 | 1.75E-12 | -0.00893936 | 0.7678351 | 0.23 |
| SMOC2 | -0.376712507 | 3.52E-38 | -0.228215359 | 2.22E-14 | 0.15 |
| SMOX | 0.289484322 | 1.52E-22 | 0.041302225 | 0.1724121 | 0.25 |
| SMO | 0.223682615 | 7.34E-14 | 0.09303598 | 0.0020771 | 0.14 |
| SMPD1 | -0.362650201 | 2.60E-35 | -0.207185709 | 4.61E-12 | 0.16 |
| SMPD2 | 0.133509273 | 9.48E-06 | 0.246149828 | 1.51E-16 | 0.12 |
| FLJ22593 | -0.281004224 | 2.77E-21 | -0.228361884 | 2.13E-14 | 0.06 |
| FLJ20756 | 0.212586195 | 1.23E-12 | 0.100146733 | 0.0009149 | 0.12 |
| SMPDL3A | -0.223351045 | 8.01E-14 | -0.139957527 | 3.41E-06 | 0.09 |
| SMPDL3B | 0.210910196 | 1.87E-12 | 0.153687146 | 3.30E-07 | 0.06 |
| SMPX | 0.223755251 | 7.20E-14 | 0.047071807 | 0.1198743 | 0.18 |
| SMR3A | 0.066478768 | 0.02796755 | 0.060537102 | 0.0453994 | 0.01 |
| SMR3B | -0.025444507 | 0.40069372 | 0.018023703 | 0.551683 | 0.05 |
| SMS | 0.38379653 | 1.11E-39 | 0.261742876 | 1.40E-18 | 0.13 |
| SMTNL1 | 0.085329904 | 0.00475802 | 0.068843711 | 0.0228396 | 0.02 |
| SMTNL2 | 0.203929227 | 1.00E-11 | 0.018595857 | 0.5391235 | 0.19 |
| SMTN | 0.053568163 | 0.07668756 | 0.021907541 | 0.4693522 | 0.04 |
| LOC728623 | 0.11268167 | 0.00018925 | 0.10451594 | 0.0005384 | 0.01 |
| SMUG1 | 0.136105467 | 6.31E-06 | 0.288474651 | 2.16E-22 | 0.16 |
| LOC729290 | -0.16171844 | 7.62E-08 | -0.29015763 | 1.20E-22 | 0.13 |
| SMURF2 | 0.028872964 | 0.34025442 | -0.133787387 | 9.08E-06 | 0.17 |
| SMYD1 | -0.03802496 | 0.20906452 | -0.074193349 | 0.0141494 | 0.04 |
| SMYD2 | 0.211182156 | 1.74E-12 | 0.319689295 | 2.16E-27 | 0.11 |
| ZNFN3A1 | -0.025407576 | 0.40137797 | 0.255560272 | 9.31E-18 | 0.29 |
| SMYD4 | -0.253553391 | 1.70E-17 | -0.260270619 | 2.21E-18 | 0.01 |
| SMYD5 | 0.404625531 | 2.61E-44 | 0.319373024 | 2.44E-27 | 0.09 |
| LOC116141 | 0.193202901 | 1.19E-10 | 0.014456771 | 0.6330582 | 0.18 |
| SNAI2 | -0.142476554 | 2.26E-06 | -0.127751003 | 2.27E-05 | 0.02 |
| SNAI3 | -0.09575101 | 0.00152836 | -0.177940925 | 3.15E-09 | 0.09 |
| SNAP23 | -0.463661434 | 2.36E-59 | -0.197182787 | 4.83E-11 | 0.27 |
| SNAP25 | 0.003560568 | 0.90640045 | -0.146365288 | 1.18E-06 | 0.15 |
| SNAP29 | -0.196004695 | 6.31E-11 | -0.044790221 | 0.1389166 | 0.16 |
| LOC100130093.1 | -0.228383441 | 2.12E-14 | -0.022069663 | 0.4660695 | 0.21 |
| SNAP91 | 0.057317474 | 0.05818037 | -0.093283389 | 0.0020205 | 0.16 |
| SNAPC1 | 0.207226184 | 4.57E-12 | 0.133193257 | 9.96E-06 | 0.08 |
| SNAPC2 | -0.003232246 | 0.91499698 | 0.002792393 | 0.9265291 | 0.01 |
| SNAPC3 | -0.09039329 | 0.00277922 | -0.027778755 | 0.358877 | 0.07 |
| FLJ13451 | 0.087912189 | 0.00362897 | 0.05205949 | 0.0853756 | 0.04 |
| SNAPC5 | 0.054023143 | 0.07421348 | -0.048225806 | 0.1110538 | 0.11 |
| SNAPIN | 0.101369261 | 0.00079039 | 0.439466441 | 7.93E-53 | 0.34 |
| SNAR.A13 | 0.034332352 | 0.25676014 | 0.001958403 | 0.9484353 | 0.04 |
| SNAR.A2 | 0.041908795 | 0.16618988 | 0.020613661 | 0.4960034 | 0.03 |
| SNAR.A3 | NA | NA | NA | NA | #VALUE! |
| SNAR.A4 | NA | NA | NA | NA | #VALUE! |
| SNAR.B2 | 0.001320661 | 0.96521381 | 0.010987039 | 0.7167297 | 0.01 |
| SNAR.C2 | NA | NA | NA | NA | #VALUE! |
| SNAR.C3 | NA | NA | NA | NA | #VALUE! |
| SNAR.C4 | 0.030601472 | 0.31212145 | 0.028216925 | 0.3513442 | 0.01 |
| SNAR.D | NA | NA | NA | NA | #VALUE! |
| SNAR.E | -0.002590276 | 0.9318336 | -0.032076774 | 0.2893562 | 0.03 |
| SNAR.F | 0.042795643 | 0.15739821 | 0.004134446 | 0.8914013 | 0.04 |
| SNAR.G1 | 0.027028278 | 0.37201229 | -0.007136966 | 0.8136771 | 0.04 |
| SNAR.G2 | NA | NA | NA | NA | #VALUE! |
| SNAR.H | NA | NA | NA | NA | #VALUE! |
| SNAR.I | -0.02025629 | 0.50350412 | -0.006573087 | 0.8281588 | 0.02 |
| SNCAIP | -0.159288421 | 1.20E-07 | -0.169267067 | 1.80E-08 | 0.01 |
| PARK1 | -0.21941362 | 2.21E-13 | -0.191028388 | 1.93E-10 | 0.03 |
| SNCB | 0.103857775 | 0.00058393 | -0.006646911 | 0.8262594 | 0.12 |
| SR | -0.04560342 | 0.13187912 | 0.110425572 | 0.0002544 | 0.16 |
| SND1 | 0.181087523 | 1.64E-09 | 0.097415227 | 0.0012615 | 0.09 |
| SNED1 | -0.523623373 | 5.55E-78 | -0.296526908 | 1.27E-23 | 0.23 |
| SNF8 | 0.243182912 | 3.54E-16 | 0.284156566 | 9.53E-22 | 0.05 |
| C14orf62 | 0.285342094 | 6.36E-22 | 0.199586709 | 2.78E-11 | 0.09 |
| SNHG11 | 0.070182409 | 0.02031515 | 0.187443661 | 4.23E-10 | 0.12 |
| SNHG12 | 0.320768543 | 1.41E-27 | 0.155676138 | 2.31E-07 | 0.17 |
| SNHG1 | 0.465977777 | 5.24E-60 | 0.303032026 | 1.20E-24 | 0.17 |
| SNHG3.RCC1.1 | 0.429013651 | 3.63E-50 | 0.224540445 | 5.87E-14 | 0.21 |
| SNHG3 | 0.443591287 | 6.66E-54 | 0.23301717 | 6.07E-15 | 0.22 |
| SNHG4 | 0.201775314 | 1.67E-11 | 0.12801544 | 2.19E-05 | 0.08 |
| SNHG5 | 0.199377793 | 2.91E-11 | 0.272684967 | 4.34E-20 | 0.08 |
| SNHG6 | 0.524527307 | 2.72E-78 | 0.382929708 | 1.70E-39 | 0.15 |
| SNHG7 | 0.214350069 | 7.96E-13 | 0.184051943 | 8.77E-10 | 0.04 |
| SNHG8 | 0.070706444 | 0.01939526 | 0.185372591 | 6.61E-10 | 0.12 |
| SNHG9 | 0.26835554 | 1.75E-19 | 0.283829048 | 1.07E-21 | 0.02 |
| SNIP1 | -0.170243877 | 1.48E-08 | -0.272651088 | 4.39E-20 | 0.11 |
| SNN | 0.035779798 | 0.23723557 | -0.138057768 | 4.63E-06 | 0.18 |
| SNORA10 | 0.156603488 | 1.95E-07 | 0.12624275 | 2.84E-05 | 0.04 |
| SNORA11B | -0.001483903 | 0.96091726 | -0.007855972 | 0.7953038 | 0.01 |
| SNORA11C | NA | NA | NA | NA | #VALUE! |
| SNORA11D | 0.019509716 | 0.51936456 | 0.019004086 | 0.5302507 | 0.01 |
| SNORA11E | 0.034906838 | 0.24888157 | 0.038277453 | 0.2060545 | 0.01 |
| SNORA11 | 0.02544911 | 0.40060849 | 0.007963912 | 0.792555 | 0.02 |
| SNORA12 | 0.051977633 | 0.08586887 | 0.008072788 | 0.789785 | 0.05 |
| SNORA13 | -0.033007016 | 0.27559045 | -0.016874483 | 0.5773354 | 0.02 |
| SNORA14A | 0.044613433 | 0.14048405 | 0.029703871 | 0.326534 | 0.02 |
| SNORA14B | 0.018040665 | 0.55130862 | -0.006840314 | 0.8212882 | 0.03 |
| SNORA15 | 0.032841401 | 0.27800801 | 0.018313813 | 0.5452968 | 0.02 |
| SNORA16A | -0.002420997 | 0.9362786 | -0.003405026 | 0.9104717 | 0.01 |
| SNORA16B | 0.056554774 | 0.06161049 | 0.059538105 | 0.0490846 | 0.01 |
| SNORA18 | 0.110505643 | 0.00025181 | -0.006467224 | 0.8308843 | 0.12 |
| SNORA19 | -0.036602327 | 0.22661921 | -0.016043116 | 0.5962357 | 0.03 |
| SNORA1 | 0.037168799 | 0.21950735 | -0.01347326 | 0.6563603 | 0.06 |
| SNORA20 | 0.071117138 | 0.01869994 | 0.021504381 | 0.4775706 | 0.05 |
| SNORA21 | 0.02344805 | 0.43867958 | 0.01156652 | 0.7024831 | 0.02 |
| SNORA22 | 0.044808448 | 0.13875573 | -0.001217655 | 0.9679255 | 0.05 |
| SNORA23 | 0.052883749 | 0.08053535 | 0.020402708 | 0.5004238 | 0.04 |
| SNORA24 | 0.104746264 | 0.00052327 | 0.089053163 | 0.0032125 | 0.02 |
| SNORA25 | 0.136242826 | 6.18E-06 | 0.099579628 | 0.0009787 | 0.04 |
| SNORA26 | 0.040016874 | 0.18616961 | 0.025100158 | 0.4071009 | 0.02 |
| SNORA27 | 0.020211527 | 0.50444783 | -0.014998856 | 0.6203666 | 0.04 |
| SNORA28 | 0.027717025 | 0.35994628 | 0.005442875 | 0.8573568 | 0.03 |
| SNORA29 | -0.034619863 | 0.25279583 | 0.02852454 | 0.346116 | 0.07 |
| SNORA2A | 0.02188857 | 0.46973718 | 0.018827486 | 0.53408 | 0.01 |
| SNORA2B | -0.043430524 | 0.15132382 | -0.022989127 | 0.4476947 | 0.03 |
| SNORA30 | -0.002606032 | 0.93141998 | 0.015835452 | 0.6010004 | 0.02 |
| SNORA31 | 0.024060119 | 0.42681966 | 0.030607664 | 0.3120235 | 0.01 |
| SNORA32 | -0.009743929 | 0.74762037 | -0.019657889 | 0.5161964 | 0.01 |
| SNORA34 | 0.111334511 | 0.00022599 | 0.082885056 | 0.0061102 | 0.03 |
| SNORA35 | NA | NA | NA | NA | #VALUE! |
| SNORA36A | 0.020184239 | 0.50502357 | 0.019094797 | 0.5282892 | 0.01 |
| SNORA36B | 0.008053994 | 0.79026299 | 0.002079506 | 0.9452515 | 0.01 |
| SNORA36C | NA | NA | NA | NA | #VALUE! |
| SNORA37 | 0.011786555 | 0.69710036 | -0.009385654 | 0.7566015 | 0.03 |
| SNORA38B | 0.052349912 | 0.08364407 | 0.019924947 | 0.5105116 | 0.04 |
| SNORA38 | 0.054413856 | 0.07214121 | 0.011463874 | 0.7049992 | 0.05 |
| SNORA71E | -0.131856122 | 1.22E-05 | -0.050009255 | 0.0984376 | 0.09 |
| SNORA45A | 0.040119387 | 0.18504351 | 0.024303791 | 0.4221505 | 0.02 |
| SNORA40 | 0.135493317 | 6.95E-06 | 0.057916087 | 0.0556005 | 0.08 |
| SNORA41 | -0.002425638 | 0.9361567 | -0.029116186 | 0.3362005 | 0.03 |
| SNORA80E | 0.01885331 | 0.53351918 | 0.033887235 | 0.2629822 | 0.02 |
| SNORA44 | -0.024615245 | 0.41622635 | -0.036071098 | 0.2334363 | 0.02 |
| SNORA45B | 0.106378654 | 0.00042683 | 0.071430151 | 0.0181847 | 0.04 |
| SNORA46 | 0.048719741 | 0.10743838 | -0.014452283 | 0.6331637 | 0.07 |
| SNORA47 | 0.060609556 | 0.04514141 | 0.028004855 | 0.3549774 | 0.04 |
| SNORA48 | 0.059489789 | 0.04926901 | 0.021359883 | 0.4805352 | 0.04 |
| SNORA49 | 0.036972494 | 0.22195356 | -0.007724736 | 0.7986492 | 0.05 |
| SNORA4 | 0.030007012 | 0.32161899 | -0.006673682 | 0.8255709 | 0.04 |
| SNORA76A | 0.000427405 | 0.98873897 | -0.038152845 | 0.207536 | 0.04 |
| SNORA51 | -0.008383547 | 0.78189345 | 0.00116842 | 0.9692217 | 0.01 |
| SNORA52 | 0.084898476 | 0.00497496 | 0.014350366 | 0.6355623 | 0.08 |
| SNORA53 | 0.100994455 | 0.0008268 | 0.038300311 | 0.2057836 | 0.07 |
| SNORA54 | -0.009113208 | 0.76345326 | 0.005250955 | 0.8623354 | 0.02 |
| SNORA55 | 0.031057188 | 0.30496686 | -0.013436148 | 0.6572463 | 0.05 |
| SNORA56 | 0.015225022 | 0.61510434 | -0.035913874 | 0.2354815 | 0.06 |
| SNORA57 | 0.170583488 | 1.39E-08 | 0.13855429 | 4.27E-06 | 0.04 |
| SNORA58 | -0.006355953 | 0.83375121 | 0.003071162 | 0.9192183 | 0.01 |
| SNORA59B | 0.056951825 | 0.05980451 | -0.040105465 | 0.1851961 | 0.1 |
| SNORA5A | 0.073194316 | 0.01550675 | 0.008962422 | 0.7672534 | 0.07 |
| SNORA5B | -0.077915101 | 0.00996946 | -0.079221255 | 0.0087873 | 0.01 |
| SNORA5C | 0.070129743 | 0.02040967 | 0.023338883 | 0.4408146 | 0.05 |
| SNORA61 | 0.102747185 | 0.00066895 | 0.023590641 | 0.4358998 | 0.08 |
| SNORA62 | 0.090380879 | 0.00278298 | 0.07076609 | 0.0192929 | 0.02 |
| SNORA63 | 0.09488008 | 0.00168784 | 0.023698507 | 0.4338038 | 0.08 |
| SNORA64 | 0.020512341 | 0.49812391 | 0.020174008 | 0.5052395 | 0.01 |
| SNORA65 | 0.069068305 | 0.02239795 | 0.005019516 | 0.8683465 | 0.07 |
| SNORA66 | -0.04274148 | 0.15792485 | -0.032820749 | 0.2783105 | 0.01 |
| SNORA67 | 0.195345047 | 7.33E-11 | 0.122672222 | 4.78E-05 | 0.08 |
| SNORA68 | 0.112452772 | 0.00019507 | 0.097351743 | 0.0012709 | 0.02 |
| SNORA69 | 0.03196966 | 0.29097046 | 0.032891998 | 0.2772679 | 0.01 |
| SNORA6 | 0.031080383 | 0.30460563 | 0.015505323 | 0.6086099 | 0.02 |
| SNORA70B | -0.006548828 | 0.82878319 | -0.007013591 | 0.8168404 | 0.01 |
| SNORA70C | NA | NA | NA | NA | #VALUE! |
| SNORA70 | 0.001871502 | 0.9507204 | -0.04748109 | 0.1166853 | 0.05 |
| SNORA71A | 0.083647635 | 0.00565534 | 0.062465363 | 0.0389426 | 0.03 |
| SNORA71B | 0.001778465 | 0.95316731 | -0.009504079 | 0.7536292 | 0.02 |
| SNORA71C | 0.088425174 | 0.00343602 | 0.048062766 | 0.112268 | 0.05 |
| SNORA71D | 0.074111917 | 0.01425597 | 0.043118629 | 0.1542853 | 0.04 |
| SNORA72 | 0.195641197 | 6.86E-11 | 0.137152977 | 5.35E-06 | 0.06 |
| SNORA74A | 0.144443397 | 1.63E-06 | 0.117804588 | 9.47E-05 | 0.03 |
| SNORA74B | 0.043586662 | 0.14985762 | 0.031187939 | 0.3029343 | 0.02 |
| SNORA75 | 0.061759994 | 0.0412079 | -0.000607955 | 0.9839825 | 0.07 |
| SNORA76C | 0.204394604 | 8.99E-12 | 0.14466966 | 1.57E-06 | 0.06 |
| SNORA77 | -0.046557583 | 0.12397717 | 0.006088706 | 0.8406458 | 0.06 |
| SNORA78 | 0.101230768 | 0.00080367 | 0.058070643 | 0.0549501 | 0.05 |
| SNORA79 | 0.095714847 | 0.0015347 | 0.046337053 | 0.1257699 | 0.05 |
| SNORA7B | -0.011519143 | 0.70364401 | 0.050187649 | 0.0972412 | 0.07 |
| SNORA80A | 0.118249623 | 8.90E-05 | 0.063206522 | 0.0366771 | 0.06 |
| SNORA81 | 0.024155384 | 0.42499068 | -0.042279768 | 0.1624684 | 0.07 |
| SNORA84 | 0.098220489 | 0.00114849 | 0.037018144 | 0.221383 | 0.07 |
| SNORA8 | 0.259278416 | 3.00E-18 | 0.101816026 | 0.0007489 | 0.16 |
| SNORA9 | 0.050380521 | 0.09596082 | -0.000270183 | 0.9928812 | 0.06 |
| SNORD102 | NA | NA | NA | NA | #VALUE! |
| SNORD103A | NA | NA | NA | NA | #VALUE! |
| SNORD104 | NA | NA | NA | NA | #VALUE! |
| SNORD105B | NA | NA | NA | NA | #VALUE! |
| SNORD105 | NA | NA | NA | NA | #VALUE! |
| SNORD109B | NA | NA | NA | NA | #VALUE! |
| SNORD10 | 0.035781339 | 0.23721535 | 0.005020639 | 0.8683173 | 0.04 |
| SNORD110 | NA | NA | NA | NA | #VALUE! |
| SNORD111B | NA | NA | NA | NA | #VALUE! |
| SNORD111 | NA | NA | NA | NA | #VALUE! |
| SNORD113.1 | NA | NA | NA | NA | #VALUE! |
| SNORD113.2 | NA | NA | NA | NA | #VALUE! |
| SNORD113.4 | NA | NA | NA | NA | #VALUE! |
| SNORD113.5 | NA | NA | NA | NA | #VALUE! |
| SNORD113.6 | NA | NA | NA | NA | #VALUE! |
| SNORD113.7 | NA | NA | NA | NA | #VALUE! |
| SNORD113.9 | NA | NA | NA | NA | #VALUE! |
| SNORD114.10 | NA | NA | NA | NA | #VALUE! |
| SNORD114.11 | NA | NA | NA | NA | #VALUE! |
| SNORD114.12 | NA | NA | NA | NA | #VALUE! |
| SNORD114.13 | NA | NA | NA | NA | #VALUE! |
| SNORD114.14 | NA | NA | NA | NA | #VALUE! |
| SNORD114.15 | NA | NA | NA | NA | #VALUE! |
| SNORD114.16 | NA | NA | NA | NA | #VALUE! |
| SNORD114.17 | NA | NA | NA | NA | #VALUE! |
| SNORD114.18 | NA | NA | NA | NA | #VALUE! |
| SNORD114.19 | NA | NA | NA | NA | #VALUE! |
| SNORD114.1 | NA | NA | NA | NA | #VALUE! |
| SNORD114.20 | NA | NA | NA | NA | #VALUE! |
| SNORD114.21 | NA | NA | NA | NA | #VALUE! |
| SNORD114.22 | NA | NA | NA | NA | #VALUE! |
| SNORD114.23 | NA | NA | NA | NA | #VALUE! |
| SNORD114.24 | NA | NA | NA | NA | #VALUE! |
| SNORD114.25 | NA | NA | NA | NA | #VALUE! |
| SNORD114.26 | NA | NA | NA | NA | #VALUE! |
| SNORD114.27 | NA | NA | NA | NA | #VALUE! |
| SNORD114.28 | NA | NA | NA | NA | #VALUE! |
| SNORD114.29 | NA | NA | NA | NA | #VALUE! |
| SNORD114.2 | NA | NA | NA | NA | #VALUE! |
| SNORD114.30 | NA | NA | NA | NA | #VALUE! |
| SNORD114.31 | NA | NA | NA | NA | #VALUE! |
| SNORD114.3 | NA | NA | NA | NA | #VALUE! |
| SNORD114.4 | NA | NA | NA | NA | #VALUE! |
| SNORD114.5 | NA | NA | NA | NA | #VALUE! |
| SNORD114.6 | NA | NA | NA | NA | #VALUE! |
| SNORD114.7 | NA | NA | NA | NA | #VALUE! |
| SNORD114.8 | NA | NA | NA | NA | #VALUE! |
| SNORD114.9 | NA | NA | NA | NA | #VALUE! |
| SNORD115.10 | NA | NA | NA | NA | #VALUE! |
| SNORD115.11 | NA | NA | NA | NA | #VALUE! |
| SNORD115.13 | -0.067838697 | 0.02490956 | -0.101172983 | 0.0008093 | 0.04 |
| SNORD115.14 | NA | NA | NA | NA | #VALUE! |
| SNORD115.16 | NA | NA | NA | NA | #VALUE! |
| SNORD115.17 | NA | NA | NA | NA | #VALUE! |
| SNORD115.1 | NA | NA | NA | NA | #VALUE! |
| SNORD115.20 | NA | NA | NA | NA | #VALUE! |
| SNORD115.22 | NA | NA | NA | NA | #VALUE! |
| SNORD115.25 | NA | NA | NA | NA | #VALUE! |
| SNORD115.26 | -0.107315297 | 0.00037926 | -0.108347377 | 0.0003326 | 0.01 |
| SNORD115.2 | NA | NA | NA | NA | #VALUE! |
| SNORD115.30 | NA | NA | NA | NA | #VALUE! |
| SNORD115.31 | NA | NA | NA | NA | #VALUE! |
| SNORD115.32 | NA | NA | NA | NA | #VALUE! |
| SNORD115.33 | NA | NA | NA | NA | #VALUE! |
| SNORD115.35 | NA | NA | NA | NA | #VALUE! |
| SNORD115.37 | NA | NA | NA | NA | #VALUE! |
| SNORD115.38 | NA | NA | NA | NA | #VALUE! |
| SNORD115.39 | NA | NA | NA | NA | #VALUE! |
| SNORD115.3 | NA | NA | NA | NA | #VALUE! |
| SNORD115.40 | NA | NA | NA | NA | #VALUE! |
| SNORD115.41 | NA | NA | NA | NA | #VALUE! |
| SNORD115.44 | NA | NA | NA | NA | #VALUE! |
| SNORD115.48 | NA | NA | NA | NA | #VALUE! |
| SNORD115.4 | NA | NA | NA | NA | #VALUE! |
| SNORD115.5 | NA | NA | NA | NA | #VALUE! |
| SNORD115.6 | NA | NA | NA | NA | #VALUE! |
| SNORD115.7 | 0.007377155 | 0.80752742 | 0.072593131 | 0.0163776 | 0.07 |
| SNORD115.8 | NA | NA | NA | NA | #VALUE! |
| SNORD115.9 | NA | NA | NA | NA | #VALUE! |
| SNORD116.10 | NA | NA | NA | NA | #VALUE! |
| SNORD116.11 | NA | NA | NA | NA | #VALUE! |
| SNORD116.12 | NA | NA | NA | NA | #VALUE! |
| SNORD116.13 | NA | NA | NA | NA | #VALUE! |
| SNORD116.14 | NA | NA | NA | NA | #VALUE! |
| SNORD116.15 | NA | NA | NA | NA | #VALUE! |
| SNORD116.16 | NA | NA | NA | NA | #VALUE! |
| SNORD116.18 | NA | NA | NA | NA | #VALUE! |
| SNORD116.19 | -0.00236171 | 0.93783585 | -0.042898564 | 0.1564011 | 0.05 |
| SNORD116.1 | NA | NA | NA | NA | #VALUE! |
| SNORD116.20 | -0.481773835 | 1.35E-64 | -0.389133592 | 7.78E-41 | 0.1 |
| SNORD116.22 | NA | NA | NA | NA | #VALUE! |
| SNORD116.23 | NA | NA | NA | NA | #VALUE! |
| SNORD116.24 | NA | NA | NA | NA | #VALUE! |
| SNORD116.25 | NA | NA | NA | NA | #VALUE! |
| SNORD116.26 | NA | NA | NA | NA | #VALUE! |
| SNORD116.27 | NA | NA | NA | NA | #VALUE! |
| SNORD116.28 | -0.518144679 | 4.03E-76 | -0.3782343 | 1.69E-38 | 0.14 |
| SNORD116.29 | NA | NA | NA | NA | #VALUE! |
| SNORD116.2 | NA | NA | NA | NA | #VALUE! |
| SNORD116.3 | NA | NA | NA | NA | #VALUE! |
| SNORD116.4 | -0.469642643 | 4.74E-61 | -0.407992413 | 4.33E-45 | 0.07 |
| SNORD116.5 | NA | NA | NA | NA | #VALUE! |
| SNORD116.8 | NA | NA | NA | NA | #VALUE! |
| SNORD117 | NA | NA | NA | NA | #VALUE! |
| SNORD119 | NA | NA | NA | NA | #VALUE! |
| SNORD11B | NA | NA | NA | NA | #VALUE! |
| SNORD11 | NA | NA | NA | NA | #VALUE! |
| SNORD121A | NA | NA | NA | NA | #VALUE! |
| SNORD121B | NA | NA | NA | NA | #VALUE! |
| SNORD123 | NA | NA | NA | NA | #VALUE! |
| SNORD124 | NA | NA | NA | NA | #VALUE! |
| SNORD125 | NA | NA | NA | NA | #VALUE! |
| SNORD126 | NA | NA | NA | NA | #VALUE! |
| SNORD127 | NA | NA | NA | NA | #VALUE! |
| SNORD12B | NA | NA | NA | NA | #VALUE! |
| SNORD12C | NA | NA | NA | NA | #VALUE! |
| SNORD12 | NA | NA | NA | NA | #VALUE! |
| SNORD15A | 0.085114599 | 0.00486519 | 0.041911594 | 0.1661616 | 0.05 |
| SNORD15B | 0.145524913 | 1.36E-06 | 0.069496922 | 0.0215757 | 0.08 |
| SNORD16 | NA | NA | NA | NA | #VALUE! |
| SNORD17 | 0.194246 | 9.40E-11 | 0.088750564 | 0.0033185 | 0.11 |
| SNORD18A | NA | NA | NA | NA | #VALUE! |
| SNORD18B | NA | NA | NA | NA | #VALUE! |
| SNORD18C | NA | NA | NA | NA | #VALUE! |
| SNORD19B | NA | NA | NA | NA | #VALUE! |
| SNORD19 | NA | NA | NA | NA | #VALUE! |
| SNORD1A | NA | NA | NA | NA | #VALUE! |
| SNORD1B | NA | NA | NA | NA | #VALUE! |
| SNORD1C | 0.124899089 | 3.46E-05 | 0.009884689 | 0.7441011 | 0.12 |
| SNORD20 | NA | NA | NA | NA | #VALUE! |
| SNORD21 | NA | NA | NA | NA | #VALUE! |
| SNORD22 | 0.12985543 | 1.66E-05 | 0.071348172 | 0.0183184 | 0.06 |
| SNORD23 | -0.010041696 | 0.7401819 | 0.013961883 | 0.6447399 | 0.03 |
| SNORD24 | NA | NA | NA | NA | #VALUE! |
| SNORD25 | NA | NA | NA | NA | #VALUE! |
| SNORD26 | NA | NA | NA | NA | #VALUE! |
| SNORD27 | NA | NA | NA | NA | #VALUE! |
| SNORD28 | NA | NA | NA | NA | #VALUE! |
| SNORD29 | NA | NA | NA | NA | #VALUE! |
| SNORD2 | NA | NA | NA | NA | #VALUE! |
| SNORD30 | NA | NA | NA | NA | #VALUE! |
| SNORD31 | NA | NA | NA | NA | #VALUE! |
| SNORD32A | NA | NA | NA | NA | #VALUE! |
| SNORD32B | NA | NA | NA | NA | #VALUE! |
| SNORD33 | NA | NA | NA | NA | #VALUE! |
| SNORD34 | NA | NA | NA | NA | #VALUE! |
| SNORD35A | NA | NA | NA | NA | #VALUE! |
| SNORD35B | NA | NA | NA | NA | #VALUE! |
| SNORD36A | NA | NA | NA | NA | #VALUE! |
| SNORD36B | NA | NA | NA | NA | #VALUE! |
| SNORD36C | NA | NA | NA | NA | #VALUE! |
| SNORD37 | NA | NA | NA | NA | #VALUE! |
| SNORD38A | NA | NA | NA | NA | #VALUE! |
| SNORD38B | NA | NA | NA | NA | #VALUE! |
| SNORD41 | NA | NA | NA | NA | #VALUE! |
| SNORD42A | NA | NA | NA | NA | #VALUE! |
| SNORD42B | NA | NA | NA | NA | #VALUE! |
| SNORD43 | NA | NA | NA | NA | #VALUE! |
| SNORD44 | NA | NA | NA | NA | #VALUE! |
| SNORD45A | NA | NA | NA | NA | #VALUE! |
| SNORD45B | NA | NA | NA | NA | #VALUE! |
| SNORD45C | NA | NA | NA | NA | #VALUE! |
| SNORD46 | NA | NA | NA | NA | #VALUE! |
| SNORD47 | NA | NA | NA | NA | #VALUE! |
| SNORD48 | NA | NA | NA | NA | #VALUE! |
| SNORD49A | NA | NA | NA | NA | #VALUE! |
| SNORD49B | NA | NA | NA | NA | #VALUE! |
| SNORD4A | NA | NA | NA | NA | #VALUE! |
| SNORD4B | NA | NA | NA | NA | #VALUE! |
| SNORD50A | NA | NA | NA | NA | #VALUE! |
| SNORD50B | NA | NA | NA | NA | #VALUE! |
| SNORD51 | NA | NA | NA | NA | #VALUE! |
| SNORD52 | NA | NA | NA | NA | #VALUE! |
| SNORD53 | NA | NA | NA | NA | #VALUE! |
| SNORD54 | NA | NA | NA | NA | #VALUE! |
| SNORD55 | NA | NA | NA | NA | #VALUE! |
| SNORD56B | NA | NA | NA | NA | #VALUE! |
| SNORD56 | NA | NA | NA | NA | #VALUE! |
| SNORD57 | NA | NA | NA | NA | #VALUE! |
| SNORD58A | NA | NA | NA | NA | #VALUE! |
| U58 | NA | NA | NA | NA | #VALUE! |
| SNORD59A | NA | NA | NA | NA | #VALUE! |
| SNORD59B | NA | NA | NA | NA | #VALUE! |
| SNORD5 | NA | NA | NA | NA | #VALUE! |
| SNORD60 | NA | NA | NA | NA | #VALUE! |
| SNORD61 | NA | NA | NA | NA | #VALUE! |
| SNORD62A | NA | NA | NA | NA | #VALUE! |
| SNORD63 | NA | NA | NA | NA | #VALUE! |
| SNORD65 | NA | NA | NA | NA | #VALUE! |
| SNORD66 | NA | NA | NA | NA | #VALUE! |
| SNORD67 | -0.0266891 | 0.37804523 | 0.011090749 | 0.7141726 | 0.04 |
| SNORD68 | NA | NA | NA | NA | #VALUE! |
| SNORD69 | NA | NA | NA | NA | #VALUE! |
| SNORD6 | NA | NA | NA | NA | #VALUE! |
| SNORD70 | NA | NA | NA | NA | #VALUE! |
| MIRN768 | NA | NA | NA | NA | #VALUE! |
| SNORD72 | NA | NA | NA | NA | #VALUE! |
| SNORD73A | NA | NA | NA | NA | #VALUE! |
| SNORD74 | NA | NA | NA | NA | #VALUE! |
| SNORD75 | NA | NA | NA | NA | #VALUE! |
| SNORD76 | NA | NA | NA | NA | #VALUE! |
| SNORD77 | NA | NA | NA | NA | #VALUE! |
| SNORD78 | NA | NA | NA | NA | #VALUE! |
| SNORD79 | NA | NA | NA | NA | #VALUE! |
| SNORD7 | NA | NA | NA | NA | #VALUE! |
| SNORD80 | NA | NA | NA | NA | #VALUE! |
| SNORD81 | NA | NA | NA | NA | #VALUE! |
| SNORD82 | NA | NA | NA | NA | #VALUE! |
| SNORD84 | NA | NA | NA | NA | #VALUE! |
| SNORD85 | NA | NA | NA | NA | #VALUE! |
| SNORD86 | NA | NA | NA | NA | #VALUE! |
| SNORD87 | NA | NA | NA | NA | #VALUE! |
| SNORD88A | NA | NA | NA | NA | #VALUE! |
| SNORD88B | NA | NA | NA | NA | #VALUE! |
| SNORD88C | NA | NA | NA | NA | #VALUE! |
| SNORD89 | -0.024669718 | 0.41519527 | 0.008909586 | 0.7685863 | 0.04 |
| SNORD8 | 0.003760885 | 0.9011608 | 0.060198624 | 0.046621 | 0.06 |
| SNORD90 | NA | NA | NA | NA | #VALUE! |
| SNORD91A | NA | NA | NA | NA | #VALUE! |
| SNORD91B | NA | NA | NA | NA | #VALUE! |
| SNORD92 | NA | NA | NA | NA | #VALUE! |
| SNORD93 | NA | NA | NA | NA | #VALUE! |
| SNORD94 | 0.044876968 | 0.13815238 | 0.018826621 | 0.5340988 | 0.03 |
| SNORD95 | NA | NA | NA | NA | #VALUE! |
| SNORD96A | NA | NA | NA | NA | #VALUE! |
| SNORD96B | NA | NA | NA | NA | #VALUE! |
| SNORD97 | 0.018041306 | 0.55129446 | 0.016987152 | 0.5747959 | 0.01 |
| SNORD98 | NA | NA | NA | NA | #VALUE! |
| SNORD99 | NA | NA | NA | NA | #VALUE! |
| SNORD9 | NA | NA | NA | NA | #VALUE! |
| SNPH | -0.271420374 | 6.54E-20 | -0.163414088 | 5.54E-08 | 0.11 |
| LOC87229 | -0.43717455 | 3.10E-52 | -0.354368534 | 1.10E-33 | 0.09 |
| SNRNP200 | -0.056073594 | 0.06385931 | -0.203462388 | 1.12E-11 | 0.15 |
| SNRNP25 | 0.302630181 | 1.39E-24 | 0.348698715 | 1.33E-32 | 0.05 |
| SNRNP27 | 0.151021973 | 5.28E-07 | 0.03748712 | 0.2155819 | 0.12 |
| SNRNP35 | 0.152758057 | 3.89E-07 | 0.226078326 | 3.91E-14 | 0.08 |
| LOC127574 | 0.251865361 | 2.82E-17 | 0.087579687 | 0.0037592 | 0.17 |
| SNRNP48 | -0.094584543 | 0.00174533 | -0.011023916 | 0.7158201 | 0.09 |
| SNRNP70 | 0.088636596 | 0.00335925 | 0.093557623 | 0.0019594 | 0.01 |
| LOC88988 | 0.617977403 | 4.12E-116 | 0.397410575 | 1.14E-42 | 0.23 |
| SNRPA | 0.62766723 | 8.64E-121 | 0.43844621 | 1.46E-52 | 0.19 |
| SNRPB2 | 0.483371693 | 4.50E-65 | 0.455593389 | 4.05E-57 | 0.03 |
| SNRPB | 0.511761005 | 5.37E-74 | 0.416966934 | 3.26E-47 | 0.1 |
| SNRPC | 0.649862888 | 3.71E-132 | 0.563867713 | 9.52E-93 | 0.09 |
| SNRPD2 | 0.54615406 | 5.32E-86 | 0.481991372 | 1.16E-64 | 0.07 |
| SNRPD3 | 0.479978362 | 4.62E-64 | 0.379624417 | 8.59E-39 | 0.11 |
| SNRPF | 0.628378789 | 3.86E-121 | 0.570922452 | 1.50E-95 | 0.06 |
| SNRPG | 0.692113253 | 1.17E-156 | 0.565542122 | 2.09E-93 | 0.13 |
| LOC647795 | -0.258977017 | 3.29E-18 | -0.113619952 | 0.0001671 | 0.15 |
| SNTA1 | -0.015987685 | 0.59750585 | -0.017846488 | 0.555602 | 0.01 |
| SNTB1 | 0.016154995 | 0.59367594 | -0.135511172 | 6.93E-06 | 0.16 |
| SNTB2 | -0.335156367 | 4.23E-30 | -0.365827285 | 6.02E-36 | 0.04 |
| LOC102724295 | 0.046411308 | 0.12516403 | 0.017396353 | 0.5656171 | 0.03 |
| LOC728185 | -0.102504868 | 0.00068897 | -0.146520976 | 1.15E-06 | 0.05 |
| LOC651293 | -0.229012159 | 1.79E-14 | 0.012482115 | 0.6801859 | 0.25 |
| SNUPN | 0.270036083 | 1.02E-19 | 0.25176847 | 2.90E-17 | 0.02 |
| LOC123116 | -0.330564151 | 2.79E-29 | -0.284125845 | 9.64E-22 | 0.05 |
| SNW1 | -0.001876127 | 0.95059877 | 0.040430189 | 0.1816601 | 0.05 |
| SNX10 | 0.019694384 | 0.51541757 | -0.124790709 | 3.52E-05 | 0.15 |
| SNX11 | -0.027584909 | 0.36224157 | 0.054851976 | 0.069874 | 0.09 |
| SNX12 | -0.299257487 | 4.74E-24 | -0.369282356 | 1.20E-36 | 0.08 |
| SNX13 | -0.442627992 | 1.19E-53 | -0.294463916 | 2.64E-23 | 0.15 |
| SNX14 | -0.248934229 | 6.69E-17 | -0.037920307 | 0.2103214 | 0.22 |
| LOC116070 | 0.080752466 | 0.00756191 | -0.030134097 | 0.3195729 | 0.12 |
| SNX16 | 0.067131754 | 0.02646108 | 0.011067121 | 0.7147549 | 0.06 |
| SNX17 | 0.086804769 | 0.00407945 | 0.038407993 | 0.2045108 | 0.05 |
| SNX18 | -0.31113173 | 5.83E-26 | -0.277886223 | 7.86E-21 | 0.04 |
| SNX19 | -0.416059459 | 5.38E-47 | -0.313283744 | 2.57E-26 | 0.11 |
| SNX1 | -0.40967621 | 1.75E-45 | -0.307544723 | 2.25E-25 | 0.11 |
| SNX20 | -0.084134062 | 0.00538137 | -0.230216871 | 1.30E-14 | 0.15 |
| SNX21 | -0.103472202 | 0.00061224 | -0.003838909 | 0.8991211 | 0.1 |
| SNX22 | 0.38562125 | 4.50E-40 | 0.193784684 | 1.04E-10 | 0.2 |
| SNX24 | -0.044050481 | 0.14556597 | -0.040359619 | 0.1824243 | 0.01 |
| SNX25 | -0.368049815 | 2.14E-36 | -0.317015311 | 6.11E-27 | 0.06 |
| KIAA0488 | -0.132427642 | 1.12E-05 | 0.120484154 | 6.52E-05 | 0.26 |
| LOC146153 | -0.484497995 | 2.06E-65 | -0.390241044 | 4.46E-41 | 0.1 |
| SNX2 | -0.294124861 | 2.98E-23 | -0.185207029 | 6.85E-10 | 0.11 |
| SNX30 | -0.495504361 | 8.75E-69 | -0.423319181 | 9.36E-49 | 0.08 |
| SNX31 | -0.029780785 | 0.32528235 | -0.075438819 | 0.0126046 | 0.05 |
| SNX32 | 0.223419139 | 7.86E-14 | 0.07045351 | 0.0198346 | 0.16 |
| SNX33 | -0.325126922 | 2.51E-28 | -0.341684204 | 2.73E-31 | 0.02 |
| SNX3 | 0.191849711 | 1.61E-10 | 0.192469205 | 1.40E-10 | 0.01 |
| SNX4 | -0.084790012 | 0.00503088 | -0.070407298 | 0.0199158 | 0.02 |
| SNX5 | 0.093700607 | 0.0019282 | 0.142542194 | 2.23E-06 | 0.05 |
| SNX6 | -0.136887752 | 5.58E-06 | -0.107580707 | 0.0003667 | 0.03 |
| SNX7 | 0.055176773 | 0.06823127 | -0.061125833 | 0.0433387 | 0.12 |
| SNX8 | 0.251819699 | 2.86E-17 | 0.076854691 | 0.0110311 | 0.18 |
| SNX9 | -0.372874073 | 2.21E-37 | -0.243772169 | 2.99E-16 | 0.13 |
| SOAT1 | -0.002425537 | 0.93615935 | -0.047452143 | 0.1169086 | 0.05 |
| SOAT2 | 0.079132539 | 0.00886344 | 0.032192712 | 0.2876158 | 0.05 |
| LOC654119 | -0.080352329 | 0.0078664 | -0.124681377 | 3.57E-05 | 0.05 |
| SOCS1 | 0.224178447 | 6.45E-14 | 0.085923682 | 0.0044734 | 0.14 |
| SOCS2 | -0.244423002 | 2.48E-16 | -0.167646806 | 2.47E-08 | 0.08 |
| ATOD4 | -0.03807906 | 0.20841692 | -0.163880554 | 5.08E-08 | 0.13 |
| SOCS4 | -0.202075183 | 1.55E-11 | -0.158206679 | 1.46E-07 | 0.05 |
| SOCS5 | -0.203673735 | 1.07E-11 | -0.281935013 | 2.03E-21 | 0.08 |
| HSPC060 | -0.159420978 | 1.17E-07 | -0.183316722 | 1.03E-09 | 0.03 |
| LOC100505522 | -0.235215849 | 3.32E-15 | -0.279093565 | 5.26E-21 | 0.05 |
| SOD1 | 0.401554682 | 1.32E-43 | 0.370270677 | 7.56E-37 | 0.04 |
| MGC5618 | 0.198756069 | 3.36E-11 | 0.004443304 | 0.8833446 | 0.2 |
| SOD3 | -0.017431949 | 0.56482194 | -0.082828243 | 0.0061454 | 0.07 |
| C9orf157 | 0.118047514 | 9.16E-05 | 0.092497104 | 0.0022055 | 0.03 |
| LOC387919 | 0.022832699 | 0.45079148 | 0.130806805 | 1.44E-05 | 0.11 |
| CAPN15 | 0.126830465 | 2.61E-05 | 0.040331303 | 0.1827316 | 0.09 |
| SON | -0.411211696 | 7.62E-46 | -0.381163938 | 4.05E-39 | 0.04 |
| SORBS1 | -0.36887791 | 1.45E-36 | -0.266820952 | 2.85E-19 | 0.11 |
| SORBS2 | -0.203610561 | 1.08E-11 | -0.127284536 | 2.44E-05 | 0.08 |
| SORBS3 | -0.168357999 | 2.15E-08 | -0.125060946 | 3.38E-05 | 0.05 |
| SORCS1 | -0.258378892 | 3.95E-18 | -0.150828987 | 5.46E-07 | 0.11 |
| SORCS2 | -0.381885586 | 2.85E-39 | -0.228998933 | 1.80E-14 | 0.16 |
| SORCS3 | -0.025388072 | 0.40173963 | 0.021301723 | 0.4817313 | 0.05 |
| SORD | -0.14121672 | 2.77E-06 | -0.02441644 | 0.4200022 | 0.12 |
| SORL1 | -0.47693196 | 3.65E-63 | -0.299204615 | 4.83E-24 | 0.18 |
| SORT1 | -0.14436765 | 1.65E-06 | -0.159532538 | 1.14E-07 | 0.02 |
| GINGF | -0.037318069 | 0.21766025 | -0.241055064 | 6.49E-16 | 0.21 |
| LOC145448 | -0.403845508 | 3.94E-44 | -0.335055821 | 4.41E-30 | 0.07 |
| SOSTDC1 | 0.107627768 | 0.00036452 | -0.006486741 | 0.8303817 | 0.12 |
| VBCH | 0.083218354 | 0.00590749 | -0.040228237 | 0.1838533 | 0.13 |
| DOM | 0.129240547 | 1.82E-05 | -0.02071551 | 0.4938767 | 0.15 |
| SOX11 | 0.312524695 | 3.43E-26 | 0.211111605 | 1.78E-12 | 0.11 |
| LOC149791 | 0.069377811 | 0.02180151 | 0.213082065 | 1.09E-12 | 0.15 |
| SOX13 | -0.200607258 | 2.19E-11 | 0.279437569 | 4.69E-21 | 0.49 |
| SOX14 | 0.173340819 | 8.03E-09 | 0.036711574 | 0.225235 | 0.14 |
| SOX15 | 0.085059623 | 0.0048929 | -0.046875277 | 0.1214297 | 0.14 |
| SOX17 | -0.234268798 | 4.31E-15 | -0.110741406 | 0.0002442 | 0.13 |
| SOX18 | -0.065863956 | 0.02945285 | 0.002557158 | 0.9327031 | 0.07 |
| SOX1 | 0.023394561 | 0.43972491 | 0.006138993 | 0.8393475 | 0.02 |
| SOX25 | 0.054848358 | 0.06989252 | -0.069420378 | 0.0217206 | 0.13 |
| SOX2.OT | -0.203976944 | 9.93E-12 | -0.155746637 | 2.28E-07 | 0.05 |
| SOX2 | 0.043203173 | 0.15347824 | 0.082468593 | 0.0063722 | 0.04 |
| SOX30 | 0.107541469 | 0.00036854 | -0.080774803 | 0.0075452 | 0.19 |
| PHP | 0.055952697 | 0.06443485 | 0.001479155 | 0.9610422 | 0.06 |
| SOX4 | 0.194237965 | 9.42E-11 | 0.204199472 | 9.42E-12 | 0.01 |
| SOX5 | -0.259346605 | 2.94E-18 | -0.321469507 | 1.07E-27 | 0.07 |
| SOX6 | 0.067223913 | 0.0262542 | -0.071448242 | 0.0181553 | 0.14 |
| SOX7 | -0.196912516 | 5.13E-11 | -0.229058455 | 1.77E-14 | 0.04 |
| SOX8 | 0.213787431 | 9.16E-13 | 0.021526839 | 0.4771108 | 0.2 |
| SOX9 | 0.095692718 | 0.00153859 | 0.155116326 | 2.56E-07 | 0.06 |
| SP100 | -0.164925831 | 4.16E-08 | -0.153672997 | 3.31E-07 | 0.02 |
| IFI75 | -0.076667859 | 0.01122825 | 0.029462654 | 0.3304796 | 0.11 |
| SP140L | -0.07016691 | 0.02034292 | -0.046909605 | 0.1211569 | 0.03 |
| LOC101928789 | 0.038817535 | 0.19972202 | -0.079060008 | 0.0089261 | 0.12 |
| SP1 | -0.433146884 | 3.31E-51 | -0.292763499 | 4.82E-23 | 0.15 |
| SP2 | -0.321932948 | 8.93E-28 | -0.294206028 | 2.90E-23 | 0.03 |
| SP3 | -0.327816977 | 8.52E-29 | -0.27727701 | 9.62E-21 | 0.06 |
| SP4 | -0.254341894 | 1.34E-17 | -0.307110058 | 2.65E-25 | 0.06 |
| SP5 | -0.235271618 | 3.27E-15 | -0.011414605 | 0.7062081 | 0.23 |
| SP6 | 0.04666515 | 0.12310999 | 0.142292288 | 2.32E-06 | 0.1 |
| SP7 | 0.004878331 | 0.87201723 | -0.043095824 | 0.1545035 | 0.05 |
| LOC155456 | 0.099198385 | 0.0010238 | 0.037921256 | 0.2103099 | 0.07 |
| SP9 | 0.026274716 | 0.38549702 | -0.02444929 | 0.4193769 | 0.06 |
| LOC90953 | -0.006544831 | 0.82888606 | 0.187149478 | 4.51E-10 | 0.2 |
| SPACA1 | 0.050414197 | 0.09573865 | 0.077123809 | 0.0107526 | 0.03 |
| SPACA3 | 0.034459607 | 0.25500022 | 0.0583234 | 0.0539001 | 0.03 |
| SPACA4 | -0.180832009 | 1.73E-09 | -0.052219845 | 0.084416 | 0.13 |
| SPACA5 | -0.118460313 | 8.65E-05 | -0.031482861 | 0.2983829 | 0.09 |
| SPAG11A | -0.009774283 | 0.74686099 | -0.012971515 | 0.6683798 | 0.01 |
| LOC51756 | -0.001506374 | 0.96032592 | 0.019531312 | 0.5189022 | 0.03 |
| SPAG16 | 0.068464171 | 0.02360307 | -0.051996473 | 0.0857551 | 0.13 |
| SPAG17 | -0.214000518 | 8.69E-13 | -0.061868351 | 0.0408528 | 0.16 |
| SPAG1 | -0.087311544 | 0.00386736 | -0.140480358 | 3.13E-06 | 0.06 |
| SPAG4 | 0.182639004 | 1.18E-09 | 0.166392576 | 3.14E-08 | 0.02 |
| SPAG5 | 0.44329701 | 7.96E-54 | 0.263100563 | 9.19E-19 | 0.19 |
| SPAG6 | -0.144576522 | 1.59E-06 | 0.002716871 | 0.9285108 | 0.15 |
| SPAG7 | 0.171224419 | 1.22E-08 | 0.146471687 | 1.16E-06 | 0.03 |
| SPAG8 | -0.111360443 | 0.00022522 | -0.03030753 | 0.3167944 | 0.09 |
| SPAG9 | -0.200401304 | 2.30E-11 | -0.228175169 | 2.24E-14 | 0.03 |
| SPAM1 | 0.051313208 | 0.08995808 | 0.011389039 | 0.7068357 | 0.04 |
| LOC220879 | 0.047302875 | 0.11806563 | 0.008466463 | 0.7797916 | 0.04 |
| SPANXB2 | 0.053555586 | 0.0767569 | 0.003422048 | 0.9100261 | 0.06 |
| SPANXC | 0.064241437 | 0.03370186 | 0.028820186 | 0.3411382 | 0.04 |
| SPANXE | 0.070473796 | 0.01979906 | 0.061836431 | 0.0409572 | 0.01 |
| SPANXN1 | 0.007867659 | 0.79500607 | 0.014573606 | 0.6303135 | 0.01 |
| SPANXN2 | 0.050133888 | 0.09760054 | 0.052008104 | 0.085685 | 0.01 |
| SPANXN3 | 0.098867378 | 0.00106453 | 0.058916688 | 0.051501 | 0.04 |
| SPANXN4 | -0.017003762 | 0.57442191 | -0.046949657 | 0.1208392 | 0.03 |
| SPANXN5 | 0.05218237 | 0.08463949 | 0.065579177 | 0.0301635 | 0.02 |
| SPARCL1 | -0.439106331 | 9.83E-53 | -0.277301195 | 9.54E-21 | 0.17 |
| SPARC | -0.298307854 | 6.68E-24 | -0.130909592 | 1.41E-05 | 0.17 |
| KIAA1083 | 0.088256896 | 0.00349826 | -0.000585307 | 0.9845791 | 0.09 |
| SPATA12 | 0.233564144 | 5.23E-15 | 0.162274384 | 6.87E-08 | 0.08 |
| LOC121757 | -0.479684488 | 5.64E-64 | -0.429734992 | 2.40E-50 | 0.05 |
| SPATA16 | 0.203742566 | 1.05E-11 | 0.137390971 | 5.15E-06 | 0.07 |
| SPATA17 | -0.167101618 | 2.74E-08 | 0.223746437 | 7.22E-14 | 0.4 |
| SPATA18 | -0.336060157 | 2.90E-30 | -0.151426073 | 4.92E-07 | 0.19 |
| SPATA19 | 0.102918516 | 0.00065512 | 0.063408699 | 0.0360789 | 0.04 |
| SPATA1 | -0.276124847 | 1.41E-20 | -0.186842361 | 4.82E-10 | 0.09 |
| SPATA20 | -0.260858674 | 1.84E-18 | -0.084925841 | 0.0049609 | 0.18 |
| SPATA21 | 0.108882795 | 0.00031055 | -0.03583756 | 0.2364788 | 0.15 |
| SPATA22 | -0.012329686 | 0.68387927 | -0.050360878 | 0.0960906 | 0.04 |
| SPATA24 | 0.006355986 | 0.83375035 | 0.01093308 | 0.7180615 | 0.01 |
| SPATA2L | 0.192960227 | 1.25E-10 | 0.171491072 | 1.16E-08 | 0.03 |
| SPATA2 | -0.040235714 | 0.18377176 | -0.034504937 | 0.2543753 | 0.01 |
| SPATA3 | 0.181172352 | 1.61E-09 | 0.083054032 | 0.0060067 | 0.1 |
| SPATA4 | -0.295223283 | 2.02E-23 | -0.126237334 | 2.85E-05 | 0.17 |
| LOC148785 | 0.100868205 | 0.00083941 | 0.121711838 | 5.48E-05 | 0.03 |
| SPATA5 | -0.145262037 | 1.42E-06 | -0.24356527 | 3.18E-16 | 0.1 |
| SPATA6 | -0.500727411 | 1.99E-70 | -0.340910326 | 3.79E-31 | 0.16 |
| SPATA7 | -0.206406702 | 5.56E-12 | 0.001187117 | 0.9687295 | 0.21 |
| SPATA8 | 0.011397717 | 0.70662263 | -0.036533799 | 0.2274906 | 0.05 |
| LOC115374 | -0.084212422 | 0.00533838 | 0.038451114 | 0.2040027 | 0.13 |
| SPATC1 | -0.009671903 | 0.74942319 | -0.125938448 | 2.97E-05 | 0.12 |
| SPATS1 | 0.025351244 | 0.40242304 | -0.004060739 | 0.8933257 | 0.03 |
| SPATS2L | -0.016960481 | 0.57539654 | -0.012817314 | 0.672091 | 0.01 |
| SPATS2 | 0.078968135 | 0.00900611 | 0.087819918 | 0.0036647 | 0.01 |
| SPC24 | 0.360979109 | 5.59E-35 | 0.17553452 | 5.16E-09 | 0.19 |
| SPC25 | 0.519139252 | 1.86E-76 | 0.356840071 | 3.63E-34 | 0.17 |
| LOC94556 | -0.016089811 | 0.59516674 | 0.193624354 | 1.08E-10 | 0.21 |
| SPCS2 | 0.176427595 | 4.30E-09 | 0.222398824 | 1.03E-13 | 0.05 |
| SPCS3 | -0.169475237 | 1.73E-08 | -0.2081193 | 3.68E-12 | 0.04 |
| SPDEF | -0.368479701 | 1.75E-36 | -0.01709095 | 0.5724609 | 0.36 |
| SPDYA | 0.242642699 | 4.13E-16 | 0.212811033 | 1.17E-12 | 0.03 |
| SPDYC | 0.149397166 | 7.00E-07 | 0.107517075 | 0.0003697 | 0.05 |
| SPDYE1 | -0.193940311 | 1.01E-10 | -0.095809972 | 0.0015181 | 0.1 |
| SPDYE2 | -0.014659527 | 0.62829824 | 0.044631817 | 0.1403204 | 0.06 |
| SPDYE3 | -0.11696989 | 0.0001062 | 0.00624052 | 0.8367277 | 0.13 |
| SPDYE4 | -0.039212525 | 0.19518142 | -0.052731421 | 0.0814127 | 0.02 |
| SPDYE5 | -0.197599061 | 4.39E-11 | -0.087365992 | 0.0038452 | 0.12 |
| SPDYE6 | -0.07474275 | 0.01344848 | 0.051355899 | 0.0896907 | 0.13 |
| SPDYE7P | -0.101667807 | 0.00076247 | 0.00978478 | 0.7465985 | 0.12 |
| SPDYE8P | -0.084723092 | 0.00506567 | 0.059097083 | 0.0507895 | 0.15 |
| SPEF1 | -0.273107774 | 3.78E-20 | 0.052161322 | 0.0847652 | 0.33 |
| FLJ23164 | -0.395961631 | 2.41E-42 | -0.114639948 | 0.0001457 | 0.29 |
| LOC729871 | 0.172237721 | 1.00E-08 | 0.015028415 | 0.6196777 | 0.16 |
| SPEM1 | 0.107148391 | 0.00038736 | 0.095195921 | 0.0016283 | 0.02 |
| LOC348488 | -0.330116707 | 3.35E-29 | -0.445653649 | 1.91E-54 | 0.12 |
| SPERT | 0.141048457 | 2.85E-06 | 0.116789338 | 0.0001089 | 0.03 |
| SPESP1 | -0.106836515 | 0.00040292 | -0.06767011 | 0.0252722 | 0.04 |
| SPG11 | -0.575965437 | 1.35E-97 | -0.352299641 | 2.74E-33 | 0.23 |
| SPG20 | -0.187142032 | 4.51E-10 | -0.184467302 | 8.03E-10 | 0.01 |
| SPG21 | 0.334190401 | 6.30E-30 | 0.282030251 | 1.96E-21 | 0.06 |
| PGN | 0.121648585 | 5.53E-05 | -0.024377665 | 0.4207409 | 0.15 |
| SPHAR | 0.059305231 | 0.04997879 | 0.411016446 | 8.48E-46 | 0.36 |
| SPHK1 | 0.238651277 | 1.28E-15 | 0.106954485 | 0.000397 | 0.14 |
| SPHK2 | -0.100087421 | 0.00092142 | -0.063328834 | 0.0363142 | 0.04 |
| SPHKAP | 0.102527952 | 0.00068703 | -0.043925607 | 0.1467121 | 0.15 |
| SPI1 | 0.021831612 | 0.47089403 | -0.098111832 | 0.0011632 | 0.12 |
| SPIB | 0.192620026 | 1.35E-10 | -0.016504852 | 0.5857038 | 0.21 |
| SPIC | 0.064782235 | 0.03223088 | -0.068467894 | 0.0235955 | 0.14 |
| LOC95616 | -0.265708323 | 4.05E-19 | -0.205822131 | 6.40E-12 | 0.06 |
| SPIN2A | -0.175689993 | 5.00E-09 | -0.094001933 | 0.001864 | 0.09 |
| LOC158583 | 0.147079081 | 1.04E-06 | 0.150459442 | 5.82E-07 | 0.01 |
| SPIN3 | -0.200936216 | 2.03E-11 | -0.183034505 | 1.09E-09 | 0.02 |
| SPIN4 | 0.052742057 | 0.08135119 | -0.118860882 | 8.18E-05 | 0.18 |
| SPINK13 | 0.0562563 | 0.06299758 | 0.076334481 | 0.0115877 | 0.03 |
| SPINK14 | -0.0184905 | 0.54142546 | 0.00597309 | 0.8436323 | 0.03 |
| SPINK1 | 0.126824666 | 2.61E-05 | 0.051147098 | 0.0910045 | 0.08 |
| SPINK2 | 0.046283554 | 0.12620782 | 0.065680827 | 0.0299082 | 0.02 |
| SPINK4 | -0.04555419 | 0.13229716 | 0.019642881 | 0.5165168 | 0.07 |
| SPINK5 | 0.034680313 | 0.25196777 | -0.060179045 | 0.0466925 | 0.1 |
| SPINK6 | 0.108914762 | 0.00030928 | -0.009854098 | 0.7448655 | 0.12 |
| SPINK7 | 0.057783452 | 0.05616378 | -0.003511407 | 0.907687 | 0.07 |
| SPINK8 | -0.167494614 | 2.54E-08 | 0.064726542 | 0.0323798 | 0.24 |
| SPINK9 | -0.01199072 | 0.69211947 | 0.003282009 | 0.9136934 | 0.02 |
| EPPIN | -0.028260198 | 0.35060571 | -0.004417494 | 0.8840174 | 0.03 |
| HAI | -0.013403271 | 0.65803167 | 0.024263368 | 0.422923 | 0.04 |
| LOC100130414 | 0.069546128 | 0.02148298 | 0.120598801 | 6.41E-05 | 0.06 |
| SPINT3 | -0.016953089 | 0.57556309 | 0.0061017 | 0.8403103 | 0.03 |
| SPINT4 | 0.025895729 | 0.39239014 | 0.000932724 | 0.9754282 | 0.03 |
| SPIRE1 | 0.047434325 | 0.11704628 | -0.165579726 | 3.67E-08 | 0.22 |
| SPIRE2 | 0.150238436 | 6.05E-07 | 0.070185879 | 0.0203089 | 0.09 |
| SPNS1 | 0.233436305 | 5.41E-15 | 0.163964231 | 5.00E-08 | 0.07 |
| SPNS2 | -0.040377492 | 0.18223052 | -0.117487764 | 9.89E-05 | 0.08 |
| LOC124977 | -0.083163617 | 0.00594036 | -0.090264923 | 0.0028183 | 0.01 |
| SPN | -0.158914094 | 1.28E-07 | -0.230342347 | 1.25E-14 | 0.08 |
| SPO11 | 0.04001387 | 0.18620269 | 0.007489324 | 0.8046596 | 0.04 |
| SPOCD1 | -0.019193299 | 0.52616347 | 0.025960962 | 0.3911984 | 0.05 |
| SPOCK1 | -0.267332175 | 2.42E-19 | -0.094680367 | 0.0017265 | 0.18 |
| SPOCK2 | 0.095100219 | 0.00164614 | -0.089293561 | 0.0031305 | 0.19 |
| SPOCK3 | -0.156907913 | 1.85E-07 | -0.143752918 | 1.82E-06 | 0.02 |
| MGC10724 | -0.289707708 | 1.41E-22 | -0.128482169 | 2.04E-05 | 0.17 |
| LOC100130872.SPON2 | -0.090334195 | 0.00279714 | -0.059355486 | 0.0497847 | 0.04 |
| SPOPL | -0.497315373 | 2.37E-69 | -0.244796444 | 2.23E-16 | 0.26 |
| SPOP | -0.239805951 | 9.24E-16 | -0.08157845 | 0.0069665 | 0.16 |
| SPP1 | 0.064426976 | 0.03319085 | -0.043392532 | 0.1516822 | 0.11 |
| SPP2 | -0.057544167 | 0.05719194 | -0.049160353 | 0.1042924 | 0.01 |
| SPPL2A | -0.275588226 | 1.68E-20 | -0.164511772 | 4.50E-08 | 0.12 |
| KIAA1532 | 0.039007926 | 0.19752386 | 0.026307788 | 0.384899 | 0.02 |
| DKFZP586C1324 | 0.123448646 | 4.27E-05 | 0.094388176 | 0.0017845 | 0.03 |
| SPRED1 | -0.2300408 | 1.36E-14 | -0.20390057 | 1.01E-11 | 0.03 |
| SPRED2 | -0.404428063 | 2.90E-44 | -0.21521955 | 6.40E-13 | 0.19 |
| LOC388538 | -0.290100738 | 1.23E-22 | -0.291660561 | 7.11E-23 | 0.01 |
| LOC399831 | 0.130689377 | 1.46E-05 | 0.126060841 | 2.92E-05 | 0.01 |
| SPRR1A | 0.182410443 | 1.24E-09 | 0.085410985 | 0.0047182 | 0.1 |
| SPRR1B | 0.190939809 | 1.97E-10 | 0.032750334 | 0.2793435 | 0.16 |
| SPRR2A | 0.189616755 | 2.63E-10 | 0.011136603 | 0.713043 | 0.18 |
| SPRR2B | 0.07678713 | 0.01110205 | -0.00822744 | 0.785855 | 0.09 |
| SPRR2C | 0.144750913 | 1.54E-06 | 0.026678494 | 0.3782349 | 0.12 |
| SPRR2D | 0.180927367 | 1.70E-09 | 0.000971175 | 0.9744155 | 0.18 |
| SPRR2E | 0.153716565 | 3.28E-07 | -0.013009553 | 0.6674655 | 0.17 |
| SPRR2F | 0.184798977 | 7.48E-10 | 0.071601197 | 0.0179085 | 0.12 |
| SPRR2G | 0.134950302 | 7.57E-06 | 0.035458537 | 0.2414759 | 0.1 |
| SPRR3 | 0.089480928 | 0.00306796 | 0.085958316 | 0.0044573 | 0.01 |
| SPRR4 | 0.102877276 | 0.00065843 | -0.011857131 | 0.6953771 | 0.12 |
| LOC91129 | -0.35958573 | 1.05E-34 | -0.235197496 | 3.34E-15 | 0.13 |
| SPRY2 | -0.165888122 | 3.46E-08 | -0.255357006 | 9.90E-18 | 0.09 |
| LOC170187 | -0.055055535 | 0.06884072 | -0.154198596 | 3.01E-07 | 0.1 |
| SPRY4 | -0.262847194 | 9.94E-19 | -0.197587845 | 4.40E-11 | 0.07 |
| SPRYD3 | -0.344168022 | 9.44E-32 | -0.161551278 | 7.87E-08 | 0.19 |
| SPRYD4 | 0.039658726 | 0.19014357 | 0.082277825 | 0.0064956 | 0.05 |
| LOC144030 | 0.152845266 | 3.83E-07 | 0.110637428 | 0.0002475 | 0.05 |
| SPR | -0.00111041 | 0.97074911 | 0.054827818 | 0.0699975 | 0.06 |
| SPSB1 | 0.034737107 | 0.25119152 | -0.045797987 | 0.130237 | 0.09 |
| SPSB2 | 0.210709812 | 1.96E-12 | 0.197441017 | 4.55E-11 | 0.02 |
| SPSB3 | 0.020681256 | 0.49459142 | 0.057701591 | 0.0565138 | 0.04 |
| SPSB4 | 0.078817523 | 0.0091386 | 0.013960672 | 0.6447686 | 0.07 |
| SPTA1 | -0.162438169 | 6.66E-08 | -0.195734841 | 6.71E-11 | 0.04 |
| SPTAN1 | -0.355316703 | 7.18E-34 | -0.350367433 | 6.42E-33 | 0.01 |
| SPTBN1 | -0.309552685 | 1.06E-25 | -0.435375372 | 8.95E-52 | 0.13 |
| SPTBN2 | 0.100138428 | 0.00091584 | -0.00372976 | 0.9019747 | 0.11 |
| SPTBN4 | -0.313279901 | 2.57E-26 | -0.211291734 | 1.70E-12 | 0.11 |
| SPTBN5 | -0.126411478 | 2.77E-05 | -0.02384687 | 0.4309304 | 0.11 |
| LOC122884 | -0.170067708 | 1.54E-08 | -0.181219197 | 1.60E-09 | 0.02 |
| SPTLC1 | -0.193646258 | 1.08E-10 | -0.135023309 | 7.49E-06 | 0.06 |
| SPTLC2 | -0.469410736 | 5.53E-61 | -0.240205485 | 8.25E-16 | 0.23 |
| SPTLC3 | -0.131429175 | 1.31E-05 | 0.01624922 | 0.591524 | 0.15 |
| SPTY2D1 | -0.347125347 | 2.64E-32 | -0.302576661 | 1.42E-24 | 0.05 |
| SPZ1 | 0.160319486 | 9.89E-08 | 0.045368463 | 0.1338835 | 0.12 |
| SQLE | 0.30323427 | 1.11E-24 | 0.155690927 | 2.30E-07 | 0.15 |
| SQRDL | -0.213616908 | 9.56E-13 | 0.02863743 | 0.3442099 | 0.25 |
| OSIL | -0.027713178 | 0.36001298 | 0.044222188 | 0.1440012 | 0.08 |
| U2SURP | 0.107873357 | 0.00035332 | 0.017281844 | 0.5681785 | 0.1 |
| SRA1 | 0.131654932 | 1.26E-05 | 0.252133656 | 2.60E-17 | 0.13 |
| SRBD1 | -0.263943087 | 7.06E-19 | -0.250268955 | 4.52E-17 | 0.02 |
| SRCAP | -0.27200052 | 5.42E-20 | -0.184521377 | 7.93E-10 | 0.09 |
| SRCIN1 | -0.309531634 | 1.07E-25 | -0.056064407 | 0.0639029 | 0.26 |
| SSC4D | 0.355706108 | 6.04E-34 | 0.173093275 | 8.43E-09 | 0.19 |
| SRC | 0.222177535 | 1.09E-13 | 0.00479411 | 0.8742082 | 0.22 |
| SRD5A1 | 0.318521301 | 3.40E-27 | 0.135495906 | 6.95E-06 | 0.19 |
| SRD5A2 | 0.018577615 | 0.53952176 | 0.00908752 | 0.7641002 | 0.01 |
| SRD5A3 | 0.072050155 | 0.01720072 | 0.209106458 | 2.90E-12 | 0.14 |
| LOC124554 | -0.221172651 | 1.41E-13 | -0.060791711 | 0.0444983 | 0.17 |
| SREBF2 | -0.113498716 | 0.00016978 | -0.124094751 | 3.89E-05 | 0.02 |
| SRFBP1 | -0.294555932 | 2.56E-23 | -0.2035082 | 1.11E-11 | 0.1 |
| SRF | 0.171061497 | 1.26E-08 | 0.042474539 | 0.1605399 | 0.13 |
| SRGAP1 | -0.243618813 | 3.13E-16 | -0.352843819 | 2.16E-33 | 0.11 |
| LOC100132966 | 0.068178232 | 0.02419278 | 0.081546933 | 0.0069884 | 0.02 |
| SRGAP3 | -0.316336611 | 7.95E-27 | -0.261616545 | 1.46E-18 | 0.06 |
| SRGN | -0.013191339 | 0.66310313 | -0.147859596 | 9.12E-07 | 0.14 |
| SRI | 0.060013469 | 0.04730088 | 0.052979202 | 0.0799895 | 0.01 |
| SRL | -0.374533028 | 1.00E-37 | -0.289737103 | 1.39E-22 | 0.09 |
| C20orf148 | -0.390947765 | 3.12E-41 | -0.165893184 | 3.46E-08 | 0.23 |
| SRM | 0.457063561 | 1.60E-57 | 0.309563762 | 1.05E-25 | 0.15 |
| SRP14 | 0.062497858 | 0.03884084 | 0.178030459 | 3.10E-09 | 0.12 |
| SRP19 | 0.251354747 | 3.28E-17 | 0.347171189 | 2.59E-32 | 0.1 |
| SRP54 | -0.088475824 | 0.00341748 | -0.048519419 | 0.1088933 | 0.04 |
| SRP68 | 0.198352671 | 3.69E-11 | 0.048744688 | 0.1072583 | 0.15 |
| SRP72 | 0.018124528 | 0.54945939 | -0.034639159 | 0.2525313 | 0.06 |
| SRP9 | 0.048807479 | 0.10680602 | 0.492098165 | 9.98E-68 | 0.45 |
| SRPK1 | 0.417016063 | 3.17E-47 | 0.188796705 | 3.15E-10 | 0.23 |
| SRPK2 | -0.206404062 | 5.57E-12 | -0.161406956 | 8.08E-08 | 0.05 |
| SRPK3 | 0.113943818 | 0.00015998 | 0.183673553 | 9.50E-10 | 0.07 |
| SRPRB | 0.376293827 | 4.30E-38 | 0.298428655 | 6.40E-24 | 0.08 |
| MGC9571 | -0.028532013 | 0.34598964 | -0.061826294 | 0.0409903 | 0.04 |
| SRPX2 | -0.207707102 | 4.07E-12 | -0.078028969 | 0.009861 | 0.13 |
| SRPX | -0.151036206 | 5.26E-07 | -0.134953275 | 7.57E-06 | 0.02 |
| SRRD | 0.18499428 | 7.17E-10 | 0.202016675 | 1.58E-11 | 0.02 |
| SRRM1 | -0.039497227 | 0.19195583 | -0.202987179 | 1.25E-11 | 0.17 |
| SRRM2 | -0.416020771 | 5.50E-47 | -0.350948695 | 4.97E-33 | 0.07 |
| SRRM3 | -0.233333635 | 5.57E-15 | -0.110542621 | 0.0002506 | 0.13 |
| SRRM4 | -0.006268415 | 0.83600816 | -0.059353388 | 0.0497928 | 0.06 |
| SRRM5 | 0.083371761 | 0.00581624 | 0.040857615 | 0.1770823 | 0.05 |
| SRRT | 0.318594632 | 3.31E-27 | 0.269836925 | 1.09E-19 | 0.05 |
| SRR | -0.149535435 | 6.83E-07 | -0.07765006 | 0.0102259 | 0.08 |
| SRXN1 | 0.047180505 | 0.1190208 | 0.049218865 | 0.1038802 | 0.01 |
| SRY | 0.040640104 | 0.17940105 | 0.022483129 | 0.4577554 | 0.02 |
| SS18L1 | 0.152637705 | 3.97E-07 | -0.005557512 | 0.8543857 | 0.16 |
| SS18L2 | 0.421790886 | 2.21E-48 | 0.455098333 | 5.53E-57 | 0.04 |
| SS18 | 0.343109928 | 1.48E-31 | 0.052255168 | 0.0842058 | 0.3 |
| SSBP1 | 0.546299502 | 4.70E-86 | 0.559412882 | 5.18E-91 | 0.02 |
| SSBP2 | -0.268463858 | 1.69E-19 | -0.107678568 | 0.0003622 | 0.17 |
| SSBP3 | 0.245181832 | 1.99E-16 | 0.098721508 | 0.001083 | 0.15 |
| SSBP4 | 0.031604344 | 0.29652139 | 0.147204049 | 1.02E-06 | 0.12 |
| SSB | 0.514112433 | 8.96E-75 | 0.3761629 | 4.58E-38 | 0.14 |
| SSC5D | -0.38348754 | 1.29E-39 | -0.194569993 | 8.74E-11 | 0.19 |
| SSFA2 | -0.3075458 | 2.25E-25 | -0.286146988 | 4.83E-22 | 0.03 |
| SSH1 | -0.330388113 | 3.00E-29 | -0.406819588 | 8.11E-45 | 0.08 |
| LOC348245 | -0.25660919 | 6.77E-18 | -0.250238305 | 4.56E-17 | 0.01 |
| FLJ10928 | -0.329169233 | 4.93E-29 | -0.061069488 | 0.0435325 | 0.27 |
| SSNA1 | 0.310743887 | 6.75E-26 | 0.31035754 | 7.82E-26 | 0.01 |
| SSPN | -0.227325864 | 2.81E-14 | -0.16591471 | 3.44E-08 | 0.07 |
| FLJ45737 | -0.28242888 | 1.71E-21 | -0.127943219 | 2.21E-05 | 0.16 |
| SSR1 | 0.135215522 | 7.26E-06 | 0.094879922 | 0.0016879 | 0.05 |
| SSR2 | 0.328312901 | 6.97E-29 | 0.551563798 | 5.10E-88 | 0.23 |
| SSR3 | 0.028144024 | 0.35259046 | 0.014281655 | 0.6371815 | 0.02 |
| SSR4 | 0.308306955 | 1.69E-25 | 0.26541831 | 4.44E-19 | 0.05 |
| SSRP1 | 0.440577724 | 4.08E-53 | 0.171155252 | 1.24E-08 | 0.27 |
| SSSCA1 | 0.311993594 | 4.20E-26 | 0.313216386 | 2.64E-26 | 0.01 |
| SSTR1 | -0.304349518 | 7.37E-25 | -0.161681001 | 7.68E-08 | 0.15 |
| SSTR2 | -0.087963001 | 0.00360943 | 0.000369523 | 0.9902639 | 0.09 |
| SSTR3 | -0.02625609 | 0.38583406 | -0.187469078 | 4.21E-10 | 0.17 |
| SSTR4 | -0.052725995 | 0.0814441 | -0.072007667 | 0.0172666 | 0.02 |
| SSTR5 | -0.030874019 | 0.30782938 | -0.003917867 | 0.8970577 | 0.03 |
| SST | 0.044933458 | 0.13765647 | -0.004675793 | 0.8772879 | 0.05 |
| SSU72 | 0.433453214 | 2.76E-51 | 0.267687096 | 2.16E-19 | 0.17 |
| SSX1 | 0.084271363 | 0.00530624 | 0.006375834 | 0.8332388 | 0.08 |
| SSX2IP | 0.1214453 | 5.69E-05 | 0.023366678 | 0.4402704 | 0.1 |
| SSX2 | 0.063979501 | 0.03443469 | 0.015919188 | 0.5990771 | 0.05 |
| SSX3 | 0.022670434 | 0.45401655 | 0.017294936 | 0.5678854 | 0.01 |
| SSX4 | 0.060846968 | 0.04430474 | 0.029675683 | 0.3269935 | 0.04 |
| SSX5 | 0.043034967 | 0.1550871 | -0.01591015 | 0.5992845 | 0.06 |
| SSXP2 | 0.095133784 | 0.00163987 | 0.047019594 | 0.120286 | 0.05 |
| SSX7 | 0.004220137 | 0.88916488 | -0.0282811 | 0.3502494 | 0.04 |
| LOC90697 | 0.050564986 | 0.09474887 | -0.01678714 | 0.5793078 | 0.07 |
| P48 | 0.011204254 | 0.71137759 | -0.031491539 | 0.2982496 | 0.05 |
| PRSS14_PENDING | 0.255819068 | 8.61E-18 | 0.156418505 | 2.02E-07 | 0.1 |
| ST18 | 0.023192436 | 0.44368798 | -0.137187104 | 5.32E-06 | 0.17 |
| ST20 | 0.359413671 | 1.14E-34 | 0.278508489 | 6.39E-21 | 0.09 |
| LOC286167 | -0.115195792 | 0.0001352 | -0.09815653 | 0.0011571 | 0.02 |
| ST3GAL2 | -0.19201355 | 1.55E-10 | -0.290959113 | 9.10E-23 | 0.1 |
| MRT12 | 0.055198839 | 0.06812082 | 0.002899065 | 0.9237309 | 0.06 |
| ST3GAL4 | 0.193947131 | 1.01E-10 | 0.038789084 | 0.200052 | 0.16 |
| ST3GAL5 | -0.191366322 | 1.79E-10 | -0.095134411 | 0.0016398 | 0.1 |
| ST3GAL6 | 0.189545482 | 2.67E-10 | 0.032793195 | 0.2787144 | 0.16 |
| ST5 | -0.104995957 | 0.00050731 | -0.116944073 | 0.0001066 | 0.02 |
| LOC102723861 | -0.126468843 | 2.75E-05 | -0.094645554 | 0.0017333 | 0.04 |
| SIAT2 | -0.265495937 | 4.33E-19 | -0.104634194 | 0.0005306 | 0.17 |
| ST6GALNAC1 | -0.262060218 | 1.27E-18 | -0.141071022 | 2.84E-06 | 0.13 |
| ST6GALNAC2 | -0.206068459 | 6.03E-12 | -0.091693217 | 0.0024105 | 0.12 |
| ST6GALNAC3 | -0.179666888 | 2.21E-09 | -0.145384942 | 1.39E-06 | 0.04 |
| ST6GALNAC4 | -0.022195965 | 0.46352093 | 0.009218013 | 0.7608153 | 0.04 |
| ST6GALNAC5 | 0.016160891 | 0.59354119 | -0.057922943 | 0.0555715 | 0.08 |
| ST6GALNAC6 | -0.261400655 | 1.56E-18 | -0.2074148 | 4.36E-12 | 0.06 |
| ST7L | -0.141298174 | 2.74E-06 | -0.162594166 | 6.47E-08 | 0.03 |
| ST7.AS1 | 0.079384678 | 0.00864856 | -0.001434689 | 0.9622125 | 0.09 |
| ST7.AS2 | -0.001466439 | 0.96137688 | -0.022891291 | 0.4496301 | 0.03 |
| ST7.OT3 | -0.001615407 | 0.95745689 | -0.053883488 | 0.0749659 | 0.06 |
| ST7.OT4 | 0.024716678 | 0.41430761 | -0.03538809 | 0.2424128 | 0.07 |
| ST7 | 0.035354814 | 0.24285627 | 0.092643129 | 0.00217 | 0.06 |
| ST8SIA1 | 0.231773497 | 8.51E-15 | 0.073065887 | 0.0156893 | 0.16 |
| ST8SIA2 | -0.074190536 | 0.01415306 | -0.069694287 | 0.021206 | 0.01 |
| ST8SIA3 | 0.051217435 | 0.09056022 | 0.006998911 | 0.817217 | 0.05 |
| ST8SIA4 | -0.152307551 | 4.21E-07 | -0.225652901 | 4.38E-14 | 0.08 |
| ST8SIA5 | 0.141674656 | 2.57E-06 | 0.009100943 | 0.7637621 | 0.14 |
| ST8SIA6 | -0.41913199 | 9.80E-48 | -0.200373988 | 2.31E-11 | 0.22 |
| STAB1 | -0.219163454 | 2.36E-13 | -0.253876762 | 1.54E-17 | 0.04 |
| STAB2 | -0.258165555 | 4.22E-18 | -0.209224073 | 2.82E-12 | 0.05 |
| STAC2 | 0.075638492 | 0.01237129 | -0.039137036 | 0.1960433 | 0.12 |
| STAC3 | 0.141564405 | 2.62E-06 | 0.021551522 | 0.4766056 | 0.13 |
| STAC | 0.182674049 | 1.17E-09 | -0.040532196 | 0.1805597 | 0.23 |
| STAG1 | -0.275614144 | 1.67E-20 | -0.321428699 | 1.09E-27 | 0.05 |
| STAG2 | -0.22070776 | 1.59E-13 | -0.273891754 | 2.93E-20 | 0.06 |
| STAG3L1 | -0.176904496 | 3.90E-09 | -0.248383174 | 7.87E-17 | 0.08 |
| STAG3L2 | -0.168180996 | 2.22E-08 | -0.260624986 | 1.98E-18 | 0.1 |
| STAG3L3 | -0.131368181 | 1.32E-05 | -0.219551901 | 2.14E-13 | 0.09 |
| STAG3L4 | 0.07298609 | 0.01580366 | 0.099788509 | 0.0009547 | 0.03 |
| STAG3 | 0.200601016 | 2.19E-11 | -0.041598726 | 0.1693492 | 0.25 |
| STAM2 | -0.533630848 | 1.81E-81 | -0.359256833 | 1.22E-34 | 0.18 |
| STAMBPL1 | -0.094788795 | 0.00170541 | -0.136666743 | 5.78E-06 | 0.05 |
| STAMBP | 0.276935008 | 1.08E-20 | 0.18896794 | 3.03E-10 | 0.09 |
| STAM | 0.039593503 | 0.19087395 | -0.070823642 | 0.0191946 | 0.12 |
| STAP1 | 0.018315539 | 0.54525894 | -0.101612628 | 0.0007676 | 0.12 |
| STAP2 | 0.273640182 | 3.18E-20 | 0.250848386 | 3.81E-17 | 0.03 |
| STARD10 | -0.167618031 | 2.48E-08 | -0.029885733 | 0.3235795 | 0.14 |
| LINC00464 | -0.464407717 | 1.45E-59 | -0.329136104 | 5.00E-29 | 0.14 |
| STARD3NL | 0.024710737 | 0.41441983 | 0.134305195 | 8.38E-06 | 0.11 |
| STARD3 | -0.005428788 | 0.85772203 | 0.06055052 | 0.0453515 | 0.07 |
| STARD4 | -0.095590754 | 0.00155663 | -0.236388504 | 2.40E-15 | 0.15 |
| STARD5 | -0.213671494 | 9.43E-13 | -0.080575629 | 0.0076951 | 0.14 |
| STARD6 | 0.170362957 | 1.45E-08 | 0.137878433 | 4.76E-06 | 0.04 |
| STARD7 | 0.324728054 | 2.94E-28 | 0.086628656 | 0.0041556 | 0.24 |
| STARD8 | -0.477948834 | 1.84E-63 | -0.399776649 | 3.34E-43 | 0.08 |
| STAR | 0.167666632 | 2.46E-08 | -0.019339228 | 0.5230222 | 0.19 |
| STAT1 | 0.026324748 | 0.38459258 | -0.110290588 | 0.0002589 | 0.14 |
| STAT2 | -0.188896332 | 3.08E-10 | -0.197377859 | 4.62E-11 | 0.01 |
| STAT3 | -0.323760876 | 4.33E-28 | -0.259067344 | 3.20E-18 | 0.07 |
| STAT4 | -0.031444517 | 0.29897202 | -0.114703247 | 0.0001445 | 0.09 |
| STAT5A | -0.105306427 | 0.0004881 | -0.086562722 | 0.0041844 | 0.02 |
| STAT5B | -0.27449042 | 2.41E-20 | -0.20930603 | 2.76E-12 | 0.07 |
| STAT6 | -0.293156936 | 4.20E-23 | -0.18726614 | 4.39E-10 | 0.11 |
| STATH | 0.107571073 | 0.00036716 | 0.055820447 | 0.0650693 | 0.06 |
| STAU1 | 0.020805151 | 0.49200897 | -0.034744268 | 0.2510938 | 0.06 |
| STAU2 | -0.03469049 | 0.25182855 | -0.023646592 | 0.4348119 | 0.02 |
| STBD1 | -0.231028568 | 1.04E-14 | -0.127679116 | 2.30E-05 | 0.11 |
| STC1 | -0.203438511 | 1.13E-11 | -0.115847038 | 0.0001238 | 0.09 |
| STC2 | -0.270174579 | 9.77E-20 | -0.174166315 | 6.80E-09 | 0.1 |
| STEAP1 | -0.101850264 | 0.00074585 | 0.065667033 | 0.0299427 | 0.17 |
| STEAP2 | -0.383335813 | 1.40E-39 | -0.139599597 | 3.61E-06 | 0.25 |
| STEAP3 | 0.090900005 | 0.00262978 | 0.026135071 | 0.3880283 | 0.07 |
| STEAP4 | -0.34420361 | 9.30E-32 | -0.293167386 | 4.18E-23 | 0.06 |
| STH | -0.262426579 | 1.13E-18 | -0.12894143 | 1.90E-05 | 0.14 |
| STIL | 0.372348881 | 2.83E-37 | 0.104850455 | 0.0005166 | 0.27 |
| STIM1 | -0.105986744 | 0.00044834 | -0.261260276 | 1.63E-18 | 0.16 |
| STIM2 | -0.199943314 | 2.56E-11 | -0.211054824 | 1.80E-12 | 0.02 |
| STIP1 | 0.410168773 | 1.34E-45 | 0.221436999 | 1.32E-13 | 0.19 |
| PRO2729 | -0.188808994 | 3.14E-10 | -0.309364307 | 1.14E-25 | 0.13 |
| STK11IP | -0.034205473 | 0.25852324 | -0.006273397 | 0.8358797 | 0.03 |
| STK11 | 0.010623217 | 0.72572532 | 0.012799456 | 0.6725213 | 0.01 |
| STK16 | -0.12271924 | 4.74E-05 | 0.057009305 | 0.0595467 | 0.18 |
| STK17A | -0.026320453 | 0.38467016 | -0.100313699 | 0.0008969 | 0.08 |
| STK17B | -0.17549875 | 5.19E-09 | -0.238502038 | 1.33E-15 | 0.07 |
| LOC154190 | 0.17087109 | 1.31E-08 | 0.250554836 | 4.15E-17 | 0.08 |
| STK24 | 0.205927291 | 6.24E-12 | 0.016242399 | 0.5916796 | 0.19 |
| STK25 | 0.221789864 | 1.20E-13 | 0.105489674 | 0.0004771 | 0.12 |
| STK31 | 0.077472864 | 0.01040065 | 0.093971088 | 0.0018705 | 0.02 |
| STK32A | -0.135362252 | 7.10E-06 | -0.211416577 | 1.65E-12 | 0.08 |
| STK32B | -0.356979662 | 3.41E-34 | -0.155213448 | 2.51E-07 | 0.21 |
| STK32C | 0.204353248 | 9.08E-12 | 0.12875408 | 1.96E-05 | 0.08 |
| STK33 | 0.191188889 | 1.86E-10 | 0.019033409 | 0.5296163 | 0.18 |
| STK35 | 0.011121053 | 0.71342598 | -0.076829265 | 0.0110578 | 0.09 |
| STK36 | -0.30654849 | 3.26E-25 | -0.105637698 | 0.0004683 | 0.21 |
| STK38L | 0.018440213 | 0.5425259 | -0.089938041 | 0.00292 | 0.11 |
| STK38 | 0.306820471 | 2.95E-25 | 0.090002852 | 0.0028996 | 0.22 |
| STK39 | -0.21881957 | 2.58E-13 | 0.037082665 | 0.2205783 | 0.26 |
| LOC116036 | 0.18090421 | 1.70E-09 | 0.087318404 | 0.0038646 | 0.1 |
| STK40 | -0.008087919 | 0.78940027 | -0.14738457 | 9.89E-07 | 0.14 |
| LOC200227 | -0.181609784 | 1.47E-09 | -0.250424285 | 4.32E-17 | 0.07 |
| RNF217.AS1 | -0.0160111 | 0.59696919 | 0.018285733 | 0.5459133 | 0.04 |
| STMN1 | 0.611287937 | 5.64E-113 | 0.371892341 | 3.51E-37 | 0.24 |
| STMN2 | -0.230222667 | 1.29E-14 | -0.102436924 | 0.0006947 | 0.13 |
| LOC115848 | -0.082303442 | 0.00647893 | -0.160082767 | 1.03E-07 | 0.08 |
| STMN4 | 0.048058133 | 0.11230265 | 0.008028346 | 0.7909154 | 0.05 |
| STOML1 | 0.051450226 | 0.08910224 | 0.017973456 | 0.5527928 | 0.04 |
| STOML2 | 0.505374489 | 6.47E-72 | 0.321888283 | 9.09E-28 | 0.19 |
| STOML3 | -0.007798258 | 0.7967746 | 0.066410142 | 0.0281301 | 0.08 |
| STOM | -0.222759984 | 9.34E-14 | -0.156976585 | 1.83E-07 | 0.07 |
| STON1.GTF2A1L | -0.228696396 | 1.95E-14 | -0.237630161 | 1.70E-15 | 0.01 |
| STON1 | -0.069821721 | 0.02097018 | -0.107043347 | 0.0003925 | 0.04 |
| STON2 | -0.311758227 | 4.60E-26 | -0.220684009 | 1.60E-13 | 0.1 |
| STOX1 | -0.113113737 | 0.00017871 | -0.143579801 | 1.88E-06 | 0.04 |
| STOX2 | -0.044303287 | 0.1432667 | -0.209197647 | 2.83E-12 | 0.17 |
| STRA13 | 0.42329514 | 9.49E-49 | 0.358885897 | 1.45E-34 | 0.07 |
| STRA6 | 0.002652225 | 0.93020745 | 0.006395557 | 0.8327305 | 0.01 |
| STRA8 | 0.248297004 | 8.07E-17 | 0.063500931 | 0.0358088 | 0.19 |
| STRADA | 0.027812706 | 0.35828968 | 0.066186433 | 0.0286655 | 0.04 |
| STRADB | -0.138583432 | 4.25E-06 | 0.062461767 | 0.0389539 | 0.21 |
| STRAP | 0.294005409 | 3.11E-23 | 0.144097797 | 1.72E-06 | 0.15 |
| ILF3L | 0.021884278 | 0.46982429 | -0.004970206 | 0.8696282 | 0.03 |
| STRC | -0.117335648 | 0.000101 | -0.118175456 | 9.00E-05 | 0.01 |
| STRN3 | -0.363029312 | 2.19E-35 | -0.220540964 | 1.66E-13 | 0.15 |
| STRN4 | 0.130088662 | 1.60E-05 | -0.000859996 | 0.9773436 | 0.14 |
| STRN | -0.305226087 | 5.33E-25 | -0.372674518 | 2.42E-37 | 0.07 |
| AKU | -0.343840747 | 1.09E-31 | -0.187776297 | 3.93E-10 | 0.16 |
| STT3A | 0.094866744 | 0.00169039 | 0.096034321 | 0.0014796 | 0.01 |
| STT3B | -0.303562705 | 9.85E-25 | -0.336310875 | 2.61E-30 | 0.04 |
| STUB1 | 0.11839193 | 8.73E-05 | 0.172783431 | 8.97E-09 | 0.06 |
| STX10 | 0.275479354 | 1.74E-20 | 0.184583024 | 7.83E-10 | 0.1 |
| STX11 | -0.112339209 | 0.00019802 | -0.273026451 | 3.88E-20 | 0.17 |
| STX12 | -0.25851961 | 3.78E-18 | -0.264015027 | 6.90E-19 | 0.01 |
| STX16 | -0.00212211 | 0.94413164 | -0.074072477 | 0.0143078 | 0.08 |
| STX17 | -0.259028506 | 3.24E-18 | -0.235588768 | 3.00E-15 | 0.03 |
| STX18 | -0.116317318 | 0.00011611 | 0.068602964 | 0.0233213 | 0.19 |
| STX19 | 0.131869599 | 1.22E-05 | 0.112954119 | 0.0001825 | 0.02 |
| STX1A | 0.130765655 | 1.45E-05 | 0.101121397 | 0.0008143 | 0.03 |
| STX1B | -0.271887919 | 5.62E-20 | -0.188125449 | 3.65E-10 | 0.09 |
| STX2C | -0.116465016 | 0.00011379 | -0.132758127 | 1.06E-05 | 0.02 |
| STX3 | 0.035374947 | 0.24258791 | 0.018119744 | 0.5495648 | 0.02 |
| STX4 | 0.083701983 | 0.00562412 | 0.104558903 | 0.0005356 | 0.03 |
| LOC116078 | 0.066214587 | 0.02859768 | 0.065485936 | 0.0303993 | 0.01 |
| LOC102724791 | 0.189930555 | 2.46E-10 | 0.444363244 | 4.17E-54 | 0.26 |
| LOC134859 | -0.160708071 | 9.20E-08 | -0.107208293 | 0.0003844 | 0.06 |
| STX8 | 0.11806951 | 9.13E-05 | 0.203222922 | 1.19E-11 | 0.09 |
| STXBP1 | -0.314952808 | 1.36E-26 | -0.331306482 | 2.06E-29 | 0.02 |
| STXBP2 | -0.108039846 | 0.0003459 | 0.03575946 | 0.2375024 | 0.15 |
| UNC.18C | -0.12865956 | 1.99E-05 | -0.175030699 | 5.71E-09 | 0.05 |
| STXBP4 | -0.306947152 | 2.81E-25 | -0.165835379 | 3.50E-08 | 0.15 |
| STXBP5L | 0.073534453 | 0.01503229 | -0.03911497 | 0.1962958 | 0.12 |
| STXBP5 | -0.324942793 | 2.70E-28 | -0.296947163 | 1.09E-23 | 0.03 |
| STXBP6 | 0.089799082 | 0.00296431 | 0.01404742 | 0.6427144 | 0.08 |
| STYK1 | 0.059573528 | 0.04894977 | -0.059049611 | 0.050976 | 0.12 |
| STYXL1 | 0.117497575 | 9.88E-05 | 0.23526364 | 3.28E-15 | 0.12 |
| LOC122792 | -0.156396852 | 2.03E-07 | 0.074969896 | 0.0131678 | 0.24 |
| LOC105374713 | 0.083503818 | 0.00573871 | 0.085825047 | 0.0045196 | 0.01 |
| LOC51770 | -0.29489514 | 2.27E-23 | -0.230710033 | 1.14E-14 | 0.07 |
| SUCLG1 | 0.375787914 | 5.48E-38 | 0.319065328 | 2.75E-27 | 0.06 |
| SUCLG2 | -0.269801809 | 1.10E-19 | 0.012546215 | 0.678635 | 0.29 |
| SUCNR1 | -0.204410485 | 8.96E-12 | -0.294959959 | 2.22E-23 | 0.1 |
| SUDS3 | -0.002819592 | 0.92581555 | 0.012616048 | 0.676947 | 0.02 |
| SUFU | -0.305385825 | 5.02E-25 | -0.271064071 | 7.33E-20 | 0.04 |
| SUGT1P3 | -0.323725568 | 4.39E-28 | -0.148552106 | 8.10E-07 | 0.18 |
| SUGT1P1 | -0.287987962 | 2.56E-22 | -0.127307779 | 2.43E-05 | 0.17 |
| SUGT1 | 0.331217582 | 2.14E-29 | 0.312400281 | 3.60E-26 | 0.02 |
| SULF1 | -0.183969261 | 8.92E-10 | -0.134598691 | 8.00E-06 | 0.05 |
| SULF2 | -0.192466893 | 1.40E-10 | -0.142160654 | 2.38E-06 | 0.06 |
| SULT1A1 | -0.119796834 | 7.18E-05 | -0.010833135 | 0.7205303 | 0.11 |
| SULT1A2 | -0.125218978 | 3.30E-05 | 0.083937276 | 0.0054907 | 0.21 |
| LOC648394 | 0.252252524 | 2.51E-17 | 0.172814484 | 8.92E-09 | 0.08 |
| LOC113104 | -0.036531566 | 0.22751898 | -0.117814364 | 9.46E-05 | 0.09 |
| LOC130514 | -0.04352564 | 0.15042935 | -0.052828871 | 0.0808505 | 0.01 |
| SULT1C3 | -0.040890232 | 0.17673653 | -0.053164084 | 0.0789408 | 0.02 |
| SULT1C | -0.241355931 | 5.96E-16 | -0.253857947 | 1.55E-17 | 0.02 |
| SULT1E1 | 0.130845403 | 1.43E-05 | -0.009464043 | 0.7546336 | 0.15 |
| SULT2A1 | -0.004942399 | 0.87035115 | -0.013755991 | 0.649626 | 0.01 |
| SULT2B1 | -0.12151154 | 5.64E-05 | -0.009580334 | 0.7517172 | 0.12 |
| SULT4A1 | 0.161834402 | 7.46E-08 | 0.028652462 | 0.3439566 | 0.14 |
| SULT6B1 | 0.067786827 | 0.02502066 | -0.032431522 | 0.284053 | 0.11 |
| SUMF1 | -0.174264758 | 6.67E-09 | 0.002847503 | 0.9250834 | 0.18 |
| SUMF2 | -0.075864518 | 0.01211184 | 0.039950622 | 0.1869001 | 0.12 |
| SUMO1P1 | 0.08240614 | 0.0064124 | 0.047457557 | 0.1168668 | 0.04 |
| SUMO1P3 | 0.400423866 | 2.38E-43 | 0.433396789 | 2.86E-51 | 0.04 |
| SUMO1 | 0.452320925 | 3.14E-56 | 0.45343876 | 1.56E-56 | 0.01 |
| SUMO2 | 0.48472835 | 1.76E-65 | 0.372471199 | 2.67E-37 | 0.12 |
| SUMO3 | 0.240560817 | 7.47E-16 | 0.210577763 | 2.02E-12 | 0.03 |
| SUMO4 | 0.015988857 | 0.59747899 | -0.020887442 | 0.4902977 | 0.04 |
| FLJ12407 | -0.091029176 | 0.00259287 | -0.207199017 | 4.60E-12 | 0.12 |
| SUN2 | -0.049708915 | 0.10047833 | -0.237200879 | 1.92E-15 | 0.19 |
| SUN3 | 0.146967408 | 1.06E-06 | 0.083849391 | 0.0055402 | 0.07 |
| SUN5 | 0.029854001 | 0.32409379 | 0.044611916 | 0.1404976 | 0.02 |
| SUOX | -0.319793252 | 2.07E-27 | -0.04153607 | 0.169993 | 0.28 |
| SUPT16H | 0.075303041 | 0.01276541 | 0.000365376 | 0.9903732 | 0.08 |
| SUPT3H | 0.151623382 | 4.75E-07 | 0.128306987 | 2.09E-05 | 0.03 |
| SUPT4H1 | 0.153713646 | 3.28E-07 | 0.211839019 | 1.48E-12 | 0.06 |
| SUPT5H | 0.203218481 | 1.19E-11 | 0.077880883 | 0.0100022 | 0.13 |
| SUPT6H | -0.22267983 | 9.53E-14 | -0.166096607 | 3.33E-08 | 0.06 |
| SUPT7L | 0.172701812 | 9.12E-09 | -0.031610864 | 0.2964217 | 0.21 |
| SUPV3L1 | 0.486406588 | 5.48E-66 | 0.322492383 | 7.16E-28 | 0.17 |
| SURF1 | 0.008619745 | 0.77591021 | 0.17383044 | 7.28E-09 | 0.17 |
| SURF2 | 0.403272167 | 5.34E-44 | 0.331928715 | 1.60E-29 | 0.08 |
| LOC157889 | 0.018150255 | 0.54889272 | -0.048941147 | 0.1058483 | 0.07 |
| SURF6 | 0.225294223 | 4.81E-14 | 0.144658898 | 1.57E-06 | 0.09 |
| SUSD1 | -0.241003638 | 6.59E-16 | -0.333534899 | 8.26E-30 | 0.1 |
| SUSD2 | -0.163070521 | 5.91E-08 | -0.115397186 | 0.0001316 | 0.05 |
| SUSD3 | -0.273877676 | 2.94E-20 | -0.077208346 | 0.0106664 | 0.2 |
| SUSD4 | 0.021598847 | 0.47563792 | 0.092405619 | 0.002228 | 0.08 |
| SUSD5 | -0.003475111 | 0.90863698 | -0.038617096 | 0.2020554 | 0.04 |
| SUV39H1 | 0.509579228 | 2.79E-73 | 0.339355995 | 7.30E-31 | 0.18 |
| SUV39H2 | 0.512918386 | 2.23E-74 | 0.299098483 | 5.02E-24 | 0.22 |
| SUV420H1 | -0.14020503 | 3.27E-06 | -0.132075416 | 1.18E-05 | 0.01 |
| SUV420H2 | 0.175587783 | 5.10E-09 | 0.172418039 | 9.65E-09 | 0.01 |
| LOC100421522 | 0.252334816 | 2.45E-17 | 0.172935337 | 8.71E-09 | 0.08 |
| SUZ12 | -0.0680264 | 0.02451107 | -0.09330944 | 0.0020146 | 0.03 |
| SV2A | -0.070647162 | 0.01949746 | -0.103496123 | 0.0006104 | 0.04 |
| LOC100128403 | -0.10805931 | 0.00034505 | -0.191820459 | 1.62E-10 | 0.09 |
| SV2C | 0.00216432 | 0.94302226 | -0.011521536 | 0.7035853 | 0.02 |
| POLYDOM | -0.456313948 | 2.57E-57 | -0.324897443 | 2.75E-28 | 0.14 |
| SVIL | -0.135419771 | 7.03E-06 | -0.188768433 | 3.17E-10 | 0.06 |
| LOC203862 | 0.009685611 | 0.74907997 | 0.010397295 | 0.7313305 | 0.01 |
| SVOPL | 0.144374979 | 1.64E-06 | 0.079542366 | 0.0085165 | 0.07 |
| LOC100505997 | 0.083555784 | 0.00570846 | -0.050968068 | 0.0921432 | 0.14 |
| SWAP70 | -0.355594679 | 6.34E-34 | -0.222557266 | 9.84E-14 | 0.14 |
| SYAP1 | -0.067949963 | 0.02467267 | 0.040230632 | 0.1838272 | 0.11 |
| SYBU | -0.347755734 | 2.01E-32 | -0.16206695 | 7.14E-08 | 0.19 |
| SYCE1L | 0.177433771 | 3.50E-09 | 0.109366054 | 0.0002918 | 0.07 |
| SYCE1 | -0.122073321 | 5.20E-05 | -0.092908139 | 0.0021069 | 0.03 |
| SYCE2 | 0.237300712 | 1.86E-15 | 0.198872475 | 3.27E-11 | 0.04 |
| SYL | -0.001377403 | 0.96372025 | -0.033590171 | 0.2671921 | 0.04 |
| SYCP1 | 0.00697794 | 0.81775504 | 0.057622511 | 0.0568536 | 0.06 |
| SYCP2L | 0.158850822 | 1.30E-07 | -0.007040418 | 0.8161523 | 0.17 |
| SYCP2 | -0.076522707 | 0.01138354 | 0.039606817 | 0.1907247 | 0.12 |
| SYCP3 | 0.023650908 | 0.43472803 | -0.016319113 | 0.58993 | 0.04 |
| SYDE1 | -0.126809761 | 2.62E-05 | -0.094005776 | 0.0018632 | 0.04 |
| SYDE2 | -0.289594282 | 1.46E-22 | -0.247926042 | 8.99E-17 | 0.05 |
| SYF2 | 0.062284174 | 0.03951417 | 0.130925029 | 1.41E-05 | 0.07 |
| SYK | 0.002169355 | 0.94288993 | -0.142701327 | 2.17E-06 | 0.15 |
| SYM | 0.043007794 | 0.1553482 | 0.00825078 | 0.7852623 | 0.04 |
| SYN1 | 0.060376323 | 0.04597626 | -0.12468705 | 3.57E-05 | 0.19 |
| SYN2 | -0.035502897 | 0.24088726 | -0.075869755 | 0.0121059 | 0.05 |
| SYN3 | -0.070336408 | 0.02004095 | -0.048957888 | 0.1057288 | 0.03 |
| SYNCRIP | 0.244405292 | 2.49E-16 | 0.155398944 | 2.43E-07 | 0.09 |
| SYNC | -0.092720749 | 0.00215134 | 0.113419698 | 0.0001716 | 0.21 |
| C6orf98 | -0.457847271 | 9.75E-58 | -0.451157501 | 6.47E-56 | 0.01 |
| DKFZP434H2235 | -0.316794045 | 6.66E-27 | -0.407579368 | 5.40E-45 | 0.1 |
| SYNGAP1 | -0.271304826 | 6.79E-20 | -0.248150384 | 8.42E-17 | 0.03 |
| SYNGR1 | -0.149165021 | 7.29E-07 | -0.166549339 | 3.05E-08 | 0.02 |
| SYNGR2 | 0.081424769 | 0.007074 | 0.138094292 | 4.60E-06 | 0.06 |
| SYNGR3 | 0.104542376 | 0.00053665 | 0.05002445 | 0.0983353 | 0.06 |
| SYNGR4 | 0.164975694 | 4.12E-08 | 0.243080453 | 3.65E-16 | 0.08 |
| SYNJ1 | -0.40786736 | 4.63E-45 | -0.400467049 | 2.33E-43 | 0.01 |
| SYNJ2BP | -0.45066976 | 8.76E-56 | -0.276300181 | 1.33E-20 | 0.18 |
| SYNJ2 | -0.316370836 | 7.85E-27 | -0.350263302 | 6.72E-33 | 0.04 |
| SYNM | 0.017299441 | 0.56778453 | -0.163597717 | 5.35E-08 | 0.19 |
| SYNPO2L | -0.137235898 | 5.28E-06 | -0.130557183 | 1.49E-05 | 0.01 |
| SYNPO2 | -0.359717892 | 9.91E-35 | -0.283218701 | 1.31E-21 | 0.08 |
| SYNPO | -0.292094698 | 6.11E-23 | -0.262717994 | 1.04E-18 | 0.03 |
| SYNPR | 0.087542421 | 0.00377409 | 0.01047923 | 0.729296 | 0.08 |
| SYNRG | -0.388942294 | 8.57E-41 | -0.338549397 | 1.02E-30 | 0.06 |
| SYPL1 | -0.23145893 | 9.27E-15 | -0.015564536 | 0.6072419 | 0.22 |
| SYPL2 | -0.223282491 | 8.15E-14 | -0.210405695 | 2.11E-12 | 0.02 |
| SYP | -0.060987631 | 0.04381522 | -0.036545041 | 0.2273474 | 0.03 |
| SYS1.DBNDD2 | -0.168736345 | 2.00E-08 | -0.12479205 | 3.52E-05 | 0.05 |
| SYS1 | -0.143550606 | 1.89E-06 | -0.029880671 | 0.3236615 | 0.12 |
| SYT10 | -0.013215568 | 0.66252256 | -0.062854352 | 0.0377392 | 0.05 |
| SYT11 | -0.282337337 | 1.77E-21 | -0.298420322 | 6.41E-24 | 0.02 |
| SYT12 | -0.049365135 | 0.10285537 | -0.030484582 | 0.3139742 | 0.02 |
| LOC116033 | -0.032565964 | 0.28206053 | -0.024216723 | 0.4238154 | 0.01 |
| SYT14P1 | -0.056785626 | 0.06055506 | -0.042868408 | 0.1566928 | 0.02 |
| SYT14 | -0.067970213 | 0.02462977 | -0.164485048 | 4.52E-08 | 0.1 |
| SYT15 | -0.203915822 | 1.01E-11 | -0.283822056 | 1.07E-21 | 0.08 |
| SYT16 | 0.030087644 | 0.32031982 | -0.055273004 | 0.0677507 | 0.09 |
| SYT17 | -0.349834292 | 8.11E-33 | -0.091676774 | 0.0024149 | 0.26 |
| LOC100129021 | -0.236031897 | 2.65E-15 | -0.096365631 | 0.0014243 | 0.14 |
| LOC100127887 | -0.096810794 | 0.00135304 | -0.010879895 | 0.7193749 | 0.09 |
| SYT3 | -0.054056918 | 0.07403245 | -0.008035176 | 0.7907416 | 0.05 |
| KIAA1342 | 0.015068788 | 0.61873735 | -0.090207632 | 0.0028359 | 0.11 |
| SYT5 | 0.063784745 | 0.03498835 | -0.02625903 | 0.3857808 | 0.1 |
| SYT6 | -0.298064675 | 7.29E-24 | -0.151080918 | 5.22E-07 | 0.15 |
| SYT7 | -0.098055009 | 0.00117093 | 0.00258024 | 0.9320971 | 0.11 |
| SYT8 | 0.095192562 | 0.00162894 | -0.00199128 | 0.9475708 | 0.1 |
| SYT9 | -0.363547189 | 1.73E-35 | -0.119634086 | 7.34E-05 | 0.25 |
| SYTL1 | -0.088960632 | 0.00324461 | -0.016149865 | 0.5937932 | 0.08 |
| FLJ21219 | -0.459078105 | 4.46E-58 | -0.293291947 | 4.00E-23 | 0.17 |
| SYTL3 | -0.019698711 | 0.51532528 | -0.033533554 | 0.2679997 | 0.02 |
| SYTL4 | -0.345351345 | 5.68E-32 | -0.158854236 | 1.30E-07 | 0.19 |
| SYTL5 | -0.376918849 | 3.18E-38 | -0.154308371 | 2.95E-07 | 0.23 |
| SYVN1 | -0.116027725 | 0.00012078 | -0.064201714 | 0.0338121 | 0.06 |
| TAAR1 | 0.042022942 | 0.16503804 | 0.044899516 | 0.1379543 | 0.01 |
| TAAR2 | 0.024902354 | 0.41080886 | 0.012132403 | 0.6886707 | 0.02 |
| TAAR5 | 0.02416861 | 0.42473711 | 0.020295704 | 0.5026739 | 0.01 |
| SCZD5 | 0.012836009 | 0.67164062 | 0.017401381 | 0.5655047 | 0.01 |
| TAAR8 | 0.016264907 | 0.59116606 | 0.040706342 | 0.1786926 | 0.03 |
| TAAR9 | -0.024924377 | 0.41039504 | -0.000297735 | 0.9921553 | 0.03 |
| TAB1 | 0.040771265 | 0.17800014 | -0.01433918 | 0.6358258 | 0.06 |
| LOC101928709 | -0.133701783 | 9.20E-06 | -0.101594723 | 0.0007692 | 0.04 |
| TAB3 | -0.413366895 | 2.36E-46 | -0.287123875 | 3.45E-22 | 0.13 |
| TAC2 | -0.300528442 | 2.99E-24 | -0.189202644 | 2.88E-10 | 0.12 |
| ZNEUROK1 | 0.060756472 | 0.0446221 | 0.007069731 | 0.8154006 | 0.06 |
| TAC4 | -0.151460707 | 4.89E-07 | -0.10580623 | 0.0004586 | 0.05 |
| LOC100131277 | -0.313782905 | 2.12E-26 | -0.347850127 | 1.93E-32 | 0.04 |
| TACC2 | -0.14905965 | 7.42E-07 | -0.125550296 | 3.15E-05 | 0.03 |
| TACC3 | 0.55194263 | 3.68E-88 | 0.32293768 | 6.00E-28 | 0.23 |
| TACO1 | 0.376043993 | 4.85E-38 | 0.2910679 | 8.76E-23 | 0.09 |
| TACR1 | -0.075173681 | 0.01292033 | -0.166438864 | 3.11E-08 | 0.1 |
| TACR2 | 0.042941894 | 0.15598281 | 0.091123374 | 0.0025663 | 0.05 |
| TAC3RL | -0.112343791 | 0.0001979 | -0.091205558 | 0.0025432 | 0.03 |
| TACSTD2 | -0.069224623 | 0.02209496 | 0.00313147 | 0.9176376 | 0.08 |
| TADA1 | 0.069679519 | 0.02123343 | 0.336932453 | 2.02E-30 | 0.27 |
| TADA2A | 0.006333194 | 0.83433785 | -0.087633181 | 0.003738 | 0.1 |
| TADA2B | -0.460864381 | 1.43E-58 | -0.313973535 | 1.97E-26 | 0.15 |
| TADA3 | 0.092627851 | 0.00217367 | 0.066771217 | 0.027284 | 0.03 |
| TAF10 | 0.312614052 | 3.32E-26 | 0.391483305 | 2.38E-41 | 0.08 |
| TAF11 | 0.555564038 | 1.56E-89 | 0.403314695 | 5.22E-44 | 0.16 |
| TAF12 | 0.229281244 | 1.67E-14 | 0.185220954 | 6.83E-10 | 0.05 |
| TAF13 | -0.175428933 | 5.27E-09 | -0.270949101 | 7.61E-20 | 0.1 |
| TAF15 | 0.181033119 | 1.66E-09 | 0.105028524 | 0.0005053 | 0.08 |
| LOC116053 | -0.052470218 | 0.08293515 | 0.142378878 | 2.29E-06 | 0.2 |
| TAF1B | -0.082368347 | 0.00643681 | -0.078829369 | 0.0091281 | 0.01 |
| TAFI95 | 0.159328854 | 1.19E-07 | 0.042517252 | 0.1601193 | 0.12 |
| TAF1D | 0.392424892 | 1.47E-41 | 0.289655582 | 1.43E-22 | 0.11 |
| TAF1L | -0.431765858 | 7.39E-51 | -0.452073376 | 3.66E-56 | 0.03 |
| DYT3 | -0.388017615 | 1.36E-40 | -0.398294093 | 7.22E-43 | 0.02 |
| TAF2 | 0.050948178 | 0.09227043 | -0.141782338 | 2.53E-06 | 0.2 |
| TAF3 | -0.088386238 | 0.00345033 | -0.17376169 | 7.38E-09 | 0.09 |
| TAF4B | 0.293202495 | 4.13E-23 | -0.02297491 | 0.4479757 | 0.32 |
| TAF4 | 0.081813923 | 0.00680465 | -0.033185582 | 0.273 | 0.12 |
| TAF5L | 0.138293217 | 4.46E-06 | 0.295366973 | 1.92E-23 | 0.16 |
| TAF5 | 0.443179494 | 8.54E-54 | 0.243583425 | 3.16E-16 | 0.2 |
| FLJ11136 | 0.180707315 | 1.78E-09 | 0.067981235 | 0.0246064 | 0.12 |
| TAF6 | 0.270935412 | 7.65E-20 | 0.207095617 | 4.71E-12 | 0.07 |
| FLJ23157 | 0.290021743 | 1.26E-22 | 0.139005153 | 3.97E-06 | 0.16 |
| LOC93080 | -0.101000515 | 0.0008262 | -0.007651275 | 0.8005235 | 0.1 |
| LOC135763 | -0.006204977 | 0.83764464 | -0.120733602 | 6.29E-05 | 0.12 |
| TAF9B | -0.304797769 | 6.25E-25 | -0.057319653 | 0.0581708 | 0.25 |
| LOC51578 | 0.029379927 | 0.33183986 | 0.219014196 | 2.45E-13 | 0.19 |
| IDDM21 | -0.105898472 | 0.00045332 | -0.23039571 | 1.24E-14 | 0.13 |
| TAGLN2 | 0.357018087 | 3.35E-34 | 0.430000094 | 2.06E-50 | 0.08 |
| TAGLN3 | 0.230855292 | 1.09E-14 | 0.04828457 | 0.1106187 | 0.19 |
| TAGLN | -0.05849783 | 0.0531853 | -0.035257547 | 0.2441557 | 0.03 |
| TAL1 | -0.362586759 | 2.68E-35 | -0.278606935 | 6.18E-21 | 0.09 |
| TAL2 | -0.079299181 | 0.00872089 | -0.004166806 | 0.8905567 | 0.08 |
| TALDO1 | 0.467134477 | 2.46E-60 | 0.351816177 | 3.39E-33 | 0.12 |
| TANC1 | -0.289829617 | 1.35E-22 | -0.289560475 | 1.48E-22 | 0.01 |
| FLJ10215 | -0.329833777 | 3.76E-29 | -0.202318019 | 1.47E-11 | 0.13 |
| TANK | -0.039685771 | 0.18984133 | -0.118635322 | 8.44E-05 | 0.08 |
| LOC100506461 | -0.390304821 | 4.31E-41 | -0.394593226 | 4.87E-42 | 0.01 |
| LOC116417 | -0.286725754 | 3.96E-22 | -0.197219668 | 4.79E-11 | 0.09 |
| TAOK3 | -0.237032013 | 2.01E-15 | -0.27944358 | 4.68E-21 | 0.05 |
| LOC135672 | 0.230945422 | 1.07E-14 | 0.049719163 | 0.1004081 | 0.19 |
| LOC92048 | 0.237344609 | 1.84E-15 | -0.010224375 | 0.7356304 | 0.25 |
| TAPBPL | 0.042055394 | 0.16471168 | -0.021964085 | 0.4682058 | 0.07 |
| TAPBP | 0.160111674 | 1.03E-07 | 0.08469413 | 0.0050808 | 0.08 |
| TAPT1 | -0.320244278 | 1.74E-27 | -0.113484963 | 0.0001701 | 0.21 |
| TARBP1 | 0.02826887 | 0.35045783 | 0.169302186 | 1.79E-08 | 0.15 |
| TARBP2 | 0.194705577 | 8.47E-11 | 0.270428499 | 9.00E-20 | 0.08 |
| AF311304 | 0.44516036 | 2.57E-54 | 0.310074325 | 8.70E-26 | 0.14 |
| TARM1 | -0.004771857 | 0.87478729 | 0.001053423 | 0.9722497 | 0.01 |
| LOC155236 | -0.047483653 | 0.11666554 | -0.028204435 | 0.3515575 | 0.02 |
| TARS2 | 0.015406931 | 0.61088613 | 0.259824784 | 2.54E-18 | 0.25 |
| TARSL2 | -0.172176936 | 1.01E-08 | -0.211728745 | 1.53E-12 | 0.04 |
| MGC9344 | 0.296645536 | 1.21E-23 | 0.112407698 | 0.0001962 | 0.19 |
| TAS1R1 | 0.088142058 | 0.00354132 | -0.062419238 | 0.0390874 | 0.16 |
| TAS1R2 | 0.025616282 | 0.3975203 | -0.043505239 | 0.1506209 | 0.07 |
| TAS1R3 | -0.080823179 | 0.00750921 | -0.090967293 | 0.0026105 | 0.02 |
| TAS2R10 | -0.134281394 | 8.41E-06 | -0.105936123 | 0.0004512 | 0.03 |
| TAS2R13 | -0.023166051 | 0.44420681 | -0.021747774 | 0.4725997 | 0.01 |
| TAS2R14 | -0.055561153 | 0.06632824 | -0.106201087 | 0.0004365 | 0.06 |
| TAS2R16 | -0.01383406 | 0.64777156 | -0.028915324 | 0.3395462 | 0.02 |
| TAS2R23 | 0.027801421 | 0.35848482 | -0.063606892 | 0.0355006 | 0.1 |
| TAS2R1 | -0.073843489 | 0.01461233 | -0.062922595 | 0.0375313 | 0.02 |
| TAS2R20 | 0.151679002 | 4.70E-07 | 0.139749474 | 3.52E-06 | 0.02 |
| TAS2R30 | 0.054237713 | 0.07306951 | -0.022500536 | 0.4574072 | 0.08 |
| TAS2R31 | -0.055701799 | 0.06564292 | -0.113514238 | 0.0001694 | 0.06 |
| TAS2R38 | 0.017662183 | 0.55969214 | 0.013219732 | 0.6624228 | 0.01 |
| TAS2R39 | 0.0600858 | 0.04703431 | 0.02291456 | 0.4491694 | 0.04 |
| TAS2R3 | -0.294104162 | 3.00E-23 | -0.231009836 | 1.05E-14 | 0.07 |
| TAS2R40 | 0.139960034 | 3.41E-06 | 0.027705952 | 0.3601383 | 0.12 |
| TAS2R41 | -0.050245726 | 0.09685424 | -0.073792655 | 0.0146807 | 0.03 |
| TAS2R42 | 0.013962735 | 0.6447197 | 0.007571847 | 0.8025513 | 0.01 |
| TAS2R43 | -0.01695939 | 0.57542113 | -0.03991068 | 0.1873415 | 0.03 |
| TAS2R46 | -0.005980237 | 0.84344765 | -0.06195046 | 0.0405855 | 0.06 |
| LOC154812 | -0.270665001 | 8.34E-20 | -0.213187395 | 1.06E-12 | 0.06 |
| TAS2R50 | -0.087623908 | 0.00374167 | -0.081930484 | 0.0067258 | 0.01 |
| TAS2R5 | -0.211397324 | 1.65E-12 | -0.225801193 | 4.21E-14 | 0.02 |
| TAS2R60 | -0.049179578 | 0.10415684 | -0.053745409 | 0.0757159 | 0.01 |
| TAS2R7 | 0.005907327 | 0.8453321 | -0.023728261 | 0.4332267 | 0.03 |
| TAS2R8 | 0.003893828 | 0.8976858 | -0.001728331 | 0.9544861 | 0.01 |
| TAS2R9 | 0.007287443 | 0.80982298 | -0.02572797 | 0.3954651 | 0.04 |
| LOC105372538 | -0.201715789 | 1.69E-11 | -0.115584687 | 0.0001283 | 0.09 |
| TATDN1 | 0.46678984 | 3.08E-60 | 0.315819721 | 9.71E-27 | 0.16 |
| TATDN2 | 0.243652591 | 3.10E-16 | -0.017033653 | 0.5737493 | 0.27 |
| TATDN3 | -0.025057058 | 0.40790718 | 0.329295627 | 4.68E-29 | 0.36 |
| TAT | -0.247976341 | 8.86E-17 | -0.186830811 | 4.83E-10 | 0.07 |
| TAX1BP1 | -0.203136773 | 1.21E-11 | -0.113192355 | 0.0001769 | 0.09 |
| TAX1BP3 | 0.153096542 | 3.66E-07 | 0.206388291 | 5.59E-12 | 0.06 |
| TAZ | 0.283440095 | 1.22E-21 | 0.202523535 | 1.40E-11 | 0.09 |
| TBC1D10A | -0.203121506 | 1.22E-11 | -0.165853413 | 3.48E-08 | 0.04 |
| TBC1D10B | 0.234726857 | 3.80E-15 | 0.204680096 | 8.40E-12 | 0.04 |
| TBC1D10C | 0.00893847 | 0.76785752 | -0.075756504 | 0.0122352 | 0.09 |
| TBC1D12 | -0.364402841 | 1.16E-35 | -0.309898114 | 9.30E-26 | 0.06 |
| TBC1D13 | -0.284692034 | 7.94E-22 | -0.225184766 | 4.95E-14 | 0.06 |
| TBC1D14 | -0.301445544 | 2.14E-24 | -0.292218033 | 5.85E-23 | 0.01 |
| TBC1D15 | -0.284464094 | 8.59E-22 | -0.203825047 | 1.03E-11 | 0.09 |
| FLJ20748 | -0.262024708 | 1.28E-18 | -0.303195476 | 1.13E-24 | 0.05 |
| TBC1D17 | -0.218268397 | 2.97E-13 | -0.117293215 | 0.0001016 | 0.11 |
| TBC1D19 | -0.361063817 | 5.38E-35 | -0.180313921 | 1.93E-09 | 0.19 |
| TBC1D1 | 0.206982505 | 4.84E-12 | -0.040004244 | 0.1863087 | 0.25 |
| TBC1D20 | -0.315052286 | 1.30E-26 | -0.210028544 | 2.31E-12 | 0.11 |
| TBC1D21 | 0.0879787 | 0.00360341 | 0.026315007 | 0.3847686 | 0.07 |
| TBC1D22A | -0.032010913 | 0.29034804 | -0.079957588 | 0.0081775 | 0.05 |
| TBC1D22B | 0.227390616 | 2.76E-14 | 0.020883879 | 0.4903717 | 0.21 |
| TBC1D23 | -0.101683228 | 0.00076105 | -0.11326831 | 0.0001751 | 0.02 |
| DFNB86 | -0.122919728 | 4.61E-05 | -0.146235529 | 1.20E-06 | 0.03 |
| TBC1D25 | -0.08658794 | 0.00417337 | -0.109360367 | 0.0002921 | 0.03 |
| TBC1D26 | 0.197471664 | 4.52E-11 | 0.064609475 | 0.0326947 | 0.14 |
| TBC1D28 | 0.065349445 | 0.03074744 | -0.003947377 | 0.8962867 | 0.07 |
| TBC1D29 | -0.042762681 | 0.15771855 | -0.080057883 | 0.0080974 | 0.04 |
| FLJ20166 | -0.385265859 | 5.37E-40 | -0.447654789 | 5.61E-55 | 0.07 |
| TBC1D2 | -0.364142369 | 1.31E-35 | -0.204413272 | 8.95E-12 | 0.16 |
| LOC643947 | 0.071605525 | 0.01790151 | 0.074877792 | 0.013281 | 0.01 |
| TBC1D3C | 0.110009314 | 0.00026857 | 0.114210371 | 0.0001544 | 0.01 |
| TBC1D3G | 0.069563609 | 0.02145013 | 0.051098657 | 0.0913115 | 0.02 |
| TBC1D3H | -0.068543596 | 0.02344149 | -0.095612391 | 0.0015528 | 0.03 |
| LOC645822 | 0.119111167 | 7.90E-05 | 0.070346716 | 0.0200227 | 0.05 |
| TBC1D3 | -0.095567632 | 0.00156074 | -0.089383775 | 0.0031003 | 0.01 |
| TBC1D4 | -0.079583324 | 0.00848255 | -0.207551488 | 4.22E-12 | 0.13 |
| TBC1D5 | -0.346858412 | 2.96E-32 | -0.34432921 | 8.81E-32 | 0.01 |
| TBC1D7 | 0.544362295 | 2.43E-85 | 0.455145569 | 5.37E-57 | 0.09 |
| TBC1D8B | -0.409258198 | 2.19E-45 | -0.26391196 | 7.13E-19 | 0.15 |
| TBC1D8 | -0.311763102 | 4.59E-26 | -0.199689723 | 2.71E-11 | 0.12 |
| TBC1D9B | -0.3748027 | 8.79E-38 | -0.339875923 | 5.86E-31 | 0.04 |
| TBC1D9 | -0.509083552 | 4.05E-73 | -0.269071905 | 1.39E-19 | 0.25 |
| TBCAa | 0.350206585 | 6.89E-33 | 0.441608344 | 2.20E-53 | 0.1 |
| LOC126386 | 0.399447957 | 3.96E-43 | 0.222308849 | 1.05E-13 | 0.18 |
| TBCCD1 | 0.113940272 | 0.00016006 | -0.058420824 | 0.0534999 | 0.18 |
| TBCC | 0.344443698 | 8.39E-32 | 0.293627461 | 3.56E-23 | 0.06 |
| TBCD | 0.182670431 | 1.18E-09 | 0.061539017 | 0.0419401 | 0.13 |
| TBCEL | -0.356759853 | 3.77E-34 | -0.325115569 | 2.52E-28 | 0.04 |
| HRD | 0.340375815 | 4.75E-31 | 0.548358624 | 8.09E-87 | 0.21 |
| TBCK | -0.311497666 | 5.08E-26 | -0.132220498 | 1.16E-05 | 0.18 |
| TBK1 | -0.148120579 | 8.72E-07 | -0.1109405 | 0.0002379 | 0.04 |
| TBKBP1 | -0.10329336 | 0.0006258 | 0.008686764 | 0.7742149 | 0.12 |
| TBL1XR1 | -0.072457224 | 0.01658032 | -0.016317141 | 0.589975 | 0.06 |
| LOC159035 | -0.033161931 | 0.27334209 | -0.097432277 | 0.001259 | 0.07 |
| TBL1Y | -0.017336373 | 0.56695806 | 0.018961455 | 0.5311738 | 0.04 |
| DKFZP434N024 | 0.156330717 | 2.05E-07 | 0.164948557 | 4.14E-08 | 0.01 |
| TBL3 | 0.224095079 | 6.59E-14 | 0.221535191 | 1.28E-13 | 0.01 |
| TBPL1 | 0.378573259 | 1.43E-38 | 0.303473175 | 1.02E-24 | 0.08 |
| TBPL2 | 0.054918991 | 0.06953246 | 0.06222312 | 0.0397084 | 0.01 |
| TBP | 0.209143109 | 2.87E-12 | 0.162496259 | 6.59E-08 | 0.05 |
| TBR1 | 0.163827041 | 5.13E-08 | 0.007655841 | 0.800407 | 0.16 |
| TBRG1 | -0.276024104 | 1.45E-20 | -0.170956487 | 1.29E-08 | 0.11 |
| KIAA0948 | 0.493512261 | 3.64E-68 | 0.344812673 | 7.16E-32 | 0.15 |
| TBX10 | -0.031110466 | 0.30413756 | 0.04992825 | 0.0989847 | 0.09 |
| FLJ39077 | -0.319982945 | 1.92E-27 | -0.257981268 | 4.46E-18 | 0.07 |
| TBX18 | -0.312756694 | 3.14E-26 | -0.268015689 | 1.95E-19 | 0.05 |
| TBX19 | 0.31218374 | 3.91E-26 | 0.196259312 | 5.96E-11 | 0.12 |
| VCF | 0.081940297 | 0.00671919 | 0.03322856 | 0.2723789 | 0.05 |
| TBX20 | -0.008375671 | 0.78209319 | -0.021929423 | 0.4689084 | 0.02 |
| TBLYM | 0.033527877 | 0.26808079 | -0.078672465 | 0.0092678 | 0.12 |
| CPX | -0.02588917 | 0.39251009 | 0.135348806 | 7.11E-06 | 0.17 |
| TBX2 | -0.166901734 | 2.85E-08 | -0.133088509 | 1.01E-05 | 0.04 |
| LOC91834 | -0.306996568 | 2.76E-25 | -0.142236976 | 2.35E-06 | 0.17 |
| TBX4 | 0.094539948 | 0.00175416 | -0.074439046 | 0.0138321 | 0.17 |
| HOS | -0.243484498 | 3.25E-16 | -0.140240067 | 3.25E-06 | 0.11 |
| TBX6 | -0.052764576 | 0.08122108 | 0.043017178 | 0.155258 | 0.1 |
| TBXA2R | -0.048448671 | 0.1094108 | -0.030821894 | 0.3086472 | 0.02 |
| TBXAS1 | -0.192811433 | 1.30E-10 | -0.170539511 | 1.40E-08 | 0.03 |
| TC2N | -0.278650346 | 6.09E-21 | -0.023563259 | 0.4364329 | 0.26 |
| TCAM1P | 0.298354102 | 6.57E-24 | 0.124794736 | 3.52E-05 | 0.18 |
| LOC115728 | 0.108468621 | 0.00032748 | 0.117682501 | 9.63E-05 | 0.01 |
| TCEA1 | 0.206195452 | 5.85E-12 | 0.135200778 | 7.28E-06 | 0.08 |
| TCEA2 | -0.027753286 | 0.35931789 | 0.0470058 | 0.120395 | 0.08 |
| TCEA3 | -0.179023255 | 2.52E-09 | 0.003235353 | 0.9149156 | 0.19 |
| LOC96422 | -0.292890008 | 4.61E-23 | -0.087965329 | 0.0036085 | 0.21 |
| TCEAL2 | -0.053388812 | 0.07768111 | -0.078787798 | 0.009165 | 0.03 |
| LOC90845 | -0.199793701 | 2.65E-11 | -0.043076689 | 0.1546869 | 0.16 |
| TCEAL4 | -0.136636311 | 5.80E-06 | 0.024846192 | 0.4118653 | 0.17 |
| LOC158978 | -0.238054412 | 1.51E-15 | -0.114979701 | 0.0001392 | 0.13 |
| TCEAL6 | -0.196975858 | 5.06E-11 | -0.038724823 | 0.2007989 | 0.16 |
| MGC23947 | -0.319043632 | 2.78E-27 | -0.160994904 | 8.73E-08 | 0.16 |
| LOC92220 | -0.024517664 | 0.41807714 | 0.089198703 | 0.0031627 | 0.12 |
| TCEANC | -0.235435817 | 3.12E-15 | -0.17521671 | 5.50E-09 | 0.07 |
| TCEB1 | 0.554498404 | 3.96E-89 | 0.454267468 | 9.31E-57 | 0.11 |
| LOC91153 | 0.328100765 | 7.60E-29 | 0.380362552 | 5.99E-39 | 0.06 |
| TCEB3B | 0.140601054 | 3.07E-06 | -0.011045304 | 0.7152927 | 0.16 |
| LOC101929868 | 0.027692656 | 0.36036896 | 0.017796121 | 0.5567183 | 0.01 |
| TCEB3 | 0.068034157 | 0.02449472 | -0.101271146 | 0.0007998 | 0.17 |
| TCERG1L | -0.111666111 | 0.00021637 | -0.116855066 | 0.0001079 | 0.01 |
| TCERG1 | 0.153284024 | 3.54E-07 | 0.080306831 | 0.0079017 | 0.08 |
| TCF12 | -0.436211491 | 5.47E-52 | -0.397154799 | 1.30E-42 | 0.04 |
| TCF15 | 0.162683573 | 6.36E-08 | 0.082102452 | 0.0066109 | 0.09 |
| LOC221536 | 0.434187055 | 1.80E-51 | 0.193862106 | 1.02E-10 | 0.25 |
| SPBP | 0.015549348 | 0.60759263 | -0.173658963 | 7.53E-09 | 0.19 |
| TCF21 | -0.123138104 | 4.47E-05 | -0.10820735 | 0.0003386 | 0.02 |
| TCF23 | -0.250067385 | 4.80E-17 | -0.211010794 | 1.82E-12 | 0.04 |
| TCF25 | 0.050876218 | 0.09273185 | -0.079665117 | 0.008415 | 0.14 |
| TCF3 | 0.279483585 | 4.62E-21 | 0.217663157 | 3.46E-13 | 0.07 |
| TCF4 | -0.391918523 | 1.91E-41 | -0.291662182 | 7.11E-23 | 0.11 |
| TCF7L1 | 0.186707315 | 4.96E-10 | 0.062170197 | 0.0398773 | 0.13 |
| TCF7L2 | -0.001734847 | 0.95431467 | 0.007839663 | 0.7957194 | 0.01 |
| TCF7 | 0.045113572 | 0.13608446 | -0.13853799 | 4.28E-06 | 0.19 |
| TCFL5 | 0.267247436 | 2.49E-19 | 0.181975251 | 1.36E-09 | 0.09 |
| TCHHL1 | 0.105021763 | 0.00050569 | 0.006902808 | 0.8196834 | 0.1 |
| TCHH | 0.002050156 | 0.94602301 | -0.054021614 | 0.0742217 | 0.06 |
| TCHP | 0.122372139 | 4.99E-05 | -0.046919267 | 0.1210802 | 0.17 |
| TCIRG1 | 0.005922668 | 0.84493552 | -0.001202098 | 0.9683351 | 0.01 |
| TCL1A | 0.076485968 | 0.01142314 | -0.064358317 | 0.0333792 | 0.15 |
| TCL1B | 0.010038676 | 0.74025721 | 0.021149366 | 0.4848722 | 0.02 |
| TCL6 | 0.041825325 | 0.16703597 | -0.022496271 | 0.4574925 | 0.07 |
| TCN1 | -0.215640385 | 5.76E-13 | -0.103578561 | 0.0006043 | 0.12 |
| TCN2 | -0.012170887 | 0.68773503 | -0.101997392 | 0.0007327 | 0.09 |
| TCOF1 | 0.490535358 | 3.02E-67 | 0.302985722 | 1.22E-24 | 0.19 |
| LOC115108 | 0.043157452 | 0.15391429 | 0.013497681 | 0.6557775 | 0.03 |
| TCP10L | 0.063492369 | 0.03583377 | 0.031960274 | 0.2911122 | 0.04 |
| TCP10 | 0.074947382 | 0.01319535 | 0.015323372 | 0.6128221 | 0.06 |
| TCP11L1 | -0.007232549 | 0.81122844 | -0.198470862 | 3.59E-11 | 0.2 |
| TCP11L2 | -0.524615055 | 2.54E-78 | -0.452313835 | 3.16E-56 | 0.08 |
| TCP11 | 0.12186487 | 5.36E-05 | 0.034850946 | 0.2496406 | 0.09 |
| TCP1 | 0.416758594 | 3.66E-47 | 0.299303735 | 4.66E-24 | 0.12 |
| TCTA | -0.169398015 | 1.75E-08 | -0.103839686 | 0.0005852 | 0.07 |
| TCTE1 | -0.06671256 | 0.0274199 | -0.017820639 | 0.5561748 | 0.05 |
| LOC154641 | 0.216093451 | 5.14E-13 | 0.176312327 | 4.40E-09 | 0.04 |
| TCTEX1D1 | -0.119253691 | 7.74E-05 | -0.088122367 | 0.0035488 | 0.04 |
| TCTEX1D2 | 0.264233371 | 6.45E-19 | 0.266771778 | 2.90E-19 | 0.01 |
| TCTEX1D4 | -0.285707162 | 5.61E-22 | -0.186294522 | 5.42E-10 | 0.1 |
| TCTN1 | -0.222425245 | 1.02E-13 | -0.023622914 | 0.4352721 | 0.2 |
| TCTN2 | -0.293835285 | 3.30E-23 | -0.016022388 | 0.5967105 | 0.28 |
| TCTN3 | -0.236040276 | 2.64E-15 | -0.120539413 | 6.47E-05 | 0.12 |
| TDGF1 | -0.213728171 | 9.30E-13 | -0.143227225 | 1.99E-06 | 0.08 |
| TDGF1P3 | -0.237923467 | 1.57E-15 | -0.162924705 | 6.08E-08 | 0.08 |
| TDG | 0.18097782 | 1.68E-09 | 0.084597199 | 0.0051317 | 0.1 |
| TDH | 0.010379983 | 0.73176057 | -0.055483096 | 0.0667111 | 0.07 |
| TDO2 | 0.190610221 | 2.11E-10 | 0.026112467 | 0.388439 | 0.17 |
| TDP1 | 0.251684416 | 2.97E-17 | 0.209363571 | 2.72E-12 | 0.05 |
| TDP2 | 0.130511801 | 1.50E-05 | 0.202342387 | 1.46E-11 | 0.08 |
| TDRD10 | -0.14393343 | 1.77E-06 | 0.00079448 | 0.9790692 | 0.15 |
| TDRD12 | -0.011159812 | 0.71247148 | -0.059144434 | 0.0506041 | 0.05 |
| TDRD1 | -0.096049145 | 0.00147704 | 0.004467234 | 0.8827208 | 0.11 |
| TDRD3 | -0.066709234 | 0.02742763 | 0.002760519 | 0.9273654 | 0.07 |
| TDRD5 | -0.019197675 | 0.52606914 | 0.081495856 | 0.0070241 | 0.11 |
| TDRD6 | 0.046610653 | 0.12354874 | -0.095357441 | 0.0015986 | 0.15 |
| TDRD7 | -0.190898426 | 1.98E-10 | -0.201360194 | 1.84E-11 | 0.02 |
| TDRD9 | -0.14081066 | 2.96E-06 | -0.126758912 | 2.64E-05 | 0.02 |
| TDRG1 | 0.076482283 | 0.01142712 | 0.102337559 | 0.0007031 | 0.03 |
| TDRKH | 0.034740624 | 0.2511435 | 0.176585073 | 4.16E-09 | 0.15 |
| AA | -0.342983367 | 1.57E-31 | -0.377520637 | 2.38E-38 | 0.04 |
| TEAD2 | 0.168016343 | 2.30E-08 | 0.25653939 | 6.92E-18 | 0.09 |
| TEAD3 | 0.118908831 | 8.13E-05 | 0.08205793 | 0.0066405 | 0.04 |
| TEAD4 | 0.435333977 | 9.18E-52 | 0.256328073 | 7.38E-18 | 0.18 |
| TECPR1 | -0.325102709 | 2.53E-28 | -0.39420283 | 5.95E-42 | 0.07 |
| TECPR2 | -0.486794809 | 4.18E-66 | -0.39573104 | 2.72E-42 | 0.1 |
| TECRL | -0.014873177 | 0.62329918 | -0.020977852 | 0.4884213 | 0.01 |
| TECR | 0.143844363 | 1.80E-06 | 0.142284411 | 2.33E-06 | 0.01 |
| TECTA | -0.459281628 | 3.92E-58 | -0.329586069 | 4.16E-29 | 0.13 |
| TECTB | 0.01975956 | 0.51402828 | -0.023711682 | 0.4335482 | 0.05 |
| TEC | -0.104317441 | 0.00055178 | -0.140466748 | 3.14E-06 | 0.04 |
| TEDDM1 | -0.116218525 | 0.00011768 | -0.000137287 | 0.9963827 | 0.12 |
| TEF | -0.330006067 | 3.51E-29 | -0.289019407 | 1.79E-22 | 0.05 |
| TEKT1 | -0.048543905 | 0.1087146 | 0.078190527 | 0.009709 | 0.13 |
| TEKT2 | -0.070349507 | 0.02001778 | 0.075374327 | 0.0126807 | 0.15 |
| TEKT3 | -0.186196812 | 5.54E-10 | 0.010650935 | 0.7250386 | 0.2 |
| TEKT4 | -0.044378148 | 0.1425912 | 0.050790399 | 0.0932846 | 0.1 |
| TEKT5 | 0.06599182 | 0.02913849 | 0.199145058 | 3.07E-11 | 0.14 |
| VMCM | -0.461393139 | 1.02E-58 | -0.34247353 | 1.95E-31 | 0.12 |
| TELO2 | 0.117495838 | 9.88E-05 | 0.119822423 | 7.15E-05 | 0.01 |
| TNS2 | -0.516753798 | 1.18E-75 | -0.251932523 | 2.76E-17 | 0.27 |
| TEP1 | -0.456680635 | 2.04E-57 | -0.391657327 | 2.18E-41 | 0.07 |
| TEPP | -0.03190627 | 0.29192863 | -0.050843539 | 0.092942 | 0.02 |
| LOC100133738 | 0.19414035 | 9.63E-11 | 0.097298026 | 0.0012788 | 0.1 |
| TERF1 | 0.136351782 | 6.07E-06 | 0.078180732 | 0.0097182 | 0.06 |
| TERF2IP | 0.037625044 | 0.21389679 | -0.082588095 | 0.006296 | 0.13 |
| TERF2 | 0.090334175 | 0.00279715 | -0.0692524 | 0.0220415 | 0.16 |
| TERT | 0.396081643 | 2.27E-42 | 0.220059613 | 1.88E-13 | 0.18 |
| TESC | -0.091577469 | 0.00244143 | -0.092864488 | 0.0021172 | 0.01 |
| TESK1 | 0.215292917 | 6.29E-13 | 0.112976014 | 0.000182 | 0.11 |
| TESK2 | -0.258949271 | 3.32E-18 | -0.211585221 | 1.58E-12 | 0.05 |
| TES | 0.065190062 | 0.03115825 | -0.010716374 | 0.7234183 | 0.08 |
| LOC729351 | 0.152878015 | 3.81E-07 | -0.023942478 | 0.4290846 | 0.18 |
| KIAA1546 | -0.330087523 | 3.39E-29 | -0.297229969 | 9.84E-24 | 0.04 |
| KIAA0401 | 0.096775377 | 0.00135859 | -0.080100666 | 0.0080635 | 0.18 |
| TEX101 | 0.071948877 | 0.01735819 | -0.01052303 | 0.7282092 | 0.09 |
| TEX10 | 0.390061054 | 4.88E-41 | 0.194032992 | 9.86E-11 | 0.2 |
| TEX11 | -0.020093247 | 0.50694592 | -0.030148339 | 0.3193441 | 0.02 |
| TEX12 | 0.1154928 | 0.00012987 | 0.060943898 | 0.0439669 | 0.06 |
| TEX13A | -0.022246181 | 0.46250982 | -0.010151485 | 0.7374453 | 0.02 |
| LOC115632 | -0.027229093 | 0.36846866 | -0.001213477 | 0.9680355 | 0.03 |
| TEX14 | -0.030594984 | 0.31222411 | -0.024323417 | 0.4217758 | 0.01 |
| TEX15 | 0.245363793 | 1.89E-16 | 0.005389523 | 0.8587403 | 0.24 |
| TEX19 | 0.134013099 | 8.77E-06 | 0.122774566 | 4.71E-05 | 0.02 |
| TEX261 | 0.123635647 | 4.16E-05 | -0.052700876 | 0.0815896 | 0.18 |
| LOC132143 | 0.000573891 | 0.98487985 | 0.122005151 | 5.25E-05 | 0.13 |
| TEX28 | 0.050476128 | 0.09533114 | 0.057383681 | 0.0578902 | 0.01 |
| TEX2 | -0.189385523 | 2.77E-10 | -0.115232375 | 0.0001345 | 0.08 |
| LOC161577 | -0.329390745 | 4.50E-29 | -0.234225589 | 4.36E-15 | 0.1 |
| TFAMP1 | -0.027309008 | 0.36706431 | -0.005663762 | 0.8516337 | 0.03 |
| LOC63296 | 0.122027273 | 5.24E-05 | 0.027243004 | 0.368224 | 0.1 |
| LOC154122 | -0.0315032 | 0.29807068 | 0.012852243 | 0.6712496 | 0.05 |
| TFAP2B | -0.267307802 | 2.44E-19 | -0.020483132 | 0.4987361 | 0.25 |
| TFAP2C | 0.237838088 | 1.60E-15 | 0.144674549 | 1.56E-06 | 0.1 |
| TFAP2D | 0.138815008 | 4.10E-06 | 0.084339063 | 0.0052695 | 0.06 |
| TFAP2E | 0.131934633 | 1.21E-05 | -0.01126759 | 0.7098197 | 0.15 |
| TFAP4 | 0.4790249 | 8.84E-64 | 0.315129943 | 1.27E-26 | 0.17 |
| TFB1M | 0.231736156 | 8.60E-15 | 0.275143326 | 1.94E-20 | 0.05 |
| TFB2M | 0.166500383 | 3.08E-08 | 0.441589997 | 2.23E-53 | 0.28 |
| TFCP2L1 | 0.152455033 | 4.10E-07 | -0.085849253 | 0.0045082 | 0.24 |
| TFCP2 | -0.24779712 | 9.34E-17 | -0.129089439 | 1.86E-05 | 0.12 |
| TFDP1 | 0.293228625 | 4.09E-23 | 0.085177658 | 0.0048336 | 0.21 |
| TFDP2 | 0.225176744 | 4.96E-14 | 0.186918969 | 4.74E-10 | 0.04 |
| TFDP3 | -0.140533123 | 3.10E-06 | 0.118594449 | 8.49E-05 | 0.26 |
| RCCP2 | -0.043984881 | 0.14616718 | -0.203546274 | 1.10E-11 | 0.16 |
| TFEB | 0.01440889 | 0.63418447 | -0.115816359 | 0.0001243 | 0.14 |
| LOC102724424 | -0.143232851 | 1.99E-06 | -0.213117117 | 1.08E-12 | 0.07 |
| TFF1 | -0.360578716 | 6.71E-35 | -0.047912318 | 0.1133977 | 0.32 |
| TFF2 | 0.066157882 | 0.02873452 | 0.083356456 | 0.0058253 | 0.02 |
| TFF3 | -0.353307653 | 1.76E-33 | -0.039407171 | 0.1929719 | 0.32 |
| TFG | 0.286458856 | 4.34E-22 | 0.214505983 | 7.66E-13 | 0.08 |
| TFIP11 | -0.099060662 | 0.00104057 | -0.005636053 | 0.8523512 | 0.1 |
| TFPI2 | -0.099017903 | 0.00104583 | -0.035243548 | 0.2443432 | 0.07 |
| TFPI | -0.175873687 | 4.81E-09 | -0.138625929 | 4.22E-06 | 0.04 |
| TFPT | 0.16197733 | 7.26E-08 | 0.133604292 | 9.34E-06 | 0.03 |
| TFR2 | 0.251865614 | 2.82E-17 | 0.198580357 | 3.50E-11 | 0.06 |
| TFRC | 0.096923784 | 0.00133549 | 0.018325947 | 0.5450305 | 0.08 |
| TF | 0.056455392 | 0.06206951 | -0.040874863 | 0.1768994 | 0.1 |
| TGDS | 0.069034181 | 0.02246456 | 0.005236239 | 0.8627174 | 0.07 |
| LOC130089 | 0.128718574 | 1.97E-05 | -0.132319047 | 1.14E-05 | 0.27 |
| TGFB1I1 | -0.191326465 | 1.80E-10 | -0.127607261 | 2.32E-05 | 0.07 |
| DPD1 | -0.247480211 | 1.02E-16 | -0.23097873 | 1.06E-14 | 0.02 |
| TGFB2 | -0.109616698 | 0.00028256 | -0.019277709 | 0.5243453 | 0.1 |
| TGFB3 | -0.476466724 | 5.00E-63 | -0.209271047 | 2.78E-12 | 0.27 |
| TGFBI | 0.034957378 | 0.24819664 | -0.072041704 | 0.0172138 | 0.11 |
| TGFBR1 | -0.175253639 | 5.46E-09 | -0.089324229 | 0.0031202 | 0.09 |
| TGFBR2 | -0.445258867 | 2.42E-54 | -0.399992306 | 2.98E-43 | 0.05 |
| TGFBR3 | -0.257417997 | 5.30E-18 | -0.175268377 | 5.44E-09 | 0.09 |
| TGFBRAP1 | -0.405813333 | 1.39E-44 | -0.472379693 | 7.73E-62 | 0.07 |
| TGIF1 | 0.094610618 | 0.00174019 | 0.144923283 | 1.50E-06 | 0.06 |
| TGIF2LX | 0.048246427 | 0.11090094 | 0.002050334 | 0.9460183 | 0.05 |
| TGIF2LY | 0.031832283 | 0.29304964 | 0.011019503 | 0.7159289 | 0.03 |
| LOC116040 | 0.343656352 | 1.18E-31 | 0.185576935 | 6.33E-10 | 0.16 |
| TGM1 | 0.296749903 | 1.17E-23 | -0.013871906 | 0.6468733 | 0.32 |
| TGM2 | -0.101353085 | 0.00079193 | -0.131111363 | 1.37E-05 | 0.03 |
| TGM3 | -0.067496813 | 0.02564978 | 0.059655805 | 0.0486378 | 0.13 |
| TGM4 | 0.063985808 | 0.03441689 | 0.09713504 | 0.0013032 | 0.04 |
| LOC220875 | 0.113724806 | 0.00016474 | -0.032283588 | 0.2862565 | 0.15 |
| TGM3L | 0.030013385 | 0.32151618 | 0.008236674 | 0.7856205 | 0.03 |
| TGM7 | 0.145086648 | 1.46E-06 | 0.061645623 | 0.0415855 | 0.09 |
| TGOLN2 | -0.519929029 | 1.01E-76 | -0.439035844 | 1.03E-52 | 0.09 |
| FLJ22995 | 0.228629123 | 1.99E-14 | 0.049209535 | 0.1039458 | 0.18 |
| TG | -0.043837575 | 0.14752414 | -0.06757382 | 0.0254814 | 0.03 |
| NELFCD | 0.372059839 | 3.25E-37 | 0.263069018 | 9.28E-19 | 0.11 |
| THADA | 0.05376084 | 0.07563175 | 0.14821665 | 8.58E-07 | 0.1 |
| THAP10 | -0.106006708 | 0.00044722 | -0.087715463 | 0.0037055 | 0.02 |
| THAP11 | 0.340353359 | 4.79E-31 | 0.173078524 | 8.46E-09 | 0.17 |
| DYT6 | 0.020678358 | 0.49465191 | 0.028713358 | 0.3429316 | 0.01 |
| THAP2 | -0.09682551 | 0.00135074 | 0.000671396 | 0.9823113 | 0.1 |
| THAP3 | 0.197669544 | 4.32E-11 | 0.187154679 | 4.50E-10 | 0.02 |
| THAP4 | 0.228412716 | 2.10E-14 | 0.209459774 | 2.66E-12 | 0.02 |
| THAP5 | -0.225890506 | 4.11E-14 | -0.161066718 | 8.61E-08 | 0.07 |
| THAP6 | -0.295187044 | 2.04E-23 | -0.172045646 | 1.04E-08 | 0.13 |
| THAP7 | 0.215456069 | 6.04E-13 | 0.222270421 | 1.06E-13 | 0.01 |
| THAP8 | 0.23486228 | 3.66E-15 | 0.120967012 | 6.09E-05 | 0.12 |
| THAP9 | 0.019220747 | 0.52557191 | -0.050020713 | 0.0983604 | 0.07 |
| THBD | -0.362647248 | 2.61E-35 | -0.255437977 | 9.66E-18 | 0.11 |
| THBS1 | -0.345642355 | 5.01E-32 | -0.289584077 | 1.47E-22 | 0.06 |
| THBS2 | -0.253073333 | 1.96E-17 | -0.141051624 | 2.85E-06 | 0.12 |
| THBS3 | 0.056118188 | 0.0636481 | 0.079416113 | 0.0086221 | 0.03 |
| THBS4 | -0.352671077 | 2.33E-33 | -0.212169677 | 1.37E-12 | 0.15 |
| LOC91957 | 0.1749931 | 5.75E-09 | 0.120125747 | 6.85E-05 | 0.06 |
| THEM4 | -0.001548997 | 0.9592043 | 0.240798977 | 6.98E-16 | 0.25 |
| THEM5 | -0.025909538 | 0.39213767 | -0.091974202 | 0.0023369 | 0.07 |
| THEMIS | -0.148606063 | 8.02E-07 | -0.190004085 | 2.42E-10 | 0.05 |
| THG1L | 0.080318523 | 0.00789262 | 0.125646985 | 3.10E-05 | 0.05 |
| THNSL1 | 0.215934389 | 5.35E-13 | 0.087040086 | 0.0039797 | 0.13 |
| THNSL2 | -0.065800463 | 0.02961003 | -0.016285433 | 0.5906979 | 0.05 |
| THOC1 | 0.462003886 | 6.86E-59 | 0.314706611 | 1.49E-26 | 0.15 |
| CXorf3 | -0.11015922 | 0.0002634 | -0.160783659 | 9.08E-08 | 0.06 |
| THOC3 | 0.383440921 | 1.32E-39 | 0.310678653 | 6.92E-26 | 0.08 |
| ALYREF | 0.595354118 | 8.42E-106 | 0.334037362 | 6.72E-30 | 0.27 |
| THOC5 | 0.301179521 | 2.36E-24 | 0.153328525 | 3.52E-07 | 0.15 |
| THOC6 | 0.353773564 | 1.43E-33 | 0.339000379 | 8.48E-31 | 0.02 |
| THOC7 | 0.408264488 | 3.74E-45 | 0.460499919 | 1.80E-58 | 0.06 |
| LOC115304 | 0.326698545 | 1.34E-28 | 0.120686451 | 6.33E-05 | 0.21 |
| THPO | -0.272411861 | 4.74E-20 | 0.046268964 | 0.1263275 | 0.32 |
| THRAP3 | -0.302283856 | 1.58E-24 | -0.300206875 | 3.36E-24 | 0.01 |
| THRA | -0.212726225 | 1.19E-12 | -0.164406656 | 4.59E-08 | 0.05 |
| PRTH | -0.409055108 | 2.45E-45 | -0.240076581 | 8.56E-16 | 0.17 |
| LOC115057 | -0.196751528 | 5.33E-11 | -0.070520337 | 0.0197177 | 0.13 |
| THSD1P1 | -0.236510569 | 2.32E-15 | -0.113499622 | 0.0001698 | 0.13 |
| THSD1 | -0.387935989 | 1.42E-40 | -0.259958488 | 2.43E-18 | 0.13 |
| THSD4 | -0.522065825 | 1.89E-77 | -0.2988593 | 5.47E-24 | 0.23 |
| KIAA0960 | -0.435398638 | 8.83E-52 | -0.378585951 | 1.42E-38 | 0.06 |
| THSD7B | -0.355561461 | 6.44E-34 | -0.224283222 | 6.27E-14 | 0.14 |
| THTPA | -0.195557128 | 6.99E-11 | -0.040710741 | 0.1786456 | 0.16 |
| THUMPD1 | -0.235264757 | 3.28E-15 | -0.132924351 | 1.04E-05 | 0.11 |
| THUMPD2 | 0.357224668 | 3.06E-34 | 0.378595125 | 1.42E-38 | 0.03 |
| THUMPD3 | -0.013537445 | 0.65482904 | 0.064548047 | 0.032861 | 0.08 |
| LOC94105 | -0.123399173 | 4.30E-05 | -0.040178572 | 0.1843957 | 0.09 |
| THYN1 | 0.197253703 | 4.75E-11 | 0.277235985 | 9.75E-21 | 0.08 |
| TH | 0.152328122 | 4.20E-07 | 0.103604126 | 0.0006024 | 0.05 |
| WDM | 0.079898524 | 0.00822497 | 0.043902096 | 0.1469286 | 0.04 |
| TIAF1 | 0.303782122 | 9.09E-25 | 0.263709074 | 7.60E-19 | 0.05 |
| TIAL1 | 0.11772695 | 9.57E-05 | 0.156110246 | 2.14E-07 | 0.04 |
| TIAM1 | -0.119313565 | 7.68E-05 | -0.158552746 | 1.37E-07 | 0.04 |
| TIAM2 | 0.033226466 | 0.27240919 | -0.165770337 | 3.54E-08 | 0.2 |
| CL24751 | 0.03591703 | 0.23544035 | 0.033473701 | 0.2688553 | 0.01 |
| TICAM2 | -0.051451569 | 0.08909388 | -0.068940554 | 0.0226482 | 0.02 |
| TIE1 | -0.307390438 | 2.38E-25 | -0.219372816 | 2.24E-13 | 0.09 |
| TIFAB | 0.089359117 | 0.00310852 | -0.078215296 | 0.0096859 | 0.17 |
| TIFA | 0.092290159 | 0.00225667 | 0.052709926 | 0.0815371 | 0.04 |
| TIGD1 | 0.34840994 | 1.51E-32 | 0.181199735 | 1.60E-09 | 0.17 |
| TIGD2 | 0.295126364 | 2.09E-23 | 0.080017303 | 0.0081297 | 0.22 |
| TIGD3 | 0.145756491 | 1.30E-06 | 0.141306934 | 2.73E-06 | 0.01 |
| TIGD4 | -0.277796532 | 8.10E-21 | -0.251403289 | 3.23E-17 | 0.03 |
| TIGD5 | 0.343531671 | 1.24E-31 | 0.178545813 | 2.78E-09 | 0.17 |
| TIGD6 | -0.331232828 | 2.13E-29 | -0.037928352 | 0.2102245 | 0.3 |
| TIGD7 | -0.046603732 | 0.12360455 | -0.086425609 | 0.004245 | 0.04 |
| TIGIT | 0.092651967 | 0.00216786 | -0.067380056 | 0.0259069 | 0.17 |
| TIMD4 | 0.05259725 | 0.08219187 | -0.085997066 | 0.0044393 | 0.14 |
| TIMELESS | 0.303366995 | 1.06E-24 | 0.114264646 | 0.0001533 | 0.19 |
| TIMM10 | 0.462268172 | 5.79E-59 | 0.444190324 | 4.64E-54 | 0.02 |
| TIMM13A | 0.294660077 | 2.47E-23 | 0.291307732 | 8.05E-23 | 0.01 |
| PAM16 | 0.449511387 | 1.79E-55 | 0.416511874 | 4.19E-47 | 0.04 |
| TIMM17A | 0.404547835 | 2.72E-44 | 0.643662759 | 6.92E-129 | 0.24 |
| TIMM17B | 0.406951416 | 7.56E-45 | 0.386013564 | 3.71E-40 | 0.03 |
| LOC91792 | 0.254383859 | 1.33E-17 | 0.196387353 | 5.79E-11 | 0.06 |
| LOC93111 | 0.449157582 | 2.23E-55 | 0.252813635 | 2.12E-17 | 0.2 |
| TIMM50 | 0.508099574 | 8.48E-73 | 0.373347657 | 1.76E-37 | 0.14 |
| MGC12262 | 0.546008431 | 6.02E-86 | 0.39708635 | 1.35E-42 | 0.15 |
| LOC115225 | 0.492480359 | 7.60E-68 | 0.428187806 | 5.84E-50 | 0.07 |
| TIMM9 | 0.312947381 | 2.92E-26 | 0.428974792 | 3.72E-50 | 0.12 |
| TIMP1 | 0.032021326 | 0.29019107 | 0.080673509 | 0.0076211 | 0.05 |
| TIMP2 | -0.242727104 | 4.04E-16 | -0.199778491 | 2.66E-11 | 0.05 |
| TIMP3 | -0.335093021 | 4.34E-30 | -0.210047806 | 2.30E-12 | 0.13 |
| TIMP4 | -0.26986971 | 1.08E-19 | -0.190057511 | 2.39E-10 | 0.08 |
| TINAGL1 | 0.02982459 | 0.32457089 | -0.06443948 | 0.0331567 | 0.1 |
| TINAG | 0.186670876 | 5.00E-10 | 0.085377899 | 0.0047344 | 0.11 |
| TINF2 | -0.29037601 | 1.12E-22 | -0.186090728 | 5.67E-10 | 0.11 |
| TIPARP | -0.392988458 | 1.11E-41 | -0.243918601 | 2.87E-16 | 0.15 |
| TIPIN | 0.455342044 | 4.74E-57 | 0.233407994 | 5.45E-15 | 0.23 |
| TIPRL | 0.259008305 | 3.26E-18 | 0.442522467 | 1.27E-53 | 0.19 |
| LOC115469 | -0.406240053 | 1.10E-44 | -0.321640815 | 1.00E-27 | 0.09 |
| TJAP1 | 0.133632464 | 9.30E-06 | 0.059173371 | 0.0504911 | 0.08 |
| TJP1 | -0.405910969 | 1.32E-44 | -0.341232777 | 3.30E-31 | 0.07 |
| DFNA51 | -0.083650353 | 0.00565377 | -0.090036089 | 0.0028892 | 0.01 |
| TJP3 | -0.261858677 | 1.35E-18 | 0.009520606 | 0.7532146 | 0.28 |
| TK1 | 0.497002249 | 2.98E-69 | 0.321526085 | 1.05E-27 | 0.18 |
| TK2 | -0.432871757 | 3.88E-51 | -0.327303189 | 1.05E-28 | 0.11 |
| TKTL1 | 0.063892694 | 0.03468054 | -0.007773295 | 0.797411 | 0.08 |
| TKTL2 | 0.007210251 | 0.81179951 | -0.007366151 | 0.8078089 | 0.02 |
| TKT | 0.409731017 | 1.70E-45 | 0.19622763 | 6.00E-11 | 0.22 |
| TLCD1 | 0.376567811 | 3.77E-38 | 0.382146807 | 2.50E-39 | 0.01 |
| LOC63270 | 0.101805692 | 0.00074988 | 0.12781867 | 2.25E-05 | 0.03 |
| TLE2 | -0.106793768 | 0.0004051 | -0.014814113 | 0.6246795 | 0.1 |
| KIAA1547 | -0.366938512 | 3.59E-36 | -0.231643978 | 8.82E-15 | 0.14 |
| BCE.1 | -0.031833898 | 0.29302514 | -0.161714459 | 7.63E-08 | 0.13 |
| MGC14966 | -0.190031428 | 2.40E-10 | 0.036829008 | 0.2237539 | 0.23 |
| LOC65357 | -0.233008013 | 6.08E-15 | -0.178503402 | 2.81E-09 | 0.06 |
| TLK2 | 0.180600631 | 1.82E-09 | 0.047960552 | 0.1130345 | 0.14 |
| TLL1 | -0.403844714 | 3.94E-44 | -0.25384261 | 1.56E-17 | 0.16 |
| KIAA0932 | -0.120405587 | 6.59E-05 | -0.027635677 | 0.3613585 | 0.1 |
| KIAA1027 | -0.292823386 | 4.72E-23 | -0.389937747 | 5.19E-41 | 0.1 |
| LOC197002 | -0.322135077 | 8.24E-28 | -0.326274452 | 1.59E-28 | 0.01 |
| TLR10 | -0.194084588 | 9.75E-11 | -0.266823059 | 2.85E-19 | 0.08 |
| TIL | -0.106810177 | 0.00040426 | -0.227208464 | 2.90E-14 | 0.13 |
| TLR2 | -0.025956327 | 0.39128298 | -0.152588025 | 4.01E-07 | 0.13 |
| TLR3 | -0.44368664 | 6.29E-54 | -0.311495597 | 5.08E-26 | 0.14 |
| TLR4 | -0.337013826 | 1.95E-30 | -0.342099369 | 2.28E-31 | 0.01 |
| SLEB1 | -0.163290876 | 5.67E-08 | 0.04329594 | 0.1525964 | 0.21 |
| TLR6 | -0.129965461 | 1.63E-05 | -0.245164563 | 2.00E-16 | 0.12 |
| TLR7.like | -0.326127091 | 1.68E-28 | -0.28729937 | 3.25E-22 | 0.04 |
| LOC115806 | -0.131163537 | 1.36E-05 | -0.249765936 | 5.24E-17 | 0.12 |
| TLR9 | 0.088081956 | 0.00356405 | -0.0727713 | 0.0161151 | 0.17 |
| TLX1NB | 0.32241399 | 7.38E-28 | 0.148558189 | 8.09E-07 | 0.18 |
| TLX1 | 0.34692952 | 2.87E-32 | 0.183375357 | 1.01E-09 | 0.17 |
| NCX | 0.034920856 | 0.24869147 | 0.052235096 | 0.0843252 | 0.02 |
| TLX3 | 0.293283269 | 4.02E-23 | 0.057802462 | 0.0560828 | 0.24 |
| TM2D1 | -0.158313508 | 1.43E-07 | -0.140304708 | 3.22E-06 | 0.02 |
| TM2D2 | 0.058553584 | 0.05295849 | 0.009748012 | 0.7475182 | 0.05 |
| TM2D3 | 0.10312289 | 0.00063898 | 0.013442663 | 0.6570907 | 0.09 |
| TM4SF18 | -0.137747159 | 4.86E-06 | -0.190791303 | 2.03E-10 | 0.06 |
| TM4SF19 | 0.022908044 | 0.4492984 | -0.039236322 | 0.1949103 | 0.07 |
| TM4SF1 | 0.14258331 | 2.22E-06 | 0.036462888 | 0.2283947 | 0.11 |
| TM4SF20 | -0.050600884 | 0.09451445 | -0.052751584 | 0.0812961 | 0.01 |
| TM4SF4 | 0.136786222 | 5.67E-06 | 0.058967199 | 0.051301 | 0.08 |
| TM4SF5 | 0.077264149 | 0.01060984 | 0.012838626 | 0.6715776 | 0.07 |
| TM6SF1 | -0.385554709 | 4.65E-40 | -0.339974673 | 5.62E-31 | 0.05 |
| TM6SF2 | -0.014434435 | 0.6335835 | 0.004271291 | 0.8878302 | 0.02 |
| TM7SF2 | -0.081293586 | 0.00716692 | 0.082494045 | 0.0063559 | 0.17 |
| TM7SF3 | 0.020531086 | 0.49773125 | 0.045372766 | 0.1338466 | 0.03 |
| DCSTAMP | 0.003487242 | 0.90831943 | -0.077862644 | 0.0100198 | 0.09 |
| TM9SF1 | -0.221288265 | 1.37E-13 | -0.027228483 | 0.3684794 | 0.2 |
| TM9SF2 | -0.296287322 | 1.38E-23 | -0.2489021 | 6.76E-17 | 0.05 |
| TM9SF3 | -0.167534028 | 2.52E-08 | -0.090492255 | 0.0027494 | 0.08 |
| TM9SF4 | -0.022677708 | 0.45387171 | -0.003516435 | 0.9075554 | 0.02 |
| LOC116359 | -0.079521098 | 0.00853424 | -0.237856947 | 1.60E-15 | 0.16 |
| LOC115511 | -0.34184588 | 2.54E-31 | 0.006610693 | 0.8271911 | 0.35 |
| TMBIM6 | -0.333404511 | 8.72E-30 | -0.110660393 | 0.0002468 | 0.23 |
| DFNB11 | 0.024402725 | 0.42026338 | -0.076834273 | 0.0110525 | 0.11 |
| LOC164301 | -0.124474657 | 3.68E-05 | -0.11062529 | 0.0002479 | 0.02 |
| TMC3 | -0.345637766 | 5.02E-32 | -0.168591285 | 2.05E-08 | 0.18 |
| TMC4 | -0.33219638 | 1.43E-29 | -0.075038294 | 0.0130843 | 0.26 |
| LOC102724257 | -0.437204953 | 3.04E-52 | -0.098463593 | 0.0011162 | 0.34 |
| EV1 | 0.038662297 | 0.20152748 | 0.00451284 | 0.8815323 | 0.04 |
| TMC7 | 0.029294052 | 0.33325569 | -0.124496227 | 3.67E-05 | 0.16 |
| TMC8 | -0.067849065 | 0.0248874 | -0.149804539 | 6.52E-07 | 0.09 |
| TMCC1 | -0.215787402 | 5.55E-13 | -0.142432776 | 2.27E-06 | 0.08 |
| TMCC2 | 0.332002481 | 1.55E-29 | 0.056937097 | 0.0598707 | 0.28 |
| TMCC3 | -0.260896098 | 1.82E-18 | -0.251861948 | 2.82E-17 | 0.01 |
| TMCO1 | 0.020143393 | 0.50588604 | 0.380581397 | 5.39E-39 | 0.37 |
| TMCO2 | 0.053467422 | 0.07724435 | 0.082737839 | 0.0062017 | 0.03 |
| TMCO3 | -0.379820778 | 7.81E-39 | -0.202630363 | 1.36E-11 | 0.18 |
| TMCO4 | 0.011416336 | 0.7061656 | -0.040897457 | 0.17666 | 0.06 |
| TMCO5A | 0.123941403 | 3.98E-05 | 0.061823332 | 0.041 | 0.07 |
| TMCO6 | 0.150327199 | 5.96E-07 | 0.140982559 | 2.88E-06 | 0.01 |
| TANGO6 | -0.10710711 | 0.00038938 | -0.257041713 | 5.94E-18 | 0.15 |
| LOC90981 | -0.179732862 | 2.18E-09 | -0.164477718 | 4.53E-08 | 0.02 |
| TMED10 | -0.229511618 | 1.57E-14 | -0.123479755 | 4.25E-05 | 0.11 |
| TMED1 | 0.210501628 | 2.06E-12 | 0.133457476 | 9.56E-06 | 0.08 |
| TMED2 | 0.062751032 | 0.0380557 | 0.067189765 | 0.0263307 | 0.01 |
| TMED3 | 0.059803264 | 0.04808281 | 0.15454639 | 2.83E-07 | 0.1 |
| TMED4 | -0.24140962 | 5.87E-16 | -0.284267365 | 9.18E-22 | 0.05 |
| TMED5 | -0.225192554 | 4.94E-14 | -0.252544111 | 2.30E-17 | 0.03 |
| TMED6 | 0.150497142 | 5.78E-07 | 0.016110793 | 0.5946867 | 0.14 |
| TMED7.TICAM2 | -0.227491768 | 2.69E-14 | -0.208786355 | 3.13E-12 | 0.02 |
| TMED7 | -0.441044007 | 3.09E-53 | -0.23656509 | 2.29E-15 | 0.21 |
| FAM15B | -0.157098836 | 1.79E-07 | -0.064473805 | 0.0330629 | 0.1 |
| TMED9 | 0.196758226 | 5.32E-11 | 0.187748699 | 3.96E-10 | 0.01 |
| TMEFF1 | 0.273930813 | 2.89E-20 | 0.081201521 | 0.0072328 | 0.2 |
| TENB2 | 0.025408675 | 0.4013576 | -0.025301466 | 0.4033479 | 0.06 |
| TMEM100 | -0.121468253 | 5.67E-05 | -0.14803749 | 8.85E-07 | 0.03 |
| TMEM101 | -0.057074995 | 0.05925327 | -0.03941196 | 0.1929177 | 0.02 |
| TMEM102 | 0.03703534 | 0.2211683 | 0.045604016 | 0.1318741 | 0.01 |
| TMEM104 | 0.135270879 | 7.20E-06 | -0.051971586 | 0.0859054 | 0.19 |
| TMEM105 | 0.25657997 | 6.84E-18 | 0.0477924 | 0.1143045 | 0.21 |
| FLJ77644 | -0.326853869 | 1.26E-28 | -0.248011664 | 8.77E-17 | 0.08 |
| TMEM106B | -0.424369445 | 5.16E-49 | -0.221438328 | 1.32E-13 | 0.21 |
| TMEM106C | 0.269505991 | 1.21E-19 | 0.252944936 | 2.04E-17 | 0.02 |
| TMEM107 | 0.019055717 | 0.52913385 | 0.038714825 | 0.2009152 | 0.02 |
| TMEM108 | 0.076248498 | 0.0116821 | -0.024031289 | 0.4273741 | 0.11 |
| TMEM109 | 0.018096602 | 0.55007483 | -0.060055931 | 0.0471442 | 0.08 |
| TMEM110 | -0.260009448 | 2.40E-18 | -0.161562256 | 7.85E-08 | 0.1 |
| EMC3 | 0.067197066 | 0.02631432 | 0.184064905 | 8.75E-10 | 0.12 |
| LOC100130293 | 0.010585418 | 0.72666211 | 0.001405267 | 0.9629868 | 0.01 |
| TMEM115 | -0.043714606 | 0.14866425 | 0.044971035 | 0.1373273 | 0.09 |
| LOC732279 | -0.142549082 | 2.23E-06 | 0.037579739 | 0.2144492 | 0.19 |
| TMEM117 | 0.08742561 | 0.00382102 | -0.068721174 | 0.0230837 | 0.16 |
| TMEM119 | -0.306064404 | 3.91E-25 | -0.179997257 | 2.06E-09 | 0.13 |
| TMEM11 | 0.328269647 | 7.10E-29 | 0.29616434 | 1.44E-23 | 0.04 |
| TMEM120A | 0.097674708 | 0.00122404 | 0.229426975 | 1.60E-14 | 0.14 |
| LOC100133827 | 0.053115385 | 0.07921593 | -0.021525241 | 0.4771435 | 0.08 |
| TMEM121 | -0.074535291 | 0.01370946 | 0.055036134 | 0.0689387 | 0.13 |
| TMEM123 | 0.116410128 | 0.00011465 | 0.090894209 | 0.0026314 | 0.03 |
| TMEM125 | -0.056873741 | 0.06015617 | 0.063356572 | 0.0362323 | 0.13 |
| TMEM126A | 0.276990641 | 1.06E-20 | 0.30140906 | 2.17E-24 | 0.03 |
| TMEM126B | 0.208152476 | 3.65E-12 | 0.247948364 | 8.93E-17 | 0.04 |
| TMEM127 | -0.394596022 | 4.86E-42 | -0.349186823 | 1.08E-32 | 0.05 |
| TMEM128 | -0.221318442 | 1.36E-13 | -0.052425082 | 0.0832005 | 0.17 |
| TMEM129 | -0.096924993 | 0.0013353 | -0.069094714 | 0.0223465 | 0.03 |
| TMEM130 | -0.237275621 | 1.88E-15 | -0.174755312 | 6.04E-09 | 0.07 |
| PRO1048 | -0.361958386 | 3.57E-35 | -0.278736713 | 5.92E-21 | 0.09 |
| TMEM132A | 0.23319692 | 5.78E-15 | 0.163235611 | 5.73E-08 | 0.07 |
| TMEM132B | -0.290035131 | 1.26E-22 | -0.233934831 | 4.72E-15 | 0.06 |
| TMEM132C | -0.328865328 | 5.57E-29 | -0.227484798 | 2.70E-14 | 0.11 |
| TMEM132D | -0.194735124 | 8.42E-11 | -0.122666692 | 4.78E-05 | 0.08 |
| TMEM132E | 0.000920812 | 0.97574188 | 0.010551312 | 0.7275077 | 0.01 |
| TMEM133 | -0.132849905 | 1.05E-05 | -0.165397337 | 3.80E-08 | 0.04 |
| TMEM134 | 0.163212248 | 5.76E-08 | 0.238904603 | 1.19E-15 | 0.08 |
| TMEM135 | -0.270917554 | 7.69E-20 | -0.032261167 | 0.2865914 | 0.24 |
| TMEM136 | -0.084040824 | 0.00543294 | -0.057660966 | 0.0566881 | 0.03 |
| TMEM138 | 0.260325742 | 2.17E-18 | 0.22420123 | 6.41E-14 | 0.04 |
| TMEM139 | 0.051742552 | 0.08729819 | -0.029283396 | 0.3334316 | 0.09 |
| TMEM140 | -0.24741162 | 1.04E-16 | -0.262958631 | 9.60E-19 | 0.02 |
| TMEM141 | 0.071430957 | 0.01818341 | 0.217272503 | 3.82E-13 | 0.15 |
| TMEM143 | -0.142270567 | 2.33E-06 | -0.031548381 | 0.2973779 | 0.12 |
| TMEM144 | -0.471815792 | 1.13E-61 | -0.1986813 | 3.42E-11 | 0.28 |
| TMEM145 | -0.092788794 | 0.00213511 | -0.023382383 | 0.4399631 | 0.07 |
| CATSPERD | 0.101212812 | 0.0008054 | 0.106067624 | 0.0004438 | 0.01 |
| TMEM147 | 0.431915502 | 6.78E-51 | 0.434049025 | 1.95E-51 | 0.01 |
| U2AF1L4 | 0.163163883 | 5.81E-08 | 0.039504634 | 0.1918724 | 0.13 |
| LOC154369 | 0.203973616 | 9.93E-12 | 0.278300727 | 6.85E-21 | 0.08 |
| TMEM14B | 0.412323114 | 4.17E-46 | 0.454031375 | 1.08E-56 | 0.05 |
| TMEM14C | 0.354466362 | 1.05E-33 | 0.364477113 | 1.12E-35 | 0.02 |
| TMEM14E | 0.049775466 | 0.10002325 | 0.03543365 | 0.2418066 | 0.02 |
| LOC200551 | -0.003798603 | 0.90017469 | 0.145691418 | 1.32E-06 | 0.15 |
| TMEM150B | 0.115326258 | 0.00013283 | -0.069474187 | 0.0216186 | 0.19 |
| LOC649616 | -0.253088672 | 1.96E-17 | -0.215647514 | 5.75E-13 | 0.04 |
| TMEM151A | 0.124167144 | 3.85E-05 | 0.01357291 | 0.6539836 | 0.12 |
| TMEM151B | -0.009677184 | 0.74929097 | -0.066751351 | 0.0273299 | 0.06 |
| TMEM154 | -0.258174831 | 4.21E-18 | -0.232381358 | 7.22E-15 | 0.03 |
| TMEM155 | 0.04451825 | 0.14133358 | -0.049528464 | 0.1017205 | 0.1 |
| TMEM156 | -0.085810735 | 0.00452634 | -0.122628065 | 4.81E-05 | 0.04 |
| TMEM158 | 0.375259929 | 7.06E-38 | 0.172020301 | 1.04E-08 | 0.21 |
| TMEM159 | 0.006585121 | 0.82784912 | 0.155017305 | 2.60E-07 | 0.15 |
| TMEM160 | 0.214484266 | 7.70E-13 | 0.203133906 | 1.21E-11 | 0.02 |
| TMEM161A | 0.344892161 | 6.92E-32 | 0.30622849 | 3.67E-25 | 0.04 |
| TMEM161B | -0.359808109 | 9.52E-35 | -0.210527447 | 2.05E-12 | 0.15 |
| TMEM163 | -0.050463471 | 0.09541431 | -0.146926729 | 1.07E-06 | 0.1 |
| LOC100130886 | -0.016931327 | 0.57605351 | 0.066832486 | 0.0271426 | 0.09 |
| TMEM165 | 0.192308865 | 1.45E-10 | 0.11149342 | 0.0002213 | 0.09 |
| TMEM167A | -0.248451851 | 7.71E-17 | -2.65E-05 | 0.9993026 | 0.25 |
| LOC199887 | -0.279937159 | 3.97E-21 | -0.186676334 | 4.99E-10 | 0.1 |
| TMEM168 | -0.161838422 | 7.45E-08 | -0.075247221 | 0.0128321 | 0.09 |
| TMEM169 | 0.003856879 | 0.89865143 | -0.143509508 | 1.90E-06 | 0.15 |
| TMEM170A | 0.095580337 | 0.00155848 | -0.111033659 | 0.0002351 | 0.21 |
| TMEM170B | -0.22242677 | 1.02E-13 | -0.195547998 | 7.00E-11 | 0.03 |
| TMEM171 | 0.305094633 | 5.60E-25 | 0.170892883 | 1.31E-08 | 0.14 |
| TMEM173 | -0.248277145 | 8.12E-17 | -0.231671201 | 8.75E-15 | 0.02 |
| TMEM174 | 0.013556786 | 0.65436789 | -0.000535705 | 0.9858858 | 0.02 |
| TMEM175 | -0.158202808 | 1.46E-07 | -0.075481377 | 0.0125545 | 0.09 |
| LOC115262 | 0.011958517 | 0.69290423 | 0.025239777 | 0.4044958 | 0.02 |
| TMEM176B | 0.015750597 | 0.60295218 | 0.005962094 | 0.8439165 | 0.01 |
| TMEM177 | 0.402840945 | 6.70E-44 | 0.370008766 | 8.55E-37 | 0.04 |
| TMEM178A | -0.305449992 | 4.91E-25 | -0.167409494 | 2.58E-08 | 0.14 |
| TMEM179B | 0.185972318 | 5.81E-10 | 0.169131271 | 1.85E-08 | 0.02 |
| TMEM179 | 0.065538349 | 0.03026657 | -0.024345042 | 0.4213631 | 0.09 |
| TMEM17 | 0.193756128 | 1.05E-10 | 0.020323129 | 0.5020967 | 0.18 |
| TMEM180 | 0.310061697 | 8.74E-26 | 0.11601719 | 0.000121 | 0.2 |
| TMEM181 | -0.236990508 | 2.03E-15 | -0.173187617 | 8.28E-09 | 0.07 |
| TMEM182 | -0.044402516 | 0.14237185 | 0.009783641 | 0.7466269 | 0.06 |
| TMEM183A | 0.459074192 | 4.47E-58 | 0.75336459 | 8.02E-201 | 0.3 |
| LOC101927064 | -0.213141129 | 1.08E-12 | -0.065118951 | 0.031343 | 0.15 |
| TMEM184B | -0.245607952 | 1.76E-16 | -0.266892657 | 2.79E-19 | 0.03 |
| TMEM184C | -0.343004139 | 1.55E-31 | -0.32663532 | 1.37E-28 | 0.02 |
| FRAXF | -0.058757902 | 0.05213421 | -0.067657467 | 0.0252996 | 0.01 |
| TMEM185B | -0.076183164 | 0.01175425 | -0.045792404 | 0.1302839 | 0.04 |
| TMEM186 | 0.380139491 | 6.68E-39 | 0.364948712 | 9.04E-36 | 0.02 |
| TMEM187 | 0.048454195 | 0.10937031 | 0.156922281 | 1.84E-07 | 0.11 |
| CNEP1R1 | -0.060583167 | 0.04523522 | -0.140349764 | 3.20E-06 | 0.08 |
| TMEM189.UBE2V1 | 0.098945846 | 0.00105474 | -0.049101819 | 0.1047061 | 0.15 |
| TMEM189 | 0.403316827 | 5.21E-44 | 0.197018474 | 5.01E-11 | 0.21 |
| TMEM18 | 0.30895881 | 1.32E-25 | 0.197237413 | 4.77E-11 | 0.12 |
| TMEM190 | 0.053257375 | 0.07841585 | 0.138333153 | 4.43E-06 | 0.09 |
| DKFZP434G1017 | 0.14645549 | 1.16E-06 | 0.125304451 | 3.26E-05 | 0.03 |
| TMEM192 | -0.324727803 | 2.94E-28 | -0.237844065 | 1.60E-15 | 0.09 |
| TMEM194A | 0.104255598 | 0.000556 | -0.0603504 | 0.0460698 | 0.17 |
| TMEM194B | 0.173898968 | 7.18E-09 | -0.052245542 | 0.084263 | 0.23 |
| AGMO | -0.144835666 | 1.52E-06 | -0.15204665 | 4.41E-07 | 0.01 |
| TMEM196 | 0.026385862 | 0.38348956 | -0.10518127 | 0.0004958 | 0.14 |
| TMEM198 | 0.084230988 | 0.00532823 | -0.05136117 | 0.0896578 | 0.14 |
| TMEM199 | 0.107232507 | 0.00038326 | 0.111432928 | 0.0002231 | 0.01 |
| TMEM19 | -0.167590825 | 2.49E-08 | -0.192579206 | 1.37E-10 | 0.03 |
| TMEM200A | -0.194411975 | 9.05E-11 | -0.203687928 | 1.06E-11 | 0.01 |
| LOC90719 | -0.135190132 | 7.29E-06 | -0.217991484 | 3.18E-13 | 0.09 |
| TMEM200C | 0.005399866 | 0.85847202 | -0.170389426 | 1.44E-08 | 0.18 |
| TMEM201 | 0.440486034 | 4.31E-53 | 0.14290936 | 2.10E-06 | 0.3 |
| TMEM202 | 0.050312581 | 0.0964103 | 0.021459723 | 0.4784858 | 0.03 |
| TMEM203 | 0.269242389 | 1.32E-19 | 0.322842855 | 6.23E-28 | 0.06 |
| TMEM204 | -0.313562214 | 2.31E-26 | -0.196767802 | 5.31E-11 | 0.12 |
| TMEM205 | -0.047407614 | 0.11725285 | 0.144429703 | 1.63E-06 | 0.2 |
| TMEM206 | 0.441337444 | 2.59E-53 | 0.394112698 | 6.23E-42 | 0.05 |
| TMEM207 | 0.097729874 | 0.0012162 | 0.022362091 | 0.4601806 | 0.08 |
| TMEM208 | 0.415906485 | 5.86E-47 | 0.300314351 | 3.23E-24 | 0.12 |
| TMEM209 | -0.04389288 | 0.14701356 | 0.01413935 | 0.6405405 | 0.06 |
| SLC35G1 | 0.246987375 | 1.18E-16 | 0.033007143 | 0.2755886 | 0.22 |
| TMEM211 | 0.133809278 | 9.05E-06 | 0.01493345 | 0.621892 | 0.12 |
| TMEM212 | -0.067826464 | 0.02493572 | 0.054914943 | 0.0695531 | 0.13 |
| TMEM213 | 0.096913389 | 0.00133709 | 0.038306185 | 0.205714 | 0.06 |
| TMEM214 | 0.11498141 | 0.00013917 | 0.050444576 | 0.0955386 | 0.07 |
| TMEM215 | -0.280187536 | 3.65E-21 | -0.155321452 | 2.46E-07 | 0.13 |
| TMEM216 | 0.27892346 | 5.56E-21 | 0.259331018 | 2.95E-18 | 0.02 |
| TMEM217 | 0.133930411 | 8.88E-06 | -0.077144031 | 0.0107319 | 0.22 |
| TMEM218 | -0.171577011 | 1.14E-08 | 0.076679431 | 0.0112159 | 0.25 |
| TMEM219 | 0.102982464 | 0.00065003 | 0.249723229 | 5.31E-17 | 0.15 |
| TMEM220 | -0.057138999 | 0.05896849 | -0.118266005 | 8.88E-05 | 0.07 |
| TMEM222 | 0.100361912 | 0.00089178 | 0.051339985 | 0.0897903 | 0.05 |
| TMEM223 | 0.385398119 | 5.03E-40 | 0.332794068 | 1.12E-29 | 0.06 |
| TMEM225 | -0.021793463 | 0.47166974 | -0.039452855 | 0.192456 | 0.02 |
| LOC340344 | 0.06115253 | 0.04324718 | 0.027633713 | 0.3613926 | 0.04 |
| TMEM229B | -0.311061094 | 5.99E-26 | -0.228368468 | 2.13E-14 | 0.09 |
| SLC35G2 | -0.22593423 | 4.06E-14 | -0.208798422 | 3.12E-12 | 0.02 |
| TMEM231 | 0.149470458 | 6.91E-07 | -0.013363665 | 0.6589783 | 0.17 |
| TMEM232 | -0.320914815 | 1.33E-27 | -0.127542736 | 2.35E-05 | 0.2 |
| TMEM233 | -0.30716846 | 2.59E-25 | -0.191942712 | 1.57E-10 | 0.12 |
| TMEM25 | -0.359409835 | 1.14E-34 | -0.105485904 | 0.0004773 | 0.26 |
| TMEM26 | -0.352369726 | 2.66E-33 | -0.247834394 | 9.24E-17 | 0.11 |
| TMEM27 | 0.166947935 | 2.82E-08 | 0.089787591 | 0.002968 | 0.08 |
| TMEM2 | -0.266164596 | 3.51E-19 | -0.266562385 | 3.09E-19 | 0.01 |
| TMEM30A | -0.261838942 | 1.36E-18 | -0.208510966 | 3.35E-12 | 0.06 |
| TMEM30B | -0.36416795 | 1.30E-35 | -0.145642302 | 1.33E-06 | 0.22 |
| TMEM30C | 0.04444881 | 0.14195584 | 0.031494947 | 0.2981973 | 0.02 |
| TMEM31 | 0.209115761 | 2.89E-12 | 0.229051359 | 1.77E-14 | 0.02 |
| TMEM33 | -0.020857524 | 0.49091948 | -0.006027518 | 0.8422261 | 0.02 |
| TMEM35 | -0.111892867 | 0.00021002 | -0.083082707 | 0.0059892 | 0.03 |
| CACNG5.1 | 0.121668528 | 5.51E-05 | 0.017880309 | 0.554853 | 0.11 |
| TMEM38A | 0.099457365 | 0.00099294 | -0.126711383 | 2.65E-05 | 0.23 |
| TMEM38B | 0.088374429 | 0.00345468 | -0.091392301 | 0.0024916 | 0.18 |
| TMEM39A | -0.036532831 | 0.22750288 | -0.099994187 | 0.0009317 | 0.07 |
| TMEM39B | 0.377508071 | 2.40E-38 | 0.153986513 | 3.13E-07 | 0.23 |
| TMEM40 | 0.157408974 | 1.69E-07 | 0.055785634 | 0.0652372 | 0.11 |
| TMEM41A | 0.256564023 | 6.87E-18 | 0.204996704 | 7.79E-12 | 0.06 |
| TMEM41B | -0.23282325 | 6.40E-15 | 0.027700842 | 0.3602269 | 0.27 |
| TMEM42 | 0.099751865 | 0.00095889 | 0.185113839 | 6.99E-10 | 0.09 |
| LOC116287 | -0.023850195 | 0.43086612 | -0.161859133 | 7.43E-08 | 0.14 |
| TMEM44 | 0.000385933 | 0.98983159 | -0.012949294 | 0.6689141 | 0.02 |
| TMEM45A | 0.148148539 | 8.68E-07 | 0.069158941 | 0.0222218 | 0.08 |
| TMEM45B | -0.313437068 | 2.42E-26 | -0.079052179 | 0.0089329 | 0.24 |
| TMEM47 | -0.237865739 | 1.59E-15 | -0.200971404 | 2.01E-11 | 0.04 |
| NDC1 | 0.04371993 | 0.14861476 | -0.079029805 | 0.0089524 | 0.13 |
| VMP1 | -0.079783915 | 0.00831781 | 0.078348855 | 0.0095621 | 0.16 |
| TMEM50A | 0.058106434 | 0.05480041 | 0.084021745 | 0.0054435 | 0.03 |
| LOC654779 | -0.305124672 | 5.53E-25 | -0.09054132 | 0.0027348 | 0.22 |
| TMEM51 | 0.150892579 | 5.40E-07 | 0.035189687 | 0.2450652 | 0.12 |
| TMEM52 | 0.341600295 | 2.82E-31 | 0.210863744 | 1.89E-12 | 0.14 |
| TMEM53 | 0.008451126 | 0.78018024 | 0.131981358 | 1.20E-05 | 0.13 |
| TMEM54 | 0.322666682 | 6.68E-28 | 0.258398509 | 3.93E-18 | 0.07 |
| TMEM55A | -0.043499251 | 0.15067711 | -0.184271072 | 8.37E-10 | 0.15 |
| TMEM55B | 0.003185564 | 0.91622008 | -0.036842919 | 0.2235789 | 0.05 |
| TMEM56 | -0.017108952 | 0.57205642 | -0.058706448 | 0.0523408 | 0.05 |
| TMEM57 | -0.212512919 | 1.26E-12 | -0.026496181 | 0.3815034 | 0.19 |
| TMEM59L | -0.179944177 | 2.08E-09 | -0.041972582 | 0.1655455 | 0.14 |
| TMEM59 | -0.24494999 | 2.13E-16 | -0.145444199 | 1.37E-06 | 0.1 |
| TMEM5 | -0.026430055 | 0.38269315 | 0.028195958 | 0.3517023 | 0.06 |
| TMEM60 | 0.263749681 | 7.50E-19 | 0.328750194 | 5.84E-29 | 0.07 |
| TMEM61 | 0.067485379 | 0.02567486 | -0.009772799 | 0.7468981 | 0.08 |
| TMEM62 | -0.239870495 | 9.07E-16 | 0.028170934 | 0.3521301 | 0.27 |
| TMEM63A | -0.259778847 | 2.57E-18 | 0.041738355 | 0.167921 | 0.31 |
| TMEM63B | 0.023046741 | 0.4465572 | 0.070389116 | 0.0199479 | 0.05 |
| TMEM63C | -0.420053647 | 5.86E-48 | -0.14991604 | 6.40E-07 | 0.28 |
| TMEM64 | -0.000248012 | 0.99346538 | -0.13650159 | 5.93E-06 | 0.14 |
| LOC286052 | 0.275391568 | 1.79E-20 | 0.077212164 | 0.0106625 | 0.2 |
| SARAF | -0.381451346 | 3.52E-39 | -0.223398183 | 7.91E-14 | 0.16 |
| MKS3 | -0.139811698 | 3.49E-06 | -0.034399089 | 0.2558361 | 0.11 |
| TMEM68 | 0.171903244 | 1.07E-08 | 0.145196222 | 1.43E-06 | 0.03 |
| TMEM69 | 0.146120721 | 1.23E-06 | 0.125119954 | 3.35E-05 | 0.03 |
| TMEM70 | 0.403470087 | 4.81E-44 | 0.302106013 | 1.68E-24 | 0.11 |
| TMEM71 | 0.087213685 | 0.00390752 | -0.050388731 | 0.0959066 | 0.14 |
| LOC648109 | 0.118554376 | 8.54E-05 | -0.04260494 | 0.1592584 | 0.17 |
| TMEM74 | 0.318802321 | 3.05E-27 | 0.115910256 | 0.0001227 | 0.21 |
| TMEM79 | 0.212692518 | 1.20E-12 | 0.219681894 | 2.07E-13 | 0.01 |
| TMEM80 | -0.003955997 | 0.89606144 | 0.038217096 | 0.2067712 | 0.05 |
| TMEM81 | 0.428749974 | 4.23E-50 | 0.617702224 | 5.57E-116 | 0.19 |
| TMEM82 | -0.053491188 | 0.07711271 | -0.013558364 | 0.6543303 | 0.04 |
| EWSAT1 | -0.27378911 | 3.03E-20 | -0.073227524 | 0.0154599 | 0.21 |
| EMC4 | 0.208013688 | 3.78E-12 | 0.279207014 | 5.06E-21 | 0.08 |
| TMEM86A | -0.307766886 | 2.07E-25 | -0.118081446 | 9.11E-05 | 0.19 |
| TMEM86B | 0.211774503 | 1.51E-12 | 0.072043787 | 0.0172106 | 0.14 |
| TMEM87A | -0.311956169 | 4.26E-26 | -0.135071124 | 7.43E-06 | 0.18 |
| TMEM87B | -0.471715827 | 1.20E-61 | -0.262709921 | 1.04E-18 | 0.21 |
| TMEM88B | -0.008996873 | 0.76638464 | -0.10603192 | 0.0004458 | 0.1 |
| TMEM88 | -0.113202291 | 0.00017662 | -0.016056496 | 0.5959293 | 0.1 |
| LOC132174 | 0.055862395 | 0.06486752 | 0.025229743 | 0.4046827 | 0.04 |
| TMEM6 | 0.028135762 | 0.35273187 | 0.028809146 | 0.3413233 | 0.01 |
| TMEM8B | -0.277460786 | 9.05E-21 | -0.165207245 | 3.94E-08 | 0.12 |
| TMEM8C | 0.0151294 | 0.61732678 | 0.053230001 | 0.0785696 | 0.04 |
| TMEM90A | -0.014756356 | 0.62603047 | -0.109623154 | 0.0002823 | 0.1 |
| SYNDIG1 | -0.224261748 | 6.31E-14 | -0.040417472 | 0.1817976 | 0.19 |
| TMEM91 | -0.12736726 | 2.41E-05 | -0.013150557 | 0.6640808 | 0.12 |
| TMEM92 | 0.035498207 | 0.24094944 | 0.10656405 | 0.000417 | 0.08 |
| EMC6 | 0.436251296 | 5.34E-52 | 0.350617597 | 5.75E-33 | 0.09 |
| TMEM95 | 0.057273606 | 0.05837327 | -0.003398914 | 0.9106318 | 0.07 |
| TMEM97 | 0.358257508 | 1.92E-34 | 0.288028173 | 2.52E-22 | 0.08 |
| TMEM98 | -0.104555347 | 0.00053579 | 0.045202521 | 0.1353132 | 0.15 |
| TMEM99 | 0.016626761 | 0.58293762 | 0.201216122 | 1.90E-11 | 0.19 |
| TMEM9B | -0.251902258 | 2.79E-17 | -0.066341179 | 0.0282942 | 0.19 |
| TMEM9 | 0.161674 | 7.69E-08 | 0.514705109 | 5.69E-75 | 0.36 |
| TMF1 | -0.476530687 | 4.79E-63 | -0.377599393 | 2.29E-38 | 0.1 |
| DFNB6 | 0.14567361 | 1.32E-06 | 0.063261353 | 0.036514 | 0.09 |
| TMIGD1 | -0.018686181 | 0.53715398 | 0.000359545 | 0.9905268 | 0.02 |
| TMIGD2 | 0.092169396 | 0.00228705 | -0.00987919 | 0.7442385 | 0.11 |
| TMLHE | -0.107757382 | 0.00035857 | -0.162062322 | 7.15E-08 | 0.06 |
| TMOD1 | 0.323126537 | 5.57E-28 | 0.217637175 | 3.48E-13 | 0.11 |
| TMOD2 | -0.085503864 | 0.00467299 | -0.258991129 | 3.28E-18 | 0.18 |
| TMOD3 | -0.291599926 | 7.27E-23 | -0.261714734 | 1.41E-18 | 0.03 |
| TMOD4 | 0.143723151 | 1.83E-06 | 0.169848118 | 1.60E-08 | 0.03 |
| TMPO | 0.259676689 | 2.65E-18 | 0.075762908 | 0.0122279 | 0.19 |
| TMPPE | -0.149176256 | 7.27E-07 | -0.216564466 | 4.57E-13 | 0.07 |
| TMPRSS11A | 0.056183056 | 0.06334187 | -0.02416217 | 0.4248606 | 0.09 |
| LOC100420694 | 0.000431595 | 0.98862858 | -0.066205133 | 0.0286205 | 0.07 |
| TMPRSS11B | 0.047874781 | 0.11368093 | -0.048892782 | 0.106194 | 0.1 |
| TMPRSS11D | 0.046733131 | 0.12256439 | -0.078427478 | 0.0094898 | 0.13 |
| TMPRSS11F | -0.035450307 | 0.24158524 | -0.063678522 | 0.0352935 | 0.03 |
| TMPRSS12 | 0.280417548 | 3.38E-21 | 0.126727305 | 2.65E-05 | 0.16 |
| TMPRSS13 | 0.196444804 | 5.71E-11 | 0.210183787 | 2.23E-12 | 0.02 |
| TMPRSS15 | 0.158560595 | 1.37E-07 | 0.056365051 | 0.0624892 | 0.11 |
| PP9284 | -0.057461721 | 0.05754979 | 0.007998074 | 0.7916856 | 0.07 |
| DFNB10 | -0.087256223 | 0.00389001 | 0.036733914 | 0.2249527 | 0.13 |
| LOC51223 | 0.10457572 | 0.00053444 | 0.034131097 | 0.2595606 | 0.08 |
| TMPRSS5 | 0.22711493 | 2.97E-14 | 0.074320386 | 0.0139845 | 0.16 |
| TMPRSS6 | -0.315085283 | 1.29E-26 | -0.109703015 | 0.0002794 | 0.21 |
| TMPRSS7 | -0.16896409 | 1.91E-08 | -0.101927297 | 0.0007389 | 0.07 |
| TMPRSS9 | -0.022983896 | 0.44779808 | -0.101863189 | 0.0007447 | 0.08 |
| TMSB10 | 0.432337174 | 5.30E-51 | 0.422014716 | 1.95E-48 | 0.02 |
| TMSB15A | 0.465906937 | 5.49E-60 | 0.276403841 | 1.28E-20 | 0.19 |
| TMSB15B | 0.375944642 | 5.09E-38 | 0.157916141 | 1.54E-07 | 0.22 |
| TMSB4Y | 0.042252086 | 0.16274394 | 0.05040081 | 0.0958269 | 0.01 |
| TMSB4XP8 | 0.103755529 | 0.00059132 | 0.144527035 | 1.60E-06 | 0.05 |
| TMTC1 | -0.266361216 | 3.30E-19 | -0.199219874 | 3.02E-11 | 0.07 |
| TMTC2 | -0.405042377 | 2.09E-44 | -0.360997306 | 5.54E-35 | 0.05 |
| TMTC3 | -0.25486776 | 1.15E-17 | -0.181316436 | 1.56E-09 | 0.08 |
| TMTC4 | 0.009276224 | 0.75935132 | 0.095068831 | 0.001652 | 0.09 |
| TMUB1 | 0.215832324 | 5.49E-13 | 0.200594738 | 2.20E-11 | 0.02 |
| TMUB2 | -0.020076671 | 0.50729653 | -0.074371191 | 0.0139191 | 0.06 |
| DKFZP564E1962 | -0.166541699 | 3.05E-08 | -0.096751052 | 0.0013624 | 0.07 |
| TMX2 | 0.161487617 | 7.96E-08 | 0.125009022 | 3.41E-05 | 0.04 |
| TMX3 | -0.024161734 | 0.42486893 | -0.154159191 | 3.03E-07 | 0.13 |
| TMX4 | -0.051831391 | 0.0867558 | -0.114959751 | 0.0001396 | 0.07 |
| TNC | -0.111894666 | 0.00020997 | -0.152765702 | 3.88E-07 | 0.05 |
| TNFAIP1 | -0.156671133 | 1.93E-07 | -0.08689512 | 0.0040409 | 0.07 |
| LOC102723726 | 0.114479813 | 0.00014889 | -0.051207492 | 0.0906229 | 0.17 |
| TNFAIP3 | -0.072217404 | 0.0169434 | -0.200633076 | 2.18E-11 | 0.13 |
| TNFAIP6 | -0.124675991 | 3.58E-05 | -0.077823454 | 0.0100575 | 0.05 |
| TNFAIP8L1 | -0.223112144 | 8.52E-14 | -0.264948879 | 5.15E-19 | 0.05 |
| TNFAIP8L2 | 0.06726777 | 0.02615625 | -0.018398318 | 0.5434435 | 0.09 |
| TNFAIP8L3 | -0.164903481 | 4.18E-08 | -0.173877141 | 7.21E-09 | 0.01 |
| TNFAIP8 | 0.046488968 | 0.12453281 | -0.035334019 | 0.2431337 | 0.09 |
| TNFRSF10A | -0.170499302 | 1.41E-08 | -0.145036673 | 1.47E-06 | 0.03 |
| TNFRSF10B | -0.131175861 | 1.36E-05 | -0.101607285 | 0.0007681 | 0.03 |
| TNFRSF10C | -0.221025408 | 1.46E-13 | -0.058068624 | 0.0549586 | 0.17 |
| TNFRSF10D | -0.122051829 | 5.22E-05 | -0.156063802 | 2.15E-07 | 0.04 |
| OFE | 0.088010965 | 0.00359107 | -0.152094599 | 4.37E-07 | 0.25 |
| TNFRSF11B | -0.154627403 | 2.79E-07 | -0.128796775 | 1.95E-05 | 0.03 |
| TNFRSF12A | 0.162172228 | 7.00E-08 | 0.114429723 | 0.0001499 | 0.05 |
| TNFRSF13B | -0.009682515 | 0.74915749 | -0.051285348 | 0.0901329 | 0.05 |
| TNFRSF13C | 0.139906853 | 3.43E-06 | -0.061622204 | 0.0416632 | 0.21 |
| LOC115113 | -0.149803889 | 6.52E-07 | -0.141151589 | 2.80E-06 | 0.01 |
| TNFRSF17 | 0.032453681 | 0.28372397 | -0.024910845 | 0.4106493 | 0.06 |
| TNFRSF18 | 0.116950029 | 0.00010649 | 0.104002441 | 0.0005736 | 0.02 |
| TNFRSF19 | -0.238434598 | 1.36E-15 | -0.093636101 | 0.0019422 | 0.15 |
| LOC116290 | 0.053881096 | 0.07497881 | 0.032253177 | 0.2867109 | 0.03 |
| TNFRSF1B | -0.063791302 | 0.03496959 | -0.196701356 | 5.39E-11 | 0.14 |
| LOC51323 | 0.162898178 | 6.11E-08 | 0.081664015 | 0.0069073 | 0.09 |
| TNFRSF25 | 0.125341285 | 3.25E-05 | -0.006018964 | 0.8424471 | 0.14 |
| TNFRSF4 | 0.129592157 | 1.73E-05 | 0.033314123 | 0.2711455 | 0.1 |
| LOC115849 | 0.054804361 | 0.07011758 | -0.039114015 | 0.1963067 | 0.1 |
| TNFRSF8 | 0.063058365 | 0.03712075 | -0.087416145 | 0.0038248 | 0.16 |
| TNFRSF9 | 0.028647115 | 0.34404665 | -0.155243352 | 2.50E-07 | 0.19 |
| TNFSF10 | -0.289088374 | 1.75E-22 | -0.045214933 | 0.1352059 | 0.25 |
| TNFSF11 | -0.08899962 | 0.00323106 | -0.077228611 | 0.0106458 | 0.02 |
| TNFSF12.TNFSF13 | 0.240389758 | 7.83E-16 | 0.159107754 | 1.24E-07 | 0.09 |
| TNFSF12 | -0.242560444 | 4.23E-16 | -0.090239407 | 0.0028261 | 0.16 |
| TNFSF20 | 0.036301267 | 0.23046501 | -0.10294557 | 0.000653 | 0.14 |
| TNFSF13 | -0.346471595 | 3.50E-32 | -0.203172759 | 1.20E-11 | 0.15 |
| LOC63472 | -0.119749612 | 7.23E-05 | -0.211485225 | 1.62E-12 | 0.1 |
| TNFSF15 | -0.102706035 | 0.00067231 | -0.178225773 | 2.97E-09 | 0.08 |
| TNFSF18 | -0.129981053 | 1.63E-05 | -0.149967633 | 6.34E-07 | 0.02 |
| TNFSF4 | -0.133233834 | 9.89E-06 | -0.101419893 | 0.0007856 | 0.04 |
| TNFSF8 | -0.283840983 | 1.06E-21 | -0.323040515 | 5.76E-28 | 0.04 |
| TNFSF9 | 0.155318007 | 2.46E-07 | 0.073751179 | 0.0147367 | 0.09 |
| TNF | 0.177650206 | 3.35E-09 | 0.039485084 | 0.1920926 | 0.14 |
| TNIK | -0.398707454 | 5.83E-43 | -0.19428598 | 9.31E-11 | 0.21 |
| TNIP1 | -0.027878624 | 0.35715119 | -0.120102233 | 6.88E-05 | 0.1 |
| LOC132908 | 0.111250386 | 0.00022849 | 0.045810144 | 0.1301349 | 0.07 |
| LOC116403 | 0.134756173 | 7.81E-06 | -0.022460575 | 0.4582068 | 0.16 |
| TNK1 | -0.203941765 | 1.00E-11 | -0.053924493 | 0.0747443 | 0.16 |
| TNK2 | -0.057066182 | 0.05929257 | -0.05167279 | 0.087726 | 0.01 |
| TNKS1BP1 | -0.18639446 | 5.31E-10 | -0.05878655 | 0.0520195 | 0.13 |
| TNKS2 | -0.361651844 | 4.11E-35 | -0.36465279 | 1.04E-35 | 0.01 |
| TNKS | -0.345026779 | 6.53E-32 | -0.273526718 | 3.30E-20 | 0.08 |
| TNMD | -0.152919801 | 3.78E-07 | -0.129121487 | 1.85E-05 | 0.03 |
| TNNC1 | -0.057404441 | 0.05779951 | -0.054023848 | 0.0742097 | 0.01 |
| TNNC2 | 0.190272038 | 2.28E-10 | 0.143798793 | 1.81E-06 | 0.05 |
| TNNI1 | 0.040110193 | 0.1851443 | 0.154824979 | 2.69E-07 | 0.12 |
| TNNI2 | 0.152239958 | 4.26E-07 | 0.023970755 | 0.4285395 | 0.13 |
| TNNI3K | -0.332067237 | 1.51E-29 | -0.184437783 | 8.08E-10 | 0.15 |
| CMD2A | 0.026949525 | 0.37340774 | 0.052614689 | 0.0820903 | 0.03 |
| TNNT1 | 0.025005164 | 0.40887915 | 0.077015793 | 0.0108636 | 0.06 |
| CMD1D | 0.275737768 | 1.60E-20 | 0.248411552 | 7.80E-17 | 0.03 |
| TNNT3 | -0.198045402 | 3.96E-11 | -0.108325481 | 0.0003335 | 0.09 |
| TNN | -0.383537859 | 1.26E-39 | -0.219514944 | 2.16E-13 | 0.17 |
| TNP1 | 0.088581561 | 0.00337908 | 0.101337681 | 0.0007934 | 0.02 |
| TNP2 | 0.048062965 | 0.11226651 | 0.035002955 | 0.2475801 | 0.02 |
| LOC134282 | -0.297417207 | 9.20E-24 | -0.207273083 | 4.52E-12 | 0.1 |
| TNPO2 | 0.149008097 | 7.49E-07 | -0.065456475 | 0.0304742 | 0.22 |
| LGMD1F | -0.142171595 | 2.37E-06 | -0.227516596 | 2.67E-14 | 0.09 |
| TNRC18 | -0.33503833 | 4.44E-30 | -0.220504652 | 1.67E-13 | 0.12 |
| TNRC6A | -0.362460352 | 2.84E-35 | -0.282975287 | 1.42E-21 | 0.08 |
| TNRC6B | -0.311192867 | 5.70E-26 | -0.339749306 | 6.18E-31 | 0.03 |
| TNRC6C | -0.317067916 | 5.99E-27 | -0.235913546 | 2.74E-15 | 0.09 |
| TNR | -0.167401765 | 2.59E-08 | -0.157965356 | 1.53E-07 | 0.01 |
| FLJ10923 | -0.511977324 | 4.56E-74 | -0.450529495 | 9.55E-56 | 0.07 |
| LOC102723974 | -0.370788814 | 5.92E-37 | -0.394541754 | 5.00E-42 | 0.03 |
| TNS4 | -0.20283851 | 1.30E-11 | -0.107491771 | 0.0003709 | 0.1 |
| LOC101060681 | -0.370960025 | 5.46E-37 | -0.262280534 | 1.19E-18 | 0.11 |
| TOB1 | -0.268117361 | 1.89E-19 | -0.060075763 | 0.0470712 | 0.21 |
| TOB2 | -0.231088441 | 1.03E-14 | -0.112511919 | 0.0001935 | 0.12 |
| FLJ13949 | 0.19685411 | 5.20E-11 | 0.085908593 | 0.0044805 | 0.12 |
| TOLLIP | -0.230437535 | 1.22E-14 | -0.156359816 | 2.04E-07 | 0.08 |
| OK.KNS.CL.3 | -0.243096256 | 3.63E-16 | -0.077763936 | 0.010115 | 0.17 |
| LOC146690 | -0.513330451 | 1.63E-74 | -0.332446066 | 1.29E-29 | 0.19 |
| TOM1 | 0.005520187 | 0.85535279 | 0.005284963 | 0.8614528 | 0.01 |
| TOMM20L | 0.036117712 | 0.23283241 | 0.051043095 | 0.0916646 | 0.02 |
| TOMM20 | 0.15374794 | 3.26E-07 | 0.41308038 | 2.76E-46 | 0.26 |
| TOMM22 | 0.492357818 | 8.30E-68 | 0.403027356 | 6.07E-44 | 0.09 |
| TOMM34 | 0.403048969 | 6.00E-44 | 0.206862463 | 4.99E-12 | 0.2 |
| TOMM40L | 0.172250409 | 9.98E-09 | 0.313359404 | 2.50E-26 | 0.15 |
| TOMM40 | 0.556781386 | 5.33E-90 | 0.306199197 | 3.71E-25 | 0.26 |
| RP11.263I4.1 | 0.621860559 | 5.76E-118 | 0.499993419 | 3.39E-70 | 0.13 |
| TOMM6 | 0.532856682 | 3.40E-81 | 0.48551196 | 1.02E-65 | 0.05 |
| TOMM70A | 0.144455425 | 1.62E-06 | 0.165433022 | 3.78E-08 | 0.03 |
| TOMM7 | 0.238454828 | 1.35E-15 | 0.319799911 | 2.07E-27 | 0.09 |
| TOP1MT | 0.487849048 | 2.00E-66 | 0.268618668 | 1.61E-19 | 0.22 |
| LOC92224 | 0.076495838 | 0.01141249 | 0.128675983 | 1.98E-05 | 0.06 |
| TOP1P2 | 0.235393402 | 3.16E-15 | 0.354580965 | 9.97E-34 | 0.12 |
| TOP1 | -0.089481384 | 0.00306781 | -0.095204232 | 0.0016268 | 0.01 |
| TOP2A | 0.328612966 | 6.18E-29 | 0.151572098 | 4.79E-07 | 0.18 |
| TOP2B | -0.189476965 | 2.71E-10 | -0.164481169 | 4.53E-08 | 0.03 |
| TOP3A | -0.070980449 | 0.0189289 | -0.123513314 | 4.23E-05 | 0.06 |
| TOP3B | 0.173834524 | 7.27E-09 | 0.087703909 | 0.0037101 | 0.09 |
| TOPBP1 | 0.123459964 | 4.26E-05 | -0.025919329 | 0.3919587 | 0.15 |
| TOPORS | -0.214646248 | 7.39E-13 | -0.174674025 | 6.14E-09 | 0.04 |
| MGC3413 | -0.435721294 | 7.30E-52 | -0.090032566 | 0.0028903 | 0.35 |
| IFRG15 | -0.31472279 | 1.48E-26 | -0.191994434 | 1.56E-10 | 0.13 |
| TOR1A | -0.025169229 | 0.40581088 | -0.029186764 | 0.33503 | 0.01 |
| MGC4386 | -0.196955042 | 5.08E-11 | -0.103485979 | 0.0006112 | 0.1 |
| TOR2A | 0.001822553 | 0.95200771 | 0.126782284 | 2.63E-05 | 0.13 |
| TOR3A | 0.237333361 | 1.85E-15 | 0.451198486 | 6.31E-56 | 0.22 |
| TOX2 | 0.012780996 | 0.67296626 | -0.102687001 | 0.0006739 | 0.12 |
| TOX3 | -0.293189942 | 4.15E-23 | -0.059617133 | 0.0487842 | 0.24 |
| TOX4 | -0.308471718 | 1.59E-25 | -0.122799265 | 4.69E-05 | 0.19 |
| TOX | 0.021241366 | 0.48297428 | -0.136809455 | 5.65E-06 | 0.16 |
| TP53AIP1 | -0.161746081 | 7.58E-08 | -0.075476419 | 0.0125603 | 0.09 |
| TP53BP1 | -0.457322818 | 1.36E-57 | -0.339128218 | 8.03E-31 | 0.12 |
| TP53BP2 | 0.136225905 | 6.19E-06 | 0.116826543 | 0.0001083 | 0.02 |
| TP53I11 | -0.253109807 | 1.94E-17 | -0.125994387 | 2.95E-05 | 0.13 |
| TP53I13 | 0.130844845 | 1.43E-05 | 0.141820399 | 2.51E-06 | 0.02 |
| TP53I3 | 0.163833996 | 5.12E-08 | 0.012683834 | 0.67531 | 0.16 |
| TP53INP1 | -0.386884157 | 2.40E-40 | -0.10123364 | 0.0008034 | 0.29 |
| Corf110 | -0.217139623 | 3.95E-13 | -0.107864029 | 0.0003537 | 0.11 |
| TP53RK | 0.126383345 | 2.79E-05 | 0.141523491 | 2.64E-06 | 0.02 |
| TP53TG1 | -0.120096216 | 6.88E-05 | 0.126574519 | 2.71E-05 | 0.25 |
| TP53TG3B | -0.138333525 | 4.43E-06 | -0.075383245 | 0.0126702 | 0.07 |
| TP53TG5 | -0.094597308 | 0.00174281 | -0.070340857 | 0.0200331 | 0.03 |
| LOC146749 | 0.030993267 | 0.30596379 | 0.032649032 | 0.2808342 | 0.01 |
| KET | -0.217263714 | 3.83E-13 | -0.173679219 | 7.50E-09 | 0.05 |
| TP73 | -0.141598592 | 2.61E-06 | -0.103624776 | 0.0006009 | 0.04 |
| X5T4 | -0.291411138 | 7.76E-23 | -0.138212879 | 4.51E-06 | 0.16 |
| FLJ20612 | -0.484286005 | 2.39E-65 | -0.265116269 | 4.88E-19 | 0.22 |
| TPCN2 | 0.015537864 | 0.60785791 | -0.061587643 | 0.0417781 | 0.08 |
| TPD52L1 | 0.134679547 | 7.90E-06 | 0.178637761 | 2.73E-09 | 0.05 |
| TPD52L2 | 0.302572355 | 1.42E-24 | 0.077720147 | 0.0101575 | 0.23 |
| TPD52L3 | 0.201753593 | 1.68E-11 | 0.062663145 | 0.0383267 | 0.14 |
| TPD52 | 0.055108095 | 0.06857596 | 0.093707857 | 0.0019266 | 0.04 |
| TPH1 | -0.222601055 | 9.73E-14 | -0.182231455 | 1.29E-09 | 0.05 |
| TPH2 | -0.061607909 | 0.04171067 | -0.059083999 | 0.0508409 | 0.01 |
| TPI1P2 | 0.034423037 | 0.25550512 | -0.142407694 | 2.28E-06 | 0.18 |
| TPI1P3 | 0.203089343 | 1.22E-11 | 0.131389706 | 1.31E-05 | 0.08 |
| LOC121572 | 0.584474657 | 3.96E-101 | 0.339138431 | 8.00E-31 | 0.25 |
| TPK1 | -0.261721659 | 1.41E-18 | -0.157409812 | 1.69E-07 | 0.11 |
| C15orf13 | -0.105497521 | 0.00047661 | -0.061612364 | 0.0416959 | 0.05 |
| TPM2 | 0.11989848 | 7.08E-05 | 0.055538383 | 0.0664397 | 0.07 |
| FLJ35371 | 0.21120367 | 1.74E-12 | 0.332443175 | 1.29E-29 | 0.13 |
| TPM4 | 0.01931042 | 0.52364159 | -0.012660355 | 0.6758768 | 0.04 |
| TPMT | 0.025778124 | 0.39454425 | 0.079096876 | 0.0088942 | 0.06 |
| TPO | -0.374277097 | 1.13E-37 | -0.198920968 | 3.24E-11 | 0.18 |
| SCAR3 | -0.344198831 | 9.32E-32 | -0.204361777 | 9.06E-12 | 0.14 |
| TPP2 | -0.11192878 | 0.00020903 | -0.149886811 | 6.43E-07 | 0.04 |
| TPPP2 | 0.187050053 | 4.61E-10 | 0.020595449 | 0.4963842 | 0.17 |
| LOC51613 | -0.076717089 | 0.01117601 | -0.025544398 | 0.3988465 | 0.06 |
| TPPP | -0.258418848 | 3.90E-18 | -0.273109856 | 3.78E-20 | 0.02 |
| LOC63108 | 0.382197888 | 2.44E-39 | 0.276940965 | 1.07E-20 | 0.11 |
| TPRG1L | -0.124740146 | 3.54E-05 | -0.131853912 | 1.22E-05 | 0.01 |
| TPRG1 | -0.325235928 | 2.40E-28 | -0.134566719 | 8.04E-06 | 0.2 |
| TPRKB | 0.462154585 | 6.23E-59 | 0.504653524 | 1.10E-71 | 0.05 |
| DFNB79 | -0.173349225 | 8.01E-09 | 0.085561121 | 0.0046453 | 0.26 |
| TPRX1 | -0.008043173 | 0.79053823 | -0.051334634 | 0.0898238 | 0.05 |
| TPRXL | 0.216523391 | 4.61E-13 | 0.038661623 | 0.2015354 | 0.18 |
| TPR | -0.124999696 | 3.41E-05 | 0.043056855 | 0.154877 | 0.17 |
| TPSAB1 | -0.316664326 | 7.00E-27 | -0.133311457 | 9.78E-06 | 0.19 |
| LOC90686 | -0.302790406 | 1.31E-24 | -0.148696585 | 7.90E-07 | 0.16 |
| TPSD1 | -0.208608232 | 3.27E-12 | -0.16389121 | 5.06E-08 | 0.05 |
| TPSG1 | -0.247513907 | 1.01E-16 | -0.081214416 | 0.0072235 | 0.17 |
| LOC115093 | -0.097847664 | 0.00119962 | -0.169657485 | 1.67E-08 | 0.08 |
| TPST2 | -0.213683869 | 9.40E-13 | -0.081345592 | 0.00713 | 0.14 |
| TPT1 | -0.00658119 | 0.82795029 | 0.124605954 | 3.61E-05 | 0.14 |
| TPTE2P1 | -0.043554559 | 0.15015819 | -0.008055208 | 0.7902321 | 0.04 |
| TPTE2P3 | -0.021814843 | 0.47123491 | 0.042334984 | 0.1619199 | 0.07 |
| TPTE2 | 0.14315751 | 2.01E-06 | 0.087532849 | 0.0037779 | 0.06 |
| TPTE | -0.065049526 | 0.03152437 | -0.115818318 | 0.0001243 | 0.06 |
| FLS353 | 0.539461277 | 1.48E-83 | 0.281844967 | 2.09E-21 | 0.26 |
| TRA2A | 0.020996623 | 0.48803217 | 0.145055747 | 1.47E-06 | 0.13 |
| TRA2B | 0.409645634 | 1.78E-45 | 0.268283022 | 1.79E-19 | 0.15 |
| TRABD | 0.22098377 | 1.48E-13 | 0.114804718 | 0.0001425 | 0.11 |
| LOC123915 | 0.053694802 | 0.07599228 | 0.081004063 | 0.0073759 | 0.03 |
| TRAF1 | -0.006014049 | 0.84257404 | -0.140206164 | 3.27E-06 | 0.14 |
| LOC203234 | 0.248587153 | 7.41E-17 | 0.153294577 | 3.54E-07 | 0.1 |
| TRAF3IP1 | -0.346092087 | 4.13E-32 | -0.197431641 | 4.56E-11 | 0.15 |
| C6orf2 | 0.080059706 | 0.00809598 | 0.151529969 | 4.83E-07 | 0.08 |
| TRAF3IP3 | 0.076710875 | 0.01118259 | -0.041477741 | 0.170594 | 0.12 |
| TRAF3 | -0.037860998 | 0.21103606 | -0.214606663 | 7.47E-13 | 0.18 |
| TRAF4 | 0.310610145 | 7.10E-26 | 0.287418795 | 3.11E-22 | 0.03 |
| TRAF5 | -0.294801831 | 2.34E-23 | 0.02069239 | 0.494359 | 0.32 |
| TRAF6 | -0.336871943 | 2.07E-30 | -0.354592531 | 9.92E-34 | 0.02 |
| TRAF7 | 0.226102601 | 3.89E-14 | 0.074828539 | 0.0133418 | 0.16 |
| TRAFD1 | -0.100226715 | 0.00090627 | -0.082352718 | 0.0064469 | 0.02 |
| TRAIP | 0.499446025 | 5.06E-70 | 0.378161714 | 1.75E-38 | 0.13 |
| TRAK1 | -0.398955768 | 5.12E-43 | -0.263621567 | 7.81E-19 | 0.14 |
| KIAA0549 | -0.427338435 | 9.51E-50 | -0.335636918 | 3.46E-30 | 0.1 |
| TRAM1L1 | -0.179621948 | 2.23E-09 | -0.053247419 | 0.0784717 | 0.13 |
| LOC100130862 | 0.050923869 | 0.0924261 | -0.0317176 | 0.294793 | 0.09 |
| LOC221364 | -0.287387932 | 3.15E-22 | -0.302556295 | 1.43E-24 | 0.02 |
| TRANK1 | -0.408743695 | 2.89E-45 | -0.364185619 | 1.29E-35 | 0.05 |
| LOC51721 | 0.395380379 | 3.25E-42 | 0.338073894 | 1.25E-30 | 0.06 |
| TRAPPC10 | -0.210646697 | 1.99E-12 | -0.317878423 | 4.37E-27 | 0.11 |
| TRAPPC1 | 0.295338136 | 1.94E-23 | 0.352069798 | 3.04E-33 | 0.06 |
| TRAPPC2L | 0.328443669 | 6.61E-29 | 0.308775024 | 1.42E-25 | 0.02 |
| TRAPPC2B | 0.091045559 | 0.00258823 | 0.247738903 | 9.50E-17 | 0.16 |
| TRAPPC2 | 0.078603065 | 0.00933025 | 0.122761568 | 4.72E-05 | 0.05 |
| TRAPPC3 | 0.405639956 | 1.52E-44 | 0.216986145 | 4.11E-13 | 0.19 |
| TRAPPC4 | 0.075651707 | 0.01235598 | 0.253366633 | 1.80E-17 | 0.18 |
| TRAPPC5 | 0.277675717 | 8.43E-21 | 0.291239875 | 8.24E-23 | 0.02 |
| TRAPPC6A | 0.105912828 | 0.00045251 | 0.203898098 | 1.01E-11 | 0.1 |
| TRAPPC6B | -0.215292889 | 6.29E-13 | -0.112855112 | 0.000185 | 0.11 |
| TRAPPC9 | 0.127906657 | 2.22E-05 | 0.021073222 | 0.4864461 | 0.11 |
| HSPC062 | -0.048098369 | 0.11200196 | -0.116283014 | 0.0001167 | 0.07 |
| TRDMT1 | 0.100982569 | 0.00082798 | -0.065725242 | 0.0297972 | 0.17 |
| TRDN | -0.129419731 | 1.77E-05 | -0.103181462 | 0.0006344 | 0.03 |
| TREH | -0.133461477 | 9.55E-06 | -0.088928522 | 0.0032558 | 0.05 |
| TREM1 | 0.232066075 | 7.86E-15 | 0.116046918 | 0.0001205 | 0.12 |
| TREM2 | -0.151686922 | 4.70E-07 | -0.044588905 | 0.1407026 | 0.11 |
| TREML1 | -0.179217445 | 2.42E-09 | -0.153513234 | 3.40E-07 | 0.03 |
| TREML5P | NA | NA | NA | NA | #VALUE! |
| LOC221440 | -0.037208347 | 0.21901688 | -0.202081151 | 1.55E-11 | 0.17 |
| TREML3P | 0.09997556 | 0.00093376 | 0.037967555 | 0.2097533 | 0.07 |
| TREML4 | 0.022472222 | 0.45797365 | -0.038611798 | 0.2021174 | 0.07 |
| BCAR2 | -0.293832215 | 3.31E-23 | -0.066658554 | 0.0275456 | 0.23 |
| CRV | 0.098223385 | 0.0011481 | 0.04203463 | 0.1649205 | 0.06 |
| TREX2 | 0.174304066 | 6.61E-09 | 0.039250805 | 0.1947454 | 0.14 |
| TRHDE | -0.26769161 | 2.16E-19 | -0.204619343 | 8.52E-12 | 0.07 |
| TRHR | 0.064939776 | 0.03181284 | 0.03026427 | 0.317486 | 0.04 |
| TRH | -0.111324404 | 0.00022629 | -0.056389871 | 0.0623737 | 0.06 |
| LOC115417 | 0.464762734 | 1.15E-59 | 0.459184606 | 4.17E-58 | 0.01 |
| GIG2 | -0.049338714 | 0.1030399 | -0.039000904 | 0.1976046 | 0.02 |
| TRIB2 | -0.009423094 | 0.75566143 | -0.026703878 | 0.3777811 | 0.02 |
| TRIB3 | 0.049187475 | 0.10410118 | 0.108320025 | 0.0003338 | 0.06 |
| TRIL | -0.311571389 | 4.93E-26 | -0.086622253 | 0.0041584 | 0.23 |
| TRIM10 | 0.246047245 | 1.55E-16 | 0.014531013 | 0.6313135 | 0.24 |
| TRIM11 | 0.204707992 | 8.35E-12 | 0.402047732 | 1.02E-43 | 0.2 |
| TRIM13 | -0.185866109 | 5.95E-10 | -0.100710315 | 0.0008554 | 0.09 |
| TRIM14 | -0.173146448 | 8.35E-09 | -0.14103692 | 2.86E-06 | 0.04 |
| LOC116249 | 0.29290371 | 4.59E-23 | 0.096800502 | 0.0013547 | 0.2 |
| TRIM16L | 0.165632182 | 3.64E-08 | 0.123885662 | 4.01E-05 | 0.05 |
| TRIM16 | 0.158020061 | 1.51E-07 | 0.061236826 | 0.0429592 | 0.1 |
| TRIM17 | -0.048309152 | 0.11043708 | 0.046300441 | 0.1260695 | 0.1 |
| TRIM21 | 0.003762409 | 0.90112095 | -0.00383062 | 0.8993378 | 0.01 |
| TRIM22 | -0.178490889 | 2.82E-09 | -0.220631687 | 1.62E-13 | 0.05 |
| TRIM23 | -0.484645614 | 1.86E-65 | -0.281576992 | 2.29E-21 | 0.21 |
| TRIM24 | 0.063886563 | 0.03469796 | 0.006932475 | 0.8189218 | 0.06 |
| TRIM25 | -0.040958406 | 0.17601543 | -0.106325811 | 0.0004297 | 0.07 |
| TRIM26 | 0.170331291 | 1.46E-08 | 0.088269503 | 0.0034936 | 0.09 |
| TRIM27 | 0.276707689 | 1.16E-20 | 0.26674843 | 2.92E-19 | 0.01 |
| LOC63481 | 0.256744546 | 6.50E-18 | 0.177450181 | 3.49E-09 | 0.08 |
| TRIM29 | 0.107711386 | 0.00036067 | 0.003154817 | 0.9170258 | 0.11 |
| TRIM2 | 0.054401413 | 0.07220647 | -0.165365696 | 3.83E-08 | 0.22 |
| LOC88008 | 0.085581766 | 0.00463536 | -0.019534399 | 0.5188361 | 0.11 |
| LGMD2H | -0.291376534 | 7.86E-23 | -0.201281134 | 1.87E-11 | 0.1 |
| TRIM33 | -0.129952299 | 1.64E-05 | -0.216717391 | 4.39E-13 | 0.09 |
| TRIM34 | -0.396781429 | 1.58E-42 | -0.204592851 | 8.58E-12 | 0.2 |
| TRIM35 | -0.228448442 | 2.08E-14 | -0.244765036 | 2.25E-16 | 0.02 |
| TRIM36 | -0.033458084 | 0.26907883 | -0.122114494 | 5.17E-05 | 0.09 |
| KIAA0898 | -0.015219751 | 0.61522675 | -0.043314946 | 0.1524162 | 0.03 |
| TRIM38 | -0.07668322 | 0.01121192 | -0.000504667 | 0.9867035 | 0.08 |
| TRIM39 | 0.00620531 | 0.83763604 | 0.00802029 | 0.7911203 | 0.01 |
| TRIM3 | -0.368470626 | 1.76E-36 | -0.103324112 | 0.0006234 | 0.27 |
| TRIM40 | 0.017007288 | 0.57434254 | -0.039270088 | 0.1945261 | 0.06 |
| TRIM41 | -0.217957965 | 3.21E-13 | -0.065513667 | 0.030329 | 0.16 |
| TRIM42 | 0.024813653 | 0.41247808 | 0.027910347 | 0.3566041 | 0.01 |
| TRIM43 | 0.29461372 | 2.51E-23 | 0.083394705 | 0.0058027 | 0.22 |
| LOC116020 | -0.330209562 | 3.23E-29 | -0.39576698 | 2.67E-42 | 0.07 |
| TRIM45 | -0.132704797 | 1.07E-05 | -0.002743715 | 0.9278064 | 0.13 |
| GENEY | 0.08439877 | 0.00523737 | 0.269788405 | 1.11E-19 | 0.19 |
| TRIM47 | 0.326560875 | 1.41E-28 | 0.093819093 | 0.0019027 | 0.24 |
| LOC143718 | 0.059452354 | 0.04941229 | -0.035458548 | 0.2414758 | 0.1 |
| LOC100131392 | 0.118832824 | 8.21E-05 | 0.007849602 | 0.7954661 | 0.12 |
| LOC220486 | 0.097982707 | 0.00118086 | 0.013132421 | 0.6645158 | 0.09 |
| LOC221783 | -0.364372147 | 1.18E-35 | -0.227138651 | 2.95E-14 | 0.14 |
| TRIM50A | 0.039233432 | 0.1949432 | -0.084563094 | 0.0051497 | 0.13 |
| TRIM52 | -0.340985057 | 3.67E-31 | -0.215115621 | 6.57E-13 | 0.13 |
| TRIM53AP | 0.098238998 | 0.00114601 | 0.010350863 | 0.7324842 | 0.09 |
| TRIM54 | 0.091591475 | 0.00243767 | 0.086003131 | 0.0044365 | 0.01 |
| TRIM55 | 0.07008535 | 0.02048963 | 0.044064304 | 0.1454395 | 0.03 |
| TRIM56 | -0.466073063 | 4.92E-60 | -0.493436978 | 3.85E-68 | 0.03 |
| TRIM58 | -0.320030112 | 1.89E-27 | -0.039442172 | 0.1925765 | 0.29 |
| MRF1 | 0.184196441 | 8.50E-10 | 0.113919972 | 0.0001605 | 0.08 |
| LOC101927800 | -0.225480168 | 4.58E-14 | -0.153283577 | 3.54E-07 | 0.08 |
| TRIM6.TRIM34 | -0.160311571 | 9.91E-08 | -0.142556774 | 2.23E-06 | 0.02 |
| RNF33 | -0.031833147 | 0.29303653 | -0.010805314 | 0.7212181 | 0.03 |
| RNF35 | -0.154721665 | 2.74E-07 | -0.196735839 | 5.35E-11 | 0.05 |
| TRIM62 | -0.374031612 | 1.27E-37 | -0.271487025 | 6.40E-20 | 0.11 |
| TRIM63 | -0.232385853 | 7.21E-15 | -0.160553848 | 9.47E-08 | 0.08 |
| TRIM64 | 0.05313406 | 0.07911033 | 0.014801921 | 0.6249645 | 0.04 |
| TRIM65 | 0.381028509 | 4.33E-39 | 0.127896001 | 2.23E-05 | 0.26 |
| TRIM66 | -0.442525536 | 1.27E-53 | -0.300127094 | 3.46E-24 | 0.15 |
| TRIM67 | 0.038790141 | 0.20003975 | 0.006832991 | 0.8214763 | 0.04 |
| TRIM68 | -0.310481304 | 7.46E-26 | -0.180111059 | 2.01E-09 | 0.14 |
| TRIM69 | 0.176235645 | 4.47E-09 | -0.02129078 | 0.4819565 | 0.2 |
| TRIM6 | -0.374117394 | 1.22E-37 | -0.150187977 | 6.10E-07 | 0.23 |
| TRIM71 | 0.0623598 | 0.03927475 | -0.018838 | 0.5338516 | 0.09 |
| TRIM72 | -0.190950482 | 1.96E-10 | 0.008997412 | 0.766371 | 0.2 |
| TRIM50C | -0.08961833 | 0.0030228 | -0.100288233 | 0.0008996 | 0.02 |
| LOC120123 | 0.009692728 | 0.74890178 | 0.00717677 | 0.8126572 | 0.01 |
| TRIM78P | -0.337317448 | 1.72E-30 | -0.298088081 | 7.23E-24 | 0.04 |
| TRIM7 | 0.02748713 | 0.36394619 | -0.135832137 | 6.59E-06 | 0.17 |
| TRIM8 | -0.334535928 | 5.46E-30 | -0.154478941 | 2.86E-07 | 0.19 |
| KIAA0282 | -0.145923795 | 1.27E-06 | -0.160826566 | 9.00E-08 | 0.02 |
| TRIML1 | 0.131743066 | 1.24E-05 | 0.071393554 | 0.0182443 | 0.07 |
| TRIML2 | 0.239748605 | 9.39E-16 | 0.088719712 | 0.0033295 | 0.16 |
| DFNB28 | -0.162732015 | 6.30E-08 | -0.172481301 | 9.53E-09 | 0.01 |
| LOC100131749 | -0.415721606 | 6.48E-47 | -0.401523785 | 1.34E-43 | 0.02 |
| TRIP10 | 0.146659813 | 1.12E-06 | 0.071258603 | 0.0184655 | 0.08 |
| TRIP11 | -0.452727427 | 2.44E-56 | -0.323742551 | 4.36E-28 | 0.13 |
| TRIP12 | -0.296976011 | 1.08E-23 | -0.296019461 | 1.52E-23 | 0.01 |
| TRIP13 | 0.548231613 | 9.02E-87 | 0.284057317 | 9.86E-22 | 0.27 |
| LOC115298 | -0.103831934 | 0.00058579 | -0.030191372 | 0.3186536 | 0.08 |
| MGC10558 | 0.108386926 | 0.00033092 | 0.172078035 | 1.03E-08 | 0.07 |
| TRIT1 | 0.438786967 | 1.19E-52 | 0.290700159 | 9.96E-23 | 0.15 |
| TRMT112 | 0.395667281 | 2.81E-42 | 0.433562253 | 2.59E-51 | 0.04 |
| LOC102723353 | 0.230107689 | 1.34E-14 | 0.080659549 | 0.0076317 | 0.15 |
| TRMT12 | 0.256791843 | 6.41E-18 | 0.149305378 | 7.11E-07 | 0.11 |
| TRMT1 | 0.349065584 | 1.13E-32 | 0.225368938 | 4.72E-14 | 0.13 |
| TRMT2A | 0.220075627 | 1.87E-13 | 0.156111068 | 2.14E-07 | 0.07 |
| TRMT2B | -0.094983213 | 0.00166818 | -0.105048649 | 0.000504 | 0.02 |
| TRMT5 | 0.013930467 | 0.64548445 | 0.143460741 | 1.92E-06 | 0.13 |
| TRMT61A | 0.366357904 | 4.71E-36 | 0.258105885 | 4.30E-18 | 0.11 |
| TRMT61B | 0.113739357 | 0.00016442 | 0.068535849 | 0.0234572 | 0.05 |
| TRMT6 | 0.352967394 | 2.04E-33 | 0.209727486 | 2.49E-12 | 0.15 |
| TRMU | 0.40098602 | 1.78E-43 | 0.218644947 | 2.69E-13 | 0.19 |
| PRO1902 | 0.306729828 | 3.05E-25 | 0.189264398 | 2.84E-10 | 0.12 |
| TRNP1 | 0.193034594 | 1.23E-10 | 0.032461968 | 0.283601 | 0.17 |
| TRNT1 | 0.079126844 | 0.00886835 | 0.102849944 | 0.0006606 | 0.03 |
| TROAP | 0.556981044 | 4.47E-90 | 0.336857744 | 2.08E-30 | 0.23 |
| TROVE2 | -0.030396258 | 0.31537902 | 0.330218941 | 3.22E-29 | 0.37 |
| LOC115125 | -0.201664001 | 1.71E-11 | -0.177605124 | 3.38E-09 | 0.03 |
| TRPA1 | 0.045650673 | 0.13147884 | 0.069641845 | 0.0213037 | 0.03 |
| TRPC1 | -0.250037942 | 4.84E-17 | -0.304560009 | 6.82E-25 | 0.06 |
| TRPC2 | 0.035404731 | 0.24219128 | 0.028187415 | 0.3518483 | 0.01 |
| LOC132331 | -0.189720526 | 2.57E-10 | -0.172795791 | 8.95E-09 | 0.02 |
| LOC115576 | 0.014059609 | 0.64242601 | -0.030088219 | 0.3203106 | 0.05 |
| TRPC4 | -0.068184224 | 0.02418029 | -0.113779299 | 0.0001635 | 0.05 |
| TRPC5 | -0.11890213 | 8.13E-05 | -0.134877959 | 7.66E-06 | 0.02 |
| FSGS2 | -0.275022957 | 2.02E-20 | -0.25603376 | 8.07E-18 | 0.02 |
| TRPC7 | 0.076290661 | 0.01163574 | 0.024612758 | 0.4162734 | 0.06 |
| TRPM1 | -0.027777656 | 0.35889598 | -0.079334776 | 0.0086907 | 0.06 |
| TRPM2 | 0.163028943 | 5.96E-08 | 0.08091015 | 0.0074448 | 0.09 |
| TRPM3 | -0.088770981 | 0.00331127 | -0.157198155 | 1.75E-07 | 0.07 |
| TRPM4 | -0.359226547 | 1.24E-34 | -0.105468651 | 0.0004783 | 0.26 |
| TRPM5 | 0.13019177 | 1.58E-05 | 0.091766368 | 0.0023912 | 0.04 |
| FLJ20087 | 0.039054208 | 0.19699219 | -0.120931984 | 6.12E-05 | 0.16 |
| TRPM7 | -0.417816602 | 2.04E-47 | -0.368326794 | 1.88E-36 | 0.05 |
| TRPM8 | 0.225633566 | 4.40E-14 | 0.081533079 | 0.0069981 | 0.15 |
| TRPS1 | -0.156554119 | 1.97E-07 | -0.071552439 | 0.0179868 | 0.09 |
| LOC93089 | 0.115975304 | 0.00012164 | 0.227431152 | 2.73E-14 | 0.12 |
| TRPV1 | -0.085116983 | 0.00486399 | 0.014269671 | 0.6374641 | 0.1 |
| VRL.1 | -0.028180645 | 0.35196405 | -0.137650363 | 4.94E-06 | 0.11 |
| TRPV3 | 0.141963487 | 2.45E-06 | -0.104972485 | 0.0005088 | 0.25 |
| LOC121647 | 0.104427875 | 0.0005443 | -0.070818275 | 0.0192037 | 0.18 |
| TRPV5 | -0.079096085 | 0.0088949 | -0.067795427 | 0.0250022 | 0.02 |
| LOC392832 | 0.155418177 | 2.42E-07 | 0.138266272 | 4.48E-06 | 0.02 |
| TRRAP | -0.160308541 | 9.91E-08 | -0.286864355 | 3.77E-22 | 0.13 |
| TRUB1 | -0.235348352 | 3.20E-15 | -0.183994613 | 8.88E-10 | 0.06 |
| TRUB2 | 0.237745807 | 1.65E-15 | 0.221458772 | 1.31E-13 | 0.02 |
| PRSS3P2 | 0.074841187 | 0.01332618 | -0.041094554 | 0.1745819 | 0.12 |
| TRYX3 | -0.00774501 | 0.7981322 | -0.043073414 | 0.1547182 | 0.04 |
| TSC1 | -0.390172676 | 4.61E-41 | -0.26650805 | 3.15E-19 | 0.13 |
| TSC22D1 | -0.268823258 | 1.51E-19 | -0.138994794 | 3.98E-06 | 0.13 |
| TSC22D2 | -0.245441849 | 1.85E-16 | -0.287479695 | 3.05E-22 | 0.05 |
| GILZ | -0.334266734 | 6.11E-30 | -0.099480198 | 0.0009903 | 0.24 |
| LOC392760 | 0.180212133 | 1.97E-09 | 0.082484471 | 0.0063621 | 0.1 |
| LOC124054 | -0.209900418 | 2.39E-12 | -0.126726601 | 2.65E-05 | 0.09 |
| TSEN15 | 0.305603678 | 4.63E-25 | 0.477814236 | 2.01E-63 | 0.18 |
| MGC4440 | 0.207313578 | 4.47E-12 | 0.249122917 | 6.33E-17 | 0.05 |
| TSEN34 | 0.372200114 | 3.04E-37 | 0.230758013 | 1.12E-14 | 0.15 |
| TSEN54 | 0.36927276 | 1.21E-36 | 0.243203423 | 3.52E-16 | 0.13 |
| TSFM | 0.352961471 | 2.05E-33 | 0.35474979 | 9.25E-34 | 0.01 |
| TSG101 | -0.022987329 | 0.44773026 | 0.08330535 | 0.0058556 | 0.11 |
| LOC648432 | 0.061175885 | 0.04316723 | 0.035224244 | 0.2446018 | 0.03 |
| TSGA10IP | 0.065516459 | 0.03032196 | -0.030541405 | 0.3130727 | 0.1 |
| TSGA10 | -0.257672009 | 4.90E-18 | -0.240801753 | 6.97E-16 | 0.02 |
| TSGA13 | 0.084135381 | 0.00538065 | 0.055218676 | 0.0680217 | 0.03 |
| CEP41 | 0.015571868 | 0.60707259 | 0.076566789 | 0.0113362 | 0.07 |
| TSHB | -0.068695059 | 0.02313602 | -0.100441621 | 0.0008833 | 0.04 |
| TSHR | 0.028960632 | 0.33878963 | -0.100652487 | 0.0008614 | 0.13 |
| CAA | -0.239839478 | 9.15E-16 | -0.278618017 | 6.16E-21 | 0.04 |
| C20orf17 | -0.42470601 | 4.27E-49 | -0.408279184 | 3.71E-45 | 0.02 |
| LOC91672 | -0.407577013 | 5.41E-45 | -0.316361548 | 7.88E-27 | 0.1 |
| TSIX | 0.310820129 | 6.56E-26 | 0.333365707 | 8.86E-30 | 0.03 |
| TSKS | 0.090844958 | 0.00264565 | 0.019731322 | 0.51463 | 0.08 |
| TSKU | -0.026789968 | 0.37624484 | -0.015695052 | 0.6042314 | 0.02 |
| TSLP | -0.066183432 | 0.02867279 | -0.111111249 | 0.0002327 | 0.05 |
| TSNARE1 | 0.191907885 | 1.59E-10 | 0.070295272 | 0.0201139 | 0.13 |
| TSNAX.DISC1 | -0.006000974 | 0.84291185 | -0.009975403 | 0.7418359 | 0.01 |
| TSNAXIP1 | -0.118669588 | 8.40E-05 | 0.005104293 | 0.8661437 | 0.13 |
| TSNAX | -0.138641507 | 4.21E-06 | 0.19346042 | 1.12E-10 | 0.34 |
| TSN | 0.222266791 | 1.06E-13 | 0.149846972 | 6.48E-07 | 0.08 |
| TSPAN10 | -0.006890208 | 0.8200069 | -0.003281326 | 0.9137112 | 0.01 |
| LOC100131093 | -0.242485311 | 4.32E-16 | -0.266563516 | 3.09E-19 | 0.03 |
| TSPAN12 | -0.059595377 | 0.04886675 | -0.095612691 | 0.0015527 | 0.04 |
| TSPAN13 | -0.342665331 | 1.80E-31 | -0.069712038 | 0.021173 | 0.28 |
| LOC116315 | 0.06363636 | 0.03541527 | -0.075262927 | 0.0128133 | 0.14 |
| TSPAN15 | -0.285826667 | 5.39E-22 | 0.051072571 | 0.0914771 | 0.34 |
| LOC88787 | -0.035072016 | 0.24664788 | 0.051014009 | 0.0918499 | 0.09 |
| TSPAN17 | 0.034286078 | 0.2574022 | 0.03136683 | 0.3001681 | 0.01 |
| TSPAN18 | -0.139560084 | 3.63E-06 | -0.173804438 | 7.31E-09 | 0.04 |
| TSPAN19 | 0.100279247 | 0.00090061 | 0.035306867 | 0.2434962 | 0.07 |
| TSPAN1 | -0.39610022 | 2.25E-42 | -0.159143496 | 1.23E-07 | 0.24 |
| TSPAN2 | -0.103672961 | 0.00059734 | -0.114724201 | 0.0001441 | 0.02 |
| TSPAN31 | -0.194206481 | 9.48E-11 | -0.086844487 | 0.0040625 | 0.11 |
| TSPAN32 | -0.076193978 | 0.01174228 | -0.1581049 | 1.49E-07 | 0.09 |
| TSPAN33 | 0.329645685 | 4.06E-29 | 0.09433734 | 0.0017948 | 0.24 |
| TM4SF8 | 0.043612814 | 0.14961311 | -0.037270499 | 0.2182477 | 0.09 |
| TSPAN4 | -0.049823562 | 0.0996954 | 0.014138822 | 0.640553 | 0.07 |
| TSPAN5 | -0.288578513 | 2.09E-22 | -0.350902151 | 5.08E-33 | 0.07 |
| TSPAN6 | 0.10342535 | 0.00061576 | 0.12319406 | 4.43E-05 | 0.02 |
| MRX58 | -0.270905221 | 7.72E-20 | -0.212416966 | 1.29E-12 | 0.06 |
| TSPAN8 | -0.06596849 | 0.02919563 | -0.035213778 | 0.2447421 | 0.04 |
| PP1057 | -0.250363113 | 4.40E-17 | -0.112190429 | 0.0002019 | 0.14 |
| TSPO2 | -0.113206971 | 0.00017651 | -0.17441358 | 6.47E-09 | 0.07 |
| TSPO | 0.297953617 | 7.59E-24 | 0.213764681 | 9.21E-13 | 0.09 |
| LOC87538 | 0.044540498 | 0.14113466 | 0.079708867 | 0.0083791 | 0.04 |
| LOC220904 | 0.06144216 | 0.0422645 | 0.067953907 | 0.0246643 | 0.01 |
| LOC728412 | 0.052604864 | 0.0821475 | 0.039200285 | 0.195321 | 0.02 |
| TSPY4 | 0.060534232 | 0.04540962 | 0.028225741 | 0.3511936 | 0.04 |
| TSPYL1 | -0.427707114 | 7.70E-50 | -0.27082677 | 7.92E-20 | 0.16 |
| TSPYL2 | -0.2216248 | 1.25E-13 | -0.209765596 | 2.47E-12 | 0.02 |
| TSPY26P | -0.342796601 | 1.70E-31 | -0.367813134 | 2.39E-36 | 0.03 |
| LOC154278 | -0.324172215 | 3.67E-28 | -0.307142924 | 2.61E-25 | 0.02 |
| TSPYL5 | 0.201407274 | 1.82E-11 | -0.005764362 | 0.8490298 | 0.21 |
| TSPYL6 | -0.05279842 | 0.08102586 | -0.069772377 | 0.0210612 | 0.02 |
| TSR1 | 0.175377255 | 5.32E-09 | 0.030710136 | 0.3104055 | 0.15 |
| TSR2 | 0.184780571 | 7.51E-10 | 0.146087638 | 1.23E-06 | 0.04 |
| LOC200474 | 0.415334937 | 8.02E-47 | 0.317488681 | 5.09E-27 | 0.1 |
| TSSC4 | 0.202582575 | 1.38E-11 | 0.172806807 | 8.93E-09 | 0.03 |
| STK22D | -0.290732624 | 9.85E-23 | -0.200262381 | 2.37E-11 | 0.1 |
| TSSK2 | NA | NA | NA | NA | #VALUE! |
| TSSK3 | -0.135640833 | 6.79E-06 | -0.167411251 | 2.58E-08 | 0.04 |
| C14orf20 | -0.410638215 | 1.04E-45 | -0.368634508 | 1.63E-36 | 0.05 |
| LOC92970 | -0.134501177 | 8.12E-06 | -0.045373879 | 0.133837 | 0.09 |
| TSTA3 | 0.223660664 | 7.38E-14 | 0.311319862 | 5.43E-26 | 0.09 |
| KAT | -0.022354402 | 0.46033493 | 0.286747491 | 3.93E-22 | 0.31 |
| TSTD2 | -0.438015027 | 1.88E-52 | -0.418519224 | 1.38E-47 | 0.02 |
| TST | 0.108954931 | 0.00030769 | 0.097144971 | 0.0013017 | 0.02 |
| TTBK1 | -0.086465826 | 0.00422712 | 0.025695035 | 0.3960705 | 0.12 |
| SCA11 | -0.527330732 | 2.93E-79 | -0.504825102 | 9.73E-72 | 0.03 |
| TTC12 | -0.344612106 | 7.80E-32 | -0.11220978 | 0.0002014 | 0.24 |
| TTC13 | 0.29921096 | 4.82E-24 | 0.353331588 | 1.74E-33 | 0.06 |
| TTC14 | -0.388338544 | 1.16E-40 | -0.193957934 | 1.00E-10 | 0.2 |
| TRAPPC12 | 0.079780605 | 0.0083205 | 0.13500861 | 7.50E-06 | 0.06 |
| TTC16 | -0.229773255 | 1.46E-14 | -0.036371132 | 0.2295684 | 0.2 |
| TTC17 | -0.27792179 | 7.77E-21 | -0.340591554 | 4.33E-31 | 0.07 |
| CFAP70 | -0.337880431 | 1.36E-30 | -0.180969176 | 1.68E-09 | 0.16 |
| TTC19 | -0.100782451 | 0.00084807 | -0.036153865 | 0.2323648 | 0.07 |
| LOC100130486 | 0.1438819 | 1.79E-06 | 0.329145959 | 4.98E-29 | 0.19 |
| TTC21A | -0.230548723 | 1.19E-14 | -0.041412604 | 0.1712671 | 0.19 |
| TTC21B | -0.122853349 | 4.65E-05 | -0.1733558 | 8.00E-09 | 0.06 |
| TTC22 | -0.035936931 | 0.2351808 | -0.083621133 | 0.0056706 | 0.05 |
| TTC23L | -0.282873816 | 1.47E-21 | -0.105375431 | 0.0004839 | 0.18 |
| TTC23 | -0.062533618 | 0.03872911 | -0.070539935 | 0.0196835 | 0.01 |
| LOC648953 | 0.04636376 | 0.12555172 | -0.118402974 | 8.72E-05 | 0.17 |
| TTC25 | -0.127626785 | 2.32E-05 | -0.014410251 | 0.6341525 | 0.12 |
| TTC26 | -0.164992761 | 4.11E-08 | -0.104784844 | 0.0005208 | 0.07 |
| TTC27 | 0.259226394 | 3.05E-18 | 0.173645752 | 7.55E-09 | 0.09 |
| TTC28 | -0.497737435 | 1.75E-69 | -0.346683704 | 3.19E-32 | 0.16 |
| TTC29 | -0.07028518 | 0.02013181 | 0.028464381 | 0.3471346 | 0.1 |
| DKFZP564G196 | -0.352453447 | 2.56E-33 | -0.148736946 | 7.84E-07 | 0.21 |
| TTC30B | -0.371664576 | 3.91E-37 | -0.213732405 | 9.29E-13 | 0.16 |
| TTC31 | -0.029567251 | 0.32876493 | -0.060154424 | 0.0467826 | 0.04 |
| TTC32 | 0.192222708 | 1.48E-10 | 0.130257139 | 1.56E-05 | 0.07 |
| TTC33 | -0.216193763 | 5.01E-13 | -0.260250473 | 2.22E-18 | 0.05 |
| EMC2 | 0.306673186 | 3.11E-25 | 0.147477806 | 9.74E-07 | 0.16 |
| TTC36 | -0.336314823 | 2.61E-30 | -0.101203003 | 0.0008064 | 0.24 |
| TTC37 | -0.44034165 | 4.70E-53 | -0.346593296 | 3.32E-32 | 0.1 |
| TTC38 | -0.13799282 | 4.68E-06 | -0.088803656 | 0.0032997 | 0.05 |
| TTC39A | -0.310012003 | 8.90E-26 | -0.059711182 | 0.0484287 | 0.26 |
| TTC39B | -0.049294254 | 0.10335099 | -0.056224998 | 0.0631445 | 0.01 |
| TTC39C | -0.027158475 | 0.36971241 | -0.14167396 | 2.57E-06 | 0.12 |
| TTC3 | -0.321767471 | 9.53E-28 | -0.148137054 | 8.70E-07 | 0.18 |
| LOC100129188 | 0.347558478 | 2.19E-32 | 0.17199113 | 1.05E-08 | 0.18 |
| TTC5 | -0.16553271 | 3.71E-08 | -0.199543706 | 2.80E-11 | 0.04 |
| TTC7A | 0.170883771 | 1.31E-08 | -0.103723211 | 0.0005937 | 0.28 |
| TTC7B | 0.036687376 | 0.2255411 | -0.166572695 | 3.03E-08 | 0.21 |
| TTC8 | -0.390701258 | 3.53E-41 | -0.182743649 | 1.16E-09 | 0.21 |
| LOC126277 | 0.114426447 | 0.00014996 | 0.099302957 | 0.0010112 | 0.02 |
| TTC9C | 0.256157695 | 7.77E-18 | 0.078479454 | 0.0094423 | 0.18 |
| TTC9 | -0.17215421 | 1.02E-08 | -0.046498575 | 0.1244549 | 0.13 |
| TTF1 | -0.022599923 | 0.45542203 | -0.006994115 | 0.81734 | 0.02 |
| LOC51682 | 0.131461583 | 1.30E-05 | -0.09192378 | 0.00235 | 0.23 |
| TTK | 0.491619224 | 1.40E-67 | 0.288107665 | 2.45E-22 | 0.21 |
| TTLL10 | -0.004714496 | 0.87628024 | -0.091691928 | 0.0024109 | 0.09 |
| LOC100133996 | -0.275395311 | 1.79E-20 | -0.199930354 | 2.56E-11 | 0.08 |
| TTLL12 | 0.147427761 | 9.82E-07 | 0.111456053 | 0.0002224 | 0.04 |
| TTLL13P | 0.031631483 | 0.29610659 | -0.087019409 | 0.0039884 | 0.12 |
| HS323M22B_2 | 0.034367582 | 0.25627208 | 0.16148294 | 7.97E-08 | 0.13 |
| TTLL2 | 0.220524718 | 1.66E-13 | 0.079342269 | 0.0086844 | 0.15 |
| TTLL3 | -0.181696663 | 1.44E-09 | -0.105734284 | 0.0004627 | 0.08 |
| TTLL4 | 0.354898887 | 8.66E-34 | 0.14746459 | 9.76E-07 | 0.21 |
| TTLL5 | -0.168646111 | 2.03E-08 | -0.138683142 | 4.19E-06 | 0.03 |
| TTLL6 | -0.04300408 | 0.15538392 | -0.009737301 | 0.7477862 | 0.04 |
| TTLL7 | -0.08594907 | 0.00446161 | -0.112956223 | 0.0001825 | 0.03 |
| TTLL8 | 0.03619517 | 0.2318313 | -0.048021818 | 0.1125746 | 0.09 |
| C20orf125 | -0.133739189 | 9.15E-06 | 0.036506941 | 0.2278327 | 0.18 |
| TTL | 0.215075395 | 6.64E-13 | -0.048078733 | 0.1121486 | 0.27 |
| LOC129826 | -0.186359193 | 5.35E-10 | -0.256212752 | 7.64E-18 | 0.07 |
| LOC149705 | -0.032149609 | 0.28826198 | -0.130065604 | 1.61E-05 | 0.1 |
| TTPA | 0.06324434 | 0.03656454 | -0.001467255 | 0.9613554 | 0.07 |
| TTR | 0.050989169 | 0.09200841 | 0.110267832 | 0.0002597 | 0.06 |
| TTTY10 | -0.020985753 | 0.48825747 | 0.011606105 | 0.7015136 | 0.04 |
| TTTY11 | NA | NA | NA | NA | #VALUE! |
| TTTY12 | NA | NA | NA | NA | #VALUE! |
| TTTY13 | NA | NA | NA | NA | #VALUE! |
| NCRNA00185.1 | 0.023740952 | 0.43298064 | 0.03191437 | 0.2918061 | 0.01 |
| TTTY15 | 0.046126672 | 0.12749885 | 0.045054888 | 0.1365951 | 0.01 |
| TTTY16 | NA | NA | NA | NA | #VALUE! |
| TTTY17A | NA | NA | NA | NA | #VALUE! |
| TTTY17B | NA | NA | NA | NA | #VALUE! |
| TTTY18 | NA | NA | NA | NA | #VALUE! |
| TTTY19 | NA | NA | NA | NA | #VALUE! |
| TTTY1B | NA | NA | NA | NA | #VALUE! |
| TTTY20 | NA | NA | NA | NA | #VALUE! |
| TTTY21 | -0.020730881 | 0.49355618 | -0.026193646 | 0.3869653 | 0.01 |
| TTTY22 | NA | NA | NA | NA | #VALUE! |
| TTTY23 | NA | NA | NA | NA | #VALUE! |
| TTTY2 | 0.016375939 | 0.58863556 | 0.034265989 | 0.2576813 | 0.02 |
| TTTY3B | NA | NA | NA | NA | #VALUE! |
| TTTY4C | -0.020910458 | 0.48981963 | 0.004534723 | 0.8809621 | 0.03 |
| TTTY5 | 0.030532246 | 0.31321787 | 0.04498949 | 0.1371659 | 0.02 |
| TTTY6B | NA | NA | NA | NA | #VALUE! |
| TTTY6 | NA | NA | NA | NA | #VALUE! |
| CLONE795723 | NA | NA | NA | NA | #VALUE! |
| TTTY8 | -0.009299671 | 0.75876186 | -0.015540925 | 0.6077872 | 0.01 |
| TTTY9B | -0.009299671 | 0.75876186 | -0.015540925 | 0.6077872 | 0.01 |
| TTYH1 | 0.278679942 | 6.03E-21 | 0.105623787 | 0.0004692 | 0.18 |
| MGC4837 | -0.071209288 | 0.01854695 | -0.16635571 | 3.16E-08 | 0.1 |
| LOC392619 | 0.110183778 | 0.00026256 | -0.009554698 | 0.7523598 | 0.12 |
| TUBA1A | 0.2266898 | 3.33E-14 | 0.088003037 | 0.0035941 | 0.14 |
| LOC88851 | 0.576977719 | 5.20E-98 | 0.382035788 | 2.64E-39 | 0.2 |
| MGC10851 | 0.592131626 | 2.13E-104 | 0.377842466 | 2.04E-38 | 0.22 |
| TUBA3C | -0.139194537 | 3.85E-06 | -0.00820805 | 0.7863474 | 0.14 |
| TUBA3D | -0.233259675 | 5.68E-15 | -0.0373772 | 0.2169316 | 0.2 |
| TUBA3E | -0.235216763 | 3.32E-15 | -0.040637675 | 0.1794271 | 0.2 |
| TUBA4A | 0.231753108 | 8.56E-15 | 0.120092996 | 6.89E-05 | 0.12 |
| TUBA4B | 0.02410096 | 0.426035 | 0.118683702 | 8.38E-05 | 0.1 |
| TUBAL2 | 0.20556491 | 6.80E-12 | 0.057989771 | 0.0552897 | 0.15 |
| TUBAL3 | -0.027003502 | 0.37245096 | 0.041545512 | 0.1698959 | 0.07 |
| TUBB1 | 0.076598788 | 0.0113019 | -0.007465845 | 0.8052596 | 0.09 |
| LOC154382 | 0.077164394 | 0.01071114 | 0.049341748 | 0.1030187 | 0.03 |
| TUBB2B | 0.074292327 | 0.01402078 | -0.064601515 | 0.0327162 | 0.14 |
| TUBB4B | 0.310665043 | 6.96E-26 | 0.252682897 | 2.21E-17 | 0.06 |
| FEOM3 | 0.311484314 | 5.10E-26 | 0.129063882 | 1.87E-05 | 0.19 |
| TUBB7P | 0.039546263 | 0.19140424 | 0.056005944 | 0.0641808 | 0.02 |
| DYT4 | 0.409080007 | 2.41E-45 | 0.248421377 | 7.78E-17 | 0.17 |
| TUBB6 | 0.300114051 | 3.48E-24 | 0.061601272 | 0.0417327 | 0.24 |
| TUBB8 | 0.072370011 | 0.01671156 | 0.13144092 | 1.30E-05 | 0.06 |
| TUBBP5 | -0.011328209 | 0.7083297 | 0.040186434 | 0.1843097 | 0.06 |
| LOC157584 | 0.547548031 | 1.62E-86 | 0.324687573 | 2.99E-28 | 0.23 |
| TUBD1 | 0.176320469 | 4.39E-09 | 0.131325489 | 1.33E-05 | 0.05 |
| TUBE1 | 0.015390592 | 0.61126448 | 0.020260771 | 0.5034097 | 0.01 |
| TUBG1 | 0.346347157 | 3.69E-32 | 0.246082014 | 1.54E-16 | 0.11 |
| TUBG2 | -0.162799503 | 6.22E-08 | -0.065493838 | 0.0303793 | 0.1 |
| TUBGCP2 | -0.02626783 | 0.38562161 | -0.002258439 | 0.940549 | 0.03 |
| TUBGCP3 | -0.002224457 | 0.9414419 | -0.121345782 | 5.77E-05 | 0.12 |
| LOC116178 | -0.189085815 | 2.96E-10 | -0.296785271 | 1.15E-23 | 0.11 |
| TUBGCP5 | -0.182239832 | 1.29E-09 | -0.17394828 | 7.10E-09 | 0.01 |
| TUBGCP6 | -0.240289398 | 8.06E-16 | -0.259007797 | 3.26E-18 | 0.02 |
| TUB | -0.063547158 | 0.03567403 | -0.17607637 | 4.62E-09 | 0.12 |
| TUFM | 0.333054239 | 1.01E-29 | 0.305173796 | 5.43E-25 | 0.03 |
| TUFT1 | -0.134995482 | 7.52E-06 | 0.148196446 | 8.61E-07 | 0.29 |
| TI.227H | -0.040677495 | 0.17900085 | -0.098558593 | 0.0011039 | 0.06 |
| LOC221483 | 0.279115929 | 5.22E-21 | 0.05578655 | 0.0652328 | 0.23 |
| TULP2 | -0.010880991 | 0.7193478 | -0.040780279 | 0.1779042 | 0.03 |
| TULP3 | 0.22134163 | 1.35E-13 | 0.033056997 | 0.2748637 | 0.19 |
| TULP4 | -0.142585012 | 2.22E-06 | -0.167873167 | 2.36E-08 | 0.03 |
| LOC389708 | 0.100960964 | 0.00083013 | 0.178641224 | 2.73E-09 | 0.08 |
| TUSC2 | -0.027305029 | 0.36713417 | 0.170390239 | 1.44E-08 | 0.2 |
| MRT7 | 0.112942035 | 0.00018283 | -0.01326738 | 0.6612817 | 0.13 |
| TUSC5 | -0.259076813 | 3.19E-18 | -0.166791865 | 2.91E-08 | 0.1 |
| TUT1 | 0.113071042 | 0.00017973 | 0.060890571 | 0.0441525 | 0.06 |
| TWF1 | -0.165707273 | 3.58E-08 | -0.029359007 | 0.3321844 | 0.14 |
| TWF2 | 0.137299459 | 5.22E-06 | 0.069442156 | 0.0216793 | 0.07 |
| CRS | 0.017816094 | 0.55627552 | 0.004021557 | 0.8943489 | 0.02 |
| TWIST2 | -0.11617241 | 0.00011842 | -0.105338065 | 0.0004862 | 0.02 |
| LOC157203 | -0.0283744 | 0.34866156 | -0.040719136 | 0.1785559 | 0.02 |
| TWSG1 | -0.233897482 | 4.77E-15 | -0.302078956 | 1.70E-24 | 0.07 |
| TXK | -0.138446697 | 4.35E-06 | -0.233547793 | 5.25E-15 | 0.1 |
| TXLNA | -0.047500355 | 0.11653685 | -0.179948404 | 2.08E-09 | 0.14 |
| LST001 | -0.231807924 | 8.43E-15 | -0.1269188 | 2.57E-05 | 0.11 |
| TXLNG | -0.187379346 | 4.29E-10 | -0.272748123 | 4.25E-20 | 0.09 |
| TXN2 | 0.207202017 | 4.59E-12 | 0.231427826 | 9.35E-15 | 0.03 |
| TXNDC11 | -0.068905032 | 0.02271826 | -0.026813032 | 0.3758339 | 0.05 |
| TXNDC12 | 0.370293186 | 7.48E-37 | 0.219090715 | 2.40E-13 | 0.16 |
| TXNDC15 | -0.327909814 | 8.21E-29 | -0.189498008 | 2.70E-10 | 0.14 |
| TXNDC16 | -0.271916492 | 5.57E-20 | -0.224598641 | 5.78E-14 | 0.05 |
| TXNDC17 | 0.406203038 | 1.13E-44 | 0.489661811 | 5.59E-67 | 0.09 |
| TXNDC2 | -0.054192139 | 0.07331127 | -0.17111186 | 1.25E-08 | 0.12 |
| NME8 | -0.138676445 | 4.19E-06 | -0.038741367 | 0.2006064 | 0.1 |
| TXNDC5 | 0.004319382 | 0.88657575 | 0.016544536 | 0.5848027 | 0.02 |
| NME9 | -0.228325911 | 2.15E-14 | -0.222735526 | 9.40E-14 | 0.01 |
| TXNDC8 | NA | NA | NA | NA | #VALUE! |
| TXNDC9 | 0.180324789 | 1.92E-09 | 0.240348771 | 7.93E-16 | 0.07 |
| TXNIP | -0.382073453 | 2.60E-39 | -0.241550743 | 5.64E-16 | 0.15 |
| TXNL1 | 0.529517299 | 5.07E-80 | 0.34259715 | 1.85E-31 | 0.19 |
| TXNL4A | 0.657820833 | 1.81E-136 | 0.430342657 | 1.69E-50 | 0.23 |
| TXNL4B | 0.361983656 | 3.53E-35 | 0.160311414 | 9.91E-08 | 0.21 |
| TXNRD1 | 0.117828596 | 9.44E-05 | -0.064047445 | 0.0342433 | 0.19 |
| TXNRD2 | 0.064461317 | 0.033097 | 0.146645281 | 1.12E-06 | 0.09 |
| TXNRD3NB | 0.153004569 | 3.72E-07 | -0.003475942 | 0.9086152 | 0.16 |
| TXN | 0.519020611 | 2.04E-76 | 0.466639283 | 3.40E-60 | 0.06 |
| LOC115716 | -0.038898529 | 0.19878473 | -0.135557675 | 6.88E-06 | 0.1 |
| MNGIE | 0.056004871 | 0.06418595 | -0.012364138 | 0.6830438 | 0.07 |
| LOC115567 | 0.601696925 | 1.31E-108 | 0.218793855 | 2.59E-13 | 0.39 |
| TYRO3 | 0.235812591 | 2.82E-15 | 0.021831191 | 0.4709026 | 0.22 |
| PLOSL | 0.067593046 | 0.02543952 | 0.004008141 | 0.8946993 | 0.07 |
| TYRP1 | -0.024601814 | 0.41648081 | 0.084681597 | 0.0050874 | 0.11 |
| TYR | -0.056085249 | 0.06380406 | -0.053543687 | 0.0768225 | 0.01 |
| TYSND1 | 0.164067712 | 4.90E-08 | 0.175699245 | 4.99E-09 | 0.02 |
| LOC155153 | -0.119001313 | 8.02E-05 | -0.087725647 | 0.0037015 | 0.04 |
| LOC91331 | -0.170411015 | 1.44E-08 | -0.026496065 | 0.3815055 | 0.15 |
| FLJ40918 | -0.09102435 | 0.00259424 | -0.174154525 | 6.82E-09 | 0.09 |
| T | 0.178097293 | 3.05E-09 | 0.008306149 | 0.7838569 | 0.17 |
| U2AF1L4.1 | 0.163163883 | 5.81E-08 | 0.039504634 | 0.1918724 | 0.13 |
| U2AFBP | 0.482797313 | 6.68E-65 | 0.351521987 | 3.86E-33 | 0.14 |
| U2AF2 | 0.223710714 | 7.29E-14 | 0.102815335 | 0.0006634 | 0.13 |
| UACA | -0.212428367 | 1.28E-12 | -0.196354864 | 5.83E-11 | 0.02 |
| UAP1L1 | -0.077463252 | 0.0104102 | -0.132193879 | 1.16E-05 | 0.06 |
| UAP1 | 0.087276574 | 0.00388167 | 0.302944493 | 1.24E-24 | 0.22 |
| SMAX2 | 0.097795505 | 0.00120693 | 0.082486141 | 0.006361 | 0.02 |
| LOC100505842 | 0.335883146 | 3.12E-30 | 0.130723654 | 1.45E-05 | 0.21 |
| DKFZp566J164 | -0.019292219 | 0.52403309 | 0.075098087 | 0.0130116 | 0.1 |
| UBA52 | 0.43441814 | 1.57E-51 | 0.433370522 | 2.90E-51 | 0.01 |
| LOC100129437 | -0.070634422 | 0.01951949 | 0.083411461 | 0.0057928 | 0.16 |
| UBA6 | -0.002820247 | 0.92579836 | -0.138944649 | 4.01E-06 | 0.14 |
| UBA7 | -0.207306116 | 4.48E-12 | -0.141305635 | 2.73E-06 | 0.07 |
| UBAC1 | 0.360426949 | 7.18E-35 | 0.22709516 | 2.99E-14 | 0.14 |
| UBAC2 | 0.217016244 | 4.07E-13 | 0.191094083 | 1.90E-10 | 0.03 |
| UBAP1 | -0.029584371 | 0.32848483 | -0.069928838 | 0.0207738 | 0.05 |
| UBAP2L | 0.125313208 | 3.26E-05 | 0.233649811 | 5.10E-15 | 0.11 |
| UBAP2 | 0.170716894 | 1.35E-08 | -0.019525609 | 0.5190243 | 0.2 |
| LOC100134052 | 0.011674549 | 0.69983847 | -0.085263412 | 0.0047909 | 0.1 |
| UBASH3B | 0.123700147 | 4.12E-05 | -0.152022879 | 4.43E-07 | 0.28 |
| UBB | 0.189129632 | 2.93E-10 | 0.177650455 | 3.35E-09 | 0.02 |
| UBC | 0.095267416 | 0.00161511 | 0.121377613 | 5.74E-05 | 0.03 |
| UBD | 0.193437461 | 1.13E-10 | 0.085820999 | 0.0045215 | 0.11 |
| UBE2A | 0.208562019 | 3.31E-12 | 0.191871843 | 1.60E-10 | 0.02 |
| UBE2B | -0.084931752 | 0.00495791 | 0.081397484 | 0.0070932 | 0.17 |
| LOC105377878 | 0.363398417 | 1.85E-35 | 0.192111539 | 1.52E-10 | 0.18 |
| UBE2C | 0.62001864 | 4.40E-117 | 0.384065622 | 9.73E-40 | 0.24 |
| SFT | 0.219722424 | 2.05E-13 | 0.158221345 | 1.46E-07 | 0.07 |
| UBE2D2 | 0.265997502 | 3.70E-19 | 0.287177216 | 3.39E-22 | 0.03 |
| LOC100128180 | -0.016942992 | 0.57579059 | -0.0053604 | 0.8594956 | 0.02 |
| LOC222070 | -0.048571804 | 0.10851131 | 0.045715611 | 0.1309303 | 0.1 |
| UBE2DNL | -0.04840319 | 0.10974452 | -0.05245458 | 0.083027 | 0.01 |
| LOC94682 | 0.273018505 | 3.89E-20 | 0.22945711 | 1.59E-14 | 0.05 |
| FLJ25157 | 0.19485542 | 8.19E-11 | 0.015674076 | 0.6047147 | 0.18 |
| UBE2E3 | 0.269208003 | 1.33E-19 | 0.141357039 | 2.71E-06 | 0.13 |
| UBE2F | 0.266754156 | 2.91E-19 | 0.169714992 | 1.65E-08 | 0.1 |
| UBE2G1 | -0.042168246 | 0.16358051 | -0.085321181 | 0.0047623 | 0.05 |
| UBE2G2 | 0.207310401 | 4.48E-12 | 0.029522584 | 0.3294965 | 0.18 |
| UBE2H | -0.327052789 | 1.16E-28 | -0.293130772 | 4.24E-23 | 0.04 |
| UBE2I | 0.408745584 | 2.89E-45 | 0.375022273 | 7.92E-38 | 0.04 |
| HSPC153 | 0.009055119 | 0.76491653 | -0.024413919 | 0.4200502 | 0.04 |
| PRO2121 | 0.234359697 | 4.20E-15 | 0.138241569 | 4.49E-06 | 0.1 |
| MGC12679 | -0.032623582 | 0.28120949 | 0.001409166 | 0.9628842 | 0.04 |
| UBE2L3 | 0.315010538 | 1.33E-26 | 0.268205499 | 1.84E-19 | 0.05 |
| UBE2L6 | 0.0745306 | 0.01371542 | 0.071529691 | 0.0180235 | 0.01 |
| UBE2MP1 | 0.250833394 | 3.83E-17 | 0.120100581 | 6.88E-05 | 0.14 |
| UBE2M | 0.342875291 | 1.64E-31 | 0.253279099 | 1.85E-17 | 0.09 |
| UBE2NL | 0.062873287 | 0.03768142 | 0.041270678 | 0.1727404 | 0.03 |
| UBE2N | 0.2815503 | 2.31E-21 | 0.257754291 | 4.78E-18 | 0.03 |
| UBE2O | 0.309222659 | 1.20E-25 | 0.05440394 | 0.0721932 | 0.26 |
| UBE2Q | 0.370474461 | 6.86E-37 | 0.479154076 | 8.10E-64 | 0.11 |
| UBE2Q2P1 | -0.236905021 | 2.08E-15 | -0.346956168 | 2.84E-32 | 0.12 |
| UBE2Q2 | -0.279401904 | 4.74E-21 | -0.156801279 | 1.89E-07 | 0.13 |
| UBE2QL1 | -0.054775587 | 0.07026508 | -0.100630887 | 0.0008636 | 0.05 |
| UBE2R2 | 0.213561628 | 9.69E-13 | 0.214086008 | 8.50E-13 | 0.01 |
| LOC147743 | 0.603565276 | 1.90E-109 | 0.327340268 | 1.03E-28 | 0.28 |
| UBE2T | 0.602497309 | 5.74E-109 | 0.573254214 | 1.72E-96 | 0.03 |
| UBE2U | 0.175301571 | 5.41E-09 | 0.040182083 | 0.1843573 | 0.14 |
| UBE2V1 | 0.237369087 | 1.83E-15 | 0.228859654 | 1.87E-14 | 0.01 |
| UBE2V2 | 0.43764129 | 2.35E-52 | 0.294132112 | 2.97E-23 | 0.15 |
| UBE2W | -0.047603799 | 0.11574227 | -0.070371724 | 0.0199785 | 0.03 |
| UBE2Z | -0.019086409 | 0.52847046 | -0.005005518 | 0.8687103 | 0.02 |
| UBE3A | -0.346062112 | 4.18E-32 | -0.23248259 | 7.02E-15 | 0.12 |
| UBE3B | -0.391703232 | 2.13E-41 | -0.251890029 | 2.80E-17 | 0.14 |
| UBE3C | -0.135933225 | 6.49E-06 | -0.233965018 | 4.68E-15 | 0.1 |
| UBE4A | -0.178838026 | 2.62E-09 | -0.106246766 | 0.000434 | 0.08 |
| UBE4B | -0.089201605 | 0.00316168 | -0.239594659 | 9.80E-16 | 0.16 |
| UBFD1 | -0.127398847 | 2.40E-05 | -0.140388879 | 3.18E-06 | 0.02 |
| UBIAD1 | -0.154090387 | 3.07E-07 | -0.103458772 | 0.0006132 | 0.06 |
| DKFZP434K151 | -0.449604801 | 1.69E-55 | -0.22495915 | 5.25E-14 | 0.23 |
| UBL4A | 0.348450418 | 1.48E-32 | 0.247037316 | 1.17E-16 | 0.11 |
| UBL4B | 0.142220607 | 2.35E-06 | 0.026166641 | 0.3874551 | 0.12 |
| UBL5 | 0.393403772 | 8.95E-42 | 0.470968527 | 1.97E-61 | 0.08 |
| UBL7 | 0.238904843 | 1.19E-15 | 0.172072197 | 1.03E-08 | 0.07 |
| UBLCP1 | -0.113439344 | 0.00017113 | -0.110402402 | 0.0002552 | 0.01 |
| UBN1 | -0.189097411 | 2.95E-10 | -0.212639713 | 1.22E-12 | 0.03 |
| UBN2 | -0.273287062 | 3.57E-20 | -0.227155232 | 2.94E-14 | 0.05 |
| UBOX5 | -0.057952491 | 0.05544676 | 0.030823836 | 0.3086167 | 0.09 |
| UBP1 | -0.038046591 | 0.20880541 | -0.116553532 | 0.0001124 | 0.08 |
| UBQLN1 | -0.064749654 | 0.03231792 | -0.128500739 | 2.03E-05 | 0.07 |
| UBQLN2 | -0.17834786 | 2.90E-09 | -0.214784255 | 7.14E-13 | 0.04 |
| UBQLN3 | -0.018302907 | 0.54553625 | 0.031311374 | 0.3010238 | 0.05 |
| C1orf6 | 0.342648934 | 1.81E-31 | 0.428735261 | 4.27E-50 | 0.09 |
| UBQLNL | -0.066476335 | 0.02797329 | 0.019563116 | 0.5182216 | 0.09 |
| FLJ14897 | -0.538933443 | 2.30E-83 | -0.373103866 | 1.98E-37 | 0.17 |
| LOC255838 | -0.306418349 | 3.42E-25 | -0.260122218 | 2.31E-18 | 0.05 |
| UBR3 | -0.355340119 | 7.11E-34 | -0.32844821 | 6.60E-29 | 0.03 |
| LOC148490 | -0.278012364 | 7.54E-21 | -0.471970648 | 1.02E-61 | 0.2 |
| KIAA0896 | -0.123222547 | 4.41E-05 | -0.220679536 | 1.60E-13 | 0.1 |
| UBR7 | -0.211045005 | 1.80E-12 | -0.11509428 | 0.0001371 | 0.1 |
| UBTD1 | 0.045590874 | 0.13198556 | 0.066051991 | 0.0289916 | 0.03 |
| UBTD2 | -0.233815427 | 4.88E-15 | -0.130412387 | 1.52E-05 | 0.11 |
| UBTFL1 | 0.041663143 | 0.16868916 | -0.009023842 | 0.7657048 | 0.06 |
| UBTF | -0.00611176 | 0.84005055 | -0.001660087 | 0.9562814 | 0.01 |
| UBXN10 | -0.449754154 | 1.54E-55 | -0.164681828 | 4.36E-08 | 0.29 |
| UBXN11 | -0.047018869 | 0.12029173 | 0.051456081 | 0.0890658 | 0.1 |
| UBXN1 | 0.20197074 | 1.59E-11 | 0.14842725 | 8.27E-07 | 0.06 |
| UBXN2A | 0.282879013 | 1.47E-21 | 0.05393234 | 0.074702 | 0.23 |
| UBXN2B | -0.059079039 | 0.05086033 | -0.054653152 | 0.0708956 | 0.01 |
| UBXN4 | -0.316170617 | 8.48E-27 | -0.255222877 | 1.03E-17 | 0.07 |
| UBXN6 | -0.099712515 | 0.00096338 | -0.069480082 | 0.0216075 | 0.04 |
| UBXN7 | -0.225390795 | 4.69E-14 | -0.324337245 | 3.44E-28 | 0.1 |
| UBXN8 | -0.217831309 | 3.31E-13 | 0.079455682 | 0.0085889 | 0.3 |
| LOC100133945 | -0.015791014 | 0.60202214 | 0.025446625 | 0.4006545 | 0.05 |
| UCHL1 | 0.337629223 | 1.51E-30 | 0.150067695 | 6.23E-07 | 0.19 |
| UCHL3 | 0.358793707 | 1.51E-34 | 0.309927279 | 9.19E-26 | 0.05 |
| LOC100506438 | 0.235525114 | 3.05E-15 | 0.331228363 | 2.13E-29 | 0.1 |
| UCK1 | 0.106173111 | 0.00043799 | 0.08339814 | 0.0058007 | 0.03 |
| UCK2 | 0.58296456 | 1.71E-100 | 0.514221218 | 8.24E-75 | 0.07 |
| UCKL1.AS1 | -0.058129773 | 0.05470297 | -0.067982406 | 0.024604 | 0.01 |
| UCKL1 | 0.200807166 | 2.09E-11 | 0.093748942 | 0.0019178 | 0.11 |
| LOC118890 | 0.203630786 | 1.08E-11 | 0.135823109 | 6.60E-06 | 0.07 |
| UCN2 | 0.055923658 | 0.06457373 | 0.001807728 | 0.9523976 | 0.06 |
| UCN3 | 0.015018483 | 0.61990912 | -0.071389351 | 0.0182512 | 0.09 |
| UCN | 0.002783199 | 0.92677036 | 0.115352193 | 0.0001324 | 0.12 |
| UCP1 | -0.190053348 | 2.39E-10 | -0.082692058 | 0.0062304 | 0.11 |
| LOC119829 | -0.081986909 | 0.00668791 | 0.060585668 | 0.0452263 | 0.15 |
| UCP3 | 0.037305003 | 0.21782148 | -0.011369115 | 0.7073249 | 0.05 |
| UEVLD | -0.458829455 | 5.22E-58 | -0.324288217 | 3.51E-28 | 0.14 |
| UFC1 | 0.236609197 | 2.26E-15 | 0.556545813 | 6.56E-90 | 0.32 |
| UFD1L | 0.409966869 | 1.50E-45 | 0.324082109 | 3.81E-28 | 0.09 |
| C13orf20 | -0.139029015 | 3.96E-06 | -0.056940281 | 0.0598564 | 0.09 |
| UFSP1 | 0.206972578 | 4.86E-12 | 0.176114923 | 4.58E-09 | 0.04 |
| UFSP2 | -0.275946681 | 1.49E-20 | -0.139878343 | 3.45E-06 | 0.14 |
| UGCG | -0.499175846 | 6.16E-70 | -0.386683567 | 2.65E-40 | 0.12 |
| UGDH | -0.309976204 | 9.03E-26 | -0.139553838 | 3.64E-06 | 0.18 |
| UGGT1 | -0.15129013 | 5.04E-07 | -0.16339141 | 5.57E-08 | 0.02 |
| FLJ11485 | -0.202377571 | 1.45E-11 | -0.141894625 | 2.48E-06 | 0.07 |
| LOC105374766 | 0.227950171 | 2.38E-14 | 0.11918447 | 7.82E-05 | 0.11 |
| UGT1A10 | 0.08965266 | 0.00301161 | -0.025242636 | 0.4044425 | 0.12 |
| LOC130913 | 0.049245331 | 0.10369417 | -0.031608137 | 0.2964634 | 0.09 |
| UGT1A3 | 0.027266707 | 0.36780726 | -0.052083995 | 0.0852284 | 0.08 |
| UGT1A4 | 0.111822689 | 0.00021196 | -0.005674146 | 0.8513649 | 0.12 |
| UGT1A5 | 0.025037414 | 0.40827495 | -0.037142393 | 0.2198353 | 0.07 |
| UGT1A6 | 0.105803522 | 0.00045874 | 0.021513492 | 0.477384 | 0.09 |
| UGT1A7 | 0.03683267 | 0.22370778 | -0.033557716 | 0.2676549 | 0.08 |
| UGT1A8 | 0.074643906 | 0.01357227 | -0.00387453 | 0.8981901 | 0.08 |
| UGT1A9 | 0.078486141 | 0.00943624 | -0.038147735 | 0.207597 | 0.12 |
| UGT2A1 | 0.068027246 | 0.02450928 | -0.032153482 | 0.2882039 | 0.11 |
| UGT2A3 | 0.012329625 | 0.68388075 | 0.018799106 | 0.5346967 | 0.01 |
| UGT2B10 | -0.11811329 | 9.07E-05 | 0.021616835 | 0.4752704 | 0.14 |
| UGT2B11 | -0.228062473 | 2.31E-14 | -0.060343865 | 0.0460935 | 0.17 |
| UGT2B15 | -0.252025068 | 2.69E-17 | -0.043216315 | 0.1533531 | 0.21 |
| UGT2B28 | -0.168111162 | 2.25E-08 | -0.075046868 | 0.0130738 | 0.1 |
| UGT2B4 | -0.062384164 | 0.03919787 | -0.003884175 | 0.8979381 | 0.06 |
| UGT2B7 | 0.188348727 | 3.47E-10 | 0.076940561 | 0.0109416 | 0.12 |
| UGT3A1 | -0.010542534 | 0.7277254 | -0.014854252 | 0.6237413 | 0.01 |
| UGT3A2 | 0.121100518 | 5.97E-05 | 0.054558575 | 0.0713858 | 0.07 |
| UGT8 | 0.274080824 | 2.75E-20 | 0.033623618 | 0.2667158 | 0.25 |
| UHMK1 | -0.42700725 | 1.15E-49 | -0.345449974 | 5.44E-32 | 0.09 |
| UHRF1BP1L | -0.405728999 | 1.45E-44 | -0.295573981 | 1.78E-23 | 0.12 |
| UHRF1BP1 | -0.185974295 | 5.81E-10 | -0.228800147 | 1.90E-14 | 0.05 |
| LOC96185 | 0.453533671 | 1.47E-56 | 0.261313719 | 1.60E-18 | 0.2 |
| UHRF2 | 0.035290341 | 0.24371708 | -0.041582429 | 0.1695165 | 0.08 |
| UIMC1 | 0.036435024 | 0.22875068 | -0.06625268 | 0.0285061 | 0.11 |
| ULBP1 | 0.278217017 | 7.04E-21 | 0.134012656 | 8.77E-06 | 0.15 |
| ULBP2 | 0.227689741 | 2.55E-14 | 0.069340083 | 0.0218735 | 0.16 |
| ULBP3 | 0.156712485 | 1.92E-07 | -0.021086243 | 0.4861768 | 0.18 |
| ULK1 | -0.132229147 | 1.16E-05 | -0.109250129 | 0.0002962 | 0.03 |
| ULK2 | -0.1367182 | 5.73E-06 | -0.197928925 | 4.07E-11 | 0.07 |
| ULK3 | 0.079591372 | 0.00847588 | 0.044928805 | 0.1376973 | 0.04 |
| DKFZp434E1822 | -0.343426511 | 1.30E-31 | -0.108423769 | 0.0003294 | 0.24 |
| FLJ36335 | 0.159642862 | 1.12E-07 | 0.20948353 | 2.64E-12 | 0.05 |
| HNFJ | -0.242007084 | 4.95E-16 | -0.085859664 | 0.0045033 | 0.16 |
| LOC131725 | 0.367644544 | 2.59E-36 | 0.393996022 | 6.61E-42 | 0.03 |
| UNC119B | -0.277066238 | 1.03E-20 | -0.25037007 | 4.39E-17 | 0.03 |
| UNC119 | 0.109880728 | 0.00027308 | 0.08626451 | 0.0043171 | 0.03 |
| UNC13A | -0.004676031 | 0.87728164 | -0.173230372 | 8.21E-09 | 0.17 |
| UNC13B | -0.280560325 | 3.22E-21 | -0.21670275 | 4.41E-13 | 0.07 |
| LOC105370828 | -0.150132002 | 6.16E-07 | -0.18293765 | 1.11E-09 | 0.04 |
| UNC13D | 0.163855972 | 5.10E-08 | 0.019970972 | 0.5095352 | 0.15 |
| FLJ10043 | 0.067095477 | 0.02654289 | -0.035040627 | 0.2470713 | 0.11 |
| CMYA4 | -0.114444941 | 0.00014959 | -0.104649379 | 0.0005296 | 0.01 |
| UNC50 | -0.032273412 | 0.28640846 | 0.057238191 | 0.0585294 | 0.09 |
| UNC5A | -0.25339551 | 1.78E-17 | -0.069325146 | 0.021902 | 0.19 |
| UNC5B | -0.33882206 | 9.14E-31 | -0.221349196 | 1.35E-13 | 0.12 |
| UNC5CL | -0.033605379 | 0.2669755 | 0.062662078 | 0.03833 | 0.1 |
| UNC5C | -0.418406013 | 1.47E-47 | -0.371302175 | 4.64E-37 | 0.05 |
| UNC5D | 0.085797188 | 0.00453273 | -0.07794014 | 0.0099455 | 0.17 |
| LOC100131345 | 0.118138979 | 9.04E-05 | -0.075677525 | 0.0123261 | 0.2 |
| UNC93A | 0.209835703 | 2.43E-12 | 0.152464735 | 4.10E-07 | 0.06 |
| UNC93B1 | 0.075835594 | 0.01214477 | 0.061423946 | 0.0423257 | 0.02 |
| LOC641901 | 0.041588084 | 0.16945841 | -0.050607234 | 0.094473 | 0.1 |
| UNG | 0.420051814 | 5.87E-48 | 0.232534117 | 6.92E-15 | 0.19 |
| LOC115940 | -0.129544046 | 1.74E-05 | -0.065632896 | 0.0300283 | 0.07 |
| UNK | 0.065477943 | 0.03041963 | -0.086685834 | 0.0041307 | 0.16 |
| UOX | -0.13163942 | 1.26E-05 | -0.016324284 | 0.5898122 | 0.12 |
| UPB1 | 0.055767583 | 0.06532438 | 0.032291292 | 0.2861414 | 0.03 |
| PLEKHD1 | -0.263710835 | 7.59E-19 | -0.189653596 | 2.61E-10 | 0.08 |
| UPF1 | -0.036226366 | 0.23142898 | -0.145332759 | 1.40E-06 | 0.11 |
| UPF2 | 0.138151671 | 4.56E-06 | -0.026522303 | 0.381034 | 0.17 |
| LOC85658 | 0.078881842 | 0.00908181 | 0.055555532 | 0.0663557 | 0.03 |
| UPF3B | 0.41403631 | 1.64E-46 | 0.172139559 | 1.02E-08 | 0.25 |
| UPK1A | -0.036148047 | 0.23243997 | 0.102760922 | 0.0006678 | 0.14 |
| UPK1B | 0.161750781 | 7.58E-08 | 0.009378478 | 0.7567818 | 0.16 |
| UPK2 | 0.303206242 | 1.12E-24 | 0.188404404 | 3.43E-10 | 0.12 |
| UPK3A | -0.047488573 | 0.11662763 | 0.075315089 | 0.0127511 | 0.13 |
| LOC102724187 | 0.239053655 | 1.14E-15 | 0.159316997 | 1.19E-07 | 0.08 |
| UPK3B | 0.059486827 | 0.04928034 | -0.025726626 | 0.3954898 | 0.09 |
| UPP1 | 0.29109199 | 8.68E-23 | 0.100096187 | 0.0009205 | 0.2 |
| UPP2 | -0.107691835 | 0.00036157 | -0.107157377 | 0.0003869 | 0.01 |
| UPRT | -0.116583545 | 0.00011196 | -0.065303089 | 0.0308664 | 0.06 |
| UQCC1 | -0.043818148 | 0.14770382 | 0.005314468 | 0.8606872 | 0.05 |
| UQCR10 | 0.371999035 | 3.34E-37 | 0.406235615 | 1.11E-44 | 0.04 |
| UQCR11 | 0.229129395 | 1.74E-14 | 0.351134952 | 4.58E-33 | 0.13 |
| UQCRB | 0.549412517 | 3.27E-87 | 0.442041507 | 1.70E-53 | 0.11 |
| UQCRC1 | 0.314140053 | 1.85E-26 | 0.255982462 | 8.19E-18 | 0.06 |
| UQCRC2 | -0.024990872 | 0.40914709 | 8.50E-05 | 0.9977604 | 0.03 |
| UQCRFS1 | 0.349468298 | 9.51E-33 | 0.22175519 | 1.21E-13 | 0.13 |
| UQCRHL | 0.582914664 | 1.79E-100 | 0.356113847 | 5.03E-34 | 0.23 |
| UQCRH | 0.618581345 | 2.13E-116 | 0.428188945 | 5.84E-50 | 0.2 |
| UQCRQ | 0.084635476 | 0.00511155 | 0.371733795 | 3.79E-37 | 0.29 |
| URB1 | -0.169082271 | 1.86E-08 | -0.272870836 | 4.08E-20 | 0.11 |
| URB2 | 0.167244905 | 2.67E-08 | 0.206405993 | 5.56E-12 | 0.04 |
| LOC154699 | -0.158099098 | 1.49E-07 | -0.047736196 | 0.1147315 | 0.12 |
| URM1 | 0.103430002 | 0.00061541 | 0.168746189 | 1.99E-08 | 0.07 |
| UROC1 | 0.260803544 | 1.88E-18 | 0.117427455 | 9.97E-05 | 0.15 |
| UROD | 0.185999375 | 5.78E-10 | 0.142730259 | 2.16E-06 | 0.05 |
| UROS | 0.193441078 | 1.13E-10 | 0.277433298 | 9.13E-21 | 0.09 |
| USE1 | 0.26500237 | 5.06E-19 | 0.314913443 | 1.38E-26 | 0.05 |
| HYPLIP1 | 0.250064787 | 4.80E-17 | 0.432260287 | 5.54E-51 | 0.19 |
| USF2 | 0.13214215 | 1.17E-05 | 0.059026113 | 0.0510685 | 0.08 |
| DFNB18 | 0.259072346 | 3.20E-18 | 0.097063899 | 0.001314 | 0.17 |
| USH1G | 0.266810824 | 2.86E-19 | 0.101883343 | 0.0007429 | 0.17 |
| LOC391164 | -0.215252884 | 6.35E-13 | 0.004974049 | 0.8695283 | 0.23 |
| USHBP1 | -0.275419724 | 1.77E-20 | -0.158039492 | 1.51E-07 | 0.12 |
| USMG5 | 0.497590402 | 1.94E-69 | 0.559019241 | 7.35E-91 | 0.07 |
| USO1 | -0.148271899 | 8.50E-07 | -0.162508592 | 6.57E-08 | 0.02 |
| LOC90756 | 0.156506864 | 1.99E-07 | -0.021366759 | 0.4803939 | 0.18 |
| USP11 | -0.013872572 | 0.64685753 | -0.207037134 | 4.78E-12 | 0.2 |
| USP12 | -0.376527552 | 3.84E-38 | -0.426722227 | 1.35E-49 | 0.06 |
| USP13 | 0.1957639 | 6.67E-11 | -0.040527544 | 0.1806098 | 0.24 |
| USP14 | 0.114847429 | 0.00014171 | -0.028445948 | 0.347447 | 0.15 |
| USP15 | -0.079641055 | 0.00843484 | -0.106753729 | 0.0004071 | 0.03 |
| USP16 | -0.120591504 | 6.42E-05 | -0.136147819 | 6.27E-06 | 0.02 |
| LOC352462 | -0.18838693 | 3.44E-10 | -0.161218218 | 8.37E-08 | 0.03 |
| LOC391628 | 0.034975556 | 0.24795059 | 0.028261147 | 0.3505895 | 0.01 |
| USP17L9P | 0.001812374 | 0.95227545 | 0.040569125 | 0.1801626 | 0.04 |
| USP18 | 0.176006871 | 4.68E-09 | 0.078678378 | 0.0092625 | 0.1 |
| USP19 | -0.402637874 | 7.45E-44 | -0.29752513 | 8.85E-24 | 0.11 |
| USP1 | 0.252241819 | 2.52E-17 | -0.014966039 | 0.6211317 | 0.27 |
| USP20 | -0.185239081 | 6.80E-10 | -0.127048105 | 2.52E-05 | 0.06 |
| USP21_2 | 0.118099849 | 9.09E-05 | 0.394384628 | 5.42E-42 | 0.28 |
| USP3L | -0.200261743 | 2.37E-11 | -0.162963111 | 6.04E-08 | 0.04 |
| USP24 | -0.162243496 | 6.91E-08 | -0.275967525 | 1.48E-20 | 0.12 |
| USP25 | -0.202917487 | 1.27E-11 | -0.187880088 | 3.85E-10 | 0.02 |
| LOC105373339 | 0.01266868 | 0.67567582 | 0.116512177 | 0.0001131 | 0.11 |
| USP27X | 0.133199298 | 9.95E-06 | -0.023392353 | 0.4397681 | 0.16 |
| USP28 | -0.078977942 | 0.00899754 | -0.112303893 | 0.0001989 | 0.04 |
| USP29 | 0.004808666 | 0.87382948 | 0.031403998 | 0.2995955 | 0.03 |
| USP2 | 0.14504324 | 1.47E-06 | 0.052249463 | 0.0842397 | 0.1 |
| USP30 | -0.305853141 | 4.22E-25 | -0.159680705 | 1.11E-07 | 0.15 |
| USP31 | 0.109689575 | 0.00027991 | -0.126623085 | 2.69E-05 | 0.24 |
| USP32 | -0.198273777 | 3.76E-11 | -0.207703875 | 4.07E-12 | 0.01 |
| USP33 | -0.290620809 | 1.02E-22 | -0.279655507 | 4.36E-21 | 0.02 |
| KIAA0729 | -0.188497493 | 3.36E-10 | -0.324124437 | 3.74E-28 | 0.14 |
| USP35 | -0.214591754 | 7.49E-13 | -0.182882169 | 1.12E-09 | 0.04 |
| FLJ12851 | 0.144861467 | 1.52E-06 | -0.119830652 | 7.14E-05 | 0.27 |
| USP37 | -0.199228402 | 3.02E-11 | -0.214676911 | 7.34E-13 | 0.02 |
| USP38 | -0.252718063 | 2.18E-17 | -0.258181753 | 4.20E-18 | 0.01 |
| USP39 | 0.450892813 | 7.63E-56 | 0.296974502 | 1.08E-23 | 0.16 |
| USP3 | -0.267505935 | 2.29E-19 | -0.146266933 | 1.20E-06 | 0.13 |
| USP40 | -0.390673198 | 3.58E-41 | -0.216010427 | 5.25E-13 | 0.18 |
| USP42 | -0.138150909 | 4.56E-06 | -0.161496531 | 7.95E-08 | 0.03 |
| USP43 | 0.007046324 | 0.81600082 | -0.063233545 | 0.0365966 | 0.08 |
| USP44 | -0.12427844 | 3.79E-05 | -0.221160819 | 1.41E-13 | 0.1 |
| USP45 | -0.130915377 | 1.41E-05 | -0.101334527 | 0.0007937 | 0.03 |
| FLJ11850 | -0.19715105 | 4.86E-11 | -0.180699994 | 1.78E-09 | 0.02 |
| USP47 | -0.368440163 | 1.78E-36 | -0.290200075 | 1.19E-22 | 0.08 |
| FLJ11328 | -0.083260411 | 0.00588234 | -0.204417055 | 8.94E-12 | 0.13 |
| LOC116114 | -0.15048013 | 5.80E-07 | -0.255573369 | 9.27E-18 | 0.11 |
| USP4 | -0.327501645 | 9.68E-29 | -0.29190787 | 6.52E-23 | 0.04 |
| USP50 | -0.081968447 | 0.00670028 | -0.03673265 | 0.2249687 | 0.05 |
| USP51 | -0.332411665 | 1.31E-29 | -0.364704546 | 1.01E-35 | 0.04 |
| USP53 | -0.464211652 | 1.65E-59 | -0.361114653 | 5.25E-35 | 0.11 |
| USP54 | -0.188461574 | 3.39E-10 | -0.196877826 | 5.18E-11 | 0.01 |
| USP5 | 0.329293219 | 4.69E-29 | 0.164780734 | 4.28E-08 | 0.17 |
| USP6NL | 0.13053721 | 1.50E-05 | -0.006472755 | 0.8307418 | 0.14 |
| HRP1 | -0.058080302 | 0.05490968 | -0.068350746 | 0.0238355 | 0.02 |
| USP7 | -0.030095452 | 0.32019421 | -0.045666858 | 0.1313419 | 0.02 |
| USP8 | -0.402708519 | 7.18E-44 | -0.34071428 | 4.11E-31 | 0.07 |
| USP9X | -0.217208843 | 3.88E-13 | -0.256952767 | 6.10E-18 | 0.04 |
| USP9Y | 0.04501701 | 0.13692551 | 0.033300132 | 0.2713469 | 0.02 |
| USPL1 | -0.122855187 | 4.65E-05 | -0.069983666 | 0.0206738 | 0.06 |
| UST | -0.094219115 | 0.00181891 | -0.17003413 | 1.55E-08 | 0.08 |
| LOC196745 | -0.0197004 | 0.51528926 | -0.023489922 | 0.4378622 | 0.01 |
| UTP11L | 0.551438218 | 5.69E-88 | 0.323817619 | 4.23E-28 | 0.23 |
| UTP14A | 0.401955707 | 1.07E-43 | 0.286114723 | 4.88E-22 | 0.12 |
| UTP14C | -0.490628981 | 2.83E-67 | -0.350052293 | 7.37E-33 | 0.15 |
| UTP15 | -0.196435268 | 5.72E-11 | -0.016495314 | 0.5859205 | 0.18 |
| UTP18 | 0.153760076 | 3.26E-07 | 0.1417227 | 2.55E-06 | 0.02 |
| UTP20 | -0.046535231 | 0.12415796 | -0.091198726 | 0.0025452 | 0.05 |
| UTP23 | 0.098278054 | 0.00114078 | 0.010979213 | 0.7169228 | 0.09 |
| UTP3 | 0.114321201 | 0.0001521 | 0.084667815 | 0.0050946 | 0.03 |
| UTP6 | 0.338120114 | 1.23E-30 | 0.23614413 | 2.57E-15 | 0.11 |
| UTRN | -0.495138069 | 1.14E-68 | -0.368194703 | 2.00E-36 | 0.13 |
| UTS2B | 0.051014008 | 0.09184993 | -0.074661573 | 0.0135501 | 0.13 |
| UTS2R | -0.261425151 | 1.55E-18 | -0.218976971 | 2.48E-13 | 0.05 |
| UTS2 | 0.103087654 | 0.00064174 | -0.018052955 | 0.5510374 | 0.13 |
| UTY | 0.043774853 | 0.14810484 | 0.039567509 | 0.1911656 | 0.01 |
| UVRAG | -0.105379738 | 0.00048366 | -0.104900457 | 0.0005134 | 0.01 |
| UXS1 | 0.312333512 | 3.69E-26 | 0.100653583 | 0.0008612 | 0.22 |
| UXT | 0.4343839 | 1.60E-51 | 0.469511536 | 5.17E-61 | 0.04 |
| LOC100130369 | 0.290577412 | 1.04E-22 | 0.069769422 | 0.0210667 | 0.23 |
| VAMP1 | -0.193950843 | 1.00E-10 | -0.062312517 | 0.0394243 | 0.14 |
| VAMP2 | -0.317790898 | 4.52E-27 | -0.219866902 | 1.97E-13 | 0.1 |
| VAMP3 | -0.290584128 | 1.04E-22 | -0.201002394 | 2.00E-11 | 0.09 |
| VAMP4 | -0.261475763 | 1.52E-18 | 0.100905419 | 0.0008357 | 0.37 |
| VAMP5 | 0.071814926 | 0.01756841 | 0.071663376 | 0.0178089 | 0.01 |
| VAMP7 | -0.045698885 | 0.1310714 | -0.042165621 | 0.1636068 | 0.01 |
| VAMP8 | 0.270905713 | 7.72E-20 | 0.275630501 | 1.66E-20 | 0.01 |
| MGC5338 | -0.076682269 | 0.01121293 | -0.163832332 | 5.12E-08 | 0.09 |
| VANGL2 | 0.095744879 | 0.00152944 | 0.147838457 | 9.15E-07 | 0.06 |
| VAPA | 0.17879916 | 2.64E-09 | 0.074677409 | 0.0135302 | 0.11 |
| VAPB | 0.073440415 | 0.01516217 | -0.012221993 | 0.6864932 | 0.09 |
| VARS2 | 0.095822982 | 0.00151583 | 0.136065967 | 6.35E-06 | 0.05 |
| VARS | 0.347794679 | 1.97E-32 | 0.256314667 | 7.41E-18 | 0.1 |
| VASH1 | -0.318276708 | 3.75E-27 | -0.318702751 | 3.17E-27 | 0.01 |
| VASH2 | 0.186513533 | 5.17E-10 | 0.175836097 | 4.85E-09 | 0.02 |
| VASN | 0.165289506 | 3.88E-08 | 0.013051073 | 0.6664682 | 0.16 |
| VASP | 0.178819139 | 2.63E-09 | 0.126488279 | 2.74E-05 | 0.06 |
| VAT1L | -0.017879925 | 0.55486153 | -0.072149755 | 0.0170471 | 0.06 |
| VAT1 | -0.007615436 | 0.80143833 | -0.034399133 | 0.2558355 | 0.03 |
| VAV1 | -0.104777237 | 0.00052127 | -0.19671947 | 5.37E-11 | 0.1 |
| VAV2 | -0.001883855 | 0.95039555 | -0.20983582 | 2.43E-12 | 0.21 |
| VAV3 | -0.365750982 | 6.24E-36 | -0.092883133 | 0.0021128 | 0.28 |
| VAX1 | 0.267676053 | 2.17E-19 | 0.083595064 | 0.0056857 | 0.19 |
| VAX2 | 0.324704574 | 2.97E-28 | 0.164808091 | 4.26E-08 | 0.16 |
| VBP1 | 0.351363101 | 4.14E-33 | 0.174310472 | 6.61E-09 | 0.18 |
| VCAM1 | 0.010852593 | 0.72004944 | -0.13443052 | 8.21E-06 | 0.15 |
| VCAN | -0.315494262 | 1.10E-26 | -0.195770778 | 6.66E-11 | 0.12 |
| VCL | -0.222926179 | 8.94E-14 | -0.27248365 | 4.63E-20 | 0.05 |
| VCPIP1 | -0.092311489 | 0.00225135 | -0.14616423 | 1.22E-06 | 0.06 |
| IBMPFD | 0.214154581 | 8.36E-13 | 0.0492612 | 0.1035828 | 0.17 |
| VCX2 | 0.05204411 | 0.08546813 | 0.031008288 | 0.3057293 | 0.03 |
| VCX3A | 0.081630044 | 0.00693075 | 0.040087425 | 0.1853941 | 0.05 |
| VCX3B | 0.030931704 | 0.30692599 | -6.76E-05 | 0.9982178 | 0.04 |
| VCX | 0.077559796 | 0.0103146 | 0.043821752 | 0.1476705 | 0.04 |
| VCY | 0.041244265 | 0.17301565 | 0.014250671 | 0.6379122 | 0.03 |
| VDAC1 | 0.278777379 | 5.84E-21 | 0.314111801 | 1.87E-26 | 0.04 |
| VDAC2 | 0.403356768 | 5.10E-44 | 0.305251166 | 5.28E-25 | 0.1 |
| VDAC3 | 0.397751098 | 9.57E-43 | 0.246156556 | 1.50E-16 | 0.16 |
| VDR | -0.100131802 | 0.00091657 | -0.081727781 | 0.0068635 | 0.02 |
| VEGFA | 0.124133291 | 3.87E-05 | -0.00990055 | 0.7437049 | 0.14 |
| VEGFB | 0.040476762 | 0.18115709 | 0.072400247 | 0.016666 | 0.04 |
| VEGFC | -0.192294359 | 1.46E-10 | -0.099765123 | 0.0009574 | 0.1 |
| VENTXP1 | 0.15780751 | 1.57E-07 | 0.046178125 | 0.1270743 | 0.12 |
| VENTXP7 | 0.039929316 | 0.18713544 | 0.026806524 | 0.3759498 | 0.02 |
| VENTX | -0.231788704 | 8.48E-15 | -0.205602269 | 6.74E-12 | 0.03 |
| LOC115396 | -0.00141953 | 0.96261146 | -0.088711909 | 0.0033323 | 0.09 |
| VEZF1 | -0.369542963 | 1.06E-36 | -0.195930907 | 6.42E-11 | 0.18 |
| VEZT | -0.289311054 | 1.62E-22 | -0.112362141 | 0.0001974 | 0.18 |
| VGF | 0.408921464 | 2.63E-45 | 0.180751913 | 1.76E-09 | 0.23 |
| VGLL1 | 0.327957502 | 8.05E-29 | 0.086797456 | 0.0040826 | 0.25 |
| LOC154272 | 0.033749187 | 0.26493289 | -0.025518084 | 0.3993326 | 0.06 |
| LOC51159 | -0.23751969 | 1.75E-15 | -0.243700793 | 3.05E-16 | 0.01 |
| VGLL4 | -0.014684848 | 0.62770486 | -0.04623153 | 0.1266348 | 0.04 |
| VHLL | 0.042795886 | 0.15739585 | 0.042409267 | 0.1611842 | 0.01 |
| VHL | -0.214195247 | 8.27E-13 | -0.284323452 | 9.01E-22 | 0.08 |
| VIL1 | 0.10756364 | 0.0003675 | 0.005230586 | 0.8628642 | 0.11 |
| VILL | -0.255832498 | 8.57E-18 | -0.141710762 | 2.56E-06 | 0.12 |
| VIM | 0.009142022 | 0.76272772 | -0.076008539 | 0.011949 | 0.09 |
| VIPAS39 | -0.015875212 | 0.60008676 | -0.00991992 | 0.7432211 | 0.01 |
| VPAC1 | -0.381847564 | 2.90E-39 | -0.168891616 | 1.94E-08 | 0.22 |
| SCZD16 | -0.343440286 | 1.29E-31 | -0.149329685 | 7.08E-07 | 0.2 |
| VIP | -0.202199262 | 1.51E-11 | -0.067406865 | 0.0258477 | 0.14 |
| VIT | -0.048731442 | 0.10735387 | -0.035846075 | 0.2363674 | 0.02 |
| VKORC1L1 | -0.067708366 | 0.02518953 | -0.093862557 | 0.0018934 | 0.03 |
| VKCFD2 | 0.310771897 | 6.68E-26 | 0.346030478 | 4.24E-32 | 0.04 |
| VLDLR | -0.023222771 | 0.4430919 | -0.034675325 | 0.252036 | 0.02 |
| MEAX | 0.253928983 | 1.52E-17 | 0.142680366 | 2.18E-06 | 0.12 |
| VMAC | -0.068664418 | 0.02319753 | 0.039853856 | 0.1879708 | 0.11 |
| VMO1 | 0.120557203 | 6.45E-05 | 0.053067422 | 0.0794877 | 0.07 |
| VN1R1 | -0.116705756 | 0.00011011 | -0.050102957 | 0.0978077 | 0.07 |
| VN1R2 | -0.025437482 | 0.40082383 | -0.04099222 | 0.1756586 | 0.02 |
| VN1R4 | -0.010411569 | 0.73097591 | 0.013819648 | 0.6481137 | 0.03 |
| VN1R5 | 0.118648606 | 8.42E-05 | 0.045400913 | 0.1336053 | 0.08 |
| VNN1 | 0.003046403 | 0.91986739 | -0.083032927 | 0.0060195 | 0.09 |
| VNN2 | -0.079718882 | 0.0083709 | -0.102149144 | 0.0007194 | 0.03 |
| VNN3 | 0.070467894 | 0.0198094 | 0.100917898 | 0.0008344 | 0.04 |
| VOPP1 | -0.051280921 | 0.09016071 | 0.016532865 | 0.5850676 | 0.07 |
| VPRBP | -0.226596161 | 3.41E-14 | -0.252351218 | 2.44E-17 | 0.03 |
| VPREB1 | 0.046102845 | 0.12769583 | -0.016103919 | 0.5948439 | 0.07 |
| VPREB3 | 0.176811779 | 3.97E-09 | 0.013091648 | 0.665494 | 0.17 |
| VPS11 | -0.321965542 | 8.81E-28 | -0.229264463 | 1.68E-14 | 0.1 |
| VPS13A | -0.311014741 | 6.09E-26 | -0.337400915 | 1.66E-30 | 0.03 |
| FLJ20583 | -0.345694866 | 4.90E-32 | -0.348998265 | 1.17E-32 | 0.01 |
| FLJ10381 | -0.53676796 | 1.38E-82 | -0.524154658 | 3.65E-78 | 0.02 |
| KIAA0453 | -0.480187441 | 4.00E-64 | -0.483229755 | 4.96E-65 | 0.01 |
| VPS16 | -0.03266608 | 0.28058292 | 0.092745334 | 0.0021455 | 0.13 |
| VPS18 | -0.250486621 | 4.24E-17 | -0.159030626 | 1.26E-07 | 0.1 |
| CHMP3 | -0.131494511 | 1.29E-05 | -0.11842475 | 8.69E-05 | 0.02 |
| VPS25 | 0.134139694 | 8.60E-06 | 0.15526314 | 2.49E-07 | 0.03 |
| VPS26A | 0.109512649 | 0.00028638 | 0.051056759 | 0.0915777 | 0.06 |
| VPS26B | -0.106127771 | 0.00044049 | -0.030726804 | 0.3101429 | 0.08 |
| VPS28 | 0.252998575 | 2.01E-17 | 0.268188794 | 1.85E-19 | 0.02 |
| VPS29 | 0.297325572 | 9.51E-24 | 0.262999836 | 9.48E-19 | 0.04 |
| VPS33A | -0.062878639 | 0.0376651 | -0.029870141 | 0.3238322 | 0.04 |
| VPS33B | 0.049911774 | 0.09909631 | -0.056173261 | 0.063388 | 0.11 |
| VPS35 | 0.105772698 | 0.00046051 | -0.035703479 | 0.2382381 | 0.15 |
| VPS36 | -0.339560434 | 6.70E-31 | -0.291702856 | 7.01E-23 | 0.05 |
| PQBP2 | -0.220328503 | 1.75E-13 | -0.123444075 | 4.27E-05 | 0.1 |
| VPS37B | 0.210438508 | 2.09E-12 | 0.127910319 | 2.22E-05 | 0.09 |
| VPS37C | -0.19582066 | 6.58E-11 | -0.006713485 | 0.8245474 | 0.19 |
| VPS37D | -0.073408058 | 0.01520708 | 0.039976665 | 0.1866127 | 0.12 |
| VPS39 | -0.520427343 | 6.82E-77 | -0.405964587 | 1.28E-44 | 0.12 |
| VPS41 | -0.356029767 | 5.22E-34 | -0.218185819 | 3.03E-13 | 0.14 |
| VPS45 | 0.083856092 | 0.00553643 | 0.342346311 | 2.06E-31 | 0.26 |
| VPS4A | 0.288450275 | 2.18E-22 | 0.025360194 | 0.4022569 | 0.27 |
| VPS4B | -0.058611726 | 0.05272283 | -0.189072301 | 2.97E-10 | 0.14 |
| VPS52 | -0.009388357 | 0.75653364 | 0.087160845 | 0.0039294 | 0.1 |
| VPS53 | -0.140611704 | 3.06E-06 | -0.251247615 | 3.38E-17 | 0.12 |
| VPS54 | -0.010955477 | 0.71750859 | -0.169265559 | 1.80E-08 | 0.16 |
| VPS72 | 0.47970539 | 5.56E-64 | 0.52148838 | 2.98E-77 | 0.05 |
| VPS8 | -0.271725548 | 5.92E-20 | -0.228760542 | 1.92E-14 | 0.05 |
| VRK1 | 0.561391539 | 8.84E-92 | 0.359167938 | 1.27E-34 | 0.21 |
| VRK2 | 0.046135676 | 0.12742448 | -0.038731738 | 0.2007184 | 0.09 |
| VRK3 | -0.136385002 | 6.04E-06 | 0.017445491 | 0.5645196 | 0.16 |
| VSIG10L | 0.139565262 | 3.63E-06 | 0.013982984 | 0.64424 | 0.13 |
| LOC144305 | -0.079272308 | 0.00874374 | -0.132430213 | 1.12E-05 | 0.06 |
| VSIG1 | -0.03561346 | 0.23942447 | 0.04846255 | 0.1093091 | 0.09 |
| VSIG2 | -0.191444308 | 1.76E-10 | -0.086435902 | 0.0042404 | 0.11 |
| VSIG4 | -0.116288034 | 0.00011657 | -0.221026941 | 1.46E-13 | 0.11 |
| VSIG8 | 0.039456296 | 0.19241713 | 0.209035196 | 2.95E-12 | 0.17 |
| VSNL1 | 0.138374757 | 4.40E-06 | 0.072081312 | 0.0171525 | 0.07 |
| VSTM1 | 0.024326118 | 0.42172423 | 0.008787147 | 0.7716777 | 0.02 |
| VSTM2A | -0.020773498 | 0.49266804 | 0.013693991 | 0.6511004 | 0.04 |
| VSTM2B | 0.091492348 | 0.0024644 | -0.010247188 | 0.7350626 | 0.11 |
| VSTM2L | 0.030487766 | 0.31392369 | 0.04442507 | 0.1421691 | 0.02 |
| VSX1 | 0.017344892 | 0.56676751 | -0.002921766 | 0.9231355 | 0.03 |
| VSX2 | 0.074743501 | 0.01344754 | -0.014800221 | 0.6250043 | 0.09 |
| VTA1 | 0.157303795 | 1.72E-07 | 0.176356057 | 4.36E-09 | 0.02 |
| VTCN1 | -0.008841209 | 0.77031225 | 0.050946365 | 0.092282 | 0.06 |
| VTI1A | -0.079100416 | 0.00889116 | -0.054993182 | 0.0691559 | 0.03 |
| VTI1B | 0.05786343 | 0.05582358 | 0.177866042 | 3.20E-09 | 0.13 |
| VTN | -0.015495584 | 0.60883504 | -0.129346029 | 1.79E-05 | 0.12 |
| VTRNA1.1 | NA | NA | NA | NA | #VALUE! |
| VTRNA1.2 | NA | NA | NA | NA | #VALUE! |
| VTRNA1.3 | NA | NA | NA | NA | #VALUE! |
| VWA1 | -0.03019929 | 0.31852662 | 0.020530095 | 0.497752 | 0.06 |
| VWA2 | -0.430061178 | 1.99E-50 | -0.187428468 | 4.24E-10 | 0.25 |
| VWA3A | -0.032964105 | 0.27621546 | -0.017201834 | 0.5699715 | 0.02 |
| VWA3B | 0.006378236 | 0.8331769 | 0.021486282 | 0.4779414 | 0.02 |
| VWA5A | -0.380499269 | 5.61E-39 | -0.228560409 | 2.02E-14 | 0.16 |
| VWA5B1 | -0.128856212 | 1.93E-05 | -0.090307258 | 0.0028053 | 0.04 |
| VWA5B2 | 0.078534776 | 0.00939203 | -0.047565163 | 0.1160385 | 0.13 |
| VWC2L | 0.037881123 | 0.21079336 | -0.08080258 | 0.0075245 | 0.12 |
| VWC2 | 0.026186916 | 0.38708733 | -0.042157901 | 0.163684 | 0.07 |
| VWCE | -0.049186341 | 0.10410918 | -0.052790085 | 0.0810739 | 0.01 |
| VWDE | 0.068883168 | 0.02276145 | -0.024001283 | 0.4279515 | 0.1 |
| VWF | -0.406792334 | 8.23E-45 | -0.325034337 | 2.60E-28 | 0.09 |
| PRO1741 | -0.020473303 | 0.49894222 | -0.112947704 | 0.0001827 | 0.1 |
| WAPAL | -0.283313649 | 1.27E-21 | -0.271724658 | 5.92E-20 | 0.02 |
| WARS2 | -9.85E-05 | 0.99740428 | 0.084143283 | 0.0053763 | 0.09 |
| WARS | 0.245554112 | 1.79E-16 | 0.006660281 | 0.8259155 | 0.24 |
| WASF1 | 0.229233771 | 1.69E-14 | 0.080648319 | 0.0076401 | 0.15 |
| WASF2 | -0.234419245 | 4.13E-15 | -0.368058612 | 2.13E-36 | 0.14 |
| WASF3 | -0.067048369 | 0.02664946 | -0.208947514 | 3.01E-12 | 0.15 |
| WASH2P | 0.086557834 | 0.00418656 | 0.11313623 | 0.0001782 | 0.03 |
| WASH3P | 0.145715445 | 1.31E-06 | 0.13014343 | 1.59E-05 | 0.02 |
| WASH5P | 0.141796201 | 2.52E-06 | 0.169586716 | 1.69E-08 | 0.03 |
| WASH5P.1 | 0.141796201 | 2.52E-06 | 0.169586716 | 1.69E-08 | 0.03 |
| WASL | -0.286329969 | 4.53E-22 | -0.11444122 | 0.0001497 | 0.18 |
| THC | 0.017165203 | 0.57079336 | -0.11512162 | 0.0001366 | 0.14 |
| WBP11P1 | -0.019591822 | 0.51760775 | -0.139188012 | 3.86E-06 | 0.12 |
| WBP11 | 0.117896675 | 9.35E-05 | 0.000985459 | 0.9740394 | 0.12 |
| WBP1 | -0.005773742 | 0.84878708 | 0.187562578 | 4.12E-10 | 0.2 |
| WBP2NL | -0.188251899 | 3.55E-10 | -0.15146687 | 4.88E-07 | 0.04 |
| WBP2 | 0.122419233 | 4.95E-05 | 0.020984814 | 0.4882769 | 0.11 |
| WBP4 | -0.008020021 | 0.7911272 | -0.08595516 | 0.0044588 | 0.08 |
| WBP5 | 0.119106682 | 7.90E-05 | 0.105270504 | 0.0004903 | 0.02 |
| WBSCR16 | 0.254711582 | 1.20E-17 | 0.231053629 | 1.03E-14 | 0.03 |
| WBSCR17 | -0.102592602 | 0.00068165 | -0.133872836 | 8.96E-06 | 0.04 |
| WBMT | 0.423631343 | 7.84E-49 | 0.374912162 | 8.35E-38 | 0.05 |
| ABHD11.AS1 | -0.05899708 | 0.05118294 | 0.021772305 | 0.4721003 | 0.09 |
| WBSCR27 | 0.045552253 | 0.13231363 | 0.093684678 | 0.0019317 | 0.05 |
| WBSCR28 | 0.195476809 | 7.12E-11 | 0.161240029 | 8.34E-08 | 0.04 |
| WDFY1 | -0.335431252 | 3.77E-30 | -0.35046009 | 6.16E-33 | 0.02 |
| WDFY2 | -0.140765181 | 2.99E-06 | -0.11761131 | 9.72E-05 | 0.03 |
| WDFY3 | -0.474498013 | 1.88E-62 | -0.46217026 | 6.16E-59 | 0.02 |
| WDFY4 | -0.24172206 | 5.37E-16 | -0.309838691 | 9.51E-26 | 0.07 |
| WDHD1 | 0.248393932 | 7.84E-17 | 0.077312279 | 0.0105613 | 0.18 |
| WDR11 | -0.411693177 | 5.87E-46 | -0.321013654 | 1.28E-27 | 0.1 |
| WDR12 | 0.448881131 | 2.64E-55 | 0.305695361 | 4.48E-25 | 0.15 |
| FLJ20563 | 0.103649236 | 0.00059909 | 0.141376671 | 2.70E-06 | 0.04 |
| WDR16 | -0.022935804 | 0.44874899 | 0.02559278 | 0.3979536 | 0.05 |
| WDR17 | -0.131296751 | 1.33E-05 | -0.168055533 | 2.28E-08 | 0.04 |
| WDR18 | 0.206289754 | 5.72E-12 | 0.19307515 | 1.22E-10 | 0.02 |
| WDR19 | -0.421681024 | 2.36E-48 | -0.263676838 | 7.67E-19 | 0.16 |
| WDR1 | -0.035920381 | 0.23539663 | -0.069366276 | 0.0218235 | 0.04 |
| WDR20 | -0.340184748 | 5.15E-31 | -0.140975521 | 2.89E-06 | 0.2 |
| WDR24 | -0.136794821 | 5.66E-06 | -0.038406347 | 0.2045302 | 0.1 |
| WDR25 | -0.126054591 | 2.92E-05 | 0.080343204 | 0.0078735 | 0.21 |
| WDR26 | -0.317767034 | 4.57E-27 | 0.08924686 | 0.0031463 | 0.41 |
| WDR27 | -0.134690403 | 7.89E-06 | -0.033609055 | 0.2669232 | 0.11 |
| WDR31 | -0.458969213 | 4.78E-58 | -0.364259409 | 1.24E-35 | 0.1 |
| WDR33 | 0.06669594 | 0.02745852 | -0.018398156 | 0.5434471 | 0.09 |
| WDR34 | 0.36601335 | 5.52E-36 | 0.296294663 | 1.38E-23 | 0.07 |
| WDR35 | -0.210938464 | 1.85E-12 | -0.162241377 | 6.91E-08 | 0.05 |
| WDR36 | -0.278954286 | 5.51E-21 | -0.171340378 | 1.20E-08 | 0.11 |
| WDR37 | -0.22958329 | 1.54E-14 | -0.202730083 | 1.33E-11 | 0.03 |
| WDR38 | -0.006501325 | 0.83000614 | 0.080389329 | 0.0078378 | 0.09 |
| WDR3 | 0.150812079 | 5.47E-07 | 0.017384377 | 0.5658847 | 0.14 |
| WDR41 | 0.031436881 | 0.29908945 | 0.052962784 | 0.0800832 | 0.03 |
| WDR43 | 0.333213897 | 9.43E-30 | 0.064540602 | 0.0328812 | 0.27 |
| WDR44 | -0.462152902 | 6.23E-59 | -0.424029148 | 6.26E-49 | 0.04 |
| WDR45B | 0.230618589 | 1.16E-14 | 0.028946269 | 0.3390293 | 0.21 |
| WDR45 | 0.137712638 | 4.89E-06 | 0.089593256 | 0.003031 | 0.05 |
| WDR46 | 0.333607727 | 8.02E-30 | 0.282214872 | 1.84E-21 | 0.06 |
| WDR47 | -0.275482628 | 1.74E-20 | -0.341592425 | 2.83E-31 | 0.07 |
| WDR48 | -0.2490041 | 6.56E-17 | -0.219980469 | 1.91E-13 | 0.03 |
| WDR49 | -0.043669853 | 0.14908085 | 0.039413889 | 0.1928959 | 0.09 |
| WDR4 | 0.435087325 | 1.06E-51 | 0.230888215 | 1.08E-14 | 0.21 |
| CFAP44 | -0.36019967 | 7.97E-35 | -0.113630442 | 0.0001668 | 0.25 |
| WDR53 | 0.47450259 | 1.87E-62 | 0.307084233 | 2.67E-25 | 0.17 |
| WDR54 | 0.069112892 | 0.02231116 | 0.054765891 | 0.0703148 | 0.02 |
| WDR55 | -0.271670476 | 6.03E-20 | -0.101919034 | 0.0007397 | 0.17 |
| CDW12 | 0.125863414 | 3.01E-05 | -0.030844074 | 0.308299 | 0.16 |
| WDR5B | -0.194416221 | 9.05E-11 | -0.027129906 | 0.3702163 | 0.17 |
| WDR5 | 0.39392599 | 6.85E-42 | 0.181189125 | 1.61E-09 | 0.22 |
| WDR60 | -0.325743027 | 1.96E-28 | -0.21853309 | 2.77E-13 | 0.11 |
| WDR61 | -0.032271009 | 0.28644436 | 0.019869629 | 0.5116864 | 0.06 |
| WDR62 | 0.405825523 | 1.38E-44 | 0.15200684 | 4.44E-07 | 0.26 |
| WDR63 | -0.21104681 | 1.80E-12 | -0.133684872 | 9.23E-06 | 0.08 |
| WDR64 | -0.048999387 | 0.10543314 | -0.011156999 | 0.7125408 | 0.04 |
| VWS2 | -0.460019259 | 2.45E-58 | -0.221281237 | 1.37E-13 | 0.24 |
| WDR66 | 0.008314222 | 0.78365202 | -0.049985949 | 0.0985948 | 0.06 |
| TBC1D31 | 0.347306911 | 2.44E-32 | 0.223725519 | 7.26E-14 | 0.13 |
| DAW1 | 0.123837692 | 4.04E-05 | -0.01966269 | 0.5160939 | 0.15 |
| WDR6 | -0.165356543 | 3.83E-08 | -0.078620861 | 0.0093142 | 0.09 |
| WDR70 | 0.177227765 | 3.65E-09 | 0.264249418 | 6.41E-19 | 0.09 |
| WDR72 | -0.093255283 | 0.00202684 | -0.080325213 | 0.0078874 | 0.02 |
| WDR73 | -0.10044559 | 0.00088292 | -0.0278983 | 0.3568118 | 0.08 |
| WDR74 | 0.526602825 | 5.23E-79 | 0.373704708 | 1.49E-37 | 0.16 |
| WDR75 | 0.316810844 | 6.62E-27 | 0.291142198 | 8.53E-23 | 0.03 |
| WDR76 | 0.224920179 | 5.31E-14 | 0.075183497 | 0.0129085 | 0.15 |
| WDR77 | 0.542962024 | 7.92E-85 | 0.33766706 | 1.48E-30 | 0.21 |
| WDR78 | -0.324375053 | 3.39E-28 | -0.131632072 | 1.27E-05 | 0.2 |
| WDR7 | -0.272474707 | 4.65E-20 | -0.356910657 | 3.52E-34 | 0.09 |
| WDR81 | -0.288635212 | 2.04E-22 | -0.302185728 | 1.63E-24 | 0.02 |
| WDR82 | -0.181728367 | 1.43E-09 | -0.23715639 | 1.94E-15 | 0.06 |
| WDR83 | 0.108311527 | 0.00033412 | 0.134892415 | 7.64E-06 | 0.03 |
| DPH7 | 0.324341022 | 3.43E-28 | 0.212644313 | 1.22E-12 | 0.12 |
| WDR86 | -0.124892504 | 3.47E-05 | -0.087496655 | 0.0037924 | 0.04 |
| WDR87 | -0.000197108 | 0.99480657 | -0.044830651 | 0.13856 | 0.05 |
| WDR88 | -0.125470122 | 3.19E-05 | -0.246430395 | 1.39E-16 | 0.13 |
| WDR89 | 0.080430988 | 0.00780569 | 0.143836165 | 1.80E-06 | 0.07 |
| FLJ20430 | 0.272095744 | 5.25E-20 | 0.125753199 | 3.06E-05 | 0.15 |
| LOC197336 | -0.107956896 | 0.00034958 | 0.031822716 | 0.2931948 | 0.14 |
| WDR91 | -0.044202742 | 0.14417779 | -0.12083712 | 6.20E-05 | 0.08 |
| WDR92 | 0.147140775 | 1.03E-06 | -0.008524761 | 0.7783147 | 0.16 |
| WDR93 | -0.282563588 | 1.64E-21 | -0.070382008 | 0.0199604 | 0.22 |
| WDSUB1 | 0.014684077 | 0.62772293 | -0.007324626 | 0.8088714 | 0.03 |
| WDTC1 | -0.295902943 | 1.58E-23 | -0.362240042 | 3.14E-35 | 0.07 |
| WDYHV1 | 0.433354785 | 2.93E-51 | 0.325692905 | 2.00E-28 | 0.11 |
| WEE1 | -0.108897257 | 0.00030998 | -0.117431193 | 9.97E-05 | 0.01 |
| WEE2 | -0.109711882 | 0.0002791 | -0.092607462 | 0.0021786 | 0.02 |
| WFDC10A | -0.022708601 | 0.4532568 | 0.107801068 | 0.0003566 | 0.14 |
| WFDC10B | 0.065760553 | 0.0297092 | 0.041068374 | 0.1748569 | 0.03 |
| WFDC11 | -0.006961751 | 0.81817045 | 0.006032656 | 0.8420934 | 0.02 |
| WFDC12 | 0.116599315 | 0.00011172 | 0.054402586 | 0.0722003 | 0.07 |
| C20orf138 | -0.091451088 | 0.00247561 | -0.03277167 | 0.2790302 | 0.06 |
| WFDC1 | 0.113799407 | 0.0001631 | 0.090177787 | 0.0028451 | 0.03 |
| WFDC2 | -0.079494748 | 0.00855622 | -0.028181717 | 0.3519457 | 0.06 |
| WFDC3 | 0.176734264 | 4.04E-09 | 0.170949574 | 1.29E-08 | 0.01 |
| WFDC5 | 0.0801978 | 0.0079869 | 0.01605954 | 0.5958596 | 0.07 |
| WFDC6 | -0.100860431 | 0.00084019 | 0.080293875 | 0.0079118 | 0.19 |
| WFDC8 | 0.077435299 | 0.01043803 | -0.013421726 | 0.6575908 | 0.1 |
| WFDC9 | 0.045301295 | 0.13446081 | 0.048715005 | 0.1074726 | 0.01 |
| WFIKKN1 | -0.06922543 | 0.02209341 | 0.007710689 | 0.7990076 | 0.08 |
| WFIKKN2 | -0.06636814 | 0.02822995 | -0.049374458 | 0.1027903 | 0.02 |
| DFNA14 | -0.3307449 | 2.60E-29 | -0.109375159 | 0.0002915 | 0.23 |
| WHAMMP3 | -0.385570501 | 4.62E-40 | -0.301341655 | 2.22E-24 | 0.09 |
| LOC643444 | -0.320453062 | 1.60E-27 | -0.298350696 | 6.58E-24 | 0.03 |
| WHAMM | -0.282738868 | 1.54E-21 | -0.330633323 | 2.72E-29 | 0.05 |
| WHSC1L1 | -0.128701164 | 1.97E-05 | -0.195992619 | 6.33E-11 | 0.07 |
| WHSC1 | 0.160976861 | 8.76E-08 | -0.042326452 | 0.1620046 | 0.21 |
| NELFA | 0.153993591 | 3.12E-07 | 0.069514894 | 0.0215418 | 0.09 |
| WIBG | 0.162197898 | 6.97E-08 | 0.312418035 | 3.58E-26 | 0.16 |
| WIF1 | 0.030121022 | 0.31978303 | -0.050489099 | 0.095246 | 0.09 |
| WIPF1 | -0.185600657 | 6.30E-10 | -0.237101351 | 1.97E-15 | 0.06 |
| WIPF2 | -0.293513821 | 3.70E-23 | -0.204650512 | 8.46E-12 | 0.09 |
| LOC641823 | 0.102687286 | 0.00067385 | -0.029175954 | 0.3352091 | 0.14 |
| WIPI1 | -0.122851427 | 4.65E-05 | -0.093059301 | 0.0020717 | 0.03 |
| WIPI2 | 0.22594872 | 4.05E-14 | 0.141272347 | 2.75E-06 | 0.09 |
| WISP1 | -0.361494705 | 4.42E-35 | -0.258817076 | 3.46E-18 | 0.11 |
| WISP2 | -0.222010034 | 1.13E-13 | -0.103488543 | 0.000611 | 0.12 |
| WISP3 | 0.076994791 | 0.01088534 | 0.014617073 | 0.6292936 | 0.07 |
| WT1AS | 0.105237113 | 0.00049233 | 0.089847809 | 0.0029487 | 0.02 |
| WIZ | 0.166572579 | 3.03E-08 | 0.005628738 | 0.8525407 | 0.17 |
| WLS | -0.285775268 | 5.48E-22 | -0.164797262 | 4.26E-08 | 0.13 |
| WNK1 | -0.135503774 | 6.94E-06 | -0.242588565 | 4.20E-16 | 0.11 |
| LOC286373 | 0.052569013 | 0.08235662 | 0.022895004 | 0.4495566 | 0.03 |
| WNK3 | 0.023206151 | 0.44341842 | -0.22923256 | 1.69E-14 | 0.26 |
| WNK4 | -0.30989913 | 9.29E-26 | -0.118812779 | 8.23E-05 | 0.2 |
| WNT10A | 0.197476598 | 4.51E-11 | 0.032102593 | 0.288968 | 0.17 |
| LOC115994 | 0.165893037 | 3.46E-08 | 0.040127832 | 0.184951 | 0.13 |
| WNT11 | -0.020502767 | 0.49832454 | 0.011359565 | 0.7075594 | 0.04 |
| WNT16 | 0.001155048 | 0.96957379 | -0.091088967 | 0.002576 | 0.1 |
| WNT1 | -0.044278227 | 0.14349337 | -0.120032986 | 6.94E-05 | 0.08 |
| WNT2B | -0.135706366 | 6.72E-06 | -0.120062595 | 6.91E-05 | 0.02 |
| WNT2 | -0.042883167 | 0.15655 | -0.001709563 | 0.9549798 | 0.05 |
| WNT3A | 0.047804599 | 0.11421201 | 0.031411964 | 0.2994728 | 0.02 |
| WNT3 | -0.104933619 | 0.00051125 | -0.150620103 | 5.66E-07 | 0.05 |
| WNT4 | -0.107051145 | 0.00039215 | -0.075878524 | 0.0120959 | 0.04 |
| LOC102724616 | -0.287133543 | 3.44E-22 | -0.111207823 | 0.0002298 | 0.18 |
| WNT5B | 0.067333641 | 0.02600971 | -0.033714016 | 0.2654315 | 0.11 |
| WNT6 | 0.318480758 | 3.46E-27 | 0.10486307 | 0.0005157 | 0.22 |
| WNT7A | 0.052205488 | 0.08450157 | -0.048901244 | 0.1061335 | 0.11 |
| WNT7B | -0.109461512 | 0.00028827 | -0.043087306 | 0.1545851 | 0.07 |
| WNT8D | -0.110004265 | 0.00026874 | -0.143587651 | 1.88E-06 | 0.04 |
| WNT8B | -0.001351403 | 0.9644046 | -0.109644281 | 0.0002816 | 0.11 |
| LOC92832 | -0.291442163 | 7.68E-23 | -0.260527768 | 2.04E-18 | 0.04 |
| WNT9B | -0.3927501 | 1.25E-41 | -0.247114902 | 1.14E-16 | 0.15 |
| WRAP53 | 0.353737248 | 1.45E-33 | 0.253030866 | 1.99E-17 | 0.11 |
| LOC116258 | 0.003485496 | 0.90836513 | 0.144956656 | 1.49E-06 | 0.15 |
| WRNIP1 | 0.465925133 | 5.42E-60 | 0.312083982 | 4.06E-26 | 0.16 |
| WRN | -0.208000042 | 3.79E-12 | -0.248772838 | 7.02E-17 | 0.05 |
| WSB1 | -0.212979784 | 1.12E-12 | -0.212806434 | 1.17E-12 | 0.01 |
| WSB2 | 0.090432882 | 0.00276727 | 0.088548862 | 0.0033909 | 0.01 |
| WSCD1 | -0.289883652 | 1.32E-22 | -0.212401015 | 1.29E-12 | 0.08 |
| WSCD2 | -0.143452533 | 1.92E-06 | -0.151762189 | 4.64E-07 | 0.01 |
| WT1 | 0.052200242 | 0.08453285 | 0.048533209 | 0.1087926 | 0.01 |
| WTAP | 0.144855262 | 1.52E-06 | 0.072423395 | 0.0166311 | 0.08 |
| WTIP | 0.008427911 | 0.78076865 | -0.045084347 | 0.1363386 | 0.06 |
| WWC1 | -0.108235672 | 0.00033737 | -0.115770696 | 0.0001251 | 0.01 |
| WWC2 | -0.36592375 | 5.76E-36 | -0.322939417 | 6.00E-28 | 0.05 |
| WWC3 | -0.220403049 | 1.72E-13 | -0.165723873 | 3.57E-08 | 0.06 |
| WWOX | -0.065523315 | 0.0303046 | -0.024958821 | 0.4097483 | 0.05 |
| WWP1 | -0.323017799 | 5.81E-28 | -0.213854063 | 9.01E-13 | 0.11 |
| LOC116013 | 0.002696382 | 0.92904851 | -0.170588347 | 1.39E-08 | 0.18 |
| WWTR1 | -0.105471862 | 0.00047814 | -0.209339658 | 2.74E-12 | 0.11 |
| XAB2 | 0.130919053 | 1.41E-05 | 0.131263571 | 1.34E-05 | 0.01 |
| XAF1 | 0.043717786 | 0.14863469 | -0.027369827 | 0.3659978 | 0.08 |
| XAGE1D | 0.051154953 | 0.0909548 | -0.003605119 | 0.9052348 | 0.06 |
| LOC90526 | 0.220343646 | 1.74E-13 | 0.079247815 | 0.0087646 | 0.15 |
| LOC158935 | 0.099345349 | 0.00100618 | 0.061745563 | 0.0412554 | 0.04 |
| XAGE5 | 0.067404849 | 0.0258521 | 0.046170624 | 0.1271361 | 0.03 |
| XBP1 | -0.456075838 | 2.99E-57 | -0.128574033 | 2.01E-05 | 0.33 |
| XCL1 | 0.078875597 | 0.00908731 | -0.011131867 | 0.7131596 | 0.1 |
| XCL2 | 0.074689445 | 0.01351511 | -0.031504563 | 0.2980498 | 0.11 |
| XCR1 | -0.273287796 | 3.57E-20 | -0.348781881 | 1.28E-32 | 0.08 |
| XDH | -0.052792127 | 0.08106213 | -0.080991795 | 0.0073849 | 0.03 |
| XG | -0.128564669 | 2.01E-05 | -0.117155452 | 0.0001035 | 0.02 |
| XIAP | -0.459835323 | 2.75E-58 | -0.360020719 | 8.64E-35 | 0.1 |
| XIRP1 | 0.12608034 | 2.91E-05 | -0.066048127 | 0.029001 | 0.2 |
| XIRP2 | 0.011239762 | 0.71050404 | 0.013136834 | 0.6644099 | 0.01 |
| XIST | -0.317008616 | 6.13E-27 | -0.284851194 | 7.52E-22 | 0.04 |
| XKR3 | 0.11291523 | 0.00018348 | 0.045121928 | 0.1360119 | 0.07 |
| XKR4 | -0.081682854 | 0.00689432 | -0.179940473 | 2.08E-09 | 0.1 |
| XKR5 | -0.062824012 | 0.0378319 | -0.115768505 | 0.0001251 | 0.06 |
| XKR6 | -0.12455766 | 3.64E-05 | -0.141120467 | 2.82E-06 | 0.02 |
| XKR7 | -0.037347624 | 0.21729586 | -0.12803767 | 2.18E-05 | 0.1 |
| LOC86422 | 0.05379962 | 0.07542069 | -0.036520016 | 0.2276661 | 0.1 |
| XKR9 | 0.177829819 | 3.23E-09 | 0.108781825 | 0.0003146 | 0.07 |
| XKRX | 0.187286799 | 4.38E-10 | 0.095664912 | 0.0015435 | 0.1 |
| XKRY2 | 0.008485581 | 0.77930717 | 0.021290116 | 0.4819702 | 0.02 |
| LOC140011 | NA | NA | NA | NA | #VALUE! |
| NA. | 0.242996401 | 3.74E-16 | 0.006962839 | 0.8181425 | 0.24 |
| XPA | -0.200652264 | 2.17E-11 | -0.020254808 | 0.5035353 | 0.19 |
| XPC | -0.458594824 | 6.07E-58 | -0.343041353 | 1.53E-31 | 0.12 |
| XPNPEPL | 0.17635858 | 4.36E-09 | -0.001962795 | 0.9483198 | 0.18 |
| XPNPEP2 | -0.154439913 | 2.88E-07 | -0.171695521 | 1.11E-08 | 0.02 |
| XPNPEP3 | 0.011927611 | 0.69365769 | 0.028631106 | 0.3443165 | 0.02 |
| XPO1 | 0.184263145 | 8.38E-10 | 0.043659892 | 0.1491737 | 0.15 |
| XPO4 | -0.180391176 | 1.90E-09 | -0.162764411 | 6.27E-08 | 0.02 |
| LOC220808 | 0.388713283 | 9.61E-41 | 0.144325261 | 1.66E-06 | 0.25 |
| XPO6 | 0.255526116 | 9.40E-18 | 0.112998985 | 0.0001815 | 0.15 |
| XPO7 | -0.03623418 | 0.23132827 | -0.079597393 | 0.0084709 | 0.05 |
| XPOT | 0.251869096 | 2.81E-17 | 0.055524828 | 0.0665062 | 0.2 |
| XPR1 | -0.214732406 | 7.24E-13 | 0.023788495 | 0.4320597 | 0.24 |
| XRCC1 | 0.022305828 | 0.46131041 | 0.033039703 | 0.275115 | 0.02 |
| XRCC2 | 0.236145962 | 2.57E-15 | 0.050399055 | 0.0958385 | 0.19 |
| XRCC3 | 0.255888158 | 8.43E-18 | 0.129326732 | 1.80E-05 | 0.13 |
| XRCC4 | 0.041418248 | 0.17120867 | 0.200056305 | 2.49E-11 | 0.16 |
| XRCC5 | 0.091909795 | 0.00235363 | 0.11481114 | 0.0001424 | 0.03 |
| LOC115029 | 0.367794761 | 2.41E-36 | 0.37434429 | 1.09E-37 | 0.01 |
| LOC94359 | 0.299750102 | 3.97E-24 | 0.156333713 | 2.05E-07 | 0.15 |
| XRN1 | -0.284291553 | 9.11E-22 | -0.337892716 | 1.35E-30 | 0.06 |
| XRN2 | -0.221322658 | 1.36E-13 | -0.154590022 | 2.81E-07 | 0.07 |
| XRRA1 | -0.031834152 | 0.29302128 | 0.044642677 | 0.1402238 | 0.08 |
| XYLB | 0.081803746 | 0.00681158 | 0.018880259 | 0.5329343 | 0.07 |
| XYLT1 | -0.245861592 | 1.64E-16 | -0.358909685 | 1.43E-34 | 0.12 |
| XYLT2 | -0.212853495 | 1.16E-12 | -0.023453264 | 0.4385778 | 0.19 |
| YAF2 | -0.02208781 | 0.46570285 | 0.021178686 | 0.4842669 | 0.05 |
| YAP1 | -0.256514303 | 6.97E-18 | -0.16670149 | 2.96E-08 | 0.09 |
| YARS2 | 0.429494454 | 2.75E-50 | 0.306584008 | 3.22E-25 | 0.13 |
| CMTDIC | 0.417297528 | 2.72E-47 | 0.064446079 | 0.0331386 | 0.36 |
| YB1 | 0.548319342 | 8.37E-87 | 0.260423433 | 2.11E-18 | 0.29 |
| YBX2 | 0.240072916 | 8.57E-16 | 0.161641119 | 7.74E-08 | 0.08 |
| YDJC | 0.439036651 | 1.03E-52 | 0.364099892 | 1.34E-35 | 0.08 |
| YEATS2 | 0.226596134 | 3.41E-14 | -0.04912025 | 0.1045757 | 0.28 |
| YEATS4 | 0.170367556 | 1.45E-08 | 0.142231083 | 2.35E-06 | 0.03 |
| YES1 | 0.282149178 | 1.88E-21 | 0.063527518 | 0.0357312 | 0.22 |
| YIF1A | 0.283292565 | 1.28E-21 | 0.251417174 | 3.22E-17 | 0.04 |
| YIF1B | 0.361247706 | 4.94E-35 | 0.208449468 | 3.40E-12 | 0.16 |
| YIPF1 | -0.161562026 | 7.85E-08 | 0.061205133 | 0.0430673 | 0.23 |
| YIPF2 | 0.158116127 | 1.48E-07 | 0.145001593 | 1.48E-06 | 0.02 |
| YIPF3 | 0.077608836 | 0.01026634 | 0.157224318 | 1.75E-07 | 0.08 |
| YIPF4 | 0.204787179 | 8.19E-12 | 0.125792282 | 3.04E-05 | 0.08 |
| YIPF5 | -0.398078326 | 8.08E-43 | -0.188100421 | 3.67E-10 | 0.21 |
| YIPF6 | -0.276577605 | 1.21E-20 | -0.225543683 | 4.51E-14 | 0.06 |
| YIPF7 | 0.013010772 | 0.66743624 | -0.010460562 | 0.7297593 | 0.03 |
| YJEFN3 | 0.05158316 | 0.08827817 | 0.033748496 | 0.2649427 | 0.02 |
| YKT6 | 0.232960048 | 6.16E-15 | 0.091685141 | 0.0024127 | 0.15 |
| YLPM1 | -0.361579818 | 4.25E-35 | -0.352628344 | 2.37E-33 | 0.01 |
| LOC115724 | -0.021731523 | 0.47293071 | -0.108230772 | 0.0003376 | 0.09 |
| YOD1 | 0.136440971 | 5.99E-06 | 0.164958476 | 4.13E-08 | 0.03 |
| YPEL1 | -0.009640833 | 0.7502013 | 0.007614455 | 0.8014634 | 0.02 |
| YPEL2 | -0.499724174 | 4.13E-70 | -0.25482157 | 1.16E-17 | 0.25 |
| YPEL3 | -0.020347137 | 0.50159172 | -0.011875498 | 0.6949288 | 0.01 |
| YPEL4 | -0.136809713 | 5.65E-06 | -0.092442769 | 0.0022188 | 0.05 |
| YPEL5 | -0.248349677 | 7.94E-17 | -0.102546777 | 0.0006855 | 0.15 |
| YRDC | 0.390900007 | 3.19E-41 | 0.113248943 | 0.0001755 | 0.28 |
| MAP3K19 | -0.104559837 | 0.00053549 | -0.003746979 | 0.9015244 | 0.11 |
| YTHDC1 | -0.175019373 | 5.72E-09 | -0.129299671 | 1.80E-05 | 0.05 |
| YTHDC2 | -0.427940755 | 6.73E-50 | -0.279238553 | 5.01E-21 | 0.15 |
| YTHDF1 | 0.162895127 | 6.11E-08 | 0.066280806 | 0.0284386 | 0.1 |
| YTHDF2 | 0.183826651 | 9.20E-10 | 0.11046448 | 0.0002532 | 0.08 |
| YTHDF3 | -0.096387652 | 0.00142069 | -0.084634583 | 0.005112 | 0.02 |
| GW128 | -0.040609412 | 0.17973004 | -0.023481301 | 0.4380305 | 0.02 |
| LOC727845 | 0.275729512 | 1.60E-20 | 0.242200877 | 4.69E-16 | 0.04 |
| LOC222186 | 0.168620882 | 2.04E-08 | 0.072366623 | 0.0167167 | 0.1 |
| YWHAH | 0.165619369 | 3.64E-08 | 0.156625469 | 1.95E-07 | 0.01 |
| YWHAQ | 0.323739843 | 4.36E-28 | 0.168531965 | 2.08E-08 | 0.16 |
| YWHAD | 0.370004587 | 8.56E-37 | 0.189456806 | 2.73E-10 | 0.19 |
| YY1AP1 | -0.005747946 | 0.84945458 | 0.256574608 | 6.85E-18 | 0.27 |
| YY1 | 0.071284744 | 0.01842249 | 0.03514966 | 0.2456028 | 0.04 |
| YY2 | -0.285962688 | 5.14E-22 | -0.386448153 | 2.98E-40 | 0.11 |
| ZACN | 0.068574809 | 0.02337825 | 0.054391397 | 0.072259 | 0.02 |
| ZADH2 | -0.072470847 | 0.0165599 | -0.1051707 | 0.0004964 | 0.04 |
| ZAK | -0.283085895 | 1.37E-21 | -0.158901486 | 1.29E-07 | 0.13 |
| ZAN | -0.145786806 | 1.30E-06 | 0.041133612 | 0.1741723 | 0.19 |
| ZAP70 | 0.019499576 | 0.51958174 | -0.086632438 | 0.0041539 | 0.11 |
| ZAR1L | 0.249971802 | 4.93E-17 | 0.134320576 | 8.36E-06 | 0.12 |
| ZAR1 | 0.140569566 | 3.08E-06 | 0.065482768 | 0.0304074 | 0.08 |
| ZBBX | 0.008906716 | 0.76865869 | 0.098087845 | 0.0011664 | 0.09 |
| ZBED1 | -0.200974463 | 2.01E-11 | -0.137098164 | 5.39E-06 | 0.07 |
| ZBED2 | 0.189851207 | 2.50E-10 | 0.009468248 | 0.7545281 | 0.19 |
| ZBED3 | -0.295024848 | 2.17E-23 | -0.237732648 | 1.65E-15 | 0.06 |
| ZBED4 | 0.118283423 | 8.86E-05 | 0.002991899 | 0.9212964 | 0.12 |
| LOC101928029 | -0.153350665 | 3.50E-07 | -0.04918939 | 0.1040877 | 0.11 |
| ZBP1 | 0.095206578 | 0.00162634 | -0.032802905 | 0.278572 | 0.13 |
| ZBTB10 | -0.053318904 | 0.0780712 | -0.187116636 | 4.54E-10 | 0.14 |
| ZBTB11 | -0.296889792 | 1.11E-23 | -0.231042618 | 1.04E-14 | 0.07 |
| C6orf46 | 0.164774293 | 4.28E-08 | 0.180354972 | 1.91E-09 | 0.02 |
| PLZF | -0.415685037 | 6.61E-47 | -0.316031967 | 8.94E-27 | 0.1 |
| ZNF60 | 0.06597208 | 0.02918683 | 0.032483883 | 0.2832759 | 0.04 |
| ZBTB1 | -0.374619127 | 9.60E-38 | -0.279983084 | 3.90E-21 | 0.1 |
| LOC102723469 | -0.365025908 | 8.73E-36 | -0.345893036 | 4.50E-32 | 0.02 |
| ZBTB22 | -0.074684084 | 0.01352183 | -0.037563975 | 0.2146417 | 0.04 |
| ZBTB24 | -0.051859179 | 0.08658671 | -0.029766381 | 0.3255165 | 0.03 |
| C14orf51 | -0.299574532 | 4.23E-24 | -0.217919989 | 3.24E-13 | 0.09 |
| ZBTB26 | -0.378489229 | 1.49E-38 | -0.373642173 | 1.53E-37 | 0.01 |
| ZBTB2 | 0.021103802 | 0.48581369 | 0.05084573 | 0.0929279 | 0.03 |
| ZBTB32 | 0.1139028 | 0.00016086 | -0.100235228 | 0.0009053 | 0.22 |
| ZBTB33 | -0.078460646 | 0.0094595 | -0.056236426 | 0.0630908 | 0.03 |
| ZBTB34 | -0.275849204 | 1.54E-20 | -0.267884823 | 2.03E-19 | 0.01 |
| ZBTB37 | -0.416690986 | 3.80E-47 | -0.2174637 | 3.64E-13 | 0.2 |
| FLJ31131 | -0.515107399 | 4.18E-75 | -0.488115231 | 1.66E-66 | 0.03 |
| ZBTB39 | -0.030435055 | 0.31476146 | -0.183752998 | 9.35E-10 | 0.16 |
| ZBTB3 | -0.159843462 | 1.08E-07 | -0.171172162 | 1.24E-08 | 0.02 |
| ZBTB40 | -0.429422606 | 2.87E-50 | -0.483301106 | 4.72E-65 | 0.06 |
| FLJ36199 | -0.345430937 | 5.49E-32 | 0.053010284 | 0.0798124 | 0.4 |
| LOC732257 | -0.329420449 | 4.45E-29 | -0.07648808 | 0.0114209 | 0.26 |
| LOC115563 | -0.313274001 | 2.58E-26 | -0.359593789 | 1.05E-34 | 0.05 |
| ZBTB44 | -0.20839464 | 3.44E-12 | -0.165817368 | 3.51E-08 | 0.05 |
| ZBTB45 | -0.082458742 | 0.00637856 | 0.013070157 | 0.6660099 | 0.1 |
| ZBTB46 | -0.195064562 | 7.81E-11 | -0.27841984 | 6.58E-21 | 0.09 |
| ZBTB47 | -0.272576697 | 4.49E-20 | -0.215197796 | 6.44E-13 | 0.06 |
| ZBTB48 | 0.013007492 | 0.66751504 | 0.030843906 | 0.3083017 | 0.02 |
| ZBTB49 | -0.167510951 | 2.53E-08 | -0.123260308 | 4.39E-05 | 0.05 |
| ZBTB4 | -0.564812458 | 4.05E-93 | -0.429259097 | 3.15E-50 | 0.14 |
| ZBTB5 | 0.09629721 | 0.00143555 | -0.025937594 | 0.391625 | 0.13 |
| ZBTB6 | -0.292737984 | 4.87E-23 | -0.208920267 | 3.03E-12 | 0.09 |
| DKFZp547O146 | -0.371655499 | 3.93E-37 | -0.249027876 | 6.51E-17 | 0.13 |
| ZBTB7B | -0.26729303 | 2.45E-19 | 0.008675643 | 0.7744962 | 0.28 |
| LOC649379 | -0.218375959 | 2.89E-13 | -0.029535698 | 0.3292816 | 0.19 |
| ZBTB8 | -0.165921621 | 3.44E-08 | -0.101131264 | 0.0008133 | 0.07 |
| ZBTB8B | 0.325221534 | 2.42E-28 | 0.07760068 | 0.0102744 | 0.25 |
| ZBTB8OS | 0.424338163 | 5.26E-49 | 0.377989737 | 1.90E-38 | 0.05 |
| ZBTB9 | 0.123041878 | 4.53E-05 | 0.182535985 | 1.21E-09 | 0.06 |
| ZC3H10 | -0.186760953 | 4.90E-10 | 0.033798661 | 0.2642327 | 0.23 |
| ZC3H11A | -0.257483786 | 5.19E-18 | 0.220877689 | 1.52E-13 | 0.48 |
| ZC3H12A | -0.120754612 | 6.27E-05 | -0.105671393 | 0.0004664 | 0.02 |
| ZC3H12B | -0.186545384 | 5.14E-10 | -0.231320617 | 9.63E-15 | 0.05 |
| ZC3H12C | -0.055627652 | 0.06600348 | -0.254226921 | 1.39E-17 | 0.2 |
| C6orf95 | -0.102539554 | 0.00068607 | -0.25163721 | 3.01E-17 | 0.15 |
| ZC3H13 | -0.390892093 | 3.21E-41 | -0.376394662 | 4.10E-38 | 0.02 |
| ZC3H14 | -0.265677314 | 4.09E-19 | -0.197146327 | 4.87E-11 | 0.07 |
| ZC3H15 | 0.234050432 | 4.57E-15 | 0.152992895 | 3.73E-07 | 0.09 |
| ZC3H18 | 0.278549431 | 6.30E-21 | -0.005405537 | 0.858325 | 0.29 |
| ZC3H3 | 0.25794373 | 4.51E-18 | 0.158348614 | 1.42E-07 | 0.1 |
| ZC3H4 | -0.078092124 | 0.00980136 | -0.09618686 | 0.0014539 | 0.02 |
| ZC3H6 | -0.424853764 | 3.92E-49 | -0.311343504 | 5.38E-26 | 0.12 |
| FLJ20318 | -0.325144393 | 2.49E-28 | -0.242268739 | 4.60E-16 | 0.09 |
| DKFZp434K0920 | -0.248650679 | 7.27E-17 | -0.377317771 | 2.63E-38 | 0.13 |
| ZC3H8 | 0.323371489 | 5.05E-28 | 0.254344281 | 1.34E-17 | 0.07 |
| ZC3HAV1L | -0.204627005 | 8.51E-12 | -0.32681805 | 1.27E-28 | 0.13 |
| FLJ13288 | -0.1717216 | 1.11E-08 | -0.195835084 | 6.56E-11 | 0.03 |
| ZC3HC1 | 0.347789713 | 1.98E-32 | 0.364820472 | 9.59E-36 | 0.02 |
| WWS | 0.12622444 | 2.85E-05 | 0.103669846 | 0.0005976 | 0.03 |
| ZCCHC10 | -0.197822736 | 4.17E-11 | 0.007062795 | 0.8155785 | 0.21 |
| ZCCHC11 | 0.048789087 | 0.10693833 | -0.158052437 | 1.50E-07 | 0.21 |
| LOC257051 | -0.00852576 | 0.77828943 | -0.076075819 | 0.0118737 | 0.07 |
| LOC203530 | -0.056523692 | 0.06175375 | -0.034391573 | 0.2559401 | 0.03 |
| MGC14139 | -0.141183398 | 2.79E-06 | -0.296632025 | 1.22E-23 | 0.16 |
| ZCCHC16 | 0.07648748 | 0.01142151 | 0.024864222 | 0.411526 | 0.06 |
| LOC95373 | 0.316502743 | 7.46E-27 | 0.13968975 | 3.56E-06 | 0.18 |
| LOC647692 | 0.143377931 | 1.94E-06 | -0.00735483 | 0.8080985 | 0.16 |
| ZCCHC24 | -0.51317644 | 1.83E-74 | -0.274659245 | 2.28E-20 | 0.24 |
| C18orf49 | 0.048219953 | 0.11109719 | -0.106955037 | 0.0003969 | 0.16 |
| ZCCHC3 | 0.124424997 | 3.71E-05 | 0.11881701 | 8.23E-05 | 0.01 |
| FLJ23024 | -0.334854368 | 4.79E-30 | -0.218423387 | 2.85E-13 | 0.12 |
| ZCCHC5 | -0.286654031 | 4.05E-22 | -0.221618615 | 1.26E-13 | 0.07 |
| ZCCHC6 | -0.319332078 | 2.48E-27 | -0.313499651 | 2.37E-26 | 0.01 |
| ZCCHC7 | 0.190915549 | 1.98E-10 | 0.128293838 | 2.10E-05 | 0.07 |
| LOC144306 | -0.029533674 | 0.32931474 | -0.054125854 | 0.0736641 | 0.03 |
| ZCCHC9 | 0.078178488 | 0.00972027 | 0.17607025 | 4.62E-09 | 0.1 |
| ZCRB1 | 0.204376494 | 9.03E-12 | 0.234687223 | 3.84E-15 | 0.04 |
| ZCWPW1 | -0.021192387 | 0.48398422 | 0.023354038 | 0.4405178 | 0.05 |
| ZCWPW2 | -0.400987787 | 1.77E-43 | -0.160048744 | 1.04E-07 | 0.25 |
| ZDBF2 | -0.318894019 | 2.94E-27 | -0.348344014 | 1.55E-32 | 0.03 |
| ZDHHC11 | -0.202187189 | 1.51E-11 | -0.098274611 | 0.0011412 | 0.11 |
| ZDHHC12 | 0.273567575 | 3.26E-20 | 0.265946092 | 3.76E-19 | 0.01 |
| ZDHHC13 | 0.210744275 | 1.94E-12 | 0.057077123 | 0.0592438 | 0.16 |
| ZDHHC14 | 0.070425698 | 0.01988346 | 0.031436255 | 0.2990991 | 0.04 |
| ZDHHC15 | -0.125544291 | 3.15E-05 | -0.171858574 | 1.08E-08 | 0.05 |
| LOC196815 | 0.154540447 | 2.83E-07 | 0.247403323 | 1.05E-16 | 0.1 |
| ZDHHC17 | -0.323334771 | 5.13E-28 | -0.265159056 | 4.82E-19 | 0.06 |
| ZDHHC18 | 0.386853402 | 2.44E-40 | 0.056310843 | 0.0627422 | 0.34 |
| ZDHHC19 | 0.116784565 | 0.00010893 | -0.003060402 | 0.9195004 | 0.12 |
| C16orf1 | -0.185801701 | 6.03E-10 | -0.053228653 | 0.0785772 | 0.14 |
| ZDHHC20 | -0.317871453 | 4.39E-27 | -0.359234426 | 1.23E-34 | 0.05 |
| ZDHHC21 | -0.294231338 | 2.87E-23 | -0.253570553 | 1.69E-17 | 0.05 |
| ZDHHC22 | -0.018616106 | 0.53868168 | -0.165231964 | 3.92E-08 | 0.15 |
| ZDHHC23 | 0.026425704 | 0.38277152 | -0.009911559 | 0.7434299 | 0.04 |
| ZDHHC24 | 0.039748326 | 0.18914359 | 0.10747952 | 0.0003714 | 0.07 |
| ZDHHC2 | -0.229860811 | 1.43E-14 | -0.338357845 | 1.11E-30 | 0.11 |
| FLJ20209 | -0.239624676 | 9.72E-16 | -0.063913333 | 0.034622 | 0.18 |
| ZDHHC4 | 0.006481544 | 0.83051549 | 0.069536457 | 0.0215012 | 0.07 |
| ZDHHC5 | -0.053212017 | 0.07867072 | -0.099613542 | 0.0009747 | 0.05 |
| ZDHHC6 | 0.164536379 | 4.48E-08 | 0.21161758 | 1.57E-12 | 0.05 |
| ZDHHC7 | -0.103028543 | 0.00064639 | -0.236517407 | 2.32E-15 | 0.14 |
| ZDHHC8P1 | 0.083336704 | 0.00583698 | 0.031296379 | 0.3012555 | 0.06 |
| ZDHHC8 | -0.034638313 | 0.2525429 | -0.163050821 | 5.94E-08 | 0.13 |
| ZDHHC10 | 0.116727405 | 0.00010978 | 0.083333522 | 0.0058389 | 0.04 |
| ZEB1 | -0.497000312 | 2.98E-69 | -0.345796027 | 4.69E-32 | 0.16 |
| LOC115481 | -0.379006996 | 1.16E-38 | -0.392742499 | 1.25E-41 | 0.02 |
| ZER1 | -0.314749764 | 1.47E-26 | -0.246597709 | 1.32E-16 | 0.07 |
| ZFAND1 | 0.223527484 | 7.65E-14 | 0.06650157 | 0.0279137 | 0.16 |
| ZFAND2A | 0.265231267 | 4.71E-19 | 0.216174004 | 5.04E-13 | 0.05 |
| ZFAND2B | 0.276060043 | 1.44E-20 | 0.213496362 | 9.85E-13 | 0.07 |
| ZFAND3 | -0.238321636 | 1.40E-15 | -0.166763768 | 2.92E-08 | 0.08 |
| LOC102724516 | 0.009303775 | 0.7586587 | -0.076853337 | 0.0110325 | 0.09 |
| ZFAND6 | -0.147697754 | 9.38E-07 | 0.009821949 | 0.7456691 | 0.16 |
| ZFAT.AS1 | -0.017961574 | 0.55305541 | -0.047905051 | 0.1134525 | 0.03 |
| ZFAT | 0.085977279 | 0.00444851 | -0.094589071 | 0.0017444 | 0.19 |
| KIAA0546 | -0.264094062 | 6.73E-19 | -0.24704436 | 1.16E-16 | 0.02 |
| ZFHX3 | -0.290980728 | 9.03E-23 | -0.264066591 | 6.79E-19 | 0.03 |
| ZFHX4 | -0.419995538 | 6.06E-48 | -0.265679918 | 4.09E-19 | 0.16 |
| ZNF106 | -0.452664903 | 2.54E-56 | -0.415629215 | 6.82E-47 | 0.04 |
| LOC94538 | -0.27428661 | 2.57E-20 | -0.197201066 | 4.81E-11 | 0.08 |
| ZFP14 | -0.402534674 | 7.87E-44 | -0.267201981 | 2.53E-19 | 0.14 |
| ZBTB14 | -0.112404379 | 0.00019632 | -0.181504838 | 1.50E-09 | 0.07 |
| ZFP1 | 0.048746462 | 0.10724548 | -0.116712345 | 0.00011 | 0.17 |
| ZFP28 | -0.133105978 | 1.01E-05 | -0.160759006 | 9.12E-08 | 0.03 |
| ZFP2 | -0.24032391 | 7.98E-16 | -0.062755155 | 0.038043 | 0.18 |
| LOC148269 | -0.176816606 | 3.97E-09 | -0.176786217 | 4.00E-09 | 0.01 |
| ZFP36L1 | -0.276010245 | 1.46E-20 | -0.13158083 | 1.28E-05 | 0.15 |
| ZFP36L2 | -0.120493284 | 6.51E-05 | -0.080062992 | 0.0080934 | 0.05 |
| ZFP36 | -0.235062471 | 3.46E-15 | -0.171818344 | 1.09E-08 | 0.07 |
| ZFP37 | -0.121049374 | 6.02E-05 | -0.027609253 | 0.3618179 | 0.1 |
| ZFP3 | -0.199377986 | 2.91E-11 | -0.165871554 | 3.47E-08 | 0.04 |
| ZFP41 | 0.039217107 | 0.19512919 | -0.075226245 | 0.0128572 | 0.12 |
| ZFP42 | 0.183990606 | 8.88E-10 | 0.072474385 | 0.0165546 | 0.12 |
| LOC642028 | 0.179294806 | 2.38E-09 | 0.022399652 | 0.4594272 | 0.16 |
| ZFP62 | -0.159035802 | 1.25E-07 | 0.002144404 | 0.9435457 | 0.17 |
| ZFP64 | 0.051360824 | 0.08965991 | -0.058208006 | 0.0543774 | 0.11 |
| ZFP82 | 0.065522779 | 0.03030596 | -0.029281894 | 0.3334565 | 0.1 |
| ZFP90 | -0.15528948 | 2.48E-07 | -0.1862147 | 5.52E-10 | 0.04 |
| ZFP91.CNTF | -0.08166284 | 0.00690811 | -0.097317208 | 0.001276 | 0.02 |
| ZFP91 | -0.228281272 | 2.18E-14 | -0.277304558 | 9.53E-21 | 0.05 |
| ZFP92 | -0.363072799 | 2.15E-35 | -0.326803967 | 1.28E-28 | 0.04 |
| MCG4 | 0.209537629 | 2.61E-12 | 0.235741084 | 2.87E-15 | 0.03 |
| LOC161884 | -0.111427611 | 0.00022325 | -0.084817635 | 0.0050166 | 0.03 |
| ZFPM2 | -0.361695155 | 4.03E-35 | -0.238857676 | 1.21E-15 | 0.13 |
| ZFR2 | 0.012644966 | 0.67624845 | -0.09604536 | 0.0014777 | 0.11 |
| ZFR | -0.079712308 | 0.00837629 | -0.214418784 | 7.83E-13 | 0.14 |
| ZFX | -0.402154533 | 9.61E-44 | -0.383542516 | 1.26E-39 | 0.02 |
| ZFYVE16 | -0.478283441 | 1.46E-63 | -0.269922198 | 1.06E-19 | 0.21 |
| ZFYVE19 | -0.029920338 | 0.32301934 | 0.053708665 | 0.0759165 | 0.09 |
| ZFYVE1 | -0.577895431 | 2.18E-98 | -0.358585183 | 1.66E-34 | 0.22 |
| RBSN | -0.297551788 | 8.77E-24 | -0.24486292 | 2.19E-16 | 0.06 |
| ZFYVE21 | -0.100431539 | 0.0008844 | 0.092754265 | 0.0021433 | 0.2 |
| ZFYVE26 | -0.451533745 | 5.12E-56 | -0.379908321 | 7.48E-39 | 0.08 |
| ZFYVE27 | -0.091151793 | 0.00255828 | -0.089842089 | 0.0029505 | 0.01 |
| ZFYVE28 | -0.329263928 | 4.74E-29 | -0.31858509 | 3.32E-27 | 0.02 |
| ZFYVE9 | -0.07688412 | 0.01100037 | -0.274912798 | 2.10E-20 | 0.2 |
| LOC100130829 | -0.029994306 | 0.32182404 | -0.033693263 | 0.2657259 | 0.01 |
| ZG16B | -0.330589381 | 2.77E-29 | -0.023912921 | 0.4296547 | 0.31 |
| ZG16 | 0.044546794 | 0.14107841 | 0.017177893 | 0.5705086 | 0.03 |
| ZGLP1 | 0.096558783 | 0.00139297 | 0.089308266 | 0.0031256 | 0.01 |
| ZGPAT | 0.146162347 | 1.22E-06 | 0.080444903 | 0.007795 | 0.07 |
| ZHX1 | -0.10613409 | 0.00044014 | -0.213095956 | 1.09E-12 | 0.11 |
| ZHX2 | -0.160258127 | 1.00E-07 | -0.101031755 | 0.0008231 | 0.06 |
| ZHX3 | -0.368648145 | 1.62E-36 | -0.334905081 | 4.69E-30 | 0.04 |
| ZIC1 | 0.276281804 | 1.34E-20 | 0.114617146 | 0.0001462 | 0.17 |
| LOC115627 | 0.035713175 | 0.23811058 | 0.093321107 | 0.002012 | 0.06 |
| ZIC3 | 0.1130606 | 0.00017997 | 0.06804281 | 0.0244765 | 0.05 |
| ZIC4 | 0.220233188 | 1.79E-13 | 0.071114472 | 0.0187044 | 0.15 |
| ZIC5 | 0.164089929 | 4.88E-08 | 0.048394305 | 0.1098098 | 0.12 |
| ZIK1 | -0.135885907 | 6.54E-06 | -0.276660578 | 1.18E-20 | 0.15 |
| ZIM2 | -0.003676499 | 0.90336757 | -0.146372733 | 1.17E-06 | 0.15 |
| ZIM3 | 0.007981496 | 0.79210749 | 0.011239533 | 0.7105097 | 0.01 |
| LOC392756 | -0.501876186 | 8.56E-71 | -0.388396777 | 1.13E-40 | 0.12 |
| ZKSCAN2 | -0.087259781 | 0.00388855 | -0.166859657 | 2.87E-08 | 0.08 |
| LOC100507255 | -0.219205341 | 2.34E-13 | -0.158032575 | 1.51E-07 | 0.07 |
| P1P373C6 | -0.19459332 | 8.69E-11 | -0.144693348 | 1.56E-06 | 0.05 |
| ZKSCAN5 | -0.181114524 | 1.63E-09 | -0.097875858 | 0.0011957 | 0.09 |
| ZMAT1 | -0.3845455 | 7.68E-40 | -0.204242003 | 9.32E-12 | 0.19 |
| ZMAT2 | 0.010892984 | 0.71905158 | 0.132962278 | 1.03E-05 | 0.13 |
| ZMAT3 | -0.326124683 | 1.68E-28 | -0.315479173 | 1.11E-26 | 0.02 |
| ZMAT4 | -0.033378117 | 0.27022548 | 0.072865242 | 0.0159783 | 0.11 |
| ZMAT5 | 0.188477176 | 3.38E-10 | 0.29057108 | 1.04E-22 | 0.11 |
| ZMIZ1 | -0.346309436 | 3.76E-32 | -0.314057859 | 1.91E-26 | 0.04 |
| ZMIZ2 | 0.186504664 | 5.18E-10 | 0.035302416 | 0.2435557 | 0.16 |
| ZMPSTE24 | -0.136411083 | 6.02E-06 | -0.104530873 | 0.0005374 | 0.04 |
| ZMYM1 | -0.188529676 | 3.34E-10 | -0.209716144 | 2.50E-12 | 0.03 |
| ZMYM2 | -0.276188689 | 1.38E-20 | -0.179343048 | 2.36E-09 | 0.1 |
| ZMYM3 | -0.147296617 | 1.00E-06 | -0.1053292 | 0.0004867 | 0.05 |
| ZMYM4 | -0.151211816 | 5.10E-07 | -0.195077637 | 7.79E-11 | 0.05 |
| ZMYM5 | -0.213109202 | 1.08E-12 | -0.111580876 | 0.0002188 | 0.11 |
| LOC220665 | -0.346991764 | 2.80E-32 | -0.326907153 | 1.23E-28 | 0.03 |
| ZMYND10 | -0.313951112 | 1.99E-26 | -0.00872846 | 0.7731607 | 0.31 |
| ZMYND11 | -0.233055198 | 6.01E-15 | -0.195870741 | 6.51E-11 | 0.04 |
| ZMYND12 | -0.094389848 | 0.00178419 | 0.038767573 | 0.2003018 | 0.14 |
| ZMYND15 | 0.032390466 | 0.28466339 | -0.016549322 | 0.5846941 | 0.05 |
| MSS51 | 0.109255044 | 0.00029604 | 0.183944275 | 8.97E-10 | 0.08 |
| ZMYND19 | 0.491447663 | 1.58E-67 | 0.313073757 | 2.79E-26 | 0.18 |
| PRO2893 | -0.264643229 | 5.67E-19 | -0.234176455 | 4.42E-15 | 0.04 |
| ZNF100 | -0.229879847 | 1.42E-14 | -0.134279409 | 8.41E-06 | 0.1 |
| ZNF101 | -0.122746828 | 4.72E-05 | -0.1398145 | 3.49E-06 | 0.02 |
| LOC101929339 | -0.26704145 | 2.66E-19 | -0.214963915 | 6.83E-13 | 0.06 |
| ZNF10 | -0.090963056 | 0.0026117 | -0.077178766 | 0.0106965 | 0.02 |
| ZNF114 | 0.071369415 | 0.01828371 | -0.008915408 | 0.7684394 | 0.09 |
| ZNF117 | -0.264454781 | 6.01E-19 | -0.119538803 | 7.44E-05 | 0.15 |
| ZNF121 | -0.088463263 | 0.00342207 | -0.166379111 | 3.15E-08 | 0.08 |
| ZNF124 | 0.143954278 | 1.76E-06 | 0.13310644 | 1.01E-05 | 0.02 |
| ZNF12 | -0.375717842 | 5.67E-38 | -0.270344773 | 9.25E-20 | 0.11 |
| ZNF131 | 0.143060038 | 2.05E-06 | 0.021633515 | 0.4749297 | 0.13 |
| LOC147676 | -0.363704903 | 1.60E-35 | -0.345379609 | 5.61E-32 | 0.02 |
| ZNF150 | 0.209205367 | 2.83E-12 | 0.180046531 | 2.04E-09 | 0.03 |
| ZNF134 | -0.400536436 | 2.25E-43 | -0.373326246 | 1.78E-37 | 0.03 |
| ZNF61 | -0.097604857 | 0.00123403 | -0.147767143 | 9.27E-07 | 0.06 |
| ZNF136 | -0.364229624 | 1.26E-35 | -0.287591806 | 2.93E-22 | 0.08 |
| ZNF137P | 0.00424847 | 0.88842559 | 0.112953789 | 0.0001825 | 0.11 |
| ZNF138 | -0.134755435 | 7.81E-06 | 0.101456516 | 0.0007821 | 0.24 |
| ZNF140 | 0.010246762 | 0.73507319 | 0.056825454 | 0.0603745 | 0.05 |
| ZNF141 | -0.292089561 | 6.12E-23 | -0.197304448 | 4.69E-11 | 0.1 |
| ZNF142 | -0.209880896 | 2.40E-12 | -0.268756436 | 1.54E-19 | 0.06 |
| ZNF143 | -0.165313306 | 3.86E-08 | -0.092625888 | 0.0021741 | 0.08 |
| LOC115116 | 0.139908802 | 3.43E-06 | 0.042430606 | 0.1609734 | 0.1 |
| LOC143619 | -0.331600423 | 1.83E-29 | -0.246405921 | 1.40E-16 | 0.09 |
| ZNF14 | -0.2143302 | 8.00E-13 | -0.121833299 | 5.38E-05 | 0.1 |
| ZNF154 | -0.42344845 | 8.70E-49 | -0.36333301 | 1.90E-35 | 0.07 |
| LOC91116 | -0.137935618 | 4.72E-06 | -0.028325168 | 0.3494988 | 0.11 |
| ZNF157 | -0.049764523 | 0.10009797 | -0.162953857 | 6.05E-08 | 0.12 |
| ZNF160 | -0.338445612 | 1.07E-30 | -0.22888139 | 1.86E-14 | 0.11 |
| LOC221585 | 0.335062192 | 4.39E-30 | 0.239147707 | 1.11E-15 | 0.1 |
| ZNF64 | -0.226727968 | 3.29E-14 | -0.212700702 | 1.20E-12 | 0.02 |
| ZNF169 | -0.248698238 | 7.17E-17 | -0.166539772 | 3.05E-08 | 0.09 |
| ZNF16 | 0.22732626 | 2.81E-14 | 0.131739149 | 1.25E-05 | 0.1 |
| ZNF174 | -0.077997545 | 0.00989086 | -0.023273069 | 0.4421046 | 0.06 |
| ZNF175 | -0.418823835 | 1.16E-47 | -0.344496093 | 8.20E-32 | 0.08 |
| ZNF177 | -0.126995929 | 2.54E-05 | -0.05238284 | 0.0834496 | 0.08 |
| ZNF17 | -0.291485017 | 7.56E-23 | -0.255660493 | 9.03E-18 | 0.04 |
| ZNF180 | -0.193750401 | 1.05E-10 | -0.18656657 | 5.11E-10 | 0.01 |
| ZNF181 | -0.27001973 | 1.03E-19 | -0.158448232 | 1.40E-07 | 0.12 |
| ZNF182 | -0.249496855 | 5.67E-17 | -0.112070847 | 0.0002052 | 0.14 |
| ZNF184 | 0.028080429 | 0.35367994 | 0.001608116 | 0.9576487 | 0.03 |
| ZNF185 | -0.153135967 | 3.64E-07 | -0.124679607 | 3.57E-05 | 0.03 |
| LOC154534 | -0.225606862 | 4.43E-14 | -0.15403236 | 3.10E-07 | 0.08 |
| LOC158097 | -0.267241118 | 2.49E-19 | -0.21242901 | 1.28E-12 | 0.06 |
| ZNF18 | -0.10782257 | 0.00035561 | -0.001608001 | 0.9576518 | 0.11 |
| ZKSCAN8 | -0.399971376 | 3.02E-43 | -0.42659421 | 1.46E-49 | 0.03 |
| ZSCAN9 | -0.006031408 | 0.84212562 | 0.105243643 | 0.0004919 | 0.12 |
| ZNF195 | 0.080180968 | 0.00800012 | 0.079097586 | 0.0088936 | 0.01 |
| ZNF166 | -0.251648742 | 3.00E-17 | -0.118684204 | 8.38E-05 | 0.14 |
| ZNF19 | -0.297636517 | 8.51E-24 | -0.149095641 | 7.37E-07 | 0.15 |
| ZNF200 | 0.07575196 | 0.01224043 | 0.028934654 | 0.3392233 | 0.05 |
| ZNF202 | 0.023270449 | 0.44215597 | 0.045931678 | 0.1291178 | 0.03 |
| ZNF204P | -0.109448456 | 0.00028876 | -0.025571287 | 0.3983501 | 0.09 |
| ZNF210 | 0.11206732 | 0.00020525 | 0.14329756 | 1.97E-06 | 0.04 |
| ZNF207 | 0.070352176 | 0.02001306 | 0.045813723 | 0.1301048 | 0.03 |
| ZNF95 | -0.221604293 | 1.26E-13 | -0.155875285 | 2.23E-07 | 0.07 |
| ZNF20 | -0.239956887 | 8.85E-16 | -0.05377176 | 0.0755723 | 0.19 |
| ZNFC25 | -0.324754751 | 2.91E-28 | -0.2568783 | 6.24E-18 | 0.07 |
| ZNF212 | 0.130554954 | 1.49E-05 | 0.097482811 | 0.0012517 | 0.04 |
| ZNF213 | -0.051754518 | 0.08722498 | 0.087684866 | 0.0037176 | 0.14 |
| ZNF214 | -0.373117635 | 1.96E-37 | -0.191391994 | 1.78E-10 | 0.19 |
| ZNF215 | -0.051786687 | 0.08702839 | -0.08612729 | 0.0043794 | 0.04 |
| ZNF217 | -0.28434085 | 8.95E-22 | -0.207270037 | 4.52E-12 | 0.08 |
| ZFP219 | -0.108897968 | 0.00030995 | -0.027012421 | 0.372293 | 0.09 |
| ZNF221 | -0.38671704 | 2.61E-40 | -0.344108946 | 9.68E-32 | 0.05 |
| ZNF222 | -0.146592056 | 1.13E-06 | -0.12544382 | 3.20E-05 | 0.03 |
| LOC199729 | -0.339238078 | 7.67E-31 | -0.251598702 | 3.05E-17 | 0.09 |
| ZNF27 | -0.480603242 | 3.01E-64 | -0.37445319 | 1.04E-37 | 0.11 |
| ZNF225 | -0.43426612 | 1.72E-51 | -0.317742704 | 4.61E-27 | 0.12 |
| LOC51595 | -0.24909853 | 6.38E-17 | -0.105010532 | 0.0005064 | 0.15 |
| ZNF227 | -0.156046171 | 2.16E-07 | -0.160237181 | 1.00E-07 | 0.01 |
| FLJ34222 | -0.043106976 | 0.15439679 | -0.184171542 | 8.55E-10 | 0.15 |
| ZNF22 | 0.155593129 | 2.35E-07 | 0.060338553 | 0.0461127 | 0.1 |
| FDZF2 | -0.284479531 | 8.54E-22 | -0.153208678 | 3.59E-07 | 0.14 |
| ZNF232 | 0.402446728 | 8.24E-44 | 0.273506207 | 3.32E-20 | 0.13 |
| ZNF233 | -0.139239312 | 3.83E-06 | -0.076557472 | 0.0113462 | 0.07 |
| LOC94536 | -0.302723029 | 1.34E-24 | -0.252063286 | 2.66E-17 | 0.06 |
| ZNF270 | -0.156375761 | 2.04E-07 | -0.164919979 | 4.17E-08 | 0.01 |
| ZNF236 | -0.221501756 | 1.29E-13 | -0.306058975 | 3.91E-25 | 0.09 |
| ZBTB18 | -0.154995709 | 2.61E-07 | 0.041991351 | 0.1653562 | 0.2 |
| HOK.2 | 0.105956158 | 0.00045006 | 0.163513418 | 5.44E-08 | 0.06 |
| ZNF23 | 0.0755332 | 0.01249382 | -0.082573148 | 0.0063055 | 0.16 |
| ZNF248 | -0.068494684 | 0.02354088 | -0.082579593 | 0.0063014 | 0.02 |
| ZNF24 | -0.10729192 | 0.00038038 | -0.16076899 | 9.10E-08 | 0.06 |
| ZNF250 | 0.17862065 | 2.74E-09 | 0.011525888 | 0.7034787 | 0.17 |
| ZNF251 | 0.174750149 | 6.04E-09 | 0.044417271 | 0.1422392 | 0.14 |
| ZNF252P | -0.000111502 | 0.99706211 | -0.050037344 | 0.0982485 | 0.05 |
| ZNF253 | -0.179779714 | 2.16E-09 | -0.091073189 | 0.0025804 | 0.09 |
| LOC100996381 | -0.308608951 | 1.51E-25 | -0.24175957 | 5.32E-16 | 0.07 |
| ZNF256 | -0.040040265 | 0.18591222 | -0.039733525 | 0.1893085 | 0.01 |
| ZNF257 | -0.090828279 | 0.00265048 | -0.0313807 | 0.2999543 | 0.06 |
| ZPR1 | 0.360339123 | 7.48E-35 | 0.194313926 | 9.26E-11 | 0.17 |
| ZNF25 | -0.394243998 | 5.82E-42 | -0.310055582 | 8.76E-26 | 0.09 |
| ZNF260 | -0.091625564 | 0.00242854 | -0.051200154 | 0.0906692 | 0.05 |
| ZNF263 | -0.029555534 | 0.32895674 | 0.054642354 | 0.0709514 | 0.09 |
| ZNF264 | -0.395369682 | 3.27E-42 | -0.309828182 | 9.54E-26 | 0.09 |
| ZNF266 | -0.0340478 | 0.2607259 | -0.00756632 | 0.8026925 | 0.03 |
| HZF2 | -0.057170337 | 0.05882947 | -0.1108241 | 0.0002416 | 0.06 |
| ZNF268 | -0.282002179 | 1.98E-21 | -0.244189818 | 2.65E-16 | 0.04 |
| ZNF26 | 0.188340953 | 3.48E-10 | 0.179298503 | 2.38E-09 | 0.01 |
| ZNFEB | 0.256609977 | 6.77E-18 | 0.050919424 | 0.0924546 | 0.21 |
| MGC12518 | 0.059969398 | 0.04746393 | 0.110841669 | 0.000241 | 0.06 |
| hfb101 | -0.062836557 | 0.03779354 | -0.15420709 | 3.01E-07 | 0.1 |
| LOC286459 | -0.227904609 | 2.41E-14 | -0.225503532 | 4.55E-14 | 0.01 |
| ZNF276 | 0.084542204 | 0.0051608 | -0.047502145 | 0.1165231 | 0.14 |
| ZNF277 | -0.053468823 | 0.07723659 | 0.042815159 | 0.1572088 | 0.1 |
| ZNF280A | 0.32822553 | 7.22E-29 | 0.101807162 | 0.0007497 | 0.23 |
| ZNF280B | 0.170244612 | 1.48E-08 | -0.095599424 | 0.0015551 | 0.27 |
| ZNF280C | 0.100811008 | 0.00084518 | -0.046757731 | 0.1223674 | 0.15 |
| ZNF280D | -0.448640819 | 3.06E-55 | -0.268176 | 1.85E-19 | 0.19 |
| ZNF281 | -0.426003012 | 2.04E-49 | -0.22077683 | 1.56E-13 | 0.21 |
| LOC729976 | 0.137412697 | 5.13E-06 | 0.03430594 | 0.2571265 | 0.11 |
| ZNF283 | -0.152137564 | 4.34E-07 | -0.136698061 | 5.75E-06 | 0.02 |
| ZNF284 | -0.299327005 | 4.62E-24 | -0.211870033 | 1.47E-12 | 0.09 |
| FLJ30747 | 0.01940763 | 0.52155314 | 0.050300375 | 0.0964912 | 0.04 |
| PPARAL | 0.20190211 | 1.62E-11 | 0.033395135 | 0.2699812 | 0.17 |
| LOC100132901 | 0.256422236 | 7.17E-18 | 0.14450995 | 1.61E-06 | 0.12 |
| ZNF287 | -0.210965227 | 1.84E-12 | -0.140882197 | 2.93E-06 | 0.08 |
| ZNF28 | -0.219270244 | 2.30E-13 | -0.121001144 | 6.06E-05 | 0.1 |
| FLJ13564 | -0.204069222 | 9.71E-12 | -0.240436557 | 7.73E-16 | 0.04 |
| ZBTB21 | -0.21060896 | 2.01E-12 | -0.240897005 | 6.79E-16 | 0.04 |
| ZNF342 | 0.049453492 | 0.10224021 | 0.119579862 | 7.40E-05 | 0.08 |
| LOC129759 | -0.043601 | 0.14972353 | 0.0114833 | 0.7045228 | 0.06 |
| ZNF300 | 0.051905506 | 0.08630539 | -0.088780526 | 0.0033079 | 0.15 |
| LOC654145 | -0.191783097 | 1.63E-10 | -0.060363872 | 0.0460212 | 0.14 |
| ZNF304 | -0.335855387 | 3.16E-30 | -0.270177458 | 9.76E-20 | 0.07 |
| ZNF30 | -0.12690909 | 2.58E-05 | -0.016367614 | 0.5888251 | 0.12 |
| ZNF311 | -0.290158678 | 1.20E-22 | -0.107063873 | 0.0003915 | 0.19 |
| ZNF317 | -0.086370399 | 0.00426957 | -0.122506625 | 4.89E-05 | 0.04 |
| LOC221427 | -0.16034156 | 9.85E-08 | -0.279336544 | 4.85E-21 | 0.12 |
| ZNF319 | -0.10056458 | 0.00087046 | -0.275750674 | 1.59E-20 | 0.18 |
| LOC100419838 | -0.155029685 | 2.60E-07 | -0.088641232 | 0.0033576 | 0.07 |
| ZNF321P | -0.146865521 | 1.08E-06 | 0.030580318 | 0.3124562 | 0.18 |
| LOC158428 | -0.188186491 | 3.60E-10 | -0.104147873 | 0.0005634 | 0.09 |
| ZNF322P1 | 0.003138096 | 0.91746398 | 0.087516339 | 0.0037845 | 0.09 |
| LOC116206 | -0.101057548 | 0.00082056 | -0.11642549 | 0.0001144 | 0.02 |
| ZNF324B | -0.199499695 | 2.83E-11 | -0.168062445 | 2.27E-08 | 0.04 |
| ZNF324 | -0.180247931 | 1.96E-09 | -0.208462874 | 3.39E-12 | 0.03 |
| FLJ20403 | 0.071606453 | 0.01790002 | -0.112437584 | 0.0001955 | 0.19 |
| ZNF329 | -0.266625544 | 3.03E-19 | -0.201245728 | 1.89E-11 | 0.07 |
| ZNF32 | 0.352628144 | 2.37E-33 | 0.392621938 | 1.33E-41 | 0.04 |
| LOC116011 | -0.027616758 | 0.3616874 | -0.085438017 | 0.004705 | 0.06 |
| LOC732358 | -0.218992153 | 2.47E-13 | -0.235360965 | 3.19E-15 | 0.02 |
| ZNF333 | -0.284953965 | 7.26E-22 | -0.196157933 | 6.10E-11 | 0.09 |
| ZNF334 | -0.069612268 | 0.02135893 | -0.195715888 | 6.74E-11 | 0.13 |
| ZNF335 | -0.02897198 | 0.33860033 | -0.069790916 | 0.021027 | 0.05 |
| ZNF337 | -0.000999844 | 0.97366058 | 0.044350094 | 0.1428441 | 0.05 |
| FLJ23404 | -0.225082699 | 5.09E-14 | -0.146855916 | 1.08E-06 | 0.08 |
| ZNF33B | -0.092978099 | 0.00209055 | -0.124809547 | 3.51E-05 | 0.04 |
| ZNF341 | 0.123347597 | 4.33E-05 | 0.200031349 | 2.50E-11 | 0.08 |
| MGC20504 | 0.000151712 | 0.99600267 | -0.036408718 | 0.2290871 | 0.04 |
| ZNF345 | -0.181676203 | 1.45E-09 | -0.151007202 | 5.29E-07 | 0.04 |
| ZNF346 | -0.111604395 | 0.00021813 | -0.117131107 | 0.0001039 | 0.01 |
| ZNF347 | -0.34002877 | 5.50E-31 | -0.28002765 | 3.85E-21 | 0.07 |
| ZNF34 | 0.134873011 | 7.67E-06 | 0.034016483 | 0.2611649 | 0.11 |
| ZNF350 | -0.204007775 | 9.85E-12 | -0.051085847 | 0.0913928 | 0.16 |
| ZNF354A | 0.245696538 | 1.72E-16 | 0.165904176 | 3.45E-08 | 0.08 |
| ZNF354B | -0.223086257 | 8.58E-14 | -0.237354274 | 1.84E-15 | 0.02 |
| ZNF354C | -0.339728642 | 6.24E-31 | -0.352259713 | 2.79E-33 | 0.02 |
| FLJ10390 | 0.275296796 | 1.85E-20 | 0.191062493 | 1.91E-10 | 0.09 |
| ZNF35 | 0.080128766 | 0.00804126 | 0.091954606 | 0.002342 | 0.02 |
| ZNF362 | 0.065603903 | 0.03010121 | -0.016008111 | 0.5970377 | 0.09 |
| UAN | -0.250983057 | 3.66E-17 | -0.134097178 | 8.65E-06 | 0.12 |
| ZNF366 | -0.528349799 | 1.29E-79 | -0.511047196 | 9.22E-74 | 0.02 |
| ZNF367 | 0.316067941 | 8.82E-27 | 0.095636479 | 0.0015485 | 0.23 |
| ZNF37A | -0.20363353 | 1.08E-11 | -0.263347544 | 8.51E-19 | 0.06 |
| LOC439958 | -0.076960627 | 0.01092074 | -0.188801082 | 3.15E-10 | 0.12 |
| ZNF382 | -0.102397364 | 0.00069802 | -0.127172401 | 2.48E-05 | 0.03 |
| ZNF383 | -0.172381154 | 9.72E-09 | -0.129765623 | 1.68E-05 | 0.05 |
| ZNF384OS | 0.191846156 | 1.61E-10 | 0.050977013 | 0.0920861 | 0.15 |
| LOC144475 | -0.284403694 | 8.76E-22 | -0.054854588 | 0.0698607 | 0.23 |
| LOC102724263 | -0.226097154 | 3.89E-14 | -0.137210546 | 5.30E-06 | 0.09 |
| LOC100505836 | -0.232809416 | 6.42E-15 | -0.114852945 | 0.0001416 | 0.12 |
| ZNF192P1 | -0.046108672 | 0.12764763 | -0.02237365 | 0.4599487 | 0.03 |
| LOC222708 | -0.096855532 | 0.00134606 | -0.140767673 | 2.99E-06 | 0.05 |
| LOC56854 | 0.167921486 | 2.34E-08 | 0.184059056 | 8.76E-10 | 0.02 |
| DKFZp434K1210 | -0.354100004 | 1.24E-33 | -0.115632438 | 0.0001274 | 0.24 |
| LOC125479 | -0.272581411 | 4.49E-20 | -0.20920054 | 2.83E-12 | 0.07 |
| ZSCAN30 | -0.104192106 | 0.00056038 | -0.230200611 | 1.30E-14 | 0.13 |
| ZNF47 | -0.146804447 | 1.09E-06 | -0.260721942 | 1.92E-18 | 0.12 |
| ZNF398 | 0.010810453 | 0.72109106 | -0.049972143 | 0.098688 | 0.07 |
| FLJ20216 | 0.081902285 | 0.00674479 | 0.148365045 | 8.36E-07 | 0.07 |
| ZNF404 | -0.057316787 | 0.05818338 | 0.013999804 | 0.6438416 | 0.08 |
| FLJ13839 | -0.24260142 | 4.18E-16 | -0.333000717 | 1.03E-29 | 0.1 |
| ZNF408 | 0.172695517 | 9.13E-09 | 0.161770937 | 7.55E-08 | 0.02 |
| LOC122967 | -0.091362797 | 0.00249974 | 0.016509698 | 0.5855937 | 0.11 |
| ZNF414 | 0.091095148 | 0.00257421 | 0.115373008 | 0.000132 | 0.03 |
| ZNF415 | -0.144642615 | 1.57E-06 | -0.008321871 | 0.7834579 | 0.14 |
| ZNF416 | -0.120489237 | 6.51E-05 | -0.10513516 | 0.0004986 | 0.02 |
| ZNF417 | -0.450773362 | 8.21E-56 | -0.391650299 | 2.18E-41 | 0.06 |
| LOC730055 | -0.207171039 | 4.63E-12 | -0.107252953 | 0.0003823 | 0.1 |
| ZNF419 | -0.023581563 | 0.43607652 | -0.047391813 | 0.1173752 | 0.03 |
| ZNF41 | -0.384108024 | 9.53E-40 | -0.283418269 | 1.23E-21 | 0.11 |
| ZNF420 | -0.31374956 | 2.15E-26 | -0.255306192 | 1.00E-17 | 0.06 |
| ZNF423 | -0.475612151 | 8.90E-63 | -0.27928957 | 4.92E-21 | 0.2 |
| ZNF425 | -0.256125342 | 7.85E-18 | -0.191205052 | 1.85E-10 | 0.07 |
| ZNF426 | -0.346858548 | 2.96E-32 | -0.337419725 | 1.65E-30 | 0.01 |
| ZNF428 | 0.190255981 | 2.29E-10 | 0.100449556 | 0.0008825 | 0.09 |
| LOC388522 | -0.354010203 | 1.29E-33 | -0.184553565 | 7.88E-10 | 0.17 |
| ZNF430 | -0.36890059 | 1.44E-36 | -0.396156684 | 2.18E-42 | 0.03 |
| ZNF431 | -0.328689823 | 5.99E-29 | -0.266247947 | 3.42E-19 | 0.07 |
| ZNF432 | -0.355080486 | 7.98E-34 | -0.276497548 | 1.24E-20 | 0.08 |
| ZNF433 | -0.173622396 | 7.59E-09 | -0.060278155 | 0.0463315 | 0.12 |
| MGC4179 | -0.247079525 | 1.15E-16 | -0.107953386 | 0.0003497 | 0.14 |
| ZNF436 | -0.303673482 | 9.46E-25 | -0.326034345 | 1.75E-28 | 0.03 |
| ZNF438 | 0.136945657 | 5.53E-06 | -0.027485431 | 0.3639759 | 0.17 |
| ZNF439 | -0.082427382 | 0.00639872 | -0.09611211 | 0.0014664 | 0.02 |
| ZNF43 | -0.27812135 | 7.27E-21 | -0.115048447 | 0.0001379 | 0.17 |
| ZNF440 | -0.292503305 | 5.29E-23 | -0.210632477 | 2.00E-12 | 0.09 |
| ZNF441 | -0.460728944 | 1.55E-58 | -0.319957539 | 1.94E-27 | 0.15 |
| ZNF442 | -0.403240865 | 5.42E-44 | -0.21088301 | 1.88E-12 | 0.2 |
| ZNF443 | -0.082861561 | 0.00612471 | 0.040249775 | 0.1836185 | 0.13 |
| ZNF444 | 0.146731695 | 1.11E-06 | 0.211872324 | 1.47E-12 | 0.07 |
| ZNF445 | -0.384430496 | 8.13E-40 | -0.354570783 | 1.00E-33 | 0.03 |
| LOC125904 | -0.165767279 | 3.54E-08 | 0.007955416 | 0.7927713 | 0.18 |
| ZNF449 | -0.28882662 | 1.91E-22 | -0.274147159 | 2.69E-20 | 0.02 |
| ZNF44 | -0.307099384 | 2.66E-25 | -0.221556839 | 1.28E-13 | 0.09 |
| LOC100506156 | -0.229524216 | 1.56E-14 | -0.193284561 | 1.17E-10 | 0.04 |
| ZNF454 | 0.122186007 | 5.12E-05 | -0.053814256 | 0.0753412 | 0.18 |
| ZNF13 | -0.359228874 | 1.24E-34 | -0.318628884 | 3.27E-27 | 0.05 |
| ZNF460 | -0.106988638 | 0.00039526 | -0.227747189 | 2.51E-14 | 0.13 |
| MGC33911 | -0.176487135 | 4.25E-09 | -0.098549809 | 0.001105 | 0.08 |
| KIAA1803 | 0.070355059 | 0.02000797 | -0.150388899 | 5.89E-07 | 0.23 |
| ZNF467 | -0.27428438 | 2.58E-20 | 0.016162349 | 0.5935079 | 0.3 |
| ZNF468 | -0.206569352 | 5.35E-12 | -0.098557948 | 0.001104 | 0.11 |
| ZNF469 | -0.155405437 | 2.43E-07 | -0.176597399 | 4.15E-09 | 0.03 |
| ZNF470 | -0.100326691 | 0.00089553 | -0.119784838 | 7.19E-05 | 0.02 |
| ZNF471 | -0.172273603 | 9.93E-09 | -0.161966328 | 7.28E-08 | 0.02 |
| ZFP100 | 0.202461149 | 1.42E-11 | 0.133584672 | 9.37E-06 | 0.07 |
| ZNF474 | -0.258437294 | 3.88E-18 | -0.080797636 | 0.0075282 | 0.18 |
| ZNF479 | 0.047938911 | 0.11319735 | 0.041912146 | 0.166156 | 0.01 |
| ZNF480 | 0.040554444 | 0.18032037 | 0.112346409 | 0.0001978 | 0.08 |
| ZNF483 | -0.391346463 | 2.55E-41 | -0.350814428 | 5.28E-33 | 0.05 |
| ZNF484 | -0.475639335 | 8.73E-63 | -0.346943711 | 2.85E-32 | 0.13 |
| LOC115002 | 0.214948778 | 6.85E-13 | 0.241872298 | 5.15E-16 | 0.03 |
| ZNF486 | -0.286622121 | 4.10E-22 | -0.263588346 | 7.89E-19 | 0.03 |
| LOC647655 | -0.339664291 | 6.41E-31 | -0.240919021 | 6.75E-16 | 0.1 |
| ZNF488 | 0.251994846 | 2.71E-17 | -0.044262259 | 0.1436379 | 0.3 |
| ZNF48 | 0.101007553 | 0.0008255 | 0.077453149 | 0.0104203 | 0.03 |
| ZNF490 | -0.253280555 | 1.85E-17 | -0.1979307 | 4.07E-11 | 0.06 |
| ZNF491 | -0.381167374 | 4.05E-39 | -0.132647767 | 1.08E-05 | 0.25 |
| ZNF492 | 0.218272277 | 2.96E-13 | 0.079086712 | 0.008903 | 0.14 |
| LOC115648 | -0.391757428 | 2.07E-41 | -0.233897203 | 4.77E-15 | 0.16 |
| ZNF496 | -0.106537526 | 0.00041839 | -0.051222335 | 0.0905293 | 0.06 |
| ZNF497 | -0.18885842 | 3.11E-10 | -0.038390821 | 0.2047134 | 0.16 |
| ZSCAN25 | -0.203478179 | 1.12E-11 | -0.225749254 | 4.27E-14 | 0.03 |
| ZNF500 | -0.315468201 | 1.11E-26 | -0.18341882 | 1.00E-09 | 0.14 |
| ZNF52 | -0.123148278 | 4.46E-05 | -0.144174187 | 1.70E-06 | 0.03 |
| ZNF502 | -0.064185364 | 0.03385761 | -0.13319018 | 9.96E-06 | 0.07 |
| LOC101929189 | -0.02004792 | 0.50790494 | -0.000194825 | 0.9948667 | 0.02 |
| ZNF506 | -0.236308221 | 2.45E-15 | -0.134408402 | 8.24E-06 | 0.11 |
| ZNF507 | -0.065863211 | 0.02945469 | -0.1919085 | 1.59E-10 | 0.13 |
| ZNF510 | -0.385969152 | 3.79E-40 | -0.341031238 | 3.60E-31 | 0.05 |
| ZNF511 | 0.381777598 | 3.00E-39 | 0.317009852 | 6.13E-27 | 0.07 |
| ZNF512B | -0.008741074 | 0.77284191 | -0.130171292 | 1.58E-05 | 0.13 |
| ZNF512 | -0.061136504 | 0.04330211 | -0.073528159 | 0.0150409 | 0.02 |
| ZNF513 | 0.091301827 | 0.00251653 | 0.074376324 | 0.0139125 | 0.02 |
| ZNF514 | -0.188000641 | 3.75E-10 | -0.160582734 | 9.42E-08 | 0.03 |
| ZNF516 | -0.335802429 | 3.23E-30 | -0.309527163 | 1.07E-25 | 0.03 |
| ZNF517 | 0.144158023 | 1.71E-06 | 0.104163027 | 0.0005624 | 0.04 |
| ZNF518A | -0.226366764 | 3.63E-14 | -0.199930167 | 2.56E-11 | 0.03 |
| ZNF518B | -0.009461841 | 0.75468889 | -0.049105811 | 0.1046778 | 0.04 |
| ZNF519 | 0.240667744 | 7.24E-16 | 0.023373879 | 0.4401295 | 0.22 |
| ZNF521 | -0.042167655 | 0.16358642 | -0.14678158 | 1.10E-06 | 0.11 |
| ZNF524 | 0.108483455 | 0.00032686 | 0.157193581 | 1.76E-07 | 0.05 |
| ZNF525 | -0.060939991 | 0.0439805 | -0.038870345 | 0.1991105 | 0.03 |
| ZNF526 | 0.044612119 | 0.14049575 | -0.034746736 | 0.2510601 | 0.08 |
| ZNF527 | -0.185602236 | 6.29E-10 | -0.127831397 | 2.25E-05 | 0.06 |
| ZNF528 | -0.329122308 | 5.02E-29 | -0.124687631 | 3.57E-05 | 0.21 |
| LOC92279 | -0.047148284 | 0.11927331 | 8.83E-05 | 0.9976729 | 0.05 |
| KIAA1508 | -0.020090509 | 0.50700382 | -0.013063889 | 0.6661604 | 0.01 |
| ZNF532 | 0.002251557 | 0.9407298 | -0.163340146 | 5.62E-08 | 0.17 |
| ZNF534 | 0.156822016 | 1.88E-07 | 0.207062218 | 4.75E-12 | 0.06 |
| ZNF536 | -0.118264349 | 8.89E-05 | -0.102091056 | 0.0007244 | 0.02 |
| ZNF540 | -0.200620454 | 2.18E-11 | -0.172987277 | 8.62E-09 | 0.03 |
| ZNF541 | -0.113547252 | 0.00016869 | 0.072056314 | 0.0171912 | 0.19 |
| ZNF542P | -0.264771298 | 5.44E-19 | -0.174499818 | 6.36E-09 | 0.1 |
| ZNF543 | -0.327549984 | 9.49E-29 | -0.289496949 | 1.52E-22 | 0.04 |
| LOC125909 | 0.079308788 | 0.00871274 | 0.013012355 | 0.6673982 | 0.07 |
| ZNF49 | -0.366589025 | 4.23E-36 | -0.183716047 | 9.42E-10 | 0.19 |
| ZNF547 | -0.050499824 | 0.09517558 | -0.018670429 | 0.5374972 | 0.04 |
| ZNF548 | -0.35674743 | 3.79E-34 | -0.258847591 | 3.42E-18 | 0.1 |
| ZNF549 | -0.219538511 | 2.14E-13 | -0.258797731 | 3.48E-18 | 0.04 |
| ZNF550 | -0.249871248 | 5.08E-17 | -0.271493969 | 6.38E-20 | 0.03 |
| ZNF551 | -0.298148841 | 7.07E-24 | -0.352522771 | 2.48E-33 | 0.06 |
| ZNF552 | -0.459631162 | 3.13E-58 | -0.147338787 | 9.97E-07 | 0.32 |
| ZNF554 | -0.098713275 | 0.001084 | 0.008255087 | 0.785153 | 0.11 |
| MGC26707 | -0.234361045 | 4.20E-15 | -0.139445618 | 3.70E-06 | 0.1 |
| LOC115305 | -0.072431907 | 0.01661832 | -0.147342984 | 9.96E-07 | 0.08 |
| ZNF557 | -0.083947402 | 0.00548506 | -0.06329801 | 0.0364053 | 0.03 |
| ZNF558 | -0.090869228 | 0.00263864 | -0.107933274 | 0.0003506 | 0.02 |
| ZNF559 | -0.136489615 | 5.94E-06 | -0.116946032 | 0.0001065 | 0.02 |
| ZNF560 | 0.119256969 | 7.74E-05 | 0.032641038 | 0.280952 | 0.09 |
| ZNF561 | -0.249765117 | 5.24E-17 | -0.168882425 | 1.94E-08 | 0.09 |
| ZNF562 | -0.154279598 | 2.97E-07 | -0.136631136 | 5.81E-06 | 0.02 |
| ZNF563 | -0.246302815 | 1.44E-16 | -0.116016736 | 0.000121 | 0.14 |
| ZNF564 | -0.287578239 | 2.95E-22 | -0.139829521 | 3.48E-06 | 0.15 |
| ZNF565 | -0.012224725 | 0.68642688 | 0.164682962 | 4.36E-08 | 0.18 |
| MGC12515 | -0.015460379 | 0.60964917 | -0.029673421 | 0.3270304 | 0.02 |
| LOC105372391 | -0.014514398 | 0.63170377 | -0.098272936 | 0.0011415 | 0.09 |
| LOC654138 | -0.347259562 | 2.49E-32 | -0.23450197 | 4.04E-15 | 0.12 |
| ZNF569 | -0.066315377 | 0.02835585 | -0.040801301 | 0.1776805 | 0.03 |
| ZNF570 | -0.253507276 | 1.73E-17 | -0.271685971 | 6.00E-20 | 0.02 |
| HSPC059 | -0.181283503 | 1.57E-09 | -0.069589758 | 0.0214011 | 0.12 |
| ZNF572 | 0.091240267 | 0.00253358 | -0.037039464 | 0.2211168 | 0.13 |
| ZNF573 | -0.37674006 | 3.47E-38 | -0.283730349 | 1.10E-21 | 0.1 |
| ZNF574 | 0.138212065 | 4.51E-06 | 0.058167168 | 0.0545471 | 0.09 |
| ZNF575 | -0.029414882 | 0.33126467 | -0.026195456 | 0.3869325 | 0.01 |
| ZNF576 | 0.188701099 | 3.22E-10 | 0.147566417 | 9.59E-07 | 0.05 |
| AC006272.1 | -0.121299789 | 5.81E-05 | -0.082856333 | 0.0061279 | 0.04 |
| ZNF578 | -0.098228036 | 0.00114748 | -0.105182851 | 0.0004957 | 0.01 |
| ZNF579 | 0.227525431 | 2.67E-14 | 0.197888228 | 4.11E-11 | 0.03 |
| ZNF57 | 0.048676327 | 0.10775237 | 0.088004757 | 0.0035934 | 0.04 |
| LOC102724369 | -0.006835999 | 0.82139905 | 0.081200713 | 0.0072334 | 0.09 |
| LOC96067 | 0.163757043 | 5.20E-08 | 0.162680502 | 6.37E-08 | 0.01 |
| ZNF582 | -0.303218534 | 1.12E-24 | -0.162488542 | 6.60E-08 | 0.15 |
| ZNF583 | -0.269095287 | 1.38E-19 | -0.15911067 | 1.24E-07 | 0.11 |
| ZNF584 | -0.139974931 | 3.40E-06 | -0.054066048 | 0.0739836 | 0.09 |
| ZNF585A | -0.31273064 | 3.17E-26 | -0.268327238 | 1.77E-19 | 0.05 |
| ZNF585B | -0.450498449 | 9.74E-56 | -0.392776125 | 1.23E-41 | 0.06 |
| ZNF586 | -0.275335433 | 1.82E-20 | -0.090461315 | 0.0027587 | 0.19 |
| FLJ20813 | -0.476601069 | 4.57E-63 | -0.320908593 | 1.34E-27 | 0.16 |
| ZNF589 | -0.134271468 | 8.42E-06 | -0.052926719 | 0.0802892 | 0.09 |
| SCAR5 | -0.186560762 | 5.12E-10 | -0.3399301 | 5.73E-31 | 0.16 |
| ZNF593 | 0.368384989 | 1.83E-36 | 0.320389095 | 1.64E-27 | 0.05 |
| ZNF594 | -0.219425357 | 2.21E-13 | -0.26773633 | 2.13E-19 | 0.05 |
| ZNF595 | -0.234612151 | 3.92E-15 | -0.112372204 | 0.0001972 | 0.13 |
| ZNF596 | -0.247191178 | 1.11E-16 | -0.069130148 | 0.0222777 | 0.18 |
| ZNF597 | -0.401572038 | 1.31E-43 | -0.260006412 | 2.40E-18 | 0.15 |
| ZNF598 | 0.268042397 | 1.93E-19 | 0.089675458 | 0.0030042 | 0.18 |
| ZNF599 | -0.221146357 | 1.42E-13 | -0.196767587 | 5.31E-11 | 0.03 |
| ZNF600 | 0.047363608 | 0.11759381 | 0.085984138 | 0.0044453 | 0.04 |
| ZNF605 | -0.105040442 | 0.00050452 | -0.066184776 | 0.0286695 | 0.04 |
| ZNF606 | -0.156787643 | 1.89E-07 | -0.091603802 | 0.0024344 | 0.07 |
| LOC101927758 | 0.015045101 | 0.61928897 | 0.036688888 | 0.225522 | 0.03 |
| DKFZP434M098 | -0.269460489 | 1.23E-19 | -0.124585384 | 3.62E-05 | 0.15 |
| LOC145951 | -0.409624903 | 1.80E-45 | -0.420543331 | 4.46E-48 | 0.02 |
| ZNF610 | -0.064659668 | 0.03255936 | 0.008691655 | 0.7740913 | 0.08 |
| ZNF611 | -0.316786528 | 6.68E-27 | -0.283807155 | 1.07E-21 | 0.04 |
| ZNF613 | -0.152859228 | 3.82E-07 | -0.048446668 | 0.1094255 | 0.11 |
| LOC90346 | -0.092754205 | 0.00214334 | -0.099181872 | 0.0010258 | 0.01 |
| ZNF615 | -0.326162968 | 1.66E-28 | -0.189687293 | 2.59E-10 | 0.14 |
| MGC45556 | -0.31246393 | 3.51E-26 | -0.258368378 | 3.96E-18 | 0.06 |
| LOC286334 | -0.119533733 | 7.45E-05 | -0.203599102 | 1.09E-11 | 0.09 |
| ZNF619 | -0.467544021 | 1.88E-60 | -0.364835092 | 9.53E-36 | 0.11 |
| ZNF620 | -0.163411285 | 5.55E-08 | -0.095252678 | 0.0016178 | 0.07 |
| ZNF621 | -0.360639093 | 6.52E-35 | -0.289585136 | 1.47E-22 | 0.08 |
| LOC202068 | 0.121284125 | 5.82E-05 | 0.162043319 | 7.17E-08 | 0.05 |
| LOC401483 | -0.063303793 | 0.03638822 | -0.195912393 | 6.45E-11 | 0.14 |
| ZNF624 | -0.325576464 | 2.10E-28 | -0.219304642 | 2.28E-13 | 0.11 |
| ZNF625 | -0.360434327 | 7.16E-35 | -0.278472583 | 6.47E-21 | 0.09 |
| ZNF626 | -0.298468337 | 6.30E-24 | -0.162954486 | 6.05E-08 | 0.14 |
| LOC101928434 | -0.235954716 | 2.71E-15 | -0.11301652 | 0.000181 | 0.13 |
| ZNF628 | 0.054358379 | 0.07243254 | 0.031768722 | 0.294015 | 0.03 |
| ZNF65 | -0.132391343 | 1.13E-05 | -0.147184185 | 1.02E-06 | 0.02 |
| ZNF630 | -0.063070533 | 0.03708414 | 0.005165765 | 0.8645471 | 0.07 |
| ZNF638 | -0.118091581 | 9.10E-05 | -0.063429355 | 0.0360182 | 0.06 |
| LOC101929571 | 0.283976005 | 1.01E-21 | 0.033531688 | 0.2680264 | 0.26 |
| ZNF641 | -0.444686652 | 3.43E-54 | -0.459075801 | 4.47E-58 | 0.02 |
| LOC654213 | 0.065464289 | 0.03045432 | 0.052132853 | 0.0849355 | 0.02 |
| ZFP69B | 0.085708538 | 0.00457471 | -0.018015704 | 0.5518596 | 0.11 |
| BM.005 | -0.078895892 | 0.00906944 | -0.192368481 | 1.43E-10 | 0.12 |
| ZNF645 | -0.005968804 | 0.84374309 | -0.020046657 | 0.5079317 | 0.02 |
| ZNF646 | -0.288038507 | 2.51E-22 | -0.213955169 | 8.78E-13 | 0.08 |
| ZNF648 | -0.159789263 | 1.09E-07 | 0.102462815 | 0.0006925 | 0.27 |
| ZNF649 | -0.155927002 | 2.21E-07 | -0.156635069 | 1.94E-07 | 0.01 |
| ZNF652 | -0.237961502 | 1.55E-15 | -0.05760723 | 0.0569195 | 0.19 |
| ZNF653 | 0.160444939 | 9.66E-08 | 0.142021226 | 2.43E-06 | 0.02 |
| FLJ21142 | -0.36788117 | 2.32E-36 | -0.274631525 | 2.30E-20 | 0.1 |
| VIK | -0.31331844 | 2.54E-26 | -0.226134697 | 3.85E-14 | 0.09 |
| ZNF658 | -0.2373919 | 1.82E-15 | -0.156589238 | 1.96E-07 | 0.09 |
| ZNF660 | -0.332460418 | 1.29E-29 | -0.346606926 | 3.30E-32 | 0.02 |
| ZNF662 | -0.116804134 | 0.00010864 | -0.186394557 | 5.31E-10 | 0.07 |
| DKFZp761B128 | -0.058494432 | 0.05319915 | -0.085903839 | 0.0044827 | 0.03 |
| ZNF665 | -0.294484794 | 2.62E-23 | -0.206519359 | 5.41E-12 | 0.09 |
| ZNF667 | 0.016700242 | 0.58127321 | -0.066956851 | 0.0268576 | 0.09 |
| ZNF668 | 0.105677233 | 0.00046604 | 0.155836051 | 2.25E-07 | 0.06 |
| ZNF669 | -0.077406579 | 0.01046668 | 0.096652982 | 0.0013779 | 0.18 |
| LOC220645 | 0.112796663 | 0.00018639 | 0.090976891 | 0.0026078 | 0.03 |
| ZNF671 | -0.407430041 | 5.85E-45 | -0.304491329 | 6.99E-25 | 0.11 |
| ZNF672 | 0.235849337 | 2.79E-15 | 0.4032944 | 5.27E-44 | 0.17 |
| KRBOX4 | 0.076095218 | 0.011852 | 0.066331371 | 0.0283176 | 0.01 |
| ZNF674 | -0.148044994 | 8.83E-07 | -0.154018673 | 3.11E-07 | 0.01 |
| ZNF675 | -0.227325226 | 2.81E-14 | -0.200588569 | 2.20E-11 | 0.03 |
| ZNF676 | -0.288331163 | 2.27E-22 | -0.212247662 | 1.34E-12 | 0.08 |
| ZNF677 | -0.125950392 | 2.97E-05 | -0.15734468 | 1.71E-07 | 0.04 |
| MGC15634 | -0.419057516 | 1.02E-47 | -0.153554468 | 3.38E-07 | 0.27 |
| ZNF679 | 0.125511786 | 3.17E-05 | 0.066525907 | 0.0278564 | 0.06 |
| ZNF680 | -0.306803184 | 2.97E-25 | -0.17795118 | 3.15E-09 | 0.13 |
| ZNF681 | -0.100176641 | 0.00091169 | -0.067665329 | 0.0252826 | 0.04 |
| LOC729906 | 0.013816976 | 0.6481772 | 0.130858108 | 1.43E-05 | 0.12 |
| ZNF683 | 0.023755916 | 0.43269065 | 0.005257407 | 0.862168 | 0.02 |
| ZNF684 | -0.046997635 | 0.12045948 | -0.055850444 | 0.064925 | 0.01 |
| ZNF687 | -0.201234775 | 1.89E-11 | 0.030473163 | 0.3141556 | 0.24 |
| ZNF688 | -0.034418472 | 0.2555682 | 0.070090921 | 0.0204796 | 0.11 |
| ZNF689 | -0.349172951 | 1.08E-32 | -0.350925303 | 5.02E-33 | 0.01 |
| ZNF691 | 0.24475507 | 2.26E-16 | 0.29861005 | 5.99E-24 | 0.06 |
| ZNF692 | 0.205891262 | 6.29E-12 | 0.389713279 | 5.81E-41 | 0.19 |
| ZNF695 | 0.504754932 | 1.02E-71 | 0.414457734 | 1.30E-46 | 0.1 |
| ZNF696 | 0.269762232 | 1.12E-19 | 0.153365282 | 3.49E-07 | 0.12 |
| ZNF697 | -0.257922001 | 4.54E-18 | -0.188405568 | 3.43E-10 | 0.07 |
| ZNF699 | -0.408506175 | 3.28E-45 | -0.464895593 | 1.06E-59 | 0.06 |
| ZNF69 | -0.094738617 | 0.00171514 | 0.063404371 | 0.0360916 | 0.16 |
| LOC112990 | -0.198887524 | 3.26E-11 | -0.029360608 | 0.332158 | 0.17 |
| LOC101060177 | -0.342735869 | 1.74E-31 | -0.16775091 | 2.42E-08 | 0.18 |
| ZNF702P | -0.146303127 | 1.19E-06 | -0.088003782 | 0.0035938 | 0.06 |
| ZNF703 | -0.357251833 | 3.02E-34 | -0.195278675 | 7.44E-11 | 0.17 |
| MGC10858 | -0.359484871 | 1.10E-34 | -0.286917845 | 3.70E-22 | 0.08 |
| ZNF705A | -0.015847734 | 0.60071807 | 0.018599577 | 0.5390423 | 0.04 |
| ZNF705D | 0.050168593 | 0.09736846 | 0.005993033 | 0.843117 | 0.05 |
| ZNF706 | 0.485980193 | 7.37E-66 | 0.302512287 | 1.45E-24 | 0.19 |
| ZNF707 | 0.19325065 | 1.18E-10 | 0.052448779 | 0.0830611 | 0.15 |
| ZNF708 | -0.217194309 | 3.89E-13 | -0.204077355 | 9.69E-12 | 0.02 |
| ZNF709 | -0.269913072 | 1.06E-19 | -0.237562824 | 1.73E-15 | 0.04 |
| ZNF70 | -0.331817347 | 1.67E-29 | -0.349765326 | 8.36E-33 | 0.02 |
| ZNF710 | -0.324994324 | 2.65E-28 | -0.350887078 | 5.11E-33 | 0.03 |
| ZNF711 | 0.092831778 | 0.00212492 | -0.019551393 | 0.5184724 | 0.12 |
| ZNF713 | -0.199172464 | 3.06E-11 | -0.14709264 | 1.04E-06 | 0.06 |
| ZNF714 | -0.037285777 | 0.21805889 | -0.111025762 | 0.0002353 | 0.08 |
| ZNF716 | 0.009229474 | 0.76052699 | 0.029549095 | 0.3290622 | 0.03 |
| LOC100508721 | -0.334483821 | 5.58E-30 | -0.33027931 | 3.14E-29 | 0.01 |
| ZNF718 | -0.261641877 | 1.45E-18 | -0.201518428 | 1.77E-11 | 0.07 |
| ZNF71 | 0.171660117 | 1.12E-08 | 0.114433233 | 0.0001498 | 0.06 |
| LOC101928852 | -0.328774911 | 5.78E-29 | -0.155453457 | 2.41E-07 | 0.18 |
| LOC441003 | -0.307549783 | 2.24E-25 | -0.200702419 | 2.14E-11 | 0.11 |
| ZNF727 | -0.24875058 | 7.06E-17 | -0.253790232 | 1.59E-17 | 0.01 |
| ZNF732 | 0.024052923 | 0.42695802 | -0.081900319 | 0.0067461 | 0.11 |
| ZNF735 | -0.021804454 | 0.47144617 | -0.009653266 | 0.7498899 | 0.02 |
| ZNF737 | -0.382210456 | 2.43E-39 | -0.174482281 | 6.38E-09 | 0.21 |
| LOC100129257 | -0.009615852 | 0.7508271 | -0.013329693 | 0.6597906 | 0.01 |
| ZNF740 | -0.320538131 | 1.55E-27 | -0.202893174 | 1.28E-11 | 0.12 |
| ZNF746 | -0.024920728 | 0.4104636 | -0.080616876 | 0.0076639 | 0.06 |
| ZNF747 | -0.210407554 | 2.11E-12 | -0.142017249 | 2.43E-06 | 0.07 |
| ZNF749 | -0.234585082 | 3.95E-15 | -0.239259511 | 1.08E-15 | 0.01 |
| ZNF74 | 0.315627316 | 1.05E-26 | 0.190104685 | 2.36E-10 | 0.13 |
| ZNF750 | -0.010915805 | 0.71848798 | -0.086648011 | 0.0041472 | 0.08 |
| FLJ31529 | -0.039295035 | 0.19424254 | -0.047073119 | 0.119864 | 0.01 |
| LOC101805489 | -0.31554786 | 1.08E-26 | -0.286686578 | 4.01E-22 | 0.03 |
| FLJ35333 | -0.152424438 | 4.13E-07 | -0.064291384 | 0.0335636 | 0.09 |
| ZNF763 | -0.383721432 | 1.15E-39 | -0.177136291 | 3.72E-09 | 0.21 |
| ZNF764 | -0.077818075 | 0.01006268 | 0.031577868 | 0.2969264 | 0.11 |
| LOC107987265 | -0.105026308 | 0.0005054 | -0.107013332 | 0.000394 | 0.01 |
| ZNF766 | -0.111618125 | 0.00021774 | 0.082385127 | 0.006426 | 0.2 |
| ZNF767P | -0.041299822 | 0.1724371 | -0.021174229 | 0.4843589 | 0.03 |
| ZNF768 | 0.081763888 | 0.00683876 | 0.130793363 | 1.44E-05 | 0.05 |
| ZNF76 | 0.111854044 | 0.00021109 | 0.10274644 | 0.000669 | 0.01 |
| PRO1914 | -0.298013768 | 7.43E-24 | -0.291937765 | 6.45E-23 | 0.01 |
| ZNF771 | 0.093737718 | 0.00192018 | 0.09893985 | 0.0010555 | 0.01 |
| ZNF772 | -0.282428527 | 1.71E-21 | -0.256370133 | 7.28E-18 | 0.03 |
| ZNF773 | -0.154685192 | 2.76E-07 | -0.193014112 | 1.24E-10 | 0.04 |
| ZNF774 | -0.23260899 | 6.78E-15 | -0.2863818 | 4.45E-22 | 0.06 |
| ZNF775 | 0.082762078 | 0.00618654 | 0.056210487 | 0.0632128 | 0.03 |
| ZNF776 | -0.424540237 | 4.69E-49 | -0.371351836 | 4.54E-37 | 0.06 |
| ZNF777 | 0.149783717 | 6.55E-07 | 0.020762302 | 0.4929013 | 0.13 |
| LOC105376780 | -0.068782181 | 0.02296188 | -0.21197539 | 1.44E-12 | 0.15 |
| ZNF77 | 0.229092973 | 1.75E-14 | 0.153097604 | 3.66E-07 | 0.08 |
| ZNF780A | -0.331545272 | 1.87E-29 | -0.191664292 | 1.67E-10 | 0.14 |
| ZNF780B | -0.327205533 | 1.09E-28 | -0.291237429 | 8.25E-23 | 0.04 |
| ZNF781 | -0.141543642 | 2.63E-06 | -0.099337657 | 0.0010071 | 0.05 |
| ZNF782 | -0.340339546 | 4.82E-31 | -0.239494942 | 1.01E-15 | 0.11 |
| ZNF784 | 0.068139374 | 0.0242739 | 0.151472815 | 4.88E-07 | 0.09 |
| ZNF785 | -0.279410734 | 4.73E-21 | -0.205183696 | 7.45E-12 | 0.08 |
| ZNF786 | 0.014973913 | 0.6209481 | -0.013177935 | 0.6634244 | 0.03 |
| ZNF787 | 0.274252764 | 2.60E-20 | 0.213611576 | 9.57E-13 | 0.07 |
| ZNF788 | -0.2304764 | 1.21E-14 | -0.115183966 | 0.0001354 | 0.12 |
| LOC154950 | 0.206928365 | 4.91E-12 | 0.159416075 | 1.17E-07 | 0.05 |
| ZNF790 | -0.161959671 | 7.29E-08 | -0.056760746 | 0.0606681 | 0.11 |
| ZNF791 | -0.392608517 | 1.34E-41 | -0.401333848 | 1.48E-43 | 0.01 |
| ZNF792 | -0.348142175 | 1.70E-32 | -0.268454931 | 1.69E-19 | 0.08 |
| ZNF793 | -0.065552942 | 0.03022969 | -0.033931029 | 0.2623655 | 0.04 |
| ZNF799 | -0.243149074 | 3.58E-16 | -0.044737157 | 0.1393856 | 0.2 |
| ZNF79 | -0.30497388 | 5.85E-25 | -0.280388334 | 3.41E-21 | 0.03 |
| ZNF7 | 0.254889939 | 1.14E-17 | 0.096733666 | 0.0013651 | 0.16 |
| ZNF800 | -0.357956962 | 2.20E-34 | -0.29172483 | 6.95E-23 | 0.07 |
| ZNF804A | -0.205476279 | 6.95E-12 | -0.249562405 | 5.57E-17 | 0.05 |
| ZNF804B | -0.098720511 | 0.00108308 | -0.124638017 | 3.60E-05 | 0.03 |
| ZNF805 | -0.123106088 | 4.49E-05 | -0.089223009 | 0.0031544 | 0.04 |
| ZNF808 | -0.249030012 | 6.51E-17 | -0.157174741 | 1.76E-07 | 0.1 |
| ZNF80 | -0.004242576 | 0.88857937 | -0.143599219 | 1.87E-06 | 0.14 |
| ZNF813 | -0.30145669 | 2.13E-24 | -0.296140278 | 1.45E-23 | 0.01 |
| LOC649506 | -0.277663293 | 8.46E-21 | -0.127486346 | 2.37E-05 | 0.16 |
| ZNF815P | -0.07587451 | 0.01210048 | 0.055265095 | 0.0677901 | 0.14 |
| ZNF816 | -0.184646114 | 7.73E-10 | -0.039243401 | 0.1948297 | 0.15 |
| ZNF81 | -0.419193678 | 9.47E-48 | -0.389704208 | 5.84E-41 | 0.03 |
| ZNF821 | 0.138464121 | 4.34E-06 | 0.078459519 | 0.0094605 | 0.07 |
| ZNF823 | -0.260844717 | 1.85E-18 | -0.066504761 | 0.0279062 | 0.2 |
| LOC101929933 | -0.11037603 | 0.00025609 | 0.011078196 | 0.7144819 | 0.13 |
| ZNF827 | -0.459088075 | 4.43E-58 | -0.429600334 | 2.59E-50 | 0.03 |
| CHAMP1 | -0.204868663 | 8.03E-12 | -0.219013843 | 2.45E-13 | 0.02 |
| ZNF829 | -0.245500509 | 1.82E-16 | -0.228410206 | 2.11E-14 | 0.02 |
| ZNF830 | 0.081814322 | 0.00680438 | 0.061768465 | 0.0411801 | 0.03 |
| ZNF831 | -0.099777975 | 0.00095593 | -0.183835612 | 9.18E-10 | 0.09 |
| ZNF833P | 0.032063179 | 0.28956074 | 0.062351139 | 0.0393021 | 0.04 |
| ZNF835 | -0.275787017 | 1.57E-20 | -0.254478339 | 1.29E-17 | 0.03 |
| ZNF836 | -0.37090589 | 5.60E-37 | -0.302991537 | 1.22E-24 | 0.07 |
| ZNF837 | -0.052466555 | 0.08295666 | -0.037188825 | 0.2192589 | 0.02 |
| ZNF839 | -0.133720799 | 9.17E-06 | 0.014564891 | 0.630518 | 0.15 |
| ZNF83 | -0.175677399 | 5.01E-09 | -0.035477676 | 0.2412218 | 0.15 |
| ZNF841 | -0.308514026 | 1.56E-25 | -0.209222963 | 2.82E-12 | 0.1 |
| ZNF843 | -0.458017345 | 8.75E-58 | -0.338437986 | 1.07E-30 | 0.12 |
| ZNF844 | -0.512529165 | 3.00E-74 | -0.344587976 | 7.88E-32 | 0.17 |
| ZNF845 | -0.237805142 | 1.62E-15 | -0.180615589 | 1.81E-09 | 0.06 |
| ZNF846 | -0.331505214 | 1.90E-29 | -0.184206547 | 8.48E-10 | 0.15 |
| ZNF84 | -0.107602251 | 0.00036571 | -0.031750233 | 0.2942962 | 0.08 |
| DKFZp547K054 | -0.147595791 | 9.54E-07 | -0.207072971 | 4.74E-12 | 0.06 |
| ZNF85 | -0.021806489 | 0.4714048 | 0.033155737 | 0.2734318 | 0.06 |
| LOC651216 | -0.352644484 | 2.35E-33 | -0.274510958 | 2.39E-20 | 0.08 |
| LOC641907 | -0.231257132 | 9.79E-15 | -0.135621283 | 6.81E-06 | 0.1 |
| LOC647793 | -0.125881346 | 3.00E-05 | -0.107275421 | 0.0003812 | 0.02 |
| LOC342972 | 0.056105867 | 0.06370639 | -0.002896302 | 0.9238033 | 0.06 |
| ZNF879 | 0.017440815 | 0.56462399 | -0.006050848 | 0.8416235 | 0.03 |
| ZNF880 | -0.137722611 | 4.88E-06 | -0.111717392 | 0.0002149 | 0.03 |
| ZNF883 | 0.269053136 | 1.40E-19 | 0.163475396 | 5.48E-08 | 0.11 |
| ZNF8 | -0.044581308 | 0.14077033 | -0.092427937 | 0.0022225 | 0.05 |
| ZNF90 | 0.247535421 | 1.01E-16 | 0.172874593 | 8.81E-09 | 0.08 |
| ZNF91 | -0.427533241 | 8.51E-50 | -0.2596853 | 2.65E-18 | 0.17 |
| ZNF92 | -0.280390592 | 3.41E-21 | -0.213826638 | 9.07E-13 | 0.07 |
| ZNF93 | 0.075487645 | 0.01254717 | 0.049193415 | 0.1040593 | 0.03 |
| ZNF98 | 0.016693351 | 0.58142922 | -0.050302788 | 0.0964752 | 0.07 |
| ZNF99 | -0.170812458 | 1.33E-08 | -0.16325774 | 5.71E-08 | 0.01 |
| ZNFX1 | -0.310539923 | 7.29E-26 | -0.300204195 | 3.36E-24 | 0.02 |
| ZNHIT1 | 0.273417194 | 3.42E-20 | 0.330146412 | 3.31E-29 | 0.06 |
| ZNHIT2 | 0.18618701 | 5.55E-10 | 0.226019258 | 3.97E-14 | 0.04 |
| ZNHIT3 | 0.185593331 | 6.31E-10 | 0.281516373 | 2.33E-21 | 0.1 |
| ZNHIT6 | 0.087697421 | 0.00371263 | -0.093464483 | 0.0019799 | 0.19 |
| ZNRD1 | 0.506014911 | 4.02E-72 | 0.432711094 | 4.26E-51 | 0.08 |
| ZNRF1 | 0.359179773 | 1.27E-34 | 0.196694127 | 5.40E-11 | 0.17 |
| ZNRF2 | -0.275173935 | 1.92E-20 | -0.049110502 | 0.1046446 | 0.23 |
| ZNRF3 | -0.165271517 | 3.89E-08 | -0.2309344 | 1.07E-14 | 0.07 |
| ZNRF4 | -0.041508458 | 0.17027732 | -0.025397129 | 0.4015717 | 0.02 |
| MGC87693 | 0.089733682 | 0.00298535 | 0.097467678 | 0.0012539 | 0.01 |
| ZP2 | -0.012626552 | 0.67669324 | 0.014189269 | 0.6393614 | 0.03 |
| ZP3 | 0.262023851 | 1.28E-18 | 0.269744945 | 1.12E-19 | 0.01 |
| ZP4 | 0.240333457 | 7.96E-16 | 0.085425289 | 0.0047112 | 0.16 |
| ZPBP2 | 0.098570521 | 0.00110233 | 0.044177271 | 0.1444093 | 0.06 |
| LOC115810 | -0.040082015 | 0.18545346 | -0.056656986 | 0.0611413 | 0.02 |
| ZPLD1 | -0.08736378 | 0.00384608 | -0.010772385 | 0.7220324 | 0.08 |
| ZRANB1 | -0.090388266 | 0.00278074 | -0.195207199 | 7.57E-11 | 0.11 |
| ZRANB2 | 0.079619025 | 0.00845302 | -0.042750329 | 0.1578387 | 0.13 |
| ZRANB3 | -0.164291268 | 4.69E-08 | -0.167073912 | 2.76E-08 | 0.01 |
| ZRSR2 | 0.029764766 | 0.32554278 | 0.086477773 | 0.0042218 | 0.06 |
| ZSCAN10 | 0.016517893 | 0.58540763 | 0.024369175 | 0.4209028 | 0.01 |
| ZNF96P1 | -0.023009791 | 0.44728656 | -0.144704906 | 1.56E-06 | 0.13 |
| ZSCAN12 | -0.245317147 | 1.92E-16 | -0.213673964 | 9.42E-13 | 0.04 |
| ZSCAN16 | 0.195383293 | 7.27E-11 | 0.292839115 | 4.70E-23 | 0.1 |
| ZSCAN18 | -0.143474127 | 1.91E-06 | -0.122639295 | 4.80E-05 | 0.03 |
| ZSCAN1 | -0.133575662 | 9.38E-06 | -0.087116561 | 0.0039477 | 0.05 |
| ZSCAN20 | -0.327338455 | 1.03E-28 | -0.315394319 | 1.14E-26 | 0.02 |
| LOC115537 | 0.059886448 | 0.04777209 | 0.097418806 | 0.001261 | 0.04 |
| ZNF50 | -0.204978152 | 7.83E-12 | -0.167119551 | 2.73E-08 | 0.04 |
| ZSCAN23 | -0.351722009 | 3.54E-33 | -0.342076736 | 2.31E-31 | 0.01 |
| ZSCAN29 | -0.346409204 | 3.60E-32 | -0.291093166 | 8.68E-23 | 0.06 |
| LOC90393 | -0.063699023 | 0.03523444 | -0.154153478 | 3.04E-07 | 0.1 |
| ZSCAN4 | -0.10175705 | 0.0007543 | -0.139281805 | 3.80E-06 | 0.04 |
| ZSCAN5A | 0.202091532 | 1.55E-11 | -0.012235497 | 0.6861653 | 0.22 |
| ZSCAN5B | 0.095091377 | 0.0016478 | -0.038596488 | 0.2022964 | 0.14 |
| ZSWIM1 | 0.023452478 | 0.43859309 | 0.104093017 | 0.0005673 | 0.09 |
| ZSWIM2 | 0.029391948 | 0.33164198 | -0.004941214 | 0.870382 | 0.04 |
| ZSWIM3 | 0.124570786 | 3.63E-05 | 0.099993468 | 0.0009318 | 0.03 |
| ZSWIM4 | 0.187598076 | 4.09E-10 | 0.036421188 | 0.2289276 | 0.16 |
| ZSWIM5 | -0.34764934 | 2.10E-32 | -0.252676812 | 2.21E-17 | 0.1 |
| ZSWIM6 | -0.348239918 | 1.63E-32 | -0.355797878 | 5.79E-34 | 0.01 |
| ZSWIM7 | 0.127340176 | 2.42E-05 | 0.172262641 | 9.96E-09 | 0.05 |
| LOC154274 | 0.168444289 | 2.11E-08 | 0.143829629 | 1.80E-06 | 0.03 |
| ZW10 | 0.151495533 | 4.86E-07 | 0.069712022 | 0.021173 | 0.09 |
| ZWILCH | 0.282471612 | 1.69E-21 | 0.082864851 | 0.0061227 | 0.2 |
| ZWINT | 0.472691218 | 6.29E-62 | 0.286593602 | 4.14E-22 | 0.19 |
| ZXDA | -0.458035991 | 8.65E-58 | -0.426416999 | 1.61E-49 | 0.04 |
| ZXDB | -0.301310611 | 2.25E-24 | -0.246747845 | 1.27E-16 | 0.06 |
| LOC100130182 | -0.243444379 | 3.29E-16 | -0.211606839 | 1.57E-12 | 0.04 |
| ZYG11A | 0.211277224 | 1.70E-12 | 0.187827743 | 3.89E-10 | 0.03 |
| ZYG11B | -0.230481113 | 1.21E-14 | -0.291475373 | 7.59E-23 | 0.07 |
| ZYX | 0.028980617 | 0.33845628 | 0.030306721 | 0.3168073 | 0.01 |
| FLJ10821 | -0.425848517 | 2.23E-49 | -0.481756009 | 1.37E-64 | 0.06 |
| ZZZ3 | -0.167112452 | 2.73E-08 | -0.267561058 | 2.25E-19 | 0.11 |
| HUMRTVLH3 | -0.104015957 | 0.00057267 | -0.036600852 | 0.2266379 | 0.07 |
| AKR1C6P | 0.147613709 | 9.51E-07 | 0.042325305 | 0.162016 | 0.11 |
